# Exposure to fine particulate matter in adults is associated with immune cell gene expression related to inflammation, the electron transport chain, and cell cycle regulation

**DOI:** 10.1093/eep/dvaf008

**Published:** 2025-04-01

**Authors:** Amanda Rundblad, Siddhartha Das, Bigina N.R Ginos, Jason Matthews, Kirsten B Holven, Trudy Voortman, Stine M Ulven

**Affiliations:** Department of Nutrition, Institute of Basic Medical Sciences, University of Oslo, Oslo 0317, Norway; Department of Nutrition, Institute of Basic Medical Sciences, University of Oslo, Oslo 0317, Norway; Department of Epidemiology, Erasmus MC, University Medical Center Rotterdam, Rotterdam 3015, the Netherlands; Department of Nutrition, Institute of Basic Medical Sciences, University of Oslo, Oslo 0317, Norway; Department of Pharmacology and Toxicology, University of Toronto, Toronto, ON M5S1A8, Canada; Department of Nutrition, Institute of Basic Medical Sciences, University of Oslo, Oslo 0317, Norway; Norwegian National Advisory Unit on Familial Hypercholesterolemia, Department of Endocrinology, Morbid Obesity and Preventive Medicine, Oslo University Hospital, Oslo 0424, Norway; Department of Epidemiology, Erasmus MC, University Medical Center Rotterdam, Rotterdam 3015, the Netherlands; Department of Nutrition, Institute of Basic Medical Sciences, University of Oslo, Oslo 0317, Norway

**Keywords:** pollutant, PM_2.5_, natural spaces, gene expression, adults

## Abstract

Exposure to air pollution and an unhealthy built environment increase disease risk by impacting metabolic risk factors and inflammation, potentially via epigenetic modifications and effects on gene expression. We aimed to explore associations between fine particulate matter (PM_2.5_), black carbon, ozone, nitrogen dioxide, distance to nearest water body, normalized difference vegetation index, and impervious surface and gene expression profiles in adults. This study is a part of the LongITools project and includes cross-sectional data from the Rotterdam Study, a population-based cohort study, and NoMa, a randomized controlled trial. Environmental exposures were assigned using land-use regression (LUR) models and satellite data. Gene expression was assessed with whole blood RNA sequencing (Rotterdam Study, *n* = 758) and microarray analyses in peripheral blood mononuclear cells (NoMa, *n* = 100). We analysed transcriptomic profiles and enriched pathways associated with each of the environmental exposures. PM_2.5_ had the strongest gene expression associations, while only a few significant associations were observed for the other environmental exposures. In both populations, exposure to PM_2.5_ was associated with genes and pathways related to inflammation, oxidative stress, DNA metabolism, cell cycle regulation, histones, electron transport chain, oxidative phosphorylation, and neural signalling. This study is limited by different methods for RNA quantification, a cross-sectional design, and a small sample size. However, in both populations, exposure to PM_2.5_ resulted in the maximum number of associations with gene expression. In conclusion, PM_2.5_ is strongly associated with various gene expression profiles, which provide information about the underlying mechanisms of the detrimental health effects of exposure to PM_2.5_.

## Graphical Abstract

**Figure:**
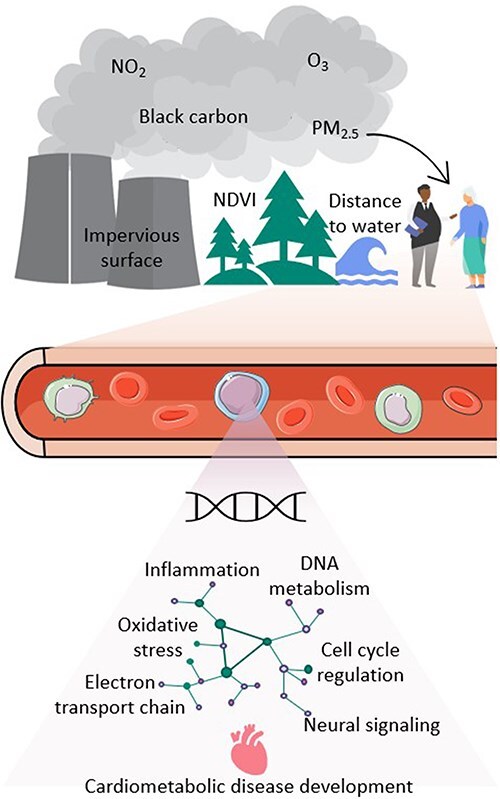

## Introduction

Among environmental exposures, exposure to air pollution is the most important cause of morbidity and premature mortality [1, 2]. Through these effects, air pollution is also a substantial contributor to health-related costs and thus a financial burden on society [1]. According to the Global Burden of Disease study, air pollution is the fourth most important risk factor for total mortality and disability adjusted life years, and this is mainly attributed to the increased risk of cardiometabolic diseases [2]. Among air pollutants such as particulate matter (PM), nitrogen dioxide (NO_2_), black carbon, and ozone (O_3_), fine particulate matter with a diameter of <2.5 µm (PM_2.5_) is most detrimental to health [3]. The Effects of Low-Level Air Pollution—A Study in Europe (ELAPSE) project found that exposure to PM_2.5_, even at levels below the 2005 World Health Organization (WHO) guideline of 10 µg/m^3^, was associated with cardiovascular disease (CVD) and mortality [4, 5]. In addition to air pollution, features of the built environment, including green space and distance to nearest water body, have been identified as important factors in determining the risk of cardiometabolic diseases [6, 7]. Living in areas that are high in green space that can be assessed by the normalized difference vegetation index (NDVI), is associated with a reduced risk of CVD mortality [6].

Exposure to air pollution and a built environment with a lack of green space have been shown to increase the risk of cardiometabolic diseases by impacting metabolic risk factors, such as inflammatory markers, hypertension, insulin resistance, and dyslipidaemia [8–12]. DNA methylation, an epigenetic modification involved in regulating gene expression, can be influenced by external factors, including air pollution and other environmental features. Exposure to pollutants and variations in the built environment can lead to changes in DNA methylation patterns and potentially also affect the expression of genes that may be implicated in cardiometabolic health [13, 14]. Studying gene expression profiles associated with environmental exposures can provide valuable insight into the downstream effects of epigenetic modifications as well as the underlying molecular mechanisms of the detrimental health effects. Although little is known about the transcriptomic profiles associated with the built environment, there is some knowledge about exposure to air pollution and gene expression associations. In addition to affecting gene expression via epigenetic modifications, exposure to air pollution may activate transcription factors via sensing pathways such as toll-like receptors, reactive oxygen species (ROS), and polycyclic aromatic hydrocarbon sensing pathways [15]. *In vitro* studies exposing human peripheral blood mononuclear cells (PBMCs) to coarse particulate matter (PM_10_) found that expression of transcription factors associated with inflammatory pathways was increased, as well as expression of genes related to proinflammatory responses [16]. Acute exposure to air pollution in adults alters expression of genes related to inflammation, oxidative stress, and coagulation in circulating cells [17–20]. Moreover, genes related to cell-signalling, apoptosis, immune response, electron transport chain (ETC), and gene expression regulation have been associated with long-term exposure to air pollution in adults [21–23]. Some of these pathways are commonly expressed in autoimmune diseases [22], linking the transcriptomic profile of air pollutants with disease risk.

PM_2.5_ is a heterogeneous mixture of particles, aerosols, metals, salts, and organic chemicals, primarily from combustion of fossil fuels, emissions from industry, and production of electricity [3, 24]. Because of the small size of the particles, after inhalation, PM_2.5_ can pass into circulation where it can exert systemic effects in addition to having detrimental effects on lungs. Immune cells, both in whole blood and the PBMC pool, are exposed to PM_2.5_ in circulation and are important drivers of the development of inflammation in cardiometabolic diseases, such as atherosclerosis [25, 26]. Hence, exposure to PM_2.5_ may induce gene expression changes that can affect disease risk. Moreover, immune cells, in particular PBMCs, may reflect systemic gene expression [27, 28]. Hence, immune cells may provide a model system for understanding systemic health. However, more knowledge about gene expression profiles in circulating immune cells associated with environmental exposures in adults is needed to further elucidate the underlying mechanisms of air pollution and the built environment on health.

The aim of this study was to explore the transcriptomic profiles associated with exposure to air pollution and the built environment in adults and identify possible links to disease development.

## Materials and methods

### Studies (recruitment, inclusion criteria, and ethics)

This study is a part of LongITools (www.longitools.org), a project funded by the European Union’s Horizon 2020 research and innovation programme and a part of the European Human Exposome Network. LongITools aims to study the relationship between environmental exposures and cardiometabolic health by leveraging data from several European cohort studies and randomized controlled trials (RCTs) [29]. This specific study investigates the association between environmental exposures and gene expression in whole blood and PBMCs using data from the Rotterdam Study and the NoMa study.

The Rotterdam Study is a prospective, population-based cohort study in Ommoord, a neighbourhood in Rotterdam, the Netherlands, aiming to investigate multifactorial diseases in mid-life and the elderly [30]. It is composed of four unique subcohorts initiated in 1990, 2000, 2006, and 2016. For the first subcohort (RS-I: *n* = 7983), all residents of Ommoord aged ≥55 years were invited. For the second subcohort (RS-II: *n* = 3011), an invitation was extended to residents who had turned 55 years or had moved in newly and were at least 55 years. Those aged ≥45 years were invited to participate in the third subcohort (RS-III: *n* = 3932) and those aged ≥40 years to participate in the fourth cohort (RS-IV: *n* = 3005). After study inclusion and baseline visits to the research centre, participants attend follow-up visits every 3–6 years. RNA sequencing was done in a random subsample of participants from the first three subcohorts during examination rounds taking place between 2009 and 2013. This random subsample corresponds to the fifth visit for RS-I (*n* = 27, years 2009–2010), third visit for RS-II (*n* = 504, years 2009–2012), and second visit for RS-III (*n* = 276, years 2010–2013). Fasting blood samples and anthropometric measures were collected at the examination centre, and data on education and smoking were collected during home interviews. The Rotterdam Study has been approved by the Medical Ethics Committee of Erasmus MC (registration number MEC 02.1015) and the Dutch Ministry of Health, Welfare and Sport (Population Screening Act WBO, license number 1071272-159521-PG). The Rotterdam Study Personal Registration Data collection is filed with the Erasmus MC Data Protection Officer under registration number EMC1712001. The Rotterdam Study has been entered into the Netherlands National Trial Register (NTR; https://onderzoekmetmensen.nl/en/trial/23050) and into the WHO International Clinical Trials Registry Platform (https://www.who.int/clinical-trials-registry-platform, search portal https://trialsearch.who.int/) under shared catalogue number NTR6831. All participants provided written informed consent to participate in the study and to have their information obtained from treating physicians.

The NoMa study is an 8-week RCT designed to investigate health effects of exchanging a few commercially available, regularly consumed food items with improved fat quality [31]. The trial recruited healthy women and men aged 25–70 years through advertisements in local newspapers and as flyer postings in the greater Oslo area between 2012 and 2014. After an initial telephone interview, participants were invited to a screening visit for clinical assessment and blood sampling. In this paper, we have used cross-sectional data collected at the screening visit. We included all participants who signed a new consent in order to use their residential addresses to obtain environmental exposure data as well as PBMC gene expression, and relevant covariates (*n* = 100). The study protocol and the procedures for collecting clinical and biochemical variables are described in detail elsewhere [31]. All participants gave written informed consent, and the study was approved by the Regional Ethics Committee for Medical Research in South East Norway (2011/1951) and registered at ClinicalTrials.gov (ClinicalTrials.gov Identifier: NCT 01679496).

### Environmental exposures

The residential addresses of the participants at the time of RNA sampling were geocoded and linked to exposure surfaces for PM_2.5_, NO_2_, annual mean and warm season O_3_, black carbon, distance to nearest water body, impervious surface, and NDVI, a measure of green space.

The ELAPSE project (http://www.elapseproject.eu) created LUR models for 2010 to estimate environmental exposure maps for annual mean PM_2.5_, NO_2_, black carbon, and O_3_ concentrations as well as warm season estimates for O_3_ [32]. The LUR models for PM_2.5_, NO_2_, and O_3_ were based on AirBase routine monitoring data and black carbon was based on ESCAPE monitoring data [33], and satellite observations, dispersion model estimates, land use, and traffic data were used to develop the models. Back-and-forward extrapolation from 2010 was performed using the Danish Eulerian Hemispheric Model with population-weighted average concentrations at the Nomenclature of territorial units for statistics spatial scale to reflect four Dutch and one Norwegian socioeconomic region [34, 35]. With this method, we could calculate individual exposure concentrations by multiplying the 2010 level with conversion factors for 2009–2013 for the Rotterdam Study and 2012-2014 for NoMa.

The Copernicus Institute created exposure maps for water bodies (EU-Hydro) and impervious surfaces. EU-Hydro consists of surface interpretation of lakes and wide rivers, and a drainage model derived from EU-DEM. The impervious surface map shows the percentage and change of soil sealing. Built-up areas are characterized by the substitution of natural land cover with an artificial impervious cover. The map provides per-pixel estimates of the degree of imperviousness, ranging from 0% to 100%. Finally, NDVI maps were created with the Terra Moderate Resolution Imaging Spectroradiometer (MODIS) Vegetation Indices (MOD13Q1), generated every 16 days at 250 m spatial resolution as a Level 3 product. Because the quality of the NDVI depends on satellite pictures, we used the NDVI in a buffer of 500 m around the residential address, where the NDVI was unvalidated if the percentage of pictures available during the year was <50%.

### Covariates

In this study, we used variables that have been harmonized within the LongITools consortium to ensure that variables are coded the same way across studies. As an example, education was classified according to the International Standard Classification of Education 97/2011 (ISCED-97/2011).

Specifically, ISCED-2011: 5-8 or ISCED-97: 5-6 was coded as high education, ISCED-2011: 3-4 or ISCED-97: 3-4 was coded as medium education, and ISCED-2011: 0-2 or ISCED-97: 0-2 was coded as low education. An overview of harmonized variables in the Rotterdam Study and the NoMa study, in addition to a description of the mapping from cohort-specific variables to harmonized variables, is available at https://data-catalogue.molgeniscloud.org/catalogue/catalogue/#/networks-catalogue/LongITools. In addition to the harmonized variables, the percentage of monocytes and lymphocytes in the PBMC pool and the percentage of monocytes, lymphocytes, and granulocytes in whole blood were calculated from white blood cell counts.

### Gene expression analysis

In the Rotterdam Study, gene expression in whole blood was analysed with RNA sequencing, as previously described [36]. In brief, total RNA was isolated from whole blood and depleted of globin transcripts with the Ambion GLOBINclear kit. Library preparation was performed with Illumina TruSeq version 2, and Illumina HiSeq 2000 was used to perform paired-end sequencing, aiming at >15 million read pairs per sample. Included reads passed the Illumina Chastity Filter. The quality of raw reads was checked using FastQC, identified adaptors were clipped using cutadapt, and low-quality ends were trimmed using Sickle. Read alignment was performed using STAR 2.3.0e. To prevent reference mapping bias, all Genome of the Netherlands single nucleotide polymorphisms with a minor allele frequency >0.01 in the reference genome were replaced with N’s. Only read pairs that had a maximum of eight mismatches and mapped to a maximum of five positions were used. SAMtools flagstat was used to get mapping statistics from BAM files, and Picard tools were used to assess 5′ and 3′ coverage bias, duplication rate, and insert sizes. Gene expression was estimated using Ensembl v.71 annotation.

In the NoMa study, gene expression was assessed in PBMCs using microarray, with a protocol that has been described in detail previously [37]. Briefly, PBMCs were isolated from fasting blood samples using BD Vacutainer Cell Preparation Tubes (Becton Dickinson, Franklin Lakes, NJ, USA), and RNA was isolated using the RNeasy mini kit with DNase I treatment (Qiagen, Hilden, Germany). The RNA quantity and quality were determined using NanoDrop (Thermo Fisher Scientific, Gothenburg, Sweden) and Agilent 2100 Bioanalyser (Agilent Technology, Santa Clara, CA, USA). The standard Illumina protocol (Illumina Inc., San Diego, CA, USA) was followed for microarray gene expression analyses. Initially, cRNA was prepared using Thermo Fisher Scientific’s Ambion® Illumina® TotalPrep RNA Amplification Kit and hybridized to HumanHT-12 Expression BeadChips that were scanned using Illumina HiScan instrument. Bead-level data were transformed into probe-level intensities and extracted using Illumina Genome Studio and BeadStudio softwares. Samples were log2-transformed, we removed batch effects due to microarray chip and plate, and we excluded genes with sufficient expression in <25 samples, leaving 13 967 transcripts for further analysis.

### Statistical analyses

All statistical analyses were performed in R (version 4.4.0), and R packages and functions are referred to as ‘package::function()’. Participant characteristics are presented as number (%) for categorical variables, mean [standard deviation (SD)] for normally distributed variables, and median [interquartile range (IQR)] for skewed variables. Skewness was assessed by visual inspection of the distribution and with ‘e1071::skewness()’.

For the RNA sequencing data in the Rotterdam Study, differential gene expression analyses were performed using the ‘edgeR’ package. First, genes located on autosomes were filtered using gene annotations from UCSG hg38 and GENCODE v38. Secondly, genes with a low expression level were filtered out using ‘edgeR::filterByExpr()’, and because this function uses the design model matrix as an argument, the number of genes filtered varied slightly among the different exposures. For PM_2.5_, 20 508 genes were included in analyses. Thirdly, the library size was normalized across samples using ‘edgeR::normLibSizes()’. Finally, before fitting a model, common, trended, and tagwise dispersions were estimated using ‘estimateGLMCommonDisp()’, ‘estimateGLMTrendedDisp()’, and ‘estimateGLMTagwiseDisp()’. For each exposure, differentially expressed genes, adjusted for age, sex, body mass index (BMI), education, smoking, RNA sequencing batch, and % monocytes, % lymphocytes, and % granulocytes were estimated using ‘glmFit()’ and a likelihood ratio test ‘glmLRT()’. Because of linear dependencies between distance to water and percent monocytes, it was not possible to adjust for percent monocytes when analysing genes associated with distance to water. Genes associated with environmental exposures at a false discovery rate (FDR) of ≤5% were considered significant.

In the NoMa study, differential gene expression analysis of the microarray data was performed using the ‘limma’ package. A total of 11 069 genes annotated to autosomes in Ensemble GRCh37 were retained for data analysis. Differentially expressed genes, adjusted for age, sex, BMI, education, smoking, % monocytes, and % lymphocytes, were estimated using ‘limma::lmFit()’. Moderated *t*-statistics and *F*-statistics, and log-odds of differential expression were calculated by empirical Bayes moderation of the standard errors using ‘limma::eBayes()’, and the mean-variance of the expression data after fitting the model was checked using ‘plotSA()’. Finally, coefficients and *P*-values were extracted using ‘topTable()’. Because of a low statistical power in NoMa compared to the Rotterdam Study, genes associated with environmental exposures at an FDR of <15% were considered statistically significant.

### Enrichment analyses

We performed enrichment analyses in Metascape (www.metascape.org) for genes associated with PM_2.5_ with a nominal *P*-value cut-off of .05. We did separate enrichment analyses for genes negatively and positively associated with PM_2.5_. Each enrichment analysis used GO Biological Processes, Hallmark Gene Sets, Reactome Gene Sets, WikiPathways, and KEGG Pathway as ontology sources, and an enrichment background gene list was provided. The enrichment factor was calculated as the ratio between the number of observed genes in a term and the number expected by chance. All terms with *P* < .01, count > 3, and an enrichment factor of >1.5 were clustered with hierarchical clustering based on membership similarities. The most significant term within a cluster was chosen to represent the cluster.

## Results

### Participant characteristics

We had available gene expression data for *n* = 807 participants from the Rotterdam Study and *n* = 239 participants in NoMa. After excluding participants with missing data on environmental exposures and covariates included in the main model, *n* = 758 participants remained in the Rotterdam Study. In NoMa, gene expression data, and air pollution data were available for *n* = 100 participants, and data on distance to nearest water body, impervious surface, and NDVI were available for *n* = 92 participants. The study population in NoMa was on average 14 years younger and was more highly educated than participants in the Rotterdam Study, while there was an even distribution of men and women and smokers in the two studies (Table 1). In both studies, the concentrations of cardiometabolic risk markers such as cholesterol and glucose were within the normal ranges. The exposures to the air pollutants PM_2.5_, black carbon, and NO_2_ were on average higher in the Rotterdam Study, while O_3_ exposures were higher in NoMa. Finally, % impervious surface was higher in the Rotterdam Study, while distance to nearest water body and NDVI were similar in the two studies.

**Table 1.** Characteristics of the participants in the Rotterdam Study and NoMa

	Rotterdam Study (*n* = 758)	NoMa (*n* = 100^a^)
Sex *n* (%)		
Female	438 (57.8)	54 (54)
Male	320 (42.2)	46 (46)
Education *n* (%)		
Lower	391 (51.6)	1 (1)
Middle	213 (28.1)	15 (15)
Higher	154 (20.3)	84 (84)
Smoking *n* (%)		
Nonsmoker	653 (86.1)	88 (88)
Occasional smoker	29 (3.8)	4 (4)
Regular smoker	76 (10)	8 (8)
Age (years), mean (SD)	67.86 (6.34)	54.41 (10.36)
BMI (kg/m^2^), mean (SD)	27.81 (4.22)	24.63 (3.43)
Total cholesterol (mmol/l), mean (SD)	5.5 (1.0)	6.4 (1.0)
LDL-cholesterol (mmol/l), mean (SD)	3.7 (1.0)^b^	3.9 (0.8)
HDL-cholesterol (mmol/l), mean (SD)	1.50 (0.44)	1.7 (0.5)
Fasting triglycerides (mmol/l), mean (SD)	1.3 (0.76)^c^	1.04 (0.70)^c^
Fasting glucose (mmol/l), mean (SD)	5.4 (1.0)^c^	5.2 (0.6)^c^
PM_2.5_ (µg/m^3^), mean (SD)	18.37 (1.47)	10.01 (1.13)
Black carbon (µg/m^3^), mean (SD)	1.49 (0.18)	0.65 (0.23)
O_3_ (annual mean, µg/m^3^), mean (SD)	57.52 (2.18)	63.50 (3.06)
O_3_ (warm season, µg/m^3^), mean (SD)	71.28 (3.34)	72.38 (2.58)
NO_2_ (µg/m^3^), mean (SD)	35.21 (3.39)	20.52 (5.06)
Distance to nearest water body (m), mean (SD)	1000 (410)	1024 (928)^c^
Impervious surface (%), mean (SD)	43.7 (23.3)	23 (22.5)^c^
NDVI, mean (SD)	0.56 (0.05)	0.58 (0.08)^c^

^a^ *n* = 92 for distance to nearest water body, impervious surface, and NDVI.

^b^LDL-C in the Rotterdam Study calculated with Friedewald’s formula.

^c^Median (IQR).

### Gene expression associations with PM_2.5_

**Figure 1.**
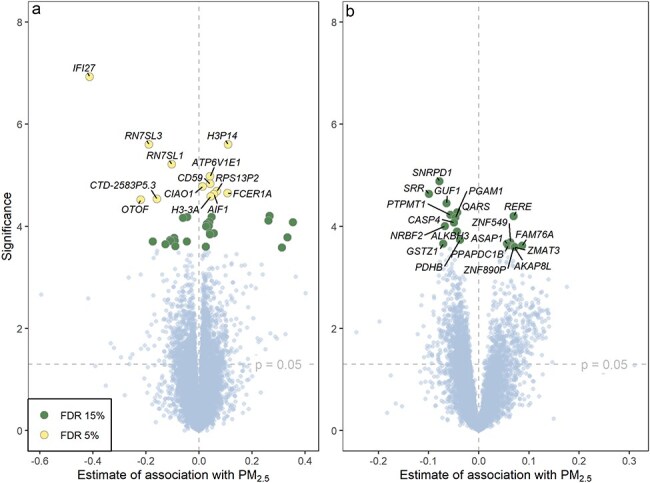
Volcano plot of all genes included in the analysis of association with PM_2.5_ in the (a) Rotterdam Study and (b) the NoMa study. The figure shows the statistical significance [−log_10_(*P*-value)] and the estimate of association with PM_2.5_, adjusted for age, sex, BMI, education, current smoking, %monocytes, and %lymphocytes. Results of the Rotterdam Study are also adjusted for RNA sequencing batch and %granulocytes. Genes associated with exposure to PM_2.5_ with an FDR of <5% were considered significant in the Rotterdam Study. Because of a lower power in the NoMa study, we considered FDR < 15% as significant. None of the genes were associated with PM_2.5_ at an FDR of <5% in NoMa. The dashed line corresponds to the *P*-value cut-off for genes to be included in pathway analyses.

PM_2.5_ was the environmental exposure with most associated genes. In the Rotterdam Study, 20 508 genes were included in analyses after filtering out low counts, and after adjusting for multiple testing (FDR < 5%), 13 genes were significantly associated with PM_2.5_ (Table 2, Fig. 1a). Of these, five were negatively associated and eight were positively associated. Most of the significantly associated genes had functions related to immune and allergic responses, histones, and translation to ER lumen (Table 2).

**Table 2.** Genes significantly associated (FDR < 5%) with exposure to PM_2.5_ in the Rotterdam Study

Gene symbol	Gene name	Estimate of association	*P*-value	FDR *q*-value	Function
*IFI27*	Interferon alpha-inducible protein 27	−0.41	1E-07	0.00	Immune response
*OTOF*	Otoferlin	−0.22	3E-05	0.05	Vesicle membrane fusion
*RN7SL3*	RNA component of signal recognition particle 7SL3	−0.19	3E-06	0.02	Translation into ER lumen
*CTD-2583P5.3*	Uncharacterized LOC105371090	−0.16	3E-05	0.05	Noncoding RNA
*RN7SL1*	RNA component of signal recognition particle 7SL1	−0.10	6E-06	0.03	Translation into ER lumen
*CIAO1*	Cytosolic iron (Fe)–sulphur (S) assembly component 1	0.01	2E-05	0.05	Delivery of Fe–S clusters to proteins
*ATP6V1E1*	ATPase H^+^ transporting V1 subunit E1	0.04	1E-05	0.04	Acidification of organelles
*CD59*	CD59 molecule	0.04	1E-05	0.05	Immune response
*H3-3A*	H3.3 histone A	0.04	3E-05	0.05	Histone
*AIF1*	Allograft inflammatory factor 1	0.06	2E-05	0.05	Immune response
*RPS13P2*	Ribosomal protein S13 pseudogene 2	0.07	2E-05	0.05	Pseudogene
*FCER1A*	Fc epsilon receptor Ia	0.11	2E-05	0.05	Allergic response
*H3P14*	H3 histone pseudogene 14	0.11	2E-06	0.02	Histone pseudogene

In NoMa, 11 069 genes were included in analyses. Because of low power, we used a less stringent threshold (FDR < 15%) and found that 19 genes were significantly associated with exposure to PM_2.5_ (Fig. 1b). Of these, 8 were positively and 11 were negatively associated. These genes have functions mainly related to regulation of histones, transcription, translation, and apoptosis (Table 3). There were no overlaps between the genes associated with PM_2.5_ in the Rotterdam Study and NoMa.

**Table 3.** Genes significantly associated (FDR < 15%) with exposure to PM_2.5_ in NoMa

Gene symbol	Gene name	Estimate of association	*P*-value	FDR *q*-value	Function
*SRR*	Serine racemase	−0.10	2E-05	0.11	Stereochemical inversion of serine
*SNRPD1*	Small nuclear ribonucleoprotein D1 polypeptide	−0.08	1E-05	0.11	mRNA splicing
*GSTZ1*	Glutathione S-transferase zeta 1	−0.07	2E-04	0.15	Detoxification
*NRBF2*	Nuclear receptor-binding factor 2	−0.07	1E-04	0.12	Autophagy
*GUF1*	GTP-binding elongation factor GUF1	−0.06	4E-05	0.11	Translation
*PTPMT1*	Protein tyrosine phosphatase mitochondrial 1	−0.06	6E-05	0.11	Apoptosis
*CASP4*	Caspase 4	−0.05	8E-05	0.12	Apoptosis
*QARS*	Glutaminyl-tRNA synthetase 1	−0.05	7E-05	0.11	Translation
*ALKBH3*	AlkB homolog 3, alpha-ketoglutarate-dependent dioxygenase	−0.04	1E-04	0.14	DNA repair
*PGAM1*	Phosphoglycerate mutase 1	−0.04	5E-05	0.11	Glycolysis
*PDHB*	Pyruvate dehydrogenase E1 subunit beta	−0.04	2E-04	0.15	PDH complex
*ASAP1*	ArfGAP with SH3 domain, ankyrin repeat, and PH domain 1	0.06	2E-04	0.15	Membrane trafficking and cytoskeleton remodelling
*PLPP5*	Phospholipid phosphatase 5	0.06	2E-04	0.15	Phospholipid dephosphorylation
*ZNF549*	Zinc finger protein 549	0.06	2E-04	0.15	Transcription
*RERE*	Arginine-glutamic acid dipeptide repeats	0.07	6E-05	0.11	Apoptosis
*ZNF890P*	ZNF890P zinc finger protein 890, pseudogene	0.07	3E-04	0.15	Pseudogene, no open reading frame
*AKAP8L*	A-kinase anchoring protein 8 like	0.07	3E-04	0.15	Histone regulation
*ZMAT3*	Zinc finger matrin-type 3	0.07	3E-04	0.15	p53-dependent growth regulation
*FAM76A*	Family with sequence similarity 76 member A	0.09	2E-04	0.15	Predicted role in transcriptional regulation

**Figure 2.**
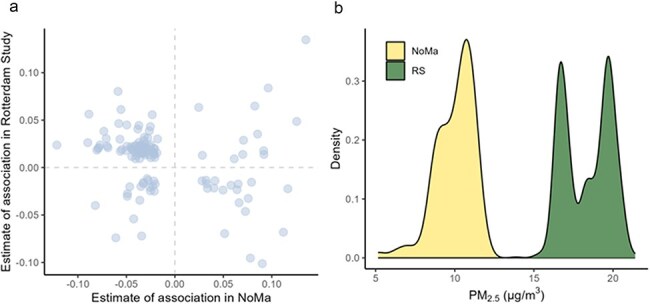
(a) Correlation plot of the estimate of association with PM_2.5_ for all genes with a significant (nominal *P* < .05) association in both the Rotterdam Study and NoMa. Most of the genes that were associated with PM_2.5_ were associated in the opposite directions. (b) The distribution of PM_2.5_ exposure in the Rotterdam Study (RS) was higher than and not overlapping with the PM_2.5_ exposure in NoMa.

In the Rotterdam Study, 1668 genes were significantly associated with exposure to PM_2.5_ at a nominal *P*-value of <.05, while 1389 genes were associated in NoMa. Of these, 126 genes were overlapping in the two studies. Surprisingly, 75% of these were associated with PM_2.5_ in the opposite direction (Fig. 2a). Because we suspected that this could be due to different exposure levels, we plotted the PM_2.5_ exposure distribution in the two studies and found that they were not overlapping, with a higher exposure in the Rotterdam Study (Fig. 2b). In sensitivity analyses of separate subcohorts of the Rotterdam Study, we found that this pattern of overlapping significant associations in opposite directions was more pronounced in RS-II than in RS-III (results not shown). The exposure to PM_2.5_ in RS-II corresponds to the highest peak at ∼20 µg/m^3^ (Fig. 2b). Of the genes significantly (nominal *P*-value < .05) associated with PM_2.5_ in both NoMa and RS-II, 62% were associated with PM_2.5_ in opposite directions. On the other hand, the exposure to PM_2.5_ was lower in RS-III, ∼16 µg/m^3^, and 42% of the genes were associated with PM_2.5_ in the opposite direction compared to NoMa. Hence, greater difference in exposure level could be a potential reason for the opposing direction of association between NoMa and the Rotterdam Study.

### Gene expression associations with all other environmental exposures

Compared to exposure to PM_2.5_, there were few significant gene expression associations with the other environmental exposures in the Rotterdam Study. Black carbon exposure was positively associated to a pseudogene (MTND2P28), related to the gene MTND2, which is involved in the ETC, while the expression of the genes encoding the growth factor midkine (*MDK*) and the cytoskeleton protein septine 4 (SEPTIN4) were negatively associated with exposure to annual mean O_3_. The greenness index NDVI was negatively associated with immunoglobulin kappa variable 2-29 and -30 (IGKV2-29 and IGKV2-30), and strongly positively associated with a gene encoding an adhesion G protein-coupled receptor (ADGRG7, estimate of association = 23.6). Finally, distance to nearest water body was negatively associated with RP11-797H7.1 with unknown function, and the pseudogene MTCYBP18 related to genes in the ETC, and positively associated to the gene encoding otoferin (OTOF) that was also negatively associated with PM_2.5_. There were no significant associations with NO_2_, warm season O_3_, or impervious surface (FDR < 5%). All gene expression associations with all environmental exposures in the Rotterdam Study can be found in Supplementary File 1.

There were no significant (FDR < 15%) gene expression associations with exposure to black carbon, NO_2_, or annual or warm season O_3_ or with distance to nearest water body, impervious surface, or NDVI in the NoMa study (Supplementary File 2).

### Pathways associated with exposure to PM_2.5_

We investigated pathways associated with PM_2.5_ by doing enrichment analysis in Metascape separately for genes negatively and positively associated with PM_2.5_ exposure (nominal *P*-value < .05). Enriched terms were clustered based on membership similarities, and each cluster is represented by the most significant term within the cluster.

**Figure 3.**
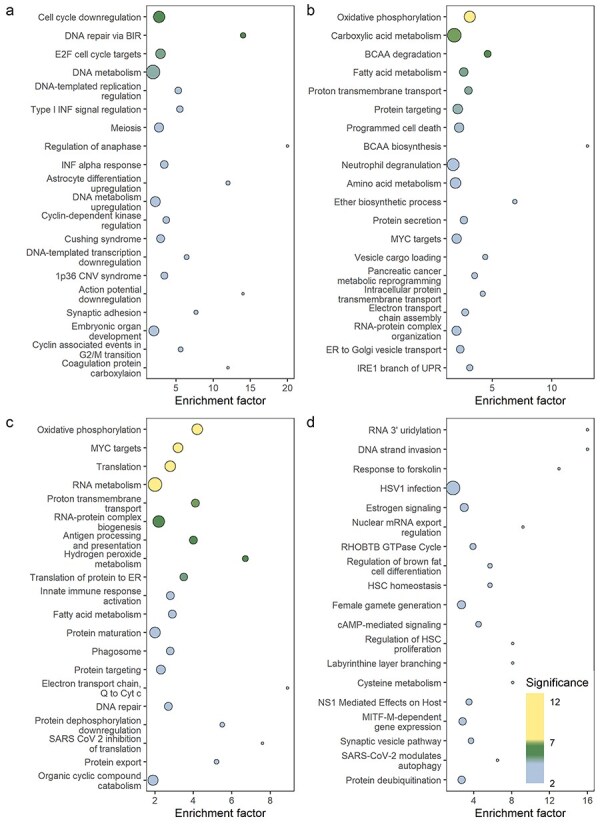
Pathways enriched among genes with a significant (*P* < .05) negative association with PM_2.5_ in the Rotterdam Study (a) and in NoMa (b) and among genes with a positive association with PM_2.5_ in the Rotterdam Study (c) and in NoMa (d). The plots show the top 20 most significant clusters of terms. Each cluster is represented with the most significant term in the cluster. The terms are ranked and coloured according to statistical significance [−log_10_(*P*-value)], and the size of the points corresponds to the number of genes in a term associated to PM_2.5_. The enrichment factor is the ratio between the number of observed genes in a term and the number expected by chance. BCAA, branched-chain amino acid, BIR, break-induced repair, cAMP, cyclic AMP, CNV, copy number variation, Cyt C, cytochrome C, ER, endoplasmic reticulum, HSC, haematopoietic stem cell, HSV1, herpes simplex virus 1, INF, interferon, Q, ubiquinol.

Most of the pathways enriched among the negatively associated genes in the Rotterdam Study were related to DNA metabolism and cell division. The top 20 enriched pathways are shown in Fig. 3a and include cell cycle downregulation, DNA repair, DNA metabolism, DNA-templated replication, downregulation of transcription, and meiosis. In addition, some of the other pathways were related to interferon (INF) response, coagulation, and the nervous system. On the other hand, enriched pathways among the genes negatively associated with PM_2.5_ in the NoMa study were related to oxidative phosphorylation, ETC, vesicle transport, branched chain amino acid metabolism, neutrophil degranulation, and apoptosis (Fig. 3b).

Of the genes positively associated with PM_2.5_ in the Rotterdam Study, many of the enriched pathways were related to translation and post-translational processing, such as translation of proteins to ER and protein maturation, targeting, and export (Fig. 3c). Other, enriched pathways included those related to oxidative phosphorylation, the ETC, and H_2_O_2_ metabolism. Finally, some pathways related to immune responses, including innate immune response and antigen processing and presentation, were also enriched. The enriched pathways among genes positively associated with PM_2.5_ in NoMa were related to signalling pathways, mRNA processing, immune response, and haematopoietic stem cell proliferation (Fig. 3d). The top 20 enriched clusters of terms, represented by the most significant term in the cluster, with full length ontology names are shown in Supplementary File 3.

Finally, we wanted to identify pathways that were enriched in both the Rotterdam Study and in NoMa. When examining overlaps between pathways in all clusters, we found that 92 enriched pathways overlapped. Interestingly, all the overlapping pathways that were positively associated with PM_2.5_ in the Rotterdam Study were negatively associated with PM_2.5_ in the NoMa study (Supplementary File 3). These overlapping pathways were related to oxidative phosphorylation, ETC, and cell cycle regulation, among others. Furthermore, pathways related to neurodegeneration, Alzheimer’s disease, and Parkinson’s disease were also overlapping.

## Discussion

In this study, we explored the gene expression profiles associated with environmental exposures and found that gene expression was primarily influenced by exposure to PM_2.5_. The other environmental exposures, such as black carbon, O_3_, NO_2_, distance to nearest water body, normalized difference vegetation index, and impervious surface, had fewer or no statistically significant gene expression associations. Exposure to PM_2.5_ was associated with genes and pathways related to inflammation, ROS metabolism, DNA metabolism, cell cycle regulation, histones, ETC, oxidative phosphorylation, and neural signalling. There were some findings that overlapped between the two study populations included in this study; however, many of the overlapping identified genes were associated in opposite directions.

The main finding in this study is that PM_2.5_ was the environmental exposure with the strongest gene expression associations. This is in line with a study by Vlaanderen *et al*. who found much stronger transcriptomic associations with PM_2.5_ compared to the weak associations for NO_2_, PM_10_, PM_coarse_, and ultrafine particles [21]. Here, we also demonstrated that PM_2.5_ is more strongly associated with transcriptomic alterations than exposure to O_3_, black carbon, distance to nearest water body, NDVI, and impervious surface. We have also observed that PM_2.5_ was the environmental exposure most strongly associated with gene expression in children residing in Rotterdam and Avon, UK [38]. These findings may indicate that PM_2.5_ has more detrimental effects at a molecular level compared to other environmental exposures, which may translate to more harmful health effects.

The harmful effects of PM_2.5_ arise from local effects in lungs after inhalation as well as systemic effects. In lungs, PM_2.5_ induces oxidative stress and inflammation [39], and our results show associations between PM_2.5_ and pathways related to inflammation and oxidative stress, as well as other pathways relevant for lung function and disease. For instance, we found PM_2.5_ to be associated with neutrophil degranulation, a process reported to be affected by air pollution and that results in the production of ROS and inflammatory mediators in the lungs, ultimately leading to pulmonary diseases [40]. Another cluster of pathways associated with PM_2.5_ in our study was embryonic development. Two of four members in that cluster were lung development and respiratory system development, indicating that this cluster of pathways is highly relevant for lung function. Exposure to PM_2.5_ is associated with increased risk of respiratory infections in adults [41]. Croft *et al*. found that exposure to air pollution was associated with pathways related to INF signalling, innate immune response, neutrophil activation, and leucocyte degranulation in patients with respiratory infection [42]. In our study, we found several immune response-related pathways to be enriched, including INF response, innate immune response activation, and antigen processing and presentation. These results are in accordance with other studies reporting that PM_2.5_ is associated with pathways related to INF signalling [21]. We found that interferon alpha-inducible protein 27 (*IFI27*) was the gene most significantly associated with exposure to PM_2.5_ in the Rotterdam Study. Gene expression of *IFI27* in peripheral blood in patients with respiratory viral infection has been shown to be associated with air pollution exposure [43]. Because *IFI27* is suggested to prevent an excessive immune response following viral infection [43], the negative association with PM_2.5_ may imply that air pollution exposure results in exaggerated immune responses following viral infections. Moreover, we have previously reported that air pollution exposure in children is negatively associated with pathways related to INF response and response to bacterial and viral infections [38]. Hence, our results indicate that air pollution exposure may result in an increased susceptibility to respiratory infections via effects on INF signalling, an effect that seems to persist from childhood to adult life.

The local inflammatory response in lungs includes the production of cytokines that can be transmitted into the blood with the subsequent induction of systemic inflammation [3]. Additionally, PM_2.5_ can pass through the six alveoli and into circulation where it can directly affect circulating immune cells and aggravate the oxidative stress and immune response [39]. These responses can ultimately accelerate the development of systemic inflammation, which can lead to atherosclerosis by sustaining the activation of the immune response. This chronic inflammatory state promotes the formation, progression, and destabilization of atherosclerotic plaques in the arteries, increasing the risk of cardiovascular events [25]. We found that PM_2.5_ was associated with immune cell expression of genes that were enriched in pathways related to DNA metabolism and cell cycle regulation, such as MYC targets. These findings may indicate that PM_2.5_ exposure induces an inflammatory response that will cause proliferation of immune cells.

Alteration of epigenetic modifications is suggested to be one of the main underlying molecular mechanisms by which air pollutants can exert their effects on human health. Particularly, DNA methylation has been given much attention as it is shown that both long-term and acute exposure to air pollution results in DNA methylation changes that may be related to disease development [14, 44]. Genes related to histones, including *H3-3A*, were among the top genes associated with PM_2.5_ exposure. Previously, air pollution exposure has been shown to affect histone modifications, and that the histone modifications were enriched in genes involved in immune activation [45, 46]. Another study found that expression of the histone gene *H3-3A* in the placenta is associated with exposure to PM_2.5_ in the second trimester of pregnancy [47]. Hence, PM_2.5_ exposure may induce epigenetic changes both through methylation and histone modifications from pregnancy and throughout the life course.

Surprisingly, of the 126 genes that were associated with PM_2.5_ in both the Rotterdam Study and in NoMa, the majority were associated in opposite directions. Furthermore, all 92 overlapping pathways were positively associated with PM_2.5_ in the Rotterdam Study and negatively associated with PM_2.5_ in NoMa. There could be several reasons for these opposing results. First, the exposure level of PM_2.5_ in the two studies was nonoverlapping, and much higher in the Rotterdam Study. This may also be because data from the Rotterdam Study are from 2009 to 2013, while the NoMa data are from 2012 to 2014 and air pollution levels overall are decreasing in Europe [48]. Sensitivity analyses also showed that the number of overlapping significant genes and the number of oppositely associated genes were higher when comparing NoMa to RS-II, the subcohort with the highest PM_2.5_ exposure levels. Hence, the results from this study may demonstrate transcriptomic profiles associated with high and low PM_2.5_ exposure. As per the US Environmental Protection Agency’s pollution standards, exposure to PM_2.5_ at ≤12 µg/m^3^ is considered to pose little to no health risk, while exposure levels between 12 and 35 µg/m^3^ are considered to pose moderate risk. Thus, some of the differences in gene expression may be due to different exposure levels from low versus moderate levels of PM_2.5_. Low sensitivity of detection of microarray relative to RNA sequencing and the lower sample size of the NoMa study relative to the RS may have also contributed to model estimates from the NoMa study that may be noisy. Secondly, the geographical distribution of groups with different socioeconomic status (SES) may differ between the studies, as people with a higher SES may live in polluted city centres in some cities, while in other cities, they may live in residential areas outside the city centre surrounded by more green space. Thirdly, in the subsample of participants with available RNA sequencing data in the Rotterdam Study, NDVI was positively correlated to PM_2.5_, while it was negatively correlated in NoMa and the entire Rotterdam Study cohort. This may imply that there is some selection bias in the Rotterdam Study participants included in this study that may affect our results. Characteristics of the two study populations may also have contributed to the opposing results. In particular, participants in the Norwegian study were more highly educated and ∼13 years younger on average compared to the Rotterdam Study. Moreover, in the NoMa study, participants were recruited to a dietary intervention study, and hence, including participants interested in nutrition and health could potentially introduce a selection bias as well. Adhering to a healthy Nordic diet is shown to modify the association between exposure to PM_2.5_ and cardiometabolic risk factors [49]. A similar moderation of the association between PM_2.5_ exposure and gene expression is thus plausible. Finally, the composition of PM_2.5_ particles may vary between the two populations. PM_2.5_ particles in the Netherlands have higher levels of sulphur, iron, zinc, and other metals compared to those in Norway [50]. These differences may arise because of greater industrial activity and traffic density. As metals on PM_2.5_ particles are associated with lung and CVDs, PM_2.5_ particle composition differences may give rise to different gene expression associations.

The pathways that were associated with PM_2.5_ in opposite directions in this study included cellular respiration, ETC, oxidative phosphorylation and ATP production, hydrogen peroxide metabolism, as well as apoptosis. This is in line with results from several other studies of transcriptomic associations with PM_2.5_ [21, 23, 51]. It is hypothesized that exposure to PM increases ROS production, which in turn leads to mitochondrial dysfunction and increased ROS production. Because of an increased cellular energy demand to repair pollution-induced damage, genes related to ETC and oxidative phosphorylation are affected [51]. Moreover, the cellular damage may also induce apoptosis [51].

Air pollution exposure has been shown in both epidemiological studies and *in vivo* studies to be adversely associated with diseases of the central nervous system, such as Alzheimer’s disease [52, 53]. Pollutants can reach the brain directly via the olfactory bulb or via blood and may result in neuroinflammation and oxidative stress in the brain [52]. In line with this, we found that pathways related to synaptic signalling, neurodegeneration, and Alzheimer’s disease were associated with PM_2.5_. Further studies are needed to get a better understanding of the underlying mechanisms of these associations.

There were gene expression associations with other environmental exposures than PM_2.5_ that may be implicated in disease development. Expression of *SEPTIN4*, a gene that may be involved in apoptosis, was associated with annual mean O_3_ and has been reported to be a part of a biomarker gene set to discriminate between chronic obstructive pulmonary disease and interstitial lung disease [54]. We found that the immunoglobulin genes *IGKV2-29* and *IGKV2-30* were negatively associated with NDVI, a measure of green space. In line with this, we have previously found that *IGKV2-30* was negatively associated with green space in 9-year-old children in the Generation R study [38]. This suggests that living close to green spaces may reduce the expression of immunoglobulin-related genes that may imply a lower activation of the adaptive immune system.

Moreover, *ADGRG7*, a G-protein-coupled receptor, was positively associated with NDVI. *In vitro* SARS-CoV-2 infection of human epithelial lung cells results in higher *ADGRG7* expression, and the gene is suggested to play a role in viral cell entry and replication [55]. In this context, our finding seems to be counter-intuitive, however, as NDVI was positively associated with PM_2.5_ in the subsample of participants from the Rotterdam Study (*n* = 758) included in this study, this result may be confounded by other environmental exposures. Although black carbon is a component of PM_2.5_, only one pseudogene was associated with exposure to black carbon. This may imply that, components of PM other than black carbon, are important for the detrimental health effects. Furthermore, black carbon exposure levels were also quite low as compared to other pollutants such as PM_2.5_. The lack of gene expression associations with NO_2_, warm season O_3_, and impervious surface may be due to a narrow distribution of these exposures in the two studies included, as well as too low power to detect associations. Previous adult studies exploring the association of gene expression with air pollutants in adults have also reported that PM_2.5_ showed the maximum number of associations with gene expression [21]. Future studies with larger sample sizes and a broader distribution in the exposure levels could provide valuable insight into the underlying molecular mechanisms of how the built environment and air pollution affect health. Finally, all the environmental exposures included in the current study could have indirect effects on immune cell gene expression, and therefore, future analyses that consider interactions with noise pollution and other lifestyle-related factors associated with an urban environment would be valuable.

This study is strengthened by the inclusion of two different European populations that enables investigations across SES, geography, and different PM_2.5_ exposure levels, although this may have also made it more difficult to interpret the findings. Moreover, the harmonized environmental exposures and covariates are a major strength of this study, making the results comparable across the included studies. This study is limited by the different methods for RNA expression quantification: RNA sequencing in the Rotterdam Study and microarray in NoMa. Additionally, gene expression was analysed in whole blood in the Rotterdam Study and in PBMC in NoMa. Therefore, although results are adjusted for cell-type composition, we cannot exclude the possibility of residual confounding by cell-type composition. The NoMa study with microarray data also had a smaller size and lower sensitivity of detection compared to the Rotterdam Study with RNA sequencing data. This resulted in the NoMa study not being sufficiently powered to detect associations for single genes, and hence, we used a more lenient cut-off. The different FDR thresholds make comparisons between the two studies included demanding. However, more advanced methods, such as pathway-level differential expression analysis methods, including single-sample GSEA that transforms a single sample’s gene expression profile to a gene set enrichment score, might also be helpful to reduce dimensionality of data with subsequent differential association analysis at the pathway level.

The LUR models utilized in our study for air pollution assessment were validated for European-wide analyses. However, extrapolation may have introduced uncertainty to our exposure assessment. Finally, although this study is a part of the LongITools consortium with available data from several population-based cohorts, the only studies with available RNA expression data in adults were the Rotterdam Study and NoMa. These studies only had cross-sectional data on gene expression, excluding the possibility of longitudinal analyses. Taken together, this limits the sample size and statistical power of the study. However, we were able to compare our results with analyses of gene expression and environmental exposures in 9-year-old children within the LongITools consortium [38]. Our findings provide evidence on how air pollution, and particularly PM_2.5_, affects human health at a molecular level. This evidence, when combined with other studies on air pollutants’ health effects, can be used by policymakers to justify stricter air quality regulations to improve public health. Because our data suggest that associations with gene expression depend on the exposure level of air pollution, these results can guide resource allocation to prioritize interventions in areas with higher pollution levels.

In conclusion, we found PM_2.5_ to be the environmental exposure with the strongest transcriptomic associations, which may partly explain the strong associations of this air pollutant with disease risk. Pathways associated with PM_2.5_ included inflammation, oxidative stress, DNA metabolism, cell cycle regulation, and ETC, which shed light on the underlying mechanisms of how PM_2.5_ induces its detrimental health effects.

## Supplementary Material

dvaf008_Supp

## Data Availability

The NoMa data analysed in this study are available from the corresponding author upon reasonable request. Access queries regarding Rotterdam Study data can be made to Trudy Voortman.

## References

[1] Landrigan PJ, Richard F, Acosta NJR et al. The Lancet Commission on pollution and health. *Lancet* 2018;391:462–512. 29056410 10.1016/S0140-6736(17)32345-0

[2] GBD 2019 Risk Factors Collaborators . Global burden of 87 risk factors in 204 countries and territories, 1990–2019: a systematic analysis for the Global Burden of Disease Study 2019. *Lancet* 2020;396:1223–49. 33069327 10.1016/S0140-6736(20)30752-2 PMC7566194

[3] Al-Kindi SG, Brook RD, Biswal S et al. Environmental determinants of cardiovascular disease: lessons learned from air pollution. *Nat Rev Cardiol* 2020;17:656–72. doi: 10.1038/s41569-020-0371-2 32382149 PMC7492399

[4] Strak M, Weinmayr G, Rodopoulou S et al. Long term exposure to low level air pollution and mortality in eight European cohorts within the ELAPSE project: pooled analysis. *BMJ* 2021;374:n1904. doi: 10.1136/bmj.n1904 PMC8409282 34470785

[5] Wolf K, Hoffmann B, Andersen ZJ et al. Long-term exposure to low-level ambient air pollution and incidence of stroke and coronary heart disease: a pooled analysis of six European cohorts within the ELAPSE project. *Lancet Planet Health* 2021;5:e620–32. doi: 10.1016/S2542-5196(21)00195-9 34508683

[6] Gascon M, Triguero-Mas M, Martínez D et al. Residential green spaces and mortality: a systematic review. *Environ Int* 2016;86:60–7. doi: 10.1016/j.envint.2015.10.013 26540085

[7] McDougall CW, Quilliam RS, Hanley N et al. Freshwater blue space and population health: an emerging research agenda. *Sci Total Environ* 2020;737:140196. doi: 10.1016/j.scitotenv.2020.140196 32783838

[8] Xu Z, Wang W, Liu Q et al. Association between gaseous air pollutants and biomarkers of systemic inflammation: a systematic review and meta-analysis. *Environ Pollut* 2022;292:118336. doi: 10.1016/j.envpol.2021.118336 34634403

[9] Gaio V, Roquette R, Dias CM et al. Ambient air pollution and lipid profile: systematic review and meta-analysis. *Environ Pollut* 2019;254:113036. doi: 10.1016/j.envpol.2019.113036 31465899

[10] Yang B-Y et al. Global association between ambient air pollution and blood pressure: a systematic review and meta-analysis. *Environ Pollut* 2018;235:576–88. 29331891 10.1016/j.envpol.2018.01.001

[11] Zhang S, Mwiberi S, Pickford R et al. Longitudinal associations between ambient air pollution and insulin sensitivity: results from the KORA cohort study. *Lancet Planet Health* 2021;5:e39–49. doi: 10.1016/S2542-5196(20)30275-8 33421408

[12] Twohig-Bennett C, Jones A. The health benefits of the great outdoors: a systematic review and meta-analysis of greenspace exposure and health outcomes. *Environ Res* 2018;166:628–37. 29982151 10.1016/j.envres.2018.06.030 PMC6562165

[13] Jeong A, Eze IC, Vienneau D et al. Residential greenness-related DNA methylation changes. *Environ Int* 2022;158:106945. doi: 10.1016/j.envint.2021.106945 34689037

[14] Wu Y, Qie R, Cheng M et al. Air pollution and DNA methylation in adults: a systematic review and meta-analysis of observational studies. *Environ Pollut* 2021;284:117152. doi: 10.1016/j.envpol.2021.117152 33895575

[15] Glencross DA, Ho T-R, Camiña N et al. Air pollution and its effects on the immune system. *Free Radic Biol Med* 2020;151:56–68. doi: 10.1016/j.freeradbiomed.2020.01.179 32007522

[16] Marín-Palma D, Fernandez GJ, Ruiz-Saenz J et al. Particulate matter impairs immune system function by up-regulating inflammatory pathways and decreasing pathogen response gene expression. *Sci Rep* 2023;13:12773. doi: 10.1038/s41598-023-39921-w PMC10406897 37550362

[17] Li S, Huff RD, Rider CF et al. Controlled human exposures to diesel exhaust or particle-depleted diesel exhaust with allergen modulates transcriptomic responses in the lung. *Sci Total Environ* 2024;945:173688. doi: 10.1016/j.scitotenv.2024.173688 38851342

[18] Cheng SL, Hedges M, Keski-Rahkonen P et al. Multiomic signatures of traffic-related air pollution in London reveal potential short-term perturbations in gut microbiome-related pathways. *Environ Sci Technol* 2024;58:8771–82. doi: 10.1021/acs.est.3c09148 38728551 PMC11112755

[19] Peretz A, Peck EC, Bammler TK et al. Diesel exhaust inhalation and assessment of peripheral blood mononuclear cell gene transcription effects: an exploratory study of healthy human volunteers. *Inhal Toxicol* 2007;19:1107–19. doi: 10.1080/08958370701665384 17987463

[20] Pettit AP, Brooks A, Laumbach R et al. Alteration of peripheral blood monocyte gene expression in humans following diesel exhaust inhalation. *Inhal Toxicol* 2012;24:172–81. doi: 10.3109/08958378.2012.654856 22369193 PMC3755508

[21] Vlaanderen J, Vermeulen R, Whitaker M et al. Impact of long-term exposure to PM_2.5_ on peripheral blood gene expression pathways involved in cell signaling and immune response. *Environ Int* 2022;168:107491. doi: 10.1016/j.envint.2022.107491 36081220

[22] Wen J, Zhang J, Zhang H et al. Large-scale genome-wide association studies reveal the genetic causal etiology between air pollutants and autoimmune diseases. *J Transl Med* 2024;22:392. doi: 10.1186/s12967-024-04928-y PMC11057084 38685026

[23] Vrijens K, Winckelmans E, Tsamou M et al. Sex-specific associations between particulate matter exposure and gene expression in independent discovery and validation cohorts of middle-aged men and women. *Environ Health Perspect* 2017;125:660–69. doi: 10.1289/EHP370 27740511 PMC5381989

[24] Dergham M, Lepers C, Verdin A et al. Temporal–spatial variations of the physicochemical characteristics of air pollution particulate matter (PM_2.5–0.3_) and toxicological effects in human bronchial epithelial cells (BEAS-2B). *Environ Res* 2015;137:256–67. doi: 10.1016/j.envres.2014.12.015 25601727

[25] Hansson GK . Inflammation, atherosclerosis, and coronary artery disease. *N Engl J Med* 2005;352:1685–95. doi: 10.1056/NEJMra043430 15843671

[26] Araujo JA, Nel AE. Particulate matter and atherosclerosis: role of particle size, composition and oxidative stress. *Part Fibre Toxicol* 2009;6:24. doi: 10.1186/1743-8977-6-24 PMC2761850 19761620

[27] Takamura T, Honda M, Sakai Y et al. Gene expression profiles in peripheral blood mononuclear cells reflect the pathophysiology of type 2 diabetes. *Biochem Biophys Res Commun* 2007;361:379–84. doi: 10.1016/j.bbrc.2007.07.006 17651698

[28] Visvikis-Siest S, Marteau J-B, Samara A et al. Peripheral blood mononuclear cells (PBMCs): a possible model for studying cardiovascular biology systems. *Clin Chem Lab Med* 2007;45:1154–68. doi: 10.1515/CCLM.2007.255 17663631

[29] Ronkainen J, Nedelec R, Atehortua A et al. LongITools: dynamic longitudinal exposome trajectories in cardiovascular and metabolic noncommunicable diseases. *Environ Epidemiol* 2022;6:e184. doi: 10.1097/EE9.0000000000000184 PMC8835657 35169663

[30] Ikram MA, Kieboom BCT, Brouwer WP et al. The Rotterdam Study. Design update and major findings between 2020 and 2024. *Eur J Epidemiol* 2024;39:183–206. doi: 10.1007/s10654-023-01094-1 38324224

[31] Ulven SM, Leder L, Elind E et al. Exchanging a few commercial, regularly consumed food items with improved fat quality reduces total cholesterol and LDL-cholesterol: a double-blind, randomised controlled trial. *Br J Nutr* 2016;116:1383–93. doi: 10.1017/S0007114516003445 27737722

[32] de Hoogh K, Chen J, Gulliver J et al. Spatial PM_2.5_, NO_2_, O_3_ and BC models for Western Europe—evaluation of spatiotemporal stability. *Environ Int* 2018;120:81–92. doi: 10.1016/j.envint.2018.07.036 30075373

[33] Eeftens M, Tsai M-Y, Ampe C et al. Spatial variation of PM_2.5_, PM_10_, PM_2.5_ absorbance and PM_coarse_ concentrations between and within 20 European study areas and the relationship with NO_2_—results of the ESCAPE project. *Atmos Environ* 2012;62:303–17. doi: 10.1016/j.atmosenv.2012.08.038

[34] Brandt J, Silver JD, Frohn LM et al. An integrated model study for Europe and North America using the Danish Eulerian Hemispheric Model with focus on intercontinental transport of air pollution. *Atmos Environ* 2012;53:156–76. doi: 10.1016/j.atmosenv.2012.01.011

[35] Chen J, Rodopoulou S, Strak M et al. Long-term exposure to ambient air pollution and bladder cancer incidence in a pooled European cohort: the ELAPSE project. *Br J Cancer* 2022;126:1499–507. doi: 10.1038/s41416-022-01735-4 35173304 PMC9090745

[36] Zhernakova DV, Deelen P, Vermaat M et al. Identification of context-dependent expression quantitative trait loci in whole blood. *Nat Genet* 2017;49:139–45. doi: 10.1038/ng.3737 27918533

[37] Christensen JJ, Ulven SM, Thoresen M et al. Associations between dietary patterns and gene expression pattern in peripheral blood mononuclear cells: a cross-sectional study. *Nutr Metab Cardiovasc Dis* 2020;30:2111–22. doi: 10.1016/j.numecd.2020.06.018 32807640

[38] Das S, Rundblad A, Marques IF et al. Air pollution exposure is associated with gene expression in children. *Environ Epigenet* 2024;10:dvae025. doi: 10.1093/eep/dvae025 PMC11668970 39723337

[39] Brook RD, Rajagopalan S, Pope CA et al. Particulate matter air pollution and cardiovascular disease: an update to the scientific statement from the American Heart Association. *Circulation* 2010;121:2331–78. doi: 10.1161/CIR.0b013e3181dbece1 20458016

[40] Valderrama A, Zapata MI, Hernandez JC et al. Systematic review of preclinical studies on the neutrophil-mediated immune response to air pollutants, 1980-2020. *Heliyon* 2022;8:e08778. doi: 10.1016/j.heliyon.2022.e08778 PMC8810373 35128092

[41] Ciencewicki J, Jaspers I. Air pollution and respiratory viral infection. *Inhal Toxicol* 2007;19:1135–46. doi: 10.1080/08958370701665434 17987465

[42] Croft DP, Burton DS, Nagel DJ et al. The effect of air pollution on the transcriptomics of the immune response to respiratory infection. *Sci Rep* 2021;11:19436. doi: 10.1038/s41598-021-98729-8 PMC8484285 34593881

[43] Villamayor L, López-García D, Rivero V et al. The IFN-stimulated gene IFI27 counteracts innate immune responses after viral infections by interfering with RIG-I signaling. *Front Microbiol* 2023;14:1176177. doi: 10.3389/fmicb.2023.1176177 PMC10175689 37187533

[44] Alfano R, Herceg Z, Nawrot TS et al. The impact of air pollution on our epigenome: how far is the evidence? (A systematic review). *Curr Environ Health Rep* 2018;5:544–78. doi: 10.1007/s40572-018-0218-8 30361985

[45] Zheng Y, Sanchez-Guerra M, Zhang Z et al. Traffic-derived particulate matter exposure and histone H3 modification: a repeated measures study. *Environ Res* 2017;153:112–19. doi: 10.1016/j.envres.2016.11.015 27918982 PMC5605137

[46] Liu C, Xu J, Chen Y et al. Characterization of genome-wide H3K27ac profiles reveals a distinct PM_2.5_-associated histone modification signature. *Environ Health* 2015;14:65. doi: 10.1186/s12940-015-0052-5 PMC4537530 26276146

[47] Enquobahrie DA, MacDonald J, Hussey M et al. Prenatal exposure to particulate matter and placental gene expression. *Environ Int* 2022;165:107310. doi: 10.1016/j.envint.2022.107310 PMC9235522 35653832

[48] Chen Z-Y, Petetin H, Méndez Turrubiates RF et al. Population exposure to multiple air pollutants and its compound episodes in Europe. *Nat Commun* 2024;15:2094. doi: 10.1038/s41467-024-46103-3 PMC10937992 38480711

[49] Healy DR, Kårlund A, Mikkonen S et al. Associations of low levels of air pollution with cardiometabolic outcomes and the role of diet quality in individuals with obesity. *Environ Res* 2024;242:117637. doi: 10.1016/j.envres.2023.117637 37993047

[50] Tsai M-Y, Hoek G, Eeftens M et al. Spatial variation of PM elemental composition between and within 20 European study areas—results of the ESCAPE project. *Environ Int* 2015;84:181–92. doi: 10.1016/j.envint.2015.04.015 26342569

[51] Winckelmans E, Nawrot TS, Tsamou M et al. Transcriptome-wide analyses indicate mitochondrial responses to particulate air pollution exposure. *Environ Health* 2017;16:87. doi: 10.1186/s12940-017-0292-7 PMC5563023 28821289

[52] Kim H, Kim W-H, Kim -Y-Y et al. Air pollution and central nervous system disease: a review of the impact of fine particulate matter on neurological disorders. *Front Public Health* 2020;8:575330. doi: 10.3389/fpubh.2020.575330 PMC7772244 33392129

[53] Jung CR, Lin YT, Hwang BF. Ozone, particulate matter, and newly diagnosed Alzheimer’s disease: a population-based cohort study in Taiwan. *J Alzheimers Dis* 2015;44:573–84. doi: 10.3233/JAD-140855 25310992

[54] Yao Y, Gu Y, Yang M et al. The gene expression biomarkers for chronic obstructive pulmonary disease and interstitial lung disease. *Front Genet* 2019;10:1154. doi: 10.3389/fgene.2019.01154 PMC6879656 31824564

[55] Žáčková S, Pávová M, Trylčová J et al. Upregulation of mRNA expression of ADGRD1/GPR133 and ADGRG7/GPR128 in SARS-CoV-2-infected lung adenocarcinoma Calu-3 cells. *Cells* 2024;13:791. doi: 10.3390/cells13100791 PMC11119037 38786015

