# Supplementary material for: Exposure to fine particulate matter in adults is associated with immune cell gene expression related to inflammation, the electron transport chain, and cell cycle regulation
Source: Environ Epigenet. 2025 Apr 1;11(1):dvaf008. doi: 10.1093/eep/dvaf008 (PMC12159804; doi:10.1093/eep/dvaf008)
Supplement: dvaf008_Supp [file dvaf008_supp.zip › suppl_data/Supplemental file 2.pdf]

Supplemental file 2:

Supplemental Table 2.1: Association between PM2.5 and gene expression in NoMa
Supplemental Table 2.2: Association between black carbon and gene expression in NoMa
Supplemental Table 2.3: Association between NO2 and gene expression in NoMa
Supplemental Table 2.4: Association between annual mean ozone and gene expression in NoMa
Supplemental Table 2.5: Association between warm season ozone and gene expression in NoMa
Supplemental Table 2.6: Association between normalized difference vegetation index and gene expression in NoMa
Supplemental Table 2.7: Association between impervious surface and gene expression in NoMa
Supplemental Table 2.8: Association between distance to nearest water body and gene expression in NoMa

All results are adjusted for age, sex, BMI, education, current smoking, %monocytes and %lymphocytes.
Genes with a significant association to the exposure (FDR < 15 %) are highlighted
Genes used in pathway analyses are shown in **bold**

Supplemental Table 2.1: Association between PM2.5 and gene expression in NoMa

name	logFC	P.Value	adj.P.Val
**SNRPD1**	-0.078404925	**1.31E-05**	0.106151448
**SRR**	-0.099322173	**2.32E-05**	0.106151448
**GUF1**	-0.064057671	**3.56E-05**	0.106151448
**PGAM1**	-0.042620864	**5.35E-05**	0.106151448
**PTPMT1**	-0.056606298	**6.01E-05**	0.106151448
**RERE**	0.069261133	**6.34E-05**	0.106151448
**QARS**	-0.046569421	**6.71E-05**	0.106151448
**CASP4**	-0.049538086	**8.41E-05**	0.116369087
**NRBF2**	-0.067585215	**9.95E-05**	0.12240604
**ALKBH3**	-0.043516906	**1.27E-04**	0.140181776
**PDHB**	-0.037217536	**1.85E-04**	0.148391464
**ZNF549**	0.062760222	**2.06E-04**	0.148391464
**GSTZ1**	-0.07063479	**2.18E-04**	0.148391464
**ASAP1**	0.056350364	**2.21E-04**	0.148391464
**PPAPDC1B**	0.058570133	**2.37E-04**	0.148391464
**FAM76A**	0.086487128	**2.40E-04**	0.148391464
**ZNF890P**	0.069415246	**2.50E-04**	0.148391464
**AKAP8L**	0.07049553	**2.53E-04**	0.148391464
**ZMAT3**	0.071782956	**2.55E-04**	0.148391464
**MRPS18A**	-0.0574396	**2.79E-04**	0.154567963
**FAM178A**	0.051240648	**3.21E-04**	0.168966141
**DDOST**	-0.034934358	**3.44E-04**	0.17132357
**SYT2**	-0.074027498	**3.57E-04**	0.17132357
**SEC24D**	-0.058890665	**3.96E-04**	0.17132357
**SSTR2**	0.059033924	**4.10E-04**	0.17132357
**CHMP2A**	-0.047868359	**4.54E-04**	0.17132357
**TEKT4**	-0.065149573	**4.66E-04**	0.17132357
**PIAS1**	0.041927408	**4.68E-04**	0.17132357
**POFUT1**	0.068520906	**4.80E-04**	0.17132357
**RPS2**	-0.077164353	**5.00E-04**	0.17132357
**ATF5**	0.054997895	**5.01E-04**	0.17132357
**DAZAP1**	0.041349697	**5.07E-04**	0.17132357
**DMC1**	0.064419049	**5.27E-04**	0.17132357
**CDC14A**	0.055692676	**5.29E-04**	0.17132357
**RGS19**	-0.053058575	**5.45E-04**	0.17132357
**TINF2**	-0.064522903	**5.77E-04**	0.17132357
**GNPNAT1**	0.067105861	**6.00E-04**	0.17132357
**OXCT2**	-0.083475144	**6.01E-04**	0.17132357
**ZNF14**	0.070792254	**6.04E-04**	0.17132357
**SNRPB**	-0.05413994	**6.42E-04**	0.172954138
**PDXDC2P**	0.134748282	**6.46E-04**	0.172954138
**TMEM17**	0.087700122	**6.61E-04**	0.172954138
**TICAM2**	-0.07437399	**6.76E-04**	0.172954138
**RPUSD1**	-0.0703094	**6.88E-04**	0.172954138
**FAM126B**	0.042688939	**7.11E-04**	0.174959729
**TBC1D16**	-0.059152964	**7.29E-04**	0.17550277
**GDAP2**	-0.053058147	**7.63E-04**	0.176946376

**EIF3E**	-0.026395686	**7.67E-04**	0.176946376
**PSMB7**	-0.050334939	**8.12E-04**	0.181090329
**NDUFAF1**	-0.056221461	**8.18E-04**	0.181090329
**AUH**	-0.058341999	**8.84E-04**	0.183167967
**CUL7**	-0.064734572	**9.10E-04**	0.183167967
**EID2B**	0.087255145	**9.13E-04**	0.183167967
**RAB5A**	-0.048421381	**9.15E-04**	0.183167967
**EVI2B**	-0.079202108	**9.25E-04**	0.183167967
**UBXN2B**	-0.058895092	**9.27E-04**	0.183167967
**DBNL**	-0.031959668	**9.56E-04**	0.184209823
**EBLN2**	0.101623147	**9.69E-04**	0.184209823
**TRIP4**	-0.043783912	**9.86E-04**	0.184209823
**SUCLA2**	-0.051466955	**0.001034128**	0.184209823
**FTSJ2**	-0.038545438	**0.001042705**	0.184209823
**DDX54**	-0.046574726	**0.001043719**	0.184209823
**SNX15**	-0.070569872	**0.001050563**	0.184209823
**NDEL1**	-0.037513778	**0.001090484**	0.184209823
**NAA20**	-0.028044056	**0.001096135**	0.184209823
**SNX29**	0.139197183	**0.001104238**	0.184209823
**MGC2752**	-0.064355291	**0.001130677**	0.184209823
**ADM2**	-0.062144849	**0.001131653**	0.184209823
**LCOR**	0.064543581	**0.001163129**	0.18658945
**PDE4C**	0.032892184	**0.001214511**	0.191786534
**TMEM63B**	0.06475985	**0.001247591**	0.191786534
**GPR141**	-0.079897783	**0.001249794**	0.191786534
**SEC11A**	-0.035941274	**0.001264831**	0.191786534
**EIF2AK2**	0.087555305	**0.00131007**	0.195476278
**PSMB6**	-0.036839358	**0.001344947**	0.195476278
**ZFC3H1**	0.046810492	**0.001346257**	0.195476278
**POLR3H**	-0.041848185	**0.001387137**	0.195476278
**DYM**	-0.040800267	**0.00138862**	0.195476278
**RPL10A**	-0.052724874	**0.001395124**	0.195476278
**MIR671**	-0.050728223	**0.001446552**	0.198650564
**HADHB**	-0.044494417	**0.00145867**	0.198650564
**SRC**	0.068870207	**0.001471619**	0.198650564
**STIP1**	0.057803888	**0.001519832**	0.199946591
**DDX10**	0.175858542	**0.001531752**	0.199946591
**HPS6**	-0.044024843	**0.001543076**	0.199946591
**ARHGEF7**	0.030100345	**0.00155399**	0.199946591
**TM9SF1**	-0.049107175	**0.001598073**	0.199946591
**CMTM7**	-0.040470587	**0.001626915**	0.199946591
**DUSP19**	0.108257034	**0.001632575**	0.199946591
**USP46**	0.071225804	**0.00163844**	0.199946591
**HIATL2**	0.107218188	**0.001643793**	0.199946591
**PQLC3**	-0.041919328	**0.001677698**	0.200375707
**MAN2B2**	-0.03922903	**0.001683525**	0.200375707
**RAB11FIP1**	0.043646643	**0.001743961**	0.205360641
**EIF3L**	-0.029660063	**0.001781316**	0.206166478
**POLDIP2**	-0.051391523	**0.001788055**	0.206166478
**EHHADH**	-0.065109521	**0.001866041**	0.212940315

**RHOBTB1**	0.12569136	**0.001922385**	0.213916945
**LRRC37BP1**	-0.055799459	**0.001954294**	0.213916945
**NKPD1**	0.123744543	**0.001985588**	0.213916945
**FER**	-0.065742314	**0.002030538**	0.213916945
**UCKL1**	0.034115349	**0.002034702**	0.213916945
**PHKB**	0.060635662	**0.002041493**	0.213916945
**ARID1B**	0.050433434	**0.002056113**	0.213916945
**PIGT**	-0.040050355	**0.00206079**	0.213916945
**THOC5**	-0.03630409	**0.002102693**	0.213916945
**SLC30A7**	0.053480529	**0.002121752**	0.213916945
**SNRNP40**	0.031590385	**0.002132696**	0.213916945
**DNAJC17**	-0.046566585	**0.002133212**	0.213916945
**CEP63**	-0.041593163	**0.002133597**	0.213916945
**BSPRY**	-0.05886573	**0.00214516**	0.213916945
**PHB**	-0.044063592	**0.00217953**	0.21540374
**PDCL3**	-0.038637537	**0.002235538**	0.216169972
**FBN2**	-0.112774967	**0.002259321**	0.216169972
**ATG10**	0.083921258	**0.002259517**	0.216169972
**SCARNA3**	0.1352995	**0.00228308**	0.216169972
**BIRC3**	0.081352847	**0.00228493**	0.216169972
**PUF60**	-0.043694225	**0.002319386**	0.216808151
**FLT3**	-0.089347869	**0.00233382**	0.216808151
**RIC3**	0.071440922	**0.002350436**	0.216808151
**SLC25A3**	-0.034362381	**0.00238513**	0.218190098
**SPTLC1**	0.070539224	**0.002431918**	0.220646726
**MAPRE1**	-0.039784677	**0.002455975**	0.221017795
**GYG1**	-0.04309821	**0.002523129**	0.225229956
**CSTA**	-0.08865253	**0.00257157**	0.227717684
**SAMM50**	-0.043367152	**0.002593405**	0.227828532
**SPATA6**	-0.054122661	**0.00263098**	0.228120947
**FAM175A**	0.11299491	**0.002639016**	0.228120947
**MFSD11**	0.075287468	**0.002675002**	0.228120947
**PGLYRP2**	-0.060948924	**0.002694792**	0.228120947
**ZNF516**	0.085939507	**0.002699778**	0.228120947
**SMAD9**	0.079224412	**0.002751526**	0.230732133
**MCM8**	0.044115141	**0.002781039**	0.231342167
**AKAP2**	0.118754715	**0.002800601**	0.231342167
**NACA**	-0.029291667	**0.002935273**	0.238996897
**ZNF876P**	0.029594458	**0.002967385**	0.238996897
**NHP2L1**	-0.034910276	**0.003038533**	0.238996897
**TMC8**	0.065256639	**0.003065594**	0.238996897
**SNRPA**	0.033742504	**0.003115405**	0.238996897
**SNAPIN**	-0.036179744	**0.003119541**	0.238996897
**HOOK2**	0.043446686	**0.003125563**	0.238996897
**DTWD2**	0.106951764	**0.003127121**	0.238996897
**IL12RB1**	-0.064983117	**0.003139369**	0.238996897
**FKBP5**	0.118756633	**0.00316153**	0.238996897
**PPA2**	0.068930518	**0.003168923**	0.238996897
**FLI1**	-0.034418989	**0.003169347**	0.238996897
**MRPS12**	-0.034526179	**0.003173958**	0.238996897

**NLRP8**	0.095263741	**0.003211126**	0.240161839
**ICMT**	-0.0689169	**0.003276394**	0.240443306
**METTL21A**	0.083021145	**0.003282825**	0.240443306
**ZDHHC7**	-0.036700988	**0.003326035**	0.240443306
**OSGIN2**	-0.05028964	**0.00333898**	0.240443306
**PCCB**	-0.038871177	**0.00343741**	0.240443306
**PRPF3**	0.039402608	**0.00345534**	0.240443306
**NSUN5P1**	0.0603884	**0.003472258**	0.240443306
**HSPA5**	-0.036780951	**0.003478197**	0.240443306
**GALM**	0.029308838	**0.003482138**	0.240443306
**C9orf47**	-0.114375612	**0.003542831**	0.240443306
**RAX2**	0.094548637	**0.003556516**	0.240443306
**GLI4**	0.054681268	**0.003584159**	0.240443306
**PRRG4**	0.106822186	**0.003596972**	0.240443306
**IL17RD**	0.108195141	**0.003597227**	0.240443306
**EWSR1**	0.028678553	**0.003602329**	0.240443306
**ESD**	-0.034726792	**0.003670487**	0.240443306
**SYNGR2**	-0.031522333	**0.003690326**	0.240443306
**SPAG5**	-0.047337642	**0.003732001**	0.240443306
**FGL2**	-0.064670906	**0.003746628**	0.240443306
**MDM4**	0.083617383	**0.003767724**	0.240443306
**PLA2G15**	-0.051291141	**0.003790378**	0.240443306
**GSTP1**	-0.040737972	**0.003795684**	0.240443306
**SSR2**	-0.058154778	**0.003810892**	0.240443306
**DPRXP4**	0.106794583	**0.003814326**	0.240443306
**SNRPC**	-0.038057651	**0.003818036**	0.240443306
**CATSPER2**	0.082364159	**0.003820245**	0.240443306
**KLHL12**	-0.030705081	**0.003850264**	0.240443306
**MTMR14**	-0.024879593	**0.003856179**	0.240443306
**PABPN1**	0.0356461	**0.003900736**	0.240443306
**ATP5A1**	-0.034822406	**0.003904356**	0.240443306
**SAP130**	-0.042931824	**0.00391727**	0.240443306
**ATPAF2**	-0.04749667	**0.003937586**	0.240443306
**GOLGA6L6**	0.104495131	**0.003958484**	0.240443306
**NKTR**	0.057733319	**0.003961515**	0.240443306
**ZNF493**	0.10226068	**0.003975167**	0.240443306
**PSEN2**	-0.046982442	**0.004073109**	0.243151058
**FKBP14**	0.120922268	**0.004102028**	0.243151058
**GABBR1**	0.077685417	**0.004114083**	0.243151058
**LAMB1**	-0.048919494	**0.004117557**	0.243151058
**OAT**	-0.05470175	**0.004150239**	0.243151058
**PFKL**	-0.057705739	**0.004183092**	0.243151058
**TMEM97**	-0.056764205	**0.004281835**	0.243151058
**ZNF430**	0.028008006	**0.004289311**	0.243151058
**ARL16**	0.060999289	**0.004310187**	0.243151058
**PLA2G4A**	-0.070793356	**0.004311596**	0.243151058
**STK17B**	0.137091724	**0.004322788**	0.243151058
**DLG4**	0.065545957	**0.004325971**	0.243151058
**DNAL1**	-0.050439403	**0.004372223**	0.243151058
**PARP12**	0.060680752	**0.004399717**	0.243151058

**PSMB10**	-0.035842238	**0.004427882**	0.243151058
**TMEM185B**	-0.043905069	**0.004460384**	0.243151058
**CXCL9**	-0.068154862	**0.004474201**	0.243151058
**SLC35D3**	0.135093935	**0.004477869**	0.243151058
**DHRS7B**	-0.036436525	**0.004478286**	0.243151058
**XRCC2**	0.128648635	**0.004485694**	0.243151058
**FUCA1**	-0.049951815	**0.004532238**	0.243151058
**ZNF577**	0.124746584	**0.004535562**	0.243151058
**THUMPD3**	-0.053398975	**0.004545051**	0.243151058
**POLA2**	-0.035464611	**0.004558907**	0.243151058
**ZNF446**	-0.040446903	**0.004569105**	0.243151058
**PARP11**	-0.043768331	**0.004635057**	0.245480592
**ANKRD35**	-0.09066674	**0.004671761**	0.246246294
**DDX51**	0.073955992	**0.004812773**	0.251586451
**BMS1P5**	0.072139161	**0.004823446**	0.251586451
**TRIM13**	0.121181363	**0.004842858**	0.251586451
**ZNF230**	-0.045163178	**0.0048807**	0.251586451
**USP32**	0.112242392	**0.004886718**	0.251586451
**RAB35**	-0.039288831	**0.004984736**	0.252807309
**MAP7D1**	-0.025821789	**0.00499922**	0.252807309
**PEX16**	-0.029900688	**0.005005972**	0.252807309
**DPF2**	-0.036080568	**0.005041678**	0.252807309
**FUT6**	0.138002256	**0.005053013**	0.252807309
**STX1A**	0.05462253	**0.005072594**	0.252807309
**EZH1**	0.139410382	**0.005087897**	0.252807309
**DHX15**	-0.021273807	**0.00512787**	0.252807309
**NAA50**	-0.049120195	**0.005140691**	0.252807309
**ZNF69**	0.111400986	**0.005160027**	0.252807309
**CREB1**	0.107438123	**0.005169514**	0.252807309
**HSD17B7**	0.092106975	**0.005195279**	0.252807309
**PKN2**	0.070299271	**0.005207342**	0.252807309
**PPM1K**	0.116494285	**0.005318295**	0.254392804
**ZNF682**	0.091390453	**0.005318672**	0.254392804
**HRSP12**	-0.045480571	**0.005355986**	0.254392804
**TMEM38A**	0.11328031	**0.005362878**	0.254392804
**ALPP**	0.08231417	**0.005367405**	0.254392804
**DOCK4**	0.118788243	**0.005380822**	0.254392804
**ZNF546**	0.019856981	**0.005441788**	0.254392804
**PDIA5**	-0.060657454	**0.005444524**	0.254392804
**SMPDL3A**	-0.090083647	**0.005466881**	0.254392804
**MRPL37**	-0.032983154	**0.005493519**	0.254392804
**NCF2**	-0.061545393	**0.005514381**	0.254392804
**TDRD1**	0.115776027	**0.005515789**	0.254392804
**ZNF77**	-0.054544141	**0.005608615**	0.255125734
**CENPA**	-0.059396292	**0.005631531**	0.255125734
**SLC15A3**	-0.048539876	**0.00564525**	0.255125734
**NMT2**	0.100772732	**0.005648808**	0.255125734
**NAT1**	-0.067787301	**0.005651938**	0.255125734
**PIGF**	-0.044407037	**0.005705927**	0.255125734
**TFAM**	-0.036540385	**0.005711755**	0.255125734

**ZNF551**	0.104992531	**0.005728016**	0.255125734
**C1orf229**	0.11789432	**0.005809169**	0.255125734
**REXO4**	-0.039226965	**0.005813323**	0.255125734
**OCIAD1**	0.061272864	**0.005818808**	0.255125734
**TEX2**	-0.033466281	**0.005862237**	0.255125734
**MRI1**	0.094462618	**0.005863836**	0.255125734
**TMPRSS15**	-0.047623001	**0.005868506**	0.255125734
**DDB1**	-0.022705724	**0.005895571**	0.255125734
**USP49**	0.097278317	**0.00590046**	0.255125734
**B3GAT3**	0.053410831	**0.005964863**	0.255710936
**NAGS**	-0.044962052	**0.005979512**	0.255710936
**AHCY**	-0.037623856	**0.005983299**	0.255710936
**RPE**	-0.04593393	**0.006019378**	0.256263449
**GPN2**	-0.034259598	**0.006067772**	0.257333989
**ZSCAN5A**	-0.050355735	**0.006197286**	0.260472493
**TNFRSF9**	0.118477705	**0.006225995**	0.260472493
**C9orf72**	0.061286029	**0.006232237**	0.260472493
**COPB2**	-0.036289375	**0.006285029**	0.260472493
**C11orf74**	-0.079372833	**0.006288855**	0.260472493
**ZNF483**	0.138812906	**0.006314018**	0.260472493
**BRPF3**	-0.03412908	**0.006339153**	0.260472493
**LEO1**	-0.039377526	**0.006339863**	0.260472493
**LTB4R2**	-0.050543574	**0.006353562**	0.260472493
**ORC4**	0.124762694	**0.006423591**	0.260773635
**AIFM2**	-0.039643698	**0.006443119**	0.260773635
**MPST**	0.059252858	**0.006474738**	0.260773635
**CCNC**	-0.064668157	**0.006512523**	0.260773635
**SULT1A1**	0.055609653	**0.006519114**	0.260773635
**EGLN3**	0.059548103	**0.006528157**	0.260773635
**GLG1**	-0.023448514	**0.006530123**	0.260773635
**CCDC88B**	0.064139337	**0.006561039**	0.260773635
**ZDHHC5**	-0.042906532	**0.006602327**	0.260773635
**DNM2**	0.046057067	**0.006654645**	0.260773635
**GHITM**	-0.026532323	**0.006697211**	0.260773635
**LEP**	0.101167905	**0.006720873**	0.260773635
**SLC25A40**	-0.067625385	**0.00673104**	0.260773635
**SERPINB8**	-0.07842815	**0.006797499**	0.260773635
**NADSYN1**	0.035540134	**0.006826672**	0.260773635
**ARHGEF1**	0.05790711	**0.00684256**	0.260773635
**VAMP4**	-0.069841236	**0.006863358**	0.260773635
**DNMT1**	-0.024530618	**0.006869804**	0.260773635
**QRFPR**	0.098135471	**0.006873558**	0.260773635
**FAM133B**	0.088961037	**0.006874982**	0.260773635
**MKLN1**	0.02828243	**0.006901245**	0.260773635
**GLB1**	-0.04082469	**0.006959028**	0.260773635
**ACTR2**	-0.030840546	**0.006978443**	0.260773635
**ZNF316**	0.04853783	**0.007015271**	0.260773635
**SLC41A3**	-0.045748554	**0.007016046**	0.260773635
**CES2**	-0.031006686	**0.007032699**	0.260773635
**ALDH2**	-0.061656038	**0.007053065**	0.260773635

**APOM**	-0.056245012	**0.007092934**	0.260773635
**BLZF1**	0.074233418	**0.007097203**	0.260773635
**TAF13**	0.061225823	**0.007108664**	0.260773635
**TNFSF15**	0.132641834	**0.007108954**	0.260773635
**PMPCB**	-0.02696233	**0.007114792**	0.260773635
**CD53**	-0.028802551	**0.007144918**	0.260984956
**ATXN10**	-0.047625085	**0.007212213**	0.260984956
**BTF3L4**	-0.044986515	**0.007228658**	0.260984956
**HINFP**	-0.039072481	**0.007249814**	0.260984956
**SUMO4**	0.08759225	**0.007318403**	0.260984956
**CTSH**	-0.047002083	**0.007337792**	0.260984956
**COG4**	0.034732316	**0.00734302**	0.260984956
**CLEC4F**	-0.197639668	**0.00738218**	0.260984956
**EIF4H**	-0.030255848	**0.007404618**	0.260984956
**C22orf34**	0.102764455	**0.007424899**	0.260984956
**GPS1**	-0.024604421	**0.007430178**	0.260984956
**NUP37**	-0.034734948	**0.007446849**	0.260984956
**LMOD3**	0.126638682	**0.007497301**	0.260984956
**ARMC7**	-0.042864955	**0.007514883**	0.260984956
**PIGK**	-0.0445322	**0.007521171**	0.260984956
**IMPDH1**	-0.040762179	**0.007533451**	0.260984956
**DENR**	0.098685022	**0.007574541**	0.260984956
**FASN**	-0.080195279	**0.007590553**	0.260984956
**ATG12**	0.028319705	**0.007610213**	0.260984956
**SETD2**	0.023437703	**0.007615502**	0.260984956
**KATNB1**	-0.034231046	**0.007615696**	0.260984956
**RNLS**	-0.038317679	**0.007641121**	0.261048038
**ANKRD28**	0.049554153	**0.007742146**	0.263685583
**FCN1**	-0.04281786	**0.00782315**	0.265366078
**HSD17B11**	-0.068954589	**0.007873778**	0.265366078
**DDX21**	-0.041147092	**0.007908824**	0.265366078
**SMARCD2**	0.056832357	**0.007916667**	0.265366078
**CD2BP2**	-0.027251894	**0.00792267**	0.265366078
**FAM73A**	0.108463638	**0.00793533**	0.265366078
**IL10**	0.062373548	**0.008021697**	0.26679683
**ZNF213**	-0.060873144	**0.008077247**	0.26679683
**CCBE1**	0.088133015	**0.008104722**	0.26679683
**TMEM115**	-0.034323606	**0.008116279**	0.26679683
**AGTRAP**	-0.064745686	**0.008118033**	0.26679683
**ARID5B**	0.044983915	**0.008167918**	0.26679683
**CPSF1**	0.056215873	**0.008191387**	0.26679683
**ABCD3**	-0.037720822	**0.008192424**	0.26679683
**MTCH1**	-0.022742964	**0.008195042**	0.26679683
**PIGY**	-0.033004087	**0.008222961**	0.266920696
**MBOAT2**	0.101762108	**0.008266639**	0.267004652
**NME1**	-0.049556923	**0.008295432**	0.267004652
**NMRAL1**	-0.044477784	**0.00830793**	0.267004652
**NDUFS2**	-0.036316066	**0.00834517**	0.267004652
**SRXN1**	-0.070624869	**0.008346157**	0.267004652
**TUBA1B**	-0.034885945	**0.008405063**	0.268114256

**ANAPC5**	0.025857755	**0.008456543**	0.268981234
**ZNF860**	0.107806032	**0.00848263**	0.269037898
**ALG11**	-0.038512408	**0.008513906**	0.269248947
**DOPEY2**	0.036006465	**0.008547965**	0.269248947
**DIRC2**	-0.058012377	**0.008611285**	0.269248947
**GDPD1**	0.109876729	**0.008638524**	0.269248947
**TBXAS1**	-0.042933973	**0.008645774**	0.269248947
**P4HA1**	-0.045215251	**0.008654434**	0.269248947
**ZNF790**	0.061008519	**0.008659556**	0.269248947
**GUSBP9**	0.0766019	**0.008712179**	0.269826192
**ZC3H15**	-0.028350619	**0.008752325**	0.269826192
**PDCL**	-0.058289528	**0.008756565**	0.269826192
**ZNF394**	0.096988365	**0.008813817**	0.269826192
**TRA2A**	0.068198089	**0.008838271**	0.269826192
**INTS3**	0.042326276	**0.008839198**	0.269826192
**RPS15**	-0.024779527	**0.008910082**	0.269826192
**UTP11L**	-0.040427255	**0.009002979**	0.269826192
**ANAPC13**	-0.036910855	**0.009008116**	0.269826192
**HS1BP3**	-0.042875376	**0.009068241**	0.269826192
**ARMC5**	-0.051295466	**0.009084773**	0.269826192
**MARCO**	-0.10997539	**0.009094953**	0.269826192
**MARK2**	0.051985652	**0.009107627**	0.269826192
**DCTN2**	-0.025699162	**0.00911839**	0.269826192
**SP3**	-0.048486832	**0.009120848**	0.269826192
**EIF3M**	-0.04160719	**0.009125062**	0.269826192
**ADCY3**	0.04214032	**0.009138359**	0.269826192
**ZNF652**	0.074558524	**0.009142867**	0.269826192
**NNT**	-0.034740945	**0.009163336**	0.269826192
**ARMC10**	-0.040085069	**0.009165656**	0.269826192
**SEMA3E**	0.139868196	**0.0092091**	0.270254476
**PRKAB2**	0.039740795	**0.009229035**	0.270254476
**CCDC126**	-0.061544137	**0.009306408**	0.271052079
**CRLS1**	-0.027453346	**0.009322587**	0.271052079
**MEI1**	-0.041027407	**0.009343357**	0.271052079
**CHMP2B**	-0.048320385	**0.009378253**	0.271052079
**ZNF319**	-0.040057351	**0.00937871**	0.271052079
**CHRNA5**	0.111877461	**0.00947173**	0.271859896
**PSMB1**	-0.026962229	**0.0095256**	0.271859896
**PSMD2**	-0.024568162	**0.009531945**	0.271859896
**SPCS2**	-0.030788586	**0.009546805**	0.271859896
**RCOR3**	0.033628387	**0.009557897**	0.271859896
**ZNF738**	0.071756513	**0.009575956**	0.271859896
**N4BP2**	0.087794978	**0.009584804**	0.271859896
**AKR1B1**	-0.029995521	**0.009603146**	0.271859896
**PPP1R12B**	-0.059756749	**0.00963727**	0.272129964
**DLG5**	-0.060272523	**0.009674051**	0.272473454
**MBD4**	0.091855301	**0.009713657**	0.272842607
**PNPT1**	0.066227923	**0.009737831**	0.272842607
**SNHG10**	0.04058069	**0.009781192**	0.272842607
**CYP20A1**	0.022706961	**0.009785754**	0.272842607

**ACOX1**	-0.053298741	**0.009873667**	0.274602054
**LMAN2**	-0.052147553	**0.009977217**	0.276318538
**ATP5SL**	-0.026620351	**0.010016144**	0.276318538
**ZC3HC1**	-0.024747699	**0.010025795**	0.276318538
**BLM**	-0.049418095	**0.010042559**	0.276318538
**DIAPH1**	0.03097724	**0.010092467**	0.276318538
**FCAR**	0.12803131	**0.0100959**	0.276318538
**TAF12**	-0.040283872	**0.010110128**	0.276318538
**BABAM1**	0.023791571	**0.010166053**	0.27670045
**SSR1**	-0.035360618	**0.010174097**	0.27670045
**PUS10**	-0.046914433	**0.01021018**	0.277001196
**ACYP1**	0.036199144	**0.010298279**	0.277765685
**ZC3H14**	-0.031004727	**0.010336035**	0.277765685
**TGS1**	-0.056537465	**0.010337148**	0.277765685
**MAPKAPK3**	-0.037294766	**0.010338735**	0.277765685
**ATP5C1**	-0.038132972	**0.010409029**	0.278102199
**SCARNA12**	0.04166624	**0.010430295**	0.278102199
**FHL3**	-0.078709485	**0.010459935**	0.278102199
**AFF4**	0.10114598	**0.010465072**	0.278102199
**TDP1**	0.110436529	**0.010510598**	0.278102199
**HADHA**	-0.042389832	**0.010536556**	0.278102199
**RAB12**	0.101365688	**0.010574201**	0.278102199
**SNX11**	-0.034276982	**0.010661361**	0.278102199
**TM9SF2**	-0.02531644	**0.010714978**	0.278102199
**DDX1**	-0.021250706	**0.010718798**	0.278102199
**EIF2A**	-0.0298955	**0.010723718**	0.278102199
**TP53I11**	-0.052790223	**0.010734664**	0.278102199
**TWISTNB**	-0.040113439	**0.010735652**	0.278102199
**PTRF**	-0.049210355	**0.010747256**	0.278102199
**MTHFD2**	-0.0529953	**0.010804956**	0.278102199
**SIL1**	-0.044515232	**0.01081677**	0.278102199
**METTL2A**	0.114498103	**0.010837559**	0.278102199
**MEAF6**	-0.04939803	**0.010838183**	0.278102199
**FAM198B**	-0.075566421	**0.010865221**	0.278102199
**SMCR5**	0.099957538	**0.010865368**	0.278102199
**SLC33A1**	-0.035010169	**0.010879379**	0.278102199
**STAG3L2**	0.069217198	**0.010903998**	0.278102199
**RPL13A**	-0.038337578	**0.010971774**	0.278630413
**YRDC**	0.096916817	**0.010975053**	0.278630413
**MTF2**	0.0373682	**0.011025599**	0.279273125
**CLTC**	-0.05480733	**0.011087361**	0.279591024
**FAAH**	0.059172179	**0.011088667**	0.279591024
**C9orf66**	-0.060909185	**0.011132143**	0.279816609
**PEX10**	-0.036004601	**0.011185495**	0.279816609
**SFMBT1**	0.03849714	**0.011209274**	0.279816609
**CNGB1**	0.096303606	**0.011220454**	0.279816609
**ARHGAP23**	-0.038733448	**0.011238772**	0.279816609
**ZNF689**	-0.032892907	**0.01124929**	0.279816609
**PNRC2**	-0.027921322	**0.011278382**	0.279911229
**VDAC2**	-0.03136968	**0.011311838**	0.280084531

**RBM7**	-0.075816099	**0.011337123**	0.280084531
**PRDX3**	-0.070008649	**0.011361275**	0.280084531
**TOR1AIP1**	-0.045339675	**0.011410131**	0.280369563
**C7orf49**	-0.03929289	**0.011454408**	0.280369563
**SNORA29**	0.059262854	**0.011464123**	0.280369563
**MYO3B**	0.130577902	**0.011513422**	0.280369563
**LSP1**	-0.062712234	**0.011517096**	0.280369563
**MTL5**	-0.074544566	**0.011556323**	0.280369563
**COPS7A**	-0.02113778	**0.011585172**	0.280369563
**HNRNPH1**	0.042637961	**0.011659085**	0.280369563
**ADCK2**	-0.043150809	**0.011660825**	0.280369563
**FAM188A**	0.033227438	**0.011672482**	0.280369563
**CCR5**	-0.059166864	**0.011696495**	0.280369563
**UBE2G2**	0.129012812	**0.011710613**	0.280369563
**TNFAIP2**	0.066931355	**0.011724095**	0.280369563
**MANEA**	-0.068240513	**0.011729702**	0.280369563
**INPP5A**	0.04066136	**0.011753324**	0.280369563
**TMEM11**	-0.045368898	**0.011778105**	0.280369563
**SLX1B-SULT1A4**	0.046788544	**0.01183278**	0.280479073
**MCEE**	-0.031349857	**0.011833384**	0.280479073
**EEF1B2**	-0.044461731	**0.011876662**	0.28090336
**PDE4DIP**	-0.243546709	**0.011953951**	0.281943682
**KRT10**	-0.043317865	**0.011981194**	0.281943682
**WDR12**	-0.030521046	**0.011997062**	0.281943682
**PNPLA3**	0.07169561	**0.012055338**	0.282409865
**MLH3**	0.091583695	**0.012067925**	0.282409865
**WWC1**	0.024585961	**0.012173511**	0.284279743
**FAM174A**	-0.05459905	**0.012227889**	0.284557326
**DPY19L4**	0.11420354	**0.012236813**	0.284557326
**AP3B1**	-0.025121546	**0.012284846**	0.284952446
**ZFAND6**	-0.075587415	**0.012305291**	0.284952446
**PCGF1**	-0.045778269	**0.012473148**	0.287722923
**SP1**	0.036351237	**0.012497418**	0.287722923
**TMEM106A**	0.11012606	**0.012502911**	0.287722923
**SBF1**	-0.026718532	**0.012535082**	0.287864785
**CX3CR1**	-0.06290276	**0.012599001**	0.288733635
**C6orf47**	-0.028446569	**0.012658022**	0.289486869
**PTGES2**	-0.062108116	**0.012687047**	0.289552415
**PSMA1**	-0.026004561	**0.01277599**	0.290152093
**GALK2**	0.060235265	**0.012790017**	0.290152093
**GRAMD1A**	-0.04083695	**0.012871847**	0.290152093
**GLT1D1**	-0.061598606	**0.012878641**	0.290152093
**PEX2**	-0.036423812	**0.01289264**	0.290152093
**MNDA**	-0.121700259	**0.012918863**	0.290152093
**SEC63**	-0.04015353	**0.012944872**	0.290152093
**COPS6**	-0.029551741	**0.012947069**	0.290152093
**LRRC42**	-0.039081803	**0.012956648**	0.290152093
**ZNF622**	-0.033290972	**0.012975453**	0.290152093
**ZNF786**	0.077725833	**0.013015672**	0.290464667
**IGSF9B**	0.071811175	**0.013050249**	0.290650319

**REEP6**	-0.05984103	**0.013076895**	0.290658942
**DEDD2**	-0.028448442	**0.013107588**	0.290757295
**ASAH1**	-0.063106503	**0.013167246**	0.291496482
**HNRNPU**	0.12119536	**0.013247333**	0.292684089
**KCNH6**	0.03159534	**0.013341776**	0.29346975
**QTRT1**	0.041645837	**0.013361269**	0.29346975
**CLEC4E**	-0.0732424	**0.01337933**	0.29346975
**CRIPT**	-0.053350471	**0.013388944**	0.29346975
**NDUFC2**	-0.056105153	**0.013427926**	0.29374251
**SCYL1**	-0.025428073	**0.013495997**	0.294649299
**UNKL**	0.067284622	**0.013525916**	0.294721184
**TIMM23**	-0.020995802	**0.013559169**	0.294865302
**BIN3**	0.06598132	**0.013620728**	0.295613492
**ARL2**	-0.03664804	**0.013646987**	0.295613492
**SCO1**	-0.042318674	**0.013745833**	0.296348334
**RIN3**	0.043421688	**0.013771527**	0.296348334
**EGR2**	-0.076905341	**0.013793939**	0.296348334
**C11orf63**	0.125699865	**0.013802004**	0.296348334
**C3orf62**	0.036909311	**0.013821473**	0.296348334
**AASDHPPT**	-0.036399735	**0.013841547**	0.296348334
**LRCH3**	0.058931964	**0.013897643**	0.296483807
**GMPR**	0.095181191	**0.013934635**	0.296483807
**IL4R**	0.049455713	**0.013959618**	0.296483807
**ATP6V1F**	-0.028702813	**0.013969437**	0.296483807
**CNST**	0.053824507	**0.014049972**	0.296483807
**CARHSP1**	-0.041669096	**0.014072195**	0.296483807
**ZNF84**	0.055033686	**0.01410397**	0.296483807
**SDSL**	-0.070097173	**0.014106968**	0.296483807
**PHAX**	0.115723281	**0.014111346**	0.296483807
**ATP5F1**	-0.028248638	**0.014115726**	0.296483807
**RPL29**	-0.022299534	**0.014180767**	0.296639227
**TIMM8B**	-0.043898903	**0.014182395**	0.296639227
**COMMD5**	-0.042559935	**0.014303402**	0.296639227
**MTA2**	0.037131267	**0.014308729**	0.296639227
**VCP**	-0.029868463	**0.014323028**	0.296639227
**ZNF653**	-0.034028119	**0.014362852**	0.296639227
**CD101**	-0.063617036	**0.014380628**	0.296639227
**CDC37**	-0.021581704	**0.014384038**	0.296639227
**PPM1M**	-0.025406041	**0.014415234**	0.296639227
**RFC1**	-0.029024824	**0.014471512**	0.296639227
**NLRP1**	0.044411001	**0.014493636**	0.296639227
**NDUFS7**	-0.032456429	**0.014528361**	0.296639227
**TRPM4**	-0.098276763	**0.014597691**	0.296639227
**SNORD83B**	0.047804017	**0.014606328**	0.296639227
**FXYD7**	0.050370841	**0.014636156**	0.296639227
**PCYOX1**	0.064488717	**0.014647181**	0.296639227
**COMMD8**	-0.050927221	**0.014654175**	0.296639227
**ZNF746**	-0.024004536	**0.01465493**	0.296639227
**FKTN**	0.020490208	**0.014675569**	0.296639227
**CCDC125**	0.089468932	**0.014678754**	0.296639227

**NUFIP1**	-0.046969515	**0.014685906**	0.296639227
**PTAR1**	0.036298277	**0.014757807**	0.296963659
**PPP1R8**	-0.040576681	**0.014760623**	0.296963659
**FMO5**	-0.052129705	**0.014782453**	0.296963659
**PECR**	-0.043290705	**0.01483686**	0.297277152
**ZBTB4**	0.029092148	**0.014851772**	0.297277152
**BRI3**	-0.052528112	**0.014887021**	0.297444822
**RNF170**	0.040812775	**0.0149423**	0.297664166
**EIF3J**	-0.033095538	**0.015010412**	0.297664166
**VIM**	-0.035274309	**0.015017665**	0.297664166
**TMEM181**	0.04648776	**0.015031023**	0.297664166
**GLIPR1**	-0.075994846	**0.015086971**	0.297664166
**C14orf105**	0.118835433	**0.015157848**	0.297664166
**MFHAS1**	0.092491135	**0.015159516**	0.297664166
**MFNG**	-0.018069127	**0.015176858**	0.297664166
**TMEM209**	-0.029057255	**0.015182046**	0.297664166
**DHX57**	0.07600986	**0.015198599**	0.297664166
**MAFF**	0.133741918	**0.015234903**	0.297664166
**PFN2**	-0.071444937	**0.015246212**	0.297664166
**ZNF239**	-0.064946711	**0.015247591**	0.297664166
**TICAM1**	-0.046912703	**0.015305723**	0.297750007
**MDH2**	-0.023525191	**0.015305787**	0.297750007
**CDRT4**	-0.03982157	**0.015407217**	0.298709184
**SPEN**	0.025499227	**0.015410201**	0.298709184
**ANKRD44**	0.10547222	**0.015436051**	0.298709184
**PLA2G2D**	0.080896522	**0.01551961**	0.299374894
**SUMF1**	-0.040962444	**0.015524545**	0.299374894
**ZW10**	-0.025299964	**0.015584836**	0.299837837
**DISP1**	-0.041189048	**0.015635152**	0.299837837
**DRG1**	-0.035444856	**0.01564594**	0.299837837
**FAM20B**	-0.020835491	**0.015663183**	0.299837837
**RAD51**	0.072763128	**0.015686853**	0.299837837
**PARK7**	-0.021297108	**0.015715998**	0.299837837
**PTGR2**	0.126779651	**0.015757561**	0.299837837
**CDKN2AIPNL**	0.025192264	**0.015765256**	0.299837837
**STAG3L1**	0.070083633	**0.015807387**	0.299889072
**FAM73B**	0.042609961	**0.015822136**	0.299889072
**RAPGEF6**	0.042959336	**0.015892668**	0.300419293
**TMEM56**	-0.052335709	**0.015923556**	0.300419293
**LIN7A**	-0.050144065	**0.015935528**	0.300419293
**UHRF2**	0.035162324	**0.015980422**	0.300419293
**MDH1**	-0.02585605	**0.016027932**	0.300419293
**HECTD3**	-0.031032558	**0.01603386**	0.300419293
**TTF1**	-0.022649503	**0.016040094**	0.300419293
**GINS3**	-0.036436495	**0.016127673**	0.301549337
**APOBEC3G**	-0.065783558	**0.016209531**	0.302113605
**PATE1**	0.049729343	**0.016225473**	0.302113605
**DGCR6L**	-0.045187586	**0.016242458**	0.302113605
**HIBADH**	-0.0340114	**0.016267026**	0.302113605
**CTSD**	0.049005413	**0.016304305**	0.302298749

**C4orf29**	0.07653796	**0.016349882**	0.302446048
**NSMCE2**	-0.051869137	**0.01640324**	0.302446048
**PITPNA**	-0.042215081	**0.016416687**	0.302446048
**RB1**	-0.060907803	**0.016421544**	0.302446048
**USP37**	0.041890538	**0.016453902**	0.302506362
**NFKB2**	0.043579573	**0.016489215**	0.302506362
**TXNDC12**	-0.029569298	**0.016506807**	0.302506362
**FRA10AC1**	-0.066731248	**0.016569627**	0.30315571
**MTMR12**	0.049381962	**0.016601798**	0.303243065
**NIP7**	-0.037492977	**0.016653405**	0.303684584
**DUSP11**	-0.032285811	**0.016716722**	0.304216166
**SRP72**	-0.027069068	**0.016742457**	0.304216166
**ZNF669**	0.083881877	**0.016834111**	0.304216166
**PRPSAP1**	-0.025139739	**0.016868821**	0.304216166
**PCDHB9**	0.075963118	**0.01688768**	0.304216166
**PCNXL3**	0.037002603	**0.016892774**	0.304216166
**PKIB**	-0.053676357	**0.016927502**	0.304216166
**PHIP**	0.030281642	**0.016962794**	0.304216166
**FAM161A**	0.111922841	**0.017013465**	0.304216166
**PTP4A2**	0.028373049	**0.017035416**	0.304216166
**KCTD11**	-0.043829227	**0.017051908**	0.304216166
**C8orf33**	-0.030636116	**0.017134445**	0.304216166
**P2RY13**	-0.050187809	**0.017138953**	0.304216166
**DYNC1I2**	-0.029893314	**0.017144347**	0.304216166
**MRPL44**	-0.034525691	**0.017147585**	0.304216166
**MRPL19**	-0.044650822	**0.017152868**	0.304216166
**TYROBP**	-0.050933361	**0.017173763**	0.304216166
**PAPD7**	0.035009368	**0.017204234**	0.304216166
**SERP1**	-0.038708323	**0.017224453**	0.304216166
**DVL2**	0.027468738	**0.017232228**	0.304216166
**DCLRE1C**	0.07525572	**0.01726525**	0.30431378
**PGAP1**	0.048848093	**0.017311882**	0.304390533
**POLR3C**	-0.020721881	**0.01736377**	0.304390533
**BTBD10**	-0.036138606	**0.017378551**	0.304390533
**ACOX2**	-0.064554296	**0.017379602**	0.304390533
**BSN-AS2**	0.093417847	**0.017415703**	0.304530345
**FCER1G**	-0.057835117	**0.017442609**	0.304530345
**ANXA2**	-0.034151403	**0.017475384**	0.304622081
**NDST2**	-0.032650951	**0.017562189**	0.30565388
**CNTNAP1**	0.078275556	**0.017620168**	0.306181542
**BCKDHA**	-0.035191288	**0.017670252**	0.30619953
**FRAT2**	-0.058929701	**0.017689899**	0.30619953
**PIK3CG**	0.051660577	**0.017704192**	0.30619953
**TMEM41A**	-0.038255126	**0.017849225**	0.308147208
**RPL31P11**	0.067679225	**0.017885604**	0.308147208
**VPS41**	0.075973471	**0.017900321**	0.308147208
**RNASET2**	-0.022818313	**0.017964086**	0.308356813
**KCTD7**	0.070017352	**0.017995958**	0.308356813
**RBM5**	0.030513428	**0.01799607**	0.308356813
**ZNF517**	-0.037704275	**0.018127949**	0.309789623

**TIMM44**	0.032226222	**0.018199032**	0.309789623
**FAR2**	-0.05169872	**0.018203919**	0.309789623
**TSHZ3**	-0.059124744	**0.018216022**	0.309789623
**CSAD**	0.067988436	**0.018219626**	0.309789623
**SOCS3**	0.066664328	**0.018368071**	0.311275252
**SNORA77**	0.051410721	**0.018388346**	0.311275252
**PLCD3**	-0.036610947	**0.018391365**	0.311275252
**NUP35**	-0.052933445	**0.018433508**	0.31151222
**PAPD5**	0.038162258	**0.018552168**	0.311627227
**FOXK1**	0.054941112	**0.018585969**	0.311627227
**ZMPSTE24**	-0.025028627	**0.018625699**	0.311627227
**FAHD2A**	-0.042076937	**0.018646001**	0.311627227
**MMACHC**	-0.044479814	**0.018697062**	0.311627227
**C16orf91**	-0.033020404	**0.018714295**	0.311627227
**SLC2A8**	-0.081725738	**0.018716416**	0.311627227
**UQCRFS1**	-0.031999635	**0.018721292**	0.311627227
**NLRX1**	-0.025200899	**0.018737269**	0.311627227
**RPPH1**	-0.034860971	**0.018757525**	0.311627227
**CD300C**	-0.089816702	**0.018759274**	0.311627227
**USP36**	0.074482345	**0.018778152**	0.311627227
**UBE2V1**	-0.041113819	**0.018963491**	0.313704196
**CD34**	-0.07247008	**0.018994475**	0.313704196
**RHBDL2**	0.098556739	**0.01902554**	0.313704196
**RAB2A**	-0.035506767	**0.019047695**	0.313704196
**UEVLD**	-0.039706497	**0.019105687**	0.313704196
**VPS13B**	0.068201258	**0.019116058**	0.313704196
**POLR1E**	-0.033268291	**0.019131334**	0.313704196
**GGTLC1**	-0.044194488	**0.019145982**	0.313704196
**LPCAT3**	-0.029306247	**0.019158373**	0.313704196
**ZNF837**	-0.044275687	**0.019248316**	0.314711392
**KIAA1328**	-0.041604388	**0.019302025**	0.314741121
**STARD7**	-0.019804598	**0.01932196**	0.314741121
**SHPK**	0.086666015	**0.01936962**	0.314741121
**GBE1**	-0.044620931	**0.019384669**	0.314741121
**PROCR**	-0.042096071	**0.019392307**	0.314741121
**SNORD31**	0.047949309	**0.019485336**	0.31578797
**DTNA**	-0.04487606	**0.019567162**	0.316307824
**KIAA0922**	0.035689174	**0.019597762**	0.316307824
**PLD3**	-0.031303841	**0.019632321**	0.316307824
**DGAT1**	-0.034738088	**0.019635401**	0.316307824
**ARL2BP**	-0.028768652	**0.0196909**	0.316307824
**GLTP**	-0.02556421	**0.019717719**	0.316307824
**KLHL21**	-0.039593724	**0.019734812**	0.316307824
**SNORA33**	0.049778828	**0.019746021**	0.316307824
**TRIM38**	0.062347037	**0.019823648**	0.316431809
**CIB1**	-0.031310799	**0.019900525**	0.316431809
**NUBPL**	0.080395837	**0.019900973**	0.316431809
**ITGA4**	-0.051520557	**0.019903889**	0.316431809
**AK1**	0.065928722	**0.019918675**	0.316431809
**CYB5RL**	0.093651679	**0.019925284**	0.316431809

**VAMP3**	-0.037041073	**0.019971016**	0.31654238
**EFEMP2**	-0.024279977	**0.020010194**	0.31654238
**PRDM8**	0.049683925	**0.020032822**	0.31654238
**DCUN1D1**	-0.050957194	**0.020046635**	0.31654238
**HSP90AB1**	0.033994992	**0.020108193**	0.316867512
**CDKN2A**	-0.04249304	**0.020124479**	0.316867512
**YBX1**	-0.025134445	**0.020231952**	0.317459992
**MOGS**	0.03289052	**0.020242405**	0.317459992
**HDAC11**	-0.049464158	**0.020248148**	0.317459992
**CYCS**	-0.047857681	**0.020316031**	0.317640721
**COX5A**	-0.034774308	**0.020317068**	0.317640721
**C19orf24**	0.044790444	**0.020462841**	0.318871721
**EHBP1L1**	0.045446795	**0.020478619**	0.318871721
**EMILIN2**	-0.040921724	**0.020512822**	0.318871721
**CDK8**	-0.038116399	**0.020522022**	0.318871721
**TNK2**	0.03547397	**0.020539844**	0.318871721
**DUSP10**	-0.047212973	**0.020618869**	0.319650226
**SYF2**	-0.034821543	**0.020671966**	0.319812382
**OCEL1**	-0.029193405	**0.020687114**	0.319812382
**TCP11L1**	-0.041501742	**0.020743336**	0.320151732
**SPINT3**	0.04735124	**0.020766912**	0.320151732
**PLAUR**	-0.056756629	**0.020800638**	0.320225681
**BID**	-0.03913105	**0.020869933**	0.320846231
**CD1B**	-0.059981715	**0.02092535**	0.321081599
**SELO**	0.042887961	**0.020960541**	0.321081599
**ZCCHC11**	0.035275272	**0.020972265**	0.321081599
**IDH3B**	-0.018000373	**0.021076995**	0.322148439
**HNRNPA0**	-0.032426112	**0.021151878**	0.322148439
**LMBRD1**	-0.035468148	**0.021164358**	0.322148439
**UBL3**	-0.037890666	**0.021204419**	0.322148439
**HNRNPR**	-0.021959073	**0.02120743**	0.322148439
**RTKN2**	0.085453015	**0.02121657**	0.322148439
**C20orf196**	-0.044202158	**0.021272073**	0.322548732
**DNTTIP1**	-0.037824752	**0.021343432**	0.323086472
**TTC18**	0.047005943	**0.021365914**	0.323086472
**TMED1**	-0.043622691	**0.02140034**	0.323165567
**ZDHHC2**	-0.045312669	**0.021467502**	0.323430477
**CRISPLD2**	-0.082095535	**0.021476321**	0.323430477
**PNPO**	-0.027978235	**0.021506938**	0.323451487
**DHRS13**	-0.040415618	**0.021568816**	0.323941957
**RBM14**	0.093642681	**0.021628933**	0.324404686
**ABHD8**	-0.032878081	**0.021702956**	0.325074449
**PIGW**	-0.037779098	**0.021774209**	0.325217089
**TNC**	0.079865767	**0.021776249**	0.325217089
**LAMC1**	-0.102301801	**0.021819084**	0.325217089
**ALKBH4**	-0.03696068	**0.021887938**	0.325217089
**UBAP2L**	0.03668108	**0.021904227**	0.325217089
**MARCH6**	0.072650404	**0.021980712**	0.325217089
**EIF2B1**	-0.029950232	**0.021982744**	0.325217089
**AKR1C2**	0.101456733	**0.022014572**	0.325217089

**EIF2AK4**	0.07794152	**0.02202547**	0.325217089
**THOC7**	-0.02922186	**0.02205439**	0.325217089
**TXNL1**	-0.049678212	**0.022071433**	0.325217089
**ALAD**	-0.043414884	**0.022099776**	0.325217089
**SRGN**	-0.047459898	**0.022154589**	0.325217089
**UPF3A**	0.046104367	**0.022210124**	0.325217089
**CCNL1**	0.030413832	**0.022251635**	0.325217089
**TACO1**	-0.025302212	**0.02233029**	0.325217089
**YWHAQ**	-0.029601369	**0.022330569**	0.325217089
**TJP1**	0.050083673	**0.022392975**	0.325217089
**NEDD9**	-0.05179567	**0.022401263**	0.325217089
**ZNF707**	-0.041776339	**0.022425166**	0.325217089
**NFXL1**	-0.055608139	**0.022430076**	0.325217089
**NEAT1**	0.055084474	**0.022445641**	0.325217089
**SH2B2**	0.044326558	**0.022469806**	0.325217089
**DSC2**	-0.097705425	**0.022472446**	0.325217089
**CD248**	-0.091327337	**0.022509597**	0.325217089
**ATE1**	-0.036299666	**0.022527884**	0.325217089
**SNAPC1**	0.087740767	**0.022541673**	0.325217089
**GGA1**	0.018080372	**0.022541876**	0.325217089
**RASSF6**	0.105656467	**0.022564525**	0.325217089
**CDC34**	0.034008797	**0.022618154**	0.325566115
**SNORD104**	0.051348478	**0.02266151**	0.325693396
**DECR1**	-0.018528324	**0.022685844**	0.325693396
**PSMD7**	-0.031701756	**0.022848473**	0.327603296
**C19orf10**	-0.046573906	**0.022990157**	0.328291414
**FUBP3**	-0.024416675	**0.023058051**	0.328291414
**ITGAV**	-0.040989031	**0.023064697**	0.328291414
**ENHO**	0.04202681	**0.023070315**	0.328291414
**IGF2R**	-0.027519719	**0.023158431**	0.328291414
**C5orf54**	-0.047585695	**0.023181993**	0.328291414
**NME7**	-0.049125517	**0.023188808**	0.328291414
**ZNF784**	-0.035400801	**0.023194209**	0.328291414
**ACIN1**	0.027710944	**0.023233117**	0.328291414
**HOXA5**	-0.058495062	**0.023240244**	0.328291414
**VPS45**	0.025482813	**0.023240916**	0.328291414
**NDUFB5**	-0.025202107	**0.023268178**	0.328291414
**R3HCC1**	-0.020440928	**0.023290926**	0.328291414
**C1orf53**	-0.05855108	**0.023367147**	0.328291414
**TMEM216**	-0.032823363	**0.023374268**	0.328291414
**ZNF586**	-0.039513711	**0.023374367**	0.328291414
**SEPHS2**	-0.033848278	**0.023426563**	0.328291414
**ARHGAP19**	-0.025352035	**0.02343032**	0.328291414
**STX16**	0.059312954	**0.023496914**	0.328411418
**RIOK2**	-0.032758657	**0.023498224**	0.328411418
**DGCR2**	0.041142064	**0.023534854**	0.32850857
**TXK**	0.070789001	**0.023603656**	0.328965176
**MRPS30**	-0.037220428	**0.023627005**	0.328965176
**ACVR1B**	-0.032501561	**0.023731335**	0.329350917
**TMEM55B**	0.026826051	**0.023753991**	0.329350917

**LIPT1**	-0.033153033	**0.02376138**	0.329350917
**ZNF671**	-0.035450792	**0.023773727**	0.329350917
**PIM3**	0.046586033	**0.023804102**	0.329359503
**KIF20B**	-0.041667117	**0.023933557**	0.330737257
**WDR74**	0.079085892	**0.024019581**	0.331351463
**UNC119B**	-0.032462167	**0.024037874**	0.331351463
**C19orf47**	0.038738845	**0.02417696**	0.332753953
**UQCR10**	-0.045186143	**0.024199741**	0.332753953
**EBNA1BP2**	-0.024318235	**0.024277613**	0.333410549
**TXNDC16**	-0.040738145	**0.024372774**	0.334114457
**P2RX6**	-0.044770834	**0.024413697**	0.334114457
**FADD**	-0.044327284	**0.024419423**	0.334114457
**TMED2**	-0.032890414	**0.024467761**	0.334132279
**TRAF7**	-0.041185664	**0.024481098**	0.334132279
**GSTTP2**	0.094134783	**0.024566519**	0.334480301
**MEIS1**	0.087019067	**0.024567033**	0.334480301
**DHRS9**	-0.103012355	**0.024617819**	0.334581976
**BTBD7**	-0.034069256	**0.024634954**	0.334581976
**IMMP1L**	-0.04322114	**0.024706356**	0.335140512
**ATP5B**	-0.022999305	**0.024827455**	0.336187848
**TNPO1**	0.046869807	**0.024892857**	0.336187848
**PACRGL**	0.050372272	**0.024910634**	0.336187848
**CAMTA1**	0.084076563	**0.024912148**	0.336187848
**TMEM219**	0.028572549	**0.024955709**	0.336187848
**C1orf122**	-0.038337	**0.024965797**	0.336187848
**RPL23AP53**	-0.023355516	**0.025057375**	0.336265882
**SEC23B**	-0.034233022	**0.025075268**	0.336265882
**DDX19B**	-0.022756084	**0.025086659**	0.336265882
**ZNHIT3**	-0.024356436	**0.025093109**	0.336265882
**AP3D1**	-0.029278882	**0.025247334**	0.337470088
**C11orf73**	-0.041297782	**0.025257747**	0.337470088
**DUSP22**	-0.030608224	**0.025284571**	0.337470088
**TSTD2**	-0.031943432	**0.025318923**	0.337470088
**MAPK3**	-0.02964464	**0.025335409**	0.337470088
**MAP4K5**	0.030805804	**0.025454488**	0.338440479
**MOCS2**	-0.057207965	**0.025624451**	0.338440479
**CCT4**	-0.031787669	**0.025632287**	0.338440479
**ANO10**	-0.038754795	**0.025699573**	0.338440479
**POLE3**	-0.022007725	**0.025713903**	0.338440479
**PDK1**	0.056210677	**0.025757594**	0.338440479
**RHEB**	-0.02796238	**0.025759205**	0.338440479
**DEK**	-0.026847256	**0.025776022**	0.338440479
**TNFSF13**	-0.031287764	**0.025780147**	0.338440479
**C9orf78**	-0.023929604	**0.025785556**	0.338440479
**ZNF428**	-0.025227728	**0.025786448**	0.338440479
**SEH1L**	-0.036776283	**0.025803279**	0.338440479
**ATP6V0E2**	-0.033583637	**0.025851505**	0.338440479
**HEBP1**	-0.030334982	**0.025859731**	0.338440479
**MPEG1**	-0.065540433	**0.025866894**	0.338440479
**IL17RB**	0.060383965	**0.025938892**	0.338599753

**PRRC2C**	0.022964871	**0.025940247**	0.338599753
**ECHDC2**	0.044365229	**0.025973371**	0.338632793
**LRRFIP1**	0.079010385	**0.026133408**	0.340158677
**ZCCHC9**	-0.033380266	**0.026151869**	0.340158677
**SNORA34**	0.036869056	**0.0262568**	0.341122676
**SLC2A9**	-0.077334701	**0.026385036**	0.342135191
**SLC25A46**	-0.040522636	**0.026466806**	0.342135191
**AGPS**	-0.029624618	**0.026471553**	0.342135191
**GUSB**	-0.0202859	**0.026491879**	0.342135191
**LMTK2**	-0.03928848	**0.026507818**	0.342135191
**RFPL2**	0.05326252	**0.026520191**	0.342135191
**ZNF317**	-0.031339994	**0.026585813**	0.3425825
**PDCD4**	0.039976239	**0.02663209**	0.342779769
**FCGR3A**	0.059422561	**0.026706523**	0.342885895
**C21orf58**	0.060706988	**0.026725499**	0.342885895
**PBX4**	0.049581031	**0.026733267**	0.342885895
**SNORD73A**	0.042446722	**0.026819386**	0.343592343
**IL18R1**	0.059798881	**0.026899493**	0.344000261
**BMP4**	-0.042354923	**0.026913382**	0.344000261
**POLR3F**	-0.03459243	**0.026989163**	0.344108393
**NOL8**	0.026348661	**0.027021658**	0.344108393
**SEC61A1**	-0.025910476	**0.027022523**	0.344108393
**WDR61**	-0.032019008	**0.027046192**	0.344108393
**DDHD1**	0.096586439	**0.027117142**	0.34415468
**PTPLAD2**	0.064663933	**0.027133281**	0.34415468
**ANXA5**	-0.043645851	**0.027143106**	0.34415468
**PDIA6**	-0.040562762	**0.027206341**	0.344561766
**SIGLEC10**	-0.066977385	**0.027258857**	0.344619215
**C17orf47**	-0.046267643	**0.027347631**	0.344619215
**SNORD89**	0.075661024	**0.027352458**	0.344619215
**GOLGA7**	-0.017930657	**0.027368059**	0.344619215
**RELL2**	0.041745217	**0.027427234**	0.344619215
**POLR2C**	-0.038806009	**0.027501893**	0.344619215
**CCDC149**	0.086939173	**0.027531595**	0.344619215
**RABEPK**	-0.018426535	**0.027593083**	0.344619215
**NDUFS8**	-0.021629568	**0.027603885**	0.344619215
**AP3M1**	-0.036182797	**0.027715684**	0.344619215
**ZDHHC17**	0.032270612	**0.027722521**	0.344619215
**PIK3R4**	-0.031477631	**0.027762158**	0.344619215
**ARHGAP30**	-0.031427394	**0.027779132**	0.344619215
**PSMB4**	-0.016682466	**0.027786478**	0.344619215
**OSBPL11**	-0.035343595	**0.027821888**	0.344619215
**CNOT2**	-0.022924741	**0.027875803**	0.344619215
**GUCA1B**	0.055789099	**0.027921115**	0.344619215
**PEX14**	-0.037784778	**0.027939599**	0.344619215
**LONP2**	0.025725632	**0.027956283**	0.344619215
**LIG1**	0.044033298	**0.02796193**	0.344619215
**HDHD2**	-0.022879327	**0.028052932**	0.344619215
**C6orf48**	-0.032500882	**0.02816846**	0.344619215
**BCYRN1**	0.070856869	**0.028171656**	0.344619215

**AZIN1**	-0.030350279	**0.028173666**	0.344619215
**RPS2P32**	-0.053113448	**0.028214072**	0.344619215
**ZNF277**	-0.030926434	**0.028239716**	0.344619215
**MFAP3L**	0.085347021	**0.028240061**	0.344619215
**PRUNE**	0.042904493	**0.028240762**	0.344619215
**TIMP2**	-0.043328863	**0.028274254**	0.344619215
**TOMM34**	-0.042270001	**0.028277517**	0.344619215
**TXNDC17**	-0.02695132	**0.028285335**	0.344619215
**UHRF1BP1L**	-0.044246491	**0.02838153**	0.344619215
**NUTF2**	0.029009781	**0.028424918**	0.344619215
**C22orf39**	-0.034022645	**0.028484786**	0.344619215
**TRIM33**	0.02204506	**0.028496699**	0.344619215
**OMG**	0.039323894	**0.028513148**	0.344619215
**ARL4C**	-0.038317359	**0.028535401**	0.344619215
**NOL7**	-0.027078565	**0.028561127**	0.344619215
**TRADD**	0.037794136	**0.02856769**	0.344619215
**POLR2J2**	0.078868416	**0.028572466**	0.344619215
**ARV1**	-0.031890111	**0.028612053**	0.344619215
**NDUFB3**	-0.025231841	**0.028654725**	0.344619215
**ASAP2**	0.083146983	**0.028663728**	0.344619215
**GRAMD3**	-0.047225331	**0.028716628**	0.344619215
**RPS6**	-0.021908227	**0.028828366**	0.344619215
**ATAD5**	0.041692889	**0.028839281**	0.344619215
**ZNF583**	-0.036380927	**0.02884749**	0.344619215
**TGOLN2**	-0.02801412	**0.028860684**	0.344619215
**TTC14**	0.055708711	**0.028885874**	0.344619215
**XPR1**	-0.025577247	**0.028910453**	0.344619215
**VPS4B**	-0.025019649	**0.028920997**	0.344619215
**E2F6**	-0.041619793	**0.02893523**	0.344619215
**MRP63**	0.052436835	**0.02896055**	0.344619215
**CXCL16**	-0.04669334	**0.02896844**	0.344619215
**ERLIN2**	-0.025427949	**0.028991092**	0.344619215
**ATP5E**	-0.01888388	**0.029037601**	0.344619215
**SLC30A1**	-0.059192849	**0.029043497**	0.344619215
**HLCS**	-0.036921156	**0.029083528**	0.344619215
**SERINC5**	0.069687888	**0.02909891**	0.344619215
**STUB1**	0.033587332	**0.029104994**	0.344619215
**PLIN5**	0.094470082	**0.029116083**	0.344619215
**DLD**	-0.038898493	**0.029177695**	0.344619215
**ZNF408**	-0.02990062	**0.02923932**	0.344619215
**ERP29**	-0.023334475	**0.029309356**	0.344619215
**QPCT**	-0.098245558	**0.029339854**	0.344619215
**CCDC14**	0.034136901	**0.029341505**	0.344619215
**TMEM167A**	-0.043696721	**0.029361555**	0.344619215
**BTBD11**	-0.055639238	**0.029378909**	0.344619215
**ASCC3**	-0.028140446	**0.029393195**	0.344619215
**UBN1**	-0.027243258	**0.029412558**	0.344619215
**KLHL28**	0.097964358	**0.029421371**	0.344619215
**COMT**	-0.040094505	**0.029492807**	0.344673106
**BST1**	-0.0759751	**0.029511306**	0.344673106

**ZDHHC24**	-0.045177453	**0.029610162**	0.344673106
**HAUS2**	0.057194895	**0.029625594**	0.344673106
**BCL2L12**	-0.02255932	**0.02967423**	0.344673106
**TMEM40**	0.081864773	**0.029680703**	0.344673106
**PEX7**	-0.040343977	**0.029706112**	0.344673106
**H2AFY2**	-0.03318023	**0.029725253**	0.344673106
**FAM105A**	-0.049644159	**0.029725922**	0.344673106
**DUSP23**	-0.025381499	**0.029737358**	0.344673106
**LIN52**	0.049809928	**0.029837805**	0.34511876
**HDAC1**	-0.026061475	**0.029838165**	0.34511876
**NUS1**	-0.02645465	**0.029956865**	0.346129996
**GNB4**	0.062126146	**0.030185345**	0.34840624
**SGK223**	-0.038365966	**0.030401634**	0.350181839
**DARS**	-0.021913118	**0.030427417**	0.350181839
**CLASRP**	0.040337153	**0.030434089**	0.350181839
**SIPA1**	-0.022847091	**0.030520461**	0.350810994
**GNS**	-0.022768489	**0.030560016**	0.350901261
**SCAPER**	-0.03602769	**0.030635619**	0.351150972
**KRTCAP2**	-0.025767986	**0.030645211**	0.351150972
**BIRC6**	-0.02884651	**0.030799476**	0.352553672
**ICA1**	0.073242881	**0.030864949**	0.352564047
**MYO10**	-0.042912296	**0.030875961**	0.352564047
**KIAA1549**	0.102434871	**0.030922568**	0.352564047
**DNAJB11**	-0.025228583	**0.030986796**	0.352564047
**SLC35A5**	-0.0355635	**0.030987419**	0.352564047
**PIM1**	0.040643855	**0.030991491**	0.352564047
**RPL21**	-0.050335965	**0.031113335**	0.353347707
**FGGY**	-0.050683088	**0.031124222**	0.353347707
**ATP5L**	-0.034800584	**0.031286878**	0.354574354
**LIME1**	-0.025441353	**0.031296336**	0.354574354
**SPHK2**	-0.021193409	**0.031449402**	0.355944198
**CTTN**	0.106321808	**0.031534114**	0.356538413
**EEF2**	-0.019230263	**0.031646788**	0.357447239
**PRPF8**	-0.03277184	**0.031679924**	0.357456752
**COX10**	-0.026549716	**0.031739778**	0.357626203
**DDX47**	-0.019614591	**0.031768189**	0.357626203
**WDR70**	0.028355325	**0.031791868**	0.357626203
**CRNKL1**	-0.046716383	**0.031871581**	0.357926794
**UFC1**	-0.02804578	**0.031900003**	0.357926794
**GCLM**	0.073646841	**0.03192896**	0.357926794
**MPND**	-0.0464252	**0.031966818**	0.357926794
**BCAT1**	-0.093024008	**0.032027839**	0.357926794
**C16orf87**	-0.044403222	**0.032034363**	0.357926794
**EVI5L**	-0.034784527	**0.032064517**	0.357926794
**MYO19**	-0.048975519	**0.032077277**	0.357926794
**SLC7A1**	-0.032185126	**0.032119055**	0.358032039
**ABHD12**	-0.079189917	**0.032227053**	0.358874493
**HNRNPA1L2**	-0.032982429	**0.032366012**	0.360059685
**TEX264**	-0.02282608	**0.032471758**	0.360368363
**WFS1**	-0.043312664	**0.032477287**	0.360368363

**ZFPL1**	-0.032192844	**0.032493378**	0.360368363
**SNX27**	-0.03003291	**0.032523985**	0.360368363
**POU2F2**	0.050486893	**0.032634814**	0.361234755
**C17orf74**	0.035944168	**0.032679626**	0.361369414
**SDHD**	-0.041591788	**0.032758986**	0.361602023
**ZNF621**	-0.029264989	**0.032800044**	0.361602023
**PGGT1B**	-0.059268074	**0.032810485**	0.361602023
**NQO2**	-0.093907425	**0.032831334**	0.361602023
**KLHL7**	-0.03216447	**0.032962565**	0.362402721
**GPR126**	-0.034358264	**0.032969513**	0.362402721
**ADH5**	-0.034065303	**0.033078288**	0.362407896
**FAM72B**	-0.047157898	**0.033236777**	0.362407896
**LCN2**	0.125695309	**0.03325298**	0.362407896
**MYO1C**	0.038124525	**0.033307013**	0.362407896
**OXR1**	-0.024016955	**0.033352028**	0.362407896
**KLHL22**	-0.031551815	**0.033377327**	0.362407896
**MGST1**	-0.081327248	**0.033401933**	0.362407896
**PPT1**	-0.033724174	**0.033406603**	0.362407896
**CPA3**	-0.058631325	**0.033429119**	0.362407896
**SRRM2**	0.033985491	**0.033431033**	0.362407896
**STK32C**	-0.055841695	**0.033434691**	0.362407896
**MEGF9**	-0.059661715	**0.033451386**	0.362407896
**PILRB**	0.047889029	**0.033479681**	0.362407896
**IRF2BP2**	-0.028512706	**0.03350872**	0.362407896
**GATA2**	0.062891976	**0.033552187**	0.362407896
**PGM1**	-0.032639275	**0.033564195**	0.362407896
**CCRL2**	-0.056351505	**0.033595626**	0.362407896
**PINX1**	-0.037051926	**0.033607815**	0.362407896
**OPA1**	-0.030229038	**0.033657679**	0.362407896
**CALM2**	-0.025040409	**0.033684423**	0.362407896
**TLR4**	-0.056680236	**0.033712751**	0.362407896
**RSRC1**	-0.043957523	**0.033765488**	0.362407896
**NUDT16L1**	-0.027025903	**0.033767903**	0.362407896
**PKP4**	-0.045315071	**0.033876592**	0.362407896
**APEX1**	-0.019735344	**0.033906095**	0.362407896
**EPG5**	0.066129958	**0.033906894**	0.362407896
**FBXO28**	-0.030737788	**0.03392511**	0.362407896
**PITPNB**	-0.027444824	**0.033927343**	0.362407896
**IGF2BP3**	0.082461455	**0.033990098**	0.362407896
**UBE2Z**	0.030377133	**0.033994427**	0.362407896
**RBBP6**	0.050365913	**0.034027294**	0.362407896
**SLC4A5**	0.059137734	**0.034064886**	0.362407896
**PRKCSH**	-0.021512605	**0.03410176**	0.362407896
**NUP62**	-0.021033871	**0.034127901**	0.362407896
**ZNF700**	0.042552441	**0.034128153**	0.362407896
**COG2**	-0.026971274	**0.034148653**	0.362407896
**RBM33**	0.044242684	**0.03427921**	0.363185971
**ALOX5**	-0.033949476	**0.034383674**	0.363185971
**IMP4**	-0.023218831	**0.034498474**	0.363185971
**RBBP5**	0.02683361	**0.034617409**	0.363185971

**PPP2R3C**	-0.035115535	**0.034617476**	0.363185971
**ACAP2**	-0.042831033	**0.034617569**	0.363185971
**NAA30**	-0.026031061	**0.034622189**	0.363185971
**ASB7**	-0.045154489	**0.034656635**	0.363185971
**PIAS4**	-0.021193156	**0.03466618**	0.363185971
**CYP1B1**	-0.100598897	**0.034738127**	0.363185971
**VPS33B**	0.01931951	**0.034746086**	0.363185971
**SF3B14**	-0.036439709	**0.034758947**	0.363185971
**NRCAM**	-0.092542182	**0.034779625**	0.363185971
**USP30**	-0.041422644	**0.034832765**	0.363185971
**SLC38A1**	0.053434006	**0.034838551**	0.363185971
**TRIM14**	0.047571344	**0.034844429**	0.363185971
**C12orf5**	-0.045217599	**0.034859635**	0.363185971
**VAC14**	-0.044656225	**0.034934888**	0.363185971
**NKIRAS2**	-0.026854599	**0.034936273**	0.363185971
**RBFA**	0.034052173	**0.034946149**	0.363185971
**CTBP1**	0.028030263	**0.034947638**	0.363185971
**ZNF320**	0.046838221	**0.034961884**	0.363185971
**LUC7L**	0.032055305	**0.035009622**	0.363185971
**ZNF22**	-0.027344743	**0.035031307**	0.363185971
**TCEANC2**	0.025889973	**0.035135524**	0.363185971
**ARRB1**	-0.033814223	**0.035220444**	0.363185971
**ANXA4**	-0.056300832	**0.035229522**	0.363185971
**TMEM59**	-0.017908028	**0.035311901**	0.363185971
**C2orf69**	0.054422772	**0.03534562**	0.363185971
**CENPJ**	0.034978164	**0.035371298**	0.363185971
**ZNF676**	0.03447339	**0.035385323**	0.363185971
**QDPR**	-0.036532322	**0.035450609**	0.363185971
**BOLA1**	-0.033376063	**0.035476144**	0.363185971
**DHRS7**	-0.030405249	**0.035490219**	0.363185971
**PSPH**	-0.04132919	**0.03549256**	0.363185971
**ATPAF1**	-0.031129486	**0.035523646**	0.363185971
**C7orf25**	-0.041878273	**0.035547504**	0.363185971
**DPH3**	-0.042316685	**0.035551063**	0.363185971
**SDHC**	-0.053943974	**0.035552963**	0.363185971
**DHDDS**	-0.036929592	**0.035564722**	0.363185971
**C19orf38**	-0.063361498	**0.035567223**	0.363185971
**STX11**	-0.047293664	**0.035850908**	0.365428041
**FIBP**	-0.032434726	**0.035852819**	0.365428041
**CEND1**	0.040229407	**0.035942723**	0.365532996
**TMEM141**	-0.027304658	**0.035962034**	0.365532996
**NUP43**	0.027784257	**0.035970738**	0.365532996
**FNDC3B**	-0.031194717	**0.036004729**	0.365532996
**CYB5R4**	-0.042759113	**0.03605949**	0.365532996
**ATP5G3**	-0.043190048	**0.036061255**	0.365532996
**EXOG**	0.037026727	**0.036115108**	0.365729029
**USP19**	0.039838313	**0.036177229**	0.365729029
**HAUS1**	-0.053022134	**0.036179717**	0.365729029
**ARF4**	-0.024001504	**0.036244194**	0.365902147
**LETM1**	0.039405727	**0.036262956**	0.365902147

**NCK2**	0.040373371	**0.03643504**	0.366604378
**CBR4**	0.031994843	**0.036435467**	0.366604378
**GIMAP5**	-0.038102382	**0.036452526**	0.366604378
**LRRC41**	-0.031323889	**0.036495997**	0.366604378
**TMEM131**	-0.019073886	**0.036507402**	0.366604378
**SLC43A3**	0.037845755	**0.03653127**	0.366604378
**AHR**	0.039233148	**0.036652398**	0.36748677
**KIAA0226**	0.033413152	**0.036698254**	0.367613548
**GMDS**	-0.032051154	**0.036783318**	0.367703292
**CRTAP**	-0.080478548	**0.03681915**	0.367703292
**GCM1**	0.035659966	**0.03683434**	0.367703292
**CD44**	-0.01929976	**0.036840089**	0.367703292
**CRYBA2**	0.047397334	**0.036901418**	0.367983605
**FNTB**	-0.109243786	**0.036990081**	0.368088518
**TMED10**	-0.049404234	**0.036990654**	0.368088518
**DCTPP1**	-0.033811549	**0.037011701**	0.368088518
**SLC25A24**	-0.056952768	**0.03721921**	0.369781467
**SUB1**	-0.03289066	**0.037269264**	0.369781467
**SIPA1L3**	0.057793366	**0.03728215**	0.369781467
**SP140L**	0.033370857	**0.037389037**	0.369973829
**ZNF557**	0.057004164	**0.037394743**	0.369973829
**AGBL5**	0.043737619	**0.037527896**	0.369973829
**IQGAP3**	-0.040202441	**0.037539655**	0.369973829
**ACYP2**	-0.039634524	**0.037547927**	0.369973829
**FASTKD3**	-0.035151919	**0.037561976**	0.369973829
**CHCHD3**	-0.034930784	**0.037594136**	0.369973829
**TMCO3**	-0.036097727	**0.037599383**	0.369973829
**TNFRSF10C**	-0.034748748	**0.037628301**	0.369973829
**NUDT21**	-0.025989839	**0.037635787**	0.369973829
**NIPA2**	-0.023574695	**0.037714214**	0.370065593
**ZBTB32**	-0.056026637	**0.037729547**	0.370065593
**BRD3**	0.038677987	**0.037767903**	0.370065593
**PART1**	-0.039543118	**0.037778853**	0.370065593
**AURKA**	-0.041603804	**0.037854551**	0.370479239
**ACOT1**	-0.048667198	**0.037888469**	0.370483626
**AHCYL2**	-0.026759444	**0.03794082**	0.370668083
**ARCN1**	-0.020368194	**0.037996612**	0.37067065
**MOAP1**	-0.025534863	**0.038008057**	0.37067065
**CHN2**	-0.036276506	**0.038100871**	0.371195924
**PFAS**	-0.023166221	**0.038128988**	0.371195924
**ERCC1**	-0.028944595	**0.038213638**	0.371578854
**DHX33**	0.027791297	**0.038235461**	0.371578854
**ILVBL**	-0.031321501	**0.03852491**	0.374063362
**PPOX**	0.023171164	**0.038561369**	0.374089212
**KCND3**	0.053821852	**0.038646252**	0.374584385
**PTMS**	-0.061157348	**0.038721593**	0.374754404
**HMGN2**	-0.020639396	**0.038731506**	0.374754404
**VPS26A**	-0.020221128	**0.038865312**	0.375720643
**B4GALNT4**	-0.03944835	**0.038982836**	0.376414416
**LDOC1L**	-0.046818346	**0.039078535**	0.376414416

**ZDHHC16**	-0.022282398	**0.039101693**	0.376414416
**INTS12**	-0.022609666	**0.039109226**	0.376414416
**FAM120B**	-0.023673475	**0.039127295**	0.376414416
**RTN4IP1**	-0.025136949	**0.03918711**	0.376414416
**HIVEP2**	0.040753787	**0.039234079**	0.376414416
**STX10**	0.026644435	**0.039245507**	0.376414416
**C8orf37**	0.032549886	**0.039290406**	0.376414416
**IFI44**	0.090186937	**0.039292256**	0.376414416
**EIF3F**	-0.030321869	**0.039311145**	0.376414416
**ZSWIM6**	0.033210798	**0.039414711**	0.376600003
**MRFAP1**	-0.021665989	**0.039421519**	0.376600003
**SAMHD1**	-0.047824186	**0.039432596**	0.376600003
**PLEKHM1P**	0.065603408	**0.039510841**	0.376852273
**TMEM169**	-0.042453012	**0.039527102**	0.376852273
**CTAGE5**	-0.035866403	**0.039610185**	0.37705135
**TRAF3**	-0.031634621	**0.039621256**	0.37705135
**WBSCR22**	0.024903973	**0.039650174**	0.37705135
**CPD**	-0.034159387	**0.039728241**	0.377469444
**PPM1D**	-0.039551905	**0.039806033**	0.377884204
**TOMM40L**	0.040262554	**0.039962159**	0.378196453
**BCAT2**	-0.038679384	**0.039978553**	0.378196453
**CD2AP**	-0.04080099	**0.03999741**	0.378196453
**GRASP**	0.067779609	**0.04003287**	0.378196453
**CD302**	-0.080112281	**0.040053222**	0.378196453
**NHP2**	-0.03246134	**0.04005469**	0.378196453
**C18orf25**	0.021009421	**0.040078095**	0.378196453
**CDC7**	0.054224295	**0.040234591**	0.37910161
**IMPA2**	-0.05100321	**0.040242514**	0.37910161
**THOC3**	-0.051291797	**0.04028991**	0.379225356
**CLDND1**	0.03244652	**0.040346876**	0.379247438
**MAP2K1**	-0.027596887	**0.040360781**	0.379247438
**RTN4**	-0.018997546	**0.040477309**	0.379967428
**HCLS1**	-0.041363947	**0.040506059**	0.379967428
**PDE7A**	0.07449693	**0.040570884**	0.380096704
**KRT8**	0.036561972	**0.040594005**	0.380096704
**VAPA**	0.052785907	**0.040622857**	0.380096704
**MMP28**	-0.081058095	**0.040703694**	0.380499984
**SLC35E1**	0.070833769	**0.040734708**	0.380499984
**ANP32A**	-0.045597852	**0.040794213**	0.380734524
**SPAG7**	-0.030690852	**0.040918409**	0.381571917
**RRP7B**	0.016679145	**0.040995212**	0.381966335
**CHRNB4**	-0.041369402	**0.04104003**	0.382062312
**SLC7A7**	-0.034750092	**0.041110394**	0.382108691
**RBM6**	0.032667622	**0.041114053**	0.382108691
**SIX5**	-0.040328275	**0.041159087**	0.382206316
**COPS8**	-0.031350463	**0.041196274**	0.382230976
**GPATCH8**	-0.030070864	**0.041326493**	0.382661086
**ATG3**	-0.038678676	**0.041336412**	0.382661086
**CCNDBP1**	-0.02119647	**0.041378445**	0.382661086
**ZFP57**	0.03769291	**0.041380912**	0.382661086

**SSSCA1**	-0.053685457	**0.041488132**	0.383332335
**MSL2**	0.028896027	**0.041568845**	0.383481999
**AP3M2**	0.037585874	**0.041587775**	0.383481999
**IGF2BP2**	0.055025667	**0.04169413**	0.383481999
**NUP93**	0.021614079	**0.041717599**	0.383481999
**KCNN4**	0.047658462	**0.041728541**	0.383481999
**DEXI**	-0.032210462	**0.041729885**	0.383481999
**SNORA13**	0.043359069	**0.041845611**	0.383481999
**JMJD4**	-0.042020761	**0.041850526**	0.383481999
**CPVL**	-0.05443859	**0.041870593**	0.383481999
**G6PC3**	-0.047683734	**0.041876713**	0.383481999
**OR1J1**	0.056133459	**0.041885422**	0.383481999
**KDM1B**	-0.04315975	**0.042079598**	0.384413341
**LRPAP1**	-0.028421288	**0.042094694**	0.384413341
**ZNF512B**	0.042917509	**0.042111054**	0.384413341
**TRAPPC4**	-0.031999488	**0.042135338**	0.384413341
**CCDC124**	0.032350473	**0.042163609**	0.384413341
**SESTD1**	-0.047041372	**0.04219552**	0.384413341
**C12orf49**	-0.028408237	**0.04226372**	0.38471802
**KNTC1**	0.03001278	**0.042446414**	0.385272359
**EPT1**	0.036414593	**0.042467049**	0.385272359
**RNFT2**	-0.05612034	**0.042468301**	0.385272359
**MED28**	-0.024357929	**0.042494752**	0.385272359
**METTL17**	0.030306501	**0.042498649**	0.385272359
**SLC35E2**	0.040749977	**0.042592089**	0.385803468
**ZNF251**	0.029748113	**0.042722398**	0.386667398
**STX2**	0.052706635	**0.042801565**	0.387067419
**IFT52**	-0.039612953	**0.042884867**	0.387238567
**FAM53A**	-0.045084444	**0.042939501**	0.387238567
**LTBP3**	0.050848064	**0.043029714**	0.387238567
**SELENBP1**	0.032536002	**0.043032877**	0.387238567
**PDXP**	-0.034270113	**0.043051053**	0.387238567
**GLRX3**	-0.03570448	**0.043061936**	0.387238567
**HYOU1**	-0.041075351	**0.04311054**	0.387238567
**UNC50**	-0.018968559	**0.043128563**	0.387238567
**PARP3**	-0.051161428	**0.043135347**	0.387238567
**RPUSD2**	-0.025632941	**0.04318892**	0.387387
**FAM111A**	0.02346067	**0.043221876**	0.387387
**RHOF**	-0.036193872	**0.043306849**	0.38749567
**UBE2J2**	-0.041769197	**0.043327509**	0.38749567
**ZNF594**	0.012332345	**0.043354033**	0.38749567
**NEK7**	-0.041490651	**0.043393596**	0.38749567
**C6orf226**	-0.031608419	**0.043440907**	0.38749567
**LATS2**	-0.046486161	**0.043456046**	0.38749567
**RAB4B-EGLN2**	-0.018174854	**0.043480396**	0.38749567
**TEX261**	-0.024889539	**0.043561526**	0.38749567
**MRPL30**	-0.042518827	**0.043600424**	0.38749567
**SGPL1**	-0.036007716	**0.043619681**	0.38749567
**KRBA1**	-0.026565947	**0.043629415**	0.38749567
**ZNF160**	0.036917436	**0.043654088**	0.38749567

**PARP6**	0.02598839	**0.043691378**	0.387515915
**ZCCHC7**	0.025305595	**0.04387152**	0.388802122
**ATHL1**	0.088290724	**0.043947755**	0.389047292
**SLC43A2**	-0.041569026	**0.043969479**	0.389047292
**QSER1**	-0.032280937	**0.044117203**	0.389693748
**TSPYL5**	0.060607753	**0.044131456**	0.389693748
**FLOT2**	-0.017909669	**0.044196825**	0.389693748
**POM121L8P**	0.061990891	**0.044208851**	0.389693748
**CTDSP2**	-0.020352006	**0.044221088**	0.389693748
**C20orf27**	-0.054250018	**0.044253776**	0.389693748
**CHRAC1**	-0.031513953	**0.04434192**	0.390159548
**PDE5A**	0.090881136	**0.04445957**	0.390884019
**CCNYL1**	-0.032970622	**0.044529075**	0.391184387
**TTC17**	0.03553745	**0.044604846**	0.391219488
**HCST**	-0.024570943	**0.044637821**	0.391219488
**LMBR1**	-0.037108424	**0.044639101**	0.391219488
**PRKAR1B**	0.045995879	**0.04471669**	0.391429808
**ZNF548**	0.039253828	**0.044733825**	0.391429808
**INO80D**	0.03296522	**0.044827077**	0.391626226
**EXOSC7**	-0.028282862	**0.044832798**	0.391626226
**MRPL3**	-0.023583846	**0.044862413**	0.391626226
**SYT11**	-0.045161299	**0.044981541**	0.392356715
**ISG15**	0.077865907	**0.045093386**	0.392840407
**DDRGK1**	-0.02688126	**0.045134463**	0.392840407
**ZSCAN2**	-0.027236958	**0.045143464**	0.392840407
**CD320**	-0.049515096	**0.045206407**	0.392985951
**TMED9**	-0.029878166	**0.045231195**	0.392985951
**ZNF142**	-0.02000344	**0.045329904**	0.393102904
**TXN**	-0.038222984	**0.045355063**	0.393102904
**HOXC4**	-0.078674719	**0.04538645**	0.393102904
**ATXN3**	0.037613145	**0.045386712**	0.393102904
**ZNF174**	-0.038333924	**0.045489285**	0.393569808
**ATP5I**	0.013920828	**0.045516202**	0.393569808
**N4BP2L2**	0.050900265	**0.045579491**	0.393569808
**MBNL2**	-0.029234637	**0.045633222**	0.393569808
**BCKDHB**	-0.049742014	**0.045649973**	0.393569808
**SLC25A11**	-0.026668293	**0.045684094**	0.393569808
**GEMIN4**	-0.023184826	**0.045707464**	0.393569808
**LDHA**	-0.025715743	**0.045751655**	0.393569808
**ZNF879**	0.041270188	**0.045794143**	0.393569808
**SLC22A16**	0.146446413	**0.04579618**	0.393569808
**L2HGDH**	0.074458158	**0.045850999**	0.393735231
**SRSF11**	0.033471505	**0.045895578**	0.393760772
**NEBL**	0.093960477	**0.04592512**	0.393760772
**RQCD1**	-0.037434992	**0.046092963**	0.39441742
**FCRL3**	0.075099837	**0.046177523**	0.39441742
**CMTM3**	-0.023847707	**0.046191423**	0.39441742
**NUDCD2**	-0.031230685	**0.046193233**	0.39441742
**CCDC84**	0.047688607	**0.046228895**	0.39441742
**SLC31A2**	-0.054115433	**0.046233073**	0.39441742

**BPNT1**	-0.031574256	**0.046275273**	0.39441742
**RNF4**	-0.026624279	**0.046294893**	0.39441742
**MIR2116**	0.045215642	**0.0463224**	0.39441742
**ARF1**	-0.023609991	**0.046419923**	0.394580834
**RPAP3**	-0.03118835	**0.046473836**	0.394580834
**ILF2**	-0.020719675	**0.046524083**	0.394580834
**HNRNPL**	0.038590419	**0.046531693**	0.394580834
**NAA25**	0.032481008	**0.046575802**	0.394580834
**NUP85**	0.017772395	**0.046590624**	0.394580834
**C12orf10**	-0.036399106	**0.046591124**	0.394580834
**ADSS**	-0.023645222	**0.046702469**	0.394658237
**ALDH9A1**	-0.021493599	**0.046782458**	0.394658237
**CUL5**	0.038197142	**0.046791969**	0.394658237
**TNFRSF1B**	-0.026187897	**0.046818283**	0.394658237
**KRT18**	0.064510578	**0.046858408**	0.394658237
**QPRT**	-0.048929811	**0.046868445**	0.394658237
**S100A4**	-0.019811095	**0.0468769**	0.394658237
**IPP**	0.064345922	**0.046979154**	0.394658237
**MAN2A1**	0.032339335	**0.047008919**	0.394658237
**IGFBP2**	0.046018883	**0.047015268**	0.394658237
**PPWD1**	0.028925422	**0.047016429**	0.394658237
**TBCA**	-0.026120207	**0.047028116**	0.394658237
**ACP1**	-0.034168946	**0.047142506**	0.394904181
**UNC13A**	0.066298983	**0.047149355**	0.394904181
**GTF2B**	-0.031416115	**0.047164453**	0.394904181
**ALG14**	-0.037124935	**0.047299605**	0.395654836
**PID1**	-0.076251772	**0.047325594**	0.395654836
**DLEU7**	-0.058219609	**0.047444135**	0.396346513
**DICER1**	0.033666596	**0.047554989**	0.396788399
**FAM86A**	-0.030718914	**0.047658156**	0.396788399
**OTX1**	-0.043904061	**0.047774931**	0.396788399
**CCR6**	0.012080227	**0.047874912**	0.396788399
**BCKDK**	-0.027323894	**0.047919305**	0.396788399
**RIMS4**	0.034883127	**0.047972132**	0.396788399
**CMTM6**	-0.036804358	**0.048195987**	0.396788399
**P2RX1**	0.074665802	**0.048200519**	0.396788399
**SLC7A5P1**	0.010553227	**0.048216485**	0.396788399
**AURKAIP1**	-0.018643947	**0.048218406**	0.396788399
**MCAT**	-0.033669362	**0.048218687**	0.396788399
**PSMD5**	-0.034857374	**0.04827173**	0.396788399
**ARHGAP15**	-0.020922593	**0.048308495**	0.396788399
**ZNF467**	-0.040358161	**0.048323185**	0.396788399
**DHX9**	-0.032339747	**0.048341737**	0.396788399
**PCYT2**	-0.027520907	**0.048358814**	0.396788399
**DAPP1**	0.02082802	**0.048388094**	0.396788399
**PDCD2**	-0.026334717	**0.048392696**	0.396788399
**IL1B**	-0.09367924	**0.048533412**	0.396788399
**BCL10**	-0.047941142	**0.048543411**	0.396788399
**LMF2**	0.035654555	**0.048547927**	0.396788399
**ERGIC1**	-0.042588217	**0.048567688**	0.396788399

**TRIAP1**	-0.022000688	**0.0485849**	0.396788399
**MECR**	0.041731376	**0.04859446**	0.396788399
**KPNB1**	-0.019177583	**0.04860968**	0.396788399
**RETSAT**	-0.030666461	**0.048632795**	0.396788399
**NOP2**	-0.025319132	**0.048638493**	0.396788399
**RSL24D1**	-0.017190882	**0.048647063**	0.396788399
**SYNJ2BP**	0.045343328	**0.048660786**	0.396788399
**SUMO1P3**	-0.023976166	**0.0486891**	0.396788399
**PAN3**	0.030984074	**0.048715388**	0.396788399
**TARBP2**	-0.033051114	**0.048724823**	0.396788399
**TBX19**	0.031746719	**0.048744388**	0.396788399
**RAPH1**	-0.042819415	**0.048786323**	0.396788399
**CS**	-0.021951146	**0.048815644**	0.396788399
**FCHO1**	-0.028797353	**0.048840827**	0.396788399
**HEBP2**	-0.040303579	**0.048862133**	0.396788399
**AGAP4**	0.063155203	**0.048871655**	0.396788399
**PSMA6**	-0.020489915	**0.048930346**	0.396788399
**UBE2H**	0.030774506	**0.048930903**	0.396788399
**FNBP4**	0.022337766	**0.048996177**	0.397026854
**LRP3**	-0.051077616	**0.049109518**	0.397654174
**GMPR2**	-0.023181033	**0.049190235**	0.397689592
**UCHL5**	-0.040576049	**0.049216017**	0.397689592
**FBXL12**	-0.02183098	**0.049256454**	0.397689592
**FOS**	0.088568207	**0.049257605**	0.397689592
**EFCAB4A**	-0.063770605	**0.049334289**	0.397853841
**YIPF3**	-0.016947358	**0.049471393**	0.397853841
**TP53BP2**	-0.02392082	**0.049487441**	0.397853841
**NUDT18**	-0.038361118	**0.049533862**	0.397853841
**SLC50A1**	0.030044801	**0.049539287**	0.397853841
**BDP1**	0.058324947	**0.049608741**	0.397853841
**MSRA**	-0.040647586	**0.049615675**	0.397853841
**CAPRIN2**	0.037996451	**0.049640054**	0.397853841
**SS18L1**	0.032269477	**0.049652582**	0.397853841
**MRPS35**	-0.035191082	**0.049687452**	0.397853841
**SLC4A7**	0.037990189	**0.049705284**	0.397853841
**DARS2**	-0.03101659	**0.049731935**	0.397853841
**FBXO9**	0.057025103	**0.04979121**	0.397853841
**RPTOR**	-0.047096357	**0.049832838**	0.397853841
**AARS**	-0.019169675	**0.049835851**	0.397853841
**SLC35C1**	-0.028867299	**0.049876926**	0.397853841
**EFHB**	-0.044305483	**0.049932414**	0.397853841
**KBTBD4**	-0.029424662	**0.049957368**	0.397853841
NACAP1	-0.044448527	0.050016642	0.397853841
FAM13A	0.049435646	0.050051424	0.397853841
WDR83	-0.022758405	0.050089724	0.397853841
GNRH1	0.037525574	0.050130373	0.397853841
PSMC3	-0.023488446	0.050137882	0.397853841
MRPS7	-0.027162323	0.050140583	0.397853841
TNFRSF10A	0.038519944	0.050202449	0.39805939
C21orf59	-0.019745564	0.050297515	0.398527699

SH3TC1	0.038217336	0.050335899	0.398543631
WDR59	0.027427367	0.050387247	0.398543631
GPN3	-0.033264359	0.050407542	0.398543631
ARSB	-0.045161913	0.050540615	0.398792279
GPR155	0.046562337	0.050556391	0.398792279
VPS54	-0.032025885	0.050575882	0.398792279
ANKRD13A	0.019278201	0.050583102	0.398792279
GSDMB	0.063413331	0.050700203	0.399430991
ECI1	-0.043989451	0.05084352	0.400275191
KIAA0930	-0.043064224	0.0509254	0.400349187
FAM78A	-0.037867126	0.05097459	0.400349187
GLRX	-0.024342816	0.050994497	0.400349187
USP35	-0.032026577	0.050997593	0.400349187
SNORD56	0.040766159	0.051302056	0.401910119
SNX10	-0.041805887	0.051312023	0.401910119
SNORA7B	0.026634545	0.051347388	0.401910119
CKAP2	-0.043293212	0.051381528	0.401910119
GOLGA1	0.028820432	0.051404915	0.401910119
PML	0.062737306	0.051443859	0.401910119
TNRC6A	0.030041086	0.051515711	0.401910119
FAM173B	-0.029639061	0.05151768	0.401910119
ZHX2	0.077531678	0.051626474	0.401910119
HIATL1	-0.032045892	0.051656955	0.401910119
GNG10	-0.061600863	0.051693447	0.401910119
PHACTR2	0.035491463	0.051713197	0.401910119
BCL2L1	0.043197927	0.051737402	0.401910119
NFIL3	-0.054859142	0.051763058	0.401910119
SGK494	0.038698662	0.051773855	0.401910119
BET1	-0.039112278	0.051777381	0.401910119
CDC42EP3	-0.060175163	0.05186655	0.402263365
PHYH	-0.031123823	0.051929698	0.402263365
MFSD3	-0.035009446	0.051931913	0.402263365
GSTA4	-0.045147632	0.052042693	0.402283603
ANAPC4	0.028329895	0.052075804	0.402283603
CCDC115	-0.038884312	0.052107588	0.402283603
C19orf52	-0.025190003	0.052111261	0.402283603
AACS	-0.033514301	0.052229206	0.402283603
SH3GLB1	-0.028539787	0.052271605	0.402283603
CSGALNACT2	-0.028772591	0.052305279	0.402283603
ELMOD1	0.080980551	0.052333257	0.402283603
TPM1	0.101403065	0.052341314	0.402283603
CCDC136	-0.032045264	0.052371887	0.402283603
SDHB	-0.026118545	0.052433247	0.402283603
SH3RF2	-0.041769782	0.052525082	0.402283603
BAMBI	0.094694803	0.052550652	0.402283603
VAV3	0.310416553	0.052622528	0.402283603
ARHGEF5	-0.027790783	0.052636716	0.402283603
EIF5B	-0.022929478	0.052650384	0.402283603
CENPN	-0.064280346	0.052654787	0.402283603
RPRD1A	0.024219761	0.052670625	0.402283603

HSH2D	0.053035004	0.052724432	0.402283603
SLC35C2	0.025038617	0.052820482	0.402283603
NFKBIE	-0.054345654	0.052852122	0.402283603
UMPS	-0.03727273	0.05286536	0.402283603
ZNF223	0.053679493	0.052871853	0.402283603
RPS8	-0.020857392	0.052879759	0.402283603
EEF2K	-0.03014719	0.052906506	0.402283603
PLBD1	-0.057082374	0.052939832	0.402283603
MFN2	0.030518127	0.052941943	0.402283603
INTU	-0.040970666	0.05298022	0.402283603
LANCL2	-0.030001527	0.053052118	0.402283603
TNFSF4	0.075799957	0.053054965	0.402283603
ARMC1	0.028418401	0.053061167	0.402283603
TIA1	0.024271615	0.05313354	0.402424811
CTSC	-0.035622737	0.053152505	0.402424811
ASNA1	-0.049242924	0.053210873	0.402591353
RBM43	0.05698902	0.053307329	0.403045646
SLC9A9	0.026301467	0.053419481	0.403324651
CCL8	0.072312992	0.053440968	0.403324651
AGAP6	0.040634795	0.053453543	0.403324651
MGAT4B	0.031493189	0.053645783	0.404499439
SIGLEC6	0.05453815	0.053765877	0.405128993
ZNF114	0.045664932	0.053811823	0.405199367
DPYSL2	-0.025065977	0.054003178	0.405376595
ZNF511	-0.019931812	0.054024657	0.405376595
ZNF365	0.079187621	0.054213025	0.405376595
LGR6	-0.07225769	0.054217778	0.405376595
NUP205	0.018257591	0.054249022	0.405376595
SNORD11	0.049150789	0.054278416	0.405376595
MMD	0.062538844	0.054284969	0.405376595
MED30	-0.027145573	0.054292555	0.405376595
C5orf30	-0.030206786	0.054294187	0.405376595
RDX	-0.038431254	0.054317474	0.405376595
LRRC20	-0.036245006	0.05434227	0.405376595
ZNF280D	-0.036101099	0.054372379	0.405376595
RAB22A	-0.028694948	0.054424962	0.405376595
CCDC66	0.031903046	0.054425374	0.405376595
UROS	-0.02312412	0.054455579	0.405376595
ATG4C	-0.029182926	0.054498556	0.405376595
PRPF31	0.026079169	0.054503289	0.405376595
CUTA	-0.023694888	0.054527498	0.405376595
SQSTM1	-0.023552138	0.054536102	0.405376595
UBN2	0.034417024	0.054613644	0.405376595
ITSN2	0.031093678	0.054642136	0.405376595
CALU	-0.032627725	0.05473598	0.405376595
MSL1	0.026572472	0.05474257	0.405376595
GRSF1	-0.017629563	0.054747114	0.405376595
ASB3	0.030650638	0.054761835	0.405376595
FBXO6	-0.047672313	0.054848912	0.405376595
ANGEL2	0.023226455	0.054851086	0.405376595

PIGX	0.047715646	0.054915775	0.405376595
CCNL2	0.046799777	0.054925371	0.405376595
ZNF439	0.047326718	0.054963146	0.405376595
GTPBP4	-0.016272439	0.054978995	0.405376595
MTCH2	-0.028350404	0.055007286	0.405376595
SNORD32A	0.038969553	0.055093606	0.4057426
KCNJ10	0.035187082	0.055136981	0.405792046
UBE2S	-0.028767247	0.055312209	0.406786653
PRELID1	-0.038974309	0.055345623	0.406786653
DCAF16	0.034681459	0.055451718	0.407296
ATL3	-0.025473331	0.055583004	0.407851314
CLNS1A	-0.023442279	0.055601015	0.407851314
PLIN2	-0.034328119	0.055666791	0.408063386
RTN4R	-0.049735951	0.055758942	0.408468381
H2AFZ	-0.019894029	0.055957032	0.409648405
POLD2	-0.024009991	0.056172455	0.410070112
ANKRD33	-0.047009811	0.056205387	0.410070112
TMPO	-0.045284041	0.056207627	0.410070112
NRAS	-0.029487207	0.056233361	0.410070112
TMBIM4	-0.015914196	0.056251082	0.410070112
DHX29	-0.016326853	0.056314997	0.410070112
FRG1B	0.048537833	0.056333381	0.410070112
GALC	-0.023712063	0.056435062	0.410070112
LRIG1	0.033333454	0.056440178	0.410070112
DNAJA2	0.01926386	0.056444456	0.410070112
KRI1	0.027320208	0.056456627	0.410070112
SORD	-0.023061319	0.056459197	0.410070112
TTC39B	0.044849506	0.056521183	0.410203759
CD300LF	-0.042718801	0.056560313	0.410203759
TRAF5	0.02979244	0.056588774	0.410203759
IL11RA	0.04196171	0.056696226	0.410283314
HIST1H2AD	0.068959803	0.056722352	0.410283314
THAP11	-0.0210215	0.056747696	0.410283314
DENND4B	0.033931008	0.056791579	0.410283314
WDR26	0.027566397	0.056803924	0.410283314
XRCC5	-0.031442652	0.056822145	0.410283314
UBE2E1	-0.019366444	0.056972364	0.411099802
PCBP1	-0.022068311	0.057013504	0.411128645
CCDC102A	-0.036933321	0.057086464	0.411352478
COASY	-0.018567625	0.057118869	0.411352478
DTD1	-0.031804223	0.057221703	0.411825118
SNORA75	0.037592339	0.057378031	0.412221065
TNNC2	0.097204159	0.057385096	0.412221065
SLC16A12	0.014174132	0.057388442	0.412221065
CEP164	0.030115423	0.057449321	0.412300782
RUFY1	0.07329923	0.057503286	0.412300782
MRPS27	-0.021205118	0.057511284	0.412300782
TMEM147	-0.021718463	0.057627653	0.412355421
ZNF330	-0.033502282	0.057665846	0.412355421
MRPL54	-0.029841056	0.05768486	0.412355421

STAMBPL1	0.029727845	0.057695853	0.412355421
ATP5J	-0.02684627	0.057705172	0.412355421
COL12A1	0.037459174	0.057802861	0.412556186
BCL2L13	-0.02538326	0.057821836	0.412556186
AGER	0.035579512	0.057936503	0.412556186
SNX13	-0.031748279	0.057944226	0.412556186
COPB1	-0.017568563	0.05796142	0.412556186
CRAMP1L	0.033438786	0.057985105	0.412556186
FRAT1	-0.041367925	0.057994166	0.412556186
BCL2L2	0.028374395	0.058131003	0.413264012
RAB5C	0.023087854	0.058219426	0.413626975
ANKRD16	-0.029955224	0.058320232	0.413732794
KIAA0368	-0.021485715	0.058343959	0.413732794
CHERP	0.028445749	0.058346453	0.413732794
SPRY2	-0.076634927	0.058426308	0.414033803
TSC22D1	0.075369729	0.058515016	0.41439713
COMMD3	-0.017808862	0.058790347	0.416080786
TMPRSS12	0.041309663	0.058835029	0.416130948
SNORD4A	0.049224904	0.059096177	0.417711102
UQCRB	-0.032767974	0.059144873	0.417788513
KIAA2013	-0.026508552	0.059248452	0.417907465
IMPAD1	-0.041309997	0.059257866	0.417907465
LTV1	0.017304662	0.059274977	0.417907465
CEP290	0.038724451	0.059404795	0.418178623
UNC5CL	0.037496988	0.059425801	0.418178623
KCNK13	-0.043567404	0.059426775	0.418178623
FBXO22	-0.032304241	0.059538173	0.418419587
MTMR11	-0.034839858	0.05961236	0.418419587
FAM115A	0.012781126	0.059634807	0.418419587
BEGAIN	0.07070345	0.05964062	0.418419587
GGH	-0.037817816	0.059684048	0.418419587
NBPF3	0.030584699	0.059687824	0.418419587
RNF13	-0.030984148	0.059727775	0.418434649
MAPK13	-0.033174951	0.059788573	0.418595647
CST3	-0.036472904	0.059890838	0.418629745
TMEM160	-0.039911003	0.059902101	0.418629745
CSPP1	0.027064636	0.059906904	0.418629745
DCUN1D5	-0.026558129	0.059977031	0.418651151
TARDBP	0.036223	0.060016904	0.418651151
OR11H1	0.035776581	0.060053653	0.418651151
RAB3GAP1	0.025258002	0.060061255	0.418651151
FAM102B	0.025994744	0.060149443	0.419002003
OR7G2	0.034419528	0.060187936	0.419006453
HTRA1	-0.063208292	0.060243682	0.419130932
ZNF76	0.035231081	0.060382529	0.419263862
RPS11	-0.014444963	0.060385549	0.419263862
DPP10	0.037797776	0.060399173	0.419263862
GOLGA8F	0.050127485	0.060425918	0.419263862
SDF2L1	-0.031033514	0.060452175	0.419263862
PTPN9	-0.036059922	0.060625816	0.419594087

FAM21B	0.035312225	0.060697274	0.419594087
PAFAH1B3	-0.03195287	0.060727765	0.419594087
GALE	-0.026706029	0.060749633	0.419594087
KLC1	0.028850789	0.060855853	0.419594087
SFT2D2	-0.036572409	0.060857259	0.419594087
CBX6	-0.026440434	0.060859642	0.419594087
EHMT1	0.0522284	0.060880493	0.419594087
LPCAT2	-0.107564009	0.060903916	0.419594087
SNRPA1	0.018765409	0.060916405	0.419594087
HAS3	-0.042182203	0.060916767	0.419594087
KIAA0247	0.02054246	0.060959914	0.419630155
SLC15A2	0.045806401	0.061154117	0.42028734
ITPKB	0.026920652	0.061194621	0.42028734
NR2C1	0.030719399	0.0613141	0.42028734
FAM184B	0.089331738	0.061321786	0.42028734
CHD4	0.022772215	0.061338242	0.42028734
MKRN2	-0.023087906	0.06135534	0.42028734
CENPT	0.038566779	0.061364189	0.42028734
GIT2	-0.025551874	0.061396679	0.42028734
KPNA2	-0.047019353	0.061397112	0.42028734
TOMM20	-0.025022176	0.061487561	0.420487482
MLH1	-0.01832742	0.061502325	0.420487482
EZH2	0.033357341	0.061696927	0.421557586
ARPC3	-0.035742879	0.061741235	0.421600081
PNN	0.037766057	0.061795124	0.421707907
C5orf28	0.063261814	0.061888782	0.422086834
TIPIN	-0.039366329	0.061961691	0.422236096
TJAP1	0.01560006	0.06198696	0.422236096
HSPA7	0.075527938	0.062100262	0.422377995
ARSA	-0.044434672	0.062172985	0.422377995
IRAK1BP1	-0.052705562	0.062192524	0.422377995
AFG3L2	-0.017538145	0.06219624	0.422377995
C4orf21	0.04448044	0.062198585	0.422377995
ATP5G2	-0.02361977	0.062270624	0.422607931
RNF123	-0.019541597	0.062397251	0.423207827
ARHGAP21	0.042134592	0.062437427	0.42322099
TRAF3IP2	-0.035447494	0.06251579	0.42349283
SAFB2	0.024222678	0.062617003	0.42374219
NBN	-0.032498952	0.062655062	0.42374219
VPS53	0.014825154	0.062667446	0.42374219
SNORA28	0.076884999	0.0628633	0.424800403
FCGR1B	-0.09958345	0.062900701	0.424800403
TFB2M	-0.024174753	0.063005344	0.424936205
MED21	-0.049816019	0.063013725	0.424936205
ASCL2	-0.057062747	0.063047308	0.424936205
NAPA	0.031669421	0.063074369	0.424936205
CORO7	0.033419092	0.063114593	0.424948561
ZNF641	0.04311238	0.063337182	0.425794215
PIK3CD	0.027775985	0.063405127	0.425794215
DAB2	0.057727413	0.063435414	0.425794215

KHSRP	-0.037701492	0.063448349	0.425794215
DDX52	-0.021879383	0.063463086	0.425794215
TRIM41	0.020330022	0.063470996	0.425794215
C11orf1	-0.037587224	0.063546327	0.425943887
COX17	-0.025366865	0.063570268	0.425943887
SRPR	-0.034550917	0.063643375	0.4259537
PIP5K1A	-0.043218174	0.063694178	0.4259537
PRKCDBP	-0.08123084	0.063705208	0.4259537
TMEM50A	-0.030673947	0.063735573	0.4259537
C17orf80	-0.033030479	0.063764141	0.4259537
PLEKHF1	-0.065859415	0.063878514	0.426460354
DYNC2LI1	-0.048417406	0.06397115	0.426821376
GATAD1	0.02471787	0.064066059	0.427045589
PIK3C2B	0.049440775	0.064081924	0.427045589
MAF1	0.028675968	0.064152643	0.427045589
RUNX3	-0.036409318	0.064185022	0.427045589
PPP1R7	0.020482875	0.064231536	0.427045589
FAU	-0.015358795	0.064236237	0.427045589
POLR2D	-0.035083277	0.064369974	0.427469494
ZNF471	0.063238524	0.064377238	0.427469494
COPS2	-0.040908285	0.064433233	0.427584805
CAMLG	-0.017201434	0.064505644	0.427690414
METTL9	-0.032503549	0.064539791	0.427690414
GPRIN3	0.047917891	0.064642012	0.427690414
LRRK2	-0.057845848	0.064669605	0.427690414
SIRT4	-0.033639297	0.064670412	0.427690414
C18orf21	-0.022063883	0.06468222	0.427690414
MZT1	-0.032976313	0.064719617	0.427690414
TBC1D23	-0.039155203	0.064972271	0.428487975
HAUS6	-0.04227333	0.064973789	0.428487975
TRAK1	-0.026434428	0.064984758	0.428487975
KCTD18	-0.022379768	0.064995149	0.428487975
NSMAF	-0.018509277	0.06504996	0.42859405
CTNNAL1	0.056948295	0.065128384	0.428607377
ANKRD36	0.057900894	0.065167846	0.428607377
SBF2	-0.031545403	0.065168147	0.428607377
HIRIP3	-0.023704243	0.065213097	0.42864023
SEC61B	-0.021286981	0.065254027	0.42864023
SCAF8	-0.020132682	0.065319452	0.42864023
ANKRD39	-0.026910166	0.065366798	0.42864023
DSCR3	-0.020354528	0.065372719	0.42864023
CAMSAP1	-0.026979098	0.065405488	0.42864023
MED10	-0.023385326	0.065561462	0.429408181
MRPL27	-0.026607948	0.065744038	0.430349355
FABP5	-0.047149591	0.06579389	0.430421142
ENTPD4	0.050579743	0.065853094	0.430553988
SETMAR	-0.035891061	0.066048266	0.43139016
PRRT2	0.043321049	0.066058932	0.43139016
PARP4	-0.023263011	0.06631318	0.432276081
COX15	-0.035596153	0.066349369	0.432276081

NANP	-0.055750364	0.066353972	0.432276081
FBXO4	-0.024806926	0.066373055	0.432276081
C14orf79	-0.026620353	0.066389858	0.432276081
GDNF	0.037642954	0.066460415	0.432481089
SDF2	-0.021486698	0.066504375	0.432512882
SNORA6	0.049999561	0.066547622	0.432540002
ATP6V1A	-0.024893128	0.066762524	0.43340842
RNMT	0.027333448	0.066795571	0.43340842
INSL3	-0.052143605	0.066804547	0.43340842
RPS5	-0.023689954	0.066837851	0.43340842
KLHL29	-0.030021327	0.066905243	0.433437037
NLRP3	-0.036809881	0.06692058	0.433437037
OVGP1	0.050505696	0.06718338	0.434399223
RNY4	0.071903325	0.067185955	0.434399223
HNRNPA1	-0.036566569	0.067186871	0.434399223
RNMTL1	-0.018805327	0.067298093	0.43468752
PPP2R1B	-0.035754319	0.067311334	0.43468752
F5	-0.069802771	0.067349272	0.43468752
ATG7	-0.031038853	0.067624126	0.436008569
NFKB1	-0.015742335	0.067682385	0.436008569
TMEM87A	-0.015560618	0.067711924	0.436008569
MCCC2	-0.031831132	0.067716264	0.436008569
SNAPC4	0.024612845	0.067800407	0.436008569
CRADD	-0.039317546	0.067826838	0.436008569
UBE2G1	-0.03951988	0.067829683	0.436008569
PPHLN1	-0.024645801	0.067953337	0.436373018
SSTR1	-0.029918667	0.067965226	0.436373018
SIAH1	-0.025776816	0.068005917	0.436381157
TMEM8A	0.063232084	0.068270153	0.437033141
MPRIP	0.025461083	0.068294889	0.437033141
IREB2	-0.041893592	0.068319559	0.437033141
ENTPD7	-0.037573528	0.06838426	0.437033141
MKNK2	0.022390485	0.068404552	0.437033141
ZNF785	0.079427861	0.068429085	0.437033141
ZBTB43	0.040349608	0.068463381	0.437033141
CDK2	0.020367069	0.068477839	0.437033141
RPL17	-0.019356762	0.068482089	0.437033141
EXOSC1	-0.012959184	0.068613145	0.437033141
SOCS6	0.040068328	0.068621506	0.437033141
CDK12	-0.047046088	0.0686607	0.437033141
PPP4R2	-0.056777028	0.068784999	0.437033141
RASSF4	-0.031534717	0.06880044	0.437033141
FUCA2	-0.029523418	0.068816778	0.437033141
C5orf22	-0.034530478	0.068873144	0.437033141
CD59	-0.036083936	0.068885224	0.437033141
KCTD3	-0.040004575	0.068895298	0.437033141
EFTUD1	-0.020825985	0.068926142	0.437033141
MDN1	0.029953527	0.068932421	0.437033141
TMEM39B	0.021691045	0.068979881	0.437033141
MPHOSPH8	0.034100106	0.069001757	0.437033141

ELANE	-0.073843655	0.069132448	0.437033141
FBXL20	-0.038506515	0.069174803	0.437033141
CENPBD1	-0.030203961	0.069192577	0.437033141
HYLS1	-0.033651682	0.069221133	0.437033141
SNORD21	0.053942619	0.069226374	0.437033141
NAA16	0.028456236	0.069313667	0.437033141
AP2A2	0.034000977	0.069322871	0.437033141
UBXN7	0.030508454	0.069365092	0.437033141
CHMP4A	-0.019059651	0.069393903	0.437033141
HARBI1	0.037807111	0.069408897	0.437033141
TIMM10	-0.079294179	0.069445879	0.437033141
GRPEL1	-0.024966075	0.069449932	0.437033141
AK4	-0.066567195	0.069665449	0.43814026
SYNCRIP	-0.023025359	0.069718218	0.438223145
KCNMB1	-0.04888521	0.069788896	0.438331284
ZNF500	0.030193167	0.069814622	0.438331284
BMP6	0.081501338	0.070047143	0.439541849
SYCE1	0.037029809	0.070159963	0.439807512
KLRC3	-0.116563337	0.070168946	0.439807512
TMTC2	-0.041430901	0.0704907	0.441098997
MACF1	0.028635429	0.070522064	0.441098997
MAGOH	-0.027431887	0.070526214	0.441098997
RAB28	-0.043470055	0.070551407	0.441098997
NMT1	-0.031284472	0.07057974	0.441098997
USP14	0.036447939	0.070614095	0.441098997
CLPX	-0.022293398	0.070821557	0.441610284
RACGAP1	0.030915855	0.070841242	0.441610284
SLC2A3	0.025795345	0.070850566	0.441610284
LRRC37B	0.024180192	0.07085553	0.441610284
TNPO3	0.038096998	0.0709142	0.441727228
SLC26A8	0.040206231	0.071102752	0.442652621
FP15737	0.033171634	0.07114833	0.442686865
TDRD6	-0.032404082	0.071188239	0.442686865
BRD4	0.036084926	0.071246447	0.44268826
NDFIP1	-0.027446146	0.071268451	0.44268826
TIGD6	-0.030518215	0.071322586	0.44270952
NSUN5	0.028492434	0.071351864	0.44270952
SUCLG1	-0.021895549	0.071625331	0.444157305
OGG1	-0.032618965	0.071672043	0.444198124
C9orf85	-0.041303733	0.071759117	0.444402995
TMEM59L	-0.031853779	0.071785397	0.444402995
MPO	-0.046728416	0.071844576	0.444520743
AMY2B	0.035819854	0.072046412	0.445520522
ATP5S	-0.031625631	0.072122256	0.445740511
NDN	-0.062588238	0.072206976	0.445787545
SLC39A9	-0.034473914	0.072210414	0.445787545
GRAMD1B	-0.032586212	0.072274003	0.445846367
TERF2IP	0.025950381	0.072361869	0.445846367
DEFA1	-0.094342776	0.072371471	0.445846367
HLA-DRB6	0.248604756	0.072381057	0.445846367

MGMT	-0.037953091	0.072435888	0.445935956
CAPRIN1	-0.033505865	0.072485878	0.445995654
SPG21	-0.049919707	0.072586905	0.44636914
PGA4	-0.054180592	0.072710016	0.446753635
GATAD2A	0.021047123	0.072730152	0.446753635
CITED4	-0.055665092	0.07310231	0.448790609
RNF220	-0.016505162	0.073171857	0.448968563
HIST1H3F	0.086021836	0.073250579	0.449112553
ASXL2	0.024165292	0.073316111	0.449112553
MX2	0.043493503	0.073350539	0.449112553
MAP4K1	-0.027395425	0.07335762	0.449112553
CHST14	-0.039316299	0.073522555	0.449602228
ZFP36L1	0.029822618	0.073537004	0.449602228
HEATR5A	0.034013205	0.073595302	0.449602228
STAP1	0.064599451	0.073600076	0.449602228
IPO5	-0.024925537	0.073679976	0.449842062
ANP32B	-0.014572445	0.073801609	0.449987576
CSNK2A1	-0.035076938	0.073855086	0.449987576
APOBEC3C	-0.034058154	0.073860313	0.449987576
TTC31	0.018960056	0.073898808	0.449987576
SNORA5C	0.028700237	0.073907075	0.449987576
CDV3	-0.02107464	0.074001237	0.450224403
IARS2	-0.016768152	0.074027321	0.450224403
TANC2	-0.031911522	0.074140658	0.450503076
BROX	-0.040995835	0.074190521	0.450503076
KIAA0020	0.021031596	0.074259053	0.450503076
IFT46	-0.02843815	0.074281288	0.450503076
EPHX4	-0.06688359	0.074308929	0.450503076
LRP5L	0.036599068	0.074353963	0.450503076
SNX20	-0.039382215	0.074358038	0.450503076
RPSAP52	-0.028453726	0.074403882	0.450534228
TMEM116	0.027912546	0.074466896	0.450669257
GFOD2	-0.028485424	0.07459041	0.450941271
PELO	-0.04702175	0.074593321	0.450941271
RPAP1	0.030789244	0.074865092	0.452073674
GLCE	-0.026731925	0.074872902	0.452073674
C10orf11	-0.05662649	0.074974439	0.452073674
RIOK1	-0.016266228	0.074976757	0.452073674
PSTK	-0.028059781	0.074984846	0.452073674
CRYGS	0.043314574	0.075073553	0.452216462
LSM10	-0.027936201	0.075090239	0.452216462
SLAIN2	-0.026694593	0.075496494	0.454281257
RSG1	-0.032555362	0.075515179	0.454281257
C12orf76	0.031511532	0.075575163	0.454395152
PRKAG1	-0.026842723	0.075761579	0.454922772
CERCAM	-0.076483019	0.075799268	0.454922772
VPS72	-0.026706598	0.075827493	0.454922772
POLR2L	-0.038993854	0.075851033	0.454922772
COX5B	-0.025865897	0.075968359	0.454922772
KCTD12	-0.044262998	0.076089185	0.454922772

CSE1L	0.01600673	0.076122081	0.454922772
RPL7L1	0.01609805	0.076152605	0.454922772
TNF	-0.068299171	0.076270131	0.454922772
GIMAP6	-0.033049231	0.076287201	0.454922772
TPP1	0.024366729	0.076288744	0.454922772
RNF213	0.05436753	0.076374334	0.454922772
ATP6V1E2	-0.036989922	0.076387705	0.454922772
CSTB	-0.029306919	0.076392939	0.454922772
CECR5	-0.02210664	0.076394171	0.454922772
WDR75	0.024086399	0.0764319	0.454922772
PEPD	-0.020167466	0.076488781	0.454922772
HRASLS	0.094717277	0.07651551	0.454922772
CNOT6L	0.025061055	0.076536125	0.454922772
COPS4	-0.021388743	0.076538175	0.454922772
TMEM186	-0.029247708	0.076562876	0.454922772
FBXO38	0.021624448	0.076578531	0.454922772
AP3S1	-0.032664719	0.076609028	0.454922772
AXL	-0.049073802	0.076823259	0.454922772
IKZF3	-0.052166066	0.076835099	0.454922772
THOC1	0.025218589	0.076878544	0.454922772
MIRLET7D	0.041596484	0.076983725	0.454922772
ZMYM1	0.027723628	0.077046742	0.454922772
USO1	-0.016086144	0.077052198	0.454922772
ZNF593	-0.029094833	0.077126663	0.454922772
PDE6B	-0.033613728	0.077138648	0.454922772
KIAA0196	-0.019611397	0.077139504	0.454922772
HLA-DOB	0.077981588	0.077184374	0.454922772
MLLT1	0.034943815	0.077190124	0.454922772
ZBTB3	-0.043272318	0.077194984	0.454922772
TSPO	-0.023539594	0.077226642	0.454922772
PSMA5	-0.018953805	0.077234929	0.454922772
EP400NL	0.0472352	0.077261355	0.454922772
LPP	0.030800153	0.07726577	0.454922772
C19orf71	-0.031249444	0.077316386	0.454978777
HOOK3	-0.039874306	0.077469591	0.455525666
USP21	0.022964316	0.077491628	0.455525666
ITFG1	-0.028916889	0.077683168	0.456203417
ALKBH1	-0.029776858	0.077689352	0.456203417
CAPNS1	-0.02799637	0.077770994	0.45622696
UTS2	0.073834169	0.077775795	0.45622696
HSPA8	-0.023214317	0.077836399	0.456340623
TNNC1	-0.028031875	0.077882375	0.456347908
MAT2A	0.024964638	0.077947871	0.456347908
LAMP3	0.062765254	0.077961324	0.456347908
RNF187	-0.028752714	0.078200583	0.45746898
ZDHHC23	0.036218154	0.078282148	0.45746898
RAB11A	0.02501685	0.078347988	0.45746898
ENDOD1	0.05628111	0.078350025	0.45746898
METTL4	0.024232983	0.078359489	0.45746898
SERPINB1	-0.037775236	0.078448966	0.457539637

CD48	-0.018261025	0.078454262	0.457539637
AFAP1L2	0.039778733	0.078562193	0.457927813
OTUD6B	-0.023214042	0.078659479	0.458252873
ITGA11	-0.037382471	0.078742307	0.458252873
FAM120AOS	-0.01948598	0.078775095	0.458252873
ZSCAN22	-0.034571266	0.078783559	0.458252873
NRP1	-0.063872634	0.078828662	0.458274404
CCDC107	-0.029710558	0.079144414	0.459107436
LMNB2	0.026153812	0.079196802	0.459107436
MTERF	-0.029828848	0.079220404	0.459107436
PLAG1	-0.038997831	0.079270485	0.459107436
IFI44L	0.12725771	0.079325555	0.459107436
TSPAN14	0.036534386	0.079412172	0.459107436
ZNF787	-0.021004348	0.0795745	0.459107436
EEFSEC	-0.031115727	0.079633811	0.459107436
PLEKHG6	0.05685031	0.079701748	0.459107436
SCARNA18	-0.027633238	0.079709438	0.459107436
MRS2	-0.028518009	0.079748823	0.459107436
FBXL15	0.020093252	0.079750689	0.459107436
DNAJC25	-0.020880545	0.07978089	0.459107436
MYSM1	-0.042533803	0.079802938	0.459107436
PROSC	-0.019831508	0.079802946	0.459107436
FMO4	-0.034657276	0.079827245	0.459107436
TBC1D3C	0.036925058	0.079835256	0.459107436
F2R	0.009931667	0.079839939	0.459107436
PDE6D	-0.022853394	0.079850538	0.459107436
ETHE1	-0.034398664	0.079858573	0.459107436
PMAIP1	-0.043345019	0.07987063	0.459107436
CYB5R2	-0.042570842	0.079896944	0.459107436
MYO1G	0.031873777	0.079925922	0.459107436
OR2AG1	0.041846687	0.08001234	0.459365451
SHOC2	-0.022448307	0.080075699	0.459411105
CYTL1	-0.048177559	0.0801033	0.459411105
ERCC8	-0.038543214	0.08019988	0.459726811
GIMAP4	-0.026890453	0.080275041	0.459919478
TUBG1	-0.029329854	0.080514044	0.460918819
DDX17	0.033128122	0.080532749	0.460918819
CDC26	-0.017616878	0.080608665	0.460987659
EFNA1	-0.057046136	0.080700513	0.460987659
FBXL17	-0.04266686	0.080717953	0.460987659
SAP30	-0.05365837	0.080734584	0.460987659
CCDC112	-0.028488392	0.080777799	0.460987659
EVI2A	-0.036112475	0.080902224	0.460987659
NCAPH2	-0.023066974	0.080903457	0.460987659
PLEKHA3	-0.037546382	0.080910875	0.460987659
SNX17	-0.020386194	0.080919597	0.460987659
TCTN3	-0.035621435	0.081340789	0.46314876
PEBP1	0.025425812	0.081390663	0.463194475
DENND4C	0.026506344	0.081440356	0.463239107
PTRH1	-0.041139768	0.081630896	0.463766372

PPP1CC	-0.017496521	0.081701121	0.463766372
MCHR2	0.050426217	0.081745769	0.463766372
CAT	-0.040549832	0.081798412	0.463766372
SMEK1	0.016846625	0.081808364	0.463766372
ETV6	0.02102732	0.081826211	0.463766372
SLC30A6	-0.030299677	0.081826337	0.463766372
KDM1A	0.016765709	0.081908442	0.46381903
PAIP2	-0.017652567	0.08202363	0.46381903
DMTF1	0.025681834	0.08203719	0.46381903
ADARB1	0.028739812	0.08213692	0.46381903
LUC7L3	0.028622434	0.082140819	0.46381903
B4GALT7	-0.022250157	0.082252084	0.46381903
SPCS1	-0.018412102	0.082357694	0.46381903
ATP6V1E1	-0.016404906	0.082370275	0.46381903
PRRC1	-0.018367577	0.082421361	0.46381903
TMED3	-0.020710558	0.08251422	0.46381903
CCDC47	-0.02706367	0.082533903	0.46381903
COPS3	-0.014969283	0.082552496	0.46381903
PROP1	-0.03285996	0.082592539	0.46381903
OSTM1	-0.025185437	0.082666466	0.46381903
PATE3	-0.035435723	0.082685176	0.46381903
IRF2	-0.034409354	0.082689305	0.46381903
DNM3	0.081838471	0.082768817	0.46381903
FAM185A	0.032930934	0.082789126	0.46381903
CTSA	0.043081831	0.082797635	0.46381903
ANP32C	-0.040251141	0.082813057	0.46381903
MEIS3	0.057965958	0.082813635	0.46381903
LY6E	0.045745109	0.082846614	0.46381903
TPI1	-0.025634673	0.082875074	0.46381903
LRRC8A	-0.03293931	0.082961263	0.46381903
PKD1P1	0.027190618	0.082975196	0.46381903
ATM	0.023715112	0.08303873	0.46381903
NLRC5	0.032750736	0.083051846	0.46381903
PCTP	-0.03074698	0.083080862	0.46381903
SUV420H1	0.022961069	0.083098295	0.46381903
WDFY3	0.038811822	0.083118917	0.46381903
OXA1L	-0.026365557	0.083134606	0.46381903
ECHS1	-0.020677889	0.083265637	0.464097803
TFPI	0.068577849	0.083301756	0.464097803
DAK	-0.027911531	0.083332807	0.464097803
TSG101	-0.018540475	0.083352284	0.464097803
SGSH	0.031931537	0.083407977	0.464174409
GPX8	0.05667281	0.083484881	0.464322445
GNPDA2	-0.025576707	0.083518474	0.464322445
ASNSD1	-0.021580395	0.083775659	0.465518459
PRKAR1A	-0.17536263	0.08384263	0.465656833
TMEM184C	-0.024000015	0.083955882	0.465754592
LRRC40	-0.040605961	0.084006879	0.465754592
MORF4L1	-0.027061247	0.084066754	0.465754592
COMMD10	-0.031660219	0.084073881	0.465754592

DNPEP	0.013109419	0.084163223	0.465754592
RAF1	0.017023976	0.084252593	0.465754592
SMAD2	0.020073985	0.084311298	0.465754592
COX7C	-0.020532166	0.084368536	0.465754592
ZMYND8	0.024458932	0.084458197	0.465754592
TSSC4	-0.028203812	0.084489199	0.465754592
RAB27A	-0.046452734	0.084529076	0.465754592
MRPS26	-0.020107307	0.084554277	0.465754592
C8orf44	0.072172986	0.084599474	0.465754592
MIR635	-0.043194209	0.084640327	0.465754592
OR7E156P	0.031825609	0.084664095	0.465754592
DCTN6	-0.02715756	0.084682617	0.465754592
UCK1	-0.033771194	0.084720842	0.465754592
TMEM184B	0.032157481	0.084735301	0.465754592
ATG16L2	0.04381597	0.084744562	0.465754592
ALOX12	0.101048103	0.084751232	0.465754592
CHCHD4	-0.019252234	0.084791864	0.465754592
SPRYD4	-0.034995941	0.084807391	0.465754592
NDUFA8	-0.019591532	0.084900796	0.465754592
RPL18	-0.014688031	0.084906841	0.465754592
CDC123	-0.022600488	0.084912166	0.465754592
RPS6KA5	0.028014753	0.084982893	0.465911662
RAB27B	0.060533425	0.085057628	0.466090539
UXS1	0.040959674	0.085123078	0.466218381
TMEM208	-0.02085203	0.085243654	0.466647876
CCT2	-0.018749475	0.085333641	0.466867662
RPL14	-0.017416009	0.085368159	0.466867662
FAM131A	-0.023845979	0.085458893	0.46713308
ZNF614	-0.043033884	0.085572894	0.467525354
PURG	0.030885635	0.085731417	0.468019952
EIF3K	-0.021428888	0.085755139	0.468019952
HEATR1	-0.053990434	0.085849925	0.468019952
NBPF8	0.0535386	0.085870274	0.468019952
NDUFB6	-0.030459406	0.085874833	0.468019952
GAK	0.016762033	0.085958689	0.46805964
MYCN	-0.040835631	0.086034817	0.46805964
DOLK	-0.032340081	0.086060436	0.46805964
TRAPPC2L	-0.023569123	0.086113711	0.46805964
USF1	0.055061586	0.086117308	0.46805964
ALDH3B1	-0.037663196	0.086236487	0.46805964
HADH	-0.023847052	0.086257301	0.46805964
CBX1	-0.039380754	0.086268224	0.46805964
HAGH	-0.019299391	0.086297841	0.46805964
UBOX5	-0.018892432	0.086304971	0.46805964
SPOCD1	0.101863289	0.086390496	0.46808842
TRIM7	-0.048658001	0.08642201	0.46808842
ARHGAP31	-0.038142631	0.086467278	0.46808842
MGEA5	0.024323156	0.086479431	0.46808842
C17orf100	-0.035953243	0.086543298	0.468205165
PELI2	-0.031716307	0.086591942	0.468239474

CACNA2D3	-0.045991482	0.086789916	0.46897046
HOXA10	-0.055173883	0.086811859	0.46897046
NDUFV3	-0.033871068	0.087054491	0.469786083
RPA2	-0.017814263	0.087192149	0.469786083
COMMD2	-0.042785133	0.087194385	0.469786083
DSTYK	-0.088516403	0.087210143	0.469786083
EXOC8	-0.036287773	0.087299206	0.469786083
FAM167A	-0.082445799	0.087337195	0.469786083
NFKBIZ	0.038566328	0.087400655	0.469786083
PSMD8	-0.025414899	0.087424382	0.469786083
TULP4	0.021390244	0.087488492	0.469786083
SDCCAG3	0.022006955	0.087532374	0.469786083
MRRF	-0.017711934	0.087562816	0.469786083
NFATC2IP	0.030140616	0.087637498	0.469786083
CINP	-0.036640855	0.087647698	0.469786083
SDCCAG8	-0.031029686	0.087668241	0.469786083
BNIP3L	-0.036511169	0.087700064	0.469786083
MRPL51	-0.02114725	0.087708092	0.469786083
EIF4A3	-0.016837655	0.087734555	0.469786083
COPE	-0.023951825	0.087796429	0.469786083
GNL1	-0.023469405	0.087913015	0.469786083
EFNA4	-0.023911406	0.087941604	0.469786083
PELI1	0.036994317	0.087960239	0.469786083
MATN2	-0.043269145	0.08796305	0.469786083
S100A11	-0.040874958	0.088052922	0.469786083
HAUS8	-0.026634789	0.088058659	0.469786083
RPL26	-0.017262377	0.088060838	0.469786083
ABCA7	0.043649732	0.088066322	0.469786083
HSCB	0.029037983	0.088269971	0.47064562
CHTOP	-0.027384175	0.088413541	0.471184153
ZNF773	0.039497835	0.088463713	0.471224659
MYL12B	-0.018440845	0.088692009	0.471978333
DDB2	0.032573507	0.088711444	0.471978333
HIST1H1D	-0.045464379	0.088763234	0.471978333
RBM47	-0.043320819	0.088825335	0.471978333
IMP3	-0.015019835	0.088844316	0.471978333
TBC1D15	0.013127853	0.088886557	0.471978333
GPBAR1	-0.038056452	0.088916616	0.471978333
SYT3	-0.031061991	0.088946319	0.471978333
DENND1B	0.032066708	0.089249358	0.473359437
ARAP1	0.033290337	0.089373974	0.473752957
MBOAT7	-0.030139881	0.089420678	0.473752957
TUBE1	0.030885964	0.089451954	0.473752957
ASB8	-0.020736359	0.089584032	0.474108259
ENTPD6	0.026524824	0.089644423	0.474108259
ASH2L	-0.015543982	0.089647537	0.474108259
MRPL40	-0.027955957	0.089746569	0.474405336
NDUFB2	-0.022198539	0.089970376	0.474855081
SCOC	-0.027166328	0.089989285	0.474855081
PPP1R2	0.023156225	0.090003367	0.474855081

ADNP	-0.018830273	0.090005078	0.474855081
MLXIPL	-0.024745062	0.090050816	0.474855081
APOBEC3A	0.073194391	0.090089048	0.474855081
PABPC1L	0.035319953	0.090137112	0.47488229
NDUFA12	-0.029623592	0.090213138	0.47505672
FIG4	-0.021012975	0.090261074	0.475083131
C4orf27	-0.030531142	0.090389161	0.475350061
MCOLN1	0.029588098	0.090397676	0.475350061
MAP7	-0.052858976	0.090562283	0.47577197
MAFB	-0.043486183	0.090563876	0.47577197
NOC2L	0.030322004	0.090647054	0.475983034
PACS2	0.029625873	0.090896412	0.476670473
FBXL5	-0.032740885	0.090904246	0.476670473
NADK	0.032443951	0.090969646	0.476670473
SRD5A3	-0.039054826	0.090988743	0.476670473
SLC25A12	-0.024608026	0.090993288	0.476670473
ELOVL7	0.073036503	0.091076543	0.476880916
SQLE	-0.03258215	0.09112681	0.476918513
CYC1	-0.016488248	0.091179125	0.476966792
GNAI3	-0.041045038	0.09131655	0.477460032
GHRL	-0.041078259	0.091440541	0.477625149
PAPOLG	-0.033386008	0.091444502	0.477625149
SDCBP	-0.043933173	0.091496137	0.477625149
FCGRT	-0.02731002	0.091539982	0.477625149
ANKS6	0.033170056	0.091566349	0.477625149
OTP	0.030885477	0.091607028	0.477625149
PARN	-0.020319276	0.091718316	0.477706703
CACNA2D4	-0.051980501	0.091721087	0.477706703
LARS2	-0.031424345	0.091752141	0.477706703
ERMAP	-0.039838647	0.091847768	0.477745256
INPP5B	0.034128664	0.09189834	0.477745256
HERC5	0.049605047	0.091958035	0.477745256
RPL23AP64	-0.025741605	0.091972203	0.477745256
MBD1	0.023068979	0.091975349	0.477745256
THYN1	0.020830656	0.092027066	0.47778968
PPFIBP2	-0.046549896	0.092189655	0.478027706
HEXB	-0.019322485	0.092296742	0.478027706
HDGF	0.025781665	0.092378977	0.478027706
CDKN2C	-0.043817567	0.092388932	0.478027706
HOXD3	0.030331457	0.09242472	0.478027706
ZNF600	0.043277105	0.09244274	0.478027706
ZNF692	0.026810552	0.092463609	0.478027706
CPXM1	-0.027614111	0.092510023	0.478027706
ENO1	-0.016197398	0.092516554	0.478027706
ACOT4	-0.052415363	0.092530837	0.478027706
MORN2	-0.034776509	0.09254796	0.478027706
LRRC2	0.03324693	0.092632949	0.478207069
UBXN8	-0.030629244	0.092669091	0.478207069
EPB41L2	0.033556676	0.092738874	0.47832108
GCA	-0.032561915	0.092777609	0.47832108

SCARNA22	-0.031157995	0.092831854	0.47837793
IGHG1	-0.023316425	0.092924013	0.478445244
MRPS23	-0.029242669	0.092931365	0.478445244
SRFBP1	-0.034272948	0.092996574	0.478468803
METTL13	-0.019830267	0.093034599	0.478468803
THAP4	0.042696876	0.093083839	0.478468803
TBRG4	0.024380246	0.093108845	0.478468803
UBR4	0.019213945	0.093207361	0.478568297
NINJ1	-0.039885106	0.093232191	0.478568297
RRP8	-0.02193417	0.093290774	0.478568297
MTHFR	0.044775349	0.093301146	0.478568297
MCFD2	-0.021789703	0.093444925	0.478938145
CNOT7	-0.021590119	0.093459788	0.478938145
ZC3H10	-0.036938837	0.093523473	0.479042723
PTPRC	-0.048000965	0.093602421	0.479139215
RNASEH2C	-0.029381762	0.093674179	0.479139215
BPHL	0.018956712	0.093774905	0.479139215
TROVE2	0.018460869	0.093780374	0.479139215
FAM203A	-0.027388002	0.09381059	0.479139215
SRD5A1	-0.030694078	0.093824758	0.479139215
CASD1	-0.029585257	0.093845317	0.479139215
SNX1	0.023876425	0.093966554	0.479537017
TNFAIP8	-0.032519317	0.094083282	0.479657412
ANKRD10	0.026045323	0.094108198	0.479657412
C20orf24	-0.020514881	0.094120146	0.479657412
TBC1D9B	-0.023844171	0.094192352	0.479804483
CHD1	0.018007639	0.094254896	0.479866994
SMAD3	0.028821541	0.094291328	0.479866994
RBPMS	-0.027790725	0.094528578	0.479978842
CTSS	-0.029302891	0.094568707	0.479978842
SNORD36A	0.028850641	0.094590304	0.479978842
HIST1H2AG	0.07520799	0.094607201	0.479978842
MRPL39	-0.02755996	0.094622231	0.479978842
CKS1B	-0.043690183	0.094623477	0.479978842
SHC1	0.021473938	0.094645879	0.479978842
EEF1A1	-0.023159506	0.094660205	0.479978842
CYTH4	-0.021258383	0.094730148	0.480113556
CD151	-0.046173205	0.094836148	0.480430809
ZNF80	0.061130297	0.09488984	0.480482908
TREML2	-0.031351752	0.095002306	0.480779315
MRPS10	0.025906952	0.095035246	0.480779315
MYO9A	0.029712371	0.095210103	0.481443869
SNX8	-0.025018593	0.095284408	0.481599594
GBGT1	-0.034313452	0.095442816	0.481889631
PYGB	-0.036460728	0.095469468	0.481889631
GFPT1	-0.032231807	0.095472397	0.481889631
VTA1	-0.025061335	0.095673201	0.482505207
SPDYE2	-0.023008902	0.095681537	0.482505207
CCNB1IP1	-0.023500718	0.095829272	0.483030152
BOP1	-0.020519808	0.095921626	0.483274626

MAD2L1BP	-0.031937571	0.095975122	0.483274626
LXN	-0.035848009	0.096025165	0.483274626
PEMT	-0.021840203	0.096052415	0.483274626
SF1	0.032836835	0.096100294	0.483295843
NUDT15	-0.036620201	0.096169027	0.48342187
CCDC88C	0.036184748	0.096285934	0.483774208
ZNF580	0.033375047	0.096375177	0.483774208
KCTD20	0.023402913	0.09637771	0.483774208
ZBTB45	-0.021558204	0.09641394	0.483774208
GBA2	-0.024314996	0.096459121	0.483781611
PIGH	-0.02134819	0.096590569	0.484221473
PPP6R3	0.028330904	0.0967585	0.484799975
PDE4D	0.04105333	0.096809054	0.484799975
CTNND1	0.055814275	0.096837361	0.484799975
DCUN1D4	0.021548359	0.096959302	0.484948848
ARFIP1	-0.028423633	0.096961883	0.484948848
MNAT1	-0.024977215	0.096998532	0.484948848
C1orf216	-0.028809826	0.097044367	0.484958959
SH3PXD2A	0.051814442	0.097124231	0.484973355
HDDC3	-0.024334801	0.097144484	0.484973355
CHMP7	0.034288152	0.097222171	0.484973355
TYSND1	-0.016737959	0.097280712	0.484973355
FAM118B	-0.029274557	0.097370361	0.484973355
VPS35	-0.021562513	0.097404591	0.484973355
DSEL	0.036650847	0.097410509	0.484973355
GOLGA6A	0.039383263	0.097412576	0.484973355
FOXC1	0.043879989	0.097456338	0.484973355
TOR1AIP2	-0.031740761	0.097485384	0.484973355
ZNF24	-0.032622178	0.097609311	0.485371726
HNRNPUL1	-0.018733263	0.097772328	0.485469113
PRKCB	0.016905138	0.097776299	0.485469113
BRD8	0.019669741	0.097827257	0.485469113
TMEM63A	0.041389931	0.097846418	0.485469113
ALKBH8	-0.038068583	0.097871963	0.485469113
GNG11	0.070133855	0.097892046	0.485469113
FAM98B	0.041996307	0.098063252	0.486012912
MBD2	-0.033294258	0.098154688	0.486012912
DFFB	0.028828097	0.098195678	0.486012912
LRP8	0.029396028	0.098205306	0.486012912
ZNF484	-0.028389598	0.098221238	0.486012912
ATXN2L	0.039835494	0.098392978	0.486152587
DBT	0.02552408	0.098410912	0.486152587
TRERF1	0.026283231	0.098420273	0.486152587
PELI3	0.036824888	0.098558432	0.486152587
DENND1A	-0.046206561	0.098560455	0.486152587
SDC4	0.043558381	0.098569554	0.486152587
MIR302C	-0.033321227	0.098622129	0.486152587
CXXC4	-0.029452098	0.098633256	0.486152587
TSPAN31	-0.020155257	0.098672547	0.486152587
EAPP	-0.043781416	0.098707949	0.486152587

NFATC3	0.026314122	0.098732588	0.486152587
ENOPH1	-0.018543437	0.098787844	0.486208378
RIMKLB	0.033222181	0.098893345	0.486442423
TMED4	-0.032629664	0.09892329	0.486442423
MYL6B	-0.03161211	0.099072577	0.486960192
TRAP1	-0.028021407	0.099186756	0.487305018
STAT3	0.019735115	0.099322808	0.487346183
SHQ1	-0.02428499	0.099351325	0.487346183
SLBP	-0.022061922	0.099394345	0.487346183
ASB13	-0.030455506	0.099413544	0.487346183
C12orf23	-0.034671218	0.099415275	0.487346183
NINJ2	-0.039824092	0.099609343	0.48808137
PPP2R3A	0.048008047	0.099716902	0.488384439
PTPN23	-0.029806384	0.099782053	0.488384439
GPR156	-0.03074447	0.099842718	0.488384439
PRPF38A	0.016779429	0.099875287	0.488384439
LYZ	-0.055137309	0.099891803	0.488384439
ITPRIP	-0.019269683	0.100045221	0.488918565
ZNF607	0.026786596	0.100104346	0.488991617
XBP1	-0.025985589	0.100354277	0.489996249
PHF23	-0.022192238	0.100656311	0.491028256
NFAT5	0.046951611	0.100724086	0.491028256
OGDH	0.03200202	0.100745547	0.491028256
SLC39A10	0.029220732	0.100770531	0.491028256
FAM32A	-0.016292825	0.100827549	0.491028256
PGM2	-0.026030301	0.100863727	0.491028256
WDSUB1	-0.031423158	0.10094203	0.491028256
CLN3	0.022470498	0.100962609	0.491028256
IL10RA	0.020445334	0.100964885	0.491028256
FTO	-0.026488407	0.101057302	0.49126187
DUSP3	0.024757811	0.101111784	0.491263595
TSPAN33	0.071417342	0.101151592	0.491263595
PTPDC1	0.030244131	0.101190803	0.491263595
CLEC4A	-0.025538749	0.101349721	0.491819406
ACRBP	0.073682025	0.10151731	0.492390393
RAPSN	0.033593055	0.101556353	0.492390393
DR1	-0.024064877	0.101842479	0.493561469
RALGPS2	0.065756105	0.101949733	0.493722222
ADIPOR2	0.014015798	0.102000094	0.493722222
LLPH	-0.025113418	0.102062457	0.493722222
CD58	-0.037415853	0.102085498	0.493722222
KIAA1009	-0.032809766	0.102098669	0.493722222
ICA1L	0.046235545	0.10216467	0.493824459
SBDS	-0.026226362	0.102216664	0.493824459
PSMC4	-0.037623834	0.102382057	0.493824459
TAF9	-0.045146344	0.102383384	0.493824459
SEMA3C	-0.041702571	0.102441674	0.493824459
TMEM14B	-0.027958756	0.102474594	0.493824459
ZNF429	-0.056225721	0.102484972	0.493824459
HCG27	0.051045092	0.102490282	0.493824459

FKBP4	-0.032064541	0.102540249	0.493824459
CD81	-0.025264704	0.102581627	0.493824459
NDUFA9	-0.020506297	0.102623041	0.493824459
DCAF11	-0.034447925	0.102716849	0.493824459
DCLK3	-0.030707978	0.102759264	0.493824459
RNASEH2A	-0.026241724	0.102769043	0.493824459
DZIP1L	0.050948878	0.102796245	0.493824459
IFFO2	0.0281699	0.102833624	0.493824459
SKAP1	-0.020863412	0.1028862	0.493862682
BTF3	-0.014882521	0.103007968	0.494210248
ACTG1	-0.034617132	0.103246738	0.494210248
TRMT112	-0.024696767	0.103287211	0.494210248
CHD8	0.030018759	0.10329547	0.494210248
NDUFB4	-0.02618762	0.103350586	0.494210248
CDK5RAP1	0.016773082	0.103391333	0.494210248
USP15	0.028348369	0.103394213	0.494210248
KLHDC9	-0.034452899	0.103407206	0.494210248
LRRFIP2	0.02320302	0.103428292	0.494210248
LENG8	0.033290973	0.103445003	0.494210248
NACC2	-0.027847565	0.103449738	0.494210248
RPL41	-0.007662085	0.10349963	0.494235291
MAP3K4	0.020712929	0.103611685	0.494557023
MED6	-0.01522234	0.103664903	0.494597762
CHIC2	-0.027324547	0.103735605	0.494721848
CD86	-0.028620768	0.103907528	0.494739219
HABP4	0.032773422	0.103960405	0.494739219
ENKUR	0.069768531	0.103963149	0.494739219
PNO1	-0.026128233	0.10405729	0.494739219
PSMG1	-0.019490934	0.104079014	0.494739219
PSMD14	-0.022592434	0.1041157	0.494739219
TNFRSF6B	0.030110605	0.104128808	0.494739219
MRPL20	-0.033482211	0.104166954	0.494739219
EXOC5	-0.019089369	0.104258414	0.494739219
PSMC5	-0.014259807	0.104309406	0.494739219
STAT5A	0.026912047	0.104360682	0.494739219
PPIA	-0.021237218	0.104361987	0.494739219
ZDHHC1	-0.047490482	0.104378288	0.494739219
ASPRV1	-0.030214741	0.104432947	0.494739219
GALK1	-0.016428383	0.104445731	0.494739219
CCT3	-0.022066919	0.104454382	0.494739219
HPCAL1	-0.023822035	0.104517558	0.494826711
LYSMD1	-0.02440266	0.104585781	0.494938014
ERP44	0.039448294	0.104639613	0.494981145
HCG11	-0.046258641	0.104885797	0.495531872
CIR1	0.016825336	0.104915532	0.495531872
NOX4	0.035972532	0.104970402	0.495531872
IFI16	-0.022319595	0.10499812	0.495531872
SNORA80	0.038360554	0.10504641	0.495531872
ZMIZ2	0.030213479	0.105058184	0.495531872
UGP2	-0.02080146	0.105069856	0.495531872

GIPC3	-0.037051818	0.105198354	0.495531872
ARPC5	-0.013442594	0.10522474	0.495531872
MTPAP	0.02586716	0.105250931	0.495531872
TXNDC15	-0.027178714	0.105298723	0.495531872
PABPC3	-0.029843279	0.105311585	0.495531872
C21orf119	-0.032677606	0.105338016	0.495531872
ACAP1	0.03138391	0.105383568	0.49553556
BLCAP	0.020084942	0.105639614	0.496528616
IFRD1	0.030506659	0.105907756	0.497425172
SCPEP1	-0.032272958	0.105920239	0.497425172
CSNK2A2	-0.018969408	0.105980391	0.497496587
TMCC1	-0.037939086	0.106097783	0.497644392
TBCC	-0.036583906	0.106225481	0.497644392
EIF2D	-0.019520534	0.106239134	0.497644392
SP2	-0.020436402	0.1062763	0.497644392
ZNF514	0.056620049	0.106287303	0.497644392
TRIM47	-0.032031313	0.106375874	0.497644392
SLC10A7	-0.027816083	0.1063866	0.497644392
WDR19	0.028818512	0.106584646	0.497644392
TDRKH	0.041289175	0.106610113	0.497644392
CYBRD1	-0.043945605	0.106617973	0.497644392
CDAN1	0.016598804	0.106624325	0.497644392
UBAP1	-0.024569692	0.106627088	0.497644392
PXK	-0.033488707	0.106644157	0.497644392
TBC1D4	0.039097722	0.106680032	0.497644392
COL7A1	0.039839043	0.106686254	0.497644392
C1orf198	0.053903771	0.106862752	0.498101755
THNSL2	0.108059932	0.10689352	0.498101755
DIDO1	0.029341858	0.106919303	0.498101755
RANBP10	0.020796413	0.106984552	0.49819605
SERPINB2	-0.060956507	0.107192358	0.498807299
CEP135	0.016189861	0.107277408	0.498807299
CCT6P1	0.01165011	0.10742181	0.498807299
HEATR5B	0.017096798	0.107478671	0.498807299
ANO9	0.040074688	0.107495195	0.498807299
TNFSF14	0.039643664	0.107549374	0.498807299
SIK1	0.054608808	0.107574987	0.498807299
AUP1	0.02207076	0.107582576	0.498807299
TFDP1	-0.023301675	0.10766815	0.498807299
JUN	-0.058381838	0.107679748	0.498807299
HNRNPC	-0.023906661	0.107716747	0.498807299
FZD1	-0.031234758	0.107730714	0.498807299
C16orf80	-0.021740361	0.107734614	0.498807299
PRG2	0.033258245	0.107746703	0.498807299
CCDC151	-0.044289858	0.107897917	0.499298514
UBE2D3	-0.022267092	0.108129619	0.500161619
PPARA	-0.024103015	0.108375242	0.50108837
ZNF362	-0.018982526	0.108424633	0.501107419
CSNK1G3	-0.011685803	0.108580128	0.50126431
RANBP3	-0.018455957	0.108592102	0.50126431

HLA-DPB1	-0.059826337	0.108634944	0.50126431
HIF1A	0.029208982	0.108639722	0.50126431
COX7A2	-0.025967056	0.108729665	0.501314943
PKNOX1	-0.025872499	0.108741275	0.501314943
EXTL2	-0.035444185	0.108823635	0.501485767
ANKRD20A4	0.109193596	0.108885974	0.501549074
DHX30	0.021671781	0.108942249	0.501549074
DLEC1	0.03465981	0.108973306	0.501549074
FNBP1	-0.013928153	0.109115573	0.501704566
GNG5	-0.030712995	0.109146802	0.501704566
CDC5L	-0.023921225	0.109174703	0.501704566
EPHA8	0.029585954	0.109344279	0.501704566
UBE2W	-0.030780925	0.109375386	0.501704566
SRSF1	-0.01367868	0.109388608	0.501704566
KIF23	-0.037128905	0.109390049	0.501704566
DNAJC8	-0.015848389	0.109394093	0.501704566
AP1M1	-0.014364517	0.109415017	0.501704566
UBB	-0.035208598	0.109539201	0.501882617
HS6ST1	-0.027101739	0.109554171	0.501882617
GABARAPL2	-0.020939596	0.109589871	0.501882617
HSDL1	0.027454897	0.109649322	0.501913615
CTLA4	0.041409846	0.109687328	0.501913615
USPL1	0.016686295	0.109747747	0.501982566
TNFRSF12A	0.028672456	0.109879799	0.502016752
GORAB	0.023022006	0.109912249	0.502016752
GTF2IRD2	-0.043185858	0.109943763	0.502016752
LY9	-0.027846985	0.109981573	0.502016752
TBC1D7	-0.030767172	0.110012286	0.502016752
TUBA3C	-0.026996039	0.110027341	0.502016752
RABGAP1	0.022160282	0.110200502	0.50246553
INPP5F	0.036713828	0.110216488	0.50246553
FAM84B	0.033658987	0.110314889	0.502549313
GTF2IRD2B	0.025656454	0.110325669	0.502549313
NSUN6	0.018569651	0.110390207	0.502615427
MPZL2	-0.041647356	0.110430998	0.502615427
KLRC1	-0.09616831	0.110488089	0.50266858
GAB1	-0.027935814	0.110653622	0.502901711
ZNF266	0.043256754	0.110665641	0.502901711
SCARNA8	0.028275796	0.11068413	0.502901711
GOSR2	-0.030523664	0.110721065	0.502901711
DNAJC13	-0.024037026	0.110842904	0.502910326
ZZEF1	0.014399625	0.110882008	0.502910326
TXNL4A	0.032969567	0.110890968	0.502910326
TCF25	-0.018680678	0.110904698	0.502910326
CYFIP1	-0.033942104	0.110981226	0.502911904
ZBTB42	-0.0272549	0.111077703	0.502911904
ZNF219	-0.027262769	0.11108403	0.502911904
THAP1	-0.036907647	0.111137828	0.502911904
PSMD6	-0.012590158	0.111149136	0.502911904
PROK2	-0.106503848	0.111177652	0.502911904

PPP2R5E	-0.02029935	0.111348142	0.503249659
RAB3IP	0.022792912	0.11135327	0.503249659
AIDA	0.014584943	0.111431606	0.503249659
SKAP2	-0.030449189	0.111434178	0.503249659
INSIG2	-0.019134094	0.11158186	0.503334179
ADSSL1	-0.033396426	0.111605394	0.503334179
RAP2A	-0.039336461	0.111642741	0.503334179
KLHL33	-0.029482081	0.11169092	0.503334179
HCG18	-0.036414311	0.111691047	0.503334179
MMADHC	-0.023005711	0.111794978	0.503334179
ANK3	0.04420344	0.11187433	0.503334179
CHMP1B	-0.024300825	0.111886225	0.503334179
DGKA	0.037281646	0.111927736	0.503334179
SMAP2	-0.02008041	0.111968529	0.503334179
UTRN	0.03742827	0.111982089	0.503334179
STAM2	-0.030793028	0.111998562	0.503334179
CTR9	-0.024577948	0.112184751	0.50390405
NSF	-0.014636321	0.112250568	0.50390405
LGALS1	-0.03116593	0.112265956	0.50390405
TTC21A	0.041012418	0.112488562	0.50390405
NDST1	-0.040987008	0.112489635	0.50390405
PLXNC1	-0.042548477	0.112513394	0.50390405
RNF144B	0.045689802	0.112516473	0.50390405
SNORD78	-0.033206438	0.112667984	0.50390405
YLPM1	-0.026541699	0.112742278	0.50390405
RPS21	-0.034454003	0.112757132	0.50390405
EIF2S1	0.018175084	0.112758022	0.50390405
TCF3	0.029759911	0.112781888	0.50390405
OLIG1	-0.078014397	0.112799813	0.50390405
TAX1BP1	-0.015035835	0.112852694	0.50390405
PRPF4B	0.028838395	0.112911374	0.50390405
SKIV2L2	-0.01886076	0.112956715	0.50390405
BACE1	-0.037059375	0.113069663	0.50390405
SNORA49	-0.026537266	0.11307848	0.50390405
AMOTL2	0.029387396	0.113138387	0.50390405
RPL32P3	0.04090695	0.113165152	0.50390405
VARS2	-0.037601745	0.113166012	0.50390405
STK38L	-0.033407094	0.113310881	0.50390405
KCNT1	-0.068458278	0.11334883	0.50390405
ZNF589	0.034757273	0.113399584	0.50390405
DNLZ	-0.040084505	0.113427506	0.50390405
MRPL35	-0.023471392	0.11343491	0.50390405
ZNF83	0.044359347	0.113464086	0.50390405
LYSMD2	-0.023452607	0.113487008	0.50390405
TMBIM1	-0.025533055	0.113521444	0.50390405
VDAC3	-0.011838682	0.113527063	0.50390405
CMTM5	0.090897674	0.113536607	0.50390405
STK38	0.02072362	0.11363694	0.503997732
CDHR3	0.043390123	0.113672974	0.503997732
TXNDC9	-0.020425447	0.113696995	0.503997732

NCBP1	-0.017895364	0.113771914	0.503997732
LYPLA1	-0.038756571	0.113818185	0.503997732
ING2	-0.027898868	0.113830909	0.503997732
PSMG3	-0.028060677	0.113961355	0.504206674
S100A12	-0.065672513	0.113969202	0.504206674
SPC25	0.060638807	0.11408075	0.504498532
GLDC	-0.07960843	0.11418069	0.504733422
SCNM1	-0.026271859	0.114225063	0.504733422
SUMO2	-0.031926685	0.114337522	0.504799065
FAM96A	-0.024890478	0.114348873	0.504799065
SNORD38A	0.032431413	0.114376733	0.504799065
HSPB3	0.042765493	0.114432785	0.504845155
CABLES1	-0.02379181	0.114559018	0.505140549
CCDC94	-0.016662027	0.114591013	0.505140549
SH2D3A	-0.030974053	0.11480104	0.50580582
VAV2	0.028348513	0.114833321	0.50580582
GNAL	0.033788636	0.114933515	0.505821488
PRMT5	-0.01998083	0.114966639	0.505821488
TSC1	0.018086859	0.114973969	0.505821488
RAB7A	-0.014005797	0.11507089	0.505918205
DHX37	-0.021479676	0.115087365	0.505918205
CBWD1	-0.037941936	0.115345402	0.506370661
MRPL36	-0.022344321	0.115379993	0.506370661
COX6A1	-0.01827169	0.115396204	0.506370661
CUTC	-0.018990238	0.115397092	0.506370661
COL9A2	-0.043358937	0.115532682	0.506370661
LRIG2	0.026065281	0.115563261	0.506370661
MRPS22	-0.01162942	0.115574012	0.506370661
ZNF26	0.023390285	0.115593587	0.506370661
ZNF567	-0.028519893	0.115602011	0.506370661
INPP1	-0.025810759	0.11571895	0.506682382
RNF126	0.017955146	0.116032	0.507688336
PIGO	-0.017988505	0.116076081	0.507688336
RAC2	-0.015554321	0.116166218	0.507688336
PSMA4	-0.017693299	0.116167041	0.507688336
GOLGA5	-0.015299571	0.116178025	0.507688336
NOTCH1	-0.026610539	0.116294149	0.507940765
ZNF282	-0.030942553	0.116327567	0.507940765
COQ10B	-0.025195932	0.11645407	0.50796352
CLEC3B	-0.036965882	0.116455128	0.50796352
GLO1	-0.029007879	0.11647045	0.50796352
SLCO4C1	-0.049457804	0.11660166	0.508335478
TARBP1	0.027015358	0.116692499	0.508459779
SOX15	-0.032387342	0.116722043	0.508459779
UBE2D4	-0.027305166	0.116823234	0.508648978
MRPL45P2	-0.040236401	0.116857381	0.508648978
HM13	0.048070893	0.116924324	0.508677214
HELZ	0.024532396	0.116955778	0.508677214
DOK1	-0.046837086	0.117075326	0.508821096
LPAR3	-0.025546583	0.117084864	0.508821096

VSTM1	-0.115100825	0.117126764	0.508821096
LILRB1	0.017350203	0.117324543	0.509480333
MED11	-0.02117185	0.117410739	0.509654694
SYMPK	0.03570366	0.117535894	0.509979074
SMC3	-0.015091206	0.117577613	0.509979074
TMEM69	-0.029325347	0.117765129	0.510592329
HIPK3	-0.038584419	0.117951518	0.510853702
HMGCL	-0.02258511	0.117961421	0.510853702
SNORD35B	0.035708846	0.117963869	0.510853702
SYS1	-0.023277824	0.118172103	0.51146634
THAP3	0.031146657	0.11819775	0.51146634
SGPP1	-0.033647834	0.118248291	0.511485084
RAP1A	0.050431153	0.118294614	0.511485578
FAM114A2	-0.022962965	0.118394967	0.511634388
YPEL5	0.022366877	0.118432871	0.511634388
TRIM61	-0.032918797	0.118503379	0.511634388
RIPK2	-0.021429345	0.118572477	0.511634388
PLCL2	0.014581982	0.118603413	0.511634388
PRKACB	-0.034141731	0.11860728	0.511634388
CENPO	0.026993191	0.118736551	0.511634388
WARS2	-0.092078367	0.118749722	0.511634388
MIR586	-0.030810762	0.118804496	0.511634388
FAM109B	0.03071813	0.118811783	0.511634388
NCLN	-0.020558072	0.118837475	0.511634388
RBL1	-0.027449849	0.118981747	0.511898628
RIOK3	0.020177263	0.118991342	0.511898628
NUDT5	-0.024498055	0.11912494	0.512274264
ELAC2	-0.020939833	0.119411204	0.513305872
OXSR1	0.011758825	0.119631614	0.5140537
RNASE10	0.026355036	0.119738837	0.51431478
PSEN1	-0.032557996	0.119855653	0.514587081
TTC8	-0.029402656	0.119911935	0.514587081
CHAF1B	-0.032949722	0.119941699	0.514587081
SNX5	-0.019796216	0.119989213	0.514591477
NLN	-0.032865909	0.12010868	0.514898018
GSR	-0.028936872	0.120225445	0.514898018
EIF3H	-0.021997703	0.120251312	0.514898018
STARD3NL	-0.040904562	0.120320959	0.514898018
MTFP1	-0.027371615	0.12038352	0.514898018
ZNF296	-0.026960628	0.12044108	0.514898018
MAP1S	-0.023751735	0.120449361	0.514898018
ARID2	-0.018809965	0.120506737	0.514898018
PRCC	0.018263244	0.120519775	0.514898018
PLK5	-0.0268563	0.12055922	0.514898018
FAM173A	-0.019473372	0.120572379	0.514898018
SLMO2	-0.031689119	0.120672929	0.515040993
GIMAP7	-0.027688398	0.120714985	0.515040993
IER3IP1	-0.032056718	0.12078623	0.515040993
CCL3	-0.066200818	0.120791979	0.515040993
MX1	0.056199724	0.120872425	0.515099565

TNRC6B	0.031652443	0.120898787	0.515099565
YIF1A	-0.021604967	0.121032071	0.515227127
IL5RA	0.038402609	0.121067783	0.515227127
DVL1	0.033244239	0.121068367	0.515227127
SERPINE2	-0.073916484	0.121150717	0.515319605
ZNF224	0.028067993	0.121183208	0.515319605
IER5	-0.028396503	0.121471365	0.516188041
EPB41	0.029683473	0.121480698	0.516188041
HIPK2	0.030942444	0.121562266	0.516336424
PUS7L	-0.0275613	0.121833016	0.517074033
PLOD1	-0.026289552	0.121836599	0.517074033
SCRN3	-0.024587873	0.121876064	0.517074033
POFUT2	0.021645241	0.122109519	0.517799051
NME3	0.017056272	0.122184779	0.517799051
TCEA1	-0.02805036	0.122187291	0.517799051
DNAJC19	-0.027899154	0.122292005	0.517860436
TBC1D3	0.040277528	0.122315469	0.517860436
KHDRBS3	0.022136583	0.12234213	0.517860436
CTIF	0.029680908	0.122438983	0.518072286
RPL15	-0.022064708	0.122683396	0.518749473
USP3	0.019092101	0.122693976	0.518749473
PYGM	0.025766778	0.122739622	0.518749473
TRIM25	0.026971125	0.122836636	0.518961345
LRRC3	-0.024908283	0.122904488	0.518989093
ZNF526	0.034055435	0.122936977	0.518989093
IKBIP	0.009768498	0.123030318	0.519185126
ACOT7	-0.033277799	0.123131215	0.519204649
RPL34	-0.024685418	0.123149142	0.519204649
C10orf32	-0.03958341	0.123201866	0.519204649
IFT88	0.023328438	0.123246115	0.519204649
APOBR	-0.025177577	0.123269475	0.519204649
SCD5	-0.056159769	0.123374958	0.519298575
BTBD1	-0.021420039	0.123385604	0.519298575
COL18A1	-0.0791412	0.123656177	0.520239538
NEDD8	-0.011167647	0.12391564	0.520620701
B4GALT3	-0.01885003	0.12396922	0.520620701
OLFML2A	-0.025678033	0.123974653	0.520620701
CHMP5	0.02290962	0.123985484	0.520620701
AP2S1	-0.014768506	0.124050662	0.520620701
HAUS5	0.026643492	0.124061707	0.520620701
DCAF15	0.027785112	0.124076015	0.520620701
C11orf95	-0.021700154	0.124317246	0.521224286
DCK	-0.031451531	0.124368928	0.521224286
RBKS	-0.022216753	0.124413809	0.521224286
RNASEK	0.01756724	0.124452571	0.521224286
GATAD2B	0.029252548	0.124455306	0.521224286
GJC2	-0.029133533	0.124508312	0.521249058
FBXO7	0.01754004	0.124717022	0.521689607
CHAF1A	-0.033901067	0.124784357	0.521689607
SACM1L	0.027304251	0.124799344	0.521689607

MESP1	-0.037924527	0.124828574	0.521689607
TRPC1	0.03632358	0.124863467	0.521689607
MIR564	-0.028562668	0.124896328	0.521689607
RPUSD3	-0.015703048	0.124957962	0.521750162
DEFB116	-0.028990073	0.125143932	0.5220822
MSX1	-0.026214858	0.125169521	0.5220822
ZNF263	0.020802241	0.125222165	0.5220822
SET	-0.016684744	0.125251652	0.5220822
PYCR2	0.021703816	0.125273315	0.5220822
TMEM158	0.0817968	0.125571836	0.52253879
STXBP5	-0.021802597	0.125604187	0.52253879
IWS1	-0.017016234	0.12569588	0.52253879
RBBP4	-0.036380747	0.125711279	0.52253879
PHF11	0.014522793	0.125711736	0.52253879
SPDYE7P	0.011863054	0.125750528	0.52253879
MIR25	0.034125007	0.125752749	0.52253879
ARGLU1	0.018387364	0.125784252	0.52253879
AGAP8	0.034526962	0.125829476	0.52253879
YOD1	0.023928677	0.125895942	0.52253879
POC1A	-0.029417695	0.125902155	0.52253879
STAG1	-0.018607057	0.126123675	0.523249845
ZNF777	-0.018803984	0.126281093	0.523249845
NOLC1	-0.024487505	0.126359325	0.523249845
TAF1D	0.055874297	0.126379279	0.523249845
AP1G1	0.019653812	0.126385894	0.523249845
CFLAR	0.031665691	0.126402908	0.523249845
APOBEC3F	-0.045661827	0.12645802	0.523249845
C11orf71	-0.032300347	0.126524589	0.523249845
LASP1	-0.01677024	0.126601082	0.523249845
RXRB	-0.01787166	0.126616524	0.523249845
RPS19BP1	-0.017289357	0.126619374	0.523249845
LYRM4	-0.034427533	0.126646727	0.523249845
ZNF292	0.036735641	0.12668801	0.523249845
TMEM212	0.050805676	0.126873971	0.523822449
DNASE2	-0.023397728	0.126947384	0.523924524
HIST2H3D	-0.038626687	0.12699336	0.523924524
TELO2	-0.050051927	0.127119192	0.524087185
NIPSNAP3A	-0.040583468	0.127127481	0.524087185
ZNF417	0.028445211	0.127267615	0.524304637
CTBS	-0.029017237	0.127290161	0.524304637
SLC44A2	-0.018098779	0.127430858	0.524304637
HMBS	-0.032940209	0.127542091	0.524304637
TRNAU1AP	-0.021894261	0.127582591	0.524304637
MARK3	0.023891965	0.127587951	0.524304637
MTDH	-0.01681925	0.127639131	0.524304637
GK5	0.038872109	0.127666009	0.524304637
AATK	-0.02819471	0.127692704	0.524304637
FAM63A	0.055555912	0.127736405	0.524304637
TTYH3	0.026037588	0.127741887	0.524304637
ARAP2	-0.024691415	0.127748632	0.524304637

ALDH7A1	-0.04380252	0.127966761	0.524967457
DAP3	-0.01516017	0.128004984	0.524967457
PCMTD2	0.020987731	0.128078607	0.525059985
RAPGEF1	-0.02312172	0.128178053	0.525059985
UAP1	-0.027572278	0.128197576	0.525059985
SLC22A31	-0.028460835	0.128251647	0.525059985
NEDD4	-0.031239266	0.128335351	0.525059985
MSH3	0.009875255	0.128344024	0.525059985
NRGN	0.06660081	0.128359592	0.525059985
NOD1	-0.031442418	0.128559871	0.525381131
CNTF	-0.011883221	0.128644857	0.525381131
PARP14	0.040037655	0.128767496	0.525381131
DOCK2	-0.017647452	0.128784455	0.525381131
SPOCK2	-0.018303953	0.128828688	0.525381131
MOV10	0.028436999	0.128832269	0.525381131
CCDC121	-0.028687332	0.128835578	0.525381131
CCT8	-0.018048245	0.12886033	0.525381131
RNF5	-0.025807763	0.128865279	0.525381131
RGMA	-0.023959882	0.128922615	0.525421363
LARP7	-0.019002459	0.129051722	0.525469912
TUBB6	0.047422972	0.129134572	0.525469912
CTDSP1	-0.016013737	0.129135034	0.525469912
USP28	0.044871516	0.129139415	0.525469912
ZNF839	0.025374632	0.129171888	0.525469912
NOD2	0.02715802	0.129313923	0.525854451
NOP16	-0.028591252	0.129509101	0.526398928
DPH2	-0.013768742	0.129542929	0.526398928
KIF2A	0.03198233	0.12961182	0.526485593
PRR13	-0.013309565	0.129714337	0.526708729
C10orf54	-0.019122038	0.129912759	0.527169689
SLC44A4	0.011142913	0.1299414	0.527169689
PIGP	-0.028282838	0.129995238	0.527169689
IFNAR1	-0.021815925	0.130018362	0.527169689
ASCC1	-0.026436615	0.130177954	0.5276235
CHKB	0.024283002	0.130330841	0.527992689
CWC27	-0.016859866	0.130364443	0.527992689
ELL2	-0.046027272	0.130447023	0.528133907
CAPZA2	0.016897736	0.130720646	0.528673826
PHGDH	-0.06084347	0.130744456	0.528673826
RAB18	-0.028912017	0.130757449	0.528673826
TRAPPC6B	0.039530976	0.130828671	0.528673826
KLRB1	-0.038844935	0.130839106	0.528673826
POLR3GL	-0.014479978	0.130866951	0.528673826
AP2B1	0.02313448	0.130939606	0.528774351
ABCF1	-0.014753285	0.131084222	0.529056625
ENGASE	0.032518028	0.131105097	0.529056625
RNF10	0.031358268	0.131208886	0.529111351
SLC17A9	0.036098573	0.131214261	0.529111351
RPSA	-0.02370815	0.131368811	0.529226963
GUK1	0.014767535	0.131415776	0.529226963

TSC22D2	-0.02507905	0.131438963	0.529226963
ETFA	-0.025675032	0.131479261	0.529226963
TRIM37	-0.021177965	0.13148199	0.529226963
ERO1LB	0.028825141	0.13159081	0.529472437
FBL	-0.020855388	0.131805535	0.529915522
KIAA1143	-0.034924209	0.131905614	0.529915522
SNORA10	0.029070749	0.131910939	0.529915522
PTBP2	0.024372998	0.131956187	0.529915522
PGAP3	-0.016515644	0.132005059	0.529915522
SBF1P1	-0.028207707	0.132093216	0.529915522
EID2	-0.025142888	0.132095766	0.529915522
S100Z	-0.042218586	0.132115028	0.529915522
EHD2	-0.023954075	0.13214388	0.529915522
DBI	-0.017412194	0.13219054	0.529915522
TONSL	0.036206564	0.132316381	0.529915522
PLEKHA8P1	0.019895399	0.132344543	0.529915522
COMMD1	-0.018289736	0.132373832	0.529915522
KDELR1	0.015979403	0.132435898	0.529915522
MLX	-0.024855793	0.132552738	0.529915522
DUSP14	-0.039301972	0.132625254	0.529915522
SQRDL	-0.028830706	0.132667358	0.529915522
ATP5O	-0.019330038	0.132667807	0.529915522
SBNO2	0.026825592	0.13268974	0.529915522
WDR33	-0.023516283	0.132767508	0.529915522
ATP8B2	0.021846544	0.132777219	0.529915522
SLC5A12	-0.030628419	0.132810714	0.529915522
CTSW	-0.039925362	0.132822983	0.529915522
CRCP	0.032967673	0.132849903	0.529915522
PAK4	-0.025739925	0.132951437	0.530129487
OSBPL1A	-0.04572311	0.133028405	0.530245379
C5orf15	-0.014224995	0.133121257	0.530424477
CHCHD1	-0.026086917	0.133216631	0.530613489
BIN2	0.021657735	0.133344243	0.530930727
TMTC4	0.02594225	0.13342294	0.531053049
RNY5	0.03668526	0.13357037	0.531359862
ALDH16A1	0.028494413	0.133615878	0.531359862
ARFRP1	-0.028688617	0.133772162	0.531359862
XAF1	0.042096108	0.1337731	0.531359862
CTSO	-0.030010085	0.133777633	0.531359862
CDADC1	0.027589845	0.133798998	0.531359862
DNAH1	0.02360105	0.133838998	0.531359862
SECISBP2	0.026312085	0.13388406	0.531359862
NCKAP1	0.041491285	0.133939386	0.531388912
NCOA5	0.022233058	0.134010271	0.531424368
RHEBL1	-0.028388119	0.134044343	0.531424368
CDS2	-0.021179773	0.134124491	0.53142729
POMT2	0.030415555	0.13414328	0.53142729
C14orf93	-0.013592928	0.134189111	0.53142729
EXOC6	0.015146015	0.134319708	0.53145286
DYSF	-0.059273049	0.134337006	0.53145286

GHDC	-0.023572466	0.134374387	0.53145286
GLMN	0.021858274	0.134387619	0.53145286
HDAC7	0.028698524	0.134512218	0.531755623
OAS3	0.06129374	0.134673654	0.531927888
ACTR10	-0.016555163	0.134675693	0.531927888
SGMS2	-0.032926407	0.134699961	0.531927888
GPR1	0.065522647	0.134787351	0.532007396
ZSCAN21	-0.023248953	0.13481622	0.532007396
AVEN	-0.02844326	0.134933652	0.53228104
GLT8D1	-0.015027855	0.135091756	0.532588672
C2orf88	0.065414392	0.135113251	0.532588672
PPP2R5B	0.026280031	0.135155983	0.532588672
KIAA1683	0.049501419	0.135237134	0.532692906
UCA1	0.024647872	0.135278684	0.532692906
COQ10A	-0.023164404	0.135427798	0.532949124
EIF3C	-0.039391694	0.135440048	0.532949124
MIR630	-0.025169541	0.135662565	0.533538762
PSENEN	-0.027636794	0.135738516	0.533538762
HIST1H2BC	0.071934927	0.135771241	0.533538762
FBXL14	-0.027413967	0.135782699	0.533538762
CDH20	0.028292583	0.135844689	0.533592925
DBP	0.023728502	0.135919289	0.533686182
SARNP	-0.017140101	0.13596486	0.533686182
THEM4	0.05291913	0.136136371	0.534169973
MED8	-0.023455832	0.136321914	0.534702052
ALOX15B	-0.034626829	0.136368587	0.534702052
RYBP	0.048784539	0.136544282	0.535038823
FFAR2	-0.042048443	0.13655115	0.535038823
GAPVD1	0.014568779	0.136616404	0.535105088
ASB6	-0.026624407	0.136784266	0.53557306
FAM179B	-0.021785334	0.136902906	0.535848043
ATP5EP2	-0.013085459	0.137205549	0.536842779
PPIAL4A	-0.018471385	0.137383957	0.537013477
QSOX2	0.024674825	0.137384139	0.537013477
ZNF616	-0.028985176	0.137394721	0.537013477
CRY1	-0.018538274	0.137594666	0.537441788
MSRB2	-0.01976889	0.137615913	0.537441788
CDK11A	0.038075122	0.137649966	0.537441788
AAGAB	0.027363395	0.137699133	0.537444182
ZFP91	0.033813998	0.137796819	0.537532089
FITM1	0.024756126	0.137936496	0.537532089
TBC1D19	-0.025892526	0.137958061	0.537532089
PDP2	0.031001844	0.137997301	0.537532089
STT3B	-0.024231587	0.138051815	0.537532089
PM20D2	0.022846702	0.138071577	0.537532089
ZKSCAN1	0.05293097	0.138096095	0.537532089
PEX5	-0.01730238	0.138135492	0.537532089
UBE3B	0.020631371	0.138159513	0.537532089
TMEM144	0.05401291	0.138207275	0.537532089
PPAP2B	-0.039079934	0.138327845	0.537812053

BSDC1	0.021583495	0.138435571	0.538041902
FAM65B	-0.022249355	0.138543989	0.538145762
THEMIS	-0.036251261	0.138559529	0.538145762
CASP7	-0.033117803	0.138740173	0.538384332
EXOC2	-0.022603853	0.138817111	0.538384332
PCBP4	-0.029145661	0.138821632	0.538384332
SNX3	-0.019056806	0.138848273	0.538384332
ELOVL1	-0.022373594	0.139017519	0.538384332
TRIM44	-0.020445781	0.139028411	0.538384332
HIBCH	-0.021123708	0.139050574	0.538384332
SEC24B	-0.019090533	0.139057245	0.538384332
EIF4ENIF1	0.015286646	0.139058705	0.538384332
GCLC	-0.020051872	0.139283359	0.539065559
RPL7A	-0.011276864	0.139499133	0.539711955
SLC25A39	-0.015391975	0.139629755	0.540028565
LRR1	-0.022450238	0.13971819	0.540181853
ABLIM3	0.071508177	0.139841724	0.540470685
CUL4A	0.013856733	0.139996378	0.540580161
PKHD1L1	0.034061287	0.140080572	0.540580161
PAPL	0.039745504	0.14008883	0.540580161
YME1L1	-0.027779409	0.140153357	0.540580161
NMNAT3	-0.033909677	0.14017575	0.540580161
ADCY6	0.023719385	0.140176124	0.540580161
PATE2	0.028075053	0.140266348	0.540580161
SRRM1	-0.011736973	0.140317	0.540580161
ZNF763	0.02892852	0.140432356	0.540580161
MAP2K5	-0.017153675	0.140438119	0.540580161
RSPRY1	0.018751823	0.140458315	0.540580161
NUP214	-0.021222856	0.140526608	0.540580161
BEST1	-0.035771239	0.140564265	0.540580161
RCAN1	-0.022014244	0.140657711	0.540580161
FXYD6	-0.045719294	0.140681944	0.540580161
B3GALT4	-0.026352724	0.140689034	0.540580161
AKT1S1	-0.021574248	0.140700284	0.540580161
TMEM145	-0.025542453	0.14084321	0.540780165
VDAC1	-0.015867903	0.140878627	0.540780165
DNAH6	0.043550867	0.140942099	0.540780165
FAM71E2	0.02554998	0.140947762	0.540780165
DMXL2	0.036282909	0.141120791	0.541075953
COX6C	-0.017756855	0.14112262	0.541075953
CAV1	-0.048200106	0.141196034	0.541169978
AAK1	0.040141483	0.141279028	0.541300645
GNE	-0.018700545	0.141384156	0.541515992
FPR1	-0.06294566	0.141505665	0.541793914
ELF1	-0.020075827	0.14163402	0.542026921
MTX3	0.028391176	0.141664458	0.542026921
OTUB1	-0.024527725	0.14179481	0.542064022
STX6	0.019157398	0.141818119	0.542064022
GNG8	0.060613482	0.141833741	0.542064022
CCPG1	-0.02451621	0.141926855	0.542064022

LGALS2	-0.092618491	0.141947998	0.542064022
SNORD69	0.024695093	0.141980702	0.542064022
GPSM2	-0.025480868	0.142033071	0.542064022
BZW1	-0.015574272	0.14212437	0.542064022
IFFO1	-0.017009259	0.142131353	0.542064022
ALDH4A1	-0.031291899	0.142198083	0.542064022
GTF2E2	-0.013510362	0.142212839	0.542064022
SELL	-0.035899891	0.142366457	0.542278827
ARHGAP17	-0.01755735	0.142472466	0.542278827
PCBD1	-0.025450983	0.142479556	0.542278827
MRPS31	0.014634183	0.142502192	0.542278827
SIRT2	-0.021490984	0.142535045	0.542278827
ARPC4	0.020520081	0.142563139	0.542278827
SNAPC5	-0.026096465	0.142656319	0.542297185
ZFP14	0.051018857	0.142700852	0.542297185
COPG2	0.042339228	0.142714943	0.542297185
MS4A14	0.043679165	0.142952631	0.543013957
TRUB2	-0.020827937	0.143134461	0.543184295
MRPL33	-0.026265932	0.14319945	0.543184295
TP53BP1	0.019988678	0.143205899	0.543184295
ABCA9	-0.022828758	0.14321361	0.543184295
FOXK2	-0.034439426	0.143329799	0.543184295
HIST1H1C	0.049127758	0.143343347	0.543184295
PTPN2	-0.019135905	0.143398741	0.543184295
POU5F1	0.033357802	0.143434286	0.543184295
PRSS45	0.028473333	0.14345714	0.543184295
PRCP	-0.019154516	0.143547656	0.543184295
CDC42EP2	-0.05136615	0.14358377	0.543184295
FAM74A4	0.02211157	0.143586344	0.543184295
TMEM14A	-0.032249555	0.143754442	0.543634408
COX8A	-0.01904623	0.143973946	0.543687365
ZNF442	-0.026556805	0.144069742	0.543687365
COX16	-0.016517399	0.144172509	0.543687365
ZNF697	-0.036561851	0.144180522	0.543687365
ENY2	-0.022437004	0.144226196	0.543687365
PFDN1	-0.020041754	0.144228878	0.543687365
RRP36	-0.013287831	0.144310838	0.543687365
SCCPDH	-0.025395271	0.144388719	0.543687365
RSBN1L	-0.021051396	0.144416145	0.543687365
MCMBP	-0.033037988	0.144472464	0.543687365
NR3C1	-0.022561095	0.144474642	0.543687365
TMCO1	-0.013891568	0.144494965	0.543687365
ELP4	-0.023408756	0.144514352	0.543687365
ZNF217	-0.027404693	0.144582932	0.543687365
LEFTY1	0.049437682	0.144691798	0.543687365
TBCK	-0.020780707	0.144704564	0.543687365
TLR1	-0.030668719	0.144706762	0.543687365
GRN	-0.027178464	0.14471017	0.543687365
MYLK	0.068815962	0.144736851	0.543687365
FH	-0.018137466	0.144771221	0.543687365

CCDC25	-0.017938769	0.144893315	0.543687365
TUBA1A	-0.015845081	0.144922101	0.543687365
MCOLN2	0.051850151	0.144929822	0.543687365
USP38	-0.022153809	0.144974545	0.543687365
ARFGAP2	-0.01800777	0.144996395	0.543687365
ADAP1	-0.016042736	0.145146324	0.544065242
IFT80	-0.023627464	0.145195506	0.544065353
SLC2A6	-0.026704796	0.14530237	0.544263135
H2AFY	-0.012934954	0.145352428	0.544263135
POLR3A	0.021735385	0.145395798	0.544263135
KIF1B	0.021439676	0.145494311	0.544398812
ACO1	-0.015856359	0.145532727	0.544398812
GTF2A2	-0.015463533	0.14557959	0.544398812
CNRIP1	-0.040444322	0.145701915	0.544672238
FAM3C	-0.030673774	0.145772522	0.544752211
EPN1	-0.014996343	0.146196881	0.546021656
B3GALNT2	-0.029165602	0.146210876	0.546021656
GAPDH	-0.024592284	0.146267062	0.546047256
ITFG3	-0.022518857	0.146373584	0.546200092
CLINT1	-0.015311108	0.146406692	0.546200092
FMO6P	0.02665394	0.146466766	0.546240105
UBC	-0.009669281	0.146635305	0.546402115
CLIC1	-0.018825062	0.146695356	0.546402115
AMY2A	0.02821782	0.146727886	0.546402115
ZNF564	-0.028142919	0.146788284	0.546402115
CNPY2	-0.01410079	0.146863845	0.546402115
NUDT14	-0.019472406	0.146890116	0.546402115
SKP1	-0.015757648	0.14689304	0.546402115
ZC3H3	-0.020446779	0.146920083	0.546402115
RCVRN	0.038993597	0.147056716	0.546402115
STK16	-0.02908655	0.147060702	0.546402115
PPM1H	-0.032523599	0.147098868	0.546402115
CYP4V2	0.036646598	0.147124672	0.546402115
AHSP	0.059370916	0.147151929	0.546402115
C16orf74	-0.039982656	0.147300315	0.54676968
ZNF626	0.049288577	0.147579171	0.547296006
SSBP3	0.030450733	0.147710914	0.547296006
RNF169	0.025398573	0.147822523	0.547296006
GSDMD	-0.021990111	0.147936777	0.547296006
ZKSCAN4	-0.027330438	0.147964954	0.547296006
RNF138	0.039496022	0.147967889	0.547296006
RSF1	-0.031008976	0.148000117	0.547296006
GOLGA8G	-0.027188203	0.148018823	0.547296006
KIDINS220	-0.016645824	0.148047345	0.547296006
BPGM	-0.035002384	0.148090549	0.547296006
SNAPC3	-0.020188636	0.148140394	0.547296006
SOD1	-0.014950936	0.148154482	0.547296006
PDRG1	-0.020995001	0.148154576	0.547296006
RASGRP1	-0.019359598	0.148214117	0.547296006
ARMC8	-0.023967166	0.148231251	0.547296006

KIAA1875	0.045714963	0.148233212	0.547296006
C10orf12	0.021056674	0.148288476	0.547317487
PRIM2	0.034697699	0.148346263	0.547348261
DDX39B	0.02410213	0.148487649	0.547481076
CLDN23	-0.049488407	0.14849021	0.547481076
ALPK2	0.052922989	0.148541082	0.547481076
VPS13A	0.025277503	0.148580102	0.547481076
LDHB	-0.024925988	0.148695905	0.547505324
SLC11A2	0.025468138	0.148770842	0.547505324
EHD1	-0.013600509	0.148773799	0.547505324
PIAS3	0.018698231	0.148794257	0.547505324
AGPAT9	-0.027181824	0.148833998	0.547505324
TACC3	0.02356959	0.148937536	0.547704182
D2HGDH	0.036147192	0.14900249	0.547761062
HERPUD1	-0.018505979	0.149113418	0.547871553
SLC7A6	0.031539445	0.149187539	0.547871553
GAMT	-0.025314148	0.149215213	0.547871553
SUGP2	0.022774837	0.149307829	0.547871553
KLHDC3	-0.010712003	0.149330771	0.547871553
PDPR	0.037880934	0.149420973	0.547871553
KDSR	0.023199903	0.149445095	0.547871553
GPR89A	0.031308393	0.149554121	0.547871553
PEA15	-0.018495107	0.149598982	0.547871553
TBK1	0.015599826	0.14966691	0.547871553
VIPR1	-0.031975816	0.14968287	0.547871553
ERCC4	0.026734303	0.149720178	0.547871553
ERMP1	-0.02693861	0.149737915	0.547871553
ST13	-0.018998496	0.149752638	0.547871553
ZNF148	0.033906744	0.149832402	0.547871553
RPL22	-0.01097492	0.149843516	0.547871553
FILIP1L	-0.028007121	0.149873978	0.547871553
COX6B1	-0.017201029	0.149996433	0.54813817
CAPN1	-0.018879363	0.150237161	0.548836678
ATP6V0C	-0.014188793	0.150350471	0.549069404
NAGPA	-0.018842437	0.150400929	0.549072521
PILRA	-0.029591878	0.150525066	0.549344529
MAPKAPK2	0.019862557	0.150744137	0.549593577
TATDN1	-0.032489996	0.150779291	0.549593577
DGKG	0.026983104	0.150783622	0.549593577
CPNE8	0.024846525	0.150791914	0.549593577
GFM1	-0.014739495	0.150918803	0.549874993
CCNT1	0.031247207	0.151076573	0.54993441
LRRC45	0.032854471	0.151078778	0.54993441
ETFB	-0.022113337	0.151091917	0.54993441
ACAD9	-0.023766716	0.15113384	0.54993441
PNPLA8	-0.022534775	0.151263824	0.550226511
ST6GALNAC3	-0.039617798	0.151522067	0.550962863
FKBP8	-0.024343691	0.151565807	0.550962863
DSN1	0.020401128	0.151684449	0.551173874
PAN2	0.021457022	0.151723443	0.551173874

GFM2	-0.023092861	0.152082085	0.552210407
HMGN1	-0.018468981	0.152108549	0.552210407
ROCK1P1	-0.028618404	0.152175651	0.55227288
SUMO1	-0.034010758	0.152303314	0.552361495
TAGLN	0.061437615	0.152344232	0.552361495
MLLT6	0.021139187	0.152349774	0.552361495
SFSWAP	0.017754657	0.152456377	0.552486797
ZNF425	-0.031280284	0.15248416	0.552486797
ZNF2	-0.03118824	0.152864856	0.553622142
EPB41L3	-0.035190103	0.153052675	0.553622142
MYCBP2	0.018399333	0.1530928	0.553622142
GTPBP10	-0.026999714	0.153093991	0.553622142
FBLN2	-0.040144689	0.153119434	0.553622142
IRF5	0.108513508	0.153122098	0.553622142
C12orf68	0.025098883	0.153147619	0.553622142
DYNLT1	0.018072389	0.153246262	0.553797868
AGL	0.021836691	0.153390574	0.554058221
LIG3	-0.027124571	0.153458838	0.554058221
ZNF613	-0.030574812	0.153527347	0.554058221
ARHGAP33	0.029471622	0.153533747	0.554058221
FOXC2	0.041450657	0.153568581	0.554058221
LYPLAL1	0.020038459	0.153729085	0.554343858
CCDC69	-0.030807039	0.153747913	0.554343858
FKBP15	-0.019403763	0.153886586	0.55466318
MRPL38	0.019787262	0.154010141	0.55487524
C10orf2	-0.02508312	0.154045678	0.55487524
AGPAT5	-0.026004984	0.154137327	0.555024747
CMC1	-0.030377281	0.154456682	0.555113444
ANKRD9	0.065564318	0.154481879	0.555113444
ZNF883	0.028268962	0.154485077	0.555113444
AMACR	-0.024472693	0.154536119	0.555113444
SUPT5H	0.02084087	0.154560084	0.555113444
PDXDC1	-0.013389886	0.154587254	0.555113444
CNPY4	0.020528004	0.154621786	0.555113444
MLF2	0.019157812	0.15467903	0.555113444
TUBA3D	0.05280963	0.154714867	0.555113444
FEZ2	0.027603944	0.154726521	0.555113444
INTS8	0.027676833	0.154735931	0.555113444
TOMM40	-0.012085792	0.154828128	0.555113444
SLC25A42	-0.018956152	0.154831909	0.555113444
DIABLO	-0.010004163	0.154864063	0.555113444
HIST1H3H	0.069529295	0.155011352	0.555164627
CNPY3	-0.031767558	0.155110651	0.555164627
SYBU	-0.028152948	0.155133519	0.555164627
PCMT1	-0.011925873	0.155209509	0.555164627
UBE2Q1	-0.020592744	0.155213989	0.555164627
SNORA72	0.039745157	0.155217408	0.555164627
ZNF92	-0.030651796	0.155229426	0.555164627
CCNG2	-0.029563302	0.155412255	0.555621114
SERAC1	-0.030853318	0.155457457	0.555621114

PIK3CB	-0.030129457	0.155543285	0.555683105
GOSR1	-0.021998391	0.155606073	0.555683105
MTMR6	-0.020711113	0.155625407	0.555683105
SIGLEC9	-0.027637615	0.155865036	0.556215192
BATF3	-0.034182356	0.155913251	0.556215192
KAT5	0.022651242	0.155929045	0.556215192
DYNC1LI2	0.019780084	0.155975423	0.556215192
BRMS1	-0.012644885	0.15604332	0.556271759
SOX4	-0.028512115	0.156091795	0.556271759
ATP5G1	-0.01909219	0.156147752	0.556292072
CD84	0.029379875	0.156250467	0.556300406
FAM53C	-0.019278739	0.156250607	0.556300406
TNIP1	-0.017110549	0.156464625	0.556864681
ATAD3A	-0.02466792	0.156543865	0.556864681
ACCS	-0.060431321	0.156578946	0.556864681
ZNF788	-0.024600152	0.156610331	0.556864681
FAM129C	0.05251043	0.15685628	0.557560105
ZBTB34	-0.020240402	0.156924865	0.557624825
MLST8	-0.023836659	0.157001963	0.557719747
FOXD4L4	0.027790169	0.157058825	0.557742744
CANX	-0.016241551	0.157307256	0.558260119
IGFBP7	-0.058746293	0.157309763	0.558260119
FBXW2	-0.017472364	0.157448091	0.558260119
TMEM126B	-0.012398935	0.157458096	0.558260119
LGALS7	0.027365466	0.157465599	0.558260119
SPPL3	0.02209058	0.157590086	0.558260119
OTUD3	0.027950011	0.157700194	0.558260119
LRRK1	-0.030914962	0.157734835	0.558260119
BRF2	-0.023592269	0.157788896	0.558260119
HAGHL	-0.025585741	0.157849157	0.558260119
USP2	-0.023295797	0.157934893	0.558260119
MIF4GD	-0.016757894	0.15800619	0.558260119
EPHX1	-0.025900182	0.158018867	0.558260119
POLR3K	-0.025025602	0.158022105	0.558260119
ZBTB40	0.028608499	0.158022126	0.558260119
HIGD1A	-0.020683376	0.158035794	0.558260119
CLC	0.050736422	0.158061904	0.558260119
TET1	-0.027269575	0.158323552	0.55862653
CCT6B	-0.026575154	0.158332312	0.55862653
EIF4EBP2	-0.021552815	0.158386975	0.55862653
ERI3	-0.019792788	0.158452959	0.55862653
SERF1B	0.036722778	0.158483797	0.55862653
ADRB2	-0.053234175	0.158569824	0.55862653
RUNDC3B	-0.036319731	0.158594607	0.55862653
HBP1	-0.015249968	0.158667465	0.55862653
PDZD8	-0.026128759	0.158720888	0.55862653
AKR7A3	-0.023762416	0.15873516	0.55862653
MPP7	-0.031960951	0.158826812	0.55862653
PSMD4	-0.019615941	0.158863355	0.55862653
NAA35	-0.017575092	0.158896827	0.55862653

NT5DC3	0.042913845	0.158906497	0.55862653
SMARCC1	-0.013796445	0.158922662	0.55862653
PPIB	-0.041758351	0.159001811	0.558687681
APBB1IP	0.018396732	0.159110722	0.558687681
SLA2	0.034190189	0.159117584	0.558687681
LILRB3	0.009281975	0.159143831	0.558687681
UBA52	0.007333411	0.159202787	0.558687681
FXR1	0.017111552	0.159297065	0.558687681
SSR3	-0.021654094	0.159339255	0.558687681
ZNF654	0.022918554	0.159374507	0.558687681
DPAGT1	-0.014193994	0.159394317	0.558687681
TRMT2A	-0.023866488	0.159553026	0.559066934
APPBP2	-0.012057452	0.15979202	0.559355639
ALDH3A2	-0.017915399	0.159801266	0.559355639
CCNE1	-0.026539426	0.159850282	0.559355639
ANKZF1	0.022367429	0.159939523	0.559355639
FBXO2	-0.036449753	0.159985114	0.559355639
PRRC2B	0.025679042	0.160006532	0.559355639
COL4A3BP	0.025354762	0.16006709	0.559355639
WDR7	-0.018347627	0.160083868	0.559355639
HSD17B4	-0.016603143	0.16010844	0.559355639
ZNF433	-0.028533058	0.160140755	0.559355639
NKD2	0.044224394	0.160233659	0.559503586
IFT27	0.023533169	0.160476064	0.559966061
KCNQ1	-0.029859684	0.160513881	0.559966061
ADAM17	0.02405107	0.160517871	0.559966061
EFHD2	-0.018247505	0.160732851	0.560390164
TM9SF3	-0.016995725	0.160740697	0.560390164
CASC4	-0.030408582	0.160887948	0.56072692
ZNF789	0.026153653	0.160951327	0.560771241
PI4K2A	-0.020800764	0.161115319	0.561104012
ZNF35	-0.036505452	0.161148221	0.561104012
TCF7L2	0.040722337	0.161201653	0.561113552
SUN1	0.015850883	0.161296535	0.561148309
ATP6V1H	-0.017207564	0.161313029	0.561148309
GP1BA	0.050531156	0.161381138	0.561208865
EXOSC3	-0.028113897	0.161565924	0.561393435
GPI	-0.02158019	0.161566965	0.561393435
MICAL2	-0.039323383	0.161685943	0.561393435
SLC4A2	0.026472543	0.161707752	0.561393435
SORL1	0.019372422	0.161725104	0.561393435
VRK3	-0.036978175	0.161738519	0.561393435
EXD2	-0.020808514	0.161856308	0.561626166
PCSK7	0.022758533	0.161952362	0.561783358
CD55	-0.035999183	0.162167448	0.562350667
MRPS11	-0.017520323	0.162217516	0.562350667
WDR82	0.017496474	0.162337255	0.562385428
LINC00290	-0.025081906	0.162358071	0.562385428
LIX1L	-0.020945847	0.162414984	0.562385428
KTN1	0.02616912	0.162430772	0.562385428

RPS15AP10	0.015621179	0.162539776	0.562446843
RGR	-0.026581531	0.162550136	0.562446843
RANBP6	-0.021419744	0.162734403	0.562908472
HIST2H2BE	0.058158745	0.162830514	0.563064967
MYH9	-0.015340126	0.162928271	0.563227055
INO80C	-0.015826089	0.163025171	0.563272353
TNFRSF14	-0.016192272	0.163075126	0.563272353
RTF1	-0.022867891	0.163200189	0.563272353
SLC16A3	-0.015184141	0.163263487	0.563272353
GNG2	-0.028192236	0.163321504	0.563272353
COQ7	0.02734816	0.163326495	0.563272353
STIM2	0.029681856	0.163350807	0.563272353
LNPEP	-0.024392693	0.163434693	0.563272353
HNMT	-0.040163981	0.163477012	0.563272353
B4GALT1	0.022118078	0.163479182	0.563272353
UBXN4	0.025163973	0.163511385	0.563272353
SNORA21	0.024015448	0.163557466	0.563272353
AK2	-0.024628217	0.163634594	0.563272353
CDC42BPA	0.022082163	0.163653798	0.563272353
ATP6V0A1	0.021155924	0.163810034	0.563351002
GRB10	-0.029819059	0.163814539	0.563351002
SUMO3	-0.015432868	0.163949302	0.563351002
SERGEF	0.017421499	0.163985221	0.563351002
SAMD15	-0.023646044	0.164009584	0.563351002
IRF3	0.02294682	0.164022692	0.563351002
PLEKHG4	-0.031520812	0.16403291	0.563351002
CD40	0.034927322	0.164126686	0.563498228
USP33	0.020899625	0.164350049	0.564090136
ZNF805	-0.022369816	0.164455234	0.564111684
SCLY	-0.017483334	0.164458253	0.564111684
EPC1	0.030314138	0.164560078	0.564154002
KIAA1324	0.027329315	0.164572524	0.564154002
ECE2	-0.032574373	0.164634114	0.564190403
GIMAP2	-0.024999381	0.164795623	0.564569095
ARFGEF2	-0.01959889	0.165049563	0.565008625
GPR124	-0.037594807	0.165102493	0.565008625
KRIT1	0.027407592	0.165127468	0.565008625
AIMP2	-0.016585448	0.165199637	0.565008625
DNAJA1	-0.01945757	0.165220177	0.565008625
RPS28	-0.015908526	0.165230185	0.565008625
IL16	-0.019986846	0.165413003	0.565459089
EXOSC10	0.012571762	0.165785556	0.566441176
SIRT7	0.018255028	0.165802639	0.566441176
SLC25A26	-0.036970053	0.166030278	0.56704386
NR4A2	0.049202048	0.166150102	0.567148174
SCAMP1	-0.011614196	0.166163296	0.567148174
VKORC1	-0.016764667	0.166334727	0.567558291
DMWD	0.030667659	0.166618423	0.567805496
NFKBIB	-0.018889691	0.166695296	0.567805496
PIK3CA	0.024250319	0.166717918	0.567805496

DBN1	-0.046993786	0.166815883	0.567805496
NCF1C	0.034148312	0.166832611	0.567805496
HBD	0.067439184	0.166844067	0.567805496
PPP2R1A	0.017378302	0.166845923	0.567805496
CMIP	-0.022665976	0.166856683	0.567805496
SATB2	-0.029169496	0.16690991	0.567805496
TPRKB	-0.031553183	0.166965163	0.567805496
ZNF354A	-0.027499398	0.167029827	0.567805496
ANXA7	-0.016459185	0.167056567	0.567805496
BAZ1A	0.01840052	0.167074036	0.567805496
FAM47E-STBD1	-0.028109483	0.167217474	0.568118545
LAMTOR3	-0.018568435	0.167317815	0.568285024
GALNT2	-0.025948623	0.167394585	0.56837137
SPATA2L	-0.020258227	0.167524893	0.568639388
GAS6	0.039972184	0.167719373	0.569091572
NMB	-0.02967426	0.167834178	0.569091572
XPNPEP1	0.016924332	0.167848683	0.569091572
HAL	-0.038101568	0.167880746	0.569091572
SLC45A1	-0.023764288	0.167940573	0.569091572
DNAJC4	0.017085866	0.167966588	0.569091572
P4HB	-0.016309489	0.168041442	0.569143604
HIST1H2AC	0.054616361	0.168084781	0.569143604
RGS10	0.02696612	0.168228574	0.569401322
LPL	-0.045108427	0.168292739	0.569401322
C15orf61	-0.026033205	0.168315216	0.569401322
SEC14L1	-0.028901413	0.16841907	0.56956598
PEX11G	-0.034001145	0.168546899	0.56956598
NLE1	-0.026162537	0.168563136	0.56956598
CA1	0.041426141	0.168634233	0.56956598
BTBD2	-0.015649206	0.168649274	0.56956598
MPV17	-0.01263011	0.168716082	0.56956598
RSBN1	0.020665735	0.168724081	0.56956598
ATP8A1	0.024468172	0.169011203	0.570205677
ZFYVE16	0.028952202	0.169016607	0.570205677
ERRFI1	-0.034287078	0.169370165	0.571224363
PLEKHM2	-0.014837715	0.169450369	0.571320783
CTDSPL	0.074690744	0.169667092	0.571877296
DCAF10	-0.018304827	0.169851876	0.572093384
PDCD6	-0.012464017	0.169938321	0.572093384
ADAM10	-0.03323278	0.169966647	0.572093384
LMO7	-0.021436309	0.170035527	0.572093384
PPIF	0.024929492	0.170067135	0.572093384
RP9P	0.022214983	0.170129466	0.572093384
SAR1B	-0.024928857	0.170262717	0.572093384
ARL15	-0.026469027	0.17030089	0.572093384
TNS1	0.040226451	0.170300913	0.572093384
DERL2	-0.014825981	0.170304185	0.572093384
RHBDD3	0.025633688	0.170330922	0.572093384
C15orf40	0.03101838	0.170351413	0.572093384
CLN5	-0.025708624	0.17055628	0.57249712

SHISA4	0.029882904	0.170656444	0.57249712
SRM	-0.024003826	0.170673487	0.57249712
MR1	-0.028272945	0.170678516	0.57249712
ALG10	-0.021256582	0.171051252	0.5727422
TOMM5	-0.033090596	0.171053111	0.5727422
TCEB3	-0.022988517	0.171120137	0.5727422
YTHDC1	0.019452953	0.171138338	0.5727422
POLDIP3	-0.012457598	0.171186159	0.5727422
PM20D1	-0.033552488	0.171331584	0.5727422
CHCHD5	-0.020748473	0.171356588	0.5727422
INO80E	0.022791053	0.171359774	0.5727422
TXNIP	-0.007347585	0.171439442	0.5727422
TSPAN18	0.036060587	0.171464524	0.5727422
ZNF17	-0.028214982	0.171514949	0.5727422
NOG	-0.033174425	0.17152655	0.5727422
CKB	-0.050766503	0.171544163	0.5727422
KCNJ15	-0.026299476	0.171573497	0.5727422
MEF2A	0.026407647	0.171579742	0.5727422
C20orf194	-0.023667329	0.171590971	0.5727422
SYNE2	0.036893694	0.171631211	0.5727422
SNTB2	0.02518909	0.171739044	0.572929318
C1orf50	-0.015706853	0.172186038	0.57424744
SNORA27	0.035005307	0.172255217	0.574261076
CLPP	-0.020739196	0.172293887	0.574261076
CTNNB1	0.022567669	0.172424578	0.574371153
YPEL3	0.016877435	0.172497166	0.574371153
CCNI	-0.011917313	0.172519989	0.574371153
SPTY2D1	-0.020716189	0.172534473	0.574371153
MIR7-1	0.03513723	0.172708373	0.574757839
HLA-E	-0.021349996	0.172754479	0.574757839
VASH1	-0.056576791	0.173027556	0.575238283
CERKL	-0.066222242	0.173035489	0.575238283
IFNGR2	-0.023429085	0.173054791	0.575238283
SS18	0.025037294	0.173162931	0.575424943
ADRM1	0.015087308	0.173327985	0.575800561
BTG2	-0.036762036	0.17341905	0.575930231
CMTM1	-0.023772518	0.173497706	0.576018628
NR2F2	0.026074827	0.173681889	0.576133989
VPS37A	-0.018223886	0.173707738	0.576133989
ZNF248	0.024231281	0.173736021	0.576133989
SLAMF8	-0.049462951	0.17374065	0.576133989
CSF1R	-0.020335275	0.173851295	0.57623724
MRPS28	-0.021833177	0.173883009	0.57623724
PSME3	-0.014585994	0.173927963	0.57623724
KIAA0101	0.00759188	0.174111601	0.576511939
TMEM174	0.031643196	0.174115043	0.576511939
XRCC1	-0.017787228	0.174240419	0.576638825
DSTN	-0.019996061	0.174257554	0.576638825
PPM1F	-0.018146922	0.174332496	0.576714405
GIGYF1	0.029692409	0.174483852	0.577042653

CACNA1I	0.044616172	0.174536249	0.577043532
FAM177A1	0.007704588	0.174624224	0.577085718
BANF1	-0.024037678	0.17465328	0.577085718
DOLPP1	-0.015374046	0.174770107	0.577198562
POLR1B	0.029563317	0.174791723	0.577198562
ABCA3	-0.031855403	0.174871954	0.577291278
NPFF	0.021418091	0.175121472	0.577775483
PSMD12	-0.017944175	0.175123023	0.577775483
PSMD3	-0.022530871	0.175282467	0.578129209
HIF1AN	0.018495042	0.175467392	0.578407546
ZNF131	0.009306085	0.175502598	0.578407546
SCML4	0.026642854	0.175593026	0.578407546
SKP2	0.01595422	0.175618873	0.578407546
COX7A2L	-0.013885609	0.175628129	0.578407546
PAK6	-0.029088749	0.175708518	0.578500175
CLTCL1	0.025020879	0.175838782	0.578648918
MUS81	-0.016888354	0.175967303	0.578648918
SPG7	0.022194336	0.17598987	0.578648918
TBC1D10A	0.018712371	0.176032211	0.578648918
PLSCR1	-0.054347733	0.176041885	0.578648918
GPR84	-0.027346629	0.176067355	0.578648918
ODF3B	0.026601828	0.176157894	0.578774629
NRG1	-0.10119238	0.176337476	0.579107252
CLEC1A	0.030970007	0.176363768	0.579107252
PSMF1	-0.01193409	0.176468574	0.579279552
RPL12	-0.014441284	0.176583184	0.579403751
RAB37	0.026305604	0.176611099	0.579403751
TMEM39A	-0.02776239	0.176785912	0.579805409
NR2E1	-0.025382603	0.176927113	0.580068871
ADO	-0.023638337	0.177011729	0.580068871
RNF144A	-0.026745652	0.177023457	0.580068871
GTF2F2	-0.016677147	0.177173152	0.580387575
MBD5	0.026300527	0.177251043	0.580470944
ARHGAP11A	-0.026066504	0.177478542	0.581044064
CD93	-0.026611243	0.177685434	0.581549398
CD8A	-0.052158791	0.177841154	0.581887002
UCHL3	-0.017495281	0.178145503	0.582697334
PPP2R2D	-0.013006049	0.1782367	0.582697334
MAD2L1	-0.030045038	0.178267958	0.582697334
VPRBP	-0.023720288	0.178299383	0.582697334
PCDH9	-0.025539753	0.17841051	0.58288841
PRKAG2	-0.015232723	0.178514172	0.583054992
FAM160A2	-0.021484888	0.178714414	0.583386828
PRRC2A	0.02913193	0.178721179	0.583386828
RRP1B	-0.018314382	0.179007584	0.583874098
C6orf120	-0.024198396	0.179037054	0.583874098
SMG7	0.025992723	0.179039467	0.583874098
MT1F	-0.026510387	0.179081449	0.583874098
NRBP1	0.01782621	0.179201408	0.58408509
CCDC93	0.01942113	0.179285685	0.58408509

FOXI3	0.02294954	0.179304466	0.58408509
MEX3D	0.024417375	0.179359584	0.584092742
NGRN	-0.021315176	0.179463213	0.584258326
RRAS2	-0.037414102	0.179657307	0.584718241
ADK	0.033159614	0.179809986	0.584888342
USP48	-0.027713159	0.179815252	0.584888342
GPM6A	-0.032761925	0.180067274	0.584981263
CAPS	0.028410705	0.180099876	0.584981263
TCF4	0.031522294	0.180124565	0.584981263
PIN1	-0.008991066	0.180178375	0.584981263
PDE8A	0.025053383	0.180236856	0.584981263
BZW2	-0.015169968	0.180290579	0.584981263
MGLL	0.077066448	0.180303113	0.584981263
LSM6	-0.036512579	0.180312286	0.584981263
PI4K2B	-0.015932826	0.180370521	0.584981263
SFT2D1	-0.021769732	0.180372306	0.584981263
DIP2B	0.016762317	0.180534616	0.585336165
MRPS21	0.018194407	0.180899938	0.586348878
TASP1	-0.021201576	0.181027492	0.586590548
DPM2	-0.028626554	0.18136492	0.587382412
NGLY1	0.014793318	0.181378	0.587382412
TSEN15	-0.015046846	0.181702971	0.588137293
RECK	-0.019666554	0.181811705	0.588137293
MBD3	-0.025114923	0.181848042	0.588137293
EIF5A2	0.025670411	0.181873268	0.588137293
HOXD11	-0.024507727	0.181876769	0.588137293
ENO2	0.028145735	0.182016239	0.588278611
TLR9	-0.035377119	0.182026763	0.588278611
CSRNP1	0.02251643	0.182188372	0.588534648
PAPSS1	-0.022308492	0.182212326	0.588534648
CRIP2	0.059653027	0.182346796	0.588723721
HPS3	-0.021598805	0.182377237	0.588723721
JAZF1	-0.028530498	0.182533266	0.588889416
PEAR1	0.060701293	0.18253497	0.588889416
HMBOX1	0.058514551	0.182679638	0.589184415
RPLP0	-0.021853107	0.18286037	0.589595524
SIRT1	0.015647449	0.183001521	0.589638626
C9orf135	-0.068700963	0.183002572	0.589638626
HIST1H2AM	0.048260131	0.183059698	0.589638626
CUEDC2	-0.014904877	0.183086815	0.589638626
ACBD4	0.023721168	0.183182081	0.589773839
GPR137	-0.017097797	0.183248934	0.589817519
AP2M1	0.015807745	0.183310817	0.589845186
MRPS5	0.014974395	0.183494445	0.59026446
CLEC1B	0.071647042	0.183583448	0.590371617
C7orf50	-0.01618098	0.183634427	0.590371617
PRMT10	-0.018814997	0.183692947	0.590388279
POLD3	-0.019854088	0.183957161	0.59106584
GAB2	-0.023113001	0.184080569	0.591290719
MEX3A	-0.022736666	0.184221944	0.591573164

ITM2B	-0.018418953	0.184341102	0.591784124
ARID5A	-0.0267226	0.184452125	0.591840272
DYDC2	-0.030933586	0.184465529	0.591840272
FANCA	0.028724015	0.184596494	0.59193327
FIGNL1	0.034856703	0.184601468	0.59193327
C19orf48	-0.020862402	0.184942292	0.592495472
LAMTOR2	-0.014323445	0.184959262	0.592495472
LRRC1	-0.02613542	0.185021572	0.592495472
AKIP1	-0.022953685	0.185047435	0.592495472
CEBPZ	0.01537222	0.185072543	0.592495472
DONSON	0.02502972	0.18522506	0.592495472
NUBP1	-0.013969083	0.185238922	0.592495472
KDM4C	0.01825468	0.185249699	0.592495472
LPCAT4	0.022292747	0.185258544	0.592495472
RILPL1	0.025465863	0.185317986	0.592514382
BSG	-0.04253468	0.185385316	0.592554875
VNN1	-0.070550162	0.185437717	0.592554875
DNAJB9	-0.030179232	0.185594706	0.592885367
SNORD33	0.025732946	0.185728163	0.592928495
SUPV3L1	0.021089049	0.185731799	0.592928495
S100A9	-0.018924422	0.185768906	0.592928495
CLP1	-0.018753745	0.185896578	0.593164953
IBA57	-0.021723578	0.186079958	0.593370318
EGLN1	0.03069895	0.186097074	0.593370318
UTP23	-0.024162777	0.186185155	0.593370318
GTF2IP1	-0.033132641	0.186206547	0.593370318
ANKRD34A	-0.025885367	0.186228972	0.593370318
EIF4E3	-0.036469415	0.186447442	0.593687628
SRSF9	-0.011844766	0.186447933	0.593687628
RNF207	0.029672254	0.186518719	0.593687628
SNX30	-0.021637336	0.1865431	0.593687628
MIR142	0.034836378	0.186694856	0.593999815
ANKRA2	0.017139856	0.186816345	0.594090609
GMFG	-0.011476502	0.186847518	0.594090609
TRRAP	0.016578398	0.186884407	0.594090609
ABCB1	-0.043147833	0.187023437	0.594204196
ATL2	0.021106651	0.187027502	0.594204196
NUDCD1	0.018189698	0.18723089	0.594477444
POC1B	-0.018554378	0.187287592	0.594477444
DENND2C	0.035188891	0.187331986	0.594477444
ARL8B	-0.012274471	0.187386199	0.594477444
COPZ1	-0.017970053	0.187417255	0.594477444
LGALS9C	-0.03159543	0.18745536	0.594477444
DPYD	-0.035319571	0.187616321	0.594477444
SMAD6	-0.023984923	0.187642665	0.594477444
NARG2	-0.036433114	0.187683581	0.594477444
AKT1	0.020337214	0.187725692	0.594477444
SLAMF7	-0.034769929	0.187747898	0.594477444
TLR5	0.02299053	0.187772742	0.594477444
CCND1	-0.030454875	0.187869977	0.594477444

UQCRQ	-0.015049412	0.187883849	0.594477444
IDE	-0.020064733	0.187919105	0.594477444
SNORA64	0.028458364	0.188042614	0.5946982
DUSP27	0.026934909	0.18815907	0.594767917
C1orf87	-0.053038948	0.188172125	0.594767917
HEMGN	0.057331356	0.188456641	0.595439142
FAM124B	-0.031982728	0.188492073	0.595439142
LZIC	-0.017139813	0.188604211	0.5956234
TTC37	-0.014667726	0.189011947	0.596740799
ATXN2	0.013919914	0.189193621	0.597136623
SRRD	-0.020213623	0.189245214	0.597136623
ZNF708	-0.030698859	0.189477083	0.597697871
SP140	0.025452448	0.189641237	0.597739226
C14orf80	-0.021131439	0.189647121	0.597739226
CENPB	-0.014034407	0.189735715	0.597739226
CDT1	-0.039441085	0.18973981	0.597739226
GSTO2	0.024524161	0.189760199	0.597739226
SNORD52	0.028646214	0.189892962	0.597827779
TMEM179B	-0.010765521	0.189911834	0.597827779
C2orf68	0.027040637	0.189950339	0.597827779
PYCARD	-0.020199268	0.19007143	0.598038844
CNNM4	-0.022754888	0.190204821	0.598229427
CAND2	-0.038879562	0.19025813	0.598229427
RAD17	-0.019917368	0.190294138	0.598229427
RAB24	0.016329368	0.190438822	0.59839053
SLC6A16	0.029302222	0.190453504	0.59839053
ISCU	-0.014345664	0.190545602	0.59851001
ADSL	-0.014059139	0.190652697	0.598667506
FAM83F	-0.025261293	0.190703914	0.598667506
MIR1323	-0.027020337	0.190839927	0.598852051
TGIF1	0.021649006	0.190870904	0.598852051
UNC13D	0.028149242	0.191051009	0.599111806
ATP2B1	0.020294041	0.191087272	0.599111806
ADAR	0.014039286	0.19116704	0.599111806
ZNF781	0.024792997	0.191170196	0.599111806
ERCC5	0.013151099	0.191246447	0.599181127
EIF5	-0.022517804	0.191368217	0.59939298
ZFAND1	-0.021866999	0.191481203	0.599577208
TMEM109	0.016468602	0.191604954	0.599795034
F2RL1	-0.046419465	0.191711137	0.599835383
PHRF1	-0.01854839	0.191789823	0.599835383
USP12	0.025598194	0.191824646	0.599835383
FAM53B	0.025367997	0.191834606	0.599835383
RPS3	-0.008876551	0.192003412	0.599962847
ADAM9	0.025669067	0.192030973	0.599962847
PLEKHA7	-0.023998798	0.192121114	0.599962847
PISD	0.01894167	0.192152662	0.599962847
POLR2A	0.013012189	0.192157979	0.599962847
PTCRA	0.063901956	0.192200583	0.599962847
APIP	-0.034416407	0.192414491	0.600376236

ZNF480	-0.019511034	0.192441493	0.600376236
TMEM60	-0.02050388	0.192628308	0.600686895
PTDSS2	-0.020437223	0.192654498	0.600686895
SCRN1	-0.026095266	0.192714799	0.600686895
PTK2	0.028597375	0.192797924	0.600686895
LGALS8	-0.017360441	0.192818443	0.600686895
COPS7B	0.013575175	0.192866675	0.600686895
TBC1D10B	0.02093836	0.192923963	0.600696301
LRP1	0.024328656	0.193011115	0.600798659
SDHAF2	-0.017156295	0.193265531	0.601421468
SHFM1	-0.01510374	0.193382031	0.601614867
WBP2	0.017962714	0.193486248	0.601669114
SRCAP	0.023272698	0.19350818	0.601669114
MYADM	-0.022749885	0.193722201	0.601942382
TRPM2	-0.02458637	0.193762027	0.601942382
GPC6	-0.021412652	0.193783318	0.601942382
PPP2CB	-0.020803845	0.193876459	0.601942382
SFXN1	0.027036369	0.193886265	0.601942382
EXT2	-0.01972949	0.194031598	0.601942382
TRAPPC6A	-0.018382681	0.19409127	0.601942382
LPIN2	0.020217359	0.194150317	0.601942382
CDC42	0.018478177	0.19417208	0.601942382
NOXA1	0.028652554	0.194180765	0.601942382
RCE1	-0.01704814	0.194221087	0.601942382
SNRPF	-0.016758022	0.194280988	0.601942382
TBX3	-0.019777645	0.194334863	0.601942382
ACN9	-0.02048834	0.194423369	0.601942382
PDCD2L	-0.024223248	0.194446157	0.601942382
SLC25A28	-0.01404342	0.194466163	0.601942382
PPP1CB	0.015505066	0.194525917	0.601959009
MPHOSPH9	-0.021039927	0.194699327	0.602189453
ALG8	-0.010341148	0.194709192	0.602189453
STAU1	-0.007141531	0.194909084	0.602475138
PLOD2	0.065955429	0.194910423	0.602475138
TRIM52	0.026300501	0.195293734	0.6031181
RASGRP3	0.043428324	0.195418264	0.6031181
FAM96B	-0.015073412	0.195449213	0.6031181
TTC9	-0.027073707	0.19545096	0.6031181
PTCD1	-0.022513445	0.195489065	0.6031181
SEC23IP	-0.016455513	0.195489099	0.6031181
MRPL50	-0.021825475	0.195499841	0.6031181
RBM39	0.014207153	0.195716861	0.603367628
ELMO3	0.020895095	0.195760712	0.603367628
CDC14B	0.046522344	0.195817136	0.603367628
ITGA3	0.028596175	0.195849139	0.603367628
GSTM2	0.075270684	0.19590342	0.603367628
CCR2	-0.0327245	0.195931634	0.603367628
WASF3	0.066356182	0.195977653	0.603367628
UQCRC2	-0.022400342	0.196054946	0.603367628
TMX1	-0.013809361	0.196110617	0.603367628

KIAA1147	0.01327827	0.196131465	0.603367628
UBE2V2	-0.014824546	0.196222175	0.603367628
DCUN1D3	-0.023163601	0.196257334	0.603367628
RRM1	0.017528783	0.196319455	0.603367628
ZSCAN12	-0.030346706	0.196343861	0.603367628
TAL1	0.046121771	0.196491877	0.603596478
RPS10	-0.006704538	0.196527392	0.603596478
ZNF770	-0.016578831	0.19667931	0.603835447
PAAF1	-0.016612821	0.1967932	0.603835447
ORMDL2	-0.02209777	0.196854892	0.603835447
TMEM88	0.032643011	0.196884919	0.603835447
MED1	0.014979313	0.196964903	0.603835447
SPA17	-0.024640733	0.197011187	0.603835447
PDCD11	-0.021873518	0.197165564	0.603835447
EFTUD2	0.012828875	0.19717577	0.603835447
LAIR1	0.007454981	0.197208813	0.603835447
NHLRC2	-0.028117862	0.197280788	0.603835447
NDC80	0.026336814	0.197297057	0.603835447
HERC2P7	-0.037392859	0.197304187	0.603835447
RABGGTB	0.01540794	0.197314375	0.603835447
SV2B	-0.021741174	0.197437389	0.604044902
FLOT1	-0.024431607	0.197679362	0.604618089
GCC1	-0.018284654	0.197884103	0.605077109
OSTCP1	-0.029555446	0.198022148	0.605242417
PDXK	-0.022336919	0.198136191	0.605242417
VILL	-0.02058021	0.198136231	0.605242417
MBIP	-0.018325533	0.198160177	0.605242417
ARHGEF2	0.016736959	0.198304527	0.605242417
SELP	0.073613207	0.198346905	0.605242417
ITGA2B	0.086775713	0.198357927	0.605242417
ZFP36L2	-0.026053217	0.198375597	0.605242417
HELB	0.035534661	0.198614904	0.605536041
LIN7C	-0.031114438	0.198623734	0.605536041
CXCR1	-0.032349707	0.198653892	0.605536041
RABIF	-0.019915563	0.198690659	0.605536041
LY86	-0.027556703	0.198752234	0.605556973
HPSE	-0.034844228	0.198817792	0.605590022
UBL5	-0.01283454	0.198996842	0.60596865
PLXNA4	0.032266239	0.199252233	0.606249437
CLDN15	0.021263177	0.19926894	0.606249437
FAM129A	-0.024538624	0.199300856	0.606249437
CUL9	0.02423977	0.199308131	0.606249437
DLAT	-0.016168474	0.19937505	0.60628638
CAMK1D	0.021684442	0.19960622	0.606731937
PLCG2	-0.017027937	0.199631197	0.606731937
PRPF4	0.010843637	0.199762796	0.606965244
PRRT1	-0.031145438	0.199875928	0.607142328
TATDN2	0.019959936	0.199953136	0.607210223
LMO2	-0.020698266	0.200252751	0.607617075
PPFIA4	0.059781957	0.200345933	0.607617075

GRAP2	0.040174863	0.200361581	0.607617075
OR9A4	0.020371299	0.200380401	0.607617075
NEIL1	0.029591736	0.200393231	0.607617075
KCTD9	-0.020995891	0.200416473	0.607617075
TBC1D3B	0.027783722	0.200491667	0.607678606
PPP1R3D	-0.030898085	0.200582083	0.607786224
CUBN	0.03414968	0.200941335	0.607820779
PPTC7	-0.023136108	0.200972073	0.607820779
WDR46	0.015619208	0.201052132	0.607820779
CIAO1	0.012763479	0.201070605	0.607820779
XPO7	-0.018554469	0.201097852	0.607820779
RELL1	-0.025381021	0.201127091	0.607820779
PSMB8	-0.018849397	0.201189892	0.607820779
VEZT	0.026650595	0.201221704	0.607820779
ADPGK	0.017018258	0.201269838	0.607820779
NCOA7	0.015293361	0.201276742	0.607820779
PSMB5	-0.014722975	0.201312814	0.607820779
OSTC	-0.0159684	0.201349467	0.607820779
ELAVL1	0.027367879	0.201446689	0.607820779
PARP15	0.030117197	0.201472976	0.607820779
MTRR	-0.028448108	0.201495164	0.607820779
NAGK	-0.021746292	0.201550045	0.607820779
NUP50	-0.02491563	0.201584801	0.607820779
LCP1	-0.011701162	0.201645848	0.607820779
RFWD3	0.017340725	0.201685808	0.607820779
GDE1	0.01888251	0.201709807	0.607820779
MBTPS1	0.013886813	0.201746638	0.607820779
UBLCP1	-0.01481626	0.201865924	0.608014669
ZNF366	-0.028904712	0.202173215	0.608517605
SEPSECS	0.018755555	0.202183977	0.608517605
SPIB	-0.048526222	0.202197827	0.608517605
ZBTB49	0.023692525	0.202331208	0.608643161
LONP1	0.016844825	0.202349519	0.608643161
UBXN11	0.027544338	0.202412009	0.608665723
RNF2	-0.02514789	0.202506057	0.60870951
RAD9A	0.023122116	0.202572174	0.60870951
RBCK1	-0.017893618	0.202591547	0.60870951
GLS2	0.025537428	0.202917465	0.608984057
MAX	0.036844202	0.202937866	0.608984057
ZMYM4	0.014704565	0.202940527	0.608984057
TMED6	0.0378898	0.20299235	0.608984057
CD1E	-0.045460537	0.203009637	0.608984057
MCRS1	-0.010371299	0.203013025	0.608984057
ACBD6	-0.018711789	0.203251575	0.609534456
CREG1	-0.028297523	0.203321068	0.609577708
ITK	0.022174083	0.203548758	0.609808205
ENOSF1	0.035507325	0.203572716	0.609808205
IFI27	0.074932903	0.203603378	0.609808205
GPR55	-0.044378534	0.203618315	0.609808205
SNORD71	0.030144195	0.203742188	0.609849616

ST3GAL1	0.021408505	0.203742333	0.609849616
SLC9A8	0.021465131	0.203813322	0.609897178
ISM1	0.045029483	0.204135197	0.610695269
GUCY1B3	0.048343061	0.204385576	0.611142259
PTCD2	0.028160893	0.204432731	0.611142259
CCNE2	-0.029694677	0.204467744	0.611142259
FBXL6	0.020257407	0.204588038	0.611142259
IL18BP	-0.015558588	0.204663883	0.611142259
MTX2	0.014559188	0.204670186	0.611142259
CEP350	0.020388279	0.204671095	0.611142259
MAP2K7	0.019318607	0.204781208	0.611306146
JPH2	0.02050123	0.204946918	0.611540309
ZEB2	-0.025761993	0.204970146	0.611540309
KLRD1	-0.046506419	0.20505578	0.611630942
ZBTB46	0.026003864	0.205248025	0.612039437
PDS5A	0.01334104	0.205426597	0.612276788
TGIF2	0.013409367	0.20543825	0.612276788
TMEM129	-0.022298802	0.205493646	0.612277031
TMEM80	0.020624411	0.205573478	0.612309057
RASGRP2	0.019791156	0.20561503	0.612309057
DDX19A	0.012308957	0.205776947	0.612397893
SCARNA7	0.020176103	0.205799729	0.612397893
APOBEC3D	0.024830766	0.205813176	0.612397893
SEL1L	-0.018229969	0.205866163	0.612397893
MYNN	-0.013411433	0.205966735	0.612429946
MAST2	-0.026397318	0.205987595	0.612429946
SF3B4	0.012151728	0.206160469	0.612660737
CPEB4	0.039812588	0.206244386	0.612660737
EIF4B	-0.014265207	0.206251731	0.612660737
VAV1	-0.016134711	0.206286617	0.612660737
RICTOR	-0.026175194	0.206446286	0.612970476
SPECC1	0.02736172	0.206563722	0.613154689
SUPT6H	0.01725354	0.206640433	0.613217949
SNRNP48	-0.024720266	0.207047263	0.614260562
CCDC91	-0.018304822	0.207179785	0.614327543
ACTL6A	0.012303824	0.207205803	0.614327543
SGOL1	-0.02147419	0.207261767	0.614327543
RPL26L1	-0.020741027	0.207339979	0.614327543
RNU11	-0.02981335	0.207347339	0.614327543
TRNT1	-0.026262358	0.207448253	0.614462058
ATP6V1B2	-0.013840874	0.207558947	0.614489359
DYRK4	-0.013968068	0.207568499	0.614489359
ARHGAP18	0.036010678	0.207820795	0.615067773
SH3BGRL2	0.067069697	0.207875015	0.615067773
EDARADD	0.033697298	0.208206541	0.61585261
CCDC85B	0.020358283	0.208251542	0.61585261
CBX2	0.023752663	0.208337241	0.615941485
CARNS1	-0.03365247	0.208483847	0.616210335
METTL5	-0.023556013	0.20867452	0.616609254
CASP8	0.033220464	0.208860714	0.616933553

RB1CC1	-0.011035478	0.208895741	0.616933553
CRBN	-0.013567273	0.209187984	0.617631847
ECHDC1	-0.02086113	0.209245113	0.617635775
PRMT6	-0.024292032	0.209442417	0.618053349
COX4I1	-0.011640595	0.209655069	0.618298141
ARID1A	0.017226489	0.209678198	0.618298141
INPP5E	0.052232644	0.209692946	0.618298141
PPAT	0.043585151	0.209923509	0.618813135
PLD1	-0.021970724	0.20999089	0.618846955
UBR7	-0.013637243	0.21011723	0.619054462
DDX5	-0.01076694	0.210283897	0.619380643
LRFN3	-0.020166134	0.210407974	0.619581237
CCNT2	0.016885517	0.210594906	0.619837594
ZMAT5	-0.02870814	0.210659676	0.619837594
UCP2	-0.033796088	0.210663025	0.619837594
GGPS1	-0.0115603	0.210810185	0.620064233
PADI4	-0.039974923	0.210852089	0.620064233
SNORA42	0.027373532	0.210938471	0.620120866
ZNF37A	-0.025109464	0.210983393	0.620120866
SAV1	0.030991007	0.211337417	0.620438897
SNCA	0.054488762	0.211374157	0.620438897
STK25	0.014332036	0.211411387	0.620438897
HNRNPUL2	0.011110634	0.211569412	0.620438897
TCEA2	-0.020245458	0.211825201	0.620438897
C19orf12	-0.022514783	0.211892168	0.620438897
PPP1R14B	0.018522303	0.211893344	0.620438897
KIAA0430	0.014655564	0.211924709	0.620438897
ZSCAN29	-0.033658541	0.211933485	0.620438897
ZBTB47	-0.027408432	0.211957788	0.620438897
STAG3L4	-0.024399338	0.211991752	0.620438897
PVRL3	0.033493089	0.212007722	0.620438897
LAT2	0.016389992	0.212023295	0.620438897
RAD51C	-0.025816638	0.212083047	0.620438897
ROMO1	-0.013196289	0.212148193	0.620438897
TSPYL4	0.020732064	0.212161173	0.620438897
RAB38	0.045210138	0.212177775	0.620438897
MRPS34	-0.024697949	0.212179051	0.620438897
PAQR3	-0.018927331	0.212256424	0.620438897
C19orf60	0.016275208	0.212290839	0.620438897
CD3D	-0.01642597	0.212367067	0.620438897
STRC	0.038283213	0.212372421	0.620438897
AEBP1	-0.037593462	0.212380791	0.620438897
EHBP1	0.01464048	0.212609277	0.62084391
SNORD100	0.027940308	0.212683636	0.62084391
BOD1	-0.014901161	0.212687696	0.62084391
PF4V1	0.099143019	0.212870176	0.620921441
FMNL3	-0.020173108	0.212888764	0.620921441
FOXJ3	0.01349806	0.212965827	0.620921441
MICALL1	-0.025477491	0.213150527	0.620921441
GTF2H1	0.020901127	0.213280334	0.620921441

AMIGO1	0.02220864	0.213304309	0.620921441
LACTB	-0.028858806	0.213324469	0.620921441
CCDC142	0.033614876	0.213404196	0.620921441
COQ2	-0.019875931	0.213419163	0.620921441
SNORD99	0.034997775	0.213548683	0.620921441
OCIAD2	0.01600532	0.213617722	0.620921441
NAP1L1	0.015661825	0.213705328	0.620921441
POMGNT1	-0.011875629	0.213713735	0.620921441
ZNF582	-0.023797596	0.213716956	0.620921441
FUS	-0.014462562	0.213817113	0.620921441
GLTSCR1	-0.01685516	0.213842287	0.620921441
COG6	-0.017785661	0.213881818	0.620921441
KIAA1033	-0.02782118	0.213887673	0.620921441
PRPF19	0.014401152	0.213913666	0.620921441
REV3L	-0.024486287	0.2139476	0.620921441
NT5E	-0.058612135	0.21407311	0.620921441
EIF3D	-0.015491891	0.214096626	0.620921441
NCALD	-0.032137466	0.214237909	0.620921441
RFC5	-0.018612487	0.214250903	0.620921441
NUMBL	-0.027156668	0.214267238	0.620921441
AP4E1	-0.012988544	0.214289031	0.620921441
LSM12	0.015460096	0.214303851	0.620921441
TMEM222	-0.017606189	0.214331407	0.620921441
POLR2F	-0.014387719	0.214360502	0.620921441
RPL36AL	-0.014353081	0.214397122	0.620921441
PPM1G	-0.010001258	0.214501954	0.621062549
KIAA0100	-0.014942816	0.214654918	0.621118791
MSLN	0.120470422	0.214668504	0.621118791
RYK	0.013167553	0.214818261	0.621118791
SHISA3	-0.023667013	0.214859447	0.621118791
CDC42EP1	-0.026164874	0.214859772	0.621118791
RAB11FIP5	-0.047509947	0.214896441	0.621118791
ATAD1	-0.019967536	0.214914172	0.621118791
PDS5B	-0.015654582	0.215130689	0.621582249
BCL11A	0.025669078	0.215319272	0.621696644
RPL13	-0.025543157	0.215430393	0.621696644
SEPHS1	-0.012793356	0.215465612	0.621696644
TECPR1	0.033854795	0.215503725	0.621696644
PON2	0.024919071	0.215505694	0.621696644
RBM38	0.024053212	0.215529293	0.621696644
SLTM	0.013781743	0.21556344	0.621696644
GTF2H2	-0.041119383	0.215624894	0.621711893
FOLR2	-0.028455104	0.215732794	0.621741564
NR1H2	0.018618507	0.215747524	0.621741564
OASL	0.048970364	0.215968645	0.622216797
PDLIM1	0.045261829	0.216079064	0.622372928
PCDH15	0.021392025	0.216177355	0.622494054
RTEL1-TNFRSF6B	0.022365245	0.216518944	0.622904619
UBTD1	0.027576701	0.216532762	0.622904619
MGAT2	-0.021004505	0.216581054	0.622904619

PRC1	-0.024863862	0.216597134	0.622904619
TSEN34	-0.013996205	0.216613475	0.622904619
CCDC71	-0.035065812	0.216657583	0.622904619
MTX1	-0.015486116	0.216759578	0.623036035
TMEM9B	-0.009799632	0.216900905	0.623155164
GXYLT1	-0.016649329	0.216925372	0.623155164
ZFYVE19	0.019330358	0.217005849	0.623155164
CPSF3L	-0.015443977	0.217026214	0.623155164
HIC2	-0.014463861	0.217109795	0.623233485
GPR18	-0.047597751	0.217323611	0.623549632
RNF11	0.038260944	0.217332594	0.623549632
WNK1	0.012062889	0.217454323	0.623583917
PRSS23	-0.053495911	0.217702779	0.623583917
GEMIN6	-0.02239941	0.217753471	0.623583917
SLC25A22	-0.016829879	0.217769362	0.623583917
SLC37A2	0.032695026	0.217815716	0.623583917
ZNF791	0.023578945	0.217846202	0.623583917
CEBPA	-0.024583751	0.217887399	0.623583917
SNHG9	0.017252173	0.217910289	0.623583917
ZNF304	-0.022105656	0.217948168	0.623583917
ARHGAP22	0.036668968	0.217969917	0.623583917
GHRHR	-0.022082802	0.218016592	0.623583917
EME1	0.02109022	0.218020576	0.623583917
FBXO5	-0.02157429	0.218329077	0.624069621
HN1	-0.023984759	0.218337945	0.624069621
RPA1	0.027855009	0.218361872	0.624069621
POLR2G	-0.00922378	0.218415911	0.624069621
PGM2L1	0.024844305	0.218483853	0.624102651
ENPP2	-0.032892629	0.218672631	0.624380028
CCDC43	-0.014581996	0.218726204	0.624380028
GJA5	0.021493679	0.218750181	0.624380028
KLHDC2	-0.017052495	0.218881767	0.624594556
FASLG	-0.057978837	0.218947931	0.624622333
CARD11	-0.017389266	0.219091783	0.62487167
MADD	-0.019129867	0.219299078	0.625297414
FBXO32	0.019218436	0.219414139	0.625297414
MCM3AP	0.014166313	0.219459471	0.625297414
MTHFD1L	-0.027172965	0.219467021	0.625297414
BBS12	-0.027087808	0.21968507	0.625462666
CCDC57	-0.025074496	0.219767117	0.625462666
STYX	0.015992417	0.2198538	0.625462666
GORASP2	-0.013194074	0.219875239	0.625462666
QRSL1	-0.032922288	0.219915152	0.625462666
ATG14	0.014927412	0.21996425	0.625462666
CBX8	-0.019743612	0.220004359	0.625462666
GOLGA7B	-0.027553379	0.220102845	0.625462666
GRB2	-0.021256977	0.220121847	0.625462666
SPRYD3	-0.020355268	0.220237675	0.625462666
POGZ	0.04523746	0.220237816	0.625462666
ALDH1A1	-0.040779267	0.220331314	0.625462666

DDX20	-0.02255781	0.220347314	0.625462666
FAM168A	-0.013888628	0.220347687	0.625462666
STRN3	0.021148686	0.220372608	0.625462666
AHCTF1	0.018101269	0.220529953	0.625580032
INTS4	0.01337671	0.220531738	0.625580032
ACACA	-0.016349898	0.220606902	0.625580032
CXCR2	-0.036867817	0.220640026	0.625580032
CIAPIN1	-0.016052347	0.221452177	0.627721933
ZNF627	-0.015435738	0.221646871	0.628112959
DNAJC3	-0.026054564	0.222067192	0.629121905
C6orf25	0.056668679	0.222116578	0.629121905
IGSF8	-0.02374725	0.222270734	0.629159473
ZNF91	0.034343989	0.222282453	0.629159473
ARL1	-0.013102693	0.222300361	0.629159473
TMCO4	-0.021242174	0.222389103	0.62924974
IGHMBP2	0.025640813	0.222616829	0.629733115
RNPC3	0.018108931	0.222744248	0.629932571
RBM23	-0.01681225	0.223020008	0.630402856
SNORD96A	0.023317744	0.223024445	0.630402856
ITGB2	-0.008748902	0.223391714	0.631056218
PEX11A	-0.025024277	0.223424894	0.631056218
C19orf54	-0.021337213	0.223426625	0.631056218
BIVM	0.022856974	0.223486186	0.631063415
CLIC3	-0.054640871	0.223657873	0.631375068
ZBTB41	-0.02246105	0.223715692	0.631375068
C19orf44	-0.024265657	0.223796019	0.631375068
HCP5	-0.028456797	0.223857955	0.631375068
METTL1	-0.018489233	0.22392887	0.631375068
VPS13C	0.03415701	0.223996522	0.631375068
OSBP2	0.071421191	0.224007786	0.631375068
TFRC	0.019004649	0.224076767	0.631375068
TMEM194A	0.026378378	0.224109914	0.631375068
DRAM2	-0.019629364	0.224280286	0.631694271
SRP54	-0.013601306	0.22436352	0.631756333
ARFGAP3	-0.016554645	0.22441647	0.631756333
SFXN5	0.024543423	0.224617413	0.632161238
CD14	-0.013174872	0.224877354	0.63235657
ZNF384	0.013208738	0.224887636	0.63235657
GRB14	0.060443157	0.224901592	0.63235657
ENSA	-0.025547836	0.224915333	0.63235657
ANKRD50	-0.021523804	0.224996692	0.632368472
ABHD13	0.02094937	0.225054014	0.632368472
CD2	-0.020399428	0.225090955	0.632368472
NUP133	-0.017100645	0.225423023	0.633140685
CES1	-0.084756239	0.225508477	0.633220023
IFT74	0.01605583	0.225609208	0.633342207
ZNHIT1	-0.015327993	0.225682569	0.633387515
JUND	0.010398967	0.225807214	0.633495431
ONECUT2	-0.01932883	0.225859133	0.633495431
PPIP5K1	0.019352952	0.225918461	0.633495431

ZNF782	0.029645452	0.226152292	0.633495431
CAMK1	0.022970509	0.226212127	0.633495431
GPR171	0.02748457	0.226246168	0.633495431
NUCB2	-0.0249158	0.226286945	0.633495431
CDK2AP2	-0.024482235	0.226338376	0.633495431
SH2B1	0.018437444	0.226385583	0.633495431
RABGEF1	0.020308575	0.226437525	0.633495431
ANAPC10	-0.022000996	0.226560061	0.633495431
SPATA7	-0.02020854	0.226590765	0.633495431
ZNF134	-0.026398364	0.22670432	0.633495431
GPC5	0.019726822	0.226802174	0.633495431
SNORD12C	0.036170672	0.22687768	0.633495431
GPR114	-0.035972825	0.226894589	0.633495431
CAMP	-0.040137765	0.226927936	0.633495431
RHOG	-0.018959627	0.227059485	0.633495431
MT1G	-0.029624979	0.227060272	0.633495431
RNF7	-0.011777818	0.227063095	0.633495431
RPS17	-0.015708701	0.22716868	0.633495431
RNF40	0.01881264	0.227215471	0.633495431
C9orf38	0.046551932	0.227227627	0.633495431
DNAJA4	0.021749768	0.227228008	0.633495431
EDEM3	0.016789045	0.227281518	0.633495431
MARCH2	0.041530203	0.227289188	0.633495431
GAS8	-0.022323134	0.227294417	0.633495431
C17orf58	-0.022470745	0.227342023	0.633495431
SLC39A6	-0.02536458	0.227380734	0.633495431
FRMD8	-0.013293257	0.227489736	0.633639628
AXIN2	0.035323225	0.227709437	0.633951093
ELMOD3	0.024603476	0.227716103	0.633951093
MAGEL2	0.018970312	0.227797252	0.634017546
MIIP	0.028232975	0.22788738	0.634022409
CLECL1	-0.058882572	0.227913557	0.634022409
KLHDC10	-0.030049368	0.228339058	0.634919702
FERMT3	0.018580679	0.228405353	0.634919702
RUNX1	-0.014682255	0.22840819	0.634919702
LENG1	-0.020036472	0.228474286	0.634943981
MAP3K1	0.014129198	0.22859371	0.635116409
SLC30A5	-0.015215241	0.228791682	0.635506932
CXXC1	-0.015492901	0.22893957	0.635555008
PRKCH	-0.018940484	0.22895426	0.635555008
C15orf57	-0.015939815	0.229077313	0.635555008
TOE1	-0.016358566	0.229097769	0.635555008
PAG1	0.007957337	0.229144007	0.635555008
MED7	-0.017593029	0.229168663	0.635555008
REPIN1	-0.0171236	0.229210913	0.635555008
CRYZ	0.036576662	0.229268814	0.635556348
JKAMP	-0.016830797	0.229349374	0.635620485
TOPORS	-0.020381739	0.229434407	0.635696984
HBS1L	0.039483262	0.229516464	0.635717775
RNF214	0.022190466	0.229556775	0.635717775

IQGAP1	-0.015995459	0.229638022	0.635783709
RPL13P5	-0.024564421	0.229782489	0.635825154
EFCAB7	0.021254544	0.229794149	0.635825154
CHAC2	-0.029806545	0.229825317	0.635825154
GMEB1	0.020668115	0.229947046	0.635871176
ST3GAL6	-0.02947137	0.229961138	0.635871176
NPRL2	-0.014635661	0.230014291	0.635871176
ANKLE2	0.014676336	0.23015761	0.63610851
SLC41A1	0.018732851	0.230361824	0.636384344
HTT	0.0225991	0.230372397	0.636384344
CADM1	-0.048240507	0.230501234	0.636581376
BATF	-0.026872381	0.230738878	0.637078733
YWHAH	0.017173264	0.230889055	0.637188839
FBLN1	0.055939602	0.23092672	0.637188839
SPATA2	-0.026502389	0.230951452	0.637188839
ABHD14A	-0.019464098	0.231416941	0.6381479
PSTPIP2	0.023322268	0.231476286	0.6381479
SMPD1	-0.021829029	0.231526391	0.6381479
EIF2B3	-0.016153205	0.231610724	0.6381479
AGA	-0.022073023	0.231716927	0.6381479
WDR6	0.016834144	0.23171732	0.6381479
DNAJB1	0.016469122	0.231861144	0.6381479
EFCAB11	-0.026054337	0.231908249	0.6381479
MON2	0.016424496	0.231938034	0.6381479
SH3GL1	-0.016135649	0.231950992	0.6381479
PDIK1L	-0.01922253	0.231968922	0.6381479
ST6GALNAC4	-0.019318252	0.231990889	0.6381479
CAPG	0.030071887	0.232126649	0.638178376
MRPL45	-0.012262433	0.232234047	0.638178376
RNF25	0.018249459	0.232259813	0.638178376
MIR555	0.031298908	0.232287829	0.638178376
TRMT1	0.011749632	0.232361504	0.638178376
ZNF585A	-0.021215386	0.232528034	0.638178376
DDX11L2	0.081504743	0.232537399	0.638178376
SNTB1	-0.022336371	0.232573735	0.638178376
SFXN3	0.017330839	0.232604145	0.638178376
RNF181	-0.013235458	0.232749156	0.638178376
RALB	-0.019719415	0.232837104	0.638178376
TMEM180	-0.020529994	0.232925662	0.638178376
HMGXB4	-0.012787284	0.233055119	0.638178376
SMG6	-0.023025223	0.233113351	0.638178376
PCGF3	0.027604455	0.233128973	0.638178376
RRP1	-0.02524226	0.233161321	0.638178376
TOM1L2	-0.0201858	0.233165216	0.638178376
PLEK2	0.018053804	0.2332153	0.638178376
TPBG	-0.027694388	0.233278082	0.638178376
STK11IP	-0.019632919	0.233315594	0.638178376
NARS2	-0.017954495	0.233419406	0.638178376
ADCY4	0.023449621	0.233493535	0.638178376
NPHP3	0.023932583	0.23353495	0.638178376

RSAD1	0.014989238	0.233546136	0.638178376
FANCF	-0.020431202	0.233554863	0.638178376
POLR2B	-0.011733159	0.233591969	0.638178376
CUL2	-0.012111023	0.233594869	0.638178376
PDK2	-0.020346388	0.233622602	0.638178376
TMEM177	-0.020175521	0.23367395	0.638178376
CALHM2	-0.023446016	0.233774833	0.638243769
KLRK1	-0.022246745	0.233813215	0.638243769
PPA1	-0.025214634	0.234006821	0.638614768
MOS	-0.024334784	0.234150807	0.638646717
STK11	0.0213803	0.234162758	0.638646717
DYRK2	0.024991906	0.234233672	0.638646717
SHMT1	-0.032381905	0.234255698	0.638646717
EXO1	-0.022282156	0.234350563	0.638646717
RPL4	-0.013670359	0.234364709	0.638646717
NMI	-0.028118753	0.234497801	0.638849956
CELF1	0.024674604	0.234554722	0.638849956
MARCH1	-0.037639845	0.234651395	0.638956037
KIF1C	-0.01861168	0.234786474	0.63916662
ARSK	-0.023350645	0.234919134	0.639230979
S100A13	-0.018183497	0.234952607	0.639230979
SRP9	-0.012084245	0.235011357	0.639230979
TOPBP1	0.016911212	0.235041114	0.639230979
ZDHHC19	0.030657155	0.235155875	0.639306669
TBC1D2	-0.024252539	0.235184457	0.639306669
SAMD10	0.0213939	0.235410076	0.63976286
TRMT5	-0.012846213	0.235494798	0.639836012
DCXR	0.02501439	0.235731161	0.639946109
RSPH4A	-0.025371894	0.235744857	0.639946109
RYR1	-0.03199456	0.235758385	0.639946109
ISOC1	-0.017447999	0.235766576	0.639946109
RPL18A	-0.0154515	0.235893144	0.640132681
CD163	-0.041608371	0.235951369	0.64013375
TSTA3	0.017908381	0.236153376	0.640524802
SNAP23	0.025388234	0.236556708	0.641461589
CHST15	-0.019869348	0.236630509	0.641504556
BCAR3	-0.024270099	0.237037477	0.642340105
LPAR6	0.023323622	0.237054777	0.642340105
C2orf49	-0.022504309	0.237343015	0.642842058
LY6G6F	0.059741785	0.237356174	0.642842058
AIF1L	-0.019888392	0.237539384	0.643047314
PAXIP1	-0.015017818	0.237586228	0.643047314
CHD2	0.017590529	0.237637802	0.643047314
ERBB2IP	0.022049009	0.237664339	0.643047314
PAWR	0.027574708	0.238034705	0.643372282
STMN1	-0.027175151	0.238105696	0.643372282
DDX46	-0.014335715	0.238135122	0.643372282
NUCB1	-0.014119306	0.238189484	0.643372282
RAB40B	-0.019212493	0.238205258	0.643372282
MLKL	0.016022856	0.238271353	0.643372282

DAZAP2	-0.012564042	0.23832208	0.643372282
ALK	-0.025386041	0.238443837	0.643372282
ELMO1	0.017232677	0.238495722	0.643372282
CCDC51	-0.019638396	0.238498196	0.643372282
UGGT2	-0.026445274	0.238534224	0.643372282
PHC3	-0.019345436	0.238609451	0.643372282
FHL2	0.052693674	0.238684107	0.643372282
PDE3B	-0.025384745	0.238705221	0.643372282
HSPBAP1	0.024032332	0.238709676	0.643372282
SUSD5	0.023581879	0.238714424	0.643372282
SLC12A2	0.022571773	0.23880289	0.643454037
CLEC12B	0.038804402	0.238873009	0.643486332
NBR2	-0.029448155	0.239087107	0.643906371
DPY19L1	-0.016613288	0.23919018	0.643997221
HRASLS2	-0.059376356	0.239237201	0.643997221
ACPP	-0.039291992	0.239354847	0.644148273
ZFAND2B	-0.011788219	0.239409702	0.644148273
SMAD7	-0.032616908	0.239580101	0.644450094
TMEM114	-0.024868769	0.239809786	0.644754967
ZBTB5	-0.016174656	0.239899617	0.644754967
ACTR3	-0.014996611	0.239972364	0.644754967
PCDH10	-0.025254052	0.239978093	0.644754967
C3AR1	-0.039284421	0.239984684	0.644754967
PNKP	-0.016233943	0.240070907	0.644808699
ODC1	0.033279248	0.240185663	0.644808699
JMJD6	-0.021083714	0.240333441	0.644808699
L3MBTL2	-0.017319667	0.240358976	0.644808699
ZNF800	-0.010890785	0.240429474	0.644808699
IRF7	0.028524306	0.240439363	0.644808699
SEPW1	0.011664423	0.240461797	0.644808699
MFF	-0.10615705	0.240470712	0.644808699
TTLL1	-0.0212484	0.240554032	0.644819653
PLA2G2C	-0.021746756	0.240598194	0.644819653
ALKBH7	-0.023919426	0.240649561	0.644819653
LSM14A	0.01154116	0.240843238	0.645160112
PPIG	0.014198516	0.240907084	0.645160112
CYP27B1	0.018449076	0.241017104	0.645160112
UBE2L6	-0.020083913	0.241032517	0.645160112
AVIL	0.020974485	0.241117164	0.645160112
BBS10	-0.027892228	0.241167062	0.645160112
HOXB2	-0.062445949	0.241241318	0.645160112
SRF	-0.012898247	0.241313412	0.645160112
TNFRSF19	0.020620193	0.241372625	0.645160112
RHBDD1	-0.015714212	0.24142731	0.645160112
EIF3A	-0.008795953	0.241445687	0.645160112
PRTFDC1	0.042824478	0.241476045	0.645160112
PPARG	-0.029920357	0.241710433	0.645276445
ARL6IP5	-0.011733296	0.241741128	0.645276445
TAPBPL	-0.023593551	0.241792351	0.645276445
EIF4E	-0.024450826	0.241827917	0.645276445

AP1G2	0.016862124	0.24185649	0.645276445
RUVBL1	-0.020149562	0.241869362	0.645276445
TPD52L2	-0.013424592	0.241980348	0.645416981
FMNL1	-0.023664842	0.242097582	0.645573995
CHCHD2	-0.016673541	0.242272943	0.645573995
ZNF202	-0.020031268	0.242311432	0.645573995
PSKH1	-0.02061842	0.242350769	0.645573995
MAP4K3	0.019348147	0.242408929	0.645573995
KANK1	0.058337481	0.242508896	0.645573995
ANG	-0.04349659	0.242547981	0.645573995
P2RY14	0.029820776	0.242552907	0.645573995
SNORA3	0.030095427	0.242618979	0.645573995
POGK	-0.01285815	0.242681461	0.645573995
PPAP2A	-0.024149889	0.242720038	0.645573995
UBA5	-0.016933141	0.242758922	0.645573995
ARF3	-0.020600172	0.242806917	0.645573995
CACNA1H	-0.03655455	0.242855734	0.645573995
CCNI2	0.027403219	0.243016324	0.645774224
H3F3C	-0.020555555	0.243047739	0.645774224
GZMB	-0.039014588	0.243184027	0.645856777
PRKD3	-0.021081716	0.243250528	0.645856777
ANKS1A	-0.01252569	0.243253853	0.645856777
NDUFA7	-0.01600329	0.24337566	0.646025224
AHSA2	0.026224877	0.243475117	0.646050096
DHX40	-0.012623512	0.243501762	0.646050096
SULT1B1	-0.032345049	0.243638478	0.646257923
RBM27	0.012761776	0.243861897	0.646524249
ATF1	-0.022373247	0.243927436	0.646524249
ZNF358	-0.01952391	0.24402333	0.646524249
IFNAR2	0.024546044	0.244071106	0.646524249
NOL12	-0.013505457	0.2440714	0.646524249
GOLGB1	-0.035183569	0.244096229	0.646524249
BBX	0.014671299	0.244147742	0.646524249
FGFR3	0.027604185	0.244318524	0.64652807
FCRLB	-0.030003877	0.244351756	0.64652807
TRAF3IP3	0.012240669	0.244354339	0.64652807
SSPN	-0.051188885	0.244382821	0.64652807
SLC38A6	0.016125017	0.244461107	0.646580644
NUP98	-0.025781932	0.244550629	0.646662904
FAIM3	-0.018677272	0.244775857	0.646937049
MAN2B1	0.021320917	0.244818812	0.646937049
CAPZA1	-0.035074964	0.244876705	0.646937049
JAM3	0.064196429	0.244888087	0.646937049
RNF41	-0.01491946	0.24507768	0.647072315
SRGAP2	0.023988485	0.2451396	0.647072315
TULP3	-0.020638188	0.245182155	0.647072315
ZKSCAN2	-0.019012422	0.245336849	0.647072315
RANGAP1	0.011766341	0.245437097	0.647072315
C8orf48	-0.019194997	0.245438215	0.647072315
NDUFAF4	-0.025885297	0.245506711	0.647072315

TAPT1	0.018258598	0.245508547	0.647072315
H2AFJ	0.024683417	0.245533143	0.647072315
CBWD5	0.030205516	0.245538045	0.647072315
NSFL1C	0.023266761	0.24580635	0.647072315
MRPS36	-0.014695669	0.245824528	0.647072315
ITGB3	0.081889722	0.245836043	0.647072315
CSRP2BP	-0.016096564	0.245916176	0.647072315
HERC6	0.024614397	0.245983953	0.647072315
PDGFD	-0.040390798	0.246059142	0.647072315
USP45	0.021397074	0.246145017	0.647072315
FAM134B	-0.026870644	0.246177258	0.647072315
ST3GAL5	0.017827291	0.246178827	0.647072315
CLASP1	0.014365705	0.24618382	0.647072315
H6PD	0.030045122	0.246295387	0.647072315
ACPT	0.026615414	0.246326246	0.647072315
GSTM3	0.090776873	0.246399053	0.647072315
PNPLA2	-0.025378833	0.246405283	0.647072315
MGRN1	-0.025511328	0.246443146	0.647072315
LTA4H	-0.020418598	0.246459199	0.647072315
CALN1	-0.018473951	0.246739502	0.647654623
SLFN13	0.031377209	0.247153522	0.648449492
PCMTD1	0.013013229	0.247159491	0.648449492
OAF	-0.022806937	0.247302844	0.648627466
KAT2B	0.013819876	0.247397179	0.648627466
SLC25A25	-0.013554477	0.247431066	0.648627466
POLR2E	0.026089833	0.247508324	0.648627466
KCTD2	0.015569553	0.247544308	0.648627466
CENPW	-0.036105204	0.247578918	0.648627466
RSL1D1	-0.014387472	0.247732043	0.648875055
ATG16L1	0.015414795	0.247898738	0.649092287
HELQ	0.015212949	0.248021518	0.649092287
MKRN1	0.00883544	0.248115238	0.649092287
ERLEC1	-0.016933597	0.248158529	0.649092287
PGAM5	-0.018555251	0.248159228	0.649092287
RPS13	-0.017510243	0.248180068	0.649092287
BRMS1L	0.016554291	0.248227206	0.649092287
WDR52	0.025299168	0.248284104	0.649092287
OTOF	0.065337231	0.248384656	0.649201833
ARL13B	-0.019589551	0.248487963	0.649318523
ZNF561	-0.021086766	0.248599481	0.649456609
HEATR2	-0.013368368	0.248937173	0.650185364
IPO9	0.019753579	0.249139012	0.650216047
CPEB3	0.016494457	0.249139584	0.650216047
BAD	0.018101983	0.24914515	0.650216047
DRD4	0.03677422	0.249183889	0.650216047
LEPREL1	0.030475845	0.249353761	0.650392199
LAX1	0.029871032	0.249368912	0.650392199
PTRH2	-0.020154046	0.249491338	0.650507785
ERBB2	-0.044010913	0.249548359	0.650507785
TMEM30A	0.017481207	0.249589535	0.650507785

INADL	0.024257002	0.249748231	0.650618312
KCNK17	0.047405267	0.249749499	0.650618312
CAPN12	-0.046547487	0.249818732	0.65064554
PHB2	0.012728077	0.249984355	0.650798663
WDR86	0.040015619	0.249995114	0.650798663
SLC22A18AS	-0.035024284	0.250169791	0.650961249
EIF1B	-0.014751502	0.250215921	0.650961249
MRPL13	-0.017164343	0.250278776	0.650961249
SLC46A2	-0.034224498	0.250292806	0.650961249
PROCA1	0.020508843	0.250398899	0.651084193
PRDX1	-0.01268929	0.250462221	0.651095895
C12orf43	-0.021918302	0.250563557	0.651206389
ATP13A3	-0.019898174	0.250694384	0.651287845
TM2D1	-0.010351688	0.250809143	0.651287845
FEM1C	0.015085118	0.250844275	0.651287845
MAPK6	-0.015620069	0.250864982	0.651287845
JAG2	-0.025698609	0.250889093	0.651287845
ASTE1	-0.023619152	0.251026097	0.651490706
TEP1	0.02575238	0.251117469	0.651521841
PPCS	-0.015292165	0.251155813	0.651521841
GALNT1	-0.016741166	0.251334788	0.651828216
ZNF729	-0.027233683	0.251507569	0.651828216
DPP7	-0.019008977	0.25158025	0.651828216
MAN1A1	-0.025622368	0.251582781	0.651828216
HACL1	-0.010052061	0.251593747	0.651828216
TRAF6	-0.02076129	0.251627244	0.651828216
ATP6V0D1	-0.025933232	0.251693033	0.651846088
C19orf43	0.014792452	0.251799487	0.651935641
DCUN1D2	-0.019228587	0.251845406	0.651935641
SURF1	-0.012076581	0.251992334	0.652078789
U2AF1L4	-0.028578437	0.25205607	0.652078789
CAP1	-0.010418453	0.252166853	0.652078789
SRGAP1	-0.020630537	0.252180087	0.652078789
CBX7	0.018568146	0.252195257	0.652078789
PREP	-0.012954478	0.252327134	0.652164987
KLHL18	-0.014850803	0.252375982	0.652164987
TGFBI	-0.019099586	0.252405349	0.652164987
TTF2	-0.016865322	0.252553356	0.652382092
FSCN1	-0.030006127	0.25260725	0.652382092
TMEM87B	-0.021480889	0.252730689	0.652511098
STAT6	-0.027743499	0.252775101	0.652511098
CAND1	-0.015672864	0.252896586	0.652663113
IL7R	0.017277711	0.252994694	0.652663113
GALNT12	0.017685061	0.253010879	0.652663113
PTPRF	0.050021538	0.253092335	0.652721123
MYO1F	0.017952564	0.2531768	0.652786862
GLRX2	-0.02128064	0.253249327	0.652821797
FARS2	0.016326204	0.253460503	0.653146256
G3BP2	-0.011087436	0.253573786	0.653146256
ACD	-0.020017097	0.253601173	0.653146256

TTC26	-0.018695095	0.253611221	0.653146256
ARL3	0.020566361	0.2537347	0.653312257
WDR36	-0.013913714	0.253958053	0.653735276
SLC25A37	0.033038172	0.254220009	0.654257447
DTWD1	-0.019956474	0.254280478	0.65426095
PSME2	-0.017836563	0.254344596	0.654273839
SENP7	0.015243955	0.254487749	0.654489986
CTCF	-0.01166968	0.254584623	0.654587038
GNGT2	-0.025897931	0.25466568	0.654616316
GCNT2	0.033768403	0.254750335	0.654616316
EXOSC2	-0.017667492	0.254786802	0.654616316
WNT10A	0.029166626	0.254832569	0.654616316
TK1	-0.029545864	0.254915788	0.654678158
AIRE	0.011472058	0.255126001	0.655035499
PVRIG	-0.021101522	0.25522978	0.655035499
ZNF222	0.02302087	0.255232461	0.655035499
TMEM156	0.023767774	0.25535742	0.655130019
DNAJC21	-0.016428773	0.255430624	0.655130019
ZNF207	-0.011696588	0.255446848	0.655130019
MTO1	-0.012793916	0.255513405	0.655148919
TREML1	0.061715567	0.255646095	0.655337339
SEC11C	-0.020819349	0.255838318	0.655606447
PPP2R2B	-0.039375457	0.255869532	0.655606447
LIPN	-0.036114736	0.256043059	0.655757734
EIF2B2	-0.020215647	0.256050061	0.655757734
ANTXR1	-0.019008884	0.256106305	0.655757734
JSRP1	-0.038128084	0.256275437	0.655767968
UQCRH	-0.013835035	0.256276915	0.655767968
EEA1	-0.017428378	0.256331671	0.655767968
CHEK2	0.015751629	0.256347276	0.655767968
SYPL1	-0.017363574	0.256424905	0.655814988
ATRN	-0.01505548	0.256523562	0.655915757
ALG3	-0.012044373	0.256643634	0.65607122
FAM89A	-0.027071948	0.256729082	0.656138123
PTPRU	-0.036649055	0.256875598	0.656276323
CLSTN1	0.016922971	0.256901735	0.656276323
RMND1	-0.022196166	0.25710219	0.656636857
AGPAT2	-0.032212239	0.257283749	0.65689858
GRAP	-0.020048026	0.257433974	0.65689858
PSMC6	-0.012352228	0.257538928	0.65689858
OSTF1	-0.013798206	0.257602886	0.65689858
ZFP2	0.020895099	0.257643344	0.65689858
CETN1	0.034084126	0.257667642	0.65689858
SELK	-0.022138393	0.257712666	0.65689858
HPS1	0.026550287	0.257783763	0.65689858
SLC9A3R1	-0.018336457	0.2578014	0.65689858
FAM193B	-0.020943571	0.257885169	0.65689858
SNORD35A	0.037866113	0.257994081	0.65689858
EIF2B5	-0.014752893	0.258016985	0.65689858
GBP2	-0.020201965	0.258037629	0.65689858

DDX60L	0.025519176	0.258130602	0.65689858
FAM50B	-0.030676688	0.258158128	0.65689858
RABL2B	0.015709021	0.258210133	0.65689858
MST1	-0.021131931	0.258213544	0.65689858
ZBTB7A	0.01847997	0.258326731	0.65703552
TAF5	0.015607899	0.25843229	0.657071244
UBA2	-0.017433819	0.258459499	0.657071244
CHD7	0.021185547	0.258601554	0.657281423
SRPRB	-0.014320767	0.25885075	0.657676327
RPRD1B	-0.016194843	0.258875757	0.657676327
LYPLA2	0.019491054	0.259003689	0.65783855
PLAU	-0.016777022	0.259058473	0.65783855
AKR7A2	-0.022573053	0.259193268	0.658016107
H1F0	0.037627612	0.259271548	0.658016107
FUK	-0.011516005	0.259313079	0.658016107
NDUFA5	-0.026071915	0.259366183	0.658016107
PLIN3	-0.015636213	0.259504399	0.658215901
TANK	0.014618263	0.259601581	0.658311545
LIPC	-0.058925175	0.259734026	0.658346567
NSUN2	0.014791781	0.259790835	0.658346567
ITGA7	0.018098229	0.260153298	0.658346567
RMND5B	-0.020201024	0.260209155	0.658346567
NT5C1B	-0.009009513	0.260237921	0.658346567
SERBP1	0.011527922	0.26031841	0.658346567
GNB1	-0.008840926	0.260320849	0.658346567
RAB10	-0.008818643	0.260459945	0.658346567
C7orf43	0.016361554	0.26047378	0.658346567
DTX1	-0.028925082	0.260700502	0.658346567
PHOSPHO2	-0.031332298	0.260758134	0.658346567
SAMD9L	0.034140369	0.260791293	0.658346567
MIB2	0.018841266	0.260831279	0.658346567
SREK1	0.015165752	0.260869096	0.658346567
ZMAT2	-0.014997267	0.260902533	0.658346567
DDX39A	0.010683772	0.260989614	0.658346567
TRIM39	-0.013561506	0.261103664	0.658346567
AQP9	-0.034071637	0.261147775	0.658346567
DEPTOR	-0.026517431	0.261155715	0.658346567
FCGR2A	-0.027073462	0.261214057	0.658346567
GLE1	-0.01107495	0.261279972	0.658346567
TRIM68	-0.01387967	0.261443516	0.658346567
TIRAP	-0.015477922	0.261493043	0.658346567
RAB6A	-0.021252154	0.261527273	0.658346567
YTHDF1	0.010145483	0.26157582	0.658346567
IGLL3P	-0.073799246	0.261644275	0.658346567
EHD4	0.019160571	0.261670897	0.658346567
MIR599	-0.020934056	0.261694899	0.658346567
BAK1	-0.025621041	0.261741405	0.658346567
ZFP42	0.031287789	0.261783812	0.658346567
ISG20L2	0.017703067	0.261807886	0.658346567
AMZ2	-0.011185393	0.261848912	0.658346567

CPT1A	-0.026660816	0.261929162	0.658346567
WASH2P	0.025682957	0.261947985	0.658346567
MOCS1	-0.026990685	0.262145357	0.658346567
NDUFB8	-0.007048312	0.26231041	0.658346567
IZUMO4	0.022772853	0.262312215	0.658346567
PIBF1	-0.020963201	0.262347933	0.658346567
PGM5	-0.050639017	0.2623713	0.658346567
WDR60	-0.019603859	0.262378087	0.658346567
KTI12	-0.01388321	0.262415345	0.658346567
SLC6A12	0.028779451	0.262456389	0.658346567
SF3A3	-0.013318549	0.262456603	0.658346567
ITFG2	-0.011955198	0.262493619	0.658346567
GBAS	-0.020230382	0.262553708	0.658346567
MAN1B1	-0.012778734	0.262555976	0.658346567
KLHL6	-0.018575017	0.262557275	0.658346567
TRIM8	-0.009920072	0.26258349	0.658346567
GCSH	-0.020428488	0.262704876	0.658346567
C1orf162	-0.011770455	0.262709728	0.658346567
ARPC2	-0.009237049	0.262768089	0.658346567
APRT	-0.017580327	0.262837847	0.658346567
CYB5A	0.014591152	0.26289372	0.658346567
MED15	0.023877162	0.262907285	0.658346567
FGD3	-0.010031563	0.262928221	0.658346567
LY6G5C	-0.027188818	0.262946081	0.658346567
RAD1	0.019103922	0.263091217	0.658474121
NCBP2	-0.010254484	0.263124114	0.658474121
SETD4	-0.022634408	0.263298086	0.658474121
IL1R2	-0.032263864	0.26331045	0.658474121
CENPH	-0.020320081	0.263317286	0.658474121
AP1B1	-0.011632985	0.263353956	0.658474121
ZNF529	0.018184445	0.263516772	0.658643255
NAT10	-0.01234942	0.263540607	0.658643255
C2CD2L	0.025766245	0.263708232	0.658655349
ZNF135	-0.03432555	0.263781059	0.658655349
ESAM	0.056020819	0.263811612	0.658655349
CCDC28B	-0.02267286	0.263815816	0.658655349
UBP1	0.010702708	0.263842969	0.658655349
RPAP2	0.020548368	0.263932093	0.658728247
TTPAL	0.014777147	0.263991192	0.658728247
RINT1	0.01633029	0.264113557	0.65888505
RNASEL	0.021252854	0.264402823	0.659458055
R3HDM2	0.018013117	0.264538147	0.659646935
DOK3	-0.021327979	0.26486336	0.660305154
HBEGF	0.033916328	0.265024175	0.660305154
ZRANB2	0.011982774	0.265099849	0.660305154
ACP5	-0.03546965	0.265166012	0.660305154
SCMH1	-0.015498504	0.26524261	0.660305154
GOLGA3	0.012301657	0.265245213	0.660305154
TROAP	-0.017942449	0.265256995	0.660305154
PDE12	-0.014638296	0.265381438	0.660305154

CCDC18	0.021844299	0.265465851	0.660305154
NAV2	0.01993759	0.265520816	0.660305154
RNF8	-0.020259103	0.265528907	0.660305154
TPRG1L	-0.014058109	0.265529846	0.660305154
MGAT1	0.014886451	0.265621504	0.660305154
RNPEPL1	-0.024202843	0.265662189	0.660305154
NOP58	0.016711197	0.265723361	0.660305154
GNPTAB	-0.022792366	0.265756569	0.660305154
STX12	0.012809328	0.265892494	0.660406405
NASP	-0.014416222	0.265952641	0.660406405
GLUL	-0.026756893	0.265976308	0.660406405
ATG5	0.013027473	0.266085944	0.660530459
SKIL	-0.019631789	0.266455774	0.661165956
FXYD1	0.021038921	0.266461409	0.661165956
WRAP53	-0.020337247	0.2666287	0.661406745
HOXB6	0.033188185	0.266677957	0.661406745
UBL7	-0.016132215	0.267145665	0.662366104
RNPEP	0.020272767	0.267184448	0.662366104
ZBTB20	0.022669978	0.267350887	0.662630311
TSPAN1	-0.02305203	0.267584371	0.662930895
DYRK3	-0.016711684	0.267591945	0.662930895
VCAM1	0.036483832	0.267763724	0.663208023
CD1C	-0.033268014	0.267999861	0.6636444
CDKN2AIP	-0.014615789	0.268133603	0.663827074
APBB3	0.019368251	0.268235109	0.663929879
FLYWCH2	-0.019602865	0.268339066	0.664038704
ASGR1	-0.018783473	0.268572832	0.664468635
PSMG2	-0.012220851	0.268826624	0.664824769
CTNNBL1	0.012009508	0.26891116	0.664824769
ALX1	-0.022375183	0.269048303	0.664824769
MARVELD1	-0.023644139	0.269057796	0.664824769
SNX4	-0.013547403	0.269080015	0.664824769
FPR2	-0.057143854	0.269093033	0.664824769
PTPRR	-0.018931394	0.269167373	0.664824769
MFAP3	-0.025824062	0.269197273	0.664824769
SLC23A2	0.01947831	0.269387844	0.664847139
SEC16A	0.01263451	0.269404179	0.664847139
MS4A6A	-0.023894562	0.26950135	0.664847139
ARID4B	0.042377513	0.269600994	0.664847139
TBC1D1	-0.020238538	0.269675802	0.664847139
SDHAP1	-0.019491635	0.26971361	0.664847139
SGK1	-0.026889018	0.269730322	0.664847139
ANKRD17	-0.010297132	0.269745881	0.664847139
RAN	-0.016032604	0.269777835	0.664847139
USP6NL	-0.022198883	0.26980697	0.664847139
NCOA1	-0.013654473	0.269878107	0.664874419
PREX1	0.01651338	0.270346185	0.66579173
RHOBTB3	0.0472152	0.27037075	0.66579173
EIF2B4	0.008082773	0.270529007	0.665892606
RNF157	0.03218887	0.270560971	0.665892606

SSH2	0.015942177	0.270592189	0.665892606
RELT	0.025713075	0.270699915	0.666009638
RABAC1	0.016323194	0.270964572	0.666413553
CISD3	0.022281859	0.270985168	0.666413553
NUDT13	0.022650801	0.271132454	0.666413553
LPXN	-0.012182909	0.27122304	0.666413553
GPAM	-0.017813764	0.271228882	0.666413553
TM4SF1	0.058022698	0.271341411	0.666413553
RALGPS1	0.021475246	0.271344187	0.666413553
GPR180	0.024127018	0.271376054	0.666413553
IFI6	0.035503562	0.271509938	0.666413553
RNASE6	-0.034672048	0.271560673	0.666413553
ECT2	-0.019793477	0.27163632	0.666413553
TLR6	-0.02273474	0.271652826	0.666413553
HIST1H2BK	0.029377649	0.271728769	0.666413553
CYFIP2	-0.014961997	0.271790533	0.666413553
LRRC8D	-0.014870051	0.271848282	0.666413553
SAMD1	0.023193698	0.271900744	0.666413553
ANXA1	-0.016151533	0.271939765	0.666413553
UTP6	0.009875004	0.271947784	0.666413553
YTHDF2	-0.011300938	0.272055435	0.666529794
TRIP6	0.02121426	0.272139362	0.666587874
CAPN5	-0.032731711	0.272307891	0.666853108
FAM184A	0.025872116	0.272418301	0.666975928
USP6	-0.022122279	0.272481348	0.666982761
HAPLN3	0.030861969	0.272572039	0.667053035
FBXL19	-0.033639473	0.27263523	0.667053035
CDC25B	0.017373511	0.272690847	0.667053035
AGFG1	0.018105521	0.272753159	0.667058046
SLC22A1	0.021057415	0.272895859	0.667259612
TMEM207	-0.023554507	0.273099557	0.667388279
TAF7	-0.015156496	0.27317639	0.667388279
NUDT3	-0.015703244	0.273210765	0.667388279
ADIPOR1	0.018963934	0.273217479	0.667388279
IGFBP6	-0.020746275	0.273311463	0.667388279
ZNF737	0.023223036	0.273427311	0.667388279
NOB1	0.014880636	0.273485072	0.667388279
SCNN1D	0.020456364	0.273577395	0.667388279
PALLD	-0.045528364	0.273593712	0.667388279
YWHAB	-0.009961824	0.273627198	0.667388279
STXBP3	-0.013864476	0.273730917	0.667388279
SLC35F5	-0.022472845	0.273831975	0.667388279
TRIOBP	-0.016369653	0.273872365	0.667388279
VMO1	-0.033985004	0.273893964	0.667388279
CAPZB	0.009577865	0.273904925	0.667388279
GIN1	-0.01721845	0.274080453	0.667388279
LRRN3	-0.063197177	0.274082219	0.667388279
PREB	0.017708097	0.274158169	0.667388279
C17orf75	-0.050270474	0.274189032	0.667388279
SNORA76	-0.021726935	0.274267491	0.667388279

EPHA1	-0.034891051	0.274316628	0.667388279
BRD9	0.010185465	0.27431907	0.667388279
GATS	0.026611408	0.274386588	0.667388279
STK32B	-0.024206674	0.274395524	0.667388279
ANK1	0.030438469	0.274740857	0.668081402
NDUFA3	-0.013109742	0.274816871	0.66811947
PABPC4	-0.010929758	0.275116755	0.66870166
GOLM1	-0.033038892	0.275333056	0.669080481
RPN2	-0.015938227	0.275593645	0.669566737
IFRD2	-0.018867751	0.27577572	0.669862068
WASF2	0.01676646	0.275999499	0.670258547
SYCE1L	0.02290627	0.276141776	0.670456967
SLC9A7P1	-0.019313848	0.27667046	0.671480867
ANKRD27	-0.019395805	0.276720963	0.671480867
PRPF6	-0.022452552	0.27674548	0.671480867
PRPF18	-0.020944785	0.276877877	0.671507888
THUMPD2	0.012650094	0.276877947	0.671507888
ALDH8A1	-0.022083636	0.277029617	0.671642899
AMT	0.025245254	0.277054971	0.671642899
TMOD3	-0.019847858	0.277218186	0.671657001
MFGE8	0.023219678	0.27723122	0.671657001
TMC4	-0.029153284	0.277289229	0.671657001
VPS8	-0.011858306	0.27734593	0.671657001
CASP1	-0.015101379	0.277364184	0.671657001
ZNF264	0.022435482	0.277425029	0.671657404
SNORD48	0.032823259	0.277527601	0.671740744
C16orf86	0.019147887	0.277580826	0.671740744
RPS25	-0.017930458	0.277701052	0.671884798
MLYCD	-0.020391912	0.277876261	0.67200148
GORASP1	0.013605353	0.277917968	0.67200148
CTNNBIP1	-0.017382597	0.278006917	0.67200148
HSPA13	-0.020656158	0.278040732	0.67200148
ZNF398	0.01502059	0.278105875	0.67200148
NOL9	0.024871897	0.278155357	0.67200148
ERH	-0.015368857	0.278174251	0.67200148
DYNLRB1	0.018582474	0.278296326	0.672029088
STAT2	0.017621197	0.278307105	0.672029088
HMGXB3	-0.010842435	0.278500942	0.672254276
RNF168	-0.019912942	0.27866065	0.672254276
IPO7	-0.019288613	0.278668152	0.672254276
IFNG	-0.070250365	0.278707018	0.672254276
ZNF133	0.01678435	0.278776001	0.672254276
LEPRE1	-0.012081526	0.278861578	0.672254276
HSPB11	-0.018337509	0.278869469	0.672254276
TCEB2	-0.011166874	0.278984228	0.672254276
EP400	0.013689244	0.279033611	0.672254276
RSAD2	0.053070431	0.27908821	0.672254276
GMPS	-0.013367203	0.279127995	0.672254276
ERICH1	-0.019178657	0.279129158	0.672254276
RAE1	-0.011953736	0.279316554	0.672559263

TUBG2	-0.017091314	0.279377708	0.67256021
TRIM65	0.020498621	0.279472218	0.672641439
C17orf96	-0.021748847	0.279649765	0.672922446
FAM172A	-0.011513727	0.279788409	0.673109737
SMAP1	-0.015769992	0.279912978	0.673263092
C16orf54	-0.023952042	0.280037769	0.673274434
JMJD1C	-0.015904041	0.280069357	0.673274434
ZNF823	-0.020943718	0.280100169	0.673274434
CDKN1C	-0.047712372	0.280299941	0.673465861
TRIM22	0.018046141	0.280428492	0.673465861
SETD3	-0.010705663	0.280448337	0.673465861
ENTPD3	-0.016174819	0.280480142	0.673465861
ALG10B	0.018519609	0.28048402	0.673465861
LMBRD2	-0.018079613	0.280625618	0.673659719
METTL8	0.02009583	0.280736871	0.673780664
SLC15A4	-0.015563505	0.28100874	0.674286958
ZNF177	-0.023444327	0.281113468	0.674392062
CKAP4	-0.023060322	0.281330458	0.674766379
PRSS53	0.020762184	0.281439097	0.674880711
RNF165	0.05897834	0.281538648	0.674973206
KIAA0947	0.017200946	0.281641374	0.675004879
SNORD34	0.030228669	0.281737949	0.675004879
HLA-G	-0.027373781	0.281783532	0.675004879
SNORD15B	0.01965229	0.281844154	0.675004879
CD244	-0.024310751	0.281856767	0.675004879
CCND3	-0.012491331	0.281977297	0.675147459
ZNF431	0.015003257	0.282271759	0.675516944
ZC3HAV1L	0.018797017	0.282404543	0.675516944
ERAL1	-0.010515681	0.282416792	0.675516944
ZNF502	0.038216429	0.28247601	0.675516944
SLC36A4	0.024699314	0.282613232	0.675516944
ZSCAN16	-0.010848673	0.282614493	0.675516944
USF2	-0.027068073	0.282677821	0.675516944
SFI1	0.020388606	0.282682794	0.675516944
BAZ1B	0.011597071	0.282708668	0.675516944
SPATC1	-0.017168592	0.282766368	0.675516944
MRPS15	-0.010940286	0.282807209	0.675516944
PPM1A	0.016587724	0.28286763	0.675516944
RAD54L2	0.021308006	0.283018181	0.675516944
HBQ1	0.060375041	0.283232204	0.675516944
MARCH3	0.030541194	0.283292745	0.675516944
LRRC14	0.019151093	0.283564876	0.675516944
POLI	-0.021445151	0.283653123	0.675516944
PIK3R2	-0.011542099	0.283682865	0.675516944
ABCC6	-0.01654432	0.283725383	0.675516944
ATXN7L3	0.014606154	0.283727647	0.675516944
TAB1	-0.016984117	0.283729648	0.675516944
COQ4	-0.027788784	0.283763574	0.675516944
MTBP	0.020253138	0.283820825	0.675516944
FBXL16	0.026109501	0.283854263	0.675516944

SMPD4	0.021919286	0.283963362	0.675516944
TOMM7	-0.010795781	0.284076078	0.675516944
CLTA	-0.014071632	0.284126154	0.675516944
TMEM218	-0.011040817	0.284167521	0.675516944
HENMT1	-0.016666307	0.284207156	0.675516944
OST4	0.018278979	0.284213921	0.675516944
SLC25A36	0.022322585	0.284261138	0.675516944
CHFR	0.012229531	0.28426777	0.675516944
MRPL12	0.016518083	0.28429973	0.675516944
CSNK1G1	0.013560091	0.284324125	0.675516944
TYK2	0.010481099	0.284531066	0.675516944
BUB3	0.008929247	0.284544879	0.675516944
INTS10	-0.010553605	0.284564504	0.675516944
PMM1	-0.013609026	0.284568678	0.675516944
HYALP1	-0.017379725	0.284597337	0.675516944
RAB43	-0.016180223	0.284620602	0.675516944
PNOC	-0.035747468	0.284740273	0.675516944
TUBB1	0.036917195	0.284801154	0.675516944
LIMS3L	0.037380218	0.28480916	0.675516944
SUPT7L	-0.020274722	0.284895483	0.675516944
CD7	-0.018914051	0.284951	0.675516944
HOMER2	0.051090191	0.284960444	0.675516944
TBP	0.011890001	0.285072328	0.675516944
FKBPL	-0.016384436	0.285196919	0.675516944
NCKIPSD	-0.017445774	0.285302213	0.675516944
DPM1	-0.020289693	0.285593892	0.675516944
MRPL32	-0.011757613	0.285623784	0.675516944
USP13	-0.016407696	0.285707951	0.675516944
THOC6	-0.015132955	0.28572118	0.675516944
GPR125	0.029428425	0.28574545	0.675516944
ADA	-0.018943228	0.285758894	0.675516944
COG5	0.020585586	0.285832803	0.675516944
NEDD4L	-0.022015812	0.285878556	0.675516944
RRN3	-0.013677779	0.285885271	0.675516944
RTBDN	-0.021475358	0.2859015	0.675516944
KCTD15	-0.019160985	0.285917111	0.675516944
BICD2	-0.018305483	0.286013245	0.675516944
RIN2	-0.024492284	0.286172911	0.675516944
SEC13	-0.017296877	0.286184256	0.675516944
BTBD6	0.013432983	0.286186548	0.675516944
CHPF	-0.021665368	0.286236484	0.675516944
TALDO1	-0.013776619	0.286258431	0.675516944
TMEM135	-0.020880686	0.286290224	0.675516944
MED4	-0.024534973	0.286310351	0.675516944
TCF19	-0.020304032	0.286394026	0.675516944
RPF1	-0.012936411	0.286461481	0.675516944
DUSP1	0.034998963	0.286464589	0.675516944
CSTF1	-0.022548056	0.286641162	0.67554252
SNF8	-0.02224656	0.286672203	0.67554252
SGSM2	0.012527375	0.286704194	0.67554252

SEPP1	0.021827465	0.286719555	0.67554252
RPP21	-0.010950964	0.286862393	0.675642282
EPHB1	0.047071157	0.286906885	0.675642282
ZNF318	0.012706495	0.286945015	0.675642282
ZNF525	0.026552457	0.287056901	0.675691662
ISCA2	-0.017040455	0.287088074	0.675691662
HNRNPH3	0.015385806	0.287257874	0.675804524
TP53RK	-0.019143537	0.287315292	0.675804524
HUS1	-0.019065397	0.287477656	0.675804524
DYNC2H1	-0.014417725	0.287527401	0.675804524
BBS9	-0.023633986	0.287602493	0.675804524
CACNA2D2	-0.034725465	0.287654854	0.675804524
FTH1	-0.018127049	0.28773831	0.675804524
BPI	-0.047045166	0.287765638	0.675804524
AGAP1	-0.046395033	0.287783957	0.675804524
ADAT2	0.017614519	0.287797753	0.675804524
TES	-0.0195914	0.287889152	0.675804524
CDKN1A	0.038438996	0.288026287	0.675804524
MCM2	0.025432491	0.288081003	0.675804524
KBTBD7	-0.019480599	0.288091619	0.675804524
SPEF2	0.020388528	0.28811862	0.675804524
BCAN	0.01812703	0.288135636	0.675804524
PIK3AP1	-0.017991584	0.288173941	0.675804524
SDR42E1	-0.023178151	0.288252086	0.675844595
EPM2A	-0.017032649	0.288452342	0.676032792
CD3G	-0.02616299	0.288502168	0.676032792
KDM4B	0.018561086	0.288515576	0.676032792
C6orf106	0.0177253	0.288624387	0.676132749
ENTPD1	-0.026842653	0.288697464	0.676132749
EID3	-0.01978414	0.288809014	0.676132749
PVRL2	0.029135119	0.288884795	0.676132749
HIST2H3A	0.051607169	0.288918823	0.676132749
GGA3	0.01830227	0.288947136	0.676132749
PPP1R16B	0.01752395	0.28898582	0.676132749
METTL21B	-0.02823251	0.289103509	0.67626516
KBTBD3	-0.021969234	0.289203401	0.67627763
ZNF93	-0.019560339	0.289231033	0.67627763
HPGD	0.038028625	0.289373025	0.676414578
GP9	0.055446239	0.289442183	0.676414578
RNF24	0.019441364	0.289472929	0.676414578
MYOM1	0.028831847	0.289655893	0.67655842
BBC3	0.015420231	0.289712593	0.67655842
STK4	0.008156227	0.289750861	0.67655842
DCAF5	-0.012213928	0.289778975	0.67655842
UBAC1	0.01087854	0.289977667	0.676760962
DDX59	0.038559907	0.289988006	0.676760962
SEMA4B	0.018316471	0.290164827	0.676819171
PTGS1	0.047122635	0.290165899	0.676819171
ZDHHC18	0.015420616	0.290196385	0.676819171
KBTBD2	0.016824494	0.290447905	0.677038633

ADAM19	0.022098038	0.2904709	0.677038633
ATP5H	-0.010869052	0.290473979	0.677038633
TRAM1	-0.00781385	0.290714551	0.677456708
STX4	-0.017254996	0.290840831	0.677608325
RAB1B	0.018472296	0.290982875	0.677756848
RNASE2	-0.044163019	0.291027039	0.677756848
DDX28	0.014582124	0.291178612	0.677886121
TAF6L	-0.014632624	0.291401197	0.677886121
PSIP1	0.013883164	0.291416653	0.677886121
PTPRA	-0.017000474	0.291417465	0.677886121
MIR1307	0.102999004	0.291465517	0.677886121
SPSB1	0.022953575	0.291492039	0.677886121
LMNA	-0.034466326	0.291511242	0.677886121
SART3	-0.015950666	0.291587074	0.677920041
DPEP2	0.016289001	0.291758573	0.677970602
HLA-B	-0.011739316	0.291854336	0.677970602
RAB11FIP2	0.012109066	0.291900736	0.677970602
NEXN	0.040166069	0.291946299	0.677970602
UBFD1	-0.017774104	0.291957779	0.677970602
F13A1	0.042949261	0.292093211	0.677970602
WHSC1L1	-0.01683818	0.292096146	0.677970602
ZNF281	0.013597094	0.292098817	0.677970602
NDUFAF3	-0.01468884	0.292224092	0.6780986
CLOCK	-0.017848711	0.292276486	0.6780986
COMMD7	-0.009923	0.292671128	0.678457715
DOCK9	0.01793601	0.292763823	0.678457715
CDH6	0.035507784	0.292827989	0.678457715
ACVR1	0.016204676	0.292840422	0.678457715
C9orf16	0.020319316	0.292854405	0.678457715
GP1BB	0.034864349	0.292869061	0.678457715
VAMP2	0.020831765	0.292881963	0.678457715
KIAA1430	-0.023732863	0.292921621	0.678457715
CCNF	-0.020001882	0.29314413	0.678831042
LRBA	0.014992789	0.293350174	0.679119425
SLC23A1	0.018573475	0.293456279	0.679119425
STAU2	-0.017354868	0.293519517	0.679119425
CNDP2	-0.013666391	0.293567193	0.679119425
NPTN	0.010622361	0.293611355	0.679119425
SLC36A1	0.024717062	0.293636784	0.679119425
BUD31	0.010404552	0.293775564	0.679298457
LILRB5	0.020074796	0.293921366	0.679493652
SNORD65	0.025948471	0.294157471	0.679897482
AP4M1	-0.017823118	0.294289972	0.680061732
TMEM50B	0.008569895	0.294559464	0.680366486
RBM18	-0.019496933	0.294570076	0.680366486
CCDC34	0.018514381	0.294633975	0.680366486
CASZ1	-0.019157684	0.294677208	0.680366486
RPL28	-0.015976049	0.294790906	0.680366486
GRTP1	0.020707174	0.294814249	0.680366486
ASF1B	-0.023735644	0.294852113	0.680366486

DACH1	-0.024876072	0.295289742	0.681234296
C9orf69	0.01690679	0.295405131	0.681246652
SCARB2	-0.012009924	0.295562663	0.681246652
IGF2BP1	0.027386802	0.295606578	0.681246652
MAK	-0.019063499	0.295670894	0.681246652
WSB1	0.019681226	0.295696338	0.681246652
PRR24	-0.019693505	0.295758428	0.681246652
SPI1	-0.023972825	0.295773623	0.681246652
GCN1L1	0.013031226	0.295787461	0.681246652
KIAA1731	0.015029196	0.296007767	0.681541512
TCP11L2	0.042594828	0.296038629	0.681541512
OSM	0.031172872	0.29619191	0.68167639
REXO2	0.010367437	0.296220384	0.68167639
ACAA1	-0.01421716	0.296427605	0.682011465
HRH1	-0.019954144	0.296814818	0.68243021
SEC31B	0.02161523	0.296823266	0.68243021
TMEM64	0.020688131	0.29686839	0.68243021
HBG2	-0.165441428	0.296936587	0.68243021
TLK1	0.024579671	0.297000862	0.68243021
LHPP	-0.021206686	0.297059221	0.68243021
DFNA5	-0.038071475	0.297134517	0.68243021
TPPP3	-0.037199983	0.297153397	0.68243021
TIAL1	0.012235824	0.297197197	0.68243021
B3GNT2	-0.024491965	0.297226131	0.68243021
COMMD6	-0.012301304	0.297295709	0.68244279
LRRCC1	0.022568383	0.29740766	0.68244279
SUN2	-0.011448493	0.29741657	0.68244279
SERPINA1	-0.024183148	0.297651714	0.682705261
SNAP47	-0.014624989	0.297708713	0.682705261
CDK13	0.012624976	0.297757687	0.682705261
ZNF562	0.016552982	0.297777668	0.682705261
MYO5C	-0.026629001	0.297864222	0.682762284
PROS1	0.070483366	0.298237266	0.683357371
LIMA1	-0.022219128	0.298247309	0.683357371
PGD	-0.019859208	0.298423995	0.683620696
C15orf41	-0.01734697	0.298655915	0.684010412
TSPAN13	0.037081195	0.298766992	0.684110024
SREK1IP1	0.018878406	0.298823016	0.684110024
N4BP1	0.018508654	0.298971223	0.684166799
NFE2L1	0.018070423	0.298974693	0.684166799
UBE2N	-0.011179244	0.299102025	0.684166799
FAM69B	0.047925293	0.299130063	0.684166799
S1PR4	0.017460872	0.299156862	0.684166799
PRR3	-0.023844252	0.299451764	0.684699767
OSBP	-0.008731892	0.29973165	0.685180932
CISH	0.038377363	0.299819884	0.685180932
BCCIP	-0.009044727	0.299847903	0.685180932
RRAS	-0.022900277	0.299970891	0.685203974
SAMD4A	-0.028874545	0.299981792	0.685203974
TBRG1	0.018379802	0.300104524	0.685342887

TMEM126A	-0.015131836	0.300221788	0.685469259
CIRBP	-0.014439992	0.300326466	0.685544081
PTK2B	0.015470766	0.300379286	0.685544081
SLFN12	-0.02254961	0.300462666	0.685544081
LTA	-0.023384805	0.300502293	0.685544081
PAPLN	0.034487401	0.300587942	0.685598173
SH3BP1	0.020803353	0.30095828	0.686113801
OSBPL3	0.016778089	0.300965332	0.686113801
EMR4P	0.06317194	0.301023276	0.686113801
ZDHHC11	0.042999195	0.301150657	0.686113801
CNTROB	0.020982884	0.301172873	0.686113801
PRAME	0.015310164	0.301237303	0.686113801
TMEM150C	0.021381556	0.301268869	0.686113801
PTDSS1	-0.011786021	0.301309891	0.686113801
MED18	-0.025373652	0.301435651	0.686155427
RNF44	0.013800276	0.301509353	0.686155427
GPR146	0.023897015	0.301514138	0.686155427
SPAG8	-0.025387344	0.301602488	0.686215403
TOR3A	-0.022951383	0.301695789	0.686286619
MAPK1	-0.013168339	0.301802046	0.686387271
NUDT19	-0.014628432	0.301874555	0.686411145
EXOC4	0.011384657	0.301969238	0.686452525
NAB1	0.017807067	0.302182481	0.686452525
NSMCE1	-0.016585575	0.302195158	0.686452525
C12orf66	-0.019522017	0.302197461	0.686452525
PPP2R2A	-0.014940569	0.302202833	0.686452525
FAM157B	0.02152367	0.302316581	0.686570011
RNF6	-0.021368289	0.302573338	0.687012159
UBE2MP1	-0.018155514	0.302900795	0.687465133
COX19	0.021012977	0.302953355	0.687465133
ZNF346	0.021879376	0.302959158	0.687465133
LILRA2	-0.020093945	0.303153956	0.687694385
MTMR10	-0.015847019	0.303227738	0.687694385
TUBGCP4	0.015551994	0.303302846	0.687694385
KBTBD6	-0.028835208	0.303328805	0.687694385
YARS	-0.011194184	0.303413321	0.687694385
PPP1R13L	-0.01645692	0.303432955	0.687694385
MIS18BP1	0.013854532	0.303499125	0.687703545
OGFR	0.016529429	0.303577623	0.687740628
GUCY1A3	0.047395198	0.303695521	0.687866937
EPHA4	0.032821454	0.303801955	0.687967233
CD79A	-0.041399635	0.303910937	0.688005344
ZNF410	0.008152325	0.303943096	0.688005344
ARHGAP32	0.021486343	0.304043631	0.688092199
TCL1B	0.044045617	0.304162843	0.688137538
SARM1	-0.01870118	0.304188	0.688137538
TMEM199	-0.014250607	0.304551034	0.688818022
HINT3	-0.017882282	0.304663009	0.68892602
GAA	0.031128292	0.304723263	0.68892602
HAUS4	-0.017550697	0.304811679	0.68898519

MS4A1	0.050682298	0.304893662	0.688993989
CPM	0.017365683	0.305090273	0.688993989
ZXDC	0.019050945	0.305189207	0.688993989
SLC19A2	0.016278955	0.30522889	0.688993989
SLC8A3	0.047533277	0.305324713	0.688993989
MT1X	-0.021720953	0.305466952	0.688993989
IDO2	0.025549891	0.305507487	0.688993989
ZBTB25	0.020423048	0.305589786	0.688993989
NTAN1	-0.017076639	0.3057372	0.688993989
SNORD114-2	0.016743571	0.305835861	0.688993989
FAM126A	-0.018153082	0.305841562	0.688993989
CDC42EP4	-0.022064684	0.305845399	0.688993989
ZNF655	0.024918428	0.305893053	0.688993989
IFITM3	0.068334014	0.305953763	0.688993989
YDJC	-0.014074527	0.306037274	0.688993989
LAPTM5	-0.010523441	0.306083599	0.688993989
EIF6	-0.014820276	0.306097146	0.688993989
BBS2	0.020041211	0.306123127	0.688993989
WIPF2	-0.019853895	0.306193316	0.688993989
ZNF45	-0.010969138	0.30625284	0.688993989
C9orf64	-0.016594845	0.306283945	0.688993989
PPP6R1	0.01511236	0.306333805	0.688993989
PPIL1	-0.015228808	0.306414481	0.688993989
SNORD46	0.025287041	0.306433467	0.688993989
GTF3C6	-0.014413154	0.306498734	0.688993989
HDGFRP2	0.018705034	0.306579375	0.688993989
DNM1L	0.018586994	0.306595128	0.688993989
TUBGCP2	0.015193827	0.306608188	0.688993989
NUB1	0.012216718	0.306731399	0.688993989
ALB	0.020441988	0.306764132	0.688993989
SLC35F3	-0.030140446	0.30676515	0.688993989
LTB4R	0.020757608	0.306807424	0.688993989
ROPN1L	-0.019416771	0.307137983	0.689596417
NR2F6	0.0285619	0.307505361	0.690041187
PAICS	-0.012831981	0.307551786	0.690041187
ATG9A	-0.015628638	0.307557209	0.690041187
FAF1	-0.011930554	0.307585439	0.690041187
FUT11	-0.025908467	0.307824205	0.690436904
SLC5A10	-0.021024841	0.307955687	0.690591876
BCL2A1	-0.025782861	0.308046913	0.690656529
NTS	0.02173717	0.308153332	0.690755211
HOXA6	0.024168074	0.308238295	0.690805767
BMPR2	-0.016735682	0.308549209	0.690853625
TCL6	0.01775848	0.308580164	0.690853625
POLR3D	-0.017235185	0.30858786	0.690853625
PVRL1	-0.01897143	0.308598238	0.690853625
MEN1	0.014942507	0.308717052	0.690853625
ABCB9	-0.043785463	0.30889829	0.690853625
PGLS	-0.012766175	0.309027556	0.690853625
FAM195B	-0.016461954	0.309075588	0.690853625

CD247	-0.0167839	0.309115793	0.690853625
KLHL9	-0.018430082	0.309128921	0.690853625
GZMH	-0.051447997	0.309146325	0.690853625
KIAA1468	0.01863304	0.309201766	0.690853625
TMEM107	0.081661146	0.309251239	0.690853625
KLHL36	-0.02220539	0.309353466	0.690853625
VHL	0.011836019	0.30954253	0.690853625
TRIM4	-0.016900683	0.309578298	0.690853625
YTHDC2	0.016216046	0.309592911	0.690853625
RRAGA	-0.011660778	0.309615915	0.690853625
TAGLN2	0.024035641	0.309616774	0.690853625
KLF4	-0.028233257	0.309625116	0.690853625
GTF3C5	0.012045861	0.309627147	0.690853625
SNIP1	-0.014748499	0.309700595	0.690853625
B4GALT5	-0.018141966	0.309708183	0.690853625
TSPAN9	0.058062649	0.30975757	0.690853625
PALB2	-0.017255085	0.310021178	0.690988761
ERP27	0.020875187	0.310074789	0.690988761
ZNF335	0.01870077	0.310160255	0.690988761
ZC3H4	0.010937472	0.310238762	0.690988761
GBF1	0.018766031	0.310239907	0.690988761
LRRC8B	0.019864401	0.310244247	0.690988761
PTCH1	-0.023173507	0.31025514	0.690988761
NOL10	0.021252725	0.310367328	0.69104541
DLG1	-0.016538678	0.310405437	0.69104541
THBS3	0.018008566	0.310504898	0.691127834
CCDC101	-0.015783407	0.310673255	0.691228569
SSPO	0.033879406	0.310787828	0.691228569
GJB7	0.024741456	0.310844076	0.691228569
MTRNR2L1	0.036982379	0.310857439	0.691228569
PSMB2	-0.014021281	0.310862392	0.691228569
AEBP2	-0.015800508	0.311116643	0.691589011
MAZ	-0.022401591	0.311162283	0.691589011
ZBTB6	-0.019652874	0.311257023	0.691589011
OR7E12P	0.01812606	0.311274411	0.691589011
MAPRE2	0.013438234	0.311447069	0.691735084
ARID3A	0.013390101	0.311465142	0.691735084
MKL1	0.017021255	0.311644945	0.691782379
MAD2L2	-0.013651286	0.311732318	0.691782379
RPGRIP1	-0.030301157	0.311782374	0.691782379
SRSF10	-0.017995157	0.311813854	0.691782379
STT3A	-0.013501037	0.311837466	0.691782379
BTBD17	-0.03520433	0.311861421	0.691782379
RAVER1	-0.016460555	0.312269314	0.692548393
STOM	0.018203319	0.312494374	0.692908699
WDFY2	0.020892724	0.312560688	0.692916934
POLG	0.011820388	0.312627747	0.692926819
DDR1	-0.021483687	0.313072635	0.693773973
UBE2K	-0.012657898	0.313308287	0.694005441
AKAP8	0.011147566	0.313420443	0.694005441

OCM2	0.026847538	0.313461588	0.694005441
TSHZ1	0.015069766	0.313476362	0.694005441
PHC1	0.018053064	0.313490578	0.694005441
CLK2	0.011211348	0.313740738	0.694314705
SNHG1	-0.013646145	0.313789845	0.694314705
ANKRD23	-0.018637639	0.313818454	0.694314705
ANXA11	0.014993621	0.314124569	0.694560993
AGGF1	-0.009867714	0.314128748	0.694560993
ZNF254	-0.016906609	0.314147329	0.694560993
SSRP1	-0.01864971	0.314228776	0.694560993
PBX1	0.039034664	0.314312036	0.694560993
RPL6	-0.009493513	0.314322938	0.694560993
EPPK1	-0.034628066	0.31436901	0.694560993
SEC31A	-0.009567957	0.314806451	0.695321974
ACAA2	-0.016133554	0.314839076	0.695321974
SETD1B	0.013623657	0.315166582	0.695906422
BST2	0.01619254	0.315372006	0.696221127
FST	0.040488227	0.3155708	0.696521075
SEC22C	0.009875574	0.315682652	0.696558303
TM2D3	-0.01671952	0.315737649	0.696558303
UBE2C	0.039388393	0.315807765	0.696558303
CPT2	0.01305928	0.315895644	0.696558303
NCEH1	-0.014391294	0.315902311	0.696558303
RBM25	0.010836539	0.316002654	0.696640784
RPS7	-0.011066091	0.316203106	0.696862151
KRT72	0.099645268	0.316256632	0.696862151
ZNF668	-0.015356252	0.316306783	0.696862151
FBXW7	0.01178532	0.31641323	0.696862151
NUDT7	-0.020572723	0.316417849	0.696862151
DENND3	0.019885338	0.316510843	0.696928291
PIGL	0.018455437	0.316681952	0.697080669
BIRC2	0.012932959	0.316714408	0.697080669
SORT1	-0.018918897	0.316776824	0.697080669
PIGB	-0.012618458	0.316831949	0.697080669
PHPT1	-0.014287554	0.316935037	0.697168905
TTC30B	-0.017208854	0.317165051	0.697424639
GMCL1	-0.017430565	0.317177309	0.697424639
ETNK1	-0.019544931	0.317334456	0.697631597
GNB5	0.031349637	0.317410539	0.697646945
PRDX5	-0.01666666	0.317467491	0.697646945
RHOBTB2	-0.021093988	0.317542846	0.697674029
TMEM120A	0.013111887	0.317610511	0.697684212
AMDHD1	-0.030661429	0.317782678	0.697923902
SAP18	-0.011130319	0.318018473	0.698089241
DBR1	-0.014222231	0.318045694	0.698089241
BARD1	0.025304143	0.31810701	0.698089241
MTHFS	-0.01648509	0.318189372	0.698089241
TRIM56	0.016733128	0.318221749	0.698089241
RPL23A	-0.008383045	0.318321749	0.698089241
GSTM1	0.086992673	0.318364457	0.698089241

BEND4	0.021530543	0.318391869	0.698089241
TMEM200A	0.029627211	0.318458245	0.698089241
CGA	0.021685856	0.318488632	0.698089241
REXO1	-0.015588944	0.318619942	0.698238792
CATSPER1	-0.021487756	0.318994478	0.698838339
SLK	-0.014230282	0.319019796	0.698838339
ASIP	-0.022983501	0.319095374	0.698865591
TSTD1	-0.019337822	0.319276986	0.69907281
OAS1	-0.041489115	0.3193163	0.69907281
IL6	0.02354326	0.319401477	0.699121011
CD5	0.02037793	0.319476612	0.699147217
JAKMIP2	-0.040505059	0.319752373	0.699501596
DERA	-0.013310007	0.319928114	0.699501596
MYH10	-0.024154921	0.319991892	0.699501596
NBPF1	-0.01916686	0.320154112	0.699501596
MFAP1	-0.020515023	0.320263738	0.699501596
RNF114	-0.006726273	0.320320758	0.699501596
CYP2S1	-0.031577995	0.320346084	0.699501596
MPP5	-0.015383399	0.320351972	0.699501596
HMGB2	-0.012996167	0.320387263	0.699501596
ISG20	-0.01414382	0.320393587	0.699501596
VARS	-0.014217611	0.320404968	0.699501596
MSX2P1	-0.024541134	0.320475501	0.699501596
MKKS	-0.011106899	0.320539085	0.699501596
PER3	-0.023106256	0.320558166	0.699501596
CHST2	-0.033142524	0.320595769	0.699501596
CLEC2B	-0.043910754	0.320649661	0.699501596
MEF2C	0.022096029	0.320882356	0.699535491
ANKHD1	-0.009705253	0.320969808	0.699535491
DBF4	-0.018423074	0.32105029	0.699535491
PPAP2C	-0.023653643	0.321058829	0.699535491
C18orf8	0.009990847	0.321080369	0.699535491
SDPR	0.034793153	0.321147755	0.699535491
OR2W3	0.039409566	0.321164311	0.699535491
PRR11	-0.020797884	0.32117078	0.699535491
RPL35	-0.01051124	0.321280468	0.699636731
TCF20	0.011458022	0.321346052	0.699641906
WDR89	0.023472624	0.321445396	0.699720567
SLC27A3	-0.014291442	0.321514503	0.699733393
HGS	-0.010852191	0.321747668	0.699845308
B3GALT2	0.042685652	0.321803538	0.699845308
PANK4	-0.013426024	0.321841127	0.699845308
PYROXD2	-0.024350131	0.321844723	0.699845308
ZNF136	0.016077473	0.322022719	0.699845308
DDX23	0.012134309	0.322048224	0.699845308
MTSS1	0.01957139	0.322112085	0.699845308
ICOS	0.021035109	0.322124281	0.699845308
EPHB4	-0.027229496	0.322201281	0.699845308
LILRA4	0.025344132	0.322251417	0.699845308
MED19	-0.01711262	0.322261409	0.699845308

PES1	-0.017228381	0.322370504	0.699944903
CCDC59	0.008850044	0.322687048	0.70049479
CCS	0.015529842	0.322765142	0.700526933
H3F3A	-0.012229629	0.322850209	0.700574195
NKIRAS1	-0.017732412	0.322983284	0.700673899
ZNF683	-0.085378665	0.3231433	0.700673899
PAFAH1B2	-0.016132873	0.323175216	0.700673899
CYB561D2	-0.009998551	0.323189399	0.700673899
TUBGCP5	-0.015814274	0.32321266	0.700673899
ASS1	0.015282018	0.323326916	0.700784341
CSRP1	0.014093965	0.323477444	0.700973341
KDM5B	0.011834473	0.323751248	0.701122141
MAT2B	-0.011372634	0.323757541	0.701122141
C17orf98	-0.022542773	0.323764703	0.701122141
ANAPC11	-0.010831812	0.323884367	0.701122141
SIKE1	-0.018079125	0.323894008	0.701122141
LAMB2	-0.025338427	0.323980272	0.701122141
TAS2R14	0.017695833	0.324044685	0.701122141
ZNF615	-0.013825967	0.324059125	0.701122141
SLC43A1	-0.023205404	0.32411618	0.701122141
C16orf58	-0.010818106	0.32424892	0.701167402
SGSM3	-0.018803002	0.324263794	0.701167402
FAM49A	-0.016500168	0.324337913	0.701190696
TEKT1	-0.015672729	0.324425744	0.701206213
PIK3IP1	-0.021221584	0.324471788	0.701206213
WDR24	0.016016977	0.324578108	0.701210964
PDSS2	0.011219669	0.324600685	0.701210964
HSF5	-0.022369474	0.324772328	0.701387197
SRSF5	-0.010812561	0.324808996	0.701387197
PIGM	-0.014837641	0.324928637	0.701409295
DDX56	0.013775768	0.325029484	0.701409295
LSM3	0.017708787	0.325053071	0.701409295
AZI1	-0.014204917	0.325072697	0.701409295
CHRNB1	-0.011696961	0.325195145	0.701536748
SCAMP4	-0.021888436	0.325578806	0.702056566
ELMOD2	-0.020470466	0.325655337	0.702056566
TMEM33	-0.011773399	0.32572884	0.702056566
FAM168B	-0.012275208	0.325755939	0.702056566
TUBA4A	0.021220287	0.325844446	0.702056566
ZC3H8	-0.012434649	0.32593967	0.702056566
TGM1	0.019148816	0.325977868	0.702056566
VPS36	0.011385139	0.325988722	0.702056566
C5AR1	-0.026315759	0.326006934	0.702056566
LY75	-0.02767685	0.326157426	0.702068705
ANKRD6	-0.018823532	0.326191821	0.702068705
ICT1	-0.009759877	0.32620285	0.702068705
LUC7L2	0.015708361	0.32635934	0.702268962
PAK2	0.009343404	0.326423732	0.702271
GAPT	-0.027850298	0.326625163	0.702514186
CLPTM1L	0.012257916	0.326663702	0.702514186

ZNF597	-0.018155166	0.326834482	0.702581444
LPA	0.019008508	0.326881502	0.702581444
C6orf136	0.009438941	0.326894253	0.702581444
RNF14	-0.013776672	0.326977736	0.702581444
NR2C2AP	-0.011457935	0.327012341	0.702581444
DET1	-0.015522842	0.327132006	0.702594524
GAS7	-0.016111199	0.327145377	0.702594524
NUDC	-0.010078226	0.327238373	0.702657914
MPL	0.055823413	0.32755479	0.702696092
PCNT	0.014563088	0.327658693	0.702696092
NAPEPLD	-0.017516125	0.327673552	0.702696092
ACAT2	-0.01437117	0.327729671	0.702696092
TLR2	-0.026875522	0.327763756	0.702696092
GDPD3	0.025102407	0.327804779	0.702696092
RSU1	-0.013609444	0.327866424	0.702696092
GTF2E1	-0.011643532	0.327928942	0.702696092
SPIRE1	0.022022683	0.327960456	0.702696092
CORO1A	-0.010968868	0.327991746	0.702696092
GATA3	0.021004817	0.328001599	0.702696092
ZNF827	-0.019823637	0.328020198	0.702696092
PI4KB	0.010996413	0.328157905	0.702696092
ZNF347	-0.020724352	0.328192189	0.702696092
CALB2	-0.019361464	0.328208402	0.702696092
EPDR1	-0.032826508	0.328374832	0.702906818
EML2	0.018368296	0.32843383	0.702906818
CST7	-0.033323705	0.328629424	0.703060298
DPH5	-0.017421581	0.328632576	0.703060298
ERI2	-0.020682189	0.32871758	0.703106259
P2RY12	0.036546986	0.329094544	0.703776566
SPATS2	0.017671066	0.329284668	0.703864882
PPIAL4G	-0.006857821	0.329375138	0.703864882
RAPGEF2	-0.0160088	0.329385134	0.703864882
RING1	-0.006885012	0.329450193	0.703864882
LARS	0.010384687	0.329453785	0.703864882
MCPH1	-0.012971058	0.329529905	0.703891648
RFESD	-0.015372009	0.329638651	0.703988081
SENP1	-0.016316748	0.329852611	0.704309135
C11orf84	-0.017144817	0.330070844	0.704606265
TSEN54	-0.019299869	0.330156971	0.704606265
DHX36	-0.012445185	0.330182735	0.704606265
CRYL1	-0.018713161	0.330381625	0.704722428
RBPMS2	-0.086682457	0.330525768	0.704722428
TMEM14C	-0.016900458	0.330548177	0.704722428
TSPAN3	-0.019543952	0.330584316	0.704722428
NCOR2	0.014213338	0.330612135	0.704722428
SUPT16H	0.011062379	0.330619168	0.704722428
VWA5A	-0.018869978	0.330687969	0.704733372
FOXO3	0.016474297	0.330764014	0.704759744
MRFAP1L1	-0.010490691	0.331024391	0.705178789
CCDC85C	-0.018892385	0.331379022	0.705553842

SPAST	0.016351198	0.331389927	0.705553842
PLXDC1	-0.02040133	0.331391673	0.705553842
LIN9	-0.016619399	0.331511915	0.705674113
ACTR3B	0.021831777	0.331662493	0.705736257
ZNF717	0.01877154	0.331703714	0.705736257
UPB1	0.024550755	0.331865067	0.705736257
JAK1	-0.016052573	0.331907185	0.705736257
PIWIL4	0.016645318	0.331922683	0.705736257
CWC15	0.008048975	0.332057117	0.705736257
PGP	0.02380387	0.332080779	0.705736257
DLST	0.013615669	0.332183291	0.705736257
UBA3	-0.019659139	0.332219612	0.705736257
CDK5R2	-0.0159451	0.332225183	0.705736257
MGST3	-0.019060267	0.332242446	0.705736257
SLC37A4	-0.013387138	0.33246361	0.70607055
FICD	-0.02449207	0.332574364	0.706121021
TWSG1	-0.013619208	0.332676594	0.706121021
CARKD	0.014205944	0.332678754	0.706121021
ALS2	-0.016361073	0.333165376	0.707018318
HBG1	-0.152758884	0.333319219	0.707072939
METTL6	-0.022239052	0.333329172	0.707072939
CDK7	-0.009630861	0.333382751	0.707072939
CPSF3	-0.008669721	0.333591187	0.707379474
YIPF4	-0.016047238	0.333769701	0.707622451
CREB3L4	-0.017450119	0.333902212	0.707747705
CPSF6	-0.019642169	0.333956659	0.707747705
DIP2A	0.037170636	0.334072965	0.707807422
LARGE	-0.034087896	0.334154975	0.707807422
E2F3	0.01390712	0.334287464	0.707807422
EGOT	0.018703596	0.334327575	0.707807422
COBL	-0.018274745	0.334426055	0.707807422
HIST1H2BH	0.047706229	0.334487268	0.707807422
XPA	0.012532644	0.334548633	0.707807422
HIST1H2BJ	0.045305485	0.334549951	0.707807422
DNAJB12	-0.015496762	0.334560343	0.707807422
NDUFB9	0.012486856	0.334794086	0.707999379
GALNT11	0.011291644	0.334845351	0.707999379
ZNF184	-0.017514848	0.334854609	0.707999379
MMAB	0.019083445	0.334906925	0.707999379
HAR1A	-0.019695179	0.335047745	0.708161826
ZKSCAN3	-0.015707549	0.335226445	0.708404262
EML4	0.012757994	0.335291207	0.708405873
RAB15	-0.026441145	0.335453781	0.708509802
ZC3H7A	-0.008252499	0.335512459	0.708509802
SOX12	0.018072698	0.335532422	0.708509802
MAGEF1	-0.020960672	0.335613794	0.708546459
ECD	-0.015059449	0.335708781	0.708611842
CLEC4D	-0.029458176	0.336004376	0.708956127
MIR611	0.018133314	0.336051673	0.708956127
PTPN7	-0.019152381	0.336083022	0.708956127

BEND7	-0.019609906	0.336138923	0.708956127
ERAP1	0.021215882	0.336192132	0.708956127
FKRP	0.018534734	0.336324484	0.709100136
EEF1G	-0.011356193	0.33654925	0.709312155
CCL4	-0.039073875	0.336568155	0.709312155
PRDX6	0.02015162	0.336617287	0.709312155
P2RX4	-0.015127602	0.336682162	0.709313829
C11orf82	-0.022239259	0.336860008	0.709553459
ZFR	-0.008062058	0.337051012	0.709701997
NAB2	0.01799737	0.337058758	0.709701997
CDYL	-0.012934515	0.337176355	0.70970379
MBOAT1	0.014469257	0.337187843	0.70970379
ZNF32	-0.01184266	0.337292807	0.709789749
LYPD3	0.01827504	0.337418063	0.709918368
AGTPBP1	-0.011770017	0.337552912	0.710067119
TMPRSS7	0.017256632	0.337728724	0.710195776
RBPJ	-0.018425867	0.337742394	0.710195776
DYNC1LI1	-0.020862421	0.337942973	0.710482578
CASP6	-0.022262371	0.338015631	0.710500384
CCBL1	0.015520819	0.338180069	0.710711065
RNF212	-0.017264878	0.338396985	0.711031934
CD33	-0.021542249	0.338523317	0.711055156
ARL17B	-0.046665324	0.3386065	0.711055156
LIMS1	0.020913062	0.338624955	0.711055156
TAF1C	0.012589794	0.338664991	0.711055156
RFX5	0.00833806	0.338817966	0.71109235
BRF1	0.017208369	0.338833365	0.71109235
KIFC1	-0.024832683	0.338875431	0.71109235
RBX1	0.013051692	0.338983254	0.711183783
SETD5	-0.017912907	0.339164238	0.711428643
COPS5	-0.008563125	0.33942401	0.711838644
TNFRSF17	-0.057668693	0.339713195	0.712037996
C9orf40	-0.025742102	0.339738223	0.712037996
UPF2	0.008033437	0.339745273	0.712037996
RPS12	-0.033436339	0.339776375	0.712037996
ZNF33B	0.011364589	0.339930489	0.71208728
JRK	0.021113438	0.339963506	0.71208728
ZNF703	-0.022360598	0.339992888	0.71208728
SNORD11B	0.020859828	0.340170562	0.712145616
TERF2	-0.014923114	0.34018296	0.712145616
TRIM28	-0.015810076	0.340271293	0.712145616
MRAP	0.031293638	0.340278089	0.712145616
ZDBF2	0.019968301	0.340468621	0.712392767
MTIF3	-0.011297194	0.340524901	0.712392767
CLEC12A	0.035925932	0.340591874	0.712398234
PLOD3	-0.014636802	0.340928527	0.712967668
SAMSN1	-0.019555555	0.34128103	0.713570026
SNTA1	-0.015498502	0.341430975	0.713748718
NCAM1	0.036324699	0.341623509	0.713904282
MED25	-0.014513659	0.341694485	0.713904282

MAD1L1	0.02612633	0.341698879	0.713904282
ERMN	0.02505359	0.341823394	0.714029656
AGPAT4	0.027207182	0.342294855	0.714625326
KBTBD8	0.019155888	0.342315247	0.714625326
ZNF415	-0.031666731	0.342363551	0.714625326
FEM1A	-0.010382057	0.3423668	0.714625326
IFIT1	0.051422244	0.342460542	0.714686226
FGFBP3	0.022833203	0.342648825	0.714805848
INVS	-0.01456333	0.342693148	0.714805848
BTBD3	0.013530815	0.342845333	0.714805848
CTGF	-0.021468614	0.343149843	0.714805848
SIRPB1	-0.033722117	0.343228572	0.714805848
LRRC56	0.016446261	0.343280802	0.714805848
BRE	-0.013792588	0.343347229	0.714805848
CCDC24	0.021159524	0.343495084	0.714805848
SIPA1L2	0.02195504	0.343523884	0.714805848
TNFRSF13B	-0.045177525	0.343535645	0.714805848
NFIB	0.065618164	0.343542473	0.714805848
GTPBP3	0.013059874	0.343584753	0.714805848
NAP1L5	-0.020184718	0.343598321	0.714805848
PPP3CC	0.010489413	0.343681828	0.714805848
SLC39A4	0.020249678	0.343841982	0.714805848
CYB5R1	0.014313564	0.343891047	0.714805848
SETBP1	0.025171807	0.343916519	0.714805848
RNF145	-0.015681734	0.343950538	0.714805848
GCHFR	-0.022619647	0.343980489	0.714805848
ASNS	0.019892632	0.344002694	0.714805848
MDC1	0.011833241	0.344069131	0.714805848
OTUD4	-0.011462008	0.344072882	0.714805848
LRRC58	-0.017834516	0.344092868	0.714805848
CACYBP	-0.023797569	0.344114759	0.714805848
TMEM140	0.033153333	0.34418232	0.714805848
MUC6	0.025461936	0.344226357	0.714805848
ATP2A3	0.018236661	0.344261449	0.714805848
STX7	-0.015721637	0.344483799	0.715133378
AKAP11	0.011872732	0.344573675	0.715180887
GPD2	0.02249954	0.344661044	0.715180887
CEP170	0.019888558	0.344700518	0.715180887
OSGEPL1	-0.016886645	0.344983449	0.715633771
KLHDC1	0.016942284	0.345081942	0.715703956
PRPF38B	0.01415787	0.345331362	0.716087083
MYBL1	0.027003277	0.345452122	0.716203323
SNORA24	0.020886853	0.345661563	0.716392503
FZD7	0.014731315	0.345707047	0.716392503
DHFRL1	0.017678578	0.345776482	0.716392503
DTL	-0.018953884	0.345802253	0.716392503
SNX2	-0.014158564	0.346200808	0.717083971
FBXO30	-0.020079287	0.346419586	0.717190504
PLSCR4	0.018278209	0.34649117	0.717190504
ZNF503	-0.025143029	0.346492438	0.717190504

BRCA1	0.019290328	0.346511412	0.717190504
DLGAP4	0.013765268	0.34672156	0.717491296
LETMD1	0.010085776	0.346825059	0.717571322
GTF3C1	0.020756386	0.346963268	0.71772312
SH3GLB2	-0.01294601	0.347368194	0.718271264
C1orf54	-0.020389516	0.347421954	0.718271264
CYP4F22	-0.043364631	0.347458438	0.718271264
FSD1L	0.020008824	0.347523794	0.718271264
RRP12	0.021215934	0.347552705	0.718271264
PLEKHH3	0.020446279	0.347714642	0.718471789
C10orf35	-0.02068504	0.347907492	0.718617997
PACS1	0.020689029	0.34807758	0.718617997
CLDN5	0.049900116	0.348085881	0.718617997
KIAA1671	-0.045287715	0.348132253	0.718617997
RBAK	-0.022502042	0.348166341	0.718617997
CCDC132	-0.010939037	0.348199337	0.718617997
SOAT1	-0.025259182	0.348239853	0.718617997
XRCC4	-0.017197323	0.348526635	0.719029851
GNB2L1	-0.004756555	0.348597855	0.719029851
CAMK1G	-0.037842787	0.348634313	0.719029851
UQCR11	-0.015121073	0.34881213	0.719233533
RNFT1	-0.022218261	0.348873759	0.719233533
NR1D2	-0.014445713	0.348928004	0.719233533
CCDC150	-0.017522718	0.349083753	0.719420604
ST6GALNAC6	0.020434455	0.349255255	0.719550092
AQP3	-0.025182914	0.349276596	0.719550092
NUDT16	-0.018478762	0.349500213	0.719876788
ABCA5	0.020150887	0.349743677	0.720130103
RPL32	0.004363482	0.349848895	0.720130103
RFTN1	-0.010778139	0.34986166	0.720130103
PAF1	-0.00913792	0.349955371	0.720130103
ETS2	-0.015950258	0.350021735	0.720130103
SNORD36C	0.022462976	0.350044093	0.720130103
HLA-C	0.064961961	0.35013233	0.720130103
PFKFB3	0.020596609	0.350193145	0.720130103
TMEM41B	0.01510669	0.350208722	0.720130103
ACACB	0.018811743	0.350460193	0.720513349
RSRC2	0.011635943	0.35059761	0.720620804
RBM26	0.013950405	0.350683387	0.720620804
TPM4	0.026555156	0.350765147	0.720620804
HSP90B1	-0.014597176	0.35077287	0.720620804
PCBD2	-0.013228329	0.350935216	0.720721054
CHMP6	-0.015364171	0.350953694	0.720721054
AFAP1	0.025879442	0.351049834	0.720721054
ZNF385D	-0.036277346	0.351168199	0.720721054
ERF	0.013057879	0.351257664	0.720721054
TOP2B	-0.007123958	0.351309051	0.720721054
NAALADL1	-0.018920404	0.351328366	0.720721054
SGCB	0.018379401	0.351342561	0.720721054
RASGRP4	-0.026892564	0.351536688	0.720912251

GIGYF2	-0.014360095	0.351566025	0.720912251
FRMD4B	-0.031355652	0.351753985	0.721091219
CWF19L1	0.010437878	0.351783592	0.721091219
C17orf62	0.012995102	0.352059093	0.721323808
ATN1	-0.02375512	0.352065487	0.721323808
KIF3B	-0.011409327	0.352121197	0.721323808
MAST3	0.012304045	0.352157725	0.721323808
SMUG1	-0.008708065	0.352305469	0.721382048
MAML1	0.013416518	0.352316501	0.721382048
KCNK6	0.012898407	0.352432658	0.721486424
MRPL4	0.013005595	0.352779694	0.721991973
RNF112	0.016044937	0.352810063	0.721991973
OTUD7B	0.018026644	0.353011646	0.722270964
WDR4	0.01517649	0.353077158	0.722271496
PAK1	-0.015949171	0.353336807	0.722635745
ADAP2	-0.017708586	0.353385788	0.722635745
SMC4	0.012393193	0.353475913	0.722686532
MEX3B	-0.015541924	0.353648418	0.722839777
SLC35A1	-0.010714156	0.353681473	0.722839777
CHRNA10	-0.017113711	0.353938797	0.723191051
PCSK6	-0.015112077	0.354006962	0.723191051
SERTAD3	0.014764471	0.35415978	0.723191051
DOCK10	0.009612966	0.354192737	0.723191051
SPON1	0.030564514	0.35420092	0.723191051
IQCC	-0.013209789	0.354245359	0.723191051
CLSPN	-0.016474836	0.354337601	0.723245972
PAQR8	0.020017847	0.354458973	0.723330892
SAFB	-0.009997983	0.354648376	0.723330892
SLC46A3	-0.011014791	0.354684173	0.723330892
PMM2	-0.013272924	0.354696694	0.723330892
PXDN	-0.052902231	0.354705943	0.723330892
REM2	0.02192785	0.354976088	0.723748446
UGCG	0.01953929	0.355350851	0.724263139
ABHD3	0.010198923	0.355504859	0.724263139
ZMYND15	-0.026782072	0.355628561	0.724263139
EIF5A	0.018628777	0.355630355	0.724263139
SH2D2A	0.025275533	0.355654352	0.724263139
STAM	0.013035135	0.35566487	0.724263139
PCCA	-0.01026306	0.35568655	0.724263139
DDX60	-0.020198995	0.355981884	0.724684038
ARHGEF12	-0.02507989	0.356072982	0.724684038
ZER1	0.014285492	0.356120894	0.724684038
IQCK	0.016532928	0.356197953	0.724684038
CD52	-0.014413311	0.356220603	0.724684038
ZFAND3	-0.014776756	0.356332783	0.724779047
SDHA	-0.010115056	0.356527191	0.725041242
HTR1E	-0.017513996	0.35663009	0.725117278
FAM117B	0.015677991	0.356823928	0.725378156
PRSS30P	-0.046889481	0.357009907	0.725622964
ARL6IP1	0.011105511	0.357131961	0.725730559

CACNA1S	-0.019753601	0.357193973	0.725730559
VDR	-0.016644808	0.357334107	0.725882039
NUP155	-0.011586983	0.357505354	0.726096653
APPL2	-0.011965135	0.35763387	0.726179375
PSMD1	0.010843808	0.357759595	0.726179375
CPNE3	-0.007691519	0.357774104	0.726179375
TTLL3	0.015071276	0.357808502	0.726179375
TMEM154	0.013759985	0.357960205	0.726354081
MVK	-0.014834921	0.358032123	0.726366858
ITGB5	0.04664864	0.35815176	0.726476421
DNM1P35	0.019037874	0.358327298	0.726627679
EIF3B	-0.00997355	0.35835762	0.726627679
PDE6G	-0.022196895	0.358501225	0.726734752
PGM3	-0.014958711	0.358544376	0.726734752
FDX1	-0.018398448	0.358683686	0.726734752
RPS27A	-0.010824244	0.358769359	0.726734752
NCOA4	-0.014248978	0.358782621	0.726734752
CSNK2B	-0.013814575	0.358903777	0.726734752
CECR1	-0.013010931	0.358965785	0.726734752
SLC45A3	0.029259752	0.3589739	0.726734752
DNAJC30	0.023564086	0.359005126	0.726734752
EAF1	0.013324317	0.359066976	0.726734752
ZYX	-0.015513666	0.359332283	0.727127785
LAG3	-0.044192227	0.359402732	0.727127785
TPRN	-0.016281544	0.359476145	0.727127785
RAB3GAP2	0.009205418	0.359523929	0.727127785
TIPRL	-0.017999373	0.359609403	0.72716779
ASAP3	-0.018338632	0.359746548	0.727186564
ST7L	-0.019606876	0.359750079	0.727186564
ACOT11	-0.017412232	0.359886867	0.727330242
ZNF256	-0.015883179	0.359982061	0.727389821
AHNAK	-0.01148343	0.360283831	0.727866713
DEAF1	0.015420084	0.360610291	0.728139464
ARID3B	-0.012829969	0.360611734	0.728139464
CUEDC1	-0.019942781	0.360616184	0.728139464
CTSZ	-0.025841517	0.360742323	0.728261312
PAOX	-0.015391546	0.360968361	0.728291941
ANKRD32	-0.06341877	0.360971667	0.728291941
KDM6B	0.015793494	0.360992929	0.728291941
NETO2	-0.020987802	0.361020678	0.728291941
VASP	0.028299166	0.361355639	0.728834834
C11orf68	0.012712463	0.361767604	0.729127234
WDR77	0.010497846	0.361801755	0.729127234
RALA	-0.009204729	0.361888869	0.729127234
YKT6	0.022252601	0.361971472	0.729127234
ING1	-0.013803681	0.362015313	0.729127234
KIFC2	0.017299173	0.362053419	0.729127234
ISY1	-0.010516428	0.362081535	0.729127234
CROCC	0.018668067	0.362091625	0.729127234
POU6F1	0.019722111	0.362093451	0.729127234

HGF	-0.016237007	0.362168161	0.72914503
DNAJC1	-0.019004673	0.36278844	0.730086487
WHAMM	0.021276145	0.362867278	0.730086487
ZNF544	-0.013572973	0.36286903	0.730086487
DBNDD1	0.039426275	0.362933207	0.730086487
MLLT11	-0.013860109	0.362965574	0.730086487
FOXD2	-0.025061023	0.363079334	0.73018262
MPV17L2	-0.015470793	0.363299506	0.730492686
SLC22A5	-0.02606004	0.363532618	0.73082865
CCT6P3	-0.008111694	0.363652282	0.730936464
TSHZ2	0.037479291	0.363731502	0.730962962
SCARNA9	-0.024853104	0.3638503	0.731012642
KYNU	-0.020630902	0.363901964	0.731012642
ZNF436	-0.017861105	0.363954347	0.731012642
ZNF324	0.016482853	0.364254893	0.731483564
TTC9C	-0.015525836	0.364363093	0.731568126
RNF130	-0.016054693	0.364567083	0.731715121
ALG1	-0.018437015	0.364575451	0.731715121
PCOLCE	0.017537106	0.36463462	0.731715121
PSME1	-0.009820633	0.364725805	0.73176544
ZNF283	-0.015330781	0.364799365	0.731780387
UCK2	-0.017859237	0.365114613	0.732280059
RPP25	0.017384024	0.365312791	0.732544797
CEP250	-0.017555426	0.365392622	0.732572167
MYB	-0.019201677	0.365462546	0.732579667
FOXO1	0.013691301	0.365646636	0.732672227
TMEM38B	-0.015937033	0.365692333	0.732672227
RAB1A	-0.01143052	0.365797692	0.732672227
MRPS9	-0.011325598	0.365823264	0.732672227
SPRN	-0.015608757	0.365839678	0.732672227
KLF11	0.016200264	0.366206231	0.733273657
MED17	-0.017770606	0.366336434	0.733401698
AXIN1	0.021571097	0.366640191	0.733665251
RIMBP3	0.01505437	0.366675173	0.733665251
FNTA	-0.006726643	0.366736971	0.733665251
PEX1	0.013127322	0.366754439	0.733665251
RPL5	-0.008386489	0.366808131	0.733665251
SEC23A	-0.018029539	0.366865766	0.733665251
ARRB2	0.014023609	0.367009004	0.733683697
WRN	0.009911227	0.367111485	0.733683697
ZNF611	0.015291802	0.367186456	0.733683697
TRIB2	0.018922838	0.367187692	0.733683697
GFI1B	0.032020301	0.367239104	0.733683697
CXCL5	0.05682185	0.367272686	0.733683697
SLMO1	0.014136566	0.367599281	0.733764253
MTMR4	0.009806934	0.367612398	0.733764253
C14orf142	-0.01087513	0.367671537	0.733764253
PRG4	-0.029474695	0.367706733	0.733764253
RASA1	0.009539576	0.367745218	0.733764253
TRABD	-0.009187524	0.367749669	0.733764253

MIER2	0.023782967	0.367790904	0.733764253
C2orf40	-0.027932473	0.367843332	0.733764253
S100PBP	0.010394286	0.367954458	0.733853674
TGFBR2	-0.010133763	0.368087871	0.73393946
KHNYN	0.017282264	0.368130082	0.73393946
TSPAN32	-0.01486098	0.368534579	0.734575717
IL2RA	0.02433317	0.368581943	0.734575717
EFCAB3	0.014929326	0.368713943	0.734706505
PCSK5	-0.019086919	0.36898599	0.735116258
RPL22L1	-0.058571528	0.369197203	0.735208342
HNRNPA3	0.020780482	0.369268055	0.735208342
NETO1	0.020418234	0.369270051	0.735208342
PIH1D1	0.011080472	0.369327351	0.735208342
MINPP1	-0.017272975	0.369364314	0.735208342
MXD4	-0.011389552	0.369672467	0.735512302
NANS	-0.01304594	0.369746906	0.735512302
CEACAM1	-0.024382165	0.369771162	0.735512302
DCTN5	0.014807893	0.369859226	0.735512302
CDC16	0.012297222	0.36992562	0.735512302
CARD14	-0.018565179	0.370001684	0.735512302
C2orf47	-0.013136794	0.370041318	0.735512302
UNC93B1	0.013226189	0.370110766	0.735512302
CABP5	0.057057041	0.370178288	0.735512302
TBC1D9	-0.022309133	0.370181501	0.735512302
PIK3R5	-0.015764922	0.370316637	0.735648753
ANKRD55	-0.046150263	0.370567263	0.735826783
ZNF74	0.015228595	0.370854617	0.735826783
LEPROTL1	-0.01493741	0.370934236	0.735826783
HIGD2A	-0.012407375	0.371052717	0.735826783
KRTAP10-11	0.016397031	0.371112645	0.735826783
ANKAR	0.021265482	0.371127812	0.735826783
AKT3	0.01769792	0.371258747	0.735826783
LILRB2	-0.014379469	0.371332314	0.735826783
YEATS4	0.020754356	0.371345822	0.735826783
BIRC5	-0.027696334	0.371381316	0.735826783
ROGDI	0.028285509	0.371394926	0.735826783
CCR7	-0.024217827	0.371470895	0.735826783
NDE1	0.014404864	0.371496271	0.735826783
ARMC6	0.014844506	0.371544018	0.735826783
TAS2R10	0.019511433	0.371555645	0.735826783
ZFAND5	-0.007982785	0.371603742	0.735826783
KRTCAP3	0.021302202	0.371628447	0.735826783
PUSL1	-0.015145932	0.371652554	0.735826783
MIR1228	-0.019246556	0.371669306	0.735826783
SEMA6B	0.018353123	0.371947329	0.736214184
MVD	-0.017832903	0.371998006	0.736214184
HIST1H2BG	0.060323122	0.372274491	0.736629663
SOAT2	0.017010397	0.372422774	0.736791365
MRM1	-0.017029856	0.372690116	0.737188508
CLTB	-0.008939136	0.372790909	0.737256132

ANKRD20A1	-0.015909595	0.372893412	0.737327112
RPP38	-0.018387971	0.37304052	0.737486251
SERF2	-0.013620164	0.373190745	0.737512378
ZNF658B	-0.015129147	0.373246699	0.737512378
WASL	-0.018785524	0.373253622	0.737512378
RPS6KA4	0.014915526	0.373371554	0.737611037
FAM103A1	-0.014949846	0.373436828	0.737611037
CCT5	0.01325407	0.37354895	0.737700862
RMI1	-0.013066821	0.373696828	0.737861255
ZNF98	-0.027469796	0.37395178	0.738064967
TIMM17A	-0.015322263	0.37396531	0.738064967
IMPA1	-0.021679577	0.374000036	0.738064967
ICOSLG	0.018171886	0.37425213	0.738214571
GCOM1	0.013229955	0.374289327	0.738214571
HCK	-0.014187333	0.374314978	0.738214571
NACA2	-0.017252049	0.374342613	0.738214571
KCNK4	-0.018572754	0.374463607	0.738321636
C3orf52	-0.013269153	0.374610796	0.73844786
CAPN11	0.032274179	0.374694302	0.73844786
GAS2L3	0.013226499	0.374727765	0.73844786
AJAP1	-0.01595522	0.374935768	0.73872624
GNAQ	0.027655866	0.375038528	0.738797201
CDC42SE1	0.01979921	0.375300582	0.739181877
HINT1	-0.011314603	0.375422496	0.739290448
ACY1	-0.014385678	0.375589507	0.739487772
ALPK1	0.02245553	0.375754482	0.739681017
TLE4	0.01023934	0.375830251	0.739698621
FUZ	0.015397409	0.375968886	0.739768935
C1QTNF6	0.021183427	0.375999641	0.739768935
SCARNA14	-0.016119215	0.376092735	0.739820594
DGKD	0.018577306	0.37617001	0.739841124
POP7	-0.01278395	0.37632229	0.740009137
TRIM16L	0.015272739	0.376422171	0.74007407
MAN2C1	0.022240338	0.376549034	0.74017233
HIST1H2BE	0.039656001	0.376605887	0.74017233
SLC35B1	-0.009235133	0.376698744	0.740223397
SENP5	0.011157734	0.376850477	0.74039012
PTPRN2	-0.020911307	0.376927476	0.740409979
WTAP	0.010352775	0.377037463	0.74049462
CALCOCO1	0.012735521	0.377143794	0.7405549
TBC1D24	-0.020862439	0.377214655	0.7405549
ZFAND2A	-0.016987362	0.377278091	0.7405549
MSRB3	0.031328311	0.37733577	0.7405549
TMEM163	-0.017876181	0.377562381	0.740634779
ESM1	-0.027123699	0.377603784	0.740634779
EMB	-0.018148045	0.377685461	0.740634779
SBNO1	-0.017471102	0.377830311	0.740634779
SUMO1P1	-0.015686316	0.37783498	0.740634779
SMAGP	-0.018126631	0.377883472	0.740634779
ZNF451	0.011384875	0.37790603	0.740634779

DNAJB14	0.028631709	0.378081115	0.740634779
RUNX2	0.026337622	0.378112085	0.740634779
PARVG	-0.013183338	0.378120739	0.740634779
SMARCB1	-0.014555235	0.378150111	0.740634779
KIF2C	-0.016313508	0.3781794	0.740634779
CHST3	-0.015552193	0.378264039	0.740669493
CDKL1	0.02144472	0.378487242	0.740867448
ZDHHC20	-0.014837441	0.378498999	0.740867448
ST3GAL3	0.024701357	0.378871231	0.741464932
TMEM132B	-0.015432353	0.378996387	0.741578754
NXNL1	0.024732616	0.379149897	0.741748005
PEX19	-0.010643406	0.379397295	0.742007694
MRPL16	-0.009048422	0.379443873	0.742007694
TRIB3	-0.021154766	0.379492328	0.742007694
TUBA1C	-0.016551568	0.379550778	0.742007694
FXR2	0.013263711	0.379736579	0.742069802
UBXN1	-0.006334846	0.379786548	0.742069802
NSL1	0.011091608	0.379859232	0.742069802
STK24	-0.006424495	0.3798748	0.742069802
MRPS17	-0.016122137	0.379917749	0.742069802
SLC25A35	0.0188906	0.380062175	0.742127109
CSDE1	0.020114884	0.38008118	0.742127109
CBLL1	0.015178968	0.380551313	0.742819308
FAM45A	-0.011729046	0.380569907	0.742819308
CYTH1	0.009570998	0.380746229	0.742949857
HMGB1	-0.010672074	0.380784306	0.742949857
FNDC3A	0.012932416	0.38083815	0.742949857
LILRB4	-0.02333486	0.380989084	0.743113334
ZNF250	-0.020817551	0.38111392	0.74322586
GGA2	0.012056344	0.381495713	0.743608019
GDI2	-0.00926118	0.381520083	0.743608019
DDX41	0.009618779	0.38153432	0.743608019
AIM2	-0.026018741	0.381596157	0.743608019
NBPF11	0.01205853	0.381645781	0.743608019
ORC2	0.011480928	0.381799046	0.743720156
C2orf44	-0.013652555	0.381939319	0.743720156
STX17	-0.013374665	0.382007024	0.743720156
TBX1	0.020544812	0.382128481	0.743720156
ACOT13	-0.012890037	0.382160423	0.743720156
BNIP2	-0.011476018	0.382206559	0.743720156
HIAT1	-0.014194659	0.382223196	0.743720156
SNORA70B	0.018723636	0.382264004	0.743720156
CHTF8	0.018590755	0.382352801	0.743720156
JAG1	-0.016317342	0.3823789	0.743720156
CDC25C	-0.020688383	0.382452612	0.743720156
TRIM27	0.008356379	0.382516165	0.743720156
ANKLE1	-0.030973956	0.382635236	0.743720156
SV2A	-0.018707513	0.382746373	0.743720156
ICAM1	-0.016987696	0.382821044	0.743720156
INCENP	-0.016968164	0.382939384	0.743720156

PPP6R2	0.014114529	0.383096301	0.743720156
KATNA1	-0.009896652	0.383190514	0.743720156
ZHX1	-0.009067144	0.383192708	0.743720156
KIAA0391	-0.010093234	0.383273594	0.743720156
NBAS	-0.011189599	0.383274743	0.743720156
HS2ST1	-0.015285867	0.383347411	0.743720156
MIR330	0.023351564	0.38339703	0.743720156
TSPAN4	-0.021022574	0.383456144	0.743720156
CD38	-0.039105964	0.383457936	0.743720156
CD47	0.01076719	0.38352859	0.743720156
DCAKD	0.010086354	0.383530236	0.743720156
UBE2E2	-0.013928006	0.383645154	0.743720156
MRPL41	0.010361335	0.383676154	0.743720156
DNAJB4	-0.021573386	0.383774209	0.743720156
MB21D2	0.023973641	0.383786207	0.743720156
DZIP3	0.01561592	0.383882227	0.743757711
BCL6	-0.016357355	0.383939973	0.743757711
MRPL52	-0.014342004	0.384038013	0.743817456
COL8A2	0.012514207	0.384469867	0.74452361
CCT7	-0.008166441	0.384766823	0.744854927
NDUFAF2	-0.015433365	0.384778548	0.744854927
CNN2	0.015624536	0.384842834	0.744854927
MRPL11	-0.010005935	0.385020686	0.745068877
UVRAG	-0.011506262	0.385190541	0.745123014
TRMT12	-0.013913189	0.385318707	0.745123014
DECR2	-0.015691266	0.385420227	0.745123014
IL1RN	-0.022249847	0.385483353	0.745123014
MRPS33	-0.013101099	0.385522688	0.745123014
ZNF426	-0.01385505	0.385551787	0.745123014
TPM2	0.034175358	0.385621704	0.745123014
DHX16	0.009434466	0.385836109	0.745123014
SLC22A15	0.021399712	0.38600397	0.745123014
SOCS4	0.013793525	0.386029421	0.745123014
EYS	-0.01293053	0.386126808	0.745123014
SAP25	0.015600136	0.386140576	0.745123014
WDR92	-0.012642607	0.386141646	0.745123014
ADCK4	0.015085016	0.386155228	0.745123014
CDK14	-0.024769041	0.386171865	0.745123014
ZNF326	0.016528791	0.38617852	0.745123014
RFX4	0.020983323	0.386297821	0.745123014
ABHD6	-0.012367855	0.386350076	0.745123014
PDGFB	0.02700634	0.386393109	0.745123014
GNAZ	0.038334033	0.386451156	0.745123014
DEFB123	0.018715604	0.386468162	0.745123014
RALGAPB	0.010251649	0.386574974	0.745123014
KLHL5	0.012663522	0.386596935	0.745123014
PGS1	0.010498053	0.387195619	0.74536782
NDRG1	0.011722562	0.387254412	0.74536782
SPINK2	-0.025830417	0.387269061	0.74536782
ARHGEF11	0.015431864	0.387330747	0.74536782

C1orf27	0.017531863	0.387417929	0.74536782
CDKN2B	-0.025765916	0.38745353	0.74536782
HSF2	-0.013080091	0.387470072	0.74536782
NIPA1	0.015240606	0.387493928	0.74536782
RAD51L3-RFFL	0.011030004	0.387497688	0.74536782
CKAP5	-0.009070265	0.387517494	0.74536782
RIPK3	0.010277682	0.38752802	0.74536782
PFKM	-0.015566326	0.387541302	0.74536782
MAP3K6	0.016032568	0.387599347	0.74536782
MTMR3	-0.010312289	0.387692599	0.745417644
NAA40	0.015371443	0.387760235	0.745418208
RASA4	0.02149764	0.387973194	0.745668892
WNT7A	0.018407221	0.388147786	0.745668892
DUSP12	-0.010214195	0.388164245	0.745668892
C17orf53	-0.015802701	0.388225911	0.745668892
FGFBP2	-0.037274977	0.388287319	0.745668892
SLCO3A1	0.011942981	0.388294832	0.745668892
UTP18	-0.014221343	0.388459004	0.745727683
MCF2L-AS1	-0.020902406	0.388467574	0.745727683
GLIPR2	-0.009536476	0.388655869	0.745727683
FLVCR1	-0.020404729	0.38866043	0.745727683
RAB14	-0.025262712	0.388745537	0.745727683
SLC6A4	0.027885293	0.388876846	0.745727683
REEP1	0.021269946	0.388909419	0.745727683
MZF1	0.013543114	0.388923485	0.745727683
MBD6	0.018297326	0.388931784	0.745727683
SPAG9	0.00765791	0.389233428	0.74609525
RTP4	-0.028159047	0.389258295	0.74609525
LEMD3	-0.01096262	0.389421104	0.746249863
NOP56	0.012440008	0.389473797	0.746249863
KLHL17	0.01747958	0.389632546	0.746424827
IGJ	-0.051561072	0.389719625	0.746462455
MYC	0.019745117	0.389946774	0.746696471
UGDH	-0.018704641	0.389976718	0.746696471
ANKMY1	0.023493223	0.390471691	0.747386129
FUBP1	0.011208226	0.390471947	0.747386129
KLF12	0.011055294	0.390710997	0.747714389
CWC25	0.014299658	0.391046581	0.748008823
FAF2	-0.008783428	0.391047808	0.748008823
FDFT1	-0.013916907	0.391197296	0.748008823
H1FX	0.014388928	0.391203724	0.748008823
ENPP4	-0.027093667	0.391312059	0.748008823
LPGAT1	-0.011188386	0.391353631	0.748008823
API5	0.008087931	0.391392303	0.748008823
DPY19L3	-0.015892025	0.391405466	0.748008823
FHIT	-0.035442095	0.391594558	0.748241009
RIC8B	0.011104471	0.391817808	0.748434441
GADD45A	0.031091379	0.391897513	0.748434441
FBLN5	0.029433177	0.391906959	0.748434441
STK17A	-0.016556461	0.391980634	0.748434441

BAG6	0.013321902	0.392054154	0.748434441
IL10RB	-0.008827827	0.392166183	0.748434441
IFI35	-0.01796854	0.392202296	0.748434441
ZNF813	-0.014995718	0.392236715	0.748434441
PPP1R12C	0.015260465	0.392579471	0.748851378
SDF4	0.013427492	0.392622821	0.748851378
MUTYH	0.010346722	0.392663006	0.748851378
TSN	-0.016445972	0.392725833	0.748851378
TAF1B	-0.013761237	0.393025009	0.74929277
VSIG10	-0.013831028	0.393105862	0.749317856
ZBBX	0.012588683	0.3932537	0.749470593
CDR2L	0.030620552	0.393520646	0.749522494
GCAT	-0.018835368	0.393590323	0.749522494
PDDC1	0.012570239	0.39359731	0.749522494
ATG2A	-0.012042215	0.39366304	0.749522494
HMGN3	-0.015318209	0.393712207	0.749522494
IL21R	-0.016573189	0.393735421	0.749522494
CLCN7	0.011608483	0.393754928	0.749522494
ZNRD1	-0.012755228	0.393982881	0.749618654
MAPK14	-0.021220267	0.394069275	0.749618654
USH1G	0.01710145	0.3940699	0.749618654
PRAMEF13	-0.018302604	0.394093584	0.749618654
PBXIP1	0.014109404	0.3943057	0.749618654
KPNA5	0.020580657	0.394309011	0.749618654
MCTP1	-0.028443324	0.394374373	0.749618654
GADD45G	-0.021575899	0.394383341	0.749618654
ZG16B	-0.024126597	0.394414946	0.749618654
CBFB	-0.011768137	0.394609751	0.749712943
SPPL2A	-0.009324345	0.394636105	0.749712943
GOLGA4	-0.01461607	0.394720465	0.749712943
SNORA26	0.021185726	0.394773407	0.749712943
DNAJC27	0.013101853	0.394841605	0.749712943
RABGGTA	0.014482879	0.394937104	0.749712943
TOLLIP	-0.01740552	0.394995423	0.749712943
BBS1	-0.010817339	0.395012118	0.749712943
ARL5B	-0.024443222	0.395074135	0.749712943
FAM200B	0.026115326	0.395163516	0.74973354
PARVB	0.037948343	0.395220454	0.74973354
SLC26A2	-0.014480473	0.395397428	0.749940736
NCOA6	0.007918727	0.395820276	0.750614123
MZT2A	-0.019988595	0.395977213	0.750783105
NDFIP2	-0.018354066	0.39621878	0.750858725
SCFD1	0.009490933	0.396253699	0.750858725
TNIP3	0.030087002	0.396279249	0.750858725
RUVBL2	-0.012509378	0.396448297	0.750858725
ZSWIM4	0.01090365	0.396474661	0.750858725
PSAP	-0.005807094	0.396482469	0.750858725
CLIP2	0.016623963	0.396537368	0.750858725
MTRF1L	0.009145725	0.396580004	0.750858725
ZNF324B	-0.015771735	0.396724094	0.750858725

HK3	0.016449603	0.396825286	0.750858725
SSBP1	-0.012003502	0.396847602	0.750858725
BAG4	-0.01345603	0.396872964	0.750858725
SNORD22	0.01638599	0.396905727	0.750858725
SLC26A11	0.012118255	0.396966778	0.750858725
C22orf46	0.010633064	0.397101371	0.750869623
IKZF4	0.016169244	0.39710821	0.750869623
DCAF6	-0.008537142	0.397261571	0.750991985
ME2	-0.0121305	0.397308616	0.750991985
CYB561D1	-0.008881488	0.397411775	0.751058723
DNAJC7	-0.009852107	0.397517849	0.751066369
CD37	-0.016166288	0.39768357	0.751066369
PLA2G6	0.015644348	0.397711769	0.751066369
GLRX5	-0.009047178	0.397713262	0.751066369
NFU1	-0.010590763	0.397828229	0.751066369
YIPF2	-0.014507551	0.397833941	0.751066369
AKAP13	-0.009741676	0.397890793	0.751066369
EXOC3	0.011263137	0.397965759	0.751079792
CTSB	0.013196201	0.398182081	0.75130622
MAP1A	0.025099169	0.398263973	0.75130622
SKI	0.011007926	0.398324594	0.75130622
NGDN	0.008689231	0.398357233	0.75130622
MRPS25	0.018813537	0.398551877	0.751367914
DCP1A	0.010142934	0.398585825	0.751367914
TRIP12	-0.007304202	0.398646377	0.751367914
RCC1	0.018258386	0.398661465	0.751367914
ANKRD26	-0.013058828	0.399039911	0.751932717
KLB	-0.011957274	0.399097002	0.751932717
DAXX	0.011974316	0.399271305	0.751992998
TCTA	-0.012875244	0.399302258	0.751992998
LRRC29	-0.012954732	0.399396361	0.751992998
PSRC1	-0.017458263	0.399414967	0.751992998
BCDIN3D	-0.01743096	0.399497326	0.751992998
HIST1H4C	-0.015086772	0.399536618	0.751992998
EGR1	-0.026087372	0.399823096	0.752404259
BCAS2	-0.020890322	0.399960183	0.752534296
C4orf32	-0.017621673	0.400224858	0.752730604
ZNF85	0.031034153	0.400353293	0.752730604
KRT73	0.059694154	0.400384658	0.752730604
IPO8	0.033382316	0.400443952	0.752730604
CHST10	0.015680014	0.400457786	0.752730604
SLC2A14	0.017910519	0.400472538	0.752730604
TBXA2R	0.020419069	0.400694236	0.75286941
RAB4B	0.020455568	0.400764959	0.75286941
ABHD14B	-0.017347857	0.400820959	0.75286941
USP5	0.011089845	0.400871252	0.75286941
PKD1	0.019110933	0.400886467	0.75286941
KIAA0556	0.010249518	0.400976405	0.752910572
JAGN1	-0.012818175	0.401125367	0.752971397
USP4	0.008247144	0.40116757	0.752971397

KCNH2	0.014486936	0.401217782	0.752971397
CNNM2	-0.017466824	0.401291848	0.752971397
XPNPEP3	0.006541419	0.401348924	0.752971397
RAD54B	-0.014514083	0.401827055	0.753740667
PRADC1	-0.015198857	0.401997796	0.753859986
ZNF768	0.01893315	0.402084163	0.753859986
TESC	-0.016959248	0.402136228	0.753859986
DYNLL1	-0.01116683	0.402163088	0.753859986
UROD	-0.007900979	0.402266063	0.753925339
PDLIM3	-0.014850928	0.402350063	0.753955113
P4HA2	0.014289152	0.40251865	0.754141547
DCTN3	-0.007750268	0.402585816	0.754141547
SMARCAL1	-0.007589891	0.402865049	0.754325415
ABAT	0.023027811	0.402875441	0.754325415
SDHAP2	0.021585124	0.402888414	0.754325415
CEP68	0.0164486	0.403178194	0.754569439
TSPAN2	-0.02287064	0.403211942	0.754569439
ADAM28	0.025771656	0.403410523	0.754569439
ZNF195	0.013683018	0.403424965	0.754569439
DGKQ	-0.019358535	0.403443355	0.754569439
SYTL2	0.030126032	0.403467634	0.754569439
ZNF385A	-0.019044955	0.403495935	0.754569439
RAB3A	-0.015290509	0.403668088	0.754763863
TMEM183A	-0.00669724	0.404186748	0.755605998
BCL7A	0.015446633	0.404287362	0.755666466
MAP2K4	-0.008437164	0.404442332	0.755828494
CHMP4B	-0.010738532	0.404627899	0.755935487
LAPTM4B	0.019895849	0.404723736	0.755935487
PRKCE	-0.016102063	0.404732176	0.755935487
CLIP4	-0.013556059	0.404772756	0.755935487
C9orf114	-0.01423402	0.40484207	0.755937393
SLC27A4	-0.015390451	0.405079035	0.756252292
NT5C	-0.007738316	0.40523891	0.756302692
CORO1B	-0.018258573	0.405266367	0.756302692
NR2C2	0.012621711	0.405353963	0.756302692
RGS18	0.023722321	0.405430048	0.756302692
AQP10	0.059439946	0.405469479	0.756302692
BRAT1	-0.007206756	0.405579937	0.756302692
SNX6	-0.013176004	0.405654863	0.756302692
EEPD1	-0.01919186	0.405659168	0.756302692
TMEM9	-0.013572074	0.405778542	0.756302692
N6AMT2	-0.013028262	0.40584325	0.756302692
PLGLB1	0.040605565	0.405903613	0.756302692
GLIS3	0.015573886	0.405978329	0.756302692
HECW2	-0.0146976	0.406031183	0.756302692
CD200	0.026064525	0.406062598	0.756302692
SIRPD	-0.01795279	0.40622798	0.75648343
NAPSA	0.037818678	0.406760339	0.757246753
ITGAX	0.017692917	0.406774703	0.757246753
SNHG5	0.03546789	0.406843847	0.757248115

POMP	-0.009271982	0.40695227	0.757322575
PSMD13	0.014623721	0.407070129	0.757414567
SLC24A1	-0.017582894	0.407348325	0.75777071
EIF4EBP1	0.013363339	0.407398454	0.75777071
SLC35A4	-0.019508076	0.40756149	0.757946595
RPL29P2	-0.014673768	0.407719048	0.758096952
FCER1A	-0.035652142	0.407841716	0.758096952
NDUFA11	-0.011888066	0.40793072	0.758096952
FURIN	0.015766472	0.407962506	0.758096952
CCDC58	-0.040219593	0.407984781	0.758096952
RBM17	0.008914333	0.408106474	0.758109943
CYBA	-0.014024934	0.408128751	0.758109943
TTC7A	0.01604505	0.408274076	0.758252642
ELL3	0.021673536	0.408403898	0.758366507
PELP1	-0.015717808	0.408751204	0.758724485
TM7SF3	0.014366673	0.408799409	0.758724485
METTL7A	-0.016881705	0.408896596	0.758724485
SMPD2	0.013607978	0.408913454	0.758724485
NEO1	0.010553023	0.409015562	0.758724485
CCNA2	-0.02633941	0.409096745	0.758724485
EMP1	-0.031325582	0.409284825	0.758724485
CCL4L2	-0.032536192	0.409289871	0.758724485
SPRED2	-0.017696632	0.409318987	0.758724485
C1orf177	-0.015157106	0.409499377	0.758724485
EOMES	-0.032191133	0.409515567	0.758724485
EPS15	0.009778063	0.409624273	0.758724485
FYCO1	-0.014466158	0.409635029	0.758724485
IQCG	-0.018478116	0.409655993	0.758724485
TARS2	-0.012093005	0.409728144	0.758724485
SNORD105B	0.013920149	0.409801174	0.758724485
CLN8	0.022254997	0.409854088	0.758724485
MN1	-0.015533274	0.409890988	0.758724485
SLC12A4	0.013514477	0.409899035	0.758724485
SAR1A	0.013241284	0.410137803	0.759039516
SMN1	0.025996577	0.410249427	0.759119175
CENPQ	-0.020330991	0.410412185	0.75929341
SNORD42B	0.014439714	0.410607363	0.759527558
GPATCH2	0.013232833	0.410742296	0.759650206
RAB8B	0.0080808	0.4108711	0.759761478
LEPROT	0.01991464	0.410969596	0.759816679
ATP13A2	-0.014674148	0.41114851	0.759935399
MAN2A2	0.013381535	0.411219351	0.759935399
RPS6KC1	-0.012244483	0.411239772	0.759935399
RPLP2	0.0125166	0.411462295	0.760059877
IK	-0.008671605	0.411558647	0.760059877
GUCA1A	-0.014512253	0.411563679	0.760059877
LPIN1	0.013278434	0.411611625	0.760059877
CLUAP1	0.005064686	0.411733149	0.760059877
AMBRA1	-0.015186775	0.411782123	0.760059877
CCNH	-0.015393476	0.411855268	0.760059877

OAS2	0.01960543	0.411929676	0.760059877
KPNA4	-0.008056164	0.411974427	0.760059877
SLC4A4	-0.023440624	0.41199379	0.760059877
TBC1D10C	-0.011830015	0.412328646	0.760135547
GOLT1B	0.012561594	0.412393922	0.760135547
CCR4	0.023673575	0.412433483	0.760135547
ZNF740	-0.014036122	0.412436825	0.760135547
FAM35A	-0.009704166	0.412568745	0.760135547
ITGAM	-0.016212186	0.412600162	0.760135547
UIMC1	-0.01040743	0.412618663	0.760135547
GNL2	0.007712708	0.412697622	0.760135547
MTFR1	-0.016804342	0.41270783	0.760135547
AASDH	-0.011691529	0.412770897	0.760135547
SAE1	-0.007505499	0.412930643	0.760135547
SFT2D3	-0.013576453	0.412989578	0.760135547
CHEK1	-0.020892558	0.413067285	0.760135547
EGF	0.031092092	0.413314844	0.760135547
ARHGEF18	-0.009634436	0.413382192	0.760135547
USP18	0.031038367	0.413382861	0.760135547
CEP70	0.0182596	0.413407668	0.760135547
GET4	0.014001528	0.413410997	0.760135547
PYGO2	-0.018093578	0.413437869	0.760135547
NFYB	0.016439728	0.413450345	0.760135547
ANKRD54	0.013743193	0.413542056	0.760135547
TP53I3	0.01923206	0.413608678	0.760135547
NQO1	-0.015157625	0.413614274	0.760135547
EMP3	-0.010242048	0.413747226	0.760253659
GPR56	-0.029838002	0.413866702	0.760282568
SERPINB6	-0.011243716	0.41390033	0.760282568
ZDHHC6	0.010566974	0.414052635	0.76043614
RNF26	-0.011920527	0.414274901	0.76069655
PRRT3	-0.019169812	0.414331873	0.76069655
GPAA1	0.012493009	0.414483837	0.760767538
ZNF701	-0.018037965	0.414507997	0.760767538
ANKRD46	-0.017861889	0.415037282	0.761612678
SNORD51	-0.014574942	0.41511921	0.761636754
TPCN2	-0.01809071	0.415690626	0.76231205
ZFHX3	-0.017943006	0.41577092	0.76231205
ACTR6	-0.011227962	0.415791034	0.76231205
SPNS3	-0.013567695	0.415938599	0.76231205
HSBP1	0.008975643	0.415985832	0.76231205
ORAI3	-0.015359059	0.415989266	0.76231205
ALDOB	0.022662259	0.416027582	0.76231205
SLC35A3	0.010803454	0.416039837	0.76231205
NOTCH2	-0.014195272	0.416153251	0.76231205
PRPF40A	0.017381699	0.416175962	0.76231205
PGRMC2	-0.009377476	0.416250727	0.762322849
APOL6	-0.021312525	0.416330966	0.762342595
RPL39L	-0.00569229	0.416399253	0.762342595
PKN1	0.013831791	0.416563509	0.762417774

ACP2	-0.01712171	0.416578073	0.762417774
RBM15B	-0.012275733	0.416678042	0.762418012
ATP5J2	-0.007365249	0.416756682	0.762418012
AMDHD2	0.015496177	0.417024866	0.762418012
ZNF649	-0.019055161	0.417063126	0.762418012
ZNF79	-0.023879191	0.417209816	0.762418012
HSPA1A	-0.013228408	0.417244631	0.762418012
PCYOX1L	0.014496264	0.417350772	0.762418012
SLC27A1	0.014651297	0.417377218	0.762418012
DOPEY1	-0.012760347	0.417381512	0.762418012
TNFAIP1	0.010007431	0.417389805	0.762418012
MMP24	-0.013347815	0.417443708	0.762418012
ATRIP	-0.011373398	0.417461352	0.762418012
NUP88	-0.008206667	0.417473626	0.762418012
MAPK1IP1L	-0.008346378	0.41756254	0.762454595
TRIM32	-0.014289183	0.417645508	0.762480311
DDX11	-0.043853062	0.417719186	0.762489061
KIAA0195	0.015182058	0.417917448	0.762663451
ACSL1	-0.017844567	0.417952525	0.762663451
PLCXD2	-0.015330636	0.418097829	0.762802846
ZUFSP	0.009845543	0.418413227	0.76316954
POLR3B	-0.012667049	0.41850425	0.76316954
VPS16	0.015162325	0.418550858	0.76316954
PRKCA	0.018031497	0.418753144	0.76316954
FBXO46	0.009906767	0.418792816	0.76316954
CHTF18	-0.014298411	0.41892758	0.76316954
DOCK5	0.015390072	0.418977161	0.76316954
AKIRIN1	0.012966216	0.41900516	0.76316954
OPTN	0.015402202	0.419020893	0.76316954
ZNF558	0.013394887	0.419021856	0.76316954
RPL37	-0.020522806	0.419057228	0.76316954
MYPOP	0.006509027	0.419317217	0.763406349
ZBTB38	-0.019604502	0.419348674	0.763406349
MXD1	0.015106354	0.419394164	0.763406349
PPP1R10	0.01624939	0.419551779	0.763528193
INO80B	-0.009844479	0.41959906	0.763528193
HEATR6	-0.01196172	0.419734511	0.763577396
PUS3	-0.011845623	0.419764067	0.763577396
SERHL2	0.016177032	0.419872325	0.763598722
ERO1L	-0.013369788	0.419913761	0.763598722
ELK4	0.01651302	0.420296067	0.764168392
NF1	0.020278414	0.420487993	0.764391788
EVI5	0.017756559	0.420598311	0.764428148
PHF21A	0.007379976	0.420661175	0.764428148
SCAMP3	-0.008310673	0.420848101	0.764428148
BCL9	-0.01552833	0.420877424	0.764428148
SLC39A3	-0.014360266	0.420928654	0.764428148
ABCB10	-0.010682812	0.420984398	0.764428148
ZNF688	0.010147296	0.421099638	0.764428148
CNIH4	-0.009790272	0.42115473	0.764428148

ANKRD12	0.012038215	0.421196713	0.764428148
WEE1	0.019701794	0.421198597	0.764428148
ZNF507	0.018185797	0.421293182	0.764474465
TKT	0.011153128	0.421459138	0.764650253
ACSBG1	0.045249884	0.421542749	0.764676613
SPOPL	-0.015345643	0.421739195	0.764907611
KIF13B	0.011866816	0.421874728	0.764923613
SP100	-0.017044292	0.421901325	0.764923613
RDH11	0.012867	0.421976184	0.764923613
MIR488	-0.014348296	0.422031565	0.764923613
GLOD4	0.006599265	0.422120888	0.764923613
RRP15	-0.014310576	0.422162648	0.764923613
POTEF	-0.005816619	0.422271531	0.764995675
ASF1A	-0.012594394	0.422665341	0.76558381
TOR1A	-0.009852957	0.422773577	0.765641036
RGMB	0.037656292	0.422866317	0.765641036
ADRA2B	0.01459046	0.422904444	0.765641036
C6orf57	0.0122959	0.423062836	0.765678493
C6orf163	-0.01793657	0.42306348	0.765678493
APOA1BP	-0.009155785	0.423132982	0.765679088
NAV1	-0.019184163	0.423795091	0.766685567
RWDD1	-0.008276184	0.423827716	0.766685567
TAP1	-0.014576978	0.423923493	0.766733521
PRKAA1	0.008279814	0.424186887	0.766925592
NAA38	-0.01621801	0.424242838	0.766925592
FBXO31	0.010589005	0.4242637	0.766925592
NOTCH4	0.021916242	0.424306832	0.766925592
IRF1	-0.015073551	0.424574736	0.767190157
ARSG	-0.010292432	0.424591824	0.767190157
FNBP1L	0.022500706	0.424926311	0.767433689
MAP2K3	-0.010864876	0.42500529	0.767433689
FAHD1	-0.014084247	0.425051176	0.767433689
FARSB	0.018583302	0.425053815	0.767433689
WTIP	-0.013464745	0.425097698	0.767433689
FRMD3	0.038661343	0.425240104	0.767433689
NEU1	-0.011546418	0.425244333	0.767433689
SPN	0.013500228	0.425341852	0.767433689
CDCA4	-0.012171464	0.425407377	0.767433689
ATOH8	0.027911861	0.425461668	0.767433689
MYO15B	0.027915341	0.425489254	0.767433689
SLC2A4RG	-0.011046643	0.425832233	0.767841426
RNF208	0.016586637	0.425854053	0.767841426
PPP2R5C	-0.012513641	0.425949126	0.767887765
CTRC	-0.014994939	0.426159783	0.768039469
SESN2	-0.018665161	0.42617205	0.768039469
HIST2H2AA4	0.02942755	0.42641753	0.768356771
STOX1	-0.029801762	0.426702682	0.76874544
SIVA1	-0.010163664	0.426983925	0.768819825
ZNF830	-0.014557228	0.426989839	0.768819825
SLC25A44	-0.008488103	0.427086639	0.768819825

C1orf85	-0.022190367	0.427094659	0.768819825
KIAA1958	0.016812364	0.427138648	0.768819825
KLHDC8B	-0.020585488	0.427211683	0.768819825
C6orf52	0.012056764	0.427231461	0.768819825
ANGPTL4	0.014129681	0.427299626	0.768819825
TTC32	0.017883688	0.427420274	0.768861836
MPP6	-0.019088092	0.427461897	0.768861836
CEACAM4	-0.018083108	0.427919236	0.769435749
HP1BP3	0.013550132	0.427919999	0.769435749
EPC2	0.017663085	0.428128721	0.769469553
PLEKHG5	0.018417269	0.428137773	0.769469553
IL2RB	-0.021749757	0.428288094	0.769469553
VTRNA1-1	0.020928904	0.428351684	0.769469553
WNT5B	-0.01665341	0.428357073	0.769469553
DEGS1	-0.010832146	0.428456687	0.769469553
TNIP2	0.01357986	0.428463169	0.769469553
SMEK2	-0.007027006	0.428558111	0.769469553
TTC19	0.010525027	0.428564441	0.769469553
MMRN1	0.041618587	0.428733768	0.769528491
MED22	-0.006865593	0.428803023	0.769528491
UBTD2	-0.011460779	0.42885165	0.769528491
NFATC1	-0.012115366	0.428968364	0.769528491
CLDN14	-0.013717945	0.428974378	0.769528491
DDX50	0.007896996	0.429014393	0.769528491
RPL7	-0.006413205	0.429285279	0.769784065
UNC5C	-0.013370559	0.429295964	0.769784065
SCAMP5	0.025114957	0.429377045	0.769804748
PAQR4	-0.014523303	0.429654765	0.769954931
POTEKP	0.013309134	0.429671655	0.769954931
ARHGAP1	-0.00781303	0.429681605	0.769954931
SMCHD1	-0.01546764	0.429755234	0.769954931
MAN1C1	-0.018560069	0.429888128	0.769954931
TUBGCP6	0.013049427	0.429954299	0.769954931
IFIH1	0.014489586	0.429982699	0.769954931
TP53INP2	-0.013140769	0.430039869	0.769954931
PRPF39	0.014766355	0.43008685	0.769954931
BIK	-0.016534471	0.430281314	0.769972141
PDZK1IP1	0.048097263	0.430288106	0.769972141
ID2	-0.01443911	0.430375403	0.769972141
ARPC1A	0.007196785	0.430512169	0.769972141
KCTD21	-0.014926255	0.430600149	0.769972141
MDM1	0.013891512	0.430603435	0.769972141
METTL14	-0.011908747	0.430605889	0.769972141
PPP3CA	-0.019076497	0.430653384	0.769972141
ABCA10	-0.016244009	0.430761177	0.769972141
MAST4	0.026542859	0.430792074	0.769972141
UBE3A	-0.006411037	0.431165645	0.77024098
MIR373	0.014441564	0.431266956	0.77024098
GINS4	0.012249257	0.431305953	0.77024098
WDR43	0.011695571	0.431330158	0.77024098

IFNGR1	-0.011273466	0.431378345	0.77024098
LRTOMT	-0.012635865	0.431470329	0.77024098
ABI2	-0.010053813	0.431476605	0.77024098
FXN	0.013596867	0.431499171	0.77024098
HLA-DOA	0.024772102	0.431572377	0.770247444
PPP3R1	-0.014013057	0.43182905	0.770435362
NSD1	0.011833015	0.431830042	0.770435362
FANCG	-0.012603082	0.431886478	0.770435362
METTL23	-0.006528312	0.431972786	0.770465158
CPPED1	0.017379304	0.432354523	0.770614559
TAS2R30	0.021345256	0.432394949	0.770614559
ARFIP2	-0.014226619	0.432421217	0.770614559
SEC24A	-0.015337291	0.432499592	0.770614559
NCKAP5	0.027253907	0.432575399	0.770614559
SNAPC2	-0.013583195	0.432576757	0.770614559
FBP1	-0.017325977	0.432582272	0.770614559
TIAM1	-0.019153511	0.432613504	0.770614559
UPK3A	-0.019247199	0.432804416	0.770830584
INSM1	0.015314519	0.433054961	0.771152729
NIPAL2	-0.013675174	0.433189169	0.771156371
INF2	0.018232961	0.433288765	0.771156371
CCDC109B	-0.008428202	0.433370459	0.771156371
MARCH7	-0.007873611	0.433444215	0.771156371
CRTC2	0.011549469	0.433469192	0.771156371
MAP3K9	0.016663159	0.433475015	0.771156371
CLIP3	0.021785837	0.433655211	0.771352969
JAK2	-0.014808115	0.434159227	0.7721254
HSF1	-0.015488418	0.434456279	0.772338462
PMCHL1	-0.011953002	0.434485338	0.772338462
VNN2	-0.018406459	0.434569676	0.772338462
TTYH2	-0.015476558	0.434587034	0.772338462
CNTNAP2	-0.062109151	0.434627905	0.772338462
GPNMB	0.015048313	0.434794537	0.772510551
FGD2	0.025516455	0.435140075	0.7730004
GRM2	0.017830025	0.435334962	0.773101356
FGFR1OP2	-0.01243369	0.435485221	0.773101356
LRRC8C	-0.017266173	0.435620913	0.773101356
INSR	0.016023032	0.435644427	0.773101356
KARS	-0.008413717	0.435654582	0.773101356
FAM117A	0.011854776	0.435669987	0.773101356
MEGF8	0.0151553	0.435685813	0.773101356
AGPAT6	0.013768445	0.436134436	0.773773373
S100A10	-0.00828089	0.436289363	0.773924191
VAMP8	-0.010478637	0.436446404	0.774078714
CTSF	0.01658541	0.436544037	0.774127835
CHML	-0.014987283	0.43678709	0.774378897
SH3BP2	0.017804664	0.436825534	0.774378897
AFTPH	0.009474074	0.437109433	0.774758096
SWAP70	-0.017718893	0.437224676	0.774838288
SCARNA17	-0.020267294	0.437340928	0.774920239

CDC45	-0.031054373	0.437811925	0.775630634
GOLGA2	0.013131335	0.438019921	0.775705169
MED9	-0.013731302	0.438074054	0.775705169
POLE4	-0.007875554	0.438219051	0.775705169
TNNT1	-0.022700656	0.438261319	0.775705169
SLC9A1	-0.009773558	0.43835837	0.775705169
SLC38A9	-0.010572508	0.438420563	0.775705169
CDK5RAP3	0.013352107	0.438450378	0.775705169
HOMER3	-0.017312544	0.4384539	0.775705169
GLYR1	0.006109452	0.438484709	0.775705169
NUDT2	-0.021661659	0.438622952	0.775825736
ACO2	-0.009922528	0.439047668	0.776155715
P2RY11	0.012897818	0.439183223	0.776155715
HIST1H4H	-0.054819851	0.439184019	0.776155715
C4orf46	-0.013396172	0.439219147	0.776155715
CUL1	0.008217853	0.439223459	0.776155715
ZNF138	0.011900699	0.439386733	0.776155715
EXOC1	0.01174117	0.439586301	0.776155715
MELK	-0.023431685	0.439684529	0.776155715
NAMPT	-0.025026735	0.439819857	0.776155715
TMX2	0.007249745	0.439872578	0.776155715
C6orf1	-0.012660354	0.439971461	0.776155715
C5orf20	-0.021236116	0.439984673	0.776155715
EPRS	-0.007350754	0.440020386	0.776155715
TPR	-0.011792613	0.440033281	0.776155715
CYB5D2	-0.011320905	0.440051064	0.776155715
PDCD5	-0.009923722	0.440082483	0.776155715
GZMA	-0.019063117	0.440210693	0.776155715
SLC35E3	-0.008870694	0.440212504	0.776155715
PDCD10	-0.011442264	0.440283127	0.776155715
HJURP	-0.016508536	0.440294898	0.776155715
NF2	-0.012750628	0.440419689	0.776155715
HLA-DPA1	-0.01630449	0.440500757	0.776155715
TADA2B	-0.00867829	0.440549478	0.776155715
MRPL42	-0.01224556	0.440597042	0.776155715
OMA1	-0.011366404	0.440613131	0.776155715
GCNT1	-0.013608196	0.440632624	0.776155715
GOLGA8B	0.01731545	0.440766432	0.776267882
PTTG1	-0.018919707	0.440882622	0.776306288
RCSD1	-0.010815102	0.440989828	0.776306288
OR2B2	0.020110358	0.440998639	0.776306288
ADPRHL2	-0.010419802	0.441209574	0.776554107
TMEM134	0.009672171	0.44163099	0.77705947
GPX1	0.018759557	0.441681817	0.77705947
LIN54	0.010852711	0.441798128	0.77705947
PPARGC1B	0.018780768	0.441895025	0.77705947
IGF1R	0.015325966	0.442038381	0.77705947
MYO5A	-0.011053109	0.4421197	0.77705947
TAF1L	0.00933675	0.442162091	0.77705947
ITPR2	-0.013553725	0.442185304	0.77705947

TACR2	-0.014142653	0.442188781	0.77705947
TMEM99	-0.014404418	0.442198717	0.77705947
DUS4L	0.010267792	0.44229128	0.777098759
MIR300	-0.015657606	0.442465004	0.777117711
TMEM191C	0.016907415	0.442496761	0.777117711
CD3EAP	-0.011746933	0.442512687	0.777117711
ZNF473	0.009839627	0.442593094	0.777135622
PICALM	-0.008973061	0.442669333	0.777146209
ZFYVE26	-0.008620356	0.442780008	0.777185239
ORAI2	0.015721853	0.442831991	0.777185239
LHFP	0.012361545	0.443152948	0.777579025
SCYL2	-0.00833697	0.443196863	0.777579025
DSCC1	-0.013078358	0.44342426	0.777838188
EIF4G3	0.011023952	0.443538662	0.777838188
FBXW5	0.011033698	0.443561931	0.777838188
HLA-DQA1	-0.051831201	0.443625664	0.777838188
RSPO2	-0.012264243	0.44385119	0.778110361
WRNIP1	0.012146007	0.44431365	0.778275796
RNF216	0.009639602	0.444316671	0.778275796
ZNF716	0.011937211	0.444411646	0.778275796
KAT2A	0.012224764	0.444411715	0.778275796
C16orf72	-0.016469015	0.444418897	0.778275796
CD97	0.007889128	0.444493683	0.778275796
MORC2	0.017305281	0.444600335	0.778275796
RCN3	0.01754621	0.444645799	0.778275796
FAM63B	-0.01802593	0.44473243	0.778275796
PYCR1	0.01291443	0.444760479	0.778275796
SERINC3	-0.012971686	0.444852631	0.778275796
ARG2	0.025341878	0.444876158	0.778275796
DDX49	0.01226519	0.444905557	0.778275796
WDR47	-0.012357295	0.44508813	0.778275796
GIT1	0.015650717	0.445098167	0.778275796
PRKAB1	-0.010538873	0.445136233	0.778275796
ABT1	-0.012324078	0.445253045	0.778275796
SEC61A2	0.017910579	0.445260702	0.778275796
SEC61G	-0.009112971	0.445301006	0.778275796
ING4	-0.00967025	0.445413356	0.778275796
KDM3A	-0.0081524	0.445422095	0.778275796
PTGS2	0.030082366	0.445712894	0.778399179
ARHGDIB	0.004320029	0.445781454	0.778399179
CCL20	-0.019656474	0.445791256	0.778399179
GRAMD4	0.014568524	0.445825174	0.778399179
ZNF592	0.013202188	0.445844322	0.778399179
DRAM1	-0.015303697	0.44597772	0.778509285
TTC23	0.015888633	0.44613577	0.778560046
SLC16A5	-0.014684721	0.446190332	0.778560046
RASL10A	-0.017358613	0.44628717	0.778560046
PNMA1	0.013824131	0.446288146	0.778560046
SH3BGRL3	-0.012950214	0.446639483	0.779050178
TCP10L	-0.013297286	0.446752624	0.77912475

SLC39A11	-0.012801119	0.446938186	0.779146232
MBP	-0.01073027	0.446947056	0.779146232
DTNBP1	0.013544964	0.447118321	0.779146232
RORC	0.037632745	0.447136479	0.779146232
KLHL8	-0.011855729	0.447218286	0.779146232
MARCKSL1	0.012034318	0.447230526	0.779146232
STAT5B	0.00944766	0.447257671	0.779146232
SAMD3	-0.018850366	0.447490063	0.779423847
OGFRL1	-0.019523359	0.447557862	0.779423847
LIMS2	-0.017121229	0.447821765	0.779760754
CBR1	-0.020283504	0.448025264	0.779992396
CELSR2	0.015365758	0.448294284	0.780103732
SIT1	-0.016009862	0.448590917	0.780103732
SLC26A6	-0.01170675	0.448665572	0.780103732
CEP120	0.016077449	0.448704946	0.780103732
HLA-A	-0.182397461	0.448772097	0.780103732
PLEKHA2	-0.008950093	0.448780086	0.780103732
ZNFX1	0.011121749	0.448869666	0.780103732
C11orf57	0.008777644	0.448874269	0.780103732
PRKRIR	-0.00823374	0.448928268	0.780103732
CLEC7A	0.030131295	0.448968804	0.780103732
ZADH2	0.020606559	0.448969109	0.780103732
REST	-0.011724695	0.448991386	0.780103732
RAB2B	0.008836925	0.449005409	0.780103732
CLMN	-0.016498977	0.449465748	0.780714945
TREM1	-0.025944934	0.449528055	0.780714945
AMPD3	-0.01475177	0.449568801	0.780714945
ZNF524	-0.012034257	0.449788011	0.780905993
LITAF	-0.013653479	0.449875252	0.780905993
NTN5	0.017392655	0.449902502	0.780905993
VCAN	-0.011372025	0.44996101	0.780905993
PRDM9	-0.013739515	0.450225613	0.781111722
FAM135A	0.011638804	0.450262703	0.781111722
CEP192	0.011561033	0.450291255	0.781111722
SIDT1	0.012584534	0.450393506	0.781166674
FBRS	0.012176625	0.450487841	0.781207882
PLA2G16	-0.027990555	0.450833753	0.781619668
FMNL2	-0.022716925	0.450866526	0.781619668
RCBTB1	0.013312693	0.451267226	0.782191814
FAM149B1	-0.007400523	0.451578766	0.782519784
SNORD114-7	-0.014066841	0.451687315	0.782519784
SCARNA2	0.023060623	0.451708434	0.782519784
MBNL1	0.030446598	0.45173922	0.782519784
SNN	0.020919696	0.451920569	0.782594502
CRB3	0.015352956	0.451923756	0.782594502
FSIP1	0.01304148	0.452029004	0.782654316
IRF9	0.012352633	0.452206156	0.78283859
GNG3	-0.016393167	0.452435707	0.782927302
MIR877	-0.014844896	0.452446141	0.782927302
PUM1	0.006250841	0.45249721	0.782927302

TRAPPC1	-0.010689753	0.452540327	0.782927302
BAX	0.014264947	0.452685843	0.783034411
MIR345	-0.012907651	0.452765544	0.783034411
ALDOC	-0.016393328	0.452821599	0.783034411
CAB39L	-0.012565179	0.452899108	0.783034411
EIF2AK1	0.00884612	0.452955943	0.783034411
SLC25A38	0.0108082	0.453094272	0.783151233
PHF10	-0.011277251	0.453517109	0.783574687
GNA15	0.011622794	0.453517141	0.783574687
APP	-0.023446611	0.453551632	0.783574687
CHI3L2	-0.033771081	0.453660492	0.783640447
MTUS1	-0.017593497	0.453790002	0.783728717
WBSCR16	-0.020965595	0.4538532	0.783728717
C14orf64	0.023524123	0.45406902	0.783979096
CCDC86	-0.013061187	0.454301296	0.784129953
FBXO3	-0.012252949	0.454363729	0.784129953
CACNG1	-0.012613075	0.454415033	0.784129953
SACS	0.011802441	0.454439755	0.784129953
C8orf82	-0.012890294	0.454809391	0.784645441
RPH3A	-0.017325407	0.454929808	0.784723209
CRK	0.011258176	0.455021888	0.784723209
SNORD87	0.014783646	0.455112757	0.784723209
NVL	0.011826831	0.455236143	0.784723209
TMEM5	-0.009309977	0.455292219	0.784723209
PDCD7	0.005331349	0.455391561	0.784723209
LYG1	0.018444977	0.455440138	0.784723209
TTC12	0.022935973	0.455461087	0.784723209
ST3GAL2	-0.013256703	0.455531711	0.784723209
RNF34	-0.010424535	0.455563406	0.784723209
PFDN6	-0.012604764	0.455885762	0.785156293
GPN1	0.008121145	0.456007487	0.785164042
CKAP2L	0.012735629	0.456088549	0.785164042
WIPI2	0.011278743	0.456178896	0.785164042
MGST2	-0.012800158	0.456282928	0.785164042
RAB7L1	0.010468931	0.456363429	0.785164042
RFPL4A	-0.015397456	0.45637321	0.785164042
BACH2	0.01919223	0.45643679	0.785164042
METAP1	0.005716129	0.45645773	0.785164042
PPP6C	-0.005882928	0.456728489	0.785391103
TBC1D5	0.01319546	0.45673164	0.785391103
DIP2C	0.015281456	0.456923121	0.785598327
C12orf77	-0.01209661	0.457184597	0.785925812
KDM2B	0.006472889	0.45743063	0.786099242
C6orf89	-0.011612011	0.457528219	0.786099242
CA13	0.014475186	0.457542127	0.786099242
NCAPD2	-0.015008399	0.457569556	0.786099242
HEXIM1	0.011810847	0.45765879	0.786107933
NLGN2	0.014240875	0.457809287	0.786107933
HTATIP2	-0.01094582	0.457812573	0.786107933
MRPS18C	-0.01297919	0.457859352	0.786107933

HOMEZ	-0.014337653	0.457959195	0.786107933
SYVN1	0.008574891	0.458144604	0.786107933
CDC20	-0.039926022	0.458145122	0.786107933
NPM3	-0.014501024	0.458167444	0.786107933
PLBD2	-0.016151582	0.458213784	0.786107933
ZNF836	0.012409644	0.458335752	0.786195326
CGGBP1	0.006671079	0.458549401	0.786439932
EIF4G1	0.009232512	0.459171636	0.787338942
SAMD8	-0.016280775	0.459215847	0.787338942
SF3B1	0.006583866	0.459449856	0.787618159
DCTN1	-0.00658732	0.459912654	0.787652795
DCP1B	-0.014107734	0.459937314	0.787652795
ADAM8	0.012327204	0.45998563	0.787652795
IRF2BPL	0.022960303	0.459993404	0.787652795
PALM	0.022171422	0.459998548	0.787652795
FRS2	0.008251087	0.460007817	0.787652795
NDUFA10	-0.013687128	0.460132537	0.787652795
TRAT1	0.018394952	0.460192405	0.787652795
DNASE1L3	0.031537483	0.460269647	0.787652795
FAM115C	0.02643956	0.460271435	0.787652795
IRAK3	-0.014349626	0.460296914	0.787652795
DNAL4	-0.007865588	0.46040132	0.787652795
GEN1	0.0110257	0.460630536	0.787652795
SUV39H2	0.016194149	0.46066741	0.787652795
NIPBL	0.007405764	0.46067653	0.787652795
PRSS8	0.014569285	0.460717743	0.787652795
PA2G4	-0.011928521	0.460718792	0.787652795
TMEM205	-0.009979901	0.460750912	0.787652795
THAP6	-0.016303939	0.460953428	0.787693793
C5orf42	-0.013210789	0.460970452	0.787693793
PI4KA	-0.009643318	0.460988381	0.787693793
GABARAP	-0.020498025	0.461178248	0.787896594
PVR	-0.010323491	0.461271495	0.787914013
FDXR	-0.014366089	0.461389679	0.787914013
PHF3	-0.00603452	0.461458596	0.787914013
SPRY1	0.026225604	0.461486321	0.787914013
GNA11	0.020369488	0.461544354	0.787914013
C4BPB	0.015286073	0.461646166	0.787966293
KDM3B	0.008414091	0.462023521	0.788488799
ITGAL	-0.01356109	0.462307142	0.788730771
HLX	-0.016910212	0.462307819	0.788730771
GTDC1	-0.012867577	0.46248928	0.788918761
CATSPER2P1	0.011666942	0.462795034	0.789318679
TRAF4	0.015863759	0.463008909	0.789494041
CXCR5	0.029039762	0.463106144	0.789494041
ORMDL3	0.020775072	0.463111826	0.789494041
RRM2	0.018765613	0.463381982	0.789832948
SNORD55	0.015248801	0.463592706	0.790017658
SLC5A6	-0.011655918	0.463762862	0.790017658
PPME1	0.01204698	0.463768511	0.790017658

GP6	0.042852446	0.463775837	0.790017658
DHX38	-0.013441066	0.463946101	0.790186089
PRICKLE1	0.024440187	0.464429618	0.790710317
RNF115	0.011293073	0.464535371	0.790710317
GOLPH3L	-0.013237451	0.464555455	0.790710317
OPA3	-0.016574555	0.46461063	0.790710317
TMC5	0.025842749	0.464659331	0.790710317
THBS1	0.048107766	0.464747769	0.790710317
MATK	-0.018283799	0.464753936	0.790710317
HOXC13	-0.013319816	0.465029533	0.790927022
RASSF2	-0.010544461	0.46507889	0.790927022
LRRC23	0.018378838	0.465095672	0.790927022
PLXDC2	-0.01623617	0.465221365	0.790959673
UFSP2	-0.008009633	0.465309425	0.790959673
MIR593	0.012861225	0.465541697	0.790959673
PIP4K2B	0.017574505	0.46555762	0.790959673
NFS1	-0.014953845	0.465595342	0.790959673
LCMT1	-0.006810522	0.465602271	0.790959673
TRAK2	0.008900339	0.465615072	0.790959673
FARP2	-0.013737828	0.465837976	0.791171562
TUBB8	-0.014257799	0.465950413	0.791171562
ST8SIA4	-0.015867387	0.466108148	0.791171562
NISCH	0.009821854	0.466299016	0.791171562
FAM169A	0.015245352	0.466430385	0.791171562
ZCCHC14	0.011454175	0.466450818	0.791171562
GSN	-0.019613978	0.466477138	0.791171562
C19orf25	0.009856229	0.466478462	0.791171562
RANBP2	-0.014001847	0.466486826	0.791171562
SAMD14	0.054133856	0.466524267	0.791171562
C1orf174	-0.009724053	0.466526044	0.791171562
P2RY2	-0.0203898	0.466658421	0.791274826
NRARP	0.010819395	0.466873212	0.791467483
ADORA2B	-0.017309077	0.466934753	0.791467483
SLC27A5	-0.012957513	0.466986551	0.791467483
CADPS	-0.013143833	0.467068975	0.79148599
PPP1R15B	-0.014374715	0.467229152	0.791555814
BTAF1	0.008872852	0.46726066	0.791555814
ZBED5	0.008071335	0.467328044	0.791555814
ZDHHC8	0.006880242	0.46742822	0.791555814
MON1A	-0.013306372	0.467467735	0.791555814
TTC27	-0.007985535	0.467641094	0.791728246
TOMM70A	0.008103644	0.467843207	0.791772918
SHMT2	0.008258527	0.467914597	0.791772918
ZNF490	-0.014143979	0.467935936	0.791772918
NAE1	-0.007399817	0.467989184	0.791772918
TMEM127	-0.011259921	0.468027189	0.791772918
ZNF397	0.011851638	0.468131253	0.791772918
PXMP2	-0.018097275	0.468178424	0.791772918
CDA	-0.021986953	0.468239725	0.791772918
TMEM86B	0.014249886	0.468350284	0.791778914

HGD	0.033573366	0.468438448	0.791778914
MESDC1	0.012416835	0.468457865	0.791778914
POLR2H	0.00599505	0.468564493	0.791806611
NUAK1	-0.036792008	0.468617319	0.791806611
ACTB	-0.010544066	0.468695752	0.791818265
SLC22A23	-0.013104152	0.469136769	0.792381802
CCZ1	-0.014524427	0.469343945	0.792381802
SEC22B	-0.00887873	0.469649358	0.792381802
DPP9	0.012974507	0.469674515	0.792381802
PWP1	-0.006003621	0.469797498	0.792381802
STK35	0.015852758	0.46986238	0.792381802
C4orf33	0.017890895	0.469874301	0.792381802
RILPL2	-0.010623458	0.469962022	0.792381802
UCN	0.013266746	0.470035718	0.792381802
LYSMD3	-0.018246033	0.47006265	0.792381802
SEC14L5	0.047999147	0.470106138	0.792381802
TNFRSF21	0.025627834	0.470245876	0.792381802
RGP1	-0.012619984	0.47031024	0.792381802
NCAN	0.011770333	0.470378881	0.792381802
TBCD	0.018833967	0.470406024	0.792381802
SMCP	0.012381088	0.470409041	0.792381802
HDC	0.04914599	0.470538417	0.792381802
REL	0.015453257	0.470643201	0.792381802
RAB34	0.017894352	0.470662687	0.792381802
IGFL3	0.014331888	0.470829742	0.792381802
FAM150B	0.024028945	0.470839664	0.792381802
ZNF18	0.010666974	0.470865202	0.792381802
CYP2D6	0.014298899	0.470919169	0.792381802
SCAF11	0.006635313	0.471039168	0.792381802
ATG4D	0.012163576	0.471047225	0.792381802
KLRG1	-0.026124944	0.471052051	0.792381802
ARHGDIA	-0.012332996	0.471149441	0.792381802
RBMS2	0.023288815	0.471183439	0.792381802
HRAS	-0.009448263	0.471200947	0.792381802
GOLPH3	-0.007618352	0.471212957	0.792381802
G3BP1	0.012054216	0.471248478	0.792381802
RNASE3	-0.030276122	0.471501006	0.792686002
NUDT6	-0.015959315	0.471628109	0.792709952
FAM110A	-0.013001314	0.471658483	0.792709952
TCEB1	-0.00802437	0.471799828	0.792806407
RNASEH1	-0.007395411	0.471949303	0.792806407
FGR	-0.006512216	0.47197638	0.792806407
SPECC1L	0.00790573	0.472016399	0.792806407
NRM	-0.012118083	0.472110469	0.792806407
ARHGAP25	-0.011103254	0.472204388	0.792806407
YARS2	-0.011050438	0.47223224	0.792806407
CCDC6	-0.007642217	0.472361927	0.792806407
FLVCR2	-0.0135525	0.472398761	0.792806407
KLHL35	0.017640608	0.472440734	0.792806407
CEMP1	-0.014943786	0.472594926	0.792806407

CSRNP2	0.010373686	0.472651015	0.792806407
NECAP2	0.009305751	0.472836557	0.792806407
ETV3	0.016231726	0.472887525	0.792806407
NPM1	-0.01252885	0.472928798	0.792806407
WDR81	0.01370805	0.47303326	0.792806407
C15orf56	0.012324989	0.473072984	0.792806407
SLC7A8	0.0159739	0.473144874	0.792806407
SNRK	-0.006819322	0.473193098	0.792806407
NIPAL3	0.01138359	0.473233875	0.792806407
APEH	-0.006640667	0.47342289	0.792806407
FTSJ3	-0.010744761	0.473620897	0.792806407
KLHL3	0.015778465	0.473630213	0.792806407
EIF4A1	-0.006458915	0.473705602	0.792806407
PIAS2	-0.012645889	0.473764998	0.792806407
NHLRC3	0.009198027	0.473799946	0.792806407
TOP3B	-0.010343302	0.473805793	0.792806407
PROK1	-0.01405958	0.473816215	0.792806407
XPOT	-0.008197429	0.473938723	0.792806407
PGBD2	-0.013390768	0.473949069	0.792806407
NKX3-1	-0.017039015	0.473998944	0.792806407
SERPINF1	-0.025222912	0.474052491	0.792806407
SCRN2	-0.012460594	0.474117402	0.792806407
SSH1	-0.014370428	0.47415109	0.792806407
IFIT3	0.037532846	0.47440114	0.793004623
TIMELESS	0.01173375	0.474452626	0.793004623
HUS1B	0.016361648	0.474554155	0.793004623
ZNRF2	-0.014542314	0.474584833	0.793004623
RBM22	-0.00475581	0.474627846	0.793004623
KIFC3	0.030770194	0.475005737	0.79332038
MANBAL	0.009095956	0.47505361	0.79332038
LRRC25	-0.038043808	0.475091869	0.79332038
CMPK1	-0.008161266	0.475219278	0.79332038
STAR	0.01665084	0.475243515	0.79332038
PCF11	-0.007884034	0.475281966	0.79332038
TUFM	-0.0107713	0.475318526	0.79332038
NAGLU	-0.010514538	0.475398114	0.793333594
NUP188	0.01172752	0.476103297	0.794390623
TSFM	0.010077111	0.47637772	0.794648466
RNF135	-0.009943159	0.476454457	0.794648466
CCAR1	0.025518623	0.476473201	0.794648466
CAPS2	0.010159368	0.476829664	0.795123162
EYA3	0.023866955	0.477069768	0.795365641
FOXD4	-0.014810837	0.477118787	0.795365641
ARHGAP24	0.031244823	0.477483206	0.795700368
RPIA	-0.0082456	0.477751838	0.795700368
ZNF444	-0.012625416	0.477785041	0.795700368
ORAOV1	0.011964276	0.47799879	0.795700368
ANTXR2	0.011356372	0.477999716	0.795700368
KCTD5	-0.011080475	0.478055953	0.795700368
AZI2	-0.011653626	0.478058531	0.795700368

RASSF5	0.007166488	0.478090703	0.795700368
RIN1	0.017738132	0.478099658	0.795700368
SUGT1	-0.009456812	0.47825346	0.795700368
ZNF350	-0.012483608	0.478274596	0.795700368
SMOX	0.031088902	0.478285067	0.795700368
ABHD10	0.01009054	0.478306449	0.795700368
AFF1	0.009918229	0.478325978	0.795700368
KIAA0586	-0.00941926	0.478487486	0.795849433
TUFT1	-0.011068389	0.47863956	0.795982765
ARRDC2	0.011973032	0.478886311	0.796273483
SLC3A2	-0.00907175	0.479131533	0.796339913
LSAMP	-0.012165962	0.479284732	0.796339913
GNPAT	0.00667577	0.479308401	0.796339913
NLRP5	0.012482548	0.479374332	0.796339913
L3MBTL4	-0.015690999	0.47943017	0.796339913
RBM11	-0.020595174	0.479495751	0.796339913
ACSL3	-0.007416025	0.479500377	0.796339913
LNX2	-0.014715436	0.479579162	0.796339913
MGA	0.01181289	0.479636594	0.796339913
NEMF	0.010603221	0.479645695	0.796339913
SMG5	-0.009005483	0.479740856	0.796378455
C9orf89	0.016562475	0.479950015	0.796606195
RDH16	0.014528933	0.480089839	0.796609262
PRDX2	0.018131219	0.480159999	0.796609262
KLF13	-0.008965516	0.480203413	0.796609262
TM2D2	-0.015316537	0.480259418	0.796609262
SUPT4H1	-0.009164107	0.4803117	0.796609262
SLC7A9	0.016003014	0.48069111	0.796935544
GJC1	0.004908668	0.480722043	0.796935544
PLXNA1	0.014268393	0.480762841	0.796935544
GTF2I	-0.009917397	0.480796419	0.796935544
TMEM128	0.033445189	0.481037136	0.797215161
METAP1D	0.01133469	0.481172501	0.797320122
HIST1H3E	-0.011136165	0.481327333	0.797343678
CD8B	-0.031393101	0.481334788	0.797343678
REC8	0.011644235	0.481402819	0.797343678
TRIB1	0.013982365	0.481574698	0.797433743
EXD3	0.014515059	0.481650056	0.797433743
EEF1A2	0.022608939	0.481673323	0.797433743
IVD	-0.014474502	0.481818263	0.797554413
RPS14	-0.008444925	0.482033222	0.797752206
ATF7IP2	0.016025188	0.482107011	0.797752206
GPX4	0.0089415	0.48217159	0.797752206
CASC3	0.009531933	0.482278057	0.797752206
FBXL3	-0.012682915	0.482298108	0.797752206
MRPL49	-0.009716911	0.482491402	0.797952686
GDAP1	-0.012062519	0.482718992	0.798209818
C1QA	-0.015095927	0.482877804	0.798353161
ATOH7	-0.012669139	0.48296647	0.798380505
ABCE1	-0.006755332	0.483103947	0.798382348

GART	0.007861815	0.483139337	0.798382348
ST14	-0.023222477	0.483185482	0.798382348
TNFRSF8	-0.014391775	0.483256097	0.798382348
DYNC1H1	0.008033549	0.483648651	0.798895824
RPL36	0.006404185	0.483746454	0.798895824
ITGA10	0.014381587	0.483783423	0.798895824
PWP2	-0.009855867	0.483889875	0.79895242
ARL4A	0.024402179	0.484052679	0.799078321
PANX1	-0.013754866	0.484110509	0.799078321
SLC29A3	-0.013249992	0.484440965	0.799504554
CA6	0.01702969	0.48462987	0.799697082
KLRC2	0.062717004	0.484724709	0.799713972
TRIM35	-0.013735257	0.484784601	0.799713972
ADD1	-0.006180775	0.485012104	0.799970047
TTC16	-0.023385936	0.485154044	0.800044159
PWWP2A	-0.009168881	0.485239773	0.800044159
NEDD1	-0.013050563	0.485298113	0.800044159
RBM15	0.007634195	0.485346149	0.800044159
PURB	0.009308938	0.485612857	0.800364609
INPP4A	-0.012689916	0.485838691	0.800617608
C4orf3	-0.008801489	0.486298613	0.801256229
CABP4	0.016559061	0.486547155	0.80146603
SERPING1	0.031849219	0.486681255	0.80146603
NTPCR	0.018555651	0.486688691	0.80146603
MAPK11	-0.010719218	0.486736628	0.80146603
CCDC28A	-0.007134142	0.486787977	0.80146603
ADCY7	-0.00806706	0.487013283	0.801493768
LDLRAP1	0.018265739	0.48724764	0.801493768
TXNRD1	-0.008585544	0.487249813	0.801493768
CTNNA1	-0.011663376	0.487278129	0.801493768
TXNL4B	-0.013112058	0.487360775	0.801493768
ZFPM1	-0.013251496	0.487366936	0.801493768
RPS29	-0.006001133	0.487464687	0.801493768
OIP5	-0.018742747	0.487467262	0.801493768
FANCI	-0.014591704	0.487470391	0.801493768
SLC38A11	0.027165598	0.487528913	0.801493768
DISC1	-0.01721066	0.487795328	0.801716746
ABCB4	0.020144659	0.487812154	0.801716746
RAB5B	0.006926584	0.487881832	0.801716746
DAGLB	-0.012668316	0.488060117	0.801845976
INPP5D	-0.010598844	0.488209342	0.801845976
VNN3	0.025080835	0.488226534	0.801845976
NCAPD3	-0.008889215	0.488250237	0.801845976
RNF38	-0.007006825	0.48833677	0.801858741
CELF2	0.009972673	0.488430404	0.801858741
PPP4C	0.006397072	0.488476363	0.801858741
RPL38	0.003637837	0.488547777	0.801858741
ADCK1	0.015671568	0.488660438	0.801924742
CHN1	-0.026784416	0.488822861	0.802072376
ZNF236	-0.011573331	0.489022005	0.802158046

C12orf57	-0.014379372	0.489065519	0.802158046
RRBP1	-0.010210631	0.489092479	0.802158046
KIAA0907	0.008745632	0.489207417	0.802227689
C16orf13	0.00925955	0.489747787	0.802994854
C1orf189	-0.011069505	0.489880299	0.803093161
U2AF2	0.004351038	0.490094469	0.803255998
NLRP7	-0.026302691	0.490124764	0.803255998
TAF4B	0.0171716	0.490415907	0.803473115
TGFBR1	0.013225867	0.490456114	0.803473115
KDELR2	-0.014048298	0.490478548	0.803473115
NFAM1	-0.015596437	0.490550065	0.803473115
PUS7	-0.014590376	0.490742001	0.803473115
FAM102A	0.013351402	0.490773401	0.803473115
DERL3	-0.016915414	0.490775132	0.803473115
C15orf26	0.044911196	0.490887406	0.803473115
CLK1	-0.018029104	0.49111437	0.803473115
FKBP1A	-0.009788927	0.491160163	0.803473115
EIF4G2	-0.004986366	0.491210207	0.803473115
METRNL	0.012927244	0.491215349	0.803473115
CCNB1	-0.015273613	0.491366552	0.803473115
KCNIP3	-0.016235768	0.491418266	0.803473115
KLF2	-0.005680314	0.491422817	0.803473115
TP53	-0.012090892	0.491424173	0.803473115
ARFGEF1	-0.006509268	0.49149759	0.803473115
GPR35	0.016708259	0.491570219	0.803473115
KIF27	0.02014451	0.491689703	0.803473115
TM7SF2	0.01097798	0.491708997	0.803473115
ZFP1	-0.013980542	0.491793112	0.803491949
SLC12A9	0.008203555	0.491882636	0.803519612
MTAP	0.011110336	0.491992478	0.803580455
GABPA	-0.012392069	0.492406469	0.804137977
SLC27A2	0.015426406	0.492612968	0.804319352
N4BP2L1	0.008206387	0.492696113	0.804319352
QSOX1	-0.00983018	0.492772684	0.804319352
LAMP1	0.006244141	0.492871041	0.804319352
CD36	-0.025203488	0.493088961	0.804319352
PAFAH2	-0.013224853	0.493130547	0.804319352
RAB11FIP4	-0.01268607	0.493164831	0.804319352
NCOA3	0.01142652	0.493176182	0.804319352
ZNF821	-0.012331416	0.493187432	0.804319352
CCDC65	-0.012025183	0.493268005	0.804319352
TFB1M	0.009491211	0.493461909	0.804319352
AQP12B	-0.016278746	0.493528181	0.804319352
RALBP1	-0.008797616	0.493530107	0.804319352
WLS	-0.021738402	0.493534831	0.804319352
IFI27L1	-0.012254215	0.493696043	0.804463639
PIGV	-0.019876922	0.493918338	0.804707402
CETN3	0.009629975	0.494095894	0.804754261
ZNF814	-0.018949154	0.494120369	0.804754261
PTOV1	-0.009750303	0.494230382	0.804754261

FAM120A	0.004564373	0.494237913	0.804754261
SNRPG	0.006851485	0.494551033	0.805110509
IL15	-0.013267293	0.494602173	0.805110509
GPD1L	0.007388394	0.494749973	0.805232679
ZHX3	-0.010024346	0.495022146	0.805346989
TRAFD1	0.009734504	0.495252833	0.805346989
ARRDC5	0.01371538	0.495263296	0.805346989
BRWD1	-0.009051757	0.495305446	0.805346989
RTN4RL1	0.012962895	0.495398844	0.805346989
ADPRH	-0.011776902	0.49543714	0.805346989
RRN3P1	0.025050188	0.49545991	0.805346989
CP	0.011096928	0.49551251	0.805346989
DDX42	0.005827196	0.495517575	0.805346989
IVNS1ABP	0.012649304	0.495547777	0.805346989
KLF10	-0.02656652	0.495847512	0.805604326
SPOCK1	0.014390597	0.495851682	0.805604326
DLGAP5	-0.025602484	0.495991334	0.805712955
FAH	0.016593361	0.496066595	0.805716969
C10orf128	0.026006399	0.496313945	0.805823934
MUC20	-0.030540898	0.49632991	0.805823934
TAGAP	0.022752387	0.496350852	0.805823934
CD72	0.025679544	0.496554218	0.806035877
F3	-0.011984342	0.496847556	0.806390253
XRN1	0.00816775	0.49696824	0.806390253
TMEM45B	0.02806561	0.497210498	0.806390253
ZBED4	0.007814342	0.497246691	0.806390253
RREB1	-0.012584501	0.497290588	0.806390253
PEF1	0.008931326	0.497326571	0.806390253
SSBP4	0.010094686	0.49736403	0.806390253
LIN7B	0.012200126	0.497371446	0.806390253
TTC39C	0.010453114	0.497491167	0.806390253
CBX4	0.009272923	0.497587935	0.806390253
PFN1	-0.008792321	0.497644762	0.806390253
CITED2	-0.012771463	0.497646745	0.806390253
SPRR1A	0.011529379	0.497986604	0.806645132
EML3	0.00802877	0.498058697	0.806645132
ADCK3	0.006864816	0.498068533	0.806645132
SIRT6	0.014390994	0.498136626	0.806645132
RWDD2B	0.011829787	0.498202451	0.806645132
ALKBH5	-0.005500101	0.498241284	0.806645132
TBL2	-0.010809891	0.498370598	0.806680486
ZNF555	0.01716033	0.498504031	0.806680486
UBE3C	-0.007726793	0.498504271	0.806680486
RABGAP1L	0.017411252	0.498586569	0.806680486
ARHGAP10	-0.012761687	0.498824122	0.806680486
PMVK	-0.009767222	0.49889182	0.806680486
CLDN7	-0.013118844	0.498920702	0.806680486
ZNF581	-0.009477884	0.498972462	0.806680486
FAM65A	-0.009069906	0.498973096	0.806680486
SNAP29	0.015439358	0.499019263	0.806680486

TIGD2	-0.012469467	0.499064773	0.806680486
CPEB2	0.012134333	0.499164278	0.80672352
B3GNT8	-0.015975144	0.499530356	0.807090045
RGPD8	-0.021940087	0.499536895	0.807090045
BSCL2	-0.016357094	0.499697145	0.807231129
SMG1	0.00756372	0.499891032	0.807295881
PARS2	-0.013789875	0.499921187	0.807295881
GMFB	-0.006911521	0.499956027	0.807295881
HLA-H	-0.007900486	0.500096927	0.807327644
PHLPP2	-0.011972677	0.500124	0.807327644
RAB40C	-0.006964532	0.500244588	0.807327644
CLIC6	-0.019218269	0.500290404	0.807327644
RNF175	0.023384712	0.500340378	0.807327644
SEMA4A	-0.012587095	0.500586126	0.807606447
PCSK4	-0.011315416	0.500832088	0.807885512
ANKRD52	-0.0145092	0.501047865	0.808115812
STK40	0.008284603	0.501355918	0.808356531
SLC11A1	0.021655675	0.501359783	0.808356531
CSTF3	-0.007119305	0.501416202	0.808356531
STIL	0.013310931	0.501757354	0.808748045
MTRF1	0.011217932	0.501807896	0.808748045
PLA2G7	-0.029045425	0.501937475	0.808748045
EXTL3	0.01234112	0.501951312	0.808748045
NUMB	-0.006435976	0.502198348	0.808923398
CRKL	-0.006875749	0.502249593	0.808923398
BAALC	-0.011885716	0.502404402	0.808923398
PIGG	0.010954055	0.502456429	0.808923398
FASTK	-0.006495173	0.502520822	0.808923398
VN1R2	0.01702623	0.502559351	0.808923398
ETFDH	-0.011240519	0.502571705	0.808923398
DCHS1	-0.015189848	0.502695385	0.80900483
CARD16	-0.014021968	0.503067948	0.809486715
ST7	0.015661414	0.503241312	0.809647978
PLEKHB2	0.018905658	0.503483377	0.809836821
MRPL10	-0.010398067	0.503679024	0.809836821
MTHFD1	-0.011580339	0.503727515	0.809836821
ZNF570	-0.011977413	0.503772702	0.809836821
ATP10A	0.012479224	0.503808076	0.809836821
ELOF1	0.008946259	0.503867858	0.809836821
ZNF383	-0.008781013	0.503937516	0.809836821
FAM46C	0.023186309	0.50394399	0.809836821
ZNF598	0.00638339	0.504027718	0.809853797
SNX24	0.013030301	0.504116512	0.809878907
SULT1A2	0.014564729	0.50475715	0.810790436
TRDMT1	0.013765401	0.5049887	0.810864576
TMEM165	0.010638504	0.505010053	0.810864576
STX1B	-0.022486858	0.505023072	0.810864576
JRKL	-0.012690659	0.505462817	0.811452925
THAP7	0.012368291	0.505949723	0.812038125
DPM3	0.009378384	0.506059111	0.812038125

FAM159A	0.013971641	0.506071315	0.812038125
C5orf24	-0.013485961	0.506192502	0.812038125
HIST1H2BN	0.019324024	0.506194151	0.812038125
POLG2	0.01007388	0.506373119	0.812107105
HEXIM2	0.02077851	0.506383886	0.812107105
MUL1	-0.007821168	0.50647551	0.812136379
RNF166	0.013406119	0.507042529	0.812927832
FEM1B	-0.014541129	0.507253627	0.812937284
FAM189B	-0.008147019	0.507263363	0.812937284
ITPA	-0.006904614	0.507268752	0.812937284
ZNF664	0.012587157	0.507491449	0.813029906
CHUK	0.006796281	0.507613509	0.813029906
UBAP2	-0.011147844	0.507707386	0.813029906
RTKN	-0.021237787	0.507756382	0.813029906
NCL	-0.00643821	0.507786396	0.813029906
DSCAM	-0.010650527	0.507834164	0.813029906
AVP	-0.013734847	0.50788671	0.813029906
FAM21C	-0.035454977	0.507914156	0.813029906
CC2D1B	0.015289951	0.508012928	0.81307043
TCTN1	0.014574433	0.508281648	0.813237471
PSD4	-0.012709546	0.508307742	0.813237471
RAB4A	-0.013365605	0.508383745	0.813237471
RPL35A	-0.005253062	0.508411175	0.813237471
PDSS1	-0.011937816	0.508587524	0.813376761
PTMA	-0.013943953	0.50877037	0.813376761
MTFMT	-0.01111248	0.50878063	0.813376761
ARHGAP5	0.012001952	0.508792185	0.813376761
CNBP	-0.009412576	0.509008909	0.813605721
MDGA1	0.022899735	0.509313149	0.813974479
TMED5	-0.00870979	0.509466882	0.81410263
SRI	-0.026562625	0.509815252	0.814508001
TAF5L	-0.020076145	0.509929244	0.814508001
TFAP4	-0.013574687	0.510246696	0.814508001
PCID2	-0.006051127	0.510276169	0.814508001
AKIRIN2	0.007298432	0.510339974	0.814508001
PRSS57	0.02619366	0.510374237	0.814508001
C21orf2	-0.014962515	0.510428954	0.814508001
NRXN2	0.015443457	0.510446325	0.814508001
LBR	-0.012817343	0.510461776	0.814508001
TOX	-0.018347849	0.510491298	0.814508001
KIAA1586	-0.01406515	0.510545204	0.814508001
PHF12	-0.008766714	0.510682522	0.814508001
HERC2P2	0.013284706	0.510690663	0.814508001
SGTB	-0.026432597	0.510750748	0.814508001
TFIP11	-0.007172441	0.510851666	0.814551583
GLYCTK	-0.011681412	0.510988377	0.814611163
GRHPR	-0.014121676	0.51103622	0.814611163
NET1	-0.017716566	0.51123763	0.81478768
SNX14	0.006779843	0.5113268	0.81478768
MRPL47	-0.010968467	0.511367785	0.81478768

HHLA3	-0.011043545	0.511637916	0.815057777
ACER3	0.013408318	0.511684569	0.815057777
SSU72	-0.007771673	0.511810539	0.815141131
TGM3	-0.012102337	0.512391179	0.815948491
C9orf62	0.013521455	0.51261043	0.815988062
NCF4	0.010913671	0.512616178	0.815988062
EHMT2	-0.010590705	0.512637184	0.815988062
KBTBD11	-0.013047143	0.512715466	0.815995326
FAM91A1	-0.012610958	0.512904879	0.816179429
TRIM66	-0.017079061	0.513030732	0.81626235
TRIM74	-0.011643865	0.513233302	0.816467293
PLD6	0.018201217	0.513379655	0.816582757
TRMT11	0.009132754	0.513546885	0.816706862
LINGO2	-0.029857503	0.513623193	0.816706862
TEX10	-0.006806458	0.51372581	0.816706862
ADCK5	0.009097042	0.513780849	0.816706862
LY96	-0.012253902	0.513826596	0.816706862
PIGU	-0.007469322	0.513942867	0.816717988
ABI1	0.006988881	0.513996776	0.816717988
RCBTB2	0.009634794	0.514054949	0.816717988
HSBP1L1	0.012037628	0.51415198	0.816754917
ARHGAP27	0.008908059	0.514401673	0.816766994
HTRA4	0.015313911	0.514448169	0.816766994
MIER1	-0.010216342	0.514476085	0.816766994
MARS	0.007607545	0.514580648	0.816766994
ICK	-0.007710333	0.514582638	0.816766994
SESN3	-0.015082666	0.514602314	0.816766994
ISOC2	-0.01144094	0.514969914	0.816911013
VPS13D	0.011415013	0.514995745	0.816911013
B3GNT1	-0.014232116	0.515018441	0.816911013
DCP2	0.009206917	0.515065952	0.816911013
EMR3	-0.02853101	0.515222749	0.816911013
DCAF4L1	-0.011449864	0.515238264	0.816911013
PSMB9	-0.016920371	0.515269337	0.816911013
ACOT2	-0.01585162	0.51532665	0.816911013
PRAM1	0.013074276	0.515365352	0.816911013
KLF9	0.012478401	0.515447522	0.816911013
IPCEF1	0.013209077	0.515504872	0.816911013
QTRTD1	-0.006954301	0.515679071	0.816995408
COLQ	0.022179941	0.515946323	0.816995408
CDH24	-0.011540093	0.516151407	0.816995408
XKR8	0.00992424	0.516421336	0.816995408
SLMAP	-0.00538886	0.516429617	0.816995408
NSUN3	-0.008884263	0.516443511	0.816995408
PLAGL1	0.014741808	0.516463482	0.816995408
CPNE5	0.019462552	0.516507825	0.816995408
CDK5	-0.012467792	0.516560721	0.816995408
WDR5B	-0.012824665	0.516619573	0.816995408
NUP210	0.013375503	0.516646803	0.816995408
TSPAN5	-0.011663396	0.516653053	0.816995408

ANXA9	0.013637576	0.516663051	0.816995408
SPTLC2	-0.012257741	0.516674316	0.816995408
RPRD2	0.008062236	0.516689246	0.816995408
IDH1	-0.00916814	0.516753965	0.816995408
CAMK2D	0.016519341	0.516812887	0.816995408
ZSWIM7	-0.031656088	0.517071613	0.817260483
ADD3	0.006403274	0.5171464	0.817260483
MCL1	0.006836221	0.517226919	0.817260483
HERC2	0.009084433	0.517275901	0.817260483
SNORD94	0.009331932	0.517469914	0.817404314
ABHD4	-0.012191133	0.517539058	0.817404314
CNTNAP3B	0.02040618	0.517588476	0.817404314
KRCC1	0.010912464	0.517741839	0.817472396
PLXNB2	-0.011514115	0.51793298	0.817472396
TRAPPC8	-0.00700009	0.518072339	0.817472396
C1orf112	-0.010925897	0.518077812	0.817472396
C17orf85	-0.006980304	0.518350939	0.817472396
CCBL2	0.006359104	0.518608349	0.817472396
HDAC5	0.01142134	0.518612479	0.817472396
LYRM2	-0.010410529	0.51877549	0.817472396
BCL7B	0.015114984	0.518787863	0.817472396
HMGCS1	-0.007810557	0.518799674	0.817472396
TMEM86A	-0.011725257	0.518851585	0.817472396
CHPT1	0.015844303	0.518914989	0.817472396
C1orf21	-0.025587101	0.519023034	0.817472396
TRPT1	-0.006763539	0.519077406	0.817472396
TGFB3	0.013087923	0.519078619	0.817472396
LYL1	-0.008246346	0.51908321	0.817472396
G2E3	-0.009185413	0.519142794	0.817472396
DTYMK	-0.014760729	0.519233507	0.817472396
SFXN4	0.01393602	0.51924554	0.817472396
IRS1	-0.015166279	0.519308169	0.817472396
CABLES2	-0.008944415	0.519331098	0.817472396
SLC24A3	0.038378893	0.519391154	0.817472396
ZNF7	0.008815894	0.519457442	0.817472396
TXNDC11	0.010599096	0.51947546	0.817472396
PDE4B	0.011480006	0.519477896	0.817472396
LILRA3	0.060905994	0.51955273	0.817473941
XRCC6BP1	-0.021137973	0.519677795	0.817554507
IMPDH2	-0.009391221	0.519844412	0.817700412
SOX13	-0.019581591	0.520088584	0.81796825
BBS4	-0.009604612	0.520263142	0.818081194
FGFR1OP	-0.010647617	0.520392685	0.818081194
C3orf17	-0.009762708	0.520443919	0.818081194
PHF20L1	0.006402691	0.520456027	0.818081194
PARP1	-0.006665961	0.520652576	0.81827394
ACVR2A	0.011006551	0.520751718	0.818313567
TMF1	-0.009783815	0.520830311	0.818320896
YIPF5	-0.017537833	0.521228672	0.818561832
DPY30	-0.008109567	0.521308228	0.818561832

SELT	-0.009724364	0.521341191	0.818561832
ALG5	-0.00915422	0.521351147	0.818561832
CLASP2	-0.011487799	0.521353412	0.818561832
C12orf4	-0.009435462	0.521635029	0.818729432
NXF1	0.00755025	0.521827385	0.818729432
PPT2	-0.012553917	0.521916259	0.818729432
ALG2	-0.010630859	0.5220237	0.818729432
GPAT2	0.027145393	0.522185631	0.818729432
IL8	0.044944605	0.522197283	0.818729432
RPS27	-0.003029773	0.522244346	0.818729432
OPLAH	0.017159778	0.522330961	0.818729432
ABCF3	0.011395209	0.522459441	0.818729432
GLTPD1	-0.010544215	0.522463222	0.818729432
FBXL8	-0.011781162	0.522589943	0.818729432
CHCHD7	-0.006293432	0.522641568	0.818729432
CYP27A1	0.025661192	0.522765919	0.818729432
NFYA	-0.009228642	0.522821929	0.818729432
FOXP4	0.012672916	0.522971521	0.818729432
SNORA1	0.013610498	0.52298282	0.818729432
FAM76B	-0.011332798	0.523104425	0.818729432
PARG	-0.008923548	0.523172886	0.818729432
KLRF1	0.023504462	0.523200339	0.818729432
FAM122A	-0.010024844	0.523209283	0.818729432
HBB	0.038077622	0.523329422	0.818729432
MRPL22	-0.006222565	0.52357096	0.818729432
PFDN4	-0.01373272	0.523629073	0.818729432
MAP4K2	0.007295102	0.523658631	0.818729432
TNPO2	0.007111101	0.523708516	0.818729432
HBA2	0.054927159	0.523710159	0.818729432
LSM7	0.008233559	0.52373658	0.818729432
ZNF629	-0.009521298	0.523852422	0.818729432
TMC6	0.010717975	0.52400612	0.818729432
ZCRB1	-0.010592385	0.524034496	0.818729432
RABEP1	0.015238584	0.524081774	0.818729432
MAVS	-0.015140237	0.524108266	0.818729432
PRKCD	-0.006131331	0.524125779	0.818729432
NCR3	0.018936529	0.524299268	0.818729432
NTSR1	-0.027916232	0.524526409	0.818729432
CAPN7	-0.008527122	0.524555455	0.818729432
SNORA58	-0.00952713	0.524645993	0.818729432
MOB2	0.010203802	0.524793985	0.818729432
ADAM23	0.016933365	0.524870265	0.818729432
PIK3C2A	-0.016751865	0.524994543	0.818729432
NRD1	0.00614152	0.525036668	0.818729432
SLC16A6	-0.015424015	0.525077085	0.818729432
TDRD12	-0.011691058	0.525131242	0.818729432
FAM46A	0.009288756	0.525350907	0.818729432
VEZF1	-0.00697515	0.525427425	0.818729432
GZMK	-0.025107742	0.525547907	0.818729432
PTPRJ	0.011812627	0.525564674	0.818729432

ALDH5A1	0.012344258	0.525567106	0.818729432
TOX2	-0.016303831	0.525569956	0.818729432
ANKRD11	0.006345705	0.525696431	0.818729432
GFI1	-0.021940814	0.525788357	0.818729432
TMEM192	-0.012794439	0.526013345	0.818729432
AATF	0.007842897	0.5260831	0.818729432
HYDIN	-0.011461174	0.526108777	0.818729432
MIR155HG	-0.016896062	0.526117245	0.818729432
FOXRED1	-0.01034987	0.52618709	0.818729432
PRIM1	-0.01116395	0.52633187	0.818729432
BCS1L	-0.008857256	0.526389455	0.818729432
UBE4A	0.00744227	0.526391406	0.818729432
EBAG9	0.006419059	0.526465129	0.818729432
STRADA	-0.006496444	0.526605945	0.818729432
DHPS	0.005380599	0.526628013	0.818729432
MTG1	-0.011074027	0.526793548	0.818729432
PRR21	-0.011200601	0.526807431	0.818729432
SLC30A3	-0.009836253	0.526863276	0.818729432
RPS6KB1	0.006315201	0.526875154	0.818729432
ZNF155	0.012818769	0.526921693	0.818729432
ELAC1	0.01126638	0.526969953	0.818729432
TAOK2	0.014723308	0.527041987	0.818729432
TGFA	0.017761114	0.52707745	0.818729432
PSMD9	-0.012152355	0.527120438	0.818729432
SPARC	0.034830652	0.527124397	0.818729432
RAP2B	-0.012738864	0.527125233	0.818729432
BASP1	-0.013002722	0.527128045	0.818729432
GPR160	-0.019440485	0.527142673	0.818729432
FBXO33	-0.007643217	0.527146332	0.818729432
PARD6A	-0.009646692	0.527155539	0.818729432
CR1	-0.018309958	0.527574491	0.819213144
TMEM91	0.012426759	0.527615006	0.819213144
SECISBP2L	0.006504466	0.527753722	0.819227137
CD9	0.036864695	0.527787815	0.819227137
LRG1	-0.017261036	0.527846051	0.819227137
COL16A1	0.011248828	0.52797111	0.819235646
CRABP2	0.012237478	0.527999557	0.819235646
BRIX1	0.007810527	0.528138002	0.819269257
ZC3H12C	0.012890208	0.528183702	0.819269257
OXTR	0.019739081	0.528243264	0.819269257
MEA1	0.006682574	0.52839269	0.819386199
UBE2R2	-0.008652412	0.528761871	0.819843837
CCNB2	-0.026384507	0.529008467	0.819976916
PRDM2	0.008983882	0.529028861	0.819976916
OAZ1	0.003626032	0.529069937	0.819976916
IER5L	0.014201976	0.529166238	0.820011352
ZNF550	-0.010652883	0.529403527	0.820264228
RRAGD	-0.013146232	0.529567991	0.820380296
ZNF513	0.009390802	0.529732531	0.820380296
FCGR1A	-0.019757202	0.529757052	0.820380296

MPPE1	-0.013373556	0.529774899	0.820380296
CASP5	-0.019888304	0.529951177	0.820538478
UBXN6	0.008215056	0.530066046	0.820544139
MUC4	0.011942	0.530103093	0.820544139
FAM109A	0.010702705	0.530255458	0.820637883
COMTD1	-0.011269857	0.530311932	0.820637883
NDUFA6	0.00973117	0.530601218	0.820740684
KIF20A	-0.011944803	0.530641609	0.820740684
HNRNPAB	0.005491407	0.530736358	0.820740684
CROT	0.012824888	0.530775244	0.820740684
TRAPPC3	-0.007773956	0.530778898	0.820740684
SAMD9	0.010031557	0.530860924	0.820740684
NAT9	-0.008652605	0.530937419	0.820740684
FAM83H	-0.01114579	0.531023675	0.820740684
RPL27	-0.004003031	0.531045693	0.820740684
CXCL10	-0.031553152	0.531447152	0.821246478
GPR183	0.013626273	0.531723569	0.821495906
B4GALT4	-0.0107475	0.531833394	0.821495906
VENTX	0.018081426	0.531890471	0.821495906
ZRANB1	0.015979087	0.531905426	0.821495906
TIMM22	0.011652241	0.53206947	0.821634621
DDHD2	0.005750522	0.532240669	0.821784344
DCLK1	0.010996333	0.532609573	0.822225768
ATF6	-0.008325744	0.532836603	0.822225768
ZBTB17	0.007584483	0.532881048	0.822225768
RHPN1	-0.013208568	0.532906645	0.822225768
MIF	-0.008553118	0.532965404	0.822225768
HHATL	0.011859647	0.532972255	0.822225768
SENP3	-0.012341566	0.533330216	0.822646817
HN1L	0.010705407	0.533562541	0.822646817
ITM2C	-0.018629199	0.533640708	0.822646817
NUSAP1	-0.015271823	0.533719131	0.822646817
SLC23A3	0.01958673	0.533727335	0.822646817
CLEC4C	-0.02492036	0.5337471	0.822646817
DNAJC15	0.016093242	0.53377988	0.822646817
OAZ2	-0.020321243	0.533922167	0.822646817
C14orf159	0.008014127	0.533932387	0.822646817
DCAF8	0.007477148	0.53398838	0.822646817
FCGR3B	-0.017963606	0.534195524	0.822730431
TNFRSF25	0.01524374	0.534303913	0.822730431
CHORDC1	0.009558765	0.534354193	0.822730431
CSNK1A1	-0.009901066	0.534357962	0.822730431
KIF24	-0.011983664	0.534414292	0.822730431
PPP2CA	0.004730363	0.534498275	0.822745294
PPP1R1B	0.010650281	0.53475827	0.822859653
CPA5	0.0288385	0.534800372	0.822859653
MRPL1	0.009289842	0.534849857	0.822859653
ITPK1	0.011129308	0.534869926	0.822859653
PERP	-0.020645512	0.534966481	0.822893827
TRPC2	0.011833105	0.535396916	0.823399235

PPARD	0.01140983	0.535443825	0.823399235
CFH	0.026388468	0.535717349	0.823551524
PPAPDC2	-0.016051229	0.535790007	0.823551524
PSMA2	-0.009063229	0.535958822	0.823551524
HES4	0.025974349	0.535982643	0.823551524
CETP	-0.025665355	0.536019085	0.823551524
CNNM3	0.008951247	0.536088914	0.823551524
RPS23	0.034427983	0.536153797	0.823551524
RALGAPA1	0.008775778	0.536154516	0.823551524
DUSP18	0.008776557	0.536257892	0.823551524
ADORA1	0.018817447	0.536286872	0.823551524
GBA	-0.008568406	0.536432395	0.82360507
ACSL6	-0.012639429	0.536470554	0.82360507
SLC38A10	0.010220229	0.536578811	0.823645722
PRAMEF7	-0.009375927	0.53667438	0.823645722
NCF1	-0.022894744	0.536720263	0.823645722
DIS3L	0.010036045	0.536865439	0.823744754
CDC23	-0.006498645	0.536933634	0.823744754
S100A14	-0.0126462	0.537029724	0.823777996
U2AF1	0.006290179	0.537211457	0.823942583
REEP5	-0.006015229	0.537398714	0.824115596
PRPS1L1	-0.009424857	0.53779845	0.824614357
ACTR1A	-0.00706017	0.538116574	0.824637158
GRINA	-0.020012328	0.538211237	0.824637158
GNPTG	-0.008690037	0.538213501	0.824637158
ANXA6	-0.009521158	0.538214815	0.824637158
INTS5	0.010432962	0.538303177	0.824637158
SOS2	-0.011393838	0.538335347	0.824637158
DHRS3	0.012352768	0.538434748	0.824637158
ITLN1	-0.013094569	0.538470913	0.824637158
KRT1	0.02811484	0.538574473	0.824637158
CFD	-0.017131492	0.538591072	0.824637158
ZNF540	-0.014879345	0.53866088	0.824637158
CIRH1A	-0.006829673	0.538763631	0.824637158
IFITM1	0.010518806	0.538781817	0.824637158
CAMKMT	-0.010156181	0.539037152	0.8249139
EDAR	-0.022287934	0.539140145	0.824957459
SCNN1A	-0.012854316	0.539531956	0.8252864
ALCAM	0.012850856	0.539545301	0.8252864
PSPC1	-0.010016368	0.539669172	0.8252864
ANKDD1A	0.038818058	0.539693939	0.8252864
MRPL34	-0.007882933	0.539727911	0.8252864
L3MBTL1	-0.010208071	0.53982175	0.825299887
WIZ	-0.009696608	0.539885851	0.825299887
MET	-0.010190904	0.539977425	0.825325893
ATF3	0.017507048	0.540458952	0.825759423
LRRC16A	0.015782507	0.540532126	0.825759423
UBE2L3	-0.01111346	0.540603605	0.825759423
C2orf82	-0.009403784	0.54062014	0.825759423
SETD6	0.010465199	0.540634072	0.825759423

C1orf115	0.015383747	0.540769145	0.825851775
NUPL2	-0.009592421	0.541146979	0.826222226
FADS1	-0.01580491	0.541161003	0.826222226
COL24A1	0.013454008	0.541291414	0.826307359
SLC25A33	-0.011888495	0.541732115	0.826866075
FAM192A	-0.00778861	0.541862978	0.826951786
FAM153C	0.022358323	0.542013093	0.827066849
GFOD1	0.018254085	0.542106633	0.827095565
GPR65	0.01249612	0.54236719	0.827273758
NSMCE4A	0.005236422	0.542388977	0.827273758
PTGER4	-0.00898581	0.542447641	0.827273758
RMND5A	-0.012583407	0.542557394	0.827327152
ARHGEF10	-0.021647677	0.542747228	0.827502626
UBE2O	0.011145803	0.542995359	0.827766924
DSC1	-0.029396757	0.54332036	0.828148315
C1orf43	-0.007626385	0.543519926	0.828247474
ADC	-0.012377	0.543535067	0.828247474
TMX4	-0.01094452	0.54387165	0.82864629
SNHG12	-0.012944248	0.544085823	0.828835493
NEIL2	-0.010243361	0.544145589	0.828835493
ING3	-0.005764485	0.544811017	0.829625119
RDH10	-0.011021055	0.544813894	0.829625119
TM9SF4	0.007333885	0.545099954	0.829839957
DFNB59	0.01078276	0.545209202	0.829839957
TP53INP1	0.011639399	0.545227369	0.829839957
MIPEP	0.009094324	0.545265193	0.829839957
HDAC4	-0.017357857	0.545329826	0.829839957
AP4B1	0.011586336	0.545466849	0.829929671
MFSD7	-0.012746028	0.545538738	0.829929671
MGP	0.010634122	0.54564148	0.829971904
CA11	-0.011815546	0.545743375	0.830012835
TNRC6C	-0.014810142	0.545826151	0.830024683
HSD17B7P2	-0.010558792	0.546030499	0.830071101
SSB	-0.00611432	0.546034907	0.830071101
ARL6IP4	0.010256185	0.546122907	0.830071101
SHCBP1	-0.005212536	0.546242913	0.830071101
HNRNPK	-0.006496823	0.546256035	0.830071101
NLRC3	-0.011471414	0.546515687	0.830071101
IRS2	-0.019679623	0.546524345	0.830071101
HVCN1	-0.011553562	0.546529108	0.830071101
OSGEP	-0.007480526	0.546531591	0.830071101
C1orf56	0.012860881	0.54675848	0.830122627
RASAL1	-0.010672115	0.546898077	0.830122627
ANKRD13D	-0.012660374	0.546945161	0.830122627
MVP	-0.009683758	0.546953524	0.830122627
NRSN2	-0.012351592	0.546975537	0.830122627
MAU2	0.011275469	0.547015488	0.830122627
SOCS2	0.012748983	0.54725111	0.830281424
CEP72	-0.010461243	0.547270148	0.830281424
RANGRF	-0.01374021	0.547364153	0.830310239

ANXA3	0.061518024	0.547506341	0.830343972
ZNF624	0.010430109	0.547536422	0.830343972
KCTD13	0.010746352	0.547932064	0.830830139
ASXL1	-0.009088732	0.548283442	0.831239734
DNMT3A	-0.010463345	0.548391706	0.831239734
TEF	-0.011381216	0.54842748	0.831239734
DNAJC12	-0.01006089	0.548637043	0.831366302
FMO2	-0.012346852	0.548674971	0.831366302
PSMA3	-0.008493561	0.548736309	0.831366302
ZNF625-ZNF20	-0.013453991	0.54881216	0.831367429
ZNF720	0.011708965	0.548937512	0.83144353
PCGF5	0.011791067	0.549098711	0.831444836
PAX7	0.012558528	0.549132751	0.831444836
ID3	-0.021479284	0.549218853	0.831444836
NEURL1B	0.008714655	0.549238833	0.831444836
HERC1	0.006964304	0.549447635	0.83156177
CC2D1A	-0.010017995	0.549516431	0.83156177
ARHGEF40	-0.012764609	0.549541454	0.83156177
EIF3I	-0.005752347	0.549806065	0.83184846
AKAP7	-0.009814714	0.550122712	0.832118337
KIAA0141	-0.007783551	0.55013479	0.832118337
TBC1D14	-0.006590382	0.550294649	0.832246409
XKR6	-0.01021976	0.550442602	0.832326446
USP16	0.006046678	0.550559848	0.832326446
IL18	0.00469068	0.550573153	0.832326446
TNFSF13B	-0.011081032	0.550750695	0.832382905
WDFY4	-0.013734849	0.550760899	0.832382905
GPATCH4	-0.008736319	0.550950559	0.832555868
CDK18	-0.013073472	0.55114174	0.832727411
COL6A2	0.017413163	0.55121454	0.832727411
STAB1	-0.017027709	0.551429803	0.832827079
SRA1	-0.008257701	0.551569209	0.832827079
EDN1	-0.011976772	0.551672301	0.832827079
MIS12	-0.008774484	0.55187567	0.832827079
FAM64A	-0.008858237	0.551941261	0.832827079
TUBB	-0.006650427	0.551983524	0.832827079
DHCR24	-0.020231888	0.55199039	0.832827079
ZNF407	0.007599529	0.552021447	0.832827079
CHRNA2	0.011146242	0.552102678	0.832827079
UBAC2	-0.00571911	0.552116002	0.832827079
RABL2A	0.010103303	0.552227539	0.832827079
KIT	-0.012217891	0.552245905	0.832827079
SCLT1	-0.014500977	0.552258629	0.832827079
RPS6KB2	0.010127583	0.552440543	0.832987927
XPO5	0.008648581	0.552550375	0.833040057
RPL19	-0.00285442	0.552640178	0.833061981
ABCC1	0.011229034	0.552722457	0.83307256
AURKAPS1	-0.00974146	0.552834105	0.833100885
CD69	0.013468896	0.553024128	0.833100885
KLKB1	0.01617093	0.553066394	0.833100885

CDKL3	0.009685934	0.553091008	0.833100885
MYH3	-0.012503619	0.553117572	0.833100885
CASP2	0.004792336	0.553427101	0.833453684
TRIM2	0.018119649	0.553600821	0.833601889
CDC27	-0.015539191	0.553710353	0.833653413
OXSM	0.007993076	0.554097237	0.833984181
RRAGC	0.005987167	0.554135766	0.833984181
RHOT2	-0.010469719	0.55415608	0.833984181
ANAPC2	0.011284425	0.554300092	0.83408751
BCL9L	-0.012223842	0.554637905	0.834286565
RNASEH2B	-0.012390723	0.554683482	0.834286565
SHOX2	0.010850353	0.554755052	0.834286565
DHX58	0.012864434	0.554759637	0.834286565
NUDT9	0.006488577	0.554843752	0.834286565
KCNJ2	-0.025836672	0.554884605	0.834286565
DHRS4L2	-0.013230094	0.554972241	0.834305003
FSTL3	0.011199824	0.555393563	0.834825006
ITGAE	0.008925287	0.555791954	0.835310405
MAPKAPK5	-0.007243433	0.55590777	0.835371043
DIS3L2	0.009443993	0.556014665	0.83541826
CALD1	0.0344626	0.556113644	0.835453572
CD96	-0.009544381	0.556436976	0.835771529
KLHL20	0.009182671	0.556497731	0.835771529
LMBR1L	0.009360796	0.556551806	0.835771529
ZDHHC12	0.009461394	0.556681039	0.835852201
ABCA11P	0.009472312	0.556805942	0.835926349
PI16	0.024807472	0.556960953	0.836045673
PRDM1	0.016014254	0.557075447	0.836063293
TMBIM6	-0.005420836	0.557123756	0.836063293
NFKBID	0.009071565	0.557249201	0.836099539
AK5	-0.019539809	0.557298979	0.836099539
SENP2	0.006415893	0.557521973	0.836210336
MTIF2	0.007932316	0.557523921	0.836210336
ENTPD5	0.011835596	0.557676462	0.836214974
TRANK1	0.011600645	0.557678104	0.836214974
IPPK	-0.010484099	0.558037108	0.83657208
NAGA	-0.011741828	0.55816346	0.83657208
HSPBP1	-0.011353146	0.55828254	0.83657208
FLYWCH1	-0.015781542	0.558291068	0.83657208
DUS3L	0.007774516	0.558294151	0.83657208
IQSEC3	-0.009209784	0.558407183	0.836628196
ATP6V0B	0.006824045	0.558535374	0.836707004
ZNF675	-0.013774807	0.558632126	0.836738701
EVL	-0.006208088	0.558990485	0.836983083
C1QB	-0.035407442	0.559002018	0.836983083
ZNF30	0.009919672	0.559022128	0.836983083
CISD1	0.008444997	0.559495985	0.837318883
C1orf86	0.013440415	0.559500463	0.837318883
FAM105B	-0.009051265	0.559504632	0.837318883
SHKBP1	-0.012973036	0.559624257	0.837318883

CTDSPL2	-0.00963967	0.559711381	0.837318883
PHLDB1	0.011013971	0.559733293	0.837318883
C19orf40	-0.011929956	0.55984159	0.837318883
IGFLR1	-0.020450935	0.559883479	0.837318883
AES	-0.009716591	0.56000218	0.837318883
HAX1	-0.00805183	0.560070208	0.837318883
JDP2	-0.014020487	0.56010266	0.837318883
DENND4A	-0.008696092	0.56015678	0.837318883
DRAP1	-0.008895501	0.560433829	0.837318883
NLK	-0.007223101	0.560460882	0.837318883
FANCC	-0.008926078	0.560551212	0.837318883
GPCPD1	0.012519435	0.560698619	0.837318883
RGL2	0.009358129	0.560775116	0.837318883
SMARCD1	0.005662788	0.560809018	0.837318883
COCH	-0.030537743	0.560821841	0.837318883
DUSP28	-0.005834642	0.560896021	0.837318883
CLIP1	0.005813822	0.560965185	0.837318883
NUMA1	0.009079317	0.560981554	0.837318883
NPC2	-0.006227255	0.560986253	0.837318883
MIR22HG	-0.015036043	0.561321043	0.837705626
MFSD8	0.010112768	0.561410059	0.837707861
ZNF33A	-0.005815857	0.561473902	0.837707861
TMEM150A	-0.006843737	0.561620264	0.837806909
COL5A1	0.011767592	0.561691668	0.837806909
GAS1	-0.018778524	0.561937553	0.837852419
FAM162A	-0.0127072	0.561980586	0.837852419
AIM1	-0.00825805	0.562020724	0.837852419
ARHGAP9	0.00685723	0.56214699	0.837852419
TADA3	-0.008252348	0.562206853	0.837852419
PSMG4	-0.009215193	0.562428631	0.837852419
XRN2	-0.072240821	0.562727235	0.837852419
TNNI2	-0.017095191	0.562750454	0.837852419
TCEA3	-0.018729849	0.562861988	0.837852419
PLEKHG2	-0.010450889	0.562910046	0.837852419
MPHOSPH6	-0.015731773	0.562963987	0.837852419
MREG	0.009906733	0.563015763	0.837852419
ROCK1	-0.011907363	0.563110304	0.837852419
ERCC6	-0.010360358	0.563206108	0.837852419
LRP10	-0.006641306	0.563249941	0.837852419
KIF16B	-0.016951316	0.563274364	0.837852419
LGMN	0.012349694	0.563285336	0.837852419
DEFB108B	-0.009572213	0.563293062	0.837852419
KRT6C	-0.012600626	0.563296891	0.837852419
PLTP	0.012949851	0.563413737	0.837852419
POP5	0.010453737	0.563487777	0.837852419
ME1	-0.019797133	0.563614409	0.837852419
IGFBP3	-0.028545493	0.563639704	0.837852419
TTK	-0.014476527	0.563721602	0.837852419
SLAIN1	-0.009092459	0.563730258	0.837852419
MRPL53	-0.009109242	0.563741964	0.837852419

GRK6	0.009844015	0.563816309	0.837852419
TMEM138	-0.005176692	0.5638416	0.837852419
BCL11B	0.008337156	0.564171094	0.83794694
C16orf93	-0.012178381	0.56418788	0.83794694
PIP4K2C	-0.007548188	0.564199371	0.83794694
CXCR4	0.019852269	0.564208017	0.83794694
CXCR6	-0.033621954	0.564310489	0.837953416
RFK	-0.013541277	0.564439174	0.837953416
MIR21	0.018073414	0.564439486	0.837953416
H2AFV	0.008992517	0.564662533	0.838172131
HDDC2	-0.007858324	0.564754572	0.838196347
GPSM1	0.013963904	0.565048738	0.838361237
PLEK	-0.008590282	0.565145128	0.838361237
BOLA3	-0.008712713	0.565259371	0.838361237
YY1	-0.006114731	0.565317799	0.838361237
NUCKS1	0.009479727	0.565368123	0.838361237
RNF31	0.009773662	0.565456433	0.838361237
GPA33	0.011306087	0.56551661	0.838361237
FOXJ2	0.004120607	0.565544389	0.838361237
NUP107	0.007067337	0.565547326	0.838361237
IPO4	-0.010019132	0.565830844	0.838591921
ASTN2	0.010454412	0.565865163	0.838591921
CRTAM	-0.019597273	0.565930224	0.838591921
C5orf34	-0.01253606	0.566264161	0.838974434
SPAG1	0.011816473	0.566423062	0.839097547
MAP6D1	-0.013294062	0.566539225	0.839157323
SLC48A1	-0.009049063	0.567021178	0.839549719
WBP4	-0.007763915	0.567170672	0.839549719
POLRMT	0.01003508	0.567174997	0.839549719
PEX13	-0.010173137	0.567183188	0.839549719
SNORD6	0.009278335	0.567183377	0.839549719
MSH5	0.011441313	0.567412306	0.839574727
KIF5B	-0.009984296	0.567492407	0.839574727
DUSP5	0.013599486	0.567517633	0.839574727
SIDT2	0.013867932	0.567578664	0.839574727
ZNF227	-0.009804557	0.56766031	0.839574727
GPT2	-0.010835498	0.567661104	0.839574727
TOR2A	-0.01040893	0.567731216	0.839574727
VWF	0.038639785	0.567811125	0.83958073
FAM21A	-0.010623209	0.568123168	0.839929924
MIB1	-0.011175312	0.568337839	0.840135088
NEK1	-0.008636285	0.568614217	0.840229237
B9D2	-0.007283418	0.568642948	0.840229237
KMO	0.014813029	0.568704129	0.840229237
ZBTB16	-0.021426523	0.568705162	0.840229237
GCH1	-0.010560339	0.569211471	0.84085149
NSA2	-0.008540543	0.569297646	0.84085149
SLCO4A1	0.008796393	0.569500124	0.84085149
OR2A9P	0.029009329	0.569594867	0.84085149
POLR3E	0.008712136	0.569746757	0.84085149

FYN	-0.008250686	0.569879971	0.84085149
FAM136A	-0.006509155	0.56993673	0.84085149
ZNF124	-0.01369875	0.569987302	0.84085149
CLCC1	-0.010301063	0.570198296	0.84085149
FBXO34	0.006902312	0.570265459	0.84085149
MAN1A2	-0.008406176	0.570269418	0.84085149
GNA12	-0.009005553	0.570283931	0.84085149
KIR3DL3	-0.038219393	0.57028408	0.84085149
TLK2	0.006549796	0.570288412	0.84085149
TCHP	0.007227806	0.570303828	0.84085149
PTPN13	-0.014988982	0.57037248	0.84085149
SNX33	0.009046505	0.570470505	0.84085149
TLR3	-0.009894836	0.570493693	0.84085149
MZB1	-0.024734096	0.570879238	0.841195089
PATL2	-0.023847067	0.570912159	0.841195089
GGT1	0.010420436	0.570954802	0.841195089
BNC2	-0.018731815	0.571135706	0.841326832
TMEM106C	0.010615204	0.571196237	0.841326832
ADAMTSL4	0.013883902	0.571314953	0.84138973
EEF1E1	-0.006788686	0.571443012	0.841466369
SETD1A	-0.008177081	0.571541099	0.841498859
PITPNM1	-0.00789653	0.571796709	0.841763236
FRY	-0.008709973	0.571906754	0.841813279
LSMD1	-0.008341525	0.572157325	0.841936387
VPS18	0.009064273	0.572217002	0.841936387
MARCH8	-0.019469289	0.572218578	0.841936387
ADRBK1	-0.005059077	0.572445458	0.842158263
MYL9	0.04066258	0.572595481	0.842267027
FBXL18	-0.00968151	0.572684277	0.842285711
ZNF416	-0.011751497	0.572814275	0.842364981
CNTLN	-0.010077981	0.57334878	0.842814836
IP6K2	-0.0281667	0.573375125	0.842814836
ZSWIM3	-0.013334838	0.573391247	0.842814836
GAL3ST4	-0.018005068	0.573424748	0.842814836
TAOK1	0.020643278	0.573515657	0.842836538
ANAPC16	0.010461068	0.573899163	0.843219341
ATF2	-0.008964593	0.573928495	0.843219341
C11orf80	0.014444106	0.574065824	0.843309171
ZNF646	-0.009969793	0.57424475	0.843375023
RAB20	-0.012319319	0.574263037	0.843375023
ZDHHC3	0.007569705	0.574431117	0.843509955
QPCTL	-0.008760804	0.574596121	0.843640331
NHLRC4	0.025321531	0.574780855	0.84379964
BECN1	-0.006165597	0.574930154	0.843906893
GSTO1	0.008220323	0.575224391	0.84416801
CNOT3	0.010277002	0.575262591	0.84416801
ELMO2	-0.007040759	0.575336839	0.84416801
MARCH9	0.009957392	0.575601173	0.844334784
STRBP	0.012248089	0.57563969	0.844334784
MARCKS	-0.011943124	0.575710121	0.844334784

HIRA	0.013303433	0.575755619	0.844334784
KIFAP3	0.009832464	0.576007704	0.844570261
NFIC	-0.009116247	0.576068793	0.844570261
CACNB3	-0.013662124	0.57624102	0.844572285
LGALS4	-0.010038505	0.576244069	0.844572285
RP9	-0.007876276	0.57631403	0.844572285
SLC25A29	-0.018894603	0.576378747	0.844572285
ITCH	0.009273531	0.576451677	0.844572285
CENPE	-0.011715025	0.576534333	0.844581595
CD68	-0.007524583	0.576616926	0.844590811
TRAF3IP1	0.010303091	0.576973847	0.84488483
URB1	-0.008691668	0.57714409	0.84488483
NUP54	-0.005300179	0.577207662	0.84488483
AIP	-0.007473088	0.577265853	0.84488483
ASPH	-0.008360656	0.577399552	0.84488483
ASPSCR1	0.006466431	0.577457179	0.84488483
GNG7	0.014027468	0.577610581	0.84488483
RFX3	-0.008944564	0.577654333	0.84488483
SIAH2	0.009121596	0.577709035	0.84488483
USP42	0.007192461	0.577740999	0.84488483
C6orf132	-0.008767885	0.577954675	0.84488483
SRP14	-0.005397816	0.577976132	0.84488483
FBXO15	0.012590745	0.577976984	0.84488483
FBXW8	-0.011384138	0.578013165	0.84488483
RGS1	0.026031486	0.578028735	0.84488483
DYRK1B	0.011779332	0.578136563	0.84488483
TJP2	0.014651687	0.578156328	0.84488483
FLNB	-0.010542476	0.578260371	0.84488483
MAP3K10	-0.008152903	0.578271411	0.84488483
CEBPB	-0.006993455	0.578344237	0.84488483
PTBP1	-0.005271055	0.578606696	0.844948111
ZBTB7B	-0.010835528	0.578612477	0.844948111
DUT	-0.01291901	0.578616558	0.844948111
FKBP1B	-0.014441759	0.578792701	0.845093841
NCS1	0.008945517	0.578956836	0.84515524
ZIM3	-0.009977626	0.579016332	0.84515524
EPB41L5	0.008976044	0.579099239	0.84515524
WFDC3	-0.015775215	0.579140166	0.84515524
C17orf89	-0.00787332	0.5793539	0.845297805
GCDH	0.007382724	0.579437819	0.845297805
DTX3	-0.01117945	0.579523809	0.845297805
JMY	-0.009710969	0.579543323	0.845297805
KIAA1524	-0.012953731	0.57963193	0.845315657
NDUFA4	-0.005951634	0.580290867	0.846108159
P2RX7	0.010856073	0.580328227	0.846108159
ILKAP	0.008879161	0.580630989	0.846203477
C11orf54	-0.006196025	0.580670982	0.846203477
SNORD84	0.012098599	0.580697759	0.846203477
BLVRB	-0.015817405	0.580699396	0.846203477
ZNF322	0.007567505	0.580978568	0.846498851

CENPK	0.038927504	0.581137782	0.846515529
RIF1	-0.007209203	0.581142967	0.846515529
LYPD1	-0.011433923	0.581343628	0.846696397
B3GNTL1	0.010673915	0.581514739	0.846823132
FAM175B	-0.005212532	0.581583653	0.846823132
BNIP3	0.008079865	0.581798577	0.846991034
LGALS9B	-0.013708455	0.581910238	0.846991034
RHOT1	0.006437781	0.582004511	0.846991034
ALDOA	-0.007025833	0.582005042	0.846991034
RPSAP58	-0.011370438	0.582201502	0.847098525
MEFV	0.014987	0.5823379	0.847098525
FANCE	0.006995755	0.582372394	0.847098525
H2AFX	-0.008605414	0.582385019	0.847098525
ECE1	0.01060848	0.582577793	0.847232517
SLC25A1	0.00976822	0.582635408	0.847232517
UBQLN1	0.004756933	0.582706763	0.847232517
WDR34	-0.010029468	0.582870131	0.847257106
URGCP	-0.006550985	0.582949687	0.847257106
TIGIT	-0.01631639	0.582953304	0.847257106
AKAP9	-0.011402527	0.583083005	0.847334355
CCM2	-0.007707675	0.583306669	0.84753796
GPR137B	-0.013649772	0.583379392	0.84753796
CRTC3	-0.006238532	0.58351076	0.84753796
IRF2BP1	-0.012951944	0.583529387	0.84753796
HLA-DRB1	0.149450518	0.583713704	0.847552678
FUT4	-0.007169844	0.583718439	0.847552678
SLC39A14	0.010297642	0.58389018	0.847552678
BAIAP2L2	-0.011540719	0.583985916	0.847552678
MT1E	-0.023099699	0.584043442	0.847552678
LHFPL2	0.01375682	0.584045847	0.847552678
LRP11	-0.010978728	0.584140612	0.847552678
HMGCR	0.005757809	0.584222243	0.847552678
C17orf51	0.010977287	0.58422865	0.847552678
GTF2H3	-0.010011842	0.584409044	0.847606797
PDE7B	-0.012783362	0.584419105	0.847606797
GINS2	-0.019322928	0.584536559	0.847666078
ZNF341	0.007626445	0.584823009	0.847694197
VIPR2	0.014453184	0.584831056	0.847694197
FZD3	-0.010268987	0.584861717	0.847694197
TTC3	0.007233948	0.584903607	0.847694197
MITD1	0.007856487	0.58496519	0.847694197
WDR49	-0.01063096	0.585015446	0.847694197
SCAP	-0.005129179	0.585200173	0.847832636
NCDN	0.009304298	0.585371895	0.847832636
MAP3K14	0.008559776	0.585389589	0.847832636
TXNRD2	-0.007411799	0.585446718	0.847832636
C1orf63	-0.010604976	0.58561874	0.847832636
IMMT	0.006098276	0.585661819	0.847832636
TTLL12	-0.008570388	0.585711636	0.847832636
ORC3	-0.00774753	0.585798539	0.847832636

TMSB10	-0.003031473	0.585827682	0.847832636
VPS25	0.012426039	0.585877042	0.847832636
C1QBP	-0.006363392	0.585953534	0.847832636
MXRA7	-0.025205734	0.58626632	0.848174343
SEMA4F	-0.008837225	0.586418219	0.848185004
FGF9	0.013452268	0.586444871	0.848185004
SLC35B4	-0.010902276	0.58650357	0.848185004
TMCC3	0.012822885	0.586758872	0.848443364
POLQ	0.013501691	0.587044742	0.848745853
RAI1	-0.007573154	0.587152096	0.848790199
SLC40A1	0.011642448	0.587501734	0.849184734
LINC00347	-0.009018127	0.587715945	0.849308315
SHPRH	0.009212308	0.587797688	0.849308315
PLAA	0.006543825	0.587817418	0.849308315
TP63	0.014627809	0.58827089	0.849852582
PTPN22	-0.011814042	0.588525006	0.849913781
POMT1	0.007426195	0.588543259	0.849913781
C1orf61	-0.012240323	0.588543602	0.849913781
C11orf31	0.011868671	0.588637616	0.84993866
SETX	-0.007391142	0.588768845	0.849946567
BMP8B	0.01547832	0.588858286	0.849946567
RPS6KL1	-0.009662392	0.588873451	0.849946567
RPN1	-0.005374994	0.589082524	0.849969461
TTLL5	-0.007787605	0.589121173	0.849969461
TBX21	-0.01106118	0.589134979	0.849969461
ZBTB39	-0.00910488	0.589254672	0.849969461
HHEX	-0.008946262	0.589312855	0.849969461
LTBP4	-0.012255572	0.589381528	0.849969461
NECAB3	0.010309777	0.589463599	0.849969461
PAQR7	0.011207615	0.589538232	0.849969461
RFX7	0.004959605	0.589594819	0.849969461
ZNF343	0.007231796	0.589657195	0.849969461
ROCK2	0.003569916	0.589794799	0.850057113
IRF8	0.008818289	0.590102075	0.850354395
STXBP2	-0.0105382	0.590361011	0.850354395
MDFIC	-0.011032796	0.590378283	0.850354395
RPS16	-0.002532773	0.59038515	0.850354395
PRKD2	-0.008985233	0.590385177	0.850354395
LMNB1	-0.015014539	0.590642921	0.850498103
TMEM170A	0.008646041	0.590691131	0.850498103
GTF2F1	0.006246343	0.590715459	0.850498103
BAP1	-0.007404685	0.591038089	0.850826916
CLPTM1	0.007268775	0.59114069	0.850826916
TPX2	-0.012632886	0.591303951	0.850826916
TMOD4	0.009687684	0.591325209	0.850826916
MRPS6	-0.005185285	0.591328165	0.850826916
ATP6V0E1	0.005850982	0.591483407	0.850838244
PRR14L	0.004176117	0.591489772	0.850838244
OSBPL2	0.005065155	0.591791951	0.851162306
UTP3	-0.008605076	0.592081373	0.851356337

ALG12	-0.007720272	0.592132498	0.851356337
RBM4B	-0.006480215	0.592157597	0.851356337
SHE	0.011432155	0.592369088	0.851549797
ZNF714	0.011018378	0.592451779	0.851558076
C6orf211	-0.011421253	0.593234552	0.852558517
NCKAP5L	-0.010785245	0.593305901	0.852558517
FCF1	-0.007881333	0.593400694	0.852558517
VPS33A	0.007367544	0.593455902	0.852558517
SIRT5	0.006192377	0.593606159	0.852663713
AFMID	0.01260085	0.593721022	0.852718048
PPP1R14A	-0.019037249	0.593921742	0.852882744
SH3RF3	-0.009827726	0.593989798	0.852882744
METTL18	-0.01416452	0.59422584	0.853111001
MAP2K6	-0.012328026	0.594684409	0.853542523
TMPRSS13	-0.010770295	0.594792105	0.853542523
OS9	-0.008211581	0.594853364	0.853542523
ST3GAL4	-0.01208194	0.594891589	0.853542523
MTHFSD	0.008639564	0.594911968	0.853542523
ATF4	-0.004690174	0.595270003	0.853945524
NBR1	-0.008327739	0.595376143	0.853987109
ZNF512	0.006426659	0.595627255	0.854207687
RUFY2	0.011928676	0.59572961	0.854207687
DPP4	0.014007666	0.595761437	0.854207687
BAG5	0.005629629	0.595873891	0.854258269
DDX31	-0.008855808	0.595954679	0.854263448
SRCIN1	-0.010037588	0.596252355	0.854352011
ZNF764	0.01180721	0.596286604	0.854352011
POLD1	0.009245366	0.596349252	0.854352011
CLEC10A	-0.01731765	0.596369501	0.854352011
DSG2	-0.007975332	0.596555133	0.854352011
FAM60A	-0.005212657	0.596606124	0.854352011
HACE1	0.008116028	0.59661929	0.854352011
MANF	-0.008418777	0.596686733	0.854352011
MALT1	0.006870748	0.596751255	0.854352011
IRF4	0.013596735	0.596788305	0.854352011
ZNF678	0.009386924	0.596923826	0.854435514
PHF20	-0.006870996	0.597080183	0.854548817
FAM49B	-0.010959444	0.597250981	0.854682755
HIST1H3D	0.013459101	0.597378682	0.854754993
HMOX1	0.012326439	0.597459094	0.85475956
SUV420H2	0.010650132	0.597761501	0.855042456
ABL1	-0.005585103	0.597973644	0.855042456
EED	-0.009943861	0.597992632	0.855042456
TMEM176A	-0.062217721	0.597993259	0.855042456
YPEL2	-0.010957507	0.598043066	0.855042456
C2orf76	-0.009535948	0.598177961	0.855124867
PTPLAD1	0.006237673	0.598662334	0.855706789
MUC2	-0.011034915	0.598994669	0.855981199
NT5M	0.021293916	0.59920109	0.855981199
C17orf66	-0.011553933	0.59928208	0.855981199

OPN3	-0.010197337	0.599300955	0.855981199
ICAM3	-0.006699227	0.599311806	0.855981199
RBM42	0.007186953	0.59939495	0.855981199
TPM3	-0.003904402	0.599465613	0.855981199
SH3RF1	-0.01163201	0.599472965	0.855981199
TLE3	0.008900275	0.600127439	0.856805188
ATXN1L	-0.004480863	0.600318045	0.856966784
THNSL1	-0.010762255	0.600480028	0.857087483
TBC1D20	0.0075866	0.600834407	0.857429548
ARNTL	0.006622225	0.600874605	0.857429548
SNORD32B	0.009933899	0.600955418	0.857434329
BMS1P4	-0.008832598	0.601306579	0.857824788
RPL31	-0.006025462	0.601555617	0.858069474
PHTF1	-0.008568088	0.601652131	0.858096564
HAT1	-0.00932785	0.601797753	0.858193678
MIA3	0.008603663	0.601989927	0.858245358
AGK	-0.007383929	0.601990175	0.858245358
TRIM24	0.009226524	0.602066601	0.858245358
RPS10P7	-0.010731186	0.602217648	0.858288608
PTGES3	-0.009255185	0.602252021	0.858288608
MANBA	-0.007322489	0.602637403	0.858658522
PNP	-0.010107715	0.602666732	0.858658522
ADH4	0.012409226	0.603696597	0.859738833
HSP90AB3P	0.008709959	0.603699986	0.859738833
ACADM	0.006227048	0.603821517	0.859738833
RPP30	-0.009546494	0.603975501	0.859738833
CD300A	0.010646976	0.604203586	0.859738833
PLA2G12A	0.016518244	0.604220613	0.859738833
ZNF658	-0.0138992	0.604299742	0.859738833
TRAPPC5	0.006909505	0.604340877	0.859738833
ZNF43	0.012184831	0.604364478	0.859738833
DNASE1	0.009838538	0.604413647	0.859738833
ANKRD24	-0.010701758	0.604499126	0.859738833
PCBP2	0.004491605	0.604540319	0.859738833
POM121C	-0.004811768	0.604654876	0.859738833
TCIRG1	0.008913112	0.604702078	0.859738833
TBC1D22A	0.008996036	0.604711397	0.859738833
CLRN1	0.008426156	0.604763472	0.859738833
ANKRD13C	0.00764128	0.604780173	0.859738833
DNAJC11	0.008108998	0.604958073	0.859738833
RPS20	-0.003188064	0.604961234	0.859738833
NEFH	0.010516641	0.604978388	0.859738833
DNAJB6	0.005114111	0.605086216	0.859781685
KPNA3	-0.007213449	0.605224866	0.859810277
IER3	-0.014663006	0.605299048	0.859810277
TPST1	0.015433339	0.60533937	0.859810277
NCK1	-0.006863415	0.605444966	0.859830489
TRA2B	-0.00504092	0.605611086	0.859830489
CTSK	0.013575627	0.605652668	0.859830489
HSPH1	0.00710776	0.605886527	0.859830489

KIAA0040	0.011717822	0.605891081	0.859830489
HSD17B12	-0.007766225	0.60606931	0.859830489
LTB	0.005923878	0.606082109	0.859830489
OSBPL7	0.008725794	0.606152324	0.859830489
PHACTR4	-0.0080329	0.606233732	0.859830489
JUP	0.029903828	0.606326394	0.859830489
TRIM3	-0.008346685	0.606363477	0.859830489
SF3B2	0.004805796	0.606574801	0.859830489
STAG3	-0.011305011	0.606577184	0.859830489
RNU4ATAC	-0.012270073	0.606622676	0.859830489
FBXO42	-0.009288573	0.606642353	0.859830489
UBE2B	0.007441559	0.606672032	0.859830489
PLD2	0.009563009	0.606828483	0.859830489
FOXH1	0.008912167	0.606904831	0.859830489
C15orf54	0.019345747	0.60695911	0.859830489
COQ3	-0.005848589	0.607035432	0.859830489
SOD2	-0.011282071	0.607090452	0.859830489
STX5	-0.005154168	0.607178561	0.859830489
RCN2	-0.008207074	0.607265196	0.859830489
CDK9	0.005882978	0.607289367	0.859830489
C11orf21	0.011214656	0.607408214	0.859830489
FLT4	0.011196432	0.60744307	0.859830489
FBXO41	-0.009814961	0.607450937	0.859830489
WWP1	0.005668704	0.607656818	0.860011931
KIF21B	0.007803515	0.607964279	0.860304776
DDX55	0.007286711	0.608019176	0.860304776
MCM6	-0.007778504	0.608124745	0.860344172
METTL10	0.007981513	0.608339829	0.860439419
MTMR2	-0.008837595	0.608475583	0.860439419
TBKBP1	-0.012013311	0.608543104	0.860439419
ZBTB2	0.008510707	0.608576942	0.860439419
TMEM161A	0.008719684	0.608604672	0.860439419
PNKD	0.014750142	0.608658474	0.860439419
ZZZ3	0.006330106	0.608916711	0.860665351
CCDC15	0.008820989	0.608976761	0.860665351
ABCC4	-0.017000748	0.609068611	0.860665351
GTSF1	-0.017294616	0.609129312	0.860665351
NOTCH2NL	-0.009594222	0.609341347	0.860797221
UBE2D2	-0.005654635	0.609620049	0.860797221
HSDL2	-0.009171471	0.609700121	0.860797221
SNW1	-0.006795703	0.609718697	0.860797221
FAHD2B	-0.007112173	0.609804323	0.860797221
APOL3	-0.008707405	0.609832633	0.860797221
MAMDC4	-0.007938761	0.609964401	0.860797221
ZAN	0.009554347	0.609968931	0.860797221
ZNF25	0.005364334	0.609975781	0.860797221
TADA1	-0.006697871	0.610000307	0.860797221
FGF23	0.009040753	0.610107364	0.860838548
VTI1B	0.007645264	0.610303663	0.860909917
NAPSB	0.02188034	0.610361944	0.860909917

PARP9	0.014176248	0.610464233	0.860909917
TTC7B	0.010791669	0.610469052	0.860909917
DNAJC14	-0.006504076	0.610561509	0.860913585
GALNT10	-0.009178397	0.610682644	0.860913585
YES1	-0.014583864	0.610891063	0.860913585
CISD2	0.00684858	0.610926492	0.860913585
COG8	-0.008462018	0.611137053	0.860913585
ZNF672	-0.006445698	0.611204588	0.860913585
TAOK3	-0.00511637	0.611233485	0.860913585
GOLIM4	-0.013861034	0.61137463	0.860913585
MEGF6	0.012602438	0.611408034	0.860913585
CALML4	-0.009907268	0.611524264	0.860913585
RDM1	-0.010609622	0.611697766	0.860913585
ESPNL	-0.014607841	0.611805371	0.860913585
TFCP2	-0.006088484	0.611875679	0.860913585
ABHD15	0.008063789	0.611925795	0.860913585
PTP4A1	-0.013621974	0.611959787	0.860913585
ZNF3	-0.008288856	0.61198839	0.860913585
CCR9	-0.01747604	0.612026968	0.860913585
UBE2T	-0.010012303	0.612057184	0.860913585
SSBP2	-0.008848622	0.612073623	0.860913585
DCAF13	-0.007810015	0.612109019	0.860913585
MIOS	-0.005382746	0.612143823	0.860913585
NEK6	-0.01014167	0.612421894	0.860913585
WWOX	0.011588477	0.612521364	0.860913585
SLC22A18	-0.014594191	0.612545927	0.860913585
ZSWIM1	0.006556635	0.612658768	0.860913585
GBP4	-0.013033417	0.612717999	0.860913585
ZNF571	-0.007757881	0.612726861	0.860913585
RC3H2	0.007518895	0.612940158	0.860913585
UBE2I	-0.005896775	0.612952441	0.860913585
GGTLC2	0.008911698	0.612962946	0.860913585
ST6GALNAC1	-0.014836804	0.613051905	0.860913585
LYN	-0.006913055	0.613157725	0.860913585
IDH3A	0.008255853	0.613330321	0.860913585
CLEC4G	0.015900861	0.613489682	0.860913585
RPA3	0.006952782	0.613504903	0.860913585
FRRS1	-0.009410463	0.613617303	0.860913585
B2M	-0.003577354	0.613675786	0.860913585
ZNF165	-0.009820313	0.613730019	0.860913585
HIST2H2BF	0.008451656	0.613742955	0.860913585
SNORD57	0.011631176	0.613948617	0.860913585
GRPEL2	0.010012788	0.614026198	0.860913585
SEMA4D	0.006184818	0.614065127	0.860913585
MTF1	0.009656127	0.614099568	0.860913585
NUAK2	-0.007993498	0.614163901	0.860913585
MUC1	-0.012617414	0.61417235	0.860913585
CARS2	0.007176592	0.614230179	0.860913585
PPP1R3E	0.01214869	0.614394452	0.860913585
IL12RB2	-0.013097669	0.61442035	0.860913585

ZYG11B	0.006600776	0.61443799	0.860913585
GTF2A1	0.013922969	0.614488656	0.860913585
ZCWPW1	0.009160048	0.614494835	0.860913585
FAM86B1	-0.012773295	0.614516057	0.860913585
NELL2	-0.014782365	0.614646499	0.860987357
ZNF799	-0.009878161	0.614820172	0.861034595
GGT7	-0.015464884	0.614886959	0.861034595
C16orf70	0.006772104	0.614913585	0.861034595
ZFP36	0.007548395	0.615242899	0.86138675
TMEM51	-0.025681522	0.615649666	0.861787477
TBPL1	0.005964397	0.615785867	0.861787477
PIP5K1C	0.009577748	0.615891364	0.861787477
SLC35E2B	0.005680293	0.615903524	0.861787477
SP4	-0.008225486	0.61594585	0.861787477
NAAA	0.010856059	0.615996253	0.861787477
MEPCE	0.00994024	0.616283247	0.862031817
SFXN2	0.00707349	0.616424019	0.862031817
ALKBH2	0.007399379	0.616542828	0.862031817
ABI3	0.009084502	0.616638389	0.862031817
PRSS21	-0.01847114	0.616719055	0.862031817
HPS5	-0.007721895	0.616761131	0.862031817
BUB1	-0.016622776	0.616844343	0.862031817
ANKMY2	0.005995946	0.616871471	0.862031817
FAIM	-0.012338442	0.616871806	0.862031817
PPFIA1	0.005661657	0.617084953	0.862057431
ECI2	-0.007029947	0.617156726	0.862057431
CD79B	-0.014057959	0.617242559	0.862057431
ZFYVE20	0.005582745	0.617275468	0.862057431
DERL1	-0.006518929	0.617440136	0.862057431
FKBP6	-0.009424227	0.617488023	0.862057431
RPS27L	-0.00559932	0.617509427	0.862057431
CD226	0.013654029	0.617623747	0.862057431
ZNF687	-0.010209836	0.617674381	0.862057431
TRMT6	-0.009538092	0.617677578	0.862057431
SMARCA2	0.004700449	0.617746819	0.862057431
PSAT1	0.012934781	0.617892473	0.862151996
E4F1	0.004662276	0.618258129	0.86241525
NUPR1	0.00849441	0.618353363	0.86241525
CPNE1	0.0072996	0.618504632	0.86241525
CCDC106	-0.010788275	0.618649624	0.86241525
IRX1	0.009029128	0.618675792	0.86241525
NFIX	-0.012785156	0.618783462	0.86241525
ADORA3	-0.018088877	0.618950564	0.86241525
ZNF543	-0.014052268	0.618967503	0.86241525
FAM129B	-0.008353059	0.619024578	0.86241525
MAGOHB	-0.00983973	0.619173651	0.86241525
PDE9A	-0.021486863	0.619325911	0.86241525
EPN2	-0.009038977	0.619345682	0.86241525
GPR162	-0.009051001	0.619403143	0.86241525
ENPP5	-0.01658027	0.619514472	0.86241525

CCDC127	-0.008399212	0.619514544	0.86241525
TRAPPC9	0.009959203	0.619535051	0.86241525
ISL2	0.009227281	0.619536291	0.86241525
NME4	-0.018059817	0.619601878	0.86241525
HCN4	0.011453929	0.619763513	0.86241525
NR1I2	-0.015499394	0.61983831	0.86241525
BTG3	0.007799713	0.619896853	0.86241525
FCER2	0.026969126	0.619933582	0.86241525
C7orf55	-0.008729469	0.620043733	0.86241525
TCN2	-0.01437386	0.620200166	0.86241525
ARRDC3	0.011166785	0.620253421	0.86241525
DYRK1A	-0.005496965	0.620256571	0.86241525
BAZ2B	0.009487786	0.620342877	0.86241525
NBEAL2	0.009230157	0.620458736	0.86241525
RBBP9	0.006545447	0.620501551	0.86241525
YWHAZ	0.004781375	0.620602126	0.86241525
CELA1	-0.023145767	0.620661627	0.86241525
ABHD11	-0.009962303	0.620687601	0.86241525
HECA	0.006820626	0.620705749	0.86241525
NME1-NME2	-0.008189343	0.620778523	0.86241525
NLRP2	0.026936287	0.62083835	0.86241525
MED24	-0.007047817	0.620885999	0.86241525
RWDD4	-0.004623336	0.621193455	0.862429912
MAPK8IP3	0.011673959	0.621296517	0.862429912
PLK3	-0.011266347	0.621393017	0.862429912
TPMT	-0.006574898	0.621412963	0.862429912
CORO1C	0.008143377	0.621508453	0.862429912
CUX2	0.015635047	0.621615277	0.862429912
MEOX1	0.016564372	0.621778666	0.862429912
C17orf70	-0.00600045	0.621784148	0.862429912
CAMK2N1	0.018567712	0.62182603	0.862429912
C6orf223	-0.009931312	0.621908729	0.862429912
VAT1	-0.007985869	0.621961786	0.862429912
RCN1	-0.008358632	0.622003462	0.862429912
FKBP11	-0.009027979	0.622069648	0.862429912
PTAFR	-0.009367726	0.622077128	0.862429912
RECQL	-0.008000186	0.622133338	0.862429912
CSNK1E	-0.008124966	0.622299283	0.862429912
ATP6AP1L	0.013201819	0.622323889	0.862429912
TRIML1	-0.008374109	0.622337476	0.862429912
JAM2	0.008063065	0.622433114	0.862429912
IP6K1	-0.006355067	0.622454835	0.862429912
RNF150	-0.022963864	0.622742657	0.86272071
CHSY1	-0.005591004	0.623031963	0.86301349
TGFB1I1	0.023123908	0.623114751	0.863020168
ANKRD36B	0.023881671	0.623287132	0.863020468
MAL	-0.012706481	0.623383774	0.863020468
THG1L	-0.015055821	0.623417215	0.863020468
AGFG2	0.009785709	0.62354311	0.863020468
NFE2L3	-0.018958924	0.623565207	0.863020468

PANK2	0.006626456	0.62362499	0.863020468
HOPX	-0.015428764	0.62370119	0.863020468
PITPNC1	0.006015154	0.623738707	0.863020468
NDRG3	0.005677223	0.623915353	0.863156985
EIF2AK3	-0.007315081	0.624007179	0.863176139
TDRD7	0.006415668	0.624405051	0.863618582
CD63	0.008859246	0.624553096	0.86371542
BRPF1	-0.004039857	0.624919634	0.864024796
AARSD1	0.00676521	0.625262696	0.864024796
GLUD1	-0.004159988	0.625275769	0.864024796
AVL9	-0.008147282	0.625315825	0.864024796
ZNF691	-0.008903381	0.62534416	0.864024796
TTC34	0.007811071	0.625378717	0.864024796
SPSB2	-0.013411342	0.6253788	0.864024796
KCNJ12	-0.010723331	0.625401271	0.864024796
POR	-0.007508673	0.625516625	0.864076317
E2F2	0.0110901	0.625773539	0.864192471
SS18L2	-0.003994294	0.625884557	0.864192471
DDIT4	0.02079722	0.625945352	0.864192471
PPM1N	-0.009804369	0.625981341	0.864192471
MYLIP	0.006448203	0.625991077	0.864192471
HYAL3	0.008577778	0.626258953	0.864297492
ARPP19	-0.005119393	0.626272047	0.864297492
FAM24B	-0.009872714	0.626301399	0.864297492
NFE2L2	-0.00904386	0.626415708	0.864347479
PICK1	-0.007093755	0.626581784	0.864468873
COL6A3	0.015588588	0.626828308	0.864701214
RNF121	-0.005491721	0.62698537	0.86471477
TUBB2A	0.019571012	0.626994376	0.86471477
ZNF469	-0.009393245	0.627161807	0.864837927
KIF15	-0.008804532	0.627263459	0.864870357
HOOK1	0.013398546	0.627603845	0.865128526
PPIH	-0.005744855	0.627607016	0.865128526
C1QC	0.013600399	0.627747435	0.865214339
RUNDC1	-0.005783134	0.627921395	0.865346354
TACSTD2	-0.06201549	0.628084619	0.865463544
RNF217	-0.009125422	0.628374101	0.865649993
TAF4	0.004844899	0.628376339	0.865649993
S1PR1	0.013200186	0.628563438	0.86570889
FAM13B	0.005516653	0.628575513	0.86570889
ZNF816	-0.011008215	0.628786589	0.8658088
RAP1GAP2	-0.009900776	0.628923643	0.8658088
BUD13	0.005406206	0.628954364	0.8658088
CD1D	-0.009681909	0.628960932	0.8658088
NLRC4	-0.010154208	0.629278619	0.866138402
FOPNL	0.005373906	0.629433528	0.86621093
FBXW11	-0.005336947	0.629487824	0.86621093
SYK	0.006674981	0.629874906	0.866543127
ACTA2	-0.023848557	0.629885807	0.866543127
NFYC	-0.005093976	0.630069274	0.866595213

ME3	0.014807945	0.630080249	0.866595213
DTHD1	-0.018000778	0.630445408	0.86692275
OLFM1	-0.03151575	0.630475032	0.86692275
NBPF14	-0.014986094	0.630623394	0.867019047
SPDYE8P	0.007948253	0.630823791	0.867061643
UNC80	-0.008512553	0.631009479	0.867061643
EDEM1	-0.005392867	0.631010241	0.867061643
C9orf156	-0.007630199	0.631159606	0.867061643
ALMS1	0.007524541	0.63117782	0.867061643
PDHX	-0.006501734	0.631201385	0.867061643
C15orf48	0.009751289	0.631288911	0.867061643
CCDC117	-0.00661936	0.631358162	0.867061643
C14orf166	-0.006247538	0.631359368	0.867061643
CLYBL	0.011152786	0.631608779	0.86719946
ANAPC1	-0.006104129	0.631743066	0.86719946
MRPL23	0.006634998	0.631781675	0.86719946
IKBKAP	-0.006398559	0.631843292	0.86719946
CBR3	-0.016662072	0.631851445	0.86719946
CCDC12	-0.004904969	0.632212608	0.867587572
NAPRT1	0.012236313	0.632580692	0.867985085
SNRPE	0.008583529	0.632851006	0.868248362
ATXN7L3B	-0.005831698	0.632990701	0.868332392
RORA	0.009793444	0.633157212	0.868453183
ERGIC2	0.009774902	0.633366661	0.868632829
ERCC3	0.005420567	0.633677857	0.868951957
HLA-DMB	-0.005985811	0.633821758	0.869041626
CAD	-0.007683796	0.633904213	0.869047032
CDKN2D	0.011220736	0.634152534	0.869265616
APLP2	-0.010217914	0.634261872	0.869265616
WDYHV1	0.009103945	0.634299248	0.869265616
CERK	-0.005680479	0.634575644	0.869351692
BOLA2B	0.006353111	0.634583202	0.869351692
SRSF7	0.006245508	0.634635593	0.869351692
SSNA1	-0.006380473	0.634736453	0.869351692
DUSP16	0.009045925	0.634837127	0.869351692
ZNF260	0.008213751	0.634911596	0.869351692
GSTT1	0.033441164	0.634911833	0.869351692
SMU1	-0.005754615	0.635105076	0.869508731
ZNF432	0.00779765	0.63528329	0.869518467
DCLRE1A	-0.013751143	0.635297706	0.869518467
PARP2	0.007061883	0.635347851	0.869518467
SLC8A1	0.015231104	0.635494963	0.869612281
C1orf109	-0.006723249	0.635694021	0.869777147
RCHY1	-0.011715572	0.635822754	0.869845763
DAPK1	-0.00295809	0.635955492	0.869919839
C14orf169	-0.005786599	0.636100197	0.870010266
RHOH	-0.008343167	0.636389095	0.870233822
IDH2	-0.00800151	0.636449534	0.870233822
DUSP2	0.014797359	0.636520174	0.870233822
CDKN3	-0.013781653	0.636586919	0.870233822

ZNF485	-0.008743496	0.636656743	0.870233822
SMYD2	-0.005026064	0.636764536	0.870273694
CD1A	-0.012317394	0.636871606	0.870312568
SAC3D1	0.00698977	0.637200306	0.870564819
P2RY6	0.010356594	0.637213494	0.870564819
DGCR6	-0.008018806	0.637322575	0.87060639
KRAS	-0.006112649	0.637715133	0.871035144
ERCC2	-0.008143401	0.637802527	0.871047029
NDUFB10	-0.004351545	0.637975423	0.871175667
MRPL24	-0.005089261	0.638059107	0.871182466
TMEM8B	-0.008758379	0.638257705	0.871346144
TJP3	0.01242985	0.638339282	0.871350045
ANGEL1	0.006948291	0.638431585	0.871368583
CDH23	-0.015419448	0.638607751	0.871379105
LAMC3	-0.020909527	0.638652549	0.871379105
PLLP	-0.010753572	0.638729485	0.871379105
SULF2	-0.012365935	0.638754183	0.871379105
RAB31	-0.006792237	0.639040666	0.871594411
TOB1	-0.010933969	0.639069495	0.871594411
MED13L	-0.008301686	0.639158373	0.871608233
N6AMT1	-0.010047949	0.639302483	0.871697362
PTGFRN	-0.008322609	0.639465208	0.871811848
PXN	0.0077674	0.639759419	0.872013313
PRDM15	0.009113943	0.639770541	0.872013313
PRUNE2	-0.024507825	0.640254379	0.872565344
POTEE	0.012596958	0.640359661	0.872601389
NAP1L4	0.007413781	0.640484892	0.872664607
ZBTB24	0.006955254	0.64068504	0.872746963
VPREB3	0.023454239	0.640773132	0.872746963
GLTSCR2	-0.003116765	0.640781874	0.872746963
GDF11	-0.008164336	0.640979263	0.872823327
ZNF844	-0.009518192	0.641006768	0.872823327
ZNF696	-0.009908488	0.641107769	0.872823327
CCND2	0.005860956	0.641215172	0.872823327
TMEM30B	-0.009857014	0.641273953	0.872823327
LSM1	-0.004191365	0.64134923	0.872823327
TRAPPC10	0.006103268	0.641405872	0.872823327
TP53I13	0.006508838	0.641468766	0.872823327
CDK20	-0.00747118	0.641599215	0.872845518
CDH26	0.008450979	0.641642785	0.872845518
ETV2	0.009071098	0.641871744	0.873049685
C9orf91	0.006872232	0.641953843	0.873054072
IQGAP2	-0.007701647	0.642140458	0.873086311
MIAT	0.021010803	0.642148859	0.873086311
TPP2	0.007718283	0.642232425	0.873086311
SNORD54	0.012624536	0.642293056	0.873086311
CCDC88A	-0.008527158	0.642470774	0.873220653
TMEM150B	-0.008124297	0.642775645	0.873527761
CRAT	0.010037095	0.643006867	0.873649688
CPS1	0.011627252	0.643094167	0.873649688

LACTB2	-0.010566127	0.643102146	0.873649688
MIR130A	-0.034741138	0.643269129	0.873769296
C21orf91	-0.008840464	0.643516166	0.873935771
LBH	0.007452174	0.643549595	0.873935771
HP	-0.035655963	0.643816224	0.874047837
FAM98A	0.007203656	0.643861649	0.874047837
LEMD2	-0.004369254	0.643945273	0.874047837
CDK5RAP2	0.012798273	0.643947973	0.874047837
LRWD1	-0.009852475	0.644284896	0.87432834
B3GALTL	0.012833214	0.644588104	0.87432834
DNAJA3	-0.004601743	0.644650284	0.87432834
PLAGL2	-0.00920825	0.64466457	0.87432834
MCOLN3	0.010015437	0.644750459	0.87432834
GALNT6	0.008300888	0.644952174	0.87432834
GABPB1	-0.007454159	0.645062952	0.87432834
C14orf28	0.006870873	0.645103987	0.87432834
STAMBP	-0.004070431	0.645110639	0.87432834
C9orf142	0.006943019	0.645111389	0.87432834
SYT15	-0.013024529	0.645140016	0.87432834
IARS	-0.004559446	0.645154721	0.87432834
DDR2	0.01159548	0.645181487	0.87432834
SNORA22	0.011832574	0.645332505	0.87442594
CLIC4	-0.011650145	0.645412196	0.874426878
ABHD16A	0.005450625	0.645644745	0.874542311
SLC25A4	-0.009676375	0.645655413	0.874542311
DNAJC24	-0.007591688	0.645809285	0.874643703
SGSM1	0.010800205	0.646368331	0.87522478
UPP1	-0.005407824	0.646396475	0.87522478
MFSD1	0.006228884	0.646476224	0.8752257
ATP2C1	0.006119899	0.646586082	0.875267377
TESK2	-0.006126802	0.646737382	0.875365136
EPHB2	-0.009534094	0.646845708	0.875404713
CD4	0.008974467	0.646937232	0.875411231
YAF2	-0.006491617	0.647077435	0.875411231
BMS1	0.00388292	0.647251515	0.875411231
FAM26F	-0.023917327	0.647253951	0.875411231
BIN1	0.007776499	0.647302963	0.875411231
ACADS	-0.008597462	0.647382214	0.875411231
TRAF2	0.010029652	0.64756005	0.875411231
AHI1	0.015310225	0.64758827	0.875411231
F12	-0.008829364	0.647607002	0.875411231
BTN2A1	-0.005802295	0.647720203	0.875411231
SLC4A8	0.013617816	0.647795473	0.875411231
LCLAT1	0.012953341	0.647799566	0.875411231
TGFBRAP1	0.008858701	0.647983322	0.875450242
CBY1	-0.007876916	0.648084512	0.875450242
QRICH1	0.003901836	0.648136473	0.875450242
PVALB	0.03583731	0.648169719	0.875450242
KCNK12	-0.010325174	0.64826921	0.875450242
BAI1	-0.009155747	0.648314493	0.875450242

GSK3A	0.011158141	0.648382065	0.875450242
OSGIN1	-0.013324147	0.648563539	0.875588464
TMEM42	0.005099829	0.64866258	0.875615378
MCM9	-0.006360514	0.649029125	0.875759934
TACC2	0.01078035	0.649130784	0.875759934
ZNF845	-0.007894984	0.649148468	0.875759934
HSPB1	0.00686876	0.649326031	0.875759934
ZBED3	-0.007810039	0.649486488	0.875759934
KPNA1	-0.004644109	0.649492936	0.875759934
UBASH3A	0.012194813	0.649497452	0.875759934
ATP5D	0.006031116	0.64956414	0.875759934
MPHOSPH10	-0.004847011	0.649616946	0.875759934
PSMB3	-0.004412964	0.649658059	0.875759934
NDNL2	-0.008562688	0.649697804	0.875759934
MICAL1	0.011197629	0.649719087	0.875759934
WIPI1	-0.009949163	0.649914517	0.875829603
DDX43	0.021931473	0.649929024	0.875829603
ABLIM1	-0.008408703	0.65005097	0.875887302
COIL	-0.004177516	0.650626311	0.876555822
EXOSC5	-0.006333629	0.650841777	0.876739397
EDC3	-0.006954425	0.650954705	0.876784817
CHST12	0.013338853	0.651670807	0.877025073
MRPS14	-0.008671822	0.651679967	0.877025073
ATG4B	-0.012039996	0.651683007	0.877025073
SLC35B3	-0.007691531	0.651704615	0.877025073
TBCB	0.005920708	0.651722908	0.877025073
PRR7	-0.011977121	0.651771347	0.877025073
CAMKK2	0.022816329	0.651847749	0.877025073
ATP6V1G2	-0.009972645	0.65189993	0.877025073
RRM2B	0.012802991	0.651909259	0.877025073
TMEM44	-0.012864492	0.651979824	0.877025073
ANPEP	0.026300417	0.652004637	0.877025073
CLDND2	0.018573648	0.652169676	0.877140479
TCP1	-0.006810997	0.652525274	0.877452168
ZNF200	-0.015045327	0.652559964	0.877452168
NFKBIA	-0.007130944	0.652677874	0.877504116
ALKBH6	0.005232464	0.652899914	0.877645767
HK1	-0.004656836	0.65294181	0.877645767
MEGF10	0.009870489	0.653217703	0.877725926
SLC20A2	-0.006349231	0.653287648	0.877725926
TNFAIP6	-0.01409564	0.65329164	0.877725926
CIZ1	-0.007788994	0.653472502	0.877725926
DCBLD2	-0.010091919	0.653521794	0.877725926
JOSD2	-0.009713297	0.65352943	0.877725926
THTPA	-0.008115006	0.653556516	0.877725926
NSUN4	-0.005894904	0.653650926	0.877746221
SH3BP5L	0.008493421	0.653838751	0.877746616
SCYL3	0.004788336	0.653904751	0.877746616
COTL1	-0.012139954	0.653983108	0.877746616
NEB	-0.009695738	0.654023577	0.877746616

AGAP3	0.00822561	0.654047709	0.877746616
DNHD1	0.009045724	0.654178069	0.87778683
SH2B3	-0.005236474	0.654236277	0.87778683
FLT3LG	0.009365299	0.654394751	0.877847009
ZNF618	0.009721912	0.65457063	0.877847009
GTF3C2	0.003911008	0.654634578	0.877847009
GNAS	-0.006121088	0.654719011	0.877847009
CD28	0.009901701	0.654785104	0.877847009
HDHD3	-0.009346243	0.654810521	0.877847009
POU2F1	0.00611476	0.654978678	0.877847009
UBA7	-0.00595824	0.65512477	0.877847009
SPHK1	0.010220099	0.655149134	0.877847009
FCGR1C	-0.020241786	0.65526594	0.877847009
MIR1224	0.009303474	0.655302755	0.877847009
BZRAP1	0.014529726	0.655364436	0.877847009
UNC45A	-0.005591449	0.655368432	0.877847009
CAPN3	0.009382936	0.65541476	0.877847009
RAD54L	-0.00789602	0.655561391	0.877847009
INPP4B	0.010579111	0.655708633	0.877847009
CDCA7	0.01261835	0.655853195	0.877847009
RAB13	-0.009636512	0.655893975	0.877847009
PDCD1	0.015574679	0.655908258	0.877847009
COMMD9	-0.00614243	0.655981134	0.877847009
MAP4K4	-0.006332248	0.656054387	0.877847009
NSUN5P2	0.011232006	0.656095779	0.877847009
SLC24A4	0.011327364	0.656105186	0.877847009
ZBTB22	-0.004591635	0.656389144	0.878112415
TUSC2	-0.005430649	0.656520768	0.878112415
HPS4	0.007230355	0.656541544	0.878112415
UPF1	-0.008077517	0.656909106	0.878441991
PSME4	0.004721162	0.657005772	0.878441991
MTR	0.007644199	0.65702604	0.878441991
RANBP1	0.00455533	0.657445625	0.878698007
ALDH6A1	-0.005474616	0.657511252	0.878698007
CREBBP	-0.00469473	0.657564136	0.878698007
TTC13	0.007048545	0.657667192	0.878698007
SUCLG2	-0.006005287	0.657817795	0.878698007
RRP7A	-0.015899772	0.657860338	0.878698007
GOPC	0.005388965	0.657871002	0.878698007
CCDC130	0.005418326	0.657947329	0.878698007
DVL3	0.005435831	0.658025856	0.878698007
LYRM1	0.003918841	0.658073018	0.878698007
UBE2J1	0.006908327	0.65810674	0.878698007
SOX8	0.01241764	0.65817013	0.878698007
TEX9	-0.011225803	0.658259478	0.878711307
PTPRCAP	0.008321175	0.65839063	0.878780403
SIRPA	0.006540354	0.658524765	0.878853463
RAD21	0.012507971	0.658855899	0.878896949
S100A8	0.003408509	0.658920105	0.878896949
APBA2	-0.010196003	0.658966276	0.878896949

TPD52	-0.010311192	0.659067314	0.878896949
SNORD68	0.010516905	0.659163133	0.878896949
KRR1	0.007963399	0.659194341	0.878896949
AAMP	0.00556516	0.659232304	0.878896949
FES	-0.007658244	0.659266597	0.878896949
TSR1	0.005347391	0.659271964	0.878896949
KIAA1407	0.010053777	0.659591101	0.87913257
NARFL	-0.008272513	0.659718215	0.87913257
URB2	-0.005609047	0.659774958	0.87913257
RARS2	-0.004785656	0.659867244	0.87913257
LONRF1	0.008109099	0.659910217	0.87913257
EIF3CL	-0.011355264	0.659936869	0.87913257
CDIPT	-0.004949254	0.660004667	0.87913257
HMOX2	-0.011176702	0.660230824	0.879255616
PPFIBP1	0.007639631	0.660255911	0.879255616
AARS2	0.005958396	0.66039355	0.879333118
IL20RB	-0.007252268	0.660518444	0.879393632
MEF2BNB	-0.008277424	0.660711134	0.879494593
C7orf26	-0.005061592	0.660753187	0.879494593
ADNP2	-0.005309048	0.660832817	0.879494824
CYP51A1	-0.010193549	0.661107452	0.879754555
BTRC	-0.006898999	0.661204349	0.87977773
INTS6	0.006342858	0.661543742	0.880123519
CDCA8	-0.008489009	0.661766232	0.880229894
TAF15	0.009395127	0.66188394	0.880229894
AGR2	-0.007542343	0.661925311	0.880229894
PPIAL4E	0.007911347	0.661941787	0.880229894
SDC1	0.008487183	0.662122375	0.880364273
ZMYND11	0.004719741	0.662252039	0.880430918
IFIT5	-0.010765979	0.662655884	0.880862013
MIR1282	-0.017325909	0.662955539	0.881061222
CDK1	-0.014019703	0.663055076	0.881061222
MRPL14	-0.006666025	0.663055832	0.881061222
ABHD5	-0.010168498	0.663124134	0.881061222
ZNF146	0.00549691	0.663393467	0.881313284
GGCX	-0.007365744	0.663726414	0.881649787
SERTAD2	0.004721603	0.664172988	0.882137125
IFT20	-0.005159503	0.664571163	0.88251574
SPATS2L	0.010625299	0.664617509	0.88251574
TTC5	0.006240534	0.664801711	0.882559505
FOXD4L1	0.009224442	0.664809934	0.882559505
TSPAN17	-0.005482655	0.665037298	0.882684812
PDE4A	-0.009142178	0.665063812	0.882684812
TBCE	-0.005282971	0.665856958	0.88363154
RBBP8	-0.008656611	0.666028052	0.883665722
TBCCD1	0.007889185	0.666042381	0.883665722
SEMA4C	-0.007729969	0.666300614	0.883874654
PHLDA1	0.012263634	0.666359562	0.883874654
CRYM	0.009067372	0.66662852	0.88412546
GEMIN5	-0.006777231	0.667003997	0.884517461

CAPN2	-0.00515606	0.66714943	0.884573816
MLEC	-0.007833674	0.667206323	0.884573816
VPS28	0.00700033	0.667445007	0.884696906
SNORD12B	0.008110625	0.667459017	0.884696906
DDX24	0.00552238	0.667802864	0.884918296
C1orf35	0.004120219	0.667816905	0.884918296
C1orf228	0.009594919	0.667910127	0.884918296
MT1A	-0.010297955	0.667945827	0.884918296
CFI	-0.008629147	0.668102459	0.88501988
ZFP30	0.009565257	0.668208654	0.885054636
SELM	-0.009810457	0.668384285	0.8851621
ATP6V1C1	-0.007011182	0.668531004	0.8851621
NPY5R	-0.007461504	0.66855283	0.8851621
SLFN11	0.00662993	0.66867763	0.8851621
FSD1	0.015827184	0.668689627	0.8851621
IL25	0.009209319	0.668882184	0.885311119
ABTB1	0.006484539	0.668970826	0.885322581
MSC	-0.016547137	0.669196267	0.88551506
SFMBT2	-0.013135232	0.669312014	0.885562357
HMGN4	0.004546524	0.669435083	0.88561933
ZNF414	-0.006787919	0.669636176	0.885703268
MGAT4A	0.007502642	0.669685412	0.885703268
FANCL	0.009390328	0.669738581	0.885703268
ABCA2	0.012592428	0.669993944	0.885809227
MFSD6L	-0.00604831	0.670096097	0.885809227
PTPN12	0.00820647	0.670157573	0.885809227
TNS3	-0.006299499	0.670179497	0.885809227
MYL6	-0.00354805	0.670390671	0.885809227
CDO1	-0.009888092	0.670392267	0.885809227
MFSD10	0.004989595	0.670466227	0.885809227
DGAT2	0.010593486	0.670572703	0.885809227
ASL	-0.010088927	0.670592669	0.885809227
SERPINA10	0.009193843	0.670618965	0.885809227
VCL	0.009660065	0.67089929	0.885951039
ATAT1	0.008169237	0.670979769	0.885951039
ATXN7L2	0.005853517	0.671038243	0.885951039
CSNK1D	-0.00416737	0.671049572	0.885951039
METTL22	0.009185178	0.671126521	0.885951039
TMEM71	0.008170925	0.671345066	0.886096682
HCFC2	0.006088029	0.671396953	0.886096682
CEP57L1	0.006359572	0.671675089	0.88632904
HPCAL4	0.016883173	0.671782939	0.88632904
SNX19	0.006429835	0.67196055	0.88632904
NIN	0.00444534	0.672005865	0.88632904
E2F4	-0.004476381	0.672049186	0.88632904
MCM10	0.012064966	0.67205345	0.88632904
WDR25	-0.008629059	0.672352338	0.886617588
ZNF576	-0.006992652	0.672671317	0.886932556
UPK1A	0.008019341	0.672793038	0.886936995
ZNF419	0.006248106	0.67283494	0.886936995

STRA13	-0.004200069	0.673201843	0.88731498
ZNF680	0.00737481	0.673329189	0.887318722
WRB	0.011276577	0.673365007	0.887318722
INTS2	-0.005264745	0.673702032	0.887565486
CMTM2	-0.015941288	0.67371264	0.887565486
ABR	-0.004623597	0.674158847	0.887973716
GPHN	-0.007235295	0.674209993	0.887973716
POLR2K	0.011102117	0.674306209	0.887973716
OR2A42	0.009411459	0.674343396	0.887973716
RPL30	0.002753573	0.674480517	0.888048632
GON4L	0.005477079	0.674632755	0.888143431
LPHN1	-0.007977774	0.674823183	0.888180951
PPBP	0.010973575	0.674935335	0.888180951
SNUPN	0.008216148	0.675111051	0.888180951
TSLP	-0.008500956	0.675137008	0.888180951
CLCN6	0.006933929	0.675205494	0.888180951
C19orf53	-0.00469036	0.675359099	0.888180951
POU2AF1	-0.018565337	0.67542225	0.888180951
DCAF4	0.01069538	0.675423392	0.888180951
NPLOC4	-0.006500232	0.675482453	0.888180951
SLC25A17	0.006067899	0.675565027	0.888180951
SOCS5	-0.013580257	0.675679255	0.888180951
FAM153B	0.024961925	0.675683825	0.888180951
YBEY	0.014747843	0.675704381	0.888180951
ZC3HAV1	-0.005606446	0.67592824	0.888329292
SERPINH1	0.009913196	0.676038701	0.888329292
PLEC	-0.007025161	0.676057996	0.888329292
OXCT1	-0.005682338	0.67627187	0.888461521
KCNQ2	0.007717722	0.676319159	0.888461521
PLCL1	0.010419172	0.67647124	0.888522237
DAD1	-0.004527359	0.676525921	0.888522237
HIST1H4E	-0.022032091	0.676785154	0.888681019
DGCR14	-0.005910354	0.676807389	0.888681019
PGBD1	-0.007153935	0.676913272	0.888714626
IER2	0.010098156	0.677016074	0.88873309
NDUFA13	-0.005404414	0.677148359	0.88873309
NCF1B	-0.013107078	0.677443594	0.88873309
DSE	-0.007257006	0.677521192	0.88873309
TADA2A	-0.007713624	0.677526398	0.88873309
CNOT10	-0.004282982	0.677638675	0.88873309
FAM193A	-0.004798608	0.677664391	0.88873309
FBXW4	0.004462748	0.677723103	0.88873309
SCGB3A2	-0.011898083	0.677891085	0.88873309
IMMP2L	-0.010401081	0.677995532	0.88873309
RAVER2	0.022660663	0.678082032	0.88873309
ATOX1	-0.007565273	0.67810156	0.88873309
FAM153A	0.019926014	0.678158198	0.88873309
SMNDC1	-0.005614068	0.678211018	0.88873309
FAM19A4	-0.007400418	0.678288822	0.88873309
IDO1	-0.010183993	0.678319407	0.88873309

RHOQ	0.006492152	0.67835443	0.88873309
ZMIZ1	-0.005519184	0.678372561	0.88873309
EPM2AIP1	0.004407424	0.678481042	0.888770018
MLLT3	-0.008577039	0.678870777	0.888935546
ADRA2A	0.007454856	0.678878865	0.888935546
MS4A4A	-0.015184911	0.678896875	0.888935546
CDON	0.007971047	0.679013208	0.888935546
HDAC3	0.003772455	0.67905817	0.888935546
TOMM6	-0.004618704	0.679123383	0.888935546
PRMT3	-0.004650387	0.679311688	0.888935546
SNORA70C	-0.008231152	0.679346631	0.888935546
RGS12	-0.009769784	0.679406336	0.888935546
SLC6A10P	-0.007199926	0.67941049	0.888935546
ADHFE1	0.008919876	0.679748459	0.889094454
PCDHGC3	0.00943196	0.679816024	0.889094454
RARRES3	-0.006470236	0.679873492	0.889094454
WDR5	0.006388303	0.679973041	0.889094454
FBXO11	0.003809213	0.679978197	0.889094454
RNF125	-0.005793838	0.680137176	0.889094454
EZR	-0.004629457	0.680173279	0.889094454
DHX35	-0.0052264	0.6802952	0.889094454
GMPPB	-0.007878927	0.680311069	0.889094454
PIGQ	-0.006816901	0.680335173	0.889094454
PARP8	-0.0104925	0.68054375	0.889262043
CRY2	-0.00486294	0.681198739	0.889954003
PBOV1	-0.007529163	0.681234101	0.889954003
AIF1	-0.005889361	0.681415628	0.890086097
ZNF143	-0.005909086	0.681700139	0.890240052
TRMT61B	0.005494185	0.681720825	0.890240052
UNCX	-0.007482672	0.68177477	0.890240052
GNB2	0.005870137	0.681998288	0.890275251
ZNF441	-0.008214212	0.682029313	0.890275251
OBFC1	-0.005310169	0.682043015	0.890275251
ZC3H6	-0.008326514	0.682151518	0.890278352
ORMDL1	0.003927928	0.682300321	0.890278352
SPATA20	-0.021595065	0.682401036	0.890278352
B3GALNT1	0.009515978	0.682459024	0.890278352
FAM134A	-0.006086117	0.68247613	0.890278352
HEXDC	0.008934103	0.68252797	0.890278352
SLC37A3	0.006505521	0.682823345	0.89055869
CFHR2	-0.006276155	0.682961022	0.890633312
C2orf74	0.013094685	0.683062112	0.890660209
LMTK3	0.007781926	0.683271865	0.890828772
BDH1	-0.008134795	0.683394874	0.890884215
DPYSL4	-0.034757663	0.683589957	0.890892252
R3HDM1	-0.007196332	0.683691642	0.890892252
C11orf83	0.006259709	0.683704897	0.890892252
ADI1	0.010510164	0.683944429	0.890892252
DEF6	-0.003888908	0.68394877	0.890892252
FAM179A	0.014941944	0.683976393	0.890892252

NCKAP1L	-0.004182043	0.684196201	0.890892252
B3GAT1	-0.019929465	0.684217472	0.890892252
MRE11A	-0.010334974	0.684269751	0.890892252
NARS	-0.003356773	0.684469931	0.890892252
TIGD5	-0.006194751	0.684495636	0.890892252
HGSNAT	0.006429528	0.684550791	0.890892252
LIAS	0.006630203	0.684668485	0.890892252
PCNA	0.005953592	0.684692822	0.890892252
GSTM4	0.016524852	0.684917925	0.890892252
DHFR	-0.002592136	0.685202328	0.890892252
TMEM110	0.008862264	0.685303856	0.890892252
RGL1	0.012290401	0.685320248	0.890892252
PTPRE	-0.006514941	0.685455721	0.890892252
SMARCA5	0.007771596	0.6854968	0.890892252
RPL9	0.003077345	0.685524316	0.890892252
GADD45B	-0.006777216	0.685795616	0.890892252
DCTN4	-0.005262066	0.685953195	0.890892252
TMTC3	0.007876528	0.686026504	0.890892252
PITRM1	0.007795749	0.686197358	0.890892252
PPP4R1	-0.004295461	0.686200311	0.890892252
SOCS7	-0.007102007	0.686276993	0.890892252
SERINC1	0.005622281	0.686287539	0.890892252
NAF1	-0.006488074	0.686316806	0.890892252
CENPF	0.007989819	0.686324572	0.890892252
LST1	-0.013486774	0.686371597	0.890892252
CALCOCO2	-0.008666253	0.686439518	0.890892252
SMARCE1	-0.007161058	0.686460344	0.890892252
KLHDC4	0.009070195	0.686564179	0.890892252
RNF185	-0.007621296	0.68662294	0.890892252
MERTK	0.015830183	0.686737045	0.890892252
JMJD7-PLA2G4B	0.006733981	0.686802946	0.890892252
SPATA24	-0.007309493	0.686916211	0.890892252
NOS3	0.00748921	0.686951397	0.890892252
FAM104A	-0.003458145	0.687041474	0.890892252
ZNF699	0.008202895	0.687058613	0.890892252
SARS	-0.004814777	0.687240455	0.890892252
SLC7A5	-0.009030512	0.687299555	0.890892252
POLR2J4	0.007684824	0.687369394	0.890892252
CLDN20	0.006777159	0.687469654	0.890892252
FIBCD1	0.006114543	0.687487688	0.890892252
WWP2	-0.008272901	0.687553866	0.890892252
TBC1D13	0.004432692	0.68761501	0.890892252
DOT1L	-0.008114431	0.687664409	0.890892252
CALM3	-0.004012055	0.687705445	0.890892252
BBIP1	0.008566967	0.687706363	0.890892252
BAG1	-0.007851723	0.687741029	0.890892252
PANK1	-0.007790326	0.687824298	0.890892252
SASS6	-0.005573229	0.687878004	0.890892252
USP10	0.005614892	0.687903885	0.890892252
SDHAF1	-0.003868508	0.687908219	0.890892252

NOM1	0.011904599	0.68832023	0.891217635
SMYD4	-0.006836681	0.688320495	0.891217635
MS4A7	-0.008294634	0.688559793	0.891400244
ARF6	-0.007629716	0.688696905	0.891400244
SNRNP70	0.005448181	0.688703125	0.891400244
TMEM206	0.005411036	0.688827757	0.891457318
IL6R	-0.008501575	0.689320394	0.891990582
ZBP1	0.008534366	0.689512018	0.892112733
RNF167	-0.006256212	0.689583638	0.892112733
TWF2	0.005167221	0.689850948	0.892112733
PSMD11	-0.005302184	0.689958853	0.892112733
PPDPF	-0.011099165	0.690033682	0.892112733
EAF2	-0.013792562	0.690037833	0.892112733
PPIE	-0.009518048	0.690128434	0.892112733
ZNF706	-0.004343506	0.690155949	0.892112733
ANKS3	-0.008512122	0.690173013	0.892112733
CDC40	-0.003452156	0.690220746	0.892112733
SEC24C	-0.005546538	0.690320698	0.892137747
CEL	0.009020811	0.690437449	0.892184464
SNPH	-0.00879112	0.690681256	0.892298657
ENC1	0.01370478	0.690687045	0.892298657
RNH1	-0.006370685	0.690836282	0.892387304
ANKRD20A5P	-0.006702323	0.691107648	0.892633671
NEURL3	-0.0078185	0.691251834	0.892715733
ACTR8	-0.005128578	0.691538175	0.89280771
DDA1	-0.003919099	0.691562362	0.89280771
CAMK4	-0.009789699	0.691592572	0.89280771
ZDHHC13	0.005381154	0.691645687	0.89280771
COL4A3	0.01306711	0.691980158	0.893135305
TNFSF10	-0.009107789	0.692348695	0.893340238
ACSF2	0.007802468	0.692367395	0.893340238
BRSK1	0.009073495	0.692443803	0.893340238
CHD1L	0.007048753	0.692461762	0.893340238
NUF2	-0.009006971	0.692646161	0.893441748
GATSL3	0.006303952	0.692701878	0.893441748
FAM69A	-0.009492652	0.692905507	0.893600263
BTD	0.008287146	0.693111013	0.89376116
NPAT	0.007427301	0.693218085	0.893779552
KIAA1279	-0.007338306	0.693327061	0.893779552
PYHIN1	-0.010513616	0.693380718	0.893779552
NAA15	-0.006575825	0.69344826	0.893779552
XRCC6	-0.00482571	0.693698313	0.893997744
CYP2R1	0.005651933	0.693802471	0.894027887
SPTAN1	0.008132587	0.694022821	0.894102393
IL1RAP	-0.005580632	0.694039096	0.894102393
ZC3H11A	-0.003721895	0.694102617	0.894102393
FAM118A	0.038950745	0.694192929	0.894114677
UGT1A1	0.007008382	0.694510061	0.894419065
CCDC135	-0.008193849	0.69485278	0.894546665
ARID4A	-0.005643016	0.694947015	0.894546665

DFFA	-0.008806971	0.695062333	0.894546665
PWWP2B	0.00736974	0.695224509	0.894546665
SYNRG	0.005160671	0.695279638	0.894546665
RFNG	-0.005455474	0.69528527	0.894546665
CKS2	-0.00951255	0.6953278	0.894546665
STMN3	0.00761079	0.695340016	0.894546665
RIC8A	0.003650653	0.695550654	0.894546665
NTN4	-0.009805868	0.695560511	0.894546665
CFL2	-0.007217496	0.695573463	0.894546665
SLU7	0.004523892	0.695578927	0.894546665
FAR1	0.006037216	0.695703117	0.894559076
SLX1A	-0.011201478	0.695840744	0.894559076
PASK	-0.013177126	0.69587664	0.894559076
SLC39A7	-0.007728099	0.695911844	0.894559076
GSTA2	0.008223784	0.696273956	0.894857755
AQP7P1	-0.008737507	0.696494171	0.894857755
SBK1	-0.006959966	0.696572476	0.894857755
TRIM17	0.006949015	0.696616112	0.894857755
KIAA0319L	0.005431173	0.696621055	0.894857755
TRAM2	0.006755956	0.696646605	0.894857755
LZTFL1	0.006271023	0.696719629	0.894857755
VAMP1	0.007417074	0.696790947	0.894857755
DAAM1	-0.006539869	0.696881158	0.894869783
SALL2	0.006609233	0.697031302	0.894882549
ROBO3	0.013405181	0.697167461	0.894882549
FANCD2	0.005625313	0.697205284	0.894882549
TRIM43	-0.007710521	0.697214482	0.894882549
MRPL48	0.005967299	0.697614212	0.895243376
HERC4	0.003664077	0.697657364	0.895243376
OTUD1	0.006476906	0.697853666	0.895391471
KCNG1	0.016408508	0.69806467	0.895520525
SPINT2	0.00682817	0.698130657	0.895520525
ARHGAP26	-0.006469953	0.698196958	0.895520525
CDK19	0.004168384	0.698304024	0.895554077
ZNF420	-0.010378553	0.698831975	0.895974425
LDLR	0.009205936	0.698852561	0.895974425
TNFRSF18	0.01038971	0.698874622	0.895974425
GRHL1	0.008134734	0.699110063	0.896048334
ENDOG	0.008331832	0.699170384	0.896048334
CYP4F3	-0.012451977	0.699175125	0.896048334
ACVRL1	-0.01370996	0.699258495	0.896051434
ARAP3	0.007305604	0.699500054	0.896100384
VPS37D	-0.006833834	0.699538399	0.896100384
RHOB	0.01065338	0.699539563	0.896100384
FABP4	0.006446276	0.699858666	0.896405412
ITPR3	0.006494077	0.700071684	0.896574507
BOK	-0.009524227	0.700317495	0.896785557
MAML2	-0.00820908	0.70069851	0.897169672
NKG7	-0.01019517	0.700872043	0.897288069
PQLC1	-0.003032386	0.700971453	0.897311554

DEPDC1B	0.00888486	0.701087135	0.897355862
CCNK	-0.003732231	0.701261462	0.897468685
IL7	0.00918473	0.70137294	0.897468685
BAIAP2	-0.008153782	0.70142001	0.897468685
CD164	-0.007545123	0.701563959	0.897468685
EMG1	0.003731412	0.701580679	0.897468685
BTLA	0.009605404	0.701733665	0.897499211
TERF1	-0.005935214	0.701766706	0.897499211
EDC4	-0.004507503	0.701981281	0.897513233
KIF19	-0.013756506	0.70199115	0.897513233
PRR5	0.008823389	0.702020921	0.897513233
SEPN1	0.006672198	0.702110181	0.897523685
MMP23B	-0.013090447	0.702230117	0.897573344
ATXN1	-0.005655952	0.702543349	0.89787003
HELLS	-0.008249801	0.7029484	0.898283981
IKBKB	0.00431298	0.703112308	0.898369314
ZNF23	-0.006595161	0.703252437	0.898369314
AMICA1	0.005249179	0.703319406	0.898369314
FAM98C	0.00571473	0.703345462	0.898369314
PLEKHA1	0.004615	0.703420981	0.898369314
TAF10	-0.004410495	0.70357408	0.898461178
NDUFV1	-0.004680833	0.703690796	0.898506566
XYLB	-0.007516968	0.704036504	0.898732141
LCMT2	-0.01000148	0.704085588	0.898732141
TCF24	0.007511833	0.704111042	0.898732141
CNFN	0.009278711	0.704197184	0.898738455
DHX32	-0.00645545	0.704363769	0.898743421
RIPK1	-0.006545015	0.704403444	0.898743421
RPL23	-0.003295998	0.704444658	0.898743421
IGLL1	-0.017885672	0.704769822	0.898804987
TNFRSF13C	-0.012237958	0.704987191	0.898804987
MBLAC2	-0.006750034	0.705155418	0.898804987
SLC4A1AP	0.004931034	0.705168606	0.898804987
PMP22	-0.013631273	0.705283702	0.898804987
C12orf29	0.004611035	0.705293258	0.898804987
NEK2	-0.010636078	0.705363695	0.898804987
INO80	0.004800643	0.7055785	0.898804987
JTB	-0.00299745	0.705590297	0.898804987
ZNF189	0.006410226	0.705597135	0.898804987
TAF3	-0.005676088	0.705617138	0.898804987
PLEKHG3	0.009657919	0.70567103	0.898804987
SMAD4	-0.004282118	0.705725054	0.898804987
RHBDL1	0.007346073	0.70602293	0.898804987
MRPS18B	0.004185099	0.706026377	0.898804987
KIAA1191	0.004040011	0.706045891	0.898804987
LSR	0.007371475	0.706117417	0.898804987
FAM57A	0.006607578	0.7062212	0.898804987
RPL37A	-0.002972758	0.706267619	0.898804987
SSFA2	-0.007026054	0.706323387	0.898804987
RWDD2A	-0.004904891	0.706346781	0.898804987

RBMS1	-0.008543425	0.706350621	0.898804987
TXNDC5	-0.013929662	0.706437006	0.898804987
TMEM167B	-0.004233636	0.706591182	0.898804987
RXRA	-0.004015984	0.706600882	0.898804987
MEG8	-0.007584739	0.706606342	0.898804987
ZWILCH	0.004712848	0.70668532	0.898804987
NPDC1	0.009018741	0.706895676	0.898969237
SF3A2	-0.004745858	0.707081512	0.899026353
PTP4A3	0.007922674	0.707123344	0.899026353
YWHAE	-0.00828451	0.707184249	0.899026353
SUSD2	-0.007475312	0.707348578	0.899101962
LRPPRC	-0.00478737	0.707406179	0.899101962
EPOR	-0.006659021	0.707733264	0.899404411
C6orf203	-0.006410137	0.707831137	0.899404411
SPR	0.007087964	0.707887906	0.899404411
C1orf101	0.007168923	0.70802777	0.899478869
BLVRA	-0.006622633	0.708116119	0.899487872
LPPR5	-0.004596528	0.708385367	0.899624746
ITGA5	-0.005070578	0.708386421	0.899624746
PTK7	-0.007765112	0.708928321	0.900209658
PIP4K2A	-0.00429793	0.70906106	0.900274934
UBR5	0.004035207	0.709329502	0.900437846
P2RX5	0.012182726	0.709352066	0.900437846
MB21D1	0.008383174	0.709687824	0.900760753
FEZ1	-0.015693952	0.709982249	0.900947221
OBSCN	0.00846423	0.709997525	0.900947221
SON	-0.002301762	0.710279465	0.90112206
GRWD1	0.006418574	0.710354201	0.90112206
MAPKAP1	0.004815173	0.710379537	0.90112206
FBXO8	-0.006226315	0.710604042	0.901222756
STRADB	0.005903186	0.710621756	0.901222756
CDCA5	-0.012530997	0.710833622	0.90133063
XCR1	0.009468144	0.710869672	0.90133063
HSPA9	0.002968572	0.711004623	0.901398484
DNAJC10	-0.003798346	0.711431886	0.901739584
MED29	0.003562146	0.711436605	0.901739584
RFXAP	-0.004871761	0.711942055	0.902245083
LFNG	-0.006659747	0.712059873	0.902245083
DNTTIP2	-0.008506672	0.712079957	0.902245083
MYD88	-0.005012511	0.712311282	0.902407548
ACSL5	0.004121384	0.712371231	0.902407548
WASH3P	-0.004847502	0.712497744	0.90246453
METTL3	0.003615124	0.712642701	0.902503864
RHOU	-0.009209677	0.712719596	0.902503864
TFAP2A	-0.005394724	0.712773401	0.902503864
TUT1	-0.006840256	0.712978086	0.902659777
HOXB5	0.007107405	0.713543055	0.903271738
CRELD1	-0.005926103	0.713626882	0.903274552
CGRRF1	0.004895213	0.713779665	0.903364637
ARHGEF35	-0.015310348	0.714068742	0.903593334

EMR2	-0.00760625	0.714147457	0.903593334
SNRPD2	-0.003348566	0.714205265	0.903593334
DGKE	-0.007925266	0.714436308	0.903729897
IRX6	-0.009112104	0.714476496	0.903729897
SETD8	0.006375335	0.714643668	0.903785408
C8orf59	-0.004373719	0.714724851	0.903785408
PIF1	-0.007917401	0.714765332	0.903785408
CUX1	-0.005606811	0.715018684	0.904002491
PC	0.006741039	0.715186658	0.904098099
FBXO18	-0.003621854	0.715257661	0.904098099
TMEM161B	0.005934739	0.715434015	0.90415793
PKD2	0.007260668	0.715514768	0.90415793
HOXB4	-0.008156737	0.715626092	0.90415793
SNORA68	0.008204945	0.715631731	0.90415793
SNRPD3	-0.007741103	0.715832701	0.904308624
DDX6	0.015115099	0.715997242	0.904413269
FBXO45	0.009350114	0.716220741	0.904592353
SLC35D2	-0.005190975	0.716346608	0.9046481
AMMECR1L	-0.005340018	0.716492269	0.904656547
APTX	-0.003772147	0.716516754	0.904656547
HNRNPA2B1	0.002832099	0.71670806	0.904697037
L1TD1	-0.013923364	0.716732003	0.904697037
GIMAP8	-0.00700718	0.716794021	0.904697037
NIF3L1	-0.00328846	0.716972861	0.904777257
ITPRIPL2	-0.009424521	0.717021059	0.904777257
MMAA	-0.005432396	0.717323788	0.904873065
WNT10B	-0.010161302	0.717403974	0.904873065
ENO3	0.006489047	0.717413779	0.904873065
SLC16A10	-0.011512112	0.717487876	0.904873065
PMEPA1	-0.009948691	0.717547488	0.904873065
CLK3	-0.004893781	0.717594269	0.904873065
FOSB	0.011272723	0.717732853	0.904873065
CCT6A	-0.003988433	0.717805234	0.904873065
PLAC8	-0.006477356	0.717881527	0.904873065
ZNF644	-0.006774683	0.717914469	0.904873065
HERPUD2	0.006055133	0.718071665	0.904873632
SIRPG	-0.009624824	0.718231353	0.904873632
FOXN2	-0.00427037	0.718246406	0.904873632
IL18RAP	0.013810953	0.718326348	0.904873632
SUFU	0.009592342	0.718451557	0.904873632
TRIM21	0.005719382	0.718532839	0.904873632
C18orf54	0.006567593	0.718613245	0.904873632
PLCB2	0.007298977	0.718824438	0.904873632
CRIPAK	0.01087916	0.718857461	0.904873632
DMXL1	-0.006190087	0.71901882	0.904873632
XPO4	0.003394929	0.719129657	0.904873632
ZFP62	-0.00665152	0.719214751	0.904873632
KIAA1161	-0.006723041	0.719220501	0.904873632
FAM101B	0.010320677	0.719298246	0.904873632
PACSIN1	-0.011096396	0.719357381	0.904873632

GM2A	-0.011821993	0.719406801	0.904873632
PACSIN2	0.003933906	0.719467814	0.904873632
SNORA57	0.008711126	0.719481558	0.904873632
PHF19	-0.004901025	0.719568419	0.904873632
MTHFD2L	0.008173479	0.71959541	0.904873632
CSK	0.00559704	0.719631636	0.904873632
EPAS1	-0.005221308	0.71987379	0.904993976
TRPM7	-0.006728929	0.719890862	0.904993976
RER1	-0.009114332	0.72010118	0.905102865
TLE1	0.011795791	0.720283484	0.905102865
TAF8	0.0054638	0.720354358	0.905102865
MAP3K11	-0.007437021	0.720366818	0.905102865
BANP	-0.004010178	0.720433924	0.905102865
LYST	0.006401416	0.720545562	0.905102865
NCAPG2	-0.01820276	0.720549864	0.905102865
LSM4	0.005258464	0.720677249	0.905160157
PDGFRB	-0.018299357	0.721029353	0.905499649
INPPL1	0.004717146	0.721236053	0.905538231
MYO1B	-0.007607202	0.721369231	0.905538231
HES6	-0.012394082	0.721446808	0.905538231
BCL7C	0.006607141	0.721513346	0.905538231
HEATR3	0.007727909	0.721547937	0.905538231
VPS4A	0.004347076	0.721579375	0.905538231
SLC25A19	0.00509442	0.721632734	0.905538231
EARS2	0.005921005	0.721730057	0.905557697
RUSC1	-0.004640321	0.721965759	0.905649084
WDR91	0.004594734	0.722051903	0.905649084
KCNA3	0.00858605	0.722218424	0.905649084
EIF1	-0.004572843	0.722379599	0.905649084
CYHR1	0.006857996	0.72240005	0.905649084
SRSF6	0.003115241	0.722440215	0.905649084
MEX3C	-0.006567737	0.72267203	0.905649084
SPTBN1	0.008412854	0.722760047	0.905649084
NBEA	-0.008210955	0.722771148	0.905649084
TRIM10	0.015294304	0.722836703	0.905649084
SLC2A1	-0.005750577	0.722866005	0.905649084
RCAN2	-0.020533539	0.72299197	0.905649084
ITGB3BP	-0.011671159	0.723042267	0.905649084
USP1	0.003899853	0.723061407	0.905649084
TMEM101	-0.004210167	0.723182664	0.905649084
LZTS2	0.005750788	0.7232987	0.905649084
TMEM143	-0.006373834	0.723380014	0.905649084
AAAS	-0.004949183	0.723424903	0.905649084
HNRNPF	0.006765994	0.723436283	0.905649084
SGTA	0.008124537	0.723439263	0.905649084
PEX6	0.014518729	0.723937849	0.906164014
COQ5	-0.004507581	0.724122724	0.906164014
SLC44A1	-0.005571636	0.724128546	0.906164014
PTEN	0.003318148	0.724178053	0.906164014
SIN3A	-0.004232205	0.724372778	0.906305219

DUS1L	0.004665485	0.724532076	0.906314807
CD200R1	-0.008782976	0.724544198	0.906314807
CBLB	-0.007037175	0.724641757	0.906334419
LCN1	-0.005791323	0.724824991	0.906394262
UNC119	-0.005143118	0.724853375	0.906394262
BCR	-0.006725121	0.724947766	0.906409897
CXXC5	0.00837002	0.725390745	0.906861324
C21orf33	0.003365836	0.725510228	0.906908268
PSMA7	-0.007167399	0.725842892	0.906937006
SOCS1	-0.010691116	0.725890703	0.906937006
SYNC	-0.008108295	0.725944491	0.906937006
ATAD2	-0.005704508	0.725977176	0.906937006
SCAMP2	0.005833948	0.726027772	0.906937006
STARD5	0.006588396	0.726043467	0.906937006
ARRDC4	-0.031434167	0.726122442	0.906937006
MAP4	-0.007252181	0.726188697	0.906937006
CRHR1	0.007021263	0.726407362	0.907074194
RINL	0.004989403	0.726576654	0.907074194
ATMIN	-0.007143576	0.72667149	0.907074194
POLR1D	-0.004652991	0.726671869	0.907074194
CREB5	0.008405778	0.726819001	0.907074194
MS4A3	0.013851127	0.726875255	0.907074194
PARP16	0.005636091	0.726961875	0.907074194
S100A1	0.00772784	0.726978883	0.907074194
TRIM23	-0.007311794	0.727071399	0.907074194
PTGDS	0.017739861	0.727118016	0.907074194
MFSD6	0.00621713	0.727326996	0.907135181
ELL	-0.004648981	0.727330809	0.907135181
PLEKHJ1	0.006541249	0.727459641	0.907193642
DCPS	-0.006324381	0.727695242	0.907250093
ZNF175	-0.007454889	0.72772046	0.907250093
MIR557	-0.00711676	0.727853456	0.907250093
MTPN	-0.003793857	0.727855295	0.907250093
C1orf159	0.006436174	0.727981468	0.907250093
MGAM	0.011521417	0.727996687	0.907250093
ECHDC3	-0.011702745	0.728262595	0.907479304
SLC19A1	0.005904689	0.72837412	0.907516111
PRSS42	-0.005728709	0.728529184	0.907607151
RNF19A	0.006057127	0.729392871	0.908479826
SMAD5	0.006750849	0.729393822	0.908479826
CCDC61	0.006299383	0.729700746	0.908551835
CWC22	0.007723065	0.729705669	0.908551835
ARPC5L	0.004617601	0.729786572	0.908551835
BTN3A1	0.006797431	0.729798058	0.908551835
PHF2	-0.003711624	0.72986204	0.908551835
PEX11B	0.003715031	0.730187567	0.908784262
POLH	-0.006075128	0.730292089	0.908784262
C11orf49	-0.006937873	0.730295059	0.908784262
RBM4	-0.005223113	0.730463476	0.908891661
WDR20	0.005050198	0.73067262	0.909040356

BHLHE40	0.006363634	0.730999107	0.909040356
LAMTOR1	-0.007344989	0.731066374	0.909040356
TMEM151B	-0.006189222	0.731113103	0.909040356
DPP3	-0.004627043	0.731163113	0.909040356
DIEXF	-0.005030698	0.731248524	0.909040356
USE1	0.005876339	0.731282486	0.909040356
WDR1	-0.003200439	0.731331053	0.909040356
GOT1	-0.003278271	0.73135278	0.909040356
AP2A1	-0.00603629	0.731404229	0.909040356
DHTKD1	0.004287637	0.731866437	0.909512696
POP1	-0.005591319	0.732031842	0.909586111
FGD4	-0.006153522	0.73208986	0.909586111
UBR3	-0.004241899	0.732346326	0.909802636
FGF18	-0.007128638	0.732556974	0.909962198
ANO8	-0.00603786	0.732796612	0.910157731
MSI2	-0.005340472	0.732897957	0.910181475
CHRDL2	-0.005594089	0.733108049	0.910276684
PDIA4	-0.007039595	0.733139094	0.910276684
LCP2	-0.004892984	0.734009592	0.91081159
MCCC1	0.003346426	0.734088912	0.91081159
KLF7	0.006482498	0.734095853	0.91081159
KIAA1107	0.005619796	0.734114121	0.91081159
C2orf42	-0.004298318	0.734136387	0.91081159
SMARCAD1	-0.004001081	0.734173175	0.91081159
USP39	-0.003828862	0.734224787	0.91081159
DHRS11	0.005156872	0.734228189	0.91081159
SIK2	-0.005772884	0.734509291	0.91105556
IL23A	-0.005886916	0.734589473	0.91105556
MGAT3	0.009874198	0.734791588	0.911160571
KRT86	-0.010909499	0.734838777	0.911160571
PCNP	-0.003110748	0.735134622	0.911425305
EDF1	-0.003655754	0.735273841	0.911495816
AOAH	0.008028902	0.735845799	0.912102704
GGCT	-0.004414511	0.736116471	0.912242328
DNAJC9	0.003845944	0.73612327	0.912242328
ZNF197	-0.004489796	0.736260036	0.912260359
TNFRSF1A	0.00441746	0.736403557	0.912260359
MXD3	0.006934251	0.736439318	0.912260359
ZNF831	0.007542246	0.736467483	0.912260359
AURKB	-0.008714763	0.736676043	0.912416596
HRK	0.011438198	0.736837951	0.91246353
SPON2	-0.016793141	0.736878805	0.91246353
GPC2	0.008547575	0.737113157	0.912651626
CCL28	-0.0070909	0.737376584	0.912875674
CD27	-0.007537611	0.737943897	0.913451647
CTC1	-0.004855897	0.738006874	0.913451647
LIMK2	0.007631164	0.738156038	0.913494346
SNRNP25	0.006007046	0.738266174	0.913494346
FASTKD1	0.005725601	0.738288953	0.913494346
ACPL2	-0.006361448	0.738977575	0.914142161

MT2A	0.011081086	0.73902969	0.914142161
SCN9A	-0.007301909	0.739066276	0.914142161
PTPRO	0.010153978	0.739142862	0.914142161
ATAD3B	-0.005194225	0.739517141	0.914427146
ITGB7	-0.006357316	0.739538514	0.914427146
RDH5	-0.007205134	0.739716393	0.914510802
LILRA6	0.010795394	0.739816685	0.914510802
USP34	0.003861639	0.739941569	0.914510802
HMMR	-0.010188953	0.740123177	0.914510802
RASD1	-0.011609887	0.740130061	0.914510802
EPSTI1	-0.013474795	0.740170874	0.914510802
CCNG1	-0.003616841	0.740289543	0.914510802
NOL11	-0.003417501	0.740390415	0.914510802
PRF1	0.011328634	0.740403548	0.914510802
FZD2	0.006720398	0.740440207	0.914510802
CCL3L3	-0.019794855	0.740514981	0.914510802
MTOR	-0.004011311	0.740922659	0.914912195
GYPB	-0.010994199	0.741455003	0.915332613
GABPB2	0.001979493	0.741463011	0.915332613
MAK16	-0.00462946	0.741511207	0.915332613
GGNBP2	0.003707018	0.741813119	0.915603191
LPAR2	0.005573954	0.74202615	0.915643187
ACBD3	-0.004010197	0.742069836	0.915643187
FGF2	0.005702878	0.742275846	0.915643187
ASGR2	-0.011055021	0.742360293	0.915643187
SLX4	0.004840743	0.742368253	0.915643187
C11orf35	0.007167055	0.742379338	0.915643187
WDR76	0.005550601	0.742471607	0.915643187
XRCC3	-0.00552544	0.742507295	0.915643187
WDR90	-0.005599274	0.742679407	0.915695656
TRPV1	-0.005649351	0.742715295	0.915695656
SMC6	-0.013664544	0.742904063	0.915701275
CLUL1	-0.005552479	0.74296721	0.915701275
PHACTR1	0.007447384	0.742968032	0.915701275
CDK6	0.005721742	0.743052939	0.915703962
TIAF1	-0.004055212	0.743276365	0.915827187
ZCCHC24	-0.006359833	0.74333556	0.915827187
LTF	-0.006651995	0.743410751	0.915827187
GPX7	0.005504206	0.743483883	0.915827187
TMEM18	-0.005032514	0.743949181	0.916298374
ZNF418	0.009654616	0.744347979	0.916687558
TNFSF9	0.006646307	0.744469474	0.916735188
COBLL1	0.010425544	0.744611719	0.91678953
SPPL2B	-0.006182524	0.744728248	0.91678953
MLC1	-0.00676093	0.744762079	0.91678953
SPAG16	0.006720553	0.744885981	0.91684009
SLC38A2	0.00437562	0.745289526	0.916892472
TPK1	-0.004418668	0.745361868	0.916892472
FCGR2B	-0.010305822	0.745375693	0.916892472
PXMP4	0.007556788	0.745456313	0.916892472

FIS1	0.005095438	0.745634508	0.916892472
RPRM	-0.01708671	0.745658875	0.916892472
PRMT2	-0.006584618	0.745691992	0.916892472
ODF2L	0.022328568	0.745700265	0.916892472
ACSS1	0.006465606	0.745715996	0.916892472
BACE2	-0.011692083	0.745756882	0.916892472
ACP6	-0.006241495	0.745864883	0.916923411
NRIP3	0.005641805	0.746047729	0.916982506
SUDS3	0.008031434	0.746078638	0.916982506
SPATA5L1	-0.005293577	0.746562539	0.917014962
C2orf43	-0.005814622	0.746600535	0.917014962
LMO4	-0.005754275	0.746631129	0.917014962
CLEC16A	0.003358543	0.746669751	0.917014962
PYROXD1	-0.010738859	0.746719347	0.917014962
XPO1	0.003049201	0.74674152	0.917014962
ATP9A	0.012299059	0.746816199	0.917014962
C21orf128	-0.009235324	0.746836239	0.917014962
FYB	-0.003299853	0.746850653	0.917014962
TGFBR3	-0.009627349	0.746951785	0.917037413
TCFL5	0.004826057	0.747485188	0.917519799
BTG1	-0.003186337	0.747510484	0.917519799
TMEM57	-0.004147341	0.747765344	0.917730857
LTN1	0.003263107	0.748061736	0.917859698
SDC2	0.006502835	0.748118616	0.917859698
SNRPN	-0.004704901	0.748224615	0.917859698
ZFP90	-0.00528632	0.748265225	0.917859698
USP7	0.005033012	0.748284932	0.917859698
PIGC	0.003637801	0.748711194	0.918280799
B9D1	-0.006052936	0.748821168	0.918313928
RNGTT	0.004361776	0.749052977	0.918326281
SLC25A34	-0.008253909	0.749068979	0.918326281
SRPK1	0.003869145	0.749178555	0.918326281
VCPIP1	0.004419145	0.749196818	0.918326281
PER2	-0.004040346	0.749250078	0.918326281
ZNF713	0.00596577	0.749453253	0.918326281
FZR1	-0.005886607	0.749474997	0.918326281
LARP4B	0.002822715	0.749546353	0.918326281
AKAP1	-0.005006353	0.749625983	0.918326281
DNAJC18	-0.004913162	0.749721437	0.918326281
RPL13AP3	-0.002389865	0.749743844	0.918326281
BLK	0.013558278	0.749976449	0.918509551
HLA-DRA	-0.004310682	0.750163336	0.918564844
CSTF2T	-0.005817365	0.750198314	0.918564844
HK2	-0.006944339	0.750270553	0.918564844
POMZP3	0.013576158	0.750547313	0.9187796
NDUFC1	-0.00437977	0.750611972	0.9187796
GPS2	0.003314892	0.750735525	0.918803073
FYTTD1	-0.003967717	0.750797162	0.918803073
TMUB2	0.004305908	0.751032229	0.91888531
EXOSC6	-0.005252012	0.751070287	0.91888531

RAB30	0.007588494	0.751191752	0.91888531
ATIC	-0.005508394	0.75119642	0.91888531
SH2D3C	-0.005685976	0.751402109	0.918963983
HIST3H2A	0.00794531	0.751454606	0.918963983
BTN3A2	-0.013076683	0.7515098	0.918963983
C18orf56	0.007752114	0.751888266	0.919325221
RNF141	-0.006161125	0.752246959	0.919662203
TIAM2	-0.008855429	0.752427077	0.919780818
CEBPD	0.005401599	0.752700413	0.920013347
DHCR7	0.005401837	0.752900338	0.920076242
TRIM36	-0.00672933	0.753049383	0.920076242
CD160	-0.014823472	0.753078269	0.920076242
BBS7	-0.004291714	0.753084357	0.920076242
PTPN11	-0.002578718	0.753264675	0.920171963
CES4A	-0.006008253	0.753328966	0.920171963
GTPBP8	0.005116956	0.753597879	0.920392422
LAT	0.004174093	0.753677412	0.920392422
CASP3	0.004443682	0.753897242	0.920392422
ANLN	-0.005772794	0.753960548	0.920392422
ERLIN1	-0.005448756	0.753968136	0.920392422
CFDP1	-0.004875223	0.754008356	0.920392422
TCERG1	0.004252652	0.754202296	0.920443448
PAPD4	-0.003221847	0.754400281	0.920443448
RARA	0.004588361	0.75441383	0.920443448
TRIP11	0.004195666	0.754500747	0.920443448
CBFA2T2	-0.004955683	0.754532555	0.920443448
GTPBP1	-0.006004286	0.754573487	0.920443448
CCDC92	0.005439753	0.754632242	0.920443448
MRPL15	-0.005330553	0.754835393	0.920589794
TCF7	-0.007815604	0.755254344	0.92096428
EID1	0.006713461	0.755308856	0.92096428
GLB1L	0.010192093	0.755705303	0.92127307
NUFIP2	-0.005620264	0.755728564	0.92127307
KRT17	0.007387124	0.755877818	0.921290593
AGMAT	-0.008734843	0.755909401	0.921290593
EMR1	-0.011052264	0.756386023	0.921659416
WDR63	-0.006644744	0.756566791	0.921659416
ECSIT	0.005322852	0.756571612	0.921659416
ESCO1	-0.005449716	0.756638729	0.921659416
SLC7A6OS	-0.005037263	0.756639451	0.921659416
FBXW9	-0.004158072	0.756827128	0.921659416
CMKLR1	0.010594263	0.756838905	0.921659416
PPP3CB	-0.002221815	0.756878136	0.921659416
IGSF11	0.005343768	0.757160509	0.921821748
SIGLEC7	-0.010432872	0.75718725	0.921821748
LYPD2	-0.012731638	0.757336364	0.921821748
TM4SF19	-0.005815412	0.757344564	0.921821748
RNF20	0.003761104	0.757780199	0.922080287
SURF2	-0.005075167	0.757790316	0.922080287
NPR2	0.005247859	0.757806881	0.922080287

LSM11	0.005189296	0.757913101	0.922105039
CYP2E1	0.007935334	0.757993834	0.922105039
ZNF606	-0.006319769	0.758144881	0.922116672
VPS39	-0.004903965	0.758190123	0.922116672
PHLDB3	0.010943719	0.758253316	0.922116672
ATP13A1	0.006157661	0.758371733	0.922159367
HSPA1L	-0.004581884	0.758584952	0.922317314
SGMS1	-0.005891712	0.758737667	0.922358941
STIM1	-0.004083275	0.758785845	0.922358941
MYADML2	-0.007215348	0.759034438	0.92255981
GPR153	-0.004883515	0.759208892	0.922593339
LARP1	0.002939365	0.759284939	0.922593339
SERPINI1	0.006063812	0.759389281	0.922593339
CDK2AP1	-0.006254188	0.759406404	0.922593339
C19orf66	-0.004364473	0.75947877	0.922593339
C2	0.008542995	0.759766183	0.922841203
CCDC104	-0.004539259	0.759925577	0.922933532
WSB2	-0.004842483	0.760251788	0.92322842
GALT	0.004681618	0.760454605	0.923373411
SOS1	0.006340315	0.760572316	0.9233746
CYP11A1	0.006156864	0.760622423	0.9233746
RPS6KA2	-0.01681951	0.760726602	0.923399798
DDX58	0.006475332	0.760812529	0.923402839
CAB39	-0.002514827	0.761048046	0.923587416
ALDH18A1	0.004977898	0.761196653	0.923666494
NOXO1	-0.005793579	0.761336303	0.923734686
TPTE	0.009345651	0.761628432	0.923836674
ZNF681	-0.010185274	0.761680644	0.923836674
PHLDB2	0.009286851	0.761711809	0.923836674
GTF3C3	0.00320052	0.761754207	0.923836674
EXT1	0.004878727	0.761857347	0.923860536
JOSD1	0.004388949	0.762175719	0.924071604
SFPQ	-0.006785567	0.762198369	0.924071604
TC2N	0.006156206	0.762398403	0.924133084
ARL5A	-0.002447408	0.762416056	0.924133084
OXNAD1	-0.00690231	0.762526564	0.924165832
MRPL46	0.0030623	0.762666658	0.924234425
CEBPG	0.003368849	0.762769053	0.924257323
NAPB	-0.006014149	0.763088439	0.924370919
CARM1	-0.003635832	0.763149682	0.924370919
VPS52	0.003873972	0.763167703	0.924370919
TBC1D8	-0.006600233	0.763196841	0.924370919
CSH2	-0.005929452	0.763447687	0.924573572
KIF21A	0.00776486	0.763738081	0.924754091
TMEM66	-0.002557638	0.763763836	0.924754091
PTGIR	0.00652468	0.763848915	0.924755949
CHCHD6	-0.005035436	0.764294709	0.92494669
FXYD5	0.004700293	0.764324454	0.92494669
C12orf73	-0.006543888	0.764500481	0.92494669
SHISA5	0.004085276	0.764512197	0.92494669

AKR1D1	0.002174187	0.764538324	0.92494669
SKA1	-0.005284732	0.764563374	0.92494669
NUDCD3	-0.005332906	0.764593187	0.92494669
PAIP1	-0.005374699	0.764674963	0.92494669
ZBTB9	-0.0068379	0.764940608	0.92506167
ATP6V1D	-0.003096463	0.764953606	0.92506167
COL13A1	-0.008285559	0.765020736	0.92506167
HIST2H2AA3	-0.008605688	0.765334696	0.925340224
RNF149	0.004333228	0.766069343	0.925933785
FNIP1	-0.003883532	0.766177456	0.925933785
NUP153	-0.003784092	0.766187869	0.925933785
CCDC138	-0.004614536	0.766238437	0.925933785
NDUFA2	-0.002523835	0.766243877	0.925933785
GANAB	-0.003710982	0.766389958	0.925965589
RASIP1	0.007046458	0.76648974	0.925965589
GPR19	0.009294799	0.766521157	0.925965589
SLC27A6	-0.006207984	0.766755052	0.926147061
SFTPD	-0.01154014	0.767084568	0.926443981
SRRT	-0.002772778	0.76743844	0.926754646
GBP5	0.007753481	0.767509245	0.926754646
GTF2H2B	-0.00971342	0.76760016	0.926763326
SARS2	0.005347599	0.767930794	0.927061398
CHCHD10	-0.004639369	0.76832901	0.927279533
ZNF302	0.004287923	0.768378662	0.927279533
CPT1B	0.009884449	0.768424693	0.927279533
PLEKHB1	-0.007092379	0.768446576	0.927279533
PNPLA6	-0.005025101	0.768626783	0.927319094
ACTR1B	-0.003044415	0.768771618	0.927319094
SLC4A1	0.01021703	0.768773187	0.927319094
SKIV2L	0.00385901	0.768814466	0.927319094
PRR5L	-0.010174021	0.768978987	0.927416475
MIR1185-1	0.004412266	0.769274633	0.927671959
DUSP8	0.009083247	0.769502579	0.927845757
GTF2H4	-0.005486684	0.769692744	0.927973966
SLC29A2	0.005087102	0.769974924	0.928213073
SWT1	0.003773844	0.770136574	0.928306843
PABPC1	0.00738059	0.770410702	0.928426003
TRPC4AP	-0.006589036	0.770415002	0.928426003
FAM160B2	0.005127617	0.770487059	0.928426003
U2SURP	0.003963729	0.770588497	0.928447162
HIST2H2AB	-0.00760404	0.770912268	0.928478085
UBE2E3	-0.004896358	0.770969267	0.928478085
KCNMB3	0.006013141	0.771023607	0.928478085
TAPBP	0.004969645	0.771103397	0.928478085
TBL1XR1	0.004282154	0.771156312	0.928478085
ATP1B3	-0.004591257	0.771192265	0.928478085
GNA13	0.003066551	0.77120133	0.928478085
PJA2	0.004557068	0.771508176	0.928746493
GPR89B	0.005630585	0.771764653	0.928954214
RUFY3	-0.00473032	0.772030718	0.929164908

KLC4	-0.00548891	0.772155374	0.929164908
CORT	-0.005516596	0.772191525	0.929164908
SND1	-0.004386525	0.772431605	0.929277518
TBC1D2B	-0.004458786	0.772453017	0.929277518
UBASH3B	0.004433179	0.772694331	0.929466806
DSTNP2	-0.003996389	0.773013565	0.929527368
ZNF273	0.005851292	0.773060325	0.929527368
ZSCAN18	-0.006001708	0.773198052	0.929527368
RHBDF2	-0.00374227	0.773270555	0.929527368
CTDP1	0.004465546	0.773288888	0.929527368
KCNK3	-0.004932263	0.77334123	0.929527368
CCDC7	0.008739546	0.773380169	0.929527368
MND1	-0.006445	0.773505962	0.929527368
TMEM202	-0.005032352	0.773560135	0.929527368
SDAD1	-0.002802527	0.773584435	0.929527368
KCNQ1OT1	0.005897577	0.773940901	0.929774303
NRBP2	0.004710142	0.774016524	0.929774303
RPS18	-0.004169406	0.774041937	0.929774303
NDUFAB1	-0.004206117	0.774477146	0.930107506
EPS15L1	-0.004706213	0.774487387	0.930107506
B4GALT6	0.007065358	0.774579018	0.930116636
MYO9B	0.004313585	0.774687374	0.930145845
PCNXL2	0.006465155	0.774873237	0.930268098
PTGES	0.004781749	0.77505492	0.930375172
SPG11	-0.003423244	0.77513053	0.930375172
DOCK3	0.005170586	0.775330454	0.930441074
PHF14	-0.003675159	0.775364875	0.930441074
RFC4	-0.004480301	0.77543761	0.930441074
PFKFB4	0.004055051	0.775614771	0.930552775
GSPT1	-0.002285517	0.776073151	0.931001811
LMF1	-0.005368799	0.776195184	0.931047301
ATP8B4	-0.006291365	0.776727911	0.931553745
FGD6	0.005507072	0.776848413	0.931553745
TMEM43	0.002919515	0.776869872	0.931553745
MMP25	0.010779967	0.776984379	0.931590131
STOML2	0.00223321	0.777206526	0.931755555
MMS19	0.002867104	0.777437908	0.931932012
ERAP2	0.025068269	0.77754983	0.931965248
CNR1	-0.005252074	0.777795529	0.932152637
HIST1H2BO	0.007829032	0.777945405	0.932152637
MED16	-0.005651391	0.777974333	0.932152637
KIAA0232	0.004680454	0.778110082	0.932152637
NMD3	0.002027466	0.778246238	0.932152637
RBM45	-0.003602747	0.77825536	0.932152637
APOL2	0.006582447	0.778295661	0.932152637
SRGAP3	0.005550815	0.778511859	0.932310697
PFKP	0.006422506	0.778897914	0.932502724
RLN1	0.007826265	0.778971821	0.932502724
PAPSS2	0.008543634	0.779111938	0.932502724
ELF2	0.003263465	0.77912127	0.932502724

RELA	-0.005262107	0.779139547	0.932502724
SNORD12	0.005543896	0.779177676	0.932502724
MED31	-0.004260559	0.77951935	0.932802254
NAPG	-0.005065116	0.7795965	0.932802254
HIST2H2AC	0.007690905	0.780166951	0.933383915
IL12A	0.006862666	0.780378527	0.933536141
CCDC90B	-0.002547133	0.780749272	0.933878722
CD180	0.00541782	0.780950041	0.934017936
ACSM5	-0.004467599	0.78107446	0.934065817
MYOF	-0.00815469	0.781227535	0.934147951
DNMT3B	0.004189387	0.781339266	0.934180637
LILRA5	0.007535734	0.781537545	0.934311672
PLK4	-0.005487166	0.781617679	0.934311672
HSPD1	0.002889954	0.781797316	0.934340645
MITF	-0.006400071	0.782072381	0.934340645
WBP11	0.002044691	0.782131346	0.934340645
MYLK4	0.007532663	0.78220376	0.934340645
NOL6	-0.003539089	0.782302165	0.934340645
HSPA14	-0.003236498	0.782322303	0.934340645
MRPL17	-0.004048681	0.782390495	0.934340645
CPSF4	0.002805395	0.782398017	0.934340645
LRFN1	-0.005797638	0.782407594	0.934340645
CDC42BPB	-0.005921431	0.782567947	0.934340645
VWA3A	0.004871809	0.782613548	0.934340645
TMOD1	0.010491415	0.782654843	0.934340645
HIST1H2BD	0.016620896	0.782796727	0.934377315
UQCRC1	0.003145888	0.782854388	0.934377315
NR3C2	0.004380953	0.782963099	0.934406019
OLFML2B	-0.004675328	0.783254074	0.934406019
PSD3	0.004449349	0.783257459	0.934406019
IFIT2	0.010167152	0.78350564	0.934406019
ESYT2	0.002982713	0.783668123	0.934406019
VMP1	-0.003726599	0.783699182	0.934406019
BCAS4	-0.00679892	0.783700968	0.934406019
IGFBPL1	0.005408464	0.783707215	0.934406019
C21orf37	0.004438525	0.783784646	0.934406019
ARHGAP11B	0.004672573	0.783794513	0.934406019
ACLY	0.002740986	0.783877302	0.934406019
TMEM203	-0.002650169	0.783925537	0.934406019
ZNF776	-0.005106072	0.784123927	0.934406019
PKDCC	0.00444067	0.784271532	0.934406019
MCF2L	0.005468471	0.784284741	0.934406019
PIK3R1	0.003239769	0.784299272	0.934406019
SRP68	-0.002933945	0.784377958	0.934406019
CIDEA	-0.006162441	0.784443016	0.934406019
METTL15	0.003769729	0.78448235	0.934406019
TMEM25	0.00888942	0.784621934	0.934471723
UBE4B	-0.002668515	0.784933041	0.934741671
EPS8	0.007906468	0.785196844	0.934948853
SKA2	0.003430452	0.785316722	0.934948853

ACAD10	0.003940958	0.785360415	0.934948853
GPATCH1	-0.004652351	0.785604058	0.935138328
PPP1R13B	-0.003895098	0.785706023	0.935159136
ADAMTSL5	0.005772228	0.78601489	0.935333656
CNOT1	-0.002569163	0.786021653	0.935333656
QKI	-0.007772501	0.78617174	0.935411694
FOXN3	0.002602981	0.78667563	0.935862829
ZFR2	-0.008024863	0.786719995	0.935862829
PPP2R4	-0.003051261	0.787013682	0.936059225
BCL2	0.004996223	0.787054224	0.936059225
MLLT10	0.002791821	0.787150208	0.936072804
MYOM2	-0.037099297	0.787250617	0.936091641
TEAD2	-0.009035433	0.787364998	0.936127085
AKAP5	-0.002115075	0.787530279	0.936175428
ZNF609	-0.004636426	0.787574811	0.936175428
HECTD1	-0.002607918	0.787845458	0.936303454
SASH1	0.009234641	0.787933984	0.936303454
NUDT8	-0.006372186	0.787980445	0.936303454
ABCB6	0.004885675	0.788057494	0.936303454
TMEM117	-0.005403446	0.788105455	0.936303454
ATP9B	-0.004446747	0.788215172	0.936317814
NMUR1	-0.010520082	0.788347263	0.936317814
S1PR5	-0.009576598	0.788452985	0.936317814
S100P	-0.017597354	0.788455899	0.936317814
ABHD2	-0.004793856	0.789161502	0.936971885
POC5	0.002771511	0.789213543	0.936971885
SUZ12	-0.00180177	0.789260625	0.936971885
TMEM19	-0.004183137	0.789690782	0.937256037
SF3A1	-0.005396684	0.789767304	0.937256037
FAM107B	-0.004002449	0.789780336	0.937256037
MIR1253	-0.004916897	0.789838677	0.937256037
LARP4	0.004258962	0.790089923	0.937453677
RIMS3	-0.005480115	0.790314848	0.937525989
ASPHD1	0.004592325	0.790402163	0.937525989
RPS26	-0.01455745	0.790459001	0.937525989
ZNF234	0.006614994	0.790492662	0.937525989
LGALS3BP	0.00870849	0.790574359	0.937525989
MUT	0.003710322	0.790858871	0.937637975
SPEG	-0.00546538	0.791021161	0.937637975
SRP19	-0.003583294	0.791112801	0.937637975
BRD2	-0.002677551	0.791152865	0.937637975
POLE	-0.004563406	0.79116958	0.937637975
FGFRL1	0.005208706	0.791192421	0.937637975
TDRD9	0.010078313	0.791261751	0.937637975
KIAA1429	-0.003070459	0.791417803	0.937701037
EHD3	0.008325104	0.791541104	0.937701037
SAT2	0.003120546	0.791569111	0.937701037
HIST1H2AE	0.014552558	0.791690519	0.937705143
ZNF566	-0.006237958	0.791752004	0.937705143
LAPTM4A	-0.004178563	0.791826721	0.937705143

PPARGC1A	-0.011720307	0.792192561	0.93783591
PARL	0.002837537	0.792307658	0.93783591
RETN	0.011147877	0.792311206	0.93783591
CDH1	-0.006307947	0.792462518	0.93783591
MIR125B2	-0.003625911	0.792481281	0.93783591
ZNF225	-0.00398517	0.79260997	0.93783591
LIMD2	0.004604353	0.792619586	0.93783591
VKORC1L1	-0.005325072	0.792697773	0.93783591
PCGF6	-0.003364818	0.792699681	0.93783591
IPO11	-0.00431881	0.792828544	0.937888122
ZNF154	0.013248843	0.792987522	0.937975944
ZGPAT	-0.002380655	0.793432254	0.938288891
IPO13	0.003959539	0.79348891	0.938288891
RTN3	-0.003493259	0.793584283	0.938288891
SLC22A4	0.00638154	0.793591165	0.938288891
CANT1	0.003286244	0.793745004	0.938354693
TOP2A	-0.010917125	0.793816365	0.938354693
RBP7	-0.006382984	0.793908583	0.938363493
CELF6	-0.005013338	0.794179084	0.938428238
DHRS12	0.004876138	0.794210416	0.938428238
LMAN1	0.004159785	0.794217701	0.938428238
LPCAT1	-0.00395294	0.794473927	0.9385317
MYL12A	0.005107659	0.794474842	0.9385317
ST6GALNAC2	-0.008048075	0.79478254	0.938750161
BAG3	-0.005210006	0.794829389	0.938750161
DCLRE1B	-0.005777465	0.794994772	0.938760855
PAK1IP1	0.002888248	0.795008064	0.938760855
RDH13	-0.006336194	0.795114978	0.938780463
PFDN5	0.002536845	0.795194292	0.938780463
CHD3	0.004855936	0.795583306	0.939139556
FOXP1	-0.00541006	0.795798915	0.939293899
PTPN18	0.005128248	0.796039495	0.939392948
EIF4A2	0.002071801	0.796052566	0.939392948
CTSG	-0.017394469	0.796534231	0.939861145
RASA3	-0.004841777	0.796637787	0.939883145
TTC38	-0.008986868	0.796783935	0.9399452
PRKCZ	0.003760401	0.797019188	0.9399452
DCBLD1	0.005756478	0.797103947	0.9399452
NCAPG	-0.009918985	0.797326288	0.9399452
FPR3	-0.010621501	0.797395886	0.9399452
ZFP3	0.00417742	0.797402475	0.9399452
ACER2	-0.004470624	0.797661016	0.9399452
METTL2B	0.00452765	0.797754235	0.9399452
AKR1C4	-0.013095519	0.797819732	0.9399452
DNAJC5	0.003345276	0.797832534	0.9399452
SIGLEC5	-0.011024746	0.797853719	0.9399452
MAPK7	0.00418409	0.797882662	0.9399452
MTA1	0.003902123	0.797919379	0.9399452
CTNS	-0.003449843	0.798145014	0.9399452
ATP6V1G1	-0.002530764	0.798149412	0.9399452

SUSD1	-0.004424741	0.798186831	0.9399452
ZMYM2	0.003561584	0.798190275	0.9399452
POLD4	0.002739064	0.798218889	0.9399452
KDM4A	0.004743198	0.798555792	0.940106125
NCSTN	-0.002139709	0.798602406	0.940106125
NARF	0.003840601	0.798698556	0.940106125
RGPD1	0.010684677	0.798737005	0.940106125
SUOX	-0.006582638	0.798780206	0.940106125
TAF2	-0.002984159	0.799254702	0.940465726
NDUFS4	-0.003766786	0.799327404	0.940465726
AKAP12	0.003798975	0.79934064	0.940465726
UST	-0.007071874	0.799459146	0.940505185
TAF6	-0.004906861	0.799680818	0.94066599
PANK3	0.003896772	0.799905317	0.940675807
COG1	0.004152002	0.799924303	0.940675807
SLFN5	-0.006680259	0.799944112	0.940675807
AQR	-0.002467636	0.800058126	0.940709943
SLC39A1	-0.003316189	0.800360913	0.94094085
SNX16	0.005503666	0.800619728	0.94094085
KCNH3	-0.013577954	0.800636133	0.94094085
HIVEP1	-0.004135324	0.800648096	0.94094085
FAM110B	-0.005305607	0.800679543	0.94094085
SPTBN4	-0.006672569	0.800855224	0.941047396
ATPIF1	-0.003041216	0.801176432	0.941084395
SCGB3A1	0.027092308	0.801399781	0.941084395
TCL1A	0.014909116	0.801502783	0.941084395
ATG2B	-0.004440331	0.801548825	0.941084395
PRKAR2A	0.004361037	0.801563254	0.941084395
STYK1	-0.005724448	0.801578475	0.941084395
ZNF684	-0.004246485	0.80158135	0.941084395
SMPD3	-0.005039441	0.801635956	0.941084395
ZNF518B	0.003390784	0.801651889	0.941084395
AKTIP	0.003363015	0.802055825	0.941458741
MPI	0.005162727	0.802380352	0.941739807
PTPN6	-0.004096461	0.802524083	0.941808638
TMEM62	0.003533031	0.802701874	0.941906166
ZNF579	-0.004626638	0.802777375	0.941906166
PODN	-0.006824472	0.803078539	0.941944058
EPHA10	-0.003723799	0.803092676	0.941944058
SH3BP4	0.00597125	0.803189701	0.941944058
PMS2CL	-0.003822134	0.803238368	0.941944058
RPL13AP6	-0.003948205	0.8033053	0.941944058
PLCD1	0.004195347	0.803368079	0.941944058
FAM26E	0.004866225	0.803405353	0.941944058
GAR1	0.002362267	0.803503743	0.941959641
TRIM62	0.004717661	0.803709332	0.942094558
ILDR1	-0.005260598	0.803838764	0.942094558
RAD51AP1	0.005632632	0.803874162	0.942094558
SNORA59A	0.004715308	0.804261736	0.94244899
DUSP6	0.004713602	0.804507698	0.942637421

ADAM15	-0.003719249	0.805137215	0.943275173
CNOT8	0.002397541	0.805330074	0.943376125
CCDC97	0.002625451	0.805498959	0.943376125
CHKA	0.004059111	0.8056521	0.943376125
SHD	0.008431385	0.805683534	0.943376125
TMEM176B	-0.022595255	0.805800633	0.943376125
IRAK2	-0.005460653	0.806058298	0.943376125
HKDC1	-0.009152043	0.806087448	0.943376125
ITPR1	-0.003663472	0.806093523	0.943376125
GDF5OS	0.00442173	0.806104016	0.943376125
ZNF226	-0.005153768	0.806133915	0.943376125
HIF3A	-0.004662385	0.806160879	0.943376125
BEND5	0.00718725	0.806335981	0.943423466
EFHD1	-0.010016447	0.806465971	0.943423466
CADM2	-0.00482679	0.806476336	0.943423466
LDB2	0.010711866	0.806635475	0.943423466
HDAC9	-0.008161657	0.806698885	0.943423466
TPT1	-0.003864933	0.806712721	0.943423466
CD74	0.003300419	0.807022351	0.943685866
TMEM204	0.007140682	0.807149138	0.943734426
GATC	-0.007088831	0.807285075	0.943793673
TRPS1	-0.005156131	0.807428799	0.94386201
ZNF404	0.006085523	0.807791949	0.94418681
SORBS3	-0.003774702	0.807981056	0.94424087
SNHG8	0.015779773	0.80800881	0.94424087
RCL1	-0.004822577	0.808257838	0.944432177
WDFY1	0.002946267	0.808431964	0.94453593
TYW1B	0.003892663	0.808608537	0.944559792
GGT3P	0.003885972	0.808662336	0.944559792
NMNAT1	-0.004261409	0.808708388	0.944559792
LGALS12	0.007067567	0.808902587	0.944686932
ASCC2	-0.002025883	0.809127259	0.944740459
PSMC3IP	-0.004621806	0.809156935	0.944740459
TINAG	-0.004487764	0.809204471	0.944740459
SREBF2	0.004367026	0.809417325	0.944889303
TUBBP5	0.005312455	0.809751372	0.945179577
SNHG11	-0.003803266	0.809912144	0.945222488
FKBP3	0.003689967	0.809958921	0.945222488
DRD3	0.007904825	0.810259099	0.945253521
MAML3	0.006222917	0.810272976	0.945253521
IGKC	0.01078295	0.810283029	0.945253521
LPAL2	0.005563419	0.810379057	0.945253521
ASRGL1	-0.006885375	0.810412496	0.945253521
ATAD2B	-0.004054561	0.810613076	0.945359804
DDTL	0.012217845	0.810730505	0.945359804
SCGB1C1	0.013978539	0.810759836	0.945359804
CBLN3	0.011584225	0.810992198	0.945401633
DAGLA	0.004528744	0.811032812	0.945401633
FECH	-0.006302442	0.811051938	0.945401633
RNF122	-0.003963881	0.811240076	0.945413509

KIF11	-0.00763293	0.811298914	0.945413509
ZNF585B	0.003995811	0.811395397	0.945413509
RXRG	-0.004496252	0.81140377	0.945413509
IFT122	0.003799832	0.812200248	0.946241926
LRMP	0.002797744	0.81236164	0.946330351
FBXO21	0.002039822	0.812504472	0.946397137
XYLT2	0.002690395	0.812636916	0.946451812
XPO6	-0.003359291	0.813064052	0.946849657
RNF19B	0.005037546	0.813181556	0.946886876
PRKDC	-0.004301372	0.813361498	0.946974519
TRAF1	0.003996892	0.813513001	0.946974519
HAVCR2	-0.003857678	0.813533466	0.946974519
SAMD11	0.004956424	0.813599032	0.946974519
ERI1	0.003238578	0.813689433	0.946980163
KIAA1467	0.003712366	0.813892237	0.947090043
MFN1	-0.003674391	0.813954971	0.947090043
STK36	0.003659145	0.814154453	0.947159921
ZNF34	0.003733826	0.814239495	0.947159921
TET2	-0.00397939	0.814287117	0.947159921
LRRC59	0.004572711	0.814357301	0.947159921
MRPL18	0.003050673	0.814656953	0.947317568
SNORA65	-0.003298538	0.814664011	0.947317568
FIZ1	0.003700855	0.81482037	0.947373226
LIG4	0.005522154	0.815063776	0.947373226
RHOC	0.004858918	0.815068483	0.947373226
RAB32	0.005191902	0.815086266	0.947373226
SLC12A6	-0.00356488	0.815216844	0.947373226
WAPAL	0.002948513	0.815338951	0.947373226
ALG9	-0.002386482	0.815342773	0.947373226
MUC7	-0.004952622	0.815396578	0.947373226
CENPM	0.005903926	0.815757281	0.947692836
SNHG7	-0.006335072	0.816030104	0.947771837
OTOGL	-0.003966474	0.816114758	0.947771837
TMEM67	-0.004031164	0.816161633	0.947771837
BNIP1	0.004207083	0.816189732	0.947771837
CYTIP	-0.004377399	0.816382	0.947771837
CREBL2	0.004194309	0.816427666	0.947771837
TST	-0.005718326	0.816805517	0.947771837
TRIM26	-0.004275309	0.816813095	0.947771837
SLC25A23	-0.004490965	0.817057204	0.947771837
MED27	-0.004810676	0.817187809	0.947771837
KIR2DL1	0.012642105	0.817193421	0.947771837
ADCY9	-0.005136856	0.817354783	0.947771837
ZNF28	0.003679683	0.817372377	0.947771837
AMY1A	-0.004050911	0.817388175	0.947771837
GOT2	0.003030918	0.817449598	0.947771837
RALGAPA2	0.004090022	0.817715894	0.947771837
LRP5	0.004247645	0.817752195	0.947771837
NXPH2	0.004431265	0.817821209	0.947771837
IL6ST	-0.005593561	0.817843768	0.947771837

ALAS1	-0.004490309	0.8180104	0.947771837
OSBPL10	-0.010996355	0.818015498	0.947771837
MIR129-2	-0.004033416	0.818020931	0.947771837
REV1	0.002876769	0.818049742	0.947771837
TMEM214	-0.003348887	0.818059825	0.947771837
DCAF12	0.003067561	0.818113112	0.947771837
SLC18A1	-0.00414277	0.818157858	0.947771837
PCNX	0.00286306	0.818189145	0.947771837
RSPH3	-0.004881142	0.81822381	0.947771837
MNX1	0.00404933	0.818308379	0.947771837
WDR54	-0.002739319	0.818645892	0.948063547
TCP10L2	-0.005291904	0.819127214	0.948276234
MXI1	0.004945284	0.819221643	0.948276234
TYW1	0.004013195	0.81938745	0.948276234
SNORA62	0.00482909	0.819423356	0.948276234
NME6	0.003037475	0.819475364	0.948276234
YEATS2	-0.002579844	0.819523864	0.948276234
SAAL1	-0.002579003	0.819532623	0.948276234
BCAP29	-0.004681957	0.819572333	0.948276234
CAV2	0.011979235	0.819680238	0.948276234
MIA-RAB4B	0.003495686	0.819686242	0.948276234
NT5DC1	-0.002652172	0.819793946	0.948301723
PTPRM	0.010640088	0.820004889	0.948446616
FCRLA	-0.011020111	0.820184345	0.948483396
SLC1A5	-0.004032818	0.820208065	0.948483396
RPL27A	-0.00134618	0.820507039	0.948635796
DSC3	-0.00417407	0.820511258	0.948635796
GARS	0.001917951	0.820780266	0.948847704
TSC22D4	0.004008785	0.820954637	0.948926906
SNX25	0.005372532	0.821021339	0.948926906
SESN1	-0.005269012	0.821105963	0.948926906
MAP3K5	-0.00379664	0.821373208	0.949090484
ENG	-0.005226933	0.821435096	0.949090484
ZNF107	0.003622649	0.821513022	0.949090484
HTR3A	-0.005201004	0.82159048	0.949090484
ORC5	-0.002311582	0.821873226	0.949212937
SIGMAR1	-0.002965759	0.821895057	0.949212937
ARHGEF10L	-0.007722002	0.821976015	0.949212937
DPEP3	-0.005959933	0.822039499	0.949212937
NUP160	0.00279997	0.822150525	0.949242116
PMF1	-0.003972347	0.822676592	0.949750437
ZFYVE27	-0.00423382	0.822762699	0.949750789
MYO18A	0.003308012	0.823163156	0.950113971
NOC4L	0.005023014	0.82330681	0.950180698
VPS26B	0.003166111	0.823482583	0.950192723
MRPS16	0.002306002	0.823488914	0.950192723
MIDN	-0.003401601	0.823768626	0.950416398
DAPK3	0.004198551	0.823941831	0.950517157
KIF22	0.002799785	0.824531814	0.95109865
PGAP2	0.00250127	0.824935119	0.95138979

SIGLEC14	-0.016087586	0.824959509	0.95138979
MCTP2	0.006342763	0.825042063	0.95138979
CENPL	-0.003767541	0.82516724	0.951435019
NPEPL1	0.004436177	0.825466775	0.951681255
DPP8	0.002017357	0.825817089	0.951739029
ADM	0.009192771	0.825882932	0.951739029
SNORA66	0.005680853	0.825909311	0.951739029
CMBL	0.003942856	0.826058766	0.951739029
YPEL1	0.005978926	0.826069487	0.951739029
C15orf52	0.006467416	0.826097114	0.951739029
GBP1	-0.007234339	0.826195686	0.951739029
LCAT	0.003979836	0.826227864	0.951739029
ICAM4	-0.009602291	0.826340721	0.951739029
BRAF	0.004055363	0.82637671	0.951739029
HARS2	-0.002879797	0.826479039	0.951739175
SLA	-0.005294397	0.826769422	0.951739175
SLC1A3	0.005564728	0.826839119	0.951739175
MIS18A	0.004515288	0.826870305	0.951739175
SLC2A11	0.003510089	0.826950852	0.951739175
MPDU1	-0.004405466	0.827115651	0.951739175
KDM5A	-0.003084508	0.827130995	0.951739175
EXOC7	-0.00242031	0.827247278	0.951739175
EXOSC4	-0.0033051	0.827291322	0.951739175
CD83	0.004705965	0.827323804	0.951739175
FSTL1	0.008416627	0.82749397	0.951739175
LCNL1	0.004144851	0.827523174	0.951739175
RTTN	-0.003023599	0.827581399	0.951739175
FDPS	-0.002735685	0.827662901	0.951739175
BRI3BP	0.003466778	0.827666573	0.951739175
ARPC1B	0.003235323	0.827765182	0.951746511
ICAM2	0.00284834	0.827844919	0.951746511
KCNH8	-0.004737146	0.828239135	0.95210084
GNAI2	-0.002747533	0.828359197	0.952139974
CCRN4L	0.005305704	0.828659302	0.952309584
SPG20	0.00351746	0.828851979	0.952309584
PCIF1	0.004651043	0.828922651	0.952309584
TOP1MT	-0.004427223	0.828930203	0.952309584
TSEN2	-0.003972604	0.828945152	0.952309584
C9orf9	0.00343646	0.829022961	0.952309584
DOK2	-0.002889913	0.829453353	0.95252848
GTF2H5	0.002674337	0.829553315	0.95252848
VPS37C	-0.003161112	0.829609856	0.95252848
DIS3	0.004297703	0.829667168	0.95252848
FCRL6	-0.012375965	0.829763829	0.95252848
RUSC2	0.004704622	0.82982489	0.95252848
PFDN2	0.005111945	0.829931741	0.95252848
NDUFB7	-0.003117628	0.829956943	0.95252848
MRPL2	-0.005494468	0.829988002	0.95252848
LIPA	-0.005129115	0.8301076	0.952542945
EFR3B	0.005111897	0.83045075	0.952542945

NOP10	-0.003418611	0.830465432	0.952542945
FHOD1	0.0030553	0.830494235	0.952542945
PHF1	0.00381042	0.830583329	0.952542945
IP6K3	-0.003932669	0.83064844	0.952542945
TMEM79	0.004134052	0.830648976	0.952542945
AIFM3	0.003852389	0.830712353	0.952542945
LRRC37A2	0.00480901	0.830775101	0.952542945
HDGFRP3	-0.007808977	0.830948672	0.952643279
KIF14	0.005401765	0.831086812	0.952648217
CCDC41	0.003445682	0.831125109	0.952648217
GDPD5	0.006986479	0.831512608	0.952993689
SPRED1	-0.006035587	0.831654814	0.953057991
TTI1	0.003652566	0.831912436	0.953254529
TRAC	0.00716237	0.832032519	0.953293442
CRIP1	-0.003317534	0.832193853	0.953379607
LYSMD4	-0.004055117	0.832280551	0.953380257
PHF13	-0.002471555	0.832657902	0.953644646
ACAD8	0.002611419	0.832683666	0.953644646
CHMP1A	0.003296052	0.832984173	0.953812458
RNF111	0.004336541	0.833002532	0.953812458
MLLT4	0.008379148	0.833133832	0.95382931
FAM72D	-0.00598149	0.833236159	0.95382931
PDE8B	0.00594259	0.833275764	0.95382931
SLC12A8	0.003709176	0.833405996	0.953879741
ITPRIPL1	0.005723147	0.833701002	0.953927715
PER1	-0.005503433	0.833962395	0.953927715
RHPN2	0.005649037	0.834231748	0.953927715
SERPINB9	0.007870347	0.834303156	0.953927715
C19orf59	-0.006271304	0.834368626	0.953927715
WDR37	-0.002630484	0.834372322	0.953927715
TRIM46	0.004570704	0.83437829	0.953927715
TSPYL6	-0.003602741	0.834543565	0.953927715
EFCAB4B	0.003258718	0.834561962	0.953927715
ARF5	-0.002361856	0.834600058	0.953927715
TFPT	0.003428455	0.834689888	0.953927715
TK2	0.003140832	0.834721342	0.953927715
GAS2L1	0.005844665	0.83484446	0.953927715
HMG20B	0.003224677	0.834847419	0.953927715
LANCL1	0.002129295	0.834868095	0.953927715
KLF3	-0.004476216	0.834919455	0.953927715
STRN	0.003326724	0.83502377	0.953927715
ZC3H18	-0.003187384	0.835042615	0.953927715
BPTF	0.003492032	0.835085334	0.953927715
PBX2	-0.002367165	0.835263205	0.954032444
CCDC50	0.00437447	0.835609948	0.954236847
MRPS2	0.002818306	0.835614578	0.954236847
PDP1	0.004336499	0.835757848	0.954302004
RSC1A1	0.003764694	0.836205269	0.954693291
SNORD59A	0.004830867	0.836273028	0.954693291
CTH	0.003922546	0.836494986	0.954848201

HYAL2	0.003422199	0.836610339	0.954881403
PRKCI	-0.002843307	0.836743733	0.954935187
PLEKHG1	0.006092181	0.836940589	0.954954266
MARCH5	0.003804974	0.836948521	0.954954266
C10orf118	-0.003200774	0.837019269	0.954954266
WRAP73	-0.002285466	0.837355545	0.955239465
YWHAG	0.002638156	0.837719683	0.955456258
PTTG1IP	-0.001881499	0.837794291	0.955456258
VEGFA	-0.004027086	0.837804539	0.955456258
NCOR1	0.002676592	0.838738329	0.95642062
NUDT1	0.003368639	0.838874812	0.95642062
NEK8	0.004176565	0.838909368	0.95642062
AKAP10	0.004332179	0.839006135	0.956432431
BCL2L11	0.004634351	0.839102113	0.956443341
CHST13	-0.012856251	0.839287022	0.956555606
FBXO43	-0.003397043	0.83938231	0.956565715
PHF5A	-0.002148379	0.839538668	0.95658
RTN1	0.004415259	0.839567685	0.95658
NICN1	-0.002761849	0.839680999	0.95661064
SPOP	-0.002204552	0.840287445	0.956944711
CYP2U1	-0.003130613	0.840401531	0.956944711
IL17D	-0.003006995	0.840404432	0.956944711
TEKT4P2	0.016670076	0.840461568	0.956944711
WDR55	0.002385459	0.840642488	0.956944711
NR1H3	-0.004639304	0.840871476	0.956944711
TMEM70	0.003664666	0.840941105	0.956944711
ACSM3	0.004086354	0.84094654	0.956944711
TOM1	0.002473315	0.841047558	0.956944711
TRIM5	-0.004924272	0.841214384	0.956944711
PMS2P3	0.003857494	0.841231272	0.956944711
URM1	0.002160179	0.841248476	0.956944711
ETAA1	-0.003668157	0.841498476	0.956944711
TFG	-0.006218964	0.841557184	0.956944711
PLXND1	-0.004623627	0.841574455	0.956944711
CDKN1B	0.002619278	0.841601222	0.956944711
ZFYVE21	-0.002554933	0.841728069	0.956944711
C4orf48	-0.002159837	0.841757841	0.956944711
SECTM1	-0.003336108	0.8417843	0.956944711
COG7	0.003574618	0.841785399	0.956944711
CRYBB2P1	0.008549246	0.841789742	0.956944711
MTA3	-0.003391291	0.842014878	0.95710235
SCARNA5	0.00519638	0.84258719	0.957654544
PLEKHA5	0.005443784	0.842874371	0.957786931
LEPR	-0.006307602	0.843046883	0.957786931
LRRC47	0.001683601	0.843083849	0.957786931
RPS9	-0.002251599	0.843193205	0.957786931
CSMD1	-0.005840536	0.843347001	0.957786931
FTL	0.001195684	0.84342958	0.957786931
ABCC10	0.003107098	0.843433974	0.957786931
DSP	0.004114524	0.843448889	0.957786931

CEACAM3	0.008137028	0.843501264	0.957786931
CYLD	0.00392887	0.843748506	0.957786931
ITGA2	0.004696092	0.843803688	0.957786931
GKAP1	-0.003634707	0.843828866	0.957786931
THRA	-0.003628706	0.843924398	0.957786931
PHKG2	-0.003162489	0.843981945	0.957786931
CYCSP52	0.003406967	0.844001601	0.957786931
DIXDC1	0.003181608	0.844477314	0.958171805
VTRNA1-3	-0.004375457	0.84451388	0.958171805
XYLT1	0.022792234	0.844670502	0.958177757
TMEM229B	-0.003802731	0.844692254	0.958177757
CPLX2	-0.003393576	0.845065312	0.958502709
TATDN3	-0.002691639	0.845319112	0.958602436
ALDH1B1	-0.003632522	0.845326442	0.958602436
ABHD1	-0.003733291	0.845493987	0.95860619
HKR1	0.003290706	0.845506789	0.95860619
SMARCC2	-0.002935435	0.845783089	0.95860619
MAF	0.005804095	0.845919392	0.95860619
VRK1	0.003093997	0.845954471	0.95860619
CABIN1	0.003352538	0.845998135	0.95860619
KCNRG	0.004423242	0.846059406	0.95860619
WDR73	-0.003787711	0.846285427	0.95860619
UFD1L	-0.002721025	0.846379746	0.95860619
BSPH1	-0.003897514	0.84641365	0.95860619
TYMP	-0.003810717	0.8464916	0.95860619
AGRN	-0.003514337	0.846538707	0.95860619
RARS	0.001621425	0.846674684	0.95860619
RBM34	0.001923473	0.846778867	0.95860619
PHLPP1	0.002568107	0.846854037	0.95860619
SCP2	0.005198672	0.846873276	0.95860619
FAS	-0.004763276	0.846883756	0.95860619
TRMT61A	-0.003399563	0.847034285	0.95860619
ILK	0.003710472	0.847051744	0.95860619
CA2	0.008741478	0.847061808	0.95860619
ANKFY1	0.002510525	0.847205935	0.958657025
AVPI1	-0.005124018	0.847590222	0.958657025
SCAND1	-0.002406058	0.84764063	0.958657025
EPHX2	-0.006378795	0.84766222	0.958657025
SNRNP27	-0.00299769	0.847787839	0.958657025
PPP1R15A	0.003156722	0.847851782	0.958657025
KLHL26	0.003237408	0.847890052	0.958657025
TRNP1	-0.004767543	0.847938587	0.958657025
RLTPR	0.003876396	0.847991993	0.958657025
CARD9	0.003568157	0.84805686	0.958657025
ZFYVE1	-0.002282456	0.848171909	0.958657025
SLC31A1	-0.003596205	0.848197292	0.958657025
MINA	0.00320672	0.848359238	0.958657025
SERPINE1	-0.003738846	0.848425542	0.958657025
MKL2	0.004030939	0.848539661	0.958657025
KIAA0513	-0.003023192	0.848545007	0.958657025

CTU1	0.003528379	0.848594977	0.958657025
TOB2	0.003301078	0.848696615	0.958657025
AQP11	-0.003421188	0.848752267	0.958657025
FLCN	-0.003763107	0.848889506	0.958705834
TLE2	-0.004375584	0.848968704	0.958705834
GPATCH3	-0.002653044	0.849361354	0.959049152
TFEC	-0.003717005	0.84944601	0.959049152
NDUFB1	-0.004785047	0.849680106	0.959199222
ORC6	0.00106562	0.849798133	0.959199222
IFI30	0.003721429	0.849888291	0.959199222
ASPHD2	0.003205458	0.849925556	0.959199222
CRELD2	0.002816947	0.85005397	0.959205127
HCFC1R1	0.002663329	0.850104101	0.959205127
SPSB3	-0.002304621	0.850286998	0.959313707
RIPK4	-0.003361032	0.850426004	0.959331547
DOCK7	-0.003838811	0.850607591	0.959331547
ATP2B4	0.003698857	0.850611477	0.959331547
DUSP15	0.003436521	0.850649484	0.959331547
ZNF552	-0.003140081	0.850792721	0.959380473
DNAH2	-0.003002296	0.850866212	0.959380473
UHMK1	0.002111258	0.850953643	0.959381328
ZNF280B	-0.003377119	0.851182027	0.959454215
CYB5R3	-0.002641389	0.851191652	0.959454215
SCAF4	0.002847323	0.851584462	0.95956199
SLC22A17	0.005424001	0.85165832	0.95956199
TRUB1	-0.003975946	0.851669513	0.95956199
ARHGEF3	0.00228011	0.851821165	0.95956199
ZNF705G	0.00423158	0.851983422	0.95956199
ORAI1	0.003745346	0.852053474	0.95956199
C15orf39	-0.003321036	0.852125641	0.95956199
SNORD17	0.004362668	0.852146584	0.95956199
ABCF2	-0.002834694	0.852246106	0.95956199
MRPL9	-0.002528314	0.852292105	0.95956199
LTBP1	-0.004562709	0.852315064	0.95956199
ACOX3	-0.003231751	0.852327535	0.95956199
ZNF286A	-0.004438337	0.852495209	0.959653154
FRG1	0.003361757	0.85268883	0.959773506
ULK2	0.003108118	0.853109891	0.96014981
NUDT22	-0.002392714	0.853308852	0.960276096
POLR2I	-0.002192508	0.853776484	0.960435302
GYS1	-0.003340171	0.853820974	0.960435302
TAB2	0.003737477	0.853820997	0.960435302
ZNF57	-0.003481622	0.853858732	0.960435302
MOSPD3	-0.002987689	0.853906653	0.960435302
CPSF2	-0.003729976	0.854009835	0.960435302
RAP1B	-0.003585153	0.8540577	0.960435302
ZNF532	-0.0043315	0.854163526	0.960456732
AKNA	0.002494342	0.854594493	0.960843722
CTU2	-0.003254185	0.854784109	0.960894736
LTBP2	0.003502994	0.854932664	0.960894736

DIAPH3	0.003174843	0.854953886	0.960894736
CYTH3	-0.003992974	0.854987104	0.960894736
YIPF1	0.001405491	0.855093129	0.96091633
SVIL	0.002377377	0.85543217	0.960998919
NOC3L	-0.002448462	0.855452878	0.960998919
CECR6	0.006536496	0.85564685	0.960998919
TBC1D22B	-0.002895206	0.855703182	0.960998919
POLE2	0.003591655	0.855740783	0.960998919
MPZL1	0.003459988	0.855762954	0.960998919
MAP3K7	0.001731496	0.855952505	0.960998919
KIAA1211	0.004642872	0.855956489	0.960998919
SPNS1	-0.00326901	0.855996969	0.960998919
SSH3	-0.002654814	0.856096408	0.960998919
TYMS	-0.006801814	0.856210441	0.960998919
PARP10	0.002904758	0.856243247	0.960998919
PCM1	0.002249349	0.856295269	0.960998919
ZBTB48	0.002319937	0.856652731	0.961302623
PRM3	-0.002815438	0.856876589	0.9613785
REPS1	0.002918868	0.856894054	0.9613785
ARHGEF19	0.00374225	0.857258239	0.961689617
ITGB1	-0.002582524	0.857548447	0.961904287
KLF6	0.002577646	0.857684704	0.961904287
ZNF274	0.001961489	0.8577103	0.961904287
P4HTM	0.002836117	0.857947414	0.961965377
KDELC2	-0.003389769	0.857953825	0.961965377
TTLL4	0.003028983	0.858045476	0.961965377
C8orf58	-0.003252465	0.858162024	0.961965377
CNOT4	0.002389241	0.858199304	0.961965377
NAIF1	-0.002262635	0.858569711	0.962260789
IRX3	0.010396338	0.858636716	0.962260789
RECQL5	0.003637645	0.859134176	0.962715213
RTEL1	-0.003125075	0.859216153	0.962715213
S100A6	-0.002095184	0.859563765	0.962898976
DRG2	0.002462722	0.859607575	0.962898976
STAT4	0.002570371	0.859755616	0.962898976
CCHCR1	0.003294233	0.859787686	0.962898976
P2RX5-TAX1BP3	0.004002124	0.859927297	0.962898976
APH1B	-0.002630537	0.85997155	0.962898976
HERC3	-0.002903417	0.860094626	0.962898976
PURA	0.003080578	0.86011873	0.962898976
CLEC11A	-0.005566407	0.860163075	0.962898976
GPSM3	0.002000052	0.860286211	0.962939435
DDIT3	0.002395471	0.860504235	0.963086085
LLGL1	-0.002585312	0.860859819	0.963386649
GPR68	0.005987803	0.861121678	0.963403107
OR2T34	0.003183035	0.861128973	0.963403107
AKR1A1	-0.001863974	0.861157596	0.963403107
PHC2	-0.003555753	0.861222671	0.963403107
EIF4E2	-0.001758836	0.861477026	0.963590258
GMNN	-0.003519286	0.861659256	0.963696706

GZMM	-0.004657517	0.861874572	0.963728151
DAP	0.003660595	0.861942038	0.963728151
APBA3	-0.002624096	0.861948567	0.963728151
CACHD1	-0.003747064	0.862156721	0.963863523
INTS7	-0.002695354	0.862546487	0.964059537
GBP3	-0.005309963	0.862583142	0.964059537
RPL23AP7	-0.001022275	0.862593337	0.964059537
HERC2P3	-0.003415668	0.862729682	0.964114574
PSMC1	0.002861207	0.862905483	0.964213688
ZBTB44	0.002639711	0.863106316	0.964310717
F11R	-0.003189017	0.863221072	0.964310717
SURF6	-0.003112521	0.863253672	0.964310717
MON1B	0.002380871	0.863420966	0.96440027
RPH3AL	-0.003337647	0.863701212	0.964615954
GSK3B	0.002669362	0.863955486	0.964669574
SRSF4	0.001523468	0.864005549	0.964669574
SEC62	-0.00274508	0.86406188	0.964669574
F2RL3	0.004911986	0.864097825	0.964669574
UNG	0.002807961	0.864201541	0.964688066
EIF1AD	-0.003877979	0.864327949	0.964731882
GMEB2	-0.001866533	0.864476857	0.9648008
ATP6V0A2	0.00196892	0.864624371	0.964815145
SHROOM1	-0.00429624	0.864749152	0.964815145
SLC45A4	0.003623294	0.864751202	0.964815145
MDP1	-0.002057364	0.8648762	0.964857353
FAM20C	0.005320992	0.865245191	0.965110852
KIR3DL1	0.010336165	0.865352174	0.965110852
NTHL1	-0.002338371	0.865425815	0.965110852
GPR132	-0.003599585	0.865511794	0.965110852
TEC	-0.002694786	0.865623837	0.965110852
RGS14	0.003309968	0.865709604	0.965110852
KREMEN2	0.002893314	0.865713764	0.965110852
PRSS36	0.002612622	0.865962605	0.965246498
SGOL2	-0.00357427	0.866014831	0.965246498
JMJD8	-0.001438015	0.866097047	0.965246498
TNFRSF4	0.00595532	0.866231438	0.965248995
UBR2	-0.00149142	0.866374966	0.965248995
FBLN7	-0.004489543	0.866487402	0.965248995
ATP1B1	-0.004783431	0.866632648	0.965248995
IFITM2	-0.004018681	0.866668053	0.965248995
CLCN3	0.002912558	0.866692372	0.965248995
PTPLA	0.004043804	0.866709708	0.965248995
KCTD6	-0.002146278	0.86696756	0.965439026
AKR1C3	-0.008377653	0.867165791	0.965562634
CDYL2	-0.002451942	0.867348731	0.965595838
PDLIM5	-0.003933819	0.867458006	0.965595838
PMPCA	-0.002145846	0.867527192	0.965595838
HSP90AA1	-0.001604659	0.867603631	0.965595838
BCLAF1	0.001586711	0.867907869	0.965595838
CD70	-0.004325102	0.868030218	0.965595838

ULK3	-0.0035761	0.868031496	0.965595838
DDAH1	0.002170857	0.868115905	0.965595838
ACOT8	0.002930282	0.86811667	0.965595838
UBIAD1	-0.002431169	0.868213334	0.965595838
AMFR	-0.006532882	0.868270506	0.965595838
RPP14	0.003224914	0.868297648	0.965595838
ZNF259	0.002161756	0.868362075	0.965595838
NANOS1	0.005596726	0.868477157	0.965595838
LARP1B	-0.002212809	0.868615823	0.965595838
RGS17	-0.006603544	0.868658987	0.965595838
RAC1	-0.001552246	0.868678594	0.965595838
MAP1LC3B	-0.002454959	0.868952775	0.965803621
NIT2	0.002400864	0.869206896	0.96598907
IFT57	0.003970958	0.869454184	0.966081487
RLF	0.00193548	0.8695269	0.966081487
AASS	-0.003737357	0.869551889	0.966081487
BMP2K	-0.003323792	0.869870986	0.966339015
HNRNPKP3	0.002764153	0.87004175	0.966364764
GIPC1	0.003241243	0.870068772	0.966364764
DDX27	0.002168693	0.870236394	0.966397235
DOCK8	-0.002510755	0.870272621	0.966397235
CCNJ	-0.003140091	0.870573487	0.96652763
VOPP1	-0.002104149	0.870646749	0.96652763
NT5DC2	-0.004983288	0.870690243	0.96652763
PLCH2	0.005752134	0.870739319	0.96652763
MYBBP1A	0.003499466	0.87101661	0.96673848
CHD6	0.001890045	0.871811752	0.96752399
SLC2A5	0.005891359	0.872134651	0.967785309
DDX18	-0.001657484	0.872362682	0.967841267
CLEC5A	0.006834997	0.872427701	0.967841267
THAP2	0.003042016	0.87244739	0.967841267
CCDC53	-0.001747535	0.872655963	0.967928212
FCGBP	0.007222476	0.872700655	0.967928212
TARSL2	-0.002947693	0.872949503	0.968107209
IFI27L2	0.002805961	0.873114969	0.968193708
IL17RA	0.00282685	0.873208119	0.968200007
ING5	-0.004480997	0.873367435	0.968269824
ZAP70	0.003262915	0.873465713	0.968269824
SP110	0.001962719	0.873541633	0.968269824
CASP10	0.002637086	0.873700538	0.968269824
ZNF438	-0.002296497	0.873708465	0.968269824
TMCO6	-0.001978917	0.873975358	0.968375846
SYNGR1	0.004480611	0.873979104	0.968375846
HLA-DMA	-0.002703309	0.874186	0.968508141
PIGS	-0.002065732	0.874388266	0.96863528
CDC42SE2	0.002336038	0.874516799	0.968680721
KIAA0355	0.001102922	0.874604577	0.968681015
RPS15A	-9.42E-04	0.874925903	0.968939951
PRMT1	0.001831691	0.87529955	0.968951359
ZNF563	0.003555916	0.875328872	0.968951359

LRIF1	-0.004112124	0.87535849	0.968951359
TMEM194B	-0.002861086	0.875513725	0.968951359
WDR41	-0.006744362	0.875524274	0.968951359
C16orf62	-0.002361587	0.875551297	0.968951359
CSRP2	0.002747036	0.875562838	0.968951359
TWF1	0.00303904	0.875636503	0.968951359
CALR	-0.002581366	0.87580937	0.969045773
SNX22	-0.003416374	0.876326782	0.969521354
GPR37	-0.002370168	0.876537714	0.969657801
SNORA5B	-0.003000781	0.876648415	0.969683352
CBFA2T3	0.003625456	0.876934925	0.969748653
BAG2	-0.003272884	0.876994574	0.969748653
RPS24	0.001387979	0.877027873	0.969748653
PGBD4	-0.002889019	0.877117595	0.969748653
GNL3	0.001511764	0.877145498	0.969748653
C12orf44	-0.00216381	0.877366244	0.969830057
FAM19A1	-0.006303456	0.877423436	0.969830057
LRFN4	0.003184755	0.877546834	0.969830057
SNORD110	0.002817977	0.87770814	0.969830057
GABARAPL1	-0.003639971	0.877731062	0.969830057
WDR18	0.002321467	0.877744829	0.969830057
RRP9	-0.002900297	0.877873683	0.969875616
ZNF8	-0.003177614	0.877990065	0.969907388
EFR3A	-0.001349421	0.878193356	0.969918471
SATB1	0.002997096	0.878305114	0.969918471
ITIH5	-8.26E-04	0.878324662	0.969918471
ZNF518A	0.001878645	0.878408549	0.969918471
DGCR8	0.004225718	0.878498669	0.969918471
CRX	0.002665767	0.878637496	0.969918471
DTX3L	-0.004791108	0.878708036	0.969918471
CDK17	0.002738909	0.878788834	0.969918471
TRIM16	-0.002942337	0.878837171	0.969918471
IKBKE	-0.002821401	0.878876345	0.969918471
ITGB1BP1	-0.003483327	0.879070108	0.970035592
C1orf123	-0.002229176	0.879231384	0.970116845
MICA	-0.003037425	0.879384198	0.970188746
PCP2	0.003227736	0.879732294	0.970476058
INPP5K	-0.002322763	0.880335015	0.971044173
PAM	0.002871463	0.880678913	0.971208262
HEMK1	0.002949382	0.880752513	0.971208262
DYTN	-0.004098821	0.880814634	0.971208262
ABCA1	-0.004827862	0.880979734	0.971208262
SLC35B2	-0.001932084	0.880987505	0.971208262
PPIL3	0.002207676	0.881010223	0.971208262
FLAD1	0.00159159	0.88120214	0.97126295
ZIK1	-0.00283537	0.881235324	0.97126295
TIFA	0.003127592	0.881427336	0.971269921
CYB561	0.003520585	0.881479942	0.971269921
CDK10	-0.003384527	0.881599391	0.971269921
FOSL2	0.002565124	0.881599588	0.971269921

HEY1	-0.002243479	0.881799067	0.971269921
IKZF1	0.005001912	0.881828357	0.971269921
TP73-AS1	0.002391098	0.881855878	0.971269921
PSTPIP1	-0.002924383	0.882119509	0.971363704
RPL8	-0.002507971	0.882136213	0.971363704
C3orf14	-0.005476009	0.882204293	0.971363704
RPAIN	-0.001380526	0.882634323	0.97160957
LRRC28	0.00272852	0.882656091	0.97160957
ESRRA	0.001838056	0.882690924	0.97160957
STK19	0.001577149	0.883201209	0.971974093
ZKSCAN5	-0.00214536	0.88332422	0.971974093
WARS	0.003409185	0.883500362	0.971974093
ZNF212	-0.001993091	0.883622191	0.971974093
CCDC137	-0.003565259	0.88363505	0.971974093
VWDE	-0.004254779	0.883795676	0.971974093
SAMD4B	0.002843108	0.883832825	0.971974093
CLCF1	-0.004627901	0.88385319	0.971974093
SDK2	-0.004029073	0.883876424	0.971974093
CSF3R	0.002643794	0.883914465	0.971974093
MSH6	-0.001656722	0.884065868	0.971974093
RALY	-0.001982228	0.884256117	0.971974093
ZNF519	-0.003524681	0.884315991	0.971974093
LIMK1	0.00306057	0.884327977	0.971974093
E2F1	-0.004147236	0.884366196	0.971974093
MFSD5	-0.002100961	0.884510198	0.971974093
INTS1	0.002082392	0.884568671	0.971974093
NFRKB	-0.002394139	0.884622438	0.971974093
TMEM55A	-0.003455727	0.884690486	0.971974093
TLN1	0.002970298	0.88484469	0.971997196
ACTN4	0.003034704	0.884972701	0.971997196
KLHL2	0.002545551	0.884974952	0.971997196
CREB3L2	-0.002056683	0.885354267	0.97221054
PDCD6IP	0.001643806	0.885452374	0.97221054
PLEKHO1	0.001690657	0.885519052	0.97221054
USP47	-0.002044606	0.885520522	0.97221054
CBX3	0.002184174	0.885699963	0.972307572
PPRC1	-0.001761371	0.885817878	0.972307572
SREBF1	-0.002917315	0.885872424	0.972307572
SYT16	-0.003416295	0.886267588	0.972422495
CDR2	-0.002349736	0.886269625	0.972422495
ANKRD49	0.00157236	0.886320689	0.972422495
C11orf58	-0.001853551	0.886328534	0.972422495
B3GNT6	0.002672299	0.886448711	0.972457956
FBXO44	0.002764986	0.886578179	0.972503604
BLMH	-0.002145709	0.886779277	0.972627806
TFEB	0.002930865	0.886993956	0.972766878
CEPT1	0.001837269	0.887086132	0.972771586
EFHC1	0.00266252	0.887189909	0.972789015
ZMYM5	0.003169046	0.887302085	0.972815648
SLC20A1	-0.002389224	0.887499622	0.972935854

KLHL24	-0.001893843	0.887804697	0.973147283
USP44	0.002514038	0.887868318	0.973147283
SART1	-0.003270586	0.888023106	0.97322057
TSGA10	-0.004780647	0.888174852	0.973290509
CDCA7L	-0.003198638	0.888396056	0.973428308
TRIP13	-0.003745307	0.888498315	0.973428308
PMS1	0.002638834	0.888662326	0.973428308
CLK4	-0.003311169	0.888744771	0.973428308
IKZF5	-0.001944775	0.888771492	0.973428308
FCHSD2	-0.002556144	0.888919964	0.973428308
DHDH	-0.00369104	0.888959505	0.973428308
SMYD3	0.00231639	0.889004135	0.973428308
RAD23B	0.003074623	0.889211258	0.97344419
ZDHHC14	-0.003447624	0.88932492	0.97344419
LIPT2	-0.002465571	0.889374414	0.97344419
C2orf15	-0.003586557	0.889518718	0.97344419
ABCC5	-0.002540489	0.889584368	0.97344419
OSBPL5	-0.004390326	0.889606529	0.97344419
C1RL	0.002545889	0.889634242	0.97344419
HIST1H4J	0.003381987	0.889968447	0.973578511
SNRNP35	0.001782376	0.889976546	0.973578511
TMEM45A	0.007579961	0.89007924	0.973578511
DMAP1	-0.00192579	0.89010882	0.973578511
SLC6A6	-0.003204226	0.890224742	0.973609096
LSS	0.003329821	0.890565356	0.973729277
ANO6	-0.003083498	0.890735537	0.973729277
POPDC2	0.002838873	0.890838535	0.973729277
ZNF395	-0.002143638	0.890865596	0.973729277
APOBEC3H	-0.005398847	0.890926321	0.973729277
LPAR1	0.007162696	0.890927444	0.973729277
CILP	-0.002419646	0.890955982	0.973729277
NFE2	0.002541038	0.891038382	0.973729277
SPIN1	0.0030389	0.8913632	0.973988081
ZNF670	0.002865682	0.891566104	0.974113631
MTERFD1	-0.001581141	0.891667475	0.974128235
RPL11	-0.001053262	0.891835142	0.974215255
TSC2	0.0022531	0.892236628	0.974223641
LRCH4	0.001897271	0.892308742	0.974223641
ELP2	0.001498968	0.892537635	0.974223641
PTPRK	0.003975309	0.892577135	0.974223641
KIAA2026	-0.001900104	0.892693	0.974223641
SNORD95	-0.003071203	0.892741904	0.974223641
MEF2D	0.001901151	0.892845892	0.974223641
PANX2	0.003565503	0.89285059	0.974223641
ATP1A1	0.001352506	0.892917488	0.974223641
KATNAL1	-0.002824907	0.893201697	0.974223641
PLEKHN1	0.002456967	0.893256049	0.974223641
TTL	0.001623	0.89327757	0.974223641
RALGDS	-0.002225086	0.893285662	0.974223641
CIITA	0.003003283	0.893552979	0.974223641

DDAH2	0.002744079	0.893629183	0.974223641
USP24	-0.001578505	0.893676197	0.974223641
RGL4	0.002293485	0.893893728	0.974223641
DOHH	0.002213864	0.893913629	0.974223641
RPL24	-0.001027632	0.893942235	0.974223641
HTRA2	-0.002029908	0.89395059	0.974223641
WAC	-0.001808526	0.893959152	0.974223641
TTC33	0.002507952	0.893973018	0.974223641
KPTN	-0.002653314	0.894001694	0.974223641
CD3E	-0.003075602	0.894040211	0.974223641
MRTO4	-0.00195835	0.894095286	0.974223641
RFC3	0.00194984	0.894219477	0.974223641
ZMYND12	0.004655913	0.894253806	0.974223641
FCHO2	-0.004379813	0.894425942	0.974223641
SIK3	-0.001227294	0.894493764	0.974223641
C17orf97	-0.014709035	0.894515461	0.974223641
PDGFC	0.003992013	0.894571243	0.974223641
FKBP2	0.001562499	0.894768806	0.974307984
ESYT1	-0.001452514	0.894896956	0.974307984
APPL1	-0.001931472	0.894928381	0.974307984
BMPR1A	-0.00265978	0.895000775	0.974307984
NPTX1	0.004361669	0.895166064	0.974392089
MTMR9	-0.00141985	0.895585866	0.974727659
ZNF510	-0.002568054	0.895742104	0.974727659
SENP6	0.001783123	0.895775711	0.974727659
POLR1C	-0.00193664	0.895826585	0.974727659
CRLF3	-0.001867272	0.895983606	0.974802686
PLD4	-0.00227192	0.896226909	0.974971563
ZNF772	0.003325552	0.896451087	0.975025409
STAC3	0.002360613	0.896452578	0.975025409
BANK1	0.004963083	0.896777506	0.975165459
ULK1	-0.001439288	0.896843683	0.975165459
RCC2	-0.001190615	0.89684833	0.975165459
XCL1	0.004213819	0.896933737	0.975165459
BTN2A2	-0.003338983	0.897282284	0.975448596
LINS	-0.003313421	0.897581151	0.975677675
H3F3B	0.003362802	0.897976526	0.976011603
IGSF6	0.003772577	0.898178444	0.976135218
FCRL5	-0.005212598	0.898374831	0.976208829
PTER	0.010303238	0.898427957	0.976208829
POLB	-0.0011457	0.898510755	0.976208829
EPB41L4A	0.00228417	0.898820687	0.976229488
SPATA13	0.003651513	0.898865831	0.976229488
DCAF17	-0.002099738	0.898938267	0.976229488
STRN4	0.002379567	0.898993989	0.976229488
RNF103	0.002150828	0.899001907	0.976229488
HIST1H2BF	0.005513175	0.899058939	0.976229488
RPL3	9.88E-04	0.899229091	0.976232673
LAIR2	0.00816207	0.899238263	0.976232673
RRS1	-0.001303094	0.899470112	0.976287848

CBL	0.001863776	0.899514554	0.976287848
ZNF605	0.002336004	0.899601278	0.976287848
BFAR	-0.001739955	0.899699532	0.976287848
ATP11B	-0.001870134	0.899730088	0.976287848
ZNF559	0.00274588	0.899862028	0.976335305
FAM171A1	-0.002997741	0.899972391	0.976359345
CPAMD8	-0.002869971	0.900169249	0.976391639
CCNY	0.002085233	0.900197239	0.976391639
DNAH12	0.005691362	0.900318097	0.976391639
UBXN2A	0.001987792	0.900400903	0.976391639
OR56B1	0.002525411	0.900484269	0.976391639
AEN	0.001891643	0.90056512	0.976391639
GPBP1L1	0.002415944	0.900619626	0.976391639
LSM5	-0.001786386	0.901129175	0.97679248
VPS37B	0.001997315	0.901165851	0.97679248
RAB11FIP3	-0.001706743	0.901313931	0.976813893
TAP2	-0.00305211	0.901401329	0.976813893
ZNF766	-0.001826877	0.90145035	0.976813893
ATF7IP	-0.002352683	0.901571727	0.976813908
BCRP2	-0.002062258	0.901626858	0.976813908
APH1A	-0.001832993	0.902223781	0.977205239
SUZ12P	0.002906038	0.902226053	0.977205239
SNORD62A	0.004058796	0.902282008	0.977205239
KIF3A	-0.002818837	0.902457635	0.977205239
EIF2S2	0.002028411	0.902516064	0.977205239
MUM1	0.001847471	0.902517766	0.977205239
ZNF765	-0.002673838	0.902644475	0.977246841
HEY2	-0.003699165	0.902928567	0.977458808
GNLY	0.005163708	0.903210061	0.977596279
PRDM10	-0.001866529	0.903232193	0.977596279
C17orf59	0.002569573	0.903610015	0.977726919
PKIA	0.003086798	0.90361527	0.977726919
C9orf169	-0.001809002	0.903617886	0.977726919
CIC	0.001740629	0.903838767	0.977840094
DACT1	-0.005038193	0.903899164	0.977840094
TNFAIP3	-0.002692344	0.90399583	0.977841452
HCN3	-0.002551306	0.9040771	0.977841452
ATF6B	0.002310536	0.904271126	0.977925709
C3orf58	0.00177978	0.904331698	0.977925709
SCFD2	-0.002241018	0.904593947	0.978022098
UBQLN4	0.001539454	0.904597546	0.978022098
KISS1R	0.005044693	0.904912567	0.978242246
C12orf65	0.00163797	0.904977921	0.978242246
NDUFV2	0.001481641	0.905153552	0.978336556
SIGIRR	-0.002035662	0.905477409	0.978543757
C12orf75	-0.003778317	0.905522062	0.978543757
UAP1L1	-0.004186217	0.905618472	0.978552408
RNF146	0.002161881	0.905865118	0.978723377
TRPV2	0.002047351	0.906408177	0.979170103
KHDRBS1	0.001098955	0.906567216	0.979170103

RAMP2	-0.002290706	0.906667496	0.979170103
WDR11	-0.011226437	0.906929786	0.979170103
SYCP2	0.00333315	0.906939017	0.979170103
WIBG	0.001257608	0.906955394	0.979170103
CNPPD1	-0.001253123	0.906970171	0.979170103
BET1L	-0.002141413	0.907080457	0.979170103
ZCCHC17	-0.001884364	0.907153335	0.979170103
FGL1	0.001754945	0.907163195	0.979170103
CLN6	0.002444345	0.907259059	0.979178094
CBX5	-0.002668047	0.90762826	0.979481058
CCDC144B	0.004780178	0.9079163	0.979568107
ZNF181	-0.002445706	0.907956227	0.979568107
IL24	-0.002044567	0.908129643	0.979568107
AMD1	-0.001394402	0.908138507	0.979568107
SMC2	0.00224452	0.908151406	0.979568107
SKA3	-0.001752961	0.908330004	0.979601844
PLCG1	0.001934142	0.908398708	0.979601844
TRMT1L	-0.0016052	0.908456689	0.979601844
GMIP	0.002006586	0.908574595	0.979601844
FUT7	-0.002865524	0.908625181	0.979601844
LCK	0.001836581	0.908746447	0.979633698
MDK	-0.002073403	0.908847193	0.979633698
RASSF1	-0.002277854	0.90898597	0.979633698
FAM138F	0.002389654	0.909023406	0.979633698
TMOD2	0.002433015	0.909186038	0.979633698
VPS29	-0.001094853	0.909239641	0.979633698
ZNF761	0.002250802	0.909304473	0.979633698
LSM2	-0.001294614	0.909456062	0.979633698
GNPDA1	-0.0015598	0.90952584	0.979633698
OAZ3	0.002433875	0.909539752	0.979633698
RBL2	-0.00199365	0.9097653	0.97968832
PITHD1	-0.001456649	0.90976748	0.97968832
SLC25A10	-0.002296173	0.910011997	0.979759206
ARIH2	-0.001359705	0.910167673	0.979759206
NCR1	-0.003194167	0.910304935	0.979759206
RRNAD1	0.00167376	0.910421166	0.979759206
TACC1	-8.15E-04	0.910514401	0.979759206
TFDP2	-0.00207129	0.910540137	0.979759206
ZNF12	-0.002107617	0.910683635	0.979759206
USP25	0.002096908	0.910738751	0.979759206
L3MBTL3	-0.0023036	0.910779503	0.979759206
FAM195A	-0.002260533	0.910804136	0.979759206
CD300LB	0.002482382	0.91089134	0.979759206
MNT	0.001109953	0.910921445	0.979759206
DNA2	0.002175433	0.911055451	0.979759206
PRKRA	-0.001290154	0.911126387	0.979759206
MAP3K12	0.001702346	0.911208034	0.979759206
C15orf38	-0.004783593	0.911310877	0.979759206
DLL1	-0.004398645	0.911390198	0.979759206
MAP3K3	0.001805527	0.911426556	0.979759206

STARD3	-0.001647898	0.911720718	0.979780757
ADAL	-0.00191083	0.91172662	0.979780757
NAIP	-0.003981338	0.911849058	0.979780757
GCC2	-0.002561922	0.91187451	0.979780757
ZCCHC3	-0.002005959	0.912035037	0.979780757
TNFAIP8L1	-0.00274531	0.912115526	0.979780757
MAP1LC3A	-0.00225942	0.912119717	0.979780757
ITIH4	-0.004201993	0.91215473	0.979780757
DAG1	0.001576822	0.912393535	0.979903693
E2F5	0.003276738	0.912446234	0.979903693
C3orf18	0.001710123	0.912550394	0.97992048
ISCA1	-0.001777373	0.912710789	0.979997645
PTGER2	0.002368278	0.912819028	0.980018799
C9orf41	-0.002398542	0.912948386	0.98004919
SLC25A20	0.002203543	0.913024415	0.98004919
SNRPB2	-9.87E-04	0.91325645	0.980061114
ABCB8	0.002501781	0.913312242	0.980061114
MAP3K2	0.00287762	0.913327734	0.980061114
TPST2	0.002170363	0.913472428	0.980061114
ISYNA1	-0.001875802	0.913537781	0.980061114
ZNF333	-0.002021656	0.913660966	0.980061114
RASA2	0.002806127	0.913794936	0.980061114
KANK3	-0.001975354	0.913823156	0.980061114
TRIM69	-0.002373375	0.91394753	0.980061114
WDHD1	0.002045877	0.913961955	0.980061114
EPHB6	-0.00250733	0.914009475	0.980061114
C17orf67	0.003121419	0.914110507	0.980074506
C19orf33	0.007413483	0.9142694	0.980149926
AQP1	0.002063593	0.9145595	0.980246617
EDEM2	-0.001114074	0.914608056	0.980246617
TPRA1	0.00177544	0.914710329	0.980246617
KIAA0895L	0.002229793	0.914713823	0.980246617
HSD17B8	-0.001845276	0.915163665	0.980562097
ACADVL	-0.00141397	0.915185385	0.980562097
PCK2	-0.001472436	0.915285139	0.980574061
ANKRD22	-0.004304685	0.915583155	0.980704645
TNFSF8	0.002563069	0.915616483	0.980704645
ECT2L	-0.001933513	0.915672826	0.980704645
ZNF331	-0.001733411	0.916068857	0.980933274
STX3	0.001603903	0.916128737	0.980933274
ST6GAL1	0.001419392	0.916230302	0.980933274
DHRS1	-0.001353164	0.916282522	0.980933274
RELB	0.001736821	0.916381907	0.980933274
TLR10	0.003642707	0.916591011	0.980933274
INSL5	0.002219192	0.916638475	0.980933274
IFT172	0.001793588	0.916769978	0.980933274
FOXRED2	-0.003440627	0.916811625	0.980933274
NOSIP	-0.001791631	0.916862491	0.980933274
GPANK1	-0.00239674	0.916978995	0.980933274
SCD	-0.002647053	0.917084761	0.980933274

APAF1	0.002183559	0.917119842	0.980933274
TGDS	-0.001959167	0.917126972	0.980933274
HLA-F	-0.00172564	0.917261226	0.980970003
CCT8L2	-0.001890517	0.917338558	0.980970003
TRIM11	-0.001452988	0.917514705	0.981019119
NRIP1	-0.001890002	0.917577554	0.981019119
ZNF211	-0.00141273	0.917650371	0.981019119
LDHC	-0.004767597	0.917899681	0.981190881
LOXHD1	0.002083514	0.918112864	0.981323995
COQ9	9.09E-04	0.918238141	0.981363134
UCP3	0.002079609	0.918742828	0.98180772
ANKIB1	9.87E-04	0.918911812	0.981809629
SMARCA4	-8.45E-04	0.918922013	0.981809629
SRSF2	6.37E-04	0.919165586	0.981881396
ROM1	0.001578829	0.919257499	0.981881396
PBRM1	-0.001659592	0.919515321	0.981881396
STEAP3	-0.003624773	0.919519649	0.981881396
AFF3	0.002874014	0.919659557	0.981881396
SNORD8	-0.00252883	0.919845113	0.981881396
PINK1	0.001363515	0.920051291	0.981881396
DPY19L2P4	-0.001936881	0.92010058	0.981881396
SLC1A7	0.00495476	0.920159325	0.981881396
KCMF1	0.001450411	0.920160287	0.981881396
TNFRSF10B	-0.001303481	0.920217147	0.981881396
DKK3	-0.00575357	0.920291149	0.981881396
HMHA1	-0.001480993	0.920318256	0.981881396
MCM5	0.001289937	0.920334683	0.981881396
DENND2D	0.001813779	0.920366324	0.981881396
CRYZL1	-0.001543351	0.92041323	0.981881396
AGPAT1	-0.002517176	0.920497177	0.981881396
C20orf197	0.002523287	0.92080534	0.982056999
ANKRD40	-0.001205796	0.920839244	0.982056999
TMEM175	-0.001127833	0.921049108	0.982186183
RPS19	5.13E-04	0.921456453	0.982525911
ZNF286B	0.002514302	0.921769822	0.982680509
PPM1B	0.001642286	0.921864824	0.982680509
TPCN1	-0.001854932	0.921867775	0.982680509
ADAT1	0.001679154	0.922065564	0.982796699
IL32	0.00280773	0.922297486	0.982889494
ATL1	0.002741818	0.922330217	0.982889494
STOML1	0.001994046	0.922673426	0.982936226
SETDB2	9.71E-04	0.922729953	0.982936226
SIGLEC16	0.003854671	0.922795624	0.982936226
TOP1	-0.001508995	0.922804225	0.982936226
ZNF48	0.001799297	0.922876277	0.982936226
C11orf48	-0.001339792	0.922906875	0.982936226
KEAP1	-0.00137577	0.923357141	0.982957857
ZBTB11	0.001817061	0.923406208	0.982957857
RNF219	-0.001283879	0.923626747	0.982957857
ART1	0.001750836	0.923904479	0.982957857

KCNMA1	-0.002899141	0.92418983	0.982957857
BEND3	-0.001940976	0.92433088	0.982957857
DUXA	0.004139409	0.924592421	0.982957857
LYAR	0.00226433	0.924656007	0.982957857
TBC1D17	-0.001818097	0.92465902	0.982957857
FN3KRP	-0.001982801	0.924690428	0.982957857
COX11	-0.00244223	0.924776837	0.982957857
SIM2	-0.00179942	0.924884032	0.982957857
MT1JP	-0.002569183	0.925033139	0.982957857
SLAMF6	-0.001627215	0.925069524	0.982957857
DEPDC5	0.001653643	0.925084531	0.982957857
ZNF638	0.001401759	0.925161938	0.982957857
TRIO	0.001496853	0.925324808	0.982957857
C1orf131	-0.001087101	0.92533778	0.982957857
SDR16C5	-0.002209277	0.925375818	0.982957857
CENPV	-0.002171663	0.925422503	0.982957857
CELSR3	-0.002970739	0.925451893	0.982957857
OCLN	-0.004757676	0.92551362	0.982957857
MCM4	-0.002057185	0.925531125	0.982957857
GTPBP2	0.001732085	0.925532548	0.982957857
PRPSAP2	-9.47E-04	0.925535184	0.982957857
ZNF10	0.001389629	0.925589274	0.982957857
NOMO1	-0.002603056	0.925620566	0.982957857
DYNLL2	-0.001680725	0.925792142	0.982957857
MSH2	-0.001867579	0.925943145	0.982957857
SHARPIN	-0.001683941	0.925999723	0.982957857
RAB21	-9.16E-04	0.926062435	0.982957857
CREB3	0.002340906	0.926067076	0.982957857
ZMYND19	-0.001088455	0.926077664	0.982957857
PUS1	-9.50E-04	0.926082457	0.982957857
NPHP4	0.001745244	0.926135714	0.982957857
BNIPL	0.002063528	0.926159198	0.982957857
MRPL28	0.001295124	0.926212887	0.982957857
ITSN1	0.002515858	0.9263933	0.982959857
JUNB	0.002853873	0.926426663	0.982959857
ANGPT1	0.003107287	0.926481181	0.982959857
SMURF1	0.001740718	0.926776447	0.983178886
ABCC2	-0.001384145	0.926937959	0.983181769
RFXANK	0.001009544	0.926975133	0.983181769
STAT1	-0.002833482	0.927045634	0.983181769
ENO1-AS1	0.002056751	0.927260593	0.983253511
ARNT	-0.001422862	0.927372381	0.983253511
CYB5B	-8.96E-04	0.927390906	0.983253511
TMEM173	-9.31E-04	0.927581845	0.983253511
SEC22A	0.001279942	0.927724078	0.983253511
MAFG	0.001445783	0.927734148	0.983253511
APCDD1	-0.002187358	0.927735086	0.983253511
TRIM64B	0.001532253	0.928070199	0.983514507
RAB8A	-9.26E-04	0.928286922	0.983610407
CXCL3	-0.001943217	0.928377022	0.983610407

MATR3	-7.90E-04	0.928495178	0.983610407
NOMO3	0.001689547	0.928516139	0.983610407
ZNF337	0.001280221	0.928693796	0.983704462
SLC25A15	-0.001150022	0.928943621	0.983826859
C18orf32	-0.001672121	0.929034403	0.983826859
ECH1	-9.88E-04	0.929147267	0.983826859
PKP3	-0.001434913	0.929281927	0.983826859
SLC14A1	0.002680604	0.929389527	0.983826859
NXT1	-9.19E-04	0.929557956	0.983826859
TMUB1	-0.001208447	0.929725177	0.983826859
NIT1	0.001188786	0.929730369	0.983826859
ZNF470	-0.001777214	0.929883364	0.983826859
MED13	0.001822484	0.930026238	0.983826859
PIP5K1B	-0.001846505	0.930363701	0.983826859
SNORD4B	-0.002196619	0.93040357	0.983826859
ASPM	0.003121887	0.930499226	0.983826859
SLC29A4	-0.001825414	0.930667477	0.983826859
ZNF101	0.002283988	0.930752777	0.983826859
DTX2	-9.66E-04	0.930795866	0.983826859
SEL1L3	0.001641351	0.930882968	0.983826859
KCNAB2	-0.001610418	0.930918172	0.983826859
AIG1	0.001441122	0.931013703	0.983826859
ACADSB	0.001281446	0.931023605	0.983826859
POTEM	-0.002300394	0.931077735	0.983826859
ETS1	-0.001074251	0.93111314	0.983826859
ZWINT	-0.00187149	0.931122278	0.983826859
PBX3	0.001141076	0.931129855	0.983826859
EIF3G	-9.84E-04	0.931142104	0.983826859
GSTA1	-0.005172052	0.931173669	0.983826859
CMTM8	0.003914083	0.931225992	0.983826859
RLN2	-0.002548968	0.931298024	0.983826859
RFX1	-0.001571423	0.931658792	0.984056048
HEG1	-0.001550181	0.931794516	0.984056048
SH2D1B	0.004367624	0.931848032	0.984056048
HIST2H4B	0.002981953	0.931870584	0.984056048
CCDC146	0.002403804	0.932118898	0.984083797
FGA	-0.001367411	0.932218912	0.984083797
CNN3	-0.002660952	0.932334585	0.984083797
EEF1D	-0.001115826	0.932390982	0.984083797
RCCD1	0.002662869	0.932436809	0.984083797
SLC25A13	-0.002539167	0.932455392	0.984083797
BMI1	-9.55E-04	0.932519193	0.984083797
COQ6	-0.001037704	0.93260985	0.984085647
RHOA	-9.28E-04	0.932788707	0.984095568
PRICKLE2	-0.001892634	0.932797064	0.984095568
THAP10	-0.001554667	0.9329289	0.984140856
ITGAD	-0.004033451	0.933112579	0.984240817
BMF	0.001336901	0.933239209	0.984280591
BLOC1S2	-0.001637103	0.933843425	0.984768887
NDUFS3	-5.39E-04	0.933880116	0.984768887

CCDC17	0.001601336	0.934050602	0.98482226
TM6SF1	0.002191876	0.934235696	0.98482226
MDM2	-0.001355757	0.934256634	0.98482226
PLB1	0.001652724	0.934286616	0.98482226
COMMD4	-0.001152191	0.934435925	0.984885855
PTPN4	0.001541434	0.934606782	0.984972148
GTF3A	-8.85E-04	0.934783922	0.984978141
PLEKHG7	0.001476415	0.934874886	0.984978141
DROSHA	-9.72E-04	0.934879424	0.984978141
SNRNP200	0.001232162	0.935551462	0.985452177
MRPL55	-9.06E-04	0.935569394	0.985452177
HMG20A	0.001315018	0.935649121	0.985452177
TMEM2	0.001415749	0.935685462	0.985452177
LYRM7	-9.88E-04	0.93605628	0.98546321
PDPK1	-0.001079884	0.936064311	0.98546321
RAB33B	0.001419989	0.936117716	0.98546321
FRYL	-0.001039645	0.936190416	0.98546321
OSBPL8	0.001171914	0.936201053	0.98546321
YIF1B	0.001038475	0.936308116	0.98546321
RANBP9	-0.00145626	0.936319142	0.98546321
FUT8	0.001925288	0.936518023	0.985578817
ZFP82	0.002058302	0.936723266	0.985701097
AP1S1	-0.001644416	0.937327655	0.985993548
SGK3	-0.001551451	0.937424776	0.985993548
CARD8	-0.001683269	0.937509437	0.985993548
WIPF1	0.001013542	0.937645857	0.985993548
FASTKD2	-0.001116595	0.937750431	0.985993548
WDR53	9.69E-04	0.937755144	0.985993548
MMS22L	0.001320433	0.937775897	0.985993548
ZCCHC6	0.001488779	0.937790318	0.985993548
ZFP37	0.002173627	0.937872975	0.985993548
C10orf105	0.001595035	0.937891956	0.985993548
MED23	-0.001274278	0.938328369	0.986213996
DDT	-0.001391113	0.938450888	0.986213996
ASB1	0.001108607	0.938515668	0.986213996
C14orf2	-0.001214387	0.938516706	0.986213996
LZTR1	1.00E-03	0.938547134	0.986213996
ZNHIT2	-0.001193842	0.938894606	0.986220375
MSTO1	-9.85E-04	0.938910762	0.986220375
PAFAH1B1	-9.02E-04	0.939023074	0.986220375
LEAP2	0.001340907	0.939125767	0.986220375
NPEPPS	7.57E-04	0.939325884	0.986220375
TMEM159	-0.001193119	0.939385443	0.986220375
MEST	-0.001336501	0.939457552	0.986220375
C19orf70	9.51E-04	0.939591038	0.986220375
PIGN	0.001337993	0.939672401	0.986220375
PLEKHM1	-9.16E-04	0.939685992	0.986220375
METAP2	-8.23E-04	0.93969951	0.986220375
TMEM168	-0.001320239	0.939750308	0.986220375
REEP4	-0.001382144	0.939752418	0.986220375

HMGA1	0.001544431	0.939970348	0.986220375
CEP152	-0.00132727	0.94003645	0.986220375
CDKAL1	0.001637531	0.940040784	0.986220375
SYTL1	0.001168816	0.940067863	0.986220375
CWF19L2	-0.001021469	0.94021784	0.986284237
DNAJB2	9.73E-04	0.940463422	0.986365023
MKS1	0.00117776	0.940473074	0.986365023
CEACAM21	-0.001644671	0.940795844	0.98654917
UHRF1BP1	-0.001349178	0.941086913	0.98654917
RPUSD4	-8.82E-04	0.941104651	0.98654917
OGFOD1	-7.86E-04	0.941276782	0.98654917
NOL3	-0.001472144	0.941471931	0.98654917
TECR	0.0010537	0.941596996	0.98654917
PYGL	-0.001467592	0.941604821	0.98654917
ARMC2	0.001100339	0.941670907	0.98654917
EMX2	-0.001347307	0.941778461	0.98654917
C2CD2	0.001294847	0.941891717	0.98654917
FAM43A	0.002624472	0.942002876	0.98654917
POT1	-0.001330081	0.942041005	0.98654917
ABCC3	0.003465773	0.942045206	0.98654917
C1orf74	-0.001720019	0.942364574	0.98654917
SF3B5	8.54E-04	0.942380364	0.98654917
CYB5D1	0.001472785	0.94239475	0.98654917
CNKSR3	-0.001179071	0.942434249	0.98654917
TIPARP	0.001037388	0.942452666	0.98654917
PPP1R12A	9.63E-04	0.942459403	0.98654917
IBTK	-9.00E-04	0.942495462	0.98654917
FKBP9L	0.002054204	0.942520325	0.98654917
VRK2	-0.001430583	0.942616657	0.986556711
MAP3K8	0.001484248	0.942761204	0.986591375
TDRD3	0.001153485	0.942828039	0.986591375
ZSWIM5	0.001765334	0.942997999	0.986675948
RGS2	0.001458645	0.943152936	0.986694647
ODF2	9.54E-04	0.943194152	0.986694647
SRSF3	7.08E-04	0.943336132	0.98674992
LPAR5	-0.001975494	0.943580179	0.986911934
HARS	-7.95E-04	0.943709193	0.986953614
TXLNA	-7.12E-04	0.944217312	0.987313198
C1orf116	0.002323263	0.944261507	0.987313198
TSSC1	8.79E-04	0.944332642	0.987313198
RFWD2	-5.38E-04	0.944470764	0.987313198
YTHDF3	7.73E-04	0.944579837	0.987313198
LTK	-0.001488306	0.944614991	0.987313198
CUL3	-0.001513123	0.944677395	0.987313198
ZNF775	-0.001164417	0.944849015	0.987399334
LIN37	-9.94E-04	0.945003962	0.987408863
KIR3DL2	0.003705199	0.945036543	0.987408863
SCARNA4	0.001489673	0.945207126	0.987493882
C5orf51	9.10E-04	0.945479664	0.987652123
MC1R	-0.002072331	0.945537044	0.987652123

FOLR3	-0.005630338	0.945725834	0.987678985
ALOX5AP	-0.001161527	0.94574122	0.987678985
RBM28	-9.30E-04	0.946052794	0.98772233
ANAPC7	0.001119679	0.946247857	0.98772233
HIP1R	0.001213012	0.946480489	0.98772233
CDK5R1	-0.002403587	0.946533329	0.98772233
TMTC1	-0.00375167	0.946570594	0.98772233
FAM41C	0.001356128	0.946620561	0.98772233
SUPT3H	0.001590993	0.946797376	0.98772233
IL27RA	0.001115501	0.947105572	0.98772233
KCTD17	-0.001062811	0.947192813	0.98772233
DPCD	-0.001357585	0.947248059	0.98772233
CORO2A	0.001297783	0.947331921	0.98772233
ACBD5	0.001347962	0.947349984	0.98772233
RHBDD2	9.72E-04	0.947394778	0.98772233
GSS	8.27E-04	0.947448468	0.98772233
ACSS3	-0.001302623	0.947487423	0.98772233
ARFGAP1	0.001365865	0.947509748	0.98772233
SHB	9.40E-04	0.947630944	0.98772233
DPY19L2P2	0.00117269	0.947759029	0.98772233
OSCAR	-0.001604812	0.947774284	0.98772233
STK10	-8.76E-04	0.947817955	0.98772233
TMEM170B	0.001326826	0.947840308	0.98772233
PLS1	0.001318008	0.94795742	0.98772233
KPNA6	8.75E-04	0.948118916	0.98772233
CARS	-0.001146407	0.94814792	0.98772233
PMS2	0.001217982	0.94822737	0.98772233
PTS	6.97E-04	0.948324903	0.98772233
TMEM217	-0.001272423	0.948342473	0.98772233
GNB1L	-0.001031374	0.948407851	0.98772233
SMARCD3	0.001172061	0.948537338	0.98772233
MINK1	-0.001387174	0.948539095	0.98772233
HLA-DQB1	0.002560787	0.948595869	0.98772233
POLR3G	-0.001997338	0.948638187	0.98772233
ACTN1	0.001537236	0.948819281	0.98774607
MAPRE3	0.00108641	0.948839458	0.98774607
CALM1	6.70E-04	0.948967278	0.987751279
STX18	-0.001100775	0.949022934	0.987751279
THUMPD1	-6.64E-04	0.949289375	0.987757949
ARHGAP12	-9.40E-04	0.949401919	0.987757949
MAP2K2	0.001478018	0.949535873	0.987757949
PPP1R11	-5.15E-04	0.949653352	0.987757949
ZNF568	-0.001113688	0.949679516	0.987757949
PIK3C3	8.09E-04	0.949730262	0.987757949
NUBP2	0.001203817	0.949862211	0.987757949
EBPL	-0.001719766	0.949915635	0.987757949
STX8	9.69E-04	0.949968908	0.987757949
GPRC5C	-0.002509038	0.949974735	0.987757949
RPS3A	7.11E-04	0.950010942	0.987757949
CAMK2G	-6.34E-04	0.950167534	0.987809891

XPC	-6.98E-04	0.950239382	0.987809891
ARIH1	7.54E-04	0.950412479	0.987816795
ELOVL5	-0.001226252	0.950424507	0.987816795
SLAMF1	0.001321647	0.950803842	0.988054072
KNCN	-0.00132019	0.951044562	0.988054072
SLC47A1	-0.001788436	0.951060965	0.988054072
TIMM13	-0.001328657	0.951165903	0.988054072
GFRA2	-0.002432352	0.951170127	0.988054072
CCNJL	-0.002205466	0.951188381	0.988054072
MYL5	0.001709149	0.951510676	0.988296112
DEF8	-0.001055058	0.951706273	0.988358464
CTRL	0.001133063	0.951749287	0.988358464
C1GALT1	0.001182363	0.951988835	0.988514486
CCDC23	0.001016066	0.952109921	0.988547483
HDAC2	-4.75E-04	0.952346765	0.988608386
KCNMB2	-0.001257718	0.952347206	0.988608386
LAMA5	0.002262651	0.952472899	0.988646147
THRAP3	8.96E-04	0.953240428	0.989350051
WDR35	8.83E-04	0.953483932	0.989509998
MICALCL	-0.00144917	0.953589968	0.989527266
UFM1	7.68E-04	0.953719739	0.989569159
PRDM4	6.48E-04	0.953974057	0.98974026
COPA	5.47E-04	0.954129959	0.989809233
CEP55	-0.001817784	0.954510554	0.990094765
EXOSC9	8.56E-04	0.954584093	0.990094765
OPRL1	-8.42E-04	0.95473103	0.990154387
GALNS	9.61E-04	0.954852528	0.990158373
TIMM9	-8.81E-04	0.954980717	0.990158373
AK3	-9.09E-04	0.955003234	0.990158373
EBF1	0.002667599	0.955180767	0.990249687
DHX34	-8.71E-04	0.955367014	0.990284487
DALRD3	-8.36E-04	0.955469032	0.990284487
DHX8	8.04E-04	0.955523701	0.990284487
MPPED2	-0.00125348	0.955572193	0.990284487
TXN2	0.001145299	0.955740415	0.990286105
TTC30A	0.001001884	0.955752684	0.990286105
MUC16	-9.51E-04	0.956486577	0.99079511
TMPRSS3	0.001311488	0.956512285	0.99079511
SNX21	-9.96E-04	0.956543775	0.99079511
C12orf45	-0.001036322	0.956718542	0.99079511
BTN3A3	8.50E-04	0.956744547	0.99079511
PDGFA	0.001647366	0.957148543	0.99079511
FAM170B	0.001506526	0.95720611	0.99079511
KIAA1522	-0.001116074	0.957240649	0.99079511
FEN1	0.001053054	0.957404258	0.99079511
PLEKHF2	-0.001285228	0.957679653	0.99079511
SYNJ1	-7.00E-04	0.957722525	0.99079511
CHD9	-7.67E-04	0.957815769	0.99079511
RNPS1	8.19E-04	0.957919945	0.99079511
SUSD3	9.86E-04	0.957999065	0.99079511

MT1H	-0.001626249	0.958039432	0.99079511
MAEA	5.60E-04	0.95812154	0.99079511
CHMP4C	8.20E-04	0.958289251	0.99079511
TMIGD2	0.00145685	0.958399346	0.99079511
ACAT1	8.10E-04	0.958568011	0.99079511
TESK1	-8.25E-04	0.958648039	0.99079511
NLRP12	0.001176408	0.95867736	0.99079511
PNPLA7	0.00155324	0.958722641	0.99079511
TDG	4.92E-04	0.958763953	0.99079511
ZNF75A	9.54E-04	0.958803583	0.99079511
MYL4	0.005484199	0.958861114	0.99079511
TTC1	5.14E-04	0.95894108	0.99079511
B3GALT6	0.001034221	0.958994721	0.99079511
LSG1	0.001077798	0.959024424	0.99079511
DBNDD2	-8.52E-04	0.959082166	0.99079511
NPL	-0.001309	0.959099736	0.99079511
CFB	-0.001219128	0.959104288	0.99079511
NEURL4	0.001082758	0.959410217	0.99079511
CREBZF	7.65E-04	0.959458713	0.99079511
RASL11A	9.74E-04	0.959484424	0.99079511
JAKMIP3	0.001075465	0.959546287	0.99079511
UBTF	9.47E-04	0.959720047	0.99079511
SCRIB	5.90E-04	0.959772073	0.99079511
CYTH2	0.001330695	0.959866497	0.99079511
AP1AR	0.001143202	0.959888598	0.99079511
BYSL	0.001279119	0.959910261	0.99079511
ZCCHC8	6.21E-04	0.959913882	0.99079511
C12orf60	9.96E-04	0.960024314	0.990802609
SLC35F2	0.001080888	0.960179748	0.990802609
FARSA	-8.50E-04	0.960261093	0.990802609
ZNF792	-8.79E-04	0.960394789	0.990802609
DEDD	4.90E-04	0.960411813	0.990802609
THADA	6.78E-04	0.960458216	0.990802609
PTPN3	7.24E-04	0.96095757	0.99121762
TRIM9	-8.95E-04	0.961089131	0.99121762
GTSE1	8.88E-04	0.961145234	0.99121762
NOP14	6.43E-04	0.961365866	0.99121762
GLS	-8.61E-04	0.961382408	0.99121762
KIAA0753	7.31E-04	0.961469886	0.99121762
IDI1	7.69E-04	0.961590496	0.99121762
CMTM4	0.001165418	0.961627818	0.99121762
SPCS3	8.98E-04	0.961722369	0.99121762
GMPPA	-7.40E-04	0.961756007	0.99121762
AHCYL1	4.37E-04	0.962183244	0.991271074
SAP30BP	7.59E-04	0.962239205	0.991271074
CASP9	-6.48E-04	0.962250885	0.991271074
LOXL3	9.80E-04	0.96226913	0.991271074
BRD1	-6.72E-04	0.962278536	0.991271074
GPR108	6.99E-04	0.962377332	0.991271074
FAM160B1	5.85E-04	0.962457901	0.991271074

RIT1	7.41E-04	0.96255581	0.991271074
MAPK9	7.39E-04	0.962613856	0.991271074
FCHSD1	8.67E-04	0.962914981	0.991487906
PDE1B	9.35E-04	0.963003567	0.991487906
RPL13AP5	-2.71E-04	0.963237879	0.991600812
POLM	8.74E-04	0.963355173	0.991600812
DND1	-0.001015516	0.963456808	0.991600812
ZNF679	-0.001668201	0.96354314	0.991600812
CTDNEP1	6.26E-04	0.963636648	0.991600812
IDUA	-8.80E-04	0.963673983	0.991600812
CPOX	5.93E-04	0.963829533	0.991600812
BRD7	-6.03E-04	0.963829897	0.991600812
ANKRD37	7.86E-04	0.963943212	0.991625224
CLEC18C	-0.001023764	0.964067426	0.991660844
BAHD1	8.96E-04	0.964193051	0.991687182
RBM12B	-0.001011703	0.964348006	0.991687182
NAT14	-7.47E-04	0.964456691	0.991687182
SLC17A3	-8.74E-04	0.964511545	0.991687182
RCAN3	9.72E-04	0.964593773	0.991687182
SELPLG	0.001386354	0.96463058	0.991687182
SEZ6L2	-8.62E-04	0.965288476	0.99217229
DGUOK	-4.93E-04	0.965339586	0.99217229
RPS6KA1	6.14E-04	0.965393353	0.99217229
PLEKHO2	-8.31E-04	0.965460993	0.99217229
TMEM223	6.11E-04	0.96615113	0.992560127
PPP1CA	-6.22E-04	0.966161436	0.992560127
HNRNPD	2.92E-04	0.966331467	0.992560127
SGCE	-0.001538695	0.966653463	0.992560127
CAST	7.51E-04	0.966658342	0.992560127
ZDHHC4	-5.67E-04	0.966658757	0.992560127
TOMM22	-3.66E-04	0.966688811	0.992560127
CLEC2D	-5.89E-04	0.966689091	0.992560127
KLHL14	0.0019093	0.966724222	0.992560127
RAP1GAP	-6.02E-04	0.966735092	0.992560127
CDK4	-3.82E-04	0.966968409	0.992560191
ATP2A2	4.91E-04	0.967104699	0.992560191
SLC37A1	5.83E-04	0.967200892	0.992560191
PARM1	0.001451954	0.967203563	0.992560191
PLRG1	-3.53E-04	0.96737043	0.992560191
USP8	-5.27E-04	0.967462295	0.992560191
MCM3	-4.27E-04	0.967538941	0.992560191
HINT2	3.78E-04	0.967789222	0.992560191
ALG6	4.41E-04	0.967986727	0.992560191
ZNF232	-6.42E-04	0.968139433	0.992560191
SRBD1	-5.04E-04	0.968231179	0.992560191
NDUFS1	-8.36E-04	0.968243103	0.992560191
NUPL1	-6.63E-04	0.968356673	0.992560191
CD6	6.40E-04	0.968381377	0.992560191
SH3BGR	-8.08E-04	0.968474383	0.992560191
PSG6	9.79E-04	0.968595147	0.992560191

CCL5	9.12E-04	0.968656476	0.992560191
THOP1	-5.81E-04	0.968683602	0.992560191
ECM1	8.79E-04	0.968769826	0.992560191
LEF1	-9.43E-04	0.968777584	0.992560191
SH3YL1	-0.001201398	0.96880967	0.992560191
USP22	6.34E-04	0.968872051	0.992560191
PRR14	3.78E-04	0.969026712	0.992560191
NIPSNAP1	-5.74E-04	0.969102801	0.992560191
SLC1A4	9.36E-04	0.969257067	0.992560191
GRIP2	-7.91E-04	0.969349453	0.992560191
CEP78	0.001022976	0.969388956	0.992560191
ELOVL6	0.001389569	0.969602839	0.992560191
CARD17	-9.44E-04	0.969628546	0.992560191
MED26	5.58E-04	0.969643764	0.992560191
PHF7	6.88E-04	0.969687549	0.992560191
RABL3	7.23E-04	0.969703605	0.992560191
SLC39A8	0.00282093	0.969843505	0.992560191
TGM2	-9.12E-04	0.969859406	0.992560191
LEPREL4	6.81E-04	0.969958789	0.992560191
PPP1R16A	7.90E-04	0.970038039	0.992560191
RAI14	8.82E-04	0.970091743	0.992560191
FAM27E3	-8.66E-04	0.970195713	0.992560191
C6orf62	4.79E-04	0.970304735	0.992560191
CLEC2L	8.98E-04	0.970321965	0.992560191
SCARNA16	0.001369611	0.970712255	0.992780356
VGLL4	-5.28E-04	0.970716577	0.992780356
C5	6.78E-04	0.971017816	0.992850465
GOLGA6L1	-2.40E-04	0.971101602	0.992850465
ZNF573	-0.001066389	0.971120266	0.992850465
FOXM1	6.16E-04	0.971207556	0.992850465
C1D	6.59E-04	0.971416818	0.992850465
STARD10	-7.32E-04	0.971662872	0.992850465
STK3	6.58E-04	0.971678275	0.992850465
NPR1	7.01E-04	0.971752192	0.992850465
IQSEC1	4.25E-04	0.971776528	0.992850465
HSPA4	3.85E-04	0.971823048	0.992850465
SLC25A16	6.97E-04	0.97187428	0.992850465
SIPA1L1	-6.11E-04	0.971898671	0.992850465
PRRG2	-7.81E-04	0.972124704	0.992850465
KCNMB4	0.001078724	0.972160988	0.992850465
ANXA8	8.52E-04	0.972183059	0.992850465
UBQLNL	8.35E-04	0.972320369	0.992850465
TOX4	4.71E-04	0.972363387	0.992850465
ADAMTS1	-0.001512996	0.972399665	0.992850465
PRKCQ	-5.73E-04	0.972574807	0.992915983
IRAK4	-4.62E-04	0.972720016	0.992915983
GLCCI1	-7.08E-04	0.97273294	0.992915983
MEG3	0.003064897	0.97288958	0.992984303
INSIG1	0.000516993	0.973000041	0.993005224
ZC3H12A	-5.84E-04	0.973409166	0.993005224

LYRM5	-3.99E-04	0.97358167	0.993005224
ZNF16	7.26E-04	0.973776838	0.993005224
UBE2M	-3.28E-04	0.973788231	0.993005224
NDRG2	6.86E-04	0.973860742	0.993005224
RASAL3	3.88E-04	0.973892814	0.993005224
TIGD7	5.90E-04	0.973912956	0.993005224
IQCB1	5.04E-04	0.974052865	0.993005224
B4GALT2	6.11E-04	0.974108403	0.993005224
RASSF7	-3.39E-04	0.974163657	0.993005224
RAP1GDS1	5.11E-04	0.974322087	0.993005224
FRS3	-4.44E-04	0.974372194	0.993005224
MYO6	6.68E-04	0.974492079	0.993005224
LLGL2	-6.83E-04	0.974502136	0.993005224
VAMP5	-6.72E-04	0.97456644	0.993005224
RAD50	-5.90E-04	0.974848368	0.993005224
RFC2	-7.08E-04	0.97485281	0.993005224
LRRC6	0.001393358	0.975011049	0.993005224
PHTF2	5.78E-04	0.975022279	0.993005224
RAB23	-9.27E-04	0.975105606	0.993005224
ELP3	-3.92E-04	0.975344973	0.993005224
TOR1B	-4.37E-04	0.975358062	0.993005224
PUM2	2.67E-04	0.975445365	0.993005224
GRK5	4.88E-04	0.975494619	0.993005224
FAM174B	-8.11E-04	0.975543841	0.993005224
ZNF565	5.36E-04	0.975567774	0.993005224
ERGIC3	3.82E-04	0.975608992	0.993005224
TNIK	6.63E-04	0.975611934	0.993005224
RPF2	4.18E-04	0.975622889	0.993005224
BMP1	-6.32E-04	0.975785136	0.993005224
GYPC	5.28E-04	0.97580629	0.993005224
SLC38A7	-6.61E-04	0.975884015	0.993005224
CCR1	8.21E-04	0.975960234	0.993005224
FCRL2	-0.001391508	0.976149579	0.993033757
MRPL21	-4.87E-04	0.976300171	0.993033757
MEMO1	-3.71E-04	0.976368742	0.993033757
SLC30A9	-3.37E-04	0.976425832	0.993033757
ARL4D	-4.82E-04	0.976436842	0.993033757
MRPL43	3.91E-04	0.976646519	0.993079507
MIR574	-5.42E-04	0.976743853	0.993079507
UBE2Q2	4.35E-04	0.97675098	0.993079507
M6PR	4.47E-04	0.976884668	0.99312421
ASH1L	-5.59E-04	0.977161826	0.993204695
RPLP1	-3.69E-04	0.977168594	0.993204695
EP300	-5.87E-04	0.977293583	0.993204695
CYP2J2	7.67E-04	0.977322752	0.993204695
LTBR	5.14E-04	0.977470631	0.993263785
CD82	3.95E-04	0.977680374	0.993286261
SERTAD1	-6.80E-04	0.977827946	0.993286261
PTGDR	0.001096321	0.978177634	0.993286261
COG3	4.26E-04	0.978254849	0.993286261

MCM7	-3.24E-04	0.978309625	0.993286261
TDP2	-5.86E-04	0.978552004	0.993286261
ZNF345	-5.42E-04	0.978722801	0.993286261
KCNA5	-7.45E-04	0.978850953	0.993286261
EXOSC8	5.57E-04	0.978964231	0.993286261
PTK6	-4.76E-04	0.978979225	0.993286261
ZNF329	-4.60E-04	0.97908287	0.993286261
BACH1	-5.39E-04	0.979116837	0.993286261
NDUFS5	-3.33E-04	0.979172931	0.993286261
ZBTB8OS	3.98E-04	0.97918074	0.993286261
AUTS2	-8.18E-04	0.979347862	0.993286261
PDK4	-8.24E-04	0.979419693	0.993286261
ITGA6	6.18E-04	0.979447911	0.993286261
SORBS2	-4.20E-04	0.979637269	0.993286261
ZNF140	-5.21E-04	0.979743077	0.993286261
IAH1	-2.68E-04	0.979812753	0.993286261
SCARNA10	4.85E-04	0.979856397	0.993286261
FAM89B	4.47E-04	0.980011176	0.993286261
C9orf37	3.92E-04	0.980067129	0.993286261
FDX1L	-3.86E-04	0.980080337	0.993286261
AGPAT3	-5.23E-04	0.980110437	0.993286261
NECAP1	-2.79E-04	0.980186689	0.993286261
RTN2	7.26E-04	0.980220584	0.993286261
DNAJC2	-6.38E-04	0.980270033	0.993286261
CMAS	2.81E-04	0.980343134	0.993286261
PRMT7	-4.84E-04	0.980458277	0.993286261
PFKFB2	-5.59E-04	0.980495728	0.993286261
GSTK1	2.25E-04	0.980513096	0.993286261
SMN2	-6.99E-04	0.980571558	0.993286261
PTPN1	3.39E-04	0.98066522	0.993286261
OSBPL9	2.32E-04	0.980709517	0.993286261
SF3B3	-3.86E-04	0.980723241	0.993286261
FAM72A	-5.28E-04	0.980953692	0.993419588
OXER1	-4.55E-04	0.981084488	0.993419588
FLII	-2.90E-04	0.981124125	0.993419588
C10orf88	4.05E-04	0.98128501	0.99349161
ST8SIA1	-6.21E-04	0.981541606	0.993660511
CTBP2	-5.02E-04	0.981865484	0.993897489
NEK3	-3.51E-04	0.982096102	0.994040028
RFPL1	4.26E-04	0.982292779	0.994148191
PLA2G4C	-0.001321231	0.982385631	0.994151266
ZNF285	5.83E-04	0.982518801	0.994163675
SSX2IP	-5.54E-04	0.982620605	0.994163675
GZF1	-3.60E-04	0.982698068	0.994163675
TMEM123	-2.83E-04	0.982833808	0.994163675
TNFRSF10D	-4.90E-04	0.982861299	0.994163675
SUMF2	-1.77E-04	0.982936784	0.994163675
ELOVL4	-9.46E-04	0.983100579	0.994191368
MORC3	-5.01E-04	0.9831438	0.994191368
SNORA12	6.80E-04	0.983287008	0.994245354

DENND1C	3.15E-04	0.983533249	0.994403502
MASTL	5.20E-04	0.984408224	0.995197244
ADCY10	-4.35E-04	0.984736753	0.99528216
TRIT1	-3.47E-04	0.984828704	0.99528216
KIAA1324L	-8.32E-04	0.98489371	0.99528216
PPP2R5D	-2.69E-04	0.984897017	0.99528216
SETDB1	2.51E-04	0.984954894	0.99528216
HNRNPM	-1.66E-04	0.985058029	0.99528216
STK39	-3.80E-04	0.985121632	0.99528216
SRPK2	3.88E-04	0.985561225	0.995339106
PI15	-3.61E-04	0.985619603	0.995339106
STYXL1	-3.59E-04	0.985657227	0.995339106
ITPKC	3.58E-04	0.985786133	0.995339106
SCN5A	-2.83E-04	0.985918224	0.995339106
ZNHIT6	1.94E-04	0.986056813	0.995339106
CD22	-5.32E-04	0.986284774	0.995339106
HIP1	-3.26E-04	0.986475477	0.995339106
KIR2DL4	-9.93E-04	0.986585178	0.995339106
FBRSL1	2.62E-04	0.986634781	0.995339106
LUZP1	-2.31E-04	0.986662774	0.995339106
MKRN3	4.00E-04	0.986723543	0.995339106
POLR2J	-5.62E-04	0.986769615	0.995339106
CD19	7.96E-04	0.986801788	0.995339106
TUBD1	3.37E-04	0.986833204	0.995339106
TMEM106B	2.72E-04	0.986838451	0.995339106
BCL3	-2.92E-04	0.986896756	0.995339106
TYW3	4.03E-04	0.986925708	0.995339106
UBR1	-2.48E-04	0.987177523	0.995339106
UNK	-2.15E-04	0.987274619	0.995339106
SURF4	-2.16E-04	0.987305608	0.995339106
FNIP2	2.61E-04	0.987327229	0.995339106
VEGFB	2.25E-04	0.987337311	0.995339106
PATL1	-1.68E-04	0.987429907	0.995339106
TOP3A	-2.73E-04	0.987446711	0.995339106
MED20	2.31E-04	0.987515951	0.995339106
YY1AP1	1.27E-04	0.987695326	0.995429261
TRIM31	-2.61E-04	0.987858684	0.995503256
TARS	-1.78E-04	0.988032907	0.995588188
LRRC57	2.48E-04	0.988469183	0.995705148
HSD11B1L	2.64E-04	0.988565931	0.995705148
FAN1	-3.18E-04	0.988608578	0.995705148
WDR27	2.81E-04	0.988774817	0.995705148
SORCS3	-3.83E-04	0.988775393	0.995705148
C11orf24	2.49E-04	0.988800237	0.995705148
PPP2R5A	1.79E-04	0.988856579	0.995705148
C21orf91-OT1	2.86E-04	0.988919034	0.995705148
SDR39U1	-2.82E-04	0.988958569	0.995705148
POMC	-4.58E-04	0.989235416	0.995728736
DCTD	-1.34E-04	0.989324374	0.995728736
KCTD10	5.43E-04	0.989380647	0.995728736

CCDC77	1.95E-04	0.9894312	0.995728736
PPP1R9B	-2.13E-04	0.989484655	0.995728736
NPC1	2.77E-04	0.989606709	0.995728736
GADD45GIP1	-2.59E-04	0.989611692	0.995728736
WBSCR27	-2.64E-04	0.98994535	0.995973921
PRNP	-1.14E-04	0.990111279	0.996050327
LAP3	-3.57E-04	0.990462552	0.996287313
SYNJ2	-2.40E-04	0.990526866	0.996287313
RAPGEFL1	2.07E-04	0.990796839	0.996384433
C10orf76	1.54E-04	0.990803456	0.996384433
CD46	-1.15E-04	0.991146376	0.996638738
PLCB1	-2.83E-04	0.991268577	0.996671076
ACTR5	-1.47E-04	0.991490267	0.99674919
CPN2	2.15E-04	0.991631816	0.99674919
XAB2	-1.53E-04	0.991673705	0.99674919
DNAJB5	-2.09E-04	0.991735599	0.99674919
ZNF276	-1.91E-04	0.991908016	0.99674919
KIAA1715	-1.55E-04	0.991963277	0.99674919
TCF12	1.38E-04	0.992107886	0.99674919
DUSP13	-1.83E-04	0.99219534	0.99674919
POLL	-1.79E-04	0.992254838	0.99674919
ADORA2A	-2.01E-04	0.992350154	0.99674919
CPSF4L	-2.16E-04	0.99242839	0.99674919
SYN2	1.71E-04	0.992472564	0.99674919
ZNF235	1.37E-04	0.992593134	0.99674919
NFIA	-1.87E-04	0.992606949	0.99674919
LRRC26	3.45E-04	0.993087065	0.997097543
AHSA1	-1.18E-04	0.993134015	0.997097543
SLC25A45	1.41E-04	0.993456356	0.997178024
NT5C2	-6.67E-05	0.993534804	0.997178024
NFX1	1.09E-04	0.993635417	0.997178024
TAF1A	1.50E-04	0.993643899	0.997178024
RAD23A	-1.07E-04	0.993664613	0.997178024
METTL16	-1.51E-04	0.994034866	0.997459155
FIP1L1	7.91E-05	0.994294213	0.997628956
CR2	2.98E-04	0.99448306	0.99766594
ILF3	9.93E-05	0.994511336	0.99766594
DENND5B	-2.38E-04	0.994653654	0.997693535
GPBP1	-6.93E-05	0.994738706	0.997693535
TBL3	1.22E-04	0.994809246	0.997693535
PDLIM7	1.56E-04	0.994960144	0.997754469
BOLA2	9.42E-05	0.995357478	0.998062499
ETF1	5.47E-05	0.995541053	0.99810552
PREPL	8.39E-05	0.995580725	0.99810552
SLC29A1	1.16E-04	0.995783241	0.99821814
PAPOLA	4.34E-05	0.99592216	0.998266991
WDR48	-1.00E-04	0.996339434	0.99859482
UBA6	-5.35E-05	0.996530588	0.998599161
DENND5A	-5.73E-05	0.996574064	0.998599161
DHRS4	4.78E-05	0.996725199	0.998599161

KIAA1598	-1.01E-04	0.996878816	0.998599161
UTP15	6.23E-05	0.997065097	0.998599161
LRSAM1	-3.94E-05	0.997110484	0.998599161
KRT3	-5.54E-05	0.997133756	0.998599161
CNOT6	3.76E-05	0.997172555	0.998599161
ACSS2	4.54E-05	0.997204332	0.998599161
RPP40	-4.67E-05	0.997245923	0.998599161
FAM8A1	4.48E-05	0.997824885	0.999088525
LGALS3	-5.70E-05	0.998043212	0.999216743
HLTF	2.68E-05	0.99828439	0.99929563
TMX3	2.65E-05	0.998302563	0.99929563
PSMC2	1.61E-05	0.99850044	0.999314438
SCARNA21	-4.49E-05	0.998501914	0.999314438
IKZF2	2.65E-05	0.998595566	0.999317812
ABCG1	-4.03E-05	0.998814177	0.99939013
ARRDC1	-2.63E-05	0.998848406	0.99939013
USP53	-3.81E-05	0.999112295	0.99956381
ASAH2B	-1.63E-05	0.999247583	0.999608812
CEP57	1.10E-05	0.999509156	0.999780124
JARID2	5.02E-06	0.999662612	0.999843268
SYTL3	3.96E-06	0.999842206	0.999902408
C21orf49	-1.83E-06	0.999902408	0.999902408

Supplemental Table 2.2: Association between black carbon and gene expression in NoMa

name	logFC	P.Value	adj.P.Val
INSIG2	-0.209087331	4.14E-04	0.974198707
TMEM150C	0.354036376	4.88E-04	0.974198707
TNFRSF10B	-0.217320589	6.59E-04	0.974198707
OXCT2	-0.418531828	6.66E-04	0.974198707
ARID5B	0.282040301	9.13E-04	0.974198707
CUL7	-0.326711401	9.22E-04	0.974198707
IFFO1	-0.187953467	0.001028843	0.974198707
RASSF4	-0.277981224	0.001219519	0.974198707
MTMR9	-0.171833052	0.001238458	0.974198707
TIMM44	0.218966896	0.001302874	0.974198707
GXYLT1	-0.213553235	0.001342144	0.974198707
WBP4	-0.213990053	0.001358378	0.974198707
GAS8	-0.292260807	0.001368717	0.974198707
C5orf54	-0.330523746	0.001573831	0.974198707
XKR8	0.235956564	0.00175485	0.974198707
ZHX2	0.618171731	0.001815278	0.974198707
PPP1R7	0.169223224	0.002115036	0.974198707
TDG	-0.143339989	0.002263601	0.974198707
CD40	0.378915019	0.002326786	0.974198707
BTN2A1	-0.18995244	0.002442005	0.974198707
CLK4	-0.351996903	0.002580677	0.974198707
ARPC4	0.206396076	0.002979594	0.974198707
ADK	0.363045841	0.003079794	0.974198707
FBXO11	-0.134944172	0.003109082	0.974198707
C10orf88	-0.250583128	0.00326981	0.974198707
MRPL9	0.196511652	0.003402294	0.974198707
KLHL2	-0.25374026	0.003522981	0.974198707
MAF1	0.224370645	0.003682308	0.974198707
CDC42	0.204700994	0.003773442	0.974198707
SETD2	0.12808229	0.003787431	0.974198707
TRIM3	-0.231695574	0.003834663	0.974198707
ZNF12	-0.267559918	0.003975642	0.974198707
ZNF77	-0.285587162	0.004044429	0.974198707
GGTLC1	-0.271650658	0.00409732	0.974198707
LTBP2	0.27012388	0.00437073	0.974198707
MUS81	-0.176019388	0.004552726	0.974198707
CLK1	-0.367922553	0.004563731	0.974198707
VEGFB	0.198884017	0.004586453	0.974198707
FBN2	-0.530212767	0.004628096	0.974198707
HSP90AB3P	0.235257619	0.004631198	0.974198707
NDNL2	0.262998723	0.004843486	0.974198707
FGFR1OP	-0.230693949	0.004942927	0.974198707
SERGEF	0.174230683	0.00515142	0.974198707
BUD31	0.137612784	0.005169843	0.974198707

DYNLL2	0.249710484	0.005187123	0.974198707
NFXL1	-0.341238242	0.005249911	0.974198707
MCEE	-0.174368798	0.005423813	0.974198707
EDF1	0.148930674	0.00543066	0.974198707
GDAP2	-0.222176816	0.005731934	0.974198707
IL1B	-0.655068078	0.005842536	0.974198707
PIGM	-0.206136581	0.005892276	0.974198707
ZC3HC1	-0.133371705	0.005905522	0.974198707
DEF6	-0.130283999	0.005935845	0.974198707
SNORD71	0.323462855	0.006122332	0.974198707
C19orf24	0.266069583	0.006143082	0.974198707
NDN	-0.476387374	0.006172156	0.974198707
ZMYM2	-0.188538681	0.006312467	0.974198707
MTRR	-0.302578726	0.006335794	0.974198707
NUP54	-0.128284582	0.006525634	0.974198707
TSHZ1	0.201989181	0.006554134	0.974198707
CTNNBL1	0.14654418	0.006668086	0.974198707
NGDN	0.13820775	0.006839111	0.974198707
BEST1	-0.327009955	0.006888013	0.974198707
FICD	-0.339340815	0.00690308	0.974198707
BAZ2B	-0.256319855	0.006982394	0.974198707
SFMBT1	0.205690809	0.007203184	0.974198707
CNNM2	0.27753805	0.007256846	0.974198707
NECAP1	-0.149343925	0.00740232	0.974198707
FAM170B	0.372593431	0.007447085	0.974198707
NETO2	-0.305222832	0.007523524	0.974198707
DVL2	0.154900006	0.007625491	0.974198707
ZNF518A	-0.162499456	0.00762986	0.974198707
TMEM39A	-0.272709852	0.007761315	0.974198707
PCGF1	-0.245902419	0.007769405	0.974198707
MPP7	-0.300804784	0.007819677	0.974198707
TRNAU1AP	-0.190469822	0.007902561	0.974198707
MYO1C	0.238475787	0.007992647	0.974198707
FASTKD2	-0.188016531	0.008027633	0.974198707
LEAP2	-0.230594419	0.008114581	0.974198707
EXOC1	-0.198897457	0.008424785	0.974198707
CD8A	-0.506932657	0.008630623	0.974198707
SKA2	0.164009772	0.00869659	0.974198707
PSMD13	0.230038142	0.008709838	0.974198707
RBM27	0.142824413	0.008828155	0.974198707
SLC25A1	0.231168316	0.008835073	0.974198707
RBM25	-0.140815707	0.008845519	0.974198707
CPOX	-0.169873652	0.008908205	0.974198707
TDRKH	0.334271694	0.008942808	0.974198707
GFRA2	-0.514971226	0.008998486	0.974198707
ZNF689	-0.171037305	0.009023555	0.974198707
TBC1D16	-0.233026103	0.009253833	0.974198707

SLC2A14	0.275542931	0.009297146	0.974198707
CNFN	0.315198041	0.009403941	0.974198707
NR1H2	0.194587498	0.009441396	0.974198707
TRIT1	-0.235230608	0.009442335	0.974198707
LSM14A	0.127139244	0.009526135	0.974198707
COPS7A	-0.109566819	0.009553903	0.974198707
B4GALT1	0.204953948	0.009670223	0.974198707
HIST3H2A	0.321475123	0.009877223	0.974198707
MPRIP	0.17997837	0.010099838	0.974198707
BAD	0.201347709	0.010103456	0.974198707
IFITM1	0.218586092	0.010196167	0.974198707
MEX3D	0.232829178	0.010285665	0.974198707
TCL6	0.222775354	0.010313208	0.974198707
PLAG1	-0.285121373	0.010390987	0.974198707
KCNMB1	-0.344092777	0.010861086	0.974198707
ZNF484	-0.21876708	0.010874778	0.974198707
EIF3E	-0.10224924	0.010918085	0.974198707
SPG11	-0.151149653	0.011149379	0.974198707
ITGAV	-0.230278397	0.01124901	0.974198707
CCDC12	0.129130689	0.011270435	0.974198707
MANEA	-0.346607354	0.011285019	0.974198707
STAB1	-0.359812366	0.011385371	0.974198707
COG4	0.165722284	0.01152829	0.974198707
SLC16A6	-0.304929213	0.011542468	0.974198707
SLC50A1	0.193638683	0.011688817	0.974198707
MTMR11	-0.233528123	0.011839952	0.974198707
ERLIN2	-0.147309887	0.01194596	0.974198707
CPN2	0.256064939	0.012125984	0.974198707
SAV1	0.310162555	0.012195839	0.974198707
WDR12	-0.153666916	0.012316121	0.974198707
VPS37C	-0.182686268	0.012363157	0.974198707
C11orf95	-0.176569498	0.012407482	0.974198707
GPR162	-0.226632309	0.012407919	0.974198707
ZNF792	0.219900816	0.012425332	0.974198707
RAB18	-0.239080463	0.012461593	0.974198707
HLA-DOB	0.552305874	0.012512311	0.974198707
LYPLA2	0.214894032	0.012592552	0.974198707
TRAF1	-0.210067393	0.012643474	0.974198707
RUFY1	0.482277994	0.0127682	0.974198707
ZNF805	-0.200105176	0.012816591	0.974198707
LRRC37BP1	-0.228372694	0.012893335	0.974198707
HHATL	0.235319426	0.012918713	0.974198707
MTCH1	-0.108320057	0.012924116	0.974198707
DPP10	0.250884388	0.012988448	0.974198707
PDLIM3	-0.219301303	0.013031146	0.974198707
MTERF	-0.21143964	0.013055616	0.974198707
THYN1	0.153717835	0.01306783	0.974198707

ZFP3	-0.200548758	0.013205045	0.974198707
BTN3A1	-0.242359996	0.013291981	0.974198707
EGR2	-0.390480539	0.013323909	0.974198707
DHX29	-0.106265349	0.013351101	0.974198707
C14orf79	-0.179940453	0.01335995	0.974198707
MASTL	-0.326924025	0.013503717	0.974198707
CES1	-0.862108345	0.013528348	0.974198707
ATN1	-0.314153596	0.013540485	0.974198707
DDX43	0.592577815	0.01371728	0.974198707
STIP1	0.229364337	0.013725452	0.974198707
TNNC2	0.632436156	0.013789561	0.974198707
SP1	0.181186507	0.013789904	0.974198707
RBM7	-0.372622462	0.013868827	0.974198707
RASSF2	-0.17660347	0.014040586	0.974198707
UCA1	0.202552404	0.014138565	0.974198707
DNAJB11	-0.144398981	0.014208149	0.974198707
CCDC34	0.215702837	0.014381693	0.974198707
TMEM109	0.153955101	0.014565114	0.974198707
FAR2	-0.269868669	0.014605278	0.974198707
SNORA64	0.263705453	0.014647716	0.974198707
RBM12B	-0.274436135	0.014800062	0.974198707
JPH2	0.196835591	0.014845715	0.974198707
RNF126	0.139357076	0.014852698	0.974198707
DTNA	-0.236142105	0.014913821	0.974198707
ZNF614	-0.305317949	0.014983998	0.974198707
CAMK1D	0.205457129	0.015005204	0.974198707
IRAK3	-0.235174065	0.015160092	0.974198707
CDCA4	0.184363293	0.015417368	0.974198707
FAM76B	-0.214065722	0.015456489	0.974198707
TEKT4	-0.231283543	0.015578287	0.974198707
ZDHHC7	-0.153858772	0.01560672	0.974198707
P2RX5	0.393385088	0.01561178	0.974198707
HSPA4	0.131157033	0.015657017	0.974198707
ALKBH3	-0.141745458	0.01574349	0.974198707
ALG6	-0.132005074	0.015816653	0.974198707
SUCLA2	-0.193571857	0.015907464	0.974198707
NAPEPLD	-0.215180976	0.015945549	0.974198707
AK3	0.193243829	0.015993294	0.974198707
NUFIP1	-0.23437348	0.016003871	0.974198707
DBP	0.191710418	0.016097516	0.974198707
SCARNA10	0.228963327	0.016515994	0.974198707
USP16	-0.120928181	0.016579666	0.974198707
SLC25A12	-0.174864505	0.016623083	0.974198707
CYB5A	0.155779228	0.016690967	0.974198707
ALS2	-0.201837322	0.016730846	0.974198707
AGBL5	0.252956277	0.016878088	0.974198707
CSGALNACT2	-0.177422345	0.017311881	0.974198707

SLC33A1	-0.165560685	0.017400644	0.974198707
ZDHHC6	-0.153439688	0.017409383	0.974198707
CDKAL1	0.25740087	0.017555705	0.974198707
GUF1	-0.191171489	0.01776359	0.974198707
AKAP8L	0.235246716	0.017862313	0.974198707
PARD6A	0.179207688	0.018446122	0.974198707
PLCL2	0.110327211	0.018500102	0.974198707
NAGK	-0.200550705	0.018533038	0.974198707
ITPRIPL2	-0.304726811	0.018655963	0.974198707
OXR1	-0.133774557	0.018694867	0.974198707
C20orf194	-0.203726153	0.018699576	0.974198707
ZNF202	-0.20123453	0.018760619	0.974198707
ZBED4	0.134879291	0.01878085	0.974198707
SART1	-0.271453358	0.018949413	0.974198707
TRIM66	-0.304867982	0.019198466	0.974198707
LRRC58	-0.22030789	0.019236614	0.974198707
TRNT1	-0.243580812	0.019410966	0.974198707
DNAJB1	0.160526201	0.019776609	0.974198707
CCDC121	-0.220436836	0.019877881	0.974198707
MRPL14	0.177434902	0.019974228	0.974198707
RTKN2	0.435906392	0.019977821	0.974198707
C19orf53	0.129855163	0.020009347	0.974198707
ATPIF1	0.139935262	0.020047228	0.974198707
MPZL2	-0.304253117	0.02006223	0.974198707
RASL11A	0.222023196	0.020075758	0.974198707
APPL2	-0.150936708	0.020120086	0.974198707
ZHX1	-0.120609241	0.020150897	0.974198707
WASL	-0.244693202	0.020169671	0.974198707
CENPA	-0.253244891	0.020206914	0.974198707
MIR671	-0.188920741	0.020410337	0.974198707
KIAA1324	0.228134712	0.02050516	0.974198707
PARVG	-0.172885336	0.020617527	0.974198707
CSRP2BP	0.160422725	0.020737451	0.974198707
PXMP2	0.287254013	0.021011794	0.974198707
ST3GAL6	-0.283159972	0.021113556	0.974198707
LSM12	0.143556348	0.021182767	0.974198707
ZNF823	-0.222573309	0.021662265	0.974198707
SNRPA	0.133408701	0.021895717	0.974198707
TMEM168	-0.199481761	0.021933875	0.974198707
SP140	0.222424252	0.02205388	0.974198707
FMO4	-0.227304476	0.022216748	0.974198707
PLXDC1	-0.239952132	0.022218939	0.974198707
TM2D2	-0.24757041	0.022240892	0.974198707
KIAA1279	-0.212065452	0.022282338	0.974198707
HUS1B	0.260832499	0.022359923	0.974198707
SRSF7	-0.149660585	0.022418908	0.974198707
ZCCHC7	0.144382358	0.022469712	0.974198707

PDXK	-0.1981971	0.022558818	0.974198707
CTNNAL1	0.35410529	0.022585489	0.974198707
LGALS8	-0.15218535	0.022601674	0.974198707
THEMIS	-0.279762988	0.022604198	0.974198707
ZNF512B	0.242217476	0.022822579	0.974198707
PQLC3	-0.155101782	0.023053998	0.974198707
FIS1	0.177926971	0.023102494	0.974198707
RAB30	0.27075673	0.023278952	0.974198707
UBE2M	0.112842212	0.0233706	0.974198707
DTNBP1	0.201585056	0.023445486	0.974198707
POTEE	0.304659502	0.023498284	0.974198707
MDN1	0.187539478	0.023535678	0.974198707
MPHOSPH9	-0.183901142	0.02360069	0.974198707
ARHGAP23	-0.175202157	0.023692053	0.974198707
SLC41A1	0.17664505	0.02381446	0.974198707
CRNKL1	-0.248081868	0.023923876	0.974198707
UBE2Q1	-0.163913366	0.02399862	0.974198707
CYBA	0.190933485	0.024179402	0.974198707
NRCAM	-0.498057437	0.024321156	0.974198707
MAF	0.335312951	0.02436471	0.974198707
HOXA10	-0.364460492	0.024367238	0.974198707
NUP133	-0.158965587	0.024371005	0.974198707
STK38L	-0.238363206	0.024377709	0.974198707
QDPR	-0.19718687	0.024424075	0.974198707
PEX14	-0.195308191	0.024445485	0.974198707
HARS2	-0.147272956	0.024585054	0.974198707
SEMA6B	0.230457094	0.024850648	0.974198707
PRKCB	0.115054426	0.024901915	0.974198707
EPC1	0.245137799	0.024929536	0.974198707
BCL7A	0.207426885	0.024963539	0.974198707
ZC3HAV1	0.149927586	0.025064787	0.974198707
DCXR	0.236501143	0.025109899	0.974198707
BCL7B	0.261662674	0.025266323	0.974198707
CCDC132	-0.130258566	0.025437286	0.974198707
CLC	0.401237486	0.025975345	0.974198707
SERPINB6	0.153077464	0.02600932	0.974198707
ZNF564	-0.216162903	0.026280268	0.974198707
BCAT1	-0.486327972	0.026400215	0.974198707
CD1D	-0.22192281	0.026491721	0.974198707
ADCY6	0.178951547	0.026500954	0.974198707
TMEM134	0.139215492	0.026637395	0.974198707
MTERFD1	-0.128370358	0.026654431	0.974198707
MGAT1	0.148305108	0.026704638	0.974198707
AMDHD1	-0.339766062	0.026865184	0.974198707
RGS12	-0.260894255	0.027012771	0.974198707
IGF2BP3	0.43398197	0.027067239	0.974198707
CD55	-0.285441068	0.027078628	0.974198707

EXOG	0.19701825	0.02711408	0.974198707
TMOD4	-0.198977105	0.027169398	0.974198707
TLR2	-0.302675251	0.027547457	0.974198707
RFC2	0.246691979	0.027758598	0.974198707
RCC1	0.237789484	0.027872963	0.974198707
SGK1	-0.268079329	0.027924349	0.974198707
PIGO	-0.126274408	0.028040684	0.974198707
MTMR3	-0.13108274	0.0280745	0.974198707
ANKRD46	-0.240510847	0.028106482	0.974198707
TRERF1	0.175486725	0.028115977	0.974198707
DCP2	-0.155082307	0.028164235	0.974198707
OPA3	-0.248123592	0.028457841	0.974198707
GCDH	0.14572638	0.028475782	0.974198707
SRR	-0.268688254	0.028520604	0.974198707
TMEM219	0.141040753	0.028604318	0.974198707
KIAA1468	-0.200659558	0.028618242	0.974198707
AATF	0.13523875	0.028626422	0.974198707
CCNE1	-0.207134726	0.028723427	0.974198707
TTC39B	0.259138624	0.028728814	0.974198707
PTPRE	-0.175638077	0.028729508	0.974198707
NDRG3	0.126390245	0.028810738	0.974198707
ATP5E	-0.095428463	0.029116205	0.974198707
BRE	0.158850131	0.029125315	0.974198707
TMEM217	0.213800583	0.029134678	0.974198707
LILRA6	-0.353781604	0.029238108	0.974198707
RHOBTB1	0.452054267	0.029248722	0.974198707
LRP10	0.125117854	0.029258643	0.974198707
ZNF212	-0.14811146	0.029281755	0.974198707
TTC16	-0.3646363	0.029393174	0.974198707
C19orf70	0.136395186	0.029407453	0.974198707
FAM185A	0.207926511	0.029429398	0.974198707
RHOT2	-0.192417962	0.029520493	0.974198707
SLC23A3	-0.342057922	0.029587572	0.974198707
FABP5	-0.280200203	0.029902553	0.974198707
CSTF3	-0.114880481	0.029932635	0.974198707
BTAF1	-0.132399125	0.029992657	0.974198707
RBCK1	-0.152661698	0.030099453	0.974198707
RARS	-0.090669796	0.030329623	0.974198707
FBXO34	0.131396951	0.030524776	0.974198707
PPAPDC2	-0.279850921	0.030722074	0.974198707
ALKBH5	0.08755387	0.03106543	0.974198707
DIS3	-0.214993492	0.031100592	0.974198707
CTSH	-0.192167413	0.031174363	0.974198707
FAR1	-0.166068916	0.031205826	0.974198707
CD248	-0.435941932	0.031380654	0.974198707
NCAM1	0.411148931	0.031468341	0.974198707
TBC1D10C	-0.155182548	0.031539693	0.974198707

ZSCAN2	-0.147329885	0.031716588	0.974198707
BIN2	0.155579776	0.031774526	0.974198707
PRCC	0.126876058	0.03179224	0.974198707
TOMM6	0.119676448	0.031900085	0.974198707
PDE4A	-0.226160065	0.032007918	0.974198707
ELOVL7	0.465539423	0.03225405	0.974198707
TOX4	0.145292923	0.032314605	0.974198707
SPAST	0.180071601	0.032581278	0.974198707
MDGA1	-0.370631244	0.032610779	0.974198707
MTA2	0.164257721	0.032651325	0.974198707
FBXL15	0.123296166	0.0326681	0.974198707
C11orf54	-0.119701837	0.032696157	0.974198707
TMOD2	-0.227307565	0.032815956	0.974198707
LTN1	-0.108251026	0.032881738	0.974198707
AMICA1	-0.146866958	0.032914914	0.974198707
MIR21	-0.334267635	0.032943177	0.974198707
CGRRF1	0.142120507	0.033009852	0.974198707
DPM3	0.150299304	0.033011003	0.974198707
C21orf2	-0.242056324	0.033170327	0.974198707
ARMC5	-0.21280157	0.033226429	0.974198707
RNF169	0.187588776	0.033240386	0.974198707
TCP11L2	0.434460926	0.033294504	0.974198707
SLC22A31	-0.199994441	0.0333594	0.974198707
SOCS3	0.304522855	0.033496446	0.974198707
ZBTB7A	0.173923953	0.033720526	0.974198707
CERK	-0.126787036	0.033755573	0.974198707
NCS1	0.170982458	0.033783419	0.974198707
RNF25	0.162524195	0.033785137	0.974198707
AP2B1	0.163238649	0.033879336	0.974198707
KLF11	0.190284826	0.033939359	0.974198707
ADO	-0.186300429	0.03396626	0.974198707
SPATA2	-0.235118904	0.033983068	0.974198707
P2RY14	0.270599378	0.034349006	0.974198707
RPL23	0.091855504	0.034436238	0.974198707
CIR1	0.110308709	0.034516128	0.974198707
TUBGCP5	-0.169068785	0.034919551	0.974198707
DCAF10	-0.1411645	0.034928367	0.974198707
SCLT1	-0.257216537	0.034937897	0.974198707
DGUOK	0.119348112	0.035073415	0.974198707
TSPAN13	0.376549658	0.035144224	0.974198707
TTF2	-0.155584207	0.035194578	0.974198707
LAMB2	-0.270879601	0.035213389	0.974198707
GRK6	0.179507572	0.035245579	0.974198707
CNN2	0.189319876	0.035387996	0.974198707
PLCD3	-0.165422646	0.03552456	0.974198707
HINFP	-0.15566899	0.035568494	0.974198707
HRK	0.357353785	0.035603672	0.974198707

ECH1	0.116596103	0.035697119	0.974198707
LIPT1	-0.155903588	0.035698329	0.974198707
TMEM41A	-0.171891166	0.035741566	0.974198707
B3GALTL	0.29162766	0.035945853	0.974198707
GPR156	-0.197090031	0.035971702	0.974198707
FAM179B	-0.153755408	0.036674813	0.974198707
C1orf189	-0.167363854	0.036835919	0.974198707
POTEM	0.277358893	0.036845626	0.974198707
GTSF1	0.352581483	0.037047422	0.974198707
P2RX6	-0.20995184	0.037072098	0.974198707
SRA1	-0.14453501	0.037141649	0.974198707
CCDC77	-0.153103937	0.037352328	0.974198707
TAF1C	-0.137168205	0.037396278	0.974198707
FAM26F	0.543687341	0.037439717	0.974198707
PRC1	-0.209961185	0.037503304	0.974198707
SRP72	-0.119265775	0.037679714	0.974198707
SIRT4	-0.190602428	0.037828291	0.974198707
EEA1	-0.159742668	0.037919878	0.974198707
NAT9	-0.143223203	0.038105791	0.974198707
RNASEK	0.119212671	0.03812829	0.974198707
NOD2	-0.186539548	0.038204556	0.974198707
NARS2	-0.156457854	0.038402366	0.974198707
NCR3	0.30796722	0.038419797	0.974198707
ENSA	0.21859366	0.038437359	0.974198707
CRLS1	-0.111104449	0.03856563	0.974198707
SLC35B4	-0.207096826	0.038807923	0.974198707
ACOT11	-0.196737083	0.038823069	0.974198707
MBD6	-0.219608261	0.038882507	0.974198707
FAM160B2	-0.181332029	0.038987935	0.974198707
DYNC1LI2	-0.144531232	0.039062684	0.974198707
C21orf119	0.209444179	0.039088347	0.974198707
COPG2	0.299381019	0.039158488	0.974198707
TBCK	-0.147609705	0.039229702	0.974198707
PLCB2	-0.208910206	0.03925657	0.974198707
C15orf40	0.234160747	0.039284919	0.974198707
SLC22A1	0.198408433	0.03929504	0.974198707
TSPAN33	0.451813248	0.03931967	0.974198707
C9orf47	-0.412955773	0.039323967	0.974198707
TCERG1	-0.13988115	0.039349416	0.974198707
IL4R	0.210410338	0.039385603	0.974198707
ARMC7	-0.168155926	0.039428522	0.974198707
TM2D3	-0.171898388	0.039529298	0.974198707
RBM47	-0.263861996	0.039554266	0.974198707
PDE1B	-0.207189218	0.039596565	0.974198707
ATP6V0B	0.120039532	0.039664579	0.974198707
B4GALT3	-0.126666842	0.039814177	0.974198707
C1orf123	0.150601943	0.039975918	0.974198707

TAOK3	0.103370007	0.040004465	0.974198707
ZNF7	-0.140598297	0.040012286	0.974198707
IL18R1	0.280622394	0.040214542	0.974198707
CREB5	-0.246556207	0.040381934	0.974198707
FUK	-0.104871395	0.040501118	0.974198707
NLRP12	-0.23245131	0.040503372	0.974198707
SNORD104	0.233665082	0.040646565	0.974198707
HABP4	0.20765189	0.040646597	0.974198707
ZKSCAN4	-0.194316637	0.040663279	0.974198707
PHB2	0.113529047	0.040768616	0.974198707
CRHR1	0.205199706	0.04079279	0.974198707
SEMA4B	0.177551013	0.040795328	0.974198707
KIAA0895L	-0.212817966	0.040822642	0.974198707
SLC35E3	-0.117630669	0.040884536	0.974198707
SERPINB9	0.384445677	0.040922177	0.974198707
ALG10	-0.159356371	0.041003711	0.974198707
MRPL48	0.156727958	0.04124959	0.974198707
NTPCR	0.272387654	0.041310873	0.974198707
CACNA2D3	-0.275701236	0.041524417	0.974198707
CXCR4	0.351047895	0.041532063	0.974198707
RNF103	-0.172612329	0.041562822	0.974198707
ENDOD1	0.328213772	0.041625135	0.974198707
RNF217	-0.191955278	0.041752627	0.974198707
MRPL32	-0.112363518	0.04182341	0.974198707
TTC23	0.212443151	0.041842915	0.974198707
DDX1	-0.08616082	0.041909549	0.974198707
RFESD	-0.160713378	0.041948321	0.974198707
NAALADL1	0.206713986	0.042098034	0.974198707
C17orf51	0.203910747	0.042132702	0.974198707
TBP	-0.113138511	0.042473433	0.974198707
FNDC3B	-0.152608116	0.042497452	0.974198707
YEATS2	-0.114668936	0.042504994	0.974198707
SNHG9	0.142518596	0.042557045	0.974198707
ZNF561	-0.185764056	0.04273305	0.974198707
NME3	0.112441428	0.0428113	0.974198707
BTBD10	-0.156133658	0.042811573	0.974198707
SYT3	-0.186276868	0.042826594	0.974198707
KIF24	0.19537404	0.042938498	0.974198707
DIS3L2	0.162322622	0.043107552	0.974198707
ACOT8	0.178070304	0.043543821	0.974198707
CEP63	-0.139937746	0.043765198	0.974198707
CLEC4F	-0.757626548	0.043796083	0.974198707
DDX55	-0.143281666	0.043814573	0.974198707
HDGFRP2	0.184869771	0.043814808	0.974198707
STRBP	0.2206858	0.043820103	0.974198707
ANKRD13A	0.100381903	0.043850291	0.974198707
EIF1	0.129758442	0.043852987	0.974198707

SYNJ1	-0.132997301	0.043956281	0.974198707
RPL32P3	-0.261672537	0.044056264	0.974198707
FOXD4L4	0.198677241	0.044113265	0.974198707
ZNF365	0.417864874	0.044190671	0.974198707
LSM7	0.12991518	0.044437223	0.974198707
SMYD3	0.166786721	0.044443661	0.974198707
HOXD3	0.182219376	0.044732318	0.974198707
VILL	-0.161137848	0.044788732	0.974198707
SEC61A1	-0.119096092	0.044790168	0.974198707
KCNJ10	0.185813243	0.044833098	0.974198707
BTBD17	-0.350056883	0.044859778	0.974198707
GPA33	0.197483808	0.044863296	0.974198707
DCAF5	-0.11604002	0.044897189	0.974198707
DHRS7B	-0.13119884	0.045051251	0.974198707
ABHD13	0.173621058	0.045132155	0.974198707
SLC4A8	-0.298636776	0.045221188	0.974198707
SIPA1L1	0.173553751	0.045271182	0.974198707
MAP3K12	-0.152677865	0.045477124	0.974198707
GNG3	0.218457027	0.045535199	0.974198707
VPS25	0.228203687	0.045553012	0.974198707
RPRD2	0.124406665	0.045618231	0.974198707
PURA	-0.174721018	0.045638067	0.974198707
AP3D1	-0.132497139	0.045642004	0.974198707
DAP	0.210154593	0.045881905	0.974198707
ACSL5	0.111539904	0.046101395	0.974198707
PRDM4	0.111896888	0.046109089	0.974198707
TSEN2	-0.183317483	0.046198505	0.974198707
SYT2	-0.213644233	0.046236915	0.974198707
RPTOR	-0.241631764	0.046342947	0.974198707
DHRS11	0.151343452	0.046369027	0.974198707
IKZF3	-0.295988145	0.046462391	0.974198707
PRR24	0.188064546	0.046484098	0.974198707
PIK3R4	-0.144220868	0.046494602	0.974198707
HERC4	-0.093936236	0.046503098	0.974198707
SEPW1	0.099267594	0.04655292	0.974198707
DAGLB	-0.182067367	0.04656125	0.974198707
CCDC124	0.160165777	0.046567554	0.974198707
TOP3A	-0.172790262	0.046596357	0.974198707
RPS24	0.089236372	0.046782943	0.974198707
NSMAF	-0.100645564	0.046811656	0.974198707
PDE4D	0.247447998	0.046923076	0.974198707
GGNBP2	-0.111768893	0.047078865	0.974198707
DENND2C	0.266173203	0.047115328	0.974198707
FBXO32	0.15588842	0.047253375	0.974198707
MRPL53	0.156694136	0.047260065	0.974198707
SLC35A5	-0.165472847	0.047403763	0.974198707
GPR137B	-0.246956389	0.047412536	0.974198707

ALKBH1	-0.168656474	0.047493806	0.974198707
CCDC17	-0.191777576	0.04750415	0.974198707
ASL	-0.235125288	0.047570205	0.974198707
MGAT4B	0.163278346	0.047587571	0.974198707
ASH1L	0.193381809	0.047666043	0.974198707
SLC9A9	0.13609173	0.047827413	0.974198707
EXOSC4	0.149919812	0.047857463	0.974198707
NPEPL1	-0.199069111	0.047894734	0.974198707
PLK3	-0.225678075	0.048050894	0.974198707
TMC8	0.222937416	0.048059167	0.974198707
LRRN3	-0.572792921	0.048177099	0.974198707
TWISTNB	-0.157947963	0.048316993	0.974198707
SRGAP1	-0.178214751	0.048709832	0.974198707
EHHADH	-0.211577294	0.04875576	0.974198707
SYCE1	0.203231971	0.048813801	0.974198707
TNFRSF25	-0.241910167	0.048872964	0.974198707
COPS7B	0.103166732	0.048878909	0.974198707
ASB8	-0.121236717	0.048918174	0.974198707
SMAD2	0.11536293	0.049072047	0.974198707
LYG1	-0.243594916	0.04908236	0.974198707
UBIAD1	0.14410165	0.049287023	0.974198707
LIN7A	-0.207682723	0.049314254	0.974198707
FCER2	0.535027746	0.049328397	0.974198707
ZNF165	0.191395696	0.049344437	0.974198707
MRPL11	0.113470941	0.049353796	0.974198707
MKL1	0.165784459	0.049430829	0.974198707
PPIH	0.11635659	0.049648039	0.974198707
MTRF1	-0.164179063	0.049663603	0.974198707
MED23	-0.161662293	0.049728166	0.974198707
OR11H1	0.188353324	0.049885327	0.974198707
PLEKHG4	-0.223282741	0.049910968	0.974198707
ZNF251	0.145504829	0.049938614	0.974198707
COQ7	0.193189721	0.050158159	0.974198707
NUP93	0.105004315	0.05044912	0.974198707
TBX3	-0.149639139	0.050623762	0.974198707
TMEM202	0.171008804	0.050666069	0.974198707
RAB4B	-0.23824119	0.050855758	0.974198707
PLAC8	0.175203323	0.050860585	0.974198707
SBF1P1	-0.184046082	0.050864048	0.974198707
CPSF1	0.211495221	0.050894377	0.974198707
DDX28	0.135278317	0.050972501	0.974198707
FBXO30	-0.208652703	0.051027953	0.974198707
FAM153A	-0.468402794	0.051292879	0.974198707
SMPDL3A	-0.322454162	0.051414595	0.974198707
HLA-DOA	0.306985091	0.051774707	0.974198707
STOX1	-0.365208162	0.051926532	0.974198707
MAK	-0.177763591	0.051927418	0.974198707

ACAD8	-0.120188608	0.05199177	0.974198707
TRDMT1	-0.200963251	0.052002811	0.974198707
TRPM2	-0.184698489	0.052060122	0.974198707
L3MBTL1	-0.161924492	0.052135358	0.974198707
TAF1D	-0.357222987	0.052223114	0.974198707
RBAK	-0.233482994	0.052231043	0.974198707
LEO1	-0.142796002	0.052348755	0.974198707
CDK5RAP3	-0.167554506	0.052357338	0.974198707
HLA-DRB6	1.353871855	0.052484801	0.974198707
PRMT1	0.11324367	0.052511567	0.974198707
TOMM22	0.085038147	0.052729573	0.974198707
CD34	-0.303208352	0.053195253	0.974198707
SRSF2	-0.060778659	0.053279567	0.974198707
ASPRV1	-0.1810855	0.053402719	0.974198707
RIN3	0.17306663	0.053468542	0.974198707
SNORA59A	-0.183635959	0.05376375	0.974198707
ANKS1A	-0.103955801	0.053791923	0.974198707
DDX52	-0.11470418	0.0539893	0.974198707
SMARCD2	0.210078421	0.05412832	0.974198707
C10orf128	0.368816383	0.054139909	0.974198707
ABCA9	-0.151047276	0.05421718	0.974198707
RGMB	0.453165602	0.054329542	0.974198707
EML2	0.181383485	0.054390363	0.974198707
CEND1	0.186738791	0.054405359	0.974198707
CHRNA10	-0.178058152	0.054460656	0.974198707
OCIAD2	0.124330848	0.05452416	0.974198707
MPPE1	-0.204955814	0.054567042	0.974198707
GLO1	0.178875435	0.054627066	0.974198707
RPL36	0.088092445	0.054652843	0.974198707
TCEB3	0.162303147	0.054674082	0.974198707
ELK4	0.197303821	0.054717618	0.974198707
THRAP3	0.146928422	0.054785485	0.974198707
SHMT2	0.109495436	0.054813553	0.974198707
FBXW4	0.10323589	0.054836753	0.974198707
MBIP	-0.137431487	0.054847008	0.974198707
SCARNA12	0.158980696	0.054877644	0.974198707
FXYD7	0.201264115	0.054964033	0.974198707
TERF2IP	0.139852015	0.055037775	0.974198707
CCNK	-0.093448026	0.055066562	0.974198707
TMEM170A	0.15430245	0.055290701	0.974198707
TMPRSS12	0.211573109	0.055399112	0.974198707
ZNF304	-0.17261476	0.055521807	0.974198707
PWWP2A	-0.126043298	0.055538176	0.974198707
SNRPN	0.140274465	0.055931921	0.974198707
VAC14	-0.204887969	0.05598366	0.974198707
CSRNP2	-0.138302491	0.056058534	0.974198707
RBFA	0.156174182	0.056088482	0.974198707

PRDX2	0.24577751	0.056154678	0.974198707
CBLL1	0.165766076	0.056201664	0.974198707
SPPL3	0.150136733	0.056237429	0.974198707
APPBP2	-0.082374526	0.056284401	0.974198707
BCL7C	0.177055736	0.05640939	0.974198707
SNORD89	0.331566563	0.056464383	0.974198707
CCR5	-0.227765534	0.056490866	0.974198707
LIMS3L	0.334506737	0.056561952	0.974198707
PFN2	-0.285064238	0.056807699	0.974198707
PVRL2	0.262608327	0.056860532	0.974198707
MAD2L1BP	-0.184136488	0.056948711	0.974198707
JOSD1	0.138106127	0.057155974	0.974198707
RPL13AP6	0.150921208	0.057217904	0.974198707
LRRC1	-0.188450766	0.057342777	0.974198707
INPP5F	0.220115338	0.057366932	0.974198707
BAG1	0.185887674	0.057373254	0.974198707
CHMP4B	0.12277741	0.057414375	0.974198707
KLHL6	-0.15818924	0.057433664	0.974198707
AUH	-0.171744293	0.057435227	0.974198707
OTUD6B	-0.12653779	0.057482632	0.974198707
YWHAG	-0.122315524	0.057850704	0.974198707
NCK2	0.185351229	0.057855742	0.974198707
TTF1	-0.090642284	0.057925247	0.974198707
GATSL3	0.151434386	0.057974944	0.974198707
TACC2	0.224836298	0.058130115	0.974198707
EIF5A	0.191681523	0.058153704	0.974198707
XPA	0.123347686	0.058384337	0.974198707
ZBTB41	-0.175565493	0.058392046	0.974198707
MINA	0.158892655	0.058398799	0.974198707
HIVEP2	0.189323849	0.058465545	0.974198707
RP9	0.133612554	0.058477532	0.974198707
CHRNB4	-0.193839559	0.058520893	0.974198707
SNRNP40	0.099839735	0.058531179	0.974198707
XPR1	-0.112221798	0.058619222	0.974198707
PPP2R5A	0.121334636	0.058657342	0.974198707
FAM126A	-0.168012765	0.058992266	0.974198707
POP7	0.136844235	0.059029513	0.974198707
TBC1D20	-0.137104548	0.059093252	0.974198707
ANKRD36B	-0.45930278	0.059302754	0.974198707
HINT2	0.088404824	0.059482039	0.974198707
PIH1D1	0.116690447	0.059483246	0.974198707
ACPT	0.217332411	0.059496567	0.974198707
SNRPD1	-0.178250854	0.059516318	0.974198707
ZBTB22	0.097366278	0.059631852	0.974198707
GLT1D1	-0.237083435	0.059908701	0.974198707
RIMS4	0.167749291	0.060041327	0.974198707
MLEC	-0.17158336	0.060063095	0.974198707

MFN2	0.149880408	0.06009749	0.974198707
HLA-DQB1	0.373728403	0.060189003	0.974198707
CACNA2D4	-0.292021158	0.060338624	0.974198707
KIAA0368	-0.107734233	0.060345984	0.974198707
TRIM9	0.172420822	0.060386471	0.974198707
MAFB	-0.24336885	0.060421989	0.974198707
DCUN1D2	-0.158242385	0.060478972	0.974198707
LAMTOR1	0.200810807	0.060606941	0.974198707
CUEDC1	-0.205247628	0.060767627	0.974198707
YTHDC2	-0.150227946	0.060777469	0.974198707
SLC35C1	-0.139529409	0.06089794	0.974198707
TBC1D22A	0.163127978	0.06099343	0.974198707
POP1	-0.153046179	0.061247624	0.974198707
JAM2	0.153457463	0.061361716	0.974198707
FARS2	0.134362559	0.061381289	0.974198707
SLC20A1	-0.158162468	0.061443941	0.974198707
DDR1	-0.199700004	0.061768025	0.974198707
EID1	0.201376893	0.061953529	0.974198707
SLC25A46	-0.172820288	0.06203334	0.974198707
RAD23B	0.206137644	0.062151033	0.974198707
FTSJ2	-0.112763744	0.062505395	0.974198707
ITGA4	-0.209343892	0.06251148	0.974198707
B4GALNT4	-0.180077328	0.062710479	0.974198707
ZBTB3	-0.230079365	0.062711316	0.974198707
ABCC1	0.176418031	0.062712688	0.974198707
ACOT2	0.227138349	0.062931071	0.974198707
SF3B5	0.109988738	0.063061002	0.974198707
UNC119B	-0.135593493	0.063248912	0.974198707
NOB1	0.126751706	0.063310099	0.974198707
SLC25A23	0.180540524	0.063317036	0.974198707
CPSF3L	-0.116778165	0.0633533	0.974198707
C17orf75	-0.428502432	0.063519197	0.974198707
FBXL12	-0.104189933	0.063563022	0.974198707
SLC22A16	0.688128563	0.063647776	0.974198707
TRIM46	-0.202985946	0.063704395	0.974198707
BOLA3	0.140792427	0.063735093	0.974198707
RABGEF1	0.15642361	0.063823267	0.974198707
BBS10	-0.221632148	0.063857617	0.974198707
WRAP73	-0.103316036	0.063894484	0.974198707
CNOT6L	0.132275149	0.064106494	0.974198707
HOXA5	-0.242021265	0.064313227	0.974198707
FITM1	0.155440017	0.06438448	0.974198707
MTMR2	0.159835248	0.064477002	0.974198707
RNF10	0.193162219	0.064971482	0.974198707
SNX16	-0.201400926	0.065022618	0.974198707
RNF145	-0.153371641	0.06520169	0.974198707
GMFB	-0.094714208	0.065264094	0.974198707

ZDHHC24	-0.194029092	0.065401628	0.974198707
VPS37A	-0.124183997	0.065470602	0.974198707
SNRPE	0.165779039	0.06555863	0.974198707
TMEM203	0.089108785	0.065705306	0.974198707
NXT1	0.095792905	0.065765063	0.974198707
CLEC1A	-0.212094772	0.065765967	0.974198707
FOXRED1	-0.150586633	0.065847204	0.974198707
SPATA5L1	-0.150936869	0.065850421	0.974198707
MMACHC	-0.176731834	0.065937284	0.974198707
FAM160B1	-0.114479905	0.065976592	0.974198707
CAND1	-0.126594908	0.066115003	0.974198707
KIF22	-0.116179816	0.066246368	0.974198707
URGCP	0.109758173	0.066470558	0.974198707
EXD2	-0.137361056	0.066612542	0.974198707
SNRNP70	-0.124907523	0.066837526	0.974198707
HECA	0.126591461	0.066873888	0.974198707
SMG5	-0.117057616	0.066998705	0.974198707
FAM72D	-0.26057174	0.067077921	0.974198707
DDHD2	0.084525919	0.067087545	0.974198707
TTL	-0.110939718	0.067127335	0.974198707
KCTD15	-0.165064636	0.067257575	0.974198707
PRPS1L1	0.14025213	0.067383841	0.974198707
CCDC14	-0.145306552	0.067447811	0.974198707
CLEC16A	0.095134286	0.067816537	0.974198707
POLR2I	0.108825886	0.067828918	0.974198707
CCDC85B	0.148525786	0.067950099	0.974198707
REM2	0.217179548	0.068014069	0.974198707
AIDA	0.084333152	0.068019164	0.974198707
CD200	0.287258793	0.068024334	0.974198707
POLR2J	0.309853862	0.068050455	0.974198707
SNHG8	0.593548643	0.068078054	0.974198707
TJP1	0.203150797	0.068136943	0.974198707
CTSD	0.189160542	0.068267704	0.974198707
CLTCL1	0.169561468	0.068337596	0.974198707
ABAT	0.251711538	0.068442129	0.974198707
CDK5R2	-0.150369719	0.068563792	0.974198707
SDHAF2	0.120854441	0.068566664	0.974198707
RRP1	-0.1938193	0.068630102	0.974198707
MRPL22	0.089047403	0.068736464	0.974198707
NIT2	0.132924621	0.068770415	0.974198707
DTX2	-0.101423068	0.068772343	0.974198707
SAP25	-0.164422631	0.068793688	0.974198707
ZNF302	-0.132713933	0.068896703	0.974198707
XPO1	-0.086048588	0.068986037	0.974198707
MGRN1	-0.201199475	0.069003289	0.974198707
GPX7	0.1532471	0.069011417	0.974198707
BAX	0.17318192	0.069175791	0.974198707

FBLN5	0.313305031	0.069399884	0.974198707
ALKBH8	-0.21068308	0.069425832	0.974198707
ACCS	-0.389953539	0.06948127	0.974198707
JMJD8	0.077542653	0.069610075	0.974198707
PRPF6	0.188109869	0.069760349	0.974198707
DHCR7	0.155894344	0.069786021	0.974198707
MUC20	-0.408072585	0.069944221	0.974198707
ERICH1	-0.161380153	0.069949162	0.974198707
GIMAP7	-0.163007043	0.06999774	0.974198707
LAMP1	0.08275113	0.070091553	0.974198707
BOK	0.22456522	0.070157265	0.974198707
TBC1D2B	-0.139913432	0.07016039	0.974198707
MUC16	0.158110067	0.070236098	0.974198707
ALDOC	0.198283988	0.070317322	0.974198707
PPP4R1	-0.096427674	0.070362479	0.974198707
GRAMD3	-0.198184416	0.070372146	0.974198707
TRIM39	-0.109748578	0.070397831	0.974198707
PAAF1	-0.11711223	0.070550025	0.974198707
GNGT2	0.20649004	0.070802481	0.974198707
ZNF239	-0.245804043	0.071071985	0.974198707
ODF2	0.121008155	0.071112444	0.974198707
ANTXR1	-0.1518255	0.071131052	0.974198707
KBTBD7	-0.166257677	0.071177028	0.974198707
TNF	-0.35110647	0.071218449	0.974198707
IGHG1	-0.126308763	0.07128454	0.974198707
STRADA	-0.09280425	0.071320348	0.974198707
TLE1	0.297366548	0.071672566	0.974198707
FASTK	-0.087481214	0.071824502	0.974198707
TMEM218	-0.093255206	0.071860626	0.974198707
BRD4	0.181790018	0.072082559	0.974198707
MGAT4A	0.158540922	0.072125975	0.974198707
PTPRF	0.395547898	0.072237088	0.974198707
ZEB2	-0.183768045	0.072337279	0.974198707
POLR2F	0.10475088	0.072361814	0.974198707
KBTBD6	-0.252880269	0.072378643	0.974198707
EXOSC9	0.135210769	0.072421366	0.974198707
GAA	0.273657137	0.072509067	0.974198707
TFAM	-0.121358157	0.072525644	0.974198707
CBX4	0.123110321	0.072629678	0.974198707
ALKBH7	0.183992793	0.072665774	0.974198707
BMS1	0.076279118	0.072997844	0.974198707
LGALS4	-0.16141538	0.073084083	0.974198707
SERPINB2	-0.342114062	0.073092945	0.974198707
SIAH1	-0.127768815	0.073525974	0.974198707
ASXL1	0.135785077	0.073727638	0.974198707
SDC4	0.23793017	0.0737869	0.974198707
MFSD8	-0.15602917	0.073993802	0.974198707

SECISBP2L	0.092284635	0.074091796	0.974198707
ZNF177	-0.195215978	0.074144164	0.974198707
ASTE1	-0.184709745	0.074218008	0.974198707
MARVELD1	-0.191943316	0.07426186	0.974198707
MSL2	0.128163032	0.07439104	0.974198707
C19orf47	0.155667312	0.074412339	0.974198707
MST1	-0.167641672	0.074426278	0.974198707
ZNF429	-0.309855495	0.074527466	0.974198707
TACR2	0.164559859	0.074759802	0.974198707
GPR108	0.132306291	0.074816704	0.974198707
VTI1B	0.133998256	0.074854639	0.974198707
TMEM64	0.177520205	0.074874597	0.974198707
EYA3	0.299981052	0.074898712	0.974198707
ABCA10	-0.1841621	0.075106834	0.974198707
PTPRJ	0.166122603	0.075119986	0.974198707
CTTN	0.446277165	0.075173838	0.974198707
OSBPL11	-0.14507434	0.075211416	0.974198707
PPP1R14B	0.132808013	0.075248422	0.974198707
ANAPC11	0.098097358	0.075353174	0.974198707
CENPB	0.09580195	0.07540429	0.974198707
PPM1B	-0.149093582	0.075600542	0.974198707
LSM4	0.130992267	0.075605153	0.974198707
C8orf37	-0.142188302	0.075650831	0.974198707
PNOC	0.298262658	0.075774518	0.974198707
DSTYK	-0.464088253	0.075785821	0.974198707
SASS6	-0.123450795	0.075882548	0.974198707
SSU72	0.10531901	0.076450119	0.974198707
HSP90AB1	0.131686002	0.07652217	0.974198707
MIR330	0.238177749	0.07662758	0.974198707
ZFYVE20	-0.099153809	0.076772728	0.974198707
VPS4A	0.108198119	0.076848461	0.974198707
C17orf89	0.125946745	0.077068137	0.974198707
MALT1	0.115120843	0.077141462	0.974198707
CCNE2	-0.208146572	0.077164401	0.974198707
OXSR1	0.067355975	0.077189608	0.974198707
CCDC135	0.184989769	0.077301078	0.974198707
EIF3G	0.100811881	0.077351287	0.974198707
DGAT1	-0.133678285	0.077365618	0.974198707
ZNF707	-0.164103561	0.0775243	0.974198707
SNORD38A	0.182880843	0.077590189	0.974198707
AMY2A	-0.172919372	0.077633088	0.974198707
DDAH2	0.181470528	0.077675184	0.974198707
IPO9	-0.152075487	0.077712133	0.974198707
ENTPD4	0.24527983	0.077719977	0.974198707
MAGEL2	0.139582814	0.077738354	0.974198707
KLHL9	-0.16061867	0.077779222	0.974198707
EBPL	0.241996839	0.077805282	0.974198707

EML4	-0.117328731	0.077812071	0.974198707
GLIS3	0.16592338	0.077818777	0.974198707
FGD3	-0.079487412	0.077839276	0.974198707
RAB8A	0.09094221	0.077889647	0.974198707
IBA57	-0.145807931	0.077929067	0.974198707
MON2	-0.121764874	0.078151526	0.974198707
ABCD3	-0.128256878	0.078197537	0.974198707
GON4L	-0.11514086	0.078416533	0.974198707
STX10	0.115272218	0.078443886	0.974198707
TSTD2	-0.127624646	0.078446017	0.974198707
PTPN12	0.169984151	0.078513976	0.974198707
IL6ST	-0.214184671	0.078518219	0.974198707
BCS1L	-0.123338259	0.078693954	0.974198707
KIAA1328	-0.158959967	0.078701427	0.974198707
MAX	0.256080276	0.078730224	0.974198707
DEFA1	-0.466477181	0.07881623	0.974198707
GOSR1	-0.137150466	0.078902297	0.974198707
PLA2G4A	-0.222942826	0.07913343	0.974198707
KIAA1147	0.090754107	0.079316996	0.974198707
SMAGP	0.181154676	0.079319164	0.974198707
METTL3	-0.086344781	0.079368406	0.974198707
TRPS1	-0.186003817	0.079418004	0.974198707
MGLL	0.507846563	0.079569304	0.974198707
NANP	-0.269185365	0.079576768	0.974198707
LY6G6F	0.445747116	0.07968906	0.974198707
IL5RA	0.218814137	0.079894157	0.974198707
FASTKD3	-0.15006215	0.079914412	0.974198707
NPEPPS	-0.087222878	0.080016425	0.974198707
ZNF789	-0.164481838	0.080140985	0.974198707
POLR1C	-0.129679746	0.080282521	0.974198707
BLCAP	0.109519152	0.080439737	0.974198707
ACOX1	-0.184164044	0.08048171	0.974198707
BANK1	0.335155449	0.080513263	0.974198707
CBX6	-0.12471132	0.080536802	0.974198707
ADM	0.366073827	0.080560926	0.974198707
KLRK1	-0.164144825	0.080780177	0.974198707
CYB5D1	0.178351637	0.080860571	0.974198707
DDX24	0.112654928	0.080877042	0.974198707
HOXB2	-0.467934554	0.080889221	0.974198707
CDKN2A	-0.162231827	0.080916731	0.974198707
TRMT2A	-0.149206515	0.080938549	0.974198707
FXYD1	0.166102183	0.081041318	0.974198707
CLN5	-0.164824714	0.081133355	0.974198707
MLLT1	0.174095716	0.081464567	0.974198707
MUC4	0.166270064	0.081472424	0.974198707
SNORD83B	0.173585851	0.081584039	0.974198707
HERC5	0.258838417	0.081674013	0.974198707

RUFY3	-0.142302067	0.082169632	0.974198707
DDX50	0.087147461	0.082189209	0.974198707
TRUB2	-0.124576233	0.082333028	0.974198707
PIGV	-0.253354095	0.082344513	0.974198707
ZNF330	-0.155145403	0.082385763	0.974198707
PDE4B	0.155315227	0.082471336	0.974198707
LRR1	-0.133079314	0.08250481	0.974198707
TTLL5	0.125547137	0.082621284	0.974198707
LIG3	-0.166150905	0.08276562	0.974198707
EEF1A2	0.279793834	0.082786246	0.974198707
STAC3	0.157731498	0.082788238	0.974198707
CCDC88A	-0.159805007	0.082821357	0.974198707
FAM195A	0.175311122	0.083100883	0.974198707
MRPL15	0.148337803	0.08310835	0.974198707
PLCG1	-0.14602692	0.083152198	0.974198707
RPS7	0.096130514	0.083326199	0.974198707
MRPS31	0.08708774	0.083480336	0.974198707
SNORD99	-0.245114449	0.083510326	0.974198707
SEC24D	-0.148926808	0.083727502	0.974198707
IL20RB	-0.14318297	0.083826348	0.974198707
MAPKAPK2	0.120434788	0.083909462	0.974198707
IFT27	0.145997173	0.084027935	0.974198707
ZNF607	0.141972742	0.084305134	0.974198707
MYL5	0.243210331	0.084353406	0.974198707
C6orf57	0.133024886	0.084363618	0.974198707
AURKB	-0.224302151	0.084373383	0.974198707
HSD17B4	-0.102771051	0.084495588	0.974198707
FGD6	0.168052325	0.084535661	0.974198707
CDK8	-0.144252353	0.084620263	0.974198707
TOMM34	-0.168704066	0.084678234	0.974198707
ENO3	-0.155037233	0.084687412	0.974198707
TBC1D8	-0.189359513	0.084768523	0.974198707
ANKRD20A5P	0.145678913	0.08506205	0.974198707
ART1	0.158231146	0.085103377	0.974198707
CHFR	-0.098899583	0.085116011	0.974198707
RSPH4A	-0.185292329	0.085289386	0.974198707
RREB1	-0.160077008	0.085429893	0.974198707
HSF5	-0.196197786	0.08575452	0.974198707
ATP6V1B2	-0.095025763	0.085776345	0.974198707
PITHD1	0.110715895	0.085825008	0.974198707
PLOD3	-0.132451031	0.086416245	0.974198707
SLA	-0.207847098	0.086677221	0.974198707
NLK	-0.106673113	0.086680305	0.974198707
SDHAP2	-0.22201393	0.086762879	0.974198707
SNRPB2	0.077843593	0.086790927	0.974198707
OMG	0.156101684	0.086817288	0.974198707
MAD1L1	0.236468424	0.086854871	0.974198707

UBXN4	0.155792361	0.086974734	0.974198707
PPCS	-0.114773572	0.086975168	0.974198707
PICALM	-0.100479462	0.086987864	0.974198707
CCDC41	-0.138575291	0.087210576	0.974198707
BCAS4	0.2123776	0.087322662	0.974198707
ATOX1	0.156269485	0.087447005	0.974198707
RIOK1	-0.07891771	0.087529423	0.974198707
CLN8	-0.231650808	0.087682138	0.974198707
SNX11	-0.116920756	0.087701163	0.974198707
STK4	0.06619509	0.087734479	0.974198707
PNPLA2	-0.188041841	0.08787508	0.974198707
GNG7	0.215740147	0.087889579	0.974198707
DYNLRB1	0.14712952	0.087914052	0.974198707
ZNF426	-0.136823314	0.088101445	0.974198707
BICD2	-0.147121076	0.088225553	0.974198707
PPARG	-0.219257595	0.088232698	0.974198707
TMEM169	-0.178183697	0.088355289	0.974198707
ATG4D	0.144380851	0.088387854	0.974198707
PYGO2	-0.189301526	0.088488408	0.974198707
UBE3C	-0.097602263	0.088612888	0.974198707
THAP3	0.17127776	0.088655452	0.974198707
PGS1	-0.103832162	0.088732965	0.974198707
GATAD2A	0.100960957	0.08875815	0.974198707
RNMT	0.128384233	0.088786473	0.974198707
PRAMEF13	0.183592068	0.088830429	0.974198707
FAN1	-0.190091602	0.088928673	0.974198707
ZC3H4	0.092200523	0.088933571	0.974198707
FMO6P	0.157320919	0.089153623	0.974198707
PPP1R16B	0.141296074	0.089155506	0.974198707
SDSL	-0.247286164	0.089187806	0.974198707
FADD	-0.1700631	0.089321696	0.974198707
NDUFS5	0.10859499	0.0896271	0.974198707
RPLP1	0.109790048	0.08968531	0.974198707
SUPT6H	0.116727721	0.089758301	0.974198707
EIF4A3	-0.084511432	0.089960029	0.974198707
PDE4DIP	-0.837156579	0.090166607	0.974198707
STX8	0.131226636	0.09022281	0.974198707
LZTR1	-0.110149997	0.090312312	0.974198707
COMMD2	-0.214087923	0.090360797	0.974198707
WDR73	-0.165944021	0.09037482	0.974198707
GIMAP8	-0.164027314	0.09055462	0.974198707
MARCH3	0.242289954	0.090612287	0.974198707
IL15	-0.165026425	0.090655218	0.974198707
FGF2	0.14714027	0.090730823	0.974198707
DNM1P35	0.176196886	0.090736764	0.974198707
MIS12	-0.125225191	0.090761779	0.974198707
HMGXB4	-0.091237239	0.090969862	0.974198707

PDIK1L	-0.136797302	0.091068145	0.974198707
NUP214	-0.122740328	0.091068717	0.974198707
GRB2	0.147447327	0.091174526	0.974198707
TMEM135	-0.16635218	0.091256346	0.974198707
C11orf57	0.098314713	0.091264584	0.974198707
CYB5R2	-0.207506146	0.091306498	0.974198707
MFSD7	-0.178729043	0.091308895	0.974198707
PPP1R11	0.069080612	0.091312109	0.974198707
HDGF	0.130630124	0.091474943	0.974198707
SPDYE2	0.117704768	0.091570826	0.974198707
CCHCR1	0.157707722	0.091760616	0.974198707
HIBADH	-0.121483893	0.091878952	0.974198707
TMEM41B	-0.13696861	0.092011054	0.974198707
SV2A	-0.18132875	0.092254131	0.974198707
C2orf82	-0.129876099	0.092263179	0.974198707
FAM124B	-0.206246803	0.092323773	0.974198707
PLA2G15	-0.152878765	0.092326582	0.974198707
ARPC1A	0.077184105	0.092410385	0.974198707
APBB3	-0.148086343	0.092515855	0.974198707
C9orf40	-0.227997705	0.092589758	0.974198707
TMEM128	0.400572459	0.092870007	0.974198707
SIK1	0.287763265	0.09311199	0.974198707
EXOSC6	0.139367132	0.09337153	0.974198707
RGL1	-0.255303419	0.093448048	0.974198707
RNF122	0.139648103	0.093484802	0.974198707
H3F3B	0.220624567	0.093541378	0.974198707
MRPL12	0.130069002	0.093757365	0.974198707
LILRA3	0.795720299	0.093796262	0.974198707
TIGD5	0.12816961	0.09385237	0.974198707
SP3	-0.159005874	0.09399888	0.974198707
NDUFB9	0.10898779	0.094109079	0.974198707
SF3A2	0.106087572	0.094133236	0.974198707
UBXN2B	-0.153606085	0.09422763	0.974198707
ZNF503	-0.224472188	0.094569905	0.974198707
USP15	-0.146971482	0.094596364	0.974198707
FAM78A	-0.164290007	0.094648185	0.974198707
STK11IP	-0.138510507	0.094896657	0.974198707
AKAP9	-0.174058401	0.095077021	0.974198707
ZNF784	-0.132300522	0.095166268	0.974198707
C8orf44	-0.353047537	0.095167455	0.974198707
CD69	0.190215703	0.095190015	0.974198707
PPM1D	-0.162776332	0.095303839	0.974198707
ZNF644	-0.156898403	0.095454926	0.974198707
KLHL36	0.183128038	0.095554189	0.974198707
NUDT18	-0.164956252	0.095586543	0.974198707
PCOLCE	0.162038523	0.095628308	0.974198707
TSSC1	0.105184796	0.095643694	0.974198707

MEA1	0.088661908	0.095748367	0.974198707
CCDC43	-0.099421139	0.095891204	0.974198707
WBSCR22	0.102201709	0.096009889	0.974198707
STUB1	0.130124593	0.096046967	0.974198707
CASP3	-0.118369079	0.096051261	0.974198707
HMOX2	0.212277038	0.09618482	0.974198707
ATG5	0.098065973	0.096214836	0.974198707
RNF146	-0.152466469	0.096411565	0.974198707
LRBA	0.119331494	0.096429629	0.974198707
RRP7B	-0.068835551	0.096550204	0.974198707
MRPS27	-0.093945799	0.09658255	0.974198707
TCF25	-0.09832537	0.096665904	0.974198707
TRIM61	-0.176764362	0.096825911	0.974198707
DUSP10	-0.172263729	0.096867557	0.974198707
POLDIP3	-0.076189045	0.096912232	0.974198707
EPB41L5	-0.134815492	0.097006207	0.974198707
FES	-0.144666962	0.097018061	0.974198707
NCLN	-0.110379328	0.097194581	0.974198707
VAMP5	0.175269883	0.097256625	0.974198707
YPEL3	0.103412552	0.097309002	0.974198707
HACE1	0.127624766	0.097316521	0.974198707
SGSM3	-0.159019221	0.097405043	0.974198707
TIMM23	-0.071870184	0.097439545	0.974198707
MEIS3	0.27998903	0.097519258	0.974198707
IWS1	-0.092935311	0.097548588	0.974198707
H2AFY2	-0.128441611	0.097581139	0.974198707
PAPD4	-0.085603728	0.097667593	0.974198707
NDEL1	-0.098116968	0.097753207	0.974198707
HNRNPKP3	0.140304472	0.097829883	0.974198707
TMEM222	-0.118198787	0.097913541	0.974198707
DCTN3	0.077027629	0.097921673	0.974198707
MIR155HG	-0.221489314	0.097965766	0.974198707
TMEM126B	-0.073208871	0.09799152	0.974198707
CASP9	0.113624052	0.09809264	0.974198707
NDUFB4	0.134308543	0.098230667	0.974198707
WDR48	0.18089013	0.098288932	0.974198707
TMEM167B	0.093284985	0.09839399	0.974198707
AMOTL2	0.15476827	0.098492741	0.974198707
PITRM1	-0.159967999	0.098569108	0.974198707
FKBP15	-0.113368447	0.098576499	0.974198707
C9orf91	0.122531455	0.098617947	0.974198707
DCTN4	-0.107667353	0.09926632	0.974198707
MAPRE2	0.110066043	0.099364717	0.974198707
DDX6	-0.343598726	0.099375584	0.974198707
CRTC3	0.09412643	0.099472867	0.974198707
RPS17	0.107917811	0.099556755	0.974198707
MRPS21	0.112906024	0.099560247	0.974198707

PGRMC2	0.09546533	0.099645705	0.974198707
KRT1	0.377973624	0.09970417	0.974198707
ZNF676	0.136876835	0.099758175	0.974198707
NAB2	-0.155168495	0.099802825	0.974198707
UBE2D2	0.091499116	0.099817427	0.974198707
ANKRD17	-0.077257119	0.099941289	0.974198707
CYB561D1	-0.086778355	0.100029945	0.974198707
CHD2	0.123286712	0.100244015	0.974198707
ADAT1	-0.141518184	0.100307385	0.974198707
CP	0.134366781	0.100355922	0.974198707
RAD54B	-0.142902094	0.100527667	0.974198707
RNPS1	0.127817724	0.100619362	0.974198707
GBA2	-0.121344244	0.10070059	0.974198707
VENTX	-0.238437817	0.100731795	0.974198707
E2F4	0.087076604	0.100910886	0.974198707
HIF1A	0.150848092	0.100955773	0.974198707
CDRT4	-0.137343613	0.101021774	0.974198707
MAGEF1	0.17944568	0.101214721	0.974198707
SNX22	0.180506677	0.101264953	0.974198707
JMJD4	-0.171556494	0.101411541	0.974198707
ZBTB44	0.125786683	0.101545191	0.974198707
CD82	0.116046501	0.101609377	0.974198707
RPL31	0.094820442	0.101824921	0.974198707
CCR7	-0.222915772	0.101833612	0.974198707
DIRC2	-0.184546872	0.101884883	0.974198707
FAM189B	0.100923076	0.102005028	0.974198707
DRAM1	-0.164977296	0.102056056	0.974198707
BFAR	-0.113234251	0.102109457	0.974198707
CAPZB	0.072002071	0.102318684	0.974198707
SCARNA18	-0.130290801	0.102344578	0.974198707
MPO	-0.214532624	0.102439772	0.974198707
COX4I1	0.076368025	0.102521582	0.974198707
CHERP	0.124244779	0.102573946	0.974198707
ADRM1	0.091162165	0.10277884	0.974198707
ZNF646	-0.145377803	0.102787264	0.974198707
SLC35D3	0.397370441	0.102807185	0.974198707
IRF2BP2	-0.111046104	0.102982321	0.974198707
RBM33	-0.173021108	0.103011686	0.974198707
DIAPH1	0.100237211	0.103035278	0.974198707
PHTF1	-0.134356981	0.103049867	0.974198707
KBTBD4	0.124002906	0.103122456	0.974198707
MAPKAP1	0.10607794	0.1031439	0.974198707
DHX15	-0.063472936	0.103147214	0.974198707
HJURP	-0.175187323	0.103181881	0.974198707
INTS2	-0.102272146	0.10322884	0.974198707
SNORD34	0.230288824	0.103244008	0.974198707
PFAS	-0.092372745	0.103270468	0.974198707

OR7G2	0.151087913	0.103288885	0.974198707
ZNF227	-0.140359066	0.103291312	0.974198707
NOL10	0.171692064	0.103331854	0.974198707
IGHMBP2	0.172490874	0.103356152	0.974198707
VIPR2	0.216311712	0.103450404	0.974198707
FAM153C	-0.299862846	0.103482072	0.974198707
EFHC1	-0.153261076	0.103664736	0.974198707
CCNY	0.135781915	0.103671999	0.974198707
TM2D1	-0.073859482	0.103751031	0.974198707
BMP8B	-0.233956871	0.10376706	0.974198707
SETDB2	-0.081761198	0.103794746	0.974198707
DTYMK	0.187046164	0.103976944	0.974198707
METTL4	0.113205553	0.104048473	0.974198707
KLHDC10	-0.203989656	0.104472686	0.974198707
AURKA	-0.165044857	0.104562668	0.974198707
VASP	0.252982949	0.104688277	0.974198707
CSNK2B	0.122810567	0.104879047	0.974198707
MARK2	0.165024542	0.105090715	0.974198707
MAP4K2	0.093145422	0.10511237	0.974198707
ZNF441	-0.163066886	0.105191878	0.974198707
COPZ1	0.111330489	0.10522335	0.974198707
MED29	0.078290141	0.105336947	0.974198707
ZNF510	0.159220281	0.10543198	0.974198707
ZNF746	-0.081162861	0.105443216	0.974198707
MZF1	-0.127964708	0.105447296	0.974198707
ATG16L2	-0.20808902	0.105518952	0.974198707
YWHAZ	0.07846608	0.105870287	0.974198707
ZRANB2	-0.087538441	0.105950359	0.974198707
CCR2	-0.206192924	0.105970626	0.974198707
ANKRD49	-0.089231627	0.105981298	0.974198707
SLC25A35	-0.174868068	0.106099195	0.974198707
KIAA0907	-0.102689675	0.106103059	0.974198707
GLOD4	0.066759095	0.1062666	0.974198707
THUMPD3	-0.155763479	0.106298802	0.974198707
SCAMP2	0.134937416	0.106400214	0.974198707
PIK3C3	0.103777231	0.106880185	0.974198707
ZC3H6	-0.164557942	0.106931183	0.974198707
RGMA	-0.128397573	0.107042208	0.974198707
CDHR3	0.223009385	0.107509748	0.974198707
C2orf69	-0.211319984	0.107561656	0.974198707
GIMAP4	-0.125181085	0.107574931	0.974198707
TMEM117	0.162371354	0.107591095	0.974198707
ZNF799	-0.158559283	0.107752339	0.974198707
PTCRA	0.397274267	0.107782436	0.974198707
AGPAT6	0.142907075	0.107825615	0.974198707
BOLA1	-0.129569229	0.107835573	0.974198707
MCAT	-0.138941102	0.107847317	0.974198707

LFNG	-0.145657973	0.107879667	0.974198707
RRP8	-0.106179042	0.108089895	0.974198707
BUB3	0.067516038	0.108103262	0.974198707
CIDEA	-0.181692291	0.108115038	0.974198707
C21orf91-OT1	-0.165861584	0.108149922	0.974198707
ELL2	-0.246768376	0.108295196	0.974198707
AARS	-0.07955495	0.108433932	0.974198707
NCAN	0.131460242	0.108642587	0.974198707
TEAD2	0.269684677	0.108744984	0.974198707
CD72	0.304343222	0.108798313	0.974198707
CXXC4	-0.144491847	0.108929563	0.974198707
PADI4	-0.257967399	0.109121096	0.974198707
MARCH1	-0.255506507	0.109279484	0.974198707
POLR3B	-0.126051482	0.109311319	0.974198707
SMC4	-0.107540623	0.109479727	0.974198707
PIM1	0.153210666	0.1095006	0.974198707
DDR2	0.202357686	0.109538781	0.974198707
ATG4B	0.214223083	0.109719291	0.974198707
WSB1	-0.151460689	0.109900235	0.974198707
RRAGC	0.081251765	0.110028581	0.974198707
SMARCAD1	-0.094484562	0.110211664	0.974198707
RPGRIP1	-0.240778914	0.110248481	0.974198707
EPB41	0.154596992	0.110300976	0.974198707
STAT6	-0.195103899	0.110302105	0.974198707
ANKHD1	-0.078531413	0.110528584	0.974198707
RPAP2	-0.147735839	0.11071646	0.974198707
C14orf28	-0.119489868	0.110725326	0.974198707
SSTR1	-0.13232893	0.110798332	0.974198707
CHMP7	0.16663248	0.110887945	0.974198707
HECTD1	-0.07756046	0.110924872	0.974198707
IER2	0.19403397	0.110964105	0.974198707
LNX2	-0.166686914	0.111069196	0.974198707
NOC2L	0.14438036	0.111110708	0.974198707
ARPC5L	0.106915419	0.111198962	0.974198707
ST8SIA4	-0.174265854	0.111274285	0.974198707
ERBB2IP	0.149744571	0.111400261	0.974198707
ANKRD32	-0.555858083	0.111455216	0.974198707
COMMD6	0.094584053	0.111467814	0.974198707
ATP2A2	-0.095103782	0.111492192	0.974198707
ABHD6	-0.114264851	0.111535281	0.974198707
ZFP57	0.14908524	0.111845537	0.974198707
PRAME	0.11849134	0.111876575	0.974198707
LPXN	-0.088663402	0.111883692	0.974198707
ZNF625-ZNF20	-0.179136391	0.112093053	0.974198707
PMS1	-0.150305425	0.11215972	0.974198707
KCNJ2	-0.349182943	0.112208786	0.974198707
HKDC1	-0.297090555	0.112453728	0.974198707

ZNF558	-0.132261238	0.112527262	0.974198707
MREG	0.136546743	0.112527982	0.974198707
WDR5B	-0.157644301	0.11262056	0.974198707
TP53I11	-0.167568844	0.112706401	0.974198707
ZMPSTE24	-0.08593424	0.11273139	0.974198707
CD226	0.217883691	0.112740398	0.974198707
H6PD	0.206991512	0.112818341	0.974198707
KIR2DL1	0.435121913	0.113024943	0.974198707
NDUFB1	0.200836042	0.11305956	0.974198707
MBD3	-0.150325664	0.113070163	0.974198707
GPN1	0.086774158	0.113127787	0.974198707
CCL3L3	-0.475083015	0.113436564	0.974198707
GCOM1	0.118540741	0.113521003	0.974198707
ZBTB2	0.132060369	0.113665379	0.974198707
DBI	0.092405041	0.113680808	0.974198707
POLR2K	0.209861498	0.113692668	0.974198707
PPIF	0.144970428	0.113716766	0.974198707
PSMD1	0.093810524	0.113792387	0.974198707
MTFR1	-0.163123912	0.113809784	0.974198707
AGAP8	-0.180146699	0.113814905	0.974198707
ADC	-0.16178126	0.114018191	0.974198707
ABCB9	-0.34216933	0.114167372	0.974198707
RHBDD3	-0.148962925	0.114202731	0.974198707
TTC8	-0.150841535	0.114241439	0.974198707
C16orf54	0.176308699	0.1142849	0.974198707
APRT	0.124854451	0.114371368	0.974198707
DNM2	0.137048038	0.114606398	0.974198707
VKORC1L1	0.160445692	0.114762273	0.974198707
RGS19	-0.12534177	0.114764783	0.974198707
SH3GLB2	-0.10926402	0.114996015	0.974198707
PKNOX1	-0.128373282	0.115356079	0.974198707
KIR2DL4	0.466655155	0.115519566	0.974198707
IVD	-0.162764781	0.115596366	0.974198707
G2E3	-0.112665321	0.11562158	0.974198707
HDHD2	-0.083327703	0.115624694	0.974198707
GLCCI1	0.163562318	0.115817762	0.974198707
PSMG4	0.125671451	0.115922783	0.974198707
ALG11	-0.117598895	0.116058027	0.974198707
ATP6V1C1	-0.129233491	0.116108162	0.974198707
SPRY2	-0.322451613	0.11610925	0.974198707
RPS27A	0.093261404	0.116160364	0.974198707
GPR141	-0.20058063	0.116273817	0.974198707
NDUFA13	0.102383895	0.116320067	0.974198707
PTPRR	-0.135431572	0.116480578	0.974198707
ARL5B	-0.226861863	0.116578253	0.974198707
ITIH5	-0.042569577	0.116643042	0.974198707
ZSCAN22	-0.156099866	0.116814289	0.974198707

ZNF684	-0.133034823	0.116894388	0.974198707
ERLEC1	-0.115797862	0.117004622	0.974198707
CYP20A1	0.070420459	0.117038246	0.974198707
PUS7	-0.166793138	0.11706013	0.974198707
NKD2	0.248854164	0.117378139	0.974198707
CIB1	-0.107246712	0.117409262	0.974198707
SFI1	-0.149527486	0.117677276	0.974198707
CDK18	-0.172417344	0.117717707	0.974198707
PTGFRN	-0.139583753	0.117770184	0.974198707
RANBP3	-0.090923099	0.117847899	0.974198707
ADIPOR2	0.067755179	0.117952726	0.974198707
TNFRSF19	0.138574051	0.118124287	0.974198707
FAF2	-0.080495213	0.118179161	0.974198707
C3orf52	-0.117368249	0.11845824	0.974198707
SLFN12	-0.171092365	0.118667986	0.974198707
SLC39A3	0.14002585	0.118669797	0.974198707
HNRNPA2B1	0.061183471	0.118788185	0.974198707
PXN	0.1300383	0.118811834	0.974198707
FBXW8	0.160381264	0.118820413	0.974198707
ZBED5	-0.08707114	0.118926878	0.974198707
NOP2	-0.101559632	0.118956161	0.974198707
DGKQ	-0.181682677	0.11902763	0.974198707
USF2	-0.197765423	0.119087126	0.974198707
RAB40C	-0.080948635	0.119116453	0.974198707
SS18L1	-0.129936838	0.119135108	0.974198707
ABCB4	0.227432906	0.119187592	0.974198707
ZNF17	-0.162190488	0.119273793	0.974198707
DSN1	0.11188564	0.119339489	0.974198707
CYP1B1	-0.37693675	0.119386596	0.974198707
TMEM174	0.18291764	0.119397265	0.974198707
LARP4	-0.125092791	0.119485874	0.974198707
ZNF219	-0.134624781	0.119520309	0.974198707
C12orf45	0.149250801	0.119527	0.974198707
NAA20	-0.069057471	0.119536954	0.974198707
ZYG11B	-0.102490246	0.119541315	0.974198707
POLG2	-0.118625679	0.119550291	0.974198707
SYS1	-0.117185027	0.119558025	0.974198707
HSD17B7P2	-0.136811923	0.119587424	0.974198707
SNORA22	-0.200976209	0.119661275	0.974198707
GSK3B	0.121642358	0.119949656	0.974198707
PIK3C2A	-0.205963352	0.119960956	0.974198707
CMTM8	0.354061843	0.119987497	0.974198707
NSUN5	0.124409419	0.119991878	0.974198707
RNF14	-0.109961107	0.120120896	0.974198707
CACNA1H	-0.245025421	0.12028945	0.974198707
CACNA1I	0.25755529	0.120304929	0.974198707
LCP2	-0.112316893	0.120418516	0.974198707

AFMID	0.184278214	0.120509989	0.974198707
PRF1	0.266601477	0.120564637	0.974198707
ALOX5AP	0.133033243	0.120608458	0.974198707
EIF2B4	-0.057360086	0.120668545	0.974198707
HSF2	-0.118106107	0.120879393	0.974198707
ZNF107	0.124977778	0.121122905	0.974198707
JMJD7-PLA2G4B	-0.130019111	0.121218492	0.974198707
TSPYL5	0.236760379	0.121272904	0.974198707
ERP44	-0.190421258	0.12130005	0.974198707
ZNF217	-0.146914765	0.121403585	0.974198707
CCDC7	-0.236079181	0.121419347	0.974198707
CCDC142	-0.210758802	0.121762634	0.974198707
C5orf20	-0.213998639	0.121776009	0.974198707
BPTF	-0.130323851	0.121810901	0.974198707
NCBP1	-0.088475632	0.121881945	0.974198707
MBD5	0.152119837	0.121886458	0.974198707
PPT2	0.152407701	0.121912788	0.974198707
PPP1CB	0.093245204	0.121934775	0.974198707
UGGT2	-0.174737674	0.1222509	0.974198707
NLRP7	-0.296095887	0.122286134	0.974198707
SBF1	-0.084390562	0.12236971	0.974198707
VPREB3	0.390141183	0.122374534	0.974198707
RABEPK	-0.065714467	0.12244024	0.974198707
LEPRE1	-0.086708689	0.122828372	0.974198707
CLEC4A	-0.12159645	0.122968594	0.974198707
RPL23A	0.065268389	0.122997257	0.974198707
FKBP1A	-0.110263257	0.122997293	0.974198707
NEFH	0.157557285	0.123016507	0.974198707
SPRED1	-0.219865452	0.123076377	0.974198707
SERPINI1	0.153293915	0.123165693	0.974198707
C15orf57	0.102924961	0.123401176	0.974198707
MBNL2	-0.11434342	0.123421582	0.974198707
H3F3C	0.136701052	0.123451433	0.974198707
FYCO1	-0.135878565	0.123671187	0.974198707
TREML1	0.421040597	0.12377175	0.974198707
ARF4	-0.089599496	0.123850901	0.974198707
L3MBTL2	-0.114396237	0.123875402	0.974198707
SARM1	-0.140998402	0.123938483	0.974198707
C20orf196	-0.150290609	0.124072779	0.974198707
DCAF16	0.1411746	0.124117777	0.974198707
PEBP1	0.113548152	0.124196739	0.974198707
BAIAP2L2	-0.162711339	0.124492485	0.974198707
MYO9A	0.138356401	0.124555305	0.974198707
PTDSS2	-0.121465755	0.124775744	0.974198707
SH3BP2	0.176724854	0.124910547	0.974198707
FAM171A1	-0.18375313	0.124971096	0.974198707
ITCH	0.128007237	0.125000019	0.974198707

POTEKP	0.130042862	0.125046308	0.974198707
SDF4	0.121173361	0.125207899	0.974198707
TOE1	-0.105109924	0.125238676	0.974198707
COPB1	-0.072019225	0.12530255	0.974198707
LTB	0.088510246	0.125338604	0.974198707
BBS7	-0.105029326	0.125367004	0.974198707
TSPAN4	-0.18600261	0.125407279	0.974198707
ZNF668	0.11817173	0.125641463	0.974198707
ZNF664	0.146214998	0.125690271	0.974198707
ASCC1	0.135123437	0.125752771	0.974198707
IPO8	0.305761336	0.125836945	0.974198707
SPATA13	-0.220865148	0.125839582	0.974198707
ERP27	-0.158466864	0.125963878	0.974198707
MBNL1	-0.311359849	0.125993256	0.974198707
SLC35E1	-0.268868121	0.126099666	0.974198707
NHLRC2	0.168239219	0.12614021	0.974198707
SLC25A16	-0.151753687	0.126174912	0.974198707
COQ6	-0.09423964	0.126219852	0.974198707
LETM1	0.146179919	0.126246462	0.974198707
GTF3C1	0.169861394	0.126253669	0.974198707
TBCB	0.100737216	0.126436068	0.974198707
HOXC13	-0.140072052	0.126549196	0.974198707
KTN1	0.144449494	0.126630951	0.974198707
FAM101B	0.22035359	0.126648475	0.974198707
PDE7A	-0.282025725	0.126803676	0.974198707
SLC38A7	-0.167512064	0.127178898	0.974198707
CD180	0.149179263	0.127389859	0.974198707
PTCD1	-0.133725406	0.127434475	0.974198707
ING1	0.116151293	0.127527952	0.974198707
RBM15B	0.115852799	0.127597553	0.974198707
BCL2L11	0.174605053	0.127717026	0.974198707
ATP5D	0.101556793	0.127890266	0.974198707
WWP2	0.157277888	0.12807027	0.974198707
CDCA7L	0.17406437	0.12838182	0.974198707
TRIM4	-0.127358258	0.128418099	0.974198707
NSMCE1	0.123057582	0.128533399	0.974198707
CA2	-0.345850654	0.128715626	0.974198707
MAPK8IP3	-0.180354997	0.128850905	0.974198707
RPS3A	0.086466904	0.128939174	0.974198707
SCLY	0.096390223	0.128962367	0.974198707
PIK3C2B	0.205394955	0.129048496	0.974198707
NRBP2	-0.125073006	0.129082357	0.974198707
LRRC20	-0.14495786	0.129172586	0.974198707
GNPDA1	0.104569124	0.129341762	0.974198707
AARS2	-0.103351955	0.129352045	0.974198707
CD8B	-0.340010871	0.129389886	0.974198707
CD37	0.145789911	0.129591314	0.974198707

GJA5	0.13350541	0.129742192	0.974198707
AGAP3	-0.139673028	0.129946709	0.974198707
CSNK1A1	0.121306593	0.129970473	0.974198707
GPS2	0.079378036	0.129987327	0.974198707
DAZAP1	0.093303475	0.130131704	0.974198707
MYH3	-0.160396729	0.130169975	0.974198707
HERC2P3	0.150161881	0.130174725	0.974198707
EPHA1	-0.243297677	0.130296293	0.974198707
ATP8A1	0.135836349	0.130317586	0.974198707
UBTD1	0.170234542	0.13032161	0.974198707
UBE2Z	0.110130927	0.130431364	0.974198707
HSDL2	0.136562714	0.130434075	0.974198707
GNA13	0.080123446	0.130478818	0.974198707
MMS19	-0.077065564	0.130486737	0.974198707
TMEM184B	0.142715253	0.130699156	0.974198707
NAV1	-0.182350871	0.130728989	0.974198707
EIF3L	-0.073947327	0.130740194	0.974198707
IQCK	0.13628945	0.130803036	0.974198707
PDE3B	-0.164078782	0.130857426	0.974198707
MTMR10	-0.117122981	0.13086685	0.974198707
RBM4	0.115039499	0.130993589	0.974198707
PTBP2	-0.123456617	0.130998949	0.974198707
IL17RB	0.208279828	0.131111013	0.974198707
TMEM150B	-0.132859102	0.131180024	0.974198707
IFNG	-0.492826401	0.131485648	0.974198707
PHF1	0.134909702	0.131557804	0.974198707
CAB39L	0.12697448	0.131564526	0.974198707
NLE1	-0.144562622	0.131747846	0.974198707
EPB41L2	0.152215987	0.131809456	0.974198707
EPB41L4A	0.135951244	0.131885126	0.974198707
MLF2	0.102412828	0.131900242	0.974198707
SUPT3H	0.180422681	0.131924728	0.974198707
RPF2	0.103557621	0.132130282	0.974198707
ARHGEF2	0.098825122	0.132218346	0.974198707
C10orf2	-0.133752102	0.132276334	0.974198707
ZNF79	-0.222905641	0.132291119	0.974198707
CWC25	-0.126277398	0.132313511	0.974198707
KIAA0513	-0.11966726	0.132370135	0.974198707
AHSA1	0.103466941	0.132389193	0.974198707
COX19	-0.154598787	0.132420652	0.974198707
HSPB3	0.206041881	0.132494943	0.974198707
WNT10B	-0.21219622	0.132518597	0.974198707
BRAT1	-0.065589372	0.132591216	0.974198707
TRAF3IP1	0.139617461	0.132633546	0.974198707
SLC25A42	0.10116553	0.132717732	0.974198707
RBBP5	0.097035138	0.13274399	0.974198707
CELSR3	0.239726631	0.132791944	0.974198707

SNX13	-0.127588705	0.132893937	0.974198707
MAP4K1	-0.116460776	0.132943303	0.974198707
UVRAG	-0.100137254	0.13321284	0.974198707
SNORD32A	0.154705631	0.133250035	0.974198707
MIER2	0.199545016	0.13331739	0.974198707
UBR7	-0.082383515	0.133361147	0.974198707
GP1BA	0.273226535	0.133683517	0.974198707
JMJD1C	-0.11124629	0.133750997	0.974198707
GNAQ	0.235305214	0.133756574	0.974198707
YLPM1	-0.126833612	0.133870645	0.974198707
FAM72B	-0.168719614	0.133961359	0.974198707
DNAH2	0.120151006	0.134125532	0.974198707
HCLS1	0.153544006	0.134363153	0.974198707
PPP1R3D	-0.182327251	0.134374258	0.974198707
THNSL2	0.50735226	0.13446381	0.974198707
RAB1B	0.131892039	0.134496071	0.974198707
NUDCD1	0.104136295	0.134515777	0.974198707
RCHY1	0.186032647	0.134637909	0.974198707
SKI	0.098109347	0.134670938	0.974198707
C12orf57	0.156376499	0.134722388	0.974198707
ABCE1	-0.072465937	0.13474471	0.974198707
BTRC	-0.118312887	0.134779745	0.974198707
ZNF613	-0.161795987	0.134799804	0.974198707
IRF5	0.573628328	0.134832277	0.974198707
WDR37	-0.094510447	0.134835139	0.974198707
TTC9C	-0.128834225	0.1348628	0.974198707
CCDC136	-0.125265009	0.134944057	0.974198707
CYP27B1	0.118556739	0.134987061	0.974198707
SUV420H1	0.100269343	0.13499183	0.974198707
FOSL2	0.129274054	0.135117426	0.974198707
WDR20	-0.110148769	0.135140743	0.974198707
KCNG1	0.317446974	0.135312745	0.974198707
TMEM19	0.117654656	0.13542276	0.974198707
RBM28	0.103092985	0.135429891	0.974198707
DCAF12	-0.100043324	0.13549405	0.974198707
ZNF513	-0.112174576	0.135494357	0.974198707
PRKAR1A	-0.766435918	0.135604789	0.974198707
PRICKLE1	0.250515205	0.136056825	0.974198707
HYOU1	-0.153673923	0.136146134	0.974198707
SKIV2L	-0.098305544	0.136192652	0.974198707
STAP1	0.272529102	0.136252011	0.974198707
SMEK2	-0.066543937	0.136308653	0.974198707
PPFIA4	-0.350810373	0.136368438	0.974198707
BST2	0.120986663	0.136416215	0.974198707
NUP88	0.075850328	0.136485448	0.974198707
C15orf48	0.152001456	0.136652972	0.974198707
ATOH7	0.135183151	0.136666581	0.974198707

SNORA28	0.311620299	0.136960593	0.974198707
PIK3AP1	0.12692189	0.137003348	0.974198707
CCL20	-0.192735011	0.137292352	0.974198707
SNRNP27	0.116587068	0.137331246	0.974198707
GRASP	0.249084595	0.137389674	0.974198707
STK35	-0.1639232	0.137404747	0.974198707
GM2A	0.245618	0.137478065	0.974198707
LRRC2	-0.148617966	0.137502529	0.974198707
CCBL2	0.073579051	0.137542001	0.974198707
RDM1	-0.155918645	0.137764062	0.974198707
DOHH	-0.123703664	0.138009489	0.974198707
KIAA0141	-0.097136345	0.138073353	0.974198707
BACE2	0.268707607	0.138102684	0.974198707
LAMB1	-0.129757031	0.138158829	0.974198707
CPT1B	-0.250095691	0.138188579	0.974198707
CNTLN	0.133355652	0.138290815	0.974198707
GPAA1	0.114194497	0.138298966	0.974198707
GET4	0.127683935	0.138315577	0.974198707
STRN	0.11893696	0.138340265	0.974198707
NFIA	-0.150071354	0.138672826	0.974198707
GPR19	-0.23290734	0.138688595	0.974198707
SLC35B3	-0.126780813	0.138813317	0.974198707
SDHA	0.081748449	0.138922686	0.974198707
MAPKAPK5	0.091523293	0.13892928	0.974198707
C9orf64	-0.120919254	0.138939252	0.974198707
STX1A	0.147845809	0.139096072	0.974198707
LRSAM1	-0.080756928	0.139140048	0.974198707
IRAK1BP1	-0.211878166	0.139213049	0.974198707
MGC2752	-0.150951934	0.139226419	0.974198707
MEI1	-0.119327466	0.139309915	0.974198707
TCF3	0.140285253	0.139370395	0.974198707
ARFGEF1	-0.070359002	0.139382814	0.974198707
TRIM62	0.140953774	0.13939955	0.974198707
SLC16A10	-0.236333423	0.139413947	0.974198707
DUSP22	-0.102918127	0.13955886	0.974198707
RAD1	-0.127119855	0.139573733	0.974198707
ITIH4	-0.282698084	0.139595924	0.974198707
ALK	-0.160390102	0.139613126	0.974198707
PNP	-0.144165343	0.139730257	0.974198707
TXNL4A	0.154432745	0.139795839	0.974198707
FAM71E2	0.129421204	0.139908385	0.974198707
NUTF2	0.099420991	0.139908582	0.974198707
RAPH1	-0.162763912	0.140002845	0.974198707
TMX3	-0.09231259	0.140023525	0.974198707
SEPHS1	-0.076862711	0.140141777	0.974198707
DNAJB9	-0.169733129	0.140208687	0.974198707
CLASRP	0.139816804	0.140209997	0.974198707

CXCR1	-0.187290395	0.140243739	0.974198707
PRPF19	0.086204224	0.140265434	0.974198707
DHX16	-0.080743255	0.140432504	0.974198707
ZNF197	-0.098649679	0.140873938	0.974198707
EXOC4	0.081825211	0.140887393	0.974198707
ZNF490	0.144353797	0.140900559	0.974198707
CDC42SE1	0.1655559	0.14090515	0.974198707
GADD45B	0.123971701	0.140928418	0.974198707
PSMA7	0.151240136	0.140935709	0.974198707
BABAM1	0.069656766	0.141054533	0.974198707
USP12	0.145662281	0.14109558	0.974198707
ZNF532	-0.174157577	0.141328663	0.974198707
RIMKLB	0.149814883	0.141375255	0.974198707
XRCC6	0.090568463	0.141442579	0.974198707
TEX2	-0.091530285	0.141455795	0.974198707
CS	0.083165475	0.141476823	0.974198707
PPP1CA	0.108342717	0.14153984	0.974198707
ARL6IP1	0.089256164	0.141703051	0.974198707
SOCS4	-0.117711902	0.141716376	0.974198707
CEPT1	-0.095476079	0.141983619	0.974198707
GOLGA6L1	0.049022167	0.141991953	0.974198707
SHB	0.105598091	0.142005314	0.974198707
SUMO3	0.082231958	0.142010118	0.974198707
NFKBIE	-0.209123787	0.142021709	0.974198707
C14orf2	0.116127277	0.142101378	0.974198707
ZNF705G	0.167269202	0.142133628	0.974198707
UBXN7	0.124883174	0.14238503	0.974198707
ENPP2	-0.197866808	0.142395255	0.974198707
LSMD1	0.108891397	0.142540766	0.974198707
PRKAB1	-0.101828083	0.142623063	0.974198707
ZSWIM6	-0.12008896	0.142664712	0.974198707
TCIRG1	-0.126847909	0.14288598	0.974198707
PKD2	-0.146701923	0.14289208	0.974198707
COPS5	0.066154039	0.14292661	0.974198707
TPM1	0.388343982	0.143095189	0.974198707
NACC2	-0.126644252	0.143304707	0.974198707
RNF34	-0.102762586	0.143831007	0.974198707
P4HB	-0.087335674	0.143840575	0.974198707
ZNF226	0.154199379	0.143972727	0.974198707
FAM103A1	-0.123554301	0.144038893	0.974198707
IAH1	0.077709879	0.144130893	0.974198707
ELOF1	0.09828368	0.144363726	0.974198707
SLC35F5	-0.151096699	0.14437793	0.974198707
COL24A1	-0.161656309	0.144452797	0.974198707
SNX19	0.111395799	0.144458198	0.974198707
CDC34	0.110864719	0.144544652	0.974198707
XRCC5	0.122111046	0.144806943	0.974198707

NR4A2	0.261649742	0.144817009	0.974198707
PSD3	0.118506432	0.144825994	0.974198707
GSTA1	0.438647773	0.14490548	0.974198707
LRRC25	0.39039419	0.145220702	0.974198707
PCBP1	0.085699685	0.145236643	0.974198707
SHISA4	0.160332224	0.145434198	0.974198707
MYPOP	-0.059032523	0.145676691	0.974198707
CYB5R3	0.102945811	0.145747912	0.974198707
MAVS	-0.173894079	0.145788138	0.974198707
PTPRU	-0.23697963	0.14590735	0.974198707
ZNF260	-0.126375081	0.146219706	0.974198707
FLT3	-0.219546351	0.146234919	0.974198707
SLC19A2	0.116195148	0.146385221	0.974198707
LYPD3	0.139349396	0.146423914	0.974198707
VIPR1	-0.162850708	0.146455547	0.974198707
RAB27B	0.258474863	0.146517494	0.974198707
ADRA2B	0.133035303	0.146558966	0.974198707
CCRL2	-0.195763115	0.146568321	0.974198707
LHPP	0.148679003	0.146866001	0.974198707
EFTUD2	0.072832645	0.14690222	0.974198707
ZNF526	-0.161928344	0.146914825	0.974198707
TRIP12	-0.063204808	0.146921497	0.974198707
DYRK2	-0.153712291	0.146923165	0.974198707
SPPL2B	-0.138400708	0.147011383	0.974198707
PSMB3	0.070822305	0.147194985	0.974198707
SMUG1	-0.068341849	0.147245161	0.974198707
CDKN2D	0.171711654	0.147524575	0.974198707
CDH20	0.138797719	0.147710782	0.974198707
AEBP2	-0.11369576	0.148043206	0.974198707
B4GALT7	-0.093797522	0.148104539	0.974198707
ARGLU1	-0.087830271	0.148186148	0.974198707
METTL8	0.135713814	0.148364278	0.974198707
DGKG	0.137093654	0.148481848	0.974198707
RNASEH2C	0.128161476	0.14864029	0.974198707
SNTA1	0.118511212	0.148677157	0.974198707
ZNF821	0.130747245	0.148728845	0.974198707
HNRNPL	0.142125525	0.148824314	0.974198707
ZNF35	-0.189970915	0.148861144	0.974198707
NAA16	-0.114635743	0.148880593	0.974198707
TMEM159	0.113795755	0.148953781	0.974198707
MTHFD1	0.12573661	0.149003945	0.974198707
ZNF2	-0.159066917	0.149014894	0.974198707
CYTH4	-0.092943176	0.149029026	0.974198707
SNORA7B	0.100094157	0.149092388	0.974198707
NEDD4	-0.1498	0.149199201	0.974198707
NUP43	-0.097133505	0.149262736	0.974198707
TARDBP	-0.140811879	0.149495116	0.974198707

ACER2	0.126308795	0.149540831	0.974198707
PCYT2	-0.101986614	0.149585444	0.974198707
SCARNA9	-0.198602702	0.149648093	0.974198707
GLRX5	0.077592468	0.149790192	0.974198707
NISCH	-0.097714844	0.14982793	0.974198707
RPS15A	0.043345651	0.14984715	0.974198707
C7orf26	-0.083466696	0.15006305	0.974198707
ZDHHC19	0.187449502	0.150108484	0.974198707
PAK1	-0.124561349	0.150121413	0.974198707
SPTY2D1	-0.110361047	0.150157015	0.974198707
TONSL	0.174945931	0.150279575	0.974198707
SLC25A10	0.146829312	0.150389129	0.974198707
ZNF879	0.150900816	0.150397078	0.974198707
IFT57	0.174595427	0.15048721	0.974198707
SEMA3C	-0.185779222	0.150540835	0.974198707
HAUS5	0.125916646	0.150602508	0.974198707
TMEM40	0.275283701	0.150730587	0.974198707
FZD3	-0.135840532	0.150730614	0.974198707
DDIT3	0.098419114	0.150835266	0.974198707
FAM175B	-0.068334422	0.150920882	0.974198707
SNORD22	0.139870343	0.150923983	0.974198707
ITGB2	-0.05209434	0.151091571	0.974198707
GGA2	0.099602447	0.15109402	0.974198707
MCM7	-0.085909009	0.151155731	0.974198707
GTPBP3	-0.099657519	0.151390523	0.974198707
NEO1	0.092300108	0.151482019	0.974198707
ACSL1	-0.159071704	0.151512968	0.974198707
BOLA2	0.116700803	0.151603095	0.974198707
LCN1	0.118542733	0.151763039	0.974198707
HS2ST1	0.126506939	0.151945791	0.974198707
BLVRA	0.127363125	0.152134894	0.974198707
PIGB	-0.090935613	0.152269025	0.974198707
LONRF1	-0.132646496	0.152269363	0.974198707
ABCB10	-0.095668784	0.152295615	0.974198707
SETDB1	-0.0957722	0.152387838	0.974198707
SLFN5	-0.189593074	0.152474705	0.974198707
TNFRSF12A	0.129817412	0.15249214	0.974198707
COX6B1	0.086395415	0.152566823	0.974198707
TMEM107	0.578835809	0.152671757	0.974198707
NAP1L5	0.153490876	0.152815024	0.974198707
PPP4R2	0.226123352	0.15282391	0.974198707
PRKCDBP	-0.317513647	0.152960561	0.974198707
ERCC6	0.128826723	0.152978418	0.974198707
DCLK1	0.126678748	0.152994459	0.974198707
TMED3	0.086346441	0.15306626	0.974198707
ABCB6	-0.130510564	0.153104235	0.974198707
GLTSCR1	0.097726474	0.153222963	0.974198707

AMMECR1L	-0.105562786	0.153323133	0.974198707
ULK1	-0.07968177	0.153329747	0.974198707
C2CD2	-0.127476621	0.153341492	0.974198707
SIPA1L3	0.201367057	0.153435046	0.974198707
FKBP9L	0.204307903	0.153615775	0.974198707
AGTPBP1	-0.088215988	0.153702898	0.974198707
VPS26A	-0.070988214	0.153724784	0.974198707
RAD17	0.109499296	0.153794034	0.974198707
NT5C2	-0.059007085	0.153811158	0.974198707
SGSM2	-0.084546449	0.153845961	0.974198707
PHGDH	-0.290277088	0.153902549	0.974198707
ANKRD9	0.331750057	0.153907586	0.974198707
ANKRD28	0.135845284	0.153915397	0.974198707
TRAPPC3	0.088910543	0.154216714	0.974198707
C17orf98	0.16400372	0.154223565	0.974198707
OSGEPL1	-0.128340112	0.154247522	0.974198707
C1orf101	-0.13710202	0.154268208	0.974198707
KLHL21	-0.123286298	0.154456613	0.974198707
PCBD2	-0.101581785	0.155051264	0.974198707
MYB	-0.151929869	0.155134743	0.974198707
SLC27A1	-0.129309321	0.155139881	0.974198707
NEDD4L	-0.147761437	0.155345174	0.974198707
ADORA2B	-0.17014647	0.15536986	0.974198707
RSG1	-0.131855566	0.155467005	0.974198707
AASDH	-0.102091441	0.155488555	0.974198707
PFKL	-0.146952854	0.155524503	0.974198707
GUK1	0.070312487	0.155530985	0.974198707
CXXC5	0.170129401	0.155602993	0.974198707
SLC37A1	0.101259275	0.155663705	0.974198707
DNTTIP1	-0.118885333	0.15574166	0.974198707
FCHO1	-0.105331568	0.155874645	0.974198707
ZNF562	-0.113702846	0.155926423	0.974198707
RNF130	0.126537072	0.15599918	0.974198707
RAB5A	-0.107108645	0.156264588	0.974198707
NDUFB7	0.103414903	0.156468541	0.974198707
CDKN2B	-0.212752227	0.156482262	0.974198707
AP2M1	0.084985399	0.156599209	0.974198707
RETSAT	-0.111824088	0.156783611	0.974198707
SH3PXD2A	0.22387403	0.156842843	0.974198707
RPL39L	0.049959828	0.156953625	0.974198707
ZFYVE26	-0.07998088	0.15722002	0.974198707
ARL15	-0.137876831	0.157338956	0.974198707
FAM13A	0.181124924	0.157358554	0.974198707
XPNPEP3	-0.055523701	0.15750326	0.974198707
ECHS1	0.085494688	0.157512751	0.974198707
MIR1307	0.695666706	0.157552013	0.974198707
UBE3A	-0.057939673	0.157704263	0.974198707

ABCC5	-0.129957472	0.157710448	0.974198707
PPP2R4	0.080209908	0.157731351	0.974198707
DDX11	-0.384884451	0.157783998	0.974198707
RNF149	-0.103446233	0.157791007	0.974198707
ARHGAP26	-0.118491459	0.157868583	0.974198707
PLEKHA8P1	0.094381217	0.157990554	0.974198707
HAUS8	-0.111659647	0.157998871	0.974198707
MAP3K5	-0.119318574	0.158119076	0.974198707
SLFN11	-0.109957808	0.158152327	0.974198707
RPL37	0.180462212	0.15819968	0.974198707
ARL13B	-0.120818471	0.158212033	0.974198707
DDX47	-0.065589094	0.158236358	0.974198707
SLC16A5	-0.13683897	0.158524284	0.974198707
ZNF473	0.0909604	0.158558352	0.974198707
ZNF418	0.209856188	0.158594314	0.974198707
CHST3	-0.125274362	0.158764636	0.974198707
UBE2D3	0.098820317	0.158923024	0.974198707
ALDH2	-0.165151442	0.158925753	0.974198707
ORAI1	0.142212186	0.158932549	0.974198707
SIVA1	0.090728378	0.159052913	0.974198707
ORMDL1	-0.067956059	0.159177686	0.974198707
CCL28	0.149651174	0.159255882	0.974198707
ZNF518B	0.095509291	0.159303029	0.974198707
PDIA5	-0.15753949	0.159308153	0.974198707
DDA1	0.069904167	0.159419561	0.974198707
SAP30BP	-0.113368537	0.159498583	0.974198707
DISC1	0.175534579	0.159733866	0.974198707
ZBTB4	0.085736295	0.159748161	0.974198707
CPNE5	0.212232438	0.159775581	0.974198707
SARS	0.084548289	0.159785345	0.974198707
EIF4E2	0.071214535	0.159948664	0.974198707
TIMM17A	-0.121976485	0.159952238	0.974198707
PHF20L1	-0.070450612	0.159997101	0.974198707
CD7	0.125304684	0.160055468	0.974198707
GNAZ	0.313190026	0.160063744	0.974198707
BAK1	-0.161730934	0.160114107	0.974198707
HBD	0.346113395	0.160218747	0.974198707
ATG9A	-0.108418528	0.160229954	0.974198707
PPRC1	-0.086589863	0.160240037	0.974198707
HIST1H2AD	0.257778336	0.160461331	0.974198707
NTS	0.150852601	0.160545467	0.974198707
FRS3	-0.097405159	0.160647719	0.974198707
CHN1	-0.273193155	0.160701249	0.974198707
PDHB	-0.072767168	0.16089874	0.974198707
RFXANK	0.077622625	0.160969853	0.974198707
SLC35A1	-0.0815958	0.160980018	0.974198707
SCARNA2	0.216235138	0.160989735	0.974198707

OSGIN1	0.206103514	0.161012286	0.974198707
ATXN7L3B	0.086101354	0.161028294	0.974198707
DHRS4L2	0.158049418	0.161031823	0.974198707
ZCCHC8	-0.086966078	0.161303895	0.974198707
ICA1L	-0.200572112	0.161334247	0.974198707
ZNF566	-0.166354417	0.161352116	0.974198707
CRCP	-0.155329503	0.161443916	0.974198707
ULK2	-0.118151426	0.161508283	0.974198707
PIWIL4	-0.120997441	0.161624855	0.974198707
MIR302C	-0.142913923	0.161764145	0.974198707
CIRBP	0.098345662	0.161794815	0.974198707
USE1	0.120372477	0.161810596	0.974198707
ID2	0.12899647	0.161814148	0.974198707
MKNK2	0.087160362	0.161907775	0.974198707
SPINT2	0.12382695	0.161948463	0.974198707
QARS	-0.085571488	0.162169039	0.974198707
STAG3L2	-0.194323625	0.162182676	0.974198707
FXYD5	0.110189698	0.16224062	0.974198707
IPO11	-0.11544863	0.16261148	0.974198707
HERPUD1	-0.090518969	0.16272644	0.974198707
PTP4A1	0.188550492	0.16277398	0.974198707
NDUFA3	0.084500612	0.1628437	0.974198707
ORMDL3	0.198898765	0.162899662	0.974198707
PLEK	0.104853886	0.162937759	0.974198707
TRAF7	-0.130115163	0.162970005	0.974198707
MARCKSL1	0.111123403	0.163345855	0.974198707
PPM1F	-0.094068994	0.163390532	0.974198707
POR	0.107859661	0.163407175	0.974198707
ALB	0.140506949	0.163429719	0.974198707
C6orf163	-0.157081408	0.163521548	0.974198707
ZNF18	-0.103756667	0.163536476	0.974198707
CYP2D6	-0.139066158	0.163606102	0.974198707
HMBOX1	0.308686492	0.163982319	0.974198707
TRIP4	-0.095650133	0.164018762	0.974198707
NDUFC2	0.161301606	0.164095272	0.974198707
MRPL23	0.096889071	0.164193653	0.974198707
C7orf43	-0.102016461	0.164224614	0.974198707
IFI27L2	0.122863878	0.164265556	0.974198707
PAK2	0.066749852	0.164277496	0.974198707
DND1	-0.154943259	0.164350281	0.974198707
SLA2	0.170624482	0.164389727	0.974198707
G3BP2	-0.068116041	0.164469584	0.974198707
VPS54	-0.115636887	0.164478923	0.974198707
SLC1A7	0.345367819	0.164617739	0.974198707
SAT2	-0.082473405	0.164836485	0.974198707
GPR18	-0.270337888	0.165087828	0.974198707
KCNMB3	-0.144215854	0.165090136	0.974198707

TTC13	-0.111038523	0.16519293	0.974198707
SAE1	0.064057652	0.165411789	0.974198707
SERF1B	0.182536472	0.165446279	0.974198707
STARD10	0.143669696	0.165605064	0.974198707
MFHAS1	0.269602966	0.165605275	0.974198707
GNB1	0.054957329	0.165610511	0.974198707
SPRED2	0.149725616	0.165689785	0.974198707
ATXN1	-0.103240734	0.165743036	0.974198707
THOP1	-0.103161971	0.16577692	0.974198707
TAPT1	0.10992644	0.16580598	0.974198707
NAB1	-0.12057662	0.165820107	0.974198707
STRADB	0.110844534	0.165983929	0.974198707
CTSK	-0.183243864	0.16599528	0.974198707
EXOSC3	-0.140582412	0.166018964	0.974198707
QTRTD1	-0.074563957	0.166132674	0.974198707
GSTT1	0.490535702	0.16618634	0.974198707
ZNF154	0.35134559	0.166352231	0.974198707
ZC3H10	-0.154393003	0.16635579	0.974198707
SH3RF3	-0.128345647	0.166427447	0.974198707
MBD1	0.095918581	0.166584054	0.974198707
ZNF189	-0.118036668	0.166627745	0.974198707
PITPNB	0.09104018	0.166664188	0.974198707
DENND4B	-0.125021422	0.16674118	0.974198707
SNORA5C	0.112559897	0.166771072	0.974198707
TFB2M	-0.091209239	0.166802449	0.974198707
CD33	-0.156809828	0.166861436	0.974198707
ZNF507	0.157332158	0.167127986	0.974198707
TLN1	0.142452149	0.167147747	0.974198707
TMEM110	-0.151912301	0.167247159	0.974198707
CHPT1	0.17072892	0.167307289	0.974198707
NR2F2	0.133620091	0.167561168	0.974198707
PFDN1	0.095744741	0.167673021	0.974198707
CYP2R1	-0.09958306	0.167849989	0.974198707
YWHAB	0.063322402	0.167864944	0.974198707
EPHA8	0.128959533	0.16791629	0.974198707
BIRC5	-0.215191027	0.168039024	0.974198707
STK36	-0.107871872	0.168168698	0.974198707
HNRNPK	0.074691932	0.1681743	0.974198707
TMEM86B	-0.13627986	0.168364819	0.974198707
HLA-DPB1	-0.259912076	0.168527594	0.974198707
SLX1B-SULT1A4	0.130832061	0.168562549	0.974198707
RAP1GDS1	0.109892228	0.168686025	0.974198707
DPM2	0.148913506	0.168698066	0.974198707
CLTB	0.069561299	0.168698201	0.974198707
NUP160	-0.086202301	0.168765048	0.974198707
TMEM127	0.107467977	0.168917709	0.974198707
C12orf4	-0.101945026	0.168936119	0.974198707

KHNYN	0.133090004	0.168964092	0.974198707
ERMAP	-0.164636288	0.169051978	0.974198707
MTO1	-0.078036213	0.169238188	0.974198707
TIMELESS	0.113519061	0.169304188	0.974198707
PITPNC1	0.08475754	0.169473454	0.974198707
HMGN3	0.124285989	0.16950538	0.974198707
ATAD3A	-0.120890707	0.169532229	0.974198707
GGT3P	0.110845172	0.169543682	0.974198707
C7orf49	-0.109108622	0.169674633	0.974198707
WASH3P	-0.090844119	0.169828361	0.974198707
TMEM71	0.132982908	0.169832979	0.974198707
RSRC2	-0.086189914	0.170114852	0.974198707
WRB	0.184570164	0.170195169	0.974198707
SNORD68	0.164479184	0.170301002	0.974198707
ZYX	0.116943629	0.170340107	0.974198707
BOLA2B	0.092190078	0.17046478	0.974198707
SNAPC3	-0.096718274	0.170586118	0.974198707
KCTD3	-0.152838968	0.170597521	0.974198707
PMPCB	-0.070351133	0.170615189	0.974198707
CSNK2A2	-0.081395181	0.170659577	0.974198707
PABPN1	0.086884443	0.171050234	0.974198707
USP30	-0.136664948	0.171079865	0.974198707
WIPF2	-0.133866625	0.171152759	0.974198707
UPK1A	-0.130693062	0.171209028	0.974198707
RGL2	-0.110760039	0.171300589	0.974198707
UTS2	0.290258858	0.171389103	0.974198707
IFITM2	0.164575523	0.17154669	0.974198707
GSPT1	0.055251967	0.171689102	0.974198707
PPP4C	0.063551866	0.171702932	0.974198707
KCTD18	-0.084064609	0.171855837	0.974198707
IL25	0.147978163	0.171914372	0.974198707
HNRNPUL1	0.078285207	0.171933053	0.974198707
AKIP1	-0.119506178	0.171934681	0.974198707
CD79B	0.193334418	0.171950406	0.974198707
SNORD35B	0.157884489	0.171963637	0.974198707
IGKC	0.308495258	0.172027399	0.974198707
KLC1	0.106656882	0.172079026	0.974198707
MED28	-0.083328592	0.172160012	0.974198707
PRPF18	0.132641041	0.172174449	0.974198707
USP46	0.159245666	0.172442054	0.974198707
FOXD4	-0.143094861	0.172462326	0.974198707
LRRC16A	0.177073898	0.172478591	0.974198707
EPHB6	-0.159265387	0.172541893	0.974198707
DDIT4	0.2926842	0.172757112	0.974198707
EXTL2	-0.152549343	0.172788409	0.974198707
CHST15	-0.11551483	0.172820797	0.974198707
SDHAF1	0.066003216	0.173067846	0.974198707

NSUN5P1	0.144728841	0.173143515	0.974198707
CHIC2	-0.1158245	0.173200379	0.974198707
CLPP	0.104455018	0.17369571	0.974198707
PIGL	0.126401792	0.173726148	0.974198707
ZFYVE27	-0.129277506	0.173816413	0.974198707
C7orf55	0.120487001	0.173834091	0.974198707
C11orf83	0.105097731	0.173900728	0.974198707
MANBA	-0.096228805	0.17396667	0.974198707
CYB5R1	0.103578151	0.174194188	0.974198707
KRT72	0.681085046	0.174258145	0.974198707
GPSM2	0.119204795	0.174354853	0.974198707
INSM1	0.133594417	0.174475868	0.974198707
RPS20	0.042152385	0.174588325	0.974198707
C9orf16	0.13224703	0.174614994	0.974198707
RBM38	0.133109656	0.174624411	0.974198707
COMMD4	0.095582908	0.174740733	0.974198707
CHORDC1	-0.105059622	0.174853627	0.974198707
FCGRT	-0.111247325	0.174910892	0.974198707
ZNF787	0.082372041	0.175023951	0.974198707
HRASLS2	-0.345143676	0.175257463	0.974198707
ATG7	-0.116789831	0.175258981	0.974198707
ADRB2	-0.258702282	0.175279236	0.974198707
RPE	-0.116253748	0.175288196	0.974198707
ZBBX	0.100676928	0.175294754	0.974198707
B3GALNT2	-0.137540881	0.175337261	0.974198707
SNORA27	0.175599001	0.175399185	0.974198707
MMP28	-0.272865372	0.175400917	0.974198707
ANGEL1	0.100780269	0.175547576	0.974198707
SWAP70	0.155591517	0.175588712	0.974198707
MED21	-0.184156243	0.175614034	0.974198707
FDPS	0.085591658	0.175627948	0.974198707
NARFL	-0.128001959	0.175681903	0.974198707
YTHDF1	0.061727387	0.175823894	0.974198707
MTG1	-0.119154102	0.176089848	0.974198707
FAM74A4	0.103413961	0.176105977	0.974198707
RNY4	0.269591331	0.176118882	0.974198707
OR7E12P	0.122096693	0.176131907	0.974198707
PLEKHJ1	0.127712487	0.176139272	0.974198707
IMMP2L	0.17046743	0.17617588	0.974198707
ETF1	-0.066633426	0.176220372	0.974198707
KIR3DL2	0.365503488	0.176253559	0.974198707
ISG15	0.266985569	0.176398808	0.974198707
HIC2	-0.079974858	0.176470078	0.974198707
OBFC1	0.088143159	0.176481383	0.974198707
LY6E	0.180694441	0.176503654	0.974198707
RASL10A	-0.155124734	0.176543071	0.974198707
SCAND1	0.085112054	0.176578363	0.974198707

RECK	-0.100526873	0.176743466	0.974198707
CENPV	0.157559892	0.176862026	0.974198707
FCRLA	0.329220845	0.176954749	0.974198707
DAP3	-0.068036237	0.177056185	0.974198707
CSTA	-0.204366711	0.177155596	0.974198707
RPS23	0.378063757	0.177161931	0.974198707
PGBD2	-0.127059369	0.17723938	0.974198707
MAN2A2	-0.110645235	0.177416265	0.974198707
FMO5	-0.14727333	0.177558385	0.974198707
RCN3	-0.155866718	0.177604228	0.974198707
NIPSNAP1	0.100519034	0.177618471	0.974198707
U2AF2	0.042809963	0.177638808	0.974198707
DHX8	0.097757221	0.177717308	0.974198707
GMPR	0.266525636	0.177762597	0.974198707
ASS1	0.105120903	0.177808642	0.974198707
TTK	-0.169926299	0.178163271	0.974198707
NPR2	-0.115234745	0.178234114	0.974198707
HDAC11	-0.146210887	0.178439085	0.974198707
SH3RF2	-0.147211077	0.178465302	0.974198707
UBAC1	0.069771473	0.17848373	0.974198707
DPP8	-0.062025773	0.178532482	0.974198707
MTX3	-0.131350569	0.178839926	0.974198707
TSTA3	0.102563296	0.178888442	0.974198707
GLYCTK	-0.120205452	0.179121514	0.974198707
PIK3R2	0.072964373	0.179124095	0.974198707
MYSM1	-0.165353421	0.179134281	0.974198707
CLEC18C	-0.153475632	0.179266267	0.974198707
TUBA3C	-0.114807941	0.17942096	0.974198707
PIK3IP1	-0.145715626	0.179666522	0.974198707
ZNF627	-0.085558723	0.179675275	0.974198707
RNF208	0.14074455	0.179790115	0.974198707
RUNX1	-0.082545912	0.179796971	0.974198707
EXOC6	0.068641398	0.179801254	0.974198707
GPAM	-0.10953951	0.179910127	0.974198707
MFAP3L	0.265579711	0.1800937	0.974198707
RAN	0.09824971	0.180145342	0.974198707
ELOVL4	-0.300880121	0.180178848	0.974198707
PGBD4	-0.125917797	0.18021435	0.974198707
PMS2	0.126386256	0.180314801	0.974198707
POFUT2	-0.094891927	0.180505024	0.974198707
DPH2	-0.061540931	0.180548188	0.974198707
MON1B	-0.093194032	0.180635487	0.974198707
ADCY3	0.110786252	0.180686064	0.974198707
SIRPB1	-0.240058999	0.18075014	0.974198707
CABLES1	-0.102125338	0.180791105	0.974198707
BACE1	-0.158417592	0.180879653	0.974198707
ST6GALNAC3	-0.186885085	0.180902725	0.974198707

VAMP2	0.133592152	0.181126516	0.974198707
ZNF230	-0.110144343	0.181204072	0.974198707
DONSON	-0.127624757	0.181213091	0.974198707
CCDC84	-0.162536622	0.181280197	0.974198707
CCNT2	-0.091047301	0.181316635	0.974198707
LRP5	-0.123882628	0.181452993	0.974198707
DAB2	0.210901629	0.181485834	0.974198707
CTSG	-0.453422717	0.181497958	0.974198707
THOC1	-0.09650791	0.181848673	0.974198707
ALDH6A1	0.082948718	0.18187584	0.974198707
RRN3	-0.08628546	0.18200894	0.974198707
ERAL1	-0.06586333	0.182018416	0.974198707
LPAR2	-0.113656693	0.182151403	0.974198707
RAB4B-EGLN2	-0.061058212	0.182255677	0.974198707
VAMP1	-0.127740131	0.182283017	0.974198707
EZH2	0.120836143	0.182351456	0.974198707
SHKBP1	0.1492629	0.182381645	0.974198707
COG8	0.111657609	0.182458357	0.974198707
BAG4	-0.106688686	0.182491067	0.974198707
ELL3	0.176084702	0.182494688	0.974198707
NIPBL	-0.06740462	0.182499769	0.974198707
TAS2R10	-0.146647161	0.182647443	0.974198707
NUS1	-0.08270412	0.182663168	0.974198707
VKORC1	0.081576767	0.182783488	0.974198707
CALHM2	-0.132408444	0.182853705	0.974198707
POGZ	-0.248001989	0.183189084	0.974198707
P2RY13	-0.143060809	0.183195526	0.974198707
ALDH3A2	-0.085755068	0.183210878	0.974198707
APOL2	0.156422783	0.183521681	0.974198707
ATE1	-0.107875446	0.183568115	0.974198707
KRCC1	-0.112925142	0.183726576	0.974198707
PAIP1	0.12006598	0.18374352	0.974198707
HK3	-0.129999923	0.183901115	0.974198707
CD59	-0.133670131	0.184070911	0.974198707
CDK5RAP2	0.185083464	0.184116986	0.974198707
GKAP1	0.123236457	0.184200196	0.974198707
AQP11	0.119793215	0.184264406	0.974198707
SLC16A3	-0.073099317	0.184354743	0.974198707
CHRAC1	0.105748723	0.184359257	0.974198707
CEBPG	-0.074523955	0.184428446	0.974198707
GLTPD1	-0.110135462	0.184563169	0.974198707
GAS6	-0.194214116	0.184823473	0.974198707
ZBTB48	-0.085661434	0.184848996	0.974198707
KIAA0226	0.107885545	0.18487002	0.974198707
MT2A	0.221866921	0.184914432	0.974198707
GRB10	-0.143527613	0.184916658	0.974198707
NRAS	-0.10395983	0.184975844	0.974198707

C16orf80	-0.090726098	0.18499984	0.974198707
CDC16	-0.091635698	0.18503191	0.974198707
TJAP1	0.05622781	0.185125741	0.974198707
NDUFAF2	0.118518951	0.185383592	0.974198707
RFWD3	0.090853683	0.185406669	0.974198707
USP4	0.065551376	0.185417105	0.974198707
LTB4R2	-0.125788495	0.185428165	0.974198707
GUCY1B3	0.254718687	0.185523265	0.974198707
UNC119	0.097297604	0.185692299	0.974198707
TRAF2	-0.146094545	0.185735187	0.974198707
ZNF529	-0.108592635	0.185781509	0.974198707
EIF3A	-0.050167044	0.185803578	0.974198707
ADH4	-0.159156993	0.185892405	0.974198707
ULK3	-0.143145851	0.186066735	0.974198707
RNF115	0.102836845	0.186070224	0.974198707
SNORD110	0.121776387	0.186152723	0.974198707
HMMR	-0.204263609	0.186262021	0.974198707
EIF2AK1	0.078477315	0.18633699	0.974198707
ZBTB11	-0.125616616	0.186357665	0.974198707
COL6A2	0.194320751	0.186499992	0.974198707
KIAA1143	0.154935206	0.186513942	0.974198707
CLASP2	-0.11913234	0.186593176	0.974198707
IZUMO4	-0.135345049	0.186751427	0.974198707
POPDC2	-0.137247232	0.187112657	0.974198707
RHOQ	-0.103920443	0.187142193	0.974198707
TP53I3	0.156304043	0.187263477	0.974198707
LRP5L	-0.137180622	0.187286576	0.974198707
NCF1B	0.209060657	0.187354234	0.974198707
PMM2	0.095282017	0.187522817	0.974198707
NDUFS3	0.04306371	0.187552713	0.974198707
DCBLD2	-0.149100727	0.187556521	0.974198707
ZFAND2A	0.127774037	0.18764353	0.974198707
IK	0.070107289	0.187707429	0.974198707
HERC2	0.093054057	0.18774293	0.974198707
RHPN1	-0.140472017	0.187774604	0.974198707
FAM118A	0.656550968	0.187828141	0.974198707
RNF216	0.083598177	0.187920004	0.974198707
RAB5C	0.081499348	0.187937422	0.974198707
PTGER2	0.143263213	0.187963005	0.974198707
TATDN3	-0.091369379	0.188010545	0.974198707
DYSF	-0.263689942	0.188030577	0.974198707
AXL	-0.185064562	0.18824492	0.974198707
GCHFR	0.158548879	0.18830048	0.974198707
ZNF33A	-0.06632263	0.18841781	0.974198707
RPS10P7	0.136268479	0.188552303	0.974198707
PIGN	-0.116906752	0.188634384	0.974198707
TSPAN18	0.175157318	0.188820968	0.974198707

HIST1H2AE	-0.364186856	0.18891318	0.974198707
PRAMEF7	0.100343852	0.189029272	0.974198707
STAU1	0.036582843	0.189066966	0.974198707
RFC1	-0.079619203	0.189247305	0.974198707
HS6ST1	0.112587568	0.18927623	0.974198707
KCNK17	0.272756825	0.189529225	0.974198707
PATL1	0.07056382	0.189557255	0.974198707
NOL6	-0.084498987	0.189775441	0.974198707
CDC42EP2	-0.232827298	0.190010736	0.974198707
DEF8	-0.114838094	0.190039184	0.974198707
CCDC92	0.114749322	0.190101421	0.974198707
OSBPL1A	-0.20177289	0.190149911	0.974198707
PDE6D	0.086652387	0.190297652	0.974198707
CD6	-0.106314241	0.19048524	0.974198707
EXOC5	-0.077905715	0.190543105	0.974198707
RCN1	-0.11169578	0.190583406	0.974198707
LPCAT4	0.11127627	0.19067465	0.974198707
NFIB	0.457058282	0.19070567	0.974198707
GCA	-0.128425647	0.190829513	0.974198707
PKIB	-0.150076641	0.190918635	0.974198707
EIF3B	-0.071586796	0.19107983	0.974198707
REXO2	0.065461951	0.191127624	0.974198707
ITPKB	0.095427659	0.191165013	0.974198707
ZBTB7B	0.128338695	0.19129339	0.974198707
TIA1	-0.0833111	0.19135403	0.974198707
PELO	-0.174765048	0.19141933	0.974198707
WFDC3	-0.187028379	0.191475105	0.974198707
RNF26	0.096070816	0.191645127	0.974198707
UBE2I	0.076620957	0.191685603	0.974198707
ELOVL5	0.129512748	0.191770787	0.974198707
ATP5I	0.046217182	0.191796201	0.974198707
RASSF1	-0.130830736	0.191828438	0.974198707
CHD9	-0.095215112	0.191879401	0.974198707
PEF1	0.086472681	0.191981781	0.974198707
LMNB2	0.098580549	0.191991936	0.974198707
STT3A	-0.087840465	0.192045418	0.974198707
ARL8B	-0.061378992	0.192104671	0.974198707
XCL1	0.213388958	0.192228251	0.974198707
GYS1	0.11884256	0.192525406	0.974198707
ETFDH	-0.109962292	0.192818704	0.974198707
TLR4	-0.176868722	0.193006522	0.974198707
ZNF316	0.120145985	0.193027665	0.974198707
ANAPC16	0.121879032	0.193176465	0.974198707
STOM	0.118300473	0.193183843	0.974198707
CTSA	0.163859136	0.193185071	0.974198707
SMCHD1	0.128427522	0.193200417	0.974198707
TFIP11	0.071479011	0.193226023	0.974198707

CUBN	-0.175497062	0.193286586	0.974198707
SH2D1B	0.334157942	0.193384377	0.974198707
ZNF544	-0.097746389	0.193684773	0.974198707
TRAPPC9	0.131217559	0.193739457	0.974198707
PRPF4B	-0.11969219	0.193846451	0.974198707
PAOX	0.11034147	0.193945717	0.974198707
SLX1A	0.187287817	0.19399443	0.974198707
RFPL2	0.158882452	0.19403761	0.974198707
SNORD35A	0.219442325	0.194039497	0.974198707
ZNF701	-0.14467359	0.194043938	0.974198707
B3GNT8	-0.154641363	0.194354128	0.974198707
UBR4	0.075280622	0.194374406	0.974198707
MIB2	0.109738624	0.194447345	0.974198707
C11orf82	-0.151499788	0.194500817	0.974198707
PGM2	-0.104262399	0.194586155	0.974198707
ZNF788	-0.1138834	0.194671479	0.974198707
MTIF2	-0.08830285	0.194745082	0.974198707
ZFPL1	-0.099328246	0.195012923	0.974198707
MARCH9	0.116034134	0.195039647	0.974198707
CCBL1	0.105883235	0.195079003	0.974198707
RDX	-0.131450379	0.195079894	0.974198707
KAT5	0.104604237	0.195084847	0.974198707
RNF19A	-0.114148814	0.195149881	0.974198707
KHDRBS3	0.093938037	0.195184265	0.974198707
C9orf156	-0.103620262	0.195241982	0.974198707
EOMES	-0.254763002	0.195277756	0.974198707
MAPK9	0.102736182	0.195298342	0.974198707
MSH2	0.130889978	0.195337107	0.974198707
HLA-DRB1	1.777363501	0.195534744	0.974198707
PIM3	0.135997405	0.195608509	0.974198707
CYB5RL	-0.265675932	0.195632842	0.974198707
TMEM170B	-0.132030738	0.195684735	0.974198707
ISG20	0.092904591	0.195719121	0.974198707
NOC3L	-0.087464612	0.195873721	0.974198707
SF3A1	0.131679136	0.19605401	0.974198707
HBP1	-0.07073303	0.196078656	0.974198707
HMGCL	-0.094542507	0.196160052	0.974198707
TMEM38B	0.114793325	0.196224838	0.974198707
CRABP2	0.126153722	0.196390808	0.974198707
FCRL3	0.247378245	0.196394082	0.974198707
ZNF383	-0.085467611	0.196531552	0.974198707
CCDC28A	-0.06674224	0.19655819	0.974198707
AURKAPS1	-0.106710814	0.196591313	0.974198707
RERE	0.1172414	0.196697356	0.974198707
HEXIM2	0.203284602	0.196804262	0.974198707
CISD1	0.094045978	0.196837425	0.974198707
TMEM57	0.083745171	0.196875133	0.974198707

NBPF1	-0.125501344	0.196902208	0.974198707
ZNF606	-0.133245918	0.196976037	0.974198707
CNTROB	0.132086917	0.196982851	0.974198707
C6orf120	-0.117344564	0.197365695	0.974198707
HNRNPAB	0.05688306	0.197389589	0.974198707
MAGOH	0.09919712	0.19746981	0.974198707
IRX1	0.117676401	0.19754006	0.974198707
SMU1	0.078645597	0.197613909	0.974198707
NCOA1	0.080469062	0.197651385	0.974198707
MPI	0.133713047	0.197653564	0.974198707
PTPRCAP	0.122024791	0.197727424	0.974198707
CUL5	-0.125753435	0.197888017	0.974198707
NT5DC3	0.198357725	0.19789318	0.974198707
NIT1	0.08730537	0.197955803	0.974198707
RUVBL1	0.111891281	0.198045294	0.974198707
MIR599	0.121169263	0.198047247	0.974198707
CBY1	-0.111781739	0.198174069	0.974198707
TCF20	0.074968851	0.198313938	0.974198707
SUGP2	-0.102776887	0.19834932	0.974198707
MAN2C1	-0.163057489	0.19844464	0.974198707
C11orf58	0.083879662	0.198530216	0.974198707
SATB1	0.126595306	0.198619172	0.974198707
IL23A	-0.112324073	0.19871192	0.974198707
TIMM9	0.100946403	0.198773284	0.974198707
ITPKC	0.129966624	0.198796878	0.974198707
PKHD1L1	0.150045361	0.198917756	0.974198707
R3HDM2	0.104653002	0.199053394	0.974198707
ASF1A	-0.101649144	0.199108251	0.974198707
FURIN	0.123259512	0.199299732	0.974198707
ALG8	-0.051705223	0.199478055	0.974198707
SH3RF1	-0.143069798	0.199479082	0.974198707
TCTN1	0.142378304	0.199483775	0.974198707
C1orf198	0.217261727	0.199495322	0.974198707
NFE2L2	-0.119972429	0.19960969	0.974198707
ATF6B	0.123979375	0.199612487	0.974198707
MTIF3	-0.076671445	0.199636314	0.974198707
SLC2A3	0.092944847	0.199663257	0.974198707
PSENEN	0.120213523	0.199735888	0.974198707
KLHL8	-0.100755215	0.199878743	0.974198707
HSPB1	0.097471532	0.199946801	0.974198707
CCT6B	-0.12210641	0.19996321	0.974198707
FAM46A	-0.094410613	0.19997429	0.974198707
FAM131A	-0.090092411	0.200010756	0.974198707
ILF3	-0.093018603	0.200062419	0.974198707
LMAN1	0.10281053	0.20013862	0.974198707
TMEM186	-0.107307632	0.200213394	0.974198707
PAFAH2	-0.124504781	0.200249764	0.974198707

CLDN5	0.343474746	0.200282865	0.974198707
TNFRSF18	0.173081542	0.200388631	0.974198707
PIGT	-0.085830919	0.200421349	0.974198707
GP9	0.338071073	0.200539368	0.974198707
GRSF1	-0.059686551	0.200540025	0.974198707
WDR70	0.086085253	0.200555775	0.974198707
HOMER3	-0.144090004	0.200642287	0.974198707
TUT1	-0.119726695	0.20078271	0.974198707
SETD8	0.112236204	0.200916734	0.974198707
SGPL1	0.116047489	0.200968278	0.974198707
PLOD2	0.328806071	0.201007321	0.974198707
FTL	0.038989531	0.201117567	0.974198707
SNORD31	0.133914321	0.201133492	0.974198707
FLYWCH2	0.114197642	0.20140331	0.974198707
UBE2B	0.092960153	0.201449725	0.974198707
COG6	-0.09233754	0.201460139	0.974198707
JMY	-0.112716654	0.201540556	0.974198707
ZC3H14	-0.079039986	0.201546232	0.974198707
ANKRD36	-0.203553142	0.201579044	0.974198707
FGFR3	0.15284436	0.201672784	0.974198707
KLF7	0.122608725	0.201712272	0.974198707
SNX15	0.142259109	0.201728124	0.974198707
RACGAP1	0.11084141	0.201841901	0.974198707
FBLN7	0.171475886	0.201881862	0.974198707
DCTD	0.064281041	0.201992442	0.974198707
RPS6KB2	0.109537861	0.202001637	0.974198707
HAL	-0.178244934	0.202009886	0.974198707
NAT1	-0.160312156	0.202330546	0.974198707
ZNF28	-0.10222155	0.20234034	0.974198707
ZNF609	-0.110381379	0.202374691	0.974198707
GCN1L1	0.080181645	0.202379335	0.974198707
CROT	-0.131361991	0.202389119	0.974198707
RPRD1B	-0.092276005	0.202531924	0.974198707
RAB11FIP3	-0.088216848	0.202737841	0.974198707
ANKAR	-0.152704363	0.202740279	0.974198707
CECR1	-0.091106133	0.202788975	0.974198707
ESAM	0.322324372	0.202792334	0.974198707
LENG8	-0.13178429	0.203271039	0.974198707
KRI1	0.092562135	0.20328609	0.974198707
FGL1	0.096325166	0.203308737	0.974198707
ESM1	-0.197146839	0.203366102	0.974198707
CAV1	-0.210645659	0.203814202	0.974198707
ASAP2	0.246029241	0.203829765	0.974198707
CDYL	-0.086364898	0.203865268	0.974198707
ACVR1B	-0.093107648	0.203940899	0.974198707
SEC31B	-0.132779578	0.204055678	0.974198707
POLR2L	0.14152278	0.204075945	0.974198707

YIPF1	-0.049183541	0.204157017	0.974198707
KLHL3	-0.140841914	0.204249187	0.974198707
KLF9	0.122694192	0.204267489	0.974198707
SAR1B	-0.116680704	0.204302252	0.974198707
PRKAR2A	0.110824067	0.204382213	0.974198707
LGR6	-0.24201435	0.204421626	0.974198707
YIPF5	-0.174761374	0.204439317	0.974198707
GGT7	-0.196386701	0.204464145	0.974198707
MMRN1	0.336309387	0.204496324	0.974198707
ZNF581	0.089551709	0.20477427	0.974198707
CATSPER2	-0.185678247	0.204835995	0.974198707
RCSD1	0.089682093	0.204862801	0.974198707
RASGRP3	0.214907228	0.204890519	0.974198707
RTN4R	-0.167422193	0.204952016	0.974198707
PDCD1	-0.223011476	0.205026742	0.974198707
WDR11	-0.612281707	0.205084897	0.974198707
ORC5	-0.065451069	0.205255982	0.974198707
ANGEL2	-0.077852283	0.205292647	0.974198707
CTU1	0.11778247	0.205332049	0.974198707
RWDD1	0.066042116	0.205508225	0.974198707
NFATC3	0.102212926	0.20570608	0.974198707
NDUFA7	0.087651577	0.205758044	0.974198707
COL9A2	-0.17650206	0.205876016	0.974198707
STK24	0.046680658	0.205933067	0.974198707
NET1	-0.171793813	0.206043723	0.974198707
FOXJ2	0.045664294	0.206181526	0.974198707
MRPS16	0.065749998	0.206307672	0.974198707
VTRNA1-3	-0.141867684	0.206320448	0.974198707
GPR37	-0.096981422	0.206399831	0.974198707
ARRDC4	-0.570686649	0.206423715	0.974198707
GIGYF2	-0.098198446	0.206542298	0.974198707
FBXL5	-0.124014811	0.20655467	0.974198707
SPAG5	-0.106037184	0.206555059	0.974198707
NCF1C	0.157778844	0.206572902	0.974198707
ZBTB25	0.127038411	0.206579275	0.974198707
KDM3A	-0.067991487	0.206658509	0.974198707
KIAA1467	0.100198739	0.20670474	0.974198707
DNAJC8	0.063318515	0.206847695	0.974198707
ADPGK	-0.084927364	0.206971587	0.974198707
MLLT11	-0.096920589	0.207123879	0.974198707
CHD1	-0.068821198	0.207134913	0.974198707
PRSS45	0.124180093	0.207284171	0.974198707
APH1A	0.094667924	0.207356703	0.974198707
EVI5	-0.14011333	0.207375595	0.974198707
POU2AF1	0.281330527	0.207524665	0.974198707
C15orf56	0.109079786	0.207538215	0.974198707
TCFL5	0.095060364	0.207678392	0.974198707

TRAPPC2L	-0.087740834	0.20771662	0.974198707
PCP2	-0.135205957	0.207802876	0.974198707
ALDH16A1	0.121111915	0.207824786	0.974198707
AP4B1	-0.121555919	0.207940127	0.974198707
MEF2BNB	-0.119536996	0.207956097	0.974198707
SPHK2	-0.063142564	0.208270478	0.974198707
DTX1	0.163341208	0.208361195	0.974198707
ANAPC4	-0.093236742	0.208428318	0.974198707
GUCA1A	-0.11203656	0.208487526	0.974198707
LEMD3	-0.080753361	0.208619506	0.974198707
WDR90	-0.10793102	0.208627643	0.974198707
RPS18	0.091857214	0.208944991	0.974198707
FEM1A	0.069298174	0.208953946	0.974198707
INPPL1	-0.083607781	0.209005866	0.974198707
LHFP	0.102026667	0.209128258	0.974198707
PEX13	0.112434155	0.209224903	0.974198707
CCDC94	0.067160835	0.209235686	0.974198707
BRIX1	0.078315694	0.209242108	0.974198707
RDH16	-0.130150274	0.209276571	0.974198707
TRIM69	0.138731031	0.209400053	0.974198707
CTDSPL	0.34519863	0.209461121	0.974198707
ESRRA	-0.078671039	0.209466383	0.974198707
IDUA	-0.121952724	0.209700239	0.974198707
H2AFJ	0.134638377	0.209735994	0.974198707
RPL13AP5	0.037142669	0.209823562	0.974198707
ADRBK1	-0.056623608	0.209866551	0.974198707
HACL1	0.05551933	0.20987356	0.974198707
FNBP4	-0.072334523	0.209909455	0.974198707
UBE2N	0.068126841	0.209922075	0.974198707
HELQ	-0.083375764	0.209940037	0.974198707
FOXC1	0.168239234	0.209945005	0.974198707
PANK4	-0.085674834	0.210170336	0.974198707
DDX39B	-0.1057633	0.210208802	0.974198707
SLC25A19	0.090140829	0.210451009	0.974198707
MICAL1	-0.155416653	0.210591731	0.974198707
GDF11	-0.110350336	0.210629199	0.974198707
ZNF682	-0.210798616	0.210662389	0.974198707
SCARNA7	0.100859765	0.210684818	0.974198707
AAGAB	0.116773013	0.210724285	0.974198707
PPP2R2A	-0.091443785	0.210818312	0.974198707
DDX54	-0.091960594	0.210997361	0.974198707
MGAM	0.208550257	0.211053765	0.974198707
TOM1L2	-0.106950459	0.211059142	0.974198707
JAKMIP2	-0.256850798	0.211104955	0.974198707
ANO8	-0.11127786	0.211279222	0.974198707
COASY	-0.061968863	0.211443042	0.974198707
TMC5	0.222535352	0.211492509	0.974198707

ZNF837	-0.120633833	0.211562527	0.974198707
ZNF136	-0.102317883	0.211595328	0.974198707
EBAG9	0.063766912	0.21168446	0.974198707
AHR	-0.119370831	0.211698662	0.974198707
SLC39A1	0.082421855	0.211748754	0.974198707
PRSS36	0.097215194	0.211960425	0.974198707
USP38	-0.096009101	0.211965807	0.974198707
CFLAR	-0.130923149	0.212020454	0.974198707
KRT17	-0.149240111	0.212059606	0.974198707
BHLHE40	0.116246555	0.212197766	0.974198707
ZNF670	-0.131944228	0.212274804	0.974198707
FUS	-0.073315344	0.212291399	0.974198707
GTF2H5	0.077986638	0.212309364	0.974198707
FAM27E3	-0.145443101	0.212359176	0.974198707
N4BP2L1	-0.075156146	0.212421463	0.974198707
ARHGAP1	0.062181147	0.212467737	0.974198707
GDPD3	-0.161282058	0.212687122	0.974198707
PPFIBP2	-0.174552619	0.212963771	0.974198707
DOLK	-0.118970397	0.213066377	0.974198707
DNAJC25	-0.07528232	0.213082509	0.974198707
BBS4	-0.093673247	0.213236698	0.974198707
SNRPF	0.081230667	0.213282039	0.974198707
PARP16	0.101081456	0.213485693	0.974198707
ZBTB9	-0.143185117	0.213553017	0.974198707
FAM50B	-0.170382963	0.213571112	0.974198707
ABHD14A	0.102178608	0.213617571	0.974198707
TCEANC2	0.077811164	0.213620664	0.974198707
JUND	0.053964586	0.213653489	0.974198707
KBTBD2	-0.09988374	0.213722541	0.974198707
NR2C2	0.095077147	0.213762681	0.974198707
SEC61G	0.074820903	0.213822885	0.974198707
ZNF573	-0.184179898	0.214053834	0.974198707
IGSF8	-0.122125142	0.2140606	0.974198707
CLK2	-0.069765504	0.214105745	0.974198707
SLC12A2	0.120226136	0.214135809	0.974198707
HBEGF	0.190821584	0.214214159	0.974198707
AFF3	0.178043506	0.21439129	0.974198707
P2RX4	-0.09861537	0.214418675	0.974198707
ARRDC2	-0.105775209	0.214456739	0.974198707
PTPN3	0.092310207	0.214512397	0.974198707
EPHB4	-0.172207751	0.214642346	0.974198707
CHD6	0.073141242	0.214710748	0.974198707
C15orf61	0.11857942	0.214716342	0.974198707
KIDINS220	-0.072249562	0.214749407	0.974198707
TMED1	0.119999721	0.214854856	0.974198707
YPEL1	0.169849092	0.214871227	0.974198707
NDUFA2	0.053011783	0.214915317	0.974198707

OR56B1	0.126019417	0.214979454	0.974198707
PSRC1	-0.129509246	0.215034333	0.974198707
TTC33	-0.117398589	0.215099977	0.974198707
MARCH5	0.11525934	0.215493259	0.975580729
CD101	-0.164512496	0.21566077	0.975584321
VDAC3	-0.046944184	0.215670326	0.975584321
TOP2B	0.047739306	0.215767703	0.975626104
RHOU	-0.155828152	0.21591011	0.975871377
ZNF282	-0.123443325	0.216141632	0.976122046
CDKN1A	0.225852562	0.216186396	0.976122046
TNFRSF4	0.220011065	0.216293101	0.976122046
SNIP1	-0.090565412	0.216409364	0.976122046
SLC6A16	0.13982656	0.216524616	0.976122046
SARNP	0.071923383	0.21653526	0.976122046
C16orf13	0.083464668	0.216619821	0.976122046
PIAS2	0.109848759	0.216903473	0.976122046
SUPT4H1	0.080791673	0.216919478	0.976122046
ARHGAP27	-0.084995542	0.216925321	0.976122046
TBCCD1	0.11361878	0.217022015	0.976122046
GPX1	0.151681891	0.217183256	0.976122046
GNLY	0.263565958	0.217477024	0.976122046
SRRM1	-0.049698156	0.217503014	0.976122046
INTS6	-0.089928494	0.217716161	0.976122046
SREBF1	-0.12609539	0.217718325	0.976122046
COX8A	0.081326751	0.217743933	0.976122046
DYDC2	-0.145233716	0.217793339	0.976122046
CNOT4	-0.082926171	0.217936465	0.976122046
C15orf38	0.266290777	0.217949518	0.976122046
DDT	0.111691706	0.218037796	0.976122046
RNF220	0.057564059	0.218266146	0.976122046
ZNF444	-0.110341564	0.218282961	0.976122046
SLC37A2	0.164998587	0.218396231	0.976122046
ZNF827	-0.125753111	0.218789515	0.976122046
FAM184B	0.298044134	0.219040475	0.976122046
PBX3	0.081675577	0.219043783	0.976122046
HIATL1	-0.10287031	0.219106283	0.976122046
CCNJ	-0.11919909	0.219123059	0.976122046
TMEM62	-0.08746146	0.219164885	0.976122046
HES4	0.259758529	0.219246293	0.976122046
REPIN1	0.088383578	0.219317239	0.976122046
SLC38A1	0.158384494	0.219321784	0.976122046
TMTC2	-0.142810676	0.219352789	0.976122046
SEC24C	-0.086068896	0.219521154	0.976122046
C15orf41	-0.103413058	0.21956819	0.976122046
TSTD1	0.120281267	0.219581522	0.976122046
ECT2L	0.112805763	0.219619439	0.976122046
RNF5	0.105592634	0.219801866	0.976122046

FBL	0.085978683	0.219844066	0.976122046
CD1B	-0.16251917	0.220197545	0.976122046
FAM153B	-0.36820115	0.22036902	0.976122046
ZNF329	0.108229731	0.220399304	0.976122046
LIPN	-0.196774292	0.220411022	0.976122046
ELL	-0.082237373	0.220534545	0.976122046
SNRNP25	0.110875372	0.220549527	0.976122046
EDARADD	-0.165767497	0.220584217	0.976122046
SLC11A2	-0.109295298	0.220800549	0.976122046
CDK20	-0.09895001	0.220887034	0.976122046
PRUNE2	-0.32323788	0.220956933	0.976122046
ZNF814	0.170908689	0.221011826	0.976122046
SET	0.067462536	0.22105439	0.976122046
CD1E	-0.220829841	0.221167743	0.976122046
TPT1	0.097269631	0.221304285	0.976122046
RNF138	0.16897461	0.221320565	0.976122046
TXNDC15	-0.103968689	0.221485421	0.976122046
PTOV1	-0.087871112	0.221524798	0.976122046
PPP1R2	0.084648788	0.221709136	0.976122046
SF3B2	0.057434775	0.221773622	0.976122046
C16orf86	0.108775838	0.221795405	0.976122046
CAST	0.110516834	0.22197325	0.976122046
TSPAN3	0.123784245	0.22197998	0.976122046
RNF214	-0.113944831	0.222029268	0.976122046
PTAR1	-0.092934926	0.222044554	0.976122046
JAK2	-0.116541739	0.222100793	0.976122046
DPAGT1	-0.0623104	0.222145066	0.976122046
NCOA7	0.073850674	0.222178593	0.976122046
ARHGAP15	-0.065782246	0.222213067	0.976122046
RABGGTA	0.104697201	0.222621162	0.976122046
PRKAA1	-0.063707748	0.222653135	0.976122046
ALOX12	0.362581631	0.22272311	0.976122046
NCAPG	-0.237052113	0.222768638	0.976122046
ZNF512	-0.074344542	0.222935898	0.976122046
NEK7	-0.127354421	0.222958357	0.976122046
TUBA3D	0.228783137	0.22308519	0.976122046
FAM65B	0.0926405	0.223158233	0.976122046
U2SURP	0.08330508	0.223468164	0.976122046
TNFRSF13C	0.198049566	0.223657353	0.976122046
DUSP2	0.191883097	0.223687387	0.976122046
NFAM1	-0.138671311	0.223820642	0.976122046
PITPNM1	-0.085574327	0.22387755	0.976122046
IGLL1	0.289063883	0.223938065	0.976122046
TUBA1C	0.115529811	0.223971789	0.976122046
PELI2	-0.114124379	0.224114912	0.976122046
TFPI	0.244181259	0.224167901	0.976122046
FGFBP3	0.147549795	0.224174671	0.976122046

RMI1	-0.090048337	0.224206114	0.976122046
WDR91	-0.07898878	0.224529145	0.976122046
CLCC1	0.110987713	0.224584205	0.976122046
FUCA1	-0.109823351	0.224753495	0.976122046
NT5C	0.056915776	0.224831443	0.976122046
SYNJ2	0.123458547	0.224849764	0.976122046
SMAD4	-0.069305307	0.22487951	0.976122046
ADAM28	0.188705687	0.225023144	0.976122046
TNFSF14	-0.151452444	0.225080471	0.976122046
LRP3	-0.160091074	0.225190214	0.976122046
FAM96B	0.071385774	0.225252014	0.976122046
RIOK3	0.07948484	0.22542365	0.976122046
ARHGAP12	-0.0903458	0.225445734	0.976122046
KLHL24	-0.081910345	0.225451108	0.976122046
ZNF142	-0.061616899	0.225500041	0.976122046
SENP2	-0.066754799	0.225769848	0.976122046
TDRD6	-0.110398018	0.225909	0.976122046
ST6GAL1	0.082246164	0.226017489	0.976122046
OGDH	0.119647333	0.226095985	0.976122046
SOCS6	0.135219839	0.226148961	0.976122046
EIF3K	0.07657168	0.22623647	0.976122046
NRP1	-0.223131043	0.226306438	0.976122046
ACOX2	-0.16773694	0.226344272	0.976122046
ANKRD34A	-0.119795778	0.226350521	0.976122046
NCAPH2	-0.081107045	0.226425088	0.976122046
AHNAK	-0.076598962	0.226516038	0.976122046
C2orf42	-0.077059855	0.226560649	0.976122046
SNORD46	0.150887829	0.226626882	0.976122046
TBC1D5	0.10802933	0.226733284	0.976122046
CTDSP1	-0.064604276	0.226745815	0.976122046
ZSCAN29	-0.164731906	0.226748362	0.976122046
NUPL2	-0.095602325	0.226762386	0.976122046
ACO2	0.078134299	0.226802802	0.976122046
C16orf72	-0.131191515	0.226890718	0.976122046
FAM134B	-0.141409131	0.226985037	0.976122046
CEP72	-0.105784017	0.227027196	0.976122046
UROS	0.073815531	0.227141674	0.976122046
UQCR11	0.098323048	0.227217435	0.976122046
PQLC1	0.048000901	0.227506088	0.976122046
LCMT1	0.056761407	0.227708373	0.976122046
ALDOA	0.077533336	0.227928982	0.976122046
SSRP1	0.112734951	0.228064833	0.976122046
ZDHHC4	0.082337897	0.228072751	0.976122046
LPCAT2	-0.351411608	0.228110436	0.976122046
KIT	-0.124804718	0.228128588	0.976122046
DDX5	-0.052381288	0.228134258	0.976122046
TBC1D10A	0.084297891	0.228147347	0.976122046

MGAT2	-0.103502289	0.228296952	0.976122046
CBLB	-0.121090225	0.228684178	0.976122046
TICAM2	-0.136610912	0.228772255	0.976122046
TCEB2	0.062685755	0.228856912	0.976122046
KIAA1191	0.064877349	0.22906263	0.976122046
TP53BP2	-0.074394727	0.229584664	0.976122046
CWC15	0.050308671	0.229672968	0.976122046
KBTBD11	-0.120633448	0.229752042	0.976122046
BBS1	-0.076988665	0.230006836	0.976122046
CDK2	0.068137989	0.230017575	0.976122046
NUBP1	0.063996536	0.230074468	0.976122046
NDUFA8	0.069234356	0.230132164	0.976122046
SGSM1	0.14225548	0.230179421	0.976122046
ST13	0.080101903	0.230218923	0.976122046
C21orf37	0.097670024	0.230375211	0.976122046
TFEB	0.124460262	0.230592086	0.976122046
ARL16	-0.131748957	0.230594523	0.976122046
PLEKHO1	0.07085328	0.230736331	0.976122046
NHLRC3	-0.077566879	0.230773938	0.976122046
ODC1	0.171548313	0.230776002	0.976122046
WHSC1L1	0.096682309	0.230827727	0.976122046
C2orf74	0.19350586	0.230899882	0.976122046
ARAP2	-0.098355074	0.230965099	0.976122046
NUBP2	0.115403525	0.231095012	0.976122046
ACTR3B	0.135881346	0.231156576	0.976122046
CMTM5	0.3485048	0.23115736	0.976122046
ZNF890P	0.118274021	0.231275329	0.976122046
QTRT1	0.103073876	0.231288507	0.976122046
TRIM23	-0.126266329	0.231323182	0.976122046
ETV6	0.073386123	0.231360297	0.976122046
SELK	0.11824262	0.231373495	0.976122046
ZNF26	-0.090102435	0.231571016	0.976122046
GDE1	0.089444463	0.231605583	0.976122046
LAMC1	-0.272304367	0.231654581	0.976122046
LCLAT1	0.170718803	0.231949177	0.976122046
PAK6	-0.129874706	0.231975347	0.976122046
MIRLET7D	-0.142678189	0.23202448	0.976122046
CCDC130	-0.073655666	0.232166609	0.976122046
HM13	-0.18559036	0.232213223	0.976122046
TYMP	-0.118344737	0.23243823	0.976122046
DDX59	0.219655732	0.232528339	0.976122046
TMEM131	-0.05546122	0.232530449	0.976122046
ZNF420	-0.161201014	0.23274683	0.976122046
RAI14	0.141222436	0.232835129	0.976122046
RICTOR	-0.12493874	0.232875225	0.976122046
TXNL4B	-0.113479222	0.233044676	0.976122046
NDUFAF1	-0.103803057	0.233229099	0.976122046

ASAP1	0.09484862	0.233305696	0.976122046
FAM168B	0.075115338	0.233447136	0.976122046
NAA30	-0.074735833	0.233661022	0.976122046
MIR555	-0.157651149	0.233756402	0.976122046
KHDRBS1	0.056128361	0.233901983	0.976122046
SEL1L3	0.113382192	0.234003277	0.976122046
HINT1	0.076629341	0.234145597	0.976122046
HOXB5	0.115974578	0.234207955	0.976122046
EVL	-0.063691607	0.234284296	0.976122046
SELP	0.344134379	0.234324598	0.976122046
MCOLN3	0.130112607	0.234340041	0.976122046
HIST2H2BF	-0.100299539	0.234351168	0.976122046
TIAM1	-0.146356634	0.234358718	0.976122046
BUB1	-0.198945462	0.234544052	0.976122046
HHEX	0.099242435	0.234592508	0.976122046
SLC46A2	-0.178725626	0.234667928	0.976122046
TAP2	-0.147381546	0.234707718	0.976122046
IP6K2	0.299429287	0.234805083	0.976122046
FAM73A	-0.248900311	0.234829903	0.976122046
LINC00290	-0.107901161	0.234841951	0.976122046
FAM72A	-0.132237574	0.234847285	0.976122046
RAB38	0.217586174	0.23488685	0.976122046
ZNF32	0.07396723	0.234997501	0.976122046
OTX1	0.133944737	0.235117397	0.976122046
CCDC150	-0.112085861	0.235162466	0.976122046
PI4K2A	-0.089144837	0.235269364	0.976122046
IRF2BP1	0.141238485	0.235356259	0.976122046
MEG8	0.120395736	0.235416384	0.976122046
KCNH3	-0.321061244	0.235662301	0.976122046
GPR84	-0.121238336	0.235686564	0.976122046
CDC42BPA	0.095095331	0.235774898	0.976122046
ZNHIT1	0.07580783	0.235781544	0.976122046
ZNF283	-0.101153832	0.235909775	0.976122046
ATAD5	-0.115238123	0.235961344	0.976122046
NANOS1	0.201624186	0.236048289	0.976122046
SAMD11	-0.125364721	0.236195922	0.976122046
EPHA4	0.190855142	0.236226739	0.976122046
FSTL3	-0.113308164	0.236393032	0.976122046
TSPYL4	0.099442691	0.236436418	0.976122046
MXD1	-0.111690686	0.236477692	0.976122046
BTLA	0.149463505	0.236698563	0.976122046
ST14	0.197540372	0.236702796	0.976122046
GAL3ST4	-0.190578524	0.236908713	0.976122046
SETBP1	0.158689855	0.237045374	0.976122046
CYP4F22	0.275341395	0.237122393	0.976122046
VPS52	-0.076534123	0.237131932	0.976122046
GALNT1	-0.087155319	0.237151265	0.976122046

CASC3	0.080839381	0.237152295	0.976122046
ZNF626	-0.203676974	0.237153492	0.976122046
CUL9	-0.112854246	0.237258763	0.976122046
MIR345	0.102427265	0.237275012	0.976122046
SFXN5	-0.120716648	0.237280967	0.976122046
FBXO7	0.068393247	0.23732076	0.976122046
BCL6	-0.111989978	0.237348766	0.976122046
SPSB2	-0.163496461	0.237382666	0.976122046
GTF3C6	0.084075829	0.237388022	0.976122046
RAB5B	0.059471379	0.237466806	0.976122046
EIF2S1	0.068600156	0.23753889	0.976122046
HOOK1	-0.164340213	0.237543595	0.976122046
JTB	0.047217817	0.237596259	0.976122046
DBN1	-0.203092871	0.237668839	0.976122046
NQO1	0.11041059	0.237670132	0.976122046
MDC1	-0.074512508	0.237764294	0.976122046
HCP5	-0.139598398	0.237783194	0.976122046
MIR557	0.121566429	0.237834375	0.976122046
WEE1	0.145788717	0.237900294	0.976122046
HGS	-0.065252492	0.237917602	0.976122046
DNASE2	0.091649578	0.237919546	0.976122046
PSMC6	-0.065047701	0.237923686	0.976122046
SMG6	-0.115109225	0.238105608	0.976315993
CPNE8	-0.103264016	0.238147365	0.976315993
KPNA6	-0.07986784	0.238322572	0.976447525
PRNP	0.054468477	0.238516582	0.976447525
UBE2J1	0.092628286	0.238833461	0.976447525
MRPS34	0.117859624	0.238900405	0.976447525
GNE	-0.075822047	0.238929701	0.976447525
TPST1	0.177178122	0.238983835	0.976447525
TELO2	-0.195634334	0.239128487	0.976447525
STAG3L1	-0.174732827	0.239194951	0.976447525
MTA1	-0.090311301	0.239261789	0.976447525
CD44	-0.055426353	0.239308189	0.976447525
GIMAP5	-0.109142386	0.239384426	0.976447525
ZNF446	-0.086241975	0.239555295	0.976447525
DYRK4	-0.065889733	0.239634637	0.976447525
FAM161A	-0.281790377	0.239711656	0.976447525
NFATC2IP	-0.105211672	0.239955521	0.976447525
CNRIP1	-0.165360901	0.239994944	0.976447525
CDC27	-0.155311445	0.24016178	0.976447525
FOXN2	-0.070015637	0.240218083	0.976447525
HCN4	0.136565091	0.240248013	0.976447525
MRPL24	0.064001754	0.240290534	0.976447525
AUTS2	0.186859537	0.240342643	0.976447525
TSFM	0.083753413	0.240349308	0.976447525
CDK10	-0.134310306	0.24044266	0.976447525

SNHG10	0.094459387	0.24049463	0.976447525
APBA3	0.089168179	0.24056275	0.976447525
RNF4	-0.079764188	0.240605983	0.976447525
SRRT	-0.055421191	0.240622422	0.976447525
ADAMTSL4	0.145000537	0.240649458	0.976447525
SPOP	-0.064628484	0.240739869	0.976456435
TXK	0.187043549	0.241174431	0.976491766
SNX30	-0.097100872	0.241212493	0.976491766
RABL3	0.11226189	0.24125809	0.976491766
RB1CC1	-0.052025009	0.241320658	0.976491766
ABCA5	-0.127392026	0.241386309	0.976491766
PHF12	-0.078691012	0.241435299	0.976491766
AZI2	-0.096962209	0.241662644	0.976491766
SLC25A45	-0.101474194	0.241731545	0.976491766
UBE2L6	0.101263428	0.242105743	0.976491766
EXOSC2	-0.091655946	0.242230013	0.976491766
RRAS2	0.164910069	0.242347713	0.976491766
MGST3	0.115995602	0.242378697	0.976491766
TFPT	0.09668399	0.242492948	0.976491766
ZFAND3	0.094480016	0.242503955	0.976491766
SLC25A13	-0.176254777	0.242604022	0.976491766
METTL14	-0.089019367	0.242681671	0.976491766
DUSP13	0.110267758	0.242845991	0.976491766
RPS10	0.030707371	0.242871269	0.976491766
KNCN	0.126338304	0.243206889	0.976491766
FAM159A	0.123584661	0.243254426	0.976491766
PSPC1	0.096036973	0.243292395	0.976491766
MLH1	-0.058088772	0.243484319	0.976491766
NRSN2	0.120522046	0.243515674	0.976491766
CCDC149	-0.2345424	0.243639853	0.976491766
EHMT2	-0.095015086	0.243854775	0.976491766
COX10	0.073412937	0.243907563	0.976491766
CENPQ	-0.145156699	0.243923886	0.976491766
NECAP2	0.076156214	0.243943644	0.976491766
TP53RK	-0.105859896	0.243963498	0.976491766
CCNG1	0.063998165	0.244262448	0.976491766
NBN	-0.103271129	0.244316373	0.976491766
AKAP5	-0.046042365	0.244328815	0.976491766
ZNF410	0.046612757	0.24436259	0.976491766
C1QBP	0.06852894	0.244393872	0.976491766
CKAP2L	-0.100209849	0.244744663	0.976491766
TNPO2	-0.065369002	0.244890521	0.976491766
MRFAP1L1	-0.063337586	0.245048502	0.976491766
TRIM36	-0.125176273	0.245173275	0.976491766
ZNF765	-0.127873626	0.245278511	0.976491766
MRPS6	0.056535014	0.245466702	0.976491766
PRPSAP2	0.059229977	0.245476258	0.976491766

NPL	-0.149245067	0.245535087	0.976491766
SLC6A6	0.135697801	0.245682219	0.976491766
PPP2R5E	-0.074963616	0.246081476	0.976491766
SPEF2	0.112379961	0.246243932	0.976491766
LSM11	0.098290938	0.246317795	0.976491766
CTSC	-0.108603439	0.246319094	0.976491766
SLC35A4	0.137747265	0.246319159	0.976491766
PAN2	-0.087849394	0.24633225	0.976491766
CBR1	0.156257891	0.246415627	0.976491766
FAM32A	0.058342874	0.246622127	0.976491766
SRBD1	0.073715315	0.247055732	0.976491766
PCBD1	0.101709827	0.2470735	0.976491766
C6orf25	0.271488973	0.247216671	0.976491766
CSDE1	-0.133784349	0.247230802	0.976491766
EXOC7	-0.064622179	0.247278352	0.976491766
LUC7L	-0.089602519	0.247354831	0.976491766
TMEM200A	0.17340197	0.247427209	0.976491766
EPHA10	0.086970732	0.24750048	0.976491766
USP6	-0.117718916	0.247628471	0.976491766
MAEA	-0.062044663	0.247724862	0.976491766
CDO1	-0.135155387	0.248141064	0.976491766
SULT1A1	-0.121135125	0.248409153	0.976491766
AQP9	-0.176798595	0.248441191	0.976491766
FUBP3	-0.063295664	0.248449393	0.976491766
NQO2	-0.258744545	0.248528728	0.976491766
ZNF517	-0.094063145	0.248566869	0.976491766
PLAU	-0.086632019	0.248600435	0.976491766
YRDC	-0.225120603	0.248669109	0.976491766
CLTA	0.07654498	0.248672214	0.976491766
SLC25A44	-0.062182299	0.248708279	0.976491766
ANGPTL4	0.103521022	0.248829778	0.976491766
GATA2	0.173843629	0.248888738	0.976491766
PRPF8	0.089640786	0.248930113	0.976491766
SNORA34	0.097546405	0.248972436	0.976491766
KDM5A	0.081995556	0.249024076	0.976491766
BET1	-0.117875744	0.249063941	0.976491766
KLF12	0.07486286	0.249296462	0.976491766
PABPC3	0.107477583	0.249964581	0.976491766
AASDHPPT	-0.087001097	0.250185408	0.976491766
LAT2	0.076331195	0.250354109	0.976491766
FAM102B	0.08079056	0.250361203	0.976491766
NCKAP1	0.161178734	0.250362839	0.976491766
RXRA	-0.061756513	0.250576066	0.976491766
PRG2	0.120419889	0.250640261	0.976491766
SERHL2	0.11618252	0.250703222	0.976491766
ECSIT	0.09933519	0.25070664	0.976491766
CHTOP	-0.093656187	0.250788501	0.976491766

CLDN14	0.100421954	0.250974662	0.976491766
NRBF2	-0.104406174	0.251044548	0.976491766
TUBG1	-0.097737367	0.251231731	0.976491766
GRHPR	0.124264568	0.25129433	0.976491766
CSF1R	-0.086772737	0.251522809	0.976491766
EIF3CL	0.149072194	0.251561197	0.976491766
CCDC138	0.089623382	0.251565542	0.976491766
FAM69A	-0.138829109	0.251584741	0.976491766
KANK3	0.105353897	0.251672434	0.976491766
FBXO45	-0.148482393	0.251784314	0.976491766
TCL1A	0.34226615	0.251814154	0.976491766
HPCAL1	0.085273181	0.251833044	0.976491766
KDM1A	0.056048033	0.251894248	0.976491766
CTIF	0.111541265	0.251962174	0.976491766
SPATA24	0.104636937	0.252036266	0.976491766
ATP6V0A2	-0.066577386	0.252295701	0.976491766
EIF2S2	0.095449383	0.252374855	0.976491766
SIGIRR	-0.098809735	0.252391962	0.976491766
CYTL1	-0.1598079	0.25277155	0.976491766
PPAP2C	-0.137625366	0.252774717	0.976491766
PPFIA1	-0.065256881	0.252782259	0.976491766
ARFGAP1	-0.11945781	0.252862303	0.976491766
NMUR1	0.225634481	0.252944183	0.976491766
PXK	0.120271681	0.253071756	0.976491766
PGM3	-0.093960235	0.253132929	0.976491766
ZNF716	0.089956041	0.253135992	0.976491766
ZSCAN16	-0.058262561	0.253267863	0.976491766
EIF4EBP1	0.092948156	0.253290437	0.976491766
CBX8	-0.09292545	0.253445278	0.976491766
BUD13	-0.064375938	0.253475999	0.976491766
PKIA	-0.146582731	0.253490292	0.976491766
MCM5	0.074164776	0.253504808	0.976491766
EXOSC5	0.080477135	0.253690804	0.976491766
LIMD2	-0.100652124	0.253786608	0.976491766
ZNF101	0.151021211	0.253866466	0.976491766
POLR2H	-0.04759052	0.253984068	0.976491766
CRIPAK	-0.173626804	0.254068971	0.976491766
FAM169A	-0.120350363	0.254190966	0.976491766
EXO1	-0.107963036	0.254223578	0.976491766
KIR3DL1	0.349980215	0.254259328	0.976491766
MIR1282	-0.228232228	0.254468914	0.976491766
EPM2AIP1	-0.061020735	0.254620286	0.976491766
PPP1R10	-0.115677919	0.254631044	0.976491766
JDP2	0.138109647	0.254779875	0.976491766
GCC1	-0.081786516	0.254823884	0.976491766
KIF27	-0.168106016	0.255071584	0.976491766
UBQLN1	0.049668357	0.255090118	0.976491766

RELT	0.134180335	0.255204024	0.976491766
IQCC	-0.081883219	0.255208921	0.976491766
FEM1C	-0.07552647	0.255262338	0.976491766
UFC1	0.075781383	0.255375693	0.976491766
OTUD3	0.113844723	0.255591989	0.976491766
FOXP4	0.113690695	0.255599303	0.976491766
SRSF1	-0.049239389	0.255833754	0.976491766
P2RX5-TAX1BP3	0.129755572	0.255939823	0.976491766
ID3	-0.205327957	0.256012857	0.976491766
NUP35	-0.130343468	0.256050125	0.976491766
STRN3	0.099090113	0.256053975	0.976491766
TRAPPC8	-0.062007957	0.25614666	0.976491766
ZNF451	-0.073978323	0.256201828	0.976491766
GPI	0.088579182	0.256337299	0.976491766
RPL22	0.043829845	0.256356004	0.976491766
ZCCHC17	0.092350713	0.256372868	0.976491766
TRAPPC10	0.074932647	0.256449735	0.976491766
PSKH1	-0.101107548	0.256583929	0.976491766
HMGCS1	-0.069233843	0.256616412	0.976491766
CCND3	0.066544161	0.256645554	0.976491766
GGCX	-0.096795762	0.256760043	0.976491766
TRIM41	-0.06313157	0.25676444	0.976491766
KIAA0232	-0.094842448	0.256946473	0.976491766
ATG2B	-0.100840644	0.25706004	0.976491766
ZNF175	-0.122205766	0.257225035	0.976491766
RIC8A	0.053244985	0.257272315	0.976491766
C12orf10	0.105435564	0.257308936	0.976491766
ADAR	0.06153889	0.257482504	0.976491766
TAF1B	-0.092022727	0.257620832	0.976491766
NUB1	0.068318925	0.257707377	0.976491766
MAN2B1	0.104829944	0.2578568	0.976491766
HPGD	0.20504629	0.258035461	0.976491766
CNTNAP2	0.453248333	0.258325846	0.976491766
FAM69B	0.263491406	0.258331417	0.976491766
CHMP1A	-0.088958419	0.258468864	0.976491766
NDUFB6	0.101669721	0.25864117	0.976491766
ALG1	0.115913626	0.258669484	0.976491766
ZNF223	-0.15924682	0.258792957	0.976491766
GOLGA8G	0.107466806	0.25879386	0.976491766
LILRB3	0.03771924	0.258853662	0.976491766
TTC26	-0.093421826	0.258857179	0.976491766
SNRPD2	0.052031577	0.258896239	0.976491766
NCKAP5L	-0.114903533	0.258914592	0.976491766
SHE	0.121478634	0.258917853	0.976491766
BRD7	0.075500976	0.259213679	0.976491766
DCPS	0.103232211	0.259224681	0.976491766
NAA35	-0.07124672	0.259243827	0.976491766

LY86	0.122313943	0.259440578	0.976491766
SLC23A1	0.100682271	0.259456618	0.976491766
PLEK2	0.086301874	0.259509052	0.976491766
CCDC125	-0.211474106	0.259513624	0.976491766
DMXL2	-0.140761474	0.259641753	0.976491766
POLR2D	0.10863768	0.259692367	0.976491766
NDUFA11	0.081676603	0.259795343	0.976491766
BZW1	-0.060558435	0.25980735	0.976491766
DYM	-0.074372715	0.259875339	0.976491766
LMNB1	0.158499821	0.259878471	0.976491766
HOPX	0.178506432	0.260007043	0.976491766
CEP250	-0.110192907	0.260265733	0.976491766
ZNF669	-0.201925324	0.260348001	0.976491766
HOXB6	0.169893298	0.260414837	0.976491766
METTL6	0.13058144	0.260498201	0.976491766
LTBR	-0.1031799	0.260620254	0.976491766
LYSMD4	-0.108468237	0.26072176	0.976491766
GOLGA8B	-0.127379526	0.260909823	0.976491766
TOR1A	0.069709947	0.260925422	0.976491766
FCN1	-0.092835124	0.261098132	0.976491766
UBXN11	-0.122804445	0.26115892	0.976491766
SF3B1	-0.050451859	0.261168666	0.976491766
PRDM15	0.110237164	0.261244795	0.976491766
FAM60A	-0.055774315	0.261251437	0.976491766
B4GALT4	-0.09735597	0.26126646	0.976491766
RAP1B	0.110247124	0.261422293	0.976491766
TRAPPC6A	0.080402765	0.261536266	0.976491766
PNPT1	-0.147386787	0.261692181	0.976491766
CTBP1	0.076010642	0.261732365	0.976491766
HSH2D	0.156227945	0.261879353	0.976491766
PDLIM1	0.207497105	0.262073325	0.976491766
RBBP9	0.074654119	0.262446471	0.976491766
PCYOX1	-0.151449419	0.262473602	0.976491766
C1orf63	-0.109856495	0.262514122	0.976491766
HYLS1	-0.105400421	0.262611571	0.976491766
ZNF514	-0.199110502	0.26269281	0.976491766
TMEM175	-0.064185126	0.26277892	0.976491766
GLS2	0.113578723	0.262824471	0.976491766
DAG1	0.080759104	0.263210311	0.976491766
RAD51L3-RFFL	0.072059715	0.263239983	0.976491766
AHI1	-0.188801191	0.263280455	0.976491766
OGFOD1	0.060079564	0.263342585	0.976491766
SIDT1	0.094031684	0.263520046	0.976491766
SEL1L	-0.081482272	0.263547792	0.976491766
YEATS4	0.130998428	0.263564861	0.976491766
TM9SF3	-0.068546315	0.263836993	0.976491766
MCM2	0.135096739	0.263915594	0.976491766

GORAB	-0.081563581	0.263991354	0.976491766
IQGAP1	0.075182778	0.264096124	0.976491766
STAR	-0.131335864	0.264101566	0.976491766
KCNK12	0.12737313	0.264102345	0.976491766
DOK1	-0.169103197	0.264305594	0.976491766
ZNF844	-0.114773281	0.264494867	0.976491766
PSMD8	0.084171435	0.264717298	0.976491766
EHD3	0.176947164	0.264739397	0.976491766
SPRYD4	-0.114932419	0.264780246	0.976491766
TIGD6	-0.095847024	0.264783254	0.976491766
PRKCE	0.108696205	0.264945	0.976491766
IPO5	-0.078898329	0.26495471	0.976491766
WDR18	0.084720757	0.265053613	0.976491766
WTAP	0.06590724	0.265188605	0.976491766
FRAT2	-0.141483566	0.265204455	0.976491766
GTF3C2	-0.049086451	0.265271944	0.976491766
DDX19B	0.057753704	0.265292341	0.976491766
FAM41C	0.113629448	0.265329137	0.976491766
ZDHHC18	0.082022452	0.265382023	0.976491766
TBC1D23	-0.120080126	0.265401192	0.976491766
DLD	-0.101202913	0.2658356	0.976491766
FYTTD1	0.070022853	0.265920779	0.976491766
UBP1	-0.053847626	0.26598944	0.976491766
ZNF10	-0.083342553	0.266064341	0.976491766
FEZ2	0.109231799	0.266104212	0.976491766
NPR1	0.110789576	0.266513963	0.976491766
WDR60	-0.098204279	0.266530461	0.976491766
HUS1	-0.100593648	0.266536791	0.976491766
ATP5B	-0.058136032	0.266547477	0.976491766
ZNF493	-0.203037596	0.266562053	0.976491766
MTMR4	-0.061040584	0.26657368	0.976491766
ADCK1	-0.126797607	0.266578184	0.976491766
MIS18BP1	-0.075545824	0.266652101	0.976491766
TNIP2	0.096096068	0.266693001	0.976491766
IQSEC3	0.088016028	0.267231606	0.976491766
C21orf58	-0.155055875	0.267382399	0.976491766
EXOSC1	-0.040094324	0.26761713	0.976491766
PTPLAD2	-0.165494184	0.267662235	0.976491766
COL6A3	0.179106456	0.267668108	0.976491766
SCRN3	-0.089250795	0.267858829	0.976491766
SNORA10	0.10830516	0.267934595	0.976491766
ARV1	-0.082283202	0.268173126	0.976491766
ANKRD6	-0.107151648	0.268351402	0.976491766
MTMR12	-0.116664241	0.268420472	0.976491766
PSMD5	0.099377876	0.268488919	0.976491766
LINC00347	0.092762365	0.268508434	0.976491766
FAM98A	-0.086886984	0.268532389	0.976491766

PSME1	0.06051992	0.268609467	0.976491766
SATB2	-0.118168264	0.268615347	0.976491766
IL18RAP	0.213405172	0.268650752	0.976491766
PRPF31	0.076303881	0.26866115	0.976491766
ZNF696	-0.118477622	0.268715492	0.976491766
NCBP2	-0.051220438	0.268742133	0.976491766
PRKCD	-0.053672991	0.268867809	0.976491766
PRTFDC1	0.204320301	0.268921026	0.976491766
CABIN1	-0.096121951	0.268927817	0.976491766
OSGEP	-0.069146978	0.268932634	0.976491766
SH2D2A	0.152708548	0.268936239	0.976491766
KHSRP	0.114127505	0.268965455	0.976491766
ELANE	-0.228125989	0.269075716	0.976491766
IRF9	-0.091596148	0.269170109	0.976491766
C11orf80	0.143125333	0.269214833	0.976491766
YWHAE	-0.122765661	0.269256029	0.976491766
ADORA2A	-0.116629708	0.269379081	0.976491766
CCDC137	-0.13547772	0.269505463	0.976491766
PRSS57	0.22153812	0.269549614	0.976491766
PLK5	-0.096747027	0.269831983	0.976491766
ABCB8	0.12771346	0.269862033	0.976491766
GSS	0.06976274	0.269893627	0.976491766
C2orf88	0.244696569	0.269924424	0.976491766
NLRX1	-0.060437161	0.269949124	0.976491766
HCG27	-0.174862531	0.270104475	0.976491766
KPNA5	0.134407559	0.270288277	0.976491766
FBXL20	-0.118659774	0.270357572	0.976491766
VARS2	0.132650574	0.270364163	0.976491766
FAM115C	-0.19904447	0.270428224	0.976491766
TOX	-0.154920259	0.270492296	0.976491766
CHTF18	-0.098307562	0.270683074	0.976491766
OSM	0.166028375	0.27069252	0.976491766
LAPTM4B	0.132696346	0.270766579	0.976491766
SNAP29	0.126792475	0.270935538	0.976491766
CDC45	-0.222255883	0.271001954	0.976491766
RPL13P5	0.11380709	0.271158686	0.976491766
EIF2B5	-0.072505271	0.271372684	0.976491766
METTL21A	-0.160015583	0.271423213	0.976491766
LGALS9B	0.137946445	0.271688717	0.976491766
DNAJB2	-0.072153548	0.271718079	0.976491766
CDC123	0.073163039	0.271905604	0.976491766
FASLG	-0.261961311	0.272047537	0.976491766
RIMBP3	0.092444827	0.272093693	0.976491766
SAMD10	0.100094623	0.272130626	0.976491766
CAMSAP1	0.081678741	0.272376309	0.976491766
RAB11FIP2	-0.063696974	0.272441957	0.976491766
RPS16	0.026077468	0.2725774	0.976491766

FAM198B	-0.166749753	0.27263735	0.976491766
PSMD4	0.077334216	0.27266944	0.976491766
RIC8B	-0.071762963	0.272828486	0.976491766
KRT73	0.392769117	0.272881441	0.976491766
CDH1	-0.132416423	0.272890064	0.976491766
FRS2	-0.06174079	0.273068195	0.976491766
SUN1	0.062786609	0.2731417	0.976491766
DEPDC1B	-0.127799613	0.273160671	0.976491766
TOMM40	0.047137011	0.273254005	0.976491766
METRNL	0.103764969	0.273269201	0.976491766
CIRH1A	0.061359359	0.273412163	0.976491766
NR3C2	0.087714386	0.2736402	0.976491766
FAM174A	-0.122146551	0.273667767	0.976491766
THRA	0.101619452	0.273698176	0.976491766
RALGAPA1	-0.078289579	0.273708526	0.976491766
BMS1P4	0.093251746	0.273785354	0.976491766
UBL7	0.080381098	0.273891765	0.976491766
APLP2	0.11841143	0.274018699	0.976491766
LRRK2	-0.174062255	0.274024758	0.976491766
DIS3L	0.089596622	0.274219217	0.976491766
LRRC41	0.083430619	0.274293674	0.976491766
RBM5	-0.072079229	0.274336865	0.976491766
PLD2	-0.102380374	0.274382632	0.976491766
DEFB116	-0.104711743	0.274407964	0.976491766
GCM1	0.095098183	0.274480603	0.976491766
CLCN6	-0.091128777	0.274490898	0.976491766
TMEM144	-0.201561301	0.274843367	0.976491766
IER5	0.101470058	0.274970726	0.976491766
MFAP1	-0.113791127	0.274995649	0.976491766
ZNF138	0.084756793	0.275004109	0.976491766
CDH23	-0.18053465	0.275269712	0.976491766
ABI3	0.099786185	0.275298514	0.976491766
CAMKMT	0.09093062	0.275371477	0.976491766
IPPK	-0.098365648	0.275633062	0.976491766
CSRNP1	0.093117681	0.275654259	0.976491766
TAGLN2	0.130232357	0.275761112	0.976491766
SNORA49	-0.092527573	0.275839392	0.976491766
ANKFY1	-0.071508381	0.275966247	0.976491766
ALDH7A1	-0.158749142	0.276342858	0.976491766
SMPD2	0.090493273	0.276439317	0.976491766
TLR10	0.19067049	0.276490889	0.976491766
FAM43A	-0.197712369	0.276577173	0.976491766
DNAJB12	-0.08823088	0.276579688	0.976491766
NFYA	0.0792229	0.276656368	0.976491766
AQP12B	0.130434353	0.276676875	0.976491766
NPTX1	-0.181222591	0.277093658	0.976491766
SNX33	0.087360407	0.277103981	0.976491766

SAMD4B	-0.106482646	0.277324266	0.976491766
DPY19L2P2	-0.097918806	0.27737758	0.976491766
RNF168	0.100840231	0.277591922	0.976491766
WDR83	-0.064164346	0.27765485	0.976491766
RABAC1	0.081294271	0.277965077	0.976491766
LRIG1	0.096396416	0.278046253	0.976491766
IKBIP	-0.034865511	0.27805678	0.976491766
AIRE	0.055289718	0.278073396	0.976491766
ABI1	0.058554618	0.278291503	0.976491766
PAPOLG	-0.108814835	0.278370633	0.976491766
EEF2K	-0.0858636	0.278481006	0.976491766
LEPR	-0.17392251	0.278500373	0.976491766
MIOS	-0.057991156	0.278523141	0.976491766
C17orf85	-0.059047734	0.278596869	0.976491766
ZFP30	-0.12185089	0.278626428	0.976491766
CX3CR1	-0.139798552	0.278865594	0.976491766
PRRT2	0.129705102	0.278872911	0.976491766
JAK1	0.090460809	0.278934697	0.976491766
MIPEP	0.082074685	0.278943536	0.976491766
PEAR1	0.24947214	0.279040569	0.976491766
DGCR14	0.077343635	0.279085127	0.976491766
ZC3H7A	-0.04683727	0.279110344	0.976491766
TMEM56	-0.120171285	0.279148373	0.976491766
C12orf68	0.096288308	0.279169003	0.976491766
SF3B4	0.052628845	0.279171114	0.976491766
HSPA13	-0.10411637	0.279218675	0.976491766
LEFTY1	0.18575892	0.279298955	0.976491766
PHLDB3	-0.193813196	0.279342102	0.976491766
ACADVL	-0.072333867	0.279538999	0.976491766
SYTL3	0.109127042	0.279572159	0.976491766
PHF7	0.098570091	0.279589313	0.976491766
DSG2	-0.082094764	0.279642737	0.976491766
NUBPL	-0.190753852	0.279647932	0.976491766
KIAA0040	0.123713207	0.279871019	0.976491766
RNF11	0.169487211	0.279920528	0.976491766
PABPC1	0.137684402	0.279941409	0.976491766
CALM3	0.054361157	0.279984819	0.976491766
TNFRSF8	-0.111857928	0.280003975	0.976491766
ATPAF2	-0.091611451	0.280037391	0.976491766
EIF6	0.079011121	0.280093126	0.976491766
WDR43	0.080984298	0.280168256	0.976491766
CENPBD1	-0.091185046	0.280314971	0.976491766
RFTN1	0.062846479	0.280386263	0.976491766
STK32C	-0.144460741	0.280424168	0.976491766
VNN3	-0.196942585	0.280550022	0.976491766
ZNF618	0.118166167	0.280562088	0.976491766
WASF3	0.280150711	0.280589117	0.976491766

MAP4K4	-0.077310021	0.2806547	0.976491766
AQR	0.052934292	0.280874871	0.976491766
SNORD59A	0.126944687	0.281034827	0.976491766
USO1	-0.049810946	0.281149997	0.976491766
FBXO43	-0.090984803	0.281180802	0.976491766
HECW2	-0.096180092	0.281236864	0.976491766
FBXW11	0.060081438	0.281287099	0.976491766
EPB41L3	-0.134389679	0.281305065	0.976491766
C1orf174	0.072582918	0.281310559	0.976491766
PTK7	-0.112864859	0.281580879	0.976491766
UTP15	-0.091863764	0.281954559	0.976491766
PNKP	-0.07512901	0.282222067	0.976491766
NOL12	-0.06301112	0.282424185	0.976491766
MAD2L1	-0.121425979	0.282462742	0.976491766
SNORD57	-0.124922925	0.282466987	0.976491766
EVI5L	0.088920321	0.282493983	0.976491766
ST6GALNAC6	0.118443626	0.282587198	0.976491766
GTF2H1	0.091272628	0.282595869	0.976491766
MIER1	-0.084923601	0.282617885	0.976491766
GTPBP1	-0.103972437	0.282632962	0.976491766
PPIG	0.065758614	0.282728328	0.976491766
OBSCN	-0.123209402	0.282733526	0.976491766
GLDC	-0.274456691	0.28281246	0.976491766
ECE1	0.10448482	0.282895928	0.976491766
LIMK2	-0.123512091	0.28291962	0.976491766
KLF10	0.211247142	0.282966217	0.976491766
ANKRD35	-0.176943032	0.283185869	0.976491766
PDK4	-0.172706444	0.283192353	0.976491766
CCM2	-0.076017241	0.283284129	0.976491766
HSD17B12	0.081457772	0.28332579	0.976491766
DYNC1I2	-0.068802854	0.283326792	0.976491766
OTUB1	-0.090716357	0.28348719	0.976491766
TSPAN9	0.309666784	0.283529475	0.976491766
COPB2	-0.073128861	0.283782059	0.976491766
MOSPD3	0.087602013	0.283853754	0.976491766
FADS1	0.139763326	0.283904348	0.976491766
KLHL12	-0.058620649	0.283910034	0.976491766
ZNF346	-0.114939773	0.284066642	0.976491766
WIZ	-0.085434414	0.284082451	0.976491766
MAFG	-0.086021435	0.284090504	0.976491766
DISP1	-0.093358956	0.284121881	0.976491766
TCF4	0.12749522	0.28413537	0.976491766
PRPF4	0.045828808	0.284164109	0.976491766
TRAPPC4	-0.085875169	0.284236477	0.976491766
PRUNE	0.106832855	0.28426251	0.976491766
ZNF211	-0.073688891	0.284386505	0.976491766
DNAJC9	0.061565385	0.284421915	0.976491766

TUBD1	-0.110080743	0.284447903	0.976491766
SNORD42B	0.094746987	0.284643482	0.976491766
APOA1BP	0.061711456	0.284690058	0.976491766
SPRY1	0.192203426	0.2847143	0.976491766
MRPL19	-0.102477047	0.284721404	0.976491766
FAM162A	0.118184208	0.284772387	0.976491766
NOL8	-0.065039736	0.28480692	0.976491766
MNT	-0.053455341	0.284855494	0.976491766
AVIL	-0.096712506	0.285020178	0.976491766
TBC1D1	-0.099058653	0.28502768	0.976491766
HIST1H4H	-0.382267482	0.285036062	0.976491766
CEP68	-0.106140962	0.285130591	0.976491766
VPS37B	0.086586892	0.28523132	0.976491766
KATNA1	0.061215194	0.285275486	0.976491766
IMP3	0.0478934	0.285312253	0.976491766
ATF3	0.154090151	0.285333816	0.976491766
CAPN7	-0.072199949	0.28534516	0.976491766
IRAK2	0.119698044	0.285474908	0.976491766
PARP2	-0.0801658	0.285542198	0.976491766
C2orf68	-0.111492131	0.28555687	0.976491766
SNX25	0.127776425	0.285566274	0.976491766
MRPS22	-0.040012764	0.285712714	0.976491766
FBXL18	-0.092345086	0.285905212	0.976491766
FFAR2	-0.152606134	0.286276462	0.976491766
PURB	-0.071801179	0.286342995	0.976491766
RPS6KC1	-0.080162777	0.286440365	0.976491766
CD244	0.121667509	0.286501925	0.976491766
ARHGEF35	0.224066367	0.287324071	0.976491766
SNX3	0.069388438	0.287605615	0.976491766
TBX21	0.109777111	0.287702683	0.976491766
ZBTB42	-0.092230953	0.287705823	0.976491766
FCRL2	0.249184755	0.28792662	0.976491766
AP1B1	-0.055856833	0.288036501	0.976491766
DCLK3	-0.101434728	0.288047436	0.976491766
ENDOG	0.115432287	0.288089848	0.976491766
NCF2	-0.12105742	0.288273626	0.976491766
PASK	0.180385539	0.288365645	0.976491766
ALPP	-0.161319965	0.288579317	0.976491766
N4BP1	-0.095527173	0.288673962	0.976491766
CKS1B	0.140747266	0.288689398	0.976491766
MS4A7	-0.110649917	0.288784416	0.976491766
FAM107B	-0.080233388	0.288928179	0.976491766
NAPB	-0.106609402	0.288993445	0.976491766
TRAF6	-0.097058246	0.289080704	0.976491766
TAPBPL	0.108020329	0.289189719	0.976491766
REEP1	0.132064599	0.289208055	0.976491766
HCG18	-0.122983325	0.289468881	0.976491766

ICAM3	0.06810949	0.289469855	0.976491766
EHD2	0.085382002	0.289552689	0.976491766
GPR171	0.121649317	0.289555371	0.976491766
BTBD6	0.067406756	0.289571307	0.976491766
IL11RA	-0.118615612	0.289585406	0.976491766
MDM4	-0.157414773	0.289620686	0.976491766
SLAMF1	-0.114279584	0.289645318	0.976491766
C9orf89	0.125240288	0.289677449	0.976491766
AGA	-0.098814631	0.289734434	0.976491766
HP1BP3	0.091277215	0.289940759	0.976491766
EGOT	0.103529759	0.28995735	0.976491766
PPP1R16A	-0.112072219	0.290148202	0.976491766
ZNF738	-0.150200697	0.290240928	0.976491766
CHD4	0.065451507	0.290242027	0.976491766
STX3	-0.081060255	0.290785441	0.976491766
NUF2	-0.121313855	0.290812026	0.976491766
YIF1A	0.074627455	0.290833528	0.976491766
CALR	0.087904529	0.290846066	0.976491766
ACIN1	0.065847736	0.290936431	0.976491766
GTF2F2	-0.066033246	0.291209656	0.976491766
KIFC1	-0.138318142	0.291488114	0.976491766
SYNE2	0.144143249	0.291489956	0.976491766
LYST	-0.095120332	0.291568669	0.976491766
CINP	0.11482875	0.291724329	0.976491766
PIGC	0.060313333	0.291966929	0.976491766
MIR1185-1	-0.079861314	0.292062737	0.976491766
SNORA42	-0.116621799	0.292073482	0.976491766
PNMA1	0.096490796	0.292092322	0.976491766
TUBG2	-0.084116273	0.292133255	0.976491766
PTGDS	0.269866786	0.292192472	0.976491766
HLA-C	0.369780184	0.292249088	0.976491766
C18orf25	0.054911513	0.292271755	0.976491766
ELMOD2	-0.110773488	0.292319064	0.976491766
PTMS	-0.158724942	0.292337901	0.976491766
DNAJC12	0.089057512	0.292350775	0.976491766
INSR	0.109202965	0.292357518	0.976491766
CRAT	0.114934779	0.292378159	0.976491766
DIP2B	0.066712731	0.292439861	0.976491766
ZNF24	0.105200041	0.292473883	0.976491766
RHBDL2	-0.226146539	0.292475457	0.976491766
CLCF1	-0.168019284	0.292499393	0.976491766
CD22	0.163993477	0.292506612	0.976491766
ZNF195	-0.086999155	0.29257415	0.976491766
PIP5K1A	0.12469267	0.292642714	0.976491766
POMGNT1	-0.050843114	0.292662069	0.976491766
METAP1D	0.085401736	0.292700559	0.976491766
DNAJC4	0.066003701	0.292850426	0.976491766

NTAN1	0.088599223	0.292892543	0.976491766
PAPSS2	0.161438493	0.292933109	0.976491766
PLEC	-0.089107969	0.293033522	0.976491766
HEXIM1	0.084358488	0.293052601	0.976491766
SNORA1	-0.112982277	0.293091557	0.976491766
ATP6V0E1	0.057798938	0.293169474	0.976491766
KLHL22	-0.079483616	0.293183967	0.976491766
MMS22L	0.08956996	0.293184057	0.976491766
CCDC50	0.111649206	0.293218651	0.976491766
RFPL1	0.101587197	0.293284571	0.976491766
ZKSCAN3	-0.086510639	0.293318734	0.976491766
WDR76	0.089396729	0.293616977	0.976491766
SRSF11	0.089622538	0.293767472	0.976491766
GNAI3	-0.129549796	0.293777581	0.976491766
FAM192A	-0.067570851	0.294006504	0.976491766
RAB11FIP1	-0.07563175	0.294044187	0.976491766
ZNF688	0.066790293	0.294054705	0.976491766
PEX11G	-0.131159154	0.294190689	0.976491766
PPP2R5C	0.083173326	0.294418904	0.976491766
TMTC3	0.102956803	0.294450397	0.976491766
C6orf62	-0.067933183	0.294500789	0.976491766
TNFSF4	0.208937077	0.294649676	0.976491766
ADNP2	0.063916142	0.29468696	0.976491766
DNAJC17	-0.08205206	0.294692046	0.976491766
OPTN	0.100784373	0.294770406	0.976491766
MRPL20	0.1095246	0.294773529	0.976491766
RASSF5	0.053398369	0.294909003	0.976491766
CYHR1	0.101887344	0.294969467	0.976491766
SRCAP	0.094844816	0.295018142	0.976491766
HMBS	-0.114725112	0.29511051	0.976491766
TRIM31	-0.090483049	0.295892822	0.976491766
TDRD3	0.084698603	0.295944062	0.976491766
OR2B2	0.13759939	0.296131202	0.976491766
CGA	0.114720008	0.296141886	0.976491766
CDAN1	-0.054539005	0.29617474	0.976491766
PRRG2	0.117608608	0.296328831	0.976491766
XRCC3	0.088474149	0.296540258	0.976491766
ARID1B	0.088197194	0.296727205	0.976491766
PIAS3	0.068450121	0.296775127	0.976491766
CHKB	-0.084895892	0.296780915	0.976491766
NTHL1	0.072534981	0.296808628	0.976491766
RPL29	-0.048559182	0.296985988	0.976491766
ANKLE1	-0.186794827	0.296994435	0.976491766
HYDIN	-0.095056721	0.297176052	0.976491766
HNRNPM	0.046517119	0.297234877	0.976491766
KCNK3	-0.089900577	0.297523791	0.976491766
BCAP29	0.107737972	0.297600511	0.976491766

C17orf70	-0.063855656	0.297789415	0.976491766
PCDH15	0.091088731	0.297819486	0.976491766
SOX15	-0.108983952	0.297890989	0.976491766
CACYBP	0.132237027	0.297916329	0.976491766
BROX	-0.121422375	0.297944131	0.976491766
SLC43A3	0.096017229	0.297945581	0.976491766
MIF	0.072019217	0.297984338	0.976491766
SLC25A11	-0.070711081	0.298039032	0.976491766
NCOA6	0.048974704	0.298270182	0.976491766
GINS4	0.081663702	0.298457334	0.976491766
MEF2A	0.101683912	0.298478199	0.976491766
XPOT	0.060047427	0.298503345	0.976491766
KIAA1033	-0.11771858	0.298543652	0.976491766
MYOM2	-0.720064686	0.298612686	0.976491766
FIGNL1	0.138192013	0.298750938	0.976491766
CCBE1	-0.177514167	0.2987522	0.976491766
SGCE	0.192657646	0.298859374	0.976491766
RNPEP	0.095941328	0.298908622	0.976491766
SEMA4A	-0.097907017	0.299003435	0.976491766
ATP6V1G1	0.051802922	0.299054964	0.976491766
CCDC117	-0.072217755	0.299142552	0.976491766
CCDC88B	0.125838457	0.299178196	0.976491766
SCNM1	0.087548197	0.299368062	0.976491766
EPHX1	-0.096417815	0.299407342	0.976491766
FAM53A	-0.117696598	0.299442269	0.976491766
KCTD9	-0.086086399	0.299541361	0.976491766
ZSCAN5A	-0.098058072	0.299578901	0.976491766
PLXND1	-0.120883716	0.299705651	0.976491766
AP3S1	0.097166603	0.299835092	0.976491766
USP19	0.100464708	0.29993501	0.976491766
DNAJB5	0.105610116	0.299977688	0.976491766
ELF2	-0.06077534	0.300048437	0.976491766
PTPN2	0.068654531	0.300183897	0.976491766
ITFG3	-0.081347639	0.300400787	0.976491766
AHCTF1	0.077370958	0.300590835	0.976491766
CXCL9	-0.127717844	0.300599774	0.976491766
FOXN3	0.050176045	0.300710677	0.976491766
NDUFA6	0.080990835	0.300806621	0.976491766
RGL4	0.089602973	0.301076247	0.976491766
KRTAP10-11	0.095671158	0.301322625	0.976491766
ZNF500	-0.087475624	0.301343545	0.976491766
ST7L	-0.11170447	0.301465145	0.976491766
HGF	0.092951622	0.301527188	0.976491766
CDH6	0.176210439	0.301583909	0.976491766
H2AFV	0.081330533	0.3016289	0.976491766
TIFA	0.109126056	0.301673229	0.976491766
EXOC8	0.111275206	0.301780982	0.976491766

FGL2	-0.118628479	0.30194706	0.976491766
ANLN	-0.095812113	0.301958645	0.976491766
PATL2	-0.219044888	0.301978429	0.976491766
ZBTB24	-0.077511095	0.302215252	0.976491766
CCNJL	0.187274245	0.302261108	0.976491766
GFPT1	-0.101202054	0.302386218	0.976491766
ILVBL	-0.07954787	0.302405017	0.976491766
CHMP2B	-0.098263987	0.302959586	0.976491766
TMEM160	0.111124721	0.302994213	0.976491766
JUNB	0.160369177	0.303113004	0.976491766
AGPAT4	-0.148995394	0.303178665	0.976491766
FASTKD1	-0.088880837	0.303460286	0.976491766
RPH3AL	0.100809909	0.303469886	0.976491766
CA1	0.156793103	0.303515555	0.976491766
CSNK1G1	0.065863334	0.30351658	0.976491766
TAF5	-0.071843076	0.30352815	0.976491766
KCNK13	-0.120918314	0.303636249	0.976491766
NXNL1	-0.146015053	0.303782257	0.976491766
C14orf93	0.047303261	0.303794228	0.976491766
SNX6	0.082211608	0.304032092	0.976491766
ZNF200	-0.173130936	0.304142927	0.976491766
SIN3A	0.062153577	0.304261444	0.976491766
ZDHHC23	0.107316587	0.304424915	0.976491766
SGK223	-0.092833183	0.304521926	0.976491766
MCM6	-0.078531408	0.304607039	0.976491766
CMPK1	-0.059184376	0.304866332	0.976491766
GINS2	-0.182845053	0.304899595	0.976491766
ZNF428	0.05926318	0.305034837	0.976491766
SUV39H2	0.113595325	0.305082681	0.976491766
FAM188A	-0.06922339	0.30517279	0.976491766
PIF1	-0.111947051	0.305263664	0.976491766
SNORD52	-0.113395055	0.305281362	0.976491766
KIAA1107	-0.085443014	0.305520014	0.976491766
FSTL1	0.199434669	0.305538307	0.976491766
ERI2	-0.109558641	0.305712583	0.976491766
RAP2A	-0.128415309	0.305933639	0.976491766
GZMM	0.138097481	0.305979644	0.976491766
UBA5	-0.075021034	0.306134188	0.976491766
DIABLO	-0.036432289	0.306635384	0.976491766
TRMT12	0.082718026	0.306671217	0.976491766
RALB	0.085442663	0.306783402	0.976491766
ATAD2B	-0.087141661	0.306802942	0.976491766
EEPD1	-0.119024529	0.306962958	0.976491766
ALG3	0.05484357	0.306986306	0.976491766
MS4A1	0.254932957	0.307105081	0.976491766
ASB1	-0.07396564	0.307121417	0.976491766
SRXN1	-0.140307413	0.307185369	0.976491766

HDGFRP3	0.188191307	0.307310674	0.976491766
GPR132	-0.10934225	0.307313286	0.976491766
COMMD9	0.070944113	0.307502728	0.976491766
NCF1	0.190722958	0.30760703	0.976491766
NELL2	-0.151013953	0.307751104	0.976491766
ZNF652	-0.149626896	0.307846975	0.976491766
C5orf30	-0.081430216	0.307916751	0.976491766
FNTB	-0.272100981	0.308062049	0.976491766
IFNGR1	-0.073711888	0.308119177	0.976491766
CPAMD8	-0.117491536	0.308123167	0.976491766
ZNF140	-0.10528456	0.308134077	0.976491766
SSBP4	0.076480984	0.308155181	0.976491766
PDCD11	-0.087471457	0.30816253	0.976491766
C19orf44	-0.102809269	0.308253724	0.976491766
SNORD95	0.116917642	0.308393431	0.976491766
IFNAR2	-0.108502681	0.308590475	0.976491766
PDE5A	0.234420795	0.30873847	0.976491766
GRPEL2	-0.101871042	0.308887304	0.976491766
TSPAN14	0.107650672	0.308957977	0.976491766
MPST	-0.113748791	0.309049435	0.976491766
SON	-0.031786351	0.309174349	0.976491766
THNSL1	-0.105374631	0.309226564	0.976491766
ANAPC1	0.065279484	0.309388354	0.976491766
GPC2	-0.130435326	0.309523331	0.976491766
PSMD2	-0.049380928	0.30967601	0.976491766
LSS	-0.123896295	0.309692447	0.976491766
POLD4	0.054847764	0.309756838	0.976491766
AMY2B	-0.102791417	0.309772144	0.976491766
OR2T34	0.093119859	0.309806808	0.976491766
TSC22D4	0.090660647	0.309838033	0.976491766
DCUN1D3	-0.092108693	0.309970107	0.976491766
C4orf21	-0.123124046	0.310074646	0.976491766
SLC24A1	0.108673219	0.310371281	0.976491766
FOXI3	0.087761958	0.310402212	0.976491766
PTPN23	-0.093212103	0.310468223	0.976491766
RRBP1	-0.075531683	0.31052133	0.976491766
PDS5A	0.054070223	0.310560686	0.976491766
DNM3	0.242916482	0.310675461	0.976491766
NECAB3	0.097666605	0.310711054	0.976491766
AHSA2	-0.115245678	0.310848877	0.976491766
ARHGAP21	0.116535912	0.310921533	0.976491766
CFD	-0.142336273	0.311140122	0.976491766
PMCHL1	-0.078170829	0.311164653	0.976491766
DGKE	-0.110479444	0.311591473	0.976491766
UBXN8	-0.093574527	0.311592508	0.976491766
LARS2	-0.095723567	0.311663757	0.976491766
TRIP11	0.068420392	0.311691371	0.976491766

OGFR	0.082137006	0.311711345	0.976491766
RUSC2	-0.111573904	0.311824103	0.976491766
TAF1L	0.062009398	0.311858335	0.976491766
ADHFE1	-0.110160339	0.311917488	0.976491766
DMTF1	-0.075834708	0.312085184	0.976491766
TUBB8	0.099749191	0.312103621	0.976491766
HIST1H4E	-0.269347586	0.312106848	0.976491766
HRASLS	0.274592464	0.3121969	0.976491766
BMS1P5	-0.133070048	0.312285392	0.976491766
PHPT1	0.072883773	0.312315699	0.976491766
RAB3A	-0.093367328	0.312431903	0.976491766
AP1G2	-0.073606995	0.312433609	0.976491766
SH3GL1	0.068955098	0.312551957	0.976491766
PHRF1	-0.072634045	0.312640539	0.976491766
TROAP	-0.082200852	0.312660559	0.976491766
MRPS18C	0.089087241	0.312668396	0.976491766
NAAA	0.110186733	0.31276098	0.976491766
UNC13A	-0.171664255	0.312795759	0.976491766
NAA15	-0.084865508	0.312935655	0.976491766
NUP153	-0.064710816	0.313047869	0.976491766
CLINT1	-0.053884764	0.313208821	0.976491766
AP1AR	0.115482496	0.31336703	0.976491766
CD83	0.10961115	0.313412377	0.976491766
MAP3K2	-0.134295272	0.313492051	0.976491766
ATP6V1A	-0.069589531	0.31349823	0.976491766
PROK2	-0.341441348	0.314121969	0.976491766
CST3	-0.099252199	0.314158087	0.976491766
SEC22B	0.062348145	0.31435055	0.976491766
SCD	0.128884746	0.314502664	0.976491766
MEIS1	0.198809127	0.314525835	0.976491766
TJP3	0.134085356	0.314629442	0.976491766
RFX4	-0.122965209	0.314658136	0.976491766
PES1	0.088423576	0.314771726	0.976491766
PPA2	-0.121073311	0.314773258	0.976491766
ADAM17	-0.087271182	0.314797174	0.976491766
SEC22A	-0.071460304	0.314941358	0.976491766
DOPEY1	-0.079795032	0.315090886	0.976491766
HOMER2	0.242526152	0.31530404	0.976491766
ATAD2	0.082345593	0.315565115	0.976491766
NDUFB2	0.066726667	0.315582443	0.976491766
ANAPC5	0.050581141	0.315599258	0.976491766
GPATCH2	-0.081489333	0.315663374	0.976491766
PRSS8	0.100003414	0.315677704	0.976491766
TNNC1	-0.080999949	0.315927341	0.976491766
TBC1D13	0.055722362	0.316055859	0.976491766
TBC1D17	-0.097114893	0.316164524	0.976491766
CHRDL2	0.082862973	0.316287234	0.976491766

LRRC45	0.116172605	0.316364851	0.976491766
EME1	0.086768806	0.316469652	0.976491766
MRPL36	0.072103634	0.316559169	0.976491766
GSDMD	-0.077131148	0.316636252	0.976491766
ATP5G1	-0.068303483	0.316646094	0.976491766
OTUD1	0.084222391	0.316675503	0.976491766
MRPS15	0.051548589	0.316778354	0.976491766
MLH3	0.187134435	0.316783064	0.976491766
TGIF2	0.053636918	0.316937254	0.976491766
CHMP1B	0.077606568	0.31696299	0.976491766
PKD1P1	0.079697508	0.317134074	0.976491766
PDDC1	-0.074418003	0.317182791	0.976491766
QSOX2	0.08419807	0.317274503	0.976491766
ARFIP2	-0.091445766	0.317419166	0.976491766
SNORA58	-0.075512416	0.317491054	0.976491766
FNBP1	-0.044106102	0.317536928	0.976491766
COX6A1	0.058814317	0.317818402	0.976491766
GRTP1	0.099743095	0.318031664	0.976491766
MAP4	-0.104231753	0.318056733	0.976491766
CD84	-0.104821341	0.318075125	0.976491766
ALKBH2	0.074371039	0.318252078	0.976491766
C11orf48	0.069639295	0.318257615	0.976491766
MYADM	-0.088433451	0.318326195	0.976491766
TM9SF2	-0.050755419	0.318383063	0.976491766
SNORA75	0.100399305	0.318427045	0.976491766
TAGAP	-0.168356224	0.318502316	0.976491766
MYNN	-0.05350273	0.318792559	0.976491766
KCNJ15	-0.097115558	0.318805224	0.976491766
REEP4	-0.09187369	0.318817461	0.976491766
ZNF69	-0.204251121	0.318829054	0.976491766
GBGT1	-0.104126377	0.318931547	0.976491766
CPA3	-0.140103122	0.318961593	0.976491766
GPATCH1	-0.085888128	0.31907799	0.976491766
PDIA4	-0.103679084	0.319199387	0.976491766
UCKL1	0.056888345	0.319427614	0.976491766
NDUFA4	0.054042387	0.319638561	0.976491766
STOML1	-0.10302928	0.319644392	0.976491766
RASAL1	0.088876432	0.3199252	0.976491766
VAT1	0.081220489	0.320020402	0.976491766
ODF2L	-0.345027306	0.320180212	0.976491766
TADA2B	-0.056459651	0.320195447	0.976491766
CANX	-0.05781754	0.320451015	0.976491766
MESDC1	0.085859142	0.32053394	0.976491766
BCYRN1	-0.163655229	0.320600534	0.976491766
INPP4A	0.091240606	0.320675096	0.976491766
HVCN1	0.095981855	0.320726484	0.976491766
DUSP16	0.095370714	0.320732594	0.976491766

ANKRD23	-0.092836685	0.320747765	0.976491766
D2HGDH	-0.126039196	0.320748217	0.976491766
TMEM129	-0.088446526	0.321065994	0.976491766
SNORD33	0.09767629	0.321147721	0.976491766
TXNIP	0.02703671	0.321172478	0.976491766
NEURL4	-0.106310615	0.321428439	0.976491766
SCN9A	-0.109557072	0.321488665	0.976491766
CALB2	0.09918084	0.321526769	0.976491766
MYO15B	-0.17528985	0.32155611	0.976491766
ITGB3	0.353512634	0.321915972	0.976491766
LDB2	0.218504774	0.321919541	0.976491766
SNORD21	0.149479625	0.321988564	0.976491766
PPP2R1A	0.063045853	0.322169755	0.976491766
B3GALT4	-0.089757735	0.322191007	0.976491766
FAM53B	0.097404651	0.322231678	0.976491766
SCP2	0.134300517	0.322282421	0.976491766
CWF19L1	-0.056050793	0.322316422	0.976491766
KIAA1324L	0.219122695	0.322380015	0.976491766
CSH2	0.098266509	0.322430985	0.976491766
NETO1	0.11359355	0.322635307	0.976491766
PSTPIP1	-0.098303055	0.322691675	0.976491766
BCKDHA	0.075027032	0.322726514	0.976491766
PTK2	0.109906148	0.32282044	0.976491766
PCGF5	0.098149994	0.322866209	0.976491766
EPPK1	-0.171925376	0.322906735	0.976491766
OTP	0.091883508	0.322924569	0.976491766
NAGS	-0.083089046	0.322996112	0.976491766
CCS	0.078417121	0.323098911	0.976491766
ILDR1	0.105479892	0.32311688	0.976491766
TMEM194A	-0.108475203	0.323124426	0.976491766
LSM3	0.089816201	0.323258774	0.976491766
MTHFD1L	-0.110600088	0.323259729	0.976491766
ZNF593	-0.082592639	0.323423214	0.976491766
GMPPB	-0.095191885	0.32349165	0.976491766
MARS	-0.058127746	0.323690473	0.976491766
KRIT1	-0.098712938	0.323692962	0.976491766
CYP11A1	0.10051983	0.323758593	0.976491766
LIAS	-0.081217681	0.323840379	0.976491766
FBXO5	-0.087481274	0.323917341	0.976491766
MPL	0.284073865	0.324117253	0.976491766
DDX18	0.051264059	0.324289295	0.976491766
BCAS2	0.123463895	0.324537732	0.976491766
ESCO1	-0.08733684	0.324549594	0.976491766
TIGD2	0.091680607	0.324658943	0.976491766
RUFY2	-0.111678457	0.324695804	0.976491766
C1orf85	-0.13892135	0.324709273	0.976491766
SNORD12C	0.149104752	0.324824095	0.976491766

MSH3	-0.032454763	0.324848353	0.976491766
NPM3	0.097126794	0.324882913	0.976491766
CRYBA2	-0.113944122	0.325051215	0.976491766
MFAP3	-0.116246797	0.325265579	0.976491766
CSNK1E	0.081821464	0.325328548	0.976491766
SCGB1C1	0.289402313	0.325394896	0.976491766
CDC14B	0.179045392	0.325401703	0.976491766
AIFM2	-0.073503485	0.32555942	0.976491766
TDP1	-0.217539094	0.325639617	0.976491766
MTA3	0.084279932	0.325731862	0.976491766
RTN4IP1	-0.061050435	0.325743958	0.976491766
PACS2	0.087413774	0.325902788	0.976491766
MGST2	0.085183797	0.32600427	0.976491766
GTPBP10	-0.09403808	0.326063458	0.976491766
SEPSECS	-0.073092248	0.326118287	0.976491766
NMT1	-0.086346272	0.326244045	0.976491766
FAM110B	0.103928452	0.32653088	0.976491766
NCAPD3	-0.06349061	0.326587369	0.976491766
VPS72	0.07498476	0.326643979	0.976491766
ANKRD40	0.059966745	0.326793507	0.976491766
PCSK6	0.080747698	0.326834612	0.976491766
NACAP1	0.11321777	0.3268443	0.976491766
PHF19	-0.067467059	0.326945888	0.976491766
ATAT1	0.095004178	0.327181632	0.976491766
RILPL2	0.072700194	0.327257158	0.976491766
ZNF30	-0.083865424	0.327467186	0.976491766
SELL	0.121405442	0.327711792	0.976491766
PINX1	0.087016942	0.327880828	0.976491766
RBM23	-0.068305894	0.327899755	0.976491766
MYO19	-0.11402067	0.327945308	0.976491766
FRA10AC1	0.139376866	0.328012115	0.976491766
FAM109B	0.097677756	0.328142243	0.976491766
FAM160A2	-0.079113829	0.328186153	0.976491766
CAMK2G	-0.049999611	0.328284481	0.976491766
RPL3	0.03847391	0.328288749	0.976491766
CCDC112	-0.081016506	0.328371712	0.976491766
ARPC1B	0.073243418	0.328561479	0.976491766
IFI30	0.096838343	0.328577273	0.976491766
FAM96A	0.078114358	0.328754059	0.976491766
CSAD	-0.143722107	0.329120689	0.976491766
UBE2E3	0.082720765	0.32931218	0.976491766
EIF4E3	0.136327766	0.329394549	0.976491766
TFAP2A	0.072038989	0.32947689	0.976491766
KIF2C	-0.091161798	0.329483786	0.976491766
SLC29A3	0.09325599	0.329518953	0.976491766
DAXX	0.069934173	0.329603643	0.976491766
LIN9	-0.084281594	0.329719947	0.976491766

PHTF2	-0.090615455	0.329766613	0.976491766
RRAGA	0.056523935	0.329849544	0.976491766
ENKUR	0.212182663	0.329922314	0.976491766
THOC5	-0.059415831	0.329959842	0.976491766
IKZF5	-0.068274623	0.33000377	0.976491766
SNORD12B	0.092683707	0.330209775	0.976491766
IKZF4	-0.093868575	0.330275747	0.976491766
RPS11	0.038115008	0.330357354	0.976491766
KIF2A	0.104151816	0.330384715	0.976491766
TECPR1	-0.134633521	0.330412918	0.976491766
ZBTB17	-0.059707489	0.330543998	0.976491766
SMARCA4	0.040706879	0.330746264	0.976491766
SLCO4A1	0.075865014	0.33076276	0.976491766
SLC7A6OS	0.079697913	0.330776169	0.976491766
KIF3A	-0.112683534	0.331033205	0.976491766
SLC25A33	0.095528321	0.33112206	0.976491766
RASGRP2	-0.076904399	0.331248406	0.976491766
USP25	-0.091576605	0.331376989	0.976491766
ISCU	0.053905427	0.331407453	0.976491766
SUOX	0.126374072	0.331647493	0.976491766
CNOT8	0.047603132	0.331771413	0.976491766
DUSP6	-0.093177187	0.331799943	0.976491766
SLC44A1	0.077248834	0.331808277	0.976491766
LARP1	0.046933386	0.331817884	0.976491766
STX5	0.04907583	0.331851686	0.976491766
MGAT3	0.142607543	0.331854067	0.976491766
TMEM191C	0.107842161	0.331869089	0.976491766
SH2B3	-0.057201499	0.332043692	0.976491766
C9orf114	0.08368835	0.332104621	0.976491766
CTRL	-0.091539519	0.332106793	0.976491766
DUSP18	-0.069437727	0.332129896	0.976491766
FOXK1	-0.115780691	0.332139743	0.976491766
MTMR14	-0.043018963	0.332217694	0.976491766
TAF7	0.067814308	0.332244719	0.976491766
LCP1	-0.045001437	0.332254829	0.976491766
EFNA4	-0.068988673	0.332374651	0.976491766
NDUFA12	0.086035991	0.332624922	0.976491766
DEAF1	0.082508152	0.332868543	0.976491766
C1GALT1	-0.095847727	0.332881787	0.976491766
TNFRSF10C	-0.08249615	0.332937754	0.976491766
FBXW5	0.070361942	0.333112131	0.976491766
UTP18	0.080594474	0.333171515	0.976491766
PREPL	-0.073887635	0.333226368	0.976491766
DOCK4	-0.21239536	0.333301304	0.976491766
TRPV1	-0.083918067	0.333424575	0.976491766
DBT	-0.075836883	0.333464078	0.976491766
CTAGE5	-0.085881401	0.333615314	0.976491766

CERCAM	-0.211623368	0.333714858	0.976491766
PTS	-0.052424079	0.333721145	0.976491766
RBM15	0.05337904	0.333746056	0.976491766
PPP3CC	0.054096763	0.333796639	0.976491766
F3	-0.086011495	0.333845336	0.976491766
ZFP14	-0.170491685	0.333883379	0.976491766
ABCC10	-0.076629258	0.333898274	0.976491766
CCDC59	0.043700708	0.333982177	0.976491766
TTC14	-0.125681498	0.33414638	0.976491766
CHST2	0.162844496	0.334201362	0.976491766
LSM10	0.077018887	0.334320206	0.976491766
EFCAB4A	-0.159450837	0.334416479	0.976491766
SSBP2	0.084937859	0.334506315	0.976491766
SRFBP1	-0.099986739	0.334511371	0.976491766
GZF1	0.080838725	0.33454959	0.976491766
IL17RA	-0.086135856	0.334729528	0.976491766
RAB20	0.106671218	0.334906263	0.976491766
CPSF4	0.049358805	0.335176171	0.976491766
SESTD1	-0.113702173	0.335202016	0.976491766
SMARCE1	0.086210782	0.335209624	0.976491766
GIMAP2	-0.087830386	0.335411536	0.976491766
SCNN1A	-0.101791229	0.335572413	0.976491766
ZFAND5	-0.043471026	0.33562528	0.976491766
C5orf28	-0.165959891	0.335627057	0.976491766
ISYNA1	0.083838539	0.335658208	0.976491766
SRCIN1	0.09200619	0.335665496	0.976491766
MIR593	0.085620299	0.335768643	0.976491766
SLC3A2	0.062276892	0.335768943	0.976491766
GSTTP2	-0.205790884	0.335960003	0.976491766
VRK3	-0.128817603	0.336022968	0.976491766
ST3GAL3	0.136398216	0.336032496	0.976491766
IRAK4	-0.065460398	0.336112947	0.976491766
ANKRD13C	0.071602483	0.336334998	0.976491766
TCEB1	0.054116728	0.336413719	0.976491766
DAD1	0.052612341	0.336449805	0.976491766
DLST	0.06818656	0.336524827	0.976491766
LILRB1	-0.054017028	0.336673107	0.976491766
METTL9	-0.085924692	0.336682348	0.976491766
PDCD4	0.088464946	0.33671386	0.976491766
NUDT16	0.095827153	0.336908419	0.976491766
MYCN	-0.116011483	0.336988354	0.976491766
B3GAT3	0.095874531	0.337039313	0.976491766
PHACTR1	0.109920617	0.337063432	0.976491766
MRPL10	0.075323735	0.337116432	0.976491766
SNORA70B	0.10388559	0.337126001	0.976491766
FAM63A	-0.177555212	0.337155902	0.976491766
RPL41	-0.022976019	0.337160126	0.976491766

TRIM27	-0.0463754	0.337286132	0.976491766
PLEKHA7	-0.089381373	0.337292915	0.976491766
STT3B	-0.079481334	0.337305164	0.976491766
USP14	-0.098317038	0.337444142	0.976491766
TAOK2	-0.112645244	0.33752463	0.976491766
NAE1	0.049364855	0.337535288	0.976491766
SLC35B1	-0.050608027	0.337562981	0.976491766
RUVBL2	0.071433567	0.337642627	0.976491766
NAIF1	0.061363026	0.337728291	0.976491766
LUZP1	0.066815565	0.337775561	0.976491766
POLI	-0.096907699	0.337891981	0.976491766
NUCB2	-0.099843115	0.337971494	0.976491766
NKTR	-0.098904105	0.338009575	0.976491766
TUBB2A	-0.194611944	0.338023023	0.976491766
NUDT13	-0.099695214	0.338116038	0.976491766
MINK1	0.103736424	0.338279205	0.976491766
ABHD11	0.097212251	0.338345096	0.976491766
ACTR6	-0.066701241	0.338406124	0.976491766
VPS16	0.090521201	0.33880262	0.976491766
SPATA6	-0.088802431	0.338939008	0.976491766
SEC14L5	0.320734095	0.338982777	0.976491766
PANK2	-0.065120592	0.339060598	0.976491766
ATAD1	-0.077902687	0.339079209	0.976491766
TSSC4	0.07937624	0.339224795	0.976491766
MRPL1	0.072184932	0.339225186	0.976491766
FUT4	-0.063075293	0.339292125	0.976491766
SDC1	0.093584014	0.339345575	0.976491766
PSMB7	-0.07453396	0.339448694	0.976491766
ZNF720	-0.094136075	0.33953643	0.976491766
SNHG7	0.131089044	0.339627816	0.976491766
KIR3DL3	0.324319327	0.339707459	0.976491766
GNPTAB	0.098856621	0.339865678	0.976491766
NSA2	0.072270585	0.339889398	0.976491766
ZNF567	-0.087714726	0.340152072	0.976491766
SIGLEC9	-0.094127071	0.340210011	0.976491766
RPL23AP7	0.028436125	0.340219204	0.976491766
PDK2	0.082382966	0.340477769	0.976491766
FAM115A	0.032930831	0.340659676	0.976491766
SLC43A1	-0.113315906	0.340695871	0.976491766
BLM	-0.093799551	0.340747621	0.976491766
CHCHD3	0.08156569	0.340945294	0.976491766
DUT	0.111742781	0.340976846	0.976491766
ETNK1	-0.094066726	0.340980231	0.976491766
RNPEPL1	-0.104709017	0.340988382	0.976491766
PSAP	0.032963247	0.341014094	0.976491766
FPR3	-0.198607781	0.341040295	0.976491766
KDELR1	0.051280151	0.341053185	0.976491766

PARS2	-0.09819177	0.341177457	0.976491766
RNH1	0.076881295	0.341221599	0.976491766
DDX56	-0.067294774	0.341451168	0.976491766
EPC2	-0.107042591	0.341549158	0.976491766
TPRKB	0.109984052	0.341683561	0.976491766
PLA2G12A	0.152807208	0.341821084	0.976491766
PA2G4	-0.07757933	0.34193604	0.976491766
HIST1H1C	0.161685722	0.342067288	0.976491766
URM1	0.051655601	0.342137824	0.976491766
F12	-0.092528268	0.342237236	0.976491766
TSPAN2	-0.131179196	0.342428012	0.976491766
PPAP2A	-0.099284302	0.342432645	0.976491766
NDST2	-0.066762444	0.342570587	0.976491766
OSCAR	-0.117199499	0.342658868	0.976491766
HDAC9	0.159577572	0.342662901	0.976491766
ZFYVE1	-0.057020021	0.342757832	0.976491766
SEC31A	-0.045642339	0.342852042	0.976491766
CASC4	0.10423322	0.342887274	0.976491766
UPK3A	0.117492002	0.342934774	0.976491766
MRPL49	0.066227137	0.342960519	0.976491766
FAM168A	-0.054387592	0.342963724	0.976491766
FARSB	-0.111547195	0.342968882	0.976491766
ZNF326	0.091357275	0.342970239	0.976491766
RPS5	0.062345619	0.343031294	0.976491766
CRB3	0.097680388	0.343108866	0.976491766
HPS6	-0.068211166	0.343118258	0.976491766
KIAA1875	0.151903027	0.343236024	0.976491766
NEBL	0.227229286	0.34331381	0.976491766
SMEK1	0.046617491	0.343317789	0.976491766
MELK	-0.145025976	0.34349293	0.976491766
ZSCAN12	-0.112621748	0.343605822	0.976491766
FKBPL	0.073389778	0.343674318	0.976491766
CCDC66	-0.079924189	0.343821702	0.976491766
SIX5	-0.095276948	0.343822199	0.976491766
DDX11L2	0.326976953	0.343878663	0.976491766
BCL9	-0.092183111	0.344013849	0.976491766
CDC14A	0.079087172	0.344051087	0.976491766
BCL2L1	0.106971101	0.344085257	0.976491766
APTX	-0.049556022	0.344160899	0.976491766
AKTIP	-0.063971736	0.344281476	0.976491766
AASS	-0.108483339	0.344337786	0.976491766
CBFA2T2	-0.075564609	0.344390124	0.976491766
MAP3K14	-0.074842015	0.344416228	0.976491766
MCMBP	-0.108493325	0.344476939	0.976491766
PRKCZ	0.069675309	0.344545557	0.976491766
CDIPT	0.053612501	0.344750621	0.976491766
RHOA	0.052413244	0.344767592	0.976491766

TUBGCP6	-0.078863989	0.344831791	0.976491766
GAB2	-0.083245903	0.344851067	0.976491766
SNORD78	0.100302732	0.345005114	0.976491766
VPS36	-0.055316146	0.345073112	0.976491766
MRPL27	-0.069436896	0.345086266	0.976491766
KARS	0.051444939	0.34516357	0.976491766
RGR	-0.091027918	0.345206515	0.976491766
CAPNS1	0.076112875	0.345476999	0.976491766
LYPLAL1	0.067152083	0.345591042	0.976491766
SLC39A14	0.089443592	0.345671266	0.976491766
ANXA6	0.073594389	0.345764965	0.976491766
TMEM30A	0.072395213	0.345919395	0.976491766
PINK1	0.064542197	0.345970463	0.976491766
FSD1L	0.101410103	0.346018673	0.976491766
ZNF830	-0.08719544	0.346062321	0.976491766
RP9P	0.077273067	0.346190364	0.976491766
NUSAP1	-0.116633014	0.346261734	0.976491766
CD63	0.086005893	0.346262111	0.976491766
SYCE1L	0.100169021	0.346292952	0.976491766
TAB2	0.096314741	0.346308363	0.976491766
THAP10	-0.087686304	0.346339592	0.976491766
PDXP	0.081272547	0.34639258	0.976491766
GADD45G	-0.120436994	0.346541155	0.976491766
PRMT2	-0.096369855	0.346545418	0.976491766
MLX	-0.078879834	0.346570639	0.976491766
BAZ1B	0.051352494	0.346832988	0.976491766
PTTG1IP	-0.043596892	0.346919869	0.976491766
PCMTD2	0.06579308	0.346962375	0.976491766
CUX1	-0.072821379	0.347029334	0.976491766
LIN7B	0.08530461	0.347123075	0.976491766
EAF1	0.068996645	0.347219131	0.976491766
TPST2	0.09465009	0.347259707	0.976491766
MRRF	-0.049498199	0.347305185	0.976491766
RAD21	0.134236639	0.347534359	0.976491766
PCSK4	-0.079654033	0.347724662	0.976491766
NSF	-0.043922949	0.347743599	0.976491766
CNBP	0.067548245	0.347788188	0.976491766
OPA1	-0.068135152	0.347962164	0.976491766
CSRP1	0.067681022	0.348198482	0.976491766
NR1D2	-0.073084174	0.348320521	0.976491766
LST1	0.157891048	0.348617992	0.976491766
IL10	-0.113236021	0.348629235	0.976491766
C22orf39	0.074301602	0.34870308	0.976491766
SF1	0.093864331	0.348895075	0.976491766
SP140L	0.076570781	0.348914618	0.976491766
GRPEL1	-0.065501634	0.348921022	0.976491766
TSLP	-0.09578575	0.3490712	0.976491766

DIEXF	-0.069144932	0.349195135	0.976491766
CEACAM1	-0.128556648	0.349233811	0.976491766
DERL2	-0.05125578	0.34942379	0.976491766
C19orf66	0.067251288	0.349504065	0.976491766
ZMAT5	0.10861358	0.349505642	0.976491766
GMPPA	-0.072774072	0.349518152	0.976491766
PAWR	0.110605376	0.349573514	0.976491766
PLIN3	0.065559007	0.350033012	0.976491766
SLC6A10P	0.082104222	0.350049589	0.976491766
CTSZ	0.133463971	0.350176533	0.976491766
AVP	-0.097769808	0.350346361	0.976491766
GTF2IRD2B	0.076146085	0.350428442	0.976491766
NDUFS4	0.069756877	0.350497307	0.976491766
APBB1IP	-0.061806385	0.350611346	0.976491766
PSME4	0.050056983	0.350667502	0.976491766
CHST12	-0.139082031	0.350720903	0.976491766
SEC23IP	-0.060062686	0.350755217	0.976491766
ANKRD12	-0.070524522	0.350784588	0.976491766
ARID5A	0.09511318	0.350953151	0.976491766
TNFSF9	0.095892631	0.351015064	0.976491766
GTPBP4	-0.040263613	0.351019943	0.976491766
BCKDHB	0.118243966	0.351038517	0.976491766
MKRN3	-0.113125156	0.351068511	0.976491766
SUPT7L	-0.08937629	0.351145527	0.976491766
UQCRH	0.057481598	0.351228119	0.976491766
KLRF1	0.173194849	0.351280047	0.976491766
METTL22	0.101685398	0.351357307	0.976491766
FANCG	-0.07545289	0.351409604	0.976491766
EFNA1	-0.15466615	0.351410214	0.976491766
C2orf15	0.121260679	0.351550255	0.976491766
KCNA3	0.113441671	0.351641172	0.976491766
PPP1R13B	0.067263757	0.351767215	0.976491766
MINPP1	-0.090495351	0.351902946	0.976491766
ELMO3	0.076124363	0.351923453	0.976491766
DNMT1	-0.043415924	0.352024643	0.976491766
FCGR1A	-0.147600085	0.35225414	0.976491766
TMCC3	-0.11064859	0.35255328	0.976491766
MTCH2	-0.069910358	0.352587835	0.976491766
ERGIC1	-0.102217182	0.352652201	0.976491766
G3BP1	0.078488437	0.352774061	0.976491766
VPS4B	-0.05432725	0.352847464	0.976491766
LIX1L	0.070581517	0.352900029	0.976491766
TCP10L2	0.108382505	0.352943995	0.976491766
ZNF274	0.051235831	0.352976273	0.976491766
AKR7A3	0.079354937	0.353045683	0.976491766
PRDX6	0.098396167	0.353243985	0.976491766
DDX27	0.062132857	0.353264677	0.976491766

TNRC6A	-0.072914466	0.353329074	0.976491766
RILPL1	-0.090370601	0.353373291	0.976491766
PLEKHG6	-0.153005268	0.353400449	0.976491766
GLTP	0.052037989	0.353441865	0.976491766
RBM34	0.04657334	0.353483251	0.976491766
ENGASE	-0.101329102	0.353620464	0.976491766
HLA-DMB	-0.058805685	0.353673785	0.976491766
PNPLA3	-0.135753945	0.353712517	0.976491766
FBXW9	-0.06279524	0.353738277	0.976491766
H1FX	0.078605192	0.353768272	0.976491766
ICA1	-0.160562871	0.353776459	0.976491766
UBOX5	0.05189164	0.353783333	0.976491766
PTGS2	-0.184655446	0.353892594	0.976491766
FAM117B	-0.079701117	0.353928781	0.976491766
ACSL3	0.049071706	0.354009343	0.976491766
KMO	0.121500308	0.354184755	0.976491766
MYL12B	0.050998253	0.354205956	0.976491766
INVS	-0.071850642	0.354245986	0.976491766
GSTA2	-0.098375043	0.354485593	0.976491766
CYTIP	-0.087984294	0.354677423	0.976491766
NMT2	-0.173276115	0.354902589	0.976491766
B4GALT5	-0.083524416	0.354905815	0.976491766
MPHOSPH6	-0.12694084	0.354969151	0.976491766
DOK2	-0.062574646	0.354977512	0.976491766
SDHB	0.063425836	0.354990681	0.976491766
GPS1	-0.043678565	0.355008874	0.976491766
CBWD1	0.113089555	0.355038214	0.976491766
PARP15	-0.110393003	0.355107403	0.976491766
HOOK2	0.070122494	0.355107878	0.976491766
RAVER1	-0.07614597	0.355122845	0.976491766
SMC3	-0.045244429	0.355150738	0.976491766
SURF2	0.07673126	0.355150961	0.976491766
ZNF417	0.087468649	0.355216588	0.976491766
JAZF1	0.100180303	0.355460803	0.976491766
C1orf115	0.117231481	0.355675317	0.976491766
SLC25A34	-0.120148248	0.355836184	0.976491766
COX5B	0.06842179	0.355850514	0.976491766
GTSE1	-0.084869162	0.355919672	0.976491766
BPHL	0.053035587	0.355931924	0.976491766
NUDT1	0.077058564	0.356281306	0.976491766
RIC3	0.111876445	0.356352434	0.976491766
KCTD10	0.189518117	0.356398032	0.976491766
TMED4	0.092592007	0.356467284	0.976491766
SLC14A1	-0.14059613	0.356601561	0.976491766
HIST2H2AA3	-0.134010064	0.356660689	0.976491766
VN1R2	0.11814297	0.3566668	0.976491766
LMO2	-0.075402868	0.356815728	0.976491766

SNRNP48	-0.091347339	0.357079845	0.976491766
NEK3	-0.072622941	0.357139372	0.976491766
U2AF1	0.047355781	0.357337317	0.976491766
TDRD9	-0.176736575	0.357343936	0.976491766
PCM1	-0.057637152	0.357458423	0.976491766
EMR1	0.165312181	0.357501147	0.976491766
ACSS2	-0.060085859	0.357504723	0.976491766
ELP2	-0.05146425	0.357621436	0.976491766
PLEKHO2	-0.089015965	0.357669754	0.976491766
SMPD1	-0.084884246	0.357917488	0.976491766
PELP1	-0.08833704	0.357976233	0.976491766
PPAP2B	-0.12289808	0.358013404	0.976491766
CUTA	0.057674817	0.358050281	0.976491766
SUMF2	0.038345045	0.358096414	0.976491766
LPHN1	0.088110212	0.358280968	0.976491766
ABLIM1	-0.085886654	0.358310046	0.976491766
ADCK3	0.046978489	0.358358963	0.976491766
NSUN6	-0.054223229	0.358383062	0.976491766
HIST1H2BO	-0.128536104	0.358541416	0.976491766
EIF4ENIF1	0.048105293	0.358590767	0.976491766
NUDC	0.047721092	0.358818928	0.976491766
TICAM1	-0.090852798	0.359010914	0.976491766
KCNA5	-0.130019615	0.359013685	0.976491766
ARHGAP24	-0.203625415	0.359140997	0.976491766
AGAP6	-0.098238546	0.359141654	0.976491766
GMEB1	0.079887092	0.359194315	0.976491766
RFNG	-0.064410174	0.359214324	0.976491766
DBR1	-0.065992747	0.359379394	0.976491766
ACER3	-0.094498856	0.359531405	0.976491766
ZNF616	-0.090650984	0.359546275	0.976491766
RXRB	-0.054374711	0.359607253	0.976491766
SNX2	0.069582934	0.359660354	0.976491766
HIST1H4C	-0.082867747	0.359671567	0.976491766
AUP1	0.063774497	0.359692393	0.976491766
SERPINF1	-0.162901566	0.359755187	0.976491766
MCOLN2	-0.165152222	0.359795115	0.976491766
DROSHA	-0.054898538	0.359855515	0.976491766
RTKN	0.148187935	0.359879045	0.976491766
FKBP4	0.091273145	0.360006739	0.976491766
MAP3K3	0.074907078	0.360015339	0.976491766
COX17	0.063664	0.360152566	0.976491766
COG3	-0.072159444	0.360321505	0.976491766
KRAS	0.059900043	0.360340715	0.976491766
GNL2	0.043505319	0.360358517	0.976491766
GTF2A2	0.049281694	0.360364983	0.976491766
AGPAT9	-0.087297984	0.360413215	0.976491766
PHB	0.067939869	0.360470161	0.976491766

SFXN4	0.09977117	0.36062317	0.976491766
GALNT11	0.054099706	0.360624427	0.976491766
PI4K2B	-0.055078312	0.360625214	0.976491766
SLC23A2	-0.08153664	0.360679764	0.976491766
SIGLEC5	0.198385809	0.360730108	0.976491766
CLRN1	0.075065604	0.360757238	0.976491766
CNNM3	-0.066722785	0.360799094	0.976491766
SLC35E2B	-0.052194144	0.361014305	0.976491766
SAMM50	-0.06788198	0.361124419	0.976491766
SDR16C5	0.108595796	0.361135983	0.976491766
CCNB2	-0.193025805	0.361500977	0.976491766
KRT6C	-0.100366546	0.361596087	0.976491766
SLC8A3	0.213870153	0.361629021	0.976491766
MC1R	-0.139522238	0.36166688	0.976491766
POLQ	-0.114405485	0.361710748	0.976491766
COG5	0.088959984	0.361730854	0.976491766
INTS4	-0.050403637	0.361799334	0.976491766
PSMC4	0.1065536	0.362248533	0.976491766
RPL37A	0.036270832	0.362275665	0.976491766
GSTO1	0.067439562	0.362323015	0.976491766
NPDC1	0.110160182	0.362461455	0.976491766
FAM105B	-0.071276073	0.362479927	0.976491766
RAB21	-0.045332015	0.362618525	0.976491766
QPCTL	0.071646055	0.362919312	0.976491766
FAM49B	0.095206448	0.362998395	0.976491766
PRKDC	0.083562719	0.363061834	0.976491766
INPP4B	0.108830925	0.363241337	0.976491766
CA13	0.089427661	0.363345851	0.976491766
ATP6V0D1	0.104034237	0.363428666	0.976491766
SLMO2	0.094157243	0.363449271	0.976491766
NTSR1	-0.20121544	0.363456235	0.976491766
WDR59	-0.06488026	0.363464254	0.976491766
ZCCHC24	0.089014473	0.363494145	0.976491766
CXCL5	0.289355423	0.363534331	0.976491766
MRPS10	0.071609764	0.363619752	0.976491766
ATAD3B	0.071550743	0.363785202	0.976491766
ZADH2	-0.124806711	0.363807614	0.976491766
CAB39	0.037887606	0.363838193	0.976491766
RALY	0.062338536	0.363915653	0.976491766
CPSF4L	-0.104015156	0.363920662	0.976491766
SLAIN2	-0.069305152	0.364036718	0.976491766
CD52	0.071665436	0.364068686	0.976491766
MTF1	-0.087690462	0.364100133	0.976491766
WDR49	-0.089138906	0.364151938	0.976491766
DENND3	0.0910743	0.364155361	0.976491766
POLR3G	-0.141864715	0.364158256	0.976491766
PTPRC	-0.13195196	0.364187288	0.976491766

BTN3A3	-0.071701979	0.364273164	0.976491766
NLN	-0.09729487	0.364471042	0.976491766
PSPH	-0.09090292	0.364541233	0.976491766
NUDT21	-0.057775788	0.364843294	0.976491766
NDST1	-0.118749948	0.364949601	0.976491766
FCGR3B	0.132126735	0.364985931	0.976491766
MAN2B2	-0.058541027	0.365002065	0.976491766
FEM1B	0.100263695	0.365015503	0.976491766
ZNF394	-0.172177101	0.365076763	0.976491766
MCFD2	-0.059757871	0.365086207	0.976491766
TANC2	-0.082282817	0.365121938	0.976491766
MARK3	-0.0719738	0.365554414	0.976491766
ERGIC3	0.057014406	0.365584246	0.976491766
POU5F1	-0.104578929	0.365625065	0.976491766
SPCS1	0.048722412	0.365830331	0.976491766
NHP2	0.072878006	0.365932777	0.976491766
RPP25	0.087702357	0.36602923	0.976491766
ICAM2	0.059699927	0.366113744	0.976491766
C4orf29	-0.147455587	0.36617773	0.976491766
EFCAB11	0.099673587	0.366184026	0.976491766
C3orf17	-0.069287467	0.366189504	0.976491766
OSGIN2	-0.079853995	0.366191972	0.976491766
HARS	0.051224181	0.366625048	0.976491766
KLRC2	0.409066612	0.366685437	0.976491766
C12orf23	-0.096447253	0.366743605	0.976491766
CENPH	0.082937913	0.3667525	0.976491766
IL2RB	0.125140897	0.366851407	0.976491766
NAGLU	-0.06710355	0.366958405	0.976491766
KRT8	0.082091588	0.367085021	0.976491766
SLC39A9	-0.087903307	0.367295937	0.976491766
NFX1	0.062007113	0.367388264	0.976491766
CHMP6	0.074969918	0.367804017	0.976491766
ANAPC10	0.082930365	0.367829402	0.976491766
C18orf21	-0.054688849	0.368093894	0.976491766
LSM2	0.051656226	0.368225166	0.976491766
KLHL18	-0.0590589	0.368294465	0.976491766
LAG3	-0.219168511	0.368402168	0.976491766
ASCC3	-0.059311304	0.368530087	0.976491766
SELENBP1	0.073674408	0.368598906	0.976491766
ERI3	0.063954531	0.36862441	0.976491766
TLE3	0.076989813	0.368784974	0.976491766
ANK1	0.126662815	0.368867701	0.976491766
PHF2	-0.048692657	0.369105026	0.976491766
ADPRH	0.078325024	0.369145099	0.976491766
BLZF1	-0.127252684	0.369163772	0.976491766
MERTK	-0.177816197	0.369167289	0.976491766
UROD	0.042797389	0.369178036	0.976491766

RDH10	-0.082476411	0.369239306	0.976491766
BTD	0.095088385	0.369333286	0.976491766
NUP37	-0.059863928	0.369343028	0.976491766
ADSS	-0.054373954	0.369373454	0.976491766
SDR39U1	0.092235655	0.369477715	0.976491766
CAMTA1	0.171819976	0.369674328	0.976491766
SPDYE8P	-0.074820801	0.369857325	0.976491766
PIGW	-0.075436349	0.370188244	0.976491766
PTPN18	0.089654975	0.370200968	0.976491766
C10orf35	-0.099788056	0.370263293	0.976491766
ZFHX3	-0.099785024	0.370265848	0.976491766
ZNF416	-0.094208787	0.370333034	0.976491766
DSC2	-0.195998339	0.370435	0.976491766
TIAM2	-0.126769457	0.370592558	0.976491766
DHX37	-0.061940518	0.370636107	0.976491766
ANKRD55	-0.233128366	0.370703524	0.976491766
BRD1	0.064095153	0.370730411	0.976491766
CPSF2	0.091496984	0.370740493	0.976491766
CWC27	0.050601057	0.370822294	0.976491766
TCTN3	-0.092938708	0.370938291	0.976491766
HOXC4	-0.179239619	0.370962233	0.976491766
TRIM11	-0.063285066	0.371014637	0.976491766
SHD	-0.154615244	0.371032922	0.976491766
PRELID1	0.092623268	0.371096854	0.976491766
SNORA12	0.146365055	0.371394222	0.976491766
PRR13	-0.039850422	0.371405488	0.976491766
FAM117A	0.068643708	0.371451073	0.976491766
VPS39	-0.071781646	0.371594829	0.976491766
UBE2L3	0.081904536	0.371604568	0.976491766
APBA2	-0.10410861	0.371700906	0.976491766
PRIM1	0.079444587	0.371712581	0.976491766
LPAR5	-0.125628009	0.372107616	0.976491766
LRRC40	-0.106578746	0.37216568	0.976491766
FAM129C	0.167771268	0.372214113	0.976491766
PMS2P3	-0.086649566	0.372230115	0.976491766
CD46	-0.046810156	0.372230398	0.976491766
ZNF296	0.078584636	0.372388574	0.976491766
RELL2	0.0861865	0.372630322	0.976491766
ATRIP	-0.063148108	0.37276815	0.976491766
SLC7A6	-0.098794908	0.372845689	0.976491766
RRN3P1	-0.165290239	0.372981054	0.976491766
PLA2G2C	0.083528737	0.373069863	0.976491766
INTS12	0.049768225	0.373161887	0.976491766
PLBD2	-0.097899233	0.373238407	0.976491766
ZNF740	-0.077008611	0.373290519	0.976491766
CD2	-0.075779878	0.373376575	0.976491766
TAF5L	0.136904209	0.373379655	0.976491766

ATP9A	0.171039409	0.373395025	0.976491766
CEMP1	-0.093483853	0.373487924	0.976491766
MT1E	-0.189459951	0.373560097	0.976491766
TULP4	0.056594158	0.373590746	0.976491766
INSL3	-0.128747737	0.37362732	0.976491766
ADM2	-0.088033231	0.373627801	0.976491766
E4F1	0.042000621	0.373695995	0.976491766
KDM1B	-0.096252683	0.373791309	0.976491766
ATP2B4	0.088077307	0.373846274	0.976491766
LYRM1	0.039705191	0.374164116	0.976491766
RIN2	-0.103208785	0.374192652	0.976491766
ZNF345	-0.090991023	0.374238595	0.976491766
FKBP14	-0.19277577	0.374503862	0.976491766
RPL27	0.028665973	0.374688101	0.976491766
ZNF585B	-0.074942201	0.37480047	0.976491766
BEND5	0.131185171	0.374936464	0.976491766
DDX42	-0.038286199	0.375170917	0.976491766
SLC7A1	-0.067967782	0.375241525	0.976491766
DENR	-0.168326212	0.375295424	0.976491766
TSEN34	0.050848279	0.375334349	0.976491766
PFDN4	-0.096332419	0.375440714	0.976491766
GP6	0.26165316	0.375673768	0.976491766
RNPC3	-0.066600282	0.375732849	0.976491766
TMEM101	-0.053093217	0.375871646	0.976491766
ALAD	0.085864581	0.37594263	0.976491766
ARHGEF1	0.097439525	0.375973324	0.976491766
ZNFX1	-0.065660677	0.375996262	0.976491766
KIF16B	0.13100634	0.37611586	0.976491766
ARHGEF40	-0.095272239	0.376147251	0.976491766
ZSCAN18	-0.092953935	0.376170709	0.976491766
MUC1	-0.111749395	0.376225656	0.976491766
PSMB1	-0.047240829	0.376234364	0.976491766
MYO10	-0.089749142	0.376406983	0.976491766
SH3BP5L	-0.084515494	0.376448063	0.976491766
PPM1H	0.100585691	0.376496016	0.976491766
DHX38	-0.081951428	0.376544433	0.976491766
CCNI2	0.105021746	0.376595967	0.976491766
HK1	-0.046197106	0.376727517	0.976491766
ZFP91	0.102110242	0.376900043	0.976491766
ZNF790	0.105415717	0.376961449	0.976491766
CLEC7A	-0.177563767	0.376969838	0.976491766
WAPAL	0.056253405	0.376981507	0.976491766
CLEC1B	0.241137502	0.376995308	0.976491766
LRP8	-0.079754605	0.377041801	0.976491766
THAP4	-0.114089327	0.377046193	0.976491766
MAP3K11	-0.092563474	0.377097321	0.976491766
MSC	-0.172562178	0.377131798	0.976491766

STAM2	-0.086834221	0.377290279	0.976491766
RAC1	0.041808049	0.377324155	0.976491766
PLXNC1	-0.120144487	0.377331468	0.976491766
C14orf64	-0.140074187	0.377367079	0.976491766
KPNA1	-0.04551074	0.37738182	0.976491766
IFITM3	0.297933263	0.377388263	0.976491766
ZNF34	0.070729498	0.377479591	0.976491766
R3HDM1	0.078636454	0.377489906	0.976491766
IL8	0.312801985	0.377605057	0.976491766
DPCD	0.091274604	0.377670221	0.976491766
GOT1	-0.042475693	0.377839859	0.976491766
UBASH3A	-0.119386511	0.377868462	0.976491766
NUDT16L1	-0.057253474	0.378130869	0.976491766
C11orf63	-0.23047396	0.378202894	0.976491766
C6orf226	0.070282286	0.378305241	0.976491766
RASSF7	0.046503398	0.37833461	0.976491766
SAC3D1	0.065864264	0.378434732	0.976491766
TRAM1	-0.032959671	0.378497008	0.976491766
EGLN3	0.099094928	0.378602771	0.976491766
ZNF777	0.054932666	0.378679518	0.976491766
IKZF1	-0.149350333	0.378783444	0.976491766
ZC3HAV1L	0.077832146	0.378811816	0.976491766
TAF15	0.095343541	0.37907489	0.976491766
SLC25A24	-0.122616172	0.379171108	0.976491766
UNC50	-0.042043817	0.37923053	0.976491766
HBA2	0.382332336	0.379279628	0.976491766
DNAJC15	0.114782292	0.379290282	0.976491766
ARHGEF7	0.043290957	0.379322195	0.976491766
PRR3	-0.102141084	0.379427152	0.976491766
CDC37	-0.039716911	0.379453243	0.976491766
BRD3	0.083446495	0.379532345	0.976491766
SELPLG	-0.138510763	0.379631077	0.976491766
ZNF324B	-0.082588046	0.379697711	0.976491766
GPAT2	0.188080872	0.379726234	0.976491766
TFDP1	0.064580406	0.379855092	0.976491766
MRPS18A	-0.072437347	0.379922303	0.976491766
KCTD17	-0.071020056	0.380096318	0.976491766
TC2N	-0.090120294	0.38023856	0.976491766
PARM1	0.156235953	0.380289812	0.976491766
TSC22D1	0.177889011	0.380398517	0.976491766
C3orf18	0.068864818	0.380468397	0.976491766
ZNF235	-0.065222337	0.380639398	0.976491766
UBR3	0.054822065	0.380885929	0.976491766
TADA1	-0.05805537	0.380947752	0.976491766
OSBPL10	0.211130728	0.381046262	0.976491766
ERMN	-0.116687688	0.381089322	0.976491766
H2AFY	-0.039469878	0.381142377	0.976491766

ZNF471	-0.152327937	0.381249871	0.976491766
BBC3	0.064486197	0.381314053	0.976491766
FRY	0.068074898	0.381337147	0.976491766
FAM175A	-0.169853208	0.38137235	0.976491766
SLC27A4	-0.081709982	0.381533243	0.976491766
MZT2A	0.10410055	0.381568911	0.976491766
ARHGAP18	0.126664433	0.381613029	0.976491766
TRANK1	0.087265216	0.382229196	0.976491766
SRP19	0.059622152	0.382262063	0.976491766
TK1	-0.114709263	0.382347486	0.976491766
DHX36	-0.056428143	0.382477132	0.976491766
CCL8	0.166492039	0.382550643	0.976491766
SNX14	-0.045504117	0.382632927	0.976491766
GMIP	-0.076921576	0.382699796	0.976491766
GABPB2	-0.026448447	0.382747464	0.976491766
QRFPR	-0.162862201	0.382862166	0.976491766
TMEM88	-0.111788919	0.38289302	0.976491766
SLC35D2	0.062882417	0.382918201	0.976491766
ZNF333	-0.08203127	0.382994143	0.976491766
FAM111A	-0.051614308	0.383002144	0.976491766
CXCR2	0.132895085	0.383164018	0.976491766
PHYH	-0.071089705	0.383378985	0.976491766
GOLGA1	-0.065681721	0.383381722	0.976491766
ANKRD26	0.068166139	0.383538155	0.976491766
CDK7	-0.043858686	0.383554098	0.976491766
BTF3L4	-0.074946283	0.383877596	0.976491766
COLQ	0.150080529	0.383932895	0.976491766
CLMN	-0.095921636	0.383946471	0.976491766
HMGCR	-0.046228678	0.384008014	0.976491766
CAPN3	-0.092350735	0.384014796	0.976491766
CITED2	0.082764116	0.38405104	0.976491766
EPRS	0.04185291	0.38414346	0.976491766
TBC1D3C	-0.093262103	0.384144752	0.976491766
ZNF14	-0.093410179	0.384154069	0.976491766
GPR126	0.071548008	0.384159851	0.976491766
ACBD6	0.0648039	0.384237492	0.976491766
NCOA3	-0.073243608	0.384347302	0.976491766
CXCR6	-0.256075865	0.384376691	0.976491766
ZHX3	0.064510359	0.384448299	0.976491766
FASN	0.134220877	0.384478778	0.976491766
PRKD3	0.079487691	0.384627366	0.976491766
DPEP3	-0.116175719	0.384664753	0.976491766
GORASP2	0.047296628	0.385054055	0.976491766
E2F5	0.130529559	0.385106867	0.976491766
DDB2	0.084450311	0.38511483	0.976491766
MND1	-0.098092506	0.38514721	0.976491766
CMKLR1	0.149859317	0.385228424	0.976491766

RORA	-0.089867483	0.385418435	0.976491766
KIFC3	0.18877695	0.385450232	0.976491766
NDUFV3	-0.08724303	0.385769449	0.976491766
ROCK2	-0.02900835	0.385833439	0.976491766
ADAM19	0.091679286	0.385900031	0.976491766
HSD17B7	-0.146924052	0.386452671	0.976491766
GPNMB	0.08422714	0.386737494	0.976491766
WWP1	0.048222345	0.386840923	0.976491766
LMTK2	-0.078257101	0.386952252	0.976491766
ICMT	0.104557622	0.387112556	0.976491766
UGP2	0.056350805	0.387286327	0.976491766
EWSR1	0.043919228	0.387335314	0.976491766
CRYZ	0.133060919	0.387610589	0.976491766
SLC38A9	-0.059536175	0.387644959	0.976491766
MED17	0.085874823	0.387684784	0.976491766
DEXI	0.069649694	0.387815638	0.976491766
AKAP8	-0.048286652	0.387840742	0.976491766
ADI1	0.112472743	0.38788057	0.976491766
ALOX5	-0.070712249	0.387885679	0.976491766
SAMD14	0.324149425	0.387934474	0.976491766
ORAOV1	-0.073516012	0.387936727	0.976491766
RCAN2	-0.252243672	0.388044852	0.976491766
STK16	-0.087798531	0.388072758	0.976491766
HYAL3	-0.076700445	0.38815218	0.976491766
ARNTL	-0.055121388	0.388221881	0.976491766
PCSK5	-0.092643616	0.388235783	0.976491766
LCAT	-0.078880531	0.388251821	0.976491766
PDZK1IP1	0.265760832	0.388282534	0.976491766
GOLGA2	0.07377634	0.388323953	0.976491766
POLD3	-0.065293841	0.388433619	0.976491766
C17orf80	-0.078193553	0.388452398	0.976491766
CARS2	-0.061945815	0.388614534	0.976491766
CHUK	0.04464828	0.388626682	0.976491766
LMTK3	-0.08293219	0.388786719	0.976491766
CAPG	0.109791757	0.388788475	0.976491766
TRIM52	-0.088647964	0.388818551	0.976491766
CHD3	-0.081469987	0.388825809	0.976491766
SUPT16H	0.049522974	0.389042245	0.976491766
POMC	0.147431462	0.389265238	0.976491766
HLA-DMA	0.074138389	0.389268175	0.976491766
SELM	0.099444497	0.389454959	0.976491766
GPCPD1	-0.093461034	0.389528661	0.976491766
SOX8	-0.121803647	0.389723331	0.976491766
RNF212	0.078382138	0.389870972	0.976491766
TGFBR2	-0.048933464	0.389892397	0.976491766
CDC40	0.03757511	0.389922905	0.976491766
C12orf29	-0.052884231	0.389925913	0.976491766

PARP12	0.094356673	0.38996805	0.976491766
TNFRSF9	-0.191470876	0.389991515	0.976491766
DBNDD2	0.072021698	0.390050555	0.976491766
POLR1E	-0.062421102	0.390222346	0.976491766
AHCY	-0.060495416	0.390234326	0.976491766
VWF	0.293316005	0.390250995	0.976491766
AQP1	-0.083313632	0.390354302	0.976491766
RARRES3	0.067962727	0.390361949	0.976491766
MAZ	-0.09605825	0.390435298	0.976491766
POLR2J4	-0.082732875	0.390459865	0.976491766
UGT1A1	0.077298708	0.390528466	0.976491766
ARPC3	0.083582087	0.390749836	0.976491766
MRTO4	0.063664808	0.390815885	0.976491766
SLC44A4	0.032042345	0.39110208	0.976491766
ZBTB32	-0.117940357	0.391147205	0.976491766
STAT3	0.052142394	0.391301529	0.976491766
NUDT22	0.055946948	0.391336035	0.976491766
CDK5RAP1	-0.044828419	0.391411986	0.976491766
SMAD6	-0.07902028	0.391480927	0.976491766
SPINK2	-0.12932974	0.391615083	0.976491766
CETN3	0.060918651	0.391620464	0.976491766
RPN1	-0.043033663	0.391623731	0.976491766
CBX1	0.099944181	0.391662175	0.976491766
THBS1	0.28465712	0.391709511	0.976491766
DFNB59	-0.077059818	0.39173425	0.976491766
COX7A2	0.070405158	0.391779242	0.976491766
GLMN	-0.063434081	0.391786321	0.976491766
AKR1C3	0.216339141	0.391789256	0.976491766
TFRC	0.067769424	0.391853493	0.976491766
TEKT4P2	0.357630918	0.391864821	0.976491766
CCDC91	0.062895966	0.392111835	0.976491766
ISL2	0.080235343	0.39221284	0.976491766
ALPK1	-0.109603811	0.392285828	0.976491766
SIK3	-0.039932335	0.392320269	0.976491766
RDH13	-0.105274048	0.392357548	0.976491766
RPS14	0.051895857	0.392375522	0.976491766
TBC1D9	-0.107601892	0.392424583	0.976491766
CAP1	0.039379601	0.392617051	0.976491766
FLNB	-0.081818005	0.392662016	0.976491766
TTC18	-0.089240944	0.392834978	0.976491766
C19orf10	-0.089472284	0.392887053	0.976491766
GCLM	-0.149717409	0.392894787	0.976491766
SLC37A4	-0.059661013	0.392938948	0.976491766
GMEB2	-0.047114166	0.393022575	0.976491766
TLR9	-0.114718029	0.393042923	0.976491766
KLKB1	-0.117516878	0.39311568	0.976491766
IL7	0.103179853	0.393161014	0.976491766

METTL2B	-0.076073133	0.393277163	0.976491766
ZNF786	-0.136931287	0.3933484	0.976491766
METTL2A	-0.19673582	0.393427082	0.976491766
TMEM79	-0.083197175	0.393473895	0.976491766
TTC39C	0.066352681	0.393731574	0.976491766
ZNF92	-0.093238587	0.39378654	0.976491766
SCARNA21	0.102916999	0.393834467	0.976491766
ORMDL2	-0.073952157	0.393839616	0.976491766
SELO	-0.08097911	0.394017279	0.976491766
TNFRSF1B	-0.057221857	0.394071011	0.976491766
HLX	-0.099015384	0.394102339	0.976491766
GRAP2	0.135404049	0.394174866	0.976491766
PRMT3	-0.048337369	0.394420389	0.976491766
CYTH2	-0.113572885	0.394440098	0.976491766
ANKS3	-0.091738039	0.394470497	0.976491766
LRRC37A2	-0.096630309	0.394555239	0.976491766
NUFIP2	0.077566433	0.394724071	0.976491766
WDFY1	-0.052169368	0.3947608	0.976491766
DNAJC2	0.110638384	0.394769273	0.976491766
DACT1	0.179089929	0.394788932	0.976491766
TATDN1	0.097522387	0.395049707	0.976491766
SERTAD1	-0.104954213	0.395309897	0.976491766
BLOC1S2	0.084536218	0.395335834	0.976491766
RPL15	0.061668509	0.395345405	0.976491766
TUBA1A	0.046858823	0.395459149	0.976491766
BCL9L	-0.088715788	0.395629044	0.976491766
SNCA	0.187508897	0.395729585	0.976491766
PLCD1	0.072167294	0.39579847	0.976491766
EDN1	-0.086229273	0.395850269	0.976491766
PPWD1	-0.063011313	0.395865771	0.976491766
INTS5	0.072663892	0.395881216	0.976491766
SGCB	0.084624228	0.395895809	0.976491766
EDC3	-0.065844014	0.39591128	0.976491766
HNRNPU	-0.21287607	0.395927276	0.976491766
C21orf91	0.081769353	0.396280369	0.976491766
TMEM205	0.057971705	0.396283522	0.976491766
LUC7L2	0.068631724	0.3963588	0.976491766
KLHL33	-0.07979411	0.396412396	0.976491766
EAF2	0.147906939	0.396652184	0.976491766
NUMB	-0.041057143	0.396680905	0.976491766
C6orf106	0.071607114	0.396725133	0.976491766
CCDC58	-0.208124679	0.396738758	0.976491766
MFSD1	-0.058070985	0.396840669	0.976491766
PRMT7	0.084425003	0.396842368	0.976491766
PTRF	-0.083846327	0.396852542	0.976491766
IDH1	-0.060489532	0.396872406	0.976491766
NSUN2	-0.056272308	0.396943784	0.976491766

EPOR	-0.07589751	0.397011661	0.976491766
FBXL17	0.105143524	0.39721999	0.976491766
DHX35	-0.054170044	0.397290145	0.976491766
CD74	0.057700244	0.397312276	0.976491766
PLIN5	0.187106197	0.397437009	0.976491766
BATF	-0.096056049	0.397438685	0.976491766
GOLGA4	-0.07342856	0.397449356	0.976491766
NOXA1	-0.094601073	0.3974502	0.976491766
TMEM151B	-0.07687242	0.39748943	0.976491766
SNHG11	0.067466364	0.397587475	0.976491766
NDUFC1	0.058796415	0.397679021	0.976491766
RABGGTB	-0.051205611	0.397690585	0.976491766
CDKL3	-0.069708634	0.397724495	0.976491766
TMEM8A	0.149212291	0.397753134	0.976491766
TRIB2	0.089683159	0.397787441	0.976491766
SNORD4B	0.107224278	0.397926613	0.976491766
BTN3A2	-0.176048537	0.398099723	0.976491766
COL8A2	0.061444142	0.398102704	0.976491766
TRIB1	-0.084778502	0.398128985	0.976491766
RRAS	0.094416321	0.39815606	0.976491766
TBC1D7	0.082576672	0.398235336	0.976491766
MAP2K7	0.065157838	0.398390285	0.976491766
ITGB5	0.21665887	0.398491317	0.976491766
CNST	-0.094786918	0.398624215	0.976491766
RHBDD1	-0.057311876	0.398631687	0.976491766
RELL1	-0.084883637	0.398647105	0.976491766
LYRM2	0.06874911	0.398710845	0.976491766
AKR1C4	0.217621903	0.398711539	0.976491766
FAM91A1	0.082099424	0.398850898	0.976491766
ZBTB47	-0.093785089	0.398986998	0.976491766
ELP4	0.068610077	0.399067653	0.976491766
ST3GAL5	-0.065608598	0.399108516	0.976491766
OXER1	-0.081496229	0.399231073	0.976491766
PAQR4	-0.078298855	0.399307118	0.976491766
TBCA	0.056465899	0.399590189	0.976491766
GPR124	-0.11563692	0.399608735	0.976491766
SLC4A5	-0.119892524	0.399845638	0.976491766
SDR42E1	-0.092938448	0.39987034	0.976491766
ZNF708	-0.099781797	0.399877222	0.976491766
LSM5	0.061050489	0.399946006	0.976491766
KCNH8	0.092695232	0.399954082	0.976491766
TMPRSS3	-0.102070491	0.400052337	0.976491766
IFIH1	0.078047937	0.400069685	0.976491766
KCMF1	0.061406241	0.400100565	0.976491766
PRKCQ	-0.070684011	0.400137293	0.976491766
AEBP1	-0.128437008	0.400176852	0.976491766
TMEM106A	-0.190158223	0.40022225	0.976491766

SLC38A10	0.070234167	0.400225369	0.976491766
HDAC2	0.033699206	0.400286287	0.976491766
HS1BP3	-0.070943783	0.400362661	0.976491766
ZNHIT6	0.047039668	0.40042903	0.976491766
MAN1C1	-0.099866086	0.400474023	0.976491766
ABHD15	0.067443872	0.400491485	0.976491766
SNORA3	0.109545826	0.400744841	0.976491766
CAPRIN2	-0.082852956	0.400818294	0.976491766
COQ3	-0.048205433	0.400952615	0.976491766
ZNF655	0.103354341	0.401067793	0.976491766
ELMOD1	-0.178363219	0.401451111	0.976491766
ZNF469	0.08186954	0.40150785	0.976491766
RPA1	0.096138413	0.401508077	0.976491766
RNF213	-0.130865186	0.401586122	0.976491766
MRPL13	0.063380616	0.401595526	0.976491766
SNORD8	-0.106284375	0.401711831	0.976491766
ACVR1	0.065345524	0.401712043	0.976491766
GFM2	-0.068549112	0.401776876	0.976491766
SLC27A6	0.088504223	0.401850285	0.976491766
TFDP2	0.077934676	0.401902703	0.976491766
FGFBP2	0.182962949	0.402054533	0.976491766
SHPRH	-0.071878396	0.402093782	0.976491766
TRPV2	-0.07356849	0.402255838	0.976491766
OSBPL5	0.133552479	0.402527131	0.976491766
C2orf47	-0.062005899	0.40253302	0.976491766
HERC2P7	-0.122980312	0.402548944	0.976491766
GMNN	-0.08523208	0.402743501	0.976491766
DDX51	-0.113030005	0.402769262	0.976491766
CLDN15	-0.070176281	0.402978796	0.976491766
RAB35	-0.060231303	0.403122185	0.976491766
RNF125	0.0592878	0.40315522	0.976491766
IL32	0.12137539	0.403170043	0.976491766
PRPF39	-0.079028637	0.403183533	0.976491766
TRPM4	-0.172330512	0.40320445	0.976491766
RHOT1	0.049349892	0.403222541	0.976491766
TRIM8	0.037444756	0.403428327	0.976491766
SH3BP1	0.0849708	0.403543241	0.976491766
MPV17	-0.038844174	0.40367208	0.976491766
LITAF	0.076194307	0.403871107	0.976491766
ATP5A1	-0.051932591	0.403883324	0.976491766
PDLIM5	0.099215045	0.403995023	0.976491766
LIMS1	0.092187249	0.404143356	0.976491766
LTF	-0.085498612	0.404266577	0.976491766
FBXO42	-0.075931119	0.404314859	0.976491766
TIAF1	-0.052081965	0.404456852	0.976491766
COL13A1	0.116571278	0.404514384	0.976491766
NOL9	-0.096713918	0.404536831	0.976491766

MAK16	-0.059009519	0.404636548	0.976491766
SLC4A1	0.146107075	0.404646048	0.976491766
LILRB2	-0.067753283	0.404657755	0.976491766
USP36	0.135140362	0.404741639	0.976491766
FKBP11	0.077009554	0.404800526	0.976491766
IL6	0.099570086	0.404966431	0.976491766
ZNF700	-0.085366369	0.404972694	0.976491766
STRA13	0.041850776	0.404978768	0.976491766
AVPI1	0.111987087	0.404994786	0.976491766
TPCN2	-0.093491895	0.405118135	0.976491766
IRS2	0.137157469	0.405181395	0.976491766
NUAK2	0.066608337	0.405214121	0.976491766
CUL1	0.044673436	0.405224822	0.976491766
NFIX	-0.1079245	0.405273079	0.976491766
MEAF6	-0.08270015	0.405715026	0.976491766
ANXA11	0.06263005	0.405746897	0.976491766
MRPL34	0.053945766	0.405871791	0.976491766
PSME2	0.065819436	0.405972651	0.976491766
PHC3	0.069047711	0.406007408	0.976491766
TIMM13	-0.090909011	0.406055107	0.976491766
SSSCA1	0.11153725	0.406163391	0.976491766
FERMT3	0.064875268	0.406224687	0.976491766
CORT	0.07983003	0.406270162	0.976491766
TRIM43	-0.08301799	0.40635817	0.976491766
OSTM1	-0.061256107	0.40639116	0.976491766
NCKIPSD	-0.068572167	0.406441051	0.976491766
TAL1	0.150149374	0.406520961	0.976491766
BIRC6	-0.056572961	0.406809352	0.976491766
ORC3	-0.059530571	0.406810457	0.976491766
SIGLEC16	0.166408608	0.406835292	0.976491766
HCK	-0.066947196	0.406876174	0.976491766
TECR	0.060167205	0.406900826	0.976491766
LHFPL2	0.105179472	0.406962886	0.976491766
VDAC1	-0.04532317	0.407098737	0.976491766
FYB	-0.042763662	0.407155848	0.976491766
LAMTOR3	-0.056486079	0.407313094	0.976491766
XYLT1	0.486249046	0.407320963	0.976491766
ELP3	-0.053069513	0.407323326	0.976491766
SMYD2	0.044494984	0.407460164	0.976491766
PPP1R15B	-0.082702748	0.40758617	0.976491766
PLTP	0.093676899	0.407683287	0.976491766
COMMD1	0.051042568	0.407882128	0.976491766
OCIAD1	-0.094613417	0.407909714	0.976491766
NEDD9	-0.095979049	0.40794049	0.976491766
UBE2W	-0.080780942	0.407982773	0.976491766
NUDT9	0.045883111	0.408158181	0.976491766
CCDC106	0.090465956	0.40822964	0.976491766

DUS4L	0.055823177	0.408290072	0.976491766
SRSF3	0.041538378	0.408298274	0.976491766
COQ9	-0.03693451	0.408392017	0.976491766
OAZ2	0.136258697	0.40876243	0.976491766
GPX4	0.053078095	0.40884856	0.976491766
MPP5	-0.064615879	0.409128243	0.976491766
INPP5B	0.084925674	0.409175619	0.976491766
CARD8	-0.089379005	0.40919292	0.976491766
PPME1	0.068543757	0.409193774	0.976491766
SRC	0.09253203	0.409345022	0.976491766
PSMB8	-0.061628659	0.409416369	0.976491766
PRPF38B	-0.062563848	0.409460368	0.976491766
C2CD2L	-0.096213951	0.409507995	0.976491766
NAPA	0.07151931	0.409541392	0.976491766
HNRNPR	0.040197777	0.409640311	0.976491766
RAD9A	-0.075739617	0.409930379	0.976491766
RSF1	-0.089553456	0.410074292	0.976491766
GMPS	0.051476346	0.410103962	0.976491766
STIL	-0.082409062	0.410197222	0.976491766
TAF10	0.048170819	0.410211071	0.976491766
CATSPER1	0.089783748	0.410297064	0.976491766
PGAP3	-0.045801261	0.410387442	0.976491766
FAHD2B	-0.057881137	0.410567452	0.976491766
SOCS2	0.087979972	0.410689501	0.976491766
SGPP1	-0.089928351	0.410785033	0.976491766
GLE1	-0.041031694	0.410923035	0.976491766
SLC35F2	0.089732273	0.411217333	0.976491766
SQLE	-0.080532928	0.411240019	0.976491766
SPN	-0.070299843	0.411322895	0.976491766
PLEKHM1	0.050150528	0.411381927	0.976491766
NAGPA	-0.054575757	0.411454232	0.976491766
RFXAP	-0.054671841	0.411498657	0.976491766
DLEU7	-0.122903591	0.411561362	0.976491766
UPB1	0.105039029	0.411588032	0.976491766
ARHGEF10L	-0.142144071	0.411622321	0.976491766
PSMB2	0.0574466	0.411697214	0.976491766
ANKRD44	-0.183109413	0.411703749	0.976491766
RIPK3	-0.04930916	0.412043078	0.976491766
FAM53C	0.056551671	0.41212649	0.976491766
DCK	0.085156161	0.412357179	0.976491766
GTF2B	-0.066107315	0.4125043	0.976491766
PPIAL4E	0.074814249	0.412558485	0.976491766
PISD	-0.060289793	0.412598338	0.976491766
PAG1	-0.027475086	0.412642752	0.976491766
BMP1	-0.086039666	0.412917654	0.976491766
FKBP2	0.048781185	0.412981434	0.976491766
HSP90AA1	0.03976107	0.413055805	0.976491766

MATK	0.103393539	0.413130526	0.976491766
PGA4	-0.125674567	0.413148818	0.976491766
CHMP2A	-0.058183764	0.413230137	0.976491766
TBC1D3B	-0.089955532	0.413236695	0.976491766
CREB3L4	0.074674001	0.413431902	0.976491766
CLEC12A	0.155889414	0.413441845	0.976491766
ARMC10	-0.064595533	0.413463341	0.976491766
ZNF579	-0.076377454	0.413745776	0.976491766
MRPS26	0.048434619	0.4137613	0.976491766
C1QC	-0.115690491	0.413767485	0.976491766
BCAR3	0.08491258	0.413804978	0.976491766
DSP	0.085879063	0.41383116	0.976491766
ISCA1	0.066829978	0.413859303	0.976491766
RPA3	0.056747557	0.414115049	0.976491766
CHAF1B	-0.087829041	0.41416705	0.976491766
SLC6A4	0.133552514	0.414171096	0.976491766
SLC30A9	-0.047012663	0.414198546	0.976491766
CCR1	-0.112216198	0.414267122	0.976491766
NTN4	-0.103197621	0.414394743	0.976491766
UBXN6	0.053907424	0.414486549	0.976491766
FAAH	0.097513395	0.414546394	0.976491766
MRPL3	-0.048901341	0.414594976	0.976491766
RNFT1	-0.097782496	0.414809239	0.976491766
MTX2	0.047413807	0.41483519	0.976491766
RIMS3	-0.084770207	0.414900743	0.976491766
PSMC2	0.035306018	0.414943677	0.976491766
FIBP	0.064249682	0.415270663	0.976491766
NDFIP2	-0.089062006	0.415292048	0.976491766
PSMC3IP	-0.078609348	0.415469823	0.976491766
C9orf135	-0.212888448	0.415514496	0.976491766
OSBPL7	-0.069543247	0.415622463	0.976491766
TMEM147	0.04742015	0.415642723	0.976491766
LRRC6	-0.182606152	0.415728181	0.976491766
HIST1H1D	-0.110478567	0.415798764	0.976491766
TGOLN2	0.053280509	0.415841736	0.976491766
ITGA2B	0.277987248	0.416024011	0.976491766
CAT	-0.096350025	0.416053531	0.976491766
PCNA	0.060174207	0.416056723	0.976491766
UNC80	0.07272574	0.416156039	0.976491766
NANS	0.05976721	0.416259791	0.976491766
C19orf40	-0.083953911	0.416337901	0.976491766
MIR635	-0.103446839	0.41656727	0.976491766
PLEKHF2	0.099238703	0.416609813	0.976491766
AFF4	-0.164679381	0.41664624	0.976491766
ELMO1	0.0601106	0.416704289	0.976491766
CASZ1	0.075107725	0.416717013	0.976491766
ZWINT	-0.088679299	0.416794036	0.976491766

GRINA	-0.13327686	0.416878286	0.976491766
ASPH	-0.061474233	0.416971501	0.976491766
SLC1A4	-0.099379282	0.417336165	0.976491766
RBM14	-0.169052033	0.417528822	0.976491766
WDYHV1	0.078311036	0.41753022	0.976491766
SLC26A8	0.091876982	0.417531106	0.976491766
SNORD87	-0.081041153	0.417630249	0.976491766
MTUS1	-0.096157376	0.417631586	0.976491766
SOCS7	-0.071895425	0.417732235	0.976491766
NLRP1	0.075421419	0.417760779	0.976491766
TMEM55B	0.049135853	0.417878515	0.976491766
HTRA1	-0.138734294	0.417892969	0.976491766
CELSR2	0.082901793	0.418016914	0.976491766
ATP5G2	0.052214809	0.418242834	0.976491766
METTL1	-0.062315638	0.418264869	0.976491766
PTGR2	-0.217676733	0.418298678	0.976491766
NAT10	-0.045240419	0.418355306	0.976491766
LUC7L3	-0.067749135	0.418379789	0.976491766
CLSPN	0.072749443	0.418466922	0.976491766
MATN2	-0.104263894	0.418565522	0.976491766
DBNDD1	0.177201243	0.418588849	0.976491766
FAM8A1	0.067051256	0.418627924	0.976491766
TRIM16L	0.070560346	0.418768877	0.976491766
NFKB2	0.07521429	0.418981885	0.976491766
ACTL6A	-0.039896977	0.419470615	0.976491766
FZD1	-0.079571257	0.41975397	0.976491766
TEX264	-0.043965083	0.419758721	0.976491766
MTHFD2	-0.08605786	0.419831053	0.976491766
ZNF671	-0.064667197	0.419863781	0.976491766
DUS1L	-0.053882296	0.419894187	0.976491766
GNG11	0.173650594	0.419903346	0.976491766
MAD2L2	0.055045481	0.419988728	0.976491766
UBAC2	0.0391657	0.420010222	0.976491766
ARHGAP9	-0.048169312	0.420048888	0.976491766
IPO7	-0.072624719	0.42017207	0.976491766
BBIP1	0.08667496	0.42030376	0.976491766
ABLIM3	0.1979158	0.420566929	0.976491766
AKR7A2	0.081538975	0.42067957	0.976491766
HN1	0.079424529	0.420890887	0.976491766
GOLGA6L6	-0.150365196	0.421229376	0.976491766
GALK1	-0.041332624	0.421322428	0.976491766
DTWD2	-0.150197089	0.421358374	0.976491766
AGPS	-0.054840759	0.421416265	0.976491766
SRGN	-0.085297582	0.421454518	0.976491766
IQGAP2	0.06724345	0.421468554	0.976491766
SYK	0.056205493	0.42147267	0.976491766
H2AFX	-0.063478258	0.421672994	0.976491766

MRPS30	0.067538853	0.421852368	0.976491766
KLHDC8B	-0.105174313	0.422067662	0.976491766
DHX40	0.043979974	0.422204958	0.976491766
MIA-RAB4B	0.062081171	0.42230839	0.976491766
ZNF540	-0.098043286	0.422350396	0.976491766
CD97	0.041832064	0.422417622	0.976491766
GHRL	-0.099174632	0.422666045	0.976491766
ZFP2	-0.074878602	0.422817041	0.976491766
CYP27A1	0.162524465	0.422820101	0.976491766
ABHD2	0.072490452	0.422857627	0.976491766
TM6SF1	-0.107383694	0.422900564	0.976491766
CENPN	-0.135420006	0.422941319	0.976491766
EEF1G	-0.047897037	0.422949755	0.976491766
PRMT6	-0.078541504	0.423056781	0.976491766
C1orf43	-0.050782732	0.423089076	0.976491766
CD9	0.236175006	0.423110337	0.976491766
CDK17	-0.072556519	0.423186741	0.976491766
SNORD105B	0.068339592	0.423214929	0.976491766
SLCO3A1	-0.056009028	0.423434368	0.976491766
DUS3L	-0.053662802	0.423552784	0.976491766
ASGR1	-0.068761564	0.423567054	0.976491766
TNFAIP3	0.090111011	0.423573417	0.976491766
ZMYND12	0.141373732	0.423656556	0.976491766
INO80	0.051295106	0.423733364	0.976491766
C20orf24	0.049810114	0.423896811	0.976491766
LDLRAP1	0.106176761	0.423997854	0.976491766
ITFG1	-0.066628584	0.424120732	0.976491766
SNAP47	0.056780298	0.424172685	0.976491766
SPTLC1	-0.09605102	0.424365428	0.976491766
IDH3A	-0.065865018	0.424488701	0.976491766
CYP51A1	0.093719743	0.424518855	0.976491766
ARF5	0.045571259	0.424564073	0.976491766
TRMT11	-0.056348784	0.424756838	0.976491766
AHSP	0.16579268	0.424821291	0.976491766
C1orf228	-0.090037929	0.425056364	0.976491766
PGM5	0.182377645	0.425119753	0.976491766
TSC2	0.066910216	0.425148986	0.976491766
HNRNPF	0.076924563	0.425270779	0.976491766
SLC30A1	0.110408884	0.425383339	0.976491766
ATP1B1	0.114437459	0.425804333	0.976491766
CLEC4D	-0.123304442	0.425817501	0.976491766
SPTAN1	-0.083076855	0.42584582	0.976491766
CLDN7	-0.078018112	0.425862372	0.976491766
MARCH2	0.13858946	0.426237911	0.976491766
SERAC1	-0.087625754	0.426293328	0.976491766
OR2W3	0.159773897	0.426469143	0.976491766
PLA2G7	-0.173624904	0.426784611	0.976491766

SPTBN1	0.095094597	0.426793222	0.976491766
USP49	-0.144414628	0.427014234	0.976491766
PPP3CA	0.097143062	0.427057052	0.976491766
RANBP1	0.041181601	0.427094717	0.976491766
PGAM5	0.064588572	0.427143927	0.976491766
DTL	-0.080715103	0.427147811	0.976491766
LYPD2	0.165084187	0.427191343	0.976491766
CYP4F3	-0.12910146	0.427228813	0.976491766
RBPJ	-0.077115627	0.427543245	0.976491766
RWDD2A	-0.052114251	0.42762214	0.976491766
ZNF430	-0.040093558	0.427771682	0.976491766
RPL29P2	-0.071019053	0.427794869	0.976491766
INO80E	-0.066975132	0.427828263	0.976491766
FAM178A	0.058915774	0.427848928	0.976491766
ANK3	0.11192567	0.427943058	0.976491766
LIPC	0.209667729	0.428189758	0.976491766
TINAG	0.074289302	0.428192881	0.976491766
PTPN7	-0.079766383	0.428215973	0.976491766
LEPROTL1	-0.066858866	0.428276357	0.976491766
KIF23	-0.093288653	0.428322789	0.976491766
MBOAT7	-0.071444247	0.428325201	0.976491766
LEF1	-0.096263751	0.428368488	0.976491766
EIF3H	0.056927546	0.428406643	0.976491766
CCDC102A	0.078221865	0.428800866	0.976491766
DPY19L4	-0.184918434	0.428945922	0.976491766
SPATC1	-0.063958823	0.428986646	0.976491766
NBPF11	-0.055096345	0.429117975	0.976491766
DSTN	0.058983822	0.429196769	0.976491766
NCOR2	-0.058379052	0.429472549	0.976491766
NBPF14	-0.124285968	0.429513095	0.976491766
SNAPC1	-0.155377195	0.429587422	0.976491766
LAMTOR2	0.043256572	0.429659287	0.976491766
HOXA6	-0.094729288	0.42978142	0.976491766
ATXN10	-0.071940162	0.429804343	0.976491766
AHCYL2	-0.051919866	0.429929919	0.976491766
C12orf44	-0.055810516	0.430163095	0.976491766
IFRD1	0.075566053	0.430421603	0.976491766
FBXL16	0.097194612	0.430430574	0.976491766
FBXO15	0.090081513	0.430449624	0.976491766
EBNA1BP2	0.0435087	0.430508022	0.976491766
SNX20	-0.088478451	0.430642637	0.976491766
TARBP1	-0.068885262	0.430785604	0.976491766
CNKSR3	0.06487458	0.430870104	0.976491766
RAP1GAP2	-0.081457837	0.430915864	0.976491766
CMTM4	-0.096242172	0.430940685	0.976491766
PLRG1	-0.034267817	0.431037306	0.976491766
C5orf42	-0.071260898	0.431116251	0.976491766

NFE2L3	0.153487829	0.431194212	0.976491766
TLR3	-0.069304719	0.431209137	0.976491766
TFEC	0.077746548	0.431257038	0.976491766
RANBP6	-0.061216839	0.431401383	0.976491766
B3GALNT1	-0.092339547	0.431492695	0.976491766
CABP5	0.253188236	0.431515359	0.976491766
PDE4C	-0.04147114	0.431565192	0.976491766
YKT6	0.097050247	0.431609043	0.976491766
TUFT1	-0.062035767	0.431775304	0.976491766
TTYH2	-0.078648699	0.431952768	0.976491766
TMEM223	0.057118424	0.431981003	0.976491766
MRPL44	-0.058244971	0.432284367	0.976491766
SNRNP200	0.060357286	0.432353602	0.976491766
SERF2	0.060693084	0.432529602	0.976491766
SLC15A3	-0.070837587	0.432615953	0.976491766
IL1R2	0.114529587	0.432731434	0.976491766
TGFBR1	-0.075991698	0.432755542	0.976491766
SLC39A8	0.295282786	0.432793916	0.976491766
MRPL16	0.040818264	0.432860855	0.976491766
INPP5A	0.064923728	0.432878286	0.976491766
SENP6	-0.053856201	0.432891701	0.976491766
PGBD1	-0.067936164	0.43298823	0.976491766
UNKL	-0.109432736	0.433293789	0.976491766
TRIM56	-0.066432516	0.433370857	0.976491766
AMFR	-0.155672573	0.433413554	0.976491766
AGER	0.074807313	0.433697051	0.976491766
FAM26E	0.077207929	0.433699193	0.976491766
GRIP2	0.081308261	0.433792165	0.976491766
CD86	0.069969115	0.433885952	0.976491766
GTF2H3	0.072317614	0.433902808	0.976491766
VPS53	-0.031747237	0.433914271	0.976491766
ZNF250	-0.094011139	0.433940954	0.976491766
PRSS21	-0.14576217	0.433966906	0.976491766
RCBTB2	-0.058332625	0.4340941	0.976491766
CDCA5	-0.133306764	0.434499216	0.976491766
ZNF773	-0.092107415	0.434518565	0.976491766
SP2	-0.05020248	0.434729408	0.976491766
KIF11	-0.125993853	0.434747853	0.976491766
HBB	0.235308991	0.434832896	0.976491766
CCDC47	0.061899518	0.434844105	0.976491766
ALX1	-0.080002102	0.434874157	0.976491766
NPAT	-0.07419529	0.434895473	0.976491766
DTX3	-0.079518396	0.435029257	0.976491766
GGT1	0.07247102	0.435047442	0.976491766
PRM3	-0.061455378	0.435156113	0.976491766
PCBP4	-0.077948103	0.435264298	0.976491766
ZNF761	0.077724477	0.435550081	0.976491766

KLRG1	-0.142745648	0.435644659	0.976491766
LCN2	0.234953352	0.43567745	0.976491766
MIR373	0.072265783	0.435705448	0.976491766
ARHGAP19	-0.044576769	0.435785683	0.976491766
COQ10B	-0.063496708	0.435920103	0.976491766
ZNF678	0.069811569	0.435926667	0.976491766
ZCCHC14	-0.061900737	0.435944343	0.976491766
HSBP1	0.043438803	0.436067295	0.976491766
GGTLC2	-0.069260108	0.436071366	0.976491766
BEND7	0.080297973	0.43607753	0.976491766
MTOR	-0.047664175	0.436263411	0.976491766
SMCR5	-0.156778289	0.43626918	0.976491766
VAV2	0.071057834	0.436345764	0.976491766
NOD1	0.081720417	0.436413587	0.976491766
PPIL1	-0.058581979	0.436719195	0.976491766
SREK1IP1	-0.071478466	0.436787435	0.976491766
DET1	-0.062300686	0.436791489	0.976491766
AP3M1	-0.065307263	0.436804961	0.976491766
YAF2	0.055641943	0.436900787	0.976491766
STK39	-0.079991134	0.436922995	0.976491766
MYC	0.090241846	0.43697572	0.976491766
QPCT	-0.178995879	0.437076531	0.976491766
RNFT2	0.109599593	0.437176146	0.976491766
BIN1	0.06664556	0.437310508	0.976491766
SMARCD3	-0.071149602	0.437373576	0.976491766
ZRANB1	0.100227527	0.437512815	0.976491766
FOXO1	0.0593958	0.437607379	0.976491766
MIAT	-0.177147708	0.43764643	0.976491766
NBEAL2	-0.07303275	0.437658891	0.976491766
KIF20B	-0.073220984	0.43766653	0.976491766
NDUFAB1	0.057481347	0.437798237	0.976491766
TRIM68	-0.048540222	0.437810546	0.976491766
SYT16	-0.093477982	0.437891472	0.976491766
RAB22A	-0.058945182	0.437992117	0.976491766
ZNF483	-0.20276074	0.438093901	0.976491766
TWF2	0.050659554	0.438249468	0.976491766
IER3	-0.11103263	0.43825517	0.976491766
NAF1	0.062844024	0.438261121	0.976491766
DCUN1D4	-0.051110523	0.438509779	0.976491766
MAP7D1	-0.036709251	0.438557007	0.976491766
ATP2A3	0.075529935	0.438566594	0.976491766
EYS	0.058405281	0.438650438	0.976491766
CNPY4	0.056636982	0.438761023	0.976491766
TPM2	-0.154166873	0.438795434	0.976491766
DSC1	-0.189066748	0.438871394	0.976491766
MEOX1	0.13119797	0.438908209	0.976491766
RPL23AP64	0.060091247	0.439036009	0.976491766

SCARNA5	0.102133488	0.439155479	0.976491766
GAK	0.038403149	0.439174718	0.976491766
ZNF845	-0.067757429	0.439215212	0.976491766
SRGAP3	0.077012832	0.439301371	0.976491766
EID3	-0.072981218	0.439328828	0.976491766
RPPH1	-0.058706429	0.439360969	0.976491766
RPL32	0.018287122	0.439418302	0.976491766
GUSBP9	0.115975952	0.439435921	0.976491766
HIP1R	0.070462932	0.439581658	0.976491766
SURF4	0.052861093	0.439946502	0.976491766
TASP1	-0.062032205	0.440032091	0.976491766
DDHD1	-0.172452053	0.440046091	0.976491766
IRF2	-0.077831147	0.440170144	0.976491766
NDUFS7	0.05252756	0.440254772	0.976491766
DNAJC30	-0.100241953	0.440268004	0.976491766
TBL2	0.062229094	0.440317908	0.976491766
IFT80	-0.063487394	0.440339694	0.976491766
COPS8	-0.06042778	0.440418368	0.976491766
CHRNA5	-0.170748182	0.44061333	0.976491766
PPM1G	0.031479317	0.440699168	0.976491766
DPYSL2	-0.051102424	0.440740667	0.976491766
ATG14	-0.047515227	0.440806416	0.976491766
SLC27A2	0.087527723	0.4408315	0.976491766
SPECC1L	0.042794823	0.440966672	0.976491766
ZNF155	-0.078832786	0.440998423	0.976491766
FABP4	0.064975303	0.441291792	0.976491766
GSTZ1	-0.076912577	0.441303649	0.976491766
EEF2	-0.03519811	0.441395842	0.976491766
SF3A3	0.046305674	0.44152807	0.976491766
C10orf105	0.079459913	0.441638487	0.976491766
NAIP	-0.139570381	0.441757484	0.976491766
HYAL2	0.06438105	0.44188069	0.976491766
TRIM7	-0.110923286	0.442010981	0.976491766
EPAS1	0.056446568	0.44225798	0.976491766
PLGLB1	-0.189695793	0.442361687	0.976491766
HNRNPUL2	0.034594997	0.442658929	0.976491766
OSBPL9	0.037119432	0.442673184	0.976491766
CDH26	0.070361049	0.442689424	0.976491766
SSX2IP	0.098548392	0.442711968	0.976491766
SRP68	0.041517505	0.442913311	0.976491766
RIF1	-0.050610702	0.443011806	0.976491766
ZMYM1	0.061181336	0.443039919	0.976491766
ZNF134	-0.084829884	0.443048308	0.976491766
TMEM33	-0.04647103	0.443163326	0.976491766
ALG2	0.064293625	0.443225871	0.976491766
TAF2	-0.045383427	0.443392242	0.976491766
MFSD5	0.05592628	0.44346506	0.976491766

SGK494	-0.077657952	0.443678137	0.976491766
TXNDC16	-0.070846659	0.443789471	0.976491766
TBC1D14	-0.042664142	0.443856286	0.976491766
KCTD11	-0.072030385	0.44389834	0.976491766
SULF2	-0.101793433	0.443977914	0.976491766
SOD2	0.084769052	0.4441104	0.976491766
CD79A	0.155861864	0.444249489	0.976491766
ANKRD20A1	-0.069046822	0.444269008	0.976491766
KEAP1	0.05516829	0.44447767	0.976491766
SCAMP4	-0.086073996	0.444672814	0.976491766
AGPAT2	-0.11002006	0.444676177	0.976491766
MYL12A	0.07559966	0.444847281	0.976491766
C8orf33	0.050262337	0.445035335	0.976491766
MKRN1	0.029594634	0.445043585	0.976491766
MLXIPL	-0.056640176	0.445210855	0.976491766
IGF2BP2	0.105205822	0.445213026	0.976491766
LMBR1	0.071927963	0.445254907	0.976491766
VPS33A	0.053188882	0.445270003	0.976491766
DIDO1	-0.070560655	0.44533744	0.976491766
FXR2	-0.058260263	0.445344163	0.976491766
KLRB1	0.099590691	0.445403253	0.976491766
SH2B1	-0.058900964	0.445424169	0.976491766
VRK2	0.076490495	0.4455566	0.976491766
C11orf1	0.078654665	0.445696888	0.976491766
ANKRD37	0.066918925	0.445708458	0.976491766
SIT1	-0.081391247	0.445806755	0.976491766
SEC23A	0.077014782	0.44582673	0.976491766
ZNF135	-0.118526191	0.445855077	0.976491766
ZNF480	-0.057818074	0.445968822	0.976491766
KCNMB2	-0.080891855	0.446161664	0.976491766
SLC2A6	0.070856079	0.446290596	0.976491766
C19orf54	-0.067570978	0.44652168	0.976491766
INTS10	-0.037986696	0.446602953	0.976491766
FAM64A	-0.057213756	0.446683575	0.976491766
VTRNA1-1	0.101596296	0.446758329	0.976491766
NOL11	-0.039607426	0.446764881	0.976491766
CCNH	0.072121986	0.446821875	0.976491766
VEZT	0.080363255	0.446893009	0.976491766
GP1BB	0.127540132	0.447002875	0.976491766
DTD1	0.064776568	0.447005807	0.976491766
NOXO1	0.073133542	0.447374963	0.976491766
GNB5	0.120513265	0.447388484	0.976491766
SEC24B	-0.049749268	0.447439602	0.976491766
TRPM7	0.071909962	0.447611903	0.976491766
PFDN6	0.064798056	0.4480663	0.976491766
CNNM4	-0.066779269	0.448074855	0.976491766
ZBTB8OS	0.058304992	0.448090625	0.976491766

NNT	-0.051939196	0.448115977	0.976491766
POFUT1	-0.077534487	0.448128232	0.976491766
PLEKHH3	0.083495006	0.448248696	0.976491766
MSRA	0.080012012	0.448296572	0.976491766
RPS9	0.043536068	0.44858807	0.976491766
FBLN1	0.179116739	0.448798107	0.976491766
ZNF717	0.07406286	0.44887929	0.976491766
FIG4	0.047724696	0.449140796	0.976491766
PXDN	-0.218678468	0.449313764	0.976491766
DNPEP	-0.029219593	0.449388489	0.976491766
PRPF3	-0.052570439	0.449597504	0.976491766
AAMP	0.048165991	0.449600774	0.976491766
RIT1	0.060213158	0.449620947	0.976491766
PDSS2	0.043542888	0.449775328	0.976491766
P2RX7	-0.074893121	0.450007416	0.976491766
C4orf27	0.069217714	0.450134325	0.976491766
GTF2IRD2	0.103617935	0.450210779	0.976491766
RHOC	0.079151286	0.450234079	0.976491766
SSH2	0.055298246	0.450271234	0.976491766
SHFM1	0.044434838	0.450296619	0.976491766
TMEM180	-0.065795472	0.450302218	0.976491766
TLK1	0.089954424	0.45066659	0.976491766
SCFD1	-0.042658817	0.450703217	0.976491766
ZNF763	-0.075077195	0.450860032	0.976491766
PSTPIP2	0.074420609	0.450902071	0.976491766
TIMM10	0.167574756	0.450914228	0.976491766
PLEKHN1	0.069631598	0.450982157	0.976491766
UHMK1	0.042723793	0.451199893	0.976491766
GFI1	-0.131569996	0.451293806	0.976491766
TNIK	-0.082442862	0.451312594	0.976491766
MARCH7	-0.038250683	0.451527185	0.976491766
RANGRF	0.086796028	0.451656506	0.976491766
QSER1	-0.061537102	0.451693494	0.976491766
STK32B	-0.084348334	0.451729531	0.976491766
C12orf43	-0.072680156	0.451736737	0.976491766
RAB40B	-0.062069025	0.451755386	0.976491766
SPHK1	0.086882109	0.4519539	0.976491766
ZNF576	-0.06282751	0.452035359	0.976491766
ZNF782	-0.093195533	0.4525005	0.976491766
ADRA2A	0.068277792	0.452505046	0.976491766
CHD7	0.071294758	0.45266692	0.976491766
PLEKHG3	0.096926273	0.452681564	0.976491766
SERTAD2	0.041222162	0.452786336	0.976491766
BSDC1	-0.055502901	0.452792738	0.976491766
ACBD5	0.077307651	0.452863592	0.976491766
RPL9	0.02881221	0.452927341	0.976491766
CARD9	-0.070517252	0.45302161	0.976491766

CLIC1	0.049371447	0.453199412	0.976491766
TXNRD2	-0.051452702	0.453323952	0.976491766
ZNF653	-0.053417942	0.453368498	0.976491766
FEZ1	0.159696259	0.453416082	0.976491766
KIFC2	-0.071949522	0.453434498	0.976491766
EXT1	0.060888369	0.45359649	0.976491766
MAP1LC3B	0.056263948	0.453636912	0.976491766
IRF8	0.061918526	0.45383345	0.976491766
CD1C	0.113868321	0.453852861	0.976491766
CCDC109B	0.040731027	0.453853472	0.976491766
PRKCA	-0.084395786	0.453984527	0.976491766
KCTD13	-0.067627829	0.454017391	0.976491766
RAB8B	0.037196375	0.454019583	0.976491766
CES4A	-0.072204883	0.454266982	0.976491766
LTBP3	0.095902367	0.454285259	0.976491766
CTNND1	-0.127807083	0.45438407	0.976491766
SNHG1	-0.051276273	0.454388345	0.976491766
SMURF1	-0.071487433	0.454415073	0.976491766
ALG5	-0.053930054	0.454501551	0.976491766
TAOK1	-0.138504269	0.454529359	0.976491766
GCC2	0.087310677	0.454725009	0.976491766
SLC44A2	0.045063616	0.454745242	0.976491766
ACACB	0.076127004	0.454964862	0.976491766
GPR180	-0.082956329	0.454966102	0.976491766
CCNYL1	-0.062514963	0.455012676	0.976491766
CTC1	-0.054726716	0.45510326	0.976491766
HSPA8	0.050026053	0.455142606	0.976491766
MAML2	-0.080490522	0.45523125	0.976491766
NAA25	-0.062124407	0.455296809	0.976491766
MB21D1	0.084855184	0.455319698	0.976491766
FAM109A	0.064249984	0.455668232	0.976491766
ATP9B	0.06231232	0.455691113	0.976491766
COQ4	-0.097846465	0.455780108	0.976491766
CIC	-0.054185411	0.456045746	0.976491766
FAM129A	-0.07219336	0.456169777	0.976491766
ASIP	-0.086950645	0.456180189	0.976491766
GJC2	-0.071712368	0.456377519	0.976491766
GINS3	-0.057773106	0.456423559	0.976491766
PGAM1	-0.041368686	0.456457107	0.976491766
NME1	-0.071840016	0.456460204	0.976491766
MRPS9	-0.047153284	0.456508257	0.976491766
KLHL28	-0.171113813	0.456521855	0.976491766
SETD3	0.037367116	0.45674563	0.976491766
PAFAH1B3	0.064560885	0.456804812	0.976491766
PIGG	-0.061397124	0.456806986	0.976491766
YIPF4	-0.062483201	0.456844503	0.976491766
GMDS	0.058282843	0.456873466	0.976491766

TNPO3	0.079861758	0.456911622	0.976491766
CD1A	0.097989881	0.456932548	0.976491766
DEPTOR	-0.088862038	0.456961546	0.976491766
PPIL3	-0.055360141	0.456981665	0.976491766
ZNF791	-0.072097367	0.457000212	0.976491766
ELF1	0.051559723	0.45701568	0.976491766
LYZ	-0.126657291	0.457024418	0.976491766
ANP32A	-0.084562583	0.457078759	0.976491766
B3GALT6	-0.075469044	0.457123416	0.976491766
PABPC4	0.037698375	0.457164938	0.976491766
PLD4	-0.065328486	0.457258528	0.976491766
PPA1	0.079693583	0.457679044	0.976491766
MRPL41	0.044657246	0.457708915	0.976491766
MDP1	0.045296914	0.457773873	0.976491766
GUCA1B	-0.096272836	0.457837987	0.976491766
FDFT1	0.060906246	0.45794556	0.976491766
SRP14	0.036372199	0.458022162	0.976491766
ARF1	0.04485625	0.458081936	0.976491766
NXPH2	0.072017114	0.458134534	0.976491766
ACAA2	0.060225268	0.458304632	0.976491766
GLCE	-0.056626823	0.458315569	0.976491766
TIAL1	-0.044038663	0.458437847	0.976491766
HAR1A	0.076586691	0.458580281	0.976491766
FANCD2	-0.054082748	0.458624643	0.976491766
RSAD1	0.047205668	0.458822993	0.976491766
GAPDH	0.063627033	0.458831014	0.976491766
GALNS	-0.063467636	0.458873617	0.976491766
SPAG16	0.07720517	0.45890009	0.976491766
MTFP1	0.066277682	0.458907934	0.976491766
MYL6B	0.072138389	0.458908631	0.976491766
NXF1	-0.044088337	0.459052943	0.976491766
PMS2CL	0.057317554	0.459054026	0.976491766
KIAA0753	-0.056502679	0.459061251	0.976491766
RPS19BP1	0.042533716	0.4591458	0.976491766
FUZ	0.065081505	0.459165087	0.976491766
SETD6	-0.063910341	0.459405544	0.976491766
RBM17	-0.040289868	0.459543165	0.976491766
EPT1	0.067673583	0.459740503	0.976491766
TMX1	-0.040025704	0.459772113	0.976491766
PSEN2	-0.062317284	0.459912744	0.976491766
MAP2K6	0.086448105	0.459942051	0.976491766
DSEL	0.083028192	0.459967201	0.976491766
ALG9	0.038090504	0.460116269	0.976491766
SLC2A8	-0.131419136	0.460201123	0.976491766
BTG2	0.101099058	0.460301762	0.976491766
CDADC1	0.068948	0.46035228	0.976491766
SIRT5	0.043258072	0.460364799	0.976491766

NUDT8	0.088231216	0.460568945	0.976491766
YIPF3	-0.032451126	0.460578484	0.976491766
CRX	0.064966099	0.460778833	0.976491766
LMO7	-0.058420095	0.46077959	0.976491766
ZNF160	-0.068812731	0.460964338	0.976491766
CDR2L	0.1337434	0.460995035	0.976491766
FCHO2	-0.122734402	0.461129582	0.976491766
KIAA0355	0.026006957	0.461249584	0.976491766
ADARB1	0.061931464	0.461410116	0.976491766
FGFRL1	-0.073109363	0.461501253	0.976491766
DCAF4L1	-0.065439021	0.461539254	0.976491766
LINS	0.095637449	0.461558236	0.976491766
RARA	-0.054493554	0.461691732	0.976491766
MRPS33	-0.056181374	0.461716512	0.976491766
LIG1	0.075323597	0.46190112	0.976491766
PNPLA6	-0.063428586	0.461912841	0.976491766
BBS12	-0.082230817	0.461997981	0.976491766
EXT2	0.056596767	0.46227569	0.976491766
ZMYM4	-0.043004349	0.462446543	0.976491766
WDR77	0.042770043	0.462494978	0.976491766
SNORA6	0.101917241	0.462550286	0.976491766
IL12A	0.091236204	0.462626912	0.976491766
PARP11	-0.058503519	0.462650438	0.976491766
FAM3C	0.078562298	0.4627789	0.976491766
XBP1	-0.059003112	0.46288477	0.976491766
TMEM176A	-0.43736823	0.462908478	0.976491766
RAPGEF6	0.066991488	0.462982282	0.976491766
PLXNA4	-0.093437018	0.463229027	0.976491766
SMN1	0.117049783	0.463261848	0.976491766
LXN	-0.080279272	0.463341056	0.976491766
PICK1	-0.053980309	0.463373563	0.976491766
VDR	-0.067049597	0.463394213	0.976491766
ATXN1L	-0.031664943	0.463416385	0.976491766
GLB1L	0.121214732	0.463465673	0.976491766
ATXN2	-0.03940124	0.463480062	0.976491766
GATC	-0.107506794	0.463516368	0.976491766
MRFAP1	-0.039348857	0.463530604	0.976491766
SLC25A17	0.053649138	0.463534659	0.976491766
AOAH	0.088053	0.463545684	0.976491766
MS4A3	0.146698036	0.463605317	0.976491766
USP2	-0.061304506	0.463749673	0.976491766
TMEM185B	-0.058301049	0.463750008	0.976491766
LBR	0.072067554	0.463848374	0.976491766
LCNL1	0.070305096	0.463917527	0.976491766
TMEM214	-0.053774025	0.464187973	0.976491766
PREX1	0.055506591	0.464205563	0.976491766
LIMS2	0.083350966	0.464606589	0.976491766

CBL	-0.054454051	0.464705607	0.976491766
PDP2	0.07753984	0.464750667	0.976491766
JAM3	0.204259444	0.464985279	0.976491766
SP110	0.045471188	0.465003637	0.976491766
NLRP8	-0.121839333	0.465318049	0.976491766
INTS8	0.072024857	0.46539443	0.976491766
SRF	0.040694602	0.465422374	0.976491766
SEC14L1	0.077650732	0.465561844	0.976491766
ACTR1A	0.042255852	0.465687857	0.976491766
DPY30	0.046599996	0.465693807	0.976491766
FGD4	0.066175804	0.465696259	0.976491766
RNF141	0.071857145	0.465712115	0.976491766
IFI6	0.11922256	0.465731402	0.976491766
MSRB3	0.130842397	0.465864919	0.976491766
ZNF675	-0.086716878	0.465950662	0.976491766
DHRS13	-0.065580475	0.466125422	0.976491766
CLPTM1	0.049797326	0.466127385	0.976491766
RNASEH1	-0.037856076	0.466189638	0.976491766
TNIP3	-0.130639526	0.466246706	0.976491766
RNASE10	0.062651021	0.466367734	0.976491766
KIF14	-0.093069434	0.466429432	0.976491766
BPI	-0.163102816	0.466472091	0.976491766
SOAT1	-0.099124083	0.466722265	0.976491766
CENPT	-0.076393182	0.466798509	0.976491766
RLN2	0.108545917	0.466874503	0.976491766
ENTPD7	-0.076336927	0.466937751	0.976491766
ARHGAP30	0.053103782	0.46693967	0.976491766
EDEM3	-0.051237322	0.466953995	0.976491766
MIR300	-0.074909915	0.467149327	0.976491766
CRYBB2P1	0.157093975	0.467341229	0.976491766
MRPS14	-0.070470378	0.467441773	0.976491766
CYP2J2	-0.098867367	0.467471016	0.976491766
CEP135	0.037100162	0.467567105	0.976491766
HOXB4	0.082082195	0.467629794	0.976491766
SNAPIN	-0.045889515	0.467721757	0.976491766
ATP10A	-0.068478837	0.467741108	0.976491766
RHOB	-0.101170781	0.467796147	0.976491766
SLC17A9	0.088143536	0.467808346	0.976491766
C9orf37	0.05741466	0.467810133	0.976491766
CD19	0.176250108	0.467856644	0.976491766
CDV3	-0.04356617	0.468009848	0.976491766
CCDC107	-0.062471535	0.468014298	0.976491766
GSR	0.068615983	0.468102854	0.976491766
RASGRP1	-0.04928574	0.468197622	0.976491766
PPP1R12A	0.048846648	0.468208769	0.976491766
CNOT6	0.038846991	0.468410027	0.976491766
GATAD2B	0.070067744	0.468586758	0.976491766

S1PR4	0.0616653	0.468706294	0.976491766
RASSF6	-0.171606636	0.468962027	0.976491766
ZNF816	-0.083257682	0.468969125	0.976491766
CABP4	0.087060967	0.46899418	0.976491766
CASD1	-0.064964238	0.469213691	0.976491766
C12orf73	-0.07978407	0.469232411	0.976491766
MET	-0.060771971	0.46931967	0.976491766
ELAVL1	-0.078544367	0.469396719	0.976491766
OXTR	-0.114363434	0.469431859	0.976491766
TMEM123	0.047953533	0.469484526	0.976491766
RTN3	0.048735817	0.46951203	0.976491766
GPR1	-0.160806598	0.469626503	0.976491766
MTPN	0.039803385	0.469637639	0.976491766
DGCR6L	0.069620913	0.469805523	0.976491766
TOR1AIP2	-0.070357958	0.469812217	0.976491766
TMTC1	-0.204124048	0.469938487	0.976491766
ZNF385D	-0.142195366	0.469943383	0.976491766
AKT1	0.056547615	0.469956731	0.976491766
RCE1	-0.048091489	0.469959721	0.976491766
PPP6R1	0.054007606	0.469989788	0.976491766
CDCA8	-0.070759309	0.470076093	0.976491766
GTF3C5	0.043331692	0.470082522	0.976491766
CYP2S1	-0.116119252	0.470105739	0.976491766
COPS4	0.044352581	0.470387674	0.976491766
TIPRL	0.071655963	0.470808252	0.976491766
SLC30A6	-0.06385368	0.470850805	0.976491766
INO80C	0.041491004	0.470862191	0.976491766
NOC4L	0.081826832	0.471021849	0.976491766
FUT11	-0.092647681	0.471073736	0.976491766
ENOSF1	-0.101974855	0.471178993	0.976491766
PGAP1	0.075737905	0.471183909	0.976491766
PRPF38A	-0.037340014	0.471210614	0.976491766
NPTN	-0.036884412	0.47126827	0.976491766
SPOCD1	0.217559861	0.471400014	0.976491766
MRPL38	0.050715007	0.471408514	0.976491766
NFIC	-0.059286497	0.471502814	0.976491766
ZNF485	-0.067255223	0.471728802	0.976491766
TRIB3	0.087541856	0.471987871	0.976491766
PIK3R1	0.042960603	0.472079747	0.976491766
VCL	0.082527072	0.472142798	0.976491766
CRYGS	-0.089023056	0.472145083	0.976491766
BTBD2	0.041435104	0.472392456	0.976491766
PF4V1	0.289645342	0.47258476	0.976491766
POM121L8P	-0.112849576	0.472628373	0.976491766
EARS2	-0.060268671	0.472645436	0.976491766
ADAP1	-0.040112621	0.472927225	0.976491766
VAV1	-0.046424374	0.4729891	0.976491766

LMOD3	-0.17470859	0.47308689	0.976491766
CDA	0.109892235	0.473097646	0.976491766
FCGR3A	0.098340591	0.473130317	0.976491766
DDX41	0.039829994	0.473592557	0.976491766
ZSWIM3	-0.085679412	0.473764545	0.976491766
AAK1	-0.099194061	0.473813466	0.976491766
ZNF48	0.067167726	0.473837097	0.976491766
SIM2	-0.068964222	0.47389567	0.976491766
DENND5A	-0.04822649	0.473915457	0.976491766
SLC12A4	0.059347755	0.47403401	0.976491766
PPP3CB	-0.025935332	0.474109131	0.976491766
ADIPOR1	0.062715375	0.474110531	0.976491766
DOK3	-0.069305661	0.474174271	0.976491766
R3HCC1	0.03297096	0.474359092	0.976491766
INSL5	-0.076514271	0.474482579	0.976491766
PATE1	0.075792366	0.474510623	0.976491766
NDUFA10	0.0669674	0.474520632	0.976491766
HELLS	-0.078086588	0.474558939	0.976491766
LACTB2	0.082318427	0.474613387	0.976491766
SNORD54	0.098110165	0.474622379	0.976491766
HSPA14	-0.042252721	0.474814532	0.976491766
C14orf166	0.04698136	0.474898482	0.976491766
ACTG1	0.077142513	0.474934172	0.976491766
SH3TC1	0.071110625	0.474963786	0.976491766
DRG1	0.053682017	0.474974925	0.976491766
ASCC2	-0.030244123	0.47502633	0.976491766
RPAP1	0.062824686	0.475026372	0.976491766
RAI1	-0.050301046	0.475143905	0.976491766
CDC42BPB	-0.077328643	0.475163307	0.976491766
NCL	-0.035069255	0.475282661	0.976491766
PARP8	0.09181458	0.475449197	0.976491766
CMTM1	0.063204374	0.475495868	0.976491766
LEP	-0.136978956	0.475613243	0.976491766
ADAM23	-0.095911041	0.475851069	0.976491766
C14orf142	-0.043504657	0.476081485	0.976491766
RFX3	0.057804486	0.476085723	0.976491766
ZNF594	0.022204857	0.476259546	0.976491766
CELF1	0.074883554	0.476294036	0.976491766
CD70	-0.093517908	0.476500222	0.976491766
SPOCK2	0.043566969	0.476547883	0.976491766
TJP2	0.09472169	0.476561644	0.976491766
APOBEC3F	0.1079796	0.476604519	0.976491766
ITGB7	-0.06869894	0.476639925	0.976491766
INSIG1	0.054844103	0.476788632	0.976491766
TIMP2	-0.071807747	0.476895119	0.976491766
ONECUT2	-0.057491156	0.476948822	0.976491766
EHMT1	-0.100884804	0.477138028	0.976491766

RCCD1	-0.112650575	0.477250919	0.976491766
SNORD32B	0.068151943	0.477307659	0.976491766
NOL3	-0.07187643	0.477441606	0.976491766
CCT8L2	-0.065278198	0.477564502	0.976491766
SMNDC1	-0.048488353	0.477831191	0.976491766
TCF19	-0.068414245	0.477845007	0.976491766
RGPD8	-0.116500236	0.47791905	0.976491766
EIF4A1	-0.032321217	0.478215561	0.976491766
KPNB1	-0.035165885	0.478268963	0.976491766
SKIL	-0.063361841	0.478618075	0.976491766
ALDH8A1	-0.072855824	0.478638493	0.976491766
SLC25A3	-0.041440023	0.478647987	0.976491766
RABGAP1L	0.092052769	0.478862192	0.976491766
DOCK2	0.04177899	0.478864988	0.976491766
SBNO1	-0.070916904	0.478958113	0.976491766
RNF175	0.124063727	0.479118858	0.976491766
SLC27A5	0.063695743	0.479174399	0.976491766
USP53	0.122382809	0.479179667	0.976491766
ASB3	-0.057518105	0.479329823	0.976491766
ATXN7L2	-0.049170188	0.479756705	0.976491766
SERPINE1	0.069727042	0.479983849	0.976491766
HPCAL4	-0.14207267	0.48005255	0.976491766
ZFAND6	0.109377735	0.480112743	0.976491766
PARN	0.043243991	0.480236567	0.976491766
SRGAP2	-0.073783371	0.480248452	0.976491766
P2RY2	-0.099914048	0.480269122	0.976491766
NFE2L1	0.062142871	0.480312807	0.976491766
ASB7	-0.077015858	0.480392541	0.976491766
LSM1	0.032065497	0.480455071	0.976491766
HIPK3	-0.088432116	0.480463268	0.976491766
HHLA3	0.059937503	0.480717517	0.976491766
REPS1	0.057596663	0.480728881	0.976491766
C1QTNF6	0.085293019	0.480858531	0.976491766
ARL5A	-0.02881583	0.480893735	0.976491766
PIP5K1C	-0.067934992	0.481003993	0.976491766
ZNF629	-0.05315418	0.4811662	0.976491766
LONP1	0.047150846	0.481341461	0.976491766
LCMT2	-0.093603374	0.48134986	0.976491766
TSGA10	0.120801083	0.481380455	0.976491766
UNC5CL	0.071324291	0.481454797	0.976491766
TMEM114	0.075409029	0.48158175	0.976491766
TGFB1I1	0.167185401	0.48164974	0.976491766
PAPL	-0.096180566	0.481744296	0.976491766
RPL22L1	-0.231963177	0.481909178	0.976491766
ZNF45	-0.038148377	0.48194546	0.976491766
LTB4R	-0.072267219	0.481990151	0.976491766
NOP10	0.05665487	0.482081008	0.976491766

RAB13	0.076750733	0.482102611	0.976491766
MTPAP	-0.05703307	0.48211784	0.976491766
BEND4	0.076731128	0.482153481	0.976491766
STK3	-0.065733224	0.482171376	0.976491766
SRSF5	0.039044625	0.482333048	0.976491766
HES6	0.1232188	0.482586735	0.976491766
TBC1D9B	0.050841227	0.482690394	0.976491766
PIGF	-0.058040628	0.482832542	0.976491766
CCDC127	0.059936655	0.482863914	0.976491766
ATF1	0.068232795	0.482873473	0.976491766
ROCK1P1	-0.07115427	0.482873667	0.976491766
WDSUB1	-0.068295607	0.483006716	0.976491766
CD53	-0.038632556	0.4830267	0.976491766
PMF1	0.062750222	0.483029359	0.976491766
COL16A1	0.063131017	0.483167889	0.976491766
KIAA1549	-0.170026024	0.483179459	0.976491766
ABCF2	0.053850773	0.483242967	0.976491766
GOLM1	-0.107474649	0.483308568	0.976491766
PCBP2	0.030701781	0.483540355	0.976491766
KIAA0586	0.047048093	0.483560386	0.976491766
RAE1	-0.039175559	0.483676543	0.976491766
CTSW	0.094444713	0.483687447	0.976491766
SREBF2	0.063991357	0.483755882	0.976491766
SLAMF7	-0.09366879	0.483924883	0.976491766
METTL21B	-0.094314296	0.484123625	0.976491766
BRSK1	-0.08100791	0.484249285	0.976491766
BNIPL	-0.078592099	0.484264213	0.976491766
CCDC115	0.071367605	0.48428907	0.976491766
RAB11A	0.050565095	0.484376152	0.976491766
TRIM24	-0.062484722	0.484389071	0.976491766
RPL17	-0.037831934	0.484398079	0.976491766
WBP2	0.048967354	0.484425003	0.976491766
PEX19	0.042811136	0.484426356	0.976491766
UPF1	-0.064187727	0.484498575	0.976491766
TNFRSF14	-0.041161213	0.48465562	0.976491766
ZNF343	0.04731203	0.484741759	0.976491766
ATP6V1G2	-0.077960886	0.484871623	0.976491766
VOPP1	0.045512465	0.485279389	0.976491766
TAS2R30	0.095892377	0.485294531	0.976491766
CRIPT	-0.077177587	0.485364201	0.976491766
MVP	-0.056662466	0.485365941	0.976491766
AKR1A1	-0.037528598	0.485405474	0.976491766
ORAI2	0.072203944	0.485572271	0.976491766
TAF3	0.052867113	0.485801798	0.976491766
MRPL30	-0.074890697	0.485916765	0.976491766
WASH2P	-0.080769976	0.485944148	0.976491766
KCNN4	0.083182397	0.486034658	0.976491766

ERAP2	0.311927251	0.486150709	0.976491766
ARHGAP11B	-0.059833683	0.486317304	0.976491766
IL21R	0.068393506	0.486365617	0.976491766
RGS10	0.069095709	0.486376593	0.976491766
SRRM2	-0.056793977	0.48648281	0.976491766
NCEH1	-0.050526755	0.486488878	0.976491766
KCND3	0.092419572	0.486539957	0.976491766
GFM1	-0.036230846	0.486567597	0.976491766
XRCC2	-0.162338051	0.486602711	0.976491766
ACTR1B	-0.036363759	0.486650727	0.976491766
DHTKD1	0.04393557	0.486697904	0.976491766
SNORD36A	0.061001764	0.486882439	0.976491766
AFTPH	0.042846569	0.486885503	0.976491766
FN3KRP	0.073598243	0.486905756	0.976491766
RER1	0.089267165	0.48693429	0.976491766
IMMP1L	-0.0684104	0.486945621	0.976491766
XPO7	0.051133852	0.486957997	0.976491766
GPN2	-0.044650349	0.487152736	0.976491766
FAM120B	-0.040689478	0.487240823	0.976491766
CSTF1	-0.074404278	0.487244139	0.976491766
CRELD2	-0.052239219	0.487298599	0.976491766
UNC45A	-0.043936961	0.487338433	0.976491766
RAB37	0.068553191	0.487411869	0.976491766
RCOR3	-0.046272905	0.487536516	0.976491766
BLK	0.149079591	0.487594989	0.976491766
RNGTT	0.047773824	0.487645868	0.976491766
GAPVD1	0.034483572	0.487662334	0.976491766
EIF2AK4	-0.120857461	0.487687419	0.976491766
C1orf21	0.139083064	0.487795119	0.976491766
CIAO1	-0.035110144	0.487827415	0.976491766
TP53	0.061595442	0.487852622	0.976491766
TM7SF2	0.055962208	0.487856989	0.976491766
XYLB	-0.069284832	0.487918166	0.976491766
CRLF3	-0.049993314	0.487981124	0.976491766
RSC1A1	0.063727788	0.488009142	0.976491766
PLXNB2	-0.062378735	0.488122549	0.976491766
HIST1H2BF	-0.152045115	0.488188141	0.976491766
CPEB3	0.050238691	0.488231206	0.976491766
TSPAN1	-0.072940539	0.488351681	0.976491766
RAB2A	0.053717118	0.488430485	0.976491766
UBXN2A	-0.0555145	0.488571655	0.976491766
MAPRE3	-0.059167039	0.488610655	0.976491766
RANBP2	-0.067280639	0.488673847	0.976491766
SIGLEC14	-0.254105185	0.488846501	0.976491766
HIATL2	-0.121990081	0.489205489	0.976491766
PON2	-0.070492846	0.489262142	0.976491766
MCM10	0.099487304	0.489304076	0.976491766

CD27	-0.078587914	0.489561302	0.976491766
PNO1	0.056434634	0.489627672	0.976491766
FAM104A	-0.029933973	0.489819734	0.976491766
SLC6A12	0.08979553	0.489845919	0.976491766
ATP5O	0.045073677	0.489858899	0.976491766
GLUD1	-0.02971025	0.489860636	0.976491766
NDUFV1	0.042899777	0.489920615	0.976491766
KPNA3	-0.048653683	0.489973651	0.976491766
APH1B	-0.051949902	0.490013861	0.976491766
TATDN2	0.054476576	0.490092297	0.976491766
TRIM74	-0.062073022	0.490232086	0.976491766
ARL4D	0.056839769	0.490374093	0.976491766
CTNNBIP1	0.055936874	0.490439635	0.976491766
PLD6	0.097026301	0.490440778	0.976491766
HLA-H	0.04082113	0.49058631	0.976491766
ROM1	0.054173868	0.490675763	0.976491766
NACA2	0.067674051	0.490739733	0.976491766
CSTF2T	0.063546628	0.49084269	0.976491766
ZNF174	-0.067326919	0.49092785	0.976491766
RTEL1-TNFRSF6B	0.063123063	0.491165584	0.976491766
FBXL3	-0.06276171	0.49141638	0.976491766
DGCR2	0.063931397	0.491459389	0.976491766
KDM5B	-0.041734609	0.491588162	0.976491766
IMPAD1	-0.076726861	0.49160013	0.976491766
KRTCAP2	0.041885075	0.491676571	0.976491766
AP2S1	0.033540848	0.491722134	0.976491766
NOTCH2NL	-0.065156224	0.491960113	0.976491766
FAM19A1	0.141670177	0.492177605	0.976491766
SYCP2	-0.09882041	0.492238974	0.976491766
PYHIN1	0.092437888	0.492335508	0.976491766
PPP1R1B	0.059492714	0.492409723	0.976491766
RALGAPA2	-0.061445462	0.492647987	0.976491766
E2F3	-0.049994286	0.49268503	0.976491766
LPAR3	-0.056751866	0.493055916	0.976491766
CSNK1G3	-0.025376842	0.49310264	0.976491766
RNF40	0.054086912	0.493154363	0.976491766
DMAP1	-0.048205819	0.493188455	0.976491766
C20orf197	-0.087752192	0.493278087	0.976491766
SPG7	-0.056962115	0.493453068	0.976491766
RRS1	0.03564748	0.493494862	0.976491766
DDAH1	-0.04515701	0.493615454	0.976491766
DDX21	-0.054504826	0.493770685	0.976491766
UBE2T	-0.068207407	0.493899081	0.976491766
PPIA	0.04547036	0.493918392	0.976491766
QSOX1	-0.049538715	0.49393963	0.976491766
CEP152	-0.060907525	0.493971196	0.976491766
DUSP8	-0.107019913	0.494004039	0.976491766

SLC26A6	-0.05339054	0.494211342	0.976491766
RNF170	-0.058754675	0.49425411	0.976491766
MXD3	-0.071080615	0.494319118	0.976491766
MFN1	0.053859954	0.494327652	0.976491766
ATXN2L	-0.083713612	0.494421118	0.976491766
NFKB1	-0.029968385	0.494650454	0.976491766
MEN1	0.050718782	0.494740166	0.976491766
POMP	0.038601299	0.494783923	0.976491766
RPS6KB1	-0.03441831	0.49488713	0.976491766
RAB1A	-0.04362473	0.494951878	0.976491766
MIF4GD	0.041093041	0.495011095	0.976491766
CARNS1	-0.092520883	0.495027288	0.976491766
NUDT3	0.049509428	0.495109773	0.976491766
ABCC2	0.051955954	0.495150512	0.976491766
ZZEF1	0.031303841	0.495164707	0.976491766
PRADC1	0.062534352	0.495247886	0.976491766
PTP4A3	0.072575965	0.495394178	0.976491766
NT5E	0.162962984	0.495419075	0.976491766
TMUB1	-0.047139776	0.495462177	0.976491766
LIN7C	-0.083625739	0.495467003	0.976491766
NRARP	0.051220793	0.495470834	0.976491766
IGF2R	-0.042263803	0.495471016	0.976491766
KTI12	0.042706919	0.495964287	0.976491766
AGAP4	-0.111298856	0.495966178	0.976491766
FGR	-0.031159731	0.496067431	0.976491766
UTP23	-0.063074823	0.496099336	0.976491766
BAALC	-0.06092672	0.496176845	0.976491766
PHLDB2	0.105203267	0.496187589	0.976491766
ZNF551	-0.133140972	0.496188763	0.976491766
RPL23AP53	0.036276286	0.496320941	0.976491766
GADD45A	0.124898885	0.496383041	0.976491766
LEMD2	-0.032463608	0.496472279	0.976491766
SS18L2	0.028143818	0.496536961	0.976491766
HIST1H2BN	-0.099824278	0.496701191	0.976491766
KIAA0319L	-0.047788758	0.496825041	0.976491766
CFI	-0.06904039	0.49685889	0.976491766
LIN54	0.048443309	0.496987509	0.976491766
ZNF37A	0.06906782	0.497140652	0.976491766
CHKA	-0.056539497	0.497162541	0.976491766
SCAMP3	0.035432828	0.497255654	0.976491766
USP33	0.051746694	0.497285184	0.976491766
UPP1	0.040400586	0.497355851	0.976491766
QKI	0.098130929	0.497422046	0.976491766
WDR74	-0.12157835	0.497572844	0.976491766
SNORD114-2	0.056114124	0.497645438	0.976491766
RPUSD3	0.035245417	0.497686723	0.976491766
RPP14	0.066547678	0.49777537	0.976491766

UCP2	-0.092742766	0.497889051	0.976491766
SMARCA2	0.032240492	0.49799949	0.976491766
DENND5B	0.121268205	0.498024998	0.976491766
DNAJC18	0.052673176	0.498061473	0.976491766
C11orf74	-0.101280416	0.498115878	0.976491766
B3GNT2	0.080559922	0.498120208	0.976491766
CORO1C	0.056409015	0.498282666	0.976491766
ARRDC3	0.077050072	0.498410417	0.976491766
RNMTL1	-0.035422931	0.498461733	0.976491766
RPL11	0.026476711	0.498533424	0.976491766
NR2E1	-0.064455048	0.498909969	0.976491766
N4BP2	-0.117703742	0.498957452	0.976491766
UBA7	-0.045545933	0.498979627	0.976491766
AKAP2	-0.138747861	0.499086832	0.976491766
SKP2	-0.040350268	0.499411033	0.976491766
SERPING1	0.156244772	0.499488597	0.976491766
RAB15	-0.093779814	0.499505315	0.976491766
SDHAP1	-0.060367831	0.499524206	0.976491766
MUL1	0.040168479	0.499526204	0.976491766
PODN	-0.093240557	0.499682772	0.976491766
CPEB2	-0.06123336	0.499723634	0.976491766
MRPS36	-0.043282592	0.499724721	0.976491766
SPG21	0.095485316	0.499849442	0.976491766
ZDHHC3	0.045937529	0.499881458	0.976491766
ALKBH6	-0.039636459	0.499883618	0.976491766
TUBA4A	0.073720642	0.499942366	0.976491766
CDK19	0.036627743	0.499963766	0.976491766
DDX20	-0.0628866	0.500071263	0.976491766
UQCRFS1	0.047004394	0.500096118	0.976491766
NOG	-0.082971744	0.500111559	0.976491766
SCYL3	-0.036360077	0.5001743	0.976491766
MPDU1	0.06863876	0.500211098	0.976491766
TAP1	-0.062128469	0.500251978	0.976491766
GMPR2	-0.040505815	0.500281462	0.976491766
SSBP1	0.048285778	0.500322886	0.976491766
ZNF563	-0.07706363	0.500497365	0.976491766
ZNF699	-0.069255354	0.500534912	0.976491766
CXCL3	0.07346529	0.500706005	0.976491766
CLEC4E	-0.102242971	0.500760654	0.976491766
SAR1A	0.054727418	0.500761132	0.976491766
POLH	-0.05988162	0.500876538	0.976491766
GEN1	-0.050827758	0.500905457	0.976491766
ZNF568	-0.059918294	0.501048905	0.976491766
SRP9	0.034686717	0.501126808	0.976491766
SPATA7	-0.056957958	0.50113685	0.976491766
ACAP1	-0.066254814	0.501153685	0.976491766
REXO4	-0.049253272	0.501173774	0.976491766

PTPRO	-0.103535253	0.501228626	0.976491766
FANCE	0.043191261	0.501459127	0.976491766
MED18	0.083479822	0.501728853	0.976491766
NPHP3	-0.068342411	0.501876794	0.976491766
CDKL1	0.082655146	0.502070564	0.976491766
PAPLN	-0.113158116	0.502147643	0.976491766
CISD2	0.045621822	0.50218259	0.976491766
IL27RA	0.056935775	0.502286873	0.976491766
XRCC4	-0.062241204	0.502354966	0.976491766
PLEKHA2	0.04006208	0.502413242	0.976491766
SIRT2	0.049887377	0.502426169	0.976491766
FRMD3	-0.16435383	0.5025359	0.976491766
MFSD10	0.03970176	0.502604916	0.976491766
FGA	0.054380961	0.502664815	0.976491766
CCDC57	-0.069385484	0.502717501	0.976491766
MSL1	0.047241189	0.502766881	0.976491766
CKAP2	-0.075907894	0.502773668	0.976491766
HEMK1	-0.066486442	0.502839357	0.976491766
COX16	0.038448683	0.503010568	0.976491766
SERPINA1	0.078677274	0.503230207	0.976491766
LRRFIP1	-0.121581182	0.50324546	0.976491766
SUZ12	-0.022786942	0.50333833	0.976491766
AP4E1	-0.03545997	0.503460021	0.976491766
FOXO3	0.057339861	0.503487958	0.976491766
GLIPR2	0.037429841	0.503547594	0.976491766
WDR55	-0.040054699	0.503558295	0.976491766
ASGR2	-0.113548337	0.503616682	0.976491766
CDC42SE2	0.049904567	0.50391349	0.976491766
GPX8	-0.111302087	0.503979768	0.976491766
PSMD3	-0.056325125	0.504021771	0.976491766
FAM135A	-0.052062864	0.504026978	0.976491766
GDPD5	-0.11072581	0.504110278	0.976491766
CTNNB1	-0.056028334	0.504188343	0.976491766
PDLIM7	0.083283174	0.504460982	0.976491766
ENTPD3	0.050638511	0.504483736	0.976491766
SLC38A2	-0.04537836	0.504591024	0.976491766
HLA-DPA1	0.071293576	0.504658219	0.976491766
PMEPA1	0.092627476	0.504702589	0.976491766
TSEN15	0.038101935	0.504835153	0.976491766
ZNF358	0.056605507	0.504868468	0.976491766
LASP1	-0.037175166	0.50497796	0.976491766
RUNDC1	-0.040173452	0.504993926	0.976491766
C6orf52	0.051167796	0.505023223	0.976491766
DENND1B	-0.0639672	0.505039018	0.976491766
RNF6	0.06990236	0.505150069	0.976491766
ICOSLG	0.068916388	0.505199609	0.976491766
VAV3	0.543911395	0.505272167	0.976491766

DNAJC1	0.070353955	0.505354129	0.976491766
AP1M1	0.030365172	0.505496246	0.976491766
PTCD2	-0.074857544	0.50568342	0.976491766
SUMO4	-0.111769471	0.50577524	0.976491766
FAM149B1	-0.033055011	0.505920207	0.976491766
SNORA72	0.094357298	0.506022645	0.976491766
ZNF548	0.066331703	0.506086231	0.976491766
ACSM5	0.053932078	0.506239136	0.976491766
MRPL55	0.037581518	0.506244416	0.976491766
XRN1	-0.040386347	0.506290634	0.976491766
NCK1	0.044583202	0.506461415	0.976491766
GDNF	0.069379385	0.506467649	0.976491766
EZH1	-0.17027954	0.50657804	0.976491766
VCP	0.041528453	0.506608507	0.976491766
ZNF546	-0.02445099	0.506669187	0.976491766
ARFRP1	-0.064496787	0.506680159	0.976491766
CADPS	-0.060625915	0.506827845	0.976491766
ZDHHC17	-0.049738256	0.506879127	0.976491766
ZNF236	-0.056095226	0.50691973	0.976491766
KCTD6	-0.042904081	0.507079372	0.976491766
CRELD1	-0.054085113	0.507081149	0.976491766
TRMT61B	-0.044862358	0.507188455	0.976491766
CROCC	-0.068708286	0.507205805	0.976491766
VASH1	-0.139662106	0.50721975	0.976491766
TRIM16	-0.064597792	0.507238631	0.976491766
RBP7	-0.081752976	0.507445122	0.976491766
FAM174B	-0.088422328	0.50770238	0.976491766
MBP	0.047241766	0.507775579	0.976491766
SMARCC1	-0.032910142	0.507815579	0.976491766
MIA3	0.055153188	0.50796338	0.976491766
CEBPB	-0.042081973	0.507989231	0.976491766
C2orf44	-0.052272471	0.508010929	0.976491766
DHFR	-0.021390978	0.50808629	0.976491766
POLR1D	0.04447731	0.508155199	0.976491766
GOLGA7	-0.027501173	0.508160764	0.976491766
CA11	0.065366385	0.508203488	0.976491766
HDAC1	0.040533162	0.508600181	0.976491766
GCNT1	0.05896267	0.508632572	0.976491766
FANCL	0.073445626	0.508842298	0.976491766
HSPBAP1	-0.068201512	0.509210274	0.976491766
TPX2	-0.0784259	0.509256175	0.976491766
DNAJC5	0.043497611	0.509365182	0.976491766
SLC7A7	-0.05728685	0.509391783	0.976491766
CASP5	0.105537372	0.509455961	0.976491766
PAICS	-0.041974288	0.509593456	0.976491766
MYO9B	-0.0501682	0.50960483	0.976491766
MRI1	-0.116368037	0.509729919	0.976491766

HKR1	-0.056184619	0.509820769	0.976491766
MIR488	0.059539425	0.509873235	0.976491766
CTNS	-0.044876698	0.509876623	0.976491766
CD4	0.065199106	0.509947847	0.976491766
KIF15	-0.060327852	0.509969981	0.976491766
ATP5EP2	-0.029451154	0.510156608	0.976491766
PHACTR2	0.061243526	0.510169256	0.976491766
MGEA5	-0.047518578	0.510312607	0.976491766
NEIL1	0.077120483	0.510314168	0.976491766
KDM4B	0.058276899	0.510322737	0.976491766
RPL36AL	0.038572988	0.510392869	0.976491766
CTR9	-0.051709002	0.510692894	0.976491766
UAP1L1	-0.117196684	0.510763316	0.976491766
SFSWAP	-0.041395672	0.510890155	0.976491766
CLDN23	-0.114275442	0.510902822	0.976491766
PRKRA	0.038341194	0.511079733	0.976491766
MRM1	-0.063485478	0.511107177	0.976491766
C16orf91	0.047255687	0.511130608	0.976491766
SH3BGRL2	0.17734494	0.511146211	0.976491766
PACSIN2	0.036325283	0.511302359	0.976491766
PVR	0.046506402	0.511385741	0.976491766
RSL1D1	0.041404091	0.511389468	0.976491766
PIGX	-0.083186699	0.511404436	0.976491766
SOCS5	0.107611871	0.511470972	0.976491766
TYK2	-0.032543843	0.511669947	0.976491766
RBM18	0.061772526	0.511781975	0.976491766
MED4	0.076405342	0.511883513	0.976491766
PROK1	0.065061351	0.511892942	0.976491766
METTL23	-0.027545372	0.512012673	0.976491766
C1QB	-0.20071125	0.51203118	0.976491766
KIFAP3	0.058224256	0.512099299	0.976491766
BCDIN3D	-0.068579673	0.512130479	0.976491766
C11orf71	-0.070344664	0.512279104	0.976491766
SETD5	0.062124987	0.512304887	0.976491766
GNPDA2	0.049247504	0.512436702	0.976491766
MRPL46	0.033532474	0.512471634	0.976491766
SLC45A1	-0.057255814	0.512473854	0.976491766
ST7	0.077428269	0.512514556	0.976491766
GTF2I	0.04655568	0.51255957	0.976491766
EIF2B1	0.043815025	0.512559847	0.976491766
CFH	0.140931025	0.512694872	0.976491766
FKRP	-0.063826905	0.512847345	0.976491766
NR1H3	0.076316844	0.512908092	0.976491766
MBOAT2	-0.129575979	0.512978753	0.976491766
FOXC2	0.096423671	0.512983411	0.976491766
AVL9	0.055118803	0.512988666	0.976491766
HIST1H2BJ	-0.155358845	0.513102454	0.976491766

ZNF586	0.058309942	0.513118584	0.976491766
HNRNPH3	-0.047853274	0.513262778	0.976491766
TIPARP	0.047417429	0.513299884	0.976491766
KCNT1	-0.143590691	0.513324736	0.976491766
MBD4	-0.119217264	0.513510858	0.976491766
POLR3A	-0.049480215	0.513726423	0.976491766
RPL7A	0.025313438	0.513795366	0.976491766
PROS1	0.223915284	0.513838583	0.976491766
ZNF768	-0.074566441	0.513983648	0.976491766
MRPL4	0.046191584	0.514128525	0.976491766
DLG5	-0.07805928	0.514160898	0.976491766
WWOX	-0.075375978	0.514216116	0.976491766
FAM150B	0.109816785	0.514354151	0.976491766
MCM3AP	-0.038120056	0.514368939	0.976491766
DUSP19	-0.116065373	0.51441852	0.976491766
CHMP5	-0.049311899	0.51447594	0.976491766
ZNF432	-0.054099791	0.514641989	0.976491766
IGFL3	0.065416711	0.514855878	0.976491766
COG1	0.053823774	0.51514726	0.976491766
CDK14	0.094063753	0.515207233	0.976491766
KCNQ2	0.060718756	0.515347364	0.976491766
ZNF557	-0.090942779	0.515394834	0.976491766
PROCR	-0.059942492	0.515551283	0.976491766
MKS1	-0.051748196	0.515596499	0.976491766
PKN1	0.055967002	0.515597711	0.976491766
CDC42EP3	-0.102574234	0.515606476	0.976491766
AGMAT	-0.092197883	0.515746066	0.976491766
SLC25A4	0.069066344	0.515776495	0.976491766
AHCYL1	-0.030232336	0.51581538	0.976491766
FAM157B	0.06861405	0.515886023	0.976491766
NPM1	0.057314111	0.51595419	0.976491766
EFCAB4B	0.051157897	0.515990741	0.976491766
MRPS17	0.060295865	0.516150404	0.976491766
HIPK2	0.065900514	0.516214821	0.976491766
MB21D2	0.090359941	0.516281832	0.976491766
LTV1	-0.030341961	0.516295118	0.976491766
MTAP	-0.053027847	0.51635784	0.976491766
NPHP4	-0.061650006	0.516433174	0.976491766
BRF1	-0.059035517	0.516610856	0.976491766
POLR3GL	0.031573168	0.516615242	0.976491766
PAIP2	-0.033502314	0.516618762	0.976491766
RPIA	0.038072727	0.516624631	0.976491766
TARSL2	0.060300635	0.516918506	0.976491766
NKIRAS1	-0.058823443	0.517038152	0.976491766
OAZ1	0.018881191	0.517123094	0.976491766
MLLT3	-0.067733961	0.517302899	0.976491766
PAQR8	-0.070812726	0.517303133	0.976491766

ADAMTSL5	-0.069469879	0.517456642	0.976491766
WNT5B	-0.068802158	0.517467543	0.976491766
ARHGEF19	0.067968719	0.517513879	0.976491766
ETS1	0.040600044	0.517557457	0.976491766
MED1	0.038074615	0.517700969	0.976491766
P4HA1	-0.057240371	0.51782099	0.976491766
PARP1	0.033911472	0.517894765	0.976491766
HDAC7	0.062920417	0.518111622	0.976491766
STK25	-0.037534894	0.518439364	0.976491766
SPI1	-0.074939313	0.518502566	0.976491766
LANCL1	-0.033287843	0.518572766	0.976491766
STAT1	-0.100820127	0.518644664	0.976491766
RCN2	0.052045732	0.51872653	0.976491766
ITGA11	-0.06978882	0.518906999	0.976491766
PSIP1	0.042931529	0.519287204	0.976491766
F11R	0.060217268	0.519291163	0.976491766
SCYL2	-0.035411019	0.519415914	0.976491766
ERCC2	0.056277171	0.519438964	0.976491766
LPIN2	0.050846315	0.519523255	0.976491766
CYCSP52	-0.056282161	0.519539538	0.976491766
RASAL3	-0.038583308	0.519677129	0.976491766
SGOL2	0.06883745	0.519724471	0.976491766
ME2	0.046642114	0.51985646	0.976491766
ZNF83	0.091583188	0.520114434	0.976491766
PATE2	-0.062147166	0.520162181	0.976491766
GALC	-0.040728248	0.520248813	0.976491766
TPM4	0.092564548	0.520272025	0.976491766
CCL3	-0.139347699	0.520273798	0.976491766
PTCH1	0.074295542	0.520343842	0.976491766
RNF144A	0.064545774	0.520633806	0.976491766
HMGN4	0.034531761	0.520884162	0.976491766
LRRC59	0.063099098	0.520938202	0.976491766
C3orf14	0.119646574	0.521276516	0.976491766
NEK6	-0.064839705	0.521327409	0.976491766
TEX9	-0.082144215	0.521557396	0.976491766
RPL31P11	0.093812829	0.521633721	0.976491766
TMEM209	-0.039312981	0.521647239	0.976491766
CCNL2	0.079622219	0.52167445	0.976491766
RSBN1L	-0.046903473	0.521738662	0.976491766
DIAPH3	-0.056151165	0.521810812	0.976491766
CAD	-0.052190213	0.52184668	0.976491766
IGSF9B	-0.095028312	0.521908148	0.976491766
CELF6	-0.062100234	0.522073736	0.976491766
MCM8	-0.048776667	0.522229665	0.976491766
EBF1	0.153291998	0.522273306	0.976491766
PIBF1	-0.060596044	0.522314052	0.976491766
MED25	-0.049402064	0.522373457	0.976491766

SNAP23	0.069503984	0.522429112	0.976491766
SAMHD1	0.075782716	0.522506594	0.976491766
VEGFA	0.06348713	0.522609849	0.976491766
SOX12	0.060673065	0.522803536	0.976491766
SENP5	-0.040803339	0.522853828	0.976491766
CCNC	-0.078132756	0.522974197	0.976491766
PWWP2B	0.060656806	0.523097715	0.976491766
EIF3C	-0.08552596	0.523174946	0.976491766
FLVCR2	-0.060842478	0.523310337	0.976491766
SLC29A2	-0.056049524	0.523366339	0.976491766
PLEKHM2	-0.034960229	0.523378898	0.976491766
EID2	0.054100432	0.523385763	0.976491766
DDRGK1	-0.043670047	0.523459922	0.976491766
WDR53	-0.039956804	0.523608564	0.976491766
TMEM173	-0.03297667	0.523727832	0.976491766
ITPR1	0.048019874	0.523899081	0.976491766
WBSCR27	0.067316506	0.524015484	0.976491766
JOSD2	-0.069627572	0.524023143	0.976491766
S100A1	0.071199641	0.524024925	0.976491766
C17orf67	-0.093073467	0.524040356	0.976491766
ATP5SL	-0.033814242	0.524180473	0.976491766
UBAP1	0.049283977	0.524226899	0.976491766
ZDHHC8	-0.030474847	0.524378045	0.976491766
FGF23	0.056996924	0.524437637	0.976491766
TMED6	0.09602587	0.524444798	0.976491766
PEX1	-0.046826424	0.52445844	0.976491766
ATP6V0E2	-0.049046848	0.524530355	0.976491766
FBXO28	-0.04709824	0.524586337	0.976491766
GALT	0.049321751	0.524657158	0.976491766
MTMR6	-0.047072051	0.524794166	0.976491766
ANXA9	0.067570758	0.524826217	0.976491766
SNHG12	-0.068530054	0.524961279	0.976491766
CAMK1	0.061128283	0.525053518	0.976491766
MRPS2	0.043553855	0.525263978	0.976491766
RNF167	0.050245077	0.525278889	0.976491766
ZDHHC14	0.079427797	0.52534013	0.976491766
CHEK2	0.044636011	0.525374685	0.976491766
FAM89B	0.057228161	0.525440628	0.976491766
ARPC2	0.02653654	0.525476309	0.976491766
RGS17	-0.127962944	0.52555938	0.976491766
ANP32C	0.074926968	0.525644207	0.976491766
FRYL	-0.041581627	0.525858316	0.976491766
ABHD8	-0.046458454	0.526207953	0.976491766
TBC1D10B	0.051676067	0.526228541	0.976491766
VPS35	0.04191608	0.526260963	0.976491766
TYW1B	-0.051411961	0.526281458	0.976491766
POMZP3	0.136618989	0.526310711	0.976491766

SNPH	-0.070682026	0.526330365	0.976491766
OAF	-0.063212696	0.526710284	0.976491766
POGK	-0.0352902	0.526797563	0.976491766
SLC24A3	0.190441813	0.526939785	0.976491766
HIST1H2BH	0.158234573	0.526971359	0.976491766
NKIRAS2	-0.041131558	0.527023634	0.976491766
MIR142	-0.084593838	0.527166405	0.976491766
EXOC3	-0.042604638	0.527224655	0.976491766
TMEM99	0.05989957	0.527304148	0.976491766
CHCHD5	0.048620548	0.527445918	0.976491766
ZNF658B	0.054298704	0.527488106	0.976491766
TBC1D3	-0.083642679	0.527571849	0.976491766
TTYH3	0.054828077	0.527705221	0.976491766
SMYD4	-0.054348932	0.527718165	0.976491766
FARP2	-0.060114375	0.527793078	0.976491766
INPP5E	-0.133208198	0.527934028	0.976491766
TES	0.058895468	0.527988607	0.976491766
ETFB	0.049316834	0.528123172	0.976491766
RBM43	0.094816015	0.528216877	0.976491766
GPR160	0.097953925	0.528311882	0.976491766
PNRC2	-0.035671793	0.528334223	0.976491766
ZNF570	-0.057076344	0.528376068	0.976491766
SMG1	-0.035725301	0.528381342	0.976491766
ZNF565	-0.055737119	0.528439108	0.976491766
DHX33	0.043154427	0.52845627	0.976491766
CST7	0.10881434	0.528460438	0.976491766
ZNF658	-0.085387438	0.528509181	0.976491766
GNRH1	-0.061541911	0.528653951	0.976491766
PDGFB	0.099308023	0.528837507	0.976491766
NME6	0.042314134	0.528843249	0.976491766
TMEM44	0.090705586	0.52890342	0.976491766
P2RY11	0.053065185	0.528996686	0.976491766
GRAMD1A	-0.053026723	0.528999392	0.976491766
OXSM	-0.042963987	0.52905054	0.976491766
MEPCE	0.063038455	0.529229476	0.976491766
EIF3J	-0.04389187	0.529229745	0.976491766
SCD5	-0.116476386	0.529249624	0.976491766
TSEN54	-0.063090637	0.529295815	0.976491766
EIF3D	0.039759058	0.529334098	0.976491766
SPATS2	0.05764976	0.529377479	0.976491766
CD300C	-0.123082756	0.529420927	0.976491766
ELAC2	-0.042956315	0.529430746	0.976491766
GSTM2	-0.185562581	0.5294337	0.976491766
C17orf53	0.058244921	0.529439643	0.976491766
RPL14	-0.032388475	0.529576742	0.976491766
PHF23	-0.043182448	0.52975522	0.976491766
UFM1	0.041973717	0.529789434	0.976491766

WDR1	0.029581329	0.529810238	0.976491766
ZC3H12C	-0.06485672	0.529921758	0.976491766
MSLN	0.309061873	0.529941637	0.976491766
STX1B	0.107062778	0.529978545	0.976491766
NTN5	0.073054601	0.530113011	0.976491766
TMEM216	-0.046495448	0.530184859	0.976491766
MTDH	-0.03519298	0.530252853	0.976491766
TMCC1	-0.074875969	0.530308205	0.976491766
ABHD3	-0.035037277	0.530387951	0.976491766
SIKE1	-0.058175706	0.530440296	0.976491766
GRHL1	-0.066664044	0.530445863	0.976491766
ZNF550	-0.053671042	0.530606122	0.976491766
VEZF1	-0.034783956	0.530928892	0.976491766
PLCL1	0.078990302	0.531015734	0.976491766
ATIC	-0.054948589	0.531058126	0.976491766
BRD8	-0.037838647	0.531075232	0.976491766
SERBP1	-0.032487724	0.53111736	0.976491766
FBXL8	-0.05830412	0.531158435	0.976491766
TXLNA	0.032159122	0.531243192	0.976491766
UXS1	0.075764805	0.531277396	0.976491766
TMEM51	0.161760346	0.531297273	0.976491766
ZSWIM4	0.040709536	0.531314823	0.976491766
COPS2	0.070494001	0.53155413	0.976491766
C17orf66	-0.069472131	0.531647934	0.976491766
KIAA0430	-0.037221825	0.531703563	0.976491766
TACSTD2	-0.40436843	0.531727312	0.976491766
RPS6KA4	-0.052999757	0.531766095	0.976491766
ZNF408	0.043828105	0.531785243	0.976491766
RBPMS2	-0.281862492	0.531797991	0.976491766
NDUFB5	-0.035530238	0.53191547	0.976491766
ZNF582	-0.060626694	0.531937817	0.976491766
COX7C	-0.037840216	0.531944257	0.976491766
TRIM26	0.05821702	0.532095746	0.976491766
ZNF207	0.032551873	0.532115329	0.976491766
PLCG2	0.042041913	0.532243512	0.976491766
LDHC	0.145805898	0.532287704	0.976491766
PI4KA	-0.041295739	0.532331703	0.976491766
AIFM3	0.056790497	0.532400613	0.976491766
AFAP1L2	0.07184578	0.53240946	0.976491766
MAPK6	-0.043019086	0.532413135	0.976491766
MRPS18B	0.034962828	0.532647754	0.976491766
CCDC53	0.034329145	0.532830575	0.976491766
PTER	0.254063215	0.532931484	0.976491766
ZCCHC9	-0.04783772	0.533122271	0.976491766
NEURL3	-0.061950211	0.533136247	0.976491766
DNAJC27	-0.048511881	0.533316698	0.976491766
DCAKD	0.036469664	0.533336626	0.976491766

YTHDF2	-0.032454709	0.533378131	0.976491766
SUMO2	0.063984812	0.533433683	0.976491766
CELF2	-0.045306183	0.533467699	0.976491766
WDR41	0.135312367	0.533569551	0.976491766
MED9	-0.05566917	0.534047854	0.976491766
LRG1	0.085897568	0.534121571	0.976491766
RNU4ATAC	0.07481069	0.534273651	0.976491766
IKZF2	-0.047141618	0.534275333	0.976491766
TIRAP	0.043381415	0.53431732	0.976491766
ZC3H3	-0.044451992	0.534431408	0.976491766
CBR3	0.109101976	0.534509447	0.976491766
KIAA2026	0.044160178	0.534554644	0.976491766
WDR92	0.045820157	0.534589211	0.976491766
GPBP1	0.032967617	0.534603886	0.976491766
ARID4A	-0.045115211	0.534697716	0.976491766
PIK3CD	0.047316075	0.534740626	0.976491766
ATP5L	0.051267332	0.53474177	0.976491766
ATG3	0.060029755	0.534979534	0.976491766
OR2AG1	0.075457375	0.535009283	0.976491766
ZSWIM5	-0.077308177	0.535036192	0.976491766
RBM11	-0.09129523	0.535148972	0.976491766
SDF2	-0.036979101	0.535203384	0.976491766
RPL24	-0.02414346	0.535229425	0.976491766
ZNF347	-0.066500668	0.535233305	0.976491766
PHACTR4	0.048777342	0.53551814	0.976491766
RPS2	-0.071556489	0.535569539	0.976491766
SUMO1	0.074691031	0.535641518	0.976491766
HTATIP2	0.046163703	0.535649519	0.976491766
PPIE	0.074655937	0.53576295	0.976491766
PDE12	-0.041205905	0.535870167	0.976491766
ATM	0.043092862	0.535934032	0.976491766
PSME3	0.033654066	0.536271073	0.976491766
C14orf105	-0.155131855	0.536297907	0.976491766
NFS1	0.064039574	0.536435096	0.976491766
IFT74	-0.041500942	0.536509679	0.976491766
SGK3	-0.061650288	0.536571279	0.976491766
MED6	-0.029384157	0.53664501	0.976491766
ARID3B	-0.043869369	0.536646201	0.976491766
RC3H2	-0.046383775	0.536691878	0.976491766
ARMC2	0.046869647	0.536889678	0.976491766
LYAR	0.074629338	0.536948534	0.976491766
GFI1B	0.110890751	0.537051039	0.976491766
TFB1M	-0.043238656	0.537051273	0.976491766
RDH11	0.050016512	0.537062487	0.976491766
NUPR1	0.053121426	0.537317897	0.976491766
PHKB	0.062761309	0.537324573	0.976491766
OASL	0.123732313	0.537340086	0.976491766

CHRNA2	0.058399686	0.537456856	0.976491766
PCID2	-0.02864689	0.537487206	0.976491766
MPHOSPH10	0.033214176	0.537680797	0.976491766
IKBKE	-0.057583667	0.537812348	0.976491766
ZNF319	0.04881018	0.537820883	0.976491766
C12orf75	0.099002556	0.537850752	0.976491766
RHBDD2	0.045800288	0.538004099	0.976491766
FCGR2A	-0.075140493	0.538110751	0.976491766
SEC61A2	-0.073018891	0.5381605	0.976491766
CXCR5	0.123122228	0.538288696	0.976491766
COL12A1	0.061901084	0.538369184	0.976491766
RINT1	-0.045565496	0.538382474	0.976491766
FECH	0.081882504	0.538408055	0.976491766
SLC9A3R1	0.050496565	0.538410746	0.976491766
SENP1	0.052119388	0.538468957	0.976491766
COMMD7	0.029364839	0.538591647	0.976491766
SLC9A8	-0.052647066	0.538669299	0.976491766
EFCAB7	0.055104695	0.538906915	0.976491766
COBLL1	0.099296872	0.538914042	0.976491766
MAT2B	-0.03582401	0.539189079	0.976491766
ZNF714	0.063837716	0.539235213	0.976491766
UBAP2	-0.052193213	0.539417541	0.976491766
SH2B2	0.060984739	0.539443923	0.976491766
NAP1L1	0.03918076	0.539446325	0.976491766
NUMBL	-0.067959073	0.539730224	0.976491766
SEMA3E	-0.16915002	0.539941382	0.976491766
CNOT3	-0.05678346	0.539994169	0.976491766
ST6GALNAC2	-0.09569475	0.540158057	0.976491766
P4HTM	-0.048983059	0.540316491	0.976491766
SYNJ2BP	-0.071817174	0.540332683	0.976491766
TSHZ2	0.127812028	0.540360867	0.976491766
PDPK1	-0.041605273	0.540393907	0.976491766
EMP3	0.038792079	0.540513385	0.976491766
RPP38	0.063904438	0.540530585	0.976491766
SNORD48	0.093635114	0.540696536	0.976491766
TMEM70	0.056377862	0.540826575	0.976491766
PAPD5	0.050754326	0.540979468	0.976491766
EGF	0.117474013	0.541043531	0.976491766
SLC11A1	-0.099411343	0.541406799	0.976491766
TMEM116	0.048605035	0.541737049	0.976491766
TAF6L	-0.042843023	0.541758927	0.976491766
CCDC15	-0.053146812	0.54176835	0.976491766
CHD1L	0.054907129	0.541808494	0.976491766
PML	-0.100170674	0.541880336	0.976491766
CLUL1	0.052124868	0.542035721	0.976491766
AQP3	-0.082999982	0.542043019	0.976491766
TMEM158	0.165353732	0.542095329	0.976491766

STIM2	0.065837197	0.542287814	0.976491766
MRPL2	-0.078706564	0.542370945	0.976491766
MRP63	-0.07474015	0.542526581	0.976491766
IFNGR2	-0.053121943	0.542576789	0.976491766
KIAA0922	0.04767943	0.542588074	0.976491766
PAPOLA	-0.026128076	0.542646187	0.976491766
FLII	0.037726377	0.542686972	0.976491766
STX17	-0.047102504	0.542693727	0.976491766
ESYT2	-0.033399625	0.542695506	0.976491766
NUDT6	-0.068217976	0.54269595	0.976491766
MLC1	-0.063818735	0.542765901	0.976491766
KIAA0556	-0.037555104	0.542858473	0.976491766
EVI2A	-0.064044648	0.542983843	0.976491766
FAM102A	-0.05956397	0.543049075	0.976491766
AAAS	-0.042945188	0.543081402	0.976491766
S100A12	-0.128325702	0.54330976	0.976491766
ALPK2	0.112984837	0.543413062	0.976491766
UCN	-0.056407577	0.543422102	0.976491766
ARL2BP	-0.038373706	0.543540469	0.976491766
LPCAT1	-0.046531054	0.543616017	0.976491766
UTRN	-0.072656655	0.543756152	0.976491766
IPO13	-0.04633841	0.543985607	0.976491766
ITGA7	0.04938032	0.544066364	0.976491766
CRYM	0.064488196	0.544071458	0.976491766
KIAA0195	0.057462553	0.544305944	0.976491766
SNRPB	0.050051153	0.544310531	0.976491766
TCHP	0.038992943	0.54445087	0.976491766
GPR35	0.074411752	0.544460209	0.976491766
SUSD2	-0.060912858	0.544628304	0.976491766
SPRR1A	0.052086087	0.544673992	0.976491766
ZNF57	-0.057766943	0.544900958	0.976491766
NMI	-0.072518707	0.545101922	0.976491766
RBM4B	0.036968604	0.545211144	0.976491766
CACHD1	0.065898908	0.545243893	0.976491766
HIP1	0.058655289	0.545270526	0.976491766
BIRC3	-0.083417163	0.545375549	0.976491766
GBP5	0.080061411	0.545464072	0.976491766
CHAC2	-0.076026495	0.545527165	0.976491766
DACH1	-0.072731175	0.545554739	0.976491766
ZZZ3	-0.037776786	0.545572599	0.976491766
TMIGD2	0.085209392	0.545584556	0.976491766
NCAPD2	-0.061728875	0.545600442	0.976491766
CREBZF	0.045911901	0.545635273	0.976491766
EID2B	-0.082611447	0.545648425	0.976491766
FAM179A	-0.11201406	0.545702971	0.976491766
RPL26	0.031103777	0.545876479	0.976491766
GLS	-0.054204746	0.546036217	0.976491766

PRPF40A	0.065250413	0.546118501	0.976491766
BRPF3	-0.038844363	0.546140952	0.976491766
SEPHS2	0.046102983	0.546299646	0.976491766
SLC39A11	0.051326242	0.546412158	0.976491766
GPR125	-0.0841839	0.546414846	0.976491766
NUP98	0.067679239	0.54651823	0.976491766
ELAC1	0.054262078	0.546525407	0.976491766
CHPF	-0.062017162	0.546540557	0.976491766
GPR114	0.090947893	0.546589544	0.976491766
HSPA1L	0.04537896	0.546624206	0.976491766
C7orf25	-0.061316987	0.546731409	0.976491766
MMAB	0.06033809	0.546832944	0.976491766
SLC35F3	-0.089936359	0.546854223	0.976491766
NAA40	-0.054212071	0.546984393	0.976491766
KIAA1407	0.069423644	0.547020083	0.976491766
FCAR	-0.153932598	0.547068849	0.976491766
PALM	0.091324079	0.547159039	0.976491766
DCP1A	0.036580496	0.54719963	0.976491766
DDX31	-0.050789709	0.547200323	0.976491766
ACAD10	-0.04397794	0.547231573	0.976491766
PAQR3	-0.046288193	0.547279082	0.976491766
NCR1	0.086112392	0.547420132	0.976491766
FCGR2B	0.096405321	0.547440313	0.976491766
SNORA26	-0.075745305	0.547455025	0.976491766
NDUFB3	-0.035459191	0.547532109	0.976491766
SPSB3	0.037071578	0.547642212	0.976491766
RHEB	-0.038565787	0.5476562	0.976491766
PBX1	0.118022871	0.547792671	0.976491766
MIR877	-0.060032567	0.54780889	0.976491766
TAF1A	-0.057012126	0.54788209	0.976491766
ZNF331	-0.049897632	0.547885459	0.976491766
PSMA2	-0.044460301	0.547939358	0.976491766
CANT1	-0.038121164	0.548046621	0.976491766
ACPP	-0.101627294	0.548071598	0.976491766
FXR1	-0.037053086	0.548141329	0.976491766
TOB1	0.07071807	0.548146027	0.976491766
CLEC11A	-0.095763867	0.548285024	0.976491766
LIPT2	-0.053717063	0.548285336	0.976491766
SSBP3	0.064095051	0.548299161	0.976491766
OSTF1	0.037064846	0.54831735	0.976491766
HDAC3	0.027642143	0.548344589	0.976491766
CEP70	-0.067716349	0.548611159	0.976491766
RNF150	-0.141410237	0.548672635	0.976491766
PLA2G2D	-0.102730621	0.54884327	0.976491766
KCTD2	0.040882083	0.548928421	0.976491766
CTSS	-0.053426707	0.548941048	0.976491766
SERINC1	0.042135646	0.548948675	0.976491766

NPY5R	0.052738124	0.549036602	0.976491766
ROPN1L	0.057555785	0.549800969	0.976491766
LETMD1	-0.032404083	0.550293703	0.976491766
TRAFD1	0.043091747	0.550313671	0.976491766
SBK1	0.053822876	0.550446476	0.976491766
UBQLNL	-0.072516842	0.550504358	0.976491766
BCL10	-0.073960834	0.550704219	0.976491766
IL17RD	-0.11443279	0.550778009	0.976491766
PHLPP2	0.053512641	0.550964124	0.976491766
PID1	-0.116925705	0.550997797	0.976491766
EIF3I	0.028981541	0.551044911	0.976491766
DHRS12	0.056266424	0.551065851	0.976491766
DIP2A	-0.116069431	0.551158558	0.976491766
TNNI2	0.088949065	0.551170384	0.976491766
SLC39A4	0.064509534	0.551180075	0.976491766
FOLR2	0.069415685	0.551281572	0.976491766
CYC1	-0.029577986	0.551368992	0.976491766
TMEM38A	-0.124829876	0.551456216	0.976491766
GPSM3	0.034162544	0.551578772	0.976491766
RNF165	0.165045321	0.551759613	0.976491766
MACF1	0.047975354	0.551809837	0.976491766
RRNAD1	-0.044689529	0.551810853	0.976491766
ADAM10	0.073106629	0.55181222	0.976491766
FLOT2	0.027006577	0.552189434	0.976491766
KLC4	0.056892967	0.55222407	0.976491766
RPL21	0.070922278	0.552305245	0.976491766
F5	-0.115489749	0.552312346	0.976491766
GLI4	0.057575031	0.552383777	0.976491766
CYFIP1	-0.064308302	0.552389253	0.976491766
ZAP70	-0.061431391	0.552578731	0.976491766
EPM2A	-0.048232147	0.552706998	0.976491766
TACC3	-0.049201822	0.552810336	0.976491766
KATNB1	-0.039153222	0.552865533	0.976491766
MRPL37	-0.03630761	0.552982438	0.976491766
RRP12	0.067799315	0.553012102	0.976491766
TCEA3	-0.097032559	0.553018691	0.976491766
SNW1	0.039893306	0.553048112	0.976491766
DPRXP4	0.112986335	0.553050722	0.976491766
HPS5	-0.046221217	0.553186095	0.976491766
FRG1	0.054202441	0.553196755	0.976491766
POLE	0.051596035	0.553201449	0.976491766
ACP2	-0.063198593	0.55321227	0.976491766
SNTB1	0.056213934	0.553254892	0.976491766
DCLRE1C	-0.09601517	0.55326826	0.976491766
STX6	-0.03923795	0.5534256	0.976491766
CDK4	0.027576419	0.553541523	0.976491766
TRABD	-0.030564804	0.553782646	0.976491766

DYNLL1	-0.039913957	0.553874203	0.976491766
TMEM184C	-0.041815756	0.553991157	0.976491766
BRI3BP	-0.047567102	0.554120459	0.976491766
SFXN3	0.043518464	0.55414557	0.976491766
LAMA5	0.113351047	0.554217959	0.976491766
KIAA2013	0.042351687	0.554249393	0.976491766
UBE2C	0.117528339	0.554297007	0.976491766
GGH	-0.060499782	0.55437406	0.976491766
GUSB	-0.027629288	0.554587065	0.976491766
ZCCHC3	0.05415605	0.554667311	0.976491766
GNG2	0.060592716	0.554895465	0.976491766
FANCF	-0.051285163	0.555079167	0.976491766
NLRC4	-0.062682672	0.555232473	0.976491766
MYH10	-0.07251059	0.555292254	0.976491766
DCP1B	0.056921545	0.555328363	0.976491766
WDR27	0.059491044	0.555333427	0.976491766
LPCAT3	-0.037782833	0.555412102	0.976491766
ARHGAP5	0.054126334	0.555449274	0.976491766
C4orf3	-0.037672888	0.555525535	0.976491766
POMT1	-0.040858659	0.55578665	0.976491766
MAP3K7	-0.028354653	0.556029011	0.976491766
COPA	0.028293616	0.556075345	0.976491766
ETAA1	-0.054510729	0.5560933	0.976491766
CDK13	-0.036117849	0.556275382	0.976491766
HAS3	-0.067455133	0.556374339	0.976491766
CD96	0.048232182	0.556420755	0.976491766
THOC6	-0.042211116	0.55645799	0.976491766
UBE2G2	-0.154424025	0.556667665	0.976491766
C17orf74	0.050519747	0.556749988	0.976491766
CNPPD1	0.031812798	0.556768911	0.976491766
METTL7A	0.060742674	0.556842074	0.976491766
TMEM59	-0.025539952	0.556843373	0.976491766
DCUN1D1	-0.065911842	0.556908358	0.976491766
ACAD9	-0.049364582	0.556913459	0.976491766
ST3GAL1	-0.05016388	0.556938915	0.976491766
ZFYVE21	-0.037945416	0.556944779	0.976491766
ARL4A	0.103506989	0.55705047	0.976491766
DNAJB14	-0.096481323	0.557103307	0.976491766
GALK2	-0.072885043	0.557143128	0.976491766
MUC7	-0.062867718	0.557195705	0.976491766
C15orf52	-0.087232845	0.557243769	0.976491766
FMNL2	-0.089397112	0.557293329	0.976491766
DFFA	-0.06655146	0.557523711	0.976491766
TBCC	0.067518863	0.557524913	0.976491766
C10orf118	-0.046068424	0.557589261	0.976491766
ZNF470	0.059780392	0.557676605	0.976491766
EP300	-0.061037896	0.557680576	0.976491766

ZNF281	0.038312341	0.557684565	0.976491766
KDSR	0.047881545	0.557814899	0.976491766
ZMIZ2	-0.05553166	0.557857822	0.976491766
LRPPRC	0.037733607	0.558011381	0.976491766
HIVEP1	0.048429399	0.558081821	0.976491766
RHOF	0.053522894	0.558127936	0.976491766
GLTSCR2	-0.019776895	0.558161648	0.976491766
GDF5OS	0.053255387	0.558170657	0.976491766
VIM	-0.043552736	0.558195929	0.976491766
RNASET2	-0.028931671	0.558272884	0.976491766
SORCS3	-0.080454749	0.558279387	0.976491766
HNRNPH1	0.050793329	0.558356455	0.976491766
FMNL1	-0.059980059	0.558416896	0.976491766
USP39	0.033323614	0.558468102	0.976491766
TNS1	0.087072233	0.558511267	0.976491766
ATP6V1E2	0.062144312	0.558636811	0.976491766
MT1A	0.07089438	0.558764848	0.976491766
SPG20	0.048016575	0.558887002	0.976491766
EDAR	-0.107136131	0.559110453	0.976491766
TUSC2	-0.0360228	0.559187956	0.976491766
PHAX	-0.141188017	0.559312408	0.976491766
PTTG1	-0.072453317	0.559415326	0.976491766
AIG1	0.049034443	0.559576518	0.976491766
ZKSCAN2	-0.048367537	0.559620581	0.976491766
CPT2	0.038428605	0.559864225	0.976491766
BCRP2	0.049103451	0.559921938	0.976491766
MICA	0.058903369	0.559973383	0.976491766
PPHLN1	0.040079046	0.560017322	0.976491766
STK11	0.053054513	0.560152397	0.976491766
GPBAR1	-0.066300309	0.560170836	0.976491766
ZNF713	0.05491841	0.560410363	0.976491766
AKT3	0.058278039	0.560563549	0.976491766
WHAMM	0.068769827	0.561035391	0.976491766
ZNF74	0.050037031	0.561104914	0.976491766
PNPO	0.036200656	0.561194321	0.976491766
DVL1	0.063288673	0.56126335	0.976491766
VNN1	0.156981859	0.561269742	0.976491766
DENND1A	0.082623972	0.561301514	0.976491766
CDK5	0.056416366	0.561400994	0.976491766
SLC1A5	0.052027203	0.561430168	0.976491766
TGM1	-0.057294194	0.5614362	0.976491766
SMARCA5	0.056259501	0.561611238	0.976491766
IFI27	0.173318333	0.561908145	0.976491766
MRPL28	0.040940784	0.561971472	0.976491766
RSPO2	-0.046953566	0.562008774	0.976491766
FOS	0.133187515	0.562054428	0.976491766
TOPORS	-0.049818589	0.562133372	0.976491766

HRAS	0.038426525	0.562134739	0.976491766
MTL5	-0.08783496	0.562139702	0.976491766
CRIP1	0.045806239	0.562347989	0.976491766
HSDL1	0.050531595	0.562366827	0.976491766
ASPHD1	0.050524734	0.56245293	0.976491766
RPS15AP10	-0.032861845	0.562737438	0.976491766
CREB1	-0.114530224	0.562945695	0.976491766
RAB34	0.072537062	0.562959579	0.976491766
WDR5	-0.045226516	0.563126866	0.976491766
SKA1	-0.051512707	0.56313912	0.976491766
RIOK2	-0.042770687	0.563267007	0.976491766
RALGDS	0.04839052	0.563405445	0.976491766
H1F0	0.097613216	0.563436204	0.976491766
HNRNPA0	-0.041593332	0.563550375	0.976491766
MYBL1	0.083649904	0.563574367	0.976491766
SLC26A2	-0.049762933	0.563647334	0.976491766
CLP1	-0.041502486	0.56380212	0.976491766
SEMA4C	-0.052248117	0.563886298	0.976491766
RBBP8	0.058458138	0.563909127	0.976491766
ASPM	-0.104258526	0.563982862	0.976491766
NRGN	0.128326162	0.564060753	0.976491766
SURF6	-0.052628913	0.56407157	0.976491766
MMP23B	-0.099732115	0.564126964	0.976491766
MYOM1	-0.079504586	0.564168301	0.976491766
THAP6	-0.064420171	0.564472144	0.976491766
CEP170	0.061355471	0.564555287	0.976491766
PPP2R3C	-0.048908649	0.564565778	0.976491766
ZNF292	0.070375465	0.564587236	0.976491766
ROMO1	0.03087823	0.564741594	0.976491766
NGLY1	-0.032325876	0.564784565	0.976491766
BMP6	0.131899413	0.564787137	0.976491766
KIF1C	-0.04568682	0.564864615	0.976491766
UBA2	0.044978967	0.564908504	0.976491766
ANAPC13	-0.041806804	0.564946288	0.976491766
HDDC2	-0.03967239	0.56515107	0.976491766
ADSSL1	-0.061344773	0.565236193	0.976491766
RBX1	0.039730387	0.565243485	0.976491766
TARS	0.034520564	0.565583647	0.976491766
C1orf27	0.058947444	0.565601716	0.976491766
ZNF691	-0.052895779	0.565758411	0.976491766
MCOLN1	0.050984221	0.566233215	0.976491766
PDCD5	0.037273789	0.566315533	0.976491766
CSTB	0.048283714	0.566326034	0.976491766
TTC1	0.028926056	0.566340151	0.976491766
TDRD7	0.037957731	0.566362453	0.976491766
MOAP1	-0.036000487	0.566737963	0.976491766
NRD1	-0.027963597	0.566961545	0.976491766

NPC2	0.030990572	0.567060333	0.976491766
TOR3A	-0.064380754	0.567160309	0.976491766
BBS2	0.056730986	0.567171773	0.976491766
DHRS4	0.033627637	0.567268244	0.976491766
NPRL2	0.035345478	0.567359085	0.976491766
TAF9	0.080368467	0.56736994	0.976491766
TRPC1	-0.068752113	0.567435385	0.976491766
TACC1	-0.020935109	0.567613541	0.976491766
RPS6KA5	-0.047274331	0.567826097	0.976491766
PTDSS1	0.032978807	0.567856094	0.976491766
NDUFB10	0.026679108	0.568041787	0.976491766
PLEKHG2	-0.052095887	0.568148595	0.976491766
ANXA3	-0.294972803	0.568182674	0.976491766
PIP4K2A	0.033211671	0.568194504	0.976491766
PLXNA1	-0.058378309	0.568202049	0.976491766
SLC4A4	0.082442332	0.568322765	0.976491766
PRRC2C	0.030092369	0.56834616	0.976491766
ZNF571	-0.044140043	0.568607587	0.976491766
SLC2A4RG	0.039988973	0.568638393	0.976491766
ANKIB1	-0.027884747	0.568998923	0.976491766
MBD2	0.058300233	0.569002518	0.976491766
MR1	0.059625424	0.569035028	0.976491766
APOBEC3C	-0.055240468	0.569044956	0.976491766
DDX60	-0.063037789	0.569145724	0.976491766
ARRB2	0.044774784	0.569169132	0.976491766
TCF7L2	0.083943045	0.569278594	0.976491766
BSCL2	-0.06969487	0.569385915	0.976491766
TRIM13	-0.126124968	0.5694568	0.976491766
FAM86A	-0.044989048	0.569614928	0.976491766
PRDM10	-0.044058914	0.569746944	0.976491766
FCGR1B	-0.155040703	0.569790228	0.976491766
PTPN4	0.053898414	0.569871282	0.976491766
UCHL3	0.037453955	0.569872006	0.976491766
CALD1	0.168110236	0.569888518	0.976491766
DUSP12	-0.034006854	0.570089085	0.976491766
THOC3	-0.072499694	0.570147186	0.976491766
TUBGCP4	0.043425568	0.570209459	0.976491766
NEXN	0.109544017	0.570284355	0.976491766
SEZ6L2	0.056734828	0.570353118	0.976491766
KISS1R	-0.121000026	0.570362988	0.976491766
YPEL5	0.041310149	0.570379444	0.976491766
C1orf109	-0.04068367	0.57039059	0.976491766
FRMD4B	-0.096684273	0.570415343	0.976491766
NCOA5	0.042751324	0.570468594	0.976491766
CARKD	-0.042107373	0.570483054	0.976491766
FIZ1	0.045262864	0.570498232	0.976491766
DEDD2	0.033380149	0.570550181	0.976491766

NSMCE2	0.062836183	0.57056848	0.976491766
SFXN2	0.040451138	0.570610952	0.976491766
SECISBP2	-0.050560315	0.570611519	0.976491766
DUSP27	0.058865212	0.570619313	0.976491766
EPN2	-0.052107044	0.570764521	0.976491766
ZUFSP	-0.03488105	0.570811819	0.976491766
TMEM176B	-0.263052355	0.570837122	0.976491766
FOXD4L1	0.060946501	0.570856082	0.976491766
GGA1	-0.023004193	0.570929451	0.976491766
CLEC4G	0.090064736	0.571182805	0.976491766
ABHD12	-0.106926087	0.571456396	0.976491766
SKAP1	-0.036796633	0.571578473	0.976491766
BRF2	-0.047949846	0.571612372	0.976491766
MECR	-0.061054312	0.571637216	0.976491766
ATP5G3	-0.059508392	0.571692761	0.976491766
EHD4	0.048883471	0.571847876	0.976491766
BDP1	-0.08564445	0.571877572	0.976491766
COMMD10	-0.052696647	0.572024026	0.976491766
ASPHD2	0.048329606	0.572058029	0.976491766
ARHGEF5	-0.041301213	0.572103112	0.976491766
CHMP4A	0.030184327	0.57233715	0.976491766
PMM1	0.036359241	0.572362031	0.976491766
HERC1	0.033189587	0.572425261	0.976491766
SEMA4F	-0.046317181	0.572516865	0.976491766
ITPRIP	-0.03359093	0.572886072	0.976491766
SNHG5	0.121930504	0.572914896	0.976491766
SLC35B2	-0.036732468	0.572917682	0.976491766
L3MBTL3	-0.058511839	0.57293498	0.976491766
SHCBP1	0.024620884	0.572979529	0.976491766
CLCN3	-0.049377493	0.573005944	0.976491766
HSPD1	-0.029701374	0.573037392	0.976491766
CD200R1	-0.070910281	0.573187004	0.976491766
FAIM	0.070188052	0.573208794	0.976491766
MRPS5	-0.032168026	0.573230222	0.976491766
SNORD17	0.066570778	0.573235396	0.976491766
HSCB	-0.048809294	0.573315169	0.976491766
LRCH4	0.039849588	0.573332291	0.976491766
ATP11B	0.042200688	0.573338058	0.976491766
SLC45A3	0.090875583	0.573384883	0.976491766
LRRCC1	-0.061730058	0.573489742	0.976491766
GDAP1	0.04889575	0.573493382	0.976491766
ZFP1	0.057865545	0.573507295	0.976491766
PDCD2	-0.038294741	0.57361921	0.976491766
CFHR2	0.043667177	0.573647498	0.976491766
BRWD1	0.037753834	0.573681128	0.976491766
CHSY1	-0.032327423	0.573752165	0.976491766
SDHD	-0.055987431	0.573800017	0.976491766

EIF2AK3	0.042388231	0.573943682	0.976491766
RABGAP1	0.039642382	0.573999701	0.976491766
OCM2	-0.075795065	0.574002908	0.976491766
RPS6KA1	0.040127205	0.574117671	0.976491766
PUS7L	-0.050863191	0.574120823	0.976491766
EP400NL	0.076472124	0.574148701	0.976491766
THAP11	0.031601592	0.574224068	0.976491766
LYSMD3	-0.07173883	0.574256858	0.976491766
EMG1	0.027617984	0.574440809	0.976491766
MRPL33	0.051131926	0.574612437	0.976491766
TOR1AIP1	-0.051639815	0.574658499	0.976491766
DCTPP1	0.046447563	0.574786301	0.976491766
EMX2	-0.05224393	0.574828015	0.976491766
MT1F	-0.05612846	0.574960895	0.976491766
HN1L	0.048720627	0.575038948	0.976491766
TRIM21	-0.044929602	0.575065353	0.976491766
SLC16A12	0.021310552	0.575376997	0.976491766
ZNF84	-0.064407651	0.575428186	0.976491766
EPS15	-0.033589828	0.575459526	0.976491766
NLGN2	0.054298052	0.575527473	0.976491766
CR2	0.121948097	0.575588114	0.976491766
RAB10	-0.02223219	0.575626444	0.976491766
PARVB	0.126365963	0.575698147	0.976491766
STX7	-0.047099751	0.575732956	0.976491766
AK4	-0.104571429	0.575758326	0.976491766
DHFRL1	-0.053074801	0.575936953	0.976491766
TNFAIP8	0.055247511	0.576005928	0.976491766
SSTR2	-0.048730117	0.57607282	0.976491766
RPL10A	-0.047840527	0.576075314	0.976491766
TTC19	0.037562849	0.576383274	0.976491766
DTX3L	0.088489152	0.576681532	0.976491766
TRIM44	-0.039163268	0.576825873	0.976491766
XPO4	-0.02658994	0.577073697	0.976491766
MYLK	0.133542937	0.57724012	0.976491766
ZBP1	-0.060118351	0.577406377	0.976491766
STX16	-0.074649892	0.577445561	0.976491766
GTF2H4	-0.052715608	0.577504026	0.976491766
ARHGAP17	0.033852409	0.577604547	0.976491766
SUV420H2	0.056787026	0.577622175	0.976491766
PTGER4	0.041506428	0.577742032	0.976491766
PCNX	0.035006348	0.577809169	0.976491766
RPL28	0.04297498	0.577864896	0.976491766
CCNB1IP1	0.039934749	0.577900429	0.976491766
SMPD4	0.057640823	0.577920536	0.976491766
NUP85	0.025332233	0.578227105	0.976491766
WDR24	-0.045740464	0.578349573	0.976491766
GRM2	0.064237844	0.57835862	0.976491766

ANG	0.104783126	0.578481758	0.976491766
SLC25A36	0.058630315	0.578638569	0.976491766
HIST2H2AA4	0.103914567	0.578665696	0.976491766
LRIG2	-0.046714899	0.57889616	0.976491766
SMAD7	0.077977059	0.578998306	0.976491766
PDCD2L	0.052524403	0.579028297	0.976491766
ANO6	0.062853446	0.579177682	0.976491766
BMF	-0.044681388	0.579213164	0.976491766
RAVER2	-0.152858726	0.579357288	0.976491766
NUDT2	-0.078387846	0.579404373	0.976491766
OR7E156P	0.052051122	0.579432297	0.976491766
SLU7	0.032355132	0.579531406	0.976491766
TACO1	-0.031403213	0.579579316	0.976491766
DYRK1A	-0.031050467	0.579629938	0.976491766
PTGES2	0.070830175	0.579649882	0.976491766
UNC93B1	-0.041357591	0.579668171	0.976491766
ETV3	-0.063290656	0.579849609	0.976491766
UBN2	-0.050533757	0.579898587	0.976491766
ERF	-0.039236798	0.579902381	0.976491766
TEKT1	-0.044554301	0.579928172	0.976491766
HAGHL	-0.050886569	0.579945342	0.976491766
DCLRE1B	-0.06209891	0.580201398	0.976491766
CILP	-0.049280873	0.580212296	0.976491766
ZNF322	-0.038293753	0.580431859	0.976491766
TRAK2	0.034082594	0.580532343	0.976491766
CD48	0.0291958	0.580571228	0.976491766
FOXM1	-0.047584686	0.580670724	0.976491766
SPTLC2	0.052754197	0.580830533	0.976491766
CTSB	-0.043628352	0.580850342	0.976491766
TNFSF13	-0.039630946	0.580897645	0.976491766
ATP8B4	-0.061835902	0.580991583	0.976491766
ADORA1	-0.084873811	0.580996013	0.976491766
CCDC18	-0.054823755	0.581121711	0.976491766
RPL7	0.022650228	0.581199535	0.976491766
C9orf66	-0.067954638	0.581249819	0.976491766
BDH1	-0.055548249	0.581364463	0.976491766
PAN3	-0.0442116	0.581372098	0.976491766
CNOT2	-0.029371275	0.581675847	0.976491766
IRS1	0.065528744	0.581701325	0.976491766
ZMAT2	0.037227097	0.581720274	0.976491766
CD68	0.03750994	0.581832034	0.976491766
VWDE	0.080933749	0.581871237	0.976491766
RRP1B	-0.038068978	0.581880296	0.976491766
PTPRK	0.081824495	0.581952797	0.976491766
CD320	0.069448942	0.581956402	0.976491766
C5	-0.051848044	0.582022714	0.976491766
TUBB6	0.087330114	0.582209754	0.976491766

ACBD3	0.033848018	0.582307513	0.976491766
FAM167A	-0.134926508	0.582377059	0.976491766
NINJ2	0.067624339	0.582384116	0.976491766
SYBU	0.055253568	0.582492149	0.976491766
LRP1	0.052104413	0.582505798	0.976491766
TMEM80	0.045408382	0.582543631	0.976491766
PTGES3	0.049307423	0.58265589	0.976491766
SIGMAR1	-0.036546276	0.582767587	0.976491766
B3GALT2	-0.119768273	0.582885202	0.976491766
RNASE6	0.087693168	0.582992592	0.976491766
RRM1	0.037768488	0.5830297	0.976491766
USP18	0.105322251	0.583160811	0.976491766
ECD	0.043446993	0.583174731	0.976491766
HDAC4	-0.079580811	0.583252847	0.976491766
ACTR2	-0.032278345	0.583303683	0.976491766
CMTM7	-0.036508966	0.583356105	0.976491766
MIDN	0.042302941	0.583380698	0.976491766
CCDC126	-0.06669465	0.583427106	0.976491766
SNORD12	0.05472152	0.583632414	0.976491766
SNORA13	0.059590223	0.583697393	0.976491766
BEND3	0.056535884	0.583703749	0.976491766
THEM4	0.098810072	0.583723915	0.976491766
SLAMF8	-0.101067535	0.583805937	0.976491766
CD164	0.054461279	0.583944452	0.976491766
KLHL7	-0.042194905	0.584021188	0.976491766
PBRM1	0.045385555	0.584155387	0.976491766
TRAF3IP3	0.029162149	0.584173465	0.976491766
ZBTB43	-0.061730347	0.584189606	0.976491766
CDC42EP4	0.059699211	0.584226982	0.976491766
CYBRD1	-0.075752751	0.584284356	0.976491766
FAM73B	0.049527869	0.584309026	0.976491766
CD300A	0.05677091	0.584353441	0.976491766
FBXO41	0.052798384	0.584374147	0.976491766
GOLGB1	0.083710185	0.584419968	0.976491766
NFKBIB	-0.037904499	0.584464068	0.976491766
ARPP19	0.029035583	0.584625372	0.976491766
ACADS	0.051893107	0.584689043	0.976491766
CHEK1	-0.070532428	0.584824105	0.976491766
EZR	-0.031001249	0.584832033	0.976491766
CRK	-0.041624897	0.584882262	0.976491766
ELMO2	-0.034691157	0.584914121	0.976491766
CXCL16	-0.059688829	0.585036694	0.976491766
C6orf136	-0.026610115	0.585058537	0.976491766
LRRC8B	0.054091979	0.585217291	0.976491766
DUSP1	0.090748673	0.585239425	0.976491766
EPHB2	-0.057368781	0.585241153	0.976491766
EFHD2	0.036037614	0.585274409	0.976491766

GNA15	0.042782129	0.585309765	0.976491766
FLOT1	0.052463466	0.58541823	0.976491766
GPR68	0.094256736	0.585481032	0.976491766
DCAF15	0.050026995	0.585734429	0.976491766
PHOSPHO2	-0.076917522	0.585756924	0.976491766
HTRA4	-0.064675659	0.585933527	0.976491766
SHISA3	0.052667151	0.586087856	0.976491766
FCGBP	0.123916894	0.586138821	0.976491766
AKR1B1	-0.032400043	0.586186643	0.976491766
MBOAT1	-0.041533612	0.586225491	0.976491766
EIF2A	-0.032749927	0.586304882	0.976491766
C15orf26	0.179284574	0.586444641	0.976491766
NKG7	0.072905397	0.586560081	0.976491766
CISD3	0.05574874	0.586580274	0.976491766
CDC26	0.027909984	0.586612501	0.976491766
IRF7	0.066954828	0.586614132	0.976491766
ACOT4	-0.086058868	0.587146114	0.976491766
KLRC1	0.166294776	0.587160913	0.976491766
PDGFD	0.095776713	0.587231747	0.976491766
CASP1	0.038213321	0.587369087	0.976491766
TMOD1	0.104192375	0.587471029	0.976491766
ASPSCR1	0.03182036	0.587501222	0.976491766
FANCA	0.05955911	0.587557044	0.976491766
NINJ1	-0.065554457	0.587559299	0.976491766
PYGB	-0.06031211	0.58765398	0.976491766
MTR	0.047155172	0.587675781	0.976491766
SPATA2L	0.04036807	0.587819024	0.976491766
DDX23	-0.033619409	0.587826288	0.976491766
YY1AP1	-0.022560603	0.587957518	0.976491766
PTK6	-0.049378943	0.588133894	0.976491766
H2AFZ	0.028741056	0.588135076	0.976491766
TBC1D2	-0.056067403	0.588139514	0.976491766
NOMO1	0.07622564	0.588211678	0.976491766
ATP5H	-0.028198638	0.588223375	0.976491766
TM7SF3	-0.047627938	0.58824834	0.976491766
IRF1	-0.051675977	0.588348554	0.976491766
ABI2	0.034980216	0.588352703	0.976491766
PPP2CA	0.020836861	0.588452222	0.976491766
SDCCAG3	0.035449518	0.588571546	0.976491766
FBXL19	0.084003038	0.588602851	0.976491766
WDFY2	-0.056630186	0.588728084	0.976491766
MAPK14	0.068096047	0.58874307	0.976491766
SLC25A29	-0.09239386	0.588756685	0.976491766
PHF5A	0.028950256	0.589005055	0.976491766
SRI	0.110031926	0.589098719	0.976491766
HSPB11	0.046322765	0.589128611	0.976491766
C9orf78	-0.029660305	0.589142324	0.976491766

C9orf169	0.040708996	0.589343289	0.976491766
COIL	-0.025152618	0.589359626	0.976491766
DENND4A	-0.040705504	0.589439063	0.976491766
TOLLIP	-0.055860001	0.589440174	0.976491766
DDX58	0.057968926	0.589460671	0.976491766
PRIM2	0.065760309	0.58948599	0.976491766
AP1G1	-0.035242805	0.589522681	0.976491766
C15orf54	0.102510302	0.589543049	0.976491766
ACBD4	-0.048765435	0.589571066	0.976491766
TRMT5	0.029601192	0.589685634	0.976491766
GIN1	-0.042998257	0.589715833	0.976491766
C1D	0.050072699	0.589756378	0.976491766
IGF1R	0.054349063	0.589819229	0.976491766
SHPK	-0.102331747	0.589852958	0.976491766
IVNS1ABP	-0.050577829	0.589865882	0.976491766
ENO2	-0.057659191	0.589917828	0.976491766
SIL1	-0.04831104	0.590197199	0.976526297
CNN3	-0.08520991	0.590228892	0.976526297
DHX32	0.046268491	0.59028672	0.976526297
TTC34	-0.043505933	0.590350167	0.976526297
FAF1	0.031867144	0.590379798	0.976526297
OAS3	0.111902839	0.590764224	0.977016166
GNB1L	0.043264075	0.59086268	0.977024726
COG2	0.034961853	0.590945934	0.977024726
FLAD1	0.028867103	0.591408516	0.977445041
FAHD1	0.047933324	0.591426256	0.977445041
RMND1	-0.053255036	0.591465072	0.977445041
CBFA2T3	-0.063427064	0.591569944	0.977472415
ZNF611	0.046011987	0.591780718	0.977592163
GGA3	-0.046852548	0.59191645	0.977592163
FLYWCH1	0.073023674	0.591959604	0.977592163
PARP4	-0.03458642	0.592047685	0.977592163
UHRF1BP1L	-0.055278848	0.592246697	0.977592163
XPO5	-0.039411716	0.592260264	0.977592163
AKAP7	0.044451992	0.592290519	0.977592163
HPS1	0.0636356	0.592381632	0.977592163
EPN1	-0.028036866	0.592667101	0.977592163
LAIR2	0.174108583	0.592696395	0.977592163
ATP6V1D	0.027981843	0.592698898	0.977592163
AFAP1	0.075075219	0.592945883	0.977592163
RASIP1	0.064030256	0.593085677	0.977592163
PIGY	0.03432063	0.593097678	0.977592163
PDK1	0.068842659	0.593424323	0.977592163
RPL34	-0.043429531	0.593459403	0.977592163
SCAF4	-0.041004823	0.593623481	0.977592163
ZNF764	0.060063732	0.593784768	0.977592163
PEX5	-0.031606188	0.593823711	0.977592163

DYNC1LI1	-0.05875492	0.593880285	0.977592163
PARP6	-0.035053956	0.593958086	0.977592163
WDR82	0.033877963	0.594021317	0.977592163
KIAA1586	0.057575258	0.594125424	0.977592163
BTBD3	0.038466304	0.594153445	0.977592163
CCNL1	-0.036289063	0.594161302	0.977592163
CTGF	0.061065933	0.594176816	0.977592163
CNTNAP3B	0.084907118	0.59427535	0.977592163
CCDC101	0.041981319	0.594295019	0.977592163
MSH6	0.030557314	0.594311335	0.977592163
SEC13	-0.043719682	0.594514167	0.977592163
FOSB	0.083823534	0.594595196	0.977592163
MON1A	0.049271964	0.594603088	0.977592163
DSCAM	-0.043231447	0.594857196	0.977592163
OS9	-0.041475741	0.594995643	0.977592163
ZNF75A	-0.049587976	0.595027748	0.977592163
ACSM3	0.054655305	0.595034425	0.977592163
RBL2	0.047209782	0.595092593	0.977592163
DAZAP2	-0.028701692	0.59516394	0.977592163
ANKZF1	-0.042916349	0.595252268	0.977592163
LRP11	-0.053837323	0.595273488	0.977592163
DNAJA3	-0.026778158	0.595302297	0.977592163
CDC7	-0.071672245	0.595351772	0.977592163
HAUS6	-0.061936686	0.595583321	0.977763382
NR2C1	-0.044396166	0.595632712	0.977763382
C5orf15	-0.025492515	0.59622615	0.977946999
GZMK	-0.105921382	0.596232793	0.977946999
ARHGAP22	0.07989893	0.596441844	0.977946999
EIF2B2	-0.047732791	0.59657918	0.977946999
PRRC2B	0.049100058	0.596581644	0.977946999
MNAT1	-0.040503957	0.596685742	0.977946999
EIF2D	0.032504602	0.596758112	0.977946999
CPD	-0.044841686	0.596971773	0.977946999
XPNPEP1	0.032926185	0.597025186	0.977946999
LRFN3	-0.043141125	0.597181252	0.977946999
ANKRD39	-0.039312409	0.597198682	0.977946999
CLEC5A	0.113524803	0.597374122	0.977946999
SRSF9	0.024024127	0.597414251	0.977946999
PTGS1	0.119109485	0.597532641	0.977946999
ABCA11P	-0.043013523	0.597534912	0.977946999
ACO1	-0.029198654	0.597597005	0.977946999
ZNF85	0.098480885	0.597799863	0.977946999
HIST1H3D	0.067921893	0.597824738	0.977946999
ATL2	0.042796859	0.597888157	0.977946999
HEATR6	-0.039544086	0.597920495	0.977946999
IFRD2	0.046241258	0.597933008	0.977946999
NCOR1	0.035021273	0.597973997	0.977946999

BNIP3L	-0.057361603	0.598007278	0.977946999
DNASE1L3	0.113855161	0.598058198	0.977946999
ISM1	0.094774404	0.598130322	0.977946999
MS4A6A	-0.057768571	0.598204616	0.977946999
SKAP2	0.051239937	0.598213902	0.977946999
PIAS1	0.032924994	0.598218369	0.977946999
RPUSD1	-0.056762451	0.598416077	0.978007395
UBE4B	-0.026007179	0.598458084	0.978007395
ZGPAT	0.02417396	0.598587879	0.978007395
TRAPPC1	0.03787092	0.598714909	0.978007395
NGRN	-0.042386871	0.598781273	0.978007395
TDP2	0.057924951	0.598785447	0.978007395
ZNF354A	-0.053111738	0.598938125	0.978112439
PRRC1	-0.028296577	0.599096293	0.978226416
ZC3H15	-0.029207888	0.599352727	0.978500788
TMEM156	0.055590087	0.599529666	0.978645314
HDHD3	-0.05542632	0.599701825	0.978781997
PEX11A	-0.054654609	0.59986462	0.978893901
TRAP1	-0.045317669	0.600037622	0.978893901
ASXL2	-0.036010633	0.600232482	0.978893901
HAUS2	-0.07043365	0.600336341	0.978893901
CFB	-0.062873396	0.600436481	0.978893901
ARHGDIA	-0.045318019	0.600502832	0.978893901
SNORA80	0.063002145	0.600546971	0.978893901
CAMK2D	0.067440945	0.600570777	0.978893901
FHIT	-0.109527598	0.600742439	0.978893901
LILRB5	-0.050675089	0.600808051	0.978893901
TNRC6B	0.054251058	0.600867177	0.978893901
UNG	0.043344961	0.601103113	0.978893901
OAS2	0.063148942	0.601351627	0.978893901
SAP18	-0.029469311	0.601512418	0.978893901
RAD51AP1	-0.05981344	0.601530724	0.978893901
IRF2BPL	-0.082048143	0.601570116	0.978893901
TMX4	0.047568393	0.601705313	0.978893901
ERH	-0.03746032	0.601795441	0.978893901
MAML3	-0.068289697	0.60187101	0.978893901
PKDCC	0.042711802	0.602051872	0.978893901
CCDC151	0.073024379	0.602069741	0.978893901
MYADML2	0.061940626	0.602077932	0.978893901
RPS8	0.028644501	0.602230691	0.978893901
ZFP36	0.03954968	0.602279876	0.978893901
BTG3	0.041404005	0.602288689	0.978893901
RAD54L	-0.046596315	0.602298314	0.978893901
TMEM8B	-0.049039261	0.602347111	0.978893901
CYFIP2	0.035913626	0.60259203	0.978893901
CLCN7	-0.035850533	0.602692032	0.978893901
SLC39A7	0.051963671	0.60286472	0.978893901

CYTH1	-0.028735562	0.603061892	0.978893901
TPCN1	-0.049641958	0.603159881	0.978893901
MBLAC2	-0.046840516	0.603197288	0.978893901
SLC25A20	0.052924148	0.603400449	0.978893901
NUDT5	0.041491871	0.603475403	0.978893901
ASRGL1	0.075275823	0.603516164	0.978893901
TMCO4	-0.04582711	0.603521079	0.978893901
FNDC3A	-0.038769202	0.603587519	0.978893901
EFR3B	-0.062582053	0.603670951	0.978893901
FAM20B	0.022947019	0.603725773	0.978893901
CCR9	-0.090352226	0.603785746	0.978893901
TBC1D24	0.062027576	0.603824673	0.978893901
GPR146	0.060768662	0.603825533	0.978893901
FIBCD1	0.039826022	0.603921149	0.978893901
HCST	-0.032395935	0.603989911	0.978893901
RNF31	-0.044573944	0.603991307	0.978893901
MED13	-0.054336997	0.604090262	0.978893901
LACTB	0.060946232	0.604197606	0.978893901
MX2	0.064106522	0.604248072	0.978893901
NEMF	0.039303334	0.604294555	0.978893901
PARL	-0.028204011	0.604339639	0.978893901
MOV10	0.049277065	0.604423182	0.978893901
TXN2	0.053965527	0.604512816	0.978893901
C14orf159	-0.033723657	0.604589617	0.978893901
ARHGDIB	0.014855254	0.604634879	0.978893901
LGALS1	0.051628245	0.604788652	0.978893901
IMMT	0.029254628	0.604842044	0.978893901
BANP	0.029287284	0.604916367	0.978893901
KRTCAP3	-0.062400345	0.60498809	0.978893901
RPS19	0.013601438	0.605216324	0.979004145
HIGD2A	-0.036275259	0.605311404	0.979004145
SIAH2	0.042786387	0.605321562	0.979004145
NDC80	0.053485795	0.605652879	0.97922496
SRPRB	0.033171122	0.605711044	0.97922496
GYG1	-0.038089957	0.605723489	0.97922496
STXBP5	0.037312185	0.605901369	0.979369488
ZMYND15	-0.075678285	0.606014526	0.979409371
KAT2A	-0.041676478	0.606184687	0.979541358
USP35	-0.043104187	0.60651824	0.979937294
SUZ12P	0.061472208	0.606798628	0.979946759
NSD1	0.039115199	0.607398661	0.979946759
DNAJC7	-0.030260329	0.607502578	0.979946759
IBTK	-0.032364051	0.607554102	0.979946759
PLA2G4C	-0.155174944	0.607583359	0.979946759
SREK1	-0.035065005	0.60787044	0.979946759
PTGES	-0.043346793	0.607953464	0.979946759
MORC3	0.061455485	0.608012709	0.979946759

WDR54	0.030933566	0.608189364	0.979946759
PDXDC1	-0.024480546	0.608202172	0.979946759
PROP1	-0.049375357	0.608283898	0.979946759
ATG2A	-0.036604675	0.608293179	0.979946759
PDE9A	-0.111993096	0.608311393	0.979946759
RYK	0.027577354	0.608381268	0.979946759
TREML2	-0.048935055	0.608396041	0.979946759
PDGFRB	-0.132520581	0.608685835	0.979946759
ANKRD54	0.043512721	0.608784115	0.979946759
AQP7P1	0.057897917	0.608860025	0.979946759
GGCT	0.03385631	0.608863035	0.979946759
ZNF876P	0.026346474	0.60888488	0.979946759
SAMD3	-0.064119949	0.609412291	0.979946759
NOP58	0.038864381	0.609423917	0.979946759
MXD4	-0.032811396	0.609589749	0.979946759
ZNF706	-0.028103605	0.60961807	0.979946759
ZKSCAN5	-0.037686033	0.609661828	0.979946759
NEU1	0.037375635	0.609841733	0.979946759
SBF2	0.044454587	0.609988743	0.979946759
VPS41	-0.083843191	0.610117772	0.979946759
MUC6	0.069468363	0.610136235	0.979946759
MRE11A	0.065458554	0.610159551	0.979946759
NOTCH1	-0.043906075	0.610165609	0.979946759
SMARCAL1	-0.02339176	0.610307314	0.979946759
RELA	0.048292654	0.610316769	0.979946759
CHCHD7	0.025354993	0.610427093	0.979946759
SERP1	-0.042426498	0.610490901	0.979946759
ADAM15	-0.038757944	0.610747793	0.979946759
ZNF580	0.051950342	0.610868398	0.979946759
RNF111	-0.05282007	0.61104388	0.979946759
TTC3	-0.034040273	0.611050784	0.979946759
PPFIBP1	0.044649883	0.611052487	0.979946759
SNX27	-0.036513451	0.611052833	0.979946759
KIAA0247	0.028414597	0.611066378	0.979946759
CMIP	-0.042300591	0.611241225	0.979946759
PGAP2	0.029022109	0.611306523	0.979946759
CKB	-0.095738977	0.611353451	0.979946759
NIPA1	-0.045340382	0.611359622	0.979946759
SNORD96A	-0.049286617	0.611388579	0.979946759
PFKP	-0.058700334	0.61140494	0.979946759
CHST14	0.056833704	0.611426734	0.979946759
SHMT1	0.070091006	0.611467207	0.979946759
POLR2C	0.045735169	0.611478137	0.979946759
TMEM165	-0.040970723	0.61156763	0.979946759
ST3GAL4	0.058262626	0.611819613	0.979946759
HMG20A	0.041726371	0.611903228	0.979946759
MYOF	0.075220946	0.611997423	0.979946759

C5AR1	0.06879455	0.612014595	0.979946759
RAPGEFL1	-0.046035537	0.612031577	0.979946759
KIAA1522	-0.053257625	0.612355578	0.979946759
RCAN1	0.038446146	0.612384995	0.979946759
FKBP5	-0.105303837	0.612418676	0.979946759
TMPO	-0.061247454	0.612452507	0.979946759
EPS8	-0.074185832	0.612595754	0.979946759
CWF19L2	0.034826588	0.61259826	0.979946759
BPGM	0.062206105	0.612685338	0.979946759
MAP3K10	-0.037515179	0.612720416	0.979946759
SCRN2	0.044527543	0.612901198	0.979946759
APOBR	-0.041988312	0.612970698	0.979946759
IL10RA	0.032060084	0.613058543	0.979946759
NBAS	0.032831603	0.613119873	0.979946759
ATP5J2	0.023197671	0.613190488	0.979946759
UNC5C	-0.043235957	0.613281966	0.979946759
TNFAIP6	-0.080055509	0.613578303	0.979946759
PRDX3	-0.071697839	0.613665408	0.979946759
SPPL2A	-0.027978309	0.613680738	0.979946759
TRIAP1	0.028721644	0.613770253	0.979946759
TTC30B	-0.043946116	0.613832682	0.979946759
ZNF776	0.047489912	0.613912639	0.979946759
LRRC3	-0.041369649	0.614038306	0.979946759
WDFY4	0.058653812	0.614172869	0.979946759
SIPA1	0.027218342	0.614218082	0.979946759
RPL38	0.013407006	0.614230645	0.979946759
PPIP5K1	0.040772713	0.614756986	0.979946759
MAP3K9	0.054137601	0.614836274	0.979946759
FKBP8	-0.04336073	0.61485797	0.979946759
PARP10	-0.04072574	0.615017907	0.979946759
C3orf58	-0.037603482	0.615048015	0.979946759
SARS2	-0.04602847	0.615057455	0.979946759
ASNS	0.053472702	0.615282079	0.979946759
MRPS28	0.040941522	0.615292354	0.979946759
ZAN	0.047550425	0.615332099	0.979946759
PRAM1	-0.051030802	0.615491338	0.979946759
MED10	0.032478192	0.615710846	0.979946759
KLHDC4	0.056978413	0.615820628	0.979946759
IER3IP1	0.052678854	0.61586707	0.979946759
GTF2IP1	0.063788337	0.61589152	0.979946759
PDZD8	-0.047194187	0.616008698	0.979946759
LTBP1	0.062050771	0.616113358	0.979946759
HIST1H2BG	0.171525307	0.61611796	0.979946759
MSX1	-0.043542329	0.616121151	0.979946759
TMEM126A	0.037079433	0.616165589	0.979946759
SH3BGR	-0.05176101	0.616168665	0.979946759
ROGDI	0.08026557	0.616184574	0.979946759

MDM2	0.041585577	0.616269123	0.979946759
LLGL2	0.054110327	0.616270622	0.979946759
BTBD1	-0.035399109	0.616302552	0.979946759
TBRG1	0.045001799	0.616340617	0.979946759
MX1	0.092251237	0.616345618	0.979946759
AGK	0.035809156	0.616706617	0.979946759
RPL35	0.026863812	0.616712118	0.979946759
RALA	-0.02557377	0.616722219	0.979946759
GANAB	0.031571868	0.616772216	0.979946759
ALDH4A1	-0.054173732	0.616824914	0.979946759
CLUAP1	-0.015635031	0.616839042	0.979946759
TTC37	-0.028331615	0.616980244	0.979946759
TP73-AS1	-0.040624174	0.617068063	0.979946759
ITM2C	-0.075639195	0.617082925	0.979946759
ANAPC2	0.048218765	0.617092605	0.979946759
SLC7A5P1	0.013646374	0.617114972	0.979946759
CAV2	-0.132610553	0.617304235	0.979946759
CSK	0.039355551	0.617344389	0.979946759
RFX1	-0.046217958	0.617434365	0.979946759
BAHD1	0.050355487	0.617462507	0.979946759
RRP36	0.023079209	0.617576831	0.979946759
COX11	0.065202413	0.61763668	0.979946759
SLC10A7	0.04370709	0.617777269	0.979946759
SCAMP1	-0.021240198	0.617873557	0.979946759
SQSTM1	0.031159527	0.617958771	0.979946759
SLC17A3	0.049473348	0.618034936	0.979946759
CHMP4C	0.039466077	0.618041339	0.979946759
P4HA2	-0.043016027	0.618332602	0.979946759
ZNHIT2	-0.039167634	0.618432679	0.979946759
PLK4	-0.049792295	0.618433441	0.979946759
HEMGN	0.110114548	0.618512616	0.979946759
TIMM22	0.046918338	0.618673461	0.979946759
FAHD2A	-0.045599941	0.618751814	0.979946759
SHARPIN	0.045556563	0.618761803	0.979946759
DZIP1L	-0.079016775	0.618782308	0.979946759
TMEM194B	0.04586086	0.618972289	0.979946759
DAK	-0.040791727	0.61898952	0.979946759
ZNF259	0.032746014	0.619078476	0.979946759
ABHD16A	0.029772824	0.619102676	0.979946759
SYPL1	0.038539022	0.619105669	0.979946759
C11orf31	-0.055082851	0.61941034	0.979946759
MTBP	-0.047530846	0.619433128	0.979946759
C2orf40	-0.077945372	0.619476262	0.979946759
ZBTB45	-0.032753169	0.61948381	0.979946759
HAUS1	0.064192184	0.619538117	0.979946759
AKIRIN1	0.040245348	0.619973392	0.980294798
RNF44	-0.033537539	0.620015963	0.980294798

TXNDC11	0.041241437	0.62002384	0.980294798
SNX17	0.029462691	0.620248548	0.980510023
GLG1	-0.021985807	0.620471635	0.980550723
DNAJC13	0.037943936	0.620525073	0.980550723
MAP1S	0.038480307	0.620546591	0.980550723
TOR2A	0.045558109	0.62069212	0.980550723
RPL4	-0.02884065	0.620717221	0.980550723
PPP2R3A	-0.073341002	0.620954623	0.980570137
NMB	0.053989084	0.620977725	0.980570137
TRIM38	-0.067729231	0.621244774	0.980570137
MAP1A	-0.074263727	0.621272052	0.980570137
ATF5	-0.040694468	0.621290791	0.980570137
TRIOBP	0.037437784	0.621307941	0.980570137
ZC3H12A	-0.043717297	0.62134962	0.980570137
ABCC6	-0.038571534	0.621652192	0.980653661
PPP1R9B	0.040253205	0.621662442	0.980653661
DOCK7	-0.050792282	0.621687825	0.980653661
TLE2	-0.057245855	0.621756924	0.980653661
DENND1C	-0.038022449	0.621998044	0.980710919
SERPINA10	-0.053835993	0.622051631	0.980710919
AIM1	-0.035455717	0.62231223	0.980710919
PRMT10	0.035362278	0.622341242	0.980710919
CALM2	-0.029680241	0.622352813	0.980710919
DHRS3	0.049982851	0.622402891	0.980710919
TMEM204	-0.072708758	0.622619409	0.980710919
CPSF6	-0.050637207	0.622666866	0.980710919
CCDC24	-0.055630312	0.62269223	0.980710919
ZNF33B	-0.029656512	0.622743595	0.980710919
C9orf9	0.039525291	0.622787973	0.980710919
ZG16B	0.07048544	0.622869944	0.980710919
NDUFV2	0.030859528	0.623157994	0.980710919
ZFP36L2	0.050478025	0.623190058	0.980710919
TOP1MT	-0.050838676	0.623215424	0.980710919
MEX3A	0.042660782	0.623329308	0.980710919
GYPB	-0.082616374	0.623541825	0.980710919
B4GALT2	-0.046630931	0.623708224	0.980710919
ADD1	-0.021958229	0.623833033	0.980710919
SF3B14	0.043237606	0.623945512	0.980710919
ZNF276	-0.046608146	0.624006917	0.980710919
CDK1	-0.079681942	0.624024983	0.980710919
CLIC3	0.111573919	0.624033554	0.980710919
GBP3	-0.07592427	0.624088617	0.980710919
TULP3	-0.044094245	0.624131235	0.980710919
MRPS12	0.029624443	0.624158524	0.980710919
SPAG7	0.037547692	0.624212831	0.980710919
RAPGEF2	-0.040692952	0.624318126	0.980710919
ZNF737	0.052571502	0.624514164	0.980710919

ACTA2	-0.122351578	0.624644231	0.980710919
PSG6	0.061431994	0.62475365	0.980710919
ADAM9	-0.048808698	0.624787572	0.980710919
ZNF43	-0.058107789	0.624843224	0.980710919
CDKN2AIP	-0.032696175	0.624853454	0.980710919
MEX3B	-0.041457852	0.624894219	0.980710919
STK38	-0.03253467	0.625152242	0.980976775
SNORA62	0.052158027	0.625394536	0.981138597
FAH	0.060165328	0.625432645	0.981138597
TMEM91	-0.048508548	0.62572988	0.981237289
DOCK3	0.044614534	0.625817884	0.981237289
HPSE	-0.067064733	0.625818875	0.981237289
CCNA2	-0.07870146	0.625850145	0.981237289
RPL8	0.041636522	0.626024554	0.981280436
IGFBP6	-0.046736976	0.62610492	0.981280436
HCN3	-0.052073162	0.626182827	0.981280436
SPSB1	0.053670661	0.62623227	0.981280436
SMOX	0.107941871	0.626343849	0.981316358
PROCA1	-0.043982891	0.626642231	0.98150048
TMEM106C	-0.046075621	0.626725387	0.98150048
MT1JP	-0.067049654	0.626760806	0.98150048
RBM45	-0.031413397	0.626883679	0.98150048
MIR574	0.04566968	0.62691614	0.98150048
VGLL4	0.035294464	0.626993396	0.98150048
FLI1	-0.029266564	0.627228063	0.981728992
NLRP3	-0.049688602	0.627350439	0.981781706
CPS1	0.061487768	0.627665326	0.982135636
DARS2	-0.039092641	0.627788967	0.982190258
TFAP4	-0.050507724	0.627935046	0.982261709
SLC8A1	-0.078581617	0.628388165	0.982261709
GOLGA3	-0.027076305	0.628420009	0.982261709
NFE2	-0.045335918	0.628421306	0.982261709
XAB2	-0.035798526	0.628596516	0.982261709
KCTD12	-0.061450439	0.628640158	0.982261709
IFI27L1	0.043759389	0.628806628	0.982261709
VWA3A	0.043104515	0.628835461	0.982261709
HLA-A	-0.588644924	0.628846725	0.982261709
GBAS	-0.044216804	0.628868651	0.982261709
CC2D1A	-0.040865685	0.629073563	0.982261709
ZBTB5	-0.033657745	0.629424036	0.982261709
TNIP1	-0.029559743	0.629654898	0.982261709
SERINC5	-0.078686269	0.629901841	0.982261709
HEY1	0.036626365	0.630686231	0.982261709
CD81	0.037812526	0.630964439	0.982261709
GPM6A	0.059559392	0.630983808	0.982261709
RANBP9	0.044180458	0.631213732	0.982261709
TPRG1L	0.030706648	0.631240193	0.982261709

PIP4K2B	-0.058453605	0.63124605	0.982261709
LRRC26	0.096535282	0.631280511	0.982261709
S100P	0.158678029	0.631925184	0.982261709
CDT1	-0.073068113	0.631991536	0.982261709
ERLIN1	-0.042049134	0.632044503	0.982261709
SNORD69	0.040889614	0.632065982	0.982261709
POT1	-0.044215763	0.6322023	0.982261709
PRR5L	-0.083720249	0.632299479	0.982261709
FBXO4	0.032938047	0.632318021	0.982261709
ZNF615	0.033944308	0.632496107	0.982261709
IDO2	0.06033918	0.632716709	0.982261709
IRF3	-0.039991637	0.632761881	0.982261709
RCL1	-0.047955288	0.632795029	0.982261709
MED11	-0.032829768	0.63285708	0.982261709
LPP	0.042396509	0.632918062	0.982261709
ATP6V0A1	0.036825172	0.633024597	0.982261709
OXNAD1	-0.055089988	0.633037615	0.982261709
ANXA4	0.065215823	0.633061966	0.982261709
LGALS7	0.046896447	0.633176637	0.982261709
TRPC2	0.046055752	0.633176968	0.982261709
UEVLD	-0.041409779	0.633316671	0.982261709
DHX30	0.032784782	0.633389286	0.982261709
MIIP	0.056603891	0.633426991	0.982261709
CMAS	0.027437286	0.633549839	0.982261709
RALBP1	0.03095766	0.633703647	0.982261709
PHF14	-0.030993065	0.633760153	0.982261709
ADCY10	-0.054755047	0.633770425	0.982261709
PRSS53	0.046509442	0.633832151	0.982261709
ATP2B1	-0.037484245	0.634046791	0.982261709
P2RX1	0.091776441	0.634103331	0.982261709
SDCBP	-0.063029979	0.634182059	0.982261709
ARRDC5	-0.048364131	0.634345784	0.982261709
RHOBTB3	0.103186731	0.634443854	0.982261709
COX5A	0.036497503	0.634468255	0.982261709
HRSP12	-0.040011379	0.634503818	0.982261709
SLC29A1	0.052833794	0.634514644	0.982261709
PTPLA	0.057839434	0.634524682	0.982261709
NFKBIA	0.03804534	0.634672572	0.982261709
TMPRSS15	-0.042302498	0.634708888	0.982261709
DYTN	0.065555742	0.634887642	0.982261709
NUPL1	-0.040048411	0.634887831	0.982261709
MEGF6	-0.059520268	0.634899109	0.982261709
ABCA1	-0.077311505	0.634954915	0.982261709
GBP4	0.061771095	0.634968336	0.982261709
CCT4	-0.034586437	0.635062562	0.982261709
DRD3	-0.078890946	0.635196455	0.982261709
ANKRD33	0.05955097	0.635203261	0.982261709

CWC22	0.053565888	0.635210985	0.982261709
PTGDR	-0.096017895	0.635216696	0.982261709
PIGU	0.027444638	0.635227767	0.982261709
BSN-AS2	-0.095518429	0.635243082	0.982261709
NARF	-0.036072576	0.635322448	0.982261709
FMNL3	-0.038920794	0.635513937	0.982261709
DPEP2	-0.037085087	0.635578007	0.982261709
HIRIP3	0.031013778	0.635895567	0.982261709
ZFP62	-0.044246511	0.635898846	0.982261709
VPS45	0.027204909	0.635979729	0.982261709
MCM4	-0.052594527	0.63607185	0.982261709
YWHAQ	-0.031385042	0.636167136	0.982261709
ZNF585A	0.042602784	0.636237484	0.982261709
BCLAF1	0.022786599	0.636338777	0.982261709
SERINC3	-0.040581557	0.636515258	0.982261709
DCTN5	0.039491455	0.636527293	0.982261709
ZNF577	-0.107084424	0.636644168	0.982261709
PM20D2	0.036955149	0.636723924	0.982261709
INPP5D	-0.036498276	0.636891472	0.982261709
ZNF114	-0.056997715	0.636915321	0.982261709
PPP1R15A	0.03922238	0.636936339	0.982261709
C1orf53	-0.06237544	0.636981253	0.982261709
TEX261	0.029703381	0.636999154	0.982261709
FTSJ3	0.035775226	0.637038936	0.982261709
RABEP1	-0.057052679	0.637069992	0.982261709
TMED5	-0.031490481	0.637131803	0.982261709
TAX1BP1	0.022733559	0.637312785	0.982261709
TRADD	0.04161642	0.637338807	0.982261709
TTC7A	0.046262109	0.637425287	0.982261709
MIR1323	-0.049361962	0.637553034	0.982261709
FAM114A2	-0.035202155	0.637613566	0.982261709
CHST10	-0.044436196	0.637631216	0.982261709
CSF3R	-0.043066959	0.637698744	0.982261709
ZNF341	-0.033219822	0.637706968	0.982261709
MAFF	-0.133099079	0.637707626	0.982261709
DOCK8	-0.036538867	0.637956288	0.982261709
CPT1A	-0.056640742	0.638069492	0.982261709
ZNF641	-0.055652239	0.638101133	0.982261709
ZNF264	0.049204146	0.638195785	0.982261709
FBXO18	0.023583624	0.638224125	0.982261709
WIPI2	-0.035988748	0.638252899	0.982261709
DYRK3	-0.03589795	0.638260459	0.982261709
CLN3	0.032755481	0.638266642	0.982261709
GCLC	0.032369488	0.63842573	0.982261709
IFT52	0.046917731	0.63848593	0.982261709
SNAPC5	0.042457293	0.638612741	0.982261709
KIAA1683	0.079019852	0.638770074	0.982261709

MAPK13	-0.042149802	0.638932309	0.982261709
HDDC3	0.035005146	0.63898564	0.982261709
LILRA2	-0.04635905	0.639008004	0.982261709
C6orf48	0.035516686	0.639113915	0.982261709
TMSB10	0.013202745	0.639146698	0.982261709
ECI1	0.053856891	0.639273893	0.982261709
NSUN3	-0.032444415	0.639281637	0.982261709
FBXW7	-0.027905195	0.639435732	0.982261709
OMA1	-0.034917839	0.639521245	0.982261709
CDC25C	-0.056057964	0.639992255	0.982261709
IL12RB2	0.061442906	0.640011408	0.982261709
SRSF4	0.021012884	0.640043315	0.982261709
SETD1A	-0.034151483	0.64012438	0.982261709
COL4A3BP	0.042829305	0.640160909	0.982261709
SLC24A4	-0.060061179	0.640285925	0.982261709
KIAA1671	-0.114039304	0.640685134	0.982261709
AQP10	0.16868643	0.640757548	0.982261709
EPS15L1	0.038712177	0.640771039	0.982261709
GABARAP	0.065558994	0.64118933	0.982261709
PMP22	0.084855681	0.641195475	0.982261709
MPND	-0.051532101	0.641362914	0.982261709
TBXAS1	-0.03915961	0.64141393	0.982261709
NUP50	-0.046082878	0.641421542	0.982261709
RPS13	0.035785697	0.641494664	0.982261709
RPH3A	-0.054575711	0.641623064	0.982261709
PTPRM	0.109895082	0.641715491	0.982261709
TMEM150A	-0.027727394	0.641753056	0.982261709
NVL	-0.037265592	0.641769145	0.982261709
POLR2E	0.053170167	0.641899008	0.982261709
PTPRA	0.037954692	0.641917405	0.982261709
EHD1	0.022220825	0.642351401	0.982261709
SENP7	-0.031483407	0.642351601	0.982261709
RBL1	-0.041545399	0.64243557	0.982261709
ZBTB34	-0.033701721	0.642465934	0.982261709
DGKA	-0.055342194	0.642542348	0.982261709
RANBP10	0.03044345	0.642596136	0.982261709
MIR630	0.039743979	0.642723914	0.982261709
HSD17B11	-0.061886155	0.642929329	0.982261709
ANKMY2	0.028072157	0.642998577	0.982261709
MAL	-0.060604846	0.643027783	0.982261709
ERCC5	-0.023659651	0.643064505	0.982261709
ZXDC	-0.043597784	0.64309637	0.982261709
IGFBP7	0.097703304	0.643159667	0.982261709
TSHZ3	-0.059442842	0.643186164	0.982261709
ASAH2B	-0.040325212	0.643254771	0.982261709
FUCA2	0.03826244	0.643416121	0.982261709
ABT1	-0.037786047	0.64353884	0.982261709

PSMD12	-0.031071153	0.643602945	0.982261709
RHOBTB2	0.049435418	0.643615897	0.982261709
COQ5	0.02983292	0.643806499	0.982261709
SLC31A2	-0.064044723	0.643891929	0.982261709
SECTM1	0.039014076	0.643906338	0.982261709
YPEL2	0.048551313	0.643914365	0.982261709
RUNDC3B	-0.060427891	0.644028925	0.982261709
SLC7A5	0.052325489	0.644336396	0.982261709
GSTA4	0.054708021	0.644377493	0.982261709
SAFB2	-0.030602086	0.644419636	0.982261709
COL4A3	0.076887392	0.644501618	0.982261709
HMG20B	0.036045111	0.64450345	0.982261709
NEDD1	0.043623307	0.644586518	0.982261709
GAS2L1	0.065297272	0.644650051	0.982261709
DNAH12	-0.105810834	0.644714514	0.982261709
E2F2	-0.052964675	0.644864544	0.982261709
TRIM25	-0.040916881	0.645071392	0.982261709
FEN1	0.045861998	0.64508019	0.982261709
BAP1	0.032076091	0.645109142	0.982261709
MEGF9	-0.066020686	0.645168358	0.982261709
ORC6	0.013101187	0.645273606	0.982261709
KIAA1524	0.054389533	0.645374716	0.982261709
RDH5	-0.050402071	0.645475248	0.982261709
MNDA	-0.115590979	0.645489958	0.982261709
LDOC1L	-0.053305583	0.645502855	0.982261709
SNORD51	-0.041610903	0.645554811	0.982261709
PRRT3	-0.054632427	0.645620982	0.982261709
SIDT2	0.056377625	0.645704316	0.982261709
ICK	-0.027493355	0.645764811	0.982261709
FOXRED2	-0.076412254	0.645992155	0.982261709
ZNF697	-0.058387046	0.64606031	0.982261709
STAU2	-0.038419623	0.646135908	0.982261709
PAFAH1B2	-0.037946659	0.646217662	0.982261709
BCL2L12	-0.024342557	0.646327017	0.982261709
POC1A	-0.044803416	0.646427127	0.982261709
C19orf43	0.030002622	0.646504805	0.982261709
RYBP	-0.076327697	0.646553762	0.982261709
UBA52	0.012141719	0.646709699	0.982261709
TYSND1	-0.023531535	0.646773627	0.982261709
MRS2	-0.03795618	0.646902354	0.982261709
KRT86	-0.074511613	0.646993451	0.982261709
PYCR2	0.032943283	0.647019919	0.982261709
SLC22A4	-0.056402923	0.647063668	0.982261709
LY96	-0.04340738	0.647368263	0.982261709
FSCN1	0.060792046	0.647398095	0.982261709
FBRSL1	-0.03617417	0.647446576	0.982261709
TRAPPC5	0.030816691	0.647479132	0.982261709

GHDC	-0.036574542	0.647489404	0.982261709
STXBP2	-0.045235088	0.647530964	0.982261709
SLC9A1	-0.029141161	0.647807633	0.982261709
TRIM17	-0.041123568	0.647843699	0.982261709
C19orf71	-0.041117263	0.648004069	0.982261709
RCC2	-0.021178397	0.648022124	0.982261709
CUTC	-0.027978595	0.648164469	0.982261709
TAF13	0.05337028	0.648462084	0.982261709
ABHD10	-0.032794365	0.648611544	0.982261709
POLR3E	0.035281675	0.648799303	0.982261709
PUS3	-0.033823978	0.64881501	0.982261709
PLEKHA3	0.049831838	0.649067733	0.982261709
PTBP1	-0.021834472	0.649074206	0.982261709
TMEM207	-0.049541226	0.649093379	0.982261709
C16orf87	0.048165118	0.649122837	0.982261709
PELI3	-0.051585341	0.649143461	0.982261709
GALNT6	-0.041390142	0.649312604	0.982261709
CENPJ	0.038615673	0.649358473	0.982261709
ECI2	0.032304754	0.649445074	0.982261709
RPLP0	-0.03783592	0.649503072	0.982261709
PCYOX1L	-0.041059108	0.649772323	0.982261709
ZNF442	-0.041919188	0.649782539	0.982261709
PDCD6	0.020932243	0.649812608	0.982261709
THADA	0.031350377	0.649937795	0.982261709
POM121C	-0.02131562	0.650048562	0.982261709
RNF166	-0.046351494	0.650053792	0.982261709
KPNA2	0.058113392	0.650104408	0.982261709
PTRH1	-0.054521936	0.650308972	0.982261709
CTDSPL2	-0.03786559	0.650352945	0.982261709
GDPD1	-0.097514857	0.650389643	0.982261709
NF1	0.057672559	0.650615821	0.982261709
DARS	-0.023432174	0.6508484	0.982261709
LIMK1	0.048054762	0.651036774	0.982261709
MED27	0.047534575	0.651106223	0.982261709
TMEM106B	-0.037609432	0.651260467	0.982261709
PYCARD	0.035335576	0.651404528	0.982261709
MTHFS	-0.037779805	0.651428717	0.982261709
PLAGL2	-0.045576159	0.651460128	0.982261709
SCFD2	0.042626678	0.651672682	0.982261709
SRPK1	0.027596882	0.651685863	0.982261709
PTPRN2	0.053992003	0.652111566	0.982261709
SSR2	0.046763129	0.652206263	0.982261709
RAD51C	0.047241758	0.652495689	0.982261709
AGAP1	0.099541926	0.65252387	0.982261709
ALCAM	0.047656827	0.65267148	0.982261709
TOX2	0.058435424	0.652690284	0.982261709
SAAL1	-0.02569432	0.652699141	0.982261709

INO80B	0.027761047	0.652763491	0.982261709
EXOSC10	0.020710697	0.652929093	0.982261709
C9orf85	-0.052538903	0.652937322	0.982261709
CFDP1	0.035316726	0.653168657	0.982261709
NDUFS1	0.047664595	0.65327725	0.982261709
WDR4	-0.037157849	0.653300317	0.982261709
FAM126B	-0.029473227	0.653400583	0.982261709
USP45	-0.041974697	0.653474004	0.982261709
NR2F6	0.063629786	0.653481849	0.982261709
SUSD1	0.039236859	0.65351716	0.982261709
FBXO33	0.027423776	0.653600715	0.982261709
MLLT10	-0.023434038	0.653659886	0.982261709
GIPC1	0.044903492	0.653665623	0.982261709
S100A6	0.026840206	0.653682057	0.982261709
C18orf54	0.041306234	0.653724031	0.982261709
C11orf24	0.040132025	0.653838808	0.982261709
HLA-DQA1	-0.153457559	0.653849677	0.982261709
CDKN3	-0.06609297	0.653854264	0.982261709
C9orf41	-0.049650857	0.654073104	0.982261709
AP4M1	-0.03856026	0.65412962	0.982261709
OAS1	0.094504534	0.654159452	0.982261709
POC5	-0.023459062	0.654175402	0.982261709
RAB3GAP2	-0.0227777	0.654219432	0.982261709
KIAA1731	-0.032625197	0.65422539	0.982261709
DIXDC1	0.03668258	0.654251461	0.982261709
TTLL12	-0.035587695	0.654289743	0.982261709
EGR1	-0.070200362	0.654311718	0.982261709
SMAP1	-0.033096724	0.654407979	0.982261709
SGTA	0.05191622	0.654435707	0.982261709
SORBS3	-0.035110157	0.654482543	0.982261709
FOPNL	-0.025184245	0.654546198	0.982261709
EFEMP2	0.02392354	0.654657103	0.982261709
LAPTM5	0.023291988	0.65474014	0.982261709
GSTP1	-0.03248815	0.654790929	0.982261709
NUP155	0.028489933	0.654885038	0.982261709
SLC39A10	-0.040464933	0.654995834	0.982261709
CSRP2	0.039583394	0.654998956	0.982261709
BNIP3	-0.033128392	0.655038659	0.982261709
ACOT13	0.033336235	0.655139536	0.982261709
PUF60	0.033149301	0.655183529	0.982261709
RPUSD2	-0.028899689	0.655202134	0.982261709
PDPR	-0.059578989	0.655242269	0.982261709
MFF	-0.204664762	0.65525177	0.982261709
CSNK2A1	0.044615984	0.655282007	0.982261709
MFGE8	-0.048300274	0.655492171	0.982261709
SETMAR	0.044378124	0.655564077	0.982261709
SLC12A6	-0.034360116	0.655611773	0.982261709

PRKRIR	0.024528369	0.655669173	0.982261709
GTPBP2	-0.04171977	0.655741685	0.982261709
DGAT2	0.056089256	0.655778553	0.982261709
ARCN1	-0.022347513	0.655859446	0.982261709
AMPD3	-0.043965352	0.655901128	0.982261709
CDC5L	0.03381511	0.656052495	0.982261709
OXA1L	0.034495252	0.656065921	0.982261709
AKAP12	0.033607026	0.656119203	0.982261709
PPP2R1B	-0.044317836	0.656225373	0.982261709
ERGIC2	-0.046093352	0.656244355	0.982261709
GAS7	-0.037049916	0.656287106	0.982261709
SLC2A9	-0.079277326	0.656369949	0.982261709
MIR22HG	0.058203591	0.656437745	0.982261709
ZNF415	0.075089657	0.656485325	0.982261709
TOMM70A	0.025108619	0.656491073	0.982261709
TRNP1	-0.05583748	0.65656488	0.982261709
PRKAG2	-0.025536834	0.656706591	0.982261709
LRRC57	-0.038441018	0.656739447	0.982261709
SNN	-0.062507911	0.656766013	0.982261709
GABARAPL1	0.053102308	0.656767313	0.982261709
C12orf65	0.030788586	0.656834472	0.982261709
TRPC4AP	-0.050666686	0.656873633	0.982261709
HLA-DRA	0.0303847	0.656900123	0.982261709
COX7A2L	0.023112614	0.656944062	0.982261709
HMGXB3	-0.022507475	0.657005798	0.982261709
NRG1	-0.168598095	0.657095436	0.982261709
RPL19	0.010805251	0.657171415	0.982261709
PNPLA7	0.067251408	0.657246969	0.982261709
NEDD8	0.016373491	0.657338237	0.982261709
SYT11	-0.051000546	0.657349965	0.982261709
ANKRD11	-0.022416611	0.657504216	0.982261709
LEPREL4	0.040507222	0.657582215	0.982261709
FAM20C	0.070195311	0.657582544	0.982261709
GART	0.025128554	0.657599826	0.982261709
SYNC	0.051784333	0.657651235	0.982261709
SLC12A9	-0.026725978	0.657933644	0.982370086
ZNF425	-0.049138187	0.65798911	0.982370086
SERPINH1	0.053059706	0.657990046	0.982370086
SUCLG1	-0.027385604	0.65824964	0.982485824
ATP6AP1L	-0.059898993	0.658309812	0.982485824
FAM21C	-0.119657806	0.658536857	0.982485824
ALDOB	-0.06229031	0.658560788	0.982485824
ZFR2	0.066194898	0.658594566	0.982485824
ZFPM1	0.042629323	0.658617789	0.982485824
FBRS	-0.03605254	0.658688888	0.982485824
USP3	-0.027747005	0.658958301	0.982542884
RHBDF2	-0.028960374	0.658982453	0.982542884

ABHD14B	-0.046075216	0.659199448	0.982542884
LRRK1	0.048992027	0.659222006	0.982542884
SNORA68	0.050163408	0.659293589	0.982542884
FTH1	0.038082286	0.659313015	0.982542884
IGJ	0.133689035	0.659413787	0.982542884
ZC3H18	0.034058465	0.659537824	0.982542884
TCP10L	-0.038923113	0.659677581	0.982542884
EMR3	-0.097607905	0.659711312	0.982542884
C1orf131	-0.025797771	0.659715333	0.982542884
STOML2	0.017551728	0.659792326	0.982542884
TSPAN5	-0.040010406	0.659924992	0.982608251
XPC	-0.024855519	0.660063704	0.982682602
COL7A1	0.055205727	0.660187247	0.982734351
ABR	-0.02441867	0.66037408	0.982855904
RFC3	0.032533986	0.660446492	0.982855904
ZNF23	0.038403741	0.660626124	0.982951678
SCO1	-0.038672632	0.660820796	0.982951678
GPR155	-0.053300574	0.660828797	0.982951678
OSBPL8	0.032441618	0.660866057	0.982951678
PLD1	-0.038972551	0.661028886	0.983061768
GTF2F1	0.025724603	0.661227767	0.983137393
TNS3	-0.032748219	0.661257376	0.983137393
ARL6IP4	0.037608339	0.661521671	0.98339825
TTLL3	0.036282606	0.661837174	0.983479718
IL1RAP	0.031342068	0.661898289	0.983479718
TCTA	0.033768389	0.66218278	0.983479718
SMARCC2	-0.033299393	0.662233875	0.983479718
USP13	-0.033996494	0.662338357	0.983479718
MPP6	0.053129711	0.66238314	0.983479718
WDR75	-0.03021914	0.662476247	0.983479718
SBDS	0.035625869	0.662498017	0.983479718
PIGS	0.028801618	0.662543642	0.983479718
TRRAP	0.027808828	0.662599375	0.983479718
TEF	-0.04181263	0.662728882	0.983479718
GPC6	-0.036454756	0.662746377	0.983479718
GSTM1	-0.192460272	0.66286532	0.983479718
GIGYF1	0.048371591	0.662890791	0.983479718
MYO3B	-0.115669453	0.662964856	0.983479718
DHRS7	0.032186907	0.66312505	0.983479718
PPM1A	0.03408335	0.663141155	0.983479718
RBM6	0.03557173	0.663175772	0.983479718
ZNF350	0.038718134	0.663640718	0.983757434
RAP1GAP	0.031667042	0.663709807	0.983757434
C7orf50	0.026849782	0.663719317	0.983757434
ZNF559	0.04790534	0.663803758	0.983757434
CTU2	0.039025917	0.663858479	0.983757434
TRA2B	0.021435324	0.664030801	0.983757434

LAIR1	-0.012753138	0.664073122	0.983757434
SLC38A11	0.085933791	0.66407404	0.983757434
SEC11A	-0.025103882	0.664404054	0.983982659
VAMP3	-0.035373524	0.664453996	0.983982659
NFYC	0.023173101	0.664618165	0.983982659
IPP	-0.07166059	0.664632737	0.983982659
SETD1B	0.029771531	0.664670552	0.983982659
MLST8	-0.037051226	0.664808854	0.984055791
SPIN1	0.048656435	0.665025945	0.984245512
SCGB3A2	0.062642207	0.665160852	0.984313565
ECT2	-0.039436715	0.665518877	0.984468544
THOC7	0.028256608	0.665525082	0.984468544
TUBB1	0.075559032	0.665638802	0.984468544
SNORD114-7	-0.040845776	0.665669129	0.984468544
MIR130A	0.163762769	0.665729152	0.984468544
ERCC4	0.040701648	0.665799216	0.984468544
TEP1	-0.049070411	0.666089091	0.984484052
C1orf162	-0.0229814	0.666173335	0.984484052
UBR5	-0.023594675	0.666177849	0.984484052
PSMD11	0.02895952	0.666297418	0.984484052
CARD14	0.045193466	0.666331903	0.984484052
SFT2D1	0.035513155	0.666737218	0.984484052
ING5	0.061132549	0.666827252	0.984484052
AMZ2	0.021749589	0.666839017	0.984484052
POLD2	0.027596015	0.66689923	0.984484052
VMP1	-0.029516853	0.666935282	0.984484052
TPBG	-0.050681616	0.666964095	0.984484052
NUDT19	-0.030862994	0.66707465	0.984484052
UBE2E2	0.034799201	0.667105938	0.984484052
TGIF1	0.03608346	0.667396733	0.984484052
PERP	-0.07227902	0.667445506	0.984484052
SIK2	0.036930974	0.667572117	0.984484052
CKAP5	-0.022806411	0.667672354	0.984484052
CEP164	-0.034691495	0.667737184	0.984484052
UBTF	0.040649487	0.667758988	0.984484052
HNRNPC	0.032436982	0.667780664	0.984484052
C6orf203	0.037084878	0.667814335	0.984484052
FAM49A	-0.036377506	0.667821327	0.984484052
TMBIM1	-0.035196237	0.667855339	0.984484052
DNAJC14	0.027656627	0.668293662	0.984976419
MSTO1	0.027803533	0.668367322	0.984976419
C1orf159	-0.040003072	0.668710809	0.984992257
AIMP2	0.025957309	0.668754982	0.984992257
SLC43A2	0.045069441	0.66876137	0.984992257
SNORD4A	0.056807395	0.669182656	0.984992257
CREBBP	-0.022852729	0.669405321	0.984992257
IL18	-0.016961608	0.669616459	0.984992257

CLEC12B	0.071215004	0.669791397	0.984992257
CDH24	0.038312737	0.669824826	0.984992257
CRYZL1	-0.033261466	0.669862801	0.984992257
PRRT1	-0.052538093	0.669867291	0.984992257
ZCRB1	0.035832068	0.669883217	0.984992257
INPP1	-0.035558783	0.669890148	0.984992257
ERO1LB	-0.041400959	0.669904344	0.984992257
CRIP2	0.096744812	0.669942931	0.984992257
RNF38	-0.021794522	0.669983505	0.984992257
BZRAP1	0.070089673	0.670055924	0.984992257
CNGB1	-0.083090901	0.670142671	0.984992257
PIGH	-0.027836205	0.670191024	0.984992257
PCNP	0.019788105	0.670211586	0.984992257
EDC4	-0.025345014	0.670241138	0.984992257
BNIP1	0.038864444	0.670742571	0.984992257
GNPNAT1	-0.043264295	0.670992045	0.984992257
MVD	-0.042941614	0.671009073	0.984992257
CRY1	-0.026939605	0.671018072	0.984992257
C15orf39	0.038217268	0.671066306	0.984992257
HEY2	0.065050652	0.671086829	0.984992257
ECHDC1	0.035738782	0.671451394	0.984992257
BID	-0.036777822	0.671606245	0.984992257
FNTA	-0.015994866	0.671619984	0.984992257
ZNF516	0.06279283	0.67162164	0.984992257
IL2RA	0.058054656	0.671678357	0.984992257
TRAT1	0.053381899	0.671758355	0.984992257
ITFG2	-0.022903223	0.67184058	0.984992257
C18orf56	0.052461937	0.671901941	0.984992257
DOCK9	0.036565177	0.671970531	0.984992257
PACS1	0.047244496	0.672095416	0.984992257
LYRM4	-0.048471159	0.672147972	0.984992257
POLG	-0.025086826	0.672173849	0.984992257
OLIG1	-0.105839886	0.672186801	0.984992257
RNF157	0.062630504	0.672216701	0.984992257
KCNK4	0.044753808	0.672382393	0.984992257
GPR137	0.027567534	0.672396562	0.984992257
SLAIN1	0.03365893	0.672460839	0.984992257
NAPSB	-0.091708506	0.672603105	0.984992257
ZFYVE16	0.045148963	0.672614336	0.984992257
EFHD1	-0.087264775	0.672632171	0.984992257
TMF1	-0.03253557	0.672803433	0.984992257
C12orf5	-0.046244057	0.672810126	0.984992257
MED13L	-0.037781532	0.67283098	0.984992257
ACD	-0.037484968	0.673132282	0.984992257
BMPR1A	0.042930171	0.67319007	0.984992257
RMND5B	0.038344379	0.673208257	0.984992257
KCTD20	0.03019753	0.673249094	0.984992257

TP63	0.057590493	0.673257733	0.984992257
HIST2H2BE	-0.089203731	0.673272329	0.984992257
MRPL18	0.027707749	0.673410967	0.985064887
VPS33B	0.019698988	0.673544643	0.985099192
PCIF1	0.045785225	0.673650405	0.985099192
RPL13	0.04403079	0.673701408	0.985099192
CENPF	0.042067823	0.673850159	0.985138936
SCGB3A1	0.228907554	0.673954508	0.985138936
DNAJB4	-0.052729278	0.673995588	0.985138936
HSPA5	-0.027332059	0.674181051	0.985152929
PTP4A2	0.025636824	0.674187418	0.985152929
S100A14	0.043526814	0.674272165	0.985152929
C1orf87	-0.085798767	0.674780191	0.985371387
RBPMS	-0.035469501	0.674877293	0.985371387
SCARNA22	-0.039559505	0.674919009	0.985371387
HAPLN3	-0.059764094	0.674964495	0.985371387
KNTC1	0.031653931	0.67508928	0.985371387
USH1G	0.042549286	0.675164976	0.985371387
LMBRD2	0.035572052	0.675172948	0.985371387
SNORA66	-0.054668581	0.675175997	0.985371387
GALE	-0.030409528	0.675269227	0.985371387
SPARC	0.116640048	0.675311893	0.985371387
EPDR1	-0.071180698	0.675526379	0.985493494
CLDND2	-0.087088837	0.675773989	0.985493494
IGFBPL1	0.041611554	0.675875038	0.985493494
ADAT2	0.035073271	0.676043833	0.985493494
SBNO2	-0.037860498	0.676182154	0.985493494
NEK2	-0.059362762	0.676183016	0.985493494
CD302	-0.083187133	0.676329318	0.985493494
UBE2O	0.038659009	0.676429902	0.985493494
SAMD4A	0.058849512	0.676618113	0.985493494
HERC3	-0.03468663	0.67676126	0.985493494
SH2D3A	-0.041595162	0.676973256	0.985493494
TKT	0.029235872	0.677082648	0.985493494
COMMD3	-0.020009661	0.677135981	0.985493494
UBA3	0.042734989	0.677185925	0.985493494
ALDH18A1	0.03444615	0.67719948	0.985493494
SLC30A5	-0.026670347	0.677343202	0.985493494
SSNA1	0.028240379	0.677351197	0.985493494
NAPRT1	0.053815985	0.677361809	0.985493494
CACNG1	-0.035470679	0.677428822	0.985493494
PCF11	-0.023218344	0.677628544	0.985493494
SLC15A2	0.051831228	0.677663529	0.985493494
POLL	-0.038689048	0.677823391	0.985493494
MAPK7	-0.034285072	0.67784082	0.985493494
PRDM2	0.029968656	0.67788572	0.985493494
SMPD3	-0.042080645	0.677900513	0.985493494

CHST13	0.132963321	0.677963804	0.985493494
CLTC	-0.045963735	0.678376963	0.985493494
MOB2	0.033638167	0.678382775	0.985493494
PI4KB	0.023611827	0.678388094	0.985493494
HBS1L	-0.069064188	0.678448553	0.985493494
CBX2	0.039672351	0.678595403	0.985493494
EHBP1L1	-0.041623924	0.678614272	0.985493494
RFX7	0.019257824	0.678632171	0.985493494
PBOV1	0.038364162	0.678670595	0.985493494
DLGAP5	-0.078759088	0.678751054	0.985493494
ATP1A1	-0.021022923	0.67876453	0.985493494
APPL1	-0.030585124	0.678830401	0.985493494
VSIG10	-0.033919459	0.678916936	0.985493494
ACADSB	0.030948422	0.678916981	0.985493494
CLOCK	-0.035528618	0.678974584	0.985493494
SNRPD3	-0.044444241	0.679114538	0.985493494
SNRPA1	-0.021115977	0.679154144	0.985493494
C1orf122	0.036187723	0.679223946	0.985493494
PM20D1	0.051440192	0.67944807	0.985665402
RAX2	-0.069177183	0.67961093	0.985665402
NRIP1	0.038094185	0.679632478	0.985665402
H3F3A	-0.025856965	0.679848303	0.985665402
KLF13	0.02649308	0.680009577	0.985665402
SNORD6	-0.033797661	0.680089309	0.985665402
LMF1	-0.039328823	0.680138461	0.985665402
ACSF2	-0.041066655	0.680238271	0.985665402
DNAL1	-0.03761914	0.680315804	0.985665402
SOCS1	-0.063446792	0.68046748	0.985665402
TTC12	0.064010395	0.680482187	0.985665402
PSMB5	-0.024066105	0.680506368	0.985665402
ISCA2	-0.033374824	0.680645044	0.985665402
MRPL50	-0.035200474	0.680681063	0.985665402
LARP4B	-0.018379836	0.68077947	0.985665402
TMEM154	-0.031161679	0.680898244	0.985665402
CDK9	-0.023763512	0.68125585	0.985665402
GJB7	0.05077873	0.681267513	0.985665402
DAGLA	-0.039295397	0.681282173	0.985665402
KDM4A	-0.038539613	0.681414607	0.985665402
FUT7	0.051757366	0.68148411	0.985665402
EIF4G2	-0.015039347	0.68160768	0.985665402
PTMA	0.043773929	0.681656718	0.985665402
ZNF600	-0.053676958	0.681696464	0.985665402
RNLS	-0.030307651	0.681772387	0.985665402
INTS3	0.034077508	0.681861185	0.985665402
DAPK3	-0.039064156	0.681944298	0.985665402
CDK2AP2	0.042025195	0.682095173	0.985665402
TNFRSF6B	-0.038593454	0.68209634	0.985665402

WDR33	-0.032520451	0.682430375	0.985665402
TESK2	0.027633448	0.682496035	0.985665402
DNAJC19	-0.037531232	0.682540058	0.985665402
MYH9	0.02283196	0.682579593	0.985665402
NSUN5P2	0.052120126	0.68259656	0.985665402
RPP21	0.021302723	0.682624251	0.985665402
PHLPP1	-0.027456569	0.682624254	0.985665402
EBLN2	-0.065412378	0.682766512	0.985665402
ABCA2	-0.061021428	0.682771347	0.985665402
MEF2C	0.046029105	0.683015381	0.985665402
PPT1	-0.033087989	0.683065885	0.985665402
PSMA6	-0.021672991	0.683200131	0.985665402
ASF1B	-0.046794322	0.683477301	0.985665402
POLR3H	0.027680109	0.683502037	0.985665402
GPR183	-0.04488922	0.683669655	0.985665402
KPTN	0.040976812	0.683693285	0.985665402
RBBP6	0.049461445	0.683771728	0.985665402
STMN1	0.047525658	0.684004957	0.985665402
SRP54	-0.023101766	0.684037462	0.985665402
BNIP2	0.027049448	0.684043833	0.985665402
ACYP2	0.039604673	0.684140937	0.985665402
HIRA	-0.048867432	0.684250043	0.985665402
TGFBRAP1	-0.039868547	0.684296742	0.985665402
RPS27	0.00975008	0.684429417	0.985665402
DICER1	0.035241557	0.684433393	0.985665402
ZNF263	0.028009892	0.68458773	0.985665402
POLR2G	0.015441441	0.684630907	0.985665402
FCF1	-0.030284129	0.684818598	0.985665402
NRBP1	0.027333126	0.684847503	0.985665402
SDHC	0.053220233	0.684847781	0.985665402
AMY1A	0.035950478	0.685013155	0.985665402
CDR2	0.033660743	0.685016159	0.985665402
AMIGO1	-0.036688886	0.685148248	0.985665402
LOXHD1	-0.041494643	0.685169179	0.985665402
ENHO	0.038376799	0.685259152	0.985665402
PFKFB3	0.045204661	0.685462115	0.985665402
ATF6	-0.027331671	0.685516919	0.985665402
GAPT	-0.058177407	0.685720142	0.985665402
SSB	0.020722301	0.685729668	0.985665402
UCK2	-0.040382579	0.685769768	0.985665402
CCDC6	0.02176323	0.685775639	0.985665402
MAP3K4	-0.026178314	0.685889521	0.985665402
RFK	-0.048032233	0.685940892	0.985665402
ZNF266	-0.055754103	0.685945326	0.985665402
CCDC90B	-0.018694278	0.685985719	0.985665402
PSMD14	0.028582111	0.686020982	0.985665402
MSH5	0.040849047	0.686266657	0.985691976

PPP6R3	0.035051703	0.686303654	0.985691976
TLR6	0.042301223	0.686444809	0.985691976
SNORD15B	-0.037325648	0.686586266	0.985691976
RNF219	-0.027285725	0.686675341	0.985691976
TXN	-0.039290926	0.68692781	0.985691976
CCL4	0.082941231	0.687036025	0.985691976
KLRD1	0.074988405	0.687052364	0.985691976
GLB1	-0.031361841	0.687167689	0.985691976
ABHD4	-0.038351141	0.687205245	0.985691976
PTPN22	-0.04441741	0.687407379	0.985691976
ADCK2	-0.035345588	0.687474945	0.985691976
AIP	0.027256912	0.687560467	0.985691976
ROBO3	-0.069997953	0.687568245	0.985691976
EPHX4	-0.076749258	0.687611584	0.985691976
SNORD56	0.042899732	0.687653852	0.985691976
DHRS9	-0.094314549	0.687671455	0.985691976
EIF4EBP2	0.031185007	0.687718182	0.985691976
ARID1A	0.027972795	0.68798059	0.985691976
NPLOC4	-0.031503335	0.688036314	0.985691976
DNM1L	0.036967318	0.688061998	0.985691976
OLFML2B	0.034456202	0.688161194	0.985691976
C11orf68	0.028310359	0.688240889	0.985691976
C19orf38	0.061786795	0.68833222	0.985691976
PFKFB4	-0.028814126	0.688489313	0.985691976
HECTD3	-0.026492985	0.688492407	0.985691976
SESN2	-0.047561462	0.688554696	0.985691976
MAN2A1	0.033298281	0.688622554	0.985691976
RGP1	0.035403422	0.688803831	0.985691976
NUDT14	-0.027301659	0.688818858	0.985691976
PIK3CB	-0.043136446	0.688825076	0.985691976
GALM	-0.020752091	0.688889071	0.985691976
KLHL26	0.034108779	0.689071373	0.985751666
LPPR5	-0.024834886	0.689182906	0.985751666
CD300LF	0.04568228	0.689268215	0.985751666
CRTAM	-0.068997717	0.689287008	0.985751666
TSC1	-0.023263706	0.690069739	0.986442688
NDUFAF3	0.028163071	0.690188161	0.986442688
GATA3	0.043338605	0.690207001	0.986442688
ACRBP	0.091136014	0.690487668	0.986442688
NMD3	-0.014491757	0.690552305	0.986442688
RAB14	-0.059045514	0.690576892	0.986442688
SLC18A1	-0.036228606	0.690586976	0.986442688
BECN1	0.022118699	0.690648918	0.986442688
IFT172	-0.034495723	0.690658781	0.986442688
PPOX	-0.022766851	0.690661382	0.986442688
MAP4K5	0.028050312	0.690943967	0.986546486
CALN1	-0.032133523	0.690998184	0.986546486

MLLT6	-0.029814977	0.691001437	0.986546486
RAB43	-0.030412014	0.69137595	0.986772967
PPAT	0.069992236	0.691381146	0.986772967
LTA4H	-0.035432001	0.691563749	0.986772967
ITGAX	0.042773515	0.691848458	0.986772967
RPRM	-0.105477654	0.691858225	0.986772967
IPO4	0.034956807	0.691868596	0.986772967
HAX1	0.027663601	0.692084233	0.986772967
ITPRIPL1	0.054487737	0.692305071	0.986772967
HIST1H3E	0.031638921	0.692388096	0.986772967
GAB1	-0.035205219	0.692531846	0.986772967
BRCA1	-0.041011951	0.692587953	0.986772967
AKR1C2	0.08968532	0.692595809	0.986772967
NAPG	0.03613892	0.692662324	0.986772967
SERPINB8	-0.058952586	0.692733131	0.986772967
FBLN2	-0.056368538	0.692821274	0.986772967
CEP290	0.041337037	0.693046098	0.986772967
PEX11B	-0.021483729	0.693057943	0.986772967
EMILIN2	-0.035712629	0.693071131	0.986772967
SLC22A18	-0.057484886	0.693078628	0.986772967
STK17B	-0.097813791	0.693137932	0.986772967
AKR1D1	-0.014479616	0.693192889	0.986772967
C22orf46	0.025063143	0.693251286	0.986772967
ANKS6	-0.039450871	0.69331356	0.986772967
UHRF1BP1	-0.036346504	0.693450574	0.986772967
FAM129B	-0.033451861	0.693602498	0.986772967
IL1RN	-0.051085765	0.693790327	0.986772967
FAM134A	0.029584785	0.693918293	0.986772967
CPSF3	-0.017867573	0.693945149	0.986772967
MOGS	0.028549872	0.694005848	0.986772967
ALG14	0.037523673	0.694407927	0.986772967
NIPSNAP3A	0.053101516	0.694479338	0.986772967
BTBD11	-0.051307504	0.69447979	0.986772967
SLC2A1	0.032176615	0.694521004	0.986772967
UGDH	0.043220289	0.694680261	0.986772967
CRKL	0.020338156	0.694772759	0.986772967
SCAF8	0.021829633	0.694952754	0.986772967
GPC5	0.032444731	0.69497429	0.986772967
TXNDC17	-0.024646689	0.694998353	0.986772967
KRT18	-0.064945574	0.695002845	0.986772967
PGD	0.037922933	0.69506357	0.986772967
JAGN1	0.030273994	0.695141569	0.986772967
ATXN3	-0.037574421	0.695330801	0.986772967
GRWD1	0.034196259	0.695358557	0.986772967
ORAI3	0.037398207	0.695450761	0.986772967
CES2	-0.023153826	0.695828147	0.986772967
MAMDC4	-0.030723635	0.696048395	0.986772967

CYB561D2	0.020018582	0.6960955	0.986772967
ZDHHC20	0.033294662	0.69616649	0.986772967
DEFB108B	-0.032691334	0.696193129	0.986772967
PGM2L1	-0.039962565	0.696288792	0.986772967
MYO1G	0.036173825	0.696327986	0.986772967
FMO2	-0.040606509	0.696404579	0.986772967
DHX57	-0.062658665	0.696436397	0.986772967
CHI3L2	-0.08892564	0.696485742	0.986772967
ZNF280D	-0.037315898	0.696607581	0.986772967
UAP1	-0.035903426	0.696678499	0.986772967
ATP13A1	0.039406591	0.696787796	0.986772967
BNC2	-0.065136844	0.696825742	0.986772967
SACS	0.031091151	0.696858092	0.986772967
SLC22A15	-0.048653846	0.696948853	0.986772967
PDRG1	0.028696801	0.697138339	0.986772967
MICALL1	0.04038764	0.697158186	0.986772967
RPL5	0.01831356	0.697158266	0.986772967
AXIN1	0.047041455	0.69731824	0.986772967
EIF5	-0.033994139	0.697456331	0.986772967
TWSG1	0.027657099	0.697629845	0.986772967
FER	-0.042819292	0.697914065	0.986772967
AIF1L	0.033116859	0.698020005	0.986772967
FAIM3	0.031579223	0.698063706	0.986772967
COMMD5	0.034578016	0.698116163	0.986772967
GNB4	-0.056824654	0.698219318	0.986772967
PDS5B	0.024822747	0.698327535	0.986772967
PPP1R8	0.033090455	0.698329104	0.986772967
EMB	-0.040340177	0.698427146	0.986772967
MYO18A	0.028964354	0.698493721	0.986772967
SLC41A3	0.033834716	0.698518728	0.986772967
WRN	0.02154156	0.698556908	0.986772967
KIAA1715	-0.030011127	0.698586243	0.986772967
FGD2	-0.064005366	0.698797564	0.986772967
SOX13	0.059529252	0.698965705	0.986772967
FBXO44	0.037849111	0.699086377	0.986772967
COL18A1	-0.100963242	0.699164828	0.986772967
AGPAT5	0.035770688	0.699473783	0.986772967
PEPD	0.02237609	0.699573645	0.986772967
ZNF366	-0.044353581	0.699625245	0.986772967
PLEKHG1	0.057681516	0.69967335	0.986772967
GIMAP6	-0.036601653	0.69989553	0.986772967
HNRNPD	-0.013485693	0.699944389	0.986772967
EIF1AD	0.044176497	0.699982047	0.986772967
KIF20A	-0.037108323	0.700009234	0.986772967
FAM13B	-0.022203174	0.700105385	0.986772967
MRPS7	0.027250161	0.700124874	0.986772967
YDJC	0.026818018	0.700177815	0.986772967

PTGIR	-0.042203521	0.700487815	0.986772967
TMEM120A	0.025555601	0.700515384	0.986772967
PPM1K	-0.082858685	0.70055498	0.986772967
PBX4	0.044035529	0.700558885	0.986772967
SPNS1	-0.034992019	0.700583331	0.986772967
PLCXD2	0.036831109	0.700605948	0.986772967
AGFG1	0.032161621	0.700618764	0.986772967
C6orf47	0.022520931	0.70063166	0.986772967
BBS9	-0.043284628	0.700638239	0.986772967
WDR63	-0.041628426	0.700661783	0.986772967
STXBP3	-0.02467106	0.700701563	0.986772967
C1orf116	0.064512221	0.700707053	0.986772967
CCNT1	-0.042467215	0.700797047	0.986772967
SPDYE7P	0.01513148	0.700979055	0.986772967
CNR1	0.036091846	0.701060932	0.986772967
ATP6V0C	0.019238603	0.701076336	0.986772967
RARS2	0.02108549	0.701197646	0.986772967
GCAT	0.042837632	0.701396917	0.986772967
AK5	-0.06452874	0.701453561	0.986772967
PALLD	-0.080772868	0.701469489	0.986772967
IFFO2	0.033669865	0.70147692	0.986772967
FAM19A4	-0.034540598	0.701594385	0.986772967
PCDHB9	-0.062428547	0.70175593	0.986772967
MLLT4	0.076893123	0.701919557	0.986772967
GNPAT	-0.018257142	0.702087927	0.986772967
ZNF860	-0.080534267	0.702240957	0.986772967
MED20	-0.028454729	0.702280998	0.986772967
ELMOD3	-0.03952349	0.702326454	0.986772967
MT1G	-0.047504639	0.702428976	0.986772967
RFX5	0.016860705	0.702506864	0.986772967
DALRD3	-0.028862785	0.702529915	0.986772967
ARID4B	0.074284281	0.702535805	0.986772967
PIAS4	0.019578684	0.702611348	0.986772967
TCN2	-0.055964234	0.702639741	0.986772967
HIST1H4J	-0.047127716	0.702718435	0.986772967
TBC1D22B	-0.030680473	0.702825144	0.986772967
C2orf76	0.03480877	0.703504309	0.986772967
TOP2A	-0.080215452	0.70384795	0.986772967
PYROXD1	-0.063852843	0.703877738	0.986772967
ITGAL	-0.035466466	0.703918067	0.986772967
TMEM17	-0.050956674	0.703939634	0.986772967
MED26	0.028120771	0.704134	0.986772967
RPL18A	0.025101824	0.704145163	0.986772967
EIF3M	0.031140811	0.704320674	0.986772967
RAB4A	0.038780235	0.704336884	0.986772967
CEP120	0.040708985	0.704537692	0.986772967
RNF112	0.0331313	0.704669691	0.986772967

GNS	-0.020408848	0.704687309	0.986772967
PARP14	-0.050745102	0.704805552	0.986772967
CCDC28B	0.038953833	0.704806486	0.986772967
HTR1E	-0.036430311	0.704837322	0.986772967
NFRKB	0.031561395	0.704881837	0.986772967
NOX4	-0.042732669	0.704941255	0.986772967
MYL9	0.137911826	0.705023753	0.986772967
MTX1	0.024064433	0.705070788	0.986772967
ABCF1	0.018794674	0.705088905	0.986772967
AFG3L2	-0.018138114	0.705175018	0.986772967
MORC2	-0.043297109	0.705372225	0.986772967
PLBD1	-0.056880544	0.705381633	0.986772967
BARD1	0.048522423	0.705413412	0.986772967
URB2	-0.024329765	0.705534891	0.986772967
ABCF3	-0.034035817	0.705573848	0.986772967
RNASEL	-0.036434102	0.705771963	0.986772967
GPR153	0.030382	0.705800935	0.986772967
TGS1	0.042749349	0.705933757	0.986772967
SSPO	-0.063875103	0.705941376	0.986772967
UCP3	-0.038836718	0.706031151	0.986772967
WARS	-0.044294682	0.706256208	0.986772967
PAQR7	0.039570124	0.706303534	0.986772967
OTOF	0.108023662	0.706544252	0.986772967
EEFSEC	-0.034028602	0.706545753	0.986772967
MEF2D	0.026826691	0.706710693	0.986772967
LAX1	0.04942413	0.706912153	0.986772967
TDRD1	-0.080842045	0.706921585	0.986772967
PAPSS1	-0.031883791	0.70715167	0.986772967
SYNCRIP	-0.024292865	0.707236999	0.986772967
PPARD	-0.034954147	0.707287224	0.986772967
POLM	0.036077173	0.707318424	0.986772967
MDH1	-0.020669321	0.707425613	0.986772967
OST4	0.032452933	0.70748767	0.986772967
CPLX2	0.032906385	0.707542572	0.986772967
ZNF785	-0.083292303	0.707648492	0.986772967
TXNDC9	-0.024599578	0.707895502	0.986772967
NDUFA9	0.023935811	0.7079889	0.986772967
EIF3F	-0.028145433	0.70804708	0.986772967
GLRX	0.023835095	0.70806102	0.986772967
DSE	-0.033015049	0.708127598	0.986772967
NME7	0.041472058	0.708140751	0.986772967
BMP4	-0.036649488	0.708207769	0.986772967
CHTF8	0.040307109	0.708217407	0.986772967
FAM84B	0.040102397	0.708309306	0.986772967
ADAMTS1	0.082551041	0.708601487	0.986772967
SMAP2	0.024011838	0.708629948	0.986772967
POLR3C	-0.016675555	0.708857196	0.986772967

CLK3	0.025529459	0.708858451	0.986772967
SFT2D3	-0.031323543	0.708934798	0.986772967
CREB3	0.047558201	0.708972839	0.986772967
UFSP2	0.020701132	0.709103283	0.986772967
LIMA1	-0.040363768	0.709113512	0.986772967
TMCO3	0.033085468	0.709126681	0.986772967
DUXA	-0.082389418	0.709174928	0.986772967
UBE3B	-0.02636476	0.709184629	0.986772967
CBX5	-0.043317133	0.709191117	0.986772967
SNRPC	0.025324626	0.709270054	0.986772967
TNFSF10	0.043363271	0.709304096	0.986772967
PDCL3	-0.024387348	0.7093115	0.986772967
TAF8	-0.028737888	0.709382698	0.986772967
EIF5A2	0.036356895	0.709459952	0.986772967
TXNDC12	-0.023563613	0.709486889	0.986772967
LONP2	0.02229292	0.709653247	0.986772967
TUFM	-0.028409347	0.709716755	0.986772967
RBM39	-0.020739438	0.709719074	0.986772967
ANKRD13D	-0.039551566	0.709727739	0.986772967
JKAMP	-0.026415059	0.709842611	0.986772967
CENPM	0.0475878	0.710057957	0.986772967
ZNF511	0.019600465	0.710318336	0.986772967
GNA12	0.029773245	0.710473378	0.986772967
SYNGR1	0.052957797	0.710552851	0.986772967
PVALB	0.147232105	0.710685762	0.986772967
BCCIP	-0.016389083	0.710768959	0.986772967
C17orf100	0.039575451	0.710908844	0.986772967
PPIAL4G	0.013206932	0.710915826	0.986772967
CMBL	-0.033588505	0.710915851	0.986772967
CLEC2B	-0.082979727	0.710946723	0.986772967
SYT15	-0.052966183	0.710963224	0.986772967
ZNRF2	-0.038093738	0.711125151	0.986772967
FBXO6	-0.046895812	0.711135067	0.986772967
PGM1	0.029056983	0.7112737	0.986772967
EEF1D	0.024583423	0.711405828	0.986772967
ACTR3	-0.023934515	0.711463746	0.986772967
TM9SF1	-0.029833486	0.711479661	0.986772967
ACSBG1	0.105355575	0.711489375	0.986772967
TMEM45B	-0.077321276	0.711502196	0.986772967
UBE2E1	0.019183536	0.711630913	0.986772967
GSK3A	-0.04570242	0.711686364	0.986772967
F2RL3	-0.053575729	0.711693025	0.986772967
HAUS4	0.032016681	0.711694941	0.986772967
ERRFI1	0.0467763	0.711870199	0.986772967
TMED9	-0.028109842	0.712087202	0.986772967
KBTBD3	0.038733399	0.712225369	0.986772967
FKBP1B	-0.048502525	0.712251578	0.986772967

FDX1	-0.037423811	0.712257347	0.986772967
TPMT	-0.024808739	0.712380488	0.986772967
ANO9	-0.046678583	0.71239041	0.986772967
S1PR5	0.066472407	0.712392158	0.986772967
NAA50	-0.033356356	0.712466307	0.986772967
HDC	0.126805458	0.71280002	0.986842139
C10orf32	-0.048028555	0.712891682	0.986842139
KCNMB4	0.057448425	0.712911088	0.986842139
XPO6	-0.026396573	0.713021707	0.986842139
MAN1A2	-0.02753321	0.713029579	0.986842139
MORF4L1	0.029327318	0.713080709	0.986842139
CLN6	-0.038962934	0.713140326	0.986842139
ZER1	0.028789327	0.713346533	0.98684373
ADD3	0.01835995	0.713492074	0.98684373
HEATR5B	-0.01980515	0.713906503	0.98684373
PLIN2	-0.03353145	0.713988147	0.98684373
TCEA2	0.030129419	0.714097703	0.98684373
ZNF772	-0.047269841	0.714193317	0.98684373
GOLGA5	-0.018107566	0.714575797	0.98684373
PRDM8	0.039996145	0.714788361	0.98684373
CISH	-0.068514352	0.714799805	0.98684373
FILIP1L	-0.03608148	0.714886844	0.98684373
DDX17	-0.035259046	0.714913516	0.98684373
RPUSD4	-0.022025191	0.714923896	0.98684373
PCMT1	0.015563108	0.714999115	0.98684373
PSMD7	-0.026033131	0.715111238	0.98684373
RNF8	0.033652103	0.715139368	0.98684373
SEC63	-0.030263726	0.715198257	0.98684373
ANXA5	0.036876446	0.715221576	0.98684373
PPP2R5B	0.032605472	0.715228989	0.98684373
C20orf27	-0.050207411	0.715349114	0.98684373
RRP15	0.032895761	0.715360237	0.98684373
EIF2AK2	0.051553727	0.715371507	0.98684373
UTP11L	-0.029021345	0.715388955	0.98684373
SNX21	-0.033664844	0.715440951	0.98684373
ROCK1	0.037942312	0.715523428	0.98684373
NICN1	-0.025119589	0.715694244	0.98684373
TRAF4	-0.039794612	0.715867469	0.98684373
BRPF1	0.015195922	0.716065326	0.98684373
SNX5	0.023523223	0.71628535	0.98684373
CDC20	-0.098884583	0.716422127	0.98684373
RSBN1	-0.027673124	0.716578211	0.98684373
AGPAT3	0.038463376	0.716658751	0.98684373
LRRC47	0.015593191	0.71668181	0.98684373
PCGF3	0.042562839	0.716881881	0.98684373
ARFIP1	-0.03156101	0.717186186	0.98684373
SQRDL	0.035288305	0.717200447	0.98684373

PRR14	0.017817427	0.717291378	0.98684373
ZNF770	0.023579364	0.717405141	0.98684373
RTP4	-0.059906668	0.717419285	0.98684373
TTC21A	-0.047539981	0.71748817	0.98684373
STAM	0.025844593	0.717544406	0.98684373
LAMC3	-0.081408749	0.717544637	0.98684373
TMEM229B	0.03544969	0.717713793	0.98684373
TAGLN	0.078753157	0.717901088	0.98684373
WRNIP1	0.029011036	0.717999088	0.98684373
SEC24A	0.035693573	0.71805895	0.98684373
CEBPZ	0.02124939	0.718129004	0.98684373
KIAA1958	-0.038653537	0.718199831	0.98684373
MPEG1	0.054302225	0.718284007	0.98684373
CD2AP	-0.03659504	0.718349192	0.98684373
SMARCD1	0.017752021	0.71838177	0.98684373
VMO1	0.05673526	0.718484879	0.98684373
RTN4RL1	0.03465886	0.718505044	0.98684373
MED7	-0.026734163	0.718559143	0.98684373
MTHFSD	-0.029593658	0.718580601	0.98684373
POLDIP2	-0.030718796	0.718611636	0.98684373
SPEN	0.019454745	0.718627157	0.98684373
PDCD10	0.02701705	0.718738977	0.98684373
RAB11FIP4	-0.033694586	0.718920781	0.98684373
CLEC10A	-0.059465206	0.719016436	0.98684373
TAB1	-0.028874435	0.719063874	0.98684373
SCNN1D	-0.034051679	0.719093128	0.98684373
HYALP1	0.029589701	0.719122933	0.98684373
CEBPD	0.031151505	0.719138855	0.98684373
MAML1	-0.026226586	0.719490012	0.98684373
TST	0.044783245	0.719515726	0.98684373
MRPL51	0.02262996	0.719616657	0.98684373
LRCH3	-0.044132731	0.719627554	0.98684373
MMADHC	0.02640837	0.719632406	0.98684373
NHP2L1	0.021846493	0.719749442	0.98684373
ACYP1	0.026011742	0.719793645	0.98684373
ITPA	0.018887566	0.719842866	0.98684373
HEXB	-0.020954835	0.719843711	0.98684373
CORO1B	0.039809372	0.719911535	0.98684373
TXNL1	0.03982282	0.720104385	0.98684373
GYPC	0.031531251	0.720130069	0.98684373
PCDH9	-0.034508365	0.720135566	0.98684373
TBCD	-0.047282176	0.720154116	0.98684373
IQCB1	-0.028048397	0.720272229	0.98684373
RGS18	0.051669635	0.720285997	0.98684373
PIK3CG	0.039961042	0.720433203	0.98684373
GOLGA8F	0.04870014	0.720481459	0.98684373
LATS2	0.042053279	0.720484764	0.98684373

CDON	-0.034819457	0.720541244	0.98684373
IGFBP3	-0.089316485	0.720810692	0.986987319
NASP	-0.023468632	0.720824418	0.986987319
NCKAP5	0.062736567	0.721042448	0.987163743
GPR65	0.036990012	0.721341451	0.987450967
STAG3L4	-0.035331382	0.721579457	0.987600195
CREBL2	0.032516494	0.721661271	0.987600195
PTRH2	-0.031583325	0.721827085	0.987600195
TP53INP2	-0.029973618	0.721994415	0.987600195
PIN1	0.012109078	0.722192214	0.987600195
MAPK1IP1L	0.018519038	0.722248745	0.987600195
NR1I2	0.05606408	0.722575333	0.987600195
SHC1	0.02320308	0.722674906	0.987600195
SSH1	-0.036035863	0.722687011	0.987600195
METAP2	-0.019497062	0.722808599	0.987600195
ENY2	0.027672307	0.722948113	0.987600195
MMP25	-0.068159221	0.722982101	0.987600195
CD151	-0.049845549	0.723058157	0.987600195
F2RL1	-0.06388762	0.723244984	0.987600195
SMAD9	-0.048408024	0.723487694	0.987600195
SURF1	-0.018904105	0.723516292	0.987600195
C1orf86	0.041178427	0.723603637	0.987600195
DCUN1D5	-0.025454197	0.723644799	0.987600195
ZNF248	-0.031960101	0.723735792	0.987600195
ATP2C1	0.023843359	0.723745511	0.987600195
SLC12A8	-0.031478985	0.723797396	0.987600195
ZNF146	0.022554101	0.723839619	0.987600195
TNFRSF1A	-0.02342879	0.72385309	0.987600195
ACTN1	0.042730628	0.72392209	0.987600195
AGTRAP	-0.044435633	0.723998986	0.987600195
MFSD6L	-0.025330821	0.72400351	0.987600195
GEMIN5	0.028073733	0.724321093	0.987600195
DECR2	-0.032265305	0.724365011	0.987600195
MIR125B2	0.024544054	0.7244404	0.987600195
TMEM140	-0.062518411	0.724612286	0.987600195
PPARGC1A	-0.07917664	0.724617236	0.987600195
C5orf34	-0.038918443	0.724669959	0.987600195
CLSTN1	0.026648632	0.724672442	0.987600195
PSMB6	0.020997941	0.724734711	0.987600195
AP1S1	0.037200067	0.724736846	0.987600195
SPECC1	-0.038691337	0.724754095	0.987600195
EIF2B3	-0.024094748	0.724898738	0.987600195
PSAT1	0.046119468	0.724921425	0.987600195
TOPBP1	0.025407745	0.724930128	0.987600195
DNMT3A	0.031004739	0.725020665	0.987601986
BAMBI	0.087593473	0.725201128	0.987612868
SH3BP4	0.042519262	0.725384509	0.987612868

MRPL43	0.023728635	0.725400484	0.987612868
PCCB	-0.024096181	0.725527605	0.987612868
PCNXL3	0.027879657	0.725543998	0.987612868
TOM1	-0.021864121	0.725652885	0.987612868
NDUFA5	-0.041109068	0.725653217	0.987612868
PGLYRP2	0.036846848	0.725748879	0.987621631
SIRT7	0.023446558	0.725887206	0.987629427
LRRC14	-0.031718782	0.725933058	0.987629427
CTBS	-0.033882963	0.726078239	0.9876433
TTC7B	-0.037503279	0.726149937	0.9876433
TMEM208	0.02158678	0.726352129	0.9876433
REV1	-0.022103241	0.726433259	0.9876433
TMEM39B	0.021261124	0.726468025	0.9876433
DTWD1	0.031032955	0.726523672	0.9876433
BLMH	-0.026619314	0.726590112	0.9876433
PYGM	0.029656029	0.726707333	0.9876433
RABL2B	0.02460923	0.726746289	0.9876433
UQCRQ	0.02023923	0.727134093	0.987972697
RECQL	-0.028624964	0.727195382	0.987972697
GSTM3	-0.138403403	0.727366334	0.987972697
NFIL3	0.050154875	0.727415238	0.987972697
PANX1	0.03464716	0.72748028	0.987972697
ACVR2A	-0.030197071	0.727524208	0.987972697
WDR86	0.061356694	0.727855225	0.988300968
CDC42EP1	-0.037196972	0.728072729	0.988475044
DHDDS	-0.031205307	0.728177563	0.98849613
ZNF467	-0.03623834	0.728291164	0.98852911
AACS	0.030542408	0.728739691	0.988623277
FANCI	-0.036854151	0.728785772	0.988623277
ABCC4	-0.058245303	0.728878488	0.988623277
SUGT1	-0.0233768	0.728920069	0.988623277
STEAP3	-0.062775316	0.729037952	0.988623277
TSR1	-0.021210833	0.729297824	0.988623277
DPP9	-0.031399014	0.729409108	0.988623277
RPSAP58	0.036117557	0.729552018	0.988623277
FCGR1C	-0.079190672	0.729644869	0.988623277
COBL	0.03312736	0.72966004	0.988623277
RPRD1A	0.022035268	0.729682115	0.988623277
HSPA9	-0.01399312	0.72970366	0.988623277
COMMD8	-0.036958644	0.729925203	0.988623277
SPCS2	-0.021082709	0.729966847	0.988623277
GPATCH4	0.025555655	0.730082937	0.988623277
OLFML2A	0.029237482	0.730361431	0.988623277
METTL18	0.046305883	0.730461359	0.988623277
GOLT1B	-0.026696066	0.730658702	0.988623277
HMOX1	0.040585686	0.730890086	0.988623277
CCDC144B	-0.071765264	0.731021011	0.988623277

ERP29	0.018832092	0.731022672	0.988623277
TBPL1	0.020652102	0.731040103	0.988623277
MFSD3	0.031560541	0.731256479	0.988623277
ANKRD10	0.027188074	0.73128703	0.988623277
PSMA3	-0.024594964	0.731289196	0.988623277
DAPK1	0.010855653	0.731362103	0.988623277
NSUN4	0.022787701	0.731453595	0.988623277
AKAP10	0.036965951	0.7314774	0.988623277
ZNF649	-0.040760893	0.731539118	0.988623277
PYGL	-0.034719035	0.731559971	0.988623277
RAPGEF1	0.026497169	0.731583911	0.988623277
TNFSF15	-0.086999956	0.731646782	0.988623277
VPS28	-0.028215063	0.731864728	0.988623277
PYROXD2	-0.042593787	0.732220854	0.988623277
IRF4	0.044455124	0.732227682	0.988623277
FDX1L	0.026711254	0.732386396	0.988623277
GRB14	0.086339878	0.732457984	0.988623277
SCCPDH	-0.030211768	0.732459731	0.988623277
DNAJC11	0.027072092	0.73258411	0.988623277
XKR6	0.029547966	0.732754844	0.988623277
AEN	0.026106658	0.732832344	0.988623277
SLC19A1	0.029325425	0.732834625	0.988623277
ITGA5	-0.023379607	0.732899725	0.988623277
FOXP1	0.036033825	0.73297752	0.988623277
MXI1	-0.037260822	0.733208823	0.988623277
MMP24	0.028385643	0.733261316	0.988623277
PRRG4	-0.064558155	0.733334673	0.988623277
MNX1	-0.030326618	0.733416609	0.988623277
FAM47E-STBD1	-0.035166259	0.733563217	0.988623277
VWA5A	-0.033433811	0.733597612	0.988623277
TMEM30B	-0.036367166	0.73376657	0.988623277
CCNB1	-0.038176872	0.733778764	0.988623277
EP400	0.021783388	0.733900139	0.988623277
FBXO31	-0.022781623	0.734046531	0.988623277
RNASE3	0.072257112	0.734057163	0.988623277
C1orf177	0.031590696	0.734065147	0.988623277
MAPK11	-0.026474309	0.734103801	0.988623277
LGALS9C	-0.041283285	0.734297111	0.988623277
CUX2	-0.054331376	0.734365263	0.988623277
CD36	-0.063091796	0.734431449	0.988623277
BMP2K	-0.034760622	0.734497529	0.988623277
CLNS1A	-0.021176481	0.734614583	0.988623277
FCRLB	0.044274694	0.734705122	0.988623277
BIRC2	-0.022164433	0.734786227	0.988623277
EEF1B2	0.03072897	0.734981175	0.988623277
TUBE1	-0.031322052	0.735079014	0.988623277
ODF3B	-0.033737448	0.735374371	0.988623277

KANK1	0.08550747	0.735431042	0.988623277
LLGL1	0.025178405	0.735431971	0.988623277
ZNF8	0.035305801	0.735607693	0.988623277
PRDM1	-0.046561533	0.735617019	0.988623277
RPS2P32	0.041786744	0.735782367	0.988623277
JRK	0.037790126	0.735873231	0.988623277
LMNA	0.055816121	0.735985343	0.988623277
APEH	-0.015796711	0.736032388	0.988623277
ADAP2	-0.032567568	0.736039972	0.988623277
NDUFS8	-0.016933306	0.736108441	0.988623277
SLC31A1	-0.031984763	0.736117685	0.988623277
SETD4	0.034554092	0.736137283	0.988623277
RTEL1	0.029976426	0.736259629	0.988623277
CADM1	-0.068684546	0.736345755	0.988623277
TRIM32	0.030006203	0.736598501	0.988623277
SLC5A6	0.027061296	0.736669785	0.988623277
RLF	-0.020012989	0.736710031	0.988623277
TRIM5	0.04166023	0.737251761	0.988623277
PACSIN1	0.052353678	0.73725428	0.988623277
KIF21B	0.025794088	0.737291709	0.988623277
FAM203A	0.027875882	0.737498447	0.988623277
ARIH1	0.020533659	0.737508778	0.988623277
ST8SIA1	-0.045401259	0.737618499	0.988623277
RBKS	0.02460129	0.737810789	0.988623277
TOR1B	0.023923923	0.737958102	0.988623277
YTHDF3	-0.018797826	0.737962234	0.988623277
PSMD9	0.032499512	0.738021163	0.988623277
GPRC5C	-0.067569059	0.738022695	0.988623277
CFL2	-0.031171038	0.738035903	0.988623277
VPS8	-0.018493163	0.738127656	0.988623277
AK1	0.048511167	0.738153053	0.988623277
STRC	0.052043658	0.738196129	0.988623277
SPOCK1	0.035715318	0.738213969	0.988623277
NSMCE4A	-0.01452585	0.738276102	0.988623277
MAP4K3	0.028033496	0.738296025	0.988623277
C9orf69	-0.027344249	0.738346718	0.988623277
RUNX3	-0.033483351	0.738443476	0.988623277
DAPP1	-0.017985656	0.738464912	0.988623277
NT5C1B	-0.013545685	0.738470522	0.988623277
NMRAL1	-0.028945433	0.738481746	0.988623277
KLHDC9	-0.035917603	0.738535443	0.988623277
SLK	0.024131959	0.738644088	0.988623277
LPIN1	-0.0272934	0.738772331	0.988623277
IGSF6	0.049653646	0.738835547	0.988623277
TRIM22	0.028219777	0.739063462	0.988623277
IGLL3P	-0.110947147	0.739161486	0.988623277
MORN2	0.035026154	0.739196318	0.988623277

IKBKAP	-0.022462957	0.739274559	0.988623277
ZNF80	0.061973735	0.739328538	0.988623277
CHCHD1	-0.029363371	0.739429819	0.988623277
FBXO3	-0.027526555	0.739706916	0.988623277
TYW1	0.029493941	0.739773983	0.988623277
AKNA	-0.022836339	0.739832461	0.988623277
RB1	-0.043223598	0.739863761	0.988623277
SHROOM1	-0.042294125	0.739933024	0.988623277
PPP1CC	0.016982978	0.740068223	0.988623277
CACNB3	-0.040995309	0.740102845	0.988623277
NME1-NME2	0.027746457	0.740139176	0.988623277
TMEM43	-0.017257233	0.740264703	0.988623277
CPXM1	-0.027689155	0.740266741	0.988623277
RHOG	0.026376318	0.740365846	0.988623277
ZDHHC2	0.033439541	0.740392361	0.988623277
DDX49	0.02689384	0.740554597	0.988623277
RFPL4A	0.034624285	0.740563327	0.988623277
GEMIN4	-0.019612043	0.740576633	0.988623277
PDCD7	-0.011973208	0.74057704	0.988623277
UNCX	0.030524803	0.740631075	0.988623277
DUSP14	0.043941636	0.740741192	0.988623277
CBWD5	-0.043617627	0.740801043	0.988623277
TAF4	0.016734879	0.740844964	0.988623277
ZNF589	-0.036913835	0.740907248	0.988623277
TMEM143	-0.03007351	0.741018256	0.988623277
NOP14	0.022169756	0.74106292	0.988623277
ZNF692	-0.026793597	0.741093204	0.988623277
COG7	-0.029882583	0.741163563	0.988623277
GFOD1	0.050005794	0.741189852	0.988623277
IL18BP	0.020550341	0.741221842	0.988623277
AGRN	0.030259727	0.741498703	0.988873391
NAA38	-0.033858319	0.741630307	0.988929752
INF2	0.038770103	0.741932506	0.989163825
FZD2	-0.033748164	0.741984573	0.989163825
UQCRC1	0.018981419	0.74207576	0.989166255
RAD51	-0.050804558	0.742243962	0.989271332
DNASE1	-0.031540634	0.742473101	0.989286221
LSM6	0.045432691	0.742558226	0.989286221
PLEKHG5	0.038619238	0.742607796	0.989286221
CCT2	0.018220273	0.74261263	0.989286221
TEX10	0.017293817	0.742829574	0.989313169
GJC1	-0.011560708	0.742882143	0.989313169
POLR2A	-0.01660849	0.74290099	0.989313169
MDM1	0.029233209	0.743051722	0.98935239
ZNF502	-0.059042522	0.743126192	0.98935239
S1PR1	-0.045160028	0.743353122	0.98935239
PNPLA8	-0.026100839	0.743465245	0.98935239

MRPL45P2	-0.042671404	0.743602803	0.98935239
SH3YL1	-0.050722028	0.743822656	0.98935239
HIST1H2AC	0.065693386	0.743996807	0.98935239
AKAP13	-0.0190416	0.74408187	0.98935239
FAM76A	-0.040186886	0.744285043	0.98935239
PIGK	-0.027962344	0.744363246	0.98935239
HIST2H3D	0.041932733	0.744448464	0.98935239
METTL16	-0.033171704	0.744637833	0.98935239
HTR3A	0.037951219	0.744642078	0.98935239
PTPLAD1	-0.01949423	0.744903949	0.98935239
POLA2	0.020969912	0.745090154	0.98935239
SART3	-0.024892172	0.745270184	0.98935239
TK2	-0.024704859	0.745285564	0.98935239
PAPD7	0.02443658	0.745702197	0.98935239
POU2F2	0.039174025	0.745729338	0.98935239
DRAP1	-0.025051186	0.745743604	0.98935239
RAF1	-0.016259247	0.746034301	0.98935239
GUCY1A3	0.075599522	0.746035632	0.98935239
LRRC29	-0.025191705	0.746072611	0.98935239
TMEM9B	0.013031263	0.746171864	0.98935239
AURKAIP1	0.015592506	0.746216134	0.98935239
PLAGL1	-0.037176755	0.746278904	0.98935239
USP7	0.02562979	0.746386921	0.98935239
MRPS35	0.029562569	0.74662314	0.98935239
TCEA1	0.029804402	0.746641432	0.98935239
MFSD11	-0.041894016	0.746648801	0.98935239
ABHD5	0.038085008	0.74683794	0.98935239
REV3L	-0.032246926	0.746924406	0.98935239
TRAC	0.055039386	0.746991574	0.98935239
PRKCI	-0.022459904	0.74730744	0.98935239
LAPTM4A	0.025771038	0.747312711	0.98935239
CDKN2C	0.042682823	0.747363647	0.98935239
ARFGAP3	0.022240569	0.747578998	0.98935239
BAG6	-0.02533946	0.747713436	0.98935239
LTK	-0.034775937	0.747935565	0.98935239
RCAN3	0.035529885	0.748023764	0.98935239
ZNF414	0.025815065	0.748136705	0.98935239
PCDHGC3	-0.037057964	0.748317919	0.98935239
CHD8	-0.030067552	0.748393926	0.98935239
HIST2H4B	-0.056512083	0.748419934	0.98935239
CYP2U1	0.025178738	0.748514598	0.98935239
MN1	0.03057727	0.748517988	0.98935239
PDSS1	-0.029278094	0.748536673	0.98935239
MGMT	0.03449703	0.748596227	0.98935239
PSMA4	-0.018349108	0.748679916	0.98935239
ZNF324	-0.029464132	0.748690489	0.98935239
SLC26A11	-0.023185088	0.748811616	0.98935239

LAP3	0.048246563	0.748860794	0.98935239
ZNF181	-0.03421013	0.748863703	0.98935239
PEX16	-0.017582051	0.748958612	0.98935239
RECQL5	0.033120094	0.749065038	0.98935239
MYD88	0.021964569	0.749158858	0.98935239
RFC5	0.024304454	0.749162228	0.98935239
NDE1	-0.026086902	0.749201843	0.98935239
HSP90B1	0.025323954	0.749212035	0.98935239
CALU	0.027694703	0.749220747	0.98935239
UNC13D	0.034919413	0.749245128	0.98935239
MIR1253	0.029789802	0.749254207	0.98935239
SNORD11	0.041628116	0.749300417	0.98935239
GNL1	0.022364696	0.749376804	0.98935239
TDRD12	0.029669749	0.749776078	0.98935239
DDOST	-0.016261386	0.74977884	0.98935239
DLG4	0.037804485	0.749791269	0.98935239
C18orf32	0.030240502	0.749851091	0.98935239
SLC40A1	-0.034591907	0.749875351	0.98935239
RPLP2	0.024577035	0.749994552	0.98935239
RPAIN	-0.015037206	0.75027963	0.98935239
BOD1	-0.019291883	0.750423814	0.98935239
ARMC6	0.02674466	0.750452795	0.98935239
RWDD2B	0.028084191	0.750522778	0.98935239
PRKAR1B	0.037191919	0.750528425	0.98935239
ALG12	0.023157597	0.750545935	0.98935239
RPS26	0.087998131	0.750562315	0.98935239
ITGA3	0.035656607	0.750568944	0.98935239
EMR4P	0.098274705	0.750783923	0.98935239
CARD17	0.039757124	0.751021191	0.98935239
TIGD7	-0.028926254	0.751026896	0.98935239
CNDP2	-0.020918317	0.751037813	0.98935239
MAP2K5	-0.01875509	0.751047616	0.98935239
SRD5A3	-0.037295566	0.751131936	0.98935239
CPNE3	0.013430202	0.751169319	0.98935239
SLC46A3	-0.01909918	0.751267797	0.98935239
RNF20	-0.019533201	0.751275593	0.98935239
KCTD21	-0.030365158	0.751317067	0.98935239
RORC	-0.079363094	0.751336983	0.98935239
PCNXL2	-0.036187365	0.751355751	0.98935239
UBR2	0.014181744	0.751466732	0.98935239
SEC61B	-0.018641369	0.751488309	0.98935239
MT1X	-0.033980413	0.751637942	0.98935239
PHF11	-0.015253559	0.751755539	0.98935239
CLIP4	-0.026041353	0.751818074	0.98935239
LRRC37B	0.021567319	0.7518436	0.98935239
CAMK4	0.039416131	0.751960276	0.98935239
ACP5	-0.05098416	0.751985408	0.98935239

CCT7	-0.015025635	0.752108154	0.98935239
ARRDC1	0.029037485	0.752272065	0.98935239
RELB	0.026369506	0.752337141	0.98935239
PPIAL4A	0.019927321	0.752498564	0.98935239
IKBKB	-0.018030602	0.752558324	0.98935239
ETV2	0.031068525	0.752645948	0.98935239
ANKDD1A	-0.100857463	0.752674549	0.98935239
TTC38	0.055562725	0.752677815	0.98935239
SNORD65	-0.039486452	0.752678359	0.98935239
TESK1	-0.025309804	0.752702608	0.98935239
VCAM1	0.052543154	0.752808221	0.98935239
MCCC2	0.027954151	0.752868928	0.98935239
NRM	-0.026829341	0.752970527	0.98935239
SLC25A37	0.046225993	0.752976742	0.98935239
DSCC1	-0.027109838	0.753499005	0.98935239
QPRT	-0.039416702	0.753763521	0.98935239
ITGB3BP	-0.052100661	0.754226897	0.98935239
TBC1D15	-0.012297133	0.754395176	0.98935239
RSAD2	0.077674476	0.754562184	0.98935239
TOMM40L	0.031296135	0.754590989	0.98935239
C19orf52	0.020679629	0.754636908	0.98935239
CTDNEP1	0.021679182	0.754698424	0.98935239
NAPSA	-0.07206113	0.754771323	0.98935239
SEPP1	0.032415583	0.75483231	0.98935239
LILRA4	0.040471601	0.754962344	0.98935239
SHQ1	-0.023396462	0.754990001	0.98935239
UFD1L	0.022137789	0.75501817	0.98935239
DPH5	0.028167924	0.755137024	0.98935239
HSPA1A	0.025727527	0.755228102	0.98935239
PAK4	-0.027129169	0.755305717	0.98935239
SNORD84	0.034481947	0.75549251	0.98935239
ST6GALNAC1	-0.046163705	0.755580166	0.98935239
TBKBP1	0.036875509	0.755793754	0.98935239
XCR1	0.040130466	0.755860156	0.98935239
MZB1	-0.068589851	0.755909891	0.98935239
DDX19A	0.015333249	0.756031825	0.98935239
NME4	-0.057131382	0.756056711	0.98935239
MLKL	-0.021402608	0.756063717	0.98935239
GPANK1	0.036046569	0.756290951	0.98935239
ITGB1BP1	0.035891342	0.756327004	0.98935239
IL12RB1	0.035248835	0.756554873	0.98935239
PDGFC	-0.047161387	0.756631663	0.98935239
MMAA	-0.023494071	0.756675246	0.98935239
SND1	-0.023744573	0.756704868	0.98935239
PPM1N	0.031496481	0.756731127	0.98935239
PTPN1	0.021863252	0.756811906	0.98935239
C12orf60	-0.031040638	0.75707003	0.98935239

ZNF654	-0.025577819	0.757118203	0.98935239
ATF2	0.024922288	0.757172414	0.98935239
MCRS1	0.012776104	0.757283706	0.98935239
EPSTI1	-0.063440185	0.757337783	0.98935239
DNAL4	0.016653829	0.757358472	0.98935239
CLEC3B	0.036961556	0.75753251	0.98935239
PRKCSH	0.016010548	0.757713355	0.98935239
ACTR10	-0.0173396	0.757842633	0.98935239
RANGAP1	-0.015832561	0.757885537	0.98935239
C19orf12	0.028199462	0.757895774	0.98935239
CRADD	0.033809884	0.757971183	0.98935239
NEK1	-0.023593012	0.758053612	0.98935239
GEMIN6	-0.028390191	0.758079825	0.98935239
ICT1	0.015505081	0.758101928	0.98935239
HLTF	-0.019401604	0.75810885	0.98935239
DPYD	-0.041882153	0.758167339	0.98935239
ASB6	-0.027983945	0.758248659	0.98935239
DUSP5	-0.036998334	0.758365887	0.98935239
SIGLEC7	-0.052436358	0.758435008	0.98935239
WDFY3	-0.035062153	0.758489639	0.98935239
DHX9	0.025694794	0.758566004	0.98935239
EXOC2	-0.023829059	0.758750105	0.98935239
METTL15	-0.021384235	0.758807705	0.98935239
AMBRA1	-0.028739245	0.758885367	0.98935239
ORC2	-0.020392204	0.758927997	0.98935239
LRRC42	0.024778925	0.758936711	0.98935239
DCAF17	0.02562043	0.759016013	0.98935239
POLE4	0.015754706	0.759284912	0.98935239
AKAP1	-0.0242886	0.759286062	0.98935239
NADK	0.029919603	0.759407122	0.98935239
STX2	0.040678499	0.75948568	0.98935239
BCL11B	0.022376826	0.75954437	0.98935239
GNAL	0.033348699	0.759600159	0.98935239
C14orf80	-0.02500099	0.759756785	0.98935239
SFXN1	0.032289256	0.759769725	0.98935239
YARS2	-0.023765971	0.759890246	0.98935239
CLIC6	-0.044036079	0.760078925	0.98935239
CEP350	-0.024898291	0.76013148	0.98935239
MAGOHB	0.030539816	0.760229197	0.98935239
CAMP	-0.051363618	0.76042669	0.98935239
GIPC3	0.035460126	0.760490965	0.98935239
SLX4	0.022677218	0.760558013	0.98935239
HTT	-0.029091176	0.760762475	0.98935239
RAD54L2	-0.03061804	0.760790007	0.98935239
HCFC1R1	0.021675744	0.760798038	0.98935239
NOMO3	0.028957389	0.760856682	0.98935239
TMC6	-0.025875783	0.760996189	0.98935239

FOXH1	0.026613471	0.761162117	0.98935239
THAP2	0.029089947	0.761185758	0.98935239
STX4	-0.025153233	0.761198939	0.98935239
UCK1	0.030301075	0.761253772	0.98935239
STAT4	-0.022333099	0.761255667	0.98935239
NIPA2	-0.017603533	0.761387118	0.98935239
CRISPLD2	-0.05547779	0.761610369	0.98935239
LEPROT	-0.037160696	0.761773464	0.98935239
ALG10B	-0.026343771	0.761914823	0.98935239
CAPN5	0.0457464	0.762081033	0.98935239
ZNF836	-0.025626619	0.762097937	0.98935239
LMBR1L	0.024365513	0.762112459	0.98935239
SKA3	-0.023263126	0.762248147	0.98935239
SLC38A6	-0.021240058	0.762339781	0.98935239
SFTPD	0.059518085	0.762405793	0.98935239
LIME1	-0.01826162	0.762504595	0.98935239
GOT2	-0.020031825	0.762698391	0.98935239
BIVM	-0.028746724	0.7627406	0.98935239
IDO1	-0.037447369	0.762820791	0.98935239
INCENP	0.029700399	0.762862696	0.98935239
INTS7	0.023729291	0.762869699	0.98935239
EAPP	-0.040703355	0.762883958	0.98935239
FAM110A	0.027564735	0.762906472	0.98935239
SUN2	0.01679697	0.76291219	0.98935239
ZNF672	0.019330369	0.762975569	0.98935239
C9orf142	0.022976361	0.763020056	0.98935239
NLRP5	-0.026905914	0.763082234	0.98935239
GALNT12	0.023628846	0.763189224	0.98935239
DLG1	-0.024878799	0.763214325	0.98935239
COQ10A	0.023750287	0.763232277	0.98935239
STAG3	0.033418071	0.76330738	0.98935239
XRCC1	0.020001602	0.763429717	0.98935239
WDHD1	-0.028781045	0.763496035	0.98935239
LARGE	-0.053713736	0.763785898	0.98935239
TNFAIP1	-0.018760799	0.76384984	0.98935239
GNB2	-0.021752444	0.76385062	0.98935239
MRPL21	-0.02484192	0.763987211	0.98935239
CCNDBP1	0.015937308	0.764015102	0.98935239
ACY1	-0.024670324	0.764033117	0.98935239
CRTC2	-0.022380772	0.764197651	0.98935239
RNF207	-0.034186014	0.764250045	0.98935239
OGG1	0.027663408	0.764315552	0.98935239
OPRL1	0.022457802	0.764374537	0.98935239
MEMO1	0.018979283	0.7645066	0.98935239
ARFGAP2	0.018790492	0.764664093	0.98935239
PRPSAP1	0.016148207	0.764717098	0.98935239
ARAP3	-0.028611443	0.764846911	0.98935239

TMEM192	0.030519241	0.764864962	0.98935239
JMJD6	-0.027221325	0.764930429	0.98935239
RTBDN	0.030481898	0.764978411	0.98935239
SLC4A2	0.028705061	0.765070913	0.98935239
GSDMB	-0.049467528	0.765153371	0.98935239
ZNF317	0.021597886	0.765218422	0.98935239
ARSG	-0.019462458	0.765400206	0.98935239
FAM120AOS	-0.016846088	0.765412451	0.98935239
TPR	0.023057214	0.76542025	0.98935239
LRWD1	-0.032178831	0.765441339	0.98935239
BAG3	0.030196513	0.765477188	0.98935239
UQCRC2	0.026198954	0.76570099	0.98935239
VSTM1	-0.111272669	0.765755059	0.98935239
S100A11	0.036342904	0.765762945	0.98935239
OR2A42	0.033721657	0.765774983	0.98935239
SMC2	-0.029273448	0.765821057	0.98935239
ZNF831	-0.033715123	0.765931699	0.98935239
NAGA	0.030181108	0.765947886	0.98935239
DUSP28	0.015099344	0.765990603	0.98935239
OSTC	-0.018860817	0.76615297	0.989430189
ATG16L1	-0.02008834	0.76645299	0.989430189
DENND4C	-0.023009098	0.766455324	0.989430189
SNRK	0.014281373	0.766557977	0.989430189
SACM1L	-0.026836502	0.766635152	0.989430189
FAM24B	0.030413766	0.766654072	0.989430189
RNF19B	0.031933522	0.766844141	0.989430189
B9D2	0.019152929	0.766860747	0.989430189
GPR89A	0.032716561	0.766866991	0.989430189
PLEKHA1	-0.018153129	0.767005501	0.989430189
PTK2B	-0.02241117	0.767130398	0.989430189
CHAF1A	-0.033226477	0.767244452	0.989430189
SRD5A1	0.02757295	0.767407795	0.989430189
HARBI1	0.031359684	0.767549851	0.989430189
MITD1	0.021494657	0.767589064	0.989430189
C5orf51	-0.019867978	0.76767835	0.989430189
TRIML1	0.025384502	0.767691587	0.989430189
ENG	0.034559274	0.767717504	0.989430189
GNA11	0.04132358	0.767783221	0.989430189
C1orf54	-0.032436732	0.767838587	0.989430189
SGMS2	-0.033003887	0.767938068	0.989443193
AZIN1	-0.020860232	0.768111473	0.989551431
TMEM97	-0.030218333	0.768248387	0.989555072
FUT8	-0.035942518	0.768506206	0.989555072
EIF4A2	-0.011921337	0.768634655	0.989555072
FLCN	-0.029350048	0.768635322	0.989555072
VAMP4	0.039123368	0.768690302	0.989555072
PPP1R3E	-0.035830792	0.768802868	0.989555072

RQCD1	0.028151006	0.768896002	0.989555072
CENPO	0.025805148	0.769230036	0.989555072
B3GNTL1	-0.028718172	0.76927172	0.989555072
CCDC71	0.042211585	0.769356319	0.989555072
KIAA1211	0.037865197	0.769491056	0.989555072
SAMD9	-0.023695167	0.769748529	0.989555072
ARHGAP32	0.030970067	0.769948332	0.989555072
USP32	-0.060118161	0.770078273	0.989555072
ING3	-0.014068399	0.770098815	0.989555072
MRPL47	0.02468102	0.770146218	0.989555072
DCHS1	-0.033451471	0.770396063	0.989555072
ZNF800	-0.013727326	0.7704221	0.989555072
AZI1	-0.021327182	0.770494728	0.989555072
MYL4	0.156411139	0.77088885	0.989555072
TM4SF19	0.027685231	0.770982598	0.989555072
MAP2K1	-0.020009589	0.771063011	0.989555072
HIST1H2BK	0.039410048	0.771110529	0.989555072
RNF144B	0.042590599	0.771128993	0.989555072
C21orf33	-0.014094458	0.771143571	0.989555072
ATG4C	0.022508263	0.771256433	0.989555072
COPS3	0.012763178	0.771329121	0.989555072
SLC25A28	-0.015962481	0.771364131	0.989555072
TREM1	-0.05043005	0.771419143	0.989555072
UBL5	0.014724147	0.771538071	0.989555072
ANKRD50	-0.026106556	0.771663727	0.989555072
PAM	0.028033779	0.771765271	0.989555072
C12orf76	0.026199858	0.771788621	0.989555072
SYNRG	0.01930071	0.771912577	0.989555072
CEL	0.033170149	0.771984293	0.989555072
MDFIC	-0.030008876	0.772120761	0.989555072
PARP3	0.037403022	0.772164272	0.989555072
CDCA7	0.041429866	0.772210576	0.989555072
ASB13	0.027214287	0.77228918	0.989555072
LIPA	-0.034951748	0.772292002	0.989555072
COTL1	-0.039606053	0.772341426	0.989555072
USP37	0.025911517	0.772348014	0.989555072
UGCG	-0.030965028	0.772359693	0.989555072
VPS29	-0.014033636	0.772461894	0.989555072
GPATCH8	-0.021760045	0.772518165	0.989555072
MYO1F	0.023021161	0.772545982	0.989555072
ANP32B	0.011997099	0.77276497	0.989555072
HMHA1	0.021579067	0.772968825	0.989555072
PAK1IP1	-0.016195097	0.773097823	0.989555072
M6PR	0.022425373	0.773362424	0.989555072
MAPKAPK3	-0.021525239	0.773401498	0.989555072
AGPAT1	0.036684046	0.773410296	0.989555072
APEX1	-0.013683361	0.77360904	0.989555072

TLK2	0.016781065	0.773626272	0.989555072
ITSN1	0.039553797	0.773728688	0.989555072
HBQ1	0.081923067	0.773841526	0.989555072
IGFLR1	0.050887671	0.774184932	0.989555072
MIS18A	0.029921832	0.774211638	0.989555072
ACTR8	-0.01871077	0.774546909	0.989555072
ARHGEF10	-0.051493056	0.774636004	0.989555072
CECR5	-0.018192834	0.774668104	0.989555072
TSN	0.027879394	0.774678506	0.989555072
UBQLN4	-0.018571167	0.774735267	0.989555072
DENND2D	-0.026232392	0.774735843	0.989555072
ACAT1	0.022548057	0.774788825	0.989555072
CDC23	-0.015208194	0.775086696	0.989555072
MED22	0.012542589	0.77517322	0.989555072
SOX4	-0.029142663	0.775298426	0.989555072
KIAA1430	0.032622088	0.775386313	0.989555072
CAMK1G	-0.058250461	0.775708192	0.989555072
CLDND1	-0.023032459	0.775720221	0.989555072
WIPI1	0.031562052	0.775748736	0.989555072
NBPF3	-0.02358772	0.775796703	0.989555072
FAM173A	0.01816004	0.775835	0.989555072
HLA-F	0.023886116	0.775942084	0.989555072
LPAL2	-0.033340987	0.775946556	0.989555072
EPG5	-0.045343558	0.776008586	0.989555072
MYO6	-0.030030883	0.77606008	0.989555072
SCOC	-0.023197086	0.776090584	0.989555072
LPAR1	-0.075045999	0.776112367	0.989555072
GTF2A1	0.039701243	0.776320852	0.989555072
DMC1	-0.027515933	0.776393458	0.989555072
JUP	0.083302866	0.776440746	0.989555072
CEP192	0.021997584	0.77648467	0.989555072
ADA	-0.025519734	0.776561358	0.989555072
PREB	-0.023288344	0.776577922	0.989555072
L1TD1	-0.055020325	0.776625917	0.989555072
CALCOCO2	0.030770214	0.776713567	0.989555072
HBG1	0.226672663	0.776821339	0.989555072
TBX19	0.023287989	0.776957246	0.989555072
LIN52	-0.03321858	0.776998397	0.989555072
TP53I13	-0.020007121	0.777072446	0.989555072
TMEM55A	-0.034078134	0.777116854	0.989555072
CUL3	0.031176787	0.777151032	0.989555072
FGFR1OP2	-0.022820384	0.777280386	0.989555072
SFT2D2	0.028136003	0.777298221	0.989555072
DGCR6	-0.024279075	0.777625206	0.989555072
IARS	-0.014128785	0.777704269	0.989555072
POLR3K	0.025412957	0.777716339	0.989555072
SLC35A3	0.018963628	0.777865354	0.989555072

CDK5R1	0.051076604	0.777881959	0.989555072
SIRPD	-0.030850122	0.777937224	0.989555072
UBE2MP1	0.025162769	0.778038956	0.989555072
CCDC69	-0.030908616	0.778117016	0.989555072
OXCT1	0.019377695	0.778142256	0.989555072
TMEM163	0.028880628	0.77821992	0.989555072
BLVRB	0.040763472	0.778288179	0.989555072
TAPBP	0.024294717	0.778365619	0.989555072
ESYT1	-0.015628133	0.778479217	0.989555072
APOBEC3A	0.061793427	0.778623405	0.989555072
SH2D3C	0.025474843	0.778808638	0.989555072
ANKMY1	0.038892463	0.778814064	0.989555072
HNRNPA1	0.028576942	0.778978493	0.989555072
HLA-G	-0.036168729	0.778983481	0.989555072
ZNF839	-0.023848178	0.779031277	0.989555072
DPP4	0.037416116	0.77925479	0.989555072
SUB1	-0.022610582	0.779318216	0.989555072
RHPN2	0.038212468	0.779325541	0.989555072
C1orf50	-0.016364401	0.779346112	0.989555072
UIMC1	-0.018013122	0.779346912	0.989555072
CRAMP1L	-0.025184618	0.77949178	0.989555072
PSMB10	-0.018191759	0.779582223	0.989555072
MIR129-2	-0.024781212	0.779618889	0.989555072
EIF5B	-0.016888544	0.779668894	0.989555072
TPK1	-0.019232217	0.779695879	0.989555072
GATS	0.034497371	0.779790044	0.989555072
ITGAD	0.06787009	0.779822583	0.989555072
SPATA20	0.074552678	0.779859133	0.989555072
CMTM3	-0.017055591	0.780040459	0.989555072
CCDC85C	-0.027489647	0.780183315	0.989555072
SHOC2	-0.018199321	0.780541985	0.989555072
NAV2	-0.025242897	0.780879846	0.989555072
SMN2	-0.040314185	0.780988179	0.989555072
POLD1	-0.024530045	0.781029618	0.989555072
PCTP	-0.025108222	0.781057708	0.989555072
SPATS2L	0.034380614	0.781357471	0.989555072
SLC48A1	-0.022168041	0.781503929	0.989555072
ADORA3	-0.050979333	0.781572882	0.989555072
LYL1	-0.017930465	0.78165488	0.989555072
ENC1	0.048226251	0.781734156	0.989555072
RTTN	0.019430206	0.781764763	0.989555072
TWF1	-0.027172091	0.781793879	0.989555072
EMP1	-0.05320139	0.781855799	0.989555072
ARL1	-0.015069713	0.781966157	0.989555072
DUSP11	-0.019133361	0.782156639	0.989555072
NEIL2	-0.023594126	0.782324319	0.989555072
LSAMP	-0.024016001	0.782468574	0.989555072

ACP1	-0.024232632	0.782723132	0.989555072
AMT	-0.032447427	0.782794956	0.989555072
GNPTG	-0.019679354	0.782828639	0.989555072
FCRL6	-0.079989251	0.783288512	0.989555072
UST	-0.038680639	0.783309009	0.989555072
CRY2	-0.016445738	0.783398095	0.989555072
SRPR	-0.026097414	0.783467313	0.989555072
SEPN1	-0.024226618	0.783477188	0.989555072
ZCCHC11	0.021493721	0.783653901	0.989555072
GZMB	0.046533927	0.783667292	0.989555072
MRPL52	-0.022891938	0.78371856	0.989555072
RIN1	0.034671077	0.784011151	0.989555072
MCPH1	-0.018464837	0.784022995	0.989555072
TMC4	0.037245134	0.784161159	0.989555072
C8orf58	0.025172332	0.784249687	0.989555072
MED19	0.023973957	0.784252543	0.989555072
CREG1	0.030892129	0.784292432	0.989555072
MRPL40	0.022951536	0.784348041	0.989555072
ALDH1B1	0.02574379	0.784351321	0.989555072
LBH	0.022276749	0.784362319	0.989555072
MYLIP	0.018295103	0.784453767	0.989555072
DDX10	-0.078693389	0.784549315	0.989555072
CTDSP2	-0.014112813	0.784704667	0.989555072
CACNA2D2	0.045205942	0.78471647	0.989555072
ZNF683	0.119563076	0.784720657	0.989555072
LYRM7	-0.016982715	0.784880856	0.989555072
KLHL17	-0.028061003	0.784963629	0.989555072
MSX2P1	-0.034126957	0.785036822	0.989555072
CCPG1	-0.02310654	0.785283118	0.989555072
TMEM115	-0.018174571	0.785305533	0.989555072
GADD45GIP1	0.027390199	0.785514002	0.989555072
TTC32	-0.031024651	0.785570157	0.989555072
RNF41	0.017679673	0.785857476	0.989555072
PHF20	-0.01785047	0.785903565	0.989555072
USP34	0.015967451	0.78593421	0.989555072
NIPAL2	-0.023971178	0.785980978	0.989555072
NAP1L4	-0.021788317	0.785998829	0.989555072
AP3M2	0.025555125	0.78619353	0.989555072
TRIM10	-0.059110597	0.786201556	0.989555072
CLIP3	-0.038184194	0.786233143	0.989555072
OSBPL3	-0.022278628	0.786308883	0.989555072
ALDH5A1	0.026655416	0.786360849	0.989555072
APCDD1	-0.033011463	0.786431855	0.989555072
PAFAH1B1	-0.016130241	0.786524114	0.989555072
PKN2	-0.035114693	0.786655124	0.989555072
CCZ1	0.027483112	0.78667573	0.989555072
ETHE1	-0.027024685	0.787000883	0.989555072

GBP2	0.024443991	0.787193568	0.989555072
CIZ1	-0.023670579	0.787201407	0.989555072
RPL26L1	0.022515832	0.787304929	0.989555072
HPS3	0.022174035	0.787378308	0.989555072
CCDC146	0.038440426	0.787449976	0.989555072
RTN2	-0.039890001	0.787536294	0.989555072
SSH3	-0.019924693	0.78763504	0.989555072
AJAP1	0.024516033	0.787675473	0.989555072
FAM83H	0.024228963	0.787692134	0.989555072
TMEM45A	0.074616622	0.78771193	0.989555072
ARFGEF2	-0.01928253	0.787909127	0.989555072
RINL	-0.01938848	0.788017581	0.989555072
ASAP3	0.027243749	0.788131302	0.989555072
HGSNAT	0.021490568	0.78815765	0.989555072
HIF1AN	0.018619135	0.788172308	0.989555072
KIF3B	0.016678257	0.788214445	0.989555072
PIK3CA	0.023913729	0.788261767	0.989555072
UBAP2L	-0.022012334	0.788294318	0.989555072
ADNP	-0.0151777	0.788352875	0.989555072
LPAR6	0.026830262	0.78851963	0.989555072
OSTCP1	-0.031243534	0.78855708	0.989555072
CCDC93	-0.019674181	0.788691263	0.989555072
FAM172A	0.014466061	0.788737592	0.989555072
ACSL6	0.027692795	0.788824881	0.989555072
C16orf93	-0.028579978	0.788985917	0.989555072
MRPL39	-0.022448845	0.789063988	0.989555072
FAM46C	0.046924784	0.789160518	0.989555072
TRUB1	0.028717268	0.78921312	0.989555072
LSP1	-0.034079409	0.78926179	0.989555072
ARHGAP10	0.025504356	0.789285002	0.989555072
TANK	-0.01755872	0.7893698	0.989555072
WDR35	0.020426023	0.789417755	0.989555072
SERPINB1	0.029199452	0.789423908	0.989555072
CHCHD6	-0.022654368	0.789480163	0.989555072
KRR1	-0.024365979	0.789480294	0.989555072
CCDC23	0.022815343	0.789526714	0.989555072
TMEM14A	0.029887088	0.789715043	0.989555072
GOLPH3L	-0.024419042	0.789716104	0.989555072
DOT1L	0.027197156	0.789754832	0.989555072
DR1	0.019909366	0.790217262	0.989555072
MTRNR2L1	-0.049161074	0.790288666	0.989555072
LGALS12	-0.039250319	0.790380644	0.989555072
C9orf62	0.027743489	0.790505713	0.989555072
TLR5	0.023528342	0.790539801	0.989555072
SLC7A9	-0.030457214	0.790751352	0.989555072
RAP1A	-0.043556264	0.790759853	0.989555072
IL6R	-0.028508877	0.790815956	0.989555072

CLDN20	0.022573428	0.790896421	0.989555072
SLC25A22	0.018363081	0.790914332	0.989555072
MUM1	-0.020199203	0.790960928	0.989555072
KREMEN2	0.022907008	0.790989147	0.989555072
STYXL1	0.026742045	0.790989623	0.989555072
LRRC28	0.024725805	0.791202633	0.989555072
ZNF234	-0.033307198	0.791214028	0.989555072
IFI35	-0.028117326	0.791387971	0.989555072
CRTAP	0.052094721	0.791425581	0.989555072
ZNF398	-0.018550233	0.791537296	0.989555072
FAM118B	0.023721265	0.791787188	0.989555072
PHC2	0.027098985	0.792007302	0.989555072
PLA2G6	0.024679214	0.792087213	0.989555072
IGSF11	-0.023007143	0.792180496	0.989555072
ME3	0.040941433	0.792228146	0.989555072
SORBS2	-0.021897821	0.792248614	0.989555072
GLRX3	0.023705454	0.792526757	0.989555072
RPAP3	0.021027428	0.792580446	0.989555072
ITLN1	0.028303824	0.792595019	0.989555072
WIBG	0.014291021	0.792629358	0.989555072
LPGAT1	0.017368264	0.792646311	0.989555072
MVK	-0.021470372	0.792738779	0.989555072
ACAT2	-0.019520872	0.792953615	0.989555072
ZNF679	-0.048369888	0.793065681	0.989555072
MED30	0.018855334	0.793298577	0.989555072
SIGLEC10	0.040668712	0.793338271	0.989555072
FKTN	-0.011294366	0.793343426	0.989555072
S100A10	0.014104692	0.793361967	0.989555072
C19orf33	-0.091096845	0.793444223	0.989555072
ANXA7	0.01583192	0.793529384	0.989555072
TMEM199	-0.018398657	0.793582921	0.989555072
CLECL1	-0.064747775	0.793755495	0.989555072
POLR1B	-0.028913872	0.793756683	0.989555072
ENTPD1	0.033459282	0.794089431	0.989555072
CARD11	0.018725128	0.794177605	0.989555072
PHF21A	-0.01210056	0.794206651	0.989555072
CD28	-0.02919689	0.794221292	0.989555072
PUM1	0.010983144	0.79422734	0.989555072
RMND5A	0.027241936	0.794312224	0.989555072
PIP5K1B	-0.027820008	0.794381822	0.989555072
ARHGAP11A	-0.02556489	0.794420626	0.989555072
HERC6	0.028019457	0.794489545	0.989555072
KIAA0391	0.015255638	0.794617282	0.989555072
TMEM63B	0.027102301	0.794694138	0.989555072
P2RY6	-0.028884296	0.794698846	0.989555072
BACH1	0.027056497	0.7947145	0.989555072
ATP5C1	0.019900921	0.794778755	0.989555072

ATXN7L3	-0.017951945	0.794860275	0.989555072
RAMP2	0.025650841	0.794954573	0.989555072
TNK2	0.020379906	0.795089377	0.989555072
YOD1	0.020616318	0.795321608	0.989555072
FAM184A	0.030983193	0.795371363	0.989555072
STAT5B	0.01631037	0.795392308	0.989555072
SLTM	-0.014636879	0.795429629	0.989555072
TMEM60	0.020688538	0.79560539	0.989555072
SYN2	0.02370913	0.795942388	0.989555072
COX15	0.025553219	0.795948776	0.989555072
TMEM18	-0.020114449	0.796142259	0.989555072
SNX29	0.057231485	0.796222353	0.989555072
CD3G	-0.032231839	0.796360294	0.989555072
ACTB	0.019000234	0.796370397	0.989555072
AKIRIN2	0.014470802	0.796377067	0.989555072
MAST3	0.017272495	0.796461253	0.989555072
CMTM2	-0.049299437	0.796693137	0.989555072
EXTL3	0.023933773	0.796840753	0.989555072
TPRN	-0.023154947	0.796869433	0.989555072
ZNF320	0.029220466	0.796880378	0.989555072
VTA1	0.019686034	0.797032979	0.989555072
GBP1	-0.042802209	0.797085021	0.989555072
MIR2116	-0.029782534	0.797116212	0.989555072
BCL2	0.024025152	0.797131659	0.989555072
TTC30A	0.023447182	0.797137916	0.989555072
DHX34	0.020196586	0.797296331	0.989555072
APAF1	-0.027207115	0.797459584	0.989555072
ACVRL1	0.046021376	0.797494908	0.989555072
EHBP1	-0.01528716	0.797515253	0.989555072
MRPL35	-0.01934475	0.79753643	0.989555072
F13A1	0.052986517	0.797550695	0.989555072
DUSP3	0.019698319	0.797670414	0.989555072
GLIPR1	0.041011264	0.798252898	0.989555072
IFI44L	0.09430157	0.798500457	0.989555072
NUDT7	0.026538049	0.798622869	0.989555072
CDS2	0.018326065	0.798677603	0.989555072
CTH	0.024489716	0.798691042	0.989555072
TCP11L1	-0.023425974	0.798883992	0.989555072
MTSS1	-0.025507487	0.798911543	0.989555072
MADD	-0.020118975	0.798949587	0.989555072
FAM133B	-0.043156055	0.799021277	0.989555072
GPHN	0.022145102	0.799055529	0.989555072
CDKN2AIPNL	-0.013625845	0.799141063	0.989555072
EPHB1	0.056954015	0.799271623	0.989555072
ATOH8	-0.045055379	0.799319887	0.989555072
MYO5C	0.032943959	0.799333475	0.989555072
ZBTB6	-0.02496214	0.799568141	0.989555072

PDHX	-0.017383443	0.799577969	0.989555072
NEURL1B	0.018667639	0.799725128	0.989555072
SPIRE1	-0.028919556	0.79978218	0.989555072
PCNT	0.019106219	0.799847711	0.989555072
DPY19L1	0.018124303	0.800083068	0.989555072
C1orf216	-0.022351271	0.800278763	0.989555072
FRRS1	0.023828698	0.800334531	0.989555072
LZTFL1	0.020558737	0.800407513	0.989555072
ZBTB20	-0.026181999	0.800466554	0.989555072
ZFAND1	-0.021467864	0.800510207	0.989555072
TNPO1	-0.02701832	0.800592559	0.989555072
YME1L1	0.024164266	0.800615552	0.989555072
SCN5A	0.020447354	0.800795616	0.989555072
KCNMA1	0.038824722	0.800855095	0.989555072
SCPEP1	-0.025607854	0.800896857	0.989555072
SIGLEC6	0.036361816	0.800989933	0.989555072
CCT6P1	0.009278975	0.801022853	0.989555072
C4orf46	-0.022077392	0.801088147	0.989555072
TOMM20	0.017189557	0.801101682	0.989555072
FUBP1	0.016627059	0.801284519	0.989555072
TMEM161B	-0.020678711	0.801528614	0.989555072
RNF185	0.023997749	0.801539006	0.989555072
SETX	-0.017373209	0.80155262	0.989555072
JAG1	-0.023764142	0.801560623	0.989555072
SALL2	-0.021558593	0.801592318	0.989555072
AGL	-0.019519854	0.801622674	0.989555072
LEPREL1	0.033694434	0.801647202	0.989555072
RNF114	-0.00861535	0.80167821	0.989555072
EFHB	-0.028950169	0.80178234	0.989555072
SRM	-0.022327697	0.801809152	0.989555072
SUSD3	0.023730207	0.801932397	0.989555072
C19orf25	0.017175875	0.801935368	0.989555072
ZNF91	-0.035785663	0.802022318	0.989555072
CLEC2D	0.017866495	0.802070924	0.989555072
UBC	0.008481993	0.802291335	0.989555072
GSTM4	0.051519747	0.802359302	0.989555072
KIAA0101	0.007106098	0.802405303	0.989555072
EIF4E	0.026495596	0.802477046	0.989555072
ZNF184	-0.02300063	0.802531201	0.989555072
BATF3	0.030587504	0.802586652	0.989555072
BCAT2	-0.024013948	0.802872591	0.989555072
DCTN2	-0.012649399	0.802957875	0.989555072
ZNF395	0.019683714	0.803078691	0.989555072
AP3B1	0.012842533	0.803205645	0.989555072
DZIP3	-0.022615188	0.803251676	0.989555072
HRH1	0.024139806	0.803259011	0.989555072
RPS12	0.044160506	0.803399243	0.989555072

KLHL5	0.018421348	0.80351581	0.989555072
HMGB1	0.01532752	0.803656418	0.989555072
FAM98B	-0.032090545	0.803802006	0.989555072
SV2B	0.021259332	0.803817472	0.989555072
ALOX15B	-0.0293409	0.803848894	0.989555072
SNORA5B	0.02424116	0.8039782	0.989555072
C2orf49	-0.023967666	0.803986547	0.989555072
ZNF621	-0.01739291	0.804042625	0.989555072
PBX2	-0.014262584	0.804155811	0.989555072
NR3C1	-0.01947611	0.804158907	0.989555072
KIF21A	-0.032367007	0.804181093	0.989555072
RBMS1	-0.028412455	0.804200045	0.989555072
PELI1	0.027351833	0.804291754	0.989555072
TMEM183A	0.010070615	0.804319442	0.989555072
APOBEC3G	0.034752275	0.804435973	0.989555072
KLHL20	-0.019525591	0.804691492	0.989555072
PEX2	-0.018587106	0.804801545	0.989555072
PVRIG	-0.023235262	0.804812991	0.989555072
PRG4	0.040921574	0.804893675	0.989555072
METTL10	0.019428705	0.805097296	0.989555072
ZDHHC16	-0.013616488	0.805099523	0.989555072
BTF3	-0.011459158	0.805185021	0.989555072
STAT2	-0.020310585	0.805256536	0.989555072
TBC1D4	0.030374761	0.805378359	0.989555072
DNAJC10	0.012784561	0.805414189	0.989555072
DDX39A	-0.011865587	0.805504439	0.989555072
YARS	-0.013568267	0.805534797	0.989555072
CAPN2	-0.014916864	0.80556127	0.989555072
TNRC6C	0.030513973	0.805607008	0.989555072
TMEM25	-0.04044206	0.805630352	0.989555072
PTEN	0.011696292	0.805637877	0.989555072
FRG1B	0.031898189	0.805772049	0.989555072
FZD7	0.019438435	0.805898247	0.989555072
AGGF1	-0.01219673	0.805997441	0.989555072
CNPY3	-0.027865427	0.806045144	0.989555072
ADAL	-0.021377106	0.806078507	0.989555072
RRP9	0.023391052	0.806212058	0.989555072
SNORD62A	0.040971544	0.806213419	0.989555072
TMX2	-0.011651735	0.806215906	0.989555072
ANPEP	0.072278376	0.806313413	0.989555072
SNORA33	0.026813739	0.806434148	0.989555072
ZNF337	-0.017706607	0.806472957	0.989555072
ATP6V1F	0.014681561	0.806520602	0.989555072
APOL3	0.021123173	0.806566513	0.989555072
MTFMT	-0.02082686	0.806585274	0.989555072
STK19	-0.013279595	0.806592934	0.989555072
PIP4K2C	0.016194348	0.806720967	0.989555072

HLA-B	0.013806634	0.806782333	0.989555072
DEPDC5	-0.021723463	0.806840016	0.989555072
LCOR	0.025226308	0.806905443	0.989555072
NFYB	-0.02485096	0.807013356	0.989555072
KCNK6	-0.017150859	0.807091511	0.989555072
UBE2Q2	-0.01843518	0.807097545	0.989555072
SWT1	-0.015926818	0.807193819	0.989555072
SUDS3	0.030569944	0.807304697	0.989555072
TGFB3	-0.025030394	0.807369144	0.989555072
ZNF362	-0.014654386	0.807507038	0.989555072
JAG2	-0.027618022	0.807684696	0.989555072
CTDP1	0.019055086	0.807800983	0.989555072
ZNF813	-0.021579768	0.807817886	0.989555072
CLPX	0.015288025	0.807903242	0.989555072
HERC2P2	-0.024831766	0.807925359	0.989555072
KCTD5	0.019204513	0.807968464	0.989555072
KIF5B	0.021458951	0.807986154	0.989555072
UBE4A	-0.014419921	0.808241137	0.98965493
TALDO1	0.015884619	0.808319104	0.98965493
METAP1	-0.00942222	0.808341357	0.98965493
ARF3	0.021677949	0.808462915	0.98965493
FSIP1	0.021261072	0.808514729	0.98965493
ZNF213	-0.028597383	0.808916201	0.989705868
STAMBP	-0.010789636	0.80918333	0.989705868
MRPL17	0.017884151	0.809211501	0.989705868
S100A9	-0.017529975	0.809213058	0.989705868
IFI16	-0.016901819	0.809331673	0.989705868
CYB5R4	-0.025065052	0.809995824	0.989705868
TMEM87A	-0.01043254	0.810153967	0.989705868
NCOA4	-0.018881544	0.810235726	0.989705868
AARSD1	-0.016804807	0.810341117	0.989705868
TSPAN17	0.015334595	0.810667408	0.989705868
POLRMT	0.021230651	0.810783515	0.989705868
HMGN1	-0.015677017	0.810892737	0.989705868
TOB2	0.020904708	0.811014216	0.989705868
TRIO	-0.019286513	0.811065184	0.989705868
DKK3	-0.06943399	0.811088605	0.989705868
ERMP1	0.022688854	0.811236619	0.989705868
PITPNA	0.021547348	0.811264909	0.989705868
CELA1	-0.056432913	0.811347621	0.989705868
TTI1	0.020749711	0.811382534	0.989705868
HSF1	0.02392146	0.811443754	0.989705868
OR2A9P	0.061534466	0.811455675	0.989705868
TERF2	-0.018888548	0.811581677	0.989705868
DYNC1H1	-0.013825668	0.811666477	0.989705868
GRN	-0.022548628	0.811759164	0.989705868
CSPP1	0.017462091	0.811838927	0.989705868

ARL4C	-0.021309497	0.811852186	0.989705868
SNAPC4	0.016342177	0.811943322	0.989705868
FAM177A1	-0.00686408	0.811959926	0.989705868
PECR	0.021658391	0.812238459	0.989705868
PTAFR	0.022813918	0.81230674	0.989705868
NFATC1	0.018401551	0.812346145	0.989705868
DNAJA1	-0.016894264	0.812457861	0.989705868
ARHGEF3	0.014632128	0.812499808	0.989705868
IFIT3	0.062890668	0.812711415	0.989705868
PDXDC2P	0.048735467	0.81272574	0.989705868
SESN1	0.027890369	0.81274944	0.989705868
HIST2H3A	-0.058407044	0.812762121	0.989705868
RSU1	0.016678883	0.812864378	0.989705868
RPS25	0.019818592	0.812878149	0.989705868
MAP2K3	-0.016311499	0.812926887	0.989705868
WDR36	-0.014630512	0.812949636	0.989705868
ZC3H11A	-0.011316528	0.813004355	0.989705868
SEH1L	-0.019972644	0.813026138	0.989705868
LRRC23	-0.030092513	0.81310851	0.989705868
TPD52L2	0.013749361	0.813193046	0.989705868
ZNF286A	-0.028493158	0.813235386	0.989705868
PSMB9	0.03106654	0.813283647	0.989705868
ZNF397	0.019500054	0.813455367	0.989705868
RPL35A	0.00948236	0.813553709	0.989705868
HNMT	0.034503063	0.813641351	0.989705868
DRG2	0.016561212	0.813888383	0.989705868
RYR1	-0.032186858	0.814035663	0.989705868
TOMM5	-0.028831777	0.814283831	0.989705868
GATAD1	0.015976918	0.81437242	0.989705868
ITPR2	-0.02094634	0.814425987	0.989705868
SNORA57	-0.028769759	0.814438354	0.989705868
VARS	-0.017004275	0.814514121	0.989705868
TFCP2	-0.014229566	0.814549814	0.989705868
KDM6B	0.020517796	0.814681817	0.989705868
SLC29A4	0.024807092	0.814946471	0.989705868
IP6K3	-0.021725613	0.815097887	0.989705868
IMP4	0.013128654	0.815120921	0.989705868
MAP7	-0.037149557	0.815241433	0.989705868
SDAD1	-0.011498543	0.815339445	0.989705868
DYRK1B	0.024985373	0.815529252	0.989705868
USP22	0.019150677	0.81554831	0.989705868
NCDN	-0.020113549	0.81555018	0.989705868
ELOVL6	0.042970984	0.81557177	0.989705868
SYF2	0.017966161	0.815770108	0.989705868
RIPK1	-0.020312615	0.815789569	0.989705868
THTPA	-0.021286376	0.815813951	0.989705868
LANCL2	-0.018423504	0.815883377	0.989705868

ADCK4	-0.020516449	0.815890137	0.989705868
WTIP	-0.019880828	0.815963256	0.989705868
SUMF1	0.020208307	0.815996903	0.989705868
TCF24	-0.023241357	0.816143895	0.989705868
IMPDH2	-0.017154903	0.816202118	0.989705868
ATL1	0.032996418	0.816350779	0.989705868
ZNF583	0.019752978	0.816574103	0.989705868
ZNRD1	0.017563962	0.816629042	0.989705868
PLCH2	-0.041397098	0.816712874	0.989705868
HGD	0.054257772	0.816850591	0.989705868
MICAL2	-0.033046657	0.816856662	0.989705868
UBXN1	0.008460308	0.816857028	0.989705868
UBE2D4	0.02050108	0.816881926	0.989705868
PRDX5	-0.019550083	0.816946678	0.989705868
ADCY7	0.013586143	0.817009506	0.989705868
RASA3	0.02196255	0.817056004	0.989705868
EXOSC8	-0.024688333	0.817134216	0.989705868
C1orf229	-0.050912395	0.817218771	0.989705868
WWC1	0.011646186	0.817220279	0.989705868
ZFAND2B	-0.011741591	0.817254754	0.989705868
XRCC6BP1	-0.038356616	0.817320162	0.989705868
TNFRSF17	0.070659833	0.817324661	0.989705868
GAMT	0.020590421	0.817357886	0.989705868
PSEN1	0.02456753	0.817442147	0.989705868
THUMPD1	-0.012166901	0.817469857	0.989705868
DVL3	0.014323852	0.817507972	0.989705868
ZNF222	-0.023648873	0.81765427	0.989705868
GAS1	0.03774472	0.817674816	0.989705868
PPP6C	0.009221543	0.817705369	0.989705868
HIST1H3H	-0.057232094	0.817735978	0.989705868
RAP2B	-0.023472458	0.817776569	0.989705868
LZTS2	0.018893644	0.817943978	0.989705868
UBR1	0.017915983	0.817944646	0.989705868
ESPNL	0.033453039	0.818175578	0.989795464
PLB1	0.023276344	0.818197534	0.989795464
VPS18	0.018624373	0.818476134	0.989815516
BACH2	0.029900844	0.818622165	0.989815516
CALML4	0.022604746	0.818701065	0.989815516
GZMH	-0.058685241	0.818877421	0.989815516
UBE2G1	0.025247698	0.818945181	0.989815516
HBG2	0.183788669	0.819112805	0.989815516
AKAP11	-0.014544649	0.819156818	0.989815516
SLC35E2	0.023458509	0.819197863	0.989815516
MARCKS	0.024661068	0.81922849	0.989815516
ACSS1	0.023012952	0.819335143	0.989815516
LILRA5	0.031349213	0.8194182	0.989815516
SP4	-0.018917479	0.819505673	0.989815516

AK2	-0.020485332	0.819522663	0.989815516
GCNT2	0.034291957	0.819539151	0.989815516
BTN2A2	0.029788546	0.819682091	0.989815516
ABCC3	0.054903116	0.819686355	0.989815516
C19orf60	0.015085909	0.819734288	0.989815516
ASCL2	-0.035585131	0.82013748	0.989922206
ILK	0.022097632	0.820170414	0.989922206
HINT3	-0.020058625	0.820215814	0.989922206
IFNAR1	0.016640357	0.820263878	0.989922206
L3MBTL4	0.02549375	0.820307925	0.989922206
HEATR1	0.036341758	0.820359237	0.989922206
CYLD	-0.022832465	0.820660617	0.990094798
HCFC2	0.016439978	0.820701067	0.990094798
TLE4	0.013259363	0.820807975	0.990094798
DHX58	-0.024923041	0.820981239	0.990094798
NDUFB8	0.00720871	0.82119673	0.990094798
LYPD1	0.023670996	0.821366158	0.990094798
POC1B	0.016123167	0.82138475	0.990094798
C5orf22	-0.021833003	0.821425226	0.990094798
TRMT61A	0.020063125	0.821726661	0.990094798
PLS1	-0.022977654	0.821787737	0.990094798
NDUFAF4	0.025440243	0.821892072	0.990094798
SNF8	0.023806357	0.821982668	0.990094798
GBE1	-0.021972862	0.822257378	0.990094798
ZDHHC12	0.018281576	0.82229555	0.990094798
SUPT5H	-0.016695223	0.82234894	0.990094798
CMTM6	0.02131556	0.822637026	0.990094798
GTF3C3	0.01195293	0.822717676	0.990094798
PDP1	0.023669323	0.822792656	0.990094798
TRIM37	-0.015981227	0.822799889	0.990094798
ITK	0.019815655	0.822809648	0.990094798
PPDPF	0.031476501	0.822938475	0.990094798
SLC4A7	-0.022104173	0.822968774	0.990094798
SNORD55	-0.023520446	0.823178991	0.990094798
SLC20A2	0.015955651	0.82328372	0.990094798
CASP8	0.029949989	0.823284695	0.990094798
LY9	0.019788754	0.823302778	0.990094798
LARP7	-0.014192836	0.823519106	0.990094798
NLRC5	0.021456774	0.823541224	0.990094798
SLC37A3	0.017926706	0.823713877	0.990094798
ARMC8	-0.01874626	0.823840615	0.990094798
SCMH1	0.015686869	0.823950533	0.990094798
NIP7	-0.017850395	0.824116981	0.990094798
ING4	-0.014251258	0.824119846	0.990094798
RPL13A	0.017204857	0.824233282	0.990094798
SLC25A26	0.030100122	0.824248349	0.990094798
TGFBI	-0.018774129	0.824404456	0.990094798

PPP2R2B	-0.038970816	0.82445237	0.990094798
IRX3	0.065393921	0.824525361	0.990094798
INADL	-0.023685388	0.824560793	0.990094798
USP10	-0.0156522	0.82467011	0.990094798
S100Z	-0.031567712	0.824679301	0.990094798
CCDC88C	-0.024527881	0.824687996	0.990094798
NEK8	0.022992499	0.824713138	0.990094798
USP6NL	0.022582806	0.824713464	0.990094798
HPS4	-0.018201116	0.824733643	0.990094798
ARL2	0.01688735	0.824795893	0.990094798
CHRNB1	-0.013316511	0.824987632	0.990094798
PCSK7	0.018265917	0.825074497	0.990094798
WAC	-0.015118697	0.825469813	0.990094798
NUCB1	0.01338525	0.825507244	0.990094798
CASP10	0.018475115	0.825530495	0.990094798
PXMP4	-0.025924239	0.825574706	0.990094798
TSC22D2	-0.018603844	0.825749382	0.990094798
ISY1	-0.012861691	0.825767696	0.990094798
TTC27	0.012244612	0.825773332	0.990094798
C12orf66	0.021093786	0.82582995	0.990094798
CNTF	0.008749083	0.82585673	0.990094798
THBS3	-0.019784193	0.825925575	0.990094798
MEST	-0.01953948	0.826019125	0.990094798
ALDH9A1	0.012094243	0.826576439	0.990094798
RASGRP4	-0.032008193	0.826669276	0.990094798
PAXIP1	-0.014118685	0.826677173	0.990094798
ATF7IP	-0.021029002	0.826794117	0.990094798
HEBP1	-0.015245946	0.826818385	0.990094798
PRKACB	0.024291646	0.827134286	0.990094798
API5	0.010427724	0.827214876	0.990094798
CDK2AP1	0.022521133	0.827260776	0.990094798
QRSL1	-0.029681513	0.82738783	0.990094798
DCAF6	-0.011125741	0.827481438	0.990094798
DUSP15	0.02006287	0.827759415	0.990094798
SULT1B1	0.030599525	0.827784232	0.990094798
SAMD1	-0.023254114	0.827937348	0.990094798
SCARNA4	-0.023786522	0.828035172	0.990094798
ZNF76	-0.020764964	0.828157231	0.990094798
RAB32	0.024342238	0.828215407	0.990094798
DCLRE1A	-0.031803049	0.828222779	0.990094798
SYTL2	0.03958216	0.828315815	0.990094798
KIF19	-0.039400095	0.828337808	0.990094798
WDR26	0.015997106	0.82843693	0.990094798
FHL2	0.049129407	0.828482727	0.990094798
ZNF318	0.013080592	0.828741327	0.990094798
WDR52	-0.024027263	0.828767849	0.990094798
CBX3	-0.016587614	0.828940844	0.990094798

ZCCHC6	-0.02081453	0.829010068	0.990094798
BBX	-0.013794817	0.829011386	0.990094798
RFWD2	-0.008427273	0.82906829	0.990094798
ARHGAP31	-0.024451816	0.829098157	0.990094798
SPEG	0.022492211	0.829125292	0.990094798
SOD1	0.011337984	0.829143496	0.990094798
ENTPD5	0.022027412	0.829145599	0.990094798
AVEN	0.020843881	0.829323977	0.990094798
UBE2K	0.013700454	0.829430468	0.990094798
GARS	-0.009217834	0.829431973	0.990094798
MAPK3	-0.014605725	0.829594974	0.990094798
CD247	0.017992643	0.829619146	0.990094798
STK17A	-0.021001475	0.830134002	0.990094798
SLC1A3	-0.027571098	0.830148592	0.990094798
PLEKHB1	-0.026103278	0.830219657	0.990094798
TMEM87B	0.020406842	0.830284414	0.990094798
RALGAPB	0.012834013	0.830448823	0.990094798
CTSO	-0.021774593	0.83050344	0.990094798
PER1	0.028394416	0.830506646	0.990094798
RAB23	-0.032099676	0.830621124	0.990094798
DBF4	0.020106063	0.830704164	0.990094798
CEACAM21	-0.023910743	0.830764765	0.990094798
ERCC3	-0.012288544	0.830773267	0.990094798
SNORA24	0.023963605	0.83082068	0.990094798
HIST2H2AC	-0.029694999	0.831133758	0.990094798
CYP4V2	0.027377659	0.831180932	0.990094798
BRMS1	0.009648764	0.831287057	0.990094798
HLCS	-0.018425261	0.831407299	0.990094798
FNIP2	-0.017658839	0.831483049	0.990094798
TSPAN31	0.013215607	0.831503045	0.990094798
ZNF148	0.025445423	0.831527704	0.990094798
RAPSN	0.022194717	0.831615317	0.990094798
RABIF	0.016711982	0.831668119	0.990094798
C4orf33	-0.02660506	0.831781264	0.990094798
ABCA3	-0.025318599	0.831792668	0.990094798
SEC62	0.017172135	0.83213459	0.990094798
ANAPC7	0.017765398	0.832313329	0.990094798
PDGFA	0.032787823	0.832357513	0.990094798
TIMM8B	-0.019439566	0.832415538	0.990094798
LTA	0.02421446	0.832420961	0.990094798
RPS6	-0.010854638	0.832422657	0.990094798
ALDH1A1	0.035683333	0.832546914	0.990094798
IL24	-0.018925246	0.832551713	0.990094798
RAB11FIP5	-0.041015619	0.832838686	0.990094798
SVIL	-0.013914956	0.832857497	0.990094798
ISG20L2	0.016853474	0.833090027	0.990094798
PRR21	0.018860923	0.83309232	0.990094798

MKKS	-0.011935308	0.833110211	0.990094798
AMACR	0.018395137	0.833126452	0.990094798
GORASP1	0.013383127	0.833204593	0.990094798
TTLL1	0.019324804	0.833274867	0.990094798
TROVE2	0.011796465	0.833388486	0.990094798
CPM	0.018000066	0.833786367	0.990094798
ZMYND19	0.01243231	0.833860997	0.990094798
S100PBP	-0.012254027	0.833957594	0.990094798
KIAA0196	-0.011847596	0.83397173	0.990094798
C10orf12	-0.015505939	0.834023714	0.990094798
SOAT2	0.020228942	0.834038162	0.990094798
CREB3L2	0.015091584	0.834143616	0.990094798
WDR81	0.020232406	0.834185867	0.990094798
RRAGD	0.02212617	0.834285601	0.990094798
TPP1	0.014647693	0.834328262	0.990094798
TMPRSS7	0.019036504	0.834577742	0.990094798
LRRFIP2	0.015136312	0.83460758	0.990094798
SSR1	-0.01475849	0.834733044	0.990094798
CTRC	-0.019890759	0.834766338	0.990094798
MAN1A1	0.023600433	0.834996373	0.990094798
STX12	0.012152783	0.835040038	0.990094798
SMCP	-0.018042531	0.835279517	0.990094798
TXNRD1	0.012995096	0.835342616	0.990094798
ZDHHC13	-0.014250904	0.835372453	0.990094798
GPN3	0.018024426	0.835439554	0.990094798
ZBTB49	-0.019552872	0.83572008	0.990094798
PRKCH	0.016558822	0.835726189	0.990094798
CENPE	-0.02197835	0.835846356	0.990094798
GRAMD1B	-0.019114239	0.836062516	0.990094798
ZSCAN21	-0.016332017	0.836240978	0.990094798
NRXN2	-0.024521975	0.836308001	0.990094798
CNTNAP1	0.03493327	0.836335694	0.990094798
CPVL	-0.028204832	0.836480716	0.990094798
QRICH1	0.008922221	0.8364857	0.990094798
BAG5	-0.011069137	0.83662134	0.990094798
C17orf59	0.022096243	0.836703366	0.990094798
WDR47	-0.01686799	0.836789843	0.990094798
BCL2L13	-0.014049289	0.836906579	0.990094798
GHRHR	-0.018712584	0.836946015	0.990094798
MDH2	0.01024641	0.836958418	0.990094798
NADSYN1	0.013922735	0.836972933	0.990094798
C6orf132	-0.016391719	0.837002755	0.990094798
VPS26B	0.014754418	0.837006296	0.990094798
SAP30	-0.032181995	0.837056172	0.990094798
TUBGCP2	0.015484797	0.837057105	0.990094798
ZNF592	-0.018015052	0.837097412	0.990094798
KIF13B	-0.015370818	0.837133952	0.990094798

ZBTB40	0.021139665	0.837263662	0.990094798
TET2	0.017578193	0.837281895	0.990094798
YBEY	-0.036574177	0.837381441	0.990094798
PSMD6	0.008253175	0.837388597	0.990094798
UQCRB	0.018170609	0.837424766	0.990094798
MCM3	0.010866391	0.837462921	0.990094798
KLHDC2	0.014429528	0.837502628	0.990094798
RNY5	0.025468964	0.837627946	0.990094798
FAM193B	-0.019219727	0.837717472	0.990094798
HAVCR2	-0.016914234	0.837847477	0.990094798
IQGAP3	0.020211362	0.837854534	0.990094798
FAM173B	-0.015887248	0.837933532	0.990094798
STIM1	0.013740988	0.837977645	0.990094798
RGPD1	0.043271076	0.83807908	0.990094798
ARID2	-0.012585206	0.838105934	0.990094798
AKT1S1	-0.015194388	0.838176583	0.990094798
SPA17	0.019785384	0.838240318	0.990094798
COX6C	0.012516938	0.838267424	0.990094798
MRPL42	0.016391381	0.838356053	0.990094798
DNAJC3	-0.022077816	0.838356951	0.990094798
MCTP1	-0.034478025	0.838390478	0.990094798
SLC5A12	0.021118962	0.838401432	0.990094798
OVGP1	-0.028679961	0.83841052	0.990094798
U2AF1L4	0.025782317	0.838499156	0.990094798
TARS2	-0.015123171	0.838592434	0.990094798
PAX7	-0.021576809	0.838713197	0.990094798
PC	-0.018980111	0.838897345	0.990094798
OGFRL1	-0.026419051	0.839049245	0.990094798
MIR7-1	-0.026556724	0.839141587	0.990094798
BTG1	-0.010157583	0.839152083	0.990094798
EFR3A	0.009030673	0.839178434	0.990094798
CD14	0.011176084	0.839207774	0.990094798
WNK1	0.010061101	0.839326415	0.990094798
HERPUD2	0.017181257	0.839375696	0.990094798
AIF1	-0.014669566	0.839705406	0.990228735
RPL18	0.008775755	0.839886889	0.990228735
CCDC25	-0.012626497	0.839950778	0.990228735
MIR611	0.019251096	0.840175601	0.990228735
SCAP	0.009583173	0.840191438	0.990228735
ALDH3B1	-0.022518422	0.840342788	0.990228735
DTHD1	-0.038095485	0.840386294	0.990228735
ZDHHC5	-0.016377477	0.840477828	0.990228735
RPL13AP3	0.007626622	0.840489062	0.990228735
ISOC1	-0.015016578	0.840527009	0.990228735
C14orf169	-0.012437866	0.840561428	0.990228735
GNAS	-0.013921925	0.84056276	0.990228735
ERBB2	0.03888945	0.840949551	0.99057897

ZNF124	0.024452361	0.841060867	0.990604676
HIF3A	-0.019201886	0.841469794	0.990708134
CPEB4	-0.031944176	0.841569392	0.990708134
CTLA4	0.026298895	0.84169864	0.990708134
ZNF439	0.025082582	0.841966651	0.990708134
PI16	0.04253528	0.842145873	0.990708134
GLRX2	-0.018766178	0.842476647	0.990708134
ZNF93	0.018583647	0.842479945	0.990708134
DSTNP2	0.013909939	0.842526866	0.990708134
RBMS2	-0.032483661	0.842531578	0.990708134
TNFRSF10D	-0.022884104	0.842552239	0.990708134
CMC1	-0.021517055	0.842595568	0.990708134
TMEM69	-0.018928181	0.84261467	0.990708134
RNASE2	-0.042069961	0.842658357	0.990708134
RAB24	-0.012565743	0.842666785	0.990708134
RING1	-0.007096804	0.842755313	0.990708134
CASP2	0.008113696	0.842792199	0.990708134
RWDD4	0.0093794	0.842814673	0.990708134
LARS	0.010694601	0.842831843	0.990708134
NUP107	-0.012330501	0.842849263	0.990708134
TRA2A	0.026516014	0.84312343	0.990839617
RPL7L1	0.009146495	0.843282521	0.990839617
MAST4	0.033683159	0.843389356	0.990839617
DPY19L3	-0.018535774	0.843442674	0.990839617
CD300LB	-0.022125164	0.843497188	0.990839617
CCT5	0.014877878	0.843598373	0.990839617
VHL	-0.011641669	0.843622924	0.990839617
WDR25	0.020337019	0.843677241	0.990839617
GDI2	0.010544852	0.844012389	0.991128064
SLMAP	0.008258926	0.844152721	0.991187702
LY6G5C	-0.024186624	0.844254256	0.991201778
TMEM138	-0.00890215	0.84440076	0.99126864
CEACAM3	-0.040818579	0.844616117	0.991402483
CSE1L	0.009005782	0.844718164	0.991402483
TRIM2	0.030283733	0.844783545	0.991402483
DCAF11	-0.021004449	0.844942966	0.991402483
GHITM	0.009845471	0.845165096	0.991402483
GNG8	-0.040904484	0.845314402	0.991402483
DHDH	0.026053756	0.845346051	0.991402483
ZNHIT3	0.010859502	0.845353903	0.991402483
FBXO2	-0.025684304	0.845422158	0.991402483
STYX	0.012854891	0.845862509	0.991402483
ADAM8	-0.016402707	0.845934659	0.991402483
C4orf48	0.010615246	0.846061661	0.991402483
TRIM33	-0.00998835	0.846222535	0.991402483
SMC6	-0.040821681	0.846260744	0.991402483
CCDC65	-0.017211711	0.846294206	0.991402483

LPA	0.019034939	0.846296415	0.991402483
TTLL4	0.016581751	0.84633885	0.991402483
CDK11A	-0.025218641	0.846571168	0.991402483
ITPK1	0.017539453	0.846661214	0.991402483
PPP2CB	0.01570948	0.846713344	0.991402483
FYN	-0.014175808	0.846916105	0.991402483
AIM2	0.029057024	0.846952974	0.991402483
ZDHHC1	0.028711066	0.846974533	0.991402483
SPC25	0.037645073	0.847050659	0.991402483
HNRNPA1L2	-0.015188737	0.847208389	0.991402483
ACPL2	-0.018555667	0.847495548	0.991402483
PANX2	-0.025715088	0.847526171	0.991402483
IFT46	-0.015600674	0.84758636	0.991402483
WIPF1	0.012580179	0.847619431	0.991402483
PEX6	0.039914799	0.847644651	0.991402483
USP24	-0.011445409	0.847908167	0.991402483
TGDS	0.018241767	0.847936636	0.991402483
ZNF384	-0.010579571	0.848012841	0.991402483
RGS1	0.045350056	0.848017467	0.991402483
CEACAM4	0.022116441	0.848056982	0.991402483
PVRL3	0.026064047	0.848173996	0.991402483
PLEKHB2	-0.027361113	0.848203928	0.991402483
CLEC2L	0.02333303	0.848220793	0.991402483
FAM138F	0.020217803	0.848242264	0.991402483
TMEM132B	0.01697556	0.848393683	0.991402483
C1orf112	0.016340758	0.848436279	0.991402483
PFDN2	0.022979096	0.848461088	0.991402483
PPP1R12B	-0.022660319	0.848597277	0.991402483
FHL3	-0.030165656	0.848604493	0.991402483
EIF4B	-0.010926849	0.848709283	0.991402483
ARL3	-0.017416494	0.848793322	0.991402483
IER5L	0.021756014	0.848832538	0.991402483
ADCY9	-0.021415711	0.848882473	0.991402483
VPRBP	-0.017019303	0.849113191	0.991402483
MCHR2	-0.028064034	0.849138645	0.991402483
TMEM59L	-0.017135933	0.849231031	0.991402483
TNFRSF13B	0.045903756	0.849279255	0.991402483
PTPN11	-0.007877215	0.849332041	0.991402483
TFG	0.029813514	0.849581525	0.991402483
FAM193A	-0.011063851	0.849626299	0.991402483
HOOK3	-0.021792679	0.849755824	0.991402483
ZMAT3	0.01944536	0.84980764	0.991402483
ARF6	0.018223022	0.849842668	0.991402483
CETN1	0.028874778	0.849967013	0.991402483
BPNT1	0.01529305	0.850035251	0.991402483
MRPL54	-0.01515157	0.850138716	0.991402483
DLAT	-0.012076288	0.850173305	0.991402483

WBSCR16	0.026743352	0.850229414	0.991402483
LMF2	-0.017419729	0.850246976	0.991402483
DEDD	-0.009408124	0.850536627	0.991635762
YWHAH	-0.013684144	0.850713651	0.991638885
CTBP2	-0.021004761	0.850816872	0.991638885
CD160	0.044770729	0.850875883	0.991638885
SNAPC2	0.01646277	0.850913185	0.991638885
FAM65A	0.012748818	0.85098724	0.991638885
TMED10	-0.022720427	0.851110154	0.991677715
CORO2A	0.018593692	0.851411081	0.99192393
SLC47A1	0.027544072	0.851588781	0.992026544
DNAH6	0.028044088	0.851986749	0.992385702
ACACA	-0.01260698	0.852284422	0.992526992
SNORA70C	0.018736799	0.852317183	0.992526992
ALKBH4	0.015364955	0.852416387	0.992526992
ZW10	-0.009981468	0.852466719	0.992526992
PROSC	-0.010704751	0.852728174	0.992726984
ZNF3	0.01531378	0.852942725	0.992872334
TRIM35	-0.018400993	0.85321786	0.992927457
SNUPN	0.018317009	0.853339503	0.992927457
EPHX2	-0.030997392	0.853429574	0.992927457
TMEM14C	0.016231028	0.853636489	0.992927457
NUMA1	-0.014567917	0.853649566	0.992927457
SIPA1L2	-0.021626077	0.8538304	0.992927457
WBP11	0.006880013	0.853939807	0.992927457
PPARA	-0.014056384	0.853940216	0.992927457
ACN9	0.014742008	0.854033545	0.992927457
CBR4	-0.014379763	0.854037931	0.992927457
ZNF552	0.015512021	0.854070257	0.992927457
NUDCD3	-0.01654737	0.8541215	0.992927457
RPS27L	0.01042228	0.854156224	0.992927457
INPP5K	0.014318908	0.854268813	0.992954058
CPPED1	0.020558277	0.854381608	0.99296968
ZDBF2	0.01943262	0.854655149	0.99296968
IDH2	0.015676454	0.854659282	0.99296968
TGM3	0.017090767	0.854861183	0.99296968
PSMG2	0.010245023	0.854911834	0.99296968
TMEM181	0.017932718	0.854964725	0.99296968
LRTOMT	0.014855085	0.854972644	0.99296968
PTPMT1	-0.013575265	0.855077377	0.99296968
THAP7	0.017168695	0.855159822	0.99296968
FGF9	-0.022814809	0.855215303	0.99296968
RAB2B	-0.010774696	0.855269033	0.99296968
FAM89A	-0.022035716	0.855505977	0.993056478
NKX3-1	-0.021918887	0.85553673	0.993056478
RPS15	0.008871224	0.85561294	0.993056478
SGOL1	0.015677674	0.8559792	0.993279274

RNF2	-0.018146614	0.856167411	0.993279274
PRDM9	0.0166853	0.856189922	0.993279274
CDKN1C	-0.040547775	0.85633524	0.993279274
C17orf97	-0.101402869	0.856439538	0.993279274
TMEM67	-0.015813374	0.856760774	0.993279274
ZFP42	0.025503526	0.856796856	0.993279274
MOS	-0.018700335	0.856920875	0.993279274
ARSA	0.02190037	0.856933134	0.993279274
TMEM50B	-0.007463493	0.857057361	0.993279274
RNF123	-0.009627995	0.857099831	0.993279274
GABARAPL2	-0.011985787	0.857130796	0.993279274
TPRA1	0.015077692	0.857142448	0.993279274
HEATR3	-0.019706809	0.857296477	0.993279274
HTRA2	0.013794677	0.857706482	0.993279274
NAMPT	0.029382299	0.857764036	0.993279274
LOXL3	-0.018750884	0.857789323	0.993279274
MANF	-0.014406036	0.857878675	0.993279274
CCNF	0.017245919	0.85803327	0.993279274
PANK3	0.013889546	0.858088949	0.993279274
ZNF16	-0.019948768	0.858098927	0.993279274
PGLS	0.011360696	0.858190261	0.993279274
SLC5A10	0.018652322	0.858300197	0.993279274
SH3BGRL3	-0.015369451	0.858350427	0.993279274
MFNG	-0.00681705	0.858363748	0.993279274
USP8	0.011636697	0.858469041	0.993279274
RAB12	0.036260037	0.85878407	0.993279274
MTHFD2L	-0.020458742	0.858885366	0.993279274
PNKD	0.025890336	0.858924063	0.993279274
HP	0.069176441	0.85915896	0.993279274
ITGAE	-0.013584345	0.859279127	0.993279274
NOTCH2	0.015649083	0.859425598	0.993279274
CD3E	-0.020637282	0.859602991	0.993279274
RNF13	-0.014833448	0.859709237	0.993279274
ASNA1	0.022969098	0.859717116	0.993279274
ISOC2	-0.015695685	0.859821533	0.993279274
UBE2S	-0.013519461	0.859866063	0.993279274
RASA2	-0.023107922	0.859955686	0.993279274
PALB2	-0.015189476	0.859978984	0.993279274
DDX46	-0.010866226	0.860041308	0.993279274
MTHFR	-0.023914391	0.860218654	0.993279274
NSL1	-0.011253789	0.860315473	0.993279274
HEBP2	0.018369632	0.860385592	0.993279274
TNNT1	-0.026063221	0.860397015	0.993279274
NT5DC1	-0.01034604	0.860401676	0.993279274
LENG1	0.014833918	0.860448467	0.993279274
ZNF598	-0.008490785	0.86054191	0.993279274
SLC4A1AP	-0.011561419	0.860677195	0.993279274

PFN1	0.011503971	0.860783998	0.993279274
ZNF280B	0.015943668	0.860838946	0.993279274
PRKAB2	-0.01375992	0.860867281	0.993279274
RNASEH2B	0.018572039	0.861010303	0.993279274
XRN2	-0.1105092	0.861013035	0.993279274
ZNF433	0.018061603	0.861018891	0.993279274
RUNX2	-0.026459972	0.861161673	0.993279274
RAB33B	-0.01565044	0.861208807	0.993279274
MED31	-0.013426231	0.861418953	0.993279274
RNF7	-0.008631327	0.861476595	0.993279274
LMBRD1	0.013755135	0.861499398	0.993279274
IFIT1	0.047861005	0.861502674	0.993279274
GTF2E2	-0.008150116	0.861702725	0.993279274
TTC17	-0.015653265	0.862462251	0.993279274
RPS6KA2	-0.048340983	0.862504172	0.993279274
DCAF13	-0.013481698	0.862553459	0.993279274
SNX24	-0.017050284	0.862799202	0.993279274
CD58	-0.020105122	0.86292926	0.993279274
PGGT1B	0.024509746	0.862948005	0.993279274
SELT	0.013239959	0.862949745	0.993279274
CUEDC2	-0.009807858	0.862983776	0.993279274
HAGH	0.009877739	0.863122378	0.993279274
FSD1	0.032202259	0.863240843	0.993279274
STAMBPL1	-0.013760335	0.863249255	0.993279274
KDELR2	0.017743435	0.863314643	0.993279274
C2	0.024288132	0.863413008	0.993279274
KCNQ1OT1	0.017847185	0.863432006	0.993279274
MUT	0.01215466	0.86350644	0.993279274
PLSCR1	-0.0350544	0.863516625	0.993279274
PVRL1	-0.016212972	0.863622851	0.993279274
POMT2	-0.017704415	0.863780887	0.993279274
ZC3H8	-0.010996139	0.863832775	0.993279274
NOLC1	0.013957069	0.863917865	0.993279274
GOPC	-0.010524751	0.864149432	0.993279274
CSMD1	-0.025550672	0.864150796	0.993279274
SCRIB	0.010111576	0.864180667	0.993279274
ATG10	0.024334476	0.86419411	0.993279274
PARP9	-0.024063908	0.864203762	0.993279274
GMFG	-0.007549028	0.864235606	0.993279274
EEF1A1	0.012061153	0.864243783	0.993279274
TUBA1B	0.011640117	0.864328897	0.993279274
SDPR	0.030337392	0.86441013	0.993279274
KIAA1009	0.017426277	0.86449827	0.993279274
SOS2	-0.015977088	0.864527239	0.993279274
SLC25A40	-0.021920027	0.864587532	0.993279274
PRR11	-0.018098895	0.864666853	0.993279274
SYNGR2	0.009553214	0.864765312	0.993279274

KIAA0020	-0.01020675	0.864992772	0.993279274
ZNF622	-0.011690911	0.86509149	0.993279274
FAM35A	0.010177802	0.865177533	0.993279274
RNF187	0.014112261	0.865230968	0.993279274
GLYR1	0.006772738	0.865236772	0.993279274
FGGY	0.020387256	0.865381817	0.993279274
DEK	-0.01044291	0.865482423	0.993279274
AP2A1	-0.015051834	0.865513276	0.993279274
FAM120A	0.005723956	0.865522127	0.993279274
COPS6	-0.010331964	0.865631557	0.993279274
MAP2K2	-0.019962737	0.86565269	0.993279274
HK2	-0.018637342	0.865780974	0.993279274
SERPINE2	-0.040968841	0.865830146	0.993279274
WDR19	0.015347046	0.865853199	0.993279274
ABCB1	0.028036888	0.865866538	0.993279274
LNPEP	0.014998708	0.866003961	0.993279274
MIR25	0.019117003	0.866011523	0.993279274
G6PC3	-0.020192767	0.866034477	0.993279274
HIGD1A	0.012546807	0.866117741	0.993279274
ATPAF1	0.012759735	0.866124452	0.993279274
CARS	-0.014976391	0.866469795	0.993400805
ZNF25	-0.008938631	0.866496521	0.993400805
PER3	-0.019808783	0.866499663	0.993400805
PLAA	-0.010249054	0.866673685	0.993497413
SPIB	0.032366758	0.866868615	0.993512632
RFC4	-0.013305465	0.866868796	0.993512632
HIST1H2BC	0.041056972	0.86695623	0.993512632
TRAF3	-0.013125857	0.86727221	0.993522226
PSMG3	-0.015082327	0.867330334	0.993522226
EXOSC7	-0.012016968	0.867421248	0.993522226
RHEBL1	-0.016061628	0.867522268	0.993522226
PHF10	0.012690902	0.867601443	0.993522226
FAM195B	0.01366011	0.867684846	0.993522226
EFTUD1	-0.0097091	0.867856044	0.993522226
ARL17B	0.041063948	0.867953798	0.993522226
CERKL	0.041017927	0.867984594	0.993522226
CNOT10	0.008621351	0.868519967	0.993522226
B3GNT6	0.015637042	0.868637892	0.993522226
PRKAG1	-0.012712583	0.868879439	0.993522226
MAN1B1	-0.009534156	0.869032088	0.993522226
C17orf62	0.011652228	0.869122711	0.993522226
NFKBIZ	-0.018922733	0.869171641	0.993522226
LRIF1	0.021816952	0.869175517	0.993522226
LRPAP1	-0.011762265	0.869187545	0.993522226
MTF2	0.012429339	0.869319487	0.993522226
PLAUR	0.020665868	0.869522187	0.993522226
ARID3A	0.011004894	0.869580986	0.993522226

CORO1A	-0.009320158	0.869692593	0.993522226
TRAK1	-0.011959976	0.869910603	0.993522226
C21orf59	-0.008427295	0.869977175	0.993522226
BCKDK	0.01154024	0.870007584	0.993522226
REST	-0.01281988	0.870033028	0.993522226
DDB1	0.006946395	0.870214857	0.993522226
DPF2	-0.010823701	0.870434919	0.993522226
POLR2B	0.008135981	0.870635351	0.993522226
DOCK10	0.008555986	0.87067407	0.993522226
CNOT7	-0.010663886	0.870704738	0.993522226
CTNNA1	0.013824131	0.870723911	0.993522226
DBNL	-0.008186261	0.870743318	0.993522226
SNORA65	-0.011566734	0.870755732	0.993522226
MCCC1	-0.008098782	0.870789004	0.993522226
FBXL14	0.015164779	0.871007305	0.993522226
TLR1	0.017343568	0.87104551	0.993522226
GPR56	0.029973813	0.871173309	0.993522226
OTOGL	0.013953841	0.871382382	0.993522226
SHISA5	0.011153222	0.871457754	0.993522226
SH3GLB1	-0.012135543	0.871520815	0.993522226
NCALD	-0.021206248	0.871664814	0.993522226
EIF4G1	-0.010194823	0.871671816	0.993522226
SLC30A7	-0.014546923	0.871799476	0.993522226
POLE3	-0.008141845	0.871965426	0.993522226
TSG101	0.008785986	0.872000848	0.993522226
IL10RB	-0.008409171	0.872111346	0.993522226
GRAP	-0.014424603	0.87231741	0.993522226
PRICKLE2	0.018173488	0.872673223	0.993522226
C4orf32	0.016988254	0.872716006	0.993522226
KPNA4	0.007961506	0.872748481	0.993522226
LRMP	-0.009527737	0.872910688	0.993522226
C22orf34	0.031593705	0.872982865	0.993522226
CCT8	0.009653138	0.873073776	0.993522226
DRAM2	0.013083488	0.873127634	0.993522226
TTC31	0.008631055	0.873138	0.993522226
CD38	-0.036251843	0.873207922	0.993522226
RTN4	0.007560312	0.873228748	0.993522226
ADCK5	0.011243278	0.873231303	0.993522226
RTN1	0.017555902	0.873434196	0.993522226
TMEM206	0.010875301	0.873492361	0.993522226
SLC22A17	-0.023314806	0.873607377	0.993522226
SRSF6	0.007054634	0.87361124	0.993522226
MS4A14	-0.024079214	0.873724566	0.993522226
CALCOCO1	-0.011601255	0.873743649	0.993522226
ASNSD1	0.010089061	0.873878524	0.993522226
CUL2	-0.008180855	0.873945911	0.993522226
SSR3	-0.012357056	0.874368663	0.993522226

ABCA7	-0.020569991	0.874554925	0.993522226
WDR89	0.018891157	0.874825399	0.993522226
CD3D	-0.01052339	0.874890155	0.993522226
TOMM7	0.008037523	0.875008393	0.993522226
DDX60L	0.017991838	0.875037987	0.993522226
C21orf49	0.011896774	0.87513659	0.993522226
DNAJC21	0.01150711	0.875146524	0.993522226
ASAH1	0.020518692	0.875250897	0.993522226
PRMT5	-0.010109172	0.875392189	0.993522226
VPS13A	-0.013934423	0.875445073	0.993522226
TP53BP1	0.010842011	0.875813851	0.993522226
CITED4	0.024726364	0.875845844	0.993522226
TBXA2R	0.01920985	0.875879006	0.993522226
S100A8	0.00610086	0.87589754	0.993522226
LAT	0.010499637	0.875899628	0.993522226
TNC	-0.027833581	0.875973317	0.993522226
MEGF8	0.015352487	0.875995517	0.993522226
PLXDC2	-0.017549906	0.876025706	0.993522226
GNB2L1	-0.0040103	0.876212453	0.993522226
ATP6V1H	-0.009711665	0.87630772	0.993522226
COPE	0.011114258	0.876365785	0.993522226
CCDC104	0.011674094	0.876424666	0.993522226
CR1	-0.022773097	0.876551168	0.993522226
CCL4L2	-0.030973002	0.876675851	0.993522226
C11orf21	-0.017112132	0.876781405	0.993522226
BCL11A	-0.01626632	0.877001106	0.993522226
ANXA2	-0.011405035	0.87702287	0.993522226
LZIC	0.010237011	0.877049336	0.993522226
CASP7	-0.01757234	0.877150032	0.993522226
TNFRSF21	0.027756455	0.877160648	0.993522226
FNIP1	0.010191168	0.877281551	0.993522226
ARHGEF18	0.009206425	0.877301645	0.993522226
TPTE	0.024034622	0.877319918	0.993522226
TMOD3	0.014287939	0.877343603	0.993522226
MCM9	0.010894933	0.877445001	0.993522226
DNTTIP2	0.017960158	0.8774777	0.993522226
KCNJ12	-0.017108404	0.877551933	0.993522226
TRPT1	0.008174433	0.877577146	0.993522226
ZFP36L1	0.013068618	0.877696637	0.993522226
TRAF5	0.012262339	0.877748502	0.993522226
SLC25A25	0.00911581	0.878075121	0.993522226
DLL1	-0.030633056	0.878095049	0.993522226
CLYBL	0.018029213	0.878154224	0.993522226
MARCH6	0.024901077	0.878187587	0.993522226
LGALS3	0.017975131	0.878315781	0.993522226
ARRB1	-0.01256044	0.878384958	0.993522226
DCTN6	0.012266479	0.878454038	0.993522226

RABL2A	-0.013131038	0.878583869	0.993522226
MARCO	0.033101938	0.878677761	0.993522226
PPP2R2D	-0.007481757	0.878765566	0.993522226
BASP1	0.015858823	0.878801955	0.993522226
MGA	-0.012873253	0.878979295	0.993522226
FBXO46	0.009439191	0.879025042	0.993522226
ASTN2	-0.014010453	0.879051546	0.993522226
SPAG8	-0.018941195	0.879093957	0.993522226
ADPRHL2	-0.010400899	0.879257559	0.993522226
TYMS	-0.028801931	0.879308676	0.993522226
XAF1	0.021656357	0.879375307	0.993522226
ZNF681	0.025746852	0.879428522	0.993522226
GTF3A	0.008293131	0.879436145	0.993522226
ACOX3	-0.013274384	0.879726049	0.993522226
RAB27A	-0.02073798	0.879797908	0.993522226
UBB	0.016903354	0.879957139	0.993522226
PPIB	-0.022692436	0.880217847	0.993522226
HAT1	-0.013616164	0.880255745	0.993522226
PCCA	0.008465747	0.880408921	0.993522226
SCARNA16	0.028346163	0.880455533	0.993522226
TBRG4	-0.011113619	0.880466195	0.993522226
ARSK	0.014987984	0.880496435	0.993522226
BZW2	0.00862349	0.880742883	0.993522226
CCDC51	0.012672255	0.880767186	0.993522226
EDEM1	0.008508761	0.880852235	0.993522226
FCER1G	0.018686977	0.880940295	0.993522226
TNFAIP2	-0.020432517	0.880952553	0.993522226
SORL1	0.010523272	0.881039902	0.993522226
APP	0.023664218	0.881163279	0.993522226
RRP7A	-0.027107905	0.881268987	0.993522226
LYSMD2	-0.011254885	0.881296488	0.993522226
ACAP2	-0.015469827	0.881339686	0.993522226
SYMPK	0.01731304	0.881355773	0.993522226
HSD17B8	0.01306257	0.881360215	0.993522226
PPBP	0.019739616	0.881372461	0.993522226
E2F6	-0.014544884	0.881392224	0.993522226
STX11	-0.017176711	0.881462998	0.993522226
FBXO22	-0.013035637	0.88148284	0.993522226
NOP16	0.014290603	0.881498727	0.993522226
OCLN	-0.038313181	0.881550347	0.993522226
DEGS1	0.010311043	0.881620563	0.993522226
AATK	-0.01399419	0.88176549	0.993522226
ZNF543	0.021237115	0.881824264	0.993522226
BEGAIN	0.028420306	0.881987401	0.993522226
CCDC61	-0.013651574	0.882215277	0.993522226
RPS21	-0.016355747	0.882328026	0.993522226
MAPK1	0.00955041	0.882508615	0.993522226

SORT1	0.014147279	0.882523603	0.993522226
RPSA	0.011799851	0.882573954	0.993522226
PDE8B	0.021065382	0.882601246	0.993522226
SUFU	-0.019844209	0.882680463	0.993522226
UBE2V1	-0.013251075	0.882724817	0.993522226
PGP	-0.018327625	0.882784718	0.993522226
ZNF729	0.017750212	0.882802937	0.993522226
MEFV	-0.020304086	0.882856968	0.993522226
MOCS2	0.01933241	0.882867734	0.993522226
ZNF224	-0.013544951	0.883058753	0.993522226
SAMD8	-0.016332895	0.883328413	0.993522226
RASA4	0.018485082	0.883422581	0.993522226
HELB	0.020554248	0.883504512	0.993522226
DNMT3B	-0.011150704	0.883748643	0.993522226
GTDC1	0.012953441	0.883776023	0.993522226
ATP5S	-0.01309414	0.883828654	0.993522226
GPT2	0.013995067	0.883944702	0.993522226
GLUL	-0.017797231	0.883949064	0.993522226
CD163	-0.025953389	0.884104179	0.993522226
DDTL	-0.037577177	0.88411427	0.993522226
SAFB	0.007961597	0.88428581	0.993522226
ANKRD22	0.029862945	0.884290005	0.993522226
PMAIP1	-0.018326885	0.884405423	0.993522226
C6orf211	0.015703526	0.884515329	0.993522226
C8orf59	0.008768007	0.88474979	0.993522226
PSMA1	-0.007772789	0.884794205	0.993522226
RAB7A	0.006542522	0.884957957	0.993522226
NOTCH4	0.020081698	0.884983776	0.993522226
ATP5J	0.010435135	0.885014289	0.993522226
DNLZ	0.018619672	0.885049354	0.993522226
NARS	-0.006031703	0.885182991	0.993522226
LILRB4	0.019466464	0.885221468	0.993522226
POU6F1	0.015804103	0.88531811	0.993522226
MED15	-0.015582064	0.885401973	0.993522226
SCARNA3	0.033078338	0.885448661	0.993522226
ANXA8	-0.017792969	0.885449368	0.993522226
F2R	-0.004163861	0.885520848	0.993522226
YES1	0.02086054	0.885546485	0.993522226
SEMA4D	-0.008917539	0.88567238	0.993522226
STARD5	0.01366025	0.885702786	0.993522226
C11orf73	-0.013581298	0.885715349	0.993522226
TCF12	0.010158732	0.885772161	0.993522226
CAPRIN1	0.013655333	0.885778251	0.993522226
AMDHD2	0.013875403	0.885830572	0.993522226
ANGPT1	0.024428925	0.885837626	0.993522226
SIRT1	0.008563028	0.88585942	0.993522226
GGPS1	-0.006714785	0.886096919	0.993522226

TAF6	-0.013989062	0.886155222	0.993522226
ZFP90	0.01191858	0.886162192	0.993522226
STARD3	-0.010748766	0.886193144	0.993522226
BSG	0.023296348	0.886371496	0.993522226
MAP3K6	-0.01341718	0.886432652	0.993522226
HOXD11	0.013292401	0.886565515	0.993522226
OSBP2	0.042488131	0.886599703	0.993522226
CARM1	-0.008691819	0.886655021	0.993522226
KIAA0947	-0.011538176	0.886686248	0.993522226
CAPN1	-0.009489849	0.886809924	0.993522226
GBF1	-0.013319903	0.886951714	0.993522226
PJA2	0.01127488	0.886957944	0.993522226
FXYD6	-0.022412251	0.886980814	0.993522226
PARG	-0.010025367	0.887223087	0.993522226
HIAT1	0.011662875	0.887230722	0.993522226
GPR89B	0.013895961	0.887353753	0.993522226
BRMS1L	0.010285579	0.887476839	0.993522226
LGALS3BP	0.023438071	0.887526691	0.993522226
ATRN	-0.00950801	0.887599876	0.993522226
METTL17	-0.010775946	0.887695081	0.993522226
BTBD7	-0.010955341	0.887768706	0.993522226
FNBP1L	0.020091532	0.88804664	0.993522226
FCHSD2	-0.013012569	0.888095096	0.993522226
TTC9	0.014923277	0.88817012	0.993522226
MCL1	0.007506783	0.888217399	0.993522226
RPL30	0.004659797	0.888296179	0.993522226
SLC36A4	-0.016360689	0.888317877	0.993522226
PRSS42	0.011710909	0.888344341	0.993522226
ZFP82	0.018378129	0.888430738	0.993522226
PCDH10	-0.015284877	0.888457441	0.993522226
UBE2J2	-0.014769554	0.888742626	0.993522226
CDYL2	-0.010373862	0.888758508	0.993522226
SDCCAG8	0.012922644	0.888897418	0.993522226
TXNDC5	0.026111255	0.888913401	0.993522226
MUC2	0.014813604	0.888960649	0.993522226
C10orf54	-0.00895668	0.88903361	0.993522226
C8orf82	0.012168919	0.889095506	0.993522226
MARCH8	0.024303626	0.889117468	0.993522226
ZFC3H1	-0.010544941	0.88933744	0.993522226
PABPC1L	-0.014758025	0.889380516	0.993522226
LDLR	-0.016718618	0.889453846	0.993522226
TAF4B	-0.017496387	0.889526786	0.993522226
IL17D	0.010462912	0.889696033	0.993522226
PFKFB2	-0.016009441	0.889755175	0.993522226
PWP2	-0.009870534	0.889770619	0.993522226
IDE	0.010713076	0.889834213	0.993522226
C16orf58	-0.007699962	0.889852683	0.993522226

VPS37D	-0.012373015	0.890035604	0.993626233
FKBP6	0.013176613	0.890146087	0.993649358
PDE6G	0.016866187	0.890409324	0.99376442
NUP62	-0.006994675	0.890428721	0.99376442
CCDC97	-0.007414428	0.890555121	0.993782787
REEP6	-0.017026221	0.89062474	0.993782787
FTO	0.011273553	0.890909891	0.993991839
CAMKK2	-0.035035842	0.890991691	0.993991839
TARBP2	0.011695125	0.891321227	0.994259263
CCR6	0.004259049	0.891439435	0.994290921
TRMT112	0.010504379	0.891686864	0.994299695
PWP1	0.005720354	0.891740674	0.994299695
GIT2	0.009470832	0.891847099	0.994299695
REEP5	-0.006699896	0.891990472	0.994299695
N6AMT1	-0.014710548	0.892038965	0.994299695
MAPRE1	-0.009227441	0.892045192	0.994299695
PLEKHG7	-0.012371316	0.892197431	0.994299695
C8orf48	-0.011342142	0.892326165	0.994299695
PKP4	0.014781922	0.892327747	0.994299695
NUAK1	0.034762849	0.892345575	0.994299695
CAPS	0.014547795	0.892450981	0.994317052
ARHGAP33	-0.014154979	0.892662673	0.994379233
ZNF285	-0.018139712	0.892747268	0.994379233
HMGN2	-0.006873808	0.89285931	0.994379233
MEG3	-0.061370428	0.892866131	0.994379233
PRSS30P	-0.034688673	0.892959921	0.994383639
PRR14L	0.005286009	0.893153619	0.994409636
MED16	0.013580107	0.89335406	0.994409636
RPF1	0.008235493	0.893485202	0.994409636
IFT122	0.010801159	0.893683036	0.994409636
C16orf74	0.018709215	0.893822006	0.994409636
CARD16	-0.01411351	0.893999778	0.994409636
DCAF4	0.017197484	0.894043978	0.994409636
COMT	0.012534872	0.894176852	0.994409636
HLA-E	0.010576455	0.89419332	0.994409636
DEFB123	0.014545813	0.894224361	0.994409636
RPL6	-0.00634582	0.894436348	0.994409636
BCR	-0.0128177	0.894446183	0.994409636
POP5	0.012131638	0.894539203	0.994409636
CACNA1S	-0.014342155	0.894969204	0.994409636
POLR3F	0.010554808	0.895099126	0.994409636
MPZL1	-0.012669072	0.895208098	0.994409636
LMAN2	-0.013697484	0.895270908	0.994409636
RNU11	-0.015788782	0.895292178	0.994409636
FUT6	-0.033398556	0.89533745	0.994409636
SDF2L1	-0.011086276	0.895370239	0.994409636
GBA	0.009218286	0.895379036	0.994409636

TTPAL	-0.008802394	0.895573995	0.994409636
SPON1	-0.021915629	0.895632288	0.994409636
RSL24D1	0.005822388	0.895955003	0.994409636
SLAMF6	0.011419019	0.896085812	0.994409636
KRT10	0.011548477	0.896249759	0.994409636
ATP6V1E1	0.006270925	0.896290483	0.994409636
CKAP4	0.014139944	0.896298274	0.994409636
PFKM	0.011881295	0.896348371	0.994409636
KCNH2	0.011378522	0.896356585	0.994409636
OIP5	0.017775461	0.896434488	0.994409636
TMEM50A	-0.010978678	0.896446164	0.994409636
GPD2	0.015691521	0.896460068	0.994409636
TMEM11	0.012039733	0.896480397	0.994409636
NOS3	0.012210854	0.896565254	0.994409636
ATF7IP2	-0.014967979	0.896747701	0.994409636
GRAMD4	-0.012540576	0.89681494	0.994409636
KCNIP3	0.015477668	0.89683757	0.994409636
PRCP	0.008626386	0.896865498	0.994409636
KCNH6	0.00849882	0.896878817	0.994409636
WFS1	-0.013415909	0.896934245	0.994409636
APIP	-0.017356328	0.896948756	0.994409636
PRRC2A	0.014230045	0.897055577	0.994409636
CCR4	0.01889041	0.897189363	0.994409636
SCARB2	-0.007514742	0.897242975	0.994409636
SLC30A3	-0.010103181	0.897735205	0.994409636
ZNF286B	-0.016620794	0.897769965	0.994409636
DHPS	0.0055217	0.897799602	0.994409636
GABPB1	0.010503505	0.897840461	0.994409636
PKD1	0.014779353	0.89788056	0.994409636
NPC1	-0.013769137	0.89804039	0.994409636
IFIT5	-0.015975242	0.898106617	0.994409636
CAMK2N1	0.024368701	0.898116197	0.994409636
CCDC86	-0.011280837	0.898372173	0.994409636
PCMTD1	-0.00728002	0.898411265	0.994409636
RAB6A	0.012204186	0.898793136	0.994409636
DOLPP1	0.007311161	0.898854406	0.994409636
YIPF2	-0.011029636	0.89894411	0.994409636
TGFBR3	0.019139686	0.899005634	0.994409636
TMEM145	-0.011183564	0.899017005	0.994409636
ENPP5	-0.021416531	0.899055735	0.994409636
ABL1	0.006791253	0.899091343	0.994409636
SMAD5	0.012505877	0.899107949	0.994409636
FCHSD1	-0.011925719	0.899253197	0.994409636
BOP1	-0.007936442	0.899322382	0.994409636
PIGQ	-0.010572775	0.899406709	0.994409636
TMEM179B	-0.005267881	0.899470712	0.994409636
NMNAT3	-0.014748989	0.89952562	0.994409636

GABBR1	-0.017642221	0.899563039	0.994409636
GTPBP8	0.010388397	0.899658666	0.994409636
JAKMIP3	-0.01349983	0.899716534	0.994409636
MUTYH	0.007717113	0.899783116	0.994409636
LMO4	-0.011335131	0.899797281	0.994409636
NBEA	0.014693011	0.900046089	0.994409636
IQCG	-0.014245955	0.900063018	0.994409636
FLVCR1	0.015037242	0.900134914	0.994409636
SNORA21	0.010959191	0.900367882	0.994409636
RAD23A	-0.008543539	0.900537194	0.994409636
CENPK	-0.044589235	0.900544095	0.994409636
MMD	0.020717158	0.900560517	0.994409636
ZBED3	-0.010854184	0.900560665	0.994409636
NDRG2	0.013206166	0.900582624	0.994409636
WDR6	0.008903684	0.90075483	0.994409636
C11orf35	-0.013736459	0.900793505	0.994409636
CAPZA1	-0.019065487	0.900796106	0.994409636
ILKAP	0.010115681	0.900945708	0.994409636
LYN	0.008606164	0.90094895	0.994409636
KBTBD8	0.012704902	0.901036133	0.994409636
TMEM161A	-0.010702319	0.901062966	0.994409636
ZFP37	-0.017494513	0.901191036	0.994409636
CAPS2	-0.008963304	0.901268969	0.994409636
TIPIN	-0.013328659	0.901359475	0.994409636
NUDCD2	0.009910153	0.901392145	0.994409636
PLCB1	0.016124283	0.90181909	0.994409636
NT5DC2	0.019053352	0.901969296	0.994409636
PI15	-0.012459568	0.901982151	0.994409636
NIF3L1	0.005645215	0.902026374	0.994409636
ADCY4	-0.012279329	0.902094502	0.994409636
SAMSN1	-0.012774007	0.902317763	0.994409636
TMEM177	-0.010542672	0.902335806	0.994409636
BMI1	0.006991722	0.902387767	0.994409636
MPPED2	0.013924561	0.902510145	0.994409636
INTU	-0.013214469	0.902642081	0.994409636
C11orf49	-0.012435522	0.902692731	0.994409636
OPN3	0.011994586	0.902722283	0.994409636
NDFIP1	0.009476874	0.902728582	0.994409636
ANKRD20A4	0.042340199	0.902737429	0.994409636
VAMP8	-0.008320408	0.902834575	0.994409636
RNF121	0.006963551	0.902991885	0.994409636
CGGBP1	0.005541098	0.903186575	0.994409636
DNAH1	-0.009731768	0.903201107	0.994409636
TAS2R14	-0.011045861	0.903268025	0.994409636
ANKRA2	0.007999738	0.903396328	0.994409636
SORD	0.007485418	0.903402792	0.994409636
MKLN1	0.006540639	0.903458183	0.994409636

LYRM5	0.007379492	0.903474455	0.994409636
SLC25A39	-0.006419114	0.903525378	0.994409636
VPS13C	-0.017247795	0.903652763	0.994409636
TMEM14B	0.010543838	0.903673912	0.994409636
SUMO1P1	0.010875416	0.903868779	0.994431329
MBTPS1	-0.0066608	0.903962413	0.994431329
TRIP13	-0.016285684	0.903963143	0.994431329
SCAF11	-0.005604049	0.904258283	0.994537883
CNPY2	-0.005903196	0.904864341	0.994537883
UBE2R2	-0.008302231	0.904880209	0.994537883
BET1L	0.011067826	0.904963309	0.994537883
SLFN13	0.016390559	0.905097836	0.994537883
PCK2	0.008338469	0.905100962	0.994537883
WDR7	0.007906255	0.905128862	0.994537883
EMR2	0.012506593	0.9051545	0.994537883
ECHDC3	-0.020283833	0.905160347	0.994537883
DPH3	0.012217873	0.905489669	0.994537883
DNHD1	-0.01211201	0.905549918	0.994537883
ANKRD52	-0.012936186	0.905598073	0.994537883
TYROBP	0.012990306	0.905674445	0.994537883
WASF2	-0.009242826	0.905688826	0.994537883
ATMIN	-0.012234953	0.905710518	0.994537883
RBBP4	0.014305469	0.905726615	0.994537883
TGM2	-0.01442645	0.905835718	0.994537883
ZNF524	-0.009528142	0.905870328	0.994537883
PLSCR4	0.011598393	0.906099354	0.994537883
THG1L	-0.018284687	0.906111443	0.994537883
COCH	0.031252858	0.906304968	0.994537883
NCKAP1L	0.006115989	0.906338834	0.994537883
ERAP1	-0.013144129	0.906354989	0.994537883
FAM200B	-0.018243325	0.906595236	0.994537883
BRD9	0.005535527	0.906711724	0.994537883
MEX3C	0.010956846	0.906753775	0.994537883
B3GNT1	-0.012885425	0.907234295	0.994537883
SRRD	0.009102785	0.907273616	0.994537883
DNAJC24	-0.009700923	0.907520531	0.994537883
SNORD94	0.008467024	0.907560903	0.994537883
C1orf35	-0.005634366	0.907578923	0.994537883
CNIH4	-0.00713463	0.907804017	0.994537883
MED8	0.009255787	0.907922484	0.994537883
FBXO21	-0.005022623	0.908003265	0.994537883
TUBBP5	0.012882111	0.908027322	0.994537883
ZNF438	-0.008430025	0.908071438	0.994537883
SNX1	0.008374674	0.908120233	0.994537883
RRM2	-0.014941915	0.908129585	0.994537883
STK40	0.007187938	0.908203915	0.994537883
SPNS3	-0.009711715	0.908409043	0.994537883

PTPN9	0.011260568	0.908530049	0.994537883
HEG1	0.010508392	0.908585461	0.994537883
MCTP2	0.016630048	0.908682701	0.994537883
GCSH	0.010603516	0.908700201	0.994537883
CARHSP1	0.009952341	0.909044661	0.994537883
TBL3	0.010842037	0.909087204	0.994537883
ARAP1	-0.011391397	0.909103065	0.994537883
ELOVL1	0.008772418	0.909121383	0.994537883
PPP1R13L	-0.009236861	0.90922662	0.994537883
C6orf89	0.008998405	0.909411811	0.994537883
DPY19L2P4	0.011096755	0.909442866	0.994537883
REXO1	-0.009002165	0.909488894	0.994537883
LIG4	0.013552602	0.909558165	0.994537883
OTUD4	0.006969293	0.909562825	0.994537883
CALM1	0.006006784	0.909630165	0.994537883
MT1H	0.017709194	0.909717302	0.994537883
FBXO9	0.016823012	0.909722148	0.994537883
MKL2	0.012092587	0.909725642	0.994537883
MAP3K8	-0.011836955	0.909774067	0.994537883
ATP5F1	0.006695751	0.909788903	0.994537883
RASA1	-0.00607489	0.90981884	0.994537883
USP48	-0.011880569	0.90981927	0.994537883
TGFA	-0.016070682	0.909915909	0.994537883
ENOPH1	0.006463251	0.909988506	0.994537883
CD93	0.011322686	0.910064443	0.994537883
DYNLT1	-0.007260754	0.910078869	0.994537883
LCK	0.009126558	0.910250393	0.994537883
RBM26	0.00852635	0.910324768	0.994537883
PDE8A	-0.01068466	0.910367999	0.994537883
MYL6	0.004740629	0.910487857	0.994537883
NT5M	0.023014719	0.910555925	0.994537883
RAB28	-0.013761069	0.910562678	0.994537883
YTHDC1	-0.00810091	0.910644059	0.994537883
FBP1	0.012530348	0.910709331	0.994537883
UNK	-0.007630739	0.910798674	0.994537883
CEP55	0.017967411	0.911148189	0.994785086
CASP4	-0.007390526	0.911204805	0.994785086
MCF2L-AS1	0.013651502	0.911414557	0.994802224
TRMT6	-0.010730708	0.911562452	0.994802224
BCL2A1	-0.014228366	0.911594898	0.994802224
TMEM42	0.006280095	0.911632969	0.994802224
KLF6	-0.008053145	0.911732321	0.994802224
S100A4	-0.005634222	0.911887254	0.994802224
RRM2B	-0.015867844	0.911938718	0.994802224
EML3	0.006630479	0.911939485	0.994802224
PRR7	0.014786556	0.912240858	0.995032919
HIST1H2BD	0.033535501	0.912361385	0.995066329

SCAMP5	-0.017643874	0.912648671	0.995137509
SLC9A7P1	-0.009842725	0.912893699	0.995137509
CDK6	-0.009647184	0.912920131	0.995137509
AFF1	0.007720477	0.913137287	0.995137509
RGS14	-0.010760672	0.913360218	0.995137509
CHCHD2	0.007856148	0.913495624	0.995137509
DYNC2H1	-0.007461311	0.913507918	0.995137509
GNAI2	-0.006953815	0.913545314	0.995137509
KDM2B	0.004785956	0.913558767	0.995137509
MAP2K4	-0.005557068	0.913616425	0.995137509
SEC22C	0.005395719	0.913831207	0.995137509
NFU1	0.00685961	0.913854795	0.995137509
ITGA6	0.013101427	0.913914499	0.995137509
RPSAP52	-0.008775691	0.914014271	0.995137509
TYW3	-0.013402211	0.91404912	0.995137509
TNFSF8	0.013187062	0.914083994	0.995137509
HSPH1	-0.007499462	0.914267769	0.995137509
LRFN4	0.011242199	0.914276282	0.995137509
TRMT1L	0.007583941	0.91438049	0.995137509
KLHL14	0.024860059	0.914391548	0.995137509
MTRF1L	0.005858332	0.914581915	0.995137509
ARHGEF12	0.014737044	0.914720126	0.995137509
ZNF766	-0.00795669	0.915000315	0.995137509
TEC	-0.008584461	0.915031695	0.995137509
SNORD11B	-0.011815271	0.915046231	0.995137509
LRRC56	-0.009371597	0.91508336	0.995137509
PPP1R14A	0.01924256	0.91512495	0.995137509
TMEM141	0.007091979	0.915126228	0.995137509
ICAM4	-0.023561162	0.915161947	0.995137509
SIRPA	0.007944414	0.915441996	0.995137509
INTS1	0.007693136	0.915471132	0.995137509
SP100	-0.011379496	0.915630029	0.995137509
PRKD2	0.008933557	0.915710734	0.995137509
TESC	-0.010829658	0.915831554	0.995137509
IL16	0.00771839	0.915997211	0.995137509
FZR1	-0.009814716	0.916093822	0.995137509
CBFB	0.007372966	0.916097882	0.995137509
PSMF1	0.004719703	0.916160981	0.995137509
NLRC3	-0.010126982	0.91617653	0.995137509
C17orf58	0.009920918	0.916310224	0.995137509
MPHOSPH8	0.010034334	0.916395022	0.995137509
BST1	-0.01871278	0.916543928	0.995137509
RNASEH2A	0.00856847	0.916611084	0.995137509
C1orf56	-0.011281926	0.916752349	0.995137509
RIPK2	-0.007295623	0.916780412	0.995137509
ZDHHC11	-0.022004715	0.916831271	0.995137509
CAMLG	-0.004953945	0.91685635	0.995137509

IQSEC1	-0.00634206	0.916878992	0.995137509
SNX10	0.011423579	0.916895443	0.995137509
STYK1	-0.011996956	0.916991442	0.995137509
SCARNA14	-0.009606807	0.917011708	0.995137509
CHCHD4	-0.005904071	0.917335034	0.995327547
ZBTB38	0.012728289	0.917494691	0.995327547
DRD4	0.016756807	0.917519288	0.995327547
FST	-0.021134947	0.917637749	0.995327547
ZNF431	-0.007312767	0.917639359	0.995327547
C21orf128	-0.014935742	0.917726348	0.995327547
PPP6R2	0.008446145	0.917884209	0.995401225
ZBTB39	0.008781133	0.917977015	0.995404347
KIAA1429	-0.006020883	0.918265975	0.995613912
NBPF8	-0.016262967	0.918390265	0.995613912
ZMYND11	-0.005594371	0.918440117	0.995613912
CYP2E1	-0.013272596	0.918778708	0.995829578
C1orf74	-0.012243823	0.918870495	0.995829578
DCBLD1	0.01151911	0.918908963	0.995829578
C3AR1	-0.017162073	0.91937214	0.995884115
NMNAT1	-0.008988919	0.919506075	0.995884115
C2orf43	-0.009188337	0.91952822	0.995884115
RGS2	-0.010431256	0.919619262	0.995884115
ECHDC2	-0.01027955	0.919725292	0.995884115
DPYSL4	-0.043336127	0.919926673	0.995884115
TMPRSS13	-0.010281846	0.92000344	0.995884115
HSBP1L1	0.009360991	0.920110625	0.995884115
BRI3	0.011072016	0.920333748	0.995884115
PLOD1	0.008637064	0.920377084	0.995884115
ANO10	-0.008882057	0.920443354	0.995884115
TBX1	-0.011869473	0.920554241	0.995884115
SRSF10	-0.008982607	0.920623162	0.995884115
HIBCH	-0.007216193	0.92078671	0.995884115
CCRN4L	0.012292615	0.920957591	0.995884115
GSTO2	0.009415696	0.920981193	0.995884115
PER2	0.00633724	0.920986702	0.995884115
CADM2	0.00987407	0.92099999	0.995884115
PTPN13	-0.01324481	0.921006621	0.995884115
APOBEC3D	0.009870185	0.921050464	0.995884115
IFIT2	-0.018533454	0.921058078	0.995884115
MAP6D1	0.011620448	0.921084102	0.995884115
MSI2	-0.007813942	0.921296011	0.995884115
TMEM5	-0.006227632	0.921379675	0.995884115
NCSTN	0.004182556	0.92140395	0.995884115
CA6	0.012157423	0.921439166	0.995884115
GTF2E1	0.005934691	0.921584522	0.995884115
TSPAN32	-0.008227415	0.921689392	0.995884115
SCARNA8	-0.008853376	0.921774885	0.995884115

NIPAL3	0.007849611	0.922133143	0.995884115
ATL3	0.006634273	0.922150563	0.995884115
TTC5	0.007105177	0.922260135	0.995884115
BIN3	0.013396029	0.922279208	0.995884115
B2M	-0.003495491	0.922349112	0.995884115
MCF2L	0.009829863	0.922429414	0.995884115
STRN4	-0.009205366	0.922584355	0.995884115
SPINT3	-0.010167034	0.922812358	0.995884115
ACP6	0.009423172	0.922871995	0.995884115
SLC25A38	0.007055831	0.92289128	0.995884115
KATNAL1	0.010286464	0.92292516	0.995884115
NBR2	0.012221878	0.923241038	0.995884115
FDXR	-0.009506991	0.92325042	0.995884115
PDCL	-0.011017548	0.923271646	0.995884115
CENPW	-0.015234757	0.923347107	0.995884115
ZNF624	-0.008433355	0.923394486	0.995884115
PTPDC1	0.00900067	0.923626019	0.995884115
HEXDC	-0.010582789	0.923644984	0.995884115
AES	-0.008081221	0.923646549	0.995884115
FPR2	0.025102059	0.923702669	0.995884115
INO80D	0.008033714	0.92372272	0.995884115
MKRN2	-0.006024059	0.923739167	0.995884115
IFT88	0.007363066	0.92377424	0.995884115
DOPEY2	0.006731958	0.923878873	0.995884115
FOXK2	0.011423239	0.923888271	0.995884115
LLPH	0.007465593	0.923907668	0.995884115
RHBDL1	-0.009386805	0.924025753	0.995914417
DSC3	0.008822013	0.924392852	0.996213074
SLC2A11	-0.007687358	0.924512849	0.996245398
CHML	0.009209575	0.924770753	0.996254442
SLMO1	-0.007498816	0.924791109	0.996254442
CLEC4C	-0.019107058	0.924844589	0.996254442
SUCLG2	-0.006462916	0.924881258	0.996254442
SNORA77	-0.010511432	0.925086795	0.996378879
DNAJB6	-0.004675475	0.925530422	0.996491788
CD2BP2	0.004931879	0.92562299	0.996491788
ERCC1	-0.006661397	0.925633789	0.996491788
VCPIP1	-0.006517556	0.925674843	0.996491788
ABHD1	-0.009027262	0.925701716	0.996491788
ACTN4	-0.009880824	0.925731778	0.996491788
SERTAD3	0.007482059	0.926128618	0.996654443
RASD1	0.016355371	0.926341797	0.996654443
MYO1B	0.009964412	0.926357766	0.996654443
GOLPH3	-0.004942954	0.926394505	0.996654443
NPFF	-0.007404917	0.926426296	0.996654443
MIR1224	-0.009725847	0.926445581	0.996654443
PDCD6IP	-0.005311902	0.926599809	0.996654443

TMEM9	-0.007612426	0.926603204	0.996654443
RSPH3	0.009852529	0.926856986	0.996787233
FBXO8	-0.007776599	0.926949514	0.996787233
ITGB1	-0.006650505	0.927128683	0.996787233
IFT20	0.00549562	0.927206577	0.996787233
RETN	0.019483289	0.927445258	0.996787233
FARSA	-0.0078447	0.927455663	0.996787233
PART1	-0.00884857	0.92752015	0.996787233
SYTL1	0.007117061	0.927808098	0.996787233
GZMA	-0.011316406	0.927864794	0.996787233
ETFA	-0.007816398	0.928025972	0.996787233
PBXIP1	0.007532773	0.928418736	0.996787233
GIT1	0.009310523	0.928471942	0.996787233
SNORA76	0.009023124	0.928621044	0.996787233
TRIM14	-0.010297892	0.928797599	0.996787233
ECE2	0.010621964	0.928899717	0.996787233
HSPBP1	-0.008750088	0.928907335	0.996787233
PEX10	0.006497019	0.929015675	0.996787233
BSPRY	0.008838539	0.929084428	0.996787233
USP28	-0.013358357	0.929185475	0.996787233
ICOS	0.009557838	0.929236841	0.996787233
KRT3	-0.006914418	0.929322108	0.996787233
HENMT1	0.006993421	0.92935111	0.996787233
WDR61	-0.006562302	0.929455977	0.996787233
ZNF335	-0.008232684	0.929727762	0.996787233
PLEKHF1	-0.015968392	0.929782342	0.996787233
NFAT5	0.012809702	0.929877206	0.996787233
STK10	0.005945291	0.929912216	0.996787233
ICAM1	-0.008665976	0.929928254	0.996787233
CD3EAP	0.006777354	0.93022056	0.996787233
ARSB	-0.01031244	0.930288294	0.996787233
UBTD2	0.006399816	0.930436777	0.996787233
ZNF703	-0.010350133	0.930508242	0.996787233
MZT1	-0.007937085	0.930508417	0.996787233
SLC27A3	-0.00635916	0.930618045	0.996787233
FAM136A	-0.005043875	0.930630061	0.996787233
HEATR5A	0.008428594	0.930663671	0.996787233
DFFB	-0.007713861	0.930714868	0.996787233
HADHB	-0.006288967	0.930904844	0.996787233
FAU	0.003668194	0.930962643	0.996787233
MAP1LC3A	-0.008952653	0.931033201	0.996787233
FGF18	0.009118201	0.931080655	0.996787233
LRRC8C	-0.00969178	0.931094397	0.996787233
PDE7B	-0.010204825	0.931189711	0.996787233
MED24	0.006214261	0.931265072	0.996787233
CNOT1	-0.004126118	0.931267797	0.996787233
LYSMD1	-0.006577036	0.931473652	0.996787233

ATP13A2	0.00774408	0.931712539	0.996787233
RHOH	0.007641032	0.931753722	0.996787233
DGKD	-0.009099195	0.931778105	0.996787233
VPS13D	-0.007586235	0.931831134	0.996787233
NARG2	-0.012003951	0.931850926	0.996787233
OLFM1	-0.028301303	0.931909376	0.996787233
CORO7	0.007828745	0.931959741	0.996787233
ARHGAP25	-0.006663017	0.932057365	0.996787233
PSMG1	-0.005196303	0.932129832	0.996787233
UBLCP1	0.005016897	0.932139154	0.996787233
LSR	-0.008407995	0.932184197	0.996787233
FRMD8	-0.004736515	0.932443439	0.996787233
CETP	0.017762067	0.932519727	0.996787233
CCL5	-0.00991799	0.932590944	0.996787233
PHLDB1	-0.008067594	0.932672885	0.996787233
DLGAP4	-0.006254521	0.932722796	0.996787233
FAM21B	-0.008103694	0.932728653	0.996787233
RLTPR	-0.008611954	0.932839544	0.996787233
C18orf8	0.004295144	0.932901713	0.996787233
TM4SF1	0.022361888	0.933348018	0.996787233
ATF4	0.003725585	0.933526457	0.996787233
OAT	-0.008192791	0.933725073	0.996787233
MATR3	0.003697329	0.933818351	0.996787233
UBE2V2	-0.004832867	0.933842012	0.996787233
MIR564	-0.007845424	0.933921403	0.996787233
MXRA7	0.019378724	0.934052505	0.996787233
PHKG2	0.006695128	0.934289622	0.996787233
GPBP1L1	-0.008045069	0.934411411	0.996787233
TMEM167A	-0.008412377	0.934664486	0.996787233
ST6GALNAC4	0.006716788	0.9346953	0.996787233
KLF2	-0.003422613	0.934730247	0.996787233
ALMS1	0.006489328	0.934731315	0.996787233
IFI44	-0.018231971	0.935007162	0.996787233
PSD4	-0.007890761	0.935297187	0.996787233
DYNC2LI1	-0.010801948	0.935396891	0.996787233
KLHDC3	0.0030567	0.935476088	0.996787233
GOLGA6A	0.009787012	0.935494785	0.996787233
DHRS1	-0.005263214	0.935514254	0.996787233
ZIM3	0.007353688	0.935544546	0.996787233
NUP205	-0.00390645	0.93567157	0.996787233
VAPA	-0.01062355	0.935716573	0.996787233
FAM122A	-0.006381956	0.935960494	0.996787233
CLIP1	0.004058841	0.936038552	0.996787233
TBC1D19	-0.007107294	0.93613676	0.996787233
FBXW2	0.005015241	0.936325004	0.996787233
ACOT1	0.009548207	0.936470478	0.996787233
ZFYVE19	-0.00632327	0.936553592	0.996787233

GPR55	-0.01408595	0.936617624	0.996787233
MIR586	-0.007945671	0.936946507	0.996787233
MICALCL	0.009944834	0.936997957	0.996787233
ACLY	-0.003988529	0.937062677	0.996787233
TRAM2	0.006907619	0.937147319	0.996787233
FAM63B	-0.009410176	0.937160897	0.996787233
RSRC1	0.008328163	0.937300806	0.996787233
RNF135	0.00555221	0.937367143	0.996787233
TOP3B	0.005728022	0.937509125	0.996787233
DNAJA4	-0.007160209	0.937531638	0.996787233
FIP1L1	-0.004380397	0.937541648	0.996787233
SYVN1	-0.004583114	0.937543401	0.996787233
BSPH1	0.00794116	0.937744804	0.996787233
LRRC8A	0.007553441	0.93777634	0.996787233
RAC2	0.003928521	0.937835321	0.996787233
CCND1	-0.009144037	0.937907173	0.996787233
KYNU	0.008955285	0.937965004	0.996787233
ENTPD6	-0.006189467	0.937994183	0.996787233
ZNF680	-0.006864542	0.938125345	0.996787233
SFPQ	0.008789015	0.938186931	0.996787233
CLASP1	0.004869199	0.938213439	0.996787233
OCEL1	0.005007612	0.938268309	0.996787233
GFOD2	-0.006303093	0.938279856	0.996787233
ZMYM5	0.008717005	0.938507938	0.996787233
OTUD7B	-0.007578263	0.938540401	0.996787233
DCAF8	-0.004686111	0.938578599	0.996787233
NEB	-0.008422366	0.938611719	0.996787233
ITSN2	-0.006355246	0.938628041	0.996787233
POLR2J2	-0.014166468	0.938740257	0.996787233
HOMEZ	0.007509776	0.938756114	0.996787233
ZNF555	-0.009853513	0.938760989	0.996787233
PAF1	0.003798376	0.938852731	0.996787233
ZFR	0.003262852	0.9388646	0.996787233
PARK7	0.003466429	0.938941903	0.996787233
ANXA1	-0.00570435	0.938995423	0.996787233
FANCC	0.005933899	0.939006598	0.996787233
TMUB2	0.005248158	0.939013927	0.996787233
GPSM1	-0.009379033	0.939089408	0.996787233
C1RL	-0.00707532	0.939164771	0.996787233
AGR2	-0.006639846	0.93929654	0.996787233
KLB	-0.005463232	0.939324449	0.996787233
GOLGA7B	-0.008665456	0.939398056	0.996787233
PIGP	0.007206006	0.939508539	0.996787233
CCND2	-0.004818209	0.939610582	0.996787233
HIST2H2AB	0.00998317	0.939702163	0.996787233
YBX1	-0.004195658	0.939724002	0.996787233
ZNF519	-0.009250672	0.939756878	0.996787233

TMEM63A	-0.009564084	0.940087889	0.996787233
NCAPG2	0.019238297	0.94037465	0.996787233
SUSD5	0.007550926	0.940694632	0.996787233
MYCBP2	0.004856509	0.940829832	0.996787233
SRPK2	0.00802596	0.940887626	0.996787233
ZMIZ1	0.00498437	0.940947687	0.996787233
CBLN3	-0.018114251	0.941012841	0.996787233
PEMT	-0.004935135	0.941098832	0.996787233
BCL2L2	0.005641735	0.941123554	0.996787233
MAU2	0.006976798	0.94126686	0.996787233
SPAG1	0.00767426	0.941304814	0.996787233
MOCS1	0.008984155	0.94130634	0.996787233
SLC22A5	0.010682338	0.941363843	0.996787233
RPP30	-0.006836951	0.941429789	0.996787233
NFKBID	0.005738326	0.941475822	0.996787233
UBASH3B	-0.005693208	0.941490684	0.996787233
TRIM65	-0.007048708	0.941507155	0.996787233
RPN2	-0.005425166	0.941635639	0.996787233
WLS	0.011758741	0.941640642	0.996787233
CYB5D2	0.005429929	0.941664991	0.996787233
GOLIM4	-0.01007631	0.941763087	0.996787233
PURG	-0.006682563	0.941791512	0.996787233
DHCR24	0.012532659	0.941925505	0.996787233
SNX4	0.004525485	0.941941387	0.996787233
GCH1	0.006831916	0.941952339	0.996787233
REC8	0.006087175	0.942009696	0.996787233
C1orf61	0.008319562	0.942033021	0.996787233
C6orf223	-0.007400458	0.942055707	0.996787233
TADA2A	-0.006814531	0.94206333	0.996787233
CD5	0.007535694	0.942069685	0.996787233
PLLP	0.008394029	0.942213216	0.996787233
CCAR1	-0.013140908	0.942232212	0.996787233
CHCHD10	0.005741015	0.942506612	0.996787233
DPM1	-0.006941165	0.942539745	0.996787233
FLT3LG	-0.007613938	0.942610039	0.996787233
C12orf77	0.005921536	0.942658323	0.996787233
GPRIN3	0.009508999	0.942665892	0.996787233
SNORD36C	-0.008730522	0.942828063	0.996863485
TMCO6	-0.004504165	0.943040801	0.996912097
MYBBP1A	0.007779148	0.943054167	0.996912097
GRK5	-0.005685802	0.943485012	0.997026034
MYLK4	-0.009753835	0.943531774	0.997026034
ITGAM	-0.007093964	0.943537846	0.997026034
APOL6	-0.009392122	0.943582512	0.997026034
HSD11B1L	-0.00658433	0.943620324	0.997026034
MDK	-0.006457851	0.943732435	0.997026034
VNN2	0.008405498	0.943792464	0.997026034

GNG5	0.006834125	0.944133725	0.997073051
RAB3GAP1	0.004794613	0.944206611	0.997073051
KAT2B	-0.004227563	0.944375488	0.997073051
PMVK	0.005084799	0.944520393	0.997073051
TCL1B	-0.015105884	0.944551209	0.997073051
LDHB	-0.006099542	0.944552088	0.997073051
BYSL	0.008937772	0.944582261	0.997073051
ZNF256	0.006102682	0.944627997	0.997073051
OPLAH	-0.009419554	0.944649667	0.997073051
COMTD1	0.006296553	0.944737751	0.997073051
RNF181	-0.003874493	0.945094489	0.997338771
UBE2H	0.005486201	0.945171836	0.997338771
ZNF225	0.005259229	0.945259829	0.997338771
TMCO1	-0.003313659	0.945396962	0.997388388
GSN	-0.009279217	0.945715609	0.997503691
MIR1228	-0.007401197	0.945904225	0.997503691
LY75	-0.009681177	0.945944613	0.997503691
PNN	-0.00699023	0.945951791	0.997503691
BIK	-0.007172892	0.946095935	0.997503691
TRAF3IP2	0.006559458	0.946108628	0.997503691
KCNRG	-0.007767858	0.946203568	0.997503691
NUP210	-0.006998248	0.946519324	0.997503691
MGP	0.005966289	0.946547149	0.997503691
GPATCH3	-0.004724618	0.946633269	0.997503691
TNFAIP8L1	0.008398244	0.946725943	0.997503691
C5orf24	0.00685168	0.946767803	0.997503691
KCNQ1	0.007196877	0.946902623	0.997503691
UBL3	-0.005601508	0.946993378	0.997503691
REL	-0.007190806	0.947101592	0.997503691
RCBTB1	0.005931691	0.947111205	0.997503691
B4GALT6	-0.008263545	0.947154833	0.997503691
SKP1	-0.003652961	0.947234692	0.997503691
N6AMT2	0.005233713	0.94740469	0.997503691
SEC11C	-0.006123961	0.947442095	0.997503691
DPP3	0.004484039	0.947476047	0.997503691
DSCR3	0.003703305	0.947569973	0.997503691
KLHL29	0.005491272	0.947578943	0.997503691
MYO5A	0.00477164	0.947722877	0.997560339
CIAPIN1	0.004346149	0.947967988	0.99767547
TCP1	-0.00498674	0.948012521	0.99767547
RPL27A	-0.001946275	0.948310216	0.997893886
DNA2	-0.00635873	0.94847497	0.997907701
ZNF98	0.010103787	0.948503652	0.997907701
SLC2A5	0.011885054	0.948767027	0.997999421
TPD52	-0.007590834	0.94877296	0.997999421
ACAA1	0.004420562	0.948918333	0.997999421
ITPR3	-0.005454718	0.948964422	0.997999421

ACADM	0.003878238	0.949041639	0.997999421
ITM2B	-0.00448431	0.949245004	0.99804082
NOL7	-0.004008105	0.949506455	0.99804082
SLC25A15	0.004113155	0.94967085	0.99804082
ENO1	-0.00308293	0.949865724	0.99804082
FPR1	0.013664283	0.949922631	0.99804082
USP47	-0.004505055	0.949941786	0.99804082
GTF2H2B	0.010407167	0.9500476	0.99804082
PPP1R12C	0.00565252	0.950122992	0.99804082
KDM3B	-0.003616455	0.950186216	0.99804082
WNT10A	0.008105581	0.950230754	0.99804082
C10orf76	-0.0042021	0.95042212	0.99804082
LGALS2	-0.019926302	0.950423045	0.99804082
IMPDH1	-0.004864894	0.950605283	0.99804082
PLD3	0.004255025	0.950663019	0.99804082
STAT5A	0.005207192	0.950755757	0.99804082
METTL13	0.00369997	0.950913203	0.99804082
WARS2	-0.018460749	0.950956955	0.99804082
ECM1	-0.006952929	0.951152623	0.99804082
RSPRY1	0.003952912	0.951244053	0.99804082
SMARCB1	-0.005110837	0.951264577	0.99804082
FAM86B1	0.007830475	0.95130744	0.99804082
DERL1	-0.004016856	0.951466056	0.99804082
FBXL6	0.004927697	0.95150647	0.99804082
BAZ1A	0.004107035	0.951579631	0.99804082
YIF1B	0.003985455	0.95160556	0.99804082
JSRP1	-0.010326097	0.95164445	0.99804082
GNG10	0.009781023	0.951729324	0.99804082
MAT2A	-0.004362369	0.951791672	0.99804082
TP53INP1	-0.005874918	0.95186301	0.99804082
GNL3	0.002979623	0.951922603	0.99804082
PSMC5	0.002693208	0.95193657	0.99804082
E2F1	0.008679846	0.951966301	0.99804082
ZNF597	0.005628882	0.952143696	0.998114298
PLEKHM1P	-0.009749733	0.952246538	0.998114298
HIST1H2AM	-0.010995092	0.952352297	0.998114298
UBN1	-0.003820708	0.952397074	0.998114298
GOSR2	0.005785665	0.952607242	0.99823632
SLC35C2	0.003906761	0.952786701	0.99823632
CCNG2	0.006251639	0.952839683	0.99823632
PPM1M	0.003149721	0.952882696	0.99823632
ABCG1	0.008079707	0.952994041	0.99823632
SNORD73A	0.00576471	0.953149613	0.99823632
SHOX2	0.005454361	0.953180596	0.99823632
DOCK5	0.005652137	0.953234972	0.99823632
MS4A4A	0.010842506	0.953372255	0.998285639
ZNF404	-0.007371751	0.953498122	0.998316761

APOM	-0.006254429	0.95363575	0.998316761
EXD3	0.00606281	0.953672548	0.998316761
PUS1	-0.002991773	0.953908983	0.998422126
METTL5	-0.005488134	0.953966082	0.998422126
TUBB	0.003258047	0.95406381	0.998422126
ITGA2	0.006927081	0.954134001	0.998422126
ZBTB16	0.010889285	0.954324623	0.998455442
UTP6	0.002606258	0.954393872	0.998455442
TBK1	0.003143357	0.954436447	0.998455442
USP42	-0.003705625	0.954773336	0.99858064
FRAT1	-0.006305157	0.954826538	0.99858064
RTF1	-0.004712634	0.954876592	0.99858064
SCAPER	-0.00479818	0.955115231	0.99858064
CASP6	0.006620246	0.955142704	0.99858064
S100A13	0.004360319	0.95521703	0.99858064
DGCR8	-0.007840717	0.955235782	0.99858064
PEA15	-0.003655919	0.955296335	0.99858064
ARPC5	-0.00236406	0.955368053	0.99858064
ZNF232	0.004531514	0.955486053	0.99860968
CDKN1B	0.00368889	0.955588046	0.998621987
USPL1	-0.002943065	0.955780157	0.998728122
CAPZA2	-0.003126785	0.956137155	0.998728122
ZNF22	0.003637879	0.956254575	0.998728122
PIK3R5	-0.004874105	0.956374216	0.998728122
PILRA	-0.005718852	0.956387162	0.998728122
SPOPL	0.005229964	0.956857935	0.998728122
ZNF775	0.004593743	0.956923716	0.998728122
C3orf62	0.00415535	0.956944134	0.998728122
DNAJA2	0.002778827	0.956991555	0.998728122
SESN3	-0.006310328	0.957013434	0.998728122
FAM57A	-0.00477214	0.957044225	0.998728122
AGFG2	0.005406139	0.957227517	0.998728122
PHLDA1	0.007683073	0.957387213	0.998728122
SCARNA17	0.007039569	0.957487991	0.998728122
L2HGDH	0.010050453	0.95790323	0.998728122
CCT6A	-0.002940583	0.957961464	0.998728122
NAT14	-0.004457572	0.958010189	0.998728122
PFDN5	-0.002597676	0.958074226	0.998728122
RPS6KL1	-0.004740761	0.958173486	0.998728122
SLCO4C1	-0.008378487	0.958312973	0.998728122
DFNA5	0.009657743	0.958374852	0.998728122
DCTN1	0.002350714	0.958442683	0.998728122
PYCR1	-0.00445575	0.958443911	0.998728122
IPCEF1	0.005317763	0.958725205	0.998728122
PHF13	0.003048681	0.958867741	0.998728122
C9orf72	-0.005947281	0.958917795	0.998728122
NUCKS1	0.004271767	0.959151098	0.998728122

SSPN	-0.011336206	0.959453815	0.998728122
CPNE1	-0.003757578	0.959567493	0.998728122
NDRG1	0.003477484	0.959598541	0.998728122
RAB7L1	0.003599495	0.959637935	0.998728122
CAPN11	0.009307214	0.959676202	0.998728122
CSNK1D	-0.002505374	0.959721043	0.998728122
TIGIT	0.007585937	0.959738704	0.998728122
AP2A2	0.004813035	0.959767613	0.998728122
SCYL1	-0.002664293	0.959799192	0.998728122
CDK12	0.006636347	0.95981493	0.998728122
FHOD1	0.003630436	0.959855996	0.998728122
THAP1	0.005921744	0.959922956	0.998728122
CIITA	-0.005696032	0.959944216	0.998728122
FP15737	-0.004681191	0.960148659	0.998728122
ZNF277	-0.003579588	0.960422828	0.998728122
LIN37	-0.003611902	0.960441807	0.998728122
FAM105A	0.005777109	0.960564912	0.998728122
RBM42	0.003403504	0.960765301	0.998728122
ARIH2	-0.002993669	0.96079408	0.998728122
RPL12	0.002667846	0.960798199	0.998728122
MSRB2	0.003325586	0.960805994	0.998728122
FOXD2	-0.006844867	0.960874137	0.998728122
CRBN	-0.002688365	0.960891729	0.998728122
PPAPDC1B	-0.00408095	0.960974389	0.998728122
CABLES2	0.003434096	0.960990068	0.998728122
MAST2	-0.005179747	0.960992162	0.998728122
DAAM1	-0.004149772	0.96100102	0.998728122
MAP3K1	0.002897979	0.96117836	0.998728122
UCHL5	0.00511931	0.961229686	0.998728122
EVI2B	0.006044149	0.961230297	0.998728122
SLC15A4	-0.003553727	0.961266154	0.998728122
MGST1	-0.009485964	0.961298673	0.998728122
EFCAB3	-0.004052212	0.961565328	0.998728122
CLPTM1L	0.003042835	0.961666306	0.998728122
CXCL10	0.01225137	0.961685781	0.998728122
ANTXR2	0.003882639	0.961761835	0.998728122
PEX7	0.004549023	0.961783701	0.998728122
PSTK	0.003831323	0.961939976	0.998728122
LYPLA1	-0.005892953	0.962297128	0.998728122
DIP2C	0.004911493	0.962310433	0.998728122
KIAA0100	-0.002883432	0.962355988	0.998728122
URB1	0.003717542	0.96238583	0.998728122
STARD3NL	0.006294381	0.962506559	0.998728122
PPARGC1B	-0.005804712	0.962539626	0.998728122
TMBIM4	-0.001995224	0.962595486	0.998728122
IDI1	-0.003781738	0.962608015	0.998728122
FAM98C	0.003551039	0.962661724	0.998728122

CCNI	0.00207303	0.962745227	0.998728122
PKP3	-0.003808648	0.962812172	0.998728122
PMPCA	-0.003018011	0.962972064	0.998728122
ETS2	0.004004406	0.96304979	0.998728122
ZNF883	-0.004670289	0.963063026	0.998728122
RCVRN	0.006323158	0.963087516	0.998728122
C10orf11	0.007439998	0.963377127	0.998728122
ESD	-0.002833297	0.963407529	0.998728122
LSG1	0.004860245	0.963428443	0.998728122
P2RY12	-0.008675058	0.96352439	0.998728122
CLIP2	0.004517969	0.963696594	0.998728122
NACA	-0.002317474	0.963717151	0.998728122
SCRN1	-0.004618455	0.963771286	0.998728122
C19orf48	-0.003611349	0.963942698	0.998728122
TRIM64B	0.003866091	0.964044145	0.998728122
TOP1	0.003503133	0.964489728	0.998728122
BANF1	0.00399325	0.964577198	0.998728122
TMBIM6	-0.002066051	0.964724069	0.998728122
ZNF254	0.003760332	0.964753028	0.998728122
PCGF6	0.002845719	0.964920653	0.998728122
EDEM2	-0.002303901	0.964997372	0.998728122
PRDX1	0.002454552	0.965036913	0.998728122
IRX6	-0.005516045	0.965050243	0.998728122
UPF2	0.001856154	0.96527181	0.998728122
USF1	0.007092245	0.965373399	0.998728122
ERI1	0.003003084	0.965507976	0.998728122
CYTH3	-0.004755155	0.965644943	0.998728122
ZKSCAN1	-0.007811063	0.965654404	0.998728122
KIAA0930	0.004819308	0.965857702	0.998728122
FH	-0.002702674	0.965891878	0.998728122
ZNF687	0.004421539	0.965896449	0.998728122
CD47	-0.002668938	0.965974332	0.998728122
SLC39A6	0.004543901	0.965993427	0.998728122
CUL4A	-0.002030254	0.966065835	0.998728122
ERCC8	0.004765013	0.966128942	0.998728122
WDR34	0.003920346	0.966134918	0.998728122
LTBP4	-0.004864903	0.966204633	0.998728122
FAM45A	0.002863245	0.966280843	0.998728122
UPF3A	0.004361606	0.966315298	0.998728122
IMPA1	0.00520513	0.966375952	0.998728122
CAND2	-0.006309216	0.966596871	0.998728122
CHN2	0.0037403	0.966626085	0.998728122
ST3GAL2	-0.00372325	0.966962577	0.998728122
MRPS11	-0.002624922	0.967113887	0.998728122
SS18	0.003842078	0.967165356	0.998728122
SDC2	0.004197667	0.967283046	0.998728122
CXXC1	-0.002679018	0.967287057	0.998728122

SEC16A	0.002374717	0.967337874	0.998728122
ZNF781	-0.003939158	0.967359368	0.998728122
USP44	0.003684288	0.967377875	0.998728122
STX18	-0.003546718	0.967478232	0.998728122
SGMS1	0.003895743	0.967945016	0.998728122
MIB1	0.003979333	0.967957871	0.998728122
CTCF	0.002082823	0.968008912	0.998728122
PUM2	-0.001754407	0.968048806	0.998728122
PSMC1	-0.00333549	0.968226618	0.998728122
PDIA6	0.003738737	0.968272908	0.998728122
RPA2	-0.002107943	0.968301057	0.998728122
HMGB2	-0.002632949	0.968306628	0.998728122
TRIP6	0.003889783	0.968313807	0.998728122
POLB	-0.001799412	0.968380915	0.998728122
WDR46	0.002457026	0.968385296	0.998728122
SPCS3	0.00374506	0.968401373	0.998728122
MLYCD	-0.003760064	0.968510578	0.998728122
FAM21A	0.00370145	0.968627434	0.998728122
NF2	0.003285003	0.968667566	0.998728122
HNRNPA3	0.004599102	0.968697259	0.998728122
POTEF	0.001432183	0.968884874	0.998728122
GAS2L3	-0.002913614	0.969189471	0.998728122
SSFA2	-0.003634983	0.969236065	0.998728122
IREB2	-0.00451579	0.969249925	0.998728122
OSBPL2	0.001840402	0.969266941	0.998728122
THUMPD2	-0.002268886	0.969307038	0.998728122
ENO1-AS1	-0.004373453	0.969352973	0.998728122
FAS	0.004782338	0.969397473	0.998728122
ILF2	-0.00203899	0.969399077	0.998728122
DERL3	-0.004754251	0.969462273	0.998728122
SPRN	-0.003340617	0.969509472	0.998728122
RAB3IP	-0.002776988	0.969580665	0.998728122
TMEM212	0.00643498	0.969661899	0.998728122
LARP1B	0.002557139	0.969824348	0.998728122
SCML4	0.003748841	0.97005712	0.998728122
KLHL35	0.004649554	0.970142486	0.998728122
CLIC4	0.004778136	0.970223531	0.998728122
LINGO2	0.008615916	0.970275528	0.998728122
JRKL	-0.003586477	0.970321939	0.998728122
KIAA1161	-0.003511438	0.970364085	0.998728122
SPRYD3	0.003125464	0.970401614	0.998728122
FOXJ3	0.002038499	0.970430545	0.998728122
SPON2	-0.009346511	0.970486916	0.998728122
ARHGEF11	-0.003332885	0.970582226	0.998733352
TMED2	-0.002746287	0.970737771	0.998800556
KLRC3	-0.011995463	0.970833324	0.998806029
ZNF131	0.001269366	0.970994779	0.998872487

HADHA	-0.003088702	0.971078402	0.998872487
JUN	-0.006626271	0.971370211	0.999003028
KDM4C	0.002506119	0.971418465	0.999003028
PRSS23	-0.007872629	0.971476067	0.999003028
SF3B3	0.002855443	0.971775095	0.999217699
IL7R	-0.00269157	0.971987775	0.99926521
UMPS	0.003447733	0.972001854	0.99926521
IDH3B	-0.001363745	0.972801604	0.999774718
ZNF419	-0.002524525	0.973077299	0.999774718
SIRT6	-0.003598823	0.97328485	0.999774718
TRIM47	-0.003377024	0.973304563	0.999774718
HIST1H3F	0.008098858	0.973598451	0.999774718
ZNF385A	0.003773281	0.973917042	0.999774718
NSFL1C	0.003318386	0.973959369	0.999774718
ABTB1	-0.002502316	0.97396559	0.999774718
VPS13B	0.004861839	0.974004375	0.999774718
NKPD1	-0.006723981	0.974126282	0.999774718
TBCE	0.002003495	0.974157591	0.999774718
RAD50	-0.003051086	0.974269903	0.999774718
CYCS	0.003389647	0.974416228	0.999774718
GSTK1	-0.001490242	0.974462544	0.999774718
SLC22A23	-0.002926093	0.974514463	0.999774718
ARNT	0.002504495	0.974669567	0.999774718
HSPA7	-0.006442035	0.975106844	0.999774718
ZNF525	-0.003939505	0.975136802	0.999774718
SNORD100	0.003544092	0.975144185	0.999774718
TRMT1	0.001545445	0.975293884	0.999774718
CDC25B	-0.002486278	0.975297441	0.999774718
CEP57	-0.002785355	0.975365458	0.999774718
BAIAP2	0.003303278	0.975485717	0.999774718
KCNAB2	0.002867241	0.975629838	0.999774718
EIF4G3	-0.002217825	0.975700176	0.999774718
ACOT7	0.00334075	0.97571594	0.999774718
NCF4	0.002564298	0.975738406	0.999774718
PPP2R5D	-0.002177374	0.975785191	0.999774718
PHIP	-0.001954574	0.976032514	0.999774718
SAP130	-0.002306186	0.976061217	0.999774718
DUSP23	0.001789738	0.976102963	0.999774718
CEBPA	-0.003023585	0.976169769	0.999774718
GALNT2	-0.002839572	0.976264188	0.999774718
ZNF638	0.002235103	0.976353068	0.999774718
MRPS25	0.003339237	0.976387236	0.999774718
POLR3D	0.002533207	0.976434627	0.999774718
ANKRD24	-0.003080365	0.97647723	0.999774718
UBA6	0.001812679	0.976727516	0.999774718
COQ2	-0.00235315	0.976838019	0.999774718
USP5	0.001937538	0.976871034	0.999774718

ZNF436	0.002880989	0.976929099	0.999774718
DPP7	-0.002427966	0.97696018	0.999774718
MRAP	0.004767842	0.97711493	0.999774718
ZSWIM7	-0.007004914	0.977390696	0.999774718
SIRPG	-0.003810733	0.977452281	0.999774718
ZIK1	0.002708704	0.977465759	0.999774718
GLT8D1	0.001426988	0.977728313	0.999774718
C4BPB	0.002920851	0.977821234	0.999774718
KRBA1	-0.001869391	0.977822194	0.999774718
SMG7	-0.002729406	0.97782833	0.999774718
PANK1	-0.00271665	0.977884482	0.999774718
ATP13A3	-0.00242872	0.977938068	0.999774718
EIF1B	0.001794079	0.977999965	0.999774718
BCAN	-0.002380423	0.978039014	0.999774718
BAI1	-0.002791342	0.978055348	0.999774718
SLC45A4	0.002955467	0.978065305	0.999774718
CEP57L1	-0.002058134	0.978349115	0.999774718
ATP1B3	-0.002164688	0.978358338	0.999774718
TRAPPC6B	-0.00360326	0.97838731	0.999774718
C16orf70	0.001832739	0.978517341	0.999774718
ME1	-0.004655461	0.978577543	0.999774718
BMPR2	-0.002209218	0.978834783	0.999774718
TPM3	-9.98E-04	0.978843087	0.999774718
SASH1	-0.004571768	0.978978092	0.999774718
WRAP53	-0.002432615	0.979088441	0.999774718
NOSIP	-0.00224539	0.979347057	0.999774718
PSMB4	0.001000361	0.979438629	0.999774718
NIN	-0.001368105	0.979438925	0.999774718
CC2D1B	-0.002997652	0.979533688	0.999774718
OSBP	0.001093793	0.979552444	0.999774718
SNX8	-0.00190652	0.980075212	0.999774718
ZNF605	0.002334766	0.980090074	0.999774718
SENP3	0.002495276	0.980132792	0.999774718
CKS2	0.003044652	0.980216735	0.999774718
EED	0.002364311	0.980219589	0.999774718
CPA5	0.005797035	0.980319772	0.999774718
SPR	0.002343167	0.980447503	0.999774718
SMAD3	0.002132824	0.980594138	0.999774718
VRK1	0.001952014	0.980648623	0.999774718
PPTC7	-0.002226866	0.98064979	0.999774718
UTP3	0.001964504	0.980700998	0.999774718
PHC1	-0.002189458	0.980750677	0.999774718
NHLRC4	0.005468207	0.980886655	0.999774718
GTF2H2	-0.003996357	0.981074891	0.999774718
PREP	-0.001357997	0.981118649	0.999774718
COL5A1	0.002426457	0.981121487	0.999774718
TRIM28	-0.00198047	0.98118369	0.999774718

CCT6P3	0.001066452	0.981187589	0.999774718
ACTR5	-0.001636972	0.981294004	0.999774718
CTSF	-0.002511867	0.981424257	0.999774718
RBM22	-7.80E-04	0.981530842	0.999774718
BRD2	-0.001181053	0.981565639	0.999774718
ASH2L	0.001076397	0.98158452	0.999774718
ZNF143	-0.001674319	0.981659114	0.999774718
SEC23B	0.001795921	0.981686699	0.999774718
C1QA	-0.002480905	0.981814828	0.999774718
HADH	0.001613427	0.981816205	0.999774718
C16orf62	0.001713094	0.982063199	0.999774718
GMCL1	0.00195904	0.982296051	0.999774718
LRRC8D	0.001520709	0.982313494	0.999774718
ZNF407	0.001429187	0.982356446	0.999774718
SUPV3L1	0.001788184	0.982364688	0.999774718
SLC22A18AS	-0.003378494	0.98254621	0.999774718
KLF4	0.003029923	0.982830831	0.999774718
NR2C2AP	0.001273739	0.982839361	0.999774718
ARL6IP5	-0.001090708	0.982885911	0.999774718
KLHDC1	0.001939183	0.982974867	0.999774718
DERA	-0.001445503	0.982990405	0.999774718
NUDT15	-0.002374989	0.983086281	0.999774718
SGTB	-0.004296445	0.983140135	0.999774718
EGLN1	-0.002487039	0.983163948	0.999774718
PTPN6	0.001704058	0.983578285	0.999774718
PDE6B	-0.00199005	0.983611565	0.999774718
MPV17L2	-0.001745122	0.983841359	0.999774718
KIAA1598	-0.002643623	0.983874409	0.999774718
BRAF	-0.001883163	0.983917347	0.999774718
ALAS1	0.001985671	0.983934499	0.999774718
DMWD	-0.002238708	0.98413735	0.999774718
TMEM86A	-0.001812317	0.984271537	0.999774718
ARMC1	-0.001477468	0.984275904	0.999774718
PHF3	-8.10E-04	0.984418967	0.999774718
ZNF273	-0.001983682	0.984565889	0.999774718
SLC7A8	0.002174962	0.984597327	0.999774718
LAMP3	0.003501453	0.984599945	0.999774718
EEF1E1	0.001147937	0.984903369	0.999774718
ARG2	0.003175675	0.984906314	0.999774718
FXN	0.001652834	0.984924377	0.999774718
SGSH	0.00177181	0.984951305	0.999774718
FBXO38	-0.001154675	0.985192335	0.999774718
TADA3	-0.001322514	0.985347709	0.999774718
MITF	-0.002126129	0.98549229	0.999774718
UBFD1	0.00151742	0.985834668	0.999774718
RPS29	-7.77E-04	0.985834907	0.999774718
NUP188	0.001478304	0.985835753	0.999774718

N4BP2L2	-0.00228718	0.985963445	0.999774718
UQCR10	-0.001781762	0.986152411	0.999774718
FKBP3	-0.001334935	0.986265514	0.999774718
RNF24	-0.001574845	0.986491189	0.999774718
SNRPG	8.49E-04	0.986652288	0.999774718
SPAG9	7.52E-04	0.986667027	0.999774718
C9orf38	-0.003230805	0.986817966	0.999774718
NLRP2	0.004529444	0.986874562	0.999774718
GK5	0.002132953	0.986881752	0.999774718
B3GAT1	-0.004016265	0.987063772	0.999774718
GAR1	-7.71E-04	0.987179757	0.999774718
SFMBT2	-0.002467279	0.987338589	0.999774718
NOP56	-0.00115373	0.987423382	0.999774718
XYLT2	8.96E-04	0.98753157	0.999774718
OR9A4	-0.001255973	0.987587145	0.999774718
SPTBN4	0.00207098	0.987639935	0.999774718
TMEM2	0.001370928	0.987662305	0.999774718
USP21	-0.001021393	0.987702442	0.999774718
ANKLE2	-9.56E-04	0.987706826	0.999774718
ZNF133	0.001207534	0.987733866	0.999774718
TSPO	-0.001034151	0.987845201	0.999774718
KIF1B	-0.001126803	0.987987503	0.999774718
DLEC1	0.001650837	0.988026313	0.999774718
SULT1A2	-0.001634948	0.988191022	0.999774718
MFSD6	0.001304481	0.988451679	0.999774718
APOBEC3H	0.002877463	0.988459933	0.999774718
CECR6	0.002613938	0.988513903	0.999774718
ANKRD27	0.001276836	0.988728638	0.999774718
SOS1	0.00146226	0.988905138	0.999774718
HCG11	0.001999428	0.989008883	0.999774718
MRPS23	0.001195029	0.989238604	0.999774718
ITGA10	-0.001393615	0.989299013	0.999774718
SLC36A1	-0.001595871	0.989324491	0.999774718
EIF4H	-7.70E-04	0.989434686	0.999774718
CYB5B	-6.56E-04	0.989460931	0.999774718
SLBP	8.57E-04	0.989962011	0.999774718
B9D1	0.001170861	0.990221591	0.999774718
FOLR3	-0.005003459	0.990447801	0.999774718
SAMD9L	-0.001799282	0.990673115	0.999774718
FCER1A	-0.002541246	0.9907011	0.999774718
ORC4	-0.002747773	0.990704887	0.999774718
SUMO1P3	-7.17E-04	0.990788874	0.999774718
ATHL1	0.002481451	0.99115429	0.999774718
HIST1H2BE	0.002502065	0.991209349	0.999774718
TET1	0.001079036	0.991230896	0.999774718
IP6K1	7.17E-04	0.991231995	0.999774718
HELZ	8.73E-04	0.991243304	0.999774718

IGF2BP1	-0.001448069	0.991292434	0.999774718
USP1	-6.06E-04	0.991303158	0.999774718
STAG1	-6.73E-04	0.991320451	0.999774718
TPP2	-9.08E-04	0.991376455	0.999774718
BCL3	-9.63E-04	0.991444138	0.999774718
POU2F1	7.23E-04	0.991658	0.999774718
CBX7	-8.48E-04	0.991768803	0.999774718
TNFSF13B	9.38E-04	0.992037115	0.999774718
CCT3	6.90E-04	0.992039002	0.999774718
RIPK4	-8.95E-04	0.992071041	0.999774718
NBR1	-7.78E-04	0.992173047	0.999774718
CATSPER2P1	7.79E-04	0.992265995	0.999774718
C12orf49	6.85E-04	0.992352242	0.999774718
RUSC1	-6.30E-04	0.992374827	0.999774718
TCF7	-0.001206836	0.992401281	0.999774718
PUS10	8.90E-04	0.992440053	0.999774718
ZMYND8	6.78E-04	0.992506502	0.999774718
CRYL1	-8.97E-04	0.992650993	0.999774718
NEAT1	0.001129936	0.9927061	0.999774718
RPS28	-5.29E-04	0.992756897	0.999774718
ATG12	4.95E-04	0.992774888	0.999774718
FLT4	-9.97E-04	0.992779012	0.999774718
NRIP3	-7.86E-04	0.99287549	0.999774718
LPL	-0.001454042	0.993022284	0.999774718
MANBAL	5.58E-04	0.993088047	0.999774718
KLF3	9.12E-04	0.99329391	0.999774718
TMTC4	7.32E-04	0.993351256	0.999774718
C19orf59	0.001252889	0.993404451	0.999774718
AXIN2	-0.00121958	0.993448728	0.999774718
ZNF549	-7.26E-04	0.993456119	0.999774718
TPPP3	-0.001481089	0.993464301	0.999774718
ATP8B2	5.81E-04	0.993724727	0.999774718
CYB561	-9.36E-04	0.993739176	0.999774718
GALNT10	-7.09E-04	0.993795081	0.999774718
PATE3	7.95E-04	0.993902706	0.999774718
PSMA5	4.16E-04	0.993927251	0.999774718
RPS3	-2.58E-04	0.994036751	0.999774718
PSMC3	-4.50E-04	0.994132901	0.999774718
HIST1H2AG	-0.001677541	0.994155627	0.999774718
SKIV2L2	4.32E-04	0.994301438	0.999774718
MEGF10	7.81E-04	0.994389174	0.999774718
BAG2	-7.33E-04	0.994528681	0.999774718
ZBTB46	7.13E-04	0.994536408	0.999774718
LDHA	4.49E-04	0.994550564	0.999774718
AMD1	-4.17E-04	0.994550734	0.999774718
CENPL	5.70E-04	0.994721052	0.999774718
C6orf1	-5.36E-04	0.994841997	0.999774718

PLEKHA5	8.97E-04	0.994842103	0.999774718
DMXL1	5.51E-04	0.994942173	0.999774718
POLE2	-6.32E-04	0.994952108	0.999774718
IGFBP2	7.29E-04	0.995082008	0.999774718
FAM83F	5.99E-04	0.995124128	0.999774718
HDAC5	5.10E-04	0.995456037	0.999774718
VDAC2	3.50E-04	0.995610891	0.999774718
SAMD15	4.74E-04	0.995621071	0.999774718
TMEM66	2.36E-04	0.995625518	0.999774718
PILRB	6.29E-04	0.995640663	0.999774718
YY1	-2.94E-04	0.995642775	0.999774718
KDELC2	5.09E-04	0.995756407	0.999774718
KCTD7	-8.00E-04	0.995792642	0.999774718
ZSWIM1	-3.44E-04	0.995813474	0.999774718
RXRG	4.99E-04	0.995818891	0.999774718
STARD7	-2.25E-04	0.995863125	0.999774718
IMPA2	6.58E-04	0.995864804	0.999774718
NDUFS2	-3.65E-04	0.995894758	0.999774718
ERO1L	-4.30E-04	0.995908584	0.999774718
FCRL5	0.001038311	0.995983077	0.999774718
DECR1	2.02E-04	0.996123342	0.999774718
TPI1	3.60E-04	0.996189302	0.999774718
ADSL	-2.59E-04	0.996205788	0.999774718
TERF1	3.68E-04	0.996256145	0.999774718
RPP40	-3.19E-04	0.996271577	0.999774718
TM9SF4	-2.80E-04	0.996356639	0.999774718
ADH5	-3.73E-04	0.996360725	0.999774718
ZCWPW1	4.16E-04	0.996387275	0.999774718
OAZ3	-4.86E-04	0.996418929	0.999774718
ENPP4	6.90E-04	0.996557776	0.999774718
WNT7A	-4.65E-04	0.996560506	0.999774718
UHRF2	-3.18E-04	0.99660978	0.999774718
SNORA29	-5.05E-04	0.996652927	0.999774718
VCAN	-3.14E-04	0.996709599	0.999774718
TBL1XR1	2.95E-04	0.996832356	0.999774718
RALGPS1	3.93E-04	0.996834139	0.999774718
RAB31	2.83E-04	0.996915057	0.999774718
TSPYL6	-3.30E-04	0.996975862	0.999774718
PACRGL	-4.32E-04	0.997005548	0.999774718
C11orf84	-3.02E-04	0.997296719	0.999774718
HMGA1	-3.44E-04	0.997352746	0.999774718
IARS2	-1.57E-04	0.997385772	0.999774718
PLA2G16	-5.95E-04	0.997473698	0.999774718
CEP78	4.25E-04	0.99748366	0.999774718
C17orf96	-3.19E-04	0.997501114	0.999774718
STMN3	-2.86E-04	0.997674303	0.999774718
WSB2	-2.25E-04	0.997765685	0.999774718

SDK2	3.67E-04	0.997897978	0.999774718
PRR5	-2.96E-04	0.997973564	0.999774718
ING2	-2.12E-04	0.998116447	0.999774718
GABPA	1.99E-04	0.998258985	0.999774718
NOM1	-3.14E-04	0.998330823	0.999774718
SNRNP35	-1.32E-04	0.99838655	0.999774718
SNTB2	-1.86E-04	0.99841276	0.999774718
RALGPS2	-3.99E-04	0.998443618	0.999774718
MESP1	-2.24E-04	0.998578352	0.999774718
TNFRSF10A	1.70E-04	0.998651412	0.999774718
MRPL45	-8.74E-05	0.998661382	0.999774718
LRFN1	-1.70E-04	0.998718979	0.999774718
ACSS3	1.59E-04	0.998732507	0.999774718
HEATR2	-8.78E-05	0.998808034	0.999774718
GPD1L	7.34E-05	0.998930205	0.999774718
TINF2	1.25E-04	0.998980592	0.999774718
TAF12	-7.89E-05	0.999218455	0.999774718
RLN1	-1.34E-04	0.999241299	0.999774718
PPP3R1	8.33E-05	0.999263171	0.999774718
OR1J1	1.30E-04	0.999266086	0.999774718
LGMN	8.70E-05	0.999357699	0.999774718
ZWILCH	-4.67E-05	0.999410784	0.999774718
PUSL1	6.31E-05	0.99941343	0.999774718
ANKRD16	4.74E-05	0.999531322	0.999802295
C17orf47	-3.17E-06	0.999976455	0.999995887
CAPN12	4.13E-06	0.999983939	0.999995887
JARID2	-3.09E-07	0.999995887	0.999995887

Supplemental Table 2.3: Association between NO2 and gene expression in NoMa

name	logFC	P.Value	adj.P.Val
RASSF4	-0.014357149	1.48E-04	0.766268089
CUL7	-0.016447323	1.55E-04	0.766268089
OXCT2	-0.020165598	2.09E-04	0.766268089
GDAP2	-0.012818924	2.77E-04	0.766268089
ZDHHC7	-0.009900539	3.70E-04	0.818777649
ARID5B	0.013121727	5.04E-04	0.904593084
GUF1	-0.012025812	6.62E-04	0.904593084
TBC1D16	-0.013302707	7.23E-04	0.904593084
ZNF77	-0.014796897	7.36E-04	0.904593084
ALKBH3	-0.008586857	8.55E-04	0.904645487
LRRC37BP1	-0.013316007	9.67E-04	0.904645487
SETD2	0.00644393	9.81E-04	0.904645487
MIR671	-0.011661693	0.001105074	0.936293664
IFFO1	-0.008229439	0.001245048	0.936293664
TEKT4	-0.013345833	0.00151197	0.936293664
ARMC7	-0.011332505	0.001533672	0.936293664
SLC33A1	-0.00968256	0.00156456	0.936293664
PCGF1	-0.012696039	0.001865518	0.936293664
EIF3E	-0.005507334	0.00188385	0.936293664
SUCLA2	-0.010876586	0.002106482	0.936293664
TIMM44	0.009304883	0.002181287	0.936293664
HIVEP2	0.013386829	0.002262193	0.936293664
SNRPD1	-0.01262506	0.002320598	0.936293664
PQLC3	-0.009118027	0.002401621	0.936293664
AKAP8L	0.01314278	0.002687353	0.936293664
FBN2	-0.024844469	0.002784891	0.936293664
KCNMB1	-0.017779316	0.002907312	0.936293664
COPS7A	-0.005560853	0.002944768	0.936293664
MCEE	-0.008212845	0.003155912	0.936293664
RHOBTB1	0.026931897	0.003173185	0.936293664
ZHX2	0.026068521	0.003185744	0.936293664
ITGAV	-0.01178381	0.00335799	0.936293664
BCAT1	-0.028249913	0.003418884	0.936293664
DNAJB11	-0.00760706	0.00346303	0.936293664
COG4	0.008477081	0.003482272	0.936293664
SP1	0.009478392	0.003558596	0.936293664
ZCCHC7	0.008103768	0.003680257	0.936293664
NDN	-0.022382519	0.003735874	0.936293664
SNX11	-0.008695095	0.003776875	0.936293664
ZNF689	-0.008357955	0.003979915	0.936293664
SAV1	0.015747705	0.004027596	0.936293664
C5orf54	-0.01340256	0.004125566	0.936293664
RTKN2	0.023593829	0.004349738	0.936293664
MANEA	-0.017272603	0.004351243	0.936293664

IGF2BP3	0.024636825	0.004439217	0.936293664
DVL2	0.007301565	0.004591211	0.936293664
BEST1	-0.015220114	0.004611004	0.936293664
CLEC4F	-0.046860989	0.004615651	0.936293664
PPP1R7	0.006934703	0.004790502	0.936293664
MTCH1	-0.005441059	0.004796554	0.936293664
IL4R	0.012617808	0.005048553	0.936293664
SFMBT1	0.009526698	0.005064205	0.936293664
DTNA	-0.012025464	0.005089892	0.936293664
NANP	-0.018869073	0.005150553	0.936293664
TMEM39A	-0.012692418	0.005256125	0.936293664
SRR	-0.015094041	0.005309484	0.936293664
CRLS1	-0.006598543	0.005310077	0.936293664
HINFP	-0.009097759	0.005338098	0.936293664
STIP1	0.011452747	0.005461574	0.936293664
DHRS7B	-0.008010824	0.005470733	0.936293664
MYO1C	0.011024776	0.005761094	0.936293664
THYN1	0.007570117	0.005809565	0.936293664
IL18R1	0.016619517	0.005852371	0.936293664
IL1B	-0.029100145	0.005905585	0.936293664
UBXN2B	-0.011092986	0.005936841	0.936293664
WDR12	-0.007450359	0.006199725	0.936293664
GAS8	-0.011146839	0.006430739	0.936293664
NDEL1	-0.00708369	0.006515487	0.936293664
QARS	-0.007302648	0.006519056	0.936293664
RERE	0.010817718	0.006556265	0.936293664
SEC24D	-0.010279991	0.006630148	0.936293664
RBM7	-0.018211952	0.00666494	0.936293664
FLT3	-0.017884709	0.006990095	0.936293664
ATP5E	-0.005191738	0.007230034	0.936293664
GPR141	-0.015035406	0.00736652	0.936293664
ANKRD28	0.011172467	0.007541365	0.936293664
CDKN2A	-0.010902926	0.00769039	0.936293664
GGTLC1	-0.011250621	0.007698149	0.936293664
SYT2	-0.012557821	0.007922236	0.936293664
CTSH	-0.010464542	0.007934628	0.936293664
DGAT1	-0.008841958	0.00796591	0.936293664
AUH	-0.010559135	0.008053168	0.936293664
ZC3HC1	-0.005716347	0.008076717	0.936293664
MTA2	0.009000424	0.008079621	0.936293664
CD40	0.014659445	0.008474606	0.936293664
EXOG	0.010341684	0.008753078	0.936293664
AGBL5	0.012303969	0.0087701	0.936293664
ZNF512B	0.012348669	0.008773366	0.936293664
C11orf95	-0.008211836	0.008831115	0.936293664
NFXL1	-0.014254268	0.008922994	0.936293664
HLA-DOB	0.025634466	0.00905506	0.936293664

CSGALNACT2	-0.008627093	0.0090636	0.936293664
ADCY6	0.009310753	0.009101141	0.936293664
RGS19	-0.009107013	0.009174841	0.936293664
SEC61A1	-0.006827486	0.009187365	0.936293664
LSM14A	0.00568047	0.009196415	0.936293664
ARMC5	-0.011487632	0.00933846	0.936293664
BTBD10	-0.008832707	0.009487415	0.936293664
SDSL	-0.016559286	0.009752279	0.936293664
TDRKH	0.01469409	0.009805725	0.936293664
ACCS	-0.024392215	0.010015265	0.936293664
OXR1	-0.006481772	0.010205104	0.936293664
SNRPA	0.006614839	0.010347426	0.936293664
PDE4DIP	-0.055792094	0.01035476	0.936293664
CENPA	-0.0123885	0.010401297	0.936293664
FAR2	-0.012561826	0.010479514	0.936293664
GBA2	-0.008330226	0.010492864	0.936293664
INSIG2	-0.00683773	0.01057139	0.936293664
EGR2	-0.017877165	0.010768963	0.936293664
EHHADH	-0.012069707	0.010934895	0.936293664
MGC2752	-0.011406057	0.011077967	0.936293664
ARPC4	0.007887368	0.011218975	0.936293664
MAF1	0.00873495	0.011455798	0.936293664
MUS81	-0.006996674	0.011590998	0.936293664
PDHB	-0.005756852	0.011646184	0.936293664
C20orf194	-0.009697257	0.011670949	0.936293664
DIRC2	-0.012487937	0.011989913	0.936293664
TSHZ1	0.008318181	0.012073074	0.936293664
DAZAP1	0.006802389	0.012176654	0.936293664
LIPT1	-0.008229232	0.012255922	0.936293664
ZNF484	-0.009572912	0.012277019	0.936293664
NUFIP1	-0.010816543	0.012334695	0.936293664
SKA2	0.006961517	0.012431202	0.936293664
LGALS8	-0.007379375	0.012693076	0.936293664
MTRR	-0.012310451	0.012813067	0.936293664
ACOT11	-0.010486536	0.012831194	0.936293664
RNMT	0.008274861	0.012866558	0.936293664
NRBF2	-0.009925817	0.012962946	0.936293664
TNNC2	0.028321079	0.013136233	0.936293664
C19orf24	0.010702124	0.013556293	0.936293664
OMG	0.009927359	0.01355707	0.936293664
SMPDL3A	-0.018059076	0.013601176	0.936293664
SLC35D3	0.026484038	0.013640865	0.936293664
ARID1B	0.009142272	0.013748119	0.936293664
CCDC34	0.00965119	0.01379248	0.936293664
TICAM2	-0.012278849	0.013829378	0.936293664
MPRIP	0.007663192	0.013939602	0.936293664
ELOVL7	0.023658901	0.014054478	0.936293664

HSP90AB3P	0.00910614	0.014232212	0.936293664
AIDA	0.005002238	0.014259368	0.936293664
C9orf47	-0.021716678	0.014376682	0.936293664
NGDN	0.005576095	0.014497729	0.936293664
RPE	-0.009211497	0.014655454	0.936293664
MTMR11	-0.010078341	0.014699921	0.936293664
PLA2G15	-0.009772312	0.014732698	0.936293664
PEX14	-0.00939195	0.014757808	0.936293664
TMEM219	0.006949077	0.015024358	0.936293664
TERF2IP	0.007831383	0.015118287	0.936293664
RABGEF1	0.00905908	0.015146107	0.936293664
CRNKL1	-0.011839408	0.015173978	0.936293664
RUFY1	0.020915881	0.015261841	0.936293664
CTNNAL1	0.016722289	0.01528715	0.936293664
SMG5	-0.00683841	0.015453144	0.936293664
MEIS3	0.018031428	0.015572135	0.936293664
SRP72	-0.006145336	0.015640484	0.936293664
ACOX2	-0.014729391	0.015688066	0.936293664
FICD	-0.013544317	0.015734185	0.936293664
TMEM41A	-0.008752356	0.015832135	0.936293664
SNORD104	0.012188465	0.015909484	0.936293664
BTN2A1	-0.006778806	0.015944628	0.936293664
MEI1	-0.008554752	0.016153488	0.936293664
TRIP4	-0.007277417	0.016188356	0.936293664
FNDC3B	-0.008010835	0.01619069	0.936293664
QDPR	-0.009350602	0.016229995	0.936293664
ZBTB4	0.006453143	0.016265554	0.936293664
ARF4	-0.006165915	0.016300801	0.936293664
ARHGAP23	-0.008249101	0.016460561	0.936293664
FTSJ2	-0.006420006	0.016461776	0.936293664
CACNA2D3	-0.014342331	0.016675593	0.936293664
MPO	-0.013865546	0.016747909	0.936293664
TRIM3	-0.008563465	0.017029578	0.936293664
DUSP10	-0.010923433	0.017070476	0.936293664
THOC5	-0.006384666	0.017176727	0.936293664
SIRT4	-0.00968204	0.01730599	0.936293664
PAAF1	-0.006812767	0.017314435	0.936293664
C14orf79	-0.007707175	0.017332672	0.936293664
ENTPD4	0.014596541	0.017503209	0.936293664
NCK2	0.01026946	0.017526349	0.936293664
TMPRSS12	0.011605455	0.017557954	0.936293664
PFKL	-0.01080189	0.017815923	0.936293664
FAM174A	-0.011621659	0.01784206	0.936293664
AP3D1	-0.006948902	0.017952957	0.936293664
SLC50A1	0.008098627	0.017971989	0.936293664
DHX15	-0.004061324	0.018150708	0.936293664
STK38L	-0.011105279	0.018219205	0.936293664

AP2B1	0.008056603	0.01824119	0.936293664
ALDH2	-0.012191796	0.01826824	0.936293664
LTB4R2	-0.009869181	0.018306888	0.936293664
CACNA2D4	-0.016183999	0.01863484	0.936293664
NAGK	-0.008900121	0.018804979	0.936293664
PRKCB	0.005352818	0.018819515	0.936293664
VAC14	-0.01114133	0.018877688	0.936293664
RAB5A	-0.007810065	0.018997665	0.936293664
GXYLT1	-0.007034478	0.018999055	0.936293664
ZDHHC24	-0.010923206	0.019028693	0.936293664
DBP	0.008312743	0.019056871	0.936293664
LIN7A	-0.010967809	0.019072227	0.936293664
EIF3L	-0.005056284	0.019139998	0.936293664
MGAT4B	0.008545006	0.019263145	0.936293664
ZNF614	-0.01307792	0.0192765	0.936293664
WDR70	0.006929098	0.019299962	0.936293664
SERGEF	0.006520756	0.019313329	0.936293664
FMO6P	0.009560637	0.01937124	0.936293664
RPUSD1	-0.011056095	0.019384649	0.936293664
CDC42	0.007400753	0.019464449	0.936293664
PARVG	-0.007752572	0.01957403	0.936293664
MDN1	0.008586422	0.019645339	0.936293664
PPM1D	-0.010054905	0.019659483	0.936293664
TJP1	0.011490339	0.019674654	0.936293664
RIMS4	0.009206978	0.019697275	0.936293664
RAB18	-0.009935252	0.019823715	0.936293664
TNFRSF10B	-0.006717211	0.019841435	0.936293664
PSMD13	0.009112096	0.019950266	0.936293664
TMEM150C	0.010694402	0.019959629	0.936293664
SERPINB2	-0.019642635	0.019967309	0.936293664
CCR5	-0.012297173	0.020024268	0.936293664
OTUD6B	-0.006848479	0.020218384	0.936293664
SNORD32A	0.010554683	0.02023343	0.936293664
CDK8	-0.00857478	0.02038232	0.936293664
MRPS18A	-0.008393381	0.020609031	0.936293664
SH3PXD2A	0.01612843	0.020701788	0.936293664
ACOX1	-0.010775373	0.020713457	0.936293664
DIAPH1	0.006278658	0.020769526	0.936293664
NTPCR	0.01367746	0.020855154	0.936293664
FMO4	-0.010210684	0.020885322	0.936293664
TTF1	-0.00488545	0.020989301	0.936293664
SYT3	-0.009394245	0.021236984	0.936293664
PLCL2	0.004793079	0.021500794	0.936293664
PDIA5	-0.01132054	0.021824207	0.936293664
SEMA6B	0.010469477	0.021845804	0.936293664
MCAT	-0.008749659	0.021873649	0.936293664
NAA20	-0.004482497	0.022103956	0.936293664

CXCL9	-0.012422164	0.022109421	0.936293664
LEO1	-0.007459905	0.022157252	0.936293664
CTTN	0.025362691	0.022257766	0.936293664
ZMPSTE24	-0.0054598	0.022516998	0.936293664
TCL6	0.008841507	0.022568368	0.936293664
UNC119B	-0.007368033	0.022630755	0.936293664
RBM27	0.005553691	0.022647018	0.936293664
MSL2	0.007238507	0.022757801	0.936293664
TSPAN33	0.022141048	0.022837171	0.936293664
PPFIBP2	-0.014053394	0.022846232	0.936293664
BUD31	0.005013713	0.022922374	0.936293664
CDC14A	0.008359288	0.022974917	0.936293664
JMJD4	-0.010514417	0.023096146	0.936293664
SOCS3	0.014439124	0.02319096	0.936293664
ZNF564	-0.009812109	0.023270903	0.936293664
SLC16A6	-0.012220586	0.023293376	0.936293664
SRGAP1	-0.009090916	0.023323841	0.936293664
CD248	-0.020384924	0.023472587	0.936293664
FAM185A	0.009611362	0.023481004	0.936293664
PIGT	-0.006691337	0.023529705	0.936293664
SMARCD2	0.010934535	0.02369948	0.936293664
EIF4A3	-0.004983418	0.023751373	0.936293664
SLC35C1	-0.00745466	0.023775546	0.936293664
DENND2C	0.013430977	0.0238761	0.936293664
TMC8	0.011291593	0.02393591	0.936293664
ZNF230	-0.008201712	0.023949659	0.936293664
TSPYL5	0.015227955	0.024041148	0.936293664
ZSCAN2	-0.006873995	0.024073322	0.936293664
PFN2	-0.014950846	0.024154657	0.936293664
CHD2	0.007469736	0.024261753	0.936293664
CCRL2	-0.013381092	0.024553014	0.936293664
PSMB7	-0.007711384	0.024687616	0.936293664
MTERF	-0.008529923	0.0247792	0.936293664
CDRT4	-0.00830274	0.024924006	0.936293664
NCF2	-0.011251442	0.024972224	0.936293664
FCN1	-0.008147773	0.025123956	0.936293664
ATG5	0.005819982	0.025584624	0.936293664
FMO5	-0.010752651	0.025634517	0.936293664
RIN3	0.008861224	0.025730176	0.936293664
C19orf47	0.008614493	0.02573825	0.936293664
DCAF16	0.009039084	0.025831818	0.936293664
FAM72B	-0.011071687	0.025956104	0.936293664
SNRPE	0.00888017	0.02596068	0.936293664
GSTZ1	-0.009778259	0.025996143	0.936293664
ADCY3	0.00812408	0.026056354	0.936293664
EWSR1	0.004968539	0.026214377	0.936293664
GPR162	-0.008984096	0.026365432	0.936293664

KLC1	0.007641961	0.02659446	0.936293664
EPC1	0.010782044	0.026629551	0.936293664
SNHG9	0.006914626	0.026635394	0.936293664
BIN2	0.007138482	0.026637507	0.936293664
MAFB	-0.012723883	0.026747837	0.936293664
NRCAM	-0.021798028	0.026757349	0.936293664
DHX29	-0.004243755	0.026803525	0.936293664
ANKS1A	-0.005292071	0.026828441	0.936293664
BRD4	0.009887679	0.027179629	0.936293664
CEP63	-0.00679246	0.027454542	0.936293664
ANKRD35	-0.016011299	0.027474003	0.936293664
MMACHC	-0.009385362	0.027523244	0.936293664
SGK1	-0.011941046	0.027689685	0.936293664
MEX3D	0.008920841	0.027773747	0.936293664
PLXDC1	-0.010280722	0.027776928	0.936293664
RPTOR	-0.011840994	0.027805923	0.936293664
ZNF330	-0.00869686	0.027833721	0.936293664
ATP5B	-0.005068863	0.027902005	0.936293664
ZNF823	-0.009489628	0.027905529	0.936293664
RSG1	-0.009007296	0.027963975	0.936293664
SP140	0.009499138	0.02810067	0.936293664
SNORD71	0.011597272	0.028225584	0.936293664
HPS6	-0.006949271	0.028244478	0.936293664
CTSD	0.010076627	0.028370369	0.936293664
KIAA1147	0.005014053	0.028572749	0.936293664
B4GALT1	0.007751487	0.028581991	0.936293664
HSP90AB1	0.007204395	0.028664157	0.936293664
SNORD89	0.016865205	0.028674252	0.936293664
NSMAF	-0.004916061	0.028676813	0.936293664
TIMM23	-0.004197248	0.028685744	0.936293664
TRERF1	0.007762357	0.029017191	0.936293664
GIMAP4	-0.007508839	0.02906522	0.936293664
PFAS	-0.005473718	0.029074111	0.936293664
PIK3C2B	0.013049446	0.029096625	0.936293664
TRNAU1AP	-0.007001179	0.029109078	0.936293664
CNOT6L	0.006905746	0.029204287	0.936293664
ADK	0.012021124	0.029222676	0.936293664
ENDOD1	0.015588698	0.029340569	0.936293664
PGAM1	-0.005322295	0.029415869	0.936293664
RNF126	0.005554665	0.02954724	0.936293664
ZHX1	-0.00502822	0.029705921	0.936293664
NCS1	0.007780851	0.029784334	0.936293664
NAGS	-0.008043802	0.029829265	0.936293664
NAPEPLD	-0.008648677	0.029876696	0.936293664
C15orf40	0.010959099	0.029891362	0.936293664
ASAP1	0.007618125	0.029932425	0.936293664
GLT1D1	-0.012126424	0.029986081	0.936293664

CIR1	0.00503202	0.03005211	0.936293664
STUB1	0.007505919	0.030053975	0.936293664
NUTF2	0.006455925	0.030057478	0.936293664
FAM178A	0.007089825	0.030143205	0.936293664
ABHD13	0.008338579	0.030223228	0.936293664
ABCD3	-0.006986015	0.030325009	0.936293664
DPAGT1	-0.004874452	0.030370287	0.936293664
TFAM	-0.006481471	0.030421036	0.936293664
SNRNP40	0.005062005	0.030578971	0.936293664
CD55	-0.012427517	0.03058048	0.936293664
ZNF239	-0.013047356	0.030607629	0.936293664
ATAD3A	-0.00839578	0.030723583	0.936293664
DCXR	0.01014043	0.031003231	0.936293664
NETO2	-0.011022091	0.031090481	0.936293664
KCTD15	-0.008615258	0.031188999	0.936293664
NME3	0.005312379	0.031196143	0.936293664
MARK2	0.009702746	0.031265381	0.936293664
DDX43	0.023109355	0.03133085	0.936293664
NCLN	-0.006338523	0.031402313	0.936293664
SPATA6	-0.008798553	0.031473525	0.936293664
FADD	-0.009526298	0.031597787	0.936293664
MFN2	0.007599714	0.031605168	0.936293664
CEND1	0.009252757	0.031710461	0.936293664
ALKBH1	-0.008111972	0.031796288	0.936293664
FGL2	-0.010871576	0.031809266	0.936293664
LYPLA2	0.008257474	0.031835002	0.936293664
DCAF10	-0.006383389	0.031939807	0.936293664
HOXA10	-0.015462048	0.031973746	0.936293664
PIGM	-0.00719204	0.03209051	0.936293664
CXXC4	-0.00854352	0.032167922	0.936293664
ZNF707	-0.008818168	0.032286587	0.936293664
DNAJC17	-0.007383959	0.032389226	0.936293664
ATP6V1B2	-0.005240599	0.032402136	0.936293664
RNF10	0.009926893	0.032448914	0.936293664
ZNF890P	0.009313594	0.03261021	0.936293664
TRPM2	-0.009016789	0.032626798	0.936293664
DDX1	-0.004020181	0.032641356	0.936293664
CD34	-0.014867975	0.032674651	0.936293664
FBXL20	-0.010137506	0.032691199	0.936293664
SLC38A1	0.01214944	0.032722663	0.936293664
XPR1	-0.005620599	0.032774674	0.936293664
B4GALNT4	-0.009156094	0.032862645	0.936293664
TDG	-0.004511256	0.032864351	0.936293664
ITGA4	-0.010635481	0.032874972	0.936293664
ERLIN2	-0.005586548	0.032894074	0.936293664
CYTH4	-0.006071641	0.032918973	0.936293664
PLA2G4A	-0.011994361	0.033016764	0.936293664

DTNBP1	0.008444609	0.033091634	0.936293664
SNORD31	0.009850181	0.033154853	0.936293664
CD8A	-0.018398531	0.033175894	0.936293664
RAPGEF6	0.008552785	0.033261517	0.936293664
KLHL21	-0.008126003	0.033675543	0.936293664
CHMP2A	-0.006647491	0.03375301	0.936293664
ZNF805	-0.007624797	0.033774398	0.936293664
ZNF788	-0.008223551	0.033913924	0.936293664
FUCA1	-0.008465569	0.033950345	0.936293664
OR7G2	0.008698407	0.03410198	0.936293664
MFAP3L	0.018539117	0.034113601	0.936293664
MPZL2	-0.012355535	0.034188101	0.936293664
UBE2Q1	-0.006846627	0.034322117	0.936293664
RBCK1	-0.00662942	0.034328912	0.936293664
CHST15	-0.007919219	0.034377016	0.936293664
MGRN1	-0.010379603	0.034381258	0.936293664
CPSF1	0.010168052	0.034516691	0.936293664
TMEM134	0.005910501	0.0345607	0.936293664
ZNF365	0.019497866	0.03457045	0.936293664
UCA1	0.007789984	0.03459616	0.936293664
FBXO32	0.007369852	0.034710927	0.936293664
RAB35	-0.006700133	0.034712352	0.936293664
RRP1	-0.009963493	0.034792612	0.936293664
HECA	0.006465752	0.034827063	0.936293664
PTPRF	0.020579109	0.03490518	0.936293664
FASTKD3	-0.008008413	0.034965117	0.936293664
ATG7	-0.008021962	0.035064099	0.936293664
DNAJB1	0.006473657	0.035066056	0.936293664
NDRG3	0.005423259	0.035116852	0.936293664
SNORA64	0.010160497	0.035192331	0.936293664
ARHGEF7	0.004553604	0.035823921	0.936293664
RANBP3	-0.005398185	0.035890022	0.936293664
MYO9A	0.00836044	0.035917063	0.936293664
HIST3H2A	0.011693629	0.035934926	0.936293664
RETSAT	-0.007319953	0.035992144	0.936293664
KIAA0368	-0.005329412	0.036295166	0.936293664
PEBP1	0.006836791	0.036421519	0.936293664
ERICH1	-0.008261079	0.036465948	0.936293664
NUP133	-0.006581392	0.036466992	0.936293664
MRPS27	-0.005228306	0.0368193	0.936293664
HOXD3	0.008420411	0.036877779	0.936293664
SNORA5C	0.007507133	0.036925795	0.936293664
ST3GAL6	-0.011417496	0.037052761	0.936293664
CLC	0.016697448	0.037541051	0.936293664
MTMR9	-0.00499806	0.037635779	0.936293664
DDX52	-0.00549311	0.037715245	0.936293664
SP3	-0.008733362	0.037877406	0.936293664

RASSF2	-0.00666864	0.037903812	0.936293664
ATPAF2	-0.007759855	0.038062227	0.936293664
ALG11	-0.006870307	0.038069656	0.936293664
WBP4	-0.006257626	0.038115594	0.936293664
RBM47	-0.011818295	0.038159397	0.936293664
FAM179B	-0.006784842	0.038220141	0.936293664
SLC22A31	-0.008662667	0.038380058	0.936293664
LIMS3L	0.016130096	0.038389854	0.936293664
ZNF879	0.009605519	0.038435949	0.936293664
TMEM185B	-0.007251837	0.038524528	0.936293664
LRBA	0.006572535	0.0387612	0.936293664
CD200	0.01442852	0.038842501	0.936293664
PDLIM3	-0.008155269	0.038860833	0.936293664
TP53BP2	-0.005645188	0.038936925	0.936293664
SELENBP1	0.007457533	0.038942825	0.936293664
NOP2	-0.005953274	0.038949176	0.936293664
KLHL12	-0.004982154	0.038987364	0.936293664
GMPR	0.018038166	0.039030759	0.936293664
PKNOX1	-0.007445664	0.039129566	0.936293664
NR1H2	0.006921548	0.039172837	0.936293664
PDXK	-0.00799046	0.039183781	0.936293664
SCARNA12	0.007578222	0.039349035	0.936293664
IWS1	-0.005115525	0.039551373	0.936293664
PSMD2	-0.004412839	0.039567188	0.936293664
NDNL2	0.008628969	0.039567882	0.936293664
IL5RA	0.011402794	0.039572859	0.936293664
MLLT1	0.009117718	0.039598289	0.936293664
SLC9A9	0.006289527	0.039602693	0.936293664
ZKSCAN4	-0.008686488	0.039636712	0.936293664
TOMM34	-0.008921453	0.03971433	0.936293664
PIK3R4	-0.006620401	0.039770795	0.936293664
FCGRT	-0.007450867	0.039821722	0.936293664
CSTF3	-0.00484393	0.039843905	0.936293664
PABPN1	0.005765677	0.039893205	0.936293664
SLC25A12	-0.006697867	0.039949753	0.936293664
FAM76B	-0.008103806	0.040157812	0.936293664
GFRA2	-0.018108895	0.040254185	0.936293664
RAB30	0.01090928	0.040342196	0.936293664
FRAT2	-0.01148838	0.040372051	0.936293664
CYB5A	0.00595523	0.040472596	0.936293664
SBF1	-0.0049525	0.040559148	0.936293664
FAM124B	-0.01112078	0.040571459	0.936293664
FGFR1OP	-0.007541485	0.040766575	0.936293664
CCDC132	-0.005315468	0.040770518	0.936293664
SLC15A3	-0.008137294	0.040778768	0.936293664
SLC16A3	-0.004976338	0.040887814	0.936293664
IRAK3	-0.008831048	0.041243894	0.936293664

THUMPD3	-0.008718251	0.041282017	0.936293664
TRIM66	-0.011851758	0.041388789	0.936293664
RNASEK	0.00521445	0.041518484	0.936293664
RNY4	0.017948398	0.041619406	0.936293664
TSPAN13	0.016206561	0.041645686	0.936293664
STAB1	-0.012946577	0.041833808	0.936293664
AURKA	-0.009164033	0.041927058	0.936293664
VEGFB	0.006405646	0.042164375	0.936293664
COPB2	-0.006115705	0.042242477	0.936293664
HSD17B4	-0.005365378	0.042252059	0.936293664
PRKCDBP	-0.019944182	0.042423281	0.936293664
ZNF653	-0.006364493	0.042630514	0.936293664
ZNF746	-0.004501919	0.042679351	0.936293664
TAPT1	0.007110029	0.042682132	0.936293664
SDC4	0.011963301	0.042763184	0.936293664
PDE4D	0.011213474	0.042839172	0.936293664
FXYD7	0.009434989	0.042871927	0.936293664
ZNF784	-0.007111618	0.04300342	0.936293664
ZBTB3	-0.011107431	0.043018322	0.936293664
KIAA1324	0.008887521	0.043112292	0.936293664
HIATL1	-0.007478798	0.043142921	0.936293664
B4GALT3	-0.005544396	0.043150479	0.936293664
TRAPPC2L	-0.00622176	0.043198389	0.936293664
PLAG1	-0.010064988	0.043315372	0.936293664
BTBD17	-0.015677613	0.043365421	0.936293664
VPS37C	-0.006598846	0.043378559	0.936293664
TTC39B	0.010663523	0.043403245	0.936293664
KCNJ10	0.008317948	0.043441233	0.936293664
ANAPC5	0.004490541	0.043461205	0.936293664
GPS1	-0.004204793	0.043509655	0.936293664
NSUN5P1	0.009484654	0.04351381	0.936293664
PHKB	0.00904261	0.043623694	0.936293664
CAMK1D	0.007617426	0.043624161	0.936293664
MTHFD2	-0.009486881	0.043646986	0.936293664
CCDC121	-0.008521003	0.043735181	0.936293664
KCTD11	-0.00835954	0.043793364	0.936293664
OCIAD2	0.005790903	0.043877766	0.936293664
GPN2	-0.005704346	0.04394606	0.936293664
MAPRE2	0.005963333	0.043964192	0.936293664
IRF2BP2	-0.006077025	0.044056986	0.936293664
P2RX6	-0.009022082	0.044133668	0.936293664
MAX	0.013004288	0.044183531	0.936293664
PHB2	0.004966902	0.044262165	0.936293664
TUBG1	-0.007564931	0.044329041	0.936293664
PRRT2	0.010621542	0.044566744	0.936293664
HS1BP3	-0.007467098	0.044694223	0.936293664
TMEM40	0.017012767	0.044757038	0.936293664

SCLT1	-0.010895106	0.044821921	0.936293664
CTSC	-0.00829675	0.044930565	0.936293664
HOXA5	-0.011647502	0.044976273	0.936293664
RHOT2	-0.007896902	0.045020642	0.936293664
ITPKB	0.006469142	0.045033116	0.936293664
IL17RB	0.012235732	0.045160733	0.936293664
FKBP15	-0.006093956	0.045183032	0.936293664
PLOD3	-0.006862378	0.045190272	0.936293664
PRC1	-0.008998619	0.045194486	0.936293664
METTL4	0.006180756	0.045229862	0.936293664
TLR4	-0.012015661	0.045463051	0.936293664
AMDHD1	-0.013688726	0.045468188	0.936293664
CXCR1	-0.01124012	0.045541921	0.936293664
RBFA	0.007262762	0.04558902	0.936293664
COASY	-0.004378013	0.045771964	0.936293664
DYNC1I2	-0.005649586	0.045998296	0.936293664
CCDC124	0.007141674	0.046012328	0.936293664
RGMA	-0.00704249	0.046085158	0.936293664
RIC3	0.01067264	0.0460917	0.936293664
CYP1B1	-0.021384069	0.046096144	0.936293664
VILL	-0.007124027	0.046118887	0.936293664
AKIP1	-0.007704913	0.046479428	0.936293664
CPSF3L	-0.005559396	0.04660039	0.936293664
CHMP7	0.00921528	0.046636159	0.936293664
TONSL	0.010705659	0.046699536	0.936293664
ZBED4	0.005098414	0.046723526	0.936293664
C19orf10	-0.009180432	0.046840233	0.936293664
PDCD4	0.008064688	0.047179384	0.936293664
PTPRR	-0.007582886	0.047295982	0.936293664
TMEM109	0.005589613	0.047302764	0.936293664
RPS2	-0.010093274	0.047352962	0.936293664
MTMR3	-0.00527595	0.047395603	0.936293664
RAB27B	0.015623836	0.047402574	0.936293664
VPS26A	-0.004366798	0.047403984	0.936293664
SIAH1	-0.006283399	0.047434558	0.936293664
NAT1	-0.011017057	0.047497003	0.936293664
TM9SF1	-0.007030049	0.047805505	0.936293664
SNX13	-0.007439571	0.047854401	0.936293664
SRSF11	0.007453665	0.047967359	0.936293664
CHRNB4	-0.009004066	0.047994951	0.936293664
PHTF1	-0.007221187	0.04811076	0.936293664
TRMT2A	-0.007494682	0.048223329	0.936293664
PKIB	-0.010018876	0.048345578	0.936293664
ATN1	-0.011235637	0.048366268	0.936293664
LSM12	0.005487299	0.048465806	0.936293664
RRP8	-0.005777885	0.048542091	0.936293664
TCF25	-0.005173235	0.048737503	0.936293664

FAM13A	0.011172261	0.04875905	0.936293664
P2RX5	0.014326146	0.048888777	0.936293664
TMEM131	-0.004042054	0.048974773	0.936293664
CD53	-0.004776172	0.049175239	0.936293664
BAD	0.0068938	0.049312589	0.936293664
GPR137B	-0.010891553	0.049350966	0.936293664
ZSCAN22	-0.008663962	0.049506625	0.936293664
KIAA1328	-0.007881732	0.049528617	0.936293664
HERPUD1	-0.005627746	0.049879561	0.936293664
AASDHPPT	-0.006555313	0.049891614	0.936293664
CDHR3	0.01204057	0.049975714	0.936293664
TM2D2	-0.009472333	0.050047815	0.936293664
GSTP1	-0.006271606	0.050086844	0.936293664
SYCE1	0.008988307	0.050129413	0.936293664
HIST1H2AD	0.015922856	0.050141944	0.936293664
ZNF177	-0.0095054	0.05020524	0.936293664
SIPA1L3	0.012223363	0.050300168	0.936293664
MFHAS1	0.016841715	0.050341175	0.936293664
TBX3	-0.006654445	0.05066954	0.936293664
CES1	-0.030506802	0.050810132	0.936293664
ZNF316	0.007972436	0.050846354	0.936293664
TWISTNB	-0.006947942	0.050892212	0.936293664
DEF6	-0.004144978	0.051336069	0.936293664
NUP214	-0.006280095	0.051336198	0.936293664
NFKBIE	-0.012290531	0.051350357	0.936293664
OSBPL11	-0.007054599	0.051372905	0.936293664
HSPA5	-0.005579475	0.05142354	0.936293664
KRI1	0.006266338	0.051475759	0.936293664
MTMR14	-0.003814224	0.051486703	0.936293664
LRRC20	-0.008241102	0.051524441	0.936293664
LIG3	-0.008274835	0.051590765	0.936293664
DOLK	-0.008223273	0.051601445	0.936293664
DNAJB9	-0.009906138	0.051939855	0.936293664
TCP11L2	0.017655196	0.052277857	0.936293664
UBXN4	0.007835857	0.052413477	0.936293664
CABLES1	-0.006549487	0.05241876	0.936293664
SAMM50	-0.006360964	0.052526949	0.936293664
DYM	-0.005653708	0.052596608	0.936293664
SNORA7B	0.005950703	0.052762829	0.936293664
STK11IP	-0.007126508	0.052769165	0.936293664
RNF169	0.007605211	0.052773533	0.936293664
TTF2	-0.006374471	0.052800532	0.936293664
TRNT1	-0.009015206	0.052816406	0.936293664
P2RY14	0.011034748	0.052832187	0.936293664
CLTCL1	0.008001554	0.052860817	0.936293664
MST1	-0.008071376	0.053120199	0.936293664
IFITM1	0.007367658	0.053311479	0.936293664

CIB1	-0.005867139	0.053319535	0.936293664
C20orf196	-0.008365865	0.053408868	0.936293664
ASAP2	0.016525463	0.053650735	0.936293664
LRRC58	-0.008114389	0.05365545	0.936293664
TSTD2	-0.006204118	0.054052029	0.936293664
FABP5	-0.011082705	0.054121117	0.936293664
MAGEL2	0.006765871	0.054185602	0.936293664
HABP4	0.008697211	0.054196207	0.936293664
ZNF35	-0.011218967	0.054242749	0.936293664
FGD3	-0.003851674	0.054344978	0.936293664
COPG2	0.012430742	0.054527522	0.936293664
IL20RB	-0.007067351	0.054584906	0.936293664
NECAP1	-0.0048068	0.054803626	0.936293664
ZSCAN5A	-0.008017913	0.054842581	0.936293664
ALS2	-0.00724185	0.054887615	0.936293664
PIM1	0.008149596	0.054902044	0.936293664
CLK1	-0.011201862	0.054910803	0.936293664
HNRNPL	0.008366503	0.055014134	0.936293664
SBF1P1	-0.008041035	0.05519309	0.936293664
SLC12A2	0.008206876	0.055204974	0.936293664
SEPW1	0.004256407	0.055208406	0.936293664
TNF	-0.016572304	0.055286287	0.936293664
DUSP22	-0.005913297	0.05536382	0.936293664
TBC1D10C	-0.006165702	0.055368222	0.936293664
TRIM39	-0.005163323	0.055393933	0.936293664
SNORD83B	0.008470117	0.055651288	0.936293664
SPAST	0.007186459	0.055736901	0.936293664
TTC16	-0.014280608	0.05581171	0.936293664
BTF3L4	-0.007264021	0.055875654	0.936293664
SLC35E3	-0.004897574	0.055965855	0.936293664
P2RY13	-0.009091816	0.055981327	0.936293664
FXYD1	0.008078062	0.056043869	0.936293664
SPHK2	-0.004241034	0.056074526	0.936293664
MAN2A1	0.006995425	0.056111716	0.936293664
MTCH2	-0.00634211	0.056149743	0.936293664
CLK4	-0.010061525	0.056192768	0.936293664
INPP5F	0.009828029	0.056393934	0.936293664
MRPL44	-0.006244555	0.056396575	0.936293664
ANKRD13A	0.004229833	0.056483157	0.936293664
SLC25A46	-0.007852203	0.05652074	0.936293664
CSTA	-0.012777111	0.056641257	0.936293664
GLI4	0.00814191	0.056712698	0.936293664
CD33	-0.009566646	0.05682312	0.936293664
C7orf49	-0.006698559	0.056889089	0.936293664
TNFRSF12A	0.007650454	0.056961515	0.936293664
LCOR	0.008644886	0.057318185	0.936293664
ZFPL1	-0.0064455	0.057404091	0.936293664

ZSCAN29	-0.011452011	0.057430902	0.936293664
VASP	0.013139382	0.057494136	0.936293664
PDE5A	0.019311456	0.057694835	0.936293664
MRPL9	0.005737851	0.057714053	0.936293664
MIR330	0.011336616	0.057816457	0.936293664
GBGT1	-0.008750833	0.057950375	0.936293664
ATP5A1	-0.005204696	0.058069175	0.936293664
ACPT	0.009717248	0.058146638	0.936293664
UBE2B	0.00609975	0.058194989	0.936293664
THEMIS	-0.0103799	0.058228303	0.936293664
SERF1B	0.011037675	0.058241447	0.936293664
BACE1	-0.009923929	0.058297702	0.936293664
PSMB1	-0.004462969	0.058311747	0.936293664
MGLL	0.024357845	0.058343008	0.936293664
LTBP2	0.008068146	0.058578146	0.936293664
ZNF607	0.00690604	0.058646636	0.936293664
FCRL3	0.016019366	0.058712522	0.936293664
NDUFB9	0.005459565	0.058859989	0.936293664
HIBADH	-0.006042231	0.058970063	0.936293664
NDST2	-0.005864579	0.059086523	0.936293664
CST3	-0.008220762	0.059163661	0.936293664
EFNA4	-0.005931622	0.059196785	0.936293664
MRPS21	0.005738462	0.059269689	0.936293664
CHERP	0.006369386	0.05929703	0.936293664
ANKRD9	0.019427793	0.059447316	0.936293664
RAPH1	-0.009212481	0.059458899	0.936293664
SEC11A	-0.004804912	0.059636189	0.936293664
SPATA2	-0.00931217	0.059652181	0.936293664
SRA1	-0.00581926	0.05974674	0.936293664
NCBP1	-0.004770761	0.059979049	0.936293664
PTCD1	-0.007315721	0.059996177	0.936293664
MRPL27	-0.006109175	0.060021982	0.936293664
JAM2	0.006856751	0.060163257	0.936293664
RPRD2	0.005209101	0.060184148	0.936293664
ZNF12	-0.00786272	0.060193164	0.936293664
CNN2	0.007535529	0.060333532	0.936293664
DYNLL2	0.007545659	0.060400924	0.936293664
BCL7A	0.007755747	0.060477034	0.936293664
HSPB3	0.011394176	0.060578068	0.936293664
DNAJC25	-0.005018293	0.060695549	0.936293664
KLHL2	-0.007349326	0.060715181	0.936293664
PRUNE	0.008270342	0.060745665	0.936293664
SNAPC3	-0.005858267	0.060916843	0.936293664
PIGO	-0.004807819	0.060939991	0.936293664
CDC37	-0.003735939	0.061186739	0.936293664
STX10	0.005448007	0.061220894	0.936293664
EIF5A	0.008425474	0.06122463	0.936293664

CNFN	0.010190539	0.061230049	0.936293664
TPM1	0.021998502	0.061250191	0.936293664
ASPRV1	-0.007808267	0.061274278	0.936293664
NUDT18	-0.008220651	0.061356457	0.936293664
NUS1	-0.005140709	0.06138478	0.936293664
RAB4B-EGLN2	-0.003791475	0.061400712	0.936293664
MALT1	0.005413125	0.061431462	0.936293664
NDUFAF1	-0.007201394	0.061551452	0.936293664
SNORA28	0.017361126	0.061602677	0.936293664
ABCA9	-0.006525895	0.061635977	0.936293664
NUP93	0.004465917	0.061652179	0.936293664
QTRT1	0.007116744	0.061723297	0.936293664
ASB8	-0.005120363	0.061759411	0.936293664
CD44	-0.003889853	0.061901812	0.936293664
PADI4	-0.013333083	0.062004579	0.936293664
USP46	0.009645784	0.062039794	0.936293664
ALOX12	0.024546422	0.062088699	0.936293664
MPP7	-0.009472103	0.062119179	0.936293664
RBBP5	0.005336973	0.062191279	0.936293664
TINF2	-0.008022254	0.062437962	0.936293664
ADARB1	0.006912481	0.06252799	0.936293664
NRAS	-0.006467262	0.062551684	0.936293664
FBXL15	0.004794566	0.0625769	0.936293664
CDC42EP2	-0.014643432	0.062643148	0.936293664
MINA	0.006953594	0.062648157	0.936293664
HRASLS	0.022351498	0.062678937	0.936293664
AK3	0.006681121	0.062719925	0.936293664
REM2	0.009846434	0.062766505	0.936293664
CYP20A1	0.003708849	0.062768008	0.936293664
SLC41A1	0.006495227	0.062831893	0.936293664
ZEB2	-0.00845511	0.062886239	0.936293664
MARCH1	-0.013166153	0.062927543	0.936293664
STRBP	0.009063059	0.06312969	0.936293664
LRP3	-0.010852605	0.063143805	0.936293664
PIGW	-0.006908792	0.063158973	0.936293664
FCER2	0.022515637	0.06318142	0.936293664
WASL	-0.008747104	0.06321642	0.936293664
PLEKHG4	-0.009417587	0.063252497	0.936293664
PSPH	-0.008221981	0.063359183	0.936293664
ZBTB41	-0.007661586	0.063397957	0.936293664
MASTL	-0.010990131	0.063732691	0.936293664
ANKRD46	-0.009057741	0.063957715	0.936293664
EEA1	-0.00635462	0.063996811	0.936293664
BRD3	0.007766834	0.064057127	0.936293664
TP53I11	-0.008680025	0.064072183	0.936293664
RNF25	0.006322785	0.064119117	0.936293664
CD300C	-0.015984545	0.064176422	0.936293664

FBXL12	-0.004623835	0.06417677	0.936293664
TACC2	0.009769625	0.06428252	0.936293664
IPO5	-0.005788971	0.064391174	0.936293664
MED28	-0.004996841	0.064577625	0.936293664
CPOX	-0.005386421	0.064606327	0.936293664
HLA-DRB6	0.057405422	0.064759443	0.936293664
SIGLEC9	-0.008051094	0.06486263	0.936293664
TLR2	-0.011321133	0.064867588	0.936293664
PPP1R2	0.005658395	0.064927403	0.936293664
DDX54	-0.006006009	0.064954822	0.936293664
USP30	-0.008159007	0.06509796	0.936293664
MTO1	-0.004635085	0.065335871	0.936293664
TXNL4A	0.008543126	0.065428999	0.936293664
GRASP	0.013692154	0.065511865	0.936293664
GTF2H1	0.006891158	0.066570242	0.936293664
MAP7D1	-0.003838886	0.066571625	0.936293664
CLASRP	0.007705719	0.066746202	0.936293664
TTC23	0.008526139	0.066870055	0.936293664
C21orf2	-0.009285942	0.067028452	0.936293664
LAMB1	-0.007104366	0.067150882	0.936293664
PNPLA2	-0.008960532	0.067159914	0.936293664
GCOM1	0.006084996	0.067177414	0.936293664
DNTTIP1	-0.006794005	0.067208111	0.936293664
JPH2	0.006620914	0.067264717	0.936293664
CCDC136	-0.006799927	0.067280185	0.936293664
SRC	0.009061301	0.067362047	0.936293664
SNAPIN	-0.005101719	0.067465032	0.936293664
NLRX1	-0.004430057	0.067527303	0.936293664
OSGIN2	-0.007132805	0.067735726	0.936293664
GP1BA	0.014756669	0.067802764	0.936293664
TMEM150B	-0.007129389	0.06782554	0.936293664
LY6G6F	0.020633874	0.06797469	0.936293664
ADO	-0.007153971	0.067982132	0.936293664
LEAP2	-0.007137561	0.068055668	0.936293664
OAT	-0.007922825	0.06816717	0.936293664
PGAP1	0.008461215	0.068268141	0.936293664
NMT1	-0.007088325	0.068278601	0.936293664
NEBL	0.019317515	0.068323391	0.936293664
LETM1	0.007729656	0.068375075	0.936293664
PELO	-0.010793337	0.068505583	0.936293664
ZMAT3	0.008243511	0.068679023	0.936293664
PDS5A	0.00429149	0.068706255	0.936293664
PPP1CB	0.004869252	0.068714028	0.936293664
FAM115A	0.002780205	0.068731754	0.936293664
SRXN1	-0.011053353	0.068839796	0.936293664
CLEC4A	-0.006363643	0.068865065	0.936293664
SP2	-0.005162861	0.068927238	0.936293664

ZNF792	0.00716924	0.069017622	0.936293664
MARCH3	0.011561172	0.069142913	0.936293664
ZBTB32	-0.011041998	0.069171849	0.936293664
PTCRA	0.019925613	0.069222544	0.936293664
CYP27B1	0.006392098	0.069224655	0.936293664
NKD2	0.012814425	0.069228812	0.936293664
ZNF517	-0.006553191	0.069268513	0.936293664
HERC5	0.011999077	0.069385031	0.936293664
C1orf198	0.013613034	0.069487186	0.936293664
CDC42BPA	0.006441302	0.069599135	0.936293664
TNFSF13	-0.005746743	0.06967528	0.936293664
POLDIP3	-0.003698134	0.069675332	0.936293664
LRRK2	-0.012768004	0.069689778	0.936293664
BUB3	0.003382783	0.069780826	0.936293664
RPPH1	-0.00607276	0.070124742	0.936293664
B3GALNT2	-0.008142108	0.070202643	0.936293664
NDST1	-0.01048499	0.070275905	0.936293664
IGHG1	-0.005637379	0.070300209	0.936293664
ZNF790	0.009541376	0.070334319	0.936293664
LRIG1	0.007112095	0.070412852	0.936293664
EXD2	-0.006025147	0.070533411	0.936293664
TMEM186	-0.00670967	0.070533747	0.936293664
GLB1	-0.00621289	0.070556374	0.936293664
UGGT2	-0.009066596	0.070766186	0.936293664
MRPL48	0.006185866	0.070784968	0.936293664
PTPRE	-0.00647618	0.070924191	0.936293664
GINS3	-0.006184847	0.071027342	0.936293664
FAM78A	-0.007881509	0.07108945	0.936293664
SLC22A1	0.007745855	0.071170409	0.936293664
DGKQ	-0.009324869	0.0714319	0.936293664
XKR8	0.006149669	0.071482767	0.936293664
BABAM1	0.003782446	0.071550731	0.936293664
FAM102B	0.005596704	0.07197251	0.936293664
PATE1	0.008412656	0.07225609	0.936293664
GUSBP9	0.01190864	0.072290052	0.936293664
CRIPT	-0.008779044	0.072294171	0.936293664
CCNE1	-0.007599188	0.072367115	0.936293664
KLRK1	-0.007508965	0.072368126	0.936293664
BCL7B	0.009390244	0.072369891	0.936293664
NOB1	0.005454885	0.072539648	0.936293664
CX3CR1	-0.010254813	0.072563484	0.936293664
SLX1B-SULT1A4	0.007561489	0.072602706	0.936293664
FBXO30	-0.008550707	0.072644454	0.936293664
TXK	0.012675174	0.072746813	0.936293664
ZNF518A	-0.004908935	0.07286692	0.936293664
FCHO1	-0.005900445	0.072992053	0.936293664
CUEDC1	-0.008734519	0.073016139	0.936293664

BAZ2B	-0.007655275	0.073084419	0.936293664
HMGXB4	-0.004297566	0.073189173	0.936293664
RPL29	-0.003687664	0.073351	0.936293664
TOX4	0.005423165	0.073488668	0.936293664
CMTM7	-0.005253993	0.073739979	0.936293664
RBM38	0.007771207	0.073744342	0.936293664
ZNF837	-0.00764523	0.073820778	0.936293664
MPHOSPH9	-0.006491795	0.073866146	0.936293664
CASP4	-0.005223748	0.073926799	0.936293664
ARHGEF1	0.008692604	0.073934545	0.936293664
DPP10	0.0080895	0.073962185	0.936293664
PDCL3	-0.00515513	0.074116397	0.936293664
TNFSF4	0.015749166	0.074128568	0.936293664
XPA	0.005178941	0.07428134	0.936293664
FBXO34	0.004839499	0.07438325	0.936293664
KIAA0141	-0.005184636	0.07439042	0.936293664
MIR302C	-0.008077792	0.07446289	0.936293664
NUP35	-0.00905769	0.07453168	0.936293664
PMPCB	-0.004058285	0.074558433	0.936293664
COL9A2	-0.011018293	0.074615404	0.936293664
UCKL1	0.00450384	0.074689778	0.936293664
NSF	-0.003684046	0.074859492	0.936293664
PTPRJ	0.007392885	0.074957077	0.936293664
RPL31P11	0.011509405	0.074995291	0.936293664
PIGF	-0.00650207	0.075083637	0.936293664
SPG11	-0.004755466	0.075085435	0.936293664
CLINT1	-0.004205637	0.075126764	0.936293664
CTNNBL1	0.004322583	0.075154185	0.936293664
LRRC1	-0.00785656	0.075161753	0.936293664
RDX	-0.007997913	0.075177794	0.936293664
HIF1A	0.007268067	0.075237826	0.936293664
TMTC2	-0.009159045	0.075277124	0.936293664
SMAD2	0.004646387	0.075289728	0.936293664
TIMM17A	-0.006845429	0.075304358	0.936293664
P4HB	-0.004712376	0.07542224	0.936293664
MAN2B2	-0.005076135	0.075586965	0.936293664
FAM131A	-0.005532432	0.075677802	0.936293664
ABAT	0.01091745	0.075724353	0.936293664
ANKRD17	-0.003705509	0.075727527	0.936293664
MLH3	0.014681974	0.075749925	0.936293664
CDC34	0.005985592	0.075756493	0.936293664
PGM2	-0.00632223	0.075862363	0.936293664
HIC2	-0.004655398	0.075873738	0.936293664
NAT9	-0.0054685	0.075936312	0.936293664
DNM3	0.018803213	0.076026379	0.936293664
TUBGCP5	-0.006346525	0.076046583	0.936293664
INPP5A	0.006485368	0.076209934	0.936293664

BBS10	-0.009436153	0.076301121	0.936293664
LGR6	-0.014974306	0.076308333	0.936293664
CCDC126	-0.009513744	0.076407915	0.936293664
PRAME	0.005861774	0.076552197	0.936293664
TMEM64	0.007848232	0.076672245	0.936293664
B3GALTL	0.010983618	0.076678625	0.936293664
ACVR1B	-0.005747787	0.076683616	0.936293664
USF2	-0.009969514	0.076763256	0.936293664
PPIG	0.004788992	0.077066141	0.936293664
PDE4B	0.00703081	0.077083352	0.936293664
CALHM2	-0.00778568	0.077196914	0.936293664
CBX6	-0.005607355	0.077241713	0.936293664
DEFA1	-0.020850883	0.077315435	0.936293664
GUCY1B3	0.015058989	0.077375922	0.936293664
TUBA3D	0.014674408	0.077641624	0.936293664
HDHD2	-0.004147986	0.077763351	0.936293664
RGS12	-0.009297059	0.077822092	0.936293664
MTMR10	-0.006067498	0.077842659	0.936293664
CCDC85B	0.006385118	0.077862339	0.936293664
ZNF212	-0.005351131	0.077919348	0.936293664
HAUS8	-0.00618058	0.078049599	0.936293664
PLCD3	-0.006187968	0.078068262	0.936293664
PWWP2A	-0.005166016	0.078122688	0.936293664
ZNF676	0.00650661	0.078238519	0.936293664
MFSD7	-0.008284142	0.0782785	0.936293664
SLC35A5	-0.006549848	0.078305878	0.936293664
LIPN	-0.012517961	0.078392763	0.936293664
COQ7	0.007735633	0.078434575	0.936293664
NCR3	0.011679562	0.078437191	0.936293664
PRPF19	0.004563745	0.078473814	0.936293664
DAB2	0.01230752	0.078546517	0.936293664
RABEPK	-0.003321291	0.078690667	0.936293664
SLC25A1	0.006967583	0.07892634	0.936293664
FAM120B	-0.004544464	0.079024571	0.936293664
GRAMD3	-0.00855759	0.07911913	0.936293664
BTN3A1	-0.007703085	0.079230251	0.936293664
AATF	0.004844639	0.079296823	0.936293664
ALOX5	-0.006347533	0.079543369	0.936293664
AHCTF1	0.005796351	0.079646463	0.936293664
CHD4	0.00479822	0.079763095	0.936293664
ZNF593	-0.006479037	0.079922343	0.936293664
CPN2	0.008010758	0.080203941	0.936293664
C11orf74	-0.011551335	0.080273479	0.936293664
ZNF138	0.006010047	0.080317724	0.936293664
FUK	-0.003993765	0.080338757	0.936293664
STX1A	0.007756055	0.080357361	0.936293664
EXOSC9	0.005856324	0.080361793	0.936293664

PPP1R14B	0.005807282	0.08039757	0.936293664
DDX28	0.005398645	0.080521669	0.936293664
GPR156	-0.00732839	0.080621491	0.936293664
OSBPL1A	-0.011925478	0.080625082	0.936293664
TNFRSF1B	-0.005185344	0.080634411	0.936293664
PIM3	0.008134139	0.080706386	0.936293664
NUP37	-0.005150106	0.080707791	0.936293664
CMTM5	0.022509655	0.080775052	0.936293664
CBLL1	0.00675232	0.080811157	0.936293664
BCL7C	0.007214435	0.080971771	0.936293664
LASP1	-0.004298386	0.080986293	0.936293664
PPP1R3D	-0.009432339	0.0809935	0.936293664
MRPS31	0.003902853	0.081139773	0.936293664
GUSB	-0.003601764	0.081143308	0.936293664
CAND1	-0.005348538	0.081161001	0.936293664
TCEANC2	0.004832951	0.081246665	0.936293664
SECISBP2L	0.004008091	0.081287838	0.936293664
ZNF142	-0.003924409	0.081393022	0.936293664
MAD2L1BP	-0.007507517	0.081444481	0.936293664
KLF11	0.006977159	0.081569876	0.936293664
ZC3H4	0.004195295	0.081722827	0.936293664
CHIC2	-0.006564103	0.081723002	0.936293664
HMBOX1	0.017115699	0.081853457	0.936293664
DERL2	-0.00421484	0.081915269	0.936293664
C16orf80	-0.005278503	0.081924293	0.936293664
B3GAT3	0.007682175	0.082020074	0.936293664
KLHL6	-0.006449513	0.082044459	0.936293664
PRDX2	0.0099652	0.082187504	0.936293664
TEX2	-0.004801298	0.082212204	0.936293664
VIPR1	-0.008647788	0.082227291	0.936293664
PSEN2	-0.006471745	0.082441521	0.936293664
KIAA0226	0.006264337	0.082449759	0.936293664
NSUN5	0.006167437	0.082629837	0.936293664
CA1	0.011699252	0.082741377	0.936293664
TM2D3	-0.006464277	0.082751884	0.936293664
VPS37A	-0.005207322	0.082772442	0.936293664
LPCAT2	-0.022398386	0.082876392	0.936293664
RASL11A	0.007409608	0.0830011	0.936293664
RTN4IP1	-0.004764238	0.083020471	0.936293664
SPRY2	-0.015796576	0.083072962	0.936293664
GSDMD	-0.005905747	0.083104193	0.936293664
EXOSC3	-0.007800661	0.083111678	0.936293664
AIFM2	-0.005726323	0.083428557	0.936293664
ALG10	-0.006017889	0.083698598	0.936293664
PLOD2	0.019700813	0.083868499	0.936293664
GPR84	-0.007827949	0.083882459	0.936293664
SLC39A9	-0.007452433	0.083935389	0.936293664

ITPRIPL2	-0.0100226	0.083997723	0.936293664
HSPA4	0.004200438	0.084133418	0.936293664
CECR1	-0.005475559	0.084134317	0.936293664
NEDD4	-0.007957936	0.084200345	0.936293664
DDOST	-0.003885976	0.084251438	0.936293664
L3MBTL1	-0.006416035	0.084263215	0.936293664
HMBS	-0.008370499	0.084353311	0.936293664
SIL1	-0.006836571	0.084420377	0.936293664
CXCL16	-0.008328205	0.084521252	0.936293664
ASTE1	-0.007942538	0.084529856	0.936293664
USO1	-0.003526805	0.08475361	0.936293664
RACGAP1	0.006634449	0.08477367	0.936293664
PTK2	0.008477355	0.084789701	0.936293664
GATAD2A	0.004543293	0.085022103	0.936293664
SNORD38A	0.007938817	0.085109673	0.936293664
VTI1B	0.005760854	0.085276605	0.936293664
C1orf189	-0.006152987	0.085581134	0.936293664
MYO19	-0.008864318	0.085631603	0.936293664
C9orf66	-0.009352766	0.085672157	0.936293664
DNAL1	-0.006925102	0.085829524	0.936293664
FUBP3	-0.00417108	0.085948831	0.936293664
YPEL3	0.004760635	0.085986536	0.936293664
MRPL37	-0.004641553	0.085994931	0.936293664
FBXW4	0.004113595	0.086012298	0.936293664
HSF5	-0.00871632	0.086124911	0.936293664
AGPS	-0.005173131	0.086280302	0.936293664
MTX2	0.004409688	0.086303917	0.936293664
ZBTB2	0.006360881	0.086331313	0.936293664
EFTUD2	0.003821783	0.086343126	0.936293664
EIF2AK2	0.010704819	0.086346651	0.936293664
TRAF3IP1	0.007067728	0.086423778	0.936293664
RAB5C	0.004703341	0.086567331	0.936293664
PCYT2	-0.005380179	0.086724151	0.936293664
DAP3	-0.003828119	0.086793191	0.936293664
TIGD6	-0.006516071	0.086884787	0.936293664
CTDSPL	0.020864013	0.086976605	0.936293664
ZNF446	-0.005559696	0.086980018	0.936293664
TMED9	-0.005755562	0.087000435	0.936293664
EPB41L2	0.007674247	0.087049366	0.936293664
SARM1	-0.006964181	0.087094957	0.936293664
ST8SIA4	-0.00831566	0.087219779	0.936293664
DLEU7	-0.01131377	0.087343761	0.936293664
ZNF671	-0.006054614	0.087606329	0.936293664
RNF217	-0.007184277	0.087692707	0.936293664
PRKAB1	-0.005264442	0.087725745	0.936293664
ATG9A	-0.005848114	0.087777139	0.936293664
HOOK2	0.005725484	0.08787063	0.936293664

DDX47	-0.003517873	0.08808623	0.936293664
ITGB2	-0.002743112	0.088477174	0.936293664
ZNF429	-0.013171703	0.088532955	0.936293664
RIOK1	-0.003498688	0.088601234	0.936293664
GOSR1	-0.005911739	0.088765555	0.936293664
KIAA0895L	-0.007902356	0.088798476	0.936293664
TREML1	0.020671088	0.088891523	0.936293664
YEATS2	-0.004290779	0.088929563	0.936293664
STAP1	0.013812398	0.088992415	0.936293664
LYPLAL1	0.005355259	0.089133295	0.936293664
R3HDM2	0.006139899	0.0892227	0.936293664
ERBB2IP	0.007102388	0.089289642	0.936293664
STRADB	0.00603187	0.089306351	0.936293664
RNASET2	-0.003709762	0.089317016	0.936293664
LPCAT4	0.006405281	0.089367149	0.936293664
ABCB4	0.011006079	0.089628225	0.936293664
ST6GAL1	0.005108694	0.089690373	0.936293664
USP38	-0.005785336	0.089720136	0.936293664
NLRP7	-0.014440201	0.089788801	0.936293664
TBXAS1	-0.00629664	0.089988614	0.936293664
EIF2S1	0.004362125	0.090047228	0.936293664
ACSL5	0.004228846	0.090048908	0.936293664
QPRT	-0.009404863	0.090063391	0.936293664
MRPS22	-0.002812373	0.090149379	0.936293664
SLC2A14	0.008065921	0.090180265	0.936293664
SGSM3	-0.007224791	0.090321083	0.936293664
OTUB1	-0.006342774	0.090341891	0.936293664
IKZF3	-0.011229433	0.090348557	0.936293664
AP3M1	-0.006288308	0.090376902	0.936293664
CERCAM	-0.016402342	0.09043858	0.936293664
DDX50	0.003776629	0.090507469	0.936293664
RPL36	0.003458491	0.090548386	0.936293664
KCNG1	0.015969291	0.090651638	0.936293664
HYOU1	-0.007746761	0.090661774	0.936293664
POLR2K	0.009971808	0.090748681	0.936293664
HIST1H1C	0.012736469	0.090769337	0.936293664
SERPINB8	-0.011141934	0.090821854	0.936293664
STT3B	-0.006193174	0.091128539	0.936293664
ADORA2B	-0.008978656	0.091140462	0.936293664
FBXO28	-0.005523724	0.091298059	0.936293664
RREB1	-0.006990398	0.091364312	0.936293664
SNORA49	-0.00634677	0.091434633	0.936293664
SLC7A7	-0.006474293	0.09157752	0.936293664
SPPL2B	-0.007146424	0.091637407	0.936293664
PDXDC2P	0.015319435	0.091669024	0.936293664
EGLN3	0.008396081	0.091720629	0.936293664
UTS2	0.015876032	0.091739252	0.936293664

DRAM1	-0.007563563	0.091795479	0.936293664
EPB41	0.007250335	0.091986416	0.936293664
GCDH	0.005010148	0.092060782	0.936293664
HLA-DPB1	-0.014097039	0.092327703	0.936293664
TRUB2	-0.005369801	0.092411055	0.936293664
SLA2	0.009161664	0.092460362	0.936293664
ADRM1	0.004176362	0.092732422	0.936293664
C1orf123	0.005502181	0.092762577	0.936293664
HHATL	0.007138721	0.092809276	0.936293664
CXCR4	0.012911838	0.093035136	0.936293664
ERAL1	-0.003675643	0.093155822	0.936293664
SLC25A3	-0.004341204	0.093178707	0.936293664
BET1	-0.007605845	0.093216986	0.936293664
PTRF	-0.007348042	0.093225452	0.936293664
IRAK1BP1	-0.010676536	0.093335337	0.936293664
ADRB2	-0.014207001	0.093374888	0.936293664
ZNF251	0.005552146	0.093473259	0.936293664
P2RY2	-0.010495045	0.093505019	0.936293664
ZC3H14	-0.004601718	0.093562468	0.936293664
CTBP1	0.0050303	0.09360449	0.936293664
HNRNPH1	0.006429041	0.093655983	0.936293664
TRAF7	-0.006938127	0.093662526	0.936293664
PIH1D1	0.004625259	0.093705022	0.936293664
IBA57	-0.006171055	0.093717857	0.936293664
ADM2	-0.007336863	0.0937462	0.936293664
DBNL	-0.003722255	0.093814616	0.936293664
TICAM1	-0.00734228	0.093886599	0.936293664
DPRXP4	0.014096255	0.093994635	0.936293664
ADCK2	-0.006497979	0.094063411	0.936293664
SNX27	-0.005310057	0.094131036	0.936293664
AGTPBP1	-0.004594679	0.094155285	0.936293664
ARL8B	-0.003493384	0.09415536	0.936293664
GRSF1	-0.003460865	0.094185288	0.936293664
ATG10	0.010510607	0.094309879	0.936293664
KCTD3	-0.008279678	0.094326786	0.936293664
ANXA2	-0.005444106	0.094345083	0.936293664
TCF20	0.004322444	0.094431984	0.936293664
PCCB	-0.005066121	0.094680852	0.936293664
DDR1	-0.007957469	0.094838104	0.936293664
AAGAB	0.006905768	0.095029657	0.936293664
MEIS1	0.014605284	0.095117497	0.936293664
SPTY2D1	-0.005678322	0.095376302	0.936293664
PRRC2C	0.003887502	0.095381345	0.936293664
FCGR3A	0.01010693	0.095397119	0.936293664
HUS1B	0.0085211	0.095502557	0.936293664
CPA3	-0.010370381	0.095650093	0.936293664
SLC22A16	0.027545768	0.095656242	0.936293664

UBXN7	0.006296743	0.095775701	0.936293664
RFWD3	0.005066536	0.095955807	0.936293664
KLHL9	-0.006746163	0.096111786	0.936293664
SPAG5	-0.006193253	0.096164658	0.936293664
H2AFY2	-0.005736339	0.096194033	0.936293664
ELL2	-0.011363269	0.096225491	0.936293664
PEAR1	0.016975197	0.096314335	0.936293664
MED1	0.004326822	0.096315395	0.936293664
SLC46A2	-0.011083325	0.096341658	0.936293664
TMEM222	-0.005281157	0.096379565	0.936293664
LRR1	-0.005670213	0.096472555	0.936293664
PVRL2	0.01021716	0.096498848	0.936293664
BOLA1	-0.005951033	0.096670443	0.936293664
GLYCTK	-0.006592184	0.096789397	0.936293664
HDGF	0.005716478	0.096908718	0.936293664
MLH1	-0.00366344	0.096967904	0.936293664
ATM	0.00510745	0.096972485	0.936293664
CCR2	-0.0094079	0.097128226	0.936293664
GLOD4	0.003048207	0.097187957	0.936293664
TCF3	0.006988587	0.097314207	0.936293664
PIGB	-0.004675632	0.097338771	0.936293664
IGF2BP2	0.010096999	0.097582297	0.936293664
DNMT1	-0.00341846	0.097793682	0.936293664
PDE4A	-0.007803741	0.097832551	0.936293664
GATA2	0.011055777	0.097965516	0.936293664
EEF2K	-0.005807087	0.097995148	0.936293664
WASH3P	-0.004857475	0.098127801	0.936293664
ILVBL	-0.005648974	0.098171931	0.936293664
ZNF17	-0.007649292	0.098295375	0.936293664
CGRRF1	0.004923205	0.098424696	0.936293664
C17orf75	-0.017002616	0.098480108	0.936293664
TMEM218	-0.003811766	0.098555661	0.936293664
RGMB	0.017347246	0.098562027	0.936293664
C9orf16	0.007136817	0.098754549	0.936293664
TMEM217	0.007232994	0.098825393	0.936293664
PPM1F	-0.00494353	0.098913433	0.936293664
COPS7B	0.00385595	0.098930868	0.936293664
MKNK2	0.004563016	0.099025771	0.936293664
NCF1C	0.009136042	0.099110735	0.936293664
PICALM	-0.004308162	0.099159672	0.936293664
ARL15	-0.007136771	0.099196453	0.936293664
TAF13	0.008526192	0.099282915	0.936293664
PPAPDC1B	0.006070033	0.099339848	0.936293664
ZFAND5	-0.003292322	0.099513464	0.936293664
CENPBD1	-0.006162907	0.099516204	0.936293664
SLC2A8	-0.01296096	0.0995757	0.936293664
NNT	-0.004986986	0.099616756	0.936293664

IMPAD1	-0.008120296	0.099728543	0.936293664
SNHG10	0.00587172	0.099734778	0.936293664
KCTD18	-0.004491539	0.100009402	0.936293664
RXRB	-0.004320169	0.100077369	0.936293664
TRIM61	-0.007783649	0.100252874	0.936293664
TRAF6	-0.006663697	0.100335527	0.936293664
CNRIP1	-0.010246629	0.100454032	0.936293664
PTDSS2	-0.00577219	0.10069405	0.936293664
PSMB10	-0.004712643	0.100738885	0.936293664
IL15	-0.007117408	0.101030287	0.936293664
FARS2	0.0052474	0.101196724	0.936293664
ZBTB44	0.005597885	0.101355437	0.936293664
OR11H1	0.007020048	0.101382331	0.936293664
CNPY4	0.005299853	0.101438891	0.936293664
CANX	-0.004220175	0.101483392	0.936293664
GNS	-0.003895734	0.101537087	0.936293664
MMP28	-0.01463037	0.101602036	0.936293664
TMEM56	-0.008051738	0.101679511	0.936293664
STK32C	-0.009699939	0.101849864	0.936293664
SIX5	-0.007288578	0.101885235	0.936293664
SCARNA7	0.005844537	0.101950739	0.936293664
ALG6	-0.004012187	0.101961148	0.936293664
GIMAP7	-0.006552909	0.102036423	0.936293664
MBD3	-0.00689376	0.102170788	0.936293664
DDX21	-0.005755334	0.102249614	0.936293664
ANKHD1	-0.003575104	0.102298563	0.936293664
GALK1	-0.003715486	0.102299992	0.936293664
PRPF31	0.004992895	0.102366053	0.936293664
LEFTY1	0.012415816	0.10263688	0.936293664
PI4K2B	-0.004352154	0.102711792	0.936293664
DCAF5	-0.004211357	0.102972797	0.936293664
SCARNA10	0.00698285	0.102984773	0.936293664
MAP4K1	-0.005613321	0.103098354	0.936293664
FAM184B	0.017516431	0.103321873	0.936293664
ATE1	-0.005861822	0.103432237	0.936293664
FAM198B	-0.01096737	0.103442208	0.936293664
EXTL2	-0.008085584	0.103527615	0.936293664
LCP2	-0.005232751	0.103584056	0.936293664
KRT1	0.016622807	0.103616848	0.936293664
MTIF3	-0.004315553	0.103664326	0.936293664
SYNE2	0.009847455	0.103781511	0.936293664
BID	-0.00623611	0.103801609	0.936293664
CHTF18	-0.006432272	0.103849955	0.936293664
PDK1	0.009268561	0.103926121	0.936293664
ATPIF1	0.004383694	0.103947124	0.936293664
STK4	0.002805673	0.1039599	0.936293664
TMPRSS15	-0.006397322	0.104014114	0.936293664

RSPH4A	-0.007791725	0.104049116	0.936293664
DPYSL2	-0.004765443	0.104143842	0.936293664
SNX20	-0.008071152	0.104175832	0.936293664
TRAF1	-0.006145183	0.104206286	0.936293664
SERPINB9	0.013639524	0.104351015	0.936293664
PARP12	0.007888049	0.104401345	0.936293664
RFC1	-0.00437004	0.104402362	0.936293664
KCNK13	-0.008452752	0.104494377	0.936293664
DSN1	0.005179999	0.104779976	0.936293664
MRPL32	-0.003997945	0.104879085	0.936293664
GNAZ	0.016048123	0.104960111	0.936293664
IPO8	0.014393065	0.104961898	0.936293664
PARP4	-0.004625048	0.105006467	0.936293664
PSD3	0.005849561	0.105185038	0.936293664
C2orf88	0.015923326	0.105211819	0.936293664
TEX264	-0.003906115	0.105233353	0.936293664
KATNB1	-0.004725277	0.105245682	0.936293664
RTN4R	-0.00948218	0.105582088	0.936293664
BLCAP	0.004513484	0.10569205	0.936293664
LSM7	0.004666098	0.105882737	0.936293664
TJAP1	0.003043536	0.106052037	0.936293664
NETO1	0.008220081	0.106076215	0.936293664
DDRGK1	-0.004891119	0.106108592	0.936293664
MFAP3	-0.008447144	0.106518344	0.936293664
PRCC	0.004263572	0.106620646	0.936293664
MBIP	-0.005149954	0.1067324	0.936293664
SNORD34	0.010137159	0.106896342	0.936293664
NKIRAS2	-0.004633623	0.107098338	0.936293664
RIOK3	0.004685043	0.107134116	0.936293664
CCDC59	0.003226895	0.107203752	0.936293664
STOX1	-0.013489612	0.107578276	0.936293664
LMTK2	-0.006441463	0.107653998	0.936293664
RBAK	-0.008632657	0.107680025	0.936293664
SLC25A11	-0.00484191	0.107746305	0.936293664
TNFRSF10C	-0.006068739	0.107766753	0.936293664
SRGN	-0.007550695	0.107801131	0.936293664
COMMD2	-0.009040832	0.108123603	0.936293664
DDHD2	0.00330442	0.108260997	0.936293664
FBXL5	-0.006991987	0.108441975	0.936293664
MBNL2	-0.005296121	0.108466983	0.936293664
HDAC11	-0.007741132	0.108504774	0.936293664
P4HA1	-0.006280788	0.108608849	0.936293664
DAGLB	-0.006551911	0.108617964	0.936293664
NRP1	-0.013121689	0.108624052	0.936293664
IFT80	-0.005839094	0.108741892	0.936293664
GALC	-0.004490833	0.108942101	0.936293664
LYG1	-0.008855569	0.108946423	0.936293664

MIR635	-0.009031736	0.108998531	0.936293664
AMICA1	-0.004931269	0.109169999	0.936293664
UBE2Z	0.005183527	0.109249131	0.936293664
MBD5	0.007001728	0.109261375	0.936293664
SLC25A24	-0.009874691	0.109638551	0.936293664
RAB38	0.012993318	0.109676756	0.936293664
FOXD4L4	0.00704774	0.109777652	0.936293664
ZNF567	-0.006512392	0.109787427	0.936293664
MAP3K12	-0.005448984	0.109843116	0.936293664
RNF14	-0.00502875	0.109898045	0.936293664
WDR83	-0.004187267	0.109925242	0.936293664
REEP6	-0.008742349	0.11001671	0.936293664
EDF1	0.003857216	0.110221336	0.936293664
L3MBTL2	-0.005278235	0.110278181	0.936293664
TXNDC15	-0.006023576	0.110280137	0.936293664
AUP1	0.00492223	0.110362008	0.936293664
FBXO7	0.004097496	0.110373755	0.936293664
TULP4	0.004495446	0.110412497	0.936293664
MAPKAPK2	0.00495238	0.110465805	0.936293664
PKHD1L1	0.008271513	0.110496745	0.936293664
ERGIC1	-0.007771859	0.110510471	0.936293664
C12orf4	-0.005250734	0.110556897	0.936293664
TBCK	-0.005102271	0.110685406	0.936293664
NAGPA	-0.004691129	0.110697776	0.936293664
ADIPOR2	0.003073655	0.110738327	0.936293664
NACC2	-0.006125447	0.111110301	0.936293664
C12orf5	-0.007711164	0.111148655	0.936293664
CLEC4E	-0.010698777	0.11122047	0.936293664
VPS54	-0.005881563	0.111337199	0.936293664
C11orf54	-0.003990284	0.111342586	0.936293664
ABI1	0.003808453	0.111607895	0.936293664
UHRF1BP1L	-0.007259755	0.111661617	0.936293664
CD180	0.006915218	0.111912274	0.936293664
MRRF	-0.003707199	0.111984949	0.936293664
HCG18	-0.00817544	0.112050214	0.936293664
ZC3HAV1	0.004759711	0.112130176	0.936293664
ZNF616	-0.006958354	0.112233488	0.936293664
ATP5G1	-0.004798473	0.112296607	0.936293664
ADC	-0.007222211	0.112674557	0.936293664
QPCT	-0.016162109	0.112829186	0.936293664
DDX59	0.012935571	0.11290734	0.936293664
B4GALT7	-0.004565474	0.113105099	0.936293664
FRAT1	-0.00779203	0.113114996	0.936293664
PRTFDC1	0.012976915	0.113184399	0.936293664
GP9	0.018558558	0.113319812	0.936293664
BAIAP2L2	-0.007456793	0.113321137	0.936293664
TLR9	-0.009413997	0.113369466	0.936293664

TMEM169	-0.007370387	0.113381386	0.936293664
ADAP1	-0.003913472	0.113519334	0.936293664
MAPKAPK3	-0.005227946	0.113581047	0.936293664
ZNF213	-0.008265005	0.113768178	0.936293664
CTSA	0.008837832	0.113820169	0.936293664
TM9SF2	-0.003563282	0.113847492	0.936293664
ZNF219	-0.006080001	0.113864687	0.936293664
ACTR2	-0.004112056	0.114036591	0.936293664
KIF2A	0.007490459	0.114046278	0.936293664
MARVELD1	-0.007571593	0.11405314	0.936293664
UBE3A	-0.002878374	0.114102513	0.936293664
ARHGAP21	0.008050936	0.114108774	0.936293664
ODC1	0.010027237	0.114280764	0.936293664
NR2F2	0.006790206	0.114286192	0.936293664
ETFDH	-0.005917593	0.114348263	0.936293664
FOXD4	-0.007350209	0.11451368	0.936293664
OPA1	-0.005073122	0.114617658	0.936293664
ADAR	0.003801168	0.11466902	0.936293664
NEDD4L	-0.007285682	0.1147699	0.936293664
ABHD12	-0.013176517	0.1148252	0.936293664
EXOSC1	-0.002528404	0.114834101	0.936293664
EBAG9	0.003571164	0.114943721	0.936293664
AGPAT9	-0.006662813	0.114981923	0.936293664
MARCO	-0.015102107	0.115040352	0.936293664
SYNGR2	-0.003906268	0.115058945	0.936293664
CD72	0.013300666	0.115184053	0.936293664
PSIP1	0.004642454	0.11518625	0.936293664
PIAS1	0.004352089	0.115226373	0.936293664
SLC35F5	-0.007245099	0.115238876	0.936293664
CHN1	-0.013624993	0.115256201	0.936293664
FAM126A	-0.006250668	0.115273166	0.936293664
ADAT1	-0.006033287	0.115429935	0.936293664
MNDA	-0.017484234	0.115522591	0.936293664
PRICKLE1	0.011750844	0.115698053	0.936293664
DHRS11	0.005334095	0.115879442	0.936293664
DLD	-0.006339647	0.115900753	0.936293664
ZNF282	-0.006960581	0.115974161	0.936293664
ITCH	0.005831958	0.11597494	0.936293664
NFIB	0.024369893	0.116012137	0.936293664
CLN5	-0.006614367	0.116121638	0.936293664
SCARNA18	-0.005574003	0.116316039	0.936293664
IGF2R	-0.004308036	0.116330953	0.936293664
FBXO11	-0.003238746	0.116529672	0.936293664
RNF208	0.007305466	0.116850082	0.936293664
ABCB10	-0.004656436	0.116854284	0.936293664
BCS1L	-0.004897708	0.117077158	0.936293664
CYB5R2	-0.008573741	0.117124381	0.936293664

ARMC10	-0.005477863	0.117213783	0.936293664
RNF146	-0.006394597	0.117257102	0.936293664
FKBP1A	-0.004979011	0.11732318	0.936293664
HOXC13	-0.00638197	0.117450779	0.936293664
LPCAT3	-0.004436522	0.117509468	0.936293664
SNORA80	0.008325487	0.117675199	0.936293664
ALG8	-0.002798265	0.117742249	0.936293664
KIF23	-0.008152901	0.117868537	0.936293664
PPP2R2A	-0.005071104	0.117875241	0.936293664
GPX7	0.005872297	0.118052473	0.936293664
C1orf53	-0.009133727	0.118103432	0.936293664
CDC14B	0.012596128	0.118398726	0.936293664
C10orf32	-0.009009601	0.118431486	0.936293664
RB1	-0.008984431	0.118468715	0.936293664
SUV420H1	0.00465504	0.118538366	0.936293664
GYG1	-0.005092577	0.118641385	0.936293664
ZNF594	0.002152503	0.118655572	0.936293664
USP6	-0.007045516	0.118712809	0.936293664
ETV6	0.004243411	0.118753344	0.936293664
TBC1D5	0.006183317	0.118864685	0.936293664
PTMS	-0.010418498	0.118900364	0.936293664
ZNF549	0.006104033	0.118911901	0.936293664
CHRNA10	-0.006439801	0.119092768	0.936293664
TUBG2	-0.00551604	0.119104363	0.936293664
IMPDH1	-0.005408993	0.119134449	0.936293664
GAB2	-0.006082021	0.11924982	0.936293664
FAM50B	-0.009469819	0.119265633	0.936293664
AMOTL2	0.006493873	0.119381912	0.936293664
SNHG8	0.022585961	0.11941462	0.936293664
CHMP2B	-0.006582456	0.119473495	0.936293664
TMEM174	0.008133671	0.119494814	0.936293664
WRB	0.009294582	0.119922511	0.936293664
BCL10	-0.008525678	0.119973186	0.936293664
HCP5	-0.00815418	0.120049384	0.936293664
DCUN1D3	-0.006248425	0.12015012	0.936293664
YTHDF1	0.003143932	0.120555248	0.936293664
MBOAT7	-0.006198229	0.120615731	0.936293664
LIG1	0.007032547	0.120671791	0.936293664
EID1	0.007467701	0.120684997	0.936293664
TTC8	-0.006593629	0.120694304	0.936293664
YIPF3	-0.003019506	0.120860481	0.936293664
KHDRBS3	0.004993424	0.120880179	0.936293664
GCA	-0.006759544	0.120941577	0.936293664
C10orf88	-0.005971978	0.121036781	0.936293664
CYTL1	-0.009606674	0.121106563	0.936293664
FES	-0.006015622	0.121136829	0.936293664
IVD	-0.007130235	0.121265082	0.936293664

CCDC43	-0.004117549	0.121410866	0.936293664
PIGK	-0.005868465	0.121646392	0.936293664
EIF2A	-0.004117653	0.121907542	0.936293664
ACSL1	-0.007618877	0.122160137	0.936293664
TMEM207	-0.007437854	0.122406679	0.936293664
APPBP2	-0.002974367	0.122487883	0.936293664
PRKAR1A	-0.035236718	0.122775586	0.936293664
VPS4B	-0.003997295	0.122816444	0.936293664
PITPNM1	-0.004816752	0.122868672	0.936293664
FAM170B	0.00967165	0.122969918	0.936293664
RASL10A	-0.007862605	0.122991332	0.936293664
ANAPC16	0.006413019	0.123037647	0.936293664
AKR1B1	-0.004060263	0.123038339	0.936293664
ZC3H10	-0.007641278	0.123095163	0.936293664
ZDHHC19	0.008923118	0.123195862	0.936293664
C11orf82	-0.007989225	0.123315366	0.936293664
STAT6	-0.008378166	0.123318686	0.936293664
CCNE2	-0.008086992	0.123362119	0.936293664
RPS23	0.019168312	0.12340836	0.936293664
STT3A	-0.004602852	0.123662314	0.936293664
C6orf163	-0.007709938	0.123731783	0.936293664
EPHA8	0.006393007	0.12385553	0.936293664
NT5DC3	0.010522283	0.123923181	0.936293664
KIFC1	-0.008937797	0.124155689	0.936293664
NAA30	-0.004278315	0.124383527	0.936293664
HPGD	0.012347487	0.124619532	0.936293664
SNORD33	0.006700313	0.124630325	0.936293664
DNM2	0.005933367	0.124715841	0.936293664
THRAP3	0.005242329	0.124719816	0.936293664
TMEM126B	-0.003024223	0.124730419	0.936293664
AARS	-0.003385772	0.124763856	0.936293664
C1orf85	-0.009595487	0.124792738	0.936293664
FAM76A	0.008360632	0.124836252	0.936293664
EEFSEC	-0.00613398	0.124839578	0.936293664
CAMTA1	0.013019907	0.124881062	0.936293664
THAP3	0.006875069	0.124960317	0.936293664
HGS	-0.003763273	0.124989853	0.936293664
SP140L	0.005555093	0.125084289	0.936293664
CYB561D1	-0.003601425	0.125231549	0.936293664
RFC2	0.007691636	0.12536555	0.936293664
KLHL22	-0.00513746	0.125389984	0.936293664
POP1	-0.005590308	0.125411516	0.936293664
TIMP2	-0.006846313	0.125485444	0.936293664
PCMTD2	0.004748799	0.125504204	0.936293664
RIMKLB	0.006938144	0.125509111	0.936293664
ATP5SL	-0.003599183	0.125540562	0.936293664
HNRNPUL2	0.003056075	0.125580305	0.936293664

FASTKD2	-0.004890899	0.125644974	0.936293664
SLC35B1	-0.003579252	0.125877435	0.936293664
FOXRED1	-0.005587379	0.125943312	0.936293664
MEAF6	-0.006740313	0.125963153	0.936293664
PPAP2B	-0.009061064	0.126107065	0.936293664
GRK6	0.005831323	0.126172744	0.936293664
ZNF217	-0.006444194	0.126700437	0.936293664
PAWR	0.007999161	0.126801867	0.936293664
SHMT2	0.003883834	0.127020871	0.936293664
SGPP1	-0.007385541	0.127173013	0.936293664
NARS2	-0.00515223	0.127236075	0.936293664
FIGNL1	0.0089915	0.127238768	0.936293664
SPPL3	0.00535185	0.127308069	0.936293664
SF3B4	0.003287642	0.127391628	0.936293664
GGA2	0.004699691	0.127493458	0.936293664
RCC1	0.007376295	0.127601854	0.936293664
POTEE	0.009175601	0.127854573	0.936293664
MPV17	-0.003134852	0.12811371	0.936293664
EVI2B	-0.00836299	0.12815265	0.936293664
P2RX4	-0.005361449	0.128359516	0.936293664
ADRBK1	-0.003047721	0.128419307	0.936293664
ENPP2	-0.009115593	0.128497107	0.936293664
DCUN1D2	-0.005716576	0.128538321	0.936293664
H2AFY	-0.003032158	0.128808553	0.936293664
WASF3	0.017480719	0.128945404	0.936293664
UBIAD1	0.004969554	0.128967578	0.936293664
ZNF799	-0.006659619	0.129098182	0.936293664
UNC50	-0.003214536	0.129122335	0.936293664
SLC35A1	-0.003924769	0.12920213	0.936293664
INTS8	0.006623031	0.129461951	0.936293664
MDGA1	-0.011760554	0.129665347	0.936293664
ZDHHC5	-0.005451758	0.129675045	0.936293664
ERCC1	-0.004778934	0.129880548	0.936293664
DDX24	0.004355798	0.129911115	0.936293664
MACF1	0.005402377	0.129971337	0.936293664
MPL	0.019332877	0.130028084	0.936293664
VPREB3	0.017002006	0.130204789	0.936293664
ZNF2	-0.007414285	0.130267322	0.936293664
STK32B	-0.007508348	0.130298534	0.936293664
KIF20B	-0.006317143	0.130489981	0.936293664
FAM71E2	0.005893668	0.130598343	0.936293664
SYS1	-0.005061829	0.130748149	0.936293664
CSF1R	-0.005073086	0.13076885	0.936293664
WBSCR22	0.004130767	0.130893636	0.936293664
UBE2W	-0.006530867	0.130951586	0.936293664
EPHX1	-0.006220295	0.131063606	0.936293664
MEA1	0.003578915	0.131099566	0.936293664

CD101	-0.008894397	0.131561604	0.936293664
HLCS	-0.005768995	0.131685773	0.936293664
SPEF2	0.006481772	0.131737288	0.936293664
SOX15	-0.006993147	0.13195082	0.936293664
DDB2	0.006488026	0.132027283	0.936293664
PIK3CG	0.00743781	0.132048072	0.936293664
RNF4	-0.004541018	0.132197335	0.936293664
VAMP3	-0.005430813	0.132201281	0.936293664
TMEM30A	0.005121862	0.132414636	0.936293664
ZNF561	-0.006160729	0.132687381	0.936293664
COPB1	-0.003142485	0.13273597	0.936293664
LRPAP1	-0.004746986	0.132808387	0.936293664
MKL1	0.005663871	0.132867944	0.936293664
PNRC2	-0.003762519	0.13293789	0.936293664
DOK1	-0.010091694	0.133166495	0.936293664
DLG4	0.007872601	0.133190425	0.936293664
C6orf25	0.015631163	0.133223621	0.936293664
BLM	-0.006551268	0.133277921	0.936293664
CCDC112	-0.005515851	0.133298826	0.936293664
MAP4K2	0.003841375	0.133322933	0.936293664
ZNF410	0.002666821	0.133391711	0.936293664
AGTRAP	-0.008349766	0.133562075	0.936293664
FCGR1B	-0.018107917	0.133599251	0.936293664
HADHA	-0.005649802	0.133647242	0.936293664
HTRA1	-0.011376196	0.133677923	0.936293664
UBE2H	0.005290133	0.133682521	0.936293664
EFCAB4A	-0.010978784	0.133756924	0.936293664
DIS3L2	0.005373275	0.134114577	0.936293664
C6orf57	0.005143059	0.134307418	0.936293664
DGKG	0.006315452	0.134360936	0.936293664
PAPD5	0.005499921	0.134461858	0.936293664
KBTBD7	-0.006148246	0.13460761	0.936293664
TMEM87A	-0.002873371	0.134622812	0.936293664
CDH6	0.011312425	0.134738881	0.936293664
ALDH3A2	-0.004278822	0.134957402	0.936293664
NLE1	-0.006371911	0.135307282	0.936293664
DHRS13	-0.005949828	0.135374195	0.936293664
FAM84B	0.00707988	0.135385109	0.936293664
RNF11	0.010388124	0.135406047	0.936293664
SUPT6H	0.0045761	0.13546682	0.936293664
ZNF516	0.009780801	0.135535876	0.936293664
RPGRIP1	-0.010014981	0.135629158	0.936293664
EXOC5	-0.003944221	0.135715991	0.936293664
SNORD46	0.008264476	0.135716218	0.936293664
TBC1D22A	0.005796893	0.135744396	0.936293664
PLCB2	-0.006756628	0.135941811	0.936293664
TMEM63B	0.006868118	0.135950006	0.936293664

DYNLRB1	0.005727518	0.135993356	0.936293664
NDUFB5	-0.003750556	0.136047798	0.936293664
PLEKHA7	-0.006154904	0.136057824	0.936293664
ISG15	0.013077137	0.13613412	0.936293664
TRIM4	-0.005553058	0.136206908	0.936293664
CDC42SE1	0.007449866	0.136252313	0.936293664
RAB40C	-0.003443506	0.136302611	0.936293664
PROK2	-0.022402282	0.136325119	0.936293664
PDLIM1	0.012225973	0.136367258	0.936293664
PTPN23	-0.006068773	0.13640973	0.936293664
GDE1	0.004941872	0.13648332	0.936293664
NAV1	-0.007992502	0.136494572	0.936293664
RNLS	-0.004864468	0.136842766	0.936293664
CCNY	0.005525896	0.136946429	0.936293664
HCST	-0.004110507	0.136954687	0.936293664
MUC20	-0.014936419	0.136961686	0.936293664
C10orf128	0.012717173	0.1370009	0.936293664
CRHR1	0.006668898	0.137013647	0.936293664
BRIX1	0.004118252	0.137020802	0.936293664
AHSP	0.013682123	0.137029804	0.936293664
EMILIN2	-0.005948979	0.137224484	0.936293664
PHRF1	-0.004740314	0.137235451	0.936293664
RFESD	-0.005247288	0.137392585	0.936293664
DCUN1D1	-0.007376839	0.137429767	0.936293664
SPCS2	-0.004013369	0.137431059	0.936293664
TTL	-0.004015552	0.137440675	0.936293664
VAV1	-0.00425427	0.137521954	0.936293664
KBTBD6	-0.009321378	0.137531671	0.936293664
YEATS4	0.007718533	0.137606117	0.936293664
HADHB	-0.004761574	0.137676849	0.936293664
DLG5	-0.007862237	0.137745363	0.936293664
AGPAT6	0.005871429	0.137836624	0.936293664
FAM160B2	-0.005827754	0.13792275	0.936293664
PHIP	0.004267933	0.13795182	0.936293664
ELL3	0.008703902	0.138022128	0.936293664
BRPF3	-0.004224851	0.13807662	0.936293664
BTLA	0.008308721	0.138337555	0.936293664
EIF3B	-0.003604611	0.138348015	0.936293664
TPST1	0.009892921	0.138505116	0.936293664
HNRNPKP3	0.005592043	0.138522003	0.936293664
DIS3L	0.005384239	0.138562826	0.936293664
FASLG	-0.01566937	0.138615557	0.936293664
DPM3	0.004670674	0.138777862	0.936293664
HSD17B11	-0.008743643	0.13877872	0.936293664
ATP6V1A	-0.004531602	0.138815978	0.936293664
AKAP9	-0.006873448	0.138962821	0.936293664
SCRN3	-0.005284206	0.139242934	0.936293664

SHISA4	0.007241191	0.139259725	0.936293664
DGCR2	0.006083189	0.13928661	0.936293664
MAP2K1	-0.004495856	0.139389686	0.936293664
ENKUR	0.014269938	0.139419453	0.936293664
MED21	-0.008929922	0.139421249	0.936293664
SASS6	-0.004583209	0.139436317	0.936293664
VPS25	0.007537688	0.139449086	0.936293664
CCHCR1	0.006155417	0.139649941	0.936293664
PAPOLG	-0.006575085	0.139804271	0.936293664
SNORD35B	0.007582732	0.13990893	0.936293664
RPL23	0.002867863	0.140026813	0.936293664
ZNF518B	0.004450907	0.140117628	0.936293664
SLC16A5	-0.006363262	0.140230467	0.936293664
UVRAG	-0.004375663	0.140257977	0.936293664
TSPAN18	0.008731577	0.140340585	0.936293664
MGAT4A	0.005798035	0.140341516	0.936293664
MCMBP	-0.007499186	0.140457821	0.936293664
ST6GALNAC3	-0.00914802	0.140482598	0.936293664
C17orf80	-0.005920204	0.140596263	0.936293664
GOLGA7	-0.002711316	0.140647904	0.936293664
SCARNA22	-0.006147391	0.14074726	0.936293664
PPARG	-0.008440119	0.140861628	0.936293664
MRPL1	0.004929347	0.140988114	0.936293664
PGAP3	-0.003628572	0.141019897	0.936293664
RAD23B	0.007260453	0.141032346	0.936293664
IFNG	-0.021396901	0.141053413	0.936293664
SV2A	-0.007062428	0.141096128	0.936293664
ARL2BP	-0.004115606	0.141097032	0.936293664
HRK	0.011193875	0.14121895	0.936293664
UBE3C	-0.003756274	0.141434722	0.936293664
SELP	0.018887067	0.141450576	0.936293664
POLD3	-0.004932204	0.141507118	0.936293664
OSTM1	-0.004805965	0.141509166	0.936293664
DSTYK	-0.017122155	0.141808403	0.936293664
SNX30	-0.005402221	0.141845281	0.936293664
SIRPB1	-0.011706272	0.141872011	0.936293664
LY6E	0.008725507	0.141917823	0.936293664
HNRNPA2B1	0.002563635	0.141987331	0.936293664
RBM12B	-0.007426487	0.142043521	0.936293664
ESAM	0.016499839	0.142128598	0.936293664
PEX16	-0.003567392	0.142188498	0.936293664
TRAPPC4	-0.005219371	0.142330355	0.936293664
TOR1AIP1	-0.005974641	0.142402505	0.936293664
DYSF	-0.013044881	0.142700855	0.936293664
ACIN1	0.004053609	0.142757321	0.936293664
HAUS5	0.005708321	0.142817281	0.936293664
MLEC	-0.0059691	0.142827084	0.936293664

SCARNA3	0.014880186	0.142850071	0.936293664
MTF2	0.004894956	0.142957516	0.936293664
PROCR	-0.005978372	0.143021306	0.936293664
PAK1	-0.00563709	0.143048299	0.936293664
SNRNP48	-0.006436918	0.143186693	0.936293664
CDKAL1	0.007122125	0.143216865	0.936293664
VPS13B	0.009657586	0.143314659	0.936293664
HLA-DMB	-0.004111207	0.143528885	0.936293664
DPF2	-0.004291991	0.143681289	0.936293664
TCP10L	-0.005719055	0.14369582	0.936293664
HJURP	-0.00700074	0.143699964	0.936293664
ZFP91	0.007487581	0.143743738	0.936293664
MAK	-0.005969341	0.1439677	0.936293664
HLA-DOA	0.010299934	0.144046078	0.936293664
CCNG1	0.003563942	0.144061854	0.936293664
SNORA13	0.007031003	0.144092558	0.936293664
CCBL1	0.005302498	0.144129615	0.936293664
ARHGAP19	-0.003700862	0.144157257	0.936293664
LFNG	-0.005891133	0.144212542	0.936293664
OXSR1	0.002482059	0.144225035	0.936293664
NCAPH2	-0.004346639	0.144289821	0.936293664
PRMT1	0.00380808	0.1443944	0.936293664
PRR13	-0.002883375	0.144513976	0.936293664
KAT5	0.005233492	0.144518062	0.936293664
TTC9C	-0.005592587	0.144607661	0.936293664
CTSS	-0.005756949	0.144626472	0.936293664
PARP11	-0.005146497	0.144634742	0.936293664
AHCYL2	-0.004250048	0.144734596	0.936293664
SEC63	-0.005351352	0.144779401	0.936293664
PLAU	-0.004859572	0.144810981	0.936293664
RBM15	0.003569411	0.144921304	0.936293664
GCM1	0.005627957	0.144954476	0.936293664
CBX8	-0.00526374	0.14496707	0.936293664
SOCS6	0.007227461	0.145117264	0.936293664
GIMAP5	-0.005999245	0.145169091	0.936293664
OSGEPL1	-0.005835409	0.145184275	0.936293664
GMFB	-0.003339957	0.145224962	0.936293664
DDR2	0.008200696	0.145283321	0.936293664
C18orf25	0.003367279	0.145447159	0.936293664
MKLN1	0.003471401	0.145568314	0.936293664
VDAC3	-0.002450115	0.14565274	0.936293664
EYA3	0.010925597	0.145829885	0.936293664
MYSM1	-0.007955312	0.145850064	0.936293664
TNFRSF19	0.005740534	0.145890917	0.936293664
SNX29	0.014256711	0.145907371	0.936293664
APOBR	-0.005342079	0.145944316	0.936293664
SART1	-0.007547585	0.145973054	0.936293664

SCO1	-0.005670135	0.146021693	0.936293664
PTPMT1	-0.004778244	0.146145486	0.936293664
TRPM4	-0.01327626	0.146147482	0.936293664
PELI2	-0.006057657	0.146178708	0.936293664
NEFH	0.006607075	0.146218295	0.936293664
NFKB2	0.005990315	0.146303172	0.936293664
UEVLD	-0.005580853	0.146304965	0.936293664
TSPAN9	0.018609269	0.146324151	0.936293664
IFT57	0.007840074	0.146571304	0.936293664
ZNF613	-0.006988334	0.146579963	0.936293664
HYLS1	-0.006060917	0.146626211	0.936293664
SLFN12	-0.007085028	0.1466332	0.936293664
MGAT1	0.004352566	0.146672105	0.936293664
ASS1	0.005032105	0.146673168	0.936293664
ANKRD34A	-0.006379564	0.146791665	0.936293664
SAP130	-0.00493202	0.146832612	0.936293664
SLC25A40	-0.008248072	0.146880102	0.936293664
GGH	-0.006572106	0.146881839	0.936293664
ART1	0.005942549	0.146898964	0.936293664
TFPI	0.012938795	0.146918969	0.936293664
GTF2B	-0.005184414	0.14695244	0.936293664
BST2	0.005240837	0.147066993	0.936293664
TNPO3	0.006894637	0.147089049	0.936293664
PLXNC1	-0.0087441	0.147165827	0.936293664
ALX1	-0.006578524	0.147244695	0.936293664
SLC2A9	-0.01142774	0.147274311	0.936293664
GAA	0.009850234	0.147308981	0.936293664
TCTN1	0.007142855	0.147368371	0.936293664
ZNF107	0.005196175	0.147676236	0.936293664
TSC22D1	0.013008435	0.147840713	0.936293664
EPHA1	-0.01035601	0.147857595	0.936293664
TBC1D8	-0.007087114	0.147876935	0.936293664
USP2	-0.005361874	0.147987795	0.936293664
HDGFRP2	0.005931461	0.147993725	0.936293664
THNSL2	0.021817128	0.148032936	0.936293664
ZC3H6	-0.006575418	0.148068892	0.936293664
NBN	-0.005695129	0.148082992	0.936293664
CAV1	-0.010645394	0.148265901	0.936293664
AHCY	-0.004510879	0.148316262	0.936293664
ASL	-0.007665876	0.14837816	0.936293664
MPPE1	-0.006880919	0.148489214	0.936293664
WNT10B	-0.009069553	0.148498517	0.936293664
MRPL19	-0.00613763	0.148714789	0.936293664
DEF8	-0.005621645	0.14882528	0.936293664
USE1	0.005521839	0.148920628	0.936293664
SLC2A3	0.004644644	0.14913656	0.936293664
NUP54	-0.003067533	0.149271161	0.936293664

PCNXL3	0.00506779	0.149272549	0.936293664
RIN2	-0.007424216	0.149277262	0.936293664
PRPF4	0.002737632	0.149345071	0.936293664
NEK7	-0.006689267	0.149465125	0.936293664
C11orf57	0.003738742	0.14961561	0.936293664
TMEM184B	0.006050308	0.149940711	0.936293664
FITM1	0.005400737	0.149961289	0.936293664
TMEM107	0.025911923	0.15000177	0.936293664
PLK5	-0.005600344	0.150060435	0.936293664
LRRN3	-0.018645188	0.150127721	0.936293664
KLHL18	-0.004186059	0.150312426	0.936293664
ZBTB7A	0.005273875	0.150323981	0.936293664
EGOT	0.006241545	0.150454548	0.936293664
FGF2	0.005574867	0.150455661	0.936293664
TMEM170A	0.005170792	0.150458274	0.936293664
HYDIN	-0.005816371	0.15046869	0.936293664
FGD6	0.00624516	0.150494143	0.936293664
FYCO1	-0.005647202	0.150602316	0.936293664
HRSP12	-0.005354426	0.15064006	0.936293664
GALE	-0.004616327	0.150739748	0.936293664
TRAPPC10	0.004211353	0.150762562	0.936293664
TOR1AIP2	-0.00619527	0.150798159	0.936293664
NDUFV3	-0.006404475	0.150882408	0.936293664
EXOC1	-0.004884943	0.150917324	0.936293664
CLEC1B	0.01737773	0.150928482	0.936293664
FAM160A2	-0.005153837	0.150931031	0.936293664
CHTOP	-0.005198702	0.150992863	0.936293664
SRCAP	0.005767113	0.151225205	0.936293664
NEO1	0.004107693	0.15125415	0.936293664
NKPD1	0.01316036	0.151291005	0.936293664
HECTD3	-0.004196644	0.151314556	0.936293664
PKN2	0.008236033	0.151360948	0.936293664
SGCB	0.006338327	0.151376984	0.936293664
C10orf2	-0.005671703	0.151507329	0.936293664
TNFRSF14	-0.003741086	0.151587943	0.936293664
ZNF510	0.006279574	0.151706997	0.936293664
RAVER1	-0.005233465	0.151747495	0.936293664
NAA35	-0.004015961	0.151959852	0.936293664
IRF5	0.024455451	0.151968699	0.936293664
ALDH16A1	0.006118997	0.151995276	0.936293664
ERMAP	-0.007623516	0.15204085	0.936293664
TMEM59	-0.002756968	0.152051216	0.936293664
PPP2R3C	-0.00538323	0.152135622	0.936293664
KLHDC8B	-0.008311778	0.152207837	0.936293664
ZCCHC11	0.004958284	0.152314287	0.936293664
ITGA11	-0.006858166	0.152351302	0.936293664
GRAP2	0.010080787	0.152366727	0.936293664

GPR18	-0.012391015	0.152433023	0.936293664
UXS1	0.007666579	0.152566948	0.936293664
LAPTM4B	0.007649438	0.152577667	0.936293664
SMAD6	-0.005846591	0.152597232	0.936293664
MIPEP	0.004811882	0.152599806	0.936293664
FIS1	0.005023579	0.152623829	0.936293664
SLC38A7	-0.006990464	0.152698435	0.936293664
CTDSP1	-0.003394157	0.15272336	0.936293664
LIMS1	0.007002449	0.152778193	0.936293664
ARHGAP18	0.009173651	0.152866947	0.936293664
EEF1A2	0.010280624	0.152870531	0.936293664
RASGRP3	0.01076139	0.153032621	0.936293664
CDCA7L	0.007279791	0.153085082	0.936293664
LPXN	-0.003547602	0.153198091	0.936293664
TXNDC12	-0.003998155	0.153204624	0.936293664
C5orf30	-0.005059758	0.153253926	0.936293664
CPNE5	0.009587572	0.153263119	0.936293664
SLC43A3	0.005842591	0.153426144	0.936293664
CHAF1B	-0.006804573	0.153427943	0.936293664
PRSS8	0.006305292	0.153808413	0.936293664
ZNF876P	0.003250615	0.15387293	0.936293664
TLK1	0.007532149	0.15401002	0.936293664
SDF2	-0.003764712	0.154027082	0.936293664
ADSS	-0.003827808	0.154064765	0.936293664
PNKP	-0.00441667	0.15428789	0.936293664
SH3RF3	-0.005874362	0.154334026	0.936293664
HARS2	-0.004183368	0.154379738	0.936293664
NOC2L	0.005748183	0.154389724	0.936293664
C22orf34	0.012449697	0.154479962	0.936293664
TMEM209	-0.003868443	0.15453287	0.936293664
PEX10	-0.00459383	0.154539299	0.936293664
ACACB	0.00642432	0.154708678	0.936293664
CLASP2	-0.005705149	0.154719522	0.936293664
MTL5	-0.00954818	0.154722523	0.936293664
AQP9	-0.009677507	0.154746537	0.936293664
GNG11	0.013575981	0.154762351	0.936293664
ANK3	0.008901697	0.154823336	0.936293664
C19orf70	0.003990283	0.154922404	0.936293664
PIGV	-0.00924693	0.155071862	0.936293664
MAPRE1	-0.004275081	0.155275749	0.936293664
STOM	0.005739786	0.155418035	0.936293664
LRRC40	-0.007521296	0.1554586	0.936293664
OGDH	0.006234361	0.155569666	0.936293664
TAS2R30	0.008644605	0.155592221	0.936293664
FCGR2A	-0.007673867	0.155675085	0.936293664
LEPRE1	-0.00355103	0.155805156	0.936293664
ZNF83	0.008950894	0.155822957	0.936293664

ORC4	0.014809201	0.155847363	0.936293664
SAR1B	-0.005794805	0.155933794	0.936293664
TOMM22	0.002782622	0.156027287	0.936293664
CD1D	-0.00635794	0.156072659	0.936293664
FEZ2	0.006181961	0.156214166	0.936293664
CDKN2B	-0.009466751	0.156303987	0.936293664
TMEM168	-0.00553185	0.156596826	0.936293664
PPIF	0.005781934	0.156637971	0.936293664
KCNK17	0.01308702	0.156669379	0.936293664
FUZ	0.005515573	0.156824803	0.936293664
VAMP2	0.006285526	0.156943431	0.936293664
GATAD2B	0.006062099	0.156976177	0.936293664
PI4K2A	-0.004720009	0.15703975	0.936293664
GPBAR1	-0.007131448	0.157046038	0.936293664
DHX33	0.00428885	0.157233117	0.936293664
IFRD1	0.006006485	0.157307198	0.936293664
ALB	0.006339948	0.157356474	0.936293664
TMED10	-0.007567948	0.157668358	0.936293664
GLG1	-0.002777481	0.157676042	0.936293664
HOMER3	-0.007066566	0.157835369	0.936293664
COMMD3	-0.003004564	0.157836013	0.936293664
SH3GLB2	-0.004359373	0.15795527	0.936293664
SLC6A4	0.010234814	0.157985132	0.936293664
CD302	-0.012452997	0.157993886	0.936293664
EPB41L3	-0.007815676	0.158000741	0.936293664
ITPRIP	-0.003722295	0.158296391	0.936293664
ARHGEF2	0.004122199	0.158312221	0.936293664
SLC20A1	-0.005327366	0.158314494	0.936293664
PITRM1	-0.006086816	0.158431004	0.936293664
RHEB	-0.004005688	0.158505042	0.936293664
C1orf162	-0.003323745	0.158697489	0.936293664
LILRB2	-0.005079373	0.15872227	0.936293664
ZMYM1	0.004981567	0.158722647	0.936293664
C5orf20	-0.00867792	0.158760955	0.936293664
RIOK2	-0.004617894	0.158790881	0.936293664
DDX5	-0.002719353	0.158934246	0.936293664
CACNA1H	-0.009892685	0.158941906	0.936293664
SNAP23	0.006778784	0.159029304	0.936293664
PAK2	0.003005637	0.15908183	0.936293664
EOMES	-0.012309754	0.159102659	0.936293664
TCIRG1	-0.005424066	0.159149235	0.936293664
RGR	-0.006020425	0.159238759	0.936293664
CCDC77	-0.004632062	0.159312124	0.936293664
CCNC	-0.007620371	0.159579466	0.936293664
FAM117A	0.004789553	0.159603799	0.936293664
TIAM1	-0.007685731	0.159681923	0.936293664
NFATC3	0.005046623	0.159688551	0.936293664

TMX1	-0.00337357	0.159703944	0.936293664
ABCA10	-0.006490777	0.159741414	0.936293664
DIABLO	-0.002222808	0.159820198	0.936293664
SESTD1	-0.00735413	0.159950713	0.936293664
NCAN	0.005128718	0.160023374	0.936293664
UTP11L	-0.00494907	0.16008568	0.936293664
PRDX3	-0.008833276	0.160096266	0.936293664
NLRP12	-0.007128206	0.160265212	0.936293664
ARHGAP15	-0.003361501	0.160265754	0.936293664
GUCA1A	-0.005556932	0.160324424	0.936293664
APOM	-0.006681618	0.160397163	0.936293664
CD69	0.007130698	0.160503956	0.936293664
EZH2	0.00565278	0.160532177	0.936293664
TMEM115	-0.004143189	0.160570101	0.936293664
GTF2F2	-0.003895532	0.160603225	0.936293664
TTYH2	-0.006222705	0.16064639	0.936293664
ITGB3	0.022209469	0.160684754	0.936293664
MRPL12	0.004850966	0.160872743	0.936293664
HEMGN	0.013726934	0.160917902	0.936293664
JAM3	0.017359221	0.161097756	0.936293664
ZW10	-0.003327964	0.161180778	0.936293664
ACYP1	0.004496653	0.161214791	0.936293664
CDK5R2	-0.00516303	0.161222863	0.936293664
NUDT16L1	-0.004035261	0.161237415	0.936293664
PAPD7	0.004672066	0.161248544	0.936293664
TMEM175	-0.003564411	0.161284352	0.936293664
MLF2	0.004238407	0.161294746	0.936293664
RELL2	0.006003764	0.161327496	0.936293664
RCE1	-0.004128941	0.161518775	0.936293664
NIT2	0.004564226	0.161519721	0.936293664
TMEM128	0.014876548	0.161520215	0.936293664
ZNF174	-0.006061674	0.161569916	0.936293664
MLXIPL	-0.004601062	0.161715598	0.936293664
RAE1	-0.003467919	0.161716883	0.936293664
ALK	-0.006760242	0.16179882	0.936293664
MLX	-0.005199917	0.161814959	0.936293664
CSNK1G1	0.003972319	0.161974097	0.936724806
SLC23A3	-0.009848577	0.162079169	0.936842986
ATP8A1	0.005578216	0.162856448	0.938172392
UPF3A	0.006377399	0.162897199	0.938172392
CRISPLD2	-0.011290954	0.163032251	0.938172392
ST3GAL3	0.008770089	0.163177762	0.938172392
ZNF701	-0.006903754	0.163226121	0.938172392
CERK	-0.003728593	0.163236982	0.938172392
ZNF202	-0.005359221	0.163287426	0.938172392
CNNM2	0.006496771	0.163413062	0.938172392
CLEC16A	0.003240913	0.163441594	0.938172392

ATP5G3	-0.006489583	0.163744072	0.938172392
ITFG3	-0.004853723	0.163814928	0.938172392
LAMB2	-0.008018053	0.163820633	0.938172392
C19orf53	0.003488426	0.163857071	0.938172392
NR2C2	0.004730174	0.163894058	0.938172392
G3BP2	-0.003033312	0.163968937	0.938172392
TMEM135	-0.006112278	0.16399408	0.938172392
MIR586	-0.006185504	0.164031062	0.938172392
VIM	-0.004584556	0.16405008	0.938172392
AXL	-0.008697256	0.164217688	0.938172392
GRTP1	0.006163301	0.164341979	0.938172392
GNG7	0.007837739	0.164406593	0.938172392
STK16	-0.006271059	0.16441206	0.938172392
SRRM1	-0.002489356	0.164457987	0.938172392
GMPR2	-0.003700918	0.164524658	0.938172392
RUNX1	-0.003803067	0.164559214	0.938172392
HECW2	-0.005506644	0.164572923	0.938172392
SLC38A9	-0.004245798	0.164699596	0.938172392
RCN1	-0.005270022	0.164770502	0.938172392
ETNK1	-0.006087092	0.164797337	0.938172392
CNTROB	0.006318284	0.16498091	0.938172392
PDZD8	-0.005785013	0.165060024	0.938172392
DIP2B	0.003904195	0.165084006	0.938172392
PPP1R16B	0.005144577	0.165138721	0.938172392
PLEK2	0.004716515	0.165190893	0.938172392
HBD	0.015211712	0.165285512	0.938228375
ZNF165	0.006037368	0.165462259	0.938349632
FBLN5	0.010681829	0.165476419	0.938349632
FZD1	-0.006061219	0.165611308	0.938633675
CCBL2	0.003056407	0.165813294	0.939158389
C19orf44	-0.006203869	0.165886012	0.939158389
DCAKD	0.003591922	0.166082675	0.939158389
CDK5RAP3	-0.005344286	0.16617209	0.939158389
USP12	0.00609853	0.166223635	0.939158389
ZKSCAN3	-0.005057503	0.166302221	0.939158389
MIR155HG	-0.008253442	0.166546237	0.939158389
AK1	0.00888223	0.166753084	0.939158389
NTSR1	-0.013577201	0.166767942	0.939158389
TAGLN2	0.007333254	0.166837363	0.939158389
COL6A3	0.009916405	0.166885903	0.939158389
OR7E156P	0.005748692	0.166977661	0.939158389
SEMA4B	0.005366146	0.16705702	0.939158389
SERAC1	-0.006746331	0.167060207	0.939158389
DOPEY2	0.004307668	0.167128082	0.939158389
SEMA4A	-0.005779938	0.16712887	0.939158389
PIK3C2A	-0.008149373	0.167152759	0.939158389
PUF60	-0.00453928	0.167263475	0.939158389

SPEN	0.003299674	0.167493124	0.939158389
PKD1P1	0.004877662	0.167657382	0.939158389
GIGYF2	-0.004765285	0.167829006	0.939158389
QSER1	-0.004997533	0.167902377	0.939158389
G2E3	-0.00439773	0.167982456	0.939158389
TBC1D2B	-0.004755155	0.167984672	0.939158389
FNTB	-0.016324045	0.168211722	0.939158389
ZNF532	-0.007259262	0.168374289	0.939158389
BCAS4	0.007625872	0.168660579	0.939158389
HOXC4	-0.012226559	0.168798227	0.939158389
H2AFJ	0.00656419	0.168807825	0.939158389
ALKBH8	-0.007127712	0.168833124	0.939158389
CCDC88B	0.007399357	0.168990496	0.939158389
RASSF1	-0.006128492	0.169048479	0.939158389
TMEM216	-0.004512038	0.169143895	0.939158389
UCP2	-0.008335891	0.16915717	0.939158389
TYMP	-0.006050722	0.169342034	0.939158389
ZNF625-ZNF20	-0.006902719	0.1693787	0.939158389
MREG	0.005267213	0.169437718	0.939158389
CBX2	0.005823386	0.16952577	0.939158389
EXOC6	0.003125898	0.169557135	0.939158389
IER2	0.007449242	0.169626412	0.939158389
ACOT8	0.005419733	0.169658287	0.939158389
AURKB	-0.007960978	0.169675862	0.939158389
HSDL1	0.005305921	0.169677159	0.939158389
PHGDH	-0.012437746	0.169720586	0.939158389
GFM2	-0.004976996	0.169776821	0.939158389
HIPK3	-0.007620243	0.17008732	0.939158389
ATP6V0E2	-0.004680681	0.17046095	0.939158389
HSPB1	0.004632268	0.170583874	0.939158389
MYL5	0.008605719	0.170967428	0.939158389
SEL1L	-0.004430739	0.170968512	0.939158389
SNRPN	0.004489452	0.170975286	0.939158389
BCL2L11	0.006987221	0.171060351	0.939158389
ZNF227	-0.005255395	0.171126743	0.939158389
PRPS1L1	0.004685227	0.171170057	0.939158389
IQCK	0.005494931	0.171266576	0.939158389
BANK1	0.011705129	0.171287511	0.939158389
U2AF2	0.001931655	0.171423343	0.939158389
ZCCHC8	-0.003777615	0.171446047	0.939158389
ALDH4A1	-0.006556694	0.171514326	0.939158389
MMRN1	0.01610224	0.17168242	0.939158389
NOTCH1	-0.005212488	0.171748892	0.939158389
TFB2M	-0.004010375	0.171769359	0.939158389
BBC3	0.004463933	0.171788678	0.939158389
LRP10	0.003514059	0.171885587	0.939158389
DHCR7	0.005243373	0.171908776	0.939158389

WWP1	0.003375724	0.171951945	0.939158389
PPM1M	-0.003222579	0.171988859	0.939158389
POLR3B	-0.004789423	0.17212942	0.939158389
SPOCD1	0.018279894	0.172139692	0.939158389
CEP250	-0.005931702	0.172377775	0.939158389
ZBTB25	0.006100382	0.172390792	0.939158389
GPR124	-0.008302909	0.172598513	0.939158389
CD59	-0.00610268	0.172627006	0.939158389
DCLK3	-0.005777516	0.172843342	0.939158389
PPAPDC2	-0.00790824	0.172983282	0.939158389
ARV1	-0.00449638	0.173000188	0.939158389
OPA3	-0.006916007	0.173059243	0.939158389
REXO4	-0.004420877	0.17306591	0.939158389
PPP1R12B	-0.007158195	0.173076863	0.939158389
TOM1L2	-0.005177168	0.173142243	0.939158389
RNPEPL1	-0.006645543	0.173155624	0.939158389
SSR1	-0.004261009	0.173594602	0.939158389
USP37	0.005393885	0.173732975	0.939158389
CYB5R1	0.004606977	0.174195732	0.939158389
TMEM17	0.008066409	0.17428802	0.939158389
SNORA6	0.008353867	0.17429286	0.939158389
ABHD6	-0.004346254	0.174338658	0.939158389
GPA33	0.00598104	0.174429884	0.939158389
DYDC2	-0.007111307	0.174496393	0.939158389
ARCN1	-0.003014981	0.174603958	0.939158389
FAM129C	0.011320981	0.174614105	0.939158389
RPL13AP6	0.004814107	0.174630031	0.939158389
ZMYM2	-0.004229845	0.174633678	0.939158389
PPIH	0.003597862	0.174640856	0.939158389
LRRC37B	0.004098446	0.174723894	0.939158389
PAK6	-0.006551114	0.17477212	0.939158389
IFT27	0.005115268	0.174801981	0.939158389
LSM3	0.0054749	0.174834039	0.939158389
MTERFD1	-0.003523069	0.174883473	0.939158389
RPS24	0.00272226	0.175045794	0.939158389
SRSF2	-0.001906404	0.175060118	0.939158389
FAM69B	0.014034347	0.175222585	0.939158389
ARAP2	-0.004943946	0.175359696	0.939158389
ERCC4	0.005655154	0.175381758	0.939158389
TAL1	0.010867974	0.175467941	0.939158389
ZNF684	-0.005121107	0.175486725	0.939158389
SMG6	-0.005871237	0.175592544	0.939158389
ZBTB42	-0.005216372	0.1756065	0.939158389
PDGFB	0.009461981	0.175666961	0.939158389
HRASLS2	-0.015335423	0.175703478	0.939158389
BCL2L1	0.006789802	0.175889102	0.939158389
CCDC88A	-0.005563034	0.17593623	0.939158389

DNAJA1	-0.004264453	0.176127946	0.939158389
TELO2	-0.009987068	0.176156034	0.939158389
CDK7	-0.003019202	0.176258473	0.939158389
GLIS3	0.005673468	0.176649179	0.939158389
GRAMD1A	-0.005041723	0.176761808	0.939158389
DDX11	-0.016374835	0.176831126	0.939158389
EIF2AK1	0.003565743	0.176937909	0.939158389
CELSR3	0.009588454	0.176945574	0.939158389
TRAF3	-0.004694074	0.176985279	0.939158389
RNF138	0.008289034	0.177002116	0.939158389
RPL10A	-0.005116509	0.177076346	0.939158389
MYCN	-0.007235734	0.177152816	0.939158389
CHD6	0.003535961	0.177170426	0.939158389
MRPS10	0.004717936	0.177260757	0.939158389
GRPEL1	-0.004184602	0.177431971	0.939158389
GHDC	-0.004777909	0.177443792	0.939158389
ZDHHC23	0.00625388	0.177572683	0.939158389
C3orf52	-0.004515432	0.177578065	0.939158389
MYH3	-0.006362982	0.177604563	0.939158389
LXN	-0.006541697	0.177629397	0.939158389
SLC35B4	-0.006047656	0.177697323	0.939158389
MAD2L1	-0.006757767	0.177918141	0.939158389
UNC5CL	0.006049142	0.177978778	0.939158389
RUFY3	-0.004919239	0.178079787	0.939158389
NT5C2	-0.002479586	0.178108725	0.939158389
SPRED1	-0.008552085	0.178138409	0.939158389
EPT1	0.005462292	0.178287065	0.939158389
ABCB9	-0.012990561	0.17829689	0.939158389
KPNA5	0.007287643	0.178307708	0.939158389
BAK1	-0.006895146	0.178345786	0.939158389
FAM26F	0.015742604	0.178370547	0.939158389
SRFBP1	-0.006186708	0.178413378	0.939158389
WDR5B	-0.005956834	0.178515813	0.939158389
F3	-0.005312597	0.17858917	0.939158389
EIF4H	-0.003463976	0.178595278	0.939158389
NUPL2	-0.004726644	0.178683305	0.939158389
CYBRD1	-0.008244706	0.178894227	0.939158389
TOP3A	-0.005215778	0.179310923	0.939158389
DGKE	-0.006506807	0.179384044	0.939158389
C9orf40	-0.008114419	0.179537319	0.939158389
DCTN4	-0.003907244	0.179696206	0.939158389
BATF	-0.006754456	0.179714274	0.939158389
CAPZB	0.002635868	0.179745621	0.939158389
PGA4	-0.009133541	0.17986234	0.939158389
CCL3	-0.012881262	0.179888722	0.939158389
NOL12	-0.00349079	0.179896974	0.939158389
CCDC107	-0.005115748	0.180053222	0.939158389

YWHAQ	-0.003938984	0.180112461	0.939158389
ABLIM3	0.014593847	0.180461191	0.939158389
NTS	0.006405018	0.180598649	0.939158389
EIF2B5	-0.003919715	0.180599085	0.939158389
ELMO3	0.004857359	0.18067953	0.939158389
KTN1	0.005636905	0.180804116	0.939158389
SLC23A1	0.005305781	0.180927567	0.939158389
DNM1P35	0.006212023	0.181210437	0.939158389
RPL41	-0.001420206	0.181229414	0.939158389
KIAA0513	-0.004733518	0.18135667	0.939158389
SNORD42B	0.005255131	0.181390073	0.939158389
RNF216	0.003772084	0.181561444	0.939158389
HINT3	-0.005218489	0.181744454	0.939158389
TSPAN1	-0.006229648	0.181828575	0.939158389
BRF2	-0.005013967	0.181871015	0.939158389
COQ10B	-0.004822678	0.182033963	0.939158389
ARPC1A	0.002729746	0.182094804	0.939158389
PTPRU	-0.009679724	0.182124059	0.939158389
RGS10	0.005867643	0.182349176	0.939158389
MCFD2	-0.003903511	0.18237025	0.939158389
C1orf216	-0.005215574	0.182452779	0.939158389
PIF1	-0.006461677	0.182531829	0.939158389
FFAR2	-0.008470441	0.182550043	0.939158389
ASB7	-0.006447107	0.182659751	0.939158389
PRSS21	-0.011006986	0.182710799	0.939158389
LRSAM1	-0.003239507	0.182727573	0.939158389
ZNF622	-0.004058833	0.182780564	0.939158389
EIF3J	-0.004118759	0.182783409	0.939158389
GHITM	-0.002973076	0.182874807	0.939158389
RBPMS	-0.004988281	0.182895938	0.939158389
APPL2	-0.003883169	0.183003737	0.939158389
FAM159A	0.006264948	0.183058501	0.939158389
HCK	-0.00476623	0.183064798	0.939158389
REXO2	0.002964882	0.183074678	0.939158389
DDX10	0.016964673	0.183168543	0.939158389
C16orf72	-0.006418642	0.183409856	0.939158389
CDK2	0.003355702	0.183467539	0.939158389
XBP1	-0.004738697	0.183565885	0.939158389
AKR1C2	0.013360156	0.183613526	0.939158389
TSHZ3	-0.007556636	0.183697073	0.939158389
LTBP3	0.00755379	0.183730207	0.939158389
TCP11L1	-0.005409648	0.183919278	0.939158389
DMC1	0.005698639	0.184050263	0.939158389
PCSK4	-0.004998825	0.18418614	0.939158389
RXRA	-0.003169738	0.18438427	0.939158389
LRRC16A	0.007664125	0.184422843	0.939158389
CFD	-0.008279923	0.184430447	0.939158389

C15orf41	-0.004968393	0.184513216	0.939158389
TCL1A	0.017604894	0.184572857	0.939158389
NCKAP1	0.008263669	0.184660791	0.939158389
MDH1	-0.003235092	0.184724106	0.939158389
PANK4	-0.004030583	0.184821831	0.939158389
HUS1	-0.005328835	0.185133569	0.939158389
CYC1	-0.002914543	0.185313465	0.939158389
GDNF	0.006129959	0.185412267	0.939158389
ZFYVE26	-0.003332315	0.185474951	0.939158389
PPCS	-0.003961081	0.185513574	0.939158389
AP2M1	0.003534968	0.185550233	0.939158389
PYGO2	-0.00656182	0.185654895	0.939158389
DDX11L2	0.020287719	0.185705934	0.939158389
INPP5B	0.00603361	0.186152447	0.939158389
ZNF697	-0.007444402	0.186232577	0.939158389
AFF3	0.008424473	0.186318776	0.939158389
CD2AP	-0.005938398	0.186347798	0.939158389
SATB1	0.005785827	0.186424041	0.939158389
IDUA	-0.005710386	0.186446862	0.939158389
PLEKHM2	-0.00320794	0.186461215	0.939158389
JMJD8	0.002521511	0.186463108	0.939158389
H1FX	0.004969393	0.186466676	0.939158389
ENTPD7	-0.006146002	0.186486066	0.939158389
BBS2	0.005801879	0.186619588	0.939158389
GTPBP4	-0.002530171	0.186632068	0.939158389
FGFR3	0.00702841	0.18669068	0.939158389
NCBP2	-0.002716343	0.18669426	0.939158389
HSPA13	-0.005642135	0.186716922	0.939158389
RBM43	0.008795035	0.186814542	0.939158389
PLEKHA8P1	0.003927417	0.186836789	0.939158389
FAM109B	0.005852139	0.186860593	0.939158389
BTBD1	-0.004130308	0.186947817	0.939158389
CEP290	0.00612052	0.187000908	0.939158389
TMEM180	-0.00509654	0.187250934	0.939158389
RPLP1	0.003804702	0.187405565	0.939158389
PLBD1	-0.008784464	0.187415858	0.939158389
DSEL	0.006564476	0.187645448	0.939158389
SMYD3	0.004892328	0.187671297	0.939158389
AK4	-0.010908948	0.187696475	0.939158389
ZBTB34	-0.004236372	0.187741359	0.939158389
RAB3A	-0.0054053	0.187750318	0.939158389
THBS1	0.019412943	0.187915104	0.939158389
CCDC12	0.003020854	0.187974941	0.939158389
ZFP3	-0.004793423	0.188029731	0.939158389
DICER1	0.005056129	0.188045243	0.939158389
SH3RF2	-0.006407059	0.188060908	0.939158389
PRDM8	0.006380699	0.188088149	0.939158389

PRR3	-0.006784277	0.188210316	0.939158389
B3GALT4	-0.00529567	0.188251471	0.939158389
TBC1D23	-0.006300414	0.188345939	0.939158389
BAG4	-0.004679233	0.18871902	0.939158389
SLC30A7	0.005245723	0.188774341	0.939158389
CAPG	0.007424135	0.188973523	0.939158389
PCOLCE	0.005690841	0.189457603	0.939158389
GNB5	0.009226201	0.189617749	0.939158389
IGSF8	-0.005730283	0.189743976	0.939158389
EFNA1	-0.009656888	0.189838533	0.939158389
POTEKP	0.004951787	0.189842839	0.939158389
ZNF507	0.006637162	0.190321213	0.939158389
HOXB6	0.008782438	0.190350015	0.939158389
SLC8A3	0.013623106	0.19035508	0.939158389
RBM34	0.002917039	0.190371544	0.939158389
HAL	-0.008134	0.190430515	0.939158389
POLR2J	0.009930802	0.190442371	0.939158389
ARL5B	-0.008434804	0.190449103	0.939158389
PROS1	0.019903539	0.190465755	0.939158389
RNFT1	-0.006962053	0.190541415	0.939158389
RNASE10	0.004990918	0.19064995	0.939158389
MARCKSL1	0.004641186	0.190837979	0.939158389
UBTD1	0.006554093	0.191032629	0.939158389
TSPYL4	0.004879695	0.191133835	0.939158389
IGFBP2	0.006846611	0.19113545	0.939158389
TM9SF3	-0.003562013	0.191211999	0.939158389
SAR1A	0.004708737	0.191301599	0.939158389
LCP1	-0.002691704	0.191366043	0.939158389
COG6	-0.004199611	0.191401069	0.939158389
CNOT2	-0.003086145	0.191452118	0.939158389
FBLN1	0.013698745	0.191458014	0.939158389
MIER2	0.007733448	0.191506459	0.939158389
SLA	-0.007070491	0.191527066	0.939158389
FBXO6	-0.007325787	0.19153422	0.939158389
SEC23B	-0.004523059	0.191690495	0.939158389
BTAF1	-0.003570358	0.191749151	0.939158389
CYB5D1	0.005951926	0.191826148	0.939158389
CASD1	-0.005192167	0.192038995	0.939158389
SPATA5L1	-0.004782111	0.192064372	0.939158389
TACO1	-0.003275693	0.192242417	0.939158389
FAF2	-0.002993577	0.1922873	0.939158389
MYLK	0.013840103	0.192365876	0.939158389
GNAQ	0.009110149	0.192459103	0.939158389
PRDX6	0.006130342	0.192501564	0.939158389
GFM1	-0.003008041	0.19262005	0.939158389
TM2D1	-0.002637197	0.192658577	0.939158389
RRN3	-0.003746859	0.192694657	0.939158389

USP19	0.005606178	0.192704355	0.939158389
LGALS4	-0.005238488	0.192725258	0.939158389
EIF4ENIF1	0.003028718	0.192780994	0.939158389
FBXO15	0.006599042	0.192837691	0.939158389
NMRAL1	-0.005000219	0.192883408	0.939158389
TXN	-0.005623002	0.192906357	0.939158389
AP1B1	-0.003040026	0.192936184	0.939158389
PSMA6	-0.003062933	0.192940365	0.939158389
PCGF3	0.006767874	0.193002189	0.939158389
CACNA1I	0.009615006	0.193105746	0.939158389
SNX19	0.004422869	0.193177165	0.939158389
INTU	-0.006223855	0.193239051	0.939158389
ACAD9	-0.004845398	0.193273805	0.939158389
RPL31	0.003361508	0.19336947	0.939158389
UBAP2	-0.004901387	0.193531703	0.939158389
MGAT2	-0.004964659	0.193579474	0.939158389
PUS10	-0.00540446	0.193617503	0.939158389
ZNF197	-0.003877718	0.1936992	0.939158389
SLC31A2	-0.007975108	0.193864721	0.939158389
NLN	-0.006185304	0.193887416	0.939158389
EXOC4	0.003214803	0.193943184	0.939158389
SEC23IP	-0.00371093	0.193959455	0.939158389
NAA50	-0.005207292	0.194004779	0.939158389
C17orf74	0.004947568	0.194104599	0.939158389
CALM2	-0.003465697	0.194309519	0.939158389
HEBP1	-0.00400394	0.194500016	0.939158389
EBPL	0.007947807	0.194509056	0.939158389
KDSR	0.004696982	0.19457306	0.939158389
BRAT1	-0.002519743	0.194638788	0.939158389
FCER1G	-0.007164053	0.194752875	0.939158389
SRPR	-0.005452977	0.194830433	0.939158389
SNX22	0.00636448	0.195094031	0.939158389
LZTR1	-0.003758789	0.195181352	0.939158389
C11orf80	0.007450745	0.195420347	0.939158389
ACTR3B	0.006530382	0.195448629	0.939158389
SNORD36A	0.005036168	0.195464903	0.939158389
GHRL	-0.00710241	0.195483764	0.939158389
FAM53B	0.005654858	0.195521013	0.939158389
PPP4R1	-0.003079855	0.195681127	0.939158389
FAM73B	0.005190782	0.195726555	0.939158389
MAD1L1	0.007972672	0.195788981	0.939158389
USP35	-0.004797141	0.195831547	0.939158389
ALDH7A1	-0.008378866	0.195859277	0.939158389
NDUFA6	0.004495914	0.195867645	0.939158389
TRA2A	0.007674662	0.195884788	0.939158389
SLC6A16	0.006506192	0.195890978	0.939158389
VENTX	-0.008376121	0.196059609	0.939158389

RAD51L3-RFFL	0.003697924	0.196291024	0.939158389
CYFIP1	-0.006198105	0.196347713	0.939158389
CLDN5	0.015410227	0.196349166	0.939158389
TNNC1	-0.004632198	0.196472218	0.939158389
PBX4	0.006552211	0.196473766	0.939158389
CDC42EP3	-0.009034298	0.196483142	0.939158389
EPHB4	-0.007968548	0.196558418	0.939158389
PSMB6	-0.003409041	0.196850231	0.939158389
C17orf51	0.005796922	0.196862482	0.939158389
FAM103A1	-0.004859918	0.196925277	0.939158389
TMEM117	0.005804894	0.196965874	0.939158389
ZNF503	-0.007726966	0.197049951	0.939158389
HOXB2	-0.01543593	0.197078598	0.939158389
STARD7	-0.002479311	0.197166311	0.939158389
FERMT3	0.00446908	0.197169734	0.939158389
EME1	0.00495916	0.197242336	0.939158389
DUSP11	-0.003949485	0.197537386	0.939158389
VAV3	0.046613553	0.197815054	0.939158389
KLF9	0.005531981	0.198080421	0.939158389
TP53RK	-0.005195801	0.198185177	0.939158389
KDM1A	0.002796976	0.198228999	0.939158389
STAM2	-0.005614683	0.198278087	0.939158389
HAS3	-0.006538155	0.198337794	0.939158389
RPRD1B	-0.004141984	0.198383468	0.939158389
LMNB2	0.00432139	0.198549509	0.939158389
RABGGTA	0.00490636	0.198610968	0.939158389
PHF12	-0.003837845	0.198655196	0.939158389
SEC24C	-0.004005041	0.198790337	0.939158389
PNP	-0.005581534	0.199251856	0.939158389
TBP	-0.003202994	0.199499391	0.939158389
RARS	-0.002407705	0.199604426	0.939158389
TRABD	-0.002934757	0.199642572	0.939158389
GTSF1	0.009714027	0.199648533	0.939158389
SUV39H2	0.00630739	0.199729439	0.939158389
DUS4L	0.003838678	0.199970702	0.939158389
BROX	-0.006637934	0.200123621	0.939158389
SLC25A10	0.005824443	0.200124314	0.939158389
PLIN2	-0.005192274	0.200180319	0.939158389
DGUOK	0.003249764	0.200282459	0.939158389
TSPO	-0.003848382	0.200501798	0.939158389
ELK4	0.00587781	0.200609428	0.939158389
ATG4D	0.004837956	0.200741514	0.939158389
QSOX2	0.00478221	0.200829105	0.939158389
TMEM181	0.005558303	0.20083372	0.939158389
EIF3C	-0.007595735	0.200901695	0.939158389
SEPHS1	-0.002967762	0.200969109	0.939158389
H6PD	0.007441577	0.200996702	0.939158389

SEMA3C	-0.007357953	0.201001876	0.939158389
TLN1	0.005868095	0.2010742	0.939158389
MAP4K5	0.003996395	0.201091288	0.939158389
LRRC45	0.006579059	0.20129854	0.939158389
NFAM1	-0.006474195	0.201472372	0.939158389
SHB	0.004091278	0.201503411	0.939158389
H3F3B	0.007497024	0.201507896	0.939158389
GSTO1	0.004196608	0.201541754	0.939158389
LIN7C	-0.006946657	0.201613557	0.939158389
MKRN1	0.002194882	0.20162649	0.939158389
HNRNPA0	-0.004075939	0.201649024	0.939158389
SGK223	-0.005124331	0.20187906	0.939158389
SNORD12C	0.008580363	0.201923571	0.939158389
DHX57	0.009079312	0.201973929	0.939158389
CEBPZ	0.003327527	0.202011899	0.939158389
DNAJB12	-0.004596039	0.202046963	0.939158389
ACD	-0.005021846	0.202137253	0.939158389
ZNF134	-0.006250533	0.202596301	0.939158389
IGKC	0.012806967	0.202692086	0.939158389
LAMTOR3	-0.003852846	0.202715117	0.939158389
VAPA	0.00742833	0.20288697	0.939158389
MARCH9	0.005071539	0.202961104	0.939158389
C16orf86	0.005037543	0.203179906	0.939158389
CHD7	0.005357063	0.203304286	0.939158389
TP53I3	0.006708081	0.203361432	0.939158389
MRPS16	0.002943241	0.203399117	0.939158389
BTBD11	-0.007361199	0.203411786	0.939158389
OSGEP	-0.003533873	0.203526702	0.939158389
PRKCD	-0.002742049	0.203627688	0.939158389
JMJD6	-0.005124523	0.203848012	0.939158389
COMMD10	-0.005252482	0.203859863	0.939158389
APOBEC3C	-0.005463119	0.203907698	0.939158389
VPS4A	0.003468787	0.204020601	0.939158389
TUBA3C	-0.004833409	0.204042292	0.939158389
SLC35B3	-0.004845403	0.204093745	0.939158389
SPINK2	-0.008503917	0.204290413	0.939158389
ENHO	0.005325455	0.204328687	0.939158389
GEMIN4	-0.003330908	0.204354737	0.939158389
SMARCA2	0.002677249	0.204456154	0.939158389
KCNT1	-0.012358836	0.204483524	0.939158389
KCNJ2	-0.012434047	0.204587569	0.939158389
C1orf50	-0.003282316	0.204615848	0.939158389
ZC3H3	-0.004023443	0.204690783	0.939158389
PDIK1L	-0.004580815	0.204792036	0.939158389
MIB2	0.004769286	0.204947675	0.939158389
ZNF544	-0.004240661	0.204975979	0.939158389
PLAUR	-0.007061232	0.205095024	0.939158389

LNX2	-0.005908437	0.205249274	0.939158389
SEC61B	-0.003302965	0.2053227	0.939158389
PITPNC1	0.003476337	0.205532184	0.939158389
TNC	0.0099942	0.205782506	0.939158389
TAOK3	0.002852	0.20581555	0.939158389
PIAS3	0.003686661	0.205829717	0.939158389
DOK3	-0.00543255	0.205897238	0.939158389
LMAN2	-0.005829394	0.205938293	0.939158389
ITFG1	-0.004674245	0.206275635	0.939158389
DBN1	-0.009658101	0.206515191	0.939158389
SETDB1	-0.003764204	0.20654395	0.939158389
TMEM158	0.015183704	0.206696974	0.939158389
DDX55	-0.004017787	0.206703537	0.939158389
ANKRD39	-0.004162666	0.206819011	0.939158389
SLC25A23	0.005485138	0.206850191	0.939158389
NEDD9	-0.00649605	0.206857339	0.939158389
FAM195A	0.005699907	0.206910505	0.939158389
CXCL5	0.017832486	0.20710216	0.939158389
RAP2A	-0.007023947	0.20737547	0.939158389
CDCA4	0.004314979	0.207497151	0.939158389
SNORD21	0.008447144	0.207553151	0.939158389
NUDT21	-0.003566299	0.207633863	0.939158389
CTIF	0.005452895	0.207646787	0.939158389
KIAA1279	-0.005249758	0.207671235	0.939158389
GUK1	0.002777158	0.207754267	0.939158389
MRPL15	0.004814043	0.207769098	0.939158389
DARS2	-0.004500817	0.20785698	0.939158389
EVL	-0.002998228	0.207886065	0.939158389
TGIF2	0.002997445	0.207926249	0.939158389
SDF2L1	-0.004702075	0.207935567	0.939158389
EFCAB7	0.005006393	0.207985441	0.939158389
BMS1	0.002392589	0.208080166	0.939158389
MEGF9	-0.007998267	0.208101608	0.939158389
PUS7	-0.005971674	0.208150244	0.939158389
STYX	0.003686284	0.208227875	0.939158389
RAB22A	-0.004242277	0.20831491	0.939158389
LSM11	0.004740878	0.208392598	0.939158389
C8orf48	-0.004669111	0.208398286	0.939158389
ATAD1	-0.004551442	0.208411442	0.939158389
ELL	-0.003755071	0.208412358	0.939158389
CCNK	-0.002740086	0.208506637	0.939158389
CAT	-0.006610986	0.20851083	0.939158389
MPHOSPH8	0.005324165	0.208575646	0.939158389
HOMER2	0.01347913	0.20887877	0.939158389
KLHL33	-0.005246499	0.208944053	0.939158389
SLC26A6	-0.004351654	0.208970159	0.939158389
PIGL	0.005195894	0.209006852	0.939158389

COPS8	-0.004365116	0.209041109	0.939158389
TSEN2	-0.005169954	0.209066234	0.939158389
DSC2	-0.012199491	0.209128972	0.939158389
PHACTR2	0.005177173	0.209319688	0.939158389
FUS	-0.00328121	0.209449766	0.939158389
FAM49A	-0.004714641	0.209465759	0.939158389
IFNGR2	-0.004854185	0.209485642	0.939158389
PPAP2C	-0.006712201	0.209508526	0.939158389
CTU1	0.005190938	0.209532346	0.939158389
ELMOD2	-0.005863346	0.209582637	0.939158389
SNORA59A	-0.00534199	0.209707762	0.939158389
TBC1D17	-0.005396958	0.209739352	0.939158389
DNAH2	0.004482208	0.209766907	0.939158389
UBR4	0.003236661	0.209819787	0.939158389
DTX2	-0.00312186	0.209962055	0.939158389
ZNF426	-0.004487698	0.210113383	0.939158389
DHDDS	-0.004984828	0.210208243	0.939158389
PEX2	-0.004172329	0.210493548	0.939158389
NFE2L2	-0.005209162	0.210611516	0.939158389
COMT	-0.005225227	0.210646254	0.939158389
DHX35	-0.003554653	0.210715635	0.939158389
KIAA0922	0.004345299	0.210726506	0.939158389
LILRB3	0.001857883	0.210798901	0.939158389
RBM23	-0.003879254	0.210856866	0.939158389
SNX33	0.004468628	0.210870128	0.939158389
PGBD2	-0.005243316	0.210920929	0.939158389
ATP5D	0.003718851	0.210951626	0.939158389
WDR82	0.003524361	0.210974102	0.939158389
TYROBP	-0.006074232	0.210984284	0.939158389
CTAGE5	-0.00493325	0.211028327	0.939158389
BOLA3	0.004245229	0.211043624	0.939158389
TRIM33	0.002853244	0.2110974	0.939158389
ANKLE1	-0.00994974	0.211107582	0.939158389
SNORD11	0.007213391	0.211379279	0.939158389
VIPR2	0.007402676	0.211552446	0.939158389
TRPS1	-0.005914834	0.211631504	0.939158389
GET4	0.00479429	0.211636682	0.939158389
POLR2I	0.003326105	0.211670744	0.939158389
NCAM1	0.010702407	0.211721838	0.939158389
STRADA	-0.002871008	0.211755642	0.939158389
FOXJ2	0.002005467	0.212036213	0.939158389
FOXO1	0.004235468	0.212171001	0.939158389
YIPF5	-0.007642761	0.212189661	0.939158389
DAP	0.005877647	0.212241376	0.939158389
EML2	0.005259153	0.212397039	0.939158389
KIFC3	0.0120371	0.212449902	0.939158389
FUT6	0.014018695	0.21257366	0.939158389

FOXP4	0.005539887	0.212643605	0.939158389
ROCK1P1	-0.005605513	0.212651746	0.939158389
SMUG1	-0.002617662	0.21267898	0.939158389
CD9	0.016298615	0.212815097	0.939158389
ZBTB22	0.002879443	0.212858188	0.939158389
SMC3	-0.002705704	0.212973345	0.939158389
TPM4	0.007948936	0.213118198	0.939158389
CBX4	0.003814014	0.213176259	0.939158389
SNORD114-2	0.004567046	0.213292791	0.939158389
FDX1	-0.005595424	0.213334915	0.939158389
GLCE	-0.004214698	0.213397773	0.939158389
TBC1D10A	0.003869624	0.213472363	0.939158389
STRN3	0.004824009	0.213507931	0.939158389
ASGR1	-0.004740442	0.213744384	0.939158389
CPT1B	-0.009343324	0.213811754	0.939158389
NCOA7	0.003344291	0.213852846	0.939158389
PEX11G	-0.006904089	0.213904199	0.939158389
CSNK2A2	-0.003286984	0.213934687	0.939158389
STAT3	0.003354033	0.214092044	0.939158389
FAR1	-0.004293936	0.214129454	0.939158389
ITGA2B	0.018838477	0.214196361	0.939158389
HMGCL	-0.004042213	0.21420908	0.939158389
POGK	-0.003070882	0.214263106	0.939158389
DCP2	-0.00393641	0.214265523	0.939158389
RRBP1	-0.004106684	0.214479432	0.939158389
PDP2	0.005839944	0.214540085	0.939158389
CASP7	-0.006250274	0.214553373	0.939158389
NLRP1	0.005125018	0.21466536	0.939158389
FGGY	-0.0066099	0.21472389	0.939158389
MBD6	-0.005908924	0.214767203	0.939158389
GP6	0.016263653	0.214790946	0.939158389
CYHR1	0.00535963	0.214960324	0.939158389
PPM1A	0.004300185	0.214994276	0.939158389
L2HGDH	0.0104584	0.214994395	0.939158389
KIF24	0.005358605	0.214999867	0.939158389
SNORD59A	0.006487094	0.215063999	0.939158389
CENPN	-0.009294019	0.215197004	0.939158389
SPAG16	0.005730788	0.215241326	0.939158389
GGNBP2	-0.003120929	0.215472141	0.939158389
SSTR1	-0.004581004	0.215780583	0.939158389
IGF1R	0.005531451	0.215856969	0.939158389
PDCD11	-0.004718543	0.2158779	0.939158389
SKIL	-0.004908241	0.215967363	0.939158389
CEBPB	-0.003488451	0.215967693	0.939158389
GLDC	-0.014049137	0.215970128	0.939158389
FAM175B	-0.002621246	0.216125025	0.939158389
FGR	-0.002511185	0.21614838	0.939158389

NAGLU	-0.004083769	0.216185068	0.939158389
RBBP9	0.003661914	0.216189499	0.939158389
CYCS	-0.005790504	0.216200001	0.939158389
PPRC1	-0.00339562	0.216226533	0.939158389
TSFM	0.003920752	0.216375532	0.939158389
UBQLN1	0.002397472	0.216534916	0.939158389
KCNJ15	-0.005345025	0.216709285	0.939158389
POLR1E	-0.003983429	0.216714915	0.939158389
METTL8	0.00516584	0.216801078	0.939158389
SH3GLB1	-0.004105341	0.216862556	0.939158389
TMPO	-0.006618449	0.216881007	0.939158389
TMED2	-0.004095339	0.216898418	0.939158389
COMMD8	-0.005855237	0.217121959	0.939158389
ENO3	-0.004953928	0.217242786	0.939158389
PABPC1	0.006986923	0.217248627	0.939158389
KRT72	0.027529282	0.217334241	0.939158389
NRBP2	-0.004530536	0.217389982	0.939158389
DCTN2	-0.002770477	0.217426557	0.939158389
RAB12	0.01113312	0.217557573	0.939158389
ATP6V1C1	-0.004521674	0.217582809	0.939158389
DPH2	-0.002522288	0.217587691	0.939158389
BIRC6	-0.003731844	0.217602206	0.939158389
MYPOP	-0.00222853	0.217615284	0.939158389
GATSL3	0.004402155	0.217813817	0.939158389
ARPC5L	0.003689018	0.217830039	0.939158389
PPP2R1A	0.003484429	0.217984114	0.939158389
CDKN1A	0.010005257	0.218040728	0.939158389
TIMELESS	0.004529383	0.218042212	0.939158389
KLF12	0.003558224	0.218062921	0.939158389
PSME4	0.002932606	0.218182971	0.939158389
IPO11	-0.004532802	0.218252309	0.939158389
MPI	0.00568571	0.218270924	0.939158389
MSL1	0.003848257	0.218410027	0.939158389
MAF	0.008228675	0.218458236	0.939158389
ZDHHC16	-0.003008729	0.218506955	0.939158389
INPP1	-0.004548022	0.218636307	0.939158389
IRF2	-0.005502862	0.218699914	0.939158389
NCKAP5L	-0.005564264	0.218724397	0.939158389
YLPM1	-0.004637549	0.218731342	0.939158389
NR3C2	0.004379262	0.218765597	0.939158389
BBS7	-0.003754781	0.21902034	0.939158389
EP400NL	0.007416132	0.219024	0.939158389
PACRGL	0.006261036	0.219052475	0.939158389
ACAD8	-0.003401146	0.219084254	0.939158389
DDX20	-0.005081031	0.219280098	0.939158389
NOL10	0.005774451	0.219438301	0.939158389
MYOM2	-0.037792115	0.219480459	0.939158389

USP36	0.008838743	0.219515887	0.939158389
DYRK4	-0.003059145	0.219516037	0.939158389
USP16	-0.00278704	0.219542016	0.939158389
SSTR2	0.004743896	0.219575488	0.939158389
ZZEF1	0.002497809	0.219827115	0.939158389
CES2	-0.003219912	0.219934574	0.939158389
ANTXR1	-0.004605061	0.220581615	0.939158389
METTL3	-0.002692804	0.220588168	0.939158389
SNRNP70	-0.003730445	0.220704136	0.939158389
SUCLG1	-0.00336014	0.220806023	0.939158389
DBNDD1	0.011902232	0.220862468	0.939158389
ABCE1	-0.002643262	0.220903404	0.939158389
FAM101B	0.007871128	0.220943336	0.939158389
ZFC3H1	0.00410979	0.220956958	0.939158389
ESM1	-0.008440072	0.220962799	0.939158389
MFAP1	-0.005666632	0.22125764	0.939158389
ABCC1	0.005181368	0.22145498	0.939158389
USP33	0.004132937	0.221649016	0.939158389
ADAM28	0.008453043	0.221707432	0.939158389
WFDC3	-0.00778595	0.221743835	0.939158389
SPRYD4	-0.005595374	0.221928463	0.939158389
UBAC1	0.002819409	0.22197364	0.939158389
KIDINS220	-0.003163468	0.222046394	0.939158389
RPS3A	0.003099421	0.222084885	0.939158389
EAF1	0.003977332	0.222390963	0.939158389
NXT1	0.002839373	0.222435579	0.939158389
MBD1	0.003767012	0.222463538	0.939158389
ZNF304	-0.004919366	0.222509018	0.939158389
MAPK3	-0.003666808	0.22269303	0.939158389
B4GALT5	-0.004888503	0.222706367	0.939158389
MTHFD1L	-0.006063075	0.222808139	0.939158389
ZSCAN12	-0.006437392	0.222851623	0.939158389
FURIN	0.00520754	0.222961641	0.939158389
GNAI3	-0.006680427	0.223065312	0.939158389
CPVL	-0.007375428	0.223194461	0.939158389
ZNF444	-0.004854441	0.223369157	0.939158389
LPIN2	0.004263092	0.22340668	0.939158389
HBP1	-0.002964251	0.223437676	0.939158389
YWHAZ	0.00263521	0.223467265	0.939158389
SNORD110	0.004990612	0.223501192	0.939158389
ZNF627	-0.003454449	0.223630252	0.939158389
RAP1B	0.005309712	0.223697971	0.939158389
BMP6	0.012360573	0.223701432	0.939158389
C9orf91	0.004027653	0.223774155	0.939158389
ARHGAP31	-0.006105927	0.223802902	0.939158389
CSNK1G3	-0.001997974	0.223817539	0.939158389
MUC4	0.005184569	0.223831176	0.939158389

SATB2	-0.005775226	0.223838275	0.939158389
CEP135	0.00275472	0.22410297	0.939158389
KCND3	0.007162099	0.224162191	0.939158389
ASCC3	-0.003558513	0.224330824	0.939158389
TUBA4A	0.005889026	0.224434295	0.939158389
EEF2	-0.002463868	0.224591169	0.939158389
ABHD8	-0.003948135	0.224600296	0.939158389
SPC25	0.010501234	0.224630202	0.939158389
PTPN7	-0.005424387	0.224753866	0.939158389
SNORD35A	0.009128513	0.224755657	0.939158389
ITIH5	-0.00146871	0.224761156	0.939158389
PDE3B	-0.005874479	0.224944808	0.939158389
TNK2	0.004218356	0.225104154	0.939158389
C20orf27	-0.007402002	0.225105737	0.939158389
IQCC	-0.003880898	0.225142372	0.939158389
TAF12	-0.004327029	0.22519845	0.939158389
RNF103	-0.004599646	0.225383344	0.939158389
PGM3	-0.004430009	0.225560253	0.939158389
PCGF5	0.005344531	0.22556986	0.939158389
C9orf72	0.006198174	0.225639797	0.939158389
TBC1D2	-0.005562031	0.225680113	0.939158389
NEAT1	0.006632696	0.22583995	0.939158389
BIRC5	-0.00841667	0.225947443	0.939158389
C7orf26	-0.003127566	0.226052528	0.939158389
PBX1	0.010540773	0.226099349	0.939158389
ZFYVE27	-0.005122419	0.226135534	0.939158389
PLD1	-0.004768268	0.226185946	0.939158389
CCDC150	-0.005081151	0.226222064	0.939158389
ORMDL3	0.00768101	0.226277773	0.939158389
DCAF11	-0.005757552	0.226343781	0.939158389
GNA13	0.00285615	0.226540495	0.939158389
POMGNT1	-0.002595777	0.22667958	0.939158389
F5	-0.010416784	0.226776126	0.939158389
HP1BP3	0.004631757	0.226890468	0.939158389
PACS2	0.004775426	0.226970224	0.939158389
C19orf54	-0.004758608	0.227020219	0.939158389
HIST1H1D	-0.007278613	0.227097795	0.939158389
NAP1L1	0.003420126	0.227141351	0.939158389
MRFAP1	-0.002876314	0.227188446	0.939158389
TNFRSF8	-0.005556128	0.227289695	0.939158389
ATF5	0.004408206	0.22741825	0.939158389
ANO10	-0.004755966	0.227449988	0.939158389
SH3RF1	-0.005987975	0.227536595	0.939158389
SUDS3	0.00669948	0.227740724	0.939158389
SRSF7	-0.003550765	0.227839337	0.939158389
GIMAP8	-0.005216871	0.227904414	0.939158389
CCL3L3	-0.016139419	0.227931502	0.939158389

EPN1	-0.002801351	0.22802621	0.939158389
GAPVD1	0.00265645	0.228172751	0.939158389
ORAI1	0.005418556	0.228186556	0.939158389
C2orf82	-0.004148372	0.228346846	0.939158389
AATK	-0.005022378	0.228347204	0.939158389
FASTK	-0.002615787	0.228389133	0.939158389
ZKSCAN2	-0.004426714	0.228599148	0.939158389
E2F6	-0.00519699	0.228931958	0.939158389
KLHDC10	-0.00674444	0.228936068	0.939158389
GPR171	0.006134977	0.229407294	0.939158389
CENPJ	0.004525584	0.229412938	0.939158389
FAM192A	-0.003437893	0.229584804	0.939158389
TRAM1	-0.001996376	0.229596957	0.939158389
NCEH1	-0.003869839	0.229621438	0.939158389
ZNF79	-0.007927603	0.229783663	0.939158389
FAM8A1	0.004416117	0.230091807	0.939158389
ST6GALNAC6	0.005878092	0.230203013	0.939158389
PDCL	-0.006081579	0.230215787	0.939158389
NDUFS2	-0.003766028	0.230248248	0.939158389
SLC45A3	0.008585209	0.230323483	0.939158389
UBE2M	0.002681065	0.230360622	0.939158389
KRT8	0.004846827	0.230434274	0.939158389
C1orf229	0.011706775	0.230439842	0.939158389
INPPL1	-0.003551018	0.23045344	0.939158389
GPX1	0.006557237	0.230458469	0.939158389
UBE2V1	-0.004771845	0.230606926	0.939158389
SIK1	0.009169895	0.230640691	0.939158389
ZBTB48	-0.003447092	0.230677677	0.939158389
HERC4	-0.002531495	0.230846208	0.939158389
DHRS9	-0.012458282	0.23093538	0.939158389
JOSD1	0.003889675	0.231090616	0.939158389
RPS18	0.003895099	0.231137382	0.939158389
CCT6B	-0.005076832	0.231148264	0.939158389
C9orf64	-0.004359791	0.231250001	0.939158389
HK1	-0.002777959	0.231264482	0.939158389
SNRPB	-0.004382852	0.2312917	0.939158389
ZNF84	0.006104401	0.231314594	0.939158389
SNORD73A	0.005203199	0.231403106	0.939158389
METTL9	-0.004753521	0.231475462	0.939158389
FOXI3	0.004597503	0.23170176	0.939158389
CD1B	-0.007052067	0.231754732	0.939158389
TRIM7	-0.007656374	0.231803865	0.939158389
LYZ	-0.009035484	0.231822238	0.939158389
C6orf120	-0.004843502	0.23187201	0.939158389
SLC39A4	0.005739146	0.23189404	0.939158389
ATL3	-0.003595977	0.231947338	0.939158389
PLK3	-0.006106775	0.232116719	0.939158389

HERC2P7	-0.007792809	0.232146266	0.939158389
SMAD9	0.007245552	0.232203231	0.939158389
SLAIN2	-0.004050792	0.232207137	0.939158389
TOE1	-0.003651734	0.232230575	0.939158389
ELANE	-0.010960708	0.232271621	0.939158389
ANKRD23	-0.004961411	0.232312473	0.939158389
ULK1	-0.002969475	0.232313176	0.939158389
SCARNA9	-0.007335321	0.232324713	0.939158389
PDE1B	-0.005385208	0.23256932	0.939158389
PPP1R11	0.00218037	0.232597275	0.939158389
DAK	-0.004341664	0.232605506	0.939158389
CLTC	-0.005865282	0.232618849	0.939158389
GNPNAT1	0.005389116	0.232652477	0.939158389
APOBEC3G	-0.007422746	0.232734237	0.939158389
GCC1	-0.00381074	0.232768473	0.939158389
ASH1L	0.005216692	0.232778386	0.939158389
FAM114A2	-0.003952801	0.232896781	0.939158389
BPI	-0.011847237	0.233232809	0.939158389
CHST3	-0.004719927	0.233273544	0.939158389
SCPEP1	-0.005362189	0.233453616	0.939158389
BIRC3	0.00728712	0.233659525	0.939158389
NDUFB3	-0.003114254	0.233673854	0.939158389
PRRG4	0.010001698	0.233737931	0.939158389
KIAA1324L	0.011713023	0.233781867	0.939158389
TRIM74	-0.004752676	0.2338001	0.939158389
HDAC2	0.002117748	0.233827368	0.939158389
TSPAN4	-0.006442725	0.23384358	0.939158389
KMO	0.006933736	0.233847044	0.939158389
CCDC17	-0.005157847	0.233861689	0.939158389
BNIP3L	-0.005742801	0.233889677	0.939158389
CD151	-0.007421435	0.233924894	0.939158389
TSSC1	0.003353247	0.234021953	0.939158389
B3GNT8	-0.006311438	0.234029496	0.939158389
IGHMBP2	0.005623617	0.234031892	0.939158389
CHFR	-0.00305163	0.234220644	0.939158389
NQO2	-0.011860768	0.234230627	0.939158389
OPTN	0.005083525	0.234387632	0.939158389
PNMA1	0.004839358	0.234470193	0.939158389
LONP2	0.003154808	0.23461219	0.939158389
ANXA11	0.003972969	0.234833706	0.939158389
C9orf156	-0.004229667	0.234902418	0.939158389
OR2AG1	0.006406399	0.235070451	0.939158389
RPL17	-0.002850193	0.235115238	0.939158389
FAM105A	-0.006143367	0.235413525	0.939158389
ZFP57	0.004962341	0.235486319	0.939158389
GPR19	-0.008316257	0.23552865	0.939158389
SNORD48	0.008051385	0.235658625	0.939158389

ARFGAP1	-0.005507942	0.235768787	0.939158389
SEL1L3	0.005024028	0.235781896	0.939158389
TRIT1	-0.004845866	0.235812584	0.939158389
PUS7L	-0.004758489	0.235826522	0.939158389
BSPRY	-0.005214602	0.236074125	0.939158389
SNAPC4	0.003606769	0.23611657	0.939158389
SH3BP2	0.006083422	0.236163177	0.939158389
MCTP1	-0.008871823	0.236345329	0.939158389
SDCCAG3	0.003441282	0.236417886	0.939158389
LDOC1L	-0.006083111	0.236420581	0.939158389
POLDIP2	-0.00447382	0.236509057	0.939158389
MRPL38	0.003699093	0.236511232	0.939158389
XCL1	0.008617061	0.236809297	0.939158389
PTP4A2	0.003195881	0.237166597	0.939158389
PPM1K	0.011287709	0.237297957	0.939158389
KDM1B	-0.00567735	0.23746954	0.939158389
PPM1B	-0.004429112	0.237502944	0.939158389
CGA	0.00576429	0.237529002	0.939158389
PIK3CA	0.004658468	0.237858105	0.939158389
PSMC6	-0.002892683	0.23801739	0.939158389
PDIA6	-0.004915004	0.238038426	0.939158389
MVP	-0.004250742	0.23817147	0.939158389
TXNL4B	-0.004991261	0.238316495	0.939158389
MFSD11	0.006777317	0.238393	0.939158389
C3orf62	0.004020427	0.238518345	0.939158389
TMEM167B	0.00296829	0.238744098	0.939158389
ALPK2	0.009716381	0.238771695	0.939158389
NLRP3	-0.005346604	0.238795258	0.939158389
BIN3	0.007167757	0.238817003	0.939158389
PRG2	0.005490571	0.238852073	0.939158389
ACOT1	-0.006251238	0.238981505	0.939158389
OR2W3	0.010496569	0.239208931	0.939158389
FCGR1A	-0.008294773	0.239214669	0.939158389
RP9	0.00371766	0.23926588	0.939158389
NME1	-0.00503781	0.239326946	0.939158389
CRABP2	0.00511389	0.239375714	0.939158389
ZNF664	0.005008248	0.239476243	0.939158389
PCBD2	-0.003743831	0.239502125	0.939158389
RANBP6	-0.00406216	0.239526547	0.939158389
AGAP3	-0.004836677	0.23953328	0.939158389
POLA2	-0.003362306	0.23961566	0.939158389
P2RX1	0.010054039	0.239646018	0.939158389
IFNGR1	-0.003777837	0.239817162	0.939158389
SLC39A14	0.004949386	0.240103678	0.939158389
PRKAR2A	0.004564394	0.240192018	0.939158389
GFOD2	-0.004236853	0.240217607	0.939158389
MS4A1	0.013025932	0.240267185	0.939158389

TBC1D9	-0.006560158	0.240281277	0.939158389
GALNT11	0.003085695	0.24041459	0.939158389
STK36	-0.004092381	0.240461247	0.939158389
KCNH6	0.003411012	0.240507734	0.939158389
C2orf47	-0.003859549	0.240571618	0.939158389
TBC1D1	-0.004832383	0.240680129	0.939158389
SPINT3	0.00545571	0.240726235	0.939158389
RFPL2	0.006388676	0.240734447	0.939158389
JUND	0.00226485	0.240829171	0.939158389
TMEM8A	0.009188648	0.240851721	0.939158389
FRS3	-0.003627623	0.240864151	0.939158389
PTPRCAP	0.004945351	0.240886822	0.939158389
TMEM11	-0.004808995	0.240955374	0.939158389
LCLAT1	0.007449571	0.240965043	0.939158389
SDR42E1	-0.00574703	0.241009484	0.939158389
PAFAH2	-0.005072615	0.241020835	0.939158389
PYGM	0.004408357	0.241222397	0.939158389
BACH2	0.006771717	0.241314778	0.939158389
NRGN	0.011559042	0.241448137	0.939158389
CWC15	0.002182541	0.241457967	0.939158389
FSD1L	0.005597622	0.241620175	0.939158389
ACVR1	0.004051263	0.241631637	0.939158389
PTPN12	0.005054463	0.241645562	0.939158389
SRRT	-0.002459696	0.241654858	0.939158389
RHBDD1	-0.003529973	0.241658477	0.939158389
FKBP8	-0.004474746	0.241659601	0.939158389
VN1R2	0.006665532	0.241676244	0.939158389
MYO10	-0.005275415	0.241694745	0.939158389
TMEM97	-0.005320183	0.241868139	0.939158389
LARS2	-0.004920082	0.241909403	0.939158389
SLAMF7	-0.006948072	0.241951259	0.939158389
AMMECR1L	-0.003853646	0.242052401	0.939158389
MON1B	-0.003625837	0.242095287	0.939158389
ZFP36L1	0.004402248	0.242150969	0.939158389
FNBP1	-0.002293068	0.24221359	0.939158389
SH2B3	-0.003063969	0.242214322	0.939158389
HIST1H2AC	0.010425587	0.242365541	0.939158389
LAMP1	0.002387526	0.242382023	0.939158389
MYB	-0.005565347	0.242483017	0.939158389
KLHL8	-0.004088824	0.242508535	0.939158389
PLEKHF1	-0.009386339	0.242511182	0.939158389
POTEM	0.006960344	0.242574201	0.939158389
NAALADL1	0.005320369	0.242947517	0.940212958
SLC15A2	0.006454507	0.243047784	0.940212958
SQLE	-0.005078325	0.243172264	0.940212958
MYNN	-0.002782522	0.243300486	0.940212958
RBM26	0.003914233	0.243369377	0.940212958

EXO1	-0.004912517	0.243380743	0.940212958
KBTBD11	-0.005210288	0.243616185	0.940212958
ODF2	0.003494294	0.24367339	0.940212958
KIF2C	-0.004842256	0.243691437	0.940212958
RNF157	0.007649572	0.243820471	0.940212958
SNCA	0.011425098	0.243859593	0.940212958
MED29	0.002513115	0.244069869	0.940212958
TXNDC17	-0.003246556	0.24408371	0.940212958
MTFR1	-0.00535874	0.244255827	0.940212958
MAP7	-0.008203028	0.244407143	0.940212958
BCL2L13	-0.003520105	0.244583817	0.940212958
SDHAP1	-0.004615477	0.244648002	0.940212958
MSH2	0.00523487	0.244657066	0.940212958
MIOS	-0.002767742	0.244709769	0.940212958
SNRPC	-0.003503267	0.244716497	0.940212958
LMO2	-0.004227707	0.244787314	0.940212958
DISP1	-0.004506506	0.244803738	0.940212958
ZNF320	0.005852869	0.244813814	0.940212958
CD63	0.004717186	0.244885171	0.940212958
CDKN2D	0.006142395	0.24501239	0.940294261
MS4A6A	-0.005650326	0.245171259	0.940294261
SPECC1L	0.002865042	0.24518182	0.940294261
PLA2G12A	0.008296851	0.245325447	0.940294261
GOLGA5	-0.002548659	0.245657138	0.940294261
EFHB	-0.005933853	0.245725726	0.940294261
CHTF8	0.005542632	0.245749648	0.940294261
BICD2	-0.004473018	0.245805489	0.940294261
RASSF5	0.002629815	0.245837661	0.940294261
CREB5	-0.006254832	0.245873934	0.940294261
EFHC1	-0.0048771	0.24591963	0.940294261
BBS1	-0.003310978	0.245925728	0.940294261
KIAA1033	-0.005836696	0.246188675	0.94064581
TROVE2	0.002881998	0.246292181	0.94064581
MARCH5	0.004800289	0.246375171	0.94064581
CDH20	0.004952826	0.24640132	0.94064581
DNAJC9	0.002963472	0.24644359	0.94064581
CLMN	-0.005669799	0.246527554	0.94064581
TMC5	0.009178238	0.246879789	0.940927818
NOD2	-0.004669977	0.247030556	0.940927818
EPHA4	0.008296315	0.24710008	0.940927818
TXNDC16	-0.00475198	0.247210956	0.940927818
MYADM	-0.004556294	0.247272603	0.940927818
TRAF5	0.004088074	0.247282327	0.940927818
LILRA6	-0.00842413	0.247344636	0.940927818
DHX30	0.003524539	0.247605298	0.940927818
ITGB5	0.013169949	0.247759345	0.940927818
FAM193B	-0.004807234	0.247809261	0.940927818

MRPL3	-0.003074832	0.247944306	0.940927818
NUDT14	-0.003491796	0.248065878	0.940927818
SLC30A5	-0.003282708	0.248111629	0.940927818
TCF4	0.006110313	0.248224829	0.940927818
CBY1	-0.004464556	0.248297542	0.940927818
TMEM200A	0.007697658	0.248345418	0.940927818
INO80D	0.004293123	0.248353657	0.940927818
MIS12	-0.003816185	0.248408212	0.940927818
PTRH1	-0.006153287	0.248619058	0.940927818
RNF175	0.008973502	0.248739635	0.940927818
ZFHX3	-0.005704194	0.248852587	0.940927818
CARHSP1	-0.004451673	0.248955457	0.940927818
PCTP	-0.004616297	0.249036411	0.940927818
IMPA2	-0.006489736	0.249073985	0.940927818
ZNF7	-0.003536362	0.24910496	0.940927818
POLR1C	-0.003817132	0.249224671	0.940927818
SYNJ1	-0.00340777	0.249236658	0.940927818
SETBP1	0.006877928	0.249323609	0.940927818
RABAC1	0.003837659	0.249359596	0.940927818
KIAA1191	0.002764367	0.24939067	0.940927818
PDK4	-0.008239976	0.249436981	0.940927818
ARSB	-0.006017246	0.24946621	0.940927818
TCTN3	-0.005312376	0.249559931	0.940927818
RAB3GAP1	0.003496258	0.249589636	0.940927818
TDRD1	0.010971915	0.249772682	0.940927818
NUP88	0.002613555	0.249821767	0.940927818
CYP2S1	-0.008210557	0.249851512	0.940927818
ZNF441	-0.005168357	0.249899789	0.940927818
PYGB	-0.005676213	0.249948379	0.940927818
RNGTT	0.003514081	0.250001691	0.940927818
BBIP1	0.005490292	0.250246681	0.94112699
UBE2D2	0.002853393	0.250293387	0.94112699
COPS6	-0.003111596	0.250309681	0.94112699
ATP8B4	-0.005707977	0.250745359	0.942285192
PRKAR1B	0.005955906	0.250799628	0.942285192
SEH1L	-0.004297476	0.250961888	0.942285192
C9orf85	-0.005946137	0.251130731	0.942285192
FAM118A	0.025470528	0.251179047	0.942285192
MED25	-0.003931795	0.251228962	0.942285192
GTF3C1	0.005682954	0.251354719	0.942285192
IL12RB1	-0.005780932	0.251477887	0.942285192
RNF34	-0.003592269	0.251674	0.942285192
TBC1D20	-0.003724383	0.25180933	0.942285192
C17orf89	0.003648307	0.251827186	0.942285192
HDGFRP3	0.009387743	0.251881875	0.942285192
SF3A1	0.005194575	0.251960039	0.942285192
PRMT6	-0.004983416	0.252271418	0.942285192

RAD54B	-0.004444672	0.252517821	0.942285192
GPAM	-0.004160424	0.252764671	0.942285192
ERLEC1	-0.003769038	0.252797309	0.942285192
PPP2R5E	-0.003286534	0.252958671	0.942285192
AEBP1	-0.007749425	0.253006416	0.942285192
ITPKC	0.005144591	0.253223149	0.942285192
RAB4B	-0.006240833	0.253243985	0.942285192
MTR	0.004405751	0.253457635	0.942285192
EIF3A	-0.001928181	0.253480106	0.942285192
JMJD1C	-0.003777497	0.253493439	0.942285192
GBE1	-0.004951354	0.253508281	0.942285192
HSH2D	0.007068858	0.253662222	0.942285192
CDC45	-0.01024371	0.253900048	0.942285192
PXN	0.004242692	0.253971547	0.942285192
ZMYND15	-0.007422757	0.254082143	0.942285192
MCM2	0.006131192	0.254247202	0.942285192
PTPRC	-0.007364513	0.25425205	0.942285192
PARK7	-0.002286755	0.254298332	0.942285192
C10orf54	-0.003243684	0.254326909	0.942285192
ST7	0.005977532	0.254548037	0.942285192
EIF5A2	0.004927079	0.254836562	0.942285192
FER	-0.005568638	0.254990774	0.942285192
TM7SF2	0.004074035	0.255170902	0.942285192
RAP1GAP2	-0.00522478	0.255187674	0.942285192
MOAP1	-0.003171347	0.25534754	0.942285192
TK1	-0.006632292	0.255461995	0.942285192
RABGAP1L	0.006560333	0.255532219	0.942285192
GPN1	0.002778611	0.255576579	0.942285192
SH3BGRL2	0.013614091	0.255698101	0.942285192
SUPT3H	0.006069835	0.255702336	0.942285192
C17orf47	-0.005404647	0.255855478	0.942285192
SNORD105B	0.00430456	0.255927689	0.942285192
MSRB3	0.009045739	0.256062085	0.942285192
POLR3F	-0.004029422	0.256159575	0.942285192
PMCHL1	-0.003895364	0.256184895	0.942285192
HMGXB3	-0.002550769	0.256525957	0.942285192
JAKMIP2	-0.010371202	0.256604591	0.942285192
GKAP1	0.004688032	0.256658905	0.942285192
CCDC92	0.00442336	0.256668035	0.942285192
RPAP1	0.004427383	0.256751614	0.942285192
PHF23	-0.003457963	0.256809083	0.942285192
RNF122	0.004215694	0.256861583	0.942285192
ACER2	0.004428617	0.256924665	0.942285192
BCKDK	-0.003544075	0.256928466	0.942285192
ARFIP2	-0.004608582	0.256934217	0.942285192
HEXIM2	0.007949426	0.256936634	0.942285192
LAMC1	-0.01148323	0.256947824	0.942285192

C2orf15	0.006555754	0.256962274	0.942285192
WHAMM	0.005949976	0.256962692	0.942285192
MPND	-0.005561308	0.257061151	0.942285192
RAB1B	0.004453248	0.257107123	0.942285192
SPRY1	0.009052017	0.257136504	0.942285192
HIST1H2BH	0.012576676	0.257149864	0.942285192
ZBTB47	-0.005595697	0.257158853	0.942285192
DEAF1	0.004288857	0.257329924	0.942285192
PRIM2	0.006123884	0.257345153	0.942285192
CIDEA	-0.00571252	0.257680708	0.942285192
AGFG1	0.004197863	0.257776968	0.942285192
UBE2J1	0.003958611	0.257860589	0.942285192
F2RL1	-0.00904945	0.257968919	0.942285192
SLC35E2	0.00514675	0.257988728	0.942285192
SGCE	0.009321976	0.2581511	0.942285192
EXOSC7	-0.003608268	0.258202561	0.942285192
PAPD4	-0.002608734	0.258299725	0.942285192
INSR	0.005210573	0.258440022	0.942285192
MAP3K5	-0.004256487	0.258522145	0.942285192
MTG1	-0.004432211	0.258567411	0.942285192
SETDB2	-0.002534444	0.258643702	0.942285192
MAZ	-0.005610402	0.258741876	0.942285192
IFFO2	0.004398437	0.258815963	0.942285192
SLC25A16	-0.004996697	0.258898428	0.942285192
GSPT1	0.002032864	0.258971145	0.942285192
ATP6V0B	0.002952176	0.259024078	0.942285192
ATP5I	0.001779341	0.259092318	0.942285192
ALKBH5	0.002056268	0.259105996	0.942285192
BST1	-0.008926748	0.259475095	0.942285192
CD226	0.006912036	0.259527021	0.942285192
ZNF705G	0.005727876	0.25952819	0.942285192
AP3M2	0.00470959	0.259540359	0.942285192
MYC	0.005811643	0.259544109	0.942285192
WDR77	0.002913385	0.259582158	0.942285192
BFAR	-0.003485769	0.259649576	0.942285192
RPRD1A	0.003187324	0.259682664	0.942285192
KCTD12	-0.006350163	0.259850639	0.942285192
ABCC5	-0.004617438	0.259980783	0.942285192
TMEM55B	0.003033691	0.260036243	0.942285192
PTGS1	0.011271072	0.260092661	0.942285192
CSPP1	0.003662443	0.260116042	0.942285192
GGT7	-0.007757922	0.260163623	0.942285192
WWC1	0.00251474	0.260317764	0.942285192
HIST1H2AG	0.011420605	0.260619566	0.942285192
TAB2	0.005111136	0.26069302	0.942285192
ARHGAP22	0.007528573	0.260710854	0.942285192
DDB1	-0.002119535	0.260802898	0.942285192

RNF145	-0.0041836	0.260809541	0.942285192
COX7C	-0.003020508	0.26087269	0.942285192
NCAPD3	-0.003233752	0.260908261	0.942285192
KCTD7	0.007562005	0.260989973	0.942285192
FHL3	-0.007874232	0.261018856	0.942285192
TOMM40L	0.004988767	0.261123334	0.942285192
TANC2	-0.004535522	0.261155152	0.942285192
CSRP2BP	0.003503788	0.261205232	0.942285192
ALKBH2	0.003721055	0.261228204	0.942285192
ZNF442	-0.00459966	0.261315568	0.942285192
SSBP3	0.005321205	0.261363353	0.942285192
FAN1	-0.005605771	0.261426464	0.942285192
ARL6IP1	0.003041097	0.26143345	0.942285192
CNOT6	0.002669964	0.261630515	0.942285192
SNORD4A	0.006619191	0.261644233	0.942285192
WDR43	0.003742652	0.261706826	0.942285192
ARMC1	0.003729976	0.261711534	0.942285192
NDUFS5	0.003205714	0.261971496	0.942285192
WDR11	-0.024125908	0.262030583	0.942285192
FKBP5	0.010340326	0.262095379	0.942285192
NDFIP2	-0.005444156	0.262156735	0.942285192
POC1A	-0.004857372	0.262184158	0.942285192
EID2B	0.006801201	0.262212998	0.942285192
ZNF580	0.005077921	0.262217661	0.942285192
CDYL	-0.003392573	0.262244902	0.942285192
KRT10	-0.004401639	0.262251258	0.942285192
ZNF135	-0.007737767	0.262313621	0.942285192
AEBP2	-0.003926476	0.262442466	0.942285192
ZNF765	-0.005486506	0.262482112	0.942285192
NDUFA13	0.003258807	0.262620325	0.942285192
TIMM8B	-0.004562639	0.262633078	0.942285192
RDH10	-0.00457009	0.262746695	0.942285192
METAP1D	0.004041299	0.262758064	0.942285192
PLIN5	0.010997448	0.262782888	0.942285192
TMEM170B	-0.005087811	0.262791073	0.942285192
EVI2A	-0.005226787	0.263239351	0.942296828
LCN2	0.014966652	0.263435261	0.942296828
KLF7	0.004781358	0.263464598	0.942296828
FILIP1L	-0.004896629	0.263619388	0.942296828
GNG3	0.00547103	0.263629722	0.942296828
WDR73	-0.004891882	0.26373721	0.942296828
ILF2	-0.002633376	0.263756686	0.942296828
SSBP4	0.003726655	0.263975661	0.942296828
SERP1	-0.004125406	0.264222745	0.942296828
CCL20	-0.006456981	0.26423733	0.942296828
C2orf74	0.008024329	0.264240174	0.942296828
MEF2A	0.004854005	0.264272448	0.942296828

SLC24A3	0.014911143	0.264313539	0.942296828
WNK1	0.002455342	0.264429702	0.942296828
TDRD9	-0.009520687	0.264437034	0.942296828
NPEPL1	-0.005029422	0.264469505	0.942296828
GLCCI1	0.005178364	0.264521731	0.942296828
PARD6A	0.003815001	0.264759932	0.942296828
LSP1	-0.006303785	0.264872323	0.942296828
GRB10	-0.005375088	0.264949421	0.942296828
SNIP1	-0.003635373	0.2649621	0.942296828
LGALS9C	-0.006012664	0.26496604	0.942296828
SNORD32B	0.00474432	0.265107657	0.942296828
MRPS36	-0.003172151	0.26510934	0.942296828
STIM2	0.005342316	0.265175046	0.942296828
NUP205	0.002390523	0.265271322	0.942296828
GFPT1	-0.004859449	0.265316506	0.942296828
POLI	-0.005002006	0.265675066	0.942296828
THOP1	-0.003691771	0.265683265	0.942296828
MARCH2	0.008606616	0.265843834	0.942296828
SLAMF8	-0.009104186	0.266023528	0.942296828
YTHDF2	-0.00257187	0.266066359	0.942296828
KLHL7	-0.0038028	0.266072654	0.942296828
FAM72D	-0.007076968	0.266156468	0.942296828
SDCBP	-0.006532194	0.266261051	0.942296828
TRIP12	-0.002159293	0.266342057	0.942296828
C17orf70	-0.003029288	0.266596797	0.942296828
WRAP73	-0.00277118	0.266602049	0.942296828
ZNF101	0.0065407	0.266634297	0.942296828
GAK	0.002448638	0.26664798	0.942296828
GPR89A	0.005434138	0.266785815	0.942296828
BOP1	-0.003088525	0.266790653	0.942296828
HEXB	-0.002877269	0.26679877	0.942296828
ZBTB40	0.005066552	0.266892763	0.942296828
PIK3IP1	-0.005367698	0.267164829	0.942296828
PHF2	-0.002672656	0.267189061	0.942296828
RAB11A	0.003561328	0.267227693	0.942296828
PRKAG1	-0.003787572	0.267297736	0.942296828
ZNF480	-0.00373637	0.267333799	0.942296828
SNORA29	0.005921996	0.267396314	0.942296828
RPF2	0.003401148	0.267507392	0.942296828
DUSP19	0.008757528	0.2676988	0.942296828
BCL2L12	-0.002607465	0.267860788	0.942296828
ACP1	-0.004316368	0.267970656	0.942296828
STARD3NL	-0.006569872	0.268418789	0.942296828
NIT1	0.003341672	0.268469125	0.942296828
DHX37	-0.003401458	0.268478054	0.942296828
FKBP9L	0.007061368	0.268544193	0.942296828
SLC25A28	-0.00269461	0.268644695	0.942296828

SMEK2	-0.002203534	0.268675104	0.942296828
TUBGCP4	0.003754125	0.268683784	0.942296828
CCL28	0.00524054	0.268723148	0.942296828
CENPO	0.00431283	0.268851539	0.942296828
SF1	0.004923193	0.268884572	0.942296828
NIPSNAP1	0.00367075	0.269114444	0.942296828
IFT46	-0.003977107	0.269139207	0.942296828
FAM133B	0.008304316	0.269200837	0.942296828
CHPT1	0.006084752	0.269369054	0.942296828
PM20D2	0.003833798	0.269396322	0.942296828
RFX7	0.002276463	0.269520076	0.942296828
UBE2J2	-0.005168064	0.269569533	0.942296828
WTAP	0.002903952	0.269732518	0.942296828
LIME1	-0.002957326	0.269792228	0.942296828
EIF3F	-0.003677643	0.269913076	0.942296828
CCNL2	0.006084038	0.269960174	0.942296828
SYT11	-0.00562617	0.269967913	0.942296828
ZCCHC9	-0.003757506	0.270015355	0.942296828
ARID4B	0.009515194	0.270051106	0.942296828
WDSUB1	-0.004766426	0.270117717	0.942296828
MICAL1	-0.006093751	0.27019272	0.942296828
TNRC6B	0.005072853	0.270214216	0.942296828
BEND5	0.007242051	0.270245888	0.942296828
CLDN7	-0.00479369	0.270592298	0.942296828
STX2	0.006490562	0.270664196	0.942296828
ZNF606	-0.005065806	0.270666045	0.942296828
APBB3	-0.004330955	0.270690198	0.942296828
PPP3CC	0.002740061	0.270717217	0.942296828
BOLA2B	0.003300554	0.270726306	0.942296828
SMPD2	0.004071918	0.270842566	0.942296828
MRPS11	-0.003108083	0.270882863	0.942296828
S1PR4	0.004158978	0.271023381	0.942296828
C2CD2	-0.004380212	0.271050947	0.942296828
GP1BB	0.008195797	0.271122283	0.942296828
LRP5	-0.004543648	0.27122183	0.942296828
UBA5	-0.003585112	0.271341209	0.942296828
NEXN	0.009415771	0.271536382	0.942296828
HIPK2	0.0049534	0.271609532	0.942296828
KIAA0040	0.00559717	0.271628984	0.942296828
YBX1	-0.002704072	0.271694341	0.942296828
CCDC41	-0.003977945	0.271835817	0.942296828
MMS19	-0.002497309	0.271957781	0.942296828
SGSM1	0.005794522	0.272116539	0.942296828
ATXN1	-0.003645186	0.27213279	0.942296828
HBB	0.014690503	0.272248954	0.942296828
INSM1	0.004812106	0.272252318	0.942296828
CPD	-0.0041296	0.272434164	0.942296828

GAB1	-0.004332642	0.27258676	0.942296828
HMGN4	0.002619232	0.272612134	0.942296828
OAS3	0.010129109	0.272650694	0.942296828
PCP2	-0.005240691	0.272812233	0.942296828
ADA	-0.004372572	0.272854415	0.942296828
WIPF2	-0.004777072	0.273063748	0.942296828
SF3B5	0.002901815	0.273067552	0.942296828
GLS2	0.004945235	0.273088662	0.942296828
EXOC7	-0.002723014	0.273220942	0.942296828
MTMR2	0.004237444	0.27324444	0.942296828
MFNG	-0.001855738	0.273277749	0.942296828
LHFP	0.003962107	0.273299213	0.942296828
LTB	0.002821731	0.273377998	0.942296828
NGRN	-0.003914274	0.273486407	0.942296828
TNFSF15	0.012314902	0.273730955	0.942296828
RGL1	-0.007438454	0.273866472	0.942296828
SRP54	-0.002754752	0.273958181	0.942296828
RECK	-0.003625783	0.274132958	0.942296828
AFTPH	0.002991573	0.274200174	0.942296828
NCOA5	0.003655496	0.274302225	0.942296828
PRUNE2	-0.012848947	0.274376738	0.942296828
SNORA27	0.006309063	0.274499424	0.942296828
CEACAM1	-0.006668525	0.274562909	0.942296828
SLC22A18	-0.007058469	0.274584928	0.942296828
TMOD2	-0.005219918	0.274708913	0.942296828
PSMD1	0.002891408	0.274813946	0.942296828
TTK	-0.006132703	0.275387748	0.942296828
FAM105B	-0.003793509	0.275400255	0.942296828
AKT3	0.004846646	0.275421285	0.942296828
PAIP2	-0.002500141	0.275549961	0.942296828
NADSYN1	0.003271524	0.275566992	0.942296828
SAMSN1	-0.00503117	0.275628505	0.942296828
LSM4	0.003593127	0.275672721	0.942296828
SAP25	-0.004405055	0.275695978	0.942296828
RHPN1	-0.005176831	0.275794448	0.942296828
ZNF513	-0.003652441	0.275802572	0.942296828
C12orf73	-0.005334182	0.275807014	0.942296828
CXCR6	-0.014245898	0.276049643	0.942296828
LDHA	-0.003174785	0.276101412	0.942296828
SBNO1	-0.004843343	0.276125513	0.942296828
ZNF646	-0.00433206	0.276241758	0.942296828
PSMA4	-0.002765607	0.276258878	0.942296828
ANKRD50	-0.004343452	0.276301783	0.942296828
C12orf45	0.004657121	0.276336611	0.942296828
EIF5	-0.004221778	0.276403039	0.942296828
INTS3	0.004013613	0.27642178	0.942296828
MAP3K11	-0.00506717	0.276484097	0.942296828

ZSCAN16	-0.002469226	0.276514068	0.942296828
EIF1	0.003138368	0.276709456	0.942296828
ADD3	0.002412279	0.276763877	0.942296828
ACADVL	-0.003235477	0.276809204	0.942296828
ACRBP	0.011040109	0.276839332	0.942296828
TMEM202	0.004261247	0.276898216	0.942296828
CHPF	-0.004961719	0.277077549	0.942296828
C16orf13	0.0032683	0.277140991	0.942296828
FAAH	0.005767403	0.277281849	0.942296828
ESRRA	-0.003032287	0.277288238	0.942296828
EP400	0.003087517	0.277344205	0.942296828
ULK3	-0.005237966	0.277377393	0.942296828
JAK2	-0.004614993	0.277413606	0.942296828
CRYZ	0.00743072	0.27748265	0.942296828
PTPN9	-0.004719544	0.277488646	0.942296828
G3BP1	0.004076562	0.277490284	0.942296828
NFX1	0.003319301	0.277534317	0.942296828
ATP5F1	-0.002845333	0.277543984	0.942296828
SLC27A3	-0.003516488	0.277559345	0.942296828
ZC3H7A	-0.002089346	0.277720705	0.942296828
PSMA1	-0.002581884	0.277750049	0.942296828
TMEM203	0.002350683	0.277832945	0.942296828
PHF1	0.004329681	0.277942922	0.942296828
LYPD3	0.004641555	0.277956708	0.942296828
CTSG	-0.01640109	0.277962849	0.942296828
LAT2	0.003205112	0.278032473	0.942296828
INTS2	-0.003039668	0.278119725	0.942304022
SOCS2	0.005150431	0.278268158	0.942518433
HBA2	0.020937891	0.278495448	0.942520337
MAGEF1	0.00529509	0.278788775	0.942520337
PPT1	-0.003892266	0.278948227	0.942520337
SERHL2	0.004871409	0.27907874	0.942520337
ZMYND8	0.00346461	0.279116718	0.942520337
CCR7	-0.006583212	0.279250555	0.942520337
TMCO4	-0.004236472	0.279260776	0.942520337
CECR5	-0.003049164	0.279333394	0.942520337
AZI2	-0.003986312	0.279407209	0.942520337
MS4A7	-0.005017094	0.279460114	0.942520337
NR4A2	0.008653252	0.279494347	0.942520337
OTP	0.004468953	0.279500753	0.942520337
STX11	-0.005523969	0.279503013	0.942520337
SLFN11	-0.003755364	0.279545261	0.942520337
POU2F2	0.005793917	0.279545963	0.942520337
VRK3	-0.00643445	0.279647585	0.94257586
KLHDC9	-0.005158234	0.279797646	0.942794566
REEP4	-0.004425863	0.280051536	0.943090053
TREML2	-0.004577782	0.280055742	0.943090053

DHX38	-0.004442096	0.280710546	0.943540841
MAEA	-0.002576395	0.280748767	0.943540841
MAP4	-0.005005796	0.280779133	0.943540841
MGST1	-0.00934926	0.280790005	0.943540841
ZNF644	-0.004533441	0.280798358	0.943540841
JMJD7-PLA2G4B	-0.004035938	0.28103349	0.943540841
TRIM46	-0.005280242	0.281038496	0.943540841
KCNN4	0.005714176	0.281105772	0.943540841
SRSF1	-0.002077971	0.281146158	0.943540841
DNLZ	-0.006154965	0.281168191	0.943540841
LIN9	-0.004140879	0.281371116	0.943540841
PTBP1	-0.00229327	0.281371511	0.943540841
TMEM167A	-0.00490116	0.281442878	0.943540841
KHDRBS1	0.002260093	0.281517985	0.943540841
FAM27E3	-0.005590232	0.281671513	0.943540841
HIST1H4C	-0.004326968	0.281815065	0.943540841
GLTPD1	-0.00397973	0.281821574	0.943540841
GLIPR1	-0.007655718	0.2819779	0.943540841
MED23	-0.0039699	0.282034756	0.943540841
ABCC6	-0.003730374	0.282078124	0.943540841
DEFB116	-0.004583465	0.282144037	0.943540841
DET1	-0.003826889	0.282158116	0.943540841
ATP2A3	0.004656268	0.282279429	0.943540841
CSTF1	-0.005112432	0.282327742	0.943540841
FAM160B1	-0.002992991	0.282642147	0.943540841
UQCR10	-0.004890032	0.282726207	0.943540841
NISCH	-0.003248355	0.282933713	0.943540841
MSX1	-0.004137897	0.282942263	0.943540841
ASCL2	-0.007451582	0.283019056	0.943540841
ZNF45	-0.002585037	0.283223147	0.943540841
WFS1	-0.004926776	0.283401132	0.943540841
PMS1	-0.004530151	0.283461622	0.943540841
COL12A1	0.004789484	0.283491875	0.943540841
PID1	-0.009333786	0.28354231	0.943540841
LIN54	0.00339447	0.283722476	0.943540841
CSRNP1	0.004072877	0.283763524	0.943540841
ERI2	-0.005097998	0.283789363	0.943540841
MAPK8IP3	-0.005678028	0.28386814	0.943540841
ZNF280D	-0.00454929	0.283936116	0.943540841
TMED5	-0.00317372	0.283963609	0.943540841
TUFT1	-0.003753167	0.284245847	0.943540841
TAF1B	-0.003872488	0.28432506	0.943540841
TOMM70A	0.002679837	0.284668824	0.943540841
PLEKHJ1	0.00449852	0.284911619	0.943540841
TUBB6	0.007531949	0.284987463	0.943540841
C21orf91-OT1	-0.004927601	0.285043765	0.943540841
GIMAP2	-0.004330259	0.285359034	0.943540841

TDRD3	0.003850113	0.285387815	0.943540841
SPSB1	0.005221432	0.285596515	0.943540841
ZNF860	0.009977011	0.28569214	0.943540841
LPAR3	-0.003922173	0.285996048	0.943540841
ATP5EP2	-0.002118137	0.286004001	0.943540841
ZDHHC6	-0.003096035	0.286403298	0.943540841
RAB5B	0.002387837	0.286576121	0.943540841
FBXO5	-0.004201352	0.286618358	0.943540841
DNAJC15	0.00618143	0.286625827	0.943540841
CRB3	0.004879876	0.286649888	0.943540841
YPEL5	0.003441471	0.286786108	0.943540841
SDF4	0.003755448	0.286892096	0.943540841
FAM162A	0.005233152	0.286938926	0.943540841
C7orf25	-0.004808745	0.286972513	0.943540841
MLLT11	-0.003642107	0.28709341	0.943540841
ZNF577	0.010706754	0.287110465	0.943540841
GJB7	0.005838386	0.2871183	0.943540841
SPPL2A	-0.002617646	0.287177595	0.943540841
TAF2	-0.002798474	0.287279273	0.943540841
MED11	-0.003244746	0.28731057	0.943540841
ATXN10	-0.004305138	0.287467326	0.943540841
BZW1	-0.002542979	0.287576467	0.943540841
SPINT2	0.004196853	0.287599951	0.943540841
C9orf78	-0.002591086	0.28773509	0.943540841
SNORD68	0.005678184	0.288142913	0.943540841
UBAP2L	0.003860963	0.288290756	0.943540841
TRIM44	-0.003307618	0.288290835	0.943540841
VASH1	-0.009924579	0.288479328	0.943540841
GINS4	0.003707469	0.288509331	0.943540841
SNRPB2	0.002155973	0.288536194	0.943540841
ARL13B	-0.004050761	0.288563556	0.943540841
S100Z	-0.006707146	0.288575907	0.943540841
GIMAP6	-0.00447047	0.288587707	0.943540841
RB1CC1	-0.002096468	0.288778639	0.943540841
USP32	0.009678125	0.288793219	0.943540841
MRPL35	-0.003547631	0.288833758	0.943540841
GPAA1	0.003644924	0.288860089	0.943540841
STX8	0.003670637	0.2888744	0.943540841
INSL3	-0.00682017	0.288887387	0.943540841
DUSP23	-0.002808968	0.289085552	0.943540841
SLC25A45	-0.004088696	0.289224558	0.943540841
GALNT1	-0.003477304	0.289317382	0.943540841
LTN1	-0.002412861	0.28934593	0.943540841
ZNF175	-0.005084294	0.289451114	0.943540841
TSHZ2	0.009812982	0.289613623	0.943540841
MMD	0.007785138	0.2896973	0.943540841
BCL6	-0.0044663	0.289712628	0.943540841

CYB5R4	-0.004894292	0.289836699	0.943540841
TOPORS	-0.004037417	0.289877633	0.943540841
RP9P	0.003859178	0.289887894	0.943540841
MRPL30	-0.0050502	0.289950431	0.943540841
GCN1L1	0.002964076	0.290007895	0.943540841
TP53INP2	-0.003954012	0.29005569	0.943540841
GRAMD1B	-0.004330595	0.290481963	0.943540841
SCARNA2	0.007267691	0.290560637	0.943540841
PBX3	0.003127025	0.290580425	0.943540841
KLRC2	0.021282747	0.290614381	0.943540841
NAT10	-0.002623882	0.29072355	0.943540841
MAPK13	-0.004211263	0.29076819	0.943540841
SLC25A19	0.003385979	0.29080539	0.943540841
EBLN2	0.007501096	0.290813682	0.943540841
TMOD4	-0.004273472	0.290868265	0.943540841
POFUT1	0.004794748	0.290898058	0.943540841
CASP3	-0.003355187	0.29095907	0.943540841
RAB8B	0.002329673	0.291036615	0.943540841
FAM53A	-0.005326236	0.291040598	0.943540841
TIMM9	0.003694228	0.291049255	0.943540841
ATG12	0.002557952	0.291092811	0.943540841
R3HDM1	0.004179334	0.291207604	0.943540841
FBXO22	-0.004091988	0.291315905	0.943540841
PRKAB2	0.003674269	0.291315989	0.943540841
RAB10	-0.001859792	0.291412547	0.943540841
IL25	0.005093115	0.291420926	0.943540841
RPL7L1	0.002164608	0.291469304	0.943540841
DOCK9	0.004041492	0.291493508	0.943540841
CDADC1	0.004373961	0.291629986	0.943540841
MT2A	0.007861316	0.291713271	0.943540841
IL6ST	-0.005738721	0.291735924	0.943540841
TMEM159	0.003705378	0.291945684	0.943540841
TRIB2	0.004963186	0.29225098	0.943540841
TMEM110	-0.005161561	0.292419603	0.943540841
ETF1	-0.00231148	0.292430174	0.943540841
TNIP2	0.004052566	0.292445294	0.943540841
TMBIM4	-0.001985714	0.29261314	0.943540841
SNORD22	0.004569816	0.292623428	0.943540841
NPEPPS	-0.002344043	0.292681244	0.943540841
SERPINI1	0.004668307	0.292797968	0.943540841
ADAM19	0.004942624	0.292811188	0.943540841
HLX	-0.005430214	0.292876432	0.943540841
TAF1L	0.002865143	0.293353997	0.943540841
GJC2	-0.004493335	0.293360357	0.943540841
ARRB1	-0.003824909	0.293435622	0.943540841
AGA	-0.004361636	0.293444062	0.943540841
FPR3	-0.009742176	0.293447748	0.943540841

TCERG1	-0.00319792	0.293541723	0.943540841
ZNF326	0.004498016	0.293576444	0.943540841
UBN1	-0.002980884	0.293642392	0.943540841
STEAP3	-0.008441752	0.293693499	0.943540841
PGGT1B	-0.006610687	0.293828442	0.943540841
NFKBIB	-0.00322922	0.293860389	0.943540841
ELAC1	0.004193043	0.293895734	0.943540841
S100A12	-0.009837135	0.293901628	0.943540841
SNORA33	0.005090683	0.294284047	0.943540841
SLC9A7P1	-0.004184095	0.294420447	0.943540841
ACAP2	-0.004819087	0.294438426	0.943540841
PEF1	0.003095279	0.29448482	0.943540841
IFITM3	0.015722897	0.294526939	0.943540841
ABCA3	-0.005541311	0.294601175	0.943540841
SH2B2	0.004622398	0.29476376	0.943540841
YWHAG	-0.003024834	0.294769316	0.943540841
TBCB	0.003081428	0.294808542	0.943540841
BOLA2	0.003801957	0.294812076	0.943540841
FAHD2A	-0.004259989	0.294854654	0.943540841
PIGN	-0.004149772	0.294908315	0.943540841
CD2BP2	-0.002453817	0.294988569	0.943540841
NFIL3	-0.006684509	0.295024828	0.943540841
GNL2	0.002213652	0.295067735	0.943540841
EEPD1	-0.005424341	0.29513582	0.943540841
TAP1	-0.00428368	0.295220405	0.943540841
G6PC3	-0.005557026	0.295226085	0.943540841
CD2	-0.003958827	0.295388217	0.943540841
SUMF1	-0.004030397	0.295429679	0.943540841
RAX2	0.007775607	0.295450713	0.943540841
TRAK1	-0.003388018	0.295570018	0.943540841
VPS45	0.002667809	0.295592687	0.943540841
TEKT1	-0.003736408	0.295679401	0.943540841
TDRD6	-0.004244329	0.295700979	0.943540841
MRPL41	0.002792766	0.295735714	0.943540841
QTRTD1	-0.002509767	0.29574447	0.943540841
C12orf44	-0.003285614	0.295803328	0.943540841
LANCL2	-0.003669172	0.295820876	0.943540841
GCC2	0.0054224	0.295888187	0.943540841
NAPA	0.004027377	0.295908013	0.943540841
GPC6	-0.003876208	0.295988752	0.943540841
CTNS	-0.003159808	0.295992777	0.943540841
MRS2	-0.003842378	0.296031072	0.943540841
ASF1A	-0.003684321	0.296080313	0.943540841
PAQR4	-0.004312512	0.296132152	0.943540841
RNMTL1	-0.00242774	0.296142039	0.943540841
GMPPA	-0.003612407	0.296184751	0.943540841
BMP4	-0.004538282	0.296215053	0.943540841

DDIT4	0.00998559	0.296621779	0.943667649
ZBTB45	-0.003054873	0.296665144	0.943667649
AZIN1	-0.003275368	0.296670631	0.943667649
FXYD5	0.003667688	0.296708736	0.943667649
THOC6	-0.00332243	0.297059234	0.943667649
WAPAL	0.002950698	0.297082691	0.943667649
RELT	0.00547204	0.297102449	0.943667649
SDHD	-0.004605863	0.297207408	0.943667649
U2SURP	0.003176109	0.297224913	0.943667649
KANK1	0.011701341	0.297259393	0.943667649
RPS21	-0.005104873	0.297595393	0.943667649
SLC22A18AS	-0.007133794	0.29762002	0.943667649
RBX1	0.003193974	0.297656746	0.943667649
TRIM68	-0.002894333	0.297660536	0.943667649
TIAM2	-0.006552583	0.297733702	0.943667649
PRDM4	0.002618366	0.297745321	0.943667649
RMI1	-0.003432477	0.298093175	0.943667649
PTOV1	-0.003330112	0.298116246	0.943667649
DDIT3	0.003178483	0.29815613	0.943667649
C11orf71	-0.004958682	0.2981848	0.943667649
SIDT1	0.003892858	0.298223822	0.943667649
ARHGAP27	-0.003187884	0.298351506	0.943667649
NLRP8	0.007704839	0.298537523	0.943667649
BBS12	-0.005161149	0.29859645	0.943667649
FAM153A	-0.011188117	0.298695032	0.943667649
FAM126B	0.003026062	0.298818248	0.943667649
CC2D1A	-0.003900799	0.298833198	0.943667649
KDELR1	0.002487697	0.298863588	0.943667649
OR2B2	0.006084959	0.298930079	0.943667649
NCF1B	0.007336841	0.299040039	0.943667649
ARFGEF1	-0.002205154	0.299051955	0.943667649
VAV2	0.004210329	0.299169094	0.943667649
RPUSD2	-0.002981462	0.299209321	0.943667649
SERINC1	0.00323951	0.299299832	0.943667649
SLC36A1	0.005490055	0.299346818	0.943667649
CHCHD4	-0.002618245	0.299402668	0.943667649
HPSE	-0.00633331	0.29945102	0.943667649
MEPCE	0.00461389	0.299634817	0.943667649
HSD17B7P2	-0.004069363	0.299636766	0.943667649
ANAPC13	-0.003344222	0.299705004	0.943667649
ACAA1	-0.003172457	0.299921752	0.943667649
OLIG1	-0.011504489	0.299940553	0.943667649
ANKRD13C	0.00343218	0.299956857	0.943667649
SAMD14	0.017292127	0.300006004	0.943667649
BTBD7	-0.003566777	0.300248679	0.94416268
TROAP	-0.003747945	0.300602603	0.944360873
GABBR1	0.006417441	0.300626389	0.944360873

SRM	-0.004084375	0.300633938	0.944360873
ZNF467	-0.004789033	0.300767285	0.944360873
AASDH	-0.003315587	0.300881308	0.944360873
ANKRD32	-0.01613089	0.300889597	0.944360873
SMEK1	0.002262406	0.300942038	0.944360873
CNTNAP1	0.007756191	0.301118485	0.944360873
KRT73	0.016479478	0.301155047	0.944360873
FAM129A	-0.004447376	0.301378895	0.944360873
CD1E	-0.00830551	0.301430361	0.944360873
PSMD7	-0.003270009	0.301454801	0.944360873
TAF6L	-0.003220981	0.301504398	0.944360873
TMEM150A	-0.002732199	0.301601253	0.944360873
POLR3H	-0.003111656	0.301684882	0.944360873
TRIM23	-0.004845943	0.302156566	0.944360873
BACE2	0.008339541	0.302232504	0.944360873
ZNF621	-0.003206812	0.302436327	0.944360873
CELF1	0.004814335	0.302507174	0.944360873
NUCKS1	0.003814093	0.302512092	0.944360873
KIF1C	-0.003631539	0.302683639	0.944360873
SMN1	0.007304912	0.302724105	0.944360873
WDR19	0.004152886	0.302760631	0.944360873
UHRF2	0.003422216	0.30278393	0.944360873
TLE1	0.007608015	0.302931671	0.944360873
RPL13AP5	0.001358751	0.302975052	0.944360873
CP	0.003762616	0.302995448	0.944360873
HDAC7	0.004452634	0.3030272	0.944360873
YIPF4	-0.003841906	0.303031131	0.944360873
DHX16	-0.002516391	0.303057157	0.944360873
MIR1185-1	-0.003472404	0.303089156	0.944360873
MUT	0.003229412	0.303148266	0.944360873
GDPD1	0.009826795	0.303400028	0.944360873
TUBA1B	-0.003107874	0.3037235	0.944360873
AIRE	0.002332335	0.303760391	0.944360873
SURF1	-0.002437999	0.303839528	0.944360873
MX2	0.005644654	0.303856956	0.944360873
NOP58	0.003470836	0.303966547	0.944360873
UPB1	0.005842868	0.303979361	0.944360873
FBXO9	0.006761358	0.304218167	0.944360873
ZNF200	-0.00769678	0.30437489	0.944360873
WDR18	0.003473332	0.304516599	0.944360873
BTBD6	0.002906848	0.30460019	0.944360873
SMARCAD1	-0.002711381	0.304686845	0.944360873
TATDN2	0.003597997	0.304708424	0.944360873
REEP1	0.005689342	0.304720806	0.944360873
C11orf73	-0.004300662	0.304868156	0.944360873
RDH11	0.003689868	0.305067878	0.944360873
CDR2L	0.008264713	0.305070692	0.944360873

ADRA2B	0.004191097	0.305152349	0.944360873
CMTM3	-0.002777104	0.305381626	0.944360873
TADA2B	-0.002588532	0.305460907	0.944360873
TESC	-0.004655952	0.305664964	0.944360873
OCEL1	-0.002937532	0.30573353	0.944360873
CDK5RAP2	0.006358222	0.305872392	0.944360873
HIST2H2BF	-0.003844679	0.305881456	0.944360873
COL4A3BP	0.004162421	0.305987155	0.944360873
PDCD2	-0.003092142	0.30605514	0.944360873
TRANK1	0.004542093	0.306166143	0.944360873
FSTL1	0.008856923	0.306275983	0.944360873
ZNF14	0.004879619	0.306352086	0.944360873
KPNB1	-0.002252919	0.306394114	0.944360873
EHD3	0.007221031	0.306432495	0.944360873
ZC3H15	-0.00252395	0.306464526	0.944360873
ANK1	0.006406873	0.306512879	0.944360873
NMI	-0.005437517	0.306814331	0.944360873
PRAMEF7	0.003478275	0.306881726	0.944360873
CNDP2	-0.002988768	0.306913822	0.944360873
GJA5	0.004017097	0.306967696	0.944360873
NIPAL3	0.003636333	0.307338778	0.944360873
LAMTOR1	0.004890634	0.307370389	0.944360873
DAZAP2	-0.00244704	0.307441254	0.944360873
STX17	-0.003506069	0.307473633	0.944360873
MIER1	-0.003588612	0.307497208	0.944360873
RAC2	-0.00227929	0.307664438	0.944360873
IDH3B	-0.001809744	0.307732215	0.944360873
TNIP1	-0.00277516	0.307735731	0.944360873
TSC22D4	0.004048828	0.307863618	0.944360873
AFMID	0.00540198	0.307906758	0.944360873
CLNS1A	-0.002825874	0.30795436	0.944360873
LILRA3	0.02163063	0.308009312	0.944360873
MYO3B	0.012006099	0.308035066	0.944360873
TBC1D13	0.002519649	0.308047609	0.944360873
RNY5	0.005619665	0.308063958	0.944360873
WBP2	0.003169926	0.308138909	0.944360873
ACPP	-0.007651632	0.308397693	0.944360873
ZNF317	-0.003267833	0.308485263	0.944360873
YOD1	0.003589701	0.308635413	0.944360873
TRAPPC8	-0.002474002	0.308719319	0.944360873
C18orf21	-0.002748324	0.308914106	0.944360873
FBXL16	0.005572693	0.308921084	0.944360873
GALM	0.002339857	0.309016972	0.944360873
MAP3K1	0.002686403	0.309045334	0.944360873
FZD3	-0.004288291	0.309092833	0.944360873
MAVS	-0.005423041	0.309160559	0.944360873
CPPED1	0.005050861	0.309415115	0.944360873

DSCR3	-0.002538489	0.309560644	0.944360873
RRM1	0.003103463	0.309570012	0.944360873
CPT1A	-0.00542867	0.309716726	0.944360873
CA2	-0.010319183	0.30976632	0.944360873
TSPAN14	0.004778856	0.309896852	0.944360873
CDK20	-0.003655067	0.30991349	0.944360873
SGOL2	0.004820388	0.310014397	0.944360873
RASGRP4	-0.006582927	0.31002896	0.944360873
CETN3	0.003207829	0.310105642	0.944360873
PSMC3	-0.002755309	0.310195102	0.944360873
ARID3B	-0.003199229	0.310196524	0.944360873
C14orf80	-0.003680907	0.31022816	0.944360873
RABGAP1	0.003176646	0.310245858	0.944360873
PEA15	-0.002933814	0.310524354	0.944360873
XPNPEP1	0.002802495	0.310731698	0.944360873
SNORA10	0.004409956	0.310753239	0.944360873
ANKRD16	-0.003628084	0.310822042	0.944360873
PDXDC1	-0.002148321	0.310847658	0.944360873
SMAD4	-0.002576985	0.310876992	0.944360873
HOXB5	0.004398463	0.31090871	0.944360873
ZNF618	0.004936956	0.310949497	0.944360873
HLA-C	0.01582408	0.311013281	0.944360873
ETHE1	-0.004494977	0.311061117	0.944360873
KRTAP10-11	0.004170574	0.311101265	0.944360873
RPN1	-0.002261203	0.311148339	0.944360873
AVP	-0.00471406	0.311149254	0.944360873
DCUN1D5	-0.003234324	0.311290132	0.944360873
SOX4	-0.004585623	0.311366903	0.944360873
KCTD20	0.003216482	0.311490264	0.944360873
COIL	-0.002093388	0.311585748	0.944360873
LSM5	0.003260838	0.31171677	0.944360873
PNN	0.004627092	0.311725085	0.944360873
MARCH6	0.007289902	0.311789677	0.944360873
TRRAP	0.002860854	0.311821378	0.944360873
PAK4	-0.003906065	0.311869404	0.944360873
ZNF92	-0.004913388	0.311950236	0.944360873
CRTAP	-0.008836611	0.311961682	0.944360873
TOMM6	0.002532019	0.311973782	0.944360873
PHYH	-0.003665062	0.31197824	0.944360873
TYSND1	-0.002304325	0.312003487	0.944360873
PITPNA	-0.004047056	0.312043654	0.944360873
LRRC8B	0.004447535	0.31208958	0.944360873
LRRC8A	-0.004337962	0.312170606	0.944360873
CALD1	0.013267945	0.312429253	0.944775763
TNS1	0.00667401	0.312478459	0.944775763
SLCO4A1	0.003501656	0.312753141	0.94477881
VDR	-0.00409952	0.312849635	0.94477881

JKAMP	-0.003179309	0.312901699	0.94477881
TMX3	-0.002816103	0.312958357	0.94477881
TSPAN31	-0.00278033	0.312982693	0.94477881
SIGLEC10	-0.006949074	0.312991589	0.94477881
ZNF226	0.004747133	0.313272672	0.944921413
VEZT	0.004732487	0.313359033	0.944921413
SLC25A44	-0.002418041	0.313643958	0.944921413
KIR2DL1	0.012353494	0.313644678	0.944921413
PAIP1	0.00405507	0.313743775	0.944921413
PCNA	0.003312044	0.313766791	0.944921413
WIZ	-0.003574191	0.313843878	0.944921413
C17orf85	-0.002442222	0.313905753	0.944921413
ZDHHC18	0.003300677	0.313910654	0.944921413
RIMBP3	0.003770402	0.314146486	0.944921413
POT1	-0.004127275	0.314150231	0.944921413
RCN3	-0.005186293	0.314207592	0.944921413
CDCA8	-0.004378544	0.314288214	0.944921413
PRSS45	0.004413648	0.31432623	0.944921413
TPD52L2	-0.002596999	0.314399588	0.944921413
CCDC135	0.004711293	0.314445553	0.944921413
CD6	-0.003639466	0.314538718	0.944921413
CCL8	0.008521333	0.31466503	0.944921413
SHQ1	-0.003344461	0.314728947	0.944921413
ZNF283	-0.00381759	0.315053265	0.944921413
BTRC	-0.003546454	0.315078288	0.944921413
CCDC57	-0.004617367	0.315262902	0.944921413
ZNF250	-0.005360904	0.315267221	0.944921413
EZH1	0.011431779	0.31536908	0.944921413
VPS33B	0.002083014	0.31552302	0.944921413
ADNP2	0.002723341	0.315587069	0.944921413
C6orf106	0.003767617	0.315725013	0.944921413
USP21	0.00294888	0.315799983	0.944921413
RNF149	-0.00327443	0.316032128	0.944921413
C1orf122	-0.00389384	0.316059676	0.944921413
TUBB1	0.007778984	0.316131077	0.944921413
IRX1	0.00407844	0.316169334	0.944921413
YWHAE	-0.004956826	0.316185156	0.944921413
MYCBP2	0.002909039	0.316220561	0.944921413
KIAA1467	0.003542094	0.316344308	0.944921413
TSTD1	0.004371328	0.316458223	0.944921413
SS18	0.004147693	0.316468153	0.944921413
TMEM86B	-0.004417912	0.316528128	0.944921413
MATN2	-0.00573614	0.31659433	0.944921413
MAPK6	-0.003064288	0.316612113	0.944921413
LPP	0.003944795	0.316729225	0.944921413
RPLP2	0.003426524	0.316730605	0.944921413
MYO1G	0.004117674	0.316792593	0.944921413

NEIL1	0.005207987	0.316794956	0.944921413
INPP4B	0.005325851	0.316915145	0.944928741
MFSD3	-0.004080144	0.317017553	0.944928741
SCRN1	-0.004510804	0.317293868	0.944928741
TMCO3	-0.003935233	0.317408305	0.944928741
TATDN3	-0.003092064	0.317451948	0.944928741
ATP6V1F	-0.002658778	0.317472736	0.944928741
DUSP16	0.00426932	0.317598652	0.944928741
ARHGAP32	0.004695908	0.317625113	0.944928741
DAXX	0.003188004	0.317647604	0.944928741
SLC35F2	0.004849275	0.317651084	0.944928741
TMEM129	-0.003959336	0.317935508	0.945520725
HLA-DQB1	0.008886164	0.318262042	0.945613297
TTC19	0.002979079	0.318301481	0.945613297
PGBD4	-0.004175192	0.318800864	0.945613297
BAX	0.004251096	0.318828857	0.945613297
GTPBP1	-0.004292173	0.318865673	0.945613297
BCAT2	-0.004251433	0.3192185	0.945613297
FMNL1	-0.004532906	0.319240418	0.945613297
TMEM39B	0.002685081	0.31947201	0.945613297
ONECUT2	-0.003574509	0.319512987	0.945613297
MAPKAPK5	0.002746327	0.319656014	0.945613297
CABP5	0.014230191	0.319754806	0.945613297
SCAMP4	-0.004976309	0.319947924	0.945613297
ZNF525	0.005576434	0.319996919	0.945613297
PLA2G7	-0.009653662	0.320035752	0.945613297
HECTD1	-0.002160615	0.320090344	0.945613297
TNS3	-0.003297284	0.320231436	0.945613297
IFIH1	0.004096688	0.3203228	0.945613297
TCFL5	0.003339628	0.320405117	0.945613297
ANKRD6	-0.004280644	0.320443934	0.945613297
NUMB	-0.002138547	0.320590199	0.945613297
CYP11A1	0.004497848	0.320838181	0.945613297
ZSWIM4	0.002866947	0.320853584	0.945613297
FAM189B	0.002737016	0.320902822	0.945613297
SLC44A4	0.001647937	0.321034428	0.945613297
SLC37A1	0.003155766	0.321133247	0.945613297
CYB5R3	0.003132069	0.321138584	0.945613297
CD300LF	-0.005029551	0.321238081	0.945613297
IL17RD	0.008444866	0.321434338	0.945613297
C14orf2	0.003499327	0.321442662	0.945613297
ZNF716	0.003473337	0.321594901	0.945613297
PCDH15	0.003858058	0.32161042	0.945613297
RNPEP	0.004072218	0.321667174	0.945613297
HS2ST1	0.003903055	0.321686431	0.945613297
IPPK	-0.003978689	0.321754188	0.945613297
CARD9	-0.004136307	0.321838635	0.945613297

OAF	-0.004392454	0.321936622	0.945613297
MDH2	-0.002186391	0.322325749	0.945613297
SEC13	-0.003608787	0.322333807	0.945613297
CALN1	-0.003550395	0.322383907	0.945613297
HERC2	0.003115015	0.32244969	0.945613297
THOC3	-0.005606368	0.322686639	0.945613297
CKAP2	-0.004975368	0.322704784	0.945613297
COG5	0.004287808	0.322782012	0.945613297
POLR3C	-0.001959759	0.322797967	0.945613297
POMC	0.00752572	0.322809377	0.945613297
TMEM33	-0.002661944	0.322853562	0.945613297
FTL	0.001343368	0.322855267	0.945613297
PSRC1	-0.004599186	0.322925073	0.945613297
JUN	-0.008095868	0.322948016	0.945613297
UBA7	-0.00295621	0.323171876	0.945613297
CCR9	-0.007635898	0.323187389	0.945613297
SKIV2L	-0.002907961	0.323264044	0.945613297
TTC26	-0.003637942	0.323274626	0.945613297
TRIM9	0.004060191	0.3233418	0.945613297
AHNAK	-0.002785123	0.323454423	0.945613297
OGG1	-0.0040417	0.323570572	0.945613297
PSMG3	-0.0039541	0.323591654	0.945613297
SLC7A5P1	0.001195808	0.323702902	0.945613297
MICAL2	-0.006247804	0.323740143	0.945613297
UCK2	-0.004369214	0.323976037	0.945613297
SNRNP200	0.003368248	0.324114539	0.945613297
CREB1	0.008666094	0.324247315	0.945613297
FMNL2	-0.006665677	0.324402506	0.945613297
ARMC8	-0.003681596	0.32447762	0.945613297
GOLGA8F	0.005950144	0.324578143	0.945613297
RPP25	0.004248837	0.32463598	0.945613297
DNAJC2	0.005690929	0.32472569	0.945613297
ANGEL1	0.003266226	0.324731245	0.945613297
PTTG1	-0.005427712	0.32474088	0.945613297
SFXN4	0.004778396	0.324758023	0.945613297
NTN4	-0.005532643	0.324826569	0.945613297
LMBRD1	-0.003443648	0.324895839	0.945613297
CHCHD1	-0.003855083	0.325116049	0.945613297
VCL	0.005016278	0.325234751	0.945613297
AKAP2	0.008966768	0.325384763	0.945613297
NUP50	-0.004319424	0.325427236	0.945613297
PRKAG2	-0.002507737	0.32547663	0.945613297
PLEKHH3	0.00480999	0.325500232	0.945613297
NARFL	-0.004141089	0.325740795	0.945613297
RPA1	0.005003969	0.325888916	0.945613297
PLAGL2	-0.004399371	0.325934002	0.945613297
PPAP2A	-0.004562136	0.326616269	0.945613297

GALK2	0.005407648	0.326628141	0.945613297
H1F0	0.007358188	0.326696957	0.945613297
TPT1	0.003473396	0.326807661	0.945613297
KRBA1	-0.002924887	0.32690747	0.945613297
STXBP2	-0.004305624	0.326918464	0.945613297
HNRNPK	0.002369143	0.326918663	0.945613297
METTL14	-0.003325048	0.327048614	0.945613297
MRPS12	-0.002630066	0.327165274	0.945613297
DYNLL1	-0.002933427	0.32722628	0.945613297
SNORD69	0.003714026	0.327234947	0.945613297
ARID1A	0.003028753	0.327243529	0.945613297
CXXC5	0.005234325	0.32730992	0.945613297
KCTD9	-0.003616324	0.327334009	0.945613297
AFAP1L2	0.005004735	0.32744638	0.945613297
MAFF	0.01228566	0.327492715	0.945613297
ZNF830	-0.004027164	0.327775079	0.945613297
EFEMP2	-0.002322418	0.327963347	0.945613297
NUDCD1	0.003037888	0.327965176	0.945613297
ZBBX	0.003239232	0.327985749	0.945613297
CLDN23	-0.007549754	0.328129543	0.945613297
BAZ1B	0.002373532	0.328201057	0.945613297
SLC35C2	0.002862055	0.328259065	0.945613297
PAQR3	-0.003337695	0.328466628	0.945613297
BUB1	-0.007282491	0.328489913	0.945613297
PVRIG	-0.004076765	0.328498788	0.945613297
MIR300	-0.004472191	0.328527192	0.945613297
VKORC1L1	0.004438744	0.328553503	0.945613297
DOHH	-0.00363556	0.328639902	0.945613297
MS4A3	0.008685097	0.328736187	0.945613297
KIAA1468	-0.00402243	0.328788622	0.945613297
HBEGF	0.006682134	0.32888012	0.945613297
NEK3	-0.003423684	0.328931677	0.945613297
GSTT1	0.015421331	0.328983022	0.945613297
THAP10	-0.004041165	0.329096243	0.945613297
ZFYVE16	0.004627886	0.329157433	0.945613297
THEM4	0.007810197	0.329333539	0.945613297
TMEM38A	0.009077982	0.329396039	0.945613297
HIST1H2AM	0.007961455	0.329457636	0.945613297
SLC30A6	-0.003836032	0.32952778	0.945613297
NBPF1	-0.004225384	0.329571149	0.945613297
PPP2R5A	0.002801661	0.329656371	0.945613297
SKI	0.002853232	0.329659448	0.945613297
ST7L	-0.004688233	0.329693852	0.945613297
NKIRAS1	-0.00393025	0.329733599	0.945613297
ADIPOR1	0.00379275	0.329868463	0.945613297
TUT1	-0.004062826	0.329914849	0.945613297
DNM1L	0.003981417	0.329961657	0.945613297

SUMO4	0.007267979	0.329976629	0.945613297
PLAC8	0.00391694	0.329986503	0.945613297
GPR146	0.005064908	0.329994257	0.945613297
C3orf17	-0.003321022	0.329999465	0.945613297
GLE1	-0.002160416	0.330024464	0.945613297
SMPD3	-0.00437993	0.330100069	0.945613297
GNG10	-0.006980946	0.330147411	0.945613297
TSEN54	-0.00433735	0.330175634	0.945613297
PRPSAP1	-0.002331559	0.330186879	0.945613297
GON4L	-0.002849518	0.33019385	0.945613297
SNORA75	0.004358639	0.330213292	0.945613297
MEF2C	0.004870047	0.330363387	0.945613297
SLC37A4	-0.003021369	0.330435251	0.945613297
PYCR2	0.003108186	0.330446697	0.945613297
SEC14L5	0.014517364	0.330479924	0.945613297
CACNB3	-0.005336326	0.330578847	0.945613297
SLC43A1	-0.005146084	0.330584894	0.945613297
ZNF80	0.008041284	0.330632876	0.945613297
RNF13	-0.003622326	0.330691054	0.945613297
F12	-0.004214044	0.330745226	0.945613297
LINC00290	-0.003933649	0.330791591	0.945613297
STAU2	-0.003612028	0.330866411	0.945613297
C11orf83	0.003349914	0.330984648	0.945658499
NAPB	-0.004346281	0.331334096	0.945658499
ITIH4	-0.008294003	0.331398637	0.945658499
RAB8A	0.002241064	0.331422686	0.945658499
RAD51	0.006657262	0.331516818	0.945658499
PARM1	0.007684424	0.331719525	0.945658499
PXMP2	0.005435145	0.331789159	0.945658499
AP4E1	-0.002284918	0.331798811	0.945658499
FAM120AOS	-0.002431429	0.331848741	0.945658499
DOK2	-0.002918256	0.332013547	0.945658499
TNFRSF25	-0.005338955	0.332117109	0.945658499
UBXN8	-0.003989052	0.332235759	0.945658499
MX1	0.007923816	0.332372258	0.945658499
ZNF696	-0.004619937	0.332577673	0.945658499
UBE2S	-0.003291553	0.332702786	0.945658499
DSG2	-0.003273661	0.332707126	0.945658499
ZNF366	-0.004942294	0.332737754	0.945658499
TMEM59L	-0.003873263	0.332867077	0.945658499
IFI16	-0.00300886	0.333058789	0.945658499
NAAA	0.004699351	0.333180529	0.945658499
ZNF146	0.002741395	0.333234421	0.945658499
DIS3	-0.004333214	0.333333315	0.945658499
CTBS	-0.004152237	0.333370327	0.945658499
IKZF5	-0.003015268	0.333457947	0.945658499
SNX25	0.005149832	0.333468674	0.945658499

IL7	0.005193945	0.333572928	0.945658499
TMTC3	0.004223867	0.333738592	0.945658499
AKT1	0.003360866	0.333764651	0.945658499
HCG11	-0.006223679	0.333814932	0.945658499
CARNS1	-0.005820316	0.3339111	0.945658499
MUC1	-0.005424531	0.333989472	0.945658499
PDCD1	-0.007573796	0.334045386	0.945658499
FMNL3	-0.003520889	0.334138539	0.945658499
CCNYL1	-0.003590861	0.334169359	0.945658499
BAG1	0.004231107	0.3341745	0.945658499
BHLHE40	0.004008883	0.334193886	0.945658499
ELOVL5	0.004269899	0.334231972	0.945658499
PARVB	0.00968085	0.334252144	0.945658499
ZNF579	-0.004009973	0.334325999	0.945658499
ECE1	0.004180109	0.334382123	0.945658499
RBM25	-0.002345524	0.334388935	0.945658499
MRPL22	0.002113433	0.334470415	0.945658499
ATF6B	0.004156351	0.334563212	0.945679314
GUCY1A3	0.009992808	0.334748037	0.945860497
KHNYN	0.004161188	0.334798214	0.945860497
SLC25A13	-0.006472498	0.335221449	0.946271794
SPATS2	0.003923048	0.335231982	0.946271794
PRRG2	0.004828711	0.335238515	0.946271794
PSMA7	0.004416535	0.335292695	0.946271794
ADM	0.009026451	0.335371239	0.946271794
SF3B2	0.002016842	0.335470928	0.946311852
GDF5OS	0.003886985	0.335864739	0.946602518
SLC19A2	0.003434597	0.335933665	0.946602518
ZNF688	0.00272528	0.335990322	0.946602518
TRIM37	-0.003045422	0.336212844	0.946602518
FOXJ3	0.002345	0.336539854	0.946602518
CENPQ	-0.005330653	0.33655872	0.946602518
C6orf47	-0.002498645	0.336570605	0.946602518
ACSM3	0.004386943	0.336616691	0.946602518
FPR1	-0.00927534	0.336643458	0.946602518
CTSK	-0.005667881	0.336667848	0.946602518
C21orf119	0.004375766	0.336697095	0.946602518
ARFIP1	-0.003714905	0.336768689	0.946602518
PPME1	0.003545154	0.336897762	0.946602518
STK11	0.003882529	0.337030892	0.946602518
ECSIT	0.00369612	0.337109154	0.946602518
FAM173B	-0.00330846	0.337127376	0.946602518
CDC42SE2	0.003183104	0.33723611	0.946602518
RNASEH2A	-0.003484189	0.337299243	0.946602518
ZNF264	0.004456005	0.337466493	0.946602518
PIK3CD	0.003246915	0.337505409	0.946602518
ZNF439	0.005353305	0.337589482	0.946602518

DVL1	0.004638439	0.337671318	0.946602518
DPEP3	-0.005695675	0.337734885	0.946602518
WDR90	-0.003665698	0.337844526	0.946602518
LYSMD2	-0.003205109	0.337895292	0.946602518
SEC31B	-0.004463549	0.337978139	0.946602518
MZT1	-0.00386943	0.338000072	0.946602518
PKD2	-0.004280276	0.338073949	0.946602518
SSR3	-0.003321279	0.338216506	0.946602518
FRMD4B	-0.00724623	0.338229493	0.946602518
ARHGEF40	-0.004582736	0.338337687	0.946602518
AQP1	-0.004127525	0.338456069	0.946602518
MED6	-0.002021853	0.338468429	0.946602518
ATP6V0E1	0.002341993	0.338481594	0.946602518
RFX5	0.001875046	0.338639681	0.946732862
OSM	0.006419268	0.338699262	0.946732862
RBBP6	0.005156196	0.338788532	0.946740578
ZSCAN21	-0.003353356	0.338873084	0.946740578
CLP1	-0.003052303	0.339144192	0.946986932
IL18BP	-0.002639927	0.339179387	0.946986932
SNORA58	-0.003210688	0.339393893	0.946986932
CALU	-0.003672829	0.339492052	0.946986932
CLEC12B	0.007081056	0.339500328	0.946986932
DLST	0.003013454	0.33964096	0.946986932
SEPP1	0.004398192	0.339756799	0.946986932
METTL22	0.004630348	0.339965137	0.946986932
UBXN6	0.002801877	0.339996259	0.946986932
FAHD2B	-0.00298321	0.340021432	0.946986932
HIST1H3F	0.010359179	0.34005958	0.946986932
RAB3IP	0.003081687	0.340230884	0.946986932
GOLGA6L1	0.001420437	0.340340794	0.946986932
GPN3	-0.003671422	0.340343616	0.946986932
RALGPS2	0.008654103	0.340432693	0.946986932
UCHL5	-0.004454439	0.340467693	0.946986932
PSMB8	-0.003165074	0.340552748	0.946986932
HIST1H4H	-0.01516088	0.340741789	0.946986932
FLI1	-0.002549187	0.340745069	0.946986932
MRI1	0.007468422	0.340859134	0.946986932
LONP1	0.002833303	0.340936623	0.946986932
HIATL2	0.007462858	0.340949734	0.946986932
ZNF624	0.003705801	0.340970994	0.946986932
PLXNB2	-0.003805616	0.34108479	0.946986932
SIGIRR	-0.003656756	0.341444856	0.946986932
MVD	-0.004269148	0.341478638	0.946986932
AP2A2	0.004027048	0.341500699	0.946986932
MIR21	-0.006693691	0.34155818	0.946986932
IL2RA	0.005782555	0.341608097	0.946986932
NCAPG	-0.008236524	0.341617397	0.946986932

SETD1B	0.00289925	0.341678656	0.946986932
PDE4C	0.002227803	0.341775626	0.946986932
SHE	0.004553166	0.341893801	0.946986932
CHAF1A	-0.004735161	0.341912202	0.946986932
PDE9A	-0.009218844	0.342073652	0.946986932
TRAF2	-0.004675275	0.342118456	0.946986932
RDM1	-0.00445399	0.342133676	0.946986932
PRPF40A	0.004560103	0.342248898	0.946986932
PF4V1	0.017013667	0.342331154	0.946986932
KCTD17	-0.003414926	0.342520245	0.946986932
RELL1	-0.004242768	0.342630213	0.946986932
TSTA3	0.003228217	0.342644737	0.946986932
ZNF566	-0.005027317	0.342648043	0.946986932
IDO1	-0.005220393	0.343193618	0.946986932
SLC7A1	-0.003228293	0.343560083	0.946986932
PPP6R1	0.003144798	0.343751014	0.946986932
COMTD1	-0.003815467	0.343857271	0.946986932
GFI1	-0.007346534	0.343911625	0.946986932
C19orf38	-0.006471494	0.343937551	0.946986932
VTRNA1-3	-0.004733442	0.34401887	0.946986932
GNB4	0.00615338	0.344238518	0.946986932
SLC25A36	0.004432709	0.344408368	0.946986932
B4GALT4	-0.003647326	0.344496997	0.946986932
FRG1B	0.005442456	0.34450597	0.946986932
AQP10	0.01516925	0.344584327	0.946986932
UBR7	-0.002315594	0.344602721	0.946986932
VCP	-0.002625056	0.344688358	0.946986932
MRPL14	0.003243159	0.344700221	0.946986932
GMEB1	0.003660629	0.344789996	0.946986932
C20orf24	-0.002614769	0.344942233	0.946986932
MTDH	-0.002351953	0.345045759	0.946986932
AGER	0.004009139	0.345166251	0.946986932
WWP2	0.004352461	0.345540599	0.946986932
BSCL2	-0.005130016	0.345694006	0.946986932
ILF3	-0.003050333	0.345758325	0.946986932
AP3B1	-0.002156181	0.345836794	0.946986932
C5orf15	-0.002014046	0.34588018	0.946986932
PGRMC2	0.002442254	0.345926196	0.946986932
TMCC1	-0.00499455	0.346011598	0.946986932
AFG3L2	-0.0020055	0.346023898	0.946986932
PLD6	0.005889563	0.346111112	0.946986932
S100A4	-0.0021277	0.346332409	0.946986932
IFNAR1	-0.003060833	0.346361557	0.946986932
XRCC2	0.009761424	0.346416241	0.946986932
SNORD95	0.004808451	0.346458411	0.946986932
AK5	-0.007031899	0.346661285	0.946986932
CCDC138	0.003275619	0.346663012	0.946986932

COQ6	-0.002589255	0.346712934	0.946986932
HNRNPAB	0.001850631	0.346742259	0.946986932
TRIM13	0.009264841	0.346761903	0.946986932
ALDH8A1	-0.004298918	0.346777407	0.946986932
CORO1C	0.003481036	0.346901836	0.946986932
RAB43	-0.003198745	0.346923062	0.946986932
CD82	0.002978924	0.347027086	0.946986932
ARHGAP26	-0.003519266	0.347061423	0.946986932
DSCAM	-0.003391235	0.347523694	0.946986932
POLR2E	0.004768768	0.34752424	0.946986932
IDH1	-0.002981818	0.347543679	0.946986932
TXNRD2	-0.002863969	0.3476191	0.946986932
LUC7L2	0.003378798	0.34764441	0.946986932
TEAD2	0.007054705	0.347672841	0.946986932
DEK	-0.00256886	0.347740083	0.946986932
PALLD	-0.008782365	0.347773476	0.946986932
NFAT5	0.006062354	0.347983409	0.946986932
GTF2IRD2B	0.003403811	0.348010472	0.946986932
PLEKHF2	0.005095908	0.348053529	0.946986932
GTPBP10	-0.003997359	0.348057178	0.946986932
TRAP1	-0.003600375	0.348201049	0.946986932
MIR129-2	-0.003686998	0.348256158	0.946986932
SHC1	0.002721779	0.348350624	0.946986932
PILRB	0.004792601	0.348354953	0.946986932
NUB1	0.002519571	0.348523585	0.946986932
TNPO1	0.004449979	0.348596158	0.946986932
PROP1	-0.004008871	0.348709151	0.946986932
SLC43A2	-0.00438231	0.348735412	0.946986932
ZNF548	0.004151096	0.348937874	0.946986932
DNAJA2	0.002141222	0.349056167	0.946986932
ABCC10	-0.003303184	0.349099843	0.946986932
SGMS2	-0.004648134	0.349133208	0.946986932
EGF	0.007991586	0.349208527	0.946986932
CROT	-0.004298793	0.349235855	0.946986932
RPL32P3	-0.005454868	0.349332112	0.946986932
PSMA5	-0.002269766	0.349390902	0.946986932
HINT2	0.001966172	0.349418978	0.946986932
FBLN7	0.00560224	0.349493871	0.946986932
KIAA0196	-0.002346617	0.349561318	0.946986932
ECI1	-0.004772021	0.349607997	0.946986932
ORC5	-0.002153166	0.349755339	0.946986932
SNORD56	0.004428768	0.34983543	0.946986932
PAPSS1	-0.003521688	0.349918829	0.946986932
SOCS7	-0.003686278	0.349949466	0.946986932
METTL13	-0.002490847	0.35037315	0.946986932
PCDHB9	0.006757656	0.350417683	0.946986932
FAM98B	0.00535333	0.350428686	0.946986932

GINS2	-0.00740382	0.350505863	0.946986932
RGL2	-0.003369252	0.35073886	0.946986932
HIGD2A	-0.002908758	0.350748347	0.946986932
NEK6	-0.004190941	0.350785853	0.946986932
ZNF329	0.003671331	0.35078976	0.946986932
RHEBL1	-0.003985868	0.350950243	0.946986932
HCN4	0.004830118	0.351025964	0.946986932
CDV3	-0.002487453	0.351112358	0.946986932
KIAA1875	0.006646114	0.351200484	0.946986932
SERTAD2	0.002274824	0.351215716	0.946986932
CD47	0.002588319	0.351220769	0.946986932
CTR9	-0.003255813	0.351234091	0.946986932
RNF181	-0.00232719	0.351277325	0.946986932
HMMR	-0.006420038	0.351421019	0.946986932
TOPBP1	0.002985779	0.351595609	0.946986932
HAGHL	-0.003802531	0.351737317	0.946986932
MTOR	-0.002533986	0.351773443	0.946986932
UBE2E3	0.00351137	0.351969616	0.946986932
KRT86	-0.006724264	0.351973511	0.946986932
PPARA	-0.00315404	0.351982132	0.946986932
SDHC	-0.005417059	0.352109981	0.946986932
ALDH3B1	-0.004615887	0.352136593	0.946986932
SCD	0.005303796	0.352247481	0.946986932
CYTH2	-0.005515547	0.352282108	0.946986932
SIPA1L1	0.00361595	0.352298108	0.946986932
PICK1	-0.003042605	0.352335127	0.946986932
TASP1	-0.003320831	0.352370252	0.946986932
KNTC1	0.003116041	0.352679653	0.946986932
MBNL1	-0.008444051	0.35270748	0.946986932
APEX1	-0.00196163	0.352870242	0.946986932
WDR60	-0.003654472	0.353051439	0.946986932
ANKRD20A4	0.014278759	0.35306941	0.946986932
MCM7	-0.002479698	0.353091202	0.946986932
PCBP2	0.001808003	0.353126914	0.946986932
RPH3AL	0.004045181	0.353475887	0.946986932
RPL37	0.00529305	0.353505939	0.946986932
C11orf58	0.002697811	0.353514885	0.946986932
HEXIM1	0.003312718	0.353554836	0.946986932
SUPT16H	0.002371134	0.353666488	0.946986932
PTGFRN	-0.003694155	0.354000887	0.946986932
CA13	0.004054482	0.35410042	0.946986932
FGFBP3	0.005011136	0.35412297	0.946986932
GTF2E2	-0.001923788	0.354186307	0.946986932
EPHA10	0.003102353	0.354298666	0.946986932
CDKL1	0.005066952	0.354375988	0.946986932
MTA3	0.003533161	0.354447367	0.946986932
C1QB	-0.012591318	0.354525558	0.946986932

TRIM14	0.004734983	0.354535802	0.946986932
SAP30	-0.00642637	0.354798717	0.946986932
COMMD4	0.002906168	0.354810307	0.946986932
TEF	-0.00393822	0.354853802	0.946986932
RSF1	-0.004471501	0.354866273	0.946986932
HIST2H2AA3	-0.005981682	0.354943079	0.946986932
LYRM4	-0.004703881	0.354956282	0.946986932
LBH	0.003342085	0.355022924	0.946986932
BSN-AS2	0.008269236	0.355049068	0.946986932
PXDN	-0.011880087	0.35507828	0.946986932
TFEB	0.004276567	0.35510757	0.946986932
CNGB1	0.008008253	0.355150744	0.946986932
IFI44L	0.01515681	0.355252193	0.946986932
C21orf37	0.003353025	0.355321888	0.946986932
SPTLC1	0.004939225	0.355410466	0.946986932
HACE1	0.003179589	0.355445108	0.946986932
MBD4	0.007490349	0.355480341	0.946986932
INTS10	-0.002049703	0.355513802	0.946986932
GDF11	-0.003627345	0.35578948	0.946986932
ICOSLG	0.00424248	0.355888187	0.946986932
CLCN6	-0.003427386	0.355898473	0.946986932
RABL2B	0.002885302	0.356042915	0.946986932
AHSA1	0.002833097	0.356056289	0.946986932
RUSC2	-0.004529894	0.356109874	0.946986932
CCDC106	0.004484453	0.356472403	0.946986932
IFI27L2	0.003632568	0.35657466	0.946986932
PCID2	-0.001902585	0.356601251	0.946986932
DPH3	-0.004208849	0.356744228	0.946986932
SAT2	-0.002440843	0.35674632	0.946986932
STX3	-0.003146381	0.356923087	0.946986932
NELL2	-0.006069366	0.356955261	0.946986932
GADD45A	0.007515418	0.356976961	0.946986932
SYNCRIP	-0.00264512	0.356991739	0.946986932
MAP2K5	-0.002416554	0.357130657	0.946986932
PREX1	0.003103198	0.357177149	0.946986932
SH3TC1	0.004072678	0.357180426	0.946986932
WDR86	0.007206741	0.357203441	0.946986932
ZNF582	-0.003966783	0.357264407	0.946986932
KIF22	-0.002606748	0.357358167	0.946986932
TPI1	-0.003075217	0.357375219	0.946986932
YKT6	0.005048666	0.357377526	0.946986932
TMBIM1	-0.003350786	0.357546858	0.946986932
DOPEY1	-0.003250784	0.357705003	0.946986932
SAMD10	0.003731298	0.357825418	0.946986932
TREM1	-0.007084711	0.357850556	0.946986932
TMEM191C	0.004545831	0.357889619	0.946986932
CEP72	-0.003586116	0.357957356	0.946986932

TSC22D2	-0.003447596	0.357993906	0.946986932
MANBA	-0.002900756	0.358102232	0.946986932
CD8B	-0.009197997	0.358174796	0.946986932
ELOF1	0.002760939	0.35819677	0.946986932
PHAX	0.009869052	0.358196885	0.946986932
ELOVL4	-0.009194915	0.358337509	0.946986932
MINK1	0.004426716	0.358341987	0.946986932
LPAR2	-0.003488843	0.358358998	0.946986932
ASB6	-0.003706196	0.358476312	0.946986932
MRPS23	-0.003610665	0.358481545	0.946986932
TGIF1	0.003423081	0.358552917	0.946986932
CD163	-0.00725211	0.358741	0.947201997
PTP4A1	0.005528965	0.358850344	0.947201997
IMMT	0.002303674	0.358891063	0.947201997
RHOU	-0.005146057	0.359070628	0.947444534
ALDOC	0.00449649	0.359209881	0.947444534
GABPB2	-0.00123535	0.359239742	0.947444534
LARP7	-0.002587613	0.359633268	0.947638924
CISD1	0.002975471	0.359641371	0.947638924
SSX2IP	0.005227283	0.359643617	0.947638924
CEPT1	-0.002657426	0.359808931	0.947638924
NR2E1	-0.003878288	0.35982589	0.947638924
WDR20	-0.003013388	0.359860004	0.947638924
ANXA5	-0.004107986	0.359927201	0.947638924
SSR2	-0.004217042	0.360022586	0.947638924
MRFAP1L1	-0.002220569	0.360165914	0.947638924
KIAA0247	0.002270622	0.360169568	0.947638924
DPP4	0.005420562	0.360305546	0.947771408
CYTIP	-0.003866835	0.360451433	0.947841878
IGFL3	0.004080338	0.360534706	0.947841878
ARHGDIA	-0.003513765	0.360589226	0.947841878
TLR3	-0.003575982	0.360907459	0.94845315
CXXC1	-0.002647072	0.361216553	0.94874839
WDR47	-0.003316829	0.361447629	0.94874839
CCT6P1	0.001491329	0.361523403	0.94874839
DEDD2	-0.002384986	0.361539836	0.94874839
PALB2	-0.003486154	0.361681692	0.94874839
NUSAP1	-0.005021426	0.361998928	0.94874839
ZBTB9	-0.004674982	0.362107501	0.94874839
NLGN2	0.003925426	0.362115014	0.94874839
PECR	-0.003687573	0.362218656	0.94874839
IFI27	0.012093288	0.36224947	0.94874839
HTR1E	-0.003889082	0.362391533	0.94874839
SH2D2A	0.005601344	0.362423459	0.94874839
SCAMP1	-0.001722139	0.362436969	0.94874839
THAP1	-0.004763191	0.36243872	0.94874839
MEG8	0.004115847	0.362517822	0.94874839

PSMD3	-0.003410398	0.362555415	0.94874839
CLEC4D	-0.006266036	0.362636993	0.94874839
TMEM141	-0.002688671	0.362641678	0.94874839
CDCA5	-0.006902485	0.362648336	0.94874839
CELSR2	0.004141743	0.362775063	0.948855664
MIR1307	0.019980792	0.362882963	0.94891366
RAB27A	-0.005536332	0.363024965	0.949012857
SIGLEC6	0.005823093	0.363140139	0.949012857
IRAK4	-0.00275326	0.363178107	0.949012857
S100A13	-0.003132896	0.363287831	0.949075526
WBSCR27	0.004263404	0.363727091	0.949706098
ASIP	-0.004710246	0.363774932	0.949706098
PDE6G	-0.004933658	0.363822086	0.949706098
COL6A2	0.005955249	0.363872397	0.949706098
SLC26A8	0.004572767	0.364100026	0.949767555
RPL14	-0.002077202	0.364138167	0.949767555
SLC5A10	-0.004206944	0.364276141	0.949767555
ZNF655	0.004964912	0.364296952	0.949767555
CNPY3	-0.00456945	0.364378787	0.949767555
YARS	-0.002217359	0.364748095	0.949767555
CLEC18C	-0.004618478	0.364774041	0.949767555
TMED6	0.006073515	0.364867659	0.949767555
TNFAIP2	0.005486459	0.3649207	0.949767555
TRPC4AP	-0.004588563	0.364928094	0.949767555
IFI44	0.008990189	0.364930224	0.949767555
PLD4	-0.003538526	0.364985853	0.949767555
RBPJ	-0.003914145	0.365011399	0.949767555
EFTUD1	-0.002345556	0.365164504	0.949942632
EXOSC10	0.001851325	0.365347072	0.950056518
SUPT7L	-0.003859216	0.365451619	0.950056518
AURKAPS1	-0.003333261	0.365543698	0.950056518
VARS	-0.002910543	0.365654699	0.950056518
UBE2G2	0.010556931	0.365682628	0.950056518
CCDC117	-0.002800242	0.365723265	0.950056518
RICTOR	-0.004217813	0.365973954	0.950484678
IL32	0.005834141	0.366145547	0.950488998
RSAD1	0.002559416	0.366193724	0.950488998
CRTC3	0.002307727	0.366233226	0.950488998
PDIA4	-0.004183231	0.366425316	0.950764608
PELP1	-0.003858403	0.366725972	0.95132172
GADD45G	-0.005133863	0.367069144	0.951809585
SHPK	0.007604322	0.36713546	0.951809585
PIK3R1	0.00239441	0.367172005	0.951809585
ADSSL1	-0.004272231	0.36727026	0.951841375
FCAR	0.010236607	0.367383202	0.95186666
FXYD6	-0.006307491	0.367496592	0.95186666
RAB1A	-0.002559376	0.367600902	0.95186666

TOX	-0.005639612	0.367623992	0.95186666
USP49	0.007277545	0.367913311	0.951977659
SSH2	0.002931436	0.367923677	0.951977659
BRI3	-0.004424007	0.367924874	0.951977659
KRT18	0.006618906	0.368150773	0.952033632
CCNDBP1	-0.002120432	0.368262708	0.952033632
C12orf43	-0.003862528	0.368270012	0.952033632
CHRNB1	-0.002404974	0.368290542	0.952033632
RTEL1-TNFRSF6B	0.003663669	0.368564173	0.95217607
PHF19	-0.002753352	0.368616082	0.95217607
GIT2	-0.002779205	0.36870016	0.95217607
C14orf105	0.010016745	0.368766848	0.95217607
NASP	-0.002619746	0.368944214	0.95217607
UBL3	-0.003359499	0.368992818	0.95217607
ELOVL1	-0.003063786	0.369093094	0.95217607
ZNF292	0.004874001	0.369140216	0.95217607
ZBTB5	-0.00278203	0.369146872	0.95217607
XPO1	-0.001902159	0.369262381	0.95217607
FOXN3	0.001937446	0.369293477	0.95217607
IMP4	-0.002237279	0.36938336	0.95217607
CD70	-0.005239317	0.369463928	0.95217607
PRF1	0.006880458	0.369724462	0.952318075
LRP1	0.00377606	0.369776125	0.952318075
SEMA4C	-0.003606807	0.369843781	0.952318075
ASCC2	-0.001687153	0.369948735	0.952318075
SPTBN1	0.004768594	0.370057453	0.952318075
TMEM176A	-0.023728941	0.370229694	0.952318075
ERP29	-0.002179221	0.370254907	0.952318075
NUP85	0.001811651	0.370758864	0.952318075
ID2	0.003680667	0.370921066	0.952318075
TPRN	-0.003572446	0.371005472	0.952318075
FAM46A	-0.002937603	0.371007282	0.952318075
PSTK	-0.003187928	0.371012994	0.952318075
SLCO4C1	-0.006364266	0.371019227	0.952318075
ARHGAP11A	-0.003896042	0.371029153	0.952318075
MOV10	0.003778484	0.371136512	0.952318075
ANKFY1	-0.002611893	0.371475067	0.952318075
RFXAP	-0.002644829	0.37158206	0.952318075
FBXL18	-0.003441371	0.371689576	0.952318075
HIST1H2BG	0.01357254	0.371728933	0.952318075
HMGCS1	-0.00242694	0.37191516	0.952318075
OR7E12P	0.003593805	0.371974277	0.952318075
SNHG1	-0.002719983	0.371977389	0.952318075
SLC4A8	-0.005970114	0.372106149	0.952318075
NIP7	-0.003182138	0.372107264	0.952318075
FAM86A	-0.003136303	0.372217629	0.952318075
FANCA	0.004351792	0.37229636	0.952318075

TAF1C	-0.002639231	0.372350494	0.952318075
MCM10	0.005704134	0.372373437	0.952318075
SPATC1	-0.003206101	0.372510825	0.952318075
DCBLD2	-0.004498209	0.372570638	0.952318075
OGFOD1	0.002132806	0.372589708	0.952318075
PLEKHM1P	0.00644301	0.372591577	0.952318075
CYP27A1	0.008034631	0.372789672	0.952318075
HMGN2	-0.002017523	0.37313943	0.952318075
DACH1	-0.004759779	0.373277719	0.952318075
ZNF800	-0.001859321	0.373313387	0.952318075
EIF3G	0.002273786	0.373355479	0.952318075
HIST1H2BC	0.009683851	0.373369362	0.952318075
NFKB1	-0.001735218	0.373577215	0.952318075
DNAJB4	-0.004952256	0.373616035	0.952318075
PMVK	-0.002884816	0.373746491	0.952318075
ICMT	-0.00478098	0.373878563	0.952318075
AK2	-0.003542962	0.374050904	0.952318075
SLC27A2	0.004486897	0.374097276	0.952318075
CORT	0.00379893	0.37412541	0.952318075
LCN1	0.003279811	0.374194261	0.952318075
SLC38A11	0.007806608	0.374302024	0.952318075
EIF4EBP1	0.003219498	0.374307506	0.952318075
IL10RA	0.002500887	0.374454069	0.952318075
COL8A2	0.002871902	0.374458814	0.952318075
TPCN2	-0.004433721	0.374609386	0.952318075
CENPW	-0.006238961	0.374682078	0.952318075
PEMT	-0.002631819	0.374691776	0.952318075
ZNF678	0.003536075	0.374729166	0.952318075
EHD4	0.003409002	0.374824171	0.952318075
ATP2A2	-0.002367248	0.375316098	0.952318075
RIPK2	-0.002747333	0.375370768	0.952318075
COX15	-0.003887538	0.375519613	0.952318075
FBXO38	0.002446667	0.37562352	0.952318075
IGLL1	0.009381235	0.375752804	0.952318075
SCAND1	0.002487531	0.375870271	0.952318075
RPS6KC1	-0.002964462	0.37591753	0.952318075
MAP2K7	0.003036977	0.376088648	0.952318075
CHD9	-0.002878077	0.376291624	0.952318075
WDR33	-0.003120964	0.376569423	0.952318075
SON	-0.001229954	0.376648151	0.952318075
PRRC2B	0.003642101	0.376735376	0.952318075
GATAD1	0.002669886	0.376802956	0.952318075
BOD1	-0.00237975	0.376848736	0.952318075
KIAA0930	-0.004416339	0.376852487	0.952318075
PIGC	0.002251311	0.37691465	0.952318075
SCGB1C1	0.0115668	0.377081111	0.952318075
KCTD2	0.002675977	0.37722659	0.952318075

C19orf40	-0.004054255	0.3773693	0.952318075
DDX17	0.003783394	0.377395407	0.952318075
NTN5	0.004561609	0.377620966	0.952318075
SETD8	0.003450418	0.377783149	0.952318075
CHMP4B	0.002553694	0.377795365	0.952318075
MAPK9	0.003119226	0.377812415	0.952318075
TULP3	-0.003523482	0.378009544	0.952318075
MESDC1	0.003390319	0.378100506	0.952318075
FOXC2	0.00577234	0.378142965	0.952318075
IRF1	-0.003737858	0.378150766	0.952318075
STK17B	0.009699691	0.378197558	0.952318075
MIR373	0.00363146	0.378300735	0.952318075
C10orf12	0.002892323	0.378611813	0.952318075
TACSTD2	-0.025292751	0.378668195	0.952318075
EIF4A1	-0.001783206	0.37868816	0.952318075
AGPAT2	-0.005632288	0.378770106	0.952318075
C5orf22	-0.003779064	0.378831378	0.952318075
SNORA22	-0.005076675	0.378867433	0.952318075
ARHGAP30	-0.00285517	0.378889792	0.952318075
OAZ1	0.001139658	0.378891773	0.952318075
PCSK5	-0.004201252	0.378980319	0.952318075
CKB	-0.007361652	0.379036822	0.952318075
SMYD2	0.002100874	0.379115492	0.952318075
DCAF4L1	-0.003473381	0.379284334	0.952318075
EEF1B2	-0.003543542	0.379311814	0.952318075
KIR2DL4	0.011642306	0.379473337	0.952318075
MITD1	0.00283761	0.379544628	0.952318075
COMMD5	-0.003478233	0.379606587	0.952318075
BBS4	-0.00294687	0.379635137	0.952318075
JAG2	-0.004425307	0.379644316	0.952318075
CMIP	-0.003247069	0.379705385	0.952318075
SLC25A37	0.005728206	0.37974094	0.952318075
ZNF433	-0.004022513	0.379747406	0.952318075
TRIM11	-0.002763585	0.379826647	0.952318075
C2orf42	-0.00249251	0.380064643	0.952318075
ZNF737	0.004187311	0.380083787	0.952318075
ANKRD20A1	-0.003521017	0.380209578	0.952318075
KIT	-0.00404801	0.380330599	0.952318075
VAMP5	0.004145307	0.380367343	0.952318075
FAM175A	0.007569876	0.380408327	0.952318075
F11R	0.003640381	0.380634892	0.952318075
C16orf93	-0.004156445	0.380658188	0.952318075
PNPO	-0.002426441	0.380684711	0.952318075
EIF2B3	-0.002664894	0.380723546	0.952318075
COG2	-0.002532819	0.380757236	0.952318075
ZNF551	0.007620467	0.380808879	0.952318075
LSAMP	-0.003383762	0.380908981	0.952318075

AP1AR	0.004466923	0.380921503	0.952318075
CCPG1	-0.00329903	0.380959599	0.952318075
PPP3CB	-0.001409992	0.381326545	0.952318075
DCLK1	0.003462558	0.381439003	0.952318075
SUGT1	-0.002621002	0.38153081	0.952318075
SORL1	0.002731315	0.381578657	0.952318075
TFRC	0.003079869	0.381589228	0.952318075
LRRFIP1	0.007057929	0.381896614	0.952318075
SLC39A8	0.014633303	0.381929385	0.952318075
SMARCA5	0.003764464	0.382005596	0.952318075
SUB1	-0.003131188	0.382117516	0.952318075
WDYHV1	0.003753612	0.382164393	0.952318075
RRAGC	0.001984874	0.382368487	0.952318075
DND1	-0.004341744	0.382450646	0.952318075
CNOT8	0.001906234	0.382466348	0.952318075
GLRX5	0.002099527	0.382570173	0.952318075
LGALS7	0.003808946	0.382804701	0.952318075
DEPTOR	-0.004634124	0.382857694	0.952318075
SERPINE2	-0.009390486	0.382996981	0.952318075
ELMO1	0.002870612	0.383089336	0.952318075
PNPLA6	-0.003342093	0.383183664	0.952318075
GATC	-0.00568365	0.383282439	0.952318075
TMEM19	0.003067214	0.383355696	0.952318075
KCNMB3	-0.004040477	0.383358595	0.952318075
RANBP10	0.002539156	0.383502944	0.952318075
CD320	-0.004882926	0.38358425	0.952318075
OXER1	-0.003745993	0.383594751	0.952318075
C12orf57	0.004064484	0.383930638	0.952318075
SLC17A9	0.004698477	0.383934508	0.952318075
MEX3B	-0.003278208	0.384083721	0.952318075
RSBN1L	-0.002830026	0.384363676	0.952318075
DEFB123	0.004223485	0.384477792	0.952318075
NSMCE2	-0.004279265	0.38458558	0.952318075
AACS	-0.003399986	0.38461035	0.952318075
DHRS7	-0.002852848	0.384657025	0.952318075
C12orf68	0.003443871	0.384700311	0.952318075
COQ4	-0.005069636	0.384707955	0.952318075
CRYM	0.004105359	0.384737325	0.952318075
PLSCR1	-0.007868072	0.384770137	0.952318075
CCNI2	0.004591576	0.384811806	0.952318075
ZNF483	0.010101751	0.384886469	0.952318075
SEMA3E	0.010642477	0.38546651	0.952318075
RPS28	-0.002243106	0.38557491	0.952318075
WDR37	-0.00244742	0.385842565	0.952318075
TGM3	-0.003596821	0.385861525	0.952318075
ORMDL2	-0.003344592	0.385900747	0.952318075
OSTCP1	-0.004484831	0.385903536	0.952318075

GOLGA6L6	0.007207088	0.385956905	0.952318075
LYSMD4	-0.003723708	0.385994819	0.952318075
RPL9	0.001478726	0.386220054	0.952318075
FGL1	0.002924271	0.386307362	0.952318075
MTRF1L	0.002100014	0.386426709	0.952318075
IMMP1L	-0.003788133	0.38644799	0.952318075
SLC25A39	-0.002036136	0.386452416	0.952318075
CRIP2	0.008726087	0.386683425	0.952318075
TUBE1	0.003557632	0.386738534	0.952318075
ZNF281	0.002512939	0.386771419	0.952318075
ATP6V1H	-0.002395518	0.387133814	0.952318075
MB21D2	0.005350002	0.387152439	0.952318075
NLK	-0.002408861	0.387160123	0.952318075
PYCARD	-0.003003632	0.387199275	0.952318075
DCAF12	-0.002587518	0.38722244	0.952318075
DNAJB5	0.003920197	0.387445279	0.952318075
FAU	-0.001624326	0.387476094	0.952318075
OR56B1	0.003914391	0.387498542	0.952318075
TMEM69	-0.003657067	0.3875414	0.952318075
RIPK1	-0.003344807	0.387562676	0.952318075
OTUD3	0.003852692	0.387720092	0.952318075
COL7A1	0.004813292	0.388086434	0.952318075
RAI1	-0.002701879	0.388167308	0.952318075
HSF2	-0.002935073	0.388219648	0.952318075
RPL3	0.001511199	0.388228927	0.952318075
ZNF609	-0.003330515	0.38824938	0.952318075
HAGH	-0.002194587	0.388262141	0.952318075
ATL2	0.003109824	0.388279076	0.952318075
C12orf49	-0.002736241	0.388292643	0.952318075
TGFB1I1	0.00911076	0.388303626	0.952318075
NUF2	-0.004407784	0.388647152	0.952318075
NIPA2	-0.002219034	0.388650203	0.952318075
LAG3	-0.00934381	0.388676969	0.952318075
ZNF473	0.002481639	0.388686341	0.952318075
SDR16C5	0.004559228	0.388807789	0.952318075
RFNG	-0.002693243	0.388847896	0.952318075
DECR1	-0.001593422	0.388967441	0.952318075
ATG2A	-0.002733302	0.388969058	0.952318075
GPR37	-0.002947391	0.389016158	0.952318075
SCYL1	-0.002019598	0.389403105	0.952318075
SMARCD1	0.001881432	0.389428386	0.952318075
CD79B	0.005432684	0.389549395	0.952318075
FYTTD1	0.002411682	0.389613607	0.952318075
LYPLA1	-0.004760754	0.38963499	0.952318075
HDAC3	0.001759764	0.389814885	0.952318075
AURKAIP1	-0.001839737	0.389824197	0.952318075
SIKE1	-0.003543637	0.389836369	0.952318075

OASL	0.007664672	0.389838774	0.952318075
ATPAF1	-0.002888918	0.389928844	0.952318075
PMAIP1	-0.004811063	0.389944617	0.952318075
SNORA77	0.004265849	0.390013736	0.952318075
PGLYRP2	-0.004008674	0.390112764	0.952318075
MYH10	-0.004694134	0.390117251	0.952318075
BCL9L	-0.003991699	0.390146859	0.952318075
LATS2	-0.004484145	0.390237303	0.952318075
IL23A	-0.003346821	0.390381582	0.952318075
TRIM16L	0.003333125	0.390387078	0.952318075
TRIM62	0.003655517	0.390599176	0.952318075
CCDC28A	-0.001977816	0.390736009	0.952318075
PLBD2	-0.004197617	0.390756748	0.952318075
TPBG	-0.004488995	0.390796412	0.952318075
NIN	0.002022119	0.390848718	0.952318075
SLC41A3	-0.003328671	0.390876148	0.952318075
CLIP4	-0.003136667	0.390984058	0.952318075
WDR26	0.002810886	0.391084451	0.952318075
TIFA	0.00403344	0.391138512	0.952318075
ATRIP	-0.002702214	0.391246574	0.952318075
RPL34	-0.003096843	0.391531518	0.952318075
CNTNAP3B	0.006067028	0.391589715	0.952318075
IL6	0.00455435	0.39171628	0.952318075
SNORD114-7	-0.003594216	0.391948444	0.952318075
SLC4A1	0.006672379	0.392143522	0.952318075
CWC22	0.00429087	0.392250283	0.952318075
EXOSC4	0.002907337	0.392272999	0.952318075
NRM	-0.003237605	0.392382227	0.952318075
ZNF708	-0.004508213	0.392443274	0.952318075
FBXW2	-0.002382845	0.392550075	0.952318075
PSMG4	0.003053786	0.39256899	0.952318075
RBL1	-0.003397993	0.392582288	0.952318075
TMCC3	-0.00452636	0.392704604	0.952318075
FANCF	-0.003300007	0.392746301	0.952318075
PLEKHG2	-0.003465776	0.392752256	0.952318075
TMEM212	0.006417943	0.392847628	0.952318075
COQ2	-0.003073738	0.392951113	0.952318075
EID3	-0.003584614	0.392951442	0.952318075
EPHB6	-0.004448955	0.393014422	0.952318075
TMEM79	-0.003703686	0.393059788	0.952318075
NR2F6	0.005376262	0.393101946	0.952318075
ZNF417	0.003594491	0.39315648	0.952318075
SERINC3	-0.003256684	0.393222228	0.952318075
INSL5	-0.004059696	0.393293466	0.952318075
IDH3A	-0.003129871	0.393400955	0.952318075
GGCX	-0.003244006	0.393511793	0.952318075
SETMAR	-0.003770054	0.39358562	0.952318075

MAMDC4	-0.002979009	0.393706214	0.952318075
TM6SF1	-0.00508157	0.393753506	0.952318075
EIF3M	-0.003107594	0.393796496	0.952318075
MINPP1	-0.003687704	0.393898846	0.952318075
C15orf56	0.003289118	0.393931856	0.952318075
ASXL1	0.002896285	0.394195233	0.952318075
TGFBR2	-0.002156815	0.394206642	0.952318075
F13A1	0.00781262	0.394295976	0.952318075
MZF1	-0.00300841	0.394495793	0.952318075
C16orf87	-0.004002701	0.394626827	0.952318075
GTF3C3	0.002016392	0.39463133	0.952318075
RPS15	-0.001842034	0.394755393	0.952318075
FAM72A	-0.004217288	0.395181253	0.952318075
ZNF540	-0.004619094	0.395291468	0.952318075
FABP4	0.00318912	0.39532317	0.952318075
CCS	0.003001236	0.395505846	0.952318075
C3AR1	-0.006395039	0.395527987	0.952318075
PSMD12	-0.002534108	0.395604535	0.952318075
GPR155	0.004583196	0.395634769	0.952318075
NPR1	0.003771702	0.395705359	0.952318075
MRPL45P2	-0.004919804	0.395745807	0.952318075
SYNGR1	0.005381272	0.395794671	0.952318075
FAM43A	-0.006870057	0.395888623	0.952318075
IFT88	0.002898522	0.396210505	0.952318075
WDR91	-0.002458661	0.396283016	0.952318075
SF3A2	0.002404228	0.396289159	0.952318075
ARRDC4	-0.01708078	0.396291732	0.952318075
GSTA1	0.011389913	0.396492293	0.952318075
SCOC	-0.003069709	0.396651806	0.952318075
MBOAT2	0.007460801	0.396686562	0.952318075
NDUFB1	0.004800437	0.396687578	0.952318075
ZNF576	-0.003147877	0.396729861	0.952318075
CTRL	-0.003559677	0.396739456	0.952318075
C19orf71	-0.003389939	0.396782515	0.952318075
PPIL1	-0.002837869	0.39682104	0.952318075
ADI1	0.004908813	0.39686826	0.952318075
PPFIBP1	0.00330386	0.396943883	0.952318075
HNRNPF	0.003633141	0.397094619	0.952318075
ADPRHL2	-0.002572788	0.397346551	0.952318075
C1orf87	-0.007685289	0.397424672	0.952318075
CXCR5	0.007523392	0.39743175	0.952318075
C6orf52	0.002886931	0.397444842	0.952318075
CEBPG	-0.002119021	0.397481959	0.952318075
FP15737	0.003518951	0.397508475	0.952318075
ZSCAN18	-0.003950965	0.397800049	0.952318075
SYF2	-0.002894328	0.397914013	0.952318075
PIK3CB	-0.00404345	0.398014861	0.952318075

PI4KB	0.002137097	0.398117901	0.952318075
TAGLN	0.00817742	0.398279657	0.952318075
ERLIN1	-0.003294752	0.398310354	0.952318075
CSRNP2	-0.002740526	0.398317139	0.952318075
SCN9A	-0.004153787	0.398388306	0.952318075
IFI6	0.006134095	0.398553756	0.952318075
ZNF274	0.002072068	0.398569223	0.952318075
RNF115	0.00292775	0.398629254	0.952318075
SLC5A12	-0.00388039	0.398688922	0.952318075
SNX16	-0.004124171	0.398956257	0.952318075
IFI35	-0.003980779	0.399016096	0.952318075
NR1D2	-0.002922414	0.399358338	0.952318075
DCAF15	0.003435925	0.399493657	0.952318075
API5	0.001787011	0.399545194	0.952318075
MRPL53	0.002984514	0.399557713	0.952318075
SHOC2	-0.002442444	0.399758166	0.952318075
MCM6	-0.002867354	0.399804606	0.952318075
H2AFV	0.002950668	0.39992502	0.952318075
SAC3D1	0.002799565	0.400039219	0.952318075
CCM2	-0.002654704	0.400082678	0.952318075
EPB41L4A	0.003390481	0.400151709	0.952318075
NFIA	-0.00380698	0.400209127	0.952318075
RYK	0.002011476	0.400227457	0.952318075
ECT2L	0.003445583	0.400227594	0.952318075
UNC5C	-0.00319623	0.400352393	0.952318075
LMF2	0.003444364	0.400454406	0.952318075
SNORD11B	0.004134376	0.400470269	0.952318075
CMTM8	0.008551656	0.400605379	0.952318075
TBX1	0.004439453	0.400776464	0.952318075
C2orf44	-0.002948775	0.400841134	0.952318075
RFXANK	0.002075492	0.400969547	0.952318075
CRAT	0.004081578	0.401031341	0.952318075
UBE2G1	-0.004112484	0.401066462	0.952318075
STXBP3	-0.002393719	0.401080228	0.952318075
EIF5B	-0.002250826	0.401126152	0.952318075
ALDH5A1	0.00366642	0.401140558	0.952318075
SNHG5	0.008066066	0.401266011	0.952318075
APOBEC3A	0.008192425	0.401290588	0.952318075
ZNF48	0.003497302	0.401527437	0.952318075
SLC16A10	-0.00598846	0.401618118	0.952318075
EPPK1	-0.006492621	0.401641053	0.952318075
CDK18	-0.004128877	0.40178665	0.952318075
SLC27A6	0.003936916	0.40179901	0.952318075
CDT1	-0.005679316	0.402040295	0.952318075
RTN4	-0.001762898	0.402044604	0.952318075
VAMP4	-0.004946321	0.402420753	0.952318075
COL18A1	-0.00971381	0.402502565	0.952318075

STK39	-0.003830363	0.402502715	0.952318075
CD46	-0.001953749	0.402537521	0.952318075
ATAT1	0.003612593	0.402540053	0.952318075
RUNX3	-0.003727582	0.402546553	0.952318075
SNORA62	0.003972451	0.402580454	0.952318075
DOLPP1	-0.002137523	0.402595126	0.952318075
IPCEF1	0.003818032	0.402618242	0.952318075
CD58	-0.004327094	0.402651935	0.952318075
TMEM50A	-0.003134092	0.402679726	0.952318075
RPS27A	0.002219587	0.402713687	0.952318075
SAMD15	-0.003205546	0.402720488	0.952318075
TMTC4	0.003263396	0.402819394	0.952318075
SERPINF1	-0.006620696	0.402843124	0.952318075
ZNF211	-0.002564025	0.402891748	0.952318075
LCMT1	0.001753683	0.403032116	0.952318075
FAM120A	0.001254855	0.403065378	0.952318075
EGLN1	0.004367532	0.403877073	0.952318075
C8orf33	-0.002442603	0.403901182	0.952318075
ASF1B	-0.004254293	0.403960651	0.952318075
ALKBH4	-0.003060302	0.40398487	0.952318075
TCL1B	0.008046538	0.40401058	0.952318075
BSG	-0.006038958	0.404104955	0.952318075
PGS1	-0.002276152	0.404254615	0.952318075
SLC37A2	0.004983393	0.404293632	0.952318075
GPR55	-0.006558505	0.404295865	0.952318075
CCL4L2	-0.007389556	0.404340613	0.952318075
ATP2B4	0.003673782	0.404418084	0.952318075
ASH2L	-0.001726216	0.404424535	0.952318075
PA2G4	-0.003028775	0.404443941	0.952318075
PSTPIP2	0.003659007	0.404471552	0.952318075
DSCC1	-0.003195913	0.404490183	0.952318075
TPK1	-0.002544344	0.40466089	0.952318075
RPS6KB2	0.00318891	0.404814537	0.952318075
ACO1	-0.002047299	0.404839625	0.952318075
MPEG1	-0.00556673	0.405007153	0.952318075
PI4KA	-0.00244693	0.405093681	0.952318075
ACOT4	-0.005862745	0.405109311	0.952318075
ARL2	-0.002819494	0.405176093	0.952318075
LRRFIP2	0.002679025	0.405250915	0.952318075
IK	0.001975434	0.405256006	0.952318075
CHN2	-0.003302747	0.405320665	0.952318075
LTBR	-0.003398187	0.4055712	0.952318075
COQ3	-0.002122674	0.405706362	0.952318075
DPYD	-0.005020105	0.405957106	0.952318075
ZNF611	0.003168036	0.405968394	0.952318075
CHSY1	-0.002121805	0.406020401	0.952318075
BIRC2	0.002414315	0.40603439	0.952318075

CNNM4	-0.003252031	0.406054795	0.952318075
FAM173A	-0.002353051	0.406061009	0.952318075
ATP6V1E1	-0.001774613	0.406087316	0.952318075
COG8	0.003103487	0.406110822	0.952318075
CMC1	-0.003996422	0.406178016	0.952318075
STRC	0.005742669	0.406268604	0.952318075
PROSC	-0.002126179	0.406314086	0.952318075
ZNF91	0.005262722	0.406315918	0.952318075
MLST8	-0.003152606	0.406553829	0.952318075
H2AFX	-0.002915902	0.406574223	0.952318075
ESD	-0.002275543	0.406585679	0.952318075
PHACTR1	0.004229047	0.40660489	0.952318075
GATS	0.004545345	0.406773496	0.952318075
ANKS3	-0.003975452	0.406780671	0.952318075
ANLN	-0.003427958	0.406819869	0.952318075
C14orf28	-0.002777408	0.406858102	0.952318075
EFCAB4B	0.00290332	0.406903522	0.952318075
CDK10	-0.004223795	0.407396485	0.952318075
TRAPPC9	0.00372942	0.407523565	0.952318075
DCTPP1	-0.003046606	0.407588521	0.952318075
PPP1R12A	0.002478554	0.407739747	0.952318075
TRIM69	0.00407911	0.407759246	0.952318075
MSLN	0.018103707	0.407767193	0.952318075
WRNIP1	0.002951849	0.408042187	0.952318075
EIF4E2	0.001871172	0.408066063	0.952318075
SH3BP1	0.003742997	0.408096362	0.952318075
CUL2	-0.001893851	0.408165325	0.952318075
GOLM1	-0.005634714	0.408398097	0.952318075
RPL22L1	-0.012120605	0.408478313	0.952318075
DYNC2LI1	-0.004890153	0.408522848	0.952318075
MYL9	0.013371023	0.408608268	0.952318075
GOLGA4	-0.003189605	0.408618917	0.952318075
C9orf89	0.00435215	0.40863109	0.952318075
CSE1L	0.001686615	0.40876632	0.952318075
RNF19A	-0.003243139	0.409169083	0.952318075
MIR593	0.003266565	0.409307191	0.952318075
DCTN6	-0.002938573	0.409312192	0.952318075
SNX1	0.002657147	0.409536843	0.952318075
DNAJC7	-0.002157723	0.409751221	0.952318075
CUL1	0.001967981	0.409764642	0.952318075
RCHY1	0.004574146	0.410135838	0.952318075
FOXO3	0.003137545	0.410199566	0.952318075
IPO7	-0.003299665	0.410215492	0.952318075
TMEM116	0.002913786	0.410466279	0.952318075
AP4B1	-0.003540915	0.410650157	0.952318075
EDC3	-0.002837393	0.410849209	0.952318075
ABR	-0.002030222	0.410852652	0.952318075

RAI14	0.004337537	0.410924102	0.952318075
CEP350	0.00297647	0.411166323	0.952318075
NLRC5	0.003511481	0.411169717	0.952318075
PKP4	-0.003984067	0.411271738	0.952318075
PPP1R8	-0.003114231	0.411523856	0.952318075
CALM3	0.001839753	0.411660239	0.952318075
HSD17B7	0.006195406	0.411690245	0.952318075
COPS5	0.001655215	0.411704269	0.952318075
C12orf76	0.00329264	0.411774417	0.952318075
NOL7	-0.002306381	0.411803002	0.952318075
DNAJC4	0.002292539	0.411945038	0.952318075
BEND4	0.003982092	0.411952721	0.952318075
CD81	-0.002868696	0.412026407	0.952318075
SPATA24	0.003337922	0.412147891	0.952318075
ETS2	-0.003146385	0.412313171	0.952318075
PCBP4	-0.00364226	0.412331204	0.952318075
C15orf26	0.012004159	0.412366023	0.952318075
S100A9	-0.002642216	0.412486518	0.952318075
STX7	-0.003064789	0.412508008	0.952318075
MIR2116	0.004215569	0.41253921	0.952318075
MRPL4	0.002578456	0.412601836	0.952318075
YTHDC2	-0.002940795	0.412720365	0.952318075
NF1	0.004633099	0.412768013	0.952318075
VPS52	-0.002362612	0.412838394	0.952318075
OBFC1	0.002381788	0.412976114	0.952318075
MCM8	0.002773176	0.413043873	0.952318075
CADPS	-0.003322467	0.413151225	0.952318075
PURA	-0.003209721	0.413161813	0.952318075
PRSS57	0.007313592	0.413175713	0.952318075
VWF	0.012427837	0.413181228	0.952318075
PEX11A	-0.003787919	0.413194521	0.952318075
C3orf18	0.002856887	0.413470505	0.952318075
GPR132	-0.003897081	0.41378915	0.952318075
VAMP8	-0.00247247	0.413910925	0.952318075
ANP32A	-0.004130706	0.413941044	0.952318075
THRA	0.003378125	0.413957572	0.952318075
MRPL39	-0.003043903	0.414021092	0.952318075
GAPT	-0.005216198	0.414022068	0.952318075
ZNF418	0.005425452	0.414139826	0.952318075
KIAA1683	0.006106311	0.414196462	0.952318075
SNORA34	0.003078187	0.414220385	0.952318075
KCNH3	-0.009849229	0.414300169	0.952318075
TCP10L2	0.004237711	0.414415176	0.952318075
IL18RAP	0.007012127	0.414499371	0.952318075
GIN1	-0.002890738	0.414569986	0.952318075
ATG14	0.002235964	0.414685772	0.952318075
TMEM214	-0.002664008	0.414757414	0.952318075

WDFY1	-0.002224001	0.414771308	0.952318075
MRPS18B	0.002030526	0.414892126	0.952318075
ACP5	-0.005841682	0.414892171	0.952318075
GRN	-0.003427818	0.414905407	0.952318075
SPATA7	-0.003068534	0.414926464	0.952318075
DTWD2	0.006770096	0.415128043	0.952318075
GPSM3	0.002077503	0.415240941	0.952318075
MRPL50	-0.003094969	0.415315581	0.952318075
BANF1	-0.003252045	0.415321075	0.952318075
METTL1	-0.002788745	0.415401048	0.952318075
MAN1A2	-0.002707644	0.415468732	0.952318075
SIRT1	0.0021561	0.415662756	0.952318075
PPP2R4	0.002062544	0.415719511	0.952318075
CSNK1D	-0.001791902	0.41593432	0.952318075
STAC3	0.003309893	0.415991566	0.952318075
ZNF324B	-0.003401607	0.416086328	0.952318075
LRRC42	-0.0029157	0.416182429	0.952318075
BAMBI	0.008997174	0.416256857	0.952318075
JOSD2	-0.00394783	0.416347511	0.952318075
TTC33	-0.003430323	0.416500328	0.952318075
ZC3HAV1L	0.003196321	0.416595911	0.952318075
KCNMB2	-0.003830595	0.417212481	0.952318075
ZDHHC2	-0.003636317	0.41722822	0.952318075
RPIA	0.002115922	0.417444788	0.952318075
CYBA	0.003089753	0.417532953	0.952318075
TADA1	-0.002389694	0.417606169	0.952318075
TTC39C	0.002805255	0.417639414	0.952318075
SKA1	-0.003209072	0.417652329	0.952318075
PCSK7	0.002974062	0.417716753	0.952318075
E4F1	0.001702052	0.417836067	0.952318075
FUT4	-0.002379489	0.417994273	0.952318075
SFXN3	0.002647782	0.418024221	0.952318075
PTRH2	-0.00319085	0.418054738	0.952318075
TOMM5	-0.004411723	0.418278848	0.952318075
MR1	-0.003765314	0.418379164	0.952318075
STARD10	0.003742008	0.418400583	0.952318075
SEC31A	-0.001732931	0.418426101	0.952318075
UPF1	-0.003302931	0.41843855	0.952318075
LOXL3	-0.003758423	0.418607588	0.952318075
NRARP	0.002701585	0.418685369	0.952318075
HNRNPA1L2	-0.002830663	0.418704786	0.952318075
MRPL2	-0.004641271	0.418901281	0.952318075
PLCG1	-0.003048755	0.418909388	0.952318075
MT1G	-0.004464114	0.418942834	0.952318075
PARS2	-0.0037108	0.418971022	0.952318075
HERC1	0.002111711	0.418986923	0.952318075
PIP4K2C	-0.002375614	0.418999848	0.952318075

BATF3	-0.004390497	0.419056079	0.952318075
DNAJC3	-0.003882862	0.419133213	0.952318075
ACSBG1	0.010214078	0.419505106	0.952318075
RPS7	0.002005488	0.41952029	0.952318075
CRIPAK	-0.005475202	0.419528075	0.952318075
MT1X	-0.00384889	0.419595926	0.952318075
GTF2H5	0.002249654	0.419682182	0.952318075
WDR61	-0.002655759	0.419739367	0.952318075
TMEM8B	-0.003373333	0.419891749	0.952318075
CHKA	-0.002985799	0.419936797	0.952318075
UMPS	-0.003517302	0.420011699	0.952318075
HPS3	-0.00294377	0.420090574	0.952318075
SP110	0.002231147	0.420093271	0.952318075
XRCC6	0.002217553	0.420103587	0.952318075
PLEKHA3	-0.003922017	0.420139625	0.952318075
ZNF28	-0.002882263	0.420164837	0.952318075
TPMT	-0.002409171	0.420253727	0.952318075
PLEKHO2	-0.003470557	0.420323161	0.952318075
NINJ2	-0.004401214	0.420590605	0.952318075
GZMK	-0.007153586	0.4206504	0.952318075
LMOD3	0.008717566	0.42071351	0.952318075
CBR4	0.002794798	0.420713662	0.952318075
PDSS2	0.002062829	0.420736392	0.952318075
PATE3	-0.003717247	0.420814365	0.952318075
EPB41L5	-0.002924713	0.420837176	0.952318075
DNAH6	0.005372114	0.420881608	0.952318075
BCRP2	0.003012497	0.42097104	0.952318075
ISG20L2	0.002856487	0.421211539	0.952318075
PMS2P3	-0.003474918	0.421270738	0.952318075
SNX17	-0.002124375	0.421342799	0.952318075
MRPS9	-0.002263884	0.42143717	0.952318075
ECI2	0.002538377	0.421447472	0.952318075
RPS17	0.002354518	0.42166628	0.952318075
VPS41	0.005870492	0.421674423	0.952318075
MAN1C1	-0.004245697	0.421674554	0.952318075
ZNF827	-0.003662066	0.421789486	0.952318075
HIBCH	-0.002588419	0.421805616	0.952318075
MAP3K2	-0.004765124	0.421805933	0.952318075
SLC16A12	0.001357884	0.421870193	0.952318075
PLD3	-0.002452426	0.421899078	0.952318075
ZNF318	0.002156405	0.421970869	0.952318075
ANO8	-0.003186656	0.42203425	0.952318075
FPR2	-0.009342573	0.422122954	0.952318075
USF1	0.005821707	0.422241177	0.952318075
SPG20	0.002928856	0.422434657	0.952318075
SHD	-0.006167412	0.42260091	0.952318075
PARP3	-0.004596565	0.423018909	0.952318075

CDO1	-0.004176355	0.423204624	0.952318075
WEE1	0.004408022	0.423281913	0.952318075
FCRLA	0.00871066	0.42330697	0.952318075
CHAC2	-0.004475302	0.423370966	0.952318075
CORO7	0.003259254	0.423425952	0.952318075
FAM69A	-0.004318544	0.423465449	0.952318075
HEATR6	-0.002666855	0.423469987	0.952318075
C17orf66	-0.003951172	0.423510275	0.952318075
RNF2	-0.003557476	0.423608492	0.952318075
PTGES2	-0.004547036	0.423618662	0.952318075
POU2F1	0.002457848	0.42363793	0.952318075
ZNF154	0.009063551	0.423690422	0.952318075
TTYH3	0.003085384	0.42400448	0.952318075
ZNF416	-0.003740865	0.424037138	0.952318075
EZR	-0.002015805	0.424041097	0.952318075
WDR75	0.002458346	0.424121345	0.952318075
PPIB	-0.005343315	0.424248907	0.952318075
RCVRN	0.004847222	0.424305684	0.952318075
TTC30B	-0.003091594	0.424330736	0.952318075
NUDT6	-0.003976749	0.424635748	0.952318075
ARFGAP3	-0.002450482	0.424665972	0.952318075
FAM26E	0.003497281	0.425254649	0.952318075
PRR11	-0.00375819	0.425472571	0.952318075
RNF123	-0.001892332	0.425491055	0.952318075
PVRL3	0.004817847	0.425506681	0.952318075
USP48	-0.003711536	0.42553004	0.952318075
ATOH7	0.003232493	0.42555644	0.952318075
MSC	-0.00692689	0.425575422	0.952318075
ERCC6	0.003206399	0.42560214	0.952318075
NLRC4	-0.003762226	0.425634823	0.952318075
NAE1	0.001824384	0.42588268	0.952318075
TMEM184C	-0.00249989	0.426025714	0.952318075
CYP2D6	-0.003545491	0.426172958	0.952318075
UBA52	9.36E-04	0.426276133	0.952318075
SCD5	-0.006545851	0.426350915	0.952318075
DNAJB2	-0.002325936	0.426393724	0.952318075
MARCH7	-0.001796899	0.426444162	0.952318075
LARP1	0.001711677	0.426586744	0.952318075
LMF1	-0.003368776	0.42665379	0.952318075
LTF	-0.003624707	0.426712127	0.952318075
BNC2	-0.00589993	0.426944621	0.952318075
KLB	-0.002531716	0.426981313	0.952318075
MAN2C1	-0.004491145	0.427064795	0.952318075
PSMC3IP	-0.003409077	0.427252789	0.952318075
SLC46A3	-0.002124603	0.427294767	0.952318075
SMG7	0.003461125	0.427390514	0.952318075
PPP1R16A	-0.00374453	0.427433184	0.952318075

BIN1	0.003028072	0.427476513	0.952318075
AASS	-0.004050495	0.427622837	0.952318075
TMEM156	0.003731799	0.427639493	0.952318075
TXNDC11	0.002930423	0.427849463	0.952318075
MYL12A	0.003488673	0.427893107	0.952318075
SDCCAG8	-0.00325642	0.427925491	0.952318075
PDZK1IP1	0.010856811	0.428264125	0.952318075
PPP2R2B	-0.006179025	0.428381464	0.952318075
HARBI1	0.003731757	0.428396465	0.952318075
FST	0.007183163	0.428663859	0.952318075
AKAP5	-0.001394588	0.42875042	0.952318075
DCTD	0.001777489	0.428886638	0.952318075
ECHDC2	0.003583077	0.428901179	0.952318075
C19orf43	0.002299241	0.428911961	0.952318075
MON2	-0.00244778	0.4289644	0.952318075
ISCA1	0.002877424	0.429002981	0.952318075
PDE6B	-0.003401995	0.42906017	0.952318075
ABCB6	-0.003224818	0.429107909	0.952318075
IRF4	0.00456288	0.429143074	0.952318075
RAPGEF2	-0.00291895	0.429169948	0.952318075
CD22	0.005484659	0.429228909	0.952318075
RSC1A1	0.003229094	0.429341022	0.952318075
SFI1	-0.003375475	0.429357031	0.952318075
ZBTB8OS	0.002700863	0.429433966	0.952318075
ULK2	-0.002976401	0.429438539	0.952318075
CEMP1	-0.003693248	0.429445186	0.952318075
NACA	-0.001787068	0.429546298	0.952318075
WDR27	0.003540801	0.429646223	0.952318075
PELI1	0.00387095	0.42974248	0.952318075
NCKAP5	0.006162228	0.429777703	0.952318075
GPAT2	0.007521997	0.429799593	0.952318075
PLSCR4	0.003447091	0.429831242	0.952318075
PHF7	0.003205405	0.429886088	0.952318075
KIAA1009	-0.003579471	0.429920327	0.952318075
C18orf8	0.001787981	0.429966037	0.952318075
NSD1	0.002669758	0.430010123	0.952318075
ATP6V0C	-0.001755973	0.430272393	0.952318075
TFPT	0.00290681	0.430412616	0.952318075
FBLN2	-0.00499633	0.430582485	0.952318075
NINJ1	-0.00423256	0.430689609	0.952318075
KDM4C	0.002447521	0.430702918	0.952318075
SPARC	0.009745931	0.430805954	0.952318075
CLOCK	-0.003004466	0.430818058	0.952318075
MOGS	0.002539554	0.430835614	0.952318075
PSKH1	-0.003127967	0.430857751	0.952318075
OR1J1	0.004928543	0.430926798	0.952318075
QSOX1	-0.002535888	0.430936946	0.952318075

PSTPIP1	-0.003485362	0.430940507	0.952318075
SMOX	0.00775762	0.430981675	0.952318075
KCTD10	0.007200865	0.431066038	0.952318075
NRG1	-0.013285289	0.431070341	0.952318075
NHP2L1	-0.002129425	0.431108517	0.952318075
EIF2B2	-0.003154307	0.431201143	0.952318075
FAM60A	-0.001740286	0.431416808	0.952318075
PGAP2	0.001996539	0.43142	0.952318075
AIF1	-0.002533776	0.43146364	0.952318075
MRTO4	0.002596248	0.431553557	0.952318075
RAB2A	-0.002710463	0.431732322	0.952318075
GDPD3	-0.004536599	0.431759752	0.952318075
MGA	0.0029514	0.431821251	0.952318075
TIGD5	0.002689354	0.431960548	0.952318075
KPNA1	-0.001802821	0.431981289	0.952318075
TARBP2	-0.0029867	0.432021365	0.952318075
KCNA3	0.004258758	0.432080257	0.952318075
MEOX1	0.005921743	0.432141505	0.952318075
SCARNA5	0.004611138	0.432266937	0.952318075
H3F3A	-0.002185875	0.43229046	0.952318075
USP4	0.001734195	0.432292829	0.952318075
ITK	0.003085411	0.432393508	0.952318075
CFH	0.007513231	0.432419466	0.952318075
THAP6	-0.003900072	0.432513002	0.952318075
NECAP2	0.002285169	0.432731228	0.952318075
CCNB2	-0.007384384	0.432795628	0.952318075
CRELD1	-0.002842783	0.432861519	0.952318075
GADD45B	0.002948995	0.432911735	0.952318075
SLC18A1	-0.003169775	0.433058334	0.952318075
ABI3	0.003193949	0.43310569	0.952318075
ZNF761	0.003475416	0.433105945	0.952318075
TMEM62	-0.002487504	0.43312657	0.952318075
RAP1GDS1	0.002792895	0.433129123	0.952318075
SFXN1	0.003676022	0.433179367	0.952318075
GOSR2	-0.003387632	0.4332444	0.952318075
FAM184A	0.004156148	0.433460191	0.952318075
TMEM41B	-0.002848369	0.433526552	0.952318075
ARL4D	0.002869389	0.433571522	0.952318075
ZNF263	0.002398314	0.433685977	0.952318075
PDE12	-0.002316173	0.43373675	0.952318075
TNFSF9	0.003582231	0.433761466	0.952318075
SACM1L	0.003143142	0.433876426	0.952318075
KNCN	0.00377543	0.433900021	0.952318075
HACL1	0.001544946	0.433935877	0.952318075
UQCRFS1	-0.002424815	0.433954244	0.952318075
CD83	0.003788048	0.433955111	0.952318075
FRG1	0.003177552	0.434153829	0.952318075

POMZP3	0.007494409	0.434249494	0.952318075
CENPV	0.004070478	0.434265766	0.952318075
PRKCZ	0.002564994	0.434363672	0.952318075
CD5	0.003599866	0.434371611	0.952318075
ATP5C1	-0.002656285	0.434394659	0.952318075
TMF1	-0.002675218	0.434465669	0.952318075
LILRA2	-0.003430408	0.434679865	0.952318075
TJP2	0.004618997	0.435007112	0.952318075
HNRNPA3	0.004062581	0.435032082	0.952318075
ZBED5	-0.0019474	0.435199293	0.952318075
CDK12	-0.004563918	0.435207565	0.952318075
ARFRP1	-0.003368998	0.435293343	0.952318075
DAPP1	0.001866579	0.435333973	0.952318075
GOT1	-0.001671838	0.435383789	0.952318075
SNORA3	0.004523116	0.435472718	0.952318075
WDFY3	0.00394874	0.435640199	0.952318075
FOXC1	0.004662928	0.435822985	0.952318075
NFE2L1	0.003050481	0.435877229	0.952318075
SLC27A1	-0.003163847	0.435911011	0.952318075
NRSN2	0.00358705	0.436068122	0.952318075
VPRBP	-0.003093911	0.436128569	0.952318075
CDKN1C	-0.007745774	0.436134326	0.952318075
KIF16B	0.0051283	0.436212692	0.952318075
KCTD21	-0.003313599	0.436247609	0.952318075
MRP63	0.004247132	0.436249343	0.952318075
PTPRM	0.008169231	0.436290883	0.952318075
NR2C1	0.002894114	0.436300631	0.952318075
MC1R	-0.005298962	0.436300672	0.952318075
PNPLA8	-0.002757445	0.436361564	0.952318075
MAN2B1	0.00321156	0.436595115	0.952318075
TGFBI	-0.002923207	0.436631997	0.952318075
NAA25	0.002877366	0.43682917	0.952318075
AGL	0.002682626	0.436911438	0.952318075
PSMD11	0.002319111	0.437002514	0.952318075
MOCS2	-0.004529009	0.437026462	0.952318075
GMIP	-0.003047422	0.437036937	0.952318075
SEZ6L2	0.003452235	0.437163113	0.952318075
DUSP27	0.003584124	0.437189531	0.952318075
KLRF1	0.006422887	0.437372749	0.952318075
NXF1	-0.002056542	0.437397273	0.952318075
AIG1	0.002900442	0.437461387	0.952318075
MAP3K9	0.003711376	0.437586171	0.952318075
LINC00347	0.002898867	0.437656118	0.952318075
AGAP4	0.005640862	0.437683508	0.952318075
NPDC1	0.004178116	0.437686746	0.952318075
RAB15	-0.004792036	0.437696077	0.952318075
FCHO2	-0.00574701	0.437748455	0.952318075

TACR2	0.003208192	0.437872602	0.952318075
ZNHIT6	0.001930792	0.437907724	0.952318075
LTV1	0.001611985	0.437974644	0.952318075
IKZF1	-0.005854521	0.438132992	0.952318075
YTHDC1	0.002485167	0.438189847	0.952318075
SAP30BP	-0.002788917	0.43819363	0.952318075
ECM1	-0.003907112	0.438236079	0.952318075
CCNA2	-0.005559467	0.438271105	0.952318075
MRPL54	-0.00276026	0.438322081	0.952318075
PLCL1	0.004343627	0.438378841	0.952318075
ACTN1	0.004163894	0.438436765	0.952318075
MIR1323	-0.003607042	0.438467462	0.952318075
TRAF3IP2	-0.003338665	0.438500215	0.952318075
RANGRF	0.003973223	0.438515875	0.952318075
LRRC3	-0.002823869	0.438538276	0.952318075
PLXND1	-0.004021158	0.438550208	0.952318075
ZFAND2A	0.003350856	0.438607767	0.952318075
CYP2R1	-0.002495875	0.438651113	0.952318075
TTC13	-0.002765615	0.438672257	0.952318075
ARSA	-0.004177656	0.438728942	0.952318075
ASAH1	-0.004492502	0.438940814	0.952318075
DSTNP2	0.002406824	0.438952008	0.952318075
FDPS	0.002182469	0.438999995	0.952318075
HEATR1	-0.005499686	0.439078675	0.952318075
RAPSN	0.003586677	0.439125821	0.952318075
TRIM35	-0.003417125	0.439158145	0.952318075
ACOT2	0.004234153	0.439176782	0.952318075
SRD5A3	-0.00404064	0.439206383	0.952318075
NR3C1	-0.002698362	0.439228675	0.952318075
LMBR1	-0.003240965	0.439321395	0.952318075
FYB	-0.001774664	0.439396096	0.952318075
ATXN7L3B	0.00212007	0.43944381	0.952318075
MCOLN3	0.003769273	0.439570071	0.952318075
MCM5	0.002236361	0.439712005	0.952318075
HLA-DRB1	0.047322237	0.439733852	0.952318075
WDR7	-0.002275699	0.439841883	0.952318075
MRM1	-0.003317678	0.439876395	0.952318075
ASNS	0.003649826	0.440168851	0.952318075
CRYL1	-0.003337905	0.440231395	0.952318075
BLZF1	0.004866203	0.440302673	0.952318075
ZNF703	-0.004066803	0.440364893	0.952318075
RGS17	-0.00691116	0.440572785	0.952318075
PRM3	-0.002701031	0.440638071	0.952318075
PLCD1	0.002915184	0.440746417	0.952318075
GLUD1	-0.001474858	0.440761376	0.952318075
ATP2B1	0.002697206	0.440788105	0.952318075
PHKG2	-0.002779651	0.440827868	0.952318075

E2F4	0.001829151	0.441020795	0.952318075
IPO13	-0.002613612	0.441343629	0.952318075
CUEDC2	-0.00194196	0.441677023	0.952318075
NDUFS8	-0.001716737	0.441815602	0.952318075
SV2B	-0.002923608	0.441820166	0.952318075
UBB	-0.003822546	0.441884415	0.952318075
OTUD1	0.002879875	0.441925128	0.952318075
PARP16	0.002785091	0.442068594	0.952318075
FAM115C	-0.006184558	0.442114044	0.952318075
PRR14L	0.001342962	0.442295636	0.952318075
ADHFE1	-0.003724832	0.442570775	0.952318075
CCDC51	-0.00288052	0.442656743	0.952318075
UBTF	0.003229507	0.442778354	0.952318075
ZNF362	-0.002049364	0.443021777	0.952318075
SOX12	0.003237855	0.443032491	0.952318075
FKBP14	0.007407491	0.443185453	0.952318075
TMEM101	-0.002045816	0.443237074	0.952318075
HERC6	0.003663282	0.443241133	0.952318075
FANCG	-0.002763291	0.4432555	0.952318075
SCAPER	-0.002902198	0.443329726	0.952318075
OGFR	0.002771384	0.443355624	0.952318075
MUC16	0.002998146	0.443448035	0.952318075
CABIN1	-0.002968691	0.443454171	0.952318075
RNF7	-0.001683533	0.443484177	0.952318075
AMFR	-0.00677333	0.443515117	0.952318075
B2M	-0.001220008	0.443629595	0.952318075
ETFA	-0.002943255	0.443744478	0.952318075
HERC2P3	0.003394898	0.443777189	0.952318075
MTA1	-0.002618774	0.443909466	0.952318075
GPR108	0.002544999	0.444118046	0.952318075
SLC4A7	0.003357594	0.444186847	0.952318075
FOXK2	-0.004062652	0.444222838	0.952318075
OVGP1	0.004777838	0.444328791	0.952318075
UIMC1	-0.002183744	0.444478892	0.952318075
CAPRIN1	-0.003228175	0.444489878	0.952318075
RPL23AP53	-0.001812126	0.444694726	0.952318075
ZDHHC17	0.002546192	0.444767899	0.952318075
MRPS33	-0.002593704	0.444841795	0.952318075
ARL6IP5	-0.001725062	0.444893573	0.952318075
RAB11FIP3	-0.002358781	0.445077362	0.952318075
USP28	0.005097689	0.445079944	0.952318075
TNFRSF10A	0.003404334	0.445156563	0.952318075
KCNK3	-0.002934267	0.445193667	0.952318075
SLC1A4	-0.004160672	0.445293572	0.952318075
ENSA	0.003619489	0.445335836	0.952318075
TTLL5	0.002470013	0.445556547	0.952318075
PEX5	-0.002009269	0.445566262	0.952318075

SMC4	-0.00229147	0.445601889	0.952318075
DUSP13	0.003209389	0.445657141	0.952318075
NCOA6	0.001599284	0.445683489	0.952318075
LEP	0.006509748	0.445833887	0.952318075
HES4	0.007186553	0.445869633	0.952318075
ARHGAP12	-0.002532883	0.445903308	0.952318075
MMS22L	0.002891269	0.446172137	0.952318075
PTPN3	0.002526243	0.446184257	0.952318075
CTC1	-0.002482111	0.446246101	0.952318075
HN1L	0.002941245	0.446365223	0.952318075
SDPR	0.006008152	0.446382712	0.952318075
LPAR6	0.003382753	0.446436994	0.952318075
CPM	0.002900152	0.446519543	0.952318075
TIPIN	-0.003635418	0.446541501	0.952318075
NUP62	-0.001716204	0.446616996	0.952318075
DGCR14	0.002422408	0.446669919	0.952318075
PHOSPHO2	-0.004773262	0.446679741	0.952318075
CDK17	-0.003065673	0.446838036	0.952318075
SAMD11	-0.003588477	0.446849994	0.952318075
MGMT	-0.003635619	0.447047451	0.952318075
GFI1B	0.006071888	0.447065889	0.952318075
VDAC2	-0.00214895	0.44708717	0.952318075
MYBL1	0.00488906	0.447605047	0.952318075
EIF2B1	-0.002258982	0.447634129	0.952318075
TNFRSF9	0.007524924	0.447701663	0.952318075
RBM6	0.002754676	0.447811157	0.952318075
GMPPB	-0.003258049	0.447887917	0.952318075
H2AFZ	-0.001789755	0.448001999	0.952318075
MAP1S	-0.002620322	0.44808957	0.952318075
CHORDC1	-0.002621005	0.448166422	0.952318075
BUD13	-0.001905461	0.448181339	0.952318075
C1GALT1	-0.003342164	0.448203271	0.952318075
PHF20L1	-0.001697006	0.448379041	0.952318075
LRRC23	0.004286119	0.448380572	0.952318075
SAMD3	-0.004228267	0.448384853	0.952318075
KIAA0907	-0.002153619	0.448528592	0.952318075
ZNF319	-0.002669091	0.448538536	0.952318075
BAALC	-0.003016382	0.448630573	0.952318075
ADAP2	-0.003251193	0.448732478	0.952318075
ZNF148	0.004023553	0.448819886	0.952318075
ZNF586	-0.003001366	0.448975293	0.952318075
AP4M1	-0.002895157	0.449024658	0.952318075
VPS8	-0.001859991	0.449103521	0.952318075
LDLRAP1	0.004471694	0.449103546	0.952318075
PRR24	0.003207973	0.449176952	0.952318075
PPP1R15B	-0.0033623	0.449201875	0.952318075
TCF7L2	0.004960877	0.449237947	0.952318075

DAPK3	-0.003203065	0.449476013	0.952318075
BMS1P5	0.004435046	0.449489666	0.952318075
TRPC2	0.003242843	0.449521297	0.952318075
CLEC1A	-0.00390498	0.449613808	0.952318075
SNORA70B	0.00364213	0.449710529	0.952318075
UPF2	0.001431154	0.449733945	0.952318075
MEN1	0.002496609	0.449736418	0.952318075
WSB1	-0.003201726	0.449747827	0.952318075
BCL2	0.003135714	0.45004781	0.952318075
B3GALNT1	-0.003943604	0.450107983	0.952318075
GZMM	0.004536905	0.450136188	0.952318075
RAB7A	-0.001516573	0.450166067	0.952318075
DDX27	0.002249366	0.450270218	0.952318075
SLC44A1	0.002675049	0.450346885	0.952318075
EIF2S2	0.002804501	0.450463	0.952318075
ASNA1	-0.004355776	0.450490357	0.952318075
GPR65	0.003476719	0.45049698	0.952318075
SSPN	-0.007471071	0.450589757	0.952318075
ALG5	-0.002419516	0.450590912	0.952318075
ZNF189	-0.002873547	0.450634874	0.952318075
HIST2H2AA4	0.006269899	0.450928103	0.952318075
ADAT2	0.002811412	0.450934953	0.952318075
ZNF383	-0.002224781	0.451062691	0.952318075
SNHG7	0.00460339	0.45130576	0.952318075
TRIP11	0.002268383	0.451343506	0.952318075
PYROXD2	-0.004160785	0.451814771	0.952318075
ITGAL	-0.003119299	0.451858504	0.952318075
DUSP2	0.005288779	0.451919945	0.952318075
ZIM3	-0.003037813	0.451922346	0.952318075
KPNA6	-0.002270984	0.451934739	0.952318075
DPY19L4	0.007822387	0.451939479	0.952318075
SCARB2	-0.00194383	0.451968345	0.952318075
SLC45A1	-0.002922477	0.452095868	0.952318075
TSG101	-0.001820889	0.452180612	0.952318075
RPS10P7	0.003475908	0.452193121	0.952318075
LMAN1	0.002690781	0.45221149	0.952318075
WDR89	0.004003649	0.452234585	0.952318075
UTP23	-0.003096718	0.452348676	0.952318075
PATL2	-0.007102352	0.452361398	0.952318075
CLEC2B	-0.007474724	0.452424242	0.952318075
SMPD1	-0.003088001	0.45242453	0.952318075
CCDC58	-0.008209545	0.452487575	0.952318075
STX16	0.004472618	0.452662928	0.952318075
TRADD	0.002945179	0.452798416	0.952318075
SLAMF1	-0.003608772	0.452837156	0.952318075
HYAL2	0.002794519	0.452999318	0.952318075
SNORD54	0.004579201	0.453021538	0.952318075

EAPP	-0.004496698	0.453078816	0.952318075
SRGAP3	0.003323141	0.453121669	0.952318075
FOSL2	0.002899475	0.453143284	0.952318075
STAU1	9.32E-04	0.453350759	0.952318075
UNG	0.002762622	0.453410124	0.952318075
CDK1	-0.005415751	0.453466192	0.952318075
RBM15B	0.002546271	0.453608702	0.952318075
MEF2BNB	-0.003172135	0.453704355	0.952318075
SKAP2	-0.003237908	0.453712027	0.952318075
IP6K2	0.008417311	0.45372402	0.952318075
MIR1282	-0.006686966	0.453727685	0.952318075
PEX7	-0.003156787	0.454051994	0.952318075
LY75	-0.004747772	0.454059694	0.952318075
C2orf49	-0.003211204	0.454095112	0.952318075
ZNF131	0.001160382	0.454201288	0.952318075
ELMOD3	0.00343816	0.454285667	0.952318075
DBR1	-0.002397863	0.454346836	0.952318075
ENO1	-0.001629139	0.454391358	0.952318075
CAMK2G	-0.001703642	0.454415448	0.952318075
CREBL2	0.003032084	0.454641892	0.952318075
PHLPP1	-0.002230842	0.454651517	0.952318075
MTRF1	-0.002805164	0.45475153	0.952318075
WDR76	0.002834068	0.454775385	0.952318075
RANBP1	0.001724201	0.454814415	0.952318075
EXOSC2	-0.002610948	0.454839439	0.952318075
POPDC2	-0.003469045	0.45486545	0.952318075
KIAA1731	0.002418041	0.45503497	0.95249283
ZKSCAN1	0.006014478	0.455346928	0.952531083
FLVCR2	-0.003162878	0.455509031	0.952531083
ATP6V0A1	0.002557259	0.455596066	0.952531083
SEC22C	0.001651825	0.455782782	0.952531083
ADCY7	-0.001944661	0.45589855	0.952531083
RALA	-0.001692546	0.456079214	0.952531083
TRIM38	0.004539836	0.456142442	0.952531083
ORAI2	0.003430958	0.456174029	0.952531083
FAM171A1	-0.003987991	0.45621198	0.952531083
TDP2	0.003645534	0.456250191	0.952531083
TOP2B	0.001281218	0.45629236	0.952531083
PSPC1	0.002731339	0.456579214	0.952531083
ALKBH7	0.00341777	0.456643419	0.952531083
CYP4V2	0.004244881	0.456776611	0.952531083
SFT2D2	-0.003287795	0.456867543	0.952531083
NOC3L	-0.002244081	0.456947623	0.952531083
NDUFS3	0.001084532	0.456981952	0.952531083
REV3L	-0.003301528	0.457065495	0.952531083
TMCO1	-0.001598066	0.45708011	0.952531083
MIR25	0.003742399	0.457086401	0.952531083

SUN1	0.001897271	0.457352613	0.952531083
RAB37	0.003262394	0.457394381	0.952531083
WNT5B	-0.003511282	0.45752625	0.952531083
TSC1	0.001925825	0.457529521	0.952531083
NEMF	0.002504126	0.457547274	0.952531083
DEFB108B	-0.002763515	0.457553145	0.952531083
YIPF2	-0.002865196	0.45762983	0.952531083
ATP5J	-0.002380022	0.457718415	0.952531083
RAPGEF1	-0.002548399	0.457743227	0.952531083
DENND1A	-0.004693032	0.457927914	0.952531083
NDUFS1	0.003499539	0.457963967	0.952531083
CSNK2A1	-0.003292649	0.4584331	0.952531083
KRAS	0.002161124	0.458449228	0.952531083
RAB21	-0.001643027	0.458524855	0.952531083
PRMT2	-0.00337837	0.458537154	0.952531083
DPY19L3	-0.003090162	0.458549519	0.952531083
MTHFD1	0.002882772	0.458781194	0.952531083
C9orf38	0.006433916	0.458801462	0.952531083
GNA11	0.004604125	0.458957985	0.952531083
DDX51	0.004451391	0.458969669	0.952531083
SDHAP2	-0.004295205	0.459099353	0.952531083
ITM2C	-0.0049755	0.45928264	0.952531083
CBFA2T2	-0.002632776	0.459308811	0.952531083
ZNF354A	-0.003320989	0.459365695	0.952531083
TARS2	-0.002440024	0.459375675	0.952531083
CPSF6	-0.003382617	0.459521514	0.952531083
ANKRD33	-0.004125165	0.459605367	0.952531083
ADNP	-0.001856731	0.459757822	0.952531083
PPIAL4E	0.003003072	0.459759299	0.952531083
BARD1	0.004215376	0.459817891	0.952531083
INPP5D	-0.002539395	0.459883222	0.952531083
NSUN5P2	0.004185369	0.459890856	0.952531083
CUL4A	0.001566399	0.459909979	0.952531083
MPP5	-0.002573746	0.45995094	0.952531083
FAM64A	-0.002470619	0.460081861	0.952531083
CHMP1A	-0.00259099	0.460183539	0.952531083
RPA2	-0.001739841	0.460220345	0.952531083
CRY1	-0.002081178	0.460249235	0.952531083
ZNF816	-0.003775242	0.460332402	0.952531083
NPL	-0.004227864	0.460480457	0.952531083
ARHGAP33	0.003438116	0.460577918	0.952531083
CD37	0.003171053	0.460627999	0.952531083
DCP1A	0.00199284	0.460737189	0.952531083
C10orf118	-0.002575968	0.460748372	0.952531083
LIN7B	0.002978775	0.460838439	0.952531083
LY6G5C	-0.004032344	0.460903517	0.952531083
ZNF276	-0.003115683	0.460936872	0.952531083

ECH1	0.001838454	0.461090167	0.952531083
POLR2B	-0.001635611	0.461098451	0.952531083
LGALS2	-0.01048811	0.46122569	0.952531083
GTSE1	-0.003015059	0.461267925	0.952531083
NDC80	0.003391957	0.461320853	0.952531083
MXD1	-0.003097794	0.461399196	0.952531083
ZNF845	-0.002867525	0.461803073	0.952531083
SLC25A17	0.002395375	0.461819929	0.952531083
DDX31	-0.002760206	0.461820673	0.952531083
TBC1D4	0.004028382	0.461964226	0.952531083
DDX60	-0.003620242	0.46202906	0.952531083
CRYBB2P1	0.007070049	0.462059561	0.952531083
HCN3	-0.003493715	0.46212034	0.952531083
SMARCE1	0.002928273	0.462235079	0.952531083
CLN8	-0.004462814	0.46228009	0.952531083
KIFAP3	0.002902934	0.462293555	0.952531083
STAMBPL1	0.002608343	0.462322755	0.952531083
ALDH6A1	0.002037595	0.46241641	0.952531083
ZNF69	0.006703605	0.462509126	0.952531083
MMP23B	-0.005647621	0.462539938	0.952531083
UBE2V2	-0.001898987	0.462726683	0.952577036
NOLC1	-0.002656174	0.462759027	0.952577036
GGTLC2	-0.002903461	0.462987604	0.952577036
LYRM1	0.001459478	0.462993991	0.952577036
TBCCD1	0.003011431	0.463113636	0.952577036
CXCL10	-0.008310894	0.463126645	0.952577036
PAN3	0.002614709	0.463191904	0.952577036
EIF4G2	-0.00119474	0.463259337	0.952577036
BTF3	-0.001512748	0.463576603	0.952577036
RPS14	0.001978657	0.463663445	0.952577036
PREP	-0.001867513	0.463715615	0.952577036
TGS1	-0.003688352	0.463744289	0.952577036
NEURL4	-0.003497687	0.463815939	0.952577036
BOK	0.004069182	0.463887307	0.952577036
ANKRD36B	-0.007995099	0.464008539	0.952577036
N6AMT1	-0.003524961	0.464043783	0.952577036
PHB	-0.002420525	0.464222824	0.952577036
CPT2	0.002144115	0.464321919	0.952577036
CCT7	-0.001545846	0.464516777	0.952577036
LSMD1	0.002425509	0.464629947	0.952577036
UPK1A	-0.00311543	0.46491986	0.952577036
DDA1	0.001620515	0.46506364	0.952577036
ENTPD3	-0.002464552	0.465076748	0.952577036
SLC9A1	-0.002070497	0.465188216	0.952577036
FBXO46	0.002011653	0.465235439	0.952577036
SNRPD2	0.001499302	0.46538336	0.952577036
UBE2T	-0.003236151	0.46545631	0.952577036

PUM1	0.001365318	0.465488909	0.952577036
ZDHHC3	0.002208913	0.465679906	0.952577036
DOCK2	-0.001914128	0.465706183	0.952577036
SPON2	-0.008184149	0.46575795	0.952577036
KCNA5	-0.004601337	0.465860836	0.952577036
FOXN2	-0.001937864	0.465886197	0.952577036
TCF19	-0.003125542	0.465943765	0.952577036
RNPS1	0.002538207	0.4660803	0.952577036
HELB	0.004541294	0.466091413	0.952577036
POLR2J2	0.005966997	0.466114549	0.952577036
FRY	0.002522075	0.466156869	0.952577036
TMEM71	0.003150788	0.46621374	0.952577036
ZNF658B	0.002784162	0.466216042	0.952577036
HSPA8	-0.002169268	0.466555341	0.952577036
ZFAND6	-0.005015464	0.466589116	0.952577036
PPT2	0.003205	0.466768605	0.952577036
USP18	0.006205643	0.466974179	0.952577036
RAB2B	0.001908105	0.467120165	0.952577036
SLC12A4	0.002680067	0.46727929	0.952577036
SAE1	0.001498409	0.46734485	0.952577036
PRMT5	-0.002081142	0.467376091	0.952577036
PLEKHO1	0.001915009	0.467522757	0.952577036
NCAPD2	-0.003299022	0.467531223	0.952577036
SEC16A	0.001871398	0.467541792	0.952577036
HHEX	0.002703803	0.467611702	0.952577036
SAMD4B	-0.003171675	0.467683877	0.952577036
N4BP2	0.00562182	0.467714738	0.952577036
MFF	-0.014792557	0.467736495	0.952577036
ALG9	0.001665529	0.467784512	0.952577036
HIST1H2BK	0.004368961	0.467861464	0.952577036
XPNPEP3	-0.001273066	0.467920139	0.952577036
MUC6	0.004394974	0.467979895	0.952577036
NECAB3	0.003113552	0.468067469	0.952577036
TNFRSF18	0.004371336	0.46848228	0.952577036
MSX2P1	-0.004029366	0.468496171	0.952577036
STRN	0.002599036	0.46850473	0.952577036
IRF2BP1	0.003846341	0.468518122	0.952577036
URGCP	0.00194162	0.468776867	0.952577036
NOD1	-0.003383903	0.468821837	0.952577036
HIST1H2AE	-0.008957915	0.468901268	0.952577036
RPS6	-0.001650116	0.468986594	0.952577036
TAF1D	-0.005977137	0.469000684	0.952577036
SMARCD3	-0.002950877	0.469045884	0.952577036
ST8SIA1	-0.004359352	0.469065092	0.952577036
MAPK1	-0.002077789	0.469159581	0.952577036
RCAN2	-0.009413461	0.469174976	0.952577036
ZMIZ1	-0.002163152	0.469194455	0.952577036

ING3	-0.001547916	0.46922214	0.952577036
MRPS7	-0.002275118	0.469265965	0.952577036
SSSCA1	-0.004323839	0.469266813	0.952577036
MFSD6L	-0.002306562	0.469274783	0.952577036
RHOG	-0.002557938	0.469497417	0.952684546
TALDO1	-0.002103737	0.469530177	0.952684546
BPHL	0.001848899	0.469740845	0.952684546
FCRL2	0.007548211	0.469932956	0.952684546
SNRNP27	0.002532962	0.469959878	0.952684546
DHFR	-0.001038265	0.470048108	0.952684546
TMEM179B	-0.001338066	0.470055606	0.952684546
KIAA0947	0.002598122	0.470070381	0.952684546
CDH26	0.002944477	0.470209409	0.952684546
MGAM	0.005370506	0.470214914	0.952684546
PLA2G2D	0.005500484	0.47030203	0.952684546
RGL4	0.002785101	0.470519858	0.952684546
METTL21A	0.004678072	0.470549274	0.952684546
HIP1	0.003110993	0.470557688	0.952684546
HDDC2	-0.002211967	0.470653132	0.952684546
NBPF3	0.002653827	0.470758711	0.952684546
ZFYVE20	-0.001809647	0.4707909	0.952684546
GPRIN3	0.004232519	0.47109124	0.952845402
BCL9	-0.003125855	0.471095087	0.952845402
WRN	0.001781251	0.471128637	0.952845402
CLEC3B	-0.003830964	0.471387208	0.953161182
RIC8A	0.001508231	0.471533708	0.953161182
IL1RN	-0.004151035	0.471545429	0.953161182
HIF1AN	0.002216104	0.471629216	0.953161182
CABP4	0.003847609	0.471839261	0.953201474
RPL32	7.57E-04	0.471859744	0.953201474
TXNDC9	-0.002098589	0.471925166	0.953201474
SYTL2	0.005831656	0.471993611	0.953201474
DOCK4	0.007028112	0.472150732	0.953319874
SPATA13	-0.004635623	0.472224489	0.953319874
PPAT	0.005628832	0.472468681	0.953481146
GCNT2	0.004796586	0.47255163	0.953481146
ANKRA2	0.002101784	0.472831336	0.953481146
DNAJC19	-0.002926892	0.472896266	0.953481146
NMT2	0.005982311	0.47308853	0.953481146
COX5A	-0.002446695	0.473285909	0.953481146
RAB11FIP1	0.002302026	0.473401818	0.953481146
PRCP	-0.002119325	0.473422553	0.953481146
UQCRB	-0.002820283	0.473425358	0.953481146
P2RX5-TAX1BP3	0.0036493	0.473449928	0.953481146
SARS	0.001925006	0.473473998	0.953481146
SIRPD	-0.003482651	0.473630681	0.953481146
PPP1CA	0.002358215	0.473808412	0.953481146

ASB1	-0.002310028	0.473929516	0.953481146
PRSS23	-0.007002194	0.473983272	0.953481146
ARL1	-0.001732043	0.474016033	0.953481146
NDUFAB1	0.002359332	0.474076936	0.953481146
CNST	0.003576408	0.474197364	0.953481146
RRP12	0.003635412	0.474303564	0.953481146
ANKRD49	-0.001766305	0.474340554	0.953481146
GRAP	-0.002852325	0.474345738	0.953481146
ASB13	-0.002988944	0.474375406	0.953481146
MAST2	-0.003363124	0.474659265	0.953481146
QRFPR	0.005937664	0.474715131	0.953481146
ID3	-0.005757687	0.474824172	0.953481146
DHRS3	0.003223547	0.474886098	0.953481146
CHRNA5	0.007037943	0.47492336	0.953481146
ING1	0.002433569	0.474959589	0.953481146
RPLP0	-0.002642643	0.475091235	0.953481146
ACOT7	-0.003480911	0.475190235	0.953481146
SMARCAL1	-0.001456699	0.475247471	0.953481146
GYS1	0.00290443	0.475350974	0.953481146
CASC3	0.00217612	0.475370748	0.953481146
CCR1	-0.00436482	0.475433801	0.953481146
DTHD1	-0.005993747	0.475654171	0.953481146
DCUN1D4	0.002093636	0.475661153	0.953481146
PIK3R2	0.001728927	0.475682178	0.953481146
ROGDI	0.005075029	0.475805381	0.953481146
DHTKD1	0.002002537	0.475958801	0.953481146
FBXL8	-0.002950763	0.475976656	0.953481146
IAH1	0.001693723	0.476066005	0.953481146
LYAR	0.003829417	0.47612269	0.953481146
LRCH3	0.003891743	0.476226388	0.953481146
C17orf58	-0.002986894	0.476323067	0.953481146
RUNDC3B	-0.004136343	0.47666583	0.953481146
C19orf52	-0.002091276	0.476875561	0.953481146
ACAT2	-0.00234988	0.476966021	0.953481146
CCDC142	-0.00432859	0.477085798	0.953481146
S100A11	-0.003851413	0.477320287	0.953481146
SLC25A34	-0.004116409	0.477340615	0.953481146
NCKAP1L	-0.001640352	0.477462721	0.953481146
PPA2	0.003810501	0.477466832	0.953481146
NUP153	-0.002029448	0.477476404	0.953481146
RPL38	8.40E-04	0.47751726	0.953481146
CAMLG	-0.001496491	0.477748129	0.953481146
IPP	0.005214248	0.477817887	0.953481146
POLR3D	-0.002703561	0.477874254	0.953481146
HADH	-0.00223131	0.477948893	0.953481146
DUSP3	0.002421704	0.478065546	0.953481146
MNAT1	-0.002413284	0.478155035	0.953481146

ZNF557	0.004409656	0.478162434	0.953481146
INTS5	0.002701397	0.478189348	0.953481146
PDDC1	-0.002349557	0.478268602	0.953481146
CLECL1	-0.007799703	0.478394692	0.953481146
RPL29P2	-0.002824638	0.478418629	0.953481146
ARID4A	-0.00229002	0.478488597	0.953481146
ISYNA1	0.002748071	0.478494632	0.953481146
PIK3AP1	0.002702648	0.478494985	0.953481146
RAB28	-0.003856713	0.478499317	0.953481146
CCDC130	-0.001947617	0.478587882	0.953481146
TBC1D3C	0.003379568	0.478593268	0.953481146
STAT5B	0.001979831	0.478643249	0.953481146
BRE	0.00231978	0.478710319	0.953481146
DENR	0.00598464	0.478830134	0.953481146
SPDYE7P	0.001239753	0.478927713	0.953481146
TLR10	0.005522864	0.479302186	0.953481146
MAN2A2	-0.0025902	0.479306653	0.953481146
HNRNPU	0.007893967	0.479341042	0.953481146
MIF	0.002180007	0.479435234	0.953481146
CSH2	0.003126659	0.479600453	0.953481146
CES4A	-0.003033735	0.479704331	0.953481146
CLSTN1	0.002375593	0.479794142	0.953481146
DHX8	0.002287393	0.479822457	0.953481146
LPHN1	0.00301682	0.479858149	0.953481146
TET1	-0.003080948	0.479892249	0.953481146
NCKIPSD	-0.002596827	0.479893147	0.953481146
OSBPL9	0.001519661	0.479930453	0.953481146
ANKRD44	0.007010104	0.480028403	0.953481146
CASP9	0.002170394	0.480050354	0.953481146
GEMIN6	-0.002892231	0.480056954	0.953481146
MECR	0.00338714	0.480271494	0.953736127
FBXW5	0.002283418	0.480590402	0.954123712
OSBPL8	0.002317795	0.480639065	0.954123712
PLEKHM1	0.001914219	0.481059788	0.954195309
PLEC	-0.002659989	0.48107909	0.954195309
SNAP29	0.003615724	0.481111316	0.954195309
NUDT15	-0.003505752	0.481127743	0.954195309
GABARAPL2	-0.002081832	0.481503847	0.954195309
TOR3A	-0.003520275	0.48156176	0.954195309
PGM2L1	0.003201754	0.481587666	0.954195309
ATP9A	0.006018407	0.481648678	0.954195309
DLAT	-0.001997759	0.48180487	0.954195309
AKT1S1	-0.002324791	0.481829542	0.954195309
SLC27A4	-0.002922501	0.481942846	0.954195309
SMCR5	0.006299615	0.481987572	0.954195309
LCMT2	-0.004156591	0.482055401	0.954195309
SLC7A6OS	0.002565206	0.482088458	0.954195309

ITGA5	-0.002139158	0.482229128	0.954195309
RUNDC1	-0.001883083	0.482285159	0.954195309
SLFN5	-0.004157547	0.482324452	0.954195309
PIAS4	-0.001601004	0.482334681	0.954195309
STAT5A	0.002630911	0.482406156	0.954195309
PWWP2B	0.002966143	0.482538397	0.954195309
AZI1	-0.002280669	0.482595077	0.954195309
EXOC2	-0.002420104	0.482672647	0.954195309
FAM134B	-0.003663352	0.482740085	0.954195309
LAMP3	0.00565574	0.482744035	0.954195309
HSDL2	0.002831225	0.482909803	0.954352546
METTL2A	0.007194363	0.483159419	0.954675403
CDC25B	0.002499556	0.483421439	0.955015057
PRPSAP2	0.001592827	0.483552611	0.955015057
GTPBP8	0.002563525	0.48365175	0.955015057
MED13	-0.003262589	0.483676431	0.955015057
SMARCB1	-0.002600921	0.483807214	0.955090868
VAT1	0.002545909	0.484000818	0.955090868
RHBDF2	-0.002040605	0.484131438	0.955090868
ZNF764	0.003502049	0.484206848	0.955090868
DNASE2	0.002421954	0.484216959	0.955090868
ZRANB1	0.004017624	0.484232537	0.955090868
TRAK2	0.001916509	0.484581606	0.955255241
RNASE2	-0.006580259	0.484637383	0.955255241
C1orf115	0.003949042	0.484640847	0.955255241
C1QBP	0.001833752	0.484728138	0.955255241
NPR2	-0.002669399	0.484747375	0.955255241
MXD4	-0.001994716	0.484931957	0.955448884
SLC6A12	0.00403598	0.485272367	0.955651659
FNTA	-0.001169854	0.485412636	0.955651659
NXPH2	0.003011476	0.485551901	0.955651659
RSAD2	0.007697804	0.485611777	0.955651659
C18orf32	0.00293751	0.485785776	0.955651659
MRPL24	0.001693398	0.485883668	0.955651659
MAFG	-0.002493116	0.485945935	0.955651659
EPM2AIP1	-0.001663615	0.485954328	0.955651659
PAOX	0.002640422	0.486017489	0.955651659
LEMD3	-0.001995506	0.486084555	0.955651659
DHX9	-0.002587206	0.48609979	0.955651659
MRAP	0.005141109	0.486218133	0.955651659
SYNJ2	0.003158514	0.486227281	0.955651659
RAD54L	-0.002767766	0.486243576	0.955651659
DAD1	0.001695972	0.486543254	0.955719967
PRKRA	0.001804679	0.486732669	0.955719967
TMEM147	-0.001802984	0.486745069	0.955719967
XRCC4	-0.002869929	0.486768742	0.955719967
GPATCH1	-0.002670025	0.486772979	0.955719967

HINT1	0.001997059	0.486796384	0.955719967
KCNH8	0.003405567	0.487151828	0.955925503
MLYCD	-0.002939254	0.487256686	0.955925503
SCYL2	-0.001697996	0.487273209	0.955925503
NOP10	0.002489957	0.487311273	0.955925503
GNAL	0.003363128	0.48734639	0.955925503
AARS2	-0.002114652	0.487463759	0.955925503
SLC15A4	-0.002256826	0.487505598	0.955925503
USH1G	0.003131099	0.487746012	0.956198291
CRADD	-0.00338217	0.48783627	0.956198291
ZNF430	0.001560217	0.48795985	0.956198291
PLOD1	-0.002661368	0.488090377	0.956198291
SNORD99	-0.004395618	0.488189741	0.956198291
POLH	-0.002741938	0.488307595	0.956198291
LNPEP	-0.002736346	0.488348093	0.956198291
EMR3	-0.006826432	0.488386759	0.956198291
ZNF394	0.005863059	0.488422182	0.956198291
SIT1	-0.003287252	0.488852918	0.956435445
SYNJ2BP	0.003609689	0.488855567	0.956435445
ATP6V1G1	0.001537971	0.488891662	0.956435445
HENMT1	-0.002424245	0.489030667	0.956435445
TNFRSF13B	-0.007420389	0.489258624	0.956435445
OTUD4	-0.001884233	0.489384101	0.956435445
SRBD1	0.001961122	0.489707134	0.956435445
GPT2	-0.002941862	0.48975536	0.956435445
BBX	0.001959988	0.489774489	0.956435445
SEC24B	-0.002012207	0.489790285	0.956435445
SNORD17	0.003628088	0.489876218	0.956435445
UBC	-0.001039147	0.489917984	0.956435445
DDT	0.002791496	0.490033335	0.956435445
CD48	-0.001621153	0.490115265	0.956435445
PMS2	0.00290547	0.490144659	0.956435445
RPP14	0.00301158	0.490282568	0.956435445
OSCAR	-0.003790355	0.490686348	0.956435445
RAD54L2	0.003077458	0.49103026	0.956435445
TMEM57	0.001993791	0.491077395	0.956435445
TRIM43	-0.003063742	0.491108947	0.956435445
DCTN3	0.001433891	0.491122158	0.956435445
SLC10A7	-0.00268065	0.491141194	0.956435445
PRIM1	0.002725781	0.491151378	0.956435445
DIAPH3	-0.002683603	0.491199884	0.956435445
RPL23AP7	9.14E-04	0.491350587	0.956435445
SOS2	-0.002862691	0.491387171	0.956435445
SAMD8	-0.003401472	0.491483113	0.956435445
TPST2	0.00308449	0.491501101	0.956435445
RALGAPA2	-0.002737973	0.491856793	0.956435445
ADCK3	0.001564632	0.49203781	0.956435445

DYRK3	-0.002331591	0.49210048	0.956435445
HAUS6	-0.003563198	0.49217334	0.956435445
PIBF1	-0.002892902	0.492192349	0.956435445
ZNF844	-0.003146937	0.492422741	0.956435445
ATAD2	0.002508655	0.492433114	0.956435445
DENND1C	-0.002352518	0.492569981	0.956435445
RPP30	-0.002836018	0.492677769	0.956435445
TMEM145	-0.002684946	0.492836854	0.956435445
NBEA	0.00356391	0.492843856	0.956435445
RNF114	-0.001045154	0.492851526	0.956435445
APLP2	0.003306378	0.493092882	0.956435445
GTF3C5	0.00182898	0.49310662	0.956435445
HMOX2	0.003912843	0.493154105	0.956435445
BLVRA	0.002722076	0.493177588	0.956435445
RNF144B	0.004456959	0.493270633	0.956435445
METTL6	0.003543346	0.493321987	0.956435445
TTPAL	0.002040756	0.493367895	0.956435445
MAPKAP1	0.001994256	0.49339735	0.956435445
SKIV2L2	-0.001840825	0.493580249	0.956435445
NAIP	-0.005526592	0.493595556	0.956435445
PPP2R1B	-0.003028924	0.493671483	0.956435445
MRPS14	-0.002952572	0.493728815	0.956435445
CLN3	0.002119368	0.493768932	0.956435445
ANKRD20A5P	0.00259182	0.493779078	0.956435445
DRG1	-0.0022872	0.493810066	0.956435445
PTPDC1	0.00285318	0.493928086	0.956435445
ARHGEF5	-0.00222171	0.494272487	0.956435445
GPX4	0.001954808	0.494292008	0.956435445
SLC35A3	0.002041257	0.494358598	0.956435445
TAF3	0.002305598	0.494368635	0.956435445
HIST1H3D	0.003911348	0.494391202	0.956435445
LIN52	0.003561032	0.494392027	0.956435445
RAB40B	-0.002507663	0.494396064	0.956435445
GCLM	0.005327457	0.49457197	0.956435445
ZNF10	-0.002281238	0.494583125	0.956435445
TNFAIP6	-0.004813172	0.494619333	0.956435445
RPSAP52	-0.002465252	0.494735501	0.956435445
TRIM36	-0.003277981	0.494834573	0.956435445
ATP5H	-0.001580398	0.494906459	0.956435445
PDE8A	0.002877542	0.49495214	0.956435445
INO80	0.00194692	0.495040122	0.956435445
SERPINB6	0.002111471	0.495073895	0.956435445
ZNF347	-0.003253754	0.495100254	0.956435445
DTX3	-0.003090737	0.495301947	0.956435445
METTL17	0.002310701	0.495475347	0.956435445
SERTAD1	-0.003745491	0.495518579	0.956435445
VRK2	0.003040137	0.495579928	0.956435445

CLCF1	-0.004846004	0.495588081	0.956435445
GOLGA7B	-0.003450446	0.495591355	0.956435445
SPIRE1	0.003451252	0.495723018	0.956435445
LSS	-0.003700922	0.495724483	0.956435445
GRK5	0.002426933	0.49578815	0.956435445
FANCE	0.001945483	0.496033643	0.956435445
FARP2	-0.002882278	0.496042197	0.956435445
CMTM6	-0.002875809	0.496077713	0.956435445
XYLB	-0.003021628	0.496454302	0.956435445
WAC	-0.0020715	0.496475586	0.956435445
TSPAN32	-0.002528069	0.496532156	0.956435445
BPTF	-0.002559897	0.496616715	0.956435445
PABPC1L	0.003203239	0.496798463	0.956435445
ZER1	0.002365817	0.49693716	0.956435445
FAM193A	-0.001760161	0.49720775	0.956435445
IGLL3P	-0.010049105	0.497336675	0.956435445
HDC	0.010389587	0.497349137	0.956435445
ING2	-0.002703721	0.497659972	0.956435445
PSMB5	-0.001761563	0.497679946	0.956435445
MAP2K3	-0.002076375	0.497767749	0.956435445
TRPM7	0.002855915	0.497852992	0.956435445
SMARCA4	0.001263541	0.497862579	0.956435445
VDAC1	-0.00164931	0.497871551	0.956435445
RTP4	-0.004985979	0.497890057	0.956435445
TOLLIP	-0.003119241	0.497894295	0.956435445
CERKL	-0.007425609	0.498204437	0.956435445
C15orf48	0.003090086	0.498313756	0.956435445
FAM157B	0.003180342	0.498325322	0.956435445
ARHGEF35	0.00635097	0.498495636	0.956435445
CTCF	-0.001560885	0.49871191	0.956435445
ZNF277	-0.002167509	0.498809044	0.956435445
TEKT4P2	0.012573699	0.498821095	0.956435445
CDK19	0.001633197	0.498904773	0.956435445
DNAJC13	-0.002303351	0.498980913	0.956435445
FAM41C	0.003073768	0.499008807	0.956435445
TMEM123	0.001993693	0.499040235	0.956435445
BCL11B	0.002195649	0.49911794	0.956435445
TTC7A	0.002948416	0.499200263	0.956435445
MRPS26	-0.001782082	0.499237169	0.956435445
CIRBP	0.002120778	0.499266136	0.956435445
SRPK1	0.001835717	0.499324259	0.956435445
ATP5S	-0.002689153	0.49939287	0.956435445
TERF2	-0.002376577	0.49964008	0.956435445
ARPC1B	0.002253753	0.499656818	0.956435445
VPS13C	0.004272143	0.499745842	0.956435445
ITSN2	0.002474145	0.499860236	0.956435445
GYPC	0.002638728	0.499866816	0.956435445

C9orf135	-0.007850153	0.499882758	0.956435445
EPHX4	-0.005720662	0.500075231	0.956435445
RGS14	-0.002962806	0.500115221	0.956435445
MELK	-0.004596631	0.500146542	0.956435445
RPA3	0.002084752	0.500210165	0.956435445
PSMB4	-0.001162474	0.500229031	0.956435445
GAS7	-0.002492545	0.500562905	0.956435445
C10orf11	-0.004848437	0.500598237	0.956435445
HIP1R	0.002732071	0.500624015	0.956435445
DCLRE1C	0.004849055	0.50071397	0.956435445
FBXW8	0.003095528	0.500736573	0.956435445
FIBP	-0.002363181	0.500756089	0.956435445
MAP3K14	-0.002372975	0.500819913	0.956435445
MED30	-0.002152384	0.500839595	0.956435445
GOLGA2	0.00256159	0.50101047	0.956435445
TIGD2	0.002789406	0.501059644	0.956435445
PRR5L	-0.005231703	0.501156896	0.956435445
HSPA14	-0.001769096	0.501184685	0.956435445
MCHR2	0.004408969	0.501186802	0.956435445
NUPR1	0.00257488	0.501339474	0.956435445
ERMP1	-0.002837583	0.501423845	0.956435445
CCNJ	-0.002907321	0.50158489	0.956435445
GYPB	-0.005027394	0.501638564	0.956435445
NUDT19	-0.002142652	0.501649598	0.956435445
NUDT9	0.001658625	0.501706276	0.956435445
EPDR1	-0.005077179	0.501741858	0.956435445
ATF6	-0.002014988	0.501771577	0.956435445
CDC25C	-0.003578392	0.501800519	0.956435445
UBE2C	0.005935769	0.501807288	0.956435445
PPP2CA	0.001149118	0.502122041	0.956435445
AKAP13	-0.001739556	0.502144848	0.956435445
VHL	0.001759748	0.502146453	0.956435445
TAP2	-0.00371148	0.502149774	0.956435445
SRSF3	0.001499804	0.502327414	0.956435445
KCNQ1	-0.003220325	0.502365625	0.956435445
UNKL	0.004168133	0.502399637	0.956435445
GOLGA6A	0.003601911	0.502562682	0.956435445
PPP2R2D	-0.001460855	0.502644802	0.956435445
ITPR1	0.002245556	0.50275849	0.956435445
SLC39A3	0.002688693	0.502785365	0.956435445
CHEK1	-0.003846098	0.50280042	0.956435445
CDK2AP2	-0.003054989	0.502890244	0.956441944
C10orf35	-0.003319706	0.503158059	0.956619134
SNRNP25	0.002702374	0.503207151	0.956619134
DEPDC1B	-0.00347846	0.503335495	0.956619134
NBPF8	0.00471904	0.503349895	0.956619134
CAPN7	-0.002014733	0.503460345	0.956619134

RBMS1	-0.003406459	0.503525831	0.956619134
CDC20	-0.008089947	0.5036811	0.956619134
NDUFA9	-0.001898988	0.503728712	0.956619134
FRMD8	-0.001658912	0.503955934	0.956619134
SLAIN1	0.002363926	0.50411392	0.956619134
SNX10	-0.003248597	0.504188865	0.956619134
SNORD84	0.003286434	0.504194502	0.956619134
CDK11A	0.003867277	0.504230261	0.956619134
ATF3	0.004289058	0.504514308	0.956619134
UBN2	0.002708989	0.5045222	0.956619134
ABHD14B	-0.003098754	0.504583142	0.956619134
EXOSC6	0.002480169	0.50467466	0.956619134
C11orf63	0.007767472	0.504680566	0.956619134
GBAS	-0.002713152	0.504684086	0.956619134
ROCK2	-9.93E-04	0.504852678	0.956619134
ASGR2	-0.005035564	0.504870514	0.956619134
EP300	-0.003085689	0.505057946	0.956619134
PPP6R3	0.002571036	0.505071069	0.956619134
SLC1A7	0.007396051	0.50508967	0.956619134
GALNT12	0.002322337	0.505159945	0.956619134
STOML1	-0.00307235	0.505243206	0.956619134
SLC35F3	-0.004421251	0.505382729	0.956619134
CCT4	-0.002157495	0.505403261	0.956619134
TMEM106A	0.006697445	0.505617902	0.95686178
AKIRIN1	0.002401074	0.505730015	0.956910349
KIAA0355	0.001044833	0.505824948	0.956926397
ARFGEF2	-0.002118641	0.505913809	0.956930956
RSPRY1	0.001909615	0.506135843	0.957187366
TDRD7	0.001956295	0.506288648	0.957312785
POLQ	-0.003710466	0.506425064	0.957407179
IGF2BP1	0.003912238	0.506891375	0.957925651
ERH	-0.002118192	0.506894656	0.957925651
KBTBD8	0.003012103	0.506958936	0.957925651
ZNF717	0.002885737	0.507230014	0.957981435
OR2A9P	0.007599723	0.507290878	0.957981435
SNF8	-0.003116508	0.507393169	0.957981435
CDKN2AIPNL	0.001576933	0.507483107	0.957981435
FAM19A4	-0.002655266	0.507484663	0.957981435
C9orf37	0.002331534	0.507507736	0.957981435
FAM74A4	0.002260641	0.507676823	0.958090825
CMTM2	-0.005631829	0.50774665	0.958090825
BCAS2	0.003696451	0.507825356	0.958090825
LHFPL2	0.003736604	0.508025422	0.958304941
PIAS2	0.002625922	0.50813105	0.958340875
SPOP	-0.001624858	0.508305249	0.958425906
CYP4F3	-0.004786027	0.508349309	0.958425906
ZNF395	0.002317601	0.508764345	0.958536087

TTC31	0.001586939	0.508771334	0.958536087
SORT1	-0.002809724	0.50889958	0.958536087
AAAS	-0.002073609	0.509048481	0.958536087
PFDN4	-0.003194329	0.509143541	0.958536087
GOLPH3	-0.001568657	0.509334868	0.958536087
ZBTB43	0.003308944	0.509387311	0.958536087
SEC11C	-0.002722725	0.509459416	0.958536087
OXA1L	-0.002270964	0.509514917	0.958536087
FOXD2	-0.004088035	0.509588766	0.958536087
DACT1	0.006173517	0.509880616	0.958536087
ZNF76	0.002800924	0.509922502	0.958536087
FBXO2	-0.003853616	0.510308421	0.958536087
UBE2E1	-0.001518275	0.510347156	0.958536087
FAM20C	0.004632449	0.510481831	0.958536087
NAA15	-0.002465338	0.510511225	0.958536087
SLC30A3	-0.002298214	0.510525091	0.958536087
MTUS1	-0.003473979	0.510546503	0.958536087
ZNF524	-0.002355327	0.510592957	0.958536087
NOX4	0.003297522	0.510892711	0.958536087
ACP2	-0.003115307	0.510991046	0.958536087
DYNC1LI1	-0.003219571	0.511063586	0.958536087
ERCC8	-0.0032754	0.511178877	0.958536087
OCIAD1	0.003342321	0.511268991	0.958536087
WBP11	0.001090599	0.511348087	0.958536087
MMAB	0.00292466	0.511358426	0.958536087
TRPV2	-0.002567342	0.511370043	0.958536087
NCALD	-0.003829126	0.511489792	0.958536087
OST4	0.002522813	0.511610769	0.958536087
CCDC88C	0.003228949	0.511628453	0.958536087
CD14	-0.001606019	0.511665834	0.958536087
RNF40	0.002303634	0.511724254	0.958536087
C15orf38	0.006325518	0.511762809	0.958536087
OS9	-0.00227518	0.51189195	0.958536087
PIK3R5	-0.002595909	0.511967458	0.958536087
GSTA4	-0.003454592	0.512018422	0.958536087
PILRA	-0.003046122	0.5120487	0.958536087
PITHD1	0.001891764	0.512108069	0.958536087
C1orf112	-0.002490384	0.512130074	0.958536087
UNC45A	-0.001844629	0.512150488	0.958536087
NEDD8	-0.00107546	0.512162652	0.958536087
URM1	0.001586316	0.512435132	0.958536087
HEBP2	-0.003038318	0.512634386	0.958536087
TBC1D14	-0.001623025	0.51272191	0.958536087
NUDCD2	-0.002325994	0.512767914	0.958536087
SNX15	-0.003253417	0.512885339	0.958536087
KLC4	0.002783685	0.51308842	0.958536087
FUCA2	-0.002402747	0.513145349	0.958536087

TTC17	0.002625088	0.513191282	0.958536087
KIAA0195	0.002755641	0.513227929	0.958536087
HERPUD2	0.002462238	0.513279506	0.958536087
OSBP2	0.008653674	0.513281182	0.958536087
FUT11	-0.003737943	0.513357767	0.958536087
ATG3	-0.002812342	0.513387763	0.958536087
RPS2P32	-0.003596486	0.513420118	0.958536087
RSPO2	-0.002353033	0.513441968	0.958536087
BRMS1L	0.002110395	0.513479191	0.958536087
GNPTG	-0.00207288	0.513530385	0.958536087
NAB1	-0.002538132	0.513590817	0.958536087
HKDC1	-0.005468175	0.513603535	0.958536087
GRB14	0.007330064	0.513714797	0.958582112
RIPK3	-0.001746029	0.514005092	0.958698798
ECT2	-0.002644105	0.514355016	0.958698798
TBC1D19	-0.002569753	0.51435537	0.958698798
NUBP2	0.002802208	0.514376489	0.958698798
ZNF546	0.001067856	0.514396574	0.958698798
FAM65A	-0.001965656	0.514481871	0.958698798
NMNAT3	-0.003382773	0.514541294	0.958698798
ST6GALNAC4	-0.002372687	0.514739627	0.958698798
FNBP1L	0.004130583	0.514787254	0.958698798
FKTN	0.001246349	0.515318138	0.958698798
KLHL28	0.006651666	0.515347288	0.958698798
NSMCE1	0.002354088	0.515388204	0.958698798
ALOX15B	-0.003413704	0.515492699	0.958698798
SQSTM1	-0.001806123	0.51552119	0.958698798
SNHG12	-0.003116693	0.515654057	0.958698798
CSDE1	-0.003350616	0.515705702	0.958698798
ADAM15	-0.002199118	0.515944572	0.958698798
HEATR5B	0.001559611	0.515978077	0.958698798
ANGPTL4	0.002599383	0.516033744	0.958698798
RBPMS2	-0.013019753	0.516068098	0.958698798
EPG5	0.004598779	0.516096723	0.958698798
NDUFS7	-0.001966374	0.516175558	0.958698798
RBM28	0.002003437	0.516190726	0.958698798
GNPDA1	0.002000651	0.516216227	0.958698798
UTP6	0.001313848	0.516416696	0.958698798
KIAA0020	0.001730815	0.516421683	0.958698798
SCMH1	-0.002032618	0.516520563	0.958698798
FRRS1	-0.002715491	0.516590695	0.958698798
PLTP	0.00326581	0.51664277	0.958698798
DTL	-0.002932271	0.516837215	0.958698798
CUTC	-0.00176681	0.516852162	0.958698798
PRELID1	-0.002988602	0.516853417	0.958698798
PPP2R3A	0.004273243	0.516928347	0.958698798
INTS6	-0.00210855	0.517048003	0.958698798

EEF1A1	-0.00203049	0.517105515	0.958698798
SIN3A	0.001746042	0.51713627	0.958698798
CRELD2	-0.002167105	0.517149584	0.958698798
HK3	-0.002827326	0.517326789	0.958698798
TMEM143	-0.002618236	0.517335951	0.958698798
AUTS2	0.004594024	0.51738924	0.958698798
ZNF114	0.003474898	0.5174218	0.958698798
BEGAIN	0.005505483	0.517480607	0.958698798
ALPP	0.004383905	0.51750161	0.958698798
HELZ	0.002287166	0.517742982	0.958951751
XRCC3	0.002442228	0.517823906	0.958951751
LDB2	0.006347437	0.518295309	0.958951751
RBM4	0.002199152	0.518346482	0.958951751
IL10	0.003472467	0.518655881	0.958951751
SNORA12	0.004704892	0.518690688	0.958951751
GTF2H4	-0.002714924	0.518816853	0.958951751
RBL2	0.002547798	0.518867336	0.958951751
PTGR2	0.007721238	0.518876664	0.958951751
TAF4B	0.003608558	0.519073411	0.958951751
ISM1	0.005154273	0.519101278	0.958951751
RPL23A	0.001219779	0.519109264	0.958951751
CTLA4	0.003771312	0.519210186	0.958951751
VPS13A	0.002545725	0.519255872	0.958951751
ZFAND2B	-0.001454349	0.519512971	0.958951751
GNRH1	0.002797734	0.51956848	0.958951751
MED8	-0.002289116	0.519711782	0.958951751
COQ10A	-0.002255621	0.519724172	0.958951751
UBFD1	-0.002444373	0.519756524	0.958951751
MFSD1	-0.001962572	0.520044427	0.958951751
GMNN	-0.002916548	0.520062189	0.958951751
SREBF1	-0.002935303	0.520068864	0.958951751
GZMA	-0.003570564	0.520280635	0.958951751
TECR	0.002075986	0.520288712	0.958951751
KLRC3	-0.009370619	0.520305108	0.958951751
USP8	0.00186408	0.520306902	0.958951751
BCDIN3D	-0.002991208	0.520354496	0.958951751
VSIG10	-0.00234113	0.520385608	0.958951751
TRAT1	0.003597642	0.520577275	0.958951751
LZTS2	0.002343003	0.520612373	0.958951751
PHLDB3	-0.005129212	0.520612778	0.958951751
ABCA1	-0.004650426	0.520619547	0.958951751
ACTA2	-0.007137824	0.52083933	0.958951751
MESP1	-0.003582379	0.520977058	0.958951751
SH2D1B	0.007359059	0.520981406	0.958951751
SEPHS2	-0.002181155	0.520997816	0.958951751
ATAD2B	-0.002438193	0.521000502	0.958951751
HAUS2	0.003835701	0.52105818	0.958951751

AMZ2	-0.001441034	0.521108998	0.958951751
CD38	-0.00647572	0.521144874	0.958951751
ADD1	-0.001276971	0.521190147	0.958951751
BAHD1	0.002874834	0.521292551	0.958980762
CCNF	-0.002746496	0.521445966	0.959103589
DPP7	-0.002393325	0.521692204	0.959116536
TTC12	0.004426104	0.521772995	0.959116536
VPS39	-0.002290893	0.521877367	0.959116536
SIGLEC14	-0.010459481	0.521889783	0.959116536
TRAF3IP3	0.001517116	0.521967175	0.959116536
SPI1	-0.00330557	0.521974203	0.959116536
CKAP5	-0.001511653	0.522059547	0.959116536
GGA1	0.001155158	0.522210344	0.959200454
SYNRG	0.001893282	0.522278538	0.959200454
RPAP3	-0.002271901	0.522600462	0.959349376
NFIX	-0.003690375	0.522601754	0.959349376
ATG4C	-0.002199584	0.52262168	0.959349376
HIST1H3H	0.007052351	0.522773619	0.959349376
RMND1	-0.002818796	0.522882982	0.959349376
TBX19	0.00233333	0.523018148	0.959349376
PRMT7	0.002832755	0.52305166	0.959349376
CRAMP1L	0.00255256	0.523111799	0.959349376
EHMT2	-0.002321241	0.523139654	0.959349376
FBXO43	-0.002401696	0.523278862	0.959445705
METTL23	-0.001192455	0.523384813	0.959481035
VNN1	-0.007664557	0.523569327	0.959492109
HIRIP3	-0.001857422	0.523608915	0.959492109
KIF21B	0.002179481	0.523650901	0.959492109
MADD	-0.002237726	0.523797896	0.9595744
MDM4	0.004222138	0.523869193	0.9595744
LYSMD1	-0.002163893	0.524293823	0.960193304
ACTR10	-0.001590377	0.524590058	0.9604859
ERO1L	-0.002370732	0.524802747	0.9604859
WDR52	0.003138043	0.52498394	0.9604859
CAMK2D	0.003640456	0.52500179	0.9604859
ARL4C	-0.00252752	0.525197857	0.9604859
CAPZA2	0.001604818	0.525199361	0.9604859
PPOX	0.001615254	0.52531361	0.9604859
APH1B	-0.002126297	0.525371609	0.9604859
GABPA	-0.002576719	0.525414976	0.9604859
GAS2L1	0.003995313	0.525568348	0.9604859
EDC4	-0.00167903	0.525701723	0.9604859
CREBZF	0.002144015	0.525731482	0.9604859
SKAP1	-0.001835157	0.525765221	0.9604859
IL17RA	-0.002523097	0.525823243	0.9604859
PTPRA	-0.002301487	0.525898231	0.9604859
SNRPA1	0.001439283	0.525931174	0.9604859

ADH4	-0.003405129	0.526044714	0.9604859
C19orf59	-0.004269643	0.526058255	0.9604859
FAM46C	0.004943892	0.526102269	0.9604859
PSMD6	-0.001132399	0.5262756	0.960533974
AP2S1	-0.001375108	0.526302155	0.960533974
RNF170	0.00242156	0.526543268	0.9608156
MED9	-0.002520447	0.526727997	0.960939941
PACS1	0.003139656	0.526869466	0.960939941
OSTC	-0.001783279	0.526902743	0.960939941
FAM167A	-0.006902342	0.526958663	0.960939941
KPNA3	-0.001981716	0.527351237	0.961211428
C1orf54	-0.003086153	0.527389985	0.961211428
ALG14	-0.002681318	0.527652989	0.961211428
SLC39A10	0.002542224	0.527726033	0.961211428
FTO	-0.002306152	0.527765465	0.961211428
MYO1B	0.003024048	0.527837317	0.961211428
KIR3DL2	0.007614688	0.52798812	0.961211428
PIWIL4	-0.00243572	0.528132266	0.961211428
ACY1	-0.00230304	0.528329765	0.961211428
APTX	-0.001470498	0.528529872	0.961211428
FCGR1C	-0.006417269	0.528614687	0.961211428
TTLL12	-0.002226063	0.528632035	0.961211428
GRB2	0.00246139	0.528751614	0.961211428
MRPL11	0.001632166	0.528785728	0.961211428
BAIAP2	-0.003007654	0.528886147	0.961211428
PTGER2	0.003058244	0.528895111	0.961211428
VAMP1	-0.002690965	0.529081386	0.961211428
SNAPC1	0.005509103	0.529147608	0.961211428
PIK3C3	0.001811441	0.529288346	0.961211428
NME7	-0.003096883	0.529397029	0.961211428
OLFML2A	-0.00236949	0.529725006	0.961211428
ELP3	-0.001791525	0.529735499	0.961211428
ITGA7	0.002274496	0.529805594	0.961211428
ARPC5	-0.001178671	0.529992965	0.961211428
CNOT7	-0.001827296	0.530232977	0.961211428
C1QC	-0.003954035	0.530289211	0.961211428
TFDP2	0.002596838	0.530366614	0.961211428
SUZ12P	0.00333192	0.530410478	0.961211428
APOL6	-0.00369875	0.530506197	0.961211428
IL12A	0.003465509	0.530630698	0.961211428
DENND3	0.002801334	0.530755846	0.961211428
COX19	-0.002878857	0.530761033	0.961211428
LEPROTL1	-0.002353398	0.530981521	0.961211428
C20orf197	-0.003569224	0.531062274	0.961211428
FAM118B	-0.002500606	0.531179009	0.961211428
ERAP2	0.012476757	0.531206525	0.961211428
HSD17B12	0.002118463	0.531267992	0.961211428

IGFBP7	-0.005869258	0.531304956	0.961211428
GLB1L	0.004604648	0.531359651	0.961211428
TDP1	0.00616782	0.531553448	0.961211428
ZMYND12	0.004919614	0.531595647	0.961211428
DSP	0.002925718	0.531672518	0.961211428
RWDD1	0.001455653	0.53172639	0.961211428
EFHD2	-0.001836118	0.531800427	0.961211428
LMO7	-0.002203263	0.531878528	0.961211428
PRMT3	-0.001578662	0.532129146	0.961211428
PLEKHA2	0.001659732	0.532225094	0.961211428
SAMD4A	-0.003917425	0.532249736	0.961211428
TAB1	-0.002227181	0.532478513	0.961211428
MET	-0.002331957	0.532607091	0.961211428
SPATS2L	0.003434269	0.532729518	0.961211428
EHBP1L1	0.002785108	0.532824573	0.961211428
SLC14A1	-0.004235634	0.532857221	0.961211428
C10orf76	-0.001872738	0.532873812	0.961211428
ELMOD1	0.005899914	0.532973439	0.961211428
ASPH	-0.002101475	0.533028766	0.961211428
PDRG1	-0.002042981	0.53307748	0.961211428
FSTL3	-0.002659816	0.533126152	0.961211428
UBE2N	0.001510653	0.533217131	0.961211428
TUBGCP6	-0.002315861	0.533383048	0.961211428
TSPAN2	-0.003832936	0.533389543	0.961211428
GPBP1	0.001470514	0.533447219	0.961211428
ST13	0.001853663	0.533610452	0.961211428
CAMP	-0.004659876	0.533649256	0.961211428
DARS	-0.001432677	0.53365436	0.961211428
CCDC85C	-0.002724617	0.533674909	0.961211428
BTD	0.002935101	0.533730864	0.961211428
TNFRSF4	0.004940007	0.533733467	0.961211428
PQLC1	0.001104066	0.533747998	0.961211428
SELPLG	-0.004367482	0.5337959	0.961211428
DFFB	0.002452596	0.533850912	0.961211428
VCAM1	0.004612071	0.53393895	0.961211428
GSTTP2	0.005924442	0.53400842	0.961211428
TMEM199	-0.001943332	0.534017705	0.961211428
HEMK1	-0.002744985	0.534042271	0.961211428
CFB	-0.003318977	0.534059902	0.961211428
WDR49	-0.002719801	0.534062458	0.961211428
VPS37B	0.002245194	0.534102501	0.961211428
SIPA1	-0.001492687	0.534131153	0.961211428
GSTO2	0.002621524	0.534226461	0.961211428
ANKRD40	0.001692875	0.534319859	0.961211428
PLA2G16	-0.005185613	0.534422817	0.961211428
KCNH2	0.00241059	0.534553922	0.961211428
SIM2	-0.002660986	0.534559966	0.961211428

RABL3	0.002653135	0.534569984	0.961211428
RBP7	-0.00340745	0.534575621	0.961211428
ANKRD54	0.002345061	0.535025542	0.961795373
PARP2	-0.002075014	0.535092559	0.961795373
TMEM176B	-0.012798507	0.535161053	0.961795373
PRKCE	0.002694589	0.535306389	0.961900392
BDH1	-0.002775647	0.535531366	0.962148465
COPA	0.001323554	0.535793413	0.962348073
SHARPIN	0.002516921	0.536365719	0.962348073
CLEC12A	0.005244338	0.536488933	0.962348073
CKAP4	-0.002978973	0.536596003	0.962348073
MIR557	0.00283788	0.536645153	0.962348073
CNOT4	-0.001854237	0.536899272	0.962348073
UAP1	-0.002527813	0.536963633	0.962348073
EML4	-0.00183922	0.537177058	0.962348073
C8orf82	-0.00239261	0.537219831	0.962348073
C16orf62	0.002088997	0.537236811	0.962348073
KIAA0100	-0.001674656	0.53730603	0.962348073
PCNT	0.002065915	0.537311291	0.962348073
RHOT1	0.001621036	0.537377545	0.962348073
ZNF713	0.002586188	0.537559024	0.962348073
EDN1	-0.002786815	0.537638716	0.962348073
RDH16	-0.00285085	0.537639767	0.962348073
RSRC1	-0.002898841	0.537756984	0.962348073
DENND5B	0.00490506	0.537839904	0.962348073
ICAM2	0.001811948	0.537890419	0.962348073
ZNHIT3	-0.001524065	0.537895983	0.962348073
PTGDR	-0.005543067	0.537899001	0.962348073
FLOT1	-0.00263387	0.537999362	0.962348073
FN3KRP	0.002899351	0.538153792	0.962348073
MSRB2	-0.001850873	0.538211942	0.962348073
SMAD3	0.002397312	0.538353698	0.962348073
C14orf142	-0.00167128	0.538377228	0.962348073
SDHAF1	0.001330899	0.538409473	0.962348073
CCDC151	-0.003828677	0.538668779	0.962348073
C16orf91	-0.001965986	0.538878411	0.962348073
ICAM3	0.001760142	0.539039332	0.962348073
C17orf98	0.003156714	0.539285299	0.962348073
PCDH10	-0.002972545	0.539323233	0.962348073
TLR5	0.002414731	0.539631016	0.962348073
RNASEL	0.002630772	0.539690422	0.962348073
GRHL1	-0.002898796	0.539695627	0.962348073
SNORD51	-0.002466474	0.539730033	0.962348073
RFC3	0.002018775	0.539776336	0.962348073
SAP18	-0.00153825	0.539865872	0.962348073
VSTM1	-0.01017141	0.540027534	0.962348073
TRAPPC3	0.00170754	0.540189036	0.962348073

MZT2A	0.003244312	0.540220097	0.962348073
LRRC29	-0.002117602	0.5402562	0.962348073
RTF1	-0.002266333	0.540277026	0.962348073
KRT17	-0.003266762	0.540377062	0.962348073
TMUB1	-0.001883639	0.54038727	0.962348073
FIG4	-0.001717955	0.540405147	0.962348073
OR2T34	0.002500333	0.540500271	0.962348073
CBLB	-0.002746073	0.540522247	0.962348073
NUDT2	-0.0038466	0.540798433	0.962348073
SPAG9	0.001223756	0.540817681	0.962348073
SPRYD3	-0.002287326	0.541127868	0.962348073
TCHP	0.001748357	0.541204272	0.962348073
SLC25A22	-0.001880354	0.54129914	0.962348073
PWP2	-0.001932718	0.541398858	0.962348073
PALM	0.004120046	0.541424625	0.962348073
HK2	-0.002991553	0.541517795	0.962348073
GRM2	0.003137219	0.541620845	0.962348073
RGS18	0.003914419	0.541659343	0.962348073
WNT10A	0.003521675	0.54165977	0.962348073
LUC7L3	0.002274079	0.541695879	0.962348073
KIAA1549	0.006582841	0.541739021	0.962348073
GPATCH8	-0.002040984	0.541813075	0.962348073
CWF19L2	0.001864807	0.542013168	0.962348073
TMX4	0.002470725	0.542074486	0.962348073
C15orf54	0.005150113	0.54217736	0.962348073
EPM2A	-0.002201955	0.542244526	0.962348073
RBBP4	-0.003269114	0.542488392	0.962348073
RWDD2A	-0.00178057	0.542568219	0.962348073
SCCPDH	-0.002391905	0.542583513	0.962348073
INSIG1	0.002088222	0.542628676	0.962348073
ANKRD55	-0.007060114	0.542649517	0.962348073
ZNF555	0.003469216	0.542698757	0.962348073
PDGFRB	-0.007005228	0.54277226	0.962348073
SNORA1	-0.002914253	0.54283356	0.962348073
ELMO2	-0.001717635	0.543090361	0.962348073
KIAA0101	7.67E-04	0.543205797	0.962348073
PSD4	-0.002625147	0.543317092	0.962348073
F2R	7.81E-04	0.543371313	0.962348073
PPFIA1	-0.00154608	0.543417368	0.962348073
C19orf25	0.001848766	0.543504627	0.962348073
RNF187	-0.002244775	0.543517383	0.962348073
SND1	-0.00206939	0.543527882	0.962348073
TFCP2	-0.00163712	0.543706751	0.962348073
CCDC137	-0.003320777	0.543743409	0.962348073
ZNF485	-0.002524533	0.543775091	0.962348073
RQCD1	-0.002584743	0.543858924	0.962348073
WARS2	-0.0080912	0.544070521	0.962348073

FOLR3	0.011262225	0.544203016	0.962348073
TCEB3	0.002298291	0.544241953	0.962348073
FAM104A	-0.001169195	0.5443444	0.962348073
RASSF6	0.006390814	0.544450543	0.962348073
FAM110A	-0.002460961	0.54453829	0.962348073
CLEC10A	-0.004451473	0.544581533	0.962348073
CENPB	0.001462491	0.544667244	0.962348073
RDH5	-0.002950593	0.544669341	0.962348073
MANF	-0.00216559	0.544670484	0.962348073
DHRS4L2	0.00305006	0.54479314	0.962348073
HNMT	-0.0039385	0.544856289	0.962348073
TBC1D10B	0.002195884	0.544893079	0.962348073
DLGAP5	-0.005117886	0.544907408	0.962348073
SLC6A10P	0.002368309	0.545025169	0.962348073
CEBPA	-0.002721596	0.545111871	0.962348073
CDH23	-0.004461964	0.545222245	0.962348073
APOL2	0.003176173	0.545225251	0.962348073
ARSG	-0.001753267	0.545315935	0.962348073
MERTK	-0.005331874	0.545363118	0.962348073
BNIPL	-0.00302285	0.545400951	0.962348073
TOP3B	-0.001963041	0.545411058	0.962348073
BLOC1S2	0.002677171	0.54542611	0.962348073
PTK6	-0.002450903	0.545552082	0.962348073
MMADHC	-0.001976295	0.545630542	0.962348073
STK24	9.95E-04	0.545778965	0.962348073
YIPF1	-0.001044107	0.545823647	0.962348073
DTYMK	0.003109299	0.545828284	0.962348073
KPNA2	-0.003440209	0.545843431	0.962348073
APH1A	0.002022764	0.546006103	0.962348073
CDC16	-0.001863404	0.546008126	0.962348073
CPS1	0.003402993	0.546032775	0.962348073
IARS2	-0.001283285	0.546038084	0.962348073
VNN3	-0.004910506	0.546075368	0.962348073
PBXIP1	0.002247932	0.546312417	0.962602085
TAF4	0.001356277	0.546493867	0.962602085
NFE2	-0.002511266	0.546566163	0.962602085
SGK3	-0.002675011	0.546602628	0.962602085
HIST1H4E	-0.007156417	0.546654324	0.962602085
RIMS3	-0.002788971	0.546775831	0.962662903
PATL1	0.001445948	0.547024806	0.962867101
DAGLA	-0.002559836	0.547276371	0.962867101
PIP5K1C	-0.002581382	0.54730435	0.962867101
CFHR2	0.002076893	0.547329287	0.962867101
HPS1	0.00318007	0.547403469	0.962867101
ACTR6	-0.001866544	0.547563672	0.962867101
HOOK3	-0.003073776	0.54768815	0.962867101
CORO1A	-0.001517761	0.547733794	0.962867101

SACS	0.002132626	0.547772697	0.962867101
CD7	0.002394239	0.547857848	0.962867101
SSH3	-0.001975589	0.547859916	0.962867101
TFAP4	-0.002784288	0.547935664	0.962867101
NIPSNAP3A	-0.003610054	0.548043523	0.96290377
MCPH1	-0.001797397	0.548323631	0.962939897
FGFRL1	-0.002651204	0.548446692	0.962939897
TPM3	-0.001003086	0.54847187	0.962939897
STX4	-0.002207016	0.548545279	0.962939897
AQP11	0.002416218	0.548585111	0.962939897
NUMBL	-0.002957045	0.54858605	0.962939897
STK3	-0.002494769	0.548843638	0.963034565
TNPO2	-0.001502271	0.549007947	0.963034565
KIF20A	-0.002565513	0.549008765	0.963034565
PPP2R5B	0.002380252	0.549060278	0.963034565
CTRC	-0.002537285	0.549352641	0.963034565
SOAT1	-0.003627787	0.549428108	0.963034565
PIN1	-9.06E-04	0.549506088	0.963034565
ZNF385A	-0.00306902	0.549529052	0.963034565
SPG21	-0.003767869	0.549534865	0.963034565
DHRS4	0.001564451	0.549562246	0.963034565
LTBP4	-0.003050121	0.549976454	0.963034565
AP1S1	0.002807114	0.549981081	0.963034565
PPIE	0.003204519	0.550136521	0.963034565
KCNK12	0.003038058	0.55019798	0.963034565
BDP1	0.004025093	0.550256468	0.963034565
SLC25A35	-0.002892149	0.550276265	0.963034565
FAM168A	-0.001525665	0.550359839	0.963034565
KLHDC4	0.003014637	0.550469512	0.963034565
MT1F	-0.00265776	0.550471918	0.963034565
PNPLA7	0.004020764	0.55071004	0.963034565
APRT	0.002110463	0.550736881	0.963034565
CNTNAP2	0.010666544	0.550750198	0.963034565
ENY2	-0.002069667	0.55083822	0.963034565
MIA3	0.002210316	0.550936185	0.963034565
FAM153C	-0.004913395	0.551030104	0.963034565
CEP57L1	0.002008455	0.551188223	0.963034565
POP7	0.001937031	0.551316381	0.963034565
SLC25A25	-0.001572986	0.551398966	0.963034565
CCNI	-0.001173999	0.551653449	0.963034565
GZMB	-0.004483677	0.551717508	0.963034565
GM2A	0.004398437	0.551785694	0.963034565
CCDC71	-0.003807066	0.551847786	0.963034565
ATP9B	0.002211243	0.55195161	0.963034565
LZTFL1	0.002149508	0.552009055	0.963034565
ISCA2	-0.002143041	0.552197703	0.963034565
CAMK1G	-0.005401638	0.552211905	0.963034565

PUSL1	-0.002267221	0.552249664	0.963034565
CCR6	8.24E-04	0.552298153	0.963034565
ATP5L	-0.002182845	0.552368034	0.963034565
SNUPN	0.002616583	0.552371861	0.963034565
SOX8	-0.003745613	0.552466423	0.963034565
C4orf29	0.004315993	0.552545084	0.963034565
NDUFB2	-0.001759203	0.552615945	0.963034565
LRRC25	0.007111238	0.552657649	0.963034565
DDX18	0.001375402	0.552782988	0.963034565
IDE	-0.002040201	0.552789186	0.963034565
TTC9	-0.002799044	0.552839426	0.963034565
LRRK1	-0.002931324	0.552864397	0.963034565
GUCA1B	0.003424047	0.552947643	0.963034565
HEATR5A	0.002553433	0.553061026	0.963034565
PPP4C	0.00123169	0.553077128	0.963034565
BEND3	0.002720515	0.55322485	0.963064695
SETX	-0.001821189	0.553314471	0.963064695
SH3BGRL3	-0.002268029	0.553424175	0.963064695
CTDSP2	-0.001360531	0.553478944	0.963064695
RGPD8	-0.004327584	0.553551759	0.963064695
EIF2AK4	0.004587894	0.553776133	0.963064695
CREG1	-0.002969129	0.553893805	0.963064695
PSEN1	-0.002799844	0.553896402	0.963064695
BTBD3	0.001900212	0.553932442	0.963064695
SMAP2	-0.001690165	0.554031961	0.963064695
ANKRD10	0.002082313	0.554051493	0.963064695
RPL39L	9.32E-04	0.554604478	0.963739612
HIGD1A	-0.001953816	0.554685463	0.963739612
HIST1H2BE	0.005959958	0.554788679	0.963739612
KIR3DL1	0.008083988	0.554796608	0.963739612
RAD9A	-0.002415821	0.554917025	0.963739612
FBXO42	-0.002392937	0.554962172	0.963739612
UBAP1	-0.002031	0.555220045	0.963799034
C7orf55	0.002333566	0.555283098	0.963799034
MAML2	-0.002829152	0.555341714	0.963799034
GPX8	0.004368933	0.555465023	0.963799034
LARP4	-0.002118336	0.555501096	0.963799034
AEN	0.002003007	0.555732866	0.963799034
IL7R	0.002005857	0.555923773	0.963799034
C9orf41	-0.002899961	0.556081497	0.963799034
ESCO1	-0.002323459	0.55634951	0.963799034
C12orf65	0.001812277	0.556407082	0.963799034
BET1L	0.00242287	0.556436256	0.963799034
RPL4	-0.00152398	0.556502044	0.963799034
TMEM106C	0.002476922	0.556548136	0.963799034
SMAGP	0.002719026	0.556612753	0.963799034
FLOT2	-0.001187574	0.556718341	0.963799034

CCAR1	0.00473594	0.556734448	0.963799034
MTPN	0.001439388	0.556791486	0.963799034
ATG4B	0.00351899	0.557008573	0.963799034
GNE	-0.001686306	0.557042718	0.963799034
RPL18	-0.001133175	0.557186131	0.963799034
RPS20	8.14E-04	0.557191147	0.963799034
ZNF398	0.001830939	0.557232342	0.963799034
GSK3B	0.00205294	0.557330881	0.963799034
C11orf68	0.001840561	0.55735702	0.963799034
ITM2B	-0.001836273	0.557490621	0.963799034
PTPN4	0.002474335	0.557515371	0.963799034
CPSF3	-0.001183508	0.557684046	0.963799034
ZNF420	-0.003532388	0.557712501	0.963799034
RNF150	-0.006145116	0.557909021	0.963799034
STK17A	-0.002548908	0.557976961	0.963799034
CSRP1	0.001881721	0.558176623	0.963799034
TCEB1	0.001468297	0.55822471	0.963799034
PARP1	0.001365873	0.558275511	0.963799034
SFT2D1	-0.002145989	0.558347261	0.963799034
NSUN4	0.001727568	0.558350151	0.963799034
IPO9	-0.002259619	0.558514249	0.963799034
RFC5	-0.001976001	0.558664837	0.963799034
SF3B1	-0.001170422	0.558794366	0.963799034
RPS15A	7.86E-04	0.558820053	0.963799034
CFL2	-0.002420979	0.558966002	0.963799034
MAP3K6	0.002438998	0.559089077	0.963799034
HDAC4	-0.003768287	0.559117825	0.963799034
HSF1	-0.002603271	0.559126499	0.963799034
SPNS3	-0.00219048	0.559286052	0.963799034
MAN1B1	-0.001500231	0.559353053	0.963799034
PRKCSH	-0.001346298	0.559514021	0.963799034
GATA3	0.002820886	0.559518632	0.963799034
IZUMO4	-0.002670649	0.559519297	0.963799034
LCNL1	0.002492402	0.559579617	0.963799034
PRRC2A	0.00284991	0.559844344	0.963799034
TBRG4	0.001914777	0.559902058	0.963799034
SESN2	-0.003074946	0.5599644	0.963799034
NUCB2	-0.002703951	0.560206939	0.963799034
PHF20	-0.001701406	0.56023547	0.963799034
KIF3A	-0.003007931	0.560269212	0.963799034
S100PBP	0.00151251	0.560453822	0.963799034
CEP192	0.002004688	0.560508141	0.963799034
TRDMT1	-0.002701327	0.560594838	0.963799034
ZNF821	0.002354043	0.560662211	0.963799034
ZNF124	-0.00315286	0.560699481	0.963799034
CEP170	0.002755824	0.560722585	0.963799034
LRWD1	-0.002789075	0.560726435	0.963799034

CCBE1	0.004428086	0.560805744	0.963799034
EIF4B	-0.001480288	0.560900056	0.963799034
ANP32B	-0.0010735	0.56102854	0.963799034
CNNM3	-0.001890119	0.561070048	0.963799034
EIF2B4	-9.61E-04	0.561094712	0.963799034
MED13L	-0.002311538	0.56118083	0.963799034
BAZ1A	0.001746305	0.561239109	0.963799034
PYCR1	0.002207727	0.561263683	0.963799034
USP14	0.002651361	0.561445303	0.963799034
MED7	-0.001914868	0.561467985	0.963799034
MAPK7	-0.00213056	0.561483999	0.963799034
KIF27	-0.003823871	0.561517065	0.963799034
PKN1	0.002223444	0.561526784	0.963799034
BCAP29	0.002675473	0.56164387	0.963850543
TLE3	0.00221277	0.561889363	0.963870131
FAM129B	-0.002189467	0.5619003	0.963870131
GDPD5	-0.004276498	0.561964965	0.963870131
C16orf74	-0.00361227	0.562003598	0.963870131
RFX3	0.002091383	0.562318688	0.964015494
ARMC6	0.002165444	0.562376092	0.964015494
POLG2	-0.001974109	0.562434041	0.964015494
DDX6	-0.00540543	0.562436721	0.964015494
UBXN11	-0.002820583	0.562702404	0.964233187
AMACR	-0.002245154	0.562858183	0.964233187
DENND5A	-0.001733204	0.563022221	0.964233187
ATP6V0A2	-0.001499864	0.563042501	0.964233187
MTIF2	-0.001757407	0.563168072	0.964233187
CEL	0.00294074	0.563270968	0.964233187
COLQ	0.004435588	0.563329499	0.964233187
ZNF652	0.003778486	0.563329712	0.964233187
PIGX	0.003256542	0.563394021	0.964233187
ADAMTSL4	0.003184271	0.563434841	0.964233187
TMEM87B	-0.002442371	0.563814225	0.964278626
CD200R1	-0.003230679	0.563874114	0.964278626
RDH13	-0.003162253	0.563900042	0.964278626
MIR564	-0.002425952	0.563998313	0.964278626
LEPR	-0.00412654	0.564067902	0.964278626
PRICKLE2	-0.002905934	0.564195683	0.964278626
UBR3	0.001606379	0.564211779	0.964278626
CPEB3	0.001859433	0.564248625	0.964278626
CATSPER2	0.003764272	0.564631001	0.964278626
MFSD5	0.001870491	0.564703066	0.964278626
SFXN2	0.001826806	0.564707264	0.964278626
HIVEP1	0.002116441	0.564988935	0.964278626
NKTR	0.002645931	0.565026358	0.964278626
JTB	0.001025775	0.565138473	0.964278626
ZAP70	-0.002645947	0.565232392	0.964278626

CPAMD8	-0.00295405	0.565259623	0.964278626
AFF4	0.005189434	0.565302667	0.964278626
TARS	0.001536402	0.565341776	0.964278626
LRRC56	0.002244819	0.56547665	0.964278626
ELAC2	-0.001746234	0.565520365	0.964278626
HEY1	0.001946665	0.56554094	0.964278626
TNFAIP3	0.002880163	0.565568247	0.964278626
SCAF8	-0.001420771	0.565924609	0.964278626
SDR39U1	0.002625566	0.566206475	0.964278626
ATG16L2	-0.0032995	0.566249751	0.964278626
HNRNPA1	-0.002594559	0.566461579	0.964278626
SPECC1	0.002799252	0.56660499	0.964278626
CANT1	-0.001617665	0.56662371	0.964278626
LRP11	-0.002582507	0.566665585	0.964278626
CYB561D2	-0.001305398	0.566717443	0.964278626
SUSD5	0.002583743	0.566758405	0.964278626
PCMTD1	0.001451533	0.566800095	0.964278626
NPM3	0.002517189	0.566871502	0.964278626
PTMA	0.00271617	0.566978656	0.964278626
RSL24D1	-0.001132671	0.567027782	0.964278626
UTP18	0.002123803	0.567029214	0.964278626
BRD1	0.001825236	0.567073955	0.964278626
CLPX	-0.001599224	0.567185246	0.964278626
TOB2	0.002222946	0.567266645	0.964278626
DDAH2	0.002635488	0.567397632	0.964278626
FRA10AC1	-0.003630106	0.567442633	0.964278626
ZNF33A	-0.001287277	0.5674477	0.964278626
SBF2	-0.002215731	0.567518867	0.964278626
SIGLEC7	-0.004332775	0.567537018	0.964278626
ZNF600	0.003325402	0.567642702	0.964278626
C1orf228	-0.002871591	0.567650969	0.964278626
BRD7	0.001705288	0.567661148	0.964278626
JSRP1	-0.004324278	0.567694731	0.964278626
SLC30A1	-0.003520657	0.567991604	0.964278626
RHOQ	-0.002007353	0.568181246	0.964278626
RNF24	0.0023587	0.568247696	0.964278626
MPST	0.002841374	0.568526311	0.964278626
ATP5G2	-0.001637325	0.568545668	0.964278626
POR	0.001970341	0.568696247	0.964278626
ZNF738	0.003607561	0.568732831	0.964278626
NFE2L3	0.004945245	0.568839779	0.964278626
PSAT1	0.003319338	0.568868812	0.964278626
RARA	-0.001876673	0.568954483	0.964278626
SNX8	-0.001930708	0.569284888	0.964278626
SPAG7	-0.00193955	0.56931445	0.964278626
NPLOC4	-0.001984544	0.569413297	0.964278626
KPNA4	-0.001256798	0.569432373	0.964278626

MRPL10	0.001988509	0.569476036	0.964278626
RIF1	-0.001670124	0.569536145	0.964278626
NSUN3	-0.001749687	0.569736422	0.964278626
MBTPS1	0.001393668	0.570005561	0.964278626
L1TD1	-0.004895426	0.570085338	0.964278626
PAXIP1	-0.001627268	0.570169152	0.964278626
C21orf59	-0.001299263	0.570169561	0.964278626
GGT3P	0.002045879	0.570247103	0.964278626
RCAN3	0.002790637	0.570296353	0.964278626
UAP1L1	-0.004499919	0.570329566	0.964278626
MIAT	-0.005764482	0.570413104	0.964278626
RNF26	0.001863442	0.570481536	0.964278626
IER5L	0.002877621	0.570540617	0.964278626
ATP8B2	0.001861831	0.570650597	0.964278626
C2orf40	-0.00395767	0.570704895	0.964278626
KCNMB4	0.003935001	0.570764821	0.964278626
HSD17B8	0.00220378	0.571024595	0.964278626
SMPD4	0.00260939	0.571157638	0.964278626
CD4	0.002492847	0.571222762	0.964278626
TNFRSF13C	0.004111272	0.571253139	0.964278626
ARPP19	0.001337499	0.571290711	0.964278626
PTER	0.010260865	0.571331272	0.964278626
PAFAH1B2	-0.002080749	0.571357565	0.964278626
C4BPB	0.002642373	0.571445263	0.964278626
WDR74	0.004511207	0.571616165	0.964278626
UNCX	-0.002318243	0.571717145	0.964278626
FMO2	-0.002615726	0.571845671	0.964278626
HDAC1	-0.001542333	0.571849573	0.964278626
GAL3ST4	-0.004063034	0.571857198	0.964278626
CLIC3	-0.005722093	0.571882702	0.964278626
C5	-0.00236797	0.571922139	0.964278626
ZNF740	-0.002177163	0.57192929	0.964278626
SETD3	-0.00126296	0.571960732	0.964278626
IER5	-0.002341074	0.572089866	0.964278626
THOC7	-0.001641775	0.572134117	0.964278626
ZYX	0.002150834	0.572315787	0.964278626
UBAC2	0.001220803	0.57233841	0.964278626
ATP11B	0.001880818	0.572562504	0.964278626
RHOBTB2	-0.002680336	0.572667263	0.964278626
TPP1	0.001755585	0.572748394	0.964278626
CFI	-0.002549679	0.572802926	0.964278626
DPP8	-0.001160456	0.57300621	0.964278626
ARL4A	0.00441877	0.573030529	0.964278626
DPCD	0.002596048	0.573127676	0.964278626
TTC37	-0.001419364	0.573152345	0.964278626
TMEM9B	-0.001008061	0.573262994	0.964278626
PABPC4	0.00127082	0.573321658	0.964278626

NUCB1	-0.00151914	0.573460617	0.964278626
RPL13P5	0.002595328	0.573523939	0.964278626
TTLL3	0.002075995	0.573547881	0.964278626
COPE	-0.001786166	0.573730656	0.964278626
ASPSCR1	0.001467618	0.573752389	0.964278626
CITED4	-0.003953784	0.574033344	0.964278626
MSRA	-0.002639638	0.574091624	0.964278626
CNTF	-9.93E-04	0.574166638	0.964278626
C9orf169	0.001884746	0.574181454	0.964278626
BCLAF1	0.001204057	0.574220512	0.964278626
ZYG11B	-0.001654089	0.574303811	0.964278626
CD97	0.001303609	0.574392971	0.964278626
MRPS6	0.001218522	0.574608664	0.964278626
SNAPC2	-0.002184509	0.574700878	0.964278626
GBA	-0.001747747	0.574814245	0.964278626
ZNF493	0.004570006	0.574881952	0.964278626
ZNF302	-0.001833278	0.575115937	0.964278626
LRIF1	0.003299735	0.575134797	0.964278626
BSPH1	-0.002532419	0.575173658	0.964278626
APEH	-0.001167481	0.575185073	0.964278626
SRP19	0.001702783	0.575234605	0.964278626
TRNP1	-0.003128129	0.575312868	0.964278626
THG1L	-0.003859109	0.57539887	0.964278626
KDM3A	-0.001345468	0.575531683	0.964278626
INTS4	-0.001378316	0.57555615	0.964278626
COMMD6	0.001488086	0.575667769	0.964278626
SAMD1	0.002660996	0.575768407	0.964278626
DLG1	-0.00205396	0.575808375	0.964278626
PTEN	0.001181855	0.575917164	0.964278626
RCOR3	0.001658889	0.575952862	0.964278626
BCL11A	0.002612431	0.575966181	0.964278626
LPAR1	-0.006559525	0.576011491	0.964278626
AIM2	-0.003740856	0.576113048	0.964278626
AIFM3	0.002261544	0.576243526	0.964278626
NPFF	0.001991494	0.576298094	0.964278626
RHBDL1	0.002444113	0.576357865	0.964278626
MCOLN1	0.002208862	0.576365233	0.964278626
AGPAT5	-0.002301395	0.576412675	0.964278626
NQO1	0.002329821	0.576422086	0.964278626
FAM203A	-0.002064862	0.576518837	0.964278626
DNHD1	0.002533438	0.576549463	0.964278626
CR1	-0.003637693	0.576626896	0.964278626
FAM83F	-0.002430595	0.576727507	0.964278626
FAM179A	-0.004600794	0.576921314	0.964278626
KRT6C	-0.002730821	0.577300927	0.964278626
CPSF4	0.001271094	0.57748096	0.964278626
PHTF2	-0.002307403	0.577520803	0.964278626

TRMT1	0.001235149	0.577583923	0.964278626
CLIC6	-0.00356744	0.577847258	0.964278626
DYNC1LI2	-0.001750969	0.578034785	0.964278626
PRPF3	0.001720797	0.578181379	0.964278626
UGDH	-0.002722086	0.578189688	0.964278626
RYBP	0.004115407	0.578193943	0.964278626
TMEM80	0.002042709	0.578247419	0.964278626
KDM4B	0.002187752	0.578574741	0.964278626
LTA4H	-0.002204856	0.57869933	0.964278626
PDXP	-0.002133142	0.579032005	0.964278626
AP2A1	-0.002190959	0.579125477	0.964278626
SNORD62A	0.004117203	0.579164761	0.964278626
CLCC1	0.002260523	0.57930405	0.964278626
KCNQ2	0.002301744	0.579391239	0.964278626
MRPL23	0.001724262	0.579433607	0.964278626
EPHB1	0.005515723	0.579548824	0.964278626
KIAA0319L	-0.001733786	0.579566176	0.964278626
ADORA3	-0.004527236	0.579618814	0.964278626
RHOB	-0.003434789	0.579650058	0.964278626
PCMT1	-0.001049001	0.579855097	0.964278626
PARG	-0.001739025	0.57992287	0.964278626
GPR68	0.004251715	0.580170106	0.964278626
SNORD12B	0.002345033	0.580297617	0.964278626
SNORD94	0.001791916	0.580319542	0.964278626
KIF3B	-0.001525587	0.580416345	0.964278626
ANXA9	0.002612151	0.5805591	0.964278626
SPRR1A	0.002113456	0.580586745	0.964278626
ZNF638	0.001851396	0.580636599	0.964278626
TRAPPC5	0.001655563	0.580643629	0.964278626
ATF7IP	-0.002359075	0.580766078	0.964278626
EIF4EBP2	-0.001904245	0.580923664	0.964278626
TSLP	-0.002515414	0.58092454	0.964278626
PSMB3	0.001205241	0.580962488	0.964278626
PPBP	0.003244377	0.581041715	0.964278626
MAT2A	0.001769715	0.581046572	0.964278626
IP6K3	-0.002278578	0.581089659	0.964278626
SPSB2	-0.003405328	0.581144752	0.964278626
CAMK1	0.002360449	0.58119276	0.964278626
CCNT2	-0.001677754	0.5811998	0.964278626
CCDC25	-0.001532824	0.581233058	0.964278626
NARF	-0.00186527	0.581321442	0.964278626
AMBRA1	-0.002293978	0.581532811	0.964278626
HMGCR	-0.001303257	0.581571553	0.964278626
NUDT13	-0.002554172	0.581725659	0.964278626
PGLS	-0.001556665	0.581749825	0.964278626
CR2	0.005333606	0.581975846	0.964278626
BBS9	-0.002755266	0.58201462	0.964278626

ATHL1	0.005474204	0.582093138	0.964278626
CBX7	0.002010994	0.58211095	0.964278626
GPR1	0.00544521	0.582192029	0.964278626
MYLK4	-0.003366441	0.582268682	0.964278626
CHUK	0.001268128	0.58241648	0.964278626
ZNF260	-0.002136501	0.582550775	0.964278626
TBL1XR1	0.00181751	0.58258039	0.964278626
SERINC5	0.003990823	0.582610453	0.964278626
BRMS1	-0.001106042	0.582702928	0.964278626
SRRD	-0.001906914	0.582998393	0.964278626
MED19	-0.00213688	0.583020542	0.964278626
CENPF	0.002439556	0.58306418	0.964278626
FYN	-0.001791297	0.583107791	0.964278626
YPEL1	0.003352725	0.583243776	0.964278626
LPL	-0.00405307	0.583338271	0.964278626
CAPZA1	-0.003728534	0.583339571	0.964278626
DCLRE1B	-0.002738883	0.583384306	0.964278626
PPIP5K1	0.001975949	0.583385901	0.964278626
ATXN1L	-0.001053919	0.583480992	0.964278626
DDX19A	0.001202925	0.583498145	0.964278626
CREB3	0.003105785	0.583526814	0.964278626
ERBB2	-0.00472102	0.583620526	0.964278626
POU2AF1	0.005459834	0.583624452	0.964278626
BCKDHA	-0.001851293	0.583960732	0.964278626
ANKRD37	0.002138621	0.584016321	0.964278626
CSTB	-0.002050338	0.584057439	0.964278626
NRBP1	0.001639347	0.58405995	0.964278626
PRRC1	-0.00131064	0.584061386	0.964278626
RASIP1	0.002917302	0.584099217	0.964278626
NOL8	0.001483701	0.58414116	0.964278626
ANXA4	-0.003324462	0.584186066	0.964278626
MRPL40	-0.002039142	0.584411158	0.964278626
SELM	0.002813595	0.584509636	0.964278626
CDC42EP1	-0.002600504	0.584539557	0.964278626
SPA17	-0.002355188	0.584568243	0.964278626
POLR2A	0.001229783	0.584840237	0.964278626
TAF15	0.002637006	0.584869483	0.964278626
RHOA	0.001350044	0.584869623	0.964278626
HAVCR2	-0.002006818	0.584876922	0.964278626
CCT3	-0.001678806	0.584913333	0.964278626
GMFG	-0.001071772	0.584947135	0.964278626
DDHD1	0.005425107	0.585284077	0.964278626
FUBP1	0.001602216	0.585338093	0.964278626
JRKL	-0.002337282	0.58537774	0.964278626
TCN2	-0.003555041	0.585443409	0.964278626
NDUFA2	0.001039915	0.585640369	0.964278626
FAM49B	0.002539609	0.585896447	0.964278626

PRPF6	0.002533005	0.58594606	0.964278626
RPL13A	-0.001874538	0.586146523	0.964278626
POLE3	-0.001222393	0.586172997	0.964278626
CMPK1	-0.001399092	0.586274004	0.964278626
ZFP42	0.003417907	0.586368567	0.964278626
CPXM1	-0.002020262	0.586400574	0.964278626
GAS6	-0.003556612	0.586525538	0.964278626
MED20	-0.001797796	0.587004609	0.964278626
ST3GAL1	0.002062941	0.587111833	0.964278626
CHEK2	0.001697773	0.587176149	0.964278626
LHPP	0.002486724	0.587181768	0.964278626
NDUFC1	0.001680523	0.587204696	0.964278626
SGSM2	-0.001438026	0.587232575	0.964278626
ARHGEF10L	-0.004183213	0.587326609	0.964278626
C1orf63	-0.00237141	0.587527133	0.964278626
CYP51A1	0.002833415	0.587581539	0.964278626
DERA	-0.001634064	0.587626353	0.964278626
L3MBTL3	-0.00250358	0.5876294	0.964278626
RER1	0.003098217	0.587675632	0.964278626
GNB2L1	-6.20E-04	0.587820122	0.964278626
ORMDL1	-0.001168801	0.58783093	0.964278626
NEU1	-0.001765341	0.587860556	0.964278626
MPHOSPH6	-0.003312171	0.587934435	0.964278626
ETS1	0.001511855	0.588092659	0.964278626
MAT2B	-0.001404597	0.588390238	0.964278626
PFKFB4	-0.001728843	0.588511074	0.964278626
BMP1	-0.00253107	0.58851342	0.964278626
DDX42	-0.00104016	0.588534238	0.964278626
GZMH	-0.006162177	0.588543388	0.964278626
SEC14L1	-0.002561577	0.588593036	0.964278626
ZNF785	0.005338832	0.588741656	0.964278626
GLRX3	-0.002165543	0.588820216	0.964278626
SCNN1A	-0.00254536	0.588873067	0.964278626
EMX2	-0.002238767	0.58890924	0.964278626
DCAF4	0.003100908	0.588966138	0.964278626
SSU72	0.001439326	0.588988416	0.964278626
UHMK1	0.001363473	0.589064169	0.964278626
RNU11	-0.002880226	0.589071507	0.964278626
QRICH1	0.001037938	0.589098473	0.964278626
SURF6	-0.002191942	0.589158161	0.964278626
ZDBF2	0.002545368	0.589318329	0.964278626
DDX60L	0.002744684	0.589361072	0.964278626
UBE2D4	-0.002123356	0.589510684	0.964278626
GPR153	0.001927915	0.590040115	0.964278626
SRRM2	0.001956674	0.590042588	0.964278626
ZNF786	0.003848616	0.59005362	0.964278626
PODN	-0.00331009	0.590220086	0.964278626

HSPA1A	-0.001974876	0.590367021	0.964278626
ZCCHC3	0.002195036	0.590385452	0.964278626
UNC13D	0.002614103	0.590408509	0.964278626
SDHA	0.00132888	0.590490677	0.964278626
LRFN3	-0.001953438	0.590578695	0.964278626
KCNQ1OT1	0.002480787	0.590631941	0.964278626
PAF1	-0.00118366	0.590690602	0.964278626
KIAA1407	0.002758119	0.59069755	0.964278626
FOS	0.005493315	0.590861295	0.964278626
LYPD1	-0.002504954	0.590893713	0.964278626
DSC1	-0.005844036	0.590903971	0.964278626
BTN3A2	-0.004985293	0.590990631	0.964278626
PGM1	-0.001874435	0.591230864	0.964278626
SORD	-0.001472033	0.591288734	0.964278626
HIST2H3A	0.005885539	0.591291558	0.964278626
IGSF11	-0.002083309	0.591466524	0.964278626
PTP4A3	0.002542241	0.591497461	0.964278626
PLEKHG5	0.002804247	0.591689602	0.964278626
SRSF10	-0.002147137	0.592014313	0.964278626
SESN1	0.002804647	0.592036274	0.964278626
SNORA21	0.002084327	0.59214741	0.964278626
PLD2	-0.002235975	0.592358267	0.964278626
FAM153B	-0.007175673	0.59245864	0.964278626
KIF1B	0.001780147	0.592530462	0.964278626
ERP27	-0.002477363	0.592687373	0.964278626
MIR599	0.002247352	0.592789646	0.964278626
ENPP4	-0.003802502	0.59286212	0.964278626
LIMD2	-0.002102768	0.592933366	0.964278626
SOCS4	-0.001913873	0.592993276	0.964278626
CDK4	0.001106807	0.592993643	0.964278626
PRPF18	0.002319327	0.592997371	0.964278626
DOCK7	-0.002446151	0.593087276	0.964278626
CHST12	-0.003545767	0.593293123	0.964278626
IQGAP3	-0.002344118	0.593373426	0.964278626
CTNND1	0.004056723	0.593683369	0.964278626
ZNF550	-0.002031399	0.593712048	0.964278626
ISL2	0.002225155	0.594131713	0.964278626
TEX261	-0.001491147	0.594246326	0.964278626
ZNF649	-0.002813579	0.594264048	0.964278626
KDM2B	0.001043674	0.594326772	0.964278626
COL16A1	0.002133748	0.594339546	0.964278626
U2AF1	0.001220451	0.594364235	0.964278626
GPS2	0.001248248	0.594388516	0.964278626
CALB2	0.002372239	0.594649855	0.964278626
GPR137	-0.001542513	0.594655931	0.964278626
MAST3	0.001583455	0.594780033	0.964278626
GTF3C6	0.001688084	0.594863872	0.964278626

ZNF682	0.003996756	0.594864099	0.964278626
XYLT1	0.013893091	0.59491163	0.964278626
PPARGC1A	-0.005312737	0.594932218	0.964278626
NPY5R	0.002081556	0.59498719	0.964278626
GOLGA8B	-0.002684815	0.595149879	0.964278626
MYO9B	-0.001798694	0.595169946	0.964278626
ZNF343	0.001600682	0.59530107	0.964278626
TGM1	-0.002330133	0.595481793	0.964278626
IGFBP3	-0.005898912	0.595497688	0.964278626
HVCN1	0.002286507	0.595527898	0.964278626
DNASE1L3	0.005097575	0.595629329	0.964278626
LSM10	-0.001886158	0.595654578	0.964278626
TXNL1	-0.002621325	0.595762092	0.964278626
SLC26A2	-0.002033268	0.595852228	0.964278626
CD86	-0.00211101	0.595857103	0.964278626
NFIC	-0.001942758	0.596010369	0.964278626
FKRP	-0.002299827	0.596152952	0.964278626
NGLY1	0.00132346	0.596195485	0.964278626
CTSF	0.002540648	0.596202031	0.964278626
PCSK6	0.001944269	0.59621814	0.964278626
PNOC	0.003987255	0.596315167	0.964278626
CCDC53	0.001296785	0.596436048	0.964278626
HSPA7	0.004858076	0.596488164	0.964278626
INTS12	-0.001317366	0.596622014	0.964278626
BCL2L2	0.001796731	0.596658724	0.964278626
RNASEH1	-0.001223037	0.596854522	0.964278626
NHLRC4	0.005365299	0.596880902	0.964278626
ALG10B	0.002044333	0.596906466	0.964278626
HDDC3	-0.001753731	0.597166479	0.964278626
SNORD8	-0.002982497	0.597172718	0.964278626
LTB4R	-0.002417086	0.597208217	0.964278626
C1RL	0.00217716	0.597235778	0.964278626
POLR1B	0.002597453	0.59727209	0.964278626
NCL	-0.0011547	0.597384913	0.964278626
CSNK1A1	0.0018918	0.597420052	0.964278626
THNSL1	-0.002438405	0.597514369	0.964278626
TAF5	-0.001642706	0.597611636	0.964278626
CDKN2AIP	-0.001569525	0.597649981	0.964278626
IKBKE	-0.002193876	0.597765927	0.964278626
SDHB	-0.001611576	0.597784259	0.964278626
IREB2	-0.002745685	0.597947268	0.964278626
ZNF408	-0.001643658	0.598157436	0.964278626
GALNT2	-0.002235409	0.598200088	0.964278626
DZIP1L	0.00372075	0.598317032	0.964278626
CLYBL	0.002753242	0.598385457	0.964278626
AMY2A	-0.002312772	0.598414364	0.964278626
LY96	-0.002222404	0.598446952	0.964278626

RRNAD1	-0.001759297	0.598499638	0.964278626
HSBP1	0.001307217	0.598544189	0.964278626
SSBP2	0.00206446	0.59856463	0.964278626
ADH5	-0.001912167	0.598649098	0.964278626
ZNF470	0.002385575	0.598935567	0.964278626
SERPINA1	-0.002749692	0.599050308	0.964278626
CMTM1	-0.002072455	0.599076142	0.964278626
ZNF558	-0.001960065	0.599139847	0.964278626
C14orf166	0.001537687	0.599257271	0.964278626
FBXW11	0.001305999	0.599362346	0.964278626
DERL1	-0.001539383	0.599721935	0.964278626
LILRB4	-0.003145594	0.599724679	0.964278626
FKBP3	0.001808714	0.599743038	0.964278626
NCF1	0.004371921	0.599748978	0.964278626
ZNF425	-0.002589507	0.599847151	0.964278626
ZCCHC17	0.001903666	0.599851217	0.964278626
PRSS30P	-0.006008976	0.599983987	0.964278626
POLR3K	-0.002098421	0.600037548	0.964278626
C6orf132	-0.001857	0.600053269	0.964278626
ZNF562	-0.001877086	0.600112339	0.964278626
SREBF2	0.002131728	0.600149432	0.964278626
FAM24B	0.002387632	0.600252798	0.964278626
SQRDL	-0.002269792	0.600284386	0.964278626
TUSC2	-0.001437266	0.600409802	0.964278626
ATP6V1E2	-0.002475017	0.600547044	0.964278626
ORC2	0.001545561	0.600847573	0.964278626
SCP2	0.003162073	0.600990757	0.964278626
SNTA1	0.001916356	0.601285274	0.964278626
ABHD15	0.001866616	0.601286256	0.964278626
IER3	-0.003331159	0.601387009	0.964278626
SORBS3	-0.001822463	0.601399882	0.964278626
SPCS3	-0.002193993	0.601577665	0.964278626
LILRA4	0.003007396	0.601900656	0.964278626
DALRD3	-0.001752323	0.602041162	0.964278626
RCCD1	-0.003677849	0.602099608	0.964278626
RPSA	-0.001851037	0.602158996	0.964278626
RPS6KA5	0.001918699	0.602230776	0.964278626
CHI3L2	-0.005282773	0.602254218	0.964278626
ZDHHC4	0.001587283	0.602517106	0.964278626
VWDE	0.003404538	0.602541998	0.964278626
IL21R	0.002276794	0.602572964	0.964278626
STAT1	-0.003618259	0.602649633	0.964278626
VPS16	0.002194787	0.602683036	0.964278626
UBE4A	0.001374708	0.602735693	0.964278626
UCN	-0.002149429	0.60275166	0.964278626
ISOC1	-0.001726447	0.602762619	0.964278626
HLA-B	-0.001305824	0.60280019	0.964278626

MRPS35	-0.002116759	0.602813874	0.964278626
ZNF776	0.002177597	0.602977672	0.964278626
ZNF236	-0.001955597	0.603112615	0.964278626
SPIN1	0.00259689	0.603286321	0.964278626
FXN	0.002020411	0.603289695	0.964278626
CLIP1	0.001168056	0.603359578	0.964278626
XAF1	0.003294047	0.603528201	0.964278626
IL27RA	0.001959342	0.603878391	0.964278626
WDR34	-0.002128426	0.604037502	0.964278626
SRD5A1	-0.002148957	0.604103521	0.964278626
LAT	0.001548827	0.604271956	0.964278626
KLRG1	-0.004221733	0.604521786	0.964278626
TMEM161B	-0.001893337	0.604647085	0.964278626
C6orf48	-0.001743746	0.604667082	0.964278626
ST6GALNAC2	-0.003597942	0.604682455	0.964278626
ITGAM	-0.002304269	0.604747746	0.964278626
SAMHD1	-0.002728983	0.60476875	0.964278626
SNORD12	0.002297536	0.60492354	0.964278626
FASN	-0.003555239	0.604979049	0.964278626
ACAD10	-0.001680915	0.605078809	0.964278626
ZNF160	0.002147348	0.605233476	0.964278626
CMKLR1	0.003972877	0.605236114	0.964278626
CSAD	0.003391654	0.605263449	0.964278626
SLC36A4	0.002675563	0.605379867	0.964278626
GPD2	0.00276842	0.605479043	0.964278626
IRF9	-0.001909145	0.605497195	0.964278626
ZNF74	0.001977908	0.605554181	0.964278626
AMIGO1	0.002076922	0.605737471	0.964278626
GTPBP3	-0.001602266	0.605757375	0.964278626
ZNF641	0.002715487	0.605813753	0.964278626
MIR877	-0.002292715	0.605881604	0.964278626
ZBTB11	-0.002189777	0.605930156	0.964278626
LONRF1	-0.002136074	0.605940065	0.964278626
ZNF207	-0.001195886	0.605943522	0.964278626
DERL3	-0.002846484	0.606070754	0.964278626
TBXA2R	0.002818626	0.60613252	0.964278626
ALDH1B1	0.002156089	0.606172133	0.964278626
TARSL2	0.002134341	0.606175213	0.964278626
CDC42BPB	-0.002483934	0.606279976	0.964278626
AFF1	0.001621322	0.60629743	0.964278626
SIVA1	0.001483469	0.606306622	0.964278626
EGR1	-0.003592901	0.606307334	0.964278626
ZADH2	-0.0031544	0.606355204	0.964278626
UCP3	-0.002358026	0.606505916	0.964278626
SCRN2	0.00201477	0.606761758	0.964278626
C12orf60	-0.002296207	0.6067652	0.964278626
AMDHD2	0.002210441	0.60683472	0.964278626

ASXL2	0.00157268	0.606862561	0.964278626
GLTP	-0.001285454	0.606871694	0.964278626
ZNF571	-0.001771662	0.606987093	0.964278626
LIMK2	-0.002637361	0.607041814	0.964278626
MDFIC	-0.002366233	0.607494927	0.964278626
UHRF1BP1	-0.002105887	0.607519397	0.964278626
CLDND1	0.001845754	0.60756087	0.964278626
ZNF629	-0.0017244	0.607618095	0.964278626
CCDC125	0.004294704	0.607621572	0.964278626
SLC22A5	-0.003313245	0.607752847	0.964278626
PTK7	-0.002397935	0.607855387	0.964278626
KLHL36	0.002523854	0.607902913	0.964278626
PRRT3	-0.002708982	0.608138425	0.964278626
KDELR2	-0.002348722	0.608182145	0.964278626
STAMBP	-0.001018193	0.60823701	0.964278626
PPP2CB	-0.001850918	0.608382317	0.964278626
NEURL3	-0.002265981	0.6084044	0.964278626
USP34	0.001338666	0.60853269	0.964278626
CEP120	0.002444697	0.608546432	0.964278626
VPS33A	0.001587599	0.608784372	0.964278626
PDPR	0.003036261	0.608883234	0.964278626
PABPC3	0.002131797	0.608932575	0.964278626
PPARGC1B	0.002810118	0.608964928	0.964278626
IGFBP6	-0.002181896	0.609057962	0.964278626
NUDT1	0.001902729	0.609131987	0.964278626
OSBPL7	-0.001944577	0.609196819	0.964278626
RNF38	-0.001162296	0.609296388	0.964278626
ADAM23	-0.003058986	0.609395206	0.964278626
ZRANB2	-0.001237981	0.609451109	0.964278626
MGAT3	0.003343651	0.609613607	0.964278626
ZNF670	-0.00241064	0.609616015	0.964278626
OR9A4	0.001831244	0.609809523	0.964278626
PART1	-0.002206356	0.609845233	0.964278626
C12orf75	0.003648067	0.609890265	0.964278626
GK5	0.002941271	0.609974776	0.964278626
RPAP2	-0.002114077	0.609998733	0.964278626
LIG4	0.002704494	0.610055402	0.964278626
UNC119	0.001674122	0.610084362	0.964278626
SLC30A9	-0.001307291	0.610104788	0.964278626
MTMR6	-0.001679319	0.610115923	0.964278626
RNF219	-0.001533328	0.610223518	0.964278626
FGF23	0.002030966	0.610232772	0.964278626
RRP1B	-0.001567523	0.610267511	0.964278626
NAV2	0.002054834	0.61050972	0.964278626
CAMKMT	0.001892868	0.610574993	0.964278626
ORC3	-0.001627268	0.61060111	0.964278626
CCL4	-0.004660341	0.610689683	0.964278626

LEMD2	-0.001081193	0.610811491	0.964278626
FIBCD1	0.001737419	0.610890049	0.964278626
USPL1	0.001200354	0.610898507	0.964278626
TMPRSS3	-0.002747764	0.610939394	0.964278626
ANG	-0.004265566	0.611134754	0.964278626
C1orf43	-0.001435069	0.611144929	0.964278626
RCBTB2	-0.001687677	0.611222181	0.964278626
NUFIP2	0.002063033	0.611239388	0.964278626
LGALS1	-0.002255415	0.611243456	0.964278626
TRIB1	-0.002271036	0.61125029	0.964278626
ANAPC2	0.002179971	0.611303455	0.964278626
RUSC1	-0.001489034	0.61130745	0.964278626
SLC4A5	0.00322155	0.611444577	0.964278626
MDM2	0.001874774	0.611482272	0.964278626
ZNF254	-0.00192114	0.611504601	0.964278626
KIAA2013	-0.001618169	0.61152038	0.964278626
ACTR8	-0.001474216	0.611719946	0.964278626
RRS1	0.001176034	0.611733316	0.964278626
RAF1	0.001132996	0.611742441	0.964278626
SYTL3	0.002283445	0.611826915	0.964278626
LACTB	-0.002653514	0.611865631	0.964278626
MBD2	-0.002310686	0.611867593	0.964278626
GZF1	0.001892418	0.61216061	0.964278626
FHL2	0.005109898	0.612222608	0.964278626
NBEAL2	-0.00212339	0.612228782	0.964278626
TMPRSS13	-0.002306421	0.612271715	0.964278626
VTRNA1-1	0.00301238	0.612318207	0.964278626
GIGYF1	0.002500125	0.612394058	0.964278626
LDHC	0.005262227	0.612451017	0.964278626
TXLNA	0.001156981	0.612685204	0.964278626
BMP8B	-0.003257336	0.612812781	0.964278626
INVS	-0.001748323	0.61286211	0.964278626
EFR3B	-0.002713083	0.612874228	0.964278626
NDUFV2	0.001413185	0.612902551	0.964278626
ZNF93	-0.002103114	0.612914619	0.964278626
CRY2	-0.001345257	0.61300982	0.964278626
IQGAP1	0.001518373	0.613029966	0.964278626
FBXW7	0.001338807	0.613294948	0.964363657
HMGN1	-0.001471481	0.613411872	0.964363657
ITGA3	0.002519371	0.613421033	0.964363657
PTGDS	0.00576652	0.613626268	0.964363657
FASTKD1	-0.001943778	0.613631144	0.964363657
PATE2	0.002169204	0.61393895	0.964363657
ZNF184	-0.002062627	0.613943223	0.964363657
P2RY11	0.001891698	0.613998913	0.964363657
ZNF573	-0.003335094	0.614169	0.964363657
SEPSECS	-0.00167213	0.614179339	0.964363657

FBRSL1	-0.001773249	0.614217013	0.964363657
CTNNA1	-0.001902474	0.614378689	0.964363657
RPSAP58	0.002339106	0.614558561	0.964363657
RNF165	0.006212082	0.614622736	0.964363657
RHPN2	0.003052432	0.614641838	0.964363657
PNPLA3	0.003282845	0.614663975	0.964363657
ALDH9A1	-0.001234976	0.614774134	0.964363657
PISD	-0.00164852	0.614874255	0.964363657
C15orf57	0.001503302	0.614893052	0.964363657
ABCA7	0.002913484	0.614929284	0.964363657
CARD8	-0.002425512	0.614975719	0.964363657
VPS28	-0.001841409	0.615000728	0.964363657
SPOPL	-0.002159531	0.615286499	0.964648753
LAMTOR2	-0.001225204	0.615356838	0.964648753
AVEN	-0.002157361	0.615736117	0.964897006
AJAP1	-0.00203101	0.615747375	0.964897006
NUP98	-0.002506051	0.615776714	0.964897006
IRF7	0.002747882	0.615895952	0.964947246
CCND1	-0.002616221	0.616065529	0.965076329
SHISA5	0.001535008	0.616386567	0.965344115
SH2D3A	-0.002223643	0.616498138	0.965344115
RNF212	0.002032663	0.616592173	0.965344115
NT5C1B	-9.03E-04	0.616815711	0.965344115
CCT8	-0.001343647	0.616902907	0.965344115
RSL1D1	-0.001403366	0.616952934	0.965344115
ARHGDIB	6.38E-04	0.617036845	0.965344115
CHKB	-0.001813052	0.617170863	0.965344115
SBK1	0.002003928	0.617303083	0.965344115
B4GALT2	-0.002111523	0.617444582	0.965344115
ABT1	-0.001813826	0.617448896	0.965344115
KAT2B	0.001344972	0.617511284	0.965344115
ING4	-0.001423534	0.617515189	0.965344115
NHLRC3	-0.001441715	0.617633811	0.965344115
MUC7	-0.002378413	0.617656444	0.965344115
PDSS1	-0.002025547	0.617908413	0.965344115
EPHB2	-0.00233246	0.617919595	0.965344115
PTGIR	-0.002433248	0.617951325	0.965344115
SIDT2	0.002719656	0.618007298	0.965344115
GOLGA3	0.001240424	0.618148866	0.965344115
NDUFA7	0.001541269	0.618170333	0.965344115
SLC2A5	0.004097676	0.618197729	0.965344115
DCP1B	0.002139286	0.618319424	0.965344115
TMEM45A	0.006137421	0.618329549	0.965344115
TCEA1	-0.002041751	0.618639173	0.965619026
TRIM16	-0.002157394	0.618706956	0.965619026
COBL	-0.002120805	0.618782665	0.965619026
PTPRK	0.003289085	0.618854582	0.965619026

SENP6	-0.001520424	0.618983424	0.965683935
DENND4C	0.00171188	0.619167373	0.965691399
CD36	-0.004108277	0.619315123	0.965691399
RALBP1	0.001435551	0.619315946	0.965691399
RCBTB1	0.0019746	0.61933718	0.965691399
ALCAM	0.002337551	0.619633547	0.965729083
P2RX7	-0.002190469	0.619673125	0.965729083
ZNF839	0.001875127	0.619736103	0.965729083
CCDC115	-0.002253506	0.619740605	0.965729083
UQCRQ	-0.00127953	0.619797579	0.965729083
BPNT1	-0.001782595	0.620056069	0.965947881
SLC38A10	0.00184301	0.620112534	0.965947881
LINGO2	-0.005092458	0.620250371	0.966026644
FBXO21	9.57E-04	0.620472409	0.966236507
YWHAB	0.001015291	0.620610536	0.966245948
NOG	-0.002710135	0.620653057	0.966245948
CD3D	-0.001469782	0.620805608	0.966288551
SWAP70	0.002536578	0.621032244	0.966288551
FAM73A	0.004618138	0.621258208	0.966288551
XRCC1	-0.001459177	0.621407653	0.966288551
PHF11	0.001057776	0.621753578	0.966288551
GNLY	0.004703693	0.621885115	0.966288551
SEC22A	-0.001562696	0.621965692	0.966288551
CDC27	-0.002908417	0.621974937	0.966288551
SLC19A1	0.001882853	0.622067432	0.966288551
ATP6V0D1	-0.002512035	0.62226617	0.966288551
TLR1	-0.002337894	0.622540317	0.966288551
ZWINT	-0.002393472	0.62258096	0.966288551
ITGA6	0.00265053	0.622716032	0.966288551
RRAS2	0.00309491	0.622821047	0.966288551
AXIN2	0.003243533	0.623225943	0.966288551
RBM11	-0.003217838	0.623237621	0.966288551
SF3B14	-0.001926238	0.623382509	0.966288551
NOL6	-0.001412898	0.623411548	0.966288551
CPNE8	-0.001917517	0.6234336	0.966288551
DUSP8	-0.003418315	0.623512899	0.966288551
SLC35B2	-0.001422228	0.623676665	0.966288551
FAM109A	0.001880573	0.623774265	0.966288551
RPL26L1	-0.001819732	0.623792367	0.966288551
ARID2	-0.001343019	0.623815182	0.966288551
MITF	0.002548838	0.623837984	0.966288551
VWA5A	-0.002141784	0.623887814	0.966288551
PPP3CA	0.002670102	0.623910205	0.966288551
IRS1	0.002594119	0.623958504	0.966288551
DFNA5	-0.004031663	0.624008983	0.966288551
NPRL2	-0.001347378	0.624082762	0.966288551
TWF2	0.001425565	0.624173856	0.966288551

CNOT3	-0.002019482	0.624249834	0.966288551
ZNF706	-0.001198973	0.624323544	0.966288551
ECHDC3	-0.003706062	0.624325378	0.966288551
SFXN5	-0.002231471	0.624367076	0.966288551
CCNL1	0.001482881	0.624523524	0.966288551
C1orf74	-0.002614829	0.624582139	0.966288551
HBS1L	0.003624687	0.624638981	0.966288551
GPM6A	-0.002698273	0.624646751	0.966288551
SGOL1	-0.001878439	0.624715227	0.966288551
PPP1CC	-0.001113145	0.624794787	0.966288551
TUBGCP2	0.001636856	0.624827066	0.966288551
CDH1	-0.002631974	0.625027513	0.966288551
CDKN2C	-0.002879021	0.625073646	0.966288551
FXR1	0.001341116	0.625171449	0.966288551
PPIL3	-0.001618496	0.625183075	0.966288551
BRD8	0.001312745	0.625295913	0.966288551
ATF7IP2	0.002503345	0.625371255	0.966288551
GIPC1	0.002172601	0.625463534	0.966288551
ASNSD1	-0.001379091	0.625482877	0.966288551
STOML2	8.65E-04	0.625529283	0.966288551
MXD3	-0.002258838	0.625535973	0.966288551
TAF7	0.001520223	0.625705627	0.966288551
THAP7	0.002039165	0.625757694	0.966288551
TBK1	0.001192537	0.625803308	0.966288551
LRRC57	-0.001875861	0.62580676	0.966288551
TMEM91	-0.002157019	0.625816194	0.966288551
PTPLAD1	0.001298934	0.625844783	0.966288551
PRAMEF13	0.002356244	0.625848806	0.966288551
SLX1A	0.003137592	0.626004809	0.966288551
C5AR1	-0.002940272	0.626005529	0.966288551
CPEB2	-0.001966289	0.626267465	0.966558083
ZNF333	-0.002037969	0.626532299	0.966832011
YARS2	-0.00168097	0.626832863	0.966847213
KCMF1	0.001579835	0.626890539	0.966847213
MZB1	-0.004767793	0.626967123	0.966847213
SGSH	0.002029049	0.627009258	0.966847213
PINK1	0.00148273	0.627020679	0.966847213
ALOX5AP	0.001862694	0.627066234	0.966847213
PMEPA1	0.003001034	0.627197265	0.966914558
TIAF1	-0.001349765	0.62755397	0.967329744
CENPM	0.002760444	0.62768129	0.967391284
CTU2	0.001936044	0.627828456	0.967418001
TBC1D15	8.47E-04	0.627873423	0.967418001
SNORD57	-0.002509347	0.62822633	0.967509416
EIF1AD	0.002468322	0.628252089	0.967509416
EMG1	0.001059212	0.628343693	0.967509416
PARP14	0.002883033	0.628362877	0.967509416

ANXA1	-0.001603133	0.628477112	0.967509416
C16orf54	0.002416945	0.62849219	0.967509416
TPX2	-0.002558215	0.628544603	0.967509416
STAM	0.001535531	0.628854485	0.967784365
EPAS1	0.001580579	0.628898088	0.967784365
ZBTB17	-0.001320698	0.629116112	0.967985299
MAST4	0.003659315	0.629238011	0.968038296
NOL3	-0.00217357	0.62933208	0.96804847
ZBTB20	0.002220195	0.629752263	0.968323719
NT5M	0.004389104	0.629838891	0.968323719
SLFN13	0.002944457	0.629936027	0.968323719
CD164	0.002131124	0.630011896	0.968323719
CHST10	-0.002020661	0.630115289	0.968323719
RALGPS1	0.002117706	0.630232093	0.968323719
OMA1	-0.001595954	0.630297308	0.968323719
RPS19	5.63E-04	0.630308987	0.968323719
NOL11	-0.001115268	0.630372081	0.968323719
SUMF2	8.94E-04	0.630450272	0.968323719
UBTD2	-0.001567728	0.630473308	0.968323719
FCGR3B	0.003121776	0.630884699	0.968341811
NOP56	0.001562142	0.631122215	0.968341811
GSN	-0.002907536	0.631259687	0.968341811
GLTSCR2	-7.21E-04	0.631347389	0.968341811
TAPBP	0.00184094	0.631451229	0.968341811
IQGAP2	0.001785843	0.631607404	0.968341811
CTSB	-0.001685	0.631688905	0.968341811
FOXP1	0.002251131	0.631697837	0.968341811
KLF4	-0.00299932	0.631767511	0.968341811
NOXO1	0.002053574	0.63177147	0.968341811
POLR2C	-0.001919045	0.631818636	0.968341811
ESPNL	-0.003099082	0.631902499	0.968341811
EHBP1	0.001268435	0.632072706	0.968341811
PIGG	-0.001758506	0.632111032	0.968341811
TOP2A	-0.004491595	0.632176297	0.968341811
MEX3A	-0.001848294	0.632371303	0.968341811
PTTG1IP	-9.88E-04	0.632400625	0.968341811
RASD1	-0.003760107	0.63255455	0.968341811
WWOX	-0.002458689	0.632562242	0.968341811
WDR25	-0.002192089	0.632568306	0.968341811
CAD	-0.001733601	0.632575118	0.968341811
REXO1	-0.001682773	0.632615703	0.968341811
LZIC	-0.001405905	0.632685361	0.968341811
ZNF669	0.003823341	0.632753907	0.968341811
OAS2	0.002568189	0.632891841	0.968341811
CTDP1	0.001663194	0.632944422	0.968341811
GSDMB	0.003514287	0.633142402	0.968341811
AFAP1	0.002981455	0.633197537	0.968341811

RPL7	8.72E-04	0.633225039	0.968341811
LPCAT1	-0.00162666	0.633233676	0.968341811
KANK3	0.001954825	0.633482246	0.968341811
TSPAN3	0.002155723	0.633649208	0.968341811
FOXM1	-0.001825822	0.63375878	0.968341811
TRUB1	0.002274652	0.633879357	0.968341811
TCEA2	-0.001741679	0.633892596	0.968341811
TKT	0.001486867	0.633896264	0.968341811
CWC25	-0.001786764	0.633914551	0.968341811
POLR1D	0.001424493	0.633918935	0.968341811
CHCHD7	0.001054144	0.633962706	0.968341811
CAND2	-0.003188658	0.63398438	0.968341811
TAGAP	-0.003579966	0.63411147	0.968402298
C5orf42	-0.001916606	0.63438063	0.968679706
GNG5	-0.002060373	0.634599595	0.968880403
SDHAF2	0.001412917	0.634899028	0.968980346
PLCG2	-0.001422048	0.634948269	0.968980346
NSUN2	-0.001404561	0.634964637	0.968980346
SUN2	-0.001174737	0.635140107	0.968980346
FNIP2	-0.001749539	0.635336066	0.968980346
COQ5	0.001360079	0.635537115	0.968980346
HNRNPD	-7.37E-04	0.635603455	0.968980346
FAM19A1	0.004348182	0.635742028	0.968980346
E2F5	0.003169133	0.635899074	0.968980346
TAS2R14	0.001912713	0.635918264	0.968980346
RNF125	0.001494515	0.636065797	0.968980346
HARS	0.00119566	0.636186033	0.968980346
FBXO3	-0.001742652	0.636188157	0.968980346
C6orf89	-0.001662244	0.636346805	0.968980346
UBASH3A	-0.002851134	0.636361289	0.968980346
GPC5	0.00173858	0.636576942	0.968980346
HLA-E	-0.001669122	0.63680818	0.968980346
ZNF235	-0.001564461	0.636840056	0.968980346
N4BP2L2	0.002727978	0.636885396	0.968980346
H3F3C	0.001873447	0.636947237	0.968980346
ELP2	-0.001175996	0.637060478	0.968980346
PAPSS2	0.003227898	0.637148691	0.968980346
ZNF511	-0.001106798	0.63718234	0.968980346
S100A1	0.002344714	0.637268497	0.968980346
SPRN	-0.001831277	0.637375476	0.968980346
CDC7	0.002829324	0.637429435	0.968980346
CPLX2	0.001837708	0.637535945	0.968980346
STK35	-0.002324246	0.637572266	0.968980346
EPOR	-0.001879844	0.637619112	0.968980346
MVK	-0.001711157	0.637634784	0.968980346
DGKA	0.002497437	0.637689609	0.968980346
TMTC1	-0.005921664	0.637694522	0.968980346

RPS19BP1	-0.001204463	0.637729362	0.968980346
GTF2A1	0.002924996	0.637783083	0.968980346
HNRNPM	9.36E-04	0.637881362	0.968980346
MIA-RAB4B	0.001620673	0.638040639	0.968980346
AQR	0.001029651	0.638068046	0.968980346
CEP57	0.001885784	0.638129836	0.968980346
WDR48	0.002303344	0.638232136	0.968980346
VRK1	0.001681652	0.63829953	0.968980346
RABIF	-0.001643002	0.638315342	0.968980346
CLPTM1	0.001429637	0.638341737	0.968980346
ZNF654	0.001726118	0.638790443	0.969282715
NDUFB8	-6.66E-04	0.638853365	0.969282715
PTS	-0.001134155	0.638871258	0.969282715
GSTK1	-9.71E-04	0.6388912	0.969282715
LIAS	-0.001720718	0.639058644	0.969403882
CETN1	0.003178568	0.639502647	0.969708959
NAMPT	-0.003413184	0.639561018	0.969708959
SUPV3L1	0.001683913	0.639598986	0.969708959
RAD21	0.002981141	0.639624276	0.969708959
NUP210	0.002171027	0.639697789	0.969708959
EIF1B	-0.001353051	0.639888119	0.969733277
ATL1	0.002951374	0.640326318	0.969733277
YY1AP1	-8.65E-04	0.640356318	0.969733277
ARL16	0.002290654	0.640407941	0.969733277
ZNF18	-0.001554289	0.640411932	0.969733277
SERPINB1	-0.002270538	0.640446504	0.969733277
ADSL	-0.001132189	0.640505009	0.969733277
TM9SF4	0.001272168	0.640617862	0.969733277
CPNE1	0.001537928	0.640666483	0.969733277
CCT5	0.001564853	0.64066834	0.969733277
MAP3K3	0.001701203	0.640778792	0.969733277
LAMA5	0.003978276	0.640823341	0.969733277
RNF214	-0.001942207	0.640922833	0.969733277
HSPBAP1	-0.002144355	0.640977348	0.969733277
CISD3	0.002126337	0.641051314	0.969733277
ACTR3	-0.001340763	0.641247272	0.969733277
DPY19L2P2	-0.001872962	0.641254161	0.969733277
DBNDD2	0.001737728	0.641485978	0.969733277
UBLCP1	-0.001218791	0.641678737	0.969733277
RCAN1	-0.001570714	0.641685687	0.969733277
SLC39A1	0.001369482	0.642044619	0.969733277
PRSS53	0.002018753	0.642048145	0.969733277
AQP3	-0.002814188	0.642210148	0.969733277
RPL12	-0.001120396	0.64238878	0.969733277
CD19	0.005017699	0.642433564	0.969733277
SFT2D3	-0.001731948	0.64254975	0.969733277
YWHAH	0.001500017	0.642600248	0.969733277

ARNTL	-0.001320259	0.642653843	0.969733277
ACOX3	-0.001809216	0.642711777	0.969733277
FOLR2	-0.002403652	0.642860287	0.969733277
N4BP2L1	-0.001247344	0.642909896	0.969733277
MND1	-0.002331637	0.643036938	0.969733277
ATXN7L2	-0.001435047	0.643107081	0.969733277
SPNS1	-0.001874906	0.643139271	0.969733277
NAP1L5	0.002222252	0.643267313	0.969733277
CCDC24	-0.002329083	0.643275903	0.969733277
RAB34	0.002583861	0.643277342	0.969733277
TBRG1	0.001850252	0.643282752	0.969733277
PSMF1	-9.23E-04	0.64330229	0.969733277
ACAT1	0.001621504	0.643537416	0.969733277
NUBPL	0.003640437	0.643565017	0.969733277
ZNF581	0.001457976	0.643752866	0.969733277
CHMP4A	-0.001099846	0.643789148	0.969733277
GTF3A	0.00112372	0.643844637	0.969733277
DDX46	-0.00126635	0.643935512	0.969733277
TTC34	-0.001661723	0.643944656	0.969733277
SCGB3A1	0.011181653	0.643999889	0.969733277
C1orf177	-0.001909825	0.644170643	0.969733277
TPTE	0.003196497	0.644213898	0.969733277
SDC2	0.002100428	0.64430329	0.969733277
PSMC2	8.90E-04	0.644468048	0.969733277
ALAD	-0.001993106	0.644529424	0.969733277
TMEM5	-0.001293954	0.64459286	0.969733277
LSM2	0.001179568	0.644637076	0.969733277
ZNF75A	0.001913697	0.64470405	0.969733277
ZNF583	-0.001744846	0.64490716	0.969733277
DPY30	0.001309729	0.645099486	0.969733277
ATOX1	0.001886551	0.645102708	0.969733277
FOXK1	0.002449337	0.645235279	0.969733277
MCM9	-0.001445939	0.645257574	0.969733277
GSR	-0.001936577	0.645498808	0.969733277
FHOD1	-0.001474405	0.645630126	0.969733277
TSC2	0.001717422	0.645718349	0.969733277
CACNA2D2	-0.003381145	0.645842372	0.969733277
TRAC	0.003485633	0.645890284	0.969733277
LTBP1	0.002528738	0.645999386	0.969733277
CTH	0.001960671	0.646052218	0.969733277
MFSD8	-0.001797738	0.64608562	0.969733277
ARHGAP25	-0.001594755	0.646214753	0.969733277
CIRH1A	0.001146823	0.64621763	0.969733277
CDKN3	-0.003007503	0.646396345	0.969733277
UTP3	0.00165618	0.646408228	0.969733277
ZFYVE21	-0.00131781	0.646618147	0.969733277
TSPAN17	-0.00130434	0.64672678	0.969733277

SCML4	0.002034688	0.646736876	0.969733277
FOXD4L1	0.002192237	0.64678269	0.969733277
FNDC3A	0.001521803	0.646830789	0.969733277
MARCKS	-0.002197805	0.646871183	0.969733277
RFPL1	0.00197422	0.646876003	0.969733277
NOXA1	-0.002280225	0.646984769	0.969733277
ZNF345	-0.00208863	0.647045805	0.969733277
DBI	0.001196525	0.647131316	0.969733277
CASP8	0.002728246	0.64726084	0.969733277
MIF4GD	-0.001226463	0.647266401	0.969733277
IMP3	9.14E-04	0.647355108	0.969733277
SAFB	-0.001112432	0.647373822	0.969733277
GPR56	-0.003757571	0.647455983	0.969733277
CAPNS1	-0.001643354	0.647575091	0.969733277
HIST1H2BO	-0.002851154	0.647577175	0.969733277
TINAG	0.001908261	0.647631484	0.969733277
RAP1A	0.003334359	0.647792783	0.969733277
MCCC2	-0.001803012	0.647875431	0.969733277
AKR1A1	-0.001093513	0.647896705	0.969733277
YRDC	0.003969878	0.648371606	0.969733277
GNG8	0.004250422	0.648383716	0.969733277
CIC	-0.001475659	0.648418196	0.969733277
SRSF4	9.11E-04	0.648551161	0.969733277
STAG1	-0.00125306	0.648567922	0.969733277
ARFGAP2	-0.001271851	0.648610857	0.969733277
NEK1	-0.001551803	0.648638511	0.969733277
IKBKB	-0.001158512	0.648713731	0.969733277
ROPN1L	-0.00195012	0.648817695	0.969733277
RRP7A	-0.003673851	0.648867048	0.969733277
SUGP2	-0.001623757	0.648998293	0.969733277
CALM1	0.001070679	0.649034288	0.969733277
KBTBD2	-0.001631799	0.649040145	0.969733277
EMB	-0.002106502	0.649152618	0.969733277
FAM47E-STBD1	-0.002089848	0.649175498	0.969733277
CEP164	0.001634507	0.649301546	0.969790692
STX12	0.00117913	0.649509156	0.969903255
CAPS	0.002172608	0.649688782	0.969903255
SEC62	0.001635807	0.649716114	0.969903255
THTPA	-0.001845001	0.649756884	0.969903255
GPR126	-0.001662497	0.649847499	0.969903255
SAFB2	0.001338511	0.649938182	0.969903255
ENDOG	0.002198428	0.649990274	0.969903255
C11orf48	0.001410698	0.650117124	0.969947667
BTN3A3	-0.001596122	0.650316608	0.969947667
ENO1-AS1	0.00229221	0.650566916	0.969947667
TACC1	-7.38E-04	0.650674977	0.969947667
MT1E	-0.00429481	0.650683381	0.969947667

ZBTB6	-0.001979286	0.650686258	0.969947667
ARAP1	0.002007397	0.650861569	0.969947667
NAPRT1	0.002602382	0.650986042	0.969947667
FLT3LG	0.002126773	0.651023569	0.969947667
IL10RB	-0.001050117	0.651144838	0.969947667
TRIP6	0.001965903	0.651547568	0.969947667
GABARAPL1	0.002399315	0.651678283	0.969947667
ANKRD22	-0.004114085	0.651994162	0.969947667
ADRA2A	0.001823776	0.652186423	0.969947667
RHOBTB3	0.004350734	0.652192292	0.969947667
PRKCH	-0.00159993	0.652239241	0.969947667
COL24A1	-0.002231113	0.652301658	0.969947667
MIS18A	0.002089468	0.652326709	0.969947667
LMBRD2	-0.001700772	0.652349274	0.969947667
SETD1A	-0.001463647	0.65237541	0.969947667
ITGB7	-0.001935302	0.652409251	0.969947667
RAP1GAP	0.001458801	0.652466907	0.969947667
CENPE	-0.002123149	0.65254557	0.969947667
KCNJ12	-0.002221917	0.65264061	0.969947667
TRIM17	-0.001801776	0.652762241	0.969947667
FANCI	-0.002125939	0.652854863	0.969947667
FBXO45	-0.002599472	0.65287563	0.969947667
GNB1	7.97E-04	0.652887121	0.969947667
FAM161A	0.004807908	0.652988574	0.969947667
MYL12B	-0.001102578	0.653027875	0.969947667
UFSP2	0.001109311	0.653056488	0.969947667
PELI3	0.002266792	0.653091926	0.969947667
ARHGAP1	0.001000446	0.653147814	0.969947667
GADD45GIP1	0.00201044	0.653249977	0.969947667
CELF6	-0.001938985	0.653331414	0.969947667
FAH	0.002462079	0.653363589	0.969947667
PPP2R5D	0.001431823	0.653435752	0.969947667
TMEM177	-0.001714387	0.653534773	0.969947667
MTHFS	-0.001669069	0.653624373	0.969947667
RSBN1	0.001520758	0.653703279	0.969947667
SLC12A8	-0.001776923	0.653734731	0.969947667
STARD3	-0.001497647	0.653752913	0.969947667
CLEC4G	0.00317134	0.654014893	0.969947667
CCDC50	0.002122117	0.654081956	0.969947667
DGCR6	-0.001712583	0.654138144	0.969947667
FLT4	0.002193431	0.654341495	0.969947667
TUFM	-0.001519633	0.654346719	0.969947667
TMEM173	-0.001030417	0.654383977	0.969947667
COX4I1	9.37E-04	0.654442049	0.969947667
EIF3CL	0.002595014	0.654564823	0.969947667
C1orf27	0.002041601	0.654707657	0.969947667
GIPC3	-0.00231284	0.654768765	0.969947667

PPM1H	-0.002264825	0.654863706	0.969947667
NFKBIZ	0.002282524	0.654948378	0.969947667
C1QTNF6	0.002406785	0.654968495	0.969947667
TMEM154	0.001505379	0.655099108	0.969947667
CDS2	-0.001426462	0.655228473	0.969947667
DMAP1	-0.001397989	0.655258748	0.969947667
PRKCI	0.001383595	0.655347443	0.969947667
CCNG2	-0.002097365	0.655364722	0.969947667
PNPT1	0.002614031	0.655365309	0.969947667
HDAC9	0.003340098	0.655792605	0.970450313
CDK6	0.001746666	0.656055117	0.970709008
NDUFA4	0.001077518	0.65617902	0.970762573
C22orf46	0.001257547	0.656312732	0.970830634
GLO1	0.001858274	0.656483033	0.97084041
MIR22HG	-0.002586092	0.656770412	0.97084041
ANO6	0.002240265	0.656830874	0.97084041
TPCN1	-0.001887124	0.656874682	0.97084041
DNMT3B	0.001505313	0.657046344	0.97084041
NEK2	-0.002803547	0.657386303	0.97084041
HOMEZ	-0.001925962	0.657589362	0.97084041
ERRFI1	-0.002496326	0.657596025	0.97084041
ZNF222	0.00202132	0.657596274	0.97084041
DUS1L	-0.001318367	0.657638718	0.97084041
COX6C	-0.001208224	0.657659549	0.97084041
CSNK1E	0.001642589	0.657741693	0.97084041
CCDC109B	0.001072211	0.657860098	0.97084041
HIAT1	-0.00161879	0.657959789	0.97084041
MIR1224	-0.002072964	0.65804942	0.97084041
C18orf54	0.00181241	0.658105883	0.97084041
ATP13A2	-0.001777475	0.65818531	0.97084041
NET1	-0.002681558	0.658389651	0.97084041
NBR1	-0.001557814	0.658518188	0.97084041
FAM117B	-0.00169305	0.65852106	0.97084041
ZNF385D	-0.003871578	0.658528728	0.97084041
ZC3H12A	-0.001739641	0.658596841	0.97084041
DNAJC21	-0.00143835	0.658662165	0.97084041
RNF168	0.001828725	0.658822146	0.97084041
C2orf68	-0.002055014	0.658887048	0.97084041
MPV17L2	-0.001690961	0.658890932	0.97084041
CCDC69	-0.00215213	0.659038085	0.97084041
SYT16	-0.002365894	0.659239361	0.97084041
CC2D1B	0.002290446	0.659268222	0.97084041
SLC9A3R1	-0.001610359	0.659289056	0.97084041
CLDND2	-0.004081728	0.659399034	0.97084041
BTG1	-9.80E-04	0.659432852	0.97084041
GALT	0.001520195	0.659497271	0.97084041
FBXO41	0.001892035	0.65950991	0.97084041

NAB2	-0.001859622	0.659547815	0.97084041
MTFMT	-0.001665849	0.659618115	0.97084041
PIGY	-0.0012581	0.659729852	0.97084041
CRX	0.001726386	0.659753544	0.97084041
QPCTL	0.001544135	0.659850071	0.97084041
ARPC2	-8.18E-04	0.659891125	0.97084041
CLASP1	0.001228835	0.659915371	0.97084041
PRNP	9.07E-04	0.660012084	0.970853655
DIXDC1	0.001602108	0.660107269	0.970864651
PRKCQ	-0.001643217	0.660542077	0.971111041
LYN	-0.001348109	0.660992227	0.971111041
TJP3	0.002603419	0.661375359	0.971111041
USP47	0.00139689	0.661480872	0.971111041
RCSD1	0.001382415	0.661571666	0.971111041
CFLAR	-0.002049313	0.661650821	0.971111041
TPP2	0.001633961	0.661686611	0.971111041
CHMP4C	0.001540596	0.661713671	0.971111041
SULT1A1	0.002047482	0.66188287	0.971111041
EDARADD	-0.002641117	0.661896099	0.971111041
TRIM2	0.003007215	0.661912871	0.971111041
EVI5L	-0.001612757	0.662012791	0.971111041
LRRC6	-0.004367517	0.66203469	0.971111041
PKD1	0.002237468	0.662085652	0.971111041
BAG5	0.001043004	0.662090289	0.971111041
HEG1	-0.001777496	0.662202447	0.971111041
TAOK1	-0.003600647	0.662324418	0.971111041
MNT	-9.73E-04	0.662368229	0.971111041
RUVBL1	0.00169447	0.662434204	0.971111041
ABCA5	-0.002117847	0.662455786	0.971111041
AHI1	-0.003283287	0.662716351	0.971111041
KRTCAP2	-0.001182462	0.662738707	0.971111041
RHBDL2	0.004177003	0.66275989	0.971111041
WDR53	-0.001215453	0.6628827	0.971111041
CADM1	-0.003953476	0.662968385	0.971111041
RPS3	-6.70E-04	0.663040826	0.971111041
FCF1	-0.001445859	0.663042626	0.971111041
FAM122A	-0.001538035	0.663157047	0.971111041
MRPL18	0.001273462	0.66317928	0.971111041
RPUSD3	-0.0010064	0.663548501	0.971111041
PSMC5	-8.64E-04	0.663665992	0.971111041
MFSD10	0.001145743	0.663798352	0.971111041
RHOF	-0.001767548	0.66382509	0.971111041
RAB11FIP4	-0.001809127	0.663907581	0.971111041
DISC1	0.002423247	0.664046565	0.971111041
NDFIP1	-0.001497085	0.664096577	0.971111041
RAB11FIP5	-0.003750038	0.66426591	0.971111041
FBXO8	-0.001636628	0.664274921	0.971111041

RAPGEFL1	-0.001752454	0.664290905	0.971111041
NAA38	-0.001981077	0.664430645	0.971111041
TK2	-0.001466809	0.664473701	0.971111041
RSU1	-0.00135849	0.664536594	0.971111041
AAK1	0.002673155	0.664562826	0.971111041
ABHD16A	0.00115485	0.664686236	0.971111041
PDPK1	-0.001310782	0.664700997	0.971111041
NADK	0.001882877	0.664704201	0.971111041
TOX2	-0.002502864	0.664759508	0.971111041
PTGS2	-0.003845314	0.664809884	0.971111041
EPSTI1	-0.003955386	0.66484808	0.971111041
CEACAM4	-0.002223311	0.664857481	0.971111041
GMCL1	-0.00170023	0.664864132	0.971111041
ARID3A	0.001289607	0.665186655	0.971111041
IFITM2	0.0023269	0.665224786	0.971111041
TNFRSF6B	0.001812758	0.665320141	0.971111041
PPP1R13B	0.001392274	0.665338692	0.971111041
PIP5K1B	0.002052541	0.6654102	0.971111041
DRAP1	-0.00148537	0.665482323	0.971111041
INF2	0.002263317	0.66552619	0.971111041
CASP2	7.86E-04	0.665604615	0.971111041
SUPT4H1	0.001262038	0.665654542	0.971111041
SLC8A1	0.003119522	0.665792383	0.971111041
ZNF438	-0.001401199	0.665952396	0.971111041
ZFP36L2	-0.001972769	0.665987162	0.971111041
ITLN1	-0.002064538	0.666215903	0.971111041
RMND5B	-0.001743862	0.666288078	0.971111041
ILDR1	0.002051376	0.66636846	0.971111041
LLPH	-0.001498182	0.666369598	0.971111041
IL6R	-0.002059966	0.666434192	0.971111041
VNN2	-0.002284338	0.66646935	0.971111041
CAMK2N1	0.003646565	0.666484477	0.971111041
RRN3P1	-0.003561488	0.666539993	0.971111041
AIMP2	-0.001162211	0.666688731	0.971111041
UBQLNL	-0.002327049	0.66680918	0.971111041
SOS1	-0.002011771	0.666944434	0.971111041
UPK3A	0.002373773	0.667206353	0.971111041
KLRB1	-0.002497222	0.667370404	0.971111041
PGBD1	-0.001657382	0.667499328	0.971111041
XPO6	-0.001370197	0.667709774	0.971111041
BRD9	9.01E-04	0.667732905	0.971111041
DONSON	-0.001829946	0.667773956	0.971111041
ARAP3	-0.00182539	0.667801552	0.971111041
HIST1H4J	-0.002356407	0.667861141	0.971111041
VMP1	-0.001308754	0.667913819	0.971111041
ZNF768	-0.002180698	0.668029231	0.971111041
TRIM31	-0.001654889	0.668074112	0.971111041

DAG1	0.001380458	0.668090287	0.971111041
PMM2	0.001384169	0.668146695	0.971111041
FBXW9	-0.001293154	0.668218933	0.971111041
BACH1	0.001981538	0.66824098	0.971111041
USP25	-0.001799894	0.668381062	0.971111041
RASAL3	-0.001141776	0.668574315	0.971111041
TP53BP1	0.001320254	0.668629596	0.971111041
DGCR6L	-0.00183501	0.668696557	0.971111041
CITED2	0.001811799	0.668817883	0.971111041
WDR55	-0.001139448	0.6690117	0.971111041
KIF11	-0.003068952	0.669175359	0.971111041
PPP2R5C	0.001511206	0.669182549	0.971111041
ARRB2	0.001495216	0.669198815	0.971111041
LPAR5	-0.002677909	0.669336122	0.971111041
RAB31	-0.001389314	0.669534158	0.971111041
ARF3	-0.001696855	0.669564193	0.971111041
ZNF814	0.002659594	0.669592573	0.971111041
SULF2	-0.002524071	0.669887059	0.971111041
ARL6IP4	0.00162831	0.669984849	0.971111041
FAM21B	0.001818675	0.670020847	0.971111041
COG7	-0.001713543	0.670158674	0.971111041
SERPING1	0.004385853	0.670195255	0.971111041
ZFP2	-0.001771236	0.670278259	0.971111041
GHRHR	-0.001720337	0.67044364	0.971111041
SLC7A8	0.002130711	0.670530237	0.971111041
TNNT1	-0.002802167	0.67062599	0.971111041
CALR	0.001577956	0.670657937	0.971111041
CUTA	-0.001188094	0.670888962	0.971111041
ISG20	0.001361958	0.67100141	0.971111041
P2RY12	0.00358166	0.671050517	0.971111041
MAN1A1	-0.002139347	0.671072422	0.971111041
CCNT1	0.002087376	0.671079789	0.971111041
PCIF1	0.002052934	0.671158007	0.971111041
CCDC86	-0.001666447	0.671298185	0.971111041
SSNA1	-0.001281039	0.671312253	0.971111041
HGD	0.004419403	0.671370604	0.971111041
CTSO	-0.001918766	0.671393961	0.971111041
C1orf86	0.002196174	0.671481052	0.971111041
NSFL1C	0.001916176	0.671573194	0.971111041
NDUFB4	0.001542119	0.671574502	0.971111041
TTC18	0.001971859	0.671653794	0.971111041
MARCH8	-0.003284474	0.6717087	0.971111041
CA11	0.001862013	0.671963609	0.971111041
NRIP1	0.001737261	0.671981359	0.971111041
SOD2	0.002088071	0.672028801	0.971111041
UFD1L	0.001334613	0.672282353	0.971111041
HAUS1	-0.002431317	0.672492954	0.971111041

IFIT1	0.005154215	0.672600771	0.971111041
ANKS6	0.001879915	0.672638072	0.971111041
GCNT1	0.001677541	0.67265582	0.971111041
MAP1LC3B	0.001412226	0.672693524	0.971111041
DDX41	0.001044871	0.672813482	0.971111041
OAS1	-0.00396164	0.672873815	0.971111041
PKIA	-0.002416801	0.672991273	0.971111041
CPEB4	0.00299858	0.673016926	0.971111041
C21orf91	0.001811793	0.673038674	0.971111041
LRP5L	-0.001960454	0.673059867	0.971111041
PTAR1	0.001432423	0.673249878	0.971111041
HMGN3	0.001704043	0.673331914	0.971111041
METRNL	0.001780497	0.673377583	0.971111041
GLRX	-0.001192758	0.673500534	0.971111041
ENTPD1	-0.002401015	0.673595848	0.971111041
SNORA72	0.00265938	0.673664757	0.971111041
KIAA1671	-0.004576326	0.673705819	0.971111041
METTL21B	-0.002526051	0.673765695	0.971111041
TAOK2	-0.002203184	0.673817421	0.971111041
GPHN	0.00162808	0.673823059	0.971111041
ITPR2	-0.001669892	0.673867674	0.971111041
IDO2	0.002363408	0.673897895	0.971111041
RAD51AP1	-0.002143945	0.673955553	0.971111041
ICAM1	-0.001841709	0.674227735	0.971111041
CAST	0.001697709	0.674231514	0.971111041
COCH	-0.004961821	0.674254507	0.971111041
USP15	-0.001654113	0.674424693	0.971111041
POLRMT	0.001656121	0.674471781	0.971111041
SURF2	0.001552823	0.674520281	0.971111041
SCARNA21	0.002256727	0.674656927	0.971111041
ABCC4	-0.003135152	0.674823387	0.971111041
PAICS	-0.001188403	0.674855559	0.971111041
CDC40	8.17E-04	0.674868077	0.971111041
ZNF436	-0.001857719	0.674888473	0.971111041
HPCAL4	-0.003755841	0.674926121	0.971111041
ARRDC2	-0.001594027	0.675144993	0.971224617
IKZF4	-0.001800267	0.675261628	0.971224617
HIST2H2BE	0.00394187	0.675268286	0.971224617
LGMN	0.00201154	0.675428359	0.971328636
UST	-0.00261695	0.675634078	0.971441037
HSBP1L1	0.001735482	0.675777442	0.971441037
PAQR7	0.001952499	0.675891334	0.971441037
NDE1	0.001516845	0.675939912	0.971441037
CAPN3	-0.001975384	0.675945331	0.971441037
SNORD4B	0.002360664	0.676066743	0.971489391
CNKSR3	0.001531769	0.676196403	0.971516755
ATRN	-0.001248711	0.676306685	0.971516755

SLC24A1	0.001993525	0.676349093	0.971516755
DNAJA3	-9.36E-04	0.6765947	0.971743446
TUBA1C	0.001767016	0.676874406	0.971747991
GGPS1	-8.68E-04	0.677113829	0.971747991
CIAO1	9.38E-04	0.677137876	0.971747991
TDRD12	-0.001722325	0.677168243	0.971747991
PCYOX1	0.002508322	0.677353334	0.971747991
SUFU	0.002485073	0.677657741	0.971747991
RHBDD2	0.0013754	0.677729738	0.971747991
SSFA2	-0.001740145	0.677936073	0.971747991
KCTD13	-0.001669145	0.678102611	0.971747991
SRGAP2	-0.001931114	0.678121923	0.971747991
RPL21	-0.002203821	0.678134341	0.971747991
NUP160	-0.001161284	0.67819525	0.971747991
GNB1L	0.00148568	0.678201456	0.971747991
NDUFS4	0.001381013	0.678290801	0.971747991
TXN2	0.00192277	0.678301211	0.971747991
IRF2BPL	-0.002899257	0.67834917	0.971747991
FBXL17	-0.002293878	0.67838684	0.971747991
NCOA4	-0.001449559	0.678439761	0.971747991
LYL1	-0.001192617	0.678450475	0.971747991
ZFAND1	-0.001565133	0.678650372	0.971747991
PCNP	8.56E-04	0.678774273	0.971747991
RHBDD3	-0.001747642	0.678793484	0.971747991
ARHGAP5	0.001690952	0.678914791	0.971747991
C9orf62	0.00192216	0.678933799	0.971747991
CARM1	-0.001122119	0.67896075	0.971747991
NT5DC2	-0.002845531	0.679050344	0.971747991
TESK1	-0.001475826	0.679505455	0.971747991
SPEG	0.001912942	0.679735551	0.971747991
RRP9	0.001749442	0.679881999	0.971747991
ZNF155	-0.001879765	0.679889361	0.971747991
KIAA2026	0.00130541	0.679951076	0.971747991
NPHP4	-0.00174431	0.679986869	0.971747991
SIRPG	-0.002471969	0.680055246	0.971747991
UNK	-0.001247765	0.68031553	0.971747991
RPN2	-0.001357391	0.680319565	0.971747991
GCH1	-0.00171863	0.680331037	0.971747991
KYNU	-0.002106899	0.680446336	0.971747991
GCLC	-0.001261857	0.680499193	0.971747991
PLA2G4C	-0.005534618	0.680559273	0.971747991
MOSPD3	0.001500562	0.680583532	0.971747991
DTD1	-0.001560776	0.680714786	0.971747991
SLC37A3	0.001472131	0.68076068	0.971747991
COPS3	-8.03E-04	0.680797501	0.971747991
HNRNPR	-8.93E-04	0.680888558	0.971747991
DUSP6	-0.001759275	0.680968601	0.971747991

SMNDC1	-0.001249318	0.681217864	0.971747991
C21orf128	-0.002639995	0.681304529	0.971747991
VPS37D	-0.001634048	0.68130485	0.971747991
IL8	0.006486057	0.68131039	0.971747991
PHC2	-0.001875967	0.681407666	0.971747991
GLRX2	-0.00172352	0.681450501	0.971747991
LUZP1	0.001274927	0.681504397	0.971747991
VTA1	-0.001396572	0.681570543	0.971747991
EMR2	-0.001914804	0.681572409	0.971747991
CRBN	-0.001000142	0.681577293	0.971747991
LPAL2	-0.00213712	0.681621898	0.971747991
HERC2P2	0.001863215	0.681638338	0.971747991
MAP4K3	0.001530459	0.681689687	0.971747991
GTF2IRD2	-0.002502589	0.682116623	0.972174071
ARSK	-0.001815158	0.682164243	0.972174071
MKRN2	-0.00114478	0.682429807	0.972427334
CFDP1	0.001429326	0.682652946	0.97249511
PDP1	0.001921307	0.682653086	0.97249511
CHCHD3	-0.001559222	0.682916117	0.972744627
KDM6B	0.001588546	0.683184246	0.97300134
CYLD	0.001827017	0.683302727	0.973044884
TMBIM6	-8.47E-04	0.683417636	0.97308333
MRPL33	-0.001651277	0.683737778	0.973232521
NSUN6	-0.001071408	0.683804224	0.973232521
RAB20	0.002007735	0.683836492	0.973232521
GALNS	-0.001552979	0.683955327	0.973232521
DGKD	0.001923377	0.683995954	0.973232521
APBA2	-0.002112972	0.684049961	0.973232521
MIR630	-0.001548641	0.684490931	0.973625852
DTWD1	-0.001603013	0.684570576	0.973625852
NLRP5	0.001611438	0.684762112	0.973625852
AKAP11	0.001148113	0.68483679	0.973625852
PARL	-9.83E-04	0.684904995	0.973625852
POM121L8P	0.002837113	0.685046432	0.973625852
SLC11A1	-0.002937377	0.685129096	0.973625852
GLTSCR1	0.001240025	0.685157882	0.973625852
SUMO3	0.00101439	0.685400088	0.973625852
ARL3	0.001645393	0.685402606	0.973625852
SNN	0.002534306	0.685446632	0.973625852
PPFIA4	-0.004264015	0.685594168	0.973625852
IFNAR2	-0.001923159	0.68561305	0.973625852
POLR3G	-0.002819596	0.685619203	0.973625852
GNA15	0.001412098	0.685645814	0.973625852
PTPN18	0.001801554	0.686096914	0.973991643
ZSWIM5	-0.002239417	0.686377782	0.973991643
ECE2	-0.002136103	0.686501852	0.973991643
ZDHHC12	0.001460753	0.686514898	0.973991643

CYTH3	-0.001980736	0.686550733	0.973991643
BCCIP	-7.93E-04	0.68659884	0.973991643
ZNF559	0.001977824	0.686619629	0.973991643
DSE	-0.00158206	0.686683776	0.973991643
TET2	-0.001535041	0.686695346	0.973991643
ADCK1	-0.002051374	0.686972936	0.974260528
MRPL51	-0.001128981	0.687191655	0.974445866
BMPR2	-0.001490103	0.687329341	0.974516264
DDAH1	0.001180981	0.68754585	0.974698388
MTF1	-0.001730469	0.687707187	0.97480226
NPM1	0.001577665	0.687871384	0.974860298
DFFA	-0.002025439	0.688319248	0.974860298
PRDX5	-0.001506304	0.688352063	0.974860298
KRCC1	-0.001521665	0.688389174	0.974860298
REPS1	0.001457416	0.688571116	0.974860298
ALDH1A1	-0.003007259	0.688602507	0.974860298
PIGS	0.001176493	0.688605919	0.974860298
CAPN11	0.00327955	0.688626481	0.974860298
RBM14	0.003720568	0.688689048	0.974860298
LAX1	0.002341756	0.688737956	0.974860298
CHRAC1	0.00142489	0.688827727	0.974860298
DDX56	-0.001262416	0.688834539	0.974860298
ATIC	-0.001562691	0.688958829	0.974860298
SMARCC1	-8.85E-04	0.689154989	0.974860298
CASP1	-0.001253245	0.689167375	0.974860298
ACSS2	-0.001162708	0.689462025	0.974860298
ATP1B1	0.002556926	0.68947438	0.974860298
NIPBL	-9.02E-04	0.689496781	0.974860298
PHF3	-7.37E-04	0.689516486	0.974860298
HSCB	0.001540491	0.689553946	0.974860298
EIF4E	-0.001881178	0.689597627	0.974860298
CLK2	-0.001000155	0.68992368	0.975086311
ABCB8	0.002058888	0.690032754	0.975086311
SLC17A3	0.0017598	0.690126528	0.975086311
PRDM15	0.001744325	0.690248765	0.975086311
ZMYND11	9.68E-04	0.690282889	0.975086311
KIAA1143	0.002086184	0.690459619	0.975086311
NME6	0.001190674	0.690508036	0.975086311
RNF213	0.002766296	0.690576249	0.975086311
HLA-DQA1	-0.006059108	0.690606729	0.975086311
METTL2B	-0.001579765	0.690645278	0.975086311
PTPLA	0.002153531	0.690729362	0.975086311
NAA16	0.001411252	0.690990352	0.975086311
ACTG1	-0.001910051	0.691095755	0.975086311
RPL11	6.92E-04	0.691263785	0.975086311
SHCBP1	-7.72E-04	0.691310889	0.975086311
ERGIC3	0.001114725	0.691348863	0.975086311

GMDS	-0.001384764	0.691356275	0.975086311
RAN	0.001299676	0.691435978	0.975086311
MGEA5	0.001274773	0.691517512	0.975086311
SH3BP5L	-0.001688757	0.69159895	0.975086311
FGD2	-0.002917948	0.691645534	0.975086311
DNA2	0.001732748	0.692051627	0.975086311
SIK2	0.001514809	0.692079418	0.975086311
STRA13	8.87E-04	0.692083259	0.975086311
ANO9	0.002230121	0.692099727	0.975086311
SIRT5	0.001033007	0.692147105	0.975086311
SOCS1	-0.002712041	0.692274627	0.975086311
TBCC	-0.002027225	0.69230228	0.975086311
CDK5	-0.001710594	0.692312161	0.975086311
TTC32	0.002004108	0.692679515	0.975182765
PPTC7	-0.001612839	0.692763385	0.975182765
SYMPK	0.002037753	0.692771255	0.975182765
EMR1	0.00316098	0.692949758	0.975182765
OTOF	0.005037458	0.693018572	0.975182765
SIRT2	-0.001306657	0.693111684	0.975182765
PANX1	-0.001743442	0.693370506	0.975182765
FAM188A	0.001186527	0.693417264	0.975182765
HSPA1L	0.001320115	0.693538824	0.975182765
MLC1	-0.001838783	0.693546085	0.975182765
TADA3	-0.001261186	0.693649334	0.975182765
ZBTB38	-0.002151043	0.693766643	0.975182765
HS6ST1	-0.001507014	0.694008096	0.975182765
PURB	-0.001181379	0.694038381	0.975182765
C7orf43	-0.001289224	0.694047866	0.975182765
WLS	-0.002808824	0.694079816	0.975182765
CHCHD2	-0.001264376	0.694154008	0.975182765
MFGE8	0.001893144	0.694237735	0.975182765
CACNG1	-0.001490455	0.694350551	0.975182765
C6orf203	0.001508805	0.694673615	0.975182765
HNRNPH3	0.001278789	0.694683388	0.975182765
ATG2B	-0.001556776	0.694901296	0.975182765
CORO2A	0.001730764	0.694954438	0.975182765
PHLDA1	0.002506342	0.695020682	0.975182765
ZC3H18	0.001348194	0.695030244	0.975182765
C12orf23	-0.001866249	0.695069507	0.975182765
FANCD2	-0.001273213	0.69516811	0.975182765
B4GALT6	-0.002171344	0.695267164	0.975182765
EHMT1	0.002474363	0.695309551	0.975182765
TMEM132B	-0.001546085	0.695374662	0.975182765
MYH9	-9.72E-04	0.69548668	0.975182765
CDKL3	-0.001437095	0.695502219	0.975182765
NOTCH2NL	-0.001651288	0.695625852	0.975182765
HHLA3	0.001480246	0.695666214	0.975182765

RNF166	-0.001777757	0.695676082	0.975182765
ROMO1	-9.33E-04	0.695680246	0.975182765
KIAA0430	0.001035894	0.695741873	0.975182765
ARIH1	0.001065355	0.695807671	0.975182765
TRMT12	0.001409679	0.695982173	0.975182765
MCF2L	0.001753795	0.695986357	0.975182765
POLE	0.001512714	0.696049125	0.975182765
TOM1	-0.001082197	0.696143615	0.975182765
LCAT	-0.001589904	0.696258341	0.975182765
RPS12	-0.00307815	0.696358822	0.975182765
NUP188	0.001443902	0.696529649	0.975182765
ZNF714	0.001804228	0.696607212	0.975182765
AMPD3	-0.001710555	0.696743795	0.975182765
BIVM	0.00165002	0.69680309	0.975182765
LCK	0.001402551	0.696823625	0.975182765
LRRCC1	0.001900639	0.696901339	0.975182765
CPA5	0.004067914	0.697022364	0.975182765
PLEKHG1	0.002588764	0.69708646	0.975182765
KCTD5	-0.001367488	0.697131055	0.975182765
TCTA	-0.001338068	0.697139688	0.975182765
HMGB2	-0.001146307	0.697226164	0.975182765
ACSS3	-0.001726863	0.697513493	0.975261187
NOP14	0.001158137	0.697910311	0.975261187
PPP1R9B	0.001408467	0.697922057	0.975261187
GAPDH	-0.001484136	0.697957464	0.975261187
DCAF17	0.001441252	0.697979245	0.975261187
SMAD5	0.001701781	0.697986283	0.975261187
C22orf39	-0.001370877	0.698031391	0.975261187
ARRDC3	0.001965377	0.698063247	0.975261187
CDK9	-9.98E-04	0.698075199	0.975261187
JAG1	-0.001630524	0.69816966	0.975270061
TAF9	-0.002421959	0.698513906	0.975457923
INO80C	-9.92E-04	0.698566191	0.975457923
FGD4	-0.001564026	0.69857552	0.975457923
ZGPAT	7.90E-04	0.69916338	0.975457923
CDC26	-8.82E-04	0.699219781	0.975457923
ZNF23	0.001503188	0.699254917	0.975457923
CD93	-0.001721599	0.699283723	0.975457923
RGS1	-0.004062123	0.69944273	0.975457923
ZNF22	-0.001138032	0.699532866	0.975457923
ACSF2	-0.001710565	0.699538645	0.975457923
FBP1	-0.001915607	0.699797382	0.975457923
ZSWIM3	-0.002052138	0.699945347	0.975457923
SLC31A1	-0.001626453	0.700001683	0.975457923
MIR125B2	0.001193009	0.700034189	0.975457923
TBKBP1	-0.002031509	0.700034923	0.975457923
B3GALT6	-0.001740857	0.7000672	0.975457923

SLC4A2	0.001645166	0.700149225	0.975457923
ETV2	0.001688196	0.700189973	0.975457923
MIR7-1	0.002239522	0.700252043	0.975457923
PCF11	-9.56E-04	0.700299982	0.975457923
THUMPD2	0.001009031	0.700339597	0.975457923
SENP1	0.001450936	0.70040624	0.975457923
COX17	-0.00119198	0.700608575	0.975457923
RGP1	-0.001511664	0.700652647	0.975457923
PTPN13	-0.002281077	0.700886777	0.975457923
GABARAP	0.002402029	0.701101407	0.975457923
POLR2F	0.001003369	0.701103704	0.975457923
FAM134A	-0.001283279	0.701139737	0.975457923
C10orf105	0.001764788	0.701156538	0.975457923
RTBDN	-0.00174003	0.70117307	0.975457923
FANCL	0.001898859	0.701183975	0.975457923
SCARNA14	-0.001572714	0.701233603	0.975457923
STK38	0.001136412	0.701289088	0.975457923
ARPC3	-0.001661901	0.701657829	0.975457923
ZNF772	-0.002198386	0.701754616	0.975457923
ZNF57	-0.001625621	0.701851265	0.975457923
DECR2	-0.001557728	0.70187894	0.975457923
RPS6KA2	-0.004750125	0.701915653	0.975457923
CSK	0.001341278	0.701922993	0.975457923
CSRP2	0.001508225	0.701924652	0.975457923
LYPD2	0.003540112	0.702214749	0.975457923
SFSWAP	0.001071378	0.702262976	0.975457923
UBA6	0.001055848	0.702336666	0.975457923
REC8	0.001421669	0.702376737	0.975457923
MAP3K4	0.001100137	0.702382937	0.975457923
TFEC	0.001680823	0.702403818	0.975457923
CKAP2L	-0.001468481	0.70244603	0.975457923
AAMP	0.001083077	0.702605438	0.975526867
RTTN	0.001190631	0.702675101	0.975526867
MOCS1	-0.002070246	0.702760072	0.975526867
JRK	0.001900039	0.702944464	0.975660473
RABEP1	-0.002050674	0.703059316	0.975664329
PEX13	0.001523199	0.703180569	0.975664329
SNX21	-0.001564783	0.703211674	0.975664329
MAK16	-0.001201274	0.703300558	0.975665357
ISOC2	-0.001503457	0.703618119	0.975983579
SOD1	-8.88E-04	0.703736623	0.976025645
TGDS	0.001607908	0.703863463	0.976079262
RLN2	0.002523813	0.703974801	0.976111371
ADORA1	-0.002597789	0.704201564	0.976303495
NOTCH2	-0.00149076	0.704369038	0.976413386
ZNF341	-0.001189508	0.704671696	0.976461757
ZNRF2	-0.001733977	0.70467495	0.976461757

MTHFD2L	0.001938928	0.704724783	0.976461757
LRIG2	0.0014191	0.704770721	0.976461757
OSBP	-7.18E-04	0.705026894	0.976461757
GLT8D1	-8.60E-04	0.705040135	0.976461757
TIGD7	-0.00153466	0.705055616	0.976461757
NAT14	-0.001424403	0.705132902	0.976461757
UBE2O	0.00155863	0.705231464	0.976461757
OSBPL2	8.03E-04	0.705286092	0.976461757
ZNF322	-0.001164325	0.705659116	0.976831899
SLU7	9.81E-04	0.705770291	0.976831899
MKL2	0.001789943	0.705827252	0.976831899
FAM136A	-9.72E-04	0.705906438	0.976831899
DENND1B	0.001610936	0.706082501	0.976927656
FZD7	0.001326305	0.70615577	0.976927656
AMY2B	0.001700564	0.706240411	0.976927656
BRSK1	-0.001942539	0.706342916	0.976947361
SMARCC2	-0.001276793	0.706528689	0.977082217
MYL6	-7.06E-04	0.706666111	0.977150179
AP1G1	0.001093392	0.706785611	0.977193346
CREB3L4	-0.001527637	0.707177109	0.977612517
TRIM28	-0.001401811	0.70731609	0.977682542
ODF2L	-0.005801996	0.707655217	0.977936188
GLYR1	6.65E-04	0.707697293	0.977936188
MYADML2	0.001981161	0.707787527	0.977936188
ANKMY2	0.001009557	0.707892425	0.977936188
GPR35	0.002046948	0.707941339	0.977936188
MYD88	-0.001144024	0.708057893	0.977975146
CYP2E1	0.002166369	0.708152717	0.977984083
SLC6A6	0.001951771	0.708241208	0.977984274
RFC4	0.001319881	0.708416545	0.978104371
DPYSL4	-0.007164814	0.708551017	0.978120109
TBC1D3B	0.001830087	0.708604675	0.978120109
ACSM5	0.001349298	0.708709975	0.978122092
MIS18BP1	-0.001132819	0.708842589	0.978122092
FECH	0.002211659	0.70887121	0.978122092
EIF2AK3	0.001250979	0.709229123	0.978139557
TGM2	-0.002021753	0.70926517	0.978139557
UROD	7.92E-04	0.709347265	0.978139557
SLC25A38	0.001207263	0.709543394	0.978139557
POMT2	0.001708827	0.709587784	0.978139557
SENP7	0.00112313	0.709593024	0.978139557
KATNA1	9.51E-04	0.709697131	0.978139557
TBCA	-0.001111565	0.709699629	0.978139557
USP6NL	-0.001687466	0.709753223	0.978139557
TRAFD1	0.00119466	0.709835761	0.978139557
VCPIP1	0.001155259	0.709973825	0.978139557
USP5	0.00110428	0.710169257	0.978139557

CLEC2L	0.002014337	0.710206682	0.978139557
HLA-A	-0.020111799	0.71048659	0.978139557
IQCG	-0.001871524	0.710604536	0.978139557
TNFSF8	0.00201565	0.710720572	0.978139557
QRSL1	-0.002244686	0.710753218	0.978139557
OSBPL10	0.003981864	0.710771449	0.978139557
GRHPR	0.001790345	0.711041083	0.978139557
ADAM10	-0.002025039	0.711107033	0.978139557
FSCN1	-0.002189713	0.711134931	0.978139557
ZNF681	0.002794274	0.711191965	0.978139557
ACTL6A	8.15E-04	0.711268639	0.978139557
DDX58	0.001768605	0.711383394	0.978139557
ZNF431	0.001163274	0.711418529	0.978139557
N4BP1	-0.001484293	0.711610077	0.978139557
PLK4	-0.001644273	0.711617781	0.978139557
CREB3L2	-0.001184802	0.711622965	0.978139557
KLHL29	-0.001371955	0.711797243	0.978139557
AGAP8	-0.001882635	0.711952805	0.978139557
NMUR1	0.003250835	0.711990713	0.978139557
TRIM21	-0.001316293	0.712065606	0.978139557
C1orf35	7.96E-04	0.71209894	0.978139557
C5orf28	0.002834987	0.712107688	0.978139557
CASP5	-0.002627562	0.712144754	0.978139557
FAM13B	9.46E-04	0.712160843	0.978139557
UCK1	-0.001634573	0.712468545	0.978139557
APOL3	-0.001413787	0.712473781	0.978139557
RLN1	0.002308909	0.712505028	0.978139557
CABLES2	-0.001150257	0.712529341	0.978139557
VPS72	-0.001255378	0.71256955	0.978139557
ZAN	0.001548058	0.713113042	0.978139557
RPL27	5.29E-04	0.713294506	0.978139557
ABCF3	-0.00147224	0.713309424	0.978139557
PMS2CL	0.001266376	0.713332356	0.978139557
NRXN2	-0.001938333	0.71340639	0.978139557
ZNF8	0.001706811	0.71358668	0.978139557
POU5F1	0.00188967	0.713711605	0.978139557
ROBO3	-0.002838931	0.713854023	0.978139557
PML	0.002677817	0.714078511	0.978139557
FAM65B	0.00124364	0.714092206	0.978139557
SLC45A4	-0.001750062	0.714238943	0.978139557
LGALS3BP	0.002696948	0.714356201	0.978139557
UBE4B	-8.04E-04	0.714411552	0.978139557
PER1	0.002156744	0.714640935	0.978139557
XPO7	-0.001197272	0.714659078	0.978139557
RAP2B	-0.001656066	0.714692945	0.978139557
POLR2J4	-0.001568794	0.714708502	0.978139557
PSMB9	-0.00213746	0.714782699	0.978139557

TOR2A	-0.001496729	0.714796666	0.978139557
ARL5A	-6.65E-04	0.714857979	0.978139557
HEATR3	-0.001780198	0.714885673	0.978139557
SGK494	0.001649072	0.714908487	0.978139557
SMCHD1	0.001609956	0.714943071	0.978139557
ZNF773	0.001916327	0.715041762	0.978139557
CCDC91	0.001194789	0.715169966	0.978139557
HOPX	0.002578853	0.715284944	0.978139557
RNF130	0.0014542	0.715301447	0.978139557
FEM1A	8.97E-04	0.71569384	0.978139557
SKA3	-0.001245385	0.715733647	0.978139557
MCF2L-AS1	-0.00198684	0.715737227	0.978139557
USP1	9.01E-04	0.715796293	0.978139557
MIRLET7D	-0.001939441	0.715846413	0.978139557
POC5	-8.48E-04	0.715947644	0.978139557
MYO18A	0.001209828	0.716048982	0.978139557
GMEB2	-8.94E-04	0.716053838	0.978139557
C15orf52	-0.002402722	0.71640179	0.978139557
TECPR1	0.002239771	0.71641293	0.978139557
C21orf58	0.002264562	0.716500074	0.978139557
RUVBL2	0.001205158	0.71665181	0.978139557
ZBTB16	-0.003067924	0.716652279	0.978139557
FAM102A	0.001579421	0.717077812	0.978139557
TNFRSF10D	-0.001855755	0.717174934	0.978139557
TNFRSF1A	-0.001068321	0.717190202	0.978139557
SNORD36C	0.001960473	0.717210204	0.978139557
CCDC66	0.001362248	0.717333358	0.978139557
DLEC1	0.001770364	0.717377231	0.978139557
SEC23A	-0.001628078	0.717408169	0.978139557
SH3BP4	0.001947939	0.717483119	0.978139557
TM4SF1	0.00429863	0.717667208	0.978139557
DHPS	6.91E-04	0.717681755	0.978139557
DDX49	0.001305934	0.71771335	0.978139557
MOS	-0.001667189	0.717739237	0.978139557
TMEM60	-0.001283827	0.717752155	0.978139557
PFDN2	0.001931173	0.717996532	0.978139557
RPS16	3.83E-04	0.718091862	0.978139557
NANOS1	0.002741168	0.718113208	0.978139557
ZNF248	0.00145123	0.718203774	0.978139557
KBTBD4	0.001229086	0.718244105	0.978139557
WDR59	0.001147811	0.718245668	0.978139557
TLR6	-0.001681464	0.718293838	0.978139557
UBE2K	-0.001019792	0.718398532	0.978139557
PPIAL4A	-0.00101295	0.718524492	0.978139557
GGT1	0.001489884	0.718593203	0.978139557
TMOD3	-0.001482468	0.718748016	0.978139557
GPNMB	0.001560111	0.718948161	0.978139557

SS18L1	-0.001342442	0.718991242	0.978139557
IGFLR1	-0.002837314	0.719068167	0.978139557
PASK	0.002724984	0.719104666	0.978139557
HLA-DRA	-0.001094411	0.71910547	0.978139557
PTAFR	-0.001536143	0.719193898	0.978139557
DNAL4	-8.61E-04	0.719463574	0.978139557
RPL6	-7.64E-04	0.719483875	0.978139557
PLEKHB1	-0.00194283	0.719680656	0.978139557
PTPN6	-0.001320936	0.71970975	0.978139557
DR1	-0.001193951	0.719794206	0.978139557
SDC1	0.001566011	0.719800299	0.978139557
NEB	-0.001744029	0.719845265	0.978139557
RPL22	6.18E-04	0.719991879	0.978139557
ZBP1	-0.001720992	0.720016613	0.978139557
PFDN1	0.001111321	0.720065772	0.978139557
MRPS18C	0.001409455	0.720124963	0.978139557
CD247	-0.001331607	0.720235947	0.978139557
FHIT	-0.003333882	0.720334335	0.978139557
KLF6	-0.001156003	0.7204359	0.978139557
C6orf1	-0.001318841	0.720709703	0.978139557
ACTR1B	-8.32E-04	0.720778359	0.978139557
CLIP3	0.002237349	0.720845749	0.978139557
C4orf33	0.001989475	0.720939678	0.978139557
YME1L1	-0.001519698	0.720974855	0.978139557
DMWD	0.001787657	0.721039874	0.978139557
RASGRP1	-0.001080009	0.721080058	0.978139557
ANXA6	0.001241565	0.721171831	0.978139557
PBX2	-9.12E-04	0.72130496	0.978139557
ABCF2	0.001219482	0.721383718	0.978139557
RTN1	-0.001747568	0.721384107	0.978139557
XKR6	-0.001372485	0.721386756	0.978139557
TOB1	0.001868836	0.721397289	0.978139557
MRPL16	8.26E-04	0.721448546	0.978139557
ZNF836	0.001342048	0.721489899	0.978139557
CUX1	-0.001229412	0.721675784	0.978139557
TGOLN2	-0.001038565	0.721781833	0.978139557
PIGP	-0.001501856	0.722057724	0.978139557
ZNF34	0.001270062	0.722074889	0.978139557
PITPNB	-0.00104632	0.722081427	0.978139557
PSG6	0.001987351	0.722100232	0.978139557
NDRG1	0.001084946	0.72225376	0.978139557
SP100	-0.001696033	0.722510914	0.978139557
SLC25A15	0.001028303	0.722669671	0.978139557
C3orf14	0.002946027	0.722739553	0.978139557
PLAA	9.63E-04	0.722739916	0.978139557
SUV420H2	0.001609884	0.722768414	0.978139557
ARL17B	0.003895282	0.722831461	0.978139557

ZNF432	-0.001310678	0.722846	0.978139557
NAA40	-0.001419217	0.723135495	0.978139557
SH3BGR	-0.001627571	0.723136306	0.978139557
RRP36	-7.28E-04	0.723305652	0.978139557
FAIM	0.001962866	0.723323154	0.978139557
CADM2	-0.00156719	0.723334529	0.978139557
CNN3	-0.002492409	0.723347995	0.978139557
DNAJC11	0.001247084	0.72342251	0.978139557
POMT1	-0.001091707	0.723578492	0.978139557
COQ9	-7.04E-04	0.72359615	0.978139557
ANAPC11	8.74E-04	0.723711881	0.978139557
SENP2	-8.70E-04	0.723726591	0.978139557
C1D	0.001459564	0.723935124	0.978139557
C1orf101	-0.001519478	0.723935495	0.978139557
TUBB8	0.001553831	0.723953688	0.978139557
CHD1L	0.001412657	0.724338993	0.978139557
TRIM65	0.001505807	0.724437067	0.978139557
TRA2B	7.74E-04	0.724444747	0.978139557
CACNA1S	-0.001702813	0.724458634	0.978139557
CLK3	-0.001071923	0.724490023	0.978139557
TCF24	0.00156611	0.724601481	0.978139557
MPHOSPH10	8.45E-04	0.724616813	0.978139557
TBC1D22B	-0.001260026	0.724651526	0.978139557
XYLT2	8.98E-04	0.724792887	0.978139557
DGAT2	0.00197051	0.724827328	0.978139557
CLEC11A	-0.0024989	0.72484809	0.978139557
NTHL1	0.001091086	0.724885419	0.978139557
UBE2I	9.22E-04	0.724946854	0.978139557
DCK	-0.001626027	0.725276142	0.978139557
CCDC97	8.42E-04	0.725298514	0.978139557
RCN2	0.00126101	0.725400477	0.978139557
GOLGA8G	0.001490883	0.725474666	0.978139557
LRFN1	-0.001655915	0.725542052	0.978139557
EVI5	-0.001740338	0.725784085	0.978139557
SCAF4	-0.001199209	0.72581524	0.978139557
BLK	0.003352604	0.725859811	0.978139557
CATSPER2P1	0.001252227	0.726113081	0.978139557
ARF1	-9.43E-04	0.726174722	0.978139557
KIF5B	-0.001374518	0.72630904	0.978139557
FBXO4	-0.00107169	0.726411878	0.978139557
KCTD6	-0.00100695	0.726523748	0.978139557
RAD1	-0.001345775	0.726531566	0.978139557
CD27	-0.001769582	0.726713805	0.978139557
HIST1H2BD	0.004733903	0.726783421	0.978139557
RCL1	-0.001559681	0.726878072	0.978139557
MDP1	9.49E-04	0.726950034	0.978139557
PDLIM7	0.001939379	0.726963117	0.978139557

C1orf109	-0.001113971	0.726971741	0.978139557
ETV3	-0.001775828	0.727040863	0.978139557
ALDH18A1	0.001284434	0.727130963	0.978139557
RTEL1	0.001381257	0.727138971	0.978139557
ZNF32	9.70E-04	0.727157549	0.978139557
POLR3A	0.001176588	0.727203314	0.978139557
AKAP7	-0.001288208	0.727265961	0.978139557
C6orf223	0.001578137	0.727390168	0.978139557
SMAP1	-0.001146401	0.727439639	0.978139557
LINS	0.002015238	0.727515385	0.978139557
MRPL43	0.001046901	0.727529404	0.978139557
RAVER2	-0.004273185	0.72766385	0.9782015
CCDC7	-0.002373449	0.727836392	0.978245113
FBXL14	-0.001445003	0.727873047	0.978245113
NLRC3	-0.001487416	0.728093605	0.978281506
RMND5A	-0.001613831	0.728368074	0.978281506
CHD8	0.001447735	0.728402821	0.978281506
GTPBP2	0.001445204	0.728494057	0.978281506
PER3	-0.001818616	0.728531477	0.978281506
NDUFB7	0.00113047	0.728936911	0.978281506
FEM1C	-0.001026205	0.728997281	0.978281506
GART	8.74E-04	0.729099976	0.978281506
WIPF1	0.001007869	0.729196866	0.978281506
TRIM47	-0.001553212	0.729229946	0.978281506
SSH1	-0.001563165	0.729276506	0.978281506
ASTN2	0.001416795	0.729297157	0.978281506
PDCD2L	-0.001456901	0.729479769	0.978281506
ZNF85	0.002872192	0.729498091	0.978281506
NR2C2AP	-9.10E-04	0.729524934	0.978281506
ZNF691	-0.001416948	0.729573015	0.978281506
OR2A42	0.001739516	0.729693612	0.978281506
UBE2MP1	-0.001371309	0.729787348	0.978281506
C11orf84	-0.001367069	0.73021925	0.978281506
TTLL4	-0.001311935	0.730247309	0.978281506
TMEM14A	-0.001718157	0.730296341	0.978281506
ARHGAP17	-9.32E-04	0.730312338	0.978281506
PCCA	-8.62E-04	0.730418178	0.978281506
ZNF687	-0.001584348	0.730429442	0.978281506
CRIP1	0.001212162	0.730467994	0.978281506
WDR35	0.00117176	0.730476294	0.978281506
DUT	0.001799989	0.730732465	0.978281506
GRIP2	0.001591266	0.73087924	0.978281506
ST3GAL4	-0.001756742	0.730921828	0.978281506
DEGS1	-0.001058749	0.730945026	0.978281506
OGFRL1	-0.001988462	0.731014674	0.978281506
DEPDC5	-0.00135669	0.731322586	0.978281506
ZMAT2	-0.001032331	0.731388414	0.978281506

TEC	-0.001227955	0.731427436	0.978281506
MYOM1	0.002105867	0.731433915	0.978281506
COG1	0.001263327	0.731464418	0.978281506
ZFP62	-0.001425612	0.731702136	0.978281506
ZNF256	-0.001338785	0.731850561	0.978281506
THAP11	-8.57E-04	0.731973621	0.978281506
RAD17	0.001175219	0.732031317	0.978281506
ZNF133	0.001195642	0.73208703	0.978281506
TMEM194B	0.001404015	0.732213086	0.978281506
CCDC47	-0.001206443	0.732534191	0.978281506
FAM89A	-0.001837008	0.732816478	0.978281506
ZKSCAN5	-0.001120636	0.732951099	0.978281506
POLR3E	0.001175926	0.732959734	0.978281506
IL16	-0.001110126	0.732975062	0.978281506
HSP90B1	-0.001201985	0.732985284	0.978281506
WDR36	-9.38E-04	0.73307078	0.978281506
SRSF5	-8.44E-04	0.733126412	0.978281506
RIC8B	-9.95E-04	0.733153067	0.978281506
TMEM43	-7.89E-04	0.733298236	0.978281506
BMI1	8.64E-04	0.733351332	0.978281506
POLR2H	-6.34E-04	0.733352408	0.978281506
DUSP14	-0.002011991	0.733387276	0.978281506
TIGIT	-0.00227223	0.733807322	0.978281506
FKBPL	0.001173749	0.734013034	0.978281506
ARGLU1	-9.23E-04	0.734047526	0.978281506
HEATR2	-8.88E-04	0.734058786	0.978281506
KLRD1	-0.002812305	0.734122989	0.978281506
DUSP28	-7.66E-04	0.73420835	0.978281506
P4HTM	-0.00120831	0.734330605	0.978281506
CRK	-0.001149312	0.734651885	0.978281506
NBPF14	-0.002374022	0.734739	0.978281506
IL24	-0.001348661	0.734749953	0.978281506
MAPRE3	-0.001288157	0.734870661	0.978281506
TRMT5	-8.27E-04	0.735002581	0.978281506
DUS3L	-0.001010831	0.735029109	0.978281506
WDHD1	-0.001438985	0.735171922	0.978281506
ZNF565	-0.001331199	0.735175142	0.978281506
HAR1A	0.001556156	0.735206263	0.978281506
CCDC93	0.00110338	0.735386231	0.978281506
RNF20	9.26E-04	0.735496797	0.978281506
CCDC127	0.001284688	0.735503175	0.978281506
FOSB	0.002367615	0.735504628	0.978281506
DCAF8	9.13E-04	0.735516044	0.978281506
ANKRD24	-0.001568772	0.735576115	0.978281506
VPS29	-7.29E-04	0.73569986	0.978281506
C11orf24	0.001343505	0.735782138	0.978281506
TRPV1	-0.00130352	0.736071475	0.978281506

KLHL26	0.001276749	0.73635451	0.978281506
PLCB1	0.001955873	0.73647524	0.978281506
DHX36	-9.69E-04	0.736487376	0.978281506
EXOC8	0.001616767	0.736511029	0.978281506
MDM1	0.001334474	0.736530774	0.978281506
CYP4F22	0.003496517	0.73657293	0.978281506
CPSF4L	-0.001717147	0.736601789	0.978281506
THAP4	0.001935246	0.736649407	0.978281506
TUBD1	-0.001542285	0.736680995	0.978281506
ZNF43	0.001777605	0.736707121	0.978281506
YES1	-0.002165609	0.736832852	0.978281506
AHCYL1	-6.96E-04	0.736839017	0.978281506
KIAA1522	-0.001571327	0.736871565	0.978281506
NOMO1	0.002104481	0.736930726	0.978281506
PLEK	0.001128165	0.736949864	0.978281506
EBF1	0.003581845	0.736962278	0.978281506
SCARNA8	0.001346529	0.736963161	0.978281506
GTF2E1	-9.00E-04	0.736995187	0.978281506
DNTTIP2	0.001738482	0.737179009	0.978281506
PUM2	6.54E-04	0.737218651	0.978281506
CCDC104	0.001120099	0.737231083	0.978281506
PSMA2	-0.00110474	0.737291692	0.978281506
COX16	-8.57E-04	0.737342759	0.978281506
CYP2J2	-0.002031675	0.737372582	0.978281506
PRSS36	0.001165278	0.737550138	0.978281506
SIK3	-6.97E-04	0.737668545	0.978281506
ZNF414	-0.001197235	0.737765484	0.978281506
ARHGEF10	-0.002678007	0.73776613	0.978281506
BNIP3	0.001104495	0.737767862	0.978281506
RAB14	-0.002209111	0.737768926	0.978281506
SSBP1	0.001067791	0.737781276	0.978281506
VEZF1	-8.27E-04	0.737798718	0.978281506
RBM22	-5.01E-04	0.737944069	0.978309378
MED10	-9.62E-04	0.738176001	0.978309378
GNL1	-0.0010404	0.738283109	0.978309378
PPWD1	0.0011045	0.73833615	0.978309378
ZNF585B	-0.001256725	0.738366568	0.978309378
PFN1	-9.73E-04	0.738635989	0.978309378
CARKD	-0.001101878	0.738681746	0.978309378
UBR1	0.001154198	0.738788057	0.978309378
FOXH1	0.001298439	0.738810566	0.978309378
NUDT5	-0.001184566	0.738943042	0.978309378
APOBEC3D	0.001475562	0.738973783	0.978309378
FAM107B	-0.001123904	0.739061175	0.978309378
CARD11	-0.001062915	0.73914213	0.978309378
POMP	8.38E-04	0.739319855	0.978309378
ADAMTSL5	-0.001589703	0.739328865	0.978309378

CIZ1	-0.001297476	0.739354461	0.978309378
USP10	0.001044972	0.739446806	0.978309378
EIF3H	-0.001064742	0.73946773	0.978309378
OSGIN1	0.002184747	0.739563674	0.978309378
NARG2	-0.002074724	0.739587395	0.978309378
ABLIM1	-0.001383759	0.739811275	0.978488589
UCHL3	-9.73E-04	0.740021552	0.978495472
ZNF469	0.001442522	0.740025976	0.978495472
VCAN	-0.001123453	0.740166366	0.978495472
CCT8L2	-0.001356654	0.740258221	0.978495472
CCDC65	-0.001307575	0.740522529	0.978495472
PTPLAD2	0.002204399	0.740656408	0.978495472
SCARNA17	-0.001941761	0.74088722	0.978495472
METAP1	5.71E-04	0.74089039	0.978495472
ALDOA	9.49E-04	0.740960877	0.978495472
NOTCH4	0.002040558	0.740983489	0.978495472
ACYP2	-0.001431459	0.740992673	0.978495472
ZNF720	0.00145156	0.74108911	0.978495472
OTX1	0.001663391	0.741126261	0.978495472
MRPL42	-0.001180455	0.741129907	0.978495472
SNORD100	0.001670818	0.741142473	0.978495472
IGSF9B	0.002180934	0.741259309	0.978525502
C19orf12	-0.001343044	0.741342024	0.978525502
CXCR2	-0.002238785	0.741595733	0.978634504
SERBP1	7.61E-04	0.741662597	0.978634504
LPIN1	0.0011997	0.741731766	0.978634504
LIMS2	0.001672209	0.741790573	0.978634504
PCDH9	-0.001410155	0.742042855	0.978634504
MICALL1	-0.001519384	0.742066074	0.978634504
MED15	0.001582069	0.742099045	0.978634504
HN1	-0.001445707	0.742215933	0.978634504
HLA-DPA1	-0.001564649	0.742220314	0.978634504
PREPL	-0.00111878	0.742390551	0.978742379
QKI	0.002114384	0.74260238	0.978894034
ZNF568	-0.001301562	0.742682456	0.978894034
SNTB2	0.001364108	0.743077109	0.979260045
HSPD1	-7.68E-04	0.743319479	0.979260045
LIMK1	0.001547702	0.743328835	0.979260045
TTC3	9.75E-04	0.743378557	0.979260045
FADS1	0.001904198	0.74340249	0.979260045
MAPK1IP1L	7.58E-04	0.743758787	0.979426697
GOLGB1	0.002225964	0.743799195	0.979426697
WARS	-0.001708407	0.743825374	0.979426697
ITGAE	0.001113008	0.74393126	0.979426697
C1orf116	0.002438371	0.743971422	0.979426697
TBX21	0.00150246	0.74421852	0.979626148
PROCA1	-0.001311946	0.744330949	0.979626148

POM121C	-6.82E-04	0.74438843	0.979626148
USP24	-8.65E-04	0.744515272	0.979658776
COL5A1	0.001483622	0.744891706	0.979658776
NCAPG2	-0.003719281	0.745022785	0.979658776
DNAH1	0.001157106	0.745038034	0.979658776
ETFB	-0.001131478	0.745052742	0.979658776
C4orf3	-9.24E-04	0.745417854	0.979658776
CLPTM1L	9.14E-04	0.745470006	0.979658776
ST3GAL5	-0.001125219	0.745470959	0.979658776
CLTB	7.33E-04	0.745611505	0.979658776
TRMT112	-0.001112677	0.745660738	0.979658776
XCR1	0.001861246	0.745767253	0.979658776
SYCP2	-0.002076944	0.745784612	0.979658776
CCR4	0.002107545	0.745794052	0.979658776
FAM195B	-0.001182054	0.745795533	0.979658776
TIPRL	-0.001433829	0.745874332	0.979658776
RSPH3	-0.001545605	0.746043208	0.979658776
DHRS12	0.001358508	0.746389264	0.979658776
PCYOX1L	0.001300951	0.746404668	0.979658776
TMC6	0.00122307	0.746501675	0.979658776
SGTB	-0.002919108	0.746772409	0.979658776
ANKIB1	-7.03E-04	0.746880129	0.979658776
SRPK2	0.001553133	0.746913193	0.979658776
GTF3C2	-6.34E-04	0.747063696	0.979658776
RING1	-5.13E-04	0.747078797	0.979658776
SLC22A4	-0.001767148	0.747132511	0.979658776
LACTB2	0.00165269	0.747155153	0.979658776
DLL1	-0.002861878	0.747262922	0.979658776
NSL1	9.15E-04	0.747611873	0.979658776
MT1A	0.00173486	0.747847366	0.979658776
TTC21A	0.001878855	0.74784889	0.979658776
ZNF700	0.001467595	0.747918517	0.979658776
ANAPC4	-0.00106355	0.747946405	0.979658776
MORF4L1	-0.001139738	0.748005008	0.979658776
WDR1	6.73E-04	0.748063875	0.979658776
MAP3K10	-0.001058801	0.748158059	0.979658776
CXCL3	0.001558519	0.748300765	0.979658776
PARP6	9.38E-04	0.748421576	0.979658776
CLPP	0.001100283	0.748437153	0.979658776
EMR4P	0.004412158	0.74851536	0.979658776
MGP	0.00126858	0.748529742	0.979658776
FCRLB	-0.001862713	0.74856537	0.979658776
C19orf66	0.001026817	0.748591358	0.979658776
DCHS1	-0.00163299	0.748708925	0.979658776
CTDSPL2	-0.001189821	0.748868664	0.979658776
MKRN3	-0.001730668	0.748889694	0.979658776
TOP1MT	-0.001472449	0.749115624	0.979658776

PIGH	-9.30E-04	0.749146301	0.979658776
CELA1	-0.003362147	0.749149203	0.979658776
XRN1	8.65E-04	0.749217212	0.979658776
SAMD9L	0.002187775	0.749227925	0.979658776
LARGE	-0.00253972	0.749366786	0.979658776
ANKRD36	0.002273872	0.749380135	0.979658776
GRINA	-0.002332804	0.749710558	0.979658776
BCL3	-0.001273082	0.749788348	0.979658776
GSTM3	0.005630112	0.749847146	0.979658776
CCT6P3	-6.41E-04	0.750025926	0.979658776
CLEC4C	-0.002869406	0.750039976	0.979658776
MED31	-0.001089453	0.750083499	0.979658776
FAS	-0.001764703	0.750207927	0.979658776
ARHGAP9	-8.47E-04	0.75022788	0.979658776
NT5C	6.66E-04	0.750272513	0.979658776
ZNF615	-0.001005124	0.750290214	0.979658776
DFNB59	-0.001275603	0.750315144	0.979658776
STAG3L1	0.002107315	0.75043242	0.979658776
TMEM14C	-0.001243703	0.750581341	0.979658776
NUP107	8.79E-04	0.75075396	0.979658776
CNTLN	0.001278106	0.750769015	0.979658776
CSMD1	-0.002108874	0.750822041	0.979658776
KDM5A	0.001007858	0.75084466	0.979658776
GORASP1	8.97E-04	0.75091485	0.979658776
CCDC28B	-0.001449392	0.751333436	0.979658776
TMED4	-0.001417661	0.751397608	0.979658776
HDHD3	-0.00148733	0.751628891	0.979658776
GOPC	8.66E-04	0.751643041	0.979658776
ANKMY1	0.001946386	0.751972163	0.979658776
WIPI1	-0.001556628	0.752087542	0.979658776
POLM	0.001349995	0.752122584	0.979658776
DCAF6	-7.17E-04	0.752155252	0.979658776
BLVRB	-0.00203298	0.75221665	0.979658776
ZNF658	-0.001903108	0.75231436	0.979658776
IMMP2L	0.001776726	0.752362201	0.979658776
BRAF	0.001310694	0.752366813	0.979658776
KIAA1524	0.00165821	0.752494346	0.979658776
MTPAP	0.001138936	0.752566559	0.979658776
C11orf21	-0.001546341	0.752703658	0.979658776
FSIP1	0.0012288	0.752809211	0.979658776
FBXO31	9.37E-04	0.753376023	0.979658776
POC1B	9.97E-04	0.753512054	0.979658776
ACACA	-9.45E-04	0.753516663	0.979658776
CLCN7	-9.62E-04	0.753670534	0.979658776
ZC3H12C	-0.001441562	0.753789275	0.979658776
EFR3A	-6.21E-04	0.753800167	0.979658776
CDKN1B	9.24E-04	0.75386789	0.979658776

AIF1L	-0.001190494	0.753868482	0.979658776
PHLDB2	0.00215688	0.75399495	0.979658776
ENOPH1	-7.97E-04	0.754050209	0.979658776
CHMP6	0.001161077	0.754284294	0.979658776
ABHD3	-7.77E-04	0.754457333	0.979658776
ZFP14	0.002459738	0.754463727	0.979658776
COBLL1	0.002249239	0.754508202	0.979658776
ZNF384	7.67E-04	0.754665032	0.979658776
PGD	-0.001343879	0.754836806	0.979658776
AP1M1	-6.34E-04	0.754877794	0.979658776
MXI1	0.001517527	0.754964132	0.979658776
MSH6	7.97E-04	0.755058653	0.979658776
TFIP11	7.66E-04	0.755074195	0.979658776
NEK8	0.001439484	0.75519106	0.979658776
PRPF38A	-7.19E-04	0.755255545	0.979658776
EDEM1	-7.87E-04	0.755339699	0.979658776
LIMA1	-0.001499739	0.755346182	0.979658776
TMEM45B	0.002897769	0.755347569	0.979658776
CDK2AP1	0.001430024	0.75536786	0.979658776
APOBEC3F	-0.002103977	0.755435509	0.979658776
NOSIP	0.001200187	0.7556618	0.979658776
FBXL3	-0.001262082	0.755975874	0.979658776
MFSD6	0.001244827	0.756090522	0.979658776
SCAF11	6.43E-04	0.756201466	0.979658776
ASAH2B	-0.001202546	0.756232347	0.979658776
ERI1	-9.59E-04	0.756246415	0.979658776
HIRA	0.001659073	0.756287203	0.979658776
EHD1	-6.61E-04	0.756303441	0.979658776
SGPL1	-0.00125742	0.756355302	0.979658776
RPL24	-5.38E-04	0.756404003	0.979658776
TXNRD1	-8.62E-04	0.75660199	0.979658776
LMBR1L	0.001109108	0.756714735	0.979658776
ROM1	0.001084121	0.756718158	0.979658776
BASP1	-0.001432462	0.756763808	0.979658776
RSRC2	-8.69E-04	0.756913328	0.979658776
EMP3	-8.73E-04	0.756963713	0.979658776
AKTIP	-9.33E-04	0.757053543	0.979658776
SCAMP2	0.001157411	0.757082499	0.979658776
PLCXD2	0.001317758	0.757128726	0.979658776
OCM2	0.0018549	0.757218305	0.979658776
PURG	0.001258007	0.7572294	0.979658776
SNORD55	0.001446497	0.757312206	0.979658776
C19orf48	-0.001097492	0.757358988	0.979658776
P2RY6	0.001524964	0.757398957	0.979658776
FIZ1	0.001096161	0.757542289	0.979658776
KIAA0556	8.48E-04	0.757597457	0.979658776
SYT15	-0.001960116	0.757864585	0.979658776

HPS5	-0.00106909	0.757960094	0.979658776
FRMD3	-0.003362192	0.758002522	0.979658776
CSNK2B	0.001044676	0.758023969	0.979658776
HBQ1	0.00390447	0.758138478	0.979658776
ZNF266	0.001888192	0.758206559	0.979658776
AKR7A2	0.001387895	0.758303496	0.979658776
SHROOM1	-0.001742851	0.758443258	0.979658776
ZNF471	0.002382777	0.758542479	0.979658776
WDR6	9.74E-04	0.758907813	0.979658776
HIST1H2BJ	0.003245267	0.758928754	0.979658776
CALML4	-0.00134534	0.75903709	0.979658776
ZNF225	-0.001044205	0.759173986	0.979658776
ZBTB49	0.001285443	0.759185237	0.979658776
SECTM1	0.001150779	0.759263591	0.979658776
SLC25A42	9.22E-04	0.759463376	0.979658776
ZNF766	-0.001014669	0.75954374	0.979658776
ZFYVE1	-8.20E-04	0.759544847	0.979658776
CRYZL1	-0.00106131	0.759776524	0.979658776
FKBP1B	-0.00178799	0.759862854	0.979658776
ECHS1	8.26E-04	0.759962672	0.979658776
APP	-0.002150142	0.760023521	0.979658776
AKAP8	-7.61E-04	0.760094288	0.979658776
ASPHD1	0.001184174	0.760357595	0.979658776
VPS53	5.51E-04	0.760440235	0.979658776
ZNF598	6.55E-04	0.76063696	0.979658776
CD3G	-0.001691921	0.760685026	0.979658776
GNAS	-9.37E-04	0.760805634	0.979658776
WDR81	-0.001307897	0.760911627	0.979658776
IRX6	0.001702889	0.76097408	0.979658776
ABL1	-7.24E-04	0.760999538	0.979658776
CHCHD5	-0.001041728	0.761013997	0.979658776
CDC42EP4	-0.001476641	0.761014198	0.979658776
CRTC2	0.001008883	0.761114658	0.979658776
ITPK1	0.001226035	0.761159718	0.979658776
TMEM127	0.001060652	0.761270424	0.979658776
TPPP3	-0.002440468	0.761479811	0.979658776
PIP4K2B	-0.001643907	0.761603064	0.979658776
MTMR12	0.001426178	0.76168201	0.979658776
CAPN5	-0.002037392	0.761774763	0.979658776
DNAJC12	0.001142365	0.761996672	0.979658776
MEGF10	-0.001495539	0.762013674	0.979658776
SCNN1D	0.001274925	0.762092558	0.979658776
MRPS2	9.24E-04	0.76209523	0.979658776
MIR1253	-0.00125382	0.762312767	0.979658776
TRIM22	0.001139085	0.76244345	0.979658776
ASRGL1	-0.001949545	0.762447688	0.979658776
BSDC1	9.95E-04	0.762508928	0.979658776

SGTA	0.001557823	0.762737972	0.979658776
ICOS	0.001444658	0.762765445	0.979658776
VPS35	-8.89E-04	0.762797348	0.979658776
SMCP	0.001163047	0.763114155	0.979658776
GSTA2	-0.001426873	0.763157055	0.979658776
TRPC1	0.001611771	0.763252777	0.979658776
ATP6AP1L	-0.001814986	0.76326104	0.979658776
TBL3	-0.001271605	0.76328465	0.979658776
GTF2F1	7.86E-04	0.763334035	0.979658776
ANGEL2	8.26E-04	0.763421849	0.979658776
OPLAH	0.00181561	0.763461938	0.979658776
MRPL49	9.37E-04	0.763492577	0.979658776
GNAI2	-8.57E-04	0.763607384	0.979658776
FOXRED2	-0.002225656	0.763636008	0.979658776
PRKCA	0.001507974	0.763845599	0.979658776
OSTF1	-8.25E-04	0.76393461	0.979658776
FAM83H	-0.001201488	0.763973574	0.979658776
C3orf58	-9.99E-04	0.764018112	0.979658776
TMEM88	-0.001712625	0.764158556	0.979658776
CHST14	-0.001493436	0.764167142	0.979658776
CRYGS	0.001652608	0.764367586	0.979658776
PMF1	0.001193452	0.764466373	0.979658776
SREK1	9.11E-04	0.764552888	0.979658776
FLVCR1	0.001595846	0.764569321	0.979658776
SPAG8	-0.0016568	0.764780057	0.979658776
CUL5	0.001304667	0.764862128	0.979658776
APPL1	9.83E-04	0.764865985	0.979658776
ARHGEF11	0.001201712	0.764923679	0.979658776
FCGBP	0.003028285	0.76495705	0.979658776
EAF2	0.002323526	0.765031819	0.979658776
PANX2	-0.001777259	0.765080128	0.979658776
C19orf60	8.79E-04	0.765191505	0.979658776
CTNNBIP1	-0.001078279	0.765268702	0.979658776
ADCY4	-0.001324975	0.765327169	0.979658776
HIST2H3D	-0.001705287	0.765682078	0.979658776
GLUL	-0.001615135	0.765796071	0.979658776
DUXA	-0.002925259	0.765940874	0.979658776
PHLDB1	0.001263851	0.765991682	0.979658776
DCTN5	0.001105588	0.766211477	0.979658776
PSMC4	-0.001549412	0.766252282	0.979658776
ZNF679	0.002437525	0.766283779	0.979658776
GGCT	8.75E-04	0.766314706	0.979658776
ANTXR2	0.001069809	0.766416248	0.979658776
ITGA10	-0.001372575	0.766419765	0.979658776
MEF2D	9.42E-04	0.766458057	0.979658776
CNBP	9.51E-04	0.766677801	0.979658776
MIR345	0.001147663	0.766700529	0.979658776

TAX1BP1	-6.36E-04	0.766791659	0.979658776
RUNX2	0.001995164	0.766813435	0.979658776
RASA3	-0.001251678	0.766892537	0.979658776
TMEM38B	0.001175877	0.766923715	0.979658776
TMEM44	0.001899879	0.767008659	0.979658776
WASF2	0.00102781	0.767027527	0.979658776
CD1A	0.001737731	0.767046313	0.979658776
MAD2L2	-8.99E-04	0.767368039	0.979658776
CBX5	-0.001528349	0.767380969	0.979658776
SUMO1P1	-0.001184369	0.767404652	0.979658776
NUMA1	0.001038779	0.76740897	0.979658776
PMM1	-8.48E-04	0.767458678	0.979658776
TNFRSF17	-0.004022655	0.767471571	0.979658776
C1orf131	-7.70E-04	0.76763263	0.979658776
GJC1	-4.63E-04	0.767666149	0.979658776
PPP1R10	-0.001337855	0.767788623	0.979658776
RFX1	-0.001214554	0.768005814	0.979658776
MIR611	0.001252076	0.76804645	0.979658776
MRPS34	0.001317218	0.768052217	0.979658776
PLEKHB2	0.001874444	0.768098953	0.979658776
ITFG2	-7.09E-04	0.768134047	0.979658776
RGS2	0.001354717	0.768208642	0.979658776
C1orf56	0.00141442	0.768233655	0.979658776
SRSF9	-5.96E-04	0.768288226	0.979658776
ABCF1	-6.50E-04	0.768427274	0.979658776
SHKBP1	0.00147217	0.768468048	0.979658776
SLC4A1AP	8.62E-04	0.768601137	0.979658776
PROK1	-0.001298993	0.768638677	0.979658776
NCOA1	8.20E-04	0.76873567	0.979658776
RPL37A	5.22E-04	0.768768569	0.979658776
POLD4	7.08E-04	0.768811773	0.979658776
CHRDL2	0.00108333	0.768875927	0.979658776
SERPINE1	0.001291501	0.768894842	0.979658776
HLA-G	-0.001683744	0.768955885	0.979658776
RBM18	-0.001230583	0.769081475	0.979658776
ADPGK	-8.82E-04	0.769215464	0.979658776
UTP15	-0.001117285	0.769229944	0.979658776
TMIGD2	0.001839249	0.769449781	0.979658776
CYB5RL	0.002687304	0.769467357	0.979658776
CBFA2T3	-0.001541569	0.769488061	0.979658776
TP63	0.001780139	0.769536649	0.979658776
RPS9	7.50E-04	0.769573135	0.979658776
TMED1	-0.001264472	0.769733686	0.979658776
SLC29A1	0.001447344	0.769764376	0.979658776
C2orf43	-0.001183504	0.769807433	0.979658776
ADAMTS1	-0.00287392	0.769910724	0.979658776
SAMD9	0.001053008	0.769941747	0.979658776

SNX5	-8.42E-04	0.770068425	0.979658776
KLHL24	-8.82E-04	0.770089607	0.979658776
MMP24	-0.001082565	0.770144504	0.979658776
MAGOH	0.001004235	0.770191602	0.979658776
ZC3H8	-8.33E-04	0.770250069	0.979658776
EML3	7.78E-04	0.770407997	0.979658776
NOP16	-0.001244069	0.770427551	0.979658776
UBQLN4	-8.42E-04	0.770483979	0.979658776
ACBD3	7.99E-04	0.770536141	0.979658776
TMEM229B	0.001271934	0.770595496	0.979658776
FAM32A	-6.55E-04	0.770640094	0.979658776
EBNA1BP2	-7.16E-04	0.770752857	0.979658776
TM7SF3	0.001140691	0.770781791	0.979658776
TYMS	-0.002456677	0.770863868	0.979658776
CAMK4	0.001615136	0.770925614	0.979658776
RNF112	0.001132121	0.770941835	0.979658776
CCT2	-7.18E-04	0.771009173	0.979658776
LIPC	0.003426812	0.771261344	0.979658776
TMEM63A	0.001644546	0.771338126	0.979658776
NIPA1	0.001153497	0.771425399	0.979658776
TMPRSS7	0.001177452	0.771479477	0.979658776
TSPYL6	-0.001125525	0.771501875	0.979658776
MS4A14	0.001956417	0.771534868	0.979658776
BCAN	0.001116453	0.771559537	0.979658776
GCHFR	0.001562854	0.771579909	0.979658776
SNX14	-6.73E-04	0.771954237	0.979658776
HLA-H	7.64E-04	0.771967988	0.979658776
SIGLEC16	0.002589624	0.772002796	0.979658776
SECISBP2	0.001149596	0.772012151	0.979658776
CYP2U1	0.001011534	0.772153787	0.979658776
CMBL	-0.001167195	0.77218264	0.979658776
BRF1	-0.001173148	0.772187966	0.979658776
IQCB1	-0.001008674	0.772203706	0.979658776
PCNX	8.10E-04	0.772311282	0.979682968
ZNF136	-0.00105761	0.772445464	0.979701686
BCYRN1	0.002123772	0.772503055	0.979701686
PEPD	-7.45E-04	0.772677509	0.979810672
SOCS5	0.002106349	0.772835866	0.979850221
METTL5	-0.001219019	0.773083764	0.979850221
SIRT7	8.57E-04	0.773187797	0.979850221
GDAP1	0.001113871	0.773246325	0.979850221
MB21D1	0.001458324	0.773260427	0.979850221
RASSF7	6.78E-04	0.773298992	0.979850221
SYK	8.97E-04	0.773328352	0.979850221
KIAA0391	7.50E-04	0.77349365	0.979947489
PACSIN1	-0.001996598	0.773622504	0.979998569
AQP7P1	-0.001447425	0.773748598	0.980046142

GRWD1	0.001116109	0.773883665	0.980071631
ZBTB46	0.001330314	0.773945806	0.980071631
AGFG2	0.001286214	0.774151472	0.980172503
UGT1A1	0.001150037	0.774323722	0.980172503
RFK	-0.001514724	0.774385101	0.980172503
EARS2	-0.001070994	0.774414334	0.980172503
GNA12	0.001022115	0.774512084	0.980172503
RASAL1	0.001141093	0.77455677	0.980172503
SUSD3	0.001204337	0.774693942	0.980234024
BTBD2	7.34E-04	0.774907051	0.980242121
NR1H3	-0.001484467	0.774963275	0.980242121
PSMD14	-8.99E-04	0.774999157	0.980242121
TC2N	-0.001307575	0.775094812	0.980242121
LRRC8D	-8.71E-04	0.775165584	0.980242121
FBXO44	0.001243421	0.775289664	0.980242121
SART3	-9.73E-04	0.775320243	0.980242121
C21orf49	-9.60E-04	0.775479493	0.980331488
ARNT	1.00E-03	0.775633796	0.980378041
DGCR8	0.00176977	0.775703617	0.980378041
IGFBPL1	0.001260991	0.775805672	0.980378041
DPP9	-0.00114954	0.775877213	0.980378041
IRAK2	0.001422492	0.775959167	0.980378041
ZNF407	8.17E-04	0.776077003	0.980415013
TMEM126A	-9.35E-04	0.776464745	0.980574654
C17orf100	-0.001348281	0.776523663	0.980574654
PARN	-7.74E-04	0.776595084	0.980574654
FAM35A	7.56E-04	0.776614022	0.980574654
USP13	-9.83E-04	0.776646309	0.980574654
UBE2L3	0.001157217	0.776960902	0.980782189
ZFYVE19	0.001000349	0.777035736	0.980782189
USP3	7.92E-04	0.777143688	0.980782189
RYR1	-0.001720803	0.777360697	0.980782189
CDK5RAP1	6.59E-04	0.777364249	0.980782189
PRKDC	0.001157579	0.777389742	0.980782189
HGSNAT	0.001005523	0.777430927	0.980782189
PLEKHG3	0.001623954	0.777545686	0.980815179
EXOC3	-8.47E-04	0.777706181	0.980872602
CCDC102A	-0.001242767	0.777768437	0.980872602
WDR54	7.57E-04	0.777960284	0.980894247
PRKRIR	6.89E-04	0.778203156	0.980894247
ENTPD6	9.96E-04	0.77839098	0.980894247
RFPL4A	-0.001308777	0.778428543	0.980894247
MIR1228	-0.001364566	0.778470565	0.980894247
ANKRD27	-0.00113024	0.778550201	0.980894247
KHSRP	-0.001294973	0.778586572	0.980894247
PPP1R1B	0.001083945	0.778775528	0.980894247
PLS1	0.001274405	0.778789847	0.980894247

AIM1	-9.00E-04	0.778826036	0.980894247
RBM39	6.95E-04	0.779213235	0.980894247
RNASE6	-0.001992456	0.779252087	0.980894247
B3GAT1	-0.003086403	0.779324937	0.980894247
TFG	0.001955752	0.779671344	0.980894247
MAP6D1	-0.001459061	0.779694249	0.980894247
C8orf59	7.52E-04	0.779700482	0.980894247
VPS26B	-8.92E-04	0.779748969	0.980894247
KDM5B	7.55E-04	0.779871772	0.980894247
SPDYE2	8.73E-04	0.779960553	0.980894247
NIPAL2	-0.001096937	0.779964448	0.980894247
POTEF	-4.56E-04	0.780002028	0.980894247
SMU1	7.62E-04	0.780014098	0.980894247
AHR	0.001191853	0.780039659	0.980894247
NDUFC2	-0.001445507	0.780259377	0.980894247
ZNF782	0.00154048	0.780371527	0.980894247
INTS1	-8.99E-04	0.780416051	0.980894247
KIAA1211	0.001602118	0.780429395	0.980894247
DPM1	-0.001193462	0.780484362	0.980894247
PMP22	-0.002255735	0.780681181	0.980894247
RPL15	9.00E-04	0.780686698	0.980894247
SNORA68	0.00140914	0.780730549	0.980894247
CDH24	0.001112775	0.780738037	0.980894247
EXOSC5	8.75E-04	0.780891033	0.980894247
FOPNL	6.97E-04	0.780895702	0.980894247
PRDM2	8.93E-04	0.780934676	0.980894247
DMXL2	-0.001548867	0.781016044	0.980894247
PHLPP2	-0.001109924	0.781109056	0.980894247
ATP1A1	-6.27E-04	0.781153021	0.980894247
FTSJ3	-9.36E-04	0.781552692	0.981100461
STX6	8.17E-04	0.781664575	0.981100461
DIEXF	-9.12E-04	0.781745373	0.981100461
NSA2	9.35E-04	0.781835265	0.981100461
PTGES3	0.001105702	0.78186931	0.981100461
NDUFAF2	0.001106766	0.78191298	0.981100461
PSMA3	-8.82E-04	0.781937688	0.981100461
BZRAP1	0.002022952	0.782234163	0.981108367
OAZ2	-0.002031034	0.78224169	0.981108367
RIT1	9.80E-04	0.782285579	0.981108367
SPOCK1	0.001313495	0.782298532	0.981108367
DDTL	0.003166678	0.782392238	0.981114725
ELF2	-7.22E-04	0.78262262	0.981292453
SLC27A5	0.001104284	0.782921074	0.981412609
SNRPF	8.03E-04	0.78295841	0.981412609
CCDC23	-0.001046111	0.783186737	0.981412609
GPBP1L1	0.001195958	0.783236589	0.981412609
CAPRIN2	0.001208221	0.783315982	0.981412609

IMPA1	-0.001508908	0.783472987	0.981412609
LGALS3	0.001433787	0.783615695	0.981412609
TMSB10	3.44E-04	0.783682015	0.981412609
RPP40	8.33E-04	0.783782292	0.981412609
ZDHHC13	-8.37E-04	0.783866556	0.981412609
DYNLT1	7.83E-04	0.784052402	0.981412609
EPS8	-0.00178587	0.784134104	0.981412609
GABPB1	-9.96E-04	0.784201443	0.981412609
CLN6	-0.001291068	0.784201857	0.981412609
RNF185	-0.001160906	0.784553388	0.981412609
TRIB3	0.00148089	0.784673068	0.981412609
SUMO2	-0.001249449	0.784704256	0.981412609
DPH5	-0.001097078	0.78479326	0.981412609
POLR2D	-0.001174032	0.784856908	0.981412609
WDR92	8.96E-04	0.785013462	0.981412609
GPSM1	0.001488807	0.785033079	0.981412609
TYW1	0.001077365	0.785038747	0.981412609
MAL	-0.001586202	0.785138576	0.981412609
NBAS	-7.88E-04	0.785163243	0.981412609
ORAOV1	-0.001034191	0.785174877	0.981412609
GOLT1B	9.40E-04	0.785249378	0.981412609
MUC2	-0.001285669	0.785254107	0.981412609
INTS7	-9.53E-04	0.785265947	0.981412609
SLMO1	9.62E-04	0.785289681	0.981412609
CLDN20	0.001029391	0.785739357	0.981863733
CUL9	-0.001157911	0.785864051	0.981871284
DUSP5	-0.001452595	0.785982608	0.981871284
PRDX1	-6.76E-04	0.786017828	0.981871284
FZD2	-0.001237417	0.786100218	0.981871284
BMF	-9.71E-04	0.786466942	0.981980393
LPGAT1	-7.96E-04	0.786499509	0.981980393
MYBBP1A	0.001310554	0.786689258	0.981980393
BTG3	9.57E-04	0.786708096	0.981980393
DENND2D	-0.001102947	0.786754809	0.981980393
PPM1G	4.92E-04	0.786858969	0.981980393
RNU4ATAC	0.00144858	0.786896562	0.981980393
SLX4	8.95E-04	0.786897289	0.981980393
JAGN1	-9.28E-04	0.787051146	0.982050977
RTN3	8.10E-04	0.787169122	0.982050977
SYVN1	7.01E-04	0.787420471	0.982050977
UGCG	0.001282938	0.787568681	0.982050977
FAM98A	-9.44E-04	0.787580602	0.982050977
MOB2	9.72E-04	0.787655504	0.982050977
PHF10	-9.11E-04	0.787794964	0.982050977
CCNB1	-0.00134353	0.787876405	0.982050977
COX5B	-8.88E-04	0.787968168	0.982050977
BIK	-0.001268618	0.788011265	0.982050977

UFM1	8.00E-04	0.788025711	0.982050977
CAPN2	-7.24E-04	0.788211525	0.982050977
MRPS30	-0.001005631	0.788330039	0.982050977
DNAJB14	0.00196105	0.788601849	0.982050977
KIAA1598	-0.00155908	0.788662103	0.982050977
CHCHD6	-0.001011622	0.788665008	0.982050977
GOLGA1	9.00E-04	0.788716591	0.982050977
RANBP2	-0.001159284	0.78873639	0.982050977
DRD3	-0.00198105	0.788896557	0.982050977
ENPP5	-0.002009809	0.788937456	0.982050977
HIST1H3E	-9.52E-04	0.789041332	0.982050977
SNRNP35	-7.74E-04	0.78912216	0.982050977
SEC61A2	-0.001411302	0.789244623	0.982050977
KIAA1107	-9.94E-04	0.789273249	0.982050977
EFCAB3	9.99E-04	0.789342472	0.982050977
BNIP1	0.001086077	0.789447383	0.982050977
NARS	-4.96E-04	0.789484497	0.982050977
BRPF1	4.96E-04	0.789505888	0.982050977
ATP13A3	-0.001042498	0.789526754	0.982050977
MYOF	-0.001759638	0.78974459	0.982160254
ZNF526	-0.001330753	0.78979207	0.982160254
IFT20	7.12E-04	0.790032414	0.982294061
YAF2	8.48E-04	0.79020358	0.982294061
RBKS	-8.69E-04	0.790326916	0.982294061
FLYWCH2	0.001061352	0.790347036	0.982294061
FAM169A	-0.001251264	0.790493519	0.982294061
TNRC6A	9.29E-04	0.79068452	0.982294061
GPATCH2	-9.61E-04	0.79074136	0.982294061
HMGB1	7.26E-04	0.791064354	0.982294061
HCFC1R1	8.38E-04	0.791292939	0.982294061
SNORD6	-9.64E-04	0.791446064	0.982294061
WDR41	0.00255485	0.791730684	0.982294061
MYL6B	-0.001144338	0.791920713	0.982294061
C16orf58	-6.52E-04	0.792001688	0.982294061
IQSEC1	-7.12E-04	0.792082148	0.982294061
KLHDC3	-4.42E-04	0.792209756	0.982294061
ELF1	-8.13E-04	0.792302938	0.982294061
BAG3	0.001185611	0.792313054	0.982294061
TANK	7.70E-04	0.792330176	0.982294061
RNF31	-0.001006797	0.792355094	0.982294061
VPS13D	0.001038078	0.792388441	0.982294061
ICA1	0.002031308	0.792389658	0.982294061
STAG3L4	-0.001160625	0.792438937	0.982294061
PPHLN1	-8.05E-04	0.792595877	0.982294061
SNAP47	8.32E-04	0.792706319	0.982294061
CALCOCO2	-0.001267845	0.792767754	0.982294061
B9D2	-7.55E-04	0.792863279	0.982294061

AGMAT	-0.001658025	0.792912009	0.982294061
GPR180	0.001297585	0.79299794	0.982294061
SET	6.46E-04	0.793109308	0.982294061
TBL2	-9.42E-04	0.793170448	0.982294061
TAS2R10	-0.001288504	0.793261012	0.982294061
ACBD5	0.001201355	0.79338017	0.982294061
GTDC1	-0.001031785	0.793456524	0.982294061
B3GNT2	-0.001386038	0.793487851	0.982294061
HIST1H2BF	-0.002556629	0.793490427	0.982294061
ZFR2	0.001744794	0.79350245	0.982294061
FH	-7.35E-04	0.793638627	0.982294061
TMEM223	8.47E-04	0.793731574	0.982294061
APCDD1	-0.001416531	0.793788377	0.982294061
TBC1D9B	-8.43E-04	0.79385965	0.982294061
GPC2	-0.001495012	0.793929536	0.982294061
SMC2	-0.001141449	0.794016851	0.982294061
ALKBH6	-6.83E-04	0.794125811	0.982294061
HCFC2	8.42E-04	0.794182385	0.982294061
CNIH4	-7.15E-04	0.794194449	0.982294061
KIFC2	-0.001114492	0.794284964	0.982294061
ST3GAL2	-0.001042286	0.794303964	0.982294061
RANGAP1	5.96E-04	0.794330908	0.982294061
LYRM2	9.46E-04	0.794361597	0.982294061
C14orf93	5.35E-04	0.794380144	0.982294061
GTF2H2B	0.001924961	0.794425552	0.982294061
C14orf159	-7.54E-04	0.794688747	0.982509744
CTGF	0.001325827	0.794918412	0.982619495
INADL	0.001234945	0.794955062	0.982619495
ASCC1	0.001025601	0.795085668	0.982671199
ACVRL1	-0.002069338	0.795325249	0.982744763
RNF135	-8.15E-04	0.795371997	0.982744763
SALL2	9.89E-04	0.795457106	0.982744763
TAF1A	-0.001094495	0.795500323	0.982744763
MTHFR	0.001561367	0.796006317	0.983167988
GALNT10	-0.001047938	0.796020553	0.983167988
MYO5C	-0.0014877	0.796328319	0.983195975
CHMP5	8.69E-04	0.796367832	0.983195975
SLC39A6	-0.001222826	0.796407052	0.983195975
NXNL1	-0.001633315	0.796443471	0.983195975
EEF1G	-6.86E-04	0.796557463	0.983195975
RNH1	-9.28E-04	0.796638322	0.983195975
ASPM	-0.002071553	0.796752235	0.983195975
RNFT2	0.00161817	0.796753808	0.983195975
DUSP15	0.001055412	0.796912868	0.983270771
SUSD1	1.00E-03	0.797103091	0.983270771
TRIAP1	-6.51E-04	0.797141126	0.983270771
AGAP6	0.001227142	0.797169744	0.983270771

TMEM151B	-0.001039462	0.797341835	0.983373457
UBXN2A	-9.16E-04	0.797442151	0.983386674
TTLL1	-0.001047304	0.797561413	0.983386674
SS18L2	4.72E-04	0.797733693	0.983386674
PMPCA	-7.40E-04	0.79795074	0.983386674
SLC29A2	-0.001000743	0.797993919	0.983386674
UQCRH	7.03E-04	0.79802978	0.983386674
SAAL1	-6.50E-04	0.798069284	0.983386674
FLCN	-0.001135109	0.798131236	0.983386674
ZDHHC20	-9.70E-04	0.798152126	0.983386674
HAX1	-7.94E-04	0.798282046	0.983437281
DPM2	0.001234754	0.798443846	0.983510103
HEXDC	0.001253456	0.798518863	0.983510103
B3GNT6	0.001072061	0.798755983	0.983564256
SPDYE8P	-9.48E-04	0.798762643	0.983564256
ATG16L1	7.67E-04	0.798890439	0.983564256
TMEM138	-5.14E-04	0.798918261	0.983564256
DPP3	-7.69E-04	0.799405011	0.983602238
TAF6	-0.001104145	0.799421841	0.983602238
MED4	-0.001317633	0.799442578	0.983602238
C12orf29	-6.96E-04	0.799543914	0.983602238
DPY19L2P4	0.001101228	0.799640631	0.983602238
DUSP18	-8.10E-04	0.799691234	0.983602238
SERPINH1	0.001352904	0.799733165	0.983602238
GBF1	0.001056642	0.799819776	0.983602238
CBX3	8.66E-04	0.799862436	0.983602238
PPP1R13L	-9.13E-04	0.799898883	0.983602238
WDR5	-8.82E-04	0.799987398	0.983602238
FKBP11	0.001042371	0.800188565	0.983602238
ACTB	-8.28E-04	0.800291774	0.983602238
EDAR	-0.00206491	0.800303954	0.983602238
MTX3	0.001104222	0.800325703	0.983602238
KLHDC1	0.00102188	0.800371916	0.983602238
TIMM22	0.001059711	0.80055505	0.983602238
RFTN1	6.56E-04	0.800597375	0.983602238
NEURL1B	8.25E-04	0.800880625	0.983602238
ICK	-6.71E-04	0.801034245	0.983602238
TMEM106B	-9.33E-04	0.801072556	0.983602238
GSK3A	0.001385955	0.801083634	0.983602238
PLXDC2	-0.001259887	0.801172569	0.983602238
SNORD15B	0.001036194	0.801216889	0.983602238
TIAL1	6.66E-04	0.801256271	0.983602238
TNFSF13B	-0.001051453	0.801278931	0.983602238
SUMO1P3	-6.94E-04	0.80142144	0.983602238
TBCD	-0.001476529	0.80143722	0.983602238
SNX24	0.001101698	0.801758828	0.98374821
MXRA7	-0.002614816	0.801760524	0.98374821

SLC25A33	0.001099862	0.80182278	0.98374821
KDM3B	6.45E-04	0.8021631	0.984005478
DUSP12	-6.67E-04	0.80224767	0.984005478
UFC1	-7.44E-04	0.802299164	0.984005478
NACA2	0.001092597	0.802683962	0.984242093
UBR5	6.08E-04	0.802696347	0.984242093
C17orf96	-0.001132211	0.802811263	0.984242093
CDIPT	6.31E-04	0.802919869	0.984242093
SNORA24	0.001244015	0.803072532	0.984242093
FAM21A	-0.00104288	0.80322407	0.984242093
NMNAT1	-9.85E-04	0.803264051	0.984242093
SLC48A1	-8.86E-04	0.80327313	0.984242093
POFUT2	-7.89E-04	0.803292354	0.984242093
COL13A1	-0.001550874	0.803418647	0.984287881
TWSG1	-7.86E-04	0.803942238	0.984311929
C12orf10	-0.001030946	0.803958564	0.984311929
PRRT1	-0.00136017	0.804076016	0.984311929
RECQL	-9.05E-04	0.804179929	0.984311929
RPRM	-0.002935525	0.804184722	0.984311929
PVR	7.80E-04	0.804517518	0.984311929
ME2	-7.99E-04	0.804535031	0.984311929
RARS2	-6.05E-04	0.804549938	0.984311929
ZNF232	8.93E-04	0.804558504	0.984311929
LYRM7	6.85E-04	0.804563009	0.984311929
GALNT6	-0.001002132	0.804573641	0.984311929
RHOH	9.82E-04	0.804605747	0.984311929
PLAGL1	-0.001263367	0.804772308	0.984311929
SLC2A6	-0.001023388	0.804925506	0.984311929
CISH	0.002058438	0.805073532	0.984311929
IQSEC3	8.73E-04	0.805079985	0.984311929
MLLT3	-0.001148582	0.805194636	0.984311929
GGA3	-9.59E-04	0.805207006	0.984311929
PPARD	-0.001021041	0.805250576	0.984311929
MCRS1	-4.53E-04	0.805267504	0.984311929
YIF1B	7.20E-04	0.805305703	0.984311929
DHRS1	-7.12E-04	0.805558526	0.984333663
MYO1F	8.72E-04	0.805582863	0.984333663
RABL2A	9.40E-04	0.805753201	0.984333663
PPP1R15A	9.09E-04	0.805817132	0.984333663
ANXA7	-6.61E-04	0.806033115	0.984333663
FEZ1	0.002328551	0.806099663	0.984333663
BPGM	-0.001341926	0.806141003	0.984333663
CTSW	-0.001471936	0.806348747	0.984333663
NR1I2	0.00172136	0.806432257	0.984333663
SPIB	-0.002103587	0.806473206	0.984333663
SF3A3	6.56E-04	0.80650005	0.984333663
MKKS	-6.17E-04	0.806539988	0.984333663

CMTM4	-0.001332224	0.806649616	0.984333663
EPS15L1	-9.03E-04	0.806692989	0.984333663
MAP1LC3A	-0.001124413	0.806908315	0.984333663
GBP4	-0.00141466	0.806967074	0.984333663
NDUFA11	7.89E-04	0.807154191	0.984333663
TNFAIP8	-0.001072775	0.807247796	0.984333663
PSMG1	-6.62E-04	0.807332902	0.984333663
PRR14	5.33E-04	0.807622017	0.984333663
APOA1BP	6.26E-04	0.807650985	0.984333663
GSS	6.87E-04	0.807701164	0.984333663
SEMA4D	6.71E-04	0.807726548	0.984333663
ZNF729	-0.001303066	0.80774369	0.984333663
D2HGDH	-0.001375806	0.807937285	0.984333663
SUOX	0.001408931	0.808177976	0.984333663
BZW2	-6.20E-04	0.808289589	0.984333663
ZNF668	8.37E-04	0.808342484	0.984333663
RARRES3	8.55E-04	0.808395375	0.984333663
TACC3	8.95E-04	0.808395488	0.984333663
POLR2G	-4.10E-04	0.808395667	0.984333663
PRMT10	-7.74E-04	0.808512126	0.984333663
LYRM5	6.55E-04	0.808618365	0.984333663
ARG2	0.001807895	0.808638342	0.984333663
C9orf69	8.81E-04	0.808792571	0.984333663
TUBA1A	-5.94E-04	0.808883637	0.984333663
ASPHD2	9.20E-04	0.808971129	0.984333663
PTDSS1	-6.20E-04	0.809278577	0.984333663
HTRA2	8.26E-04	0.809298508	0.984333663
ACADM	6.51E-04	0.809328747	0.984333663
HLA-F	9.00E-04	0.809505174	0.984333663
RBM17	5.85E-04	0.809538611	0.984333663
PPP6C	4.29E-04	0.809630824	0.984333663
ACTN4	-0.001135042	0.809725313	0.984333663
COG3	-8.46E-04	0.809738802	0.984333663
MLKL	7.37E-04	0.809933728	0.984333663
ATXN2L	0.00131182	0.8099602	0.984333663
PBOV1	9.90E-04	0.81004403	0.984333663
KLRC1	-0.003275255	0.810112628	0.984333663
DDX39B	-9.04E-04	0.81039976	0.984333663
ZNF358	9.06E-04	0.810595472	0.984333663
NFYC	5.69E-04	0.810898254	0.984333663
PKP3	8.69E-04	0.810972883	0.984333663
HSPBP1	-0.001043125	0.810978886	0.984333663
DBT	8.36E-04	0.811038131	0.984333663
REST	-8.32E-04	0.811226681	0.984333663
MLLT6	7.96E-04	0.8115589	0.984333663
C4orf46	-9.29E-04	0.811671723	0.984333663
MS4A4A	-0.001964362	0.811716597	0.984333663

MAPK11	-8.26E-04	0.811798219	0.984333663
IL18	-4.21E-04	0.811902216	0.984333663
ZNF490	0.001043154	0.811955896	0.984333663
C8orf37	-8.53E-04	0.812132362	0.984333663
MIR130A	0.004004836	0.812303679	0.984333663
ECHDC1	-8.90E-04	0.812325492	0.984333663
CCNJL	0.001920562	0.812479801	0.984333663
KLF2	-4.41E-04	0.81257288	0.984333663
ERF	-7.48E-04	0.812589347	0.984333663
SEMA4F	-8.65E-04	0.812791341	0.984333663
INCENP	-0.001036386	0.812896067	0.984333663
TIMM13	-0.001153004	0.813024612	0.984333663
KLHL5	7.79E-04	0.813060377	0.984333663
CAPS2	7.60E-04	0.813114659	0.984333663
SIPA1L2	0.001233048	0.813289473	0.984333663
SCARNA16	0.001977924	0.813464513	0.984333663
ZSWIM1	6.87E-04	0.813484272	0.984333663
PEX11B	-5.71E-04	0.813503381	0.984333663
PHF21A	4.87E-04	0.813596089	0.984333663
XRCC6BP1	-0.001738992	0.813851795	0.984333663
FAM45A	-7.08E-04	0.814269134	0.984333663
LRPPRC	6.73E-04	0.81430529	0.984333663
TYK2	-5.18E-04	0.81445103	0.984333663
ADCY10	-0.001200216	0.814451032	0.984333663
NFU1	6.61E-04	0.814705667	0.984333663
DROSHA	-6.26E-04	0.814746105	0.984333663
JUNB	0.001625206	0.814974864	0.984333663
JUP	0.003051394	0.815105964	0.984333663
GBP2	-9.41E-04	0.815233895	0.984333663
KLF13	-6.67E-04	0.815572992	0.984333663
SLC5A6	-8.34E-04	0.815838304	0.984333663
LLGL1	-7.72E-04	0.815859899	0.984333663
UPP1	6.17E-04	0.815986014	0.984333663
IPO4	9.13E-04	0.816043714	0.984333663
TMEM208	-6.38E-04	0.816118481	0.984333663
DNAJC24	-8.64E-04	0.816129912	0.984333663
CMAS	5.95E-04	0.816287434	0.984333663
CAB39L	8.75E-04	0.816291734	0.984333663
CNPPD1	5.59E-04	0.816470726	0.984333663
NAPG	9.43E-04	0.81661552	0.984333663
E2F1	-0.001484867	0.816757209	0.984333663
MAU2	9.75E-04	0.816923758	0.984333663
ERO1LB	9.99E-04	0.81711526	0.984333663
PPP6R2	8.42E-04	0.81718374	0.984333663
FBL	7.23E-04	0.81721412	0.984333663
TMC4	-0.001397747	0.817243482	0.984333663
LDHB	-9.01E-04	0.81726724	0.984333663

DOCK10	5.40E-04	0.817348674	0.984333663
SCFD2	9.68E-04	0.817710714	0.984333663
YTHDF3	-5.76E-04	0.81776534	0.984333663
DNAJA4	9.36E-04	0.817805989	0.984333663
NMD3	-3.73E-04	0.817828819	0.984333663
ZNF143	7.46E-04	0.817888939	0.984333663
DMXL1	8.90E-04	0.817992154	0.984333663
TRIML1	-8.80E-04	0.818013205	0.984333663
RPS29	-4.48E-04	0.81803433	0.984333663
PRKACB	-0.001137101	0.818221158	0.984333663
LYST	-9.24E-04	0.818297491	0.984333663
CD300LB	-0.001144667	0.818366408	0.984333663
ELAVL1	0.001108717	0.818622042	0.984333663
EDEM2	-5.35E-04	0.818719119	0.984333663
RIN1	0.001289146	0.818773546	0.984333663
ERAP1	0.001138152	0.818836695	0.984333663
NUPL1	-8.59E-04	0.818977166	0.984333663
USP22	8.34E-04	0.819254226	0.984333663
RAB3GAP2	5.17E-04	0.819258726	0.984333663
RNF41	-6.61E-04	0.819288303	0.984333663
LMTK3	9.79E-04	0.819361427	0.984333663
MUTYH	6.22E-04	0.819410092	0.984333663
EID2	-8.61E-04	0.819454144	0.984333663
COPS2	-0.001144411	0.81953057	0.984333663
KCNAB2	-9.52E-04	0.819587497	0.984333663
PRSS42	-8.46E-04	0.819648461	0.984333663
NBR2	-0.001284452	0.819886318	0.984333663
RINT1	7.50E-04	0.819946403	0.984333663
PBRM1	8.40E-04	0.819948723	0.984333663
DHFRL1	-9.61E-04	0.820072163	0.984333663
TRIM24	9.05E-04	0.820087137	0.984333663
RPS27L	-5.73E-04	0.820117217	0.984333663
CTBP2	-0.001129107	0.820180046	0.984333663
DOCK5	-9.74E-04	0.820192798	0.984333663
COMMD1	-6.24E-04	0.820285027	0.984333663
LAIR2	0.00328937	0.820359693	0.984333663
AKNA	-6.93E-04	0.820755227	0.984333663
PLLP	0.001166778	0.820761131	0.984333663
ARID5A	-0.001029558	0.820803822	0.984333663
NICN1	-6.94E-04	0.821027875	0.984333663
MYLIP	6.72E-04	0.821281238	0.984333663
SLC1A5	9.00E-04	0.821352896	0.984333663
CHCHD10	-7.99E-04	0.821465661	0.984333663
AGR2	-8.75E-04	0.821549123	0.984333663
TBC1D24	-0.001199617	0.82158688	0.984333663
MARK3	-7.99E-04	0.821635663	0.984333663
MORN2	-0.001054728	0.821701291	0.984333663

RPL36AL	5.88E-04	0.821775315	0.984333663
PLEKHA5	-0.001388603	0.821987132	0.984333663
PARP10	-8.10E-04	0.82209415	0.984333663
TRIO	8.06E-04	0.822190217	0.984333663
DEXI	-8.08E-04	0.822193564	0.984333663
ZNF286A	-0.001205002	0.822263226	0.984333663
METTL10	7.87E-04	0.822272048	0.984333663
CATSPER1	-0.001091126	0.822290338	0.984333663
LRP8	-9.04E-04	0.822302176	0.984333663
METTL16	-0.00101627	0.822505947	0.984333663
C2orf76	-9.12E-04	0.822566003	0.984333663
NDUFA12	-8.87E-04	0.822728195	0.984333663
RFX4	-0.001221219	0.822797148	0.984333663
TP53INP1	9.69E-04	0.822832508	0.984333663
ABCA11P	8.12E-04	0.822855437	0.984333663
PHF14	6.48E-04	0.822966046	0.984333663
TSGA10	0.001708869	0.823012735	0.984333663
IKZF2	-7.55E-04	0.82307634	0.984333663
FAM110B	0.001055912	0.823085881	0.984333663
TSSC4	-8.28E-04	0.823152145	0.984333663
STAR	-0.001172724	0.823155417	0.984333663
STX1B	0.001694177	0.823349092	0.984333663
ACVR2A	-8.60E-04	0.823437572	0.984333663
FAM149B1	-4.93E-04	0.823527938	0.984333663
CBWD5	0.001307832	0.823563498	0.984333663
ZNF789	-9.40E-04	0.823572625	0.984333663
VOPP1	6.47E-04	0.823639708	0.984333663
PERP	-0.001667755	0.823679022	0.984333663
LYSMD3	-0.001266197	0.823706755	0.984333663
NRIP3	8.71E-04	0.823908636	0.984333663
PXK	-0.001044885	0.823921139	0.984333663
DNASE1	-9.50E-04	0.82399892	0.984333663
PHACTR4	7.79E-04	0.824016758	0.984333663
NKX3-1	-0.00119073	0.824023928	0.984333663
HNRNPC	-7.47E-04	0.824184946	0.984333663
NFYB	0.001005146	0.824208293	0.984333663
KLHL3	-0.001100381	0.824245166	0.984333663
ACN9	-7.91E-04	0.824316423	0.984333663
SNORD96A	9.58E-04	0.824324665	0.984333663
NUAK1	-0.002535091	0.824378875	0.984333663
JMY	-8.74E-04	0.824597995	0.984333663
WDR4	8.15E-04	0.824828926	0.984333663
PVALB	0.003907645	0.824863494	0.984333663
DYRK1A	-5.52E-04	0.824875636	0.984333663
IBTK	6.20E-04	0.82509245	0.984333663
RPP21	-5.11E-04	0.825427194	0.984333663
ABHD11	9.98E-04	0.825428282	0.984333663

UBE2E2	-7.94E-04	0.825528402	0.984333663
PAPOLA	-4.21E-04	0.825528962	0.984333663
SLK	7.09E-04	0.825538364	0.984333663
RABGGTB	-5.94E-04	0.825584658	0.984333663
SLC12A9	-5.91E-04	0.825697841	0.984333663
IL2RB	-0.001360502	0.825771171	0.984333663
CBLN3	0.002396452	0.825773152	0.984333663
SHMT1	-0.001351282	0.825821827	0.984333663
GAMT	-8.73E-04	0.825847802	0.984333663
ACAA2	-7.96E-04	0.825874388	0.984333663
ICAM4	-0.002162809	0.825960972	0.984333663
RAMP2	9.65E-04	0.826003359	0.984333663
ZMYM4	5.73E-04	0.82610791	0.984333663
C6orf136	4.76E-04	0.826253198	0.984333663
LRRC41	-7.47E-04	0.826283602	0.984333663
MYO6	-0.001030563	0.826297563	0.984333663
RTN2	-0.001444085	0.826378266	0.984333663
CCND3	5.74E-04	0.826401398	0.984333663
CNPY2	-4.81E-04	0.826544459	0.984333663
FRYL	-6.39E-04	0.82657162	0.984333663
NDUFAF4	0.001100788	0.826654277	0.984333663
SENP3	9.76E-04	0.826668111	0.984333663
SORBS2	-8.10E-04	0.826674361	0.984333663
ELP4	7.94E-04	0.82671804	0.984333663
HCG27	0.00154828	0.826753694	0.984333663
S1PR1	0.001342875	0.826757049	0.984333663
RPS6KA4	-8.25E-04	0.826942554	0.984333663
TMED3	5.90E-04	0.827104815	0.984333663
MSTO1	6.31E-04	0.827130485	0.984333663
SOX13	0.001495496	0.827142066	0.984333663
ZNF419	7.26E-04	0.827186108	0.984333663
PPIA	-6.46E-04	0.827275794	0.984333663
DDX19B	-5.04E-04	0.827403419	0.984333663
ZNF296	-8.56E-04	0.827509959	0.984333663
AKR1C3	0.002452027	0.827556685	0.984333663
DDX23	6.01E-04	0.827559559	0.984333663
TMEM194A	0.001066231	0.827570278	0.984333663
LSG1	0.001026691	0.827590405	0.984333663
LENG8	-0.001007428	0.827609837	0.984333663
ADCY9	-0.00108833	0.827649237	0.984333663
C17orf97	-0.005427437	0.827654208	0.984333663
MORC2	0.001108459	0.827768083	0.984333663
ILK	9.41E-04	0.827770038	0.984333663
UQCRC1	5.57E-04	0.828264381	0.984333663
EPS15	5.79E-04	0.828340032	0.984333663
SVIL	6.36E-04	0.828374913	0.984333663
MRPL55	5.46E-04	0.828432319	0.984333663

ZNF286B	-0.00124525	0.828648379	0.984333663
PGM5	0.002202843	0.828788927	0.984333663
CARS2	-6.92E-04	0.828921045	0.984333663
PANK3	-7.46E-04	0.828990994	0.984333663
PAQR8	0.001051568	0.829008593	0.984333663
TPM2	-0.001915169	0.8290427	0.984333663
TRMT1L	6.77E-04	0.829218919	0.984333663
GTF2H2	-0.001614761	0.829359347	0.984333663
FBXO18	-4.80E-04	0.829618511	0.984333663
SLC22A23	-8.76E-04	0.829653806	0.984333663
XPO4	4.57E-04	0.829691929	0.984333663
MLLT4	0.001922547	0.829694475	0.984333663
MIIP	0.001135116	0.829829023	0.984333663
AGRN	-8.77E-04	0.829855903	0.984333663
CRLF3	-6.90E-04	0.829914024	0.984333663
FARSA	-8.22E-04	0.830178751	0.984333663
FAM177A1	2.75E-04	0.830251125	0.984333663
C8orf58	8.76E-04	0.830374515	0.984333663
APBA3	7.26E-04	0.83037855	0.984333663
TYW3	0.001182867	0.830395646	0.984333663
PINX1	-8.49E-04	0.830430262	0.984333663
SLMAP	4.00E-04	0.830581375	0.984333663
FLNB	-9.12E-04	0.830608881	0.984333663
PRDM9	-8.76E-04	0.830686009	0.984333663
GTF2I	6.77E-04	0.83069911	0.984333663
FAM20B	-4.21E-04	0.830725604	0.984333663
WTIP	-8.12E-04	0.830813714	0.984333663
CD96	7.78E-04	0.831134182	0.984333663
ARF6	9.12E-04	0.831290613	0.984333663
NKG7	-0.001270747	0.831344197	0.984333663
WHSC1L1	7.67E-04	0.831362679	0.984333663
PFKFB3	0.001057412	0.831373533	0.984333663
ERP44	-0.001171275	0.831374162	0.984333663
ZNF3	7.82E-04	0.831528102	0.984333663
MAP1A	-0.001422712	0.831571453	0.984333663
HLA-DMA	8.16E-04	0.831586105	0.984333663
SIRT6	0.001016304	0.831599129	0.984333663
SRF	-5.28E-04	0.831713328	0.984333663
ENO2	0.001011201	0.831817547	0.984333663
PEX19	5.79E-04	0.831820915	0.984333663
TM4SF19	8.98E-04	0.831980899	0.984333663
EIF2D	-5.80E-04	0.832009027	0.984333663
USP53	0.00163298	0.83208292	0.984333663
IDI1	-7.60E-04	0.832305985	0.984333663
NEIL2	-8.04E-04	0.832354017	0.984333663
R3HCC1	-4.34E-04	0.832356913	0.984333663
CD3EAP	-7.28E-04	0.832549272	0.984333663

DOCK8	-7.30E-04	0.832588668	0.984333663
FAM63A	0.00174267	0.832645569	0.984333663
NT5E	0.002249178	0.832646604	0.984333663
PTPN22	-0.001037547	0.832687859	0.984333663
NCDN	8.09E-04	0.832879201	0.984333663
SLC2A4RG	6.58E-04	0.832999189	0.984333663
MANBAL	6.04E-04	0.833015802	0.984333663
OXCT1	6.45E-04	0.8330194	0.984333663
POLE4	-4.82E-04	0.833021492	0.984333663
CNOT10	-4.88E-04	0.833186528	0.984333663
WSB2	-7.51E-04	0.833337786	0.984333663
GPR114	-0.001412261	0.833431587	0.984333663
FEN1	9.32E-04	0.833438883	0.984333663
NPAT	8.90E-04	0.833484483	0.984333663
SETD5	-8.87E-04	0.83365469	0.984333663
ZNRD1	-7.06E-04	0.834083233	0.984333663
DPEP2	-7.29E-04	0.834106047	0.984333663
MRPL46	4.77E-04	0.834211425	0.984333663
TMEM50B	3.86E-04	0.834260416	0.984333663
MUL1	5.54E-04	0.834349328	0.984333663
LENG1	-7.85E-04	0.834370066	0.984333663
VKORC1	5.72E-04	0.834374259	0.984333663
IFIT5	-0.001159822	0.834419152	0.984333663
PFKM	-8.48E-04	0.834447986	0.984333663
IRF8	7.69E-04	0.834507436	0.984333663
HELLS	-0.001015861	0.83452818	0.984333663
MON1A	-8.61E-04	0.834581064	0.984333663
SMC6	0.001955443	0.834585966	0.984333663
C17orf67	-0.00135803	0.834595273	0.984333663
C4orf21	0.001129107	0.834648177	0.984333663
RC3H2	6.98E-04	0.834655457	0.984333663
COL4A3	0.001547078	0.834723754	0.984333663
BCL2A1	-0.001188828	0.834771239	0.984333663
MTX1	-5.90E-04	0.834859128	0.984333663
KLKB1	-0.001277302	0.834985375	0.984333663
LRTOMT	-7.53E-04	0.835024499	0.984333663
KCNK4	9.80E-04	0.835146987	0.984333663
RALY	6.37E-04	0.835187457	0.984333663
PAN2	-7.02E-04	0.83545855	0.984333663
LAIR1	2.71E-04	0.83547704	0.984333663
CSF3R	-8.45E-04	0.835497327	0.984333663
SLC38A6	6.48E-04	0.835652375	0.984333663
CYFIP2	6.36E-04	0.835744825	0.984333663
GAS1	0.001509733	0.835763842	0.984333663
AGGF1	-4.58E-04	0.835791516	0.984333663
SMURF1	-8.82E-04	0.835800164	0.984333663
JAZF1	0.001001521	0.835812294	0.984333663

SMYD4	-7.94E-04	0.835933559	0.984333663
ACADS	8.75E-04	0.835963487	0.984333663
CILP	-8.20E-04	0.836238786	0.984333663
OSBPL3	7.55E-04	0.836299335	0.984333663
PFDN6	-7.86E-04	0.836420144	0.984333663
RBM4B	-5.62E-04	0.836471097	0.984333663
MPZL1	-8.83E-04	0.836484111	0.984333663
ABHD14A	7.57E-04	0.836516514	0.984333663
TPD52	-0.001084142	0.836536083	0.984333663
EXT2	-7.07E-04	0.836605334	0.984333663
GSTM4	0.001887982	0.836625811	0.984333663
SYCE1L	9.77E-04	0.836818875	0.984456173
ZNF428	-5.29E-04	0.837213745	0.984789456
ITGAD	0.002216755	0.837364051	0.984789456
ADCK4	8.04E-04	0.837429485	0.984789456
PAPL	0.001248243	0.837549674	0.984789456
CNR1	8.58E-04	0.837567773	0.984789456
HKR1	-7.77E-04	0.837812709	0.984789456
ASB3	7.41E-04	0.837881922	0.984789456
GPR125	0.001271013	0.837912862	0.984789456
CKS1B	-0.001209534	0.838029909	0.984789456
ABCB1	-0.001507227	0.838224831	0.984789456
SELK	9.00E-04	0.838241048	0.984789456
PAFAH1B1	-5.41E-04	0.83832038	0.984789456
RASGRP2	-7.20E-04	0.838406166	0.984789456
ZNF680	8.01E-04	0.83857685	0.984789456
ACOT13	-6.77E-04	0.83857925	0.984789456
TXNIP	2.47E-04	0.838610881	0.984789456
CD74	6.18E-04	0.838688358	0.984789456
FAM150B	0.00152626	0.838703605	0.984789456
ARHGAP11B	-7.78E-04	0.838924248	0.984859419
LRRC59	8.90E-04	0.838941139	0.984859419
NCR1	0.001293384	0.839140149	0.984937087
SNORD52	-0.001001047	0.839185262	0.984937087
KIAA0586	-6.04E-04	0.8398386	0.985428172
TBPL1	5.40E-04	0.839844286	0.985428172
SEC61G	5.43E-04	0.839870754	0.985428172
IMPDH2	6.63E-04	0.840056808	0.985442284
TRAF4	9.81E-04	0.840091948	0.985442284
ZFP1	9.21E-04	0.840468046	0.985442284
YBEY	0.001594993	0.840511868	0.985442284
MRPL52	-7.46E-04	0.840726588	0.985442284
ANAPC10	8.24E-04	0.840810115	0.985442284
HIF3A	-8.58E-04	0.840813727	0.985442284
TOMM20	-6.10E-04	0.84081946	0.985442284
RPL13	9.34E-04	0.840955468	0.985442284
WDFY4	0.001038907	0.84097541	0.985442284

PI16	0.001906067	0.840976276	0.985442284
UTRN	0.001068345	0.841055981	0.985442284
TLE4	5.22E-04	0.841072011	0.985442284
SPATA20	-0.002377533	0.84117938	0.985442284
UQCR11	7.28E-04	0.841334342	0.985442284
NPTX1	-0.001488969	0.84134557	0.985442284
CARD17	0.001115401	0.841396244	0.985442284
SUMO1	0.001073034	0.841566531	0.985537445
ZFP30	-0.001001973	0.841714701	0.985606689
THADA	6.12E-04	0.842067138	0.985915078
TSPAN5	-8.05E-04	0.842210663	0.985978829
SNHG11	7.07E-04	0.842396512	0.98609211
MAGOHB	8.84E-04	0.842562582	0.98609721
ANKAR	-0.001062814	0.842658445	0.98609721
KIF13B	-6.60E-04	0.842668128	0.98609721
BRWD1	5.92E-04	0.842929867	0.986299228
TNNI2	-0.001314702	0.843127616	0.986400798
FRS2	-4.97E-04	0.843306142	0.986400798
CBX1	0.001027358	0.84335098	0.986400798
FKBP2	5.24E-04	0.843417626	0.986400798
FIP1L1	-4.91E-04	0.843466394	0.986400798
ITGB1BP1	0.001015056	0.843608084	0.986400798
FUT7	-0.001101857	0.844308325	0.986400798
SCAMP5	0.001403843	0.844413244	0.986400798
ZNF285	0.001174264	0.84441837	0.986400798
SCLY	5.57E-04	0.844447724	0.986400798
ADCK5	-6.14E-04	0.844710173	0.986400798
ANGPT1	0.001482084	0.844728682	0.986400798
ST6GALNAC1	-0.00129021	0.844942086	0.986400798
LPA	8.54E-04	0.844962698	0.986400798
PHF5A	-4.66E-04	0.844965534	0.986400798
ZNF224	8.01E-04	0.845010249	0.986400798
CD28	9.73E-04	0.845021228	0.986400798
TIRAP	-6.07E-04	0.845027148	0.986400798
ABHD4	-8.28E-04	0.845064047	0.986400798
FCER1A	-0.001891736	0.845328306	0.986400798
SNORA26	-0.001091923	0.845540478	0.986400798
METTL15	-6.03E-04	0.845631641	0.986400798
PRKD2	-7.31E-04	0.845672147	0.986400798
PDGFA	0.001340758	0.845698066	0.986400798
CDR2	7.17E-04	0.845958473	0.986400798
UBE3B	6.10E-04	0.846121389	0.986400798
PTK2B	6.53E-04	0.846165173	0.986400798
TCF7	0.001093163	0.846196265	0.986400798
SNORA42	9.58E-04	0.846200174	0.986400798
EIF4G1	5.44E-04	0.846300212	0.986400798
DENND4B	-7.83E-04	0.846320754	0.986400798

TMEM70	7.95E-04	0.846334257	0.986400798
AKR1C4	0.002226656	0.846336555	0.986400798
SEPN1	7.60E-04	0.846377365	0.986400798
RGPD1	0.001823962	0.846438632	0.986400798
RHOC	9.04E-04	0.84647086	0.986400798
ATP2C1	5.81E-04	0.846475927	0.986400798
ACSL3	4.57E-04	0.846487342	0.986400798
MEGF8	8.47E-04	0.846571152	0.986400798
MN1	8.21E-04	0.846657813	0.986400798
ZNF350	-7.66E-04	0.846670339	0.986400798
PDLIM5	0.001021223	0.847126538	0.986642486
IFT52	-8.57E-04	0.847141932	0.986642486
FGF9	0.001071189	0.847257859	0.986642486
TIPARP	6.21E-04	0.847520186	0.986642486
ATP10A	-8.07E-04	0.847625275	0.986642486
SLC2A11	-6.93E-04	0.847733659	0.986642486
TMEM204	-0.001262097	0.847770947	0.986642486
PACSIN2	4.72E-04	0.84791296	0.986642486
RPUSD4	5.14E-04	0.848049156	0.986642486
NUDT22	5.57E-04	0.848129931	0.986642486
XAB2	6.31E-04	0.848146784	0.986642486
PTGES	-7.20E-04	0.848189183	0.986642486
PTPRN2	-0.001018903	0.848370904	0.986642486
ZNF781	8.19E-04	0.848381762	0.986642486
STXBP5	-6.15E-04	0.84840475	0.986642486
MBLAC2	-7.66E-04	0.848439828	0.986642486
ZBTB39	-7.25E-04	0.848456793	0.986642486
SPOCK2	5.20E-04	0.848569124	0.986642486
ZCCHC14	-6.76E-04	0.848571367	0.986642486
GNPTAB	-8.81E-04	0.848688587	0.986675136
ZFR	-3.61E-04	0.848849505	0.986746493
MIR555	-0.001125815	0.848928255	0.986746493
ZNF552	-7.14E-04	0.849039457	0.986772128
MARS	-4.99E-04	0.849400845	0.987088499
MED24	-6.07E-04	0.84986559	0.987423869
ZNF672	-5.39E-04	0.850000676	0.987423869
IKBIP	2.71E-04	0.850157955	0.987423869
MAPK14	-0.001058816	0.850187395	0.987423869
PRPF38B	6.38E-04	0.850287482	0.987423869
ZC3H11A	4.02E-04	0.850344404	0.987423869
STK19	-4.55E-04	0.850392995	0.987423869
GLMN	6.23E-04	0.850403085	0.987423869
IFIT3	0.002219749	0.850871497	0.987543722
PAM	8.07E-04	0.851071739	0.987543722
DHDH	0.00111536	0.851077055	0.987543722
SERPINA10	-9.12E-04	0.85107888	0.987543722
CAB39	3.49E-04	0.851238956	0.987543722

MRPS17	7.75E-04	0.851247287	0.987543722
ANKRD13D	-8.86E-04	0.851253468	0.987543722
TTC14	0.001087152	0.851358949	0.987543722
ANAPC1	5.37E-04	0.851373093	0.987543722
TMEM18	-6.47E-04	0.851776899	0.987543722
RNF144A	-8.35E-04	0.85193375	0.987543722
RPS11	-3.26E-04	0.851984594	0.987543722
SPATA2L	-6.18E-04	0.852022622	0.987543722
ENOSF1	0.001175543	0.852038912	0.987543722
ABCC3	0.001997747	0.852072457	0.987543722
MRPS15	4.28E-04	0.852154888	0.987543722
SSB	4.24E-04	0.852299048	0.987543722
PSMG2	-4.64E-04	0.852432418	0.987543722
CGGBP1	-3.76E-04	0.852633515	0.987543722
CCDC14	-6.62E-04	0.852703485	0.987543722
EXT1	6.72E-04	0.852709202	0.987543722
TCP1	6.31E-04	0.852759522	0.987543722
SHPRH	7.09E-04	0.852769926	0.987543722
MYO15B	-0.001462234	0.852877172	0.987543722
ZNF33B	4.97E-04	0.852881328	0.987543722
NDUFV1	-5.13E-04	0.853034968	0.987543722
STMN3	8.09E-04	0.853061087	0.987543722
NEDD1	7.79E-04	0.853080109	0.987543722
TBC1D7	-8.06E-04	0.853209812	0.987543722
RBM33	-8.79E-04	0.853250542	0.987543722
TYW1B	-6.68E-04	0.85327909	0.987543722
HM13	-0.001281786	0.853361253	0.987543722
U2AF1L4	-0.001037777	0.853665719	0.987736349
CYCSP52	-7.17E-04	0.853706175	0.987736349
TIA1	-5.24E-04	0.853921468	0.987882184
HAT1	-7.39E-04	0.854053781	0.987931999
SLC22A15	-0.001021643	0.854169981	0.987963168
CARS	-7.27E-04	0.854328279	0.988043018
CACYBP	0.001038503	0.854575317	0.988067082
FAM96A	6.54E-04	0.854592413	0.988067082
ZDHHC8	-3.90E-04	0.854662544	0.988067082
IKBKAP	-5.49E-04	0.854813853	0.988067082
RPS10	-2.15E-04	0.854894045	0.988067082
DCBLD1	-9.20E-04	0.854894542	0.988067082
LARP4B	-3.63E-04	0.855075075	0.988067082
CDK13	4.99E-04	0.85508803	0.988067082
SNORA70C	8.17E-04	0.855152466	0.988067082
SLC35E2B	-4.65E-04	0.855248189	0.988074544
ZIK1	-7.77E-04	0.855399054	0.98810211
CLUAP1	2.53E-04	0.855450585	0.98810211
TRIM8	-3.62E-04	0.856282044	0.988959302
AQP12B	9.66E-04	0.856718589	0.989234355

GCAT	-8.96E-04	0.856939614	0.989234355
LRRC2	-8.06E-04	0.857181052	0.989234355
ANP32C	-9.43E-04	0.857540977	0.989234355
ACAP1	7.87E-04	0.857610217	0.989234355
CLEC2D	5.69E-04	0.857635443	0.989234355
HP	-0.003107375	0.857773489	0.989234355
NAIF1	5.11E-04	0.857820445	0.989234355
NFS1	8.26E-04	0.857931678	0.989234355
COX11	0.001040364	0.857952493	0.989234355
GBP3	-0.001233786	0.857976903	0.989234355
RBM45	-5.14E-04	0.858087412	0.989234355
STAG3L2	0.001110979	0.858124082	0.989234355
MICA	8.04E-04	0.85818256	0.989234355
SEC22B	4.94E-04	0.858258767	0.989234355
ZNF585A	-7.16E-04	0.858324076	0.989234355
AGPAT1	0.00101064	0.858476702	0.989234355
SHOX2	-7.34E-04	0.859081079	0.989234355
PNO1	-6.45E-04	0.859272443	0.989234355
SUZ12	-2.69E-04	0.859273995	0.989234355
FBXL6	6.38E-04	0.859569337	0.989234355
TPRA1	-6.59E-04	0.859663585	0.989234355
CDC5L	-5.97E-04	0.859699462	0.989234355
SRCIN1	7.53E-04	0.859706785	0.989234355
UBA3	-8.07E-04	0.859734434	0.989234355
USP45	-7.35E-04	0.859795946	0.989234355
IFT172	-6.80E-04	0.859951566	0.989234355
SPSB3	4.84E-04	0.859997239	0.989234355
RPS27	1.88E-04	0.860026878	0.989234355
RNASEH2B	8.32E-04	0.860027404	0.989234355
PRG4	-0.001299009	0.860070427	0.989234355
ERCC5	4.00E-04	0.860164644	0.989234355
TMX2	3.72E-04	0.860175976	0.989234355
C1orf174	5.29E-04	0.860192815	0.989234355
HRH1	-7.58E-04	0.860363409	0.989234355
MAP2K6	-9.16E-04	0.860452478	0.989234355
IDH2	-6.69E-04	0.86050397	0.989234355
HTR3A	9.11E-04	0.860546222	0.989234355
PCNXL2	-8.92E-04	0.860606736	0.989234355
TARDBP	7.66E-04	0.860756139	0.989234355
SULT1B1	-0.001097188	0.860789687	0.989234355
SRP9	4.02E-04	0.860922878	0.989234355
ZNF273	7.99E-04	0.860942816	0.989234355
C7orf50	4.81E-04	0.860976666	0.989234355
SPON1	0.001299918	0.861121357	0.989234355
PRPF8	-6.07E-04	0.861181865	0.989234355
CAPN12	-0.00159493	0.861193641	0.989234355
CA6	9.58E-04	0.861338408	0.989234355

OPRL1	5.82E-04	0.861362579	0.989234355
E2F2	-8.93E-04	0.861427716	0.989234355
MAP4K4	-5.58E-04	0.861504604	0.989234355
UQCRC2	-6.82E-04	0.861540747	0.989234355
SKP1	-4.28E-04	0.861571056	0.989234355
EED	7.39E-04	0.861623947	0.989234355
LSM6	-0.00107234	0.861652641	0.989234355
TAF10	4.54E-04	0.861830228	0.989234355
SUPT5H	5.75E-04	0.861945293	0.989234355
PCBD1	6.82E-04	0.861957657	0.989234355
SIAH2	6.40E-04	0.862032184	0.989234355
PON2	7.89E-04	0.862056529	0.989234355
LEF1	-9.41E-04	0.862081985	0.989234355
PAFAH1B3	-6.71E-04	0.862162039	0.989234355
COX7A2	-6.35E-04	0.862333243	0.989234355
TRMT6	7.45E-04	0.862377455	0.989234355
ZNF512	-4.72E-04	0.86246618	0.989234355
IFT74	5.18E-04	0.862514914	0.989234355
SNORA76	7.76E-04	0.862540245	0.989234355
REL	8.33E-04	0.86272547	0.989234355
SWT1	-5.02E-04	0.86277908	0.989234355
AHSA2	-8.76E-04	0.862900118	0.989234355
FAM3C	-8.23E-04	0.862902788	0.989234355
RALGDS	6.43E-04	0.863026559	0.989234355
YY1	4.13E-04	0.863044193	0.989234355
UBP1	-3.71E-04	0.863588095	0.98946304
ZNF529	-6.30E-04	0.863592449	0.98946304
ITPRIPL1	0.001052172	0.863633842	0.98946304
XRN2	-0.00481371	0.863853904	0.98946304
CD52	6.03E-04	0.864052161	0.98946304
CHML	-7.42E-04	0.86412648	0.98946304
CLIC4	9.73E-04	0.864226705	0.98946304
PSMD5	-6.85E-04	0.864251441	0.98946304
ARF5	4.35E-04	0.864274157	0.98946304
GNGT2	8.76E-04	0.864341201	0.98946304
ACER3	-7.84E-04	0.864558688	0.98946304
PSME1	4.16E-04	0.864604155	0.98946304
IGSF6	0.00112955	0.864631256	0.98946304
GORAB	-5.55E-04	0.864685646	0.98946304
CD300A	7.87E-04	0.864781265	0.98946304
USP39	4.32E-04	0.864788247	0.98946304
VMO1	-0.001190967	0.86494029	0.98946304
TSN	7.36E-04	0.865020835	0.98946304
MAP3K8	-7.89E-04	0.865220018	0.98946304
CRCP	-8.41E-04	0.865302762	0.98946304
ZNF415	0.001274235	0.865310883	0.98946304
GPI	5.91E-04	0.865334873	0.98946304

ENGASE	-8.26E-04	0.865381043	0.98946304
CEP68	7.50E-04	0.865467217	0.98946304
SDK2	0.001050151	0.86548299	0.98946304
EIF4G3	5.48E-04	0.865612935	0.98946304
HTT	7.19E-04	0.865657248	0.98946304
NPC1	-8.07E-04	0.865842235	0.989541949
TPR	-5.80E-04	0.865905079	0.989541949
TFAP2A	5.56E-04	0.866002621	0.989551256
LMNB1	0.001055981	0.866413993	0.989744394
ZNF570	-6.78E-04	0.866456247	0.989744394
PTPRO	-0.001152353	0.866494907	0.989744394
FXR2	-5.71E-04	0.866719971	0.989744394
KLHL20	5.89E-04	0.866791791	0.989744394
FAF1	-4.42E-04	0.866823435	0.989744394
BAP1	-5.19E-04	0.866965291	0.989744394
HMG20B	5.82E-04	0.867032874	0.989744394
NFATC2IP	6.69E-04	0.867064432	0.989744394
PHF13	-4.40E-04	0.867186722	0.989744394
PLEKHG7	-6.78E-04	0.86738493	0.989744394
ACLY	-3.75E-04	0.86742152	0.989744394
LEPREL4	6.78E-04	0.867525192	0.989744394
NDRG2	7.84E-04	0.867631585	0.989744394
POGZ	-0.001387378	0.867652081	0.989744394
SYPL1	5.74E-04	0.867785678	0.989744394
NOMO3	7.03E-04	0.868064191	0.989744394
SERTAD3	-5.96E-04	0.868144717	0.989744394
MAP2K2	-8.71E-04	0.868177489	0.989744394
ATP13A1	7.47E-04	0.868181487	0.989744394
ARRDC1	6.79E-04	0.868237882	0.989744394
LRG1	-0.001019872	0.868306358	0.989744394
KLHL35	9.16E-04	0.868366457	0.989744394
RPL26	-3.79E-04	0.868543288	0.989744394
RNF121	-4.21E-04	0.868555301	0.989744394
ZNF543	-0.00105154	0.868555891	0.989744394
CYTH1	4.07E-04	0.868665284	0.989744394
PUS3	-5.46E-04	0.86867529	0.989744394
POLD2	-4.71E-04	0.868833865	0.989795866
USP7	5.82E-04	0.868899307	0.989795866
ZNHIT1	-4.71E-04	0.869002414	0.989811455
ARHGEF19	-7.70E-04	0.869187279	0.989878285
AOAH	8.80E-04	0.869376828	0.989878285
CLRN1	6.02E-04	0.869460983	0.989878285
NFKBIA	5.85E-04	0.869479867	0.989878285
RPS6KA1	5.22E-04	0.869560705	0.989878285
FARSB	8.59E-04	0.869834937	0.989878285
POLD1	-6.43E-04	0.869844464	0.989878285
CTDNEP1	5.06E-04	0.869913938	0.989878285

SLBP	-4.96E-04	0.869934857	0.989878285
TMEM160	-7.88E-04	0.869955367	0.989878285
KIR3DL3	0.002473275	0.870267989	0.990056821
CDC23	-3.86E-04	0.870391035	0.990056821
NACAP1	8.40E-04	0.870421274	0.990056821
C11orf1	-7.49E-04	0.870487535	0.990056821
IRS2	0.001196295	0.870559494	0.990056821
XRCC5	6.10E-04	0.870692318	0.990060461
ZNF699	-7.45E-04	0.870757712	0.990060461
CHST13	0.002316673	0.870831028	0.990060461
OXSM	-4.93E-04	0.871135822	0.99030527
TRIM32	6.43E-04	0.871274854	0.99036161
DHCR24	-0.001238652	0.871393025	0.990380295
CINP	-7.85E-04	0.871628677	0.990380295
SETD6	6.21E-04	0.871730454	0.990380295
NMB	-7.84E-04	0.871747832	0.990380295
NHP2	-5.79E-04	0.871867048	0.990380295
GTF2IP1	-9.12E-04	0.871891415	0.990380295
EFHD1	-0.001481548	0.871917605	0.990380295
TMEM86A	-6.58E-04	0.872096652	0.99047983
JAK1	5.99E-04	0.872299937	0.99047983
JARID2	4.28E-04	0.872358773	0.99047983
ZNFX1	-5.31E-04	0.872363164	0.99047983
FKBP6	-6.81E-04	0.872565628	0.990489577
CCL5	8.36E-04	0.872639605	0.990489577
DNAJC14	4.60E-04	0.872640198	0.990489577
ARHGAP24	-0.001580801	0.873106477	0.990917214
TCF12	5.02E-04	0.873290183	0.991024096
RNF141	6.99E-04	0.873481445	0.991027907
NOC4L	8.05E-04	0.873565579	0.991027907
CCDC146	0.00100682	0.873862649	0.991027907
TESK2	4.77E-04	0.87398412	0.991027907
EYS	5.33E-04	0.874003	0.991027907
HOXA6	-8.46E-04	0.874175425	0.991027907
VGLL4	5.11E-04	0.874292011	0.991027907
ZNF331	5.84E-04	0.874362327	0.991027907
KCNRG	8.09E-04	0.874377456	0.991027907
TOR1A	4.37E-04	0.874563119	0.991027907
NLRP2	0.001932331	0.874611157	0.991027907
PIGU	-4.06E-04	0.874656286	0.991027907
SOAT2	6.77E-04	0.874675949	0.991027907
CTSZ	0.001004062	0.874717588	0.991027907
ABCC2	5.34E-04	0.87488746	0.991027907
CD1C	0.001066078	0.874897768	0.991027907
ZBTB24	-5.27E-04	0.875099605	0.991027907
SCNM1	-5.89E-04	0.875503835	0.991027907
ZFPM1	6.72E-04	0.875552563	0.991027907

SLC25A29	-0.001191154	0.875562306	0.991027907
ZNF223	9.85E-04	0.875652278	0.991027907
C2CD2L	-8.13E-04	0.875694695	0.991027907
SLC9A8	5.96E-04	0.87572795	0.991027907
SLC20A2	-4.97E-04	0.875798015	0.991027907
ADORA2A	-7.36E-04	0.875908983	0.991027907
RNF111	-7.22E-04	0.875934921	0.991027907
PPA1	7.45E-04	0.876106275	0.991027907
POP5	6.33E-04	0.876443754	0.991027907
ACP6	-6.73E-04	0.87648557	0.991027907
RNF207	7.88E-04	0.876493509	0.991027907
TATDN1	7.93E-04	0.876622742	0.991027907
HRAS	-4.58E-04	0.876691678	0.991027907
STYK1	-7.94E-04	0.876739661	0.991027907
C8orf44	-0.001468331	0.876889965	0.991027907
SMAD7	-9.69E-04	0.876922227	0.991027907
WDFY2	-7.22E-04	0.876958684	0.991027907
SYN2	-6.31E-04	0.876982406	0.991027907
METAP2	-3.78E-04	0.877004118	0.991027907
GLIPR2	-3.86E-04	0.877033098	0.991027907
OAZ3	7.45E-04	0.877046087	0.991027907
RAD23A	-4.70E-04	0.877095198	0.991027907
ALG2	5.76E-04	0.877371629	0.991027907
UBE2R2	-4.77E-04	0.877379959	0.991027907
ZNF140	-7.09E-04	0.877611229	0.991027907
SLC25A4	7.28E-04	0.877718023	0.991027907
XPC	3.87E-04	0.877823982	0.991027907
RWDD2B	6.04E-04	0.877848345	0.991027907
KLHL14	0.001580188	0.877896886	0.991027907
ZNF324	-6.28E-04	0.877942993	0.991027907
RASA1	3.66E-04	0.877977176	0.991027907
DYRK2	-7.27E-04	0.878135778	0.991027907
AGAP1	-0.001507873	0.878180338	0.991027907
TUBB2A	-0.001387072	0.878266175	0.991027907
C21orf33	-3.30E-04	0.878322438	0.991027907
TSEN15	-3.89E-04	0.878374676	0.991027907
TMEM144	-0.001259214	0.878471642	0.991027907
CUBN	-9.21E-04	0.87859695	0.991027907
DNMT3A	-5.99E-04	0.878636677	0.991027907
USP44	6.12E-04	0.878648152	0.991027907
ZFP37	-9.57E-04	0.878665451	0.991027907
AMY1A	6.01E-04	0.878831239	0.991053276
GORASP2	3.70E-04	0.878867012	0.991053276
SCRIB	3.99E-04	0.8794638	0.991146945
DHX32	5.79E-04	0.879613439	0.991146945
SURF4	4.62E-04	0.879621828	0.991146945
TNRC6C	-8.35E-04	0.879656228	0.991146945

SP4	5.58E-04	0.879726707	0.991146945
UNC80	6.02E-04	0.879992162	0.991146945
C5orf34	-7.42E-04	0.88004677	0.991146945
PARP8	8.65E-04	0.880067978	0.991146945
CAP1	-3.09E-04	0.880234538	0.991146945
TMEM163	-6.87E-04	0.880235956	0.991146945
UNC13A	0.001142269	0.880281925	0.991146945
CARD16	-7.09E-04	0.880423511	0.991146945
NDUFA3	4.06E-04	0.8806693	0.991146945
GLS	-6.00E-04	0.880748288	0.991146945
INO80E	-5.64E-04	0.880811315	0.991146945
TRIOBP	-5.06E-04	0.880829876	0.991146945
RASA2	8.73E-04	0.880834403	0.991146945
CACHD1	7.25E-04	0.881097065	0.991146945
SNORD65	8.34E-04	0.881125337	0.991146945
TRIM10	-0.001447373	0.8813115	0.991146945
KDM4A	-6.24E-04	0.881326927	0.991146945
DHX34	-5.22E-04	0.881448717	0.991146945
RWDD4	3.14E-04	0.881499328	0.991146945
DENND4A	-5.00E-04	0.881503441	0.991146945
PYGL	-6.71E-04	0.881551097	0.991146945
MYO5A	-4.82E-04	0.881565583	0.991146945
ZNF775	-5.64E-04	0.881565969	0.991146945
TRMT61B	4.49E-04	0.881599359	0.991146945
IRF3	5.55E-04	0.881606677	0.991146945
NTAN1	5.59E-04	0.881783785	0.991146945
MAML1	-4.83E-04	0.881784436	0.991146945
HYALP1	-5.44E-04	0.881815666	0.991146945
TRAPPC6B	8.78E-04	0.882025152	0.991146945
FAHD1	5.90E-04	0.882081298	0.991146945
DUSP1	0.001097809	0.882084069	0.991146945
SPTAN1	-6.89E-04	0.882212166	0.991190262
OSBPL5	-0.001051968	0.882354584	0.991249659
LRCH4	4.65E-04	0.882592908	0.991366074
CWF19L1	-3.73E-04	0.882667215	0.991366074
SLC38A2	-4.47E-04	0.882726897	0.991366074
URB2	-4.22E-04	0.882866207	0.991421938
B3GALT2	0.001428106	0.883038073	0.991514347
KIAA0753	-4.96E-04	0.883986085	0.991952904
CLIC1	-4.28E-04	0.883998772	0.991952904
ARMC2	4.93E-04	0.884003238	0.991952904
ZCWPW1	5.96E-04	0.88415471	0.991952904
TTI1	5.63E-04	0.884164802	0.991952904
PFKP	-7.48E-04	0.884313322	0.991952904
SLC35E1	0.001143044	0.884456885	0.991952904
LRRC47	2.77E-04	0.884661001	0.991952904
OCLN	0.001658296	0.884710104	0.991952904

BECN1	3.58E-04	0.884816613	0.991952904
TMEM2	5.71E-04	0.884817071	0.991952904
POLR3GL	3.14E-04	0.884855772	0.991952904
ASAP3	6.53E-04	0.884868839	0.991952904
ZFP36	4.89E-04	0.884910294	0.991952904
CAMSAP1	-4.80E-04	0.884946745	0.991952904
C6orf62	-4.18E-04	0.885138739	0.991952904
FGF18	-6.77E-04	0.885250388	0.991952904
SELL	7.98E-04	0.885292791	0.991952904
TMEM120A	4.26E-04	0.885325201	0.991952904
MTSS1	6.42E-04	0.885385125	0.991952904
C9orf142	4.88E-04	0.885562039	0.991952904
MRPS5	-3.66E-04	0.885616094	0.991952904
CUL3	7.05E-04	0.885652668	0.991952904
TNIK	-7.00E-04	0.885754289	0.991952904
NBPF11	-4.46E-04	0.885792403	0.991952904
NF2	-5.34E-04	0.885898572	0.991952904
C1orf159	-5.97E-04	0.885939762	0.991952904
C18orf56	7.90E-04	0.886031467	0.991952904
TAF8	-4.92E-04	0.886082075	0.991952904
LIPA	-7.70E-04	0.886117112	0.991952904
ZNF181	-6.79E-04	0.886507392	0.992175563
SH3GL1	4.34E-04	0.886663125	0.992175563
UBL5	-3.21E-04	0.886711841	0.992175563
ME1	-0.001097493	0.886817967	0.992175563
RNPC3	-4.76E-04	0.887005587	0.992175563
MCOLN2	-0.001142238	0.887006092	0.992175563
KEAP1	4.56E-04	0.887069393	0.992175563
ATXN3	6.06E-04	0.887108617	0.992175563
FCRL5	0.00130132	0.887181702	0.992175563
IFRD2	-5.54E-04	0.88721237	0.992175563
DRG2	-4.43E-04	0.887342112	0.99222041
ITGB3BP	0.001046971	0.887540215	0.992256195
MRPL45	-3.27E-04	0.887642201	0.992256195
PHPT1	4.55E-04	0.887643043	0.992256195
LITAF	5.73E-04	0.887923611	0.9924696
MMP25	-0.001204564	0.888020911	0.992478137
JDP2	-7.61E-04	0.888243414	0.992526224
BTN2A2	-8.15E-04	0.888444358	0.992526224
ADPRH	-5.45E-04	0.888459328	0.992526224
PYROXD1	-0.001048283	0.888462677	0.992526224
PARP15	-7.44E-04	0.888801429	0.992526224
CD79A	0.001268506	0.888833153	0.992526224
FTH1	-5.35E-04	0.889201353	0.992526224
PCM1	-3.89E-04	0.889289496	0.992526224
RAB33B	5.54E-04	0.889404818	0.992526224
TOMM7	-3.16E-04	0.889442495	0.992526224

LST1	0.001042098	0.889632432	0.992526224
DPY19L1	-4.41E-04	0.889780546	0.992526224
ZNF335	5.75E-04	0.889785665	0.992526224
HNRNPUL1	-3.55E-04	0.889828272	0.992526224
EEF1D	4.09E-04	0.88987976	0.992526224
BCAR3	6.41E-04	0.889909472	0.992526224
FDXR	6.07E-04	0.889955428	0.992526224
SH2D3C	-5.58E-04	0.890009146	0.992526224
MCM4	-6.83E-04	0.890128255	0.992526224
TMEM9	-5.08E-04	0.890154763	0.992526224
ATMIN	6.34E-04	0.890220455	0.992526224
LAPTM5	-3.20E-04	0.890231174	0.992526224
THBS3	5.52E-04	0.890280139	0.992526224
PIP5K1A	-7.29E-04	0.890301555	0.992526224
IP6K1	-4.00E-04	0.890524306	0.992526224
C16orf70	-4.16E-04	0.890606838	0.992526224
POLG	-3.62E-04	0.890825229	0.992526224
SREK1IP1	5.62E-04	0.890920695	0.992526224
RTN4RL1	5.86E-04	0.891038625	0.992526224
SLC47A1	-8.96E-04	0.891207835	0.992526224
PI15	6.15E-04	0.891259671	0.992526224
SCARNA4	6.66E-04	0.891261115	0.992526224
CEP152	-5.42E-04	0.891288683	0.992526224
PRDM10	-4.71E-04	0.891311974	0.992526224
ERMN	8.11E-04	0.891340277	0.992526224
OPN3	-5.95E-04	0.891497163	0.992526224
EPHX2	0.001016385	0.891671251	0.992526224
FAM138F	6.39E-04	0.891810706	0.992526224
MEFV	-8.32E-04	0.892002568	0.992526224
SLC26A11	4.37E-04	0.892088789	0.992526224
ERGIC2	6.25E-04	0.892099778	0.992526224
DCTN1	-2.72E-04	0.892297182	0.992526224
DCLRE1A	-8.82E-04	0.892336844	0.992526224
MIDN	4.65E-04	0.89233935	0.992526224
NUDT16	-6.02E-04	0.892353299	0.992526224
CPNE3	2.55E-04	0.892357613	0.992526224
PLA2G2C	5.66E-04	0.892361288	0.992526224
MIB1	-5.96E-04	0.892448861	0.992526224
ANKDD1A	-0.001925009	0.89245763	0.992526224
ZHX3	4.45E-04	0.892987064	0.992920371
RXRG	-5.69E-04	0.893053519	0.992920371
KAT2A	-4.80E-04	0.89378508	0.992920371
OIP5	-8.11E-04	0.893804957	0.992920371
APIP	-7.95E-04	0.893938574	0.992920371
XPO5	-4.37E-04	0.893962627	0.992920371
SUCLG2	-4.06E-04	0.894078742	0.992920371
ZNF404	7.47E-04	0.894234756	0.992920371

CLEC5A	0.001269948	0.894390263	0.992920371
DOCK3	5.40E-04	0.89455017	0.992920371
CREBBP	-3.16E-04	0.894583383	0.992920371
RBBP8	5.97E-04	0.894669146	0.992920371
SNORD87	-5.90E-04	0.894692377	0.992920371
PGAM5	-4.80E-04	0.8946977	0.992920371
SLC39A7	5.87E-04	0.89485475	0.992920371
ERI3	4.19E-04	0.894931628	0.992920371
AVL9	4.95E-04	0.894939963	0.992920371
TGFA	-8.34E-04	0.894947838	0.992920371
GPATCH3	-4.14E-04	0.89502713	0.992920371
PPIAL4G	2.09E-04	0.895032785	0.992920371
ANKRD11	2.96E-04	0.895161559	0.992920371
SETD4	-6.01E-04	0.895243347	0.992920371
ALG1	6.03E-04	0.895249928	0.992920371
PHC1	5.31E-04	0.895321776	0.992920371
ZNF519	7.16E-04	0.895370427	0.992920371
PRADC1	-5.36E-04	0.895532312	0.992920371
INPP5E	0.001233655	0.895546944	0.992920371
HELQ	-3.89E-04	0.89579604	0.992920371
ABHD10	4.19E-04	0.895863788	0.992920371
CCDC90B	-2.69E-04	0.896056834	0.992920371
FANCC	4.51E-04	0.896067576	0.992920371
RRP7B	-2.42E-04	0.896119131	0.992920371
RPS13	4.46E-04	0.896161479	0.992920371
NFRKB	-4.84E-04	0.896175777	0.992920371
TMOD1	0.001113104	0.896400125	0.992920371
LARP1B	3.91E-04	0.896474179	0.992920371
EIF3D	-3.65E-04	0.896729216	0.992920371
TUBB	-3.26E-04	0.896974391	0.992920371
RAB7L1	4.09E-04	0.897128356	0.992920371
ANKLE2	3.57E-04	0.89713529	0.992920371
PRR7	7.71E-04	0.897150885	0.992920371
ZCCHC24	5.64E-04	0.897268647	0.992920371
C17orf59	6.14E-04	0.897510075	0.992920371
EHD2	4.63E-04	0.897565628	0.992920371
SLC29A4	6.07E-04	0.897586818	0.992920371
CS	3.25E-04	0.897935153	0.992920371
BCR	5.51E-04	0.897963251	0.992920371
ABHD1	-5.51E-04	0.898205877	0.992920371
MUM1	-4.33E-04	0.898433989	0.992920371
BMPR1A	-5.78E-04	0.898491651	0.992920371
ZNF337	-4.10E-04	0.898521165	0.992920371
ISY1	-3.31E-04	0.898681665	0.992920371
EEF1E1	-3.44E-04	0.898694374	0.992920371
OXNAD1	-6.54E-04	0.898710126	0.992920371
SNRPG	2.86E-04	0.89914346	0.992920371

COPZ1	3.90E-04	0.89914884	0.992920371
ODF3B	-5.63E-04	0.899191472	0.992920371
PLXNA1	5.77E-04	0.899209847	0.992920371
IL12RB2	7.39E-04	0.899354439	0.992920371
PRR21	-5.03E-04	0.899393711	0.992920371
MT1JP	7.76E-04	0.899427213	0.992920371
RINL	4.05E-04	0.899547688	0.992920371
CORO1B	-6.23E-04	0.899704157	0.992920371
UROS	-3.44E-04	0.899731273	0.992920371
PGP	-6.96E-04	0.899888932	0.992920371
ANKRD52	-6.08E-04	0.900204511	0.992920371
SFTPD	-0.00109793	0.900236105	0.992920371
THUMPD1	2.94E-04	0.900277935	0.992920371
C14orf169	-3.45E-04	0.900286799	0.992920371
DIP2A	0.001085494	0.9003561	0.992920371
DMTF1	4.19E-04	0.900390052	0.992920371
PRKD3	-5.10E-04	0.900393548	0.992920371
NT5DC1	-3.28E-04	0.900394652	0.992920371
MGST2	-4.83E-04	0.900579874	0.992920371
EIF3I	-2.70E-04	0.900692561	0.992920371
AKAP12	4.18E-04	0.901012372	0.992920371
CENPK	0.001972621	0.901072628	0.992920371
MAML3	7.24E-04	0.901102328	0.992920371
CCDC94	2.97E-04	0.901102583	0.992920371
AGK	3.95E-04	0.901277472	0.992920371
PPM1N	5.61E-04	0.901297704	0.992920371
FDX1L	4.31E-04	0.901323491	0.992920371
AP1G2	-4.03E-04	0.901373776	0.992920371
RPAIN	-2.60E-04	0.901488921	0.992920371
VWA3A	-4.91E-04	0.901591587	0.992920371
TNFAIP8L1	-6.91E-04	0.901639269	0.992920371
PSENEN	-5.17E-04	0.901670664	0.992920371
SCAMP3	2.87E-04	0.901901028	0.992920371
TARBP1	4.80E-04	0.901950871	0.992920371
CLEC7A	-0.001101688	0.902110271	0.992920371
CARD14	-5.73E-04	0.902218033	0.992920371
CBR3	9.60E-04	0.902255165	0.992920371
SLC40A1	-5.93E-04	0.902298173	0.992920371
LARS	2.94E-04	0.902355098	0.992920371
ATP6V1G2	-6.10E-04	0.90236347	0.992920371
ALPK1	-7.00E-04	0.902478745	0.992920371
C11orf49	-5.54E-04	0.902586723	0.992920371
OBSCN	-6.26E-04	0.902631207	0.992920371
MRPS25	-6.13E-04	0.902748448	0.992920371
TTC1	2.74E-04	0.902935067	0.992920371
HBG2	-0.004355339	0.903032118	0.992920371
RPS8	-2.98E-04	0.903032852	0.992920371

IFI27L1	-4.90E-04	0.903135452	0.992920371
STAT2	-4.46E-04	0.903157708	0.992920371
PER2	3.46E-04	0.903226734	0.992920371
CYB561	6.45E-04	0.903336128	0.992920371
ZNF597	-5.06E-04	0.903390952	0.992920371
NDUFA10	5.06E-04	0.903422352	0.992920371
MCM3	2.86E-04	0.903488197	0.992920371
RIPK4	4.85E-04	0.903544071	0.992920371
CCDC6	-2.90E-04	0.903552345	0.992920371
CD3E	-6.29E-04	0.903595053	0.992920371
MED16	5.46E-04	0.903630235	0.992920371
C1orf21	0.00107859	0.903830231	0.992920371
MCM3AP	3.14E-04	0.903882655	0.992920371
F2RL3	-7.77E-04	0.904060605	0.992920371
ADAM9	-5.35E-04	0.904187113	0.992920371
PDE7B	-6.32E-04	0.904247053	0.992920371
PWP1	-2.25E-04	0.904366031	0.992920371
PAPLN	9.02E-04	0.904370246	0.992920371
GPR89B	5.22E-04	0.904786296	0.992920371
BEND7	-5.49E-04	0.904814333	0.992920371
ORAI3	5.08E-04	0.904834381	0.992920371
ITPA	-2.80E-04	0.904847014	0.992920371
NFKBID	4.15E-04	0.905058215	0.992920371
S100A14	-5.50E-04	0.905068348	0.992920371
LMO4	-4.77E-04	0.905218109	0.992920371
SASH1	-9.18E-04	0.905274667	0.992920371
DCPS	4.86E-04	0.905337307	0.992920371
METTL18	7.11E-04	0.905362877	0.992920371
COX6B1	3.21E-04	0.90538784	0.992920371
NME1-NME2	-4.42E-04	0.905417865	0.992920371
CASP10	4.42E-04	0.905576745	0.992920371
SESN3	-6.18E-04	0.905591089	0.992920371
SPHK1	6.10E-04	0.905597764	0.992920371
DVL3	-3.27E-04	0.905698415	0.992920371
CASZ1	-4.88E-04	0.905799128	0.992920371
HEY2	-8.07E-04	0.905806989	0.992920371
COTL1	-7.20E-04	0.905822733	0.992920371
PPP3R1	-4.74E-04	0.906044722	0.992920371
UBA2	4.10E-04	0.906080511	0.992920371
FAM63B	-6.25E-04	0.906272774	0.992920371
LY86	5.69E-04	0.906377049	0.992920371
DKK3	-0.00151718	0.906533431	0.992920371
TMEM66	-2.25E-04	0.90661391	0.992920371
SNRPD3	5.60E-04	0.906709026	0.992920371
TERF1	-4.08E-04	0.906802215	0.992920371
RPL5	-2.45E-04	0.906864948	0.992920371
SARNP	3.04E-04	0.9069591	0.992920371

PFKFB2	-6.00E-04	0.90701348	0.992920371
NSMCE4A	2.26E-04	0.907051523	0.992920371
DAAM1	4.41E-04	0.907084166	0.992920371
PIGQ	-4.34E-04	0.907115123	0.992920371
DHX40	-2.84E-04	0.907270096	0.992920371
STRN4	4.90E-04	0.907339609	0.992920371
ABCG1	7.09E-04	0.907363136	0.992920371
ITGAX	5.59E-04	0.907368286	0.992920371
NOM1	7.76E-04	0.907406536	0.992920371
EXD3	-5.40E-04	0.907443394	0.992920371
MCTP2	-7.49E-04	0.907564427	0.992920371
INO80B	-3.19E-04	0.907597269	0.992920371
RELA	-4.89E-04	0.90769433	0.992920371
NCK1	-3.46E-04	0.907747225	0.992920371
ITGB1	-3.75E-04	0.907772137	0.992920371
TRIM5	-6.40E-04	0.907823193	0.992920371
ROCK1	5.36E-04	0.907951517	0.992920371
PCDHGC3	5.94E-04	0.907976684	0.992920371
AGPAT4	7.44E-04	0.908186307	0.992920371
BLMH	3.90E-04	0.908385705	0.992920371
CLDN14	4.49E-04	0.908417521	0.992920371
PPP1R3E	-6.24E-04	0.908461524	0.992920371
CENPH	-4.71E-04	0.908522525	0.992920371
C19orf33	-0.001777756	0.908548893	0.992920371
GBP1	-8.51E-04	0.908553866	0.992920371
DTX1	6.66E-04	0.908559187	0.992920371
VEGFA	5.07E-04	0.908680821	0.992920371
GAS2L3	-3.85E-04	0.908750529	0.992920371
LRRC28	4.76E-04	0.908779138	0.992920371
MRPL34	3.31E-04	0.908887065	0.99294028
RLF	-3.02E-04	0.909127898	0.993039439
ESYT2	-2.79E-04	0.909184778	0.993039439
APOBEC3H	-0.001007402	0.909353069	0.993039439
GRPEL2	-5.08E-04	0.9094075	0.993039439
ATF4	-2.26E-04	0.909426397	0.993039439
ABHD5	-5.95E-04	0.909687405	0.993126533
PDE7A	-9.37E-04	0.909699412	0.993126533
CNOT1	2.41E-04	0.909775322	0.993126533
WRAP53	-4.65E-04	0.910220587	0.993418745
CCNH	4.76E-04	0.910333576	0.993418745
PCBP1	2.96E-04	0.910341036	0.993418745
CCNB1IP1	-3.59E-04	0.910402001	0.993418745
RAD51C	-5.23E-04	0.910766777	0.993587879
BCKDHB	-6.33E-04	0.910804445	0.993587879
PLEKHN1	4.61E-04	0.910832127	0.993587879
ZNF675	-5.92E-04	0.911011086	0.993587879
HOXB4	5.62E-04	0.911140162	0.993587879

LMNA	-8.22E-04	0.91114171	0.993587879
TMEM140	-8.79E-04	0.911381239	0.993587879
REEP5	-2.44E-04	0.911393697	0.993587879
PXMP4	5.81E-04	0.911566994	0.993587879
B9D1	-4.72E-04	0.91156854	0.993587879
TMEM165	-3.98E-04	0.911753647	0.993587879
IGJ	-0.001494872	0.911818232	0.993587879
HSPB11	-4.22E-04	0.911892985	0.993587879
NPC2	-2.66E-04	0.911970476	0.993587879
ATF1	4.78E-04	0.91203639	0.993587879
DYNC1H1	2.85E-04	0.912194227	0.993587879
ZZZ3	-3.06E-04	0.912323223	0.993587879
MDK	4.48E-04	0.912427121	0.993587879
CCZ1	-4.96E-04	0.912491633	0.993587879
TTC7B	-5.23E-04	0.912573644	0.993587879
DNAH12	0.001121069	0.912579734	0.993587879
RNF44	-3.29E-04	0.912850117	0.993587879
FKBP4	-4.85E-04	0.913100955	0.993587879
SFMBT2	-7.54E-04	0.913155719	0.993587879
HSD11B1L	-4.52E-04	0.913165851	0.993587879
HTATIP2	3.62E-04	0.913209908	0.993587879
HLTF	-3.05E-04	0.913427973	0.993587879
SHFM1	-2.85E-04	0.913451229	0.993587879
GAR1	2.32E-04	0.913510949	0.993587879
CDON	4.70E-04	0.913514913	0.993587879
MSH5	4.88E-04	0.913602205	0.993587879
ENG	-5.64E-04	0.913716138	0.993587879
NAPSB	-0.001045152	0.913813656	0.993587879
ZFAND3	3.91E-04	0.913818084	0.993587879
ORC6	1.37E-04	0.913824816	0.993587879
SNTB1	-4.54E-04	0.914349155	0.993587879
ZNF883	4.82E-04	0.914426436	0.993587879
ARHGEF3	2.95E-04	0.9144477	0.993587879
FLAD1	2.57E-04	0.914508262	0.993587879
DLGAP4	3.54E-04	0.914529707	0.993587879
LIN37	-3.48E-04	0.914532661	0.993587879
NCSTN	-2.02E-04	0.914533669	0.993587879
CHD3	-4.52E-04	0.914545424	0.993587879
PLEKHG6	7.88E-04	0.914565367	0.993587879
NCOR1	3.17E-04	0.914599627	0.993587879
SRSF6	2.11E-04	0.914762628	0.993587879
FCHSD1	4.48E-04	0.914821183	0.993587879
SENP5	-3.04E-04	0.914948009	0.993587879
FCRL6	-0.00138084	0.915002898	0.993587879
FAM57A	-4.20E-04	0.915140995	0.993587879
ZNF813	-4.20E-04	0.915163726	0.993587879
ZNF683	0.002070023	0.915330776	0.993587879

TTC5	3.43E-04	0.915568983	0.993587879
TIMM10	-0.001049289	0.915599463	0.993587879
CRKL	-2.44E-04	0.915682129	0.993587879
ICT1	-2.37E-04	0.915764539	0.993587879
SNORA65	3.34E-04	0.915834343	0.993587879
NOL9	5.46E-04	0.915949919	0.993587879
ZNF589	5.23E-04	0.916132003	0.993587879
CASC4	-5.16E-04	0.916138137	0.993587879
CCND2	2.98E-04	0.916175089	0.993587879
UBR2	2.09E-04	0.916445734	0.993587879
MCCC1	2.32E-04	0.916499629	0.993587879
TFB1M	3.27E-04	0.916540278	0.993587879
POU6F1	5.10E-04	0.916683336	0.993587879
LIX1L	-3.53E-04	0.916894851	0.993587879
RANBP9	4.27E-04	0.917042977	0.993587879
UBASH3B	3.59E-04	0.917044516	0.993587879
RPS25	3.88E-04	0.917063432	0.993587879
RRAS	-5.18E-04	0.91710797	0.993587879
REPIN1	3.34E-04	0.917164068	0.993587879
UBOX5	-2.59E-04	0.917180141	0.993587879
KRR1	-4.22E-04	0.917259279	0.993587879
LGALS9B	5.82E-04	0.91728265	0.993587879
SNX4	-2.87E-04	0.917377151	0.993587879
BANP	-2.61E-04	0.917485747	0.993587879
TP53I13	-3.25E-04	0.917555745	0.993587879
MKS1	-3.67E-04	0.917558524	0.993587879
UBL7	3.38E-04	0.917909851	0.993754455
APAF1	4.85E-04	0.918035844	0.993754455
NPHP3	4.66E-04	0.918134889	0.993754455
RCC2	-2.12E-04	0.918179138	0.993754455
MBOAT1	3.48E-04	0.918311233	0.993754455
PDE6D	3.02E-04	0.918571149	0.993754455
STARD5	4.31E-04	0.918859505	0.993754455
E2F3	3.30E-04	0.918919859	0.993754455
STK10	-3.06E-04	0.918923712	0.993754455
MGST3	4.48E-04	0.919342876	0.993754455
GANAB	-2.84E-04	0.919399777	0.993754455
RTKN	-7.29E-04	0.91946344	0.993754455
GNB2	3.25E-04	0.919526671	0.993754455
CROCC	4.66E-04	0.919572433	0.993754455
DNAJC27	3.50E-04	0.919600757	0.993754455
MTAP	-3.67E-04	0.919657542	0.993754455
SNW1	3.01E-04	0.919844758	0.993754455
ZNF30	3.84E-04	0.919848744	0.993754455
RALB	-3.75E-04	0.919870845	0.993754455
NUBP1	2.39E-04	0.919999792	0.993754455
ANAPC7	3.74E-04	0.920194388	0.993754455

SSPO	-7.55E-04	0.920200991	0.993754455
ZDHHC1	-6.62E-04	0.920268359	0.993754455
TST	5.55E-04	0.920282783	0.993754455
RPS6KB1	2.24E-04	0.920383189	0.993754455
C4orf32	-4.71E-04	0.920478014	0.993754455
KIAA1161	-4.19E-04	0.920537919	0.993754455
CD160	-0.001056525	0.920549418	0.993754455
CDCA7	6.35E-04	0.920596656	0.993754455
AVPI1	5.96E-04	0.920718041	0.993754455
HAUS4	-3.84E-04	0.920718576	0.993754455
C4orf48	2.42E-04	0.920748798	0.993754455
ATXN7L3	3.06E-04	0.920762189	0.993754455
ERCC3	-2.54E-04	0.920772118	0.993754455
CST7	-7.63E-04	0.920911978	0.993754455
ABCA2	-6.59E-04	0.920944367	0.993754455
PAG1	1.48E-04	0.921114518	0.993841174
ITPR3	3.74E-04	0.921426595	0.994025451
CCDC84	5.35E-04	0.921514954	0.994025451
GEMIN5	-3.49E-04	0.921584383	0.994025451
TADA2A	-4.10E-04	0.921746535	0.994025451
DSC3	4.06E-04	0.921856913	0.994025451
HMHA1	-3.26E-04	0.921989235	0.994025451
PLEKHA1	2.66E-04	0.922137319	0.994025451
SLC44A2	2.62E-04	0.922142775	0.994025451
CD68	2.96E-04	0.92225196	0.994025451
GTF2A2	-2.34E-04	0.922296269	0.994025451
BMS1P4	3.71E-04	0.922305386	0.994025451
MED18	-5.39E-04	0.922362943	0.994025451
NUDCD3	-3.89E-04	0.922550676	0.994113915
CWC27	-2.45E-04	0.922713223	0.994113915
ANXA3	-0.002231987	0.92271446	0.994113915
ZNF346	4.64E-04	0.922806389	0.994116197
PRKAA1	-2.25E-04	0.923090158	0.994325123
PSMD4	3.03E-04	0.923290963	0.99444465
DNAJC18	3.32E-04	0.923573382	0.994624308
CBFB	-2.98E-04	0.92363748	0.994624308
NAPSA	9.79E-04	0.924035094	0.994654216
RPL7A	1.65E-04	0.924046317	0.994654216
RAC1	2.01E-04	0.924155373	0.994654216
TBCE	2.62E-04	0.924174862	0.994654216
TNFSF14	-5.30E-04	0.924257034	0.994654216
KTI12	-2.65E-04	0.924364387	0.994654216
MRPS28	-3.44E-04	0.924479274	0.994654216
REV1	-2.66E-04	0.924496204	0.994654216
C9orf114	3.63E-04	0.924797719	0.994654216
FAM98C	3.18E-04	0.924887951	0.994654216
NAGA	-4.25E-04	0.924889578	0.994654216

CHST2	-7.07E-04	0.925081165	0.994654216
LRRC26	8.41E-04	0.925177548	0.994654216
ZWILCH	-2.64E-04	0.925195394	0.994654216
BRD2	-2.13E-04	0.925235073	0.994654216
HAPLN3	5.95E-04	0.925246973	0.994654216
WDR63	-4.51E-04	0.925343102	0.994654216
TRMT11	-2.94E-04	0.925511584	0.994654216
AIP	-2.82E-04	0.925577523	0.994654216
GCSH	3.84E-04	0.92559788	0.994654216
KCNK6	-2.91E-04	0.925697013	0.994654216
CBR1	5.61E-04	0.925715789	0.994654216
SNORD78	-4.41E-04	0.925732021	0.994654216
MPDU1	-4.19E-04	0.926284096	0.994899374
RAB23	-6.17E-04	0.926357175	0.994899374
B3GNT1	-4.55E-04	0.92635746	0.994899374
JAKMIP3	4.40E-04	0.926378864	0.994899374
TMEM192	-4.19E-04	0.926453532	0.994899374
HIST2H2AB	5.42E-04	0.926499481	0.994899374
IARS	-2.05E-04	0.926621026	0.994933372
NUDT3	2.97E-04	0.926848051	0.99493575
TNFAIP1	2.55E-04	0.926931373	0.99493575
IL1RAP	2.92E-04	0.927084541	0.99493575
HIST1H2BN	-5.97E-04	0.927319659	0.99493575
SPG7	-3.38E-04	0.927340693	0.99493575
UBE2Q2	3.06E-04	0.927357562	0.99493575
TLK2	2.37E-04	0.92735769	0.99493575
CIAPIN1	-2.70E-04	0.927386157	0.99493575
IL1R2	-5.91E-04	0.927645237	0.99493575
STYXL1	-4.08E-04	0.927649593	0.99493575
TWF1	-3.96E-04	0.927675123	0.99493575
SLTM	2.27E-04	0.928067461	0.99493575
ANKRD12	3.04E-04	0.928085358	0.99493575
GPCPD1	-4.37E-04	0.928120919	0.99493575
MYL4	0.002154424	0.928154706	0.99493575
BAI1	4.07E-04	0.928177029	0.99493575
CCDC61	3.69E-04	0.928286684	0.99493575
PDCD7	1.45E-04	0.92842761	0.99493575
WNT7A	4.31E-04	0.928449846	0.99493575
STAG3	4.43E-04	0.928464505	0.99493575
MED26	-2.96E-04	0.928510823	0.99493575
DNAJC10	-2.06E-04	0.928843909	0.994983591
HIST2H4B	-6.99E-04	0.928916398	0.994983591
NDUFAF3	-2.80E-04	0.929032629	0.994983591
ALDOB	-5.58E-04	0.92916531	0.994983591
ZNF777	2.47E-04	0.929274649	0.994983591
COX10	-2.49E-04	0.92933196	0.994983591
OXTR	-6.23E-04	0.929446126	0.994983591

AARSD1	-2.75E-04	0.929561839	0.994983591
MED17	-3.91E-04	0.929646112	0.994983591
LY9	-3.48E-04	0.929654454	0.994983591
EMP1	7.54E-04	0.929667648	0.994983591
INPP4A	-3.61E-04	0.929710652	0.994983591
SELT	3.01E-04	0.929724029	0.994983591
PCK2	-2.74E-04	0.929895343	0.994987022
ZSWIM7	-9.66E-04	0.929965834	0.994987022
MTMR4	2.15E-04	0.929996904	0.994987022
PANK1	-3.81E-04	0.930317372	0.995209955
PTBP2	3.18E-04	0.930697104	0.995209955
FUT8	4.71E-04	0.930846967	0.995209955
CSTF2T	3.56E-04	0.930944951	0.995209955
PTPN1	2.72E-04	0.931034065	0.995209955
CEP55	6.20E-04	0.931037765	0.995209955
EXOSC8	4.09E-04	0.931334791	0.995209955
SFPQ	4.34E-04	0.931388727	0.995209955
EIF3K	2.43E-04	0.931457744	0.995209955
ZMIZ2	3.62E-04	0.93168901	0.995209955
CEBPD	3.30E-04	0.931852175	0.995209955
DNPEP	-1.47E-04	0.931855177	0.995209955
KRT3	-2.95E-04	0.932159249	0.995209955
KIAA1429	-2.22E-04	0.932276209	0.995209955
TRIM52	3.90E-04	0.932283045	0.995209955
GNPAT	1.80E-04	0.932432312	0.995209955
DNAJC5	2.49E-04	0.932451604	0.995209955
WASH2P	-4.37E-04	0.93248689	0.995209955
MTHFSD	3.09E-04	0.932646231	0.995209955
P4HA2	3.25E-04	0.932692845	0.995209955
AES	-3.17E-04	0.932698728	0.995209955
PLB1	-3.79E-04	0.932912078	0.995209955
CCDC101	-2.95E-04	0.933054037	0.995209955
TOR1B	-2.67E-04	0.933120246	0.995209955
TNFRSF21	6.70E-04	0.933120641	0.995209955
CD84	-3.92E-04	0.933211854	0.995209955
INPP5K	2.91E-04	0.933231254	0.995209955
NUDT7	3.87E-04	0.93326559	0.995209955
STIL	-3.73E-04	0.933349005	0.995209955
ADAL	3.24E-04	0.93336074	0.995209955
SCAP	1.76E-04	0.933535421	0.995209955
KIF15	-3.39E-04	0.933724345	0.995209955
WDR46	2.28E-04	0.934081645	0.995209955
GBP5	4.88E-04	0.934087374	0.995209955
KBTBD3	-3.86E-04	0.934157923	0.995209955
ARRDC5	-3.73E-04	0.934344974	0.995209955
FAM111A	2.17E-04	0.934433804	0.995209955
C6orf226	-2.92E-04	0.934536166	0.995209955

PKDCC	-2.99E-04	0.934569176	0.995209955
S100A6	2.18E-04	0.934645297	0.995209955
KLF3	3.96E-04	0.934706158	0.995209955
SPCS1	1.96E-04	0.934808854	0.995209955
FBRS	2.96E-04	0.934998968	0.995209955
SLMO2	-3.75E-04	0.935258984	0.995209955
PSMC1	3.02E-04	0.935282626	0.995209955
IRX3	0.001063192	0.935409027	0.995209955
FLII	2.23E-04	0.935442345	0.995209955
HCLS1	-3.71E-04	0.935495495	0.995209955
C1orf61	-4.11E-04	0.935577504	0.995209955
PAK1IP1	-2.02E-04	0.935636907	0.995209955
CEP70	4.04E-04	0.935856052	0.995209955
RPF1	2.20E-04	0.935877699	0.995209955
ZUFSP	-2.20E-04	0.936038272	0.995209955
AXIN1	4.32E-04	0.936047169	0.995209955
CPSF2	3.65E-04	0.936173497	0.995209955
KLHDC2	2.51E-04	0.936196552	0.995209955
FNBP4	-2.06E-04	0.936327856	0.995209955
ZNF98	-5.55E-04	0.936464014	0.995209955
PFDN5	1.75E-04	0.936667034	0.995209955
SMN2	5.10E-04	0.936946635	0.995209955
FAM96B	2.08E-04	0.937017042	0.995209955
RPL27A	-1.05E-04	0.937077374	0.995209955
TTC30A	3.20E-04	0.937154018	0.995209955
TBC1D3	4.64E-04	0.937285149	0.995209955
TP73-AS1	-2.84E-04	0.937305305	0.995209955
ACPL2	-3.38E-04	0.937316323	0.995209955
SNORA57	4.29E-04	0.937331305	0.995209955
ACO2	2.26E-04	0.937520253	0.995209955
FDFT1	2.86E-04	0.937577148	0.995209955
CAV2	-9.22E-04	0.937778446	0.995209955
FAM174B	-4.63E-04	0.937863085	0.995209955
TRAM2	3.03E-04	0.937946211	0.995209955
RPL8	2.95E-04	0.938117674	0.995209955
POLE2	-3.44E-04	0.938174513	0.995209955
HSPH1	-2.39E-04	0.93841695	0.995209955
CHMP1B	-2.67E-04	0.938520435	0.995209955
LBR	-3.38E-04	0.938552088	0.995209955
PDHX	-2.35E-04	0.938592843	0.995209955
DEDD	-1.71E-04	0.938631508	0.995209955
ZFP90	-2.85E-04	0.938699229	0.995209955
LILRA5	4.69E-04	0.938769021	0.995209955
PUS1	-1.77E-04	0.938787923	0.995209955
CLUL1	-2.92E-04	0.938789478	0.995209955
ENC1	5.93E-04	0.938928502	0.995209955
PRR5	-3.96E-04	0.939075096	0.995209955

SPR	-3.25E-04	0.939130069	0.995209955
STAT4	2.48E-04	0.939452322	0.995209955
ZBTB7B	3.33E-04	0.939531135	0.995209955
PLRG1	-1.47E-04	0.939735875	0.995209955
ZNF234	4.23E-04	0.939810478	0.995209955
TMCO6	-2.11E-04	0.939958263	0.995209955
CELF2	-2.44E-04	0.940067232	0.995209955
C11orf35	3.68E-04	0.940067829	0.995209955
SUSD2	-3.36E-04	0.940112176	0.995209955
CYB5B	-1.66E-04	0.940115676	0.995209955
OLFM1	-0.00110207	0.940369039	0.995209955
TGFBRAP1	-3.26E-04	0.940395182	0.995209955
SYBU	-3.34E-04	0.940484901	0.995209955
RPH3A	-3.88E-04	0.940709713	0.995209955
TRMT61A	-2.94E-04	0.940753543	0.995209955
TMEM30B	-3.54E-04	0.94075585	0.995209955
SPTBN4	4.41E-04	0.940909199	0.995209955
NDUFB10	-1.54E-04	0.941042096	0.995209955
DNAJC30	4.28E-04	0.941058546	0.995209955
MNX1	-2.93E-04	0.941128578	0.995209955
KREMEN2	2.83E-04	0.941257185	0.995209955
FAM89B	2.94E-04	0.941605448	0.995209955
PYHIN1	4.39E-04	0.941614297	0.995209955
USP42	-2.13E-04	0.941632486	0.995209955
GSTM1	-0.00143694	0.941689503	0.995209955
MDC1	2.06E-04	0.941834989	0.995209955
EPN2	2.98E-04	0.941960333	0.995209955
ZXDC	3.02E-04	0.942460604	0.995209955
DIDO1	2.97E-04	0.942576549	0.995209955
DNAJC1	3.39E-04	0.942601903	0.995209955
SLC29A3	3.07E-04	0.942682668	0.995209955
MSI2	2.53E-04	0.942704226	0.995209955
FAM135A	-2.48E-04	0.942908541	0.995209955
SCGB3A2	-4.61E-04	0.942918222	0.995209955
VPS18	2.58E-04	0.942961053	0.995209955
HERC3	-2.65E-04	0.942986902	0.995209955
TAPBPL	3.24E-04	0.943178338	0.995209955
TGFB3	-3.25E-04	0.943325185	0.995209955
UNC93B1	-2.36E-04	0.943364072	0.995209955
ALG3	1.70E-04	0.943481766	0.995209955
PSMB2	-2.21E-04	0.943544928	0.995209955
CCDC149	6.36E-04	0.943548669	0.995209955
CDYL2	2.33E-04	0.94364465	0.995209955
RUFY2	3.57E-04	0.943648739	0.995209955
LEPREL1	4.21E-04	0.943707775	0.995209955
COMMD7	-1.50E-04	0.943776617	0.995209955
SYTL1	-2.46E-04	0.943827168	0.995209955

AKR7A3	-2.68E-04	0.943908952	0.995209955
RPP38	3.27E-04	0.943934086	0.995209955
MTFP1	-2.80E-04	0.943976637	0.995209955
LDLR	3.76E-04	0.944029141	0.995209955
HDAC5	-2.79E-04	0.944167419	0.995209955
CENPT	3.27E-04	0.94424714	0.995209955
ILKAP	-2.53E-04	0.944250819	0.995209955
CEACAM21	-3.47E-04	0.944373415	0.995209955
HIST2H2AC	-4.31E-04	0.944551965	0.995209955
VPS36	1.82E-04	0.944581975	0.995209955
DOT1L	3.15E-04	0.944622613	0.995209955
SCFD1	-1.75E-04	0.944624284	0.995209955
ADAM8	-2.60E-04	0.944855244	0.995209955
TRAPPC1	2.21E-04	0.944888797	0.995209955
SPRED2	3.34E-04	0.944911764	0.995209955
C6orf211	3.32E-04	0.944955614	0.995209955
ZSWIM6	2.53E-04	0.944967215	0.995209955
RAB11FIP2	-1.79E-04	0.945026498	0.995209955
KIAA1715	-2.38E-04	0.945076902	0.995209955
ITSN1	4.22E-04	0.945078343	0.995209955
SPN	-2.63E-04	0.945106761	0.995209955
PC	-2.86E-04	0.945156145	0.995209955
RPL23AP64	-2.38E-04	0.94522114	0.995209955
NANS	-2.25E-04	0.945341438	0.995209955
SBDS	-2.49E-04	0.945409849	0.995209955
RBMS2	4.98E-04	0.945434727	0.995209955
RBM5	2.01E-04	0.945492896	0.995209955
GSTM2	-8.97E-04	0.945552967	0.995209955
ZNF24	-3.04E-04	0.945602069	0.995209955
KATNAL1	3.22E-04	0.945647502	0.995209955
METTL7A	3.13E-04	0.945767007	0.995209955
FAM200B	4.70E-04	0.945801833	0.995209955
PVRL1	-2.85E-04	0.945821118	0.995209955
FAM172A	1.62E-04	0.946205429	0.995209955
AMD1	-1.83E-04	0.946305606	0.995209955
DSTN	-2.23E-04	0.946401709	0.995209955
CRTAM	-5.16E-04	0.946431263	0.995209955
ITGA2	3.60E-04	0.94647666	0.995209955
SDAD1	-1.47E-04	0.946480026	0.995209955
PANK2	2.03E-04	0.946612383	0.995209955
RNASE3	-6.31E-04	0.946840744	0.995209955
S100P	9.82E-04	0.94686586	0.995209955
MIR488	-2.68E-04	0.946961641	0.995209955
LUC7L	-2.30E-04	0.946989729	0.995209955
KLF10	-5.83E-04	0.947064425	0.995209955
TOP1	2.32E-04	0.947142121	0.995209955
ATP5J2	1.35E-04	0.947146943	0.995209955

HMGA1	3.04E-04	0.947357813	0.995209955
TRIM27	-1.42E-04	0.947507587	0.995209955
HPS4	-2.40E-04	0.94774639	0.995209955
MED22	-1.28E-04	0.947809248	0.995209955
SLC7A6	3.23E-04	0.947826623	0.995209955
LRMP	1.73E-04	0.947842464	0.995209955
MMAA	-2.21E-04	0.94784569	0.995209955
KLHL17	2.99E-04	0.947865984	0.995209955
MCL1	1.55E-04	0.947874763	0.995209955
DCAF13	-2.26E-04	0.947942028	0.995209955
SLC12A6	-2.24E-04	0.94795774	0.995209955
IFIT2	-5.43E-04	0.947992451	0.995209955
SLC1A3	-3.72E-04	0.948144984	0.995209955
NUP155	1.83E-04	0.948421351	0.995209955
FAIM3	2.34E-04	0.94854129	0.995209955
RPL30	9.50E-05	0.94864233	0.995209955
ZNF770	-1.86E-04	0.94884586	0.995209955
SMG1	1.62E-04	0.948876688	0.995209955
MTRNR2L1	-5.26E-04	0.949005767	0.995209955
PDGFC	-4.31E-04	0.949247686	0.995209955
RAB4A	2.89E-04	0.94924867	0.995209955
MFN1	2.23E-04	0.949260723	0.995209955
SERF2	2.19E-04	0.949321514	0.995209955
HSPA9	-1.14E-04	0.949473212	0.995209955
LAP3	4.25E-04	0.949506763	0.995209955
LGALS12	-4.15E-04	0.949617852	0.995209955
ALAS1	-2.77E-04	0.949681666	0.995209955
NRD1	-1.37E-04	0.949687613	0.995209955
OTUD7B	2.76E-04	0.949743751	0.995209955
COPS4	-1.72E-04	0.949825597	0.995209955
LTA	3.20E-04	0.949842923	0.995209955
KIAA1958	-2.99E-04	0.949939761	0.995209955
ARHGEF18	-1.66E-04	0.950088592	0.995209955
AP3S1	-2.61E-04	0.950143577	0.995209955
MRE11A	3.57E-04	0.950148487	0.995209955
ERCC2	2.43E-04	0.950228317	0.995209955
TOMM40	1.20E-04	0.950230605	0.995209955
MEG3	-0.001261859	0.950357505	0.995209955
SKP2	-1.66E-04	0.950383857	0.995209955
ZNF259	-1.82E-04	0.950405648	0.995209955
SIGLEC5	5.97E-04	0.950816302	0.995209955
YPEL2	-2.88E-04	0.950902156	0.995209955
SLC4A4	3.96E-04	0.950961244	0.995209955
S100A10	1.47E-04	0.950965217	0.995209955
MEST	2.43E-04	0.951038231	0.995209955
LRFN4	2.85E-04	0.951073113	0.995209955
GARS	-1.17E-04	0.951113429	0.995209955

ZNF692	2.21E-04	0.951147482	0.995209955
NFATC1	-2.11E-04	0.951297944	0.995209955
RILPL2	2.02E-04	0.951325335	0.995209955
PRPF4B	-2.51E-04	0.951356709	0.995209955
SIRPA	2.02E-04	0.951645387	0.995209955
TAF5L	4.15E-04	0.951685161	0.995209955
ZDHHC11	5.67E-04	0.951721214	0.995209955
CCRN4L	3.33E-04	0.95177471	0.995209955
SPTLC2	2.57E-04	0.95184695	0.995209955
KIAA1586	-2.90E-04	0.951915512	0.995209955
RBM42	1.85E-04	0.952159322	0.995209955
STK40	1.66E-04	0.952195579	0.995209955
PEX1	-1.96E-04	0.952253517	0.995209955
ZNF195	-2.21E-04	0.952281474	0.995209955
SSRP1	2.49E-04	0.952387705	0.995209955
ATP5O	1.74E-04	0.952397033	0.995209955
RAB13	2.90E-04	0.952413079	0.995209955
TMEM161A	-2.28E-04	0.952430867	0.995209955
GOLPH3L	2.43E-04	0.952430869	0.995209955
TES	2.46E-04	0.952716642	0.995209955
TRIM64B	-2.26E-04	0.952732925	0.995209955
TMEM206	1.79E-04	0.952992543	0.995209955
C12orf77	-2.16E-04	0.95304359	0.995209955
MTBP	2.50E-04	0.9531028	0.995209955
RPL18A	-1.73E-04	0.953112234	0.995209955
KIAA1430	2.99E-04	0.953151027	0.995209955
CHD1	-1.43E-04	0.953216326	0.995209955
HOOK1	3.64E-04	0.953275187	0.995209955
SLC25A20	2.65E-04	0.953466321	0.995209955
CCT6A	1.45E-04	0.953507026	0.995209955
MAP2K4	-1.33E-04	0.953528015	0.995209955
NUAK2	-2.07E-04	0.953670721	0.995209955
TNFSF10	-3.01E-04	0.953681585	0.995209955
NFYA	1.88E-04	0.953820103	0.995209955
HPCAL1	-1.92E-04	0.953871772	0.995209955
ZNF280B	2.33E-04	0.953973207	0.995209955
EFCAB11	2.84E-04	0.953979158	0.995209955
MIR574	-2.41E-04	0.954017604	0.995209955
ABI2	-1.66E-04	0.954031334	0.995209955
ATAD5	-2.48E-04	0.954365563	0.995405658
PPP1R14A	-4.59E-04	0.954411306	0.995405658
HOXD11	-2.37E-04	0.954491274	0.995405658
ANKZF1	2.05E-04	0.954578648	0.995405658
MATK	3.20E-04	0.954713501	0.995452501
ZNF592	-2.20E-04	0.955078887	0.995739681
ZNF787	-1.51E-04	0.955512812	0.996077307
PSME3	-1.35E-04	0.955582702	0.996077307

PDCD5	1.60E-04	0.955786061	0.996144428
COX8A	-1.63E-04	0.955827082	0.996144428
C9orf9	1.96E-04	0.956223393	0.996183628
ATF2	-1.96E-04	0.956356475	0.996183628
FBXL19	-3.78E-04	0.95636772	0.996183628
APBB1IP	1.61E-04	0.956399067	0.996183628
POLL	-2.26E-04	0.956468808	0.996183628
RNF19B	2.61E-04	0.956534862	0.996183628
ZNF25	1.29E-04	0.95656363	0.996183628
AMT	2.85E-04	0.956607901	0.996183628
RAB32	2.71E-04	0.956762456	0.996183628
ZNF763	2.40E-04	0.956815428	0.996183628
KISS1R	-5.13E-04	0.956883691	0.996183628
S100A8	-9.36E-05	0.95705307	0.996183628
TRIP13	-3.23E-04	0.957077711	0.996183628
CBL	-1.78E-04	0.957252636	0.996183628
RAD50	-2.25E-04	0.957397794	0.996183628
PLA2G6	-2.22E-04	0.957488862	0.996183628
SRPRB	1.52E-04	0.957500478	0.996183628
TEP1	2.69E-04	0.957662651	0.996183628
TUBBP5	2.63E-04	0.957720132	0.996183628
RRAGA	1.37E-04	0.957773703	0.996183628
EPC2	-2.66E-04	0.957795144	0.996183628
CTNNB1	1.97E-04	0.957857885	0.996183628
FZR1	2.19E-04	0.957934844	0.996183628
MIR142	-3.13E-04	0.958130213	0.996183628
COMMD9	-1.63E-04	0.958153584	0.996183628
NDUFA8	-1.35E-04	0.958306579	0.996183628
WIBG	1.26E-04	0.95853071	0.996183628
LRRC37A2	2.63E-04	0.958546495	0.996183628
CKS2	-2.84E-04	0.958555296	0.996183628
RNASEH2C	2.06E-04	0.958564623	0.996183628
NDUFA5	-2.69E-04	0.958810299	0.996190263
SLC7A9	-2.62E-04	0.959042728	0.996190263
MICALCL	-2.87E-04	0.959138955	0.996190263
RRP15	2.05E-04	0.959229597	0.996190263
ATOH8	-4.02E-04	0.959318208	0.996190263
ALMS1	1.80E-04	0.959329637	0.996190263
SNX6	-1.82E-04	0.959334818	0.996190263
LRRC8C	2.54E-04	0.959424481	0.996190263
SBNO2	2.05E-04	0.959435816	0.996190263
RORA	-2.34E-04	0.95947099	0.996190263
SRI	-4.58E-04	0.95972132	0.996289292
GOLIM4	3.09E-04	0.9597857	0.996289292
TMEM99	-2.12E-04	0.959898171	0.996289292
SNX2	1.70E-04	0.959926398	0.996289292
PRPF39	-2.11E-04	0.960020168	0.996291614

ZNF791	2.16E-04	0.960169153	0.996291614
TRAPPC6A	1.59E-04	0.960413286	0.996291614
C14orf64	-3.50E-04	0.960510938	0.996291614
CLDN15	1.84E-04	0.960720308	0.996291614
TPRKB	2.54E-04	0.960783763	0.996291614
SELO	2.08E-04	0.960818852	0.996291614
MORC3	2.62E-04	0.96084999	0.996291614
POLR2L	-2.44E-04	0.960900327	0.996291614
FGA	1.77E-04	0.960900816	0.996291614
FCHSD2	-2.01E-04	0.9609521	0.996291614
NIF3L1	9.97E-05	0.961008724	0.996291614
ABTB1	-1.66E-04	0.961106146	0.9962993
ZFP82	-2.83E-04	0.961233055	0.996337549
UBXN1	7.85E-05	0.961466863	0.996465723
MRPL13	1.62E-04	0.961660447	0.996465723
ATXN2	-1.15E-04	0.961664874	0.996465723
PDS5B	1.36E-04	0.961818602	0.996465723
SCN5A	-1.72E-04	0.96204735	0.996465723
BAG6	-1.65E-04	0.962361073	0.996465723
PDCD6IP	1.21E-04	0.962401782	0.996465723
ICA1L	-3.01E-04	0.962560944	0.996465723
KCNIP3	2.49E-04	0.962573616	0.996465723
SLC25A26	2.83E-04	0.962588877	0.996465723
MLLT10	-1.09E-04	0.962726949	0.996465723
PTPN2	-1.38E-04	0.96276112	0.996465723
NVL	1.66E-04	0.962912612	0.996465723
CEACAM3	-4.31E-04	0.962920208	0.996465723
ZNF605	1.93E-04	0.962949119	0.996465723
GPANK1	-2.40E-04	0.962972958	0.996465723
LAMC3	-4.64E-04	0.963040213	0.996465723
PREB	1.69E-04	0.963046105	0.996465723
C5orf51	1.38E-04	0.963092925	0.996465723
TGFBR3	3.10E-04	0.963157175	0.996465723
CEP78	2.75E-04	0.963424353	0.996583297
ETAA1	-1.89E-04	0.963450886	0.996583297
PRAM1	-2.06E-04	0.963697218	0.996744955
AKIRIN2	1.12E-04	0.964047183	0.996794861
UGP2	-1.31E-04	0.964082346	0.996794861
LILRB5	1.94E-04	0.96418283	0.996794861
PDE8B	2.85E-04	0.964183957	0.996794861
ANKRD26	1.56E-04	0.964195734	0.996794861
DRD4	3.22E-04	0.96431805	0.996828212
IVNS1ABP	-1.86E-04	0.964461676	0.996883237
SLC39A11	1.67E-04	0.964783036	0.996883237
GPR183	-2.16E-04	0.964895925	0.996883237
IL17D	-1.48E-04	0.964917259	0.996883237
SNAPC5	1.77E-04	0.964972511	0.996883237

RETN	-4.17E-04	0.965033163	0.996883237
MRPL21	-1.61E-04	0.965061693	0.996883237
VARS2	2.34E-04	0.96522617	0.996883237
GOT2	-1.28E-04	0.965478422	0.996883237
LIPT2	-1.72E-04	0.965489733	0.996883237
RRAGD	0.000202996	0.965583068	0.996883237
ZCRB1	-1.61E-04	0.965767988	0.996883237
FAM168B	1.21E-04	0.96580618	0.996883237
WIPI2	-1.46E-04	0.965809954	0.996883237
MPPED2	-2.17E-04	0.965812693	0.996883237
RPS26	5.27E-04	0.965890076	0.996883237
SLC35A4	2.27E-04	0.965902315	0.996883237
SARS2	1.72E-04	0.966246768	0.996956933
N6AMT2	1.49E-04	0.966378933	0.996956933
UBE2D3	1.32E-04	0.966387071	0.996956933
ZDHHC14	2.34E-04	0.966487111	0.996956933
LTK	-2.01E-04	0.966717256	0.996956933
DTX3L	2.92E-04	0.966950517	0.996956933
ME3	2.85E-04	0.967070195	0.996956933
ECD	-1.45E-04	0.967276923	0.996956933
FAM53C	-1.26E-04	0.967387056	0.996956933
KIF14	-2.32E-04	0.967440419	0.996956933
OLFML2B	-1.55E-04	0.96767455	0.996956933
COX7A2L	-9.37E-05	0.967739126	0.996956933
DYRK1B	1.92E-04	0.96784661	0.996956933
SNX3	-1.17E-04	0.967904685	0.996956933
C11orf31	1.98E-04	0.967927485	0.996956933
PLXNA4	2.28E-04	0.967949098	0.996956933
HBG1	-0.001428793	0.967962271	0.996956933
C2orf69	2.36E-04	0.968004099	0.996956933
C5orf24	-1.83E-04	0.968034917	0.996956933
PSAP	6.18E-05	0.968066753	0.996956933
TTC27	-9.88E-05	0.968137556	0.996956933
ZNF397	1.46E-04	0.968248125	0.996956933
LPPR5	-1.10E-04	0.968295455	0.996956933
HGF	1.59E-04	0.968322065	0.996956933
PDK2	1.52E-04	0.9684367	0.996956933
MRPL17	1.29E-04	0.96878371	0.996956933
SORCS3	-2.39E-04	0.968888494	0.996956933
SEC24A	-1.71E-04	0.968926735	0.996956933
SLC23A2	-1.55E-04	0.968934628	0.996956933
GNL3	8.55E-05	0.968964006	0.996956933
POLB	-7.84E-05	0.969034247	0.996956933
STK25	-9.99E-05	0.969200047	0.996956933
NCOR2	-1.27E-04	0.969210386	0.996956933
TRIM41	-9.56E-05	0.969309124	0.996956933
SNORA66	-2.23E-04	0.969400104	0.996956933

CLTA	-1.13E-04	0.969480156	0.996956933
GMPS	-1.06E-04	0.969492075	0.996956933
SYNC	-1.99E-04	0.969525638	0.996956933
MRPL20	1.78E-04	0.969580196	0.996956933
DRAM2	-1.39E-04	0.969664279	0.996956933
SH2B1	-1.31E-04	0.969688006	0.996956933
DAPK1	5.32E-05	0.969826351	0.996956933
TNIP3	3.01E-04	0.969947591	0.996956933
ATP1B3	-1.34E-04	0.96995376	0.996956933
C12orf66	-1.60E-04	0.970043611	0.996956933
PDCD10	1.25E-04	0.970187717	0.996956933
ARIH2	-1.01E-04	0.970206892	0.996956933
SLC7A5	-1.87E-04	0.970370988	0.997019345
RPL13AP3	-6.24E-05	0.970447775	0.997019345
RPS6KL1	-1.48E-04	0.97057572	0.997052987
YIF1A	-1.14E-04	0.971270883	0.997052987
LOXHD1	1.63E-04	0.971406877	0.997052987
HYAL3	1.41E-04	0.971504605	0.997052987
YDJC	1.10E-04	0.97180284	0.997052987
RPS15AP10	8.93E-05	0.971814291	0.997052987
ESYT1	8.73E-05	0.971832488	0.997052987
THOC1	-1.14E-04	0.971867917	0.997052987
C15orf61	-1.50E-04	0.971893472	0.997052987
ZMAT5	-1.82E-04	0.97193137	0.997052987
MPP6	1.90E-04	0.971958168	0.997052987
MT1H	2.43E-04	0.972104109	0.997052987
ACTR1A	-9.02E-05	0.972123471	0.997052987
B3GNTL1	1.52E-04	0.972147355	0.997052987
SULT1A2	1.71E-04	0.972164208	0.997052987
BAG2	-1.66E-04	0.972165253	0.997052987
SRP14	-7.61E-05	0.972186642	0.997052987
ENTPD5	1.58E-04	0.972195886	0.997052987
IER3IP1	1.62E-04	0.972266337	0.997052987
CRYBA2	-1.79E-04	0.97230863	0.997052987
SCYL3	8.29E-05	0.972476808	0.997052987
C17orf62	-1.08E-04	0.972511071	0.997052987
NUP43	-1.03E-04	0.972693691	0.997052987
DNAJB6	7.52E-05	0.973021752	0.997052987
LILRB1	-8.42E-05	0.973197025	0.997052987
TMEM42	8.45E-05	0.973226732	0.997052987
CDA	-2.28E-04	0.973384327	0.997052987
ACBD4	1.34E-04	0.973393966	0.997052987
GPR160	-2.30E-04	0.973437756	0.997052987
AKR1D1	5.42E-05	0.973513607	0.997052987
GRAMD4	1.42E-04	0.973570756	0.997052987
HTRA4	-1.75E-04	0.973644322	0.997052987
BMP2K	1.51E-04	0.97365451	0.997052987

FNIP1	-9.67E-05	0.97371208	0.997052987
RECQL5	-1.52E-04	0.97372275	0.997052987
GFOD1	-2.21E-04	0.973865079	0.997052987
MBP	1.04E-04	0.973888242	0.997052987
SH3YL1	2.25E-04	0.974062271	0.997052987
CLSPN	1.30E-04	0.974106614	0.997052987
OTOGL	1.24E-04	0.974145471	0.997052987
STMN1	1.68E-04	0.974272331	0.997052987
LSR	1.42E-04	0.974273832	0.997052987
GIT1	1.48E-04	0.974353795	0.997052987
RNF8	-1.30E-04	0.974630617	0.997113291
NAF1	-1.15E-04	0.974636709	0.997113291
TRPT1	-7.49E-05	0.974700674	0.997113291
PJA2	-1.11E-04	0.974787432	0.997113291
ZNF563	1.61E-04	0.974863135	0.997113291
FEM1B	1.54E-04	0.975062977	0.997172679
TMEM55A	-1.67E-04	0.975101371	0.997172679
RILPL1	-1.34E-04	0.975342938	0.997286072
SNORA5B	-1.34E-04	0.975392449	0.997286072
DHX58	1.50E-04	0.975546732	0.997351693
RALGAPA1	9.68E-05	0.975796384	0.997431173
RNF220	-6.31E-05	0.975898157	0.997431173
ING5	1.89E-04	0.976082927	0.997431173
ARHGEF12	1.83E-04	0.976099178	0.997431173
BRI3BP	-1.07E-04	0.976143982	0.997431173
ACBD6	-9.86E-05	0.976296635	0.997431173
S1PR5	2.37E-04	0.976408622	0.997431173
PIP4K2A	7.62E-05	0.976529925	0.997431173
LLGL2	1.40E-04	0.976713981	0.997431173
CISD2	8.79E-05	0.976829263	0.997431173
CLIP2	-1.28E-04	0.976956755	0.997431173
PSMD8	-9.70E-05	0.977024714	0.997431173
GDI2	6.83E-05	0.977158493	0.997431173
ST14	-2.13E-04	0.977223688	0.997431173
SLC11A2	-1.14E-04	0.977241046	0.997431173
FAM86B1	-1.62E-04	0.977291356	0.997431173
NUDC	6.59E-05	0.977320728	0.997431173
TSEN34	-7.26E-05	0.977334447	0.997431173
TMEM51	-3.27E-04	0.977347953	0.997431173
TEX10	6.64E-05	0.977426681	0.997431173
LAPTM4A	9.94E-05	0.97771045	0.997516153
ADAM17	1.08E-04	0.97776866	0.997516153
CALCOCO1	8.89E-05	0.978159111	0.997516153
SLC3A2	7.88E-05	0.978204524	0.997516153
SNRK	5.83E-05	0.978254805	0.997516153
AGPAT3	-1.28E-04	0.97827489	0.997516153
MRPL47	-1.02E-04	0.978320035	0.997516153

AKAP10	1.30E-04	0.978335822	0.997516153
ARHGAP10	1.15E-04	0.978342401	0.997516153
BNIP2	8.00E-05	0.978411137	0.997516153
TFDP1	8.81E-05	0.978559381	0.997575408
CHRNA2	1.12E-04	0.978891273	0.997695151
GPSM2	-1.03E-04	0.978983869	0.997695151
NCOA3	-9.88E-05	0.97898475	0.997695151
TEX9	-1.49E-04	0.97917721	0.997695151
PTGER4	8.59E-05	0.979360751	0.997695151
C4orf27	-1.05E-04	0.979412202	0.997695151
KCNMA1	1.76E-04	0.979462363	0.997695151
EDEM3	-8.07E-05	0.979475115	0.997695151
C2	1.61E-04	0.97954773	0.997695151
L3MBTL4	-1.27E-04	0.979665527	0.997695151
RPL35A	4.55E-05	0.979719334	0.997695151
DIP2C	1.17E-04	0.979857969	0.997695151
ZNF37A	1.14E-04	0.979912129	0.997695151
TXNDC5	2.08E-04	0.980088432	0.997695151
TRIM26	-1.03E-04	0.980217905	0.997695151
PAX7	1.16E-04	0.980399934	0.997695151
NPTN	5.60E-05	0.980408234	0.997695151
IFT122	-8.77E-05	0.980528027	0.997695151
FCGR2B	-1.74E-04	0.980538146	0.997695151
SLC35D2	7.75E-05	0.98074481	0.997695151
HMOX1	1.25E-04	0.980936236	0.997695151
SLC2A1	8.62E-05	0.981135366	0.997695151
ZBED3	-9.11E-05	0.981193959	0.997695151
ZNF626	-1.81E-04	0.981229681	0.997695151
EXTL3	9.70E-05	0.981281282	0.997695151
ALG12	7.60E-05	0.981283609	0.997695151
ZNF500	8.86E-05	0.981284573	0.997695151
CCDC15	9.08E-05	0.981328406	0.997695151
ZNF514	1.84E-04	0.981466093	0.997695151
TCEA3	1.69E-04	0.981497016	0.997695151
DYNC2H1	-7.08E-05	0.981515418	0.997695151
ISCU	-5.67E-05	0.981711931	0.997695151
SIGMAR1	6.75E-05	0.981803504	0.997695151
BRCA1	-1.05E-04	0.98183423	0.997695151
GNG2	-1.04E-04	0.981891664	0.997695151
CAMKK2	-2.58E-04	0.98192167	0.997695151
RAB6A	-9.62E-05	0.98201911	0.997702573
COX6A1	-5.84E-05	0.982266202	0.997845868
CAPN1	6.56E-05	0.982359685	0.997845868
PPDPF	-1.38E-04	0.982430597	0.997845868
LRRC14	8.82E-05	0.9825228	0.997847956
SLAMF6	8.34E-05	0.982884454	0.998009371
CDC123	-6.36E-05	0.982927733	0.998009371

PDCD6	-4.38E-05	0.982978943	0.998009371
DDX39A	4.56E-05	0.983042386	0.998009371
HES6	-1.65E-04	0.983176717	0.998054208
CDK14	1.33E-04	0.983474596	0.998148525
PLCH2	-1.64E-04	0.983533832	0.998148525
ANPEP	-2.68E-04	0.98372161	0.998148525
STX5	-4.59E-05	0.983747254	0.998148525
SLC22A17	1.33E-04	0.98376887	0.998148525
ACSS1	9.08E-05	0.983836279	0.998148525
KIAA0232	7.53E-05	0.983900854	0.998148525
TGFBR1	-8.62E-05	0.984065723	0.99818182
NAP1L4	7.11E-05	0.984114031	0.99818182
RRM2	-1.14E-04	0.984245441	0.998205559
EIF4E3	1.21E-04	0.98451494	0.998205559
ACADSB	6.43E-05	0.984576012	0.998205559
CLCN3	-7.54E-05	0.984578464	0.998205559
NME4	1.58E-04	0.984588337	0.998205559
AKAP1	6.72E-05	0.984803023	0.998331775
PPP1R12C	-7.61E-05	0.984894417	0.998332995
DZIP3	-7.58E-05	0.985020202	0.9983347
KRTCAP3	-9.93E-05	0.985249117	0.9983347
ATP6V1D	-4.29E-05	0.985306452	0.9983347
SLCO3A1	5.72E-05	0.98535835	0.9983347
PHC3	6.75E-05	0.985448442	0.9983347
TMEM67	7.09E-05	0.985483838	0.9983347
FGFR1OP2	-6.47E-05	0.985611727	0.9983347
RLTPR	-8.15E-05	0.985698793	0.9983347
KPTN	-8.01E-05	0.985726921	0.9983347
CASP6	9.32E-05	0.985798019	0.9983347
ZMYM5	8.85E-05	0.985952459	0.998381581
PPP4R2	1.24E-04	0.986062738	0.998381581
URB1	6.02E-05	0.986291419	0.998381581
C17orf53	6.92E-05	0.98661638	0.998381581
MEGF6	9.26E-05	0.986760456	0.998381581
MEX3C	-6.84E-05	0.986874686	0.998381581
RALGAPB	4.38E-05	0.986886364	0.998381581
RASA4	-9.15E-05	0.986982701	0.998381581
ZMYND19	4.29E-05	0.98702118	0.998381581
KIF21A	-9.44E-05	0.98703489	0.998381581
LEPROT	-8.80E-05	0.987132484	0.998381581
SF3B3	-5.77E-05	0.98717029	0.998381581
RELB	-5.91E-05	0.987314188	0.998381581
ZNF26	5.32E-05	0.987377675	0.998381581
PARP9	-9.86E-05	0.987432601	0.998381581
CDAN1	-3.66E-05	0.987475875	0.998381581
C15orf39	-6.23E-05	0.987584637	0.998381581
IFI30	-6.87E-05	0.987589636	0.998381581

CUX2	-1.10E-04	0.987647317	0.998381581
CENPL	5.93E-05	0.987648235	0.998381581
NDUFB6	-6.10E-05	0.987886298	0.998430719
RPL28	5.20E-05	0.987939944	0.998430719
TMEM14B	-5.84E-05	0.987967446	0.998430719
TMUB2	4.52E-05	0.98819036	0.998564825
CCDC18	-6.44E-05	0.988381397	0.998666698
TPRG1L	3.95E-05	0.988918767	0.999020201
RFWD2	2.41E-05	0.988943502	0.999020201
TP53	-5.41E-05	0.989078063	0.999020201
TMEM25	-1.00E-04	0.989092274	0.999020201
MED27	6.34E-05	0.989183854	0.99902154
C1QA	6.51E-05	0.989276329	0.999023783
UBE2L6	5.04E-05	0.989589166	0.999248538
XPOT	3.33E-05	0.989693304	0.999250327
TMEM114	6.12E-05	0.989771486	0.999250327
SGMS1	-5.36E-05	0.990083428	0.999474096
PTCH1	-6.32E-05	0.990205853	0.999506528
PEX6	-1.10E-04	0.990508702	0.999665147
MAP3K7	-2.53E-05	0.990586361	0.999665147
CD244	5.94E-05	0.990690135	0.999665147
CCDC144B	1.08E-04	0.990724244	0.999665147
AVIL	-4.64E-05	0.990817334	0.999667949
FAM21C	1.31E-04	0.991311158	0.999915263
ZNHIT2	3.72E-05	0.991521224	0.999915263
GTF2H3	4.34E-05	0.991589275	0.999915263
RPL35	2.49E-05	0.991672555	0.999915263
FAM91A1	-4.52E-05	0.991675954	0.999915263
ELOVL6	-8.39E-05	0.991835355	0.999915263
RAB24	2.86E-05	0.991883265	0.999915263
TCEB2	2.36E-05	0.991899013	0.999915263
SNPH	-4.93E-05	0.992075667	0.999915263
DYTN	5.84E-05	0.992418535	0.999915263
PSME2	-3.34E-05	0.992449093	0.999915263
PNKD	6.03E-05	0.992568721	0.999915263
EPRS	-1.99E-05	0.992596551	0.999915263
FLYWCH1	5.48E-05	0.992793503	0.999915263
ZG16B	-5.75E-05	0.992810015	0.999915263
RPL19	-9.54E-06	0.992973949	0.999915263
TSR1	2.34E-05	0.993164092	0.999915263
THAP2	3.63E-05	0.99319804	0.999915263
NCF4	-3.12E-05	0.993371907	0.999915263
PTPN11	-1.49E-05	0.993542856	0.999915263
WDR24	2.95E-05	0.993565864	0.999915263
ZNF451	-2.33E-05	0.993596743	0.999915263
SRP68	-1.92E-05	0.993657726	0.999915263
KARS	-1.84E-05	0.993969955	0.999915263

FGFBP2	-7.29E-05	0.994020484	0.999915263
FSD1	-6.04E-05	0.994208129	0.999915263
PCGF6	-2.08E-05	0.994224445	0.999915263
CBWD1	3.92E-05	0.994265902	0.999915263
GPD1L	1.74E-05	0.994305414	0.999915263
ACSL6	3.26E-05	0.994337686	0.999915263
PES1	2.73E-05	0.994441696	0.999915263
CECR6	5.58E-05	0.994487751	0.999915263
HMG20A	2.40E-05	0.994772105	0.999915263
CYB5D2	-2.16E-05	0.994785084	0.999915263
ZNF831	3.29E-05	0.994787706	0.999915263
ZNF16	3.24E-05	0.994789082	0.999915263
LETMD1	1.56E-05	0.994842018	0.999915263
STX18	2.38E-05	0.995090837	0.999915263
MRPL28	-1.83E-05	0.995361977	0.999915263
SLC24A4	-3.25E-05	0.995468768	0.999915263
MRPL36	-1.81E-05	0.995493243	0.999915263
NHLRC2	-2.73E-05	0.995568104	0.999915263
TRIM56	-2.04E-05	0.995701671	0.999915263
CIITA	-2.71E-05	0.995718963	0.999915263
TRIM25	-2.08E-05	0.995795517	0.999915263
M6PR	1.71E-05	0.996059823	0.999915263
NUDT8	2.61E-05	0.996085504	0.999915263
MEMO1	1.36E-05	0.996137432	0.999915263
LANCL1	1.07E-05	0.996276493	0.999915263
PLIN3	-1.45E-05	0.996297671	0.999915263
RNF5	1.75E-05	0.996369203	0.999915263
BYSL	-2.57E-05	0.996414044	0.999915263
RPS5	-1.30E-05	0.996456896	0.999915263
STIM1	-1.33E-05	0.99646158	0.999915263
EIF6	-1.43E-05	0.996511251	0.999915263
CETP	3.89E-05	0.996674874	0.999915263
TLE2	-2.14E-05	0.996691484	0.999915263
RNF167	1.45E-05	0.996705326	0.999915263
RNF6	-1.92E-05	0.996714785	0.999915263
EIF4A2	7.07E-06	0.996872944	0.999915263
HSP90AA1	7.85E-06	0.997106531	0.999915263
KDELC2	-1.52E-05	0.997144984	0.999915263
WBSCR16	-2.18E-05	0.997244711	0.999915263
ZNF502	-2.50E-05	0.997508776	0.999915263
TMEM183A	5.54E-06	0.997557602	0.999915263
PDGFD	-2.11E-05	0.997855484	0.999915263
KIF19	-2.12E-05	0.997903113	0.999915263
FBXO33	-6.96E-06	0.99795796	0.999915263
TMEM205	-6.94E-06	0.998180996	0.999915263
ABHD2	8.93E-06	0.998231195	0.999915263
KRIT1	-9.45E-06	0.998309587	0.999915263

RORC	-2.31E-05	0.998347769	0.999915263
LSM1	4.18E-06	0.998352686	0.999915263
SHISA3	-8.69E-06	0.998389653	0.999915263
GNPDA2	6.62E-06	0.998421622	0.999915263
ACTR5	5.97E-06	0.998465873	0.999915263
PTCD2	9.50E-06	0.99848575	0.999915263
GEN1	6.34E-06	0.998494758	0.999915263
ANXA8	-9.80E-06	0.998576381	0.999915263
ATAD3B	-5.93E-06	0.998652367	0.999915263
MATR3	-3.01E-06	0.99878738	0.999915263
SPAG1	-6.39E-06	0.998899639	0.999915263
GPATCH4	-4.54E-06	0.998901531	0.999915263
IL11RA	-5.91E-06	0.999055975	0.999915263
PLGLB1	1.29E-05	0.999061602	0.999915263
TTC38	7.10E-06	0.999277812	0.999915263
PM20D1	-4.79E-06	0.999310493	0.999915263
MSH3	1.26E-06	0.999316828	0.999915263
GPRC5C	-7.68E-06	0.999317862	0.999915263
DNAJC8	-1.85E-06	0.999342682	0.999915263
ZCCHC6	2.90E-06	0.999460624	0.999915263
BTG2	-2.97E-06	0.999611997	0.999915263
NOS3	1.92E-06	0.999633268	0.999915263
RRM2B	2.89E-06	0.99963925	0.999915263
DBF4	-1.01E-06	0.999807983	0.999915263
CDK5R1	1.89E-06	0.999813283	0.999915263
PSMD9	9.49E-07	0.999824928	0.999915263
PRDM1	-2.00E-07	0.999974015	0.999974015

Supplemental Table 2.4: Association between annual mean ozone and gene expression in NoMa

name	logFC	P.Value	adj.P.Val
SETD2	-0.011342625	4.71E-04	0.993552802
MCEE	0.015642436	6.68E-04	0.993552802
SLC33A1	0.01700127	8.22E-04	0.993552802
C5orf54	0.025440243	9.93E-04	0.993552802
TEKT4	0.022980857	0.001011312	0.993552802
NFXL1	0.029519985	0.001014828	0.993552802
TBC1D16	0.021331373	0.001158637	0.993552802
OXCT2	0.029171532	0.001413692	0.993552802
CUL7	0.023263511	0.001479976	0.993552802
GGTLC1	0.022114132	0.001521717	0.993552802
GDAP2	0.018718234	0.001564319	0.993552802
RASSF4	0.02015215	0.001564462	0.993552802
GUF1	0.018648498	0.001587756	0.993552802
DTNA	0.022302349	0.00170921	0.993552802
ZC3HC1	0.011106797	0.001854118	0.993552802
DNAJB11	0.013421888	0.001890876	0.993552802
RTKN2	-0.042449263	0.00198073	0.993552802
ARPC4	-0.015788427	0.002121008	0.993552802
ARID5B	-0.019305635	0.002273327	0.993552802
AGBL5	-0.023493737	0.002497575	0.993552802
IGF2BP3	-0.043306092	0.002599774	0.993552802
SUCLA2	0.017642191	0.002774657	0.993552802
MYO1C	-0.019804023	0.002797484	0.993552802
MANEA	0.029538627	0.003361767	0.993552802
WDR12	0.013238505	0.003397187	0.993552802
COPS7A	0.009113132	0.003450733	0.993552802
C19orf24	-0.020890567	0.003584899	0.993552802
IFFO1	0.012406036	0.00363467	0.993552802
RUFY1	-0.04125837	0.003814168	0.993552802
ATP5E	0.009278122	0.003832802	0.993552802
ZDHHC7	0.01337494	0.004296608	0.993552802
TIMM44	-0.014432383	0.004439207	0.993552802
PQLC3	0.014254252	0.00448637	0.993552802
NDN	0.036247635	0.00484659	0.993552802
BTN2A1	0.013097643	0.004918375	0.993552802
MGAT4B	-0.016933729	0.005062779	0.993552802
ZCCHC7	-0.0130098	0.005174142	0.993552802
ZNF823	0.019929871	0.00518991	0.993552802
NCS1	-0.016479278	0.005333453	0.993552802
AKAP8L	-0.020201386	0.005762831	0.993552802
RHOBTB1	-0.042051127	0.005794703	0.993552802
TCL6	-0.017592596	0.006103403	0.993552802
RBM7	0.030585218	0.00618646	0.993552802
HINFP	0.014856521	0.006328774	0.993552802

HPGD	-0.036022976	0.006392401	0.993552802
ZHX2	-0.040191039	0.006489784	0.993552802
IL1B	0.047795563	0.006639897	0.993552802
PAAF1	0.012865691	0.006700688	0.993552802
ZSCAN2	0.013670092	0.006718369	0.993552802
ACOT11	0.018927576	0.006817198	0.993552802
CD40	-0.024964018	0.007038662	0.993552802
HIVEP2	-0.019754629	0.007106195	0.993552802
C14orf79	0.014430538	0.007211922	0.993552802
BEST1	0.02406567	0.007256722	0.993552802
RERE	-0.017773901	0.007330151	0.993552802
CTSD	-0.020355744	0.007446399	0.993552802
ITGAV	0.017948587	0.007516921	0.993552802
MPHOSPH9	0.015896164	0.00792028	0.993552802
GFRA2	0.03871804	0.007962825	0.993552802
SNRPD1	0.018415813	0.007977833	0.993552802
TNNC2	-0.050294806	0.00801448	0.993552802
SAV1	-0.024222625	0.008087686	0.993552802
ZNF689	0.012828119	0.008149502	0.993552802
LYPLA2	-0.016835526	0.008170589	0.993552802
BCAT1	0.042642074	0.00821423	0.993552802
IL4R	-0.019826863	0.008285426	0.993552802
SMG5	0.012356498	0.008401246	0.993552802
LRRC37BP1	0.017857075	0.008519032	0.993552802
MTERF	0.016480457	0.008861344	0.993552802
SLC35A5	0.016045057	0.008898418	0.993552802
KCNMB1	0.026095065	0.009032767	0.993552802
CD8A	0.0372505	0.009174951	0.993552802
CTSH	0.017068849	0.009350806	0.993552802
B4GALT3	0.011763974	0.009459725	0.993552802
MAF1	-0.014880991	0.009616868	0.993552802
SLC50A1	-0.01469438	0.009709922	0.993552802
STIP1	-0.017773287	0.009800922	0.993552802
SLC9A9	-0.013058292	0.009807856	0.993552802
MFN2	-0.015114914	0.009842672	0.993552802
TMEM219	-0.012255964	0.009850308	0.993552802
GPR162	0.017295796	0.009894722	0.993552802
ALKBH3	0.011168198	0.010038655	0.993552802
SP1	-0.013985881	0.010150777	0.993552802
OTUD6B	0.012561494	0.010278773	0.993552802
CRLS1	0.010141526	0.010309262	0.993552802
ZHX1	0.009829627	0.010319972	0.993552802
ZNF614	0.023782943	0.010364684	0.993552802
ADCY6	-0.015223629	0.010474221	0.993552802
TNFRSF10B	0.012231318	0.010623338	0.993552802
C19orf47	-0.016328327	0.010802041	0.993552802
TMEM64	-0.018553311	0.011231006	0.993552802

TDG	0.008864033	0.011398545	0.993552802
DVL2	-0.010868369	0.011637077	0.993552802
CACNA2D4	0.028819494	0.01167295	0.993552802
NR1H2	-0.013980438	0.011884027	0.993552802
TRPM2	0.01759339	0.01188413	0.993552802
PPP1R7	-0.010338774	0.011898382	0.993552802
MTCH1	0.008081051	0.012251298	0.993552802
UCA1	-0.015290726	0.012317071	0.993552802
CD34	0.028838366	0.012436442	0.993552802
AMDHD1	0.028281071	0.012534161	0.993552802
DHX15	0.007122948	0.012679524	0.993552802
ENDOD1	-0.029524534	0.012845204	0.993552802
CSGALNACT2	0.013700678	0.012951313	0.993552802
YPEL3	-0.011363067	0.013056489	0.993552802
GXYLT1	0.012372531	0.013061573	0.993552802
MYO9A	-0.016381024	0.013132082	0.993552802
PIM3	-0.019073413	0.013140989	0.993552802
ARMC7	0.014913286	0.013175541	0.993552802
LGALS8	0.012219874	0.013198662	0.993552802
CLEC4F	0.068575899	0.013224048	0.993552802
DHRS7B	0.011938401	0.013277616	0.993552802
FLT3	0.027403156	0.013326641	0.993552802
GMPR	-0.035837332	0.013328049	0.993552802
RIN3	-0.016308659	0.013408205	0.993552802
DBP	-0.014549709	0.013570257	0.993552802
SDSL	0.026319403	0.013763903	0.993552802
DIAPH1	-0.011101149	0.013897867	0.993552802
FAR2	0.020091797	0.014065527	0.993552802
LRRC20	0.017193692	0.014120293	0.993552802
ACOX2	0.02488181	0.014183424	0.993552802
TRNT1	0.018894205	0.014204479	0.993552802
EIF4A3	0.008958556	0.014408396	0.993552802
RAB30	-0.021560966	0.014480811	0.993552802
MCAT	0.015451372	0.014852451	0.993552802
TMC8	-0.020230169	0.014880121	0.993552802
EPC1	-0.019666341	0.014884568	0.993552802
COG4	-0.011834918	0.014965283	0.993552802
FABP5	0.023164133	0.015027965	0.993552802
SLC35E3	0.010308924	0.015073074	0.993552802
SOCS3	-0.025605893	0.015405477	0.993552802
FBN2	0.033708803	0.015625544	0.993552802
CCR5	0.021243505	0.015690095	0.993552802
TDRKH	-0.022934997	0.015695722	0.993552802
SLC35D3	-0.043160187	0.015832675	0.993552802
ADIPOR2	-0.00767528	0.015841994	0.993552802
MEIS3	-0.029920178	0.015932385	0.993552802
LEO1	0.013062869	0.01596918	0.993552802

SNRPA	-0.010365642	0.016026461	0.993552802
MIR671	0.014510131	0.016061673	0.993552802
NDEL1	0.010476413	0.016102393	0.993552802
SLC16A6	0.021537786	0.016151603	0.993552802
TSPAN33	-0.038804291	0.016409715	0.993552802
ZNF77	0.017747807	0.016545229	0.993552802
FMO4	0.017592165	0.016713276	0.993552802
CENPA	0.019303171	0.016726825	0.993552802
CNN2	-0.015889357	0.016728994	0.993552802
TRIM3	0.014293597	0.016744613	0.993552802
GRK6	-0.015045579	0.016797665	0.993552802
FGFR1OP	0.014608105	0.016859377	0.993552802
SMARCD2	-0.019194351	0.016914197	0.993552802
DUSP10	0.018187188	0.017066903	0.993552802
CTSA	-0.021965659	0.017304163	0.993552802
GPR156	0.016507228	0.017318219	0.993552802
BRD4	-0.017694111	0.017355662	0.993552802
ACCS	0.037552944	0.017535773	0.993552802
RNF10	-0.018271552	0.017738621	0.993552802
RASL11A	-0.016746838	0.017815997	0.993552802
CES1	0.061311801	0.017847253	0.993552802
TMEM150C	-0.018093755	0.017984352	0.993552802
DNAJC17	0.013554526	0.018018015	0.993552802
CXCL9	0.021342178	0.018092158	0.993552802
MEI1	0.013987321	0.01822952	0.993552802
CCDC121	0.016480737	0.018688889	0.993552802
NANP	0.026564129	0.018703709	0.993552802
NETO2	0.019966338	0.018717505	0.993552802
LSM14A	-0.008552543	0.018898088	0.993552802
HLA-DPB1	0.032473112	0.01901442	0.993552802
GUCY1B3	-0.033080696	0.019034429	0.993552802
MTRR	0.019330109	0.019126041	0.993552802
RPE	0.014728929	0.019222939	0.993552802
C11orf95	0.012243567	0.019473158	0.993552802
AIDA	-0.007923787	0.019914395	0.993552802
FXYD1	-0.016294156	0.020044736	0.993552802
TTF2	0.01268124	0.020129232	0.993552802
DHX29	0.007385721	0.02048107	0.993552802
RBM27	-0.009391633	0.020554903	0.993552802
LIPT1	0.012695702	0.020592935	0.993552802
CCDC136	0.014244906	0.020644539	0.993552802
SEC61A1	0.010118473	0.020886047	0.993552802
MARK2	-0.017286339	0.020955931	0.993552802
VAMP2	-0.016915224	0.021005738	0.993552802
NAA20	0.007521044	0.02106791	0.993552802
STX10	-0.0111215	0.021107653	0.993552802
HOXA10	0.027612252	0.02117865	0.993552802

PFN2	0.025428235	0.021187046	0.993552802
HOXA5	0.022212746	0.021207451	0.993552802
ITPKB	-0.012318716	0.021444583	0.993552802
INSIG2	0.010274601	0.021458946	0.993552802
ZKSCAN4	0.016111309	0.021538387	0.993552802
THUMPD3	0.016291338	0.021615415	0.993552802
NOP2	0.010996635	0.021632137	0.993552802
SNRPE	-0.015232051	0.021673421	0.993552802
MUS81	0.010623378	0.021706661	0.993552802
CCDC132	0.009871545	0.022122444	0.993552802
ST3GAL6	0.020805442	0.022165441	0.993552802
DAB2	-0.026480127	0.022247946	0.993552802
EIF3E	0.006823311	0.022248489	0.993552802
C10orf88	0.014544567	0.022325798	0.993552802
SEMA6B	-0.017363363	0.022376136	0.993552802
LRBA	-0.012061854	0.022390056	0.993552802
AP3D1	0.011166819	0.022497993	0.993552802
STAT3	-0.010155451	0.022538931	0.993552802
NSMAF	0.008523714	0.022555274	0.993552802
CTNNAL1	-0.026226369	0.02256112	0.993552802
GP1BA	-0.030526265	0.022589437	0.993552802
ENTPD4	-0.023292699	0.022935912	0.993552802
ZNF484	0.014504853	0.02306832	0.993552802
PKIB	0.019134113	0.023072147	0.993552802
TSTD2	0.012137266	0.023076842	0.993552802
SKA2	-0.01055665	0.023196189	0.993552802
NCBP1	0.009530594	0.023431973	0.993552802
ASAP1	-0.013223425	0.023458701	0.993552802
UBXN2B	0.015303154	0.023499797	0.993552802
ARID1B	-0.014026341	0.023524195	0.993552802
BCL7A	-0.015510899	0.023542158	0.993552802
CTTN	-0.041816512	0.023625979	0.993552802
PIK3C2B	-0.022492733	0.023743457	0.993552802
SIRT4	0.015336204	0.023748993	0.993552802
SRP72	0.009587053	0.023786005	0.993552802
CAMK1D	-0.014146564	0.024031431	0.993552802
STK32C	0.022101025	0.024261659	0.993552802
SFMBT1	-0.012831063	0.024386128	0.993552802
TMEM40	-0.031653048	0.024523423	0.993552802
CHMP2A	0.011701804	0.024584602	0.993552802
TSPAN13	-0.029699098	0.024611175	0.993552802
MMACHC	0.015894622	0.024901032	0.993552802
HSP90AB3P	-0.013877525	0.025223654	0.993552802
ALOX12	-0.048656011	0.025751776	0.993552802
NUFIP1	0.01609198	0.025844877	0.993552802
LSM12	-0.010272196	0.026096259	0.993552802
TFAM	0.011069033	0.026300813	0.993552802

TRAF1	0.013896042	0.02641575	0.993552802
PRC1	0.016555315	0.026524597	0.993552802
TUBG1	0.013856792	0.026590949	0.993552802
CD300C	0.031753118	0.026615017	0.993552802
BIN2	-0.011880844	0.026694445	0.993552802
ABHD13	-0.01418421	0.026734029	0.993552802
RAB18	0.015749868	0.026748265	0.993552802
GAS8	0.015185746	0.026750953	0.993552802
CRABP2	-0.015876643	0.026809954	0.993552802
FASTKD3	0.013981538	0.026835898	0.993552802
NAGK	0.013968266	0.027035047	0.993552802
THEMIS	0.020102612	0.027059051	0.993552802
TMEM134	-0.010279134	0.027102792	0.993552802
CLC	-0.029490888	0.027133702	0.993552802
SNX11	0.011128534	0.027381523	0.993552802
ASB8	0.010030411	0.027399256	0.993552802
CCRL2	0.021865918	0.027407955	0.993552802
MTMR11	0.015201647	0.027527041	0.993552802
DENND2C	-0.021827797	0.027538552	0.993552802
ATPAF2	0.013686844	0.027774591	0.993552802
MPO	0.021273873	0.02784632	0.993552802
DDX52	0.009658984	0.027986923	0.993552802
PFAS	0.009166057	0.028137281	0.993552802
RRP8	0.010673482	0.028240554	0.993552802
C16orf80	0.011031028	0.028250937	0.993552802
ACOX1	0.017018794	0.028434951	0.993552802
MAPRE2	-0.010775191	0.028492263	0.993552802
PCGF1	0.0150732	0.028502055	0.993552802
SLA2	-0.019710162	0.028867734	0.993552802
MDN1	-0.013412271	0.028886371	0.993552802
SNRNP40	-0.008500214	0.029147496	0.993552802
HOXD3	-0.014620964	0.029338186	0.993552802
ABCA9	0.012625304	0.029346371	0.993552802
PLA2G4A	0.020381585	0.029481668	0.993552802
TMEM39A	0.016609121	0.029541136	0.993552802
TCP11L2	-0.032849538	0.029670009	0.993552802
MAPKAPK2	-0.011163897	0.029750093	0.993552802
LRRC1	0.015909718	0.029765873	0.993552802
MTMR9	0.008684267	0.029883218	0.993552802
EHHADH	0.017217589	0.029979546	0.993552802
ALG11	0.0119465	0.030123366	0.993552802
SGK1	0.01955602	0.030403315	0.993552802
RSG1	0.014762582	0.030568086	0.993552802
CHMP7	-0.01664391	0.030588437	0.993552802
CRNKL1	0.017602439	0.030659956	0.993552802
FGD3	0.007185762	0.030665509	0.993552802
CLK1	0.020942214	0.03068878	0.993552802

DNAJB1	-0.011044583	0.030698024	0.993552802
AUH	0.014395047	0.03105601	0.993552802
ERICH1	0.01416893	0.031056432	0.993552802
MTCH2	0.011885786	0.031129115	0.993552802
ZNF805	0.012883674	0.0311527	0.993552802
ASAP2	-0.030624772	0.031317421	0.993552802
MTA2	-0.012255918	0.031325293	0.993552802
RBM38	-0.015511881	0.031401974	0.993552802
CHN1	0.030812808	0.031439641	0.993552802
LEAP2	0.013961865	0.031472567	0.993552802
EGR2	0.0252134	0.031581859	0.993552802
RAB27B	-0.028113284	0.031821033	0.993552802
CLTCL1	-0.014723124	0.031996749	0.993552802
UBE2Q1	0.011522653	0.0323757	0.993552802
CHD2	-0.011826158	0.032390068	0.993552802
MIR155HG	0.021102881	0.032402677	0.993552802
MTIF3	0.00939036	0.032556263	0.993552802
FBXL20	0.016875088	0.032722983	0.993552802
BTBD10	0.012166864	0.032845421	0.993552802
RNMT	-0.011864397	0.032927283	0.993552802
SRR	0.019377866	0.033045556	0.993552802
SP140	-0.015343407	0.033274645	0.993552802
MFHAS1	-0.03043476	0.033327864	0.993552802
FADD	0.015700653	0.033396626	0.993552802
TRAPPC2L	0.010882918	0.033461967	0.993552802
SBF1P1	0.014800144	0.033645754	0.993552802
FAM13A	-0.020017371	0.033647436	0.993552802
MPRIP	-0.011062078	0.033728705	0.993552802
RIMS4	-0.013985404	0.033796814	0.993552802
CDKN2A	0.014547397	0.0338741	0.993552802
NCK2	-0.015315169	0.033881548	0.993552802
SEC24D	0.013469322	0.033958659	0.993552802
TSHZ1	-0.011753252	0.033995738	0.993552802
HSPB3	-0.021365424	0.034101469	0.993552802
COPG2	-0.022746589	0.034218706	0.993552802
SMPDL3A	0.025902993	0.034258581	0.993552802
B4GALT1	-0.012489244	0.034273435	0.993552802
SYCE1	-0.016134598	0.03438542	0.993552802
ZNF784	0.012359145	0.034480569	0.993552802
SNORA5C	-0.012660106	0.034503469	0.993552802
ELOVL7	-0.034042465	0.03452862	0.993552802
CIB1	0.010665015	0.034537182	0.993552802
PDXK	0.013624415	0.034584888	0.993552802
AP2B1	-0.012042041	0.034587204	0.993552802
RIOK1	0.007198742	0.034608532	0.993552802
CPOX	0.010223731	0.034643685	0.993552802
PPAPDC2	0.020261953	0.034738691	0.993552802

TREML1	-0.042494885	0.034921614	0.993552802
TOMM34	0.015210667	0.035088344	0.993552802
H1FX	-0.0131011	0.03513855	0.993552802
HDGFRP2	-0.014284375	0.035257158	0.993552802
DNM3	-0.036989538	0.035430992	0.993552802
NAT1	0.019414448	0.035699553	0.993552802
TRIT1	0.014165961	0.035905621	0.993552802
PLCL2	-0.007293166	0.036065256	0.993552802
ARV1	0.011438868	0.036083555	0.993552802
PIK3R4	0.011228609	0.036132472	0.993552802
ZNF746	0.007741688	0.036179255	0.993552802
B4GALNT4	0.014965453	0.036275864	0.993552802
CDRT4	0.012924849	0.036325874	0.993552802
BACE1	0.018220913	0.036399135	0.993552802
SRA1	0.010744757	0.036401345	0.993552802
SBF1	0.008399088	0.036890035	0.993552802
LY6G6F	-0.039155452	0.036989033	0.993552802
PTK2	-0.017010353	0.037092964	0.993552802
TMPRSS12	-0.017019076	0.037096545	0.993552802
MGLL	-0.044556169	0.037148577	0.993552802
LYG1	0.019078301	0.037172196	0.993552802
SPRY2	0.031510871	0.037174454	0.993552802
HOXC13	0.014072364	0.037184846	0.993552802
B3GALTL	-0.021449229	0.037249555	0.993552802
RNY4	-0.030534453	0.037269412	0.993552802
TMEM186	0.012824374	0.037352501	0.993552802
ZNF2	0.016893023	0.037365998	0.993552802
GPR141	0.019584442	0.03743085	0.993552802
DGKE	0.016673793	0.037503139	0.993552802
KIAA1147	-0.007939289	0.037568564	0.993552802
ANKRD9	-0.035607323	0.037602499	0.993552802
MKL1	-0.012963702	0.037778875	0.993552802
PLXDC1	0.016177928	0.037811663	0.993552802
DTNBP1	-0.013710049	0.037825547	0.993552802
CDK8	0.012818478	0.037839306	0.993552802
ZNF792	-0.013583611	0.038016503	0.993552802
NUTF2	-0.010282329	0.038174638	0.993552802
TERF2IP	-0.011162367	0.038343103	0.993552802
RRP1	0.016283726	0.038349357	0.993552802
C20orf194	0.013315304	0.038582152	0.993552802
ITGA4	0.01717509	0.038638426	0.993552802
ENKUR	-0.033023118	0.038867881	0.993552802
KLHL2	0.013449099	0.038871238	0.993552802
CTSC	0.014196721	0.039187805	0.993552802
COMMD2	0.019200237	0.039554059	0.993552802
PDE4D	-0.018967051	0.039562107	0.993552802
SLC25A3	0.008825075	0.039642117	0.993552802

CCDC124	-0.012252362	0.03965097	0.993552802
QARS	0.009270158	0.039675733	0.993552802
PDE4B	-0.013576228	0.03975391	0.993552802
DNM2	-0.013166309	0.039754201	0.993552802
CD200	-0.023898423	0.039880407	0.993552802
PPP1CB	-0.00912478	0.040007988	0.993552802
CSTF3	0.008056778	0.040051832	0.993552802
PPM1D	0.014777933	0.040102567	0.993552802
FARS2	-0.010890518	0.040233592	0.993552802
CMTM5	-0.043827808	0.040529467	0.993552802
RAB5C	-0.009328397	0.040532495	0.993552802
DDX47	0.007004737	0.040699807	0.993552802
COPS8	0.011753351	0.04080471	0.993552802
HIST3H2A	-0.018990804	0.040855335	0.993552802
C6orf25	-0.035258481	0.040894456	0.993552802
C17orf51	-0.015187818	0.040925427	0.993552802
SLC2A14	-0.016146211	0.040925566	0.993552802
DNAL1	0.013673183	0.040981478	0.993552802
ATP8A1	-0.013513797	0.041103057	0.993552802
NCF1C	-0.018765719	0.041112424	0.993552802
CTNNBL1	-0.008229463	0.041337014	0.993552802
AK3	-0.012163312	0.041465833	0.993552802
HIF1A	-0.013828655	0.041517098	0.993552802
C3orf52	0.011292711	0.041584479	0.993552802
TNFSF4	-0.029827363	0.041693066	0.993552802
DEF6	0.007199796	0.041874739	0.993552802
OXR1	0.008602587	0.041893514	0.993552802
PTPRF	-0.033050454	0.04203374	0.993552802
UNC119B	0.010956859	0.042319962	0.993552802
FAM72B	0.016842505	0.042333047	0.993552802
P2RX6	0.015136399	0.042496523	0.993552802
PHTF1	0.012332933	0.042511031	0.993552802
MLLT1	-0.01495737	0.042658712	0.993552802
ZNF177	0.016361925	0.042764938	0.993552802
CEND1	-0.014547549	0.042788833	0.993552802
CDC42	-0.010717662	0.04285292	0.993552802
CRHR1	-0.01504563	0.042866488	0.993552802
ERLIN2	0.008833011	0.043020712	0.993552802
DDX11	0.040597296	0.043052311	0.993552802
NUP93	-0.008034389	0.043130734	0.993552802
SERPINB2	0.028513815	0.043190243	0.993552802
MALT1	-0.009726486	0.043211735	0.993552802
NDNL2	-0.014110961	0.043300269	0.993552802
FCER2	-0.040683843	0.043412127	0.993552802
FAM178A	-0.011005441	0.043571146	0.993552802
HDGF	-0.011528693	0.043616806	0.993552802
GATAD2A	-0.008827752	0.043831462	0.993552802

SNORA28	-0.03110844	0.043941049	0.993552802
PDS5A	-0.007889894	0.044011354	0.993552802
GATAD2B	-0.014267801	0.044272504	0.993552802
FAM179B	0.010960869	0.04447561	0.993552802
PLAG1	0.016659348	0.044580985	0.993552802
NTPCR	-0.019859069	0.044622456	0.993552802
ZBTB3	0.018346538	0.044730689	0.993552802
ZMPSTE24	0.008020933	0.044756844	0.993552802
HSP90AB1	-0.011020388	0.044821829	0.993552802
THOC5	0.008986029	0.0448949	0.993552802
POLR1C	0.010982359	0.044933958	0.993552802
TMEM109	-0.009402749	0.045010767	0.993552802
MED28	0.009006092	0.045086385	0.993552802
PRKCDBP	0.032780014	0.045196806	0.993552802
HSD17B4	0.008810062	0.045235836	0.993552802
NECAP1	0.008335583	0.045297524	0.993552802
RGS19	0.01172097	0.045557253	0.993552802
TRIM66	0.019338108	0.045714109	0.993552802
CTDSPL	-0.040435726	0.045721601	0.993552802
FAM76B	0.013139047	0.045797835	0.993552802
DHCR7	-0.012692075	0.04580213	0.993552802
SH3PXD2A	-0.023260162	0.045848709	0.993552802
MAX	-0.021482123	0.045897547	0.993552802
RABGEF1	-0.012456664	0.04593912	0.993552802
EIF3L	0.007200572	0.045939598	0.993552802
ANKRD35	0.02418232	0.045995107	0.993552802
ANKRD28	-0.014006278	0.046000881	0.993552802
C1orf198	-0.024861815	0.046062851	0.993552802
PRCC	-0.008739084	0.046199801	0.993552802
MRPS18A	0.012072915	0.046281939	0.993552802
NDST2	0.01029655	0.046306055	0.993552802
IL18R1	-0.020194581	0.046320304	0.993552802
NUP37	0.009754382	0.046406113	0.993552802
TAGLN2	-0.017487423	0.046504599	0.993552802
TPM1	-0.038878074	0.04668048	0.993552802
ARHGEF7	-0.00719232	0.046724745	0.993552802
FRAT2	0.018557518	0.046858035	0.993552802
PPIF	-0.013427143	0.047058189	0.993552802
TRERF1	-0.011775671	0.047145436	0.993552802
C9orf47	0.029465711	0.047243574	0.993552802
ZBTB7A	-0.012044469	0.047401247	0.993552802
NGDN	-0.007566571	0.047501431	0.993552802
ANAPC5	-0.007338121	0.047586001	0.993552802
HLA-DRB6	-0.102343206	0.047659842	0.993552802
LIG3	0.01401103	0.047670892	0.993552802
PRKAR1A	0.074899082	0.047905561	0.993552802
ANKRD46	0.016068276	0.048121966	0.993552802

PTCRA	-0.035989311	0.048353339	0.993552802
ZNF330	0.013023279	0.048436412	0.993552802
STUB1	-0.011385151	0.048588546	0.993552802
DCAF10	0.009784311	0.048705856	0.993552802
DPAGT1	0.007402778	0.048742693	0.993552802
PRICKLE1	-0.024411697	0.048822308	0.993552802
C20orf196	0.014200139	0.048833509	0.993552802
ZNF365	-0.030305136	0.048846827	0.993552802
ZNF788	0.012725896	0.049037158	0.993552802
PIGM	0.01101659	0.049049361	0.993552802
PSMB1	0.007711764	0.049250967	0.993552802
ARF4	0.008441605	0.049366659	0.993552802
STK38L	0.015453467	0.049459055	0.993552802
OMG	-0.013228916	0.049476515	0.993552802
ARHGAP23	0.011303416	0.049498161	0.993552802
VPS37C	0.010685701	0.049580372	0.993552802
SP3	0.01376217	0.049678633	0.993552802
PDLIM1	-0.026698377	0.04976134	0.993552802
DCXR	-0.015384735	0.04982232	0.993552802
SNX22	-0.015950479	0.049824999	0.993552802
BUD31	-0.007219112	0.049955297	0.993552802
XKR8	-0.011111971	0.050097948	0.993552802
MTERFD1	0.008424524	0.050145565	0.993552802
STAB1	0.020748412	0.050294953	0.993552802
ARHGAP21	-0.016544322	0.050349281	0.993552802
RPS2	0.016577001	0.050494312	0.993552802
GPN2	0.009225511	0.050497807	0.993552802
AKIP1	0.012603442	0.050531803	0.993552802
LTBP2	-0.013876625	0.050572317	0.993552802
OR7G2	-0.013386058	0.050573728	0.993552802
DIRC2	0.016273156	0.050738229	0.993552802
VILL	0.011624163	0.05074302	0.993552802
GTF3C1	-0.016008276	0.050758372	0.993552802
TM2D2	0.015720968	0.050788727	0.993552802
FAM131A	0.010108926	0.050840784	0.993552802
ARHGEF1	-0.015788964	0.050848382	0.993552802
GIMAP5	0.013320636	0.051001867	0.993552802
ADRM1	-0.008040844	0.051293641	0.993552802
PRUNE	-0.014290904	0.051441003	0.993552802
SLC38A1	-0.018472434	0.051626052	0.993552802
RXRB	0.008488141	0.051639093	0.993552802
MEX3D	-0.013170431	0.051668983	0.993552802
HARS2	0.009468685	0.051761074	0.993552802
CYTL1	0.019994084	0.051794088	0.993552802
TUBGCP5	0.011559636	0.05188892	0.993552802
GPR137B	0.017932848	0.051971624	0.993552802
PPAP2C	0.017199167	0.052069619	0.993552802

PRPS1L1	-0.011011695	0.052208483	0.993552802
BAD	-0.011337374	0.052216738	0.993552802
CNNM2	-0.014984519	0.052384715	0.993552802
SYNE2	-0.019472672	0.052647922	0.993552802
ZNF790	-0.016982155	0.052732076	0.993552802
PDHB	0.007407378	0.052832938	0.993552802
RPPH1	0.010794649	0.052872062	0.993552802
CNOT6L	-0.010233895	0.052876861	0.993552802
MFAP3L	-0.028253233	0.05291209	0.993552802
TRIP4	0.009803477	0.052957517	0.993552802
DDX43	-0.034653597	0.053048801	0.993552802
PDE4DIP	0.07060813	0.053055151	0.993552802
TFPI	-0.028590408	0.053187987	0.993552802
CALHM2	0.014136694	0.053567576	0.993552802
MSL2	-0.010253615	0.053569853	0.993552802
CCDC85B	-0.01161864	0.053584698	0.993552802
ALS2	0.01212078	0.05359437	0.993552802
ADCY3	-0.01176083	0.053763577	0.993552802
RNF169	-0.012608674	0.053801755	0.993552802
CCDC34	-0.012657921	0.053847252	0.993552802
CHRNB4	0.014622338	0.053879324	0.993552802
TTC23	-0.014905559	0.054103108	0.993552802
HPS6	0.01018369	0.054177024	0.993552802
PLOD2	-0.036444596	0.054307806	0.993552802
CEP63	0.009897134	0.054371826	0.993552802
NRBF2	0.012877412	0.054384091	0.993552802
GUSB	0.006602709	0.054405774	0.993552802
ZNF561	0.013072619	0.054410983	0.993552802
WASL	0.015069292	0.054419174	0.993552802
ZNF316	-0.013062686	0.054714171	0.993552802
MPP7	0.016223228	0.054848721	0.993552802
DPP10	-0.01445064	0.054902647	0.993552802
CFD	0.019837731	0.054903792	0.993552802
COPS7B	-0.00744891	0.054943929	0.993552802
CIR1	-0.007432145	0.05494748	0.993552802
RNASEK	-0.008184731	0.054954004	0.993552802
ZBED4	-0.008196618	0.054986011	0.993552802
DAZAP1	-0.008722603	0.055152026	0.993552802
PDE5A	-0.032467758	0.055231624	0.993552802
ANKS1A	0.007646087	0.055485464	0.993552802
TEX2	0.008790233	0.055575854	0.993552802
NME3	-0.007878497	0.05558865	0.993552802
ELMO1	-0.010397996	0.055865545	0.993552802
ESAM	-0.035641088	0.055871672	0.993552802
GNAZ	-0.031424931	0.055875509	0.993552802
ST6GAL1	-0.009562847	0.055895754	0.993552802
FMO5	0.015383305	0.055999732	0.993552802

B3GAT3	-0.014031106	0.056011451	0.993552802
NKD2	-0.022409755	0.056104889	0.993552802
MFAP3	0.016598703	0.05610868	0.993552802
HLA-DOB	-0.031492142	0.05613005	0.993552802
SLC35C1	0.010525945	0.056139404	0.993552802
POTEKP	-0.01194577	0.056224349	0.993552802
CCNY	-0.011762493	0.05631038	0.993552802
TTF1	0.006755642	0.056324313	0.993552802
ABCC5	0.012926325	0.056673474	0.993552802
TUBA3D	-0.026344635	0.05674054	0.993552802
FICD	0.017892946	0.056745404	0.993552802
THYN1	-0.008796025	0.05676541	0.993552802
STX1A	-0.014025248	0.057172395	0.993552802
UCKL1	-0.007991189	0.057221758	0.993552802
MAP4K2	-0.008072772	0.057232869	0.993552802
QDPR	0.012383406	0.057273159	0.993552802
ITPRIPL2	0.018327193	0.057307121	0.993552802
ARMC5	0.01409645	0.057411785	0.993552802
FUK	0.007212018	0.057515145	0.993552802
ZNF653	0.009940647	0.057521791	0.993552802
MARCH3	-0.020090191	0.057632261	0.993552802
CDC34	-0.010634916	0.057784288	0.993552802
RABEPK	0.005959186	0.057784886	0.993552802
ZNF219	0.012111432	0.057811448	0.993552802
MREG	-0.012051737	0.05785942	0.993552802
CLK4	0.016636956	0.057929123	0.993552802
TCF3	-0.013275959	0.057961882	0.993552802
RASSF2	0.010157279	0.058019432	0.993552802
SPAST	-0.011854116	0.058088728	0.993552802
LIN7A	0.014829055	0.058204343	0.993552802
SAMM50	0.010348103	0.058285308	0.993552802
SART1	0.016279616	0.058650471	0.993552802
PCYT2	0.009870638	0.058667612	0.993552802
PLA2G15	0.012689852	0.058701584	0.993552802
FNDC3B	0.010545977	0.058708624	0.993552802
HIST1H2AD	-0.025585379	0.058876052	0.993552802
NTS	-0.01496782	0.059015185	0.993552802
LIMS3L	-0.024529182	0.05905834	0.993552802
CPSF1	-0.015147666	0.05915072	0.993552802
JMJD4	0.014597089	0.0592341	0.993552802
FERMT3	-0.010825007	0.059350202	0.993552802
SNX20	0.015532363	0.059743587	0.993552802
ZNF383	0.009180528	0.059766355	0.993552802
SMUG1	0.006545363	0.059924711	0.993552802
CYP20A1	-0.006239811	0.0600201	0.993552802
PRKCB	-0.007172029	0.060023814	0.993552802
PSMD13	-0.012311988	0.060187846	0.993552802

MRPL32	0.007694552	0.06024559	0.993552802
IRAK3	0.013555292	0.060295534	0.993552802
TMEM218	0.007199524	0.060401156	0.993552802
TFB2M	0.009133278	0.060465192	0.993552802
NCR3	-0.020714294	0.060550198	0.993552802
OR11H1	-0.013359539	0.060570288	0.993552802
NCLN	0.009229206	0.060587949	0.993552802
CACNA2D3	0.018824683	0.060618098	0.993552802
MARCKSL1	-0.01102933	0.060657608	0.993552802
ATP5G1	0.009412965	0.060674758	0.993552802
SLC25A46	0.01286334	0.060682457	0.993552802
RBCK1	0.009801023	0.06087859	0.993552802
C11orf74	0.020583466	0.060894815	0.993552802
MARCH1	0.022085028	0.060938279	0.993552802
ZDHHC24	0.014608867	0.060939956	0.993552802
MGAT1	-0.00931968	0.061007171	0.993552802
ZNF837	0.0133264	0.061040708	0.993552802
GUSBP9	-0.020647959	0.061053917	0.993552802
OSBPL11	0.011297929	0.061109152	0.993552802
THRAP3	-0.010611404	0.061187048	0.993552802
PSD3	-0.011224991	0.061244818	0.993552802
CDC42EP2	0.024498768	0.061348933	0.993552802
FBXO30	0.014827039	0.061355598	0.993552802
WBP4	0.009415472	0.061480628	0.993552802
ASPRV1	0.012988662	0.061507501	0.993552802
ZBTB32	0.018895722	0.061646984	0.993552802
TMEM150A	0.008176964	0.061661627	0.993552802
FASTKD2	0.009899593	0.061841018	0.993552802
PIGW	0.011557648	0.06188612	0.993552802
DGAT1	0.010454682	0.061896922	0.993552802
ELANE	0.028363649	0.061940191	0.993552802
DDX1	0.005863152	0.062027857	0.993552802
RAPGEF6	-0.012514412	0.062063558	0.993552802
EXOSC1	0.004968037	0.062093208	0.993552802
CHERP	-0.010492155	0.0620978	0.993552802
NEBL	-0.032883619	0.062244824	0.993552802
EFTUD2	-0.006896277	0.062710313	0.993552802
RCN1	0.011693039	0.063059265	0.993552802
STOX1	0.025871298	0.063185806	0.993552802
THAP3	-0.013815197	0.063293649	0.993552802
MTO1	0.007771274	0.063439768	0.993552802
TNFRSF10C	0.011626165	0.063632497	0.993552802
MAFB	0.01780203	0.063682493	0.993552802
JPH2	-0.011167709	0.063697821	0.993552802
KCTD15	0.012379942	0.063769019	0.993552802
SNORD83B	-0.013667124	0.063792071	0.993552802
AATF	-0.00850296	0.06409547	0.993552802

PTPRJ	-0.012783041	0.064227467	0.993552802
RASSF5	-0.006942938	0.064277691	0.993552802
GHRL	0.016822604	0.064296442	0.993552802
EXOSC3	0.013835671	0.064588297	0.993552802
DDX24	-0.008821849	0.064649513	0.993552802
PIH1D1	-0.008470423	0.064809932	0.993552802
MPPE1	0.014589816	0.064816087	0.993552802
GRASP	-0.022836095	0.065030821	0.993552802
PLEK2	-0.010389521	0.06528985	0.993552802
KCNG1	-0.02892188	0.065309729	0.993552802
DDX28	-0.00946653	0.065437139	0.993552802
EFNA4	0.009631793	0.065879898	0.993552802
GIMAP4	0.010572158	0.065903647	0.993552802
SNORA64	-0.014805634	0.066024994	0.993552802
GP9	-0.035777699	0.066104568	0.993552802
ZNF613	0.014668756	0.066307767	0.993552802
LSM11	-0.011466117	0.066352425	0.993552802
SIAH1	0.009701673	0.066396688	0.993552802
LGR6	0.025803579	0.06640471	0.993552802
ANKRD13A	-0.006780001	0.066524128	0.993552802
SYT2	0.014574789	0.06675694	0.993552802
ZNF593	0.011277015	0.066942145	0.993552802
GLT1D1	0.017103197	0.066948139	0.993552802
RDX	0.013697992	0.067049555	0.993552802
ARHGAP19	0.007698201	0.067178741	0.993552802
SV2A	0.014571141	0.067332596	0.993552802
PSMB7	0.010496619	0.067504753	0.993552802
CD55	0.017549823	0.067574754	0.993552802
KIAA0226	-0.010967647	0.06758469	0.993552802
PRTFDC1	-0.024858181	0.06777494	0.993552802
TBC1D22A	-0.011781117	0.067800724	0.993552802
AASDH	0.009685041	0.067867331	0.993552802
DHRS11	-0.01028966	0.067877275	0.993552802
ALG8	0.005424068	0.067950079	0.993552802
SERHL2	-0.013599574	0.067960537	0.993552802
FBXO34	-0.008238824	0.067986406	0.993552802
PEAR1	-0.030966019	0.068029705	0.993552802
NUS1	0.008352667	0.068030338	0.993552802
C7orf49	0.010689379	0.068208209	0.993552802
NOL12	0.007869636	0.068282029	0.993552802
NR2C2	-0.010267304	0.068536167	0.993552802
SLC25A12	0.009907341	0.068646474	0.993552802
SLC23A3	0.021262892	0.068837264	0.993552802
HEXIM2	-0.02112376	0.068907384	0.993552802
ODC1	-0.019184543	0.068958596	0.993552802
RPL32P3	0.017527551	0.069096916	0.993552802
ADK	-0.016748334	0.069098674	0.993552802

OPA3	0.015304309	0.069101075	0.993552802
CD72	-0.025485539	0.069229971	0.993552802
C17orf80	0.012117314	0.06923106	0.993552802
L3MBTL1	0.011231661	0.069250393	0.993552802
AASDHPPT	0.010124493	0.069305067	0.993552802
MAD2L1BP	0.013020279	0.06933512	0.993552802
ZNF12	0.012650476	0.069519797	0.993552802
RASL10A	0.015364736	0.069579974	0.993552802
SMAD6	0.012308067	0.069587302	0.993552802
VPREB3	-0.033817014	0.069889668	0.993552802
EXD2	0.010048896	0.070040047	0.993552802
ABCD3	0.00976365	0.070087485	0.993552802
PUS7	0.0142439	0.070096643	0.993552802
SPATA5L1	0.010999374	0.070353547	0.993552802
IFNG	0.043662324	0.07040783	0.993552802
CBX4	-0.009186478	0.070437265	0.993552802
ABCB9	0.028944241	0.070565732	0.993552802
TJP1	-0.014900182	0.070853884	0.993552802
CLASRP	-0.012642572	0.070885032	0.993552802
RCC1	-0.014521435	0.070910512	0.993552802
ZNF627	0.008493832	0.070983269	0.993552802
RBBP5	-0.008607392	0.071003784	0.993552802
SDC4	-0.017777373	0.071186973	0.993552802
EWSR1	-0.006742755	0.071237908	0.993552802
BRD3	-0.012595547	0.071405902	0.993552802
ALKBH1	0.011377975	0.071503573	0.993552802
SRC	-0.014865262	0.071546693	0.993552802
PWWP2A	0.008790955	0.071669537	0.993552802
SUPT6H	-0.009165947	0.071738269	0.993552802
TELO2	0.022040114	0.071913533	0.993552802
FBXO7	-0.007674314	0.071989785	0.993552802
TRNAU1AP	0.009639977	0.072289799	0.993552802
ZSCAN5A	0.012508577	0.072327886	0.993552802
SLC46A2	0.019916948	0.072335186	0.993552802
PLEKHG4	0.015169367	0.072505563	0.993552802
SYT3	0.012257174	0.072563045	0.993552802
SERGEF	-0.008377647	0.072714278	0.993552802
TTC16	0.022330227	0.072786601	0.993552802
TULP4	-0.008395772	0.072845126	0.993552802
TMEM41A	0.010907506	0.072855073	0.993552802
ATG7	0.011399447	0.07295532	0.993552802
JAM2	-0.010898674	0.072985333	0.993552802
ABHD12	0.024886271	0.073094878	0.993552802
TICAM2	0.014994014	0.073114477	0.993552802
YWHAZ	-0.00642829	0.073185612	0.993552802
LONP2	-0.007870808	0.073552689	0.993552802
PPCS	0.008878675	0.073577802	0.993552802

SLC22A1	-0.012789135	0.073590801	0.993552802
CXCR1	0.016762742	0.073876028	0.993552802
COMMD3	0.006309685	0.073930151	0.993552802
MASTL	0.017643042	0.074005605	0.993552802
KIAA0368	0.007594877	0.074035395	0.993552802
MPZL2	0.017401769	0.074177986	0.993552802
ZNF230	0.010842104	0.074457305	0.993552802
ERMAP	0.015752804	0.074587171	0.993552802
MPL	-0.03780983	0.074649899	0.993552802
BCL2L1	-0.014831944	0.074721037	0.993552802
PHB2	-0.00734125	0.074801323	0.993552802
IL5RA	-0.016479545	0.074808098	0.993552802
TIMM23	0.005715147	0.07480996	0.993552802
KCTD2	-0.008930938	0.074881616	0.993552802
CLN5	0.012459128	0.074917819	0.993552802
PABPN1	-0.008342638	0.074929071	0.993552802
SLC35B3	0.011247119	0.075441224	0.993552802
MAP7D1	0.006195063	0.075543951	0.993552802
HERPUD1	0.008499701	0.075789313	0.993552802
CD1E	0.023619843	0.075926789	0.993552802
SHMT2	-0.007504803	0.075927718	0.993552802
MTHFD2	0.013926995	0.075963082	0.993552802
FURIN	-0.012569338	0.075980556	0.993552802
KIAA1328	0.011876678	0.076021253	0.993552802
ZNF564	0.01284093	0.076035944	0.993552802
MIS12	0.009718524	0.076075157	0.993552802
GNA13	-0.006944807	0.076075592	0.993552802
EIF5A	-0.01330322	0.076139647	0.993552802
PARVG	0.00986551	0.076140563	0.993552802
B4GALT7	0.008492639	0.076207221	0.993552802
NLRX1	0.007157163	0.076229573	0.993552802
NUP35	0.014990511	0.076304204	0.993552802
TAL1	-0.023578867	0.076463578	0.993552802
ERBB2IP	-0.012318571	0.076519444	0.993552802
DOLK	0.012473751	0.076671821	0.993552802
HRK	-0.0223595	0.076784454	0.993552802
TOX4	-0.008928102	0.076828963	0.993552802
IGHG1	0.009177574	0.076875162	0.993552802
CYB5R2	0.016058749	0.077413222	0.993552802
KLRK1	0.01228702	0.077495378	0.993552802
ATG5	-0.007698411	0.077572823	0.993552802
FBXL12	0.007346737	0.077578481	0.993552802
ADO	0.01152368	0.077595698	0.993552802
ZZEF1	-0.00595574	0.07763786	0.993552802
BBS10	0.015637544	0.077695992	0.993552802
OR2W3	-0.026075564	0.077707824	0.993552802
HERC5	-0.01941436	0.077751622	0.993552802

NDUFAF1	0.011323643	0.077754442	0.993552802
RBM47	0.016791742	0.077778011	0.993552802
MLF2	-0.008853629	0.077951475	0.993552802
CD248	0.026531138	0.07810926	0.993552802
HSD17B7P2	0.011435684	0.078404786	0.993552802
PPP2R1A	-0.00825188	0.078485184	0.993552802
EXOC6	-0.006645563	0.078490805	0.993552802
PTCD1	0.011405714	0.078542523	0.993552802
C15orf40	-0.014842871	0.078572314	0.993552802
PFKL	0.013433911	0.078700408	0.993552802
DHRS13	0.011637839	0.078702609	0.993552802
SARM1	0.011909112	0.078738796	0.993552802
WDR83	0.007651098	0.078962233	0.993552802
ADAR	-0.007036133	0.078984379	0.993552802
NAPEPLD	0.011696176	0.079002354	0.993552802
SCLT1	0.01591818	0.079034568	0.993552802
AGPAT6	-0.011538741	0.079157142	0.993552802
EPB41L2	-0.013101087	0.079211711	0.993552802
POTEE	-0.017574889	0.079223733	0.993552802
TM2D3	0.010885113	0.079254323	0.993552802
CCDC43	0.007751757	0.079350889	0.993552802
CLEC4A	0.01021831	0.079508021	0.993552802
ZC3H4	-0.007032781	0.07962359	0.993552802
WDR70	-0.008694101	0.079745635	0.993552802
TBX3	0.009952436	0.079761379	0.993552802
PVRL2	-0.017916206	0.079779309	0.993552802
SLC2A3	-0.009360525	0.079994546	0.993552802
NSUN5P1	-0.013718757	0.080264638	0.993552802
DNAJB9	0.014866658	0.080343611	0.993552802
WBP2	-0.009006066	0.080406952	0.993552802
CD33	0.014641518	0.080418343	0.993552802
RETSAT	0.010192497	0.080529784	0.993552802
ABCB4	-0.018857254	0.08054187	0.993552802
SECISBP2L	-0.006688584	0.0805874	0.993552802
P2RX5	-0.021191388	0.08079467	0.993552802
TXNL4A	-0.013490136	0.080857519	0.993552802
ZNF512B	-0.013829899	0.080914468	0.993552802
MRPS27	0.007301877	0.080942294	0.993552802
PROCR	0.011828833	0.081039811	0.993552802
DNAH2	-0.010339153	0.081091598	0.993552802
ANKRD6	0.012439035	0.081252496	0.993552802
IKZF3	0.019236049	0.081285547	0.993552802
SAR1B	0.011819086	0.08135496	0.993552802
TMEM185B	0.010201993	0.081379163	0.993552802
HSPA4	-0.007054308	0.08143292	0.993552802
AP3M1	0.010765536	0.0814988	0.993552802
RPS23	-0.036014208	0.081574485	0.993552802

P2RY14	-0.016550838	0.081667232	0.993552802
WDR5B	0.012793139	0.081688485	0.993552802
TMEM217	-0.012689476	0.08174166	0.993552802
MYOM2	0.088764377	0.081998723	0.993552802
ZNF251	-0.009574725	0.08215591	0.993552802
C17orf75	0.029753964	0.082206899	0.993552802
RAB35	0.009214652	0.082250332	0.993552802
ACIN1	-0.007982438	0.082316455	0.993552802
SMG6	0.012499881	0.08231855	0.993552802
CDHR3	-0.017802795	0.082381112	0.993552802
PROS1	-0.043809891	0.082422641	0.993552802
KLHL12	0.00700009	0.082499896	0.993552802
IL20RB	0.010646436	0.082551851	0.993552802
UGGT2	0.014511402	0.082571004	0.993552802
DAGLB	0.011785681	0.082657909	0.993552802
G2E3	0.009189367	0.082672207	0.993552802
ASTE1	0.013300188	0.082716515	0.993552802
USP12	-0.01268759	0.082753534	0.993552802
ABAT	-0.017739009	0.083164516	0.993552802
FCN1	0.010550429	0.083166963	0.993552802
FAM174A	0.014247872	0.083188778	0.993552802
CYB561D1	0.006760363	0.083363696	0.993552802
KCNA3	-0.015520842	0.083536269	0.993552802
MARCH2	-0.022174092	0.08366255	0.993552802
MBD5	-0.012572549	0.08377512	0.993552802
KLHL9	0.011648986	0.084207472	0.993552802
C1orf85	0.017926344	0.084489006	0.993552802
METTL8	-0.011967586	0.084503855	0.993552802
AGPS	0.008659269	0.084578878	0.993552802
TRPS1	0.013546107	0.084600934	0.993552802
SLC22A31	0.012052547	0.084612299	0.993552802
SNHG9	-0.008996615	0.084781476	0.993552802
SUV420H1	-0.008542833	0.084900039	0.993552802
LRP3	0.016774646	0.084901943	0.993552802
SOX15	0.013291272	0.08491073	0.993552802
VN1R2	-0.016255952	0.085025801	0.993552802
FGL2	0.014578124	0.085135306	0.993552802
BLCAP	-0.007984662	0.08532594	0.993552802
LIMS1	-0.013993447	0.085380566	0.993552802
SCRN3	0.010215007	0.085382448	0.993552802
MBIP	0.009134369	0.085430832	0.993552802
ZSCAN22	0.012658442	0.085501993	0.993552802
SCARNA9	0.017486429	0.085973992	0.993552802
FAM124B	0.01557446	0.085974985	0.993552802
GGH	0.012922355	0.0859835	0.993552802
NRBP2	0.010459187	0.086016441	0.993552802
RFESD	0.010073518	0.086115043	0.993552802

TWISTNB	0.010182568	0.086408754	0.993552802
EIF2AK1	-0.007515175	0.086484617	0.993552802
RTN4R	0.016699804	0.086642614	0.993552802
SNORA49	0.010721595	0.086718111	0.993552802
CERK	0.007604415	0.086831912	0.993552802
HABP4	-0.012899717	0.086962064	0.993552802
LASP1	0.007017269	0.087178733	0.993552802
NRCAM	0.028167422	0.087209858	0.993552802
GSDMD	0.009703731	0.087287795	0.993552802
TMEM135	0.012465382	0.087411124	0.993552802
DEFA1	0.033595794	0.087514021	0.993552802
ZNF17	0.013147106	0.087741227	0.993552802
SERF1B	-0.016590609	0.087859718	0.993552802
RPUSD1	0.013525806	0.087904787	0.993552802
CCDC41	0.010239653	0.087912875	0.993552802
SNORD71	-0.0150912	0.087935413	0.993552802
ZFP3	0.010307471	0.088103898	0.993552802
TMEM126B	0.00558211	0.088311804	0.993552802
EIF4ENIF1	-0.006577167	0.088368754	0.993552802
TMEM56	0.013945964	0.088425695	0.993552802
TXNDC15	0.01068851	0.088465259	0.993552802
GSTP1	0.009101315	0.088470823	0.993552802
HDAC11	0.013665874	0.088524774	0.993552802
MAN2B2	0.008105026	0.088586706	0.993552802
ZNF671	0.010039665	0.088917069	0.993552802
EXOC1	0.009600853	0.089285501	0.993552802
CNTROB	-0.012825688	0.089664316	0.993552802
PAK6	0.013595015	0.089873277	0.993552802
PKN1	-0.010738308	0.090001461	0.993552802
NOB1	-0.008580483	0.090083661	0.993552802
SNORD89	-0.021853164	0.090206896	0.993552802
AZI2	0.010349729	0.090207529	0.993552802
SMAD2	-0.007374178	0.090235532	0.993552802
MMRN1	-0.033137352	0.090276062	0.993552802
KRT72	-0.062687802	0.090401362	0.993552802
CDC14A	-0.010422114	0.090546799	0.993552802
ATP5B	0.006530053	0.090559738	0.993552802
NAGS	0.010481002	0.090672845	0.993552802
TAPT1	-0.009912492	0.090713959	0.993552802
VAC14	0.01345717	0.090722268	0.993552802
PIM1	-0.01196913	0.091082339	0.993552802
BABAM1	-0.005911437	0.091085944	0.993552802
RGL1	0.01903777	0.091141957	0.993552802
FBXL15	-0.007246659	0.091466684	0.993552802
MYCN	0.015017033	0.091631934	0.993552802
ZNF879	-0.013079222	0.091661295	0.993552802
KCTD18	0.007665501	0.091679947	0.993552802

GPX1	-0.015295911	0.091723585	0.993552802
MAD2L1	0.014043548	0.091775721	0.993552802
TTL	0.007575946	0.091798167	0.993552802
SRSF2	0.00393486	0.091907627	0.993552802
GMPR2	0.007451949	0.091978562	0.993552802
TRIM39	0.007574615	0.092119906	0.993552802
JAKMIP2	0.025523515	0.092229679	0.993552802
CDC42BPA	-0.009942622	0.092922532	0.993552802
SNX13	0.010543075	0.09307881	0.993552802
SCARNA12	-0.010319837	0.093142581	0.993552802
PKNOX1	0.010126405	0.093203775	0.993552802
MGC2752	0.012669135	0.093217409	0.993552802
UBR4	-0.007188913	0.093264589	0.993552802
ASL	0.014790266	0.093336742	0.993552802
NFKBIE	0.017666826	0.093346569	0.993552802
TMEM97	0.012651607	0.093352351	0.993552802
BAZ2B	0.011943184	0.093395707	0.993552802
NCKAP1	-0.017345743	0.09352736	0.993552802
RNF208	-0.012995414	0.093540783	0.993552802
SLC25A24	0.017215923	0.093661678	0.993552802
CPA3	0.017367564	0.093678994	0.993552802
SLX1B-SULT1A4	-0.011764577	0.093758294	0.993552802
REXO4	0.009027078	0.093918492	0.993552802
DNAJC25	0.007474842	0.093953712	0.993552802
SLC27A6	-0.013020158	0.094001906	0.993552802
RNF14	0.008762778	0.094080939	0.993552802
ORAI1	-0.01248937	0.094090829	0.993552802
TMEM30A	-0.009472691	0.094174127	0.993552802
PDCD4	-0.011352254	0.094325959	0.993552802
TSPAN1	0.012959554	0.094383163	0.993552802
QTRT1	-0.010617259	0.09471847	0.993552802
VPS26A	0.006145245	0.094763132	0.993552802
DDX55	0.00882278	0.094793652	0.993552802
CDC14B	-0.022415445	0.094797771	0.993552802
ZKSCAN3	0.010133658	0.094993248	0.993552802
SSBP4	-0.009236109	0.095007569	0.993552802
SNORD104	-0.014154926	0.09521926	0.993552802
SRGAP1	0.011201813	0.095264676	0.993552802
ITGA2B	-0.041978703	0.095334113	0.993552802
ZNF518A	0.007608403	0.095351894	0.993552802
CAMTA1	-0.023527091	0.095422875	0.993552802
NRGN	-0.027288849	0.095480539	0.993552802
PLEKHA7	0.011443415	0.095505723	0.993552802
ART1	-0.011350082	0.095510926	0.993552802
CENPBD1	0.010379682	0.095598145	0.993552802
DGKG	-0.011687423	0.095625551	0.993552802
LRRC45	-0.014215065	0.096252207	0.993552802

COL6A3	-0.019814749	0.096370368	0.993552802
CDKN2D	-0.014571328	0.096385656	0.993552802
SLC35B4	0.012388179	0.09641521	0.993552802
FAM101B	-0.017739034	0.096430807	0.993552802
TNF	0.023986133	0.096467074	0.993552802
H2AFY2	0.009541447	0.096519944	0.993552802
FGF2	-0.010707563	0.096544608	0.993552802
ST3GAL3	-0.017360848	0.096600691	0.993552802
ZNF890P	-0.012108506	0.096768995	0.993552802
PCNXL3	-0.009698728	0.0967917	0.993552802
ZNF239	0.016767233	0.096803739	0.993552802
EFCAB4A	0.020196442	0.097120796	0.993552802
KCTD11	0.01148984	0.097192911	0.993552802
CNRIP1	0.017216283	0.097325008	0.993552802
LPCAT2	0.035670495	0.097367077	0.993552802
SNX30	0.010131665	0.097464629	0.993552802
KIAA1467	-0.009705254	0.097612976	0.993552802
PDIA5	0.013697723	0.097761266	0.993552802
SLC25A1	-0.010940539	0.097825504	0.993552802
SIPA1L1	-0.010661186	0.097864775	0.993552802
SLC45A3	-0.019653632	0.097924741	0.993552802
SNORD32A	-0.01261179	0.097935499	0.993552802
MRPL27	0.008958199	0.09836544	0.993552802
KIAA0895L	0.012783182	0.098383793	0.993552802
LCOR	-0.012542525	0.098398247	0.993552802
PPP1R16B	-0.010172196	0.098562664	0.993552802
RAB5A	0.00922254	0.098622184	0.993552802
CERCAM	0.026644021	0.098675096	0.993552802
RNF11	-0.01909469	0.098815465	0.993552802
ITGB3	-0.043398425	0.099009593	0.993552802
BET1	0.012444337	0.099097367	0.993552802
NCF2	0.013858854	0.099234355	0.993552802
TIMM17A	0.010571899	0.099376311	0.993552802
HYLS1	0.011436604	0.099404347	0.993552802
BLM	0.011956415	0.099416875	0.993552802
LRRN3	0.035471793	0.099514456	0.993552802
DGCR2	-0.011266953	0.09956054	0.993552802
MDGA1	0.021250094	0.099667701	0.993552802
PSPH	0.012157842	0.099803033	0.993552802
NARS2	0.009246299	0.099861074	0.993552802
ATP2A3	-0.011814674	0.099900208	0.993552802
TNPO3	-0.013005599	0.09990136	0.993552802
GLOD4	-0.005030111	0.100248452	0.993552802
ARMC10	0.00955954	0.100363363	0.993552802
AURKA	0.012364165	0.100531441	0.993552802
STT3B	0.010021848	0.100718699	0.993552802
CHD4	-0.007488025	0.100878105	0.993552802

C2orf88	-0.026837313	0.100932654	0.993552802
MST1	0.011425522	0.101053345	0.993552802
ALDH2	0.014206542	0.101123545	0.993552802
STRBP	-0.013337059	0.101171752	0.993552802
GPAM	0.009890093	0.101248995	0.993552802
GMEB1	-0.010522091	0.101267737	0.993552802
PTPRR	0.010462987	0.101301739	0.993552802
MRPL44	0.008950192	0.101343379	0.993552802
EIF3A	0.004588883	0.101393942	0.993552802
POP1	0.009936483	0.101538691	0.993552802
FAM73B	-0.01090791	0.101561055	0.993552802
IFITM1	-0.010421486	0.101587644	0.993552802
WNT10B	0.017076058	0.101738858	0.993552802
CHORDC1	0.009352136	0.102156581	0.993552802
PMPCB	0.006197762	0.102382429	0.993552802
GET4	-0.010406429	0.102432146	0.993552802
NRAS	0.009460357	0.102506879	0.993552802
ACOT8	-0.010702867	0.102632299	0.993552802
ZNF212	0.008256527	0.102719236	0.993552802
HRASLS	-0.032676477	0.102868343	0.993552802
DIABLO	0.004284047	0.103105962	0.993552802
VTRNA1-3	0.013512301	0.103107024	0.993552802
PEBP1	-0.008903258	0.103296496	0.993552802
ZNF304	0.01090002	0.103434119	0.993552802
TMEM216	0.008876314	0.103551871	0.993552802
HERC4	0.005708047	0.10356353	0.993552802
EID1	-0.013031577	0.103601946	0.993552802
GP1BB	-0.020094918	0.103783009	0.993552802
TPBG	0.014090966	0.103922065	0.993552802
CHRNA10	0.011180053	0.103922218	0.993552802
CASP4	0.007923101	0.104023048	0.993552802
PARP12	-0.013146656	0.104031885	0.993552802
CD8B	0.026963017	0.104056846	0.993552802
MPI	-0.012461324	0.104062243	0.993552802
VASP	-0.018760781	0.104177262	0.993552802
TMEM87A	0.005190595	0.104179341	0.993552802
HNRNPL	-0.011826777	0.104231229	0.993552802
PRPF31	-0.008267603	0.104274576	0.993552802
CCDC88A	0.011090958	0.104364875	0.993552802
CSNK1G1	-0.007665885	0.104408088	0.993552802
PPP1R3D	0.014627016	0.104473734	0.993552802
HIBADH	0.008669257	0.104503884	0.993552802
PTRF	0.011831034	0.104683361	0.993552802
NDRG3	-0.006985817	0.104712957	0.993552802
MYNN	0.006419181	0.104790496	0.993552802
WDR11	0.057798156	0.105288851	0.993552802
IBA57	0.009926196	0.105556586	0.993552802

KCTD3	0.01333627	0.105710222	0.993552802
EXTL2	0.013376965	0.105752021	0.993552802
INPP5A	-0.00986361	0.10577397	0.993552802
ZBTB22	-0.006207325	0.105780629	0.993552802
EPB41	-0.011591941	0.105845073	0.993552802
MIR330	-0.016129605	0.105871143	0.993552802
LETM1	-0.01143851	0.105898408	0.993552802
TP53BP2	0.007391684	0.105908366	0.993552802
ANTXR1	0.010088595	0.105966791	0.993552802
PIAS1	-0.007429156	0.106203766	0.993552802
TONSL	-0.014526519	0.106237153	0.993552802
TMEM150B	0.010518581	0.106269773	0.993552802
FKBP9L	-0.017099959	0.106273283	0.993552802
MRPL37	0.007275736	0.106308673	0.993552802
ATP6V1C1	0.009829165	0.106435573	0.993552802
CDKN1A	-0.021768121	0.106471073	0.993552802
ITCH	-0.0099645	0.106592891	0.993552802
RAB4B-EGLN2	0.005458508	0.106600461	0.993552802
NAT9	0.008285385	0.106802383	0.993552802
WAC	0.008130559	0.106920388	0.993552802
MTMR14	0.005272411	0.107028142	0.993552802
THBS1	-0.03943771	0.107316215	0.993552802
FOXO1	-0.009076247	0.107359069	0.993552802
UBXN7	-0.010138785	0.107369148	0.993552802
PGAP1	-0.012463292	0.10740589	0.993552802
LY6E	-0.015900909	0.107526216	0.993552802
AP2M1	-0.007138555	0.107565428	0.993552802
TSPAN9	-0.034278952	0.107566048	0.993552802
BOLA1	0.009598777	0.107698311	0.993552802
SIL1	0.010618227	0.107812631	0.993552802
KIAA1279	0.011108962	0.108360536	0.993552802
FGD6	-0.011593875	0.108416909	0.993552802
CHIC2	0.010089487	0.10845126	0.993552802
CSTA	0.017960222	0.108533978	0.993552802
TXK	-0.018894126	0.108740397	0.993552802
PEX14	0.01036788	0.109059017	0.993552802
GRAMD3	0.013020279	0.109067935	0.993552802
WASF3	-0.030684762	0.109185466	0.993552802
FAM115A	-0.004081823	0.109207331	0.993552802
FAM160B1	0.007400286	0.109271624	0.993552802
GPS1	0.005574479	0.109355816	0.993552802
DTX2	0.006620403	0.109385525	0.993552802
SEMA4B	-0.01033284	0.109428097	0.993552802
ALG6	0.006539777	0.109470752	0.993552802
HECA	-0.008202919	0.109569614	0.993552802
OSGEP	0.007384388	0.109633733	0.993552802
C9orf85	0.01375098	0.109764424	0.993552802

EXOG	-0.010622423	0.109781425	0.993552802
ZCCHC11	-0.009208054	0.109875336	0.993552802
SCARNA22	0.011096017	0.109917714	0.993552802
PRDX2	-0.015270449	0.110083385	0.993552802
SPATA2	0.013168776	0.110601719	0.993552802
TRAPPC9	-0.01189651	0.110785484	0.993552802
BBS7	0.008087303	0.110848983	0.993552802
KLHL22	0.008893469	0.11087791	0.993552802
SLC8A3	-0.027535359	0.111103992	0.993552802
OXSR1	-0.004504065	0.111130106	0.993552802
INTS10	0.005856761	0.111234111	0.993552802
DCAF5	0.006850805	0.111234229	0.993552802
PAK2	-0.005651541	0.111251334	0.993552802
MAP3K12	0.009036382	0.111274402	0.993552802
SLC37A1	-0.008398798	0.111296736	0.993552802
TM9SF3	0.00720917	0.11133082	0.993552802
LRR1	0.009047792	0.111362819	0.993552802
CX3CR1	0.015158722	0.111534769	0.993552802
EEF2K	0.009300255	0.111659048	0.993552802
DERL2	0.006423479	0.111763721	0.993552802
IPO8	-0.023502959	0.111897611	0.993552802
LSM4	-0.008687258	0.112161384	0.993552802
TLR4	0.015939651	0.112359652	0.993552802
KRT1	-0.027005737	0.112361949	0.993552802
SPEN	-0.006308007	0.112417568	0.993552802
ANKRD34A	0.011607508	0.112425536	0.993552802
RNF25	-0.009031965	0.113058174	0.993552802
RBFA	-0.009620213	0.113120232	0.993552802
ZNF202	0.01011957	0.113321756	0.993552802
BTF3L4	0.010043012	0.113427624	0.993552802
SDF2	0.006950262	0.113522646	0.993552802
GBA2	0.008666163	0.113596889	0.993552802
SLC6A4	-0.019066611	0.113667017	0.993552802
TRANK1	-0.011644485	0.113707535	0.993552802
PBX1	-0.022863202	0.113810297	0.993552802
SELP	-0.033756316	0.114133433	0.993552802
RNFT1	0.013956361	0.114264239	0.993552802
ADRB2	0.022272997	0.114423072	0.993552802
GRAP2	-0.018487123	0.114487714	0.993552802
PHKB	-0.011824884	0.114514965	0.993552802
CPNE5	-0.017615959	0.114585981	0.993552802
C9orf16	-0.011362747	0.11461973	0.993552802
TRIM61	0.012441017	0.114788449	0.993552802
HEMGN	-0.02566465	0.114893397	0.993552802
BTN3A1	0.011536436	0.114941248	0.993552802
DYNLL2	-0.01057389	0.114972162	0.993552802
FBXO32	-0.009204771	0.115034052	0.993552802

CAND1	0.008058116	0.115066872	0.993552802
TSC22D4	-0.010376817	0.115101822	0.993552802
NMRAL1	0.010052672	0.11512181	0.993552802
TMEM184B	-0.011011272	0.115142112	0.993552802
CTBP1	-0.007875334	0.115195742	0.993552802
HOXB2	0.031313456	0.115233222	0.993552802
SH3BP2	-0.0134232	0.11538457	0.993552802
IGF2BP2	-0.015983554	0.115465848	0.993552802
ZW10	0.006214611	0.1155874	0.993552802
KIFC3	-0.025219904	0.115741451	0.993552802
PPP2R3C	0.009831637	0.11580659	0.993552802
CD180	-0.011389111	0.115883607	0.993552802
CLEC1B	-0.031623478	0.116054139	0.993552802
RIOK3	-0.007607948	0.116194616	0.993552802
C16orf86	-0.010330638	0.116202029	0.993552802
TCF20	-0.006765213	0.116220794	0.993552802
TAOK3	-0.005882175	0.116239175	0.993552802
FBXW4	-0.006268018	0.116606226	0.993552802
BATF	0.013124934	0.11674737	0.993552802
POLR3B	0.009144132	0.11683679	0.993552802
UNC50	0.005527389	0.116905158	0.993552802
CD226	-0.015948416	0.117006681	0.993552802
PGM2	0.009306679	0.117186494	0.993552802
KCTD10	-0.023738918	0.117203581	0.993552802
KIAA1324	-0.011505682	0.117352428	0.993552802
PIGT	0.007757619	0.117353655	0.993552802
TMEM59L	0.010386775	0.11736425	0.993552802
TBC1D10A	-0.008085206	0.117598444	0.993552802
PKHD1L1	-0.013506483	0.117613039	0.993552802
LPP	-0.010225516	0.117644336	0.993552802
SPPL3	-0.009139095	0.117719162	0.993552802
FAM53B	-0.0113537	0.117781175	0.993552802
ACSL5	-0.006501617	0.11792208	0.993552802
C5orf20	0.01600388	0.118077085	0.993552802
KIAA0247	-0.006429881	0.118079204	0.993552802
CLDN5	-0.030967593	0.118131939	0.993552802
CCNE2	0.013648194	0.118251826	0.993552802
TTC8	0.01104806	0.118306704	0.993552802
SNHG8	-0.037713488	0.118318769	0.993552802
WBSCR22	-0.00710258	0.118606518	0.993552802
SOCS6	-0.012878459	0.118608241	0.993552802
HUS1B	-0.01329204	0.118641089	0.993552802
ARL6IP1	-0.007012533	0.118724558	0.993552802
GSTZ1	0.011481334	0.118828175	0.993552802
NOL10	-0.012181005	0.118830197	0.993552802
RNASET2	0.005676999	0.118831134	0.993552802
TMEM63B	-0.011950716	0.11895825	0.993552802

PCGF5	-0.011418926	0.118977005	0.993552802
NUP214	0.008394711	0.119015629	0.993552802
GPA33	-0.011411001	0.119074347	0.993552802
ANKLE1	0.020593835	0.119190704	0.993552802
EOMES	0.022650412	0.119221588	0.993552802
RNF122	-0.009614203	0.119289947	0.993552802
ALDH3A2	0.007419585	0.11931839	0.993552802
KHNYN	-0.011144275	0.119333911	0.993552802
KLF11	-0.010405029	0.119339956	0.993552802
STRADB	-0.009215619	0.119408428	0.993552802
GRPEL1	0.008030707	0.119454083	0.993552802
TLR2	0.015935104	0.119523605	0.993552802
MRPS22	0.004304987	0.119692917	0.993552802
GALE	0.008315012	0.119713314	0.993552802
CNFN	-0.014138809	0.119838011	0.993552802
DAP3	0.005796011	0.119889954	0.993552802
FTSJ2	0.006990866	0.119938931	0.993552802
FAM72D	0.016419945	0.120082113	0.993552802
HOMER2	-0.027695576	0.1201138	0.993552802
ACAD8	0.007141727	0.120273028	0.993552802
ZC3H6	0.011748747	0.120290429	0.993552802
COPB2	0.007822151	0.120339868	0.993552802
MARVELD1	0.0123863	0.120581907	0.993552802
SSBP3	-0.012200718	0.120794	0.993552802
IL15	0.011218972	0.120800429	0.993552802
XPR1	0.006838831	0.120814626	0.993552802
TOX	0.016099262	0.120842133	0.993552802
RANBP3	0.006679489	0.120860674	0.993552802
VDAC3	0.004345478	0.120883046	0.993552802
ETV6	-0.007019045	0.121204596	0.993552802
MBD1	-0.007947339	0.121233944	0.993552802
STT3A	0.007709528	0.121392762	0.993552802
KIF2A	-0.012225253	0.121445335	0.993552802
PIGV	0.016752549	0.121445506	0.993552802
RREB1	0.010688474	0.12147821	0.993552802
CYP27B1	-0.00909103	0.121522306	0.993552802
SDR16C5	-0.013578754	0.121540942	0.993552802
UBE2B	-0.008327245	0.121543904	0.993552802
AFMID	-0.013605836	0.121622515	0.993552802
NSF	0.005340102	0.121709474	0.993552802
DDX11L2	-0.039432618	0.121717404	0.993552802
PRRC2C	-0.006011641	0.121781328	0.993552802
SATB1	-0.011261695	0.12180066	0.993552802
SNRPN	-0.008433209	0.121920651	0.993552802
HMBS	0.012498966	0.122205074	0.993552802
NFKB2	-0.010602247	0.12224149	0.993552802
TMEM203	-0.005554361	0.122353342	0.993552802

ZBTB4	-0.006966801	0.122503699	0.993552802
ATP6V1B2	0.00632854	0.12287624	0.993552802
PDK4	0.018323726	0.122930908	0.993552802
HHATL	-0.01092051	0.12294955	0.993552802
CLASP2	0.010283487	0.123057278	0.993552802
POMGNT1	0.005498711	0.123115281	0.993552802
CHPT1	-0.014096773	0.123162621	0.993552802
LTB4R2	0.010825662	0.123192048	0.993552802
MACF1	-0.009150174	0.123378509	0.993552802
FER	0.012509317	0.123507152	0.993552802
HDDC2	0.007823284	0.123566101	0.993552802
EYA3	-0.019243522	0.123635886	0.993552802
PRPF40A	-0.012260775	0.123639111	0.993552802
ACVR1B	0.008339798	0.123642797	0.993552802
MKNK2	-0.00709433	0.123807227	0.993552802
DCAF16	-0.010459943	0.123915282	0.993552802
ABI1	-0.006133607	0.123970549	0.993552802
YEATS2	0.006466872	0.124123784	0.993552802
TNFRSF19	-0.010098789	0.124157537	0.993552802
RARS	0.00479638	0.124157671	0.993552802
DNTTIP1	0.009524371	0.124261895	0.993552802
MMD	-0.018745316	0.124475983	0.993552802
BMP4	0.011070153	0.124652824	0.993552802
CLEC4E	0.017179719	0.12467612	0.993552802
C19orf10	0.011848342	0.124924336	0.993552802
SAMD14	-0.04247812	0.124938779	0.993552802
ELK4	-0.011708156	0.125012262	0.993552802
PDCL3	0.007388727	0.125022964	0.993552802
KLF7	-0.01088857	0.125033848	0.993552802
PTPRE	0.009180212	0.125039016	0.993552802
FOXD4L4	-0.011260617	0.125062833	0.993552802
PTGS1	-0.025480226	0.125143201	0.993552802
USP30	0.011323122	0.12519278	0.993552802
SNORA80	-0.013582085	0.12534765	0.993552802
ADORA2B	0.013578693	0.125385748	0.993552802
LGALS4	0.010235786	0.125696664	0.993552802
BTAF1	0.006961038	0.125709201	0.993552802
SLC35F5	0.011727967	0.125808448	0.993552802
KRI1	-0.008232148	0.125812361	0.993552802
CCDC126	0.013710717	0.125874884	0.993552802
ZNF567	0.010382885	0.125899089	0.993552802
PPIG	-0.006916353	0.125909711	0.993552802
C10orf128	-0.021763902	0.126244058	0.993552802
VPS4A	-0.006939146	0.126260306	0.993552802
FCGR3A	-0.015443199	0.126347138	0.993552802
MTMR2	-0.009813504	0.126374969	0.993552802
PPFIBP2	0.01582406	0.126546638	0.993552802

ACRBP	-0.025737429	0.126683298	0.993552802
FZD1	0.011098937	0.126886796	0.993552802
ZC3HAV1	-0.007617382	0.126929851	0.993552802
CLEC16A	-0.005903044	0.126989451	0.993552802
SP140L	-0.009199854	0.127123224	0.993552802
SLFN12	0.012388811	0.127169297	0.993552802
PSMD2	0.005471657	0.127223438	0.993552802
RDM1	0.011853569	0.12746436	0.993552802
SLC35B1	0.005934297	0.127496491	0.993552802
MAGEL2	-0.008948244	0.127541939	0.993552802
EDF1	-0.00612806	0.12772364	0.993552802
SLC26A6	0.008766031	0.127733335	0.993552802
SRP54	0.006365448	0.127812207	0.993552802
LMNB2	-0.008492944	0.128439439	0.993552802
SPTY2D1	0.008614515	0.129090794	0.993552802
GNG11	-0.024078366	0.129286196	0.993552802
DYNLRB1	-0.009700192	0.129294974	0.993552802
MRPL50	0.009552316	0.129377571	0.993552802
DRAM1	0.011339244	0.129515767	0.993552802
UBXN8	0.010346	0.129554559	0.993552802
SLC16A3	0.006172191	0.129603558	0.993552802
LPCAT1	0.008554517	0.129652603	0.993552802
EML2	-0.010620041	0.129669855	0.993552802
GLB1	0.008689772	0.129706807	0.993552802
ATP6V0E2	0.00860418	0.129755471	0.993552802
TICAM1	0.011070106	0.12976092	0.993552802
CCDC112	0.009265328	0.129835131	0.993552802
SPATA6	0.010377681	0.129882565	0.993552802
IRF2BP2	0.007645158	0.129911353	0.993552802
CSRNP1	-0.009550493	0.129966341	0.993552802
CDH20	-0.010741265	0.130115233	0.993552802
P2RY13	0.012026526	0.130270163	0.993552802
RGMB	-0.02645686	0.130676564	0.993552802
RPL29	0.005194811	0.130702674	0.993552802
CBX6	0.008000837	0.130808122	0.993552802
FAM185A	-0.010751903	0.130816953	0.993552802
SF3B4	-0.005425247	0.130848798	0.993552802
UBE3A	0.004584948	0.130859014	0.993552802
TSEN2	0.010332941	0.130859196	0.993552802
TMEM167B	-0.006320798	0.130956715	0.993552802
MED21	0.015187917	0.130995055	0.993552802
NOL7	0.00703502	0.131052095	0.993552802
SH3BGRL2	-0.030031957	0.131064966	0.993552802
IFT80	0.009159087	0.131070848	0.993552802
C7orf25	0.011312009	0.131304499	0.993552802
ATP5A1	0.006923281	0.131317768	0.993552802
SPSB1	-0.01224695	0.13133597	0.993552802

ZC3H10	0.012451046	0.131384486	0.993552802
GTF2F2	0.006966552	0.131491331	0.993552802
IFT57	-0.01355427	0.131512811	0.993552802
TMEM168	0.009795896	0.131616162	0.993552802
ZNF765	0.012257601	0.131636148	0.993552802
PTPRU	0.018186373	0.131650092	0.993552802
TEX264	0.006058008	0.131654868	0.993552802
PMS1	0.010569484	0.131683923	0.993552802
MYH3	0.011831416	0.131692314	0.993552802
ZNF701	0.012413762	0.131771432	0.993552802
EPT1	-0.010169816	0.131798311	0.993552802
ATAD3A	0.009807982	0.131934635	0.993552802
TBCK	0.008025159	0.132118599	0.993552802
PPP1R14B	-0.008343544	0.13216284	0.993552802
LCP1	0.005156937	0.132166514	0.993552802
ELL2	0.017140375	0.132300426	0.993552802
CYB5R3	-0.007883223	0.132354136	0.993552802
C12orf4	0.008249472	0.132415876	0.993552802
SMEK1	-0.005461033	0.132488548	0.993552802
ST6GALNAC3	0.015539717	0.132511023	0.993552802
QSOX2	-0.009344234	0.132651671	0.993552802
RNF115	-0.008644201	0.132854528	0.993552802
DYM	0.007325211	0.132996897	0.993552802
TNFRSF12A	-0.010085936	0.133110845	0.993552802
DYNC1I2	0.007107946	0.133345488	0.993552802
CA1	-0.016882909	0.13339425	0.993552802
DYDC2	0.013066769	0.133552908	0.993552802
FAM120B	0.006475625	0.133640025	0.993552802
NLRP1	-0.010293917	0.133682057	0.993552802
COQ7	-0.010999308	0.133710897	0.993552802
B3GALNT2	0.011257796	0.133719051	0.993552802
NDFIP2	0.012085183	0.133887959	0.993552802
MYSM1	0.013643434	0.133969414	0.993552802
CAPG	-0.014080091	0.134023814	0.993552802
SKIL	0.009876169	0.134084709	0.993552802
ALG10	0.008695363	0.134103558	0.993552802
REM2	-0.013237398	0.134185574	0.993552802
TCF25	0.00657237	0.134279208	0.993552802
TMEM169	0.011613678	0.134309849	0.993552802
STK32B	0.01237132	0.13437309	0.993552802
METTL4	-0.007728808	0.134388158	0.993552802
MRPS36	0.007076234	0.134394699	0.993552802
GRSF1	0.005161317	0.134406035	0.993552802
C5orf30	0.00882289	0.134522181	0.993552802
ZNF625-ZNF20	0.012497649	0.134712596	0.993552802
PPARG	0.014269232	0.13475105	0.993552802
SERPINB9	-0.020924426	0.134928255	0.993552802

TXNDC17	0.00692073	0.134953256	0.993552802
DDOST	0.005612655	0.134965004	0.993552802
EHD3	-0.017514874	0.13500486	0.993552802
TRIM46	0.012148408	0.135212519	0.993552802
CYB5R1	-0.008423105	0.135247607	0.993552802
PHACTR2	-0.010232589	0.135454251	0.993552802
GLYCTK	0.009880464	0.135473195	0.993552802
P2RX1	-0.021197907	0.135589264	0.993552802
CHRNB1	0.006617276	0.135610589	0.993552802
ONECUT2	0.008889131	0.135779377	0.993552802
P2RY2	0.015549363	0.136169161	0.993552802
PITRM1	0.010695069	0.136396268	0.993552802
H2AFY	0.00495126	0.136436192	0.993552802
H1F0	-0.018525865	0.136728963	0.993552802
ARHGAP31	0.012408031	0.136842684	0.993552802
ZNF684	0.009351566	0.136913199	0.993552802
RGS10	-0.010882035	0.136968424	0.993552802
CCL3L3	0.033067435	0.13708575	0.993552802
IRF2BP1	-0.013069703	0.137274783	0.993552802
CRYM	-0.011636643	0.137420616	0.993552802
LPCAT4	-0.009342021	0.137423258	0.993552802
PSMA6	0.005809325	0.137520785	0.993552802
KBTBD6	0.015509186	0.137787954	0.993552802
RPL17	0.00591817	0.137870215	0.993552802
SEC23IP	0.007046982	0.137932447	0.993552802
KIAA0922	-0.008558995	0.138032695	0.993552802
MYL5	-0.015506576	0.138069316	0.993552802
SCGB1C1	-0.032208147	0.138121012	0.993552802
SPRED1	0.015658612	0.13825594	0.993552802
RBM43	-0.016416386	0.138417222	0.993552802
DCUN1D3	0.009923527	0.138438388	0.993552802
POTEM	-0.014657399	0.138528911	0.993552802
RGMA	0.008745293	0.138680434	0.993552802
MYLK	-0.026136076	0.138723537	0.993552802
NEK7	0.011433657	0.138805521	0.993552802
RGS12	0.01302183	0.138878637	0.993552802
PDIK1L	0.008877416	0.139356364	0.993552802
MINA	-0.009220205	0.139495887	0.993552802
SLC43A3	-0.010064973	0.13950539	0.993552802
SNHG1	0.007466471	0.139587732	0.993552802
ATF6B	-0.010555242	0.139748936	0.993552802
EIF1	-0.007075041	0.139757767	0.993552802
CXCR4	-0.018910746	0.140200424	0.993552802
TMEM202	-0.009595375	0.140371759	0.993552802
PIAS3	-0.00714254	0.140390545	0.993552802
NUP133	0.007766875	0.140460351	0.993552802
CDC37	0.004913851	0.140545981	0.993552802

OSGEPL1	0.009826017	0.140613217	0.993552802
TSSC1	-0.006898869	0.140623894	0.993552802
DUSP23	0.006481941	0.140651397	0.993552802
UEVLD	0.009419318	0.140783367	0.993552802
PLCD3	0.008630637	0.14090118	0.993552802
SASS6	0.007601788	0.140967749	0.993552802
OGDH	-0.010754934	0.141006142	0.993552802
LAMB1	0.009535072	0.14124778	0.993552802
SLC25A16	0.010811938	0.14136274	0.993552802
NDUFB9	-0.007100219	0.141487033	0.993552802
SNAPIN	0.006849553	0.141574399	0.993552802
ITGB2	0.003948957	0.141580547	0.993552802
KRT73	-0.038889101	0.141623894	0.993552802
TTC9C	0.009378104	0.141714438	0.993552802
RAD51L3-RFFL	-0.006987205	0.142011301	0.993552802
PICALM	0.006396246	0.14204127	0.993552802
PLAU	0.008138384	0.142439881	0.993552802
FMO6P	-0.010067879	0.142600751	0.993552802
GPR18	0.021123083	0.142756962	0.993552802
AAGAB	-0.010110428	0.14276383	0.993552802
RIMKLB	-0.011049597	0.142997293	0.993552802
ADARB1	-0.009081737	0.14301229	0.993552802
PRPF19	-0.006339261	0.143098756	0.993552802
EFHC1	0.010221877	0.143268772	0.993552802
ELMOD2	0.011368447	0.143365473	0.993552802
C18orf21	0.00656129	0.14339944	0.993552802
GNG7	-0.013719267	0.143575815	0.993552802
RFC2	-0.012226429	0.143610672	0.993552802
MLH3	-0.020176204	0.143759042	0.993552802
GP6	-0.031832468	0.144114791	0.993552802
ADM2	0.010657072	0.14440864	0.993552802
C2orf47	0.007975281	0.144452516	0.993552802
RPGRIP1	0.016310039	0.144475186	0.993552802
NEDD4	0.01122168	0.144486373	0.993552802
HIC2	0.006388599	0.144512874	0.993552802
EBPL	-0.014864872	0.144529516	0.993552802
IWS1	0.006070628	0.144553076	0.993552802
HDHD2	0.005727149	0.144590332	0.993552802
SYS1	0.008138956	0.144619406	0.993552802
PNRC2	0.006083258	0.144658469	0.993552802
TRIM62	-0.010302546	0.144665443	0.993552802
TRMT2A	0.009253316	0.144821423	0.993552802
SNORD31	-0.011288833	0.145090466	0.993552802
PDK1	-0.013845957	0.145100055	0.993552802
UBE2H	-0.008557506	0.145212827	0.993552802
ATP9A	-0.02065044	0.145284084	0.993552802
KIFC1	0.014102603	0.145324037	0.993552802

KIT	0.011143427	0.145478868	0.993552802
TBP	0.006043138	0.145487678	0.993552802
SIRPB1	0.019315988	0.145499057	0.993552802
CSTF1	0.011489567	0.145696885	0.993552802
GCDH	-0.007217769	0.145699354	0.993552802
GPR84	0.01099282	0.145758241	0.993552802
DUSP22	0.007499733	0.146059605	0.993552802
HSH2D	-0.014954096	0.146077067	0.993552802
ZNF35	0.014154712	0.146332735	0.993552802
RAB4B	0.013176182	0.146533991	0.993552802
RASSF1	0.010761705	0.146684126	0.993552802
GSTA1	-0.032340169	0.146686284	0.993552802
NAA30	0.006729474	0.146864679	0.993552802
TIMELESS	-0.00886772	0.14687101	0.993552802
RBAK	0.012976662	0.146884874	0.993552802
NOC2L	-0.009743417	0.146990181	0.993552802
CYTH4	0.006915363	0.147094503	0.993552802
FAM50B	0.014683691	0.147156343	0.993552802
ATG4D	-0.009114722	0.147182364	0.993552802
NLRP7	0.02058174	0.147220698	0.993552802
POLR1E	0.007769593	0.147236058	0.993552802
HLA-DMB	0.006782849	0.147237297	0.993552802
SLC43A1	0.012716833	0.147483881	0.993552802
SPG11	0.006460682	0.147521923	0.993552802
DDHD2	-0.004964946	0.147762441	0.993552802
REEP6	0.013197214	0.147821474	0.993552802
GPN1	-0.005875214	0.147931485	0.993552802
FSTL1	-0.020789583	0.148077566	0.993552802
ZFPL1	0.00820056	0.14809169	0.993552802
GTF2B	0.00860506	0.148215831	0.993552802
SMARCAL1	0.004890884	0.14825832	0.993552802
MED23	0.008856585	0.148454937	0.993552802
ADRA2B	-0.009804731	0.148512691	0.993552802
C9orf91	-0.007949247	0.148616091	0.993552802
GUCY1A3	-0.024830623	0.148728576	0.993552802
TMEM174	-0.012569751	0.148779445	0.993552802
ADCK2	0.009346107	0.148817108	0.993552802
NFIB	-0.03730403	0.148842573	0.993552802
MLEC	0.009787294	0.149056653	0.993552802
ATP6V0B	-0.006269839	0.149069758	0.993552802
RNF145	0.008914858	0.149219505	0.993552802
EP400	-0.006805957	0.149403155	0.993552802
LRRK2	0.016947133	0.149435354	0.993552802
GPATCH1	0.009171169	0.149646034	0.993552802
SIPA1L3	-0.015047847	0.149667769	0.993552802
HRSP12	0.008935739	0.149671502	0.993552802
RAD23B	-0.011828819	0.149853555	0.993552802

JMJD1C	0.007914201	0.149910785	0.993552802
MRRF	0.005597594	0.149979537	0.993552802
GGNBP2	0.006029559	0.150054265	0.993552802
EIF3B	0.005830608	0.150068266	0.993552802
SYNJ1	0.007073226	0.150087815	0.993552802
FAM71E2	-0.009346043	0.150130576	0.993552802
CDK2	-0.006038773	0.150205559	0.993552802
TADA1	0.007035243	0.150259158	0.993552802
ADIPOR1	-0.009295709	0.150261116	0.993552802
TSC22D1	-0.021520916	0.150443312	0.993552802
FAM170B	-0.015023845	0.150450329	0.993552802
HSF5	0.012186305	0.1504743	0.993552802
ZNF549	-0.009378179	0.150546777	0.993552802
PIF1	0.011591773	0.150607719	0.993552802
ANKHD1	0.005244866	0.150632696	0.993552802
MIA3	-0.008829037	0.150655975	0.993552802
UBAC1	-0.005513196	0.150853316	0.993552802
PPRC1	0.00655809	0.150861771	0.993552802
TRUB2	0.007645527	0.150954354	0.993552802
HADHB	0.007672156	0.150970874	0.993552802
ABCB10	0.007106638	0.151019454	0.993552802
TSPAN18	-0.014159822	0.151053153	0.993552802
PELO	0.014208204	0.151117721	0.993552802
SPARC	-0.029452417	0.151165753	0.993552802
C9orf40	0.014436809	0.151212686	0.993552802
FAM69B	-0.024723661	0.151219024	0.993552802
FAM103A1	0.008985204	0.151463259	0.993552802
FUZ	-0.009297552	0.151662807	0.993552802
ZNF517	0.008638642	0.151665094	0.993552802
TBC1D13	-0.005883786	0.151735158	0.993552802
PGAM1	0.005873056	0.151776513	0.993552802
PTTG1	0.013114638	0.151787422	0.993552802
ZSCAN29	0.014430854	0.15208731	0.993552802
ABCA10	0.011002793	0.152216604	0.993552802
BRAT1	0.004625926	0.152299869	0.993552802
TP53RK	0.00961261	0.152339876	0.993552802
PGBD2	0.009974498	0.152360587	0.993552802
ZNF429	0.018466382	0.152426855	0.993552802
GPR108	-0.007892823	0.152445066	0.993552802
EFNA1	0.017529928	0.152512905	0.993552802
SNX27	0.007566316	0.152769512	0.993552802
SLC41A1	-0.008341581	0.152806635	0.993552802
CCHCR1	-0.00992124	0.152878318	0.993552802
TM9SF2	0.00536508	0.15325991	0.993552802
TGIF2	-0.00565044	0.153449816	0.993552802
VEGFB	-0.007529304	0.153684552	0.993552802
BCS1L	0.00743031	0.153748145	0.993552802

GADD45B	-0.00889528	0.153839987	0.993552802
SPINT2	-0.00934291	0.154083837	0.993552802
ACPT	-0.012212642	0.154347959	0.993552802
CHD7	-0.009974691	0.154359806	0.993552802
ZNF280D	0.01004447	0.154365952	0.993552802
AMMECR1L	0.007797599	0.154378148	0.993552802
C1orf189	0.008506349	0.154431842	0.993552802
GINS3	0.008147786	0.154497274	0.993552802
MX2	-0.012980399	0.154506028	0.993552802
ZNF213	0.012400031	0.154635512	0.993552802
H3F3B	-0.013888569	0.154824979	0.993552802
DCUN1D2	0.00891534	0.154844061	0.993552802
UBAP2L	-0.008590953	0.154883074	0.993552802
MIR302C	0.010757177	0.154902512	0.993552802
SNX29	-0.02321393	0.155079076	0.993552802
MYL9	-0.038157216	0.155199714	0.993552802
GEMIN4	0.006203777	0.15521336	0.993552802
PLOD3	0.008150472	0.155215595	0.993552802
PIGK	0.008975874	0.155434972	0.993552802
NAALADL1	-0.010754551	0.155580047	0.993552802
HJURP	0.01131724	0.15582784	0.993552802
PRKAR2A	-0.009162336	0.15606902	0.993552802
MKRN1	-0.004055148	0.156099506	0.993552802
KCNJ10	-0.009777592	0.156169517	0.993552802
FRS3	0.007290544	0.156170461	0.993552802
LAPTM4B	-0.012625275	0.156202396	0.993552802
PKD2	0.010516523	0.156252932	0.993552802
GAA	-0.016048173	0.156274556	0.993552802
ZNF282	0.01046194	0.156386842	0.993552802
DLD	0.009524329	0.156492657	0.993552802
TCEANC2	-0.006557342	0.156498254	0.993552802
OSBPL1A	0.016141702	0.156641104	0.993552802
TSPAN14	-0.011064142	0.156865634	0.993552802
ENTPD7	0.010958208	0.156865723	0.993552802
SHB	-0.007540041	0.157003472	0.993552802
CAT	0.012364909	0.157235382	0.993552802
MRPL19	0.01001098	0.157236719	0.993552802
RNF126	-0.006050632	0.15750014	0.993552802
DDB2	-0.010143822	0.157547151	0.993552802
TRPC2	-0.010052409	0.157576662	0.993552802
EIF2S2	-0.008710004	0.157660937	0.993552802
SEC11A	0.006024471	0.157770209	0.993552802
SEC63	0.008632652	0.157831827	0.993552802
NFKBIB	0.007216553	0.157879623	0.993552802
TMOD2	0.011210934	0.157948514	0.993552802
RNF34	0.007352091	0.158063194	0.993552802
BCL7B	-0.012320654	0.15812272	0.993552802

SAMD10	-0.009499784	0.158438062	0.993552802
ARFGEF1	0.004974237	0.158453093	0.993552802
ATXN1	0.007776968	0.15850548	0.993552802
FKBP15	0.007178613	0.158648636	0.993552802
ZNF174	0.010157703	0.158838127	0.993552802
DNMT1	0.004851922	0.158965954	0.993552802
PLXND1	0.012126597	0.159057831	0.993552802
ACSL1	0.011566724	0.15912271	0.993552802
MMP28	0.020983277	0.159209975	0.993552802
MEA1	-0.005560266	0.159223041	0.993552802
RBM12B	0.011848362	0.159638742	0.993552802
EIF2B3	0.007091749	0.159870052	0.993552802
EXOSC2	0.008143307	0.159941263	0.993552802
PPP1R2	-0.007197054	0.160088687	0.993552802
ZNF622	0.007121056	0.160137235	0.993552802
PARVB	-0.023380314	0.160161847	0.993552802
MYB	0.011116375	0.160177444	0.993552802
TOP3A	0.00907569	0.160289926	0.993552802
ZNF707	0.009687546	0.160625463	0.993552802
FBLN5	-0.017985305	0.160694677	0.993552802
PNPLA7	-0.015664051	0.160863272	0.993552802
CMTM7	0.006880771	0.160896828	0.993552802
WRB	-0.013964772	0.161003107	0.993552802
ARHGAP32	-0.010928448	0.161382731	0.993552802
SELENBP1	-0.008470299	0.161481296	0.993552802
ENO3	0.009345154	0.161617764	0.993552802
SNCA	-0.0228073	0.161648985	0.993552802
BBC3	-0.007606851	0.161839401	0.993552802
PTPRCAP	-0.009801987	0.162020894	0.993552802
EEA1	0.00801729	0.162074353	0.993552802
CCT6B	0.009850958	0.162289208	0.993552802
BCL2L12	0.00546467	0.16230942	0.993552802
MAP2K7	-0.007955533	0.162441958	0.993552802
MFAP1	0.010759562	0.162514567	0.993552802
GIMAP7	0.009336977	0.162516464	0.993552802
DDR2	-0.013094154	0.162679933	0.993552802
VENTX	0.015045137	0.16276444	0.993552802
FAM104A	0.00446302	0.162808941	0.993552802
RP9P	-0.008453044	0.162890825	0.993552802
CALM3	-0.005188005	0.162976113	0.993552802
PCMTD2	-0.007206169	0.163043409	0.993552802
HSPA13	0.009918705	0.163050544	0.993552802
USP46	-0.012051559	0.163109241	0.993552802
MRPL30	0.011055555	0.163182578	0.993552802
CORO7	-0.009419797	0.163206577	0.993552802
RTN4IP1	0.006398206	0.163229939	0.993552802
CHTF18	0.009195846	0.163304381	0.993552802

TRIM7	0.01483967	0.163349211	0.993552802
EIF3C	0.01376841	0.163436104	0.993552802
CCDC135	-0.010838056	0.163521627	0.993552802
CD1D	0.010398391	0.163592668	0.993552802
NCF1B	-0.016342556	0.163756373	0.993552802
EIF2B5	0.006784061	0.163809535	0.993552802
TUBA4A	-0.011223503	0.163827637	0.993552802
TTC39B	-0.012305801	0.163872215	0.993552802
CUEDC1	0.011330629	0.163877053	0.993552802
PLEKHJ1	-0.009729853	0.163893208	0.993552802
BANK1	-0.019824401	0.163936994	0.993552802
UBE2W	0.010026912	0.164067893	0.993552802
SUB1	0.008271675	0.164212653	0.993552802
MSX1	0.008905433	0.164281166	0.993552802
VIPR1	0.011554849	0.164291706	0.993552802
RANBP6	0.007983044	0.164433879	0.993552802
GIMAP2	0.009359185	0.16458963	0.993552802
PITPNC1	-0.00634726	0.164686229	0.993552802
UBE2M	-0.005164061	0.164725668	0.993552802
PPM1F	0.006944238	0.164729603	0.993552802
CCR2	0.013144657	0.164774726	0.993552802
KCNK13	0.012062947	0.164838103	0.993552802
TRRAP	-0.006523616	0.164977265	0.993552802
SCARNA7	-0.008277475	0.164990494	0.993552802
ZDHHC16	0.005646046	0.165016238	0.993552802
USO1	0.004742609	0.16502281	0.993552802
DUSP11	0.007070537	0.165582716	0.993552802
C10orf32	0.013330634	0.165829617	0.993552802
LCP2	0.007430605	0.165865483	0.993552802
BTBD17	0.018004322	0.165869201	0.993552802
ZNF876P	-0.005260243	0.165890194	0.993552802
ZMAT3	-0.010483168	0.16590842	0.993552802
TIAM1	0.012604251	0.166098006	0.993552802
POR	-0.007936583	0.166133637	0.993552802
MBNL2	0.007618543	0.166224004	0.993552802
GATA2	-0.015430814	0.16627689	0.993552802
NF1	-0.012994297	0.166280772	0.993552802
GFOD2	0.008302366	0.166291506	0.993552802
TOR1AIP2	0.009939962	0.166361776	0.993552802
NPR2	0.008766747	0.166549825	0.993552802
MFSD7	0.010861779	0.166875222	0.993552802
FAR1	0.007947185	0.166875887	0.993552802
ZNF580	-0.010403584	0.166960487	0.993552802
PUS7L	0.009221962	0.167048931	0.993552802
DPH2	0.004701076	0.16709354	0.993552802
ZNF217	0.009716662	0.167093735	0.993552802
RPTOR	0.012475424	0.167110353	0.993552802

TUBG2	0.008151298	0.167149954	0.993552802
LPHN1	-0.009783606	0.167179124	0.993552802
CES4A	0.009829995	0.167398013	0.993552802
TBC1D5	-0.009124288	0.167485468	0.993552802
RPP25	-0.009887527	0.167549036	0.993552802
KBTBD7	0.009447304	0.167699984	0.993552802
BCL2L11	-0.011722841	0.167748557	0.993552802
ARL5B	0.014784394	0.167881606	0.993552802
ACTR3B	-0.011569345	0.168074675	0.993552802
KCTD20	-0.007275978	0.168117151	0.993552802
BMP6	-0.023284386	0.168135139	0.993552802
PPAP2B	0.013605729	0.16821307	0.993552802
ACOT1	0.012162352	0.168231403	0.993552802
ZBTB2	-0.00853064	0.168289375	0.993552802
RAB11A	-0.007348843	0.168361203	0.993552802
ZEB2	0.010458213	0.168765191	0.993552802
PHF23	0.006972282	0.168988974	0.993552802
CSNK1G3	0.003756668	0.169092813	0.993552802
PRG2	-0.010656116	0.169174839	0.993552802
MEIS1	-0.02007403	0.169258603	0.993552802
ADC	0.010436502	0.169272261	0.993552802
SIDT1	-0.008544551	0.169346962	0.993552802
EIF3J	0.007067417	0.169622798	0.993552802
JMJD8	-0.004362538	0.169662228	0.993552802
NEFH	-0.010398324	0.169847581	0.993552802
CLEC18C	0.011611852	0.1699192	0.993552802
HLCS	0.00874875	0.170159167	0.993552802
SCAND1	-0.006395191	0.170273378	0.993552802
KIAA1468	0.009380663	0.170304427	0.993552802
DCAF15	-0.009279155	0.170423057	0.993552802
MIER1	0.008004014	0.170658663	0.993552802
H6PD	-0.013261858	0.170806435	0.993552802
SDF2L1	0.008508065	0.170809316	0.993552802
HLA-DOA	-0.016086233	0.170849657	0.993552802
TLN1	-0.010457577	0.170885412	0.993552802
BACH2	-0.01315756	0.170923562	0.993552802
ADAP1	0.005646726	0.170928162	0.993552802
SMARCA2	-0.004806018	0.170968899	0.993552802
CDK7	0.005087927	0.171018031	0.993552802
ST7	-0.011936261	0.171123206	0.993552802
HYOU1	0.010458343	0.171199958	0.993552802
TSC2	-0.008472557	0.171269215	0.993552802
GPX7	-0.008570344	0.171294494	0.993552802
SRSF1	0.004383145	0.171340849	0.993552802
SNORA27	-0.013121637	0.171465677	0.993552802
SPINK2	0.015238406	0.171561377	0.993552802
LEFTY1	-0.017350706	0.171571813	0.993552802

ITGB5	-0.025898145	0.171599521	0.993552802
C11orf82	0.011814561	0.171644221	0.993552802
SPRYD4	0.01041464	0.171676842	0.993552802
GAS2L1	-0.014264919	0.171760688	0.993552802
ZBTB41	0.009426267	0.17189385	0.993552802
PUS10	0.009447855	0.172073177	0.993552802
FAF2	0.005217065	0.172145636	0.993552802
CALM2	0.006065837	0.172230445	0.993552802
ZNF92	0.011015478	0.172383396	0.993552802
IGHMBP2	-0.010721599	0.172462143	0.993552802
ANK1	-0.014191618	0.172761756	0.993552802
HIST1H1D	0.013663604	0.17277516	0.993552802
BAG4	0.008076917	0.172836677	0.993552802
CECR5	0.006384706	0.172878143	0.993552802
TRAF7	0.009405715	0.173334112	0.993552802
CDKN2B	0.015142459	0.17338626	0.993552802
STOM	-0.009159998	0.173464964	0.993552802
HIPK2	-0.010192624	0.173503024	0.993552802
USP38	0.007746464	0.173512406	0.993552802
WAPAL	-0.006397999	0.173615279	0.993552802
GALNT11	-0.005944972	0.173786027	0.993552802
ANP32A	0.011413918	0.173788451	0.993552802
OR7E156P	-0.009421243	0.173791503	0.993552802
EGLN3	-0.011303475	0.173799066	0.993552802
ARL15	0.009820947	0.173944314	0.993552802
RNLS	0.007408273	0.174002851	0.993552802
AARS	0.00499652	0.174214728	0.993552802
NCBP2	0.004652689	0.174228648	0.993552802
SDHD	0.009973751	0.174289011	0.993552802
IGF1R	-0.010099635	0.174344851	0.993552802
TMEM180	0.008735175	0.174480053	0.993552802
ARAP2	0.008241986	0.174795511	0.993552802
HNRNPH1	-0.008685186	0.17484246	0.993552802
TNFRSF14	0.005896613	0.174871305	0.993552802
VPS54	0.008358495	0.174913679	0.993552802
KLHL21	0.00870191	0.174918993	0.993552802
BRE	-0.007369848	0.174951255	0.993552802
ASH1L	-0.009860906	0.17503043	0.993552802
RPS29	0.004371799	0.175145787	0.993552802
N6AMT1	0.010828441	0.17521912	0.993552802
CDH6	-0.017084421	0.175319568	0.993552802
FXYD7	-0.010574536	0.175391584	0.993552802
VAV3	-0.081607444	0.175471488	0.993552802
LYPLAL1	-0.007122908	0.175632901	0.993552802
TSPAN3	-0.010154575	0.175689428	0.993552802
PIGF	0.008264289	0.175708944	0.993552802
SNORD42B	-0.008862836	0.175741261	0.993552802

C1orf123	-0.007397597	0.175758285	0.993552802
FOXC1	-0.013443431	0.175805839	0.993552802
ZNF544	0.007534965	0.175896349	0.993552802
CD53	0.005498234	0.176117537	0.993552802
GAB1	0.008876613	0.176282113	0.993552802
NRSN2	-0.010331939	0.176450299	0.993552802
PHF1	-0.00895634	0.176922223	0.993552802
TTYH2	0.009972053	0.177077385	0.993552802
TUBA3C	0.008545309	0.177188076	0.993552802
SNORD114-2	-0.008238551	0.177203409	0.993552802
ESRRA	0.006259001	0.17728387	0.993552802
ZNF79	0.014811187	0.177344822	0.993552802
MRPS10	-0.007852107	0.177421403	0.993552802
HECW2	0.008896247	0.177582998	0.993552802
MLH1	0.004966471	0.177631683	0.993552802
SLC15A3	0.008982071	0.177682985	0.993552802
DDIT4	-0.02142662	0.177744848	0.993552802
ZBED5	0.005580346	0.177891095	0.993552802
PAPOLG	0.009994716	0.17805022	0.993552802
NPR1	-0.009928644	0.178069965	0.993552802
TTC26	0.008239747	0.178080626	0.993552802
PLXNC1	0.013532339	0.178155288	0.993552802
TRAPPC4	0.007979906	0.178270113	0.993552802
BUB3	-0.004193363	0.178688134	0.993552802
ULK2	0.008407417	0.178709328	0.993552802
TSPYL5	-0.015238043	0.178754782	0.993552802
SLC35A1	0.005796844	0.17887784	0.993552802
FAM109B	-0.009920938	0.178878742	0.993552802
ZDHHC18	-0.007319705	0.178900754	0.993552802
CDCA4	-0.007656386	0.17891713	0.993552802
THEM4	-0.017875672	0.178949015	0.993552802
PRPSAP1	0.005345759	0.178967858	0.993552802
RSC1A1	-0.009112481	0.179034373	0.993552802
CBLL1	-0.008678971	0.179097869	0.993552802
EIF2S1	-0.005771812	0.179150213	0.993552802
SHC1	-0.00647549	0.179162405	0.993552802
ATG9A	0.007683278	0.179185542	0.993552802
EIF3G	-0.005696916	0.179214112	0.993552802
FUBP3	0.00544846	0.179220039	0.993552802
PSMB6	0.005904906	0.179226255	0.993552802
POLA2	0.006386821	0.179234906	0.993552802
SDR42E1	0.010944027	0.17941585	0.993552802
PPP2R5A	-0.006409799	0.179426559	0.993552802
YWHAG	0.006438309	0.179630263	0.993552802
IVD	0.010297016	0.179631116	0.993552802
TOMM40L	-0.009903194	0.179679213	0.993552802
TBC1D10C	0.00722283	0.179761725	0.993552802

GFPT1	0.009727088	0.179857875	0.993552802
GALK1	0.005089803	0.179863695	0.993552802
MRS2	0.008194369	0.17991551	0.993552802
ADAT1	0.008570283	0.180005006	0.993552802
TTYH3	-0.008587759	0.180033809	0.993552802
ATAD1	0.008070829	0.180068461	0.993552802
POC1A	0.009654605	0.180094462	0.993552802
HSPA5	0.006423144	0.180301079	0.993552802
SUCLG1	0.006116142	0.180374237	0.993552802
SEL1L	0.007217675	0.180489833	0.993552802
TSPAN4	0.012052314	0.180574405	0.993552802
PKD1P1	-0.007880145	0.180744971	0.993552802
PIK3CG	-0.011018331	0.180767945	0.993552802
TLE1	-0.016422694	0.180825443	0.993552802
NUP54	0.004741774	0.180944691	0.993552802
CHAF1B	0.010624043	0.180954369	0.993552802
PIK3C2A	0.013141265	0.18104218	0.993552802
SAMD11	0.010471277	0.181072141	0.993552802
HINT3	0.008699182	0.181195111	0.993552802
EEF1A2	-0.016020108	0.181212799	0.993552802
RAB8A	-0.005123377	0.18136409	0.993552802
COASY	0.004905069	0.181421792	0.993552802
NR4A2	-0.01776722	0.18159126	0.993552802
AMOTL2	-0.00928403	0.181860591	0.993552802
GOT1	0.004748703	0.181949448	0.993552802
SLC24A3	-0.029642887	0.181958733	0.993552802
UBE3C	0.00568181	0.182010206	0.993552802
NPEPL1	0.00999655	0.182185585	0.993552802
CCDC92	-0.008649579	0.182217406	0.993552802
POLDIP3	0.004545119	0.182294263	0.993552802
SLC37A4	0.006877974	0.182332715	0.993552802
TRAPPC10	-0.00651115	0.182443682	0.993552802
SGSM3	0.009490334	0.182709491	0.993552802
NEXN	-0.018962021	0.182868528	0.993552802
DDX21	0.007828111	0.182930653	0.993552802
UBTD1	-0.011111193	0.182984345	0.993552802
OR2AG1	-0.011948586	0.183041766	0.993552802
BAIAP2L2	0.01045839	0.183082454	0.993552802
SLC25A44	0.005308718	0.183090774	0.993552802
PDLIM3	0.00880767	0.183120927	0.993552802
CHST15	0.00835338	0.183221264	0.993552802
PRPF4	-0.004210627	0.183246923	0.993552802
SYT11	0.011282861	0.183288016	0.993552802
TXN	0.00956127	0.183507651	0.993552802
RPL13AP5	-0.002914953	0.183665892	0.993552802
ILVBL	0.007580271	0.183676609	0.993552802
RPL10A	0.008391663	0.183678604	0.993552802

OTP	-0.0091317	0.183734033	0.993552802
XPNPEP3	0.003866244	0.184093249	0.993552802
MRPL12	-0.007651722	0.184285712	0.993552802
TACC2	-0.011719551	0.184407576	0.993552802
AGPAT9	0.009358043	0.184459007	0.993552802
SEMA4A	0.009245304	0.184595775	0.993552802
SLC39A9	0.009554878	0.184627758	0.993552802
IQCC	0.007060917	0.184677081	0.993552802
PDE4A	0.010434145	0.184824448	0.993552802
DNAJB5	-0.009986541	0.18486914	0.993552802
TBCCD1	-0.009031158	0.184879013	0.993552802
SLC27A3	0.007135334	0.185058955	0.993552802
TM7SF2	-0.007889558	0.18513492	0.993552802
LRP10	-0.00567437	0.18530478	0.993552802
PLK3	0.011255815	0.185504703	0.993552802
MRPL3	0.005858964	0.185600799	0.993552802
PAPD5	-0.008106473	0.185723722	0.993552802
OTUB1	0.008272616	0.185894696	0.993552802
USP37	-0.008733364	0.185991947	0.993552802
CBX8	0.007958221	0.186081211	0.993552802
BCL10	0.012091996	0.186084517	0.993552802
AKT1	-0.007638158	0.1860968	0.993552802
PNP	0.00956787	0.186155389	0.993552802
HIST2H2BF	0.008249485	0.186170924	0.993552802
SRRM1	0.003942886	0.186218551	0.993552802
ZSCAN21	0.007697969	0.186317537	0.993552802
CABP5	-0.031402983	0.186356082	0.993552802
RNMTL1	0.005103659	0.186375042	0.993552802
AEBP2	0.007697901	0.186407153	0.993552802
FSD1L	-0.010504743	0.186447957	0.993552802
RABGAP1	-0.006872373	0.186506357	0.993552802
CD2	0.008306505	0.186515276	0.993552802
VAMP1	0.009368157	0.186575609	0.993552802
GFI1B	-0.017503493	0.186666084	0.993552802
DPM3	-0.006944775	0.186670689	0.993552802
HAUS8	0.007735219	0.186885225	0.993552802
WDR73	0.009605207	0.186923018	0.993552802
COQ10B	0.007939903	0.186998246	0.993552802
RNF175	-0.01707171	0.187028548	0.993552802
ZNF518B	-0.006634614	0.18704447	0.993552802
MAP2K1	0.006687833	0.187150603	0.993552802
NICN1	0.006707801	0.187197219	0.993552802
C9orf64	0.007989522	0.187262152	0.993552802
FAM117A	-0.007479192	0.187421169	0.993552802
BBS2	-0.009640742	0.187488813	0.993552802
FAM159A	-0.010326629	0.1875033	0.993552802
PID1	0.019060375	0.187710663	0.993552802

KIAA0040	-0.011151564	0.18772095	0.993552802
BAZ1B	-0.005313146	0.187761008	0.993552802
C21orf2	0.011164553	0.187841438	0.993552802
PRDX3	0.013795464	0.187871572	0.993552802
IGF2R	0.006023489	0.188096802	0.993552802
VCL	-0.011149714	0.188175569	0.993552802
ADORA3	0.017832928	0.188338302	0.993552802
ETNK1	0.00960222	0.188353809	0.993552802
GLIS3	-0.009198708	0.188354408	0.993552802
VPS37A	0.006595329	0.188414776	0.993552802
GPR19	0.01532941	0.188691027	0.993552802
ZCCHC8	0.006047354	0.188706664	0.993552802
ERGIC1	0.010680009	0.188710669	0.993552802
PSEN2	0.008181394	0.188778915	0.993552802
FAM27E3	0.011343094	0.188838606	0.993552802
ST8SIA4	0.010670712	0.188841979	0.993552802
TMEM110	0.010702682	0.189063641	0.993552802
RPH3AL	-0.009508543	0.18919156	0.993552802
KIF23	0.011418023	0.18920624	0.993552802
ZMYM2	0.006813195	0.189227862	0.993552802
NCAM1	-0.018712384	0.189498815	0.993552802
CTSG	0.032975142	0.189509829	0.993552802
TNNC1	0.007831794	0.189565474	0.993552802
EIF2B2	0.008726198	0.18961846	0.993552802
CAV1	0.01609535	0.18965299	0.993552802
LRRC40	0.011568166	0.189773027	0.993552802
RNF4	0.00659358	0.189837843	0.993552802
RUFY3	0.007973589	0.189941416	0.993552802
SCD	-0.012411149	0.190060814	0.993552802
TTK	0.012248571	0.190097839	0.993552802
NACC2	0.008405127	0.190112578	0.993552802
ANKRD32	0.033952644	0.190155282	0.993552802
LONP1	-0.006474499	0.190250985	0.993552802
GFM2	0.007908404	0.190293889	0.993552802
KLC1	-0.007577111	0.190366994	0.993552802
PTPMT1	0.007174111	0.190472883	0.993552802
YIPF3	0.00425062	0.190502752	0.993552802
PDSS2	-0.005568464	0.190518527	0.993552802
DLG4	-0.011440664	0.190584693	0.993552802
TAS2R30	-0.013274992	0.190616469	0.993552802
SLC4A8	0.014538294	0.190661757	0.993552802
PDE3B	0.010540335	0.190673894	0.993552802
PEA15	0.00629146	0.190684796	0.993552802
DARS2	0.007779053	0.190911962	0.993552802
FAM43A	0.017566442	0.191118738	0.993552802
FAM26F	-0.025462418	0.191189529	0.993552802
FUCA1	0.008749569	0.191255319	0.993552802

LAMB2	0.012535791	0.19131593	0.993552802
FDX1	0.00977515	0.191438954	0.993552802
STAP1	-0.017717467	0.191462844	0.993552802
CACNB3	0.011903476	0.191495723	0.993552802
SCO1	0.008489178	0.191599886	0.993552802
AHSP	-0.020027428	0.191696457	0.993552802
CDC42SE1	-0.01088329	0.191708771	0.993552802
ARL8B	0.004549169	0.191755157	0.993552802
CYP2R1	0.006982748	0.191829555	0.993552802
FASLG	0.023018559	0.191875691	0.993552802
SLC23A1	-0.008618213	0.191904034	0.993552802
PPP6R1	-0.007200698	0.191951098	0.993552802
FYCO1	0.008542858	0.191962893	0.993552802
DLST	-0.006841238	0.191984576	0.993552802
NUSAP1	0.011937392	0.192032365	0.993552802
OAT	0.009473093	0.192140418	0.993552802
TLR9	0.012934702	0.192295454	0.993552802
C15orf41	0.008128145	0.192338467	0.993552802
FPR3	0.020095731	0.192338494	0.993552802
ERI2	0.010307956	0.192371593	0.993552802
FAM198B	0.014651097	0.192463789	0.993552802
PCSK4	0.008168306	0.192534074	0.993552802
THAP10	0.008965502	0.192570994	0.993552802
SRSF7	0.006379958	0.192824333	0.993552802
IQCK	-0.008710082	0.192933145	0.993552802
EME1	-0.008334007	0.193039058	0.993552802
HTRA1	0.016460491	0.193085571	0.993552802
NFATC3	-0.00778161	0.193162431	0.993552802
HCG18	0.011170651	0.193169453	0.993552802
ACSBG1	-0.027321296	0.193409823	0.993552802
CIDEA	0.010912535	0.193564488	0.993552802
SNRNP48	0.009527358	0.193612269	0.993552802
FAM102B	-0.006759075	0.193652191	0.993552802
EIF2AK2	-0.013552534	0.193656367	0.993552802
KLHL7	0.007390704	0.193681743	0.993552802
GOLGA7	0.003986854	0.193752146	0.993552802
FASTK	0.004692904	0.194043096	0.993552802
USP16	0.004905641	0.194098974	0.993552802
PEX16	0.005261094	0.194137575	0.993552802
SNORD57	0.011158691	0.194192266	0.993552802
LPCAT3	0.006137347	0.194299138	0.993552802
TMEM128	-0.022979513	0.194302207	0.993552802
CANX	0.00558302	0.194328437	0.993552802
CDCA7L	-0.011018092	0.194430774	0.993552802
NFE2L2	0.008986321	0.19444531	0.993552802
NLE1	0.00922778	0.194555355	0.993552802
FAM175B	0.004574324	0.194657224	0.993552802

ARPC1B	-0.007184867	0.194732206	0.993552802
CSF1R	0.007259481	0.194735556	0.993552802
RECK	0.0071455	0.194937607	0.993552802
PRKCE	-0.009346809	0.194983311	0.993552802
ZDHHC12	-0.007777131	0.195065556	0.993552802
HAL	0.013400931	0.195142249	0.993552802
DPRXP4	-0.018205131	0.195233945	0.993552802
POLD3	0.007243826	0.195236553	0.993552802
UBIAD1	-0.007069981	0.195275057	0.993552802
NAB2	0.009064796	0.195375083	0.993552802
MUT	-0.006749706	0.195412757	0.993552802
DDX54	0.007041673	0.195759059	0.993552802
EPHB4	0.013289203	0.195798259	0.993552802
EAPP	0.012867078	0.19590914	0.993552802
PTPN12	-0.009282425	0.196161554	0.993552802
P4HA1	0.008443726	0.196185581	0.993552802
CD69	-0.010942388	0.196189308	0.993552802
ALDH7A1	0.01393836	0.196234462	0.993552802
DNAJA1	0.006785521	0.196250079	0.993552802
PSMB10	0.006194439	0.196263284	0.993552802
DSN1	-0.006886825	0.196349768	0.993552802
AURKB	0.012475668	0.196411487	0.993552802
PRAME	-0.007144684	0.196510741	0.993552802
TAF3	-0.007230679	0.19659926	0.993552802
DCLK3	0.009114545	0.196713214	0.993552802
ICOSLG	-0.00985557	0.196721153	0.993552802
GGT7	0.014791725	0.196750506	0.993552802
ARL2BP	0.006017782	0.196774254	0.993552802
PLIN2	0.008709762	0.19678418	0.993552802
ARHGAP18	-0.013803839	0.196817528	0.993552802
PRR3	0.011079631	0.19685898	0.993552802
C11orf71	0.010225283	0.19687927	0.993552802
OGFR	-0.007743669	0.197019384	0.993552802
CYB5A	-0.006284157	0.197059755	0.993552802
TRAF6	0.008730666	0.197192786	0.993552802
PSMC3	0.005819647	0.197210678	0.993552802
CXCL16	0.010397871	0.197345702	0.993552802
MAP4K1	0.007410667	0.197349257	0.993552802
PLK5	0.008359913	0.197368605	0.993552802
CRB3	-0.009815297	0.197393601	0.993552802
GTF3C5	-0.005707928	0.197502046	0.993552802
PHRF1	0.006852912	0.197541417	0.993552802
GSK3B	-0.007475398	0.197801584	0.993552802
WASH3P	0.006313124	0.197926514	0.993552802
TMEM39B	-0.005769797	0.198051691	0.993552802
UBE2Z	-0.006951499	0.198105352	0.993552802
ZNF473	-0.00615094	0.198333914	0.993552802

NEDD4L	0.00991335	0.198413062	0.993552802
SKI	-0.006255258	0.198463707	0.993552802
RSPRY1	-0.006127607	0.198626149	0.993552802
NAPB	0.009556484	0.198681434	0.993552802
TRIM33	-0.00488136	0.198694417	0.993552802
TGFB1I1	-0.022537211	0.198842149	0.993552802
CRAT	-0.010368154	0.198974037	0.993552802
WNK1	-0.004700295	0.198977872	0.993552802
HP1BP3	-0.00818997	0.199138685	0.993552802
KRT10	0.008380477	0.199367645	0.993552802
CD70	0.012442764	0.199481033	0.993552802
ZNF621	0.006629383	0.199709979	0.993552802
JUND	-0.004120003	0.199712407	0.993552802
CCDC12	-0.004899754	0.199739609	0.993552802
HMGCL	0.006945967	0.19975142	0.993552802
SNORD34	-0.01345575	0.199768667	0.993552802
CSNK2A2	0.005644026	0.199822824	0.993552802
MDH1	0.005204803	0.200102518	0.993552802
DNAJB12	0.007684771	0.20011096	0.993552802
BAK1	0.010933057	0.200125571	0.993552802
ZBTB42	0.008219354	0.200138427	0.993552802
PRKAB1	0.006596488	0.200275289	0.993552802
SCARNA18	0.007582086	0.200455335	0.993552802
HNRNPK	-0.005140938	0.200559163	0.993552802
GALNT1	0.006982027	0.200681552	0.993552802
FAN1	0.010618248	0.200922146	0.993552802
CCNG1	-0.005199931	0.20108136	0.993552802
GFM1	0.004915357	0.201082125	0.993552802
TUBGCP4	-0.007216614	0.201084702	0.993552802
VTI1B	-0.007150237	0.201105644	0.993552802
RHOT2	0.008435158	0.201255303	0.993552802
C11orf73	0.008904438	0.201271094	0.993552802
SQLE	0.009247909	0.201443186	0.993552802
DIP2B	-0.005984557	0.201585255	0.993552802
LRP1	-0.008928005	0.201796289	0.993552802
MAPK13	0.008459216	0.201847647	0.993552802
IPO7	0.008492537	0.201873043	0.993552802
C21orf91-OT1	0.009775832	0.202131888	0.993552802
FCGR1B	0.02567583	0.202145176	0.993552802
ANKRD23	0.008814162	0.202249884	0.993552802
FOXN3	-0.004570914	0.202342436	0.993552802
RAB12	-0.019158816	0.202354884	0.993552802
CCNE1	0.009014251	0.202381304	0.993552802
SH2D2A	-0.013018713	0.202635313	0.993552802
STX3	0.00722955	0.202671231	0.993552802
WRAP73	0.005285386	0.202700455	0.993552802
OGG1	0.008666452	0.202835754	0.993552802

ALDH16A1	-0.009063905	0.202996418	0.993552802
CCDC17	0.009178215	0.202997437	0.993552802
MSRB3	-0.016860147	0.20316298	0.993552802
ZNF664	-0.009015058	0.203178139	0.993552802
ABCE1	0.0045728	0.203260273	0.993552802
INSR	-0.009757671	0.203285792	0.993552802
NMT1	0.00827132	0.203340061	0.993552802
RAB1B	-0.008314994	0.203362246	0.993552802
NR3C2	-0.007537946	0.203446345	0.993552802
RAP2A	0.011793668	0.203502572	0.993552802
LAMP1	-0.004318912	0.203743388	0.993552802
CIRBP	-0.006621052	0.203799094	0.993552802
TUBB1	-0.0163855	0.20397907	0.993552802
TCL1A	-0.028063893	0.204121171	0.993552802
SFI1	0.009002572	0.20434599	0.993552802
FAM120AOS	0.005282409	0.20463259	0.993552802
HIST1H1C	-0.01595479	0.204648312	0.993552802
SRRT	0.004426917	0.205360818	0.993552802
ADM	-0.01972001	0.205379914	0.993552802
PACS1	-0.010418474	0.205794803	0.993552802
ASGR1	0.008027628	0.205938573	0.993552802
HCST	0.005829277	0.205968658	0.993552802
TACO1	0.00529078	0.206005386	0.993552802
GCA	0.009194425	0.20610981	0.993552802
STAT6	0.011466917	0.206149176	0.993552802
DDRGK1	0.00638765	0.206171558	0.993552802
AIFM2	0.006987536	0.206190598	0.993552802
USP33	-0.007113877	0.206285594	0.993552802
SCOC	0.007602612	0.206306654	0.993552802
MAN2B1	-0.008663189	0.206323447	0.993552802
PCBD2	0.006693283	0.206324016	0.993552802
RIN2	0.010844619	0.206367594	0.993552802
XPNPEP1	-0.005805467	0.206544739	0.993552802
TSFM	-0.006667393	0.206638378	0.993552802
ATPIF1	-0.005683961	0.206658968	0.993552802
EPB41L5	0.007619836	0.206670951	0.993552802
CABLES1	0.007139295	0.20668653	0.993552802
MED29	-0.004531555	0.206830142	0.993552802
TM9SF1	0.007511146	0.206834909	0.993552802
AFF3	-0.013399633	0.206937825	0.993552802
WTAP	-0.005522908	0.206961299	0.993552802
AQP10	-0.0336351	0.207283112	0.993552802
PPP1R15B	0.009302278	0.207353823	0.993552802
GSTT1	-0.033095504	0.207521248	0.993552802
TIGD7	0.008475359	0.207560013	0.993552802
GUK1	-0.004625694	0.207577199	0.993552802
SEC61B	0.005472245	0.207577967	0.993552802

ZNF708	0.011035509	0.207594348	0.993552802
DAP	-0.009884608	0.207621776	0.993552802
HOXB6	-0.014070946	0.207748726	0.993552802
PXN	-0.007794086	0.207799421	0.993552802
MTIF2	0.006353961	0.207804737	0.993552802
GATC	0.013639587	0.207869873	0.993552802
PDE1B	0.009454734	0.207891627	0.993552802
POLG2	0.00712006	0.208062445	0.993552802
TUFT1	0.007337287	0.208100389	0.993552802
PGAP3	0.005173738	0.208155055	0.993552802
ABCC1	-0.008878283	0.208160418	0.993552802
RAB37	-0.00917052	0.208352068	0.993552802
ZNF227	0.008047943	0.208449202	0.993552802
PFDN4	0.010096978	0.20870042	0.993552802
PRMT1	-0.00546834	0.208728011	0.993552802
SIK1	-0.016002128	0.208786577	0.993552802
RNPS1	-0.007263666	0.209018904	0.993552802
ATRIP	0.006576831	0.209083927	0.993552802
GLCCI1	-0.009697136	0.209092658	0.993552802
TPST1	-0.013987535	0.209113578	0.993552802
TNFRSF1B	0.006230654	0.209132544	0.993552802
PREX1	-0.007033944	0.209134777	0.993552802
H2AFJ	-0.009980735	0.209248552	0.993552802
MCMBP	0.01065177	0.209252492	0.993552802
TSPYL4	-0.007806015	0.209272021	0.993552802
PROK2	0.031460648	0.209704027	0.993552802
P4HB	0.005558566	0.20971722	0.993552802
MUC4	-0.008894529	0.209944462	0.993552802
RNF157	-0.013691559	0.209965375	0.993552802
PCCB	0.006347277	0.209995872	0.993552802
ECHDC3	0.015738138	0.210015832	0.993552802
VWF	-0.031614	0.210105834	0.993552802
RPRD2	-0.005813163	0.210109889	0.993552802
LARP1	-0.004479131	0.21034472	0.993552802
KRT17	0.011092347	0.210358448	0.993552802
PRR13	0.004127168	0.210359393	0.993552802
COMMD8	0.009892171	0.210362561	0.993552802
HIST1H2AC	-0.018586877	0.210362943	0.993552802
SERPINF1	0.016463399	0.210452896	0.993552802
TJP2	-0.01229849	0.210714904	0.993552802
AXL	0.013041145	0.21072135	0.993552802
ZNF799	0.009160383	0.210778101	0.993552802
TCF4	-0.011017353	0.210839926	0.993552802
TMEM59	0.004014602	0.210910508	0.993552802
AGTPBP1	0.005735777	0.210942836	0.993552802
OSTC	0.005851159	0.211015628	0.993552802
LYSMD2	0.006952444	0.211042928	0.993552802

DDX50	-0.004661359	0.211098922	0.993552802
BCL7C	-0.00864026	0.21114606	0.993552802
SPHK2	0.004646317	0.211219123	0.993552802
JAM3	-0.025796016	0.21148136	0.993552802
ATN1	0.011912306	0.211495456	0.993552802
EPB41L3	0.01152875	0.211503287	0.993552802
C16orf93	0.009840163	0.21158652	0.993552802
TBC1D2B	0.00717634	0.211808404	0.993552802
R3HDM2	-0.007536009	0.211846865	0.993552802
FBXL5	0.009076735	0.211924436	0.993552802
PSMC6	0.005092148	0.211992205	0.993552802
CENPO	-0.008090159	0.212444926	0.993552802
PI4KB	-0.005238294	0.212497851	0.993552802
SLC35C2	-0.006066856	0.212528043	0.993552802
SLC18A1	0.008371042	0.212542063	0.993552802
HSPB1	-0.007019618	0.212699084	0.993552802
IMPAD1	0.010264587	0.212748785	0.993552802
SNORA10	-0.009011659	0.212824844	0.993552802
SCARNA3	-0.021084496	0.213096272	0.993552802
DIS3L2	-0.007448652	0.213120157	0.993552802
NUCB2	0.009585358	0.213391112	0.993552802
NOD2	0.008352729	0.21339933	0.993552802
SRCAP	-0.008332401	0.21357422	0.993552802
ZDHHC5	0.007464183	0.21360674	0.993552802
OPA1	0.006672774	0.213732847	0.993552802
SYNJ2	-0.009362876	0.213778613	0.993552802
VPS33B	-0.004289626	0.213807785	0.993552802
RMI1	0.006817984	0.213906622	0.993552802
FAM160B2	0.008140711	0.214022245	0.993552802
B4GALT5	0.008291503	0.214086435	0.993552802
SYNGR2	0.005140155	0.214096622	0.993552802
KATNB1	0.006050065	0.214157977	0.993552802
UNC13D	-0.010010749	0.214190378	0.993552802
AHNAK	0.005821344	0.21445771	0.993552802
TBC1D9	0.011540916	0.214474317	0.993552802
SRGN	0.009729545	0.214521763	0.993552802
HPS1	-0.010888413	0.214541981	0.993552802
FAM184B	-0.022284083	0.214571503	0.993552802
WDR33	0.007278197	0.214601902	0.993552802
ODF2	-0.006188648	0.214697746	0.993552802
MGAT2	0.007890853	0.214750937	0.993552802
SLC12A2	-0.008889509	0.214839405	0.993552802
LXN	0.010027765	0.214867842	0.993552802
GTPBP10	0.008780777	0.214904214	0.993552802
TUBB8	-0.009049715	0.214946616	0.993552802
UTS2	-0.019503642	0.214964836	0.993552802
RAB5B	-0.004621293	0.214966622	0.993552802

RGR	0.008840024	0.214979953	0.993552802
PGM3	0.007543453	0.215100968	0.993552802
RNF146	0.00844464	0.215179272	0.993552802
CD2BP2	0.004829757	0.215182153	0.993552802
DCTN4	0.006013713	0.215188579	0.993552802
DSEL	-0.010285092	0.215245835	0.993552802
ZSCAN16	0.004677564	0.215261867	0.993552802
SCARNA10	-0.008861046	0.215303054	0.993552802
RFXAP	0.006093255	0.215432213	0.993552802
OCIAD2	-0.005964501	0.215547354	0.993552802
PEF1	-0.006078352	0.215761149	0.993552802
SRFBP1	0.009480763	0.215836782	0.993552802
PUF60	0.00677761	0.215976072	0.993552802
CDK20	0.007405372	0.215988521	0.993552802
EPHA8	-0.008576221	0.216032611	0.993552802
C9orf78	0.005013592	0.216166682	0.993552802
NLK	0.005722393	0.216312507	0.993552802
RBM25	0.004993704	0.216343025	0.993552802
OR56B1	-0.009304103	0.216365153	0.993552802
PPT1	0.007391601	0.216424103	0.993552802
STAM2	0.008984688	0.216436642	0.993552802
SNAP23	-0.009916849	0.216455482	0.993552802
TATDN2	-0.007202984	0.216573461	0.993552802
CPN2	-0.00945715	0.216626027	0.993552802
DNAJC19	0.008364869	0.216689183	0.993552802
ST6GALNAC6	-0.010071797	0.216795547	0.993552802
BNIPL	0.010248365	0.216808507	0.993552802
CYBA	-0.007817832	0.216873103	0.993552802
MFF	0.041767535	0.216914072	0.993552802
GNAL	-0.009926498	0.216957222	0.993552802
RAB22A	0.006930546	0.217049578	0.993552802
UBXN4	-0.008348431	0.217203784	0.993552802
LINC00290	0.008296616	0.217251402	0.993552802
ARL13B	0.00783144	0.217336423	0.993552802
VAMP8	0.006196115	0.217745471	0.993552802
AKAP5	0.003606412	0.217981793	0.993552802
BOD1	0.005511743	0.218055997	0.993552802
AP1B1	0.004790748	0.218057812	0.993552802
CDKAL1	-0.009992385	0.218098872	0.993552802
YTHDF1	-0.004161112	0.218258576	0.993552802
RPL41	0.002179464	0.218270085	0.993552802
LRSAM1	0.004984134	0.218551524	0.993552802
ELMO3	-0.00743621	0.218695453	0.993552802
TM2D1	0.004146151	0.218872018	0.993552802
MBNL1	0.018561098	0.219035002	0.993552802
SDF4	-0.007208242	0.219087853	0.993552802
PLTP	-0.010271035	0.219225305	0.993552802

ITPKC	-0.009200924	0.219448365	0.993552802
EPM2AIP1	0.00486799	0.219510832	0.993552802
PAQR3	0.006967745	0.219804707	0.993552802
VPS37B	-0.007355372	0.219811442	0.993552802
TRIM9	-0.00838676	0.219821411	0.993552802
SH3BP1	-0.009222154	0.219874101	0.993552802
CARHSP1	0.007883462	0.219879882	0.993552802
CPSF3L	0.005743517	0.219889197	0.993552802
PHIP	-0.005887276	0.219890125	0.993552802
ZNF688	-0.005776788	0.219914503	0.993552802
ZNF507	-0.01035653	0.219924093	0.993552802
TMEM161B	0.007444722	0.219934236	0.993552802
MRPL9	-0.006205163	0.220215858	0.993552802
TAF1L	-0.005559565	0.220231945	0.993552802
PAQR4	0.008415318	0.220257741	0.993552802
C11orf54	0.005128802	0.220273222	0.993552802
B3GALNT1	0.010626397	0.220454889	0.993552802
NCAPG	0.017640704	0.220468066	0.993552802
SLC4A1	-0.015873417	0.220483694	0.993552802
C10orf2	0.008077817	0.220541801	0.993552802
DENND3	-0.009088084	0.220564234	0.993552802
GYG1	0.006670635	0.220686193	0.993552802
YWHAQ	0.005996487	0.220706304	0.993552802
SNORA75	-0.009112173	0.220797047	0.993552802
GMFB	0.004681729	0.22083678	0.993552802
SLC7A1	0.006935397	0.220921971	0.993552802
JOSD1	-0.006618341	0.220924657	0.993552802
CENPV	-0.01058183	0.221042698	0.993552802
C6orf120	0.008252689	0.22105262	0.993552802
ETF1	0.004468163	0.221121592	0.993552802
FAM160A2	0.007319977	0.2211801	0.993552802
CEP135	-0.004615022	0.221219884	0.993552802
INPPL1	0.00603008	0.221222085	0.993552802
KRT8	-0.008229803	0.221239604	0.993552802
LPIN2	-0.007128676	0.221378352	0.993552802
ZC3H14	0.00560209	0.221755038	0.993552802
WDSUB1	0.00878313	0.221917683	0.993552802
TDRD9	0.017332307	0.222067615	0.993552802
MFSD6L	0.006460689	0.222259986	0.993552802
MTMR3	0.005439472	0.222487294	0.993552802
TRDMT1	0.009393751	0.22277043	0.993552802
MTL5	0.013638469	0.222782178	0.993552802
DPYSL2	0.005964142	0.22327142	0.993552802
SMOX	-0.019919103	0.223489167	0.993552802
LCLAT1	-0.012870416	0.22355405	0.993552802
GLI4	-0.008706816	0.223592066	0.993552802
POLR3C	0.004010732	0.223641684	0.993552802

TCTN1	-0.010003606	0.223676851	0.993552802
FOXP4	-0.009003336	0.223850886	0.993552802
NDUFB3	0.005294025	0.223921854	0.993552802
ZNF107	-0.007278855	0.223931276	0.993552802
PPP1R11	-0.003697943	0.223951629	0.993552802
CRX	-0.007910535	0.224079609	0.993552802
ATG10	-0.012755327	0.224247411	0.993552802
DHX30	-0.006165266	0.224300549	0.993552802
IL17RB	-0.012437168	0.224417944	0.993552802
EGF	-0.01723232	0.22461567	0.993552802
RNF217	0.008534148	0.224821665	0.993552802
LAMTOR1	-0.009669528	0.224911807	0.993552802
ZSCAN12	0.010668919	0.224979607	0.993552802
PLEKHA8P1	-0.006014138	0.225055818	0.993552802
STK11IP	0.007476759	0.225057832	0.993552802
GNAI3	0.011074706	0.225065171	0.993552802
CD82	-0.006387647	0.225139533	0.993552802
LPAR3	0.007416067	0.225224832	0.993552802
C16orf13	-0.006066888	0.225290976	0.993552802
SLC16A5	0.008724436	0.225372006	0.993552802
SHE	-0.009654795	0.225401968	0.993552802
AGAP3	0.008298156	0.225405265	0.993552802
GGTLC2	0.007963647	0.225490188	0.993552802
HBP1	0.004911371	0.225717275	0.993552802
PIGB	0.005704832	0.225948227	0.993552802
RANBP10	-0.005858385	0.22633089	0.993552802
NETO1	-0.010276144	0.22635051	0.993552802
CYC1	0.004435045	0.226411542	0.993552802
SH3RF3	0.008317638	0.226447241	0.993552802
TPM4	-0.012859201	0.226477455	0.993552802
PRAMEF7	-0.00684593	0.226575216	0.993552802
NCOA7	-0.005415993	0.226730961	0.993552802
RPS24	-0.004043272	0.226832	0.993552802
NNT	0.006113342	0.226868887	0.993552802
MAP3K9	-0.009596594	0.226918519	0.993552802
KIF20B	0.008413544	0.227419015	0.993552802
OSTCP1	0.010373317	0.227503666	0.993552802
SMYD3	-0.007466101	0.227531888	0.993552802
SORL1	-0.006260038	0.227544806	0.993552802
NGRN	0.0071759	0.227582515	0.993552802
TARBP2	0.007618577	0.227630929	0.993552802
TMTC2	0.010387771	0.227703496	0.993552802
TYSND1	0.004570734	0.227878025	0.993552802
AKR1B1	0.005298369	0.227914328	0.993552802
RPL34	0.007241245	0.227949633	0.993552802
PRDX6	-0.009448093	0.227968083	0.993552802
C14orf80	0.00727078	0.228115274	0.993552802

SLC38A9	0.006139567	0.228116241	0.993552802
PYGO2	0.009953351	0.228244182	0.993552802
PCOLCE	-0.008705202	0.228300866	0.993552802
USP2	0.007446489	0.228319706	0.993552802
CGA	-0.009786111	0.228343482	0.993552802
COMMD5	0.007923164	0.228484813	0.993552802
NCAN	-0.007329064	0.2284916	0.993552802
TRIP12	0.003890234	0.228820889	0.993552802
TMEM158	-0.024098464	0.228875114	0.993552802
SIGLEC14	0.032624709	0.228925017	0.993552802
TNRC6B	-0.009206726	0.229118633	0.993552802
TMEM55B	-0.005390515	0.229139366	0.993552802
TCTN3	0.009234366	0.229157996	0.993552802
KCNT1	0.019506377	0.229210642	0.993552802
AKAP9	0.009313992	0.229386215	0.993552802
GRB14	-0.022399011	0.229415884	0.993552802
MKLN1	-0.004781273	0.229436224	0.993552802
EIF2A	0.00533817	0.229585437	0.993552802
ANKRD39	0.006598308	0.229613453	0.993552802
SNX19	-0.006801064	0.229749935	0.993552802
FBXO5	0.007876569	0.229812702	0.993552802
FBXW9	0.006011831	0.229866092	0.993552802
AMICA1	0.00617194	0.229874034	0.993552802
RABAC1	-0.006653639	0.230179952	0.993552802
ARHGEF2	-0.005844922	0.230248769	0.993552802
PI4K2A	0.006672312	0.230252349	0.993552802
CAPZB	-0.003928301	0.230272027	0.993552802
PPIL1	0.006676369	0.23031717	0.993552802
ANK3	-0.012513656	0.230368675	0.993552802
KIDINS220	0.005173912	0.230391644	0.993552802
CENPQ	0.011056163	0.23061459	0.993552802
SETDB2	0.004473905	0.230866598	0.993552802
PATE1	-0.009377502	0.230995831	0.993552802
FAM129C	-0.016646668	0.231043363	0.993552802
GTF2E2	0.004133796	0.23110382	0.993552802
PCIF1	-0.009606814	0.231209667	0.993552802
GNG3	-0.009748166	0.231346206	0.993552802
HAUS6	0.010308262	0.23149514	0.993552802
NLRP12	0.010121489	0.231751449	0.993552802
C19orf70	-0.005593774	0.231801589	0.993552802
IRF5	-0.034036645	0.231832464	0.993552802
BBS1	0.005678062	0.231942896	0.993552802
FBLN1	-0.020883011	0.232003722	0.993552802
PDCD11	0.007588805	0.232032	0.993552802
RFC1	0.005371576	0.232066104	0.993552802
RSPO2	0.007145406	0.232069441	0.993552802
DCBLD2	0.010020364	0.232074221	0.993552802

CACNA1H	0.01399036	0.232169304	0.993552802
HERC1	-0.005186617	0.232224926	0.993552802
LIPN	0.014205256	0.232314012	0.993552802
DEAF1	-0.007526678	0.232317909	0.993552802
C18orf25	-0.004607264	0.232408489	0.993552802
UBE2J2	0.009299882	0.232499817	0.993552802
CCDC88B	-0.010705803	0.232537515	0.993552802
SLFN11	0.006897975	0.232556859	0.993552802
SEC14L5	-0.029607225	0.232643996	0.993552802
CHRDL2	-0.007299174	0.232733442	0.993552802
STRADA	0.004567859	0.232921597	0.993552802
CST3	0.008700546	0.232941649	0.993552802
MCM2	-0.010668345	0.233299851	0.993552802
MRPS16	-0.004594403	0.233309246	0.993552802
SF3B2	-0.004149669	0.233356905	0.993552802
SAP30	0.013758956	0.233375406	0.993552802
TMPO	0.01063652	0.233394557	0.993552802
DONSON	0.00842986	0.233488237	0.993552802
PTRH1	0.010575709	0.233548724	0.993552802
PF4V1	-0.035487172	0.233550733	0.993552802
FCGRT	0.007241521	0.233591008	0.993552802
LARP7	0.005592953	0.233633178	0.993552802
LRP5	0.008183981	0.233669541	0.993552802
CXXC4	0.007973027	0.23370425	0.993552802
DPF2	0.005827746	0.233954604	0.993552802
GCC1	0.006327538	0.23403837	0.993552802
CPS1	-0.01113287	0.23421415	0.993552802
SIGLEC9	0.008680456	0.234312265	0.993552802
TMPRSS15	0.00781718	0.23431841	0.993552802
MEAF6	0.008734655	0.234782449	0.993552802
CELF1	-0.009223302	0.235020372	0.993552802
GPR37	0.006753045	0.235028825	0.993552802
NDST1	0.011508184	0.235033872	0.993552802
SFT2D2	0.008716051	0.235085863	0.993552802
SCN9A	0.009704398	0.235141224	0.993552802
OSTM1	0.006473458	0.235203614	0.993552802
ZBBX	-0.006537001	0.235204083	0.993552802
FCRL3	-0.016835328	0.235431826	0.993552802
TCF7L2	-0.01292131	0.235465219	0.993552802
FAAH	-0.010478668	0.235495175	0.993552802
EMILIN2	0.007921652	0.235555881	0.993552802
FANCF	0.007611579	0.235579578	0.993552802
LDOC1L	0.010144854	0.235626913	0.993552802
FAM129A	0.008489507	0.235629957	0.993552802
HYAL2	-0.007336827	0.235632257	0.993552802
LRRC42	0.007057995	0.236238955	0.993552802
TMEM207	0.009511245	0.236650316	0.993552802

GINS2	0.015602108	0.236674163	0.993552802
PRRG2	-0.009859866	0.236693489	0.993552802
ZNF395	-0.006891971	0.236730474	0.993552802
CLINT1	0.004676166	0.236813029	0.993552802
CRIPT	0.009666421	0.23684421	0.993552802
DISP1	0.007628437	0.236949357	0.993552802
COL12A1	-0.008787202	0.23694987	0.993552802
RABGGTB	0.005292684	0.236976926	0.993552802
MUC20	0.019815662	0.236988907	0.993552802
CCDC77	0.006485207	0.237332572	0.993552802
ERP27	0.00908428	0.237343384	0.993552802
PTPRC	0.012701198	0.237484575	0.993552802
ITIH4	0.016773417	0.237504041	0.993552802
GSTO1	-0.006465312	0.237575126	0.993552802
SSR3	0.006807957	0.237776155	0.993552802
UBA5	0.006402542	0.237824812	0.993552802
CAMK1G	0.017806893	0.237894361	0.993552802
DEK	0.005367851	0.237979546	0.993552802
HADHA	0.007416254	0.238053067	0.993552802
NOL11	0.004538501	0.238108411	0.993552802
ZBTB47	0.009695972	0.238143174	0.993552802
C19orf43	-0.005695818	0.238245762	0.993552802
DOPEY2	-0.006130904	0.238258264	0.993552802
SRXN1	0.011988271	0.238272259	0.993552802
MYH10	0.010704122	0.238360023	0.993552802
CLPTM1	-0.005952148	0.238414236	0.993552802
KIF3A	0.01010763	0.238472473	0.993552802
WDR82	-0.005532026	0.238494803	0.993552802
TYMP	0.008651647	0.238500134	0.993552802
C9orf89	-0.010315034	0.238601886	0.993552802
INO80	-0.005585162	0.238602637	0.993552802
TEKT1	0.007004607	0.238635253	0.993552802
CGRRF1	-0.005865018	0.238691627	0.993552802
TUBD1	0.0089655	0.238802096	0.993552802
NLN	0.009343815	0.238853319	0.993552802
FBXO11	0.004057237	0.238979702	0.993552802
MOV10	-0.008268753	0.239057024	0.993552802
S1PR4	-0.007402715	0.239079592	0.993552802
CCNC	0.010633868	0.239185975	0.993552802
IGKC	-0.019712543	0.239189752	0.993552802
SEPW1	-0.004375482	0.23934705	0.993552802
NEDD9	0.010086221	0.239388469	0.993552802
PDXDC2P	-0.017856132	0.239473245	0.993552802
HMGXB4	0.004720409	0.239478331	0.993552802
IMP4	0.004873903	0.239482939	0.993552802
PARP4	0.00560304	0.239727506	0.993552802
UBA7	0.005850322	0.239764245	0.993552802

SNORD33	-0.008561543	0.2397824	0.993552802
MAP3K1	-0.005162784	0.239900477	0.993552802
AP2A2	-0.008272779	0.239940506	0.993552802
ARHGAP15	0.004690244	0.240031021	0.993552802
APPBP2	0.003777266	0.240054154	0.993552802
MEF2A	-0.008501951	0.240059857	0.993552802
GTPBP4	0.003750842	0.240121893	0.993552802
CYP2S1	0.013952034	0.240136385	0.993552802
EPHA1	0.014019704	0.240193765	0.993552802
CSRP1	-0.006266926	0.240194871	0.993552802
IL17RA	0.007756916	0.240209632	0.993552802
LAMTOR3	0.005917673	0.240237216	0.993552802
GAB2	0.00765286	0.24032273	0.993552802
RAB38	-0.015927427	0.24034238	0.993552802
TOR1AIP1	0.007976397	0.240444796	0.993552802
ASB1	0.006289999	0.240469434	0.993552802
GLG1	0.003847895	0.240499781	0.993552802
UTP11L	0.006893992	0.240597685	0.993552802
C9orf156	0.006957149	0.240637832	0.993552802
RHEB	0.005558464	0.240646487	0.993552802
LTBP3	-0.011111971	0.240649786	0.993552802
ADAM28	-0.013515865	0.240705818	0.993552802
INPP1	0.007223942	0.240725573	0.993552802
HIST1H2AG	-0.019822203	0.240762763	0.993552802
LBH	-0.00704351	0.24105134	0.993552802
CYB5D1	-0.008907955	0.241089029	0.993552802
CALD1	-0.025603967	0.241223787	0.993552802
ZNF264	-0.009053482	0.241227467	0.993552802
C2orf42	0.005531507	0.24124901	0.993552802
TP53I3	-0.010290718	0.241471884	0.993552802
CDK5RAP2	-0.012099229	0.24147468	0.993552802
NAPA	-0.007507321	0.241547478	0.993552802
HMGCS1	0.005290587	0.241663701	0.993552802
INPP5F	-0.010100703	0.241681087	0.993552802
MOGS	-0.006271626	0.241688134	0.993552802
SEC11C	0.008024697	0.241729326	0.993552802
CDH1	0.010458734	0.242011403	0.993552802
CCNL2	-0.010730864	0.242354103	0.993552802
LSM7	-0.005633587	0.242514019	0.993552802
ZNF676	-0.007222372	0.242517389	0.993552802
HAS3	0.009896203	0.242597293	0.993552802
RPL31P11	-0.012634067	0.242652929	0.993552802
VPS25	-0.009940271	0.242784778	0.993552802
NUDT16L1	0.005609855	0.242843547	0.993552802
ENPP2	0.011688693	0.242884047	0.993552802
CHMP4B	-0.005620647	0.242918601	0.993552802
CNOT6	-0.004622967	0.242928628	0.993552802

DHDDS	0.007737779	0.242978027	0.993552802
MIR1307	-0.042615471	0.243104648	0.993552802
ATP5G3	0.00906976	0.243122439	0.993552802
MTFR1	0.008941401	0.243217019	0.993552802
RELL2	-0.008340403	0.243282496	0.993552802
THNSL2	-0.029347443	0.243421991	0.993552802
ZNF83	-0.0122686	0.243441685	0.993552802
C2orf82	0.006687744	0.243554083	0.993552802
FOXO3	-0.007383019	0.243594522	0.993552802
STK11	-0.007838965	0.243785644	0.993552802
ABLIM3	-0.021164826	0.243882362	0.993552802
CYP1B1	0.020943462	0.243912471	0.993552802
GCOM1	-0.006483048	0.243920043	0.993552802
STK4	-0.003359039	0.243947296	0.993552802
IFRD1	-0.008252544	0.243993586	0.993552802
COG1	-0.007116884	0.244032862	0.993552802
SDHAP1	0.007689886	0.24430935	0.993552802
MAP4K5	-0.006063742	0.244330661	0.993552802
NAV1	0.010426714	0.244397042	0.993552802
PPAPDC1B	-0.007160884	0.244588132	0.993552802
CTIF	-0.008387407	0.244664854	0.993552802
CD59	0.008678517	0.244690862	0.993552802
NIPSNAP1	-0.006430756	0.244739137	0.993552802
TRIM68	0.005376909	0.244882944	0.993552802
MAPK3	0.005823467	0.244929482	0.993552802
SLC25A34	0.011189472	0.244930178	0.993552802
PTDSS2	0.006830157	0.245025907	0.993552802
ZFP91	-0.009930994	0.245125844	0.993552802
PSIP1	-0.005719109	0.245180412	0.993552802
NLGN2	-0.008322431	0.245192575	0.993552802
MCM7	0.00515917	0.245273527	0.993552802
TAF13	-0.01004098	0.245331645	0.993552802
SLC6A16	-0.009738239	0.245341621	0.993552802
NDUFS2	0.006073006	0.245357759	0.993552802
ADCK3	-0.004393888	0.245424058	0.993552802
MAK	0.007914975	0.245531516	0.993552802
ZNF705G	-0.009817799	0.245637357	0.993552802
ATP5F1	0.005059658	0.24595067	0.993552802
SLC30A5	0.005490354	0.245957031	0.993552802
NECAP2	-0.005614728	0.246052712	0.993552802
C1orf216	0.007563078	0.246095512	0.993552802
MGRN1	0.009551456	0.246156577	0.993552802
TSTA3	-0.00655997	0.246230996	0.993552802
IGFL3	-0.008602746	0.2462857	0.993552802
AGPAT2	0.012330385	0.24641875	0.993552802
CLEC10A	0.014139494	0.246482142	0.993552802
WFS1	0.008856388	0.246543953	0.993552802

TRPM4	0.017659211	0.246569649	0.993552802
INTS2	0.005403468	0.246673891	0.993552802
MRPL35	0.006447036	0.246699218	0.993552802
MAN2A1	-0.007104976	0.24676803	0.993552802
MINPP1	0.008327394	0.246774678	0.993552802
CACNA1S	0.009278599	0.247111521	0.993552802
RHEBL1	0.008221344	0.247280797	0.993552802
HCP5	0.010131955	0.2473046	0.993552802
RPL13AP6	-0.006836026	0.247582112	0.993552802
KDSR	-0.006975054	0.247704989	0.993552802
FAM49A	0.007232759	0.24780786	0.993552802
MNDA	0.021438266	0.247832699	0.993552802
CASP9	-0.005899632	0.247875261	0.993552802
ZBTB9	0.009854749	0.247917903	0.993552802
ALG5	0.006156594	0.247950903	0.993552802
PPP2R5B	-0.007620049	0.248067685	0.993552802
XBP1	0.006855682	0.248392398	0.993552802
HGS	0.004724972	0.248578279	0.993552802
MCM5	-0.005549104	0.248595548	0.993552802
BTLA	-0.010792196	0.248669757	0.993552802
CDCA8	0.008348898	0.248744043	0.993552802
ZDHHC6	0.005574202	0.248750827	0.993552802
SNRPB2	-0.003898879	0.24875721	0.993552802
XRCC6	-0.005270542	0.248917015	0.993552802
PRDM8	-0.009313754	0.249000828	0.993552802
HBD	-0.021065903	0.249191042	0.993552802
DUS4L	-0.005749405	0.249332632	0.993552802
TMX1	0.004609676	0.249334364	0.993552802
NTN5	-0.009901092	0.249364659	0.993552802
CYBRD1	0.011775929	0.249446061	0.993552802
MRPS7	0.006015156	0.249481722	0.993552802
KCNJ2	0.01880268	0.249575769	0.993552802
RPL36	-0.003931412	0.249790098	0.993552802
DIS3	0.008572753	0.249840314	0.993552802
NCOA5	-0.006403674	0.249883883	0.993552802
DMC1	-0.008221797	0.250224172	0.993552802
BTBD6	-0.005413664	0.250486908	0.993552802
IRX1	-0.007776696	0.250588718	0.993552802
IFITM3	-0.028665612	0.250732611	0.993552802
ZNF148	-0.010134225	0.250949051	0.993552802
IGFBP2	-0.010017173	0.251141061	0.993552802
TMED10	0.010251255	0.251162723	0.993552802
NDUFV3	0.008534939	0.251174605	0.993552802
JKAMP	0.006009892	0.251524845	0.993552802
FOXD4L1	-0.009106504	0.251531486	0.993552802
LRIG1	-0.007536629	0.251943241	0.993552802
TAF1B	0.006895819	0.251954271	0.993552802

F13A1	-0.017464966	0.25197377	0.993552802
CRTC3	-0.004865031	0.251976842	0.993552802
SLC25A10	-0.008676701	0.252054311	0.993552802
C21orf119	-0.008675231	0.252136323	0.993552802
ZNF432	0.007022849	0.25223281	0.993552802
ATP8B4	0.009472492	0.252291858	0.993552802
FAM126A	0.007587848	0.252298878	0.993552802
MAP4	0.008841081	0.252371847	0.993552802
SMPD2	-0.007040959	0.252672322	0.993552802
C22orf34	-0.016679862	0.252760284	0.993552802
PATE3	0.008775308	0.252866966	0.993552802
TINF2	0.008242172	0.252935584	0.993552802
TNS1	-0.012567419	0.252940513	0.993552802
ALKBH5	-0.003467965	0.252962573	0.993552802
GPSM3	-0.004844905	0.253005328	0.993552802
PDCD1	0.014901219	0.253172547	0.993552802
PMCHL1	0.00652592	0.253215709	0.993552802
ORAI2	-0.008740777	0.253252366	0.993552802
PIK3CD	-0.006430873	0.253405238	0.993552802
TAPBP	-0.007276174	0.253464954	0.993552802
NEO1	-0.005452517	0.253506014	0.993552802
RNF138	-0.011686553	0.253581033	0.993552802
DLEU7	0.012631204	0.253645216	0.993552802
SNRNP70	0.005791952	0.253682237	0.993552802
PARD6A	-0.006499791	0.253712855	0.993552802
TNC	-0.015020173	0.253772004	0.993552802
FEZ2	-0.008296304	0.253951958	0.993552802
UBXN6	-0.005571438	0.25397436	0.993552802
FAM195A	-0.008583285	0.254012595	0.993552802
DDX41	-0.004684022	0.254136127	0.993552802
NAA50	0.007618819	0.254192668	0.993552802
G3BP2	0.004143801	0.254252023	0.993552802
POLR2B	0.004202903	0.25438446	0.993552802
NRP1	0.015569397	0.254449642	0.993552802
BARD1	-0.010798244	0.254490741	0.993552802
ADAM19	-0.008908031	0.254555074	0.993552802
RIC8A	-0.003964516	0.254703005	0.993552802
TAF15	-0.009125209	0.254944175	0.993552802
APH1A	-0.006334036	0.254984527	0.993552802
KIAA1033	0.009540012	0.254986621	0.993552802
GCM1	-0.007330663	0.25518344	0.993552802
EGOT	-0.008238467	0.255258689	0.993552802
CD1B	0.011172638	0.255306148	0.993552802
GIMAP8	0.008196001	0.255402273	0.993552802
ZNF576	0.007022885	0.255402362	0.993552802
C6orf163	0.009508525	0.255532627	0.993552802
NUPR1	-0.007229667	0.255775612	0.993552802

BIRC5	0.013157058	0.255793366	0.993552802
MARCH6	-0.013625599	0.255813481	0.993552802
MIR1323	0.00879099	0.255861113	0.993552802
RB1CC1	0.003735547	0.256071966	0.993552802
DHRS9	0.01966875	0.256144418	0.993552802
DSTYK	0.022081814	0.256208645	0.993552802
DSG2	0.006382085	0.256249473	0.993552802
DOK1	0.012733895	0.256262172	0.993552802
TMOD4	0.007643837	0.25627526	0.993552802
HECTD3	0.00553819	0.256365175	0.993552802
TXNDC9	0.005500977	0.256444513	0.993552802
FAM78A	0.008292926	0.256466646	0.993552802
POLR2J	-0.014346097	0.256474683	0.993552802
DUSP16	-0.008063226	0.256560614	0.993552802
MMP23B	0.014484932	0.256797	0.993552802
RAP1GDS1	-0.006714202	0.256869129	0.993552802
AKR1C2	-0.018985639	0.256990628	0.993552802
TRIM4	0.007048003	0.257161265	0.993552802
C17orf74	-0.007195522	0.2571717	0.993552802
MT1G	0.010396768	0.257419398	0.993552802
PAXIP1	0.005390073	0.257436853	0.993552802
SPCS2	0.005106595	0.257446052	0.993552802
XPA	-0.005496693	0.257585351	0.993552802
TCP11L1	0.007682192	0.257661356	0.993552802
DBR1	0.006027723	0.257726883	0.993552802
DDX6	0.017536632	0.257840478	0.993552802
ZNF729	0.010049825	0.258116188	0.993552802
MED1	-0.004914651	0.258439978	0.993552802
ZFAND3	-0.00676545	0.25853048	0.993552802
RAB27A	0.01143372	0.258617859	0.993552802
SUPT7L	0.008009773	0.258659299	0.993552802
FGFBP3	-0.010155027	0.25885406	0.993552802
F12	0.00813038	0.259295604	0.993552802
STRN	-0.006713738	0.259779133	0.993552802
GHITM	0.00419362	0.259781559	0.993552802
CDR2L	-0.015108178	0.259841855	0.993552802
TUBGCP2	-0.006256715	0.260269809	0.993552802
TRIM37	0.005929915	0.260298485	0.993552802
SEC22A	0.005923296	0.26035403	0.993552802
DDX31	0.007014437	0.260456785	0.993552802
WFDC3	0.011944979	0.260466287	0.993552802
KLHL6	0.006980772	0.260468974	0.993552802
GPAA1	-0.006435332	0.260528895	0.993552802
CDYL	0.005667265	0.260653584	0.993552802
GNA11	-0.011620623	0.260673127	0.993552802
RNF103	0.007108093	0.260728964	0.993552802
APBB3	0.0073614	0.260735958	0.993552802

C19orf44	0.008397628	0.260785442	0.993552802
C11orf57	-0.004867923	0.260850245	0.993552802
SNHG10	-0.006697202	0.261256702	0.993552802
RPS6KC1	0.006251863	0.261447815	0.993552802
CXXC5	-0.009982657	0.261451037	0.993552802
IDH3B	0.003314791	0.26156346	0.993552802
BZW1	0.004465493	0.261869573	0.993552802
CTR9	0.006515355	0.26203655	0.993552802
PAFAH2	0.008079967	0.262137475	0.993552802
GGCX	0.007087241	0.262197599	0.993552802
NEK3	0.006539988	0.262293247	0.993552802
ANKRD17	0.003912393	0.262442031	0.993552802
TRIM23	0.008753473	0.262699982	0.993552802
TSHZ3	0.010612722	0.262735756	0.993552802
ABHD6	0.005974896	0.262770798	0.993552802
TOE1	0.00570026	0.262923648	0.993552802
USP21	-0.005475241	0.262975594	0.993552802
DCTD	-0.004178111	0.263272131	0.993552802
DGKQ	0.009684667	0.263279471	0.993552802
CTSK	0.010975331	0.263390849	0.993552802
IFI27	-0.024684668	0.263460316	0.993552802
SLC22A16	-0.030890939	0.263613184	0.993552802
SATB2	0.008839811	0.263732536	0.993552802
IFT27	-0.007023969	0.26383311	0.993552802
APLP2	-0.008955104	0.26387482	0.993552802
MSC	0.01614422	0.263903555	0.993552802
ACP1	0.007245246	0.264044775	0.993552802
RPRD1B	0.005994391	0.264088079	0.993552802
HIATL1	0.006925261	0.264250428	0.993552802
EHD4	-0.007130895	0.264270371	0.993552802
SLAMF8	0.015211721	0.264305915	0.993552802
EEF2	0.003772355	0.264401302	0.993552802
ACTR2	0.004850819	0.264419522	0.993552802
WDR20	0.006107435	0.264500094	0.993552802
IPO5	0.005846239	0.264674011	0.993552802
LFNG	0.007507338	0.264802943	0.993552802
KLF12	-0.005365616	0.264958598	0.993552802
ACP5	0.013277424	0.264960684	0.993552802
TMEM19	-0.006521929	0.264967201	0.993552802
PTP4A3	-0.008767663	0.265075006	0.993552802
ZNF516	-0.01218762	0.265174148	0.993552802
L2HGDH	-0.015656309	0.265259032	0.993552802
OSGIN2	0.007279682	0.265431602	0.993552802
ZNF510	-0.008138186	0.265505207	0.993552802
CD5	-0.008522517	0.265518217	0.993552802
CASD1	0.007385924	0.265593529	0.993552802
DCAF11	0.00882475	0.2656574	0.993552802

BROX	0.009609686	0.265765853	0.993552802
MIB2	-0.006977326	0.265818124	0.993552802
PRRC2B	-0.007624155	0.26583081	0.993552802
ELL3	-0.010898467	0.26585332	0.993552802
MLXIPL	0.006101986	0.265911088	0.993552802
PAPD7	-0.006189236	0.26591525	0.993552802
WARS2	0.024641047	0.266025316	0.993552802
ALKBH8	0.009607826	0.26609816	0.993552802
CLTC	0.009099687	0.266249169	0.993552802
MRPS12	0.004959081	0.266802871	0.993552802
RABGAP1L	-0.010667867	0.266846073	0.993552802
UNC5C	0.007015358	0.266904255	0.993552802
CDK5R2	0.006824991	0.266916968	0.993552802
ARPC1A	-0.003784214	0.267271007	0.993552802
SMNDC1	0.005597923	0.267573691	0.993552802
COG6	0.005940691	0.267604603	0.993552802
AHCY	0.005770416	0.267812745	0.993552802
MAZ	0.009161954	0.268034379	0.993552802
MEPCE	-0.008199598	0.268042038	0.993552802
SHISA4	-0.009049169	0.268292834	0.993552802
GNB5	-0.012977988	0.268379504	0.993552802
HNRNPKP3	-0.006973576	0.268422101	0.993552802
PQLC1	-0.003262059	0.268425505	0.993552802
SEL1L3	-0.007811349	0.268441625	0.993552802
MZT1	0.00743445	0.2684777	0.993552802
ZNF618	-0.008971503	0.268512135	0.993552802
GRTP1	-0.008178803	0.268587256	0.993552802
PPP4R1	0.004389364	0.268599906	0.993552802
LIPC	-0.02164168	0.268675507	0.993552802
CHMP2B	0.007810106	0.268685611	0.993552802
FIS1	-0.006481557	0.268713445	0.993552802
HDAC2	-0.003277113	0.268761547	0.993552802
STARD3NL	0.010929606	0.268796855	0.993552802
RASGRP3	-0.013891736	0.268932826	0.993552802
CCL3	0.017704787	0.268954002	0.993552802
C8orf48	0.006834996	0.269153027	0.993552802
PTS	0.004434264	0.269209817	0.993552802
MFSD11	-0.010578311	0.269232324	0.993552802
CXCL5	-0.026022355	0.269295379	0.993552802
CENPW	0.01291022	0.269325101	0.993552802
MOCS2	0.010698961	0.269338939	0.993552802
FAM76A	-0.010046264	0.269416707	0.993552802
MNAT1	0.006240734	0.26971604	0.993552802
ZFYVE20	0.004601122	0.269873421	0.993552802
WDR77	-0.004747455	0.269931614	0.993552802
HDAC7	-0.007936704	0.269968049	0.993552802
AURKAPS1	0.006758514	0.269979864	0.993552802

MRPL4	-0.005771664	0.269981796	0.993552802
NUDT18	0.008116569	0.270082725	0.993552802
FBXL18	0.007065183	0.270132838	0.993552802
PPFIA1	0.004660906	0.270155153	0.993552802
ELL	0.005481903	0.270494266	0.993552802
BID	0.007058997	0.270541376	0.993552802
BTRC	0.006472216	0.270555521	0.993552802
C18orf8	-0.004148626	0.270636213	0.993552802
C9orf66	0.010024842	0.270709283	0.993552802
RAB40C	0.004250198	0.270713975	0.993552802
FBXO6	0.010297631	0.270780294	0.993552802
C12orf5	0.008904814	0.270848955	0.993552802
MPV17	0.003787905	0.270879622	0.993552802
CEP350	-0.006627654	0.271029464	0.993552802
ZER1	-0.006369218	0.271065026	0.993552802
CD101	0.010837095	0.271116803	0.993552802
PPM1B	0.006872553	0.271168732	0.993552802
CCDC150	0.007697175	0.271185702	0.993552802
SERPINB8	0.012118435	0.271346795	0.993552802
BCAT2	0.007812118	0.271350293	0.993552802
TMC5	-0.014518186	0.271422925	0.993552802
PEX2	0.006106367	0.271462003	0.993552802
ZNF830	0.007528557	0.27152682	0.993552802
RABGGTA	-0.006994628	0.2715667	0.993552802
MICAL2	0.011576271	0.271820737	0.993552802
CHD6	-0.004800428	0.271938696	0.993552802
RELL1	0.008170947	0.271941357	0.993552802
PRPF6	-0.008484146	0.271993749	0.993552802
JMJD6	0.007383488	0.272011263	0.993552802
PCID2	0.003769706	0.272181747	0.993552802
POU2F2	-0.009794366	0.272187823	0.993552802
PLCB2	0.008304838	0.272263325	0.993552802
SCGB3A2	-0.011732204	0.272392327	0.993552802
SH3TC1	-0.008069841	0.272712873	0.993552802
APOBEC3G	0.011364074	0.272763618	0.993552802
SAP130	0.006221183	0.272835511	0.993552802
ZNF200	0.013672067	0.272960662	0.993552802
HMMR	0.012563099	0.272987779	0.993552802
RSBN1L	0.005928086	0.273205767	0.993552802
KIF22	0.005159745	0.273406546	0.993552802
NKPD1	-0.016758579	0.273477186	0.993552802
MED11	0.005558946	0.273486539	0.993552802
PAWR	-0.009579588	0.27351849	0.993552802
INO80D	-0.006781193	0.273612784	0.993552802
GOLGA6L1	-0.002712709	0.273770664	0.993552802
RAE1	0.004524745	0.273787091	0.993552802
PHF10	0.006149558	0.273875662	0.993552802

FAM105B	0.006335396	0.27391477	0.993552802
TMEM131	0.003765624	0.274004696	0.993552802
NELL2	0.011985699	0.27411069	0.993552802
LMAN2	0.008400712	0.274122247	0.993552802
SNORA59A	0.007760821	0.274189854	0.993552802
PKN2	-0.010473752	0.274213373	0.993552802
MIAT	0.018448018	0.274277328	0.993552802
NUP85	-0.003680471	0.274292222	0.993552802
ATXN10	0.007364243	0.274377747	0.993552802
TNFSF13	0.005794364	0.274595897	0.993552802
DSCAM	0.006559744	0.274704583	0.993552802
NUPL2	0.006400787	0.27480626	0.993552802
LEPRE1	0.004558383	0.274872737	0.993552802
FAM134B	0.009469886	0.274909185	0.993552802
SUPT16H	-0.004642545	0.274978556	0.993552802
AFAP1	-0.011325459	0.275001437	0.993552802
RAB3GAP1	-0.005518302	0.275237561	0.993552802
SCAMP2	-0.00677588	0.275375865	0.993552802
FRAT1	0.008961399	0.275436907	0.993552802
RACGAP1	-0.007021891	0.27544875	0.993552802
CA13	-0.007936095	0.275545662	0.993552802
FAM168B	-0.005092593	0.275646509	0.993552802
IL6ST	0.009874783	0.27575928	0.993552802
FAHD2A	0.007377102	0.275795207	0.993552802
MPZL1	0.00773819	0.275814624	0.993552802
GATSL3	-0.006486538	0.275843569	0.993552802
MED6	0.003828787	0.275951581	0.993552802
TUBA1C	-0.007669759	0.276000762	0.993552802
BBS4	0.006075016	0.276145936	0.993552802
ADRBK1	0.003645528	0.276147858	0.993552802
RUNDC1	0.004850042	0.276193026	0.993552802
ICAM3	-0.005183549	0.276227121	0.993552802
HSD17B11	0.010734932	0.276254299	0.993552802
DNAJC4	-0.005058041	0.276278563	0.993552802
POLI	0.008146576	0.276291438	0.993552802
CHML	0.007837212	0.276384461	0.993552802
NADK	-0.00785097	0.276443927	0.993552802
DCUN1D5	0.00578469	0.276534448	0.993552802
SSX2IP	-0.010327583	0.276578436	0.993552802
MRPL45P2	0.010481302	0.276582566	0.993552802
GUCA1A	0.007184281	0.276609179	0.993552802
TOMM6	-0.00453159	0.276900543	0.993552802
POLR2A	-0.004064088	0.277080405	0.993552802
UNC45A	0.005082134	0.277108003	0.993552802
GLS2	-0.008165012	0.277117491	0.993552802
ALDOA	-0.005178418	0.277189056	0.993552802
CD302	0.015989128	0.27726146	0.993552802

CHST3	0.007166594	0.277320284	0.993552802
C2orf40	0.012593645	0.277401537	0.993552802
HSPA14	0.004746007	0.277536769	0.993552802
NSUN6	0.004742136	0.277668857	0.993552802
RAD54B	0.007021456	0.277772491	0.993552802
YIPF4	0.006736288	0.277919098	0.993552802
RAB1A	0.005126467	0.277941948	0.993552802
TLK1	-0.009565569	0.277957948	0.993552802
UVRAG	0.005371553	0.27811275	0.993552802
RIC3	-0.009733007	0.278134697	0.993552802
TFPT	-0.006645445	0.278244497	0.993552802
E2F6	0.007801399	0.278371638	0.993552802
AASS	0.009200391	0.278452252	0.993552802
QPCT	0.018454854	0.278515226	0.993552802
RCAN2	0.023415765	0.278675006	0.993552802
SDCCAG3	-0.005242263	0.278967394	0.993552802
MTMR10	0.006233752	0.278980858	0.993552802
NIP7	0.006417698	0.279151093	0.993552802
HADH	0.00565568	0.279162372	0.993552802
ISOC1	0.005962974	0.279164554	0.993552802
TNFRSF10A	-0.008020264	0.279223856	0.993552802
DCAKD	-0.004681351	0.27927472	0.993552802
HES4	-0.016947665	0.27948901	0.993552802
SLC38A11	-0.015805031	0.279491131	0.993552802
TP53I11	0.008488507	0.279606563	0.993552802
ELOF1	-0.005404014	0.279667501	0.993552802
ZNF48	-0.007487693	0.280102737	0.993552802
LSAMP	0.00693688	0.280124786	0.993552802
GBGT1	0.008351065	0.280183062	0.993552802
SGK223	0.007227434	0.280222013	0.993552802
IDO1	0.009892247	0.280325039	0.993552802
KCNN4	-0.009527065	0.280420283	0.993552802
SLC7A7	0.006925545	0.280500038	0.993552802
ZC3H8	0.005109122	0.280501079	0.993552802
KANK1	-0.020165533	0.280519683	0.993552802
BST2	-0.006509126	0.280592681	0.993552802
KIR2DL1	-0.022013837	0.280632061	0.993552802
CPT1A	0.009594833	0.280700739	0.993552802
RNF216	-0.005079051	0.280736982	0.993552802
CYCS	0.008416815	0.280737629	0.993552802
VPS13D	-0.007061954	0.280774531	0.993552802
MIPEP	-0.006054398	0.28082536	0.993552802
C2CD2	0.007144293	0.280985517	0.993552802
CPT1B	0.013502714	0.281033363	0.993552802
FES	0.00698808	0.281047054	0.993552802
TBC1D23	0.008608526	0.281048065	0.993552802
CNOT2	0.004245675	0.281116603	0.993552802

JAK2	0.007621847	0.281315259	0.993552802
KMO	-0.010453508	0.281337301	0.993552802
SFXN3	-0.005855637	0.281416793	0.993552802
FLI1	0.004793292	0.281638931	0.993552802
ZC3H15	0.004421255	0.281803746	0.993552802
REV1	0.00502064	0.281894765	0.993552802
SLC17A9	-0.009657348	0.281916764	0.993552802
AARS2	0.00544409	0.282125022	0.993552802
MBD3	0.007581777	0.282211921	0.993552802
UHRF1BP1L	0.008199095	0.282284645	0.993552802
VASH1	0.016733653	0.282349272	0.993552802
SLC16A10	0.01276267	0.28240588	0.993552802
AURKAIP1	0.003824695	0.282415005	0.993552802
XCL1	-0.013043289	0.282442757	0.993552802
TMX4	-0.007236317	0.282496562	0.993552802
SGPP1	0.008689992	0.282520418	0.993552802
MLLT11	0.006121182	0.282534292	0.993552802
UBQLN1	-0.00347454	0.282551768	0.993552802
APPL2	0.005225998	0.282563989	0.993552802
ZNF616	0.007864353	0.282699293	0.993552802
PLIN5	-0.017565833	0.282791497	0.993552802
CCL20	0.01034442	0.28281169	0.993552802
GNAQ	-0.012516028	0.282840681	0.993552802
PYGM	-0.006727337	0.283023432	0.993552802
MARCO	0.017185888	0.28308452	0.993552802
VEZT	-0.008382624	0.283270215	0.993552802
NT5C2	0.003294689	0.283383537	0.993552802
ALDH4A1	0.008580391	0.283392545	0.993552802
ERLEC1	0.0058881	0.283451998	0.993552802
GJB7	-0.009788577	0.283728401	0.993552802
MFNG	0.003023662	0.283756364	0.993552802
MRPL38	-0.00557913	0.283885947	0.993552802
CCDC59	-0.003586502	0.284061853	0.993552802
RALY	-0.005442134	0.28406372	0.993552802
SGCB	-0.007898177	0.284073407	0.993552802
ZNF7	0.00547377	0.284188915	0.993552802
ACVR1	-0.006172158	0.284243402	0.993552802
UBE2J1	-0.006236714	0.284484169	0.993552802
MUC16	-0.006959853	0.284559804	0.993552802
RIC8B	0.005187004	0.284568165	0.993552802
DCP1A	-0.00480441	0.284725589	0.993552802
P2RX5-TAX1BP3	-0.009051374	0.284732814	0.993552802
KIF2C	0.007401571	0.284750542	0.993552802
ZBTB48	0.005125842	0.284758138	0.993552802
METTL1	0.006085695	0.285151951	0.993552802
TBC1D19	0.006997744	0.285283596	0.993552802
GIPC1	-0.007898859	0.28531776	0.993552802

SSR1	0.005579899	0.285396436	0.993552802
PTMS	0.011921399	0.285465997	0.993552802
C19orf53	-0.004464043	0.285609486	0.993552802
IL18BP	0.004907304	0.285631525	0.993552802
ENDOG	-0.008590937	0.285659719	0.993552802
EIF4E2	-0.004015136	0.285751376	0.993552802
ITGA11	0.008535818	0.285786631	0.993552802
HMBOX1	-0.017563858	0.285885299	0.993552802
COMT	0.007423837	0.285960214	0.993552802
TNFRSF8	0.008181708	0.285981932	0.993552802
MYO1G	-0.007302745	0.286083883	0.993552802
CCR7	0.010806457	0.286098664	0.993552802
PDIA6	0.007402575	0.286130537	0.993552802
MRPS23	0.006977333	0.286142297	0.993552802
PPP4C	-0.00367912	0.286268746	0.993552802
APOM	0.008467569	0.286355106	0.993552802
FITM1	-0.006673558	0.286486441	0.993552802
IK	-0.004206914	0.286523719	0.993552802
SERAC1	0.008681278	0.286532779	0.993552802
DNLZ	0.010131356	0.286684792	0.993552802
GSPT1	-0.003195738	0.286698617	0.993552802
PRF1	-0.013590926	0.286808166	0.993552802
PRSS45	-0.007775341	0.286878963	0.993552802
ZBTB25	-0.00793944	0.287037981	0.993552802
USP19	-0.007639284	0.287078498	0.993552802
NUDCD1	-0.005499601	0.287267168	0.993552802
SPOCD1	-0.023762619	0.28732229	0.993552802
ANXA2	0.005788826	0.287442952	0.993552802
MAPKAP1	-0.005145465	0.287672466	0.993552802
ZNF441	0.00795238	0.287871413	0.993552802
PLCG1	0.006664205	0.287976682	0.993552802
SLC2A9	0.013984034	0.288026883	0.993552802
OR7E12P	-0.007110119	0.288318735	0.993552802
EXOSC9	-0.005949361	0.288444767	0.993552802
RPRD1A	-0.004998864	0.288529835	0.993552802
ATP5D	-0.005258014	0.288645594	0.993552802
ASIP	0.009153622	0.288725681	0.993552802
PSMA2	0.005799295	0.288936986	0.993552802
ZNF594	-0.002444076	0.288964327	0.993552802
ORMDL2	0.006803231	0.288975944	0.993552802
PLEKHO1	-0.004645927	0.289009853	0.993552802
UCK2	0.00781568	0.289083553	0.993552802
KRBA1	0.005263096	0.289147525	0.993552802
C5orf24	-0.008031449	0.289155918	0.993552802
PSMB5	0.00457488	0.289308566	0.993552802
ATP6V1A	0.0054146	0.28945926	0.993552802
ERH	0.005617539	0.289569351	0.993552802

TMEM175	0.004494019	0.289874462	0.993552802
DEFB116	0.007509664	0.289920103	0.993552802
NBN	0.006954467	0.290009646	0.993552802
QPRT	0.009818493	0.290053438	0.993552802
C9orf72	-0.009018417	0.29008059	0.993552802
BRIX1	-0.004891472	0.290167836	0.993552802
FCF1	0.005828015	0.290306884	0.993552802
DDR1	0.008422575	0.290380545	0.993552802
RPS6	0.004003285	0.29061037	0.993552802
FANCG	0.006332129	0.290638022	0.993552802
B2M	0.00279782	0.29070717	0.993552802
MX1	-0.014369252	0.290822744	0.993552802
CDK5RAP3	0.006801326	0.290901627	0.993552802
SPATA7	0.0066096	0.29097746	0.993552802
CD9	-0.023023719	0.291027578	0.993552802
MIR1185-1	0.005925624	0.291116254	0.993552802
FBXO22	0.006815628	0.291120671	0.993552802
TOMM5	0.009566988	0.291180425	0.993552802
NEAT1	-0.009631854	0.291235234	0.993552802
SPRY1	-0.014036192	0.291458598	0.993552802
PRCP	0.005183768	0.291476551	0.993552802
PHLDB3	0.013994932	0.291550102	0.993552802
MUC1	0.009852895	0.291658876	0.993552802
NOC3L	0.005287009	0.291800111	0.993552802
ZNF189	0.006676127	0.291807858	0.993552802
MRPS9	0.004936037	0.291847092	0.993552802
GNE	0.005028415	0.291944831	0.993552802
ERAL1	0.003858622	0.29194617	0.993552802
FBXL8	0.007251531	0.291981914	0.993552802
KIAA0907	0.004978548	0.291991396	0.993552802
SLC25A40	0.010001115	0.292004507	0.993552802
PPARA	0.005941259	0.292011005	0.993552802
C15orf54	-0.014793217	0.292044819	0.993552802
PPP6R3	-0.006752388	0.292218974	0.993552802
TOM1L2	0.006677492	0.292255979	0.993552802
FGGY	0.009352118	0.292269135	0.993552802
STX4	0.006436161	0.292380506	0.993552802
LIME1	0.004697775	0.292533666	0.993552802
MAGEF1	-0.00856608	0.292719111	0.993552802
NDUFB5	0.004419694	0.29281503	0.993552802
DDIT3	-0.00535053	0.292818111	0.993552802
KCNH6	-0.005090115	0.293152915	0.993552802
GADD45G	0.00994882	0.293410929	0.993552802
ATL3	0.005266874	0.293416246	0.993552802
RPS6KB2	-0.006688503	0.293443674	0.993552802
CXCR6	0.022879208	0.293490668	0.993552802
PTGFRN	0.006965277	0.293571302	0.993552802

UBE2T	0.007741477	0.293581736	0.993552802
AP4B1	0.007516415	0.293639667	0.993552802
SMPD3	0.007859663	0.293660498	0.993552802
GKAP1	-0.00722855	0.293715213	0.993552802
HIST1H4H	0.027804318	0.293725302	0.993552802
UBE2E3	-0.006590184	0.293769159	0.993552802
LRRC25	-0.020886211	0.293913204	0.993552802
RHOU	0.009795631	0.294037633	0.993552802
PRR24	-0.007393277	0.294159123	0.993552802
ISG20L2	-0.006194193	0.294255782	0.993552802
SBNO1	0.007766388	0.294333229	0.993552802
SNRPB	0.006397576	0.294380845	0.993552802
COMMD4	-0.005476653	0.294644364	0.993552802
SAP30BP	0.006268528	0.294662765	0.993552802
ATP2B4	-0.007679338	0.294683976	0.993552802
PDE6G	0.009469414	0.294803641	0.993552802
TXNDC12	0.004894213	0.294853358	0.993552802
SETD8	-0.006815859	0.294951521	0.993552802
MRFAP1L1	0.004225574	0.295311495	0.993552802
VDAC2	0.00491671	0.295470245	0.993552802
FRMD4B	0.013172095	0.295513919	0.993552802
FAM53A	0.008785413	0.29556765	0.993552802
KIAA0141	0.005087343	0.295669878	0.993552802
ARRB2	-0.006078279	0.295705296	0.993552802
SEPHS1	0.004044479	0.295923272	0.993552802
RGS17	0.015572055	0.29597455	0.993552802
IL25	-0.00840031	0.295982347	0.993552802
ZWINT	0.008441464	0.296031339	0.993552802
PEX10	0.005627693	0.296041207	0.993552802
KLF9	-0.007488782	0.296054989	0.993552802
LMF2	-0.007119428	0.296061387	0.993552802
C1GALT1	0.007655325	0.296106864	0.993552802
ASF1A	0.006132296	0.29622435	0.993552802
TNFAIP3	-0.008695805	0.296451037	0.993552802
SNORD59A	-0.009106104	0.296463757	0.993552802
PBX4	-0.008828682	0.296590805	0.993552802
C21orf37	-0.006299943	0.296605895	0.993552802
BAX	-0.007407018	0.29671107	0.993552802
DBNDD1	-0.016903895	0.296852951	0.993552802
PRKAR1B	-0.009011429	0.296943252	0.993552802
ZSWIM5	0.00960717	0.29696578	0.993552802
CAMK2D	-0.009924962	0.297057826	0.993552802
BTBD11	0.010061289	0.297085171	0.993552802
FAM189B	-0.004784582	0.297200304	0.993552802
LPAR2	0.00658716	0.297287354	0.993552802
CPAMD8	0.008895621	0.297336251	0.993552802
PHYH	0.006287697	0.297404885	0.993552802

ZNF366	0.008846964	0.297493368	0.993552802
TCP10L2	-0.008995819	0.297522386	0.993552802
STYX	-0.005087036	0.297547196	0.993552802
MTHFD1	-0.006737502	0.297666934	0.993552802
STK36	0.006048243	0.297787506	0.993552802
IFT46	0.006241201	0.297790789	0.993552802
COX5A	0.00590481	0.297833195	0.993552802
SLC37A2	-0.010345959	0.297870685	0.993552802
RNPEP	-0.007118784	0.297943583	0.993552802
CPEB3	-0.005576973	0.298034316	0.993552802
POPDC2	0.008031351	0.298045844	0.993552802
UBE2O	-0.007121183	0.29805582	0.993552802
SERPINI1	-0.00768725	0.298146281	0.993552802
MESDC1	-0.006655864	0.298277566	0.993552802
MAPKAPK3	0.005742181	0.298358376	0.993552802
SLC6A6	-0.009005906	0.298475784	0.993552802
ARSA	0.009323979	0.298642248	0.993552802
PYCR2	-0.005524169	0.298683847	0.993552802
SLC25A11	0.005228531	0.298784047	0.993552802
KLHL18	0.005047166	0.298827856	0.993552802
EIF2B4	0.002853842	0.298840175	0.993552802
SH2B2	-0.007630445	0.298913933	0.993552802
FAM133B	-0.012999808	0.299034245	0.993552802
SSTR1	0.006406056	0.299071234	0.993552802
MRPL48	-0.005953067	0.299074507	0.993552802
DIXDC1	-0.006283287	0.299120982	0.993552802
ACTR6	0.005354264	0.299202921	0.993552802
CD37	-0.007417711	0.299231201	0.993552802
BBX	-0.004896749	0.29925495	0.993552802
USE1	-0.006628198	0.299335136	0.993552802
GLTSCR2	0.00259007	0.29949475	0.993552802
ETFDH	0.006497915	0.29950681	0.993552802
TMEM199	0.00538738	0.299568892	0.993552802
PIGO	0.004459144	0.299636674	0.993552802
TBXA2R	-0.009417778	0.299754277	0.993552802
PRSS8	-0.007651039	0.299811099	0.993552802
ZFP57	-0.007225907	0.299886655	0.993552802
UBE2G1	0.008443224	0.299968373	0.993552802
SF1	-0.007686501	0.300012047	0.993552802
ECE1	-0.007463476	0.30038508	0.993552802
GMNN	0.007801378	0.300557121	0.993552802
ZNF138	-0.005954089	0.300577997	0.993552802
CNTNAP3B	-0.012193946	0.300585921	0.993552802
SLC30A7	-0.006889807	0.300683523	0.993552802
DCUN1D1	0.008582317	0.300714974	0.993552802
RSPH4A	0.008290502	0.300872847	0.993552802
IER2	-0.009360675	0.300968228	0.993552802

ZCCHC9	0.005865401	0.301247644	0.993552802
NOTCH1	0.006576359	0.301405474	0.993552802
ATP6V1F	0.004574868	0.301461572	0.993552802
KIAA1683	-0.012846761	0.301650995	0.993552802
PHF7	-0.006974744	0.301662028	0.993552802
TCERG1	0.005234714	0.301791968	0.993552802
PRDM4	-0.004322351	0.301901294	0.993552802
TAF1C	0.005081376	0.301917886	0.993552802
RGS18	-0.010997943	0.302164795	0.993552802
S100A12	0.01609621	0.302326544	0.993552802
RICTOR	0.008006006	0.302376552	0.993552802
HUS1	0.00691652	0.302469782	0.993552802
PPIH	-0.004559059	0.302540325	0.993552802
TMEM167A	0.007811521	0.302622923	0.993552802
FGFR3	-0.00915149	0.302624232	0.993552802
NFE2L1	-0.00671001	0.302700585	0.993552802
PTPN23	0.0070131	0.302744849	0.993552802
BTBD1	0.005378925	0.302826535	0.993552802
MLX	0.006390508	0.302901767	0.993552802
PPM1A	-0.005954099	0.303058773	0.993552802
TRIM14	-0.008763683	0.303117988	0.993552802
MRPL54	0.006098084	0.303169591	0.993552802
MED30	0.005470858	0.303321724	0.993552802
C5orf15	0.003660006	0.303352374	0.993552802
SDPR	-0.013500081	0.303491978	0.993552802
NR2F2	-0.007392827	0.303564648	0.993552802
DCTPP1	0.006294978	0.303588742	0.993552802
ZNF410	-0.003053191	0.303596628	0.993552802
TIGD6	0.006549571	0.303703036	0.993552802
SEMA4C	0.006882511	0.303722327	0.993552802
ERCC1	0.005419646	0.303806821	0.993552802
UCN	0.007055002	0.303880382	0.993552802
TMEM80	-0.006277243	0.303881163	0.993552802
HNRNPAB	-0.003364329	0.304037088	0.993552802
KCTD21	0.007273847	0.304088684	0.993552802
PI4K2B	0.004581734	0.304201587	0.993552802
NPEPPS	0.003811285	0.304209945	0.993552802
TNFRSF25	0.00941285	0.304257446	0.993552802
HLA-H	-0.004497847	0.30427863	0.993552802
VIM	0.005645995	0.304445287	0.993552802
DDX5	0.003308564	0.304497906	0.993552802
HEATR6	0.0056874	0.304719437	0.993552802
ZC3H3	0.005425026	0.305063031	0.993552802
MRPL40	0.006352849	0.305125701	0.993552802
ZNF426	0.00612212	0.305137781	0.993552802
SLC25A22	0.005245473	0.305217344	0.993552802
CDV3	0.004551287	0.305273832	0.993552802

JUNB	-0.011816007	0.305553523	0.993552802
RPL21	0.009036294	0.305625496	0.993552802
TMEM8B	0.007122769	0.305819447	0.993552802
SMC4	0.005115634	0.305820487	0.993552802
RNASEH2A	0.006186537	0.306008416	0.993552802
PTP4A2	-0.004611018	0.306104254	0.993552802
AZIN1	0.005348046	0.306141192	0.993552802
ARFIP1	0.006587048	0.306145347	0.993552802
HERC2	-0.005362993	0.306157391	0.993552802
TMED2	0.005656494	0.306258947	0.993552802
SLC20A1	0.006446331	0.306410049	0.993552802
SNAPC3	0.005356368	0.306523167	0.993552802
URM1	-0.004115709	0.306581413	0.993552802
CLN8	0.010315966	0.306800158	0.993552802
CHCHD1	0.006664085	0.306846404	0.993552802
MPND	0.008351085	0.306983704	0.993552802
TAF12	0.00607499	0.306984045	0.993552802
SF3A2	-0.004815813	0.307062986	0.993552802
CCL8	-0.014408345	0.307136983	0.993552802
MT1F	0.007554224	0.307191826	0.993552802
VIPR2	-0.010083884	0.307363539	0.993552802
TFEB	-0.007854016	0.307471039	0.993552802
PSME4	-0.00405057	0.307612425	0.993552802
WWP1	-0.004206693	0.307655527	0.993552802
ORC4	-0.017764482	0.307772534	0.993552802
PEX11G	0.009441834	0.307929634	0.993552802
BDH1	0.007590123	0.308015366	0.993552802
GDPD3	0.009781906	0.308052639	0.993552802
DBN1	0.012991747	0.308144174	0.993552802
TRIM74	0.006779022	0.308200349	0.993552802
KIR2DL4	-0.022452266	0.308326168	0.993552802
MIR300	0.007760199	0.308411504	0.993552802
TOR3A	0.00846526	0.30868997	0.993552802
DNAJC9	-0.004336417	0.308763133	0.993552802
LYZ	0.012818371	0.308775808	0.993552802
NSUN5	-0.00605015	0.308989921	0.993552802
RPL22L1	0.024807552	0.309111217	0.993552802
SNORD38A	-0.007845025	0.309265462	0.993552802
ZFYVE26	0.004267868	0.309338741	0.993552802
LCN1	-0.006241699	0.309441788	0.993552802
JMJD7-PLA2G4B	0.006337138	0.309479986	0.993552802
MESP1	0.00942269	0.309789596	0.993552802
MRFAP1	0.004031459	0.309880953	0.993552802
LRRC16A	-0.009778759	0.31000915	0.993552802
SNORA7B	-0.005230066	0.310032725	0.993552802
ZNF135	0.011672095	0.310101885	0.993552802
PMS2P3	0.007293653	0.31015857	0.993552802

NLRC4	0.007968724	0.310301845	0.993552802
DAXX	-0.005387725	0.310383031	0.993552802
DDX10	-0.021567116	0.310388833	0.993552802
FAM192A	0.004837368	0.310474938	0.993552802
KLHDC8B	0.009824672	0.310688676	0.993552802
ACP6	0.0072845	0.310873278	0.993552802
HOOK2	-0.005687483	0.310935197	0.993552802
METTL22	-0.008183007	0.310962716	0.993552802
ZNF644	0.007089436	0.311219519	0.993552802
AHCTF1	-0.005605154	0.311271822	0.993552802
PADI4	0.012121613	0.311278064	0.993552802
CYFIP1	0.008100342	0.311304507	0.993552802
METTL3	0.003709112	0.3114774	0.993552802
MSL1	-0.005273982	0.31164283	0.993552802
SIGLEC6	-0.010780365	0.311660452	0.993552802
CES2	0.004426467	0.311739695	0.993552802
ZNF467	0.007794959	0.311767779	0.993552802
LMAN1	-0.006020103	0.311834984	0.993552802
RNF149	0.005498648	0.311859147	0.993552802
DDB1	0.003175644	0.311901857	0.993552802
PDGFB	-0.011787293	0.311954841	0.993552802
GYS1	-0.006840219	0.311987578	0.993552802
PSAT1	-0.00978868	0.312027706	0.993552802
RPLP0	0.006219307	0.312043043	0.993552802
WSB1	0.007118444	0.312238148	0.993552802
ZBTB45	0.004925205	0.312309874	0.993552802
ANKS6	-0.007467834	0.312312665	0.993552802
MRPS31	-0.003784001	0.312375298	0.993552802
ORMDL1	0.0036183	0.312665629	0.993552802
GPR124	0.010255255	0.312685638	0.993552802
ZMYND8	-0.005382941	0.312694776	0.993552802
TM6SF1	0.010006761	0.312737889	0.993552802
SAP25	0.006794825	0.312759159	0.993552802
KLHDC3	0.002813554	0.313052839	0.993552802
C1orf53	0.009851806	0.313130341	0.993552802
ARL2	0.005680922	0.313341346	0.993552802
CTSS	0.006642937	0.313415076	0.993552802
COIL	0.003472096	0.313460003	0.993552802
SNORD94	-0.005426795	0.31363598	0.993552802
PPP1CA	-0.005515531	0.313660374	0.993552802
RPL29P2	0.006675585	0.313664892	0.993552802
NLRP3	0.00761991	0.313741728	0.993552802
ALOX5	0.006104692	0.313763916	0.993552802
SCAMP4	0.008390586	0.313793403	0.993552802
CRELD2	0.005600348	0.313940198	0.993552802
GPBAR1	0.008468939	0.314064147	0.993552802
GNB1	-0.002962394	0.314068637	0.993552802

PXDN	0.021521778	0.31408451	0.993552802
NIPA2	0.004311009	0.314120118	0.993552802
ASXL1	-0.00569087	0.314274378	0.993552802
HIVEP1	-0.006150939	0.314277802	0.993552802
CEP72	0.00653252	0.314309632	0.993552802
TRAFD1	-0.005362703	0.314668084	0.993552802
ISG15	-0.014735319	0.314673836	0.993552802
GIN1	0.005925094	0.314738864	0.993552802
LTB	-0.004313577	0.314812401	0.993552802
ENHO	-0.007031026	0.314914954	0.993552802
ZNF532	0.008836595	0.315012146	0.993552802
PLA2G12A	-0.011951665	0.315236373	0.993552802
DENND4A	0.00559894	0.3152668	0.993552802
C1orf50	0.004333129	0.315277051	0.993552802
WDR60	0.006574861	0.315413636	0.993552802
LRP8	0.006707732	0.315444182	0.993552802
OAS3	-0.015436847	0.315553999	0.993552802
MIR373	-0.00688233	0.315621033	0.993552802
NISCH	0.00505636	0.315622783	0.993552802
CHAC2	0.009329836	0.315650621	0.993552802
SON	0.002322379	0.315807275	0.993552802
WHAMM	-0.008770985	0.315879992	0.993552802
XPO1	0.003535174	0.315987042	0.993552802
SKAP1	0.004819733	0.31612847	0.993552802
GNPNAT1	-0.007543079	0.316192381	0.993552802
BAG1	-0.007308938	0.316252313	0.993552802
SIX5	0.007466031	0.316338085	0.993552802
CCT3	0.005117756	0.316354466	0.993552802
SPHK1	-0.008558605	0.316474888	0.993552802
PRRC2A	-0.008136555	0.316530059	0.993552802
FAHD2B	0.005211607	0.3166458	0.993552802
BCKDK	0.005215339	0.316696243	0.993552802
HSDL2	-0.00671654	0.316788074	0.993552802
GDE1	-0.005549216	0.316806003	0.993552802
NTSR1	0.016402814	0.31686449	0.993552802
GEMIN6	0.00681549	0.316885519	0.993552802
MANF	0.005946423	0.316957671	0.993552802
SLC38A10	-0.006181896	0.317007705	0.993552802
CD79B	-0.010511144	0.317015054	0.993552802
HEBP1	0.005150104	0.317065	0.993552802
TSR1	0.004530129	0.317146363	0.993552802
CROT	0.007644204	0.317261483	0.993552802
KIR3DL2	-0.020055269	0.317350562	0.993552802
MIR586	0.007416387	0.317441904	0.993552802
HRASLS2	0.018882904	0.317660344	0.993552802
SYK	-0.005169062	0.317671488	0.993552802
RBL2	-0.006557747	0.317884242	0.993552802

KAT5	-0.00598151	0.31794102	0.993552802
UCHL5	0.007767647	0.317968069	0.993552802
TEF	0.007073325	0.318053594	0.993552802
PNMA1	-0.006772192	0.318175526	0.993552802
SLC44A1	-0.00588169	0.318293816	0.993552802
RRM1	-0.005071409	0.318660127	0.993552802
USP6	0.007524639	0.318740609	0.993552802
HAX1	0.005144208	0.318934546	0.993552802
TRIM17	0.006628483	0.319130546	0.993552802
PSMA4	0.00421511	0.319173709	0.993552802
SETBP1	-0.009908644	0.319276778	0.993552802
FUT6	-0.018662351	0.319617849	0.993552802
EPB41L4A	-0.006669821	0.319810699	0.993552802
MAF	-0.011090193	0.319813771	0.993552802
OLFML2A	0.006234603	0.319881243	0.993552802
TPM3	0.002763148	0.320048579	0.993552802
ANKRD16	0.005926278	0.320113243	0.993552802
CD4	-0.007273172	0.320196871	0.993552802
AK2	0.006592351	0.320244964	0.993552802
SNORA22	0.009541441	0.320246574	0.993552802
HPS5	0.00572628	0.320364007	0.993552802
MTHFD1L	0.008236025	0.320541805	0.993552802
CD44	0.003468119	0.32060786	0.993552802
TMCC1	0.008761313	0.320643213	0.993552802
HBEGF	-0.011314747	0.320658969	0.993552802
HVCN1	-0.007104579	0.32093757	0.993552802
RIMBP3	-0.006189209	0.321035755	0.993552802
PDZD8	0.006901647	0.321048353	0.993552802
FCER1G	0.009144383	0.321061453	0.993552802
PANK4	0.005030916	0.321153233	0.993552802
PIN1	0.002496217	0.321307766	0.993552802
CSRP2BP	-0.005152829	0.321341939	0.993552802
STRN3	-0.006411695	0.321390005	0.993552802
CDKL1	-0.009029139	0.321402726	0.993552802
USP36	-0.011899472	0.321449864	0.993552802
NAT10	0.00410063	0.321507002	0.993552802
SCP2	-0.009960538	0.321527593	0.993552802
SNORA34	-0.00621358	0.321809868	0.993552802
GTF2F1	-0.004298084	0.321818751	0.993552802
ZNF260	0.006399408	0.321863437	0.993552802
SNORD35B	-0.008497277	0.321967095	0.993552802
PRPF39	0.00692737	0.322051293	0.993552802
RPA2	0.003878564	0.322220383	0.993552802
INPP5B	-0.007536051	0.322276087	0.993552802
PLEKHH3	-0.008059708	0.322382116	0.993552802
HACE1	-0.005666213	0.322464666	0.993552802
IFFO2	-0.006420173	0.322566168	0.993552802

C2orf43	0.006645117	0.322578	0.993552802
SDCCAG8	0.006758186	0.322626424	0.993552802
GALK2	-0.009077264	0.322643979	0.993552802
DNASE2	-0.005692953	0.322718597	0.993552802
GDNF	-0.007634219	0.322875724	0.993552802
AGA	0.006834606	0.322923044	0.993552802
RPL23	-0.003208017	0.323010933	0.993552802
DPEP3	0.009773927	0.323036439	0.993552802
OGFOD1	-0.003932991	0.323151795	0.993552802
SLA	0.008916123	0.323470233	0.993552802
TRMT11	0.005156362	0.323548251	0.993552802
GTPBP2	-0.006826715	0.323598601	0.993552802
NPY5R	-0.006422943	0.323611474	0.993552802
GPC6	0.006096955	0.323639434	0.993552802
RUNX1	0.004507739	0.323648182	0.993552802
MIOS	0.003913679	0.323654011	0.993552802
RAI14	-0.008660949	0.323676015	0.993552802
C6orf106	-0.006170959	0.32368912	0.993552802
ALX1	0.007479453	0.323739273	0.993552802
DHX16	0.004013489	0.323958039	0.993552802
HGD	-0.017069895	0.323963743	0.993552802
USP5	-0.004866286	0.324237573	0.993552802
TJAP1	-0.003104234	0.324241844	0.993552802
CCDC28A	0.003779512	0.324282776	0.993552802
FUS	0.004295731	0.324473577	0.993552802
F3	0.006489928	0.324732944	0.993552802
RBBP6	-0.008836029	0.324760149	0.993552802
CPSF6	0.007488072	0.324859172	0.993552802
PDCD2	0.004952166	0.32494233	0.993552802
ZNF446	0.005350203	0.325033625	0.993552802
LTBP1	-0.008999654	0.325198193	0.993552802
FAM84B	-0.007791475	0.325244037	0.993552802
TMEM159	-0.005760309	0.325294285	0.993552802
MAEA	0.003912831	0.325416121	0.993552802
HNRNPA2B1	-0.002867424	0.325438518	0.993552802
AVIL	0.006589541	0.325498033	0.993552802
SNF8	0.00768508	0.325507557	0.993552802
ZNF281	-0.00474881	0.325653091	0.993552802
C9orf135	0.019014676	0.325662084	0.993552802
INTS7	0.005711119	0.325684335	0.993552802
C12orf44	0.005144144	0.325703959	0.993552802
PPM1M	0.003869876	0.325710716	0.993552802
BRD1	-0.005207036	0.325920118	0.993552802
DHX38	0.006738395	0.325936244	0.993552802
TTC7A	-0.007126419	0.325939815	0.993552802
POLDIP2	0.006186289	0.32609289	0.993552802
C10orf12	-0.005368053	0.326115468	0.993552802

MRPS21	-0.005007494	0.326162441	0.993552802
ANKRD49	0.004031139	0.326174297	0.993552802
RAF1	-0.003642076	0.326191716	0.993552802
CDC26	0.003724339	0.326344662	0.993552802
ADNP	0.004099113	0.326445952	0.993552802
ZNF629	0.005475991	0.326509296	0.993552802
ZNF33A	0.003671339	0.326533856	0.993552802
DPY19L2P2	0.006550974	0.326701731	0.993552802
MTRF1	0.00612053	0.326762265	0.993552802
ANO10	0.006440345	0.326893629	0.993552802
LNPEP	0.006436686	0.326957974	0.993552802
TMEM181	-0.007103545	0.327000616	0.993552802
YIPF5	0.010011801	0.327052358	0.993552802
NR2F6	-0.010264857	0.327105357	0.993552802
SNORD110	-0.006697508	0.327164603	0.993552802
C2orf49	0.006989689	0.327220207	0.993552802
C6orf52	-0.005561362	0.327231559	0.993552802
ZNF611	-0.006213213	0.327290846	0.993552802
CAPZA2	-0.00411232	0.327441334	0.993552802
APOBR	0.006008424	0.327447359	0.993552802
ZNF566	0.008634166	0.32755855	0.993552802
CDK1	0.011761017	0.3276609	0.993552802
KIF27	0.010713115	0.327779257	0.993552802
ZNF165	-0.007110307	0.327783539	0.993552802
SNORD48	-0.011074154	0.327794135	0.993552802
UBE2D4	0.006398808	0.327926923	0.993552802
ERGIC3	-0.004563413	0.327944422	0.993552802
ACACB	-0.007372144	0.328019175	0.993552802
NT5DC3	-0.011176532	0.328117296	0.993552802
H2AFV	-0.005703431	0.328133611	0.993552802
SHQ1	0.005415385	0.328277764	0.993552802
RFX5	-0.003188688	0.328366152	0.993552802
CSNK1D	0.003581769	0.32838024	0.993552802
GLDC	0.018502039	0.328443462	0.993552802
OR2T34	-0.006636189	0.328470904	0.993552802
AGFG1	-0.006038787	0.328594965	0.993552802
FANCD2	0.005273656	0.328620183	0.993552802
SF3B5	-0.004308971	0.328628669	0.993552802
ARPC5L	-0.004875991	0.328638691	0.993552802
SLC25A35	0.007860418	0.328738428	0.993552802
IFNGR2	0.006299499	0.328757592	0.993552802
EXOC5	0.004312203	0.328870663	0.993552802
TMEM115	0.004812128	0.328954657	0.993552802
SNORD99	0.010290162	0.329112965	0.993552802
GPAT2	-0.015460709	0.329278621	0.993552802
ARRDC1	-0.006629821	0.329370647	0.993552802
SRSF4	-0.003238583	0.329514201	0.993552802

TCP10L	0.00636958	0.329528537	0.993552802
ACOT4	0.011422244	0.329612795	0.993552802
BCRP2	-0.006068635	0.329821126	0.993552802
THAP1	0.008482496	0.329830966	0.993552802
ITPR1	-0.005428176	0.329920177	0.993552802
SGSM2	0.00428935	0.329952547	0.993552802
CCDC107	0.006203155	0.329973049	0.993552802
PARK7	0.003256395	0.330015137	0.993552802
ZNF34	-0.00577708	0.330130419	0.993552802
MAK16	0.005103127	0.330132724	0.993552802
CD200R1	0.009061188	0.330173311	0.993552802
C3orf62	-0.005536187	0.330191912	0.993552802
TNFSF9	-0.007411736	0.330241782	0.993552802
SNORA58	0.005446669	0.330298504	0.993552802
GOLGA4	0.006250883	0.330387286	0.993552802
COPB1	0.003399098	0.330395424	0.993552802
NAA35	0.004555624	0.330427655	0.993552802
CD47	-0.004496935	0.330558118	0.993552802
RNF24	-0.006684493	0.330601162	0.993552802
EIF5	0.006286546	0.330618633	0.993552802
TRA2A	-0.00962845	0.33067797	0.993552802
TMED9	0.00547482	0.330694348	0.993552802
FAM105A	0.008394118	0.330757373	0.993552802
CMTM3	0.004388519	0.330775552	0.993552802
MIR635	0.009159673	0.330988665	0.993552802
PTPDC1	-0.006742035	0.330994682	0.993552802
ZNF444	0.006461169	0.331043662	0.993552802
DGKD	-0.007631127	0.331082078	0.993552802
NFAM1	0.008215898	0.331112961	0.993552802
LPXN	0.004029963	0.331193219	0.993552802
SERP1	0.005979784	0.331430692	0.993552802
FILIP1L	0.007087463	0.331519733	0.993552802
LTF	0.007365351	0.331614976	0.993552802
SERPINB6	-0.004995399	0.331698244	0.993552802
DCTN6	0.005751838	0.33169993	0.993552802
SLC14A1	0.010958979	0.33175047	0.993552802
KCNK17	-0.014971977	0.331779056	0.993552802
NPDC1	-0.008686814	0.332016644	0.993552802
ZNF844	0.007393846	0.332079273	0.993552802
DDX18	-0.003734682	0.332260092	0.993552802
MAPK6	0.004940181	0.332272742	0.993552802
LIN7C	0.00879629	0.332321883	0.993552802
TIMM8B	0.006577082	0.332512922	0.993552802
TRPV1	0.006224549	0.332630646	0.993552802
LAG3	0.017472248	0.332705205	0.993552802
COL4A3BP	-0.006559578	0.332709104	0.993552802
MRPL39	0.006003822	0.332851179	0.993552802

SLC26A8	-0.008120022	0.33290226	0.993552802
YARS	0.003942089	0.332998031	0.993552802
GRB2	-0.006288715	0.333030757	0.993552802
S100A4	0.003640057	0.333162231	0.993552802
UQCR10	0.007338579	0.3331822	0.993552802
GALC	0.004531427	0.333386495	0.993552802
MAPRE1	0.004858092	0.333436173	0.993552802
PLAUR	0.008989194	0.333439829	0.993552802
PGA4	0.010983551	0.333778497	0.993552802
TLR3	0.00629426	0.333971753	0.993552802
BIN1	-0.006132971	0.334000556	0.993552802
HLA-C	-0.025128274	0.334012869	0.993552802
SEC23B	0.005583266	0.334030987	0.993552802
POLQ	0.008967268	0.334193371	0.993552802
ATM	-0.004971415	0.334205068	0.993552802
RCSD1	-0.005066018	0.334353139	0.993552802
MAD1L1	-0.009923801	0.334404944	0.993552802
ANO6	-0.008087441	0.334427767	0.993552802
PELI2	0.006718703	0.334513736	0.993552802
DYNLL1	0.004811105	0.334531364	0.993552802
AK4	0.013329972	0.334612588	0.993552802
EIF4EBP1	-0.005818844	0.334644398	0.993552802
PMF1	-0.006382903	0.334741173	0.993552802
RNF2	0.007133325	0.334825525	0.993552802
CLDN7	0.006991316	0.334869175	0.993552802
DOHH	0.00597569	0.33488432	0.993552802
ARRDC4	0.032304993	0.334966474	0.993552802
UXS1	-0.00862662	0.33505532	0.993552802
C10orf35	0.007947535	0.335063641	0.993552802
FCGR1A	0.011320679	0.335223042	0.993552802
ZNF318	-0.00430446	0.335320923	0.993552802
HELZ	-0.005663466	0.33539653	0.993552802
ZDHHC19	-0.009317458	0.335486376	0.993552802
SRSF11	-0.006090877	0.335502711	0.993552802
SLC2A8	0.012677377	0.335532629	0.993552802
TESC	0.007289678	0.335550829	0.993552802
TMX3	0.004475141	0.335622612	0.993552802
NAP1L1	-0.004544961	0.33573201	0.993552802
DCP2	0.005086918	0.335856241	0.993552802
MRPS33	0.005432871	0.335996503	0.993552802
RMND1	0.007056609	0.336057309	0.993552802
UGT1A1	-0.006409342	0.336094116	0.993552802
EXO1	0.006751554	0.336106946	0.993552802
ZNF607	-0.005888894	0.336201763	0.993552802
CLCN6	0.00593746	0.336665384	0.993552802
TANC2	0.006461038	0.336769604	0.993552802
RAB23	0.010649238	0.336787149	0.993552802

CCDC142	0.009724353	0.336832982	0.993552802
CPNE8	0.006233444	0.336858069	0.993552802
DEPDC1B	0.008297776	0.337005467	0.993552802
CHFR	0.00410237	0.337445347	0.993552802
EIF3F	0.005327744	0.337447633	0.993552802
ANAPC2	-0.00683759	0.337509939	0.993552802
DUSP2	-0.011213141	0.337654283	0.993552802
NT5C1B	0.002874211	0.337751486	0.993552802
RFX7	-0.00329269	0.337835644	0.993552802
TMEM41B	0.00579861	0.337868212	0.993552802
MAT2B	0.004134136	0.337935663	0.993552802
C1orf122	0.006197309	0.337967184	0.993552802
NMI	0.008486315	0.3382406	0.993552802
SLC7A6OS	-0.005809851	0.338413575	0.993552802
CDC27	0.009384194	0.338451526	0.993552802
ANLN	0.0065805	0.338570266	0.993552802
RALA	0.003614903	0.338577442	0.993552802
SGSH	-0.006639949	0.338645576	0.993552802
FAM20C	-0.011198461	0.338696902	0.993552802
DDX59	-0.013052254	0.338701968	0.993552802
MTDH	0.003967439	0.338704178	0.993552802
FNTB	0.018909137	0.338941081	0.993552802
DPYSL4	0.030427231	0.339358495	0.993552802
NUP50	0.006987738	0.339390391	0.993552802
WIPF2	0.006935969	0.339529261	0.993552802
TSC22D2	0.005962617	0.339568726	0.993552802
MAP3K7	-0.003401039	0.339594485	0.993552802
EBAG9	-0.003616312	0.339684894	0.993552802
MGAT4A	-0.006270177	0.339787499	0.993552802
SWAP70	-0.008136994	0.339962809	0.993552802
IDH1	0.005041403	0.340093137	0.993552802
UQCR11	-0.005760151	0.340122979	0.993552802
C16orf72	0.007679068	0.340190103	0.993552802
ZNF197	0.004747531	0.340199183	0.993552802
SPTBN1	-0.008444057	0.340273439	0.993552802
SPC25	-0.013749967	0.340295187	0.993552802
BCAS4	-0.008817789	0.340316797	0.993552802
SOAT1	0.009606336	0.340352702	0.993552802
ROCK1P1	0.0071515	0.340383675	0.993552802
REEP1	-0.008801944	0.340400026	0.993552802
S100Z	0.010036155	0.340458967	0.993552802
METTL23	0.002961295	0.340573726	0.993552802
CCNI2	-0.008378223	0.340663544	0.993552802
MIR593	-0.006275275	0.340782281	0.993552802
SOD2	-0.007807197	0.340793835	0.993552802
ICT1	0.003543855	0.340844291	0.993552802
NSMCE2	0.007801347	0.340870477	0.993552802

CHMP4A	0.003764788	0.340892168	0.993552802
OLIG1	0.017602758	0.340991777	0.993552802
BFAR	0.004906311	0.341082201	0.993552802
DEF8	0.006190263	0.34115633	0.993552802
CDK10	0.008074077	0.341208225	0.993552802
SRBD1	-0.004493423	0.341250969	0.993552802
IMMP1L	0.006924883	0.34144132	0.993552802
GDF11	0.00621976	0.341529052	0.993552802
KCNJ12	0.007799432	0.34174636	0.993552802
FCGR3B	-0.010264117	0.341829004	0.993552802
CXCL10	0.017904313	0.341954686	0.993552802
KRTAP10-11	-0.006515843	0.342012743	0.993552802
SLC19A2	-0.005646878	0.342042667	0.993552802
MTRF1L	-0.003834001	0.342054713	0.993552802
KCNH8	-0.007740855	0.342276096	0.993552802
COMMD10	0.006547381	0.342342407	0.993552802
RTN3	-0.004732546	0.342434842	0.993552802
S100A1	-0.007845175	0.342471345	0.993552802
MYPOP	0.002861275	0.342541323	0.993552802
SNX16	0.007719574	0.342738958	0.993552802
EVL	0.003764824	0.343081093	0.993552802
PHACTR1	-0.008039392	0.343085913	0.993552802
DPM1	0.006739902	0.343460048	0.993552802
SPAG5	0.005898637	0.343596703	0.993552802
TOMM22	-0.003102063	0.343633987	0.993552802
ADA	0.006291338	0.343718164	0.993552802
CDC42EP3	0.011047824	0.343746376	0.993552802
GGA2	-0.004877983	0.343863883	0.993552802
ZNF816	0.008049414	0.343956368	0.993552802
NCKAP5	-0.012286057	0.344037962	0.993552802
NUDT6	0.007840366	0.344099494	0.993552802
ACO1	0.003869174	0.344127122	0.993552802
RCVRN	-0.00954841	0.344165594	0.993552802
ACTR3	0.004522979	0.344256891	0.993552802
THRA	-0.00650656	0.34431981	0.993552802
RAB3A	0.006472937	0.344472542	0.993552802
NRD1	-0.00341391	0.344540829	0.993552802
ZBTB34	0.005072322	0.344545167	0.993552802
ACAA1	0.004818394	0.344567921	0.993552802
ESM1	0.010868009	0.34458663	0.993552802
SEC24B	0.004578919	0.34467202	0.993552802
USF2	0.008914346	0.344702434	0.993552802
TTC33	0.006637399	0.344736414	0.993552802
SYNGR1	-0.009964398	0.344737134	0.993552802
CARD9	0.006568719	0.344792016	0.993552802
ZNF250	0.008398832	0.344814893	0.993552802
HDAC4	0.010130868	0.344836716	0.993552802

RPL4	0.0040692	0.344959185	0.993552802
CLECL1	0.017280814	0.345001521	0.993552802
RXRA	0.003763082	0.34501041	0.993552802
CCT8L2	0.006419499	0.345104301	0.993552802
CALM1	-0.003689438	0.345375589	0.993552802
DHRS4L2	-0.00790106	0.345408193	0.993552802
C2orf74	-0.011300084	0.34546226	0.993552802
WWP2	-0.007245449	0.345608039	0.993552802
ATF5	-0.005742468	0.345677434	0.993552802
CSK	-0.00549107	0.345713844	0.993552802
ZNF581	-0.004939812	0.345796593	0.993552802
ZNF678	-0.00625038	0.345864297	0.993552802
YTHDF2	0.003631317	0.346050047	0.993552802
PKP3	-0.00568644	0.346076305	0.993552802
DBNL	0.00350249	0.346089064	0.993552802
E2F4	-0.003720841	0.346163218	0.993552802
PLAC8	-0.006307495	0.346171062	0.993552802
GLYR1	-0.002777269	0.34631671	0.993552802
RYBP	-0.011587269	0.346318673	0.993552802
DHRS3	-0.007065761	0.346362226	0.993552802
CCDC24	0.007869003	0.346461631	0.993552802
RAP1B	-0.006850678	0.346475405	0.993552802
CD22	-0.010862124	0.346711237	0.993552802
MT1E	0.014829009	0.346826998	0.993552802
SNIP1	0.005110465	0.347050274	0.993552802
ECT2	0.006340204	0.347082922	0.993552802
NFIL3	0.009994782	0.347263616	0.993552802
CD58	0.008086985	0.347268863	0.993552802
CLDN23	0.012078137	0.347486782	0.993552802
YPEL5	-0.005055805	0.347589278	0.993552802
SAP18	0.003918019	0.347712242	0.993552802
IFNGR1	0.005030916	0.347760383	0.993552802
SMN1	-0.011078786	0.348051945	0.993552802
F11R	-0.00648511	0.348057233	0.993552802
ADSS	0.004207532	0.348165173	0.993552802
BUD13	0.003918917	0.348435292	0.993552802
KLRG1	0.012704257	0.348441372	0.993552802
DHTKD1	-0.004379983	0.348597222	0.993552802
TMEM151B	0.006299458	0.348908313	0.993552802
SP2	0.004453757	0.349063716	0.993552802
ZNF45	0.003757043	0.349252685	0.993552802
CELSR2	-0.007090448	0.349315654	0.993552802
LRRC8B	-0.00685762	0.349377684	0.993552802
DDX27	-0.004636229	0.349601559	0.993552802
TRIM36	0.007468492	0.349696112	0.993552802
PSMD1	-0.004125691	0.349735043	0.993552802
ATAT1	-0.006715589	0.349751223	0.993552802

CCDC58	0.016994513	0.349848598	0.993552802
ZNF697	0.008785198	0.349908947	0.993552802
GLCE	0.005278895	0.350107789	0.993552802
C12orf43	0.006677244	0.350120764	0.993552802
RTEL1-TNFRSF6B	-0.006336933	0.350153072	0.993552802
CCNB2	0.014625078	0.350424025	0.993552802
ZMYM1	-0.005510923	0.350447173	0.993552802
C17orf89	-0.004954961	0.350465199	0.993552802
ANTXR2	-0.005586182	0.350506037	0.993552802
MBD6	0.007415355	0.350588335	0.993552802
KHDRBS3	-0.00502315	0.350617675	0.993552802
MRPS26	0.004094003	0.350646669	0.993552802
MMS22L	-0.005891284	0.350793212	0.993552802
MIR129-2	0.006106446	0.350797578	0.993552802
LYSMD4	0.006669896	0.350845881	0.993552802
EXOSC4	-0.005276064	0.350917813	0.993552802
SNORA70B	-0.007476576	0.350954386	0.993552802
ASS1	-0.00539958	0.351155038	0.993552802
ADHFE1	0.007522718	0.35126556	0.993552802
ACTN1	-0.008333937	0.351266093	0.993552802
ATE1	0.005612144	0.351316272	0.993552802
RPS28	0.004008336	0.351579517	0.993552802
SF3A1	-0.007038291	0.351740943	0.993552802
MRPS14	0.006680902	0.35179777	0.993552802
ADRA2A	-0.006260067	0.351924741	0.993552802
SMAD3	-0.006028266	0.352105056	0.993552802
MAPK9	-0.005477925	0.352118462	0.993552802
PACS2	-0.006130734	0.352281483	0.993552802
PRMT7	-0.006857937	0.352490294	0.993552802
PTGDR	0.013909232	0.352575993	0.993552802
P2RX4	0.005476556	0.352696295	0.993552802
HAUS5	-0.006046099	0.352845962	0.993552802
NDUFS8	0.00344994	0.352895037	0.993552802
ST7L	0.007438181	0.353072291	0.993552802
CHMP6	-0.005723507	0.353107632	0.993552802
POLR3H	0.004660206	0.353120975	0.993552802
SNORD35A	-0.01163884	0.353311764	0.993552802
GIGYF2	0.005353796	0.353329878	0.993552802
RPL14	0.003536747	0.353338762	0.993552802
KLHL8	0.005413238	0.353343555	0.993552802
MRPS34	-0.006886366	0.353422687	0.993552802
SSH2	-0.005029418	0.353438108	0.993552802
CYP27A1	-0.013922094	0.353534089	0.993552802
TRAPPC5	-0.004622191	0.353539264	0.993552802
CEBPG	0.003866203	0.353631656	0.993552802
PLA2G7	0.014993175	0.353767332	0.993552802
TMEM14A	0.00767917	0.353805658	0.993552802

UAP1	0.006311445	0.353890274	0.993552802
FAM179A	0.012704624	0.35410787	0.993552802
KIAA0195	-0.006494452	0.354152171	0.993552802
PVRIG	0.006437534	0.35416936	0.993552802
ASCC3	0.004524764	0.354203035	0.993552802
LRRC58	0.006534109	0.354302488	0.993552802
VAV2	-0.006253847	0.354601007	0.993552802
MED7	0.005075387	0.354623872	0.993552802
CTU1	-0.006383066	0.354946037	0.993552802
NUDT15	0.007655837	0.355006015	0.993552802
PHC3	-0.005688801	0.355030145	0.993552802
ZNF540	0.008363857	0.355055271	0.993552802
C14orf142	0.004176683	0.355134692	0.993552802
ANGEL1	-0.005107716	0.355136002	0.993552802
SCARNA2	-0.010592386	0.355187989	0.993552802
SNX8	0.005213029	0.355372727	0.993552802
TMEM170B	0.006998215	0.355391539	0.993552802
RBM15B	-0.005223874	0.35540709	0.993552802
TMEM17	-0.009157118	0.355527301	0.993552802
CACNG1	0.005823357	0.355560014	0.993552802
HSP90B1	0.005408729	0.355565687	0.993552802
PRNP	-0.003164814	0.355753245	0.993552802
RPL11	-0.002671727	0.355927564	0.993552802
PSRC1	0.007153274	0.355954173	0.993552802
SEH1L	0.005759522	0.356019177	0.993552802
B4GALT4	0.005927335	0.356208922	0.993552802
QRICH1	-0.002947285	0.356250008	0.993552802
RSF1	0.007419106	0.356528399	0.993552802
MSLN	-0.033543131	0.356694885	0.993552802
ARL4C	0.006094574	0.356916548	0.993552802
SDHA	-0.003781582	0.356999305	0.993552802
IPPK	0.00615911	0.357068915	0.993552802
RB1	0.00886062	0.357075116	0.993552802
SLC15A2	-0.008488848	0.357084666	0.993552802
ZNF283	0.005827769	0.357164191	0.993552802
COX19	0.007030158	0.357298864	0.993552802
CDT1	0.010379587	0.357451369	0.993552802
SSH3	0.005029119	0.357554404	0.993552802
TSEN54	0.006824128	0.35759725	0.993552802
TMEM71	-0.006617363	0.357606532	0.993552802
TLR5	-0.006020026	0.357783603	0.993552802
DPH3	0.006988058	0.358077522	0.993552802
AGER	-0.006498456	0.358127943	0.993552802
SNORA29	-0.008175063	0.358274497	0.993552802
CDC42EP1	0.007261613	0.358434931	0.993552802
PRR7	-0.009101991	0.358534065	0.993552802
TMEM45A	-0.018799409	0.35858398	0.993552802

GNS	0.003653559	0.358678306	0.993552802
MSX2P1	0.008486659	0.35870222	0.993552802
DSP	-0.007137612	0.358842043	0.993552802
SLAMF7	0.009083652	0.358846457	0.993552802
ITFG1	0.005660504	0.358897754	0.993552802
ALKBH7	-0.00700746	0.358939953	0.993552802
DYRK4	0.003809941	0.359238698	0.993552802
PCDH15	-0.005943103	0.359297898	0.993552802
GTPBP3	0.004729436	0.359440423	0.993552802
INTS3	-0.005631456	0.359458727	0.993552802
PRKAB2	-0.005314486	0.359626886	0.993552802
VPS13B	-0.010094696	0.35981889	0.993552802
ZNF761	-0.006754865	0.359852386	0.993552802
ZNF142	0.003453016	0.359861538	0.993552802
OASL	-0.013583677	0.359878707	0.993552802
LIN9	0.005863814	0.359953236	0.993552802
UBAP2	0.005757969	0.360005347	0.993552802
FKTN	-0.002915567	0.360174029	0.993552802
PLEKHF1	0.012252006	0.360174378	0.993552802
ST3GAL1	-0.005777871	0.360292969	0.993552802
C17orf66	0.007514729	0.36032609	0.993552802
POLR2I	-0.004062778	0.360418926	0.993552802
ZNF225	0.00517637	0.360423394	0.993552802
FOXJ2	-0.00245095	0.360576404	0.993552802
AGPAT1	-0.008603095	0.360908869	0.993552802
ERCC4	-0.006364029	0.360961089	0.993552802
PRRG4	-0.012788047	0.361198567	0.993552802
NSFL1C	-0.006855493	0.361414275	0.993552802
N4BP2L1	0.004081465	0.361420514	0.993552802
RPL13P5	-0.006996341	0.361445566	0.993552802
HNRNPA0	0.004859816	0.361507736	0.993552802
LPL	0.011207612	0.361609447	0.993552802
SENP1	-0.005717092	0.361632667	0.993552802
ZNF28	0.005426201	0.361786252	0.993552802
RNF19A	0.005963097	0.361844024	0.993552802
PRMT6	0.006614181	0.362030596	0.993552802
SLC30A6	0.005971756	0.362069885	0.993552802
RHBDD1	0.004580636	0.362165841	0.993552802
WDR86	-0.011873959	0.362236914	0.993552802
ZSCAN18	0.007080961	0.362566884	0.993552802
ZNF302	0.004948329	0.362841627	0.993552802
SPRYD3	0.005661259	0.363057246	0.993552802
SDCBP	0.008905809	0.363141208	0.993552802
RPS15A	-0.002032037	0.363583849	0.993552802
UBE2V1	0.006030856	0.363588992	0.993552802
PSMG4	-0.005401546	0.363595108	0.993552802
KDM1B	0.007278785	0.36370407	0.993552802

GAPT	0.009652993	0.363711468	0.993552802
RSRC2	0.004237005	0.363810141	0.993552802
PTPN3	-0.005010697	0.363817344	0.993552802
ARL1	0.003653863	0.363939148	0.993552802
MTG1	0.005936655	0.363968938	0.993552802
ING2	0.006019833	0.363985528	0.993552802
NXT1	-0.003523426	0.364013208	0.993552802
BRPF3	0.00431943	0.364214077	0.993552802
MYO19	0.007832762	0.364305661	0.993552802
ARHGEF5	0.004904167	0.364367079	0.993552802
ALDH8A1	0.006901382	0.364373475	0.993552802
COQ6	0.004155715	0.364440255	0.993552802
MPHOSPH6	0.009217009	0.364460863	0.993552802
CLIC6	0.009663589	0.364558091	0.993552802
RPS21	0.007403408	0.36456966	0.993552802
L3MBTL3	0.006959044	0.364612858	0.993552802
TAF1D	0.012447043	0.364713725	0.993552802
CEP192	-0.005190324	0.364753593	0.993552802
AKT3	-0.006709408	0.364946543	0.993552802
RPS12	0.011876511	0.364955502	0.993552802
ZNF558	0.005616059	0.364995006	0.993552802
SEPHS2	0.005116709	0.365319816	0.993552802
PRSS21	0.012481378	0.365332968	0.993552802
GPX4	-0.004306128	0.365381972	0.993552802
COX11	0.008740155	0.365461426	0.993552802
THOP1	0.005003143	0.365469187	0.993552802
SMC3	0.003277137	0.365866511	0.993552802
DNM1P35	-0.007011901	0.365909262	0.993552802
DHX33	-0.004578625	0.365931965	0.993552802
C3AR1	0.011326926	0.365934248	0.993552802
PDDC1	0.004983041	0.365993825	0.993552802
RNGTT	-0.0046033	0.366059713	0.993552802
LILRB2	0.005440251	0.36606101	0.993552802
CAMK2G	0.003423515	0.366265885	0.993552802
SLC26A2	0.005757407	0.366369593	0.993552802
SHKBP1	-0.007505095	0.366459003	0.993552802
C12orf77	0.005493678	0.366496602	0.993552802
ELAC1	-0.006008672	0.366672588	0.993552802
TPMT	0.004489782	0.366762406	0.993552802
UBFD1	0.005700027	0.36676869	0.993552802
HIP1R	-0.006089609	0.366775425	0.993552802
AMY2A	0.006588193	0.366836865	0.993552802
MYCBP2	-0.004362966	0.366849133	0.993552802
PRUNE2	0.017675102	0.366942682	0.993552802
MCOLN3	-0.007320609	0.366975206	0.993552802
PNPLA8	0.005316314	0.367150441	0.993552802
FOXD4	0.00702306	0.367186211	0.993552802

GABPB2	0.002023026	0.367280713	0.993552802
VAV1	0.004315828	0.367286561	0.993552802
SETD1B	-0.004575797	0.367521904	0.993552802
DDA1	-0.003325655	0.367533611	0.993552802
PHTF2	0.00620576	0.367601306	0.993552802
MELK	0.01021286	0.367775538	0.993552802
ARID4A	0.004841036	0.36779379	0.993552802
FP15737	-0.006234506	0.367816477	0.993552802
ZNF583	0.005666648	0.367929559	0.993552802
TMEM141	0.004427585	0.367931617	0.993552802
UPK3A	-0.008261203	0.367963933	0.993552802
C16orf58	0.003697975	0.368119344	0.993552802
LCN2	-0.020057495	0.368581779	0.993552802
ZFC3H1	-0.005034528	0.368682245	0.993552802
BSCL2	0.008142949	0.368738295	0.993552802
COL9A2	0.009305505	0.368784794	0.993552802
LRP5L	0.006940284	0.368813115	0.993552802
WDR48	-0.007318489	0.368827913	0.993552802
PIK3AP1	-0.005699994	0.368917374	0.993552802
TTC30B	0.005785724	0.368958191	0.993552802
RAPH1	0.00736267	0.369051797	0.993552802
DAGLA	0.006352793	0.369101666	0.993552802
MCFD2	0.004386708	0.369312619	0.993552802
ZNF740	0.005750127	0.369314014	0.993552802
COX7C	0.004020232	0.369336192	0.993552802
SAT2	0.003958678	0.36938746	0.993552802
WDR61	0.00491318	0.369754964	0.993552802
SUGP2	0.005318759	0.369779093	0.993552802
FBXO28	0.004910886	0.36980056	0.993552802
GGT1	-0.00616105	0.36993998	0.993552802
EEF1E1	0.004019003	0.370010539	0.993552802
CTNS	0.004515878	0.370044455	0.993552802
CORT	-0.006378719	0.370058955	0.993552802
NKIRAS1	0.006020275	0.370061007	0.993552802
TMTC1	0.018734402	0.370120547	0.993552802
RNY5	-0.008230387	0.370207587	0.993552802
TBX19	-0.005442574	0.370396133	0.993552802
RIPK3	0.003985209	0.370501629	0.993552802
TREML2	0.006323548	0.370595515	0.993552802
PFKM	0.006034043	0.370632896	0.993552802
AIRE	-0.003382226	0.370732437	0.993552802
KBTBD11	0.006668951	0.370740912	0.993552802
MYO1F	-0.005268694	0.37078879	0.993552802
PPP1R8	0.005644148	0.371195914	0.993552802
CWC15	-0.002775272	0.371743199	0.993552802
FARP2	0.006289567	0.371860246	0.993552802
ZMYND15	0.009685566	0.372037759	0.993552802

SLC27A4	0.006171493	0.372072374	0.993552802
CRY1	0.004184516	0.372205886	0.993552802
NBPF3	-0.005460152	0.372423434	0.993552802
SHD	0.011411008	0.372643038	0.993552802
TMEM8A	-0.011646043	0.372658948	0.993552802
LSM3	-0.006005176	0.372695553	0.993552802
KCNJ15	0.00643395	0.37270547	0.993552802
RUNX3	0.006607155	0.372714575	0.993552802
PMVK	0.004812786	0.37277546	0.993552802
NQO2	0.014818543	0.372823153	0.993552802
EIF4A1	0.003004632	0.372977272	0.993552802
NUP210	-0.006867283	0.373075008	0.993552802
TRADD	-0.005813372	0.373140079	0.993552802
HENMT1	0.005184731	0.373755912	0.993552802
NACA	0.003347088	0.373986996	0.993552802
NOS3	-0.006170756	0.374020662	0.993552802
HIST2H2AA3	0.009566349	0.374311408	0.993552802
CLASP1	-0.004123843	0.374367906	0.993552802
USP13	0.005112844	0.374408364	0.993552802
TBL1XR1	-0.004882728	0.374550103	0.993552802
MED13	0.006880913	0.374577098	0.993552802
API5	-0.003134604	0.374698314	0.993552802
DCAF13	0.005108519	0.374745232	0.993552802
YKT6	-0.008107031	0.374783325	0.993552802
CLSTN1	-0.004963976	0.374788837	0.993552802
PPP2R2B	0.011523137	0.374825344	0.993552802
UQCRB	0.005806824	0.374956251	0.993552802
APEX1	0.003119438	0.375001353	0.993552802
SYTL3	-0.006633975	0.375208485	0.993552802
KIAA1324L	-0.014547419	0.375212095	0.993552802
SMARCD1	-0.003226764	0.375270131	0.993552802
SMEK2	0.002943517	0.375397866	0.993552802
C14orf28	0.004939727	0.37548102	0.993552802
EFCAB4B	-0.005164038	0.375491607	0.993552802
GAS6	0.009635924	0.375511481	0.993552802
FOXRED1	0.005407973	0.375622788	0.993552802
CABP4	-0.007882237	0.375655364	0.993552802
NFIA	0.006673109	0.375691302	0.993552802
ANKRD50	0.005890226	0.375723557	0.993552802
MSH2	-0.006645682	0.375733158	0.993552802
DPCD	-0.006783487	0.375942791	0.993552802
DGUOK	-0.003748707	0.375971172	0.993552802
ISCA1	-0.005359692	0.376046233	0.993552802
GAPVD1	-0.003253738	0.376179267	0.993552802
IMPA2	0.008306805	0.376231753	0.993552802
RALGPS2	-0.013378002	0.376232897	0.993552802
MATN2	0.008442756	0.376240275	0.993552802

VAMP3	0.005334398	0.376305934	0.993552802
EXOC7	0.003663986	0.376419305	0.993552802
C1orf131	0.003831884	0.376425055	0.993552802
ICAM2	-0.004323829	0.376787062	0.993552802
TSPAN2	0.009043829	0.37690956	0.993552802
NUDT21	0.004171936	0.376971068	0.993552802
EAF1	-0.004803341	0.377002125	0.993552802
DYNC1LI1	0.007198298	0.377055805	0.993552802
BBS9	0.007350568	0.377164933	0.993552802
C12orf45	-0.006295217	0.377304098	0.993552802
DHX8	-0.004753863	0.377424127	0.993552802
TCF19	0.006288931	0.377936237	0.993552802
SLC27A1	0.005956481	0.378096539	0.993552802
CMC1	0.007055929	0.378289831	0.993552802
ADORA1	0.010018837	0.378359653	0.993552802
LRRC23	-0.008286304	0.378429653	0.993552802
FIGNL1	-0.008679634	0.378507869	0.993552802
DHRS1	0.004232753	0.378622137	0.993552802
ACD	0.005781434	0.378863529	0.993552802
PATL2	0.013836921	0.37893742	0.993552802
FNBP1	0.002876907	0.378946036	0.993552802
HOXC4	0.013050579	0.379056953	0.993552802
FAM83F	0.00636747	0.379217636	0.993552802
NFKB1	0.002855243	0.379235209	0.993552802
WDR90	0.005600381	0.379324432	0.993552802
CREB5	0.007900901	0.379349416	0.993552802
FAM184A	-0.007762569	0.379361024	0.993552802
ERCC8	0.00728966	0.37937896	0.993552802
CCDC85C	0.006398838	0.379481298	0.993552802
CSRNP2	0.004746563	0.379522191	0.993552802
METTL5	0.006169286	0.379903838	0.993552802
MYC	-0.007541862	0.380131114	0.993552802
LRCH3	-0.007974385	0.380290585	0.993552802
RNF114	0.002224127	0.380409431	0.993552802
CCNT2	0.004436026	0.380432059	0.993552802
RCN3	0.007528284	0.380536714	0.993552802
C9orf41	0.007183439	0.380589234	0.993552802
LIMK2	0.00747195	0.380851875	0.993552802
PCSK6	-0.005346657	0.380883225	0.993552802
CCDC91	-0.00476708	0.381045718	0.993552802
CTAGE5	0.00576369	0.381110829	0.993552802
AXIN1	-0.007830656	0.381170368	0.993552802
TROVE2	-0.003628794	0.381320485	0.993552802
MRPL23	-0.004529272	0.381330673	0.993552802
TNPO1	-0.006921711	0.381337733	0.993552802
ZDHHC3	-0.004407547	0.381668877	0.993552802
MS4A6A	0.007091493	0.381723707	0.993552802

SOX8	0.009171027	0.381786373	0.993552802
TSPAN31	0.004015052	0.381815164	0.993552802
RPLP1	-0.004213063	0.381825615	0.993552802
CHCHD4	0.003675875	0.381948067	0.993552802
RIOK2	0.004785635	0.381954597	0.993552802
CLDND2	0.013459919	0.381965225	0.993552802
PHF2	0.003510424	0.381993496	0.993552802
LARP4	0.00522348	0.382067365	0.993552802
MC1R	0.009901278	0.38210094	0.993552802
SESTD1	0.007637723	0.382230695	0.993552802
C2orf15	-0.00842096	0.38244574	0.993552802
TAB2	-0.006616092	0.382486752	0.993552802
FBXO43	0.005465497	0.382517309	0.993552802
ZNF571	0.005001105	0.382528093	0.993552802
SAMSN1	0.006717019	0.382532349	0.993552802
GNG10	0.010426086	0.382585038	0.993552802
DCAF4L1	0.005743053	0.382591649	0.993552802
LRRFIP2	-0.004678402	0.382598141	0.993552802
GBAS	0.005905342	0.382726168	0.993552802
KLHL5	-0.004775582	0.382889354	0.993552802
SCAMP1	0.002747765	0.382891451	0.993552802
CYTH1	-0.003566118	0.382905835	0.993552802
PSMC5	0.002879753	0.383204552	0.993552802
ZNF80	-0.01200278	0.383337855	0.993552802
SETMAR	0.006413248	0.383344368	0.993552802
ACADVL	0.004322145	0.383428665	0.993552802
ATP5SL	0.00342302	0.383531568	0.993552802
B3GNT8	0.007704454	0.3837362	0.993552802
RAB28	0.007886011	0.383755852	0.993552802
LAMC1	0.014708995	0.383768797	0.993552802
PRRT3	0.007648395	0.383959302	0.993552802
CP	-0.005298741	0.384002922	0.993552802
TBC1D1	0.005978587	0.384058112	0.993552802
UHRF2	-0.004816676	0.384063802	0.993552802
RPSAP52	0.005226876	0.384199842	0.993552802
TCFL5	-0.004872442	0.384200878	0.993552802
YTHDC2	0.005199984	0.384230148	0.993552802
HIPK3	0.008068823	0.384325301	0.993552802
VPS4B	0.003767702	0.38439107	0.993552802
CWC25	0.005425934	0.384396603	0.993552802
FOSL2	-0.005592211	0.384587816	0.993552802
CLP1	0.004624518	0.384593766	0.993552802
LHFPL2	-0.008162263	0.384748049	0.993552802
DUSP3	-0.004935672	0.384845714	0.993552802
CDC16	0.004460748	0.384857887	0.993552802
CHD8	-0.006021408	0.38488246	0.993552802
GAK	-0.003193069	0.384902545	0.993552802

CCL4L2	0.012816936	0.384912756	0.993552802
EIF5A2	-0.006268364	0.384919402	0.993552802
C1orf115	-0.008166343	0.385044512	0.993552802
RAB10	0.002551735	0.385086232	0.993552802
AKAP11	-0.004083132	0.385226929	0.993552802
CHTOP	0.005250163	0.385356836	0.993552802
TMEM191C	-0.007145386	0.385506925	0.993552802
RRN3P1	0.011920135	0.385645348	0.993552802
[truncated: 2,659,132 more chars]
