# Supplementary material for: Exposure to fine particulate matter in adults is associated with immune cell gene expression related to inflammation, the electron transport chain, and cell cycle regulation
Source: Environ Epigenet. 2025 Apr 1;11(1):dvaf008. doi: 10.1093/eep/dvaf008 (PMC12159804; doi:10.1093/eep/dvaf008)
Supplement: dvaf008_Supp [file dvaf008_supp.zip › suppl_data/Supplemental file 3.pdf]

| GO | Category | Description | Clean description (in figure) | LogP | Enrichment | Z-score | Genes in GO | Genes in GO and hit list | Percent in GO | Percent genes in GO in hit list |
|---|---|---|---|---|---|---|---|---|---|---|
| Pathways enriched among genes that are negatively associated with exposure to PM2.5 | | | | | | | | | | |
| GO:0045786 | GO Biological Processes | negative regulation of cell cycle | Cell cycle downregulation | -6.2 | 2.7 | 5.9 | 311 | 31 | 5.1 | 10.0 |
| GO:0000727 | GO Biological Processes | double-strand break repair via break-induced replication | DNA repair via BIR | -5.7 | 14 | 8.5 | 12 | 6 | 0.99 | 50.0 |
| M5925 | Hallmark Gene Sets | HALLMARK E2F TARGETS | E2F cell cycle targets | -5 | 2.9 | 5.3 | 193 | 21 | 3.5 | 10.9 |
| GO:0006259 | GO Biological Processes | DNA metabolic process | DNA metabolism | -4.8 | 1.9 | 4.7 | 685 | 48 | 7.9 | 7.0 |
| GO:0090329 | GO Biological Processes | regulation of DNA-templated DNA replication | DNA-templated replication regulation | -4.4 | 5.3 | 5.7 | 46 | 9 | 1.5 | 19.6 |
| GO:0060338 | GO Biological Processes | regulation of type I interferon-mediated signaling pathway | Type I INF signal regulation | -4.1 | 5.5 | 5.6 | 39 | 8 | 1.3 | 20.5 |
| GO:0051321 | GO Biological Processes | meiotic cell cycle | Meiosis | -4.1 | 2.7 | 4.6 | 190 | 19 | 3.1 | 10.0 |
| GO:1905784 | GO Biological Processes | regulation of anaphase-promoting complex-dependent catabolic process | Regulation of anaphase | -3.7 | 20 | 7.6 | 4 | 3 | 0.49 | 75.0 |
| GO:0048711 | GO Biological Processes | positive regulation of astrocyte differentiation | Astrocyte differentiation upregulation | -3.7 | 12 | 6.5 | 9 | 4 | 0.66 | 44.4 |
| M5911 | Hallmark Gene Sets | HALLMARK INTERFERON ALPHA RESPONSE | INF alpha response | -3.7 | 3.4 | 4.6 | 96 | 12 | 2 | 12.5 |
| GO:0051054 | GO Biological Processes | positive regulation of DNA metabolic process | DNA metabolism upregulation | -3.3 | 2.2 | 3.9 | 270 | 22 | 3.6 | 8.1 |
| hsa04934 | KEGG Pathway | Cushing syndrome | Cushing syndrome | -3.2 | 2.9 | 4.1 | 123 | 13 | 2.1 | 10.6 |
| GO:0000079 | GO Biological Processes | regulation of cyclin-dependent protein serine/threonine kinase activity | Cyclin-dependent kinase regulation | -3.2 | 3.7 | 4.3 | 66 | 9 | 1.5 | 13.6 |
| WP5345 | WikiPathways | 1p36 copy number variation syndrome | 1p36 CNV syndrome | -3.1 | 3.4 | 4.2 | 80 | 10 | 1.6 | 12.5 |
| GO:0032785 | GO Biological Processes | negative regulation of DNA-templated transcription, elongation | DNA-templated transcription downregulation | -3.1 | 6.4 | 4.9 | 21 | 5 | 0.82 | 23.8 |
| GO:0045759 | GO Biological Processes | negative regulation of action potential | Action potential downregulation | -3 | 14 | 6 | 6 | 3 | 0.49 | 50.0 |
| R-HSA-8849932 | Reactome Gene Sets | Synaptic adhesion-like molecules | Synaptic adhesion | -2.9 | 7.7 | 4.9 | 14 | 4 | 0.66 | 28.6 |
| R-HSA-159740 | Reactome Gene Sets | Gamma-carboxylation of protein precursors | Coagulation protein carboxylaion | -2.8 | 12 | 5.5 | 7 | 3 | 0.49 | 42.9 |
| R-HSA-69273 | Reactome Gene Sets | Cyclin A/B1/B2 associated events during G2/M transition | Cyclin associated events in G2/M transition | -2.8 | 5.6 | 4.4 | 24 | 5 | 0.82 | 20.8 |
| GO:0048568 | GO Biological Processes | embryonic organ development | Embryonic organ development | -2.8 | 2 | 3.4 | 298 | 22 | 3.6 | 7.4 |
| Pathways enriched among genes that are positively associated with exposure to PM2.5 | | | | | | | | | | |
| M5936 | Hallmark Gene Sets | HALLMARK OXIDATIVE PHOSPHORYLATION | Oxidative phosphorylation | -13 | 4.2 | 9.8 | 188 | 37 | 4.8 | 19.7 |
| M5926 | Hallmark Gene Sets | HALLMARK MYC TARGETS V1 | MYC targets | -7.6 | 3.2 | 6.8 | 193 | 29 | 3.8 | 15.0 |
| R-HSA-72766 | Reactome Gene Sets | Translation | Translation | -7.5 | 2.8 | 6.6 | 277 | 36 | 4.7 | 13.0 |
| R-HSA-8953854 | Reactome Gene Sets | Metabolism of RNA | RNA metabolism | -7.4 | 2 | 6.1 | 681 | 65 | 8.4 | 9.5 |
| GO:1902600 | GO Biological Processes | proton transmembrane transport | Proton transmembrane transport | -6.8 | 4.1 | 6.8 | 100 | 19 | 2.5 | 19.0 |
| GO:0022613 | GO Biological Processes | ribonucleoprotein complex biogenesis | RNA-protein complex biogenesis | -6.4 | 2.2 | 5.8 | 429 | 45 | 5.8 | 10.5 |
| R-HSA-1236975 | Reactome Gene Sets | Antigen processing-Cross presentation | Antigen processing and presentation | -6.4 | 4 | 6.5 | 96 | 18 | 2.3 | 18.8 |
| GO:0042743 | GO Biological Processes | hydrogen peroxide metabolic process | Hydrogen peroxide metabolism | -5.9 | 6.7 | 7.1 | 32 | 10 | 1.3 | 31.3 |
| R-HSA-1799339 | Reactome Gene Sets | SRP-dependent cotranslational protein targeting to membrane | Translation of protein to ER | -5.1 | 3.5 | 5.6 | 105 | 17 | 2.2 | 16.2 |
| M5935 | Hallmark Gene Sets | HALLMARK FATTY ACID METABOLISM | Fatty acid metabolism | -4.4 | 2.9 | 4.9 | 131 | 18 | 2.3 | 13.7 |
| GO:0002218 | GO Biological Processes | activation of innate immune response | Innate immune response activation | -4.4 | 2.8 | 4.9 | 144 | 19 | 2.5 | 13.2 |
| GO:0051604 | GO Biological Processes | protein maturation | Protein maturation | -4.3 | 2 | 4.5 | 395 | 37 | 4.8 | 9.4 |
| hsa04145 | KEGG Pathway | Phagosome | Phagosome | -4 | 2.8 | 4.6 | 129 | 17 | 2.2 | 13.2 |
| GO:0006605 | GO Biological Processes | protein targeting | Protein targeting | -3.9 | 2.3 | 4.4 | 221 | 24 | 3.1 | 10.9 |
| M5898 | Hallmark Gene Sets | HALLMARK DNA REPAIR | DNA repair | -3.9 | 2.7 | 4.5 | 142 | 18 | 2.3 | 12.7 |
| GO:0006122 | GO Biological Processes | mitochondrial electron transport, ubiquinol to cytochrome c | ETC, Q to Cyt c | -3.9 | 8.9 | 6.1 | 12 | 5 | 0.65 | 41.7 |
| GO:0035308 | GO Biological Processes | negative regulation of protein dephosphorylation | Protein dephosphorylation downregulation | -3.7 | 5.5 | 5.2 | 27 | 7 | 0.91 | 25.9 |
| GO:1901361 | GO Biological Processes | organic cyclic compound catabolic process | Organic cyclic compound catabolism | -3.5 | 1.9 | 3.9 | 354 | 32 | 4.2 | 9.0 |
| hsa03060 | KEGG Pathway | Protein export | Protein export | -3.5 | 5.2 | 5 | 29 | 7 | 0.91 | 24.1 |
| WP5027 | WikiPathways | nsp1 from SARS CoV 2 inhibits translation initiation in the host cell | SARS CoV 2 inhibition of translation | -3.5 | 7.6 | 5.5 | 14 | 5 | 0.65 | 35.7 |

| GO | Category | Description | Clean description (in figure) | LogP | Enrichment | Z-score | Genes in GO | Genes in GO and hit list | Percent in GO | Percent genes in GO in hit list |
|---|---|---|---|---|---|---|---|---|---|---|
| Pathways enriched among genes that are negatively associated with exposure to PM2.5 | | | | | | | | | | |
| M5936 | Hallmark Gene Sets | HALLMARK OXIDATIVE PHOSPHORYLATION | Oxidative phosphorylation | -11 | 3.1 | 8.4 | 184 | 45 | 5.2 | 24.5 |
| GO:0019752 | GO Biological Processes | carboxylic acid metabolic process | Carboxylic acid metabolism | -6.8 | 1.8 | 5.7 | 518 | 75 | 8.6 | 14.5 |
| hsa00280 | KEGG Pathway | Valine, leucine and isoleucine degradation | BCAA degradation | -6.6 | 4.6 | 6.8 | 41 | 15 | 1.7 | 36.6 |
| M5935 | Hallmark Gene Sets | HALLMARK FATTY ACID METABOLISM | Fatty acid metabolism | -5 | 2.6 | 5.1 | 123 | 25 | 2.9 | 20.3 |
| GO:1902600 | GO Biological Processes | proton transmembrane transport | Proton transmembrane transport | -4.9 | 3 | 5.2 | 81 | 19 | 2.2 | 23.5 |
| GO:0006605 | GO Biological Processes | protein targeting | Protein targeting | -4.7 | 2.1 | 4.7 | 194 | 33 | 3.8 | 17.0 |
| R-HSA-5357801 | Reactome Gene Sets | Programmed Cell Death | Programmed cell death | -4.5 | 2.2 | 4.7 | 180 | 31 | 3.6 | 17.2 |
| GO:0009082 | GO Biological Processes | branched-chain amino acid biosynthetic process | BCAA biosynthesis | -4.4 | 13 | 6.8 | 4 | 4 | 0.46 | 100.0 |
| R-HSA-6798695 | Reactome Gene Sets | Neutrophil degranulation | Neutrophil degranulation | -4.3 | 1.7 | 4.3 | 396 | 54 | 6.2 | 13.6 |
| R-HSA-71291 | Reactome Gene Sets | Metabolism of amino acids and derivatives | Amino acid metabolism | -4.2 | 1.9 | 4.3 | 268 | 40 | 4.6 | 14.9 |
| GO:1901503 | GO Biological Processes | ether biosynthetic process | Ether biosynthetic process | -4.1 | 6.9 | 5.7 | 11 | 6 | 0.69 | 54.5 |
| M5910 | Hallmark Gene Sets | HALLMARK PROTEIN SECRETION | Protein secretion | -3.8 | 2.6 | 4.4 | 81 | 17 | 1.9 | 21.0 |
| M5926 | Hallmark Gene Sets | HALLMARK MYC TARGETS V1 | MYC targets | -3.7 | 2 | 4 | 191 | 30 | 3.4 | 15.7 |
| GO:0035459 | GO Biological Processes | vesicle cargo loading | Vesicle cargo loading | -3.6 | 4.4 | 4.8 | 23 | 8 | 0.92 | 34.8 |
| WP5220 | WikiPathways | Metabolic reprogramming in pancreatic cancer | Pancreatic cancer metabolic reprogramming | -3.5 | 3.5 | 4.4 | 36 | 10 | 1.1 | 27.8 |
| GO:0065002 | GO Biological Processes | intracellular protein transmembrane transport | Intracellular protein transmembrane transport | -3.5 | 4.2 | 4.6 | 24 | 8 | 0.92 | 33.3 |
| GO:0033108 | GO Biological Processes | mitochondrial respiratory chain complex assembly | Electron transport chain assembly | -3.4 | 2.7 | 4.1 | 71 | 15 | 1.7 | 21.1 |
| GO:0071826 | GO Biological Processes | protein-RNA complex organization | RNA-protein complex organization | -3.3 | 2 | 3.8 | 181 | 28 | 3.2 | 15.5 |
| GO:0006888 | GO Biological Processes | endoplasmic reticulum to Golgi vesicle-mediated transport | ER to Golgi vesicle transport | -3.3 | 2.3 | 3.9 | 97 | 18 | 2.1 | 18.6 |
| R-HSA-381070 | Reactome Gene Sets | IRE1alpha activates chaperones | IRE1 branch of UPR | -3.2 | 3.1 | 4.1 | 45 | 11 | 1.3 | 24.4 |
| Pathways enriched among genes that are positively associated with exposure to PM2.5 | | | | | | | | | | |
| GO:0042148 | GO Biological Processes | DNA strand invasion | DNA strand invasion | -3.4 | 16 | 6.7 | 4 | 3 | 0.59 | 75.0 |
| GO:0071076 | GO Biological Processes | RNA 3' uridylation | RNA 3' uridylation | -3.4 | 16 | 6.7 | 4 | 3 | 0.59 | 75.0 |
| GO:1904321 | GO Biological Processes | response to forskolin | Response to forskolin | -3 | 13 | 5.9 | 5 | 3 | 0.59 | 60.0 |
| hsa05168 | KEGG Pathway | Herpes simplex virus 1 infection | HSV1 infection | -2.9 | 1.8 | 3.4 | 372 | 31 | 6.1 | 8.3 |
| hsa04915 | KEGG Pathway | Estrogen signaling pathway | Estrogen signaling | -2.7 | 3 | 3.7 | 73 | 10 | 2 | 13.7 |
| GO:0010793 | GO Biological Processes | regulation of mRNA export from nucleus | Nuclear mRNA export regulation | -2.5 | 9.2 | 4.8 | 7 | 3 | 0.59 | 42.9 |
| R-HSA-9706574 | Reactome Gene Sets | RHOBTB GTPase Cycle | RHOBTB GTPase Cycle | -2.4 | 3.9 | 3.7 | 33 | 6 | 1.2 | 18.2 |
| GO:0007292 | GO Biological Processes | female gamete generation | Female gamete generation | -2.4 | 2.7 | 3.3 | 81 | 10 | 2 | 12.3 |
| GO:0061484 | GO Biological Processes | hematopoietic stem cell homeostasis | HSC homeostasis | -2.4 | 5.7 | 4.1 | 15 | 4 | 0.78 | 26.7 |
| GO:0090335 | GO Biological Processes | regulation of brown fat cell differentiation | Regulation of brown fat cell differentiation | -2.4 | 5.7 | 4.1 | 15 | 4 | 0.78 | 26.7 |
| GO:0019933 | GO Biological Processes | cAMP-mediated signaling | cAMP-mediated signaling | -2.4 | 4.5 | 3.8 | 24 | 5 | 0.98 | 20.8 |
| GO:0060670 | GO Biological Processes | branching involved in labyrinthine layer morphogenesis | Labyrinthine layer branching | -2.3 | 8.1 | 4.4 | 8 | 3 | 0.59 | 37.5 |
| GO:1902033 | GO Biological Processes | regulation of hematopoietic stem cell proliferation | Regulation of HSC proliferation | -2.3 | 8.1 | 4.4 | 8 | 3 | 0.59 | 37.5 |
| GO:0006534 | GO Biological Processes | cysteine metabolic process | Cysteine metabolism | -2.3 | 8.1 | 4.4 | 8 | 3 | 0.59 | 37.5 |
| R-HSA-168276 | Reactome Gene Sets | NS1 Mediated Effects on Host Pathways | NS1 Mediated Effects on Host | -2.2 | 3.5 | 3.4 | 37 | 6 | 1.2 | 16.2 |
| R-HSA-9856651 | Reactome Gene Sets | MITF-M-dependent gene expression | MITF-M-dependent gene expression | -2.2 | 2.8 | 3.2 | 61 | 8 | 1.6 | 13.1 |
| R-HSA-9754560 | Reactome Gene Sets | SARS-CoV-2 modulates autophagy | SARS-CoV-2 modulates autophagy | -2 | 6.5 | 3.8 | 10 | 3 | 0.59 | 30.0 |
| WP2267 | WikiPathways | Synaptic vesicle pathway | Synaptic vesicle pathway | -2 | 3.7 | 3.2 | 29 | 5 | 0.98 | 17.2 |
| GO:0016579 | GO Biological Processes | protein deubiquitination | Protein deubiquitination | -2 | 2.7 | 2.9 | 65 | 8 | 1.6 | 12.3 |

Pathways enriched among genes positively associated with PM2.5 in the Rotterdam Study and negatively associated with PM2.5 in NoMa

| Pathway | Pathway name |
|---|---|
| M5936 | HALLMARK OXIDATIVE PHOSPHORYLATION |
| GO:0045333 | cellular respiration |
| GO:0009060 | aerobic respiration |
| R-HSA-163200 | Respiratory electron transport, ATP synthesis by chemiosmotic coupling, and heat production by uncoupling proteins. |
| R-HSA-1428517 | Aerobic respiration and respiratory electron transport |
| hsa05012 | Parkinson disease |
| GO:0006119 | oxidative phosphorylation |
| hsa00190 | Oxidative phosphorylation |
| GO:0015980 | energy derivation by oxidation of organic compounds |
| WP111 | Electron transport chain OXPHOS system in mitochondria |
| GO:0022904 | respiratory electron transport chain |
| R-HSA-611105 | Respiratory electron transport |
| GO:0042773 | ATP synthesis coupled electron transport |
| GO:0042775 | mitochondrial ATP synthesis coupled electron transport |
| GO:0015986 | proton motive force-driven ATP synthesis |
| GO:0006091 | generation of precursor metabolites and energy |
| hsa05020 | Prion disease |
| GO:0019646 | aerobic electron transport chain |
| GO:0006754 | ATP biosynthetic process |
| GO:0009206 | purine ribonucleoside triphosphate biosynthetic process |
| GO:0009145 | purine nucleoside triphosphate biosynthetic process |
| GO:0009205 | purine ribonucleoside triphosphate metabolic process |
| GO:0009201 | ribonucleoside triphosphate biosynthetic process |
| GO:0009144 | purine nucleoside triphosphate metabolic process |
| GO:0009199 | ribonucleoside triphosphate metabolic process |
| GO:0009152 | purine ribonucleotide biosynthetic process |
| GO:0009260 | ribonucleotide biosynthetic process |
| GO:0055086 | nucleobase-containing small molecule metabolic process |
| GO:0046390 | ribose phosphate biosynthetic process |
| GO:0046034 | ATP metabolic process |
| hsa05208 | Chemical carcinogenesis - reactive oxygen species |
| GO:0009142 | nucleoside triphosphate biosynthetic process |
| hsa04714 | Thermogenesis |
| GO:0072521 | purine-containing compound metabolic process |
| GO:0009141 | nucleoside triphosphate metabolic process |
| GO:0072522 | purine-containing compound biosynthetic process |
| WP623 | Oxidative phosphorylation |
| GO:0006753 | nucleoside phosphate metabolic process |
| GO:0009150 | purine ribonucleotide metabolic process |
| GO:0006164 | purine nucleotide biosynthetic process |
| GO:0009259 | ribonucleotide metabolic process |
| GO:0042776 | proton motive force-driven mitochondrial ATP synthesis |
| GO:0009117 | nucleotide metabolic process |
| hsa05016 | Huntington disease |
| GO:0006163 | purine nucleotide metabolic process |
| GO:0022900 | electron transport chain |
| GO:0019693 | ribose phosphate metabolic process |
| hsa05010 | Alzheimer disease |
| hsa05415 | Diabetic cardiomyopathy |
| GO:0006120 | mitochondrial electron transport, NADH to ubiquinone |
| hsa05022 | Pathways of neurodegeneration - multiple diseases |
| hsa05014 | Amyotrophic lateral sclerosis |
| GO:0033108 | mitochondrial respiratory chain complex assembly |
| M5926 | HALLMARK MYC TARGETS V1 |
| GO:1902600 | proton transmembrane transport |
| R-HSA-163210 | Formation of ATP by chemiosmotic coupling |
| R-HSA-8949613 | Cristae formation |
| R-HSA-1592230 | Mitochondrial biogenesis |
| R-HSA-1236975 | Antigen processing-Cross presentation |
| R-HSA-9762114 | GSK3B and BTRC:CUL1-mediated-degradation of NFE2L2 |
| R-HSA-2871837 | FCERI mediated NF-kB activation |
| R-HSA-1236978 | Cross-presentation of soluble exogenous antigens (endosomes) |
| R-HSA-180534 | Vpu mediated degradation of CD4 |
| R-HSA-349425 | Autodegradation of the E3 ubiquitin ligase COP1 |
| R-HSA-8854050 | FBXL7 down-regulates AURKA during mitotic entry and in early mitosis |
| R-HSA-1169091 | Activation of NF-kappaB in B cells |
| R-HSA-174113 | SCF-beta-TrCP mediated degradation of Emi1 |
| R-HSA-9604323 | Negative regulation of NOTCH4 signaling |
| R-HSA-450408 | AUF1 (hnRNP D0) binds and destabilizes mRNA |
| R-HSA-69541 | Stabilization of p53 |
| R-HSA-5610783 | Degradation of GLI2 by the proteasome |
| hsa03050 | Proteasome |
| R-HSA-5610780 | Degradation of GLI1 by the proteasome |
| R-HSA-5610785 | GLI3 is processed to GLI3R by the proteasome |
| R-HSA-5678895 | Defective CFTR causes cystic fibrosis |
| R-HSA-8852276 | The role of GTSE1 in G2/M progression after G2 checkpoint |
| R-HSA-211733 | Regulation of activated PAK-2p34 by proteasome mediated degradation |
| R-HSA-350562 | Regulation of ornithine decarboxylase (ODC) |
| R-HSA-69601 | Ubiquitin Mediated Degradation of Phosphorylated Cdc25A |
| R-HSA-69610 | p53-Independent DNA Damage Response |
| R-HSA-69613 | p53-Independent G1/S DNA damage checkpoint |
| R-HSA-75815 | Ubiquitin-dependent degradation of Cyclin D |
| R-HSA-169911 | Regulation of Apoptosis |
| R-HSA-69563 | p53-Dependent G1 DNA Damage Response |
| R-HSA-69580 | p53-Dependent G1/S DNA damage checkpoint |
| R-HSA-71291 | Metabolism of amino acids and derivatives |
| M5935 | HALLMARK FATTY ACID METABOLISM |
| GO:0006099 | tricarboxylic acid cycle |
| hsa00020 | Citrate cycle (TCA cycle) |
| GO:0006605 | protein targeting |
| R-HSA-9609507 | Protein localization |
| hsa03060 | Protein export |
