# Supplementary material for: Exposure to fine particulate matter in adults is associated with immune cell gene expression related to inflammation, the electron transport chain, and cell cycle regulation
Source: Environ Epigenet. 2025 Apr 1;11(1):dvaf008. doi: 10.1093/eep/dvaf008 (PMC12159804; doi:10.1093/eep/dvaf008)
Supplement: dvaf008_Supp [file Supplemental file 1.pdf › suppl_data/Supplemental file 1.pdf]

Supplemental file 1:

Supplemental Table 1.1: Association between PM2.5 and gene expression in the Rotterdam Study
Supplemental Table 1.2: Association between black carbon and gene expression in the Rotterdam Study
Supplemental Table 1.3: Association between NO2 and gene expression in the Rotterdam Study
Supplemental Table 1.4: Association between annual mean ozone and gene expression in the Rotterdam Study
Supplemental Table 1.5: Association between warm season ozone and gene expression in the Rotterdam Study
Supplemental Table 1.6: Association between normalized difference vegetation index and gene expression in the Rotterdam Study
Supplemental Table 1.7: Association between impervious surface and gene expression in the Rotterdam Study
Supplemental Table 1.8: Association between distance to nearest water body and gene expression in the Rotterdam Study

All results are adjusted for age, sex, BMI, education, current smoking, RNA sequencing batch, %monocytes*, %lymphocytes and %granulocytes.

Genes with a significant association to the exposure (FDR < 5 %) are highlighted

Genes used in pathway analyses are shown in **bold**

* Associations with distance to water is not adjusted for % monocytes because of linear dependencies.

Supplemental Table 1.1: Association between PM2.5 and gene expression in the Rotterdam Study

Geneid	logFC	P-value	Adjusted p-value	Gene name
ENSG00000165949.12	-0.411760862	1.18E-07	0.002426832	**IFI27**
ENSG00000250966.2	0.110175983	2.50E-06	0.017130655	**H3P14**
ENSG00000278771.1	-0.187892425	2.51E-06	0.017130655	**RN7SL3**
ENSG00000276168.1	-0.102545802	6.14E-06	0.031487852	**RN7SL1**
ENSG00000131100.13	0.041523057	1.06E-05	0.043435657	**ATP6V1E1**
ENSG00000085063.17	0.042792013	1.47E-05	0.047761188	**CD59**
ENSG00000144021.3	0.013947352	1.67E-05	0.047761188	**CIAO1**
ENSG00000228929.1	0.067643054	2.06E-05	0.047761188	**RPS13P2**
ENSG00000179639.10	0.108118426	2.24E-05	0.047761188	**FCER1A**
ENSG00000204472.13	0.056519992	2.37E-05	0.047761188	**AIF1**
ENSG00000163041.13	0.044562831	2.62E-05	0.047761188	**H3-3A**
ENSG00000261158.1	-0.158024223	2.93E-05	0.047761188	**CTD-2583P5.3**
ENSG00000115155.19	-0.219379366	3.03E-05	0.047761188	**OTOF**
ENSG00000240409.1	0.26626034	6.28E-05	0.082739993	**MTATP8P1**
ENSG00000243147.8	0.047713859	6.61E-05	0.082739993	**MRPL33**
ENSG00000166965.12	-0.044760784	6.64E-05	0.082739993	**RCCD1**
ENSG00000177606.8	-0.059919145	6.86E-05	0.082739993	**JUN**
ENSG00000196565.15	0.262498341	7.79E-05	0.085545984	**HBG2**
ENSG00000116459.11	0.040137173	8.13E-05	0.085545984	**ATP5PB**
ENSG00000143194.13	0.355006513	8.34E-05	0.085545984	**MAEL**
ENSG00000116209.12	0.030085818	9.05E-05	0.088411761	**TMEM59**
ENSG00000111229.16	0.037359703	9.76E-05	0.090281841	**ARPC3**
ENSG00000163933.10	0.026865274	1.01E-04	0.090281841	**RFT1**
ENSG00000163597.15	0.041577291	1.34E-04	0.112519684	**SNHG16**
ENSG00000231043.3	0.055796195	1.37E-04	0.112519684	**AC007238.1**
ENSG00000100554.12	0.041797148	1.44E-04	0.113276062	**ATP6V1D**
ENSG00000140254.13	0.334251842	1.66E-04	0.123441295	**DUOXA1**
ENSG00000213638.6	-0.092464959	1.69E-04	0.123441295	**ADAT3**
ENSG00000116014.10	-0.108142178	1.87E-04	0.123441295	**KISS1R**
ENSG00000167608.12	-0.090766364	1.91E-04	0.123441295	**TMC4**
ENSG00000236278.2	-0.10624057	1.93E-04	0.123441295	**PEBP1P3**
ENSG00000286190.2	-0.045097366	1.98E-04	0.123441295	**RP11-807C20.3**
ENSG00000196639.7	-0.174076272	1.99E-04	0.123441295	**HRH1**
ENSG00000160932.11	-0.125689318	2.24E-04	0.135263296	**LY6E**
ENSG00000243678.12	-0.089221809	2.45E-04	0.143372245	**NME2**
ENSG00000126653.18	0.025824336	2.53E-04	0.144049009	**NSRP1**
ENSG00000140279.13	0.313603317	2.61E-04	0.144786456	**DUOX2**
ENSG00000259295.6	-0.157479763	3.04E-04	0.158034527	**CSPG4P12**
ENSG00000138942.16	0.024621569	3.19E-04	0.158034527	**RNF185**
ENSG00000138433.16	0.037259124	3.22E-04	0.158034527	**CIR1**
ENSG00000188483.8	-0.076436434	3.32E-04	0.158034527	**IER5L**
ENSG00000204262.14	-0.159270118	3.44E-04	0.158034527	**COL5A2**
ENSG00000173369.17	0.141823646	3.53E-04	0.158034527	**C1QB**
ENSG00000176619.13	-0.029230445	3.68E-04	0.158034527	**LMNB2**

ENSG00000072210.19	0.019926628	3.68E-04	0.158034527	**ALDH3A2**
ENSG00000106028.11	0.030804607	3.69E-04	0.158034527	**SSBP1**
ENSG00000105245.9	-0.038218656	3.72E-04	0.158034527	**NUMBL**
ENSG00000160058.19	0.01407748	3.73E-04	0.158034527	**BSDC1**
ENSG00000100603.14	0.019402164	3.83E-04	0.158034527	**SNW1**
ENSG00000285774.3	0.101396932	3.85E-04	0.158034527	**RP11-724F15.2**
ENSG00000267065.2	-0.170972623	4.21E-04	0.169136863	**LINC02080**
ENSG00000121067.19	0.013739941	4.37E-04	0.172254279	**SPOP**
ENSG00000135736.6	-0.043749669	4.54E-04	0.175814586	**CCDC102A**
ENSG00000197471.12	-0.033613112	5.09E-04	0.19348767	**SPN**
ENSG00000183347.15	0.123206814	5.30E-04	0.197600938	**GBP6**
ENSG00000006042.12	0.236154465	5.64E-04	0.200348233	**TMEM98**
ENSG00000243607.3	0.122786049	5.67E-04	0.200348233	**RPL35AP26**
ENSG00000235098.9	0.173181944	5.78E-04	0.200348233	**ANKRD65**
ENSG00000239887.6	0.160973872	5.94E-04	0.200348233	**C1orf226**
ENSG00000137310.12	-0.041783855	6.04E-04	0.200348233	**TCF19**
ENSG00000229759.1	0.095724168	6.07E-04	0.200348233	**MRPS18AP1**
ENSG00000133315.12	-0.052471039	6.08E-04	0.200348233	**MACROD1**
ENSG00000185049.16	-0.026107718	6.15E-04	0.200348233	**NELFA**
ENSG00000186468.13	0.048392552	6.48E-04	0.206147674	**RPS23**
ENSG00000204136.12	0.069986913	6.53E-04	0.206147674	**GGTA1**
ENSG00000249353.2	0.051822772	6.86E-04	0.207738331	**NPM1P27**
ENSG00000104375.17	0.038190675	6.86E-04	0.207738331	**STK3**
ENSG00000286136.2	-0.090359737	6.89E-04	0.207738331	**RP11-348N12.2**
ENSG00000129932.10	-0.044561398	7.31E-04	0.217413014	**DOHH**
ENSG00000079246.16	0.015981738	8.43E-04	0.228101588	**XRCC5**
ENSG00000130254.12	-0.024007786	8.45E-04	0.228101588	**SAFB2**
ENSG00000123201.14	0.190099391	8.45E-04	0.228101588	**GUCY1B2**
ENSG00000196655.12	0.028439441	8.56E-04	0.228101588	**TRAPPC4**
ENSG00000205155.8	0.03985293	8.56E-04	0.228101588	**PSENEN**
ENSG00000104936.20	-0.042099779	8.75E-04	0.228101588	**DMPK**
ENSG00000204625.10	-0.138837593	8.78E-04	0.228101588	**HCG9**
ENSG00000146755.11	-0.219638902	8.78E-04	0.228101588	**TRIM50**
ENSG00000142973.14	0.240884199	8.80E-04	0.228101588	**CYP4B1**
ENSG00000272841.1	-0.078461602	8.98E-04	0.228101588	**MAP3K4-AS1**
ENSG00000172927.8	0.100835508	9.13E-04	0.228101588	**MYEOV**
ENSG00000181588.17	-0.049037329	9.16E-04	0.228101588	**MEX3D**
ENSG00000136932.14	0.01770357	9.16E-04	0.228101588	**TRMO**
ENSG00000140749.9	0.026182259	9.51E-04	0.228101588	**IGSF6**
ENSG00000263276.1	-0.04925535	9.60E-04	0.228101588	**RP11-96D1.10**
ENSG00000172339.10	0.044349099	9.61E-04	0.228101588	**ALG14**
ENSG00000164756.13	0.365221576	9.67E-04	0.228101588	**SLC30A8**
ENSG00000114850.7	0.020783454	9.80E-04	0.228101588	**SSR3**
ENSG00000143222.12	0.031261802	0.001003603	0.228101588	**UFC1**
ENSG00000167674.15	-0.028445934	0.00100868	0.228101588	**HDGFL2**
ENSG00000235272.2	-0.26478954	0.001009132	0.228101588	**RAMACL**
ENSG00000140740.11	0.018749783	0.001017542	0.228101588	**UQCRC2**

ENSG00000124596.17	0.024000279	0.001029406	0.228101588	**OARD1**
ENSG00000136213.10	-0.040517631	0.001034399	0.228101588	**CHST12**
ENSG00000204611.7	0.024502806	0.001109534	0.237425126	**ZNF616**
ENSG00000189241.8	-0.016090701	0.001109847	0.237425126	**TSPYL1**
ENSG00000165672.7	0.022604535	0.001111411	0.237425126	**PRDX3**
ENSG00000188290.11	-0.121390637	0.001134931	0.239950206	**HES4**
ENSG00000197798.9	0.024705403	0.001166393	0.241238308	**FAM118B**
ENSG00000165502.7	0.04895592	0.001173517	0.241238308	**RPL36AL**
ENSG00000241837.7	0.047585489	0.00117639	0.241238308	**ATP5PO**
ENSG00000232874.1	-0.133127092	0.001188076	0.241238308	**RP11-135A1.2**
ENSG00000168288.13	0.023638617	0.001206125	0.241730885	**MMADHC**
ENSG00000250334.6	0.066023075	0.001214077	0.241730885	**LINC00989**
ENSG00000274259.2	-0.088975513	0.001241355	0.243691758	**SYNGAP1-AS1**
ENSG00000274525.1	0.127995317	0.001256947	0.243691758	**CTC-338M12.13**
ENSG00000245008.4	0.128701629	0.001259573	0.243691758	**RP11-744N12.3**
ENSG00000230565.1	0.074891546	0.001286398	0.246555643	**ZNF32-AS2**
ENSG00000231312.7	0.061736727	0.001326915	0.249491951	**MAP4K3-DT**
ENSG00000168653.11	0.055813832	0.001361032	0.249491951	**NDUFS5**
ENSG00000118971.9	-0.02447331	0.001373782	0.249491951	**CCND2**
ENSG00000126705.15	-0.035106054	0.001381186	0.249491951	**AHDC1**
ENSG00000196456.13	-0.042740552	0.001381637	0.249491951	**ZNF775**
ENSG00000148229.13	0.015872828	0.001382463	0.249491951	**POLE3**
ENSG00000168374.11	0.017741986	0.001386877	0.249491951	**ARF4**
ENSG00000229852.4	-0.040558719	0.00144649	0.256300165	**RP11-398K22.12**
ENSG00000259150.6	0.19686369	0.001466712	0.256300165	**LINC00929**
ENSG00000227678.8	-0.052327903	0.001487954	0.256300165	**RP11-73O6.3**
ENSG00000164096.13	0.030606314	0.001497501	0.256300165	**C4orf3**
ENSG00000277476.1	-0.030025396	0.001513493	0.256300165	**RP11-147L13.13**
ENSG00000168282.6	-0.094670778	0.001525436	0.256300165	**MGAT2**
ENSG00000235782.1	0.177431951	0.001535178	0.256300165	**RP3-333A15.1**
ENSG00000253720.2	-0.095813082	0.0015492	0.256300165	**RP11-473O4.3**
ENSG00000101363.13	0.022195595	0.00157783	0.256300165	**MANBAL**
ENSG00000257218.6	0.017587099	0.001578908	0.256300165	**GATC**
ENSG00000273112.1	0.089333106	0.001595654	0.256300165	**RP11-25K21.6**
ENSG00000158869.11	0.046371342	0.001597812	0.256300165	**FCER1G**
ENSG00000009307.16	0.013082218	0.001599956	0.256300165	**CSDE1**
ENSG00000108861.9	0.028416372	0.001601464	0.256300165	**DUSP3**
ENSG00000141933.9	-0.06242872	0.001616487	0.256300165	**TPGS1**
ENSG00000284931.1	0.215250205	0.001638838	0.256300165	**CTD-2643I7.4**
ENSG00000148834.13	0.036610063	0.001653267	0.256300165	**GSTO1**
ENSG00000198618.5	0.036201494	0.001663357	0.256300165	**PPIAP22**
ENSG00000034510.6	0.043446786	0.001664794	0.256300165	**TMSB10**
ENSG00000159063.14	0.022567305	0.001674674	0.256300165	**ALG8**
ENSG00000167657.14	-0.030030535	0.00171135	0.259973019	**DAPK3**
ENSG00000110911.17	0.017177273	0.001753197	0.263135365	**SLC11A2**
ENSG00000103035.11	0.015269246	0.001757828	0.263135365	**PSMD7**
ENSG00000185862.7	0.020006259	0.00178543	0.263621381	**EVI2B**

ENSG00000154262.13	0.141599532	0.001802635	0.263621381	**ABCA6**
ENSG00000101608.13	0.027176394	0.001809956	0.263621381	**MYL12A**
ENSG00000103522.16	-0.026348962	0.001828101	0.263621381	**IL21R**
ENSG00000286001.1	-0.114282272	0.001848865	0.263621381	**CTD-2546F11.1**
ENSG00000111729.15	0.043261561	0.001862195	0.263621381	**CLEC4A**
ENSG00000099822.3	-0.060029708	0.001883074	0.263621381	**HCN2**
ENSG00000113558.19	0.020985929	0.001892473	0.263621381	**SKP1**
ENSG00000232434.2	-0.061921381	0.001903586	0.263621381	**AJM1**
ENSG00000139725.8	-0.034532651	0.001933841	0.263621381	**RHOF**
ENSG00000136758.19	0.013749803	0.001937126	0.263621381	**YME1L1**
ENSG00000223855.2	0.082222276	0.001937954	0.263621381	**HRAT92**
ENSG00000220008.3	-0.056451861	0.001938874	0.263621381	**LINGO3**
ENSG00000285077.2	-0.074703688	0.001941039	0.263621381	**ARHGAP11B**
ENSG00000112208.11	-0.038672802	0.001992198	0.268007779	**BAG2**
ENSG00000117586.11	0.047618136	0.001999473	0.268007779	**TNFSF4**
ENSG00000224616.3	0.053308611	0.002018304	0.268775129	**RTCA-AS1**
ENSG00000110077.15	0.03710797	0.002038068	0.269656104	**MS4A6A**
ENSG00000225542.2	0.402048528	0.002058541	0.270092949	**ZNF385D-AS1**
ENSG00000058335.16	0.167380597	0.002072218	0.270092949	**RASGRF1**
ENSG00000092531.10	0.020403316	0.00208088	0.270092949	**SNAP23**
ENSG00000197296.6	0.024712643	0.002158721	0.278434242	**FITM2**
ENSG00000144820.8	-0.594322254	0.00218565	0.280145661	**ADGRG7**
ENSG00000141582.15	-0.024003749	0.002220152	0.282800538	**CBX4**
ENSG00000156261.13	0.020721437	0.002247277	0.283544757	**CCT8**
ENSG00000122420.10	-0.172088906	0.002263151	0.283544757	**PTGFR**
ENSG00000255737.2	-0.062082691	0.002275745	0.283544757	**AGAP2-AS1**
ENSG00000254717.2	0.119186244	0.002290677	0.283544757	**GLYATL1P2**
ENSG00000096401.8	0.018667778	0.002302436	0.283544757	**CDC5L**
ENSG00000168461.13	0.025623467	0.002330718	0.283544757	**RAB31**
ENSG00000211796.1	-0.067974746	0.002368159	0.283544757	**TRAV16**
ENSG00000204287.14	0.036040373	0.002372111	0.283544757	**HLA-DRA**
ENSG00000174130.12	0.035046323	0.002372821	0.283544757	**TLR6**
ENSG00000050130.18	0.024058853	0.002376652	0.283544757	**JKAMP**
ENSG00000272379.1	-0.054868915	0.002378082	0.283544757	**RP1-257A7.5**
ENSG00000213462.5	0.057779506	0.002426962	0.287700163	**ERV3-1**
ENSG00000105856.14	0.028237611	0.002443251	0.287966665	**HBP1**
ENSG00000120656.13	0.023621915	0.002521142	0.292328141	**TAF12**
ENSG00000160633.13	-0.021967139	0.002532886	0.292328141	**SAFB**
ENSG00000105499.14	0.125574791	0.002544135	0.292328141	**PLA2G4C**
ENSG00000105229.7	-0.024935744	0.002547866	0.292328141	**PIAS4**
ENSG00000164048.14	0.030611059	0.002551528	0.292328141	**ZNF589**
ENSG00000259129.6	-0.417364923	0.002591594	0.293840226	**LINC00648**
ENSG00000165678.21	0.013367729	0.002596085	0.293840226	**GHITM**
ENSG00000063169.11	-0.029247668	0.002612535	0.293840226	**BICRA**
ENSG00000259826.1	0.056009564	0.002626619	0.293840226	**RP11-467D6.1**
ENSG00000139684.15	0.023887627	0.00265097	0.293840226	**ESD**
ENSG00000064651.14	-0.032859917	0.002675141	0.293840226	**SLC12A2**

ENSG00000274294.1	-0.083292515	0.002675157	0.293840226	**RP11-20G13.5**
ENSG00000249709.8	-0.055451685	0.002691582	0.293840226	**ZNF564**
ENSG00000166803.14	-0.115467785	0.002693679	0.293840226	**PCLAF**
ENSG00000226002.1	0.122261976	0.00274928	0.295326425	**GTF2IP14**
ENSG00000184389.9	-0.129444354	0.002765958	0.295326425	**A3GALT2**
ENSG00000173175.15	-0.05149073	0.002773588	0.295326425	**ADCY5**
ENSG00000113811.11	0.043859652	0.00279367	0.295326425	**SELENOK**
ENSG00000236977.1	-0.074379638	0.002797715	0.295326425	**ANKRD44-IT1**
ENSG00000279140.1	-0.116340075	0.002799198	0.295326425	**RP11-477I4.4**
ENSG00000103429.11	0.013773316	0.002820221	0.295326425	**BFAR**
ENSG00000211807.3	-0.064950309	0.002822507	0.295326425	**TRAV26-1**
ENSG00000269843.1	-0.063461454	0.002846066	0.296279802	**CTC-490E21.10**
ENSG00000181852.18	0.012413583	0.002958967	0.305514633	**RNF41**
ENSG00000061337.15	0.104898604	0.002973448	0.305514633	**LZTS1**
ENSG00000170264.13	-0.068125654	0.002979468	0.305514633	**FAM161A**
ENSG00000113068.10	0.021443953	0.00300864	0.306971058	**PFDN1**
ENSG00000261455.1	-0.036596123	0.003036963	0.307455477	**LINC01003**
ENSG00000182747.5	0.13465729	0.003043371	0.307455477	**SLC35D3**
ENSG00000133265.11	-0.034171109	0.00306342	0.307963813	**HSPBP1**
ENSG00000184979.10	-0.121419245	0.003101036	0.309811201	**USP18**
ENSG00000174796.12	0.027450834	0.003137481	0.309811201	**THAP6**
ENSG00000126698.11	0.022543917	0.00314002	0.309811201	**DNAJC8**
ENSG00000286305.1	0.078912402	0.003142224	0.309811201	**RP4-808A1.2**
ENSG00000078668.14	0.018954571	0.003165252	0.310588411	**VDAC3**
ENSG00000185803.12	-0.025996543	0.003182107	0.310755522	**SLC52A2**
ENSG00000055147.19	0.018027211	0.003306472	0.320224541	**FAM114A2**
ENSG00000280183.1	-0.106014101	0.003310299	0.320224541	**RP11-400F19.12**
ENSG00000196081.10	-0.095885595	0.003343204	0.321889363	**ZNF724**
ENSG00000165264.11	0.031580544	0.003399898	0.325818253	**NDUFB6**
ENSG00000170412.18	0.106956194	0.003421076	0.326322922	**GPRC5C**
ENSG00000163541.12	0.019726148	0.003447654	0.32673012	**SUCLG1**
ENSG00000185787.15	0.015670963	0.003457209	0.32673012	**MORF4L1**
ENSG00000135439.12	-0.020047025	0.003482928	0.327650829	**AGAP2**
ENSG00000164976.9	-0.04938238	0.003511334	0.328814743	**MYORG**
ENSG00000175602.4	-0.062736668	0.003535752	0.329596396	**CCDC85B**
ENSG00000170684.9	-0.038033724	0.003569731	0.329649673	**ZNF296**
ENSG00000136930.13	0.017894524	0.003579431	0.329649673	**PSMB7**
ENSG00000274105.1	-0.079212471	0.003584546	0.329649673	**RP11-278C7.3**
ENSG00000215283.3	-0.066081072	0.003613598	0.330837784	**HMGB3P24**
ENSG00000174996.12	-0.025304241	0.00364667	0.33238179	**KLC2**
ENSG00000120254.16	-0.038267088	0.003693685	0.333607132	**MTHFD1L**
ENSG00000162959.14	-0.035070128	0.003706267	0.333607132	**MEMO1**
ENSG00000150337.14	0.087441778	0.003720565	0.333607132	**FCGR1A**
ENSG00000268401.2	-0.080284915	0.003725182	0.333607132	**CTC-344H19.4**
ENSG00000114491.14	0.020445274	0.003765189	0.334683254	**UMPS**
ENSG00000233586.1	-0.100848559	0.003769838	0.334683254	**RP11-763B22.3**
ENSG00000271821.1	0.047263006	0.00379387	0.335364989	**XXbac-BPG299F13.14**

ENSG00000199753.1	-0.06959155	0.003848399	0.336877705	**SNORD104**
ENSG00000204936.10	0.185407417	0.003856872	0.336877705	**CD177**
ENSG00000130511.16	-0.03915113	0.003860262	0.336877705	**SSBP4**
ENSG00000204394.13	-0.027002076	0.003942308	0.34257989	**VARS1**
ENSG00000163170.12	0.044357476	0.003959075	0.342585312	**BOLA3**
ENSG00000163568.15	0.066359378	0.003992465	0.34275917	**AIM2**
ENSG00000198019.13	0.071261473	0.00401085	0.34275917	**FCGR1B**
ENSG00000129055.13	0.026565734	0.004023618	0.34275917	**ANAPC13**
ENSG00000143799.14	-0.022852169	0.004027938	0.34275917	**PARP1**
ENSG00000106803.10	0.026577238	0.004056971	0.343803143	**SEC61B**
ENSG00000119231.11	0.011805037	0.004157758	0.349364755	**SENP5**
ENSG00000073803.14	0.030585626	0.004171165	0.349364755	**MAP3K13**
ENSG00000116044.16	0.016586902	0.004173706	0.349364755	**NFE2L2**
ENSG00000108576.10	0.070798074	0.004205219	0.350571707	**SLC6A4**
ENSG00000131495.9	0.030946775	0.004326534	0.355104617	**NDUFA2**
ENSG00000218283.2	0.021077552	0.004330118	0.355104617	**MORF4L1P1**
ENSG00000105507.3	0.084320012	0.004337448	0.355104617	**CABP5**
ENSG00000234585.6	0.041893377	0.004341576	0.355104617	**CCT6P3**
ENSG00000108788.12	0.014375508	0.00434617	0.355104617	**MLX**
ENSG00000163737.4	0.079064321	0.004395168	0.356362397	**PF4**
ENSG00000260060.1	-0.057263255	0.004396318	0.356362397	**RP11-388M20.1**
ENSG00000139668.9	0.023076232	0.004419838	0.35685844	**WDFY2**
ENSG00000255135.4	0.050892242	0.004468086	0.359241295	**RP11-111M22.3**
ENSG00000223350.2	-0.122665473	0.00448671	0.359241295	**IGLV9-49**
ENSG00000173372.17	0.088827281	0.004501902	0.359241295	**C1QA**
ENSG00000152942.19	0.017516576	0.004555318	0.359469927	**RAD17**
ENSG00000270587.1	-0.059433494	0.004569277	0.359469927	**RP11-51F16.9**
ENSG00000286885.1	-0.059208064	0.004581702	0.359469927	**RP11-14H3.7**
ENSG00000166428.14	-0.050794049	0.004611701	0.359469927	**PLD4**
ENSG00000185838.14	-0.032322417	0.004627819	0.359469927	**GNB1L**
ENSG00000275198.1	0.075139455	0.004633654	0.359469927	**RP11-471B22.3**
ENSG00000152213.4	0.022131549	0.004651525	0.359469927	**ARL11**
ENSG00000117262.19	0.030847243	0.004698402	0.359469927	**GPR89A**
ENSG00000115762.16	0.012679773	0.004718373	0.359469927	**PLEKHB2**
ENSG00000146007.11	0.03342073	0.004725559	0.359469927	**ZMAT2**
ENSG00000231889.7	-0.035792541	0.004743001	0.359469927	**TRAF3IP2-AS1**
ENSG00000150782.12	0.033780539	0.004754449	0.359469927	**IL18**
ENSG00000117450.15	0.030178074	0.00477859	0.359469927	**PRDX1**
ENSG00000006015.18	-0.036024841	0.004786236	0.359469927	**REX1BD**
ENSG00000101367.9	0.012728299	0.004786796	0.359469927	**MAPRE1**
ENSG00000243155.1	-0.04390657	0.00479434	0.359469927	**RP11-46A10.5**
ENSG00000106399.11	0.035966106	0.004802748	0.359469927	**RPA3**
ENSG00000153015.16	0.030791223	0.004823182	0.359686599	**CWC27**
ENSG00000262332.1	-0.075331781	0.004879183	0.36045112	**RP11-517A5.7**
ENSG00000162735.19	0.012726336	0.00488919	0.36045112	**PEX19**
ENSG00000100575.14	0.025990779	0.004889818	0.36045112	**TIMM9**
ENSG00000125746.18	-0.018244567	0.004969476	0.36045112	**EML2**

ENSG00000142168.15	0.029040306	0.004979412	0.36045112	**SOD1**
ENSG00000244398.1	0.099134602	0.004991676	0.36045112	**RP11-466H18.1**
ENSG00000160953.17	-0.018316162	0.005035111	0.36045112	**PWWP3A**
ENSG00000123143.13	-0.02556439	0.005047717	0.36045112	**PKN1**
ENSG00000099940.12	0.018838474	0.0050592	0.36045112	**SNAP29**
ENSG00000118181.11	0.042021794	0.005082561	0.36045112	**RPS25**
ENSG00000132963.8	0.032304767	0.005088348	0.36045112	**POMP**
ENSG00000197766.8	-0.106820957	0.005089113	0.36045112	**CFD**
ENSG00000082196.21	-0.033854847	0.005150342	0.36045112	**C1QTNF3**
ENSG00000120533.13	0.022025116	0.005157396	0.36045112	**ENY2**
ENSG00000110700.7	0.034382269	0.005196663	0.36045112	**RPS13**
ENSG00000008838.20	-0.019083687	0.005249373	0.36045112	**MED24**
ENSG00000179038.8	-0.110628817	0.005255081	0.36045112	**RP11-157K17.5**
ENSG00000130479.11	-0.035319795	0.005270822	0.36045112	**MAP1S**
ENSG00000111087.10	-0.045516253	0.005285829	0.36045112	**GLI1**
ENSG00000110455.14	0.067270007	0.005293844	0.36045112	**ACCS**
ENSG00000147889.18	-0.054109914	0.005311114	0.36045112	**CDKN2A**
ENSG00000266852.2	-0.167099508	0.005313689	0.36045112	**MIR4482**
ENSG00000273156.2	-0.04721497	0.005329217	0.36045112	**RP11-127B20.2**
ENSG00000033011.14	-0.020663271	0.005348291	0.36045112	**ALG1**
ENSG00000169020.10	0.051467219	0.00535572	0.36045112	**ATP5ME**
ENSG00000150764.14	0.064065446	0.005366514	0.36045112	**DIXDC1**
ENSG00000275202.1	-0.054964698	0.005370051	0.36045112	**RP11-156K23.3**
ENSG00000138495.7	0.03612122	0.0053714	0.36045112	**COX17**
ENSG00000272555.1	-0.086512851	0.005397099	0.36045112	**RP11-459I19.1**
ENSG00000140307.11	0.024738321	0.005418358	0.36045112	**GTF2A2**
ENSG00000211792.2	-0.077053708	0.005437563	0.36045112	**TRAV14DV4**
ENSG00000265241.7	0.013439173	0.005439151	0.36045112	**RBM8A**
ENSG00000179253.3	0.086773682	0.005468914	0.36045112	**RP11-429E11.3**
ENSG00000189013.14	0.095932055	0.005474714	0.36045112	**KIR2DL4**
ENSG00000237637.1	0.09569403	0.00549736	0.36045112	**FRY-AS1**
ENSG00000180329.14	0.02143451	0.005498963	0.36045112	**CCDC43**
ENSG00000260316.2	-0.06493698	0.005505193	0.36045112	**LA16c-306A4.2**
ENSG00000226479.4	0.019104607	0.005536988	0.36045112	**TMEM185B**
ENSG00000065621.15	-0.0473301	0.005582579	0.36045112	**GSTO2**
ENSG00000176454.14	-0.018931889	0.005588053	0.36045112	**LPCAT4**
ENSG00000132478.10	-0.015547238	0.005602262	0.36045112	**UNK**
ENSG00000170142.12	0.028049524	0.005602885	0.36045112	**UBE2E1**
ENSG00000099783.12	-0.021714228	0.005610669	0.36045112	**HNRNPM**
ENSG00000213937.4	-0.039629039	0.005618542	0.36045112	**CLDN9**
ENSG00000139168.9	0.025915434	0.005672965	0.36045112	**ZCRB1**
ENSG00000237877.7	-0.083207627	0.005686321	0.36045112	**LINC01473**
ENSG00000224805.2	0.080115383	0.005692265	0.36045112	**LINC00853**
ENSG00000113272.14	0.022414084	0.005700768	0.36045112	**THG1L**
ENSG00000188157.15	-0.085829651	0.005708219	0.36045112	**AGRN**
ENSG00000100926.15	-0.030236959	0.005713289	0.36045112	**TM9SF1**
ENSG00000235508.3	0.061252003	0.005758707	0.36045112	**RPS2P7**

ENSG00000259820.1	0.033639285	0.005765799	0.36045112	**AC083843.1**
ENSG00000067334.14	0.030842721	0.005795216	0.36045112	**DNTTIP2**
ENSG00000254162.1	-0.104322601	0.005800013	0.36045112	**RP11-48B3.3**
ENSG00000116032.5	-0.064539709	0.005835153	0.36045112	**GRIN3B**
ENSG00000109113.20	0.037478252	0.005835795	0.36045112	**RAB34**
ENSG00000168291.13	0.01746508	0.005846844	0.36045112	**PDHB**
ENSG00000104408.11	0.038587512	0.005853201	0.36045112	**EIF3E**
ENSG00000145592.14	0.038225041	0.005870425	0.36045112	**RPL37**
ENSG00000013392.8	0.029361598	0.005931788	0.363131683	**RWDD2A**
ENSG00000180096.12	-0.024244646	0.005962496	0.363925194	**SEPTIN1**
ENSG00000144713.13	0.035454435	0.005992028	0.364642441	**RPL32**
ENSG00000036565.15	-0.109217103	0.006017987	0.365138716	**SLC18A1**
ENSG00000228327.3	0.051521842	0.006085963	0.367489463	**RP11-206L10.2**
ENSG00000062582.14	-0.052269499	0.006116075	0.367489463	**MRPS24**
ENSG00000231731.7	-0.038964596	0.006150066	0.367489463	**AC010976.2**
ENSG00000166272.18	0.013059052	0.006197378	0.367489463	**WBP1L**
ENSG00000129460.16	0.026621756	0.006234881	0.367489463	**NGDN**
ENSG00000188070.9	-0.025852795	0.006273597	0.367489463	**C11orf95**
ENSG00000127920.6	0.069717194	0.006280798	0.367489463	**GNG11**
ENSG00000177000.13	0.022345441	0.00628214	0.367489463	**MTHFR**
ENSG00000047849.22	-0.026095029	0.006283748	0.367489463	**MAP4**
ENSG00000106355.10	0.033567925	0.006313427	0.367489463	**LSM5**
ENSG00000253719.4	0.010440568	0.006317701	0.367489463	**ATXN7L3B**
ENSG00000224745.1	-0.051828073	0.006324714	0.367489463	**RP11-380G5.2**
ENSG00000164032.12	0.025395849	0.006337352	0.367489463	**H2AZ1**
ENSG00000250535.1	0.205848787	0.006350474	0.367489463	**STK19B**
ENSG00000262692.1	-0.033939125	0.006351696	0.367489463	**CTD-3195I5.3**
ENSG00000115541.11	0.038066328	0.006375617	0.367489463	**HSPE1**
ENSG00000272361.2	-0.094490768	0.00637797	0.367489463	**GS1-166A23.2**
ENSG00000149547.15	0.014644697	0.006380549	0.367489463	**EI24**
ENSG00000135211.6	0.026908891	0.006431253	0.367489463	**TMEM60**
ENSG00000198189.11	0.020931421	0.006452856	0.367489463	**HSD17B11**
ENSG00000286071.1	-0.086623845	0.006467211	0.367489463	**DISC2**
ENSG00000200087.1	-0.13020016	0.006492319	0.367489463	**SNORA73B**
ENSG00000180354.16	0.029031399	0.006497426	0.367489463	**MTURN**
ENSG00000112096.19	0.029942085	0.006504736	0.367489463	**SOD2**
ENSG00000144115.18	0.141187288	0.006511644	0.367489463	**THNSL2**
ENSG00000111716.14	0.043948022	0.006540196	0.367489463	**LDHB**
ENSG00000276649.1	0.035025087	0.006540553	0.367489463	**RP5-858L17.1**
ENSG00000211789.2	-0.058197017	0.006570314	0.367844595	**TRAV12-2**
ENSG00000170989.10	-0.021529791	0.006588011	0.367844595	**S1PR1**
ENSG00000106952.8	0.026009279	0.0066106	0.367844595	**TNFSF8**
ENSG00000135740.17	-0.037836018	0.006631359	0.367844595	**SLC9A5**
ENSG00000280734.3	0.034227704	0.006645644	0.367844595	**LINC01232**
ENSG00000229453.2	0.100962383	0.00667634	0.367844595	**SPINK8**
ENSG00000160888.7	-0.027845937	0.006690889	0.367844595	**IER2**
ENSG00000129562.11	0.024856703	0.006692368	0.367844595	**DAD1**

ENSG00000070159.14	-0.133161627	0.006708303	0.367844595	**PTPN3**
ENSG00000233268.3	-0.105177329	0.006792887	0.368935263	**RP11-558F24.2**
ENSG00000287063.1	0.12167993	0.006798496	0.368935263	**RP11-384P7.10**
ENSG00000205302.7	0.028995733	0.006821396	0.368935263	**SNX2**
ENSG00000122406.14	0.031369179	0.006831473	0.368935263	**RPL5**
ENSG00000166831.9	-0.12167705	0.006846447	0.368935263	**RBPMS2**
ENSG00000140553.18	-0.020177029	0.006857597	0.368935263	**UNC45A**
ENSG00000213453.3	0.052196791	0.006864582	0.368935263	**FTH1P3**
ENSG00000260368.1	-0.061004083	0.00689013	0.368935263	**RP11-521I2.3**
ENSG00000128011.5	-0.039905903	0.006922259	0.368935263	**LRFN1**
ENSG00000180900.20	-0.029960273	0.006933945	0.368935263	**SCRIB**
ENSG00000114023.15	0.025087817	0.006941127	0.368935263	**FAM162A**
ENSG00000235750.10	0.02190414	0.006944071	0.368935263	**KIAA0040**
ENSG00000186231.17	0.052119762	0.006965607	0.369121165	**KLHL32**
ENSG00000134697.13	0.020341556	0.007010391	0.369121165	**GNL2**
ENSG00000181163.14	0.026105541	0.007019693	0.369121165	**NPM1**
ENSG00000234337.4	0.081127196	0.007049167	0.369121165	**RP11-297L17.6**
ENSG00000114391.13	0.038766373	0.0070509	0.369121165	**RPL24**
ENSG00000273802.2	0.064123942	0.007065417	0.369121165	**H2BC8**
ENSG00000186834.4	-0.01317269	0.007073562	0.369121165	**HEXIM1**
ENSG00000183762.13	0.064115758	0.00712214	0.370368227	**KREMEN1**
ENSG00000197099.8	0.103782294	0.00713358	0.370368227	**RP11-573D15.8**
ENSG00000196262.15	0.022830421	0.00717197	0.371421118	**PPIA**
ENSG00000144118.14	0.022954465	0.007206327	0.371470365	**RALB**
ENSG00000076864.20	-0.160880427	0.007221036	0.371470365	**RAP1GAP**
ENSG00000086061.16	0.036416754	0.007227261	0.371470365	**DNAJA1**
ENSG00000169764.16	0.01588576	0.007265591	0.372506827	**UGP2**
ENSG00000143933.19	0.024125645	0.00730534	0.373196571	**CALM2**
ENSG00000171992.13	-0.059303793	0.00732611	0.373196571	**SYNPO**
ENSG00000197483.10	-0.038296127	0.007333637	0.373196571	**ZNF628**
ENSG00000224165.6	0.058698896	0.007383784	0.37477179	**DNAJC27-AS1**
ENSG00000111639.8	0.040533625	0.00740114	0.37477179	**MRPL51**
ENSG00000273763.2	-0.113042003	0.007434835	0.375550707	**RP11-420C9.1**
ENSG00000178184.16	-0.05808406	0.007457416	0.375765824	**PARD6G**
ENSG00000138385.16	0.03070664	0.007544536	0.379223877	**SSB**
ENSG00000281571.2	-0.073770974	0.007565386	0.379342129	**CH17-195P21.2**
ENSG00000126231.15	-0.065196344	0.007604334	0.379712292	**PROZ**
ENSG00000130772.14	0.020968444	0.007609799	0.379712292	**MED18**
ENSG00000272599.2	-0.05335101	0.007725864	0.384568	**RP11-152N13.16**
ENSG00000162032.16	-0.055249145	0.007835411	0.384722613	**SPSB3**
ENSG00000157551.19	0.041149507	0.007870893	0.384722613	**KCNJ15**
ENSG00000126243.9	-0.03871288	0.007891983	0.384722613	**LRFN3**
ENSG00000273796.1	0.158112556	0.007895431	0.384722613	**LL21NC02-21A1.1**
ENSG00000169247.14	0.067868621	0.007896041	0.384722613	**SH3TC2**
ENSG00000105329.11	-0.028499648	0.007906277	0.384722613	**TGFB1**
ENSG00000082213.18	0.01751855	0.007921099	0.384722613	**C5orf22**
ENSG00000198522.14	0.016441076	0.007988767	0.384722613	**GPN1**

ENSG00000062716.13	0.021192872	0.007995063	0.384722613	**VMP1**
ENSG00000204397.9	0.045479793	0.00799817	0.384722613	**CARD16**
ENSG00000158623.14	0.031713887	0.008035544	0.384722613	**COPG2**
ENSG00000171490.13	0.017553548	0.008043696	0.384722613	**RSL1D1**
ENSG00000235381.1	-0.039782485	0.008047018	0.384722613	**RP11-477D19.2**
ENSG00000253352.10	0.017140856	0.008056117	0.384722613	**TUG1**
ENSG00000133895.18	-0.019862973	0.008068604	0.384722613	**MEN1**
ENSG00000071626.17	-0.013858976	0.008072457	0.384722613	**DAZAP1**
ENSG00000111206.13	-0.036060057	0.008089796	0.384722613	**FOXM1**
ENSG00000127463.15	-0.016650738	0.008091878	0.384722613	**EMC1**
ENSG00000265982.1	-0.080330954	0.008107959	0.384722613	**RP11-927P21.4**
ENSG00000113231.14	-0.061174668	0.008119275	0.384722613	**PDE8B**
ENSG00000058453.17	-0.030093263	0.008140966	0.384722613	**CROCC**
ENSG00000235257.9	0.065518799	0.008141682	0.384722613	**ITGA9-AS1**
ENSG00000124713.6	-0.039676389	0.008207426	0.386937697	**GNMT**
ENSG00000175467.15	-0.024958154	0.008296238	0.38931073	**SART1**
ENSG00000137947.12	0.031966867	0.008307535	0.38931073	**GTF2B**
ENSG00000183751.15	-0.02519587	0.008314711	0.38931073	**TBL3**
ENSG00000113732.9	0.024198335	0.008385407	0.391726466	**ATP6V0E1**
ENSG00000162066.16	-0.023808532	0.008434423	0.39297354	**AMDHD2**
ENSG00000254485.5	-0.178529629	0.008451859	0.39297354	**RP11-380O24.1**
ENSG00000139835.14	-0.045781784	0.008485608	0.39297354	**GRTP1**
ENSG00000107960.11	0.019782054	0.00848875	0.39297354	**STN1**
ENSG00000280351.2	-0.044285701	0.008558339	0.393380017	**CTD-2561B21.8**
ENSG00000065268.11	-0.032814756	0.008559866	0.393380017	**WDR18**
ENSG00000105122.13	-0.023410225	0.0085912	0.393380017	**RASAL3**
ENSG00000168002.12	0.019831963	0.008612625	0.393380017	**POLR2G**
ENSG00000182117.6	0.040038332	0.008628843	0.393380017	**NOP10**
ENSG00000125870.11	0.026169612	0.008638991	0.393380017	**SNRPB2**
ENSG00000149575.9	0.133826936	0.00864106	0.393380017	**SCN2B**
ENSG00000112078.14	0.016460317	0.008680696	0.393380017	**KCTD20**
ENSG00000150316.12	0.022686176	0.008687471	0.393380017	**CWC15**
ENSG00000127616.20	-0.022150002	0.008689348	0.393380017	**SMARCA4**
ENSG00000169224.13	0.089862592	0.008737756	0.394700211	**GCSAML**
ENSG00000272047.3	0.037931888	0.008852408	0.395852952	**GTF2H5**
ENSG00000170092.14	-0.05035583	0.00886768	0.395852952	**SPDYE5**
ENSG00000286264.2	-0.042001569	0.008869321	0.395852952	**RP11-783K16.16**
ENSG00000253341.1	0.025623661	0.008887347	0.395852952	**PCBP2P2**
ENSG00000129071.10	0.013837599	0.008890442	0.395852952	**MBD4**
ENSG00000236529.2	0.100728306	0.008912914	0.395852952	**RP13-254B10.1**
ENSG00000050628.20	0.102350579	0.008937811	0.395852952	**PTGER3**
ENSG00000239672.8	0.044882124	0.008955537	0.395852952	**NME1**
ENSG00000147592.9	0.026873198	0.008966547	0.395852952	**LACTB2**
ENSG00000169683.8	-0.027796024	0.008974731	0.395852952	**LRRC45**
ENSG00000230989.7	0.020259542	0.008975601	0.395852952	**HSBP1**
ENSG00000153495.11	-0.068373073	0.009048641	0.39749587	**TEX29**
ENSG00000185052.13	0.053986228	0.009078029	0.39749587	**SLC24A3**

ENSG00000278311.5	0.01317451	0.009093803	0.39749587	**GGNBP2**
ENSG00000106443.17	0.021992964	0.009097068	0.39749587	**PHF14**
ENSG00000125482.13	0.016382834	0.009109765	0.39749587	**TTF1**
ENSG00000140406.4	-0.023427635	0.00915914	0.398801797	**TLNRD1**
ENSG00000123064.13	-0.020274991	0.009228556	0.399542311	**DDX54**
ENSG00000213131.3	0.040727632	0.009254661	0.399542311	**YWHAZP4**
ENSG00000229769.2	-0.123185689	0.009261681	0.399542311	**TRBV10-2**
ENSG00000177646.20	-0.01856077	0.009267122	0.399542311	**ACAD9**
ENSG00000243404.2	-0.109712472	0.009279385	0.399542311	**RPL35AP32**
ENSG00000103769.10	0.01406337	0.009293041	0.399542311	**RAB11A**
ENSG00000119684.16	0.017675348	0.00931658	0.399713554	**MLH3**
ENSG00000104859.15	-0.021604508	0.009336005	0.399713554	**CLASRP**
ENSG00000166523.8	0.038269741	0.009372693	0.400205988	**CLEC4E**
ENSG00000180787.6	0.022851969	0.009445035	0.400205988	**ZFP3**
ENSG00000131373.15	0.018786502	0.009448146	0.400205988	**HACL1**
ENSG00000136717.15	-0.024193249	0.009462715	0.400205988	**BIN1**
ENSG00000143106.13	0.020835013	0.009496801	0.400205988	**PSMA5**
ENSG00000196421.9	-0.081023406	0.009505284	0.400205988	**C20orf204**
ENSG00000233360.4	-0.047804247	0.009542414	0.400205988	**PDXP-DT**
ENSG00000229056.2	0.064742578	0.009549932	0.400205988	**HECW2-AS1**
ENSG00000154518.10	0.023485606	0.009552923	0.400205988	**ATP5MC3**
ENSG00000110057.8	-0.030725372	0.009588651	0.400205988	**UNC93B1**
ENSG00000159322.18	0.013735571	0.009598178	0.400205988	**ADPGK**
ENSG00000265808.4	0.015208363	0.009626085	0.400205988	**SEC22B**
ENSG00000137575.12	0.022749231	0.009630578	0.400205988	**SDCBP**
ENSG00000146872.19	0.011541156	0.009658933	0.400205988	**TLK2**
ENSG00000189253.7	0.181281488	0.009667075	0.400205988	**TRIM64B**
ENSG00000264773.1	0.084511568	0.009678245	0.400205988	**MIR4420**
ENSG00000141569.12	-0.014711936	0.009679255	0.400205988	**TRIM65**
ENSG00000162062.15	-0.071571453	0.009724725	0.400656796	**TEDC2**
ENSG00000069275.13	0.014674637	0.009788641	0.400656796	**NUCKS1**
ENSG00000004455.17	0.01095626	0.009802831	0.400656796	**AK2**
ENSG00000176915.15	-0.009245422	0.009813509	0.400656796	**ANKLE2**
ENSG00000279296.1	-0.092596995	0.009814774	0.400656796	**PRAL**
ENSG00000240925.1	-0.104082625	0.009815002	0.400656796	**RPS20P31**
ENSG00000169021.6	0.02237084	0.009826915	0.400656796	**UQCRFS1**
ENSG00000105655.19	-0.029691399	0.009855066	0.401007313	**ISYNA1**
ENSG00000159596.7	0.014904336	0.009945775	0.403896951	**TMEM69**
ENSG00000256897.1	-0.076909029	0.010070089	0.408137145	**RP11-17G12.2**
ENSG00000261221.4	-0.032007802	0.010115158	0.408444247	**ZNF865**
ENSG00000124172.10	0.039581706	0.010117499	0.408444247	**ATP5F1E**
ENSG00000198720.13	-0.046644155	0.01019846	0.410903752	**ANKRD13B**
ENSG00000163131.12	0.020537705	0.01028198	0.413030394	**CTSS**
ENSG00000117614.10	0.017945884	0.010294201	0.413030394	**SYF2**
ENSG00000107949.17	0.019953419	0.010311662	0.413030394	**BCCIP**
ENSG00000108100.18	0.014649172	0.010356759	0.413377775	**CCNY**
ENSG00000233297.4	0.094706951	0.010360648	0.413377775	**RASA4DP**

ENSG00000272693.4	-0.076688198	0.010502588	0.417653644	**RP11-479O9.4**
ENSG00000068024.17	0.0189891	0.010508547	0.417653644	**HDAC4**
ENSG00000244242.2	-0.08543279	0.010609833	0.419982169	**IFITM10**
ENSG00000286633.1	0.101887334	0.010617651	0.419982169	**RP11-367G6.4**
ENSG00000107872.14	-0.04452693	0.010628572	0.419982169	**FBXL15**
ENSG00000166170.11	0.012024371	0.010726917	0.421544598	**BAG5**
ENSG00000241360.2	-0.072135641	0.010745008	0.421544598	**PDXP**
ENSG00000132017.10	-0.017538351	0.01078062	0.421544598	**DCAF15**
ENSG00000251706.1	-0.099809981	0.010783993	0.421544598	**RNU7-61P**
ENSG00000088930.8	0.015373856	0.010798338	0.421544598	**XRN2**
ENSG00000261236.8	-0.024784202	0.010801782	0.421544598	**BOP1**
ENSG00000177410.13	0.029131959	0.010840107	0.421544598	**ZFAS1**
ENSG00000101224.18	-0.023039709	0.010850414	0.421544598	**CDC25B**
ENSG00000287812.1	0.154337232	0.010871649	0.421544598	**CTD-3247F14.3**
ENSG00000144455.14	0.022310337	0.010911607	0.421544598	**SUMF1**
ENSG00000153975.10	0.032641727	0.010953451	0.421544598	**ZUP1**
ENSG00000211776.2	-0.057417626	0.010955423	0.421544598	**TRAV2**
ENSG00000198856.13	0.026884901	0.010987121	0.421544598	**OSTC**
ENSG00000033170.17	0.02583174	0.011001794	0.421544598	**FUT8**
ENSG00000167900.12	-0.052737835	0.0110195	0.421544598	**TK1**
ENSG00000170545.17	-0.027289178	0.011027359	0.421544598	**SMAGP**
ENSG00000260428.3	-0.074955103	0.011048452	0.421544598	**SCX**
ENSG00000163961.4	-0.018254273	0.01104959	0.421544598	**RNF168**
ENSG00000205090.9	-0.067105572	0.01105866	0.421544598	**TMEM240**
ENSG00000048544.6	0.015579278	0.011092801	0.421564319	**MRPS10**
ENSG00000156467.10	0.046428002	0.011100289	0.421564319	**UQCRB**
ENSG00000251330.3	-0.079331002	0.011143162	0.422410282	**CTD-2283N19.1**
ENSG00000176383.9	-0.056651524	0.011205047	0.423250195	**B3GNT4**
ENSG00000211778.2	-0.058177738	0.011206595	0.423250195	**TRAV4**
ENSG00000137959.17	-0.135661808	0.011256978	0.424371498	**IFI44L**
ENSG00000102897.10	0.022142358	0.011309039	0.425551875	**LYRM1**
ENSG00000276058.1	0.105893778	0.011417017	0.428828171	**STMN1P1**
ENSG00000060069.18	-0.020734544	0.011485551	0.430183572	**CTDP1**
ENSG00000162851.8	0.026079659	0.011507706	0.430183572	**TFB2M**
ENSG00000189409.14	-0.085876926	0.011536276	0.430183572	**MMP23B**
ENSG00000287735.1	-0.096989757	0.011540375	0.430183572	**RP11-203M5.10**
ENSG00000274184.1	-0.066080052	0.011565041	0.430183572	**RP11-158H5.8**
ENSG00000112651.12	-0.027082435	0.011578961	0.430183572	**MRPL2**
ENSG00000088827.13	-0.123893557	0.01170791	0.433978467	**SIGLEC1**
ENSG00000146005.4	-0.087860733	0.01173198	0.433978467	**PSD2**
ENSG00000159363.19	-0.022253599	0.01174459	0.433978467	**ATP13A2**
ENSG00000245149.4	-0.026783264	0.011832086	0.436175235	**RNF139-AS1**
ENSG00000279092.1	-0.194174688	0.011846577	0.436175235	**RP11-1H8.5**
ENSG00000205791.3	0.0851362	0.011940153	0.438832738	**LOH12CR2**
ENSG00000131236.18	0.013983105	0.011990354	0.43945164	**CAP1**
ENSG00000287115.1	0.072956718	0.012023487	0.43945164	**RP11-107E5.5**
ENSG00000117594.10	0.10211665	0.0120541	0.43945164	**HSD11B1**

ENSG00000227775.3	0.055896425	0.012064792	0.43945164	**RP1-283E3.4**
ENSG00000255449.1	-0.069003557	0.01206965	0.43945164	**RP11-91P24.6**
ENSG00000180992.7	0.029880136	0.012085563	0.43945164	**MRPL14**
ENSG00000136869.16	0.029861044	0.012130972	0.440322064	**TLR4**
ENSG00000118855.21	0.017143152	0.012256958	0.443762141	**MFSD1**
ENSG00000159199.14	0.025367887	0.012269023	0.443762141	**ATP5MC1**
ENSG00000119689.15	0.008388865	0.01233866	0.445030279	**DLST**
ENSG00000215158.9	0.039510003	0.012384093	0.445030279	**RP11-1023L17.1**
ENSG00000229000.1	-0.09847549	0.012411086	0.445030279	**SEPTIN7P8**
ENSG00000226102.3	0.269429458	0.012542035	0.445030279	**SEPTIN7P3**
ENSG00000118680.14	0.025401202	0.012542349	0.445030279	**MYL12B**
ENSG00000233461.6	0.035003862	0.012555656	0.445030279	**RP11-295G20.2**
ENSG00000227165.9	0.056392114	0.012573019	0.445030279	**WDR11-AS1**
ENSG00000178950.17	-0.019895521	0.01258655	0.445030279	**GAK**
ENSG00000254477.2	0.039656367	0.01262326	0.445030279	**AP000640.10**
ENSG00000167964.13	-0.085254411	0.012628167	0.445030279	**RAB26**
ENSG00000255189.1	0.095143643	0.012640723	0.445030279	**GLYATL1P1**
ENSG00000235890.2	-0.09466601	0.012650434	0.445030279	**TSPEAR-AS1**
ENSG00000126464.14	-0.028884663	0.012681018	0.445030279	**PRR12**
ENSG00000269161.1	-0.046383576	0.012703137	0.445030279	**CTD-3131K8.3**
ENSG00000166548.17	0.019074941	0.012714348	0.445030279	**TK2**
ENSG00000172732.12	-0.016767852	0.012738921	0.445030279	**MUS81**
ENSG00000144677.15	0.055005785	0.012744943	0.445030279	**CTDSPL**
ENSG00000170604.5	-0.028202334	0.012795527	0.445030279	**IRF2BP1**
ENSG00000266412.6	0.024621151	0.012820194	0.445030279	**NCOA4**
ENSG00000119655.11	0.028266227	0.012878116	0.445030279	**NPC2**
ENSG00000182093.16	0.034286857	0.012926459	0.445030279	**GET1**
ENSG00000129559.13	0.026242055	0.012932721	0.445030279	**NEDD8**
ENSG00000170310.15	0.02735349	0.012939301	0.445030279	**STX8**
ENSG00000259865.1	-0.047010829	0.01296223	0.445030279	**RP11-488L18.10**
ENSG00000108953.17	0.015281203	0.012970244	0.445030279	**YWHAE**
ENSG00000279332.1	-0.048036419	0.012979125	0.445030279	**RP11-799B12.1**
ENSG00000244227.7	0.327285024	0.013005456	0.445030279	**LRRC77P**
ENSG00000257086.1	0.076599151	0.013006203	0.445030279	**RP11-783K16.13**
ENSG00000076928.18	-0.018653252	0.013025782	0.445030279	**ARHGEF1**
ENSG00000236308.1	-0.056307414	0.013040396	0.445030279	**RP11-316M21.7**
ENSG00000180712.4	-0.063267131	0.013050705	0.445030279	**LINC02363**
ENSG00000237214.3	0.058255072	0.013072184	0.445030279	**RP11-12M9.3**
ENSG00000152766.6	0.091269985	0.013077245	0.445030279	**ANKRD22**
ENSG00000277151.1	-0.048277465	0.013097695	0.445030279	**RP11-380B4.3**
ENSG00000167513.9	-0.05707127	0.013102046	0.445030279	**CDT1**
ENSG00000121766.16	0.024452043	0.013140472	0.445030279	**ZCCHC17**
ENSG00000238150.1	-0.082474322	0.013140713	0.445030279	**AC008753.3**
ENSG00000169908.12	0.143514719	0.013151845	0.445030279	**TM4SF1**
ENSG00000182636.8	0.098498796	0.013166323	0.445030279	**NDN**
ENSG00000160124.9	0.033826514	0.013172098	0.445030279	**MIX23**
ENSG00000083844.10	-0.019532406	0.013221167	0.445953434	**ZNF264**

ENSG00000111540.16	0.012739034	0.013255466	0.446376195	**RAB5B**
ENSG00000143612.21	0.014319522	0.013296556	0.446961058	**C1orf43**
ENSG00000120675.6	0.037748239	0.013330186	0.446961058	**DNAJC15**
ENSG00000178449.9	0.030818229	0.013397166	0.446961058	**COX14**
ENSG00000161031.13	-0.074007866	0.013410506	0.446961058	**PGLYRP2**
ENSG00000284707.2	-0.034337988	0.013424819	0.446961058	**RP5-1090P18.1**
ENSG00000284194.3	-0.073377942	0.013433054	0.446961058	**SCO2**
ENSG00000121552.4	0.056313897	0.013452979	0.446961058	**CSTA**
ENSG00000166145.15	-0.035384333	0.013467344	0.446961058	**SPINT1**
ENSG00000146729.10	0.023204201	0.013468984	0.446961058	**NIPSNAP2**
ENSG00000259869.2	0.084556612	0.013547021	0.44809035	**AL022344.7**
ENSG00000211873.1	-0.092710924	0.013568474	0.44809035	**TRAJ16**
ENSG00000169612.4	0.022106511	0.013609957	0.44809035	**RAMAC**
ENSG00000182307.14	0.013343238	0.013612855	0.44809035	**C8orf33**
ENSG00000105298.14	-0.018954163	0.013632056	0.44809035	**CACTIN**
ENSG00000143420.19	0.016779052	0.013636316	0.44809035	**ENSA**
ENSG00000062822.15	-0.022709786	0.013655962	0.44809035	**POLD1**
ENSG00000089723.10	0.055278935	0.013719462	0.448676703	**OTUB2**
ENSG00000119280.17	0.035833908	0.013770999	0.448676703	**C1orf198**
ENSG00000266714.9	0.03407816	0.013786713	0.448676703	**MYO15B**
ENSG00000143947.15	0.03763364	0.013818857	0.448676703	**RPS27A**
ENSG00000184897.6	-0.031111755	0.013842708	0.448676703	**H1-10**
ENSG00000166401.15	0.021031186	0.013852629	0.448676703	**SERPINB8**
ENSG00000115520.8	0.020648106	0.01390324	0.448676703	**COQ10B**
ENSG00000224137.1	0.099276854	0.01390853	0.448676703	**LINC01857**
ENSG00000239779.7	-0.018801205	0.013912504	0.448676703	**WBP1**
ENSG00000049245.13	0.015163149	0.013964138	0.448676703	**VAMP3**
ENSG00000177733.7	-0.015704631	0.014024171	0.448676703	**HNRNPA0**
ENSG00000108651.10	0.016327244	0.014030267	0.448676703	**UTP6**
ENSG00000177556.12	0.023114326	0.014056264	0.448676703	**ATOX1**
ENSG00000159189.12	0.105429373	0.014075728	0.448676703	**C1QC**
ENSG00000272807.1	-0.035963438	0.014116696	0.448676703	**RP11-260M2.1**
ENSG00000137285.11	-0.186463097	0.014145629	0.448676703	**TUBB2B**
ENSG00000179144.5	0.031424287	0.014161193	0.448676703	**GIMAP7**
ENSG00000149201.10	-0.066000409	0.014164868	0.448676703	**CCDC81**
ENSG00000157796.18	0.024619991	0.014176752	0.448676703	**WDR19**
ENSG00000125733.18	-0.025683984	0.014183715	0.448676703	**TRIP10**
ENSG00000252916.1	-0.073653211	0.014189404	0.448676703	**RNU6-762P**
ENSG00000111335.14	-0.067140732	0.014198331	0.448676703	**OAS2**
ENSG00000134717.18	0.020487032	0.014215979	0.448676703	**BTF3L4**
ENSG00000272483.1	-0.084543051	0.014219298	0.448676703	**RP11-169K17.3**
ENSG00000135245.10	0.031283905	0.014220785	0.448676703	**HILPDA**
ENSG00000108406.10	0.015451241	0.014258378	0.449171755	**DHX40**
ENSG00000007392.17	-0.013339077	0.014382982	0.452227185	**LUC7L**
ENSG00000281128.2	0.103764653	0.014399471	0.452227185	**PTENP1-AS**
ENSG00000155760.3	-0.08060028	0.014458088	0.452490854	**FZD7**
ENSG00000269416.6	0.288740696	0.014486743	0.452490854	**LINC01224**

ENSG00000251323.3	0.086131771	0.014495628	0.452490854	**LINC02728**
ENSG00000171658.8	-0.102097774	0.014496123	0.452490854	**NMRAL2P**
ENSG00000168096.15	-0.020727296	0.014550243	0.452886595	**ANKS3**
ENSG00000180398.13	0.015345827	0.014553244	0.452886595	**MCFD2**
ENSG00000106034.18	0.04543086	0.014592843	0.452886595	**CPED1**
ENSG00000119698.12	0.107569479	0.014603294	0.452886595	**PPP4R4**
ENSG00000226564.1	0.039654797	0.01466449	0.452886595	**FTH1P20**
ENSG00000179564.4	-0.087226614	0.014664749	0.452886595	**LSMEM2**
ENSG00000266708.1	0.077982574	0.014670849	0.452886595	**RP11-661O13.1**
ENSG00000155438.12	0.02902871	0.014685468	0.452886595	**NIFK**
ENSG00000163736.4	0.075189984	0.014749952	0.453370309	**PPBP**
ENSG00000131981.16	0.035112191	0.014765673	0.453370309	**LGALS3**
ENSG00000146063.20	-0.014419334	0.014778076	0.453370309	**TRIM41**
ENSG00000143207.21	0.015353763	0.014809479	0.453370309	**COP1**
ENSG00000172965.17	0.028374904	0.014815287	0.453370309	**MIR4435-2HG**
ENSG00000103671.10	0.019164291	0.014852066	0.453370309	**TRIP4**
ENSG00000026103.22	0.033220558	0.014855902	0.453370309	**FAS**
ENSG00000145685.14	0.037578523	0.014900254	0.454048145	**LHFPL2**
ENSG00000130305.17	-0.019789857	0.014948153	0.454831942	**NSUN5**
ENSG00000167641.11	-0.064699842	0.014982437	0.454868595	**PPP1R14A**
ENSG00000123136.15	-0.013943936	0.015021508	0.454868595	**DDX39A**
ENSG00000236304.1	0.089762341	0.015034396	0.454868595	**AP001189.4**
ENSG00000159479.17	0.014300247	0.015038078	0.454868595	**MED8**
ENSG00000108590.11	0.024818698	0.015082511	0.454880616	**MED31**
ENSG00000079435.10	-0.025123896	0.015082837	0.454880616	**LIPE**
ENSG00000115486.12	-0.016146458	0.015136692	0.455834492	**GGCX**
ENSG00000075618.18	-0.039194497	0.015164902	0.456014393	**FSCN1**
ENSG00000226337.3	0.071185859	0.015201101	0.456433651	**TMEM252-DT**
ENSG00000134283.18	0.013699742	0.015239724	0.45692435	**PPHLN1**
ENSG00000265100.1	-0.058417123	0.01526284	0.45694938	**RP11-147L13.2**
ENSG00000100865.15	0.015356515	0.01533953	0.457930336	**CINP**
ENSG00000177303.10	-0.06804576	0.015340264	0.457930336	**CASKIN2**
ENSG00000151729.11	-0.025055616	0.015451236	0.460168031	**SLC25A4**
ENSG00000230593.3	-0.090861056	0.015468563	0.460168031	**PPIAP40**
ENSG00000196531.14	0.021131434	0.015482541	0.460168031	**NACA**
ENSG00000196188.12	0.089249276	0.015541191	0.460246268	**CTSE**
ENSG00000275294.4	-0.111849433	0.015563514	0.460246268	**LINC02340**
ENSG00000278668.1	-0.069289173	0.015577923	0.460246268	**RP11-227G15.11**
ENSG00000167680.17	-0.078846106	0.015587787	0.460246268	**SEMA6B**
ENSG00000134882.16	0.010831796	0.015598173	0.460246268	**UBAC2**
ENSG00000173621.9	-0.040395989	0.015619827	0.460246268	**LRFN4**
ENSG00000162896.6	0.220562318	0.015703123	0.461458663	**PIGR**
ENSG00000148204.12	-0.073910854	0.015705976	0.461458663	**CRB2**
ENSG00000232006.9	0.294951707	0.015770823	0.462701063	**AC005537.2**
ENSG00000146223.16	0.01174505	0.015826394	0.463668129	**RPL7L1**
ENSG00000243063.1	0.099452329	0.015931735	0.465535989	**IGKV3-7**
ENSG00000240487.1	0.100835424	0.015972898	0.465535989	**FAM214BP1**

ENSG00000160716.6	-0.067181967	0.015976704	0.465535989	**CHRNB2**
ENSG00000254207.1	-0.149245568	0.015995376	0.465535989	**RP11-43A14.1**
ENSG00000103266.11	-0.021933069	0.016035575	0.465535989	**STUB1**
ENSG00000148498.16	0.062118683	0.016045736	0.465535989	**PARD3**
ENSG00000148019.14	-0.034151396	0.016060313	0.465535989	**CEP78**
ENSG00000115592.11	-0.101296849	0.016071752	0.465535989	**PRKAG3**
ENSG00000143387.14	-0.028115228	0.016115861	0.466155265	**CTSK**
ENSG00000183621.15	0.027169864	0.016164122	0.466392024	**ZNF438**
ENSG00000287896.1	-0.074986966	0.016189076	0.466392024	**RP11-43N16.7**
ENSG00000165125.22	0.070647993	0.016192272	0.466392024	**TRPV6**
ENSG00000259856.1	0.033007982	0.016236746	0.467017104	**RAB43P1**
ENSG00000138035.15	-0.037070383	0.016274045	0.467434322	**PNPT1**
ENSG00000164077.14	-0.021056429	0.01636842	0.469487487	**MON1A**
ENSG00000170891.11	0.087939449	0.016506666	0.472791495	**CYTL1**
ENSG00000232940.6	-0.037879611	0.016586559	0.473369286	**HCG25**
ENSG00000067365.15	0.015382895	0.016597528	0.473369286	**METTL22**
ENSG00000105711.14	0.045207816	0.016618235	0.473369286	**SCN1B**
ENSG00000232388.5	0.028767247	0.016619167	0.473369286	**SMIM26**
ENSG00000132204.14	0.23389317	0.016666155	0.474049236	**LINC00470**
ENSG00000088205.13	0.013694932	0.016693729	0.474175889	**DDX18**
ENSG00000118508.5	0.024519109	0.016721983	0.474321487	**RAB32**
ENSG00000074201.9	0.019922091	0.016783148	0.474594832	**CLNS1A**
ENSG00000071575.12	-0.025587563	0.016792201	0.474594832	**TRIB2**
ENSG00000111846.19	0.038066686	0.016801046	0.474594832	**GCNT2**
ENSG00000232815.1	-0.043179026	0.016832334	0.47460154	**DUX4L50**
ENSG00000227232.5	-0.045092177	0.016857826	0.47460154	**WASH7P**
ENSG00000107951.15	-0.014221626	0.016885326	0.47460154	**MTPAP**
ENSG00000120333.4	0.017619213	0.016893852	0.47460154	**MRPS14**
ENSG00000259719.6	0.055290267	0.017003242	0.477021184	**LINC02284**
ENSG00000176903.5	-0.019517387	0.017101065	0.477651233	**PNMA1**
ENSG00000164764.11	-0.036210697	0.017103532	0.477651233	**SBSPON**
ENSG00000259717.1	0.056001868	0.017107606	0.477651233	**LINC00677**
ENSG00000104671.8	0.039620288	0.01713481	0.477651233	**DCTN6**
ENSG00000028116.18	0.031922465	0.017142155	0.477651233	**VRK2**
ENSG00000170128.5	-0.077579195	0.017223112	0.479255889	**GPR25**
ENSG00000285420.1	-0.030199793	0.017251566	0.479397163	**PDGFRL2P**
ENSG00000135472.9	0.132799077	0.017277398	0.479465337	**FAIM2**
ENSG00000185267.10	0.047971984	0.017382367	0.481726462	**CDNF**
ENSG00000130749.10	-0.01760972	0.017450507	0.482962205	**ZC3H4**
ENSG00000235776.2	0.073597346	0.017535115	0.484649775	**AC000089.3**
ENSG00000205213.14	0.061266565	0.017594003	0.485152335	**LGR4**
ENSG00000134440.12	0.013079162	0.017600611	0.485152335	**NARS1**
ENSG00000260565.7	0.029435985	0.017692832	0.487039738	**ERVK13-1**
ENSG00000204311.15	-0.041324302	0.017723196	0.48722158	**PJVK**
ENSG00000118420.17	0.0270609	0.017961164	0.487376693	**UBE3D**
ENSG00000163106.11	0.094226569	0.017981005	0.487376693	**HPGDS**
ENSG00000279656.1	0.074653092	0.018006216	0.487376693	**RP11-298I3.6**

ENSG00000115275.14	-0.017143633	0.018009615	0.487376693	**MOGS**
ENSG00000213373.7	-0.046388917	0.018017618	0.487376693	**LINC00671**
ENSG00000183155.5	0.011152802	0.018024845	0.487376693	**RABIF**
ENSG00000103196.12	-0.039970428	0.018029836	0.487376693	**CRISPLD2**
ENSG00000211950.2	-0.083914538	0.018050952	0.487376693	**IGHV1-24**
ENSG00000196954.14	0.022410302	0.018073409	0.487376693	**CASP4**
ENSG00000170275.15	0.018411178	0.018101865	0.487376693	**CRTAP**
ENSG00000163249.13	0.015044379	0.018172656	0.487376693	**CCNYL1**
ENSG00000105708.9	0.028764211	0.018172914	0.487376693	**ZNF14**
ENSG00000223482.8	-0.040731245	0.018189972	0.487376693	**NUTM2A-AS1**
ENSG00000240710.1	0.054228608	0.018204628	0.487376693	**RP11-430C7.4**
ENSG00000073169.14	-0.021719383	0.018231	0.487376693	**SELENOO**
ENSG00000188322.8	-0.02675255	0.018243071	0.487376693	**SBK1**
ENSG00000090554.13	-0.029883669	0.018246977	0.487376693	**FLT3LG**
ENSG00000123989.14	-0.036651609	0.018250985	0.487376693	**CHPF**
ENSG00000246250.3	-0.081608991	0.018279009	0.487376693	**RP11-613D13.5**
ENSG00000244405.8	-0.051884488	0.018284755	0.487376693	**ETV5**
ENSG00000128262.8	0.225131578	0.018343434	0.487376693	**POM121L9P**
ENSG00000134056.12	0.023604544	0.018372787	0.487376693	**MRPS36**
ENSG00000132361.18	-0.022941207	0.018388754	0.487376693	**CLUH**
ENSG00000277938.1	-0.045382014	0.018394551	0.487376693	**RP5-965G21.3**
ENSG00000225450.1	-0.042740141	0.018407297	0.487376693	**RP3-508I15.14**
ENSG00000127580.17	-0.023177827	0.018426343	0.487376693	**WDR24**
ENSG00000148600.15	-0.049879405	0.018440934	0.487376693	**CDHR1**
ENSG00000130748.7	-0.043801695	0.018463681	0.487376693	**TMEM160**
ENSG00000141378.15	0.022132492	0.018480558	0.487376693	**PTRH2**
ENSG00000235119.1	0.054474975	0.01849695	0.487376693	**RP11-9M16.2**
ENSG00000261136.1	-0.025803023	0.018502474	0.487376693	**RP11-37C7.3**
ENSG00000173660.12	0.032146679	0.018502582	0.487376693	**UQCRH**
ENSG00000126461.15	-0.024520608	0.018552304	0.487376693	**SCAF1**
ENSG00000131153.9	-0.057872068	0.018571605	0.487376693	**GINS2**
ENSG00000169919.17	-0.017428073	0.018614115	0.487376693	**GUSB**
ENSG00000106245.11	0.018089056	0.018614604	0.487376693	**BUD31**
ENSG00000261644.2	0.024629756	0.018673509	0.487376693	**CYLD-AS1**
ENSG00000140932.10	0.041960149	0.018696501	0.487376693	**CMTM2**
ENSG00000248641.1	-0.091002448	0.018704353	0.487376693	**HMGA1P2**
ENSG00000280286.1	-0.092382175	0.018714921	0.487376693	**FRG1KP**
ENSG00000138801.9	0.031513722	0.018722708	0.487376693	**PAPSS1**
ENSG00000244273.1	0.10379988	0.018737078	0.487376693	**PGBD4P1**
ENSG00000250687.6	0.076265355	0.018769024	0.487376693	**NAIPP1**
ENSG00000214975.4	0.055020746	0.018797184	0.487376693	**PPIAP29**
ENSG00000135929.9	-0.055505265	0.018798272	0.487376693	**CYP27A1**
ENSG00000272799.1	0.060902911	0.018872364	0.48797525	**RP11-474N24.6**
ENSG00000137720.8	0.037856794	0.018879636	0.48797525	**C11orf1**
ENSG00000088247.18	-0.017465896	0.018892742	0.48797525	**KHSRP**
ENSG00000185839.3	0.075403571	0.018919897	0.488061938	**RP4-592A1.2**
ENSG00000131475.7	0.019172805	0.018966582	0.48865158	**VPS25**

ENSG00000271204.1	-0.102841577	0.019025035	0.489436921	**RP11-138A9.1**
ENSG00000246363.3	0.079472478	0.019044795	0.489436921	**LINC02458**
ENSG00000174151.15	0.01009863	0.019152135	0.491207026	**CYB561D1**
ENSG00000078142.13	0.012455969	0.01923219	0.491207026	**PIK3C3**
ENSG00000169032.10	-0.013601901	0.019241729	0.491207026	**MAP2K1**
ENSG00000284735.1	-0.065069528	0.019252979	0.491207026	**RP4-736L20.4**
ENSG00000158717.11	-0.020659286	0.019270812	0.491207026	**RNF166**
ENSG00000184602.6	0.017582181	0.019276695	0.491207026	**SNN**
ENSG00000265625.1	-0.047283844	0.019283116	0.491207026	**RP11-68I3.11**
ENSG00000174276.7	-0.034857999	0.019306925	0.491207026	**ZNHIT2**
ENSG00000119632.4	0.032531525	0.019354818	0.491207026	**IFI27L2**
ENSG00000112984.12	-0.074173713	0.019360524	0.491207026	**KIF20A**
ENSG00000169228.14	-0.028753829	0.019390063	0.491207026	**RAB24**
ENSG00000163513.19	0.01441154	0.019401097	0.491207026	**TGFBR2**
ENSG00000213939.4	0.062613155	0.019499914	0.492173873	**RP11-314A20.1**
ENSG00000188000.6	-0.332826612	0.019540593	0.492173873	**OR7D2**
ENSG00000065057.9	-0.026268905	0.019554464	0.492173873	**NTHL1**
ENSG00000239523.6	0.040173585	0.01955911	0.492173873	**MYLK-AS1**
ENSG00000011132.12	-0.019609033	0.019561906	0.492173873	**APBA3**
ENSG00000109971.14	0.016181064	0.019600973	0.492173873	**HSPA8**
ENSG00000273265.1	0.042723659	0.019640962	0.492173873	**CNNM3-DT**
ENSG00000215030.5	0.041955831	0.019648418	0.492173873	**RPL13P12**
ENSG00000170482.17	-0.038890997	0.019665384	0.492173873	**SLC23A1**
ENSG00000138772.13	0.054855173	0.019711987	0.492173873	**ANXA3**
ENSG00000172260.15	-0.079115944	0.019754536	0.492173873	**NEGR1**
ENSG00000083223.18	0.023715681	0.019759924	0.492173873	**TUT7**
ENSG00000269836.1	-0.075565079	0.019772861	0.492173873	**CTD-3032J10.4**
ENSG00000111142.14	0.02171072	0.019775272	0.492173873	**METAP2**
ENSG00000134326.12	-0.103410815	0.019819091	0.492619852	**CMPK2**
ENSG00000162063.13	-0.026452791	0.019841233	0.492619852	**CCNF**
ENSG00000145741.16	0.024409241	0.019921609	0.494017364	**BTF3**
ENSG00000233013.10	0.053860529	0.019949799	0.494118945	**FAM157B**
ENSG00000154930.15	-0.012797006	0.020074584	0.496362193	**ACSS1**
ENSG00000281195.1	-0.06596445	0.020088776	0.496362193	**RP11-236P2.2**
ENSG00000136045.12	0.015176715	0.020245455	0.49963152	**PWP1**
ENSG00000112118.20	-0.015947542	0.020300042	0.500376512	**MCM3**
ENSG00000127586.17	-0.025784821	0.02036557	0.50050627	**CHTF18**
ENSG00000115561.16	0.014900809	0.020369197	0.50050627	**CHMP3**
ENSG00000213178.3	0.069386872	0.020406339	0.50050627	**RPL22P1**
ENSG00000091640.8	0.024474727	0.020411396	0.50050627	**SPAG7**
ENSG00000168994.14	0.0432873	0.020427333	0.50050627	**PXDC1**
ENSG00000226976.3	0.042396079	0.02051415	0.501048246	**COX6A1P2**
ENSG00000169087.11	0.019427156	0.020514856	0.501048246	**HSPBAP1**
ENSG00000185187.13	-0.02466268	0.020522749	0.501048246	**SIGIRR**
ENSG00000118308.15	0.018902876	0.020590503	0.501565308	**IRAG2**
ENSG00000211898.8	-0.074592114	0.020592841	0.501565308	**IGHD**
ENSG00000260417.1	-0.092414993	0.020660267	0.502610623	**CTD-2542L18.1**

ENSG00000136810.13	0.040629838	0.020740958	0.503666588	**TXN**
ENSG00000126709.16	-0.095425013	0.020752792	0.503666588	**IFI6**
ENSG00000155465.20	0.022652523	0.020793244	0.503733184	**SLC7A7**
ENSG00000240682.10	0.018674135	0.02083533	0.503733184	**ISY1**
ENSG00000136286.16	-0.021420974	0.020846687	0.503733184	**MYO1G**
ENSG00000130338.13	-0.017926624	0.020878559	0.503733184	**TULP4**
ENSG00000143476.18	-0.066490603	0.020893107	0.503733184	**DTL**
ENSG00000140612.14	0.018196044	0.020902913	0.503733184	**SEC11A**
ENSG00000148773.14	-0.057494345	0.020966371	0.504669415	**MKI67**
ENSG00000151304.6	0.02711396	0.021045265	0.505974558	**SRFBP1**
ENSG00000061794.13	0.015061962	0.021076416	0.506130138	**MRPS35**
ENSG00000253636.1	-0.042618503	0.021140166	0.506796452	**RP11-531A24.5**
ENSG00000167136.7	-0.040506688	0.021167591	0.506796452	**ENDOG**
ENSG00000140395.9	0.015682067	0.021178299	0.506796452	**WDR61**
ENSG00000223609.11	0.100411823	0.021256776	0.50751239	**HBD**
ENSG00000136231.14	0.064837853	0.021305396	0.50751239	**IGF2BP3**
ENSG00000181524.6	0.048586211	0.021357735	0.50751239	**RPL24P4**
ENSG00000251393.4	0.038866222	0.02136103	0.50751239	**RP1-240K6.3**
ENSG00000070831.17	0.011594173	0.021378495	0.50751239	**CDC42**
ENSG00000162227.8	-0.01856349	0.021386304	0.50751239	**TAF6L**
ENSG00000263766.5	-0.039405815	0.021401496	0.50751239	**KPNB1-DT**
ENSG00000163430.12	0.071371814	0.021407174	0.50751239	**FSTL1**
ENSG00000107485.18	-0.024394914	0.021457716	0.50751239	**GATA3**
ENSG00000138669.10	0.068714608	0.021503278	0.50751239	**PRKG2**
ENSG00000113845.10	0.016342887	0.021543382	0.50751239	**TIMMDC1**
ENSG00000260193.1	-0.039460575	0.021561547	0.50751239	**LINC02846**
ENSG00000279203.1	-0.05323295	0.021563106	0.50751239	**AC005785.5**
ENSG00000187135.7	-0.20437679	0.021597383	0.50751239	**VSTM2B**
ENSG00000179840.5	-0.029594785	0.021600267	0.50751239	**PIK3CD-AS1**
ENSG00000119421.7	0.02316358	0.02160417	0.50751239	**NDUFA8**
ENSG00000286873.1	0.046762456	0.021637182	0.507706324	**RP11-395A13.3**
ENSG00000198729.5	-0.119432162	0.021703543	0.508681435	**PPP1R14C**
ENSG00000145391.14	0.023231942	0.021767681	0.509500531	**SETD7**
ENSG00000284830.1	-0.066749602	0.021813477	0.509500531	**RP4-773A18.8**
ENSG00000073331.18	0.02323813	0.021849183	0.509500531	**ALPK1**
ENSG00000271590.1	0.101086005	0.021851689	0.509500531	**RP11-181E10.3**
ENSG00000149499.12	-0.018210565	0.021866594	0.509500531	**EML3**
ENSG00000233608.4	-0.16779406	0.021887554	0.509500531	**TWIST2**
ENSG00000163357.11	-0.044137651	0.02191973	0.509670997	**DCST1**
ENSG00000161618.10	-0.029550189	0.021999331	0.510942558	**ALDH16A1**
ENSG00000076513.17	0.013841279	0.022134437	0.513207931	**ANKRD13A**
ENSG00000140319.11	0.027312082	0.022146919	0.513207931	**SRP14**
ENSG00000164904.18	0.061835011	0.022241029	0.513284574	**ALDH7A1**
ENSG00000196453.8	-0.02176081	0.022259966	0.513284574	**ZNF777**
ENSG00000267454.6	-0.036154789	0.022270767	0.513284574	**ZNF582-DT**
ENSG00000141428.17	0.020861413	0.022293109	0.513284574	**C18orf21**
ENSG00000109606.13	0.013568549	0.02230043	0.513284574	**DHX15**

ENSG00000258301.3	0.04074448	0.022349897	0.513284574	**VASH1-AS1**
ENSG00000117691.10	0.02852936	0.022361088	0.513284574	**NENF**
ENSG00000123739.11	0.025133663	0.022362789	0.513284574	**PLA2G12A**
ENSG00000164930.12	-0.05214245	0.022375483	0.513284574	**FZD6**
ENSG00000163191.6	0.028057871	0.022704925	0.516073992	**S100A11**
ENSG00000012171.20	-0.048214971	0.022708186	0.516073992	**SEMA3B**
ENSG00000237172.4	-0.028226397	0.022713673	0.516073992	**B3GNT9**
ENSG00000119446.14	0.013066865	0.022728353	0.516073992	**RBM18**
ENSG00000262663.1	-0.115740648	0.022730181	0.516073992	**RP11-497H17.1**
ENSG00000271851.1	-0.037896004	0.022751119	0.516073992	**RP11-565F19.2**
ENSG00000240972.2	-0.079537383	0.022766121	0.516073992	**MIF**
ENSG00000108551.5	-0.060799592	0.022789681	0.516073992	**RASD1**
ENSG00000273076.1	-0.044630176	0.022790936	0.516073992	**RP3-508I15.22**
ENSG00000120437.9	0.019467662	0.022821886	0.516073992	**ACAT2**
ENSG00000265354.4	0.019907311	0.022861061	0.516073992	**TIMM23**
ENSG00000226608.3	0.039868069	0.022887317	0.516073992	**FTLP3**
ENSG00000197381.17	-0.019194103	0.022906659	0.516073992	**ADARB1**
ENSG00000089127.15	-0.067568697	0.022909236	0.516073992	**OAS1**
ENSG00000275395.6	-0.058094781	0.022922426	0.516073992	**FCGBP**
ENSG00000211794.3	-0.057101819	0.022923066	0.516073992	**TRAV12-3**
ENSG00000125900.13	0.035930688	0.022945541	0.516073992	**SIRPD**
ENSG00000222009.8	-0.03230229	0.022959798	0.516073992	**BTBD19**
ENSG00000213214.5	-0.09812998	0.02297795	0.516073992	**ARHGEF35**
ENSG00000231851.6	-0.052756763	0.023081375	0.516073992	**MRPS9-AS1**
ENSG00000162623.16	0.023728541	0.023091357	0.516073992	**TYW3**
ENSG00000254691.1	-0.073361043	0.023102229	0.516073992	**RP11-91P24.5**
ENSG00000123374.11	-0.015025219	0.023121988	0.516073992	**CDK2**
ENSG00000231113.2	-0.018059465	0.02312541	0.516073992	**RP3-475N16.1**
ENSG00000249437.8	0.054066526	0.023126195	0.516073992	**NAIP**
ENSG00000169299.14	0.019009944	0.02321704	0.516856941	**PGM2**
ENSG00000287978.1	0.06132255	0.023228165	0.516856941	**CH17-408M7.2**
ENSG00000188152.13	-0.053947898	0.023236888	0.516856941	**NUTM2G**
ENSG00000272402.1	-0.050868596	0.023298912	0.517675074	**RP1-30M3.6**
ENSG00000156110.15	-0.03376171	0.023373734	0.5181596	**ADK**
ENSG00000260742.1	0.052763214	0.023383483	0.5181596	**RP11-366L5.1**
ENSG00000241370.6	-0.024315629	0.023396518	0.5181596	**RPP21**
ENSG00000124383.9	0.015133779	0.02346045	0.518210095	**MPHOSPH10**
ENSG00000123700.5	0.035251327	0.023477022	0.518210095	**KCNJ2**
ENSG00000197935.7	0.065562359	0.023477205	0.518210095	**ZNF311**
ENSG00000151778.11	0.075838807	0.023524667	0.518210095	**SERP2**
ENSG00000187730.9	0.157204633	0.023525141	0.518210095	**GABRD**
ENSG00000169122.11	-0.036689817	0.023564082	0.518510942	**FAM110B**
ENSG00000197043.14	-0.020773478	0.023706096	0.520586656	**ANXA6**
ENSG00000213625.9	0.016109823	0.023754454	0.520586656	**LEPROT**
ENSG00000272650.1	-0.049803417	0.023776525	0.520586656	**RP11-571I18.4**
ENSG00000105373.19	-0.027782153	0.023778965	0.520586656	**NOP53**
ENSG00000155463.14	0.014353937	0.023785337	0.520586656	**OXA1L**

ENSG00000166024.14	0.012095482	0.023825236	0.520903995	**R3HCC1L**
ENSG00000128342.5	-0.072452498	0.023924062	0.522507621	**LIF**
ENSG00000118432.13	-0.08117223	0.023975656	0.523077397	**CNR1**
ENSG00000182013.18	-0.319693594	0.024007613	0.523217984	**PNMA8A**
ENSG00000178685.14	-0.032259526	0.024084133	0.523453546	**PARP10**
ENSG00000115317.12	-0.015644333	0.024106706	0.523453546	**HTRA2**
ENSG00000169756.16	0.019582671	0.024129638	0.523453546	**LIMS1**
ENSG00000162441.12	0.010103106	0.024161343	0.523453546	**LZIC**
ENSG00000170734.12	-0.01565714	0.02416343	0.523453546	**POLH**
ENSG00000278997.1	-0.028167881	0.024171568	0.523453546	**RP11-131M11.2**
ENSG00000065534.19	0.043455987	0.024201359	0.523545847	**MYLK**
ENSG00000126524.10	0.020022939	0.024247659	0.523575581	**SBDS**
ENSG00000254363.6	-0.038958461	0.024337769	0.523575581	**CTB-131B5.5**
ENSG00000160255.18	-0.023381385	0.024341926	0.523575581	**ITGB2**
ENSG00000065154.12	0.018981643	0.02437248	0.523575581	**OAT**
ENSG00000152784.16	-0.023716615	0.024391442	0.523575581	**PRDM8**
ENSG00000203760.9	0.050468294	0.024392199	0.523575581	**CENPW**
ENSG00000224897.8	0.071303932	0.024406815	0.523575581	**POT1-AS1**
ENSG00000237017.1	-0.041026612	0.024406976	0.523575581	**AC012314.8**
ENSG00000181284.3	-0.023521221	0.024511217	0.525262327	**TMEM102**
ENSG00000280388.1	-0.045879101	0.024590396	0.525638717	**RP11-229D13.3**
ENSG00000117597.18	0.016587669	0.024592378	0.525638717	**UTP25**
ENSG00000231128.6	-0.066782293	0.024641981	0.525638717	**RP5-1073O3.2**
ENSG00000149823.9	-0.022015708	0.024646021	0.525638717	**VPS51**
ENSG00000256673.1	-0.083481833	0.024712047	0.525638717	**RP11-599J14.2**
ENSG00000286430.1	0.057863537	0.024719589	0.525638717	**RP11-524F11.6**
ENSG00000136436.15	0.013202998	0.024725759	0.525638717	**CALCOCO2**
ENSG00000143486.16	0.013315577	0.024773285	0.525638717	**EIF2D**
ENSG00000108379.10	0.124127227	0.024779881	0.525638717	**WNT3**
ENSG00000251000.1	-0.050990257	0.024785091	0.525638717	**GGCTP1**
ENSG00000130402.13	-0.024295473	0.024885158	0.526540827	**ACTN4**
ENSG00000008988.11	0.031470089	0.024893281	0.526540827	**RPS20**
ENSG00000055211.14	0.015850979	0.024904652	0.526540827	**GINM1**
ENSG00000250222.2	0.070065314	0.024993603	0.527877255	**CTC-338M12.5**
ENSG00000023697.13	0.016045205	0.025048975	0.528502441	**DERA**
ENSG00000278599.6	-0.218505933	0.025157332	0.530243124	**TBC1D3E**
ENSG00000205238.10	-0.066140517	0.02519384	0.530369341	**SPDYE2**
ENSG00000204370.13	0.01650294	0.025215043	0.530369341	**SDHD**
ENSG00000105327.18	-0.026697474	0.025267955	0.530937717	**BBC3**
ENSG00000121749.16	0.024026871	0.025379747	0.531132201	**TBC1D15**
ENSG00000141391.14	-0.072782058	0.025390067	0.531132201	**PRELID3A**
ENSG00000137494.14	-0.020268207	0.025405576	0.531132201	**ANKRD42**
ENSG00000131779.11	0.01397658	0.025415506	0.531132201	**PEX11B**
ENSG00000260711.2	-0.026986421	0.025428719	0.531132201	**RP11-747H7.3**
ENSG00000118946.12	-0.191845576	0.025442457	0.531132201	**PCDH17**
ENSG00000111665.12	-0.045669777	0.025472033	0.531132201	**CDCA3**
ENSG00000152147.11	0.017275271	0.025484401	0.531132201	**GEMIN6**

ENSG00000238035.8	0.065306413	0.025566272	0.531402334	**AC138035.2**
ENSG00000251600.8	0.063346926	0.0255836	0.531402334	**RP11-673E1.1**
ENSG00000135898.10	0.04379567	0.02560383	0.531402334	**GPR55**
ENSG00000215006.4	-0.074694897	0.025648473	0.531402334	**CHCHD2P2**
ENSG00000104979.9	0.021790717	0.025691334	0.531402334	**C19orf53**
ENSG00000279759.1	-0.038300744	0.025693932	0.531402334	**CTC-425O23.5**
ENSG00000232648.4	-0.086241451	0.02572016	0.531402334	**RP11-367N14.2**
ENSG00000267305.2	0.251208967	0.025726325	0.531402334	**RP11-378J18.10**
ENSG00000171084.15	0.063376078	0.025737412	0.531402334	**FAM86JP**
ENSG00000143398.20	0.01151368	0.025756481	0.531402334	**PIP5K1A**
ENSG00000163617.11	0.026635707	0.025853121	0.532860107	**CCDC191**
ENSG00000103495.14	-0.017474354	0.025883319	0.5329469	**MAZ**
ENSG00000164406.8	-0.027569683	0.025947517	0.533732875	**LEAP2**
ENSG00000115526.11	-0.028697041	0.025979984	0.533865249	**CHST10**
ENSG00000122870.12	0.096519807	0.02607539	0.533972332	**BICC1**
ENSG00000139641.13	-0.019122145	0.026084693	0.533972332	**ESYT1**
ENSG00000173812.11	0.018017096	0.026085218	0.533972332	**EIF1**
ENSG00000117399.14	-0.068927461	0.026096634	0.533972332	**CDC20**
ENSG00000116288.13	0.020191892	0.026115382	0.533972332	**PARK7**
ENSG00000173137.12	-0.022603898	0.026231059	0.535569192	**ADCK5**
ENSG00000164283.13	-0.089396903	0.026245711	0.535569192	**ESM1**
ENSG00000228775.8	0.047467076	0.026332538	0.53680685	**WEE2-AS1**
ENSG00000261159.1	-0.080643245	0.026374874	0.53713596	**RP11-723O4.9**
ENSG00000161036.13	-0.02745456	0.02640454	0.537206644	**LRWD1**
ENSG00000163644.15	-0.022383865	0.02648653	0.538340683	**PPM1K**
ENSG00000171223.6	-0.027811179	0.026590576	0.538761659	**JUNB**
ENSG00000101255.12	-0.030146546	0.026647648	0.538761659	**TRIB3**
ENSG00000213513.3	-0.108433302	0.026661629	0.538761659	**IMPDH1P5**
ENSG00000142409.6	-0.025295417	0.026695334	0.538761659	**ZNF787**
ENSG00000111331.14	-0.092768324	0.026707151	0.538761659	**OAS3**
ENSG00000228474.6	0.02828859	0.026710063	0.538761659	**OST4**
ENSG00000260947.1	0.086425473	0.02672926	0.538761659	**RP11-384P7.7**
ENSG00000004864.14	0.016034667	0.026767428	0.538761659	**SLC25A13**
ENSG00000180448.11	-0.019993586	0.026793677	0.538761659	**ARHGAP45**
ENSG00000146731.11	0.010249196	0.026812383	0.538761659	**CCT6A**
ENSG00000184432.11	0.00943223	0.026814042	0.538761659	**COPB2**
ENSG00000161677.12	-0.031952083	0.026822491	0.538761659	**JOSD2**
ENSG00000267198.1	0.143096673	0.02687338	0.539255655	**RP11-798G7.6**
ENSG00000162913.10	0.040775866	0.026934182	0.539284705	**OBSCN-AS1**
ENSG00000125952.20	0.013714026	0.026937899	0.539284705	**MAX**
ENSG00000143622.11	0.017813875	0.026953717	0.539284705	**RIT1**
ENSG00000266677.1	0.060945246	0.027007964	0.539843402	**RP11-258F1.1**
ENSG00000175110.13	0.024888627	0.027073213	0.5406207	**MRPS22**
ENSG00000177301.16	0.037560563	0.0271012	0.540647657	**KCNA2**
ENSG00000168872.17	-0.016081588	0.027127289	0.540647657	**DDX19A**
ENSG00000140093.10	0.119665941	0.027163308	0.540839918	**SERPINA10**
ENSG00000165898.14	0.020945927	0.027222602	0.541460865	**ISCA2**

ENSG00000105926.16	-0.037598964	0.027267801	0.541460865	**MPP6**
ENSG00000287884.1	-0.072672057	0.027412418	0.541460865	**LLNLR-229A6.1**
ENSG00000158715.6	0.068134599	0.027436799	0.541460865	**SLC45A3**
ENSG00000167700.9	-0.027700432	0.027437623	0.541460865	**MFSD3**
ENSG00000225972.1	0.101858304	0.027454457	0.541460865	**MTND1P23**
ENSG00000125901.6	-0.020442792	0.027484097	0.541460865	**MRPS26**
ENSG00000267369.1	-0.025135274	0.027502795	0.541460865	**TAF5LP1**
ENSG00000287839.1	0.030840748	0.027531383	0.541460865	**RP11-29H23.8**
ENSG00000161513.12	-0.025871088	0.027571477	0.541460865	**FDXR**
ENSG00000237980.2	0.091716866	0.027621522	0.541460865	**LINC02773**
ENSG00000279570.1	-0.066701166	0.027642935	0.541460865	**CTD-2357A8.2**
ENSG00000159692.16	-0.014017079	0.027676409	0.541460865	**CTBP1**
ENSG00000167595.15	-0.018648798	0.02768666	0.541460865	**PROSER3**
ENSG00000152404.15	0.030438806	0.027694308	0.541460865	**CWF19L2**
ENSG00000100429.18	-0.024436969	0.027694661	0.541460865	**HDAC10**
ENSG00000118939.18	0.042440561	0.027699836	0.541460865	**UCHL3**
ENSG00000255240.6	0.052037965	0.027727907	0.541460865	**RP11-142C4.6**
ENSG00000166851.15	-0.032046726	0.027774165	0.541460865	**PLK1**
ENSG00000222005.9	0.067138074	0.027785388	0.541460865	**LINC01118**
ENSG00000129083.13	0.01296206	0.027794883	0.541460865	**COPB1**
ENSG00000154330.13	0.10842467	0.027862841	0.541460865	**PGM5**
ENSG00000167034.10	-0.131147737	0.027899227	0.541460865	**NKX3-1**
ENSG00000105613.10	-0.05252987	0.027902726	0.541460865	**MAST1**
ENSG00000227252.2	-0.05198364	0.027905167	0.541460865	**AC105760.2**
ENSG00000163803.13	-0.041377347	0.027907116	0.541460865	**PLB1**
ENSG00000258940.2	0.06987569	0.02790736	0.541460865	**RP11-407N17.5**
ENSG00000129518.9	0.017377647	0.028051402	0.543297957	**EAPP**
ENSG00000196873.16	-0.035247975	0.028055029	0.543297957	**CBWD3**
ENSG00000136870.11	0.016140522	0.028097602	0.543609078	**ZNF189**
ENSG00000131381.12	0.010115266	0.028129739	0.543717888	**RBSN**
ENSG00000198857.3	-0.104590053	0.02816644	0.543914649	**HSD3BP5**
ENSG00000116871.16	-0.020808024	0.028227682	0.543948946	**MAP7D1**
ENSG00000109911.19	0.015204904	0.028229292	0.543948946	**ELP4**
ENSG00000175505.11	-0.030992163	0.028247788	0.543948946	**CLCF1**
ENSG00000169398.19	0.028255303	0.028374419	0.545301712	**PTK2**
ENSG00000148925.11	0.018989721	0.028393438	0.545301712	**BTBD10**
ENSG00000205078.6	-0.050964637	0.028404845	0.545301712	**SYCE1L**
ENSG00000131941.8	-0.057133592	0.028424397	0.545301712	**RHPN2**
ENSG00000166946.14	0.014482147	0.028504135	0.546320368	**CCNDBP1**
ENSG00000181778.5	0.051258296	0.028591458	0.546802067	**TMEM252**
ENSG00000121542.12	0.017430758	0.028593365	0.546802067	**SEC22A**
ENSG00000163751.4	0.080242367	0.028670228	0.546802067	**CPA3**
ENSG00000259366.1	0.053141256	0.02870861	0.546802067	**CTD-2647L4.4**
ENSG00000143226.15	0.021693441	0.028710156	0.546802067	**FCGR2A**
ENSG00000251075.2	-0.083195195	0.028734449	0.546802067	**HTT-AS**
ENSG00000253522.6	-0.03233244	0.028744482	0.546802067	**MIR3142HG**
ENSG00000134321.13	-0.129569155	0.028832041	0.546802067	**RSAD2**

ENSG00000131016.17	0.087370232	0.028853888	0.546802067	**AKAP12**
ENSG00000154269.15	0.074601428	0.028911578	0.546802067	**ENPP3**
ENSG00000242114.6	-0.032637999	0.028928715	0.546802067	**MTFP1**
ENSG00000229344.1	0.133914005	0.028942459	0.546802067	**MTCO2P12**
ENSG00000149483.12	0.01246172	0.028962605	0.546802067	**TMEM138**
ENSG00000143319.17	0.010476721	0.028993886	0.546802067	**ISG20L2**
ENSG00000204179.11	0.110597431	0.029029413	0.546802067	**PTPN20**
ENSG00000234338.1	0.118675642	0.02905308	0.546802067	**RP11-797H7.1**
ENSG00000272625.1	0.043291799	0.029061278	0.546802067	**RP11-737O24.5**
ENSG00000137965.11	-0.101253929	0.029067493	0.546802067	**IFI44**
ENSG00000278356.1	0.076085889	0.029071279	0.546802067	**RP11-372B4.3**
ENSG00000133275.16	-0.019507785	0.029075152	0.546802067	**CSNK1G2**
ENSG00000254473.1	0.062395127	0.029104934	0.546802067	**RP11-522I20.3**
ENSG00000110697.13	-0.017890234	0.02911585	0.546802067	**PITPNM1**
ENSG00000159884.12	-0.036997443	0.02917072	0.547192218	**CCDC107**
ENSG00000169359.16	-0.019960463	0.02919377	0.547192218	**SLC33A1**
ENSG00000059573.9	-0.016431105	0.029278917	0.547192218	**ALDH18A1**
ENSG00000117133.11	0.022935931	0.029285487	0.547192218	**RPF1**
ENSG00000254427.2	0.05802039	0.029294152	0.547192218	**LINC02696**
ENSG00000074319.13	0.012691156	0.029296716	0.547192218	**TSG101**
ENSG00000087302.9	0.018287022	0.029339849	0.5474992	**RTRAF**
ENSG00000091106.19	0.020451171	0.029394514	0.548020633	**NLRC4**
ENSG00000198464.14	0.025701237	0.029434758	0.548037711	**ZNF480**
ENSG00000131899.11	-0.014168123	0.029448876	0.548037711	**LLGL1**
ENSG00000185641.6	0.050115933	0.029571883	0.549827894	**CTD-2287O16.1**
ENSG00000171763.20	-0.020941492	0.02963486	0.550499739	**SPATA5L1**
ENSG00000130299.17	-0.018541665	0.029668534	0.550596898	**GTPBP3**
ENSG00000168071.22	-0.02329696	0.029693786	0.550596898	**CCDC88B**
ENSG00000211952.3	-0.098220068	0.029743038	0.55066423	**IGHV4-28**
ENSG00000111319.13	0.036728013	0.02975112	0.55066423	**SCNN1A**
ENSG00000166938.13	0.014122422	0.029880231	0.552126279	**DIS3L**
ENSG00000253005.1	-0.056268075	0.029932854	0.552126279	**RNU6-176P**
ENSG00000276071.1	-0.058379753	0.029938672	0.552126279	**CTD-3234P18.6**
ENSG00000100721.11	-0.070718154	0.029949225	0.552126279	**TCL1A**
ENSG00000128789.21	0.014682223	0.029964723	0.552126279	**PSMG2**
ENSG00000286340.1	-0.080378362	0.030085913	0.553861674	**RP11-477E3.3**
ENSG00000173626.10	0.073416879	0.030136977	0.554304153	**TRAPPC3L**
ENSG00000260851.7	0.088965579	0.030244417	0.554545538	**RP11-403P17.5**
ENSG00000137393.10	0.024761636	0.030269866	0.554545538	**RNF144B**
ENSG00000135900.4	0.015661286	0.030303031	0.554545538	**MRPL44**
ENSG00000145700.10	0.078074202	0.030308696	0.554545538	**ANKRD31**
ENSG00000230068.2	-0.069892396	0.03031557	0.554545538	**CDC42-IT1**
ENSG00000128050.9	0.015948415	0.030350879	0.554545538	**PAICS**
ENSG00000141577.14	-0.023500186	0.030384348	0.554545538	**CEP131**
ENSG00000185532.20	0.090253347	0.030406967	0.554545538	**PRKG1**
ENSG00000203910.9	-0.087198203	0.030430575	0.554545538	**C1orf146**
ENSG00000231752.6	0.062568491	0.030431887	0.554545538	**EMBP1**

ENSG00000063245.15	-0.022414588	0.030454106	0.554545538	**EPN1**
ENSG00000104866.11	-0.02086925	0.030506407	0.554545538	**PPP1R37**
ENSG00000250786.2	-0.107512385	0.030551268	0.554545538	**SNHG18**
ENSG00000084072.17	0.026299747	0.030553113	0.554545538	**PPIE**
ENSG00000152253.9	-0.099035251	0.030555708	0.554545538	**SPC25**
ENSG00000198604.11	0.02505813	0.030628479	0.554682029	**BAZ1A**
ENSG00000250850.2	-0.077162912	0.030659565	0.554682029	**RP11-297B17.3**
ENSG00000106113.19	-0.095510404	0.030661515	0.554682029	**CRHR2**
ENSG00000150967.18	-0.0388219	0.030671417	0.554682029	**ABCB9**
ENSG00000004766.17	0.022893513	0.030703146	0.554766624	**VPS50**
ENSG00000255776.1	-0.084991702	0.030851753	0.556257431	**VDAC2P2**
ENSG00000168497.5	0.042723392	0.030890768	0.556257431	**CAVIN2**
ENSG00000103472.10	-0.042544864	0.030893658	0.556257431	**RRN3P2**
ENSG00000168259.17	0.011664338	0.030894149	0.556257431	**DNAJC7**
ENSG00000165527.7	-0.010492667	0.031083251	0.558471204	**ARF6**
ENSG00000258515.1	-0.04091183	0.0310853	0.558471204	**RP11-203M5.7**
ENSG00000182196.14	-0.030470205	0.031098796	0.558471204	**ARL6IP4**
ENSG00000223508.5	0.055690119	0.031144009	0.558793824	**RPL23AP53**
ENSG00000224093.5	0.084784017	0.03119044	0.558894834	**BCAR3-AS1**
ENSG00000134910.14	-0.013922204	0.031204144	0.558894834	**STT3A**
ENSG00000225632.1	0.082998885	0.031253144	0.559284006	**RP5-997D24.3**
ENSG00000155368.17	0.038833811	0.031283176	0.559311743	**DBI**
ENSG00000068697.7	0.014725355	0.03135891	0.559311743	**LAPTM4A**
ENSG00000213445.10	-0.019616505	0.031359823	0.559311743	**SIPA1**
ENSG00000265975.1	0.02766793	0.031363785	0.559311743	**CTB-41I6.2**
ENSG00000214226.9	-0.024683973	0.031404004	0.559542418	**C17orf67**
ENSG00000138029.14	0.011658796	0.03143488	0.559606356	**HADHB**
ENSG00000111669.15	0.018229184	0.031463779	0.559635025	**TPI1**
ENSG00000086102.19	0.010167097	0.03160404	0.559892144	**NFX1**
ENSG00000109927.11	0.042051144	0.031606042	0.559892144	**TECTA**
ENSG00000171943.12	-0.027846439	0.031661162	0.559892144	**SRGAP2C**
ENSG00000125037.12	0.027582432	0.031668564	0.559892144	**EMC3**
ENSG00000119969.15	-0.031244382	0.031713823	0.559892144	**HELLS**
ENSG00000104325.7	0.015514084	0.031730178	0.559892144	**DECR1**
ENSG00000070423.18	-0.019372527	0.031743146	0.559892144	**RNF126**
ENSG00000237080.3	-0.04389622	0.031744981	0.559892144	**EHMT2-AS1**
ENSG00000125912.11	-0.020128362	0.031796708	0.559892144	**NCLN**
ENSG00000164142.16	0.049861682	0.031801781	0.559892144	**FAM160A1**
ENSG00000225331.1	-0.083105618	0.03180498	0.559892144	**LINC01678**
ENSG00000168003.18	-0.018764235	0.03181033	0.559892144	**SLC3A2**
ENSG00000276317.1	0.030065945	0.031833924	0.559892144	**RP11-93B14.10**
ENSG00000139746.16	-0.017221469	0.031903119	0.559892144	**RBM26**
ENSG00000102854.16	-0.250644637	0.03190761	0.559892144	**MSLN**
ENSG00000105146.13	-0.046894087	0.031915053	0.559892144	**AURKC**
ENSG00000211850.1	-0.079780084	0.031954996	0.560088045	**TRAJ39**
ENSG00000090263.16	0.031587368	0.031980842	0.560088045	**MRPS33**
ENSG00000225920.2	-0.065814167	0.032051397	0.560844761	**RIMKLBP2**

ENSG00000005238.20	0.015532001	0.032106448	0.561329098	**FAM214B**
ENSG00000130811.12	-0.018919913	0.032180626	0.561864034	**EIF3G**
ENSG00000126934.14	-0.019825271	0.032191839	0.561864034	**MAP2K2**
ENSG00000186615.11	0.044306515	0.03231586	0.562470112	**KTN1-AS1**
ENSG00000242252.2	-0.041326437	0.032325231	0.562470112	**BGLAP**
ENSG00000275432.1	-0.0699436	0.032325387	0.562470112	**CTD-3093B17.2**
ENSG00000137806.10	0.017138136	0.032405069	0.562470112	**NDUFAF1**
ENSG00000229474.8	-0.037224182	0.032407659	0.562470112	**PATL2**
ENSG00000167470.13	-0.020075215	0.032423183	0.562470112	**MIDN**
ENSG00000158941.16	-0.015946968	0.032429908	0.562470112	**CCAR2**
ENSG00000179965.12	-0.041804434	0.032477599	0.562470112	**ZNF771**
ENSG00000261783.1	-0.111997108	0.032506724	0.562470112	**RP11-252K23.2**
ENSG00000142552.8	-0.031084479	0.03252463	0.562470112	**RCN3**
ENSG00000014641.21	0.02191351	0.032530406	0.562470112	**MDH1**
ENSG00000286975.1	-0.062036323	0.032566555	0.562470112	**RP11-715F3.4**
ENSG00000120129.6	0.029472919	0.032609153	0.562470112	**DUSP1**
ENSG00000167895.15	-0.017341217	0.03261054	0.562470112	**TMC8**
ENSG00000145945.7	-0.027954958	0.032656649	0.562720339	**FAM50B**
ENSG00000174446.13	0.014513118	0.032700435	0.562720339	**SNAPC5**
ENSG00000077782.22	-0.042169658	0.032707365	0.562720339	**FGFR1**
ENSG00000259330.3	0.01614077	0.032827505	0.564313884	**INAFM2**
ENSG00000109321.11	0.110810345	0.032861655	0.564427819	**AREG**
ENSG00000157734.14	-0.04585639	0.032958261	0.565613398	**SNX22**
ENSG00000072422.17	0.048617196	0.0330079	0.565799874	**RHOBTB1**
ENSG00000246731.3	0.032044908	0.033024305	0.565799874	**MGC16275**
ENSG00000247137.9	0.033732875	0.033211709	0.567588902	**RP11-727A23.5**
ENSG00000170871.12	0.017171357	0.033240774	0.567588902	**KIAA0232**
ENSG00000146555.19	0.097249089	0.033249922	0.567588902	**SDK1**
ENSG00000214544.7	0.08864726	0.033255863	0.567588902	**GTF2IRD2P1**
ENSG00000211964.3	-0.088822797	0.033273617	0.567588902	**IGHV3-48**
ENSG00000039123.16	0.019952262	0.033324088	0.567588902	**MTREX**
ENSG00000279518.1	-0.050300763	0.033348194	0.567588902	**AC083843.4**
ENSG00000247271.7	0.023596867	0.03336065	0.567588902	**ZBED5-AS1**
ENSG00000169291.10	-0.08102739	0.033394186	0.567588902	**SHE**
ENSG00000104814.13	-0.019189782	0.033405491	0.567588902	**MAP4K1**
ENSG00000271554.1	-0.059914407	0.033520325	0.567989104	**RP4-665N4.8**
ENSG00000278267.1	-0.0818115	0.033566328	0.567989104	**MIR6859-1**
ENSG00000285006.1	0.050920124	0.033575431	0.567989104	**RP1-240K6.5**
ENSG00000142507.10	0.022156801	0.033577125	0.567989104	**PSMB6**
ENSG00000133943.21	0.017280673	0.03367862	0.567989104	**DGLUCY**
ENSG00000137364.5	0.021847274	0.033682983	0.567989104	**TPMT**
ENSG00000115539.14	0.020566404	0.033689457	0.567989104	**PDCL3**
ENSG00000178026.13	-0.040669469	0.033701665	0.567989104	**LRRC75B**
ENSG00000276832.2	-0.084984144	0.033739625	0.567989104	**CDRT15P6**
ENSG00000173915.16	0.024286994	0.033741459	0.567989104	**ATP5MK**
ENSG00000259042.3	0.071138715	0.033760715	0.567989104	**AE000661.50**
ENSG00000093009.11	-0.062301133	0.033789473	0.567989104	**CDC45**

ENSG00000104881.16	-0.027795708	0.033813592	0.567989104	**PPP1R13L**
ENSG00000073111.14	-0.029766612	0.033816788	0.567989104	**MCM2**
ENSG00000134780.10	0.03150453	0.033906066	0.568559393	**DAGLA**
ENSG00000176945.17	-0.062493585	0.033907806	0.568559393	**MUC20**
ENSG00000101290.14	0.015635839	0.033938288	0.568559393	**CDS2**
ENSG00000271795.1	-0.033917008	0.03399442	0.568559393	**CTC-251D13.1**
ENSG00000165660.8	0.010915882	0.034009097	0.568559393	**ABRAXAS2**
ENSG00000063244.13	-0.012089827	0.034022214	0.568559393	**U2AF2**
ENSG00000115758.13	0.025031117	0.034065839	0.568559393	**ODC1**
ENSG00000236320.4	0.035105996	0.034072532	0.568559393	**SLFN14**
ENSG00000279476.1	-0.090220821	0.034167789	0.569685372	**RP11-391L3.3**
ENSG00000197272.2	0.047850054	0.034269855	0.570533524	**IL27**
ENSG00000213533.13	0.023511773	0.034281572	0.570533524	**STIMATE**
ENSG00000100209.11	0.024242078	0.034322412	0.570533524	**HSCB**
ENSG00000138722.10	0.058254874	0.034330878	0.570533524	**MMRN1**
ENSG00000132341.12	0.015936991	0.034357758	0.570533524	**RAN**
ENSG00000125430.9	-0.021581817	0.034421163	0.570766717	**HS3ST3B1**
ENSG00000087206.17	0.012519664	0.034434583	0.570766717	**UIMC1**
ENSG00000136444.10	-0.011019247	0.034455295	0.570766717	**RSAD1**
ENSG00000258384.1	-0.062169084	0.034504491	0.570942205	**RCCD1-AS1**
ENSG00000157216.16	-0.023239314	0.03454422	0.570942205	**SSBP3**
ENSG00000197114.12	-0.018846465	0.034572527	0.570942205	**ZGPAT**
ENSG00000154118.13	-0.075143273	0.034577249	0.570942205	**JPH3**
ENSG00000177294.7	-0.094955943	0.034683342	0.571253329	**FBXO39**
ENSG00000111325.16	-0.027204757	0.034779829	0.571253329	**OGFOD2**
ENSG00000138185.20	0.016317427	0.034792315	0.571253329	**ENTPD1**
ENSG00000187608.10	-0.09543992	0.034832955	0.571253329	**ISG15**
ENSG00000162512.16	0.053352529	0.03484503	0.571253329	**SDC3**
ENSG00000151465.14	0.016784519	0.034886821	0.571253329	**CDC123**
ENSG00000198478.8	0.047941703	0.034889808	0.571253329	**SH3BGRL2**
ENSG00000227036.8	0.079556555	0.034902404	0.571253329	**LINC00511**
ENSG00000114315.4	-0.044928931	0.034917319	0.571253329	**HES1**
ENSG00000172586.8	0.021911923	0.034933197	0.571253329	**CHCHD1**
ENSG00000162783.11	-0.019644846	0.034937617	0.571253329	**IER5**
ENSG00000136816.16	-0.027540966	0.034941665	0.571253329	**TOR1B**
ENSG00000109332.20	0.012314188	0.034965176	0.571253329	**UBE2D3**
ENSG00000114742.14	0.00989722	0.035161197	0.571253329	**WDR48**
ENSG00000100604.13	-0.070052536	0.035162041	0.571253329	**CHGA**
ENSG00000183291.17	0.014774075	0.03519815	0.571253329	**SELENOF**
ENSG00000090266.13	0.025507002	0.035237878	0.571253329	**NDUFB2**
ENSG00000138668.19	-0.010970784	0.03523833	0.571253329	**HNRNPD**
ENSG00000011028.14	0.061123093	0.03525772	0.571253329	**MRC2**
ENSG00000212232.1	-0.112446779	0.03527535	0.571253329	**SNORD17**
ENSG00000168264.11	-0.011648377	0.035295455	0.571253329	**IRF2BP2**
ENSG00000197496.6	0.083333566	0.035298201	0.571253329	**SLC2A10**
ENSG00000132432.14	0.025363394	0.03534832	0.571253329	**SEC61G**
ENSG00000261346.1	-0.054662095	0.035349381	0.571253329	**RP11-297C4.2**

ENSG00000159259.8	-0.025298025	0.035371734	0.571253329	**CHAF1B**
ENSG00000248333.9	-0.016173383	0.035395012	0.571253329	**CDK11B**
ENSG00000128191.16	-0.015178142	0.035428111	0.571253329	**DGCR8**
ENSG00000287971.1	0.216203284	0.035433949	0.571253329	**RP11-341N2.6**
ENSG00000211880.1	-0.090888718	0.035450947	0.571253329	**TRAJ9**
ENSG00000165181.17	-0.044236659	0.035497966	0.571253329	**SHOC1**
ENSG00000187609.16	-0.023037557	0.035549167	0.571253329	**EXD3**
ENSG00000258273.1	-0.102015288	0.035581698	0.571253329	**RP11-370I10.4**
ENSG00000157933.10	-0.018449736	0.035598434	0.571253329	**SKI**
ENSG00000108691.9	-0.124252411	0.035615163	0.571253329	**CCL2**
ENSG00000211828.1	0.082845486	0.035667056	0.571253329	**TRDJ3**
ENSG00000163528.13	0.01674171	0.03567173	0.571253329	**CHCHD4**
ENSG00000237788.1	0.072565796	0.035683814	0.571253329	**RP5-1041C10.3**
ENSG00000070047.13	-0.019144608	0.035718623	0.571253329	**PHRF1**
ENSG00000213399.3	0.062699193	0.035727304	0.571253329	**RPS2P17**
ENSG00000279432.1	-0.047525459	0.035740099	0.571253329	**RP11-135N5.3**
ENSG00000287195.1	-0.052528209	0.035758906	0.571253329	**RP11-1146N6.3**
ENSG00000225889.9	-0.031886621	0.035766007	0.571253329	**AC074289.1**
ENSG00000204576.12	0.012260565	0.035930437	0.572700568	**PRR3**
ENSG00000141232.5	-0.019859504	0.035988542	0.572700568	**TOB1**
ENSG00000261609.8	-0.020659886	0.036039413	0.572700568	**GAN**
ENSG00000198400.13	0.070923182	0.036075407	0.572700568	**NTRK1**
ENSG00000106404.14	-0.017244334	0.036090684	0.572700568	**CLDN15**
ENSG00000092068.20	0.079061471	0.036146127	0.572700568	**SLC7A8**
ENSG00000129968.17	-0.022230771	0.036173524	0.572700568	**ABHD17A**
ENSG00000114902.14	0.015656286	0.03619502	0.572700568	**SPCS1**
ENSG00000082068.9	0.012945421	0.036218563	0.572700568	**WDR70**
ENSG00000211725.3	-0.063061803	0.036224855	0.572700568	**TRBV5-5**
ENSG00000154124.5	0.011185829	0.036242753	0.572700568	**OTULIN**
ENSG00000233493.4	-0.054142845	0.036246591	0.572700568	**TMEM238**
ENSG00000130312.7	-0.022350578	0.036283065	0.572700568	**MRPL34**
ENSG00000167578.18	-0.021523857	0.036294063	0.572700568	**RAB4B**
ENSG00000144580.14	0.009613768	0.036315693	0.572700568	**CNOT9**
ENSG00000255495.1	-0.099656188	0.03633931	0.572700568	**AC145124.2**
ENSG00000116711.10	0.030529591	0.036372455	0.572700568	**PLA2G4A**
ENSG00000213999.17	-0.035837331	0.036402139	0.572700568	**MEF2B**
ENSG00000228079.2	-0.05792324	0.036431277	0.572700568	**AC012368.1**
ENSG00000174125.8	0.01970008	0.036432412	0.572700568	**TLR1**
ENSG00000169508.7	0.028631614	0.036443058	0.572700568	**GPR183**
ENSG00000179044.16	-0.074173935	0.036528764	0.573607878	**EXOC3L1**
ENSG00000278175.4	0.048118203	0.036596484	0.573634019	**GLIDR**
ENSG00000184076.13	0.022043015	0.036597036	0.573634019	**UQCR10**
ENSG00000163563.8	0.023681001	0.036621711	0.573634019	**MNDA**
ENSG00000184983.11	0.026786097	0.036642314	0.573634019	**NDUFA6**
ENSG00000211800.3	-0.046311863	0.03670171	0.574125612	**TRAV20**
ENSG00000140525.19	-0.018294057	0.03674994	0.574441894	**FANCI**
ENSG00000137675.5	-0.294420531	0.036788515	0.574606902	**MMP27**

ENSG00000070669.17	-0.039249065	0.036830071	0.574818191	**ASNS**
ENSG00000164237.9	0.091596732	0.036865659	0.574936071	**CMBL**
ENSG00000134825.16	0.024781189	0.036906693	0.575138641	**TMEM258**
ENSG00000087076.9	0.080453214	0.036954591	0.575378337	**HSD17B14**
ENSG00000255274.9	-0.033409069	0.036978186	0.575378337	**SMIM35**
ENSG00000156171.15	0.019495917	0.037096305	0.576539341	**DRAM2**
ENSG00000133874.2	-0.020691435	0.037112165	0.576539341	**RNF122**
ENSG00000269821.1	0.019411465	0.037222099	0.576539341	**KCNQ1OT1**
ENSG00000071564.17	-0.018145259	0.037227149	0.576539341	**TCF3**
ENSG00000051180.17	-0.046241828	0.037234136	0.576539341	**RAD51**
ENSG00000105835.13	0.026689444	0.037238481	0.576539341	**NAMPT**
ENSG00000229001.1	0.087475076	0.037249592	0.576539341	**ACTBP14**
ENSG00000011007.12	0.013019939	0.037324978	0.576845079	**ELOA**
ENSG00000145113.22	-0.097486487	0.037325601	0.576845079	**MUC4**
ENSG00000167193.8	0.017655609	0.037386973	0.577294731	**CRK**
ENSG00000287710.1	-0.048663899	0.037410996	0.577294731	**RP11-400F19.19**
ENSG00000184925.12	-0.044548394	0.037495373	0.578025966	**LCN12**
ENSG00000224046.2	0.057771995	0.037514753	0.578025966	**AC005076.5**
ENSG00000211695.2	-0.077784585	0.037566081	0.578134712	**TRGV9**
ENSG00000279283.1	-0.046201319	0.037578192	0.578134712	**RP11-417L19.5**
ENSG00000254395.1	-0.109360813	0.037699372	0.579197971	**IGHV4-55**
ENSG00000024862.18	0.013503187	0.037703788	0.579197971	**CCDC28A**
ENSG00000142937.12	0.024458461	0.037752079	0.579456839	**RPS8**
ENSG00000157077.16	0.022936932	0.037780147	0.579456839	**ZFYVE9**
ENSG00000187672.14	-0.258870494	0.037805405	0.579456839	**ERC2**
ENSG00000258210.1	0.062226095	0.037908193	0.580166039	**RP11-478C19.2**
ENSG00000198089.16	-0.018141341	0.037914781	0.580166039	**SFI1**
ENSG00000070731.11	0.03132782	0.037936545	0.580166039	**ST6GALNAC2**
ENSG00000267191.2	-0.078936579	0.037977781	0.58036388	**ZNF45-AS1**
ENSG00000123094.15	0.088814267	0.038048076	0.580943515	**RASSF8**
ENSG00000258057.6	-0.036416355	0.038099255	0.580943515	**BCDIN3D-AS1**
ENSG00000161888.11	-0.053449482	0.038100694	0.580943515	**SPC24**
ENSG00000198795.11	0.067055098	0.038152966	0.581308334	**ZNF521**
ENSG00000170448.12	0.061164124	0.038249578	0.581435699	**NFXL1**
ENSG00000090006.18	-0.026724703	0.038285547	0.581435699	**LTBP4**
ENSG00000120727.13	0.015024636	0.038306545	0.581435699	**PAIP2**
ENSG00000174233.12	0.056311455	0.038332603	0.581435699	**ADCY6**
ENSG00000100075.10	-0.022334836	0.038348951	0.581435699	**SLC25A1**
ENSG00000040608.14	-0.039605404	0.038369795	0.581435699	**RTN4R**
ENSG00000137815.14	0.012000122	0.038388371	0.581435699	**RTF1**
ENSG00000167562.13	0.018511333	0.038395647	0.581435699	**ZNF701**
ENSG00000270760.1	-0.053886023	0.038423849	0.581435699	**AD001527.7**
ENSG00000105404.11	-0.024742055	0.038512611	0.581435699	**RABAC1**
ENSG00000104853.16	-0.016484946	0.038593383	0.581435699	**CLPTM1**
ENSG00000204406.14	0.014293114	0.038632145	0.581435699	**MBD5**
ENSG00000161057.13	0.020744352	0.038688288	0.581435699	**PSMC2**
ENSG00000174173.7	0.022382601	0.038724364	0.581435699	**TRMT10C**

ENSG00000166046.11	0.028852873	0.038761276	0.581435699	**TCP11L2**
ENSG00000120217.14	0.057709548	0.038776247	0.581435699	**CD274**
ENSG00000116017.11	-0.017533499	0.038777585	0.581435699	**ARID3A**
ENSG00000140374.16	0.017884337	0.038802151	0.581435699	**ETFA**
ENSG00000261040.7	0.084297844	0.038826614	0.581435699	**WFDC21P**
ENSG00000154719.14	0.027987251	0.038835867	0.581435699	**MRPL39**
ENSG00000015475.19	0.016428503	0.038838839	0.581435699	**BID**
ENSG00000214753.4	-0.01527082	0.038869011	0.581435699	**HNRNPUL2**
ENSG00000213609.3	0.052534833	0.038896471	0.581435699	**RPL7AP50**
ENSG00000185201.17	0.029327901	0.038909249	0.581435699	**IFITM2**
ENSG00000083454.22	-0.043564855	0.038922317	0.581435699	**P2RX5**
ENSG00000186141.9	0.01062571	0.038952039	0.581435699	**POLR3C**
ENSG00000232729.8	-0.084708576	0.038985947	0.581435699	**AC083884.8**
ENSG00000284395.2	0.074584304	0.039015061	0.581435699	**PERCC1**
ENSG00000177692.12	-0.047887733	0.039016732	0.581435699	**DNAJC28**
ENSG00000281538.1	-0.037831031	0.039019805	0.581435699	**RP4-669P10.20**
ENSG00000166228.9	0.023723278	0.039067488	0.581435699	**PCBD1**
ENSG00000198948.12	0.035280237	0.039068578	0.581435699	**MFAP3L**
ENSG00000109736.15	-0.021806796	0.03911716	0.581736568	**MFSD10**
ENSG00000169246.16	-0.046506722	0.039158721	0.581932651	**NPIPB3**
ENSG00000222046.3	-0.045359504	0.039216608	0.582057491	**DCDC2B**
ENSG00000287269.1	-0.059312663	0.03926859	0.582057491	**RP1-68O2.5**
ENSG00000077097.16	0.013560368	0.039303769	0.582057491	**TOP2B**
ENSG00000211968.3	-0.087589577	0.039340015	0.582057491	**IGHV1-58**
ENSG00000234432.4	-0.053245455	0.039349483	0.582057491	**RP11-1275H24.1**
ENSG00000166171.13	0.03371605	0.039390273	0.582057491	**DPCD**
ENSG00000141664.10	-0.043025404	0.039438166	0.582057491	**ZCCHC2**
ENSG00000185896.11	-0.018638701	0.039458876	0.582057491	**LAMP1**
ENSG00000130669.17	-0.020383976	0.039473504	0.582057491	**PAK4**
ENSG00000127831.11	0.061028048	0.039483997	0.582057491	**VIL1**
ENSG00000187621.15	-0.064690977	0.039490054	0.582057491	**TCL6**
ENSG00000267078.1	0.046431517	0.039507706	0.582057491	**RP11-666A8.9**
ENSG00000279233.1	0.042765567	0.039669054	0.582799701	**RP11-158L12.4**
ENSG00000212283.1	-0.032548522	0.039725493	0.582799701	**SNORD89**
ENSG00000090615.15	-0.011349575	0.039753896	0.582799701	**GOLGA3**
ENSG00000182318.6	-0.015472239	0.039754238	0.582799701	**ZSCAN22**
ENSG00000143776.19	0.047416479	0.039803353	0.582799701	**CDC42BPA**
ENSG00000109667.12	0.023796711	0.039810694	0.582799701	**SLC2A9**
ENSG00000014914.21	0.024331683	0.039837493	0.582799701	**MTMR11**
ENSG00000231424.3	0.184699169	0.039838774	0.582799701	**RP1-45C12.1**
ENSG00000103253.19	-0.031215827	0.039853943	0.582799701	**HAGHL**
ENSG00000229780.1	0.054701285	0.03987551	0.582799701	**UBE2Q1-AS1**
ENSG00000164181.14	0.045933041	0.039908666	0.582799701	**ELOVL7**
ENSG00000232759.1	-0.0742647	0.039956343	0.582799701	**AC002480.3**
ENSG00000088367.23	0.075793868	0.039973506	0.582799701	**EPB41L1**
ENSG00000113269.14	0.012910701	0.039989924	0.582799701	**RNF130**
ENSG00000005249.13	0.043926531	0.040020663	0.582799701	**PRKAR2B**

ENSG00000126254.12	-0.022966538	0.040030764	0.582799701	**RBM42**
ENSG00000138442.10	0.017110969	0.04012077	0.582799701	**WDR12**
ENSG00000197818.12	0.016021925	0.040177322	0.582799701	**SLC9A8**
ENSG00000264112.1	-0.016129304	0.040183441	0.582799701	**RP11-159D12.2**
ENSG00000164010.15	0.015655754	0.040209097	0.582799701	**ERMAP**
ENSG00000135506.16	-0.016272099	0.040215769	0.582799701	**OS9**
ENSG00000103381.12	0.020383309	0.040219819	0.582799701	**CPPED1**
ENSG00000175305.18	-0.025053677	0.040231712	0.582799701	**CCNE2**
ENSG00000144043.12	0.010406313	0.04024012	0.582799701	**TEX261**
ENSG00000156970.13	-0.060073864	0.040319093	0.582991465	**BUB1B**
ENSG00000152229.18	0.02628651	0.040326518	0.582991465	**PSTPIP2**
ENSG00000174227.16	0.009886793	0.040364562	0.582991465	**PIGG**
ENSG00000256020.1	0.091580151	0.04036707	0.582991465	**RP5-1154L15.2**
ENSG00000264575.1	-0.033299896	0.040423911	0.583201026	**LINC00526**
ENSG00000120306.11	0.030858961	0.040438456	0.583201026	**CYSTM1**
ENSG00000211642.4	0.117026652	0.040476104	0.583333762	**IGLV10-54**
ENSG00000258891.1	-0.074100175	0.040625217	0.585071589	**RP5-1021I20.5**
ENSG00000118217.7	0.015443011	0.040675001	0.585086704	**ATF6**
ENSG00000003056.8	0.014240409	0.040685862	0.585086704	**M6PR**
ENSG00000167850.4	0.026234848	0.040711855	0.585086704	**CD300C**
ENSG00000125534.10	-0.03872698	0.040760496	0.585127197	**PPDPF**
ENSG00000068831.19	-0.013502931	0.040787094	0.585127197	**RASGRP2**
ENSG00000145216.16	-0.011099698	0.040810382	0.585127197	**FIP1L1**
ENSG00000119138.5	0.02638211	0.040852504	0.585127197	**KLF9**
ENSG00000215811.6	0.076088535	0.040857331	0.585127197	**BTNL10**
ENSG00000021762.20	-0.033828488	0.040927542	0.585266005	**OSBPL5**
ENSG00000109684.15	0.086101331	0.040959829	0.585266005	**CLNK**
ENSG00000128266.9	0.044276837	0.040969986	0.585266005	**GNAZ**
ENSG00000215067.10	0.014077744	0.040981177	0.585266005	**ALOX12-AS1**
ENSG00000170835.17	-0.047013837	0.041050393	0.585504525	**CEL**
ENSG00000257122.5	-0.017312499	0.041054979	0.585504525	**RRN3P3**
ENSG00000196839.13	-0.02386303	0.041098151	0.58552529	**ADA**
ENSG00000093167.18	0.012595815	0.041140874	0.58552529	**LRRFIP2**
ENSG00000228998.4	-0.057165873	0.041142088	0.58552529	**RP11-697E2.7**
ENSG00000108187.16	0.023120466	0.04120278	0.585881068	**PBLD**
ENSG00000188976.11	-0.015188947	0.041224224	0.585881068	**NOC2L**
ENSG00000119013.10	0.043177418	0.04127204	0.586154431	**NDUFB3**
ENSG00000262814.8	-0.034011629	0.041362386	0.586504133	**MRPL12**
ENSG00000214114.9	0.014755017	0.04137201	0.586504133	**MYCBP**
ENSG00000222112.1	0.050306726	0.041392354	0.586504133	**RN7SKP16**
ENSG00000130684.14	0.028516797	0.041411058	0.586504133	**ZNF337**
ENSG00000225936.1	0.053433051	0.041502431	0.587392579	**RP11-501J20.5**
ENSG00000188486.3	-0.02609991	0.041561986	0.587829794	**H2AX**
ENSG00000138642.15	-0.041140159	0.041593425	0.587869029	**HERC6**
ENSG00000278730.1	0.011985239	0.041634399	0.588042877	**RP11-147L13.11**
ENSG00000243335.10	0.013827126	0.041783393	0.589716711	**KCTD7**
ENSG00000113645.15	0.063393173	0.041843213	0.589716711	**WWC1**

ENSG00000277978.1	-0.022988333	0.041850981	0.589716711	**RP11-403P17.6**
ENSG00000214562.15	-0.044428037	0.041869847	0.589716711	**NUTM2D**
ENSG00000135144.7	-0.04475272	0.041906855	0.589716711	**DTX1**
ENSG00000138621.12	0.020317242	0.041987172	0.589716711	**PPCDC**
ENSG00000122565.19	0.021253353	0.042001032	0.589716711	**CBX3**
ENSG00000218891.5	-0.031770912	0.04202396	0.589716711	**ZNF579**
ENSG00000131116.12	-0.02454082	0.042052075	0.589716711	**ZNF428**
ENSG00000134001.15	0.013870061	0.042074383	0.589716711	**EIF2S1**
ENSG00000115183.15	0.063726492	0.042078401	0.589716711	**TANC1**
ENSG00000120705.13	0.009124708	0.042097975	0.589716711	**ETF1**
ENSG00000074276.10	-0.032078262	0.042172979	0.590186548	**CDHR2**
ENSG00000171056.8	0.08149775	0.042208791	0.590186548	**SOX7**
ENSG00000183734.5	-0.04109289	0.04221785	0.590186548	**ASCL2**
ENSG00000117877.11	0.027741207	0.042299689	0.590357979	**POLR1G**
ENSG00000100478.16	0.014878682	0.042329489	0.590357979	**AP4S1**
ENSG00000145244.12	0.079442915	0.04237602	0.590357979	**CORIN**
ENSG00000173020.11	-0.013927607	0.042468211	0.590357979	**GRK2**
ENSG00000118707.11	0.01372307	0.042519817	0.590357979	**TGIF2**
ENSG00000099821.14	-0.017387159	0.042524554	0.590357979	**POLRMT**
ENSG00000168298.7	-0.092500287	0.042534498	0.590357979	**H1-4**
ENSG00000183779.7	-0.035805052	0.042540339	0.590357979	**ZNF703**
ENSG00000256590.2	-0.150028888	0.042549195	0.590357979	**TRDV3**
ENSG00000121858.11	0.030839094	0.042592883	0.590357979	**TNFSF10**
ENSG00000115368.10	0.017076816	0.042596188	0.590357979	**WDR75**
ENSG00000262408.1	-0.084333593	0.042603944	0.590357979	**RP11-670E13.3**
ENSG00000233527.9	0.024405683	0.042635952	0.590357979	**ZNF529-AS1**
ENSG00000079739.17	-0.016869617	0.042644457	0.590357979	**PGM1**
ENSG00000111644.8	0.035255933	0.042661914	0.590357979	**ACRBP**
ENSG00000054690.14	-0.051603132	0.042782386	0.591569029	**PLEKHH1**
ENSG00000074416.15	0.03873101	0.042831318	0.591569029	**MGLL**
ENSG00000285872.1	-0.060195227	0.042835967	0.591569029	**RP11-83M8.1**
ENSG00000243273.1	0.073967084	0.042909881	0.592191008	**RP11-166N6.2**
ENSG00000270885.2	0.078259398	0.042948359	0.592283956	**RASL10B**
ENSG00000166025.18	0.034380553	0.042980675	0.592283956	**AMOTL1**
ENSG00000185567.7	-0.049276578	0.043003258	0.592283956	**AHNAK2**
ENSG00000143228.13	-0.051871595	0.043093771	0.592325444	**NUF2**
ENSG00000214867.3	-0.059694035	0.043138241	0.592325444	**SRSF9P1**
ENSG00000106078.19	0.079334919	0.043208264	0.592325444	**COBL**
ENSG00000215305.10	-0.012901701	0.043224936	0.592325444	**VPS16**
ENSG00000185298.13	-0.014177167	0.043249234	0.592325444	**CCDC137**
ENSG00000075914.13	0.017415573	0.043257737	0.592325444	**EXOSC7**
ENSG00000151023.17	0.046972191	0.043272748	0.592325444	**ENKUR**
ENSG00000132716.19	0.008500482	0.043301846	0.592325444	**DCAF8**
ENSG00000263961.8	0.076570055	0.04332033	0.592325444	**RHEX**
ENSG00000200090.1	0.061622867	0.043322636	0.592325444	**Y_RNA**
ENSG00000168038.11	0.03054644	0.04334618	0.592325444	**ULK4**
ENSG00000125977.7	0.013321117	0.043371207	0.592325444	**EIF2S2**

ENSG00000104897.10	-0.022113845	0.043450695	0.592325444	**SF3A2**
ENSG00000197557.7	0.034249521	0.043452191	0.592325444	**TTC30A**
ENSG00000166508.18	-0.012637882	0.043527603	0.592325444	**MCM7**
ENSG00000145996.11	-0.012966833	0.043534509	0.592325444	**CDKAL1**
ENSG00000159055.4	0.02329987	0.043555949	0.592325444	**MIS18A**
ENSG00000113494.17	0.048170343	0.043567679	0.592325444	**PRLR**
ENSG00000227992.1	0.075225627	0.043568495	0.592325444	**AC108463.2**
ENSG00000085999.13	-0.051585843	0.043621455	0.592325444	**RAD54L**
ENSG00000228314.1	-0.13012255	0.043631322	0.592325444	**CYP4F29P**
ENSG00000269403.1	-0.064039231	0.043694382	0.592325444	**CTD-2616J11.11**
ENSG00000182048.11	0.055459129	0.043694985	0.592325444	**TRPC2**
ENSG00000259287.2	0.06792045	0.043702169	0.592325444	**RP11-3D4.2**
ENSG00000224080.1	0.056533959	0.043730981	0.592325444	**UBE2FP1**
ENSG00000111897.7	0.021093394	0.043757219	0.592325444	**SERINC1**
ENSG00000173163.10	0.020178278	0.043850515	0.59261022	**COMMD1**
ENSG00000278009.1	0.090146862	0.04386069	0.59261022	**RP11-649E7.8**
ENSG00000133316.16	-0.015483678	0.04389867	0.59261022	**WDR74**
ENSG00000135723.14	-0.015393947	0.043998613	0.59261022	**FHOD1**
ENSG00000100906.11	0.021117383	0.044009432	0.59261022	**NFKBIA**
ENSG00000100749.9	0.018706047	0.044032014	0.59261022	**VRK1**
ENSG00000277791.5	0.020996054	0.044058307	0.59261022	**PSMB3**
ENSG00000125247.16	0.028469171	0.044064549	0.59261022	**TMTC4**
ENSG00000248571.1	-0.056640783	0.044083813	0.59261022	**RP11-768B22.2**
ENSG00000263069.6	-0.04492114	0.04409413	0.59261022	**RNF213-AS1**
ENSG00000256683.7	0.017425938	0.044114757	0.59261022	**ZNF350**
ENSG00000157212.19	-0.015033761	0.044125015	0.59261022	**PAXIP1**
ENSG00000197149.5	0.061162054	0.044162025	0.592719119	**RP11-452G18.2**
ENSG00000262700.1	-0.082068633	0.044243091	0.593418774	**RP11-266L9.3**
ENSG00000142444.7	-0.015198869	0.044325289	0.593914292	**TIMM29**
ENSG00000177548.13	-0.01933893	0.044337955	0.593914292	**RABEP2**
ENSG00000260747.1	0.068689228	0.04438682	0.594180753	**RP11-421N8.1**
ENSG00000166250.12	-0.04666472	0.044419607	0.594231762	**CLMP**
ENSG00000233057.1	0.074024105	0.044511781	0.59490131	**EEF1A1P14**
ENSG00000250348.1	0.067106537	0.044563934	0.59490131	**RP11-466P24.6**
ENSG00000250848.1	-0.074407569	0.044570517	0.59490131	**CTD-2083E4.5**
ENSG00000183283.16	0.011402359	0.044608028	0.59490131	**DAZAP2**
ENSG00000276568.1	0.074531641	0.044614697	0.59490131	**Metazoa_SRP**
ENSG00000135469.14	-0.015698139	0.044797601	0.595794778	**COQ10A**
ENSG00000170468.8	-0.013598463	0.04481897	0.595794778	**RIOX1**
ENSG00000100445.18	-0.016765255	0.044845834	0.595794778	**SDR39U1**
ENSG00000093217.11	0.035252736	0.044863769	0.595794778	**XYLB**
ENSG00000163093.12	-0.035612469	0.044877361	0.595794778	**BBS5**
ENSG00000129173.13	-0.075857751	0.044942579	0.595794778	**E2F8**
ENSG00000050555.19	0.075189431	0.044958736	0.595794778	**LAMC3**
ENSG00000262999.1	-0.068274863	0.044972609	0.595794778	**CTD-3088G3.6**
ENSG00000228985.1	0.084832017	0.044976085	0.595794778	**TRDD3**
ENSG00000197872.11	0.013786932	0.04499241	0.595794778	**CYRIA**

ENSG00000149527.18	-0.034665603	0.045022142	0.595794778	**PLCH2**
ENSG00000248643.5	-0.026205705	0.045037208	0.595794778	**RBM14-RBM4**
ENSG00000135116.9	-0.089986514	0.045059377	0.595794778	**HRK**
ENSG00000257964.1	-0.094714137	0.045139081	0.596293881	**RP11-133N21.10**
ENSG00000236264.5	0.093576447	0.045178838	0.596293881	**RPL26P30**
ENSG00000244479.7	-0.061890262	0.045212705	0.596293881	**OR2A1-AS1**
ENSG00000237586.3	-0.067603341	0.045268671	0.596293881	**RP11-383J24.2**
ENSG00000163312.11	0.016246201	0.045273918	0.596293881	**HELQ**
ENSG00000221843.4	0.033877846	0.045286689	0.596293881	**C2orf16**
ENSG00000170627.11	0.049180139	0.045300657	0.596293881	**GTSF1**
ENSG00000175221.15	-0.023673088	0.045379023	0.59661861	**MED16**
ENSG00000161243.9	0.088625822	0.045427344	0.59661861	**FBXO27**
ENSG00000173786.17	-0.020044921	0.045463932	0.59661861	**CNP**
ENSG00000157426.14	0.018369318	0.045475863	0.59661861	**AASDH**
ENSG00000163634.12	0.020128077	0.045489315	0.59661861	**THOC7**
ENSG00000273387.1	0.056897946	0.045517144	0.59661861	**RP3-412A9.16**
ENSG00000162645.13	0.028394099	0.045545886	0.59661861	**GBP2**
ENSG00000104885.18	-0.015647004	0.045581658	0.59661861	**DOT1L**
ENSG00000232284.7	-0.059174858	0.045591631	0.59661861	**GNG12-AS1**
ENSG00000211935.3	0.101177422	0.045616246	0.59661861	**IGHV1-3**
ENSG00000117650.13	-0.071535069	0.045668065	0.596915665	**NEK2**
ENSG00000243746.1	0.060722973	0.04598171	0.599614237	**EEF1A1P10**
ENSG00000280194.1	-0.024038169	0.046000343	0.599614237	**AD000864.6**
ENSG00000275833.1	-0.086812257	0.046025159	0.599614237	**RP11-479O9.3**
ENSG00000130813.18	-0.022563679	0.046063871	0.599614237	**SHFL**
ENSG00000174586.11	-0.043497515	0.04607192	0.599614237	**ZNF497**
ENSG00000242299.1	0.035986375	0.046102119	0.599614237	**RP11-234A1.1**
ENSG00000278619.5	-0.019013709	0.046198882	0.599614237	**MRM1**
ENSG00000113083.15	-0.061677771	0.046199899	0.599614237	**LOX**
ENSG00000272279.1	-0.070043596	0.046226971	0.599614237	**RP11-157J24.2**
ENSG00000183864.5	-0.015081548	0.046230658	0.599614237	**TOB2**
ENSG00000267279.2	0.054073354	0.046241973	0.599614237	**RP11-879F14.2**
ENSG00000103485.19	-0.027489531	0.046251755	0.599614237	**QPRT**
ENSG00000124701.6	0.054034826	0.046254619	0.599614237	**APOBEC2**
ENSG00000277089.4	0.095944835	0.046383374	0.6009035	**CCL3-AS1**
ENSG00000085872.15	-0.018990628	0.04645641	0.601206917	**CHERP**
ENSG00000100632.11	0.030306207	0.046483869	0.601206917	**ERH**
ENSG00000176884.16	-0.08022154	0.046527789	0.601206917	**GRIN1**
ENSG00000109519.13	0.011602295	0.0465623	0.601206917	**GRPEL1**
ENSG00000080824.19	0.029700955	0.046583337	0.601206917	**HSP90AA1**
ENSG00000243811.12	-0.024963846	0.046592599	0.601206917	**APOBEC3D**
ENSG00000161847.14	-0.028321543	0.046655664	0.601206917	**RAVER1**
ENSG00000241431.1	0.068841627	0.04668194	0.601206917	**RPL37P6**
ENSG00000143157.12	0.009266964	0.046691837	0.601206917	**POGK**
ENSG00000157181.16	0.023998112	0.046699952	0.601206917	**ODR4**
ENSG00000103647.13	-0.079790813	0.046772984	0.601769353	**CORO2B**
ENSG00000182095.15	-0.021695246	0.046843338	0.602238872	**TNRC18**

ENSG00000161558.11	-0.016107422	0.046875824	0.602238872	**TMEM143**
ENSG00000154359.13	0.021731006	0.046914099	0.602238872	**LONRF1**
ENSG00000183624.14	-0.014596335	0.046980218	0.602238872	**HMCES**
ENSG00000008513.16	0.012451633	0.047003312	0.602238872	**ST3GAL1**
ENSG00000280287.1	-0.029208622	0.047009955	0.602238872	**RP13-554M15.7**
ENSG00000088832.18	0.014219902	0.04701504	0.602238872	**FKBP1A**
ENSG00000187554.14	0.028383919	0.04708918	0.60235378	**TLR5**
ENSG00000235111.1	-0.031125421	0.047095186	0.60235378	**RP1-29C18.8**
ENSG00000259659.1	-0.05797613	0.047112125	0.60235378	**RP11-151N17.1**
ENSG00000144354.14	-0.04069979	0.047151924	0.602487019	**CDCA7**
ENSG00000214309.5	-0.035928634	0.047201007	0.602738636	**MBLAC1**
ENSG00000113318.11	0.021675582	0.047239798	0.602822806	**MSH3**
ENSG00000128989.11	0.012572667	0.047266387	0.602822806	**ARPP19**
ENSG00000173338.13	-0.031082344	0.047321301	0.603148065	**KCNK7**
ENSG00000227508.6	0.033493127	0.047477282	0.604398888	**LINC01624**
ENSG00000235174.1	0.061398334	0.04747838	0.604398888	**RPL39P3**
ENSG00000249109.1	0.08017373	0.047563771	0.605109767	**RP11-1026M7.2**
ENSG00000170873.19	-0.024036939	0.047596278	0.605109767	**MTSS1**
ENSG00000278129.2	-0.014950326	0.04762281	0.605109767	**ZNF8**
ENSG00000275569.1	-0.047157302	0.047652247	0.605109767	**RP11-930O11.3**
ENSG00000010165.20	-0.011827351	0.047740654	0.605857262	**EEF1AKNMT**
ENSG00000084623.12	0.013886063	0.04778605	0.605869999	**EIF3I**
ENSG00000233730.1	0.073831839	0.047800744	0.605869999	**LINC01765**
ENSG00000196214.11	0.013487746	0.048018632	0.608255778	**ZNF766**
ENSG00000087095.13	0.01412946	0.04808791	0.608406054	**NLK**
ENSG00000105401.9	-0.014220162	0.048105454	0.608406054	**CDC37**
ENSG00000104142.11	-0.017259696	0.048133163	0.608406054	**VPS18**
ENSG00000137944.18	0.013408726	0.048149163	0.608406054	**KYAT3**
ENSG00000100242.15	-0.013010173	0.048241855	0.609201951	**SUN2**
ENSG00000180574.3	0.063069508	0.048327579	0.609908922	**EIF2S3B**
ENSG00000136811.16	-0.011755666	0.048399276	0.610438097	**ODF2**
ENSG00000212664.5	0.079693607	0.048522304	0.611247106	**RP11-592N21.1**
ENSG00000135451.13	-0.051845826	0.048545175	0.611247106	**TROAP**
ENSG00000163811.12	0.017917676	0.048552835	0.611247106	**WDR43**
ENSG00000171848.16	-0.054943004	0.048649165	0.611366544	**RRM2**
ENSG00000164897.14	-0.021338991	0.048661649	0.611366544	**TMUB1**
ENSG00000278845.5	0.016058219	0.048687076	0.611366544	**MRPL45**
ENSG00000066044.15	-0.010722504	0.048716436	0.611366544	**ELAVL1**
ENSG00000117751.18	0.009334954	0.048742831	0.611366544	**PPP1R8**
ENSG00000211951.2	-0.072971882	0.048790255	0.611366544	**IGHV2-26**
ENSG00000109180.15	0.009211381	0.04879966	0.611366544	**OCIAD1**
ENSG00000196363.10	-0.012123084	0.048802199	0.611366544	**WDR5**
ENSG00000144381.18	0.01169966	0.048830622	0.611366544	**HSPD1**
ENSG00000023572.10	-0.020607894	0.048915309	0.611636865	**GLRX2**
ENSG00000224203.2	-0.078636144	0.04894712	0.611636865	**RPS23P10**
ENSG00000108515.18	-0.020312551	0.048966469	0.611636865	**ENO3**
ENSG00000107959.17	-0.014454878	0.048989677	0.611636865	**PITRM1**

ENSG00000132004.13	-0.022060082	0.049001335	0.611636865	**FBXW9**
ENSG00000125898.13	-0.026272643	0.049038566	0.611729262	**FAM110A**
ENSG00000180537.13	0.155004826	0.049114842	0.61230831	**RNF182**
ENSG00000142765.18	-0.018181009	0.049240645	0.612947712	**SYTL1**
ENSG00000070729.14	0.083790476	0.049268747	0.612947712	**CNGB1**
ENSG00000148730.7	0.009442363	0.049318741	0.612947712	**EIF4EBP2**
ENSG00000251297.1	0.21336007	0.049321987	0.612947712	**TUBB7P**
ENSG00000177173.5	-0.068816694	0.049359498	0.612947712	**NAP1L4P1**
ENSG00000127564.17	-0.04680287	0.049377024	0.612947712	**PKMYT1**
ENSG00000206762.1	-0.0618592	0.049458683	0.612947712	**RNU6-418P**
ENSG00000278881.2	-0.059081351	0.049461043	0.612947712	**RP3-477J10.1**
ENSG00000164953.17	0.033552794	0.049463791	0.612947712	**TMEM67**
ENSG00000285928.1	-0.075999146	0.049516177	0.612947712	**RP11-386I14.5**
ENSG00000021355.13	0.016709907	0.049523544	0.612947712	**SERPINB1**
ENSG00000140287.11	0.079094703	0.049524788	0.612947712	**HDC**
ENSG00000260220.7	-0.083219978	0.049555137	0.612953404	**CCDC187**
ENSG00000272886.6	0.011687513	0.049603516	0.613181982	**DCP1A**
ENSG00000269102.1	-0.085234983	0.049724396	0.614305974	**CTD-2525I3.5**
ENSG00000250742.4	0.087877405	0.049761407	0.614382253	**LINC02381**
ENSG00000171222.11	-0.027829088	0.049807317	0.614382253	**SCAND1**
ENSG00000169230.10	0.018582298	0.049842862	0.614382253	**PRELID1**
ENSG00000171408.14	0.051000005	0.04987543	0.614382253	**PDE7B**
ENSG00000171840.13	0.029115425	0.049923009	0.614382253	**NINJ2**
ENSG00000164542.13	-0.056009117	0.049949786	0.614382253	**KIAA0895**
ENSG00000134248.14	0.017944435	0.049982553	0.614382253	**LAMTOR5**
ENSG00000074370.18	-0.015472837	0.049987694	0.614382253	**ATP2A3**
ENSG00000261360.1	-0.044336383	0.050000194	0.614382253	OTULIN-DT
ENSG00000160447.7	-0.03510084	0.050080084	0.614995424	PKN3
ENSG00000141522.12	-0.018643871	0.050206877	0.616093659	ARHGDIA
ENSG00000112787.14	-0.019101754	0.050251855	0.616093659	FBRSL1
ENSG00000142396.10	0.019073689	0.05025964	0.616093659	ERVK3-1
ENSG00000166796.12	-0.263467678	0.05031199	0.616366963	LDHC
ENSG00000265206.5	-0.025311668	0.050596805	0.618572758	RP5-1171I10.5
ENSG00000089685.15	-0.058357843	0.050630089	0.618572758	BIRC5
ENSG00000189366.10	0.100031228	0.050638816	0.618572758	ALG1L
ENSG00000130758.8	-0.021099182	0.050673521	0.618572758	MAP3K10
ENSG00000234571.2	0.096171938	0.050687076	0.618572758	H2BP2
ENSG00000119333.12	-0.030431898	0.050694216	0.618572758	DYNC2I2
ENSG00000253314.7	0.167909821	0.05070318	0.618572758	LINC00293
ENSG00000284879.1	-0.06113646	0.050831277	0.618934338	RP13-511M20.1
ENSG00000186409.18	-0.021000992	0.050854473	0.618934338	CCDC30
ENSG00000180764.13	0.052849226	0.050884244	0.618934338	PIPSL
ENSG00000127989.14	0.02166805	0.050901372	0.618934338	MTERF1
ENSG00000151846.9	0.040736625	0.050905888	0.618934338	PABPC3
ENSG00000184986.11	-0.060170601	0.050925835	0.618934338	TMEM121
ENSG00000146453.13	0.085012581	0.050970198	0.618934338	PNLDC1
ENSG00000137628.18	-0.050001647	0.050974259	0.618934338	DDX60

ENSG00000167985.7	0.018617928	0.051009865	0.619000181	SDHAF2
ENSG00000070087.14	0.03672936	0.05107426	0.619211477	PFN2
ENSG00000167543.16	-0.018534969	0.051087664	0.619211477	TP53I13
ENSG00000078098.14	-0.12713066	0.051124315	0.619216924	FAP
ENSG00000112031.16	0.027692666	0.051181133	0.619216924	MTRF1L
ENSG00000248483.7	-0.066513146	0.051191127	0.619216924	POU5F2
ENSG00000180263.14	0.02845331	0.051210492	0.619216924	FGD6
ENSG00000261790.2	0.03207452	0.051251333	0.619216924	AC005606.14
ENSG00000261173.1	-0.071106292	0.051280826	0.619216924	DNAJA2-DT
ENSG00000138395.17	0.08391172	0.051323954	0.619216924	CDK15
ENSG00000172955.17	0.072968197	0.05136854	0.619216924	ADH6
ENSG00000173678.14	-0.131378739	0.051403888	0.619216924	SPDYE2B
ENSG00000228663.1	-0.04549708	0.051477085	0.619216924	PSMD10P1
ENSG00000227946.1	-0.029988293	0.051492234	0.619216924	AC007383.3
ENSG00000063176.16	-0.017938625	0.051499308	0.619216924	SPHK2
ENSG00000110047.18	-0.018746751	0.05152217	0.619216924	EHD1
ENSG00000253676.1	0.034299943	0.051544374	0.619216924	TAGLN2P1
ENSG00000144283.22	-0.023354711	0.051585563	0.619216924	PKP4
ENSG00000226469.1	-0.044195411	0.051614732	0.619216924	ADAM1B
ENSG00000135537.17	0.027465289	0.0517	0.619216924	AFG1L
ENSG00000211770.1	-0.047944149	0.051741578	0.619216924	TRBJ2-6
ENSG00000110768.12	0.009813439	0.051791944	0.619216924	GTF2H1
ENSG00000249915.8	0.022092621	0.051915687	0.619216924	PDCD6
ENSG00000255031.5	-0.020953233	0.051935133	0.619216924	RP11-802E16.3
ENSG00000114302.16	0.009765928	0.051989139	0.619216924	PRKAR2A
ENSG00000079257.8	0.02985939	0.052007172	0.619216924	LXN
ENSG00000115919.15	0.027236351	0.052012454	0.619216924	KYNU
ENSG00000169598.17	0.012107981	0.052014931	0.619216924	DFFB
ENSG00000224631.4	0.040843313	0.052042918	0.619216924	RPS27AP16
ENSG00000272667.1	-0.03796509	0.052052282	0.619216924	RP11-395A13.2
ENSG00000198258.11	0.023168032	0.052097135	0.619216924	UBL5
ENSG00000204872.4	0.057979209	0.052174297	0.619216924	NAT8B
ENSG00000072195.15	0.041695607	0.052210964	0.619216924	SPEG
ENSG00000188011.5	-0.066514059	0.052219848	0.619216924	RTP5
ENSG00000064545.15	-0.018491182	0.052224025	0.619216924	TMEM161A
ENSG00000130816.17	-0.014481236	0.052237507	0.619216924	DNMT1
ENSG00000225178.5	0.081867497	0.052274031	0.619216924	RPSAP58
ENSG00000185215.11	0.015858964	0.052400507	0.619216924	TNFAIP2
ENSG00000269976.1	-0.051422772	0.052435954	0.619216924	RP11-130L8.2
ENSG00000214688.6	-0.030754863	0.052453794	0.619216924	C10orf105
ENSG00000050820.17	-0.166782128	0.052506354	0.619216924	BCAR1
ENSG00000166949.17	-0.013065834	0.05251216	0.619216924	SMAD3
ENSG00000197008.10	0.027138081	0.052517502	0.619216924	ZNF138
ENSG00000261556.10	-0.021722873	0.0525466	0.619216924	SMG1P7
ENSG00000181467.5	-0.01282996	0.052554641	0.619216924	RAP2B
ENSG00000161179.14	-0.02234422	0.052562911	0.619216924	YDJC
ENSG00000163666.10	-0.091409109	0.052693276	0.619216924	HESX1

ENSG00000145949.11	0.038330192	0.052744523	0.619216924	MYLK4
ENSG00000164330.17	-0.051509767	0.052748527	0.619216924	EBF1
ENSG00000263731.1	-0.031084738	0.052776251	0.619216924	RP11-498C9.15
ENSG00000257433.6	0.03477275	0.052784029	0.619216924	RP1-197B17.3
ENSG00000204371.11	-0.014138829	0.052788343	0.619216924	EHMT2
ENSG00000197150.13	-0.013353076	0.05283619	0.619216924	ABCB8
ENSG00000227355.3	0.054580967	0.052838012	0.619216924	RP11-162D16.2
ENSG00000164587.13	0.025844625	0.052877683	0.619216924	RPS14
ENSG00000139547.8	-0.025204631	0.052892726	0.619216924	RDH16
ENSG00000167105.8	-0.064793432	0.052939203	0.619216924	TMEM92
ENSG00000174944.9	0.047274582	0.052953744	0.619216924	P2RY14
ENSG00000272498.1	0.040428594	0.052974476	0.619216924	RP11-415F23.3
ENSG00000273319.1	-0.070868503	0.052981672	0.619216924	RP11-138A9.2
ENSG00000156463.18	-0.109323556	0.053049398	0.619216924	SH3RF2
ENSG00000131037.15	-0.037323727	0.053059923	0.619216924	EPS8L1
ENSG00000020633.19	-0.022966646	0.053069733	0.619216924	RUNX3
ENSG00000287991.1	0.059512163	0.053091787	0.619216924	CTD-3104H21.1
ENSG00000134463.15	0.032897733	0.053091814	0.619216924	ECHDC3
ENSG00000240247.8	0.204092425	0.05310412	0.619216924	DEFA1B
ENSG00000088726.16	0.043261075	0.053105749	0.619216924	TMEM40
ENSG00000122882.11	0.009653039	0.053135642	0.619216924	ECD
ENSG00000215867.4	-0.07561885	0.053136807	0.619216924	KRT18P57
ENSG00000184319.16	0.023927359	0.053171402	0.619216924	RPL23AP82
ENSG00000122026.11	0.041550535	0.053184234	0.619216924	RPL21
ENSG00000287049.1	0.068017893	0.053208818	0.619216924	RP11-27M15.2
ENSG00000266401.2	0.053583065	0.053226777	0.619216924	RP11-874J12.4
ENSG00000111728.11	-0.035611228	0.053294942	0.619216924	ST8SIA1
ENSG00000167987.11	0.017300467	0.053318681	0.619216924	VPS37C
ENSG00000211947.2	0.065184649	0.053373307	0.619216924	IGHV3-21
ENSG00000082996.20	0.017517186	0.053374258	0.619216924	RNF13
ENSG00000180817.12	0.026889504	0.053389608	0.619216924	PPA1
ENSG00000184635.16	0.025623483	0.053412796	0.619216924	ZNF93
ENSG00000162888.4	0.029844616	0.053413046	0.619216924	C1orf147
ENSG00000234476.3	0.065841194	0.053451028	0.619307168	LINC02765
ENSG00000086288.12	-0.032287861	0.053578492	0.619870687	NME8
ENSG00000175538.11	0.013910019	0.053632332	0.619870687	KCNE3
ENSG00000184361.13	-0.055047216	0.053632588	0.619870687	SPATA32
ENSG00000142556.19	0.020971482	0.053736624	0.619870687	ZNF614
ENSG00000095485.18	0.012363691	0.053736982	0.619870687	CWF19L1
ENSG00000092841.19	0.027300263	0.053777013	0.619870687	MYL6
ENSG00000162298.19	-0.014903854	0.053781531	0.619870687	SYVN1
ENSG00000061936.10	-0.010218152	0.05379124	0.619870687	SFSWAP
ENSG00000273373.1	-0.017479121	0.053797603	0.619870687	RP5-1074L1.4
ENSG00000259845.1	0.109886486	0.053801922	0.619870687	HERC2P10
ENSG00000239246.3	0.046333775	0.053870507	0.620155484	RP11-464D20.2
ENSG00000275056.1	-0.069155346	0.053887121	0.620155484	RP11-127I20.8
ENSG00000228495.2	-0.095420794	0.053931097	0.620282039	LINC01013

ENSG00000100938.18	0.010500772	0.053984002	0.620282039	GMPR2
ENSG00000213640.3	-0.094940177	0.054005809	0.620282039	EEF1DP4
ENSG00000115661.14	0.014135476	0.054069273	0.620282039	STK16
ENSG00000254901.8	0.01418782	0.054071469	0.620282039	BORCS8
ENSG00000006025.12	-0.017426497	0.054079593	0.620282039	OSBPL7
ENSG00000182870.13	-0.059018708	0.054203704	0.620702698	GALNT9
ENSG00000181135.16	-0.013990081	0.054244114	0.620702698	ZNF707
ENSG00000132394.11	-0.017411051	0.054298441	0.620702698	EEFSEC
ENSG00000187764.12	-0.015234461	0.054341113	0.620702698	SEMA4D
ENSG00000145936.9	0.027010359	0.054365214	0.620702698	KCNMB1
ENSG00000107863.19	0.021979332	0.054382274	0.620702698	ARHGAP21
ENSG00000254952.1	-0.04645603	0.054392942	0.620702698	LINC02705
ENSG00000037241.7	0.041378516	0.054394905	0.620702698	RPL26L1
ENSG00000167487.12	-0.017241651	0.054467107	0.620702698	KLHL26
ENSG00000196465.10	-0.029261602	0.054480497	0.620702698	MYL6B
ENSG00000146670.10	-0.042147218	0.054493287	0.620702698	CDCA5
ENSG00000250420.8	-0.389999227	0.054527786	0.620702698	AACSP1
ENSG00000158985.14	0.013416829	0.054554824	0.620702698	CDC42SE2
ENSG00000231439.4	-0.092875078	0.054585904	0.620702698	WASIR2
ENSG00000110060.9	0.015264663	0.054609227	0.620702698	PUS3
ENSG00000110375.3	-0.062061928	0.054647843	0.620702698	UPK2
ENSG00000227376.1	0.032955321	0.054656604	0.620702698	FTH1P16
ENSG00000050426.16	0.009549856	0.054677023	0.620702698	LETMD1
ENSG00000020181.18	0.037712228	0.054691329	0.620702698	ADGRA2
ENSG00000272100.1	-0.049421339	0.054755525	0.62108756	RP11-155O18.6
ENSG00000253188.1	0.117436359	0.0548248	0.621214484	TRBV6-7
ENSG00000150977.11	0.015546656	0.054840847	0.621214484	RILPL2
ENSG00000077514.9	0.012195029	0.054860841	0.621214484	POLD3
ENSG00000166123.14	0.041254347	0.05488788	0.621214484	GPT2
ENSG00000109854.14	0.01421281	0.054957558	0.621283042	HTATIP2
ENSG00000117016.10	0.020893538	0.054999403	0.621283042	RIMS3
ENSG00000198551.10	0.01958563	0.055041607	0.621283042	ZNF627
ENSG00000256987.1	-0.064280397	0.055048408	0.621283042	RP11-428G5.7
ENSG00000228695.10	0.184126883	0.055059466	0.621283042	CES1P1
ENSG00000259363.6	0.038565317	0.055075706	0.621283042	CTD-2054N24.2
ENSG00000226328.7	0.025620139	0.055194769	0.622283847	NUP50-DT
ENSG00000188033.10	-0.017244741	0.05526047	0.622682263	ZNF490
ENSG00000229700.1	-0.064188344	0.055332977	0.623156889	RP1-130G2.1
ENSG00000198130.16	0.014836641	0.055491266	0.624556378	HIBCH
ENSG00000286691.1	-0.054552463	0.055548571	0.624556378	RP11-181P4.2
ENSG00000075188.9	0.019716345	0.055548607	0.624556378	NUP37
ENSG00000180644.8	-0.040586159	0.055585697	0.624630941	PRF1
ENSG00000134256.13	-0.035570968	0.055617001	0.624640446	CD101
ENSG00000172115.9	0.015286749	0.055650099	0.624644817	CYCS
ENSG00000253873.6	0.092211246	0.055680855	0.624644817	PCDHGA11
ENSG00000149179.14	-0.015575916	0.055747914	0.624644817	C11orf49
ENSG00000140830.9	0.011126372	0.055756966	0.624644817	TXNL4B

ENSG00000166091.21	0.054904596	0.05581903	0.624644817	CMTM5
ENSG00000182541.18	0.019034171	0.055891075	0.624644817	LIMK2
ENSG00000152939.17	0.060165477	0.055917697	0.624644817	MARVELD2
ENSG00000268157.1	0.170028945	0.055980427	0.624644817	AC010524.4
ENSG00000132405.19	0.029068265	0.055988022	0.624644817	TBC1D14
ENSG00000128595.17	0.010851272	0.05600717	0.624644817	CALU
ENSG00000118523.6	-0.102422622	0.056037656	0.624644817	CCN2
ENSG00000088325.16	-0.044885179	0.056041152	0.624644817	TPX2
ENSG00000101343.15	0.009269665	0.056042674	0.624644817	CRNKL1
ENSG00000279024.1	0.051649768	0.05604381	0.624644817	RP11-1193F23.1
ENSG00000198155.5	0.052738909	0.056090911	0.624830203	ZNF876P
ENSG00000110931.19	0.013270026	0.056210778	0.625825531	CAMKK2
ENSG00000256269.10	0.031352768	0.056271279	0.626091989	HMBS
ENSG00000227776.1	0.075649571	0.056309895	0.626091989	AKR1D1P1
ENSG00000164182.12	0.024532824	0.056434554	0.626091989	NDUFAF2
ENSG00000279166.1	-0.045290201	0.056466732	0.626091989	RP11-107E5.3
ENSG00000211767.1	-0.032836812	0.056481883	0.626091989	TRBJ2-3
ENSG00000133392.18	-0.032250612	0.056484954	0.626091989	MYH11
ENSG00000228205.1	0.041008674	0.056510272	0.626091989	RP11-778D9.4
ENSG00000176890.16	-0.046979447	0.056550824	0.626091989	TYMS
ENSG00000228329.3	-0.036486486	0.056557851	0.626091989	LINC01890
ENSG00000272604.1	-0.031075705	0.056611695	0.626091989	RP11-251G23.5
ENSG00000204930.10	0.080214449	0.056648773	0.626091989	FAM221B
ENSG00000259933.6	-0.064522891	0.05679301	0.626091989	RP11-304L19.1
ENSG00000121039.10	-0.022347537	0.056798925	0.626091989	RDH10
ENSG00000101265.16	0.013752184	0.056807123	0.626091989	RASSF2
ENSG00000140451.13	-0.039881147	0.056815662	0.626091989	PIF1
ENSG00000047315.17	0.011691172	0.056819523	0.626091989	POLR2B
ENSG00000240445.6	-0.027187218	0.056833448	0.626091989	FOXO3B
ENSG00000258311.5	-0.026012109	0.056849249	0.626091989	RP11-644F5.10
ENSG00000161800.13	-0.015240043	0.05690932	0.626091989	RACGAP1
ENSG00000131459.13	-0.095232034	0.056962233	0.626091989	GFPT2
ENSG00000182087.14	-0.015446788	0.056991903	0.626091989	TMEM259
ENSG00000170236.16	-0.033579755	0.05699434	0.626091989	USP50
ENSG00000187210.14	-0.019056948	0.057025448	0.626091989	GCNT1
ENSG00000132286.12	0.009794945	0.057029775	0.626091989	TIMM10B
ENSG00000167207.14	0.028154147	0.057056136	0.626091989	NOD2
ENSG00000177889.10	0.014472138	0.057094023	0.626091989	UBE2N
ENSG00000148908.15	0.027801221	0.057104515	0.626091989	RGS10
ENSG00000155660.11	-0.015452323	0.057147843	0.626091989	PDIA4
ENSG00000258667.2	-0.052519399	0.057178581	0.626091989	HIF1A-AS3
ENSG00000236352.1	0.079218222	0.057207078	0.626091989	AC005220.3
ENSG00000161970.15	0.048377913	0.057211617	0.626091989	RPL26
ENSG00000123472.12	0.010933162	0.057212742	0.626091989	ATPAF1
ENSG00000178977.3	0.013954899	0.057242173	0.626091989	LINC00324
ENSG00000276997.4	0.076599901	0.057382332	0.627290443	RP11-378J18.9
ENSG00000265298.1	-0.013943518	0.057477911	0.628000529	RP13-104F24.3

ENSG00000121236.21	-0.067730575	0.057609061	0.628985343	TRIM6
ENSG00000242766.1	-0.192380722	0.057629387	0.628985343	IGKV1D-17
ENSG00000225643.1	-0.045578789	0.057673302	0.629129824	RP11-70P17.1
ENSG00000278090.3	-0.046219716	0.057770718	0.629857456	LUNAR1
ENSG00000111012.10	-0.050753403	0.057876942	0.630680306	CYP27B1
ENSG00000113643.9	0.014323502	0.057942706	0.630720184	RARS1
ENSG00000135441.7	0.021920229	0.05796286	0.630720184	BLOC1S1
ENSG00000167553.16	0.017320075	0.057972867	0.630720184	TUBA1C
ENSG00000059122.17	-0.032139589	0.058012318	0.630814754	FLYWCH1
ENSG00000099797.15	-0.018877347	0.058076156	0.631159853	TECR
ENSG00000110321.19	0.010438976	0.058107016	0.631159853	EIF4G2
ENSG00000111725.11	0.010575349	0.05816555	0.631159853	PRKAB1
ENSG00000283390.1	0.058660269	0.058169763	0.631159853	RP11-134F2.7
ENSG00000244513.7	-0.029218632	0.058197936	0.631159853	CTD-2013N24.2
ENSG00000251593.1	0.049146616	0.058262583	0.631203508	MSNP1
ENSG00000148843.15	-0.01349239	0.058284571	0.631203508	PDCD11
ENSG00000165688.12	-0.011458242	0.058294297	0.631203508	PMPCA
ENSG00000182912.6	-0.070430852	0.058330581	0.631263093	TSPEAR-AS2
ENSG00000009335.18	0.009628817	0.058535084	0.632422297	UBE3C
ENSG00000039560.14	-0.078721185	0.058569696	0.632422297	RAI14
ENSG00000272386.1	0.051505308	0.058630883	0.632422297	RP11-666A8.12
ENSG00000276045.3	-0.017947244	0.058643718	0.632422297	ORAI1
ENSG00000198286.10	-0.016042866	0.05864831	0.632422297	CARD11
ENSG00000260884.1	-0.05141208	0.058672894	0.632422297	AC009120.5
ENSG00000006712.15	-0.016701988	0.058677395	0.632422297	PAF1
ENSG00000159247.13	0.090637864	0.05876767	0.632422297	TUBBP5
ENSG00000284738.1	-0.031073359	0.058768293	0.632422297	RP11-422P24.13
ENSG00000164258.12	0.042892216	0.058775203	0.632422297	NDUFS4
ENSG00000117410.14	0.017809028	0.058796878	0.632422297	ATP6V0B
ENSG00000104774.13	-0.017218521	0.058891987	0.632422297	MAN2B1
ENSG00000243659.1	-0.043851514	0.058897427	0.632422297	RP11-4K3__A.3
ENSG00000240288.8	-0.038307204	0.0589178	0.632422297	GHRLOS
ENSG00000159202.18	0.009332842	0.058929296	0.632422297	UBE2Z
ENSG00000214999.3	-0.030848533	0.058931101	0.632422297	AC129492.6
ENSG00000112799.9	0.026030434	0.05896888	0.632425414	LY86
ENSG00000211769.1	-0.059733231	0.058993067	0.632425414	TRBJ2-5
ENSG00000049247.14	0.098173308	0.059035042	0.632498041	UTS2
ENSG00000170006.12	0.01664864	0.059120398	0.632498041	TMEM154
ENSG00000173281.5	0.020897295	0.059136701	0.632498041	PPP1R3B
ENSG00000123080.12	-0.025183994	0.059158435	0.632498041	CDKN2C
ENSG00000260285.1	0.020791578	0.059180767	0.632498041	RP11-600F24.7
ENSG00000157538.14	0.006314148	0.059210072	0.632498041	VPS26C
ENSG00000161547.17	-0.011221598	0.059263999	0.632498041	SRSF2
ENSG00000104356.11	-0.014625887	0.059273392	0.632498041	POP1
ENSG00000265531.3	0.075143144	0.059277415	0.632498041	FCGR1CP
ENSG00000244486.9	-0.030329896	0.059333505	0.632625359	SCARF2
ENSG00000259612.1	0.074993117	0.059375151	0.632625359	EEF1A1P22

ENSG00000104613.12	0.009317951	0.059381891	0.632625359	INTS10
ENSG00000224505.2	-0.037760179	0.059419135	0.632659868	AC002117.1
ENSG00000279568.1	-0.0603442	0.059466699	0.632659868	RP11-20I23.5
ENSG00000175294.6	0.026816943	0.059492221	0.632659868	CATSPER1
ENSG00000129810.15	-0.040949523	0.059544725	0.632659868	SGO1
ENSG00000253570.1	0.095195699	0.05955284	0.632659868	RNF5P1
ENSG00000285994.1	-0.071044429	0.059570226	0.632659868	RP11-495F22.1
ENSG00000102904.15	-0.048234685	0.059635214	0.632719928	TSNAXIP1
ENSG00000124615.20	0.066999386	0.059637586	0.632719928	MOCS1
ENSG00000244124.1	-0.055157895	0.05975498	0.633191048	ATP1B3-AS1
ENSG00000124635.9	0.040319688	0.059767917	0.633191048	H2BC11
ENSG00000285881.1	-0.096092078	0.059774618	0.633191048	CTD-2530N21.6
ENSG00000140939.14	-0.028094863	0.059848844	0.633626003	NOL3
ENSG00000143126.8	-0.027057535	0.059877472	0.633626003	CELSR2
ENSG00000120262.10	0.03285277	0.059945085	0.633692091	CCDC170
ENSG00000158435.8	-0.009058187	0.06003704	0.633692091	CNOT11
ENSG00000197070.14	-0.016387395	0.060041054	0.633692091	ARRDC1
ENSG00000176946.12	-0.014894537	0.06004381	0.633692091	THAP4
ENSG00000127419.17	-0.015066802	0.06004989	0.633692091	TMEM175
ENSG00000185090.15	-0.032774135	0.060080651	0.633692091	MANEAL
ENSG00000205517.13	-0.044897399	0.060138242	0.633692091	RGL3
ENSG00000160957.14	-0.02181736	0.060175174	0.633692091	RECQL4
ENSG00000137168.8	0.014933764	0.06021318	0.633692091	PPIL1
ENSG00000025156.13	0.019002371	0.060251155	0.633692091	HSF2
ENSG00000085741.13	0.053837188	0.060301955	0.633692091	WNT11
ENSG00000235016.1	-0.015468167	0.060304116	0.633692091	SEMA3F-AS1
ENSG00000171311.13	0.012507282	0.060307108	0.633692091	EXOSC1
ENSG00000228509.6	0.057815815	0.060316314	0.633692091	AC006460.2
ENSG00000197956.10	0.022292175	0.06037589	0.633993217	S100A6
ENSG00000196470.12	-0.020430911	0.060429418	0.634137051	SIAH1
ENSG00000106785.15	-0.01717616	0.06045143	0.634137051	TRIM14
ENSG00000065320.9	-0.053875468	0.060502826	0.63435172	NTN1
ENSG00000085415.16	0.012255479	0.060574698	0.634780738	SEH1L
ENSG00000090487.11	0.013979373	0.06060916	0.634817494	SPG21
ENSG00000066027.12	0.018895668	0.060700057	0.635445006	PPP2R5A
ENSG00000089558.9	-0.059494048	0.060767752	0.635614794	KCNH4
ENSG00000100350.15	-0.039733539	0.060814052	0.635614794	FOXRED2
ENSG00000185798.8	0.014324601	0.060816164	0.635614794	WDR53
ENSG00000236279.7	0.077757819	0.060855486	0.635614794	CLEC2L
ENSG00000224870.7	-0.011513724	0.060890052	0.635614794	MRPL20-AS1
ENSG00000119725.20	-0.023130047	0.060914977	0.635614794	ZNF410
ENSG00000203778.8	0.058542204	0.06093323	0.635614794	FAM229B
ENSG00000213188.3	-0.073392444	0.061004927	0.635782084	YBX1P4
ENSG00000239415.1	-0.030105929	0.061011271	0.635782084	AP001469.9
ENSG00000100836.11	-0.011139413	0.061134829	0.636272987	PABPN1
ENSG00000196141.14	-0.059850836	0.061159453	0.636272987	SPATS2L
ENSG00000116962.15	0.037647587	0.06116621	0.636272987	NID1

ENSG00000168234.13	-0.013406889	0.061182482	0.636272987	TTC39C
ENSG00000228013.1	0.043199963	0.061220135	0.636341878	IL6R-AS1
ENSG00000183570.17	-0.043436456	0.061289972	0.636674939	PCBP3
ENSG00000159069.14	-0.018729982	0.061324686	0.636674939	FBXW5
ENSG00000286546.1	-0.077773307	0.061417574	0.636674939	RP5-841K13.1
ENSG00000264546.1	-0.092917413	0.061433852	0.636674939	RP11-464D20.6
ENSG00000285665.1	-0.042521971	0.061446378	0.636674939	TOP1P1
ENSG00000271959.1	-0.053514916	0.061452639	0.636674939	CTD-3064M3.7
ENSG00000115446.12	0.012641053	0.061469494	0.636674939	UNC50
ENSG00000133606.11	0.023553609	0.061563423	0.637325938	MKRN1
ENSG00000174574.16	0.011532665	0.061596052	0.63734199	AKIRIN1
ENSG00000224387.1	-0.059007414	0.061661531	0.637697772	RP3-395M20.2
ENSG00000170638.10	-0.018953804	0.061781229	0.638613629	TRABD
ENSG00000204767.4	-0.045450983	0.061824068	0.638734506	INSYN2B
ENSG00000137507.11	0.050802596	0.061872847	0.638916594	LRRC32
ENSG00000106991.14	-0.022130393	0.06192079	0.639049503	ENG
ENSG00000213985.4	0.049724834	0.062009649	0.639049503	AC078899.1
ENSG00000268618.1	-0.063797521	0.062081849	0.639049503	AC092316.1
ENSG00000247134.7	0.055759554	0.062087947	0.639049503	RP11-11N9.4
ENSG00000104825.17	-0.017361766	0.062109846	0.639049503	NFKBIB
ENSG00000185684.15	-0.017099707	0.062127131	0.639049503	EP400P1
ENSG00000197622.13	0.009549163	0.062132874	0.639049503	CDC42SE1
ENSG00000163328.14	0.017621851	0.062135006	0.639049503	GPR155
ENSG00000287218.1	-0.055084939	0.062175091	0.63914124	RP11-104L21.4
ENSG00000240418.1	-0.066177548	0.062264438	0.63928465	CTD-2013N17.1
ENSG00000187838.17	-0.02471619	0.062300378	0.63928465	PLSCR3
ENSG00000229222.1	-0.054468074	0.062339058	0.63928465	KRT18P4
ENSG00000131188.12	-0.024805403	0.062430991	0.63928465	PRR7
ENSG00000138175.9	0.017963677	0.062445897	0.63928465	ARL3
ENSG00000129757.15	-0.04511398	0.062484027	0.63928465	CDKN1C
ENSG00000173992.9	-0.0184808	0.062503151	0.63928465	CCS
ENSG00000129535.13	0.022270296	0.062595263	0.63928465	NRL
ENSG00000168758.11	-0.016290699	0.0626278	0.63928465	SEMA4C
ENSG00000117791.16	0.051738999	0.062640344	0.63928465	MTARC2
ENSG00000264522.6	-0.019363862	0.062647046	0.63928465	OTUD7B
ENSG00000254986.7	-0.016570607	0.062691263	0.63928465	DPP3
ENSG00000157601.15	-0.074541501	0.06269449	0.63928465	MX1
ENSG00000279356.1	0.055311224	0.062702627	0.63928465	RP11-429P3.8
ENSG00000244509.4	-0.018752698	0.062706919	0.63928465	APOBEC3C
ENSG00000171603.18	-0.017730632	0.062721718	0.63928465	CLSTN1
ENSG00000136478.8	0.014425268	0.062766618	0.63928465	TEX2
ENSG00000130348.11	0.017545358	0.062785835	0.63928465	QRSL1
ENSG00000257322.6	0.075933778	0.062792639	0.63928465	RP11-511B23.2
ENSG00000182871.16	-0.041375099	0.062812491	0.63928465	COL18A1
ENSG00000101412.13	-0.038867551	0.062891251	0.639555548	E2F1
ENSG00000223496.3	-0.016943424	0.062901479	0.639555548	EXOSC6
ENSG00000167088.11	0.024309094	0.062969635	0.639633332	SNRPD1

ENSG00000121152.10	-0.035220315	0.062971509	0.639633332	NCAPH
ENSG00000166800.10	-0.055233773	0.063082092	0.64009367	LDHAL6A
ENSG00000123689.6	-0.052488602	0.063146696	0.64009367	G0S2
ENSG00000211678.2	-0.08835328	0.063161791	0.64009367	IGLJ3
ENSG00000280670.3	-0.071337082	0.063182927	0.64009367	CCDC163
ENSG00000130755.13	0.018164431	0.063190572	0.64009367	GMFG
ENSG00000242324.1	0.029550235	0.0632041	0.64009367	RP4-576H24.2
ENSG00000156052.11	0.018099541	0.063257741	0.640121806	GNAQ
ENSG00000211649.3	0.099400087	0.063290141	0.640121806	IGLV7-46
ENSG00000120903.13	0.06156422	0.063300518	0.640121806	CHRNA2
ENSG00000100028.12	0.010247246	0.063431922	0.64113448	SNRPD3
ENSG00000079387.14	0.012131167	0.063568342	0.641681599	SENP1
ENSG00000100092.24	-0.024221115	0.063586992	0.641681599	SH3BP1
ENSG00000122692.9	0.008293882	0.06360201	0.641681599	SMU1
ENSG00000215704.10	-0.065456877	0.063637322	0.641681599	CELA2B
ENSG00000143847.15	0.050435881	0.063655361	0.641681599	PPFIA4
ENSG00000069345.12	0.008977088	0.063687425	0.641681599	DNAJA2
ENSG00000144036.16	0.01413659	0.063727388	0.641681599	EXOC6B
ENSG00000109272.4	0.080273657	0.063749447	0.641681599	PF4V1
ENSG00000228305.2	0.045647949	0.063809224	0.641681599	PRELID1P6
ENSG00000230266.1	0.057259155	0.063813531	0.641681599	XXYLT1-AS2
ENSG00000263326.1	0.067514673	0.063856056	0.641681599	RP11-266L9.6
ENSG00000104884.17	-0.016778532	0.063891082	0.641681599	ERCC2
ENSG00000234617.2	-0.031112914	0.063906808	0.641681599	SNRK-AS1
ENSG00000222020.3	-0.048791369	0.063994397	0.641681599	HDAC4-AS1
ENSG00000162976.13	0.01581553	0.064002245	0.641681599	SLC66A3
ENSG00000114124.3	-0.064535618	0.064059434	0.641681599	GRK7
ENSG00000175800.5	0.057622857	0.064074154	0.641681599	OR52B3P
ENSG00000104164.12	0.019763547	0.064103953	0.641681599	BLOC1S6
ENSG00000223959.8	-0.011080468	0.06413175	0.641681599	AFG3L1P
ENSG00000069329.18	0.009306607	0.064200196	0.641681599	VPS35
ENSG00000050730.16	-0.060619604	0.064341146	0.641681599	TNIP3
ENSG00000051523.11	-0.018771707	0.064403934	0.641681599	CYBA
ENSG00000257167.2	0.022168354	0.064416643	0.641681599	TMPO-AS1
ENSG00000237356.7	-0.041046848	0.064433772	0.641681599	AL163953.3
ENSG00000101558.14	0.014510589	0.06444073	0.641681599	VAPA
ENSG00000069702.11	-0.03566917	0.064460722	0.641681599	TGFBR3
ENSG00000261359.2	-0.064411207	0.064466464	0.641681599	PYCARD-AS1
ENSG00000185507.21	-0.037915017	0.064466895	0.641681599	IRF7
ENSG00000018189.13	0.014072803	0.064479566	0.641681599	RUFY3
ENSG00000167775.11	-0.028992716	0.064534558	0.641681599	CD320
ENSG00000229728.1	0.042285258	0.064635616	0.641681599	RP11-314N13.3
ENSG00000249264.1	0.031474298	0.064684158	0.641681599	EEF1A1P9
ENSG00000206557.6	-0.067824033	0.064705481	0.641681599	TRIM71
ENSG00000129084.18	0.014247159	0.064773028	0.641681599	PSMA1
ENSG00000225138.8	-0.054598892	0.064782425	0.641681599	SLC9A3-AS1
ENSG00000185721.13	0.014217096	0.064785998	0.641681599	DRG1

ENSG00000134684.12	-0.013897942	0.064794066	0.641681599	YARS1
ENSG00000167771.6	-0.072832758	0.064799997	0.641681599	RCOR2
ENSG00000115718.18	-0.035712402	0.064809089	0.641681599	PROC
ENSG00000196365.12	-0.015091223	0.064815564	0.641681599	LONP1
ENSG00000263990.1	-0.027932972	0.064833192	0.641681599	CTC-542B22.2
ENSG00000165684.5	-0.016120849	0.064846561	0.641681599	SNAPC4
ENSG00000231607.13	-0.018179866	0.06484667	0.641681599	DLEU2
ENSG00000178974.11	0.012066318	0.064891712	0.641681599	FBXO34
ENSG00000259005.1	0.084353334	0.064894072	0.641681599	RP3-449M8.6
ENSG00000258527.1	-0.086568853	0.064986761	0.641810578	ASB9P1
ENSG00000270157.1	0.047460326	0.064999865	0.641810578	RP5-894A10.6
ENSG00000203875.13	0.034984753	0.065001003	0.641810578	SNHG5
ENSG00000203497.2	0.018831315	0.065154322	0.642609691	PDCD4-AS1
ENSG00000142675.18	-0.033176561	0.06518195	0.642609691	CNKSR1
ENSG00000246889.2	0.038016404	0.065191471	0.642609691	AP000487.5
ENSG00000172731.15	-0.023125886	0.065230247	0.642609691	LRRC20
ENSG00000203414.2	-0.078110722	0.065238608	0.642609691	BTBD7P1
ENSG00000182481.10	-0.015255841	0.065312988	0.642687564	KPNA2
ENSG00000196498.13	-0.021990397	0.06533875	0.642687564	NCOR2
ENSG00000273001.1	-0.06429653	0.06536845	0.642687564	PFKP-DT
ENSG00000104147.9	-0.053294775	0.065371868	0.642687564	OIP5
ENSG00000065328.17	-0.063225357	0.065440604	0.643055057	MCM10
ENSG00000197548.12	0.012868751	0.065481576	0.643149501	ATG7
ENSG00000117322.18	-0.044088619	0.065627269	0.643683208	CR2
ENSG00000241134.3	-0.069941446	0.06563792	0.643683208	BET1P1
ENSG00000185596.16	-0.033061272	0.065662938	0.643683208	WASH3P
ENSG00000260891.1	-0.044738312	0.065676314	0.643683208	RP11-96D1.5
ENSG00000115946.8	0.013586193	0.065745885	0.643683208	PNO1
ENSG00000118564.15	0.014445069	0.0657832	0.643683208	FBXL5
ENSG00000229337.2	-0.045669617	0.065812653	0.643683208	AC079305.8
ENSG00000108010.12	0.014249247	0.065833657	0.643683208	GLRX3
ENSG00000027697.15	0.019035239	0.06583709	0.643683208	IFNGR1
ENSG00000109775.11	0.013368512	0.0658537	0.643683208	UFSP2
ENSG00000100883.13	0.009106076	0.065892521	0.643683208	SRP54
ENSG00000154723.13	0.027357226	0.065924889	0.643683208	ATP5PF
ENSG00000248794.1	-0.081653857	0.065973748	0.643683208	CTD-2248H3.1
ENSG00000143797.12	0.018960369	0.066006347	0.643683208	MBOAT2
ENSG00000173486.14	-0.042414124	0.066006809	0.643683208	FKBP2
ENSG00000105516.11	-0.018084669	0.066061288	0.643683208	DBP
ENSG00000214087.9	0.027718267	0.066126177	0.643683208	ARL16
ENSG00000104951.16	-0.04274505	0.066174952	0.643683208	IL4I1
ENSG00000237649.8	-0.04236657	0.066176807	0.643683208	KIFC1
ENSG00000120158.12	-0.015064255	0.066219672	0.643683208	RCL1
ENSG00000231329.8	-0.049591423	0.066228918	0.643683208	RP1-225E12.2
ENSG00000267796.8	-0.016612383	0.066237938	0.643683208	LIN37
ENSG00000087269.16	-0.011477754	0.066257814	0.643683208	NOP14
ENSG00000172531.16	-0.015211424	0.066303707	0.643824067	PPP1CA

ENSG00000149357.10	0.016209096	0.066452742	0.644175419	LAMTOR1
ENSG00000231393.1	-0.076825048	0.066494661	0.644175419	RP11-331F9.3
ENSG00000138430.16	0.016151919	0.066503799	0.644175419	OLA1
ENSG00000140264.21	0.025738409	0.066543466	0.644175419	SERF2
ENSG00000116586.12	0.018652414	0.066557296	0.644175419	LAMTOR2
ENSG00000255163.1	0.039243471	0.06656927	0.644175419	HSPE1P18
ENSG00000254332.1	0.047571595	0.066589582	0.644175419	GS1-44D20.1
ENSG00000197894.12	0.016041111	0.066613453	0.644175419	ADH5
ENSG00000153815.17	-0.015773545	0.066668003	0.644175419	CMIP
ENSG00000213222.3	0.074745825	0.066708481	0.644175419	TOMM40P4
ENSG00000125122.16	-0.020115683	0.06674013	0.644175419	LRRC29
ENSG00000277142.1	-0.037398993	0.066775811	0.644175419	LINC00235
ENSG00000286410.1	0.063263727	0.06678175	0.644175419	RP11-798P24.2
ENSG00000100336.18	0.070898104	0.066824549	0.644175419	APOL4
ENSG00000154370.16	-0.012122709	0.066825694	0.644175419	TRIM11
ENSG00000183255.12	0.010102827	0.066850794	0.644175419	PTTG1IP
ENSG00000136261.15	0.012271074	0.066878044	0.644175419	BZW2
ENSG00000224051.7	-0.020069798	0.066936103	0.644175419	CPTP
ENSG00000157224.16	-0.041747331	0.066936699	0.644175419	CLDN12
ENSG00000213793.5	0.021466555	0.066979035	0.644280513	ZNF888
ENSG00000230453.9	0.196675439	0.067093576	0.644498238	ANKRD18B
ENSG00000152953.13	0.039956565	0.067132925	0.644498238	STK32B
ENSG00000185522.9	-0.027395593	0.067139447	0.644498238	LMNTD2
ENSG00000174915.12	-0.015644056	0.067162026	0.644498238	PTDSS2
ENSG00000151611.17	0.015281596	0.067189139	0.644498238	MMAA
ENSG00000230623.6	-0.072589132	0.067220122	0.644498238	RP11-469A15.2
ENSG00000110696.10	0.014527646	0.067221656	0.644498238	C11orf58
ENSG00000287837.1	-0.038957568	0.067272995	0.644680053	RP11-471M10.3
ENSG00000148187.18	0.010213297	0.067311962	0.644680053	MRRF
ENSG00000168594.15	0.166122334	0.067431266	0.644680053	ADAM29
ENSG00000256280.1	-0.045204098	0.067444432	0.644680053	ATP5MGP1
ENSG00000171469.11	0.012966831	0.067445475	0.644680053	ZNF561
ENSG00000271751.1	-0.069750642	0.067485398	0.644680053	RP11-110I1.14
ENSG00000256433.3	-0.036357371	0.067528431	0.644680053	RP1-102E24.8
ENSG00000250959.2	-0.022337456	0.067552608	0.644680053	GLUD1P3
ENSG00000206838.1	0.049142665	0.06760434	0.644680053	SNORA5A
ENSG00000260487.1	-0.05960194	0.067630095	0.644680053	RP11-297C4.3
ENSG00000205045.11	-0.021717415	0.067638723	0.644680053	SLFN12L
ENSG00000116251.11	0.01589018	0.067762731	0.644680053	RPL22
ENSG00000093183.14	0.008705673	0.067784113	0.644680053	SEC22C
ENSG00000180035.13	-0.015689037	0.067788721	0.644680053	ZNF48
ENSG00000235652.9	0.030413334	0.0677918	0.644680053	FBXO30-DT
ENSG00000061938.21	-0.016314236	0.06780284	0.644680053	TNK2
ENSG00000132912.13	0.011601798	0.067842463	0.644680053	DCTN4
ENSG00000173436.15	0.015929124	0.067873671	0.644680053	MICOS10
ENSG00000173762.8	-0.027035166	0.067886202	0.644680053	CD7
ENSG00000188107.15	-0.033179798	0.067925461	0.644680053	EYS

ENSG00000135205.15	0.02820447	0.067926433	0.644680053	CCDC146
ENSG00000260792.1	0.071507701	0.067945838	0.644680053	LINC02280
ENSG00000112763.17	-0.012304936	0.067963637	0.644680053	BTN2A1
ENSG00000164898.13	0.026630327	0.068021976	0.644935124	FMC1
ENSG00000226243.1	0.057153549	0.068132995	0.645390393	RPL37AP1
ENSG00000198000.12	0.012498087	0.068208441	0.645390393	NOL8
ENSG00000065802.12	0.009677117	0.068240438	0.645390393	ASB1
ENSG00000224295.3	-0.071844271	0.06826858	0.645390393	OLFM5P
ENSG00000135049.16	0.016813196	0.068313924	0.645390393	AGTPBP1
ENSG00000089876.12	0.0165144	0.068349662	0.645390393	DHX32
ENSG00000175581.14	0.024338865	0.068353538	0.645390393	MRPL48
ENSG00000173213.9	0.164486895	0.068391569	0.645390393	TUBB8B
ENSG00000165312.7	-0.016625008	0.068431343	0.645390393	OTUD1
ENSG00000273521.1	-0.057710716	0.068464265	0.645390393	RP11-45A17.4
ENSG00000170417.16	0.031488179	0.068473677	0.645390393	TMEM182
ENSG00000205323.9	0.037695556	0.068479609	0.645390393	SARNP
ENSG00000135077.9	0.02099204	0.068502512	0.645390393	HAVCR2
ENSG00000245205.3	0.056957367	0.068530204	0.645390393	EEF1A1P4
ENSG00000142544.7	-0.024465485	0.068542046	0.645390393	CTU1
ENSG00000099949.21	-0.014272491	0.068754383	0.646119131	LZTR1
ENSG00000269480.1	-0.056774258	0.068754975	0.646119131	CTD-3032J10.3
ENSG00000178209.16	-0.021328166	0.068796787	0.646119131	PLEC
ENSG00000055957.11	0.038844741	0.068797455	0.646119131	ITIH1
ENSG00000160299.17	-0.015632396	0.068901358	0.646119131	PCNT
ENSG00000188738.15	0.091482954	0.068905564	0.646119131	FSIP2
ENSG00000226015.2	0.031852473	0.068959389	0.646119131	CCT8P1
ENSG00000086544.3	0.014911051	0.068983454	0.646119131	ITPKC
ENSG00000008735.14	-0.057606126	0.068990857	0.646119131	MAPK8IP2
ENSG00000118640.11	0.021157187	0.06900268	0.646119131	VAMP8
ENSG00000007264.15	-0.033015807	0.069028577	0.646119131	MATK
ENSG00000161920.11	0.014159742	0.06903815	0.646119131	MED11
ENSG00000241157.1	-0.104301797	0.069050119	0.646119131	RPL32P32
ENSG00000166136.16	0.01763375	0.069106234	0.646119131	NDUFB8
ENSG00000160214.13	-0.015696654	0.069114503	0.646119131	RRP1
ENSG00000139197.11	-0.012859104	0.069194439	0.646119131	PEX5
ENSG00000197558.13	0.028382788	0.06921897	0.646119131	SSPOP
ENSG00000260874.5	-0.084342822	0.06932195	0.646119131	RP11-715J22.4
ENSG00000177685.17	-0.042527965	0.069326872	0.646119131	CRACR2B
ENSG00000171566.12	0.014727826	0.069351654	0.646119131	PLRG1
ENSG00000138463.9	0.01729404	0.069360877	0.646119131	SLC49A4
ENSG00000213347.10	-0.020802153	0.069415925	0.646119131	MXD3
ENSG00000183484.12	-0.010845233	0.069455685	0.646119131	GPR132
ENSG00000196684.12	-0.020534728	0.069462707	0.646119131	HSH2D
ENSG00000179978.11	0.04197895	0.069463131	0.646119131	NAIPP2
ENSG00000260876.6	0.078754832	0.069472258	0.646119131	LINC01229
ENSG00000254910.1	-0.044344296	0.069509079	0.646119131	RP11-326C3.7
ENSG00000105289.15	-0.027343255	0.069513517	0.646119131	TJP3

ENSG00000105058.12	0.011084857	0.069533105	0.646119131	FAM32A
ENSG00000272181.1	-0.050662421	0.069579242	0.646122686	RP11-245J9.6
ENSG00000130590.14	-0.022399695	0.0695965	0.646122686	SAMD10
ENSG00000116198.14	-0.013818175	0.069684971	0.64645822	CEP104
ENSG00000164038.16	-0.016907891	0.069705314	0.64645822	SLC9B2
ENSG00000088356.6	0.013378547	0.069763096	0.64645822	PDRG1
ENSG00000256196.2	0.061084529	0.069773653	0.64645822	RP11-881M11.4
ENSG00000274272.1	-0.020622652	0.069841501	0.64645822	RP11-44M6.7
ENSG00000152413.15	-0.045464413	0.069901595	0.64645822	HOMER1
ENSG00000127325.19	0.109935379	0.069901855	0.64645822	BEST3
ENSG00000178904.19	0.020110693	0.069917761	0.64645822	DPY19L3
ENSG00000125995.16	0.025382047	0.069925075	0.64645822	ROMO1
ENSG00000084652.16	-0.008452232	0.069947864	0.64645822	TXLNA
ENSG00000005206.17	-0.018187426	0.070019269	0.646826655	SPPL2B
ENSG00000105953.15	-0.015079336	0.070134677	0.647601065	OGDH
ENSG00000169714.17	0.010447121	0.070229904	0.647796297	CNBP
ENSG00000088808.18	-0.014977687	0.07027067	0.647796297	PPP1R13B
ENSG00000125910.6	-0.022015612	0.07027759	0.647796297	S1PR4
ENSG00000204424.10	0.055100111	0.070298345	0.647796297	LY6G6F
ENSG00000244627.5	-0.019783457	0.070313758	0.647796297	TPTEP2
ENSG00000231131.8	0.080381666	0.070410692	0.648398057	LNCAROD
ENSG00000258521.1	0.048103307	0.070480362	0.648653159	RP11-638I2.9
ENSG00000224557.7	0.079849111	0.070501653	0.648653159	HLA-DPB2
ENSG00000117984.15	-0.025453429	0.070539502	0.648694468	CTSD
ENSG00000166326.7	0.010244791	0.070647518	0.648694468	TRIM44
ENSG00000106477.20	0.017792343	0.070657389	0.648694468	CEP41
ENSG00000234630.1	-0.066613413	0.070687359	0.648694468	LL22NC03-2H8.4
ENSG00000165533.19	0.023004621	0.070699234	0.648694468	TTC8
ENSG00000222041.11	0.024397093	0.07080011	0.648694468	CYTOR
ENSG00000140986.8	0.073828578	0.070810297	0.648694468	RPL3L
ENSG00000261888.1	-0.032468447	0.070813381	0.648694468	AC144831.1
ENSG00000179889.19	0.009605794	0.070827193	0.648694468	PDXDC1
ENSG00000257354.2	0.01720243	0.070846138	0.648694468	RP11-631N16.2
ENSG00000087470.19	0.011679507	0.070882167	0.648694468	DNM1L
ENSG00000074855.11	-0.020349655	0.070885718	0.648694468	ANO8
ENSG00000247774.7	0.018371481	0.070936394	0.648756411	PCED1B-AS1
ENSG00000276533.1	-0.054533822	0.070955755	0.648756411	RP11-139H15.5
ENSG00000130595.19	-0.037989367	0.071038269	0.648943929	TNNT3
ENSG00000234750.1	-0.060098528	0.071039551	0.648943929	CTD-2666L21.2
ENSG00000132005.9	-0.019017387	0.071164809	0.649662214	RFX1
ENSG00000198517.10	-0.01715855	0.071181539	0.649662214	MAFK
ENSG00000100425.18	-0.013378469	0.071315739	0.649758902	BRD1
ENSG00000169710.9	-0.020469416	0.071352119	0.649758902	FASN
ENSG00000177885.15	0.01068542	0.071399636	0.649758902	GRB2
ENSG00000163154.6	0.015077899	0.071402101	0.649758902	TNFAIP8L2
ENSG00000250486.5	0.07124674	0.071405547	0.649758902	FAM218A
ENSG00000156564.9	-0.082967345	0.071406013	0.649758902	LRFN2

ENSG00000104765.16	0.01787924	0.071413915	0.649758902	BNIP3L
ENSG00000148344.11	0.069939806	0.071542235	0.650309338	PTGES
ENSG00000107625.14	0.016041997	0.071554069	0.650309338	DDX50
ENSG00000119661.15	0.01602636	0.071620467	0.650309338	DNAL1
ENSG00000141040.15	0.017681827	0.071642002	0.650309338	ZNF287
ENSG00000155393.13	0.016770978	0.071660046	0.650309338	HEATR3
ENSG00000160753.16	-0.012773942	0.071664672	0.650309338	RUSC1
ENSG00000104852.15	-0.01480672	0.071721686	0.650538847	SNRNP70
ENSG00000228063.2	0.048776969	0.071804706	0.650754591	LYPLAL1-DT
ENSG00000288610.1	0.126297803	0.071831568	0.650754591	RP11-736N17.12
ENSG00000267263.1	-0.086084239	0.071864935	0.650754591	RP11-75C10.7
ENSG00000151117.9	0.024686094	0.071892715	0.650754591	TMEM86A
ENSG00000197756.10	0.021837193	0.071934244	0.650754591	RPL37A
ENSG00000234851.4	0.027116403	0.071949604	0.650754591	RPL23AP42
ENSG00000110917.8	0.009389554	0.071988268	0.650754591	MLEC
ENSG00000228451.4	0.036720267	0.072034237	0.650754591	SDAD1P1
ENSG00000262712.1	-0.035991976	0.072058497	0.650754591	RP11-295D4.1
ENSG00000170222.12	0.023395167	0.07211265	0.650754591	ADPRM
ENSG00000267758.1	-0.08500156	0.072120873	0.650754591	RP11-358B23.5
ENSG00000254598.3	0.052271845	0.072137865	0.650754591	CSNK2A3
ENSG00000182612.10	-0.055160331	0.072157984	0.650754591	TSPAN10
ENSG00000101096.20	-0.018100633	0.07228455	0.65160947	NFATC2
ENSG00000082781.12	0.042449136	0.072489487	0.653053792	ITGB5
ENSG00000167460.17	0.012275411	0.072508459	0.653053792	TPM4
ENSG00000180834.8	0.062790806	0.072662028	0.653656046	MAP6D1
ENSG00000167842.16	0.015441323	0.072662939	0.653656046	MIS12
ENSG00000117595.12	-0.064773697	0.072698108	0.653656046	IRF6
ENSG00000125445.11	0.014097973	0.072722412	0.653656046	MRPS7
ENSG00000119408.17	0.023122329	0.07277396	0.653656046	NEK6
ENSG00000180953.11	-0.029167826	0.072801524	0.653656046	ST20
ENSG00000115596.4	-0.05070817	0.072843866	0.653656046	WNT6
ENSG00000272894.5	-0.032487305	0.072877054	0.653656046	MIOS-DT
ENSG00000108561.8	0.013472611	0.072884266	0.653656046	C1QBP
ENSG00000282034.1	-0.032587982	0.072916719	0.653656046	RP11-2C24.9
ENSG00000101109.12	0.010534407	0.072925933	0.653656046	STK4
ENSG00000160972.10	-0.028989561	0.073016616	0.654108712	PPP1R16A
ENSG00000260810.1	-0.036734951	0.073040226	0.654108712	CTD-2547L24.4
ENSG00000159128.15	0.013211703	0.073093542	0.654258385	IFNGR2
ENSG00000171793.16	-0.012799309	0.073120744	0.654258385	CTPS1
ENSG00000164346.10	0.019730076	0.073180043	0.65450341	NSA2
ENSG00000006007.12	0.012479731	0.073231135	0.654674859	GDE1
ENSG00000215156.5	-0.071328448	0.073326645	0.654832184	RP11-1023L17.2
ENSG00000186300.12	0.017434643	0.073382851	0.654832184	ZNF555
ENSG00000274180.1	0.020885971	0.073396958	0.654832184	NATD1
ENSG00000166526.18	0.00743483	0.073399776	0.654832184	ZNF3
ENSG00000271428.1	-0.081675176	0.073413444	0.654832184	MPHOSPH6P1
ENSG00000177666.17	-0.017819879	0.073440317	0.654832184	PNPLA2

ENSG00000112695.12	0.028925436	0.07347875	0.654890141	COX7A2
ENSG00000126749.16	0.014281315	0.073512077	0.654902557	EMG1
ENSG00000087495.17	0.05821452	0.073560777	0.655051851	PHACTR3
ENSG00000213212.3	-0.050056258	0.073661943	0.655112914	NCLP1
ENSG00000104154.7	-0.021621905	0.073664573	0.655112914	SLC30A4
ENSG00000235681.1	-0.0457257	0.073684606	0.655112914	AC008746.5
ENSG00000158825.6	0.027554225	0.073724078	0.655112914	CDA
ENSG00000277767.1	0.031627802	0.073741835	0.655112914	RP11-365P13.5
ENSG00000244480.1	-0.018347854	0.073761372	0.655112914	AC005154.7
ENSG00000179528.16	-0.04982068	0.073791244	0.655112914	LBX2
ENSG00000173039.19	-0.013319957	0.073981939	0.655536797	RELA
ENSG00000232748.3	-0.044409481	0.073985336	0.655536797	RP11-196G11.6
ENSG00000261126.7	-0.080906432	0.074015184	0.655536797	RBFADN
ENSG00000164039.15	-0.027996194	0.074039768	0.655536797	BDH2
ENSG00000258429.2	-0.046485789	0.074044699	0.655536797	PDF
ENSG00000276141.4	0.026153203	0.074077453	0.655536797	WHAMMP3
ENSG00000161040.16	-0.038184362	0.074111537	0.655536797	FBXL13
ENSG00000117748.10	0.011631398	0.074125194	0.655536797	RPA2
ENSG00000186687.16	0.016571745	0.074126674	0.655536797	LYRM7
ENSG00000126088.14	0.014147389	0.074165899	0.655600974	UROD
ENSG00000183617.5	0.024343752	0.074296995	0.656476848	MRPL54
ENSG00000174720.16	0.026688851	0.074380817	0.656639238	LARP7
ENSG00000168778.13	-0.025143867	0.074421552	0.656639238	TCTN2
ENSG00000285933.1	-0.061749606	0.07444325	0.656639238	RP11-167J8.5
ENSG00000122203.15	0.006906028	0.074488844	0.656639238	KIAA1191
ENSG00000073861.3	-0.03308803	0.074509883	0.656639238	TBX21
ENSG00000136897.8	0.024847142	0.074517421	0.656639238	MRPL50
ENSG00000009830.13	-0.013878052	0.074539504	0.656639238	POMT2
ENSG00000204248.12	0.035838402	0.074594082	0.656837882	COL11A2
ENSG00000241464.3	-0.057386766	0.074629183	0.656864926	RPL39P38
ENSG00000011304.22	-0.011166543	0.074729145	0.657462596	PTBP1
ENSG00000255397.1	-0.06504789	0.074885015	0.657797612	NASPP1
ENSG00000279406.1	-0.03007978	0.074891412	0.657797612	RP11-327E2.5
ENSG00000168569.8	-0.014168115	0.074906091	0.657797612	TMEM223
ENSG00000124191.18	-0.034639219	0.074908562	0.657797612	TOX2
ENSG00000164713.10	0.018540824	0.07494747	0.657797612	BRI3
ENSG00000133773.12	0.015849533	0.074959675	0.657797612	CCDC59
ENSG00000090470.15	-0.009527872	0.075020632	0.657867109	PDCD7
ENSG00000161911.11	0.051318422	0.075128802	0.657867109	TREML1
ENSG00000215196.5	0.041744089	0.075129338	0.657867109	BASP1-AS1
ENSG00000247317.3	-0.059764568	0.075171308	0.657867109	LY6E-DT
ENSG00000182866.17	-0.014508418	0.075176471	0.657867109	LCK
ENSG00000143061.18	0.084666601	0.075188897	0.657867109	IGSF3
ENSG00000110108.10	-0.013409513	0.075210862	0.657867109	TMEM109
ENSG00000135047.16	0.036300487	0.075239074	0.657867109	CTSL
ENSG00000160087.21	-0.01261713	0.075256302	0.657867109	UBE2J2
ENSG00000165092.13	0.028391201	0.075433014	0.658134668	ALDH1A1

ENSG00000168918.14	-0.013852995	0.075461696	0.658134668	INPP5D
ENSG00000288649.2	-0.034092707	0.075462518	0.658134668	ACTL10
ENSG00000257698.2	0.032530244	0.075465367	0.658134668	GIHCG
ENSG00000231365.6	-0.018671877	0.075491857	0.658134668	WARS2-AS1
ENSG00000100601.10	0.010666174	0.075502095	0.658134668	ALKBH1
ENSG00000010318.21	0.016598798	0.075532281	0.658134668	PHF7
ENSG00000151422.13	0.020040083	0.075543642	0.658134668	FER
ENSG00000167491.18	-0.012666187	0.075604418	0.658384464	GATAD2A
ENSG00000168329.14	-0.025963447	0.075856159	0.659447059	CX3CR1
ENSG00000184922.14	-0.016072203	0.075860646	0.659447059	FMNL1
ENSG00000181804.15	0.014511479	0.075874847	0.659447059	SLC9A9
ENSG00000213719.8	0.018305156	0.075901575	0.659447059	CLIC1
ENSG00000116106.12	-0.030265928	0.075904084	0.659447059	EPHA4
ENSG00000270816.6	-0.086775954	0.075933623	0.659447059	LINC00221
ENSG00000272369.1	0.07191653	0.075951529	0.659447059	RP11-446N19.1
ENSG00000265656.1	-0.059833437	0.076076059	0.660248763	RP11-13N13.6
ENSG00000053108.17	0.056463878	0.076147866	0.660497773	FSTL4
ENSG00000204850.4	-0.086970057	0.0762204	0.660497773	CTB-158D10.3
ENSG00000174946.7	0.027345515	0.076223315	0.660497773	GPR171
ENSG00000288719.1	-0.084798719	0.076245083	0.660497773	RP4-669P10.21
ENSG00000158457.6	0.032159727	0.076285121	0.660497773	TSPAN33
ENSG00000125726.11	-0.039192403	0.076297992	0.660497773	CD70
ENSG00000100918.13	-0.024740366	0.076353105	0.660695978	REC8
ENSG00000203666.12	0.033276065	0.076450151	0.66075475	EFCAB2
ENSG00000287437.1	-0.07388236	0.076495622	0.66075475	RP11-439L12.4
ENSG00000092421.17	-0.022958569	0.076500014	0.66075475	SEMA6A
ENSG00000244161.1	0.059374433	0.076574132	0.66075475	FLNB-AS1
ENSG00000117000.9	0.016832836	0.076575803	0.66075475	RLF
ENSG00000151413.17	0.018019353	0.076592449	0.66075475	NUBPL
ENSG00000100299.18	-0.019133418	0.076606429	0.66075475	ARSA
ENSG00000107140.16	-0.014788324	0.076643433	0.66075475	TESK1
ENSG00000008018.9	0.01764072	0.076661548	0.66075475	PSMB1
ENSG00000125485.17	-0.01470118	0.07672111	0.66075475	DDX31
ENSG00000184221.13	-0.035839429	0.076802765	0.66075475	OLIG1
ENSG00000179455.10	-0.076155379	0.076821953	0.66075475	MKRN3
ENSG00000228393.4	-0.021322673	0.07682208	0.66075475	LINC01004
ENSG00000127946.17	0.041940472	0.076850977	0.66075475	HIP1
ENSG00000268858.2	0.013901406	0.076892078	0.66075475	RP4-591C20.9
ENSG00000177191.2	-0.023948085	0.076947123	0.66075475	B3GNT8
ENSG00000267121.6	-0.013314606	0.076960307	0.66075475	FMNL1-DT
ENSG00000260861.6	0.034567054	0.076964786	0.66075475	RP4-576H24.4
ENSG00000110799.14	0.047705263	0.076972064	0.66075475	VWF
ENSG00000272356.1	-0.024973457	0.077039025	0.661052859	RP5-1112D6.8
ENSG00000171051.9	0.020723618	0.077084732	0.661168419	FPR1
ENSG00000231419.6	0.104842425	0.077117691	0.661174586	LINC00689
ENSG00000079616.13	-0.012495592	0.077169516	0.661342428	KIF22
ENSG00000030582.18	-0.022331836	0.077231767	0.661599451	GRN

ENSG00000160654.11	-0.018929199	0.077286085	0.661758426	CD3G
ENSG00000165392.11	0.015622897	0.077321031	0.661758426	WRN
ENSG00000184220.12	0.019932149	0.07734713	0.661758426	CMSS1
ENSG00000152778.10	-0.042133276	0.077384317	0.661800489	IFIT5
ENSG00000137547.9	0.020363259	0.077585465	0.662863409	MRPL15
ENSG00000271581.1	0.052011348	0.077587407	0.662863409	XXbac-BPG248L24.12
ENSG00000276014.1	-0.09588041	0.077612439	0.662863409	RN7SL301P
ENSG00000141456.16	-0.015574559	0.077737873	0.662863409	PELP1
ENSG00000168661.15	-0.020329173	0.077750901	0.662863409	ZNF30
ENSG00000174943.11	-0.010246018	0.077765074	0.662863409	KCTD13
ENSG00000228330.1	-0.083343981	0.077771933	0.662863409	RP11-730A19.5
ENSG00000125731.13	-0.018313032	0.077861174	0.662863409	SH2D3A
ENSG00000135315.12	0.019098138	0.077934051	0.662863409	CEP162
ENSG00000204614.9	0.067416124	0.077952704	0.662863409	TRIM40
ENSG00000167552.15	0.019956452	0.077960255	0.662863409	TUBA1A
ENSG00000135521.9	0.015373042	0.07798103	0.662863409	LTV1
ENSG00000176022.7	-0.016775884	0.078041004	0.662863409	B3GALT6
ENSG00000101413.12	0.009276918	0.078055262	0.662863409	RPRD1B
ENSG00000267904.1	-0.038941126	0.078096043	0.662863409	CTC-429P9.5
ENSG00000100346.18	0.035439358	0.078109132	0.662863409	CACNA1I
ENSG00000084090.14	0.009123876	0.078122869	0.662863409	STARD7
ENSG00000260267.1	-0.026277749	0.078152006	0.662863409	RP11-452L6.5
ENSG00000160208.13	-0.011126492	0.078163826	0.662863409	RRP1B
ENSG00000175463.12	-0.014744623	0.078178045	0.662863409	TBC1D10C
ENSG00000249896.2	-0.058529085	0.078203757	0.662863409	LINC02495
ENSG00000150540.14	0.031442895	0.078219692	0.662863409	HNMT
ENSG00000254718.6	0.050927249	0.078263677	0.662962204	CTD-2184C24.2
ENSG00000114416.18	0.014136056	0.078348908	0.663072958	FXR1
ENSG00000170954.12	0.058667294	0.078374674	0.663072958	ZNF415
ENSG00000286909.1	-0.051094918	0.07839115	0.663072958	RP11-496H1.3
ENSG00000107566.14	0.013776946	0.078406082	0.663072958	ERLIN1
ENSG00000114021.12	0.012627814	0.078457084	0.663196198	NIT2
ENSG00000042286.15	-0.017271027	0.078485331	0.663196198	AIFM2
ENSG00000149091.15	-0.014300443	0.078549378	0.663292202	DGKZ
ENSG00000114013.16	0.017707179	0.078593096	0.663292202	CD86
ENSG00000186395.9	0.019348962	0.078601892	0.663292202	KRT10
ENSG00000117013.17	0.039408318	0.078626065	0.663292202	KCNQ4
ENSG00000107937.19	0.009825952	0.078709283	0.663680005	GTPBP4
ENSG00000277072.5	-0.021942032	0.078741863	0.663680005	STAG3L2
ENSG00000138160.7	-0.030400921	0.078769121	0.663680005	KIF11
ENSG00000262049.1	-0.053658054	0.078832392	0.663940327	RP13-1032I1.7
ENSG00000102871.16	-0.018247123	0.078879386	0.663987571	TRADD
ENSG00000187546.14	0.075224054	0.078911407	0.663987571	AGMO
ENSG00000179115.11	-0.015670011	0.078964861	0.663987571	FARSA
ENSG00000160233.8	0.030347777	0.07896751	0.663987571	LRRC3
ENSG00000101216.11	-0.011864527	0.079088901	0.664535836	GMEB2
ENSG00000185761.11	-0.029979625	0.079097522	0.664535836	ADAMTSL5

ENSG00000165355.7	0.016849362	0.079130472	0.664540422	FBXO33
ENSG00000167397.15	0.018433875	0.079235618	0.664618835	VKORC1
ENSG00000108960.9	0.030375447	0.07923765	0.664618835	MMD
ENSG00000230202.1	-0.048506929	0.079247383	0.664618835	RP11-632C17__A.1
ENSG00000166900.17	0.014074098	0.07926944	0.664618835	STX3
ENSG00000172292.15	0.022273561	0.07943828	0.665613627	CERS6
ENSG00000114098.18	0.00887957	0.079479501	0.665613627	ARMC8
ENSG00000241490.1	-0.052473659	0.079504759	0.665613627	RP11-553L6.2
ENSG00000100568.11	0.011560687	0.079531417	0.665613627	VTI1B
ENSG00000135372.9	-0.01285682	0.079557382	0.665613627	NAT10
ENSG00000167476.10	-0.047209193	0.079611674	0.665613627	JSRP1
ENSG00000182326.15	-0.035560161	0.079615283	0.665613627	C1S
ENSG00000106327.13	-0.025254534	0.079677411	0.665849524	TFR2
ENSG00000105204.14	-0.01698253	0.079747487	0.665849524	DYRK1B
ENSG00000139239.7	0.021005361	0.079764302	0.665849524	RPL14P1
ENSG00000183323.13	0.017439831	0.07977337	0.665849524	CCDC125
ENSG00000211643.2	-0.096273023	0.07988653	0.666139486	IGLV5-52
ENSG00000214013.9	0.013387325	0.079950823	0.666139486	GANC
ENSG00000223799.1	0.017125583	0.079954819	0.666139486	IL10RB-DT
ENSG00000087157.19	0.015549538	0.079970086	0.666139486	PGS1
ENSG00000123178.15	-0.018295957	0.079991717	0.666139486	SPRYD7
ENSG00000204209.13	-0.010960986	0.080003002	0.666139486	DAXX
ENSG00000034533.12	0.009552031	0.080100446	0.666680174	ASTE1
ENSG00000213876.4	0.025767573	0.080151027	0.666830535	RPL7AP64
ENSG00000251503.8	-0.075123924	0.08026393	0.666985146	CENPS-CORT
ENSG00000267453.7	0.113407353	0.080299672	0.666985146	CLEC4O
ENSG00000273189.1	-0.037600591	0.080316224	0.666985146	CTD-3148I10.15
ENSG00000179933.6	0.011663318	0.080345503	0.666985146	C14orf119
ENSG00000133661.17	-0.043532288	0.080350213	0.666985146	SFTPD
ENSG00000148426.13	0.031591428	0.0803795	0.666985146	PROSER2
ENSG00000263740.2	-0.056401774	0.080397273	0.666985146	RN7SL4P
ENSG00000165507.9	0.047913165	0.080487984	0.667236338	DEPP1
ENSG00000186994.11	-0.034872136	0.080535559	0.667236338	KANK3
ENSG00000125743.11	0.028041465	0.080537588	0.667236338	SNRPD2
ENSG00000198945.8	-0.020096131	0.08058025	0.667236338	L3MBTL3
ENSG00000179698.14	-0.036273481	0.080590229	0.667236338	WDR97
ENSG00000100567.13	0.020871409	0.080755592	0.668335626	PSMA3
ENSG00000164308.17	0.063109621	0.080869862	0.669011347	ERAP2
ENSG00000152492.15	0.022105397	0.08090719	0.669050267	CCDC50
ENSG00000162627.17	0.106618396	0.081002321	0.669202227	SNX7
ENSG00000264357.1	-0.062473536	0.081041825	0.669202227	MIR4648
ENSG00000135114.13	-0.058413729	0.081055638	0.669202227	OASL
ENSG00000223396.4	0.021910011	0.081056092	0.669202227	RPS10P7
ENSG00000233820.2	-0.064487478	0.081174363	0.669748168	RP11-535M15.2
ENSG00000171813.14	-0.021912764	0.081187534	0.669748168	PWWP2B
ENSG00000243199.1	0.031265921	0.081271551	0.670171684	RP11-408P14.1
ENSG00000184838.15	0.062497817	0.081389725	0.670681598	PRR16

ENSG00000142303.14	-0.030700787	0.081398795	0.670681598	ADAMTS10
ENSG00000187653.11	0.027837624	0.081434141	0.670703355	TMSB4XP8
ENSG00000100652.5	-0.051590094	0.081689127	0.67253337	SLC10A1
ENSG00000204624.8	0.052294497	0.081767459	0.672908124	DISP3
ENSG00000099381.19	-0.015289318	0.081885923	0.673612722	SETD1A
ENSG00000162734.13	0.013897955	0.081935825	0.673668697	PEA15
ENSG00000002919.15	0.012500344	0.081983183	0.673668697	SNX11
ENSG00000254860.6	-0.037162741	0.081991275	0.673668697	TMEM9B-AS1
ENSG00000185745.10	-0.084395696	0.082150353	0.674549954	IFIT1
ENSG00000162542.14	-0.013450209	0.082197581	0.674549954	TMCO4
ENSG00000160961.12	-0.014312494	0.082219902	0.674549954	ZNF333
ENSG00000244459.2	-0.024811604	0.0822301	0.674549954	RP11-1398P2.1
ENSG00000213189.5	0.044862743	0.082291873	0.674786779	BTF3L4P2
ENSG00000141564.15	-0.020006509	0.082373171	0.674822886	RPTOR
ENSG00000111348.9	0.013424359	0.082391846	0.674822886	ARHGDIB
ENSG00000163697.17	0.050393869	0.082425697	0.674822886	APBB2
ENSG00000204564.12	-0.014450706	0.082427898	0.674822886	C6orf136
ENSG00000267470.6	0.043273441	0.082624683	0.675959195	ZNF571-AS1
ENSG00000127152.18	-0.016061306	0.082632617	0.675959195	BCL11B
ENSG00000197530.13	-0.025612095	0.082681333	0.67608803	MIB2
ENSG00000158806.14	-0.057402791	0.082793409	0.676282843	NPM2
ENSG00000162676.12	-0.032513992	0.082804917	0.676282843	GFI1
ENSG00000104899.8	-0.049260012	0.082826149	0.676282843	AMH
ENSG00000069696.7	-0.04559377	0.082837064	0.676282843	DRD4
ENSG00000234009.1	0.050949462	0.082903276	0.676554072	RPL5P34
ENSG00000223553.6	0.067577549	0.082936785	0.676558306	SMPD4P1
ENSG00000188958.9	0.096917282	0.083030991	0.677057482	UTS2B
ENSG00000160766.14	-0.028876244	0.083103397	0.677133738	GBAP1
ENSG00000106333.13	-0.021596558	0.083106379	0.677133738	PCOLCE
ENSG00000225465.10	-0.043266931	0.083163577	0.677330671	RFPL1S
ENSG00000205903.7	-0.016964694	0.083268309	0.677559276	ZNF316
ENSG00000248714.7	-0.028830926	0.083285913	0.677559276	RP11-1079K10.3
ENSG00000213889.11	-0.022931341	0.083310862	0.677559276	PPM1N
ENSG00000158710.15	0.021395298	0.083327763	0.677559276	TAGLN2
ENSG00000224543.4	-0.063192801	0.083356839	0.677559276	SNRPGP15
ENSG00000180628.15	0.02818165	0.083422959	0.677828071	PCGF5
ENSG00000151725.12	-0.030961845	0.083534666	0.678078389	CENPU
ENSG00000223519.8	0.051947284	0.083567958	0.678078389	KIF28P
ENSG00000157353.17	-0.011916748	0.083587165	0.678078389	FCSK
ENSG00000286646.1	0.024542036	0.083608733	0.678078389	RP11-549D23.1
ENSG00000157637.13	-0.017121641	0.083646595	0.678078389	SLC38A10
ENSG00000171314.9	0.012004064	0.083666909	0.678078389	PGAM1
ENSG00000254789.3	0.069585279	0.083697667	0.678078389	RP11-531H8.2
ENSG00000171530.15	0.019790477	0.08371828	0.678078389	TBCA
ENSG00000167962.14	-0.015152506	0.083768585	0.678217981	ZNF598
ENSG00000243175.1	-0.071767661	0.083913108	0.679119976	RPSAP36
ENSG00000211946.3	0.086609723	0.084006661	0.679353795	IGHV3-20

ENSG00000102978.13	0.010174569	0.084038037	0.679353795	POLR2C
ENSG00000204390.10	0.015444296	0.084041378	0.679353795	HSPA1L
ENSG00000280355.1	-0.059103259	0.084173148	0.680150872	RP11-119F19.5
ENSG00000145736.14	0.08375422	0.084273628	0.68054985	GTF2H2
ENSG00000205744.10	-0.013565644	0.084288893	0.68054985	DENND1C
ENSG00000269696.2	-0.044166338	0.084371073	0.680685846	ZNF470-DT
ENSG00000136098.17	0.017820633	0.084372119	0.680685846	NEK3
ENSG00000162585.17	-0.018216824	0.084407469	0.680703256	FAAP20
ENSG00000214530.10	-0.023728875	0.084482452	0.681040142	STARD10
ENSG00000124532.15	0.010478407	0.084735576	0.682305345	MRS2
ENSG00000168386.19	0.049045328	0.084792996	0.682305345	FILIP1L
ENSG00000111906.18	0.019861766	0.084800689	0.682305345	HDDC2
ENSG00000124380.11	0.015318809	0.084851157	0.682305345	SNRNP27
ENSG00000164117.14	0.018441873	0.084856489	0.682305345	FBXO8
ENSG00000211809.2	0.039843007	0.084865308	0.682305345	TRAV27
ENSG00000260082.1	-0.061076684	0.084883229	0.682305345	RP11-2C24.5
ENSG00000269044.2	-0.015123899	0.084927049	0.682305345	CTC-429P9.3
ENSG00000238045.9	-0.018419697	0.084942694	0.682305345	AC009133.12
ENSG00000101335.10	0.066075429	0.084972101	0.682305345	MYL9
ENSG00000169045.17	-0.010210067	0.085040828	0.682498763	HNRNPH1
ENSG00000160050.16	-0.014961472	0.085127636	0.682498763	CCDC28B
ENSG00000277402.1	-0.137527365	0.085170846	0.682498763	MIR6891
ENSG00000205790.1	-0.029484533	0.085204469	0.682498763	DPP9-AS1
ENSG00000164733.22	0.017563859	0.085271882	0.682498763	CTSB
ENSG00000168070.12	0.086130857	0.085366504	0.682498763	MAJIN
ENSG00000173530.6	0.017988896	0.085369524	0.682498763	TNFRSF10D
ENSG00000167862.10	0.018260831	0.08537536	0.682498763	MRPL58
ENSG00000284526.1	0.027558959	0.085417541	0.682498763	RP11-666A8.13
ENSG00000131732.12	0.012899767	0.085431991	0.682498763	ZCCHC9
ENSG00000122085.17	0.01335009	0.085444979	0.682498763	MTERF4
ENSG00000249763.1	-0.073947263	0.085446088	0.682498763	RP11-618I10.1
ENSG00000235271.6	-0.054885092	0.085447828	0.682498763	RP1-90L6.3
ENSG00000235081.1	-0.050786404	0.085490684	0.682498763	AC010492.2
ENSG00000107331.17	-0.021286985	0.085495383	0.682498763	ABCA2
ENSG00000005007.13	-0.014172562	0.085626409	0.682743737	UPF1
ENSG00000261226.1	0.031515319	0.085666595	0.682743737	RP11-830F9.7
ENSG00000076650.7	0.012328075	0.085698841	0.682743737	GPATCH1
ENSG00000104964.15	-0.015473012	0.08572374	0.682743737	TLE5
ENSG00000172543.9	-0.034988561	0.085735386	0.682743737	CTSW
ENSG00000116793.16	0.016042899	0.085802538	0.682743737	PHTF1
ENSG00000277200.1	0.073071834	0.085830871	0.682743737	RP11-74E22.8
ENSG00000196449.4	0.012097603	0.085843571	0.682743737	YRDC
ENSG00000196544.8	-0.017494476	0.0858703	0.682743737	BORCS6
ENSG00000148200.17	0.017862247	0.085875682	0.682743737	NR6A1
ENSG00000228526.7	-0.053635648	0.085924835	0.682743737	MIR34AHG
ENSG00000172493.23	0.019348886	0.085931699	0.682743737	AFF1
ENSG00000189180.16	0.015132211	0.085963138	0.682743737	ZNF33A

ENSG00000166734.20	-0.017904312	0.086021829	0.682743737	GOLM2
ENSG00000083535.16	0.0216005	0.086070141	0.682743737	PIBF1
ENSG00000122694.16	0.013086915	0.086135837	0.682743737	GLIPR2
ENSG00000256745.1	0.036922366	0.086149333	0.682743737	ARPC3P3
ENSG00000119328.12	0.013003684	0.086149699	0.682743737	ABITRAM
ENSG00000087365.16	-0.013776771	0.086207491	0.682743737	SF3B2
ENSG00000106397.12	-0.015886521	0.086266794	0.682743737	PLOD3
ENSG00000211810.3	0.032085477	0.086268915	0.682743737	TRAV29DV5
ENSG00000130175.10	-0.014364408	0.086375737	0.682743737	PRKCSH
ENSG00000161914.10	-0.018571144	0.086388938	0.682743737	ZNF653
ENSG00000177374.13	-0.027222639	0.086474371	0.682743737	HIC1
ENSG00000159166.15	0.157670581	0.086474914	0.682743737	LAD1
ENSG00000099364.18	-0.015600577	0.086491721	0.682743737	FBXL19
ENSG00000197258.5	0.022566082	0.086516576	0.682743737	EIF4BP6
ENSG00000138399.18	0.020112692	0.086542896	0.682743737	FASTKD1
ENSG00000205937.12	-0.009026027	0.086566307	0.682743737	RNPS1
ENSG00000230897.1	0.068292849	0.086582277	0.682743737	RPS18P12
ENSG00000133466.14	0.020465928	0.086584327	0.682743737	C1QTNF6
ENSG00000120008.16	0.014085909	0.086610781	0.682743737	WDR11
ENSG00000250658.1	0.20164955	0.086624693	0.682743737	RP11-138B4.1
ENSG00000217930.8	-0.016996835	0.086734918	0.683234185	PAM16
ENSG00000234477.1	-0.062321884	0.08676067	0.683234185	AC004231.2
ENSG00000144597.14	0.010971871	0.086786866	0.683234185	EAF1
ENSG00000242110.8	-0.02843126	0.086861251	0.683358996	AMACR
ENSG00000255557.1	-0.050740844	0.086913019	0.683358996	RP11-770G2.2
ENSG00000136156.15	0.013522373	0.08692041	0.683358996	ITM2B
ENSG00000168575.10	-0.010425426	0.08696301	0.683358996	SLC20A2
ENSG00000174564.13	-0.0294172	0.08700266	0.683358996	IL20RB
ENSG00000119537.18	0.013865826	0.087031415	0.683358996	KDSR
ENSG00000211820.1	0.04402167	0.087035971	0.683358996	TRAV41
ENSG00000105509.11	-0.077941354	0.087087133	0.683374893	HAS1
ENSG00000274124.1	-0.040024289	0.087104641	0.683374893	RP11-474C8.8
ENSG00000164609.10	0.015478612	0.087166363	0.683597621	SLU7
ENSG00000198301.12	0.008709181	0.087330861	0.68413008	SDAD1
ENSG00000256073.3	0.031822134	0.087379645	0.68413008	URB1-AS1
ENSG00000026559.14	-0.06065599	0.087433813	0.68413008	KCNG1
ENSG00000249855.1	0.030501747	0.087472369	0.68413008	EEF1A1P19
ENSG00000151914.21	0.026390567	0.087489271	0.68413008	DST
ENSG00000156453.14	-0.048926856	0.087555479	0.68413008	PCDH1
ENSG00000284649.1	0.039508998	0.087575868	0.68413008	RP11-231C14.9
ENSG00000215440.12	-0.015968178	0.087577813	0.68413008	NPEPL1
ENSG00000164442.10	-0.011612497	0.087638887	0.68413008	CITED2
ENSG00000059145.19	-0.018437192	0.087644252	0.68413008	UNKL
ENSG00000108352.13	0.025054099	0.087694827	0.68413008	RAPGEFL1
ENSG00000279357.1	0.036430713	0.087716781	0.68413008	RP11-475D10.4
ENSG00000198498.10	0.016805165	0.087719452	0.68413008	TMA16
ENSG00000231841.1	-0.042615893	0.087721203	0.68413008	FAM192BP

ENSG00000179041.4	-0.016085328	0.087753408	0.68413008	RRS1
ENSG00000274929.1	0.046267799	0.087768005	0.68413008	RP11-165D7.5
ENSG00000267213.4	0.060114204	0.087843261	0.684206948	DPY19L3-DT
ENSG00000007080.11	-0.01883932	0.087911053	0.684206948	CCDC124
ENSG00000198959.12	0.048441767	0.087935855	0.684206948	TGM2
ENSG00000261620.1	-0.06181739	0.087942236	0.684206948	HMGN2P41
ENSG00000248275.2	0.023959451	0.087944681	0.684206948	TRIM52-AS1
ENSG00000268756.1	0.03762805	0.088066733	0.684896685	AC104534.2
ENSG00000140534.14	-0.057037791	0.088135786	0.684988148	TICRR
ENSG00000128918.15	-0.035143962	0.088145296	0.684988148	ALDH1A2
ENSG00000151461.20	0.013073506	0.088213695	0.685260024	UPF2
ENSG00000171823.7	-0.013872924	0.088362817	0.685599605	FBXL14
ENSG00000111832.13	0.024895055	0.088457811	0.685599605	RWDD1
ENSG00000279369.1	0.014828372	0.088466035	0.685599605	RP11-51F16.1
ENSG00000214900.11	-0.015893889	0.088505116	0.685599605	LINC01588
ENSG00000168894.10	0.016217357	0.088516309	0.685599605	RNF181
ENSG00000112062.11	0.01645988	0.088557052	0.685599605	MAPK14
ENSG00000287601.1	0.055214398	0.08859359	0.685599605	RP11-11N7.6
ENSG00000257285.6	0.027502318	0.088600212	0.685599605	RP11-298I3.1
ENSG00000167283.8	0.020915823	0.088607887	0.685599605	ATP5MG
ENSG00000100003.18	-0.034704783	0.08868365	0.685599605	SEC14L2
ENSG00000183914.14	-0.099891573	0.088732304	0.685599605	DNAH2
ENSG00000237276.8	-0.049894495	0.088790236	0.685599605	ANO7L1
ENSG00000275055.1	-0.059694262	0.088826194	0.685599605	CTC-471J1.11
ENSG00000119688.21	-0.007934274	0.088868997	0.685599605	ABCD4
ENSG00000160051.12	-0.019376293	0.08890072	0.685599605	IQCC
ENSG00000273240.1	-0.047643444	0.088911085	0.685599605	RP11-455J20.3
ENSG00000267938.1	-0.060509834	0.088981214	0.685599605	EIF1P6
ENSG00000287938.1	0.070677877	0.089066403	0.685599605	CTD-2249K22.3
ENSG00000139620.13	0.010227139	0.089070969	0.685599605	KANSL2
ENSG00000254093.9	0.016630318	0.089077809	0.685599605	PINX1
ENSG00000211818.1	0.047113562	0.089091007	0.685599605	TRAV39
ENSG00000233030.2	0.050934372	0.089104558	0.685599605	RP11-196G18.3
ENSG00000232973.13	0.0315996	0.089117142	0.685599605	CYP1B1-AS1
ENSG00000120659.15	0.065792339	0.089131383	0.685599605	TNFSF11
ENSG00000185009.13	0.009064467	0.08914029	0.685599605	AP3M1
ENSG00000260464.1	0.048688722	0.089155459	0.685599605	RP4-561L24.3
ENSG00000157379.14	-0.015394454	0.089160042	0.685599605	DHRS1
ENSG00000237298.10	0.02189384	0.089277994	0.685757458	TTN-AS1
ENSG00000262678.1	-0.045774369	0.089307051	0.685757458	CAMTA2-AS1
ENSG00000014257.16	0.019705583	0.089342838	0.685757458	ACP3
ENSG00000173614.14	0.012234442	0.089472303	0.685757458	NMNAT1
ENSG00000178665.16	0.039758289	0.089478565	0.685757458	ZNF713
ENSG00000182004.13	0.026601203	0.089516055	0.685757458	SNRPE
ENSG00000251230.6	0.040118912	0.089524917	0.685757458	MIR3945HG
ENSG00000175874.10	0.049640183	0.089574527	0.685757458	CREG2
ENSG00000184661.14	-0.057381378	0.089606843	0.685757458	CDCA2

ENSG00000288611.1	-0.098548049	0.08961473	0.685757458	NPBWR1
ENSG00000167863.12	0.01645056	0.08961627	0.685757458	ATP5PD
ENSG00000198658.4	0.053644598	0.089671678	0.685757458	ABHD17AP1
ENSG00000184752.14	0.018443031	0.089680398	0.685757458	NDUFA12
ENSG00000103932.12	-0.013221416	0.089700934	0.685757458	RPAP1
ENSG00000269318.1	-0.030683561	0.089730534	0.685757458	AC007292.3
ENSG00000110203.9	0.08188967	0.089739734	0.685757458	FOLR3
ENSG00000198740.9	-0.012250734	0.089749026	0.685757458	ZNF652
ENSG00000103326.12	-0.022691446	0.089871567	0.686197169	CAPN15
ENSG00000189091.13	-0.012851596	0.089873493	0.686197169	SF3B3
ENSG00000234420.7	-0.012903913	0.089976999	0.686625293	ZNF37BP
ENSG00000163993.7	0.058339734	0.089996528	0.686625293	S100P
ENSG00000269194.1	-0.036002396	0.090101519	0.687170676	AC006942.4
ENSG00000138434.17	0.017995673	0.090171421	0.687323939	ITPRID2
ENSG00000114383.10	0.011615312	0.090258933	0.687323939	TUSC2
ENSG00000114316.13	0.008511674	0.090271509	0.687323939	USP4
ENSG00000221914.11	0.00764109	0.090282256	0.687323939	PPP2R2A
ENSG00000172009.15	-0.015784298	0.090289189	0.687323939	THOP1
ENSG00000182372.10	0.014690689	0.090383625	0.687787525	CLN8
ENSG00000136630.13	-0.023546945	0.090470665	0.688019718	HLX
ENSG00000153253.19	-0.058249627	0.090538826	0.688019718	SCN3A
ENSG00000179348.12	0.063577582	0.090555952	0.688019718	GATA2
ENSG00000117222.14	0.008471537	0.090581244	0.688019718	RBBP5
ENSG00000180739.14	-0.035021997	0.090581882	0.688019718	S1PR5
ENSG00000099219.14	-0.017912634	0.090637439	0.688186816	ERMP1
ENSG00000211885.1	-0.0611733	0.090701756	0.688420287	TRAJ4
ENSG00000172765.18	0.01601505	0.090896559	0.689461288	TMCC1
ENSG00000009844.16	0.013048388	0.09090615	0.689461288	VTA1
ENSG00000163938.17	0.019950987	0.091029603	0.690142362	GNL3
ENSG00000211956.2	-0.063292745	0.091065694	0.690160851	IGHV4-34
ENSG00000064270.13	0.046796692	0.091151423	0.690291659	ATP2C2
ENSG00000251411.1	0.047955537	0.091174642	0.690291659	RP11-397E7.4
ENSG00000249502.3	-0.055974973	0.091183965	0.690291659	AC006160.5
ENSG00000081721.12	0.01346896	0.091217593	0.690291659	DUSP12
ENSG00000230204.1	0.039461638	0.091294592	0.690570169	FTH1P5
ENSG00000153823.19	0.026288035	0.091343543	0.690570169	PID1
ENSG00000148690.12	-0.018809555	0.091393519	0.690570169	FRA10AC1
ENSG00000288675.1	-0.037332234	0.091450293	0.690570169	PANO1
ENSG00000129437.11	-0.042787372	0.091453062	0.690570169	KLK14
ENSG00000166979.13	-0.022062946	0.091485875	0.690570169	EVA1C
ENSG00000164818.16	-0.011854553	0.091511932	0.690570169	DNAAF5
ENSG00000265491.5	0.007976801	0.091523782	0.690570169	RNF115
ENSG00000136731.13	-0.011639041	0.091592785	0.690820336	UGGT1
ENSG00000177669.4	0.034160707	0.091649589	0.690820336	MBOAT4
ENSG00000196155.13	0.019773951	0.091678652	0.690820336	PLEKHG4
ENSG00000134317.18	0.040146323	0.091691679	0.690820336	GRHL1
ENSG00000257261.6	0.038301119	0.091754385	0.691038901	RP11-96H19.1

ENSG00000267532.5	-0.059622211	0.091826083	0.691300954	MIR497HG
ENSG00000224536.2	0.021058239	0.091856597	0.691300954	RP11-134G8.7
ENSG00000133574.10	0.013557795	0.091894785	0.691334647	GIMAP4
ENSG00000165309.14	0.062518376	0.091937524	0.691402543	ARMC3
ENSG00000162408.11	-0.011189372	0.092034078	0.691874951	NOL9
ENSG00000125775.15	-0.026711538	0.092096755	0.692005954	SDCBP2
ENSG00000213742.7	0.030969349	0.09211899	0.692005954	ZNF337-AS1
ENSG00000149927.18	0.055840373	0.09223125	0.692361312	DOC2A
ENSG00000211942.3	0.070769047	0.092279263	0.692361312	IGHV3-13
ENSG00000085563.15	-0.028435916	0.092297094	0.692361312	ABCB1
ENSG00000256028.2	-0.024695258	0.092310839	0.692361312	RP11-197N18.2
ENSG00000183250.12	0.022428224	0.092335098	0.692361312	LINC01547
ENSG00000092978.11	0.019494932	0.09237047	0.692373388	GPATCH2
ENSG00000162600.12	0.014117921	0.092429024	0.692559159	OMA1
ENSG00000275178.1	-0.056410975	0.092540384	0.692970838	RP11-4B16.3
ENSG00000272906.1	-0.029070787	0.092591345	0.692970838	RP11-533E19.7
ENSG00000133328.4	0.08853457	0.09259196	0.692970838	PLAAT2
ENSG00000136274.9	0.079992856	0.092619128	0.692970838	NACAD
ENSG00000130475.14	-0.015508292	0.092735008	0.693221786	FCHO1
ENSG00000184524.6	-0.047460433	0.092806731	0.693221786	CEND1
ENSG00000119574.13	-0.016238017	0.09282599	0.693221786	ZBTB45
ENSG00000244682.7	0.045231319	0.092835311	0.693221786	FCGR2C
ENSG00000126814.7	0.014450673	0.092886126	0.693221786	TRMT5
ENSG00000151445.16	0.009243055	0.092958022	0.693221786	VIPAS39
ENSG00000049323.16	0.043279733	0.092979469	0.693221786	LTBP1
ENSG00000184178.16	0.013220532	0.093003432	0.693221786	SCFD2
ENSG00000075292.19	0.0133932	0.093012568	0.693221786	ZNF638
ENSG00000204103.4	-0.025268042	0.093019661	0.693221786	MAFB
ENSG00000122861.16	-0.038464203	0.093036233	0.693221786	PLAU
ENSG00000107731.12	-0.086599409	0.093059386	0.693221786	UNC5B
ENSG00000211654.2	0.109688358	0.093117922	0.693221786	IGLV5-37
ENSG00000213316.10	-0.027004679	0.093125903	0.693221786	LTC4S
ENSG00000286825.1	0.067705404	0.09318975	0.693433056	RP11-934B9.9
ENSG00000182327.8	-0.042287806	0.093250623	0.693433056	GLTPD2
ENSG00000277246.1	0.042020589	0.093255723	0.693433056	RP11-153I24.4
ENSG00000239445.6	0.060407947	0.093319967	0.693516477	ST3GAL6-AS1
ENSG00000090971.5	-0.023584483	0.093337604	0.693516477	NAT14
ENSG00000163806.16	-0.034768275	0.093368392	0.693516477	SPDYA
ENSG00000047662.5	-0.024925793	0.093573052	0.694784993	FAM184B
ENSG00000143110.12	0.015970481	0.093732964	0.694996938	C1orf162
ENSG00000287200.1	-0.035036	0.093784784	0.694996938	RP11-181L23.11
ENSG00000134375.11	0.015324223	0.093791785	0.694996938	TIMM17A
ENSG00000167100.15	0.059482261	0.093817328	0.694996938	SAMD14
ENSG00000182310.15	-0.030293513	0.093845021	0.694996938	SPACA6
ENSG00000203392.3	0.048069791	0.093931481	0.694996938	CTD-2026K11.6
ENSG00000164934.14	0.01995922	0.093946573	0.694996938	DCAF13
ENSG00000225111.1	-0.065374683	0.093950365	0.694996938	AC083900.1

ENSG00000136560.14	0.016542099	0.093992191	0.694996938	TANK
ENSG00000167693.17	-0.041883008	0.094014262	0.694996938	NXN
ENSG00000140479.18	0.04207266	0.094078382	0.694996938	PCSK6
ENSG00000144460.12	-0.13926076	0.094087018	0.694996938	NYAP2
ENSG00000255435.7	-0.052324781	0.094090714	0.694996938	RP11-770J1.3
ENSG00000185088.14	-0.022577339	0.094159979	0.694996938	RPS27L
ENSG00000185829.18	0.035162183	0.094171944	0.694996938	ARL17A
ENSG00000270039.1	-0.032039451	0.094244055	0.694996938	RP11-571M6.17
ENSG00000235151.1	-0.078803546	0.094246844	0.694996938	AC114730.2
ENSG00000271614.1	-0.022847675	0.094248544	0.694996938	ATP2B1-AS1
ENSG00000011198.10	0.016341258	0.094320909	0.694996938	ABHD5
ENSG00000142494.13	-0.048374625	0.094348733	0.694996938	SLC47A1
ENSG00000260404.3	0.027262945	0.094473031	0.694996938	RP11-384K6.6
ENSG00000065518.8	0.014870929	0.094491824	0.694996938	NDUFB4
ENSG00000115998.8	0.01033186	0.09449929	0.694996938	C2orf42
ENSG00000156265.16	0.030274498	0.094516917	0.694996938	MAP3K7CL
ENSG00000153790.12	0.012455446	0.094523514	0.694996938	C7orf31
ENSG00000267023.5	0.020561996	0.094528348	0.694996938	LRRC37A16P
ENSG00000164066.13	0.072499145	0.094545782	0.694996938	INTU
ENSG00000158874.11	0.055947322	0.09455049	0.694996938	APOA2
ENSG00000069966.19	0.014276757	0.0946026	0.695121215	GNB5
ENSG00000103254.10	-0.026514263	0.094668642	0.695121215	ANTKMT
ENSG00000279069.1	-0.048012895	0.094669083	0.695121215	RP11-159D12.6
ENSG00000278740.1	-0.046801882	0.094797714	0.695816581	RP11-147L13.14
ENSG00000211716.2	0.040955378	0.094863534	0.695873366	TRBV9
ENSG00000271228.1	-0.051285458	0.094912653	0.695873366	CTC-336P14.1
ENSG00000153179.13	0.012115847	0.095055462	0.695873366	RASSF3
ENSG00000167968.13	-0.029824422	0.095059342	0.695873366	DNASE1L2
ENSG00000143162.9	0.01892311	0.095070658	0.695873366	CREG1
ENSG00000197498.13	0.018497066	0.095083886	0.695873366	RPF2
ENSG00000251957.1	-0.059772458	0.09510084	0.695873366	RNU6-1095P
ENSG00000002587.10	-0.044584062	0.095180772	0.695873366	HS3ST1
ENSG00000075624.17	-0.016076959	0.095213793	0.695873366	ACTB
ENSG00000103569.10	0.018187995	0.095248298	0.695873366	AQP9
ENSG00000163701.19	0.050406937	0.095271597	0.695873366	IL17RE
ENSG00000118690.13	-0.018254955	0.095334011	0.695873366	ARMC2
ENSG00000171806.12	0.028248351	0.095344573	0.695873366	METTL18
ENSG00000268205.1	-0.01332496	0.095357881	0.695873366	CTC-444N24.11
ENSG00000267731.1	-0.04150246	0.095391211	0.695873366	RP11-147L13.8
ENSG00000155792.10	0.033898866	0.095394167	0.695873366	DEPTOR
ENSG00000173210.20	0.04520741	0.095419225	0.695873366	ABLIM3
ENSG00000279641.1	-0.042765918	0.095466917	0.695873366	CTD-2545G14.6
ENSG00000196778.4	-0.035641098	0.09549341	0.695873366	OR52K1
ENSG00000134330.19	0.015988937	0.095501361	0.695873366	IAH1
ENSG00000163141.20	-0.020614508	0.095614295	0.695873366	BNIPL
ENSG00000010030.14	0.068717538	0.095638869	0.695873366	ETV7
ENSG00000138646.9	-0.067825124	0.095708093	0.695873366	HERC5

ENSG00000140961.14	-0.028455326	0.095783643	0.695873366	OSGIN1
ENSG00000274987.1	-0.049090934	0.095787472	0.695873366	RP11-295I5.3
ENSG00000115956.10	0.01318226	0.095791827	0.695873366	PLEK
ENSG00000187961.14	-0.015102479	0.095814717	0.695873366	KLHL17
ENSG00000109158.11	-0.205961199	0.095847442	0.695873366	GABRA4
ENSG00000286481.1	0.073576911	0.095943525	0.695873366	RP11-640F22.1
ENSG00000141376.23	-0.01388658	0.095946908	0.695873366	BCAS3
ENSG00000230383.1	0.060107752	0.095953428	0.695873366	AC009245.3
ENSG00000248554.1	0.053462988	0.096060143	0.695873366	C5orf34-AS1
ENSG00000101052.13	0.012356069	0.096096512	0.695873366	IFT52
ENSG00000211938.2	0.060646978	0.096138591	0.695873366	IGHV3-7
ENSG00000132003.9	-0.02207014	0.096151922	0.695873366	ZSWIM4
ENSG00000176248.9	-0.011275795	0.096167056	0.695873366	ANAPC2
ENSG00000110871.15	0.018385005	0.096168847	0.695873366	COQ5
ENSG00000280303.2	-0.054497131	0.096203805	0.695873366	ERICD
ENSG00000111269.3	0.010152112	0.096211142	0.695873366	CREBL2
ENSG00000118246.14	0.011039489	0.096227175	0.695873366	FASTKD2
ENSG00000177614.11	0.073344011	0.096229757	0.695873366	PGBD5
ENSG00000091164.13	0.010356113	0.096230586	0.695873366	TXNL1
ENSG00000122033.15	0.01170049	0.096291603	0.696069154	MTIF3
ENSG00000261924.1	-0.059786309	0.096458397	0.696704732	CTD-2561B21.5
ENSG00000274964.1	-0.0491931	0.096565439	0.696704732	RP11-817I4.1
ENSG00000184060.11	0.019214606	0.096595244	0.696704732	ADAP2
ENSG00000130513.6	-0.052305924	0.096628991	0.696704732	GDF15
ENSG00000257906.3	-0.067000689	0.096633499	0.696704732	LINC02156
ENSG00000250073.3	-0.040400826	0.096640713	0.696704732	RP11-677M14.3
ENSG00000245937.8	0.013640519	0.096662709	0.696704732	LINC01184
ENSG00000173846.13	0.014474044	0.09670407	0.696704732	PLK3
ENSG00000171649.12	0.021423933	0.096711443	0.696704732	ZIK1
ENSG00000128298.18	-0.025656366	0.096808786	0.696704732	BAIAP2L2
ENSG00000139531.13	0.01507279	0.09681358	0.696704732	SUOX
ENSG00000173728.11	-0.046215717	0.096824007	0.696704732	C1orf100
ENSG00000168438.15	0.015165571	0.096846503	0.696704732	CDC40
ENSG00000106009.16	-0.015829762	0.096871453	0.696704732	BRAT1
ENSG00000166484.20	-0.012881036	0.096893631	0.696704732	MAPK7
ENSG00000133997.12	0.011356431	0.09694051	0.696704732	MED6
ENSG00000278156.2	-0.024996076	0.097082034	0.696704732	TSC22D1-AS1
ENSG00000232354.8	0.029147429	0.097145748	0.696704732	VIPR1-AS1
ENSG00000138002.16	-0.013936818	0.097147737	0.696704732	IFT172
ENSG00000118596.12	-0.023304162	0.097158692	0.696704732	SLC16A7
ENSG00000168913.7	0.039168506	0.097165628	0.696704732	ENHO
ENSG00000268006.1	-0.041955498	0.097171815	0.696704732	PTOV1-AS1
ENSG00000119616.11	0.01207901	0.097224572	0.696704732	FCF1
ENSG00000172301.11	0.021534869	0.097235464	0.696704732	COPRS
ENSG00000121410.12	0.037718935	0.097236226	0.696704732	A1BG
ENSG00000105705.16	-0.010651606	0.097296493	0.696704732	SUGP1
ENSG00000166169.17	0.023568	0.097296779	0.696704732	POLL

ENSG00000116752.6	0.0129136	0.097399524	0.696892632	BCAS2
ENSG00000160785.14	0.013059727	0.097416763	0.696892632	SLC25A44
ENSG00000255046.1	0.049464695	0.097424965	0.696892632	RP11-297N6.4
ENSG00000083817.9	0.014317341	0.097551684	0.697365791	ZNF416
ENSG00000008283.16	0.022287952	0.097559121	0.697365791	CYB561
ENSG00000148154.10	0.019960694	0.097602555	0.697397467	UGCG
ENSG00000185122.11	-0.013279436	0.09764504	0.697397467	HSF1
ENSG00000127774.7	-0.031776956	0.097665571	0.697397467	EMC6
ENSG00000255561.7	-0.019467089	0.097737331	0.697666961	FDXACB1
ENSG00000067057.18	-0.018401212	0.097782101	0.697743675	PFKP
ENSG00000255197.6	0.013924077	0.097909111	0.698259822	RP11-750H9.5
ENSG00000236756.4	0.0382325	0.09793811	0.698259822	DNAJC9-AS1
ENSG00000127955.17	0.041399069	0.098068163	0.698259822	GNAI1
ENSG00000235106.10	-0.011991281	0.098083624	0.698259822	BRD3OS
ENSG00000269086.3	0.021777127	0.098114795	0.698259822	CTC-523E23.5
ENSG00000182022.18	0.014909315	0.098132047	0.698259822	CHST15
ENSG00000163684.12	0.011581782	0.098148576	0.698259822	RPP14
ENSG00000183048.12	-0.031229036	0.098175458	0.698259822	SLC25A10
ENSG00000224097.5	0.049429141	0.098255553	0.698259822	RP11-472B18.1
ENSG00000145687.16	0.012634378	0.098269284	0.698259822	SSBP2
ENSG00000269378.1	-0.032693634	0.098276895	0.698259822	ITGB1P1
ENSG00000143552.9	-0.094327772	0.098287992	0.698259822	NUP210L
ENSG00000198673.11	-0.034168737	0.09829706	0.698259822	TAFA2
ENSG00000205784.4	-0.0222606	0.098410167	0.698626975	ARRDC5
ENSG00000165185.15	-0.027259507	0.098423913	0.698626975	KIAA1958
ENSG00000083807.10	-0.022168839	0.098450944	0.698626975	SLC27A5
ENSG00000125633.11	0.009877618	0.098489302	0.698657422	CCDC93
ENSG00000111481.10	0.011897201	0.098585305	0.699096622	COPZ1
ENSG00000262482.1	-0.048501369	0.098686466	0.699572086	LA16c-321D4.2
ENSG00000187122.17	-0.034313494	0.098780731	0.699998352	SLIT1
ENSG00000186001.14	-0.008201518	0.098873771	0.700074302	LRCH3
ENSG00000179832.18	-0.015243763	0.098877278	0.700074302	MROH1
ENSG00000122545.20	0.01260577	0.098917073	0.700074302	SEPTIN7
ENSG00000232259.1	-0.048942079	0.098932853	0.700074302	RP11-4C20.3
ENSG00000249119.1	-0.09673038	0.09898674	0.700074302	MTND6P4
ENSG00000272144.1	-0.024833353	0.098996269	0.700074302	CTD-2035E11.5
ENSG00000275004.4	-0.022712667	0.099088282	0.700111982	ZNF280B
ENSG00000271736.2	0.061733104	0.099106215	0.700111982	LINC02772
ENSG00000129355.7	0.015926786	0.099116724	0.700111982	CDKN2D
ENSG00000086598.11	0.010447923	0.099138151	0.700111982	TMED2
ENSG00000198690.10	0.016974844	0.099188429	0.700225924	FAN1
ENSG00000198563.14	0.011304314	0.099290144	0.700583374	DDX39B
ENSG00000135452.10	0.010250631	0.099336242	0.700583374	TSPAN31
ENSG00000174684.7	-0.017613349	0.099354671	0.700583374	B4GAT1
ENSG00000283050.2	0.029116829	0.099434567	0.700583374	GTF2IP12
ENSG00000165630.14	0.024296225	0.099454886	0.700583374	PRPF18
ENSG00000168899.5	0.031001694	0.099483583	0.700583374	VAMP5

ENSG00000171617.15	-0.045027612	0.099539118	0.700583374	ENC1
ENSG00000185115.6	-0.009330339	0.099551107	0.700583374	NSMCE3
ENSG00000166278.15	0.052752915	0.09957659	0.700583374	C2
ENSG00000170037.14	-0.010953476	0.099580677	0.700583374	CNTROB
ENSG00000204583.10	0.080515844	0.099624424	0.700650783	LRCOL1
ENSG00000160209.19	-0.013039854	0.099702836	0.700817767	PDXK
ENSG00000131196.18	-0.015515246	0.099716513	0.700817767	NFATC1
ENSG00000161647.19	0.043714316	0.099875713	0.701028502	MPP3
ENSG00000182732.18	0.03861347	0.099926406	0.701028502	RGS6
ENSG00000256235.3	0.015660498	0.099944392	0.701028502	SMIM3
ENSG00000110442.12	0.012443486	0.09996596	0.701028502	COMMD9
ENSG00000103494.16	0.022979011	0.100009687	0.701028502	RPGRIP1L
ENSG00000138755.6	0.050651073	0.100064358	0.701028502	CXCL9
ENSG00000197182.14	-0.022796529	0.100089221	0.701028502	MIRLET7BHG
ENSG00000246982.7	-0.041201142	0.100099958	0.701028502	RP1-179N16.6
ENSG00000241399.7	0.021371675	0.100128138	0.701028502	CD302
ENSG00000205659.11	0.016430262	0.100128966	0.701028502	LIN52
ENSG00000111231.9	0.020735105	0.100229986	0.701028502	GPN3
ENSG00000114204.15	0.054954806	0.100457354	0.701028502	SERPINI2
ENSG00000165837.12	0.037054064	0.100470594	0.701028502	ERICH6B
ENSG00000151366.13	0.017736073	0.100471787	0.701028502	NDUFC2
ENSG00000139610.2	0.065048017	0.100481375	0.701028502	CELA1
ENSG00000285848.1	-0.065982332	0.100492469	0.701028502	RP11-254F19.5
ENSG00000143674.11	0.028994108	0.100497067	0.701028502	MAP3K21
ENSG00000261456.6	0.188113397	0.100506276	0.701028502	TUBB8
ENSG00000163879.11	0.06210932	0.100531817	0.701028502	DNALI1
ENSG00000171794.4	-0.072053337	0.10053286	0.701028502	UTF1
ENSG00000198721.13	0.012849855	0.100560383	0.701028502	ECI2
ENSG00000079805.19	-0.010625237	0.100567104	0.701028502	DNM2
ENSG00000128928.10	0.010909227	0.10058671	0.701028502	IVD
ENSG00000103024.8	-0.02103646	0.100618346	0.701028502	NME3
ENSG00000121690.11	0.038961395	0.100654838	0.701028502	DEPDC7
ENSG00000153982.11	0.020169637	0.100683323	0.701028502	GDPD1
ENSG00000220842.6	0.056449325	0.100831328	0.701028502	RPL21P16
ENSG00000113648.16	0.008582871	0.10083154	0.701028502	MACROH2A1
ENSG00000166311.10	-0.015518413	0.100834293	0.701028502	SMPD1
ENSG00000233930.4	-0.064786565	0.10086582	0.701028502	KRTAP5-AS1
ENSG00000188676.13	0.0508158	0.100871228	0.701028502	IDO2
ENSG00000032742.18	0.018646373	0.100872543	0.701028502	IFT88
ENSG00000166402.9	0.03155347	0.100985595	0.701028502	TUB
ENSG00000198001.14	0.009045487	0.101045438	0.701028502	IRAK4
ENSG00000131470.15	0.021308102	0.101123671	0.701028502	PSMC3IP
ENSG00000103363.15	0.020768161	0.101151622	0.701028502	ELOB
ENSG00000221909.3	0.014661633	0.10117104	0.701028502	FAM200A
ENSG00000136521.13	0.015348729	0.101171674	0.701028502	NDUFB5
ENSG00000133056.14	-0.018122846	0.10118945	0.701028502	PIK3C2B
ENSG00000105472.13	-0.040860984	0.101205928	0.701028502	CLEC11A

ENSG00000002822.16	-0.027523885	0.101272106	0.701028502	MAD1L1
ENSG00000227070.1	0.053784512	0.101315599	0.701028502	EPS15-AS1
ENSG00000163754.18	0.018047773	0.101327689	0.701028502	GYG1
ENSG00000034693.15	0.015467661	0.101328512	0.701028502	PEX3
ENSG00000233654.2	-0.056062457	0.101334953	0.701028502	AC093388.3
ENSG00000227191.8	-0.049805299	0.101336031	0.701028502	TRGC2
ENSG00000006715.16	0.00950939	0.101353106	0.701028502	VPS41
ENSG00000198963.11	-0.093936999	0.101392928	0.701067489	RORB
ENSG00000279799.1	-0.043071135	0.101462378	0.701094228	AC006077.3
ENSG00000142798.20	0.037348946	0.101466879	0.701094228	HSPG2
ENSG00000266490.1	-0.028865413	0.10151179	0.701094228	CTD-2349P21.9
ENSG00000251562.8	0.018062654	0.101552606	0.701094228	MALAT1
ENSG00000177453.7	-0.049729303	0.101580885	0.701094228	NIM1K
ENSG00000090861.17	-0.013775807	0.101601914	0.701094228	AARS1
ENSG00000234511.10	0.025617299	0.101686119	0.701285776	C5orf58
ENSG00000198064.13	0.11609148	0.101698064	0.701285776	NPIPB13
ENSG00000147872.10	0.015579182	0.101782469	0.701435131	PLIN2
ENSG00000183801.8	-0.068168501	0.101789073	0.701435131	OLFML1
ENSG00000165685.9	-0.096978276	0.101822332	0.701435131	TMEM52B
ENSG00000155097.12	0.013165929	0.101870507	0.701531349	ATP6V1C1
ENSG00000168310.11	0.010905113	0.101906806	0.701545744	IRF2
ENSG00000143036.17	0.067331184	0.102035879	0.701805867	SLC44A3
ENSG00000143256.5	0.020136535	0.102067037	0.701805867	PFDN2
ENSG00000273142.1	0.023108266	0.102077213	0.701805867	LINC02604
ENSG00000161955.17	0.016197015	0.102081476	0.701805867	TNFSF13
ENSG00000128203.7	0.018478801	0.102154081	0.702069666	ASPHD2
ENSG00000167702.13	-0.019889132	0.102207695	0.702194589	KIFC2
ENSG00000141627.14	0.007624456	0.102257363	0.702194589	DYM
ENSG00000175471.19	0.017056331	0.102274977	0.702194589	MCTP1
ENSG00000092820.18	-0.012610449	0.102338601	0.702396259	EZR
ENSG00000188404.10	0.019225508	0.102407503	0.702634018	SELL
ENSG00000115233.12	0.015227874	0.102453765	0.702716324	PSMD14
ENSG00000104228.13	-0.010489376	0.102661456	0.70372466	TRIM35
ENSG00000160111.15	-0.037636529	0.102680216	0.70372466	CPAMD8
ENSG00000125434.11	-0.015237432	0.102738181	0.70372466	SLC25A35
ENSG00000196683.11	0.035345723	0.102756003	0.70372466	TOMM7
ENSG00000228863.8	-0.028231014	0.10277235	0.70372466	RP11-404F10.2
ENSG00000234945.8	-0.046837056	0.102831811	0.703896787	GTF3C2-AS1
ENSG00000186806.6	-0.033476765	0.10286736	0.703905175	VSIG10L
ENSG00000053254.16	0.013847743	0.10301698	0.704193988	FOXN3
ENSG00000266993.3	-0.03404083	0.103083617	0.704193988	RP4-657D16.3
ENSG00000248719.2	0.040131843	0.103151195	0.704193988	RP11-377G16.2
ENSG00000089094.19	-0.008517569	0.103208894	0.704193988	KDM2B
ENSG00000227061.2	0.082573459	0.103213925	0.704193988	AC079779.7
ENSG00000157823.17	0.013260987	0.103214059	0.704193988	AP3S2
ENSG00000080947.15	-0.015947619	0.103237744	0.704193988	CROCCP3
ENSG00000147804.10	-0.02376947	0.103258524	0.704193988	SLC39A4

ENSG00000126267.11	0.019047222	0.103265714	0.704193988	COX6B1
ENSG00000257094.1	-0.058891849	0.103323217	0.704193988	RP11-50I19.1
ENSG00000124549.14	-0.028861692	0.103327877	0.704193988	BTN2A3P
ENSG00000211783.3	0.181492032	0.10335031	0.704193988	TRAV9-1
ENSG00000246526.2	-0.028891957	0.103447011	0.704193988	LINC02481
ENSG00000182183.15	-0.019963317	0.103465332	0.704193988	SHISAL2A
ENSG00000136315.4	0.047282616	0.103476692	0.704193988	RP11-84C10.2
ENSG00000181392.17	-0.024200975	0.103496201	0.704193988	SYNE4
ENSG00000205746.9	-0.051008968	0.103497199	0.704193988	PKD1P4
ENSG00000243244.7	-0.023689435	0.103571887	0.704193988	STON1
ENSG00000233334.3	-0.04662507	0.103583763	0.704193988	FAM53B-AS1
ENSG00000226221.1	0.0821706	0.103596317	0.704193988	RPL26P19
ENSG00000263307.1	-0.053326783	0.10372031	0.704587268	RSL1D1-DT
ENSG00000248858.7	0.061433923	0.103722887	0.704587268	FLJ46284
ENSG00000197959.14	0.029825407	0.103758413	0.704595208	DNM3
ENSG00000172572.7	0.05670896	0.103793812	0.704602285	PDE3A
ENSG00000203896.10	-0.018579138	0.103946791	0.705284311	LIME1
ENSG00000112699.11	-0.014951924	0.103988722	0.705284311	GMDS
ENSG00000137166.17	-0.017919124	0.103997788	0.705284311	FOXP4
ENSG00000251442.6	0.027883013	0.104032695	0.705284311	LINC01094
ENSG00000157693.15	0.018353755	0.104066234	0.705284311	TMEM268
ENSG00000258730.1	-0.049246672	0.104149414	0.705614859	ITPK1-AS1
ENSG00000134262.13	-0.008719128	0.104236114	0.705835326	AP4B1
ENSG00000258904.1	-0.04730758	0.104273747	0.705835326	RP11-638I2.2
ENSG00000154274.15	-0.034194111	0.104285208	0.705835326	C4orf19
ENSG00000278058.1	0.036894475	0.104395953	0.705899818	RP11-70D24.2
ENSG00000235552.4	0.026269584	0.104413682	0.705899818	RPL6P27
ENSG00000266896.1	-0.02689293	0.104428303	0.705899818	RP1-266L20.9
ENSG00000117758.14	0.00874939	0.104452468	0.705899818	STX12
ENSG00000199082.1	0.048463472	0.10446684	0.705899818	MIR342
ENSG00000279841.1	0.034778379	0.104592638	0.706343301	RP11-274A11.4
ENSG00000226508.1	-0.077323963	0.104627188	0.706343301	LINC01918
ENSG00000139631.19	-0.013392009	0.104688252	0.706343301	CSAD
ENSG00000149930.18	-0.010772702	0.104700799	0.706343301	TAOK2
ENSG00000100239.16	-0.009568843	0.104704683	0.706343301	PPP6R2
ENSG00000213228.5	0.088079287	0.104771171	0.706539869	RPL12P38
ENSG00000268903.1	-0.030817595	0.1048296	0.706539869	RP11-34P13.15
ENSG00000112773.16	-0.019170716	0.104837177	0.706539869	TENT5A
ENSG00000258754.8	-0.08919396	0.104903772	0.706756422	LINC01579
ENSG00000038945.15	0.055420113	0.104975796	0.706831499	MSR1
ENSG00000281530.1	0.038565596	0.104983848	0.706831499	DGCR12
ENSG00000287445.1	-0.074191249	0.105021917	0.706851871	RP11-61J19.7
ENSG00000211976.2	-0.075202243	0.105086102	0.706851871	IGHV3-73
ENSG00000154451.14	0.043333032	0.105103254	0.706851871	GBP5
ENSG00000140284.11	0.040584896	0.105124742	0.706851871	SLC27A2
ENSG00000163807.6	0.010759253	0.105195085	0.707034497	KIAA1143
ENSG00000144848.11	0.014403775	0.105268409	0.707034497	ATG3

ENSG00000174695.10	0.016937839	0.105333272	0.707034497	TMEM167A
ENSG00000112902.12	-0.057394992	0.105335393	0.707034497	SEMA5A
ENSG00000254233.1	0.126460494	0.105352954	0.707034497	LINC02365
ENSG00000184616.9	-0.037711527	0.105364218	0.707034497	SPDYE12P
ENSG00000167202.12	0.011784533	0.105393235	0.707034497	TBC1D2B
ENSG00000184014.9	0.012117553	0.105523822	0.707457686	DENND5A
ENSG00000205918.9	0.057859966	0.10552531	0.707457686	PDPK2P
ENSG00000184677.18	0.010967416	0.105573823	0.707551624	ZBTB40
ENSG00000231645.3	0.147799997	0.105633842	0.707663664	KRT17P6
ENSG00000145375.9	-0.015440779	0.105686614	0.707663664	SPATA5
ENSG00000075142.14	0.011983812	0.105694061	0.707663664	SRI
ENSG00000139174.12	-0.033796442	0.105786504	0.707884795	PRICKLE1
ENSG00000109536.12	0.015882014	0.105796123	0.707884795	FRG1
ENSG00000100345.22	-0.020785343	0.105919013	0.708021997	MYH9
ENSG00000099992.16	-0.011058072	0.105944805	0.708021997	TBC1D10A
ENSG00000132141.14	0.032864238	0.106029887	0.708021997	CCT6B
ENSG00000198108.4	0.114284086	0.106129793	0.708021997	CHSY3
ENSG00000109685.19	-0.009572844	0.106200101	0.708021997	NSD2
ENSG00000135476.12	-0.032845476	0.106200225	0.708021997	ESPL1
ENSG00000166105.16	-0.027896981	0.10620684	0.708021997	GLB1L3
ENSG00000230872.2	-0.045670469	0.106301126	0.708021997	MFSD13B
ENSG00000222011.9	-0.019727502	0.106313911	0.708021997	FAM185A
ENSG00000106635.8	0.009343586	0.106376358	0.708021997	BCL7B
ENSG00000172159.16	0.038080236	0.106514816	0.708021997	FRMD3
ENSG00000241741.1	-0.034282725	0.106557856	0.708021997	RPL7AP30
ENSG00000267370.1	-0.034221684	0.106736173	0.708021997	CTD-2623N2.3
ENSG00000241244.1	0.074813943	0.106792531	0.708021997	IGKV1D-16
ENSG00000138592.14	0.011525359	0.106810417	0.708021997	USP8
ENSG00000163818.19	0.018116808	0.106838001	0.708021997	LZTFL1
ENSG00000176268.5	-0.046194237	0.106894794	0.708021997	CYCSP34
ENSG00000170088.14	-0.010309012	0.106906318	0.708021997	TMEM192
ENSG00000268912.1	-0.031139204	0.106919002	0.708021997	CTD-2619J13.17
ENSG00000267649.1	-0.041091489	0.107009545	0.708021997	CTD-2587H24.10
ENSG00000235888.3	0.037656203	0.107054277	0.708021997	AF064858.8
ENSG00000110660.15	-0.01773613	0.107069367	0.708021997	SLC35F2
ENSG00000103091.15	-0.007218329	0.107096796	0.708021997	WDR59
ENSG00000235532.1	0.035213444	0.10710359	0.708021997	LINC00402
ENSG00000278931.1	-0.101433478	0.10710372	0.708021997	bP-2189O9.2
ENSG00000123600.21	0.018845339	0.107225956	0.708021997	METTL8
ENSG00000172974.11	0.028082234	0.107303914	0.708021997	VDAC2P5
ENSG00000232112.3	0.02653162	0.107359567	0.708021997	TMA7
ENSG00000272858.1	0.036759946	0.107380143	0.708021997	CTA-292E10.8
ENSG00000285884.1	0.037529048	0.107382939	0.708021997	AL022345.12
ENSG00000104081.14	0.014187484	0.107385332	0.708021997	BMF
ENSG00000127415.13	-0.018720619	0.107398779	0.708021997	IDUA
ENSG00000108370.17	-0.038222533	0.107426324	0.708021997	RGS9
ENSG00000270120.1	0.039466979	0.107433169	0.708021997	RP11-327F22.6

ENSG00000065559.15	0.012423004	0.107454894	0.708021997	MAP2K4
ENSG00000271424.1	0.048273179	0.107511415	0.708021997	GLRX5P3
ENSG00000271109.2	0.020099077	0.107524459	0.708021997	CTC-523E23.11
ENSG00000163221.9	0.041959911	0.107546203	0.708021997	S100A12
ENSG00000179218.15	-0.014821903	0.107550875	0.708021997	CALR
ENSG00000285737.1	-0.043265571	0.107551482	0.708021997	LINC02680
ENSG00000136866.14	0.033738206	0.107581592	0.708021997	ZFP37
ENSG00000115840.14	0.009702114	0.107582759	0.708021997	SLC25A12
ENSG00000169184.6	-0.0364981	0.107586265	0.708021997	MN1
ENSG00000163626.17	0.010284169	0.107663664	0.708021997	COX18
ENSG00000123179.14	0.022671128	0.107713107	0.708021997	EBPL
ENSG00000232024.2	0.024470979	0.107759322	0.708021997	LSM12P1
ENSG00000090316.16	-0.010296491	0.107785587	0.708021997	MAEA
ENSG00000283602.1	0.025075314	0.10779058	0.708021997	AC011380.8
ENSG00000224712.12	-0.128276333	0.107810291	0.708021997	NPIPA3
ENSG00000225630.1	0.054047707	0.107830091	0.708021997	MTND2P28
ENSG00000275481.1	0.032313495	0.107840565	0.708021997	RP11-474P2.6
ENSG00000132024.18	-0.014287978	0.107862989	0.708021997	CC2D1A
ENSG00000227766.1	-0.057946963	0.107889317	0.708021997	HCG4P5
ENSG00000286242.1	-0.049552793	0.107905195	0.708021997	RP11-10L12.8
ENSG00000205771.6	-0.037937462	0.107937725	0.708021997	CATSPER2P1
ENSG00000267472.1	-0.052019868	0.108003127	0.708021997	ARHGAP27P2
ENSG00000136933.17	0.013093765	0.108010943	0.708021997	RABEPK
ENSG00000100647.8	0.01316463	0.108011228	0.708021997	SUSD6
ENSG00000138756.19	0.015406859	0.108119251	0.708021997	BMP2K
ENSG00000238018.2	0.041834373	0.108129675	0.708021997	AC093110.3
ENSG00000154813.10	0.01461339	0.108196342	0.708021997	DPH3
ENSG00000187800.14	0.041330394	0.108197134	0.708021997	PEAR1
ENSG00000131126.19	0.083008538	0.108239405	0.708021997	TEX101
ENSG00000287387.1	-0.029381714	0.108286877	0.708021997	RP11-339H12.2
ENSG00000059378.13	-0.026614827	0.10830045	0.708021997	PARP12
ENSG00000263368.1	-0.053654913	0.108304732	0.708021997	RP11-720N19.1
ENSG00000127957.18	0.017267918	0.10837566	0.708021997	PMS2P3
ENSG00000276248.1	-0.038452354	0.108470173	0.708021997	RP11-230F18.5
ENSG00000143156.14	0.020757464	0.108474861	0.708021997	NME7
ENSG00000137941.17	0.050214626	0.108482048	0.708021997	TTLL7
ENSG00000134058.12	0.010666353	0.108488745	0.708021997	CDK7
ENSG00000169684.13	0.067639684	0.108491658	0.708021997	CHRNA5
ENSG00000252965.1	0.03908335	0.108499899	0.708021997	Y_RNA
ENSG00000102595.20	0.029780282	0.108509044	0.708021997	UGGT2
ENSG00000213658.12	-0.019294779	0.108567804	0.708021997	LAT
ENSG00000198346.11	0.017814554	0.108647005	0.708021997	ZNF813
ENSG00000118197.14	0.014996747	0.108709492	0.708021997	DDX59
ENSG00000065427.15	0.011252422	0.108713852	0.708021997	KARS1
ENSG00000105963.15	-0.022635033	0.108713873	0.708021997	ADAP1
ENSG00000184967.7	-0.02015515	0.108752408	0.708021997	NOC4L
ENSG00000101162.4	0.036100036	0.108795451	0.708021997	TUBB1

ENSG00000091436.17	0.019911213	0.108825611	0.708021997	MAP3K20
ENSG00000286782.1	-0.032575945	0.108935801	0.708021997	RP11-392A14.12
ENSG00000186432.10	0.007881864	0.108987041	0.708021997	KPNA4
ENSG00000109771.16	-0.026496807	0.108996825	0.708021997	LRP2BP
ENSG00000260400.1	-0.029634881	0.109036826	0.708021997	RP11-119F7.5
ENSG00000076258.10	0.018381939	0.109115255	0.708021997	FMO4
ENSG00000132600.17	-0.01155583	0.109145273	0.708021997	PRMT7
ENSG00000285581.1	-0.064606973	0.109197904	0.708021997	RP11-371C18.3
ENSG00000110841.14	-0.028122349	0.109209158	0.708021997	PPFIBP1
ENSG00000166289.6	-0.025792708	0.109228719	0.708021997	PLEKHF1
ENSG00000272777.1	-0.04178541	0.109272203	0.708021997	RP11-571L19.8
ENSG00000183506.17	0.025433046	0.109319276	0.708021997	PI4KAP2
ENSG00000088305.18	0.03010299	0.109323776	0.708021997	DNMT3B
ENSG00000029364.12	0.007772062	0.109344971	0.708021997	SLC39A9
ENSG00000183562.3	-0.046847699	0.10934837	0.708021997	CTC-343N3.1
ENSG00000136048.14	0.015449272	0.109409758	0.708021997	DRAM1
ENSG00000112941.14	-0.008475777	0.109448881	0.708021997	TENT4A
ENSG00000267096.1	-0.031501917	0.109467012	0.708021997	CTD-2537I9.13
ENSG00000272140.2	-0.032532162	0.109475226	0.708021997	RP11-574K11.29
ENSG00000185730.8	-0.013502472	0.109481798	0.708021997	ZNF696
ENSG00000135845.10	0.013776328	0.109490408	0.708021997	PIGC
ENSG00000187109.15	0.012546311	0.10952708	0.708021997	NAP1L1
ENSG00000142459.8	-0.014251756	0.109567122	0.708021997	EVI5L
ENSG00000109062.12	-0.015681321	0.109593778	0.708021997	SLC9A3R1
ENSG00000165983.15	0.017645831	0.1096515	0.708021997	PTER
ENSG00000053524.13	-0.032136434	0.109654941	0.708021997	MCF2L2
ENSG00000213096.11	0.027102952	0.109684945	0.708021997	ZNF254
ENSG00000143119.14	0.008901245	0.109693581	0.708021997	CD53
ENSG00000181789.14	-0.010859966	0.109729937	0.708021997	COPG1
ENSG00000166233.15	0.008384715	0.109746431	0.708021997	ARIH1
ENSG00000075240.17	-0.014479975	0.109754027	0.708021997	GRAMD4
ENSG00000153395.10	-0.014831175	0.109770218	0.708021997	LPCAT1
ENSG00000124019.10	0.028669661	0.109886909	0.708021997	FAM124B
ENSG00000243566.7	-0.040600199	0.109924145	0.708021997	UPK3B
ENSG00000162552.15	0.02820895	0.109931277	0.708021997	WNT4
ENSG00000166035.11	0.049215277	0.110007149	0.708021997	LIPC
ENSG00000184363.10	-0.074657739	0.110051269	0.708021997	PKP3
ENSG00000107317.13	-0.043460898	0.110132319	0.708021997	PTGDS
ENSG00000206417.8	-0.028167258	0.110211514	0.708021997	H1-10-AS1
ENSG00000146242.9	0.065854822	0.110247254	0.708021997	TPBG
ENSG00000152782.17	0.036263588	0.110284226	0.708021997	PANK1
ENSG00000263482.3	0.036843521	0.110310031	0.708021997	ANTXRLP1
ENSG00000115268.10	-0.020448406	0.110328015	0.708021997	RPS15
ENSG00000158321.18	-0.031139547	0.110371192	0.708021997	AUTS2
ENSG00000104728.16	0.078208091	0.110436247	0.708021997	ARHGEF10
ENSG00000188368.11	-0.053318754	0.110437236	0.708021997	PRR19
ENSG00000029725.17	0.014524132	0.110443826	0.708021997	RABEP1

ENSG00000174842.17	-0.017793172	0.110452138	0.708021997	GLMN
ENSG00000173726.11	0.010281788	0.110487889	0.708021997	TOMM20
ENSG00000137757.11	0.039163997	0.11051131	0.708021997	CASP5
ENSG00000220305.1	0.054119248	0.11051429	0.708021997	HNRNPH1P1
ENSG00000168000.16	-0.017578232	0.110517988	0.708021997	BSCL2
ENSG00000166454.10	0.007909688	0.110536428	0.708021997	ATMIN
ENSG00000135953.11	0.027042749	0.110596707	0.708021997	MFSD9
ENSG00000147421.18	-0.010598185	0.110627967	0.708021997	HMBOX1
ENSG00000142235.13	-0.024027594	0.110742158	0.708021997	LMTK3
ENSG00000115085.14	-0.014389237	0.110761935	0.708021997	ZAP70
ENSG00000129467.14	-0.01720755	0.110798926	0.708021997	ADCY4
ENSG00000174501.14	-0.033464676	0.110801568	0.708021997	ANKRD36C
ENSG00000196295.12	0.012158219	0.110811563	0.708021997	GARS1-DT
ENSG00000135093.13	0.010194322	0.110847679	0.708021997	USP30
ENSG00000050393.11	0.014571485	0.110892771	0.708021997	MCUR1
ENSG00000130766.5	-0.017228961	0.110913535	0.708021997	SESN2
ENSG00000178015.5	-0.038370208	0.110957016	0.708021997	GPR150
ENSG00000101019.22	0.007703746	0.111057608	0.708021997	UQCC1
ENSG00000152475.7	-0.031706841	0.111069756	0.708021997	ZNF837
ENSG00000133639.6	0.013290861	0.111080933	0.708021997	BTG1
ENSG00000154237.13	0.015212896	0.11112518	0.708021997	LRRK1
ENSG00000198585.12	0.011773803	0.111126737	0.708021997	NUDT16
ENSG00000054282.16	0.013768708	0.111158025	0.708021997	SDCCAG8
ENSG00000085871.9	0.020535109	0.111199791	0.708021997	MGST2
ENSG00000165568.18	0.048270291	0.111205837	0.708021997	AKR1E2
ENSG00000174327.7	-0.013736903	0.111263351	0.708021997	SLC16A13
ENSG00000105497.8	0.012642023	0.11128071	0.708021997	ZNF175
ENSG00000279072.1	0.05679142	0.111287108	0.708021997	RP13-580B18.4
ENSG00000224614.2	-0.023783746	0.111299118	0.708021997	TNK2-AS1
ENSG00000144791.10	0.011716322	0.111350021	0.708021997	LIMD1
ENSG00000152767.17	0.025802173	0.111353017	0.708021997	FARP1
ENSG00000150667.8	0.048999599	0.111403437	0.708021997	FSIP1
ENSG00000129195.16	-0.056799593	0.111435769	0.708021997	PIMREG
ENSG00000124225.16	-0.030664105	0.111440721	0.708021997	PMEPA1
ENSG00000172409.7	0.009172089	0.111454104	0.708021997	CLP1
ENSG00000261019.1	-0.048556969	0.111500211	0.708021997	RP11-111K18.2
ENSG00000149084.13	-0.020832626	0.111556179	0.708021997	HSD17B12
ENSG00000198580.7	0.049594977	0.111595072	0.708021997	RP11-611L7.2
ENSG00000172530.21	-0.01295495	0.111611942	0.708021997	BANP
ENSG00000168772.11	-0.070804375	0.111616692	0.708021997	CXXC4
ENSG00000138449.11	0.017411637	0.111674512	0.708169726	SLC40A1
ENSG00000242802.9	-0.013631037	0.111732798	0.70824035	AP5Z1
ENSG00000258568.1	0.047336317	0.111754719	0.70824035	RHOQP1
ENSG00000225573.4	0.079163274	0.11184168	0.7085725	RPL35P5
ENSG00000262874.2	-0.039892987	0.111906208	0.708757384	C19orf84
ENSG00000257365.8	0.01790477	0.111939983	0.708757384	FNTB
ENSG00000019102.12	0.037734404	0.112116406	0.709279094	VSIG2

ENSG00000258525.2	0.075518785	0.112133817	0.709279094	RP11-829H16.3
ENSG00000109083.14	-0.011553285	0.112144417	0.709279094	IFT20
ENSG00000020426.11	0.018690691	0.112273975	0.709279094	MNAT1
ENSG00000232855.7	-0.037564822	0.112275712	0.709279094	AF131217.1
ENSG00000184613.11	0.027518697	0.112281355	0.709279094	NELL2
ENSG00000241678.1	-0.04336374	0.112297855	0.709279094	RP11-732A19.1
ENSG00000239791.2	-0.044237808	0.112346934	0.709279094	AC002310.7
ENSG00000131143.10	0.015283577	0.112372929	0.709279094	COX4I1
ENSG00000016391.11	0.055580233	0.112389871	0.709279094	CHDH
ENSG00000154856.13	0.041139062	0.112410297	0.709279094	APCDD1
ENSG00000170296.10	0.015170909	0.112450262	0.709279094	GABARAP
ENSG00000086570.13	0.039747499	0.112496435	0.709279094	FAT2
ENSG00000151458.12	0.021527647	0.112512465	0.709279094	ANKRD50
ENSG00000101353.15	0.027328374	0.112570559	0.709279094	MROH8
ENSG00000211715.1	0.097048335	0.112593593	0.709279094	TRBV5-3
ENSG00000265452.1	-0.046160322	0.11262738	0.709279094	MIR3682
ENSG00000213386.3	0.07370981	0.112644919	0.709279094	RP11-779O18.2
ENSG00000231064.7	-0.029455351	0.112717973	0.709359706	THBS3-AS1
ENSG00000244134.1	-0.038618284	0.112726901	0.709359706	RPS12P20
ENSG00000105220.17	-0.016277576	0.112762727	0.709367485	GPI
ENSG00000177119.17	0.013910036	0.112843814	0.709477462	ANO6
ENSG00000143614.10	-0.010147628	0.112955148	0.709477462	GATAD2B
ENSG00000119596.18	-0.014079508	0.113015531	0.709477462	YLPM1
ENSG00000127412.7	0.040455911	0.113046848	0.709477462	TRPV5
ENSG00000231747.1	0.049814661	0.113056239	0.709477462	AC079922.2
ENSG00000236144.8	0.014917824	0.113083306	0.709477462	TMEM147-AS1
ENSG00000120053.12	-0.012126244	0.113085506	0.709477462	GOT1
ENSG00000236859.7	0.014852581	0.113095421	0.709477462	NIFK-AS1
ENSG00000117543.21	0.020619233	0.113104288	0.709477462	DPH5
ENSG00000279989.1	0.050144448	0.113142314	0.709477462	RP11-158H5.2
ENSG00000125458.7	-0.016516419	0.113163071	0.709477462	NT5C
ENSG00000253200.1	-0.017425402	0.113295669	0.709477462	RP11-582J16.5
ENSG00000140320.12	-0.009726975	0.113321222	0.709477462	BAHD1
ENSG00000248455.6	0.055164437	0.113357053	0.709477462	LINC02217
ENSG00000178252.18	-0.012473549	0.113394094	0.709477462	WDR6
ENSG00000160781.17	-0.035615083	0.113396004	0.709477462	PAQR6
ENSG00000279833.1	0.020894642	0.113432927	0.709477462	RP4-742C19.13
ENSG00000139180.11	0.014207912	0.113453356	0.709477462	NDUFA9
ENSG00000141994.16	-0.01476955	0.113546882	0.709477462	DUS3L
ENSG00000265806.1	0.038372589	0.113549114	0.709477462	MIR4292
ENSG00000161981.11	-0.01880015	0.113566951	0.709477462	SNRNP25
ENSG00000276961.1	0.044143846	0.113568255	0.709477462	MIR7848
ENSG00000233806.7	-0.035689945	0.113575898	0.709477462	LINC01237
ENSG00000239559.2	0.063206405	0.11369934	0.709693793	RPL37P2
ENSG00000196263.8	0.052012802	0.113718249	0.709693793	ZNF471
ENSG00000230061.2	-0.041922219	0.113742612	0.709693793	TRPM2-AS
ENSG00000224858.5	0.043096562	0.113785135	0.709693793	RPL29P11

ENSG00000117461.15	-0.026721563	0.113789106	0.709693793	PIK3R3
ENSG00000229054.1	0.07594871	0.113838583	0.709693793	RPS29P14
ENSG00000197006.14	0.01448717	0.113885863	0.709693793	METTL9
ENSG00000184164.15	-0.013933374	0.113967905	0.709693793	CRELD2
ENSG00000226266.6	0.043057533	0.114010763	0.709693793	AC009961.3
ENSG00000287644.1	0.033931657	0.114011526	0.709693793	CTD-2267D19.8
ENSG00000051341.15	-0.040649323	0.114052604	0.709693793	POLQ
ENSG00000116497.18	0.009713734	0.114061698	0.709693793	S100PBP
ENSG00000160310.18	0.013092717	0.114098544	0.709693793	PRMT2
ENSG00000196569.13	0.037787291	0.114144923	0.709693793	LAMA2
ENSG00000003756.17	0.006406634	0.114168379	0.709693793	RBM5
ENSG00000211728.2	0.045496705	0.114208755	0.709693793	TRBV5-6
ENSG00000243066.3	-0.044437006	0.114222315	0.709693793	RN7SL842P
ENSG00000067836.13	0.023336663	0.114271428	0.709693793	ROGDI
ENSG00000143845.15	0.030117338	0.11430716	0.709693793	ETNK2
ENSG00000281383.1	-0.074198259	0.114561077	0.709693793	CH507-513H4.5
ENSG00000070756.17	0.008524387	0.114664293	0.709693793	PABPC1
ENSG00000159899.15	-0.019691973	0.114677646	0.709693793	NPR2
ENSG00000237181.1	0.020425622	0.114682957	0.709693793	PRKAR1B-AS1
ENSG00000100558.9	0.051874458	0.114683266	0.709693793	PLEK2
ENSG00000178105.11	-0.011498831	0.114759879	0.709693793	DDX10
ENSG00000280069.1	-0.056067194	0.114776134	0.709693793	CTD-2349P21.3
ENSG00000197208.6	0.019178296	0.114784269	0.709693793	SLC22A4
ENSG00000140105.18	0.027631505	0.114784967	0.709693793	WARS1
ENSG00000089163.4	-0.024600543	0.114808364	0.709693793	SIRT4
ENSG00000261408.7	-0.020045311	0.114856506	0.709693793	TEN1-CDK3
ENSG00000116120.11	0.010718888	0.114864261	0.709693793	FARSB
ENSG00000158195.11	0.00966453	0.114870814	0.709693793	WASF2
ENSG00000165669.14	0.012566252	0.114981286	0.709693793	FAM204A
ENSG00000188295.15	-0.01839645	0.115099399	0.709693793	ZNF669
ENSG00000220749.4	0.055529907	0.11511595	0.709693793	RPL21P28
ENSG00000128829.12	0.013501325	0.115124933	0.709693793	EIF2AK4
ENSG00000124205.18	-0.070557308	0.115126869	0.709693793	EDN3
ENSG00000149503.13	-0.017719683	0.115148628	0.709693793	INCENP
ENSG00000167967.16	-0.012887631	0.115227173	0.709693793	E4F1
ENSG00000228318.3	-0.129056168	0.115270157	0.709693793	AP001610.5
ENSG00000182685.8	-0.021010315	0.115271831	0.709693793	BRICD5
ENSG00000138303.18	0.009997169	0.115323122	0.709693793	ASCC1
ENSG00000228251.1	0.03925731	0.115332305	0.709693793	AC012442.6
ENSG00000162614.19	0.035092383	0.115369908	0.709693793	NEXN
ENSG00000185559.16	0.125449351	0.115397921	0.709693793	DLK1
ENSG00000273837.1	-0.051380565	0.115415458	0.709693793	LLNLR-470E3.1
ENSG00000105379.10	-0.016098105	0.11541782	0.709693793	ETFB
ENSG00000204110.6	0.080021489	0.115488946	0.709693793	LINC02520
ENSG00000166451.14	0.020392247	0.115534026	0.709693793	CENPN
ENSG00000242028.8	-0.016211561	0.115545465	0.709693793	HYPK
ENSG00000168924.15	-0.008796149	0.115554414	0.709693793	LETM1

ENSG00000285647.1	0.159549782	0.115565082	0.709693793	XXbac-BPG248L24.14
ENSG00000099904.16	-0.013318508	0.115573165	0.709693793	ZDHHC8
ENSG00000100911.16	0.021721439	0.115576218	0.709693793	PSME2
ENSG00000169495.5	-0.060106471	0.115594232	0.709693793	HTRA4
ENSG00000119801.13	0.015272936	0.115669124	0.709693793	YPEL5
ENSG00000136937.13	0.008620616	0.115727391	0.709693793	NCBP1
ENSG00000067248.10	0.010850299	0.115744409	0.709693793	DHX29
ENSG00000233968.7	0.065378353	0.115752637	0.709693793	RP11-354E11.2
ENSG00000165804.16	-0.023184824	0.115757121	0.709693793	ZNF219
ENSG00000065135.12	0.011110343	0.115838997	0.709693793	GNAI3
ENSG00000226124.8	0.034608179	0.115856401	0.709693793	FTCDNL1
ENSG00000139514.13	-0.014122465	0.115891822	0.709693793	SLC7A1
ENSG00000113140.11	0.040070534	0.115944088	0.709693793	SPARC
ENSG00000181754.7	0.01755793	0.116030504	0.709693793	AMIGO1
ENSG00000171867.18	0.012730691	0.116034604	0.709693793	PRNP
ENSG00000124785.9	0.067357664	0.116039619	0.709693793	NRN1
ENSG00000182979.18	-0.012075374	0.116048122	0.709693793	MTA1
ENSG00000117091.10	0.017392809	0.116152495	0.709693793	CD48
ENSG00000185800.12	-0.015772001	0.116241632	0.709693793	DMWD
ENSG00000155666.12	-0.011886785	0.11626795	0.709693793	KDM8
ENSG00000169583.13	-0.037791716	0.116292818	0.709693793	CLIC3
ENSG00000070010.19	0.008212378	0.116317116	0.709693793	UFD1
ENSG00000276672.1	-0.03294458	0.116326723	0.709693793	RP11-142E9.1
ENSG00000100664.11	0.009634661	0.116345241	0.709693793	EIF5
ENSG00000136114.17	-0.02998179	0.116410258	0.709693793	THSD1
ENSG00000279622.2	-0.053898075	0.116415945	0.709693793	RP11-254F19.4
ENSG00000176092.17	-0.025124893	0.116460573	0.709693793	CRYBG2
ENSG00000100441.10	0.011741069	0.11649574	0.709693793	KHNYN
ENSG00000176473.14	-0.017995595	0.116580042	0.709693793	WDR25
ENSG00000286699.1	-0.024808451	0.116583534	0.709693793	RP11-454H13.8
ENSG00000256338.2	0.070002674	0.116601902	0.709693793	RPL41P2
ENSG00000142920.17	0.018685262	0.116608871	0.709693793	AZIN2
ENSG00000170265.12	-0.01329759	0.116639287	0.709693793	ZNF282
ENSG00000105369.10	-0.041705507	0.116686514	0.709693793	CD79A
ENSG00000111254.8	0.037137457	0.116760223	0.709693793	AKAP3
ENSG00000260589.1	-0.036448787	0.116761627	0.709693793	STAM-AS1
ENSG00000224985.1	0.034366703	0.116783839	0.709693793	RP11-297K8.2
ENSG00000238287.1	-0.045506435	0.116806383	0.709693793	RP11-656D10.3
ENSG00000161091.14	-0.013019869	0.116828997	0.709693793	MFSD12
ENSG00000248309.7	-0.029785529	0.116843173	0.709693793	MEF2C-AS1
ENSG00000231789.2	-0.043466245	0.116893157	0.709693793	PIK3CD-AS2
ENSG00000263220.1	-0.054205657	0.116899902	0.709693793	RP11-420A6.2
ENSG00000171735.19	0.014295184	0.116929057	0.709693793	CAMTA1
ENSG00000237493.3	-0.05404667	0.116968004	0.709693793	RP11-603J24.7
ENSG00000136059.15	-0.01147203	0.117052139	0.709693793	VILL
ENSG00000228487.2	0.046783434	0.117065614	0.709693793	RP13-225O21.2
ENSG00000229017.7	0.044972922	0.117094996	0.709693793	LINC01277

ENSG00000258443.1	0.061595448	0.117116597	0.709693793	RP3-414A15.11
ENSG00000160282.15	-0.081640133	0.117119032	0.709693793	FTCD
ENSG00000159433.12	-0.018668106	0.117139763	0.709693793	STARD9
ENSG00000176531.10	-0.021054785	0.117152716	0.709693793	PHLDB3
ENSG00000113712.19	0.01076656	0.11722827	0.709693793	CSNK1A1
ENSG00000171621.14	-0.024958858	0.117257821	0.709693793	SPSB1
ENSG00000005108.16	0.077667177	0.117319227	0.709693793	THSD7A
ENSG00000116213.16	-0.009104455	0.117348198	0.709693793	WRAP73
ENSG00000196781.16	-0.027286749	0.117356457	0.709693793	TLE1
ENSG00000095303.17	0.026074467	0.117371088	0.709693793	PTGS1
ENSG00000231856.2	0.017630048	0.117424767	0.709693793	RP11-327P2.5
ENSG00000163449.11	0.018521845	0.117435491	0.709693793	TMEM169
ENSG00000153558.16	-0.02498423	0.117451762	0.709693793	FBXL2
ENSG00000169330.9	-0.039337087	0.117509843	0.7097735	MINAR1
ENSG00000121316.11	0.015560292	0.117557417	0.7097735	PLBD1
ENSG00000205336.14	-0.035330452	0.117568782	0.7097735	ADGRG1
ENSG00000008324.12	0.019423671	0.117639951	0.709994149	SS18L2
ENSG00000204283.4	0.042734922	0.117680845	0.710032	LINC01973
ENSG00000130702.15	-0.03603436	0.117791538	0.71049084	LAMA5
ENSG00000055332.19	-0.03394762	0.117931305	0.710510758	EIF2AK2
ENSG00000176472.10	-0.02146589	0.117965932	0.710510758	ZNF575
ENSG00000127125.9	0.009683767	0.118026302	0.710510758	PPCS
ENSG00000133250.14	-0.018192235	0.118027307	0.710510758	ZNF414
ENSG00000170634.13	0.014958723	0.118055997	0.710510758	ACYP2
ENSG00000105538.10	-0.08660053	0.118061006	0.710510758	RASIP1
ENSG00000092853.14	-0.033066908	0.11808021	0.710510758	CLSPN
ENSG00000132881.12	-0.019523966	0.118092243	0.710510758	CPLANE2
ENSG00000241560.7	-0.048152419	0.118109892	0.710510758	ZBTB20-AS1
ENSG00000235092.6	-0.032918871	0.118141295	0.710510758	ID2-AS1
ENSG00000105321.14	-0.016004909	0.118307512	0.710957826	CCDC9
ENSG00000175063.17	-0.026692543	0.11834337	0.710957826	UBE2C
ENSG00000141030.13	0.011672795	0.118358072	0.710957826	COPS3
ENSG00000165609.13	0.009814228	0.118362605	0.710957826	NUDT5
ENSG00000188710.3	0.045668762	0.118388969	0.710957826	QRFP
ENSG00000240440.1	0.061509573	0.11857146	0.711035599	AC007879.3
ENSG00000267892.1	0.040477028	0.118604568	0.711035599	CTD-2540F13.2
ENSG00000115165.10	0.009507665	0.118614568	0.711035599	CYTIP
ENSG00000131778.19	-0.020730598	0.11869442	0.711035599	CHD1L
ENSG00000275344.1	0.046678184	0.118714342	0.711035599	MIR6503
ENSG00000144306.15	0.015048562	0.118722524	0.711035599	SCRN3
ENSG00000139233.7	0.012960682	0.118753396	0.711035599	LLPH
ENSG00000108179.14	0.015152659	0.118773869	0.711035599	PPIF
ENSG00000240225.10	0.019715365	0.118777979	0.711035599	ZNF542P
ENSG00000180596.7	0.020861278	0.118807368	0.711035599	H2BC4
ENSG00000170903.11	0.015850305	0.118807474	0.711035599	MSANTD4
ENSG00000183208.13	-0.019314558	0.118846995	0.711035599	GDPGP1
ENSG00000104960.15	-0.013161279	0.118852644	0.711035599	PTOV1

ENSG00000145569.6	0.013437782	0.118937055	0.71106897	OTULINL
ENSG00000196968.11	-0.016145531	0.118954888	0.71106897	FUT11
ENSG00000162496.9	-0.020063093	0.118965005	0.71106897	DHRS3
ENSG00000215021.10	0.010814343	0.119054623	0.71106897	PHB2
ENSG00000182718.18	0.015914824	0.11907126	0.71106897	ANXA2
ENSG00000173928.4	0.01590225	0.119084465	0.71106897	SWSAP1
ENSG00000139200.14	0.029021919	0.119100932	0.71106897	PIANP
ENSG00000090020.11	-0.015261431	0.1192081	0.711434846	SLC9A1
ENSG00000113575.10	0.007804537	0.119333605	0.711434846	PPP2CA
ENSG00000100401.20	-0.013959686	0.119398507	0.711434846	RANGAP1
ENSG00000175592.9	0.037841466	0.11944007	0.711434846	FOSL1
ENSG00000268218.1	-0.018574739	0.119486208	0.711434846	AC137932.4
ENSG00000237672.1	-0.042698438	0.119510746	0.711434846	KRR1P1
ENSG00000165475.15	0.013461376	0.119527475	0.711434846	CRYL1
ENSG00000005844.19	-0.014125321	0.119531166	0.711434846	ITGAL
ENSG00000205133.12	0.017819638	0.119535333	0.711434846	TRIQK
ENSG00000069424.15	-0.014169431	0.11960586	0.711434846	KCNAB2
ENSG00000075884.14	0.012250041	0.119625112	0.711434846	ARHGAP15
ENSG00000175741.5	0.036253017	0.119655538	0.711434846	RWDD4P2
ENSG00000170837.3	-0.020893767	0.119656446	0.711434846	GPR27
ENSG00000126001.16	-0.00964275	0.119660802	0.711434846	CEP250
ENSG00000121454.6	0.031157448	0.119727769	0.711434846	LHX4
ENSG00000072803.18	0.008981704	0.119751177	0.711434846	FBXW11
ENSG00000233008.6	-0.070131259	0.119751955	0.711434846	LINC01725
ENSG00000186152.6	-0.057030742	0.119845286	0.711729932	LILRP1
ENSG00000090989.18	0.010634424	0.119871035	0.711729932	EXOC1
ENSG00000106066.15	0.017285499	0.119943826	0.711956003	CPVL
ENSG00000206560.12	0.01877305	0.1200162	0.712179466	ANKRD28
ENSG00000234072.1	-0.013743656	0.120068736	0.712256813	AC074117.10
ENSG00000186496.12	0.023543987	0.120100564	0.712256813	ZNF396
ENSG00000241106.8	-0.038051997	0.120135929	0.712256813	HLA-DOB
ENSG00000278661.1	-0.05894146	0.120168158	0.712256813	TRAJ37
ENSG00000280789.2	-0.010744795	0.120240407	0.712479129	PAGR1
ENSG00000157152.17	-0.053782537	0.120308539	0.712616414	SYN2
ENSG00000143549.21	0.007535408	0.120333072	0.712616414	TPM3
ENSG00000143621.17	0.010236988	0.120396996	0.712789144	ILF2
ENSG00000228315.12	0.0143802	0.120439911	0.712837428	GUSBP11
ENSG00000184640.20	-0.010471748	0.120563078	0.713128463	SEPTIN9
ENSG00000117118.10	0.010243719	0.120594812	0.713128463	SDHB
ENSG00000105974.13	-0.052482984	0.120646212	0.713128463	CAV1
ENSG00000171790.15	-0.03317277	0.120670629	0.713128463	SLFNL1
ENSG00000168488.19	-0.01113863	0.120736774	0.713128463	ATXN2L
ENSG00000172346.15	0.084495028	0.120748971	0.713128463	CSDC2
ENSG00000011243.19	-0.010984993	0.120761769	0.713128463	AKAP8L
ENSG00000244313.3	0.02895433	0.120777124	0.713128463	RP11-425L10.1
ENSG00000249115.9	-0.01115202	0.12080437	0.713128463	HAUS5
ENSG00000171219.9	-0.018925668	0.120851131	0.713128463	CDC42BPG

ENSG00000104218.16	-0.013051718	0.120871589	0.713128463	CSPP1
ENSG00000198736.12	0.016044452	0.12100926	0.713553632	MSRB1
ENSG00000261327.5	0.067999195	0.121028169	0.713553632	RP11-863P13.3
ENSG00000221937.4	0.049704283	0.121048034	0.713553632	TAS2R40
ENSG00000165959.12	0.015395079	0.121152988	0.713967092	CLMN
ENSG00000287853.1	0.059788883	0.121226984	0.71399365	RP1-64K7.5
ENSG00000259248.7	-0.021866576	0.121227125	0.71399365	USP3-AS1
ENSG00000279861.1	-0.02149752	0.121424362	0.714923097	RP11-43N16.4
ENSG00000164483.17	-0.023150545	0.121529223	0.714923097	SAMD3
ENSG00000275066.5	-0.011130482	0.121540494	0.714923097	SYNRG
ENSG00000167483.19	-0.035365535	0.12155374	0.714923097	NIBAN3
ENSG00000225259.4	0.051609669	0.121589171	0.714923097	ST13P6
ENSG00000167186.11	0.011002885	0.121616151	0.714923097	COQ7
ENSG00000129566.13	0.010724727	0.121641597	0.714923097	TEP1
ENSG00000117335.20	0.017372287	0.121663819	0.714923097	CD46
ENSG00000286158.1	0.054343403	0.12170142	0.714939191	RP5-1103G7.11
ENSG00000143543.15	0.015566471	0.121775424	0.714992219	JTB
ENSG00000131584.19	-0.013923901	0.121811795	0.714992219	ACAP3
ENSG00000084710.14	0.049038199	0.121815039	0.714992219	EFR3B
ENSG00000235459.5	0.089301608	0.121874851	0.715018609	RPS26P31
ENSG00000167114.13	-0.013008673	0.121889265	0.715018609	SLC27A4
ENSG00000181800.6	0.042726985	0.121976394	0.715325102	CELF2-AS1
ENSG00000082212.13	0.008575193	0.122068614	0.715661274	ME2
ENSG00000114473.14	0.01806176	0.122256845	0.716237261	IQCG
ENSG00000275854.1	-0.034128607	0.122262431	0.716237261	RP11-278C7.5
ENSG00000270001.1	0.046605148	0.122271633	0.716237261	RP11-218C14.8
ENSG00000182118.8	0.023061368	0.122362307	0.716335934	FAM89A
ENSG00000197308.9	-0.061381385	0.122430138	0.716335934	GATA3-AS1
ENSG00000237879.1	0.046769939	0.122464485	0.716335934	LINC00398
ENSG00000163359.17	0.033337816	0.122473268	0.716335934	COL6A3
ENSG00000213516.10	0.010485654	0.122502496	0.716335934	RBMXL1
ENSG00000124251.11	-0.027214078	0.12253296	0.716335934	TP53TG5
ENSG00000120334.16	-0.017212142	0.122532985	0.716335934	CENPL
ENSG00000147475.17	0.00882514	0.122689629	0.716708059	ERLIN2
ENSG00000279640.1	-0.064101745	0.122691714	0.716708059	RP13-977J11.6
ENSG00000213024.12	-0.010239375	0.122701482	0.716708059	NUP62
ENSG00000118162.14	-0.024063581	0.122818554	0.717141037	KPTN
ENSG00000118557.16	-0.025524912	0.12285689	0.717141037	PMFBP1
ENSG00000159885.14	0.01918918	0.122920487	0.717141037	ZNF222
ENSG00000265787.2	-0.035943178	0.122940497	0.717141037	CYP4F35P
ENSG00000104731.14	-0.012920993	0.122950453	0.717141037	KLHDC4
ENSG00000274750.3	-0.050271684	0.123026501	0.717380578	H3C6
ENSG00000263002.8	-0.013318719	0.123196622	0.717781129	ZNF234
ENSG00000261468.1	0.020612977	0.123215093	0.717781129	RP11-1024P17.1
ENSG00000217325.2	0.035612697	0.123282745	0.717781129	PRELID1P1
ENSG00000205929.11	0.041060316	0.123288905	0.717781129	C21orf62
ENSG00000266402.3	-0.050276193	0.123297065	0.717781129	SNHG25

ENSG00000138757.15	0.010333469	0.123310579	0.717781129	G3BP2
ENSG00000215529.12	0.025965067	0.12339241	0.717781129	EFCAB8
ENSG00000215568.9	0.103304457	0.123410237	0.717781129	GAB4
ENSG00000287543.1	0.031016682	0.123427766	0.717781129	RP11-941F15.2
ENSG00000159110.21	0.011743368	0.123481836	0.717781129	IFNAR2
ENSG00000225544.1	0.10248334	0.123521792	0.717781129	KB-1027C11.4
ENSG00000111328.7	0.016989476	0.12357171	0.717781129	CDK2AP1
ENSG00000232004.1	0.032350342	0.123588317	0.717781129	CAP1P2
ENSG00000181090.21	-0.009996373	0.123601901	0.717781129	EHMT1
ENSG00000023318.8	0.008829984	0.123641342	0.717781129	ERP44
ENSG00000134508.13	0.045548096	0.123667042	0.717781129	CABLES1
ENSG00000106537.8	-0.032247331	0.123690195	0.717781129	TSPAN13
ENSG00000287809.1	-0.040833761	0.12373218	0.717821653	CTB-31N19.7
ENSG00000100678.19	0.047195855	0.123790218	0.71784101	SLC8A3
ENSG00000245322.7	-0.036115766	0.123817059	0.71784101	H2AZ1-DT
ENSG00000166821.9	0.01865269	0.123857282	0.71784101	PEX11A
ENSG00000227012.3	-0.148701623	0.123875528	0.71784101	LINC02527
ENSG00000260997.1	-0.020364733	0.123972143	0.718184355	RP4-647J21.1
ENSG00000173546.7	0.045463059	0.124004818	0.718184355	CSPG4
ENSG00000267745.1	-0.027781898	0.12404331	0.718204458	RP11-686D22.8
ENSG00000013503.10	0.012144872	0.124093639	0.71829307	POLR3B
ENSG00000134193.15	0.067778604	0.12430467	0.718954598	REG4
ENSG00000165097.16	0.014176689	0.12430479	0.718954598	KDM1B
ENSG00000161643.13	0.023996317	0.124348901	0.718954598	SIGLEC16
ENSG00000115241.11	-0.012121062	0.124361891	0.718954598	PPM1G
ENSG00000236552.2	0.020325413	0.12442541	0.718954598	RPL13AP5
ENSG00000248734.2	0.029989964	0.12445391	0.718954598	CTD-2260A17.1
ENSG00000255337.1	-0.047178252	0.124538441	0.718954598	RP11-315O6.1
ENSG00000183813.7	-0.026334991	0.124578822	0.718954598	CCR4
ENSG00000127528.6	-0.016056811	0.124581697	0.718954598	KLF2
ENSG00000211965.4	-0.058018735	0.124601222	0.718954598	IGHV3-49
ENSG00000157992.12	-0.023029603	0.124607439	0.718954598	KRTCAP3
ENSG00000144895.12	0.015220269	0.124668621	0.718954598	EIF2A
ENSG00000211611.2	0.071112552	0.12469397	0.718954598	IGKV6-21
ENSG00000010379.16	-0.045233528	0.124708642	0.718954598	SLC6A13
ENSG00000186026.7	-0.015400157	0.124733785	0.718954598	ZNF284
ENSG00000130147.16	0.027330015	0.124777771	0.719006046	SH3BP4
ENSG00000073150.14	-0.024319942	0.124813354	0.719009065	PANX2
ENSG00000151577.13	-0.069396049	0.124859802	0.719074647	DRD3
ENSG00000183844.17	-0.139686187	0.124935646	0.719115777	FAM3B
ENSG00000167394.13	-0.019201975	0.124962102	0.719115777	ZNF668
ENSG00000119335.17	0.007330969	0.124998602	0.719115777	SET
ENSG00000171631.16	-0.028071341	0.125007204	0.719115777	P2RY6
ENSG00000287555.1	-0.057546069	0.125139825	0.719656042	RP11-555K12.4
ENSG00000130921.9	0.00971938	0.125171304	0.719656042	C12orf65
ENSG00000105251.11	-0.047928557	0.125356796	0.720520511	SHD
ENSG00000251322.9	0.062046118	0.12545842	0.720902573	SHANK3

ENSG00000117640.18	0.008952064	0.125578065	0.72125359	MTFR1L
ENSG00000217130.1	0.057047746	0.125589846	0.72125359	RP3-375P9.2
ENSG00000241258.7	0.007875173	0.125627135	0.721265757	CRCP
ENSG00000278206.1	-0.042630244	0.125728329	0.72162292	RP1-20N2.8
ENSG00000108819.11	-0.011773498	0.125759719	0.72162292	PPP1R9B
ENSG00000164054.16	-0.019310409	0.12584893	0.72188907	SHISA5
ENSG00000259935.1	-0.059671342	0.125881244	0.72188907	RP11-519C12.1
ENSG00000139266.6	-0.013571566	0.125942892	0.72188907	MARCHF9
ENSG00000005075.15	0.015379732	0.125946903	0.72188907	POLR2J
ENSG00000076924.12	-0.012066989	0.126048246	0.722081152	XAB2
ENSG00000178075.20	-0.033153311	0.126050835	0.722081152	GRAMD1C
ENSG00000151806.14	0.011301113	0.126116621	0.722199868	GUF1
ENSG00000273691.1	-0.055856343	0.126153266	0.722199868	RP11-617F23.2
ENSG00000101161.8	-0.013078288	0.126177205	0.722199868	PRPF6
ENSG00000090565.17	-0.011180061	0.126252289	0.722427995	RAB11FIP3
ENSG00000231672.8	-0.043119784	0.126316271	0.722559496	DIRC3
ENSG00000261096.1	-0.052643027	0.126345736	0.722559496	RP11-690I21.2
ENSG00000178814.17	-0.027620547	0.126416948	0.722765196	OPLAH
ENSG00000172940.13	-0.05868599	0.126489223	0.722976864	SLC22A13
ENSG00000287299.1	-0.078528427	0.126553388	0.723142065	RP11-6G22.1
ENSG00000231989.5	0.051674164	0.126600622	0.723180536	PPP1R2B
ENSG00000115548.17	0.01148117	0.126630647	0.723180536	KDM3A
ENSG00000226240.2	-0.049991312	0.126728153	0.723535902	LINC00381
ENSG00000116701.15	0.010597465	0.126868126	0.723948342	NCF2
ENSG00000164050.13	0.025178006	0.126870994	0.723948342	PLXNB1
ENSG00000138777.20	0.01742712	0.126930013	0.723956692	PPA2
ENSG00000250274.2	0.03664742	0.126948469	0.723956692	CTB-114C7.4
ENSG00000162490.7	0.045563522	0.126978361	0.723956692	DRAXIN
ENSG00000226791.7	-0.017847878	0.127035715	0.72408239	LINC02611
ENSG00000170348.9	0.007934796	0.127076004	0.724110777	TMED10
ENSG00000215458.8	0.020409838	0.127193831	0.724145961	AATBC
ENSG00000268366.1	0.046815961	0.127221496	0.724145961	SERTAD3-AS1
ENSG00000225431.1	-0.07545999	0.127237922	0.724145961	LINC01671
ENSG00000267795.6	0.073792115	0.127263864	0.724145961	SMIM22
ENSG00000233469.1	-0.039092559	0.127267196	0.724145961	ST6GALNAC4P1
ENSG00000179588.9	-0.025387294	0.127373003	0.724145961	ZFPM1
ENSG00000269961.1	-0.031987597	0.127419049	0.724145961	ERBIN-DT
ENSG00000235772.1	0.060539337	0.12744783	0.724145961	AP001625.6
ENSG00000225313.5	0.019806806	0.127455763	0.724145961	RP11-415J8.3
ENSG00000254909.1	0.032492157	0.127509204	0.724145961	RP11-110I1.5
ENSG00000235238.1	0.050383418	0.127520982	0.724145961	SUMO2P1
ENSG00000140368.13	-0.0136371	0.127537211	0.724145961	PSTPIP1
ENSG00000165526.9	0.008020031	0.127541214	0.724145961	RPUSD4
ENSG00000286485.1	0.043044795	0.127612414	0.724285197	LL22NC01-132D12.4
ENSG00000255833.2	0.030590202	0.127651195	0.724285197	TIFAB
ENSG00000282386.1	-0.039877118	0.127681546	0.724285197	RP11-422P24.12
ENSG00000228808.1	-0.060133514	0.127707877	0.724285197	HMGB3P4

ENSG00000139154.16	-0.014637279	0.127742323	0.724285197	AEBP2
ENSG00000109436.8	0.019252319	0.127779412	0.72429524	TBC1D9
ENSG00000204227.5	-0.011344712	0.127871261	0.724426507	RING1
ENSG00000055163.20	-0.010318543	0.127880565	0.724426507	CYFIP2
ENSG00000249207.2	-0.04440325	0.127908542	0.724426507	RP11-360F5.1
ENSG00000108298.12	0.015323365	0.127971327	0.724530295	RPL19
ENSG00000113384.14	-0.008760826	0.128009541	0.724530295	GOLPH3
ENSG00000100528.12	0.014233302	0.128032855	0.724530295	CNIH1
ENSG00000158477.7	0.02675703	0.128115764	0.724799473	CD1A
ENSG00000255284.2	-0.023577957	0.128178803	0.724956119	AP006621.5
ENSG00000144407.10	0.057960635	0.128269194	0.725010687	PTH2R
ENSG00000166181.13	0.008754008	0.128313555	0.725010687	API5
ENSG00000066056.14	-0.038640162	0.128349029	0.725010687	TIE1
ENSG00000105732.13	-0.013322954	0.128366692	0.725010687	ZNF574
ENSG00000164509.16	0.046548507	0.128398033	0.725010687	IL31RA
ENSG00000204301.6	0.025772569	0.128455012	0.725010687	NOTCH4
ENSG00000260231.2	0.025665664	0.128463979	0.725010687	KDM7A-DT
ENSG00000207399.1	0.055312666	0.128471271	0.725010687	RNU6-1011P
ENSG00000279649.1	0.029026888	0.128690086	0.726045747	RP11-96D1.8
ENSG00000166922.8	-0.060465284	0.128788191	0.726399402	SCG5
ENSG00000004779.10	0.014817058	0.12888427	0.726623153	NDUFAB1
ENSG00000100362.13	0.077217406	0.128898724	0.726623153	PVALB
ENSG00000182985.18	-0.0439694	0.128976485	0.726691053	CADM1
ENSG00000197054.12	0.020060724	0.128981638	0.726691053	ZNF763
ENSG00000251988.1	-0.051569056	0.129102132	0.727061991	RNU4ATAC18P
ENSG00000078808.19	-0.009857965	0.129118382	0.727061991	SDF4
ENSG00000207171.1	-0.0658175	0.129219823	0.72743347	ENSG10010134965.1
ENSG00000217555.13	0.021403962	0.129421238	0.727647573	CKLF
ENSG00000166398.14	0.01321553	0.129427686	0.727647573	GARRE1
ENSG00000163877.11	-0.007175119	0.12947674	0.727647573	SNIP1
ENSG00000100129.18	0.013722069	0.129517452	0.727647573	EIF3L
ENSG00000185347.18	-0.018072534	0.129520013	0.727647573	TEDC1
ENSG00000272223.1	-0.044479043	0.1295351	0.727647573	RP1-20C7.6
ENSG00000147789.16	0.007514041	0.129540823	0.727647573	ZNF7
ENSG00000223668.1	0.053587825	0.129541705	0.727647573	EEF1A1P24
ENSG00000220614.1	-0.04490805	0.129585853	0.727696243	RP11-480N24.4
ENSG00000243964.1	0.043786581	0.129691181	0.72793615	RPL23AP65
ENSG00000249249.2	0.033651271	0.129699566	0.72793615	AC010226.4
ENSG00000286207.1	0.030477737	0.129785635	0.728219918	RP11-17G11.2
ENSG00000168517.11	-0.01503993	0.129959316	0.728465293	HEXIM2
ENSG00000148356.15	-0.010354058	0.129961539	0.728465293	LRSAM1
ENSG00000100353.18	0.009089861	0.130066615	0.728465293	EIF3D
ENSG00000173545.5	0.011016055	0.130080146	0.728465293	ZNF622
ENSG00000099995.19	-0.013826135	0.130137484	0.728465293	SF3A1
ENSG00000090273.14	-0.013375313	0.130151819	0.728465293	NUDC
ENSG00000112874.10	0.04797245	0.13015725	0.728465293	NUDT12
ENSG00000060491.17	-0.014724059	0.130180876	0.728465293	OGFR

ENSG00000109943.9	-0.028060537	0.130187558	0.728465293	CRTAM
ENSG00000213171.3	0.079684791	0.13019826	0.728465293	LINGO4
ENSG00000091140.14	0.012794241	0.130220098	0.728465293	DLD
ENSG00000281181.1	-0.18780995	0.130357008	0.728520382	CH507-513H4.3
ENSG00000267062.1	-0.044389862	0.130366233	0.728520382	CTD-2659N19.10
ENSG00000121101.16	0.045717197	0.130380105	0.728520382	TEX14
ENSG00000038274.17	0.009436125	0.130405399	0.728520382	MAT2B
ENSG00000198553.9	-0.042747272	0.130420281	0.728520382	KCNRG
ENSG00000263370.1	-0.045061284	0.130451501	0.728520382	RP11-68I3.5
ENSG00000197579.8	0.015097855	0.130624387	0.728520382	TOPORS
ENSG00000062194.16	0.013612266	0.130639026	0.728520382	GPBP1
ENSG00000170175.11	-0.014000573	0.130697962	0.728520382	CHRNB1
ENSG00000198876.13	0.031734054	0.130711749	0.728520382	DCAF12
ENSG00000124216.4	-0.037055652	0.130767732	0.728520382	SNAI1
ENSG00000198732.11	0.052188761	0.130828227	0.728520382	SMOC1
ENSG00000286086.1	-0.069076785	0.130831505	0.728520382	RP11-110C15.5
ENSG00000265407.1	-0.054355591	0.130851403	0.728520382	MIR4324
ENSG00000124145.6	0.02260595	0.130869437	0.728520382	SDC4
ENSG00000106524.9	0.012354264	0.130878136	0.728520382	ANKMY2
ENSG00000249042.6	-0.036854407	0.130888649	0.728520382	CTD-2015H6.3
ENSG00000211752.3	0.037297772	0.130952723	0.728520382	TRBV27
ENSG00000100258.18	-0.012997174	0.131042218	0.728520382	LMF2
ENSG00000167920.10	0.020501256	0.131056664	0.728520382	TMEM99
ENSG00000070371.16	0.024283439	0.131076313	0.728520382	CLTCL1
ENSG00000153187.20	-0.008524808	0.131132583	0.728520382	HNRNPU
ENSG00000275807.1	0.038439324	0.13115665	0.728520382	RP11-1348G14.8
ENSG00000172995.17	0.08005806	0.131170558	0.728520382	ARPP21
ENSG00000227345.9	0.009795113	0.131191297	0.728520382	PARG
ENSG00000251513.2	-0.051582775	0.131204467	0.728520382	LIX1-AS1
ENSG00000141076.18	0.011105347	0.131246649	0.728520382	UTP4
ENSG00000250764.1	-0.033966493	0.131253161	0.728520382	CTD-2152M20.2
ENSG00000286406.1	-0.078756612	0.131279555	0.728520382	RP11-1277N13.1
ENSG00000196961.13	-0.012683615	0.131312772	0.728520382	AP2A1
ENSG00000010219.13	0.013123271	0.131331181	0.728520382	DYRK4
ENSG00000204613.11	0.046260577	0.131447247	0.72866247	TRIM10
ENSG00000151693.11	0.034073512	0.131458891	0.72866247	ASAP2
ENSG00000065485.20	0.01806525	0.131463387	0.72866247	PDIA5
ENSG00000261305.2	0.064249168	0.131528838	0.728828266	RP4-584D14.7
ENSG00000136522.14	0.031238979	0.131603424	0.729044573	MRPL47
ENSG00000185432.12	0.013900569	0.131651901	0.729116172	METTL7A
ENSG00000256417.1	-0.351122901	0.131690605	0.72913362	RP11-1038A11.3
ENSG00000279198.1	-0.028003833	0.131820226	0.729654305	CTD-2231E14.2
ENSG00000248278.1	0.045089032	0.13186973	0.72973136	SUMO2P17
ENSG00000150054.19	0.018893883	0.132044443	0.730229699	MPP7
ENSG00000285693.1	-0.014270921	0.132055989	0.730229699	RP11-475M20.2
ENSG00000235958.5	-0.026659952	0.132066606	0.730229699	UBOX5-AS1
ENSG00000214575.10	0.062035096	0.132302633	0.730596897	CPEB1

ENSG00000257108.2	0.020840982	0.132348524	0.730596897	NHLRC4
ENSG00000164296.7	0.018448148	0.132351955	0.730596897	TIGD6
ENSG00000154814.14	0.017568113	0.132362529	0.730596897	OXNAD1
ENSG00000185883.12	-0.020816452	0.132372134	0.730596897	ATP6V0C
ENSG00000279196.1	0.03524603	0.132383261	0.730596897	RP11-1072A3.3
ENSG00000103197.18	-0.013533072	0.132395043	0.730596897	TSC2
ENSG00000287032.1	-0.052030257	0.132443674	0.730596897	RP11-379H18.2
ENSG00000277425.1	-0.052719665	0.132470473	0.730596897	RP5-1116H23.3
ENSG00000150594.7	0.054170123	0.132534923	0.730596897	ADRA2A
ENSG00000100211.10	0.015582719	0.132554621	0.730596897	CBY1
ENSG00000105819.14	0.006843437	0.132560516	0.730596897	PMPCB
ENSG00000096872.17	0.024189269	0.132667845	0.730608534	IFT74
ENSG00000158470.5	0.017426737	0.132696648	0.730608534	B4GALT5
ENSG00000058729.11	0.01694127	0.132741842	0.730608534	RIOK2
ENSG00000132635.17	-0.012425503	0.132758355	0.730608534	PCED1A
ENSG00000133424.22	-0.036666734	0.132766996	0.730608534	LARGE1
ENSG00000116030.17	0.011852555	0.13277638	0.730608534	SUMO1
ENSG00000178397.13	-0.009930694	0.132902474	0.730757113	FAM220A
ENSG00000142173.16	-0.033545782	0.132950747	0.730757113	COL6A2
ENSG00000189266.13	0.014387348	0.132962313	0.730757113	PNRC2
ENSG00000105829.13	0.014141534	0.132984401	0.730757113	BET1
ENSG00000147465.12	-0.039821296	0.133022789	0.730757113	STAR
ENSG00000089818.18	0.009852664	0.133080441	0.730757113	NECAP1
ENSG00000160993.4	-0.012537994	0.13310544	0.730757113	ALKBH4
ENSG00000023892.11	-0.011894812	0.133115057	0.730757113	DEF6
ENSG00000271270.7	0.022401359	0.133131389	0.730757113	TMCC1-DT
ENSG00000163026.12	-0.009440724	0.133179577	0.730757113	WDCP
ENSG00000134548.11	0.04953536	0.133195343	0.730757113	SPX
ENSG00000267321.3	0.016510114	0.133381619	0.731370981	SNHG30
ENSG00000279766.1	-0.050394034	0.133412553	0.731370981	RP11-642A1.2
ENSG00000138617.16	-0.013008981	0.133414221	0.731370981	PARP16
ENSG00000184545.11	-0.031863256	0.133478793	0.731529417	DUSP8
ENSG00000204348.10	-0.011426983	0.133517574	0.731546464	DXO
ENSG00000168256.18	0.009611787	0.133599346	0.731798982	NKIRAS2
ENSG00000277999.1	0.030856013	0.133678913	0.73203929	RP11-426C22.8
ENSG00000229719.5	-0.0420102	0.133878961	0.732289692	MIR194-2HG
ENSG00000158406.6	-0.02647344	0.133962866	0.732289692	H4C8
ENSG00000257275.6	-0.061505161	0.133994489	0.732289692	RP11-164H13.1
ENSG00000252835.1	-0.062510265	0.134027116	0.732289692	SCARNA21
ENSG00000171960.12	0.010661249	0.134055471	0.732289692	PPIH
ENSG00000185483.12	0.107494291	0.134063686	0.732289692	ROR1
ENSG00000226686.8	0.073604375	0.134083642	0.732289692	LINC01535
ENSG00000251201.8	-0.051213542	0.1341616	0.732289692	TMED7-TICAM2
ENSG00000205758.12	0.010131226	0.13419987	0.732289692	CRYZL1
ENSG00000272183.1	-0.045500548	0.134230452	0.732289692	RP11-523H20.3
ENSG00000163947.12	-0.012112736	0.134290326	0.732289692	ARHGEF3
ENSG00000124507.11	-0.026799431	0.134301213	0.732289692	PACSIN1

ENSG00000196460.14	0.051950103	0.134328384	0.732289692	RFX8
ENSG00000180422.3	-0.042748349	0.134367409	0.732289692	LINC00304
ENSG00000174840.9	-0.008572222	0.134411689	0.732289692	PDE12
ENSG00000265992.1	-0.100688481	0.134454551	0.732289692	ESRG
ENSG00000100908.14	-0.015763836	0.134454678	0.732289692	EMC9
ENSG00000105619.14	-0.014597651	0.134486081	0.732289692	TFPT
ENSG00000167720.13	-0.020140462	0.134532876	0.732289692	SRR
ENSG00000150756.14	0.015007824	0.134533647	0.732289692	ATPSCKMT
ENSG00000112996.11	-0.009004714	0.134555593	0.732289692	MRPS30
ENSG00000135643.5	0.032263706	0.134616937	0.732289692	KCNMB4
ENSG00000198791.12	0.010416845	0.134624134	0.732289692	CNOT7
ENSG00000170606.15	0.009044122	0.1346268	0.732289692	HSPA4
ENSG00000127993.16	-0.007729658	0.134639289	0.732289692	RBM48
ENSG00000104983.8	-0.013747686	0.134719413	0.732289692	CCDC61
ENSG00000130935.10	0.008566018	0.134730557	0.732289692	NOL11
ENSG00000094804.12	-0.034611806	0.134735294	0.732289692	CDC6
ENSG00000204316.13	-0.014838813	0.134829991	0.732289692	MRPL38
ENSG00000226491.1	-0.020501902	0.13488403	0.732289692	FTOP1
ENSG00000143793.13	-0.014318722	0.134890058	0.732289692	C1orf35
ENSG00000170860.4	0.024122775	0.134928158	0.732289692	LSM3
ENSG00000196357.12	0.020465664	0.134932318	0.732289692	ZNF565
ENSG00000277462.2	0.025032137	0.134938694	0.732289692	ZNF670
ENSG00000002330.14	-0.019619073	0.135010864	0.732487516	BAD
ENSG00000185414.20	0.011460861	0.135090741	0.732727036	MRPL30
ENSG00000238072.1	0.089576632	0.135312991	0.733738452	CDC26P1
ENSG00000177432.8	-0.024471708	0.135401255	0.734022984	NAP1L5
ENSG00000284428.1	-0.01913638	0.135590351	0.734685117	RP11-15H20.6
ENSG00000218357.3	0.067127079	0.13560261	0.734685117	LINC01644
ENSG00000252010.1	-0.116337451	0.13569464	0.734685117	SCARNA5
ENSG00000172893.17	-0.015674798	0.135704518	0.734685117	DHCR7
ENSG00000168405.17	0.019406409	0.135735858	0.734685117	CMAHP
ENSG00000181029.9	-0.022177328	0.135746657	0.734685117	TRAPPC5
ENSG00000126062.4	-0.011859065	0.135787303	0.734685117	TMEM115
ENSG00000156170.13	-0.014484581	0.135865602	0.734685117	NDUFAF6
ENSG00000276600.5	0.041537705	0.135873005	0.734685117	RAB7B
ENSG00000224607.4	0.141575775	0.135908182	0.734685117	IGKV1D-27
ENSG00000244879.8	-0.042084859	0.13593639	0.734685117	GABPB1-AS1
ENSG00000141510.18	-0.011649025	0.135986828	0.734685117	TP53
ENSG00000005020.13	0.019035686	0.135989112	0.734685117	SKAP2
ENSG00000157617.17	0.012119069	0.136026359	0.734692803	C2CD2
ENSG00000277959.1	-0.023078012	0.136121773	0.734849337	RP11-45A17.2
ENSG00000180279.7	0.057193979	0.136127006	0.734849337	LINC01869
ENSG00000258839.4	-0.023051401	0.136222082	0.734945357	MC1R
ENSG00000225873.4	0.022210572	0.136255957	0.734945357	C3orf86
ENSG00000136143.16	0.013640532	0.136282053	0.734945357	SUCLA2
ENSG00000183137.15	0.016458528	0.13632278	0.734945357	CEP57L1
ENSG00000006638.13	-0.030063268	0.136323978	0.734945357	TBXA2R

ENSG00000185989.11	-0.009881519	0.13644327	0.735395158	RASA3
ENSG00000102890.16	-0.015150348	0.136689156	0.736526855	ELMO3
ENSG00000109265.15	0.047695566	0.136747448	0.736597198	CRACD
ENSG00000115145.10	0.013613143	0.136833359	0.736597198	STAM2
ENSG00000225802.3	-0.065365127	0.136853796	0.736597198	WARS1P1
ENSG00000256043.5	0.010489222	0.136887331	0.736597198	CTSO
ENSG00000271888.1	0.041075232	0.136911985	0.736597198	JARID2-DT
ENSG00000287077.1	-0.029846603	0.136917716	0.736597198	RP11-322M19.5
ENSG00000268480.3	0.063327807	0.136959595	0.736629261	NFILZ
ENSG00000109674.4	-0.062270883	0.137018526	0.736752996	NEIL3
ENSG00000155096.15	0.012465881	0.137075777	0.736867636	AZIN1
ENSG00000213977.8	-0.016698508	0.137146401	0.736970342	TAX1BP3
ENSG00000231767.4	0.044644379	0.137179841	0.736970342	RPS27AP5
ENSG00000131351.15	-0.012209934	0.137229034	0.736970342	HAUS8
ENSG00000100335.15	0.006403131	0.137281026	0.736970342	MIEF1
ENSG00000286488.1	-0.020062425	0.137289827	0.736970342	RP11-761I4.6
ENSG00000107742.13	-0.014192283	0.137360748	0.736970342	SPOCK2
ENSG00000223522.2	-0.026350099	0.137448751	0.736970342	AC093690.1
ENSG00000108312.15	-0.008216813	0.137475946	0.736970342	UBTF
ENSG00000085514.16	0.014702898	0.137492948	0.736970342	PILRA
ENSG00000104833.12	-0.033652501	0.137610913	0.736970342	TUBB4A
ENSG00000277998.1	0.045733324	0.137650693	0.736970342	RP11-452N17.1
ENSG00000272129.1	-0.052196724	0.137667957	0.736970342	RP11-250B2.6
ENSG00000116350.17	-0.007850895	0.137679789	0.736970342	SRSF4
ENSG00000151348.16	0.011060939	0.137840076	0.736970342	EXT2
ENSG00000118965.16	0.015499835	0.137855509	0.736970342	WDR35
ENSG00000167508.12	-0.017260313	0.137861599	0.736970342	MVD
ENSG00000184786.6	-0.019712614	0.137919838	0.736970342	DYNLT2
ENSG00000288061.1	0.036816817	0.137924416	0.736970342	RP11-762B21.7
ENSG00000187950.8	0.100939325	0.137948944	0.736970342	OVCH1
ENSG00000112164.6	0.094672871	0.137989282	0.736970342	GLP1R
ENSG00000231680.1	0.028607228	0.13822927	0.736970342	LINC02723
ENSG00000217716.3	0.078197406	0.138259841	0.736970342	RPS10P3
ENSG00000278000.1	-0.034269851	0.13826366	0.736970342	AC139100.4
ENSG00000246067.8	-0.019297328	0.138302086	0.736970342	RAB30-DT
ENSG00000196189.13	-0.019074638	0.138306647	0.736970342	SEMA4A
ENSG00000276805.2	0.025220244	0.138313104	0.736970342	RP11-291L22.9
ENSG00000287920.1	-0.032922924	0.138333478	0.736970342	RP3-510L9.2
ENSG00000268278.1	-0.077032139	0.138352072	0.736970342	RP11-420K14.1
ENSG00000100439.10	0.012487575	0.13839448	0.736970342	ABHD4
ENSG00000105607.13	-0.011618931	0.13840317	0.736970342	GCDH
ENSG00000162065.14	-0.010521872	0.138534645	0.736970342	TBC1D24
ENSG00000171132.14	-0.018750228	0.138603474	0.736970342	PRKCE
ENSG00000139083.11	0.009242505	0.138632187	0.736970342	ETV6
ENSG00000273980.1	-0.071043821	0.138649218	0.736970342	RP13-49I15.6
ENSG00000270127.2	-0.038989394	0.138652985	0.736970342	RP11-526I2.5
ENSG00000272449.2	-0.023829311	0.138659861	0.736970342	RP3-395M20.12

ENSG00000132670.21	-0.00702627	0.138673357	0.736970342	PTPRA
ENSG00000184678.10	0.022102304	0.138682777	0.736970342	H2BC21
ENSG00000137267.7	-0.076390025	0.138685639	0.736970342	TUBB2A
ENSG00000136492.10	-0.029819362	0.138697063	0.736970342	BRIP1
ENSG00000168487.20	-0.020534979	0.138699018	0.736970342	BMP1
ENSG00000164037.17	-0.031497429	0.138743114	0.736970342	SLC9B1
ENSG00000250602.6	0.052100229	0.138767337	0.736970342	RP11-517I3.1
ENSG00000185112.6	-0.017791936	0.138789066	0.736970342	FAM43A
ENSG00000121966.7	0.013255114	0.138809364	0.736970342	CXCR4
ENSG00000148572.16	0.012891145	0.138814479	0.736970342	NRBF2
ENSG00000273604.2	-0.033972561	0.138823751	0.736970342	EPOP
ENSG00000179899.8	0.034286275	0.138823856	0.736970342	PHC1P1
ENSG00000096968.14	0.020633884	0.138855734	0.736970342	JAK2
ENSG00000164902.14	0.011836481	0.139043865	0.737510686	PHAX
ENSG00000066279.19	-0.046127778	0.139045338	0.737510686	ASPM
ENSG00000196296.14	-0.021138299	0.139065429	0.737510686	ATP2A1
ENSG00000265908.1	-0.05660002	0.139106688	0.737538769	RP11-20B24.6
ENSG00000088836.14	-0.039955192	0.139167119	0.737668462	SLC4A11
ENSG00000233547.1	-0.056953779	0.13923714	0.737848904	RP11-57H14.2
ENSG00000268170.2	0.034987869	0.139354671	0.73828096	RP11-556I13.2
ENSG00000154258.17	0.069696201	0.139417352	0.738318877	ABCA9
ENSG00000170445.16	-0.00713392	0.13945225	0.738318877	HARS1
ENSG00000108479.13	-0.017626445	0.139469833	0.738318877	GALK1
ENSG00000197763.20	0.031724271	0.139575156	0.738359523	TXNRD3
ENSG00000143653.10	0.017585954	0.139633476	0.738359523	SCCPDH
ENSG00000175611.12	0.024148596	0.139633843	0.738359523	LINC00476
ENSG00000198046.12	-0.046300703	0.139671509	0.738359523	ZNF667
ENSG00000151552.12	0.017951361	0.139694193	0.738359523	QDPR
ENSG00000272463.1	-0.038993658	0.139710659	0.738359523	RP11-532F6.3
ENSG00000168397.17	-0.010580752	0.139729535	0.738359523	ATG4B
ENSG00000229754.1	0.03133957	0.139792508	0.738460736	CXCR2P1
ENSG00000148343.19	0.009189505	0.139828347	0.738460736	MIGA2
ENSG00000225760.2	0.165250321	0.139856714	0.738460736	LINC00431
ENSG00000203363.2	0.070066572	0.140019681	0.739117355	GPR160P1
ENSG00000167325.15	-0.010969469	0.140065753	0.739117355	RRM1
ENSG00000276564.1	-0.049958538	0.140089192	0.739117355	CTA-276F8.1
ENSG00000136379.12	-0.025478622	0.140211992	0.739470534	ABHD17C
ENSG00000197852.12	0.016212991	0.140230368	0.739470534	INKA2
ENSG00000142330.20	-0.010782315	0.140284783	0.739470534	CAPN10
ENSG00000274963.1	-0.03624092	0.140300363	0.739470534	RN7SL600P
ENSG00000122025.15	0.027201756	0.14040488	0.739722044	FLT3
ENSG00000261441.1	-0.052151112	0.140420222	0.739722044	POLG-DT
ENSG00000167447.13	0.008347844	0.140468502	0.739786349	SMG8
ENSG00000128524.5	0.014871864	0.140596343	0.740269524	ATP6V1F
ENSG00000285799.1	0.036929333	0.14082795	0.740931563	MICF
ENSG00000079337.16	0.034709756	0.140830697	0.740931563	RAPGEF3
ENSG00000248727.6	0.022922277	0.140914249	0.740931563	LINC01948

ENSG00000160336.17	0.018787224	0.140928123	0.740931563	ZNF761
ENSG00000142611.17	0.089905274	0.140944891	0.740931563	PRDM16
ENSG00000167588.13	-0.029257494	0.140951629	0.740931563	GPD1
ENSG00000134285.11	-0.013178121	0.140996978	0.740931563	FKBP11
ENSG00000236948.2	-0.072295529	0.141076972	0.740931563	RP11-154H17.1
ENSG00000136872.20	-0.05927691	0.141121553	0.740931563	ALDOB
ENSG00000102996.5	-0.032270797	0.141131774	0.740931563	MMP15
ENSG00000148362.11	-0.017822833	0.1412063	0.740931563	PAXX
ENSG00000128607.14	0.007415472	0.14122411	0.740931563	KLHDC10
ENSG00000187922.14	-0.045052161	0.141291595	0.740931563	LCN10
ENSG00000072849.11	0.008278628	0.141301132	0.740931563	DERL2
ENSG00000172831.14	0.00885766	0.141397423	0.740931563	CES2
ENSG00000145781.9	0.0188863	0.14139824	0.740931563	COMMD10
ENSG00000197744.5	0.031264305	0.141408221	0.740931563	PTMAP2
ENSG00000145050.19	0.014199968	0.141415657	0.740931563	MANF
ENSG00000175899.15	-0.035843051	0.141428516	0.740931563	A2M
ENSG00000221025.1	0.044662757	0.141471201	0.740931563	MIR1250
ENSG00000211978.2	-0.042889356	0.141480788	0.740931563	IGHV5-78
ENSG00000150753.12	0.007588987	0.14154866	0.740986308	CCT5
ENSG00000169255.15	0.034929589	0.14158424	0.740986308	B3GALNT1
ENSG00000243560.3	-0.054957717	0.141638189	0.740986308	RN7SL364P
ENSG00000095787.24	0.007811255	0.141665199	0.740986308	WAC
ENSG00000105877.18	-0.055138115	0.141671899	0.740986308	DNAH11
ENSG00000129636.13	0.008163957	0.141750201	0.741130722	ITFG1
ENSG00000204843.13	-0.012076795	0.141797781	0.741130722	DCTN1
ENSG00000286219.1	-0.039833155	0.141815384	0.741130722	NOTCH2NLC
ENSG00000141524.17	-0.010385837	0.141878056	0.741130722	TMC6
ENSG00000072201.14	-0.023247351	0.14188073	0.741130722	LNX1
ENSG00000185024.17	-0.011289023	0.141916342	0.741130722	BRF1
ENSG00000269656.1	-0.039855792	0.141992054	0.741337332	NOP53-AS1
ENSG00000173638.19	-0.015853052	0.142110698	0.741545811	SLC19A1
ENSG00000271918.1	-0.024223657	0.142186458	0.741545811	CTD-2287O16.5
ENSG00000150776.18	0.013144705	0.142186842	0.741545811	NKAPD1
ENSG00000226499.1	0.052834417	0.142203334	0.741545811	RP5-882O7.1
ENSG00000127951.8	0.017946841	0.142227987	0.741545811	FGL2
ENSG00000167306.20	0.030507667	0.142293047	0.741545811	MYO5B
ENSG00000240563.2	-0.06500285	0.142310804	0.741545811	L1TD1
ENSG00000005513.10	0.034250288	0.142321256	0.741545811	SOX8
ENSG00000103257.9	-0.022155935	0.142617443	0.74280366	SLC7A5
ENSG00000251247.11	-0.016893741	0.142650772	0.74280366	ZNF345
ENSG00000272419.6	-0.017263547	0.142671329	0.74280366	LINC01145
ENSG00000107281.10	-0.033292887	0.142715898	0.742847116	NPDC1
ENSG00000137970.7	0.047394213	0.14276285	0.742902953	RPL7P9
ENSG00000196504.19	0.013194179	0.142840819	0.743120122	PRPF40A
ENSG00000089048.15	0.024229088	0.142880372	0.743137373	ESF1
ENSG00000090863.12	-0.013147866	0.142979665	0.743306071	GLG1
ENSG00000163608.15	0.00789053	0.142985296	0.743306071	NEPRO

ENSG00000273356.1	-0.01753827	0.143078279	0.743600947	LINC02019
ENSG00000162222.14	0.007778051	0.143182828	0.743955773	TTC9C
ENSG00000183576.13	0.007676063	0.143245098	0.744037475	SETD3
ENSG00000125304.10	0.008117727	0.143355463	0.744037475	TM9SF2
ENSG00000215251.4	0.007808705	0.143398507	0.744037475	FASTKD5
ENSG00000123329.20	-0.012740514	0.143400805	0.744037475	ARHGAP9
ENSG00000149262.17	-0.008400039	0.143492039	0.744037475	INTS4
ENSG00000169951.10	-0.009769109	0.14352713	0.744037475	ZNF764
ENSG00000213362.3	0.042156771	0.143540888	0.744037475	FTH1P12
ENSG00000100368.14	0.016135026	0.143576728	0.744037475	CSF2RB
ENSG00000259772.6	-0.01910497	0.143602213	0.744037475	RP11-16E12.2
ENSG00000135829.17	0.007025584	0.143616868	0.744037475	DHX9
ENSG00000250251.6	0.024756424	0.143634417	0.744037475	PKD1P6
ENSG00000144535.20	-0.008808414	0.143695242	0.744037475	DIS3L2
ENSG00000141556.22	-0.011003123	0.143718827	0.744037475	TBCD
ENSG00000260992.1	0.0288029	0.143742961	0.744037475	DOCK9-DT
ENSG00000227953.7	0.042655547	0.143758051	0.744037475	LINC01341
ENSG00000263001.7	-0.019469493	0.143785203	0.744037475	GTF2I
ENSG00000214194.9	0.029976513	0.143825724	0.744037475	SMIM30
ENSG00000177946.7	0.012233101	0.14387271	0.744037475	CENPBD1
ENSG00000254827.6	0.034048822	0.143893851	0.744037475	SLC22A18AS
ENSG00000130522.6	-0.01928571	0.144020271	0.744037475	JUND
ENSG00000112306.8	0.020941243	0.144023393	0.744037475	RPS12
ENSG00000137411.19	-0.026909966	0.144040728	0.744037475	VARS2
ENSG00000106948.17	-0.012372667	0.144054062	0.744037475	AKNA
ENSG00000250182.3	0.023952962	0.144101746	0.744037475	EEF1A1P13
ENSG00000065183.16	0.009241307	0.144105561	0.744037475	WDR3
ENSG00000273145.1	0.033099974	0.144161933	0.744141182	CITF22-92A6.1
ENSG00000225556.1	-0.041342863	0.14420078	0.744154403	C2CD4D
ENSG00000172264.18	-0.066582231	0.144272287	0.744336114	MACROD2
ENSG00000100023.20	-0.008848287	0.144441563	0.744839087	PPIL2
ENSG00000213463.5	-0.014748509	0.144442415	0.744839087	SYNJ2BP
ENSG00000172971.7	-0.110879842	0.144586162	0.74529872	UNC93B3
ENSG00000240024.5	-0.024816636	0.144659198	0.74529872	LINC00888
ENSG00000085644.14	-0.013166776	0.144662432	0.74529872	ZNF213
ENSG00000103994.18	0.011321204	0.144676916	0.74529872	ZNF106
ENSG00000063180.9	-0.014984666	0.144735266	0.745412064	CA11
ENSG00000103534.17	0.037978146	0.144842715	0.745771296	TMC5
ENSG00000211782.2	-0.038627491	0.144934032	0.745771296	TRAV8-1
ENSG00000204323.5	0.031909501	0.14494791	0.745771296	SMIM5
ENSG00000129925.11	-0.014432461	0.145026505	0.745771296	PGAP6
ENSG00000164889.15	-0.011399507	0.145043709	0.745771296	SLC4A2
ENSG00000187902.13	-0.058335908	0.145050801	0.745771296	SHISA7
ENSG00000230042.1	-0.052252096	0.145059572	0.745771296	AK3P3
ENSG00000130529.16	0.03262125	0.145116438	0.745861502	TRPM4
ENSG00000251050.1	0.048101684	0.145149856	0.745861502	RBX1P2
ENSG00000177675.9	0.051983089	0.14523342	0.745904626	CD163L1

ENSG00000137210.14	0.015192825	0.145277041	0.745904626	TMEM14B
ENSG00000104267.10	0.030600486	0.145321934	0.745904626	CA2
ENSG00000151006.7	-0.017235025	0.145364653	0.745904626	PRSS53
ENSG00000272831.1	0.039550665	0.145441237	0.745904626	RP11-792A8.4
ENSG00000243660.11	0.016917873	0.145513868	0.745904626	ZNF487
ENSG00000229314.6	0.053614145	0.145528236	0.745904626	ORM1
ENSG00000257599.3	0.078693827	0.145529145	0.745904626	OVCH1-AS1
ENSG00000235724.10	0.049617854	0.145545863	0.745904626	AC009299.2
ENSG00000143641.10	-0.011498475	0.145551295	0.745904626	GALNT2
ENSG00000235488.1	-0.04082482	0.145558753	0.745904626	JARID2-AS1
ENSG00000212123.4	-0.023346613	0.145638531	0.745904626	PRR22
ENSG00000261253.2	0.036036242	0.145660961	0.745904626	AC137932.6
ENSG00000163945.19	-0.016658413	0.145667448	0.745904626	UVSSA
ENSG00000263563.6	0.055186572	0.145706196	0.745915305	UBBP4
ENSG00000151062.15	0.018479168	0.145791724	0.745915305	CACNA2D4
ENSG00000252448.1	0.061471799	0.145839214	0.745915305	SNORA63
ENSG00000261587.2	-0.04985891	0.14589236	0.745915305	TMEM249
ENSG00000165282.14	-0.008060359	0.145893813	0.745915305	PIGO
ENSG00000259262.1	-0.153772002	0.145914875	0.745915305	NDUFA3P4
ENSG00000066032.19	0.095992626	0.145924137	0.745915305	CTNNA2
ENSG00000275325.4	0.131521861	0.14611386	0.746120294	PDCD6IPP1
ENSG00000164669.13	-0.071102498	0.14616117	0.746120294	INTS4P1
ENSG00000106484.16	0.023966266	0.146173797	0.746120294	MEST
ENSG00000130699.19	-0.011374063	0.146247256	0.746120294	TAF4
ENSG00000108559.12	0.010685301	0.146247877	0.746120294	NUP88
ENSG00000286787.1	-0.033272477	0.146278883	0.746120294	RP4-545L17.11
ENSG00000250751.1	-0.038188832	0.14631499	0.746120294	RP11-613C6.2
ENSG00000163251.4	0.029955337	0.14631521	0.746120294	FZD5
ENSG00000172062.17	0.022715982	0.146356391	0.746120294	SMN1
ENSG00000143158.11	0.012442831	0.146386073	0.746120294	MPC2
ENSG00000119004.16	-0.01009618	0.146400671	0.746120294	CYP20A1
ENSG00000160007.19	-0.012001964	0.146400822	0.746120294	ARHGAP35
ENSG00000100220.12	0.010616841	0.146472801	0.746301667	RTCB
ENSG00000134028.14	0.043911165	0.146671315	0.746959951	ADAMDEC1
ENSG00000198382.9	0.0081039	0.146674845	0.746959951	UVRAG
ENSG00000129951.19	-0.079621062	0.146816584	0.747306799	PLPPR3
ENSG00000137767.14	0.016136663	0.14683156	0.747306799	SQOR
ENSG00000196693.15	0.012476058	0.146852272	0.747306799	ZNF33B
ENSG00000254098.1	0.080432935	0.146939229	0.747501948	IGKV2-26
ENSG00000010310.9	-0.020534321	0.147018651	0.747501948	GIPR
ENSG00000257703.6	-0.068648683	0.147018779	0.747501948	RP11-328J6.1
ENSG00000185015.8	0.019385943	0.147056805	0.747501948	CA13
ENSG00000104177.18	-0.029244104	0.147084275	0.747501948	MYEF2
ENSG00000188428.20	0.011714372	0.147159099	0.747501948	BLOC1S5
ENSG00000171163.16	-0.01137613	0.147185781	0.747501948	ZNF692
ENSG00000158482.10	-0.061848884	0.147190227	0.747501948	SNX29P1
ENSG00000268516.2	-0.020916894	0.147258917	0.747501948	ZNF8-DT

ENSG00000282431.1	-0.024689298	0.147277975	0.747501948	TRBD1
ENSG00000088038.19	-0.010840302	0.147318034	0.747501948	CNOT3
ENSG00000129474.16	-0.02708373	0.147359052	0.747501948	AJUBA
ENSG00000234336.7	-0.04622538	0.147364462	0.747501948	JAZF1-AS1
ENSG00000144840.10	0.012288594	0.147408516	0.747540517	RABL3
ENSG00000130270.16	-0.016454451	0.14748746	0.747750856	ATP8B3
ENSG00000266904.6	0.021652076	0.147550033	0.747750856	LINC00663
ENSG00000167699.13	0.007948802	0.147614556	0.747750856	GLOD4
ENSG00000159214.13	0.024987785	0.147754228	0.747750856	CCDC24
ENSG00000167711.14	0.026694516	0.147757984	0.747750856	SERPINF2
ENSG00000244041.7	0.019075344	0.14776787	0.747750856	LINC01011
ENSG00000236194.3	-0.051632171	0.147835351	0.747750856	AC003104.1
ENSG00000107789.16	-0.011259575	0.147855758	0.747750856	MINPP1
ENSG00000178952.11	-0.014225784	0.147900161	0.747750856	TUFM
ENSG00000000457.14	0.00909523	0.147939199	0.747750856	SCYL3
ENSG00000268584.1	-0.052185362	0.147980235	0.747750856	RP11-464F9.20
ENSG00000122970.16	-0.030488983	0.147983274	0.747750856	IFT81
ENSG00000229413.2	-0.024014406	0.147994075	0.747750856	RP11-274B21.12
ENSG00000203801.8	0.058846261	0.148080054	0.747750856	LINC00222
ENSG00000176407.18	0.00677034	0.14808691	0.747750856	KCMF1
ENSG00000237429.1	-0.028512051	0.148110659	0.747750856	RP1-159A19.4
ENSG00000174004.6	-0.014263064	0.148121659	0.747750856	NRROS
ENSG00000164983.8	-0.01498336	0.148202085	0.747750856	TMEM65
ENSG00000106351.13	-0.012256577	0.148222068	0.747750856	AGFG2
ENSG00000179750.16	0.083587417	0.148409013	0.747750856	APOBEC3B
ENSG00000145198.14	-0.050647771	0.148486984	0.747750856	VWA5B2
ENSG00000284600.1	0.123897585	0.148510422	0.747750856	RP11-755H23.1
ENSG00000120697.10	0.013159814	0.148535018	0.747750856	ALG5
ENSG00000225200.2	0.03170501	0.148622962	0.747750856	AB019441.29
ENSG00000221890.6	-0.023237513	0.148634792	0.747750856	NPTXR
ENSG00000196642.19	-0.011464893	0.148670091	0.747750856	RABL6
ENSG00000101665.10	-0.017534622	0.148671477	0.747750856	SMAD7
ENSG00000149485.19	0.021914425	0.148707659	0.747750856	FADS1
ENSG00000244556.1	-0.045871946	0.148750364	0.747750856	ODCP
ENSG00000090924.15	-0.012551611	0.14876484	0.747750856	PLEKHG2
ENSG00000279148.1	-0.035120032	0.148764849	0.747750856	RP11-135F9.4
ENSG00000149532.16	0.008528596	0.148768776	0.747750856	CPSF7
ENSG00000129167.10	0.031376461	0.148899807	0.747750856	TPH1
ENSG00000162576.17	0.025380626	0.148919084	0.747750856	MXRA8
ENSG00000136146.15	0.009380711	0.14898999	0.747750856	MED4
ENSG00000113356.13	-0.028990471	0.149097167	0.747750856	POLR3G
ENSG00000004478.8	-0.012428575	0.149133302	0.747750856	FKBP4
ENSG00000034713.8	0.011434661	0.149135242	0.747750856	GABARAPL2
ENSG00000125971.17	0.017243429	0.149139171	0.747750856	DYNLRB1
ENSG00000224597.10	0.01346711	0.149179364	0.747750856	SVIL-AS1
ENSG00000147853.17	-0.011904409	0.149231919	0.747750856	AK3
ENSG00000103174.13	-0.012607837	0.149248649	0.747750856	NAGPA

ENSG00000187583.11	-0.029404896	0.149252318	0.747750856	PLEKHN1
ENSG00000250067.12	-0.021540045	0.149283544	0.747750856	YJEFN3
ENSG00000124641.16	0.010493233	0.14928501	0.747750856	MED20
ENSG00000231090.2	-0.063175157	0.149288529	0.747750856	MIR4422HG
ENSG00000236878.1	0.051609746	0.14930468	0.747750856	MTATP6P26
ENSG00000272049.1	0.065118589	0.149320114	0.747750856	RP11-480D4.6
ENSG00000166825.15	-0.021784852	0.149361362	0.747750856	ANPEP
ENSG00000159423.17	0.015632617	0.149401403	0.747750856	ALDH4A1
ENSG00000105717.14	-0.016020837	0.14945897	0.747750856	PBX4
ENSG00000180769.10	0.030591422	0.149465906	0.747750856	WDFY3-AS2
ENSG00000286512.1	-0.049356796	0.149505259	0.747750856	RP1-80B9.4
ENSG00000272040.1	-0.021514384	0.149715137	0.747750856	CTC-366B18.4
ENSG00000284691.1	-0.015657352	0.149724669	0.747750856	RP11-511P7.5
ENSG00000286104.1	0.035423193	0.149756114	0.747750856	LLNLR-271E8.1
ENSG00000085831.16	0.029534327	0.149761431	0.747750856	TTC39A
ENSG00000103264.18	-0.010338324	0.14977564	0.747750856	FBXO31
ENSG00000025434.19	0.016686603	0.149797541	0.747750856	NR1H3
ENSG00000149600.12	0.011921985	0.149803154	0.747750856	COMMD7
ENSG00000158545.16	-0.011814504	0.149837604	0.747750856	ZC3H18
ENSG00000234975.6	-0.023647613	0.149839417	0.747750856	FTH1P2
ENSG00000129028.9	0.054185345	0.149844985	0.747750856	THAP10
ENSG00000011260.14	0.009526301	0.149910114	0.747750856	UTP18
ENSG00000273218.1	0.032813061	0.149930032	0.747750856	LLNLR-246C6.1
ENSG00000248124.8	-0.015052093	0.14993795	0.747750856	RRN3P1
ENSG00000169682.18	-0.018369413	0.149974289	0.747750856	SPNS1
ENSG00000229808.1	-0.024111328	0.149982832	0.747750856	RP11-456P18.2
ENSG00000234156.3	0.11504032	0.150009301	0.747750856	RP11-542K23.7
ENSG00000282951.1	-0.023461767	0.150040563	0.747750856	CTC-490E21.14
ENSG00000156253.7	0.01438775	0.15005795	0.747750856	RWDD2B
ENSG00000134107.5	-0.016067129	0.150142231	0.747750856	BHLHE40
ENSG00000111671.10	-0.019931413	0.150149166	0.747750856	SPSB2
ENSG00000246430.7	0.030087265	0.150152176	0.747750856	LINC00968
ENSG00000118520.15	0.046159529	0.150228107	0.747750856	ARG1
ENSG00000139318.8	0.014440085	0.150229839	0.747750856	DUSP6
ENSG00000232450.1	0.031212667	0.150257523	0.747750856	RP4-730K3.3
ENSG00000241755.1	0.058760573	0.150296733	0.747764534	IGKV1-9
ENSG00000162747.12	0.031678391	0.150489039	0.748303251	FCGR3B
ENSG00000019169.11	0.039835313	0.150499697	0.748303251	MARCO
ENSG00000262339.5	-0.05059547	0.150544423	0.748303251	RP11-1197K16.2
ENSG00000141574.8	0.021875209	0.15060855	0.748303251	SECTM1
ENSG00000286654.1	-0.042557483	0.150611911	0.748303251	RP11-353K11.2
ENSG00000131061.14	-0.011469665	0.150774023	0.748303251	ZNF341
ENSG00000173209.23	-0.012291378	0.150793454	0.748303251	AHSA2P
ENSG00000137764.20	-0.012212266	0.150813463	0.748303251	MAP2K5
ENSG00000142945.13	-0.030065247	0.150885255	0.748303251	KIF2C
ENSG00000186868.17	-0.027032296	0.150896051	0.748303251	MAPT
ENSG00000167840.13	0.012219283	0.150921671	0.748303251	ZNF232

ENSG00000266913.1	-0.029011233	0.150934023	0.748303251	LINC01841
ENSG00000186185.14	-0.046195037	0.150945759	0.748303251	KIF18B
ENSG00000266947.1	0.019683802	0.150958496	0.748303251	RP11-799D4.4
ENSG00000198832.10	-0.027683664	0.15098274	0.748303251	SELENOM
ENSG00000226287.8	-0.01884537	0.15100384	0.748303251	TMEM191A
ENSG00000228925.1	0.046640191	0.151025315	0.748303251	AC016722.4
ENSG00000053900.11	-0.010913172	0.151079889	0.748392843	ANAPC4
ENSG00000119714.11	-0.020271948	0.151287308	0.748993797	GPR68
ENSG00000275413.1	-0.039654977	0.151293202	0.748993797	CTC-529I10.1
ENSG00000144596.13	-0.101799228	0.151310772	0.748993797	GRIP2
ENSG00000279982.1	-0.050188241	0.151399455	0.749164629	RP11-45A17.3
ENSG00000224578.5	0.022164055	0.151418343	0.749164629	HNRNPA1P48
ENSG00000140839.12	0.043082967	0.151518996	0.749481806	CLEC18B
ENSG00000146425.11	0.01604294	0.15165362	0.74961418	DYNLT1
ENSG00000287975.1	-0.032061628	0.151674672	0.74961418	RP11-33I11.4
ENSG00000287168.1	-0.03128665	0.151678081	0.74961418	RP11-262H14.14
ENSG00000169372.13	0.012367032	0.151716324	0.74961418	CRADD
ENSG00000135241.17	0.019308775	0.151728519	0.74961418	PNPLA8
ENSG00000277734.8	-0.01677593	0.151779487	0.749685383	TRAC
ENSG00000145014.18	-0.020788823	0.151849173	0.749775096	TMEM44
ENSG00000155754.15	-0.054891146	0.15187077	0.749775096	C2CD6
ENSG00000092036.19	0.01834914	0.151937905	0.749889876	HAUS4
ENSG00000010244.19	-0.006581992	0.152031763	0.749889876	ZNF207
ENSG00000100151.16	-0.013614173	0.152037774	0.749889876	PICK1
ENSG00000187796.15	-0.01751009	0.152040282	0.749889876	CARD9
ENSG00000134146.12	0.015495096	0.152125485	0.750102805	DPH6
ENSG00000082074.19	0.013242286	0.152168968	0.750102805	FYB1
ENSG00000248213.3	-0.050557669	0.152308392	0.750102805	CICP16
ENSG00000137522.18	0.00782307	0.152334771	0.750102805	RNF121
ENSG00000165948.11	0.018530138	0.152355075	0.750102805	IFI27L1
ENSG00000185278.15	0.015219632	0.15235848	0.750102805	ZBTB37
ENSG00000138085.17	0.011455962	0.152395805	0.750102805	ATRAID
ENSG00000274211.6	0.008694459	0.152454305	0.750102805	SOCS7
ENSG00000170312.16	-0.045804708	0.152454657	0.750102805	CDK1
ENSG00000139718.11	-0.012889955	0.152520349	0.750102805	SETD1B
ENSG00000165905.18	-0.026697252	0.152568407	0.750102805	LARGE2
ENSG00000146386.8	0.015927019	0.152597491	0.750102805	ABRACL
ENSG00000106636.9	0.006713405	0.152677809	0.750102805	YKT6
ENSG00000213339.9	-0.01313176	0.152709229	0.750102805	QTRT1
ENSG00000092929.12	-0.01190435	0.152741427	0.750102805	UNC13D
ENSG00000220804.9	0.02608438	0.152783951	0.750102805	LINC01881
ENSG00000134014.17	-0.009633578	0.152801214	0.750102805	ELP3
ENSG00000277406.2	0.028656738	0.15281873	0.750102805	SEC22B4P
ENSG00000213866.3	0.021029563	0.152861616	0.750102805	YBX1P10
ENSG00000168286.3	-0.00882417	0.152886016	0.750102805	THAP11
ENSG00000151092.18	0.007110274	0.15302296	0.750102805	NGLY1
ENSG00000079313.15	-0.011166403	0.153059976	0.750102805	REXO1

ENSG00000144504.15	-0.00869211	0.153105506	0.750102805	ANKMY1
ENSG00000133243.10	-0.011984792	0.15317904	0.750102805	BTBD2
ENSG00000136193.17	0.01656232	0.153200234	0.750102805	SCRN1
ENSG00000248810.2	0.044623076	0.153293234	0.750102805	LINC02432
ENSG00000228278.5	0.022793939	0.153320723	0.750102805	ORM2
ENSG00000281404.2	-0.018166233	0.153347354	0.750102805	LINC01176
ENSG00000035928.16	0.007521315	0.153370061	0.750102805	RFC1
ENSG00000102962.5	-0.078278667	0.15338299	0.750102805	CCL22
ENSG00000171298.13	-0.01924902	0.153400133	0.750102805	GAA
ENSG00000143436.11	0.009913426	0.153535451	0.750102805	MRPL9
ENSG00000072952.20	0.019522414	0.153546259	0.750102805	IRAG1
ENSG00000147677.11	0.009942734	0.153546799	0.750102805	EIF3H
ENSG00000134215.16	0.013916299	0.15355465	0.750102805	VAV3
ENSG00000254759.1	0.041304081	0.153570824	0.750102805	NAP1L1P1
ENSG00000158109.15	0.01063154	0.15380679	0.750102805	TPRG1L
ENSG00000285667.1	-0.019571546	0.153838103	0.750102805	RP11-565O12.1
ENSG00000168701.19	0.014695208	0.153839344	0.750102805	TMEM208
ENSG00000175265.18	-0.024015316	0.153840078	0.750102805	GOLGA8A
ENSG00000102967.12	-0.009615722	0.153890173	0.750102805	DHODH
ENSG00000126581.13	0.007225723	0.153928696	0.750102805	BECN1
ENSG00000188342.12	0.015467203	0.154037977	0.750102805	GTF2F2
ENSG00000135069.14	-0.022453552	0.154091517	0.750102805	PSAT1
ENSG00000206531.10	0.0887866	0.154104226	0.750102805	CD200R1L
ENSG00000230666.5	0.125410638	0.154145722	0.750102805	CEACAM22P
ENSG00000007237.19	0.014790079	0.154190339	0.750102805	GAS7
ENSG00000024048.11	0.010765709	0.154196714	0.750102805	UBR2
ENSG00000176623.12	0.010298244	0.154224048	0.750102805	RMDN1
ENSG00000223547.10	0.017975011	0.154278624	0.750102805	ZNF844
ENSG00000270923.1	-0.051940181	0.154425375	0.750102805	TAS2R6P
ENSG00000100330.16	-0.009796036	0.154471387	0.750102805	MTMR3
ENSG00000162757.4	-0.011512431	0.15448018	0.750102805	C1orf74
ENSG00000236936.1	0.039854921	0.154517863	0.750102805	RP3-329E20.2
ENSG00000167792.13	-0.011441697	0.154581461	0.750102805	NDUFV1
ENSG00000117448.14	0.012227629	0.154595471	0.750102805	AKR1A1
ENSG00000267990.1	0.021726171	0.154615771	0.750102805	CTD-3187F8.15
ENSG00000106263.18	-0.011151498	0.154686414	0.750102805	EIF3B
ENSG00000114867.22	-0.012745833	0.154708711	0.750102805	EIF4G1
ENSG00000181722.17	0.025589705	0.154713627	0.750102805	ZBTB20
ENSG00000163431.13	0.06702907	0.154713697	0.750102805	LMOD1
ENSG00000280828.1	-0.041529772	0.15472354	0.750102805	RP11-274B21.1
ENSG00000145365.11	0.019993683	0.154740909	0.750102805	TIFA
ENSG00000197050.11	0.014664546	0.1547733	0.750102805	ZNF420
ENSG00000167994.13	0.050142443	0.154868256	0.750102805	RAB3IL1
ENSG00000168079.17	0.058765964	0.15487791	0.750102805	SCARA5
ENSG00000005448.17	-0.01453385	0.154967281	0.750102805	WDR54
ENSG00000081377.17	0.017507208	0.154977567	0.750102805	CDC14B
ENSG00000259687.2	-0.041130876	0.154996357	0.750102805	LINC01220

ENSG00000260196.1	-0.024928077	0.155007693	0.750102805	RP1-239B22.5
ENSG00000247081.8	0.038995917	0.155022538	0.750102805	BAALC-AS1
ENSG00000205869.2	-0.036974938	0.155037188	0.750102805	KRTAP5-1
ENSG00000229809.9	-0.015788263	0.155073495	0.750102805	ZNF688
ENSG00000262587.2	-0.021388901	0.155123224	0.750102805	RP11-266L9.4
ENSG00000161904.12	0.006914558	0.155150159	0.750102805	LEMD2
ENSG00000177125.5	0.014508296	0.155154403	0.750102805	ZBTB34
ENSG00000014216.16	-0.012836859	0.155175513	0.750102805	CAPN1
ENSG00000213626.13	-0.010642752	0.155186088	0.750102805	LBH
ENSG00000164379.7	0.123451137	0.155238551	0.750102805	FOXQ1
ENSG00000196923.14	-0.01720144	0.155248146	0.750102805	PDLIM7
ENSG00000230587.2	-0.028193707	0.155249555	0.750102805	LINC02580
ENSG00000175215.11	0.009575958	0.155263934	0.750102805	CTDSP2
ENSG00000280953.2	-0.030690301	0.155275746	0.750102805	LINC01163
ENSG00000111144.10	0.016311512	0.155317778	0.750102805	LTA4H
ENSG00000230982.1	0.03037545	0.155359681	0.750102805	DSTNP1
ENSG00000220848.5	0.019315948	0.155393286	0.750102805	RPS18P9
ENSG00000065548.18	0.018156248	0.155479453	0.750102805	ZC3H15
ENSG00000203867.8	0.052360575	0.155565033	0.750102805	RBM20
ENSG00000140854.13	-0.010241421	0.155634777	0.750102805	KATNB1
ENSG00000168818.10	0.008013628	0.155659976	0.750102805	STX18
ENSG00000180884.10	0.010853504	0.155709983	0.750102805	ZNF792
ENSG00000227014.6	0.043569911	0.155728193	0.750102805	FKBP14-AS1
ENSG00000188659.10	-0.084616701	0.155756872	0.750102805	SAXO2
ENSG00000166295.9	0.008429275	0.155794386	0.750102805	ANAPC16
ENSG00000196652.12	0.007818124	0.155914071	0.750102805	ZKSCAN5
ENSG00000211817.2	0.035308667	0.155919585	0.750102805	TRAV38-2DV8
ENSG00000137842.7	-0.011447332	0.155972106	0.750102805	TMEM62
ENSG00000159147.18	-0.012249664	0.156003912	0.750102805	DONSON
ENSG00000198598.7	-0.027550915	0.156018625	0.750102805	MMP17
ENSG00000157881.16	-0.009232725	0.156026261	0.750102805	PANK4
ENSG00000135678.12	-0.018955965	0.156031241	0.750102805	CPM
ENSG00000219545.12	0.012283121	0.15605761	0.750102805	UMAD1
ENSG00000203416.3	-0.038612227	0.156131879	0.750102805	FAM32BP
ENSG00000169564.7	-0.011064116	0.156143868	0.750102805	PCBP1
ENSG00000267980.1	-0.029078321	0.156172025	0.750102805	AC007292.6
ENSG00000125246.15	0.016499026	0.156189058	0.750102805	CLYBL
ENSG00000165121.11	0.019272666	0.156196555	0.750102805	RP11-213G2.3
ENSG00000052841.15	0.006081608	0.156223759	0.750102805	TTC17
ENSG00000174485.16	0.012479751	0.156240287	0.750102805	DENND4A
ENSG00000204590.13	-0.010239535	0.156294897	0.750102805	GNL1
ENSG00000154556.18	0.069779275	0.156420901	0.750102805	SORBS2
ENSG00000120688.9	0.014980308	0.156428538	0.750102805	WBP4
ENSG00000112137.18	-0.02013726	0.156431947	0.750102805	PHACTR1
ENSG00000174473.16	0.067015929	0.156483184	0.750102805	GALNTL6
ENSG00000232810.4	-0.015365558	0.156508524	0.750102805	TNF
ENSG00000283709.1	-0.031650894	0.156511347	0.750102805	FAM238C

ENSG00000088053.11	0.035399388	0.156645969	0.750102805	GP6
ENSG00000005882.12	0.013440916	0.156659571	0.750102805	PDK2
ENSG00000035141.8	0.008717252	0.156666019	0.750102805	FAM136A
ENSG00000150048.10	0.023616944	0.156676609	0.750102805	CLEC1A
ENSG00000226715.4	0.074059279	0.156738913	0.750102805	LINC01709
ENSG00000173166.18	0.052290908	0.156744453	0.750102805	RAPH1
ENSG00000134057.15	-0.024423128	0.156782267	0.750102805	CCNB1
ENSG00000166971.17	-0.012540164	0.156791846	0.750102805	AKTIP
ENSG00000066379.15	0.013957131	0.156792954	0.750102805	POLR1H
ENSG00000270629.6	-0.0140845	0.156820548	0.750102805	NBPF14
ENSG00000070950.10	-0.009366852	0.156842162	0.750102805	RAD18
ENSG00000172053.18	0.008407616	0.156843562	0.750102805	QARS1
ENSG00000279277.2	-0.019529293	0.156848399	0.750102805	RP11-861A13.2
ENSG00000261656.6	0.037167214	0.156907583	0.750102805	BEAN1-AS1
ENSG00000225339.4	0.032766397	0.15692008	0.750102805	RP11-513I15.6
ENSG00000248592.7	0.017653601	0.156959991	0.750102805	STIMATE-MUSTN1
ENSG00000158481.13	0.022019507	0.156960917	0.750102805	CD1C
ENSG00000167889.13	0.065396699	0.156984652	0.750102805	MGAT5B
ENSG00000282965.1	0.150733159	0.157050448	0.750242393	RP11-683L23.7
ENSG00000139914.7	-0.020040661	0.15717869	0.750589272	FITM1
ENSG00000287878.1	0.022742407	0.157221129	0.750589272	RP11-326O24.1
ENSG00000260643.2	0.052314643	0.157245423	0.750589272	RP11-303E16.8
ENSG00000168476.12	-0.013753996	0.157269461	0.750589272	REEP4
ENSG00000143369.15	0.032493086	0.157403082	0.750961813	ECM1
ENSG00000283929.1	-0.038510963	0.157424384	0.750961813	MIR5010
ENSG00000225806.8	0.041020525	0.157488626	0.750961813	RP1-309F20.3
ENSG00000110651.12	-0.015333164	0.157516783	0.750961813	CD81
ENSG00000132530.17	-0.041433273	0.157530608	0.750961813	XAF1
ENSG00000104043.15	0.020827665	0.157698452	0.751266566	ATP8B4
ENSG00000244187.8	0.014712486	0.157748056	0.751266566	TMEM141
ENSG00000198865.10	0.035867645	0.157763083	0.751266566	CCDC152
ENSG00000127530.4	-0.04037076	0.157763644	0.751266566	OR7C1
ENSG00000187049.10	0.012680297	0.15787658	0.751266566	TMEM216
ENSG00000267480.1	0.025104727	0.157887727	0.751266566	RP11-703I16.1
ENSG00000257495.5	0.044479408	0.157911884	0.751266566	KRT73-AS1
ENSG00000175756.13	-0.016196142	0.157954808	0.751266566	AURKAIP1
ENSG00000272832.1	-0.05996004	0.157964465	0.751266566	RP11-91K8.5
ENSG00000126091.21	0.015463601	0.157981129	0.751266566	ST3GAL3
ENSG00000009724.17	-0.022674034	0.157997498	0.751266566	MASP2
ENSG00000111642.16	-0.016511503	0.158059413	0.751315728	CHD4
ENSG00000174177.13	-0.013005876	0.158176037	0.751315728	CTU2
ENSG00000077463.15	-0.013285364	0.158206699	0.751315728	SIRT6
ENSG00000186431.19	0.018705799	0.158218435	0.751315728	FCAR
ENSG00000106004.5	0.070541705	0.15822474	0.751315728	HOXA5
ENSG00000103496.15	-0.010250596	0.158227649	0.751315728	STX4
ENSG00000234616.9	0.008321911	0.158270106	0.751343363	JRK
ENSG00000130589.16	-0.025776443	0.158345106	0.75152544	HELZ2

ENSG00000271855.1	-0.033383306	0.158441722	0.751810002	RP11-214N9.1
ENSG00000269246.1	0.04637112	0.15858754	0.752279505	CTC-246B18.10
ENSG00000144045.14	0.051082462	0.15862804	0.752279505	DQX1
ENSG00000181631.7	0.012547807	0.158650715	0.752279505	P2RY13
ENSG00000073605.19	-0.018044913	0.158752192	0.752517121	GSDMB
ENSG00000269365.2	-0.099908684	0.158834501	0.752517121	RP11-380M21.4
ENSG00000198839.10	0.015239461	0.158853222	0.752517121	ZNF277
ENSG00000204261.9	0.009075035	0.158854404	0.752517121	PSMB8-AS1
ENSG00000183527.12	-0.013945925	0.158884296	0.752517121	PSMG1
ENSG00000105996.7	0.043134258	0.159004937	0.752778698	HOXA2
ENSG00000132155.13	0.015694221	0.159016565	0.752778698	RAF1
ENSG00000167807.15	0.038156981	0.159055538	0.752778698	CTD-2369P2.10
ENSG00000113716.13	0.006893211	0.159086351	0.752778698	HMGXB3
ENSG00000107551.21	0.013748046	0.159214676	0.75321213	RASSF4
ENSG00000163406.11	-0.017702287	0.159256728	0.753237311	SLC15A2
ENSG00000149932.17	0.014781251	0.159390197	0.753598632	TMEM219
ENSG00000285730.1	-0.059281205	0.159410628	0.753598632	RP1-167A14.3
ENSG00000211599.2	-0.055164071	0.159468109	0.753598632	IGKV5-2
ENSG00000149294.17	-0.032483466	0.159480108	0.753598632	NCAM1
ENSG00000056050.7	0.016696111	0.159607378	0.753694059	HPF1
ENSG00000106459.15	0.005745732	0.159625878	0.753694059	NRF1
ENSG00000273456.1	-0.02891715	0.159647556	0.753694059	RP11-686O6.2
ENSG00000164022.18	0.011264319	0.159662925	0.753694059	AIMP1
ENSG00000011143.19	-0.010562691	0.159688151	0.753694059	MKS1
ENSG00000241411.1	0.044866885	0.159735356	0.753694059	RP11-192C21.1
ENSG00000224397.7	0.016455029	0.159757562	0.753694059	PELATON
ENSG00000178988.11	0.012005396	0.159856451	0.75394672	MRFAP1L1
ENSG00000106330.12	-0.015610618	0.159896622	0.75394672	MOSPD3
ENSG00000180902.18	-0.016503033	0.159921408	0.75394672	D2HGDH
ENSG00000006625.18	0.014950827	0.159975649	0.7540291	GGCT
ENSG00000145247.12	0.016517962	0.160021544	0.754072109	OCIAD2
ENSG00000112812.16	-0.041345785	0.160090081	0.754124623	PRSS16
ENSG00000122224.18	-0.010710996	0.160143474	0.754124623	LY9
ENSG00000256347.1	-0.056761329	0.160158568	0.754124623	OR8R1P
ENSG00000141526.18	-0.014135851	0.160179776	0.754124623	SLC16A3
ENSG00000145491.12	0.019198434	0.160336909	0.754126861	ROPN1L
ENSG00000229659.1	0.053865148	0.160340089	0.754126861	RPL26P6
ENSG00000279382.1	0.017885498	0.160356179	0.754126861	RP11-449J21.3
ENSG00000176024.18	0.018181676	0.160358691	0.754126861	ZNF613
ENSG00000143951.16	0.013670537	0.160381867	0.754126861	WDPCP
ENSG00000174099.12	0.020410315	0.160400886	0.754126861	MSRB3
ENSG00000225265.2	0.043135551	0.1605039	0.754438225	TAF1A-AS1
ENSG00000075856.12	-0.007275126	0.16067028	0.755034715	SART3
ENSG00000148290.10	0.011923591	0.160704434	0.755034715	SURF1
ENSG00000288064.1	-0.061058643	0.16081158	0.755223958	RP11-629B4.1
ENSG00000160072.20	-0.012874459	0.160824997	0.755223958	ATAD3B
ENSG00000256029.6	0.040602105	0.160855191	0.755223958	RP11-190A12.7

ENSG00000198677.12	0.0123634	0.160901203	0.755267078	TTC37
ENSG00000226252.1	0.043443973	0.161106088	0.756021724	RP1-18D14.7
ENSG00000057608.17	0.008120318	0.161208908	0.756021724	GDI2
ENSG00000287315.1	0.028367235	0.161236883	0.756021724	RP11-245P10.9
ENSG00000070501.12	0.016074023	0.161248727	0.756021724	POLB
ENSG00000143390.18	0.007953754	0.161273852	0.756021724	RFX5
ENSG00000229097.1	0.030591204	0.16128316	0.756021724	CALM2P2
ENSG00000172819.17	0.011784422	0.161339145	0.756086809	RARG
ENSG00000023041.12	0.006499409	0.161430146	0.756086809	ZDHHC6
ENSG00000147443.13	-0.013867625	0.161451864	0.756086809	DOK2
ENSG00000243517.1	-0.04885585	0.161478292	0.756086809	RP11-627K11.1
ENSG00000151923.18	-0.005526087	0.161489263	0.756086809	TIAL1
ENSG00000287126.1	-0.024379984	0.161522766	0.756086809	RP13-512J5.2
ENSG00000248980.1	0.085853169	0.16155512	0.756086809	SPCS3-AS1
ENSG00000281207.1	-0.022945573	0.161649262	0.756354796	SLFNL1-AS1
ENSG00000154016.14	0.01845093	0.161718368	0.756468047	GRAP
ENSG00000274227.1	0.026257802	0.16184154	0.756468047	RP1-267L14.6
ENSG00000185168.5	-0.028167657	0.161854153	0.756468047	LINC00482
ENSG00000152315.5	0.023815235	0.162068605	0.756468047	KCNK13
ENSG00000108094.16	0.008759877	0.162109698	0.756468047	CUL2
ENSG00000252206.1	-0.048104079	0.162164418	0.756468047	RNU7-40P
ENSG00000271550.1	-0.038362691	0.16216503	0.756468047	BNIP3P11
ENSG00000169877.10	0.057877679	0.162243676	0.756468047	AHSP
ENSG00000169220.18	-0.010207196	0.162248087	0.756468047	RGS14
ENSG00000262434.1	-0.043252254	0.162303912	0.756468047	RP11-676J12.8
ENSG00000137154.13	0.018653911	0.1623061	0.756468047	RPS6
ENSG00000107362.14	-0.009856241	0.16232672	0.756468047	ABHD17B
ENSG00000104976.12	-0.013835059	0.162367999	0.756468047	SNAPC2
ENSG00000080819.9	-0.01247868	0.162416337	0.756468047	CPOX
ENSG00000060339.14	0.011739132	0.162458314	0.756468047	CCAR1
ENSG00000197063.11	0.010558107	0.16253203	0.756468047	MAFG
ENSG00000175155.11	0.01087613	0.16256491	0.756468047	YPEL2
ENSG00000197265.9	0.010158665	0.162569716	0.756468047	GTF2E2
ENSG00000130726.12	-0.012534446	0.162570847	0.756468047	TRIM28
ENSG00000240053.8	-0.015415746	0.162577082	0.756468047	LY6G5B
ENSG00000200534.1	-0.030082971	0.16259883	0.756468047	SNORA33
ENSG00000273247.5	0.02082306	0.162611003	0.756468047	RAB33B-AS1
ENSG00000113916.18	0.018807308	0.162620405	0.756468047	BCL6
ENSG00000198075.10	-0.055351347	0.162634745	0.756468047	SULT1C4
ENSG00000236498.1	-0.056660115	0.162693847	0.756468047	AC107081.5
ENSG00000181924.7	0.010943785	0.162718344	0.756468047	COA4
ENSG00000108846.16	0.027439836	0.162808112	0.756468047	ABCC3
ENSG00000117151.13	0.013534069	0.162830581	0.756468047	CTBS
ENSG00000103111.15	0.007464814	0.162845143	0.756468047	MON1B
ENSG00000114270.18	-0.026289474	0.162947163	0.756468047	COL7A1
ENSG00000243716.10	-0.034525552	0.163007608	0.756468047	NPIPB5
ENSG00000105722.10	-0.014644705	0.16300976	0.756468047	ERF

ENSG00000253882.6	0.030578086	0.163031684	0.756468047	RP11-61L23.2
ENSG00000135414.10	-0.010350965	0.16304173	0.756468047	GDF11
ENSG00000140497.16	-0.010440094	0.163148322	0.756468047	SCAMP2
ENSG00000091317.8	0.011654691	0.163164077	0.756468047	CMTM6
ENSG00000270921.1	0.067079848	0.16319767	0.756468047	CICP23
ENSG00000149925.22	-0.01778312	0.163220278	0.756468047	ALDOA
ENSG00000163060.8	-0.080741587	0.16322387	0.756468047	TEKT4
ENSG00000234911.2	-0.029348036	0.163258318	0.756468047	TEX21P
ENSG00000187688.15	-0.010306539	0.163368239	0.756468047	TRPV2
ENSG00000282458.1	0.058116017	0.163371684	0.756468047	WASH5P
ENSG00000131652.14	-0.013078621	0.163383458	0.756468047	THOC6
ENSG00000116741.8	0.017785319	0.163427887	0.756468047	RGS2
ENSG00000276728.1	-0.02111725	0.16349002	0.756468047	AC142472.6
ENSG00000198843.13	0.015800729	0.16350904	0.756468047	SELENOT
ENSG00000146083.12	-0.010556446	0.163570071	0.756468047	RNF44
ENSG00000149929.16	-0.013893868	0.163670069	0.756468047	HIRIP3
ENSG00000183978.8	0.014912261	0.16367046	0.756468047	COA3
ENSG00000253764.3	0.054149215	0.163695728	0.756468047	RP11-439C15.4
ENSG00000155749.13	-0.022529692	0.163710753	0.756468047	FLACC1
ENSG00000146250.7	-0.081527494	0.163734347	0.756468047	PRSS35
ENSG00000260816.2	0.070276687	0.163800183	0.756468047	RP11-319G9.3
ENSG00000280193.1	0.04597193	0.163815346	0.756468047	RP13-923O23.7
ENSG00000161929.15	0.015386837	0.16382895	0.756468047	SCIMP
ENSG00000213290.4	0.049948148	0.163849953	0.756468047	PGK1P2
ENSG00000225886.3	-0.068987464	0.163863047	0.756468047	RP11-288L9.4
ENSG00000214803.3	-0.053054499	0.16386359	0.756468047	RP11-37N22.1
ENSG00000175224.17	0.008761074	0.163867047	0.756468047	ATG13
ENSG00000276471.1	0.046702032	0.163991328	0.756468047	RP11-398A8.5
ENSG00000281990.1	-0.147629631	0.164034436	0.756468047	IGHV1-69-2
ENSG00000154059.11	0.011970876	0.164068352	0.756468047	IMPACT
ENSG00000185359.14	-0.014002123	0.164079876	0.756468047	HGS
ENSG00000011600.12	0.013917895	0.164090521	0.756468047	TYROBP
ENSG00000257727.6	0.012652906	0.164102126	0.756468047	CNPY2
ENSG00000267309.1	-0.025845041	0.164107975	0.756468047	ZNF566-AS1
ENSG00000155876.6	0.007550543	0.164170776	0.756587476	RRAGA
ENSG00000279488.1	-0.036056253	0.164237336	0.756688742	AC004623.3
ENSG00000213337.9	-0.011943245	0.164266544	0.756688742	ANKRD39
ENSG00000106829.20	-0.008983943	0.164314999	0.756741973	TLE4
ENSG00000286796.1	-0.02134563	0.164385306	0.756762806	RP11-339H12.1
ENSG00000134759.14	0.008385741	0.164393325	0.756762806	ELP2
ENSG00000225851.1	-0.115669817	0.164494467	0.757058466	HLA-S
ENSG00000107798.18	0.019152423	0.164569504	0.757233876	LIPA
ENSG00000213859.8	-0.01129428	0.164692731	0.757566386	KCTD11
ENSG00000100084.14	-0.012192542	0.164735581	0.757566386	HIRA
ENSG00000126214.21	-0.009880091	0.164755133	0.757566386	KLC1
ENSG00000166710.21	0.022218812	0.164789528	0.757566386	B2M
ENSG00000106415.13	-0.015538283	0.164835275	0.757606864	GLCCI1

ENSG00000287125.1	-0.030171187	0.164925455	0.757767297	RP11-568J23.10
ENSG00000250479.9	0.018248252	0.164975815	0.757767297	CHCHD10
ENSG00000226318.1	-0.043381994	0.165032413	0.757767297	RPS3AP38
ENSG00000239332.6	0.050214727	0.165040002	0.757767297	LINC01119
ENSG00000225062.1	-0.052473114	0.165065091	0.757767297	CATIP-AS1
ENSG00000109079.10	0.006317213	0.16509188	0.757767297	TNFAIP1
ENSG00000117228.11	0.03647957	0.165175141	0.757979814	GBP1
ENSG00000286831.1	-0.033189026	0.16521884	0.758010734	CH17-112M04.1
ENSG00000175920.18	-0.037774722	0.165294944	0.758076177	DOK7
ENSG00000259728.5	0.057538765	0.165389097	0.758076177	LINC00933
ENSG00000265590.10	-0.031057583	0.165401406	0.758076177	CFAP298-TCP10L
ENSG00000079101.16	0.122881416	0.165411848	0.758076177	CLUL1
ENSG00000007908.16	-0.078743229	0.165464213	0.758076177	SELE
ENSG00000156508.19	0.016535886	0.16549159	0.758076177	EEF1A1
ENSG00000168685.15	0.017911327	0.165497131	0.758076177	IL7R
ENSG00000131368.8	0.008903085	0.165553821	0.758076177	MRPS25
ENSG00000168275.16	0.017285009	0.16557389	0.758076177	COA6
ENSG00000272979.1	0.037073964	0.165602754	0.758076177	RP11-647K16.1
ENSG00000165548.11	-0.046930976	0.165732608	0.758365871	TMEM63C
ENSG00000149428.19	-0.012539284	0.165739996	0.758365871	HYOU1
ENSG00000282133.1	0.027477604	0.165813348	0.758385763	TRBJ1-3
ENSG00000117411.18	0.020925729	0.165818303	0.758385763	B4GALT2
ENSG00000188585.9	-0.078878701	0.165865165	0.758416283	CLEC20A
ENSG00000226352.2	-0.021301293	0.165898939	0.758416283	PSPC1-AS2
ENSG00000260461.1	-0.04213189	0.166015431	0.75877219	RP11-541N10.3
ENSG00000279765.3	-0.026472045	0.166100148	0.75877219	RP11-437B10.1
ENSG00000105186.16	-0.007335378	0.166145304	0.75877219	ANKRD27
ENSG00000126226.22	-0.009072884	0.166150298	0.75877219	PCID2
ENSG00000143942.5	-0.025607456	0.166209419	0.75877219	CHAC2
ENSG00000163516.14	-0.008887567	0.166276948	0.75877219	ANKZF1
ENSG00000272914.1	0.025470576	0.166315993	0.75877219	RP11-330O11.3
ENSG00000105737.9	0.035177543	0.166333928	0.75877219	GRIK5
ENSG00000141858.12	-0.014306068	0.166379014	0.75877219	SAMD1
ENSG00000274576.2	-0.062697656	0.1663915	0.75877219	IGHV2-70
ENSG00000105726.17	-0.011516784	0.166419628	0.75877219	ATP13A1
ENSG00000206127.11	0.05507899	0.166464964	0.75877219	GOLGA8O
ENSG00000113312.12	0.009570541	0.16651138	0.75877219	TTC1
ENSG00000138594.14	-0.008976324	0.166516607	0.75877219	TMOD3
ENSG00000103512.15	-0.01627324	0.166578853	0.75877219	NOMO1
ENSG00000143858.12	0.024207715	0.166601442	0.75877219	SYT2
ENSG00000104320.14	0.014970155	0.166610794	0.75877219	NBN
ENSG00000150593.18	0.008255056	0.166648295	0.75877219	PDCD4
ENSG00000038002.9	0.014510541	0.16667977	0.75877219	AGA
ENSG00000189319.14	-0.010777396	0.166743566	0.758894152	FAM53B
ENSG00000255139.2	-0.023118044	0.166846825	0.759195628	AP000442.1
ENSG00000112339.15	0.011514246	0.166922644	0.759289827	HBS1L
ENSG00000214263.2	0.117111408	0.166963167	0.759289827	RPSAP53

ENSG00000255121.2	0.022344608	0.166981132	0.759289827	CENATAC-DT
ENSG00000254481.1	0.039714063	0.167052542	0.759289827	PTP4A2P2
ENSG00000105520.10	-0.015337065	0.167052648	0.759289827	PLPPR2
ENSG00000228137.1	-0.040902525	0.167097104	0.7593236	AP001469.7
ENSG00000220472.1	0.049931676	0.167161795	0.759372694	RP11-69L16.5
ENSG00000156689.7	0.138234957	0.167233552	0.759372694	GLYATL2
ENSG00000134871.19	0.079800467	0.167254316	0.759372694	COL4A2
ENSG00000168216.13	0.011147415	0.16725602	0.759372694	LMBRD1
ENSG00000162929.14	-0.015029238	0.167411047	0.75990831	KIAA1841
ENSG00000268279.4	0.024086153	0.167588554	0.760503939	RP11-434D12.1
ENSG00000225981.1	0.057411729	0.167616433	0.760503939	AC102953.4
ENSG00000105287.13	-0.014160251	0.167739184	0.760554112	PRKD2
ENSG00000259804.1	-0.038542556	0.167748931	0.760554112	CTD-2012K14.7
ENSG00000171105.14	-0.015630717	0.167853406	0.760554112	INSR
ENSG00000255484.2	-0.082269536	0.167864664	0.760554112	LINC02764
ENSG00000136888.8	0.020142964	0.16787775	0.760554112	ATP6V1G1
ENSG00000116830.12	-0.012572857	0.167881955	0.760554112	TTF2
ENSG00000099849.15	-0.014517089	0.167912163	0.760554112	RASSF7
ENSG00000197847.12	0.034360555	0.167924177	0.760554112	SLC22A20P
ENSG00000177981.11	0.006307924	0.16803459	0.760886151	ASB8
ENSG00000128512.23	0.028692935	0.168146545	0.76122502	DOCK4
ENSG00000128915.12	0.011961041	0.16822344	0.761269782	ICE2
ENSG00000256582.1	-0.035813769	0.168253279	0.761269782	LINC02390
ENSG00000117395.13	0.010492707	0.16845046	0.761269782	EBNA1BP2
ENSG00000198093.11	0.016077693	0.168475853	0.761269782	ZNF649
ENSG00000176597.12	0.024412854	0.16849077	0.761269782	B3GNT5
ENSG00000211685.3	-0.064621614	0.168503496	0.761269782	IGLC7
ENSG00000198431.16	0.010321193	0.168506281	0.761269782	TXNRD1
ENSG00000243789.11	0.042901226	0.168537838	0.761269782	JMJD7
ENSG00000163864.17	0.021475182	0.168551847	0.761269782	NMNAT3
ENSG00000227905.1	-0.054275338	0.168593797	0.761269782	MED6P1
ENSG00000144635.9	0.01056773	0.168611939	0.761269782	DYNC1LI1
ENSG00000115816.15	0.017743914	0.168622585	0.761269782	CEBPZ
ENSG00000180581.7	0.047904931	0.168740346	0.761269782	SRP9P1
ENSG00000248098.12	-0.016692791	0.168755484	0.761269782	BCKDHA
ENSG00000168993.15	-0.045655586	0.168755682	0.761269782	CPLX1
ENSG00000005175.10	0.012638574	0.168769845	0.761269782	RPAP3
ENSG00000132718.9	-0.016146256	0.168790743	0.761269782	SYT11
ENSG00000151466.12	0.01242118	0.168824604	0.761269782	SCLT1
ENSG00000128656.15	0.033988368	0.168880831	0.76135592	CHN1
ENSG00000096384.20	-0.011209291	0.168946377	0.761484024	HSP90AB1
ENSG00000119636.16	0.031218732	0.169129872	0.762124501	BBOF1
ENSG00000104886.12	-0.012112558	0.169162801	0.762124501	PLEKHJ1
ENSG00000268030.1	0.016189766	0.169253041	0.762363576	AC005253.2
ENSG00000081923.15	-0.023842522	0.169423454	0.76283224	ATP8B1
ENSG00000285696.1	0.056909072	0.16948051	0.76283224	RP11-144G7.4
ENSG00000242474.3	0.016938826	0.169490396	0.76283224	AC093627.9

ENSG00000259479.6	0.037725224	0.169505877	0.76283224	SORD2P
ENSG00000247473.2	0.047329978	0.169651945	0.763166995	CARS1-AS1
ENSG00000260661.1	-0.03691855	0.169654688	0.763166995	RP11-152L20.3
ENSG00000165821.12	0.017972812	0.169743009	0.763177744	SALL2
ENSG00000074800.16	-0.013635607	0.1697478	0.763177744	ENO1
ENSG00000162367.11	0.032005276	0.169768718	0.763177744	TAL1
ENSG00000134461.16	-0.013819404	0.169832029	0.763177992	ANKRD16
ENSG00000233799.1	-0.044067635	0.169843201	0.763177992	AC139887.4
ENSG00000273302.1	-0.029870943	0.169882282	0.763186383	RP11-493E12.2
ENSG00000267041.6	-0.016982369	0.169970287	0.763414508	ZNF850
ENSG00000099917.17	-0.010616736	0.170047883	0.763587437	MED15
ENSG00000144741.18	0.011047918	0.170083256	0.763587437	SLC25A26
ENSG00000257337.7	-0.020969248	0.170176923	0.763840739	RP11-983P16.4
ENSG00000140987.20	0.005967916	0.170312167	0.764224784	ZSCAN32
ENSG00000167851.15	0.013334763	0.170348576	0.764224784	CD300A
ENSG00000166478.10	0.007262633	0.170396844	0.764224784	ZNF143
ENSG00000282420.1	0.031696687	0.170424548	0.764224784	TRBJ1-2
ENSG00000272800.3	-0.032918305	0.170479959	0.764224784	RP11-438L19.1
ENSG00000260923.7	0.050864662	0.170495237	0.764224784	LINC02193
ENSG00000188807.13	-0.012478424	0.170558652	0.764224784	TMEM201
ENSG00000111602.12	-0.01387624	0.170560603	0.764224784	TIMELESS
ENSG00000105821.15	0.010498039	0.17067071	0.764418758	DNAJC2
ENSG00000287104.1	0.016275507	0.170678442	0.764418758	RP11-326F6.1
ENSG00000164649.20	-0.023779111	0.170753222	0.764506384	CDCA7L
ENSG00000253772.1	-0.055993086	0.170772564	0.764506384	RP11-34P1.1
ENSG00000165629.20	0.021320777	0.170916391	0.764978928	ATP5F1C
ENSG00000116478.12	-0.006149116	0.170952722	0.764978928	HDAC1
ENSG00000168255.20	0.027568442	0.171065036	0.765314521	POLR2J3
ENSG00000166582.10	0.013566226	0.171226742	0.765367345	CENPV
ENSG00000249849.1	0.060311274	0.171251185	0.765367345	RP11-1101H11.1
ENSG00000276663.1	-0.046084147	0.171304079	0.765367345	RP11-407G23.7
ENSG00000162438.12	-0.032133487	0.171304356	0.765367345	CTRC
ENSG00000159648.13	-0.032787843	0.17130971	0.765367345	TEPP
ENSG00000082153.18	0.00950366	0.171315958	0.765367345	BZW1
ENSG00000188761.13	-0.024003162	0.171338087	0.765367345	BCL2L15
ENSG00000197815.4	0.030566316	0.17162285	0.766335133	RP1-253P7.4
ENSG00000286035.1	-0.027737835	0.171636634	0.766335133	RP11-429O22.1
ENSG00000152223.15	0.010635422	0.171666842	0.766335133	EPG5
ENSG00000188612.12	0.009134975	0.17176801	0.76650719	SUMO2
ENSG00000111445.14	0.008557239	0.171780137	0.76650719	RFC5
ENSG00000272518.1	0.036239673	0.171845531	0.766632185	RP11-26J3.3
ENSG00000185338.7	0.03060149	0.171886557	0.766648435	SOCS1
ENSG00000124574.15	-0.009830513	0.171932836	0.766688106	ABCC10
ENSG00000132744.8	0.032252408	0.171981827	0.766702471	ACY3
ENSG00000091879.14	-0.048239946	0.172010828	0.766702471	ANGPT2
ENSG00000232021.7	0.033757429	0.172112119	0.766987253	LEF1-AS1
ENSG00000213722.10	-0.014959105	0.172263763	0.767159207	DDAH2

ENSG00000254614.2	-0.032808264	0.172266109	0.767159207	AP003068.23
ENSG00000279549.1	0.048133575	0.172327084	0.767159207	AP000437.3
ENSG00000110107.9	-0.010995389	0.172385045	0.767159207	PRPF19
ENSG00000159625.15	0.033136542	0.172427831	0.767159207	DRC7
ENSG00000196922.10	0.011917815	0.172523824	0.767159207	ZNF252P
ENSG00000144792.9	0.028905203	0.172558221	0.767159207	ZNF660
ENSG00000268615.1	0.049492412	0.172591277	0.767159207	RP11-65J3.15
ENSG00000165490.13	0.020467603	0.172592477	0.767159207	DDIAS
ENSG00000224914.4	-0.02647731	0.172617434	0.767159207	LINC00863
ENSG00000164916.11	-0.010641692	0.172654447	0.767159207	FOXK1
ENSG00000270557.1	-0.028617947	0.172762053	0.767159207	RP11-546J1.1
ENSG00000259407.1	0.032853309	0.172763658	0.767159207	RP11-158M2.3
ENSG00000198846.6	-0.01981142	0.172780206	0.767159207	TOX
ENSG00000180357.9	0.01282819	0.172801768	0.767159207	ZNF609
ENSG00000154146.13	0.031593411	0.172839348	0.767159207	NRGN
ENSG00000140859.16	0.023205064	0.172877343	0.767159207	KIFC3
ENSG00000164535.15	-0.010622627	0.172904613	0.767159207	DAGLB
ENSG00000184611.12	0.024911332	0.17291207	0.767159207	KCNH7
ENSG00000279539.1	0.04271038	0.173027578	0.767159207	AC006486.10
ENSG00000170946.15	0.012820541	0.173069921	0.767159207	DNAJC24
ENSG00000180233.11	-0.015004998	0.173142877	0.767159207	ZNRF2
ENSG00000157191.20	0.009006149	0.173168409	0.767159207	NECAP2
ENSG00000125459.17	-0.010593068	0.173178009	0.767159207	MSTO1
ENSG00000103888.17	0.021483888	0.17320327	0.767159207	CEMIP
ENSG00000261754.2	-0.027438081	0.173204439	0.767159207	CTC-523E23.1
ENSG00000113520.11	0.049083556	0.173218675	0.767159207	IL4
ENSG00000234782.3	0.065360712	0.17322702	0.767159207	TPT1P9
ENSG00000231177.4	-0.018650235	0.173235889	0.767159207	LINC00852
ENSG00000060688.13	0.008454012	0.17327294	0.767159207	SNRNP40
ENSG00000083290.20	0.011104471	0.173322285	0.76721205	ULK2
ENSG00000146021.15	0.017824216	0.173420151	0.767479596	KLHL3
ENSG00000135540.11	-0.024415031	0.17351379	0.767640571	NHSL1
ENSG00000176390.12	0.010149758	0.173531387	0.767640571	CRLF3
ENSG00000185591.10	0.007552817	0.173644846	0.767916565	SP1
ENSG00000258744.2	-0.033069987	0.173668667	0.767916565	RP11-80A15.1
ENSG00000135324.6	-0.140621333	0.173732134	0.768031602	MRAP2
ENSG00000224067.2	-0.035741826	0.173785786	0.768103209	RP11-430K21.2
ENSG00000168175.15	-0.006549983	0.173930338	0.768301225	MAPK1IP1L
ENSG00000092199.18	0.008075119	0.173933945	0.768301225	HNRNPC
ENSG00000157322.17	-0.052015016	0.173971319	0.768301225	CLEC18A
ENSG00000121851.13	0.013084139	0.173980441	0.768301225	POLR3GL
ENSG00000265871.1	-0.05202213	0.174062051	0.768394483	MIR3174
ENSG00000167645.17	-0.01371265	0.174076495	0.768394483	YIF1B
ENSG00000268154.2	-0.031700647	0.174175933	0.768667966	Metazoa_SRP
ENSG00000235579.1	-0.0326983	0.174220197	0.76869789	RPL38P5
ENSG00000175084.13	0.044407551	0.174402617	0.76933725	DES
ENSG00000184588.18	0.013706043	0.174543726	0.769768658	PDE4B

ENSG00000232618.2	0.025521998	0.174619312	0.769768658	RP11-439L18.1
ENSG00000107404.20	-0.012239263	0.174687115	0.769768658	DVL1
ENSG00000107164.16	-0.010010411	0.174687651	0.769768658	FUBP3
ENSG00000092098.17	-0.013656563	0.174688089	0.769768658	RNF31
ENSG00000161654.10	-0.011169478	0.174786584	0.769801123	LSM12
ENSG00000162885.14	-0.019738017	0.17480288	0.769801123	B3GALNT2
ENSG00000139278.10	0.011317022	0.174808067	0.769801123	GLIPR1
ENSG00000072818.12	-0.011015239	0.174911158	0.769840932	ACAP1
ENSG00000166863.13	0.061743791	0.174936537	0.769840932	TAC3
ENSG00000225921.7	0.009224868	0.174980581	0.769840932	NOL7
ENSG00000105982.17	0.02000358	0.17499348	0.769840932	RNF32
ENSG00000162571.14	-0.058842976	0.175004799	0.769840932	TTLL10
ENSG00000280007.1	-0.021772983	0.175088679	0.769911194	AC008079.10
ENSG00000227077.3	0.031627615	0.175095856	0.769911194	AC107983.4
ENSG00000146677.7	0.033428259	0.175252075	0.770184664	RPL32P18
ENSG00000116981.4	-0.069285827	0.175300401	0.770184664	NT5C1A
ENSG00000280378.1	0.01990152	0.175303333	0.770184664	RP4-758J24.6
ENSG00000086967.10	-0.056405366	0.17530827	0.770184664	MYBPC2
ENSG00000270504.1	0.045496457	0.175363456	0.770262103	RP11-420L9.5
ENSG00000178053.20	0.041542392	0.175403795	0.77027431	MLF1
ENSG00000233175.2	-0.022218908	0.175529274	0.77032123	CTD-2020K17.3
ENSG00000272186.1	0.053190573	0.175541892	0.77032123	VPS11-DT
ENSG00000143127.13	-0.017155952	0.175556775	0.77032123	ITGA10
ENSG00000256171.1	0.037434812	0.175564727	0.77032123	GCSHP4
ENSG00000047056.16	-0.005990365	0.175803676	0.771049905	WDR37
ENSG00000010295.20	-0.008379681	0.175809625	0.771049905	IFFO1
ENSG00000159753.14	-0.014464823	0.17585525	0.771049905	CARMIL2
ENSG00000105855.10	0.059518446	0.175898789	0.771049905	ITGB8
ENSG00000036257.13	0.008534077	0.17592428	0.771049905	CUL3
ENSG00000172671.20	0.011100046	0.175956386	0.771049905	ZFAND4
ENSG00000227782.2	-0.019822512	0.176025127	0.771112513	CTC-529I10.2
ENSG00000173269.14	0.050997265	0.176045874	0.771112513	MMRN2
ENSG00000178966.17	0.015088858	0.176107918	0.771219557	RMI1
ENSG00000160285.15	-0.012702204	0.176340126	0.771330316	LSS
ENSG00000064989.13	0.03198097	0.176380898	0.771330316	CALCRL
ENSG00000240204.3	-0.02632187	0.176427268	0.771330316	SMKR1
ENSG00000156042.18	0.022062594	0.176434961	0.771330316	CFAP70
ENSG00000253214.2	0.040919	0.176477987	0.771330316	RP11-1149M10.2
ENSG00000274979.1	-0.03168566	0.17649259	0.771330316	RP11-1143G9.5
ENSG00000285954.1	-0.025914137	0.176494072	0.771330316	RP11-117D22.3
ENSG00000101236.17	0.018635631	0.176511858	0.771330316	RNF24
ENSG00000033030.15	-0.005956853	0.176548329	0.771330316	ZCCHC8
ENSG00000170004.18	-0.009684405	0.17656875	0.771330316	CHD3
ENSG00000113658.18	0.017423388	0.176578917	0.771330316	SMAD5
ENSG00000111775.3	0.015027194	0.176618331	0.771330316	COX6A1
ENSG00000165732.13	0.009358457	0.176642478	0.771330316	DDX21
ENSG00000276754.1	0.059806254	0.176674399	0.771330316	RP11-461A8.5

ENSG00000118046.16	-0.011464911	0.176697378	0.771330316	STK11
ENSG00000165060.15	0.011859226	0.176778555	0.771520454	FXN
ENSG00000105939.14	-0.012726565	0.176908892	0.771574653	ZC3HAV1
ENSG00000250091.3	0.021802853	0.176911765	0.771574653	DNAH10OS
ENSG00000072163.20	-0.026656133	0.176952005	0.771574653	LIMS2
ENSG00000249835.2	-0.070606453	0.176961415	0.771574653	VCAN-AS1
ENSG00000163479.14	0.011891718	0.17697909	0.771574653	SSR2
ENSG00000226026.6	0.070569743	0.177145917	0.772137823	RP11-57H12.3
ENSG00000166192.15	0.025161735	0.177421964	0.773014853	SENP8
ENSG00000253330.1	0.036589727	0.177424653	0.773014853	RP11-697N18.3
ENSG00000249274.1	0.027499353	0.177460207	0.773014853	PDLIM1P4
ENSG00000274808.6	0.059347477	0.177499621	0.773022347	TBC1D3B
ENSG00000154316.16	0.045191973	0.177677871	0.773252673	TDH
ENSG00000176994.11	0.011076164	0.17772783	0.773252673	SMCR8
ENSG00000253476.1	0.02559266	0.177771444	0.773252673	RP11-395I14.2
ENSG00000144857.15	-0.109097337	0.177804082	0.773252673	BOC
ENSG00000128268.12	0.02627939	0.177825471	0.773252673	MGAT3
ENSG00000274213.1	-0.028102641	0.177849458	0.773252673	RP11-670E13.6
ENSG00000268056.5	-0.055489342	0.177862825	0.773252673	CTD-3032J10.2
ENSG00000274372.5	-0.044328032	0.17786668	0.773252673	LINC02804
ENSG00000273061.1	-0.038185027	0.177891852	0.773252673	CDC37L1-DT
ENSG00000241889.1	-0.032296476	0.178063101	0.773833032	RP11-435F17.3
ENSG00000123358.20	-0.029569556	0.178126029	0.773915526	NR4A1
ENSG00000214455.4	-0.037416347	0.178157558	0.773915526	RCN1P2
ENSG00000099326.9	-0.011904574	0.178218904	0.77401806	MZF1
ENSG00000104129.10	0.009960164	0.178608702	0.774839036	DNAJC17
ENSG00000111247.15	-0.039398706	0.178638318	0.774839036	RAD51AP1
ENSG00000177732.9	-0.017486998	0.178644842	0.774839036	SOX12
ENSG00000003509.16	0.011480705	0.178677187	0.774839036	NDUFAF7
ENSG00000151090.20	0.035300404	0.178696551	0.774839036	THRB
ENSG00000139537.11	-0.014274566	0.178728345	0.774839036	CCDC65
ENSG00000280800.1	0.233725941	0.178732207	0.774839036	CH507-513H4.6
ENSG00000108424.11	0.009561243	0.178743397	0.774839036	KPNB1
ENSG00000168062.10	0.052721755	0.178747975	0.774839036	BATF2
ENSG00000042445.14	-0.010581271	0.178829891	0.774901784	RETSAT
ENSG00000221164.1	-0.044175002	0.178838021	0.774901784	SNORA11F
ENSG00000179082.3	0.02988051	0.178878153	0.774911947	C9orf106
ENSG00000233975.2	-0.086740343	0.178980252	0.775174212	LINC02574
ENSG00000103089.9	-0.055128634	0.179045154	0.775174212	FA2H
ENSG00000246477.3	-0.042880387	0.179087137	0.775174212	AF131216.6
ENSG00000270589.1	-0.019170084	0.179125726	0.775174212	RP11-348N5.7
ENSG00000137221.14	-0.007674797	0.179150407	0.775174212	TJAP1
ENSG00000135775.14	0.009709989	0.179256017	0.775174212	COG2
ENSG00000267724.1	0.053521248	0.179278641	0.775174212	RP11-49K24.8
ENSG00000270325.1	0.062430052	0.179282428	0.775174212	BNIP3P9
ENSG00000196372.13	0.012224278	0.179296063	0.775174212	ASB13
ENSG00000143919.15	0.013863008	0.179382019	0.775174212	CAMKMT

ENSG00000162426.16	-0.024405402	0.179399592	0.775174212	SLC45A1
ENSG00000270890.1	0.044427916	0.179587646	0.775174212	RP3-468K18.6
ENSG00000162078.11	0.036468927	0.179593428	0.775174212	ZG16B
ENSG00000105784.15	0.061969346	0.179596238	0.775174212	RUNDC3B
ENSG00000249602.1	-0.034928821	0.179605813	0.775174212	RP11-98D18.3
ENSG00000251578.1	0.037594557	0.179609469	0.775174212	TRBV21-1
ENSG00000102760.13	0.018915146	0.179740699	0.775174212	RGCC
ENSG00000260063.1	0.036273065	0.179782224	0.775174212	RP5-968P14.2
ENSG00000269970.1	0.036322153	0.179912855	0.775174212	RP11-498E2.9
ENSG00000180875.5	0.042757279	0.179980857	0.775174212	GREM2
ENSG00000162949.16	0.065413773	0.18000029	0.775174212	CAPN13
ENSG00000268581.1	0.034211167	0.180004476	0.775174212	SIGLEC18P
ENSG00000162882.15	0.01726162	0.180078522	0.775174212	HAAO
ENSG00000141527.18	-0.019074705	0.180080608	0.775174212	CARD14
ENSG00000171729.14	0.042342035	0.1800817	0.775174212	TMEM51
ENSG00000135046.14	0.024509816	0.180166095	0.775174212	ANXA1
ENSG00000052126.15	-0.029845334	0.180167989	0.775174212	PLEKHA5
ENSG00000171798.19	-0.040204031	0.180172815	0.775174212	KNDC1
ENSG00000211961.3	0.064355383	0.18023676	0.775174212	IGHV1-45
ENSG00000174444.15	0.014833979	0.180283312	0.775174212	RPL4
ENSG00000138028.16	-0.055300018	0.180286098	0.775174212	CGREF1
ENSG00000112419.14	-0.01346259	0.180286219	0.775174212	PHACTR2
ENSG00000263366.2	-0.059091616	0.180336084	0.775174212	ABHD17AP5
ENSG00000164114.19	-0.027074025	0.180336681	0.775174212	MAP9
ENSG00000012124.17	-0.03330909	0.180374553	0.775174212	CD22
ENSG00000143184.5	0.044438103	0.180444223	0.775174212	XCL1
ENSG00000105464.4	-0.027082166	0.180456555	0.775174212	GRIN2D
ENSG00000126456.16	-0.011198453	0.180471107	0.775174212	IRF3
ENSG00000235688.2	0.058250227	0.180472723	0.775174212	SNTG2-AS1
ENSG00000176340.4	0.015645713	0.180473533	0.775174212	COX8A
ENSG00000227896.2	0.022020818	0.180488437	0.775174212	RP11-77P6.2
ENSG00000134698.11	0.010333587	0.18058671	0.775433888	AGO4
ENSG00000083444.17	-0.016560553	0.18069321	0.775728774	PLOD1
ENSG00000165280.18	-0.010994637	0.180731746	0.775731824	VCP
ENSG00000279187.1	-0.033833668	0.18099497	0.776314455	RP11-455O6.5
ENSG00000256771.4	0.015488975	0.180997682	0.776314455	ZNF253
ENSG00000125319.15	-0.023889332	0.181006862	0.776314455	HROB
ENSG00000260517.3	-0.028024973	0.181051741	0.776314455	RP11-426C22.5
ENSG00000236137.1	-0.050231948	0.181098927	0.776314455	RP11-27K13.3
ENSG00000176490.5	-0.042668649	0.181153614	0.776314455	DIRAS1
ENSG00000122644.13	0.017912385	0.18116818	0.776314455	ARL4A
ENSG00000168273.8	0.016008115	0.181170323	0.776314455	SMIM4
ENSG00000287631.1	-0.019627837	0.181265887	0.776561688	CTB-161A2.8
ENSG00000133104.14	0.011028786	0.18136048	0.776614155	SPART
ENSG00000075234.17	-0.022370154	0.181363864	0.776614155	TTC38
ENSG00000136999.5	-0.025796711	0.181402171	0.776614155	CCN3
ENSG00000172340.15	0.010166807	0.181447833	0.776614155	SUCLG2

ENSG00000102780.17	-0.02109725	0.181522439	0.776614155	DGKH
ENSG00000130881.14	-0.021034518	0.181582218	0.776614155	LRP3
ENSG00000131480.9	-0.019277905	0.181583167	0.776614155	AOC2
ENSG00000279349.1	-0.034414487	0.181609137	0.776614155	RP11-147G7.3
ENSG00000160949.17	-0.014593758	0.181618953	0.776614155	TONSL
ENSG00000105370.8	-0.034539507	0.181698751	0.776768966	LIM2
ENSG00000170271.11	0.037326017	0.181750369	0.776768966	FAXDC2
ENSG00000283839.1	-0.016637504	0.181768786	0.776768966	RP11-363K21.1
ENSG00000176485.13	0.025698888	0.181909095	0.777080388	PLAAT3
ENSG00000288109.1	-0.08252367	0.181937218	0.777080388	RP11-360N9.5
ENSG00000124181.14	-0.011603256	0.181955336	0.777080388	PLCG1
ENSG00000116670.15	-0.01300577	0.182078899	0.777446192	MAD2L2
ENSG00000165406.16	0.018227499	0.18215007	0.777447515	MARCHF8
ENSG00000231389.7	0.01713701	0.182155028	0.777447515	HLA-DPA1
ENSG00000119392.16	0.005688853	0.182386886	0.778159169	GLE1
ENSG00000173947.14	-0.044951352	0.182425264	0.778159169	PIFO
ENSG00000100201.23	0.010187225	0.1824356	0.778159169	DDX17
ENSG00000150991.15	0.010834895	0.182584499	0.778498449	UBC
ENSG00000196090.12	-0.194677874	0.182591064	0.778498449	PTPRT
ENSG00000125651.14	-0.009251725	0.182686953	0.778567282	GTF2F1
ENSG00000102753.10	-0.010183222	0.182741749	0.778567282	KPNA3
ENSG00000257267.3	0.013858181	0.182755532	0.778567282	ZNF271P
ENSG00000143155.13	0.009438359	0.182759064	0.778567282	TIPRL
ENSG00000245534.7	-0.033171549	0.18297027	0.779305149	RORA-AS1
ENSG00000175970.11	0.007576654	0.183166398	0.779756886	UNC119B
ENSG00000100241.21	-0.010197136	0.1832237	0.779756886	SBF1
ENSG00000140943.17	0.005497354	0.183262626	0.779756886	MBTPS1
ENSG00000283063.1	-0.040114522	0.183299342	0.779756886	TRBV6-2
ENSG00000171160.18	0.031243754	0.183330144	0.779756886	MORN4
ENSG00000130787.14	-0.014728407	0.183333036	0.779756886	HIP1R
ENSG00000183020.14	-0.008545194	0.183516478	0.779756886	AP2A2
ENSG00000184384.14	0.013818903	0.183531012	0.779756886	MAML2
ENSG00000106993.12	0.01457134	0.183581034	0.779756886	CDC37L1
ENSG00000187626.9	0.009244314	0.183617676	0.779756886	ZKSCAN4
ENSG00000103356.18	0.007853959	0.183702959	0.779756886	EARS2
ENSG00000160284.15	-0.031947662	0.183706563	0.779756886	SPATC1L
ENSG00000254325.2	-0.05737186	0.183712636	0.779756886	RP11-318K15.2
ENSG00000122257.20	0.00845119	0.183830666	0.779756886	RBBP6
ENSG00000112992.18	-0.010589963	0.183847247	0.779756886	NNT
ENSG00000087086.15	0.016322353	0.183850152	0.779756886	FTL
ENSG00000151176.8	-0.013912249	0.183867748	0.779756886	PLBD2
ENSG00000164961.16	0.007902084	0.183887762	0.779756886	WASHC5
ENSG00000139890.10	-0.016990412	0.183903803	0.779756886	REM2
ENSG00000198873.12	-0.009045273	0.183989031	0.779756886	GRK5
ENSG00000173567.15	-0.024990744	0.184000909	0.779756886	ADGRF3
ENSG00000139437.18	-0.007656863	0.184007325	0.779756886	TCHP
ENSG00000071127.17	-0.008627675	0.184071056	0.779756886	WDR1

ENSG00000176396.11	-0.00979724	0.184083782	0.779756886	EID2
ENSG00000211966.2	-0.045978422	0.184103346	0.779756886	IGHV5-51
ENSG00000156206.14	0.043358114	0.184120929	0.779756886	CFAP161
ENSG00000116001.17	-0.014196477	0.184137548	0.779756886	TIA1
ENSG00000274471.2	-0.036039971	0.184169629	0.779756886	HERC2P7
ENSG00000243403.1	0.066387748	0.184178972	0.779756886	RP11-330L19.1
ENSG00000130640.15	-0.009376929	0.184238283	0.779818717	TUBGCP2
ENSG00000255769.7	0.031739662	0.184274666	0.779818717	GOLGA2P10
ENSG00000279926.1	-0.026780533	0.184307652	0.779818717	RP3-406P24.5
ENSG00000167912.6	-0.020859326	0.18435151	0.779843394	RP11-25K19.1
ENSG00000101194.18	-0.015812642	0.184411709	0.779937167	SLC17A9
ENSG00000173848.19	0.021830611	0.184602652	0.780407194	NET1
ENSG00000097046.13	-0.020147006	0.184605273	0.780407194	CDC7
ENSG00000155761.14	0.054823631	0.184644378	0.780407194	SPAG17
ENSG00000270339.3	-0.065053951	0.184675059	0.780407194	PPIAL4H
ENSG00000272142.4	0.022912232	0.184731236	0.780483762	LYRM4-AS1
ENSG00000145908.13	0.026381335	0.184829657	0.780714925	ZNF300
ENSG00000285554.1	-0.02295369	0.184890736	0.780714925	CH17-67K23.2
ENSG00000142961.15	0.010692475	0.184900155	0.780714925	MOB3C
ENSG00000160229.13	0.032020851	0.184958772	0.780801666	ZNF66
ENSG00000180979.10	0.007732208	0.185067318	0.780976013	LRRC57
ENSG00000187904.3	-0.054142036	0.185076235	0.780976013	AC097382.5
ENSG00000196290.16	0.011393135	0.185157562	0.781158463	NIF3L1
ENSG00000250361.9	0.069241233	0.185211816	0.781226641	GYPB
ENSG00000207445.1	-0.056513602	0.185398034	0.781575398	SNORD15B
ENSG00000128285.4	0.057561102	0.185419469	0.781575398	MCHR1
ENSG00000260005.6	-0.015684732	0.185432608	0.781575398	AC027601.1
ENSG00000066294.15	0.013345376	0.18545178	0.781575398	CD84
ENSG00000135334.9	-0.009507095	0.185485053	0.781575398	AKIRIN2
ENSG00000089041.17	0.021445343	0.185720291	0.782405862	P2RX7
ENSG00000146410.12	-0.018740567	0.185898669	0.782935802	MTFR2
ENSG00000173262.12	-0.074673386	0.185932646	0.782935802	SLC2A14
ENSG00000221883.4	0.019160514	0.185967044	0.782935802	ARIH2OS
ENSG00000135336.16	0.011307822	0.185998792	0.782935802	ORC3
ENSG00000135318.12	-0.029198017	0.186095955	0.783184044	NT5E
ENSG00000156958.15	0.011714494	0.186200307	0.783395057	GALK2
ENSG00000162909.18	-0.009155095	0.186222494	0.783395057	CAPN2
ENSG00000242970.2	0.064584074	0.18629252	0.783528918	AC068522.4
ENSG00000117477.12	-0.032920398	0.186528654	0.784361212	CCDC181
ENSG00000184916.9	-0.025521643	0.186613942	0.784558982	JAG2
ENSG00000119414.12	0.006408011	0.186704438	0.784718803	PPP6C
ENSG00000118200.14	0.023029215	0.18675028	0.784718803	CAMSAP2
ENSG00000119929.13	0.013248803	0.186766748	0.784718803	CUTC
ENSG00000173230.15	0.009264082	0.186901919	0.785125883	GOLGB1
ENSG00000162004.19	-0.019354065	0.186985419	0.785315785	CCDC78
ENSG00000254064.1	-0.022542098	0.18712535	0.785504135	CTD-2530N21.4
ENSG00000229205.5	-0.113085979	0.187150075	0.785504135	LINC00200

ENSG00000285732.1	0.049523119	0.187178751	0.785504135	RP11-242C24.4
ENSG00000151718.16	0.023180293	0.187251856	0.785504135	WWC2
ENSG00000234608.8	0.01091889	0.187289155	0.785504135	MAPKAPK5-AS1
ENSG00000201217.1	-0.062640766	0.187301515	0.785504135	Y_RNA
ENSG00000160226.16	-0.016051012	0.187359519	0.785504135	CFAP410
ENSG00000170525.21	0.01627064	0.187362595	0.785504135	PFKFB3
ENSG00000134108.14	0.008748292	0.187375637	0.785504135	ARL8B
ENSG00000120709.11	0.011868719	0.187495417	0.785504135	FAM53C
ENSG00000115091.12	0.008007214	0.187499265	0.785504135	ACTR3
ENSG00000259954.1	0.027538708	0.187561129	0.785504135	IL21R-AS1
ENSG00000124562.10	0.012737807	0.187572356	0.785504135	SNRPC
ENSG00000247735.2	-0.039232752	0.187613676	0.785504135	CTD-2574D22.2
ENSG00000228261.1	-0.05376263	0.187625769	0.785504135	ITPRIP-AS1
ENSG00000163704.12	-0.015025559	0.187643103	0.785504135	PRRT3
ENSG00000232303.2	-0.040940722	0.18772163	0.785528799	DFFBP1
ENSG00000158864.13	0.008956783	0.187726398	0.785528799	NDUFS2
ENSG00000101003.10	-0.031615222	0.187763905	0.785528799	GINS1
ENSG00000182165.18	0.017140169	0.18787397	0.785587483	TP53TG1
ENSG00000250859.1	0.026601362	0.187919619	0.785587483	HNRNPKP1
ENSG00000211692.1	0.056943519	0.187940222	0.785587483	TRGJP1
ENSG00000242375.1	-0.067299553	0.187966031	0.785587483	RP11-498P14.3
ENSG00000135249.8	0.009203218	0.188151078	0.785587483	RINT1
ENSG00000198218.11	-0.005872377	0.188168519	0.785587483	QRICH1
ENSG00000069869.17	-0.023732004	0.188170323	0.785587483	NEDD4
ENSG00000233937.7	-0.018257739	0.188198957	0.785587483	CTC-338M12.4
ENSG00000158113.13	-0.04073853	0.188234371	0.785587483	LRRC43
ENSG00000231822.1	-0.048800432	0.18823699	0.785587483	SMC3P1
ENSG00000279495.1	0.02186046	0.188240993	0.785587483	RP11-521B24.4
ENSG00000186222.5	-0.011543123	0.18827399	0.785587483	BLOC1S4
ENSG00000129187.15	0.007063101	0.188310047	0.785587483	DCTD
ENSG00000272988.1	0.03556827	0.188339786	0.785587483	RP11-150D20.5
ENSG00000196204.12	0.010854877	0.188352528	0.785587483	RNF216P1
ENSG00000274275.1	0.059386294	0.188522524	0.785619696	RP11-326K13.5
ENSG00000116983.13	0.031575068	0.188649524	0.785619696	HPCAL4
ENSG00000260456.7	0.037479907	0.188695672	0.785619696	C16orf95
ENSG00000127922.9	0.019605217	0.188710583	0.785619696	SEM1
ENSG00000136449.15	-0.040916258	0.188759353	0.785619696	MYCBPAP
ENSG00000138785.15	0.008957385	0.188925236	0.785619696	INTS12
ENSG00000105364.14	-0.01255281	0.189101764	0.785619696	MRPL4
ENSG00000283020.1	-0.037096209	0.189132438	0.785619696	DUX4L37
ENSG00000143147.15	0.043560775	0.189152996	0.785619696	GPR161
ENSG00000050438.17	0.015323703	0.189205915	0.785619696	SLC4A8
ENSG00000135404.12	0.013167511	0.18923619	0.785619696	CD63
ENSG00000257702.3	-0.021764755	0.189240478	0.785619696	LBX2-AS1
ENSG00000141028.6	-0.032053713	0.189248553	0.785619696	CDRT15P1
ENSG00000161277.11	-0.012232565	0.189343787	0.785619696	THAP8
ENSG00000124787.14	0.019590541	0.189356178	0.785619696	RPP40

ENSG00000186575.19	-0.009326741	0.18936387	0.785619696	NF2
ENSG00000225067.4	0.030381338	0.189363971	0.785619696	RPL23AP2
ENSG00000243679.1	-0.01833155	0.189392584	0.785619696	RP11-274B21.3
ENSG00000235910.2	-0.037860524	0.189393787	0.785619696	APOA1-AS
ENSG00000114388.14	-0.008826043	0.189444029	0.785619696	NPRL2
ENSG00000211701.2	-0.053770474	0.189498814	0.785619696	TRGV1
ENSG00000083814.13	-0.00942391	0.18951867	0.785619696	ZNF671
ENSG00000229663.1	-0.051244637	0.189565202	0.785619696	DPY19L4P1
ENSG00000274104.1	0.032877938	0.189614889	0.785619696	CTD-2553L13.10
ENSG00000132842.14	0.009174343	0.189627696	0.785619696	AP3B1
ENSG00000123342.16	0.03239289	0.189647834	0.785619696	MMP19
ENSG00000241280.1	0.059310082	0.189687094	0.785619696	RP11-221J22.2
ENSG00000169860.7	0.028987016	0.189709695	0.785619696	P2RY1
ENSG00000144369.13	0.051837422	0.189759904	0.785619696	FAM171B
ENSG00000102900.13	-0.010629516	0.189779835	0.785619696	NUP93
ENSG00000237883.2	-0.016088196	0.189800874	0.785619696	DGUOK-AS1
ENSG00000284052.1	0.031047596	0.189827311	0.785619696	RP11-284E5.1
ENSG00000223060.1	0.039040239	0.189841626	0.785619696	Y_RNA
ENSG00000140526.18	0.015413616	0.189853575	0.785619696	ABHD2
ENSG00000246528.3	-0.053836526	0.189891361	0.785619696	RP11-159H10.3
ENSG00000275092.1	-0.050587892	0.189894487	0.785619696	LA16c-329F2.2
ENSG00000134460.18	0.019577974	0.189901824	0.785619696	IL2RA
ENSG00000212125.2	0.051658946	0.189915082	0.785619696	TAS2R15P
ENSG00000132256.20	-0.01697338	0.189927269	0.785619696	TRIM5
ENSG00000087274.18	-0.009018052	0.189946755	0.785619696	ADD1
ENSG00000236540.7	-0.012805408	0.189968481	0.785619696	AC006547.13
ENSG00000279662.1	-0.046061333	0.190064223	0.785619696	RP11-609N14.4
ENSG00000285888.1	-0.055320183	0.190079785	0.785619696	RP3-431A14.7
ENSG00000175832.13	-0.049129267	0.190094511	0.785619696	ETV4
ENSG00000206177.7	-0.044025958	0.190128002	0.785619696	HBM
ENSG00000260807.7	0.032803197	0.190212937	0.785619696	CEROX1
ENSG00000267491.1	-0.043470582	0.190297797	0.785619696	CTD-2373H9.5
ENSG00000247950.7	0.019498914	0.190332418	0.785619696	SEC24B-AS1
ENSG00000109572.14	0.0089351	0.190349004	0.785619696	CLCN3
ENSG00000117475.14	0.015771034	0.190354071	0.785619696	BLZF1
ENSG00000168477.19	0.021929013	0.190371694	0.785619696	TNXB
ENSG00000108963.19	-0.010686053	0.190382888	0.785619696	DPH1
ENSG00000204574.14	-0.010113801	0.190410435	0.785619696	ABCF1
ENSG00000235481.3	-0.040639023	0.190458234	0.785619696	UBE2R2-AS1
ENSG00000124222.22	0.007489631	0.190480457	0.785619696	STX16
ENSG00000163421.9	0.026063951	0.190505498	0.785619696	PROK2
ENSG00000279453.1	-0.01347635	0.190575361	0.7857498	RP3-425C14.4
ENSG00000251364.7	0.021162976	0.190696029	0.786089281	CTD-2516F10.2
ENSG00000287733.1	0.028877887	0.190774735	0.78625568	RP11-134L10.2
ENSG00000203993.6	-0.008839132	0.190858572	0.786443157	ARRDC1-AS1
ENSG00000183977.14	-0.031136254	0.190967279	0.786733017	PP2D1
ENSG00000261655.1	-0.037146558	0.191108666	0.787155921	CTD-3064M3.3

ENSG00000226935.6	0.05366671	0.191160593	0.787155921	LINC00161
ENSG00000245213.7	-0.019808107	0.191252212	0.787155921	RP11-10K16.1
ENSG00000236829.9	-0.02722301	0.19127901	0.787155921	Z97634.3
ENSG00000176974.20	-0.01703674	0.191304504	0.787155921	SHMT1
ENSG00000147419.19	0.010940897	0.191386064	0.787155921	CCDC25
ENSG00000179023.9	0.074007158	0.191410429	0.787155921	KLHDC7A
ENSG00000148219.18	-0.017996363	0.191528344	0.787155921	ASTN2
ENSG00000228476.2	-0.078044084	0.191536697	0.787155921	CSTP1
ENSG00000119787.14	0.011981445	0.191554534	0.787155921	ATL2
ENSG00000230330.1	-0.044744271	0.191590242	0.787155921	HMGN2P3
ENSG00000280213.1	-0.023890482	0.191593664	0.787155921	UCKL1-AS1
ENSG00000123545.6	0.01510745	0.191598247	0.787155921	NDUFAF4
ENSG00000169955.7	0.00976877	0.191607293	0.787155921	ZNF747
ENSG00000122862.5	0.019210789	0.191709621	0.787402615	SRGN
ENSG00000254838.5	0.018867373	0.191856592	0.787402615	GVINP1
ENSG00000228705.2	0.037414261	0.191868632	0.787402615	LINC00659
ENSG00000273382.1	-0.022874681	0.191885114	0.787402615	TMEM167B-DT
ENSG00000117054.15	0.015329406	0.191910515	0.787402615	ACADM
ENSG00000225528.3	0.026295179	0.191923372	0.787402615	RP3-370M22.8
ENSG00000204685.7	0.009687193	0.191982537	0.787402615	STARD7-AS1
ENSG00000230424.1	0.030759968	0.192027787	0.787402615	EMC1-AS1
ENSG00000186174.13	-0.017870388	0.192030162	0.787402615	BCL9L
ENSG00000265874.1	-0.025926812	0.192051291	0.787402615	MIR4489
ENSG00000163220.11	0.020062076	0.192096126	0.787429013	S100A9
ENSG00000245662.4	0.129724478	0.192227077	0.787595009	LINC02211
ENSG00000240809.1	0.016905668	0.192231268	0.787595009	CAP1P1
ENSG00000198851.10	-0.015290822	0.192251834	0.787595009	CD3E
ENSG00000171853.16	-0.008997131	0.19240588	0.788068663	TRAPPC12
ENSG00000226057.7	0.042883473	0.192445269	0.788072601	PHF2P2
ENSG00000105887.11	0.009726815	0.192638493	0.788620752	MTPN
ENSG00000166342.19	-0.048221415	0.192656035	0.788620752	NETO1
ENSG00000260805.2	-0.02573942	0.192700303	0.788644545	RP11-61J19.5
ENSG00000123119.12	-0.034267687	0.192874781	0.789124088	NECAB1
ENSG00000163453.11	0.021387722	0.192894434	0.789124088	IGFBP7
ENSG00000262652.1	0.049153011	0.192982929	0.78917895	RP13-638C3.2
ENSG00000165689.17	0.008733724	0.193063274	0.78917895	ENTR1
ENSG00000115128.7	0.018693218	0.193079717	0.78917895	SF3B6
ENSG00000226806.1	0.104687028	0.193090286	0.78917895	LCT-AS1
ENSG00000167874.7	-0.021183255	0.193100252	0.78917895	TMEM88
ENSG00000151651.16	-0.013306378	0.19319386	0.789279649	ADAM8
ENSG00000106682.16	0.005340649	0.193219164	0.789279649	EIF4H
ENSG00000169397.4	0.059134457	0.193240351	0.789279649	RNASE3
ENSG00000186940.6	-0.044925244	0.193313153	0.789419783	CHCHD2P9
ENSG00000261997.1	0.024171382	0.193398917	0.789492765	RP11-212I21.4
ENSG00000279789.1	-0.02123895	0.193408019	0.789492765	CTD-2574D22.3
ENSG00000134297.6	-0.012378392	0.193489352	0.789667589	PLEKHA8P1
ENSG00000258366.10	0.022051593	0.193577197	0.789868914	RTEL1

ENSG00000116809.12	-0.009031686	0.193775933	0.790449502	ZBTB17
ENSG00000267283.1	-0.029616221	0.193838936	0.790449502	AC005306.3
ENSG00000287970.1	-0.105112295	0.193848434	0.790449502	CH17-98J9.1
ENSG00000200164.1	0.113798591	0.193873659	0.790449502	Y_RNA
ENSG00000101457.13	0.010614291	0.193913774	0.790455909	DNTTIP1
ENSG00000279561.3	-0.037967346	0.194178838	0.791348711	RP11-4L24.4
ENSG00000145979.18	0.015068201	0.194233875	0.791348711	TBC1D7
ENSG00000153363.13	0.015722289	0.194297073	0.791348711	LINC00467
ENSG00000258559.2	-0.018620702	0.194301066	0.791348711	AC005519.4
ENSG00000268743.1	-0.039058782	0.194325732	0.791348711	CTD-2538G9.5
ENSG00000148120.18	0.013080799	0.194544395	0.791497365	AOPEP
ENSG00000121594.12	0.043635304	0.19469957	0.791497365	CD80
ENSG00000267710.9	-0.030051203	0.194751166	0.791497365	EDDM13
ENSG00000176155.19	0.010408328	0.194758855	0.791497365	CCDC57
ENSG00000153029.16	0.008732575	0.194791285	0.791497365	MR1
ENSG00000103202.13	0.022987629	0.194791385	0.791497365	NME4
ENSG00000103852.13	0.031045549	0.194888229	0.791497365	TTC23
ENSG00000168209.6	0.02260178	0.194916536	0.791497365	DDIT4
ENSG00000108666.10	0.01060328	0.194952243	0.791497365	C17orf75
ENSG00000171202.7	0.019032623	0.194958767	0.791497365	TMEM126A
ENSG00000170113.16	-0.013551087	0.194998123	0.791497365	NIPA1
ENSG00000272078.1	0.021137049	0.195007029	0.791497365	RP4-734G22.3
ENSG00000091009.8	-0.011703929	0.19507024	0.791497365	RBM27
ENSG00000260000.2	-0.046701403	0.195137742	0.791497365	RP3-467N11.1
ENSG00000146834.14	-0.010228841	0.195184027	0.791497365	MEPCE
ENSG00000260828.1	-0.027254737	0.195203395	0.791497365	HMGB3P32
ENSG00000218713.1	0.048844138	0.195204857	0.791497365	RP1-34L19.1
ENSG00000174307.7	-0.033224146	0.195218732	0.791497365	PHLDA3
ENSG00000197785.14	-0.013745573	0.195265989	0.791497365	ATAD3A
ENSG00000276250.1	0.042799361	0.195268895	0.791497365	CTD-2349P21.12
ENSG00000089280.19	-0.008522101	0.195293899	0.791497365	FUS
ENSG00000236305.1	0.044744774	0.195306512	0.791497365	SLC12A9-AS1
ENSG00000161328.11	-0.020018591	0.195346189	0.791497365	LRRC56
ENSG00000107816.17	-0.010711428	0.195349743	0.791497365	LZTS2
ENSG00000138398.17	0.018509709	0.195371619	0.791497365	PPIG
ENSG00000205593.12	0.015900841	0.195417886	0.791497365	DENND6B
ENSG00000267648.1	-0.035070233	0.19542515	0.791497365	RP11-686D22.3
ENSG00000242615.1	-0.034292792	0.195442884	0.791497365	CTC-359D24.3
ENSG00000225131.2	0.027337796	0.195528611	0.79160872	PSME2P2
ENSG00000276842.1	-0.022478561	0.195576161	0.79160872	RP11-713N11.6
ENSG00000182944.18	-0.005245939	0.19558618	0.79160872	EWSR1
ENSG00000115159.16	0.014610895	0.195676534	0.79166757	GPD2
ENSG00000171451.15	-0.037268245	0.195677926	0.79166757	DSEL
ENSG00000169814.16	0.006971988	0.195736524	0.79174845	BTD
ENSG00000169252.6	-0.017323066	0.19583024	0.791879843	ADRB2
ENSG00000152133.16	0.015708425	0.195846234	0.791879843	GPATCH11
ENSG00000226571.2	0.060475209	0.195977287	0.79225354	RP11-445F6.2

ENSG00000162729.14	-0.012869135	0.196020807	0.792273299	IGSF8
ENSG00000129347.21	-0.008606812	0.196159021	0.792484504	KRI1
ENSG00000237310.2	0.03097441	0.196205245	0.792484504	GS1-124K5.4
ENSG00000178585.15	0.009654887	0.196216099	0.792484504	CTNNBIP1
ENSG00000134470.21	0.016058944	0.196227634	0.792484504	IL15RA
ENSG00000204623.9	0.013920909	0.196312933	0.792672892	ZNRD1ASP
ENSG00000179029.15	0.009348571	0.196362889	0.79271853	TMEM107
ENSG00000120265.19	0.013154955	0.196580414	0.793440491	PCMT1
ENSG00000155893.13	-0.016790423	0.196744492	0.793634942	PXYLP1
ENSG00000151612.18	-0.015942906	0.196835583	0.793634942	ZNF827
ENSG00000163393.13	0.014876454	0.197023415	0.793634942	SLC22A15
ENSG00000137486.17	0.010220276	0.197118928	0.793634942	ARRB1
ENSG00000284642.1	-0.029342998	0.197133728	0.793634942	RP4-736L20.5
ENSG00000100580.8	0.012892134	0.197137863	0.793634942	TMED8
ENSG00000234684.7	0.014163489	0.197144887	0.793634942	SDCBP2-AS1
ENSG00000225698.4	0.062111948	0.197153559	0.793634942	IGHV3-72
ENSG00000260265.1	-0.157693514	0.197182525	0.793634942	LINC02562
ENSG00000228203.7	-0.039115513	0.197203117	0.793634942	GRASLND
ENSG00000132549.20	0.01070572	0.197249933	0.793634942	VPS13B
ENSG00000109929.10	0.014790735	0.197276478	0.793634942	SC5D
ENSG00000149187.18	0.007552628	0.197346198	0.793634942	CELF1
ENSG00000279800.2	0.041762429	0.19738377	0.793634942	BCLAF1P2
ENSG00000205426.10	0.052217339	0.197400936	0.793634942	KRT81
ENSG00000115216.14	0.00723992	0.197412706	0.793634942	NRBP1
ENSG00000089199.10	-0.082458777	0.197422447	0.793634942	CHGB
ENSG00000170959.15	0.080544176	0.197481307	0.793634942	DCDC1
ENSG00000131759.18	-0.012202724	0.197508625	0.793634942	RARA
ENSG00000111802.14	0.012093802	0.197513434	0.793634942	TDP2
ENSG00000259318.1	-0.059484263	0.197610646	0.793634942	HMGN1P1
ENSG00000131069.20	0.009988435	0.197628493	0.793634942	ACSS2
ENSG00000255052.5	0.041606313	0.197739281	0.793634942	FAM66D
ENSG00000072958.9	-0.010048408	0.197755585	0.793634942	AP1M1
ENSG00000103274.11	0.011232412	0.197768211	0.793634942	NUBP1
ENSG00000117569.19	-0.014891224	0.197821806	0.793634942	PTBP2
ENSG00000280145.3	0.117441992	0.197884376	0.793634942	CH507-154B10.1
ENSG00000187260.16	0.018784911	0.197944329	0.793634942	WDR86
ENSG00000223768.2	0.015675933	0.197944663	0.793634942	LINC00205
ENSG00000287825.1	0.021863676	0.197968399	0.793634942	RP3-475N16.9
ENSG00000089177.19	0.020592586	0.198038553	0.793634942	KIF16B
ENSG00000260641.1	0.035818688	0.198046835	0.793634942	RP11-1299A16.3
ENSG00000154832.16	-0.009555443	0.198164564	0.793634942	CXXC1
ENSG00000103423.14	-0.006678382	0.198269023	0.793634942	DNAJA3
ENSG00000215630.6	0.060730855	0.198315804	0.793634942	GUSBP9
ENSG00000232629.9	0.065320109	0.19832752	0.793634942	HLA-DQB2
ENSG00000272918.1	-0.021662273	0.198341646	0.793634942	CTB-152G17.6
ENSG00000075399.14	0.015038307	0.198369413	0.793634942	VPS9D1
ENSG00000023608.5	0.020932931	0.198407468	0.793634942	SNAPC1

ENSG00000004939.16	0.050225412	0.198477796	0.793634942	SLC4A1
ENSG00000242076.3	-0.047269164	0.198641658	0.793634942	IGKV1-33
ENSG00000164975.15	-0.010599162	0.198664923	0.793634942	SNAPC3
ENSG00000087087.20	-0.008215772	0.198694729	0.793634942	SRRT
ENSG00000125508.4	0.052290399	0.19876261	0.793634942	SRMS
ENSG00000071794.16	0.020478199	0.198843413	0.793634942	HLTF
ENSG00000143575.15	0.010481859	0.198878707	0.793634942	HAX1
ENSG00000144824.21	-0.02375352	0.1988837	0.793634942	PHLDB2
ENSG00000250644.3	-0.035465434	0.198884765	0.793634942	RP11-295K3.1
ENSG00000205763.13	0.025629176	0.198922405	0.793634942	RP9P
ENSG00000258919.1	-0.040296937	0.198930439	0.793634942	RP11-1029J19.4
ENSG00000239906.1	0.039275769	0.198950855	0.793634942	RP11-34P13.14
ENSG00000214063.11	0.018419271	0.198965246	0.793634942	TSPAN4
ENSG00000135220.11	0.179241226	0.199039935	0.793634942	UGT2A3
ENSG00000159792.10	0.008163834	0.199041418	0.793634942	PSKH1
ENSG00000172548.15	-0.037924548	0.19917152	0.793634942	NIPAL4
ENSG00000211840.1	0.054451435	0.199178756	0.793634942	TRAJ49
ENSG00000110583.13	-0.00902893	0.199208284	0.793634942	NAA40
ENSG00000171606.18	0.00724949	0.199257814	0.793634942	ZNF274
ENSG00000131355.15	0.016250737	0.199364744	0.793634942	ADGRE3
ENSG00000166813.16	-0.041986303	0.199376077	0.793634942	KIF7
ENSG00000279928.2	0.052742755	0.199384697	0.793634942	DDX11L17
ENSG00000211698.2	-0.043789423	0.199396195	0.793634942	TRGV4
ENSG00000114942.14	0.020474161	0.199465431	0.793634942	EEF1B2
ENSG00000214770.3	-0.048003428	0.199519087	0.793634942	RP11-544I20.2
ENSG00000277452.1	-0.033365825	0.199605262	0.793634942	RN7SL473P
ENSG00000171858.18	0.021858414	0.199662408	0.793634942	RPS21
ENSG00000185418.16	0.009152211	0.19967344	0.793634942	TARS3
ENSG00000213260.3	0.023999275	0.199689292	0.793634942	YWHAZP5
ENSG00000197870.12	0.065498784	0.199724507	0.793634942	PRB3
ENSG00000166439.6	0.019958805	0.199759927	0.793634942	RNF169
ENSG00000075785.14	0.006907889	0.199817853	0.793634942	RAB7A
ENSG00000279880.1	-0.042066609	0.199880726	0.793634942	RP11-855A2.2
ENSG00000240395.1	0.035697636	0.200007057	0.793634942	RPL5P23
ENSG00000221968.9	-0.017156432	0.200023467	0.793634942	FADS3
ENSG00000164929.17	-0.042631622	0.200027752	0.793634942	BAALC
ENSG00000031823.14	-0.007821629	0.20004235	0.793634942	RANBP3
ENSG00000130706.13	-0.011558042	0.200089755	0.793634942	ADRM1
ENSG00000205927.5	-0.038949891	0.200176504	0.793634942	OLIG2
ENSG00000175634.15	-0.008737636	0.200191903	0.793634942	RPS6KB2
ENSG00000278963.1	-0.043754103	0.20026557	0.793634942	RP11-294J22.7
ENSG00000279080.1	-0.028995062	0.200273398	0.793634942	CTA-228A9.4
ENSG00000110497.15	-0.01012105	0.200276472	0.793634942	AMBRA1
ENSG00000163683.12	0.011339142	0.200378466	0.793634942	SMIM14
ENSG00000116396.15	0.014831532	0.200391308	0.793634942	KCNC4
ENSG00000251093.1	0.042238341	0.200412078	0.793634942	RP11-414H23.3
ENSG00000169905.13	0.006697034	0.20043466	0.793634942	TOR1AIP2

ENSG00000104921.15	-0.038977808	0.200515836	0.793634942	FCER2
ENSG00000166925.9	-0.012150871	0.200537103	0.793634942	TSC22D4
ENSG00000140323.6	0.025870971	0.200571078	0.793634942	DISP2
ENSG00000171135.15	0.010784887	0.200579874	0.793634942	JAGN1
ENSG00000181097.5	-0.023197259	0.200589736	0.793634942	LINC02878
ENSG00000163956.13	-0.011140037	0.200676339	0.793634942	LRPAP1
ENSG00000140463.14	0.009329606	0.200746657	0.793634942	BBS4
ENSG00000226233.1	0.075454115	0.200750875	0.793634942	RP1-151B14.9
ENSG00000179846.10	-0.028001772	0.200754631	0.793634942	NKPD1
ENSG00000162636.16	0.012886104	0.200796977	0.793634942	FAM102B
ENSG00000167523.15	-0.011994966	0.200805017	0.793634942	SPATA33
ENSG00000286588.1	0.044640883	0.200824143	0.793634942	RP11-475G3.1
ENSG00000133794.20	0.009972153	0.200827261	0.793634942	ARNTL
ENSG00000136874.11	0.009416432	0.200836266	0.793634942	STX17
ENSG00000137996.13	0.007942493	0.200890872	0.793634942	RTCA
ENSG00000139737.22	-0.010231735	0.200935796	0.793634942	SLAIN1
ENSG00000143878.10	-0.014621304	0.201047821	0.793634942	RHOB
ENSG00000286177.1	-0.020891387	0.201058338	0.793634942	CTC-435M10.13
ENSG00000129038.16	0.025415599	0.2010801	0.793634942	LOXL1
ENSG00000144029.12	0.006788871	0.201096802	0.793634942	MRPS5
ENSG00000156113.24	0.065106915	0.201234849	0.793634942	KCNMA1
ENSG00000223901.2	-0.045989983	0.201284264	0.793634942	AP001469.5
ENSG00000100994.12	-0.012631855	0.201317104	0.793634942	PYGB
ENSG00000114993.17	-0.02602546	0.201333592	0.793634942	RTKN
ENSG00000103168.17	-0.010358467	0.201336307	0.793634942	TAF1C
ENSG00000224848.2	-0.043135841	0.201337379	0.793634942	RP11-535M15.1
ENSG00000006695.12	0.007720471	0.201343329	0.793634942	COX10
ENSG00000115084.15	-0.011679771	0.201350703	0.793634942	SLC35F5
ENSG00000147650.12	0.027370952	0.201375115	0.793634942	LRP12
ENSG00000234286.1	0.062599174	0.201422521	0.793634942	AC006026.13
ENSG00000211721.2	-0.029046208	0.201430094	0.793634942	TRBV6-5
ENSG00000139626.16	-0.013648513	0.201520323	0.793634942	ITGB7
ENSG00000142864.15	0.005439982	0.201549385	0.793634942	SERBP1
ENSG00000125498.20	0.04657762	0.201553639	0.793634942	KIR2DL1
ENSG00000167815.12	-0.017713033	0.201594265	0.793634942	PRDX2
ENSG00000260943.1	0.039173327	0.201631823	0.793634942	LINC02555
ENSG00000137825.11	0.051543554	0.20165544	0.793634942	ITPKA
ENSG00000168228.15	0.010866771	0.201732893	0.793634942	ZCCHC4
ENSG00000150627.16	-0.038269671	0.201740582	0.793634942	WDR17
ENSG00000232520.2	0.063344214	0.201786631	0.793634942	GCSIR
ENSG00000092108.22	0.009804917	0.201836196	0.793634942	SCFD1
ENSG00000272839.1	-0.030797085	0.201869751	0.793634942	RP11-452C13.1
ENSG00000213199.8	-0.021959227	0.201875015	0.793634942	ASIC3
ENSG00000261740.6	-0.038048741	0.201960883	0.793634942	BOLA2-SMG1P6
ENSG00000114541.15	0.0143581	0.201965418	0.793634942	FRMD4B
ENSG00000105063.19	-0.010268642	0.202014645	0.793634942	PPP6R1
ENSG00000229980.5	-0.018132933	0.20204329	0.793634942	TOB1-AS1

ENSG00000275454.1	-0.02500582	0.20205123	0.793634942	CTD-2026K11.4
ENSG00000211825.1	0.03762734	0.202058689	0.793634942	TRDJ1
ENSG00000236015.1	0.027697103	0.202059053	0.793634942	AC011290.5
ENSG00000119878.6	0.009541598	0.202135696	0.793634942	CRIPT
ENSG00000240731.1	0.013648149	0.20214083	0.793634942	RP5-890O3.9
ENSG00000204104.12	-0.012459406	0.202220998	0.793634942	TRAF3IP1
ENSG00000198612.11	0.010479107	0.202272516	0.793634942	COPS8
ENSG00000262223.8	-0.062652753	0.202325814	0.793634942	RP11-1055B8.3
ENSG00000184497.13	-0.025896844	0.202338733	0.793634942	TMEM255B
ENSG00000272092.1	-0.039624796	0.202341057	0.793634942	RP11-350N15.5
ENSG00000158089.15	0.035952088	0.202345614	0.793634942	GALNT14
ENSG00000233966.1	0.052219196	0.202400434	0.793634942	UBE2SP1
ENSG00000141002.20	-0.008684757	0.202535084	0.793634942	TCF25
ENSG00000223660.1	-0.035293003	0.202538217	0.793634942	AC012314.20
ENSG00000285518.1	0.032375272	0.202552107	0.793634942	RP4-816G1.2
ENSG00000280604.1	-0.047368035	0.202603454	0.793634942	PCBP3-OT1
ENSG00000271092.5	0.038477385	0.202617073	0.793634942	TLCD4-RWDD3
ENSG00000260669.2	-0.015094246	0.202628313	0.793634942	AL136419.6
ENSG00000149809.17	-0.013783297	0.202641417	0.793634942	TM7SF2
ENSG00000120925.16	0.008716794	0.202671798	0.793634942	RNF170
ENSG00000260388.2	-0.030123021	0.202674768	0.793634942	LINC00562
ENSG00000164967.10	0.012921704	0.202675429	0.793634942	RPP25L
ENSG00000227097.5	0.066123103	0.202679815	0.793634942	RPS28P7
ENSG00000211665.3	-0.054308411	0.202704302	0.793634942	IGLV3-16
ENSG00000205611.5	-0.048172123	0.202882547	0.794137381	LINC01597
ENSG00000280027.1	-0.028404584	0.202910078	0.794137381	RP11-44F14.10
ENSG00000242574.9	0.013151837	0.203020122	0.794238059	HLA-DMB
ENSG00000165066.13	-0.089461294	0.203093932	0.794238059	NKX6-3
ENSG00000153046.18	0.007262629	0.203124979	0.794238059	CDYL
ENSG00000205765.9	0.006919976	0.203132737	0.794238059	C5orf51
ENSG00000233261.3	-0.024417524	0.20323079	0.794238059	FAM238A
ENSG00000278259.5	-0.009129994	0.203319555	0.794238059	MYO19
ENSG00000087258.16	-0.02975551	0.203327445	0.794238059	GNAO1
ENSG00000268785.1	-0.05151014	0.203327555	0.794238059	RPL7P50
ENSG00000267736.2	-0.040165895	0.203332631	0.794238059	HMGB2P1
ENSG00000259668.6	0.031088283	0.203376204	0.794238059	RP11-707P17.2
ENSG00000216809.1	0.048719742	0.203411387	0.794238059	RP11-57K17.1
ENSG00000280614.1	-0.27957588	0.203428911	0.794238059	CH507-513H4.4
ENSG00000170836.12	0.007424858	0.203444645	0.794238059	PPM1D
ENSG00000103227.19	-0.013346727	0.203526993	0.794238059	LMF1
ENSG00000153487.13	-0.007714359	0.203573114	0.794238059	ING1
ENSG00000122378.14	0.019142637	0.203588634	0.794238059	PRXL2A
ENSG00000074657.14	0.019512591	0.203685596	0.794238059	ZNF532
ENSG00000180198.17	0.010651144	0.203862082	0.794238059	RCC1
ENSG00000247853.2	-0.026373506	0.203901655	0.794238059	RP5-940J5.6
ENSG00000148935.11	-0.023310518	0.203924165	0.794238059	GAS2
ENSG00000138032.21	-0.015193905	0.204039072	0.794238059	PPM1B

ENSG00000286545.1	-0.028117528	0.204047904	0.794238059	RP11-399B17.2
ENSG00000141736.14	-0.024719857	0.204068648	0.794238059	ERBB2
ENSG00000162413.16	0.009241656	0.20407364	0.794238059	KLHL21
ENSG00000267270.6	-0.032080991	0.204152554	0.794238059	PARD6G-AS1
ENSG00000133703.13	-0.010505624	0.20417321	0.794238059	KRAS
ENSG00000007384.15	-0.044930332	0.204210184	0.794238059	RHBDF1
ENSG00000080815.19	0.007339907	0.204222676	0.794238059	PSEN1
ENSG00000285646.2	-0.035418	0.204245849	0.794238059	RP5-934G17.7
ENSG00000168439.17	-0.010066012	0.204302986	0.794238059	STIP1
ENSG00000105612.9	-0.015356031	0.204306169	0.794238059	DNASE2
ENSG00000253451.1	-0.074314902	0.20431861	0.794238059	IGLV2-28
ENSG00000133321.11	0.017659135	0.204325211	0.794238059	PLAAT4
ENSG00000077454.16	-0.012604396	0.204346815	0.794238059	LRCH4
ENSG00000051382.9	0.008622318	0.204357828	0.794238059	PIK3CB
ENSG00000064201.16	-0.013879638	0.204407056	0.794238059	TSPAN32
ENSG00000185917.14	-0.007385365	0.204412806	0.794238059	SETD4
ENSG00000151893.15	0.008337765	0.204438948	0.794238059	CACUL1
ENSG00000145246.14	0.014004784	0.204446202	0.794238059	ATP10D
ENSG00000176732.7	-0.024560513	0.204611697	0.794508824	PFN4
ENSG00000272240.1	0.031461169	0.204630794	0.794508824	RP5-855D21.1
ENSG00000231858.5	-0.018515594	0.204632125	0.794508824	AC067945.4
ENSG00000124224.17	0.013481014	0.204710496	0.794662663	PPP4R1L
ENSG00000071205.12	-0.015712776	0.204763757	0.794718986	ARHGAP10
ENSG00000265458.2	0.018588934	0.204835533	0.794771601	NARF-AS2
ENSG00000224846.3	0.049654377	0.204854822	0.794771601	NQO2-AS1
ENSG00000112658.8	0.007150584	0.204905398	0.79481746	SRF
ENSG00000141068.14	-0.014052834	0.204978118	0.794842249	KSR1
ENSG00000229951.5	-0.050857893	0.205018832	0.794842249	FLJ31356
ENSG00000279673.1	-0.021951388	0.205116197	0.794842249	RP11-185E8.2
ENSG00000178761.15	0.011081283	0.205123278	0.794842249	FAM219B
ENSG00000113441.16	-0.008765502	0.205149186	0.794842249	LNPEP
ENSG00000272345.1	0.018485551	0.205218109	0.794842249	RP1-30M3.5
ENSG00000270000.1	0.056511375	0.205225371	0.794842249	RP3-449M8.9
ENSG00000137337.15	-0.010205133	0.205248528	0.794842249	MDC1
ENSG00000261794.2	0.026802173	0.205260608	0.794842249	GOLGA8H
ENSG00000164332.8	0.009068716	0.205303392	0.794857839	UBLCP1
ENSG00000211707.3	0.040485044	0.205423981	0.795022443	TRBV7-1
ENSG00000178764.8	-0.009492086	0.205457194	0.795022443	ZHX2
ENSG00000133106.15	-0.045125315	0.205468678	0.795022443	EPSTI1
ENSG00000273160.1	-0.046774946	0.205501665	0.795022443	RP11-104L21.3
ENSG00000184451.5	-0.032950586	0.20553974	0.795022443	CCR10
ENSG00000149196.16	0.011152836	0.205637493	0.795181355	HIKESHI
ENSG00000168434.13	-0.006907354	0.205658373	0.795181355	COG7
ENSG00000174516.15	-0.013845555	0.205728781	0.795183516	PELI3
ENSG00000231305.4	-0.03243014	0.20573648	0.795183516	RP11-723O4.2
ENSG00000164244.21	-0.009774914	0.205796314	0.795264898	PRRC1
ENSG00000260507.1	0.042764139	0.205916617	0.79546877	RP11-356C4.3

ENSG00000112304.11	0.008266882	0.205926648	0.79546877	ACOT13
ENSG00000227155.7	-0.026851081	0.206036851	0.795744584	RP11-165F24.3
ENSG00000156966.7	-0.028278847	0.206148295	0.795845475	B3GNT7
ENSG00000117036.12	-0.007906861	0.206171963	0.795845475	ETV3
ENSG00000243650.3	-0.032216368	0.206179394	0.795845475	RN7SL834P
ENSG00000253106.1	-0.013316659	0.20627684	0.795896959	RP11-158K1.3
ENSG00000138663.9	0.014147691	0.20632137	0.795896959	COPS4
ENSG00000123473.17	-0.021844701	0.206331063	0.795896959	STIL
ENSG00000189114.8	-0.009617831	0.206347968	0.795896959	BLOC1S3
ENSG00000273192.1	-0.022991946	0.206414606	0.796004277	CITF22-1A6.3
ENSG00000172262.12	0.010844272	0.20655449	0.79639396	ZNF131
ENSG00000135722.9	-0.01685096	0.206655733	0.796551648	FBXL8
ENSG00000255801.1	0.028436899	0.206673189	0.796551648	RP11-561P12.5
ENSG00000177602.5	-0.031957068	0.206711911	0.796551648	HASPIN
ENSG00000155657.28	0.018031454	0.206793786	0.796717444	TTN
ENSG00000198780.12	0.025252605	0.206852997	0.796795879	FAM169A
ENSG00000064995.18	0.009988376	0.207007733	0.797162274	TAF11
ENSG00000287593.1	0.050431328	0.207025857	0.797162274	RP3-413H6.3
ENSG00000131747.15	-0.031926433	0.207136669	0.797439234	TOP2A
ENSG00000181929.13	-0.018522741	0.207296343	0.79783156	PRKAG1
ENSG00000001561.7	-0.023552291	0.207316383	0.79783156	ENPP4
ENSG00000137504.15	-0.016576348	0.207364407	0.797866653	CREBZF
ENSG00000125997.5	0.035993921	0.207426216	0.797868663	BPIFB9P
ENSG00000279598.1	-0.050559188	0.20749104	0.797868663	RP11-65L3.3
ENSG00000244921.2	0.135614063	0.207507496	0.797868663	MTCYBP18
ENSG00000271868.1	-0.043235263	0.207535954	0.797868663	RP11-1293J14.1
ENSG00000275700.6	-0.010450766	0.207559456	0.797868663	AATF
ENSG00000272079.2	-0.017153131	0.207665826	0.798127954	LA16c-380H5.5
ENSG00000092470.12	-0.012389993	0.207774727	0.798396871	WDR76
ENSG00000149922.11	-0.021650418	0.207839589	0.798496497	TBX6
ENSG00000233578.1	0.032734918	0.207915524	0.798638616	EIF4EP1
ENSG00000135218.19	0.017065416	0.208011486	0.798715357	CD36
ENSG00000153563.16	-0.033856502	0.208038803	0.798715357	CD8A
ENSG00000106479.11	0.007972706	0.208052342	0.798715357	ZNF862
ENSG00000102879.16	-0.009828876	0.208323792	0.798917916	CORO1A
ENSG00000163001.12	0.01374073	0.208341959	0.798917916	CFAP36
ENSG00000260855.1	0.039730109	0.208366553	0.798917916	RP11-439E19.10
ENSG00000060762.19	0.01001421	0.208466496	0.798917916	MPC1
ENSG00000147679.12	0.011182912	0.208469919	0.798917916	UTP23
ENSG00000167995.17	0.011774682	0.208487377	0.798917916	BEST1
ENSG00000198502.6	0.033025554	0.208488771	0.798917916	HLA-DRB5
ENSG00000205846.4	0.037251591	0.208497069	0.798917916	CLEC6A
ENSG00000251602.7	0.045019439	0.20854828	0.798917916	RP11-521B24.3
ENSG00000186666.6	0.010215206	0.208586094	0.798917916	BCDIN3D
ENSG00000153404.14	0.057367593	0.208615824	0.798917916	PLEKHG4B
ENSG00000241127.8	0.018992424	0.208645438	0.798917916	YAE1
ENSG00000145386.11	-0.024063917	0.208650319	0.798917916	CCNA2

ENSG00000105618.14	-0.011663893	0.208743043	0.798917916	PRPF31
ENSG00000213438.2	-0.041313976	0.208796211	0.798917916	YBX2P1
ENSG00000138180.16	-0.036905316	0.208819139	0.798917916	CEP55
ENSG00000100596.7	0.010429068	0.208861812	0.798917916	SPTLC2
ENSG00000143363.17	0.007135649	0.208905931	0.798917916	PRUNE1
ENSG00000164741.15	-0.035318378	0.208909345	0.798917916	DLC1
ENSG00000254165.1	-0.039321073	0.208921793	0.798917916	RP11-503E24.2
ENSG00000279430.1	0.044486151	0.208993701	0.798917916	RP11-190A12.9
ENSG00000062650.20	0.010058012	0.209062396	0.798917916	WAPL
ENSG00000172590.18	0.009232757	0.209089576	0.798917916	MRPL52
ENSG00000167315.18	0.010531522	0.209105675	0.798917916	ACAA2
ENSG00000184731.6	-0.037630015	0.209120533	0.798917916	FAM110C
ENSG00000125538.12	0.017651174	0.209139217	0.798917916	IL1B
ENSG00000121895.8	0.014742132	0.209177455	0.798917916	TMEM156
ENSG00000204983.14	0.064850874	0.209209674	0.798917916	PRSS1
ENSG00000253998.3	-0.077641344	0.209234841	0.798917916	IGKV2-29
ENSG00000128578.10	-0.027224807	0.209322472	0.799103734	STRIP2
ENSG00000285938.1	0.031056725	0.209388538	0.799207173	BCL6-AS1
ENSG00000137434.12	0.046950447	0.209519827	0.799413212	C6orf52
ENSG00000102931.8	-0.021129956	0.20952048	0.799413212	ARL2BP
ENSG00000116171.19	0.009680935	0.209574572	0.799470858	SCP2
ENSG00000122367.20	0.060336442	0.209754026	0.800006616	LDB3
ENSG00000156639.12	0.008124247	0.209813433	0.800084397	ZFAND3
ENSG00000272502.1	-0.019460451	0.210016462	0.800578098	RP11-713M15.2
ENSG00000287235.1	0.050860546	0.210020976	0.800578098	RP11-116G8.8
ENSG00000264695.1	0.043275762	0.210091365	0.800697586	RP11-178F10.2
ENSG00000197816.16	-0.016570453	0.210155007	0.800782187	CCDC180
ENSG00000178752.16	-0.062987106	0.210191658	0.800782187	ERFE
ENSG00000117174.11	0.008599086	0.210242199	0.800824793	ZNHIT6
ENSG00000224186.8	0.023250398	0.21028094	0.800824793	C5orf66
ENSG00000278383.1	-0.043780523	0.210450426	0.800851753	RP4-694B14.8
ENSG00000147439.13	0.010777866	0.210495044	0.800851753	BIN3
ENSG00000089006.17	0.00674176	0.210513816	0.800851753	SNX5
ENSG00000089639.11	-0.010459546	0.210566712	0.800851753	GMIP
ENSG00000250326.2	-0.023629071	0.210694685	0.800851753	RP11-284M14.1
ENSG00000280181.1	-0.037284792	0.210718964	0.800851753	RP11-629N8.4
ENSG00000178860.9	-0.038045168	0.21076983	0.800851753	MSC
ENSG00000005187.12	0.020689245	0.21079979	0.800851753	ACSM3
ENSG00000117305.15	0.008922249	0.210833676	0.800851753	HMGCL
ENSG00000033800.14	0.0073131	0.210861383	0.800851753	PIAS1
ENSG00000165410.15	-0.017056661	0.210875185	0.800851753	CFL2
ENSG00000143164.15	0.010000301	0.210902239	0.800851753	DCAF6
ENSG00000266967.7	-0.013981642	0.210904459	0.800851753	AARSD1
ENSG00000163157.15	-0.01157264	0.210939376	0.800851753	TMOD4
ENSG00000280114.1	0.076087516	0.210974601	0.800851753	RP5-875O13.7
ENSG00000228784.9	0.013758853	0.211036535	0.800851753	LINC00954
ENSG00000213757.3	0.057483469	0.211038581	0.800851753	RPS3AP20

ENSG00000129515.20	0.009641377	0.211116926	0.800851753	SNX6
ENSG00000093000.19	0.009793801	0.211123938	0.800851753	NUP50
ENSG00000175701.11	-0.019013154	0.211136375	0.800851753	MTLN
ENSG00000048342.18	-0.018000651	0.211172214	0.800851753	CC2D2A
ENSG00000253669.4	0.028188522	0.211289806	0.800851753	GASAL1
ENSG00000119608.15	0.023799545	0.211300033	0.800851753	PROX2
ENSG00000276593.1	-0.043166912	0.211318784	0.800851753	RP11-295H24.5
ENSG00000214837.8	0.039013984	0.211331478	0.800851753	LINC01347
ENSG00000272456.1	0.061314346	0.211350019	0.800851753	CTD-2342N23.3
ENSG00000220517.2	-0.03603086	0.211374088	0.800851753	ASS1P1
ENSG00000140675.13	0.029976827	0.211381438	0.800851753	SLC5A2
ENSG00000237054.9	0.032001757	0.211436124	0.800910977	PRMT5-AS1
ENSG00000158042.9	0.012570358	0.211566505	0.801256858	MRPL17
ENSG00000273055.1	-0.038071138	0.211684867	0.801557099	CTB-13F3.1
ENSG00000122729.19	-0.013384508	0.211831305	0.801883336	ACO1
ENSG00000168890.14	0.009419235	0.211849225	0.801883336	TMEM150A
ENSG00000142733.17	-0.016266476	0.211956643	0.80206765	MAP3K6
ENSG00000119541.10	0.008842126	0.212023815	0.80206765	VPS4B
ENSG00000138448.12	-0.022186917	0.212100382	0.80206765	ITGAV
ENSG00000072736.19	-0.012345072	0.2121219	0.80206765	NFATC3
ENSG00000130227.17	0.006803091	0.212123831	0.80206765	XPO7
ENSG00000110955.9	0.007715308	0.212132579	0.80206765	ATP5F1B
ENSG00000163808.17	-0.031331578	0.212242596	0.802230954	KIF15
ENSG00000263412.1	-0.02853517	0.212254006	0.802230954	NFE2L1-DT
ENSG00000156976.17	0.010222665	0.21236456	0.802500901	EIF4A2
ENSG00000170852.11	0.009233261	0.212456726	0.802701278	KBTBD2
ENSG00000142751.15	-0.006387816	0.212570489	0.802812858	GPN2
ENSG00000202533.1	0.020088259	0.212626012	0.802812858	Y_RNA
ENSG00000269148.1	-0.026305192	0.212636434	0.802812858	AC092301.3
ENSG00000261737.1	0.029558912	0.212642844	0.802812858	CLCA4-AS1
ENSG00000237984.3	0.016713793	0.212718662	0.802914946	PTENP1
ENSG00000263004.2	0.028314305	0.21283209	0.802914946	AKAP1-DT
ENSG00000152969.20	-0.033196585	0.212927639	0.802914946	JAKMIP1
ENSG00000255837.2	-0.022123627	0.212942827	0.802914946	TAS2R20
ENSG00000272989.1	-0.034883868	0.212950955	0.802914946	LINC02012
ENSG00000198554.12	0.018222331	0.212997032	0.802914946	WDHD1
ENSG00000272109.2	-0.035179916	0.212998389	0.802914946	CTD-2260A17.3
ENSG00000197165.11	-0.037316066	0.213036828	0.802914946	SULT1A2
ENSG00000119953.14	0.008549497	0.213038251	0.802914946	SMNDC1
ENSG00000229994.1	0.041284757	0.213061397	0.802914946	RPL5P4
ENSG00000240859.3	-0.018435614	0.213130279	0.803026965	AC093627.10
ENSG00000253838.1	0.072887055	0.213183214	0.803078867	RP11-44K6.2
ENSG00000224429.8	0.018715433	0.213248953	0.803123094	LINC00539
ENSG00000178301.4	-0.020382357	0.213281389	0.803123094	AQP11
ENSG00000117242.7	0.011415299	0.213312439	0.803123094	PINK1-AS
ENSG00000228157.4	0.135892562	0.213403408	0.803318115	AC007952.5
ENSG00000254873.1	-0.043886362	0.213484397	0.803399391	RP11-770J1.5

ENSG00000140521.17	-0.007087288	0.213503349	0.803399391	POLG
ENSG00000204455.7	0.058994315	0.213604596	0.80363292	TRIM51BP
ENSG00000211887.2	-0.042385079	0.213665166	0.80368695	TRAJ2
ENSG00000143374.17	-0.008230003	0.213697335	0.80368695	TARS2
ENSG00000185527.12	0.020188799	0.21382364	0.804014522	PDE6G
ENSG00000164924.18	0.006712569	0.213889004	0.804112866	YWHAZ
ENSG00000048052.23	0.017234133	0.213992002	0.804182836	HDAC9
ENSG00000269888.1	0.048334304	0.21400536	0.804182836	RP11-3P17.5
ENSG00000275720.1	0.035261359	0.214049984	0.804182836	CTB-75G16.3
ENSG00000286044.1	0.053370959	0.214092529	0.804182836	RP11-344F5.1
ENSG00000172466.16	0.010549966	0.214121464	0.804182836	ZNF24
ENSG00000267272.5	0.038887838	0.214142894	0.804182836	LINC01140
ENSG00000249825.6	-0.022204098	0.214277647	0.804362895	THBS4-AS1
ENSG00000080854.16	-0.020094044	0.21435235	0.804362895	IGSF9B
ENSG00000237264.4	0.022641215	0.214375157	0.804362895	FTH1P11
ENSG00000100526.20	-0.041249063	0.214378983	0.804362895	CDKN3
ENSG00000164941.14	0.008767108	0.214418761	0.804362895	INTS8
ENSG00000165525.18	0.013472324	0.214433294	0.804362895	NEMF
ENSG00000228146.8	0.035954616	0.21454043	0.804362895	CASP16P
ENSG00000272168.8	0.044022324	0.214573237	0.804362895	CASC15
ENSG00000168795.5	0.008206233	0.214574079	0.804362895	ZBTB5
ENSG00000159618.16	-0.018999315	0.214590849	0.804362895	ADGRG5
ENSG00000267387.1	-0.028743115	0.214643098	0.804362895	CTD-2240E14.4
ENSG00000152455.16	-0.01565699	0.214767782	0.804362895	SUV39H2
ENSG00000166166.13	-0.012795366	0.214779259	0.804362895	TRMT61A
ENSG00000180190.13	0.03351838	0.214781298	0.804362895	TDRP
ENSG00000162961.14	0.017268277	0.214863307	0.804362895	DPY30
ENSG00000232117.2	-0.066108521	0.214869029	0.804362895	LINC00384
ENSG00000252103.1	-0.030827867	0.214876273	0.804362895	RNU6-917P
ENSG00000214389.2	0.038444761	0.214924175	0.804362895	RPS3AP26
ENSG00000186111.11	-0.011147112	0.214936057	0.804362895	PIP5K1C
ENSG00000116678.20	0.025902209	0.215067243	0.804706992	LEPR
ENSG00000259520.6	0.049003742	0.21514713	0.804859056	CTD-2651B20.3
ENSG00000275180.1	0.021923327	0.215209989	0.804874393	RP11-631N16.4
ENSG00000136925.15	0.014968565	0.215229724	0.804874393	TSTD2
ENSG00000206965.1	0.033207928	0.215310706	0.804906432	RNU6-5P
ENSG00000197562.10	-0.010543257	0.215316788	0.804906432	RAB40C
ENSG00000041353.10	0.02031753	0.215433522	0.805048459	RAB27B
ENSG00000246763.7	-0.058631937	0.215455993	0.805048459	RGMB-AS1
ENSG00000187266.14	-0.012621459	0.215515926	0.805048459	EPOR
ENSG00000242220.9	-0.04341555	0.215568989	0.805048459	TCP10L
ENSG00000256039.2	0.039184001	0.21560207	0.805048459	LINC02446
ENSG00000070019.5	-0.033298092	0.215690597	0.805048459	GUCY2C
ENSG00000138193.16	-0.016093519	0.215706227	0.805048459	PLCE1
ENSG00000198483.13	-0.032998722	0.215749823	0.805048459	ANKRD35
ENSG00000163050.18	-0.017509914	0.215753885	0.805048459	COQ8A
ENSG00000234912.12	0.010451474	0.215820556	0.805048459	SNHG20

ENSG00000243896.4	0.053438107	0.215822263	0.805048459	OR2A7
ENSG00000165985.10	-0.055959522	0.215825845	0.805048459	C1QL3
ENSG00000272146.6	-0.052800762	0.216216152	0.805973596	ARF4-AS1
ENSG00000278709.2	-0.043607982	0.216273776	0.805973596	NKILA
ENSG00000235065.1	0.053398785	0.216292197	0.805973596	RPL24P2
ENSG00000239704.11	0.022674014	0.216322279	0.805973596	CDRT4
ENSG00000117153.16	0.006174332	0.216358507	0.805973596	KLHL12
ENSG00000060709.15	-0.147294826	0.216411583	0.805973596	RIMBP2
ENSG00000230489.1	-0.054350862	0.216449943	0.805973596	VAV3-AS1
ENSG00000279672.2	-0.023256901	0.216463815	0.805973596	CMB9-55F22.1
ENSG00000108039.18	0.007238329	0.216562221	0.805973596	XPNPEP1
ENSG00000025293.17	0.007271604	0.216603359	0.805973596	PHF20
ENSG00000225603.3	0.111166303	0.216608701	0.805973596	RP11-325P15.1
ENSG00000103653.17	-0.010671015	0.216620233	0.805973596	CSK
ENSG00000152377.14	0.02426723	0.21664608	0.805973596	SPOCK1
ENSG00000260630.7	-0.011841264	0.216734502	0.805973596	SNAI3-AS1
ENSG00000035499.13	-0.036684149	0.216779462	0.805973596	DEPDC1B
ENSG00000065615.14	0.009994067	0.216792214	0.805973596	CYB5R4
ENSG00000105223.20	-0.01307952	0.216798651	0.805973596	PLD3
ENSG00000091527.16	0.00624711	0.216905753	0.805973596	CDV3
ENSG00000068001.14	0.012771568	0.216935066	0.805973596	HYAL2
ENSG00000095906.17	-0.012211283	0.21693859	0.805973596	NUBP2
ENSG00000266338.6	-0.020628569	0.216940394	0.805973596	NBPF15
ENSG00000232533.1	0.014755962	0.216941987	0.805973596	AC093673.5
ENSG00000121058.5	0.007588425	0.217001819	0.805973596	COIL
ENSG00000124571.18	-0.007615443	0.217111839	0.805973596	XPO5
ENSG00000287725.1	0.038667968	0.217133981	0.805973596	RP11-554A11.11
ENSG00000136908.18	0.021264359	0.217138659	0.805973596	DPM2
ENSG00000251215.1	0.033067077	0.217199882	0.805973596	GOLGA5P1
ENSG00000227827.3	0.089284315	0.217202334	0.805973596	PKD1P2
ENSG00000162775.17	-0.008980144	0.217213578	0.805973596	RBM15
ENSG00000176049.16	-0.025964367	0.217322753	0.806203214	JAKMIP2
ENSG00000142002.18	-0.007917202	0.217400546	0.806203214	DPP9
ENSG00000242960.1	0.026714033	0.217428525	0.806203214	FTH1P23
ENSG00000163472.19	-0.010247768	0.217432708	0.806203214	TMEM79
ENSG00000173276.14	-0.013652706	0.217540118	0.806222151	ZBTB21
ENSG00000127366.5	0.030959795	0.217597075	0.806222151	TAS2R5
ENSG00000228363.2	0.037995445	0.217603927	0.806222151	AC015971.2
ENSG00000151500.15	0.009833155	0.217645545	0.806222151	THYN1
ENSG00000172296.13	0.039676409	0.217669482	0.806222151	SPTLC3
ENSG00000286388.1	-0.022238958	0.217673691	0.806222151	RP11-325I22.4
ENSG00000141968.8	-0.008325364	0.21776289	0.806406889	VAV1
ENSG00000256282.2	0.047569366	0.217853328	0.80642685	RP11-504G3.4
ENSG00000251417.2	-0.024823915	0.21787953	0.80642685	RP11-1348G14.4
ENSG00000250938.6	0.047608249	0.217886248	0.80642685	RP11-679C8.2
ENSG00000141699.11	-0.007551824	0.217943806	0.80649374	RETREG3
ENSG00000167703.15	-0.012831373	0.217982973	0.80649374	SLC43A2

ENSG00000265218.1	0.069964688	0.21808321	0.806719061	RP11-927P21.1
ENSG00000173264.15	-0.011131284	0.218141922	0.80679072	GPR137
ENSG00000064102.15	0.009563156	0.21828589	0.806983785	INTS13
ENSG00000273619.1	-0.029153432	0.218297373	0.806983785	RP5-908M14.9
ENSG00000204531.20	-0.035039713	0.218312173	0.806983785	POU5F1
ENSG00000240344.9	0.016148505	0.218373813	0.807019248	PPIL3
ENSG00000147905.18	0.011018066	0.218400469	0.807019248	ZCCHC7
ENSG00000163535.18	-0.024517708	0.218533257	0.807258422	SGO2
ENSG00000273599.1	0.020295755	0.2185808	0.807258422	RP11-59C5.3
ENSG00000263301.1	0.047574626	0.218583285	0.807258422	RP11-104H15.8
ENSG00000259421.1	-0.062571438	0.21863424	0.807301224	CTA-150C2.20
ENSG00000100852.13	0.022043912	0.21885736	0.807950746	ARHGAP5
ENSG00000226029.1	-0.01839506	0.218966538	0.807950746	LINC01772
ENSG00000173917.11	0.026955598	0.218981864	0.807950746	HOXB2
ENSG00000092020.11	0.01341854	0.218993651	0.807950746	PPP2R3C
ENSG00000185813.11	-0.010300922	0.219007129	0.807950746	PCYT2
ENSG00000255320.1	-0.016723716	0.219164103	0.808173463	RP11-755F10.1
ENSG00000243970.3	-0.021833961	0.219248557	0.808173463	PPIEL
ENSG00000144130.11	-0.025100981	0.219249012	0.808173463	NT5DC4
ENSG00000169607.13	-0.036536292	0.219274762	0.808173463	CKAP2L
ENSG00000209042.1	0.050002112	0.219294508	0.808173463	SNORD12C
ENSG00000253819.2	0.05149973	0.219324974	0.808173463	LINC01151
ENSG00000065150.21	0.006201168	0.219358741	0.808173463	IPO5
ENSG00000168118.12	0.009943432	0.219382761	0.808173463	RAB4A
ENSG00000140829.12	-0.007997402	0.219475989	0.808371693	DHX38
ENSG00000106052.14	0.0172873	0.219536394	0.808447867	TAX1BP1
ENSG00000131183.11	0.040354521	0.219575513	0.808447867	SLC34A1
ENSG00000255302.5	0.013324122	0.219687954	0.808602007	EID1
ENSG00000127220.6	-0.013658441	0.219696235	0.808602007	ABHD8
ENSG00000225217.1	0.03517296	0.21975227	0.808663119	HSPA7
ENSG00000163466.16	0.008460606	0.219808714	0.808725709	ARPC2
ENSG00000242550.6	-0.05527044	0.219920663	0.808867602	SERPINB10
ENSG00000136940.14	0.00770938	0.220057978	0.808867602	PDCL
ENSG00000169105.8	-0.011009369	0.220080947	0.808867602	CHST14
ENSG00000238058.2	-0.0240556	0.220096865	0.808867602	RP11-432J22.2
ENSG00000183765.23	-0.012044798	0.22012609	0.808867602	CHEK2
ENSG00000100348.10	0.010442159	0.220145692	0.808867602	TXN2
ENSG00000226904.1	-0.050960097	0.220187193	0.808867602	RP11-561O23.8
ENSG00000099308.10	-0.011908905	0.220246271	0.808867602	MAST3
ENSG00000144566.11	0.008681815	0.220319841	0.808867602	RAB5A
ENSG00000251259.1	0.020768261	0.220333832	0.808867602	AC004069.2
ENSG00000225370.1	0.038514371	0.220346433	0.808867602	AC011515.2
ENSG00000149136.9	-0.007812099	0.22034727	0.808867602	SSRP1
ENSG00000260136.6	-0.034798381	0.220376354	0.808867602	CTD-2270L9.4
ENSG00000161912.18	-0.014034966	0.220399462	0.808867602	ADCY10P1
ENSG00000177738.4	-0.009077109	0.220767556	0.810073545	CTD-2201E18.3
ENSG00000177051.6	-0.009790864	0.220882984	0.810352098	FBXO46

ENSG00000231130.1	-0.089032263	0.220977381	0.81042189	HLA-T
ENSG00000099250.18	0.035175839	0.220981042	0.81042189	NRP1
ENSG00000198208.12	0.026838582	0.221025104	0.810438553	RPS6KL1
ENSG00000235237.1	-0.038121555	0.221130387	0.810550039	RP1-151B14.6
ENSG00000238086.5	-0.047846475	0.221134556	0.810550039	PPP1R26P1
ENSG00000177076.6	-0.027816579	0.221224969	0.810736539	ACER2
ENSG00000070476.15	-0.007491392	0.221322858	0.810747961	ZXDC
ENSG00000203685.10	0.101038731	0.221416303	0.810747961	STUM
ENSG00000042088.14	0.006899245	0.221417512	0.810747961	TDP1
ENSG00000245146.7	0.020942548	0.221428126	0.810747961	MALINC1
ENSG00000227512.1	-0.029046528	0.221470651	0.810747961	RP11-413M3.4
ENSG00000198929.13	0.073393959	0.22149687	0.810747961	NOS1AP
ENSG00000120885.22	0.032138628	0.221504819	0.810747961	CLU
ENSG00000207736.1	0.04504737	0.221621569	0.810929107	MIR657
ENSG00000089053.13	0.004932014	0.22167241	0.810929107	ANAPC5
ENSG00000066230.12	-0.023612883	0.221672936	0.810929107	SLC9A3
ENSG00000111341.10	0.037051012	0.221751534	0.811071958	MGP
ENSG00000253209.1	0.105972643	0.222000273	0.811433123	IGHV3-65
ENSG00000100479.13	-0.021000251	0.222004569	0.811433123	POLE2
ENSG00000211460.12	0.006575474	0.222051301	0.811433123	TSN
ENSG00000261215.1	-0.045269812	0.222130603	0.811433123	RP11-195F19.30
ENSG00000143093.15	-0.005593925	0.222176401	0.811433123	STRIP1
ENSG00000130311.11	-0.008149786	0.222189505	0.811433123	DDA1
ENSG00000141959.17	-0.01037609	0.222193825	0.811433123	PFKL
ENSG00000205930.10	-0.018416334	0.22220943	0.811433123	C21orf62-AS1
ENSG00000083099.11	0.013343527	0.222228922	0.811433123	LYRM2
ENSG00000160813.7	-0.013044106	0.222297183	0.811433123	PPP1R35
ENSG00000251188.1	-0.045461466	0.222341891	0.811433123	RP11-478C6.6
ENSG00000286931.1	0.039009741	0.22235931	0.811433123	RP11-76P17.4
ENSG00000273474.1	-0.042825984	0.222364646	0.811433123	RP11-295P9.12
ENSG00000133103.17	0.014999663	0.22245611	0.811593256	COG6
ENSG00000287261.1	0.029740587	0.222487677	0.811593256	RP11-439D8.4
ENSG00000130396.21	0.029989641	0.222677866	0.812142571	AFDN
ENSG00000102921.8	0.009111985	0.222751926	0.812268227	N4BP1
ENSG00000112167.10	0.009979848	0.222825035	0.812390366	SAYSD1
ENSG00000136305.11	-0.018719366	0.222913962	0.812464487	CIDEB
ENSG00000288075.1	0.032497336	0.222924599	0.812464487	RP11-1396O13.23
ENSG00000148672.10	0.006102278	0.222995899	0.81257994	GLUD1
ENSG00000116147.17	0.047587048	0.223124937	0.812657885	TNR
ENSG00000198576.4	0.051581483	0.223138322	0.812657885	ARC
ENSG00000272468.1	0.033794069	0.223185688	0.812657885	RP1-86C11.7
ENSG00000285219.2	0.032519005	0.223219113	0.812657885	HULC
ENSG00000071282.12	-0.033698104	0.223248657	0.812657885	LMCD1
ENSG00000276289.5	0.048533099	0.223296203	0.812657885	KCNE1B
ENSG00000283633.1	0.032401345	0.223340819	0.812657885	AC005301.9
ENSG00000235162.9	-0.020608067	0.223369558	0.812657885	C12orf75
ENSG00000277969.1	-0.014415825	0.223424961	0.812657885	CTB-58E17.1

ENSG00000118898.16	-0.023609989	0.223437863	0.812657885	PPL
ENSG00000215717.7	0.008814722	0.223460636	0.812657885	TMEM167B
ENSG00000175182.15	0.007911198	0.223533953	0.812657885	FAM131A
ENSG00000260448.6	-0.02432694	0.223639954	0.812657885	LCMT1-AS1
ENSG00000105246.6	-0.044172728	0.223679938	0.812657885	EBI3
ENSG00000133028.12	0.006500453	0.223682461	0.812657885	SCO1
ENSG00000214140.11	0.023433655	0.223685632	0.812657885	PRCD
ENSG00000211888.2	0.03298969	0.223690938	0.812657885	TRAJ1
ENSG00000178425.14	0.007968148	0.223852683	0.81297081	NT5DC1
ENSG00000183662.11	0.032809708	0.223928357	0.81297081	TAFA1
ENSG00000185252.19	-0.008607522	0.223969433	0.81297081	ZNF74
ENSG00000175387.16	0.005010152	0.223984333	0.81297081	SMAD2
ENSG00000236876.3	0.039712643	0.224051604	0.81297081	TMSB4XP1
ENSG00000279342.2	0.02449162	0.224068808	0.81297081	RP11-677M14.8
ENSG00000081870.12	0.012847773	0.22417229	0.81297081	HSPB11
ENSG00000197461.13	0.034608944	0.224174427	0.81297081	PDGFA
ENSG00000171045.16	0.011882704	0.224225308	0.81297081	TSNARE1
ENSG00000169762.17	-0.010317759	0.224276503	0.81297081	TAPT1
ENSG00000204920.11	0.022201848	0.224280578	0.81297081	ZNF155
ENSG00000072786.13	-0.009441167	0.224286746	0.81297081	STK10
ENSG00000278987.1	-0.042541121	0.224341942	0.81297081	LA16c-431H6.7
ENSG00000158486.13	0.036612419	0.224350915	0.81297081	DNAH3
ENSG00000287643.1	0.052186791	0.224640578	0.81297081	RP1-292L20.4
ENSG00000154655.16	0.022419918	0.224676453	0.81297081	L3MBTL4
ENSG00000170632.14	0.008473374	0.22470038	0.81297081	ARMC10
ENSG00000059691.12	0.009890105	0.224791029	0.81297081	GATB
ENSG00000184730.11	-0.017664225	0.224796065	0.81297081	APOBR
ENSG00000155085.16	0.013338075	0.224823595	0.81297081	AK9
ENSG00000247903.1	-0.026456231	0.224841444	0.81297081	RP11-421F16.3
ENSG00000211632.4	0.053810551	0.224873751	0.81297081	IGKV3D-11
ENSG00000150347.17	-0.012723117	0.224886	0.81297081	ARID5B
ENSG00000129473.11	0.010030072	0.224906537	0.81297081	BCL2L2
ENSG00000184436.12	-0.011478348	0.224940172	0.81297081	THAP7
ENSG00000103942.13	0.020372664	0.224946697	0.81297081	HOMER2
ENSG00000251441.3	0.035014785	0.224965353	0.81297081	RTEL1P1
ENSG00000078795.17	0.032134007	0.225016036	0.81297081	PKD2L2
ENSG00000169925.17	-0.011034936	0.22503515	0.81297081	BRD3
ENSG00000104973.19	-0.015612161	0.225038984	0.81297081	MED25
ENSG00000058799.15	0.010055245	0.225055689	0.81297081	YIPF1
ENSG00000095059.16	-0.011071659	0.225081072	0.81297081	DHPS
ENSG00000083635.8	0.010861744	0.225085248	0.81297081	NUFIP1
ENSG00000286835.1	-0.025144362	0.225159806	0.813096902	RP11-172F4.7
ENSG00000123091.5	0.017066449	0.225236839	0.813231882	RNF11
ENSG00000243836.5	0.028152512	0.225315548	0.813372866	WDR86-AS1
ENSG00000178741.12	0.011344222	0.225454645	0.813457684	COX5A
ENSG00000083838.16	-0.010769007	0.225457042	0.813457684	ZNF446
ENSG00000211871.1	-0.03336254	0.225464713	0.813457684	TRAJ18

ENSG00000099864.18	-0.05638707	0.225497705	0.813457684	PALM
ENSG00000023734.11	0.005538805	0.225622145	0.813763444	STRAP
ENSG00000278949.1	-0.021804659	0.225751873	0.8138658	RP11-46H11.11
ENSG00000188986.9	-0.008791533	0.225766403	0.8138658	NELFB
ENSG00000102886.15	-0.012488101	0.225769579	0.8138658	GDPD3
ENSG00000173809.18	0.0627478	0.225895802	0.814099426	TDRD12
ENSG00000113522.14	0.018288193	0.226143036	0.814099426	RAD50
ENSG00000135373.13	0.047544332	0.226181363	0.814099426	EHF
ENSG00000163293.12	0.053124146	0.226202918	0.814099426	NIPAL1
ENSG00000268117.1	0.083163255	0.226231028	0.814099426	VN1R84P
ENSG00000276023.5	-0.015339348	0.226240633	0.814099426	DUSP14
ENSG00000125652.8	-0.017150847	0.226274426	0.814099426	ALKBH7
ENSG00000125755.19	-0.009429535	0.226276943	0.814099426	SYMPK
ENSG00000089195.15	0.008566182	0.226290618	0.814099426	TRMT6
ENSG00000186765.12	-0.032001135	0.226322445	0.814099426	FSCN2
ENSG00000200095.1	-0.053860824	0.22633474	0.814099426	RNU6-181P
ENSG00000057294.16	0.02927688	0.226377144	0.814099426	PKP2
ENSG00000099377.14	0.026277403	0.226385437	0.814099426	HSD3B7
ENSG00000260286.4	-0.029436335	0.226390142	0.814099426	ARMH2
ENSG00000145241.11	0.010577907	0.226445858	0.814157021	CENPC
ENSG00000105767.3	-0.016395825	0.226492598	0.814182333	CADM4
ENSG00000136720.7	-0.01047056	0.22655807	0.814274958	HS6ST1
ENSG00000145494.12	-0.015453562	0.226852161	0.814828272	NDUFS6
ENSG00000129480.13	0.016430193	0.226890089	0.814828272	DTD2
ENSG00000279700.1	0.023254799	0.226914598	0.814828272	RP13-554M15.2
ENSG00000099810.21	0.007991425	0.227011697	0.814828272	MTAP
ENSG00000025796.14	0.007232816	0.227090105	0.814828272	SEC63
ENSG00000188305.6	-0.01546819	0.2271525	0.814828272	PEAK3
ENSG00000223969.6	-0.021329887	0.22734665	0.814828272	AC002456.2
ENSG00000248240.1	-0.049330178	0.227352802	0.814828272	RP11-159F24.5
ENSG00000143252.16	0.008133109	0.227366178	0.814828272	SDHC
ENSG00000280022.1	-0.051601654	0.22738849	0.814828272	RP11-707O23.1
ENSG00000213885.4	0.026872754	0.227426652	0.814828272	RPL13AP7
ENSG00000189182.10	-0.165773242	0.22743899	0.814828272	KRT77
ENSG00000272970.3	-0.046704391	0.227488138	0.814828272	RP11-329B9.4
ENSG00000179918.19	0.006349442	0.227504335	0.814828272	SEPHS2
ENSG00000151883.19	0.01064277	0.227511623	0.814828272	PARP8
ENSG00000179088.15	-0.020069387	0.22753474	0.814828272	C12orf42
ENSG00000135314.12	-0.023799869	0.227547238	0.814828272	KHDC1
ENSG00000226121.2	0.03935422	0.227568639	0.814828272	AHCTF1P1
ENSG00000250366.3	-0.067272165	0.227602032	0.814828272	TUNAR
ENSG00000125551.19	0.044755196	0.227605134	0.814828272	PLGLB2
ENSG00000031081.11	0.014247147	0.227688806	0.814828272	ARHGAP31
ENSG00000177576.12	0.00829833	0.227755134	0.814828272	C18orf32
ENSG00000235618.8	0.039883242	0.227760684	0.814828272	FAM21EP
ENSG00000176978.14	-0.012128955	0.227766447	0.814828272	DPP7
ENSG00000228399.1	0.054752872	0.227776099	0.814828272	RP4-575N6.2

ENSG00000007312.13	-0.02428007	0.227781786	0.814828272	CD79B
ENSG00000100427.16	-0.023806278	0.227840079	0.814828272	MLC1
ENSG00000140983.14	-0.009939189	0.227842168	0.814828272	RHOT2
ENSG00000174748.22	0.011210669	0.227872996	0.814828272	RPL15
ENSG00000124459.12	0.010072604	0.227903987	0.814828272	ZNF45
ENSG00000173930.9	-0.019571009	0.227954249	0.81486591	SLCO4C1
ENSG00000135763.10	-0.010952977	0.2281309	0.815355262	URB2
ENSG00000152104.12	-0.034404486	0.228233035	0.815578164	PTPN14
ENSG00000280107.1	-0.04194685	0.228327967	0.815775254	AL022393.9
ENSG00000158816.16	-0.051179096	0.228553683	0.816031927	VWA5B1
ENSG00000113389.16	0.041194476	0.228589485	0.816031927	NPR3
ENSG00000100722.20	0.007205806	0.228648648	0.816031927	ZC3H14
ENSG00000211450.10	-0.012721503	0.228721412	0.816031927	SELENOH
ENSG00000231205.11	0.032942926	0.228743853	0.816031927	ZNF826P
ENSG00000162300.13	-0.011589975	0.228746589	0.816031927	ZFPL1
ENSG00000227939.1	0.030510513	0.228748198	0.816031927	RPL3P2
ENSG00000280128.1	-0.011539412	0.228757171	0.816031927	XXbac-BPG283O16.9
ENSG00000111647.13	0.012700862	0.228757926	0.816031927	UHRF1BP1L
ENSG00000143353.12	0.012693576	0.228820332	0.816112586	LYPLAL1
ENSG00000119471.15	0.010304466	0.228950367	0.81633019	HSDL2
ENSG00000234329.1	0.030858693	0.228975527	0.81633019	RP11-767N6.2
ENSG00000107864.15	-0.018208721	0.22900076	0.81633019	CPEB3
ENSG00000084754.12	0.00732076	0.229155529	0.816739934	HADHA
ENSG00000103152.12	-0.011399495	0.229340785	0.816962451	MPG
ENSG00000099769.6	-0.034533816	0.229372452	0.816962451	IGFALS
ENSG00000196511.14	0.010188955	0.229393483	0.816962451	TPK1
ENSG00000132467.4	0.008421583	0.229430644	0.816962451	UTP3
ENSG00000184500.16	0.038742879	0.229443969	0.816962451	PROS1
ENSG00000109743.11	0.011465427	0.229483463	0.816962451	BST1
ENSG00000159445.13	0.015859458	0.229516074	0.816962451	THEM4
ENSG00000215475.5	0.049855508	0.229541694	0.816962451	SIAH3
ENSG00000090238.12	-0.013513794	0.229717006	0.816962451	YPEL3
ENSG00000280088.1	0.01052271	0.229722442	0.816962451	RP11-135F9.3
ENSG00000286084.2	-0.048734943	0.229802782	0.816962451	RP3-359N14.3
ENSG00000118777.12	0.042515991	0.229838769	0.816962451	ABCG2
ENSG00000259207.9	0.03711683	0.229861141	0.816962451	ITGB3
ENSG00000279738.1	-0.013366651	0.229863087	0.816962451	RP5-1014D13.2
ENSG00000213020.10	0.013071138	0.229973078	0.816962451	ZNF611
ENSG00000285868.1	0.031853447	0.229981502	0.816962451	CTB-109A12.2
ENSG00000138375.13	-0.008676354	0.229988281	0.816962451	SMARCAL1
ENSG00000213075.7	-0.044874741	0.230126435	0.816962451	RPL31P11
ENSG00000095951.17	0.012572194	0.230130385	0.816962451	HIVEP1
ENSG00000152234.16	0.006441742	0.230133222	0.816962451	ATP5F1A
ENSG00000236830.7	0.02051801	0.230143087	0.816962451	CBR3-AS1
ENSG00000235150.1	-0.040405237	0.230158342	0.816962451	RCBTB2P1
ENSG00000124813.23	-0.012897115	0.230172775	0.816962451	RUNX2
ENSG00000223509.8	-0.020057343	0.230221707	0.816962451	RP11-632K20.7

ENSG00000272954.1	0.045653088	0.230294965	0.816962451	KB-1440D3.13
ENSG00000213523.12	0.01157521	0.23029725	0.816962451	SRA1
ENSG00000284690.3	-0.039152311	0.230299098	0.816962451	CD300H
ENSG00000111252.11	0.008627924	0.230333377	0.816962451	SH2B3
ENSG00000270175.1	0.026608823	0.230626682	0.817329342	RP11-793H13.11
ENSG00000254056.1	0.081399892	0.230635714	0.817329342	IGHV3-71
ENSG00000198853.12	0.014760923	0.230697453	0.817329342	RUSC2
ENSG00000108798.9	-0.015097601	0.230707167	0.817329342	ABI3
ENSG00000115204.15	0.007184967	0.230735292	0.817329342	MPV17
ENSG00000121578.13	0.012092516	0.230797932	0.817329342	B4GALT4
ENSG00000166793.13	0.027180126	0.230815765	0.817329342	YPEL4
ENSG00000276454.1	-0.049017158	0.230916111	0.817329342	RP11-493L12.7
ENSG00000124074.12	-0.013621871	0.230949094	0.817329342	ENKD1
ENSG00000227963.1	-0.023986581	0.230975493	0.817329342	RBM15-AS1
ENSG00000115355.17	0.015967929	0.231067033	0.817329342	CCDC88A
ENSG00000171522.6	-0.01133836	0.231067479	0.817329342	PTGER4
ENSG00000149782.13	-0.011667852	0.231075484	0.817329342	PLCB3
ENSG00000240890.2	0.04045009	0.231089663	0.817329342	RP11-65E22.2
ENSG00000111596.14	0.004780088	0.23111879	0.817329342	CNOT2
ENSG00000214106.9	0.012500506	0.231122732	0.817329342	PAXIP1-AS2
ENSG00000177479.20	0.00518669	0.231178286	0.817329342	ARIH2
ENSG00000112290.13	-0.025213066	0.231285005	0.817329342	WASF1
ENSG00000105397.14	-0.007902072	0.231318572	0.817329342	TYK2
ENSG00000160877.6	-0.009393247	0.231356769	0.817329342	NACC1
ENSG00000184047.20	-0.012875538	0.231411287	0.817329342	DIABLO
ENSG00000231991.4	0.015066758	0.231426054	0.817329342	ANXA2P2
ENSG00000129244.9	0.049477876	0.231430201	0.817329342	ATP1B2
ENSG00000188641.14	0.012115928	0.231515716	0.817329342	DPYD
ENSG00000198752.11	0.014854262	0.231538124	0.817329342	CDC42BPB
ENSG00000183496.6	-0.030293645	0.231540739	0.817329342	MEX3B
ENSG00000249863.2	-0.019565815	0.231581354	0.817329342	RP11-177C12.1
ENSG00000179935.10	-0.03426445	0.231603912	0.817329342	LINC00652
ENSG00000105556.12	-0.010816489	0.231628334	0.817329342	MIER2
ENSG00000287431.1	0.029023124	0.231650034	0.817329342	RENO1
ENSG00000264024.2	-0.04368921	0.231700463	0.817329342	MIR4507
ENSG00000142632.17	-0.013149614	0.231712151	0.817329342	ARHGEF19
ENSG00000226334.1	-0.05824147	0.231881544	0.817405551	RP11-217B7.2
ENSG00000128683.14	-0.113151068	0.231888457	0.817405551	GAD1
ENSG00000249637.2	-0.023866207	0.231916085	0.817405551	CTC-329D1.2
ENSG00000240671.4	-0.047614674	0.231951637	0.817405551	IGKV1-8
ENSG00000278983.1	-0.06304402	0.231990128	0.817405551	RP11-426J5.3
ENSG00000231133.8	0.040581711	0.232024584	0.817405551	HAR1B
ENSG00000124593.16	-0.018903203	0.232035341	0.817405551	RP11-298J23.10
ENSG00000197780.10	0.015016675	0.232052619	0.817405551	TAF13
ENSG00000250992.2	0.045863876	0.232146453	0.817431826	RP11-826N14.1
ENSG00000232850.3	-0.02809514	0.232174092	0.817431826	PTGES2-AS1
ENSG00000011105.14	0.028851022	0.232218035	0.817431826	TSPAN9

ENSG00000159164.10	-0.017327279	0.232231397	0.817431826	SV2A
ENSG00000255299.5	0.041576869	0.232259374	0.817431826	RP11-655C2.3
ENSG00000111796.4	0.023774173	0.232356337	0.817528512	KLRB1
ENSG00000136628.18	0.006235277	0.232366574	0.817528512	EPRS1
ENSG00000287900.1	-0.116122331	0.232438363	0.817640815	AC061961.2
ENSG00000144843.11	0.013347816	0.232553306	0.817753476	ADPRH
ENSG00000172428.11	0.01530874	0.232575707	0.817753476	COPS9
ENSG00000162390.18	0.025222445	0.232590015	0.817753476	ACOT11
ENSG00000161944.16	-0.023653733	0.232754275	0.817892177	ASGR2
ENSG00000179051.14	-0.007654114	0.232786022	0.817892177	RCC2
ENSG00000169855.20	-0.060267183	0.232787064	0.817892177	ROBO1
ENSG00000065613.15	0.012901241	0.23286072	0.817892177	SLK
ENSG00000198039.11	-0.018724103	0.232914518	0.817892177	ZNF273
ENSG00000260001.7	0.04411983	0.232916142	0.817892177	TGFBR3L
ENSG00000140090.18	-0.017671462	0.232939685	0.817892177	SLC24A4
ENSG00000180730.5	0.056714957	0.232950865	0.817892177	SHISA2
ENSG00000258695.2	0.050480716	0.233023873	0.817892177	RP3-414A15.2
ENSG00000178951.9	-0.008418835	0.233028281	0.817892177	ZBTB7A
ENSG00000246016.2	0.027001022	0.233157582	0.8179517	LINC01513
ENSG00000141401.12	0.014193399	0.233168692	0.8179517	IMPA2
ENSG00000196588.20	-0.009977949	0.233193159	0.8179517	MRTFA
ENSG00000204177.10	0.034326667	0.233204778	0.8179517	BMS1P1
ENSG00000284734.1	-0.022051145	0.233260284	0.817977933	RP4-620F22.3
ENSG00000162543.6	-0.027283361	0.233377628	0.817977933	UBXN10
ENSG00000104880.19	-0.010182309	0.233395353	0.817977933	ARHGEF18
ENSG00000140941.14	0.007289497	0.233435926	0.817977933	MAP1LC3B
ENSG00000113924.12	0.024432464	0.233452641	0.817977933	HGD
ENSG00000244151.1	0.021913213	0.233478602	0.817977933	RP11-148K1.12
ENSG00000196235.14	-0.009391823	0.23349959	0.817977933	SUPT5H
ENSG00000181396.13	-0.009905998	0.233552826	0.817977933	OGFOD3
ENSG00000150656.15	0.047347963	0.23357123	0.817977933	CNDP1
ENSG00000182473.22	0.009291635	0.233704453	0.818304749	EXOC7
ENSG00000103723.17	0.051767129	0.233842766	0.818517058	AP3B2
ENSG00000172728.16	-0.029502694	0.233852268	0.818517058	FUT10
ENSG00000106686.17	-0.027119334	0.233884823	0.818517058	SPATA6L
ENSG00000115271.11	0.013714413	0.23399343	0.818577231	GCA
ENSG00000029534.21	0.033904001	0.234002538	0.818577231	ANK1
ENSG00000174776.12	0.035216524	0.234021762	0.818577231	WDR49
ENSG00000106462.12	-0.010637724	0.234092914	0.818686475	EZH2
ENSG00000137752.24	0.011381251	0.234212013	0.818963336	CASP1
ENSG00000064547.14	-0.013488352	0.234311681	0.81908541	LPAR2
ENSG00000265972.6	0.009424505	0.234326804	0.81908541	TXNIP
ENSG00000080503.24	-0.008556116	0.234600277	0.819851641	SMARCA2
ENSG00000260274.1	0.017889716	0.234643936	0.819851641	RP11-817O13.8
ENSG00000128708.13	0.021798466	0.234665942	0.819851641	HAT1
ENSG00000047621.12	0.011468885	0.234884421	0.819885645	C12orf4
ENSG00000235159.1	0.044018334	0.23488505	0.819885645	RP6-109B7.4

ENSG00000185499.16	0.02491523	0.234895856	0.819885645	MUC1
ENSG00000212802.4	0.022154079	0.234931346	0.819885645	RPL15P3
ENSG00000049541.11	0.008391211	0.234946652	0.819885645	RFC2
ENSG00000132514.14	0.021694616	0.234947245	0.819885645	CLEC10A
ENSG00000185453.13	-0.014190367	0.234989839	0.819885645	ZSWIM9
ENSG00000187556.8	0.049247224	0.235080201	0.819885645	NANOS3
ENSG00000178440.8	-0.035486645	0.235082785	0.819885645	TIMM23B-AGAP6
ENSG00000229390.1	0.05125118	0.235150681	0.819885645	MICD
ENSG00000115507.10	0.028213078	0.235154518	0.819885645	OTX1
ENSG00000145022.4	0.008199982	0.235183993	0.819885645	TCTA
ENSG00000169116.11	-0.026787669	0.235209132	0.819885645	PARM1
ENSG00000100804.19	0.010183412	0.235289146	0.819885645	PSMB5
ENSG00000255569.1	-0.037439421	0.23529705	0.819885645	TRAV1-1
ENSG00000153879.9	0.008849118	0.235328723	0.819885645	CEBPG
ENSG00000134243.12	0.01265646	0.235385253	0.819885645	SORT1
ENSG00000261172.1	0.048127951	0.235557426	0.819885645	RP11-356C4.5
ENSG00000174469.23	-0.053484376	0.235569233	0.819885645	CNTNAP2
ENSG00000269737.2	0.032812604	0.235611338	0.819885645	RP11-345P4.6
ENSG00000118705.17	-0.0073687	0.235649771	0.819885645	RPN2
ENSG00000267501.1	0.037030683	0.235664232	0.819885645	RP11-108P20.2
ENSG00000176261.16	0.01048987	0.235668002	0.819885645	ZBTB8OS
ENSG00000159399.10	-0.014442347	0.235673917	0.819885645	HK2
ENSG00000225793.3	0.027207953	0.235757067	0.819885645	TMEM30A-DT
ENSG00000068784.13	0.011642231	0.235812495	0.819885645	SRBD1
ENSG00000119737.6	-0.030204865	0.235916748	0.819885645	GPR75
ENSG00000013583.10	0.013207686	0.236017968	0.819885645	HEBP1
ENSG00000250731.1	0.034459733	0.236092644	0.819885645	TPM3P6
ENSG00000068796.19	0.009730947	0.23612833	0.819885645	KIF2A
ENSG00000171877.21	-0.04913937	0.236186147	0.819885645	FRMD5
ENSG00000241547.1	-0.040498964	0.236196825	0.819885645	ACTG1P20
ENSG00000170382.12	0.01843924	0.236333127	0.819885645	LRRN2
ENSG00000104723.21	-0.125373752	0.236386883	0.819885645	TUSC3
ENSG00000140386.13	0.010897373	0.236406412	0.819885645	SCAPER
ENSG00000260256.1	-0.049828126	0.236411997	0.819885645	AP001063.1
ENSG00000126562.17	-0.037687235	0.236443878	0.819885645	WNK4
ENSG00000127252.7	0.049919718	0.236452483	0.819885645	PLAAT1
ENSG00000168395.16	-0.007761911	0.236464821	0.819885645	ING5
ENSG00000268144.1	-0.048001755	0.236511833	0.819885645	NIFKP6
ENSG00000101542.10	-0.050268983	0.236557012	0.819885645	CDH20
ENSG00000004777.18	-0.01494854	0.236641337	0.819885645	ARHGAP33
ENSG00000119943.13	-0.012752953	0.236652017	0.819885645	PYROXD2
ENSG00000166897.16	0.030925051	0.236672719	0.819885645	ELFN2
ENSG00000223622.2	0.062705566	0.236672933	0.819885645	GSTA6P
ENSG00000092758.18	-0.040846403	0.236708844	0.819885645	COL9A3
ENSG00000089847.12	-0.025875281	0.236867627	0.819885645	ANKRD24
ENSG00000255987.1	-0.022518312	0.236939527	0.819885645	TOMM20P2
ENSG00000213279.2	0.025418888	0.23696309	0.819885645	RP1-29C18.9

ENSG00000137942.18	0.020224642	0.236977093	0.819885645	FNBP1L
ENSG00000178385.15	0.010864825	0.237017383	0.819885645	PLEKHM3
ENSG00000277161.2	-0.012364404	0.237036117	0.819885645	PIGW
ENSG00000068878.15	0.006911852	0.237107078	0.819885645	PSME4
ENSG00000240382.3	0.045149884	0.23711185	0.819885645	IGKV1-17
ENSG00000258634.3	-0.009664414	0.237159348	0.819885645	RP4-773N10.4
ENSG00000238009.6	-0.05788937	0.237175974	0.819885645	RP11-34P13.7
ENSG00000153443.13	-0.012367297	0.237201455	0.819885645	UBALD1
ENSG00000049618.24	-0.009300428	0.23726036	0.819885645	ARID1B
ENSG00000157870.17	-0.010749458	0.237338611	0.819885645	PRXL2B
ENSG00000129103.19	-0.007108051	0.237399762	0.819885645	SUMF2
ENSG00000166188.3	-0.009407401	0.237431794	0.819885645	ZNF319
ENSG00000070444.15	-0.008735743	0.237465275	0.819885645	MNT
ENSG00000095139.14	0.006972339	0.237467626	0.819885645	ARCN1
ENSG00000278196.3	-0.047137039	0.237500098	0.819885645	IGLV2-8
ENSG00000143595.13	0.042130444	0.237506519	0.819885645	AQP10
ENSG00000215199.3	0.036874264	0.237588267	0.819885645	YWHAZP6
ENSG00000158246.8	-0.040387547	0.237593606	0.819885645	TENT5B
ENSG00000183520.12	0.007873899	0.237609007	0.819885645	UTP11
ENSG00000274767.1	-0.059811106	0.237640181	0.819885645	AC131056.3
ENSG00000159339.13	-0.01766257	0.237654625	0.819885645	PADI4
ENSG00000211673.2	0.043195911	0.237721638	0.819885645	IGLV3-1
ENSG00000105447.13	-0.008789186	0.237772576	0.819885645	GRWD1
ENSG00000196123.13	-0.015783345	0.237805673	0.819885645	KIAA0895L
ENSG00000236499.2	0.028390597	0.237836271	0.819885645	LINC00896
ENSG00000228436.3	0.029796802	0.237900572	0.819885645	RP5-864K19.4
ENSG00000278897.1	-0.047959855	0.237939246	0.819885645	AC020951.1
ENSG00000251623.2	0.044465924	0.237944736	0.819885645	FAM153B
ENSG00000248559.1	-0.03495774	0.237994336	0.819885645	RP11-215P8.3
ENSG00000288524.1	0.089181252	0.238001119	0.819885645	RP11-505O20.1
ENSG00000213145.10	-0.015410795	0.238043341	0.819885645	CRIP1
ENSG00000272576.1	-0.051200112	0.238058668	0.819885645	RP11-365H22.2
ENSG00000204188.7	0.036199683	0.238087584	0.819885645	GGNBP1
ENSG00000109861.17	0.010768263	0.23813478	0.819885645	CTSC
ENSG00000198755.11	0.012857849	0.238135543	0.819885645	RPL10A
ENSG00000261573.1	-0.044194694	0.238158771	0.819885645	RP11-553K8.5
ENSG00000162849.16	0.030672259	0.238182147	0.819885645	KIF26B
ENSG00000055070.17	0.006493312	0.238215525	0.819885645	SZRD1
ENSG00000063660.9	-0.02204826	0.238226673	0.819885645	GPC1
ENSG00000135604.10	0.013001052	0.23823379	0.819885645	STX11
ENSG00000272391.6	-0.009645986	0.23828251	0.819902204	POM121C
ENSG00000153832.12	0.022688724	0.238363276	0.819902204	FBXO36
ENSG00000120159.13	-0.012478919	0.238394307	0.819902204	CAAP1
ENSG00000258704.6	-0.021230415	0.238446988	0.819902204	SRP54-AS1
ENSG00000116544.12	-0.021862691	0.238553408	0.819902204	DLGAP3
ENSG00000117394.24	-0.013771199	0.238622675	0.819902204	SLC2A1
ENSG00000204305.15	-0.013139954	0.238688116	0.819902204	AGER

ENSG00000277369.1	0.025593429	0.238699312	0.819902204	RP11-486I11.2
ENSG00000259881.1	0.018883593	0.238748016	0.819902204	RP11-830F9.5
ENSG00000010017.13	0.006897757	0.238762757	0.819902204	RANBP9
ENSG00000248896.3	-0.041061102	0.238782248	0.819902204	CTD-2135J3.3
ENSG00000148153.14	0.006372016	0.238808107	0.819902204	INIP
ENSG00000188522.15	-0.013836263	0.238859072	0.819902204	FAM83G
ENSG00000159377.11	0.009577417	0.238930437	0.819902204	PSMB4
ENSG00000287580.1	0.027598869	0.238936537	0.819902204	RP11-797H7.8
ENSG00000273841.5	0.015190205	0.238953511	0.819902204	TAF9
ENSG00000173818.17	0.016766924	0.238956611	0.819902204	ENDOV
ENSG00000165891.16	-0.042629719	0.238975089	0.819902204	E2F7
ENSG00000115652.15	0.007923947	0.238998214	0.819902204	UXS1
ENSG00000130810.20	-0.012775405	0.239084579	0.820061306	PPAN
ENSG00000180957.18	-0.004804151	0.23916809	0.820083617	PITPNB
ENSG00000219607.4	-0.03954113	0.239173435	0.820083617	PPP1R3G
ENSG00000154839.10	-0.032871889	0.239211049	0.820083617	SKA1
ENSG00000133612.19	0.010336728	0.239263819	0.820127429	AGAP3
ENSG00000202461.1	-0.039732376	0.23936253	0.820189167	Y_RNA
ENSG00000123485.12	-0.033278168	0.23942189	0.820189167	HJURP
ENSG00000162746.14	-0.018951405	0.239423906	0.820189167	FCRLB
ENSG00000279970.1	0.042191554	0.239450666	0.820189167	RP11-299G20.5
ENSG00000259015.1	0.034893873	0.239526964	0.820189167	RP11-109N23.6
ENSG00000234127.9	-0.009527565	0.23953323	0.820189167	TRIM26
ENSG00000184900.16	0.007413382	0.239561786	0.820189167	SUMO3
ENSG00000137831.15	0.024520185	0.239739077	0.820633927	UACA
ENSG00000278023.7	-0.032011039	0.239781579	0.820633927	RDM1
ENSG00000121053.6	0.02731744	0.239822721	0.820633927	EPX
ENSG00000234568.3	0.020079241	0.239851753	0.820633927	BIN2P1
ENSG00000272442.2	0.039447343	0.239909739	0.820695401	RP11-444E17.6
ENSG00000285545.1	-0.046579974	0.239977333	0.820710573	RP11-452G18.4
ENSG00000264456.1	0.015056483	0.239994212	0.820710573	RP11-848P1.2
ENSG00000124160.12	-0.005977112	0.240203017	0.821120289	NCOA5
ENSG00000139618.17	-0.020774202	0.24023481	0.821120289	BRCA2
ENSG00000166246.14	-0.027171567	0.240338502	0.821120289	C16orf71
ENSG00000254810.1	0.051677026	0.240341957	0.821120289	RP11-672A2.4
ENSG00000156026.14	0.008454047	0.240386759	0.821120289	MCU
ENSG00000146197.9	-0.025649637	0.2404069	0.821120289	SCUBE3
ENSG00000155066.16	-0.032264342	0.240409641	0.821120289	PROM2
ENSG00000163902.12	0.007410282	0.240475987	0.821120289	RPN1
ENSG00000185236.12	-0.010276357	0.240526687	0.821120289	RAB11B
ENSG00000162738.6	-0.085697226	0.240580276	0.821120289	VANGL2
ENSG00000267293.1	-0.037951546	0.240619701	0.821120289	RP11-8H2.1
ENSG00000157335.21	0.077382481	0.2406563	0.821120289	CLEC18C
ENSG00000264577.1	-0.013453539	0.240677462	0.821120289	AC010761.8
ENSG00000259922.1	-0.044366006	0.240714584	0.821120289	RP11-322D14.1
ENSG00000154529.15	0.074036439	0.24084918	0.821120289	CNTNAP3B
ENSG00000254101.7	-0.101195837	0.240857833	0.821120289	LINC02055

ENSG00000138074.15	-0.008631524	0.240877491	0.821120289	SLC5A6
ENSG00000234284.7	0.014706072	0.240887029	0.821120289	ZNF879
ENSG00000285476.1	0.048162007	0.2408988	0.821120289	RP11-826N14.7
ENSG00000078246.17	-0.009529417	0.240943741	0.821120289	TULP3
ENSG00000173540.14	-0.00822833	0.240954842	0.821120289	GMPPB
ENSG00000074966.11	0.018958985	0.241018235	0.821199862	TXK
ENSG00000123838.11	-0.101595224	0.241118207	0.821404018	C4BPA
ENSG00000103335.22	-0.009636604	0.241212089	0.821587364	PIEZO1
ENSG00000270049.3	-0.01032228	0.24131506	0.821638259	ATP6V0D1-DT
ENSG00000076382.17	-0.015003697	0.241340016	0.821638259	SPAG5
ENSG00000280138.1	-0.014702082	0.241407085	0.821638259	RP11-463O12.5
ENSG00000130487.9	-0.020453319	0.241465946	0.821638259	KLHDC7B
ENSG00000005339.15	-0.013352615	0.241511175	0.821638259	CREBBP
ENSG00000266173.7	-0.007738708	0.241571961	0.821638259	STRADA
ENSG00000189136.9	-0.036577211	0.241598286	0.821638259	UBE2Q2P1
ENSG00000138413.14	0.008680211	0.241732967	0.821638259	IDH1
ENSG00000115902.11	-0.015524548	0.24174008	0.821638259	SLC1A4
ENSG00000184992.13	-0.009968147	0.241770143	0.821638259	BRI3BP
ENSG00000267033.1	-0.032435071	0.241782442	0.821638259	CTD-2562J15.4
ENSG00000236559.1	0.025143379	0.241783391	0.821638259	BCL2L1-AS1
ENSG00000136573.14	-0.027325838	0.241815193	0.821638259	BLK
ENSG00000167535.8	-0.015724474	0.24194046	0.821638259	CACNB3
ENSG00000101057.16	-0.036846433	0.241999999	0.821638259	MYBL2
ENSG00000262246.6	-0.011803132	0.24201401	0.821638259	CORO7
ENSG00000047617.16	0.065421474	0.242035858	0.821638259	ANO2
ENSG00000105559.12	-0.024576737	0.242046574	0.821638259	PLEKHA4
ENSG00000168564.6	-0.009510455	0.242063784	0.821638259	CDKN2AIP
ENSG00000131148.9	0.008051365	0.242078125	0.821638259	EMC8
ENSG00000100376.12	0.041919095	0.242090978	0.821638259	FAM118A
ENSG00000170396.9	0.022266358	0.242114303	0.821638259	ZNF804A
ENSG00000174788.10	-0.0194812	0.242167511	0.821638259	PCP2
ENSG00000272462.3	-0.013441367	0.242227851	0.821638259	U91328.19
ENSG00000101464.11	0.009869862	0.242257117	0.821638259	PIGU
ENSG00000212743.2	-0.02195694	0.242290773	0.821638259	LINC02656
ENSG00000105983.23	0.007829163	0.242382885	0.821638259	LMBR1
ENSG00000272578.5	-0.0192953	0.242387617	0.821638259	AP000347.2
ENSG00000130023.16	-0.007796277	0.242486583	0.821638259	ERMARD
ENSG00000105088.9	-0.01509457	0.242538674	0.821638259	OLFM2
ENSG00000164091.12	0.005633481	0.242571304	0.821638259	WDR82
ENSG00000066084.13	-0.009817611	0.242626177	0.821638259	DIP2B
ENSG00000203999.9	-0.024083363	0.242653595	0.821638259	LINC01270
ENSG00000263934.5	-0.128556736	0.242661385	0.821638259	SNORD3A
ENSG00000185710.9	0.102404141	0.242663057	0.821638259	SMG1P4
ENSG00000253549.6	0.039763414	0.242669345	0.821638259	CA3-AS1
ENSG00000250317.8	-0.015164967	0.242783407	0.821756221	SMIM20
ENSG00000172771.12	-0.018175833	0.242795658	0.821756221	EFCAB12
ENSG00000233029.3	0.038428601	0.242824395	0.821756221	RP11-439A17.9

ENSG00000287038.1	-0.076084081	0.242881533	0.821813971	RP11-373A6.2
ENSG00000091073.20	-0.012535909	0.243033946	0.822043023	DTX2
ENSG00000187699.10	0.020485631	0.243058445	0.822043023	C2orf88
ENSG00000160194.18	0.00807631	0.243175268	0.822043023	NDUFV3
ENSG00000124767.7	0.009080347	0.243182127	0.822043023	GLO1
ENSG00000173597.9	0.01634265	0.243202834	0.822043023	SULT1B1
ENSG00000111850.11	-0.015019039	0.243210642	0.822043023	SMIM8
ENSG00000165655.17	-0.025762751	0.243229816	0.822043023	ZNF503
ENSG00000197121.15	-0.024440917	0.243347866	0.822181419	PGAP1
ENSG00000230521.1	-0.027189395	0.243386542	0.822181419	HCG4P7
ENSG00000159307.19	0.042261959	0.243416476	0.822181419	SCUBE1
ENSG00000221829.10	-0.009274959	0.243514628	0.822181419	FANCG
ENSG00000104738.18	-0.014020734	0.243579747	0.822181419	MCM4
ENSG00000224546.2	0.022975777	0.243596093	0.822181419	EIF4BP3
ENSG00000249242.8	-0.042364049	0.243704399	0.822181419	TMEM150C
ENSG00000287672.1	-0.047410089	0.243726006	0.822181419	CTB-104F4.3
ENSG00000087460.29	-0.012582894	0.243753838	0.822181419	GNAS
ENSG00000134909.19	-0.017305792	0.243789924	0.822181419	ARHGAP32
ENSG00000257543.1	-0.043920849	0.243895865	0.822181419	RP11-321F8.4
ENSG00000160999.10	-0.015174904	0.243941979	0.822181419	SH2B2
ENSG00000177788.6	0.021246176	0.243956276	0.822181419	RP5-1061H20.4
ENSG00000280502.1	0.034313146	0.244158882	0.822181419	Metazoa_SRP
ENSG00000154582.17	0.01149039	0.244164949	0.822181419	ELOC
ENSG00000118096.10	0.009387738	0.244183905	0.822181419	IFT46
ENSG00000117592.9	0.018393912	0.244226819	0.822181419	PRDX6
ENSG00000164136.17	0.016613512	0.244280199	0.822181419	IL15
ENSG00000184232.9	0.012826637	0.244322539	0.822181419	OAF
ENSG00000185219.17	-0.008618251	0.244366835	0.822181419	ZNF445
ENSG00000105583.11	0.043423184	0.24437448	0.822181419	WDR83OS
ENSG00000184307.17	0.016717104	0.2443777	0.822181419	ZDHHC23
ENSG00000197122.12	0.014552933	0.244385805	0.822181419	SRC
ENSG00000199691.1	0.04074726	0.244407506	0.822181419	RN7SKP173
ENSG00000278017.1	-0.04537728	0.24446708	0.822181419	RP11-120K19.4
ENSG00000241990.5	0.021529114	0.244490858	0.822181419	PRR34-AS1
ENSG00000125347.15	0.011923219	0.244508611	0.822181419	IRF1
ENSG00000114650.20	-0.010541677	0.244526252	0.822181419	SCAP
ENSG00000261512.2	0.014455614	0.244545548	0.822181419	RP11-46D6.1
ENSG00000154165.5	-0.045734424	0.244546063	0.822181419	GPR15
ENSG00000267571.1	0.012596972	0.24455411	0.822181419	AC104532.4
ENSG00000082146.13	0.034870721	0.244587225	0.822181419	STRADB
ENSG00000145063.15	0.049928124	0.244604422	0.822181419	AC062028.1
ENSG00000165417.12	-0.010531438	0.244637532	0.822181419	GTF2A1
ENSG00000211787.1	0.02116588	0.244684536	0.822181419	TRAV8-3
ENSG00000214711.10	-0.026703535	0.24476844	0.822181419	CAPN14
ENSG00000165506.15	-0.011879447	0.244787099	0.822181419	DNAAF2
ENSG00000260948.1	0.01909547	0.244832669	0.822181419	RP11-552M11.8
ENSG00000164162.14	-0.0254462	0.244849357	0.822181419	ANAPC10

ENSG00000169740.14	0.014528101	0.244874396	0.822181419	ZNF32	
ENSG00000008952.17	0.011905863	0.24496496	0.822230247	SEC62	
ENSG00000175866.16	-0.018646324	0.245075911	0.822230247	BAIAP2	
ENSG00000107862.6	-0.009601937	0.245082648	0.822230247	GBF1	
ENSG00000211816.2	-0.037181167	0.245086839	0.822230247	TRAV38-1	
ENSG00000267481.1	0.012002643	0.245089404	0.822230247	CTC-559E9.5	
ENSG00000204482.11	0.011814739	0.245251529	0.822275642	LST1	
ENSG00000146376.11	0.012058026	0.245260723	0.822275642	ARHGAP18	
ENSG00000175213.3	-0.00944714	0.245295017	0.822275642	ZNF408	
ENSG00000119203.14	0.00666287	0.245364766	0.822275642	CPSF3	
ENSG00000101040.19	-0.00811884	0.245501219	0.822275642	ZMYND8	
ENSG00000143753.13	0.007674132	0.245510279	0.822275642	DEGS1	
ENSG00000259673.6	0.014791423	0.245517202	0.822275642	IQCH-AS1	
ENSG00000207034.1	-0.043598477	0.245520564	0.822275642	Y_RNA	
ENSG00000087111.21	-0.007013727	0.245562696	0.822275642	PIGS	
ENSG00000224043.8	0.015795868	0.245612488	0.822275642	CCNT2-AS1	
ENSG00000157110.16	0.014880464	0.24561902	0.822275642	RBPMS	
ENSG00000145817.17	0.007186003	0.245644049	0.822275642	YIPF5	
ENSG00000010278.15	0.03033853	0.245685619	0.822275642	CD9	
ENSG00000157087.20	-0.054088704	0.24574681	0.822275642	ATP2B2	
ENSG00000087502.18	0.015485352	0.245787162	0.822275642	ERGIC2	
ENSG00000284948.1	-0.037309295	0.245792797	0.822275642	RP11-875O11.5	
ENSG00000260018.1	-0.042790191	0.245799898	0.822275642	RP11-505K9.1	
ENSG00000163935.14	0.010748787	0.245829751	0.822275642	SFMBT1	
ENSG00000173207.13	0.018437114	0.245864747	0.822275642	CKS1B	
ENSG00000231304.2	-0.024041052	0.245926385	0.822347679	SGO1-AS1	
ENSG00000267834.1	-0.033676603	0.246019612	0.822420854	RP11-167N5.5	
ENSG00000260179.1	-0.028484856	0.246049672	0.822420854	RP5-902P8.12	
ENSG00000248514.1	0.03981021	0.246074236	0.822420854	CTC-338M12.1	
ENSG00000165494.11	0.009027021	0.246186518	0.822420854	PCF11	
ENSG00000144199.12	-0.014924796	0.246225005	0.822420854	FAHD2B	
ENSG00000230138.2	0.020457897	0.246250724	0.822420854	LINC02812	
ENSG00000168612.5	0.008981111	0.246257222	0.822420854	ZSWIM1	
ENSG00000148985.20	-0.008834699	0.246269088	0.822420854	PGAP2	
ENSG00000138798.13	0.032681327	0.246321106	0.822427736	EGF	
ENSG00000166986.15	-0.005812364	0.246365319	0.822427736		mar.01
ENSG00000206559.8	0.029814117	0.246391457	0.822427736	ZCWPW2	
ENSG00000149657.20	-0.006617321	0.246480587	0.822591354	LSM14B	
ENSG00000104419.17	-0.009086586	0.246598832	0.822771088	NDRG1	
ENSG00000162642.14	0.009261367	0.246614681	0.822771088	C1orf52	
ENSG00000114026.22	-0.006371271	0.246761989	0.822981079	OGG1	
ENSG00000139734.19	-0.048712004	0.246875942	0.822981079	DIAPH3	
ENSG00000287769.1	-0.027388153	0.246929499	0.822981079	RP11-317B17.5	
ENSG00000219395.2	-0.048088336	0.246938236	0.822981079	HSPA8P15	
ENSG00000023171.19	0.01663546	0.246979845	0.822981079	GRAMD1B	
ENSG00000196132.14	-0.068220655	0.246990151	0.822981079	MYT1	
ENSG00000131471.7	-0.018019008	0.247034275	0.822981079	AOC3	

ENSG00000169245.6	0.05082181	0.247061023	0.822981079	CXCL10
ENSG00000130313.7	-0.011553552	0.24711307	0.822981079	PGLS
ENSG00000251453.1	0.038547217	0.247171541	0.822981079	HAUS1P1
ENSG00000008323.16	-0.024922853	0.24717983	0.822981079	PLEKHG6
ENSG00000160683.5	-0.027119325	0.247188229	0.822981079	CXCR5
ENSG00000187166.1	-0.034353656	0.24719931	0.822981079	H1-7
ENSG00000132793.12	-0.038230641	0.247308742	0.823211766	LPIN3
ENSG00000287079.1	-0.044116275	0.247381002	0.823257226	RP5-1153D9.6
ENSG00000187595.16	0.029924986	0.247402686	0.823257226	ZNF385C
ENSG00000110080.20	0.014208154	0.247486148	0.823401351	ST3GAL4
ENSG00000184154.15	0.011215898	0.247608255	0.82360012	LRTOMT
ENSG00000123933.17	-0.008630678	0.247662196	0.82360012	MXD4
ENSG00000219747.1	0.056916235	0.247666371	0.82360012	RPL32P16
ENSG00000174652.20	-0.014876944	0.247791745	0.823883448	ZNF266
ENSG00000220875.1	0.028850798	0.247860601	0.823978796	H3C9P
ENSG00000197170.10	0.006337229	0.247976764	0.824231359	PSMD12
ENSG00000140948.13	-0.009843414	0.248043475	0.824311319	ZCCHC14
ENSG00000198711.5	-0.020412583	0.24808121	0.824311319	SSBP3-AS1
ENSG00000213619.11	0.011497801	0.248173843	0.824335385	NDUFS3
ENSG00000281021.1	-0.043753037	0.248207777	0.824335385	RP1-12G14.9
ENSG00000138604.10	0.011971778	0.248209041	0.824335385	GLCE
ENSG00000239653.1	0.02339246	0.248410967	0.824449542	PSMD6-AS2
ENSG00000128602.11	0.040399698	0.248413833	0.824449542	SMO
ENSG00000211943.2	-0.038750924	0.248416533	0.824449542	IGHV3-15
ENSG00000273723.1	-0.019373298	0.248425568	0.824449542	SUGT1-DT
ENSG00000186827.11	-0.024864167	0.24844442	0.824449542	TNFRSF4
ENSG00000096092.6	-0.014516488	0.248629929	0.824601724	TMEM14A
ENSG00000211799.3	-0.032116544	0.248633864	0.824601724	TRAV19
ENSG00000105409.19	-0.025887592	0.24863828	0.824601724	ATP1A3
ENSG00000125503.13	-0.00857394	0.248671694	0.824601724	PPP1R12C
ENSG00000258035.2	-0.040424186	0.248809096	0.824601724	RP11-74K11.2
ENSG00000111358.14	0.009376657	0.248810601	0.824601724	GTF2H3
ENSG00000134851.13	0.012068021	0.248897812	0.824601724	TMEM165
ENSG00000282499.1	-0.034196796	0.248916522	0.824601724	TRBV25-1
ENSG00000213830.3	-0.038498859	0.248936593	0.824601724	CFL1P5
ENSG00000272994.1	-0.017634143	0.249005612	0.824601724	C2orf49-DT
ENSG00000185947.15	0.02035629	0.249022458	0.824601724	ZNF267
ENSG00000122550.18	0.009214394	0.249073362	0.824601724	KLHL7
ENSG00000132581.10	0.008754305	0.249119113	0.824601724	SDF2
ENSG00000148158.17	0.009950832	0.249121886	0.824601724	SNX30
ENSG00000225422.4	0.025668368	0.249178267	0.824601724	RBMS1P1
ENSG00000204427.12	-0.008414929	0.249182253	0.824601724	ABHD16A
ENSG00000196912.12	0.0316767	0.249306345	0.824601724	ANKRD36B
ENSG00000275582.1	-0.018404581	0.249450826	0.824601724	RP4-681N20.5
ENSG00000141385.11	0.005843038	0.249469562	0.824601724	AFG3L2
ENSG00000176444.19	-0.005417141	0.249486867	0.824601724	CLK2
ENSG00000162086.16	0.008009703	0.249531188	0.824601724	ZNF75A

ENSG00000204389.10	-0.015154973	0.249539401	0.824601724	HSPA1A
ENSG00000273096.1	-0.013786846	0.249544731	0.824601724	RP3-508I15.20
ENSG00000277692.1	-0.028614174	0.249558911	0.824601724	RP11-358N2.2
ENSG00000059758.8	-0.014913108	0.249613358	0.824601724	CDK17
ENSG00000143384.14	0.008335837	0.249613634	0.824601724	MCL1
ENSG00000225137.1	0.030798379	0.249657892	0.824601724	DYNC1I2P1
ENSG00000235105.1	0.04211783	0.249675345	0.824601724	RP11-329A14.1
ENSG00000143554.14	-0.012712169	0.249702689	0.824601724	SLC27A3
ENSG00000155011.9	-0.073809374	0.249715204	0.824601724	DKK2
ENSG00000059915.17	-0.017439732	0.249766656	0.824601724	PSD
ENSG00000272632.1	0.029429293	0.249776961	0.824601724	RP11-362F19.3
ENSG00000064666.15	-0.011778459	0.249932793	0.824983375	CNN2
ENSG00000162444.12	0.016091604	0.250010284	0.825106358	RBP7
ENSG00000143977.14	0.021580312	0.250104434	0.825181436	SNRPG
ENSG00000158615.9	0.008910671	0.250149412	0.825181436	PPP1R15B
ENSG00000204176.14	0.043522878	0.250198769	0.825181436	SYT15
ENSG00000164751.15	0.013167345	0.250213465	0.825181436	PEX2
ENSG00000187097.13	0.013213339	0.250234218	0.825181436	ENTPD5
ENSG00000279204.1	0.051078454	0.250345848	0.825241313	RP11-175K6.2
ENSG00000153774.9	0.013879243	0.250387949	0.825241313	CFDP1
ENSG00000197774.14	-0.010465114	0.250396725	0.825241313	EME2
ENSG00000160392.14	-0.009358231	0.250413336	0.825241313	C19orf47
ENSG00000279908.1	0.021687487	0.250517936	0.825270065	RP11-325I22.3
ENSG00000174021.11	-0.012581633	0.250522514	0.825270065	GNG5
ENSG00000070404.10	-0.017265404	0.250542785	0.825270065	FSTL3
ENSG00000112357.14	0.014915227	0.250634369	0.825357708	PEX7
ENSG00000146426.19	-0.015643427	0.250702623	0.825357708	TIAM2
ENSG00000100403.12	-0.006640271	0.250718745	0.825357708	ZC3H7B
ENSG00000188873.4	0.041539193	0.250730374	0.825357708	RPL10AP2
ENSG00000198663.17	0.005550538	0.25080688	0.825434792	C6orf89
ENSG00000272980.4	0.041443454	0.25083429	0.825434792	RP11-517H2.6
ENSG00000135482.7	0.005754516	0.250875196	0.825436953	ZC3H10
ENSG00000230847.4	0.084447886	0.250959048	0.825580389	OCLNP1
ENSG00000164073.11	-0.009378897	0.251042111	0.82559909	MFSD8
ENSG00000229153.5	0.042487124	0.251050712	0.82559909	EPHA1-AS1
ENSG00000258120.2	0.055110165	0.25110583	0.82559909	KRT128P
ENSG00000125505.17	-0.013476387	0.251125762	0.82559909	MBOAT7
ENSG00000288096.1	-0.029198914	0.251219566	0.8257751	CTD-3065J16.12
ENSG00000124256.15	-0.022071661	0.251460467	0.826303834	ZBP1
ENSG00000243238.1	-0.048426148	0.251480947	0.826303834	IGKV2-30
ENSG00000104093.14	0.014586959	0.251556506	0.826303834	DMXL2
ENSG00000145287.11	0.014542658	0.251634735	0.826303834	PLAC8
ENSG00000205683.12	-0.022704837	0.251653011	0.826303834	DPF3
ENSG00000196850.6	0.010223717	0.251667754	0.826303834	PPTC7
ENSG00000132694.19	0.010208919	0.251704581	0.826303834	ARHGEF11
ENSG00000272916.5	0.031236849	0.251794274	0.826303834	RP11-574K11.31
ENSG00000269834.6	0.018581607	0.251810113	0.826303834	ZNF528-AS1

ENSG00000215241.4	0.027531326	0.251843862	0.826303834	LINC02449
ENSG00000072864.16	0.01119976	0.251845597	0.826303834	NDE1
ENSG00000089009.16	0.01319898	0.25186392	0.826303834	RPL6
ENSG00000164946.20	0.066277957	0.252016704	0.826672834	FREM1
ENSG00000156256.15	0.013827287	0.252058671	0.82667827	USP16
ENSG00000130202.10	-0.062043142	0.252137658	0.826738269	NECTIN2
ENSG00000104522.16	-0.01911493	0.252157591	0.826738269	GFUS
ENSG00000236283.5	-0.051063536	0.252247509	0.826834716	AC013463.2
ENSG00000274641.2	-0.041439176	0.25232362	0.826834716	H2BC17
ENSG00000198874.13	0.007546522	0.252386509	0.826834716	TYW1
ENSG00000135317.14	-0.012150614	0.252413888	0.826834716	SNX14
ENSG00000011638.11	0.009536546	0.25242577	0.826834716	TMEM159
ENSG00000261857.7	-0.039755405	0.252428914	0.826834716	MIA
ENSG00000196542.8	0.033018168	0.252627961	0.827318677	SPTSSB
ENSG00000198417.7	-0.018630026	0.252708196	0.827318677	MT1F
ENSG00000119705.10	0.013919861	0.252716704	0.827318677	SLIRP
ENSG00000162613.17	-0.007753233	0.252753471	0.827318677	FUBP1
ENSG00000127418.15	-0.012815785	0.252797593	0.827318677	FGFRL1
ENSG00000269427.1	-0.023875357	0.252818712	0.827318677	CTC-429P9.1
ENSG00000267048.1	-0.028796506	0.252863615	0.827333603	RP11-566K11.7
ENSG00000198830.11	-0.008215924	0.252916411	0.827374344	HMGN2
ENSG00000238184.1	-0.03914214	0.253037037	0.827625416	CD81-AS1
ENSG00000156603.19	0.01224757	0.253097932	0.827625416	MED19
ENSG00000041357.16	0.016255432	0.25312515	0.827625416	PSMA4
ENSG00000231721.7	-0.010885105	0.253154585	0.827625416	LINC-PINT
ENSG00000160791.13	-0.022402969	0.253367966	0.828190985	CCR5
ENSG00000198225.5	0.019859064	0.253436912	0.828284333	FKBP1C
ENSG00000137500.9	0.010002934	0.25353215	0.828437428	CCDC90B
ENSG00000248360.7	0.054932093	0.253565516	0.828437428	LINC00504
ENSG00000100418.8	0.007141166	0.25364512	0.828437428	DESI1
ENSG00000073350.14	-0.014716245	0.25371817	0.828437428	LLGL2
ENSG00000125734.15	-0.009171718	0.253772983	0.828437428	GPR108
ENSG00000085978.22	0.004758788	0.253896873	0.828437428	ATG16L1
ENSG00000278390.5	-0.024922863	0.253912367	0.828437428	RP11-74J13.8
ENSG00000143546.10	0.028885267	0.25396874	0.828437428	S100A8
ENSG00000120063.10	0.010969157	0.253982904	0.828437428	GNA13
ENSG00000172878.14	-0.012615732	0.253993926	0.828437428	METAP1D
ENSG00000239486.1	-0.030523841	0.254159333	0.828437428	RP11-163E9.1
ENSG00000065911.13	0.014006311	0.254200762	0.828437428	MTHFD2
ENSG00000107829.15	-0.009163867	0.254203498	0.828437428	FBXW4
ENSG00000236383.8	-0.0157629	0.254295985	0.828437428	CCDC200
ENSG00000261270.1	0.02228238	0.254319134	0.828437428	RP11-325K4.3
ENSG00000204923.4	0.01081896	0.254336567	0.828437428	FBXO48
ENSG00000254760.1	-0.018994131	0.254404288	0.828437428	CTD-2616J11.3
ENSG00000070814.22	-0.009708411	0.254487019	0.828437428	TCOF1
ENSG00000230185.5	-0.032011716	0.254502037	0.828437428	C9orf147
ENSG00000071994.11	0.006347644	0.254504057	0.828437428	PDCD2

ENSG00000256357.1	-0.042160505	0.254516571	0.828437428	RP11-349I1.2
ENSG00000231682.1	0.032889831	0.254572438	0.828437428	LINC01891
ENSG00000143303.12	-0.00748958	0.254572762	0.828437428	RRNAD1
ENSG00000163631.17	0.055867554	0.254591883	0.828437428	ALB
ENSG00000214029.6	-0.014963925	0.254625135	0.828437428	ZNF891
ENSG00000285589.1	-0.010308723	0.254660803	0.828437428	CTD-2192J16.27
ENSG00000101084.18	0.009518714	0.254713317	0.828437428	RAB5IF
ENSG00000284957.1	0.067788527	0.254837474	0.828437428	AF131216.8
ENSG00000232578.4	0.041430007	0.254838366	0.828437428	CTC-303L1.1
ENSG00000183741.12	-0.007194229	0.25484272	0.828437428	CBX6
ENSG00000278276.1	-0.041729315	0.254960911	0.828437428	RP3-324O17.8
ENSG00000117115.13	0.02436896	0.2549684	0.828437428	PADI2
ENSG00000154174.8	0.007128268	0.254978024	0.828437428	TOMM70
ENSG00000232485.3	0.027870089	0.254978672	0.828437428	RPL37A-DT
ENSG00000280832.1	0.018130755	0.255051367	0.828437428	GSEC
ENSG00000117593.12	-0.008135894	0.255098287	0.828437428	DARS2
ENSG00000273216.1	-0.038416459	0.255131602	0.828437428	AC002059.10
ENSG00000188933.16	0.083898096	0.255153013	0.828437428	USP32P1
ENSG00000142192.21	-0.013735272	0.2552122	0.828437428	APP
ENSG00000197540.8	-0.018865483	0.255310701	0.828437428	GZMM
ENSG00000115866.11	0.008336713	0.255333545	0.828437428	DARS1
ENSG00000271680.1	0.024426712	0.255341991	0.828437428	RP11-564A8.8
ENSG00000255443.1	-0.032852485	0.255357921	0.828437428	CD44-AS1
ENSG00000170190.16	0.009492768	0.255436282	0.828437428	SLC16A5
ENSG00000273137.1	-0.013273031	0.255463049	0.828437428	RP3-402G11.28
ENSG00000157184.7	0.007239706	0.255493253	0.828437428	CPT2
ENSG00000104635.15	-0.011443143	0.255572309	0.828437428	SLC39A14
ENSG00000108671.11	0.005606894	0.255577573	0.828437428	PSMD11
ENSG00000153339.15	0.010348359	0.255586018	0.828437428	TRAPPC8
ENSG00000167723.15	-0.028252551	0.255598186	0.828437428	TRPV3
ENSG00000163659.13	0.009812839	0.255598734	0.828437428	TIPARP
ENSG00000127399.15	0.016919054	0.255612753	0.828437428	LRRC61
ENSG00000096093.16	0.012023294	0.255718625	0.828437428	EFHC1
ENSG00000095319.14	-0.008886558	0.255742932	0.828437428	NUP188
ENSG00000237772.2	-0.018553689	0.25576049	0.828437428	LINC02631
ENSG00000265791.1	-0.024463036	0.255769668	0.828437428	CTD-2349P21.10
ENSG00000007402.12	-0.018747958	0.255798406	0.828437428	CACNA2D2
ENSG00000002016.18	-0.009856007	0.255851434	0.828437428	RAD52
ENSG00000179168.15	-0.032166192	0.255867109	0.828437428	GGN
ENSG00000122012.14	0.043693266	0.25596423	0.828621061	SV2C
ENSG00000185250.16	-0.038248924	0.256066131	0.828820111	PPIL6
ENSG00000280239.1	-0.013190127	0.25613223	0.828903229	CTB-50L17.8
ENSG00000100320.23	0.027778688	0.256309481	0.829004143	RBFOX2
ENSG00000221869.5	-0.018733562	0.256327215	0.829004143	CEBPD
ENSG00000101017.14	0.018873518	0.256406797	0.829004143	CD40
ENSG00000128039.13	-0.025439355	0.256411865	0.829004143	SRD5A3
ENSG00000234965.3	-0.052160981	0.256430518	0.829004143	SHISA8

ENSG00000134198.10	0.01873482	0.256503595	0.829004143	TSPAN2
ENSG00000258727.1	-0.011796109	0.25653635	0.829004143	RP11-66N24.3
ENSG00000113734.18	0.01248337	0.256537369	0.829004143	BNIP1
ENSG00000197312.12	0.007507015	0.256561584	0.829004143	DDI2
ENSG00000116584.20	-0.007046875	0.25658122	0.829004143	ARHGEF2
ENSG00000176160.11	-0.022577377	0.25660807	0.829004143	HSF5
ENSG00000274015.2	-0.027460907	0.256730568	0.82922475	CTD-2302E22.6
ENSG00000214391.3	0.037785375	0.256783895	0.82922475	TUBAP2
ENSG00000196388.8	0.011840245	0.256833245	0.82922475	INCA1
ENSG00000165480.16	-0.041248773	0.256896531	0.82922475	SKA3
ENSG00000270426.1	0.016349694	0.256900717	0.82922475	RP11-326I11.5
ENSG00000161204.11	-0.007209668	0.256928902	0.82922475	ABCF3
ENSG00000186010.19	0.012481606	0.256959396	0.82922475	NDUFA13
ENSG00000234506.5	0.022887587	0.257021082	0.829293321	LINC01506
ENSG00000276957.1	-0.035677918	0.25709479	0.829314954	RP11-29B2.6
ENSG00000285676.1	0.026256782	0.25714079	0.829314954	RP11-431N8.1
ENSG00000197951.10	-0.008898778	0.257149102	0.829314954	ZNF71
ENSG00000224041.3	-0.080555	0.257260533	0.829442361	IGKV3D-15
ENSG00000152056.17	0.019708468	0.257396869	0.829442361	AP1S3
ENSG00000177595.19	-0.012378079	0.257407445	0.829442361	PIDD1
ENSG00000235621.9	0.031368346	0.257435078	0.829442361	LINC00494
ENSG00000225791.7	0.016375913	0.257445344	0.829442361	TRAM2-AS1
ENSG00000023191.17	-0.011874589	0.257466972	0.829442361	RNH1
ENSG00000232470.1	0.015307371	0.257508013	0.829442361	RP11-313D6.3
ENSG00000267475.1	0.021332282	0.257512166	0.829442361	NUDT19-DT
ENSG00000233006.8	0.022705643	0.257624982	0.82967543	MIR3936HG
ENSG00000165061.15	-0.060233173	0.257697265	0.829675971	ZMAT4
ENSG00000196935.9	-0.024173058	0.257742481	0.829675971	SRGAP1
ENSG00000214783.9	-0.015483224	0.257746519	0.829675971	POLR2J4
ENSG00000088538.13	0.017008205	0.25787579	0.829863051	DOCK3
ENSG00000197694.18	-0.011961792	0.257926972	0.829863051	SPTAN1
ENSG00000116285.13	0.023208729	0.257962916	0.829863051	ERRFI1
ENSG00000144218.21	-0.02351609	0.257966498	0.829863051	AFF3
ENSG00000132254.13	0.007060647	0.258072042	0.829954013	ARFIP2
ENSG00000080031.10	-0.04573487	0.258190711	0.829954013	PTPRH
ENSG00000138964.17	-0.007670021	0.258249096	0.829954013	PARVG
ENSG00000144712.12	0.026492705	0.258254102	0.829954013	CAND2
ENSG00000187980.7	0.031940562	0.2582663	0.829954013	PLA2G2C
ENSG00000159592.11	0.004752155	0.258299874	0.829954013	GPBP1L1
ENSG00000244733.5	-0.031751111	0.258383017	0.829954013	RP11-506M13.3
ENSG00000075218.19	-0.025179581	0.25838636	0.829954013	GTSE1
ENSG00000196862.10	-0.049726975	0.25838736	0.829954013	RGPD4
ENSG00000136367.14	-0.029887394	0.258438798	0.829954013	ZFHX2
ENSG00000270673.1	0.038393945	0.258439942	0.829954013	YTHDF3-DT
ENSG00000063127.16	-0.0109651	0.258577275	0.83026503	SLC6A16
ENSG00000214954.8	0.019317992	0.258673059	0.830351848	LRRC69
ENSG00000174837.15	0.018966145	0.258738381	0.830351848	ADGRE1

ENSG00000204758.8	0.032970107	0.258794488	0.830351848	CTC-308K20.1
ENSG00000254999.4	0.010106231	0.258799501	0.830351848	BRK1
ENSG00000196323.14	-0.009100963	0.259051931	0.830351848	ZBTB44
ENSG00000142867.14	-0.007030808	0.259057001	0.830351848	BCL10
ENSG00000246922.9	-0.010073619	0.259085067	0.830351848	UBAP1L
ENSG00000269463.1	0.040485249	0.259186313	0.830351848	RP11-727F15.13
ENSG00000211644.3	-0.044390219	0.259216263	0.830351848	IGLV1-51
ENSG00000138031.15	0.010937376	0.259224225	0.830351848	ADCY3
ENSG00000103126.15	-0.008236498	0.259257083	0.830351848	AXIN1
ENSG00000081014.11	-0.009775047	0.259325788	0.830351848	AP4E1
ENSG00000179094.16	0.020938566	0.25935298	0.830351848	PER1
ENSG00000175564.13	-0.011019937	0.259357076	0.830351848	UCP3
ENSG00000092964.18	-0.012479913	0.259451743	0.830351848	DPYSL2
ENSG00000183682.8	0.021605152	0.25948357	0.830351848	BMP8A
ENSG00000103044.11	-0.014159665	0.25952021	0.830351848	HAS3
ENSG00000225523.2	0.074364718	0.259520376	0.830351848	IGKV6D-21
ENSG00000066135.13	-0.007561484	0.259555624	0.830351848	KDM4A
ENSG00000276953.1	-0.033731867	0.25955992	0.830351848	TRBV12-4
ENSG00000104889.7	-0.035067396	0.25971243	0.830351848	RNASEH2A
ENSG00000226210.3	-0.030456132	0.259736157	0.830351848	WASH8P
ENSG00000186470.14	-0.023495483	0.259791383	0.830351848	BTN3A2
ENSG00000137809.17	-0.038789737	0.259796536	0.830351848	ITGA11
ENSG00000207248.1	0.026549404	0.259933598	0.830351848	RNU6-1005P
ENSG00000278784.1	-0.011707453	0.259954816	0.830351848	RP11-468E2.11
ENSG00000076108.12	0.010093448	0.259961229	0.830351848	BAZ2A
ENSG00000279968.3	-0.041242209	0.260036367	0.830351848	CCDC28A-AS1
ENSG00000234705.2	-0.030407876	0.26013946	0.830351848	HMGA1P4
ENSG00000270765.7	-0.040767993	0.260237687	0.830351848	GAS2L2
ENSG00000055044.11	0.011473724	0.260279252	0.830351848	NOP58
ENSG00000035664.11	-0.013619044	0.26032055	0.830351848	DAPK2
ENSG00000010810.17	-0.006967348	0.260362867	0.830351848	FYN
ENSG00000269925.1	0.02032307	0.260385389	0.830351848	RP3-467L1.6
ENSG00000140265.13	-0.010085002	0.260396702	0.830351848	ZSCAN29
ENSG00000162076.13	-0.012103492	0.260408773	0.830351848	FLYWCH2
ENSG00000133318.14	0.008974053	0.260411063	0.830351848	RTN3
ENSG00000133065.11	-0.007688631	0.260464704	0.830351848	SLC41A1
ENSG00000211663.2	-0.040742817	0.260480886	0.830351848	IGLV3-19
ENSG00000276384.1	0.040109505	0.260594555	0.830351848	RP11-186B7.7
ENSG00000126790.12	-0.015660027	0.260621002	0.830351848	L3HYPDH
ENSG00000251023.1	0.01895519	0.260630788	0.830351848	RP11-549J18.1
ENSG00000147416.11	0.007690512	0.260644097	0.830351848	ATP6V1B2
ENSG00000169862.19	0.053932195	0.260646244	0.830351848	CTNND2
ENSG00000223697.3	0.034396476	0.260658185	0.830351848	AF230666.2
ENSG00000283525.1	-0.026106144	0.260739793	0.830351848	LLNLR-271C9.1
ENSG00000237854.3	0.017255879	0.260743649	0.830351848	LINC00674
ENSG00000169750.9	-0.023739452	0.260751371	0.830351848	RAC3
ENSG00000113838.13	0.007647367	0.260786988	0.830351848	TBCCD1

ENSG00000161016.18	-0.012735887	0.260822712	0.830351848	RPL8
ENSG00000286365.1	-0.039842138	0.260833582	0.830351848	KB-1440D3.21
ENSG00000185585.20	-0.027418655	0.260847568	0.830351848	OLFML2A
ENSG00000119685.20	0.009618796	0.260877694	0.830351848	TTLL5
ENSG00000131873.7	0.014245507	0.260908908	0.830351848	CHSY1
ENSG00000197536.11	0.013682383	0.260942802	0.830351848	IRF1-AS1
ENSG00000197168.13	0.025849771	0.260970228	0.830351848	NEK5
ENSG00000111707.12	0.005123981	0.261001109	0.830351848	SUDS3
ENSG00000104695.13	-0.008478872	0.26100196	0.830351848	PPP2CB
ENSG00000274943.1	-0.032023418	0.261004581	0.830351848	RP11-351C21.2
ENSG00000268575.1	0.020512393	0.261033663	0.830351848	RP1-283E3.8
ENSG00000275342.5	-0.014109108	0.261095066	0.830418364	PRAG1
ENSG00000036549.13	0.012494807	0.261138808	0.8304287	ZZZ3
ENSG00000261971.8	0.015595226	0.261190053	0.830444012	MMP25-AS1
ENSG00000100485.12	0.010939037	0.261242885	0.830444012	SOS2
ENSG00000286555.1	0.012224962	0.26126914	0.830444012	RP11-31L22.8
ENSG00000143412.10	-0.011765773	0.261308668	0.830444012	ANXA9
ENSG00000186132.15	0.021003897	0.261346092	0.830444012	C2orf76
ENSG00000172183.16	0.01477219	0.261591063	0.831093652	ISG20
ENSG00000286781.1	-0.043201125	0.261732761	0.831415035	RP11-256B12.1
ENSG00000254501.1	-0.038124594	0.261854262	0.831672172	AP003068.9
ENSG00000219200.12	-0.009997198	0.261920619	0.831681833	RNASEK
ENSG00000139540.12	-0.037239501	0.261938412	0.831681833	SLC39A5
ENSG00000225828.1	-0.011343173	0.262013183	0.831705398	FAM229A
ENSG00000172650.15	-0.032389522	0.262047977	0.831705398	AGAP5
ENSG00000189298.14	-0.008984297	0.262082162	0.831705398	ZKSCAN3
ENSG00000135070.15	0.01140153	0.262108055	0.831705398	ISCA1
ENSG00000184719.12	0.014090474	0.262190953	0.831839736	RNLS
ENSG00000154710.18	-0.015637489	0.262304549	0.831951252	RABGEF1
ENSG00000186583.12	-0.031469465	0.26232908	0.831951252	SPATC1
ENSG00000261471.1	0.01783443	0.262347803	0.831951252	RP11-61F12.1
ENSG00000116273.6	-0.008302101	0.262441936	0.831983907	PHF13
ENSG00000205710.4	-0.0268516	0.26244547	0.831983907	C17orf107
ENSG00000200204.1	0.042244913	0.262483037	0.831983907	RNU1-22P
ENSG00000281406.2	0.042952848	0.262520376	0.831983907	BLACAT1
ENSG00000163319.11	0.014266698	0.262722285	0.832216741	MRPS18C
ENSG00000198914.5	-0.16509122	0.262730653	0.832216741	POU3F3
ENSG00000223705.9	-0.01287203	0.26287088	0.832216741	NSUN5P1
ENSG00000102934.10	-0.013090028	0.262913575	0.832216741	PLLP
ENSG00000165071.15	0.00952233	0.262917426	0.832216741	TMEM71
ENSG00000173085.15	0.011894613	0.262959912	0.832216741	COQ2
ENSG00000150477.15	0.010218903	0.262992243	0.832216741	KIAA1328
ENSG00000135541.22	-0.023699828	0.263010225	0.832216741	AHI1
ENSG00000116922.15	-0.008002903	0.263027501	0.832216741	C1orf109
ENSG00000088876.13	0.008800737	0.263089377	0.832216741	ZNF343
ENSG00000277224.2	0.033278135	0.26316004	0.832216741	H2BC7
ENSG00000153551.14	0.009750865	0.263204732	0.832216741	CMTM7

ENSG00000114062.22	0.010072755	0.263256854	0.832216741	UBE3A
ENSG00000240403.5	-0.037167078	0.263282839	0.832216741	KIR3DL2
ENSG00000184385.2	-0.083767379	0.26329841	0.832216741	UMODL1-AS1
ENSG00000274011.1	-0.043479836	0.263299081	0.832216741	Metazoa_SRP
ENSG00000100280.17	-0.009490606	0.263332925	0.832216741	AP1B1
ENSG00000165219.22	0.007092813	0.263378976	0.832216741	GAPVD1
ENSG00000134690.11	-0.01467017	0.263397974	0.832216741	CDCA8
ENSG00000106133.18	0.029529921	0.263405445	0.832216741	NSUN5P2
ENSG00000250144.1	0.044466316	0.263570648	0.832610422	RP11-553P9.1
ENSG00000225411.3	-0.0249812	0.26364817	0.832722754	RP11-764K9.1
ENSG00000204152.14	0.016358203	0.263717808	0.832722754	TIMM23B
ENSG00000240038.7	-0.009271475	0.26379233	0.832722754	AMY2B
ENSG00000270149.5	-0.032220038	0.26380678	0.832722754	RP11-544M22.13
ENSG00000168385.18	0.008184426	0.263817949	0.832722754	SEPTIN2
ENSG00000012660.14	0.008561783	0.263936212	0.832722754	ELOVL5
ENSG00000262943.7	0.028573383	0.26394019	0.832722754	ALOX12P2
ENSG00000187569.3	0.068613408	0.263955715	0.832722754	DPPA3
ENSG00000185621.11	0.014662903	0.263971651	0.832722754	LMLN
ENSG00000164815.11	0.008177029	0.264067437	0.83281353	ORC5
ENSG00000106366.9	0.032420288	0.264099329	0.83281353	SERPINE1
ENSG00000146143.19	0.008499957	0.264144032	0.83281353	PRIM2
ENSG00000077420.16	-0.010882626	0.264162864	0.83281353	APBB1IP
ENSG00000188763.5	-0.051869974	0.264347837	0.833007147	FZD9
ENSG00000260793.2	0.011940056	0.264417472	0.833007147	RP5-882C2.2
ENSG00000126838.10	-0.027584421	0.264462969	0.833007147	PZP
ENSG00000115993.13	0.008567034	0.264470429	0.833007147	TRAK2
ENSG00000151690.15	-0.009877377	0.264530754	0.833007147	MFSD6
ENSG00000173933.21	0.005959701	0.264578131	0.833007147	RBM4
ENSG00000138674.17	-0.008642241	0.264581709	0.833007147	SEC31A
ENSG00000204852.17	0.013986732	0.264609111	0.833007147	TCTN1
ENSG00000162383.13	-0.041615346	0.264611973	0.833007147	SLC1A7
ENSG00000237641.1	0.03695256	0.264635918	0.833007147	RPL28P2
ENSG00000196369.11	-0.020554452	0.264691064	0.833007147	SRGAP2B
ENSG00000186298.12	0.006695572	0.264746621	0.833007147	PPP1CC
ENSG00000127191.18	-0.009096206	0.26482857	0.833007147	TRAF2
ENSG00000187498.16	0.048392346	0.264840365	0.833007147	COL4A1
ENSG00000181472.5	-0.006338106	0.26486391	0.833007147	ZBTB2
ENSG00000262777.1	-0.031693767	0.264905991	0.833007147	RP11-818O24.3
ENSG00000268670.1	0.022515575	0.26502088	0.833007147	CTD-2622I13.3
ENSG00000183615.6	0.029544692	0.265039733	0.833007147	FAM167B
ENSG00000196312.15	-0.009342412	0.265052079	0.833007147	MFSD14C
ENSG00000108106.14	-0.015777617	0.265137758	0.833007147	UBE2S
ENSG00000170049.10	-0.012434274	0.265145552	0.833007147	KCNAB3
ENSG00000238160.1	0.019270049	0.265155664	0.833007147	LINC02863
ENSG00000226312.7	-0.020057202	0.265160283	0.833007147	CFLAR-AS1
ENSG00000156017.13	-0.014127891	0.265295273	0.833007147	CARNMT1
ENSG00000211973.2	-0.062867845	0.265301251	0.833007147	IGHV1-69

ENSG00000146701.12	-0.009338453	0.265304459	0.833007147	MDH2
ENSG00000287817.1	-0.022394449	0.265320981	0.833007147	RP11-247I13.12
ENSG00000197794.2	-0.052478349	0.265407324	0.833071636	IGKV7-3
ENSG00000008311.16	-0.022446546	0.26550143	0.833071636	AASS
ENSG00000144821.10	-0.050672368	0.265520701	0.833071636	MYH15
ENSG00000111860.14	0.017461637	0.265559728	0.833071636	CEP85L
ENSG00000196743.9	0.019264668	0.265607442	0.833071636	GM2A
ENSG00000166833.22	-0.033838858	0.2656086	0.833071636	NAV2
ENSG00000162892.16	-0.015542787	0.265625874	0.833071636	IL24
ENSG00000272734.1	0.017933426	0.265739377	0.833300175	ADIRF-AS1
ENSG00000278831.1	-0.017866878	0.265858525	0.833483902	RP11-12J10.4
ENSG00000041515.16	-0.057253183	0.265879251	0.833483902	MYO16
ENSG00000249700.9	-0.037430105	0.265984051	0.833610475	SRD5A3-AS1
ENSG00000133742.14	0.053069608	0.266000924	0.833610475	CA1
ENSG00000272568.6	0.047972396	0.266053062	0.833646477	CTB-113D17.1
ENSG00000268758.7	-0.027058912	0.266162183	0.833817603	ADGRE4P
ENSG00000174175.17	0.02120402	0.266237408	0.833817603	SELP
ENSG00000105997.23	-0.047245727	0.266243828	0.833817603	HOXA3
ENSG00000115525.18	0.00890586	0.266270308	0.833817603	ST3GAL5
ENSG00000224358.1	0.04130235	0.266335224	0.833893553	TMCO1-AS1
ENSG00000155265.11	-0.013410659	0.26662278	0.834444901	GOLGA7B
ENSG00000143198.13	0.013479144	0.266623622	0.834444901	MGST3
ENSG00000143416.21	0.044797671	0.266633384	0.834444901	SELENBP1
ENSG00000287454.1	-0.035509605	0.26668818	0.834489045	RP11-394J1.3
ENSG00000108433.17	0.004432662	0.266785069	0.834528365	GOSR2
ENSG00000271122.1	0.01410126	0.266802067	0.834528365	HERPUD2-AS1
ENSG00000205702.11	-0.01658818	0.26687602	0.834528365	CYP2D7
ENSG00000103642.12	0.009924006	0.266897536	0.834528365	LACTB
ENSG00000256053.9	0.01158577	0.266916693	0.834528365	COA8
ENSG00000226706.1	0.021688296	0.26694883	0.834528365	RP11-426A6.5
ENSG00000007376.8	-0.011538356	0.267071075	0.834528365	RPUSD1
ENSG00000142583.18	-0.029892689	0.267112249	0.834528365	SLC2A5
ENSG00000100029.18	-0.00938113	0.267140907	0.834528365	PES1
ENSG00000072042.13	0.006082306	0.267157208	0.834528365	RDH11
ENSG00000280014.1	-0.027451177	0.267180104	0.834528365	AC006129.3
ENSG00000246790.2	0.026384037	0.267206452	0.834528365	RP11-730K11.1
ENSG00000178573.7	-0.013176779	0.267310868	0.834528365	MAF
ENSG00000174950.11	-0.080446241	0.267402187	0.834528365	CD164L2
ENSG00000132359.15	-0.00868424	0.267516035	0.834528365	RAP1GAP2
ENSG00000260378.1	-0.04115648	0.267519957	0.834528365	CTD-2583P5.1
ENSG00000237989.1	0.027765457	0.267600027	0.834528365	LINC01679
ENSG00000280157.1	0.0191345	0.267600616	0.834528365	RP11-520H14.7
ENSG00000242880.2	0.035203585	0.26770108	0.834528365	RP11-190P13.2
ENSG00000284669.1	0.030720867	0.267712062	0.834528365	RP11-331G2.8
ENSG00000139651.11	0.004735552	0.267773635	0.834528365	ZNF740
ENSG00000196586.16	-0.026387486	0.26784041	0.834528365	MYO6
ENSG00000196917.6	0.050994869	0.267844118	0.834528365	HCAR1

ENSG00000141298.19	0.010282233	0.267870433	0.834528365	SSH2
ENSG00000279246.1	-0.038911363	0.267920097	0.834528365	RP11-286N22.14
ENSG00000149534.9	0.043523567	0.267923833	0.834528365	MS4A2
ENSG00000274460.1	0.012839658	0.267952895	0.834528365	CTD-2649C14.2
ENSG00000140876.11	0.022700643	0.267958447	0.834528365	NUDT7
ENSG00000271971.1	-0.017151511	0.267965506	0.834528365	CTD-2006H14.2
ENSG00000233223.3	-0.014989336	0.267993707	0.834528365	AC113189.5
ENSG00000211788.2	-0.021675111	0.268024184	0.834528365	TRAV13-1
ENSG00000189403.15	0.012344638	0.268042268	0.834528365	HMGB1
ENSG00000125843.11	-0.008345282	0.268187532	0.834528365	AP5S1
ENSG00000132681.17	0.102035888	0.268254838	0.834528365	ATP1A4
ENSG00000162139.10	0.0058008	0.268256	0.834528365	NEU3
ENSG00000103249.18	-0.011489269	0.26826853	0.834528365	CLCN7
ENSG00000087191.13	-0.008300113	0.268290258	0.834528365	PSMC5
ENSG00000286694.1	-0.04724992	0.268292038	0.834528365	RP11-472N13.6
ENSG00000138459.9	0.008982721	0.268298016	0.834528365	SLC35A5
ENSG00000013375.16	0.009557697	0.268328459	0.834528365	PGM3
ENSG00000276334.1	-0.036198376	0.268459928	0.83458784	AL133243.1
ENSG00000095917.14	-0.094786043	0.268464434	0.83458784	TPSD1
ENSG00000197860.10	0.0110038	0.268469669	0.83458784	SGTB
ENSG00000196422.11	0.013946048	0.268626107	0.834857803	PPP1R26
ENSG00000133958.13	0.036044603	0.268637929	0.834857803	UNC79
ENSG00000162244.12	0.011537474	0.268775912	0.835043396	RPL29
ENSG00000108984.15	0.01194255	0.268808981	0.835043396	MAP2K6
ENSG00000183317.17	-0.059405458	0.268819802	0.835043396	EPHA10
ENSG00000188603.22	0.008505082	0.268890122	0.835135337	CLN3
ENSG00000215765.3	0.138622994	0.269042676	0.835273318	CTB-61M7.1
ENSG00000116819.9	-0.01033921	0.269073724	0.835273318	TFAP2E
ENSG00000164151.12	0.008079435	0.26908927	0.835273318	ICE1
ENSG00000163904.13	0.00517635	0.269117224	0.835273318	SENP2
ENSG00000283288.2	-0.037464399	0.269181756	0.835273318	SMIM33
ENSG00000114978.18	0.006193884	0.269184768	0.835273318	MOB1A
ENSG00000186660.15	0.005489721	0.26925577	0.835273318	ZFP91
ENSG00000101365.21	-0.008118794	0.269297222	0.835273318	IDH3B
ENSG00000182853.12	-0.041698094	0.269335054	0.835273318	VMO1
ENSG00000122873.12	0.015067851	0.269367588	0.835273318	CISD1
ENSG00000280211.1	-0.017644307	0.269419899	0.835273318	RP11-2C24.3
ENSG00000255730.5	-0.033244644	0.269423298	0.835273318	CTC-435M10.3
ENSG00000266783.1	0.033120402	0.269533382	0.8354883	RP11-715F3.2
ENSG00000164603.12	0.013141423	0.269635779	0.835515318	BMT2
ENSG00000260259.1	-0.039622107	0.269639189	0.835515318	LINC02166
ENSG00000156860.16	-0.009185844	0.269746065	0.835515318	FBRS
ENSG00000134480.16	0.00731055	0.269747745	0.835515318	CCNH
ENSG00000229519.2	-0.035275338	0.26976911	0.835515318	RP11-58A11.2
ENSG00000121621.7	-0.026410958	0.269874831	0.835515318	KIF18A
ENSG00000162873.15	0.038581124	0.269878194	0.835515318	KLHDC8A
ENSG00000104907.13	-0.008714597	0.269885514	0.835515318	TRMT1

ENSG00000186115.13	-0.057460062	0.269910524	0.835515318	CYP4F2
ENSG00000172992.12	0.009357068	0.269949507	0.835515318	DCAKD
ENSG00000269934.3	0.015552759	0.270151148	0.835836059	RP5-1139B12.2
ENSG00000251867.4	0.02337611	0.270169409	0.835836059	RP11-48B3.5
ENSG00000013016.16	0.016441874	0.270175406	0.835836059	EHD3
ENSG00000112029.10	-0.013998963	0.270313763	0.835890824	FBXO5
ENSG00000139116.19	-0.02050459	0.270322838	0.835890824	KIF21A
ENSG00000278662.5	-0.052962724	0.270421141	0.835890824	GOLGA6L10
ENSG00000148835.11	-0.007595778	0.270432067	0.835890824	TAF5
ENSG00000054116.12	0.006830983	0.270433907	0.835890824	TRAPPC3
ENSG00000287377.1	-0.025351086	0.270437664	0.835890824	RP11-415F23.5
ENSG00000106780.9	0.011382252	0.270483252	0.835905747	MEGF9
ENSG00000277678.1	-0.026305455	0.270553491	0.835996835	RNU1-153P
ENSG00000260097.3	0.025108864	0.270619737	0.836005282	SPDYE6
ENSG00000266017.1	-0.028350993	0.270641919	0.836005282	MIR4477B
ENSG00000271553.1	-0.019439047	0.270710536	0.836005282	RP11-274B21.10
ENSG00000119042.17	-0.031695349	0.270719284	0.836005282	SATB2
ENSG00000272540.1	0.036923928	0.271004345	0.836715192	XXbac-BPG252P9.9
ENSG00000184574.10	-0.011577907	0.27103077	0.836715192	LPAR5
ENSG00000160117.16	-0.014491647	0.271072515	0.836718111	ANKLE1
ENSG00000180938.6	0.02013273	0.271187605	0.836798817	ZNF572
ENSG00000172987.13	-0.066222465	0.271217151	0.836798817	HPSE2
ENSG00000198142.5	-0.019593158	0.271221162	0.836798817	SOWAHC
ENSG00000112473.18	-0.00747457	0.271261875	0.836798817	SLC39A7
ENSG00000162511.8	0.007280028	0.271327292	0.83684877	LAPTM5
ENSG00000279536.1	-0.037914498	0.27135968	0.83684877	RP11-445N20.2
ENSG00000240303.8	0.025220649	0.271501484	0.837083579	ACAD11
ENSG00000121570.12	0.02938232	0.271518561	0.837083579	DPPA4
ENSG00000260086.3	0.022550349	0.271615471	0.837083579	RP11-42I10.1
ENSG00000144136.11	-0.00877487	0.271635311	0.837083579	SLC20A1
ENSG00000177917.11	-0.010804484	0.271639907	0.837083579	ARL6IP6
ENSG00000015532.10	-0.007572723	0.271708303	0.837168553	XYLT2
ENSG00000161905.13	-0.042308558	0.271773956	0.837245048	ALOX15
ENSG00000269489.1	-0.036420287	0.271879076	0.837389912	RP11-98D18.17
ENSG00000204264.12	0.011835796	0.271967099	0.837389912	PSMB8
ENSG00000167613.16	-0.009005203	0.271982031	0.837389912	LAIR1
ENSG00000225361.4	0.029737363	0.27210233	0.837389912	PPP1R26-AS1
ENSG00000144746.7	0.007421109	0.272116665	0.837389912	ARL6IP5
ENSG00000235138.1	0.034793551	0.272118497	0.837389912	RP11-374P20.4
ENSG00000124226.11	0.005570452	0.272142748	0.837389912	RNF114
ENSG00000285668.1	-0.073115139	0.27224794	0.837389912	RP11-707O23.6
ENSG00000259319.1	0.01968419	0.272289091	0.837389912	RP11-293M10.6
ENSG00000205339.10	0.009343726	0.272297759	0.837389912	IPO7
ENSG00000223725.6	0.031180734	0.272312005	0.837389912	AC007879.5
ENSG00000248516.1	0.044256225	0.272320567	0.837389912	RP11-265O12.1
ENSG00000268201.1	-0.031839597	0.272351927	0.837389912	CTD-3138B18.6
ENSG00000135976.20	-0.021695738	0.272392632	0.837389912	ANKRD36

ENSG00000206567.10	-0.012693808	0.272698208	0.83776413	AC022007.5
ENSG00000284024.4	0.007607273	0.272706014	0.83776413	RP11-398C13.8
ENSG00000116977.19	0.007811718	0.272706045	0.83776413	LGALS8
ENSG00000152082.15	-0.018467456	0.272713716	0.83776413	MZT2B
ENSG00000135828.11	0.00850309	0.272718614	0.83776413	RNASEL
ENSG00000285672.1	0.037740936	0.272835857	0.83797618	RP11-569D9.7
ENSG00000137968.16	0.086540944	0.272869365	0.83797618	SLC44A5
ENSG00000135709.13	0.009580389	0.272972798	0.83816831	KIAA0513
ENSG00000185664.15	-0.022214693	0.273033906	0.838230442	PMEL
ENSG00000187736.13	-0.010628386	0.273190929	0.838497397	NHEJ1
ENSG00000197451.12	-0.006834997	0.273223414	0.838497397	HNRNPAB
ENSG00000130255.13	0.013818168	0.273248702	0.838497397	RPL36
ENSG00000156858.12	-0.008835463	0.273284406	0.838497397	PRR14
ENSG00000189134.4	0.034031469	0.273407871	0.838643843	NKAPL
ENSG00000131686.15	-0.034114596	0.273413923	0.838643843	CA6
ENSG00000236165.1	-0.037732457	0.273470047	0.838690554	PRADC1P1
ENSG00000260778.5	-0.017662565	0.273661048	0.838824597	RP11-304L19.13
ENSG00000279933.1	-0.016484389	0.273661841	0.838824597	RP4-671O14.5
ENSG00000100246.13	0.008466454	0.273675622	0.838824597	DNAL4
ENSG00000165632.8	-0.008865197	0.273677364	0.838824597	TAF3
ENSG00000287823.1	0.029397171	0.273763853	0.838960538	RP11-216B13.1
ENSG00000175354.20	0.008425973	0.27393076	0.838960538	PTPN2
ENSG00000004838.14	-0.015066897	0.273938387	0.838960538	ZMYND10
ENSG00000187607.16	-0.011202736	0.273944383	0.838960538	ZNF286A
ENSG00000178404.10	-0.025115837	0.273987097	0.838960538	CEP295NL
ENSG00000284719.1	-0.019261741	0.274012037	0.838960538	RP1-118J21.27
ENSG00000166664.14	0.043166797	0.274028116	0.838960538	CHRFAM7A
ENSG00000273975.1	-0.040923544	0.274048988	0.838960538	Metazoa_SRP
ENSG00000196352.16	0.014441425	0.274154197	0.839031576	CD55
ENSG00000145423.5	0.04296465	0.274155025	0.839031576	SFRP2
ENSG00000171403.10	-0.159032525	0.274195633	0.839031576	KRT9
ENSG00000072121.17	0.008445235	0.274366491	0.839031576	ZFYVE26
ENSG00000219665.8	-0.012815281	0.274544472	0.839031576	ZNF433-AS1
ENSG00000227632.2	0.073359812	0.274611954	0.839031576	AC018804.6
ENSG00000130340.17	0.010983784	0.27461445	0.839031576	SNX9
ENSG00000124657.1	-0.033604536	0.274639475	0.839031576	OR2B6
ENSG00000181652.19	0.018517509	0.274706915	0.839031576	ATG9B
ENSG00000184226.15	-0.037220527	0.274886647	0.839031576	PCDH9
ENSG00000286292.1	-0.02254868	0.274909937	0.839031576	RP11-326I11.7
ENSG00000101282.9	-0.044840348	0.274922018	0.839031576	RSPO4
ENSG00000169507.9	-0.040120957	0.274986827	0.839031576	SLC38A11
ENSG00000067601.8	-0.019542438	0.274997137	0.839031576	PMS2P4
ENSG00000197958.13	0.011824737	0.275044454	0.839031576	RPL12
ENSG00000248099.4	-0.023749233	0.275052301	0.839031576	INSL3
ENSG00000090659.18	0.029763279	0.275072529	0.839031576	CD209
ENSG00000229122.1	0.021566189	0.275094246	0.839031576	AGBL5-IT1
ENSG00000196301.3	-0.062093081	0.275107379	0.839031576	HLA-DRB9

ENSG00000083720.13	0.008524581	0.275133695	0.839031576	OXCT1
ENSG00000117859.19	0.006242323	0.275152863	0.839031576	OSBPL9
ENSG00000133114.18	0.008581173	0.275194507	0.839031576	GPALPP1
ENSG00000186951.16	0.010041237	0.275196114	0.839031576	PPARA
ENSG00000101342.10	-0.014191627	0.275213468	0.839031576	TLDC2
ENSG00000113851.15	0.012952324	0.275217066	0.839031576	CRBN
ENSG00000112232.10	-0.035685858	0.275242305	0.839031576	KHDRBS2
ENSG00000185238.13	-0.009520441	0.275286129	0.839031576	PRMT3
ENSG00000237765.8	0.011056225	0.275339364	0.839031576	FAM200B
ENSG00000091262.17	-0.015421143	0.275429988	0.839031576	ABCC6
ENSG00000125869.10	0.025407817	0.275430595	0.839031576	LAMP5
ENSG00000273325.1	0.067820828	0.275440399	0.839031576	RP1-90G24.11
ENSG00000234287.1	0.034355845	0.27545899	0.839031576	RP11-761N21.2
ENSG00000204525.17	-0.015836759	0.275459216	0.839031576	HLA-C
ENSG00000136111.14	0.016313034	0.275481053	0.839031576	TBC1D4
ENSG00000108465.15	-0.009019069	0.275520416	0.839031576	CDK5RAP3
ENSG00000134489.7	0.028573545	0.275545039	0.839031576	HRH4
ENSG00000142655.13	-0.008972931	0.275588246	0.839038561	PEX14
ENSG00000122884.13	0.009212911	0.27573565	0.839362731	P4HA1
ENSG00000136877.15	-0.009014324	0.275804077	0.839446426	FPGS
ENSG00000121274.14	-0.011863364	0.275961649	0.839673147	TENT4B
ENSG00000164951.16	0.013956684	0.275965264	0.839673147	PDP1
ENSG00000218426.5	0.01488489	0.276001399	0.839673147	RP11-475C16.1
ENSG00000255893.1	0.04958008	0.276067086	0.839748412	RP11-685N10.1
ENSG00000142166.13	0.008632213	0.276205162	0.839789703	IFNAR1
ENSG00000280383.1	-0.02465116	0.276224515	0.839789703	CTA-941F9.10
ENSG00000237111.1	0.031106427	0.276232586	0.839789703	IGHJ3P
ENSG00000171448.9	0.013631074	0.276307124	0.839789703	ZBTB26
ENSG00000204713.11	0.007465863	0.27632249	0.839789703	TRIM27
ENSG00000139211.7	-0.010538976	0.276343627	0.839789703	AMIGO2
ENSG00000233830.2	0.024285919	0.276367306	0.839789703	EIF4HP1
ENSG00000112679.15	0.007185694	0.276490648	0.839878969	DUSP22
ENSG00000204420.10	0.02820101	0.276597965	0.839878969	MPIG6B
ENSG00000271993.1	0.022400082	0.276608133	0.839878969	RP11-285J16.1
ENSG00000253710.5	0.014563083	0.276676675	0.839878969	ALG11
ENSG00000115042.10	0.007956023	0.276698678	0.839878969	FAHD2A
ENSG00000105707.15	-0.035743926	0.276737105	0.839878969	HPN
ENSG00000184619.8	0.011714967	0.276753888	0.839878969	KRBA2
ENSG00000273080.1	0.037644448	0.276767663	0.839878969	RP11-301O19.1
ENSG00000272173.1	-0.025921213	0.276802924	0.839878969	U47924.31
ENSG00000102580.15	0.009685249	0.276884451	0.839878969	DNAJC3
ENSG00000099624.8	-0.015012574	0.276917448	0.839878969	ATP5F1D
ENSG00000221962.5	-0.051597579	0.276935391	0.839878969	TMEM14EP
ENSG00000166140.17	-0.007726921	0.277025878	0.839878969	ZFYVE19
ENSG00000100298.16	-0.023502761	0.277036334	0.839878969	APOBEC3H
ENSG00000168676.11	-0.03263959	0.277040951	0.839878969	KCTD19
ENSG00000197712.12	0.015152589	0.277074226	0.839878969	FAM114A1

ENSG00000164087.8	0.015298706	0.277092896	0.839878969	POC1A
ENSG00000134864.11	0.01152034	0.277233849	0.840038002	GGACT
ENSG00000120963.12	0.00614331	0.27725403	0.840038002	ZNF706
ENSG00000277299.1	0.023075393	0.277268248	0.840038002	RP11-837J7.4
ENSG00000182325.11	-0.009397027	0.277341559	0.840046067	FBXL6
ENSG00000172197.11	-0.007468953	0.277417293	0.840046067	MBOAT1
ENSG00000132952.12	0.007670428	0.277424012	0.840046067	USPL1
ENSG00000142208.18	-0.007339155	0.277434758	0.840046067	AKT1
ENSG00000132153.15	-0.006781093	0.27751644	0.840122749	DHX30
ENSG00000231503.4	0.028648974	0.277542014	0.840122749	PTMAP4
ENSG00000217644.5	-0.028305684	0.277653936	0.840337502	RP4-803A2.1
ENSG00000133706.19	-0.006587743	0.277801921	0.840511534	LARS1
ENSG00000174844.14	-0.038228975	0.277840008	0.840511534	DNAH12
ENSG00000256238.1	0.033046456	0.277845413	0.840511534	SUPT16HP1
ENSG00000184489.13	-0.017325239	0.277875375	0.840511534	PTP4A3
ENSG00000079459.13	0.008313496	0.27795216	0.840619806	FDFT1
ENSG00000196208.14	0.033123209	0.2780315	0.840625571	GREB1
ENSG00000170469.11	-0.015173582	0.278051519	0.840625571	SPATA24
ENSG00000281469.1	-0.029078154	0.278077037	0.840625571	RP11-567F11.1
ENSG00000234076.1	0.03586275	0.278241985	0.840842115	TPRG1-AS1
ENSG00000042429.12	0.00659649	0.278248588	0.840842115	MED17
ENSG00000163885.12	-0.027766007	0.278271671	0.840842115	CFAP100
ENSG00000144524.18	-0.005992078	0.278392475	0.841083216	COPS7B
ENSG00000198912.12	-0.0072758	0.278455401	0.841149413	C1orf174
ENSG00000124491.16	0.020384083	0.278531285	0.841254725	F13A1
ENSG00000114956.20	0.009041253	0.278657038	0.841482304	DGUOK
ENSG00000268471.6	-0.014297507	0.278704699	0.841482304	MIR4453HG
ENSG00000184371.14	-0.016380803	0.278771656	0.841482304	CSF1
ENSG00000213995.12	-0.006424206	0.278787298	0.841482304	NAXD
ENSG00000238105.7	0.011004301	0.278811793	0.841482304	GOLGA2P5
ENSG00000274717.1	0.02516914	0.278902604	0.841632518	RP1-47A17.1
ENSG00000055118.17	-0.023177091	0.279020816	0.841697277	KCNH2
ENSG00000238164.6	-0.013398292	0.279032931	0.841697277	TNFRSF14-AS1
ENSG00000182899.17	0.01519389	0.279047191	0.841697277	RPL35A
ENSG00000197375.13	0.013590278	0.279138343	0.841784182	SLC22A5
ENSG00000177335.11	-0.038938612	0.279158095	0.841784182	C8orf31
ENSG00000116679.16	0.008880221	0.279206537	0.841806477	IVNS1ABP
ENSG00000167779.9	-0.032479591	0.279273034	0.841883197	IGFBP6
ENSG00000112796.10	-0.022605786	0.279345389	0.841962817	ENPP5
ENSG00000270580.5	-0.025828199	0.279381557	0.841962817	PKD1P6-NPIPP1
ENSG00000155957.18	0.015060084	0.279598633	0.842262679	TMBIM4
ENSG00000198589.14	-0.010549632	0.279737561	0.842262679	LRBA
ENSG00000008256.16	-0.011895139	0.279760496	0.842262679	CYTH3
ENSG00000163344.6	-0.011520209	0.27976678	0.842262679	PMVK
ENSG00000184898.7	-0.014221471	0.27979224	0.842262679	RBM43
ENSG00000203907.10	-0.058489091	0.279851947	0.842262679	OOEP
ENSG00000248385.8	0.046463212	0.279859585	0.842262679	TARM1

ENSG00000287356.1	-0.024518291	0.279884415	0.842262679	RP11-181G12.8
ENSG00000167323.12	-0.006903146	0.27988446	0.842262679	STIM1
ENSG00000211597.2	-0.030917199	0.279899111	0.842262679	IGKJ1
ENSG00000095002.15	0.01190588	0.280014049	0.842262679	MSH2
ENSG00000213741.11	0.02184451	0.280025537	0.842262679	RPS29
ENSG00000240877.3	-0.039406156	0.280043821	0.842262679	RN7SL521P
ENSG00000254469.9	-0.014487204	0.280056037	0.842262679	RP11-849H4.2
ENSG00000123124.14	0.009681724	0.280214462	0.842600513	WWP1
ENSG00000257221.4	0.022614685	0.280250541	0.842600513	RP11-689B22.2
ENSG00000136694.9	-0.047591999	0.280311781	0.842661097	IL36A
ENSG00000268034.1	0.031802308	0.280434613	0.842906792	AC005795.1
ENSG00000163888.4	0.049600889	0.280483969	0.8429316	CAMK2N2
ENSG00000250645.1	-0.151305637	0.280542574	0.842984193	CTD-2228K2.1
ENSG00000221949.6	-0.02821665	0.28063536	0.843124469	LINC01465
ENSG00000104131.13	0.00703045	0.280714505	0.843124469	EIF3J
ENSG00000147852.17	-0.030798169	0.280757681	0.843124469	VLDLR
ENSG00000136052.10	-0.020642728	0.280817245	0.843124469	SLC41A2
ENSG00000236770.1	0.071444811	0.280913358	0.843124469	AC079325.5
ENSG00000110925.7	0.005255745	0.280941262	0.843124469	CSRNP2
ENSG00000205786.8	-0.025868382	0.280999931	0.843124469	LINC01531
ENSG00000197808.12	-0.010833561	0.281002123	0.843124469	ZNF461
ENSG00000131969.15	-0.018381982	0.281124302	0.843124469	ABHD12B
ENSG00000265743.1	0.020622189	0.281139713	0.843124469	RP11-848P1.3
ENSG00000286256.2	0.025713882	0.281150211	0.843124469	RP11-701P16.6
ENSG00000254703.2	0.015036511	0.281190181	0.843124469	SENCR
ENSG00000028203.18	0.009746168	0.281224484	0.843124469	VEZT
ENSG00000256312.1	-0.038569478	0.281358383	0.843124469	RP13-977J11.8
ENSG00000181126.13	0.069619646	0.281395688	0.843124469	HLA-V
ENSG00000214797.3	-0.030329492	0.281425089	0.843124469	RP11-1036E20.9
ENSG00000287868.1	0.025892705	0.281443137	0.843124469	RP11-392A14.13
ENSG00000129691.16	0.005046311	0.281451054	0.843124469	ASH2L
ENSG00000287037.1	-0.046736559	0.281451438	0.843124469	RP11-19B4.2
ENSG00000076201.16	-0.009895827	0.281462254	0.843124469	PTPN23
ENSG00000258424.1	-0.016771361	0.281555096	0.843124469	RP11-471B22.2
ENSG00000151135.10	-0.012301875	0.281569565	0.843124469	TMEM263
ENSG00000129993.15	-0.01135074	0.28163822	0.843124469	CBFA2T3
ENSG00000278238.1	0.042087764	0.281673716	0.843124469	RP11-245D16.4
ENSG00000272843.1	-0.039126794	0.281716714	0.843124469	RP11-313P13.5
ENSG00000169962.5	-0.02098788	0.281722205	0.843124469	TAS1R3
ENSG00000198792.13	-0.008911662	0.281724759	0.843124469	TMEM184B
ENSG00000162804.14	0.016801364	0.281748797	0.843124469	SNED1
ENSG00000114670.14	0.019741778	0.281819655	0.843124469	NEK11
ENSG00000109670.16	0.008381364	0.281831319	0.843124469	FBXW7
ENSG00000127948.16	-0.01084749	0.281863729	0.843124469	POR
ENSG00000152503.10	0.022489619	0.281930182	0.84320026	TRIM36
ENSG00000137101.13	-0.024944891	0.282038943	0.843252767	CD72
ENSG00000221970.4	-0.047811324	0.282087114	0.843252767	OR2A1

ENSG00000162928.9	-0.008333864	0.282100855	0.843252767	PEX13
ENSG00000140379.9	0.028160827	0.282146721	0.843252767	BCL2A1
ENSG00000279942.1	0.055124915	0.282162707	0.843252767	RP1-153P14.7
ENSG00000226332.2	-0.013952985	0.282241935	0.843252767	RP11-157P1.4
ENSG00000142233.14	-0.024127443	0.282296946	0.843252767	NTN5
ENSG00000140199.13	0.008386306	0.28231416	0.843252767	SLC12A6
ENSG00000178460.18	0.02160907	0.28250435	0.843252767	MCMDC2
ENSG00000131504.17	-0.010140629	0.282599798	0.843252767	DIAPH1
ENSG00000196313.12	-0.009745519	0.28263773	0.843252767	POM121
ENSG00000164053.22	-0.007438331	0.282643735	0.843252767	ATRIP
ENSG00000203757.2	0.058313265	0.282684278	0.843252767	OR6K3
ENSG00000106070.20	0.017061984	0.28278043	0.843252767	GRB10
ENSG00000118412.13	0.016076498	0.282781845	0.843252767	CASP8AP2
ENSG00000202538.1	-0.128898002	0.282786141	0.843252767	RNU4-2
ENSG00000119650.13	0.01163538	0.282787896	0.843252767	IFT43
ENSG00000151963.4	0.063759523	0.282800226	0.843252767	ZNF37CP
ENSG00000123454.12	0.02868854	0.282800605	0.843252767	DBH
ENSG00000279689.1	-0.012193862	0.2828075	0.843252767	RP11-574K11.32
ENSG00000122417.15	-0.012264766	0.282811221	0.843252767	ODF2L
ENSG00000070388.11	-0.033204382	0.282964017	0.843585704	FGF22
ENSG00000198331.11	0.009496274	0.283105726	0.843885498	HYLS1
ENSG00000133961.21	0.008298075	0.283166412	0.843943727	NUMB
ENSG00000164236.12	0.014393413	0.2832855	0.844050052	ANKRD33B
ENSG00000154079.6	0.013182437	0.28330882	0.844050052	SDHAF4
ENSG00000175376.9	-0.004948882	0.283358504	0.844050052	EIF1AD
ENSG00000148339.13	0.007879644	0.28340758	0.844050052	SLC25A25
ENSG00000106018.14	-0.033801071	0.283407873	0.844050052	VIPR2
ENSG00000105698.16	-0.008111813	0.283473306	0.844104535	USF2
ENSG00000234409.7	-0.021028485	0.283677781	0.844104535	CCDC188
ENSG00000245466.1	-0.03719504	0.28367855	0.844104535	CTD-2540L5.6
ENSG00000269439.6	-0.013362577	0.283682141	0.844104535	PGLS-DT
ENSG00000138346.15	-0.01677587	0.283706754	0.844104535	DNA2
ENSG00000148384.14	-0.007651197	0.283722972	0.844104535	INPP5E
ENSG00000137078.9	-0.011944468	0.283732049	0.844104535	SIT1
ENSG00000148803.12	0.015296516	0.283755445	0.844104535	FUOM
ENSG00000266983.1	-0.036720871	0.283822868	0.844141231	RANBP3-DT
ENSG00000254893.6	0.022987158	0.283913094	0.844141231	RAP1BL
ENSG00000137497.18	-0.008958597	0.28393956	0.844141231	NUMA1
ENSG00000126070.20	-0.008909343	0.284042212	0.844141231	AGO3
ENSG00000108511.10	0.080118589	0.284108875	0.844141231	HOXB6
ENSG00000204657.4	-0.044614662	0.284143363	0.844141231	OR2H2
ENSG00000261499.2	-0.072747237	0.284158332	0.844141231	CH17-260O16.1
ENSG00000205352.11	0.009754291	0.284218765	0.844141231	PRR13
ENSG00000253831.3	0.043777733	0.28426998	0.844141231	ETV3L
ENSG00000180304.14	0.007845796	0.284302313	0.844141231	OAZ2
ENSG00000267046.1	-0.025601032	0.284314325	0.844141231	E2F3P1
ENSG00000008382.16	-0.010379203	0.284372859	0.844141231	MPND

ENSG00000100034.14	0.010045485	0.284483845	0.844141231	PPM1F
ENSG00000177542.11	-0.009269156	0.28462443	0.844141231	SLC25A22
ENSG00000259294.1	0.047992487	0.284626194	0.844141231	CTA-339C12.1
ENSG00000143952.20	0.009713481	0.284637976	0.844141231	VPS54
ENSG00000011021.23	0.008604051	0.284640856	0.844141231	CLCN6
ENSG00000198216.12	0.021380126	0.284648958	0.844141231	CACNA1E
ENSG00000215271.9	-0.006817951	0.284673645	0.844141231	HOMEZ
ENSG00000130940.15	-0.012352384	0.28468108	0.844141231	CASZ1
ENSG00000115073.8	0.005303529	0.284694866	0.844141231	ACTR1B
ENSG00000104381.14	0.013817904	0.28470584	0.844141231	GDAP1
ENSG00000157014.11	-0.007857388	0.284714497	0.844141231	TATDN2
ENSG00000228446.2	0.045036095	0.284774001	0.844150035	ANKRD49P1
ENSG00000172081.14	-0.009746682	0.28483949	0.844150035	MOB3A
ENSG00000174500.13	0.014483094	0.284840952	0.844150035	GCSAM
ENSG00000173801.17	-0.041151739	0.285002465	0.844506654	JUP
ENSG00000104835.15	0.013254615	0.285086085	0.844602356	SARS2
ENSG00000232216.1	-0.04401006	0.28511713	0.844602356	IGHV3-43
ENSG00000109381.20	0.007881096	0.285177475	0.844650367	ELF2
ENSG00000160408.16	-0.016802225	0.285215711	0.844650367	ST6GALNAC6
ENSG00000006555.11	-0.011341767	0.285361932	0.844961378	TTC22
ENSG00000154914.17	0.031992763	0.285460286	0.844991524	USP43
ENSG00000261170.2	-0.027124257	0.285494582	0.844991524	RP11-252A24.5
ENSG00000245667.2	-0.020097574	0.28551937	0.844991524	RP5-940J5.8
ENSG00000163002.13	0.012815821	0.285536925	0.844991524	NUP35
ENSG00000013275.8	-0.008239995	0.285601402	0.845031117	PSMC4
ENSG00000146674.16	-0.025123702	0.285632714	0.845031117	IGFBP3
ENSG00000214812.3	-0.037193635	0.285729486	0.845195485	RP5-1053E7.3
ENSG00000115694.15	-0.005928589	0.285836905	0.845275492	STK25
ENSG00000260698.1	0.015410431	0.285838967	0.845275492	RP11-439E19.9
ENSG00000187446.12	0.007366099	0.286085428	0.845765042	CHP1
ENSG00000186162.10	0.007927087	0.286107955	0.845765042	CIDECP1
ENSG00000103855.18	0.05653953	0.286128236	0.845765042	CD276
ENSG00000255552.7	0.034278292	0.286221399	0.845784353	LY6G6E
ENSG00000248569.1	-0.025881171	0.286309634	0.845784353	CTC-512J14.5
ENSG00000095066.12	-0.00985596	0.286349441	0.845784353	HOOK2
ENSG00000213326.4	0.041884344	0.286375489	0.845784353	RPS7P11
ENSG00000105325.15	-0.007399249	0.286410456	0.845784353	FZR1
ENSG00000249141.1	0.03445328	0.286412176	0.845784353	RP11-514O12.4
ENSG00000063046.18	0.006550166	0.286435122	0.845784353	EIF4B
ENSG00000135119.15	-0.019857785	0.286474754	0.845784353	RNFT2
ENSG00000119979.18	0.007369568	0.286505944	0.845784353	DENND10
ENSG00000250569.1	0.030982289	0.286594542	0.845834984	NTAN1P2
ENSG00000261308.4	-0.02520178	0.286605584	0.845834984	FIGNL2
ENSG00000272562.2	-0.021383892	0.286660093	0.845874127	H3-3A-DT
ENSG00000211661.2	0.086946367	0.286703062	0.845879211	IGLV3-22
ENSG00000224144.1	-0.054957502	0.286894244	0.846082655	ASH2LP1
ENSG00000204959.4	-0.033799917	0.286981019	0.846082655	ARHGEF34P

ENSG00000259891.1	-0.012261025	0.286981328	0.846082655	CTA-204B4.2
ENSG00000249936.3	-0.015036472	0.28700603	0.846082655	RAC1P2
ENSG00000139597.18	0.007706023	0.287008105	0.846082655	N4BP2L1
ENSG00000080200.10	0.018785491	0.287060801	0.846082655	CRYBG3
ENSG00000267221.3	0.018156115	0.287126364	0.846082655	C17orf113
ENSG00000234742.1	-0.036546248	0.28713657	0.846082655	AC144530.1
ENSG00000213277.3	-0.040537774	0.287143323	0.846082655	MARCKSL1P1
ENSG00000162688.17	-0.014377165	0.287236674	0.84612873	AGL
ENSG00000247796.3	-0.019599967	0.287241477	0.84612873	CTD-2366F13.1
ENSG00000225975.7	0.015042325	0.287437202	0.846500254	LINC01534
ENSG00000132825.7	0.007755685	0.287450155	0.846500254	PPP1R3D
ENSG00000126882.13	-0.005780672	0.287504737	0.846506357	FAM78A
ENSG00000167984.18	-0.008189566	0.287593982	0.846506357	NLRC3
ENSG00000225460.1	0.02325667	0.287613451	0.846506357	RP13-93L13.1
ENSG00000185875.13	0.012869504	0.287617335	0.846506357	THNSL1
ENSG00000183856.11	-0.044673744	0.287746958	0.846766338	IQGAP3
ENSG00000148688.14	0.006925829	0.287862015	0.846834073	RPP30
ENSG00000272432.1	-0.035142357	0.287873505	0.846834073	RP3-465N24.6
ENSG00000122966.17	-0.020525174	0.287918238	0.846834073	CIT
ENSG00000234147.2	0.073062255	0.287935147	0.846834073	RP11-515I12.1
ENSG00000259792.1	0.032559087	0.288039653	0.846938688	RP11-114H24.6
ENSG00000167642.13	-0.011566471	0.288053313	0.846938688	SPINT2
ENSG00000234282.1	0.037292439	0.288151889	0.847085797	RP4-673D20.4
ENSG00000198824.7	-0.009711285	0.288209337	0.847085797	CHAMP1
ENSG00000164821.5	0.01741535	0.288227262	0.847085797	DEFA4
ENSG00000260007.3	-0.021002571	0.288395822	0.847269682	RP11-315D16.2
ENSG00000113048.17	0.005296573	0.288484296	0.847269682	MRPS27
ENSG00000261812.6	0.105476674	0.288497835	0.847269682	TUBB8P7
ENSG00000132792.19	-0.008309739	0.288632348	0.847269682	CTNNBL1
ENSG00000237190.4	0.009500241	0.288654553	0.847269682	CDKN2AIPNL
ENSG00000257446.4	-0.027276753	0.288677959	0.847269682	ZNF878
ENSG00000174945.14	0.028210569	0.288685127	0.847269682	AMZ1
ENSG00000286132.1	0.041182989	0.288765922	0.847269682	CTC-359D24.5
ENSG00000115947.14	0.009712112	0.28877458	0.847269682	ORC4
ENSG00000215210.3	0.032227343	0.288775533	0.847269682	RBMXP2
ENSG00000166947.15	0.038109873	0.288794952	0.847269682	EPB42
ENSG00000270362.1	-0.030640182	0.288882141	0.847269682	HMGN3-AS1
ENSG00000135549.15	0.038807188	0.288920605	0.847269682	PKIB
ENSG00000168427.9	0.054171294	0.288939376	0.847269682	KLHL30
ENSG00000211845.1	-0.045831785	0.28896748	0.847269682	TRAJ44
ENSG00000284070.1	-0.052338567	0.288998761	0.847269682	KB-1995A5.5
ENSG00000256720.1	-0.029051394	0.289010159	0.847269682	BTG1P1
ENSG00000278861.1	0.051925824	0.289033484	0.847269682	RP11-338K17.6
ENSG00000251034.2	-0.01129544	0.289231385	0.8475843	RP11-582J16.4
ENSG00000110944.9	0.014561024	0.28923236	0.8475843	IL23A
ENSG00000286159.1	-0.021562581	0.2892648	0.8475843	RP5-998C11.1
ENSG00000133477.17	0.032891986	0.289390729	0.847674569	FAM83F

ENSG00000205464.13	-0.030187461	0.289448032	0.847674569	ATP6AP1L
ENSG00000147687.19	0.014855321	0.289509389	0.847674569	TATDN1
ENSG00000101470.10	0.031577219	0.289544011	0.847674569	TNNC2
ENSG00000163510.14	0.009375834	0.289552091	0.847674569	CWC22
ENSG00000093144.19	0.011888427	0.289580051	0.847674569	ECHDC1
ENSG00000174672.16	-0.02666649	0.289594944	0.847674569	BRSK2
ENSG00000182175.14	-0.041618175	0.289706631	0.847674569	RGMA
ENSG00000075275.18	-0.027949959	0.289808054	0.847674569	CELSR1
ENSG00000131375.10	0.011084079	0.28982569	0.847674569	CAPN7
ENSG00000113282.14	0.008157148	0.289827737	0.847674569	CLINT1
ENSG00000171291.9	0.016138566	0.289848752	0.847674569	ZNF439
ENSG00000213139.8	-0.009204304	0.289896432	0.847674569	CRYGS
ENSG00000136273.13	0.006595067	0.289926507	0.847674569	HUS1
ENSG00000132965.10	0.015473473	0.289948146	0.847674569	ALOX5AP
ENSG00000183397.6	-0.012089774	0.289988741	0.847674569	C19orf71
ENSG00000246339.6	-0.023153165	0.290047904	0.847674569	EXTL3-AS1
ENSG00000287913.1	0.043685877	0.290064307	0.847674569	RP11-723P16.4
ENSG00000254842.6	-0.04317763	0.29008095	0.847674569	LINC02551
ENSG00000143365.19	0.026247375	0.290199538	0.847900289	RORC
ENSG00000149781.13	-0.009633909	0.290357523	0.84809826	FERMT3
ENSG00000060749.16	0.010866112	0.290369521	0.84809826	QSER1
ENSG00000267769.1	0.026659851	0.290392749	0.84809826	CTB-50L17.9
ENSG00000276234.5	0.006691369	0.290438042	0.84809826	TADA2A
ENSG00000119866.22	-0.015139025	0.290474068	0.84809826	BCL11A
ENSG00000261123.1	0.040009943	0.290597928	0.848308841	RP11-304L19.3
ENSG00000241058.4	0.011452141	0.290631959	0.848308841	NSUN6
ENSG00000164430.17	-0.010248536	0.290689453	0.848308841	CGAS
ENSG00000064489.23	0.035991023	0.290711651	0.848308841	BORCS8-MEF2B
ENSG00000154957.14	0.006283332	0.290840833	0.848548078	ZNF18
ENSG00000133687.16	0.051149001	0.290886894	0.848548078	TMTC1
ENSG00000166501.14	0.006982019	0.290917765	0.848548078	PRKCB
ENSG00000287504.1	0.047086478	0.29116494	0.848734511	RP11-18H21.5
ENSG00000171101.13	-0.025416747	0.291208188	0.848734511	SIGLEC17P
ENSG00000154310.17	-0.009306546	0.291291765	0.848734511	TNIK
ENSG00000131966.14	0.008943642	0.291302014	0.848734511	ACTR10
ENSG00000128609.16	0.01915621	0.291352256	0.848734511	NDUFA5
ENSG00000115266.12	-0.012102075	0.291367169	0.848734511	APC2
ENSG00000180846.8	-0.022192088	0.291379166	0.848734511	CSNK1G2-AS1
ENSG00000173894.11	0.024767139	0.291510794	0.848734511	CBX2
ENSG00000288721.1	0.039113861	0.291516223	0.848734511	RP5-973N23.5
ENSG00000100162.15	-0.025910984	0.291520243	0.848734511	CENPM
ENSG00000143067.5	0.015504964	0.291541455	0.848734511	ZNF697
ENSG00000104221.13	0.007377822	0.291549873	0.848734511	BRF2
ENSG00000183785.16	-0.029031204	0.291667464	0.848734511	TUBA8
ENSG00000182247.10	0.010382926	0.291698697	0.848734511	UBE2E2
ENSG00000155906.20	0.008042112	0.291719013	0.848734511	RMND1
ENSG00000178199.14	-0.012553076	0.291747422	0.848734511	ZC3H12D

ENSG00000273032.3	-0.022701034	0.291748156	0.848734511	DGCR5
ENSG00000126264.10	-0.016952003	0.291754833	0.848734511	HCST
ENSG00000261211.1	0.030495038	0.291778598	0.848734511	RP1-80N2.3
ENSG00000271631.1	0.02121116	0.291852329	0.848734511	RP11-408O19.5
ENSG00000197024.9	-0.00627401	0.291861091	0.848734511	ZNF398
ENSG00000102678.7	0.032826814	0.291924973	0.848734511	FGF9
ENSG00000011523.14	0.008824855	0.292041957	0.848734511	CEP68
ENSG00000272106.1	0.010035489	0.292049461	0.848734511	RP11-345P4.9
ENSG00000115286.20	-0.011362327	0.292088641	0.848734511	NDUFS7
ENSG00000115468.12	-0.030346194	0.292097487	0.848734511	EFHD1
ENSG00000161542.17	-0.005766118	0.292099092	0.848734511	PRPSAP1
ENSG00000181035.14	-0.00796392	0.292255412	0.848747381	SLC25A42
ENSG00000163508.13	-0.019667109	0.292279392	0.848747381	EOMES
ENSG00000286423.1	-0.06238806	0.292300061	0.848747381	RP6-91H8.6
ENSG00000120913.23	-0.007673304	0.292367947	0.848747381	PDLIM2
ENSG00000288596.2	-0.010331306	0.292370851	0.848747381	C8orf44
ENSG00000213341.11	0.011021546	0.292448328	0.848747381	CHUK
ENSG00000199477.1	-0.031719661	0.292469187	0.848747381	SNORA31
ENSG00000151715.8	0.022294304	0.292520371	0.848747381	TMEM45B
ENSG00000271380.1	-0.037302716	0.292561283	0.848747381	RP11-307C12.12
ENSG00000198466.12	-0.011811821	0.292567846	0.848747381	ZNF587
ENSG00000090661.12	-0.014945461	0.292627256	0.848747381	CERS4
ENSG00000275052.5	0.007895372	0.292678501	0.848747381	PPP4R3B
ENSG00000120458.13	-0.007472573	0.292681684	0.848747381	MSANTD2
ENSG00000167081.18	0.007658152	0.292710803	0.848747381	PBX3
ENSG00000274824.1	0.032087592	0.292736111	0.848747381	MIR7152
ENSG00000224660.2	0.009502273	0.2927657	0.848747381	SH3BP5-AS1
ENSG00000135390.21	0.010154393	0.292849385	0.848869991	ATP5MC2
ENSG00000262870.1	-0.033141976	0.29289465	0.848881214	CYCSP40
ENSG00000097033.14	0.008041067	0.293002404	0.849050233	SH3GLB1
ENSG00000223797.6	0.017191969	0.293133714	0.849050233	ENTPD3-AS1
ENSG00000165934.13	0.006647987	0.293150541	0.849050233	CPSF2
ENSG00000063322.15	0.004463688	0.293164836	0.849050233	MED29
ENSG00000143079.15	0.016688125	0.293181168	0.849050233	CTTNBP2NL
ENSG00000108061.12	0.009527948	0.293201372	0.849050233	SHOC2
ENSG00000240322.3	-0.032569468	0.293467069	0.849694752	RN7SL481P
ENSG00000132436.12	-0.014941471	0.293547066	0.849694752	FIGNL1
ENSG00000145780.8	0.01135848	0.293633416	0.849694752	FEM1C
ENSG00000253948.2	-0.016654688	0.293663497	0.849694752	VPS13B-DT
ENSG00000204392.11	0.010265932	0.293665743	0.849694752	LSM2
ENSG00000126561.18	0.007453091	0.293705077	0.849694752	STAT5A
ENSG00000241316.8	-0.029307398	0.29371397	0.849694752	SUCLG2-AS1
ENSG00000090339.9	0.012475828	0.293755404	0.849694756	ICAM1
ENSG00000270344.3	0.012949002	0.294007722	0.850017951	POC1B-AS1
ENSG00000105393.16	0.010931423	0.294015852	0.850017951	BABAM1
ENSG00000205808.7	-0.01027032	0.294026598	0.850017951	PLPP6
ENSG00000160075.12	-0.007436552	0.294032931	0.850017951	SSU72

ENSG00000196951.11	-0.025442817	0.294160216	0.850226676	SCOC-AS1
ENSG00000155849.16	-0.008500805	0.294188048	0.850226676	ELMO1
ENSG00000231249.1	-0.031693483	0.294408797	0.850744766	ITPR1-DT
ENSG00000151353.15	0.006401333	0.294515445	0.850933044	TMEM18
ENSG00000226525.5	0.045561905	0.294610085	0.850936775	RPS7P10
ENSG00000123505.19	0.007143401	0.294616085	0.850936775	AMD1
ENSG00000197016.12	0.01484961	0.294641215	0.850936775	ZNF470
ENSG00000167182.15	-0.008129306	0.29471107	0.850941211	SP2
ENSG00000285492.1	0.030549223	0.294725737	0.850941211	RP1-111C20.3
ENSG00000273759.1	0.015787975	0.294777508	0.850949214	RP4-563E14.1
ENSG00000111786.9	-0.005389067	0.294866197	0.850949214	SRSF9
ENSG00000170949.18	0.008408582	0.294883834	0.850949214	ZNF160
ENSG00000271730.1	-0.035689753	0.294894483	0.850949214	RP11-787I22.3
ENSG00000126246.10	-0.017305603	0.29494919	0.850951377	IGFLR1
ENSG00000136867.11	0.015700561	0.29497822	0.850951377	SLC31A2
ENSG00000128872.10	0.013123828	0.295079602	0.851124117	TMOD2
ENSG00000174080.12	-0.014068709	0.295278774	0.851578834	CTSF
ENSG00000100399.16	0.02546508	0.295332246	0.851588437	CHADL
ENSG00000166482.12	-0.027056237	0.29537015	0.851588437	MFAP4
ENSG00000188895.12	0.007944208	0.295442218	0.851588437	MSL1
ENSG00000253736.3	0.027510751	0.295448202	0.851588437	RP11-779O18.3
ENSG00000171703.17	-0.010528757	0.295553113	0.851771113	TCEA2
ENSG00000154511.12	0.01007251	0.295630362	0.851816663	DIPK1A
ENSG00000181908.5	0.013630035	0.29565199	0.851816663	LINC02724
ENSG00000230725.6	-0.041541089	0.295725776	0.851909567	RP4-738P15.1
ENSG00000124920.14	-0.028820114	0.295850709	0.852149768	MYRF
ENSG00000175550.8	-0.013872821	0.296039146	0.852572786	DRAP1
ENSG00000065618.21	0.052679052	0.296143277	0.852752925	COL17A1
ENSG00000215817.7	0.029026491	0.296240927	0.852914351	ZC3H11B
ENSG00000188315.8	0.007951491	0.296331018	0.852959637	C3orf62
ENSG00000163017.14	-0.058361504	0.296339839	0.852959637	ACTG2
ENSG00000066697.15	0.011876039	0.296403924	0.85302437	MSANTD3
ENSG00000268336.1	0.027260577	0.296499939	0.853180965	SIGLEC20P
ENSG00000228403.1	-0.035714492	0.296639656	0.853298022	RP11-563N6.6
ENSG00000175348.11	0.006623966	0.296648048	0.853298022	TMEM9B
ENSG00000177731.16	-0.008186032	0.296698208	0.853298022	FLII
ENSG00000225830.16	0.007615197	0.296707051	0.853298022	ERCC6
ENSG00000263266.2	0.037934458	0.29679762	0.853438811	RPS7P1
ENSG00000104980.8	-0.007616336	0.297103475	0.853749897	TIMM44
ENSG00000134686.19	0.011583151	0.29713567	0.853749897	PHC2
ENSG00000185261.15	0.016941613	0.297197909	0.853749897	KIAA0825
ENSG00000169733.12	-0.010043281	0.297201221	0.853749897	RFNG
ENSG00000277855.1	-0.026704469	0.297205437	0.853749897	RP11-154H23.4
ENSG00000275719.1	-0.036413141	0.297237116	0.853749897	CTB-147N14.6
ENSG00000213064.10	-0.006524327	0.297237493	0.853749897	SFT2D2
ENSG00000172379.21	0.08567481	0.297264222	0.853749897	ARNT2
ENSG00000198561.16	0.01299348	0.297308742	0.853749897	CTNND1

ENSG00000109790.18	0.010129303	0.297388096	0.853749897	KLHL5
ENSG00000217165.1	0.017410004	0.297436913	0.853749897	ANKRD18EP
ENSG00000215883.11	0.010555779	0.297444706	0.853749897	CYB5RL
ENSG00000162510.6	-0.02788194	0.297446997	0.853749897	MATN1
ENSG00000054983.17	-0.00848723	0.297517758	0.853833499	GALC
ENSG00000273117.1	0.015809821	0.297574885	0.853877954	INSIG1-DT
ENSG00000167904.15	-0.012578667	0.297618231	0.853882858	TMEM68
ENSG00000146109.5	0.006671038	0.297826086	0.85391461	ABT1
ENSG00000119912.18	0.007120596	0.297827978	0.85391461	IDE
ENSG00000129245.12	-0.007012769	0.297846033	0.85391461	FXR2
ENSG00000234664.1	0.018556503	0.297856545	0.85391461	HMGN2P5
ENSG00000265749.6	-0.029649131	0.297871915	0.85391461	RP11-849F2.5
ENSG00000160908.15	0.005005546	0.29789838	0.85391461	ZNF394
ENSG00000164465.19	0.017029549	0.29793794	0.85391461	DCBLD1
ENSG00000233431.1	0.026344853	0.298031068	0.85391461	LINC02596
ENSG00000237082.1	0.032447289	0.298039068	0.85391461	COX5BP6
ENSG00000226856.7	0.057229432	0.29816535	0.85391461	THORLNC
ENSG00000234332.1	-0.0295615	0.298174297	0.85391461	BCAS2P2
ENSG00000204856.12	0.010870871	0.298439848	0.85391461	FAM216A
ENSG00000257270.1	0.013164892	0.298472789	0.85391461	RP11-521B24.5
ENSG00000232653.8	-0.022068245	0.298476991	0.85391461	GOLGA8N
ENSG00000089692.9	-0.028747633	0.298615844	0.85391461	LAG3
ENSG00000226942.2	-0.025385014	0.298716784	0.85391461	IL9RP3
ENSG00000163815.6	-0.032615936	0.298723742	0.85391461	CLEC3B
ENSG00000143995.20	0.02246335	0.298754611	0.85391461	MEIS1
ENSG00000240893.1	0.056110277	0.298758233	0.85391461	LINC02042
ENSG00000121774.18	-0.004522698	0.298760448	0.85391461	KHDRBS1
ENSG00000229119.3	0.02297716	0.298802762	0.85391461	CTB-63M22.1
ENSG00000274419.6	0.170394523	0.298823025	0.85391461	TBC1D3D
ENSG00000085733.16	0.024430485	0.298843968	0.85391461	CTTN
ENSG00000039068.20	-0.035466759	0.298855594	0.85391461	CDH1
ENSG00000187017.17	0.03289073	0.298872339	0.85391461	ESPN
ENSG00000233355.7	0.026894831	0.298876032	0.85391461	CHRM3-AS2
ENSG00000228817.4	-0.021154815	0.298880973	0.85391461	BACH1-IT2
ENSG00000174013.8	0.009565742	0.298961357	0.85391461	FBXO45
ENSG00000147586.10	0.021503238	0.298999631	0.85391461	MRPS28
ENSG00000182230.12	0.047786374	0.299029187	0.85391461	FAM153B
ENSG00000258352.1	-0.012299819	0.299037679	0.85391461	IFITM3P6
ENSG00000070785.17	0.009551348	0.299076447	0.85391461	EIF2B3
ENSG00000223387.6	0.071729317	0.299114494	0.85391461	LINC02068
ENSG00000164548.11	-0.006939037	0.299190941	0.85391461	TRA2A
ENSG00000231704.6	-0.041068726	0.299191884	0.85391461	AC004895.4
ENSG00000133246.12	-0.011852113	0.299213947	0.85391461	PRAM1
ENSG00000116729.14	-0.024031201	0.299302005	0.85391461	WLS
ENSG00000131019.11	-0.028814771	0.299312829	0.85391461	ULBP3
ENSG00000211972.2	0.046895877	0.299313396	0.85391461	IGHV3-66
ENSG00000176387.7	0.037494176	0.299359437	0.85391461	HSD11B2

ENSG00000213988.11	0.020929289	0.299433334	0.85391461	ZNF90
ENSG00000006451.8	0.007036054	0.299442094	0.85391461	RALA
ENSG00000234426.3	-0.097492618	0.299453418	0.85391461	RP11-459O1.2
ENSG00000134183.13	-0.021992862	0.299461375	0.85391461	GNAT2
ENSG00000162194.12	-0.011044613	0.299590563	0.854019476	LBHD1
ENSG00000001460.18	0.011862952	0.299647738	0.854019476	STPG1
ENSG00000273464.1	-0.03050861	0.299697614	0.854019476	RP11-313P22.1
ENSG00000166529.15	-0.009574741	0.29970445	0.854019476	ZSCAN21
ENSG00000152465.18	-0.009714662	0.29974625	0.854019476	NMT2
ENSG00000224940.9	-0.03460112	0.29974801	0.854019476	PRRT4
ENSG00000181555.21	0.007351702	0.299812654	0.854084999	SETD2
ENSG00000189223.15	-0.055034416	0.300043022	0.854324927	PAX8-AS1
ENSG00000136868.11	0.010392612	0.300049173	0.854324927	SLC31A1
ENSG00000102575.13	-0.017602226	0.30007433	0.854324927	ACP5
ENSG00000235308.1	-0.021902707	0.300094555	0.854324927	RP11-82K18.2
ENSG00000106624.11	-0.02213275	0.300105167	0.854324927	AEBP1
ENSG00000112210.12	-0.026319531	0.300249218	0.854335138	RAB23
ENSG00000100599.16	-0.009345542	0.300272623	0.854335138	RIN3
ENSG00000236609.4	-0.012393249	0.300294135	0.854335138	ZNF853
ENSG00000099899.15	-0.007675304	0.300317521	0.854335138	TRMT2A
ENSG00000128585.18	0.010420401	0.300334244	0.854335138	MKLN1
ENSG00000111605.18	-0.007096329	0.300459486	0.854335138	CPSF6
ENSG00000275580.1	-0.019623772	0.300540546	0.854335138	RP11-295H24.4
ENSG00000147684.10	0.010553039	0.300557957	0.854335138	NDUFB9
ENSG00000111052.8	0.01335642	0.300635638	0.854335138	LIN7A
ENSG00000154548.9	-0.028756754	0.300693011	0.854335138	SRSF12
ENSG00000153113.24	0.008539116	0.300694009	0.854335138	CAST
ENSG00000066336.12	-0.011277757	0.300737347	0.854335138	SPI1
ENSG00000160318.6	-0.022842591	0.300760949	0.854335138	CLDND2
ENSG00000186197.15	-0.021030997	0.300778034	0.854335138	EDARADD
ENSG00000154222.15	-0.005287077	0.300832295	0.854335138	CC2D1B
ENSG00000140853.16	0.010245652	0.300848432	0.854335138	NLRC5
ENSG00000100916.14	0.01567137	0.300922241	0.854335138	BRMS1L
ENSG00000130714.19	-0.007839592	0.301025388	0.854335138	POMT1
ENSG00000211815.3	0.029230013	0.301041178	0.854335138	TRAV36DV7
ENSG00000259540.1	-0.052075137	0.301084453	0.854335138	RP11-526I2.1
ENSG00000279168.2	0.037272844	0.301136729	0.854335138	RP11-514P8.10
ENSG00000229558.2	-0.046172659	0.301162503	0.854335138	SACS-AS1
ENSG00000137343.18	-0.008319182	0.301211736	0.854335138	ATAT1
ENSG00000102743.15	0.007855049	0.301215619	0.854335138	SLC25A15
ENSG00000268442.1	0.054316091	0.301257652	0.854335138	CTD-2027I19.2
ENSG00000165695.10	-0.039317081	0.301327329	0.854335138	AK8
ENSG00000237781.3	-0.019389723	0.30133151	0.854335138	ADAMTSL4-AS2
ENSG00000276291.5	0.012094911	0.301453903	0.854335138	FRG1HP
ENSG00000100142.16	0.009631552	0.301461349	0.854335138	POLR2F
ENSG00000163975.12	-0.02443825	0.301474657	0.854335138	MELTF
ENSG00000007047.15	-0.012219626	0.301501738	0.854335138	MARK4

ENSG00000103184.12	0.036037492	0.301512053	0.854335138	SEC14L5
ENSG00000196787.3	0.019997388	0.301551614	0.854335138	H2AC11
ENSG00000134955.12	-0.012170761	0.301557506	0.854335138	SLC37A2
ENSG00000162702.8	0.012316123	0.301595765	0.854335138	ZNF281
ENSG00000058600.16	-0.005954292	0.301612905	0.854335138	POLR3E
ENSG00000130592.17	-0.01132622	0.301685159	0.854335138	LSP1
ENSG00000279520.1	0.009569738	0.301691782	0.854335138	RP11-20I23.2
ENSG00000253276.4	-0.012838242	0.301798794	0.854399327	CCDC71L
ENSG00000150779.12	0.02249164	0.301889291	0.854399327	TIMM8B
ENSG00000149506.11	-0.037179552	0.301933362	0.854399327	ZP1
ENSG00000167614.14	-0.026030078	0.302009549	0.854399327	TTYH1
ENSG00000125449.7	-0.00867038	0.302027657	0.854399327	ARMC7
ENSG00000123609.11	0.012010231	0.302047451	0.854399327	NMI
ENSG00000259307.1	-0.023770835	0.302055607	0.854399327	PLCB2-AS1
ENSG00000178386.13	0.021389867	0.302089249	0.854399327	ZNF223
ENSG00000223711.1	-0.024639109	0.302089405	0.854399327	AC091633.3
ENSG00000185864.17	-0.022214284	0.302167937	0.854503593	NPIPB4
ENSG00000128394.17	-0.010565079	0.302214435	0.854517252	APOBEC3F
ENSG00000170298.16	0.046537439	0.302306932	0.854552885	LGALS9B
ENSG00000116095.11	-0.008885246	0.302310375	0.854552885	PLEKHA3
ENSG00000259182.5	0.028299138	0.30239874	0.854646686	RP11-424I19.2
ENSG00000280046.1	-0.016682932	0.302426907	0.854646686	RP11-1099M24.6
ENSG00000135905.20	0.009268884	0.302579028	0.854958762	DOCK10
ENSG00000168404.13	0.008631037	0.302620847	0.85495913	MLKL
ENSG00000104872.11	-0.0081078	0.30266626	0.85496965	PIH1D1
ENSG00000175445.17	0.044134841	0.302736917	0.855051467	LPL
ENSG00000149679.11	-0.008539655	0.302817867	0.855155385	CABLES2
ENSG00000092850.12	-0.041230442	0.302857107	0.855155385	TEKT2
ENSG00000164169.13	0.009611259	0.302963976	0.855310805	PRMT9
ENSG00000152380.10	-0.018226784	0.303026773	0.855310805	FAM151B
ENSG00000173418.12	0.009126275	0.303037269	0.855310805	NAA20
ENSG00000187193.9	0.015362704	0.303277649	0.855696963	MT1X
ENSG00000198885.10	-0.016943116	0.303281412	0.855696963	ITPRIPL1
ENSG00000215256.4	0.008056092	0.30329926	0.855696963	DHRS4-AS1
ENSG00000176920.13	-0.018096465	0.303363664	0.855732594	FUT2
ENSG00000274602.5	-0.036161508	0.303395343	0.855732594	PI4KAP1
ENSG00000205456.11	-0.056747497	0.303446273	0.85575855	TP53TG3D
ENSG00000165006.14	0.006829431	0.303572097	0.855823412	UBAP1
ENSG00000255920.3	-0.026884647	0.303590869	0.855823412	CCND2-AS1
ENSG00000184557.4	0.019219857	0.303635131	0.855823412	SOCS3
ENSG00000196547.15	0.008696684	0.303636198	0.855823412	MAN2A2
ENSG00000250240.6	-0.045551708	0.30378064	0.856034631	CTD-2154I11.2
ENSG00000204536.14	-0.009066695	0.303794619	0.856034631	CCHCR1
ENSG00000245025.3	-0.010813095	0.303882693	0.85616517	RP11-875O11.1
ENSG00000115750.17	-0.011046553	0.303961469	0.856255669	TAF1B
ENSG00000170858.10	-0.030741547	0.304051606	0.856255669	LILRP2
ENSG00000100297.16	-0.008817727	0.304062077	0.856255669	MCM5

ENSG00000184349.14	-0.035165597	0.304081823	0.856255669	EFNA5
ENSG00000215039.7	0.008431745	0.304304678	0.856690465	CD27-AS1
ENSG00000211896.7	0.046554325	0.304319779	0.856690465	IGHG1
ENSG00000169896.18	-0.010070099	0.304432544	0.856745505	ITGAM
ENSG00000115657.14	-0.013331279	0.304432989	0.856745505	ABCB6
ENSG00000113621.15	0.006238516	0.304482671	0.856745505	TXNDC15
ENSG00000121350.16	-0.012670667	0.304506436	0.856745505	PYROXD1
ENSG00000126603.9	-0.020105418	0.304656128	0.856946496	GLIS2
ENSG00000130449.6	0.009975207	0.304734238	0.856946496	ZSWIM6
ENSG00000129235.11	0.012149797	0.304755796	0.856946496	TXNDC17
ENSG00000249923.1	-0.0949771	0.304760853	0.856946496	LINC02891
ENSG00000173531.15	0.01077322	0.304786802	0.856946496	MST1
ENSG00000141458.13	-0.010145119	0.304916584	0.856996072	NPC1
ENSG00000214888.3	0.084724639	0.304921886	0.856996072	XXyac-YM21GA2.7
ENSG00000008394.13	0.014583337	0.304984634	0.856996072	MGST1
ENSG00000102977.17	-0.009042372	0.30509763	0.856996072	ACD
ENSG00000253483.1	0.046233103	0.305107797	0.856996072	CTC-756D1.1
ENSG00000162630.6	0.033199377	0.305150059	0.856996072	B3GALT2
ENSG00000111877.18	0.007200469	0.305168378	0.856996072	MCM9
ENSG00000185669.6	-0.011224518	0.305197361	0.856996072	SNAI3
ENSG00000163719.20	0.006627468	0.305242917	0.856996072	MTMR14
ENSG00000259536.5	-0.031919591	0.305267666	0.856996072	RP11-111A22.1
ENSG00000154803.13	0.008660011	0.30530922	0.856996072	FLCN
ENSG00000100726.15	-0.010021806	0.305347061	0.856996072	TELO2
ENSG00000125618.18	-0.053062702	0.305347684	0.856996072	PAX8
ENSG00000165650.12	0.012923607	0.30545208	0.857163175	PDZD8
ENSG00000226415.1	0.016685943	0.305517337	0.857163175	TPI1P1
ENSG00000182362.14	0.01684565	0.305550894	0.857163175	YBEY
ENSG00000184903.10	0.016144449	0.305574409	0.857163175	IMMP2L
ENSG00000115568.16	-0.006963218	0.305699591	0.85733756	ZNF142
ENSG00000224635.2	-0.03328566	0.30574309	0.85733756	RP4-564F22.5
ENSG00000103148.17	-0.02421095	0.305766271	0.85733756	NPRL3
ENSG00000180189.10	-0.041745092	0.305871748	0.85733756	HMGB1P14
ENSG00000138639.18	0.015763809	0.30588431	0.85733756	ARHGAP24
ENSG00000005884.18	-0.014553593	0.305997716	0.85733756	ITGA3
ENSG00000113758.13	-0.0146432	0.306017317	0.85733756	DBN1
ENSG00000065609.14	-0.176100043	0.306069785	0.85733756	SNAP91
ENSG00000259834.1	-0.011917529	0.306126013	0.85733756	RP11-284N8.3
ENSG00000253552.8	0.030056881	0.306224252	0.85733756	HOXA-AS2
ENSG00000006453.14	-0.01832041	0.306260342	0.85733756	BAIAP2L1
ENSG00000266604.1	-0.034279468	0.306369197	0.85733756	LINC01887
ENSG00000166839.17	-0.021139745	0.306379329	0.85733756	ANKDD1A
ENSG00000116688.18	-0.012963146	0.306389996	0.85733756	MFN2
ENSG00000188818.12	0.022380351	0.306410933	0.85733756	ZDHHC11
ENSG00000163932.15	-0.007491134	0.306414959	0.85733756	PRKCD
ENSG00000279145.2	-0.023181334	0.30648305	0.85733756	RP11-547D13.1
ENSG00000238201.1	0.033348913	0.306490059	0.85733756	AC114752.3

ENSG00000153898.13	-0.017864413	0.306498734	0.85733756	MCOLN2
ENSG00000128284.19	0.010269972	0.306516637	0.85733756	APOL3
ENSG00000220267.1	-0.093926756	0.306532719	0.85733756	ACTBP8
ENSG00000233387.1	0.022386834	0.306602154	0.85733756	IATPR
ENSG00000169504.15	0.013040753	0.306614826	0.85733756	CLIC4
ENSG00000255458.7	-0.031389861	0.306676226	0.85733756	RP11-539G18.3
ENSG00000287539.1	-0.026109167	0.306699025	0.85733756	RP11-323N12.6
ENSG00000135299.17	0.010395997	0.306723507	0.85733756	ANKRD6
ENSG00000099814.16	0.026096448	0.306790609	0.857408259	CEP170B
ENSG00000230325.1	0.028241425	0.306893157	0.85757799	RP11-385F5.4
ENSG00000179913.11	0.043933043	0.306945834	0.857608334	B3GNT3
ENSG00000137274.13	0.010673044	0.307164575	0.858008745	BPHL
ENSG00000165682.14	0.018799697	0.307172821	0.858008745	CLEC1B
ENSG00000186020.13	-0.010141272	0.307363738	0.858331067	ZNF529
ENSG00000214113.11	0.012261683	0.30740908	0.858331067	LYRM4
ENSG00000221821.4	-0.014958255	0.307413774	0.858331067	C6orf226
ENSG00000114923.17	0.022462545	0.307558346	0.858570561	SLC4A3
ENSG00000275713.2	0.022547779	0.30758328	0.858570561	H2BC9
ENSG00000127952.17	0.012847715	0.307735983	0.858865453	STYXL1
ENSG00000283429.1	0.038855434	0.307796829	0.858865453	MIR1244-3
ENSG00000273445.1	0.032408668	0.307814564	0.858865453	RP11-1399P15.1
ENSG00000130544.12	0.007030693	0.307876745	0.858922091	ZNF557
ENSG00000159733.14	-0.012120384	0.308119339	0.858958718	ZFYVE28
ENSG00000223946.1	-0.038639213	0.30816574	0.858958718	RP11-533O20.2
ENSG00000162654.9	0.021552738	0.308203058	0.858958718	GBP4
ENSG00000138738.11	0.025460697	0.308380476	0.858958718	PRDM5
ENSG00000211784.2	-0.026556193	0.308380611	0.858958718	TRAV10
ENSG00000229833.10	0.012861777	0.308439679	0.858958718	PET100
ENSG00000142453.12	0.017818142	0.308472893	0.858958718	CARM1
ENSG00000271976.1	-0.010494125	0.308483013	0.858958718	RP11-884K10.7
ENSG00000125735.11	0.010471362	0.308519523	0.858958718	TNFSF14
ENSG00000198682.13	-0.013045222	0.308552488	0.858958718	PAPSS2
ENSG00000077380.16	0.00687011	0.308622769	0.858958718	DYNC1I2
ENSG00000128951.14	-0.011467424	0.308631091	0.858958718	DUT
ENSG00000188487.12	0.033652509	0.308663894	0.858958718	INSC
ENSG00000245571.7	-0.009330134	0.308674686	0.858958718	FAM111A-DT
ENSG00000092010.15	0.011533839	0.308722895	0.858958718	PSME1
ENSG00000226393.1	-0.029615469	0.308789228	0.858958718	IFNA20P
ENSG00000182054.10	-0.009768525	0.308792132	0.858958718	IDH2
ENSG00000281501.1	0.018060933	0.308803554	0.858958718	SEPSECS-AS1
ENSG00000152795.18	-0.005380207	0.308806444	0.858958718	HNRNPDL
ENSG00000166343.10	-0.008687397	0.308830065	0.858958718	MSS51
ENSG00000178726.7	-0.018828931	0.308838093	0.858958718	THBD
ENSG00000104918.8	-0.028809588	0.308931988	0.858958718	RETN
ENSG00000151657.12	0.007365102	0.308951021	0.858958718	KIN
ENSG00000103056.12	-0.020870832	0.308954606	0.858958718	SMPD3
ENSG00000159958.7	-0.024403909	0.308992825	0.858958718	TNFRSF13C

ENSG00000196793.14	0.016722776	0.309015952	0.858958718	ZNF239
ENSG00000287064.1	0.031817329	0.309020744	0.858958718	RP11-274N19.4
ENSG00000149564.12	0.02301312	0.309118774	0.859114761	ESAM
ENSG00000196865.4	-0.010645164	0.309173165	0.859149493	NHLRC2
ENSG00000276216.1	0.027907386	0.309235984	0.859207636	CH17-373J23.1
ENSG00000211594.2	-0.033591265	0.309294094	0.85925268	IGKJ4
ENSG00000238062.6	0.047775343	0.309379566	0.859295374	SPATA3-AS1
ENSG00000012963.15	0.004815505	0.309432757	0.859295374	UBR7
ENSG00000103160.12	0.007428458	0.309438338	0.859295374	HSDL1
ENSG00000172554.12	0.045560559	0.309513925	0.859295374	SNTG2
ENSG00000211813.2	-0.039809854	0.309570129	0.859295374	TRAV34
ENSG00000177169.10	-0.009614724	0.309599813	0.859295374	ULK1
ENSG00000117724.13	-0.019485606	0.309625852	0.859295374	CENPF
ENSG00000105053.11	0.007339434	0.309644666	0.859295374	VRK3
ENSG00000135090.14	0.006591739	0.309769801	0.859526328	TAOK3
ENSG00000237575.4	-0.033391655	0.309881296	0.859719375	PYY2
ENSG00000154319.16	-0.034612225	0.310021106	0.859990916	FAM167A
ENSG00000225783.8	-0.019985964	0.310098157	0.860027227	MIAT
ENSG00000249087.7	0.01406174	0.310118068	0.860027227	ZNF436-AS1
ENSG00000141295.14	-0.010627472	0.310193339	0.860119658	SCRN2
ENSG00000108826.16	0.010338933	0.310361568	0.86039286	MRPL27
ENSG00000183401.12	-0.008966806	0.310393874	0.86039286	CCDC159
ENSG00000269191.1	-0.015096294	0.310417728	0.86039286	AC005387.2
ENSG00000261505.1	0.013892558	0.31046522	0.860408207	LA16c-358B7.3
ENSG00000214353.8	0.033548576	0.310559285	0.860552602	VAC14-AS1
ENSG00000140691.18	-0.010848219	0.310609886	0.860576538	ARMC5
ENSG00000263818.5	0.034211567	0.310693318	0.860576906	RDM1P5
ENSG00000172375.14	0.007386036	0.310693944	0.860576906	C2CD2L
ENSG00000167074.15	0.009150553	0.310764968	0.86061105	TEF
ENSG00000270948.1	-0.034982362	0.310790201	0.86061105	MTDHP1
ENSG00000235863.4	-0.009132552	0.310920782	0.860679384	B3GALT4
ENSG00000286214.1	-0.03238933	0.310937289	0.860679384	COPG2IT1
ENSG00000181915.5	0.006597546	0.310958483	0.860679384	ADO
ENSG00000109220.11	0.007953612	0.311036052	0.860679384	CHIC2
ENSG00000275910.1	0.032357721	0.311043684	0.860679384	RP11-680G24.6
ENSG00000111845.5	0.009639944	0.311073203	0.860679384	PAK1IP1
ENSG00000201441.1	0.041022892	0.311123803	0.860679384	RNU6-646P
ENSG00000090376.11	0.011656368	0.31117991	0.860679384	IRAK3
ENSG00000232956.9	0.007789329	0.31119259	0.860679384	SNHG15
ENSG00000130203.10	-0.036884423	0.311324364	0.860684323	APOE
ENSG00000211648.2	-0.034695378	0.311395873	0.860684323	IGLV1-47
ENSG00000165338.17	0.027114889	0.311414622	0.860684323	HECTD2
ENSG00000111186.13	-0.023880015	0.31144814	0.860684323	WNT5B
ENSG00000138363.15	-0.008086942	0.311508596	0.860684323	ATIC
ENSG00000277954.1	-0.039345901	0.311517245	0.860684323	RP11-679B19.1
ENSG00000272758.6	0.016695596	0.311553137	0.860684323	WDR5B-DT
ENSG00000197321.15	-0.010473271	0.311597223	0.860684323	SVIL

ENSG00000267809.1	0.031193123	0.311680922	0.860684323	NDUFV2P1
ENSG00000172954.14	0.012866944	0.311740202	0.860684323	LCLAT1
ENSG00000276445.1	-0.02112603	0.311756185	0.860684323	LLNLR-268E12.1
ENSG00000155846.17	0.013732235	0.311776114	0.860684323	PPARGC1B
ENSG00000121931.16	0.00934953	0.311806312	0.860684323	LRIF1
ENSG00000167281.19	-0.034068073	0.311821291	0.860684323	RBFOX3
ENSG00000187514.17	-0.005609466	0.311823899	0.860684323	PTMA
ENSG00000088035.18	0.007773526	0.311965847	0.860750205	ALG6
ENSG00000104231.11	0.012601754	0.312018347	0.860750205	ZFAND1
ENSG00000283761.1	-0.033438237	0.31206658	0.860750205	RP4-714D9.5
ENSG00000143437.21	-0.006942126	0.312080235	0.860750205	ARNT
ENSG00000205531.13	0.00601578	0.312106499	0.860750205	NAP1L4
ENSG00000257509.1	-0.027217509	0.3121495	0.860750205	RP11-762I7.4
ENSG00000108599.15	0.00728054	0.312175168	0.860750205	AKAP10
ENSG00000133247.14	-0.010424808	0.312184052	0.860750205	KMT5C
ENSG00000135365.16	0.007558457	0.312225511	0.860750205	PHF21A
ENSG00000169221.14	-0.008109393	0.312332434	0.86086658	TBC1D10B
ENSG00000120948.19	-0.0038914	0.31238556	0.86086658	TARDBP
ENSG00000170619.10	0.006760841	0.312411408	0.86086658	COMMD5
ENSG00000245532.9	-0.010762376	0.312456471	0.86086658	NEAT1
ENSG00000008294.21	0.008445496	0.31247761	0.86086658	SPAG9
ENSG00000215252.12	-0.020095105	0.312599076	0.861085541	GOLGA8B
ENSG00000127863.16	-0.04516008	0.312643264	0.861091601	TNFRSF19
ENSG00000160097.20	0.022840426	0.312727906	0.861135611	FNDC5
ENSG00000175536.7	0.015572133	0.312743224	0.861135611	LIPT2
ENSG00000176714.10	0.01531552	0.312791346	0.861152494	CCDC121
ENSG00000171161.13	-0.007705381	0.312839328	0.861154541	ZNF672
ENSG00000118263.15	0.011089441	0.312882081	0.861154541	KLF7
ENSG00000139351.15	-0.02692642	0.313035338	0.861154541	SYCP3
ENSG00000285572.2	-0.032428609	0.313103814	0.861154541	RP11-19D22.1
ENSG00000268043.7	-0.007049417	0.313124235	0.861154541	NBPF12
ENSG00000267342.1	-0.016030632	0.313205168	0.861154541	RP11-552F3.10
ENSG00000280080.2	-0.033038698	0.313219176	0.861154541	TBC1D22A-AS1
ENSG00000109475.17	0.029785362	0.31325826	0.861154541	RPL34
ENSG00000100292.18	-0.01303938	0.313296354	0.861154541	HMOX1
ENSG00000183044.12	-0.01301162	0.313301364	0.861154541	ABAT
ENSG00000134452.20	0.006471542	0.313359513	0.861154541	FBH1
ENSG00000177045.10	-0.028666794	0.313450346	0.861154541	SIX5
ENSG00000181026.15	0.007811106	0.313463295	0.861154541	AEN
ENSG00000035115.22	0.014964614	0.31349036	0.861154541	SH3YL1
ENSG00000123159.16	-0.010717497	0.31349616	0.861154541	GIPC1
ENSG00000006016.12	-0.015707204	0.313507184	0.861154541	CRLF1
ENSG00000226677.3	0.036694608	0.313537768	0.861154541	IGBP1P1
ENSG00000288082.1	0.029701686	0.31354793	0.861154541	RP11-546M8.2
ENSG00000178913.8	0.011793877	0.313626483	0.861254942	TAF7
ENSG00000204560.10	-0.00489543	0.313741894	0.861424714	DHX16
ENSG00000178852.16	-0.019594743	0.313772314	0.861424714	EFCAB13

ENSG00000117682.17	-0.004805351	0.313827438	0.861460727	DHDDS
ENSG00000189058.9	0.125155308	0.313971743	0.861479388	APOD
ENSG00000103549.22	-0.01029604	0.313975669	0.861479388	RNF40
ENSG00000169972.12	-0.010399328	0.314002028	0.861479388	PUSL1
ENSG00000268751.1	-0.03277035	0.314006957	0.861479388	SCGB1B2P
ENSG00000203876.10	0.020185791	0.31406788	0.861479388	ADD3-AS1
ENSG00000115423.19	-0.028480215	0.3141141	0.861479388	DNAH6
ENSG00000069943.10	0.010388555	0.314237598	0.861479388	PIGB
ENSG00000163636.11	0.004277639	0.314330116	0.861479388	PSMD6
ENSG00000204934.10	-0.016163885	0.31437062	0.861479388	ATP6V0E2-AS1
ENSG00000151164.19	0.017312593	0.314423862	0.861479388	RAD9B
ENSG00000169258.7	-0.019083639	0.314443123	0.861479388	GPRIN1
ENSG00000228923.1	0.035971479	0.314469454	0.861479388	AP000355.2
ENSG00000168852.13	0.019683844	0.314471746	0.861479388	TPTE2P5
ENSG00000110400.11	-0.013381418	0.314477474	0.861479388	NECTIN1
ENSG00000242852.7	-0.029810123	0.314527829	0.861479388	ZNF709
ENSG00000110719.10	-0.01008155	0.314536214	0.861479388	TCIRG1
ENSG00000075702.19	-0.01349227	0.314570629	0.861479388	WDR62
ENSG00000229043.3	-0.017441885	0.314628342	0.861479388	ZFAND2A-DT
ENSG00000238109.1	0.044783631	0.314658723	0.861479388	RNF14P3
ENSG00000224272.2	-0.065164659	0.314674376	0.861479388	AC114730.3
ENSG00000215790.8	0.015077269	0.314836343	0.861628064	SLC35E2A
ENSG00000153250.20	0.007723377	0.31491805	0.861628064	RBMS1
ENSG00000231799.2	-0.026935443	0.315003838	0.861628064	PA2G4P6
ENSG00000042062.13	0.016604252	0.315048984	0.861628064	RIPOR3
ENSG00000105402.8	-0.01233308	0.315123705	0.861628064	NAPA
ENSG00000170955.10	-0.040094647	0.315150883	0.861628064	CAVIN3
ENSG00000261367.1	0.022265694	0.31516958	0.861628064	RP11-455F5.4
ENSG00000249786.7	0.028511617	0.315182329	0.861628064	EAF1-AS1
ENSG00000188321.14	0.011011774	0.315266521	0.861628064	ZNF559
ENSG00000163812.15	0.005579011	0.315287981	0.861628064	ZDHHC3
ENSG00000246263.3	-0.015728461	0.315300097	0.861628064	UBR5-AS1
ENSG00000131462.9	-0.010068261	0.315390874	0.861628064	TUBG1
ENSG00000236577.1	-0.033479691	0.315409967	0.861628064	SNRPGP14
ENSG00000154025.16	0.014254326	0.315410482	0.861628064	SLC5A10
ENSG00000256937.1	0.042906948	0.315455144	0.861628064	KRT17P8
ENSG00000275160.1	-0.033753845	0.31549809	0.861628064	RP11-187C18.3
ENSG00000120889.13	0.007981678	0.315523457	0.861628064	TNFRSF10B
ENSG00000089057.15	0.008047907	0.315585022	0.861628064	SLC23A2
ENSG00000014824.14	0.010418578	0.31560926	0.861628064	SLC30A9
ENSG00000122386.11	-0.025026167	0.315621565	0.861628064	ZNF205
ENSG00000140274.14	0.115745974	0.315699613	0.861628064	DUOXA2
ENSG00000166226.13	0.010375482	0.315727303	0.861628064	CCT2
ENSG00000259343.7	0.020458367	0.315733483	0.861628064	TMC3-AS1
ENSG00000272263.1	0.038054853	0.315737024	0.861628064	RP11-767C1.2
ENSG00000236438.7	0.016087265	0.315797874	0.861654601	FAM157A
ENSG00000132780.17	0.006430678	0.31583078	0.861654601	NASP

ENSG00000153944.12	-0.006685162	0.315895201	0.861714977	MSI2
ENSG00000197912.16	-0.005368775	0.315936947	0.861714977	SPG7
ENSG00000231616.9	-0.041603525	0.316059621	0.861826003	RP11-575L7.4
ENSG00000027869.12	-0.016438549	0.316061701	0.861826003	SH2D2A
ENSG00000112335.15	0.009303465	0.316131177	0.861900849	SNX3
ENSG00000070018.9	0.024754848	0.316230494	0.861985344	LRP6
ENSG00000163126.15	-0.012178142	0.316246232	0.861985344	ANKRD23
ENSG00000154781.16	0.004171856	0.316300814	0.862019546	CCDC174
ENSG00000106077.19	0.01084369	0.316489333	0.8624143	ABHD11
ENSG00000075886.11	-0.023764322	0.316579022	0.8624143	TUBA3D
ENSG00000261824.7	0.013276567	0.316710168	0.8624143	LINC00662
ENSG00000264937.1	-0.018337179	0.316719841	0.8624143	AC100830.5
ENSG00000278224.7	-0.022868675	0.316722457	0.8624143	PRICKLE4
ENSG00000280347.1	0.012441917	0.316729118	0.8624143	AC000123.2
ENSG00000198756.12	0.032316869	0.316776676	0.8624143	COLGALT2
ENSG00000263465.5	-0.006429549	0.316782081	0.8624143	SRSF8
ENSG00000180573.10	0.011302269	0.31683686	0.862439357	H2AC6
ENSG00000228109.1	-0.024302226	0.316875393	0.862439357	MELTF-AS1
ENSG00000198919.13	0.012168856	0.316988389	0.862502597	DZIP3
ENSG00000235568.7	0.008834214	0.317005713	0.862502597	NFAM1
ENSG00000145868.17	0.006695619	0.317036269	0.862502597	FBXO38
ENSG00000103355.14	-0.032413579	0.317070714	0.862502597	PRSS33
ENSG00000283384.1	0.037206061	0.317132526	0.862502597	RP11-153I24.5
ENSG00000196466.10	0.014109722	0.31715364	0.862502597	ZNF799
ENSG00000106608.17	-0.00693117	0.317220196	0.862502597	URGCP
ENSG00000130770.18	-0.006294179	0.317245029	0.862502597	ATP5IF1
ENSG00000131238.18	0.01314998	0.31727714	0.862502597	PPT1
ENSG00000139613.12	-0.007494128	0.317368271	0.862635985	SMARCC2
ENSG00000130713.16	0.00621068	0.317486682	0.862838832	EXOSC2
ENSG00000121380.12	0.042338225	0.317527046	0.862838832	BCL2L14
ENSG00000157456.8	-0.028820907	0.317631023	0.862851947	CCNB2
ENSG00000231412.2	0.058778753	0.317633098	0.862851947	CTC-490G23.2
ENSG00000215068.8	0.011295642	0.317658094	0.862851947	AC025171.1
ENSG00000258725.2	-0.025266417	0.317703787	0.862861775	PRC1-AS1
ENSG00000179163.12	0.010065749	0.317770997	0.862930033	FUCA1
ENSG00000087116.16	-0.067107961	0.318081448	0.863378566	ADAMTS2
ENSG00000153107.13	0.008864341	0.318115197	0.863378566	ANAPC1
ENSG00000128891.16	0.009150036	0.318172419	0.863378566	CCDC32
ENSG00000171766.17	0.022299403	0.318226892	0.863378566	GATM
ENSG00000246130.1	-0.034120423	0.318250131	0.863378566	RP11-875O11.2
ENSG00000023287.13	0.015029525	0.318259336	0.863378566	RB1CC1
ENSG00000204070.10	0.005170551	0.318330093	0.863378566	SYS1
ENSG00000214402.7	-0.030504975	0.31840596	0.863378566	LCNL1
ENSG00000269609.6	0.007119695	0.318502223	0.863378566	C10orf95-AS1
ENSG00000106804.8	0.013190228	0.318514715	0.863378566	C5
ENSG00000006062.18	0.007212424	0.318521289	0.863378566	MAP3K14
ENSG00000111788.10	-0.028995514	0.318579014	0.863378566	RP11-22B23.1

ENSG00000233058.2	-0.018858469	0.318601795	0.863378566	LINC00884
ENSG00000254750.1	0.021573709	0.31862495	0.863378566	CASP1P2
ENSG00000272941.1	0.014005457	0.318715263	0.863378566	RP11-134L10.1
ENSG00000114529.13	0.017849864	0.318718339	0.863378566	C3orf52
ENSG00000178896.9	-0.012681139	0.318731617	0.863378566	EXOSC4
ENSG00000064961.19	-0.007950193	0.318741659	0.863378566	HMG20B
ENSG00000260806.1	-0.028214706	0.318796673	0.863378566	PAPOLA-DT
ENSG00000159904.11	-0.049610758	0.318810063	0.863378566	ZNF890P
ENSG00000141905.19	-0.009544814	0.318820259	0.863378566	NFIC
ENSG00000005059.16	0.008421112	0.318894397	0.863465315	MCUB
ENSG00000169189.17	0.009678217	0.319062686	0.863662755	NSMCE1
ENSG00000269293.3	0.01366812	0.319135477	0.863662755	ZSCAN16-AS1
ENSG00000103540.16	0.011115901	0.319156702	0.863662755	CCP110
ENSG00000134324.12	-0.008567992	0.319158659	0.863662755	LPIN1
ENSG00000055955.17	0.015139146	0.319177883	0.863662755	ITIH4
ENSG00000227189.2	0.036775761	0.319276334	0.863697275	AC092535.3
ENSG00000112309.11	0.019379636	0.319340794	0.863697275	B3GAT2
ENSG00000041880.15	0.010700165	0.319372829	0.863697275	PARP3
ENSG00000228409.6	-0.01034965	0.319464442	0.863697275	CCT6P1
ENSG00000125895.6	0.035972751	0.319541787	0.863697275	TMEM74B
ENSG00000164105.4	0.01468852	0.319610335	0.863697275	SAP30
ENSG00000264235.6	-0.022552165	0.319713326	0.863697275	MYL12-AS1
ENSG00000155367.15	0.020627329	0.31973038	0.863697275	PPM1J
ENSG00000235169.11	0.054026358	0.319748641	0.863697275	SMIM1
ENSG00000165409.18	-0.034982381	0.319778463	0.863697275	TSHR
ENSG00000115137.12	0.0097594	0.319827423	0.863697275	DNAJC27
ENSG00000204592.9	0.007739444	0.31984811	0.863697275	HLA-E
ENSG00000272602.6	0.02495507	0.319882769	0.863697275	ZNF595
ENSG00000243264.3	0.055149095	0.319937274	0.863697275	IGKV2D-29
ENSG00000255741.1	0.031628185	0.320107725	0.863697275	RP11-757G1.5
ENSG00000065883.16	-0.005712777	0.320112082	0.863697275	CDK13
ENSG00000255874.2	-0.039064453	0.320113749	0.863697275	PRECSIT
ENSG00000270083.1	0.020666396	0.320115945	0.863697275	RP1-257I20.14
ENSG00000185666.15	-0.041463187	0.320178215	0.863697275	SYN3
ENSG00000154928.19	0.015071969	0.320181282	0.863697275	EPHB1
ENSG00000138658.16	-0.014300681	0.320196912	0.863697275	ZGRF1
ENSG00000267074.1	-0.015106579	0.320199528	0.863697275	RP11-1094M14.5
ENSG00000275936.1	-0.030782732	0.32021159	0.863697275	RP1-278C19.8
ENSG00000104904.12	0.012518341	0.320243186	0.863697275	OAZ1
ENSG00000107036.12	0.009386885	0.320243519	0.863697275	RIC1
ENSG00000286061.1	0.021798533	0.320324238	0.863713002	RCAN3AS
ENSG00000087074.8	0.009816384	0.320360179	0.863713002	PPP1R15A
ENSG00000156983.17	-0.006214094	0.320375698	0.863713002	BRPF1
ENSG00000177338.14	0.036132409	0.320452569	0.863720402	LINC00469
ENSG00000093134.15	-0.013642456	0.320462675	0.863720402	VNN3
ENSG00000152219.5	0.011735599	0.320603988	0.863765803	ARL14EP
ENSG00000286918.1	0.034611084	0.32061541	0.863765803	RP4-592A1.5

ENSG00000275898.1	-0.032685406	0.320670639	0.863765803	RP11-497G19.7
ENSG00000141012.13	-0.007204756	0.320733087	0.863765803	GALNS
ENSG00000168566.13	0.007957015	0.320809544	0.863765803	SNRNP48
ENSG00000162231.14	0.004246886	0.32081302	0.863765803	NXF1
ENSG00000175793.12	-0.02690142	0.320848614	0.863765803	SFN
ENSG00000089597.18	-0.00701235	0.320863914	0.863765803	GANAB
ENSG00000273973.1	0.034563784	0.320876371	0.863765803	RP11-973D8.5
ENSG00000272853.1	-0.023644329	0.320905142	0.863765803	SUV39H2-DT
ENSG00000250056.8	-0.03114536	0.320942823	0.863765803	LINC01018
ENSG00000163964.18	0.009605074	0.321039338	0.863912181	PIGX
ENSG00000151948.12	0.009459259	0.321106611	0.863979845	GLT1D1
ENSG00000276241.1	0.054189605	0.321165377	0.864024603	CTB-91J4.1
ENSG00000091844.8	-0.023036784	0.321388088	0.864510349	RGS17
ENSG00000284946.1	0.017466151	0.321568163	0.864700426	AC068831.18
ENSG00000242472.1	0.029998601	0.321585567	0.864700426	IGHJ5
ENSG00000275613.2	0.040304471	0.3215971	0.864700426	CTD-3194G12.2
ENSG00000179886.6	-0.009457699	0.321658795	0.864700426	TIGD5
ENSG00000155755.19	-0.014285178	0.321699615	0.864700426	TMEM237
ENSG00000172809.13	0.011119027	0.321711734	0.864700426	RPL38
ENSG00000141084.11	0.0123912	0.321870211	0.864803674	RANBP10
ENSG00000230074.3	0.023244796	0.321913123	0.864803674	RP11-195F19.9
ENSG00000010270.14	0.010847069	0.321959312	0.864803674	STARD3NL
ENSG00000152894.15	-0.024555554	0.322190848	0.864803674	PTPRK
ENSG00000158156.9	0.009041219	0.3222034	0.864803674	XKR8
ENSG00000184182.19	0.009382958	0.322205276	0.864803674	UBE2F
ENSG00000183647.11	0.008476838	0.322234226	0.864803674	ZNF530
ENSG00000223960.7	-0.014326306	0.322273223	0.864803674	CHROMR
ENSG00000223991.1	0.032181179	0.322330031	0.864803674	AC104809.2
ENSG00000263120.1	0.032162498	0.322339014	0.864803674	RP5-1107A17.4
ENSG00000143190.23	-0.007071829	0.322347616	0.864803674	POU2F1
ENSG00000183066.14	-0.018023965	0.322444949	0.864803674	WBP2NL
ENSG00000146842.17	-0.009793835	0.322491066	0.864803674	TMEM209
ENSG00000286799.1	0.033083053	0.322528795	0.864803674	RP11-135L13.7
ENSG00000010438.17	0.045250879	0.322529156	0.864803674	PRSS3
ENSG00000218048.2	0.076321406	0.322537762	0.864803674	RP3-407E4.4
ENSG00000100351.17	0.009306796	0.322543032	0.864803674	GRAP2
ENSG00000170677.6	0.014064709	0.32261334	0.864803674	SOCS6
ENSG00000160179.19	-0.017468872	0.32262211	0.864803674	ABCG1
ENSG00000264739.1	-0.037136033	0.322635341	0.864803674	RP11-138I1.2
ENSG00000254231.3	0.023962013	0.322711147	0.864803674	CTD-2284J15.1
ENSG00000165030.4	0.009477296	0.32277995	0.864803674	NFIL3
ENSG00000236088.10	-0.011994848	0.322819711	0.864803674	COX10-AS1
ENSG00000159842.16	-0.006681829	0.32284165	0.864803674	ABR
ENSG00000255760.2	0.016246516	0.322883552	0.864803674	LINC02422
ENSG00000127884.5	0.009352273	0.322960803	0.864803674	ECHS1
ENSG00000172578.12	0.008021092	0.322975729	0.864803674	KLHL6
ENSG00000237758.1	-0.032915061	0.323124248	0.864803674	BANF1P3

ENSG00000106526.11	-0.019599711	0.323217234	0.864803674	ACTR3C
ENSG00000285130.2	-0.020037677	0.323250176	0.864803674	RP11-16N10.2
ENSG00000285813.1	-0.016490273	0.323324868	0.864803674	RP11-822P4.2
ENSG00000188554.15	0.005586059	0.32339681	0.864803674	NBR1
ENSG00000101210.13	-0.041399353	0.323417399	0.864803674	EEF1A2
ENSG00000119599.17	-0.011942524	0.323471957	0.864803674	DCAF4
ENSG00000118363.12	0.00750668	0.323483834	0.864803674	SPCS2
ENSG00000198590.12	-0.016700578	0.323486766	0.864803674	C3orf35
ENSG00000138768.15	0.008415233	0.323532774	0.864803674	USO1
ENSG00000181061.14	0.013852035	0.323544773	0.864803674	HIGD1A
ENSG00000253230.9	-0.11132233	0.323559042	0.864803674	MIR124-1HG
ENSG00000176209.12	0.009629351	0.323560822	0.864803674	SMIM19
ENSG00000182107.7	-0.018783817	0.32364504	0.864803674	TMEM30B
ENSG00000187987.9	0.053494633	0.323651147	0.864803674	ZSCAN23
ENSG00000214432.9	-0.039310537	0.323659614	0.864803674	VPS33B-DT
ENSG00000180138.7	0.022888264	0.323690297	0.864803674	CSNK1A1L
ENSG00000131473.17	-0.007459451	0.323694374	0.864803674	ACLY
ENSG00000268222.1	0.033289576	0.323704515	0.864803674	EEF1A1P7
ENSG00000187790.11	0.015295314	0.323811422	0.864803674	FANCM
ENSG00000165102.15	0.007145452	0.323823644	0.864803674	HGSNAT
ENSG00000159713.11	0.020958229	0.323889879	0.864803674	TPPP3
ENSG00000267288.2	-0.018950888	0.324005057	0.864803674	RP13-890H12.2
ENSG00000133816.18	0.011831962	0.32409254	0.864803674	MICAL2
ENSG00000189376.12	0.009885484	0.324102008	0.864803674	C8orf76
ENSG00000162526.7	0.02162865	0.324110449	0.864803674	TSSK3
ENSG00000256862.3	0.023707441	0.324136152	0.864803674	PARP11-AS1
ENSG00000136044.12	0.006647836	0.324140964	0.864803674	APPL2
ENSG00000139946.10	0.008222264	0.324179268	0.864803674	PELI2
ENSG00000227057.10	-0.0075195	0.324228937	0.864803674	WDR46
ENSG00000260973.1	-0.136184673	0.324253181	0.864803674	RP11-105C19.2
ENSG00000076554.16	-0.013782412	0.324350687	0.864803674	TPD52
ENSG00000236814.1	0.037413755	0.324393684	0.864803674	RP11-446E9.1
ENSG00000181458.10	-0.04660194	0.324401241	0.864803674	TMEM45A
ENSG00000054179.12	-0.037603965	0.324425437	0.864803674	ENTPD2
ENSG00000288033.1	0.025878273	0.324533429	0.864803674	RP13-46H24.3
ENSG00000145416.13	0.013800154	0.324538309	0.864803674	MARCHF1
ENSG00000204860.5	-0.022878142	0.324599732	0.864803674	FAM201A
ENSG00000147535.17	-0.016084594	0.324603828	0.864803674	PLPP5
ENSG00000134900.12	0.007729534	0.324613259	0.864803674	TPP2
ENSG00000188389.11	-0.020933925	0.324683034	0.864803674	PDCD1
ENSG00000227598.1	-0.023397638	0.324716784	0.864803674	RP1-167A14.2
ENSG00000140459.18	0.04834293	0.324782605	0.864803674	CYP11A1
ENSG00000165501.17	0.010946867	0.324842416	0.864803674	LRR1
ENSG00000157895.11	0.006203875	0.324865681	0.864803674	C12orf43
ENSG00000133937.5	-0.063999746	0.324866101	0.864803674	GSC
ENSG00000101150.18	0.006080897	0.324886677	0.864803674	TPD52L2
ENSG00000245573.9	-0.022404011	0.324924781	0.864803674	BDNF-AS

ENSG00000258581.2	0.031862681	0.32500961	0.864803674	RP11-638I2.10
ENSG00000146433.10	-0.011298448	0.325012587	0.864803674	TMEM181
ENSG00000255882.1	0.036009207	0.325039337	0.864803674	RP11-290C10.1
ENSG00000164989.17	-0.022078934	0.325168581	0.864971608	CCDC171
ENSG00000211974.3	-0.063898988	0.325194719	0.864971608	IGHV2-70D
ENSG00000225636.1	-0.037124596	0.325228987	0.864971608	HNRNPA3P15
ENSG00000147437.10	-0.008499607	0.325340599	0.865156251	GNRH1
ENSG00000123416.15	0.009029636	0.325653521	0.865851922	TUBA1B
ENSG00000109756.9	0.011286981	0.325686646	0.865851922	RAPGEF2
ENSG00000272899.4	-0.018846948	0.325871759	0.866119994	ATP6V1FNB
ENSG00000162598.13	-0.105348902	0.325871946	0.866119994	C1orf87
ENSG00000271828.1	0.027793901	0.326017037	0.866320533	CTD-2310F14.1
ENSG00000237892.1	-0.037757445	0.326031884	0.866320533	KLF7-IT1
ENSG00000177359.20	-0.063680811	0.326091372	0.866366349	OVOS2
ENSG00000152495.11	0.014050208	0.326319314	0.866857676	CAMK4
ENSG00000130045.16	-0.059041918	0.326412084	0.866857676	NXNL2
ENSG00000226874.1	-0.041869051	0.326459145	0.866857676	AC005154.8
ENSG00000141551.14	-0.006122782	0.326506202	0.866857676	CSNK1D
ENSG00000177192.14	-0.007949777	0.326506326	0.866857676	PUS1
ENSG00000154645.14	0.042537536	0.326529917	0.866857676	CHODL
ENSG00000227825.4	0.014896116	0.326609963	0.866863436	SLC9A7P1
ENSG00000153904.21	-0.058571378	0.326636557	0.866863436	DDAH1
ENSG00000279483.2	-0.030156468	0.326658938	0.866863436	RP11-84E17.1
ENSG00000243243.5	-0.028837027	0.32675942	0.866863436	AC073130.3
ENSG00000089157.16	0.011560445	0.326785871	0.866863436	RPLP0
ENSG00000285578.1	0.033054715	0.326789766	0.866863436	RP3-416J7.6
ENSG00000198648.11	-0.008456625	0.326843592	0.866863436	STK39
ENSG00000134627.12	-0.013087159	0.326870243	0.866863436	PIWIL4
ENSG00000255559.1	-0.01655593	0.326976328	0.866991249	ZNF252P-AS1
ENSG00000174680.10	-0.03633242	0.327049596	0.866991249	GRIK1-AS1
ENSG00000177728.17	-0.00638982	0.327079463	0.866991249	TMEM94
ENSG00000167123.19	0.025893392	0.327206957	0.866991249	CERCAM
ENSG00000124786.12	0.008686066	0.32723499	0.866991249	SLC35B3
ENSG00000273004.1	0.030015575	0.327270803	0.866991249	GS1-279B7.2
ENSG00000099985.4	0.016743627	0.327271725	0.866991249	OSM
ENSG00000166669.13	0.008984359	0.327337444	0.866991249	ATF7IP2
ENSG00000267534.4	-0.011472049	0.327383476	0.866991249	S1PR2
ENSG00000261423.1	-0.022342852	0.327530125	0.866991249	TMEM202-AS1
ENSG00000184708.18	-0.005247194	0.327545113	0.866991249	EIF4ENIF1
ENSG00000183458.14	0.02308267	0.327591281	0.866991249	PKD1P3
ENSG00000177238.14	0.038530378	0.327600449	0.866991249	TRIM72
ENSG00000174137.13	-0.019050857	0.327602122	0.866991249	FAM53A
ENSG00000064490.14	-0.009326156	0.327684731	0.866991249	RFXANK
ENSG00000124164.16	0.004279545	0.327719247	0.866991249	VAPB
ENSG00000166676.17	-0.010442236	0.32774313	0.866991249	TVP23A
ENSG00000144228.9	0.012368131	0.327793235	0.866991249	SPOPL
ENSG00000165272.16	-0.013693679	0.327822335	0.866991249	AQP3

ENSG00000038532.16	-0.00638894	0.327850757	0.866991249	CLEC16A
ENSG00000185504.17	-0.007688367	0.327858104	0.866991249	FAAP100
ENSG00000277075.2	0.026809246	0.327881048	0.866991249	H2AC8
ENSG00000111554.15	0.010121024	0.3279222	0.866991249	MDM1
ENSG00000101004.15	-0.030925222	0.327955967	0.866991249	NINL
ENSG00000120549.18	0.059444023	0.328043899	0.866991249	KIAA1217
ENSG00000259674.1	0.027415339	0.328068561	0.866991249	RP11-356M20.1
ENSG00000118515.12	-0.010780541	0.328087629	0.866991249	SGK1
ENSG00000213694.6	0.015374333	0.328102159	0.866991249	S1PR3
ENSG00000272255.1	0.034766619	0.328179036	0.867018982	CTD-3224K15.3
ENSG00000264386.1	0.039026886	0.32822117	0.867018982	MIR4513
ENSG00000086015.22	-0.009488339	0.328304194	0.867018982	MAST2
ENSG00000279443.1	-0.033246672	0.328308616	0.867018982	RP4-669K10.8
ENSG00000079950.14	0.009121367	0.328339561	0.867018982	STX7
ENSG00000176014.13	-0.020901047	0.328366317	0.867018982	TUBB6
ENSG00000204344.15	-0.006982023	0.328447499	0.86704442	STK19
ENSG00000185304.15	0.04793329	0.328460508	0.86704442	RGPD2
ENSG00000211955.2	-0.033445007	0.32855207	0.867151944	IGHV3-33
ENSG00000285417.1	0.022481142	0.328585808	0.867151944	CH17-84K15.2
ENSG00000176208.9	-0.011752517	0.328672032	0.867261768	ATAD5
ENSG00000175048.17	-0.012529384	0.328712001	0.867261768	ZDHHC14
ENSG00000160200.18	-0.052216702	0.328865243	0.867452576	CBS
ENSG00000263916.1	-0.023294784	0.328868918	0.867452576	RP11-110H1.4
ENSG00000262903.1	-0.020675602	0.329004027	0.867697348	RP11-235E17.6
ENSG00000113368.12	0.010955905	0.3291524	0.867786253	LMNB1
ENSG00000167930.17	-0.008544315	0.329183271	0.867786253	FAM234A
ENSG00000087586.18	-0.014009985	0.32920545	0.867786253	AURKA
ENSG00000164144.16	0.010348011	0.329206995	0.867786253	ARFIP1
ENSG00000129472.15	0.007245884	0.329276758	0.867792501	RAB2B
ENSG00000178217.15	0.026536223	0.329358402	0.867792501	SH2D4B
ENSG00000163728.11	-0.010945716	0.329384604	0.867792501	TTC14
ENSG00000188649.15	-0.017200644	0.32938574	0.867792501	CC2D2B
ENSG00000172232.10	-0.045108497	0.329484469	0.867792501	AZU1
ENSG00000211779.3	-0.026829417	0.329504282	0.867792501	TRAV5
ENSG00000101346.14	-0.006099438	0.329505569	0.867792501	POFUT1
ENSG00000266075.2	0.030937787	0.329706744	0.868204554	RN7SL574P
ENSG00000269981.1	-0.016777477	0.329746697	0.868204554	RP11-34P13.16
ENSG00000188566.15	-0.008708088	0.329806065	0.868249395	NDOR1
ENSG00000119682.18	0.005838382	0.32989641	0.868307809	AREL1
ENSG00000219626.9	0.010695486	0.329912934	0.868307809	FAM228B
ENSG00000196937.11	-0.015153099	0.330018911	0.868367946	FAM3C
ENSG00000138794.10	0.008751402	0.330020469	0.868367946	CASP6
ENSG00000196715.7	0.006034471	0.33018794	0.868697149	VKORC1L1
ENSG00000092201.10	0.004334882	0.330303252	0.868889058	SUPT16H
ENSG00000115415.20	0.017677285	0.330354606	0.868889533	STAT1
ENSG00000105662.16	-0.008213944	0.330388169	0.868889533	CRTC1
ENSG00000246705.4	0.010027276	0.330506586	0.869018758	H2AJ

ENSG00000217083.1	-0.032080802	0.330522055	0.869018758	MTCO2P33
ENSG00000105854.13	0.010736008	0.330660155	0.86912937	PON2
ENSG00000105254.12	-0.008552307	0.330738865	0.86912937	TBCB
ENSG00000161960.16	-0.009458031	0.330773816	0.86912937	EIF4A1
ENSG00000168356.13	0.037676709	0.330814058	0.86912937	SCN11A
ENSG00000228140.2	-0.017055575	0.330822812	0.86912937	RP3-467K16.4
ENSG00000149100.13	0.006384873	0.330835633	0.86912937	EIF3M
ENSG00000277290.1	-0.02552764	0.330945318	0.86912937	RP11-326C3.16
ENSG00000164604.14	-0.056995363	0.330970091	0.86912937	GPR85
ENSG00000167461.12	-0.007688241	0.33101944	0.86912937	RAB8A
ENSG00000109063.15	0.010616075	0.33103578	0.86912937	MYH3
ENSG00000186654.21	-0.012903025	0.331070716	0.86912937	PRR5
ENSG00000088833.18	-0.007641532	0.331103908	0.86912937	NSFL1C
ENSG00000251580.3	-0.026983006	0.331140884	0.86912937	LINC02482
ENSG00000115825.11	0.008744425	0.331168701	0.86912937	PRKD3
ENSG00000197283.18	-0.010066897	0.331225632	0.86912937	SYNGAP1
ENSG00000272677.1	0.028359956	0.331258013	0.86912937	HNRNPD-DT
ENSG00000230747.1	-0.021814236	0.331284586	0.86912937	AC021188.4
ENSG00000198060.10	0.005831431	0.331379449	0.869267044	MARCHF5
ENSG00000130177.16	0.007469754	0.331617564	0.86972763	CDC16
ENSG00000279457.4	0.017523005	0.331664533	0.86972763	WASH9P
ENSG00000274173.1	0.034980356	0.33168226	0.86972763	RP4-568C11.4
ENSG00000114423.23	-0.008569549	0.331767763	0.869840614	CBLB
ENSG00000251179.1	-0.02916592	0.331849018	0.869882098	TMEM92-AS1
ENSG00000108958.4	-0.02586596	0.331868419	0.869882098	AC016292.3
ENSG00000247627.2	0.084332946	0.331980829	0.870065539	MTND4P12
ENSG00000182257.8	0.015472483	0.332047741	0.870129706	PRR34
ENSG00000101444.13	0.007482771	0.332246994	0.870484602	AHCY
ENSG00000239494.2	-0.036219105	0.332268064	0.870484602	RN7SL333P
ENSG00000155729.13	0.005673035	0.33235745	0.870550855	KCTD18
ENSG00000170242.19	0.009966547	0.332378252	0.870550855	USP47
ENSG00000109805.10	-0.029555784	0.332455876	0.87064297	NCAPG
ENSG00000145107.16	-0.035881899	0.33252585	0.870715031	TM4SF19
ENSG00000181215.15	-0.031146846	0.332618509	0.870846469	C4orf50
ENSG00000105205.7	0.028881272	0.332766462	0.870992946	CLC
ENSG00000070759.17	0.007797681	0.332809347	0.870992946	TESK2
ENSG00000061918.14	0.01897269	0.332825971	0.870992946	GUCY1B1
ENSG00000198799.12	-0.005690368	0.332869063	0.870992946	LRIG2
ENSG00000120915.14	0.017645892	0.332895587	0.870992946	EPHX2
ENSG00000283294.1	-0.034245112	0.332969716	0.870992946	RP11-681B3.4
ENSG00000183597.16	0.007357311	0.332971752	0.870992946	TANGO2
ENSG00000274403.1	-0.024707299	0.333139295	0.871165907	CTD-2036P10.6
ENSG00000155090.15	-0.010731697	0.333187815	0.871165907	KLF10
ENSG00000185624.16	-0.007053455	0.33320311	0.871165907	P4HB
ENSG00000165304.8	-0.03083164	0.333252303	0.871165907	MELK
ENSG00000096996.16	0.010175158	0.333306462	0.871165907	IL12RB1
ENSG00000257277.2	-0.041266752	0.333331502	0.871165907	RP11-434H14.1

ENSG00000149516.14	0.034392759	0.333397498	0.871165907	MS4A3
ENSG00000168453.16	0.071774995	0.33344297	0.871165907	HR
ENSG00000180776.16	0.011955067	0.333448063	0.871165907	ZDHHC20
ENSG00000227006.2	-0.03553866	0.333462667	0.871165907	RP5-956O18.2
ENSG00000244198.7	-0.023522009	0.333528072	0.871177967	ARHGEF35-AS1
ENSG00000108306.13	-0.007108391	0.333552243	0.871177967	FBXL20
ENSG00000211777.2	0.022319766	0.333812696	0.871383129	TRAV3
ENSG00000154917.11	0.022606223	0.333922307	0.871383129	RAB6B
ENSG00000285292.1	-0.033993243	0.334081234	0.871383129	ABCF2-H2BE1
ENSG00000163602.11	0.007728542	0.334118644	0.871383129	RYBP
ENSG00000228078.1	0.049985491	0.334131317	0.871383129	HLA-U
ENSG00000132967.9	0.016048406	0.3341548	0.871383129	HMGB1P5
ENSG00000142949.17	0.028150383	0.33416235	0.871383129	PTPRF
ENSG00000132680.11	0.004505431	0.334178142	0.871383129	KHDC4
ENSG00000197497.11	-0.01561918	0.334199896	0.871383129	ZNF665
ENSG00000168802.14	-0.010763	0.334235888	0.871383129	CHTF8
ENSG00000167716.19	-0.0083604	0.334257809	0.871383129	WDR81
ENSG00000183386.10	-0.018718178	0.334276573	0.871383129	FHL3
ENSG00000129990.15	-0.082595179	0.334298762	0.871383129	SYT5
ENSG00000169570.10	0.010905191	0.334431071	0.871383129	DTWD2
ENSG00000184923.12	0.03152505	0.33443464	0.871383129	NUTM2A
ENSG00000170345.10	0.012775109	0.334440013	0.871383129	FOS
ENSG00000241852.10	-0.010233802	0.334442846	0.871383129	C8orf58
ENSG00000274051.1	-0.026312295	0.334520798	0.871383129	ENSG10010136777.1
ENSG00000145864.14	0.13330332	0.33454153	0.871383129	GABRB2
ENSG00000100360.15	0.006513897	0.33457453	0.871383129	IFT27
ENSG00000067955.15	-0.007257196	0.334582649	0.871383129	CBFB
ENSG00000260708.1	-0.019427596	0.334589966	0.871383129	CTA-29F11.1
ENSG00000236782.7	-0.032035267	0.334608062	0.871383129	ZNF593OS
ENSG00000268583.2	-0.016891446	0.33465455	0.871393539	CTC-453G23.8
ENSG00000175267.15	0.024190316	0.334758262	0.871552931	VWA3A
ENSG00000228010.6	0.030129985	0.335022318	0.872040502	AC073343.13
ENSG00000143107.10	-0.040912656	0.335053015	0.872040502	FNDC7
ENSG00000284914.1	-0.048794151	0.335076056	0.872040502	RP11-517H2.7
ENSG00000270681.1	0.024085568	0.335153907	0.872040502	RP11-372K14.2
ENSG00000266445.1	-0.022151238	0.335158145	0.872040502	NARF-AS1
ENSG00000100767.17	0.011806162	0.335271714	0.87210043	PAPLN
ENSG00000198569.10	-0.032917615	0.335290839	0.87210043	SLC34A3
ENSG00000188375.5	-0.035235633	0.335308752	0.87210043	H3-5
ENSG00000130204.13	-0.007945787	0.335403411	0.872235414	TOMM40
ENSG00000233746.2	-0.030424404	0.335445714	0.872235414	LINC00656
ENSG00000172456.18	0.0130932	0.335594168	0.872505829	FGGY
ENSG00000138382.15	0.009879417	0.335803865	0.872505829	METTL5
ENSG00000205544.4	0.017609858	0.335863825	0.872505829	TMEM256
ENSG00000160570.14	-0.007995619	0.335893223	0.872505829	DEDD2
ENSG00000145103.15	-0.030159504	0.335959713	0.872505829	ILDR1
ENSG00000013523.11	0.0060467	0.336002685	0.872505829	ANGEL1

ENSG00000212694.8	0.010596084	0.336020257	0.872505829	LINC01089
ENSG00000109654.16	0.021143294	0.336038897	0.872505829	TRIM2
ENSG00000149541.10	-0.009151319	0.336051233	0.872505829	B3GAT3
ENSG00000256591.5	-0.015280909	0.336194604	0.872505829	RP11-286N22.8
ENSG00000162889.11	0.006749126	0.336353984	0.872505829	MAPKAPK2
ENSG00000137802.14	-0.006792665	0.336360034	0.872505829	MAPKBP1
ENSG00000250575.1	0.049934536	0.336407113	0.872505829	RP4-669L17.8
ENSG00000249348.1	0.020726914	0.336408256	0.872505829	UGDH-AS1
ENSG00000185899.1	0.061015529	0.336425273	0.872505829	TAS2R60
ENSG00000169758.13	-0.019573448	0.336429215	0.872505829	TMEM266
ENSG00000172057.10	-0.010763554	0.336451876	0.872505829	ORMDL3
ENSG00000131507.11	0.006767398	0.336460269	0.872505829	NDFIP1
ENSG00000219481.10	-0.014997873	0.336463596	0.872505829	NBPF1
ENSG00000110987.9	-0.019262839	0.336480243	0.872505829	BCL7A
ENSG00000271032.1	-0.012754937	0.336544717	0.872505829	CTD-2527I21.14
ENSG00000135480.16	-0.037090083	0.336555704	0.872505829	KRT7
ENSG00000217648.1	-0.034757081	0.336642784	0.872505829	RP1-95L4.4
ENSG00000181007.9	-0.010634987	0.336657507	0.872505829	ZFP82
ENSG00000230084.6	0.019595038	0.336851364	0.872505829	RP4-613B23.1
ENSG00000108771.13	-0.01930577	0.33687132	0.872505829	DHX58
ENSG00000241506.1	0.018606985	0.336873804	0.872505829	PSMC1P1
ENSG00000287005.1	-0.022078334	0.336966173	0.872505829	RP11-1D19.1
ENSG00000148737.17	-0.013721408	0.336995136	0.872505829	TCF7L2
ENSG00000214176.9	0.009003901	0.337003561	0.872505829	PLEKHM1P1
ENSG00000163374.20	0.007389775	0.337081798	0.872505829	YY1AP1
ENSG00000159860.7	0.036726139	0.337127918	0.872505829	TCAF2P1
ENSG00000280119.1	-0.024795299	0.337166968	0.872505829	AC093642.1
ENSG00000133983.16	0.014444871	0.337279173	0.872505829	COX16
ENSG00000106153.13	0.012808468	0.337301524	0.872505829	CHCHD2
ENSG00000225733.6	0.00720512	0.337391765	0.872505829	FGD5-AS1
ENSG00000197892.13	-0.008860795	0.337410964	0.872505829	KIF13B
ENSG00000153827.14	0.006277393	0.337425786	0.872505829	TRIP12
ENSG00000181274.7	0.010465686	0.337430139	0.872505829	FRAT2
ENSG00000228252.9	-0.032855166	0.337519447	0.872505829	COL6A4P2
ENSG00000178234.13	-0.006587075	0.337527781	0.872505829	GALNT11
ENSG00000171189.17	0.019190001	0.337582761	0.872505829	GRIK1
ENSG00000207425.1	0.026176408	0.337611637	0.872505829	Y_RNA
ENSG00000204677.13	0.029078119	0.337734857	0.872505829	FAM153CP
ENSG00000140030.6	0.011743386	0.337745527	0.872505829	GPR65
ENSG00000258551.6	0.047452503	0.337815484	0.872505829	CRAT37
ENSG00000165644.11	-0.014682537	0.337890754	0.872505829	COMTD1
ENSG00000272825.1	0.014307807	0.337914114	0.872505829	LL21NC02-1C16.2
ENSG00000088766.12	-0.014129868	0.337954726	0.872505829	CRLS1
ENSG00000134318.14	0.008590371	0.337986383	0.872505829	ROCK2
ENSG00000143633.13	0.006671451	0.337989077	0.872505829	C1orf131
ENSG00000169727.12	-0.007525027	0.33800754	0.872505829	GPS1
ENSG00000119559.17	0.007248895	0.338009413	0.872505829	C19orf25

ENSG00000105519.18	-0.010408755	0.338045594	0.872505829	CAPS
ENSG00000135127.11	-0.008707361	0.33807335	0.872505829	BICDL1
ENSG00000067596.12	0.006984282	0.338184131	0.872505829	DHX8
ENSG00000079393.21	0.029050484	0.338232928	0.872505829	DUSP13
ENSG00000140332.17	-0.010144997	0.338277212	0.872505829	TLE3
ENSG00000231628.1	0.020815496	0.338278399	0.872505829	RP3-355L5.4
ENSG00000100711.14	-0.006437048	0.338330225	0.872505829	ZFYVE21
ENSG00000250081.1	-0.055824705	0.338352052	0.872505829	CTD-2116N20.1
ENSG00000257176.3	-0.020142427	0.338373705	0.872505829	RP11-996F15.2
ENSG00000160959.8	-0.006369261	0.338392904	0.872505829	LRRC14
ENSG00000204568.12	0.007086389	0.338442921	0.872505829	MRPS18B
ENSG00000223551.1	0.019432304	0.338464203	0.872505829	TMSB4XP4
ENSG00000211814.1	-0.022278894	0.338464656	0.872505829	TRAV35
ENSG00000165914.15	0.016182518	0.338482697	0.872505829	TTC7B
ENSG00000105991.10	-0.020621632	0.338488217	0.872505829	HOXA1
ENSG00000254802.1	0.020400675	0.338517129	0.872505829	AC022182.3
ENSG00000138095.20	0.006358705	0.338527837	0.872505829	LRPPRC
ENSG00000286733.1	0.052935232	0.338683348	0.872784122	LINC02348
ENSG00000064655.19	0.056770059	0.33872093	0.872784122	EYA2
ENSG00000287684.1	0.039683364	0.338798191	0.872873531	RP11-452F19.4
ENSG00000213087.3	0.047888432	0.338994254	0.873268956	RP11-613F7.1
ENSG00000230626.4	-0.03322407	0.339052077	0.873308213	RP11-286H14.4
ENSG00000260032.2	0.00756445	0.339106292	0.873338169	NORAD
ENSG00000225338.1	-0.036971355	0.339263327	0.873632888	RPL23AP18
ENSG00000119729.12	0.007092205	0.339426808	0.873810167	RHOQ
ENSG00000235847.2	0.047380584	0.339441983	0.873810167	LDHAP7
ENSG00000196152.10	-0.008725375	0.339459996	0.873810167	ZNF79
ENSG00000166546.14	0.023504347	0.339650826	0.874190206	BEAN1
ENSG00000078140.14	0.005075379	0.339692888	0.874190206	UBE2K
ENSG00000258546.1	-0.026679212	0.339806764	0.874262745	CENPUP2
ENSG00000181222.19	-0.011154083	0.339866683	0.874262745	POLR2A
ENSG00000162073.14	-0.012406975	0.339891024	0.874262745	PAQR4
ENSG00000154920.15	-0.020322028	0.339891597	0.874262745	EME1
ENSG00000089022.14	-0.00551669	0.340155457	0.874452533	MAPKAPK5
ENSG00000100426.7	-0.006387334	0.340201086	0.874452533	ZBED4
ENSG00000182606.17	-0.006407792	0.340217093	0.874452533	TRAK1
ENSG00000255860.3	0.120184642	0.340250127	0.874452533	RP11-807H22.6
ENSG00000145708.11	0.020542351	0.340284393	0.874452533	CRHBP
ENSG00000070190.13	0.011245681	0.340297253	0.874452533	DAPP1
ENSG00000112742.10	-0.029801784	0.3403286	0.874452533	TTK
ENSG00000169385.3	0.029196943	0.340328935	0.874452533	RNASE2
ENSG00000101181.18	0.006354809	0.340349138	0.874452533	MTG2
ENSG00000077235.18	-0.007791309	0.34046602	0.874613985	GTF3C1
ENSG00000173805.16	0.04844852	0.340552084	0.874613985	HAP1
ENSG00000100014.20	-0.007361609	0.3405806	0.874613985	SPECC1L
ENSG00000136149.6	0.018301563	0.340582567	0.874613985	RPL13AP25
ENSG00000215012.9	0.006847075	0.340630999	0.874628838	RTL10

ENSG00000272501.1	-0.012565703	0.340730061	0.874654837	XXbac-BPG299F13.17
ENSG00000272143.1	-0.030414644	0.34079497	0.874654837	FGF14-AS2
ENSG00000205056.8	-0.029159726	0.340845601	0.874654837	LINC02397
ENSG00000132010.16	-0.025918944	0.341018823	0.874654837	ZNF20
ENSG00000277435.1	0.024304192	0.34102412	0.874654837	RP11-1180F24.1
ENSG00000157445.15	0.014381664	0.341036736	0.874654837	CACNA2D3
ENSG00000260490.2	-0.030345545	0.341090314	0.874654837	MYL12BP1
ENSG00000253364.2	0.044114159	0.341188726	0.874654837	COPDA1
ENSG00000114030.13	0.005189465	0.341198091	0.874654837	KPNA1
ENSG00000171055.15	0.007614426	0.341200285	0.874654837	FEZ2
ENSG00000279089.1	-0.017982358	0.341213486	0.874654837	RP11-481C4.2
ENSG00000267416.1	0.034137725	0.341343124	0.874654837	HEATR6-DT
ENSG00000139055.7	-0.009497173	0.341359556	0.874654837	ERP27
ENSG00000235079.1	-0.023388628	0.341372971	0.874654837	ZRANB2-AS1
ENSG00000261067.7	-0.011680521	0.341380492	0.874654837	RP11-264B17.3
ENSG00000115594.12	-0.013095862	0.341391589	0.874654837	IL1R1
ENSG00000189144.15	-0.020445122	0.341423826	0.874654837	ZNF573
ENSG00000106086.20	0.009947552	0.341464509	0.874654837	PLEKHA8
ENSG00000127314.18	0.00982471	0.341515795	0.874654837	RAP1B
ENSG00000137265.15	-0.012923276	0.341606525	0.874654837	IRF4
ENSG00000180370.10	0.006692235	0.341618088	0.874654837	PAK2
ENSG00000082438.18	0.022723845	0.341639326	0.874654837	COBLL1
ENSG00000149582.16	-0.012078703	0.341725354	0.874654837	TMEM25
ENSG00000161664.7	0.011660741	0.341765332	0.874654837	ASB16
ENSG00000100629.17	0.012716425	0.341834004	0.874654837	CEP128
ENSG00000120616.16	0.004870071	0.341842193	0.874654837	EPC1
ENSG00000280136.2	-0.088976181	0.341855768	0.874654837	RP11-1228E12.2
ENSG00000143761.16	0.005536824	0.341871711	0.874654837	ARF1
ENSG00000054793.14	0.021230112	0.341937312	0.874654837	ATP9A
ENSG00000213088.12	0.033923827	0.341975858	0.874654837	ACKR1
ENSG00000111667.14	-0.007590403	0.341985094	0.874654837	USP5
ENSG00000231079.7	0.030053975	0.342005907	0.874654837	KIF5C-AS1
ENSG00000122376.12	0.01218287	0.342195874	0.874847037	SHLD2
ENSG00000286640.1	-0.033830413	0.34220162	0.874847037	RP11-399E6.5
ENSG00000120860.11	0.009471927	0.342215898	0.874847037	WASHC3
ENSG00000254966.1	-0.029484008	0.342273145	0.874847037	RP11-1081L13.4
ENSG00000170791.18	0.006582683	0.342339619	0.874847037	CHCHD7
ENSG00000164125.16	0.016036039	0.342376639	0.874847037	GASK1B
ENSG00000173068.18	0.027400013	0.342379672	0.874847037	BNC2
ENSG00000136542.9	0.044149836	0.342580884	0.875099384	GALNT5
ENSG00000271580.1	-0.031940287	0.342607899	0.875099384	RP11-536L3.4
ENSG00000262228.3	-0.020297987	0.342616023	0.875099384	RP11-676J12.4
ENSG00000189051.5	0.029231889	0.342741205	0.875099384	RNF222
ENSG00000180385.8	-0.017558139	0.342769478	0.875099384	EMC3-AS1
ENSG00000137449.17	-0.016516093	0.342775282	0.875099384	CPEB2
ENSG00000260293.2	0.026475066	0.342777129	0.875099384	RP11-715J22.6
ENSG00000147604.14	0.023687255	0.342820926	0.875102259	RPL7

ENSG00000177879.17	0.008252868	0.342890665	0.875171344	AP3S1
ENSG00000081041.9	-0.035472606	0.342938594	0.875184754	CXCL2
ENSG00000169718.18	-0.007034547	0.343111781	0.875517781	DUS1L
ENSG00000175928.6	0.02231724	0.343248165	0.875756826	LRRN1
ENSG00000234981.1	0.033290205	0.34331614	0.875821296	RP11-534L20.4
ENSG00000132781.20	-0.009890897	0.343384776	0.875887436	MUTYH
ENSG00000158714.11	0.023523357	0.343464978	0.875942462	SLAMF8
ENSG00000091157.14	-0.008854602	0.343580845	0.875942462	WDR7
ENSG00000141971.13	-0.009186617	0.3435813	0.875942462	MVB12A
ENSG00000268119.6	0.028002114	0.343609043	0.875942462	CTD-2561J22.5
ENSG00000181408.5	-0.05460853	0.343635273	0.875942462	UTS2R
ENSG00000283312.1	-0.036146617	0.343721902	0.875942462	RP11-562I5.1
ENSG00000142731.11	-0.019015705	0.343802159	0.875942462	PLK4
ENSG00000199804.1	-0.018559704	0.343854533	0.875942462	RNA5SP383
ENSG00000272205.1	-0.023735079	0.34391021	0.875942462	POU2F1-DT
ENSG00000117362.13	0.005752568	0.343934753	0.875942462	APH1A
ENSG00000134516.18	-0.007686655	0.344012528	0.875942462	DOCK2
ENSG00000167705.12	0.018854456	0.344026625	0.875942462	RILP
ENSG00000188687.17	0.013595821	0.344055849	0.875942462	SLC4A5
ENSG00000170421.12	0.026161065	0.344085026	0.875942462	KRT8
ENSG00000272382.1	-0.015114769	0.344090833	0.875942462	CTD-2035E11.4
ENSG00000010072.16	0.007983621	0.344195159	0.875942462	SPRTN
ENSG00000114744.9	0.008407385	0.344210683	0.875942462	COMMD2
ENSG00000257103.9	-0.004249582	0.344221457	0.875942462	LSM14A
ENSG00000240065.8	0.013989034	0.344244347	0.875942462	PSMB9
ENSG00000037757.14	-0.009361614	0.344291466	0.875942462	MRI1
ENSG00000136371.11	-0.012451011	0.344344642	0.875942462	MTHFS
ENSG00000164342.13	-0.022471681	0.344346017	0.875942462	TLR3
ENSG00000215492.6	0.024632267	0.344393465	0.875954505	HNRNPA1P7
ENSG00000149806.11	0.012426861	0.344791228	0.876857454	FAU
ENSG00000224470.9	0.006828597	0.345109719	0.876928356	ATXN1L
ENSG00000237149.5	0.025871779	0.345146877	0.876928356	ZNF503-AS2
ENSG00000131389.18	0.009390544	0.345166966	0.876928356	SLC6A6
ENSG00000265728.1	-0.029199625	0.345197034	0.876928356	RP11-883A18.3
ENSG00000117713.20	-0.009017236	0.345221697	0.876928356	ARID1A
ENSG00000105376.5	0.019771331	0.345235323	0.876928356	ICAM5
ENSG00000268088.1	0.040924736	0.34536922	0.876928356	AC093063.2
ENSG00000237424.1	-0.011223204	0.34541053	0.876928356	FOXD2-AS1
ENSG00000133641.18	-0.013772122	0.34546648	0.876928356	C12orf29
ENSG00000159761.15	0.010992176	0.345534693	0.876928356	C16orf86
ENSG00000218018.3	0.009159088	0.345555202	0.876928356	RBM38-AS1
ENSG00000085274.16	-0.013747761	0.345560549	0.876928356	MYNN
ENSG00000126217.21	0.017099289	0.34557701	0.876928356	MCF2L
ENSG00000255200.1	-0.017344148	0.345588178	0.876928356	PGAM1P8
ENSG00000140471.17	0.008923549	0.345601718	0.876928356	LINS1
ENSG00000163624.6	0.023367768	0.34565416	0.876928356	CDS1
ENSG00000123395.15	-0.009066627	0.345662515	0.876928356	ATG101

ENSG00000250802.7	-0.029153393	0.345685371	0.876928356	ZBED3-AS1
ENSG00000221923.9	0.010294001	0.345689886	0.876928356	ZNF880
ENSG00000260317.1	-0.021420873	0.345704199	0.876928356	RP11-48B3.4
ENSG00000227848.1	0.031516046	0.345717074	0.876928356	SUCLA2-AS1
ENSG00000184163.3	-0.023266507	0.345817457	0.876931796	C1QTNF12
ENSG00000204241.8	-0.021897186	0.345899855	0.876931796	LINC02731
ENSG00000168495.13	0.005316765	0.346012696	0.876931796	POLR3D
ENSG00000234222.6	-0.01322437	0.346030472	0.876931796	LIX1L-AS1
ENSG00000267508.6	-0.020553194	0.346039438	0.876931796	ZNF285
ENSG00000265399.1	-0.016695031	0.346039467	0.876931796	RP13-270P17.2
ENSG00000165714.11	-0.007321067	0.34605449	0.876931796	BORCS5
ENSG00000184292.7	0.054085695	0.346081089	0.876931796	TACSTD2
ENSG00000225697.13	-0.007819355	0.346111845	0.876931796	SLC26A6
ENSG00000274659.2	-0.01863756	0.3461806	0.876931796	LINC02371
ENSG00000128272.17	0.00575987	0.346188795	0.876931796	ATF4
ENSG00000250909.1	-0.02072181	0.346284443	0.877049535	RP11-843P14.2
ENSG00000233559.2	-0.0258429	0.346432716	0.877049535	LINC00513
ENSG00000270820.6	-0.01218537	0.346466017	0.877049535	USP34-DT
ENSG00000127663.15	-0.008516788	0.346547268	0.877049535	KDM4B
ENSG00000286904.1	-0.037395428	0.346569743	0.877049535	RP11-592G13.2
ENSG00000205022.9	-0.020947482	0.346605566	0.877049535	PABPN1L
ENSG00000196628.20	0.016835179	0.346654691	0.877049535	TCF4
ENSG00000284930.1	0.012912123	0.346690913	0.877049535	RP1-240K6.4
ENSG00000051128.19	-0.013045959	0.346715925	0.877049535	HOMER3
ENSG00000287608.1	-0.029664348	0.346775091	0.877049535	CTD-2224J9.11
ENSG00000198920.11	-0.006630085	0.346809195	0.877049535	KIAA0753
ENSG00000249565.2	0.025996457	0.346830875	0.877049535	SERBP1P5
ENSG00000278239.1	0.048721189	0.346870998	0.877049535	AC020956.3
ENSG00000119771.15	-0.018807895	0.346871688	0.877049535	KLHL29
ENSG00000281420.1	-0.031058999	0.346876769	0.877049535	ENSG00000281420
ENSG00000242314.1	-0.028979369	0.346925718	0.877065165	RPL12P32
ENSG00000143771.12	0.009464526	0.347086047	0.877074367	CNIH4
ENSG00000178342.4	-0.04538368	0.347116449	0.877074367	KCNG2
ENSG00000178562.18	0.012380002	0.347184776	0.877074367	CD28
ENSG00000119922.11	-0.026098992	0.347214114	0.877074367	IFIT2
ENSG00000124570.21	0.009514182	0.347251527	0.877074367	SERPINB6
ENSG00000159346.13	0.019908154	0.347411074	0.877074367	ADIPOR1
ENSG00000222808.1	0.033394277	0.347436667	0.877074367	RNU4-47P
ENSG00000151655.19	-0.018166261	0.347452014	0.877074367	ITIH2
ENSG00000124299.15	-0.008154195	0.347504834	0.877074367	PEPD
ENSG00000259959.1	0.015705872	0.347590887	0.877074367	RP11-121C2.2
ENSG00000119396.11	-0.005203286	0.347593277	0.877074367	RAB14
ENSG00000259456.4	-0.01432498	0.347599243	0.877074367	ADNP-AS1
ENSG00000125450.11	-0.005884957	0.347641652	0.877074367	NUP85
ENSG00000138750.15	0.007409508	0.347686126	0.877074367	NUP54
ENSG00000175220.12	-0.006606535	0.347707605	0.877074367	ARHGAP1
ENSG00000225476.1	0.047562851	0.347762046	0.877074367	MTCO3P5

ENSG00000112139.17	-0.0599369	0.347821335	0.877074367	MDGA1
ENSG00000150459.12	0.005767039	0.347831597	0.877074367	SAP18
ENSG00000145020.16	-0.01455148	0.347838748	0.877074367	AMT
ENSG00000163817.16	0.045851389	0.347840464	0.877074367	SLC6A20
ENSG00000132823.11	0.007309516	0.347863576	0.877074367	OSER1
ENSG00000267751.5	-0.021732055	0.347870241	0.877074367	BSG-AS1
ENSG00000188931.4	0.015415572	0.348114827	0.877420429	CFAP126
ENSG00000005189.20	0.018149976	0.348145752	0.877420429	REXO5
ENSG00000148341.18	0.007158273	0.348174346	0.877420429	SH3GLB2
ENSG00000160867.15	-0.019965467	0.348178635	0.877420429	FGFR4
ENSG00000179428.2	0.042027507	0.348313055	0.877651325	IL6-AS1
ENSG00000109534.17	-0.00995073	0.348369154	0.87768484	GAR1
ENSG00000180543.5	-0.019556054	0.348520022	0.877957082	TSPYL5
ENSG00000143195.13	0.031127408	0.348566774	0.877967011	ILDR2
ENSG00000239528.1	0.060933627	0.348686466	0.878160634	RPS14P8
ENSG00000168906.13	0.006145585	0.348754756	0.878205378	MAT2A
ENSG00000171295.13	0.009138717	0.348829029	0.878205378	ZNF440
ENSG00000197857.15	0.006906455	0.3488327	0.878205378	ZNF44
ENSG00000136002.20	-0.017884347	0.348905781	0.878276754	ARHGEF4
ENSG00000157578.14	0.027557022	0.348971319	0.878276754	LCA5L
ENSG00000141934.10	0.054753795	0.349188499	0.878276754	PLPP2
ENSG00000171786.6	-0.01908837	0.349200634	0.878276754	NHLH1
ENSG00000260859.1	-0.041244905	0.349258164	0.878276754	RP11-254F19.2
ENSG00000143578.16	0.008750597	0.349299306	0.878276754	CREB3L4
ENSG00000185958.10	0.033109915	0.349338112	0.878276754	FAM186A
ENSG00000103064.15	0.009006133	0.349370793	0.878276754	SLC7A6
ENSG00000285509.1	0.046079244	0.349387701	0.878276754	TBCEL-TECTA
ENSG00000283930.1	0.027843666	0.349399028	0.878276754	PLD5P1
ENSG00000186283.14	0.006491964	0.349458922	0.878276754	TOR3A
ENSG00000116266.11	0.0088606	0.349528626	0.878276754	STXBP3
ENSG00000286127.1	-0.038020651	0.349541489	0.878276754	KB-1440D3.20
ENSG00000125779.24	-0.00561966	0.349619975	0.878276754	PANK2
ENSG00000145919.11	0.007434738	0.349659391	0.878276754	BOD1
ENSG00000183735.11	0.010345831	0.349667216	0.878276754	TBK1
ENSG00000197415.12	0.025988882	0.349789523	0.878276754	VEPH1
ENSG00000230021.10	0.035790798	0.349797324	0.878276754	RP11-206L10.17
ENSG00000148296.7	-0.007087103	0.349800029	0.878276754	SURF6
ENSG00000260423.2	-0.032853759	0.349812903	0.878276754	LINC02367
ENSG00000117318.9	-0.01729427	0.34982272	0.878276754	ID3
ENSG00000230778.2	-0.034122774	0.349829731	0.878276754	ANKRD63
ENSG00000230836.1	0.054615122	0.349865316	0.878276754	LINC01293
ENSG00000264500.1	-0.033071482	0.349888877	0.878276754	MIR3124
ENSG00000136982.6	-0.029477323	0.350049182	0.87857161	DSCC1
ENSG00000286196.1	0.017006339	0.350146422	0.878708128	RP11-228B15.6
ENSG00000134765.10	0.033307232	0.350262913	0.878815774	DSC1
ENSG00000259030.8	-0.024019399	0.350275021	0.878815774	FPGT-TNNI3K
ENSG00000188042.8	-0.007799062	0.350467383	0.879090143	ARL4C

ENSG00000279873.2	0.017506586	0.350470109	0.879090143	LINC01126
ENSG00000002834.18	-0.006751862	0.350687581	0.879311358	LASP1
ENSG00000260249.3	0.015470646	0.35076085	0.879311358	RP11-401P9.5
ENSG00000232788.1	-0.014121956	0.350803929	0.879311358	ITGA6-AS1
ENSG00000065717.15	-0.014549408	0.350824078	0.879311358	TLE2
ENSG00000108439.11	-0.006831241	0.35086657	0.879311358	PNPO
ENSG00000266028.8	-0.010244622	0.350902835	0.879311358	SRGAP2
ENSG00000287514.2	0.041494093	0.351002296	0.879311358	RP11-344B23.4
ENSG00000122547.11	-0.010248046	0.351016266	0.879311358	EEPD1
ENSG00000159352.16	0.00782478	0.351105066	0.879311358	PSMD4
ENSG00000186919.13	-0.031431926	0.351124653	0.879311358	ZACN
ENSG00000136636.13	0.013659437	0.351132741	0.879311358	KCTD3
ENSG00000287895.1	-0.028791375	0.351187429	0.879311358	RP5-881P19.8
ENSG00000286285.1	0.028364651	0.35121498	0.879311358	RP11-57K16.1
ENSG00000052802.13	0.008491255	0.351313905	0.879311358	MSMO1
ENSG00000283160.1	-0.029477016	0.351342119	0.879311358	MIR4521
ENSG00000259781.1	0.015514757	0.351398588	0.879311358	HMGB1P6
ENSG00000187840.5	0.011620536	0.351416377	0.879311358	EIF4EBP1
ENSG00000283036.3	-0.019666922	0.351533615	0.879311358	LINC01988
ENSG00000253210.2	0.015430182	0.351549563	0.879311358	RP11-809O17.1
ENSG00000062282.15	0.010951514	0.351573614	0.879311358	DGAT2
ENSG00000204577.11	0.009524108	0.351600182	0.879311358	LILRB3
ENSG00000282199.1	0.036547815	0.351725154	0.879311358	RP11-527L4.5
ENSG00000187870.7	0.05262573	0.351727382	0.879311358	RNFT1P3
ENSG00000149480.7	-0.005816798	0.351738389	0.879311358	MTA2
ENSG00000131697.18	-0.007650538	0.35175999	0.879311358	NPHP4
ENSG00000108387.16	0.027845143	0.351822172	0.879311358	SEPTIN4
ENSG00000101639.18	0.011122254	0.35194961	0.879311358	CEP192
ENSG00000257150.3	0.077576434	0.35195031	0.879311358	PGAM1P5
ENSG00000128059.8	0.010615376	0.351951958	0.879311358	PPAT
ENSG00000108813.11	-0.019392313	0.351964273	0.879311358	DLX4
ENSG00000164047.6	0.033304084	0.351979806	0.879311358	CAMP
ENSG00000022840.17	0.015688681	0.352043341	0.879311358	RNF10
ENSG00000272505.1	-0.01576292	0.352082528	0.879311358	RP11-981G7.6
ENSG00000251002.8	0.014542672	0.352143826	0.879311358	AE000661.37
ENSG00000151746.15	-0.010883265	0.352157626	0.879311358	BICD1
ENSG00000181031.16	0.017429795	0.352161154	0.879311358	RPH3AL
ENSG00000014164.7	-0.009318633	0.352193814	0.879311358	ZC3H3
ENSG00000114446.5	0.014511387	0.352229333	0.879311358	IFT57
ENSG00000044459.15	0.013347531	0.352230811	0.879311358	CNTLN
ENSG00000231793.5	0.021090354	0.35234106	0.879311358	DOC2GP
ENSG00000013561.18	0.006298406	0.352356815	0.879311358	RNF14
ENSG00000116691.11	-0.009571794	0.352365201	0.879311358	MIIP
ENSG00000164190.19	0.008331264	0.352401992	0.879311358	NIPBL
ENSG00000267737.1	0.02559092	0.352502603	0.879455399	AC061992.2
ENSG00000157985.19	-0.024117499	0.352551891	0.879471377	AGAP1
ENSG00000156374.16	-0.012378304	0.352715683	0.879684167	PCGF6

ENSG00000111424.12	-0.009280842	0.352722982	0.879684167	VDR
ENSG00000287763.1	0.062948988	0.352917546	0.880062382	RP11-153P14.1
ENSG00000114054.14	0.006244621	0.353043377	0.880192833	PCCB
ENSG00000226986.4	-0.025890614	0.353126882	0.880192833	PRELID1P5
ENSG00000173757.10	0.006509601	0.353169764	0.880192833	STAT5B
ENSG00000112640.16	-0.007474822	0.353260303	0.880192833	PPP2R5D
ENSG00000003436.16	0.030002765	0.353352718	0.880192833	TFPI
ENSG00000278002.1	0.032027237	0.35336619	0.880192833	RP11-596C23.2
ENSG00000033327.13	0.00847885	0.353421847	0.880192833	GAB2
ENSG00000139436.21	0.005926461	0.353422497	0.880192833	GIT2
ENSG00000124564.17	0.043717643	0.353424632	0.880192833	SLC17A3
ENSG00000186352.9	-0.013235189	0.353458517	0.880192833	ANKRD37
ENSG00000147654.15	0.008254936	0.353517903	0.880192833	EBAG9
ENSG00000007516.14	0.023349265	0.353547368	0.880192833	BAIAP3
ENSG00000220323.4	0.013801086	0.353585871	0.880192833	H2BC19P
ENSG00000264057.1	0.033658317	0.353690756	0.880192833	RP11-583F2.1
ENSG00000068654.17	-0.008269858	0.353776539	0.880192833	POLR1A
ENSG00000274370.1	0.017712362	0.353854796	0.880192833	AC144831.3
ENSG00000267260.2	-0.031661477	0.354016213	0.880192833	CTD-2162K18.4
ENSG00000224177.8	0.034238376	0.354062188	0.880192833	LINC00570
ENSG00000168546.11	-0.02241165	0.354093668	0.880192833	GFRA2
ENSG00000186907.8	-0.028555066	0.354135072	0.880192833	RTN4RL2
ENSG00000249993.1	-0.036891458	0.354171167	0.880192833	BFSP2-AS1
ENSG00000120693.14	-0.01238326	0.354189306	0.880192833	SMAD9
ENSG00000178096.9	-0.009958484	0.354199769	0.880192833	BOLA1
ENSG00000158669.11	-0.004324145	0.354302568	0.880192833	GPAT4
ENSG00000251669.6	0.018585538	0.354381273	0.880192833	FAM86EP
ENSG00000183150.8	-0.016193759	0.354467162	0.880192833	GPR19
ENSG00000197046.12	0.014410627	0.354618122	0.880192833	SIGLEC15
ENSG00000135972.9	0.007261959	0.354738799	0.880192833	MRPS9
ENSG00000272338.2	-0.02815038	0.354739704	0.880192833	RP11-722E23.2
ENSG00000116815.16	0.011179207	0.354787293	0.880192833	CD58
ENSG00000158445.10	-0.015816748	0.354845445	0.880192833	KCNB1
ENSG00000186704.9	0.02743069	0.354854419	0.880192833	DTX2P1
ENSG00000226950.8	0.008894683	0.354919578	0.880192833	DANCR
ENSG00000274210.1	-0.034194574	0.354971911	0.880192833	RNVU1-27
ENSG00000120647.10	0.007948711	0.354976641	0.880192833	CCDC77
ENSG00000188234.14	0.01520648	0.355012783	0.880192833	AGAP4
ENSG00000160789.24	0.018235444	0.355047272	0.880192833	LMNA
ENSG00000100314.4	-0.01622838	0.355219683	0.880192833	CABP7
ENSG00000074660.16	0.017657521	0.355275556	0.880192833	SCARF1
ENSG00000180257.14	0.012603866	0.355291712	0.880192833	ZNF816
ENSG00000109452.12	0.014360634	0.355300849	0.880192833	INPP4B
ENSG00000229647.2	0.021706012	0.355311884	0.880192833	MYOSLID
ENSG00000267681.1	-0.019007299	0.355361865	0.880192833	CTD-3199J23.6
ENSG00000223356.1	0.022145987	0.355405634	0.880192833	RP11-66D17.5
ENSG00000205018.2	-0.017041675	0.355548467	0.880192833	RP11-830F9.6

ENSG00000213553.4	0.013727957	0.355603387	0.880192833	RPLP0P6
ENSG00000104626.15	0.007335951	0.355670414	0.880192833	ERI1
ENSG00000215417.13	-0.01615976	0.355755719	0.880192833	MIR17HG
ENSG00000120685.20	-0.006201816	0.355773917	0.880192833	PROSER1
ENSG00000185272.14	0.025363023	0.355818715	0.880192833	RBM11
ENSG00000109089.7	0.031919012	0.355828663	0.880192833	CDR2L
ENSG00000273203.1	-0.035450333	0.355844493	0.880192833	AC006946.16
ENSG00000167740.10	0.007229175	0.355964733	0.880192833	CYB5D2
ENSG00000143816.8	0.027827405	0.355985337	0.880192833	WNT9A
ENSG00000104953.20	-0.037397547	0.356008801	0.880192833	TLE6
ENSG00000271643.2	-0.016869401	0.356041895	0.880192833	PDCD6IP-DT
ENSG00000204619.8	0.006580768	0.35607526	0.880192833	PPP1R11
ENSG00000205307.12	-0.018269917	0.356080946	0.880192833	SAP25
ENSG00000162946.23	0.011940333	0.356137578	0.880192833	DISC1
ENSG00000136807.14	-0.006012006	0.356188353	0.880192833	CDK9
ENSG00000076604.15	-0.00993825	0.356233277	0.880192833	TRAF4
ENSG00000213231.13	-0.047543739	0.356238404	0.880192833	TCL1B
ENSG00000213671.3	0.054217533	0.356252914	0.880192833	OLA1P2
ENSG00000180901.11	0.005620456	0.356293167	0.880192833	KCTD2
ENSG00000200879.1	-0.028802546	0.356363949	0.880192833	SNORD14E
ENSG00000257924.1	0.056189391	0.356385579	0.880192833	LINC02416
ENSG00000092203.15	0.004211018	0.356431389	0.880192833	TOX4
ENSG00000100985.7	-0.022058504	0.356445983	0.880192833	MMP9
ENSG00000267682.1	0.037191461	0.35648204	0.880192833	CTD-3220F14.2
ENSG00000175792.12	-0.006848415	0.356511445	0.880192833	RUVBL1
ENSG00000281691.1	-0.024866334	0.356582099	0.880192833	RBM5-AS1
ENSG00000076003.5	0.007728515	0.356599314	0.880192833	MCM6
ENSG00000211672.2	-0.044187329	0.356641851	0.880192833	IGLV4-3
ENSG00000270277.1	-0.016812515	0.35668907	0.880192833	RP11-65L3.2
ENSG00000235101.1	-0.026105195	0.356762192	0.880192833	SETP9
ENSG00000163611.11	-0.015379258	0.356774825	0.880192833	SPICE1
ENSG00000106771.13	0.007767772	0.356780124	0.880192833	TMEM245
ENSG00000175573.7	-0.008552426	0.356851838	0.880192833	C11orf68
ENSG00000255571.9	-0.017244738	0.356859512	0.880192833	MIR9-3HG
ENSG00000266916.7	0.029543148	0.356867093	0.880192833	ZNF793-AS1
ENSG00000230280.2	0.023621755	0.356871274	0.880192833	HNRNPA1P59
ENSG00000144648.16	-0.026693244	0.356874183	0.880192833	ACKR2
ENSG00000152684.11	0.008471967	0.356912525	0.880192833	PELO
ENSG00000141543.12	0.006633863	0.356916661	0.880192833	EIF4A3
ENSG00000130997.16	0.020682898	0.356928599	0.880192833	POLN
ENSG00000082014.17	0.009493049	0.356950019	0.880192833	SMARCD3
ENSG00000204604.13	0.010887023	0.356953277	0.880192833	ZNF468
ENSG00000176463.14	-0.007028278	0.356986402	0.880192833	SLCO3A1
ENSG00000182324.7	0.016467665	0.357044077	0.880192833	KCNJ14
ENSG00000095739.11	-0.032134412	0.35710246	0.880192833	BAMBI
ENSG00000149256.16	0.070451646	0.357126662	0.880192833	TENM4
ENSG00000272374.1	0.010356826	0.357132063	0.880192833	RP3-329A5.8

ENSG00000229635.1	-0.023054769	0.357151222	0.880192833	RP4-713B5.2
ENSG00000160991.17	0.010020821	0.35715151	0.880192833	ORAI2
ENSG00000228589.3	-0.016526031	0.357277644	0.880192833	SPCS2P4
ENSG00000073849.16	-0.006478695	0.357279818	0.880192833	ST6GAL1
ENSG00000251229.1	-0.029283744	0.357397221	0.880192833	RP11-503N18.5
ENSG00000205853.12	0.018215612	0.357417748	0.880192833	RFPL3S
ENSG00000003400.15	0.006291648	0.357424086	0.880192833	CASP10
ENSG00000067606.17	-0.008881688	0.357477395	0.880192833	PRKCZ
ENSG00000116329.11	0.023993329	0.357481992	0.880192833	OPRD1
ENSG00000281398.4	0.01058241	0.357522175	0.880192833	SNHG4
ENSG00000272034.1	0.0269386	0.357615481	0.880192833	SNORD14A
ENSG00000250474.2	0.022780951	0.357626873	0.880192833	WBP1LP2
ENSG00000202314.1	0.026336301	0.357652107	0.880192833	SNORD6
ENSG00000287166.1	0.032640577	0.357674898	0.880192833	CTC-756D1.4
ENSG00000134250.20	0.009478977	0.357688586	0.880192833	NOTCH2
ENSG00000188060.8	-0.024480717	0.357704053	0.880192833	RAB42
ENSG00000237522.1	0.017849319	0.357705947	0.880192833	NONOP2
ENSG00000122507.21	0.007010326	0.357711363	0.880192833	BBS9
ENSG00000141552.18	0.009366491	0.357733921	0.880192833	ANAPC11
ENSG00000182621.18	0.016817227	0.357843764	0.880357475	PLCB1
ENSG00000273010.1	0.034271533	0.357904534	0.880401366	RP11-96K19.5
ENSG00000160172.10	0.015129437	0.358001687	0.880472326	FAM86C2P
ENSG00000146859.6	0.012664212	0.358019247	0.880472326	TMEM140
ENSG00000100285.10	-0.016772356	0.358111031	0.880544449	NEFH
ENSG00000225880.5	-0.015993367	0.358134447	0.880544449	LINC00115
ENSG00000242142.1	-0.051637188	0.358451909	0.881187708	SERBP1P3
ENSG00000261542.1	-0.018697867	0.358482009	0.881187708	RP11-16E18.3
ENSG00000138778.13	-0.024218161	0.358611777	0.881401045	CENPE
ENSG00000033867.16	-0.014696744	0.358669115	0.881436334	SLC4A7
ENSG00000087152.16	0.004285563	0.358797081	0.881634279	ATXN7L3
ENSG00000136011.15	-0.03031903	0.358894781	0.881634279	STAB2
ENSG00000130768.15	0.017981678	0.358900079	0.881634279	SMPDL3B
ENSG00000179859.10	0.008307673	0.358921621	0.881634279	RNF227
ENSG00000176788.9	0.012353755	0.359127372	0.882034028	BASP1
ENSG00000232333.1	0.040889575	0.35926788	0.882169864	RPS27AP2
ENSG00000181234.10	0.039953253	0.359287538	0.882169864	TMEM132C
ENSG00000162545.6	-0.018435098	0.359339176	0.882169864	CAMK2N1
ENSG00000237217.1	-0.043349676	0.359354742	0.882169864	AC008074.5
ENSG00000147912.13	-0.01310496	0.359432292	0.882205729	FBXO10
ENSG00000133216.17	-0.023264561	0.359455387	0.882205729	EPHB2
ENSG00000116786.13	0.006667498	0.359535831	0.882297574	PLEKHM2
ENSG00000163399.16	-0.007881126	0.359619354	0.882396951	ATP1A1
ENSG00000138468.16	-0.015208082	0.359770159	0.882411264	SENP7
ENSG00000272564.1	0.033609906	0.359829468	0.882411264	RP11-548P2.2
ENSG00000180479.14	-0.011969148	0.359927714	0.882411264	ZNF571
ENSG00000280159.1	0.020584394	0.359969863	0.882411264	CTD-2591A1.1
ENSG00000229422.2	-0.042337715	0.360006312	0.882411264	RP11-262H14.13

ENSG00000100060.18	0.006507672	0.360030998	0.882411264	MFNG
ENSG00000168528.12	0.025236303	0.36006936	0.882411264	SERINC2
ENSG00000270550.1	-0.032967443	0.360083126	0.882411264	IGHV3-30
ENSG00000070614.15	0.012012707	0.360117078	0.882411264	NDST1
ENSG00000066382.17	0.037498033	0.360136421	0.882411264	MPPED2
ENSG00000227081.5	0.027219981	0.360159867	0.882411264	RP11-543P15.1
ENSG00000132323.9	-0.00548893	0.360196321	0.882411264	ILKAP
ENSG00000201772.1	0.015888699	0.360213402	0.882411264	SNORA5C
ENSG00000163485.17	0.038704123	0.360249493	0.882411264	ADORA1
ENSG00000214719.13	-0.020315069	0.36028433	0.882411264	AC005562.1
ENSG00000271538.6	-0.030934355	0.360342503	0.882411264	LINC02427
ENSG00000282508.2	0.014429188	0.360395523	0.882411264	LINC01002
ENSG00000100422.14	0.004774485	0.360438496	0.882411264	CERK
ENSG00000180089.6	0.015522201	0.360442713	0.882411264	TMEM86B
ENSG00000211679.2	0.035925973	0.360542085	0.882465882	IGLC3
ENSG00000214659.4	-0.031155716	0.360594861	0.882465882	KRT8P26
ENSG00000112511.18	-0.006909269	0.360606417	0.882465882	PHF1
ENSG00000164116.17	0.017874826	0.360637144	0.882465882	GUCY1A1
ENSG00000164978.18	0.013333021	0.360734404	0.882512885	NUDT2
ENSG00000120071.15	0.005521133	0.360742418	0.882512885	KANSL1
ENSG00000204257.15	0.010578036	0.360938951	0.882747015	HLA-DMA
ENSG00000286194.1	0.019623324	0.360964175	0.882747015	RP11-848P1.10
ENSG00000244300.3	0.038081347	0.36099591	0.882747015	GATA2-AS1
ENSG00000281358.1	-0.015448955	0.361056857	0.882747015	RASSF1-AS1
ENSG00000100097.12	0.011876994	0.361176546	0.882747015	LGALS1
ENSG00000153767.10	0.008375506	0.361200747	0.882747015	GTF2E1
ENSG00000114126.18	-0.008598388	0.361266482	0.882747015	TFDP2
ENSG00000116151.14	-0.011413951	0.36128465	0.882747015	MORN1
ENSG00000287665.1	-0.026500292	0.361331319	0.882747015	RP11-32P3.1
ENSG00000200170.1	0.023869097	0.361349239	0.882747015	Y_RNA
ENSG00000107020.10	0.010950171	0.361382653	0.882747015	PLGRKT
ENSG00000133193.12	0.008258177	0.361394612	0.882747015	FAM104A
ENSG00000153233.13	0.056170763	0.361473565	0.882747015	PTPRR
ENSG00000161249.21	0.023156718	0.36153045	0.882747015	DMKN
ENSG00000245954.8	0.028364244	0.361573107	0.882747015	LINC02273
ENSG00000287934.1	0.023650008	0.361623874	0.882747015	RP11-157K17.7
ENSG00000282608.2	0.023752531	0.36162471	0.882747015	ADORA3
ENSG00000104450.13	-0.014713889	0.361663119	0.882747015	SPAG1
ENSG00000163900.11	0.00491997	0.361679955	0.882747015	TMEM41A
ENSG00000229117.9	0.017869651	0.361700074	0.882747015	RPL41
ENSG00000164620.9	-0.010319906	0.361817135	0.882747015	RELL2
ENSG00000269815.1	-0.020995733	0.361829154	0.882747015	CTD-2278I10.4
ENSG00000221988.13	0.010096017	0.361924252	0.882747015	PPT2
ENSG00000109066.14	-0.006812356	0.361929588	0.882747015	TMEM104
ENSG00000100307.13	0.006130568	0.362024895	0.882747015	CBX7
ENSG00000092871.17	0.006611509	0.362041518	0.882747015	RFFL
ENSG00000288558.2	-0.008405089	0.362115534	0.882747015	DUS4L-BCAP29

ENSG00000066923.18	-0.01057679	0.362143607	0.882747015	STAG3
ENSG00000280039.1	0.027419576	0.362154672	0.882747015	RN7SKP23
ENSG00000119965.13	-0.009342924	0.362174403	0.882747015	C10orf88
ENSG00000270110.2	-0.028963364	0.362183543	0.882747015	RP5-1139B12.4
ENSG00000037474.15	-0.005345872	0.362215532	0.882747015	NSUN2
ENSG00000270060.1	-0.011010722	0.362278282	0.882795034	RP11-390K5.6
ENSG00000253641.6	-0.021689214	0.362423572	0.883029893	LINCR-0001
ENSG00000136040.9	0.009182428	0.362461242	0.883029893	PLXNC1
ENSG00000117632.23	-0.010839103	0.362537469	0.883029893	STMN1
ENSG00000111817.19	0.010201498	0.362572385	0.883029893	DSE
ENSG00000230606.11	0.031387253	0.362635172	0.883029893	AC159540.1
ENSG00000284959.2	0.022617031	0.362658627	0.883029893	RP11-114N19.3
ENSG00000077254.14	0.007043117	0.362676067	0.883029893	USP33
ENSG00000129226.14	-0.020460573	0.362776855	0.883130563	CD68
ENSG00000010803.16	0.006391123	0.36280354	0.883130563	SCMH1
ENSG00000128218.8	-0.026000678	0.362854201	0.883130569	VPREB3
ENSG00000276814.1	0.016843403	0.362889668	0.883130569	RP1-228P16.8
ENSG00000267249.1	-0.020093143	0.363141414	0.883253614	RP11-973H7.3
ENSG00000114378.17	-0.013450179	0.363151354	0.883253614	HYAL1
ENSG00000255438.2	-0.028913963	0.363275511	0.883253614	CTD-2653D5.1
ENSG00000087995.16	0.006259776	0.363295575	0.883253614	METTL2A
ENSG00000173821.19	-0.013389927	0.363299295	0.883253614	RNF213
ENSG00000157800.18	0.01267971	0.363323672	0.883253614	SLC37A3
ENSG00000136929.13	0.025105898	0.363367971	0.883253614	HEMGN
ENSG00000104763.20	0.007146478	0.363368089	0.883253614	ASAH1
ENSG00000261546.1	0.020841778	0.363422099	0.883253614	CTD-2555A7.3
ENSG00000229990.3	-0.030156224	0.363424179	0.883253614	RP11-574K11.8
ENSG00000275371.1	-0.013353482	0.363447144	0.883253614	RP11-455F5.6
ENSG00000262362.1	-0.019560449	0.363491602	0.883253614	LA16c-380H5.2
ENSG00000197978.9	0.021489541	0.363502877	0.883253614	GOLGA6L9
ENSG00000179950.14	-0.007313599	0.363685583	0.883253614	PUF60
ENSG00000162869.16	0.004571663	0.363688538	0.883253614	PPP1R21
ENSG00000125149.12	0.004717187	0.36369304	0.883253614	C16orf70
ENSG00000181938.14	-0.010561831	0.363732199	0.883253614	GINS3
ENSG00000108344.15	-0.006206812	0.363750057	0.883253614	PSMD3
ENSG00000102870.6	0.010156872	0.363758534	0.883253614	ZNF629
ENSG00000111679.17	-0.007727356	0.363820203	0.883298771	PTPN6
ENSG00000140836.17	0.012166591	0.363877134	0.883332417	ZFHX3
ENSG00000213714.1	-0.016242438	0.364050866	0.883444949	FAM209B
ENSG00000058804.12	0.006890646	0.36410088	0.883444949	NDC1
ENSG00000090857.14	0.013812534	0.364109743	0.883444949	PDPR
ENSG00000103978.16	0.004518444	0.364268967	0.883444949	TMEM87A
ENSG00000141013.17	0.011313431	0.364297163	0.883444949	GAS8
ENSG00000115685.15	0.006690458	0.364316952	0.883444949	PPP1R7
ENSG00000284773.1	-0.010842036	0.364329997	0.883444949	RP11-244H3.5
ENSG00000107290.14	0.006558086	0.364409723	0.883444949	SETX
ENSG00000198862.14	0.013459218	0.364432896	0.883444949	LTN1

ENSG00000175197.13	0.010466522	0.364433126	0.883444949	DDIT3
ENSG00000068137.15	-0.020354898	0.364441773	0.883444949	PLEKHH3
ENSG00000151623.15	0.010686018	0.364499045	0.883444949	NR3C2
ENSG00000057657.17	0.010091261	0.364528863	0.883444949	PRDM1
ENSG00000158517.15	-0.013163548	0.364644545	0.883444949	NCF1
ENSG00000267291.1	-0.032931441	0.364708942	0.883444949	MAP4K1-AS1
ENSG00000158201.10	-0.009601215	0.364726981	0.883444949	ABHD3
ENSG00000124614.16	0.014035306	0.364792852	0.883444949	RPS10
ENSG00000185436.12	-0.018257899	0.36479756	0.883444949	IFNLR1
ENSG00000179409.11	-0.007224175	0.364802476	0.883444949	GEMIN4
ENSG00000147408.14	0.015975962	0.364823922	0.883444949	CSGALNACT1
ENSG00000183486.14	-0.012790337	0.364877444	0.883444949	MX2
ENSG00000183340.7	0.012012494	0.364918635	0.883444949	JRKL
ENSG00000207561.1	-0.015230536	0.36494209	0.883444949	MIR635
ENSG00000105248.16	-0.00795479	0.365017152	0.883444949	YJU2
ENSG00000076053.12	0.007075978	0.365144974	0.883444949	RBM7
ENSG00000114948.13	0.030155651	0.365179002	0.883444949	ADAM23
ENSG00000162267.12	0.020049717	0.365184831	0.883444949	ITIH3
ENSG00000263753.8	-0.009728692	0.365234908	0.883444949	LINC00667
ENSG00000177494.6	-0.029273604	0.365247333	0.883444949	ZBED2
ENSG00000163794.6	-0.016379729	0.36526938	0.883444949	UCN
ENSG00000148082.10	-0.031315351	0.365303396	0.883444949	SHC3
ENSG00000144935.16	0.021779035	0.365339434	0.883444949	TRPC1
ENSG00000249601.2	0.037557341	0.365389544	0.883444949	LINC01187
ENSG00000179195.16	-0.006217241	0.36539891	0.883444949	ZNF664
ENSG00000238243.3	0.032095444	0.365453108	0.883444949	OR2W3
ENSG00000214193.11	-0.01369387	0.3654743	0.883444949	SH3D21
ENSG00000203772.7	-0.032379375	0.365719896	0.883870979	SPRN
ENSG00000244462.8	0.004172248	0.365736743	0.883870979	RBM12
ENSG00000285803.1	0.025525903	0.365854821	0.883922028	RP11-324H6.8
ENSG00000188263.10	0.040571698	0.365886393	0.883922028	IL17REL
ENSG00000279605.1	-0.013067193	0.365887171	0.883922028	RP11-661A12.8
ENSG00000178162.8	-0.041609025	0.36596937	0.883984643	FAR2P2
ENSG00000261799.1	-0.007157416	0.366059389	0.883984643	RP11-283I3.6
ENSG00000186364.12	-0.009899311	0.366068192	0.883984643	NUDT17
ENSG00000130734.10	-0.006945834	0.366085507	0.883984643	ATG4D
ENSG00000258602.1	-0.04118127	0.366131357	0.883991273	LINC01629
ENSG00000232706.3	-0.031918718	0.366380852	0.884067574	NUTM2HP
ENSG00000230295.1	0.0275512	0.366420469	0.884067574	GTF2IP23
ENSG00000243488.3	-0.033721702	0.366498594	0.884067574	RN7SL337P
ENSG00000030419.17	-0.016920977	0.36649913	0.884067574	IKZF2
ENSG00000085449.15	0.010855723	0.36657372	0.884067574	WDFY1
ENSG00000132386.11	0.010712813	0.366651699	0.884067574	SERPINF1
ENSG00000105879.12	-0.005518442	0.366732999	0.884067574	CBLL1
ENSG00000275023.5	-0.006217203	0.366787057	0.884067574	MLLT6
ENSG00000157778.9	0.009334508	0.366830598	0.884067574	PSMG3
ENSG00000258136.2	0.029009455	0.36688676	0.884067574	RP11-864J10.4

ENSG00000234494.7	-0.02171229	0.366969354	0.884067574	SP2-AS1
ENSG00000102786.15	0.006499808	0.36699759	0.884067574	INTS6
ENSG00000115540.15	-0.01303506	0.367124098	0.884067574	MOB4
ENSG00000230825.2	-0.035225028	0.367266219	0.884067574	AC005532.5
ENSG00000168710.18	0.004388704	0.36726874	0.884067574	AHCYL1
ENSG00000179152.20	0.007686541	0.367276728	0.884067574	TCAIM
ENSG00000116521.11	-0.007875926	0.367282973	0.884067574	SCAMP3
ENSG00000112195.9	0.010840675	0.367304922	0.884067574	TREML2
ENSG00000240522.1	0.033769433	0.367364694	0.884067574	RPL7AP10
ENSG00000255050.1	0.021937117	0.367377928	0.884067574	RP11-661A12.9
ENSG00000135312.7	0.048620569	0.367400184	0.884067574	HTR1B
ENSG00000275765.5	-0.017181275	0.367556065	0.884067574	RP11-54C4.3
ENSG00000130413.15	-0.030877609	0.367600184	0.884067574	STK33
ENSG00000167515.10	0.007708816	0.3676809	0.884067574	TRAPPC2L
ENSG00000253982.2	0.01994615	0.367684062	0.884067574	CTD-2336O2.1
ENSG00000288598.2	0.020676837	0.367695707	0.884067574	RP11-157L14.5
ENSG00000065989.16	-0.011916798	0.367697662	0.884067574	PDE4A
ENSG00000172058.16	0.083379759	0.367722155	0.884067574	SERF1A
ENSG00000126878.13	0.03536637	0.367804551	0.884067574	AIF1L
ENSG00000144231.11	0.004624813	0.36782605	0.884067574	POLR2D
ENSG00000285257.1	-0.022546735	0.367850814	0.884067574	RP11-322M19.4
ENSG00000072310.18	-0.010605621	0.367989235	0.884067574	SREBF1
ENSG00000287670.1	-0.023885862	0.368003399	0.884067574	RP11-1378G18.2
ENSG00000238241.2	0.026949581	0.368057912	0.884067574	CCR12P
ENSG00000183019.7	0.016983347	0.368062389	0.884067574	MCEMP1
ENSG00000105202.9	-0.008865231	0.368130056	0.884067574	FBL
ENSG00000251474.6	0.008102263	0.368140652	0.884067574	RPL32P3
ENSG00000160767.21	-0.008397043	0.368153283	0.884067574	FAM189B
ENSG00000080007.8	-0.030519456	0.368175871	0.884067574	DDX43
ENSG00000255491.3	-0.024187445	0.368226697	0.884067574	RP11-1082L8.3
ENSG00000170209.5	-0.019784583	0.368230506	0.884067574	ANKK1
ENSG00000273174.1	0.014238684	0.368257325	0.884067574	RP11-434H6.6
ENSG00000095370.20	-0.007605055	0.368325929	0.884067574	SH2D3C
ENSG00000279865.1	0.026868853	0.36834121	0.884067574	RP11-69M1.6
ENSG00000198836.11	0.007201503	0.36842303	0.884067574	OPA1
ENSG00000233757.6	-0.03466075	0.368489763	0.884067574	AC092835.2
ENSG00000254996.5	-0.007641992	0.368512567	0.884067574	ANKHD1-EIF4EBP3
ENSG00000121807.7	0.01092566	0.368595979	0.884067574	CCR2
ENSG00000205181.7	0.017974892	0.368606836	0.884067574	LINC00654
ENSG00000185246.18	-0.010568893	0.368638864	0.884067574	PRPF39
ENSG00000183281.15	-0.019453147	0.368778557	0.884067574	PLGLB1
ENSG00000127980.16	0.008014875	0.368893359	0.884067574	PEX1
ENSG00000100815.12	0.00718946	0.368942822	0.884067574	TRIP11
ENSG00000106236.4	-0.049752319	0.368960433	0.884067574	NPTX2
ENSG00000133111.4	-0.008979517	0.369003951	0.884067574	RFXAP
ENSG00000162430.18	0.007438973	0.369078818	0.884067574	SELENON
ENSG00000145982.13	-0.007806481	0.369128933	0.884067574	FARS2

ENSG00000076242.16	0.005320496	0.369190728	0.884067574	MLH1
ENSG00000160223.17	0.051386909	0.369194431	0.884067574	ICOSLG
ENSG00000172785.18	0.013899929	0.369196343	0.884067574	CBWD1
ENSG00000254166.3	-0.02824434	0.369217461	0.884067574	CASC19
ENSG00000175764.16	0.009665807	0.369221404	0.884067574	TTLL11
ENSG00000164715.6	0.01025316	0.369227955	0.884067574	LMTK2
ENSG00000286942.1	-0.023737506	0.369251778	0.884067574	RP11-562I5.4
ENSG00000236991.6	0.023878292	0.369349687	0.884067574	EDRF1-AS1
ENSG00000114841.18	0.008655998	0.369409618	0.884067574	DNAH1
ENSG00000078747.16	0.00673002	0.369420847	0.884067574	ITCH
ENSG00000155380.13	-0.00817371	0.369425355	0.884067574	SLC16A1
ENSG00000163866.9	0.005538296	0.369528476	0.884067574	SMIM12
ENSG00000172922.11	-0.007030291	0.369588516	0.884067574	RNASEH2C
ENSG00000185033.14	-0.008405294	0.369651123	0.884067574	SEMA4B
ENSG00000111300.10	0.00739937	0.369743982	0.884067574	NAA25
ENSG00000188177.14	0.007869824	0.369772435	0.884067574	ZC3H6
ENSG00000137726.17	-0.012803094	0.369804537	0.884067574	FXYD6
ENSG00000197157.11	-0.007328093	0.369815944	0.884067574	SND1
ENSG00000198468.9	0.027684631	0.369836744	0.884067574	FLVCR1-DT
ENSG00000122705.17	0.00758847	0.369863741	0.884067574	CLTA
ENSG00000134308.14	-0.006749152	0.369887645	0.884067574	YWHAQ
ENSG00000227920.3	0.046787755	0.369896756	0.884067574	RP1-153P14.5
ENSG00000133818.14	-0.013168857	0.369928357	0.884067574	RRAS2
ENSG00000270020.1	-0.031140987	0.369962842	0.884067574	RP11-463O9.9
ENSG00000170846.17	-0.007131888	0.369991691	0.884067574	AC093323.3
ENSG00000172086.9	0.013577762	0.370014176	0.884067574	KRCC1
ENSG00000250878.3	0.025278981	0.370030233	0.884067574	METTL21EP
ENSG00000136463.9	-0.007017101	0.370063475	0.884067574	TACO1
ENSG00000130377.14	-0.038441481	0.370258004	0.884067574	ACSBG2
ENSG00000105699.17	-0.013157878	0.370263443	0.884067574	LSR
ENSG00000124788.19	0.007175116	0.370322097	0.884067574	ATXN1
ENSG00000255503.1	-0.024928676	0.370330867	0.884067574	RP11-113K21.4
ENSG00000187486.7	0.048850772	0.370394697	0.884067574	KCNJ11
ENSG00000266976.3	0.118304453	0.370465067	0.884067574	AC079466.1
ENSG00000272509.1	-0.018840634	0.370545332	0.884067574	RP11-347C18.5
ENSG00000179954.16	0.027596823	0.370553836	0.884067574	SSC5D
ENSG00000102572.15	0.007058172	0.370560327	0.884067574	STK24
ENSG00000272476.1	0.0332244	0.370592354	0.884067574	RP1-191J18.66
ENSG00000174951.12	0.038916603	0.370712689	0.884067574	FUT1
ENSG00000188051.7	-0.023539212	0.370739633	0.884067574	TMEM221
ENSG00000219410.6	0.022753112	0.370752763	0.884067574	RP4-761J14.8
ENSG00000231507.2	0.022085704	0.370753337	0.884067574	LINC01353
ENSG00000265929.1	-0.030115485	0.370774828	0.884067574	MIR5195
ENSG00000118518.17	0.00666888	0.370783139	0.884067574	RNF146
ENSG00000197903.8	-0.014018759	0.370794254	0.884067574	H2BC12
ENSG00000164691.18	0.007354636	0.370833393	0.884067574	TAGAP
ENSG00000168675.19	0.006823178	0.370844669	0.884067574	LDLRAD4

ENSG00000078114.19	0.089124203	0.37084764	0.884067574	NEBL
ENSG00000279133.1	0.014687482	0.370884258	0.884067574	RP11-342K2.1
ENSG00000166902.5	-0.005692136	0.371044899	0.884067574	MRPL16
ENSG00000088543.15	-0.007378684	0.371057393	0.884067574	C3orf18
ENSG00000054523.19	0.009668018	0.371062911	0.884067574	KIF1B
ENSG00000148516.23	0.011234937	0.371222461	0.884067574	ZEB1
ENSG00000228444.2	0.020637207	0.371226217	0.884067574	RP11-173B14.4
ENSG00000161682.15	-0.033597486	0.371265456	0.884067574	FAM171A2
ENSG00000159708.18	0.023919627	0.371295071	0.884067574	LRRC36
ENSG00000261884.2	0.007298823	0.371314645	0.884067574	CTC-479C5.12
ENSG00000255545.8	-0.029146499	0.371365662	0.884067574	B3GAT1-DT
ENSG00000057019.16	0.014881118	0.371411254	0.884067574	DCBLD2
ENSG00000123965.13	-0.032458774	0.371416567	0.884067574	PMS2P5
ENSG00000229358.3	-0.034317886	0.371476009	0.884067574	DPY19L1P1
ENSG00000091542.9	-0.005929702	0.371511258	0.884067574	ALKBH5
ENSG00000258819.2	0.021060699	0.37154906	0.884067574	LINC02289
ENSG00000288612.1	-0.015158409	0.371694883	0.884067574	RP1-90J20.15
ENSG00000230319.1	-0.02544748	0.371717401	0.884067574	TTLL1-AS1
ENSG00000165637.13	0.006320938	0.371737478	0.884067574	VDAC2
ENSG00000071539.14	-0.02402961	0.371743599	0.884067574	TRIP13
ENSG00000122543.11	-0.019345355	0.371781203	0.884067574	OCM
ENSG00000164776.10	-0.012349512	0.371812039	0.884067574	PHKG1
ENSG00000120539.15	0.012077349	0.371831558	0.884067574	MASTL
ENSG00000132357.14	0.008675319	0.371834023	0.884067574	CARD6
ENSG00000181619.11	0.021758036	0.371880474	0.884067574	GPR135
ENSG00000162739.14	-0.008284504	0.371918263	0.884067574	SLAMF6
ENSG00000245648.2	-0.016842124	0.371996604	0.884067574	KLRK1-AS1
ENSG00000176148.16	0.011053699	0.371998615	0.884067574	TCP11L1
ENSG00000272720.1	-0.022330862	0.372035292	0.884067574	CTA-228A9.3
ENSG00000240875.6	0.022721248	0.372035889	0.884067574	LINC00886
ENSG00000105185.12	0.006474689	0.372051315	0.884067574	PDCD5
ENSG00000254695.2	0.036629892	0.372079217	0.884067574	RP11-396O20.1
ENSG00000272077.1	-0.016587966	0.372092316	0.884067574	RP11-348P10.2
ENSG00000153406.14	-0.0087346	0.372111922	0.884067574	NMRAL1
ENSG00000163702.20	-0.017500188	0.372178794	0.884124026	IL17RC
ENSG00000112293.15	-0.018945091	0.372231781	0.884147482	GPLD1
ENSG00000070495.15	-0.006106597	0.372286495	0.884175037	JMJD6
ENSG00000155307.19	0.012204367	0.372477681	0.884494668	SAMSN1
ENSG00000155508.14	0.004886603	0.372571214	0.884494668	CNOT8
ENSG00000254340.2	-0.033840582	0.372642052	0.884494668	RP11-10A14.3
ENSG00000286810.1	-0.023763679	0.372670097	0.884494668	RP11-301N24.6
ENSG00000186481.16	-0.024804881	0.372726517	0.884494668	ANKRD20A5P
ENSG00000100744.15	0.013316131	0.372735223	0.884494668	GSKIP
ENSG00000280081.4	-0.148296308	0.372740326	0.884494668	LINC01667
ENSG00000180113.16	0.022175098	0.372766111	0.884494668	TDRD6
ENSG00000111664.11	0.025187578	0.372940145	0.884622086	GNB3
ENSG00000203362.2	-0.024530446	0.373025123	0.884622086	POLH-AS1

ENSG00000183134.5	-0.020337196	0.373175014	0.884622086	PTGDR2
ENSG00000177943.14	-0.012039115	0.373175873	0.884622086	MAMDC4
ENSG00000157916.20	0.003441229	0.373230665	0.884622086	RER1
ENSG00000115257.15	-0.018713957	0.373245759	0.884622086	PCSK4
ENSG00000012232.9	0.008237246	0.373250502	0.884622086	EXTL3
ENSG00000227268.5	0.013939936	0.373261583	0.884622086	KLLN
ENSG00000139644.13	0.004342139	0.373297019	0.884622086	TMBIM6
ENSG00000180758.12	0.011470807	0.373372515	0.884622086	GPR157
ENSG00000236753.7	0.010621306	0.373407032	0.884622086	MKLN1-AS
ENSG00000224389.9	0.040560663	0.37346071	0.884622086	C4B
ENSG00000111215.12	0.019139154	0.373660399	0.884622086	PRR4
ENSG00000286408.1	0.023872719	0.373694164	0.884622086	RP11-485M7.4
ENSG00000101773.19	0.014156827	0.37373086	0.884622086	RBBP8
ENSG00000119865.9	-0.024921313	0.373755215	0.884622086	CNRIP1
ENSG00000141642.9	0.009581487	0.373793639	0.884622086	ELAC1
ENSG00000122477.13	-0.015058951	0.373797924	0.884622086	LRRC39
ENSG00000084676.16	0.008182472	0.373806377	0.884622086	NCOA1
ENSG00000271335.6	-0.014574362	0.37382205	0.884622086	CCNY-AS1
ENSG00000144567.11	0.006719553	0.373844168	0.884622086	RETREG2
ENSG00000256377.6	-0.151022191	0.373852725	0.884622086	RP11-1060J15.4
ENSG00000047410.14	0.007222604	0.373902035	0.884622086	TPR
ENSG00000130303.14	-0.014438277	0.373968658	0.884622086	BST2
ENSG00000172867.4	0.031038056	0.373981555	0.884622086	KRT2
ENSG00000137094.16	0.018166662	0.374054312	0.884622086	DNAJB5
ENSG00000101230.6	0.023394042	0.374087157	0.884622086	ISM1
ENSG00000237039.1	-0.024039603	0.374134527	0.884622086	RPS28P4
ENSG00000245556.3	-0.007801004	0.374273347	0.884622086	SCAMP1-AS1
ENSG00000267676.1	0.036233839	0.374299714	0.884622086	THA1P
ENSG00000170161.7	-0.026143322	0.374316222	0.884622086	RP11-262H14.4
ENSG00000004897.12	0.006544329	0.374330216	0.884622086	CDC27
ENSG00000286755.1	0.039663626	0.374346917	0.884622086	RP11-643E20.1
ENSG00000111885.7	0.012977486	0.374373052	0.884622086	MAN1A1
ENSG00000079999.14	-0.006766862	0.374417451	0.884622086	KEAP1
ENSG00000261188.1	0.019486763	0.374444118	0.884622086	TFIP11-DT
ENSG00000204498.11	-0.007806859	0.374448967	0.884622086	NFKBIL1
ENSG00000262227.1	-0.017898362	0.374501815	0.884622086	RP5-1050D4.5
ENSG00000156469.9	0.009220192	0.374502094	0.884622086	MTERF3
ENSG00000100395.15	-0.006076763	0.374639099	0.884843791	L3MBTL2
ENSG00000143452.16	-0.021696008	0.374691053	0.884864593	HORMAD1
ENSG00000242876.3	-0.033194901	0.374753149	0.884909337	RN7SL812P
ENSG00000166762.19	-0.010519374	0.374950708	0.885270243	CATSPER2
ENSG00000170291.15	0.00636076	0.375095943	0.885270243	ELP5
ENSG00000286067.1	0.014976681	0.375123479	0.885270243	RP1-278C19.9
ENSG00000168283.14	-0.010464299	0.37513778	0.885270243	BMI1
ENSG00000277586.4	0.025507688	0.375162006	0.885270243	NEFL
ENSG00000169155.10	0.005758018	0.375164993	0.885270243	ZBTB43
ENSG00000110801.14	0.005856476	0.375231933	0.885326333	PSMD9

ENSG00000259494.2	-0.008068537	0.375382259	0.885458346	MRPL46
ENSG00000064932.16	-0.009990988	0.375418373	0.885458346	SBNO2
ENSG00000163950.13	0.005652817	0.375426127	0.885458346	SLBP
ENSG00000077585.14	0.010493637	0.37546059	0.885458346	GPR137B
ENSG00000227811.3	-0.026617108	0.375535799	0.885533881	INKA2-AS1
ENSG00000239636.1	0.010945208	0.375654835	0.885556125	TFAP2E-AS1
ENSG00000281468.1	0.02106494	0.375758459	0.885556125	CTC-459F4.9
ENSG00000211797.2	-0.020554956	0.375812892	0.885556125	TRAV17
ENSG00000099331.14	-0.007571227	0.375829423	0.885556125	MYO9B
ENSG00000148143.13	0.043884248	0.375894895	0.885556125	ZNF462
ENSG00000204315.4	-0.010011788	0.37595012	0.885556125	FKBPL
ENSG00000183605.17	0.011376914	0.375996652	0.885556125	SFXN4
ENSG00000119121.22	0.014216226	0.376026457	0.885556125	TRPM6
ENSG00000223345.3	0.065391326	0.376053915	0.885556125	H2BP1
ENSG00000154065.17	0.03142451	0.37606853	0.885556125	ANKRD29
ENSG00000205837.7	-0.055461674	0.376085696	0.885556125	LINC00487
ENSG00000161048.12	-0.00691913	0.376224938	0.885556125	NAPEPLD
ENSG00000211655.3	-0.042710935	0.376236107	0.885556125	IGLV1-36
ENSG00000077312.9	-0.00767908	0.376237147	0.885556125	SNRPA
ENSG00000237988.5	0.046168751	0.376264677	0.885556125	OR2I1P
ENSG00000163322.14	0.01030887	0.376287495	0.885556125	ABRAXAS1
ENSG00000286342.1	-0.033862435	0.376290412	0.885556125	RP11-460N20.8
ENSG00000134815.19	-0.008389304	0.37634589	0.885556125	DHX34
ENSG00000261526.2	-0.010654594	0.376365671	0.885556125	CTB-31O20.2
ENSG00000234553.1	0.034400768	0.376456493	0.88556904	AC022431.3
ENSG00000165795.24	0.010534456	0.376457524	0.88556904	NDRG2
ENSG00000137804.14	-0.02356609	0.376617269	0.885749732	NUSAP1
ENSG00000151702.17	-0.005145244	0.376620717	0.885749732	FLI1
ENSG00000152700.14	0.006629646	0.377003656	0.885934439	SAR1B
ENSG00000241288.8	-0.018566101	0.377037633	0.885934439	LINC02614
ENSG00000271734.1	0.020933376	0.377220825	0.885934439	RP1-111B22.3
ENSG00000259956.2	0.00398971	0.377353241	0.885934439	RBM15B
ENSG00000172775.17	0.003954093	0.377370809	0.885934439	PSME3IP1
ENSG00000162692.12	0.051883208	0.377450681	0.885934439	VCAM1
ENSG00000058668.15	-0.009344863	0.377454639	0.885934439	ATP2B4
ENSG00000114812.13	0.010922586	0.377483826	0.885934439	VIPR1
ENSG00000158467.16	0.005565882	0.377585889	0.885934439	AHCYL2
ENSG00000148291.10	-0.008847606	0.377586323	0.885934439	SURF2
ENSG00000134827.8	0.024081478	0.377589369	0.885934439	TCN1
ENSG00000164880.16	-0.009099307	0.377731157	0.885934439	INTS1
ENSG00000141469.18	0.018287403	0.377739625	0.885934439	SLC14A1
ENSG00000240954.1	-0.022022636	0.377763732	0.885934439	RPL4P1
ENSG00000103121.9	0.010341397	0.377786177	0.885934439	CMC2
ENSG00000121089.4	0.033280153	0.37784396	0.885934439	NACA3P
ENSG00000250132.6	0.01953193	0.377874722	0.885934439	RP11-359B12.2
ENSG00000154783.12	-0.033292237	0.377920301	0.885934439	FGD5
ENSG00000205871.5	0.041120404	0.377948552	0.885934439	RPS3AP47

ENSG00000173110.8	-0.011780746	0.378016384	0.885934439	HSPA6
ENSG00000124496.12	-0.007178077	0.378050253	0.885934439	TRERF1
ENSG00000205809.10	0.041509235	0.378075371	0.885934439	KLRC2
ENSG00000258086.1	-0.021986062	0.378098397	0.885934439	RP11-753H16.5
ENSG00000253309.7	0.026810656	0.378117169	0.885934439	SERPINE3
ENSG00000038210.14	-0.012793577	0.3781849	0.885934439	PI4K2B
ENSG00000129696.13	-0.005962497	0.378186667	0.885934439	TTI2
ENSG00000118193.12	-0.035395159	0.378199234	0.885934439	KIF14
ENSG00000160191.18	-0.024023279	0.378200504	0.885934439	PDE9A
ENSG00000072858.11	-0.00631533	0.378212598	0.885934439	SIDT1
ENSG00000222267.1	-0.019238753	0.378248228	0.885934439	RNU6-892P
ENSG00000158747.15	0.022774009	0.37828996	0.885934439	NBL1
ENSG00000106853.20	0.031279987	0.378325121	0.885934439	PTGR1
ENSG00000186301.8	0.016355044	0.378331105	0.885934439	MST1P2
ENSG00000116985.12	0.010259833	0.378341971	0.885934439	BMP8B
ENSG00000196497.17	-0.013052997	0.378348817	0.885934439	IPO4
ENSG00000288253.1	-0.01986863	0.378382236	0.885934439	CTD-3018O17.7
ENSG00000117281.16	0.020387867	0.378393336	0.885934439	CD160
ENSG00000186594.14	0.008076321	0.378424397	0.885934439	MIR22HG
ENSG00000178538.10	0.029249107	0.378429451	0.885934439	CA8
ENSG00000281026.1	0.006686356	0.378449628	0.885934439	N4BP2L2-IT2
ENSG00000182393.3	0.028153529	0.378482528	0.885934439	IFNL1
ENSG00000116353.16	0.007932002	0.378513631	0.885934439	MECR
ENSG00000225850.3	-0.025212497	0.378697035	0.885986552	RP11-452K12.4
ENSG00000158428.4	-0.017136883	0.378709016	0.885986552	CATIP
ENSG00000214182.5	0.016782401	0.378812609	0.885986552	PTMAP5
ENSG00000138050.15	0.008887516	0.378837323	0.885986552	THUMPD2
ENSG00000122176.12	0.187512113	0.378883822	0.885986552	FMOD
ENSG00000173991.6	-0.014345953	0.378910103	0.885986552	TCAP
ENSG00000145425.10	0.023314338	0.378923802	0.885986552	RPS3A
ENSG00000165389.7	0.009710971	0.37899342	0.885986552	SPTSSA
ENSG00000177427.13	0.006519282	0.379007052	0.885986552	MIEF2
ENSG00000157657.14	0.013434906	0.37901772	0.885986552	ZNF618
ENSG00000211804.4	0.035783959	0.379048585	0.885986552	TRDV1
ENSG00000100385.14	-0.013981573	0.37905432	0.885986552	IL2RB
ENSG00000162407.9	-0.024836201	0.379197769	0.886220838	PLPP3
ENSG00000273891.1	0.019384503	0.379340492	0.886354598	RP11-500G22.5
ENSG00000111490.15	-0.015975992	0.37937738	0.886354598	TBC1D30
ENSG00000064726.10	0.006960512	0.379419029	0.886354598	BTBD1
ENSG00000211886.1	-0.027789435	0.379427882	0.886354598	TRAJ3
ENSG00000273311.1	0.013032225	0.379556922	0.886555053	DGCR11
ENSG00000087903.13	0.015377219	0.379692565	0.8866518	RFX2
ENSG00000168246.6	0.00829667	0.379696468	0.8866518	UBTD2
ENSG00000108786.11	-0.02979523	0.379728046	0.8866518	HSD17B1
ENSG00000073737.17	0.018780003	0.379789279	0.886693822	DHRS9
ENSG00000233614.6	-0.04822434	0.379907431	0.886705799	DDX11L10
ENSG00000103707.10	-0.006474766	0.380000131	0.886705799	MTFMT

ENSG00000264769.1	-0.024782031	0.380006261	0.886705799	RP11-498C9.12
ENSG00000105135.16	-0.007407386	0.380012931	0.886705799	ILVBL
ENSG00000156030.13	-0.007184558	0.38009641	0.886705799	MIDEAS
ENSG00000090432.7	-0.006626591	0.380113904	0.886705799	MUL1
ENSG00000188868.14	0.016738711	0.380139508	0.886705799	ZNF563
ENSG00000105849.6	0.008252094	0.380182986	0.886705799	POLR1F
ENSG00000179981.11	-0.008723074	0.380200172	0.886705799	TSHZ1
ENSG00000111321.11	0.008361479	0.38022678	0.886705799	LTBR
ENSG00000160183.17	-0.019980875	0.380316474	0.88673714	TMPRSS3
ENSG00000238057.10	0.020896008	0.380369202	0.88673714	ZEB2-AS1
ENSG00000247828.8	0.008591157	0.380392227	0.88673714	TMEM161B-AS1
ENSG00000173214.7	-0.008546592	0.380445885	0.88673714	MFSD4B
ENSG00000112297.18	0.005306359	0.380504067	0.88673714	CRYBG1
ENSG00000225871.2	0.014473981	0.380553071	0.88673714	RP11-495P10.10
ENSG00000267858.6	-0.011748787	0.380607613	0.88673714	MZF1-AS1
ENSG00000283236.1	0.017739308	0.380618632	0.88673714	AC074141.3
ENSG00000147459.18	0.008587803	0.380647002	0.88673714	DOCK5
ENSG00000124731.13	0.012076408	0.380762213	0.88673714	TREM1
ENSG00000229591.1	0.028664986	0.380822814	0.88673714	RP5-981O7.2
ENSG00000225774.2	0.056223267	0.380930612	0.88673714	SIRPAP1
ENSG00000104660.19	0.006599063	0.380949177	0.88673714	LEPROTL1
ENSG00000169313.10	0.018391652	0.380976336	0.88673714	P2RY12
ENSG00000184990.13	0.007541848	0.381005444	0.88673714	SIVA1
ENSG00000214706.12	0.006667713	0.381125737	0.88673714	IFRD2
ENSG00000273123.1	0.037935834	0.381267756	0.88673714	RP11-9N20.3
ENSG00000233056.3	0.032684351	0.381321367	0.88673714	ERVH48-1
ENSG00000170035.16	0.007216652	0.381362873	0.88673714	UBE2E3
ENSG00000277595.1	-0.018665374	0.381406262	0.88673714	RP11-946P6.6
ENSG00000276529.1	-0.01662167	0.381461808	0.88673714	AP001505.10
ENSG00000084207.18	0.010225143	0.381463163	0.88673714	GSTP1
ENSG00000133063.16	0.03062517	0.381560298	0.88673714	CHIT1
ENSG00000164944.12	0.00484613	0.38163162	0.88673714	VIRMA
ENSG00000256537.5	0.007227555	0.381640887	0.88673714	SMIM10L1
ENSG00000249673.7	0.007055321	0.381689794	0.88673714	NOP14-AS1
ENSG00000138311.18	-0.02410206	0.381713756	0.88673714	ZNF365
ENSG00000161973.11	0.02729854	0.381770372	0.88673714	CCDC42
ENSG00000185697.17	-0.017710452	0.381773523	0.88673714	MYBL1
ENSG00000234719.9	-0.03991023	0.381818967	0.88673714	NPIPB2
ENSG00000256967.1	0.019598662	0.381827625	0.88673714	RP11-273B20.1
ENSG00000246859.3	0.018584703	0.381854213	0.88673714	STARD4-AS1
ENSG00000136689.19	-0.013755948	0.381868435	0.88673714	IL1RN
ENSG00000181610.13	0.007896215	0.381873166	0.88673714	MRPS23
ENSG00000175106.17	-0.010376872	0.381876445	0.88673714	TVP23C
ENSG00000251666.1	0.019840732	0.381876989	0.88673714	ZNF346-IT1
ENSG00000272606.1	-0.03912328	0.381900794	0.88673714	PPP4R3B-DT
ENSG00000140104.14	0.012481707	0.381910119	0.88673714	CLBA1
ENSG00000171843.17	0.009434169	0.381965379	0.88673714	MLLT3

ENSG00000279537.1	0.051868063	0.381969763	0.88673714	RP11-133K1.8
ENSG00000180185.11	0.008474038	0.382021175	0.886756113	FAHD1
ENSG00000237977.1	-0.019387552	0.382087818	0.88677352	EIF4HP2
ENSG00000278985.1	-0.025200175	0.382115155	0.88677352	RP11-303E16.9
ENSG00000109099.16	0.024901609	0.382397048	0.887257277	PMP22
ENSG00000269982.1	-0.00959697	0.382435821	0.887257277	RP11-1020A11.2
ENSG00000183318.11	-0.035262407	0.3824534	0.887257277	SPDYE4
ENSG00000152558.15	-0.013539633	0.382503374	0.887272843	TMEM123
ENSG00000144120.13	-0.009103047	0.382618737	0.887440066	TMEM177
ENSG00000253854.1	-0.019563227	0.382786031	0.887485078	RP11-10N23.2
ENSG00000233913.7	0.049663966	0.382831819	0.887485078	RPL10P9
ENSG00000154265.16	-0.012505905	0.382877593	0.887485078	ABCA5
ENSG00000197119.13	0.018583053	0.382882051	0.887485078	SLC25A29
ENSG00000054219.11	-0.009333616	0.382894067	0.887485078	LY75
ENSG00000116774.12	-0.033105173	0.382905866	0.887485078	OLFML3
ENSG00000135838.14	0.007902111	0.382977144	0.887485078	NPL
ENSG00000116663.11	0.015079969	0.383023322	0.887485078	FBXO6
ENSG00000099840.14	-0.011021394	0.383040694	0.887485078	IZUMO4
ENSG00000197971.16	0.006252031	0.383070895	0.887485078	MBP
ENSG00000267655.1	-0.03247612	0.383203403	0.887638031	CTD-2286N8.2
ENSG00000269534.5	0.02759621	0.383265505	0.887638031	CTC-453G23.5
ENSG00000133048.13	0.026852451	0.383281236	0.887638031	CHI3L1
ENSG00000231770.6	0.013851916	0.383310045	0.887638031	TMEM44-AS1
ENSG00000145555.15	-0.022861586	0.38336768	0.887671264	MYO10
ENSG00000244437.1	0.026379734	0.38349116	0.887856932	IGKV3-15
ENSG00000162437.14	-0.030324253	0.383564443	0.887874461	RAVER2
ENSG00000146463.12	-0.005841728	0.383585319	0.887874461	ZMYM4
ENSG00000198961.10	0.009559663	0.383664715	0.887958016	PJA2
ENSG00000206562.12	0.006528761	0.383788938	0.888038541	METTL6
ENSG00000261795.1	0.130891522	0.383798689	0.888038541	RP11-90P13.1
ENSG00000197520.10	0.022961672	0.383837747	0.888038541	FAM177B
ENSG00000164823.11	0.01089187	0.383935488	0.888038541	OSGIN2
ENSG00000164403.15	-0.021014717	0.38394242	0.888038541	SHROOM1
ENSG00000197771.13	0.004577832	0.383981234	0.888038541	MCMBP
ENSG00000237484.6	0.019504668	0.384002622	0.888038541	LINC01684
ENSG00000166598.16	0.00630115	0.384112674	0.888052899	HSP90B1
ENSG00000118503.15	0.00887971	0.384112761	0.888052899	TNFAIP3
ENSG00000171174.15	-0.013787473	0.384144386	0.888052899	RBKS
ENSG00000212978.6	0.009677321	0.38420039	0.888052899	AC016747.3
ENSG00000102699.6	0.007083503	0.384243086	0.888052899	PARP4
ENSG00000125970.12	-0.007080064	0.384326612	0.888052899	RALY
ENSG00000198454.2	-0.036903021	0.384332873	0.888052899	LINC02692
ENSG00000126858.19	0.008391356	0.384355253	0.888052899	RHOT1
ENSG00000151240.17	0.015886163	0.384471878	0.888068468	DIP2C
ENSG00000211699.2	-0.024991214	0.38447435	0.888068468	TRGV3
ENSG00000235052.1	0.031533505	0.384491902	0.888068468	RP1-150O5.3
ENSG00000115339.14	-0.011309791	0.384679167	0.888227088	GALNT3

ENSG00000204231.11	0.004773445	0.384690185	0.888227088	RXRB
ENSG00000225434.3	-0.011845252	0.384721367	0.888227088	LINC01504
ENSG00000211694.2	-0.024030046	0.384733822	0.888227088	TRGV10
ENSG00000255158.1	-0.033915046	0.384804195	0.888289557	RP11-754B17.1
ENSG00000178537.10	0.007876554	0.384850692	0.888296428	SLC25A20
ENSG00000188677.15	0.0121805	0.384921911	0.888296428	PARVB
ENSG00000277423.1	0.032008356	0.384970072	0.888296428	RP11-173P15.9
ENSG00000181381.15	-0.014513772	0.38498043	0.888296428	DDX60L
ENSG00000094914.14	-0.006096367	0.385044803	0.888345014	AAAS
ENSG00000088986.11	0.008385226	0.385216901	0.888642094	DYNLL1
ENSG00000186448.15	0.008615595	0.385323419	0.88878784	ZNF197
ENSG00000243959.3	-0.016406455	0.385584998	0.888887533	RN7SL684P
ENSG00000178718.7	-0.011177162	0.385654278	0.888887533	RPP25
ENSG00000170425.4	0.018636048	0.385740556	0.888887533	ADORA2B
ENSG00000138814.17	-0.007081961	0.385752937	0.888887533	PPP3CA
ENSG00000164402.14	0.009976081	0.385809896	0.888887533	SEPTIN8
ENSG00000249471.8	-0.007591293	0.385827048	0.888887533	ZNF324B
ENSG00000129204.17	0.024896189	0.38583721	0.888887533	USP6
ENSG00000254709.8	-0.027250567	0.385891411	0.888887533	IGLL5
ENSG00000168010.11	-0.0069774	0.38589906	0.888887533	ATG16L2
ENSG00000164338.10	0.00774784	0.385917974	0.888887533	UTP15
ENSG00000086506.3	-0.026860606	0.38594415	0.888887533	HBQ1
ENSG00000126777.18	0.012082031	0.385951713	0.888887533	KTN1
ENSG00000135547.9	0.032023286	0.386005906	0.888887533	HEY2
ENSG00000167972.14	-0.008996973	0.386052515	0.888887533	ABCA3
ENSG00000144395.18	0.026505566	0.386119351	0.888887533	CCDC150
ENSG00000224650.2	-0.031081335	0.386157961	0.888887533	IGHV3-74
ENSG00000261596.2	0.011980328	0.386223163	0.888887533	CTB-31N19.3
ENSG00000066455.13	0.004318544	0.386253965	0.888887533	GOLGA5
ENSG00000280332.1	0.00907787	0.386334494	0.888887533	CTD-2013N17.6
ENSG00000237976.1	0.028895413	0.386354343	0.888887533	RFX5-AS1
ENSG00000119630.14	0.031987214	0.386439582	0.888887533	PGF
ENSG00000145912.9	-0.008265961	0.386449246	0.888887533	NHP2
ENSG00000267787.7	0.009054556	0.386457793	0.888887533	RP11-35G9.5
ENSG00000203791.15	0.009755257	0.386517715	0.888887533	EEF1AKMT2
ENSG00000223510.7	0.040691035	0.38652153	0.888887533	CDRT15
ENSG00000175931.13	-0.008461271	0.386531386	0.888887533	UBE2O
ENSG00000159915.13	0.016592263	0.386536913	0.888887533	ZNF233
ENSG00000275158.1	0.036771248	0.386661153	0.888924297	TRBV12-5
ENSG00000151067.23	0.013971102	0.386782294	0.888924297	CACNA1C
ENSG00000205639.12	0.02412949	0.386825633	0.888924297	MFSD2B
ENSG00000249624.10	-0.018585525	0.386834766	0.888924297	AP000295.9
ENSG00000198113.3	-0.008557684	0.386847378	0.888924297	TOR4A
ENSG00000250299.6	0.030296891	0.386901866	0.888924297	MRPS31P4
ENSG00000085840.13	-0.016007112	0.386914554	0.888924297	ORC1
ENSG00000273628.1	0.037937578	0.386938274	0.888924297	RP11-756A22.7
ENSG00000272870.3	-0.015639275	0.386969569	0.888924297	SAP30-DT

ENSG00000211640.4	0.039895887	0.387016962	0.888924297	IGLV6-57
ENSG00000137513.10	-0.006959523	0.387068768	0.888924297	NARS2
ENSG00000162755.14	-0.029467177	0.387073043	0.888924297	KLHDC9
ENSG00000143842.15	-0.017383334	0.387147422	0.888956743	SOX13
ENSG00000132464.13	-0.047683973	0.387173865	0.888956743	ENAM
ENSG00000259158.4	-0.020653761	0.387353305	0.889183291	ADAM20P1
ENSG00000247095.3	-0.020677774	0.38739956	0.889183291	MIR210HG
ENSG00000089486.17	-0.008233518	0.387421892	0.889183291	CDIP1
ENSG00000260773.1	-0.018723845	0.38745261	0.889183291	RP11-352G18.2
ENSG00000233892.1	-0.020522087	0.387514591	0.889183291	PAIP1P1
ENSG00000211668.2	0.03055241	0.387532683	0.889183291	IGLV2-11
ENSG00000286313.1	-0.020998424	0.387598242	0.889234226	RP11-394G3.3
ENSG00000123612.16	0.021463377	0.387758192	0.889417875	ACVR1C
ENSG00000188735.13	-0.006200551	0.387835971	0.889417875	TMEM120B
ENSG00000257913.2	-0.029182071	0.387921938	0.889417875	DDN-AS1
ENSG00000260518.2	0.129291799	0.38798495	0.889417875	BMS1P8
ENSG00000111271.15	0.004981122	0.387992234	0.889417875	ACAD10
ENSG00000275549.1	-0.025416302	0.388077785	0.889417875	STPG3-AS1
ENSG00000131142.14	-0.052594229	0.38809822	0.889417875	CCL25
ENSG00000187531.14	-0.005566928	0.388162169	0.889417875	SIRT7
ENSG00000143869.7	0.034669127	0.388165941	0.889417875	GDF7
ENSG00000136709.12	-0.003848525	0.388251641	0.889417875	WDR33
ENSG00000151849.16	-0.008104564	0.388267278	0.889417875	CENPJ
ENSG00000257135.7	-0.011128203	0.38830224	0.889417875	ODC1-DT
ENSG00000237940.3	-0.017095927	0.388322397	0.889417875	LINC01238
ENSG00000228623.6	0.022650224	0.388446908	0.889417875	ZNF883
ENSG00000108021.20	0.009422629	0.388473555	0.889417875	TASOR2
ENSG00000004700.16	0.011860624	0.388520821	0.889417875	RECQL
ENSG00000163637.13	0.035162604	0.38854898	0.889417875	PRICKLE2
ENSG00000055130.17	0.006711466	0.388578551	0.889417875	CUL1
ENSG00000163467.12	0.028887891	0.388611169	0.889417875	TSACC
ENSG00000114859.16	-0.011052076	0.388630609	0.889417875	CLCN2
ENSG00000261801.6	-0.010968531	0.388638095	0.889417875	LOXL1-AS1
ENSG00000109103.12	0.005905786	0.388666042	0.889417875	UNC119
ENSG00000287737.1	-0.014044279	0.388861155	0.889417875	RP13-516M14.11
ENSG00000278948.1	-0.012830196	0.388865239	0.889417875	RP5-1039K5.12
ENSG00000251131.2	-0.019457465	0.388878473	0.889417875	CTD-2035E11.3
ENSG00000271105.1	-0.030712233	0.388888439	0.889417875	SCML2P2
ENSG00000110046.13	-0.010847848	0.388900461	0.889417875	ATG2A
ENSG00000237133.1	0.025883997	0.388911397	0.889417875	RASGRP3-AS1
ENSG00000143321.19	0.008485822	0.388937735	0.889417875	HDGF
ENSG00000287742.1	-0.033407781	0.388987825	0.889417875	RP11-314L11.2
ENSG00000134769.23	0.023095564	0.389057127	0.889417875	DTNA
ENSG00000259417.3	0.027806251	0.389093299	0.889417875	CTXND1
ENSG00000159556.10	-0.020346964	0.38916792	0.889417875	ISL2
ENSG00000119917.15	-0.032163959	0.389274716	0.889417875	IFIT3
ENSG00000163159.14	-0.006379831	0.389295753	0.889417875	VPS72

ENSG00000169994.19	-0.017316618	0.389304609	0.889417875	MYO7B
ENSG00000135002.12	0.009654035	0.38942232	0.889417875	RFK
ENSG00000287595.1	0.021569336	0.389422334	0.889417875	RP11-624D20.3
ENSG00000241468.8	0.008898637	0.389447936	0.889417875	ATP5MF
ENSG00000088682.14	0.004155567	0.389454633	0.889417875	COQ9
ENSG00000239830.1	0.037106099	0.389494731	0.889417875	RPS4XP22
ENSG00000176871.9	0.00803997	0.389502482	0.889417875	WSB2
ENSG00000239203.1	-0.020051833	0.389571093	0.889417875	AC093484.4
ENSG00000274712.2	-0.011434635	0.389586541	0.889417875	RP11-147L13.15
ENSG00000260027.5	0.027887786	0.389722626	0.889478727	HOXB7
ENSG00000150687.12	-0.020734051	0.389764722	0.889478727	PRSS23
ENSG00000235917.1	0.03647922	0.389798516	0.889478727	MTCO2P11
ENSG00000273154.3	-0.012430037	0.389839369	0.889478727	RP4-583P15.15
ENSG00000188827.11	-0.008167349	0.390053525	0.889478727	SLX4
ENSG00000152382.6	0.007174	0.390085259	0.889478727	TADA1
ENSG00000272842.1	-0.02283012	0.390095224	0.889478727	RP11-513M16.7
ENSG00000180530.11	0.011939011	0.390111181	0.889478727	NRIP1
ENSG00000114547.10	0.041946648	0.390119268	0.889478727	ROPN1B
ENSG00000268833.1	0.039956067	0.390152331	0.889478727	AC011513.4
ENSG00000115590.14	0.013020649	0.390167165	0.889478727	IL1R2
ENSG00000268555.2	0.033217865	0.390172084	0.889478727	RP11-678G14.3
ENSG00000189233.12	-0.020300771	0.390234885	0.889478727	NUGGC
ENSG00000246082.2	0.010889646	0.390253964	0.889478727	NUDT16L2P
ENSG00000237899.1	0.031593489	0.390263779	0.889478727	RP4-739H11.3
ENSG00000284968.1	-0.017128144	0.390361186	0.889576147	RP11-397E7.5
ENSG00000037749.13	0.007321759	0.390444798	0.889576147	MFAP3
ENSG00000177380.14	-0.013958421	0.390466887	0.889576147	PPFIA3
ENSG00000118894.15	-0.008252061	0.390480031	0.889576147	EEF2KMT
ENSG00000169169.15	-0.032376894	0.390692839	0.889717581	CPT1C
ENSG00000110002.16	0.008345264	0.390752799	0.889717581	VWA5A
ENSG00000111785.21	-0.005443723	0.390768713	0.889717581	RIC8B
ENSG00000216937.13	-0.010791196	0.390783618	0.889717581	CCDC7
ENSG00000269220.1	-0.007877298	0.390823027	0.889717581	LINC00528
ENSG00000211669.3	-0.041936516	0.390922107	0.889717581	IGLV3-10
ENSG00000240889.1	-0.014698206	0.390958191	0.889717581	NDUFB2-AS1
ENSG00000266962.2	-0.008141367	0.390963974	0.889717581	HSD17B1-AS1
ENSG00000264954.2	-0.029308677	0.390984512	0.889717581	PRR29-AS1
ENSG00000235172.8	0.017750174	0.391056913	0.889717581	LINC01366
ENSG00000012048.23	0.00867319	0.391076727	0.889717581	BRCA1
ENSG00000156482.11	0.013152536	0.391175039	0.889717581	RPL30
ENSG00000281912.1	0.019825095	0.391259459	0.889717581	LINC01144
ENSG00000125814.17	-0.007677418	0.391269517	0.889717581	NAPB
ENSG00000113593.12	0.008348475	0.391277679	0.889717581	PPWD1
ENSG00000213983.12	-0.006766905	0.391292579	0.889717581	AP1G2
ENSG00000178177.16	-0.010499365	0.391312547	0.889717581	LCORL
ENSG00000134852.15	-0.008670502	0.391323024	0.889717581	CLOCK
ENSG00000168101.14	-0.007848937	0.39139695	0.889787013	NUDT16L1

ENSG00000233639.7	-0.1233286	0.391636375	0.890103417	PANTR1
ENSG00000116473.14	0.006022239	0.391645806	0.890103417	RAP1A
ENSG00000286407.1	-0.03528212	0.391666337	0.890103417	RP4-708P22.1
ENSG00000136754.18	0.005359706	0.391829916	0.890269212	ABI1
ENSG00000067900.8	0.01229951	0.391845849	0.890269212	ROCK1
ENSG00000197928.11	0.017550273	0.391894452	0.890269212	ZNF677
ENSG00000100031.19	0.014705667	0.391912934	0.890269212	GGT1
ENSG00000255152.8	-0.014690795	0.391963818	0.890286186	MSH5-SAPCD1
ENSG00000149089.13	0.013300438	0.392061255	0.890408883	APIP
ENSG00000255398.3	0.015344199	0.392121688	0.890447523	HCAR3
ENSG00000115020.17	-0.009476349	0.392176218	0.890472751	PIKFYVE
ENSG00000162994.16	-0.020214785	0.392247658	0.890536362	CLHC1
ENSG00000233016.7	0.010277998	0.392393441	0.890680382	SNHG7
ENSG00000171466.10	0.005118568	0.392397955	0.890680382	ZNF562
ENSG00000232774.8	-0.021543756	0.392573572	0.890769237	RP11-47I22.3
ENSG00000132561.14	-0.021955079	0.392597248	0.890769237	MATN2
ENSG00000008405.12	-0.007719648	0.392602497	0.890769237	CRY1
ENSG00000139146.14	0.0083779	0.392610841	0.890769237	SINHCAF
ENSG00000270882.2	0.027406592	0.392669162	0.890803005	H4C14
ENSG00000151882.11	-0.007832989	0.392824523	0.890844673	CCL28
ENSG00000153922.11	0.009373268	0.392831641	0.890844673	CHD1
ENSG00000143382.16	-0.011175652	0.392845816	0.890844673	ADAMTSL4
ENSG00000147536.12	-0.011731464	0.392871885	0.890844673	GINS4
ENSG00000015133.19	-0.006101949	0.392986675	0.890844673	CCDC88C
ENSG00000078804.13	-0.014541617	0.393000082	0.890844673	TP53INP2
ENSG00000187860.11	-0.010691615	0.393082492	0.890844673	CCDC157
ENSG00000183207.14	-0.007503183	0.393105898	0.890844673	RUVBL2
ENSG00000063438.20	-0.052712919	0.393112291	0.890844673	AHRR
ENSG00000264745.1	-0.024368233	0.39323369	0.890844673	TTC39C-AS1
ENSG00000234515.1	0.023851507	0.39325192	0.890844673	PPP1R2P1
ENSG00000177112.8	0.018024327	0.393261578	0.890844673	IRAG1-AS1
ENSG00000213090.2	0.030465291	0.393294013	0.890844673	SCYL2P1
ENSG00000267040.6	-0.008302709	0.393295673	0.890844673	RP11-35G9.3
ENSG00000282021.1	-0.04005267	0.393348412	0.890865736	CTD-2336O2.3
ENSG00000272902.2	0.022654318	0.393443794	0.890982133	TBC1D8-AS1
ENSG00000090104.12	-0.026738996	0.39350367	0.890982133	RGS1
ENSG00000254428.1	0.016298223	0.393534338	0.890982133	RP11-110I1.11
ENSG00000225093.1	-0.030739301	0.393618627	0.890982133	RPL3P7
ENSG00000233328.3	-0.025230005	0.393629399	0.890982133	PFN1P1
ENSG00000112299.8	-0.021476633	0.393660479	0.890982133	VNN1
ENSG00000159905.15	0.029933733	0.393715835	0.891009086	ZNF221
ENSG00000247516.8	0.021972294	0.393832352	0.891174432	MIR4458HG
ENSG00000128165.9	-0.015174714	0.393903857	0.891186465	ADM2
ENSG00000141562.18	0.007446508	0.394002208	0.891186465	NARF
ENSG00000167646.14	0.080439289	0.394092528	0.891186465	DNAAF3
ENSG00000185909.15	0.016338093	0.394184463	0.891186465	KLHDC8B
ENSG00000118276.12	-0.019652412	0.394206588	0.891186465	B4GALT6

ENSG00000187391.22	0.024042146	0.394218717	0.891186465	MAGI2
ENSG00000104823.9	-0.008323059	0.394219166	0.891186465	ECH1
ENSG00000111912.20	-0.010749329	0.39426256	0.891186465	NCOA7
ENSG00000087237.12	0.015809549	0.394268899	0.891186465	CETP
ENSG00000069020.18	-0.012253335	0.394272225	0.891186465	MAST4
ENSG00000273270.1	0.009541978	0.394315858	0.891186866	RP11-212P7.2
ENSG00000160469.17	0.013138353	0.394426308	0.891213506	BRSK1
ENSG00000268521.1	0.035141025	0.394474353	0.891213506	VN1R83P
ENSG00000170160.17	0.062927264	0.394482499	0.891213506	CCDC144A
ENSG00000226471.6	-0.015246268	0.394501473	0.891213506	CTA-292E10.6
ENSG00000090447.12	-0.0073753	0.394575234	0.891281959	TFAP4
ENSG00000151748.15	0.009002135	0.394683003	0.891397373	SAV1
ENSG00000101190.13	-0.007872356	0.39471326	0.891397373	TCFL5
ENSG00000088970.16	0.005194661	0.394780011	0.891449953	KIZ
ENSG00000268062.1	-0.023747211	0.394854091	0.891519068	SRP14P4
ENSG00000213015.9	-0.009055927	0.395012786	0.891576905	ZNF580
ENSG00000078699.22	-0.004363401	0.395018631	0.891576905	CBFA2T2
ENSG00000115524.17	0.008177905	0.395056772	0.891576905	SF3B1
ENSG00000239213.6	-0.010650923	0.395135545	0.891576905	NCK1-DT
ENSG00000171860.5	0.01650897	0.395224579	0.891576905	C3AR1
ENSG00000133302.13	-0.010908577	0.395254084	0.891576905	SLF1
ENSG00000151364.17	-0.050476744	0.39527502	0.891576905	KCTD14
ENSG00000255182.2	-0.015571857	0.39533447	0.891576905	CTD-2517M22.14
ENSG00000272917.1	-0.035757764	0.395356649	0.891576905	RP11-705C15.5
ENSG00000108669.16	-0.003833028	0.395405859	0.891576905	CYTH1
ENSG00000165816.13	0.047124639	0.395416742	0.891576905	VWA2
ENSG00000275437.1	0.013506886	0.395424266	0.891576905	RP5-908M14.10
ENSG00000133401.16	0.013825108	0.395476723	0.891576905	PDZD2
ENSG00000144040.13	-0.006209538	0.39549368	0.891576905	SFXN5
ENSG00000224988.1	0.022751467	0.395531826	0.891576905	RP11-409K20.7
ENSG00000148335.15	0.006999814	0.395641291	0.89172564	NTMT1
ENSG00000253328.2	-0.019475677	0.395830026	0.891738757	SUMO2P19
ENSG00000238231.2	0.032157899	0.395831295	0.891738757	RP11-460I13.6
ENSG00000135269.18	0.00528119	0.395857717	0.891738757	TES
ENSG00000239998.6	-0.008266825	0.395860166	0.891738757	LILRA2
ENSG00000214174.8	-0.013527838	0.395946685	0.891738757	AMZ2P1
ENSG00000255062.1	-0.010646781	0.39596654	0.891738757	RP11-712L6.5
ENSG00000130803.15	0.004207484	0.396008515	0.891738757	ZNF317
ENSG00000124406.16	-0.012671258	0.396086865	0.891738757	ATP8A1
ENSG00000233436.7	-0.02397382	0.396123041	0.891738757	BTBD18
ENSG00000178429.9	0.038050394	0.396146201	0.891738757	RPS3AP5
ENSG00000236772.1	0.023345977	0.39622814	0.891738757	RP5-1184F4.5
ENSG00000099203.7	0.007657525	0.396237898	0.891738757	TMED1
ENSG00000279456.1	0.033252089	0.396250409	0.891738757	RP11-87H9.5
ENSG00000261150.3	-0.026423658	0.39628174	0.891738757	EPPK1
ENSG00000267174.5	0.023819859	0.396299348	0.891738757	CTC-510F12.4
ENSG00000130717.13	0.005333968	0.396394512	0.891855037	UCK1

ENSG00000141580.16	0.00396167	0.396865337	0.892728772	WDR45B
ENSG00000134905.17	0.006896519	0.396869915	0.892728772	CARS2
ENSG00000152926.15	0.017200155	0.396966072	0.892755031	ZNF117
ENSG00000018408.15	-0.019216982	0.397041484	0.892755031	WWTR1
ENSG00000101445.10	0.007100269	0.397100335	0.892755031	PPP1R16B
ENSG00000253326.2	0.029770411	0.397111617	0.892755031	RP11-261C10.7
ENSG00000133026.13	-0.011559685	0.397184173	0.892755031	MYH10
ENSG00000168916.16	-0.02288307	0.397215715	0.892755031	ZNF608
ENSG00000145782.13	0.006672034	0.397223073	0.892755031	ATG12
ENSG00000231586.2	0.052770372	0.397256932	0.892755031	RPS23P9
ENSG00000233953.1	0.04251495	0.397273377	0.892755031	AC009970.1
ENSG00000224729.6	0.022623218	0.397362371	0.892857183	PCOLCE-AS1
ENSG00000259211.1	0.025257215	0.397493792	0.892973176	RP11-64K12.8
ENSG00000270959.1	-0.012442111	0.397513149	0.892973176	LPP-AS2
ENSG00000168887.11	0.003728131	0.397555984	0.892973176	C2orf68
ENSG00000275418.1	-0.025604224	0.397598679	0.892973176	RP11-126O1.6
ENSG00000286089.1	0.019554719	0.397631707	0.892973176	RP11-395I6.4
ENSG00000115163.15	-0.033084082	0.397800921	0.893050807	CENPA
ENSG00000085760.15	0.00560422	0.397811561	0.893050807	MTIF2
ENSG00000111678.11	0.012510076	0.397909533	0.893050807	C12orf57
ENSG00000167280.17	0.009475222	0.397912238	0.893050807	ENGASE
ENSG00000232888.4	0.01571035	0.397944647	0.893050807	RPS11P5
ENSG00000136878.14	-0.005594799	0.397997197	0.893050807	USP20
ENSG00000232545.1	0.029928012	0.398023552	0.893050807	KB-318B8.7
ENSG00000101082.14	0.008393366	0.39818636	0.893050807	SLA2
ENSG00000087250.9	-0.046217072	0.398254089	0.893050807	MT3
ENSG00000237101.1	0.026591481	0.398262003	0.893050807	RP11-365O16.6
ENSG00000109107.14	-0.009202747	0.398264828	0.893050807	ALDOC
ENSG00000285752.1	0.009339739	0.398279815	0.893050807	CDC42-AS1
ENSG00000138678.11	0.00915071	0.39841873	0.893050807	GPAT3
ENSG00000100181.22	0.024362136	0.398461019	0.893050807	TPTEP1
ENSG00000111913.20	0.006667495	0.398465217	0.893050807	RIPOR2
ENSG00000248508.8	-0.009983092	0.398470919	0.893050807	SRP14-AS1
ENSG00000184271.17	0.010061348	0.398512798	0.893050807	POU6F1
ENSG00000283828.1	-0.030428737	0.39855948	0.893050807	RP11-98F14.12
ENSG00000119915.5	-0.017824189	0.398562336	0.893050807	ELOVL3
ENSG00000123353.10	0.007276315	0.398572379	0.893050807	ORMDL2
ENSG00000106344.8	-0.004384512	0.398602306	0.893050807	RBM28
ENSG00000092621.13	0.019610814	0.398624297	0.893050807	PHGDH
ENSG00000162631.20	0.04184884	0.39867277	0.893061843	NTNG1
ENSG00000119718.11	0.006207514	0.39882131	0.893133549	EIF2B2
ENSG00000234219.1	-0.02860171	0.398843792	0.893133549	CDCA4P4
ENSG00000183648.11	0.007947677	0.398937859	0.893133549	NDUFB1
ENSG00000278053.5	0.004607733	0.398947907	0.893133549	DDX52
ENSG00000227456.9	-0.026650688	0.399005995	0.893133549	LINC00310
ENSG00000143512.13	0.028418327	0.399117407	0.893133549	HHIPL2
ENSG00000138336.9	0.017235581	0.399154866	0.893133549	TET1

ENSG00000238260.1	-0.01182291	0.399199787	0.893133549	RP11-46F15.2
ENSG00000198440.9	-0.011520647	0.399249824	0.893133549	ZNF583
ENSG00000196118.12	-0.014356876	0.399259836	0.893133549	CCDC189
ENSG00000173889.16	-0.009101584	0.399282639	0.893133549	PHC3
ENSG00000205356.10	-0.006894981	0.399389511	0.893133549	TECPR1
ENSG00000140455.17	0.004307491	0.399398349	0.893133549	USP3
ENSG00000265511.1	0.01189565	0.399411432	0.893133549	RP11-524F11.1
ENSG00000276547.2	-0.03925738	0.399428828	0.893133549	PCDHGB5
ENSG00000124444.16	-0.006259708	0.399437472	0.893133549	ZNF576
ENSG00000261614.1	-0.044182524	0.399445139	0.893133549	YBX3P1
ENSG00000120910.15	-0.005149432	0.399582465	0.893235115	PPP3CC
ENSG00000057935.14	0.006463127	0.39965235	0.893235115	MTA3
ENSG00000215912.12	0.014433349	0.399756756	0.893235115	TTC34
ENSG00000198380.13	-0.006591236	0.399775367	0.893235115	GFPT1
ENSG00000258344.1	0.028663022	0.399808537	0.893235115	RP11-968A15.8
ENSG00000160803.8	-0.00551106	0.399875227	0.893235115	UBQLN4
ENSG00000167094.16	-0.018471903	0.399880456	0.893235115	TTC16
ENSG00000171595.14	0.018711188	0.399888129	0.893235115	DNAI2
ENSG00000247373.3	0.009784929	0.399939126	0.893235115	TMED2-DT
ENSG00000227582.2	-0.091086339	0.399986686	0.893235115	ADGRF5P1
ENSG00000276571.1	0.022081808	0.400011864	0.893235115	AC002550.6
ENSG00000095564.14	0.011887006	0.400019572	0.893235115	BTAF1
ENSG00000239282.8	0.011363965	0.400115311	0.893235115	CASTOR1
ENSG00000254682.1	0.013848718	0.400141994	0.893235115	RP11-660L16.2
ENSG00000068028.18	-0.007866941	0.400143895	0.893235115	RASSF1
ENSG00000270179.1	0.014324142	0.400249662	0.893238411	RP11-159N11.4
ENSG00000268573.1	0.012770536	0.400258917	0.893238411	RP11-158H5.7
ENSG00000154380.17	-0.02946932	0.400360655	0.893238411	ENAH
ENSG00000100823.12	0.00596638	0.400412696	0.893238411	APEX1
ENSG00000239951.1	0.027063104	0.400425319	0.893238411	IGKV3-20
ENSG00000109390.13	0.008056951	0.400458932	0.893238411	NDUFC1
ENSG00000204308.8	0.007785598	0.400470402	0.893238411	RNF5
ENSG00000277349.1	0.034543092	0.400510965	0.893238411	CICP25
ENSG00000006634.8	-0.0071178	0.400537372	0.893238411	DBF4
ENSG00000197746.14	-0.008498616	0.400608556	0.893300017	PSAP
ENSG00000197302.10	0.007457263	0.400704413	0.893416624	ZNF720
ENSG00000132773.12	-0.005695575	0.400853543	0.893651969	TOE1
ENSG00000172500.13	-0.007330947	0.401070092	0.894037548	FIBP
ENSG00000263464.2	-0.036811251	0.401248956	0.894315337	PPIAL4C
ENSG00000108828.16	-0.008130223	0.401281926	0.894315337	VAT1
ENSG00000253755.1	0.036924321	0.401599982	0.894899796	IGHGP
ENSG00000006459.11	0.007060418	0.401657838	0.894899796	KDM7A
ENSG00000151687.15	0.010448183	0.401675084	0.894899796	ANKAR
ENSG00000141096.6	-0.014135536	0.401739234	0.894945494	DPEP3
ENSG00000144426.19	-0.012878542	0.40181337	0.895013425	NBEAL1
ENSG00000196418.13	0.008688151	0.401980339	0.895288097	ZNF124
ENSG00000250659.2	-0.024683121	0.402104289	0.895394259	RP11-864I4.3

ENSG00000160752.15	-0.005431568	0.402115327	0.895394259	FDPS
ENSG00000185245.8	-0.012279565	0.402213285	0.895514786	GP1BA
ENSG00000163297.18	-0.007034044	0.402277564	0.895514786	ANTXR2
ENSG00000279141.4	-0.016723633	0.402300455	0.895514786	LINC01451
ENSG00000244306.11	-0.068055834	0.402381745	0.895556785	DUXAP10
ENSG00000206337.12	0.006616293	0.40240666	0.895556785	HCP5
ENSG00000263968.2	-0.032602276	0.402474446	0.895610452	RN7SL381P
ENSG00000278739.1	0.038537175	0.402659975	0.895811423	MIR6859-4
ENSG00000262152.7	-0.013903608	0.402684711	0.895811423	LINC00514
ENSG00000012061.16	0.006184519	0.402695802	0.895811423	ERCC1
ENSG00000140682.19	-0.02268803	0.402893034	0.895937624	TGFB1I1
ENSG00000132170.24	0.029590113	0.40290859	0.895937624	PPARG
ENSG00000273729.1	-0.007520142	0.402918289	0.895937624	RP11-7F17.8
ENSG00000131013.4	0.008246201	0.402965189	0.895937624	PPIL4
ENSG00000113328.19	0.006993535	0.403000027	0.895937624	CCNG1
ENSG00000248323.7	-0.009796946	0.403014657	0.895937624	LUCAT1
ENSG00000054654.19	-0.009555802	0.403109353	0.896051009	SYNE2
ENSG00000152952.12	0.03119673	0.403270647	0.896108202	PLOD2
ENSG00000282122.1	-0.083801527	0.403284815	0.896108202	IGHV7-4-1
ENSG00000196611.6	0.050491021	0.40331899	0.896108202	MMP1
ENSG00000148680.16	0.022743334	0.403321305	0.896108202	HTR7
ENSG00000085231.14	0.014949816	0.40335356	0.896108202	AK6
ENSG00000236397.3	0.039761268	0.403530375	0.896345685	DDX11L2
ENSG00000181045.15	-0.007441666	0.40354787	0.896345685	SLC26A11
ENSG00000253239.1	-0.043383489	0.403617968	0.896404299	IGLVI-70
ENSG00000105011.9	-0.011311083	0.403748345	0.896596757	ASF1B
ENSG00000164707.16	-0.012381008	0.403839948	0.896678453	SLC13A4
ENSG00000164082.15	-0.01860983	0.403908073	0.896678453	GRM2
ENSG00000223478.1	0.010108012	0.403916303	0.896678453	RP11-545E17.3
ENSG00000152439.13	0.007877586	0.403981618	0.89672638	ZNF773
ENSG00000255478.1	0.02483917	0.404089515	0.8968368	RP11-867O8.5
ENSG00000123191.16	0.014859541	0.404118825	0.8968368	ATP7B
ENSG00000135622.13	-0.006356294	0.404264836	0.897063759	SEMA4F
ENSG00000260942.1	0.007495682	0.404419371	0.897068541	CAPN10-DT
ENSG00000156411.10	0.012381162	0.404451489	0.897068541	ATP5MJ
ENSG00000164163.11	0.008564195	0.404501522	0.897068541	ABCE1
ENSG00000127603.31	-0.008155956	0.4045026	0.897068541	MACF1
ENSG00000214654.8	-0.013791184	0.404546776	0.897068541	B3GALT9
ENSG00000259235.2	-0.086019497	0.404564783	0.897068541	RP11-605F22.2
ENSG00000142627.13	-0.021576123	0.404655909	0.897068541	EPHA2
ENSG00000172239.14	0.008723183	0.404662677	0.897068541	PAIP1
ENSG00000137747.16	0.010699721	0.404679008	0.897068541	TMPRSS13
ENSG00000178229.8	-0.009689489	0.404729504	0.897068541	ZNF543
ENSG00000130347.13	-0.008057225	0.404751697	0.897068541	RTN4IP1
ENSG00000104133.16	-0.006153909	0.404828839	0.897068541	SPG11
ENSG00000163512.14	0.010234123	0.404868727	0.897068541	AZI2
ENSG00000188372.15	-0.018602945	0.404920811	0.897068541	ZP3

ENSG00000167118.11	0.006788663	0.404957343	0.897068541	URM1
ENSG00000268204.1	0.015240353	0.404966869	0.897068541	CTD-3214H19.6
ENSG00000111276.12	-0.005181703	0.405060072	0.897141386	CDKN1B
ENSG00000142512.15	-0.012250411	0.405087246	0.897141386	SIGLEC10
ENSG00000157368.11	-0.027450769	0.405210356	0.897266518	IL34
ENSG00000119640.9	-0.008844117	0.405232053	0.897266518	ACYP1
ENSG00000238042.5	0.042732751	0.405275003	0.897266518	LINC02257
ENSG00000282980.1	-0.023445402	0.405360932	0.897291814	RP11-807C20.2
ENSG00000106603.20	0.007255851	0.405373935	0.897291814	COA1
ENSG00000173578.9	-0.028820187	0.405777754	0.898014178	XCR1
ENSG00000231358.4	0.041052829	0.405787858	0.898014178	RP11-442J17.3
ENSG00000270441.1	-0.012555731	0.405921882	0.898170666	LAMB2P1
ENSG00000196597.13	0.009600885	0.405946163	0.898170666	ZNF782
ENSG00000100228.14	0.013424676	0.406074297	0.898267591	RAB36
ENSG00000189238.6	-0.022186217	0.406077571	0.898267591	LINC00943
ENSG00000141665.13	-0.02179313	0.406217044	0.8984792	FBXO15
ENSG00000156515.24	-0.007243312	0.406268251	0.898495555	HK1
ENSG00000133393.13	0.008237666	0.406464258	0.898704175	CEP20
ENSG00000141026.6	0.005350991	0.406475464	0.898704175	MED9
ENSG00000235576.1	0.02127324	0.406510554	0.898704175	LINC01871
ENSG00000164746.14	-0.039235276	0.40653787	0.898704175	C7orf57
ENSG00000211771.1	-0.013271263	0.406628923	0.898808575	TRBJ2-7
ENSG00000211785.1	0.018193421	0.406773466	0.899031171	TRAV12-1
ENSG00000171502.15	0.025474424	0.406878664	0.899107702	COL24A1
ENSG00000184465.16	-0.008739247	0.406914322	0.899107702	WDR27
ENSG00000129351.17	-0.003739136	0.407020377	0.899107702	ILF3
ENSG00000175029.17	-0.006070199	0.407029272	0.899107702	CTBP2
ENSG00000146592.17	0.011643149	0.407037695	0.899107702	CREB5
ENSG00000175544.14	0.013709159	0.407071143	0.899107702	CABP4
ENSG00000171462.15	-0.011118186	0.407251831	0.899117147	DLK2
ENSG00000067798.16	-0.032432225	0.407320208	0.899117147	NAV3
ENSG00000148848.15	0.026251129	0.407352157	0.899117147	ADAM12
ENSG00000141293.16	-0.006827177	0.407362675	0.899117147	SKAP1
ENSG00000206199.11	0.022528919	0.40737878	0.899117147	ANKUB1
ENSG00000235297.3	0.02820491	0.407379469	0.899117147	FAUP1
ENSG00000170234.13	-0.005751105	0.407410817	0.899117147	PWWP2A
ENSG00000103995.14	0.009818469	0.407426158	0.899117147	CEP152
ENSG00000130167.14	-0.01512306	0.407538812	0.899268986	TSPAN16
ENSG00000235010.1	-0.071932821	0.407618789	0.899271647	RP11-140A10.3
ENSG00000177465.5	0.017819922	0.40765745	0.899271647	ACOT4
ENSG00000219392.1	-0.030802589	0.407811777	0.899271647	ZNF602P
ENSG00000158691.15	0.009755077	0.407831331	0.899271647	ZSCAN12
ENSG00000240137.6	-0.01337024	0.407857206	0.899271647	ERICH6-AS1
ENSG00000131650.14	-0.026173106	0.407938031	0.899271647	KREMEN2
ENSG00000184304.16	-0.061614701	0.407961761	0.899271647	PRKD1
ENSG00000232626.3	-0.031128942	0.408016577	0.899271647	RP11-175B9.2
ENSG00000285796.1	0.008668455	0.408038839	0.899271647	RP11-465L10.14

ENSG00000138326.21	0.011178715	0.408075771	0.899271647	RPS24
ENSG00000196167.10	-0.039327298	0.408088618	0.899271647	COLCA1
ENSG00000132932.19	0.017006066	0.408114584	0.899271647	ATP8A2
ENSG00000279452.1	-0.014281399	0.408159257	0.899271647	AC006277.3
ENSG00000141429.14	0.007694437	0.408192454	0.899271647	GALNT1
ENSG00000137054.16	0.006279149	0.408266195	0.899271647	POLR1E
ENSG00000140398.14	-0.011822635	0.408306839	0.899271647	NEIL1
ENSG00000007255.10	-0.008971572	0.408335054	0.899271647	TRAPPC6A
ENSG00000120278.16	-0.018660417	0.408354557	0.899271647	PLEKHG1
ENSG00000226696.6	-0.010065956	0.408373164	0.899271647	LENG8-AS1
ENSG00000091138.13	-0.036358085	0.408469288	0.899297431	SLC26A3
ENSG00000166963.13	-0.016907813	0.408472575	0.899297431	MAP1A
ENSG00000080493.18	-0.019953666	0.408535277	0.89931823	SLC4A4
ENSG00000257345.2	0.034202287	0.408654347	0.89931823	LINC02413
ENSG00000153933.10	-0.007730073	0.408760213	0.89931823	DGKE
ENSG00000177455.15	-0.022217073	0.408800922	0.89931823	CD19
ENSG00000226360.5	-0.018280371	0.408816766	0.89931823	RPL10AP6
ENSG00000209482.1	0.02570029	0.408853416	0.89931823	SNORD83A
ENSG00000106341.11	0.035618988	0.408881904	0.89931823	PPP1R17
ENSG00000198933.9	-0.013007166	0.408892071	0.89931823	TBKBP1
ENSG00000239862.1	0.046017875	0.408899392	0.89931823	IGKV1-37
ENSG00000279412.1	0.021457192	0.408920543	0.89931823	RP11-394B2.5
ENSG00000145293.16	0.006755815	0.409040729	0.899454914	ENOPH1
ENSG00000223561.7	-0.026953713	0.409107492	0.899454914	AC003090.1
ENSG00000105671.12	0.006519831	0.409114269	0.899454914	DDX49
ENSG00000275228.1	-0.033973291	0.409229876	0.89959159	RP11-370I10.10
ENSG00000181450.18	0.010011914	0.409264167	0.89959159	ZNF678
ENSG00000139899.11	0.022085958	0.40942776	0.899854731	CBLN3
ENSG00000139410.15	0.01739913	0.409510353	0.899939811	SDSL
ENSG00000176102.13	-0.004858003	0.40966583	0.900185026	CSTF3
ENSG00000145284.12	-0.023056676	0.409809581	0.900332895	SCD5
ENSG00000116005.12	-0.006304106	0.409820927	0.900332895	PCYOX1
ENSG00000168522.13	0.005075263	0.409891311	0.900391067	FNTA
ENSG00000171421.13	0.010311473	0.410030713	0.90060082	MRPL36
ENSG00000171103.11	0.007732356	0.410099229	0.90065485	TRMT61B
ENSG00000100216.6	0.006346822	0.410448646	0.901263902	TOMM22
ENSG00000167996.16	-0.008936117	0.410493863	0.901263902	FTH1
ENSG00000278420.1	0.02533094	0.410508392	0.901263902	MIR6819
ENSG00000261490.1	-0.008502507	0.410614131	0.901284464	RP11-448G15.3
ENSG00000075239.14	0.010131147	0.41064698	0.901284464	ACAT1
ENSG00000180481.11	0.03323241	0.410649602	0.901284464	GLIPR1L2
ENSG00000027075.16	-0.00613525	0.410796232	0.901380798	PRKCH
ENSG00000163635.20	0.00840186	0.410796725	0.901380798	ATXN7
ENSG00000235586.1	0.034050168	0.410870228	0.901380798	AC011247.3
ENSG00000261575.2	-0.059914934	0.410889651	0.901380798	RP11-259G18.1
ENSG00000159873.10	0.00614816	0.410913257	0.901380798	CCDC117
ENSG00000129484.14	-0.006771912	0.410989607	0.901451857	PARP2

ENSG00000205885.7	0.012680063	0.411042481	0.90145811	C1RL-AS1
ENSG00000260269.5	0.025179264	0.411131324	0.90145811	CTD-2323K18.1
ENSG00000181218.5	-0.016914333	0.41113767	0.90145811	H2AW
ENSG00000163162.9	0.006825531	0.411168284	0.90145811	RNF149
ENSG00000198718.13	0.011103632	0.411276044	0.901585279	TOGARAM1
ENSG00000285851.1	-0.012203731	0.411336815	0.901585279	RP11-118B23.9
ENSG00000188549.12	0.009422682	0.411411409	0.901585279	CCDC9B
ENSG00000164221.13	0.011191452	0.411426182	0.901585279	CCDC112
ENSG00000279573.1	-0.035151495	0.4114461	0.901585279	RP11-855A2.1
ENSG00000108389.10	0.005111479	0.411587087	0.901722025	MTMR4
ENSG00000166689.16	-0.015375085	0.411596444	0.901722025	PLEKHA7
ENSG00000059377.18	0.006546074	0.411716497	0.901868276	TBXAS1
ENSG00000179598.6	0.012866665	0.411793096	0.901868276	PLD6
ENSG00000099998.19	0.029912335	0.411795131	0.901868276	GGT5
ENSG00000239480.1	-0.020650234	0.411967628	0.901968675	RP11-514P8.2
ENSG00000196411.10	0.014448382	0.411995584	0.901968675	EPHB4
ENSG00000164074.16	-0.007557122	0.412005577	0.901968675	ABHD18
ENSG00000227201.2	-0.023182522	0.412058707	0.901968675	CNN2P1
ENSG00000244045.13	0.004426229	0.412119742	0.901968675	TMEM199
ENSG00000116260.17	-0.008626067	0.412135762	0.901968675	QSOX1
ENSG00000255582.1	0.048043968	0.412173018	0.901968675	OR10G2
ENSG00000108578.15	-0.005572543	0.412198176	0.901968675	BLMH
ENSG00000187098.17	0.013262792	0.412236805	0.901968675	MITF
ENSG00000133800.9	0.01707926	0.41237221	0.902043135	LYVE1
ENSG00000100056.12	-0.006500013	0.412559475	0.902043135	ESS2
ENSG00000196705.8	-0.00929473	0.412611416	0.902043135	ZNF431
ENSG00000118507.18	0.010074087	0.412701218	0.902043135	AKAP7
ENSG00000271659.1	0.026464963	0.412758832	0.902043135	RP11-435O5.4
ENSG00000263413.2	-0.024672509	0.412830703	0.902043135	MIR4538
ENSG00000261094.2	0.008422473	0.412933967	0.902043135	RP11-355O1.11
ENSG00000228634.1	-0.027488314	0.413043623	0.902043135	RP4-534N18.2
ENSG00000169660.17	-0.008073844	0.413058516	0.902043135	HEXD
ENSG00000158793.14	0.004463861	0.413065735	0.902043135	NIT1
ENSG00000183049.13	0.009118342	0.413085502	0.902043135	CAMK1D
ENSG00000247240.8	0.008754209	0.413156977	0.902043135	UBL7-AS1
ENSG00000196535.17	-0.008160603	0.41316451	0.902043135	MYO18A
ENSG00000235927.4	0.028866693	0.413166738	0.902043135	NEXN-AS1
ENSG00000204160.12	0.007423424	0.413189203	0.902043135	ZDHHC18
ENSG00000213465.8	0.009702739	0.413310944	0.902043135	ARL2
ENSG00000122008.15	0.012232464	0.413313716	0.902043135	POLK
ENSG00000228547.5	0.06946816	0.413322012	0.902043135	OR7E26P
ENSG00000286288.1	-0.011536041	0.41332581	0.902043135	RP5-968J1.1
ENSG00000277758.6	0.034155479	0.413338994	0.902043135	ABC7-42404400C24.1
ENSG00000197111.16	0.006248149	0.41335934	0.902043135	PCBP2
ENSG00000165716.11	-0.016227378	0.413385099	0.902043135	DIPK1B
ENSG00000188167.9	0.011405669	0.413392982	0.902043135	TMPPE
ENSG00000180104.16	0.005621754	0.413402106	0.902043135	EXOC3

ENSG00000108785.7	-0.01193068	0.413430681	0.902043135	HSD17B1P1
ENSG00000187186.15	-0.022523136	0.413446654	0.902043135	RP11-195F19.5
ENSG00000268996.3	-0.014237666	0.413529063	0.902043135	MAN1B1-DT
ENSG00000287255.1	0.016125347	0.413539534	0.902043135	RP11-206M19.1
ENSG00000226752.9	-0.027054643	0.413546399	0.902043135	CUTALP
ENSG00000122965.11	-0.004609391	0.413596633	0.902056763	RBM19
ENSG00000225855.7	-0.007165573	0.413702226	0.902099848	RUSC1-AS1
ENSG00000214279.13	-0.016796102	0.413704362	0.902099848	SCART1
ENSG00000152904.11	0.006366265	0.41388671	0.902238554	GGPS1
ENSG00000151229.13	-0.01369216	0.414003117	0.902238554	SLC2A13
ENSG00000184956.16	0.034870173	0.414009191	0.902238554	MUC6
ENSG00000132964.12	-0.007099077	0.414022635	0.902238554	CDK8
ENSG00000150510.17	0.043454812	0.414108118	0.902238554	FAM124A
ENSG00000101557.15	0.005073116	0.414158142	0.902238554	USP14
ENSG00000232677.9	-0.018641119	0.414187389	0.902238554	LINC00665
ENSG00000287095.1	-0.017876189	0.414219194	0.902238554	CTC-215C12.2
ENSG00000092931.11	-0.004767311	0.414305682	0.902238554	MFSD11
ENSG00000168309.18	-0.040772211	0.414358925	0.902238554	FAM107A
ENSG00000111364.16	0.005494341	0.414414641	0.902238554	DDX55
ENSG00000228794.10	0.0074979	0.41444789	0.902238554	LINC01128
ENSG00000211653.2	-0.028591237	0.414486165	0.902238554	IGLV1-40
ENSG00000141977.9	-0.020906137	0.414496961	0.902238554	CIB3
ENSG00000118762.8	-0.010562689	0.414501157	0.902238554	PKD2
ENSG00000149792.9	0.00384011	0.414586894	0.902238554	MRPL49
ENSG00000111196.10	0.008954687	0.414612308	0.902238554	MAGOHB
ENSG00000173473.11	-0.004933229	0.414613801	0.902238554	SMARCC1
ENSG00000136603.14	-0.012788382	0.414715219	0.902238554	SKIL
ENSG00000152284.5	0.010040655	0.414722448	0.902238554	TCF7L1
ENSG00000142039.4	-0.005590833	0.414843186	0.902238554	CCDC97
ENSG00000175356.14	0.028241472	0.414844175	0.902238554	SCUBE2
ENSG00000160917.15	-0.00469498	0.414862302	0.902238554	CPSF4
ENSG00000267496.4	0.027082913	0.41486422	0.902238554	FAM215A
ENSG00000257335.8	0.013433883	0.414867835	0.902238554	MGAM
ENSG00000112851.15	0.009167839	0.414919103	0.90225437	ERBIN
ENSG00000239571.1	-0.062301285	0.415017522	0.902295359	IGKV2D-30
ENSG00000259863.1	0.0310155	0.415065781	0.902295359	SH3RF3-AS1
ENSG00000263874.2	-0.009643504	0.415069944	0.902295359	LINC00672
ENSG00000188501.11	0.02776156	0.415123398	0.902307547	LCTL
ENSG00000043462.13	0.005063134	0.415163546	0.902307547	LCP2
ENSG00000242193.11	0.009522719	0.415248241	0.902395986	CRYZL2P
ENSG00000273261.1	0.013283257	0.415328986	0.902453861	RP11-531F16.4
ENSG00000170293.9	-0.018832198	0.415362883	0.902453861	CMTM8
ENSG00000105419.18	-0.043633994	0.415507994	0.902521047	MEIS3
ENSG00000271789.1	-0.023193392	0.415548468	0.902521047	RP5-1112D6.7
ENSG00000147533.17	0.007432094	0.415605887	0.902521047	GOLGA7
ENSG00000198105.16	-0.006804221	0.415608988	0.902521047	ZNF248
ENSG00000110011.13	-0.007416522	0.415693333	0.902521047	DNAJC4

ENSG00000214199.3	0.015182991	0.415713625	0.902521047	EEF1A1P12
ENSG00000247982.6	-0.020846527	0.415802411	0.902521047	LINC00926
ENSG00000134531.10	0.019224788	0.415832056	0.902521047	EMP1
ENSG00000187522.16	0.009619261	0.41585161	0.902521047	HSPA14
ENSG00000225978.3	0.016809908	0.416010542	0.902521047	HAR1A
ENSG00000152457.18	0.005662871	0.416028832	0.902521047	DCLRE1C
ENSG00000084093.19	-0.006175915	0.416036765	0.902521047	REST
ENSG00000211856.1	-0.037311863	0.416066091	0.902521047	TRAJ33
ENSG00000261349.1	0.04844055	0.416134507	0.902521047	RP3-465N24.5
ENSG00000170456.16	-0.018389388	0.416207679	0.902521047	DENND5B
ENSG00000116731.22	0.00532497	0.416214128	0.902521047	PRDM2
ENSG00000285802.1	0.037138135	0.416215748	0.902521047	RP1-150O5.4
ENSG00000256278.1	-0.031090459	0.416230561	0.902521047	RP11-182J1.5
ENSG00000132259.13	-0.027689693	0.416366867	0.902521047	CNGA4
ENSG00000176845.13	-0.011211344	0.416402969	0.902521047	METRNL
ENSG00000264247.2	0.00720604	0.416403562	0.902521047	ZNF407-AS1
ENSG00000183722.9	0.030042159	0.416436912	0.902521047	LHFPL6
ENSG00000186564.6	-0.017745229	0.416453474	0.902521047	FOXD2
ENSG00000251381.8	0.11401134	0.4164822	0.902521047	LINC00958
ENSG00000073969.18	0.004759374	0.416494011	0.902521047	NSF
ENSG00000185055.11	0.02469081	0.416711255	0.902855021	EFCAB10
ENSG00000154277.13	0.053598481	0.416798515	0.902855021	UCHL1
ENSG00000111684.11	0.008833131	0.416873031	0.902855021	LPCAT3
ENSG00000044115.21	0.009534736	0.416878318	0.902855021	CTNNA1
ENSG00000051108.15	0.004752204	0.416892315	0.902855021	HERPUD1
ENSG00000142207.7	0.007729229	0.41691228	0.902855021	URB1
ENSG00000264188.1	-0.022456297	0.416976885	0.902899583	RP11-13N13.5
ENSG00000266840.1	-0.051712482	0.417060204	0.902980714	RP11-543H23.2
ENSG00000283403.1	0.029598324	0.417181342	0.902980714	AC005307.5
ENSG00000272375.1	-0.02214411	0.417210184	0.902980714	RP11-51J9.6
ENSG00000188997.8	0.005543724	0.417263154	0.902980714	KCTD21
ENSG00000257595.3	0.029550981	0.417315914	0.902980714	LINC02356
ENSG00000145860.12	0.00539833	0.417342996	0.902980714	RNF145
ENSG00000261613.2	-0.068035184	0.417402057	0.902980714	RP11-20I23.13
ENSG00000276405.1	-0.028584901	0.417422542	0.902980714	TRBV13
ENSG00000164758.7	0.007912601	0.417463724	0.902980714	MED30
ENSG00000089737.17	-0.00551584	0.417481396	0.902980714	DDX24
ENSG00000196812.5	0.007290375	0.417510367	0.902980714	ZSCAN16
ENSG00000286872.1	-0.020611837	0.417542721	0.902980714	RP11-382A20.8
ENSG00000213380.16	-0.005358422	0.417666779	0.9029972	COG8
ENSG00000143322.21	0.006058367	0.41768034	0.9029972	ABL2
ENSG00000175216.15	-0.007059066	0.417682438	0.9029972	CKAP5
ENSG00000102974.16	-0.004945245	0.417732606	0.903010466	CTCF
ENSG00000176919.13	-0.01781363	0.417812355	0.903087666	C8G
ENSG00000001631.16	-0.007973372	0.417871646	0.903120635	KRIT1
ENSG00000105270.15	-0.012982229	0.418047201	0.903242358	CLIP3
ENSG00000157654.19	-0.011163291	0.418054668	0.903242358	PALM2AKAP2

ENSG00000135744.9	-0.013524668	0.41806068	0.903242358	AGT
ENSG00000257715.1	-0.020879062	0.41810414	0.903242358	RP11-256L6.2
ENSG00000139160.13	-0.010427986	0.418190184	0.903261956	ETFBKMT
ENSG00000184863.11	0.005325013	0.418236489	0.903261956	RBM33
ENSG00000211710.3	-0.025289783	0.418284285	0.903261956	TRBV4-1
ENSG00000185164.15	-0.013010219	0.418295689	0.903261956	NOMO2
ENSG00000130725.8	-0.007620245	0.418485329	0.903261956	UBE2M
ENSG00000167685.15	-0.007770488	0.418523533	0.903261956	ZNF444
ENSG00000059769.20	0.011433101	0.418532259	0.903261956	DNAJC25
ENSG00000003249.15	0.020026828	0.418644724	0.903261956	DBNDD1
ENSG00000141644.18	-0.00271468	0.418661117	0.903261956	MBD1
ENSG00000070882.13	-0.006947134	0.418719036	0.903261956	OSBPL3
ENSG00000075035.10	0.040639169	0.418748103	0.903261956	WSCD2
ENSG00000168763.16	-0.004567528	0.418772557	0.903261956	CNNM3
ENSG00000272541.1	0.037962455	0.41880552	0.903261956	XXbac-BPGBPG55C20.1
ENSG00000001461.17	0.005743232	0.418827065	0.903261956	NIPAL3
ENSG00000144959.11	0.00941157	0.418843922	0.903261956	NCEH1
ENSG00000149634.5	0.015310537	0.418864312	0.903261956	SPATA25
ENSG00000187605.16	0.00752583	0.418870593	0.903261956	TET3
ENSG00000162419.13	-0.005062038	0.418913305	0.903261956	GMEB1
ENSG00000286707.1	0.015981902	0.419045853	0.903261956	RP11-499P9.1
ENSG00000186399.10	-0.030211263	0.419062774	0.903261956	GOLGA8R
ENSG00000135631.17	-0.016113245	0.419063462	0.903261956	RAB11FIP5
ENSG00000212242.1	-0.030542126	0.419082188	0.903261956	RNA5SP219
ENSG00000114784.4	0.006775951	0.419144337	0.903286777	EIF1B
ENSG00000197442.10	-0.005718338	0.419181795	0.903286777	MAP3K5
ENSG00000120049.20	-0.014643099	0.41924432	0.903326594	KCNIP2
ENSG00000134453.16	0.003385558	0.419339177	0.903436059	RBM17
ENSG00000076248.11	-0.007161447	0.419528015	0.903600764	UNG
ENSG00000205913.7	0.012081242	0.419541649	0.903600764	SRRM2-AS1
ENSG00000196653.12	0.018912453	0.419555042	0.903600764	ZNF502
ENSG00000077147.16	0.006372294	0.419609868	0.903600764	TM9SF3
ENSG00000236675.1	-0.011045282	0.419635931	0.903600764	MTX1P1
ENSG00000078081.8	-0.03240892	0.41972151	0.903690154	LAMP3
ENSG00000260277.2	-0.017506256	0.419794511	0.903702493	RP11-101E7.2
ENSG00000144867.13	0.00553575	0.419815372	0.903702493	SRPRB
ENSG00000188599.17	0.0142	0.419924665	0.903721743	NPIPP1
ENSG00000157978.12	0.010866072	0.4200584	0.903721743	LDLRAP1
ENSG00000286886.1	-0.049140149	0.420093866	0.903721743	AJ011931.2
ENSG00000234484.1	0.021452237	0.420142485	0.903721743	RP1-55C23.7
ENSG00000186104.11	0.006895412	0.420182666	0.903721743	CYP2R1
ENSG00000278607.1	0.017407709	0.420188319	0.903721743	RP11-405M12.3
ENSG00000245869.2	-0.026951699	0.42019148	0.903721743	RP11-158I9.5
ENSG00000231292.6	0.047241394	0.420215001	0.903721743	IGKV1OR2-108
ENSG00000185722.18	-0.007156704	0.420234688	0.903721743	ANKFY1
ENSG00000224903.2	-0.016043996	0.42031121	0.903721743	RNF32-AS1
ENSG00000261087.1	0.007865426	0.420323032	0.903721743	ZNNT1

ENSG00000258920.1	0.014454121	0.420411629	0.903721743	FOXN3-AS1
ENSG00000169242.12	0.019779514	0.420418462	0.903721743	EFNA1
ENSG00000135605.13	-0.011321632	0.420467768	0.903721743	TEC
ENSG00000127804.13	-0.005049057	0.420551799	0.903721743	METTL16
ENSG00000273348.1	-0.020876585	0.420767416	0.903721743	RP11-535A5.1
ENSG00000250942.1	-0.04085679	0.420962391	0.903721743	ENPP7P11
ENSG00000237398.1	0.047595206	0.420973406	0.903721743	HLA-DPA3
ENSG00000273824.1	-0.028010239	0.421024441	0.903721743	RP11-81H14.1
ENSG00000235821.1	-0.04161826	0.421156211	0.903721743	IFITM4P
ENSG00000196981.4	0.008837572	0.421204969	0.903721743	WDR5B
ENSG00000177873.14	-0.006586381	0.421221041	0.903721743	ZNF619
ENSG00000168754.15	0.018006762	0.421242721	0.903721743	FAM178B
ENSG00000117862.13	0.009611361	0.421243756	0.903721743	TXNDC12
ENSG00000146535.14	0.010519274	0.421259375	0.903721743	GNA12
ENSG00000007923.16	-0.006017074	0.421261316	0.903721743	DNAJC11
ENSG00000127540.12	0.010101565	0.421263771	0.903721743	UQCR11
ENSG00000260910.2	0.019078659	0.421282357	0.903721743	LINC00565
ENSG00000242689.3	-0.02340546	0.42130422	0.903721743	CNTF
ENSG00000285437.1	-0.020444137	0.421343866	0.903721743	POLR2J3
ENSG00000269072.1	-0.014202509	0.421397911	0.903721743	CTD-3187F8.14
ENSG00000136122.18	0.010077021	0.421516109	0.903721743	BORA
ENSG00000266970.1	-0.031451471	0.421518755	0.903721743	SOCS3-DT
ENSG00000277053.4	0.012958054	0.42157088	0.903721743	GTF2IP1
ENSG00000177103.15	0.033913516	0.42162763	0.903721743	DSCAML1
ENSG00000143811.19	0.005121136	0.421643711	0.903721743	PYCR2
ENSG00000285865.1	-0.018984979	0.421702808	0.903721743	CTC-563A5.5
ENSG00000281106.4	0.0159851	0.421812199	0.903721743	TMEM272
ENSG00000123146.20	-0.00948932	0.421836293	0.903721743	ADGRE5
ENSG00000166167.18	0.004490013	0.421976495	0.903721743	BTRC
ENSG00000129292.21	0.010040925	0.422169876	0.903721743	PHF20L1
ENSG00000115053.17	-0.005675678	0.422174109	0.903721743	NCL
ENSG00000141837.22	-0.015507553	0.422201203	0.903721743	CACNA1A
ENSG00000163482.12	0.005417103	0.422315891	0.903721743	STK36
ENSG00000143553.11	0.005805366	0.422340797	0.903721743	SNAPIN
ENSG00000153064.12	-0.01745642	0.422371903	0.903721743	BANK1
ENSG00000144550.13	0.023560776	0.422407503	0.903721743	CPNE9
ENSG00000221510.1	0.033330003	0.422466142	0.903721743	MIR548O
ENSG00000112667.13	-0.011575532	0.422480035	0.903721743	DNPH1
ENSG00000106610.15	0.008631519	0.422496426	0.903721743	STAG3L4
ENSG00000172322.14	0.026312107	0.422543774	0.903721743	CLEC12A
ENSG00000269877.3	-0.020751051	0.422575937	0.903721743	CTD-3022G6.1
ENSG00000113391.19	0.007418919	0.422651853	0.903721743	FAM172A
ENSG00000234694.1	0.019186628	0.422664093	0.903721743	RP1-92O14.3
ENSG00000235314.1	-0.007821086	0.422728593	0.903721743	LINC00957
ENSG00000225579.2	0.02633713	0.422816734	0.903721743	EDNRB-AS1
ENSG00000273486.1	-0.012168157	0.422839582	0.903721743	RP11-731C17.2
ENSG00000277481.2	-0.029685312	0.422851776	0.903721743	PKD1L3

ENSG00000137098.14	0.011626009	0.422863059	0.903721743	SPAG8
ENSG00000032444.17	-0.007475651	0.422904728	0.903721743	PNPLA6
ENSG00000204745.4	-0.015823861	0.422906723	0.903721743	AC083899.3
ENSG00000169641.13	-0.007841083	0.422911464	0.903721743	LUZP1
ENSG00000246228.6	0.046446455	0.422942316	0.903721743	CASC8
ENSG00000205041.1	0.021000469	0.422959765	0.903721743	CTC-425O23.2
ENSG00000031003.10	0.006970825	0.422967985	0.903721743	FAM13B
ENSG00000227855.3	0.024771055	0.423007226	0.903721743	DPY19L2P3
ENSG00000238279.1	-0.013238891	0.423009104	0.903721743	BX470102.3
ENSG00000215788.11	-0.010489369	0.423088107	0.903721743	TNFRSF25
ENSG00000272523.1	0.020664546	0.423100231	0.903721743	LINC01023
ENSG00000072954.7	0.011217592	0.423124526	0.903721743	TMEM38A
ENSG00000269940.1	0.011489628	0.423138542	0.903721743	RP11-73M18.7
ENSG00000185043.12	-0.00827763	0.423145205	0.903721743	CIB1
ENSG00000163534.15	-0.022831781	0.423169219	0.903721743	FCRL1
ENSG00000080608.10	0.008208921	0.423206466	0.903721743	PUM3
ENSG00000166126.11	-0.016474917	0.423215908	0.903721743	AMN
ENSG00000183726.11	0.005500178	0.423229207	0.903721743	TMEM50A
ENSG00000148700.15	0.008468645	0.423240337	0.903721743	ADD3
ENSG00000232265.8	0.041353813	0.423261525	0.903721743	LINC02805
ENSG00000225891.2	-0.012851027	0.423528069	0.903901605	DHDDS-AS1
ENSG00000198901.14	-0.016709933	0.423545872	0.903901605	PRC1
ENSG00000273045.7	0.015341227	0.423549727	0.903901605	C2orf15
ENSG00000112983.18	0.003826878	0.423551576	0.903901605	BRD8
ENSG00000028839.10	0.005804011	0.423651524	0.903901605	TBPL1
ENSG00000132613.15	0.019319668	0.423819414	0.903901605	MTSS2
ENSG00000126870.16	-0.00771627	0.423844675	0.903901605	DYNC2I1
ENSG00000169314.15	-0.017037317	0.424005042	0.903901605	C22orf15
ENSG00000113580.15	0.007432535	0.424029209	0.903901605	NR3C1
ENSG00000215397.4	0.033805171	0.424040282	0.903901605	SCRT2
ENSG00000235945.1	-0.031829829	0.424081178	0.903901605	AC002543.2
ENSG00000172602.11	0.029574729	0.424151989	0.903901605	RND1
ENSG00000271943.2	0.020056415	0.424187711	0.903901605	AC098614.2
ENSG00000272333.7	-0.00699645	0.424211616	0.903901605	KMT2B
ENSG00000113742.14	0.011707701	0.424228196	0.903901605	CPEB4
ENSG00000261338.2	-0.010450473	0.424270143	0.903901605	RP11-378A13.1
ENSG00000137269.15	-0.012597315	0.424290768	0.903901605	LRRC1
ENSG00000090520.12	0.0050894	0.424427403	0.903901605	DNAJB11
ENSG00000182376.2	-0.024356115	0.424453218	0.903901605	RP5-1142A6.8
ENSG00000222037.5	-0.034830941	0.424517054	0.903901605	IGLC6
ENSG00000234745.13	0.008428216	0.424543041	0.903901605	HLA-B
ENSG00000159593.15	0.007380683	0.424574531	0.903901605	NAE1
ENSG00000182993.5	0.015623248	0.424653534	0.903901605	C12orf60
ENSG00000076944.16	-0.008203374	0.424699938	0.903901605	STXBP2
ENSG00000174871.11	-0.015739865	0.424759923	0.903901605	CNIH2
ENSG00000211812.1	-0.021067972	0.42477464	0.903901605	TRAV26-2
ENSG00000279118.1	-0.023343419	0.424811902	0.903901605	RP11-517I3.2

ENSG00000101220.18	-0.013275831	0.424831876	0.903901605	C20orf27
ENSG00000273014.1	0.034906636	0.424853892	0.903901605	RP11-225B17.2
ENSG00000011258.16	0.007615407	0.42488469	0.903901605	MBTD1
ENSG00000254577.1	-0.024035656	0.42489071	0.903901605	RP11-484D2.4
ENSG00000123297.20	-0.010297118	0.424964631	0.903901605	TSFM
ENSG00000101333.18	-0.043601235	0.424967752	0.903901605	PLCB4
ENSG00000100490.10	0.008522972	0.424984259	0.903901605	CDKL1
ENSG00000145217.14	-0.010715856	0.424992218	0.903901605	SLC26A1
ENSG00000114573.10	0.007238451	0.425033267	0.903901605	ATP6V1A
ENSG00000227627.3	0.025033898	0.425036061	0.903901605	RP1-101K10.6
ENSG00000276255.2	-0.030354541	0.425098831	0.903901605	LINC02809
ENSG00000258659.7	-0.018345104	0.425124954	0.903901605	TRIM34
ENSG00000256294.8	0.007502896	0.425132592	0.903901605	ZNF225
ENSG00000156011.17	0.017397877	0.425184194	0.903901605	PSD3
ENSG00000057757.10	0.006051993	0.425224718	0.903901605	PITHD1
ENSG00000272707.1	-0.026811235	0.425341263	0.903901605	RP11-534C12.1
ENSG00000078237.7	-0.007526596	0.425375715	0.903901605	TIGAR
ENSG00000073711.11	-0.011115448	0.425442076	0.903901605	PPP2R3A
ENSG00000276085.1	-0.03794263	0.425447387	0.903901605	CCL3L1
ENSG00000177098.9	0.024849574	0.425455156	0.903901605	SCN4B
ENSG00000256706.1	-0.017625588	0.42546139	0.903901605	RP5-1096D14.3
ENSG00000140025.16	0.010992708	0.425564234	0.903989179	EFCAB11
ENSG00000272097.2	0.022325503	0.425590771	0.903989179	TMEM14B-DT
ENSG00000272732.1	0.022144435	0.425787953	0.904314347	RP5-1159O4.2
ENSG00000166557.14	0.007019874	0.425840849	0.904333037	TMED3
ENSG00000197301.7	-0.025268269	0.425908579	0.904340783	HMGA2-AS1
ENSG00000261476.1	-0.030176628	0.426005743	0.904340783	RP11-77K12.3
ENSG00000237476.1	0.017144694	0.426088809	0.904340783	LINC01637
ENSG00000184984.10	-0.018304768	0.426142739	0.904340783	CHRM5
ENSG00000132676.16	0.003983573	0.426157774	0.904340783	DAP3
ENSG00000260290.2	-0.019580633	0.42616822	0.904340783	CTD-2033A16.2
ENSG00000278376.1	-0.015044951	0.42621752	0.904340783	RP11-158I9.8
ENSG00000159200.18	0.006222346	0.426251442	0.904340783	RCAN1
ENSG00000241721.1	0.01711041	0.426280879	0.904340783	SUMO1P1
ENSG00000286244.1	0.023791277	0.426285467	0.904340783	RP11-51I5.2
ENSG00000188242.4	-0.013998011	0.426421542	0.904355271	PP7080
ENSG00000241015.2	-0.014134311	0.426421836	0.904355271	TPM3P9
ENSG00000242198.1	-0.021972491	0.426431259	0.904355271	CTD-2235C13.1
ENSG00000123349.15	-0.004995623	0.426531646	0.904355271	PFDN5
ENSG00000185614.7	-0.013260542	0.426535045	0.904355271	INKA1
ENSG00000211664.3	0.039085462	0.426596793	0.904355271	IGLV2-18
ENSG00000277687.1	-0.018756019	0.426659989	0.904355271	RP11-427L15.2
ENSG00000161055.4	0.05805741	0.426715423	0.904355271	SCGB3A1
ENSG00000205476.9	0.006073576	0.426776563	0.904355271	CCDC85C
ENSG00000183889.12	0.031176111	0.42678991	0.904355271	AC138969.4
ENSG00000167434.10	-0.013995045	0.426811389	0.904355271	CA4
ENSG00000259120.3	-0.025860291	0.426821468	0.904355271	SMIM6

ENSG00000173456.5	-0.00611861	0.426884989	0.904396422	RNF26
ENSG00000179967.11	0.011055033	0.426967353	0.904406637	PPP1R14BP3
ENSG00000141540.11	0.006688996	0.426978011	0.904406637	TTYH2
ENSG00000272086.1	-0.012167482	0.427042488	0.904414004	GOLPH3-DT
ENSG00000254528.7	0.026504592	0.42706969	0.904414004	RP11-728F11.4
ENSG00000232118.2	-0.017337634	0.427114387	0.904415267	BACH1-AS1
ENSG00000123268.9	-0.007238918	0.427162416	0.904420209	ATF1
ENSG00000131791.8	0.007260082	0.427204923	0.904420209	PRKAB2
ENSG00000184584.13	-0.006688899	0.427423102	0.904788706	STING1
ENSG00000160094.15	-0.005827314	0.427593457	0.905044745	ZNF362
ENSG00000122484.9	0.006737772	0.42764348	0.905044745	RPAP2
ENSG00000087884.14	0.011535376	0.427676449	0.905044745	AAMDC
ENSG00000078674.18	0.006363154	0.42780888	0.905093896	PCM1
ENSG00000100997.20	-0.006211949	0.427860902	0.905093896	ABHD12
ENSG00000140464.20	-0.013194888	0.427928185	0.905093896	PML
ENSG00000117560.8	-0.013825498	0.427965745	0.905093896	FASLG
ENSG00000276103.1	-0.026834748	0.427972882	0.905093896	U4
ENSG00000245750.10	-0.013977738	0.427992383	0.905093896	DRAIC
ENSG00000164300.17	0.00749528	0.428040062	0.905093896	SERINC5
ENSG00000138411.13	0.014423299	0.42806595	0.905093896	HECW2
ENSG00000147894.17	0.0117138	0.428154546	0.905093896	C9orf72
ENSG00000102878.18	-0.011355415	0.428181024	0.905093896	HSF4
ENSG00000103479.17	0.007339135	0.428188166	0.905093896	RBL2
ENSG00000233231.1	-0.020154998	0.42822928	0.905093896	HNRNPA1P49
ENSG00000147601.15	0.005457072	0.428429758	0.905110508	TERF1
ENSG00000241294.1	-0.029619271	0.428459632	0.905110508	IGKV2-24
ENSG00000117360.13	0.003209343	0.428501554	0.905110508	PRPF3
ENSG00000279345.1	-0.02382121	0.428513946	0.905110508	RP3-522J7.7
ENSG00000158711.14	-0.007259711	0.42858357	0.905110508	ELK4
ENSG00000109686.19	0.020564424	0.428703645	0.905110508	SH3D19
ENSG00000274114.2	0.012621202	0.428712354	0.905110508	ALOX15P1
ENSG00000181481.14	0.006277021	0.428712692	0.905110508	RNF135
ENSG00000101115.13	0.034395879	0.428723968	0.905110508	SALL4
ENSG00000198743.7	-0.009961811	0.428742704	0.905110508	SLC5A3
ENSG00000100065.15	0.030641307	0.428754489	0.905110508	CARD10
ENSG00000109016.17	0.00655501	0.428801337	0.905110508	DHRS7B
ENSG00000270379.6	-0.017204531	0.42881785	0.905110508	HEATR9
ENSG00000198915.12	-0.01094745	0.428870336	0.905110508	RASGEF1A
ENSG00000119185.13	0.006322173	0.428899157	0.905110508	ITGB1BP1
ENSG00000277229.1	0.041247773	0.429074018	0.905321324	RP11-355N15.3
ENSG00000095932.7	-0.027149992	0.429087345	0.905321324	SMIM24
ENSG00000126261.13	0.004879014	0.429192538	0.905450114	UBA2
ENSG00000234028.3	0.011794285	0.429284815	0.905456498	EIF2AK3-DT
ENSG00000285448.1	0.030242704	0.429294523	0.905456498	RP11-178C10.4
ENSG00000115267.9	-0.015677298	0.429328018	0.905456498	IFIH1
ENSG00000227288.3	0.020000567	0.429408536	0.905533188	ARID3BP1
ENSG00000127124.16	-0.010289809	0.429540645	0.905622641	HIVEP3

ENSG00000157483.9	0.013243156	0.429567664	0.905622641	MYO1E
ENSG00000189350.13	-0.013020771	0.429583434	0.905622641	TOGARAM2
ENSG00000172071.15	-0.006029774	0.429638858	0.905646387	EIF2AK3
ENSG00000063241.8	-0.009595232	0.429715573	0.905715002	ISOC2
ENSG00000185640.6	0.049873867	0.429853225	0.905896885	KRT79
ENSG00000177463.15	0.008650148	0.429890213	0.905896885	NR2C2
ENSG00000276644.5	-0.014544072	0.429973898	0.905980139	DACH1
ENSG00000132589.16	0.007898181	0.430040379	0.90602713	FLOT2
ENSG00000243544.3	-0.020933028	0.43024036	0.906355347	RN7SL172P
ENSG00000123685.9	0.014102197	0.43029433	0.906375937	BATF3
ENSG00000141198.16	0.027562628	0.430421132	0.906423117	TOM1L1
ENSG00000198624.13	0.004805892	0.430438276	0.906423117	CCDC69
ENSG00000214894.6	0.017408108	0.430449324	0.906423117	LINC00243
ENSG00000283787.2	-0.006797222	0.430497414	0.906431312	PRR33
ENSG00000128335.14	0.009530772	0.430572361	0.90645236	APOL2
ENSG00000124875.10	-0.019166265	0.430595811	0.90645236	CXCL6
ENSG00000167637.18	0.00948455	0.430705076	0.906536608	ZNF283
ENSG00000171988.19	0.010973859	0.43072424	0.906536608	JMJD1C
ENSG00000232453.7	0.019330436	0.430843104	0.906596299	LINC02777
ENSG00000106617.15	-0.005653312	0.430887605	0.906596299	PRKAG2
ENSG00000102837.7	0.049594261	0.430888866	0.906596299	OLFM4
ENSG00000172673.11	-0.012212005	0.431098897	0.906596299	THEMIS
ENSG00000146733.14	-0.009765369	0.43113617	0.906596299	PSPH
ENSG00000179820.16	-0.010944121	0.431262717	0.906596299	MYADM
ENSG00000133835.18	0.005552526	0.431277811	0.906596299	HSD17B4
ENSG00000255823.5	0.021192608	0.431332829	0.906596299	MTRNR2L8
ENSG00000155850.8	-0.008634372	0.43133838	0.906596299	SLC26A2
ENSG00000026508.20	0.005320375	0.431411524	0.906596299	CD44
ENSG00000111641.12	-0.009048243	0.431541211	0.906596299	NOP2
ENSG00000116690.13	-0.01951195	0.431581485	0.906596299	PRG4
ENSG00000189337.17	0.022333868	0.431654363	0.906596299	KAZN
ENSG00000273340.1	-0.016346633	0.431670811	0.906596299	MICE
ENSG00000185946.16	0.009355594	0.431704716	0.906596299	RNPC3
ENSG00000196739.15	-0.023744454	0.431712936	0.906596299	COL27A1
ENSG00000197403.5	0.044661032	0.431723386	0.906596299	OR6N1
ENSG00000165733.8	-0.005181701	0.431787656	0.906596299	BMS1
ENSG00000275457.1	0.017926005	0.431807738	0.906596299	RP1-198K11.5
ENSG00000211931.1	0.03729965	0.431860457	0.906596299	IGHD2-2
ENSG00000070413.20	-0.005557613	0.431917811	0.906596299	DGCR2
ENSG00000065491.8	0.005877339	0.431932062	0.906596299	TBC1D22B
ENSG00000182240.16	0.017350819	0.431937191	0.906596299	BACE2
ENSG00000162129.14	-0.006401981	0.431946804	0.906596299	CLPB
ENSG00000158186.13	0.012722437	0.431970156	0.906596299	MRAS
ENSG00000262312.2	-0.0142277	0.431982467	0.906596299	LA16c-390H2.4
ENSG00000187815.10	0.00786606	0.431983867	0.906596299	ZFP69
ENSG00000175782.11	-0.006245104	0.431990396	0.906596299	SLC35E3
ENSG00000013810.21	0.007255898	0.432098019	0.906685582	TACC3

ENSG00000143819.13	-0.0071365	0.432121361	0.906685582	EPHX1
ENSG00000134996.12	0.005445359	0.432166538	0.906687608	OSTF1
ENSG00000182405.6	0.011048244	0.432317803	0.906770596	PGBD4
ENSG00000179119.15	0.006553713	0.432432895	0.906770596	SPTY2D1
ENSG00000109586.12	-0.008039547	0.432438741	0.906770596	GALNT7
ENSG00000162402.14	-0.004939017	0.432449703	0.906770596	USP24
ENSG00000129625.13	0.006020263	0.432466189	0.906770596	REEP5
ENSG00000273812.3	-0.016995394	0.432471387	0.906770596	WI2-87327B8.2
ENSG00000271344.1	-0.017343116	0.432596168	0.906873059	RP11-274B21.9
ENSG00000139354.11	0.018642916	0.432649328	0.906873059	GAS2L3
ENSG00000211900.2	0.022801947	0.432661718	0.906873059	IGHJ6
ENSG00000183354.12	0.007102247	0.432726406	0.906873059	KIAA2026
ENSG00000269951.1	-0.027185393	0.432741357	0.906873059	RP11-797A18.6
ENSG00000107874.11	0.007281387	0.43294838	0.9072142	CUEDC2
ENSG00000135835.12	0.012106949	0.433032593	0.907297958	KIAA1614
ENSG00000198744.5	-0.089706019	0.433142746	0.907412469	MTCO3P12
ENSG00000228300.14	-0.009834134	0.433202297	0.907412469	FAM174C
ENSG00000106714.17	0.021268696	0.433221107	0.907412469	CNTNAP3
ENSG00000196605.7	0.009039851	0.433264234	0.907412469	ZNF846
ENSG00000237541.4	0.052016786	0.433333294	0.907464434	HLA-DQA2
ENSG00000261592.1	-0.023744665	0.433450909	0.907539775	RP11-178L8.3
ENSG00000229692.3	-0.012656945	0.433465112	0.907539775	SOS1-IT1
ENSG00000111261.14	0.010286074	0.43350203	0.907539775	MANSC1
ENSG00000081791.9	0.00396477	0.433552355	0.907551673	DELE1
ENSG00000121057.13	0.005461786	0.433598462	0.907551673	AKAP1
ENSG00000229320.3	0.007210584	0.433831926	0.907551673	KRT8P12
ENSG00000133704.10	0.005185279	0.433862652	0.907551673	IPO8
ENSG00000110693.19	0.023572347	0.433897443	0.907551673	SOX6
ENSG00000213563.7	-0.007983752	0.433934951	0.907551673	C8orf82
ENSG00000227741.1	-0.016814352	0.433955407	0.907551673	RP11-536C5.7
ENSG00000172663.9	-0.006387462	0.434009383	0.907551673	TMEM134
ENSG00000259642.2	0.008257348	0.434022288	0.907551673	ST20-AS1
ENSG00000186281.12	0.032411508	0.43407369	0.907551673	GPAT2
ENSG00000169609.14	0.006498471	0.434155211	0.907551673	C15orf40
ENSG00000073910.23	0.008030564	0.434219212	0.907551673	FRY
ENSG00000259033.2	0.03184379	0.434270824	0.907551673	RP11-718G2.5
ENSG00000164292.13	-0.015372694	0.434310617	0.907551673	RHOBTB3
ENSG00000172348.15	0.03837537	0.434310909	0.907551673	RCAN2
ENSG00000205581.11	-0.005670087	0.434335111	0.907551673	HMGN1
ENSG00000166704.12	0.006807411	0.434374077	0.907551673	ZNF606
ENSG00000119900.9	-0.009209254	0.434394615	0.907551673	OGFRL1
ENSG00000198406.7	0.014812174	0.434420327	0.907551673	BZW1P2
ENSG00000056586.16	0.005367122	0.434458684	0.907551673	RC3H2
ENSG00000172890.13	0.004380506	0.434462935	0.907551673	NADSYN1
ENSG00000272764.1	-0.025694902	0.434511572	0.907551673	RP11-318E3.9
ENSG00000214517.10	0.004772402	0.434550626	0.907551673	PPME1
ENSG00000261220.2	0.011137748	0.434644976	0.907551673	ST3GAL1-DT

ENSG00000183742.13	-0.022440557	0.434876168	0.907551673	MACC1
ENSG00000248334.6	0.011618737	0.434887787	0.907551673	WHAMMP2
ENSG00000232907.8	0.014619017	0.434888492	0.907551673	DLGAP4-AS1
ENSG00000129255.16	0.007243674	0.434890658	0.907551673	MPDU1
ENSG00000166444.19	-0.021711827	0.434980226	0.907551673	DENND2B
ENSG00000223823.1	-0.02826199	0.435091543	0.907551673	LINC01342
ENSG00000119946.11	0.032717347	0.43512672	0.907551673	CNNM1
ENSG00000136813.15	0.004785812	0.435127852	0.907551673	ECPAS
ENSG00000148337.21	-0.005787465	0.435155175	0.907551673	CIZ1
ENSG00000243368.2	-0.017296329	0.435186167	0.907551673	MCCC1-AS1
ENSG00000038427.16	0.010889087	0.435194095	0.907551673	VCAN
ENSG00000167083.7	0.010454518	0.435214614	0.907551673	GNGT2
ENSG00000272008.1	0.026987941	0.435219056	0.907551673	RP11-393I2.4
ENSG00000141424.13	-0.006208053	0.435223509	0.907551673	SLC39A6
ENSG00000260528.5	0.018370359	0.435233602	0.907551673	FAM157C
ENSG00000232133.1	-0.0260425	0.435305071	0.907608419	IMPDH1P10
ENSG00000272908.1	-0.015911572	0.435429857	0.907689753	RP11-121A8.1
ENSG00000119125.17	-0.113744077	0.435432601	0.907689753	GDA
ENSG00000103005.12	0.0065893	0.435532068	0.907764932	USB1
ENSG00000227544.10	0.027737497	0.435557194	0.907764932	AC018647.3
ENSG00000153896.19	0.008256021	0.435724713	0.907944985	ZNF599
ENSG00000081985.13	0.017305342	0.435732131	0.907944985	IL12RB2
ENSG00000173482.17	-0.019280843	0.435856411	0.907957966	PTPRM
ENSG00000235194.9	0.007336244	0.435863707	0.907957966	PPP1R3E
ENSG00000163444.12	0.004289708	0.435939754	0.907957966	TMEM183A
ENSG00000125844.16	-0.008837706	0.435973612	0.907957966	RRBP1
ENSG00000159208.16	0.023374636	0.435985348	0.907957966	CIART
ENSG00000186166.9	-0.005740505	0.436004001	0.907957966	CENATAC
ENSG00000084234.18	-0.006912431	0.436206009	0.908250994	APLP2
ENSG00000140470.15	0.015523199	0.436304883	0.908250994	ADAMTS17
ENSG00000104808.8	0.019582133	0.436312716	0.908250994	DHDH
ENSG00000149131.17	0.032598594	0.436437158	0.908250994	SERPING1
ENSG00000188636.4	-0.0080325	0.436463657	0.908250994	RTL6
ENSG00000249806.1	0.021759288	0.436477827	0.908250994	RP11-223C24.1
ENSG00000278638.2	-0.028062202	0.436510066	0.908250994	CTC-268N12.2
ENSG00000105672.14	0.016766596	0.436552801	0.908250994	ETV2
ENSG00000274737.1	-0.017752332	0.436658755	0.908250994	RP5-1057I20.6
ENSG00000125375.17	0.008211924	0.436659129	0.908250994	DMAC2L
ENSG00000196378.13	-0.006950441	0.436661865	0.908250994	ZNF34
ENSG00000207547.1	0.013413483	0.436676165	0.908250994	MIR25
ENSG00000137692.12	0.010023141	0.436794293	0.908365094	DCUN1D5
ENSG00000236114.1	-0.02415966	0.436828765	0.908365094	RP11-517P14.7
ENSG00000270228.1	0.028867961	0.43689314	0.908365094	RP11-453E17.3
ENSG00000100058.12	-0.007501145	0.436912522	0.908365094	CRYBB2P1
ENSG00000114503.11	0.004223407	0.436964937	0.908365094	NCBP2
ENSG00000271797.1	-0.018855919	0.436996783	0.908365094	CTC-428G20.6
ENSG00000118873.16	0.007442397	0.437049897	0.908383428	RAB3GAP2

ENSG00000152117.17	-0.006558814	0.437203198	0.908537323	SMPD4BP
ENSG00000128626.12	-0.008608383	0.437242654	0.908537323	MRPS12
ENSG00000234211.2	0.042870052	0.437275369	0.908537323	RP11-25K21.1
ENSG00000251595.7	-0.014687658	0.437301147	0.908537323	ABCA11P
ENSG00000185022.12	0.010213715	0.437476314	0.908645058	MAFF
ENSG00000167210.17	0.023604294	0.437511918	0.908645058	LOXHD1
ENSG00000132749.11	0.007968136	0.437533578	0.908645058	TESMIN
ENSG00000166133.18	-0.006568695	0.437535415	0.908645058	RPUSD2
ENSG00000234184.6	0.028544823	0.437574536	0.908645058	LINC01781
ENSG00000262823.1	-0.011240574	0.43766613	0.90864706	RP13-580F15.2
ENSG00000269681.1	-0.020840808	0.437789863	0.90864706	CTD-3187F8.6
ENSG00000109270.13	0.010873548	0.437798562	0.90864706	LAMTOR3
ENSG00000178826.11	-0.02292818	0.437932677	0.90864706	TMEM139
ENSG00000115738.10	0.007964383	0.437952302	0.90864706	ID2
ENSG00000122432.18	-0.012432331	0.437954963	0.90864706	SPATA1
ENSG00000161791.14	-0.005558651	0.437962665	0.90864706	FMNL3
ENSG00000230267.7	-0.058591355	0.437991712	0.90864706	HERC2P4
ENSG00000075790.12	0.018502433	0.437996217	0.90864706	BCAP29
ENSG00000270953.1	0.025127121	0.43801857	0.90864706	RP11-2E11.9
ENSG00000197646.8	0.028467273	0.438099602	0.908654464	PDCD1LG2
ENSG00000136448.13	-0.003910817	0.438110754	0.908654464	NMT1
ENSG00000235328.1	-0.0219853	0.438227061	0.908746051	AC006946.12
ENSG00000081320.11	0.006542698	0.438243536	0.908746051	STK17B
ENSG00000286511.1	-0.021208659	0.438364573	0.908905131	RP11-249L21.6
ENSG00000120875.9	-0.017008659	0.438506152	0.909053431	DUSP4
ENSG00000183199.6	-0.02098352	0.438550628	0.909053431	HSP90AB3P
ENSG00000172315.6	0.004888647	0.438621238	0.909053431	TP53RK
ENSG00000229628.1	-0.029917043	0.438695809	0.909053431	AC073115.7
ENSG00000007545.16	-0.00575639	0.438752005	0.909053431	CRAMP1
ENSG00000024422.12	-0.026747723	0.438763711	0.909053431	EHD2
ENSG00000178035.13	-0.006324019	0.438800164	0.909053431	IMPDH2
ENSG00000053372.5	-0.007064698	0.438814925	0.909053431	MRTO4
ENSG00000249129.1	-0.020258298	0.438835038	0.909053431	SUDS3P1
ENSG00000163110.15	0.00612297	0.438900604	0.909097424	PDLIM5
ENSG00000261286.1	0.025776681	0.438947216	0.909102151	ATP2C2-AS1
ENSG00000213965.4	-0.006060012	0.43909635	0.909312317	NUDT19
ENSG00000066777.9	0.005448529	0.439162729	0.909312317	ARFGEF1
ENSG00000130764.10	-0.004129274	0.43921347	0.909312317	LRRC47
ENSG00000181220.17	-0.006375019	0.439252933	0.909312317	ZNF746
ENSG00000176953.12	-0.003683943	0.439280192	0.909312317	NFATC2IP
ENSG00000179941.9	0.010742352	0.439338178	0.909312317	BBS10
ENSG00000035681.9	0.004072675	0.439359067	0.909312317	NSMAF
ENSG00000112701.18	0.008360885	0.43945572	0.909420575	SENP6
ENSG00000134748.13	-0.003210863	0.439530027	0.909482575	PRPF38A
ENSG00000271732.1	0.030377114	0.439615934	0.909568561	RP5-1182A14.5
ENSG00000188725.8	-0.007830744	0.439813987	0.909784763	SMIM15
ENSG00000211937.3	-0.030188827	0.439824205	0.909784763	IGHV2-5

ENSG00000171262.11	0.006107995	0.439853517	0.909784763	FAM98B
ENSG00000230979.3	-0.015933334	0.439927218	0.90984544	AC079250.1
ENSG00000256393.1	0.023507147	0.439993241	0.909859405	RPL41P5
ENSG00000169241.19	0.005746725	0.440022703	0.909859405	SLC50A1
ENSG00000181744.9	-0.008286126	0.440099674	0.909864823	DIPK2A
ENSG00000174132.9	-0.009798538	0.440114055	0.909864823	FAM174A
ENSG00000116604.18	-0.005313125	0.44020726	0.909965118	MEF2D
ENSG00000274425.1	-0.011771251	0.440251312	0.909965118	AC114271.2
ENSG00000126522.17	-0.006556774	0.440392947	0.910048747	ASL
ENSG00000231345.3	0.037695537	0.440404762	0.910048747	BEND3P1
ENSG00000187650.4	0.00833519	0.440424898	0.910048747	VMAC
ENSG00000174851.16	-0.007486594	0.440514206	0.910141582	YIF1A
ENSG00000096063.16	0.008304589	0.440600362	0.910203306	SRPK1
ENSG00000165828.15	0.033149163	0.440650194	0.910203306	PRAP1
ENSG00000122035.7	-0.013126408	0.440710921	0.910203306	RASL11A
ENSG00000130812.11	-0.014957965	0.440833929	0.910203306	ANGPTL6
ENSG00000244063.1	0.02264832	0.440969859	0.910203306	AC024704.2
ENSG00000168066.21	-0.004848439	0.440985693	0.910203306	SF1
ENSG00000259591.2	0.059908241	0.441008297	0.910203306	RP11-554D20.1
ENSG00000084444.14	0.007415103	0.441063572	0.910203306	FAM234B
ENSG00000100342.21	0.013423744	0.441086136	0.910203306	APOL1
ENSG00000206149.10	0.008405426	0.441094798	0.910203306	HERC2P9
ENSG00000161281.11	-0.031441556	0.441115034	0.910203306	COX7A1
ENSG00000148090.12	-0.007066746	0.441133531	0.910203306	AUH
ENSG00000117601.15	0.025799209	0.4411536	0.910203306	SERPINC1
ENSG00000286271.2	0.025119113	0.441224383	0.910203306	CTD-2201E18.6
ENSG00000130598.16	-0.011938873	0.441226492	0.910203306	TNNI2
ENSG00000075131.10	-0.011085258	0.441254207	0.910203306	TIPIN
ENSG00000287277.1	-0.030135856	0.441345349	0.910222691	RP11-799O21.2
ENSG00000073008.15	-0.009604102	0.441392749	0.910222691	PVR
ENSG00000257596.1	-0.014011055	0.441396756	0.910222691	SCAT2
ENSG00000257226.2	-0.013240335	0.441469887	0.910227035	CTD-2252P21.1
ENSG00000205309.14	-0.020718192	0.44151093	0.910227035	NT5M
ENSG00000249035.7	-0.017054882	0.441676571	0.910227035	CLMAT3
ENSG00000242338.6	-0.02267496	0.441722155	0.910227035	BMS1P4
ENSG00000168411.14	-0.004952351	0.441914639	0.910227035	RFWD3
ENSG00000185760.17	0.013536824	0.441988477	0.910227035	KCNQ5
ENSG00000124370.11	-0.009324322	0.442018664	0.910227035	MCEE
ENSG00000227591.5	0.026632602	0.442021618	0.910227035	HSD11B1-AS1
ENSG00000083799.18	0.005615612	0.44203606	0.910227035	CYLD
ENSG00000244687.12	0.008344384	0.442064358	0.910227035	UBE2V1
ENSG00000086848.15	-0.006805972	0.442152314	0.910227035	ALG9
ENSG00000160471.13	-0.036132853	0.442212516	0.910227035	COX6B2
ENSG00000250365.7	-0.017284803	0.442212851	0.910227035	CTD-2213F21.2
ENSG00000178038.17	-0.013485007	0.442272177	0.910227035	ALS2CL
ENSG00000079974.18	0.004941699	0.44231725	0.910227035	RABL2B
ENSG00000283674.3	0.018699415	0.442334005	0.910227035	RP11-419I17.1

ENSG00000123908.12	-0.006225986	0.442386401	0.910227035	AGO2
ENSG00000107968.10	0.006778496	0.442396941	0.910227035	MAP3K8
ENSG00000276819.1	-0.023540935	0.442427961	0.910227035	TRBV15
ENSG00000221838.10	-0.006719395	0.442448254	0.910227035	AP4M1
ENSG00000101337.16	-0.005396383	0.442449073	0.910227035	TM9SF4
ENSG00000140577.16	0.005816115	0.44254018	0.910227035	CRTC3
ENSG00000100263.14	-0.007443466	0.442597981	0.910227035	RHBDD3
ENSG00000251194.2	0.016813552	0.442606624	0.910227035	RP1-68D18.2
ENSG00000123415.16	0.005981286	0.442616636	0.910227035	SMUG1
ENSG00000238390.1	-0.026254655	0.442652174	0.910227035	ENSG10010138890.1
ENSG00000146963.18	-0.004357322	0.442660966	0.910227035	LUC7L2
ENSG00000120093.12	0.013348741	0.442748855	0.910227035	HOXB3
ENSG00000132604.11	-0.004846336	0.442823564	0.910227035	TERF2
ENSG00000259704.2	0.016745025	0.442857386	0.910227035	CTD-3094K11.1
ENSG00000107957.16	0.011026305	0.442923543	0.910227035	SH3PXD2A
ENSG00000228649.9	0.011647625	0.442953787	0.910227035	SNHG26
ENSG00000060566.14	-0.037405172	0.442959682	0.910227035	CREB3L3
ENSG00000183822.3	-0.012793992	0.44301533	0.910227035	NCF4-AS1
ENSG00000185475.11	0.006587048	0.443104448	0.910227035	TMEM179B
ENSG00000163739.5	-0.011095898	0.443116917	0.910227035	CXCL1
ENSG00000118785.15	-0.033418361	0.44311995	0.910227035	SPP1
ENSG00000069974.16	0.006487652	0.443126205	0.910227035	RAB27A
ENSG00000273987.1	-0.024066064	0.443283952	0.910227035	RP11-585P4.6
ENSG00000196284.17	0.008725821	0.443349471	0.910227035	SUPT3H
ENSG00000119397.18	0.006626381	0.443362877	0.910227035	CNTRL
ENSG00000067191.16	-0.007107063	0.443423734	0.910227035	CACNB1
ENSG00000181004.10	0.009383661	0.443445337	0.910227035	BBS12
ENSG00000261582.1	0.01591389	0.443467478	0.910227035	RP4-614O4.11
ENSG00000211454.15	0.023831879	0.443504862	0.910227035	AKR7L
ENSG00000213862.4	0.016663884	0.443557984	0.910227035	CTD-2270N23.1
ENSG00000110711.11	-0.006369466	0.443594421	0.910227035	AIP
ENSG00000236209.1	0.056785227	0.443650824	0.910227035	AC104135.2
ENSG00000213860.4	0.032959228	0.44366262	0.910227035	RPL21P75
ENSG00000101152.11	0.004568228	0.443707411	0.910227035	DNAJC5
ENSG00000185480.12	-0.015810447	0.443761991	0.910227035	PARPBP
ENSG00000186081.12	0.028032636	0.443767909	0.910227035	KRT5
ENSG00000130545.16	0.018659176	0.443796879	0.910227035	CRB3
ENSG00000287263.1	0.007465036	0.443902751	0.910227035	CTD-2201E18.8
ENSG00000270170.2	-0.007139919	0.443951342	0.910227035	NCBP2AS2
ENSG00000230581.2	-0.022296128	0.443955614	0.910227035	ACTG1P14
ENSG00000118689.15	0.007055156	0.443962844	0.910227035	FOXO3
ENSG00000134046.12	0.004443991	0.444016491	0.910227035	MBD2
ENSG00000268804.1	0.032597752	0.444032001	0.910227035	LINC02132
ENSG00000158373.8	-0.017345556	0.444071021	0.910227035	H2BC5
ENSG00000287467.1	-0.01481027	0.44412436	0.910227035	RP11-582J16.7
ENSG00000166340.18	-0.007865397	0.444182334	0.910227035	TPP1
ENSG00000173080.6	-0.025815688	0.444223359	0.910227035	RXFP4

ENSG00000084463.8	-0.004027512	0.444246234	0.910227035	WBP11
ENSG00000147471.12	0.003925964	0.444301808	0.910227035	PLPBP
ENSG00000119321.9	0.005562936	0.444374248	0.910227035	FKBP15
ENSG00000155592.16	0.007880682	0.444448685	0.910227035	ZKSCAN2
ENSG00000198131.14	0.006312618	0.444478353	0.910227035	ZNF544
ENSG00000121743.4	0.03084394	0.444501798	0.910227035	GJA3
ENSG00000286818.1	0.057630078	0.444505742	0.910227035	CTC-431G16.4
ENSG00000168792.5	-0.005526743	0.444655594	0.910326966	ABHD15
ENSG00000264853.1	-0.028876164	0.444679539	0.910326966	RP11-16C1.2
ENSG00000159216.19	0.005218883	0.444737115	0.910326966	RUNX1
ENSG00000144320.14	-0.00685806	0.44481721	0.910326966	LNPK
ENSG00000126067.12	0.006002042	0.444821665	0.910326966	PSMB2
ENSG00000227347.1	-0.022714796	0.444837226	0.910326966	HNRNPKP2
ENSG00000188906.17	0.010533882	0.444865265	0.910326966	LRRK2
ENSG00000113812.14	-0.004195684	0.444969303	0.910429324	ACTR8
ENSG00000183421.12	-0.028243019	0.445004073	0.910429324	RIPK4
ENSG00000126773.13	-0.007149113	0.445061164	0.910455297	PCNX4
ENSG00000152422.16	0.016478725	0.445265627	0.910683061	XRCC4
ENSG00000052749.14	0.011497716	0.445293559	0.910683061	RRP12
ENSG00000279199.1	-0.021131947	0.445310737	0.910683061	RP11-749I16.3
ENSG00000256594.8	0.01083812	0.445376058	0.910683061	RP11-705C15.2
ENSG00000156968.9	0.041060207	0.445396085	0.910683061	MPV17L
ENSG00000181027.11	-0.005001389	0.44543894	0.910683061	FKRP
ENSG00000162722.9	0.027168106	0.445496721	0.910697128	TRIM58
ENSG00000214300.7	0.023656542	0.445534634	0.910697128	SPDYE3
ENSG00000260233.3	0.008134514	0.445692065	0.910918066	ZNRD2-AS1
ENSG00000243389.1	0.020113385	0.44578112	0.910918066	AC012442.5
ENSG00000182504.11	-0.009622204	0.445788982	0.910918066	CEP97
ENSG00000139438.6	-0.018009501	0.445820393	0.910918066	FAM222A
ENSG00000250415.1	-0.029888576	0.445902102	0.910984733	CTC-461F20.1
ENSG00000183696.14	0.007152969	0.446026419	0.910984733	UPP1
ENSG00000105700.11	0.004347841	0.446034409	0.910984733	KXD1
ENSG00000172345.14	0.010101592	0.446081303	0.910984733	STARD5
ENSG00000085382.12	-0.010652251	0.44618824	0.910984733	HACE1
ENSG00000147854.17	-0.005166502	0.446284421	0.910984733	UHRF2
ENSG00000115464.15	0.011231788	0.4463412	0.910984733	USP34
ENSG00000205037.2	0.025564268	0.44641725	0.910984733	RP11-863P13.4
ENSG00000240497.3	0.025089714	0.446446238	0.910984733	RP11-185E8.1
ENSG00000100319.13	-0.006809396	0.446494801	0.910984733	ZMAT5
ENSG00000188766.12	-0.01721766	0.446529096	0.910984733	SPRED3
ENSG00000114988.12	0.005159301	0.446550214	0.910984733	LMAN2L
ENSG00000229019.1	0.062541234	0.44657086	0.910984733	RP11-88I18.3
ENSG00000054598.9	-0.037726378	0.446767409	0.910984733	FOXC1
ENSG00000235823.3	-0.012891004	0.44678736	0.910984733	OLMALINC
ENSG00000197603.16	0.009643375	0.446809289	0.910984733	CPLANE1
ENSG00000125637.16	-0.007141635	0.446856071	0.910984733	PSD4
ENSG00000254521.7	-0.040962057	0.446902953	0.910984733	SIGLEC12

ENSG00000158488.16	0.018043998	0.446904828	0.910984733	CD1E
ENSG00000123843.13	0.029093705	0.446911124	0.910984733	C4BPB
ENSG00000248477.6	0.027142546	0.446942081	0.910984733	NAIPP3
ENSG00000185189.18	-0.006429226	0.447015906	0.910984733	NRBP2
ENSG00000136159.4	0.008699404	0.447069236	0.910984733	NUDT15
ENSG00000160221.18	-0.068020836	0.447075113	0.910984733	GATD3A
ENSG00000123607.16	0.006621474	0.447089571	0.910984733	TTC21B
ENSG00000229023.1	-0.030292001	0.447130502	0.910984733	RAB1AP1
ENSG00000238024.1	0.030737537	0.447130732	0.910984733	DDX39BP2
ENSG00000277218.1	0.017265634	0.447132126	0.910984733	RP11-500G10.5
ENSG00000198722.14	0.01440838	0.447141229	0.910984733	UNC13B
ENSG00000104892.17	-0.027492928	0.447463605	0.91155097	KLC3
ENSG00000141873.11	-0.007306263	0.447575364	0.911587166	SLC39A3
ENSG00000166908.18	-0.004290029	0.447610877	0.911587166	PIP4K2C
ENSG00000049656.14	-0.003812444	0.447614724	0.911587166	CLPTM1L
ENSG00000213753.11	0.004596994	0.447744647	0.911761218	CENPBD1P1
ENSG00000204859.13	-0.00516339	0.447932919	0.911917937	ZBTB48
ENSG00000278986.1	-0.017892102	0.447979202	0.911917937	RP11-723J4.3
ENSG00000118160.14	0.095354143	0.448031761	0.911917937	SLC8A2
ENSG00000116237.16	-0.004644063	0.448109448	0.911917937	ICMT
ENSG00000282692.1	0.165976958	0.448130609	0.911917937	CTD-3212A4.2
ENSG00000214787.10	0.010575695	0.44822997	0.911917937	MS4A4E
ENSG00000285103.2	0.010882565	0.448244543	0.911917937	RP11-27J8.3
ENSG00000254852.8	-0.099665043	0.448254609	0.911917937	NPIPA2
ENSG00000171861.11	-0.006840133	0.448266173	0.911917937	MRM3
ENSG00000172382.10	0.016003297	0.448266273	0.911917937	PRSS27
ENSG00000130560.9	0.004962985	0.448355397	0.912008777	UBAC1
ENSG00000260613.2	0.035589329	0.448451952	0.912114712	RP3-522J7.6
ENSG00000183723.13	0.009043787	0.44860788	0.912316492	CMTM4
ENSG00000224945.1	-0.023986371	0.448679059	0.912316492	RP11-82L18.2
ENSG00000139372.15	0.007249727	0.448711558	0.912316492	TDG
ENSG00000136861.18	-0.00595022	0.448729104	0.912316492	CDK5RAP2
ENSG00000283646.2	-0.040400283	0.448804038	0.912342087	LINC02009
ENSG00000035687.10	-0.004968337	0.448830667	0.912342087	ADSS2
ENSG00000226648.2	-0.012154085	0.448969594	0.912530078	PLCG1-AS1
ENSG00000078902.16	0.00572563	0.449019252	0.912530078	TOLLIP
ENSG00000161921.17	0.009018526	0.449056639	0.912530078	CXCL16
ENSG00000206344.7	0.009095772	0.449204097	0.912664377	HCG27
ENSG00000167566.17	-0.007382828	0.449267591	0.912664377	NCKAP5L
ENSG00000259583.3	0.022548135	0.449325609	0.912664377	RP11-66B24.4
ENSG00000180999.11	0.051798747	0.449347234	0.912664377	C1orf105
ENSG00000145850.9	-0.01928995	0.449353506	0.912664377	TIMD4
ENSG00000202441.2	0.013948084	0.449422514	0.912664377	RNY4P10
ENSG00000196072.12	0.009644163	0.449477712	0.912664377	BLOC1S2
ENSG00000215114.10	0.005873565	0.44947875	0.912664377	UBXN2B
ENSG00000005001.10	-0.028931671	0.449628934	0.912739534	PRSS22
ENSG00000152642.11	0.005439092	0.449629299	0.912739534	GPD1L

ENSG00000224596.8	0.01792826	0.449649284	0.912739534	ZMIZ1-AS1
ENSG00000049249.9	0.012476616	0.449694119	0.912740201	TNFRSF9
ENSG00000163214.21	0.005873753	0.449800221	0.912784008	DHX57
ENSG00000273033.2	0.006830953	0.449816885	0.912784008	LINC02035
ENSG00000106244.13	-0.004417768	0.449849228	0.912784008	PDAP1
ENSG00000279066.1	0.016861117	0.450004393	0.912874047	HEXD-IT1
ENSG00000166012.17	0.006800872	0.450021057	0.912874047	TAF1D
ENSG00000275106.1	0.028892899	0.450027141	0.912874047	RP11-309L24.10
ENSG00000167232.14	-0.007998675	0.45012582	0.912921519	ZNF91
ENSG00000155016.18	0.006957077	0.450143915	0.912921519	CYP2U1
ENSG00000159079.19	-0.005684108	0.45018409	0.912921519	CFAP298
ENSG00000148411.8	0.006831102	0.450397581	0.91322434	NACC2
ENSG00000198829.6	0.023259523	0.450427697	0.91322434	SUCNR1
ENSG00000198805.12	0.00667515	0.450485312	0.91322434	PNP
ENSG00000189190.10	-0.013736711	0.450511539	0.91322434	ZNF600
ENSG00000261707.1	0.027881763	0.450658765	0.913386044	RP11-264M12.2
ENSG00000215712.11	-0.005886162	0.450700143	0.913386044	TMEM242
ENSG00000196756.13	-0.007660389	0.450758863	0.913386044	SNHG17
ENSG00000176386.9	-0.008382268	0.450769463	0.913386044	CDC26
ENSG00000261840.3	-0.014715974	0.450836651	0.913431934	RP11-146F11.1
ENSG00000242086.8	0.012671006	0.451050336	0.913709652	MUC20-OT1
ENSG00000166165.13	-0.016762306	0.451127662	0.913709652	CKB
ENSG00000186019.11	-0.016912609	0.45124331	0.913709652	AC084219.4
ENSG00000241553.13	0.007797163	0.451300136	0.913709652	ARPC4
ENSG00000114767.7	-0.007307745	0.451457663	0.913709652	RRP9
ENSG00000233609.3	-0.015178361	0.451461567	0.913709652	RPL10P19
ENSG00000230092.7	-0.019369891	0.451592869	0.913709652	RP11-206L10.8
ENSG00000165300.8	0.030151111	0.451605371	0.913709652	SLITRK5
ENSG00000168004.10	-0.02195074	0.451624847	0.913709652	PLAAT5
ENSG00000264964.2	0.023847886	0.451629692	0.913709652	TWSG1-DT
ENSG00000072840.13	-0.020106701	0.451630135	0.913709652	EVC
ENSG00000155304.6	0.010841826	0.451703043	0.913709652	HSPA13
ENSG00000244055.2	-0.012059256	0.451719681	0.913709652	AC007566.10
ENSG00000179295.18	0.006558594	0.451865875	0.913709652	PTPN11
ENSG00000172817.4	0.025583279	0.451870125	0.913709652	CYP7B1
ENSG00000148303.17	0.00710592	0.451902507	0.913709652	RPL7A
ENSG00000211941.3	0.02952628	0.45200424	0.913709652	IGHV3-11
ENSG00000143494.16	-0.0139507	0.452015569	0.913709652	VASH2
ENSG00000212807.3	-0.026492621	0.452032542	0.913709652	OR2A42
ENSG00000177721.5	-0.007792401	0.452038266	0.913709652	ANXA2R
ENSG00000248780.1	-0.026567859	0.452146295	0.913709652	ARL4AP2
ENSG00000171817.17	0.009146359	0.452156922	0.913709652	ZNF540
ENSG00000137501.17	-0.013470564	0.452206395	0.913709652	SYTL2
ENSG00000272444.1	0.019726043	0.452263979	0.913709652	RP11-1017G21.6
ENSG00000269800.1	0.021824761	0.452373034	0.913709652	PLEKHA3P1
ENSG00000139908.15	0.010794714	0.452385819	0.913709652	TSSK4
ENSG00000100243.21	-0.006897259	0.452416772	0.913709652	CYB5R3

ENSG00000049167.16	0.007507944	0.452436443	0.913709652	ERCC8
ENSG00000205978.6	-0.019095332	0.452441321	0.913709652	NYNRIN
ENSG00000279692.1	-0.023228125	0.452443308	0.913709652	RP11-1055B8.1
ENSG00000083812.12	0.00499881	0.45245056	0.913709652	ZNF324
ENSG00000162191.14	-0.006759492	0.452547447	0.913709652	UBXN1
ENSG00000273899.5	-0.006361195	0.452646511	0.913709652	NOL12
ENSG00000172336.5	-0.007446534	0.452649961	0.913709652	POP7
ENSG00000233225.3	-0.014766565	0.452730094	0.913709652	SSBL3P1
ENSG00000160593.19	0.005560484	0.452786443	0.913709652	JAML
ENSG00000143545.10	0.017036704	0.452823007	0.913709652	RAB13
ENSG00000130024.15	-0.005593777	0.452928741	0.913709652	PHF10
ENSG00000196092.14	-0.020411001	0.453164436	0.913709652	PAX5
ENSG00000234263.2	-0.023862517	0.453178808	0.913709652	MAP3K5-AS1
ENSG00000176928.7	0.012425732	0.453215758	0.913709652	GCNT4
ENSG00000154727.11	-0.009701376	0.453245209	0.913709652	GABPA
ENSG00000229585.4	-0.025556351	0.453252244	0.913709652	RPL21P44
ENSG00000185900.11	-0.005016795	0.453255264	0.913709652	POMK
ENSG00000147324.11	0.007259039	0.453256907	0.913709652	MFHAS1
ENSG00000043591.6	0.031378815	0.453268965	0.913709652	ADRB1
ENSG00000177590.7	-0.023321304	0.45328894	0.913709652	GIMAP3P
ENSG00000101197.13	0.052818479	0.453323902	0.913709652	BIRC7
ENSG00000235546.2	-0.038039338	0.45335774	0.913709652	AC087499.5
ENSG00000232442.1	-0.008982419	0.453392471	0.913709652	MHENCR
ENSG00000167550.11	-0.010322859	0.453412638	0.913709652	RHEBL1
ENSG00000182179.13	0.005975191	0.453415392	0.913709652	UBA7
ENSG00000101182.15	0.005404883	0.45345432	0.913709652	PSMA7
ENSG00000108175.18	-0.008730035	0.453456517	0.913709652	ZMIZ1
ENSG00000275993.3	0.021772408	0.453505153	0.913709652	SIK1B
ENSG00000228686.2	0.01096898	0.45351132	0.913709652	RP11-492I21.1
ENSG00000197724.11	-0.00556265	0.45351329	0.913709652	PHF2
ENSG00000160678.12	0.014466232	0.453586756	0.913767897	S100A1
ENSG00000137198.10	0.023436097	0.453817472	0.914003397	GMPR
ENSG00000149054.16	0.042878067	0.453832681	0.914003397	ZNF215
ENSG00000228804.5	-0.027581242	0.453892656	0.914003397	RP11-211G3.3
ENSG00000180747.15	-0.015215708	0.453921151	0.914003397	SMG1P3
ENSG00000229515.1	0.023515151	0.453926497	0.914003397	FLT1P1
ENSG00000231500.7	0.012027846	0.4540352	0.914104307	RPS18
ENSG00000078269.15	-0.011385751	0.454065759	0.914104307	SYNJ2
ENSG00000122783.17	0.007538446	0.454121704	0.9141272	CYREN
ENSG00000108591.10	-0.004710477	0.454246242	0.91421274	DRG2
ENSG00000076321.11	0.006454667	0.454253356	0.91421274	KLHL20
ENSG00000169282.18	0.014502551	0.454604013	0.914723186	KCNAB1
ENSG00000228719.1	0.024355636	0.454621583	0.914723186	RP5-1119A7.14
ENSG00000164347.18	0.006646521	0.454658226	0.914723186	GFM2
ENSG00000135974.10	0.006549841	0.454747915	0.914723186	C2orf49
ENSG00000114455.13	-0.018335799	0.455079111	0.914723186	HHLA2
ENSG00000235027.1	0.013115086	0.455090661	0.914723186	AC068580.6

ENSG00000134138.20	-0.030559896	0.455116134	0.914723186	MEIS2
ENSG00000188981.11	-0.0147192	0.455122399	0.914723186	MSANTD1
ENSG00000283378.1	-0.039964738	0.455125475	0.914723186	CNTNAP3C
ENSG00000240463.1	-0.017489951	0.455181165	0.914723186	RPS19P3
ENSG00000250948.1	0.015966856	0.455255613	0.914723186	RP11-1079K10.2
ENSG00000214331.8	-0.008661358	0.455258477	0.914723186	PDPR2P
ENSG00000211660.3	-0.026735872	0.455270261	0.914723186	IGLV2-23
ENSG00000230071.2	0.021611049	0.455297006	0.914723186	RPL4P6
ENSG00000238113.7	-0.012198957	0.455299918	0.914723186	LINC01410
ENSG00000084733.11	0.004889395	0.455370649	0.914723186	RAB10
ENSG00000136631.15	0.004208012	0.455392199	0.914723186	VPS45
ENSG00000196366.3	0.019235008	0.455487057	0.914723186	C9orf163
ENSG00000175886.10	-0.018052451	0.455509079	0.914723186	RPL7AP66
ENSG00000180616.9	-0.029938892	0.455520267	0.914723186	SSTR2
ENSG00000245748.1	0.019315975	0.45553887	0.914723186	RP11-367J11.2
ENSG00000167107.13	-0.007966034	0.455548899	0.914723186	ACSF2
ENSG00000237719.1	-0.02351435	0.455573754	0.914723186	RP1-179N16.3
ENSG00000243797.6	0.024304429	0.455583225	0.914723186	CTB-111H14.1
ENSG00000213149.3	-0.013427713	0.455662153	0.914723186	CNN2P9
ENSG00000006071.16	0.06829664	0.455745772	0.914723186	ABCC8
ENSG00000123352.18	0.006562465	0.455803872	0.914723186	SPATS2
ENSG00000145088.9	0.019221863	0.455811056	0.914723186	EAF2
ENSG00000153310.19	0.005324019	0.455828685	0.914723186	CYRIB
ENSG00000079134.12	0.004172652	0.455845083	0.914723186	THOC1
ENSG00000286760.1	0.017472201	0.456082882	0.914780668	RP1-168L15.7
ENSG00000114686.9	0.011082915	0.456140526	0.914780668	MRPL3
ENSG00000186812.13	0.005049514	0.456256423	0.914780668	ZNF397
ENSG00000158402.21	-0.025194092	0.456264361	0.914780668	CDC25C
ENSG00000006194.10	0.003278065	0.456272936	0.914780668	ZNF263
ENSG00000275494.1	-0.023089643	0.456294943	0.914780668	RP11-266L9.8
ENSG00000135423.13	0.018774634	0.456402798	0.914780668	GLS2
ENSG00000125266.8	-0.025238469	0.456483668	0.914780668	EFNB2
ENSG00000215386.13	0.044129642	0.456495438	0.914780668	MIR99AHG
ENSG00000256084.1	-0.029495081	0.456498721	0.914780668	RP11-134N1.2
ENSG00000162819.12	-0.006361494	0.45651939	0.914780668	BROX
ENSG00000280057.1	0.023613408	0.456522037	0.914780668	RP1-168L15.6
ENSG00000267986.1	-0.031172538	0.456532865	0.914780668	AC130469.2
ENSG00000183513.9	0.00474797	0.456581221	0.914780668	COA5
ENSG00000223825.5	-0.024462471	0.456587825	0.914780668	DAZAP2P1
ENSG00000168781.22	-0.005630141	0.456621357	0.914780668	PPIP5K1
ENSG00000274925.1	-0.007230206	0.456830588	0.914780668	ZKSCAN2-DT
ENSG00000117408.11	-0.005233887	0.456836325	0.914780668	IPO13
ENSG00000250510.8	0.017395044	0.456860367	0.914780668	GPR162
ENSG00000142089.17	-0.03094918	0.456901722	0.914780668	IFITM3
ENSG00000258831.1	-0.024810702	0.456912649	0.914780668	RP11-76E17.4
ENSG00000270605.1	-0.008053383	0.45700646	0.914780668	RP5-1092A3.4
ENSG00000136950.13	-0.006652279	0.457013188	0.914780668	ARPC5L

ENSG00000113387.12	0.013298784	0.457026862	0.914780668	SUB1
ENSG00000120526.12	0.013412012	0.457071596	0.914780668	NUDCD1
ENSG00000274225.1	-0.024585011	0.457102929	0.914780668	KB-68A7.1
ENSG00000150455.14	-0.005673396	0.457121094	0.914780668	TIRAP
ENSG00000174370.11	0.014966098	0.457122698	0.914780668	C11orf45
ENSG00000266274.2	-0.019569715	0.457186511	0.914807295	RN7SL138P
ENSG00000178567.8	0.006157165	0.457270696	0.914807295	EPM2AIP1
ENSG00000138069.18	0.004195444	0.457289321	0.914807295	RAB1A
ENSG00000137955.16	0.006475438	0.457314433	0.914807295	RABGGTB
ENSG00000286311.1	-0.028997267	0.457430529	0.914909765	RP5-1184F4.8
ENSG00000126432.14	0.009810409	0.457463819	0.914909765	PRDX5
ENSG00000233392.5	-0.019263549	0.457499495	0.914909765	UICLM
ENSG00000120805.14	-0.005237912	0.457646982	0.915090613	ARL1
ENSG00000288044.1	-0.020112466	0.457715719	0.915090613	RP11-934B9.8
ENSG00000136457.10	-0.013792149	0.457723791	0.915090613	CHAD
ENSG00000172794.20	-0.005785458	0.457786644	0.915127058	RAB37
ENSG00000198121.14	0.015770357	0.45783953	0.915143576	LPAR1
ENSG00000224877.4	0.011298944	0.457934982	0.915245162	NDUFAF8
ENSG00000135596.18	-0.006019428	0.458000902	0.915272219	MICAL1
ENSG00000175166.17	-0.005049437	0.458052782	0.915272219	PSMD2
ENSG00000219928.2	0.020397575	0.458087999	0.915272219	RP11-40C6.2
ENSG00000185101.13	-0.009692513	0.458145327	0.915272219	ANO9
ENSG00000022277.13	0.004993073	0.45817167	0.915272219	RTF2
ENSG00000178776.5	0.049787531	0.458335543	0.91541566	C5orf46
ENSG00000234807.7	0.013547742	0.45837575	0.91541566	LINC01135
ENSG00000086232.13	0.008322258	0.458377385	0.91541566	EIF2AK1
ENSG00000241945.8	-0.054605083	0.458427032	0.915425664	PWP2
ENSG00000179241.13	0.009904202	0.458517812	0.915517796	LDLRAD3
ENSG00000107443.16	0.006893948	0.45869708	0.915773875	CCNJ
ENSG00000074219.14	0.021284381	0.458783577	0.915773875	TEAD2
ENSG00000186818.12	0.010511129	0.458828801	0.915773875	LILRB4
ENSG00000259341.2	0.023788341	0.458911212	0.915773875	RP11-20G13.1
ENSG00000156395.14	-0.027631675	0.458919249	0.915773875	SORCS3
ENSG00000156675.16	0.00955542	0.458958118	0.915773875	RAB11FIP1
ENSG00000161010.15	0.006695311	0.458963783	0.915773875	MRNIP
ENSG00000160741.17	-0.005158191	0.459045238	0.915773875	CRTC2
ENSG00000118579.13	-0.004611541	0.459157171	0.915773875	MED28
ENSG00000137460.10	0.012622117	0.459168783	0.915773875	FHDC1
ENSG00000229671.3	0.020080719	0.459169085	0.915773875	LINC01150
ENSG00000137574.11	0.005929822	0.45933735	0.915773875	TGS1
ENSG00000180867.11	0.008238757	0.459355436	0.915773875	PDIA3P1
ENSG00000129493.15	0.008637669	0.459360928	0.915773875	HEATR5A
ENSG00000273559.5	0.003503093	0.459367973	0.915773875	CWC25
ENSG00000143337.19	0.004401018	0.459373626	0.915773875	TOR1AIP1
ENSG00000118997.14	0.025672894	0.459434792	0.915773875	DNAH7
ENSG00000214960.11	0.02167818	0.45948519	0.915773875	CRPPA
ENSG00000169371.14	0.00546315	0.459494499	0.915773875	SNUPN

ENSG00000107614.22	0.006808949	0.459640945	0.915976727	TRDMT1
ENSG00000270264.1	-0.017113182	0.459744567	0.915995829	NDUFB8P2
ENSG00000158158.12	-0.00533683	0.459758735	0.915995829	CNNM4
ENSG00000218052.5	-0.022676719	0.459827396	0.915995829	ADAMTS7P4
ENSG00000204347.4	-0.040194985	0.459879421	0.915995829	BTBD17
ENSG00000197822.12	0.055667871	0.459899738	0.915995829	OCLN
ENSG00000131748.16	-0.005014416	0.459918522	0.915995829	STARD3
ENSG00000267521.1	0.039506039	0.460109097	0.916066813	RP11-87G24.6
ENSG00000163378.14	-0.008301469	0.46012538	0.916066813	EOGT
ENSG00000281097.1	-0.042236414	0.460188664	0.916066813	LINC01395
ENSG00000213186.8	-0.008475182	0.460205749	0.916066813	TRIM59
ENSG00000276043.5	-0.00929032	0.460216046	0.916066813	UHRF1
ENSG00000204628.12	0.005960108	0.460257488	0.916066813	RACK1
ENSG00000255221.4	0.020581136	0.46028347	0.916066813	CARD17
ENSG00000237399.7	0.014868961	0.460347117	0.916066813	PITRM1-AS1
ENSG00000227253.3	0.01955198	0.460457125	0.916066813	RP11-166N17.1
ENSG00000111530.13	0.004912904	0.460577362	0.916066813	CAND1
ENSG00000230306.1	0.022597966	0.460629923	0.916066813	BANF1P2
ENSG00000187187.14	0.008229067	0.460703364	0.916066813	ZNF546
ENSG00000197980.13	0.029470728	0.460717734	0.916066813	LEKR1
ENSG00000138111.14	0.007688431	0.460750759	0.916066813	MFSD13A
ENSG00000069493.15	-0.005458988	0.460768173	0.916066813	CLEC2D
ENSG00000165138.18	-0.006259176	0.460769947	0.916066813	ANKS6
ENSG00000127585.12	-0.011786349	0.460773089	0.916066813	FBXL16
ENSG00000162616.9	-0.013183907	0.460787082	0.916066813	DNAJB4
ENSG00000240666.2	0.032004285	0.460802869	0.916066813	MME-AS1
ENSG00000213600.3	-0.01402625	0.460855882	0.916082734	XXcos-LUCA16.1
ENSG00000286070.2	-0.020261349	0.460984623	0.916082734	KB-1995A5.6
ENSG00000238249.2	0.012736187	0.46098942	0.916082734	HMGN2P17
ENSG00000255002.2	-0.032466144	0.461030048	0.916082734	LINC02324
ENSG00000132535.22	0.007690385	0.461039979	0.916082734	DLG4
ENSG00000135387.21	0.003554484	0.461078895	0.916082734	CAPRIN1
ENSG00000175455.16	-0.008234344	0.461144462	0.91612425	CCDC14
ENSG00000226419.8	-0.009289431	0.461276944	0.916188381	SLC16A1-AS1
ENSG00000095383.20	-0.007101776	0.461453248	0.916188381	TBC1D2
ENSG00000161202.20	-0.004635843	0.4614703	0.916188381	DVL3
ENSG00000057704.13	0.008031971	0.461597706	0.916188381	TMCC3
ENSG00000252516.1	0.025524477	0.461618469	0.916188381	RNA5SP82
ENSG00000121653.11	-0.018559915	0.461631425	0.916188381	MAPK8IP1
ENSG00000183091.20	-0.015247602	0.461642422	0.916188381	NEB
ENSG00000267049.1	0.017964466	0.46164266	0.916188381	AC002398.13
ENSG00000133067.18	-0.017169147	0.461677746	0.916188381	LGR6
ENSG00000157020.18	0.004295347	0.461756512	0.916188381	SEC13
ENSG00000171049.9	0.009814518	0.461809083	0.916188381	FPR2
ENSG00000278918.1	0.008445643	0.461822475	0.916188381	CTD-2267D19.1
ENSG00000067715.14	-0.042890017	0.461824141	0.916188381	SYT1
ENSG00000104432.14	-0.019838724	0.461835282	0.916188381	IL7

ENSG00000121022.14	0.005175258	0.461846864	0.916188381	COPS5
ENSG00000172113.10	0.004415249	0.461914908	0.916234735	NME6
ENSG00000090612.22	0.007337342	0.462062787	0.916379022	ZNF268
ENSG00000198298.13	0.009091271	0.462079501	0.916379022	ZNF485
ENSG00000173226.17	0.00669987	0.462121701	0.916379022	IQCB1
ENSG00000211766.1	-0.014769196	0.462410735	0.916472006	TRBJ2-2P
ENSG00000104957.14	-0.005339889	0.462444203	0.916472006	CCDC130
ENSG00000238278.3	-0.020371462	0.462521738	0.916472006	ALG1L6P
ENSG00000131653.13	0.00465795	0.462540798	0.916472006	TRAF7
ENSG00000287786.1	0.039332175	0.462549017	0.916472006	RP11-586D19.4
ENSG00000184350.11	0.058239031	0.462597919	0.916472006	MRGPRE
ENSG00000232229.6	-0.017842964	0.462615501	0.916472006	LINC00865
ENSG00000163795.14	-0.005305976	0.462640126	0.916472006	ZNF513
ENSG00000143924.19	0.006027608	0.462645579	0.916472006	EML4
ENSG00000254126.8	0.032762125	0.462686004	0.916472006	CD8B2
ENSG00000223704.2	-0.010272898	0.462724755	0.916472006	LINC01422
ENSG00000280054.1	0.017801559	0.462733412	0.916472006	RP1-197B17.7
ENSG00000142528.16	0.004859754	0.462764989	0.916472006	ZNF473
ENSG00000201451.1	-0.023547194	0.462889993	0.916472006	Y_RNA
ENSG00000215835.2	-0.01678415	0.462922195	0.916472006	RP11-9L18.2
ENSG00000272808.4	0.036033837	0.462969693	0.916472006	RP11-66B24.7
ENSG00000244361.1	0.022645712	0.462996264	0.916472006	RPL30P7
ENSG00000154217.15	-0.004785668	0.463137141	0.916472006	PITPNC1
ENSG00000267282.1	0.031306584	0.463141207	0.916472006	CTB-129P6.4
ENSG00000181023.8	0.032619888	0.463202878	0.916472006	OR56B1
ENSG00000154485.5	-0.019901385	0.463227098	0.916472006	MMP21
ENSG00000135924.17	0.007927066	0.463240943	0.916472006	DNAJB2
ENSG00000174038.13	-0.028259311	0.46326534	0.916472006	C9orf131
ENSG00000167721.11	0.004666718	0.463308389	0.916472006	TSR1
ENSG00000146676.10	0.005972452	0.463330219	0.916472006	PURB
ENSG00000211658.2	-0.037258218	0.463330494	0.916472006	IGLV3-27
ENSG00000204387.14	-0.008760824	0.463399763	0.916520622	SNHG32
ENSG00000196526.11	0.020623311	0.463552839	0.916605203	AFAP1
ENSG00000163527.10	0.006644798	0.463642668	0.916605203	STT3B
ENSG00000198931.11	-0.007412087	0.463660052	0.916605203	APRT
ENSG00000011451.21	-0.005310343	0.463689385	0.916605203	WIZ
ENSG00000214872.8	0.02957907	0.463706752	0.916605203	SMTNL1
ENSG00000102794.10	0.038440825	0.463714784	0.916605203	ACOD1
ENSG00000165995.22	0.011386603	0.463885184	0.916605203	CACNB2
ENSG00000108641.19	-0.013139158	0.463920045	0.916605203	B9D1
ENSG00000272507.1	0.025681697	0.46402654	0.916605203	RNU6-88P
ENSG00000166265.13	-0.021709451	0.464031536	0.916605203	CYYR1
ENSG00000112561.19	-0.007033643	0.464077511	0.916605203	TFEB
ENSG00000125772.14	-0.006901558	0.464082505	0.916605203	GPCPD1
ENSG00000255857.6	0.006939246	0.464121353	0.916605203	PXN-AS1
ENSG00000140326.13	-0.005037986	0.464167174	0.916605203	CDAN1
ENSG00000175449.14	0.01303797	0.46418725	0.916605203	RFESD

ENSG00000178127.13	-0.012457754	0.464270732	0.916605203	NDUFV2
ENSG00000240184.7	-0.024651507	0.464271183	0.916605203	PCDHGC3
ENSG00000155621.15	-0.006301936	0.464305477	0.916605203	C9orf85
ENSG00000011376.12	-0.005114169	0.464318243	0.916605203	LARS2
ENSG00000239941.1	0.023284906	0.464374025	0.916605203	RP11-246A10.1
ENSG00000178033.6	0.024009268	0.46439285	0.916605203	CALHM5
ENSG00000225193.5	0.029142052	0.46451426	0.916605203	RPS12P26
ENSG00000256875.2	0.065554459	0.464535157	0.916605203	RP11-503G7.2
ENSG00000204381.12	0.019367491	0.464583863	0.916605203	LAYN
ENSG00000197448.14	0.005880448	0.464585096	0.916605203	GSTK1
ENSG00000137819.14	-0.030529443	0.464649977	0.916605203	PAQR5
ENSG00000197020.11	0.012481578	0.46467949	0.916605203	ZNF100
ENSG00000198707.17	0.010787628	0.464735286	0.916605203	CEP290
ENSG00000138867.17	0.004734908	0.464738683	0.916605203	GUCD1
ENSG00000265907.2	-0.017982403	0.464797497	0.916633049	RP11-737O24.2
ENSG00000107372.13	-0.005561267	0.464957989	0.916849723	ZFAND5
ENSG00000008226.20	0.007965868	0.46499678	0.916849723	DLEC1
ENSG00000204516.10	0.007972421	0.465079436	0.916861374	MICB
ENSG00000112146.17	0.008542391	0.465092104	0.916861374	FBXO9
ENSG00000140848.17	-0.007891298	0.465207139	0.916870814	CPNE2
ENSG00000197345.13	0.008055507	0.465210718	0.916870814	MRPL21
ENSG00000143643.13	-0.004931651	0.4652843	0.916870814	TTC13
ENSG00000067221.14	0.007719413	0.465374527	0.916870814	STOML1
ENSG00000151093.8	-0.007886023	0.465416802	0.916870814	OXSM
ENSG00000173744.18	0.004450028	0.465448951	0.916870814	AGFG1
ENSG00000272910.1	0.021080711	0.465503956	0.916870814	RP11-15L13.4
ENSG00000254034.2	0.023841529	0.4655778	0.916870814	INTS9-AS1
ENSG00000115523.17	0.019935552	0.465596108	0.916870814	GNLY
ENSG00000185324.22	-0.0081384	0.465600899	0.916870814	CDK10
ENSG00000135940.7	0.008550217	0.465619625	0.916870814	COX5B
ENSG00000261267.1	0.024025555	0.465693485	0.916870814	RP11-44I10.3
ENSG00000061676.15	0.01272346	0.465712255	0.916870814	NCKAP1
ENSG00000087128.10	0.058271304	0.465866073	0.916870814	TMPRSS11E
ENSG00000150768.17	-0.004638233	0.465923402	0.916870814	DLAT
ENSG00000260526.1	-0.019208406	0.465934408	0.916870814	RP11-73K9.2
ENSG00000067082.15	0.005772535	0.465957112	0.916870814	KLF6
ENSG00000100095.19	0.016129776	0.465964947	0.916870814	SEZ6L
ENSG00000134369.15	0.009383874	0.466029431	0.916870814	NAV1
ENSG00000255339.6	0.029587341	0.466074762	0.916870814	RP11-411B6.6
ENSG00000124171.9	-0.012379036	0.466103321	0.916870814	PARD6B
ENSG00000011009.11	0.006735161	0.466197995	0.916870814	LYPLA2
ENSG00000274751.1	0.017246644	0.466292304	0.916870814	LA16c-358B7.4
ENSG00000259162.1	0.018780283	0.466296177	0.916870814	RP11-203M5.6
ENSG00000165233.18	0.00645971	0.466300455	0.916870814	CARD19
ENSG00000088882.8	-0.028036351	0.46635171	0.916870814	CPXM1
ENSG00000181264.9	0.034823058	0.466409986	0.916870814	TLCD5
ENSG00000127311.10	-0.013644397	0.466415587	0.916870814	HELB

ENSG00000274026.2	-0.028545095	0.466430956	0.916870814	FAM27E3
ENSG00000230124.8	0.004561387	0.466438132	0.916870814	ACBD6
ENSG00000176927.15	-0.019456173	0.466498819	0.916884546	EFCAB5
ENSG00000079785.16	0.004581124	0.466534535	0.916884546	DDX1
ENSG00000177398.19	-0.022551464	0.466609004	0.916943029	UMODL1
ENSG00000271869.1	0.019308292	0.466939665	0.917057286	DCTN6-DT
ENSG00000250673.2	0.019446301	0.467018154	0.917057286	REELD1
ENSG00000256087.7	-0.007807175	0.467021009	0.917057286	ZNF432
ENSG00000164611.13	-0.01289005	0.467022672	0.917057286	PTTG1
ENSG00000127511.10	-0.005131639	0.467027863	0.917057286	SIN3B
ENSG00000112303.14	0.01166478	0.467059667	0.917057286	VNN2
ENSG00000161132.6	0.01929361	0.467090617	0.917057286	XXbac-B444P24.10
ENSG00000115828.17	0.00860376	0.467095997	0.917057286	QPCT
ENSG00000171928.14	0.010711602	0.467136268	0.917057286	TVP23B
ENSG00000272933.1	-0.012586405	0.467193732	0.917057286	RP11-47A8.5
ENSG00000267575.7	0.005513017	0.46722081	0.917057286	CTC-459F4.3
ENSG00000189159.16	0.00713766	0.46736735	0.917057286	JPT1
ENSG00000287426.1	0.034663903	0.467394439	0.917057286	RP11-507F16.5
ENSG00000104969.11	-0.005574729	0.467408135	0.917057286	SGTA
ENSG00000164081.13	0.005814267	0.467442578	0.917057286	TEX264
ENSG00000101624.11	0.007926153	0.467478753	0.917057286	CEP76
ENSG00000179262.10	-0.006354978	0.467661839	0.917057286	RAD23A
ENSG00000285531.1	0.018895787	0.467696627	0.917057286	CTA-216E10.11
ENSG00000213123.11	0.017249428	0.467703914	0.917057286	DYNLT2B
ENSG00000088826.18	0.01633875	0.467783558	0.917057286	SMOX
ENSG00000104490.18	-0.010744913	0.467826822	0.917057286	NCALD
ENSG00000179909.16	-0.017292893	0.467851731	0.917057286	ZNF154
ENSG00000104812.15	-0.004883396	0.467884448	0.917057286	GYS1
ENSG00000104524.14	-0.008180711	0.467927452	0.917057286	PYCR3
ENSG00000179085.8	0.009521464	0.467952704	0.917057286	DPM3
ENSG00000173811.11	0.017880127	0.467965177	0.917057286	CCDC13-AS1
ENSG00000148840.11	-0.005721654	0.467979902	0.917057286	PPRC1
ENSG00000287346.1	0.017641155	0.468021842	0.917057286	CTD-2134A5.3
ENSG00000286952.1	0.020384469	0.468050134	0.917057286	RP11-241K3.5
ENSG00000234810.4	0.032825071	0.468141612	0.917057286	RP11-90C4.1
ENSG00000131018.25	-0.007282998	0.468143326	0.917057286	SYNE1
ENSG00000136718.10	-0.004914922	0.468207267	0.917057286	IMP4
ENSG00000287222.1	0.016096757	0.468261123	0.917057286	ABC12-46663800M24.2
ENSG00000211805.1	-0.023885665	0.468267008	0.917057286	TRAV24
ENSG00000071243.16	0.004886276	0.468271186	0.917057286	ING3
ENSG00000270558.1	-0.017420619	0.468323961	0.917057286	CTD-2124B8.2
ENSG00000288066.1	-0.007680325	0.468389858	0.917057286	CTD-2021K4.2
ENSG00000250616.2	0.011684945	0.468431278	0.917057286	RP11-455F5.3
ENSG00000105875.15	0.00433692	0.468453824	0.917057286	WDR91
ENSG00000118900.15	-0.006932518	0.468457137	0.917057286	UBN1
ENSG00000213049.3	-0.028238497	0.46852145	0.917057286	HNRNPA1P34
ENSG00000168785.8	0.014808172	0.468592585	0.917057286	TSPAN5

ENSG00000163520.14	-0.02162291	0.468606887	0.917057286	FBLN2
ENSG00000226025.9	-0.027234673	0.46872422	0.917057286	LGALS17A
ENSG00000233325.3	0.019821093	0.468740119	0.917057286	MIPEPP3
ENSG00000115392.12	0.01086264	0.468753091	0.917057286	FANCL
ENSG00000227262.3	0.023055839	0.468768848	0.917057286	HCG4B
ENSG00000135519.8	-0.013012037	0.468983768	0.917208891	KCNH3
ENSG00000125812.16	0.004105705	0.468984063	0.917208891	GZF1
ENSG00000230701.2	-0.014789684	0.469121031	0.917208891	FBXW4P1
ENSG00000169692.13	-0.008167103	0.469132296	0.917208891	AGPAT2
ENSG00000196437.12	0.010361453	0.469225282	0.917208891	ZNF569
ENSG00000125656.11	-0.007220329	0.469236451	0.917208891	CLPP
ENSG00000213281.5	0.006746615	0.469331181	0.917208891	NRAS
ENSG00000167528.13	0.008692407	0.46933631	0.917208891	ZNF641
ENSG00000229093.1	-0.038485278	0.469341619	0.917208891	OR51AB1P
ENSG00000130876.11	-0.034573654	0.469353952	0.917208891	SLC7A10
ENSG00000139192.12	0.007280884	0.469373414	0.917208891	TAPBPL
ENSG00000138101.19	-0.006390601	0.469395695	0.917208891	DTNB
ENSG00000248671.8	-0.013980463	0.469427761	0.917208891	ALG1L9P
ENSG00000154447.15	0.013532385	0.469522973	0.917233864	SH3RF1
ENSG00000287586.1	0.018339537	0.469529993	0.917233864	RP1-77N19.1
ENSG00000225526.6	-0.020015611	0.46963571	0.917332058	MKRN2OS
ENSG00000151694.14	0.006725235	0.46966972	0.917332058	ADAM17
ENSG00000110063.10	-0.004886309	0.469756912	0.917380417	DCPS
ENSG00000121486.12	0.005750135	0.469787125	0.917380417	TRMT1L
ENSG00000106605.11	0.010569863	0.46982903	0.917380417	BLVRA
ENSG00000247596.10	-0.006213008	0.46987341	0.917380417	TWF2
ENSG00000245680.11	-0.005749815	0.469983085	0.917507198	ZNF585B
ENSG00000142634.13	-0.006892657	0.470082347	0.917609152	EFHD2
ENSG00000240449.1	0.012419048	0.470161391	0.917609152	REPIN1-AS1
ENSG00000138131.4	-0.018009509	0.470173433	0.917609152	LOXL4
ENSG00000072364.13	0.007045527	0.470214286	0.917609152	AFF4
ENSG00000255310.2	-0.009038922	0.470501029	0.917866249	AF131215.2
ENSG00000064042.18	0.026840403	0.470580335	0.917866249	LIMCH1
ENSG00000168769.14	0.009073167	0.470591566	0.917866249	TET2
ENSG00000247679.3	0.008106781	0.470685397	0.917866249	RP11-1277A3.1
ENSG00000279792.1	0.016071882	0.470707715	0.917866249	RP11-893F2.18
ENSG00000116668.13	0.006084402	0.470818361	0.917866249	SWT1
ENSG00000167881.15	-0.004252195	0.470861399	0.917866249	SRP68
ENSG00000128274.17	0.044441567	0.470879272	0.917866249	A4GALT
ENSG00000267106.8	0.009496823	0.470925004	0.917866249	ZNF561-AS1
ENSG00000240616.1	-0.017129101	0.470981307	0.917866249	RPS6P25
ENSG00000225940.7	0.021132037	0.471037981	0.917866249	C5orf67
ENSG00000142686.8	-0.007305552	0.471096183	0.917866249	C1orf216
ENSG00000139974.16	0.00751495	0.471183008	0.917866249	SLC38A6
ENSG00000234699.1	0.02177949	0.471249779	0.917866249	RP11-225H22.4
ENSG00000279328.1	-0.016952178	0.471278855	0.917866249	RP11-203H19.2
ENSG00000121940.16	0.005230117	0.47128309	0.917866249	CLCC1

ENSG00000235641.5	0.028233997	0.471497336	0.917866249	LINC00484
ENSG00000196689.13	-0.006645251	0.47152516	0.917866249	TRPV1
ENSG00000278932.4	-0.025875672	0.47158907	0.917866249	bP-21264C1.2
ENSG00000101307.16	0.01014868	0.471595016	0.917866249	SIRPB1
ENSG00000119720.18	-0.003905784	0.471664031	0.917866249	NRDE2
ENSG00000106554.13	0.004271932	0.471726471	0.917866249	CHCHD3
ENSG00000211765.1	-0.015863965	0.471740226	0.917866249	TRBJ2-2
ENSG00000215187.12	0.021722078	0.471744701	0.917866249	FAM166B
ENSG00000211731.1	-0.034937924	0.471774475	0.917866249	TRBV5-7
ENSG00000134809.9	0.014542941	0.471825259	0.917866249	TIMM10
ENSG00000099785.11	0.010519795	0.471826181	0.917866249	MARCHF2
ENSG00000284685.1	-0.01903482	0.471837652	0.917866249	RP11-426C22.12
ENSG00000183474.16	0.010260369	0.471842647	0.917866249	GTF2H2C
ENSG00000164855.16	-0.021718193	0.471923878	0.917866249	TMEM184A
ENSG00000224875.2	0.021220367	0.471998786	0.917866249	EML4-AS1
ENSG00000107672.15	0.005437681	0.472079687	0.917866249	NSMCE4A
ENSG00000134291.12	0.005924018	0.47211206	0.917866249	TMEM106C
ENSG00000181085.15	-0.040657375	0.47224209	0.917866249	MAPK15
ENSG00000181513.15	-0.006298787	0.472291618	0.917866249	ACBD4
ENSG00000136197.13	-0.020610836	0.472317213	0.917866249	C7orf25
ENSG00000278434.1	-0.020450812	0.472392508	0.917866249	RP11-709D24.8
ENSG00000100311.17	0.013435051	0.472446869	0.917866249	PDGFB
ENSG00000171916.17	-0.026270975	0.472521485	0.917866249	LGALS9C
ENSG00000166349.10	0.017912911	0.472535309	0.917866249	RAG1
ENSG00000279617.1	0.022671063	0.472590764	0.917866249	AC005796.2
ENSG00000135930.14	0.004148324	0.472627581	0.917866249	EIF4E2
ENSG00000270647.6	-0.009403035	0.472642441	0.917866249	TAF15
ENSG00000136425.14	0.013340785	0.472652745	0.917866249	CIB2
ENSG00000167065.14	0.006925086	0.472659417	0.917866249	DUSP18
ENSG00000153214.11	-0.006844338	0.472666534	0.917866249	TMEM87B
ENSG00000130164.14	0.01130418	0.472670537	0.917866249	LDLR
ENSG00000276966.2	0.019295194	0.472832613	0.917866249	H4C5
ENSG00000161405.17	-0.006799877	0.472835972	0.917866249	IKZF3
ENSG00000188199.10	0.02344869	0.472910884	0.917866249	NUTM2B
ENSG00000144802.11	0.007694469	0.472941623	0.917866249	NFKBIZ
ENSG00000272226.1	-0.016946972	0.472945423	0.917866249	RP11-63G10.3
ENSG00000099365.11	0.01394864	0.47305008	0.917866249	STX1B
ENSG00000175707.9	0.01345246	0.4730796	0.917866249	KDF1
ENSG00000220349.4	0.025939361	0.473080525	0.917866249	RP3-431A14.4
ENSG00000165819.12	-0.003401416	0.473081093	0.917866249	METTL3
ENSG00000165724.6	-0.005199267	0.473127342	0.917866249	ZMYND19
ENSG00000116539.14	0.006784615	0.473156415	0.917866249	ASH1L
ENSG00000288393.1	-0.037440011	0.473191585	0.917866249	ZNF75CP
ENSG00000182150.19	0.005108633	0.473274496	0.917866249	ERCC6L2
ENSG00000197077.14	-0.019562391	0.473398501	0.917866249	KIAA1671
ENSG00000261326.4	0.007496738	0.473441165	0.917866249	LINC01355
ENSG00000217078.1	-0.01712457	0.473482363	0.917866249	RP1-13D10.3

ENSG00000167634.12	0.014029566	0.473509503	0.917866249	NLRP7
ENSG00000146457.16	0.005089588	0.47366544	0.917866249	WTAP
ENSG00000095777.17	-0.068157688	0.473668386	0.917866249	MYO3A
ENSG00000165115.15	0.011824986	0.47367458	0.917866249	KIF27
ENSG00000102738.8	0.006133301	0.47368886	0.917866249	MRPS31
ENSG00000269845.1	0.027576958	0.473704062	0.917866249	CCNYL6
ENSG00000175414.7	0.00771781	0.473735026	0.917866249	ARL10
ENSG00000263126.1	-0.008253209	0.473794062	0.917866249	CTC-479C5.10
ENSG00000119760.16	-0.002885261	0.473794779	0.917866249	SUPT7L
ENSG00000100811.14	-0.002669917	0.4738395	0.917866249	YY1
ENSG00000167969.13	-0.006903243	0.473867751	0.917866249	ECI1
ENSG00000278231.1	0.010215398	0.473910588	0.917866249	RP5-906C1.1
ENSG00000182809.11	0.017972464	0.473935094	0.917866249	CRIP2
ENSG00000181856.15	0.02804144	0.473979785	0.917866249	SLC2A4
ENSG00000160345.13	-0.019557547	0.474006935	0.917866249	C9orf116
ENSG00000090061.17	0.00932017	0.47417001	0.917866249	CCNK
ENSG00000279339.1	-0.021202865	0.474225135	0.917866249	CTD-2373H9.3
ENSG00000229638.1	0.011360949	0.474258772	0.917866249	RPL4P4
ENSG00000124831.19	0.006107837	0.474334045	0.917866249	LRRFIP1
ENSG00000163564.15	-0.009057955	0.47439009	0.917866249	PYHIN1
ENSG00000198106.8	-0.016904647	0.474399982	0.917866249	RP11-231C14.7
ENSG00000256448.5	0.010100078	0.474421352	0.917866249	RP11-809N8.4
ENSG00000239911.2	0.009008144	0.474544671	0.917866249	PRKAG2-AS1
ENSG00000144668.12	0.023034353	0.474715792	0.917866249	ITGA9
ENSG00000183935.5	0.011257198	0.474851098	0.917866249	HTR7P1
ENSG00000258130.8	-0.027479401	0.474936176	0.917866249	RP11-347C12.3
ENSG00000246898.1	0.015004974	0.474947369	0.917866249	LINC00920
ENSG00000053438.11	0.014911549	0.474965993	0.917866249	NNAT
ENSG00000167632.18	-0.007039944	0.475111765	0.917866249	TRAPPC9
ENSG00000148655.15	0.010966273	0.475123405	0.917866249	LRMDA
ENSG00000064199.7	0.017632947	0.475129637	0.917866249	SPA17
ENSG00000225808.1	-0.016317433	0.475205795	0.917866249	DNAJC19P5
ENSG00000198026.8	0.004735594	0.475243242	0.917866249	ZNF335
ENSG00000092529.25	0.008678062	0.47525731	0.917866249	CAPN3
ENSG00000051009.11	0.004591178	0.475285842	0.917866249	FAM160A2
ENSG00000280310.1	-0.032383485	0.475289776	0.917866249	RP11-326I19.2
ENSG00000229961.3	0.017894308	0.475384035	0.917866249	RP11-71G12.1
ENSG00000001167.15	0.004751266	0.475587383	0.917866249	NFYA
ENSG00000200714.1	0.024393503	0.475599311	0.917866249	Y_RNA
ENSG00000007866.22	0.024475622	0.475617931	0.917866249	TEAD3
ENSG00000225648.5	0.008059455	0.475643972	0.917866249	SBDSP1
ENSG00000182952.5	0.005830558	0.475664561	0.917866249	HMGN4
ENSG00000132300.19	0.003730289	0.475712751	0.917866249	PTCD3
ENSG00000286613.1	-0.023199862	0.475727846	0.917866249	RP11-15A1.10
ENSG00000153048.11	-0.00640609	0.475804832	0.917866249	CARHSP1
ENSG00000288056.1	-0.022516386	0.475816876	0.917866249	RP3-466P17.4
ENSG00000205571.14	0.027871558	0.475839057	0.917866249	SMN2

ENSG00000125826.21	-0.007563941	0.475892767	0.917866249	RBCK1
ENSG00000287713.1	-0.02725217	0.475932545	0.917866249	RP11-277J24.7
ENSG00000170540.15	0.005364518	0.475968474	0.917866249	ARL6IP1
ENSG00000176182.6	-0.007442575	0.476058978	0.917866249	MYPOP
ENSG00000183060.16	0.007291125	0.476110666	0.917866249	LYSMD4
ENSG00000256812.2	0.025082723	0.476145094	0.917866249	CAPNS2
ENSG00000163082.10	-0.010292797	0.476196543	0.917866249	SGPP2
ENSG00000257681.1	0.021060931	0.476214868	0.917866249	RP11-341G23.4
ENSG00000261671.1	0.029199737	0.476255194	0.917866249	RP11-573G6.6
ENSG00000232838.4	-0.017957015	0.476318972	0.917866249	PET117
ENSG00000124602.10	-0.006974671	0.476334672	0.917866249	UNC5CL
ENSG00000162482.5	0.019466126	0.4763599	0.917866249	AKR7A3
ENSG00000178636.8	-0.029059986	0.476433364	0.917866249	RP11-455G16.1
ENSG00000104332.12	-0.054028493	0.476450084	0.917866249	SFRP1
ENSG00000211872.1	-0.025959667	0.476454238	0.917866249	TRAJ17
ENSG00000143443.10	-0.006453437	0.476467802	0.917866249	C1orf56
ENSG00000272275.1	-0.024169825	0.476501082	0.917866249	RP11-791G15.2
ENSG00000197361.8	-0.01919318	0.476533662	0.917866249	FBXL22
ENSG00000214293.9	-0.006811442	0.476624876	0.917866249	APTR
ENSG00000231551.8	-0.008500216	0.476626279	0.917866249	PDE4DIPP6
ENSG00000129128.13	0.00430018	0.476626693	0.917866249	SPCS3
ENSG00000229589.1	-0.032817909	0.476716266	0.917866249	ACVR2B-AS1
ENSG00000261716.2	0.010603888	0.476832713	0.917866249	H2BC20P
ENSG00000148308.18	-0.004768646	0.47685135	0.917866249	GTF3C5
ENSG00000287927.1	-0.038163756	0.476938952	0.917866249	CTA-392E5.2
ENSG00000131203.13	0.022068008	0.476940082	0.917866249	IDO1
ENSG00000103248.19	-0.004695335	0.476956894	0.917866249	MTHFSD
ENSG00000274270.1	-0.019135934	0.476998998	0.917866249	RP11-34F20.7
ENSG00000112212.12	0.022753408	0.477018155	0.917866249	TSPO2
ENSG00000279370.1	-0.023721286	0.477098545	0.917866249	RP1-247F3.1
ENSG00000275120.3	-0.012759969	0.477120675	0.917866249	RP11-182J1.18
ENSG00000214765.8	0.005881209	0.477127467	0.917866249	SEPTIN7P2
ENSG00000112877.8	0.007615661	0.477169441	0.917866249	CEP72
ENSG00000115129.14	0.009608842	0.47732646	0.917866249	TP53I3
ENSG00000165156.15	0.008371822	0.477327726	0.917866249	ZHX1
ENSG00000176700.20	-0.00810578	0.477338403	0.917866249	SCAND2P
ENSG00000241764.4	0.011535301	0.477498248	0.917866249	AC002467.7
ENSG00000021488.13	-0.021717281	0.477510198	0.917866249	SLC7A9
ENSG00000133812.17	0.007181679	0.477542303	0.917866249	SBF2
ENSG00000211865.1	-0.035056747	0.47758285	0.917866249	TRAJ24
ENSG00000171533.12	-0.041669686	0.477601995	0.917866249	MAP6
ENSG00000169957.10	-0.006797982	0.477615167	0.917866249	ZNF768
ENSG00000225492.6	0.035392395	0.477645249	0.917866249	GBP1P1
ENSG00000159658.15	0.003491405	0.477783166	0.917866249	EFCAB14
ENSG00000143367.16	0.00850344	0.477807964	0.917866249	TUFT1
ENSG00000099954.19	0.019988466	0.477983613	0.917866249	CECR2
ENSG00000147454.14	0.009788986	0.478024467	0.917866249	SLC25A37

ENSG00000250790.4	0.017704577	0.478032822	0.917866249	RP11-46H11.3
ENSG00000278737.1	-0.026785979	0.478067532	0.917866249	RP11-325L12.7
ENSG00000174780.17	0.005459847	0.478098593	0.917866249	SRP72
ENSG00000216588.9	-0.026935876	0.478131602	0.917866249	IGSF23
ENSG00000144559.10	-0.008111747	0.478170418	0.917866249	TAMM41
ENSG00000134333.14	0.004663004	0.478312492	0.917866249	LDHA
ENSG00000123200.17	-0.005992895	0.478343175	0.917866249	ZC3H13
ENSG00000135916.16	0.010351578	0.478353322	0.917866249	ITM2C
ENSG00000225187.1	0.021421132	0.478402571	0.917866249	AC073283.7
ENSG00000244073.1	-0.030167176	0.478439207	0.917866249	RPS4XP6
ENSG00000114933.16	-0.005658595	0.478535618	0.917866249	INO80D
ENSG00000071462.12	-0.005022355	0.478549791	0.917866249	BUD23
ENSG00000281183.1	0.020421319	0.478555353	0.917866249	NPTN-IT1
ENSG00000150527.17	0.003444474	0.478569259	0.917866249	MIA2
ENSG00000240401.9	-0.012601337	0.47858482	0.917866249	AC012358.8
ENSG00000127995.17	-0.008679528	0.478592294	0.917866249	CASD1
ENSG00000174353.17	-0.008749095	0.478604212	0.917866249	STAG3L3
ENSG00000132746.15	-0.04561002	0.478622031	0.917866249	ALDH3B2
ENSG00000182551.14	0.005406656	0.478651129	0.917866249	ADI1
ENSG00000108405.4	-0.012243364	0.478669899	0.917866249	P2RX1
ENSG00000065833.9	0.01723724	0.478678112	0.917866249	ME1
ENSG00000211904.2	0.037278463	0.478766116	0.917866249	IGHJ2
ENSG00000197753.11	0.01229716	0.478797612	0.917866249	LHFPL5
ENSG00000123552.18	-0.010440181	0.478798529	0.917866249	USP45
ENSG00000060642.11	0.00436328	0.4789054	0.917866249	PIGV
ENSG00000056558.11	0.00552352	0.478906281	0.917866249	TRAF1
ENSG00000141854.9	-0.011645889	0.478924283	0.917866249	MISP3
ENSG00000171777.16	0.005977656	0.478961323	0.917866249	RASGRP4
ENSG00000277157.2	-0.019820334	0.478998072	0.917866249	H4C4
ENSG00000121281.13	0.010720213	0.479012418	0.917866249	ADCY7
ENSG00000115935.18	-0.004361805	0.479026287	0.917866249	WIPF1
ENSG00000074590.14	-0.028921285	0.479079819	0.917866249	NUAK1
ENSG00000179148.10	-0.030575824	0.479088944	0.917866249	ALOXE3
ENSG00000138246.17	0.006200035	0.479121151	0.917866249	DNAJC13
ENSG00000125863.20	0.007225847	0.479149475	0.917866249	MKKS
ENSG00000170379.21	0.00613895	0.479164079	0.917866249	TCAF2
ENSG00000186523.14	-0.024294197	0.479168571	0.917866249	FAM86B1
ENSG00000233296.2	0.031936896	0.479195604	0.917866249	TMEM18-DT
ENSG00000136770.11	-0.007918878	0.479452298	0.917866249	DNAJC1
ENSG00000124155.18	-0.004710616	0.47951863	0.917866249	PIGT
ENSG00000259793.1	0.023839804	0.479535131	0.917866249	RP11-400N9.1
ENSG00000118961.15	-0.00589356	0.479572498	0.917866249	LDAH
ENSG00000153012.12	0.019676441	0.479594084	0.917866249	LGI2
ENSG00000164366.4	0.004456149	0.479626747	0.917866249	CCDC127
ENSG00000218358.2	-0.030479733	0.479642739	0.917866249	RAET1K
ENSG00000255363.3	0.025481376	0.479793006	0.917866249	LINC02757
ENSG00000005243.10	0.014507321	0.479804533	0.917866249	COPZ2

ENSG00000211801.3	-0.016635823	0.479854108	0.917866249	TRAV21
ENSG00000285943.1	-0.014584871	0.479912495	0.917866249	RP11-553D4.3
ENSG00000135316.19	0.004979926	0.479955372	0.917866249	SYNCRIP
ENSG00000273017.1	-0.01695267	0.48002645	0.917866249	AP000240.9
ENSG00000161958.11	0.02456434	0.480082986	0.917866249	FGF11
ENSG00000136699.20	-0.004984069	0.480121515	0.917866249	SMPD4
ENSG00000268889.1	-0.028833284	0.480133271	0.917866249	CTD-2616J11.14
ENSG00000243960.1	0.015722361	0.480141211	0.917866249	RP11-552M11.4
ENSG00000274349.5	0.014548702	0.480142543	0.917866249	ZNF658
ENSG00000276116.2	0.02187596	0.480146974	0.917866249	FUT8-AS1
ENSG00000177058.12	0.004716651	0.480201577	0.917866249	SLC38A9
ENSG00000130309.11	-0.004475645	0.480219766	0.917866249	COLGALT1
ENSG00000075826.17	-0.00654734	0.480352516	0.917866249	SEC31B
ENSG00000273828.2	0.0135676	0.480374452	0.917866249	RP11-394O2.3
ENSG00000085982.14	0.005476251	0.480410771	0.917866249	USP40
ENSG00000259994.1	-0.011185948	0.480414204	0.917866249	RP11-305E6.4
ENSG00000163040.15	0.033218241	0.480423406	0.917866249	CCDC74A
ENSG00000182180.14	0.004062976	0.480594258	0.917866249	MRPS16
ENSG00000180988.3	-0.049512581	0.480635333	0.917866249	OR52N2
ENSG00000146955.11	-0.012257416	0.480750977	0.917866249	RAB19
ENSG00000205583.13	0.018884397	0.480757766	0.917866249	STAG3L1
ENSG00000260398.1	-0.020846684	0.480798303	0.917866249	RP11-594N15.3
ENSG00000257258.2	0.022500434	0.480801674	0.917866249	RP11-946L16.1
ENSG00000021826.17	-0.025110116	0.480829319	0.917866249	CPS1
ENSG00000175906.5	-0.014283885	0.480864591	0.917866249	ARL4D
ENSG00000100150.20	-0.005040382	0.480886743	0.917866249	DEPDC5
ENSG00000211638.2	-0.033332423	0.480911679	0.917866249	IGLV8-61
ENSG00000248874.5	-0.088436418	0.48095208	0.917866249	C5orf17
ENSG00000083845.9	0.00893146	0.480988328	0.917866249	RPS5
ENSG00000244026.6	0.013778725	0.481004793	0.917866249	FAM86DP
ENSG00000132274.16	-0.012858771	0.481080045	0.917866249	TRIM22
ENSG00000267058.1	0.0255922	0.481083262	0.917866249	RP11-15A1.3
ENSG00000115761.16	0.00408456	0.481093089	0.917866249	NOL10
ENSG00000181830.9	-0.004993521	0.481260853	0.917866249	SLC35C1
ENSG00000230953.2	0.022471601	0.481328141	0.917866249	RP4-631H13.6
ENSG00000175482.9	-0.005903443	0.481333043	0.917866249	POLD4
ENSG00000278922.1	-0.017744668	0.481358089	0.917866249	AC002310.14
ENSG00000152804.11	-0.006095706	0.481365943	0.917866249	HHEX
ENSG00000286750.1	0.035360458	0.481404254	0.917866249	RP11-666A8.14
ENSG00000130638.17	0.003960811	0.481464078	0.917866249	ATXN10
ENSG00000144476.6	-0.007852361	0.481468803	0.917866249	ACKR3
ENSG00000267009.6	-0.011967328	0.481529051	0.917866249	RP11-120M18.2
ENSG00000134013.16	-0.00797678	0.481568118	0.917866249	LOXL2
ENSG00000127838.14	-0.007593285	0.48167499	0.917866249	PNKD
ENSG00000184939.16	-0.006354557	0.481682855	0.917866249	ZFP90
ENSG00000182173.14	-0.007641639	0.48170588	0.917866249	TSEN54
ENSG00000287855.1	-0.014360858	0.481822634	0.917866249	LA16c-360B4.2

ENSG00000172247.4	-0.031011951	0.481847622	0.917866249	C1QTNF4
ENSG00000108468.15	0.004129756	0.481872661	0.917866249	CBX1
ENSG00000187045.19	-0.013342451	0.481886938	0.917866249	TMPRSS6
ENSG00000105492.16	0.019651349	0.481949995	0.917866249	SIGLEC6
ENSG00000113013.16	-0.003255667	0.481980109	0.917866249	HSPA9
ENSG00000153814.13	-0.00673986	0.481984621	0.917866249	JAZF1
ENSG00000225449.3	-0.037655429	0.482001864	0.917866249	RAB6C-AS1
ENSG00000186591.13	0.004011425	0.482051339	0.917866249	UBE2H
ENSG00000279594.1	-0.026274043	0.482089904	0.917866249	RP11-950C14.10
ENSG00000108518.8	-0.007491658	0.48210519	0.917866249	PFN1
ENSG00000213654.10	0.00686939	0.482216547	0.917866249	GPSM3
ENSG00000172766.19	-0.006925365	0.4822375	0.917866249	NAA16
ENSG00000129911.9	-0.007374043	0.482319404	0.917866249	KLF16
ENSG00000100276.10	-0.017621008	0.482441009	0.917866249	RASL10A
ENSG00000211791.2	-0.01485059	0.482476783	0.917866249	TRAV13-2
ENSG00000118804.9	-0.018979157	0.482534145	0.917866249	STBD1
ENSG00000256988.2	0.022798649	0.482538313	0.917866249	RP11-234B24.4
ENSG00000130856.17	-0.007392628	0.482550728	0.917866249	ZNF236
ENSG00000141682.12	-0.013164564	0.482560635	0.917866249	PMAIP1
ENSG00000167525.15	0.007042139	0.482565883	0.917866249	PROCA1
ENSG00000229419.1	0.013799909	0.482623517	0.917866249	RALGAPA1P1
ENSG00000251432.7	-0.012406664	0.482744806	0.917866249	LINC02615
ENSG00000244607.7	0.015906884	0.482807891	0.917866249	CCDC13
ENSG00000171425.10	-0.007398263	0.482857468	0.917866249	ZNF581
ENSG00000174977.8	0.014773525	0.482860739	0.917866249	AC026271.5
ENSG00000235897.1	-0.019941749	0.482925046	0.917866249	TM4SF19-AS1
ENSG00000165810.17	0.031899643	0.482949094	0.917866249	BTNL9
ENSG00000163873.10	0.074322391	0.482957596	0.917866249	GRIK3
ENSG00000174529.7	-0.007745837	0.482957671	0.917866249	TMEM81
ENSG00000130165.10	0.007938894	0.483077059	0.917866249	ELOF1
ENSG00000136518.17	0.003969738	0.483093373	0.917866249	ACTL6A
ENSG00000226822.3	-0.016528864	0.4831348	0.917866249	LINC02785
ENSG00000118473.23	-0.033812129	0.483145306	0.917866249	SGIP1
ENSG00000229180.7	-0.004494119	0.483152494	0.917866249	GS1-124K5.12
ENSG00000147955.18	0.005061578	0.48328418	0.917866249	SIGMAR1
ENSG00000136295.15	-0.007253616	0.483299304	0.917866249	TTYH3
ENSG00000154358.23	0.011795439	0.483328656	0.917866249	OBSCN
ENSG00000136014.12	0.016271873	0.483361884	0.917866249	USP44
ENSG00000273367.1	0.025910828	0.483375437	0.917866249	RP5-827C21.6
ENSG00000164344.16	0.015178559	0.483411389	0.917866249	KLKB1
ENSG00000254788.7	0.014638082	0.483434888	0.917866249	CKLF-CMTM1
ENSG00000138231.13	0.004320317	0.483504054	0.917866249	DBR1
ENSG00000221957.8	-0.039763437	0.483542521	0.917866249	KIR2DS4
ENSG00000255883.1	-0.026032794	0.48357134	0.917866249	FUNDC2P1
ENSG00000162813.18	0.006449916	0.483593955	0.917866249	BPNT1
ENSG00000279583.1	0.018773211	0.483667215	0.917914945	RP11-368N21.5
ENSG00000007968.7	-0.010662857	0.483781006	0.917914945	E2F2

ENSG00000232995.7	0.014093629	0.483985914	0.917914945	RGS5
ENSG00000274561.1	0.008657503	0.484167868	0.917914945	RP11-147L13.12
ENSG00000105552.15	-0.006874747	0.484189679	0.917914945	BCAT2
ENSG00000178078.12	-0.015069534	0.484231667	0.917914945	STAP2
ENSG00000162377.6	0.006390904	0.484281699	0.917914945	COA7
ENSG00000078401.7	-0.013466131	0.484335753	0.917914945	EDN1
ENSG00000139714.12	-0.010584417	0.484435046	0.917914945	MORN3
ENSG00000134873.10	0.036767964	0.484561212	0.917914945	CLDN10
ENSG00000260121.1	0.008740194	0.484575283	0.917914945	RP5-1142A6.9
ENSG00000129197.15	0.005780608	0.484652113	0.917914945	RPAIN
ENSG00000067533.6	-0.006071548	0.484688418	0.917914945	RRP15
ENSG00000136828.19	0.007292773	0.48472122	0.917914945	RALGPS1
ENSG00000143479.18	0.016120911	0.484721894	0.917914945	DYRK3
ENSG00000125378.16	0.035502158	0.484765059	0.917914945	BMP4
ENSG00000166352.18	0.022016258	0.484812616	0.917914945	IFTAP
ENSG00000161509.14	-0.012286364	0.484838975	0.917914945	GRIN2C
ENSG00000223446.2	-0.021087817	0.484840808	0.917914945	RP11-274J16.5
ENSG00000180539.8	0.006471196	0.48484595	0.917914945	C9orf139
ENSG00000185811.19	-0.004093175	0.484898543	0.917914945	IKZF1
ENSG00000136010.14	0.020850174	0.484946516	0.917914945	ALDH1L2
ENSG00000228274.4	-0.010359668	0.484948812	0.917914945	RP3-508I15.9
ENSG00000137478.15	0.004600566	0.484971149	0.917914945	FCHSD2
ENSG00000233098.9	0.033550991	0.484977014	0.917914945	CCDC144NL-AS1
ENSG00000186891.14	-0.017290425	0.485017609	0.917914945	TNFRSF18
ENSG00000103489.12	-0.007152973	0.485099541	0.917914945	XYLT1
ENSG00000234776.5	-0.026537458	0.485139194	0.917914945	C11orf94
ENSG00000184857.8	-0.005122635	0.485141521	0.917914945	TMEM186
ENSG00000103657.14	0.005525363	0.485180265	0.917914945	HERC1
ENSG00000005156.12	-0.004302965	0.485229489	0.917914945	LIG3
ENSG00000178878.13	-0.013112241	0.485245847	0.917914945	APOLD1
ENSG00000124762.14	0.011331387	0.485309874	0.917914945	CDKN1A
ENSG00000218510.9	0.00889696	0.485344965	0.917914945	LINC00339
ENSG00000166747.13	0.003797502	0.485377164	0.917914945	AP1G1
ENSG00000272334.1	0.017266806	0.485394317	0.917914945	RP11-129K12.1
ENSG00000171574.18	0.007833211	0.485402634	0.917914945	ZNF584
ENSG00000036530.9	0.021817554	0.485468556	0.917914945	CYP46A1
ENSG00000259349.2	-0.020865343	0.485472991	0.917914945	RP11-15E18.1
ENSG00000188511.12	-0.008460505	0.485479518	0.917914945	C22orf34
ENSG00000211945.2	-0.024322143	0.485487374	0.917914945	IGHV1-18
ENSG00000133488.15	0.027613661	0.485510263	0.917914945	SEC14L4
ENSG00000279320.1	-0.015466214	0.485544242	0.917914945	RP11-329N15.3
ENSG00000163281.12	0.008589685	0.48574858	0.9182166	GNPDA2
ENSG00000267002.4	-0.004860159	0.485799715	0.918228623	RP11-242D8.1
ENSG00000007520.4	-0.00648624	0.485891186	0.918316877	TSR3
ENSG00000158555.15	0.010091545	0.486148766	0.91838424	GDPD5
ENSG00000013306.16	0.020242864	0.486151161	0.91838424	SLC25A39
ENSG00000172273.13	0.003836071	0.486376828	0.91838424	HINFP

ENSG00000279031.1	0.023496825	0.486519734	0.91838424	LA16c-360H6.1
ENSG00000260588.1	0.018072366	0.486583821	0.91838424	RP11-930P14.2
ENSG00000274021.1	0.008564541	0.486690541	0.91838424	RP11-823E8.3
ENSG00000163703.20	-0.006544979	0.486725763	0.91838424	CRELD1
ENSG00000110958.16	0.004382509	0.486743908	0.91838424	PTGES3
ENSG00000186628.12	0.027770776	0.486750617	0.91838424	FSD2
ENSG00000181019.13	-0.009734242	0.486767346	0.91838424	NQO1
ENSG00000155975.10	-0.004509939	0.486823368	0.91838424	VPS37A
ENSG00000139832.5	0.011728203	0.486830332	0.91838424	RAB20
ENSG00000267287.1	0.024176676	0.486849589	0.91838424	RP11-567M16.1
ENSG00000278876.1	-0.019345224	0.486857878	0.91838424	RP11-498C9.4
ENSG00000155974.13	0.009263906	0.486969893	0.91838424	GRIP1
ENSG00000213204.8	-0.021828227	0.486973828	0.91838424	RP3-382I10.7
ENSG00000232022.7	-0.018310534	0.486999656	0.91838424	FAAHP1
ENSG00000198435.4	0.022555351	0.487034552	0.91838424	NRARP
ENSG00000139163.16	-0.009812656	0.487048076	0.91838424	ETNK1
ENSG00000167664.8	0.014242153	0.487097403	0.91838424	TMIGD2
ENSG00000006607.14	0.007464411	0.487099659	0.91838424	FARP2
ENSG00000120438.12	-0.003190362	0.487100367	0.91838424	TCP1
ENSG00000274034.1	0.013388707	0.487203024	0.91838424	MIR6813
ENSG00000118454.13	-0.00702053	0.487256727	0.91838424	ANKRD13C
ENSG00000211855.1	-0.028563212	0.487266617	0.91838424	TRAJ34
ENSG00000171517.6	-0.030076997	0.487325587	0.91838424	LPAR3
ENSG00000254004.7	0.007549538	0.487368319	0.91838424	ZNF260
ENSG00000247765.2	-0.042665449	0.487415821	0.91838424	RP11-32B5.7
ENSG00000135617.4	0.00777288	0.487569011	0.91838424	PRADC1
ENSG00000184293.7	-0.017684813	0.487581419	0.91838424	CLECL1
ENSG00000249898.8	0.012492091	0.487642694	0.91838424	MCPH1-AS1
ENSG00000112599.9	-0.00869325	0.487648619	0.91838424	GUCA1B
ENSG00000173406.16	0.019823564	0.487667143	0.91838424	DAB1
ENSG00000102531.16	0.008552983	0.487680327	0.91838424	FNDC3A
ENSG00000163629.13	0.014430759	0.487818424	0.91838424	PTPN13
ENSG00000071655.18	-0.007443645	0.487824499	0.91838424	MBD3
ENSG00000122482.21	0.010459004	0.487916018	0.91838424	ZNF644
ENSG00000125245.14	0.007993194	0.48796435	0.91838424	GPR18
ENSG00000198642.7	0.00663473	0.487986619	0.91838424	KLHL9
ENSG00000229106.1	-0.015942196	0.488068833	0.91838424	BTBD6P1
ENSG00000136826.15	-0.0083291	0.48810015	0.91838424	KLF4
ENSG00000196236.13	0.004732232	0.488103027	0.91838424	XPNPEP3
ENSG00000111581.10	0.00486185	0.488110507	0.91838424	NUP107
ENSG00000266786.1	-0.035213102	0.488131044	0.91838424	LGALS9DP
ENSG00000119139.21	-0.01861332	0.488184079	0.91838424	TJP2
ENSG00000184702.20	-0.01676798	0.488324482	0.91838424	SEPTIN5
ENSG00000144182.17	0.007513953	0.4883498	0.91838424	LIPT1
ENSG00000260038.1	0.021539435	0.488417915	0.91838424	RP11-407G23.4
ENSG00000206190.12	-0.007631664	0.488456047	0.91838424	ATP10A
ENSG00000240356.6	0.018254928	0.488502806	0.91838424	RPL23AP7

ENSG00000091831.24	0.012675851	0.488516649	0.91838424	ESR1
ENSG00000148481.14	-0.007763733	0.488529099	0.91838424	MINDY3
ENSG00000273437.1	0.014610174	0.488591274	0.91838424	RP11-434H6.7
ENSG00000138092.11	0.006185386	0.488610496	0.91838424	CENPO
ENSG00000239306.5	-0.004684463	0.488626831	0.91838424	RBM14
ENSG00000115808.12	-0.005096918	0.488692757	0.91838424	STRN
ENSG00000155903.13	-0.008264893	0.488836859	0.91838424	RASA2
ENSG00000137200.13	-0.007780431	0.488873081	0.91838424	CMTR1
ENSG00000141985.9	-0.005174747	0.488887661	0.91838424	SH3GL1
ENSG00000164830.19	0.009837714	0.48891551	0.91838424	OXR1
ENSG00000167670.16	-0.006317939	0.488928766	0.91838424	CHAF1A
ENSG00000119772.18	-0.004740278	0.488989869	0.91838424	DNMT3A
ENSG00000247315.4	-0.00320748	0.489106288	0.91838424	ZCCHC3
ENSG00000273245.1	-0.01684379	0.489139397	0.91838424	RP11-434P11.2
ENSG00000198276.16	-0.004925403	0.489145978	0.91838424	UCKL1
ENSG00000129450.9	0.005755912	0.489152949	0.91838424	SIGLEC9
ENSG00000248049.7	0.006486585	0.489164917	0.91838424	UBA6-AS1
ENSG00000261000.1	-0.021715333	0.489167546	0.91838424	RP11-534L20.5
ENSG00000175691.9	0.008836971	0.489215842	0.91838424	ZNF77
ENSG00000274209.6	0.042156771	0.489295052	0.91838424	ANTXRL
ENSG00000153044.10	0.008844773	0.489369028	0.91838424	CENPH
ENSG00000197943.10	-0.00546283	0.489409242	0.91838424	PLCG2
ENSG00000232295.7	0.016747342	0.489413538	0.91838424	RP11-154D6.1
ENSG00000183248.12	0.029338003	0.489433003	0.91838424	PRR36
ENSG00000269918.1	-0.00862978	0.489499229	0.91838424	AF131215.9
ENSG00000248485.2	-0.021549327	0.489540137	0.91838424	PCP4L1
ENSG00000121413.13	0.009564375	0.489555005	0.91838424	ZSCAN18
ENSG00000182782.8	0.010219062	0.489579867	0.91838424	HCAR2
ENSG00000144485.11	-0.01553923	0.48960217	0.91838424	HES6
ENSG00000175591.12	-0.011483093	0.489611526	0.91838424	P2RY2
ENSG00000241962.9	-0.018675103	0.489636508	0.91838424	RP11-111H13.1
ENSG00000170889.14	0.008622813	0.489718081	0.91838424	RPS9
ENSG00000198680.6	0.019783182	0.489732102	0.91838424	TUSC1
ENSG00000235351.1	-0.015572618	0.489740622	0.91838424	AC114730.11
ENSG00000112486.17	0.014941439	0.489822159	0.91838424	CCR6
ENSG00000172007.7	0.00976607	0.489824021	0.91838424	RAB33B
ENSG00000111737.12	-0.004341421	0.489854845	0.91838424	RAB35
ENSG00000064787.13	-0.013750227	0.489954495	0.91838424	BCAS1
ENSG00000163823.4	-0.011550358	0.489981047	0.91838424	CCR1
ENSG00000247287.3	-0.007244452	0.489989419	0.91838424	RP11-902B17.1
ENSG00000196353.12	-0.037536714	0.490001968	0.91838424	CPNE4
ENSG00000196159.14	-0.026861356	0.490069548	0.918426967	FAT4
ENSG00000013573.17	-0.023834658	0.490136049	0.918467662	DDX11
ENSG00000228544.1	0.010846906	0.490212045	0.918526142	CCDC183-AS1
ENSG00000228956.9	0.0100623	0.490296831	0.918551884	SATB1-AS1
ENSG00000163389.12	-0.005273147	0.490360419	0.918551884	POGLUT1
ENSG00000132664.12	-0.006139199	0.490403126	0.918551884	POLR3F

ENSG00000159788.19	-0.007963355	0.490497	0.918551884	RGS12
ENSG00000140450.9	0.013620272	0.490523251	0.918551884	ARRDC4
ENSG00000242477.1	-0.021086983	0.490570332	0.918551884	CTD-2161E19.1
ENSG00000216895.9	-0.007715638	0.490619117	0.918551884	AC009403.2
ENSG00000198185.11	-0.02931754	0.490652159	0.918551884	ZNF334
ENSG00000175283.8	-0.005076022	0.490695959	0.918551884	DOLK
ENSG00000101276.18	0.051489544	0.490830615	0.918551884	SLC52A3
ENSG00000102804.15	0.008079457	0.490836046	0.918551884	TSC22D1
ENSG00000164062.13	0.004327069	0.490851763	0.918551884	APEH
ENSG00000247934.4	-0.018311065	0.49086953	0.918551884	RP11-967K21.1
ENSG00000280294.1	-0.02715867	0.49091784	0.918551884	RP11-177H2.1
ENSG00000267871.6	0.011698914	0.490941562	0.918551884	ZNF460-AS1
ENSG00000065600.13	0.005441832	0.490942423	0.918551884	PACC1
ENSG00000172215.6	0.017225601	0.491124859	0.918735114	CXCR6
ENSG00000100614.18	0.007297664	0.491129952	0.918735114	PPM1A
ENSG00000150456.11	0.009075017	0.49121125	0.918803385	EEF1AKMT1
ENSG00000271889.1	-0.019738723	0.49142986	0.919092856	RP11-493E12.1
ENSG00000144554.13	0.005633662	0.49145564	0.919092856	FANCD2
ENSG00000168496.4	-0.006170036	0.491560987	0.919143475	FEN1
ENSG00000285596.1	-0.014484248	0.491600669	0.919143475	RP5-1022I14.2
ENSG00000177963.15	-0.004282135	0.491617163	0.919143475	RIC8A
ENSG00000137824.16	-0.004644208	0.491694127	0.91920357	RMDN3
ENSG00000285444.1	0.02546942	0.491974592	0.919644055	RP11-147H23.3
ENSG00000183426.17	0.014773917	0.492068869	0.919673533	NPIPA1
ENSG00000170074.19	0.017814535	0.492089326	0.919673533	FAM153A
ENSG00000268947.1	0.011513205	0.49217088	0.919673533	AD000684.2
ENSG00000198168.9	0.009917477	0.492182529	0.919673533	SVIP
ENSG00000277117.5	-0.027957634	0.492260088	0.919673533	CH507-9B2.1
ENSG00000229539.1	0.019270848	0.492280115	0.919673533	PANK2-AS1
ENSG00000156738.18	-0.017208009	0.492340135	0.919673533	MS4A1
ENSG00000285215.2	0.027195233	0.492393501	0.919673533	CH17-118O6.7
ENSG00000157404.16	-0.013361671	0.492415299	0.919673533	KIT
ENSG00000134575.13	-0.006977661	0.492511685	0.919673533	ACP2
ENSG00000131871.15	0.005380028	0.492539801	0.919673533	SELENOS
ENSG00000137817.17	0.003203656	0.492620699	0.919673533	PARP6
ENSG00000259577.2	0.015235571	0.492630197	0.919673533	CERNA1
ENSG00000039319.17	0.010506599	0.492632307	0.919673533	ZFYVE16
ENSG00000178381.12	0.008342013	0.492693892	0.919673533	ZFAND2A
ENSG00000230113.1	-0.02320372	0.492748708	0.919673533	AC091177.1
ENSG00000151726.15	0.009496041	0.49275272	0.919673533	ACSL1
ENSG00000117143.13	0.006457724	0.492901175	0.919778934	UAP1
ENSG00000090013.11	0.013652751	0.492968296	0.919778934	BLVRB
ENSG00000167615.17	-0.005842209	0.493072851	0.919778934	LENG8
ENSG00000103269.14	-0.013487057	0.493074373	0.919778934	RHBDL1
ENSG00000154429.11	0.008672434	0.493097164	0.919778934	CCSAP
ENSG00000136141.15	-0.005722579	0.493113888	0.919778934	LRCH1
ENSG00000137185.13	-0.007076874	0.493123141	0.919778934	ZSCAN9

ENSG00000127720.8	0.005872536	0.493202	0.919792739	METTL25
ENSG00000273784.5	0.017105792	0.493230516	0.919792739	RP11-78J21.7
ENSG00000120738.8	0.01329487	0.493265094	0.919792739	EGR1
ENSG00000158161.16	0.004781982	0.493387342	0.91993705	EYA3
ENSG00000245970.2	-0.008557393	0.493538099	0.919953943	KB-1208A12.3
ENSG00000187534.6	-0.008202979	0.493698619	0.919953943	PRR13P5
ENSG00000228903.7	-0.008655313	0.493708604	0.919953943	RASA4CP
ENSG00000198825.15	0.008020434	0.493710773	0.919953943	INPP5F
ENSG00000086475.15	-0.00339997	0.493726814	0.919953943	SEPHS1
ENSG00000112077.17	0.030819187	0.493753237	0.919953943	RHAG
ENSG00000245614.4	-0.025920957	0.493772158	0.919953943	DDX11-AS1
ENSG00000137441.8	-0.016802001	0.493790499	0.919953943	FGFBP2
ENSG00000147576.17	-0.008791252	0.493800127	0.919953943	ADHFE1
ENSG00000137601.17	0.008322949	0.493909092	0.920073364	NEK1
ENSG00000254017.1	0.025186241	0.494030189	0.920080215	IGHEP2
ENSG00000276488.1	0.011373795	0.494038284	0.920080215	CTD-2537I9.18
ENSG00000125841.13	-0.011161904	0.494047363	0.920080215	NRSN2
ENSG00000273687.1	0.017496523	0.494308267	0.920427771	RP5-837J1.6
ENSG00000147485.13	0.02196452	0.494353728	0.920427771	PXDNL
ENSG00000244490.1	-0.025352713	0.494399823	0.920427771	RWDD4P1
ENSG00000123983.15	0.0049254	0.494423213	0.920427771	ACSL3
ENSG00000117625.14	-0.005716009	0.494458394	0.920427771	RCOR3
ENSG00000100577.19	0.008099493	0.494542379	0.920500554	GSTZ1
ENSG00000056736.10	-0.010401752	0.494591741	0.920508887	IL17RB
ENSG00000183579.16	0.013826138	0.494667441	0.920566233	ZNRF3
ENSG00000172932.16	-0.004752542	0.494976713	0.920939047	ANKRD13D
ENSG00000141570.11	-0.007174507	0.494994119	0.920939047	CBX8
ENSG00000083544.16	-0.004228607	0.495002492	0.920939047	TDRD3
ENSG00000086504.18	-0.006668972	0.495071692	0.920984239	MRPL28
ENSG00000166736.12	-0.022511999	0.495255602	0.921103762	HTR3A
ENSG00000286696.1	0.023655739	0.495263946	0.921103762	CTD-2311M21.6
ENSG00000136842.14	0.022895407	0.495295158	0.921103762	TMOD1
ENSG00000163694.15	0.007554623	0.495351475	0.921103762	RBM47
ENSG00000164175.15	-0.041096668	0.495499761	0.921103762	SLC45A2
ENSG00000261548.1	-0.0366437	0.495622654	0.921103762	HLA-P
ENSG00000229334.1	-0.019935145	0.495681925	0.921103762	AC046143.3
ENSG00000213762.12	0.004313149	0.495693575	0.921103762	ZNF134
ENSG00000164070.12	0.029478698	0.495728178	0.921103762	HSPA4L
ENSG00000263574.2	-0.028425474	0.495761012	0.921103762	CTD-2532D12.4
ENSG00000273306.1	-0.015429043	0.495784816	0.921103762	RP11-527J8.1
ENSG00000178445.10	0.032877328	0.495824949	0.921103762	GLDC
ENSG00000203697.12	-0.017420529	0.495839471	0.921103762	CAPN8
ENSG00000216775.3	0.02391919	0.495924054	0.921103762	RP1-152L7.5
ENSG00000169118.18	0.004509368	0.495955325	0.921103762	CSNK1G1
ENSG00000233266.1	0.023039375	0.496018153	0.921103762	HMGB1P31
ENSG00000067141.17	-0.010848341	0.496035609	0.921103762	NEO1
ENSG00000237667.7	0.07078454	0.496039001	0.921103762	LINC01115

ENSG00000198833.7	0.007521724	0.496044778	0.921103762	UBE2J1
ENSG00000125657.5	-0.019168623	0.496078294	0.921103762	TNFSF9
ENSG00000149716.13	-0.003666098	0.496192558	0.921103762	LTO1
ENSG00000099974.8	-0.006911677	0.496200418	0.921103762	DDTL
ENSG00000176125.7	-0.015107985	0.496204929	0.921103762	UFSP1
ENSG00000087253.13	0.007264519	0.496234493	0.921103762	LPCAT2
ENSG00000229598.1	-0.015799089	0.496274845	0.921103762	PRDX3P1
ENSG00000183196.10	0.047750028	0.496306323	0.921103762	CHST6
ENSG00000168056.17	0.011107176	0.496348628	0.921103762	LTBP3
ENSG00000156795.8	0.008151172	0.496426827	0.921113878	NTAQ1
ENSG00000263843.1	-0.012692534	0.496443909	0.921113878	MIF4GD-DT
ENSG00000113249.13	-0.021116558	0.496610416	0.921199664	HAVCR1
ENSG00000078687.17	-0.005824779	0.496629987	0.921199664	TNRC6C
ENSG00000279407.1	0.011613048	0.496676237	0.921199664	AC007191.4
ENSG00000231621.1	0.020480481	0.496775444	0.921199664	ANKRD44-AS1
ENSG00000233276.8	0.012454137	0.496818756	0.921199664	GPX1
ENSG00000255899.1	-0.030696791	0.496837527	0.921199664	XXbac-BPG299F13.16
ENSG00000227719.1	-0.01497358	0.496875548	0.921199664	AC006042.6
ENSG00000243433.1	-0.019320628	0.496881513	0.921199664	RP11-148K1.10
ENSG00000162148.11	-0.008131624	0.496894416	0.921199664	PPP1R32
ENSG00000006468.14	0.032692138	0.496950541	0.921220438	ETV1
ENSG00000137074.20	0.004011699	0.497047342	0.9213082	APTX
ENSG00000211696.2	-0.023842968	0.497125241	0.9213082	TRGV8
ENSG00000167377.18	-0.016647371	0.497132657	0.9213082	ZNF23
ENSG00000256751.6	0.009872401	0.497297209	0.921357412	PLBD1-AS1
ENSG00000196810.5	-0.005854893	0.497353092	0.921357412	CTBP1-DT
ENSG00000149269.10	-0.005411292	0.497434084	0.921357412	PAK1
ENSG00000160505.16	0.049438516	0.49743921	0.921357412	NLRP4
ENSG00000156097.12	0.015713838	0.497499086	0.921357412	GPR61
ENSG00000100416.15	-0.004630144	0.497560803	0.921357412	TRMU
ENSG00000230773.9	-0.017985173	0.497570341	0.921357412	AC079807.4
ENSG00000134987.12	-0.006296862	0.497598535	0.921357412	WDR36
ENSG00000283138.1	-0.028889011	0.49766398	0.921357412	RP3-488M23.3
ENSG00000228672.4	-0.00999101	0.497675599	0.921357412	PROB1
ENSG00000163600.13	0.009539984	0.497685714	0.921357412	ICOS
ENSG00000280434.1	-0.014398616	0.497773755	0.921357412	RP4-671O14.6
ENSG00000140694.17	-0.003564861	0.4977986	0.921357412	PARN
ENSG00000099284.15	-0.007399287	0.497835406	0.921357412	MACROH2A2
ENSG00000148468.17	-0.010512106	0.497865387	0.921357412	FAM171A1
ENSG00000137807.16	-0.01310924	0.49787804	0.921357412	KIF23
ENSG00000204977.10	0.004744927	0.497937285	0.921383906	TRIM13
ENSG00000176809.11	0.010342354	0.498065951	0.92150307	LRRC37A3
ENSG00000282051.1	0.022609793	0.498091551	0.92150307	LLNLF-173C4.1
ENSG00000287597.1	0.033090487	0.498161203	0.921548797	RP11-209B12.1
ENSG00000159228.13	0.007716138	0.498252328	0.921559161	CBR1
ENSG00000153574.9	0.005387624	0.498267609	0.921559161	RPIA
ENSG00000287576.1	0.027745757	0.498400894	0.921559161	RP11-661A12.15

ENSG00000186446.12	-0.011215069	0.498428865	0.921559161	ZNF501
ENSG00000237803.6	0.013028603	0.49849543	0.921559161	LINC00211
ENSG00000073060.17	0.008108139	0.498702928	0.921559161	SCARB1
ENSG00000250722.6	-0.017273383	0.49875586	0.921559161	SELENOP
ENSG00000136238.18	0.00504164	0.498767711	0.921559161	RAC1
ENSG00000178093.14	-0.008273635	0.498775489	0.921559161	TSSK6
ENSG00000253729.8	-0.00683734	0.498778761	0.921559161	PRKDC
ENSG00000257052.1	0.018533228	0.498814192	0.921559161	RP11-881M11.2
ENSG00000271425.9	0.008511317	0.498889721	0.921559161	NBPF10
ENSG00000119457.8	-0.01037459	0.498897254	0.921559161	SLC46A2
ENSG00000237914.5	0.01755712	0.498926766	0.921559161	SIRPG-AS1
ENSG00000132383.12	-0.004281747	0.498969067	0.921559161	RPA1
ENSG00000263941.2	-0.01873805	0.498980921	0.921559161	RN7SL32P
ENSG00000151490.15	0.01100096	0.498985302	0.921559161	PTPRO
ENSG00000168229.4	-0.012152841	0.49901006	0.921559161	PTGDR
ENSG00000226742.4	-0.009869789	0.499020601	0.921559161	HSBP1L1
ENSG00000159685.11	0.008980289	0.499091511	0.921607124	CHCHD6
ENSG00000053770.12	0.00585778	0.499217249	0.921737042	AP5M1
ENSG00000271780.1	0.012503514	0.499348191	0.921737042	RP11-1017G21.5
ENSG00000161180.11	0.019629527	0.499357575	0.921737042	CCDC116
ENSG00000118495.20	0.009413975	0.49940949	0.921737042	PLAGL1
ENSG00000142185.17	-0.008028815	0.499441983	0.921737042	TRPM2
ENSG00000227507.3	-0.008867837	0.499539202	0.921737042	LTB
ENSG00000165028.12	-0.01244309	0.499540354	0.921737042	NIPSNAP3B
ENSG00000185818.8	-0.022560329	0.499553797	0.921737042	NAT8L
ENSG00000082397.18	0.009943871	0.499566375	0.921737042	EPB41L3
ENSG00000198169.9	-0.005072448	0.499741718	0.921757586	ZNF251
ENSG00000128944.13	-0.00796745	0.499795907	0.921757586	KNSTRN
ENSG00000198954.9	0.004239181	0.499847254	0.921757586	KIFBP
ENSG00000177034.17	-0.007413375	0.49990717	0.921757586	MTX3
ENSG00000101246.20	-0.00586713	0.499912645	0.921757586	ARFRP1
ENSG00000267586.7	-0.023470667	0.499965173	0.921757586	LINC00907
ENSG00000286999.1	-0.01455928	0.499985119	0.921757586	RP5-1022E24.7
ENSG00000128482.16	-0.013141555	0.499990014	0.921757586	RNF112
ENSG00000267605.7	0.049568176	0.500019797	0.921757586	CTD-3220F14.1
ENSG00000268081.1	0.030281498	0.500026972	0.921757586	RP11-678G14.2
ENSG00000166068.13	-0.011238031	0.500095979	0.921801935	SPRED1
ENSG00000167173.19	-0.007400347	0.50035156	0.922055079	C15orf39
ENSG00000167658.16	0.005425313	0.50041855	0.922055079	EEF2
ENSG00000114735.10	0.004406175	0.500450745	0.922055079	HEMK1
ENSG00000149150.9	-0.00630767	0.500534217	0.922055079	SLC43A1
ENSG00000230492.1	0.024195214	0.50062569	0.922055079	RP4-753D10.5
ENSG00000272072.1	0.015889839	0.500650798	0.922055079	CTA-363E19.2
ENSG00000249138.1	-0.030656439	0.500703135	0.922055079	SLED1
ENSG00000127578.7	-0.017480677	0.500703907	0.922055079	WFIKKN1
ENSG00000286024.1	-0.024700231	0.500720498	0.922055079	CTD-2639E6.11
ENSG00000177600.9	-0.009093189	0.500736282	0.922055079	RPLP2

ENSG00000124702.18	0.004937319	0.500740476	0.922055079	KLHDC3
ENSG00000196205.8	0.011128125	0.50080395	0.922055079	EEF1A1P5
ENSG00000241556.1	0.021645588	0.500817804	0.922055079	RP11-490G8.1
ENSG00000228340.6	0.009550551	0.500940493	0.922172802	MIR646HG
ENSG00000009780.16	0.004572249	0.500971679	0.922172802	FAM76A
ENSG00000161267.12	-0.006908964	0.501154542	0.922176087	BDH1
ENSG00000177951.17	0.004976837	0.501167797	0.922176087	BET1L
ENSG00000136942.15	0.010440814	0.501203307	0.922176087	RPL35
ENSG00000196712.18	0.005813227	0.501215705	0.922176087	NF1
ENSG00000272817.1	0.026050908	0.50121791	0.922176087	RP11-402D21.2
ENSG00000058272.19	0.008825988	0.501245823	0.922176087	PPP1R12A
ENSG00000230082.1	0.019941336	0.501305305	0.922176087	PRRT3-AS1
ENSG00000213676.13	-0.004097745	0.501406446	0.922176087	ATF6B
ENSG00000182600.9	-0.013104214	0.501423762	0.922176087	SNORC
ENSG00000140153.18	-0.003239265	0.501487037	0.922176087	WDR20
ENSG00000272829.1	-0.021751816	0.501500346	0.922176087	XXbac-B135H6.18
ENSG00000095380.11	-0.004952976	0.501513063	0.922176087	NANS
ENSG00000230002.3	-0.018205714	0.501614409	0.922223823	ALMS1-IT1
ENSG00000064012.22	0.005350833	0.501636223	0.922223823	CASP8
ENSG00000133069.17	0.018557479	0.501673931	0.922223823	TMCC2
ENSG00000068305.17	0.00718571	0.501765739	0.922309919	MEF2A
ENSG00000186205.13	-0.009578846	0.501921892	0.92244068	MTARC1
ENSG00000213903.9	-0.005743131	0.501926836	0.92244068	LTB4R
ENSG00000170909.13	-0.008780323	0.502030947	0.922541306	OSCAR
ENSG00000259097.2	0.032239552	0.502071558	0.922541306	RP11-61O1.1
ENSG00000119411.11	-0.016156607	0.502126731	0.922560025	BSPRY
ENSG00000092208.19	-0.010189945	0.502204564	0.92262037	GEMIN2
ENSG00000271366.1	-0.023801366	0.502312929	0.922736792	AC002128.5
ENSG00000280254.1	0.010420986	0.502455519	0.922820231	RP11-81A22.4
ENSG00000197594.14	0.022200153	0.50255277	0.922820231	ENPP1
ENSG00000181788.4	0.009849438	0.502581941	0.922820231	SIAH2
ENSG00000162772.17	0.019481115	0.50259297	0.922820231	ATF3
ENSG00000076356.7	-0.010633655	0.502600102	0.922820231	PLXNA2
ENSG00000113763.12	0.013847579	0.502628339	0.922820231	UNC5A
ENSG00000242887.1	0.024920924	0.502754874	0.922932881	IGHJ3
ENSG00000228878.8	0.012330928	0.50292438	0.922932881	SEPTIN7-DT
ENSG00000110042.8	-0.009530124	0.502928223	0.922932881	DTX4
ENSG00000287216.1	-0.014378685	0.502948735	0.922932881	RP11-153M7.6
ENSG00000109501.15	-0.008479676	0.503009172	0.922932881	WFS1
ENSG00000287736.1	0.019880426	0.503064619	0.922932881	PTCSC1
ENSG00000275895.7	-0.048384756	0.503077954	0.922932881	U2AF1L5
ENSG00000186831.11	-0.057597433	0.50318509	0.922932881	KRT17P2
ENSG00000154114.13	0.006568409	0.503211659	0.922932881	TBCEL
ENSG00000158815.11	-0.024806684	0.503310186	0.922932881	FGF17
ENSG00000100450.13	-0.022084557	0.503344572	0.922932881	GZMH
ENSG00000268047.1	0.01712241	0.503394053	0.922932881	AC018766.6
ENSG00000230709.1	-0.046340737	0.503428226	0.922932881	AC104024.1

ENSG00000151014.6	-0.008051275	0.503453766	0.922932881	NOCT
ENSG00000267244.5	-0.009202253	0.503455324	0.922932881	CTB-31O20.4
ENSG00000185808.14	0.007890852	0.50347673	0.922932881	PIGP
ENSG00000239265.5	-0.045604785	0.503480578	0.922932881	CLRN1-AS1
ENSG00000077238.14	0.008843342	0.503629885	0.922932881	IL4R
ENSG00000175746.6	0.023425654	0.503654891	0.922932881	C15orf54
ENSG00000100139.14	-0.005979416	0.503698745	0.922932881	MICALL1
ENSG00000135045.7	-0.007665832	0.503731593	0.922932881	C9orf40
ENSG00000073921.18	0.004642126	0.503782635	0.922932881	PICALM
ENSG00000239883.8	0.013489916	0.503817336	0.922932881	PARGP1
ENSG00000204149.14	0.012423558	0.503836995	0.922932881	AGAP6
ENSG00000254503.1	-0.011848945	0.503837526	0.922932881	CTD-2521M24.4
ENSG00000142534.7	0.007140221	0.503859788	0.922932881	RPS11
ENSG00000005194.15	0.004243556	0.504004936	0.923032561	CIAPIN1
ENSG00000077616.11	0.01440368	0.504096917	0.923032561	NAALAD2
ENSG00000182158.15	0.005774424	0.504111706	0.923032561	CREB3L2
ENSG00000163625.16	0.008162244	0.504142072	0.923032561	WDFY3
ENSG00000161149.12	-0.018300342	0.504179551	0.923032561	TUBA3FP
ENSG00000274386.5	0.013287647	0.504184257	0.923032561	TMEM269
ENSG00000251287.8	0.02161487	0.504264319	0.923096729	ALG1L2
ENSG00000010610.10	-0.007013989	0.504317034	0.92311083	CD4
ENSG00000162385.11	-0.007260331	0.504469684	0.923240407	MAGOH
ENSG00000286532.1	-0.010191115	0.504500574	0.923240407	PARTICL
ENSG00000204463.13	-0.007464247	0.504586319	0.923240407	BAG6
ENSG00000129673.10	0.016048327	0.504598388	0.923240407	AANAT
ENSG00000000938.13	0.005082901	0.504612918	0.923240407	FGR
ENSG00000134109.11	0.003904982	0.504744472	0.923355265	EDEM1
ENSG00000232104.3	0.014688925	0.504767821	0.923355265	RFX3-AS1
ENSG00000219222.1	-0.023195378	0.504912109	0.923355265	RPL12P47
ENSG00000206561.14	0.014096743	0.504970241	0.923355265	COLQ
ENSG00000180425.11	-0.00628447	0.50501878	0.923355265	C11orf71
ENSG00000247556.6	-0.004681051	0.505037301	0.923355265	OIP5-AS1
ENSG00000223313.1	0.022294537	0.505039314	0.923355265	RNU6-516P
ENSG00000248923.1	0.031131591	0.505049436	0.923355265	MTND5P11
ENSG00000227487.4	-0.026460073	0.505101435	0.923355265	NCAM1-AS1
ENSG00000131149.19	-0.00634488	0.505177354	0.923355265	GSE1
ENSG00000228624.7	0.018223442	0.505189855	0.923355265	HDAC2-AS2
ENSG00000238829.1	-0.021073457	0.505215985	0.923355265	RNU7-45P
ENSG00000279696.1	0.008016256	0.50534313	0.923440641	RP11-178H8.7
ENSG00000151532.14	0.003461662	0.505352756	0.923440641	VTI1A
ENSG00000246575.2	0.010474482	0.505440824	0.923463736	AC093162.5
ENSG00000271361.1	0.01770914	0.505470055	0.923463736	HTATSF1P2
ENSG00000270562.1	0.009414449	0.505500483	0.923463736	RP11-154H23.3
ENSG00000110066.15	0.003336493	0.505628331	0.923554264	KMT5B
ENSG00000275491.1	-0.025095837	0.505817661	0.923554264	LINC01730
ENSG00000252274.1	0.020428599	0.505836786	0.923554264	ENSG10010139303.1
ENSG00000115687.14	0.009768482	0.505838767	0.923554264	PASK

ENSG00000217643.1	0.020460146	0.505966331	0.923554264	PTGES3P2
ENSG00000179021.10	0.006944966	0.505973584	0.923554264	C3orf38
ENSG00000287958.1	-0.017684481	0.505984911	0.923554264	RP11-331G2.11
ENSG00000152463.15	-0.030662248	0.506009254	0.923554264	OLAH
ENSG00000030110.13	0.008007371	0.506014689	0.923554264	BAK1
ENSG00000064933.18	0.007426371	0.506109965	0.923554264	PMS1
ENSG00000123453.18	0.014641101	0.506129453	0.923554264	SARDH
ENSG00000131469.15	0.012481078	0.506224864	0.923554264	RPL27
ENSG00000270031.1	0.019596217	0.506232069	0.923554264	RP3-426I6.6
ENSG00000108588.14	0.00413463	0.506265844	0.923554264	CCDC47
ENSG00000071189.21	0.008352935	0.506281845	0.923554264	SNX13
ENSG00000251369.9	-0.0089436	0.506321797	0.923554264	ZNF550
ENSG00000242258.1	0.01291845	0.506356611	0.923554264	LINC00996
ENSG00000140543.15	0.008795184	0.506501221	0.923554264	DET1
ENSG00000032219.19	0.008012495	0.506507012	0.923554264	ARID4A
ENSG00000279491.1	-0.014147849	0.506509904	0.923554264	RP11-810P12.7
ENSG00000253590.1	-0.034374539	0.506514114	0.923554264	IGLV3-13
ENSG00000152443.13	-0.006392488	0.506569767	0.923554264	ZNF776
ENSG00000276597.1	-0.028134656	0.506644248	0.923554264	TRBV11-3
ENSG00000187566.6	0.012059459	0.506649847	0.923554264	NHLRC1
ENSG00000280543.1	-0.023500953	0.50671949	0.923554264	ASAP1-IT2
ENSG00000243554.1	-0.017751938	0.506770084	0.923554264	AC004967.7
ENSG00000113194.13	0.002981473	0.506792071	0.923554264	FAF2
ENSG00000114796.16	0.004888815	0.506810986	0.923554264	KLHL24
ENSG00000231587.1	0.017084209	0.507001298	0.923712056	SNORD62B
ENSG00000269397.1	-0.024572416	0.507002355	0.923712056	CTB-92J24.2
ENSG00000074527.13	0.026076951	0.507048647	0.923712056	NTN4
ENSG00000104915.15	-0.006005911	0.507077741	0.923712056	STX10
ENSG00000204084.13	-0.006218903	0.507185665	0.923786443	INPP5B
ENSG00000147316.14	-0.003802392	0.507208667	0.923786443	MCPH1
ENSG00000259514.1	-0.019661779	0.507371117	0.924000254	RP11-685G9.2
ENSG00000139182.15	-0.008467075	0.507533021	0.924135331	CLSTN3
ENSG00000119669.5	-0.007398644	0.507562771	0.924135331	IRF2BPL
ENSG00000118816.11	0.005155801	0.507640461	0.924135331	CCNI
ENSG00000234797.5	0.022460423	0.50766845	0.924135331	RPS3AP6
ENSG00000126247.11	-0.006842196	0.507683248	0.924135331	CAPNS1
ENSG00000182919.15	0.00663805	0.507715661	0.924135331	C11orf54
ENSG00000226055.2	0.017195216	0.50787565	0.924285396	PAICSP1
ENSG00000167191.12	-0.022997852	0.507923553	0.924285396	GPRC5B
ENSG00000185880.13	0.006970576	0.507972001	0.924285396	TRIM69
ENSG00000172236.18	-0.035299772	0.508035074	0.924285396	TPSAB1
ENSG00000168827.15	-0.004119133	0.50807332	0.924285396	GFM1
ENSG00000249926.2	-0.047010248	0.508113436	0.924285396	RP11-495K9.6
ENSG00000255983.1	-0.131921868	0.508137446	0.924285396	RP11-1038A11.1
ENSG00000225969.2	0.024899669	0.508175808	0.924285396	ABHD11-AS1
ENSG00000139574.9	-0.009136774	0.508211641	0.924285396	NPFF
ENSG00000128408.9	-0.027287907	0.508304005	0.924285396	RIBC2

ENSG00000064687.13	-0.009364693	0.508325209	0.924285396	ABCA7
ENSG00000205572.10	0.017367259	0.50833894	0.924285396	SERF1B
ENSG00000155363.18	-0.009335692	0.508415626	0.924314677	MOV10
ENSG00000119508.18	0.022277354	0.508445186	0.924314677	NR4A3
ENSG00000101104.13	0.005097378	0.508536781	0.924344159	PABPC1L
ENSG00000263011.1	0.014744257	0.508558957	0.924344159	RP11-473M20.11
ENSG00000260083.2	0.008698916	0.50859662	0.924344159	MIR762HG
ENSG00000204519.11	0.007132162	0.508652079	0.924363033	ZNF551
ENSG00000179456.10	0.005938647	0.508808316	0.924401274	ZBTB18
ENSG00000223518.5	0.068154342	0.508914678	0.924401274	CSNK1A1P1
ENSG00000175274.19	0.008431242	0.508965123	0.924401274	TP53I11
ENSG00000105197.11	-0.004974688	0.50905752	0.924401274	TIMM50
ENSG00000110723.12	-0.015081094	0.509095488	0.924401274	EXPH5
ENSG00000196724.13	-0.014060813	0.50920573	0.924401274	ZNF418
ENSG00000196696.13	-0.010538259	0.509221027	0.924401274	PDXDC2P-NPIPB14P
ENSG00000114698.15	-0.035759975	0.509286088	0.924401274	PLSCR4
ENSG00000231453.1	0.031942238	0.509320629	0.924401274	LINC01305
ENSG00000091129.21	0.027668985	0.509345992	0.924401274	NRCAM
ENSG00000215148.11	-0.023698672	0.509402935	0.924401274	PRSS41
ENSG00000258738.1	-0.008153091	0.509410469	0.924401274	RP11-73E17.2
ENSG00000166225.9	0.006428583	0.509427419	0.924401274	FRS2
ENSG00000100796.18	-0.003533392	0.509539651	0.924401274	PPP4R3A
ENSG00000076685.19	0.008455427	0.509567288	0.924401274	NT5C2
ENSG00000169752.17	-0.020132619	0.509573749	0.924401274	NRG4
ENSG00000233672.9	0.019043405	0.509580627	0.924401274	RNASEH2B-AS1
ENSG00000231125.2	-0.012971235	0.509611704	0.924401274	AF129075.5
ENSG00000044574.9	-0.005850996	0.509615799	0.924401274	HSPA5
ENSG00000265148.6	-0.004983788	0.509689615	0.924401274	TSPOAP1-AS1
ENSG00000261448.1	-0.018377704	0.509689868	0.924401274	CTD-2576D5.4
ENSG00000018510.18	0.005651211	0.509699016	0.924401274	AGPS
ENSG00000123104.12	0.006454418	0.509810572	0.924401274	ITPR2
ENSG00000237550.5	0.02808829	0.509849453	0.924401274	RPL9P9
ENSG00000164985.15	0.005220411	0.509884569	0.924401274	PSIP1
ENSG00000223374.1	0.013584676	0.509935306	0.924401274	AC005104.3
ENSG00000100281.14	0.004859283	0.509984898	0.924401274	HMGXB4
ENSG00000152484.14	0.006971243	0.510045521	0.924401274	USP12
ENSG00000262147.1	-0.021368311	0.51012503	0.924401274	RP13-638C3.3
ENSG00000111452.13	0.011771086	0.510209368	0.924401274	ADGRD1
ENSG00000197563.11	0.004907953	0.510217361	0.924401274	PIGN
ENSG00000283317.1	-0.01743381	0.510228918	0.924401274	RP11-122G18.11
ENSG00000237094.12	0.013726727	0.510236769	0.924401274	RP4-669L17.4
ENSG00000142230.13	-0.00399227	0.510301343	0.924401274	SAE1
ENSG00000100077.15	0.005530928	0.510414973	0.924401274	GRK3
ENSG00000241269.1	-0.014866393	0.510692429	0.924401274	AC093620.5
ENSG00000121775.18	-0.005218189	0.510708657	0.924401274	TMEM39B
ENSG00000179335.20	0.003072369	0.510730716	0.924401274	CLK3
ENSG00000274220.1	-0.016351061	0.510754096	0.924401274	RP11-77K12.9

ENSG00000278970.2	0.006495612	0.510816683	0.924401274	HEIH
ENSG00000182197.13	0.006336817	0.510843479	0.924401274	EXT1
ENSG00000088280.19	0.01322423	0.510851297	0.924401274	ASAP3
ENSG00000258056.2	0.007405919	0.511060736	0.924401274	RP11-644F5.11
ENSG00000133789.15	-0.00854529	0.51112736	0.924401274	SWAP70
ENSG00000285162.1	0.037724163	0.511158415	0.924401274	RP5-1189D6.2
ENSG00000174669.12	-0.007621636	0.51121228	0.924401274	SLC29A2
ENSG00000143774.17	0.013400115	0.511418832	0.924401274	GUK1
ENSG00000159176.14	0.004556487	0.511429778	0.924401274	CSRP1
ENSG00000106615.10	0.005100816	0.511438838	0.924401274	RHEB
ENSG00000196428.12	-0.006053584	0.511537029	0.924401274	TSC22D2
ENSG00000128596.17	0.007780195	0.511584145	0.924401274	CCDC136
ENSG00000162522.11	0.010961448	0.511632358	0.924401274	KIAA1522
ENSG00000260549.2	-0.03315439	0.511636484	0.924401274	MT1L
ENSG00000101199.13	-0.004455408	0.511648761	0.924401274	ARFGAP1
ENSG00000237973.1	0.011670725	0.511653607	0.924401274	MTCO1P12
ENSG00000005471.19	-0.017799217	0.511759695	0.924401274	ABCB4
ENSG00000280433.1	-0.010384887	0.51180246	0.924401274	CH507-9B2.9
ENSG00000120696.9	0.008686781	0.511837002	0.924401274	KBTBD7
ENSG00000077157.22	0.008555446	0.511858161	0.924401274	PPP1R12B
ENSG00000105568.18	-0.005572267	0.511861526	0.924401274	PPP2R1A
ENSG00000213221.5	-0.0161876	0.51187612	0.924401274	DNLZ
ENSG00000280295.1	-0.024499008	0.511880215	0.924401274	RP11-358B23.6
ENSG00000112855.17	-0.002675546	0.51189175	0.924401274	HARS2
ENSG00000213963.6	0.008952628	0.511905776	0.924401274	AC074286.1
ENSG00000133624.13	-0.004185053	0.511934339	0.924401274	ZNF767P
ENSG00000274214.1	0.023924862	0.511940797	0.924401274	RP11-757O6.6
ENSG00000214274.10	-0.013214569	0.51199142	0.924401274	ANG
ENSG00000101421.4	0.004919229	0.512084353	0.924401274	CHMP4B
ENSG00000269404.7	-0.012795044	0.512087772	0.924401274	SPIB
ENSG00000285744.1	0.022205766	0.51220304	0.924401274	RP11-115D10.1
ENSG00000182700.5	-0.007280432	0.512211354	0.924401274	IGIP
ENSG00000135899.19	-0.006368114	0.512220749	0.924401274	SP110
ENSG00000275367.1	0.018182833	0.512235945	0.924401274	RP11-266K4.14
ENSG00000122218.16	0.0046571	0.51231615	0.924401274	COPA
ENSG00000131043.13	0.004518483	0.512369876	0.924401274	AAR2
ENSG00000099956.20	-0.005580976	0.51239709	0.924401274	SMARCB1
ENSG00000182141.10	0.009986945	0.512398153	0.924401274	ZNF708
ENSG00000114127.11	0.007895794	0.512423322	0.924401274	XRN1
ENSG00000264538.6	-0.005622277	0.512448238	0.924401274	SUZ12P1
ENSG00000149476.16	-0.005687404	0.512492635	0.924401274	TKFC
ENSG00000167785.9	0.006242663	0.512527402	0.924401274	ZNF558
ENSG00000116685.16	-0.005220281	0.512544403	0.924401274	KIAA2013
ENSG00000182957.16	-0.006907514	0.512634883	0.924401274	SPATA13
ENSG00000256518.3	-0.042161465	0.512705138	0.924401274	FOLR1P1
ENSG00000130349.10	0.006370576	0.512775783	0.924401274	MTRES1
ENSG00000286125.1	0.023675058	0.512793812	0.924401274	AC006115.7

ENSG00000230749.5	0.028931589	0.512813209	0.924401274	MEIS1-AS2
ENSG00000129116.19	-0.011907741	0.512840662	0.924401274	PALLD
ENSG00000285728.2	0.007150076	0.512871463	0.924401274	RP5-994D16.13
ENSG00000255477.1	0.04801334	0.512875668	0.924401274	RP11-63D14.1
ENSG00000163781.14	0.004325162	0.5128785	0.924401274	TOPBP1
ENSG00000186998.16	0.018152536	0.512916304	0.924401274	EMID1
ENSG00000254087.8	0.005257619	0.513024504	0.924401274	LYN
ENSG00000250990.1	-0.024391977	0.513028511	0.924401274	AC073635.5
ENSG00000169592.15	-0.005258388	0.513096878	0.924401274	INO80E
ENSG00000225695.1	-0.022370282	0.513138573	0.924401274	HNRNPA1P35
ENSG00000104611.12	0.023663275	0.513183833	0.924401274	SH2D4A
ENSG00000146856.14	-0.012336712	0.513257003	0.924401274	AGBL3
ENSG00000204147.11	-0.014511779	0.513268678	0.924401274	ASAH2B
ENSG00000137124.8	-0.005842904	0.513301678	0.924401274	ALDH1B1
ENSG00000109046.15	0.006406378	0.513348419	0.924401274	WSB1
ENSG00000214357.9	-0.017865235	0.513386801	0.924401274	NEURL1B
ENSG00000136147.18	-0.006782085	0.513394547	0.924401274	PHF11
ENSG00000230510.7	-0.020761719	0.513490782	0.924401274	PPP5D1
ENSG00000204091.8	-0.039759513	0.513526459	0.924401274	TDRG1
ENSG00000237118.3	0.016412056	0.513640245	0.924401274	CYP2F2P
ENSG00000261377.5	0.019003024	0.513743666	0.924401274	PDCD6IPP2
ENSG00000213398.8	-0.006138178	0.513743781	0.924401274	LCAT
ENSG00000105136.21	-0.005787112	0.513768108	0.924401274	ZNF419
ENSG00000019144.19	-0.012263032	0.513805126	0.924401274	PHLDB1
ENSG00000262873.2	0.018572148	0.513847438	0.924401274	CTD-2561B21.11
ENSG00000116117.18	-0.028599658	0.513886253	0.924401274	PARD3B
ENSG00000249646.2	0.017873833	0.513974718	0.924401274	OR7E94P
ENSG00000138286.15	0.004700947	0.514135744	0.924401274	FAM149B1
ENSG00000142347.19	-0.005939972	0.514149013	0.924401274	MYO1F
ENSG00000126787.13	-0.023593859	0.514176182	0.924401274	DLGAP5
ENSG00000280407.3	0.009914573	0.514244749	0.924401274	RP13-516M14.8
ENSG00000207650.1	0.017133734	0.514366255	0.924401274	MIR570
ENSG00000262500.1	-0.076481763	0.514439621	0.924401274	MAPK8IP1P1
ENSG00000278875.1	0.021790825	0.51448947	0.924401274	CTC-439O9.1
ENSG00000159348.13	0.005139546	0.514503698	0.924401274	CYB5R1
ENSG00000261451.1	-0.019283758	0.514612901	0.924401274	RP11-981G7.1
ENSG00000248469.1	0.033845077	0.514618646	0.924401274	RP11-826N14.2
ENSG00000173588.15	0.008844187	0.514634053	0.924401274	CEP83
ENSG00000223865.11	0.008391937	0.514651682	0.924401274	HLA-DPB1
ENSG00000198496.12	0.006951586	0.514655066	0.924401274	NBR2
ENSG00000285163.1	-0.025257831	0.514655459	0.924401274	RP11-542M13.4
ENSG00000166004.15	0.010169987	0.514695388	0.924401274	CEP295
ENSG00000103319.12	-0.006148467	0.514703688	0.924401274	EEF2K
ENSG00000004866.22	0.007221145	0.514738623	0.924401274	ST7
ENSG00000246548.3	0.015897079	0.514747329	0.924401274	LINC02288
ENSG00000138376.11	-0.009386824	0.514868589	0.924401274	BARD1
ENSG00000145428.15	-0.007871559	0.514875637	0.924401274	RNF175

ENSG00000161956.13	-0.004245574	0.514920356	0.924401274	SENP3
ENSG00000259004.2	0.012920833	0.514971764	0.924401274	LINC02285
ENSG00000280374.1	-0.020246895	0.51501945	0.924401274	RP11-337N6.3
ENSG00000122359.18	-0.005659358	0.515101228	0.924401274	ANXA11
ENSG00000115944.15	-0.006801593	0.515126854	0.924401274	COX7A2L
ENSG00000175054.16	-0.005085737	0.515142402	0.924401274	ATR
ENSG00000188037.13	0.028394857	0.515179384	0.924401274	CLCN1
ENSG00000132329.11	-0.019346717	0.515226584	0.924401274	RAMP1
ENSG00000100099.21	0.003515364	0.51522785	0.924401274	HPS4
ENSG00000197162.10	-0.004962334	0.51523483	0.924401274	ZNF785
ENSG00000163545.11	-0.005780125	0.515255932	0.924401274	NUAK2
ENSG00000275888.1	-0.007567733	0.515299573	0.924401274	RP13-516M14.10
ENSG00000085511.20	0.003798786	0.515352787	0.924401274	MAP3K4
ENSG00000108557.19	-0.006868897	0.515425951	0.924401274	RAI1
ENSG00000177830.18	-0.005891245	0.515429573	0.924401274	CHID1
ENSG00000211847.1	-0.026767487	0.515453981	0.924401274	TRAJ42
ENSG00000115839.18	0.004420671	0.515507816	0.924401274	RAB3GAP1
ENSG00000232053.7	-0.020326148	0.515560402	0.924401274	AC009784.3
ENSG00000092140.16	-0.008404812	0.515574374	0.924401274	G2E3
ENSG00000182796.14	0.00569415	0.515601448	0.924401274	TMEM198B
ENSG00000163257.11	0.004558144	0.515640653	0.924401274	DCAF16
ENSG00000219682.4	-0.026356636	0.51565997	0.924401274	RP3-425P12.4
ENSG00000177842.12	-0.008944409	0.515737773	0.924401274	ZNF620
ENSG00000244932.2	0.009686229	0.515815045	0.924401274	RP11-529F4.1
ENSG00000167695.15	-0.011121181	0.516034173	0.924401274	TLCD3A
ENSG00000163380.16	-0.013359997	0.516036843	0.924401274	LMOD3
ENSG00000196139.14	0.016383007	0.516055417	0.924401274	AKR1C3
ENSG00000105677.12	0.006617339	0.516063363	0.924401274	TMEM147
ENSG00000135862.6	-0.016112772	0.516138071	0.924401274	LAMC1
ENSG00000231925.12	-0.005964598	0.516208649	0.924401274	TAPBP
ENSG00000273343.1	0.010657035	0.516307152	0.924401274	XXbac-B444P24.13
ENSG00000162384.14	0.004139122	0.516361928	0.924401274	CZIB
ENSG00000174456.15	0.004484653	0.516404006	0.924401274	C12orf76
ENSG00000113749.7	0.007552608	0.516488987	0.924401274	HRH2
ENSG00000082805.20	0.006219982	0.516537932	0.924401274	ERC1
ENSG00000133119.13	-0.006542461	0.516579833	0.924401274	RFC3
ENSG00000275426.1	-0.027372587	0.516616333	0.924401274	CH17-262A2.1
ENSG00000222448.1	0.023427922	0.516684096	0.924401274	RN7SKP150
ENSG00000271816.1	0.01554402	0.516689243	0.924401274	BMS1P4
ENSG00000152217.20	0.010730397	0.5167981	0.924401274	SETBP1
ENSG00000279170.3	-0.014012694	0.516811947	0.924401274	TSTD3
ENSG00000205560.13	-0.01068668	0.516817613	0.924401274	CPT1B
ENSG00000154845.16	0.006017542	0.516832274	0.924401274	PPP4R1
ENSG00000275763.3	0.017873087	0.516834482	0.924401274	ZNF516-DT
ENSG00000277449.1	-0.010729825	0.516871719	0.924401274	CEBPB-AS1
ENSG00000149305.7	0.027702981	0.516871798	0.924401274	HTR3B
ENSG00000164168.8	0.003928908	0.516916845	0.924401274	TMEM184C

ENSG00000139194.8	0.014215892	0.516935495	0.924401274	RBP5
ENSG00000074054.20	-0.004728132	0.516938416	0.924401274	CLASP1
ENSG00000230262.8	-0.021155496	0.51694934	0.924401274	LINC02603
ENSG00000156990.14	-0.004849228	0.51696695	0.924401274	RPUSD3
ENSG00000189050.16	0.008183069	0.517215461	0.924692217	RNFT1
ENSG00000110422.12	0.008778709	0.517219837	0.924692217	HIPK3
ENSG00000013374.17	0.006459139	0.517370595	0.92473159	NUB1
ENSG00000269091.6	0.015206407	0.517384456	0.92473159	CTD-2126E3.3
ENSG00000177519.4	0.058072109	0.517492844	0.92473159	RPRM
ENSG00000137700.18	-0.004563608	0.517548805	0.92473159	SLC37A4
ENSG00000152359.15	0.006256691	0.51756209	0.92473159	POC5
ENSG00000256576.2	-0.008119908	0.517568148	0.92473159	LINC02361
ENSG00000197885.11	-0.0060355	0.51758521	0.92473159	NKIRAS1
ENSG00000131508.16	0.003649602	0.517615256	0.92473159	UBE2D2
ENSG00000235173.7	-0.005497368	0.517647682	0.92473159	HGH1
ENSG00000196557.13	-0.014668014	0.517745746	0.924826213	CACNA1H
ENSG00000261332.1	-0.023607072	0.517842307	0.924873171	RP11-297C4.1
ENSG00000200502.1	0.02732037	0.517914087	0.924873171	Y_RNA
ENSG00000197721.17	-0.021960634	0.518034724	0.924873171	CR1L
ENSG00000078967.13	-0.005145162	0.518039929	0.924873171	UBE2D4
ENSG00000183475.13	0.004652867	0.518165948	0.924873171	ASB7
ENSG00000083642.19	0.005047036	0.51818585	0.924873171	PDS5B
ENSG00000124006.15	0.019713748	0.518199213	0.924873171	OBSL1
ENSG00000250651.1	-0.018373569	0.518270476	0.924873171	PABPC1P7
ENSG00000168591.16	0.003578676	0.51831457	0.924873171	TMUB2
ENSG00000099338.23	-0.010520136	0.518322636	0.924873171	CATSPERG
ENSG00000273542.2	-0.02231912	0.518370443	0.924873171	H4C12
ENSG00000176476.9	-0.00692722	0.518403593	0.924873171	SGF29
ENSG00000168061.17	-0.008191418	0.518413746	0.924873171	SAC3D1
ENSG00000272398.6	0.016852705	0.518446099	0.924873171	CD24
ENSG00000162599.17	-0.007416763	0.518448507	0.924873171	NFIA
ENSG00000113161.17	0.005714037	0.518634998	0.925096627	HMGCR
ENSG00000052795.13	0.007487446	0.518663986	0.925096627	FNIP2
ENSG00000105866.15	-0.007684591	0.518830098	0.925233958	SP4
ENSG00000235978.6	0.01821659	0.518831213	0.925233958	AC018816.3
ENSG00000172936.16	0.004726894	0.518928678	0.925283953	MYD88
ENSG00000177868.12	0.007276695	0.518976666	0.925283953	SVBP
ENSG00000279758.1	0.016454565	0.519006137	0.925283953	RP11-752G15.10
ENSG00000243477.6	-0.006278981	0.519039721	0.925283953	NAA80
ENSG00000249236.2	0.023757345	0.519244236	0.925512348	CTD-2227I18.1
ENSG00000196517.13	0.024555568	0.519293305	0.925512348	SLC6A9
ENSG00000212993.5	0.013494677	0.519337115	0.925512348	POU5F1B
ENSG00000231050.1	-0.027965693	0.519450873	0.925512348	RP1-140A9.1
ENSG00000243290.3	0.023705714	0.519496485	0.925512348	IGKV1-12
ENSG00000260302.3	-0.017550086	0.519533096	0.925512348	LINC01882
ENSG00000152078.10	0.02271202	0.519616081	0.925512348	TLCD4
ENSG00000186792.17	0.012152447	0.519659087	0.925512348	HYAL3

ENSG00000100979.15	-0.01399391	0.519687729	0.925512348	PLTP
ENSG00000107821.15	-0.025567331	0.519822666	0.925512348	KAZALD1
ENSG00000228323.2	0.021021627	0.519837289	0.925512348	MYADM-AS1
ENSG00000198162.12	0.005675783	0.519853787	0.925512348	MAN1A2
ENSG00000185031.6	0.023184333	0.519918319	0.925512348	SLC2A3P2
ENSG00000189143.10	0.027269941	0.519954966	0.925512348	CLDN4
ENSG00000286379.1	0.023712068	0.519966822	0.925512348	RP11-268J15.6
ENSG00000173258.13	0.009705825	0.519994698	0.925512348	ZNF483
ENSG00000175334.8	0.006368364	0.520026471	0.925512348	BANF1
ENSG00000178700.9	0.006925349	0.520027789	0.925512348	DHFR2
ENSG00000196230.14	-0.004695343	0.520031287	0.925512348	TUBB
ENSG00000248476.1	-0.012809791	0.520070426	0.925512348	BACH1-IT1
ENSG00000253930.1	-0.009848652	0.52021354	0.925536747	TNFRSF10A-AS1
ENSG00000154640.15	0.008366893	0.520257349	0.925536747	BTG3
ENSG00000188820.13	0.0205654	0.520267881	0.925536747	CALHM6
ENSG00000201207.1	-0.016781288	0.520304451	0.925536747	Y_RNA
ENSG00000234292.3	-0.02194132	0.520309789	0.925536747	RP11-213H15.1
ENSG00000214253.9	0.00823578	0.52038858	0.925596618	FIS1
ENSG00000287092.1	0.037312866	0.520609615	0.925735115	RP11-537P24.1
ENSG00000124659.7	0.003928987	0.520650771	0.925735115	TBCC
ENSG00000140044.13	-0.005577057	0.520711999	0.925735115	JDP2
ENSG00000280003.1	-0.009114362	0.520714398	0.925735115	RP11-330H6.6
ENSG00000071246.11	-0.014308729	0.520721805	0.925735115	VASH1
ENSG00000235560.4	0.010165168	0.520796446	0.925735115	AC002310.12
ENSG00000198315.11	0.00498879	0.520851026	0.925735115	ZKSCAN8
ENSG00000183444.10	-0.017118898	0.52086502	0.925735115	OR7E38P
ENSG00000228395.2	0.012352348	0.52087696	0.925735115	RP11-216B9.6
ENSG00000120594.17	0.005972133	0.520957336	0.925735115	PLXDC2
ENSG00000186130.5	0.007408054	0.520967203	0.925735115	ZBTB6
ENSG00000211751.9	-0.00889969	0.521028704	0.925735115	TRBC1
ENSG00000242516.2	-0.029183844	0.521053269	0.925735115	LINC00960
ENSG00000241772.2	0.044118837	0.521188543	0.925895239	AC092620.2
ENSG00000196646.12	0.006038177	0.521297288	0.925989296	ZNF136
ENSG00000086300.16	0.007677463	0.52135041	0.925989296	SNX10
ENSG00000250506.8	0.009078069	0.521376945	0.925989296	CDK3
ENSG00000125107.19	0.006907212	0.521573096	0.92616626	CNOT1
ENSG00000167772.12	0.016937288	0.521605144	0.92616626	ANGPTL4
ENSG00000252759.1	-0.024307293	0.521612069	0.92616626	Y_RNA
ENSG00000089060.12	0.004583514	0.521850338	0.926207842	SLC8B1
ENSG00000259205.2	-0.021324097	0.521857582	0.926207842	PRKXP1
ENSG00000168490.14	0.108377199	0.52187227	0.926207842	PHYHIP
ENSG00000273444.1	0.022527043	0.521941258	0.926207842	RP5-1147A1.2
ENSG00000130818.12	-0.004723774	0.522051509	0.926207842	ZNF426
ENSG00000162704.16	0.004583881	0.5220901	0.926207842	ARPC5
ENSG00000267114.1	0.014465011	0.522101728	0.926207842	CTB-129P6.11
ENSG00000106976.21	-0.011123184	0.522118685	0.926207842	DNM1
ENSG00000166260.13	0.00387833	0.522123396	0.926207842	COX11

ENSG00000152433.15	-0.011040973	0.522227469	0.926207842	ZNF547
ENSG00000179915.24	-0.022880758	0.522391845	0.926207842	NRXN1
ENSG00000270006.2	-0.028193471	0.52243311	0.926207842	C16orf95-DT
ENSG00000264278.1	-0.009822684	0.522493395	0.926207842	ZNF236-DT
ENSG00000115896.16	0.011562117	0.522524422	0.926207842	PLCL1
ENSG00000135919.14	0.016500958	0.522698714	0.926207842	SERPINE2
ENSG00000173221.14	0.0072923	0.522795298	0.926207842	GLRX
ENSG00000140365.16	0.007164619	0.522916201	0.926207842	COMMD4
ENSG00000185065.6	-0.018977144	0.522928095	0.926207842	AC000068.5
ENSG00000227028.6	-0.023624085	0.522988888	0.926207842	SLC8A1-AS1
ENSG00000226200.7	0.006625447	0.523042466	0.926207842	SGMS1-AS1
ENSG00000156711.17	-0.005634082	0.523137289	0.926207842	MAPK13
ENSG00000197747.9	0.007298797	0.523279988	0.926207842	S100A10
ENSG00000070985.13	0.026948637	0.523286621	0.926207842	TRPM5
ENSG00000164877.19	0.009024592	0.523295702	0.926207842	MICALL2
ENSG00000136026.14	0.006714288	0.523385206	0.926207842	CKAP4
ENSG00000149289.11	0.01306956	0.52351075	0.926207842	ZC3H12C
ENSG00000204778.4	0.017393037	0.523552194	0.926207842	CBWD4P
ENSG00000253731.3	-0.018534827	0.523676702	0.926207842	PCDHGA6
ENSG00000102901.13	-0.0049213	0.523701058	0.926207842	CENPT
ENSG00000161835.11	-0.009025112	0.523703357	0.926207842	TAMALIN
ENSG00000124466.9	0.012415443	0.523732637	0.926207842	LYPD3
ENSG00000269973.1	0.008322403	0.523764568	0.926207842	RP11-95D17.1
ENSG00000272588.1	-0.019506954	0.523786471	0.926207842	RP11-440L14.4
ENSG00000166562.9	0.006917992	0.523817222	0.926207842	SEC11C
ENSG00000112592.14	-0.002783011	0.523818717	0.926207842	TBP
ENSG00000153130.18	-0.014446931	0.523936581	0.926207842	SCOC
ENSG00000278535.5	0.007765721	0.523947286	0.926207842	DHRS11
ENSG00000211821.2	-0.038766633	0.523949292	0.926207842	TRDV2
ENSG00000286813.1	-0.015266683	0.523958195	0.926207842	RP11-81A1.10
ENSG00000258944.1	0.013477921	0.523992501	0.926207842	RP4-647C14.3
ENSG00000270728.1	0.018122643	0.524003908	0.926207842	RP4-657E11.10
ENSG00000241666.2	-0.019540232	0.524010869	0.926207842	RP3-455J7.4
ENSG00000105281.12	0.009116446	0.524020209	0.926207842	SLC1A5
ENSG00000073584.20	-0.00500078	0.524130267	0.926207842	SMARCE1
ENSG00000268362.6	0.011637223	0.524153518	0.926207842	CTD-2017D11.1
ENSG00000134758.14	-0.007522899	0.524224025	0.926207842	RNF138
ENSG00000287362.1	-0.022210899	0.524241758	0.926207842	RP11-708D7.1
ENSG00000086619.14	-0.006975963	0.524280233	0.926207842	ERO1B
ENSG00000154874.15	0.025530888	0.524283716	0.926207842	CCDC144B
ENSG00000266256.3	-0.020315173	0.524305758	0.926207842	LINC00683
ENSG00000073536.18	0.006047902	0.524308443	0.926207842	NLE1
ENSG00000105366.15	-0.018545906	0.524358778	0.926207842	SIGLEC8
ENSG00000197467.17	0.022847132	0.524407691	0.926207842	COL13A1
ENSG00000166206.15	0.046405635	0.524488997	0.926207842	GABRB3
ENSG00000258511.1	0.018719216	0.524561276	0.926207842	LINC02295
ENSG00000287766.1	-0.015650645	0.524649997	0.926207842	AC006130.5

ENSG00000134153.10	0.004138024	0.524684011	0.926207842	EMC7
ENSG00000287917.1	0.010649058	0.524694695	0.926207842	RP11-867O8.11
ENSG00000197992.7	0.016492664	0.524737897	0.926207842	CLEC9A
ENSG00000165119.21	0.002811724	0.524892646	0.926207842	HNRNPK
ENSG00000189339.12	-0.007352689	0.525021776	0.926207842	SLC35E2B
ENSG00000267852.1	0.016832701	0.525102036	0.926207842	ARHGEF18-AS1
ENSG00000239557.1	-0.016002883	0.525113439	0.926207842	PPP2R5CP
ENSG00000163605.15	0.008719799	0.525161192	0.926207842	PPP4R2
ENSG00000261777.6	-0.017909847	0.52522059	0.926207842	DDX19A-DT
ENSG00000221598.3	-0.019585765	0.525223155	0.926207842	MIR1249
ENSG00000101166.16	-0.004965678	0.52523231	0.926207842	PRELID3B
ENSG00000123360.12	0.007684889	0.525310649	0.926207842	PDE1B
ENSG00000182973.19	0.002945122	0.525321011	0.926207842	CNOT10
ENSG00000134668.12	0.020388703	0.525349246	0.926207842	SPOCD1
ENSG00000106638.17	-0.004178357	0.52541963	0.926207842	TBL2
ENSG00000083828.16	0.004453628	0.525462707	0.926207842	ZNF586
ENSG00000076043.11	-0.005024394	0.52551112	0.926207842	REXO2
ENSG00000115607.9	0.01345976	0.525605951	0.926207842	IL18RAP
ENSG00000163735.7	0.020239524	0.525722471	0.926207842	CXCL5
ENSG00000111011.18	0.003497949	0.525757927	0.926207842	RSRC2
ENSG00000225792.2	0.016712319	0.525784787	0.926207842	AC004540.4
ENSG00000115350.12	0.008526691	0.525789902	0.926207842	POLE4
ENSG00000163606.11	0.009383949	0.52583726	0.926207842	CD200R1
ENSG00000086730.17	-0.004832207	0.525842387	0.926207842	LAT2
ENSG00000274373.1	-0.021057693	0.525848213	0.926207842	RP13-714J12.1
ENSG00000243181.2	0.033698438	0.52585459	0.926207842	RP11-734J24.1
ENSG00000176293.20	-0.015512821	0.525918245	0.926207842	ZNF135
ENSG00000198176.13	-0.006739974	0.525933964	0.926207842	TFDP1
ENSG00000134824.14	-0.027217113	0.525946585	0.926207842	FADS2
ENSG00000140905.11	-0.021742354	0.525971448	0.926207842	GCSH
ENSG00000111863.13	0.0169028	0.525978003	0.926207842	ADTRP
ENSG00000146574.16	-0.009981258	0.526018372	0.926207842	CCZ1B
ENSG00000164638.11	-0.016499185	0.526162461	0.926207842	SLC29A4
ENSG00000243449.7	-0.014924879	0.526242719	0.926207842	C4orf48
ENSG00000130779.22	0.006030202	0.526254994	0.926207842	CLIP1
ENSG00000124783.14	0.003259069	0.526267661	0.926207842	SSR1
ENSG00000196792.12	-0.006980738	0.526288498	0.926207842	STRN3
ENSG00000125730.17	-0.015381801	0.526301927	0.926207842	C3
ENSG00000164061.5	0.011755039	0.526354818	0.926207842	BSN
ENSG00000127533.4	0.01488773	0.52639755	0.926207842	F2RL3
ENSG00000173706.14	-0.008474485	0.526414076	0.926207842	HEG1
ENSG00000141576.16	0.009582788	0.52649683	0.926207842	RNF157
ENSG00000240583.14	0.017879356	0.526510533	0.926207842	AQP1
ENSG00000198133.9	-0.00565225	0.526527061	0.926207842	TMEM229B
ENSG00000162551.14	0.013796615	0.526529869	0.926207842	ALPL
ENSG00000254536.2	-0.010564845	0.526626926	0.926207842	RP11-108K14.8
ENSG00000170266.16	0.004900775	0.526655547	0.926207842	GLB1

ENSG00000253981.5	0.010610412	0.526684595	0.926207842	ALG1L13P
ENSG00000270240.2	-0.02073975	0.526714421	0.926207842	AC015849.2
ENSG00000263503.1	-0.105313313	0.52674278	0.926207842	MAPK8IP1P2
ENSG00000100902.11	0.010188556	0.526754642	0.926207842	PSMA6
ENSG00000182472.9	-0.011748374	0.526783828	0.926207842	CAPN12
ENSG00000109111.15	-0.005169062	0.526802074	0.926207842	SUPT6H
ENSG00000272849.1	0.00726147	0.526860541	0.926207842	RP11-347I19.8
ENSG00000229325.1	-0.017327186	0.526877192	0.926207842	ACAP2-IT1
ENSG00000242372.9	0.006229013	0.526888265	0.926207842	EIF6
ENSG00000245281.8	-0.018815264	0.526889633	0.926207842	CTD-2547L16.1
ENSG00000147874.11	0.006443397	0.526908561	0.926207842	HAUS6
ENSG00000065000.19	-0.004402039	0.526964093	0.926207842	AP3D1
ENSG00000167748.11	0.02338866	0.526986944	0.926207842	KLK1
ENSG00000164307.13	-0.008575197	0.526992401	0.926207842	ERAP1
ENSG00000258405.11	0.015193976	0.527013992	0.926207842	ZNF578
ENSG00000173239.14	0.054203391	0.527030627	0.926207842	LIPM
ENSG00000070269.14	0.005142374	0.527055077	0.926207842	TMEM260
ENSG00000068079.8	-0.013165752	0.527153063	0.926257474	IFI35
ENSG00000197057.10	0.014718249	0.527232307	0.926257474	DTHD1
ENSG00000139344.8	-0.014199254	0.527398134	0.926257474	AMDHD1
ENSG00000176909.12	-0.012709564	0.527413259	0.926257474	MAMSTR
ENSG00000223117.1	0.025043206	0.527500387	0.926257474	RN7SKP296
ENSG00000109072.14	-0.017398871	0.5275026	0.926257474	VTN
ENSG00000163155.12	-0.009187498	0.527535216	0.926257474	LYSMD1
ENSG00000156802.13	0.00757731	0.527726965	0.926257474	ATAD2
ENSG00000012779.11	-0.005486711	0.527753985	0.926257474	ALOX5
ENSG00000183963.19	-0.007571801	0.527755653	0.926257474	SMTN
ENSG00000155542.12	0.014297156	0.527774054	0.926257474	SETD9
ENSG00000205559.5	0.011243083	0.527821449	0.926257474	CHKB-DT
ENSG00000234771.3	0.00585376	0.527832216	0.926257474	SLC25A25-AS1
ENSG00000232869.2	-0.015699488	0.527883361	0.926257474	TRBV29-1
ENSG00000258230.2	0.024538828	0.527913804	0.926257474	RP11-511H9.3
ENSG00000101473.17	0.004448942	0.527954401	0.926257474	ACOT8
ENSG00000249459.11	0.010998495	0.527957926	0.926257474	ZNF286B
ENSG00000276476.3	-0.019573578	0.528008562	0.926257474	LINC00540
ENSG00000141480.18	0.005191623	0.528270136	0.926257474	ARRB2
ENSG00000176595.4	0.006486137	0.52829507	0.926257474	KBTBD11
ENSG00000103415.12	-0.005147415	0.528308699	0.926257474	HMOX2
ENSG00000140416.23	0.008501583	0.528328614	0.926257474	TPM1
ENSG00000284373.1	0.020094308	0.528358102	0.926257474	TAF11L2
ENSG00000185000.12	-0.005398907	0.5284052	0.926257474	DGAT1
ENSG00000250571.7	-0.005945896	0.528493655	0.926257474	GLI4
ENSG00000188243.14	0.015643531	0.528526512	0.926257474	COMMD6
ENSG00000232671.6	0.016487593	0.528594004	0.926257474	ZNF687-AS1
ENSG00000274695.1	0.023575747	0.528604641	0.926257474	RP11-21K12.3
ENSG00000108799.13	0.00324225	0.528686474	0.926257474	EZH1
ENSG00000120440.15	-0.024259194	0.528711865	0.926257474	TTLL2

ENSG00000259070.7	0.022317412	0.528737528	0.926257474	LINC00639
ENSG00000121297.8	0.008345132	0.528743808	0.926257474	TSHZ3
ENSG00000124228.15	-0.003351689	0.528746886	0.926257474	DDX27
ENSG00000254206.5	0.022249768	0.52879076	0.926257474	NPIPB11
ENSG00000100473.18	0.020572414	0.528868064	0.926257474	COCH
ENSG00000132382.15	-0.005493126	0.528879305	0.926257474	MYBBP1A
ENSG00000234500.1	-0.010096945	0.528979394	0.926257474	GS1-124K5.10
ENSG00000175894.18	-0.022791415	0.528995407	0.926257474	TSPEAR
ENSG00000122912.15	0.004239286	0.529009161	0.926257474	SLC25A16
ENSG00000288302.1	-0.012218319	0.529032582	0.926257474	RP11-909M7.5
ENSG00000108064.11	0.005756983	0.529056552	0.926257474	TFAM
ENSG00000144647.6	-0.006152762	0.529127163	0.926257474	POMGNT2
ENSG00000167037.19	-0.011097607	0.529177394	0.926257474	SGSM1
ENSG00000162341.18	-0.005021292	0.529180112	0.926257474	TPCN2
ENSG00000287848.1	0.024669843	0.529185128	0.926257474	RP11-445P17.9
ENSG00000258689.1	0.023653556	0.529187523	0.926257474	LINC01269
ENSG00000105971.15	0.02290688	0.52923311	0.926257474	CAV2
ENSG00000222375.1	-0.019706564	0.529317712	0.926257474	RN7SKP127
ENSG00000074317.11	-0.06100199	0.529412541	0.926257474	SNCB
ENSG00000076706.17	-0.015195672	0.529428612	0.926257474	MCAM
ENSG00000165886.5	-0.007201087	0.529465118	0.926257474	UBTD1
ENSG00000181240.14	0.031895714	0.529536235	0.926257474	SLC25A41
ENSG00000198513.14	-0.010207857	0.52957212	0.926257474	ATL1
ENSG00000175518.8	-0.01430795	0.529600752	0.926257474	UBQLNL
ENSG00000152192.8	0.027483167	0.529628094	0.926257474	POU4F1
ENSG00000225171.2	0.017330195	0.529670664	0.926257474	DUTP6
ENSG00000129946.11	0.031264323	0.529698637	0.926257474	SHC2
ENSG00000156510.13	-0.014627335	0.529802587	0.926257474	HKDC1
ENSG00000142546.14	-0.007648829	0.530022035	0.926257474	NOSIP
ENSG00000236403.2	-0.023213874	0.530040201	0.926257474	RP11-447M12.2
ENSG00000258824.2	0.014985746	0.530053754	0.926257474	CTD-2555O16.2
ENSG00000005801.18	-0.006156275	0.530066855	0.926257474	ZNF195
ENSG00000177570.15	0.015454744	0.530117016	0.926257474	SAMD12
ENSG00000130751.10	-0.019991423	0.530151702	0.926257474	NPAS1
ENSG00000074582.15	-0.004894982	0.530244123	0.926257474	BCS1L
ENSG00000283361.2	0.031663515	0.530249918	0.926257474	CFAP97D2
ENSG00000073578.17	-0.004839173	0.530286083	0.926257474	SDHA
ENSG00000141504.12	0.006105459	0.530359478	0.926257474	SAT2
ENSG00000109917.11	0.003132479	0.530407283	0.926257474	ZPR1
ENSG00000148483.9	0.018731581	0.530509803	0.926257474	TMEM236
ENSG00000263823.1	-0.012838813	0.530657841	0.926257474	RP11-326K13.4
ENSG00000115207.13	0.003324753	0.530686755	0.926257474	GTF3C2
ENSG00000080298.16	-0.007495407	0.530774139	0.926257474	RFX3
ENSG00000226278.1	0.105115093	0.530781745	0.926257474	PSPHP1
ENSG00000211892.4	-0.030332488	0.530797829	0.926257474	IGHG4
ENSG00000168300.14	0.008978511	0.530807822	0.926257474	PCMTD1
ENSG00000072134.15	0.011341569	0.530834943	0.926257474	EPN2

ENSG00000259376.1	0.017270174	0.530905713	0.926257474	RP11-505E24.3
ENSG00000121406.9	0.005062315	0.530982513	0.926257474	ZNF549
ENSG00000155980.13	0.012783816	0.531064865	0.926257474	KIF5A
ENSG00000144589.22	-0.004679448	0.531140686	0.926257474	STK11IP
ENSG00000142082.15	-0.004590623	0.531153329	0.926257474	SIRT3
ENSG00000168615.13	0.00746915	0.531155372	0.926257474	ADAM9
ENSG00000164933.12	-0.015765266	0.531175604	0.926257474	SLC25A32
ENSG00000136244.12	-0.027498983	0.53120339	0.926257474	IL6
ENSG00000217027.1	0.022844453	0.53141922	0.926257474	TPT1P4
ENSG00000242798.1	0.013127538	0.531434792	0.926257474	RP11-506M12.1
ENSG00000028310.18	-0.002620661	0.531437308	0.926257474	BRD9
ENSG00000183808.12	0.006653733	0.531465544	0.926257474	RBM12B
ENSG00000285053.1	0.010147106	0.531551385	0.926257474	TBCE
ENSG00000239382.10	-0.005857	0.531616481	0.926257474	ALKBH6
ENSG00000171453.20	-0.004925801	0.531639711	0.926257474	POLR1C
ENSG00000163909.8	0.023466595	0.531804358	0.926257474	HEYL
ENSG00000144031.12	-0.018488133	0.531842803	0.926257474	ANKRD53
ENSG00000280071.4	-0.015335681	0.531939225	0.926257474	GATD3B
ENSG00000287967.1	0.015841426	0.531946286	0.926257474	RP1-76B20.16
ENSG00000143624.14	0.004441035	0.532000089	0.926257474	INTS3
ENSG00000126460.11	-0.016795718	0.532011684	0.926257474	PRRG2
ENSG00000151773.13	0.017442371	0.532026868	0.926257474	CCDC122
ENSG00000176723.10	-0.022195096	0.532034545	0.926257474	ZNF843
ENSG00000179406.8	-0.006484045	0.532053341	0.926257474	LINC00174
ENSG00000269119.1	0.015341055	0.53210254	0.926257474	HNRNPA1P52
ENSG00000287262.1	-0.015501599	0.532154218	0.926257474	RP11-472B18.3
ENSG00000150281.7	-0.016507795	0.532214426	0.926257474	CTF1
ENSG00000183010.17	-0.017372393	0.532265659	0.926257474	PYCR1
ENSG00000188282.12	0.013978786	0.532269377	0.926257474	RUFY4
ENSG00000023445.16	0.009354092	0.532349447	0.926257474	BIRC3
ENSG00000267554.1	0.014639849	0.532367387	0.926257474	RP11-686D22.10
ENSG00000140092.15	0.01529582	0.532399055	0.926257474	FBLN5
ENSG00000254681.6	-0.034849226	0.532440509	0.926257474	PKD1P5
ENSG00000101000.6	-0.011112217	0.532441219	0.926257474	PROCR
ENSG00000154889.17	-0.006802143	0.532448158	0.926257474	MPPE1
ENSG00000112367.12	0.004090205	0.532448982	0.926257474	FIG4
ENSG00000230724.9	0.020918918	0.532468367	0.926257474	LINC01001
ENSG00000198785.7	0.010471232	0.5325443	0.926257474	GRIN3A
ENSG00000179299.17	-0.011100617	0.532557828	0.926257474	NSUN7
ENSG00000259972.3	-0.00479575	0.532573456	0.926257474	AC009120.6
ENSG00000226212.2	0.029302982	0.532617227	0.926257474	TRGV6
ENSG00000170633.16	-0.002359615	0.532653673	0.926257474	RNF34
ENSG00000168646.13	0.010925953	0.532656994	0.926257474	AXIN2
ENSG00000125744.12	-0.008672189	0.532693804	0.926257474	RTN2
ENSG00000253305.2	-0.022184271	0.53271885	0.926257474	PCDHGB6
ENSG00000157036.13	0.004418006	0.532747172	0.926257474	EXOG
ENSG00000145703.16	0.007829799	0.532829154	0.926257474	IQGAP2

ENSG00000203730.3	-0.026598236	0.532904895	0.926257474	TEDDM1
ENSG00000155252.13	-0.004818477	0.532913842	0.926257474	PI4K2A
ENSG00000137642.13	0.006665333	0.532918826	0.926257474	SORL1
ENSG00000204428.12	0.008986061	0.532946398	0.926257474	LY6G5C
ENSG00000280163.1	-0.015179293	0.532960309	0.926257474	CTC-277H1.6
ENSG00000104447.13	0.006549256	0.533011583	0.926257474	TRPS1
ENSG00000198482.14	-0.005935945	0.533025406	0.926257474	ZNF808
ENSG00000144048.10	0.003776205	0.533113111	0.926257474	DUSP11
ENSG00000006837.12	-0.015966097	0.533155208	0.926257474	CDKL3
ENSG00000286895.1	-0.017893101	0.533177608	0.926257474	RP11-90D4.7
ENSG00000153391.16	0.005906706	0.533250384	0.926257474	INO80C
ENSG00000186184.19	0.005744314	0.533255654	0.926257474	POLR1D
ENSG00000233885.7	-0.010130432	0.533291082	0.926257474	YEATS2-AS1
ENSG00000124257.7	0.014230441	0.533361833	0.926257474	NEURL2
ENSG00000129657.16	-0.006892334	0.533420995	0.926257474	SEC14L1
ENSG00000236778.8	0.007808718	0.533456106	0.926257474	INTS6-AS1
ENSG00000261207.1	-0.015622499	0.533475148	0.926257474	LA16c-361A3.3
ENSG00000181873.13	-0.005211983	0.533496844	0.926257474	IBA57
ENSG00000224307.2	0.009476361	0.533567352	0.926262136	RP11-344B5.2
ENSG00000136158.12	0.012994883	0.533608131	0.926262136	SPRY2
ENSG00000167419.11	-0.036067373	0.533763196	0.926262136	LPO
ENSG00000163125.16	-0.005049054	0.533767006	0.926262136	RPRD2
ENSG00000183431.12	0.00395475	0.533793006	0.926262136	SF3A3
ENSG00000113552.16	0.004631251	0.533801142	0.926262136	GNPDA1
ENSG00000122958.15	0.003995766	0.533815691	0.926262136	VPS26A
ENSG00000288681.1	0.027869642	0.533876708	0.926289638	RP11-326C3.17
ENSG00000254505.11	-0.005607012	0.533940176	0.926321388	CHMP4A
ENSG00000260103.2	-0.021718769	0.534011085	0.92636604	RP11-10O17.1
ENSG00000170500.13	-0.030839779	0.534166857	0.926481551	LONRF2
ENSG00000271576.1	-0.015523538	0.534168025	0.926481551	PRKACB-DT
ENSG00000283959.1	0.019747655	0.534215607	0.926485723	KB-1042C11.5
ENSG00000250771.2	-0.030084343	0.534302015	0.926500583	RP11-153M7.3
ENSG00000227195.11	-0.029311073	0.534337626	0.926500583	MIR663AHG
ENSG00000265996.1	0.015399161	0.534359708	0.926500583	MIR3671
ENSG00000168995.13	-0.007690834	0.53440895	0.926507629	SIGLEC7
ENSG00000160584.17	-0.00441886	0.534513258	0.926603579	SIK3
ENSG00000035862.12	-0.005311881	0.534595729	0.926603579	TIMP2
ENSG00000084110.11	-0.006371091	0.534599842	0.926603579	HAL
ENSG00000180447.7	-0.020085751	0.534659529	0.926628718	GAS1
ENSG00000081692.13	0.006181379	0.534745276	0.926699013	JMJD4
ENSG00000154767.15	-0.004657167	0.535265644	0.92722354	XPC
ENSG00000143971.9	-0.008658226	0.535288034	0.92722354	ETAA1
ENSG00000180773.15	-0.006718312	0.535289556	0.92722354	SLC36A4
ENSG00000189280.3	0.027390823	0.535394023	0.92722354	GJB5
ENSG00000139187.10	-0.014249091	0.535462127	0.92722354	KLRG1
ENSG00000236603.2	0.01993442	0.535483183	0.92722354	RANP1
ENSG00000134287.10	0.00484166	0.535544458	0.92722354	ARF3

ENSG00000254612.2	0.007327835	0.535547455	0.92722354	DNAJB6P1
ENSG00000157107.14	0.010392203	0.535556603	0.92722354	FCHO2
ENSG00000163013.12	0.004525592	0.53557872	0.92722354	FBXO41
ENSG00000125691.14	0.01461689	0.535623263	0.92722354	RPL23
ENSG00000164463.12	0.006556659	0.535649515	0.92722354	CREBRF
ENSG00000111837.12	-0.010718159	0.535787219	0.92722354	MAK
ENSG00000272787.1	-0.012685648	0.535961065	0.92722354	KB-226F1.2
ENSG00000237520.1	0.018053607	0.535964219	0.92722354	RP11-443B7.2
ENSG00000254595.1	0.011819484	0.535982823	0.92722354	CTD-2010I16.1
ENSG00000122952.17	0.014559924	0.536034992	0.92722354	ZWINT
ENSG00000139370.12	0.005244824	0.536054858	0.92722354	SLC15A4
ENSG00000106701.13	-0.012448797	0.536237809	0.92722354	FSD1L
ENSG00000169621.10	0.009630246	0.536254865	0.92722354	APLF
ENSG00000259081.1	-0.017227927	0.536322526	0.92722354	RP11-488C13.6
ENSG00000225342.2	0.007899022	0.536357285	0.92722354	LRRK2-DT
ENSG00000115170.16	0.005426106	0.53636587	0.92722354	ACVR1
ENSG00000111732.11	-0.021575613	0.536366156	0.92722354	AICDA
ENSG00000100030.15	0.004408792	0.53640286	0.92722354	MAPK1
ENSG00000286276.1	0.014704173	0.536404391	0.92722354	RP5-1086K13.3
ENSG00000136527.18	0.003447119	0.536424445	0.92722354	TRA2B
ENSG00000242797.3	-0.011648659	0.536474283	0.92722354	GLYCTK-AS1
ENSG00000221955.11	0.01909257	0.536514741	0.92722354	SLC12A8
ENSG00000260625.3	-0.021189898	0.536554609	0.92722354	RP11-452L6.7
ENSG00000196584.3	-0.019170913	0.536559625	0.92722354	XRCC2
ENSG00000100564.9	0.003928975	0.536563761	0.92722354	PIGH
ENSG00000243710.8	-0.025619848	0.53659752	0.92722354	CFAP57
ENSG00000248936.1	-0.012340987	0.536636073	0.92722354	RP11-36B15.1
ENSG00000154760.14	-0.006520557	0.536654861	0.92722354	SLFN13
ENSG00000164078.13	-0.013656713	0.53667561	0.92722354	MST1R
ENSG00000169413.3	0.010584026	0.536914006	0.927302743	RNASE6
ENSG00000128463.13	0.004212678	0.536970624	0.927302743	EMC4
ENSG00000150681.10	0.010814521	0.536992925	0.927302743	RGS18
ENSG00000117906.14	-0.008344249	0.537112643	0.927302743	RCN2
ENSG00000099834.19	-0.01496514	0.537208428	0.927302743	CDHR5
ENSG00000278966.2	-0.018950076	0.537241806	0.927302743	RP5-1174N9.2
ENSG00000144233.11	0.003712529	0.537265172	0.927302743	AMMECR1L
ENSG00000259146.4	0.018165924	0.537275526	0.927302743	RP1-261D10.2
ENSG00000235989.4	-0.016756151	0.537288771	0.927302743	MORC2-AS1
ENSG00000281100.1	0.008073279	0.53737459	0.927302743	RP11-640L9.2
ENSG00000090060.19	0.004704614	0.537406354	0.927302743	PAPOLA
ENSG00000285725.1	0.012913806	0.537447011	0.927302743	RP5-1111F22.1
ENSG00000161533.12	0.006425831	0.537569021	0.927302743	ACOX1
ENSG00000225231.2	0.050973278	0.537589425	0.927302743	LINC02470
ENSG00000282886.1	-0.041208571	0.537590678	0.927302743	PTMAP12
ENSG00000266472.6	0.005766004	0.537619599	0.927302743	MRPS21
ENSG00000164045.12	-0.019702139	0.537648198	0.927302743	CDC25A
ENSG00000205060.11	0.003993956	0.537700561	0.927302743	SLC35B4

ENSG00000103051.20	0.003665946	0.537717591	0.927302743	COG4
ENSG00000264920.2	0.013060309	0.537722882	0.927302743	SP2-DT
ENSG00000114405.11	-0.014239248	0.537732498	0.927302743	C3orf14
ENSG00000242485.6	0.004716824	0.537749718	0.927302743	MRPL20
ENSG00000084764.12	0.007857453	0.537761436	0.927302743	MAPRE3
ENSG00000240005.6	-0.030671329	0.537858637	0.927392376	STIM2-AS1
ENSG00000156886.12	0.021284962	0.537930891	0.927438984	ITGAD
ENSG00000226009.2	0.019522808	0.538123616	0.927630122	KCNIP2-AS1
ENSG00000123610.5	0.017307047	0.53813222	0.927630122	TNFAIP6
ENSG00000273363.1	0.019425211	0.538434661	0.927871771	RP11-285G1.14
ENSG00000172725.14	-0.005021606	0.538592903	0.927871771	CORO1B
ENSG00000137133.11	0.011101006	0.538600108	0.927871771	HINT2
ENSG00000109163.7	0.02783814	0.538635099	0.927871771	GNRHR
ENSG00000213246.7	0.005829339	0.5386407	0.927871771	SUPT4H1
ENSG00000221963.6	0.010511836	0.538645552	0.927871771	APOL6
ENSG00000204580.14	-0.009886516	0.538652093	0.927871771	DDR1
ENSG00000183023.18	0.007632481	0.538749347	0.927871771	SLC8A1
ENSG00000112238.12	0.055785521	0.538831272	0.927871771	PRDM13
ENSG00000189043.10	0.013613206	0.53884406	0.927871771	NDUFA4
ENSG00000078124.13	0.004897571	0.538864114	0.927871771	ACER3
ENSG00000149403.13	-0.043995135	0.538900301	0.927871771	GRIK4
ENSG00000164683.18	-0.022166111	0.53891545	0.927871771	HEY1
ENSG00000129219.14	0.006196422	0.538965904	0.927871771	PLD2
ENSG00000105928.16	0.01890298	0.539132177	0.927871771	GSDME
ENSG00000287781.1	-0.030555649	0.539174644	0.927871771	RP11-382A18.5
ENSG00000179914.5	0.022714829	0.539210068	0.927871771	ITLN1
ENSG00000225720.6	-0.012901795	0.539224127	0.927871771	RP4-742C19.12
ENSG00000091947.10	0.004825094	0.53923112	0.927871771	TMEM101
ENSG00000103037.12	-0.004570097	0.539233969	0.927871771	SETD6
ENSG00000198324.14	-0.006721317	0.539262259	0.927871771	PHETA1
ENSG00000115421.13	-0.004600443	0.53926778	0.927871771	PAPOLG
ENSG00000149639.15	-0.007974207	0.53936548	0.927917226	SOGA1
ENSG00000141380.14	-0.003865556	0.539470118	0.927917226	SS18
ENSG00000174744.14	0.004918802	0.539470783	0.927917226	BRMS1
ENSG00000114520.11	0.009971271	0.539494534	0.927917226	SNX4
ENSG00000104870.13	0.006125581	0.539557774	0.927917226	FCGRT
ENSG00000234773.7	-0.011453776	0.539590025	0.927917226	CTD-2666L21.1
ENSG00000266088.6	-0.011502114	0.539610925	0.927917226	RP5-1028K7.2
ENSG00000174136.13	-0.014813323	0.539859632	0.928267068	RGMB
ENSG00000198420.10	-0.00941046	0.540024719	0.928473084	TCAF1
ENSG00000285897.1	0.011356789	0.540156048	0.928621028	RP5-1171I10.8
ENSG00000145349.18	0.006537989	0.540312011	0.928761417	CAMK2D
ENSG00000159409.15	0.013225048	0.540363455	0.928761417	CELF3
ENSG00000232187.1	0.007844987	0.540381834	0.928761417	FTH1P7
ENSG00000285184.3	0.015484238	0.540438045	0.928761417	CH17-55D9.1
ENSG00000160691.19	-0.003902131	0.540481807	0.928761417	SHC1
ENSG00000262098.1	-0.02675968	0.540537893	0.928761417	CTD-2561B21.10

ENSG00000167549.19	-0.01168237	0.54056772	0.928761417	CORO6
ENSG00000155275.19	-0.003546226	0.540600011	0.928761417	TRMT44
ENSG00000164309.15	0.019305306	0.540678527	0.928818499	CMYA5
ENSG00000171611.10	0.014277193	0.540949348	0.929139669	PTCRA
ENSG00000155254.13	0.008270455	0.540956097	0.929139669	MARVELD1
ENSG00000241549.8	-0.021657374	0.541067106	0.929196804	GUSBP2
ENSG00000100146.18	0.017728345	0.54107998	0.929196804	SOX10
ENSG00000136235.17	0.021908381	0.541175935	0.929280104	GPNMB
ENSG00000246985.8	0.009907442	0.541219113	0.929280104	SOCS2-AS1
ENSG00000122490.19	-0.006847672	0.541344304	0.929415226	SLC66A2
ENSG00000286721.1	-0.019986449	0.541410286	0.929415226	CTD-2093G19.1
ENSG00000164414.18	0.008381256	0.541433768	0.929415226	SLC35A1
ENSG00000130561.17	0.027467323	0.541636023	0.929452893	SAG
ENSG00000146416.19	0.005824141	0.541667404	0.929452893	AIG1
ENSG00000072756.17	-0.006138968	0.541719873	0.929452893	TRNT1
ENSG00000110092.4	0.010206055	0.541757699	0.929452893	CCND1
ENSG00000136643.12	0.004303272	0.541802502	0.929452893	RPS6KC1
ENSG00000132849.21	0.006485458	0.541876584	0.929452893	PATJ
ENSG00000245498.7	0.011585889	0.54188426	0.929452893	RP11-677M14.7
ENSG00000118513.19	-0.009625912	0.541891537	0.929452893	MYB
ENSG00000154743.19	0.005806372	0.541905854	0.929452893	TSEN2
ENSG00000242580.1	-0.02873109	0.541908925	0.929452893	IGKV1D-43
ENSG00000172037.14	-0.015638077	0.54196786	0.929467554	LAMB2
ENSG00000163931.16	0.005436945	0.542008118	0.929467554	TKT
ENSG00000112159.12	0.005408898	0.542097656	0.929481628	MDN1
ENSG00000182379.10	0.025408558	0.542156529	0.929481628	NXPH4
ENSG00000167196.14	0.004843948	0.542171551	0.929481628	FBXO22
ENSG00000105808.18	0.014102961	0.542208481	0.929481628	RASA4
ENSG00000125868.16	0.005166219	0.542282059	0.929481628	DSTN
ENSG00000141349.9	0.005975823	0.542288262	0.929481628	G6PC3
ENSG00000273084.1	-0.016829217	0.542365564	0.929536435	RP11-1275H24.3
ENSG00000141485.17	0.025787453	0.542475859	0.929564738	SLC13A5
ENSG00000142669.15	0.007093106	0.542493724	0.929564738	SH3BGRL3
ENSG00000105968.19	-0.002612709	0.542518059	0.929564738	H2AZ2
ENSG00000180881.19	-0.011930438	0.542593102	0.929573912	CAPS2
ENSG00000214485.6	0.022688411	0.542674543	0.929573912	RPL7P1
ENSG00000197705.10	0.018345056	0.542731486	0.929573912	KLHL14
ENSG00000122435.10	-0.009528551	0.542766742	0.929573912	TRMT13
ENSG00000175073.8	0.006802382	0.542824204	0.929573912	VCPIP1
ENSG00000131401.11	0.014527909	0.542834393	0.929573912	NAPSB
ENSG00000227295.2	0.025187747	0.542840704	0.929573912	ELL2P1
ENSG00000204946.10	-0.00372577	0.543008041	0.929718606	ZNF783
ENSG00000126262.5	0.008555574	0.54301587	0.929718606	FFAR2
ENSG00000232093.1	0.014213641	0.543234196	0.929940879	DCST1-AS1
ENSG00000245080.7	0.020766692	0.543275834	0.929940879	MIR3150BHG
ENSG00000235962.5	-0.019486412	0.543281728	0.929940879	RPL7AP53
ENSG00000154589.7	0.012458096	0.543413368	0.930021545	LY96

ENSG00000187118.14	-0.00939703	0.543426983	0.930021545	CMC1
ENSG00000130958.13	0.006297393	0.543522294	0.930021545	SLC35D2
ENSG00000284688.1	0.02614364	0.543579625	0.930021545	BTN1A1P1
ENSG00000273355.1	0.019111133	0.543585206	0.930021545	RP11-672L10.6
ENSG00000119048.8	0.005690654	0.543600949	0.930021545	UBE2B
ENSG00000279048.1	-0.012732217	0.543650096	0.930028042	RP11-511H23.2
ENSG00000131781.13	-0.007186601	0.543717377	0.930065557	FMO5
ENSG00000117620.15	-0.007257446	0.54384591	0.930207833	SLC35A3
ENSG00000033122.21	0.017885157	0.543946482	0.930216692	LRRC7
ENSG00000255314.4	-0.030972092	0.543971292	0.930216692	RP11-702F3.3
ENSG00000136099.13	-0.072516234	0.544004073	0.930216692	PCDH8
ENSG00000260412.1	-0.029459153	0.544032524	0.930216692	RP11-438B23.2
ENSG00000137992.15	0.005179549	0.5441866	0.930398612	DBT
ENSG00000170509.12	0.012801155	0.544229654	0.930398612	HSD17B13
ENSG00000107954.10	-0.010909961	0.544302638	0.93044582	NEURL1
ENSG00000049860.14	0.004314163	0.544642005	0.930724233	HEXB
ENSG00000107719.9	-0.012116635	0.544695914	0.930724233	PALD1
ENSG00000148218.16	-0.003133782	0.544739419	0.930724233	ALAD
ENSG00000176170.14	-0.009645083	0.54483977	0.930724233	SPHK1
ENSG00000166794.6	-0.005007378	0.544867045	0.930724233	PPIB
ENSG00000232504.5	-0.018288709	0.544873491	0.930724233	ST3GAL5-AS1
ENSG00000112130.17	-0.004335752	0.544894501	0.930724233	RNF8
ENSG00000257242.8	-0.010132266	0.544932249	0.930724233	LINC01619
ENSG00000156304.15	-0.004690649	0.544968644	0.930724233	SCAF4
ENSG00000184007.22	0.003716148	0.545020959	0.930724233	PTP4A2
ENSG00000204599.14	-0.003546065	0.545024913	0.930724233	TRIM39
ENSG00000173465.8	0.005962467	0.545164257	0.930724233	ZNRD2
ENSG00000169136.12	0.007996155	0.545199946	0.930724233	ATF5
ENSG00000284154.1	0.016378924	0.545220175	0.930724233	MIR3605
ENSG00000172613.8	-0.004761592	0.545240839	0.930724233	RAD9A
ENSG00000081665.14	0.004446614	0.545245009	0.930724233	ZNF506
ENSG00000197363.10	-0.004773571	0.545310801	0.930724233	ZNF517
ENSG00000270903.1	0.022022014	0.545341013	0.930724233	HNRNPA3P9
ENSG00000165271.17	0.00420135	0.545368321	0.930724233	NOL6
ENSG00000223361.5	0.019965761	0.545373177	0.930724233	FTH1P10
ENSG00000178057.15	0.00685093	0.545468451	0.930809368	NDUFAF3
ENSG00000211706.2	-0.013574285	0.545520807	0.930821259	TRBV6-1
ENSG00000240342.3	0.01587142	0.545579889	0.930844623	RPS2P5
ENSG00000126391.14	-0.00412391	0.545696613	0.930872631	FRMD8
ENSG00000143183.18	0.009850435	0.545733399	0.930872631	TMCO1
ENSG00000168036.18	-0.002685067	0.545763172	0.930872631	CTNNB1
ENSG00000253816.3	-0.023976253	0.545777868	0.930872631	GUSBP14
ENSG00000204435.15	0.005762897	0.545899512	0.930975085	CSNK2B
ENSG00000198178.11	0.016919341	0.545928729	0.930975085	CLEC4C
ENSG00000077348.9	0.005664254	0.546031327	0.93106776	EXOSC5
ENSG00000121310.17	-0.005887886	0.546073875	0.93106776	ECHDC2
ENSG00000083123.15	-0.005574468	0.54617253	0.931109347	BCKDHB

ENSG00000135913.11	0.010822408	0.54618907	0.931109347	USP37
ENSG00000146112.12	0.004886853	0.546281988	0.931115253	PPP1R18
ENSG00000235522.6	-0.022890251	0.546306074	0.931115253	AC009505.2
ENSG00000272645.3	0.00833838	0.546335237	0.931115253	GTF2IP20
ENSG00000223914.4	-0.022403198	0.546374144	0.931115253	LINC02471
ENSG00000279149.1	-0.012568355	0.546594803	0.931189458	RP11-374F3.5
ENSG00000164112.14	-0.030650031	0.546609456	0.931189458	SMIM43
ENSG00000213413.2	-0.015861833	0.546640597	0.931189458	PVRIG
ENSG00000174917.9	0.006603612	0.546640708	0.931189458	MICOS13
ENSG00000253846.3	-0.023606474	0.546830839	0.931189458	PCDHGA10
ENSG00000259709.1	-0.029131232	0.546984972	0.931189458	CTD-2184D3.7
ENSG00000152749.8	-0.008447372	0.546986011	0.931189458	GPR180
ENSG00000229162.1	0.013947246	0.547036883	0.931189458	RUNX3-AS1
ENSG00000007038.11	-0.023226889	0.547084179	0.931189458	PRSS21
ENSG00000122971.9	-0.005720482	0.547101779	0.931189458	ACADS
ENSG00000124733.5	0.005057749	0.547111063	0.931189458	MEA1
ENSG00000090975.14	0.008008762	0.547190815	0.931189458	PITPNM2
ENSG00000284713.1	0.023711371	0.547193576	0.931189458	SMIM38
ENSG00000151320.11	0.016296088	0.547249328	0.931189458	AKAP6
ENSG00000004534.15	0.005314859	0.547293319	0.931189458	RBM6
ENSG00000254415.3	-0.021531843	0.547313421	0.931189458	SIGLEC14
ENSG00000048405.10	-0.003983156	0.547337791	0.931189458	ZNF800
ENSG00000162664.17	0.005663225	0.547612417	0.931189458	ZNF326
ENSG00000285043.3	0.013971309	0.547640439	0.931189458	CTD-2515O10.6
ENSG00000235082.2	-0.017168161	0.547649767	0.931189458	SUMO1P3
ENSG00000177483.12	-0.015556064	0.547651763	0.931189458	RBM44
ENSG00000228382.1	0.00962475	0.547685642	0.931189458	ITPKB-IT1
ENSG00000119041.11	0.005020393	0.547707515	0.931189458	GTF3C3
ENSG00000105479.16	-0.012008921	0.547830799	0.931189458	ODAD1
ENSG00000268510.1	0.017695731	0.547880807	0.931189458	IFNL3P1
ENSG00000223878.1	0.014711032	0.547881204	0.931189458	PPIAP53
ENSG00000177613.9	-0.002896676	0.547923454	0.931189458	CSTF2T
ENSG00000125945.15	0.004483129	0.548072517	0.931189458	ZNF436
ENSG00000185019.17	0.005246326	0.54812105	0.931189458	UBOX5
ENSG00000120451.11	0.005595541	0.548176699	0.931189458	SNX19
ENSG00000137563.13	-0.011094492	0.548218833	0.931189458	GGH
ENSG00000233230.1	-0.010079895	0.548271198	0.931189458	AC079807.2
ENSG00000224831.3	0.01029444	0.548291906	0.931189458	TMEM183B
ENSG00000273568.1	-0.013623845	0.548400674	0.931189458	RP11-417L19.6
ENSG00000204010.3	0.021166422	0.548429593	0.931189458	IFIT1B
ENSG00000010256.11	-0.00543465	0.548433404	0.931189458	UQCRC1
ENSG00000127954.13	0.008633031	0.548435713	0.931189458	STEAP4
ENSG00000060971.19	-0.003947136	0.548468431	0.931189458	ACAA1
ENSG00000164199.18	-0.019027278	0.548559921	0.931189458	ADGRV1
ENSG00000196345.13	0.00820942	0.548564071	0.931189458	ZKSCAN7
ENSG00000185963.14	0.004443145	0.548567256	0.931189458	BICD2
ENSG00000161298.18	-0.008144592	0.548648031	0.931189458	ZNF382

ENSG00000148484.18	0.004882931	0.548711023	0.931189458	RSU1
ENSG00000144134.19	-0.004483332	0.548720883	0.931189458	RABL2A
ENSG00000207697.1	-0.020048243	0.548741077	0.931189458	MIR573
ENSG00000269481.1	-0.01214839	0.54879713	0.931189458	CTD-2521M24.6
ENSG00000160339.16	-0.02237413	0.548806408	0.931189458	FCN2
ENSG00000066117.15	0.003499866	0.5488984	0.931189458	SMARCD1
ENSG00000269038.1	-0.014309418	0.548903758	0.931189458	SF1-DT
ENSG00000181982.19	0.007648835	0.548932853	0.931189458	CCDC149
ENSG00000164088.18	-0.004237599	0.548977986	0.931189458	PPM1M
ENSG00000284664.1	0.014388717	0.549085014	0.931189458	RP11-712C19.2
ENSG00000255422.3	-0.02381471	0.549091371	0.931189458	AP002954.4
ENSG00000132330.18	0.006447839	0.54910334	0.931189458	SCLY
ENSG00000240591.2	-0.01135985	0.549130846	0.931189458	RP11-247I13.11
ENSG00000081237.20	0.006121206	0.549176277	0.931189458	PTPRC
ENSG00000105738.11	-0.007142847	0.549231913	0.931189458	SIPA1L3
ENSG00000176371.14	-0.004475225	0.549234171	0.931189458	ZSCAN2
ENSG00000276791.1	-0.010534591	0.549243166	0.931189458	CTD-2270P14.5
ENSG00000205250.9	0.003380369	0.549259805	0.931189458	E2F4
ENSG00000166924.9	0.01502222	0.549310199	0.931189458	NYAP1
ENSG00000122779.18	0.004416546	0.549428709	0.931189458	TRIM24
ENSG00000175893.12	-0.01103156	0.549481636	0.931189458	ZDHHC21
ENSG00000255733.6	0.018677486	0.549499876	0.931189458	IFNG-AS1
ENSG00000130222.11	-0.011540041	0.549534637	0.931189458	GADD45G
ENSG00000181894.15	-0.005466683	0.549541435	0.931189458	ZNF329
ENSG00000215859.9	-0.010263252	0.549552867	0.931189458	PDZK1P1
ENSG00000211895.5	-0.024096295	0.549564361	0.931189458	IGHA1
ENSG00000169018.6	0.004955272	0.549570801	0.931189458	FEM1B
ENSG00000270804.1	0.008861504	0.549776182	0.931189458	CTD-2583A14.11
ENSG00000239855.1	-0.022424149	0.54980458	0.931189458	IGKV1-6
ENSG00000151327.13	-0.004393259	0.54981424	0.931189458	FAM177A1
ENSG00000163714.18	0.005736815	0.549817284	0.931189458	U2SURP
ENSG00000238098.9	0.015709649	0.549828949	0.931189458	ABCA17P
ENSG00000188396.4	-0.012084764	0.549860982	0.931189458	DYNLT4
ENSG00000128340.15	-0.005108126	0.549933329	0.931189458	RAC2
ENSG00000233673.7	-0.030299697	0.550017381	0.931189458	ANAPC1P1
ENSG00000105323.17	-0.004377662	0.55005225	0.931189458	HNRNPUL1
ENSG00000269235.2	-0.012951912	0.550074	0.931189458	ZNF350-AS1
ENSG00000285534.1	-0.028531711	0.550093123	0.931189458	RP11-358F13.1
ENSG00000211625.2	0.024101175	0.550095587	0.931189458	IGKV3D-20
ENSG00000128567.17	-0.008925105	0.550257932	0.931292532	PODXL
ENSG00000078399.19	-0.018936847	0.550304119	0.931292532	HOXA9
ENSG00000165118.15	-0.004193358	0.550343054	0.931292532	C9orf64
ENSG00000233429.9	0.012733609	0.5503459	0.931292532	HOTAIRM1
ENSG00000136021.19	0.00598511	0.550427286	0.931292532	SCYL2
ENSG00000123411.15	0.005523137	0.550428944	0.931292532	IKZF4
ENSG00000085276.19	0.030875139	0.550557361	0.931432962	MECOM
ENSG00000175606.11	0.005684794	0.55075438	0.931439592	TMEM70

ENSG00000237938.5	0.009892778	0.550757729	0.931439592	RP11-288I21.1
ENSG00000245468.4	0.017977288	0.550793949	0.931439592	LINC02447
ENSG00000250654.7	-0.011374943	0.550818037	0.931439592	RP11-834C11.7
ENSG00000267169.2	0.005307658	0.550890669	0.931439592	ADGRL1-AS1
ENSG00000100167.20	0.018475658	0.550908871	0.931439592	SEPTIN3
ENSG00000232656.9	-0.014260602	0.550931825	0.931439592	IDI2-AS1
ENSG00000267733.5	0.026586373	0.551007145	0.931439592	RP11-64C12.3
ENSG00000229656.7	-0.022621226	0.551042306	0.931439592	ITGB1-DT
ENSG00000153071.15	-0.008685455	0.551168655	0.931439592	DAB2
ENSG00000250988.7	0.010819746	0.551210353	0.931439592	SNHG21
ENSG00000236618.2	-0.010374395	0.551260325	0.931439592	PITPNA-AS1
ENSG00000162877.13	0.026253219	0.551286943	0.931439592	PM20D1
ENSG00000197136.5	-0.004231628	0.551296871	0.931439592	PCNX3
ENSG00000186862.20	-0.012523744	0.551319213	0.931439592	PDZD7
ENSG00000154229.12	0.007782104	0.551329198	0.931439592	PRKCA
ENSG00000185339.9	0.012498447	0.551393832	0.931439592	TCN2
ENSG00000071082.11	0.015631487	0.551502881	0.931439592	RPL31
ENSG00000180535.4	0.022132198	0.551558012	0.931439592	BHLHA15
ENSG00000131797.12	0.008131712	0.55158019	0.931439592	CLUHP3
ENSG00000211670.2	-0.022302139	0.55159639	0.931439592	IGLV3-9
ENSG00000110315.7	0.006356794	0.5516505	0.931439592	RNF141
ENSG00000259065.2	0.017443464	0.551676957	0.931439592	RP5-1021I20.1
ENSG00000228887.3	0.017962905	0.551701971	0.931439592	EEF1DP1
ENSG00000260361.1	0.010777472	0.551738192	0.931439592	CTD-2313J17.5
ENSG00000136152.15	-0.004127882	0.551785034	0.931439592	COG3
ENSG00000182534.14	-0.018627979	0.551787576	0.931439592	MXRA7
ENSG00000275111.5	0.003841931	0.551932543	0.931607621	ZNF2
ENSG00000183891.6	0.009466763	0.552079419	0.931778844	TTC32
ENSG00000170322.15	-0.00384736	0.552156937	0.931823741	NFRKB
ENSG00000182512.5	0.013092451	0.552214509	0.931823741	GLRX5
ENSG00000227017.1	0.02811617	0.552256914	0.931823741	AC007036.6
ENSG00000204267.16	0.007683398	0.552350284	0.931823741	TAP2
ENSG00000173653.8	-0.004942521	0.5523656	0.931823741	RCE1
ENSG00000288531.1	-0.035600986	0.552447967	0.931823741	RP11-206L10.19
ENSG00000079691.18	-0.011902622	0.552451416	0.931823741	CARMIL1
ENSG00000104903.5	-0.010945897	0.552478887	0.931823741	LYL1
ENSG00000130332.15	-0.006675208	0.552646722	0.931823741	LSM7
ENSG00000275734.2	-0.013822061	0.552737872	0.931823741	RP11-368I7.6
ENSG00000010327.10	0.008727197	0.552820538	0.931823741	STAB1
ENSG00000119782.14	0.013788874	0.552859869	0.931823741	FKBP1B
ENSG00000010292.13	-0.004756772	0.552860024	0.931823741	NCAPD2
ENSG00000215022.7	-0.01047031	0.552861603	0.931823741	RP1-257A7.4
ENSG00000288043.1	-0.034229451	0.552868778	0.931823741	RP11-69M1.9
ENSG00000258539.1	-0.014430044	0.55288376	0.931823741	RP11-12J10.3
ENSG00000275896.6	-0.0375959	0.552892183	0.931823741	PRSS2
ENSG00000171954.13	-0.017286041	0.552923888	0.931823741	CYP4F22
ENSG00000116128.12	0.006410046	0.553050588	0.931884447	BCL9

ENSG00000151247.13	0.006161447	0.55305079	0.931884447	EIF4E
ENSG00000173914.12	0.00393006	0.553213753	0.932055608	RBM4B
ENSG00000236056.1	-0.028744736	0.55324874	0.932055608	GAPDHP14
ENSG00000007314.12	-0.018176927	0.55329541	0.932055608	SCN4A
ENSG00000258794.3	-0.031416747	0.553429369	0.932055608	DUX4L27
ENSG00000103066.13	0.005877391	0.553444432	0.932055608	PLA2G15
ENSG00000011590.14	0.016361803	0.553445937	0.932055608	ZBTB32
ENSG00000260927.2	-0.010108229	0.553470509	0.932055608	RP11-459F6.3
ENSG00000215493.3	0.018175696	0.553643956	0.932271144	XXbac-B562F10.11
ENSG00000178773.15	0.016351857	0.553726447	0.932285809	CPNE7
ENSG00000166887.16	0.003062575	0.553743585	0.932285809	VPS39
ENSG00000138796.18	0.003132116	0.553812244	0.932308338	HADH
ENSG00000185518.12	-0.027798924	0.553847888	0.932308338	SV2B
ENSG00000137709.10	-0.062074063	0.553907743	0.932332568	POU2F3
ENSG00000240898.1	0.014072157	0.554016569	0.932374532	RP11-48B14.1
ENSG00000235859.5	0.005128092	0.554023603	0.932374532	AC006978.6
ENSG00000287385.1	-0.011989758	0.554081823	0.932395997	CTD-2325P2.5
ENSG00000167751.13	0.074688911	0.554279868	0.932585928	KLK2
ENSG00000256628.3	0.007067935	0.554363862	0.932585928	ZBTB11-AS1
ENSG00000260645.2	-0.018472991	0.554452344	0.932585928	RP11-250B2.5
ENSG00000174032.17	0.004379185	0.554588144	0.932585928	SLC25A30
ENSG00000175826.12	-0.005199567	0.554590415	0.932585928	CTDNEP1
ENSG00000086589.12	-0.002441233	0.554643005	0.932585928	RBM22
ENSG00000211676.2	-0.029237288	0.554715879	0.932585928	IGLJ2
ENSG00000141452.10	-0.00345524	0.554777487	0.932585928	RMC1
ENSG00000174282.12	-0.003776641	0.554796339	0.932585928	ZBTB4
ENSG00000101745.17	0.008410396	0.554827769	0.932585928	ANKRD12
ENSG00000200235.1	0.022976446	0.554837181	0.932585928	ENSG10010134471.1
ENSG00000251692.8	-0.018129562	0.554853903	0.932585928	PTX4
ENSG00000167618.10	-0.021947779	0.554928329	0.932585928	LAIR2
ENSG00000270951.1	0.020391168	0.55497251	0.932585928	RP1-309F20.4
ENSG00000170832.13	0.004961234	0.555084289	0.932585928	USP32
ENSG00000213185.7	0.008632673	0.555085775	0.932585928	FAM24B
ENSG00000125650.4	-0.006665394	0.555100272	0.932585928	PSPN
ENSG00000140563.15	0.006378584	0.555133708	0.932585928	MCTP2
ENSG00000251161.4	-0.013756297	0.555158818	0.932585928	RP11-540O11.1
ENSG00000064999.15	-0.00406624	0.555232061	0.932585928	ANKS1A
ENSG00000135362.14	-0.008570428	0.555272157	0.932585928	PRR5L
ENSG00000100442.11	-0.005344592	0.555301272	0.932585928	FKBP3
ENSG00000043143.22	-0.003749061	0.555393936	0.932585928	JADE2
ENSG00000108587.16	0.002571009	0.555405508	0.932585928	GOSR1
ENSG00000130414.13	0.002783802	0.555408696	0.932585928	NDUFA10
ENSG00000125166.13	-0.004425544	0.555586003	0.932585928	GOT2
ENSG00000074356.17	-0.002889111	0.555633096	0.932585928	NCBP3
ENSG00000079385.23	0.011210973	0.555705072	0.932585928	CEACAM1
ENSG00000203288.4	-0.015842649	0.55571177	0.932585928	TDRKH-AS1
ENSG00000056097.16	0.004851741	0.555715241	0.932585928	ZFR

ENSG00000005102.14	-0.014794289	0.555826606	0.932585928	MEOX1
ENSG00000074803.20	-0.045960249	0.555872731	0.932585928	SLC12A1
ENSG00000174928.16	-0.015216782	0.555933996	0.932585928	C3orf33
ENSG00000142599.19	0.005169385	0.555942923	0.932585928	RERE
ENSG00000170075.10	-0.019325753	0.556004909	0.932585928	GPR37L1
ENSG00000154127.10	0.00492309	0.55600817	0.932585928	UBASH3B
ENSG00000111666.11	0.010894144	0.556010171	0.932585928	CHPT1
ENSG00000200648.1	0.024710539	0.556048014	0.932585928	RNU6-226P
ENSG00000182841.12	-0.007154355	0.55605535	0.932585928	RRP7BP
ENSG00000075975.17	0.003538221	0.556056413	0.932585928	MKRN2
ENSG00000144852.20	-0.018139867	0.556072473	0.932585928	NR1I2
ENSG00000120662.16	-0.005284276	0.556104609	0.932585928	MTRF1
ENSG00000172780.17	0.008989988	0.556268148	0.932640307	RAB43
ENSG00000213799.13	0.006943951	0.556279087	0.932640307	ZNF845
ENSG00000234665.9	-0.075443044	0.556298903	0.932640307	LERFS
ENSG00000230912.1	0.019508023	0.556329859	0.932640307	RP3-508I15.10
ENSG00000141499.17	-0.004754465	0.556366663	0.932640307	WRAP53
ENSG00000250130.1	-0.023065257	0.55646381	0.932640307	GAPDHP43
ENSG00000248636.7	0.018001829	0.556639768	0.932640307	RP11-768F21.1
ENSG00000181192.12	0.004126115	0.556665218	0.932640307	DHTKD1
ENSG00000186088.16	-0.007176691	0.556720223	0.932640307	GSAP
ENSG00000103342.13	0.009524641	0.556748638	0.932640307	GSPT1
ENSG00000087301.9	0.00598725	0.556749234	0.932640307	TXNDC16
ENSG00000166263.13	-0.008286558	0.556816819	0.932640307	STXBP4
ENSG00000140350.15	0.00335263	0.556871982	0.932640307	ANP32A
ENSG00000167103.12	0.014072631	0.556891329	0.932640307	PIP5KL1
ENSG00000224739.2	0.024423079	0.556936288	0.932640307	AC016735.1
ENSG00000267898.1	-0.011286795	0.556966752	0.932640307	CTD-2639E6.9
ENSG00000269053.1	-0.012208494	0.556975497	0.932640307	CTD-2521M24.8
ENSG00000180245.7	-0.021691654	0.557119639	0.932640307	RRH
ENSG00000123213.23	0.007240677	0.557128034	0.932640307	NLN
ENSG00000203644.3	0.007516382	0.557174215	0.932640307	RP11-332M2.1
ENSG00000233483.3	0.011408148	0.557177015	0.932640307	EFCAB15P
ENSG00000204351.12	-0.004255105	0.557210235	0.932640307	SKIV2L
ENSG00000124177.16	0.005227975	0.557229293	0.932640307	CHD6
ENSG00000198231.13	-0.002751273	0.557255116	0.932640307	DDX42
ENSG00000135392.19	0.004033237	0.557273957	0.932640307	DNAJC14
ENSG00000167992.13	0.019928129	0.55740113	0.932769152	VWCE
ENSG00000271966.1	-0.014066054	0.557441911	0.932769152	ARFGEF1-DT
ENSG00000196678.14	-0.009301394	0.557552953	0.932841726	ERI2
ENSG00000126457.22	-0.004247139	0.557618789	0.932841726	PRMT1
ENSG00000237693.4	-0.013654065	0.5576899	0.932841726	IRGM
ENSG00000283597.3	0.017778341	0.557697238	0.932841726	FAM169B
ENSG00000101278.6	0.014344864	0.557962601	0.932841726	RPS10P5
ENSG00000183298.5	0.021494234	0.558065486	0.932841726	RPSAP19
ENSG00000139998.16	-0.008331181	0.558091779	0.932841726	RAB15
ENSG00000174775.18	-0.00604067	0.558170272	0.932841726	HRAS

ENSG00000196975.16	0.004925699	0.558202934	0.932841726	ANXA4
ENSG00000167395.10	-0.004492254	0.558250914	0.932841726	ZNF646
ENSG00000142178.9	-0.054160581	0.558300646	0.932841726	SIK1
ENSG00000111181.13	-0.011004594	0.55831313	0.932841726	SLC6A12
ENSG00000100968.14	0.02623679	0.558353	0.932841726	NFATC4
ENSG00000163069.13	-0.008250568	0.558366507	0.932841726	SGCB
ENSG00000179604.10	0.005784051	0.558465498	0.932841726	CDC42EP4
ENSG00000225872.4	0.014974139	0.558497703	0.932841726	LINC01529
ENSG00000243927.6	0.004530244	0.558511435	0.932841726	MRPS6
ENSG00000164120.14	0.013330406	0.558516574	0.932841726	HPGD
ENSG00000140299.13	0.004935808	0.558525335	0.932841726	BNIP2
ENSG00000263272.1	0.004577998	0.558526791	0.932841726	CTC-524C5.2
ENSG00000183814.16	0.010861977	0.558543834	0.932841726	LIN9
ENSG00000078018.21	0.022383297	0.558553684	0.932841726	MAP2
ENSG00000232926.1	0.01751104	0.558576872	0.932841726	AC000078.5
ENSG00000228960.6	-0.026753792	0.558581453	0.932841726	OR2A9P
ENSG00000264364.3	-0.00337101	0.558685815	0.932841726	DYNLL2
ENSG00000106789.13	-0.005648829	0.558710491	0.932841726	CORO2A
ENSG00000180011.7	-0.004707587	0.558713425	0.932841726	ZADH2
ENSG00000249572.1	0.023191561	0.558787519	0.932889486	CTD-2203K17.1
ENSG00000002745.13	-0.022055583	0.558929018	0.932948382	WNT16
ENSG00000121104.8	0.005490766	0.559092019	0.932948382	FAM117A
ENSG00000146066.3	0.006705038	0.559095014	0.932948382	HIGD2A
ENSG00000244649.5	-0.02957777	0.559146299	0.932948382	LINC02086
ENSG00000134352.20	0.009416251	0.559161811	0.932948382	IL6ST
ENSG00000287926.1	0.01266129	0.559182786	0.932948382	RP11-69H7.5
ENSG00000114554.12	-0.005660366	0.559225879	0.932948382	PLXNA1
ENSG00000273483.1	-0.019978211	0.559255705	0.932948382	RP4-671G15.2
ENSG00000213934.9	0.072314928	0.559262726	0.932948382	HBG1
ENSG00000235290.1	-0.024094809	0.559319793	0.932948382	HLA-W
ENSG00000129749.3	-0.006787363	0.55936002	0.932948382	CHRNA10
ENSG00000183718.5	0.006229046	0.559369274	0.932948382	TRIM52
ENSG00000205740.2	0.017424209	0.559426735	0.932948382	RP11-363N22.3
ENSG00000203747.12	0.007996002	0.559501829	0.932948382	FCGR3A
ENSG00000254319.6	0.032678221	0.559505176	0.932948382	RP11-134O21.1
ENSG00000128513.16	0.006585319	0.559617806	0.933006261	POT1
ENSG00000248015.7	-0.017426212	0.559630877	0.933006261	AC005329.7
ENSG00000278991.1	-0.025035251	0.559737526	0.933070251	RP11-797A18.5
ENSG00000164086.10	-0.003838941	0.559889063	0.933070251	DUSP7
ENSG00000095261.14	0.003218046	0.559902498	0.933070251	PSMD5
ENSG00000124134.9	-0.008778951	0.559923947	0.933070251	KCNS1
ENSG00000230359.5	0.019100712	0.560062563	0.933070251	TPI1P2
ENSG00000230539.1	0.015643273	0.560067033	0.933070251	AOAH-IT1
ENSG00000225402.1	0.017112744	0.560067619	0.933070251	AC010878.3
ENSG00000196268.12	-0.007260562	0.560139161	0.933070251	ZNF493
ENSG00000068976.14	-0.005736613	0.560143115	0.933070251	PYGM
ENSG00000009954.11	-0.004111382	0.56018359	0.933070251	BAZ1B

ENSG00000158528.12	0.020298554	0.560186344	0.933070251	PPP1R9A
ENSG00000221946.8	0.012086454	0.560215233	0.933070251	FXYD7
ENSG00000151468.11	0.029692429	0.560461147	0.933344226	CCDC3
ENSG00000177096.9	0.00700065	0.560479836	0.933344226	PHETA2
ENSG00000146166.17	0.038012426	0.560519634	0.933344226	LGSN
ENSG00000025039.15	0.005196793	0.560561773	0.933344226	RRAGD
ENSG00000110934.13	0.003347898	0.560618122	0.93336227	BIN2
ENSG00000063015.20	0.016377148	0.560730816	0.933474111	SEZ6
ENSG00000143375.15	-0.018503103	0.560838615	0.933567605	CGN
ENSG00000176124.15	-0.006097863	0.560980531	0.933567605	DLEU1
ENSG00000271447.6	0.018728525	0.561012366	0.933567605	MMP28
ENSG00000256250.1	-0.033367447	0.561014449	0.933567605	RP11-989F5.1
ENSG00000277763.1	0.010494138	0.561100736	0.933567605	CTD-2588E21.1
ENSG00000236469.1	-0.033066209	0.561153462	0.933567605	AC007040.8
ENSG00000256742.1	-0.011621932	0.561182504	0.933567605	KDM2B-DT
ENSG00000138629.16	0.006281511	0.561272892	0.933567605	UBL7
ENSG00000248905.10	0.026892876	0.561295301	0.933567605	FMN1
ENSG00000170185.11	0.004664527	0.561297678	0.933567605	USP38
ENSG00000135925.9	-0.011442259	0.561318363	0.933567605	WNT10A
ENSG00000186648.15	-0.013876	0.561376658	0.933567605	CARMIL3
ENSG00000184162.15	-0.004906396	0.561470624	0.933567605	NR2C2AP
ENSG00000215041.10	0.003797767	0.561508951	0.933567605	NEURL4
ENSG00000286809.1	0.010324181	0.561509565	0.933567605	CTD-2360A17.3
ENSG00000171714.13	0.016167309	0.561589503	0.933567605	ANO5
ENSG00000173327.8	-0.005537286	0.56159625	0.933567605	MAP3K11
ENSG00000106266.11	-0.006062872	0.561653089	0.933567605	SNX8
ENSG00000170522.10	0.009425396	0.561762252	0.933567605	ELOVL6
ENSG00000142621.20	-0.018901419	0.561785617	0.933567605	FHAD1
ENSG00000163517.15	-0.007589496	0.561813723	0.933567605	HDAC11
ENSG00000272182.1	-0.013562972	0.561892715	0.933567605	RP11-802O23.3
ENSG00000113303.12	0.013015738	0.561937921	0.933567605	BTNL8
ENSG00000215571.5	-0.021284949	0.561963854	0.933567605	GRK6P1
ENSG00000225527.1	0.021984271	0.561986059	0.933567605	RP11-383B4.4
ENSG00000107341.5	0.004323486	0.561993332	0.933567605	UBE2R2
ENSG00000171401.15	-0.02179919	0.562016074	0.933567605	KRT13
ENSG00000225964.6	-0.024905157	0.562146425	0.933575108	NRIR
ENSG00000207042.1	-0.01580516	0.562174624	0.933575108	RNU6-196P
ENSG00000133101.10	0.02499766	0.562208188	0.933575108	CCNA1
ENSG00000259692.6	0.049104749	0.562274151	0.933575108	RP11-499F3.2
ENSG00000268049.1	-0.012584172	0.562291235	0.933575108	CTD-2619J13.9
ENSG00000169403.12	0.00534472	0.562293726	0.933575108	PTAFR
ENSG00000260101.1	0.010313401	0.562357341	0.933605144	RP11-568N6.1
ENSG00000019991.18	0.007804931	0.562630397	0.933862546	HGF
ENSG00000276180.1	0.009625883	0.562683878	0.933862546	H4C9
ENSG00000240041.1	0.018275468	0.562780502	0.933862546	IGHJ4
ENSG00000283023.1	-0.02019664	0.562851039	0.933862546	FRG1GP
ENSG00000116863.11	-0.004571059	0.562886988	0.933862546	ADPRS

ENSG00000229391.7	0.021040624	0.562929908	0.933862546	HLA-DRB6
ENSG00000184988.8	0.005189543	0.562954827	0.933862546	TMEM106A
ENSG00000107130.10	-0.015667265	0.562955322	0.933862546	NCS1
ENSG00000137075.18	0.003550944	0.562960523	0.933862546	RNF38
ENSG00000006704.11	0.01747409	0.562967752	0.933862546	GTF2IRD1
ENSG00000196890.4	-0.01491889	0.563090171	0.933942817	H2BU1
ENSG00000272990.1	0.010349984	0.56312043	0.933942817	RP11-305K5.1
ENSG00000237576.2	0.016001725	0.563237112	0.933942817	LINC01888
ENSG00000198700.10	0.002814125	0.563240532	0.933942817	IPO9
ENSG00000283041.1	0.012143767	0.56335664	0.933942817	RP11-371A22.1
ENSG00000229036.7	-0.007221334	0.563375601	0.933942817	VDAC1P8
ENSG00000144744.17	0.005808988	0.563409429	0.933942817	UBA3
ENSG00000172638.13	-0.006334861	0.563423316	0.933942817	EFEMP2
ENSG00000278745.1	0.036254749	0.563432538	0.933942817	RP1-229K20.9
ENSG00000273056.1	0.020266042	0.56350436	0.933942817	RP11-77E14.2
ENSG00000048392.12	0.007382715	0.563517087	0.933942817	RRM2B
ENSG00000188015.10	-0.016690162	0.563668993	0.93397174	S100A3
ENSG00000173705.9	0.024486246	0.563691689	0.93397174	SUSD5
ENSG00000184110.15	-0.009137732	0.563696516	0.93397174	EIF3C
ENSG00000139173.10	0.009696535	0.563716706	0.93397174	TMEM117
ENSG00000272031.3	-0.006815091	0.563829214	0.934061842	ANKRD34A
ENSG00000115942.9	-0.00355981	0.563862181	0.934061842	ORC2
ENSG00000143318.13	0.028768453	0.56401518	0.934144638	CASQ1
ENSG00000157782.9	0.029495965	0.564018101	0.934144638	CABP1
ENSG00000183691.6	-0.017084909	0.564145294	0.934144638	NOG
ENSG00000171094.18	-0.023295527	0.564154216	0.934144638	ALK
ENSG00000170579.17	-0.022650883	0.564197959	0.934144638	DLGAP1
ENSG00000126453.10	-0.005854882	0.564203291	0.934144638	BCL2L12
ENSG00000196419.13	-0.004275881	0.564289574	0.934144638	XRCC6
ENSG00000088002.12	-0.017593214	0.564428781	0.934144638	SULT2B1
ENSG00000167635.12	0.005057771	0.564471671	0.934144638	ZNF146
ENSG00000183569.18	-0.008913689	0.564532201	0.934144638	SERHL2
ENSG00000158417.11	-0.004795763	0.564585854	0.934144638	EIF5B
ENSG00000129596.5	-0.044602272	0.564634805	0.934144638	CDO1
ENSG00000228674.4	-0.108156799	0.564693071	0.934144638	PPIAP59
ENSG00000148950.11	-0.011827031	0.564706238	0.934144638	IMMP1L
ENSG00000226624.1	-0.024401641	0.564848162	0.934144638	CTA-351J1.1
ENSG00000117090.15	0.005846136	0.564964912	0.934144638	SLAMF1
ENSG00000184313.20	-0.020015684	0.565039588	0.934144638	MROH7
ENSG00000260924.2	0.010397219	0.565048321	0.934144638	LINC01311
ENSG00000115457.10	-0.025763736	0.565048569	0.934144638	IGFBP2
ENSG00000204314.12	0.014812378	0.565096764	0.934144638	PRRT1
ENSG00000198858.10	0.007617821	0.565098876	0.934144638	R3HDM4
ENSG00000274678.1	-0.018124672	0.565107322	0.934144638	RP11-2C24.7
ENSG00000101280.8	0.015021043	0.565151961	0.934144638	ANGPT4
ENSG00000132819.17	0.012184907	0.565173065	0.934144638	RBM38
ENSG00000103740.10	0.013705896	0.565206863	0.934144638	ACSBG1

ENSG00000160326.14	0.006673474	0.565215057	0.934144638	SLC2A6
ENSG00000263417.4	0.020576468	0.565217244	0.934144638	GTSCR1
ENSG00000198015.14	0.004895959	0.565278711	0.934144638	MRPL42
ENSG00000125991.20	0.004299229	0.565281334	0.934144638	ERGIC3
ENSG00000284770.2	-0.007831348	0.565341037	0.934144638	TBCE
ENSG00000171827.11	-0.006348673	0.565341368	0.934144638	ZNF570
ENSG00000179988.15	0.006041454	0.565391009	0.934144638	PSTK
ENSG00000257740.1	-0.01312666	0.56546346	0.934144638	RP11-977G19.12
ENSG00000287603.1	-0.016093421	0.56547538	0.934144638	CTC-273B12.13
ENSG00000171757.17	-0.011620521	0.565542984	0.934144638	LRRC34
ENSG00000286360.1	-0.016679668	0.565571789	0.934144638	RP11-681L4.4
ENSG00000004142.12	-0.004034158	0.565614578	0.934144638	POLDIP2
ENSG00000164610.10	0.005745927	0.565679117	0.934144638	RP9
ENSG00000139190.17	-0.004764645	0.565784377	0.934144638	VAMP1
ENSG00000140807.7	0.009230439	0.565799182	0.934144638	NKD1
ENSG00000075213.11	0.024222631	0.565863268	0.934144638	SEMA3A
ENSG00000103187.8	0.005159458	0.565949386	0.934144638	COTL1
ENSG00000278540.5	0.004782483	0.566096379	0.934144638	ACACA
ENSG00000123219.13	-0.019854675	0.566119565	0.934144638	CENPK
ENSG00000236901.6	-0.010528742	0.566134254	0.934144638	MIR600HG
ENSG00000188848.16	0.017182773	0.566199611	0.934144638	BEND4
ENSG00000108639.8	0.005054906	0.566234323	0.934144638	SYNGR2
ENSG00000076351.13	-0.0077179	0.566340833	0.934144638	SLC46A1
ENSG00000173559.15	0.006390557	0.566365706	0.934144638	NABP1
ENSG00000127481.15	0.005334001	0.566397033	0.934144638	UBR4
ENSG00000215915.10	-0.01505129	0.566455118	0.934144638	ATAD3C
ENSG00000254635.6	0.005164584	0.566461279	0.934144638	WAC-AS1
ENSG00000172046.19	-0.003513772	0.566463662	0.934144638	USP19
ENSG00000183826.18	-0.00383055	0.566466657	0.934144638	BTBD9
ENSG00000279314.1	-0.014460806	0.566508199	0.934144638	RP1-257C22.2
ENSG00000124357.13	-0.006279326	0.566610042	0.934144638	NAGK
ENSG00000241679.2	0.014137967	0.566791062	0.934144638	RP11-80H8.4
ENSG00000186049.8	0.023173019	0.56680559	0.934144638	KRT73
ENSG00000184209.15	0.004806891	0.566816491	0.934144638	SNRNP35
ENSG00000135776.5	-0.006453349	0.566842198	0.934144638	ABCB10
ENSG00000231202.1	0.024966778	0.566861425	0.934144638	TRGVB
ENSG00000159840.16	-0.007249726	0.566935007	0.934144638	ZYX
ENSG00000198746.13	-0.004260169	0.566938207	0.934144638	GPATCH3
ENSG00000279434.1	0.007307921	0.566996935	0.934144638	RP11-85K15.3
ENSG00000250012.2	0.018749238	0.567055671	0.934144638	RP11-124N2.1
ENSG00000182185.18	0.006510295	0.567064027	0.934144638	RAD51B
ENSG00000262884.1	0.0185671	0.567091016	0.934144638	CTD-3060P21.1
ENSG00000059588.10	0.005055099	0.567120482	0.934144638	TARBP1
ENSG00000138613.14	0.003974361	0.567254193	0.934144638	APH1B
ENSG00000100122.7	0.018382945	0.567262692	0.934144638	CRYBB1
ENSG00000184100.6	0.014827288	0.56734911	0.934144638	BRD7P2
ENSG00000234572.1	-0.018222615	0.567354165	0.934144638	LINC01800

ENSG00000107679.14	-0.005822815	0.567371485	0.934144638	PLEKHA1
ENSG00000078487.18	0.004313357	0.567402356	0.934144638	ZCWPW1
ENSG00000187866.10	0.002503084	0.567438335	0.934144638	PABIR1
ENSG00000168811.7	-0.016467708	0.567511614	0.934144638	IL12A
ENSG00000137812.20	0.013395377	0.567581542	0.934144638	KNL1
ENSG00000240864.3	0.021017797	0.567634173	0.934144638	IGKV1-16
ENSG00000170191.5	0.006650084	0.567662548	0.934144638	NANP
ENSG00000225611.1	0.012052301	0.567677753	0.934144638	LINC02158
ENSG00000137270.11	0.011275349	0.567726392	0.934144638	GCM1
ENSG00000197183.14	-0.003938756	0.567734217	0.934144638	NOL4L
ENSG00000252690.3	-0.011144954	0.567768846	0.934144638	RP11-752G15.7
ENSG00000232528.3	0.01033538	0.567802173	0.934144638	RP4-673D20.3
ENSG00000112305.15	0.004786692	0.567846657	0.934144638	SMAP1
ENSG00000226981.2	0.022538424	0.567874026	0.934144638	ABHD17AP6
ENSG00000170471.15	0.003459882	0.567876996	0.934144638	RALGAPB
ENSG00000006118.15	0.018029812	0.567963648	0.934144638	TMEM132A
ENSG00000211650.2	-0.023267199	0.567966135	0.934144638	IGLV5-45
ENSG00000138138.14	0.006512278	0.568151322	0.934219064	ATAD1
ENSG00000136827.12	-0.003413596	0.568183543	0.934219064	TOR1A
ENSG00000142156.16	0.015758515	0.568185614	0.934219064	COL6A1
ENSG00000174255.8	-0.012936429	0.568268684	0.934219064	ZNF80
ENSG00000173320.12	0.034778181	0.568350202	0.934219064	STOX2
ENSG00000260495.1	0.01028531	0.568400891	0.934219064	RP11-55K13.1
ENSG00000197555.10	0.005659567	0.568430641	0.934219064	SIPA1L1
ENSG00000270021.1	0.021715958	0.568514451	0.934219064	CTC-203F4.2
ENSG00000178307.10	0.004610216	0.568518621	0.934219064	TMEM11
ENSG00000176225.15	0.004307116	0.56852855	0.934219064	RTTN
ENSG00000275464.5	0.01499636	0.568528951	0.934219064	CH507-9B2.5
ENSG00000137936.18	0.011918927	0.568558033	0.934219064	BCAR3
ENSG00000058063.16	0.006422914	0.56865926	0.934310535	ATP11B
ENSG00000085265.11	0.007345855	0.568867494	0.934323155	FCN1
ENSG00000228852.6	-0.01534138	0.568872463	0.934323155	RP11-57H12.5
ENSG00000274256.1	0.044676135	0.568888909	0.934323155	RP1-229K20.8
ENSG00000160551.12	0.005018407	0.568984137	0.934323155	TAOK1
ENSG00000175857.9	-0.007676639	0.569022021	0.934323155	GAPT
ENSG00000132356.11	-0.005550905	0.569085718	0.934323155	PRKAA1
ENSG00000042753.12	0.006509091	0.56909488	0.934323155	AP2S1
ENSG00000177426.22	0.004372531	0.569103827	0.934323155	TGIF1
ENSG00000100373.10	0.013387171	0.56917693	0.934323155	UPK3A
ENSG00000241170.3	-0.00603895	0.569186821	0.934323155	RP11-147I3.1
ENSG00000003137.9	0.095325749	0.569196161	0.934323155	CYP26B1
ENSG00000273007.2	-0.012271534	0.569344802	0.934323155	RP11-170N16.3
ENSG00000124207.17	0.003319483	0.569371209	0.934323155	CSE1L
ENSG00000176853.16	-0.006077341	0.569383037	0.934323155	FAM91A1
ENSG00000232818.2	0.012420696	0.569450239	0.934323155	RPS2P32
ENSG00000139842.15	-0.003089774	0.569533136	0.934323155	CUL4A
ENSG00000179593.16	0.016963251	0.56954096	0.934323155	ALOX15B

ENSG00000104946.13	-0.003965809	0.569544797	0.934323155	TBC1D17
ENSG00000244731.8	0.016908022	0.569562217	0.934323155	C4A
ENSG00000125089.18	-0.006236006	0.569622089	0.934323155	SH3TC1
ENSG00000188130.14	0.009213699	0.569637749	0.934323155	MAPK12
ENSG00000137474.22	-0.01102569	0.569669238	0.934323155	MYO7A
ENSG00000099800.8	0.006087187	0.569775688	0.934368797	TIMM13
ENSG00000217801.10	-0.010823584	0.569788189	0.934368797	RP11-465B22.3
ENSG00000237004.4	-0.026769407	0.569843794	0.934385267	ZNRF2P1
ENSG00000203286.5	-0.019460862	0.569938499	0.934416258	Metazoa_SRP
ENSG00000242607.1	0.022604954	0.569953821	0.934416258	RPS3AP34
ENSG00000099999.15	-0.004931845	0.570062874	0.934453296	RNF215
ENSG00000161265.15	-0.006206277	0.570097877	0.934453296	U2AF1L4
ENSG00000273008.1	0.014478385	0.570157704	0.934453296	CSGALNACT2-DT
ENSG00000132950.19	0.006610723	0.570170201	0.934453296	ZMYM5
ENSG00000204681.11	0.005808268	0.570209578	0.934453296	GABBR1
ENSG00000106261.17	0.003944642	0.570289989	0.934453296	ZKSCAN1
ENSG00000124789.12	0.00477183	0.57029537	0.934453296	NUP153
ENSG00000159871.15	-0.020358509	0.570387414	0.934529446	LYPD5
ENSG00000164494.12	0.003701239	0.570466466	0.934584301	PDSS2
ENSG00000142541.18	0.006391306	0.570581805	0.934585966	RPL13A
ENSG00000272911.1	0.017889955	0.570585486	0.934585966	RP11-74E22.6
ENSG00000161999.13	-0.004982482	0.570751016	0.934585966	JMJD8
ENSG00000242992.3	0.021192234	0.570761282	0.934585966	FTH1P4
ENSG00000261879.7	-0.007163575	0.57077375	0.934585966	ZNF594-DT
ENSG00000076067.13	-0.009409974	0.570777441	0.934585966	RBMS2
ENSG00000142327.13	-0.005280103	0.570786484	0.934585966	RNPEPL1
ENSG00000042317.17	0.007885781	0.570832354	0.934586454	SPATA7
ENSG00000075151.23	0.006253765	0.570914015	0.934645535	EIF4G3
ENSG00000118007.13	0.005863665	0.571068674	0.934768096	STAG1
ENSG00000164002.12	-0.005059959	0.571081607	0.934768096	EXO5
ENSG00000108924.14	0.013753171	0.571125622	0.934768096	HLF
ENSG00000234323.8	-0.024183961	0.571178091	0.93477937	LINC01505
ENSG00000140688.17	0.004412514	0.571368457	0.934967273	RUSF1
ENSG00000136715.19	-0.004469511	0.571428127	0.934967273	SAP130
ENSG00000136383.7	0.009869161	0.571444852	0.934967273	ALPK3
ENSG00000202058.1	0.013690201	0.571518523	0.934967273	RN7SKP80
ENSG00000154721.15	-0.013006406	0.571552905	0.934967273	JAM2
ENSG00000177932.7	0.008797398	0.571566448	0.934967273	ZNF354C
ENSG00000135931.19	0.015293075	0.571625001	0.934988477	ARMC9
ENSG00000188092.15	0.005366563	0.571820041	0.935084254	GPR89B
ENSG00000243304.3	-0.016530545	0.571820284	0.935084254	CTC-428G20.1
ENSG00000112033.14	0.004225332	0.571820345	0.935084254	PPARD
ENSG00000160606.11	-0.016321599	0.57195335	0.935227181	TLCD1
ENSG00000115514.12	0.009096967	0.572056338	0.935321006	TXNDC9
ENSG00000165476.14	-0.006063135	0.572157675	0.935408153	REEP3
ENSG00000171953.16	0.004465087	0.572221147	0.935408153	ATPAF2
ENSG00000105609.17	-0.019530434	0.572246474	0.935408153	LILRB5

ENSG00000154027.19	0.013151151	0.572390714	0.935522015	AK5
ENSG00000139428.12	0.005488365	0.572407365	0.935522015	MMAB
ENSG00000187231.14	0.007294923	0.572479842	0.935565909	SESTD1
ENSG00000165046.13	0.007422817	0.572548288	0.93560321	LETM2
ENSG00000120686.12	0.005936129	0.572629496	0.935606778	UFM1
ENSG00000173581.8	-0.009065284	0.57267237	0.935606778	CCDC106
ENSG00000188811.14	0.004701012	0.572737521	0.935606778	NHLRC3
ENSG00000102981.10	-0.006615687	0.572947399	0.935606778	PARD6A
ENSG00000235298.1	-0.018647191	0.572981367	0.935606778	RP11-575L7.8
ENSG00000232630.1	-0.012547429	0.573035571	0.935606778	PRPS1P2
ENSG00000272977.1	-0.009967516	0.573100883	0.935606778	CTA-390C10.10
ENSG00000232176.1	0.020255882	0.573116809	0.935606778	RP11-146N23.1
ENSG00000156787.17	-0.005326584	0.573168447	0.935606778	TBC1D31
ENSG00000230513.2	-0.012315151	0.573175532	0.935606778	THAP7-AS1
ENSG00000280402.1	0.020905197	0.573181958	0.935606778	RP11-20I23.10
ENSG00000132481.7	-0.008816121	0.573182039	0.935606778	TRIM47
ENSG00000008128.23	0.008352903	0.57332251	0.935606778	CDK11A
ENSG00000272812.1	0.014599353	0.573345603	0.935606778	RP5-855D21.3
ENSG00000102471.14	-0.009255818	0.573361883	0.935606778	NDFIP2
ENSG00000249667.1	0.011599067	0.573438308	0.935606778	LINC01259
ENSG00000158023.10	0.011406367	0.573477885	0.935606778	CFAP251
ENSG00000272733.1	-0.016469627	0.573525852	0.935606778	KB-208E9.1
ENSG00000224565.2	0.033329103	0.573529591	0.935606778	LINC01754
ENSG00000135778.12	0.006886645	0.573533363	0.935606778	NTPCR
ENSG00000105383.15	0.00554673	0.573548317	0.935606778	CD33
ENSG00000021776.11	0.003572357	0.573579204	0.935606778	AQR
ENSG00000260647.1	-0.014800062	0.573599767	0.935606778	RP1-178F10.1
ENSG00000180855.16	-0.009282931	0.573651082	0.935616064	ZNF443
ENSG00000136100.14	-0.005410523	0.573736159	0.93568041	VPS36
ENSG00000166446.15	-0.006085917	0.573810839	0.935706937	CDYL2
ENSG00000160218.13	0.006878201	0.573843678	0.935706937	TRAPPC10
ENSG00000281490.1	-0.010046353	0.573929807	0.935755221	CICP14
ENSG00000138030.13	-0.008647548	0.573987158	0.935755221	KHK
ENSG00000110514.19	-0.00387165	0.574025251	0.935755221	MADD
ENSG00000004399.13	-0.007170451	0.574091834	0.935755221	PLXND1
ENSG00000107779.14	0.007525838	0.574101433	0.935755221	BMPR1A
ENSG00000213839.4	-0.006175524	0.574294867	0.935855357	TMX2P1
ENSG00000234281.5	0.020665373	0.574295447	0.935855357	LANCL1-AS1
ENSG00000273972.1	-0.021957587	0.57433499	0.935855357	CTD-2306A12.1
ENSG00000170759.11	0.006894549	0.574345403	0.935855357	KIF5B
ENSG00000132874.15	0.017048054	0.574492567	0.93588524	SLC14A2
ENSG00000261386.2	0.012611385	0.574503992	0.93588524	CTD-2012K14.6
ENSG00000144655.15	-0.007109528	0.574633211	0.93588524	CSRNP1
ENSG00000114544.17	0.003996273	0.574649763	0.93588524	SLC41A3
ENSG00000160460.16	-0.017542009	0.574757199	0.93588524	SPTBN4
ENSG00000158882.15	-0.004716675	0.574761444	0.93588524	TOMM40L
ENSG00000272810.1	-0.015565742	0.574851213	0.93588524	U91328.22

ENSG00000152785.8	0.033216153	0.574864393	0.93588524	BMP3
ENSG00000130158.14	0.010773349	0.574944193	0.93588524	DOCK6
ENSG00000072274.13	0.00506268	0.574946394	0.93588524	TFRC
ENSG00000280649.2	-0.009048364	0.575023044	0.93588524	CH17-172B3.1
ENSG00000261959.1	0.019135285	0.575033191	0.93588524	RP11-893F2.14
ENSG00000177106.16	-0.007886563	0.575062002	0.93588524	EPS8L2
ENSG00000142186.17	-0.004719442	0.575112426	0.93588524	SCYL1
ENSG00000184227.8	0.018792605	0.575129075	0.93588524	ACOT1
ENSG00000115705.22	0.025408226	0.575496909	0.93588524	TPO
ENSG00000226688.7	-0.007073113	0.575572471	0.93588524	ENTPD1-AS1
ENSG00000279265.1	0.008687575	0.575612507	0.93588524	AC000123.3
ENSG00000227533.6	-0.016078863	0.575824654	0.93588524	SLC2A1-AS1
ENSG00000227615.1	0.018712026	0.575947863	0.93588524	RP11-864N7.2
ENSG00000181291.8	0.015900878	0.575988537	0.93588524	TMEM132E
ENSG00000225690.1	0.053100428	0.575990734	0.93588524	TREML5P
ENSG00000173114.13	-0.018713523	0.576006503	0.93588524	LRRN3
ENSG00000006744.19	-0.003821533	0.576031218	0.93588524	ELAC2
ENSG00000273893.1	-0.008578445	0.576105267	0.93588524	RP1-101A2.1
ENSG00000133317.16	-0.010629334	0.576158419	0.93588524	LGALS12
ENSG00000179242.16	-0.012711166	0.576221713	0.93588524	CDH4
ENSG00000286864.1	-0.018012661	0.576231617	0.93588524	RP11-497D24.1
ENSG00000015153.15	-0.005075729	0.576314137	0.93588524	YAF2
ENSG00000261819.1	-0.014916287	0.576377989	0.93588524	RP11-680G24.4
ENSG00000247708.8	0.004741002	0.57641974	0.93588524	STX18-AS1
ENSG00000179476.8	-0.007144022	0.576505305	0.93588524	C14orf28
ENSG00000228889.7	0.010184581	0.576578088	0.93588524	UBAC2-AS1
ENSG00000234518.2	0.007749792	0.57670422	0.93588524	PTGES3P1
ENSG00000239670.1	-0.016011179	0.576796325	0.93588524	RP4-803A2.2
ENSG00000164663.15	0.005081166	0.576870728	0.93588524	USP49
ENSG00000237419.1	0.035639598	0.576922491	0.93588524	RP11-885N19.6
ENSG00000109919.10	0.003802567	0.576985677	0.93588524	MTCH2
ENSG00000177105.10	0.005881429	0.57700189	0.93588524	RHOG
ENSG00000099937.11	0.021213128	0.577085808	0.93588524	SERPIND1
ENSG00000253497.1	0.04504715	0.577164903	0.93588524	IGKV1-13
ENSG00000055208.19	0.004484623	0.577167431	0.93588524	TAB2
ENSG00000214456.8	-0.008146131	0.577321713	0.93588524	PLIN5
ENSG00000198246.9	-0.005265427	0.577373166	0.93588524	SLC29A3
ENSG00000134222.16	0.007033189	0.577437526	0.93588524	PSRC1
ENSG00000106178.7	-0.036683481	0.577445349	0.93588524	CCL24
ENSG00000081181.8	-0.011945175	0.577479727	0.93588524	ARG2
ENSG00000186469.8	-0.005606058	0.577501848	0.93588524	GNG2
ENSG00000089012.14	0.007617618	0.577536585	0.93588524	SIRPG
ENSG00000073614.13	0.003881897	0.577548356	0.93588524	KDM5A
ENSG00000237037.9	-0.007211998	0.577563423	0.93588524	NDUFA6-DT
ENSG00000279900.1	0.017157683	0.577577088	0.93588524	RP11-113K21.6
ENSG00000256229.8	0.00836685	0.577595677	0.93588524	ZNF486
ENSG00000196227.11	0.005096482	0.577607885	0.93588524	FAM217B

ENSG00000267680.5	-0.004945061	0.577614879	0.93588524	ZNF224
ENSG00000174738.13	-0.005627972	0.577618492	0.93588524	NR1D2
ENSG00000168724.17	0.004247691	0.577669815	0.93588524	DNAJC21
ENSG00000167861.16	0.009850696	0.57769998	0.93588524	HID1
ENSG00000196209.13	0.006300204	0.57779039	0.93588524	SIRPB2
ENSG00000105374.10	-0.011697214	0.577798852	0.93588524	NKG7
ENSG00000120725.13	-0.006232215	0.577827182	0.93588524	SIL1
ENSG00000130382.9	-0.004905238	0.577828965	0.93588524	MLLT1
ENSG00000120156.22	-0.02616436	0.577832418	0.93588524	TEK
ENSG00000075415.15	0.003734107	0.577842089	0.93588524	SLC25A3
ENSG00000167077.13	0.005229379	0.577944871	0.93588524	MEI1
ENSG00000113971.21	-0.00725854	0.57798059	0.93588524	NPHP3
ENSG00000149115.14	-0.006602836	0.577985487	0.93588524	TNKS1BP1
ENSG00000230551.4	0.007529125	0.577996507	0.93588524	CTB-89H12.4
ENSG00000286331.1	-0.022113116	0.578018671	0.93588524	CTB-171P15.2
ENSG00000170145.5	0.003902025	0.578031912	0.93588524	SIK2
ENSG00000288156.1	-0.005315885	0.578037165	0.93588524	AC104530.1
ENSG00000259985.1	0.015847593	0.57805459	0.93588524	RP11-549B18.1
ENSG00000166016.6	0.010608971	0.57807038	0.93588524	ABTB2
ENSG00000163686.15	-0.004604635	0.578083475	0.93588524	ABHD6
ENSG00000149743.14	-0.004758709	0.578147003	0.93588524	TRPT1
ENSG00000137404.15	-0.005454003	0.578152115	0.93588524	NRM
ENSG00000181656.7	0.017139638	0.578182341	0.93588524	GPR88
ENSG00000148926.10	-0.010196815	0.578198193	0.93588524	ADM
ENSG00000121481.11	0.004961431	0.578199869	0.93588524	RNF2
ENSG00000064419.14	-0.003796542	0.578297977	0.93588524	TNPO3
ENSG00000176358.15	-0.010374445	0.578339192	0.93588524	TAC4
ENSG00000107263.19	-0.004604008	0.578370998	0.93588524	RAPGEF1
ENSG00000177156.11	0.005808145	0.578400087	0.93588524	TALDO1
ENSG00000169439.12	-0.01545289	0.578404194	0.93588524	SDC2
ENSG00000264006.8	0.025197385	0.578412312	0.93588524	AKR1C8P
ENSG00000261105.6	-0.015851467	0.578419694	0.93588524	LMO7-AS1
ENSG00000010319.7	-0.014293433	0.578466217	0.93588524	SEMA3G
ENSG00000233170.4	0.017539967	0.578483953	0.93588524	RP11-863K10.4
ENSG00000092607.15	0.037140974	0.578507615	0.93588524	TBX15
ENSG00000282978.1	-0.033004822	0.578563553	0.93588524	RP11-144C15.2
ENSG00000077935.17	-0.013560148	0.578570728	0.93588524	SMC1B
ENSG00000197045.13	0.007717949	0.578655581	0.93588524	GMFB
ENSG00000259940.2	-0.009068357	0.578780551	0.93588524	CTD-3203P2.1
ENSG00000104722.14	0.01778034	0.578792292	0.93588524	NEFM
ENSG00000110429.14	0.004842866	0.578809018	0.93588524	FBXO3
ENSG00000109920.13	0.003689976	0.578862936	0.93588524	FNBP4
ENSG00000134954.14	-0.005041767	0.578881832	0.93588524	ETS1
ENSG00000128487.18	0.005367607	0.578928912	0.93588524	SPECC1
ENSG00000269019.2	-0.01786846	0.57893116	0.93588524	HOMER3-AS1
ENSG00000137776.17	0.002996831	0.578991371	0.93588524	SLTM
ENSG00000286010.1	-0.01665456	0.579123744	0.93588524	RP11-1136L8.3

ENSG00000117308.15	0.004788455	0.579129397	0.93588524	GALE
ENSG00000253320.7	0.00767702	0.579132561	0.93588524	MAILR
ENSG00000121073.15	0.003282011	0.579249082	0.93588524	SLC35B1
ENSG00000189283.10	0.01323308	0.579264114	0.93588524	FHIT
ENSG00000114395.11	-0.004604849	0.579299656	0.93588524	CYB561D2
ENSG00000107771.17	0.004813262	0.579319874	0.93588524	CCSER2
ENSG00000273428.4	-0.017777698	0.579321371	0.93588524	RP4-539M6.22
ENSG00000213366.13	-0.011474799	0.579388986	0.93588524	GSTM2
ENSG00000176055.10	-0.008041575	0.579418215	0.93588524	MBLAC2
ENSG00000242154.1	0.018164833	0.579457431	0.93588524	RP4-778K6.3
ENSG00000124795.17	0.006990486	0.579506841	0.93588524	DEK
ENSG00000170921.15	-0.00650019	0.579520512	0.93588524	TANC2
ENSG00000187775.17	0.006473557	0.579667003	0.936048103	DNAH17
ENSG00000132463.14	0.002885621	0.579849231	0.936099702	GRSF1
ENSG00000174718.12	0.006820555	0.579867474	0.936099702	RESF1
ENSG00000152454.4	-0.006768078	0.579910958	0.936099702	ZNF256
ENSG00000144061.14	-0.01447693	0.579936246	0.936099702	NPHP1
ENSG00000245888.7	-0.013534337	0.580001107	0.936099702	NSMCE1-DT
ENSG00000106144.20	-0.003304961	0.580008469	0.936099702	CASP2
ENSG00000143768.13	-0.03437899	0.580018476	0.936099702	LEFTY2
ENSG00000187862.13	0.011477012	0.580215077	0.936132365	TTC24
ENSG00000051596.10	0.007580783	0.580216344	0.936132365	THOC3
ENSG00000279344.1	-0.012413376	0.580222545	0.936132365	RP11-44F14.7
ENSG00000180611.7	0.009934803	0.580228939	0.936132365	MB21D2
ENSG00000099860.9	0.006202909	0.58026695	0.936132365	GADD45B
ENSG00000279059.1	-0.006074195	0.580470699	0.936300768	RP11-257O5.2
ENSG00000213261.3	0.019039699	0.580498322	0.936300768	EEF1B2P6
ENSG00000211933.2	-0.019551778	0.580508302	0.936300768	IGHV6-1
ENSG00000258499.1	-0.026094852	0.580955651	0.936829111	LINC02287
ENSG00000232060.3	0.024163128	0.581036497	0.936829111	SLC4A1APP1
ENSG00000135686.13	-0.004054934	0.581036502	0.936829111	KLHL36
ENSG00000165140.12	0.006797703	0.581059727	0.936829111	FBP1
ENSG00000243414.6	-0.016430166	0.581064282	0.936829111	TICAM2
ENSG00000270503.1	0.009291601	0.581145832	0.936886937	YTHDF2P1
ENSG00000123500.10	0.02631244	0.581216634	0.936902505	COL10A1
ENSG00000053501.13	-0.005298955	0.581246858	0.936902505	USE1
ENSG00000182389.20	-0.014400824	0.581328487	0.936960437	CACNB4
ENSG00000228477.1	-0.016513266	0.58154481	0.937135318	RP3-342P20.2
ENSG00000177383.5	-0.005466834	0.58154994	0.937135318	MAGEF1
ENSG00000141194.7	0.010086987	0.58167627	0.937135318	OR4D1
ENSG00000133858.17	0.004376776	0.581744391	0.937135318	ZFC3H1
ENSG00000131446.17	-0.004507389	0.581751342	0.937135318	MGAT1
ENSG00000256667.6	-0.013417259	0.58177159	0.937135318	KLRA1P
ENSG00000279805.1	-0.009550609	0.581932663	0.937135318	CTA-212A2.1
ENSG00000245148.2	-0.010028699	0.581940826	0.937135318	ARAP1-AS2
ENSG00000151332.19	-0.00586714	0.581975878	0.937135318	MBIP
ENSG00000110330.9	0.006714215	0.58198139	0.937135318	BIRC2

ENSG00000171169.9	0.003603711	0.5820265	0.937135318	NAIF1
ENSG00000132185.18	-0.014694897	0.58214334	0.937135318	FCRLA
ENSG00000234436.1	-0.015225108	0.582178	0.937135318	AC008984.7
ENSG00000114738.11	0.004128919	0.582291971	0.937135318	MAPKAPK3
ENSG00000082641.16	-0.00419527	0.582308896	0.937135318	NFE2L1
ENSG00000275632.1	0.012206032	0.582351001	0.937135318	RP5-967N21.11
ENSG00000097096.9	-0.017994213	0.58235102	0.937135318	SYDE2
ENSG00000287569.1	0.017396519	0.582358919	0.937135318	RP4-760C5.1
ENSG00000169519.21	-0.005907934	0.582377177	0.937135318	METTL15
ENSG00000144681.11	-0.029265359	0.582467513	0.937135318	STAC
ENSG00000138640.15	-0.007248724	0.582483729	0.937135318	FAM13A
ENSG00000133116.8	0.015360235	0.582487147	0.937135318	KL
ENSG00000152620.13	-0.005640287	0.582488	0.937135318	NADK2
ENSG00000211924.1	0.024599066	0.582739008	0.937465609	IGHD3-9
ENSG00000108448.22	-0.009061541	0.582791811	0.937477015	TRIM16L
ENSG00000176973.8	0.006160279	0.582862855	0.937508146	FAM89B
ENSG00000139793.19	-0.007433162	0.582902592	0.937508146	MBNL2
ENSG00000146904.9	-0.008500491	0.582952411	0.937514746	EPHA1
ENSG00000128254.16	0.014036086	0.583027396	0.937561816	C22orf24
ENSG00000124201.15	0.005711249	0.583198197	0.937697111	ZNFX1
ENSG00000188582.10	0.038558755	0.583202977	0.937697111	PAQR9
ENSG00000148358.20	0.002904792	0.583289104	0.937762068	GPR107
ENSG00000188994.13	0.008640724	0.583479649	0.937994877	ZNF292
ENSG00000206077.14	0.016413437	0.583537148	0.938013783	ZDHHC11B
ENSG00000164659.15	0.015145742	0.583618237	0.938033402	ELAPOR2
ENSG00000243663.1	0.020388656	0.583645686	0.938033402	RPS4XP14
ENSG00000224261.2	-0.014429872	0.583686573	0.938033402	RPSAP18
ENSG00000153234.15	-0.010411107	0.583780587	0.938069638	NR4A2
ENSG00000197879.17	-0.006562201	0.583800604	0.938069638	MYO1C
ENSG00000278857.1	-0.039277792	0.583865251	0.938100012	IGKV1D-12
ENSG00000146243.14	0.010619562	0.583949327	0.938161598	IRAK1BP1
ENSG00000269954.2	-0.013761108	0.584127049	0.938289907	RP11-148O21.2
ENSG00000146757.14	0.009521879	0.584157568	0.938289907	ZNF92
ENSG00000234902.6	-0.013628985	0.584242602	0.938289907	AC007879.2
ENSG00000077150.20	-0.005155576	0.584284316	0.938289907	NFKB2
ENSG00000160014.17	-0.00403368	0.58433337	0.938289907	CALM3
ENSG00000214401.4	0.020154789	0.584364752	0.938289907	KANSL1-AS1
ENSG00000182986.15	0.00705159	0.584374538	0.938289907	ZNF320
ENSG00000236021.2	0.015852939	0.584395211	0.938289907	RP11-195C7.1
ENSG00000100412.17	-0.00500611	0.584461014	0.938322097	ACO2
ENSG00000169180.12	0.004978246	0.584579798	0.938385709	XPO6
ENSG00000068885.15	-0.007559464	0.584600405	0.938385709	IFT80
ENSG00000170260.9	-0.003562399	0.584637907	0.938385709	ZNF212
ENSG00000063854.13	0.010267576	0.584860076	0.938615959	HAGH
ENSG00000157326.19	0.006428243	0.584872896	0.938615959	DHRS4
ENSG00000279565.1	-0.02488949	0.58495521	0.938674605	RP4-570O12.3
ENSG00000013725.14	-0.005875406	0.585113743	0.938855538	CD6

ENSG00000261684.2	-0.013280541	0.585194975	0.938908268	RP11-265N6.1
ENSG00000166153.17	0.01668366	0.585240109	0.938908268	DEPDC4
ENSG00000114854.8	-0.012365924	0.585283953	0.938908268	TNNC1
ENSG00000179902.13	-0.021414743	0.585421165	0.939054928	C1orf194
ENSG00000119326.15	-0.014999621	0.585539945	0.939172	CTNNAL1
ENSG00000168958.20	0.003160487	0.58569021	0.939325202	MFF
ENSG00000116885.18	0.012478253	0.585751137	0.939325202	OSCP1
ENSG00000238083.8	0.011241335	0.585800341	0.939325202	LRRC37A2
ENSG00000198363.18	0.007861523	0.58583276	0.939325202	ASPH
ENSG00000136514.3	-0.013258302	0.585864475	0.939325202	RTP4
ENSG00000065457.11	-0.004607247	0.585910799	0.939326038	ADAT1
ENSG00000123836.15	0.007242162	0.586058817	0.939489895	PFKFB2
ENSG00000225079.2	-0.014188253	0.586188562	0.939594375	FTH1P22
ENSG00000159579.14	0.003086513	0.586294547	0.939594375	RSPRY1
ENSG00000268355.1	-0.021299734	0.586302445	0.939594375	AC006129.1
ENSG00000287181.1	-0.033257367	0.586316031	0.939594375	RP11-10L7.4
ENSG00000272711.1	0.008611861	0.586353073	0.939594375	HK2-DT
ENSG00000086189.11	-0.00482102	0.586458781	0.939676032	DIMT1
ENSG00000167977.9	0.002815828	0.586498804	0.939676032	KCTD5
ENSG00000116717.13	-0.006959195	0.586578182	0.939676032	GADD45A
ENSG00000127743.6	-0.015745425	0.586624286	0.939676032	IL17B
ENSG00000114631.11	-0.011159317	0.586678568	0.939676032	PODXL2
ENSG00000271161.1	-0.015014634	0.586762151	0.939676032	BOLA2P2
ENSG00000160213.9	0.006222695	0.586769039	0.939676032	CSTB
ENSG00000184305.15	-0.01496876	0.586777514	0.939676032	CCSER1
ENSG00000258227.7	0.012121183	0.586824636	0.939676032	CLEC5A
ENSG00000104219.13	-0.007093341	0.58686223	0.939676032	ZDHHC2
ENSG00000113719.16	0.004909957	0.586971113	0.939763975	ERGIC1
ENSG00000234614.1	0.012106761	0.587008803	0.939763975	C2CD4D-AS1
ENSG00000106723.17	0.004266676	0.587094291	0.939827471	SPIN1
ENSG00000170819.5	-0.019989352	0.587147436	0.939839183	BFSP2
ENSG00000196247.12	0.01077137	0.587300519	0.940010853	ZNF107
ENSG00000251819.1	-0.018700575	0.587594642	0.940096602	RNU6-322P
ENSG00000101605.13	0.007315179	0.587604834	0.940096602	MYOM1
ENSG00000139826.6	0.007884508	0.587706131	0.940096602	ABHD13
ENSG00000267197.1	-0.013796022	0.587807536	0.940096602	CTC-429L19.3
ENSG00000267711.1	0.021055288	0.587864061	0.940096602	RP11-686D22.5
ENSG00000226167.1	-0.008141382	0.587882257	0.940096602	AP4B1-AS1
ENSG00000160883.11	-0.005316703	0.58788883	0.940096602	HK3
ENSG00000074603.19	0.004084545	0.587903792	0.940096602	DPP8
ENSG00000121879.6	0.006154255	0.587910892	0.940096602	PIK3CA
ENSG00000144224.17	0.005247416	0.587910951	0.940096602	UBXN4
ENSG00000185198.12	-0.017787888	0.587951568	0.940096602	PRSS57
ENSG00000282600.2	-0.029339941	0.58797061	0.940096602	IGHV3-69-1
ENSG00000173898.15	-0.013472305	0.587976813	0.940096602	SPTBN2
ENSG00000120756.13	0.011684401	0.587995861	0.940096602	PLS1
ENSG00000259556.2	0.011342669	0.588098824	0.940119644	RP11-56B16.2

ENSG00000276968.1	0.016954057	0.588102081	0.940119644	RP11-90M2.5
ENSG00000162188.6	0.017369136	0.588147797	0.940119644	GNG3
ENSG00000277662.1	-0.013545712	0.588237887	0.940186195	RP11-74J13.9
ENSG00000274290.3	-0.013645666	0.588295664	0.940186195	H2BC6
ENSG00000077327.16	0.020404444	0.588326967	0.940186195	SPAG6
ENSG00000231346.5	0.011230775	0.588456776	0.940248901	LINC01160
ENSG00000221539.1	-0.0134404	0.58849745	0.940248901	SNORD99
ENSG00000128228.5	-0.007009241	0.588503749	0.940248901	SDF2L1
ENSG00000256806.6	-0.007364623	0.588616047	0.940287581	C17orf100
ENSG00000162378.13	0.004935935	0.588624872	0.940287581	ZYG11B
ENSG00000166147.15	0.016605356	0.588665509	0.940287581	FBN1
ENSG00000117868.17	0.003524147	0.588713447	0.940290917	ESYT2
ENSG00000254275.6	0.020467277	0.588870204	0.94035294	LINC00824
ENSG00000085998.14	0.003451867	0.588901736	0.94035294	POMGNT1
ENSG00000242736.1	0.016373418	0.588933648	0.94035294	TRBV1
ENSG00000241860.7	-0.013259189	0.58894704	0.94035294	RP11-34P13.13
ENSG00000184261.5	-0.014479782	0.589130248	0.94035294	KCNK12
ENSG00000230910.4	0.020148811	0.589345035	0.94035294	RP3-525N10.2
ENSG00000213557.4	0.016055045	0.589373383	0.94035294	RP11-240E2.2
ENSG00000179632.10	-0.005473702	0.589407822	0.94035294	MAF1
ENSG00000260077.1	0.007724123	0.589430207	0.94035294	RP11-254F7.2
ENSG00000188897.9	0.008889478	0.589451376	0.94035294	CTD-3088G3.8
ENSG00000175198.17	-0.005297183	0.589528972	0.94035294	PCCA
ENSG00000184281.15	-0.005104157	0.589640218	0.94035294	TSSC4
ENSG00000165671.21	-0.004308965	0.589720583	0.94035294	NSD1
ENSG00000135842.17	0.007226599	0.589753157	0.94035294	NIBAN1
ENSG00000285763.1	-0.012509799	0.589780057	0.94035294	RP11-421M1.9
ENSG00000184005.11	-0.009808227	0.589827836	0.94035294	ST6GALNAC3
ENSG00000110367.13	0.003046527	0.58991233	0.94035294	DDX6
ENSG00000251139.2	-0.014573602	0.590015498	0.94035294	RP11-701P16.2
ENSG00000162643.13	-0.025942375	0.590032342	0.94035294	DNAI3
ENSG00000206573.9	-0.004720023	0.590060834	0.94035294	THUMPD3-AS1
ENSG00000162852.14	0.004514781	0.590168346	0.94035294	CNST
ENSG00000134245.18	-0.007690703	0.590197993	0.94035294	WNT2B
ENSG00000226581.2	-0.019742168	0.590266698	0.94035294	LINC02848
ENSG00000287727.1	0.017099131	0.590425161	0.94035294	RP4-734G22.4
ENSG00000115970.19	-0.002852963	0.59046441	0.94035294	THADA
ENSG00000164506.14	-0.006247856	0.590504139	0.94035294	STXBP5
ENSG00000125170.11	0.010398132	0.59050854	0.94035294	DOK4
ENSG00000235453.11	0.005476764	0.590538932	0.94035294	SMIM27
ENSG00000225578.1	-0.015958547	0.590608723	0.94035294	NCBP2-AS1
ENSG00000273487.1	0.018487817	0.590658815	0.94035294	RP4-621B10.8
ENSG00000273015.2	-0.005097508	0.590742601	0.94035294	RP11-352M15.2
ENSG00000229204.3	0.010886939	0.590770879	0.94035294	PTGES3P3
ENSG00000281103.2	-0.006861248	0.59077422	0.94035294	TRG-AS1
ENSG00000087589.17	0.006153295	0.590835671	0.94035294	CASS4
ENSG00000280486.2	-0.010578388	0.59087186	0.94035294	LLNLR-307A6.1

ENSG00000106211.10	-0.008124407	0.590899014	0.94035294	HSPB1
ENSG00000228594.4	0.00776515	0.590962801	0.94035294	FNDC10
ENSG00000151116.17	0.00498506	0.5909998	0.94035294	UEVLD
ENSG00000184445.12	0.005304242	0.591141018	0.94035294	KNTC1
ENSG00000140988.16	0.005524675	0.591146651	0.94035294	RPS2
ENSG00000215014.5	-0.013752282	0.591175873	0.94035294	RP5-832C2.5
ENSG00000092445.12	-0.027102457	0.591185134	0.94035294	TYRO3
ENSG00000113448.19	0.007973877	0.591195156	0.94035294	PDE4D
ENSG00000165025.15	0.004131884	0.591241265	0.94035294	SYK
ENSG00000271947.1	-0.026680213	0.591250029	0.94035294	RP11-439M11.1
ENSG00000163959.10	-0.005973855	0.591272528	0.94035294	SLC51A
ENSG00000179912.20	-0.002668769	0.591283003	0.94035294	R3HDM2
ENSG00000260296.1	0.010179984	0.59130782	0.94035294	RP11-395I6.3
ENSG00000106591.4	0.006684613	0.591419066	0.94035294	MRPL32
ENSG00000129933.21	0.00304724	0.591561194	0.94035294	MAU2
ENSG00000115282.21	-0.002717217	0.591589011	0.94035294	TTC31
ENSG00000185834.10	0.018491838	0.591637661	0.94035294	RPL12P4
ENSG00000225756.1	0.012850439	0.591726093	0.94035294	DBH-AS1
ENSG00000173451.7	0.006487053	0.591745949	0.94035294	THAP2
ENSG00000079215.15	-0.014136905	0.591798479	0.94035294	SLC1A3
ENSG00000161714.12	-0.00769455	0.591825506	0.94035294	PLCD3
ENSG00000196381.11	0.012750911	0.591837988	0.94035294	ZNF781
ENSG00000124217.5	0.003242427	0.591838516	0.94035294	MOCS3
ENSG00000115459.18	0.00290013	0.591849636	0.94035294	ELMOD3
ENSG00000267365.1	0.009844513	0.591859594	0.94035294	KCNJ2-AS1
ENSG00000260467.1	0.010870355	0.591931063	0.94035294	RP11-405F3.4
ENSG00000219355.2	-0.015646745	0.591932925	0.94035294	RPL31P52
ENSG00000161692.18	0.006446846	0.591949464	0.94035294	DBF4B
ENSG00000178502.6	0.010776779	0.592021991	0.94035294	KLHL11
ENSG00000250138.4	0.017075558	0.592039173	0.94035294	RP11-848G14.5
ENSG00000143434.16	-0.007192104	0.592075448	0.94035294	SEMA6C
ENSG00000266733.7	-0.013586068	0.592093622	0.94035294	TBC1D29P
ENSG00000166435.16	-0.015948726	0.592139719	0.94035294	XRRA1
ENSG00000241839.10	0.00473646	0.592218259	0.94035294	PLEKHO2
ENSG00000023516.9	-0.006469057	0.592264329	0.94035294	AKAP11
ENSG00000145723.17	0.008634793	0.592302858	0.94035294	GIN1
ENSG00000211829.9	0.011942178	0.592343017	0.94035294	TRDC
ENSG00000166405.15	0.008465491	0.592352947	0.94035294	RIC3
ENSG00000128311.14	0.007035824	0.592391488	0.94035294	TST
ENSG00000262580.5	0.007588343	0.59247651	0.94035294	RP11-334C17.5
ENSG00000131051.24	0.003802808	0.592521629	0.94035294	RBM39
ENSG00000163492.15	0.013757902	0.592542237	0.94035294	CCDC141
ENSG00000204252.14	0.009062144	0.592550804	0.94035294	HLA-DOA
ENSG00000187997.11	-0.019576387	0.592576336	0.94035294	C17orf99
ENSG00000177283.8	-0.020292443	0.592616827	0.94035294	FZD8
ENSG00000068745.15	0.002181711	0.592649609	0.94035294	IP6K2
ENSG00000157470.12	-0.014544789	0.592685863	0.94035294	FAM81A

ENSG00000174123.11	-0.008017428	0.592697978	0.94035294	TLR10
ENSG00000099341.12	0.003999867	0.592741828	0.94035294	PSMD8
ENSG00000104643.10	-0.004516747	0.592793416	0.94035294	MTMR9
ENSG00000275183.2	-0.006537093	0.59281818	0.94035294	LENG9
ENSG00000156414.19	0.015082991	0.592831327	0.94035294	TDRD9
ENSG00000204444.11	0.00697195	0.592946013	0.94035294	APOM
ENSG00000172172.8	-0.011010392	0.593101277	0.94035294	MRPL13
ENSG00000033627.17	0.004793735	0.593188673	0.94035294	ATP6V0A1
ENSG00000105258.9	-0.008235968	0.593220067	0.94035294	POLR2I
ENSG00000257704.4	-0.008065024	0.593220187	0.94035294	INAFM1
ENSG00000091483.8	0.003462304	0.593242246	0.94035294	FH
ENSG00000108509.21	-0.003814381	0.593255831	0.94035294	CAMTA2
ENSG00000185986.12	0.005645076	0.593400908	0.94035294	SDHAP3
ENSG00000178464.6	0.006818455	0.593437195	0.94035294	RPL10P16
ENSG00000278816.1	0.017589051	0.593457965	0.94035294	RP5-1116H23.4
ENSG00000287496.1	-0.017880529	0.593459245	0.94035294	CTD-2528L19.7
ENSG00000270210.1	-0.009709197	0.593510063	0.94035294	RP11-373D23.3
ENSG00000226853.3	0.015642374	0.593510556	0.94035294	AC010894.3
ENSG00000087008.16	-0.004170659	0.593568548	0.94035294	ACOX3
ENSG00000134201.12	-0.029355635	0.593570075	0.94035294	GSTM5
ENSG00000277782.1	-0.012078385	0.593573243	0.94035294	RP11-775C24.5
ENSG00000100410.8	0.008676182	0.593622136	0.94035294	PHF5A
ENSG00000211713.3	0.020336005	0.593626119	0.94035294	TRBV6-4
ENSG00000138166.6	-0.008033266	0.593781569	0.94035294	DUSP5
ENSG00000255468.7	-0.008745956	0.593781714	0.94035294	RP11-867G23.8
ENSG00000268970.1	-0.006314114	0.593800794	0.94035294	CTD-3099C6.11
ENSG00000211652.2	0.02182151	0.593847027	0.94035294	IGLV7-43
ENSG00000266389.1	0.007471237	0.593876263	0.94035294	PIK3R5-DT
ENSG00000136997.21	-0.006334374	0.593876849	0.94035294	MYC
ENSG00000042980.13	-0.00938837	0.593954804	0.94035294	ADAM28
ENSG00000081148.12	0.01142353	0.593955448	0.94035294	IMPG2
ENSG00000117481.11	0.004018547	0.593979519	0.94035294	NSUN4
ENSG00000152128.13	-0.014077732	0.594141314	0.940424146	TMEM163
ENSG00000265194.1	-0.017002297	0.594150889	0.940424146	RP11-70L8.4
ENSG00000232645.5	0.01323836	0.594277198	0.940424146	LINC01431
ENSG00000280537.2	-0.014179748	0.594305453	0.940424146	RP11-33O4.2
ENSG00000105643.11	-0.003053024	0.594316736	0.940424146	ARRDC2
ENSG00000103222.20	0.003687308	0.594350993	0.940424146	ABCC1
ENSG00000119523.10	0.002683383	0.594443548	0.940424146	ALG2
ENSG00000237169.1	0.010016802	0.59447332	0.940424146	RPL12P27
ENSG00000257534.1	-0.028525184	0.59451812	0.940424146	RP11-834C11.10
ENSG00000237491.10	-0.009164795	0.594549421	0.940424146	LINC01409
ENSG00000092295.12	0.00792171	0.594569245	0.940424146	TGM1
ENSG00000177971.9	-0.004528592	0.594574774	0.940424146	IMP3
ENSG00000169026.13	0.006395078	0.594620753	0.940424339	SLC49A3
ENSG00000108091.11	-0.003370564	0.594678879	0.940439366	CCDC6
ENSG00000107201.11	-0.011832391	0.594721969	0.940439366	DDX58

ENSG00000100504.17	0.005819005	0.594859282	0.940583975	PYGL
ENSG00000066422.5	0.006667546	0.594908527	0.94058932	ZBTB11
ENSG00000272696.1	-0.012803799	0.595012411	0.940623848	RP11-339B21.13
ENSG00000171044.11	-0.007345572	0.595111109	0.940623848	XKR6
ENSG00000111843.14	0.006466078	0.595152634	0.940623848	TMEM14C
ENSG00000251022.6	-0.005293015	0.595238683	0.940623848	THAP9-AS1
ENSG00000130720.13	-0.029490123	0.595271282	0.940623848	FIBCD1
ENSG00000196172.9	-0.009476334	0.595290327	0.940623848	ZNF681
ENSG00000180098.9	-0.00335173	0.595321137	0.940623848	TRNAU1AP
ENSG00000226824.7	0.010616686	0.595389892	0.940623848	RP4-756H11.3
ENSG00000196878.15	-0.011168152	0.595414658	0.940623848	LAMB3
ENSG00000214465.3	0.017362639	0.595420007	0.940623848	SMARCE1P6
ENSG00000148450.13	0.0079788	0.595434894	0.940623848	MSRB2
ENSG00000132953.17	0.003959797	0.595576942	0.940719944	XPO4
ENSG00000287678.1	-0.018253601	0.595587466	0.940719944	RP1-34B21.7
ENSG00000111711.10	0.0066686	0.595684204	0.940726089	GOLT1B
ENSG00000165283.16	-0.00409482	0.595723437	0.940726089	STOML2
ENSG00000239763.2	-0.013488396	0.59572897	0.940726089	AC009120.3
ENSG00000119681.12	0.007682713	0.595829628	0.940812597	LTBP2
ENSG00000108256.9	0.004893023	0.595929543	0.940822947	NUFIP2
ENSG00000123975.5	0.012057919	0.595986651	0.940822947	CKS2
ENSG00000268433.1	0.024260166	0.59600686	0.940822947	MTDHP3
ENSG00000136250.12	-0.005503395	0.596019686	0.940822947	AOAH
ENSG00000149308.17	0.004595409	0.596292334	0.941043812	NPAT
ENSG00000258867.6	-0.010390191	0.596322738	0.941043812	LINC01146
ENSG00000184887.14	0.004095574	0.596325516	0.941043812	BTBD6
ENSG00000105429.13	-0.004882096	0.596396047	0.941043812	MEGF8
ENSG00000178163.8	-0.00437787	0.596403169	0.941043812	ZNF518B
ENSG00000105825.14	-0.048455037	0.596434927	0.941043812	TFPI2
ENSG00000182359.15	0.009715445	0.596631042	0.941275967	KBTBD3
ENSG00000274020.4	-0.004843986	0.596673862	0.941275967	LINC01138
ENSG00000128923.11	-0.007606102	0.596758185	0.941279573	MINDY2
ENSG00000282988.2	0.011355133	0.596767944	0.941279573	RP1-34B20.21
ENSG00000259488.2	-0.02503169	0.596878993	0.941337971	RP11-154J22.1
ENSG00000181634.8	-0.015886817	0.596896771	0.941337971	TNFSF15
ENSG00000123154.12	0.004897742	0.597068202	0.941478107	WDR83
ENSG00000178803.12	-0.008972745	0.597077446	0.941478107	ADORA2A-AS1
ENSG00000272211.1	-0.011626068	0.597137491	0.941500398	RP11-347P5.1
ENSG00000185920.17	-0.009174518	0.597195807	0.941519958	PTCH1
ENSG00000185482.8	-0.006821322	0.597521841	0.941603138	STAC3
ENSG00000154102.11	-0.009231621	0.59752981	0.941603138	C16orf74
ENSG00000110717.13	0.005102693	0.59767536	0.941603138	NDUFS8
ENSG00000175003.15	-0.009336001	0.597693053	0.941603138	SLC22A1
ENSG00000081026.19	-0.007138718	0.597698529	0.941603138	MAGI3
ENSG00000120742.11	0.003808374	0.597712167	0.941603138	SERP1
ENSG00000286753.1	-0.010515621	0.597719415	0.941603138	RP11-531A21.6
ENSG00000119535.18	0.00501455	0.597808327	0.941603138	CSF3R

ENSG00000245812.3	-0.02105335	0.597831944	0.941603138	LINC02202
ENSG00000164109.14	0.00883726	0.597896556	0.941603138	MAD2L1
ENSG00000270871.1	-0.010214539	0.597922686	0.941603138	AC015849.19
ENSG00000180071.20	0.01913179	0.597925551	0.941603138	ANKRD18A
ENSG00000264812.1	-0.019291108	0.597958658	0.941603138	RP13-20L14.4
ENSG00000127527.14	-0.004201981	0.598014553	0.941603138	EPS15L1
ENSG00000066136.20	-0.003380986	0.59805375	0.941603138	NFYC
ENSG00000101336.18	0.004858245	0.598227678	0.941603138	HCK
ENSG00000112079.9	0.002810813	0.598278814	0.941603138	STK38
ENSG00000068120.15	0.003574409	0.598286283	0.941603138	COASY
ENSG00000182903.16	0.006872492	0.598298353	0.941603138	ZNF721
ENSG00000140993.11	0.009181633	0.598327615	0.941603138	TIGD7
ENSG00000131437.16	0.006455999	0.598346882	0.941603138	KIF3A
ENSG00000163827.14	-0.02416515	0.598388832	0.941603138	LRRC2
ENSG00000213707.2	-0.013939085	0.598478083	0.941603138	HMGB1P10
ENSG00000260592.1	0.014352423	0.598629866	0.941603138	CTA-363E6.6
ENSG00000116455.14	-0.003038628	0.598674093	0.941603138	WDR77
ENSG00000267117.1	0.013312174	0.598695571	0.941603138	AC010525.4
ENSG00000226174.7	0.008141711	0.59870633	0.941603138	TEX22
ENSG00000271128.1	-0.022072415	0.598706943	0.941603138	CTB-113I20.1
ENSG00000125630.16	0.004078536	0.598740136	0.941603138	POLR1B
ENSG00000235505.7	-0.007568254	0.598757682	0.941603138	CASP4LP
ENSG00000089091.16	-0.006205255	0.598774862	0.941603138	DZANK1
ENSG00000254461.1	-0.013166957	0.598776476	0.941603138	RP11-755F10.3
ENSG00000179921.15	0.007927439	0.598842554	0.941603138	GPBAR1
ENSG00000118514.14	0.011524695	0.598848151	0.941603138	ALDH8A1
ENSG00000163382.13	-0.005177115	0.598855556	0.941603138	NAXE
ENSG00000286025.1	-0.013296937	0.599017412	0.941785426	FAM246C
ENSG00000163539.18	0.004202997	0.599111803	0.941843682	CLASP2
ENSG00000221978.12	-0.002521868	0.599146317	0.941843682	CCNL2
ENSG00000156973.14	0.003545003	0.599210283	0.941872038	PDE6D
ENSG00000185651.15	0.003822375	0.599341196	0.941879312	UBE2L3
ENSG00000130433.8	0.016307118	0.599350435	0.941879312	CACNG6
ENSG00000166037.11	0.006066967	0.599352693	0.941879312	CEP57
ENSG00000229368.1	-0.013087671	0.59955715	0.941934866	AC090587.4
ENSG00000286848.1	0.024957537	0.599583419	0.941934866	RP11-46J23.2
ENSG00000182749.6	0.006648399	0.599585181	0.941934866	PAQR7
ENSG00000110076.19	-0.016518474	0.599589705	0.941934866	NRXN2
ENSG00000029639.11	0.003899773	0.599617695	0.941934866	TFB1M
ENSG00000166341.9	0.01141574	0.599669613	0.941944273	DCHS1
ENSG00000169629.12	-0.01479227	0.599733696	0.941972784	RGPD8
ENSG00000143742.14	0.006672775	0.599792088	0.941992353	SRP9
ENSG00000273747.1	-0.016092891	0.600098392	0.942214623	RP11-90B9.3
ENSG00000167799.10	0.010106102	0.600109926	0.942214623	NUDT8
ENSG00000166377.20	0.003381799	0.600117163	0.942214623	ATP9B
ENSG00000118156.12	-0.013151247	0.600127914	0.942214623	ZNF541
ENSG00000211689.7	-0.01346961	0.600279616	0.942214623	TRGC1

ENSG00000134755.18	0.0124651	0.600295624	0.942214623	DSC2
ENSG00000254812.1	-0.020080878	0.60029579	0.942214623	RP11-661A12.12
ENSG00000226979.9	-0.007178966	0.600301164	0.942214623	LTA
ENSG00000138685.17	-0.030732119	0.600401122	0.942260519	FGF2
ENSG00000276570.1	-0.005015902	0.600460578	0.942260519	CTD-2587H24.14
ENSG00000262877.5	0.011149911	0.600468243	0.942260519	RP11-1055B8.4
ENSG00000173171.15	0.004309919	0.600545326	0.942296547	MTX1
ENSG00000260369.2	0.04122697	0.600612753	0.942296547	CTD-2526A2.2
ENSG00000143149.12	-0.003879296	0.600657989	0.942296547	ALDH9A1
ENSG00000254419.1	-0.007524864	0.600674993	0.942296547	RP11-261P9.4
ENSG00000156239.12	-0.005151662	0.600772115	0.942316886	N6AMT1
ENSG00000079332.15	-0.002278331	0.600779856	0.942316886	SAR1A
ENSG00000141161.12	0.013410561	0.600873499	0.942366482	UNC45B
ENSG00000196636.8	-0.006384446	0.600951779	0.942366482	SDHAF3
ENSG00000119318.13	0.00314372	0.60098307	0.942366482	RAD23B
ENSG00000124103.10	-0.008026753	0.601036327	0.942366482	FAM209A
ENSG00000232254.1	0.01278445	0.601131047	0.942366482	CSF2RBP1
ENSG00000268061.5	0.007786282	0.601172915	0.942366482	NAPA-AS1
ENSG00000223750.1	0.011775948	0.601249425	0.942366482	SIRPB3P
ENSG00000157557.13	0.004934596	0.601310923	0.942366482	ETS2
ENSG00000101400.6	-0.006672681	0.601416183	0.942366482	SNTA1
ENSG00000228335.1	-0.015081192	0.601451773	0.942366482	AC073063.10
ENSG00000105438.9	0.004434499	0.601454755	0.942366482	KDELR1
ENSG00000165434.8	-0.006640464	0.601465385	0.942366482	PGM2L1
ENSG00000166928.11	0.008400613	0.601594517	0.942366482	MS4A14
ENSG00000204338.8	-0.018605888	0.60162573	0.942366482	CYP21A1P
ENSG00000115504.15	0.004070622	0.601657813	0.942366482	EHBP1
ENSG00000231760.5	-0.02026832	0.601712127	0.942366482	RP11-350J20.5
ENSG00000178498.16	0.005418463	0.601765311	0.942366482	DTX3
ENSG00000264968.1	0.017763932	0.601818113	0.942366482	RP11-387H17.4
ENSG00000224715.1	-0.03577337	0.601820895	0.942366482	CITF22-49D8.1
ENSG00000204194.1	0.126012618	0.601898633	0.942366482	RPL12P1
ENSG00000268403.2	0.008286875	0.601927836	0.942366482	CTD-2371O3.3
ENSG00000005486.17	-0.004595504	0.601994314	0.942366482	RHBDD2
ENSG00000107077.19	-0.002833462	0.602003654	0.942366482	KDM4C
ENSG00000115966.17	0.006038691	0.602049503	0.942366482	ATF2
ENSG00000171488.15	-0.003881146	0.602064834	0.942366482	LRRC8C
ENSG00000119844.15	0.003013784	0.602068389	0.942366482	AFTPH
ENSG00000134278.15	0.00981337	0.602092837	0.942366482	SPIRE1
ENSG00000164484.12	-0.012661118	0.60217138	0.942366482	TMEM200A
ENSG00000166321.14	0.005808439	0.602241807	0.942366482	NUDT13
ENSG00000146192.15	-0.005480024	0.602306367	0.942366482	FGD2
ENSG00000132313.15	0.003907663	0.602345396	0.942366482	MRPL35
ENSG00000197566.10	-0.007905768	0.60241358	0.942366482	ZNF624
ENSG00000160161.9	0.023252525	0.602506716	0.942366482	CILP2
ENSG00000108622.11	0.004625365	0.602514105	0.942366482	ICAM2
ENSG00000253986.1	0.026719445	0.602611829	0.942366482	CTC-756D1.3

ENSG00000160294.11	0.003946227	0.602618523	0.942366482	MCM3AP
ENSG00000080802.19	0.003321048	0.602715757	0.942366482	CNOT4
ENSG00000116133.13	0.008835323	0.602730786	0.942366482	DHCR24
ENSG00000072571.20	-0.018485061	0.602756553	0.942366482	HMMR
ENSG00000254634.4	-0.018538005	0.602807974	0.942366482	SMG1P6
ENSG00000154822.18	0.002814955	0.602818419	0.942366482	PLCL2
ENSG00000185561.10	0.013391911	0.602822654	0.942366482	TLCD2
ENSG00000166532.16	0.005297735	0.60283183	0.942366482	RIMKLB
ENSG00000196550.11	0.015779564	0.602833327	0.942366482	FAM72A
ENSG00000243910.8	0.014722188	0.602886778	0.942378204	TUBA4B
ENSG00000271664.1	-0.01162932	0.603035253	0.942462683	RP4-800G7.3
ENSG00000180066.10	-0.020779144	0.603179075	0.942462683	LINC02870
ENSG00000221882.4	-0.028269777	0.603187646	0.942462683	OR3A2
ENSG00000134897.14	-0.006880817	0.603239705	0.942462683	BIVM
ENSG00000286248.1	0.010495511	0.603256296	0.942462683	RP11-340F14.7
ENSG00000111371.16	-0.004673252	0.603270305	0.942462683	SLC38A1
ENSG00000112759.19	-0.011937334	0.603296952	0.942462683	SLC29A1
ENSG00000159374.18	0.009860363	0.603336756	0.942462683	M1AP
ENSG00000204839.9	-0.008580945	0.603390059	0.942462683	MROH6
ENSG00000117226.12	0.011326795	0.603457663	0.942462683	GBP3
ENSG00000175470.20	0.002517045	0.603469898	0.942462683	PPP2R2D
ENSG00000175779.4	0.013787539	0.603537886	0.942462683	LINC02694
ENSG00000164919.11	0.009347365	0.603541794	0.942462683	COX6C
ENSG00000171552.14	0.01426051	0.603584719	0.942462683	BCL2L1
ENSG00000279727.1	0.008446903	0.603663327	0.942462683	LINC02033
ENSG00000283498.1	-0.011086177	0.603765571	0.942462683	MIR1244-2
ENSG00000152240.13	-0.009925011	0.603870713	0.942462683	HAUS1
ENSG00000132016.11	0.010144199	0.603902002	0.942462683	BRME1
ENSG00000167676.4	-0.007931533	0.604014112	0.942462683	PLIN4
ENSG00000113273.17	0.004804147	0.60401874	0.942462683	ARSB
ENSG00000140795.13	-0.016504204	0.604096288	0.942462683	MYLK3
ENSG00000213385.3	-0.014125441	0.604152569	0.942462683	RP11-577H5.1
ENSG00000002933.9	-0.028446623	0.60418995	0.942462683	TMEM176A
ENSG00000182463.16	0.0122183	0.604224491	0.942462683	TSHZ2
ENSG00000280160.1	0.013770003	0.604246199	0.942462683	RP11-196G11.3
ENSG00000143815.15	-0.005570458	0.604254343	0.942462683	LBR
ENSG00000072778.20	-0.004246265	0.604269484	0.942462683	ACADVL
ENSG00000187735.14	-0.00367464	0.604323892	0.942462683	TCEA1
ENSG00000124608.5	-0.003395707	0.604349035	0.942462683	AARS2
ENSG00000171307.19	-0.003299728	0.604382331	0.942462683	ZDHHC16
ENSG00000230415.1	0.010718642	0.604526043	0.942462683	LINC01786
ENSG00000263065.1	-0.017061276	0.604537187	0.942462683	AF001548.6
ENSG00000257950.3	-0.00918126	0.604582822	0.942462683	P2RX5-TAX1BP3
ENSG00000215146.5	-0.026257953	0.60458566	0.942462683	RP11-313J2.1
ENSG00000039650.12	-0.004160857	0.604591616	0.942462683	PNKP
ENSG00000284882.1	0.008529254	0.604604801	0.942462683	RP11-118B23.6
ENSG00000261218.5	0.007855233	0.604668777	0.942462683	RP11-960L18.1

ENSG00000228653.2	-0.017015281	0.604687146	0.942462683	HNRNPCP7
ENSG00000208036.1	0.015970721	0.604759243	0.942503424	MIR106B
ENSG00000257379.1	0.019008858	0.60484199	0.942560755	RP11-793H13.8
ENSG00000174514.13	0.009316736	0.604972128	0.942691923	MFSD4A
ENSG00000116667.15	-0.01049251	0.605096713	0.942783342	C1orf21
ENSG00000258745.1	0.015973677	0.605165643	0.942783342	RP11-218E20.5
ENSG00000273271.1	-0.007649162	0.60516871	0.942783342	AP000254.8
ENSG00000266998.1	0.011798594	0.605301898	0.942878252	RP11-936I5.1
ENSG00000215018.10	-0.025071709	0.605342213	0.942878252	COL28A1
ENSG00000110906.13	-0.004166119	0.605367561	0.942878252	KCTD10
ENSG00000252412.1	-0.014592524	0.605478616	0.942892137	Y_RNA
ENSG00000059804.16	0.006845008	0.605515301	0.942892137	SLC2A3
ENSG00000231711.2	0.006684024	0.605581083	0.942892137	LINC00899
ENSG00000117425.14	-0.007177681	0.605601604	0.942892137	PTCH2
ENSG00000123405.14	0.006223188	0.605768373	0.942892137	NFE2
ENSG00000182578.14	-0.006955259	0.605875361	0.942892137	CSF1R
ENSG00000167768.4	-0.024612434	0.605901382	0.942892137	KRT1
ENSG00000264350.1	0.024853939	0.605974214	0.942892137	SNRPGP2
ENSG00000166847.10	-0.004283662	0.605986182	0.942892137	DCTN5
ENSG00000252391.1	0.017027686	0.606000326	0.942892137	RNU6-638P
ENSG00000196391.11	-0.02394118	0.606023909	0.942892137	ZNF774
ENSG00000143294.15	-0.003693487	0.606059362	0.942892137	PRCC
ENSG00000268001.1	0.005783585	0.606063234	0.942892137	CARD8-AS1
ENSG00000287420.1	-0.017321074	0.606095709	0.942892137	RP11-253D19.4
ENSG00000171863.15	0.012763493	0.606115094	0.942892137	RPS7
ENSG00000163320.12	-0.005046086	0.606138323	0.942892137	CGGBP1
ENSG00000135720.13	0.006335547	0.606158081	0.942892137	DYNC1LI2
ENSG00000156931.16	0.003686446	0.60627538	0.943003071	VPS8
ENSG00000207721.1	0.017826881	0.606381326	0.943076761	MIR186
ENSG00000276523.1	0.007572367	0.606419187	0.943076761	RP11-490B18.6
ENSG00000108733.11	-0.004094905	0.606522695	0.943076761	PEX12
ENSG00000137822.13	-0.003936346	0.606524094	0.943076761	TUBGCP4
ENSG00000176393.11	-0.006811995	0.606596664	0.943076761	RNPEP
ENSG00000115310.18	-0.002806806	0.606631826	0.943076761	RTN4
ENSG00000115138.11	0.015171227	0.606740951	0.943076761	POMC
ENSG00000281357.3	-0.01046841	0.606802912	0.943076761	ARRDC3-AS1
ENSG00000272948.2	0.019015846	0.606920112	0.943076761	AP001412.1
ENSG00000162620.16	0.021989606	0.606985897	0.943076761	LRRIQ3
ENSG00000203705.11	0.004486945	0.60698642	0.943076761	TATDN3
ENSG00000088727.14	0.006940857	0.60699762	0.943076761	KIF9
ENSG00000255507.6	-0.0186706	0.607005395	0.943076761	UVRAG-DT
ENSG00000213057.5	0.013309109	0.607065053	0.943076761	C1orf220
ENSG00000255122.1	-0.015647223	0.607093531	0.943076761	RP11-303G3.6
ENSG00000166323.13	-0.011200054	0.607112502	0.943076761	C11orf65
ENSG00000271913.7	0.005912439	0.607138702	0.943076761	TAGAP-AS1
ENSG00000103490.14	0.006028211	0.607150501	0.943076761	PYCARD
ENSG00000169902.15	-0.012882825	0.6074982	0.943379455	TPST1

ENSG00000267632.1	-0.011874359	0.607508246	0.943379455	RP11-400F19.18
ENSG00000134184.13	-0.073797591	0.607563956	0.943379455	GSTM1
ENSG00000169926.11	-0.003489268	0.607694315	0.943379455	KLF13
ENSG00000172828.13	0.01779759	0.607724659	0.943379455	CES3
ENSG00000184840.12	0.00395108	0.607750619	0.943379455	TMED9
ENSG00000286656.1	-0.008770081	0.607778418	0.943379455	CTD-2201E18.7
ENSG00000166881.10	0.005101952	0.607779507	0.943379455	NEMP1
ENSG00000185085.2	-0.004533104	0.607874087	0.943379455	INTS5
ENSG00000267128.2	0.009981458	0.607893169	0.943379455	RNF157-AS1
ENSG00000099866.16	-0.012583856	0.607975582	0.943379455	MADCAM1
ENSG00000225484.7	0.006036159	0.608023083	0.943379455	NUTM2B-AS1
ENSG00000122481.17	0.008924707	0.608119638	0.943379455	RWDD3
ENSG00000273179.1	0.013027735	0.608148917	0.943379455	RP11-20I20.4
ENSG00000103876.14	-0.006032092	0.608211489	0.943379455	FAH
ENSG00000160712.13	0.004019334	0.608226902	0.943379455	IL6R
ENSG00000110169.11	-0.010843049	0.608230699	0.943379455	HPX
ENSG00000145632.15	-0.008686092	0.608236415	0.943379455	PLK2
ENSG00000278952.1	0.02187036	0.608273373	0.943379455	RP11-399J13.2
ENSG00000237702.2	-0.012708727	0.608292849	0.943379455	TRBV3-1
ENSG00000103145.11	-0.006057836	0.608311386	0.943379455	HCFC1R1
ENSG00000101213.7	-0.007480634	0.608418502	0.943474225	PTK6
ENSG00000144579.8	0.003918934	0.608489343	0.943512736	CTDSP1
ENSG00000265118.5	0.015800452	0.608712613	0.943581509	CTD-2370N5.3
ENSG00000173208.4	0.008118137	0.608738042	0.943581509	ABCD2
ENSG00000110492.16	-0.010578622	0.608779001	0.943581509	MDK
ENSG00000130508.11	-0.034057394	0.608804084	0.943581509	PXDN
ENSG00000125875.15	0.002015969	0.6088052	0.943581509	TBC1D20
ENSG00000184985.16	0.013438139	0.608809759	0.943581509	SORCS2
ENSG00000269958.1	0.003748302	0.608897041	0.943645471	RP11-73M18.8
ENSG00000106992.19	-0.006937176	0.609082338	0.94378306	AK1
ENSG00000186280.7	-0.013984561	0.609090996	0.94378306	KDM4D
ENSG00000273243.1	0.011205697	0.60914761	0.94378306	CTA-217C2.2
ENSG00000099968.18	0.002931185	0.609228352	0.94378306	BCL2L13
ENSG00000064393.16	-0.006643417	0.609262872	0.94378306	HIPK2
ENSG00000139496.16	0.004446609	0.609318402	0.94378306	NUP58
ENSG00000245849.8	0.00379175	0.609388406	0.94378306	RAD51-AS1
ENSG00000173905.9	-0.006812354	0.609436885	0.94378306	GOLIM4
ENSG00000278318.5	0.016242601	0.609447131	0.94378306	ZNF229
ENSG00000139343.10	0.005556489	0.609447293	0.94378306	SNRPF
ENSG00000100949.15	-0.004255535	0.609556627	0.94378306	RABGGTA
ENSG00000166387.14	0.00668142	0.609619182	0.94378306	PPFIBP2
ENSG00000225218.1	0.017069266	0.60962783	0.94378306	AP001628.6
ENSG00000101782.15	0.005312552	0.609630105	0.94378306	RIOK3
ENSG00000281162.2	-0.010333517	0.609719413	0.943814636	LINC01127
ENSG00000237176.4	-0.028272749	0.609813619	0.943814636	RP11-813P10.2
ENSG00000250519.7	0.012828126	0.609816746	0.943814636	RP11-680H20.2
ENSG00000112782.19	-0.007838808	0.609885842	0.943814636	CLIC5

ENSG00000058262.10	-0.003162018	0.609955553	0.943814636	SEC61A1
ENSG00000248472.8	-0.02732218	0.609995068	0.943814636	DDX11L9
ENSG00000279108.1	-0.008091893	0.609998748	0.943814636	CTC-490E21.11
ENSG00000163576.18	-0.024359269	0.610074244	0.943814636	EFHB
ENSG00000278611.2	0.018381384	0.610111515	0.943814636	ZNF426-DT
ENSG00000283994.1	0.013516346	0.610151306	0.943814636	AC079584.3
ENSG00000196510.14	-0.002505621	0.610170453	0.943814636	ANAPC7
ENSG00000181649.8	-0.019610076	0.61023509	0.943814636	PHLDA2
ENSG00000228216.1	-0.02774579	0.610276435	0.943814636	RP11-367F23.1
ENSG00000204789.4	0.010894909	0.610313372	0.943814636	ZNF204P
ENSG00000211862.1	0.02196825	0.610340828	0.943814636	TRAJ27
ENSG00000179314.15	0.021890924	0.610418233	0.943829048	WSCD1
ENSG00000127947.16	-0.00508153	0.610442193	0.943829048	PTPN12
ENSG00000079819.19	-0.006348808	0.610545908	0.943918243	EPB41L2
ENSG00000244734.4	0.016578913	0.610603035	0.943935403	HBB
ENSG00000066583.12	0.004868335	0.610720501	0.943981484	ISOC1
ENSG00000172508.10	-0.006985706	0.610775147	0.943981484	CARNS1
ENSG00000253167.1	-0.01743081	0.61083535	0.943981484	WASHC5-AS1
ENSG00000014919.13	0.002784785	0.610843895	0.943981484	COX15
ENSG00000263847.1	0.014102066	0.610880711	0.943981484	RP11-143J12.3
ENSG00000186716.21	-0.004609461	0.610909024	0.943981484	BCR
ENSG00000123870.10	0.007720122	0.611186309	0.944338794	ZNF137P
ENSG00000082293.13	-0.016312261	0.611366613	0.944475147	COL19A1
ENSG00000261366.1	0.013552683	0.611425802	0.944475147	MANEA-DT
ENSG00000248473.2	-0.009901161	0.611443988	0.944475147	LINC01962
ENSG00000261118.1	-0.008377002	0.611491851	0.944475147	RP11-104N10.1
ENSG00000102547.19	-0.005130072	0.61157111	0.944475147	CAB39L
ENSG00000232070.9	0.020645822	0.611719194	0.944475147	TMEM253
ENSG00000165917.10	-0.009830495	0.611773	0.944475147	RAPSN
ENSG00000150712.11	0.003599675	0.611809503	0.944475147	MTMR12
ENSG00000102755.12	0.015336699	0.611868445	0.944475147	FLT1
ENSG00000266709.1	-0.009732276	0.611910111	0.944475147	RP11-214O1.2
ENSG00000231010.1	-0.012076947	0.611916663	0.944475147	RP6-109B7.2
ENSG00000066739.12	0.004020104	0.611957032	0.944475147	ATG2B
ENSG00000170915.9	-0.004456529	0.611971708	0.944475147	PAQR8
ENSG00000162601.11	-0.006802055	0.612038446	0.944475147	MYSM1
ENSG00000175137.11	-0.003563333	0.612041579	0.944475147	SH3BP5L
ENSG00000168301.13	0.003545063	0.612053981	0.944475147	KCTD6
ENSG00000273297.2	-0.020603181	0.612087435	0.944475147	RP11-38M8.1
ENSG00000144357.18	-0.00444636	0.612158184	0.944475147	UBR3
ENSG00000213062.5	0.009926307	0.612191991	0.944475147	RP1-206D15.6
ENSG00000258469.1	0.021262614	0.612217776	0.944475147	CHMP4BP1
ENSG00000272953.1	0.009693635	0.612241691	0.944475147	RP11-1275H24.2
ENSG00000161681.16	0.020384003	0.612359842	0.944586358	SHANK1
ENSG00000181826.10	-0.006628559	0.612414761	0.944598957	RELL1
ENSG00000234618.1	0.012768984	0.612493414	0.944598957	RPSAP9
ENSG00000172460.17	-0.013574781	0.612534866	0.944598957	PRSS30P

ENSG00000196581.11	0.016540769	0.612610676	0.944598957	AJAP1
ENSG00000266378.1	-0.017063639	0.612652948	0.944598957	RP11-214O1.3
ENSG00000196405.13	-0.003290695	0.612673913	0.944598957	EVL
ENSG00000084774.14	-0.003936749	0.612711449	0.944598957	CAD
ENSG00000243701.7	0.011418851	0.612736489	0.944598957	DUBR
ENSG00000204642.14	0.005395686	0.612857569	0.944714599	HLA-F
ENSG00000279613.1	0.019783299	0.612916532	0.944734478	RP13-638C3.5
ENSG00000112242.15	0.00590449	0.613159991	0.944964959	E2F3
ENSG00000100784.12	-0.004900326	0.61328596	0.944964959	RPS6KA5
ENSG00000166394.15	0.016812667	0.613393379	0.944964959	CYB5R2
ENSG00000116299.17	-0.016071487	0.613474511	0.944964959	ELAPOR1
ENSG00000272666.1	0.011461184	0.613534266	0.944964959	KLHDC7B-DT
ENSG00000214708.4	-0.019555476	0.613545798	0.944964959	AC090616.2
ENSG00000118194.20	0.096143974	0.613831078	0.944964959	TNNT2
ENSG00000261222.3	-0.012435999	0.613902157	0.944964959	CTD-2006K23.1
ENSG00000268520.1	0.012332473	0.613904124	0.944964959	CTD-2616J11.10
ENSG00000137462.9	0.005319229	0.613932606	0.944964959	TLR2
ENSG00000165861.14	0.003422682	0.614048616	0.944964959	ZFYVE1
ENSG00000136167.15	0.003226261	0.614068423	0.944964959	LCP1
ENSG00000108239.9	0.008907101	0.61409271	0.944964959	TBC1D12
ENSG00000255970.1	-0.016166329	0.61411737	0.944964959	LINC02421
ENSG00000054118.15	-0.003004385	0.61415559	0.944964959	THRAP3
ENSG00000007062.12	0.022593601	0.61416418	0.944964959	PROM1
ENSG00000087088.20	-0.004600223	0.614227307	0.944964959	BAX
ENSG00000255587.9	-0.008796249	0.614307895	0.944964959	RAB44
ENSG00000198042.11	0.003538373	0.614309585	0.944964959	MAK16
ENSG00000261654.1	-0.016834138	0.614341519	0.944964959	RP11-96K19.4
ENSG00000213790.2	0.013874551	0.614362042	0.944964959	OLA1P1
ENSG00000104412.8	0.005944389	0.614449143	0.944964959	EMC2
ENSG00000067208.15	0.004890483	0.614512785	0.944964959	EVI5
ENSG00000172116.23	-0.013390377	0.614665191	0.944964959	CD8B
ENSG00000173065.14	-0.003647449	0.614688931	0.944964959	FAM222B
ENSG00000125810.10	0.00723931	0.614696206	0.944964959	CD93
ENSG00000156574.10	-0.012788822	0.614819064	0.944964959	NODAL
ENSG00000160799.12	0.005167028	0.614825317	0.944964959	CCDC12
ENSG00000166886.13	-0.004618017	0.614902558	0.944964959	NAB2
ENSG00000141219.16	0.003655889	0.614933177	0.944964959	C17orf80
ENSG00000108700.5	0.037676374	0.614952168	0.944964959	CCL8
ENSG00000280011.1	-0.008447575	0.61496727	0.944964959	RP4-671O14.7
ENSG00000275964.1	-0.009602165	0.615008173	0.944964959	RP11-61K9.3
ENSG00000101160.15	-0.004006732	0.615013271	0.944964959	CTSZ
ENSG00000116138.13	0.003152607	0.6150312	0.944964959	DNAJC16
ENSG00000256060.3	0.011038214	0.615056574	0.944964959	TRAPPC2B
ENSG00000269352.1	-0.005328166	0.615056598	0.944964959	PTOV1-AS2
ENSG00000099953.10	-0.009349377	0.615074867	0.944964959	MMP11
ENSG00000166750.10	-0.005859783	0.615076321	0.944964959	SLFN5
ENSG00000026297.17	-0.007242815	0.615254739	0.944964959	RNASET2

ENSG00000272871.1	-0.008744339	0.615275266	0.944964959	RP11-408A13.4
ENSG00000236682.2	0.014423548	0.615320868	0.944964959	MAP3K2-DT
ENSG00000131788.16	-0.002981914	0.61537359	0.944964959	PIAS3
ENSG00000159131.17	0.002635807	0.615374088	0.944964959	GART
ENSG00000054392.13	0.006467741	0.615375966	0.944964959	HHAT
ENSG00000163235.16	0.006352231	0.615470989	0.944964959	TGFA
ENSG00000267419.2	0.006183374	0.615672356	0.944964959	ZNF56
ENSG00000253967.1	0.051921106	0.615687319	0.944964959	RP11-333A23.4
ENSG00000066855.16	0.006717792	0.615689775	0.944964959	MTFR1
ENSG00000129521.15	0.009713015	0.615697123	0.944964959	EGLN3
ENSG00000197948.11	-0.003202852	0.615744805	0.944964959	FCHSD1
ENSG00000197728.11	0.019721924	0.615759095	0.944964959	RPS26
ENSG00000139625.13	-0.003658554	0.615794539	0.944964959	MAP3K12
ENSG00000100100.13	0.005179949	0.615814861	0.944964959	PIK3IP1
ENSG00000105085.11	0.0035009	0.615829773	0.944964959	MED26
ENSG00000270640.1	-0.017034672	0.615852664	0.944964959	RP11-373D23.2
ENSG00000126822.18	-0.005170188	0.615877227	0.944964959	PLEKHG3
ENSG00000182586.9	0.015944273	0.615914716	0.944964959	LINC00334
ENSG00000263834.1	-0.025579809	0.615948976	0.944964959	MIR4635
ENSG00000113638.14	0.006949179	0.615973275	0.944964959	TTC33
ENSG00000089351.15	0.004617431	0.615983045	0.944964959	GRAMD1A
ENSG00000126458.4	0.006928804	0.616112119	0.944964959	RRAS
ENSG00000270878.1	-0.019103571	0.616152051	0.944964959	GCATP1
ENSG00000038219.13	0.005829036	0.616162949	0.944964959	BOD1L1
ENSG00000167014.11	0.017516439	0.616288606	0.944964959	TERB2
ENSG00000167113.11	-0.003419633	0.616311599	0.944964959	COQ4
ENSG00000158769.18	0.004782705	0.616374771	0.944964959	F11R
ENSG00000150938.10	0.007091317	0.616415898	0.944964959	CRIM1
ENSG00000279846.1	-0.020593164	0.616448268	0.944964959	RP11-20G13.4
ENSG00000092345.14	0.030905577	0.616580158	0.944964959	DAZL
ENSG00000143632.15	0.018121546	0.616589121	0.944964959	ACTA1
ENSG00000286681.1	0.006724698	0.616657745	0.944964959	RP5-1047A19.16
ENSG00000174171.6	-0.012376465	0.616670592	0.944964959	RP11-23P13.6
ENSG00000158104.11	0.013431671	0.616684049	0.944964959	HPD
ENSG00000233478.1	0.0159865	0.616725114	0.944964959	RP1-187B23.1
ENSG00000172469.17	-0.008880849	0.6167467	0.944964959	MANEA
ENSG00000215374.6	0.014703856	0.616786788	0.944964959	FAM66B
ENSG00000185133.14	0.016717471	0.616794971	0.944964959	INPP5J
ENSG00000110075.15	0.002796028	0.616807217	0.944964959	PPP6R3
ENSG00000197037.11	0.002692364	0.616807406	0.944964959	ZSCAN25
ENSG00000133055.9	0.016099132	0.616858455	0.944964959	MYBPH
ENSG00000198342.10	0.012126468	0.616907719	0.944964959	ZNF442
ENSG00000275963.1	0.012414711	0.616908753	0.944964959	RP11-180M15.6
ENSG00000122140.11	-0.004190812	0.617018466	0.944964959	MRPS2
ENSG00000269514.3	0.007963146	0.617034788	0.944964959	RP11-370I10.12
ENSG00000005700.15	0.005004137	0.617041708	0.944964959	IBTK
ENSG00000130653.16	-0.00880174	0.617074836	0.944964959	PNPLA7

ENSG00000074696.13	0.003428421	0.617174498	0.945027499	HACD3
ENSG00000125703.15	0.004745177	0.617241458	0.945027499	ATG4C
ENSG00000136247.15	0.004091963	0.617375632	0.945027499	ZDHHC4
ENSG00000164118.13	-0.00564796	0.617412023	0.945027499	CEP44
ENSG00000105278.12	-0.010694161	0.61743076	0.945027499	ZFR2
ENSG00000167797.8	-0.005466797	0.617468504	0.945027499	CDK2AP2
ENSG00000130518.17	-0.008136697	0.617495435	0.945027499	IQCN
ENSG00000204618.10	-0.008472008	0.617509935	0.945027499	RNF39
ENSG00000146285.14	0.004497924	0.617541723	0.945027499	SCML4
ENSG00000211746.3	0.009359345	0.617576484	0.945027499	TRBV19
ENSG00000130005.13	-0.007166575	0.617740127	0.94520738	GAMT
ENSG00000277654.6	0.013634208	0.61787718	0.945232715	RP11-255M2.3
ENSG00000246375.3	0.026259212	0.617878031	0.945232715	PPM1K-DT
ENSG00000109339.24	-0.016563142	0.617894957	0.945232715	MAPK10
ENSG00000105339.11	0.003889831	0.617970813	0.945278245	DENND3
ENSG00000215845.11	-0.005068392	0.618233659	0.945609776	TSTD1
ENSG00000125845.7	-0.021891823	0.618388328	0.945686399	BMP2
ENSG00000169696.16	-0.004882624	0.618422882	0.945686399	ASPSCR1
ENSG00000145335.17	0.018821753	0.618493886	0.945686399	SNCA
ENSG00000186318.18	0.0058364	0.618554502	0.945686399	BACE1
ENSG00000188747.9	-0.007264443	0.618574618	0.945686399	NOXA1
ENSG00000121390.19	0.001935761	0.618641448	0.945686399	PSPC1
ENSG00000101292.8	0.026446142	0.618674638	0.945686399	PROKR2
ENSG00000101493.11	-0.004166205	0.618697771	0.945686399	ZNF516
ENSG00000233396.8	0.028350863	0.618740033	0.945686399	LINC01719
ENSG00000254396.1	0.023902297	0.618744885	0.945686399	RP11-56F10.3
ENSG00000103018.17	0.002539463	0.618857348	0.945787801	CYB5B
ENSG00000197147.15	0.007761302	0.619116444	0.945932924	LRRC8B
ENSG00000211959.2	-0.019854197	0.619156875	0.945932924	IGHV4-39
ENSG00000253667.2	-0.017276225	0.619198636	0.945932924	RP11-30L15.4
ENSG00000272595.2	0.036778369	0.619234834	0.945932924	OR10AH1P
ENSG00000152705.8	-0.014160849	0.619245807	0.945932924	CATSPER3
ENSG00000211891.6	-0.027023433	0.619251361	0.945932924	IGHE
ENSG00000173692.14	0.002679459	0.619314711	0.945932924	PSMD1
ENSG00000184277.13	0.003279053	0.619321308	0.945932924	TM2D3
ENSG00000177469.13	-0.01154562	0.619373285	0.945941862	CAVIN1
ENSG00000255474.1	-0.020995515	0.619620401	0.946219462	GAU1
ENSG00000168092.14	0.002424104	0.619647327	0.946219462	PAFAH1B2
ENSG00000230641.1	0.021594056	0.619813516	0.946297902	USP12-AS2
ENSG00000179344.16	-0.017371384	0.61989335	0.946297902	HLA-DQB1
ENSG00000177025.3	0.013572796	0.619963344	0.946297902	C19orf18
ENSG00000269001.2	-0.007483708	0.62002643	0.946297902	ZNF818P
ENSG00000256618.3	-0.070172157	0.620033077	0.946297902	MTRNR2L1
ENSG00000266302.6	0.036024742	0.620112603	0.946297902	RP11-219A15.1
ENSG00000263982.1	0.017333029	0.620142251	0.946297902	RP11-504I13.3
ENSG00000122741.16	0.00414627	0.620142412	0.946297902	DCAF10
ENSG00000260868.1	-0.016332535	0.62017483	0.946297902	LINC01960

ENSG00000167914.12	-0.025108885	0.620232446	0.946297902	GSDMA
ENSG00000167208.15	0.003096781	0.620330501	0.946297902	SNX20
ENSG00000141449.15	-0.015557204	0.620343625	0.946297902	GREB1L
ENSG00000284606.1	-0.018038394	0.620463602	0.946297902	RP11-556O5.7
ENSG00000134901.13	0.01276237	0.620601607	0.946297902	POGLUT2
ENSG00000197343.11	0.002718565	0.620612806	0.946297902	ZNF655
ENSG00000118257.17	-0.011178897	0.62062374	0.946297902	NRP2
ENSG00000250254.2	0.012532594	0.620667739	0.946297902	PTTG2
ENSG00000271936.1	0.008833568	0.620727142	0.946297902	RP11-443B20.1
ENSG00000135525.19	-0.010057039	0.620800089	0.946297902	MAP7
ENSG00000164284.15	0.005572944	0.620871365	0.946297902	GRPEL2
ENSG00000017797.13	-0.004904944	0.620878554	0.946297902	RALBP1
ENSG00000211667.3	-0.035756575	0.62091844	0.946297902	IGLV3-12
ENSG00000255931.2	-0.023260071	0.620922445	0.946297902	RP11-286N22.10
ENSG00000205189.12	0.006198019	0.620966841	0.946297902	ZBTB10
ENSG00000268938.2	0.008330244	0.621057253	0.946297902	AC005387.3
ENSG00000224066.1	-0.013536378	0.621182075	0.946297902	RP4-622L5.7
ENSG00000122515.15	-0.00416257	0.621256304	0.946297902	ZMIZ2
ENSG00000168904.15	0.003678623	0.621335982	0.946297902	LRRC28
ENSG00000264070.1	-0.023613176	0.621563252	0.946297902	DND1P1
ENSG00000155629.15	0.005757287	0.621590516	0.946297902	PIK3AP1
ENSG00000260751.2	-0.00918393	0.621609477	0.946297902	CTD-2196E14.6
ENSG00000000971.16	-0.015401498	0.621629563	0.946297902	CFH
ENSG00000278341.1	-0.010616513	0.621682784	0.946297902	RP5-1142A6.10
ENSG00000035403.18	0.00516352	0.621712478	0.946297902	VCL
ENSG00000234789.1	-0.014162449	0.621715309	0.946297902	RP11-483H20.4
ENSG00000163738.19	-0.006889465	0.621752679	0.946297902	MTHFD2L
ENSG00000111880.16	0.004181335	0.621808707	0.946297902	RNGTT
ENSG00000112562.19	0.046727001	0.62180921	0.946297902	SMOC2
ENSG00000128000.17	-0.005847353	0.621874905	0.946297902	ZNF780B
ENSG00000108679.13	-0.010364914	0.621895352	0.946297902	LGALS3BP
ENSG00000236039.3	-0.029892576	0.621936439	0.946297902	LINC02889
ENSG00000003147.19	-0.006803072	0.621958429	0.946297902	ICA1
ENSG00000274776.1	0.017927034	0.621994827	0.946297902	RP11-687F6.5
ENSG00000119638.14	-0.002182123	0.622009067	0.946297902	NEK9
ENSG00000187091.14	-0.00429249	0.622039131	0.946297902	PLCD1
ENSG00000137103.20	0.005021517	0.622088608	0.946297902	TMEM8B
ENSG00000125148.7	-0.013084633	0.622174951	0.946297902	MT2A
ENSG00000253738.2	0.009244306	0.622211216	0.946297902	OTUD6B-AS1
ENSG00000150636.17	0.007808779	0.622216801	0.946297902	CCDC102B
ENSG00000278959.1	0.008292845	0.622312883	0.946297902	RP11-455I9.1
ENSG00000169139.12	0.004685809	0.622326749	0.946297902	UBE2V2
ENSG00000065361.17	-0.014651747	0.622335425	0.946297902	ERBB3
ENSG00000261766.1	-0.01580168	0.622393312	0.946297902	RP11-22P6.2
ENSG00000178623.12	-0.00624079	0.622434285	0.946297902	GPR35
ENSG00000219529.2	-0.019290753	0.622438761	0.946297902	AP000580.1
ENSG00000058866.15	0.00590079	0.622550551	0.946297902	DGKG

ENSG00000136802.12	-0.003285704	0.622604559	0.946297902	LRRC8A
ENSG00000050748.18	-0.002737623	0.622611904	0.946297902	MAPK9
ENSG00000198270.13	0.004407687	0.622696356	0.946297902	TMEM116
ENSG00000165209.19	-0.006521098	0.622718075	0.946297902	STRBP
ENSG00000109466.14	0.0064936	0.622749856	0.946297902	KLHL2
ENSG00000233178.7	-0.020107114	0.6228035	0.946297902	RP11-88I18.2
ENSG00000257923.12	0.004088944	0.622814445	0.946297902	CUX1
ENSG00000255198.4	0.009748618	0.622847229	0.946297902	SNHG9
ENSG00000178802.18	-0.003827102	0.622872696	0.946297902	MPI
ENSG00000163655.16	0.002344281	0.62291553	0.946297902	GMPS
ENSG00000231246.2	0.014839777	0.622957372	0.946297902	LINC02884
ENSG00000227001.3	0.018178228	0.623036491	0.946297902	NBPF2P
ENSG00000108852.15	0.012591712	0.623046601	0.946297902	MPP2
ENSG00000189127.8	0.019036666	0.623099179	0.946297902	ANKRD34B
ENSG00000118620.13	0.006259227	0.623102635	0.946297902	ZNF430
ENSG00000198794.12	0.010309821	0.623139602	0.946297902	SCAMP5
ENSG00000175175.6	0.015377305	0.623187341	0.946297902	PPM1E
ENSG00000108244.17	0.010435468	0.623187677	0.946297902	KRT23
ENSG00000110435.13	-0.003668376	0.623350475	0.946297902	PDHX
ENSG00000267637.1	0.02207582	0.6233532	0.946297902	RP11-619I22.1
ENSG00000266524.3	-0.01914146	0.623429756	0.946297902	GDF10
ENSG00000185163.10	-0.003651517	0.623455467	0.946297902	DDX51
ENSG00000280198.1	0.008864726	0.623460754	0.946297902	RP11-384M20.1
ENSG00000123643.13	0.006176157	0.623510715	0.946297902	SLC36A1
ENSG00000138081.22	0.006022366	0.623516951	0.946297902	FBXO11
ENSG00000151650.8	-0.007695513	0.623543485	0.946297902	VENTX
ENSG00000286369.1	0.013008649	0.623549732	0.946297902	RP11-856M9.1
ENSG00000181751.10	0.005823845	0.623607714	0.946297902	MACIR
ENSG00000106049.9	-0.003882307	0.623801946	0.946297902	HIBADH
ENSG00000118402.6	0.013008914	0.623821685	0.946297902	ELOVL4
ENSG00000050327.15	-0.00735069	0.623837819	0.946297902	ARHGEF5
ENSG00000272320.1	0.020781523	0.623903113	0.946297902	RP11-506K6.4
ENSG00000136938.9	0.003767169	0.623908953	0.946297902	ANP32B
ENSG00000161217.12	0.00247599	0.623965112	0.946297902	PCYT1A
ENSG00000131398.15	0.007266927	0.624021322	0.946297902	KCNC3
ENSG00000134077.16	0.003705819	0.624090731	0.946297902	THUMPD3
ENSG00000101439.9	-0.007387383	0.624214463	0.946297902	CST3
ENSG00000286230.1	-0.016621924	0.624272418	0.946297902	CTD-2587H24.15
ENSG00000228113.7	0.014211071	0.624346531	0.946297902	AC003991.3
ENSG00000174611.12	0.008288053	0.624427249	0.946297902	KY
ENSG00000227388.3	-0.006791671	0.624429233	0.946297902	RP11-112J3.16
ENSG00000228657.1	-0.044008261	0.624436646	0.946297902	RPL23AP58
ENSG00000134504.14	-0.006576891	0.624451474	0.946297902	KCTD1
ENSG00000211772.11	0.005908294	0.6245919	0.946297902	TRBC2
ENSG00000151881.15	-0.007866132	0.624632746	0.946297902	TMEM267
ENSG00000078814.17	-0.007728386	0.624632907	0.946297902	MYH7B
ENSG00000107338.10	0.011186823	0.624686447	0.946297902	SHB

ENSG00000163554.15	0.015897539	0.624720835	0.946297902	SPTA1
ENSG00000279930.1	0.013106956	0.624783277	0.946297902	LA16c-312E8.4
ENSG00000124302.13	0.021079397	0.624784326	0.946297902	CHST8
ENSG00000124508.17	-0.004381339	0.624799954	0.946297902	BTN2A2
ENSG00000132570.15	0.003285914	0.62480801	0.946297902	PCBD2
ENSG00000273374.1	-0.018098716	0.624828306	0.946297902	RP11-383I23.2
ENSG00000161021.13	0.002951386	0.624921429	0.946297902	MAML1
ENSG00000120694.20	-0.004711095	0.624931213	0.946297902	HSPH1
ENSG00000205269.6	0.007527097	0.624951628	0.946297902	TMEM170B
ENSG00000182446.14	-0.002511973	0.62495947	0.946297902	NPLOC4
ENSG00000223891.6	0.004928062	0.624978915	0.946297902	OSER1-DT
ENSG00000146918.20	-0.012440674	0.625027917	0.946297902	NCAPG2
ENSG00000175390.15	-0.004419315	0.625051267	0.946297902	EIF3F
ENSG00000162069.16	-0.018206955	0.62511416	0.946323259	BICDL2
ENSG00000259314.1	0.018768186	0.625207731	0.946395051	CTD-3065B20.3
ENSG00000083168.11	0.005102801	0.62527374	0.946425113	KAT6A
ENSG00000230530.2	0.007332163	0.625346598	0.946438856	LIMD1-AS1
ENSG00000160948.14	0.005272817	0.625386152	0.946438856	VPS28
ENSG00000166483.12	-0.006905964	0.625421269	0.946438856	WEE1
ENSG00000105610.6	0.016714402	0.625639209	0.946698804	KLF1
ENSG00000204366.4	-0.007460525	0.6257025	0.946724721	ZBTB12
ENSG00000189057.11	0.013734412	0.625869543	0.946907605	FAM111B
ENSG00000145041.16	-0.003761021	0.625982329	0.947008381	DCAF1
ENSG00000076984.18	-0.003265106	0.62607645	0.947014	MAP2K7
ENSG00000152683.14	0.003462342	0.626078399	0.947014	SLC30A6
ENSG00000020129.16	-0.00431093	0.626174008	0.947073615	NCDN
ENSG00000031691.7	-0.01257884	0.626210173	0.947073615	CENPQ
ENSG00000174807.4	-0.015631906	0.626280541	0.947110193	CD248
ENSG00000115232.14	-0.00633465	0.626419315	0.947198698	ITGA4
ENSG00000174106.3	-0.005927908	0.626468015	0.947198698	LEMD3
ENSG00000004809.14	-0.017069007	0.626477626	0.947198698	SLC22A16
ENSG00000150990.9	-0.003339878	0.626638746	0.947372459	DHX37
ENSG00000100359.21	-0.004543932	0.626827073	0.947410216	SGSM3
ENSG00000167380.17	0.004458147	0.626909673	0.947410216	ZNF226
ENSG00000183666.17	0.006130941	0.626945052	0.947410216	GUSBP1
ENSG00000028528.15	0.001791686	0.626994132	0.947410216	SNX1
ENSG00000273654.1	0.012049724	0.62706594	0.947410216	CTB-52I2.4
ENSG00000253692.3	0.037935546	0.627066385	0.947410216	IGHEP1
ENSG00000235865.2	-0.014621726	0.627067391	0.947410216	GSN-AS1
ENSG00000163376.11	0.009470786	0.627075519	0.947410216	KBTBD8
ENSG00000177084.18	-0.003163074	0.627123696	0.947410216	POLE
ENSG00000234814.8	0.008147064	0.627165022	0.947410216	SVIL2P
ENSG00000170962.13	-0.009963735	0.627268651	0.947410216	PDGFD
ENSG00000205277.10	-0.014267226	0.627302956	0.947410216	MUC12
ENSG00000188732.11	-0.007533174	0.62730476	0.947410216	FAM221A
ENSG00000181666.18	-0.004150181	0.627356308	0.947410216	ZNF875
ENSG00000113296.14	-0.008501523	0.627391068	0.947410216	THBS4

ENSG00000171109.19	0.004056071	0.627464051	0.947410216	MFN1
ENSG00000136819.15	-0.009565326	0.627516244	0.947410216	C9orf78
ENSG00000132821.12	0.020107562	0.627518116	0.947410216	VSTM2L
ENSG00000072110.16	-0.005414849	0.627541465	0.947410216	ACTN1
ENSG00000236423.6	-0.008948595	0.627859584	0.947658568	LINC01134
ENSG00000285210.1	0.015390373	0.627888361	0.947658568	LINC02795
ENSG00000269924.1	0.014497711	0.628005721	0.947658568	RP11-697N18.4
ENSG00000078043.16	0.004440246	0.628095899	0.947658568	PIAS2
ENSG00000065978.19	-0.005781064	0.628102614	0.947658568	YBX1
ENSG00000189171.15	-0.007579872	0.628172601	0.947658568	S100A13
ENSG00000131467.11	0.003099737	0.628219293	0.947658568	PSME3
ENSG00000241644.2	0.015363702	0.628224513	0.947658568	INMT
ENSG00000185485.14	0.005141127	0.628236665	0.947658568	SDHAP1
ENSG00000275532.1	-0.016065578	0.628244982	0.947658568	CTB-58E17.3
ENSG00000120051.15	0.007723119	0.628276647	0.947658568	CFAP58
ENSG00000180228.14	0.003914042	0.628323795	0.947658568	PRKRA
ENSG00000155313.16	0.004083889	0.628406634	0.947658568	USP25
ENSG00000103591.13	0.002908774	0.628413074	0.947658568	AAGAB
ENSG00000213760.11	-0.007522083	0.628521915	0.947658568	ATP6V1G2
ENSG00000232712.6	-0.012997641	0.628571515	0.947658568	KIZ-AS1
ENSG00000115364.14	0.003275411	0.628576851	0.947658568	MRPL19
ENSG00000106105.15	-0.002817203	0.628580955	0.947658568	GARS1
ENSG00000178409.14	-0.007322014	0.62867577	0.947658568	BEND3
ENSG00000155070.8	-0.046840853	0.628864342	0.947658568	UNC93B2
ENSG00000163362.11	-0.021558629	0.628883577	0.947658568	INAVA
ENSG00000152348.16	0.007785175	0.628925277	0.947658568	ATG10
ENSG00000263033.2	-0.01225713	0.628955189	0.947658568	RP11-396B14.2
ENSG00000095627.9	-0.032808152	0.629029776	0.947658568	TDRD1
ENSG00000138080.14	-0.007246354	0.629081109	0.947658568	EMILIN1
ENSG00000122512.16	0.004486233	0.629144581	0.947658568	PMS2
ENSG00000134644.16	-0.004326544	0.629191021	0.947658568	PUM1
ENSG00000244625.7	0.004591583	0.629196684	0.947658568	MIATNB
ENSG00000265195.1	-0.020497312	0.629205734	0.947658568	MIR4312
ENSG00000197056.11	0.005248485	0.62924459	0.947658568	ZMYM1
ENSG00000163348.4	-0.002452181	0.629350846	0.947658568	PYGO2
ENSG00000287376.1	-0.042469005	0.629357497	0.947658568	RP11-556N21.6
ENSG00000196776.17	0.003863744	0.629439945	0.947658568	CD47
ENSG00000144827.9	0.004644609	0.629451053	0.947658568	ABHD10
ENSG00000159882.13	0.006245582	0.629563921	0.947658568	ZNF230
ENSG00000170027.7	0.003366708	0.629581952	0.947658568	YWHAG
ENSG00000168970.22	0.005534408	0.629683185	0.947658568	JMJD7-PLA2G4B
ENSG00000116161.18	0.00324342	0.629687807	0.947658568	CACYBP
ENSG00000275764.1	-0.004814226	0.629726535	0.947658568	RP11-582E3.6
ENSG00000163462.18	-0.005669133	0.629865724	0.947658568	TRIM46
ENSG00000090905.19	0.003377689	0.629915551	0.947658568	TNRC6A
ENSG00000280106.1	-0.006418309	0.629931815	0.947658568	CTC-523E23.3
ENSG00000262691.1	0.010805174	0.629945028	0.947658568	CTC-277H1.7

ENSG00000006327.14	-0.006591996	0.630019731	0.947658568	TNFRSF12A
ENSG00000168917.9	-0.014769106	0.630032521	0.947658568	SLC35G2
ENSG00000135437.10	-0.007689432	0.630039786	0.947658568	RDH5
ENSG00000273058.2	0.006708572	0.630086884	0.947658568	RP11-385F5.5
ENSG00000279720.1	-0.023796863	0.630099609	0.947658568	SOWAHCP2
ENSG00000093072.18	-0.005010076	0.630121552	0.947658568	ADA2
ENSG00000097007.19	-0.003784256	0.630164011	0.947658568	ABL1
ENSG00000222345.1	-0.017336473	0.630231612	0.947658568	SNORD19C
ENSG00000185897.7	-0.019619904	0.630234205	0.947658568	FFAR3
ENSG00000185158.12	0.003018793	0.63023805	0.947658568	LRRC37B
ENSG00000132434.10	0.003684425	0.630327201	0.947658568	LANCL2
ENSG00000126005.17	0.005352387	0.630339421	0.947658568	MMP24OS
ENSG00000179862.7	0.009441574	0.630372259	0.947658568	CITED4
ENSG00000163681.16	0.004809324	0.630375676	0.947658568	SLMAP
ENSG00000226416.1	-0.010660289	0.630480341	0.947658568	MRPL23-AS1
ENSG00000171385.10	0.014958239	0.630582516	0.947658568	KCND3
ENSG00000257093.7	0.004506636	0.630613021	0.947658568	DENND11
ENSG00000163930.10	0.002531004	0.630641728	0.947658568	BAP1
ENSG00000270259.2	-0.028147605	0.630794235	0.947658568	RP13-122B23.9
ENSG00000168137.19	0.002874653	0.630866651	0.947658568	SETD5
ENSG00000185736.16	0.051253107	0.630928144	0.947658568	ADARB2
ENSG00000276744.1	0.012950689	0.630939529	0.947658568	CTD-2562G15.3
ENSG00000227242.4	0.045592302	0.630979832	0.947658568	CH17-112A12.2
ENSG00000244482.10	-0.007841705	0.630982833	0.947658568	LILRA6
ENSG00000170248.15	-0.002906655	0.631042903	0.947658568	PDCD6IP
ENSG00000198467.16	-0.008764882	0.631049773	0.947658568	TPM2
ENSG00000239219.2	0.02138125	0.631104051	0.947658568	RP11-379K17.4
ENSG00000184156.17	0.021384242	0.631163664	0.947658568	KCNQ3
ENSG00000275243.1	-0.027528225	0.631249897	0.947658568	TRBV16
ENSG00000198848.13	0.015284492	0.631315896	0.947658568	CES1
ENSG00000094880.11	-0.002326536	0.631354631	0.947658568	CDC23
ENSG00000143344.16	0.007533445	0.631366482	0.947658568	RGL1
ENSG00000271420.1	-0.019995214	0.631367467	0.947658568	RP5-1057J7.7
ENSG00000279863.1	-0.009079696	0.631380718	0.947658568	RP11-521C22.2
ENSG00000181585.7	0.011217496	0.63139333	0.947658568	TMIE
ENSG00000151498.12	0.002572475	0.631402248	0.947658568	ACAD8
ENSG00000230896.1	0.007142664	0.631402705	0.947658568	RP11-767N6.7
ENSG00000157500.12	0.003954082	0.631507523	0.947678138	APPL1
ENSG00000198538.11	0.006585424	0.631508164	0.947678138	ZNF28
ENSG00000260382.1	0.029246033	0.631640942	0.94772651	RP11-540B6.2
ENSG00000155827.12	-0.002615397	0.631667676	0.94772651	RNF20
ENSG00000022556.16	0.015368019	0.631764224	0.94772651	NLRP2
ENSG00000197982.14	-0.005631139	0.631767235	0.94772651	C1orf122
ENSG00000230257.5	-0.010059759	0.631771461	0.94772651	NFE4
ENSG00000135766.9	0.006068263	0.631823149	0.947734723	EGLN1
ENSG00000211639.2	-0.02460091	0.631878	0.947747679	IGLV4-60
ENSG00000099622.14	-0.003147679	0.631969164	0.947757507	CIRBP

ENSG00000166507.19	0.006039346	0.632085924	0.947757507	NDST2
ENSG00000054267.22	0.006590915	0.632090316	0.947757507	ARID4B
ENSG00000268189.2	-0.013422733	0.632130087	0.947757507	AC005785.2
ENSG00000198937.9	-0.007572695	0.632292494	0.947757507	CCDC167
ENSG00000154328.16	-0.00447309	0.632301526	0.947757507	NEIL2
ENSG00000196182.11	0.004068662	0.632350859	0.947757507	STK40
ENSG00000131378.14	-0.002901579	0.632541034	0.947757507	RFTN1
ENSG00000165801.10	0.006816932	0.632590768	0.947757507	ARHGEF40
ENSG00000108773.11	-0.003773743	0.632597193	0.947757507	KAT2A
ENSG00000124882.4	0.018717491	0.632611055	0.947757507	EREG
ENSG00000249947.2	-0.01218951	0.632666744	0.947757507	XBP1P1
ENSG00000129538.14	0.01982889	0.632713304	0.947757507	RNASE1
ENSG00000267390.1	0.009996904	0.632779499	0.947757507	KDSR-DT
ENSG00000135164.19	0.004386196	0.632801922	0.947757507	DMTF1
ENSG00000238078.1	-0.022512782	0.632816016	0.947757507	LINC01352
ENSG00000267122.1	-0.015747491	0.63283293	0.947757507	AC004490.1
ENSG00000250982.2	-0.024421038	0.632902401	0.947757507	GAPDHP35
ENSG00000198771.11	0.002878413	0.632977534	0.947757507	RCSD1
ENSG00000149489.9	0.006768927	0.63303379	0.947757507	ROM1
ENSG00000218226.1	-0.009857012	0.633128366	0.947757507	TATDN2P2
ENSG00000144671.11	-0.01470901	0.633194968	0.947757507	SLC22A14
ENSG00000237296.9	0.014999675	0.633235484	0.947757507	SMG1P1
ENSG00000188611.17	-0.020629643	0.633328942	0.947757507	ASAH2
ENSG00000286856.1	-0.010601118	0.633537418	0.947757507	RP11-114D6.2
ENSG00000164080.14	-0.002836523	0.633660036	0.947757507	RAD54L2
ENSG00000110811.20	0.010371417	0.633691408	0.947757507	P3H3
ENSG00000170458.14	-0.006749104	0.633714089	0.947757507	CD14
ENSG00000223177.1	-0.016737727	0.633734901	0.947757507	RNA5SP39
ENSG00000248791.1	-0.017227297	0.633839056	0.947757507	CTD-2165H16.3
ENSG00000099246.18	0.005868833	0.633846282	0.947757507	RAB18
ENSG00000128309.17	-0.004928744	0.633850578	0.947757507	MPST
ENSG00000159314.11	-0.002897489	0.634065721	0.947757507	ARHGAP27
ENSG00000231245.2	0.036267629	0.634092637	0.947757507	C1DP1
ENSG00000167526.14	-0.005915208	0.634106753	0.947757507	RPL13
ENSG00000135870.12	-0.003582875	0.634111371	0.947757507	RC3H1
ENSG00000146263.12	-0.00489942	0.634131204	0.947757507	MMS22L
ENSG00000164023.14	0.007502132	0.634201386	0.947757507	SGMS2
ENSG00000240505.9	0.015517354	0.634214199	0.947757507	TNFRSF13B
ENSG00000226261.1	0.015025135	0.634248019	0.947757507	PIMREGP1
ENSG00000121289.18	0.002788136	0.634277398	0.947757507	CEP89
ENSG00000116406.19	-0.006667479	0.634299492	0.947757507	EDEM3
ENSG00000100012.12	0.021164812	0.634310231	0.947757507	SEC14L3
ENSG00000111879.20	-0.011472185	0.634319008	0.947757507	FAM184A
ENSG00000178175.12	0.010369573	0.634361307	0.947757507	ZNF366
ENSG00000127334.11	-0.003823851	0.63436447	0.947757507	DYRK2
ENSG00000262001.1	-0.006626354	0.634366443	0.947757507	DLGAP1-AS2
ENSG00000224020.1	0.020337519	0.634390192	0.947757507	MIR181A2HG

ENSG00000131374.14	0.003423679	0.63443989	0.947757507	TBC1D5
ENSG00000156587.16	0.008768938	0.634492627	0.947757507	UBE2L6
ENSG00000176058.13	-0.004796589	0.634517055	0.947757507	TPRN
ENSG00000267344.1	0.005073799	0.634540096	0.947757507	CTB-39G8.3
ENSG00000227799.1	-0.00761323	0.634557045	0.947757507	CDPF1P1
ENSG00000137841.12	-0.003803634	0.634594524	0.947757507	PLCB2
ENSG00000212127.6	0.010403916	0.634609694	0.947757507	TAS2R14
ENSG00000205482.10	0.008156636	0.634625708	0.947757507	SPDYE18
ENSG00000159111.13	0.003846895	0.634684276	0.947757507	MRPL10
ENSG00000250490.1	-0.01376494	0.634710912	0.947757507	LINC02145
ENSG00000253649.5	0.012070263	0.634754864	0.947757507	PRSS51
ENSG00000122786.20	0.014314868	0.6347591	0.947757507	CALD1
ENSG00000286525.1	0.024353344	0.634776715	0.947757507	RP11-546M8.3
ENSG00000221866.9	0.011020315	0.634825034	0.947757507	PLXNA4
ENSG00000106100.11	0.003739456	0.634828672	0.947757507	NOD1
ENSG00000224478.3	-0.010459036	0.63489459	0.947757507	RP11-13P5.1
ENSG00000267397.1	-0.016965675	0.6349087	0.947757507	RP11-873E20.1
ENSG00000006047.13	-0.028857551	0.635037244	0.947757507	YBX2
ENSG00000023839.12	0.005499229	0.635116519	0.947757507	ABCC2
ENSG00000088179.9	-0.005545211	0.635220543	0.947757507	PTPN4
ENSG00000170881.5	0.004109126	0.635288202	0.947757507	RNF139
ENSG00000196821.10	0.003113283	0.635312	0.947757507	ILRUN
ENSG00000144909.8	0.003998169	0.63532622	0.947757507	OSBPL11
ENSG00000168393.13	0.004191094	0.635363328	0.947757507	DTYMK
ENSG00000230373.8	0.020949067	0.635391218	0.947757507	GOLGA6L5P
ENSG00000145331.14	0.00694658	0.635447616	0.947757507	TRMT10A
ENSG00000105576.16	0.002292943	0.635457918	0.947757507	TNPO2
ENSG00000124126.14	-0.004189684	0.635480621	0.947757507	PREX1
ENSG00000101193.8	0.002676693	0.635488678	0.947757507	GID8
ENSG00000117602.13	0.006278328	0.635489247	0.947757507	RCAN3
ENSG00000090372.15	-0.003516677	0.635609649	0.947788587	STRN4
ENSG00000178719.17	0.005963394	0.63563857	0.947788587	GRINA
ENSG00000279917.1	-0.016742055	0.635671291	0.947788587	RP11-360N9.2
ENSG00000103154.10	0.010986315	0.635702895	0.947788587	NECAB2
ENSG00000243176.6	-0.006954519	0.635792716	0.947788587	RP11-550I24.2
ENSG00000196415.10	0.027496963	0.635824465	0.947788587	PRTN3
ENSG00000138670.18	0.005760413	0.636062224	0.947788587	RASGEF1B
ENSG00000116580.19	0.003557695	0.636087727	0.947788587	GON4L
ENSG00000254876.5	-0.007979126	0.636226771	0.947788587	STRA6LP
ENSG00000264176.1	0.01443756	0.636241983	0.947788587	MAGOH2P
ENSG00000254531.1	0.007257508	0.636306298	0.947788587	FLJ20021
ENSG00000213190.4	0.004336821	0.636373562	0.947788587	MLLT11
ENSG00000215908.10	0.00640271	0.636396405	0.947788587	CROCCP2
ENSG00000256553.1	-0.013250514	0.636406592	0.947788587	TRAV1-2
ENSG00000160256.13	-0.005358209	0.636430934	0.947788587	FAM207A
ENSG00000132698.15	-0.013585118	0.636452826	0.947788587	RAB25
ENSG00000130193.8	0.00478066	0.636478742	0.947788587	THEM6

ENSG00000160613.14	-0.004336139	0.636484725	0.947788587	PCSK7
ENSG00000246100.4	-0.016284478	0.636540156	0.947788587	LINC00900
ENSG00000120318.16	-0.005104048	0.636580821	0.947788587	ARAP3
ENSG00000166002.7	0.006816562	0.636607502	0.947788587	SMCO4
ENSG00000108474.17	0.004029916	0.636608348	0.947788587	PIGL
ENSG00000223552.1	0.014013367	0.636668931	0.947788587	CCR5AS
ENSG00000165699.15	0.002634224	0.636802678	0.947788587	TSC1
ENSG00000197457.10	-0.005591798	0.636939971	0.947788587	STMN3
ENSG00000198336.9	-0.014291388	0.637026257	0.947788587	MYL4
ENSG00000163882.9	0.003353995	0.637027593	0.947788587	POLR2H
ENSG00000203667.10	-0.007337692	0.637053451	0.947788587	COX20
ENSG00000145721.12	0.019098266	0.637109987	0.947788587	LIX1
ENSG00000100890.16	0.005013569	0.637122817	0.947788587	PRORP
ENSG00000228107.1	0.012256817	0.637134337	0.947788587	AP000692.9
ENSG00000146221.10	0.018923064	0.637192966	0.947788587	TCTE1
ENSG00000146576.13	-0.003557192	0.637216069	0.947788587	C7orf26
ENSG00000081059.20	0.00645558	0.63723285	0.947788587	TCF7
ENSG00000196199.14	-0.002236032	0.637274184	0.947788587	MPHOSPH8
ENSG00000248367.1	-0.007484864	0.637312894	0.947788587	CTB-129O4.1
ENSG00000106246.18	-0.003697682	0.637423423	0.947788587	PTCD1
ENSG00000064309.16	-0.013541033	0.637470546	0.947788587	CDON
ENSG00000287252.1	-0.009734824	0.637501434	0.947788587	RP1-97D16.9
ENSG00000135148.12	0.00507914	0.637505677	0.947788587	TRAFD1
ENSG00000091732.17	0.003307545	0.637565193	0.947788587	ZC3HC1
ENSG00000277957.1	0.019226321	0.637610813	0.947788587	SENP3-EIF4A1
ENSG00000162398.12	0.012729745	0.637618129	0.947788587	LEXM
ENSG00000096654.16	0.005340832	0.637620713	0.947788587	ZNF184
ENSG00000227788.1	0.019079095	0.637628573	0.947788587	MTCO3P43
ENSG00000260563.3	0.005234673	0.637636003	0.947788587	RP13-516M14.1
ENSG00000244167.1	0.019326721	0.637748146	0.947886576	RP4-733B9.1
ENSG00000255642.1	0.014726006	0.63783133	0.947941512	PABPC1P4
ENSG00000164111.15	-0.005036691	0.6379558	0.948057793	ANXA5
ENSG00000115604.12	-0.007337228	0.638138156	0.948161668	IL18R1
ENSG00000280417.1	-0.014099718	0.638166652	0.948161668	RP11-5O17.1
ENSG00000230224.1	-0.008975004	0.638194669	0.948161668	PHBP9
ENSG00000271952.2	0.058680001	0.638212173	0.948161668	LINC01954
ENSG00000147570.10	0.02182415	0.638314129	0.948161668	DNAJC5B
ENSG00000150457.9	0.004661888	0.638360853	0.948161668	LATS2
ENSG00000132122.12	0.006331483	0.638388449	0.948161668	SPATA6
ENSG00000160401.15	-0.006899205	0.638395568	0.948161668	CFAP157
ENSG00000060237.19	-0.004824264	0.638596705	0.948358938	WNK1
ENSG00000243406.6	-0.011279545	0.638692787	0.948358938	MRPS31P5
ENSG00000186017.15	-0.004669245	0.638699185	0.948358938	ZNF566
ENSG00000164291.17	-0.006991385	0.638713363	0.948358938	ARSK
ENSG00000106346.11	-0.003546217	0.638854098	0.948499229	USP42
ENSG00000250831.1	0.019632044	0.638970533	0.948591569	CTD-2232E5.2
ENSG00000131067.17	-0.004597075	0.639042013	0.948591569	GGT7

ENSG00000287935.1	0.014804498	0.639145015	0.948591569	CTD-2245O6.4
ENSG00000198793.13	-0.003480933	0.639147384	0.948591569	MTOR
ENSG00000163428.4	0.005151716	0.639155729	0.948591569	LRRC58
ENSG00000272221.1	-0.011522761	0.639233009	0.948591569	XXbac-BPG181B23.7
ENSG00000285533.2	-0.004897315	0.63935274	0.948591569	RELA-DT
ENSG00000145012.14	0.004139478	0.639375575	0.948591569	LPP
ENSG00000258826.6	-0.011208189	0.639535463	0.948591569	LINC01147
ENSG00000124490.14	0.029618249	0.639535793	0.948591569	CRISP2
ENSG00000223722.3	0.014830843	0.63953823	0.948591569	IFITM3P2
ENSG00000275649.1	-0.032769929	0.639564499	0.948591569	RP11-286O1.1
ENSG00000132846.6	0.006661921	0.639579841	0.948591569	ZBED3
ENSG00000157911.11	0.004216071	0.639582434	0.948591569	PEX10
ENSG00000125257.16	0.006238028	0.639610114	0.948591569	ABCC4
ENSG00000183921.7	0.013165447	0.639718679	0.948683942	SDR42E2
ENSG00000166200.15	0.00840402	0.63977672	0.948683942	COPS2
ENSG00000241057.2	0.012107454	0.639811176	0.948683942	AC004985.12
ENSG00000198855.7	-0.003784045	0.640053124	0.948921905	FICD
ENSG00000130244.12	-0.003071392	0.640087841	0.948921905	FAM98C
ENSG00000125386.16	-0.003340261	0.640110476	0.948921905	FAM193A
ENSG00000211786.3	-0.007770517	0.640168774	0.948928738	TRAV8-2
ENSG00000237513.2	-0.007744867	0.640218915	0.948928738	RP11-325F22.2
ENSG00000111058.8	0.007943821	0.640253899	0.948928738	ACSS3
ENSG00000228060.3	-0.015150406	0.640508704	0.949174593	PABPC4-AS1
ENSG00000233927.5	-0.006575344	0.640521897	0.949174593	RPS28
ENSG00000226314.8	-0.012839155	0.640566836	0.949174593	ZNF192P1
ENSG00000262484.2	0.011002108	0.640697768	0.949174593	CCER2
ENSG00000137040.10	0.005877041	0.640750554	0.949174593	RANBP6
ENSG00000163519.14	0.00919108	0.640769007	0.949174593	TRAT1
ENSG00000172661.19	-0.003931654	0.640791077	0.949174593	WASHC2C
ENSG00000138018.18	0.004506548	0.641068143	0.949174593	SELENOI
ENSG00000253829.1	-0.015642742	0.641084074	0.949174593	RP11-723D22.3
ENSG00000150787.8	-0.006874999	0.641133112	0.949174593	PTS
ENSG00000255142.1	-0.008347656	0.641217999	0.949174593	AP006621.6
ENSG00000279821.1	-0.007923564	0.641229641	0.949174593	RP11-1334A24.5
ENSG00000211645.2	-0.023719233	0.64127631	0.949174593	IGLV1-50
ENSG00000262050.1	0.016081115	0.641279237	0.949174593	RP11-74E22.3
ENSG00000070610.14	0.002809902	0.641348697	0.949174593	GBA2
ENSG00000132465.12	0.01734426	0.641362058	0.949174593	JCHAIN
ENSG00000106003.13	-0.003880204	0.641456425	0.949174593	LFNG
ENSG00000109794.13	0.024345457	0.641463462	0.949174593	FAM149A
ENSG00000125821.13	-0.004544619	0.641468702	0.949174593	DTD1
ENSG00000124772.12	0.008726495	0.641545435	0.949174593	CPNE5
ENSG00000141744.4	-0.015736425	0.641558579	0.949174593	PNMT
ENSG00000185716.12	0.005863765	0.641591223	0.949174593	MOSMO
ENSG00000279699.1	-0.01794396	0.641595411	0.949174593	RP1-102K2.9
ENSG00000167261.14	-0.00461154	0.641603956	0.949174593	DPEP2
ENSG00000215840.3	0.014458222	0.641628763	0.949174593	RP11-122G18.7

ENSG00000182253.15	0.015515451	0.641654592	0.949174593	SYNM
ENSG00000197748.13	-0.017064965	0.641699447	0.949174593	CFAP43
ENSG00000243646.10	-0.004044381	0.641824297	0.949174593	IL10RB
ENSG00000224418.1	0.015813205	0.64185463	0.949174593	STK24-AS1
ENSG00000133872.14	0.002825465	0.641856499	0.949174593	SARAF
ENSG00000279554.1	0.020044304	0.641866862	0.949174593	CTA-113A6.2
ENSG00000064763.11	0.005317112	0.64190084	0.949174593	FAR2
ENSG00000104879.5	0.018858466	0.642053105	0.949331297	CKM
ENSG00000230734.1	0.016072533	0.642174776	0.949366604	RPL10P3
ENSG00000279913.1	-0.021077671	0.642250863	0.949366604	AP001962.3
ENSG00000168005.9	-0.004156371	0.642258473	0.949366604	SPINDOC
ENSG00000069188.17	0.013660149	0.642305233	0.949366604	SDK2
ENSG00000261245.2	0.010012325	0.642308447	0.949366604	RP11-120K18.3
ENSG00000183625.16	0.010939523	0.64240399	0.949439393	CCR3
ENSG00000134551.13	-0.015103992	0.642588698	0.949638593	PRH2
ENSG00000164896.20	-0.003880212	0.642631383	0.949638593	FASTK
ENSG00000287312.1	-0.028778341	0.642893709	0.949751814	RP13-726E6.4
ENSG00000196233.14	-0.003264037	0.642900867	0.949751814	LCOR
ENSG00000137409.20	0.00245651	0.642998333	0.949751814	MTCH1
ENSG00000156471.13	0.002463899	0.643008903	0.949751814	PTDSS1
ENSG00000142687.18	0.0039457	0.643055946	0.949751814	KIAA0319L
ENSG00000274902.1	0.012775153	0.643205082	0.949751814	RP1-197B17.4
ENSG00000274765.1	-0.009332501	0.643229709	0.949751814	RP11-139H15.7
ENSG00000286186.1	-0.008395728	0.643231898	0.949751814	RP11-196B3.5
ENSG00000154227.14	0.016322447	0.643242684	0.949751814	CERS3
ENSG00000259807.2	0.00699549	0.643254569	0.949751814	RP11-426C22.4
ENSG00000141127.15	0.002752275	0.643267745	0.949751814	PRPSAP2
ENSG00000117519.16	0.010684403	0.643277387	0.949751814	CNN3
ENSG00000186106.11	0.005740386	0.643356099	0.949751814	ANKRD46
ENSG00000221817.10	-0.003647158	0.643356358	0.949751814	PPP3CB-AS1
ENSG00000132423.12	0.005996926	0.643589652	0.950027825	COQ3
ENSG00000273018.7	0.02995879	0.643717503	0.950148162	FAM106A
ENSG00000072832.15	0.013795481	0.64377135	0.95014944	CRMP1
ENSG00000005100.13	0.003446433	0.643811031	0.95014944	DHX33
ENSG00000168014.18	0.003127719	0.643872895	0.950172364	C2CD3
ENSG00000260774.1	-0.011287486	0.644018535	0.950299215	CTD-2083E4.4
ENSG00000012822.16	0.002994861	0.64405153	0.950299215	CALCOCO1
ENSG00000240253.6	-0.032138165	0.644216408	0.95043192	FAR2P3
ENSG00000213280.2	0.015938286	0.644234158	0.95043192	RP11-212P7.1
ENSG00000152582.14	0.006216802	0.644319489	0.950489431	SPEF2
ENSG00000196659.10	0.005848816	0.644481828	0.950521995	TTC30B
ENSG00000188186.10	0.005289141	0.644482626	0.950521995	LAMTOR4
ENSG00000110395.7	0.004137731	0.644512801	0.950521995	CBL
ENSG00000145354.12	-0.006526769	0.644526958	0.950521995	CISD2
ENSG00000159086.15	0.004875534	0.644669407	0.950552662	PAXBP1
ENSG00000127990.19	0.012404031	0.644669931	0.950552662	SGCE
ENSG00000234465.11	0.013149156	0.644686804	0.950552662	PINLYP

ENSG00000187116.14	0.007687586	0.644740063	0.950557816	LILRA5
ENSG00000227359.1	-0.013853054	0.644797673	0.950557816	AC017074.2
ENSG00000112053.14	-0.009523788	0.644852739	0.950557816	SLC26A8
ENSG00000141519.15	0.01515633	0.644875702	0.950557816	CCDC40
ENSG00000213588.6	-0.003105461	0.644963867	0.950577621	ZBTB9
ENSG00000096433.11	-0.00585987	0.64502656	0.950577621	ITPR3
ENSG00000163156.12	0.003920258	0.645028193	0.950577621	SCNM1
ENSG00000187676.8	0.005248761	0.645161288	0.950705447	B3GLCT
ENSG00000107185.10	0.002803757	0.645508585	0.950870067	RGP1
ENSG00000188825.14	-0.006568057	0.645534228	0.950870067	LINC00910
ENSG00000204653.10	-0.011082884	0.645580645	0.950870067	ASPDH
ENSG00000228716.7	0.006377584	0.645587592	0.950870067	DHFR
ENSG00000188393.9	0.050196878	0.645589605	0.950870067	CLEC2A
ENSG00000126467.11	-0.021355909	0.645655597	0.950870067	TSKS
ENSG00000270956.1	-0.010838616	0.645703153	0.950870067	RP11-65L3.4
ENSG00000188343.13	0.011529616	0.645737438	0.950870067	CIBAR1
ENSG00000279227.1	-0.007622644	0.645775661	0.950870067	AC009303.2
ENSG00000133561.15	0.003125394	0.64586194	0.950870067	GIMAP6
ENSG00000254837.2	0.00860263	0.645920611	0.950870067	AP001372.2
ENSG00000281958.1	0.008366191	0.645930678	0.950870067	TRBJ1-4
ENSG00000180346.4	-0.004994195	0.645957363	0.950870067	TIGD2
ENSG00000181104.7	-0.009513126	0.646027856	0.950870067	F2R
ENSG00000276778.1	-0.049288428	0.646055566	0.950870067	LINC02227
ENSG00000137656.12	-0.002404328	0.646070646	0.950870067	BUD13
ENSG00000288720.1	-0.015826897	0.646118641	0.950870067	RP11-852E15.3
ENSG00000119723.17	0.00348581	0.646190907	0.950870067	COQ6
ENSG00000180878.3	-0.014692841	0.646203742	0.950870067	C11orf42
ENSG00000175787.17	0.003979632	0.646255979	0.950870067	ZNF169
ENSG00000171604.12	-0.005948013	0.646257118	0.950870067	CXXC5
ENSG00000125741.5	-0.002701642	0.64629305	0.950870067	OPA3
ENSG00000197019.5	0.004905313	0.646420821	0.950989827	SERTAD1
ENSG00000141696.13	-0.005808873	0.646585026	0.951163168	P3H4
ENSG00000120833.14	0.006016465	0.646669417	0.951219079	SOCS2
ENSG00000119715.15	-0.020776029	0.646886888	0.951244708	ESRRB
ENSG00000005436.14	-0.004178372	0.646958939	0.951244708	GCFC2
ENSG00000288093.1	-0.014738013	0.647004716	0.951244708	RP11-122G18.13
ENSG00000175600.16	-0.01321491	0.647039612	0.951244708	SUGCT
ENSG00000197619.14	0.00525127	0.64708815	0.951244708	ZNF615
ENSG00000162695.12	-0.004571059	0.647107701	0.951244708	SLC30A7
ENSG00000267136.1	-0.016599168	0.647168359	0.951244708	RP11-53B2.3
ENSG00000110852.5	0.005659856	0.647357415	0.951244708	CLEC2B
ENSG00000144642.22	0.016142246	0.647372194	0.951244708	RBMS3
ENSG00000164056.11	-0.01289441	0.647405771	0.951244708	SPRY1
ENSG00000091622.16	0.010001793	0.647423665	0.951244708	PITPNM3
ENSG00000120949.15	0.005185691	0.647438773	0.951244708	TNFRSF8
ENSG00000285618.1	-0.010148855	0.647445344	0.951244708	CTD-2266L18.2
ENSG00000237301.1	-0.016649429	0.647539845	0.951244708	RP4-680D5.2

ENSG00000075336.12	0.003402327	0.647541556	0.951244708	TIMM21
ENSG00000259337.4	-0.046176331	0.647546015	0.951244708	IGHV1OR15-2
ENSG00000166262.16	0.007002011	0.647602503	0.951244708	FAM227B
ENSG00000183258.12	-0.003438429	0.647751058	0.951244708	DDX41
ENSG00000159496.15	0.006475524	0.647758662	0.951244708	RGL4
ENSG00000133121.21	-0.012518893	0.647934531	0.951244708	STARD13
ENSG00000123737.13	-0.003633892	0.647959018	0.951244708	EXOSC9
ENSG00000261167.1	-0.006887045	0.647999109	0.951244708	RP11-517B11.7
ENSG00000187726.9	0.007630329	0.64800949	0.951244708	DNAJB13
ENSG00000230454.1	-0.009175111	0.648118147	0.951244708	U73166.2
ENSG00000237773.7	-0.011192659	0.648188009	0.951244708	AC003075.4
ENSG00000122566.22	0.00389292	0.648189915	0.951244708	HNRNPA2B1
ENSG00000269825.2	0.009458758	0.648247616	0.951244708	CTD-3099C6.13
ENSG00000232573.1	0.007442052	0.648327456	0.951244708	RPL3P4
ENSG00000109956.13	-0.013941325	0.64836894	0.951244708	B3GAT1
ENSG00000188227.13	-0.007368142	0.648421378	0.951244708	ZNF793
ENSG00000163814.8	0.010857983	0.648476409	0.951244708	CDCP1
ENSG00000278768.2	0.00476861	0.648487322	0.951244708	BACE1-AS
ENSG00000267254.7	-0.006373328	0.648549347	0.951244708	ZNF790-AS1
ENSG00000188739.15	0.006994535	0.648568227	0.951244708	RBM34
ENSG00000106546.14	-0.005939153	0.648632423	0.951244708	AHR
ENSG00000148334.16	-0.003514254	0.648667825	0.951244708	PTGES2
ENSG00000272335.1	-0.004944139	0.648709127	0.951244708	RP11-53O19.3
ENSG00000198646.14	-0.004464761	0.648737637	0.951244708	NCOA6
ENSG00000182552.15	0.004627017	0.648760601	0.951244708	RWDD4
ENSG00000198917.13	0.005507491	0.648816218	0.951244708	SPOUT1
ENSG00000273066.5	0.007663436	0.648930579	0.951244708	RP11-216L13.19
ENSG00000204001.10	0.034658069	0.648969789	0.951244708	LCN8
ENSG00000286129.1	-0.008573702	0.648972833	0.951244708	CH17-264L24.2
ENSG00000067225.19	-0.004282708	0.648984663	0.951244708	PKM
ENSG00000277283.1	-0.005824919	0.648999654	0.951244708	RP1-267D11.6
ENSG00000167378.9	-0.002645061	0.649110678	0.951244708	IRGQ
ENSG00000271743.1	0.018894973	0.649227972	0.951244708	CTD-2541M15.3
ENSG00000213639.10	-0.005304204	0.64932125	0.951244708	PPP1CB
ENSG00000257303.2	-0.005460096	0.64932381	0.951244708	RP11-977G19.11
ENSG00000043514.17	0.003532028	0.649350577	0.951244708	TRIT1
ENSG00000163661.4	-0.00850584	0.649378784	0.951244708	PTX3
ENSG00000175854.14	0.004169996	0.649433082	0.951244708	SWI5
ENSG00000121892.15	0.003056203	0.649531297	0.951244708	PDS5A
ENSG00000100304.13	-0.003672703	0.649532506	0.951244708	TTLL12
ENSG00000168389.18	-0.005902721	0.649577214	0.951244708	MFSD2A
ENSG00000249160.4	-0.015750695	0.649617631	0.951244708	LINC02213
ENSG00000225282.1	-0.022871151	0.64970635	0.951244708	AP000350.6
ENSG00000198796.7	-0.020902256	0.649707442	0.951244708	ALPK2
ENSG00000122591.13	-0.005713244	0.649752917	0.951244708	FAM126A
ENSG00000255150.2	0.006401927	0.649774705	0.951244708	EID3
ENSG00000130559.19	0.003363799	0.649819565	0.951244708	CAMSAP1

ENSG00000146278.11	0.0037998	0.649867561	0.951244708	PNRC1	
ENSG00000127452.9	-0.002569101	0.649900077	0.951244708	FBXL12	
ENSG00000269374.1	-0.009599636	0.649932404	0.951244708	CTB-50E14.5	
ENSG00000120784.17	0.00608565	0.649933554	0.951244708	ZFP30	
ENSG00000280010.1	-0.011573985	0.65000688	0.951244708	AP001350.4	
ENSG00000247626.5	-0.003861819	0.650015469	0.951244708		mar.02
ENSG00000286482.1	0.008296072	0.650076024	0.951244708	RP3-395C13.2	
ENSG00000154001.14	-0.002935515	0.65008556	0.951244708	PPP2R5E	
ENSG00000125827.9	0.004027268	0.650099082	0.951244708	TMX4	
ENSG00000267320.5	0.051368516	0.650131502	0.951244708	AC005780.1	
ENSG00000227527.2	-0.016169014	0.650199549	0.951244708	RP11-223A3.1	
ENSG00000166261.11	0.002649457	0.650216238	0.951244708	ZNF202	
ENSG00000269929.3	0.004320658	0.650284454	0.951244708	MIRLET7A1HG	
ENSG00000168152.13	-0.00699932	0.650298395	0.951244708	THAP9	
ENSG00000143153.13	0.009358281	0.650302793	0.951244708	ATP1B1	
ENSG00000107902.14	-0.005523086	0.650335072	0.951244708	LHPP	
ENSG00000136935.14	0.002231541	0.650390057	0.951244708	GOLGA1	
ENSG00000107815.10	-0.003660088	0.650399751	0.951244708	TWNK	
ENSG00000278727.1	0.009265515	0.650400041	0.951244708	AC000403.4	
ENSG00000287151.1	0.01913329	0.650535671	0.951244708	C2orf27A	
ENSG00000166816.15	-0.011525775	0.650538045	0.951244708	LDHD	
ENSG00000090097.22	-0.004749435	0.650539062	0.951244708	PCBP4	
ENSG00000182263.14	-0.030095317	0.650583103	0.951244708	FIGN	
ENSG00000280248.1	-0.012186433	0.650691913	0.951335977	CTD-2047H16.3	
ENSG00000118292.9	-0.006590726	0.650839208	0.951483495	C1orf54	
ENSG00000237943.7	0.005425704	0.650949879	0.951575631	PRKCQ-AS1	
ENSG00000088451.11	0.004911185	0.65107596	0.951575631	TGDS	
ENSG00000175164.16	-0.020050265	0.651107067	0.951575631	ABO	
ENSG00000222057.1	-0.014972105	0.651157402	0.951575631	RNU4-62P	
ENSG00000057252.13	-0.003177143	0.651204832	0.951575631	SOAT1	
ENSG00000251301.7	-0.014546284	0.651232152	0.951575631	LINC02384	
ENSG00000166441.13	0.005569093	0.651255763	0.951575631	RPL27A	
ENSG00000198643.7	-0.0179424	0.651273433	0.951575631	FAM3D	
ENSG00000125885.13	-0.005446018	0.65135936	0.951580645	MCM8	
ENSG00000275793.1	0.008938974	0.651432991	0.951580645	RIMBP3	
ENSG00000229447.2	0.012598697	0.651459883	0.951580645	RP11-490K7.4	
ENSG00000136104.21	-0.003572193	0.651495658	0.951580645	RNASEH2B	
ENSG00000149554.14	-0.007588273	0.651508866	0.951580645	CHEK1	
ENSG00000267348.3	0.006307171	0.651593756	0.951607501	GEMIN7-AS1	
ENSG00000174891.14	0.003992114	0.651620057	0.951607501	RSRC1	
ENSG00000120162.10	0.006002624	0.651769787	0.951656191	MOB3B	
ENSG00000277232.2	-0.00858458	0.65182991	0.951656191	GTSE1-DT	
ENSG00000155115.7	0.004442392	0.651847848	0.951656191	GTF3C6	
ENSG00000145920.15	-0.017218429	0.65185796	0.951656191	CPLX2	
ENSG00000132470.14	0.013843944	0.651929518	0.951656191	ITGB4	
ENSG00000055609.21	-0.005583638	0.651931823	0.951656191	KMT2C	
ENSG00000108830.10	0.014416428	0.652042357	0.951730468	RND2	

ENSG00000204899.6	-0.006112547	0.652191829	0.951730468	MZT1
ENSG00000145907.16	-0.002016819	0.652194066	0.951730468	G3BP1
ENSG00000141756.19	0.014255295	0.652217726	0.951730468	FKBP10
ENSG00000123096.12	0.010529636	0.652343172	0.951730468	SSPN
ENSG00000275431.1	0.021174969	0.652359802	0.951730468	CTB-186H2.3
ENSG00000214212.9	0.004101164	0.652431216	0.951730468	C19orf38
ENSG00000250131.2	-0.012060915	0.652431974	0.951730468	RP11-130F10.1
ENSG00000279259.1	0.008596317	0.652466876	0.951730468	RP11-334C17.3
ENSG00000160783.19	0.004255325	0.652509358	0.951730468	PMF1
ENSG00000230730.1	-0.013156975	0.652581232	0.951730468	AC074011.2
ENSG00000240535.8	0.01008254	0.652659043	0.951730468	CTD-2313F11.1
ENSG00000172269.20	-0.003488999	0.652676727	0.951730468	DPAGT1
ENSG00000100253.13	0.018691766	0.652790064	0.951730468	MIOX
ENSG00000132938.22	-0.018092051	0.652797715	0.951730468	MTUS2
ENSG00000234425.1	-0.016538456	0.652806844	0.951730468	RP11-528G1.2
ENSG00000240230.6	-0.002103041	0.65282881	0.951730468	COX19
ENSG00000100505.14	-0.011278809	0.65284769	0.951730468	TRIM9
ENSG00000236810.6	-0.006683406	0.652864454	0.951730468	ELOA-AS1
ENSG00000138071.14	0.00349759	0.65307912	0.951975734	ACTR2
ENSG00000177565.18	0.004555981	0.653141744	0.951999352	TBL1XR1
ENSG00000214359.3	-0.018445773	0.653247029	0.952085145	RPL18P10
ENSG00000162927.14	-0.005144648	0.653449367	0.952241794	PUS10
ENSG00000170315.14	0.009380601	0.653507221	0.952241794	UBB
ENSG00000164970.15	-0.002513735	0.653525772	0.952241794	FAM219A
ENSG00000130159.14	-0.005435434	0.653548921	0.952241794	ECSIT
ENSG00000066933.16	0.003987181	0.653586673	0.952241794	MYO9A
ENSG00000203965.13	-0.007711852	0.653660212	0.952281283	EFCAB7
ENSG00000101391.21	-0.002244728	0.653780411	0.952291887	CDK5RAP1
ENSG00000175548.9	0.007362246	0.653979308	0.952291887	ALG10B
ENSG00000228201.1	-0.012121537	0.653983724	0.952291887	AL022341.3
ENSG00000116031.9	-0.017941838	0.65403917	0.952291887	CD207
ENSG00000136931.10	0.018976064	0.654079787	0.952291887	NR5A1
ENSG00000260176.1	0.028630001	0.654088338	0.952291887	CTD-3126B10.4
ENSG00000146909.8	-0.003085242	0.654099058	0.952291887	NOM1
ENSG00000245105.4	0.009745334	0.654116363	0.952291887	A2M-AS1
ENSG00000189067.14	0.004651814	0.654175583	0.952291887	LITAF
ENSG00000272316.1	0.008092554	0.65424165	0.952291887	XXbac-BPGBPG55C20.2
ENSG00000138835.22	-0.004109396	0.654370011	0.952291887	RGS3
ENSG00000267940.1	-0.005028549	0.65440846	0.952291887	RP11-290F24.6
ENSG00000225938.1	-0.007722679	0.654449826	0.952291887	RP4-575N6.4
ENSG00000177042.16	-0.003989441	0.654490023	0.952291887	TMEM80
ENSG00000273472.1	0.010560375	0.654490637	0.952291887	RP11-102N12.3
ENSG00000259153.2	0.016295156	0.654535352	0.952291887	RP6-65G23.3
ENSG00000162461.8	-0.007130738	0.654571827	0.952291887	SLC25A34
ENSG00000111057.11	0.008314109	0.654582056	0.952291887	KRT18
ENSG00000272084.1	-0.009719881	0.654637014	0.952291887	RP5-1126H10.2
ENSG00000248866.1	-0.012924214	0.654648315	0.952291887	USP46-DT

ENSG00000136710.10	0.002403651	0.654699149	0.952291887	CCDC115
ENSG00000134574.12	0.002952006	0.654750799	0.952291887	DDB2
ENSG00000128731.18	0.004897612	0.654763619	0.952291887	HERC2
ENSG00000106733.21	0.006104541	0.654781934	0.952291887	NMRK1
ENSG00000102606.19	0.002537607	0.654875585	0.952308412	ARHGEF7
ENSG00000158525.16	-0.033444907	0.654930205	0.952308412	CPA5
ENSG00000166033.13	0.014275846	0.654955934	0.952308412	HTRA1
ENSG00000175324.10	0.006256278	0.655068695	0.952308412	LSM1
ENSG00000235313.1	-0.008681024	0.655098041	0.952308412	HM13-IT1
ENSG00000139350.12	0.005340405	0.655114851	0.952308412	NEDD1
ENSG00000263956.7	0.003569585	0.655237483	0.952308412	NBPF11
ENSG00000234174.1	0.016230727	0.655266076	0.952308412	AC016683.5
ENSG00000183495.14	-0.00377391	0.655275468	0.952308412	EP400
ENSG00000188185.13	0.005213704	0.655324568	0.952308412	LINC00265
ENSG00000140511.12	0.006404092	0.655348074	0.952308412	HAPLN3
ENSG00000257231.1	0.017357272	0.655350528	0.952308412	DYNLL1P4
ENSG00000242661.1	-0.020117962	0.655511503	0.952321308	RPS3AP43
ENSG00000185104.20	-0.002743568	0.655542108	0.952321308	FAF1
ENSG00000101752.12	-0.006360777	0.655591541	0.952321308	MIB1
ENSG00000178722.13	-0.023333132	0.655670801	0.952321308	C5orf64
ENSG00000099994.11	0.009273352	0.655723604	0.952321308	SUSD2
ENSG00000130558.20	0.015765606	0.655746581	0.952321308	OLFM1
ENSG00000126353.3	0.008574024	0.655810143	0.952321308	CCR7
ENSG00000107611.16	0.007319393	0.65581754	0.952321308	CUBN
ENSG00000075391.17	-0.010685511	0.655840068	0.952321308	RASAL2
ENSG00000016864.19	0.002016787	0.655964813	0.952321308	GLT8D1
ENSG00000288674.1	-0.010872579	0.65598163	0.952321308	RP5-1087E8.6
ENSG00000259755.1	-0.016432137	0.655985718	0.952321308	RP11-505E24.2
ENSG00000144488.15	0.018813457	0.656026265	0.952321308	ESPNL
ENSG00000125835.19	0.004381031	0.656089867	0.952321308	SNRPB
ENSG00000142197.12	-0.003905856	0.65613418	0.952321308	DOP1B
ENSG00000169976.7	0.004662094	0.656148669	0.952321308	SF3B5
ENSG00000239920.2	-0.017203355	0.656148824	0.952321308	CTD-2643I7.5
ENSG00000255529.9	-0.005007325	0.656235	0.952325081	POLR2M
ENSG00000160307.10	0.027138429	0.656244297	0.952325081	S100B
ENSG00000090054.15	-0.002810455	0.656372522	0.952346218	SPTLC1
ENSG00000168806.8	-0.003393279	0.656377448	0.952346218	LCMT2
ENSG00000225693.1	-0.016826655	0.656491272	0.952346218	LAGE3P1
ENSG00000262879.6	0.005363465	0.656508983	0.952346218	RP11-156P1.3
ENSG00000128242.13	-0.016365701	0.656671231	0.952346218	GAL3ST1
ENSG00000204792.2	0.043401516	0.656848836	0.952346218	LINC01291
ENSG00000259098.1	0.035169487	0.65687973	0.952346218	RP11-603B24.2
ENSG00000107593.17	0.022364622	0.656944761	0.952346218	PKD2L1
ENSG00000040633.13	-0.003136172	0.656967854	0.952346218	PHF23
ENSG00000173040.13	-0.011358632	0.656971921	0.952346218	EVC2
ENSG00000173218.15	-0.006452181	0.657005749	0.952346218	VANGL1
ENSG00000215005.2	0.039045808	0.657024347	0.952346218	HSPD1P7

ENSG00000211455.8	0.004665904	0.657100041	0.952346218	STK38L
ENSG00000172594.13	0.006727357	0.657103207	0.952346218	SMPDL3A
ENSG00000132970.14	0.015367838	0.657130381	0.952346218	WASF3
ENSG00000077984.6	0.008634533	0.65713043	0.952346218	CST7
ENSG00000224376.1	0.009791626	0.657225601	0.952346218	AC017104.6
ENSG00000149292.17	0.006084913	0.657325887	0.952346218	TTC12
ENSG00000172354.10	-0.0042364	0.657378424	0.952346218	GNB2
ENSG00000221995.5	-0.004469515	0.65743602	0.952346218	TIAF1
ENSG00000150630.4	0.018799304	0.657445487	0.952346218	VEGFC
ENSG00000213949.10	0.010411404	0.657488787	0.952346218	ITGA1
ENSG00000110921.14	-0.003767882	0.657495	0.952346218	MVK
ENSG00000163710.9	0.028892881	0.657496147	0.952346218	PCOLCE2
ENSG00000136848.17	-0.018398285	0.657507285	0.952346218	DAB2IP
ENSG00000204345.1	-0.023641313	0.657516253	0.952346218	CD300LD
ENSG00000131044.17	-0.016671146	0.657537458	0.952346218	TTLL9
ENSG00000122642.11	-0.007203483	0.657580794	0.952346218	FKBP9
ENSG00000070031.4	-0.014926182	0.657605559	0.952346218	SCT
ENSG00000128692.8	-0.011513258	0.657689403	0.952384384	EIF2S2P4
ENSG00000254995.4	-0.005861934	0.657881263	0.952384384	STX16-NPEPL1
ENSG00000159588.15	-0.005301579	0.657906852	0.952384384	CCDC17
ENSG00000154122.14	0.004931517	0.657935969	0.952384384	ANKH
ENSG00000181036.14	-0.010356976	0.657951522	0.952384384	FCRL6
ENSG00000250075.7	0.016445194	0.657968999	0.952384384	RP11-584P21.2
ENSG00000177807.10	0.017576035	0.658054892	0.952384384	KCNJ10
ENSG00000117280.13	-0.003179514	0.658096555	0.952384384	RAB29
ENSG00000229921.7	0.048260042	0.658247183	0.952384384	KIF25-AS1
ENSG00000133884.10	-0.002878849	0.658250876	0.952384384	DPF2
ENSG00000279608.1	0.013338045	0.658262524	0.952384384	RP11-182N22.9
ENSG00000075223.14	-0.007796152	0.658318554	0.952384384	SEMA3C
ENSG00000167981.7	0.004057246	0.658360513	0.952384384	ZNF597
ENSG00000177225.17	-0.003072255	0.658382607	0.952384384	GATD1
ENSG00000224078.15	-0.005231086	0.658439426	0.952384384	SNHG14
ENSG00000105176.18	0.003008575	0.65845527	0.952384384	URI1
ENSG00000163346.17	-0.004469172	0.658456733	0.952384384	PBXIP1
ENSG00000187953.10	-0.004209468	0.658467826	0.952384384	PMS2CL
ENSG00000211879.1	-0.018481247	0.658522692	0.952396571	TRAJ10
ENSG00000281332.1	0.017452059	0.658646746	0.952508812	LINC00997
ENSG00000144674.16	0.005134953	0.658811231	0.952667717	GOLGA4
ENSG00000128322.7	-0.020776271	0.658849533	0.952667717	IGLL1
ENSG00000228506.2	0.007891101	0.658980764	0.952790292	RP11-98I9.4
ENSG00000178921.14	0.00384445	0.659035612	0.95280242	PFAS
ENSG00000007129.18	-0.006483195	0.65927005	0.952853069	CEACAM21
ENSG00000276566.1	0.040129449	0.659344347	0.952853069	IGKV1D-13
ENSG00000164879.7	-0.01522271	0.659351292	0.952853069	CA3
ENSG00000173334.4	0.004045018	0.659357753	0.952853069	TRIB1
ENSG00000175416.16	-0.004227162	0.659368468	0.952853069	CLTB
ENSG00000237753.2	-0.006709671	0.659376758	0.952853069	AC079922.3

ENSG00000226281.4	-0.008865708	0.65945399	0.952853069	RP1-80N2.2
ENSG00000286943.1	-0.016627593	0.659497411	0.952853069	RP11-147I3.4
ENSG00000150556.17	-0.022595334	0.65961771	0.952853069	LYPD6B
ENSG00000134986.14	0.006811946	0.659636139	0.952853069	NREP
ENSG00000157259.8	-0.002671438	0.659648573	0.952853069	GATAD1
ENSG00000100644.17	-0.004486289	0.659673014	0.952853069	HIF1A
ENSG00000241973.11	-0.003611722	0.659695797	0.952853069	PI4KA
ENSG00000267452.3	0.030190469	0.659744678	0.952853069	LINC02073
ENSG00000163016.10	0.012608831	0.659767582	0.952853069	ALMS1P1
ENSG00000197465.14	-0.014886938	0.659912747	0.952995607	GYPE
ENSG00000136205.17	-0.005410277	0.659986	0.953034283	TNS3
ENSG00000188215.10	-0.003486462	0.660204915	0.953139975	DCUN1D3
ENSG00000163006.12	-0.006137704	0.660243615	0.953139975	CCDC138
ENSG00000181827.16	-0.004671887	0.660285643	0.953139975	RFX7
ENSG00000109189.13	0.005316188	0.660290644	0.953139975	USP46
ENSG00000286638.1	-0.012272427	0.660294055	0.953139975	RP11-213H15.5
ENSG00000111674.9	-0.003878318	0.660423553	0.953139975	ENO2
ENSG00000237161.4	0.01628268	0.660530391	0.953139975	RP11-32B5.1
ENSG00000158887.19	-0.004539359	0.660533608	0.953139975	MPZ
ENSG00000163590.14	0.006208932	0.660597565	0.953139975	PPM1L
ENSG00000175322.12	0.00694764	0.660689246	0.953139975	ZNF519
ENSG00000275409.1	-0.009509741	0.660706599	0.953139975	RP11-131L12.4
ENSG00000272447.1	0.014136375	0.660713413	0.953139975	RP11-182L21.6
ENSG00000278828.1	0.010894698	0.660797111	0.953139975	H3C10
ENSG00000067064.12	0.005861076	0.660933203	0.953139975	IDI1
ENSG00000162817.7	0.007673089	0.660952362	0.953139975	C1orf115
ENSG00000139133.7	-0.006562431	0.660976678	0.953139975	ALG10
ENSG00000204044.6	-0.015279116	0.661074682	0.953139975	SLC12A5-AS1
ENSG00000019995.6	0.00394064	0.661115312	0.953139975	ZRANB1
ENSG00000138802.11	0.003232877	0.661216526	0.953139975	SEC24B
ENSG00000259075.6	-0.014306378	0.66122069	0.953139975	POC1B-GALNT4
ENSG00000134253.10	-0.010246995	0.661274559	0.953139975	TRIM45
ENSG00000230148.8	0.009967143	0.661350614	0.953139975	HOXB-AS1
ENSG00000273399.2	-0.016791984	0.661356126	0.953139975	RP11-408A13.3
ENSG00000141503.17	-0.003946995	0.661356959	0.953139975	MINK1
ENSG00000211593.2	-0.014279675	0.661449367	0.953139975	IGKJ5
ENSG00000272779.1	-0.012947248	0.661489392	0.953139975	LL22NC03-80A10.6
ENSG00000225663.8	0.004850249	0.661515844	0.953139975	MCRIP1
ENSG00000185262.9	0.004935807	0.661536516	0.953139975	UBALD2
ENSG00000034152.19	0.004645855	0.661646043	0.953139975	MAP2K3
ENSG00000249592.6	-0.005774907	0.661649292	0.953139975	RP11-440L14.1
ENSG00000141741.12	0.004245562	0.66164948	0.953139975	MIEN1
ENSG00000176222.9	0.008447261	0.661665248	0.953139975	ZNF404
ENSG00000198818.10	-0.003493389	0.661678853	0.953139975	SFT2D1
ENSG00000109118.14	-0.003092791	0.661679751	0.953139975	PHF12
ENSG00000256092.2	-0.00989373	0.66168587	0.953139975	SBNO1-AS1
ENSG00000273749.5	0.004815133	0.661744489	0.953152584	CYFIP1

ENSG00000197417.9	0.005945093	0.661787577	0.953152584	SHPK
ENSG00000256525.8	-0.003002987	0.661917812	0.953194932	POLG2
ENSG00000204659.5	0.014876048	0.661943223	0.953194932	CBY3
ENSG00000134030.14	0.004465648	0.661956418	0.953194932	CTIF
ENSG00000256948.1	0.027414951	0.662012303	0.953195766	RP11-598F7.3
ENSG00000143870.13	0.003074829	0.662049956	0.953195766	PDIA6
ENSG00000196747.4	-0.016875991	0.662246438	0.953299998	H2AC13
ENSG00000198574.6	0.010585879	0.662251753	0.953299998	SH2D1B
ENSG00000207554.1	-0.007839594	0.662261803	0.953299998	MIR647
ENSG00000248593.3	-0.004450158	0.66274864	0.953933823	DSTNP2
ENSG00000111275.13	-0.005314192	0.662893752	0.954061943	ALDH2
ENSG00000286887.1	0.017154088	0.662954536	0.954061943	RP3-528L19.1
ENSG00000179869.15	0.019335799	0.662983384	0.954061943	ABCA13
ENSG00000287983.1	-0.025806701	0.663023738	0.954061943	RP4-705O1.2
ENSG00000153721.19	-0.01844562	0.663183663	0.95416989	CNKSR3
ENSG00000236297.1	-0.013219964	0.663298526	0.95416989	RP11-175P19.2
ENSG00000162594.16	0.023541451	0.663342981	0.95416989	IL23R
ENSG00000167414.4	-0.016262532	0.663359157	0.95416989	GNG8
ENSG00000100055.21	0.003747129	0.663414794	0.95416989	CYTH4
ENSG00000109184.15	-0.005066538	0.663429781	0.95416989	DCUN1D4
ENSG00000107018.8	0.019237624	0.663466836	0.95416989	RLN1
ENSG00000259448.3	-0.004674782	0.663470969	0.95416989	LINC02352
ENSG00000283078.1	-0.010918868	0.663550145	0.954210085	RP11-11M20.4
ENSG00000286644.1	0.014043258	0.663609678	0.954210085	CTC-338M12.15
ENSG00000141965.5	-0.005067772	0.663638504	0.954210085	FEM1A
ENSG00000204186.10	0.009108595	0.663917782	0.954522337	ZDBF2
ENSG00000217261.4	0.018852157	0.663948758	0.954522337	POM121L4P
ENSG00000158604.15	0.001300647	0.664033274	0.954557013	TMED4
ENSG00000165029.17	-0.009062245	0.664116996	0.954557013	ABCA1
ENSG00000203326.12	0.006264269	0.664118217	0.954557013	ZNF525
ENSG00000119402.18	0.002292414	0.664159061	0.954557013	FBXW2
ENSG00000235919.4	0.006675455	0.664227352	0.954576617	ASH1L-AS1
ENSG00000152944.9	0.005634198	0.664265794	0.954576617	MED21
ENSG00000134962.7	0.016007832	0.664321961	0.954578142	KLB
ENSG00000254211.6	-0.013122312	0.664359948	0.954578142	LINC01485
ENSG00000106012.18	-0.003994038	0.66445956	0.954583609	IQCE
ENSG00000271452.1	-0.012760624	0.66447742	0.954583609	RP11-342K6.2
ENSG00000155324.10	0.005449388	0.664503394	0.954583609	GRAMD2B
ENSG00000103811.18	0.00507185	0.664561009	0.954599508	CTSH
ENSG00000225931.3	-0.008505767	0.664607777	0.954599824	RP3-395M20.7
ENSG00000230006.7	-0.016947699	0.664736199	0.954688146	ANKRD36BP2
ENSG00000119699.8	-0.005051351	0.664874425	0.954688146	TGFB3
ENSG00000163029.16	0.005626445	0.664924986	0.954688146	SMC6
ENSG00000206145.8	0.041571523	0.664937748	0.954688146	P2RX6P
ENSG00000106302.10	-0.055837962	0.664980459	0.954688146	HYAL4
ENSG00000260228.6	-0.007741121	0.665047678	0.954688146	RP11-483P21.2
ENSG00000244945.1	0.006980125	0.665071426	0.954688146	RUFY1-AS1

ENSG00000285535.2	0.018537349	0.665077561	0.954688146	RP11-316M20.3
ENSG00000137207.12	0.003642788	0.665114361	0.954688146	YIPF3
ENSG00000181322.15	0.010209841	0.665183203	0.954688146	NME9
ENSG00000141027.21	-0.003949309	0.665212025	0.954688146	NCOR1
ENSG00000243649.9	0.020219601	0.665274622	0.954688146	CFB
ENSG00000159256.13	0.004721887	0.665341257	0.954688146	MORC3
ENSG00000197880.9	0.009361925	0.665411178	0.954688146	MDS2
ENSG00000258646.1	0.012045018	0.665561805	0.954688146	RP11-950C14.3
ENSG00000125648.15	0.005035688	0.665639703	0.954688146	SLC25A23
ENSG00000152256.14	0.00506667	0.665658753	0.954688146	PDK1
ENSG00000272556.2	-0.007921609	0.66567416	0.954688146	GTF2IP13
ENSG00000163138.19	0.004890732	0.66583505	0.954688146	PACRGL
ENSG00000048740.18	-0.002486371	0.665878898	0.954688146	CELF2
ENSG00000154764.6	0.008757603	0.665929204	0.954688146	WNT7A
ENSG00000249684.6	-0.008954753	0.66596617	0.954688146	RP11-423H2.3
ENSG00000259321.1	-0.007934004	0.665997208	0.954688146	RP11-468E2.5
ENSG00000237669.1	-0.009433286	0.666005624	0.954688146	HCG4P3
ENSG00000121897.15	-0.003034596	0.6661141	0.954688146	LIAS
ENSG00000138078.16	0.004476286	0.666182564	0.954688146	PREPL
ENSG00000170876.8	-0.002835572	0.66621128	0.954688146	TMEM43
ENSG00000251429.1	0.0134128	0.666305609	0.954688146	AIDAP2
ENSG00000167548.17	0.004732903	0.666312249	0.954688146	KMT2D
ENSG00000171792.11	0.003464261	0.666396146	0.954688146	RHNO1
ENSG00000260425.1	-0.008484896	0.666445839	0.954688146	LA16c-316G12.2
ENSG00000125753.14	0.0043321	0.666508601	0.954688146	VASP
ENSG00000128383.13	-0.005361794	0.666543351	0.954688146	APOBEC3A
ENSG00000171811.14	-0.0155027	0.666560564	0.954688146	CFAP46
ENSG00000158856.18	0.01276702	0.666567644	0.954688146	DMTN
ENSG00000108839.12	0.005888736	0.666671084	0.954688146	ALOX12
ENSG00000246273.8	0.007191575	0.666682926	0.954688146	SBF2-AS1
ENSG00000230362.1	-0.012156787	0.666695948	0.954688146	ACTG1P23
ENSG00000177628.16	-0.00396925	0.666808077	0.954688146	GBA
ENSG00000100124.15	-0.003757345	0.666880175	0.954688146	ANKRD54
ENSG00000155545.20	-0.007006166	0.666948976	0.954688146	MIER3
ENSG00000197989.14	-0.003282815	0.666965374	0.954688146	SNHG12
ENSG00000176171.11	0.003038503	0.667009813	0.954688146	BNIP3
ENSG00000204791.10	-0.014811353	0.667039764	0.954688146	SMPD5
ENSG00000196367.14	-0.003672935	0.667087443	0.954688146	TRRAP
ENSG00000111412.6	0.002121988	0.667153145	0.954688146	SPRING1
ENSG00000166359.11	-0.0084077	0.667183213	0.954688146	WDR88
ENSG00000172845.18	-0.004865886	0.667228761	0.954688146	SP3
ENSG00000133627.18	0.003730083	0.667231265	0.954688146	ACTR3B
ENSG00000166333.14	-0.004738802	0.667239577	0.954688146	ILK
ENSG00000115109.14	-0.004967165	0.66726202	0.954688146	EPB41L5
ENSG00000117298.16	0.004478853	0.667262735	0.954688146	ECE1
ENSG00000168067.12	-0.002756183	0.667271214	0.954688146	MAP4K2
ENSG00000109762.16	-0.006209199	0.6674	0.954688146	SNX25

ENSG00000205578.6	0.015700756	0.667442499	0.954688146	POM121B
ENSG00000250303.4	0.008440488	0.667521714	0.954688146	LINC02762
ENSG00000141655.17	-0.010639292	0.667603671	0.954688146	TNFRSF11A
ENSG00000105617.4	-0.003964925	0.66766648	0.954688146	LENG1
ENSG00000123810.9	-0.004592349	0.66767341	0.954688146	B9D2
ENSG00000260219.2	0.006226517	0.667716761	0.954688146	CD2BP2-DT
ENSG00000163995.21	-0.00727901	0.667763272	0.954688146	ABLIM2
ENSG00000100612.14	0.00317901	0.667778639	0.954688146	DHRS7
ENSG00000171914.16	-0.0103427	0.667781779	0.954688146	TLN2
ENSG00000198909.8	0.002850612	0.667782235	0.954688146	MAP3K3
ENSG00000113456.19	0.002992187	0.667786811	0.954688146	RAD1
ENSG00000123636.18	0.005887652	0.667816136	0.954688146	BAZ2B
ENSG00000141252.21	-0.004001502	0.667840213	0.954688146	VPS53
ENSG00000262333.1	0.0096154	0.667868594	0.954688146	HNRNPA1P16
ENSG00000156735.11	-0.003825354	0.667881355	0.954688146	BAG4
ENSG00000253704.2	-0.011379716	0.667962116	0.954737041	RP11-267M23.4
ENSG00000101191.17	0.002123426	0.668114246	0.954887934	DIDO1
ENSG00000055917.16	0.003531709	0.668249047	0.954975555	PUM2
ENSG00000258987.2	0.011228983	0.668276918	0.954975555	RP11-131H24.4
ENSG00000088387.20	-0.004975538	0.668315251	0.954975555	DOCK9
ENSG00000169914.6	-0.003514833	0.66858757	0.95513182	OTUD3
ENSG00000134853.12	-0.019274503	0.668588821	0.95513182	PDGFRA
ENSG00000253047.1	-0.010577986	0.668599201	0.95513182	ENSG10010137222.1
ENSG00000182134.16	-0.003965994	0.668610904	0.95513182	TDRKH
ENSG00000169704.5	0.012118285	0.668687589	0.955159997	GP9
ENSG00000170153.11	-0.063704142	0.668748641	0.955159997	RNF150
ENSG00000268027.5	-0.005678014	0.668770353	0.955159997	AC006129.2
ENSG00000151692.15	-0.004393557	0.668872481	0.955239334	RNF144A
ENSG00000022976.16	0.002897897	0.668970885	0.955270697	ZNF839
ENSG00000240087.3	0.014640445	0.668987603	0.955270697	RPSAP12
ENSG00000205707.11	0.00481192	0.669050943	0.955294628	ETFRF1
ENSG00000108055.10	0.003556929	0.669140227	0.955316443	SMC3
ENSG00000112343.11	0.003174703	0.66924136	0.955316443	TRIM38
ENSG00000211890.4	-0.01828005	0.669281749	0.955316443	IGHA2
ENSG00000175193.14	0.005251604	0.669391625	0.955316443	PARL
ENSG00000276517.1	0.007808841	0.669537175	0.955316443	AL133243.2
ENSG00000260367.2	-0.006657055	0.669566724	0.955316443	RP11-264B17.4
ENSG00000170631.15	-0.003495781	0.66966496	0.955316443	ZNF16
ENSG00000182208.14	-0.003566544	0.66970638	0.955316443	MOB2
ENSG00000105640.13	0.005204308	0.669708995	0.955316443	RPL18A
ENSG00000275131.3	0.007047374	0.66971854	0.955316443	PDE4DIPP2
ENSG00000225399.4	0.013859882	0.669730741	0.955316443	RP11-3B7.1
ENSG00000285836.1	-0.015499311	0.669733225	0.955316443	RP11-274J15.4
ENSG00000164674.17	0.004398043	0.669782304	0.955316443	SYTL3
ENSG00000139572.4	-0.009565467	0.669803601	0.955316443	GPR84
ENSG00000273136.7	0.01643994	0.669927651	0.955316443	NBPF26
ENSG00000261560.1	-0.012279295	0.669993912	0.955316443	RP11-166B2.3

ENSG00000115806.13	0.002159631	0.670014367	0.955316443	GORASP2
ENSG00000083937.10	0.003287779	0.670048562	0.955316443	CHMP2B
ENSG00000244171.4	0.009732701	0.670051328	0.955316443	PBX2P1
ENSG00000118960.13	0.003222346	0.670068757	0.955316443	HS1BP3
ENSG00000168237.18	0.003342628	0.670082834	0.955316443	GLYCTK
ENSG00000032389.13	-0.003373114	0.670091039	0.955316443	EIPR1
ENSG00000162399.9	-0.022005446	0.670188409	0.955379736	BSND
ENSG00000126883.17	0.002608041	0.670284928	0.955379736	NUP214
ENSG00000142856.17	0.006336102	0.670304796	0.955379736	ITGB3BP
ENSG00000006740.17	-0.013129899	0.670321778	0.955379736	ARHGAP44
ENSG00000176142.13	-0.002447576	0.670680757	0.955824945	TMEM39A
ENSG00000230303.6	-0.01313356	0.670842335	0.955973109	RP11-213G2.2
ENSG00000233791.5	-0.013619054	0.670980083	0.955973109	LINC01136
ENSG00000237361.3	-0.030140323	0.670981079	0.955973109	TUSC8
ENSG00000148488.17	-0.008506159	0.670983105	0.955973109	ST8SIA6
ENSG00000123684.13	0.003552349	0.671063054	0.955973109	LPGAT1
ENSG00000182580.3	-0.010200014	0.671198024	0.955973109	EPHB3
ENSG00000179104.9	0.005253218	0.671216242	0.955973109	TMTC2
ENSG00000187824.9	-0.008171209	0.671310711	0.955973109	TMEM220
ENSG00000211734.3	0.008660032	0.671358753	0.955973109	TRBV5-1
ENSG00000156273.16	0.004875146	0.671410111	0.955973109	BACH1
ENSG00000122696.14	0.003241596	0.671426789	0.955973109	SLC25A51
ENSG00000101384.12	-0.006496823	0.671459231	0.955973109	JAG1
ENSG00000185869.15	-0.005835626	0.671513648	0.955973109	ZNF829
ENSG00000198715.13	-0.006489215	0.671572577	0.955973109	GLMP
ENSG00000182010.11	-0.010747804	0.671662525	0.955973109	RTKN2
ENSG00000134202.11	0.017568712	0.671662911	0.955973109	GSTM3
ENSG00000133460.19	0.004401221	0.671691571	0.955973109	SLC2A11
ENSG00000170515.14	0.003090757	0.671699556	0.955973109	PA2G4
ENSG00000112531.17	0.004342459	0.67170854	0.955973109	QKI
ENSG00000054277.14	-0.00508121	0.671717013	0.955973109	OPN3
ENSG00000242611.2	0.028994477	0.671791008	0.956010348	AC093627.8
ENSG00000011405.13	0.005272476	0.671836412	0.956010348	PIK3C2A
ENSG00000251414.1	-0.008896817	0.671913931	0.956018516	RP11-843P14.1
ENSG00000261183.5	-0.012481136	0.671951529	0.956018516	SPINT1-AS1
ENSG00000165923.16	-0.005640874	0.671982003	0.956018516	AGBL2
ENSG00000243417.1	0.009020856	0.672063043	0.95606628	RPS3AP18
ENSG00000004975.12	-0.002637575	0.672125909	0.95606628	DVL2
ENSG00000204060.7	0.018756722	0.672155433	0.95606628	FOXO6
ENSG00000157429.16	-0.00536511	0.672203289	0.956068039	ZNF19
ENSG00000278396.1	0.014806687	0.672272163	0.95609969	RP11-120E11.2
ENSG00000189129.14	0.006796461	0.67232326	0.956106054	PLAC9
ENSG00000273253.2	-0.007399001	0.672508811	0.956303612	RP3-402G11.26
ENSG00000272341.1	-0.006537028	0.672605045	0.956374142	RP1-151F17.2
ENSG00000224138.1	-0.00644268	0.672655259	0.956379233	AC000123.4
ENSG00000275479.1	0.01269498	0.672720984	0.956406374	RP11-334C17.6
ENSG00000204764.14	-0.020633006	0.672794142	0.956444077	RANBP17

ENSG00000103528.17	0.00893543	0.672932034	0.956562667	SYT17
ENSG00000160360.13	-0.006448074	0.672998834	0.956562667	GPSM1
ENSG00000115677.18	-0.003317136	0.673017492	0.956562667	HDLBP
ENSG00000130600.19	-0.015007718	0.673143512	0.956567624	H19
ENSG00000175262.14	0.008942386	0.67316533	0.956567624	C1orf127
ENSG00000100483.14	-0.004396478	0.673247881	0.956567624	VCPKMT
ENSG00000137571.11	0.017830534	0.673272488	0.956567624	SLCO5A1
ENSG00000224769.1	0.01897984	0.67327905	0.956567624	MUC20P1
ENSG00000230155.6	-0.005491023	0.673387678	0.956567624	CEP250-AS1
ENSG00000226125.2	0.01156795	0.673427438	0.956567624	LINC01907
ENSG00000183784.7	0.006725293	0.673476005	0.956567624	DOCK8-AS1
ENSG00000211452.11	-0.009702089	0.673485533	0.956567624	DIO1
ENSG00000283297.2	-0.006497512	0.673487416	0.956567624	TEX52
ENSG00000221983.8	-0.00689571	0.673557912	0.956569674	UBA52
ENSG00000261324.2	0.006837531	0.673582147	0.956569674	RP11-174G6.5
ENSG00000130724.9	0.004543654	0.673705916	0.956639435	CHMP2A
ENSG00000149573.9	-0.00622897	0.673724564	0.956639435	MPZL2
ENSG00000100227.18	-0.002229977	0.67388493	0.956728264	POLDIP3
ENSG00000147883.12	0.008047768	0.673946409	0.956728264	CDKN2B
ENSG00000116205.14	-0.00261892	0.673959351	0.956728264	TCEANC2
ENSG00000129534.14	0.004748706	0.673973729	0.956728264	MIS18BP1
ENSG00000269054.2	-0.011765581	0.674051293	0.956733045	ZNF497-AS1
ENSG00000100889.12	-0.003962051	0.674081485	0.956733045	PCK2
ENSG00000226816.3	0.018463439	0.674118516	0.956733045	AC005082.12
ENSG00000135679.26	0.004350333	0.674163703	0.956733045	MDM2
ENSG00000187513.9	0.019402439	0.674299069	0.956831396	GJA4
ENSG00000143994.14	-0.006824139	0.674329804	0.956831396	ABHD1
ENSG00000146247.14	-0.006348263	0.674372976	0.956831396	PHIP
ENSG00000184924.6	-0.005651543	0.674740764	0.957213606	PTRHD1
ENSG00000227218.7	0.008716991	0.674744132	0.957213606	RP11-203J24.8
ENSG00000233293.1	-0.017631953	0.674782383	0.957213606	RP11-410N8.3
ENSG00000071967.12	0.006698232	0.674957207	0.957363511	CYBRD1
ENSG00000158106.14	-0.006901908	0.675030874	0.957363511	RHPN1
ENSG00000110344.10	0.005171322	0.675056571	0.957363511	UBE4A
ENSG00000132376.20	-0.00325606	0.675086655	0.957363511	INPP5K
ENSG00000102858.13	0.004856717	0.675260837	0.957363511	MGRN1
ENSG00000273415.3	0.014428234	0.675295005	0.957363511	LINC02725
ENSG00000176083.18	-0.020578602	0.67534836	0.957363511	ZNF683
ENSG00000213700.3	0.01297095	0.675360243	0.957363511	RPL17P50
ENSG00000162366.8	0.013059073	0.675417556	0.957363511	PDZK1IP1
ENSG00000092148.13	0.003349487	0.675442019	0.957363511	HECTD1
ENSG00000279602.1	0.01506199	0.675468557	0.957363511	CTD-3014M21.1
ENSG00000134444.15	0.004731352	0.675590722	0.957363511	RELCH
ENSG00000142046.15	-0.00491298	0.675591333	0.957363511	TMEM91
ENSG00000247092.7	-0.004052886	0.675608988	0.957363511	SNHG10
ENSG00000159023.22	0.003941973	0.675644251	0.957363511	EPB41
ENSG00000286607.1	-0.012480952	0.67570579	0.957363511	CTD-2547L24.5

ENSG00000077684.16	0.002973938	0.67572425	0.957363511	JADE1
ENSG00000287808.1	-0.010422425	0.675751479	0.957363511	RP11-249O6.2
ENSG00000142102.16	-0.005677981	0.675802403	0.957363511	PGGHG
ENSG00000143178.13	0.003904051	0.675821706	0.957363511	TBX19
ENSG00000100290.3	-0.007251717	0.675886322	0.957388913	BIK
ENSG00000251405.3	-0.010466005	0.675993372	0.957470106	CTB-109A12.1
ENSG00000248971.2	0.012311017	0.676037016	0.957470106	KRT8P46
ENSG00000223629.1	-0.045615925	0.676212113	0.957616883	DEFA8P
ENSG00000260306.1	0.009423638	0.676280339	0.957616883	RP11-645C24.5
ENSG00000157423.18	-0.056845377	0.676280735	0.957616883	HYDIN
ENSG00000271725.1	0.008787463	0.676375344	0.957684725	RP11-761I4.4
ENSG00000229425.3	0.016220147	0.676512851	0.957805079	AJ006998.2
ENSG00000286629.1	-0.015805423	0.676603907	0.957805079	RP11-214C8.6
ENSG00000259031.1	0.018380086	0.676756297	0.957805079	CTD-2062F14.3
ENSG00000252464.1	0.016481113	0.676776934	0.957805079	RN7SKP70
ENSG00000281392.2	-0.023238169	0.676804811	0.957805079	LINC00506
ENSG00000155330.10	0.004686149	0.67685758	0.957805079	C16orf87
ENSG00000205726.15	-0.00601382	0.676875216	0.957805079	ITSN1
ENSG00000133739.16	0.0091183	0.676959144	0.957805079	LRRCC1
ENSG00000124782.20	-0.002983603	0.67698033	0.957805079	RREB1
ENSG00000138709.19	-0.004442173	0.677052692	0.957805079	LARP1B
ENSG00000138279.16	0.002141467	0.677061768	0.957805079	ANXA7
ENSG00000006282.21	0.009993353	0.677091697	0.957805079	SPATA20
ENSG00000023330.15	-0.002969452	0.677092661	0.957805079	ALAS1
ENSG00000242337.5	-0.015276526	0.677114201	0.957805079	INHCAP
ENSG00000074842.8	-0.004024282	0.677191267	0.957848024	MYDGF
ENSG00000256705.3	0.009059206	0.67737442	0.957989658	CTD-2017C7.1
ENSG00000186676.3	-0.023213381	0.677495009	0.957989658	EEF1GP1
ENSG00000171962.18	-0.004087714	0.677541128	0.957989658	DRC3
ENSG00000251450.2	-0.009968565	0.677552864	0.957989658	RASGRF2-AS1
ENSG00000171476.22	0.008314111	0.677570995	0.957989658	HOPX
ENSG00000170956.17	0.004393467	0.677571679	0.957989658	CEACAM3
ENSG00000076641.4	0.004702124	0.677702276	0.958082503	PAG1
ENSG00000243317.8	0.005151222	0.677730782	0.958082503	STMP1
ENSG00000081386.13	0.003581105	0.677798314	0.958111927	ZNF510
ENSG00000259381.2	0.016347554	0.677850274	0.958119334	RP11-192M23.1
ENSG00000259088.1	0.01210003	0.677937902	0.958146199	CTD-2017C7.2
ENSG00000255538.1	0.014875289	0.677962722	0.958146199	OR10V2P
ENSG00000262655.4	0.007634412	0.678011071	0.958148501	SPON1
ENSG00000166801.17	0.003676642	0.678135263	0.958230268	FAM111A
ENSG00000008710.20	-0.00471917	0.678232252	0.958230268	PKD1
ENSG00000085365.18	-0.004595254	0.678237916	0.958230268	SCAMP1
ENSG00000211949.3	0.013843526	0.678348857	0.958230268	IGHV3-23
ENSG00000108854.16	0.003405791	0.678357752	0.958230268	SMURF2
ENSG00000231852.9	0.027222377	0.678449363	0.958230268	CYP21A2
ENSG00000260236.1	-0.006577791	0.678515552	0.958230268	RP11-708J19.1
ENSG00000109881.17	0.00779126	0.678568235	0.958230268	CCDC34

ENSG00000260339.1	-0.007545844	0.678581032	0.958230268	HEXA-AS1
ENSG00000156873.16	-0.00335214	0.678607833	0.958230268	PHKG2
ENSG00000177674.16	0.0039246	0.678777157	0.958230268	AGTRAP
ENSG00000226777.7	-0.009131514	0.678783755	0.958230268	FAM30A
ENSG00000186517.14	-0.002358094	0.678810747	0.958230268	ARHGAP30
ENSG00000128886.12	-0.016376774	0.678816317	0.958230268	ELL3
ENSG00000237945.7	0.007087357	0.678855384	0.958230268	LINC00649
ENSG00000145779.8	0.0031372	0.678876971	0.958230268	TNFAIP8
ENSG00000224237.1	0.045205498	0.678907909	0.958230268	MICOS10P3
ENSG00000185271.9	0.011569317	0.678965686	0.958230268	KLHL33
ENSG00000108883.13	-0.003287275	0.679011853	0.958230268	EFTUD2
ENSG00000214944.10	-0.013738251	0.679031345	0.958230268	ARHGEF28
ENSG00000215347.4	0.012901987	0.679055738	0.958230268	SLC25A5P1
ENSG00000151929.10	-0.005657607	0.679096875	0.958230268	BAG3
ENSG00000101138.12	0.001881129	0.67916633	0.958262339	CSTF1
ENSG00000180667.11	0.006064656	0.679368324	0.958481397	YOD1
ENSG00000171772.17	0.038877915	0.679425491	0.958496111	SYCE1
ENSG00000208028.1	-0.014220178	0.679520444	0.958512415	MIR616
ENSG00000103599.20	-0.013141586	0.679571319	0.958512415	IQCH
ENSG00000279392.1	-0.009214392	0.679603088	0.958512415	RP11-665C16.9
ENSG00000153561.13	-0.002884482	0.679715978	0.958512415	RMND5A
ENSG00000162980.17	-0.005620362	0.679770445	0.958512415	ARL5A
ENSG00000235316.1	-0.007476865	0.6798398	0.958512415	DUSP8P5
ENSG00000260331.1	-0.015439601	0.679859451	0.958512415	RP11-111J6.2
ENSG00000154945.7	-0.0022665	0.679902152	0.958512415	ANKRD40
ENSG00000165457.14	-0.007336607	0.680043466	0.958512415	FOLR2
ENSG00000167604.15	-0.003558152	0.68006502	0.958512415	NFKBID
ENSG00000126249.8	-0.005385152	0.680181062	0.958512415	PDCD2L
ENSG00000259436.1	-0.004756545	0.680208936	0.958512415	CTC-378H22.2
ENSG00000249396.2	-0.013323524	0.680215089	0.958512415	LINC02212
ENSG00000155868.8	0.004289359	0.680230814	0.958512415	MED7
ENSG00000283529.1	0.015886796	0.680280515	0.958512415	RP4-791K14.2
ENSG00000028277.21	-0.003583349	0.680305599	0.958512415	POU2F2
ENSG00000196110.8	-0.004558745	0.680314364	0.958512415	ZNF699
ENSG00000143515.19	-0.003504058	0.680378203	0.958512415	ATP8B2
ENSG00000116857.17	0.004528612	0.680381613	0.958512415	TMEM9
ENSG00000231494.1	-0.013426919	0.68041201	0.958512415	RPL21P35
ENSG00000117697.15	0.003101034	0.680459512	0.958512415	NSL1
ENSG00000270954.1	0.019187151	0.680465294	0.958512415	RPSAP75
ENSG00000177425.11	-0.009307979	0.680603129	0.958597154	PAWR
ENSG00000157350.13	-0.00362949	0.680618937	0.958597154	ST3GAL2
ENSG00000070770.10	0.002281821	0.680685542	0.958625127	CSNK2A2
ENSG00000237015.1	-0.010045756	0.680744391	0.958642174	CTA-984G1.5
ENSG00000267838.2	-0.007932587	0.68100825	0.958753745	AC008746.12
ENSG00000244165.2	0.004519707	0.681029083	0.958753745	P2RY11
ENSG00000069998.12	0.003104129	0.681088204	0.958753745	HDHD5
ENSG00000113407.14	0.002423121	0.681145587	0.958753745	TARS1

ENSG00000197782.15	-0.003949908	0.681161612	0.958753745	ZNF780A
ENSG00000127589.4	0.012281899	0.681171006	0.958753745	TUBBP1
ENSG00000270419.1	0.00871107	0.681264009	0.958753745	CAHM
ENSG00000111199.12	-0.01092757	0.68130931	0.958753745	TRPV4
ENSG00000164253.14	0.003647531	0.681327389	0.958753745	WDR41
ENSG00000196407.12	-0.007195923	0.681441655	0.958753745	THEM5
ENSG00000182774.13	0.003318981	0.681462875	0.958753745	RPS17
ENSG00000238246.1	0.012211698	0.681530905	0.958753745	RP11-575A19.2
ENSG00000243064.8	0.016712855	0.681575825	0.958753745	ABCC13
ENSG00000153037.15	-0.003368129	0.681579818	0.958753745	SRP19
ENSG00000158966.16	0.013074191	0.681596177	0.958753745	CACHD1
ENSG00000278989.1	0.014952017	0.681653878	0.958753745	RP11-762L8.6
ENSG00000162881.6	-0.006679183	0.681670461	0.958753745	OXER1
ENSG00000167925.16	-0.00338879	0.681729208	0.958753745	GHDC
ENSG00000091972.18	0.011505326	0.681747972	0.958753745	CD200
ENSG00000274653.1	-0.007643967	0.681758624	0.958753745	RP11-347C12.11
ENSG00000166199.13	0.003420973	0.681810156	0.958760469	ALKBH3
ENSG00000117834.13	-0.007194346	0.682014878	0.958813049	SLC5A9
ENSG00000177954.14	0.009629258	0.682017302	0.958813049	RPS27
ENSG00000196967.11	0.004279064	0.682055213	0.958813049	ZNF585A
ENSG00000165322.18	0.005596409	0.682202512	0.958813049	ARHGAP12
ENSG00000006747.15	-0.015646971	0.68220848	0.958813049	SCIN
ENSG00000279803.1	-0.012059178	0.682237522	0.958813049	RP11-407G23.5
ENSG00000288670.1	-0.004887346	0.682271022	0.958813049	RP11-122G18.14
ENSG00000107537.14	0.004066409	0.682326228	0.958813049	PHYH
ENSG00000165813.20	-0.004970304	0.682355576	0.958813049	CCDC186
ENSG00000243943.10	0.002582836	0.682383224	0.958813049	ZNF512
ENSG00000184208.12	-0.00339892	0.682406895	0.958813049	C22orf46
ENSG00000169991.11	0.003367379	0.682462635	0.958813049	IFFO2
ENSG00000164306.11	0.004933373	0.682566965	0.958813049	PRIMPOL
ENSG00000148396.18	-0.00345695	0.682575305	0.958813049	SEC16A
ENSG00000188452.15	-0.00779253	0.682656104	0.958813049	CERKL
ENSG00000118242.16	-0.004583759	0.682688657	0.958813049	MREG
ENSG00000047578.13	0.002923327	0.682730086	0.958813049	KATNIP
ENSG00000078304.19	0.002581421	0.68276528	0.958813049	PPP2R5C
ENSG00000198416.9	-0.012951274	0.6827694	0.958813049	ZNF658B
ENSG00000066735.15	-0.019992154	0.682816132	0.958813049	KIF26A
ENSG00000244720.1	-0.014158668	0.682979842	0.958813049	NT5C3AP2
ENSG00000108774.15	-0.002794277	0.683033985	0.958813049	RAB5C
ENSG00000132622.11	-0.025457088	0.683048997	0.958813049	HSPA12B
ENSG00000239839.7	0.028394221	0.683095183	0.958813049	DEFA3
ENSG00000180423.4	0.003802333	0.683222434	0.958813049	HARBI1
ENSG00000258172.1	-0.015006572	0.68323309	0.958813049	RP11-1105G2.4
ENSG00000198053.12	0.003613865	0.683260043	0.958813049	SIRPA
ENSG00000099901.17	0.003353667	0.683355497	0.958813049	RANBP1
ENSG00000231890.8	-0.00593926	0.68337694	0.958813049	DARS1-AS1
ENSG00000244661.1	0.03206511	0.683403461	0.958813049	TRBV12-1

ENSG00000228049.7	-0.010906729	0.683409432	0.958813049	POLR2J2
ENSG00000159720.12	0.004088721	0.683451796	0.958813049	ATP6V0D1
ENSG00000166130.15	-0.004736681	0.683495663	0.958813049	IKBIP
ENSG00000197863.9	-0.005335165	0.683564403	0.958813049	ZNF790
ENSG00000138190.17	0.00428103	0.68369049	0.958813049	EXOC6
ENSG00000162999.13	-0.006972231	0.68369712	0.958813049	DUSP19
ENSG00000275484.1	-0.012233055	0.683725812	0.958813049	CTC-1337H24.4
ENSG00000112237.13	-0.005277474	0.683785063	0.958813049	CCNC
ENSG00000172426.16	-0.009084091	0.683832928	0.958813049	RSPH9
ENSG00000220201.7	0.00627008	0.683841031	0.958813049	ZGLP1
ENSG00000185156.6	0.008781527	0.683994343	0.958813049	MFSD6L
ENSG00000162894.12	-0.004629865	0.68400822	0.958813049	FCMR
ENSG00000284825.1	0.012121173	0.684053829	0.958813049	RP11-517H2.8
ENSG00000188610.12	-0.014363039	0.684095554	0.958813049	FAM72B
ENSG00000085117.12	0.004327215	0.684186566	0.958813049	CD82
ENSG00000259032.2	-0.012226446	0.684188517	0.958813049	ENSAP2
ENSG00000270977.2	-0.014270552	0.684214086	0.958813049	AC015849.16
ENSG00000255595.5	0.028401598	0.684344057	0.958813049	RP4-809F18.1
ENSG00000236409.2	0.006236775	0.684354266	0.958813049	NRADDP
ENSG00000237262.1	0.023050018	0.684384977	0.958813049	AC068492.1
ENSG00000259118.5	0.010188848	0.684431213	0.958813049	RP11-840I19.3
ENSG00000226660.2	0.010133594	0.684469467	0.958813049	TRBV2
ENSG00000126803.10	0.010661727	0.684519949	0.958813049	HSPA2
ENSG00000182057.5	0.004890923	0.684528595	0.958813049	OGFRP1
ENSG00000160446.19	0.004138357	0.684564086	0.958813049	ZDHHC12
ENSG00000174405.14	-0.006314507	0.6845848	0.958813049	LIG4
ENSG00000173950.16	-0.003631922	0.684619902	0.958813049	XXYLT1
ENSG00000155506.18	0.003126419	0.684672735	0.958813049	LARP1
ENSG00000242628.6	0.009969406	0.684689078	0.958813049	AC009228.1
ENSG00000075426.12	-0.004284357	0.684710569	0.958813049	FOSL2
ENSG00000089505.18	0.007205558	0.684722922	0.958813049	CMTM1
ENSG00000198952.8	-0.003901812	0.684781663	0.958813049	SMG5
ENSG00000196735.13	-0.011330932	0.684792994	0.958813049	HLA-DQA1
ENSG00000273402.1	-0.013815169	0.684889781	0.958883098	RP5-855D21.2
ENSG00000273284.1	0.009895974	0.684947806	0.958885203	RP11-888D10.4
ENSG00000145743.17	-0.003581603	0.685070344	0.958885203	FBXL17
ENSG00000008516.18	0.007804413	0.685090342	0.958885203	MMP25
ENSG00000011422.12	0.004431221	0.685144795	0.958885203	PLAUR
ENSG00000259291.2	0.003820902	0.685235436	0.958885203	ZNF710-AS1
ENSG00000167333.13	0.002316821	0.68528141	0.958885203	TRIM68
ENSG00000073282.14	-0.013190805	0.685352451	0.958885203	TP63
ENSG00000048471.14	0.00403735	0.68537736	0.958885203	SNX29
ENSG00000196663.16	0.005049843	0.68547322	0.958885203	TECPR2
ENSG00000162931.12	0.005437136	0.685497571	0.958885203	TRIM17
ENSG00000172667.11	-0.003319606	0.685498582	0.958885203	ZMAT3
ENSG00000187555.16	-0.00214839	0.685518558	0.958885203	USP7
ENSG00000255441.1	0.009554207	0.685556074	0.958885203	SIGLEC10-AS1

ENSG00000233393.1	-0.028657649	0.685557723	0.958885203	AP000688.29
ENSG00000134884.15	0.006395666	0.685642491	0.958885203	ARGLU1
ENSG00000121989.15	0.004720749	0.68566165	0.958885203	ACVR2A
ENSG00000101247.18	-0.004610923	0.685717054	0.958885203	NDUFAF5
ENSG00000132801.7	0.002624286	0.685732904	0.958885203	ZSWIM3
ENSG00000177599.13	0.007040834	0.685807705	0.958924416	ZNF491
ENSG00000141560.15	-0.004421549	0.685858787	0.958930461	FN3KRP
ENSG00000171700.14	-0.003431561	0.685992563	0.958958155	RGS19
ENSG00000256379.2	0.013087592	0.6860177	0.958958155	TRAV8-5
ENSG00000239388.9	-0.007122401	0.686061678	0.958958155	ASB14
ENSG00000017260.20	0.002596537	0.686096822	0.958958155	ATP2C1
ENSG00000120314.19	0.001682806	0.686112396	0.958958155	WDR55
ENSG00000157833.13	0.005503531	0.686218154	0.959040609	GAREM2
ENSG00000266541.1	-0.019182885	0.686507357	0.959379412	RP11-881L2.1
ENSG00000197320.5	-0.025291869	0.686628199	0.95939039	AC060834.2
ENSG00000159461.15	-0.002750753	0.686649323	0.95939039	AMFR
ENSG00000127054.21	-0.002643764	0.686655556	0.95939039	INTS11
ENSG00000075407.18	0.003283067	0.686776988	0.959494684	ZNF37A
ENSG00000145439.12	0.003586727	0.686862098	0.959508397	CBR4
ENSG00000068724.17	-0.003270271	0.686896269	0.959508397	TTC7A
ENSG00000254676.1	-0.007876005	0.686940434	0.959508397	RP11-727A23.4
ENSG00000259020.3	-0.016056216	0.687018864	0.959508397	RP11-529H20.3
ENSG00000156136.10	-0.003970752	0.687020739	0.959508397	DCK
ENSG00000245598.6	-0.010332446	0.687094047	0.959545435	DACT3-AS1
ENSG00000163558.13	-0.004217138	0.687164914	0.959579059	PRKCI
ENSG00000198863.7	-0.001961744	0.68727312	0.959664816	RUNDC1
ENSG00000108961.14	0.00436575	0.687390699	0.959700633	RANGRF
ENSG00000277977.1	-0.015202917	0.687392364	0.959700633	CTD-3018O17.5
ENSG00000080986.13	-0.006029419	0.687485171	0.959764867	NDC80
ENSG00000273369.1	0.010751661	0.687707699	0.959979916	RP11-700J17.1
ENSG00000172575.12	-0.003847012	0.687732832	0.959979916	RASGRP1
ENSG00000132879.14	-0.003818677	0.687863386	0.960065488	FBXO44
ENSG00000163939.18	0.003178494	0.687928429	0.960065488	PBRM1
ENSG00000135632.12	0.003547673	0.687934579	0.960065488	SMYD5
ENSG00000038358.15	0.003838819	0.687996047	0.960085938	EDC4
ENSG00000211724.3	0.008332847	0.688101008	0.96016384	TRBV6-6
ENSG00000142188.17	-0.00364153	0.68814551	0.96016384	TMEM50B
ENSG00000143458.12	0.002767183	0.688200131	0.960174725	GABPB2
ENSG00000273611.5	0.003932758	0.688284542	0.960227169	ZNHIT3
ENSG00000011485.15	0.003951386	0.688493356	0.960453149	PPP5C
ENSG00000255974.8	-0.016319537	0.688592949	0.960473184	CYP2A6
ENSG00000172175.15	0.003217339	0.688601386	0.960473184	MALT1
ENSG00000135951.16	0.007874396	0.688683082	0.960521806	TSGA10
ENSG00000121933.19	0.011312975	0.688865157	0.960710414	TMIGD3
ENSG00000172977.13	-0.00282775	0.689027415	0.960817771	KAT5
ENSG00000154240.17	0.017012483	0.689070379	0.960817771	CEP112
ENSG00000144815.16	0.003393649	0.689082689	0.960817771	NXPE3

ENSG00000172006.12	-0.00439537	0.689147127	0.960835612	ZNF554
ENSG00000236714.3	-0.162064881	0.689204908	0.960835612	LINC01844
ENSG00000204092.2	-0.024800591	0.689236039	0.960835612	LINC00951
ENSG00000253921.1	0.013953524	0.689326384	0.96089624	CTB-113P19.3
ENSG00000079308.20	0.012914622	0.689379734	0.960905294	TNS1
ENSG00000018869.17	-0.005846855	0.689506205	0.96097652	ZNF582
ENSG00000187801.15	-0.007131042	0.689601802	0.96097652	ZFP69B
ENSG00000197568.14	0.005875019	0.689610881	0.96097652	HHLA3
ENSG00000188352.12	-0.003700753	0.689674995	0.96097652	FOCAD
ENSG00000211749.1	-0.010292655	0.689789782	0.96097652	TRBV23-1
ENSG00000169062.15	0.00337125	0.689793429	0.96097652	UPF3A
ENSG00000179743.4	-0.003657377	0.689821037	0.96097652	FLJ37453
ENSG00000079150.18	-0.009781563	0.689871246	0.96097652	FKBP7
ENSG00000279019.1	0.008300817	0.689890013	0.96097652	RP11-407G23.2
ENSG00000198033.12	0.038576558	0.689908514	0.96097652	TUBA3C
ENSG00000105948.13	-0.009298871	0.689954193	0.96097652	TTC26
ENSG00000061987.16	-0.0033045	0.690061858	0.96097652	MON2
ENSG00000270076.1	0.014647998	0.690073492	0.96097652	AF131215.8
ENSG00000232065.1	0.015629451	0.69020257	0.96097652	LINC01063
ENSG00000127377.10	-0.024314965	0.690216815	0.96097652	CRYGN
ENSG00000107104.20	0.009262942	0.690259546	0.96097652	KANK1
ENSG00000143373.18	0.003057917	0.690265544	0.96097652	ZNF687
ENSG00000103260.9	-0.004926926	0.690274289	0.96097652	METRN
ENSG00000272002.1	-0.015987607	0.690350495	0.961017374	RP11-557L19.1
ENSG00000287632.1	0.018816916	0.690464332	0.961056059	RP11-792D21.3
ENSG00000176422.14	-0.003574456	0.690515246	0.961056059	SPRYD4
ENSG00000174547.13	0.004401618	0.690607078	0.961056059	MRPL11
ENSG00000276293.5	0.001719319	0.690679562	0.961056059	PIP4K2B
ENSG00000157111.13	-0.0192299	0.690812048	0.961056059	TMEM171
ENSG00000274752.1	0.008630349	0.690828733	0.961056059	TRBV12-3
ENSG00000198838.14	-0.005250319	0.690832711	0.961056059	RYR3
ENSG00000100522.10	0.003480304	0.690885309	0.961056059	GNPNAT1
ENSG00000145191.15	-0.001925325	0.69095175	0.961056059	EIF2B5
ENSG00000170779.11	-0.003651585	0.691017218	0.961056059	CDCA4
ENSG00000104517.13	0.003637576	0.691090074	0.961056059	UBR5
ENSG00000203485.14	0.006549235	0.691263557	0.961056059	INF2
ENSG00000020922.13	-0.00296287	0.691332835	0.961056059	MRE11
ENSG00000237352.4	-0.010680424	0.691341741	0.961056059	LINC01358
ENSG00000256944.1	-0.012540752	0.691352029	0.961056059	RP11-881M11.1
ENSG00000146830.11	-0.002817751	0.69136825	0.961056059	GIGYF1
ENSG00000146833.16	-0.002839334	0.691394197	0.961056059	TRIM4
ENSG00000145623.13	0.012326349	0.691404642	0.961056059	OSMR
ENSG00000211970.3	-0.018779468	0.691418601	0.961056059	IGHV4-61
ENSG00000265845.2	-0.011977517	0.691487416	0.961056059	RP11-20B24.5
ENSG00000177710.6	-0.014993768	0.691494848	0.961056059	SLC35G5
ENSG00000286381.1	-0.016861183	0.691496862	0.961056059	RP5-925J7.1
ENSG00000176659.9	0.005995004	0.691516878	0.961056059	C20orf197

ENSG00000167965.18	-0.003465893	0.691591268	0.961056059	MLST8
ENSG00000204584.1	-0.006878067	0.691693041	0.961056059	RP11-304F15.3
ENSG00000047346.13	-0.004303247	0.691756069	0.961056059	FAM214A
ENSG00000241782.1	-0.01418782	0.691763426	0.961056059	RP11-91P24.1
ENSG00000112972.15	0.003779873	0.691832803	0.961056059	HMGCS1
ENSG00000128739.23	0.003475845	0.691874802	0.961056059	SNRPN
ENSG00000117215.15	-0.013331953	0.69194764	0.961056059	PLA2G2D
ENSG00000143401.15	-0.004731407	0.691961608	0.961056059	ANP32E
ENSG00000100147.14	0.004260221	0.692043231	0.961056059	CCDC134
ENSG00000253307.1	0.014782558	0.692058339	0.961056059	RP11-10J21.4
ENSG00000233154.6	-0.008417817	0.692105187	0.961056059	LINC01762
ENSG00000213203.3	-0.002848615	0.692141574	0.961056059	GIMAP1
ENSG00000287265.1	0.006586493	0.69221971	0.961056059	RP11-328C17.2
ENSG00000227076.1	-0.009311956	0.692260481	0.961056059	RP11-4C20.4
ENSG00000228570.8	0.012943904	0.692277456	0.961056059	NUTM2E
ENSG00000166477.13	0.002373614	0.692379282	0.961056059	LEO1
ENSG00000031698.13	-0.002646742	0.692384866	0.961056059	SARS1
ENSG00000162769.13	-0.005640412	0.692408525	0.961056059	FLVCR1
ENSG00000139323.14	0.003631012	0.692473377	0.961056059	POC1B
ENSG00000233321.2	-0.018108161	0.692504796	0.961056059	LINC02669
ENSG00000257524.6	-0.004853216	0.692509618	0.961056059	RP11-203J24.9
ENSG00000176087.16	0.002790357	0.692518927	0.961056059	SLC35A4
ENSG00000105255.11	0.010450797	0.692565309	0.961056059	FSD1
ENSG00000251634.2	-0.012827684	0.692630213	0.961056059	NAIPP4
ENSG00000182400.15	0.007363603	0.692673342	0.961056059	TRAPPC6B
ENSG00000182397.14	-0.015576392	0.692674547	0.961056059	DNM1P46
ENSG00000008438.5	0.008262065	0.692758855	0.96110801	PGLYRP1
ENSG00000254226.6	0.015191716	0.692929126	0.961235262	LINC01933
ENSG00000188010.15	0.009278408	0.692984182	0.961235262	MORN2
ENSG00000252061.1	-0.014257979	0.693167212	0.961235262	RNU6-415P
ENSG00000140443.15	-0.005144431	0.693209252	0.961235262	IGF1R
ENSG00000088448.14	0.002419045	0.693256359	0.961235262	ANKRD10
ENSG00000213672.8	0.003490691	0.693311693	0.961235262	NCKIPSD
ENSG00000025772.8	0.004040764	0.693400652	0.961235262	TOMM34
ENSG00000121064.13	0.003016646	0.6934721	0.961235262	SCPEP1
ENSG00000135535.17	-0.004058358	0.693518007	0.961235262	CD164
ENSG00000143320.9	-0.007679069	0.693558966	0.961235262	CRABP2
ENSG00000279415.1	0.008042711	0.693582692	0.961235262	RP11-1102P22.1
ENSG00000156831.8	0.006322706	0.693618826	0.961235262	NSMCE2
ENSG00000074964.17	0.004536861	0.693674674	0.961235262	ARHGEF10L
ENSG00000104365.16	-0.001681312	0.693704397	0.961235262	IKBKB
ENSG00000137193.14	0.003346308	0.693739443	0.961235262	PIM1
ENSG00000137266.15	0.004660246	0.693774787	0.961235262	SLC22A23
ENSG00000180209.12	0.009016744	0.693881909	0.961235262	MYLPF
ENSG00000121964.15	0.003117138	0.69389984	0.961235262	GTDC1
ENSG00000168826.16	-0.002876518	0.693923475	0.961235262	ZBTB49
ENSG00000079432.9	-0.004202536	0.694115371	0.961235262	CIC

ENSG00000233077.1	0.013031472	0.694168771	0.961235262	LINC01271
ENSG00000267533.1	-0.017402243	0.694176771	0.961235262	RP11-815J4.7
ENSG00000111405.9	0.013386951	0.694207259	0.961235262	ENDOU
ENSG00000184056.15	0.003944684	0.694228234	0.961235262	VPS33B
ENSG00000108946.15	0.00388318	0.694252971	0.961235262	PRKAR1A
ENSG00000137100.16	0.002846742	0.694330842	0.961235262	DCTN3
ENSG00000155926.14	0.003154636	0.694351081	0.961235262	SLA
ENSG00000255568.3	-0.01481808	0.694374463	0.961235262	BRWD1-AS2
ENSG00000259116.2	-0.013784175	0.694423257	0.961235262	RP11-973N13.4
ENSG00000112081.17	0.002111686	0.694496919	0.961235262	SRSF3
ENSG00000183117.19	0.025549824	0.694541862	0.961235262	CSMD1
ENSG00000258818.4	-0.015689491	0.694582896	0.961235262	RNASE4
ENSG00000143507.18	-0.003461538	0.694583592	0.961235262	DUSP10
ENSG00000188786.10	0.003018213	0.694593332	0.961235262	MTF1
ENSG00000130304.17	0.003910324	0.694631545	0.961235262	SLC27A1
ENSG00000186866.17	0.002550081	0.694655613	0.961235262	POFUT2
ENSG00000173960.14	-0.003019079	0.694789667	0.961235262	UBXN2A
ENSG00000055732.13	0.012853765	0.694824278	0.961235262	MCOLN3
ENSG00000011478.13	-0.004103782	0.694855276	0.961235262	QPCTL
ENSG00000239736.2	0.028161122	0.695017957	0.961235262	CEACAMP3
ENSG00000130649.10	-0.009388214	0.695056893	0.961235262	CYP2E1
ENSG00000144645.15	0.010318222	0.69509615	0.961235262	OSBPL10
ENSG00000243829.1	0.008887262	0.695153604	0.961235262	CTB-33G10.1
ENSG00000154144.13	0.001747962	0.695177082	0.961235262	TBRG1
ENSG00000274818.1	0.010628744	0.69523679	0.961235262	RP1-292L20.3
ENSG00000165521.15	-0.012583193	0.695246771	0.961235262	EML5
ENSG00000111077.18	-0.0076124	0.695247968	0.961235262	TNS2
ENSG00000007168.14	0.001710421	0.695259832	0.961235262	PAFAH1B1
ENSG00000105518.14	0.004479102	0.695345235	0.961235262	TMEM205
ENSG00000185955.5	0.007107478	0.695431221	0.961235262	C7orf61
ENSG00000228168.1	-0.010425079	0.695557707	0.961235262	HNRNPA1P21
ENSG00000130176.8	0.021717405	0.695656204	0.961235262	CNN1
ENSG00000170615.15	-0.029443341	0.695658918	0.961235262	SLC26A5
ENSG00000258315.5	0.005120511	0.695702458	0.961235262	C17orf49
ENSG00000145911.6	-0.007373936	0.695732958	0.961235262	N4BP3
ENSG00000241404.7	0.006654356	0.695736785	0.961235262	EGFL8
ENSG00000182873.5	-0.004277387	0.695791181	0.961235262	PRKCZ-AS1
ENSG00000247157.7	-0.011881753	0.695885381	0.961235262	LINC01252
ENSG00000128438.10	-0.012982833	0.695892707	0.961235262	TBC1D27P
ENSG00000119927.14	0.004917262	0.695901375	0.961235262	GPAM
ENSG00000163660.12	-0.003418194	0.696070449	0.961235262	CCNL1
ENSG00000154309.9	0.003496223	0.69616215	0.961235262	DISP1
ENSG00000166889.14	0.002741412	0.696208268	0.961235262	PATL1
ENSG00000224383.8	-0.004612366	0.696243724	0.961235262	PRR29
ENSG00000115365.12	0.003198891	0.696250499	0.961235262	LANCL1
ENSG00000233932.6	-0.043633153	0.696268118	0.961235262	CTXN2
ENSG00000150867.14	-0.003146474	0.696277836	0.961235262	PIP4K2A

ENSG00000075290.8	0.014303758	0.696279848	0.961235262	WNT8B
ENSG00000254263.1	-0.014489151	0.696347495	0.961235262	RP11-473O4.4
ENSG00000178715.8	0.01678756	0.696354168	0.961235262	RP11-169K16.8
ENSG00000169490.17	-0.002248744	0.696521887	0.961235262	TM2D2
ENSG00000130520.11	-0.003148491	0.696530765	0.961235262	LSM4
ENSG00000148399.13	-0.002436793	0.696533269	0.961235262	DPH7
ENSG00000165757.9	0.015530708	0.696545151	0.961235262	JCAD
ENSG00000099251.14	-0.006802249	0.696680821	0.961235262	HSD17B7P2
ENSG00000106683.15	-0.004377059	0.696716202	0.961235262	LIMK1
ENSG00000149243.16	-0.010208834	0.696726098	0.961235262	KLHL35
ENSG00000165863.17	0.021980578	0.696779898	0.961235262	C10orf82
ENSG00000258790.1	-0.013382785	0.69681113	0.961235262	RP11-561B11.2
ENSG00000260260.1	-0.007956508	0.696856787	0.961235262	SNHG19
ENSG00000165458.14	-0.00273102	0.696875005	0.961235262	INPPL1
ENSG00000134571.11	-0.005263918	0.696982664	0.961235262	MYBPC3
ENSG00000232208.2	0.013922855	0.696984619	0.961235262	RP3-477M7.5
ENSG00000136830.12	-0.004561049	0.696985947	0.961235262	NIBAN2
ENSG00000233828.5	0.011941674	0.697040908	0.961235262	MIR4280HG
ENSG00000133059.17	0.002406242	0.697044536	0.961235262	DSTYK
ENSG00000179010.15	-0.00200301	0.697047825	0.961235262	MRFAP1
ENSG00000204815.10	-0.009826233	0.697056883	0.961235262	ODAD4
ENSG00000104756.16	0.003778425	0.69706351	0.961235262	KCTD9
ENSG00000057468.7	-0.018701338	0.697068989	0.961235262	MSH4
ENSG00000188542.10	0.002600719	0.697228936	0.961391179	DUSP28
ENSG00000182983.15	0.009094496	0.69735033	0.96147064	ZNF662
ENSG00000156299.13	0.004364624	0.697393218	0.96147064	TIAM1
ENSG00000097021.20	-0.00540598	0.697465935	0.96147064	ACOT7
ENSG00000095397.16	0.007754581	0.697510613	0.96147064	WHRN
ENSG00000174903.16	0.003300436	0.697520976	0.96147064	RAB1B
ENSG00000055483.19	-0.002351067	0.697615469	0.961531987	USP36
ENSG00000143033.18	0.003659587	0.697780077	0.961531987	MTF2
ENSG00000232719.1	0.013843028	0.697835419	0.961531987	RP11-13J8.1
ENSG00000138378.19	0.003157214	0.697869322	0.961531987	STAT4
ENSG00000258701.1	-0.007332274	0.697888214	0.961531987	LINC00638
ENSG00000243819.4	-0.0112048	0.697920411	0.961531987	RN7SL832P
ENSG00000115419.13	-0.003870204	0.69793044	0.961531987	GLS
ENSG00000153291.16	0.01221053	0.697994444	0.961531987	SLC25A27
ENSG00000255517.6	-0.005876624	0.69806361	0.961531987	CTD-3074O7.5
ENSG00000107242.20	-0.005038757	0.69806473	0.961531987	PIP5K1B
ENSG00000122557.11	-0.002201645	0.698081224	0.961531987	HERPUD2
ENSG00000211934.3	0.018920928	0.698159824	0.961539601	IGHV1-2
ENSG00000100453.13	0.008460797	0.698180524	0.961539601	GZMB
ENSG00000007541.17	0.003675426	0.698283214	0.961616448	PIGQ
ENSG00000103742.12	0.014024264	0.698364848	0.96163919	IGDCC4
ENSG00000064652.11	0.004794366	0.698393509	0.96163919	SNX24
ENSG00000261098.1	-0.006216297	0.698552682	0.961742356	RP11-819C21.1
ENSG00000228408.6	0.018669786	0.698562226	0.961742356	RP1-111D6.3

ENSG00000100591.8	-0.002656085	0.69866511	0.961819431	AHSA1
ENSG00000271151.1	0.006241718	0.698781962	0.961873439	RP11-394I13.2
ENSG00000284128.1	-0.014242945	0.69884365	0.961873439	BCRP3
ENSG00000285895.1	0.011392939	0.698849053	0.961873439	RP11-655C2.4
ENSG00000224513.2	0.03712566	0.69889195	0.961873439	AC109309.4
ENSG00000113649.13	-0.00247596	0.699038634	0.96198639	TCERG1
ENSG00000151276.23	0.033665511	0.699084553	0.96198639	MAGI1
ENSG00000266053.2	0.004159261	0.699169768	0.96198639	NDUFV2-AS1
ENSG00000115884.11	0.022450143	0.699194256	0.96198639	SDC1
ENSG00000120896.14	-0.00473531	0.699208559	0.96198639	SORBS3
ENSG00000071054.16	0.002760846	0.699294535	0.962040137	MAP4K4
ENSG00000162836.12	0.004229519	0.6995489	0.962325519	ACP6
ENSG00000092847.13	-0.002462416	0.699762742	0.962355355	AGO1
ENSG00000267801.1	-0.013752869	0.699812415	0.962355355	RP11-552F3.9
ENSG00000231560.1	-0.015393561	0.699813046	0.962355355	CLEC12A-AS1
ENSG00000181404.17	0.010776139	0.699837239	0.962355355	WASHC1
ENSG00000157653.12	0.010902622	0.699879402	0.962355355	C9orf43
ENSG00000237604.1	-0.022425069	0.699898779	0.962355355	AP001056.1
ENSG00000186665.10	0.005357059	0.699915826	0.962355355	C17orf58
ENSG00000151491.14	0.007596067	0.699967255	0.962355355	EPS8
ENSG00000033100.16	0.002316969	0.699992921	0.962355355	CHPF2
ENSG00000158473.8	0.004074284	0.70005201	0.962372075	CD1D
ENSG00000148288.13	0.004280627	0.700175195	0.962442466	GBGT1
ENSG00000198821.11	-0.004219263	0.700197074	0.962442466	CD247
ENSG00000235795.1	-0.010154094	0.700297306	0.962515726	RP11-421L21.2
ENSG00000134812.8	-0.013573426	0.700367028	0.962547046	CBLIF
ENSG00000121577.14	0.004295409	0.700443347	0.962547381	POPDC2
ENSG00000231327.1	0.012890881	0.700461143	0.962547381	LINC01816
ENSG00000279352.1	-0.026460315	0.700653066	0.962629722	RP11-411B10.7
ENSG00000053702.15	-0.003935536	0.700669748	0.962629722	NRIP2
ENSG00000267546.2	-0.006595917	0.700748122	0.962629722	RP11-666A8.8
ENSG00000172840.7	-0.003505446	0.700774406	0.962629722	PDP2
ENSG00000113810.16	0.005815874	0.700838812	0.962629722	SMC4
ENSG00000133195.11	-0.003314937	0.700867191	0.962629722	SLC39A11
ENSG00000225675.2	-0.015704116	0.700892259	0.962629722	LINC01771
ENSG00000166855.9	0.001814877	0.700943014	0.962629722	CLPX
ENSG00000141568.21	-0.001725561	0.700994801	0.962629722	FOXK2
ENSG00000137216.19	-0.004447254	0.701039062	0.962629722	TMEM63B
ENSG00000232098.3	0.003858944	0.701048236	0.962629722	CTD-2619J13.14
ENSG00000238227.8	-0.002933839	0.701084572	0.962629722	TMEM250
ENSG00000040531.16	0.002824528	0.701163818	0.962629722	CTNS
ENSG00000174607.11	0.011404576	0.70124861	0.962629722	UGT8
ENSG00000257497.2	0.006001485	0.701282318	0.962629722	RP11-585P4.5
ENSG00000070214.16	0.003263211	0.701305973	0.962629722	SLC44A1
ENSG00000159335.18	0.007231405	0.701365966	0.962629722	PTMS
ENSG00000113088.6	-0.009913239	0.70136597	0.962629722	GZMK
ENSG00000228549.5	0.013196814	0.701533991	0.962795897	RP11-108M9.3

ENSG00000152518.8	-0.004084562	0.701813537	0.963115098	ZFP36L2
ENSG00000064225.12	0.00450682	0.701960801	0.963167923	ST3GAL6
ENSG00000151632.17	-0.023586861	0.702028119	0.963167923	AKR1C2
ENSG00000229648.1	0.013610994	0.702040908	0.963167923	RPSAP22
ENSG00000229873.1	0.008577668	0.702078508	0.963167923	OGFR-AS1
ENSG00000214784.4	-0.019061281	0.702123678	0.963167923	RPS3AP21
ENSG00000070961.16	0.003783469	0.702134435	0.963167923	ATP2B1
ENSG00000230658.2	0.017857559	0.702219301	0.963167923	KLHL7-DT
ENSG00000223529.1	0.015205173	0.702227754	0.963167923	EEF1A1P8
ENSG00000117419.16	0.003311285	0.702332028	0.963246521	ERI3
ENSG00000150403.18	0.003232022	0.7023887	0.963259828	TMCO3
ENSG00000275835.5	-0.003181626	0.702532471	0.96336943	TUBGCP5
ENSG00000103034.14	0.010010787	0.70256257	0.96336943	NDRG4
ENSG00000150433.10	-0.002539892	0.702653828	0.963428351	TMEM218
ENSG00000001036.14	0.003079415	0.702764896	0.963428351	FUCA2
ENSG00000089916.18	0.002624548	0.702773646	0.963428351	GPATCH2L
ENSG00000264772.6	-0.009385616	0.702857619	0.963428351	RP11-186B7.4
ENSG00000245685.7	0.01384464	0.702905258	0.963428351	FRG1-DT
ENSG00000183665.5	0.004149565	0.702915072	0.963428351	TRMT12
ENSG00000231105.2	0.005052926	0.702940098	0.963428351	ECE1-AS1
ENSG00000176986.16	0.00455273	0.703042798	0.963428351	SEC24C
ENSG00000265293.2	-0.012496337	0.703066653	0.963428351	ARGFXP2
ENSG00000171136.7	-0.015696413	0.703075322	0.963428351	RLN3
ENSG00000146205.14	-0.015583547	0.703158249	0.963448671	ANO7
ENSG00000064601.21	-0.003869141	0.703231005	0.963448671	CTSA
ENSG00000125520.14	-0.003859183	0.703232382	0.963448671	SLC2A4RG
ENSG00000230943.3	0.015694001	0.703278068	0.963448671	LINC02541
ENSG00000169432.18	0.009746457	0.703413473	0.963557454	SCN9A
ENSG00000183307.4	-0.006840429	0.703451444	0.963557454	TMEM121B
ENSG00000112977.16	0.003293297	0.703524642	0.96357351	DAP
ENSG00000276603.1	0.012257054	0.703573038	0.96357351	RP11-425M5.7
ENSG00000225377.6	-0.007026999	0.703642225	0.96357351	NRSN2-AS1
ENSG00000255186.1	0.013488061	0.70366125	0.96357351	RP11-514F3.5
ENSG00000179818.15	0.003471153	0.703698091	0.96357351	PCBP1-AS1
ENSG00000175066.16	-0.005790817	0.703857487	0.963611723	GK5
ENSG00000092969.12	0.020760901	0.703871572	0.963611723	TGFB2
ENSG00000126602.11	0.004395915	0.703943043	0.963611723	TRAP1
ENSG00000081913.14	0.005156503	0.703995894	0.963611723	PHLPP1
ENSG00000279253.1	0.004116819	0.704099731	0.963611723	RP4-614O4.13
ENSG00000211768.1	-0.011007307	0.704138456	0.963611723	TRBJ2-4
ENSG00000156194.18	0.011624547	0.704139833	0.963611723	PPEF2
ENSG00000187051.9	-0.003532397	0.704164431	0.963611723	RPS19BP1
ENSG00000242259.9	0.001857119	0.704170194	0.963611723	C22orf39
ENSG00000237126.8	-0.006051376	0.704220099	0.963611723	AC073254.1
ENSG00000272655.2	0.003455865	0.70437798	0.963611723	POLR2J4
ENSG00000171865.10	0.001972538	0.704513037	0.963611723	RNASEH1
ENSG00000260671.2	0.021569339	0.704533794	0.963611723	RP11-278A23.2

ENSG00000286504.1	0.011863035	0.704542569	0.963611723	RP11-90D4.8
ENSG00000167986.15	-0.002457077	0.704603351	0.963611723	DDB1
ENSG00000110881.12	0.0137918	0.704607584	0.963611723	ASIC1
ENSG00000162591.16	-0.004727704	0.70461164	0.963611723	MEGF6
ENSG00000196074.13	-0.007966584	0.704629032	0.963611723	SYCP2
ENSG00000167555.14	0.004310925	0.704692524	0.963611723	ZNF528
ENSG00000100523.16	0.003723639	0.704728938	0.963611723	DDHD1
ENSG00000223534.1	0.014901189	0.704737791	0.963611723	HLA-DQB1-AS1
ENSG00000174326.14	-0.007895673	0.704766977	0.963611723	SLC16A11
ENSG00000029153.14	-0.008747872	0.704806702	0.963611723	ARNTL2
ENSG00000117400.18	0.008756455	0.70499237	0.963710476	MPL
ENSG00000181773.7	0.011916783	0.705000531	0.963710476	GPR3
ENSG00000272054.1	0.007930393	0.705022412	0.963710476	RP11-423P10.2
ENSG00000232640.1	-0.011566848	0.705092687	0.963710476	RP1-266L20.2
ENSG00000185728.17	0.003330971	0.705113891	0.963710476	YTHDF3
ENSG00000136240.10	0.002263714	0.705281486	0.96384169	KDELR2
ENSG00000168878.19	-0.015785383	0.705303893	0.96384169	SFTPB
ENSG00000254389.3	0.01328187	0.705495495	0.963900271	RHPN1-AS1
ENSG00000179611.3	-0.004380378	0.705507319	0.963900271	DGKZP1
ENSG00000285480.2	0.009775098	0.705527205	0.963900271	H2BE1
ENSG00000268069.2	-0.009872632	0.705536021	0.963900271	RP5-1057I20.4
ENSG00000158796.17	0.002244598	0.705609071	0.963900271	DEDD
ENSG00000172889.16	-0.008709164	0.705684592	0.963900271	EGFL7
ENSG00000264764.1	0.017839604	0.705726937	0.963900271	MIR4772
ENSG00000206503.13	-0.004644506	0.705816392	0.963900271	HLA-A
ENSG00000115875.19	0.002529413	0.70587454	0.963900271	SRSF7
ENSG00000165275.10	0.002490131	0.70594099	0.963900271	TRMT10B
ENSG00000189306.11	-0.00513957	0.706017878	0.963900271	RRP7A
ENSG00000201544.1	-0.013460704	0.706044761	0.963900271	SNORA16B
ENSG00000178467.18	0.002350146	0.706049931	0.963900271	P4HTM
ENSG00000233306.2	0.014164286	0.706050632	0.963900271	TRGV2
ENSG00000089693.11	0.003488621	0.706069215	0.963900271	MLF2
ENSG00000226862.1	0.008857393	0.706098779	0.963900271	RP11-569A11.1
ENSG00000234444.10	0.004535646	0.706278291	0.96408115	ZNF736
ENSG00000111203.13	-0.001904542	0.706423143	0.964173446	ITFG2
ENSG00000139880.20	0.006451432	0.706440977	0.964173446	CDH24
ENSG00000112200.17	-0.0035969	0.706531142	0.964173446	ZNF451
ENSG00000172167.8	-0.005050241	0.706533964	0.964173446	MTBP
ENSG00000120708.17	0.004687829	0.706584946	0.964178859	TGFBI
ENSG00000082516.9	0.003194868	0.706732816	0.964316473	GEMIN5
ENSG00000155256.18	0.001951126	0.706888774	0.964325801	ZFYVE27
ENSG00000165996.14	-0.010038131	0.706916646	0.964325801	HACD1
ENSG00000165832.6	0.005151084	0.706917777	0.964325801	TRUB1
ENSG00000229388.1	0.012697645	0.70692774	0.964325801	LINC01715
ENSG00000231769.3	-0.010222684	0.706992499	0.964349995	RP1-8B1.4
ENSG00000136854.24	0.005848186	0.707100718	0.96436996	STXBP1
ENSG00000161671.17	-0.00281319	0.707123311	0.96436996	EMC10

ENSG00000146776.15	-0.002680922	0.707148209	0.96436996	ATXN7L1
ENSG00000054611.14	0.002749001	0.707286292	0.964488293	TBC1D22A
ENSG00000172803.18	-0.008153376	0.707335756	0.964488293	SNX32
ENSG00000187189.11	-0.00296446	0.707376068	0.964488293	TSPYL4
ENSG00000135966.13	0.002039973	0.707559841	0.964643352	TGFBRAP1
ENSG00000107738.20	0.0033544	0.707607464	0.964643352	VSIR
ENSG00000111859.17	0.003115827	0.707640826	0.964643352	NEDD9
ENSG00000223638.3	0.045779008	0.707677942	0.964643352	RFPL4A
ENSG00000040933.16	-0.002310759	0.707767142	0.964700821	INPP4A
ENSG00000137871.21	0.006154722	0.707938493	0.964870248	ZNF280D
ENSG00000165487.14	-0.003662489	0.708121543	0.9650349	MICU2
ENSG00000272368.2	-0.007451517	0.708153414	0.9650349	RP4-605O3.4
ENSG00000251615.3	-0.00419758	0.708212135	0.965050795	RP11-774O3.3
ENSG00000176894.10	-0.005661468	0.708341436	0.965162857	PXMP2
ENSG00000160062.15	0.011828116	0.708452448	0.965249987	ZBTB8A
ENSG00000279386.1	-0.007452674	0.708509079	0.965263017	RP11-177C12.5
ENSG00000148331.12	0.001637428	0.70862376	0.965314226	ASB6
ENSG00000086065.14	-0.007045706	0.7087308	0.965314226	CHMP5
ENSG00000269243.1	-0.007073233	0.7087471	0.965314226	CTD-2231E14.8
ENSG00000245719.1	0.024884942	0.708754682	0.965314226	RP11-34F13.2
ENSG00000139722.7	-0.003292739	0.708782017	0.965314226	VPS37B
ENSG00000125124.14	-0.003630377	0.708829243	0.965314438	BBS2
ENSG00000196700.9	-0.003569347	0.708938066	0.965364559	ZNF512B
ENSG00000230207.1	0.007507991	0.708960192	0.965364559	RPL4P5
ENSG00000111670.16	0.003763165	0.709024495	0.965388019	GNPTAB
ENSG00000213613.2	-0.012267998	0.709188372	0.965547045	RPL11P3
ENSG00000185608.9	0.005193231	0.709289454	0.965549375	MRPL40
ENSG00000132000.14	-0.008098953	0.709298271	0.965549375	PODNL1
ENSG00000276775.1	-0.018148275	0.709498439	0.965549375	IGHV4-4
ENSG00000113595.15	0.005597265	0.709521179	0.965549375	TRIM23
ENSG00000242498.8	0.008961863	0.7095776	0.965549375	ARPIN
ENSG00000230731.3	-0.039394887	0.709594273	0.965549375	RP11-478K15.6
ENSG00000261143.1	0.017406021	0.709624402	0.965549375	ADAMTS7P3
ENSG00000218073.1	-0.009637839	0.709638785	0.965549375	RP1-13D10.2
ENSG00000277728.1	-0.008059361	0.7097811	0.965549375	RP11-143K11.7
ENSG00000166819.12	-0.01090748	0.709792681	0.965549375	PLIN1
ENSG00000134265.13	-0.003348994	0.709794478	0.965549375	NAPG
ENSG00000169992.10	-0.006471743	0.709837263	0.965549375	NLGN2
ENSG00000197961.12	0.003031016	0.709870516	0.965549375	ZNF121
ENSG00000163521.16	-0.008251859	0.709871778	0.965549375	GLB1L
ENSG00000166866.13	0.007193229	0.709896308	0.965549375	MYO1A
ENSG00000174953.14	0.002745562	0.709965182	0.965558469	DHX36
ENSG00000188690.15	0.00233686	0.710016757	0.965558469	UROS
ENSG00000128973.13	-0.005841398	0.710114314	0.965558469	CLN6
ENSG00000134716.11	-0.010917458	0.710142905	0.965558469	CYP2J2
ENSG00000160185.15	-0.004646994	0.710148104	0.965558469	UBASH3A
ENSG00000120798.17	0.002890223	0.710291991	0.965558469	NR2C1

ENSG00000154188.10	-0.010628523	0.710315467	0.965558469	ANGPT1
ENSG00000244560.7	0.005192622	0.710324749	0.965558469	RP4-800G7.2
ENSG00000076662.10	0.004028409	0.710427661	0.965558469	ICAM3
ENSG00000168874.13	0.017202425	0.710494914	0.965558469	ATOH8
ENSG00000267277.1	0.010254925	0.710513713	0.965558469	CTD-2342J14.6
ENSG00000256128.6	-0.010304913	0.710635306	0.965558469	LINC00944
ENSG00000185379.21	0.002135988	0.710655842	0.965558469	RAD51D
ENSG00000248008.3	-0.005030046	0.710673198	0.965558469	NRAV
ENSG00000173064.14	0.003128101	0.710685914	0.965558469	HECTD4
ENSG00000127329.16	-0.012115727	0.710695486	0.965558469	PTPRB
ENSG00000243696.5	-0.004947215	0.710703389	0.965558469	RP5-966M1.6
ENSG00000181143.15	-0.020206623	0.710760183	0.965571663	MUC16
ENSG00000179627.10	-0.004246736	0.710916613	0.965715031	ZBTB42
ENSG00000106868.17	0.00369275	0.711099289	0.965715031	SUSD1
ENSG00000134323.12	-0.017273706	0.711108686	0.965715031	MYCN
ENSG00000135837.16	0.003546008	0.711134689	0.965715031	CEP350
ENSG00000123427.17	0.006346182	0.711140523	0.965715031	EEF1AKMT3
ENSG00000153157.13	-0.020394659	0.711148254	0.965715031	SYCP2L
ENSG00000177535.9	0.008721693	0.711401758	0.965816973	OR2B11
ENSG00000106367.15	0.003746457	0.711528824	0.965816973	AP1S1
ENSG00000151689.13	0.003290387	0.711568461	0.965816973	INPP1
ENSG00000213930.12	0.002577348	0.711593181	0.965816973	GALT
ENSG00000111652.10	0.002211318	0.711609163	0.965816973	COPS7A
ENSG00000132518.7	-0.009890763	0.711687102	0.965816973	GUCY2D
ENSG00000228502.1	0.007862857	0.711708504	0.965816973	EEF1A1P11
ENSG00000267394.1	0.006765147	0.711764736	0.965816973	ATXN7L3-AS1
ENSG00000269560.1	0.015417231	0.71182118	0.965816973	CTD-2192J16.21
ENSG00000100196.11	0.010251779	0.711985576	0.965816973	KDELR3
ENSG00000258441.1	-0.004104402	0.712036257	0.965816973	LINC00641
ENSG00000258654.1	-0.0137302	0.712040814	0.965816973	RP11-509A17.3
ENSG00000172031.7	-0.016329567	0.712078458	0.965816973	EPHX4
ENSG00000100138.15	0.00265529	0.712094253	0.965816973	SNU13
ENSG00000127074.15	0.023961748	0.712251917	0.965816973	RGS13
ENSG00000247199.5	-0.00777638	0.71225894	0.965816973	RP11-373N22.3
ENSG00000151148.14	-0.001899504	0.712260622	0.965816973	UBE3B
ENSG00000006652.15	-0.005145317	0.712261344	0.965816973	IFRD1
ENSG00000280067.1	0.005478841	0.712392421	0.965816973	CTD-2600H12.2
ENSG00000141384.13	0.005294036	0.712492978	0.965816973	TAF4B
ENSG00000146067.16	-0.002625858	0.712507074	0.965816973	FAM193B
ENSG00000197647.12	0.007067832	0.712559404	0.965816973	ZNF433
ENSG00000175595.16	0.003501582	0.712609852	0.965816973	ERCC4
ENSG00000170624.14	-0.019455052	0.712617484	0.965816973	SGCD
ENSG00000228302.2	0.004044892	0.71262733	0.965816973	RP11-186N15.3
ENSG00000139926.16	-0.01194125	0.71265278	0.965816973	FRMD6
ENSG00000101407.13	0.00211054	0.712668679	0.965816973	TTI1
ENSG00000272323.1	0.011606562	0.712670061	0.965816973	TTC23L-AS1
ENSG00000232344.2	0.012222084	0.712675851	0.965816973	AC087163.2

ENSG00000132275.11	0.002110231	0.712680187	0.965816973	RRP8
ENSG00000236514.1	-0.011966103	0.712726162	0.965816973	RP11-162G10.5
ENSG00000197386.14	-0.002625368	0.712730353	0.965816973	HTT
ENSG00000182742.6	0.006128383	0.712872749	0.965907715	HOXB4
ENSG00000173585.17	0.011482998	0.712944551	0.965907715	CCR9
ENSG00000198898.14	0.00577666	0.712998455	0.965907715	CAPZA2
ENSG00000272525.1	0.01352239	0.713049508	0.965907715	BTF3-DT
ENSG00000034053.15	-0.004826561	0.71306845	0.965907715	APBA2
ENSG00000262410.1	0.012689132	0.71307991	0.965907715	RP11-388C12.8
ENSG00000242473.1	0.014417013	0.713164878	0.965959007	KIR2DP1
ENSG00000091039.17	0.006526975	0.713271419	0.966012046	OSBPL8
ENSG00000101638.14	-0.020901529	0.713298245	0.966012046	ST8SIA5
ENSG00000040341.18	-0.002954377	0.713476459	0.966172417	STAU2
ENSG00000167778.9	-0.00299044	0.713510886	0.966172417	SPRYD3
ENSG00000279086.1	-0.005588817	0.713578215	0.96618184	RP11-667F14.1
ENSG00000135801.10	0.002290617	0.713664811	0.96618184	TAF5L
ENSG00000136280.17	-0.002674815	0.713731208	0.96618184	CCM2
ENSG00000178226.11	-0.004765004	0.71373433	0.96618184	PRSS36
ENSG00000218631.1	0.010770188	0.713775548	0.96618184	RP3-395C13.1
ENSG00000227051.7	0.010647913	0.713800519	0.96618184	C14orf132
ENSG00000125531.7	0.007207427	0.713992724	0.966378219	FNDC11
ENSG00000198556.14	-0.002861569	0.714178359	0.966553567	ZNF789
ENSG00000211456.13	0.003750218	0.714245536	0.966553567	SACM1L
ENSG00000137261.15	0.006907026	0.714342777	0.966553567	KIAA0319
ENSG00000280035.1	-0.007321675	0.714424008	0.966553567	RP11-10J21.2
ENSG00000230107.1	0.011048017	0.714462094	0.966553567	CTA-126B4.7
ENSG00000261659.2	-0.006331013	0.714479597	0.966553567	LA16c-313D11.12
ENSG00000134313.16	-0.003790136	0.714551635	0.966553567	KIDINS220
ENSG00000230445.4	0.009797018	0.714554981	0.966553567	LRRC37A6P
ENSG00000139726.11	0.002538678	0.714630327	0.966553567	DENR
ENSG00000088899.16	-0.006879429	0.714644673	0.966553567	LZTS3
ENSG00000008130.15	-0.003024262	0.714645915	0.966553567	NADK
ENSG00000077063.11	-0.017206547	0.714687843	0.966553567	CTTNBP2
ENSG00000156671.15	0.004133901	0.714791221	0.966627252	SAMD8
ENSG00000176236.6	0.014726622	0.714836595	0.966627252	RPP38-DT
ENSG00000198355.5	-0.004401678	0.714906075	0.966657466	PIM3
ENSG00000213614.11	-0.002272117	0.714956213	0.966661525	HEXA
ENSG00000249992.2	0.013158698	0.715026437	0.96669274	TMEM158
ENSG00000285793.1	0.006681393	0.715214761	0.966777086	ANAPC1P2
ENSG00000177697.19	-0.007011972	0.715215857	0.966777086	CD151
ENSG00000160439.16	-0.004690475	0.715230249	0.966777086	RDH13
ENSG00000137492.9	0.003844446	0.715438705	0.966976208	THAP12
ENSG00000075568.17	0.002967107	0.715471863	0.966976208	TMEM131
ENSG00000132510.11	-0.00423364	0.715706954	0.967230195	KDM6B
ENSG00000126746.18	0.003914952	0.715835888	0.967297552	ZNF384
ENSG00000164124.11	0.006591056	0.715851128	0.967297552	TMEM144
ENSG00000279500.1	0.008396458	0.715964797	0.96734175	RP11-21K12.2

ENSG00000213906.10	0.005040381	0.715978176	0.96734175	LTB4R2
ENSG00000006432.16	-0.00474338	0.716085748	0.967370374	MAP3K9
ENSG00000227815.2	-0.016666388	0.716093702	0.967370374	RP11-195C7.3
ENSG00000119986.7	0.007100194	0.716155344	0.967389922	AVPI1
ENSG00000273295.1	-0.022297044	0.716212999	0.967404082	AP000350.5
ENSG00000112715.25	0.004523466	0.716299703	0.967443609	VEGFA
ENSG00000071537.14	0.003163839	0.71633661	0.967443609	SEL1L
ENSG00000242125.3	-0.00312234	0.716583585	0.967713431	SNHG3
ENSG00000173988.13	-0.010518972	0.716746895	0.967870239	LRRC63
ENSG00000265778.3	0.007551153	0.717045223	0.967964383	ZNF516-AS1
ENSG00000174358.16	0.028215476	0.71708021	0.967964383	SLC6A19
ENSG00000170017.12	-0.005030923	0.717082602	0.967964383	ALCAM
ENSG00000130429.15	0.003196788	0.717121533	0.967964383	ARPC1B
ENSG00000221886.4	0.007330589	0.717155578	0.967964383	ZBED8
ENSG00000239697.11	-0.003527367	0.717162878	0.967964383	TNFSF12
ENSG00000230795.3	-0.012911834	0.717233382	0.967964383	HLA-K
ENSG00000276747.1	0.021488325	0.717238489	0.967964383	PADI6
ENSG00000113369.9	-0.004864416	0.717241407	0.967964383	ARRDC3
ENSG00000270062.1	-0.006079802	0.717311522	0.967995309	RP11-248J18.3
ENSG00000175087.10	0.004655492	0.717491737	0.968105846	PDIK1L
ENSG00000197275.14	0.00944314	0.717525806	0.968105846	RAD54B
ENSG00000288009.1	-0.01275984	0.717575761	0.968105846	RP11-63L7.6
ENSG00000285651.1	-0.012535515	0.717604906	0.968105846	RP11-139D23.7
ENSG00000204388.7	0.005450575	0.717760893	0.968105846	HSPA1B
ENSG00000138182.15	0.005828912	0.717784387	0.968105846	KIF20B
ENSG00000103365.15	-0.002581448	0.717805639	0.968105846	GGA2
ENSG00000110880.11	-0.002595699	0.717886169	0.968105846	CORO1C
ENSG00000130939.20	0.00254248	0.717887326	0.968105846	UBE4B
ENSG00000241685.10	0.003423323	0.718020398	0.968105846	ARPC1A
ENSG00000272288.6	-0.006012747	0.7180825	0.968105846	ILRUN-AS1
ENSG00000265817.4	-0.010681033	0.718147422	0.968105846	FSBP
ENSG00000230131.6	-0.013350588	0.718168446	0.968105846	LINC02641
ENSG00000144230.17	0.014521542	0.718171662	0.968105846	GPR17
ENSG00000174791.11	0.005733737	0.718182764	0.968105846	RIN1
ENSG00000136875.13	-0.001859077	0.718197692	0.968105846	PRPF4
ENSG00000080345.18	-0.004159458	0.718274051	0.968105846	RIF1
ENSG00000211764.1	-0.006408705	0.718281053	0.968105846	TRBJ2-1
ENSG00000235706.8	-0.003774913	0.71838354	0.968105846	DICER1-AS1
ENSG00000257621.8	-0.00339781	0.718428326	0.968105846	PSMA3-AS1
ENSG00000145016.16	0.004308851	0.718446225	0.968105846	RUBCN
ENSG00000174437.18	0.002503798	0.718473567	0.968105846	ATP2A2
ENSG00000228242.6	0.005837465	0.718527955	0.968105846	XPC-AS1
ENSG00000111752.11	0.00409925	0.71856209	0.968105846	PHC1
ENSG00000171310.11	0.002593807	0.718666644	0.968105846	CHST11
ENSG00000269343.9	0.002785134	0.718705951	0.968105846	ZNF587B
ENSG00000250539.1	-0.010374848	0.718727929	0.968105846	KRT8P33
ENSG00000166797.11	0.004263071	0.718750514	0.968105846	CIAO2A

ENSG00000125818.18	0.004337222	0.718780197	0.968105846	PSMF1
ENSG00000170385.10	-0.004511002	0.718852843	0.968105846	SLC30A1
ENSG00000229409.1	0.012637868	0.718856827	0.968105846	RP11-494O16.3
ENSG00000173391.9	-0.018757772	0.718971182	0.968148097	OLR1
ENSG00000245848.3	0.003722128	0.718982617	0.968148097	CEBPA
ENSG00000197776.8	-0.00615754	0.719083312	0.968182409	KLHDC1
ENSG00000273108.1	-0.007809126	0.719102518	0.968182409	RP11-416N2.4
ENSG00000136856.18	-0.004461833	0.719264834	0.968337374	SLC2A8
ENSG00000255710.2	-0.01386526	0.719399541	0.968455153	RP11-667M19.9
ENSG00000169499.15	0.002470844	0.719548473	0.968575252	PLEKHA2
ENSG00000215244.3	-0.00562841	0.719712086	0.968575252	LINC02649
ENSG00000168883.20	0.001631868	0.719716465	0.968575252	USP39
ENSG00000182272.12	-0.015148299	0.719724144	0.968575252	B4GALNT4
ENSG00000106628.12	-0.003450005	0.7197249	0.968575252	POLD2
ENSG00000145675.15	0.002983472	0.719806043	0.968620888	PIK3R1
ENSG00000158773.14	0.002589422	0.719895754	0.968678048	USF1
ENSG00000280798.1	0.003646873	0.720004137	0.968760322	LINC00294
ENSG00000100271.17	-0.00360726	0.720160728	0.968861015	TTLL1
ENSG00000196911.11	-0.005948045	0.72017346	0.968861015	KPNA5
ENSG00000198546.15	-0.003345646	0.720366534	0.969057191	ZNF511
ENSG00000146072.6	-0.007875286	0.720489423	0.969158933	TNFRSF21
ENSG00000183508.5	0.008554241	0.720629035	0.969216773	TENT5C
ENSG00000168090.10	0.002713099	0.720672618	0.969216773	COPS6
ENSG00000185630.19	0.007828726	0.720674204	0.969216773	PBX1
ENSG00000101577.10	0.003471797	0.720738494	0.969239674	LPIN2
ENSG00000142676.14	0.005602803	0.720912974	0.969410746	RPL11
ENSG00000104205.16	-0.003246198	0.721128823	0.969497155	SGK3
ENSG00000224086.6	-0.003446561	0.721146574	0.969497155	PPM1F-AS1
ENSG00000201988.2	0.012021081	0.721181077	0.969497155	Y_RNA
ENSG00000253251.3	-0.007866741	0.721220639	0.969497155	SHLD3
ENSG00000113240.13	-0.005077402	0.721329408	0.969497155	CLK4
ENSG00000077044.11	0.002241585	0.721338257	0.969497155	DGKD
ENSG00000207757.1	-0.009880419	0.721366532	0.969497155	MIR93
ENSG00000125966.10	0.005538712	0.721405769	0.969497155	MMP24
ENSG00000165807.8	0.016044471	0.721410728	0.969497155	PPP1R36
ENSG00000139636.16	-0.002004073	0.721479043	0.969497155	LMBR1L
ENSG00000004660.15	-0.004477954	0.721514796	0.969497155	CAMKK1
ENSG00000127561.15	0.008923659	0.721544523	0.969497155	SYNGR3
ENSG00000229152.2	0.006094067	0.721652943	0.969526724	ANKRD10-IT1
ENSG00000139193.4	-0.005167288	0.721661081	0.969526724	CD27
ENSG00000135378.4	-0.006342728	0.721740903	0.969533755	PRRG4
ENSG00000149257.16	-0.003402904	0.721760866	0.969533755	SERPINH1
ENSG00000124766.7	0.004547891	0.721931927	0.969568723	SOX4
ENSG00000115290.10	-0.013331291	0.722001524	0.969568723	GRB14
ENSG00000277007.1	-0.00773793	0.722082763	0.969568723	RP11-392O17.2
ENSG00000227495.1	0.007592725	0.722085364	0.969568723	KIF1C-AS1
ENSG00000146281.6	-0.004618096	0.722156258	0.969568723	PM20D2

ENSG00000171747.9	0.00609077	0.722170985	0.969568723	LGALS4
ENSG00000185420.19	0.003107856	0.722237938	0.969568723	SMYD3
ENSG00000186314.12	-0.00815472	0.722250496	0.969568723	PRELID2
ENSG00000279825.1	0.010294254	0.722292873	0.969568723	CTC-457L16.1
ENSG00000167978.17	-0.003612617	0.722347943	0.969568723	SRRM2
ENSG00000163219.12	-0.002672289	0.722512141	0.969568723	ARHGAP25
ENSG00000269858.6	0.002785121	0.722533129	0.969568723	EGLN2
ENSG00000162572.21	-0.005161621	0.722683513	0.969568723	SCNN1D
ENSG00000255112.3	0.002541577	0.722710882	0.969568723	CHMP1B
ENSG00000211827.1	0.017052155	0.722728504	0.969568723	TRDJ2
ENSG00000134247.10	0.007751293	0.722745908	0.969568723	PTGFRN
ENSG00000227039.6	-0.005470141	0.722793201	0.969568723	ITGB2-AS1
ENSG00000095015.6	0.00411949	0.722938615	0.969568723	MAP3K1
ENSG00000254815.6	-0.005878122	0.722952768	0.969568723	LMNTD2-AS1
ENSG00000099337.5	-0.00295973	0.722980761	0.969568723	KCNK6
ENSG00000233365.2	0.027048876	0.723012596	0.969568723	RP4-655C5.4
ENSG00000249476.2	0.006175509	0.723020404	0.969568723	CTD-2587M2.1
ENSG00000161551.15	-0.004985329	0.723022186	0.969568723	ZNF577
ENSG00000120820.13	-0.0117839	0.723026056	0.969568723	GLT8D2
ENSG00000121741.17	0.003602378	0.723042712	0.969568723	ZMYM2
ENSG00000144847.13	-0.018015442	0.723055405	0.969568723	IGSF11
ENSG00000197355.11	0.006127994	0.72311181	0.969568723	UAP1L1
ENSG00000080546.13	0.004549313	0.723131733	0.969568723	SESN1
ENSG00000266473.1	-0.016683981	0.72324448	0.969568723	HELZ-AS1
ENSG00000049449.10	-0.00404537	0.723246765	0.969568723	RCN1
ENSG00000245112.2	0.010549702	0.723252502	0.969568723	SMARCA5-AS1
ENSG00000187741.15	-0.005795357	0.723344622	0.969570245	FANCA
ENSG00000181904.9	-0.00461648	0.723350051	0.969570245	C5orf24
ENSG00000167536.14	-0.004973797	0.723433984	0.969570245	DHRS13
ENSG00000270755.1	-0.010950206	0.723442748	0.969570245	RP11-138E2.1
ENSG00000228264.1	-0.011692481	0.723514658	0.969603255	PSMD8P1
ENSG00000211584.14	-0.004175225	0.723629811	0.969694208	SLC48A1
ENSG00000242294.6	-0.005055171	0.723746652	0.96971801	STAG3L5P
ENSG00000104368.19	-0.007072018	0.723781365	0.96971801	PLAT
ENSG00000128563.13	-0.002161878	0.723868398	0.96971801	PRKRIP1
ENSG00000189362.12	-0.002858368	0.723873261	0.96971801	NEMP2
ENSG00000198723.11	-0.015436918	0.723883997	0.96971801	TEX45
ENSG00000213144.2	-0.013652011	0.723974517	0.969761723	RP11-64B16.2
ENSG00000180720.8	0.011573489	0.724011202	0.969761723	CHRM4
ENSG00000049759.20	0.006522434	0.724121873	0.969764023	NEDD4L
ENSG00000189430.13	-0.007213089	0.724224371	0.969764023	NCR1
ENSG00000115602.17	0.010541947	0.724231381	0.969764023	IL1RL1
ENSG00000110031.13	0.002319622	0.724251106	0.969764023	LPXN
ENSG00000135932.11	0.002865389	0.724306682	0.969764023	CAB39
ENSG00000247498.10	0.015101919	0.724392714	0.969764023	GPRC5D-AS1
ENSG00000079156.17	0.0111566	0.724447652	0.969764023	OSBPL6
ENSG00000261351.2	0.003587242	0.72451535	0.969764023	CTD-3185P2.1

ENSG00000101546.13	-0.002922351	0.724562768	0.969764023	RBFA
ENSG00000137710.17	0.003919204	0.724579511	0.969764023	RDX
ENSG00000255036.6	-0.013720856	0.724590859	0.969764023	SUGT1P4-STRA6LP-CCDC180
ENSG00000073792.16	0.008059939	0.72464535	0.969764023	IGF2BP2
ENSG00000110171.20	0.002551101	0.724655751	0.969764023	TRIM3
ENSG00000145388.15	-0.003348403	0.724674939	0.969764023	METTL14
ENSG00000139117.14	0.004528903	0.724942837	0.969853883	CPNE8
ENSG00000205085.13	0.005160106	0.724960425	0.969853883	FAM71F2
ENSG00000181625.17	-0.017253334	0.724971913	0.969853883	SLX1B
ENSG00000270433.1	0.009926636	0.725048835	0.969853883	H3P37
ENSG00000134899.24	0.004357019	0.725054443	0.969853883	ERCC5
ENSG00000279078.1	-0.009649576	0.725103605	0.969853883	SND1-IT1
ENSG00000280135.1	-0.004337705	0.725119807	0.969853883	RP1-47M23.3
ENSG00000233901.6	0.004768371	0.725120421	0.969853883	LINC01503
ENSG00000198146.4	0.006128253	0.72521984	0.969893388	ZNF770
ENSG00000168936.11	-0.002307142	0.725278964	0.969893388	TMEM129
ENSG00000231062.1	0.007750198	0.725329977	0.969893388	AC103563.9
ENSG00000118492.18	-0.025480478	0.7254647	0.969893388	ADGB
ENSG00000089327.15	0.003748773	0.725565787	0.969893388	FXYD5
ENSG00000151239.14	0.005620716	0.725636153	0.969893388	TWF1
ENSG00000112110.10	0.002537986	0.725643375	0.969893388	MRPL18
ENSG00000155428.12	-0.008952626	0.725661147	0.969893388	TRIM74
ENSG00000163171.8	-0.00363598	0.72566453	0.969893388	CDC42EP3
ENSG00000138443.17	-0.002735066	0.725679359	0.969893388	ABI2
ENSG00000137414.6	-0.004379083	0.725698393	0.969893388	FAM8A1
ENSG00000158092.7	0.002696003	0.725809708	0.969893388	NCK1
ENSG00000236439.4	-0.012554082	0.725849181	0.969893388	RP11-175B9.3
ENSG00000140006.12	0.0047517	0.725939461	0.969893388	WDR89
ENSG00000113108.20	0.002568873	0.725960413	0.969893388	APBB3
ENSG00000187024.15	-0.016436201	0.726001815	0.969893388	PTRH1
ENSG00000170801.10	-0.019283606	0.726013556	0.969893388	HTRA3
ENSG00000287989.1	0.014017018	0.726141917	0.969893388	RP11-404H1.1
ENSG00000236915.2	0.021346947	0.726156087	0.969893388	CLCA4-AS1
ENSG00000287707.1	0.010744251	0.72616601	0.969893388	RP11-155D18.17
ENSG00000104783.14	0.006451193	0.726174851	0.969893388	KCNN4
ENSG00000164867.11	0.00625114	0.726230318	0.969893388	NOS3
ENSG00000102805.16	0.003307737	0.726237706	0.969893388	CLN5
ENSG00000174839.13	0.002768154	0.726320438	0.969940714	DENND6A
ENSG00000156127.8	-0.004601091	0.726386357	0.969943688	BATF
ENSG00000172538.7	-0.018440276	0.726449477	0.969943688	FAM170B
ENSG00000261485.1	-0.003980459	0.726464552	0.969943688	PAN3-AS1
ENSG00000124067.17	0.002478639	0.726542434	0.969984521	SLC12A4
ENSG00000230555.2	-0.005922589	0.726680467	0.970054675	RP11-517P14.2
ENSG00000160321.15	0.014859806	0.726727528	0.970054675	ZNF208
ENSG00000128016.7	0.002654343	0.726736885	0.970054675	ZFP36
ENSG00000213621.3	0.010348051	0.726834757	0.97005772	RPSAP54
ENSG00000246214.2	-0.012184595	0.726861577	0.97005772	RP11-260E18.1

ENSG00000188672.19	-0.013397374	0.726983008	0.97005772	RHCE
ENSG00000114648.12	0.001998247	0.727017801	0.97005772	KLHL18
ENSG00000131591.18	-0.003640506	0.727027531	0.97005772	C1orf159
ENSG00000214413.9	0.003893747	0.727033844	0.97005772	BBIP1
ENSG00000286616.1	0.012355874	0.727070276	0.97005772	RP3-487J7.4
ENSG00000132541.11	0.007274624	0.727121552	0.970063023	RIDA
ENSG00000280216.1	-0.013194388	0.727353898	0.970220055	RP3-333H23.9
ENSG00000188191.15	-0.005220524	0.727354295	0.970220055	PRKAR1B
ENSG00000236790.6	0.006068127	0.727390288	0.970220055	LINC00299
ENSG00000178295.15	-0.006567875	0.727428495	0.970220055	GEN1
ENSG00000188385.12	0.009677236	0.727496534	0.970247703	JAKMIP3
ENSG00000206052.11	0.008033657	0.727558329	0.970267018	DOK6
ENSG00000283632.4	0.011461419	0.727627062	0.970292167	EXOC3L2
ENSG00000287030.1	0.018045845	0.72772028	0.970292167	RP11-613M5.5
ENSG00000169203.16	-0.012547622	0.72783068	0.970292167	NPIPB12
ENSG00000070367.16	-0.003718922	0.727868636	0.970292167	EXOC5
ENSG00000198429.10	0.005189658	0.72795955	0.970292167	ZNF69
ENSG00000132972.19	0.0526323	0.727991809	0.970292167	RNF17
ENSG00000162434.13	0.00277562	0.728058183	0.970292167	JAK1
ENSG00000162825.16	-0.006091138	0.728192937	0.970292167	NBPF20
ENSG00000048828.17	0.002006818	0.728218257	0.970292167	FAM120A
ENSG00000170854.18	0.002449628	0.728218692	0.970292167	RIOX2
ENSG00000282939.1	-0.008263213	0.728239887	0.970292167	TRBV7-2
ENSG00000128833.13	-0.007057667	0.728245779	0.970292167	MYO5C
ENSG00000187244.12	0.01634151	0.728283907	0.970292167	BCAM
ENSG00000019485.14	0.002814652	0.728321906	0.970292167	PRDM11
ENSG00000254887.1	-0.004786322	0.728404452	0.970292167	CTC-378H22.1
ENSG00000248429.5	0.005987276	0.728459818	0.970292167	FAM198B-AS1
ENSG00000272682.1	-0.011823065	0.728497305	0.970292167	AC004471.10
ENSG00000230715.3	-0.004774935	0.72849987	0.970292167	RP11-274B21.4
ENSG00000139131.13	0.002520533	0.728537929	0.970292167	YARS2
ENSG00000128791.12	0.004995116	0.728545344	0.970292167	TWSG1
ENSG00000148346.12	0.015780963	0.728584783	0.970292167	LCN2
ENSG00000128654.14	0.004427572	0.728651327	0.970292167	MTX2
ENSG00000269388.1	-0.012984686	0.728682621	0.970292167	AC018755.16
ENSG00000253818.1	0.019511246	0.728791023	0.970292167	IGLV1-41
ENSG00000081154.12	0.003572534	0.728798083	0.970292167	PCNP
ENSG00000264743.2	0.009272277	0.728850933	0.970292167	DPRXP4
ENSG00000169410.10	0.00215043	0.728876625	0.970292167	PTPN9
ENSG00000179958.10	0.003424662	0.728901947	0.970292167	DCTPP1
ENSG00000227688.2	0.010483646	0.728962328	0.970309563	HNRNPA3P2
ENSG00000182831.12	-0.002919576	0.729187963	0.970507067	C16orf72
ENSG00000198198.17	0.002674012	0.729229909	0.970507067	SZT2
ENSG00000277511.1	-0.008690836	0.729252677	0.970507067	CTD-2095E4.5
ENSG00000250903.9	0.003339325	0.729387935	0.970575618	GMDS-DT
ENSG00000104972.16	-0.004059022	0.729434493	0.970575618	LILRB1
ENSG00000224372.1	0.049137668	0.729478382	0.970575618	HLA-N

ENSG00000248445.5	0.005180083	0.729537538	0.970575618	SEMA6A-AS1
ENSG00000236935.1	-0.008033841	0.729540821	0.970575618	AP003774.1
ENSG00000170089.15	0.005863247	0.729607493	0.970601354	RP11-423H2.1
ENSG00000183309.12	0.002188795	0.729730548	0.970648199	ZNF623
ENSG00000083457.12	-0.002657691	0.729737367	0.970648199	ITGAE
ENSG00000158483.17	-0.006776814	0.729821776	0.970697515	FAM86C1P
ENSG00000231443.2	-0.012584615	0.729968606	0.970711517	AC024937.6
ENSG00000254470.3	0.003359538	0.729983139	0.970711517	AP5B1
ENSG00000179361.18	0.002468477	0.729984772	0.970711517	ARID3B
ENSG00000157890.18	0.005387991	0.730037931	0.970711517	MEGF11
ENSG00000163755.9	0.002346184	0.73006897	0.970711517	HPS3
ENSG00000225948.2	-0.007334544	0.730303869	0.970727128	LINC02648
ENSG00000239884.3	0.007995638	0.730312434	0.970727128	RN7SL608P
ENSG00000102554.14	-0.005494495	0.730561099	0.970727128	KLF5
ENSG00000168538.16	0.002080507	0.730561462	0.970727128	TRAPPC11
ENSG00000102543.14	-0.003060565	0.730590746	0.970727128	CDADC1
ENSG00000165171.11	0.017426814	0.730641167	0.970727128	METTL27
ENSG00000106080.11	-0.004163224	0.730658266	0.970727128	FKBP14
ENSG00000255229.1	0.094040171	0.730730179	0.970727128	RP11-304M2.3
ENSG00000171824.14	0.002204449	0.730743313	0.970727128	EXOSC10
ENSG00000128536.16	-0.005054697	0.730759079	0.970727128	CDHR3
ENSG00000164329.14	0.002662633	0.730769107	0.970727128	TENT2
ENSG00000279255.1	0.011914615	0.730807083	0.970727128	LA16c-380A1.2
ENSG00000112365.5	0.002328016	0.730827247	0.970727128	ZBTB24
ENSG00000214160.10	-0.003040663	0.731032397	0.970727128	ALG3
ENSG00000233750.3	-0.008316492	0.731069612	0.970727128	CICP27
ENSG00000188765.7	0.011690671	0.731090387	0.970727128	TMSB4XP2
ENSG00000198924.8	0.004230767	0.73109429	0.970727128	DCLRE1A
ENSG00000198668.13	-0.001782181	0.731127502	0.970727128	CALM1
ENSG00000225684.4	0.015647454	0.731174379	0.970727128	FAM225B
ENSG00000260257.2	-0.00435172	0.731310957	0.970727128	RP5-1085F17.3
ENSG00000160679.13	0.001103211	0.731364376	0.970727128	CHTOP
ENSG00000175985.10	0.015906342	0.731468832	0.970727128	PLEKHD1
ENSG00000126107.15	-0.002346476	0.731540568	0.970727128	HECTD3
ENSG00000124098.10	0.009167299	0.731659111	0.970727128	FAM210B
ENSG00000183423.12	-0.008174036	0.731667253	0.970727128	LRIT3
ENSG00000272491.1	0.011166478	0.731693314	0.970727128	RP5-1024N4.4
ENSG00000288046.1	0.010213743	0.731739257	0.970727128	RP3-470L22.2
ENSG00000230615.7	-0.011317747	0.731931993	0.970727128	RP5-1198O20.4
ENSG00000137142.5	-0.029321729	0.731940371	0.970727128	IGFBPL1
ENSG00000186417.14	0.017806128	0.731990901	0.970727128	GLDN
ENSG00000042493.16	0.00475235	0.732004792	0.970727128	CAPG
ENSG00000196811.13	-0.012212239	0.732049384	0.970727128	CHRNG
ENSG00000169857.9	-0.003280819	0.732110563	0.970727128	AVEN
ENSG00000186480.13	0.003107901	0.732145816	0.970727128	INSIG1
ENSG00000131626.19	0.00250043	0.732163166	0.970727128	PPFIA1
ENSG00000107223.13	-0.003313374	0.73218584	0.970727128	EDF1

ENSG00000161640.15	0.010607055	0.732202064	0.970727128	SIGLEC11
ENSG00000188846.14	0.00357014	0.732205797	0.970727128	RPL14
ENSG00000121797.10	0.004557737	0.732222819	0.970727128	CCRL2
ENSG00000286965.1	-0.012120387	0.732227405	0.970727128	RP11-521H5.1
ENSG00000271605.6	-0.00564638	0.732290893	0.970727128	MILR1
ENSG00000249626.1	-0.009454272	0.732366857	0.970727128	RP11-496H1.1
ENSG00000066185.13	0.010120385	0.732388852	0.970727128	ZMYND12
ENSG00000162521.19	0.001370723	0.732449585	0.970727128	RBBP4
ENSG00000136068.16	0.004201058	0.732477449	0.970727128	FLNB
ENSG00000251739.1	0.009708776	0.732562093	0.970727128	RNU6-1053P
ENSG00000251039.3	-0.015803938	0.732562395	0.970727128	IGKV2D-40
ENSG00000188171.16	0.006072802	0.732574876	0.970727128	ZNF626
ENSG00000152672.8	0.019877632	0.732590677	0.970727128	CLEC4F
ENSG00000081052.14	0.007707687	0.732601088	0.970727128	COL4A4
ENSG00000106305.10	-0.003006295	0.732647223	0.970727128	AIMP2
ENSG00000279311.1	-0.010331083	0.732660376	0.970727128	RP11-170K4.2
ENSG00000153140.9	0.008475962	0.732691135	0.970727128	CETN3
ENSG00000280620.1	0.015344715	0.732830559	0.970727128	SCAANT1
ENSG00000182670.13	0.00237786	0.732834756	0.970727128	TTC3
ENSG00000124541.7	0.002103167	0.732855413	0.970727128	RRP36
ENSG00000222019.7	-0.010584539	0.732862341	0.970727128	URAHP
ENSG00000171467.16	-0.002953614	0.732876167	0.970727128	ZNF318
ENSG00000158006.14	-0.002653917	0.732905632	0.970727128	PAFAH2
ENSG00000178028.14	-0.001796534	0.732920755	0.970727128	DMAP1
ENSG00000180549.8	-0.004564792	0.733016459	0.970791188	FUT7
ENSG00000213928.9	-0.003946781	0.733222156	0.970832801	IRF9
ENSG00000072401.15	0.005052306	0.733250724	0.970832801	UBE2D1
ENSG00000159082.18	-0.003336944	0.733279488	0.970832801	SYNJ1
ENSG00000253537.3	0.012018453	0.733310776	0.970832801	PCDHGA7
ENSG00000138744.16	0.004812815	0.733313267	0.970832801	NAAA
ENSG00000186854.11	-0.00570486	0.733331915	0.970832801	TRABD2A
ENSG00000115239.24	0.002250619	0.733462178	0.970859379	ASB3
ENSG00000111676.15	-0.003565449	0.733506316	0.970859379	ATN1
ENSG00000285280.2	0.007625394	0.733514507	0.970859379	RP11-139E24.1
ENSG00000197858.11	-0.003057986	0.733643765	0.970859379	GPAA1
ENSG00000267422.1	0.007760558	0.733727402	0.970859379	CTD-2554C21.1
ENSG00000170043.12	0.003392899	0.733752653	0.970859379	TRAPPC1
ENSG00000175662.18	0.002269712	0.733833872	0.970859379	TOM1L2
ENSG00000132294.15	0.00360768	0.733884769	0.970859379	EFR3A
ENSG00000141425.18	0.002467715	0.733888931	0.970859379	RPRD1A
ENSG00000248774.1	0.008613877	0.733909328	0.970859379	RP11-798M19.3
ENSG00000082515.18	0.00461737	0.733928344	0.970859379	MRPL22
ENSG00000158716.9	0.00442348	0.733968111	0.970859379	DUSP23
ENSG00000104331.9	0.002450847	0.733975647	0.970859379	BPNT2
ENSG00000135185.12	-0.003389602	0.73407778	0.970859379	TMEM243
ENSG00000263724.2	0.012541342	0.734110927	0.970859379	DLGAP1-AS3
ENSG00000196417.13	-0.004108254	0.734166496	0.970859379	ZNF765

ENSG00000198353.8	-0.008563938	0.734215528	0.970859379	HOXC4
ENSG00000224791.1	0.015427182	0.734243188	0.970859379	KRT18P39
ENSG00000147364.17	0.002025962	0.734251461	0.970859379	FBXO25
ENSG00000135108.15	0.001858385	0.734389532	0.970911587	FBXO21
ENSG00000132466.19	0.002987947	0.734391542	0.970911587	ANKRD17
ENSG00000174804.4	-0.011766546	0.734452318	0.970911587	FZD4
ENSG00000115107.20	0.006714368	0.734480318	0.970911587	STEAP3
ENSG00000171612.7	0.00375469	0.734551056	0.97094251	SLC25A33
ENSG00000250656.2	0.018402616	0.734609927	0.970957746	ST3GAL1P1
ENSG00000206013.2	0.019579344	0.734679671	0.970987348	IFITM5
ENSG00000148634.16	0.002656503	0.734773818	0.970991306	HERC4
ENSG00000198171.13	-0.003151554	0.734777359	0.970991306	DDRGK1
ENSG00000137055.15	0.001747498	0.734960524	0.971170775	PLAA
ENSG00000246560.2	0.007958952	0.735015483	0.971180822	UBE2D3-AS1
ENSG00000196151.11	0.003307339	0.735076884	0.971199377	WDSUB1
ENSG00000154611.15	0.015004738	0.735294122	0.971299062	PSMA8
ENSG00000171067.11	-0.002979303	0.735324525	0.971299062	C11orf24
ENSG00000151151.6	-0.006058541	0.735327438	0.971299062	IPMK
ENSG00000174165.8	-0.002338068	0.735407832	0.971299062	ZDHHC24
ENSG00000155158.20	-0.004829138	0.735431634	0.971299062	TTC39B
ENSG00000203804.5	-0.006733438	0.735436505	0.971299062	ADAMTSL4-AS1
ENSG00000117533.15	-0.003414554	0.735657564	0.971436253	VAMP4
ENSG00000279278.1	-0.006467955	0.735706717	0.971436253	CH17-264L24.1
ENSG00000276698.1	-0.004648869	0.735755041	0.971436253	RP11-468E2.10
ENSG00000141101.13	-0.002261233	0.735769237	0.971436253	NOB1
ENSG00000075945.13	-0.002304113	0.735777225	0.971436253	KIFAP3
ENSG00000288101.1	0.030119827	0.735827759	0.971440432	RP11-596A13.1
ENSG00000170629.14	-0.005423318	0.736077102	0.971628065	DPY19L2P2
ENSG00000270019.1	0.010537184	0.736091866	0.971628065	RP11-141B14.1
ENSG00000283696.1	-0.007033296	0.736112017	0.971628065	RP11-122G18.12
ENSG00000198728.11	0.001858247	0.736203814	0.971666782	LDB1
ENSG00000120868.14	-0.003832306	0.736236109	0.971666782	APAF1
ENSG00000167524.16	-0.003007853	0.736375209	0.971787824	RSKR
ENSG00000269473.1	0.011981184	0.736512336	0.971794308	ZNF132-DT
ENSG00000177947.14	-0.013240509	0.736567035	0.971794308	ODF3
ENSG00000140575.13	0.003489804	0.736616304	0.971794308	IQGAP1
ENSG00000261744.1	-0.009472588	0.736666002	0.971794308	ZFPM1-AS1
ENSG00000164068.16	0.00400762	0.736690474	0.971794308	RNF123
ENSG00000169223.15	-0.002582299	0.736702749	0.971794308	LMAN2
ENSG00000121060.19	0.003113568	0.736711825	0.971794308	TRIM25
ENSG00000109458.8	-0.003884338	0.736800767	0.971819668	GAB1
ENSG00000187510.11	0.014722681	0.736825825	0.971819668	PLEKHG7
ENSG00000229436.1	0.011951325	0.736894641	0.971847928	AC073850.6
ENSG00000122986.14	0.003509779	0.737063147	0.971965565	HVCN1
ENSG00000164543.7	-0.002247219	0.737078626	0.971965565	STK17A
ENSG00000270091.1	0.00396419	0.737241721	0.972028891	RP11-78O7.2
ENSG00000226004.1	-0.019912561	0.73725783	0.972028891	LINC02528

ENSG00000198018.7	-0.002813632	0.737268841	0.972028891	ENTPD7
ENSG00000233084.2	0.011919335	0.737529531	0.972310081	RPL23AP25
ENSG00000158169.13	-0.003126031	0.737655879	0.972310353	FANCC
ENSG00000156521.14	0.002134961	0.737733956	0.972310353	TYSND1
ENSG00000257815.7	-0.003773165	0.737743244	0.972310353	PRANCR
ENSG00000134744.14	-0.002645706	0.737754781	0.972310353	TUT4
ENSG00000263786.1	-0.007025402	0.737846444	0.972310353	RP11-649A18.4
ENSG00000136279.21	-0.001993134	0.737972157	0.972310353	DBNL
ENSG00000105341.19	0.002284253	0.73798095	0.972310353	DMAC2
ENSG00000175040.6	-0.005574161	0.737985513	0.972310353	CHST2
ENSG00000153093.20	0.005677619	0.737997124	0.972310353	ACOXL
ENSG00000065029.15	0.001864102	0.73800385	0.972310353	ZNF76
ENSG00000235478.5	-0.009946681	0.738174364	0.972438068	LINC01664
ENSG00000253616.5	-0.006203035	0.738195623	0.972438068	RP11-875O11.3
ENSG00000137814.12	0.002420701	0.738340467	0.972566401	HAUS2
ENSG00000060140.9	-0.010393348	0.738653912	0.972699488	STYK1
ENSG00000133985.3	-0.005315533	0.738730568	0.972699488	TTC9
ENSG00000228058.2	-0.009352414	0.738900208	0.972699488	LINC01736
ENSG00000158411.11	0.002639593	0.739042961	0.972699488	MITD1
ENSG00000267248.3	0.006788632	0.739051213	0.972699488	CTD-2319I12.4
ENSG00000272752.6	-0.005129374	0.739079512	0.972699488	STAG3L5P-PVRIG2P-PILRB
ENSG00000233369.7	-0.004446866	0.739090326	0.972699488	GTF2IP4
ENSG00000174705.13	-0.008987182	0.739146555	0.972699488	SH3PXD2B
ENSG00000169567.13	0.004535815	0.739150852	0.972699488	HINT1
ENSG00000197608.12	0.002954091	0.739308251	0.972699488	ZNF841
ENSG00000066557.6	0.004792184	0.739335044	0.972699488	LRRC40
ENSG00000138172.11	-0.003218221	0.73942351	0.972699488	CALHM2
ENSG00000145916.19	0.002108644	0.739442415	0.972699488	RMND5B
ENSG00000277879.1	0.008090783	0.739444801	0.972699488	RP11-129M16.4
ENSG00000158122.12	0.002982804	0.739446169	0.972699488	PRXL2C
ENSG00000078369.18	0.001746175	0.739487569	0.972699488	GNB1
ENSG00000214078.13	0.003509615	0.739513492	0.972699488	CPNE1
ENSG00000107758.16	0.00188945	0.739514465	0.972699488	PPP3CB
ENSG00000136206.4	0.005419608	0.739524864	0.972699488	SPDYE1
ENSG00000155229.21	0.001609387	0.739525422	0.972699488	MMS19
ENSG00000168214.21	0.002455619	0.739610078	0.972699488	RBPJ
ENSG00000267082.1	-0.01223556	0.739636698	0.972699488	CTC-510F12.2
ENSG00000266094.8	0.00183405	0.739698319	0.972699488	RASSF5
ENSG00000159210.10	0.002373714	0.739706401	0.972699488	SNF8
ENSG00000105352.10	0.004446406	0.739850569	0.972699488	CEACAM4
ENSG00000272950.1	-0.012750276	0.739866794	0.972699488	RP11-307C18.1
ENSG00000073050.12	-0.002698294	0.739868628	0.972699488	XRCC1
ENSG00000235374.2	0.005816675	0.73987213	0.972699488	SSR4P1
ENSG00000258733.6	0.004220304	0.739924815	0.972699488	LINC02328
ENSG00000140750.17	0.002807728	0.739946121	0.972699488	ARHGAP17
ENSG00000264924.1	-0.009840595	0.73994672	0.972699488	RP11-799B12.2
ENSG00000288538.1	-0.006385916	0.740018044	0.972699488	RP11-705O3.3

ENSG00000266405.3	0.005362901	0.740066818	0.972699488	CBX3P2
ENSG00000282651.2	-0.027809522	0.740107066	0.972699488	IGHV5-10-1
ENSG00000213699.9	-0.00252399	0.74011874	0.972699488	SLC35F6
ENSG00000228727.9	-0.007449864	0.740189861	0.972699488	SAPCD1
ENSG00000182487.12	-0.004995047	0.740208953	0.972699488	NCF1B
ENSG00000273183.1	0.004661781	0.740268956	0.972699488	RP11-5C23.2
ENSG00000276557.1	0.007049213	0.740291282	0.972699488	TRBV18
ENSG00000182902.14	-0.006575162	0.74041268	0.972796671	SLC25A18
ENSG00000288025.1	0.010839401	0.740591544	0.972969339	RP11-489G11.6
ENSG00000092067.6	-0.007791106	0.740748751	0.972998651	CEBPE
ENSG00000099889.14	-0.00704401	0.740796638	0.972998651	ARVCF
ENSG00000246223.9	0.004884889	0.740813761	0.972998651	LINC01550
ENSG00000274736.5	-0.015151244	0.740828063	0.972998651	CCL23
ENSG00000080823.23	0.006787856	0.740950769	0.972998651	MOK
ENSG00000285979.1	-0.006201093	0.741046368	0.972998651	RP11-407G23.8
ENSG00000280334.1	-0.006053666	0.74105499	0.972998651	RP11-361L15.5
ENSG00000149218.5	0.003578686	0.741081143	0.972998651	ENDOD1
ENSG00000135912.11	-0.003169907	0.741093949	0.972998651	TTLL4
ENSG00000228223.3	0.004592425	0.741111414	0.972998651	HCG11
ENSG00000186047.11	0.005721436	0.741135748	0.972998651	DLEU7
ENSG00000286808.1	-0.004732992	0.741226911	0.973056043	RP11-21M24.6
ENSG00000128815.19	-0.004499376	0.741600708	0.973360119	WDFY4
ENSG00000236234.1	0.009008803	0.741715762	0.973360119	AC091132.1
ENSG00000166619.15	0.001582133	0.741732729	0.973360119	BLCAP
ENSG00000271267.1	-0.009012007	0.741747585	0.973360119	RP11-29H23.7
ENSG00000135094.11	-0.008761142	0.741753714	0.973360119	SDS
ENSG00000198373.13	-0.002800615	0.741882973	0.973360119	WWP2
ENSG00000119383.21	-0.002901575	0.741891817	0.973360119	PTPA
ENSG00000247595.4	0.008295642	0.741903109	0.973360119	SPTY2D1OS
ENSG00000156875.14	-0.005158647	0.741918291	0.973360119	MFSD14A
ENSG00000273230.1	0.004911709	0.742098051	0.973360119	RP11-1246C19.1
ENSG00000131944.10	-0.005315135	0.742127507	0.973360119	FAAP24
ENSG00000100650.16	-0.001781501	0.742162517	0.973360119	SRSF5
ENSG00000208772.1	-0.009001593	0.742186877	0.973360119	SNORD94
ENSG00000099290.17	-0.002773586	0.742222725	0.973360119	WASHC2A
ENSG00000283208.2	0.01012781	0.742280572	0.973360119	RP11-226E21.4
ENSG00000124196.6	0.013053935	0.742308226	0.973360119	GTSF1L
ENSG00000110025.13	0.003558051	0.742322897	0.973360119	SNX15
ENSG00000168303.9	-0.002579277	0.742400405	0.973360119	MPLKIP
ENSG00000073670.14	-0.00698093	0.742446188	0.973360119	ADAM11
ENSG00000110619.18	-0.001836228	0.742447554	0.973360119	CARS1
ENSG00000137076.21	-0.004217761	0.742455253	0.973360119	TLN1
ENSG00000276148.1	0.00579268	0.742527697	0.973392866	RP11-278C7.4
ENSG00000170892.13	0.00328106	0.742698283	0.97345925	TSEN34
ENSG00000272763.1	0.017079663	0.742704127	0.97345925	RP11-357H14.17
ENSG00000167767.14	-0.011671402	0.74275736	0.97345925	KRT80
ENSG00000184117.12	-0.002080471	0.742852469	0.97345925	NIPSNAP1

ENSG00000260941.1	-0.010629224	0.742854757	0.97345925	LINC00622
ENSG00000110074.11	0.002995101	0.742863139	0.97345925	FOXRED1
ENSG00000157954.15	-0.001938015	0.743012593	0.97351858	WIPI2
ENSG00000272669.1	0.004815258	0.743021057	0.97351858	RP3-508I15.21
ENSG00000006831.10	-0.001609477	0.743072486	0.97351858	ADIPOR2
ENSG00000103502.14	0.002780651	0.743107377	0.97351858	CDIPT
ENSG00000182934.12	-0.00187248	0.743145766	0.97351858	SRPRA
ENSG00000167005.14	-0.003627615	0.743262575	0.973562571	NUDT21
ENSG00000142408.7	0.009933113	0.743274291	0.973562571	CACNG8
ENSG00000185950.9	0.005184682	0.743381613	0.973640958	IRS2
ENSG00000231102.1	0.009996812	0.743502645	0.973693925	RP11-298J23.5
ENSG00000263327.6	0.004384391	0.743546563	0.973693925	TAPT1-AS1
ENSG00000143418.20	-0.001902368	0.743594994	0.973693925	CERS2
ENSG00000212719.13	0.005119024	0.743748883	0.973693925	LINC02693
ENSG00000156599.11	0.001876636	0.743789197	0.973693925	ZDHHC5
ENSG00000227500.10	-0.002800855	0.743790135	0.973693925	SCAMP4
ENSG00000236255.2	-0.004016875	0.743813069	0.973693925	AC009404.2
ENSG00000138134.12	0.003482125	0.743915581	0.973693925	STAMBPL1
ENSG00000166770.11	-0.008257262	0.743952904	0.973693925	ZNF667-AS1
ENSG00000139645.11	-0.002043044	0.743963141	0.973693925	ANKRD52
ENSG00000235908.1	-0.008353041	0.743974996	0.973693925	RHOA-IT1
ENSG00000035720.8	-0.006656087	0.743991798	0.973693925	STAP1
ENSG00000259877.2	-0.004543471	0.74421692	0.973791491	RP11-46C24.7
ENSG00000211662.2	0.014135125	0.744231901	0.973791491	IGLV3-21
ENSG00000287642.1	0.013625628	0.744257549	0.973791491	RP11-206F17.3
ENSG00000164008.16	-0.003412192	0.744307847	0.973791491	C1orf50
ENSG00000287583.1	-0.015590822	0.744360504	0.973791491	RP11-455M16.1
ENSG00000135297.17	0.001805783	0.744474224	0.973791491	MTO1
ENSG00000107938.18	-0.002361941	0.744485056	0.973791491	EDRF1
ENSG00000184402.15	-0.002896251	0.744553394	0.973791491	SS18L1
ENSG00000132109.10	0.00343189	0.744562783	0.973791491	TRIM21
ENSG00000171206.15	-0.002676724	0.744591998	0.973791491	TRIM8
ENSG00000162639.16	-0.003450521	0.744593727	0.973791491	HENMT1
ENSG00000155329.12	0.005840862	0.744711691	0.973791491	ZCCHC10
ENSG00000175575.13	0.002847548	0.744837051	0.973791491	PAAF1
ENSG00000279632.1	0.012976203	0.744938528	0.973791491	RP11-286N22.6
ENSG00000065809.14	0.002328327	0.744951122	0.973791491	FAM107B
ENSG00000214026.11	-0.003334749	0.744956753	0.973791491	MRPL23
ENSG00000110090.13	-0.003957308	0.745081902	0.973791491	CPT1A
ENSG00000136490.9	0.002778236	0.745161519	0.973791491	LIMD2
ENSG00000228192.8	-0.006853886	0.745200217	0.973791491	RP11-342M1.3
ENSG00000155970.12	0.011162631	0.745250591	0.973791491	MICU3
ENSG00000165879.9	-0.003383859	0.745495541	0.973791491	FRAT1
ENSG00000138834.14	-0.002832834	0.745753947	0.973791491	MAPK8IP3
ENSG00000259803.8	-0.013625143	0.745972299	0.973791491	SLC22A31
ENSG00000236213.2	-0.006156593	0.746067342	0.973791491	AC006369.2
ENSG00000180626.10	0.006282722	0.746116167	0.973791491	ZNF594

ENSG00000167747.15	0.003363133	0.746162481	0.973791491	C19orf48
ENSG00000127507.18	0.005365549	0.746199066	0.973791491	ADGRE2
ENSG00000123815.13	-0.002137746	0.74627445	0.973791491	COQ8B
ENSG00000115234.11	0.002445734	0.746276247	0.973791491	SNX17
ENSG00000120837.8	-0.005317655	0.746289362	0.973791491	NFYB
ENSG00000179271.3	0.003865524	0.746308041	0.973791491	GADD45GIP1
ENSG00000223803.1	0.012275355	0.746357507	0.973791491	RPS20P14
ENSG00000224114.1	0.021956021	0.746491684	0.973791491	RP11-343H5.4
ENSG00000286151.1	0.015497122	0.746506976	0.973791491	RP11-217A12.2
ENSG00000188460.4	0.011292147	0.74651291	0.973791491	ACTBP11
ENSG00000119862.13	0.006031087	0.746563538	0.973791491	LGALSL
ENSG00000185361.9	0.002494159	0.746612139	0.973791491	TNFAIP8L1
ENSG00000166224.17	-0.001936819	0.746642948	0.973791491	SGPL1
ENSG00000272636.4	-0.016542079	0.746643493	0.973791491	DOC2B
ENSG00000164850.15	0.008528238	0.746644162	0.973791491	GPER1
ENSG00000137713.16	-0.002096253	0.74665401	0.973791491	PPP2R1B
ENSG00000134294.14	0.00359686	0.746658086	0.973791491	SLC38A2
ENSG00000146350.14	-0.005188631	0.746729468	0.973791491	TBC1D32
ENSG00000278615.5	-0.006741589	0.746788374	0.973791491	C11orf98
ENSG00000272037.1	0.004181211	0.746801991	0.973791491	KB-431C1.5
ENSG00000273576.1	-0.002766607	0.746825826	0.973791491	RP11-390P24.1
ENSG00000133962.8	-0.006417738	0.746872714	0.973791491	CATSPERB
ENSG00000104047.15	0.00293521	0.746882288	0.973791491	DTWD1
ENSG00000100207.21	0.002746197	0.746905601	0.973791491	TCF20
ENSG00000095464.10	-0.005712215	0.746907223	0.973791491	PDE6C
ENSG00000272264.1	0.009082656	0.746937898	0.973791491	RP11-92K15.3
ENSG00000147862.17	0.016067438	0.746955196	0.973791491	NFIB
ENSG00000274828.1	-0.004358571	0.747169353	0.973791491	RP11-567M16.6
ENSG00000070061.16	-0.001863998	0.747179335	0.973791491	ELP1
ENSG00000174109.5	0.003318738	0.747198046	0.973791491	C16orf91
ENSG00000113790.11	0.005923775	0.747202565	0.973791491	EHHADH
ENSG00000187942.12	0.008955859	0.747240752	0.973791491	LDLRAD2
ENSG00000127903.14	0.007377002	0.747261534	0.973791491	ZNF835
ENSG00000143013.13	0.00204667	0.747295021	0.973791491	LMO4
ENSG00000257511.1	-0.005803131	0.747346086	0.973791491	RP11-278C7.1
ENSG00000114439.19	0.002668885	0.747364605	0.973791491	BBX
ENSG00000266980.1	-0.008696093	0.747380558	0.973791491	RP11-552F3.4
ENSG00000270231.4	-0.006605367	0.747384188	0.973791491	NBPF8
ENSG00000100106.22	0.002859418	0.7474787	0.973791491	TRIOBP
ENSG00000243364.8	-0.002637853	0.747489022	0.973791491	EFNA4
ENSG00000163053.11	0.009302887	0.747516721	0.973791491	SLC16A14
ENSG00000147813.16	-0.005334574	0.747554357	0.973791491	NAPRT
ENSG00000226987.1	-0.008835353	0.74771938	0.973791491	RP11-544A12.5
ENSG00000135968.21	0.004101761	0.747740961	0.973791491	GCC2
ENSG00000284657.1	0.009382297	0.747762751	0.973791491	RP3-465N24.8
ENSG00000206249.3	-0.014232278	0.747769199	0.973791491	CTD-2308N23.2
ENSG00000138780.15	-0.004618895	0.747783668	0.973791491	GSTCD

ENSG00000258760.1	0.014589398	0.747900158	0.973791491	CTD-2509G16.5
ENSG00000149761.9	-0.002778225	0.747905904	0.973791491	NUDT22
ENSG00000010626.15	0.004412317	0.747915868	0.973791491	LRRC23
ENSG00000185344.15	-0.002121534	0.747948422	0.973791491	ATP6V0A2
ENSG00000090621.15	0.002096768	0.748039922	0.973791491	PABPC4
ENSG00000269956.2	0.00547825	0.748060126	0.973791491	MKNK1-AS1
ENSG00000235781.1	-0.013072654	0.748060293	0.973791491	LINC02569
ENSG00000143333.7	-0.007478215	0.748094273	0.973791491	RGS16
ENSG00000134152.11	0.004603579	0.748121111	0.973791491	KATNBL1
ENSG00000091409.16	0.00391811	0.748195713	0.973791491	ITGA6
ENSG00000237626.2	0.029140642	0.74822014	0.973791491	RP11-406A20.1
ENSG00000119812.19	0.002418887	0.748271506	0.973791491	FAM98A
ENSG00000101132.10	-0.010023599	0.748280788	0.973791491	PFDN4
ENSG00000168081.9	0.007092036	0.748325011	0.973791491	PNOC
ENSG00000141098.13	0.002246088	0.748329841	0.973791491	GFOD2
ENSG00000108506.13	-0.00359741	0.748453312	0.973791491	INTS2
ENSG00000105993.15	0.002289109	0.748477794	0.973791491	DNAJB6
ENSG00000213585.11	0.002298883	0.748489915	0.973791491	VDAC1
ENSG00000211637.2	0.011366215	0.748497444	0.973791491	IGLV4-69
ENSG00000137727.13	-0.014477042	0.748569966	0.973791491	ARHGAP20
ENSG00000170584.11	0.003180293	0.748579064	0.973791491	NUDCD2
ENSG00000125611.15	-0.003595442	0.74864479	0.973791491	CHCHD5
ENSG00000204054.14	0.003642173	0.748730517	0.973791491	LINC00963
ENSG00000272030.1	0.009852933	0.748805124	0.973791491	RP1-178F15.4
ENSG00000168502.17	0.009225643	0.748855433	0.973791491	MTCL1
ENSG00000237471.2	0.011083704	0.748942615	0.973791491	AC073115.6
ENSG00000229644.6	0.011298188	0.748960543	0.973791491	NAMPTP1
ENSG00000179922.6	-0.003159405	0.748961462	0.973791491	ZNF784
ENSG00000013364.19	-0.002711553	0.749026263	0.973791491	MVP
ENSG00000234389.1	0.012807372	0.749083238	0.973791491	AC007278.3
ENSG00000247077.7	-0.001906217	0.749097274	0.973791491	PGAM5
ENSG00000213236.3	0.008519708	0.749147767	0.973791491	YWHAZP2
ENSG00000186260.17	-0.003593994	0.749187765	0.973791491	MRTFB
ENSG00000161860.8	0.005190296	0.749193929	0.973791491	SYCE2
ENSG00000243466.1	-0.011621987	0.749220478	0.973791491	IGKV1-5
ENSG00000176438.13	0.002506235	0.749244125	0.973791491	SYNE3
ENSG00000223745.8	0.003994594	0.749252508	0.973791491	CCDC18-AS1
ENSG00000248932.6	0.0055558	0.749289532	0.973791491	RP11-319G6.1
ENSG00000133030.22	-0.002248864	0.749529045	0.973898296	MPRIP
ENSG00000129250.12	-0.002203545	0.749539126	0.973898296	KIF1C
ENSG00000047932.14	0.003677809	0.749636517	0.973898296	GOPC
ENSG00000280091.1	0.009365422	0.749685236	0.973898296	CTC-312O10.3
ENSG00000260898.6	0.008399143	0.74975056	0.973898296	ADPGK-AS1
ENSG00000108523.16	-0.002591333	0.749803764	0.973898296	RNF167
ENSG00000156345.18	0.003291575	0.749850162	0.973898296	CDK20
ENSG00000229727.7	0.009153101	0.749878127	0.973898296	AC013460.1
ENSG00000164440.15	0.006514841	0.749926184	0.973898296	TXLNB

ENSG00000132182.12	0.002330762	0.749939567	0.973898296	NUP210
ENSG00000236200.6	-0.004217333	0.749947216	0.973898296	KDM4A-AS1
ENSG00000103047.8	-0.002106175	0.749973844	0.973898296	TANGO6
ENSG00000056972.20	0.002894951	0.750091206	0.973898296	TRAF3IP2
ENSG00000067182.8	-0.002815272	0.75014825	0.973898296	TNFRSF1A
ENSG00000107643.16	-0.002634603	0.75015457	0.973898296	MAPK8
ENSG00000140548.10	-0.003073239	0.750208211	0.973898296	ZNF710
ENSG00000197535.15	-0.002791275	0.750229171	0.973898296	MYO5A
ENSG00000151789.12	0.011048973	0.750273286	0.973898296	ZNF385D
ENSG00000260070.1	0.009810487	0.750273999	0.973898296	RP11-182J23.1
ENSG00000201499.1	-0.011741038	0.750353194	0.973939449	RNU6-312P
ENSG00000205221.12	0.009984132	0.7504292	0.973939449	VIT
ENSG00000212371.1	0.011295796	0.750637295	0.973939449	ENSG10010139899.1
ENSG00000162241.13	-0.002512877	0.750754646	0.973939449	SLC25A45
ENSG00000151575.14	-0.010975283	0.750773394	0.973939449	TEX9
ENSG00000278112.1	-0.008307999	0.750833078	0.973939449	RP11-972P1.11
ENSG00000132669.14	-0.004275516	0.750951837	0.973939449	RIN2
ENSG00000203739.4	0.003156781	0.750973169	0.973939449	PRDX6-AS1
ENSG00000163743.14	0.002997626	0.751004806	0.973939449	RCHY1
ENSG00000272931.1	-0.006611836	0.751074377	0.973939449	LRRC8D-DT
ENSG00000094975.14	0.003896795	0.75108875	0.973939449	SUCO
ENSG00000253519.1	0.009232098	0.751092469	0.973939449	AC106801.1
ENSG00000130294.18	0.018206487	0.751096435	0.973939449	KIF1A
ENSG00000233122.2	0.004909236	0.751113498	0.973939449	CTAGE7P
ENSG00000135074.16	0.00354694	0.751134597	0.973939449	ADAM19
ENSG00000054148.18	-0.003480847	0.751184706	0.973939449	PHPT1
ENSG00000214223.4	-0.009740265	0.751195431	0.973939449	HNRNPA1P10
ENSG00000213967.11	0.006502682	0.751275821	0.973939449	ZNF726
ENSG00000127445.14	-0.002303257	0.751282098	0.973939449	PIN1
ENSG00000260186.5	-0.040948031	0.751286159	0.973939449	LINC02137
ENSG00000109065.12	0.002342241	0.751309386	0.973939449	NAT9
ENSG00000132517.15	-0.008411642	0.751350499	0.973939449	SLC52A1
ENSG00000143450.19	-0.005796104	0.7514024	0.973945166	OAZ3
ENSG00000100372.15	-0.002035083	0.751528673	0.974047275	SLC25A17
ENSG00000266918.1	0.016162581	0.75159485	0.974071485	RP11-798G7.8
ENSG00000122674.12	0.004707237	0.75166228	0.974097317	CCZ1
ENSG00000260095.1	-0.013194542	0.751813022	0.974154485	RP11-715J22.3
ENSG00000163832.16	0.002271541	0.751834506	0.974154485	ELP6
ENSG00000224689.9	0.052315855	0.751848898	0.974154485	ZNF812P
ENSG00000158859.10	-0.005454666	0.751966584	0.974231995	ADAMTS4
ENSG00000279348.1	-0.004389478	0.752033772	0.974231995	RP11-566E18.1
ENSG00000143498.18	-0.004008719	0.752071823	0.974231995	TAF1A
ENSG00000114125.14	0.002696705	0.752098739	0.974231995	RNF7
ENSG00000154736.6	-0.008451948	0.752149339	0.974236004	ADAMTS5
ENSG00000164828.18	-0.00237882	0.752241621	0.974255748	SUN1
ENSG00000275441.1	-0.011411979	0.752259595	0.974255748	RP11-666O2.2
ENSG00000280202.1	-0.004366265	0.752368875	0.974335747	RP5-1180D12.1

ENSG00000245317.2	-0.00867506	0.75247081	0.974406224	CTC-241N9.1
ENSG00000005483.21	-0.003173013	0.752566576	0.974406307	KMT2E
ENSG00000285830.1	0.0085539	0.752714968	0.974406307	RP11-545M17.4
ENSG00000126106.14	-0.003383592	0.752732925	0.974406307	TMEM53
ENSG00000138688.16	-0.003748789	0.752800067	0.974406307	KIAA1109
ENSG00000221184.1	-0.011279925	0.752819238	0.974406307	ENSG00000221184
ENSG00000153560.12	0.001636056	0.752864301	0.974406307	UBP1
ENSG00000260325.2	0.009507392	0.752872003	0.974406307	HSPB9
ENSG00000175061.18	0.004304938	0.752949543	0.974406307	SNHG29
ENSG00000197256.11	0.009218169	0.752958916	0.974406307	KANK2
ENSG00000159388.6	0.002239408	0.753131677	0.974406307	BTG2
ENSG00000205268.11	0.002618753	0.753182313	0.974406307	PDE7A
ENSG00000105219.10	-0.011940193	0.753237815	0.974406307	CCNP
ENSG00000158079.16	-0.004018348	0.753391849	0.974406307	PTPDC1
ENSG00000186603.7	-0.009320965	0.753415416	0.974406307	HPDL
ENSG00000115112.8	0.012611964	0.753510121	0.974406307	TFCP2L1
ENSG00000074071.15	0.003232397	0.753512253	0.974406307	MRPS34
ENSG00000100991.12	0.001891395	0.753589602	0.974406307	TRPC4AP
ENSG00000130643.9	0.008799485	0.753633065	0.974406307	CALY
ENSG00000119522.16	-0.003159026	0.753656391	0.974406307	DENND1A
ENSG00000228496.1	0.055553519	0.753741154	0.974406307	AC106875.1
ENSG00000102984.15	-0.003258408	0.753745892	0.974406307	ZNF821
ENSG00000128908.17	-0.001864583	0.753806885	0.974406307	INO80
ENSG00000248302.3	-0.014970134	0.753818958	0.974406307	BNIP3P41
ENSG00000104497.14	0.006250606	0.753829752	0.974406307	SNX16
ENSG00000213160.10	-0.007269243	0.753872294	0.974406307	KLHL23
ENSG00000278616.1	0.010732652	0.753885761	0.974406307	BEND3P3
ENSG00000119777.20	-0.002394062	0.753886509	0.974406307	TMEM214
ENSG00000165702.15	0.00617639	0.753924931	0.974406307	GFI1B
ENSG00000225507.1	-0.004473909	0.75401158	0.974406307	AC069282.6
ENSG00000214846.4	-0.005469552	0.754039989	0.974406307	RP11-115L11.1
ENSG00000179454.14	-0.004277935	0.754058676	0.974406307	KLHL28
ENSG00000211727.3	0.008546383	0.754174722	0.974406307	TRBV7-6
ENSG00000146282.18	0.002604156	0.754180031	0.974406307	RARS2
ENSG00000166912.17	0.002587689	0.754204572	0.974406307	MTMR10
ENSG00000055950.16	0.002492026	0.754204689	0.974406307	MRPL43
ENSG00000083896.13	-0.001426463	0.754331914	0.974406307	YTHDC1
ENSG00000127554.13	-0.003143459	0.754365645	0.974406307	GFER
ENSG00000186889.10	0.012225166	0.754476495	0.974406307	TMEM17
ENSG00000288076.1	-0.022260971	0.754479481	0.974406307	RP11-16G12.1
ENSG00000090530.10	0.007964225	0.754535318	0.974406307	P3H2
ENSG00000066926.13	-0.006559871	0.754603499	0.974406307	FECH
ENSG00000278558.5	-0.018246307	0.754608337	0.974406307	TMEM191B
ENSG00000233967.7	-0.006777175	0.754640646	0.974406307	RP11-250B2.3
ENSG00000103550.14	-0.002709899	0.754664964	0.974406307	KNOP1
ENSG00000124243.19	-0.004284544	0.754685772	0.974406307	BCAS4
ENSG00000258674.5	0.007732739	0.754713151	0.974406307	CTC-260F20.3

ENSG00000211651.3	0.012519308	0.754733086	0.974406307	IGLV1-44
ENSG00000213593.10	0.002181683	0.754795319	0.974406307	TMX2
ENSG00000245275.8	-0.003082952	0.754840086	0.974406307	SAP30L-AS1
ENSG00000197632.9	-0.00705025	0.754846548	0.974406307	SERPINB2
ENSG00000169871.13	-0.002274906	0.755211872	0.974816533	TRIM56
ENSG00000261693.1	0.012803046	0.755344204	0.974925983	RP13-467H17.1
ENSG00000243708.11	-0.007522076	0.75543756	0.974937733	PLA2G4B
ENSG00000135040.16	0.002370929	0.755492903	0.974937733	NAA35
ENSG00000211798.3	0.007693198	0.755495926	0.974937733	TRAV18
ENSG00000196562.14	0.003738329	0.755552685	0.97494963	SULF2
ENSG00000260135.7	-0.027459799	0.755640498	0.975001594	MMP2-AS1
ENSG00000265413.1	0.014826546	0.755815662	0.975128271	RP11-789C17.1
ENSG00000272506.1	0.009249391	0.755857508	0.975128271	RP4-535B20.4
ENSG00000169435.14	0.012892493	0.755881321	0.975128271	RASSF6
ENSG00000134982.17	0.00312522	0.75600039	0.97522053	APC
ENSG00000121988.18	-0.003819143	0.756101176	0.975289195	ZRANB3
ENSG00000121644.19	-0.002692776	0.756291701	0.975473597	DESI2
ENSG00000185345.23	0.008172775	0.756344874	0.97548083	PRKN
ENSG00000079335.20	0.004143634	0.756735448	0.975628429	CDC14A
ENSG00000248265.2	-0.013724244	0.756821853	0.975628429	FLJ12825
ENSG00000223886.3	0.009533132	0.756996335	0.975628429	RP11-251G23.2
ENSG00000173852.15	-0.003727841	0.757052907	0.975628429	DPY19L1
ENSG00000186567.13	0.00599282	0.757108544	0.975628429	CEACAM19
ENSG00000157315.5	-0.006609157	0.757123197	0.975628429	TMED6
ENSG00000074706.13	0.002164198	0.757192357	0.975628429	IPCEF1
ENSG00000177030.18	0.002415916	0.757195439	0.975628429	DEAF1
ENSG00000065665.21	0.002626717	0.757353925	0.975628429	SEC61A2
ENSG00000070761.8	0.001975781	0.757360352	0.975628429	CFAP20
ENSG00000198911.12	-0.002298067	0.75736981	0.975628429	SREBF2
ENSG00000270392.2	-0.008406853	0.757447347	0.975628429	PFN1P2
ENSG00000179715.13	-0.00387306	0.757564253	0.975628429	PCED1B
ENSG00000186074.19	0.002548644	0.757598321	0.975628429	CD300LF
ENSG00000286389.1	0.006167267	0.75762567	0.975628429	RP3-451C13.1
ENSG00000265478.2	0.009032055	0.757791142	0.975628429	CTD-2145A24.5
ENSG00000269837.1	0.003012007	0.757840373	0.975628429	IPO5P1
ENSG00000267383.7	-0.007335014	0.757856968	0.975628429	CTC-260E6.6
ENSG00000255585.3	0.005830392	0.757857364	0.975628429	RP11-188C12.2
ENSG00000109323.11	-0.001906078	0.757870989	0.975628429	MANBA
ENSG00000213066.13	0.003081293	0.757900368	0.975628429	CEP43
ENSG00000143801.18	-0.005501388	0.757914647	0.975628429	PSEN2
ENSG00000279106.1	0.007508834	0.757926236	0.975628429	RP11-426C22.1
ENSG00000078618.22	0.001825393	0.757966753	0.975628429	NRDC
ENSG00000125864.14	-0.008401336	0.758008324	0.975628429	BFSP1
ENSG00000100116.17	-0.010472089	0.758068098	0.975628429	GCAT
ENSG00000068489.13	0.003700975	0.758091068	0.975628429	PRR11
ENSG00000249186.2	0.013009555	0.758180465	0.975628429	RP11-423H2.5
ENSG00000157349.17	-0.00215612	0.758195582	0.975628429	DDX19B

ENSG00000143740.15	-0.001966624	0.758232446	0.975628429	SNAP47
ENSG00000177462.9	-0.017179575	0.758238266	0.975628429	OR2T8
ENSG00000277491.1	0.009153484	0.758353318	0.975628429	RP11-676J12.9
ENSG00000165516.11	0.00235426	0.758409115	0.975628429	KLHDC2
ENSG00000227354.7	-0.007480228	0.758434076	0.975628429	RBM26-AS1
ENSG00000267216.1	-0.007806279	0.758462094	0.975628429	ZNF8-ERVK3-1
ENSG00000197549.9	-0.01225388	0.758521593	0.975628429	PRAMENP
ENSG00000243056.2	-0.004089868	0.758529786	0.975628429	EIF4EBP3
ENSG00000107147.14	-0.007620604	0.758534885	0.975628429	KCNT1
ENSG00000133740.11	-0.003494933	0.758537864	0.975628429	E2F5
ENSG00000142405.22	0.002897802	0.758680016	0.975628429	NLRP12
ENSG00000266718.2	0.007497084	0.75870706	0.975628429	RP11-466A19.1
ENSG00000073146.16	-0.018940131	0.758742477	0.975628429	MOV10L1
ENSG00000163463.13	-0.00237041	0.758771562	0.975628429	KRTCAP2
ENSG00000166197.17	-0.001900528	0.758792283	0.975628429	NOLC1
ENSG00000134049.6	0.003730031	0.758793533	0.975628429	IER3IP1
ENSG00000169375.16	-0.002237054	0.758797996	0.975628429	SIN3A
ENSG00000167625.11	-0.002281187	0.758823299	0.975628429	ZNF526
ENSG00000262115.1	-0.011699756	0.758976948	0.975628429	RP11-455O6.2
ENSG00000273344.1	0.003058242	0.759038081	0.975628429	PAXIP1-DT
ENSG00000115841.21	0.004153551	0.759050086	0.975628429	RMDN2
ENSG00000122641.11	-0.020080103	0.759100683	0.975628429	INHBA
ENSG00000141446.11	0.003992253	0.759183636	0.975628429	ESCO1
ENSG00000238228.1	-0.008253675	0.759254122	0.975628429	OR7E7P
ENSG00000100083.19	0.002053506	0.759256678	0.975628429	GGA1
ENSG00000185670.9	-0.00218663	0.759294447	0.975628429	ZBTB3
ENSG00000261079.1	-0.012453563	0.759294496	0.975628429	RP11-252A24.3
ENSG00000238198.2	-0.007949534	0.759409173	0.975628429	LRIG2-DT
ENSG00000064703.13	-0.001473615	0.759474012	0.975628429	DDX20
ENSG00000275401.1	-0.010232994	0.759493846	0.975628429	RP4-564F22.7
ENSG00000183684.8	-0.002899823	0.759520647	0.975628429	ALYREF
ENSG00000240376.1	0.007831928	0.759598777	0.975628429	RP11-36C20.1
ENSG00000074695.6	-0.002960211	0.759600445	0.975628429	LMAN1
ENSG00000139637.14	0.006326291	0.759611972	0.975628429	MYG1
ENSG00000174007.9	0.003806895	0.759677579	0.975628429	CEP19
ENSG00000279948.1	-0.008302447	0.759811653	0.975628429	CTD-2233K9.1
ENSG00000263235.1	-0.006917463	0.759831405	0.975628429	RP11-461A8.4
ENSG00000078403.17	0.002463743	0.759832119	0.975628429	MLLT10
ENSG00000204710.2	-0.02028626	0.759929413	0.975628429	SPDYC
ENSG00000074181.9	-0.009845518	0.75994399	0.975628429	NOTCH3
ENSG00000283064.1	-0.006838891	0.759961009	0.975628429	RP1-221C16.8
ENSG00000136319.12	0.00210143	0.76001058	0.975628429	TTC5
ENSG00000120899.18	0.002031171	0.760022435	0.975628429	PTK2B
ENSG00000129654.8	-0.009773299	0.760044537	0.975628429	FOXJ1
ENSG00000283445.1	0.021688359	0.760068971	0.975628429	RP11-63G10.4
ENSG00000115306.16	-0.003753652	0.760083861	0.975628429	SPTBN1
ENSG00000050405.13	0.003313589	0.760172595	0.975628429	LIMA1

ENSG00000198815.9	0.001809547	0.76019593	0.975628429	FOXJ3
ENSG00000174428.18	-0.002783784	0.76034152	0.975628429	GTF2IRD2B
ENSG00000108423.15	0.002521122	0.760343349	0.975628429	TUBD1
ENSG00000141867.19	-0.002317418	0.760389843	0.975628429	BRD4
ENSG00000197580.13	0.005395144	0.760410659	0.975628429	BCO2
ENSG00000135999.12	0.002643232	0.760474205	0.975628429	EPC2
ENSG00000131370.16	0.003505848	0.760611104	0.975628429	SH3BP5
ENSG00000158296.14	0.008767942	0.760619432	0.975628429	SLC13A3
ENSG00000250508.1	0.010358624	0.760634041	0.975628429	LINC02701
ENSG00000037637.11	0.001585173	0.760658347	0.975628429	FBXO42
ENSG00000188921.14	0.002764159	0.760663148	0.975628429	HACD4
ENSG00000186188.11	-0.009797688	0.760670073	0.975628429	FFAR4
ENSG00000134072.11	-0.004029396	0.760810034	0.975628429	CAMK1
ENSG00000281706.3	-0.005405503	0.76090016	0.975628429	LINC01012
ENSG00000238142.2	-0.00667566	0.760917767	0.975628429	RP11-108M9.4
ENSG00000184203.8	0.00218639	0.760933287	0.975628429	PPP1R2
ENSG00000227217.1	0.013565476	0.761022219	0.975628429	RP11-367J7.3
ENSG00000128965.13	0.012717834	0.761085082	0.975628429	CHAC1
ENSG00000177575.13	-0.004752027	0.76108518	0.975628429	CD163
ENSG00000007171.18	0.016809625	0.761086022	0.975628429	NOS2
ENSG00000138036.18	0.006361995	0.761185878	0.975628429	DYNC2LI1
ENSG00000272791.1	-0.009075981	0.761213793	0.975628429	RP11-464F9.22
ENSG00000288700.1	-0.004135572	0.761220474	0.975628429	RP11-22E12.2
ENSG00000129646.16	0.004184297	0.761226263	0.975628429	QRICH2
ENSG00000118482.12	0.002382222	0.761284762	0.975628429	PHF3
ENSG00000273432.1	0.007478036	0.76138043	0.975628429	RP5-1165K10.2
ENSG00000068650.19	-0.003215673	0.761395736	0.975628429	ATP11A
ENSG00000287236.1	-0.010948642	0.761406915	0.975628429	LL22NC01-146D10.5
ENSG00000174989.13	-0.003211654	0.76151713	0.975708691	FBXW8
ENSG00000243667.7	-0.003109181	0.761646276	0.97574193	WDR92
ENSG00000228606.3	-0.006158504	0.761661565	0.97574193	RP11-574F21.2
ENSG00000244575.3	-0.012725514	0.761692371	0.97574193	IGKV1-27
ENSG00000162723.10	-0.017021243	0.761733387	0.97574193	SLAMF9
ENSG00000287367.1	0.00980765	0.761894739	0.975775053	CTD-2591A1.2
ENSG00000272416.1	0.007947286	0.761901383	0.975775053	CTD-2081C10.7
ENSG00000164849.10	-0.007898634	0.761901986	0.975775053	GPR146
ENSG00000214851.5	-0.009452738	0.761983861	0.975818972	LINC00612
ENSG00000138614.15	-0.001614143	0.762070424	0.975863849	INTS14
ENSG00000283486.2	-0.0065153	0.762140136	0.975863849	FAM95C
ENSG00000153485.6	0.00249026	0.762161657	0.975863849	TMEM251
ENSG00000114120.14	0.003488112	0.762248222	0.975866564	SLC25A36
ENSG00000092330.18	-0.001716869	0.762258947	0.975866564	TINF2
ENSG00000267278.6	-0.003414698	0.762466635	0.97607152	MAP3K14-AS1
ENSG00000125846.16	0.001977013	0.762539952	0.976100683	ZNF133
ENSG00000053328.9	0.012078387	0.762662506	0.976100683	METTL24
ENSG00000259062.2	0.011305483	0.762678771	0.976100683	ACTN1-AS1
ENSG00000099957.16	0.008085984	0.762687292	0.976100683	P2RX6

ENSG00000141179.14	0.002760208	0.762757947	0.976100683	PCTP
ENSG00000187010.21	0.020820622	0.762788615	0.976100683	RHD
ENSG00000125384.7	-0.002654369	0.762833795	0.976100683	PTGER2
ENSG00000163682.17	0.007270515	0.762929489	0.976100683	RPL9
ENSG00000272153.1	-0.003512106	0.763005566	0.976100683	RP1-286D6.5
ENSG00000203799.13	0.008659295	0.763006248	0.976100683	CCDC162P
ENSG00000008517.19	-0.004961431	0.763026414	0.976100683	IL32
ENSG00000166743.10	-0.007069053	0.763111627	0.976100683	ACSM1
ENSG00000212607.1	0.008803573	0.763223821	0.976100683	SNORA3B
ENSG00000111834.13	0.007543591	0.763278387	0.976100683	RSPH4A
ENSG00000122223.13	0.002965742	0.763292418	0.976100683	CD244
ENSG00000151640.13	-0.01591257	0.763309008	0.976100683	DPYSL4
ENSG00000186532.12	-0.002305255	0.763329786	0.976100683	SMYD4
ENSG00000204673.11	0.002942549	0.763360363	0.976100683	AKT1S1
ENSG00000163468.15	-0.002013274	0.763393742	0.976100683	CCT3
ENSG00000110713.17	0.002207401	0.763536193	0.976188248	NUP98
ENSG00000256263.1	-0.016191497	0.763557426	0.976188248	DDX11L8
ENSG00000131558.15	-0.001862829	0.763659469	0.976226864	EXOC4
ENSG00000027847.14	0.003020536	0.763682835	0.976226864	B4GALT7
ENSG00000167701.14	0.006662606	0.763745978	0.976240564	GPT
ENSG00000167363.14	-0.00556889	0.763928193	0.976240564	FN3K
ENSG00000259431.6	-0.00269092	0.7639712	0.976240564	THTPA
ENSG00000168394.12	0.003828746	0.763995273	0.976240564	TAP1
ENSG00000132485.14	-0.004098549	0.764034434	0.976240564	ZRANB2
ENSG00000100628.12	-0.005185875	0.764040066	0.976240564	ASB2
ENSG00000225762.1	-0.008361101	0.764061	0.976240564	LINC01389
ENSG00000196242.10	-0.014864096	0.764117722	0.976240564	OR2C3
ENSG00000152818.18	-0.00330459	0.76413266	0.976240564	UTRN
ENSG00000122043.11	-0.008885169	0.764169581	0.976240564	LINC00544
ENSG00000250696.5	-0.009696637	0.76429415	0.976338883	RP11-704M14.1
ENSG00000271398.1	0.012442694	0.764375952	0.976382561	RP5-1092A3.5
ENSG00000178982.10	0.002681841	0.764441609	0.976385351	EIF3K
ENSG00000183780.13	-0.009438182	0.76449646	0.976385351	SLC35F3
ENSG00000196867.8	0.004982768	0.764536429	0.976385351	ZFP28
ENSG00000286416.1	-0.007859303	0.764568576	0.976385351	RP11-128N14.6
ENSG00000114209.15	0.006346537	0.764654058	0.976433712	PDCD10
ENSG00000072694.21	0.006641403	0.764779758	0.976533421	FCGR2B
ENSG00000233762.3	0.004805706	0.764946578	0.976611969	RPS15P4
ENSG00000170919.16	0.002168381	0.764950075	0.976611969	TPT1-AS1
ENSG00000287110.1	0.01029119	0.764984136	0.976611969	RP11-723O4.10
ENSG00000255145.3	0.01165759	0.765080328	0.976629128	STX17-AS1
ENSG00000008853.17	0.004179787	0.765092821	0.976629128	RHOBTB2
ENSG00000280279.1	-0.007975046	0.76514697	0.976637459	LINC02887
ENSG00000262319.1	-0.011154966	0.765258247	0.976693277	CTC-457L16.2
ENSG00000219201.4	-0.007788107	0.76528595	0.976693277	RP11-181C21.4
ENSG00000215861.6	-0.01055834	0.765468647	0.976710396	WI2-1896O14.1
ENSG00000062370.17	0.009764475	0.765518707	0.976710396	ZNF112

ENSG00000114853.15	0.003502595	0.765703713	0.976710396	ZBTB47
ENSG00000149418.11	-0.004539604	0.765706238	0.976710396	ST14
ENSG00000141542.11	-0.003368761	0.765761366	0.976710396	RAB40B
ENSG00000160305.18	-0.004508472	0.765781343	0.976710396	DIP2A
ENSG00000250151.9	0.006664565	0.765796279	0.976710396	ARPC4-TTLL3
ENSG00000100024.15	-0.005952272	0.765797907	0.976710396	UPB1
ENSG00000130182.8	-0.011731422	0.76586894	0.976710396	ZSCAN10
ENSG00000160145.16	0.005454143	0.765871846	0.976710396	KALRN
ENSG00000212916.6	-0.004553201	0.765882857	0.976710396	MAP10
ENSG00000177683.14	0.004199842	0.765900894	0.976710396	THAP5
ENSG00000102898.12	0.002304303	0.766007655	0.976710396	NUTF2
ENSG00000167633.18	0.010135754	0.76601574	0.976710396	KIR3DL1
ENSG00000282851.2	-0.004173418	0.766016053	0.976710396	BISPR
ENSG00000276075.1	-0.012331044	0.766070647	0.976710396	CTD-2012K14.8
ENSG00000231948.2	-0.009890058	0.766278249	0.976710396	HS1BP3-IT1
ENSG00000196083.10	-0.004677433	0.766312766	0.976710396	IL1RAP
ENSG00000188266.14	-0.007239091	0.766360664	0.976710396	HYKK
ENSG00000124356.17	-0.001382435	0.766432758	0.976710396	STAMBP
ENSG00000165633.13	0.017573184	0.766612679	0.976710396	VSTM4
ENSG00000116990.11	0.003040634	0.766635256	0.976710396	MYCL
ENSG00000188056.12	0.016922929	0.766641068	0.976710396	TREML4
ENSG00000227413.1	0.00982518	0.766669951	0.976710396	RP5-1042K10.12
ENSG00000188175.10	0.013227593	0.766697493	0.976710396	HEPACAM2
ENSG00000183793.14	0.015405151	0.766762136	0.976710396	NPIPA5
ENSG00000001630.17	-0.007018885	0.766767702	0.976710396	CYP51A1
ENSG00000162396.6	0.002976618	0.766878007	0.976710396	PARS2
ENSG00000274501.1	-0.007427253	0.766902682	0.976710396	RP4-583P15.16
ENSG00000141759.15	0.002313697	0.766918012	0.976710396	TXNL4A
ENSG00000224183.1	0.01626676	0.766939176	0.976710396	SDHDP6
ENSG00000132768.14	-0.001897067	0.766989526	0.976710396	DPH2
ENSG00000142252.11	-0.003381262	0.767017025	0.976710396	GEMIN7
ENSG00000137331.12	-0.004416953	0.767032819	0.976710396	IER3
ENSG00000178691.11	-0.003344871	0.767088285	0.976710396	SUZ12
ENSG00000249685.1	-0.008865218	0.767144959	0.976710396	RP11-360F5.3
ENSG00000204469.13	-0.002751088	0.767177008	0.976710396	PRRC2A
ENSG00000067560.14	0.001907358	0.76722703	0.976710396	RHOA
ENSG00000185100.11	-0.004748497	0.767229054	0.976710396	ADSS1
ENSG00000115977.21	0.002055914	0.767345096	0.976710396	AAK1
ENSG00000111790.14	0.003833695	0.767351114	0.976710396	FGFR1OP2
ENSG00000267102.1	-0.005506304	0.767354705	0.976710396	RP11-686D22.7
ENSG00000010704.19	-0.002855478	0.767364606	0.976710396	HFE
ENSG00000105641.4	0.006046112	0.7673949	0.976710396	SLC5A5
ENSG00000281741.2	-0.016718247	0.767455301	0.976726655	CH17-118O6.6
ENSG00000237438.7	-0.010811514	0.767656358	0.976874981	CECR7
ENSG00000164134.13	0.002462981	0.767797477	0.976874981	NAA15
ENSG00000173013.6	-0.004115897	0.767834404	0.976874981	CCDC96
ENSG00000140396.13	-0.003454658	0.767848191	0.976874981	NCOA2

ENSG00000226067.7	-0.003871915	0.767910698	0.976874981	LINC00623
ENSG00000286996.1	-0.01008365	0.767946717	0.976874981	RP11-256B12.2
ENSG00000196549.13	0.004483498	0.767955332	0.976874981	MME
ENSG00000279267.1	-0.005697958	0.768007738	0.976874981	RP11-395L14.18
ENSG00000110888.17	0.002282163	0.768121108	0.976874981	CAPRIN2
ENSG00000259429.5	-0.010995868	0.76819739	0.976874981	UBE2Q2P2
ENSG00000211747.3	0.005053993	0.768210627	0.976874981	TRBV20-1
ENSG00000277548.1	0.00504437	0.768210949	0.976874981	RP11-139H15.6
ENSG00000237547.1	0.014264186	0.768234523	0.976874981	IGHJ2P
ENSG00000232439.1	0.009621322	0.768238721	0.976874981	RPL18AP7
ENSG00000007341.19	-0.002509911	0.768407707	0.976962086	ST7L
ENSG00000197134.13	-0.007483176	0.768470246	0.976962086	ZNF257
ENSG00000152556.17	-0.002587885	0.768518967	0.976962086	PFKM
ENSG00000246877.1	0.013786159	0.768604669	0.976962086	DNM1P35
ENSG00000233452.7	0.004590956	0.768701881	0.976962086	STXBP5-AS1
ENSG00000260034.1	-0.012124014	0.768712903	0.976962086	LCMT1-AS2
ENSG00000136450.13	-0.002018811	0.768747185	0.976962086	SRSF1
ENSG00000167220.12	-0.003109766	0.768770666	0.976962086	HDHD2
ENSG00000288065.1	0.007651221	0.768946015	0.976962086	RP11-467P9.2
ENSG00000196576.16	-0.003940436	0.769075804	0.976962086	PLXNB2
ENSG00000158683.9	-0.007988874	0.76909618	0.976962086	PKD1L1
ENSG00000248746.6	-0.013848658	0.76912744	0.976962086	ACTN3
ENSG00000119673.15	-0.003222441	0.769140125	0.976962086	ACOT2
ENSG00000134242.16	-0.003662514	0.769191976	0.976962086	PTPN22
ENSG00000075711.21	-0.002525135	0.769207801	0.976962086	DLG1
ENSG00000259483.1	0.008204529	0.769221462	0.976962086	RP11-930O11.2
ENSG00000112425.16	0.002914401	0.769414792	0.976962086	EPM2A
ENSG00000146666.5	0.032384238	0.769439977	0.976962086	LINC00525
ENSG00000184260.6	0.00743505	0.769517288	0.976962086	H2AC20
ENSG00000163923.10	0.006502342	0.769580222	0.976962086	RPL39L
ENSG00000198010.13	0.035927186	0.769625929	0.976962086	DLGAP2
ENSG00000143315.8	0.002162099	0.769808776	0.976962086	PIGM
ENSG00000150760.13	0.007325364	0.769894235	0.976962086	DOCK1
ENSG00000265479.7	-0.005698039	0.770010284	0.976962086	DTX2P1-UPK3BP1-PMS2P11
ENSG00000156853.13	0.001728167	0.770013463	0.976962086	ZNF689
ENSG00000105173.14	-0.004944843	0.770067211	0.976962086	CCNE1
ENSG00000048028.11	-0.00327307	0.770121958	0.976962086	USP28
ENSG00000082512.15	-0.002397436	0.770142651	0.976962086	TRAF5
ENSG00000142677.4	-0.019902891	0.770202439	0.976962086	IL22RA1
ENSG00000204386.12	0.002044755	0.770219353	0.976962086	NEU1
ENSG00000271327.1	-0.004704014	0.770278252	0.976962086	RP11-1109F11.3
ENSG00000100079.7	0.009863417	0.770311334	0.976962086	LGALS2
ENSG00000164885.13	-0.003232355	0.770324627	0.976962086	CDK5
ENSG00000087053.20	0.002125053	0.770355648	0.976962086	MTMR2
ENSG00000276710.4	0.007258541	0.770404717	0.976962086	CSPG4P10
ENSG00000185842.16	-0.007754483	0.770438093	0.976962086	DNAH14
ENSG00000255670.1	0.010813482	0.77059726	0.976962086	RP11-253I19.3

ENSG00000169047.6	-0.005521947	0.770752652	0.976962086	IRS1
ENSG00000100600.15	-0.004034067	0.770756979	0.976962086	LGMN
ENSG00000271862.1	0.002981394	0.770779768	0.976962086	RP11-343L5.2
ENSG00000258289.10	-0.005313315	0.770812739	0.976962086	CHURC1
ENSG00000155111.15	0.002447287	0.770852283	0.976962086	CDK19
ENSG00000272583.1	0.007939044	0.770879723	0.976962086	RP11-344P13.6
ENSG00000170855.4	0.004114585	0.77093192	0.976962086	TRIAP1
ENSG00000164466.13	-0.001957543	0.771017103	0.976962086	SFXN1
ENSG00000176681.14	0.006905303	0.771055578	0.976962086	LRRC37A
ENSG00000144445.17	0.002981362	0.771109615	0.976962086	KANSL1L
ENSG00000104856.14	-0.003555044	0.771128525	0.976962086	RELB
ENSG00000263528.8	-0.002329423	0.771267997	0.976962086	IKBKE
ENSG00000188211.9	0.008306448	0.771375346	0.976962086	NCR3LG1
ENSG00000171302.17	-0.002312027	0.771384868	0.976962086	CANT1
ENSG00000124374.9	-0.003190885	0.771406938	0.976962086	PAIP2B
ENSG00000171236.10	0.004183348	0.771430256	0.976962086	LRG1
ENSG00000227671.4	-0.004952241	0.771494529	0.976962086	ZNF731P
ENSG00000167770.12	-0.002390257	0.771502861	0.976962086	OTUB1
ENSG00000099991.18	-0.002846364	0.771504314	0.976962086	CABIN1
ENSG00000107796.13	0.006343563	0.771539868	0.976962086	ACTA2
ENSG00000004799.8	-0.0049669	0.771615813	0.976962086	PDK4
ENSG00000183598.4	0.01850495	0.771663841	0.976962086	H3C13
ENSG00000243156.9	-0.005017124	0.771675854	0.976962086	MICAL3
ENSG00000245017.2	0.008042503	0.77167873	0.976962086	LINC02453
ENSG00000282828.2	0.0162778	0.771788934	0.976962086	RP11-443F4.1
ENSG00000131097.7	-0.008230229	0.771881902	0.976962086	HIGD1B
ENSG00000148110.16	0.003121129	0.771908635	0.976962086	MFSD14B
ENSG00000201151.1	-0.007237773	0.771964728	0.976962086	ENSG10010137848.1
ENSG00000225612.2	0.009618119	0.77199297	0.976962086	AC099552.2
ENSG00000110079.19	0.006244369	0.772011923	0.976962086	MS4A4A
ENSG00000259943.1	0.004589959	0.772032165	0.976962086	RP1-39G22.7
ENSG00000123106.10	0.002674892	0.772077909	0.976962086	CCDC91
ENSG00000145833.16	0.001660158	0.772085234	0.976962086	DDX46
ENSG00000160224.17	0.00835736	0.772115102	0.976962086	AIRE
ENSG00000157388.20	-0.006262549	0.772161052	0.976962086	CACNA1D
ENSG00000188277.10	0.002669101	0.772314616	0.976962086	C15orf62
ENSG00000225963.8	0.00692471	0.772350836	0.976962086	AC009950.2
ENSG00000169129.15	-0.003432736	0.772362842	0.976962086	AFAP1L2
ENSG00000140990.15	0.00289764	0.772370269	0.976962086	NDUFB10
ENSG00000256271.1	-0.005143295	0.772371866	0.976962086	CACNA1C-AS2
ENSG00000169689.15	-0.002815154	0.772448014	0.976962086	CENPX
ENSG00000197599.12	-0.005300534	0.772453439	0.976962086	CCDC154
ENSG00000101294.18	-0.002452037	0.772475603	0.976962086	HM13
ENSG00000088881.21	-0.006393023	0.772543391	0.976962086	EBF4
ENSG00000229431.1	-0.006949466	0.772560852	0.976962086	MED8-AS1
ENSG00000182885.17	0.004293674	0.772662326	0.976962086	ADGRG3
ENSG00000275759.1	0.00525926	0.772665742	0.976962086	RP11-131L12.3

ENSG00000251129.2	0.028739424	0.772672964	0.976962086	LINC02506
ENSG00000167554.15	0.007817364	0.772686974	0.976962086	ZNF610
ENSG00000128699.14	0.002369201	0.772688292	0.976962086	ORMDL1
ENSG00000242048.3	0.009155622	0.772690221	0.976962086	RP13-452N2.1
ENSG00000130783.14	0.005902025	0.772703338	0.976962086	CCDC62
ENSG00000145740.19	-0.001947695	0.772741942	0.976962086	SLC30A5
ENSG00000176533.13	-0.004399543	0.772746081	0.976962086	GNG7
ENSG00000117676.14	0.002093954	0.772778931	0.976962086	RPS6KA1
ENSG00000135473.15	-0.002557292	0.772807983	0.976962086	PAN2
ENSG00000177302.15	-0.001932155	0.772823612	0.976962086	TOP3A
ENSG00000101544.9	0.001963237	0.772905986	0.976962086	ADNP2
ENSG00000242861.1	0.003623076	0.772907077	0.976962086	RP11-285F7.2
ENSG00000249978.1	0.006597251	0.772928118	0.976962086	TRGV7
ENSG00000198925.12	0.00269971	0.773165372	0.977066909	ATG9A
ENSG00000204882.4	-0.008971177	0.773198576	0.977066909	GPR20
ENSG00000198081.11	0.002312009	0.773247052	0.977066909	ZBTB14
ENSG00000127914.19	0.00235788	0.773273342	0.977066909	AKAP9
ENSG00000134371.14	0.002220132	0.773288168	0.977066909	CDC73
ENSG00000253626.4	0.010414645	0.773296909	0.977066909	EIF5AL1
ENSG00000275302.2	0.005339877	0.773364609	0.97709225	CCL4
ENSG00000108784.10	0.002338235	0.773454861	0.977092948	NAGLU
ENSG00000229932.3	-0.005797796	0.773536998	0.977092948	YWHAZP3
ENSG00000156475.19	0.005360196	0.773599686	0.977092948	PPP2R2B
ENSG00000278601.1	-0.006582409	0.773627445	0.977092948	RP11-348N5.9
ENSG00000171723.16	-0.003143282	0.773636959	0.977092948	GPHN
ENSG00000183960.9	-0.009067241	0.773651028	0.977092948	KCNH8
ENSG00000248991.1	-0.014563333	0.773752874	0.977106353	RP11-73G16.2
ENSG00000287131.1	0.0080864	0.77376538	0.977106353	RP11-321A23.3
ENSG00000138380.18	-0.003846368	0.77386017	0.977106353	CARF
ENSG00000277247.1	0.021229336	0.773867498	0.977106353	RP11-785D18.3
ENSG00000085552.17	0.011289843	0.773899868	0.977106353	IGSF9
ENSG00000264644.1	-0.009909807	0.774021858	0.977183776	KRT18P8
ENSG00000254912.2	-0.017382613	0.774056487	0.977183776	RP11-632K20.2
ENSG00000235655.3	0.004701538	0.77419974	0.977230506	H3P6
ENSG00000125084.12	0.005161496	0.774237327	0.977230506	WNT1
ENSG00000132388.13	0.00141906	0.774240697	0.977230506	UBE2G1
ENSG00000276231.5	-0.004876437	0.774284108	0.977230506	PIK3R6
ENSG00000238186.1	0.008395663	0.774468293	0.977370811	RP11-656D10.6
ENSG00000172244.9	0.005020663	0.774595593	0.977370811	C5orf34
ENSG00000276900.1	0.003507101	0.774596842	0.977370811	RP11-467L13.7
ENSG00000277463.1	0.010392241	0.774689804	0.977370811	CTD-3035K23.6
ENSG00000277258.5	-0.008860404	0.774691968	0.977370811	PCGF2
ENSG00000153208.17	0.006684729	0.774762669	0.977370811	MERTK
ENSG00000196154.12	-0.003404807	0.774775461	0.977370811	S100A4
ENSG00000258526.7	-0.009294144	0.774824688	0.977370811	RP11-111A21.1
ENSG00000168040.5	0.002947135	0.774833493	0.977370811	FADD
ENSG00000163875.16	-0.001589794	0.774941043	0.977370811	MEAF6

ENSG00000264553.1	0.010099555	0.774963865	0.977370811	MIR4257
ENSG00000181350.12	-0.004105576	0.775013946	0.977370811	LRRC75A
ENSG00000172270.22	-0.004331199	0.775032936	0.977370811	BSG
ENSG00000135272.12	-0.003257465	0.775062488	0.977370811	MDFIC
ENSG00000114354.15	-0.001710417	0.775252676	0.977500673	TFG
ENSG00000213080.3	-0.006203551	0.775400541	0.977500673	RP11-312J18.5
ENSG00000020577.14	0.00657874	0.775410228	0.977500673	SAMD4A
ENSG00000047579.20	0.002592277	0.775460208	0.977500673	DTNBP1
ENSG00000133112.17	0.005582833	0.775534786	0.977500673	TPT1
ENSG00000184207.9	-0.002049594	0.775557333	0.977500673	PGP
ENSG00000152763.17	0.007489042	0.775607103	0.977500673	DNAI4
ENSG00000134121.10	0.013467748	0.775661799	0.977500673	CHL1
ENSG00000128534.8	-0.003461659	0.775694002	0.977500673	LSM8
ENSG00000111110.12	-0.003750388	0.775725749	0.977500673	PPM1H
ENSG00000273680.1	-0.004804772	0.775738425	0.977500673	RP11-996F15.6
ENSG00000106348.18	-0.002494489	0.775801595	0.977500673	IMPDH1
ENSG00000197226.13	0.00231199	0.77584826	0.977500673	TBC1D9B
ENSG00000227258.6	0.020542031	0.775882445	0.977500673	SMIM2-AS1
ENSG00000175352.11	0.007693846	0.775899	0.977500673	NRIP3
ENSG00000069956.13	0.003436511	0.775977326	0.977500673	MAPK6
ENSG00000173273.16	0.001841411	0.776016953	0.977500673	TNKS
ENSG00000278635.1	-0.011339435	0.776023428	0.977500673	CTD-2318O12.1
ENSG00000160963.14	-0.020985207	0.776163869	0.977560218	COL26A1
ENSG00000007202.15	0.001848349	0.776301323	0.977560218	KIAA0100
ENSG00000114745.14	-0.001850055	0.776304377	0.977560218	GORASP1
ENSG00000253865.1	0.007109956	0.776320167	0.977560218	RP11-394O4.3
ENSG00000149212.12	-0.004013218	0.776357216	0.977560218	SESN3
ENSG00000119487.17	0.001195304	0.776445561	0.977560218	MAPKAP1
ENSG00000170542.6	-0.002664027	0.776451667	0.977560218	SERPINB9
ENSG00000114450.11	0.003420068	0.776452038	0.977560218	GNB4
ENSG00000116191.18	-0.005389153	0.776552969	0.977627273	RALGPS2
ENSG00000236562.4	-0.014158088	0.776633036	0.977668057	IFITM3P1
ENSG00000185658.14	-0.003042737	0.776707272	0.977701493	BRWD1
ENSG00000189343.7	-0.007167516	0.776925302	0.977914641	RPS2P46
ENSG00000259984.1	0.009225828	0.777003671	0.977914641	RP11-335G20.7
ENSG00000139291.14	-0.002080129	0.777216664	0.977914641	TMEM19
ENSG00000100519.12	0.004387374	0.777218981	0.977914641	PSMC6
ENSG00000104312.8	-0.002226138	0.777248018	0.977914641	RIPK2
ENSG00000232027.2	0.01178139	0.777284818	0.977914641	RP11-275F13.3
ENSG00000182827.9	0.002402546	0.777301953	0.977914641	ACBD3
ENSG00000128294.16	-0.002660563	0.777311044	0.977914641	TPST2
ENSG00000282780.1	0.004742832	0.777402394	0.977914641	TRBJ1-6
ENSG00000104894.12	-0.002677311	0.777440944	0.977914641	CD37
ENSG00000236296.8	0.011329928	0.777453559	0.977914641	GUSBP5
ENSG00000226306.6	-0.010144137	0.777506605	0.977914641	NPY6R
ENSG00000282885.2	-0.006701231	0.777511318	0.977914641	RP11-596C23.6
ENSG00000213780.11	0.004386319	0.777580293	0.977914641	GTF2H4

ENSG00000109689.19	-0.001669304	0.777591869	0.977914641	STIM2
ENSG00000259250.1	0.004108689	0.777734573	0.978025122	RP11-50C13.1
ENSG00000117020.19	0.002332827	0.777890559	0.978025122	AKT3
ENSG00000236698.1	0.009413684	0.777950115	0.978025122	EIF1AXP1
ENSG00000170667.15	-0.005569334	0.777952394	0.978025122	RASA4B
ENSG00000008869.12	-0.002545194	0.777986503	0.978025122	HEATR5B
ENSG00000146802.13	0.002940396	0.778056437	0.978025122	TMEM168
ENSG00000104324.16	0.002599407	0.77808396	0.978025122	CPQ
ENSG00000151233.11	-0.002873104	0.778087809	0.978025122	GXYLT1
ENSG00000242616.4	-0.005731093	0.778133776	0.978025122	GNG10
ENSG00000256167.1	-0.009217349	0.778156618	0.978025122	ATF4P4
ENSG00000135636.15	0.003812919	0.778321924	0.978118208	DYSF
ENSG00000273720.1	0.010222898	0.778326069	0.978118208	RP11-613E4.4
ENSG00000100897.18	0.001968085	0.778395472	0.978145487	DCAF11
ENSG00000141622.14	0.007299388	0.778483868	0.978196628	RNF165
ENSG00000171475.14	0.001867162	0.77859008	0.978226205	WIPF2
ENSG00000095637.23	0.005762301	0.778602806	0.978226205	SORBS1
ENSG00000287201.1	-0.008114435	0.778651094	0.978226944	RP11-72O13.2
ENSG00000205138.4	-0.002845229	0.778772551	0.97824357	SDHAF1
ENSG00000180767.10	-0.007936628	0.778793461	0.97824357	CHST13
ENSG00000276550.4	-0.004865065	0.778858148	0.97824357	HERC2P2
ENSG00000080839.12	-0.002436071	0.778942393	0.97824357	RBL1
ENSG00000156398.13	0.002641247	0.779074024	0.97824357	SFXN2
ENSG00000074266.22	0.002384583	0.779122097	0.97824357	EED
ENSG00000103043.15	0.001960241	0.779233458	0.97824357	VAC14
ENSG00000152464.15	0.002285479	0.779258442	0.97824357	RPP38
ENSG00000132522.16	-0.002736823	0.779262808	0.97824357	GPS2
ENSG00000285165.1	0.006464097	0.779269209	0.97824357	CTC-736O2.1
ENSG00000140403.13	0.002534454	0.779277208	0.97824357	DNAJA4
ENSG00000238266.2	0.013241367	0.779305214	0.97824357	LINC00707
ENSG00000128606.13	0.008896035	0.77931229	0.97824357	LRRC17
ENSG00000138760.11	0.0028447	0.779375884	0.97824357	SCARB2
ENSG00000223612.3	0.003533478	0.779397398	0.97824357	RP11-289I10.2
ENSG00000163746.12	-0.013716528	0.779427537	0.97824357	PLSCR2
ENSG00000139324.12	-0.004106712	0.779519848	0.978265055	TMTC3
ENSG00000105486.14	-0.002255195	0.77956937	0.978265055	LIG1
ENSG00000203668.3	0.004717365	0.779617111	0.978265055	CHML
ENSG00000119227.8	0.005057089	0.779635462	0.978265055	PIGZ
ENSG00000143341.12	-0.013963888	0.779719882	0.978311125	HMCN1
ENSG00000279433.1	-0.00797242	0.779844892	0.97835808	RP11-4B16.1
ENSG00000161980.6	0.002986936	0.779881948	0.97835808	POLR3K
ENSG00000253392.3	-0.006848129	0.779900424	0.97835808	AC006277.2
ENSG00000137288.10	0.002128283	0.780073351	0.97837572	UQCC2
ENSG00000173212.5	0.008426498	0.78011468	0.97837572	MAB21L3
ENSG00000229484.1	0.011971458	0.780130456	0.97837572	LINC02766
ENSG00000164164.17	-0.002683531	0.78014218	0.97837572	OTUD4
ENSG00000278600.1	-0.002505772	0.780232513	0.97837572	RP11-81A1.6

ENSG00000274922.1	0.003737833	0.780293	0.97837572	RP11-88E10.5
ENSG00000111249.14	0.006893919	0.780318066	0.97837572	CUX2
ENSG00000228590.2	-0.006486117	0.780499122	0.97837572	MIR4432HG
ENSG00000073756.13	-0.004794618	0.780512825	0.97837572	PTGS2
ENSG00000159674.12	-0.006664364	0.780538421	0.97837572	SPON2
ENSG00000278962.1	0.007189292	0.780541769	0.97837572	RP11-399B17.1
ENSG00000171680.23	0.006038503	0.78067073	0.97837572	PLEKHG5
ENSG00000132824.14	0.001717871	0.780696601	0.97837572	SERINC3
ENSG00000213442.5	0.004070623	0.780727297	0.97837572	RPL18AP3
ENSG00000152332.16	0.002923202	0.780739684	0.97837572	UHMK1
ENSG00000234199.3	0.006512312	0.780753634	0.97837572	LINC01191
ENSG00000274276.4	0.021995011	0.780838725	0.97837572	CBSL
ENSG00000120690.16	-0.002344551	0.78088829	0.97837572	ELF1
ENSG00000081177.18	0.00208247	0.780914546	0.97837572	EXD2
ENSG00000148814.18	-0.003549083	0.780944855	0.97837572	LRRC27
ENSG00000112685.14	-0.001908254	0.780988031	0.97837572	EXOC2
ENSG00000101189.7	-0.001932521	0.78101942	0.97837572	MRGBP
ENSG00000160180.17	0.011432257	0.781113118	0.97837572	TFF3
ENSG00000146085.8	0.002438051	0.781116091	0.97837572	MMUT
ENSG00000285331.2	0.004430124	0.781244442	0.97837572	RP11-323F24.5
ENSG00000204161.14	0.005323379	0.781263707	0.97837572	TMEM273
ENSG00000237499.7	-0.00350858	0.781339691	0.97837572	WAKMAR2
ENSG00000229473.2	0.009679985	0.781665572	0.97837572	RGS17P1
ENSG00000122299.12	-0.001868118	0.781678225	0.97837572	ZC3H7A
ENSG00000253958.2	0.006383746	0.781790552	0.97837572	CLDN23
ENSG00000168140.5	-0.00364488	0.781954984	0.97837572	VASN
ENSG00000275772.1	0.007616233	0.781962211	0.97837572	LL22NC03-24A12.9
ENSG00000100053.10	-0.01309486	0.782027861	0.97837572	CRYBB3
ENSG00000269028.3	-0.008899815	0.782067184	0.97837572	MTRNR2L12
ENSG00000119431.10	0.003447108	0.78209722	0.97837572	HDHD3
ENSG00000174238.15	0.002059656	0.782097392	0.97837572	PITPNA
ENSG00000273213.4	-0.012652892	0.782161584	0.97837572	H3-2
ENSG00000186777.12	-0.009428856	0.782298941	0.97837572	ZNF732
ENSG00000160199.15	0.001646969	0.782302409	0.97837572	PKNOX1
ENSG00000120992.18	-0.003137252	0.782387813	0.97837572	LYPLA1
ENSG00000146700.9	0.007382981	0.782483072	0.97837572	SSC4D
ENSG00000115904.14	0.002207703	0.782494412	0.97837572	SOS1
ENSG00000071073.13	-0.002800605	0.782494643	0.97837572	MGAT4A
ENSG00000175206.10	0.006210155	0.782504884	0.97837572	NPPA
ENSG00000263293.2	0.013761996	0.782509666	0.97837572	EFCAB13-DT
ENSG00000162736.17	-0.002186526	0.782595272	0.97837572	NCSTN
ENSG00000253295.2	0.013152652	0.782663003	0.97837572	RP11-779O18.1
ENSG00000262097.2	-0.007746534	0.782682958	0.97837572	LINC02185
ENSG00000155100.11	-0.004621585	0.782702013	0.97837572	OTUD6B
ENSG00000267764.1	-0.010493654	0.782721323	0.97837572	RP11-484L8.1
ENSG00000171608.16	0.001937128	0.782737346	0.97837572	PIK3CD
ENSG00000107371.14	-0.002014569	0.782776223	0.97837572	EXOSC3

ENSG00000260953.1	-0.006773176	0.782803912	0.97837572	RP11-426C22.6
ENSG00000185155.12	0.013953891	0.78290131	0.97837572	MIXL1
ENSG00000204520.14	0.003913154	0.782943969	0.97837572	MICA
ENSG00000176658.17	-0.003962301	0.783068088	0.97837572	MYO1D
ENSG00000145545.12	0.002342285	0.783112648	0.97837572	SRD5A1
ENSG00000118307.20	-0.008589832	0.783136901	0.97837572	CFAP94
ENSG00000172985.11	0.004914967	0.783195398	0.97837572	SH3RF3
ENSG00000126775.9	0.001598488	0.783278101	0.97837572	ATG14
ENSG00000144560.15	-0.002595744	0.783294893	0.97837572	VGLL4
ENSG00000181690.8	-0.006303902	0.783328414	0.97837572	PLAG1
ENSG00000226012.1	-0.010755619	0.78336106	0.97837572	AP001434.2
ENSG00000198900.7	-0.001842152	0.783391556	0.97837572	TOP1
ENSG00000237470.3	0.02609023	0.783425352	0.97837572	DCLRE1CP1
ENSG00000279330.1	0.003431651	0.783425575	0.97837572	AJ003147.11
ENSG00000196505.11	-0.001852815	0.783548751	0.97837572	GDAP2
ENSG00000137731.14	-0.007000976	0.783609738	0.97837572	FXYD2
ENSG00000165997.5	0.003535302	0.783623332	0.97837572	ARL5B
ENSG00000143393.17	0.001350442	0.783668439	0.97837572	PI4KB
ENSG00000203814.6	0.005042689	0.783680709	0.97837572	H2BC18
ENSG00000134779.15	-0.004956145	0.78380547	0.97837572	TPGS2
ENSG00000261442.1	-0.007668673	0.783891503	0.97837572	RP11-709D24.6
ENSG00000116514.17	0.002280414	0.783935403	0.97837572	RNF19B
ENSG00000111224.14	0.002524028	0.783975882	0.97837572	PARP11
ENSG00000259768.6	0.004910423	0.78398169	0.97837572	RP5-991G20.1
ENSG00000164615.6	0.002030002	0.784037361	0.97837572	CAMLG
ENSG00000260804.3	0.002964875	0.784131191	0.97837572	LINC01963
ENSG00000277149.5	0.007373099	0.784153801	0.97837572	TYW1B
ENSG00000211806.2	0.006858831	0.784157461	0.97837572	TRAV25
ENSG00000100714.17	0.002174172	0.784179981	0.97837572	MTHFD1
ENSG00000276390.1	0.007509191	0.784272194	0.97837572	RP1-197B17.5
ENSG00000244620.1	0.009396191	0.784312181	0.97837572	AL122127.25
ENSG00000267749.1	-0.012368488	0.784361081	0.97837572	CTC-265F19.1
ENSG00000077942.19	-0.015659787	0.78442152	0.97837572	FBLN1
ENSG00000268798.1	0.007537592	0.784464977	0.97837572	CTB-25B13.5
ENSG00000271303.2	0.006599833	0.784467597	0.97837572	SRXN1
ENSG00000277496.1	0.002905797	0.78448289	0.97837572	RP11-93B14.9
ENSG00000104327.7	-0.006485994	0.784620085	0.97837572	CALB1
ENSG00000172939.9	0.001452837	0.784676814	0.97837572	OXSR1
ENSG00000125249.7	-0.004598293	0.784739141	0.97837572	RAP2A
ENSG00000232912.6	0.003236411	0.784756992	0.97837572	RERE-AS1
ENSG00000170100.14	0.001849565	0.78480294	0.97837572	ZNF778
ENSG00000116574.6	0.003967654	0.784881497	0.97837572	RHOU
ENSG00000214367.9	-0.00263557	0.784980266	0.97837572	HAUS3
ENSG00000201302.1	-0.006268867	0.785018394	0.97837572	SNORA65
ENSG00000198586.14	0.002616102	0.785090533	0.97837572	TLK1
ENSG00000142959.5	0.005513241	0.785140481	0.97837572	BEST4
ENSG00000280063.1	0.005291052	0.785166818	0.97837572	RP11-295D4.3

ENSG00000138758.12	-0.002558533	0.785194293	0.97837572	SEPTIN11
ENSG00000274315.1	-0.006972315	0.785202779	0.97837572	RP11-996F15.5
ENSG00000141258.13	0.00203502	0.785210679	0.97837572	SGSM2
ENSG00000260188.1	0.009736962	0.785262681	0.97837572	CTA-331P3.1
ENSG00000262728.6	-0.010903446	0.785328221	0.97837572	RP11-1000B6.8
ENSG00000267364.2	-0.004290998	0.785409785	0.97837572	RP11-47L3.1
ENSG00000281005.1	0.002202813	0.785413044	0.97837572	LINC00921
ENSG00000168172.9	-0.002085827	0.785440185	0.97837572	HOOK3
ENSG00000177369.9	-0.004818982	0.785457802	0.97837572	FLJ40194
ENSG00000025800.14	0.001311439	0.785477567	0.97837572	KPNA6
ENSG00000287691.1	0.004647467	0.785492546	0.97837572	RP5-1174N9.3
ENSG00000008441.18	0.00635711	0.785536327	0.97837572	NFIX
ENSG00000188822.8	-0.00451729	0.78567524	0.97837572	CNR2
ENSG00000179943.8	-0.002015964	0.785710204	0.97837572	FIZ1
ENSG00000213071.11	0.004200763	0.785758282	0.97837572	LPAL2
ENSG00000164331.10	-0.002897987	0.785790908	0.97837572	ANKRA2
ENSG00000241657.1	-0.007289377	0.785799566	0.97837572	TRBV11-2
ENSG00000105426.17	-0.005850171	0.785865246	0.97837572	PTPRS
ENSG00000176720.6	-0.009923044	0.785865256	0.97837572	BOK
ENSG00000287979.1	0.011313958	0.785879037	0.97837572	CH17-3B23.3
ENSG00000253111.2	-0.014777717	0.785947107	0.97837572	RP11-136O12.2
ENSG00000236349.1	-0.008393778	0.786030436	0.97837572	SUCLG2P2
ENSG00000014138.9	0.002420966	0.786069347	0.97837572	POLA2
ENSG00000101442.10	0.001762783	0.786072344	0.97837572	ACTR5
ENSG00000228981.3	-0.008209269	0.786112772	0.97837572	RP11-364L4.1
ENSG00000142606.16	0.008582773	0.786114728	0.97837572	MMEL1
ENSG00000125804.14	0.013117584	0.786163064	0.97837572	FAM182A
ENSG00000233622.3	-0.006094149	0.786196867	0.97837572	CYP2T1P
ENSG00000105771.14	-0.002119475	0.786199136	0.97837572	SMG9
ENSG00000162144.10	-0.002586958	0.786256535	0.97837572	CYB561A3
ENSG00000213713.3	-0.004287753	0.786286265	0.97837572	PIGCP1
ENSG00000262118.1	-0.014026486	0.786385511	0.97837572	MTCO1P28
ENSG00000257017.9	0.009400989	0.786388314	0.97837572	HP
ENSG00000140995.17	0.002145204	0.786468469	0.97837572	DEF8
ENSG00000124102.5	0.009054031	0.786473957	0.97837572	PI3
ENSG00000011426.11	-0.008617278	0.78652379	0.97837572	ANLN
ENSG00000269949.1	0.00904869	0.786545044	0.97837572	RP11-738E22.3
ENSG00000265190.7	-0.019631578	0.78655215	0.97837572	ANXA8
ENSG00000277632.2	-0.006674359	0.786552226	0.97837572	CCL3
ENSG00000220785.7	0.006874539	0.786571185	0.97837572	MTMR9LP
ENSG00000111696.12	0.005319686	0.786593469	0.97837572	NT5DC3
ENSG00000143199.18	-0.007814858	0.786794279	0.97852016	ADCY10
ENSG00000105639.20	-0.002439888	0.786805024	0.97852016	JAK3
ENSG00000059728.11	0.002553121	0.787056651	0.978727763	MXD1
ENSG00000104472.10	0.001641089	0.787096933	0.978727763	CHRAC1
ENSG00000239335.4	0.012351573	0.787115125	0.978727763	LLPH-DT
ENSG00000241525.4	-0.010866501	0.787212207	0.978741977	AC108004.3

ENSG00000091490.11	-0.002905503	0.787250707	0.978741977	SEL1L3
ENSG00000235954.7	0.002134373	0.78728959	0.978741977	TTC28-AS1
ENSG00000136485.15	-0.001612631	0.787388231	0.978741977	DCAF7
ENSG00000138641.18	0.004311903	0.787398899	0.978741977	HERC3
ENSG00000140391.15	-0.002941988	0.787412906	0.978741977	TSPAN3
ENSG00000143502.16	-0.006783749	0.787496981	0.978787156	SUSD4
ENSG00000183154.1	-0.006407238	0.787548998	0.978792489	RP11-863K10.7
ENSG00000268093.1	-0.005715651	0.787634306	0.978811701	AC022154.7
ENSG00000100802.15	-0.001989012	0.787659913	0.978811701	C14orf93
ENSG00000173267.14	-0.012938158	0.787727003	0.978831345	SNCG
ENSG00000177054.14	-0.002428806	0.787782284	0.978831345	ZDHHC13
ENSG00000124588.22	-0.004825064	0.787896659	0.978831345	NQO2
ENSG00000103226.19	-0.006339201	0.787900506	0.978831345	NOMO3
ENSG00000114107.9	-0.004465963	0.787914367	0.978831345	CEP70
ENSG00000124120.11	-0.001704972	0.787985378	0.978860266	TTPAL
ENSG00000133398.4	-0.00205829	0.788063885	0.978898495	MED10
ENSG00000227473.1	0.0068409	0.788290051	0.979042429	TSSK5P
ENSG00000138107.13	0.001743168	0.788306486	0.979042429	ACTR1A
ENSG00000243323.7	0.006436182	0.78836775	0.979042429	PTPRVP
ENSG00000258811.1	-0.009330339	0.788473227	0.979042429	CTD-3051D23.1
ENSG00000121691.7	0.00384465	0.788496611	0.979042429	CAT
ENSG00000054356.14	0.009790099	0.788524017	0.979042429	PTPRN
ENSG00000124721.18	0.009238986	0.788532647	0.979042429	DNAH8
ENSG00000135486.19	0.001536775	0.788614172	0.979042429	HNRNPA1
ENSG00000163687.14	-0.006728247	0.788649914	0.979042429	DNASE1L3
ENSG00000180917.18	0.003087261	0.788669114	0.979042429	CMTR2
ENSG00000255508.7	0.004021676	0.788704894	0.979042429	RP11-864I4.1
ENSG00000214776.12	-0.013957087	0.788776562	0.97907213	OVOS
ENSG00000260190.1	-0.010101901	0.788857932	0.979073693	RP11-229P13.25
ENSG00000101187.16	0.007697934	0.788899061	0.979073693	SLCO4A1
ENSG00000257556.2	-0.006513229	0.789047215	0.979073693	LINC02298
ENSG00000279254.1	-0.007721778	0.789069701	0.979073693	RP11-536C12.1
ENSG00000272305.5	0.00570396	0.789070394	0.979073693	RP11-894J14.5
ENSG00000104691.16	-0.003536261	0.78915709	0.979073693	UBXN8
ENSG00000139508.15	-0.001633323	0.789164327	0.979073693	SLC46A3
ENSG00000271819.1	0.007653035	0.789188532	0.979073693	RNU6-94P
ENSG00000080845.19	-0.001961695	0.789214686	0.979073693	DLGAP4
ENSG00000167371.21	0.004098698	0.789256913	0.979073693	PRRT2
ENSG00000262089.1	0.003455416	0.789302973	0.979073693	RP11-589P10.5
ENSG00000266265.4	0.016316573	0.789652881	0.97910921	KLF14
ENSG00000213901.11	-0.004909354	0.789792888	0.97910921	SLC23A3
ENSG00000213590.2	-0.010026963	0.789828563	0.97910921	RP11-229P13.2
ENSG00000104518.11	-0.002670829	0.789921177	0.97910921	GSDMD
ENSG00000263345.1	0.003531678	0.78996818	0.97910921	RP1-59D14.5
ENSG00000131771.14	-0.012892292	0.789985394	0.97910921	PPP1R1B
ENSG00000186235.11	-0.00908316	0.790016246	0.97910921	LINC02610
ENSG00000155287.11	-0.001958695	0.790033355	0.97910921	SLC25A28

ENSG00000165916.9	0.002152978	0.790074177	0.97910921	PSMC3
ENSG00000118985.16	0.002925887	0.790187681	0.97910921	ELL2
ENSG00000234936.1	-0.004131217	0.790215794	0.97910921	AC010883.5
ENSG00000279082.4	-0.008389398	0.790216909	0.97910921	LINC01727
ENSG00000178104.19	-0.002363554	0.790228034	0.97910921	PDE4DIP
ENSG00000117528.14	0.002661303	0.790248965	0.97910921	ABCD3
ENSG00000167130.18	-0.001836158	0.790294287	0.97910921	DOLPP1
ENSG00000114739.14	0.004280716	0.790368188	0.97910921	ACVR2B
ENSG00000171456.20	0.001347054	0.79043789	0.97910921	ASXL1
ENSG00000249307.7	-0.010880833	0.790462145	0.97910921	LINC01088
ENSG00000243753.5	-0.010629879	0.790474888	0.97910921	HLA-L
ENSG00000246451.2	0.004069564	0.790530592	0.97910921	RP11-894P9.1
ENSG00000238000.1	0.007462029	0.790531891	0.97910921	PSME2P1
ENSG00000275476.1	-0.007682826	0.790587662	0.97910921	RP11-996F15.4
ENSG00000273073.1	0.013328009	0.790612701	0.97910921	RP11-109E12.1
ENSG00000172216.6	-0.003047195	0.790634261	0.97910921	CEBPB
ENSG00000260853.2	-0.003195577	0.790793073	0.97910921	RP11-264B17.2
ENSG00000111801.16	0.002407866	0.790864481	0.97910921	BTN3A3
ENSG00000170776.22	-0.002288789	0.790866653	0.97910921	AKAP13
ENSG00000151665.13	0.002875242	0.790932724	0.97910921	PIGF
ENSG00000008083.14	-0.002446552	0.790958713	0.97910921	JARID2
ENSG00000110665.12	0.002736524	0.790967848	0.97910921	C11orf21
ENSG00000127184.13	0.005911398	0.791026343	0.97910921	COX7C
ENSG00000239264.9	0.006694311	0.791064545	0.97910921	TXNDC5
ENSG00000187742.15	0.001085505	0.791067859	0.97910921	SECISBP2
ENSG00000157764.14	0.001755129	0.791160975	0.97910921	BRAF
ENSG00000178149.17	-0.002061569	0.79121263	0.97910921	DALRD3
ENSG00000153283.13	-0.001924663	0.791243788	0.97910921	CD96
ENSG00000173890.17	-0.004276467	0.791267511	0.97910921	GPR160
ENSG00000156162.16	0.003665916	0.791268781	0.97910921	DPY19L4
ENSG00000231663.1	-0.006147865	0.791294015	0.97910921	COA6-AS1
ENSG00000149557.14	0.006133554	0.791311801	0.97910921	FEZ1
ENSG00000186153.18	0.00249607	0.791392062	0.97910921	WWOX
ENSG00000256223.6	0.003278365	0.791423011	0.97910921	ZNF10
ENSG00000211930.1	0.011067094	0.791487544	0.97910921	IGHD3-3
ENSG00000253559.1	0.006582436	0.791570646	0.97910921	OSGEPL1-AS1
ENSG00000118600.12	0.002402019	0.791621748	0.97910921	RXYLT1
ENSG00000259475.1	-0.013600685	0.791721019	0.97910921	RP11-654A16.3
ENSG00000173511.10	-0.002996554	0.791727747	0.97910921	VEGFB
ENSG00000110876.10	-0.002444495	0.791727969	0.97910921	SELPLG
ENSG00000260160.1	-0.004426748	0.79177247	0.97910921	CTC-471J1.2
ENSG00000100294.13	-0.002619444	0.791775283	0.97910921	MCAT
ENSG00000113583.8	0.002263578	0.791831926	0.97910921	C5orf15
ENSG00000110448.11	-0.003181665	0.791856341	0.97910921	CD5
ENSG00000250462.8	-0.002521197	0.791861974	0.97910921	LRRC37BP1
ENSG00000181038.14	-0.001743159	0.791970504	0.979184367	METTL23
ENSG00000148459.16	-0.003171183	0.792289875	0.979414793	PDSS1

ENSG00000138615.6	-0.004122347	0.792289907	0.979414793	CILP
ENSG00000116874.12	-0.002814051	0.792322889	0.979414793	WARS2
ENSG00000278030.1	-0.005470719	0.792471752	0.979414793	TRBV7-9
ENSG00000168952.15	-0.014281645	0.79257207	0.979414793	STXBP6
ENSG00000224950.2	0.00330048	0.792670022	0.979414793	RP5-1086K13.1
ENSG00000211753.4	-0.010503168	0.792698801	0.979414793	TRBV28
ENSG00000179523.5	-0.002102758	0.792728846	0.979414793	EIF3J-DT
ENSG00000204256.14	0.00122864	0.792810922	0.979414793	BRD2
ENSG00000001084.13	-0.002140358	0.792871763	0.979414793	GCLC
ENSG00000115307.17	-0.00166149	0.792985613	0.979414793	AUP1
ENSG00000196358.11	-0.004210962	0.793001257	0.979414793	NTNG2
ENSG00000225914.3	-0.009920025	0.793033255	0.979414793	TSBP1-AS1
ENSG00000074755.15	0.002066995	0.793084786	0.979414793	ZZEF1
ENSG00000226054.2	-0.004180668	0.793088006	0.979414793	MEMO1P1
ENSG00000272156.1	0.005719785	0.793116684	0.979414793	RP11-477N3.1
ENSG00000171368.12	0.005954233	0.793146589	0.979414793	TPPP
ENSG00000273449.1	-0.004950243	0.793173534	0.979414793	RP11-218F10.3
ENSG00000259562.2	-0.006200397	0.79321935	0.979414793	RP11-762H8.2
ENSG00000219470.1	-0.005125264	0.793258783	0.979414793	RP3-337H4.6
ENSG00000243547.1	0.005531377	0.793290909	0.979414793	HNRNPKP4
ENSG00000154734.16	-0.007363996	0.793312327	0.979414793	ADAMTS1
ENSG00000085491.17	0.003612553	0.793376834	0.979414793	SLC25A24
ENSG00000121764.11	-0.007923469	0.793382954	0.979414793	HCRTR1
ENSG00000166595.12	0.002469809	0.793472894	0.979414793	CIAO2B
ENSG00000215154.6	-0.004927789	0.793526773	0.979414793	AC141586.5
ENSG00000206113.11	-0.007706493	0.793527119	0.979414793	CFAP99
ENSG00000207523.1	0.008187682	0.793556429	0.979414793	SNORA66
ENSG00000116750.14	-0.002494196	0.793597012	0.979414793	UCHL5
ENSG00000225335.3	-0.005026808	0.793614459	0.979414793	XXbac-B476C20.9
ENSG00000287338.1	0.008502248	0.793671252	0.979414793	XXyac-YM960E7.1
ENSG00000069764.10	0.010430377	0.79368512	0.979414793	PLA2G10
ENSG00000138686.10	0.003206708	0.79375683	0.979444348	BBS7
ENSG00000272690.6	0.005849982	0.793949876	0.979560178	LINC02018
ENSG00000105227.16	-0.003637257	0.793954549	0.979560178	PRX
ENSG00000246334.2	-0.003005998	0.794007887	0.979560178	PRR7-AS1
ENSG00000287697.1	0.010699977	0.79404176	0.979560178	RP1-102G20.8
ENSG00000125740.14	0.005050779	0.794163373	0.979649439	FOSB
ENSG00000139719.11	-0.001393706	0.794221894	0.979649439	VPS33A
ENSG00000172795.17	0.00257997	0.794257423	0.979649439	DCP2
ENSG00000156535.15	0.006420837	0.794306595	0.97965117	CD109
ENSG00000130695.16	0.00186244	0.794404249	0.979712692	CEP85
ENSG00000197635.11	0.00372585	0.794457653	0.979719636	DPP4
ENSG00000168297.16	-0.002180086	0.794559444	0.979786248	PXK
ENSG00000093010.15	-0.002393934	0.794821896	0.979895396	COMT
ENSG00000137338.6	0.00318479	0.794836672	0.979895396	PGBD1
ENSG00000113597.18	0.003360111	0.794935133	0.979895396	TRAPPC13
ENSG00000278811.4	-0.007112904	0.795017421	0.979895396	LINC00624

ENSG00000271933.1	-0.008264472	0.795057769	0.979895396	RP11-338I21.1
ENSG00000267150.1	0.007910763	0.795298309	0.979895396	RP11-411B10.2
ENSG00000213079.10	0.001926457	0.795298765	0.979895396	SCAF8
ENSG00000110318.15	0.003995787	0.795330837	0.979895396	CEP126
ENSG00000258461.5	0.004472176	0.795333057	0.979895396	RP11-164J13.1
ENSG00000226828.2	-0.010933348	0.795476309	0.979895396	LINC02774
ENSG00000228801.6	0.003606665	0.795516124	0.979895396	RP11-110G21.1
ENSG00000162517.13	0.00200361	0.795540647	0.979895396	PEF1
ENSG00000228661.1	-0.007022691	0.795592717	0.979895396	STIM1-AS1
ENSG00000211790.2	-0.005264064	0.795593751	0.979895396	TRAV8-4
ENSG00000119509.13	-0.002211128	0.795634963	0.979895396	INVS
ENSG00000182154.8	-0.003609354	0.795728584	0.979895396	MRPL41
ENSG00000151952.16	0.006020364	0.795742064	0.979895396	TMEM132D
ENSG00000153094.24	-0.002241091	0.795785853	0.979895396	BCL2L11
ENSG00000079277.22	0.002443617	0.795791652	0.979895396	MKNK1
ENSG00000087448.11	-0.002217391	0.795801574	0.979895396	KLHL42
ENSG00000143376.14	-0.00160659	0.795837189	0.979895396	SNX27
ENSG00000251136.9	-0.003555491	0.795841885	0.979895396	RP11-37B2.1
ENSG00000242071.3	-0.004191633	0.795888878	0.979895396	RPL7AP6
ENSG00000141367.12	0.001954747	0.79596386	0.979895396	CLTC
ENSG00000171217.5	-0.007386699	0.795970636	0.979895396	CLDN20
ENSG00000230023.2	-0.045620695	0.796042155	0.979895396	LINC02800
ENSG00000130307.12	-0.005331536	0.796051462	0.979895396	USHBP1
ENSG00000231475.3	0.01090561	0.79607235	0.979895396	IGHV4-31
ENSG00000272282.1	-0.005554147	0.796110031	0.979895396	LINC02084
ENSG00000225177.6	-0.004311814	0.796112308	0.979895396	RP11-390P2.4
ENSG00000061656.11	-0.0067225	0.796129173	0.979895396	SPAG4
ENSG00000165416.15	-0.001492389	0.79618815	0.979909175	SUGT1
ENSG00000157540.22	-0.001804096	0.796303309	0.979957217	DYRK1A
ENSG00000262468.6	0.003144639	0.796449479	0.979957217	LINC01569
ENSG00000070718.12	0.002402269	0.796511853	0.979957217	AP3M2
ENSG00000100605.17	-0.002714335	0.796535666	0.979957217	ITPK1
ENSG00000169019.11	0.006032796	0.796575412	0.979957217	COMMD8
ENSG00000272750.1	0.004531248	0.796658314	0.979957217	RP11-378J18.8
ENSG00000120942.14	-0.00163135	0.796659596	0.979957217	UBIAD1
ENSG00000174371.17	-0.007298169	0.796692595	0.979957217	EXO1
ENSG00000107833.10	-0.003201439	0.79672491	0.979957217	NPM3
ENSG00000261114.1	0.005404933	0.796725333	0.979957217	RP11-325K4.2
ENSG00000131165.16	0.002143512	0.79675281	0.979957217	CHMP1A
ENSG00000276718.1	0.003116509	0.796808267	0.979966653	RP1-102E24.10
ENSG00000132646.11	0.00226143	0.79685918	0.9799705	PCNA
ENSG00000095585.17	-0.004851023	0.796927563	0.979989332	BLNK
ENSG00000274265.5	0.004840435	0.796989687	0.979989332	CH17-189H20.1
ENSG00000197930.13	0.00270038	0.797039507	0.979989332	ERO1A
ENSG00000267750.6	0.009180507	0.797065636	0.979989332	RUNDC3A-AS1
ENSG00000271259.1	0.006387306	0.797224287	0.980125632	RP11-1109F11.5
ENSG00000185924.7	-0.006783893	0.797278232	0.980133197	RTN4RL1

ENSG00000283444.1	0.013053658	0.79748917	0.980278421	GPR141BP
ENSG00000182511.12	-0.002335605	0.797542736	0.980278421	FES
ENSG00000137857.18	0.002828131	0.797579386	0.980278421	DUOX1
ENSG00000156650.14	0.002784029	0.79761256	0.980278421	KAT6B
ENSG00000005379.17	-0.004733263	0.797657574	0.980278421	TSPOAP1
ENSG00000258741.4	0.006308799	0.797683162	0.980278421	H2AZ2P1
ENSG00000187624.9	-0.012147434	0.797817262	0.980311	C17orf97
ENSG00000083093.10	0.002013413	0.798080631	0.980311	PALB2
ENSG00000085721.13	-0.001819171	0.798098313	0.980311	RRN3
ENSG00000258045.2	0.012445948	0.798098773	0.980311	Metazoa_SRP
ENSG00000255717.7	-0.002030518	0.798139865	0.980311	SNHG1
ENSG00000272148.1	0.004542653	0.798160091	0.980311	RP11-195B17.1
ENSG00000148429.14	0.002993247	0.798314395	0.980311	USP6NL
ENSG00000270194.1	0.006620257	0.798316275	0.980311	GOLGA4-AS1
ENSG00000050165.19	-0.010431292	0.798329288	0.980311	DKK3
ENSG00000139624.14	0.00134324	0.798343028	0.980311	CERS5
ENSG00000239799.1	-0.009111347	0.798346537	0.980311	ITIH4-AS1
ENSG00000131042.15	0.002442408	0.798355128	0.980311	LILRB2
ENSG00000143669.14	0.002528759	0.798408708	0.980311	LYST
ENSG00000232043.1	-0.005565921	0.79856712	0.980311	RP4-530I15.9
ENSG00000279741.1	0.006210161	0.798630253	0.980311	RP11-44F14.9
ENSG00000112294.14	0.003436168	0.798745239	0.980311	ALDH5A1
ENSG00000114790.13	-0.008857119	0.798761599	0.980311	ARHGEF26
ENSG00000144736.14	0.001543129	0.798775648	0.980311	SHQ1
ENSG00000233954.6	0.004238996	0.798836548	0.980311	UQCRHL
ENSG00000260252.1	-0.007113589	0.798843374	0.980311	RP11-384M15.3
ENSG00000119778.15	0.002891161	0.798863514	0.980311	ATAD2B
ENSG00000223442.1	0.008875884	0.798875075	0.980311	TH2LCRR
ENSG00000121417.14	-0.001561772	0.798883804	0.980311	ZNF211
ENSG00000226091.7	-0.005048824	0.798905244	0.980311	LINC00937
ENSG00000173442.13	-0.002261725	0.79894767	0.980311	EHBP1L1
ENSG00000143442.22	-0.001384551	0.798952509	0.980311	POGZ
ENSG00000229393.1	-0.008384098	0.799094532	0.980426603	RP3-395M20.3
ENSG00000206530.11	0.002747161	0.799208488	0.980491431	CFAP44
ENSG00000100532.13	0.003754075	0.799242991	0.980491431	CGRRF1
ENSG00000240694.9	0.020592353	0.799363892	0.980581092	PNMA2
ENSG00000024526.17	-0.011773517	0.799438753	0.980614267	DEPDC1
ENSG00000105372.8	-0.002960373	0.799507659	0.980640136	RPS19
ENSG00000243302.3	0.003413391	0.799610493	0.980664149	RP11-274B21.2
ENSG00000181638.20	-0.002212102	0.799622874	0.980664149	ZFP41
ENSG00000105127.10	9.58E-04	0.799686842	0.980683954	AKAP8
ENSG00000204438.11	-0.001937188	0.799823153	0.980764362	GPANK1
ENSG00000121957.15	-0.002563789	0.799848057	0.980764362	GPSM2
ENSG00000100462.16	0.002247016	0.80005871	0.98096401	PRMT5
ENSG00000197385.6	0.008307539	0.800258088	0.980971566	ZNF860
ENSG00000135341.18	-0.002009517	0.800277291	0.980971566	MAP3K7
ENSG00000232807.2	-0.003180379	0.800488324	0.980971566	RP11-536K7.3

ENSG00000099194.6	-0.004520438	0.800518085	0.980971566	SCD
ENSG00000173421.17	-0.006291514	0.80056322	0.980971566	IHO1
ENSG00000275318.1	0.00452701	0.800582909	0.980971566	RP11-526D8.11
ENSG00000285966.1	0.008807792	0.800672281	0.980971566	RP11-7F17.10
ENSG00000164684.13	0.009456092	0.80068767	0.980971566	ZNF704
ENSG00000068912.14	0.002288435	0.800749662	0.980971566	ERLEC1
ENSG00000271788.1	0.00508325	0.800823379	0.980971566	CTD-2201E18.5
ENSG00000175513.9	-0.008159689	0.800948263	0.980971566	TSGA10IP
ENSG00000100461.18	0.001755153	0.800968407	0.980971566	RBM23
ENSG00000102910.14	0.001743492	0.800977573	0.980971566	LONP2
ENSG00000229739.4	-0.006172547	0.801087391	0.980971566	PDC-AS1
ENSG00000274523.5	-0.001952137	0.801095773	0.980971566	RCC1L
ENSG00000249309.1	0.008022553	0.801115156	0.980971566	RP11-153M7.5
ENSG00000198890.9	-0.002517239	0.801126455	0.980971566	PRMT6
ENSG00000071909.19	0.013615437	0.801152311	0.980971566	MYO3B
ENSG00000026025.16	0.002661136	0.801310477	0.980971566	VIM
ENSG00000116698.21	-0.001408574	0.801364921	0.980971566	SMG7
ENSG00000285761.1	-0.007985576	0.801582061	0.980971566	XXbac-BPG170G13.34
ENSG00000163798.13	0.001234961	0.80159741	0.980971566	SLC4A1AP
ENSG00000180409.3	-0.00874687	0.80161819	0.980971566	OR10AA1P
ENSG00000145604.17	0.0024232	0.801668616	0.980971566	SKP2
ENSG00000180061.10	-0.003674245	0.801703988	0.980971566	TMEM150B
ENSG00000270427.1	-0.006940195	0.801730923	0.980971566	NRBF2P5
ENSG00000169744.13	0.008956486	0.801756433	0.980971566	LDB2
ENSG00000169599.13	0.00278573	0.801789954	0.980971566	NFU1
ENSG00000147457.14	0.002126018	0.801856477	0.980971566	CHMP7
ENSG00000011114.15	0.001961931	0.80197156	0.980971566	BTBD7
ENSG00000147548.17	-0.001656269	0.801988248	0.980971566	NSD3
ENSG00000231074.9	-0.001776228	0.801994064	0.980971566	HCG18
ENSG00000258082.1	-0.004388099	0.802006893	0.980971566	RP11-443B7.3
ENSG00000275557.1	-0.006257773	0.802018318	0.980971566	RP11-353N4.6
ENSG00000124203.7	0.002252959	0.802039784	0.980971566	ZNF831
ENSG00000215267.8	0.008931676	0.802077347	0.980971566	AKR1C7P
ENSG00000135960.10	0.006648282	0.802129136	0.980971566	EDAR
ENSG00000152127.9	0.002144059	0.802150709	0.980971566	MGAT5
ENSG00000171150.9	-0.002552162	0.802177051	0.980971566	SOCS5
ENSG00000155366.17	0.003514035	0.802234029	0.980971566	RHOC
ENSG00000149328.15	-0.008419459	0.802435262	0.980971566	GLB1L2
ENSG00000182308.7	0.00461885	0.802442766	0.980971566	DCAF4L1
ENSG00000271833.1	-0.00730581	0.802519889	0.980971566	RP11-356B19.11
ENSG00000167565.13	0.002045764	0.802537017	0.980971566	SERTAD3
ENSG00000145431.11	-0.004375902	0.802551482	0.980971566	PDGFC
ENSG00000164949.8	0.01099482	0.802568284	0.980971566	GEM
ENSG00000260719.1	-0.002714535	0.802581047	0.980971566	AC009133.17
ENSG00000233426.3	0.006665128	0.802590515	0.980971566	EIF3FP3
ENSG00000101049.17	0.005720537	0.802626717	0.980971566	SGK2
ENSG00000272010.1	-0.006663671	0.802633292	0.980971566	CTD-3025N20.3

ENSG00000267123.7	0.006281233	0.802661433	0.980971566	SCAT1
ENSG00000268087.1	-0.003419513	0.802667225	0.980971566	CTC-429P9.2
ENSG00000240823.3	0.005707306	0.802722185	0.980971566	RN7SL23P
ENSG00000116704.8	0.00192684	0.802759154	0.980971566	SLC35D1
ENSG00000287119.1	-0.010371055	0.80283949	0.980971566	RP11-16D24.1
ENSG00000175514.2	0.0085208	0.802857775	0.980971566	GPR152
ENSG00000139921.13	-0.003772519	0.802871605	0.980971566	TMX1
ENSG00000185689.17	-0.008146118	0.803117465	0.980971566	C6orf201
ENSG00000243107.1	-0.009908353	0.803168609	0.980971566	AC000120.7
ENSG00000107521.20	0.003129388	0.80317919	0.980971566	HPS1
ENSG00000082258.13	-0.003422931	0.803184167	0.980971566	CCNT2
ENSG00000250011.1	0.008411974	0.803309586	0.980971566	HMGB1P3
ENSG00000254726.3	-0.010175323	0.803378223	0.980971566	MEX3A
ENSG00000162971.11	0.002270619	0.803403893	0.980971566	TYW5
ENSG00000286940.1	-0.003742095	0.8034182	0.980971566	MICB-DT
ENSG00000119514.7	-0.002712506	0.803493632	0.980971566	GALNT12
ENSG00000282826.2	-0.005136811	0.803537574	0.980971566	FRG1CP
ENSG00000148225.16	0.00673758	0.803566522	0.980971566	WDR31
ENSG00000173575.22	-0.001697861	0.803638258	0.980971566	CHD2
ENSG00000126259.20	-0.011542427	0.8036578	0.980971566	KIRREL2
ENSG00000142875.19	0.003403744	0.803736586	0.980971566	PRKACB
ENSG00000240771.8	-0.008593354	0.803800102	0.980971566	ARHGEF25
ENSG00000100413.17	-0.001950661	0.803821198	0.980971566	POLR3H
ENSG00000103061.13	0.002131625	0.803837746	0.980971566	SLC7A6OS
ENSG00000284874.1	-0.007038595	0.803925572	0.980971566	SEPT5-GP1BB
ENSG00000274008.1	0.00596406	0.803987617	0.980971566	Metazoa_SRP
ENSG00000229676.3	-0.01057706	0.804012637	0.980971566	ZNF492
ENSG00000261008.7	-0.008714447	0.804069215	0.980971566	LINC01572
ENSG00000104824.18	-9.63E-04	0.804078051	0.980971566	HNRNPL
ENSG00000173295.8	0.003149218	0.804140993	0.980971566	FAM86B3P
ENSG00000106123.12	0.002850645	0.804280873	0.980971566	EPHB6
ENSG00000149016.16	-0.002198079	0.804300015	0.980971566	TUT1
ENSG00000172757.13	-0.00238044	0.804330257	0.980971566	CFL1
ENSG00000279425.1	0.004485261	0.804330674	0.980971566	CTD-2017D11.2
ENSG00000109944.11	-0.003775896	0.80433899	0.980971566	JHY
ENSG00000251455.1	-0.008239798	0.804373753	0.980971566	RP11-164P12.3
ENSG00000235674.2	-0.008626156	0.804395766	0.980971566	LDHAP2
ENSG00000129353.15	0.002191244	0.804504699	0.980971566	SLC44A2
ENSG00000146477.6	-0.008700845	0.804540515	0.980971566	SLC22A3
ENSG00000231369.1	-0.007467352	0.804562825	0.980971566	RP1-40G4P.1
ENSG00000286672.1	0.010132191	0.804571517	0.980971566	RP4-744I24.5
ENSG00000115255.12	-0.003956476	0.804574979	0.980971566	REEP6
ENSG00000126861.5	0.003524482	0.804719031	0.980971566	OMG
ENSG00000145390.11	-0.00558852	0.804722698	0.980971566	USP53
ENSG00000077549.19	-0.001723681	0.80475835	0.980971566	CAPZB
ENSG00000266124.1	0.007793693	0.804768385	0.980971566	MIR5587
ENSG00000187474.5	0.005827368	0.804811673	0.980971566	FPR3

ENSG00000267366.1	0.005575727	0.804882299	0.980971566	RP11-53B2.5
ENSG00000287721.1	-0.008057975	0.804891214	0.980971566	CTD-2008P7.11
ENSG00000214198.8	0.005969083	0.804895662	0.980971566	TTC41P
ENSG00000108375.13	0.003967834	0.804947576	0.980971566	RNF43
ENSG00000133027.18	-0.002794981	0.804971222	0.980971566	PEMT
ENSG00000163833.8	-0.006863808	0.805078911	0.980971566	FBXO40
ENSG00000151065.14	0.001904049	0.805150626	0.980971566	DCP1B
ENSG00000136451.9	-0.001619482	0.805175679	0.980971566	VEZF1
ENSG00000065675.15	-0.001783198	0.805222341	0.980971566	PRKCQ
ENSG00000130731.16	0.002899645	0.805266399	0.980971566	METTL26
ENSG00000069399.15	-0.003085092	0.805269546	0.980971566	BCL3
ENSG00000112149.10	-0.003182878	0.805278736	0.980971566	CD83
ENSG00000148297.16	0.00156077	0.805500608	0.981113045	MED22
ENSG00000153207.16	0.002457175	0.805550122	0.981113045	AHCTF1
ENSG00000078900.15	0.007254704	0.805576018	0.981113045	TP73
ENSG00000243795.1	-0.007707422	0.805586238	0.981113045	LINC02044
ENSG00000105967.16	0.005332101	0.80569067	0.981136702	TFEC
ENSG00000231528.3	-0.009001462	0.805733218	0.981136702	FAM225A
ENSG00000116984.15	-0.002219223	0.805805923	0.981136702	MTR
ENSG00000196220.16	0.004368045	0.805841518	0.981136702	SRGAP3
ENSG00000183773.16	0.004369145	0.805863227	0.981136702	AIFM3
ENSG00000197937.13	0.00321516	0.805972901	0.981136702	ZNF347
ENSG00000260912.1	0.003535205	0.806002544	0.981136702	RP11-363E7.4
ENSG00000135436.8	-0.006493847	0.806011244	0.981136702	FAM186B
ENSG00000214756.8	0.002566868	0.806040863	0.981136702	CSKMT
ENSG00000167110.18	-0.001656219	0.806192346	0.981136702	GOLGA2
ENSG00000070081.17	0.003239983	0.806242624	0.981136702	NUCB2
ENSG00000153317.15	0.002543967	0.806280024	0.981136702	ASAP1
ENSG00000114861.23	-0.002384178	0.806342583	0.981136702	FOXP1
ENSG00000177707.11	-0.006177692	0.806380666	0.981136702	NECTIN3
ENSG00000165424.7	-0.002997592	0.806402434	0.981136702	ZCCHC24
ENSG00000274070.2	-0.002630737	0.806456644	0.981136702	CASTOR2
ENSG00000139044.12	0.012104598	0.80646878	0.981136702	B4GALNT3
ENSG00000124784.9	0.00130388	0.806512152	0.981136702	RIOK1
ENSG00000100379.17	-0.002142403	0.806514654	0.981136702	KCTD17
ENSG00000143499.14	0.001962192	0.806744626	0.981358253	SMYD2
ENSG00000286974.1	0.004784592	0.806900301	0.981480041	XXbac-BPG300A18.17
ENSG00000178789.9	0.002419111	0.806988101	0.981480041	CD300LB
ENSG00000288586.1	-0.006077992	0.807068343	0.981480041	RP11-331F9.11
ENSG00000251485.1	0.006490854	0.807068819	0.981480041	NRBF2P6
ENSG00000278743.1	-0.00632742	0.807084036	0.981480041	RP11-707G18.1
ENSG00000188993.4	0.008402887	0.807184245	0.9815437	LRRC66
ENSG00000148943.12	0.002283751	0.807338737	0.981673355	LIN7C
ENSG00000107897.20	-0.002032074	0.807557394	0.981700165	ACBD5
ENSG00000149798.5	-0.004723353	0.807617145	0.981700165	CDC42EP2
ENSG00000270112.4	-0.00902582	0.807663039	0.981700165	ST8SIA5-DT
ENSG00000258181.1	-0.007942013	0.807673299	0.981700165	RP11-493L12.4

ENSG00000196730.13	-0.003319108	0.807705624	0.981700165	DAPK1
ENSG00000229124.7	0.002314375	0.807791837	0.981700165	VIM-AS1
ENSG00000112249.14	0.00208445	0.807796365	0.981700165	ASCC3
ENSG00000261187.1	0.005057232	0.807814409	0.981700165	RP11-1007O24.2
ENSG00000261371.6	0.002070035	0.807825732	0.981700165	PECAM1
ENSG00000121671.12	0.001315615	0.807892375	0.981700165	CRY2
ENSG00000232134.1	0.007969133	0.807958816	0.981700165	RPS15AP12
ENSG00000204237.5	0.002369219	0.808091669	0.981700165	OXLD1
ENSG00000161813.22	-0.002588134	0.808133667	0.981700165	LARP4
ENSG00000211897.9	-0.009360871	0.808135842	0.981700165	IGHG3
ENSG00000228692.2	-0.006525114	0.808193911	0.981700165	RP5-826L7.1
ENSG00000158850.15	-0.001981101	0.808251799	0.981700165	B4GALT3
ENSG00000232063.2	-0.008182634	0.808281209	0.981700165	RP11-307E17.8
ENSG00000179331.3	0.006179437	0.808301042	0.981700165	RAB39A
ENSG00000177990.12	-0.009014747	0.808413615	0.981700165	DPY19L2
ENSG00000233850.1	0.005990053	0.808431442	0.981700165	AC103563.8
ENSG00000173838.12	-0.008602282	0.808495428	0.981700165	MARCHF10
ENSG00000253291.1	-0.007062713	0.808527379	0.981700165	TRBV7-7
ENSG00000114904.13	-0.002372388	0.808599062	0.981700165	NEK4
ENSG00000261474.1	-0.003120612	0.808609504	0.981700165	RP11-452L6.1
ENSG00000231170.7	0.008792151	0.80869628	0.981700165	AC002451.3
ENSG00000152527.14	0.009021982	0.808816247	0.981700165	PLEKHH2
ENSG00000205485.13	-0.003195085	0.808847387	0.981700165	AC004980.7
ENSG00000271806.1	-0.005837636	0.808915127	0.981700165	RP5-892K4.1
ENSG00000137877.10	0.004950657	0.808937938	0.981700165	SPTBN5
ENSG00000254016.3	-0.010980217	0.808954429	0.981700165	ALG1L10P
ENSG00000275485.1	-0.007595603	0.809021785	0.981700165	RP11-261P13.6
ENSG00000163412.13	0.002552547	0.809028759	0.981700165	EIF4E3
ENSG00000186272.13	-0.002462195	0.809074841	0.981700165	ZNF17
ENSG00000163291.14	-0.003262767	0.809157138	0.981700165	PAQR3
ENSG00000272465.1	0.010468717	0.809173375	0.981700165	RP1-136B1.1
ENSG00000138698.15	0.001292105	0.809191514	0.981700165	RAP1GDS1
ENSG00000145332.14	0.002592363	0.809242023	0.981700165	KLHL8
ENSG00000286004.1	0.005856105	0.809303067	0.981700165	RP11-352G9.2
ENSG00000272927.1	-0.003929868	0.809333807	0.981700165	RP11-1191J2.5
ENSG00000157064.11	-0.008563032	0.809358016	0.981700165	NMNAT2
ENSG00000166822.13	-0.001455659	0.80940099	0.981700165	TMEM170A
ENSG00000170485.17	-0.00725873	0.809427329	0.981700165	NPAS2
ENSG00000176593.8	0.003117072	0.809436341	0.981700165	CTD-2368P22.1
ENSG00000040487.13	0.002107999	0.809467027	0.981700165	SLC66A1
ENSG00000141337.13	0.002098702	0.80957104	0.981768251	ARSG
ENSG00000165646.14	0.006557389	0.809733802	0.981812084	SLC18A2
ENSG00000143882.12	-0.003447799	0.809765883	0.981812084	ATP6V1C2
ENSG00000135407.10	0.003375168	0.809803607	0.981812084	AVIL
ENSG00000268658.5	-0.011741088	0.809809328	0.981812084	LINC00664
ENSG00000174243.10	0.001232233	0.809846558	0.981812084	DDX23
ENSG00000173141.5	-0.002427829	0.809914056	0.981835187	MRPL57

ENSG00000170689.10	-0.015304803	0.809998737	0.981835187	HOXB9
ENSG00000030066.13	0.001829857	0.810009241	0.981835187	NUP160
ENSG00000082701.17	0.001595227	0.810107704	0.981862787	GSK3B
ENSG00000173272.16	-0.003580773	0.810127766	0.981862787	MZT2A
ENSG00000162711.18	0.00273343	0.810239646	0.981870181	NLRP3
ENSG00000170340.11	-0.002975376	0.810255932	0.981870181	B3GNT2
ENSG00000156381.9	0.00610205	0.810361821	0.981870181	ANKRD9
ENSG00000164265.9	-0.01224268	0.810440794	0.981870181	SCGB3A2
ENSG00000158234.12	0.003327521	0.810471754	0.981870181	FAIM
ENSG00000159267.17	-0.002052959	0.810489545	0.981870181	HLCS
ENSG00000084092.7	-0.001380044	0.810562122	0.981870181	NOA1
ENSG00000272630.1	0.003032259	0.810630828	0.981870181	RP11-344N10.5
ENSG00000177082.13	-0.002074459	0.810674826	0.981870181	WDR73
ENSG00000223648.4	0.016168001	0.810683648	0.981870181	IGHV3-64
ENSG00000181016.9	-0.004130201	0.810688795	0.981870181	LSMEM1
ENSG00000259092.1	0.006860712	0.810799061	0.981870181	TRAV30
ENSG00000184470.21	0.002125343	0.810827797	0.981870181	TXNRD2
ENSG00000179761.12	0.004504999	0.81086513	0.981870181	PIPOX
ENSG00000122877.17	-0.005593836	0.810917346	0.981870181	EGR2
ENSG00000236534.1	0.007482114	0.811019351	0.981870181	H3P36
ENSG00000276007.1	-0.00454265	0.811075692	0.981870181	RP11-358L22.3
ENSG00000105810.10	0.002112906	0.811095681	0.981870181	CDK6
ENSG00000125967.17	-0.002359397	0.811160067	0.981870181	NECAB3
ENSG00000106392.11	-0.00321422	0.811162838	0.981870181	C1GALT1
ENSG00000149260.18	0.004092295	0.811183689	0.981870181	CAPN5
ENSG00000235033.7	0.013169742	0.81118717	0.981870181	DAAM2-AS1
ENSG00000128346.11	0.003980279	0.811378339	0.982043613	C22orf23
ENSG00000273674.5	0.007806377	0.811615025	0.98221678	CTD-2378E12.1
ENSG00000240498.9	-0.004704345	0.811617201	0.98221678	CDKN2B-AS1
ENSG00000110848.8	0.005579393	0.811735343	0.982301789	CD69
ENSG00000117479.15	0.003866752	0.81180597	0.982329292	SLC19A2
ENSG00000099958.15	0.00598654	0.812080169	0.982568374	DERL3
ENSG00000107736.22	0.002392907	0.812139474	0.982568374	CDH23
ENSG00000164252.13	0.001987111	0.812237018	0.982568374	AGGF1
ENSG00000221990.5	0.00469269	0.812312966	0.982568374	EXOC3-AS1
ENSG00000163349.22	-0.002088821	0.812362094	0.982568374	HIPK1
ENSG00000232884.9	0.01071948	0.812421451	0.982568374	AF127936.3
ENSG00000186350.12	0.001880851	0.812499521	0.982568374	RXRA
ENSG00000258512.1	-0.00609638	0.812544657	0.982568374	LINC00239
ENSG00000115289.14	0.001810296	0.812559539	0.982568374	PCGF1
ENSG00000161011.20	-0.001986405	0.812600199	0.982568374	SQSTM1
ENSG00000198521.12	-0.002092967	0.812613548	0.982568374	ZNF43
ENSG00000129007.16	-0.003663579	0.812675312	0.982568374	CALML4
ENSG00000105171.10	0.001657139	0.812780764	0.982568374	POP4
ENSG00000228789.9	0.014953095	0.812800161	0.982568374	HCG22
ENSG00000256424.2	-0.013945187	0.812809243	0.982568374	LINC02414
ENSG00000174606.14	0.002253566	0.812810292	0.982568374	ANGEL2

ENSG00000105750.15	0.004659887	0.812818045	0.982568374	ZNF85
ENSG00000138161.14	0.00784571	0.812919397	0.982598687	CUZD1
ENSG00000122679.8	-0.010708895	0.812938947	0.982598687	RAMP3
ENSG00000137502.10	0.003705295	0.813100207	0.982671265	RAB30
ENSG00000280120.1	0.002291193	0.81323777	0.982671265	RP11-546D6.3
ENSG00000104064.18	0.001629493	0.813294068	0.982671265	GABPB1
ENSG00000065526.12	-0.002456569	0.813302311	0.982671265	SPEN
ENSG00000136783.10	-0.003442616	0.813324791	0.982671265	NIPSNAP3A
ENSG00000085662.14	0.001949109	0.813327622	0.982671265	AKR1B1
ENSG00000117505.13	-0.001642113	0.813437936	0.982671265	DR1
ENSG00000273064.1	-0.005499918	0.813466403	0.982671265	RP11-474G23.3
ENSG00000280385.1	-0.004221749	0.813522189	0.982671265	AP000648.5
ENSG00000197249.14	0.002649176	0.813529091	0.982671265	SERPINA1
ENSG00000160588.10	0.00234947	0.813656182	0.982671265	MPZL3
ENSG00000170464.10	0.002514523	0.813701991	0.982671265	DNAJC18
ENSG00000182648.13	0.005365666	0.813731865	0.982671265	LINC01006
ENSG00000182149.21	0.00106167	0.813827131	0.982671265	IST1
ENSG00000153147.6	0.001960267	0.813867844	0.982671265	SMARCA5
ENSG00000233836.7	0.00595533	0.813870242	0.982671265	ZNF726P1
ENSG00000265205.1	0.005773435	0.813915797	0.982671265	AC010761.13
ENSG00000151503.13	0.001875008	0.813933991	0.982671265	NCAPD3
ENSG00000237991.3	-0.006470082	0.813936687	0.982671265	RPL35P1
ENSG00000276070.5	0.007562033	0.813994656	0.982671265	CCL4L2
ENSG00000160602.14	-0.002526375	0.814005239	0.982671265	NEK8
ENSG00000129158.11	-0.002300299	0.814086103	0.982711037	SERGEF
ENSG00000170917.14	-0.004815524	0.81416827	0.982752377	NUDT6
ENSG00000237972.1	0.009453737	0.814247393	0.982786519	TUBG1P
ENSG00000167522.16	0.001625205	0.814292399	0.982786519	ANKRD11
ENSG00000165238.16	-0.0070932	0.814423262	0.982855599	WNK2
ENSG00000286688.1	0.007516019	0.814488178	0.982855599	CTD-3074O7.13
ENSG00000112282.18	0.002140424	0.814525069	0.982855599	MED23
ENSG00000227053.1	0.009534238	0.814542462	0.982855599	MUC12-AS1
ENSG00000137770.14	0.002366517	0.814589263	0.982855599	CTDSPL2
ENSG00000235280.2	0.006255217	0.814658205	0.982880954	MCF2L-AS1
ENSG00000146556.14	-0.00310507	0.814840032	0.98298895	WASH2P
ENSG00000106692.15	0.00236215	0.814861221	0.98298895	FKTN
ENSG00000144026.12	0.002904485	0.814891512	0.98298895	ZNF514
ENSG00000130635.16	-0.008621124	0.815097472	0.982996273	COL5A1
ENSG00000270084.2	0.004127693	0.815128536	0.982996273	GAS5-AS1
ENSG00000150672.18	0.007541617	0.815154018	0.982996273	DLG2
ENSG00000161203.13	-0.001942207	0.81516886	0.982996273	AP2M1
ENSG00000173200.13	-0.003053199	0.815179787	0.982996273	PARP15
ENSG00000240219.1	-0.002820522	0.815185177	0.982996273	RP11-430C7.5
ENSG00000170180.22	0.012959611	0.815245429	0.983011127	GYPA
ENSG00000265752.3	-0.008521401	0.815382046	0.983118055	RP11-403A21.1
ENSG00000187105.9	0.00371647	0.815434668	0.983123703	HEATR4
ENSG00000136504.13	0.001363707	0.815564345	0.983222244	KAT7

ENSG00000149591.17	0.004448818	0.815617222	0.983228191	TAGLN
ENSG00000259408.2	-0.006578064	0.815694546	0.983252819	RP11-3D4.3
ENSG00000186529.16	0.003454593	0.815733541	0.983252819	CYP4F3
ENSG00000139433.11	0.001398556	0.815825161	0.983305459	GLTP
ENSG00000254965.1	-0.008721689	0.815975724	0.983308509	C1DP5
ENSG00000256826.1	-0.00908705	0.815983525	0.983308509	ATP5MFP4
ENSG00000227373.6	-0.007576128	0.816109469	0.983308509	RABGAP1L-DT
ENSG00000264290.1	-0.003853452	0.816144907	0.983308509	RP11-68I3.4
ENSG00000167004.13	-0.001974949	0.816149546	0.983308509	PDIA3
ENSG00000134020.8	0.01268241	0.816235133	0.983308509	PEBP4
ENSG00000285999.1	-0.004747021	0.816255082	0.983308509	AC025442.4
ENSG00000226284.1	0.006780044	0.816257585	0.983308509	ARPC3P1
ENSG00000100983.12	0.001748131	0.816259218	0.983308509	GSS
ENSG00000167264.18	-0.002150847	0.81635235	0.983314254	DUS2
ENSG00000276758.1	-0.008418787	0.816359883	0.983314254	AC010518.2
ENSG00000259383.1	9.35E-04	0.816456063	0.983372347	RP11-403B2.6
ENSG00000180336.18	-0.006859002	0.816647229	0.983397276	MEIOC
ENSG00000070182.21	0.00721116	0.816707288	0.983397276	SPTB
ENSG00000137558.9	-0.046370136	0.81677265	0.983397276	PI15
ENSG00000287562.1	0.002929284	0.816947561	0.983397276	RP5-1120P11.6
ENSG00000014123.10	0.003109784	0.817014273	0.983397276	UFL1
ENSG00000234456.9	-0.008436365	0.817066273	0.983397276	MAGI2-AS3
ENSG00000171033.13	-0.003939524	0.817079143	0.983397276	PKIA
ENSG00000218537.1	-0.010447224	0.817101221	0.983397276	MIF-AS1
ENSG00000231584.8	0.002864824	0.81726815	0.983397276	FAHD2CP
ENSG00000237840.6	0.005220529	0.817320174	0.983397276	FAM21FP
ENSG00000015479.20	-0.003949478	0.817375292	0.983397276	MATR3
ENSG00000083097.15	0.002381283	0.817433935	0.983397276	DOP1A
ENSG00000132640.15	0.002966685	0.817566914	0.983397276	BTBD3
ENSG00000228463.10	0.007106772	0.817590519	0.983397276	AP006222.1
ENSG00000206140.12	-0.004611276	0.817631977	0.983397276	TMEM191C
ENSG00000137073.24	0.001661078	0.817645784	0.983397276	UBAP2
ENSG00000249717.1	-0.048789952	0.817666532	0.983397276	PARM1-AS1
ENSG00000171984.15	0.002477671	0.817701057	0.983397276	SHLD1
ENSG00000158050.5	-0.005184787	0.817761766	0.983397276	DUSP2
ENSG00000214922.9	0.0026521	0.817830243	0.983397276	HLA-F-AS1
ENSG00000116489.13	0.002890821	0.817895013	0.983397276	CAPZA1
ENSG00000049192.15	0.006592069	0.81790108	0.983397276	ADAMTS6
ENSG00000116852.15	-0.001882146	0.817922677	0.983397276	KIF21B
ENSG00000126003.7	0.00206425	0.817933198	0.983397276	PLAGL2
ENSG00000167600.14	0.004606238	0.817981601	0.983397276	CYP2S1
ENSG00000257878.1	0.004219052	0.818007351	0.983397276	RP11-256L6.3
ENSG00000082269.17	0.003485317	0.818107186	0.983397276	FAM135A
ENSG00000270574.1	-0.004167973	0.818113716	0.983397276	RP11-171I2.2
ENSG00000263826.1	0.007999578	0.818116797	0.983397276	RP11-573D15.9
ENSG00000169193.12	-0.003288016	0.818178077	0.983397276	CCDC126
ENSG00000106290.15	-0.00191555	0.818186163	0.983397276	TAF6

ENSG00000235665.6	0.006219429	0.818279942	0.983397276	LINC00298
ENSG00000249790.3	-0.012391234	0.818281862	0.983397276	RP11-20D14.6
ENSG00000272668.2	-0.005727991	0.81839101	0.983397276	RP11-190A12.10
ENSG00000273002.1	-0.002621183	0.818414793	0.983397276	ARHGEF2-AS2
ENSG00000183087.15	0.003416905	0.81856192	0.983397276	GAS6
ENSG00000237248.5	-0.005520637	0.818566706	0.983397276	LINC00987
ENSG00000122034.17	0.001828532	0.818594503	0.983397276	GTF3A
ENSG00000189423.13	-0.00632794	0.818631824	0.983397276	USP32P3
ENSG00000071051.14	-0.001896173	0.818676179	0.983397276	NCK2
ENSG00000229604.2	0.01593488	0.818725029	0.983397276	MTATP8P2
ENSG00000229828.2	-0.018694994	0.818879478	0.983397276	PDE4DIPP1
ENSG00000112182.15	0.003697051	0.818882536	0.983397276	BACH2
ENSG00000257802.1	0.011774915	0.818890712	0.983397276	MRS2P2
ENSG00000163354.15	-0.003184379	0.818927427	0.983397276	DCST2
ENSG00000231964.1	-0.002571569	0.818932457	0.983397276	RP11-67C2.2
ENSG00000271964.1	0.004399568	0.818945952	0.983397276	RP11-415F23.2
ENSG00000218739.10	-0.001817471	0.81901799	0.983397276	CEBPZOS
ENSG00000185885.17	0.003934883	0.81906497	0.983397276	IFITM1
ENSG00000260852.1	-0.004084043	0.819122748	0.983397276	FBXL19-AS1
ENSG00000264659.2	0.004419188	0.819130518	0.983397276	CTD-2006K23.2
ENSG00000277972.2	-0.002617891	0.819304779	0.983397276	CISD3
ENSG00000255026.1	-0.004381782	0.819316957	0.983397276	RP11-326C3.2
ENSG00000101654.18	-0.001189207	0.819398744	0.983397276	RNMT
ENSG00000236333.4	-0.012883806	0.819418612	0.983397276	TRHDE-AS1
ENSG00000286966.1	-0.012512668	0.819471573	0.983397276	RP1-127C7.8
ENSG00000109819.9	0.015451018	0.819499258	0.983397276	PPARGC1A
ENSG00000204581.3	-0.006774319	0.81952119	0.983397276	ACOXL-AS1
ENSG00000270472.3	0.01518445	0.819543527	0.983397276	IGHV3OR16-9
ENSG00000198951.12	-0.002201416	0.819606396	0.983397276	NAGA
ENSG00000137106.18	-0.002512126	0.819625889	0.983397276	GRHPR
ENSG00000215375.6	0.002801193	0.819626417	0.983397276	MYL5
ENSG00000221916.4	0.005904361	0.819642546	0.983397276	C19orf73
ENSG00000167384.11	0.002395074	0.819644771	0.983397276	ZNF180
ENSG00000142794.19	-0.005292091	0.819706408	0.983397276	NBPF3
ENSG00000270147.1	-0.006542027	0.819725872	0.983397276	RP11-646I6.6
ENSG00000278126.1	0.004242461	0.819771746	0.983397276	RP11-454E5.4
ENSG00000272864.1	-0.011672394	0.819800333	0.983397276	RP11-17E13.2
ENSG00000160539.6	0.005720412	0.819852808	0.983397276	PLPP7
ENSG00000288573.1	-0.00546778	0.819867409	0.983397276	RP11-7O11.3
ENSG00000167769.5	-0.005716212	0.820007677	0.983397276	ACER1
ENSG00000250507.1	0.0105371	0.82007406	0.983397276	AC008984.6
ENSG00000162337.12	0.004484213	0.820077882	0.983397276	LRP5
ENSG00000008282.9	0.001872585	0.820116655	0.983397276	SYPL1
ENSG00000123144.11	0.002332597	0.820147746	0.983397276	TRIR
ENSG00000272567.1	0.008421627	0.820217414	0.983397276	RP11-73K9.3
ENSG00000127337.7	-0.003322903	0.82027147	0.983397276	YEATS4
ENSG00000186815.13	0.002105738	0.820283376	0.983397276	TPCN1

ENSG00000141447.18	0.003059518	0.820323736	0.983397276	OSBPL1A
ENSG00000272836.1	0.003129595	0.820344148	0.983397276	RP3-402G11.27
ENSG00000185686.18	-0.03924862	0.820360864	0.983397276	PRAME
ENSG00000168028.14	-0.002947771	0.820462492	0.983461616	RPSA
ENSG00000259268.2	0.009798903	0.820523211	0.983476914	AC007950.1
ENSG00000116254.18	0.007701344	0.820580369	0.983487943	CHD5
ENSG00000274191.1	-0.007259325	0.820713251	0.983589724	RP11-324E6.10
ENSG00000146054.18	0.003377152	0.820809564	0.983615595	TRIM7
ENSG00000010818.10	0.002633538	0.820866485	0.983615595	HIVEP2
ENSG00000167601.12	0.006339431	0.820969887	0.983615595	AXL
ENSG00000280079.1	-0.005328847	0.820993504	0.983615595	CTC-260E6.4
ENSG00000025423.11	0.006540674	0.821013389	0.983615595	HSD17B6
ENSG00000160201.12	0.010893037	0.821022613	0.983615595	U2AF1
ENSG00000156384.15	0.003042835	0.821101346	0.983652457	SFR1
ENSG00000143727.16	0.001476193	0.82146906	0.984000323	ACP1
ENSG00000267414.1	0.005625753	0.821520371	0.984000323	SETBP1-DT
ENSG00000131773.14	0.008299078	0.821637746	0.984000323	KHDRBS3
ENSG00000112406.5	0.001894582	0.821653642	0.984000323	HECA
ENSG00000269887.1	0.010241364	0.821707628	0.984000323	RP11-477H21.2
ENSG00000011454.18	0.001338089	0.82176443	0.984000323	RABGAP1
ENSG00000285863.1	-0.006708636	0.821775179	0.984000323	RP11-16B9.3
ENSG00000148300.12	-0.001373031	0.821822143	0.984000323	REXO4
ENSG00000272343.1	0.004643314	0.821855043	0.984000323	RP11-140I16.3
ENSG00000235831.7	0.003935947	0.821871539	0.984000323	BHLHE40-AS1
ENSG00000273736.1	0.008977938	0.821945046	0.984030882	CTB-186H2.2
ENSG00000157873.18	0.001772579	0.822065549	0.984040865	TNFRSF14
ENSG00000064886.14	-0.005385597	0.822095935	0.984040865	CHI3L2
ENSG00000279555.1	-0.009036595	0.822100939	0.984040865	RP11-426D19.1
ENSG00000226803.9	-0.005535244	0.822177328	0.984040865	ZNF451-AS1
ENSG00000279026.1	0.004850953	0.822193301	0.984040865	RP3-414A15.12
ENSG00000231073.1	-0.007148675	0.822257609	0.984044812	RP11-316M1.3
ENSG00000178202.13	-0.002553852	0.82233921	0.984044812	POGLUT3
ENSG00000171246.6	-0.008814485	0.822396354	0.984044812	NPTX1
ENSG00000272913.1	0.006186519	0.822413783	0.984044812	RP11-440D17.3
ENSG00000175203.17	-0.001306619	0.822436517	0.984044812	DCTN2
ENSG00000131979.20	0.00300497	0.822491578	0.98405328	GCH1
ENSG00000158292.7	0.005164041	0.822582337	0.984070693	GPR153
ENSG00000204514.10	0.002511738	0.822639864	0.984070693	ZNF814
ENSG00000259802.1	-0.00570772	0.822650086	0.984070693	CTD-2256P15.2
ENSG00000124279.12	0.001928336	0.822774141	0.984106732	FASTKD3
ENSG00000111727.12	0.002512913	0.822839581	0.984106732	HCFC2
ENSG00000232742.3	-0.005893365	0.822910037	0.984106732	RHOQP2
ENSG00000240857.2	-0.002526218	0.822925808	0.984106732	RDH14
ENSG00000175727.14	-0.00186804	0.822956057	0.984106732	MLXIP
ENSG00000127666.10	-0.002110543	0.822968132	0.984106732	TICAM1
ENSG00000165244.7	-0.003239287	0.823039628	0.984134843	ZNF367
ENSG00000115756.13	-0.00188675	0.823128496	0.984183722	HPCAL1

ENSG00000103343.13	-0.001422182	0.823275288	0.984229144	ZNF174
ENSG00000081087.15	-0.002347207	0.823284277	0.984229144	OSTM1
ENSG00000151079.7	-0.006434006	0.823310462	0.984229144	KCNA6
ENSG00000173727.12	0.003471788	0.823427726	0.984241838	CMB9-22P13.1
ENSG00000136936.11	0.001912201	0.823464126	0.984241838	XPA
ENSG00000099804.9	0.004077652	0.82346506	0.984241838	CDC34
ENSG00000224152.1	-0.003436595	0.823750586	0.984362244	AC009506.1
ENSG00000101350.8	0.001565848	0.823761541	0.984362244	KIF3B
ENSG00000219891.2	0.007117478	0.823985436	0.984362244	ZSCAN12P1
ENSG00000261594.4	0.00832157	0.824068355	0.984362244	TPBGL
ENSG00000169031.21	0.007978571	0.824070218	0.984362244	COL4A3
ENSG00000173145.11	0.003099814	0.824080798	0.984362244	NOC3L
ENSG00000163958.14	-0.007053584	0.824082743	0.984362244	ZDHHC19
ENSG00000168610.16	-0.001728019	0.824135395	0.984362244	STAT3
ENSG00000231827.3	-0.007338292	0.824144544	0.984362244	RP11-216N14.5
ENSG00000211750.2	0.007550532	0.82428923	0.984362244	TRBV24-1
ENSG00000261804.2	0.005289399	0.824323808	0.984362244	RP11-44F14.2
ENSG00000253520.1	-0.005215823	0.824356833	0.984362244	RP11-798K23.5
ENSG00000240849.11	-0.001650169	0.824420217	0.984362244	PEDS1
ENSG00000184743.13	-0.001410573	0.824577247	0.984362244	ATL3
ENSG00000241878.11	-0.002192978	0.824583224	0.984362244	PISD
ENSG00000166579.16	0.001623976	0.824628274	0.984362244	NDEL1
ENSG00000189269.12	0.006249656	0.824774173	0.984362244	DRICH1
ENSG00000227502.3	-0.012495191	0.824817952	0.984362244	MROCKI
ENSG00000139289.14	0.0034591	0.824832572	0.984362244	PHLDA1
ENSG00000174886.14	-0.002392819	0.824838041	0.984362244	NDUFA11
ENSG00000128283.7	0.006770835	0.824855491	0.984362244	CDC42EP1
ENSG00000143297.19	-0.009070979	0.824923858	0.984362244	FCRL5
ENSG00000215548.2	0.008353838	0.824956053	0.984362244	FRG1JP
ENSG00000110628.16	-0.003586097	0.824956808	0.984362244	SLC22A18
ENSG00000120645.12	-0.011796437	0.824965041	0.984362244	IQSEC3
ENSG00000197702.14	-0.006505243	0.825054489	0.984362244	PARVA
ENSG00000148841.17	0.001812277	0.82509663	0.984362244	ITPRIP
ENSG00000085377.15	0.001245383	0.825156162	0.984362244	PREP
ENSG00000025708.14	-0.003712079	0.825185335	0.984362244	TYMP
ENSG00000090581.11	-0.001450676	0.825215795	0.984362244	GNPTG
ENSG00000198087.7	-0.003198788	0.82525279	0.984362244	CD2AP
ENSG00000268500.6	0.004074645	0.82533585	0.984362244	SIGLEC5
ENSG00000287185.1	-0.005912515	0.825361717	0.984362244	RP11-69M11.1
ENSG00000164104.12	-0.003467711	0.825407789	0.984362244	HMGB2
ENSG00000171130.19	-0.002577944	0.825518449	0.984362244	ATP6V0E2
ENSG00000100105.18	-0.00132489	0.8256802	0.984362244	PATZ1
ENSG00000287892.1	0.005952568	0.825766704	0.984362244	RP11-505E24.4
ENSG00000233922.3	0.007683985	0.825821152	0.984362244	LINC01694
ENSG00000182795.13	-0.006383799	0.825822758	0.984362244	C1orf116
ENSG00000108469.15	0.001699858	0.825942589	0.984362244	RECQL5
ENSG00000128253.15	0.006577581	0.825951222	0.984362244	RFPL2

ENSG00000100068.14	0.002320501	0.82605163	0.984362244	LRP5L
ENSG00000165929.13	0.002835279	0.826075768	0.984362244	TC2N
ENSG00000166783.22	0.001648834	0.826087533	0.984362244	MARF1
ENSG00000213235.3	0.008349159	0.826098184	0.984362244	EEF1A1P16
ENSG00000226763.5	-0.004379589	0.82622523	0.984362244	SRRM5
ENSG00000101347.11	0.001742157	0.826234074	0.984362244	SAMHD1
ENSG00000274487.2	-0.004393206	0.826245716	0.984362244	NPEPPSP1
ENSG00000132334.17	-0.001389863	0.826296964	0.984362244	PTPRE
ENSG00000176715.17	-0.00143748	0.826307084	0.984362244	ACSF3
ENSG00000137845.15	-0.002113338	0.826307564	0.984362244	ADAM10
ENSG00000124193.16	0.001346842	0.826315985	0.984362244	SRSF6
ENSG00000175344.19	0.011021149	0.826340487	0.984362244	CHRNA7
ENSG00000205629.12	-0.001659512	0.826341728	0.984362244	LCMT1
ENSG00000274292.1	0.003985269	0.826448613	0.984362244	RP11-347I19.7
ENSG00000235513.2	0.003476805	0.826497247	0.984362244	L3MBTL2-AS1
ENSG00000187164.20	0.004008288	0.826504932	0.984362244	SHTN1
ENSG00000058673.17	-0.00551186	0.82658949	0.984362244	ZC3H11A
ENSG00000186522.15	-0.00352366	0.826624478	0.984362244	SEPTIN10
ENSG00000278765.1	-0.006150317	0.826657189	0.984362244	RP5-890E16.5
ENSG00000112576.13	0.001936096	0.826727484	0.984362244	CCND3
ENSG00000212864.3	-0.005344698	0.826789533	0.984362244	RNF208
ENSG00000100284.22	-0.002345203	0.826853454	0.984362244	TOM1
ENSG00000167106.12	0.002790678	0.826891191	0.984362244	FAM102A
ENSG00000178188.15	0.00146237	0.826906201	0.984362244	SH2B1
ENSG00000144401.14	-0.001804855	0.82691157	0.984362244	METTL21A
ENSG00000189042.14	0.003211692	0.826914952	0.984362244	ZNF567
ENSG00000261235.1	0.01202214	0.826956517	0.984362244	RP11-510J16.5
ENSG00000258484.4	-0.014308002	0.826962988	0.984362244	SPESP1
ENSG00000100154.14	0.004516344	0.826989241	0.984362244	TTC28
ENSG00000175170.16	0.008979098	0.827111711	0.984362244	FAM182B
ENSG00000253955.2	0.00560015	0.827119873	0.984362244	CTB-33O18.3
ENSG00000151292.17	0.00293882	0.827128413	0.984362244	CSNK1G3
ENSG00000115484.15	-0.001467383	0.827166178	0.984362244	CCT4
ENSG00000137834.15	0.006753083	0.827443673	0.984362244	SMAD6
ENSG00000244219.7	-0.005690888	0.827446283	0.984362244	TMEM225B
ENSG00000231160.10	0.004164719	0.827472011	0.984362244	KLF3-AS1
ENSG00000169738.8	-0.002994616	0.827488239	0.984362244	DCXR
ENSG00000132763.15	-0.002037744	0.827506251	0.984362244	MMACHC
ENSG00000260314.3	0.006785839	0.827531491	0.984362244	MRC1
ENSG00000170571.12	0.002010105	0.827541816	0.984362244	EMB
ENSG00000170091.11	-0.009407494	0.827564191	0.984362244	NSG2
ENSG00000176542.10	-0.002026208	0.827627004	0.984362244	USF3
ENSG00000251791.1	-0.007920775	0.827644526	0.984362244	SCARNA6
ENSG00000287074.1	-0.003509437	0.827645708	0.984362244	RP11-678N14.1
ENSG00000175567.11	0.002373148	0.827762743	0.984393756	UCP2
ENSG00000135821.19	0.001956184	0.827865751	0.984393756	GLUL
ENSG00000196150.14	-0.00154868	0.827925317	0.984393756	ZNF250

ENSG00000275703.1	0.006244762	0.827993197	0.984393756	U47924.32
ENSG00000213492.2	0.008941527	0.827999107	0.984393756	NT5C3AP1
ENSG00000205810.9	-0.008824743	0.828047534	0.984393756	KLRC3
ENSG00000272150.5	0.00464485	0.828104179	0.984393756	NBPF25P
ENSG00000198879.13	-0.002363927	0.828125091	0.984393756	SFMBT2
ENSG00000125817.8	-0.001936542	0.82812782	0.984393756	CENPB
ENSG00000122707.12	-0.002317282	0.828152208	0.984393756	RECK
ENSG00000246465.1	0.006280776	0.828256582	0.984460762	RP11-57A19.2
ENSG00000121742.19	0.008044764	0.828326699	0.984487044	GJB6
ENSG00000166780.11	0.002969433	0.828392004	0.984507604	BMERB1
ENSG00000260351.1	-0.008694281	0.828461858	0.984514571	RP11-382A20.6
ENSG00000280077.1	0.002397855	0.828493878	0.984514571	RP11-87H9.4
ENSG00000124104.19	0.00229886	0.828609024	0.984544034	SNX21
ENSG00000119711.13	-0.001850669	0.828614688	0.984544034	ALDH6A1
ENSG00000251661.3	-0.004755528	0.829004285	0.98455457	RP11-326C3.11
ENSG00000002549.13	-0.005209722	0.829052107	0.98455457	LAP3
ENSG00000105701.16	-0.005281941	0.829072945	0.98455457	FKBP8
ENSG00000180155.20	-0.005286129	0.829122774	0.98455457	LYNX1
ENSG00000108219.15	0.00123703	0.829154466	0.98455457	TSPAN14
ENSG00000275393.1	-0.005812199	0.829248335	0.98455457	RP11-568J23.8
ENSG00000099910.17	-0.001556347	0.829267748	0.98455457	KLHL22
ENSG00000177311.12	-0.002999498	0.829305001	0.98455457	ZBTB38
ENSG00000260022.1	0.0034028	0.829349535	0.98455457	LA16c-306A4.1
ENSG00000273226.1	0.00337896	0.829451778	0.98455457	RP11-513M16.8
ENSG00000163607.17	0.00179653	0.829489229	0.98455457	GTPBP8
ENSG00000275216.2	0.010825383	0.829544872	0.98455457	RP11-54H7.4
ENSG00000169826.8	0.002346758	0.829549631	0.98455457	CSGALNACT2
ENSG00000270184.1	-0.005536849	0.829556354	0.98455457	RP11-568J23.5
ENSG00000184441.4	0.002437116	0.829621197	0.98455457	AP001062.7
ENSG00000176761.7	0.007231043	0.829640083	0.98455457	ZNF285B
ENSG00000249494.6	-0.010229593	0.829643396	0.98455457	CTB-161M19.4
ENSG00000120699.13	-0.00164503	0.829714626	0.98455457	EXOSC8
ENSG00000101266.19	9.68E-04	0.829743854	0.98455457	CSNK2A1
ENSG00000186642.16	0.004013864	0.829776863	0.98455457	PDE2A
ENSG00000285816.1	0.004219432	0.82980692	0.98455457	RP11-867O8.9
ENSG00000271858.5	0.00305017	0.829889303	0.98455457	CYB561D2
ENSG00000134207.17	-0.011898669	0.82989351	0.98455457	SYT6
ENSG00000086827.9	0.001318651	0.829939962	0.98455457	ZW10
ENSG00000114331.15	-0.002125408	0.829942154	0.98455457	ACAP2
ENSG00000171791.14	0.002349575	0.830034485	0.98455457	BCL2
ENSG00000259293.1	0.010665763	0.830041955	0.98455457	LIPC-AS1
ENSG00000158062.20	0.002060957	0.830130051	0.98455457	UBXN11
ENSG00000091536.19	0.003693607	0.830204812	0.98455457	MYO15A
ENSG00000273148.1	0.003534167	0.830223112	0.98455457	LINC00653
ENSG00000261786.1	-0.009413998	0.83023894	0.98455457	RP4-555D20.2
ENSG00000162104.10	-0.00296739	0.830239495	0.98455457	ADCY9
ENSG00000286548.1	0.009449813	0.830551962	0.98455457	RP4-644F6.4

ENSG00000113569.16	-0.001251181	0.830558745	0.98455457	NUP155
ENSG00000236242.1	-0.010826418	0.830693696	0.98455457	MYO16-AS1
ENSG00000106258.15	-0.002738849	0.830702762	0.98455457	CYP3A5
ENSG00000164556.7	0.006413098	0.830732136	0.98455457	FAM183BP
ENSG00000123992.20	0.001754323	0.830810697	0.98455457	DNPEP
ENSG00000166348.19	-0.002085991	0.831050808	0.98455457	USP54
ENSG00000065054.14	-0.003527355	0.831160736	0.98455457	SLC9A3R2
ENSG00000184860.10	-0.003140988	0.831229896	0.98455457	SDR42E1
ENSG00000170085.18	-0.001676285	0.831317938	0.98455457	SIMC1
ENSG00000244116.3	-0.007163304	0.831346831	0.98455457	IGKV2-28
ENSG00000198841.4	0.002181663	0.831363144	0.98455457	KTI12
ENSG00000080371.6	-0.001557705	0.831399455	0.98455457	RAB21
ENSG00000135018.14	-0.001181754	0.831407461	0.98455457	UBQLN1
ENSG00000164845.16	0.004709434	0.831459048	0.98455457	FAM86FP
ENSG00000134970.14	-0.002260476	0.8314901	0.98455457	TMED7
ENSG00000151414.15	-0.002986496	0.831596738	0.98455457	NEK7
ENSG00000106268.16	-0.002503724	0.831662057	0.98455457	NUDT1
ENSG00000272754.1	0.006287981	0.831777376	0.98455457	AL133245.2
ENSG00000280109.3	-0.011949099	0.831799037	0.98455457	PLAC4
ENSG00000131885.17	-0.014918023	0.831859747	0.98455457	KRT17P1
ENSG00000123066.9	0.002490671	0.831872674	0.98455457	MED13L
ENSG00000174059.17	0.006297223	0.831890971	0.98455457	CD34
ENSG00000261055.1	-0.007956443	0.832013463	0.98455457	RP11-195M16.3
ENSG00000154099.18	-0.006195938	0.832037089	0.98455457	DNAAF1
ENSG00000134490.14	0.002499673	0.832042026	0.98455457	TMEM241
ENSG00000129465.16	0.001534235	0.832057539	0.98455457	RIPK3
ENSG00000235609.7	0.004864507	0.83208636	0.98455457	AF127936.9
ENSG00000189060.6	-0.004143007	0.832178571	0.98455457	H1-0
ENSG00000125834.13	0.001142827	0.832223874	0.98455457	STK35
ENSG00000163874.11	0.001656036	0.832280474	0.98455457	ZC3H12A
ENSG00000121769.8	0.00342413	0.832371326	0.98455457	FABP3
ENSG00000270964.1	-0.006201508	0.832389332	0.98455457	MAP2K5-DT
ENSG00000279532.1	-0.007693092	0.832444208	0.98455457	CTB-96E2.6
ENSG00000213904.9	-0.004049299	0.83248141	0.98455457	LIPE-AS1
ENSG00000226833.6	0.003999283	0.832486076	0.98455457	AC097724.3
ENSG00000141448.11	-0.010461864	0.83252394	0.98455457	GATA6
ENSG00000183530.14	-0.001295365	0.832541356	0.98455457	PRR14L
ENSG00000137275.16	0.001121205	0.832549885	0.98455457	RIPK1
ENSG00000151470.13	0.002641342	0.832580841	0.98455457	C4orf33
ENSG00000188707.6	0.00210775	0.832584221	0.98455457	ZBED6CL
ENSG00000117245.13	0.007053889	0.832702805	0.98455457	KIF17
ENSG00000203872.7	0.00341194	0.832729606	0.98455457	C6orf163
ENSG00000225885.7	-0.00890837	0.832786662	0.98455457	AC023590.1
ENSG00000234741.8	-0.002184706	0.832824672	0.98455457	GAS5
ENSG00000144214.10	0.003236421	0.832844424	0.98455457	LYG1
ENSG00000206384.10	0.008013178	0.832849066	0.98455457	COL6A6
ENSG00000147649.10	0.001359621	0.832989082	0.98455457	MTDH

ENSG00000139579.13	0.001858942	0.83301533	0.98455457	NABP2
ENSG00000211666.2	-0.007066582	0.833429307	0.98455457	IGLV2-14
ENSG00000204291.11	-0.007084927	0.833437631	0.98455457	COL15A1
ENSG00000162745.11	-0.004568728	0.833479193	0.98455457	OLFML2B
ENSG00000248256.1	0.007535598	0.83352044	0.98455457	OCIAD1-AS1
ENSG00000223583.1	0.006936604	0.83352553	0.98455457	RP3-476K8.4
ENSG00000267053.8	-0.011449141	0.833532523	0.98455457	CTD-3162L10.1
ENSG00000255092.1	-0.007593768	0.833555864	0.98455457	RP11-58K22.4
ENSG00000246308.2	0.004679544	0.833616123	0.98455457	RP11-685M7.3
ENSG00000104361.10	-0.003778365	0.833678932	0.98455457	NIPAL2
ENSG00000159917.17	-0.002240187	0.833723557	0.98455457	ZNF235
ENSG00000004468.13	-0.003550633	0.833748055	0.98455457	CD38
ENSG00000223343.1	0.006532803	0.833870283	0.98455457	RP13-131K19.2
ENSG00000274512.6	-0.008176149	0.83392594	0.98455457	TBC1D3L
ENSG00000106400.12	0.001843536	0.834023254	0.98455457	ZNHIT1
ENSG00000214826.5	0.003762515	0.834034205	0.98455457	DDX12P
ENSG00000144152.13	-0.003998518	0.83404197	0.98455457	FBLN7
ENSG00000168807.16	0.002069285	0.834079642	0.98455457	SNTB2
ENSG00000092051.17	0.003638969	0.834149113	0.98455457	JPH4
ENSG00000112234.9	0.001515085	0.83416064	0.98455457	FBXL4
ENSG00000197888.3	0.012202962	0.834189777	0.98455457	UGT2B17
ENSG00000273027.1	-0.00672663	0.834222917	0.98455457	LL21NC02-1C16.1
ENSG00000256139.2	0.006988502	0.83423181	0.98455457	RP11-968O1.5
ENSG00000238121.6	-0.002306989	0.834272775	0.98455457	LINC00426
ENSG00000090674.16	0.002436085	0.83434321	0.98455457	MCOLN1
ENSG00000183763.9	-0.002798936	0.834362032	0.98455457	TRAIP
ENSG00000267060.7	-0.007512412	0.83451724	0.98455457	PTGES3L
ENSG00000116918.15	0.002632983	0.834542887	0.98455457	TSNAX
ENSG00000236449.2	-0.002602012	0.834572224	0.98455457	AC018890.6
ENSG00000254946.2	0.010109055	0.834593655	0.98455457	LINC02751
ENSG00000165802.23	0.002182566	0.834675389	0.98455457	NSMF
ENSG00000166471.11	0.002042288	0.834676588	0.98455457	TMEM41B
ENSG00000163584.18	0.006075942	0.834688685	0.98455457	RPL22L1
ENSG00000106772.18	-0.007467007	0.834697231	0.98455457	PRUNE2
ENSG00000236345.1	0.003122947	0.83471046	0.98455457	SCAT8
ENSG00000109047.8	0.008548697	0.834729055	0.98455457	RCVRN
ENSG00000233846.2	0.006146578	0.834883788	0.98455457	SELENOTP1
ENSG00000110851.12	-0.001166238	0.834914619	0.98455457	PRDM4
ENSG00000149658.18	-9.24E-04	0.83493793	0.98455457	YTHDF1
ENSG00000269564.1	-0.004947357	0.83494319	0.98455457	AC008753.4
ENSG00000235408.6	0.00515891	0.835026638	0.98455457	SNORA71B
ENSG00000211659.2	0.007914412	0.83502981	0.98455457	IGLV3-25
ENSG00000198887.9	0.001963328	0.835034982	0.98455457	SMC5
ENSG00000258702.3	0.00710927	0.835086664	0.98455457	RP11-433J8.1
ENSG00000232682.2	0.01042976	0.83511005	0.98455457	RP11-388P9.2
ENSG00000109814.12	0.002166378	0.83512808	0.98455457	UGDH
ENSG00000276691.1	-0.005791432	0.835239412	0.98455457	RP5-1057I20.5

ENSG00000134363.12	0.028678403	0.835243636	0.98455457	FST
ENSG00000145882.11	0.001658244	0.83527118	0.98455457	PCYOX1L
ENSG00000171148.14	-0.001619257	0.835271924	0.98455457	TADA3
ENSG00000068615.19	-0.013215493	0.83528611	0.98455457	REEP1
ENSG00000072415.10	0.001921091	0.835306855	0.98455457	MPP5
ENSG00000198625.13	0.001356231	0.835340309	0.98455457	MDM4
ENSG00000116761.12	-0.004597868	0.835344396	0.98455457	CTH
ENSG00000176054.6	-0.005286818	0.835368348	0.98455457	RPL23P2
ENSG00000213920.9	0.005049653	0.835382718	0.98455457	MDP1
ENSG00000227403.2	0.005527836	0.835393204	0.98455457	LINC01806
ENSG00000271009.3	-0.004166068	0.835537722	0.98455457	RP11-346C20.4
ENSG00000285230.2	0.002852045	0.835695707	0.98455457	RALY-AS1
ENSG00000198835.4	-0.005331431	0.835728024	0.98455457	GJC2
ENSG00000242445.1	0.006835294	0.83577461	0.98455457	RPL7AP11
ENSG00000231826.6	-0.00485601	0.83578515	0.98455457	LINC01819
ENSG00000173542.9	0.002812462	0.835838677	0.98455457	MOB1B
ENSG00000279464.1	0.006479511	0.835999957	0.98455457	RP11-17P16.1
ENSG00000039523.20	0.00183749	0.836013233	0.98455457	RIPOR1
ENSG00000266777.1	-0.002967821	0.836024153	0.98455457	SH3GL1P1
ENSG00000112414.15	-0.006518606	0.836098192	0.98455457	ADGRG6
ENSG00000267440.2	0.007947131	0.83615045	0.98455457	LINC02594
ENSG00000286584.2	0.00295374	0.836227158	0.98455457	RP11-59M4.2
ENSG00000246582.3	0.003639174	0.836266099	0.98455457	RP11-1149O23.3
ENSG00000261996.1	-0.006163133	0.836291967	0.98455457	CTC-281F24.1
ENSG00000196843.17	-0.002047229	0.836447328	0.98455457	ARID5A
ENSG00000135655.16	0.002193656	0.836544881	0.98455457	USP15
ENSG00000167880.8	0.007590965	0.836579226	0.98455457	EVPL
ENSG00000175130.7	-0.002440915	0.83660336	0.98455457	MARCKSL1
ENSG00000130775.16	0.001651799	0.836621726	0.98455457	THEMIS2
ENSG00000233184.7	0.002717316	0.836624941	0.98455457	RP11-421L21.3
ENSG00000126368.6	-0.002746714	0.836683288	0.98455457	NR1D1
ENSG00000242247.11	0.001215478	0.836752396	0.98455457	ARFGAP3
ENSG00000213300.5	-0.004994898	0.836879245	0.98455457	HNRNPA3P6
ENSG00000267080.6	-0.001505961	0.836919782	0.98455457	ASB16-AS1
ENSG00000275234.1	0.003689813	0.836962835	0.98455457	CTD-2396E7.11
ENSG00000171503.13	0.001445078	0.836968699	0.98455457	ETFDH
ENSG00000140678.17	-0.002310799	0.836985183	0.98455457	ITGAX
ENSG00000272686.1	-0.003515946	0.836988498	0.98455457	WASL-DT
ENSG00000091136.15	-0.002944688	0.837033766	0.98455457	LAMB1
ENSG00000128641.19	-0.006149164	0.837075323	0.98455457	MYO1B
ENSG00000138439.12	-0.002321706	0.837075389	0.98455457	FAM117B
ENSG00000164631.19	0.002090903	0.837084249	0.98455457	ZNF12
ENSG00000211717.3	0.011381	0.83708833	0.98455457	TRBV10-1
ENSG00000279631.1	-0.004149368	0.837107479	0.98455457	RP11-573G6.4
ENSG00000101417.12	0.00124885	0.837134243	0.98455457	PXMP4
ENSG00000103351.13	-0.00288305	0.837189696	0.98455457	CLUAP1
ENSG00000085465.13	0.003013663	0.837220696	0.98455457	OVGP1

ENSG00000088340.17	0.006432552	0.837237498	0.98455457	FER1L4
ENSG00000278917.1	-0.006823554	0.837244388	0.98455457	RP11-15A1.4
ENSG00000112893.10	0.001861867	0.837244832	0.98455457	MAN2A1
ENSG00000273173.5	-0.005823953	0.837250574	0.98455457	SNURF
ENSG00000169217.9	-0.001310647	0.837266894	0.98455457	CD2BP2
ENSG00000240970.1	0.004666981	0.837306914	0.98455457	RPL23AP64
ENSG00000120733.14	-0.001439178	0.837316321	0.98455457	KDM3B
ENSG00000258912.2	-0.00637726	0.837367382	0.98455457	LINC02316
ENSG00000278869.1	-0.004452081	0.837426223	0.98455457	CITF22-49E9.3
ENSG00000156502.14	-0.001238779	0.837555138	0.98455457	SUPV3L1
ENSG00000100109.17	0.001839311	0.837890495	0.98455457	TFIP11
ENSG00000231084.2	0.005763443	0.838001121	0.98455457	RPL22P24
ENSG00000236519.1	-0.007584702	0.838010886	0.98455457	LINC01424
ENSG00000149474.14	-0.00119045	0.838051434	0.98455457	KAT14
ENSG00000137714.3	0.002001232	0.838077016	0.98455457	FDX1
ENSG00000006534.16	0.001982821	0.838077098	0.98455457	ALDH3B1
ENSG00000259658.5	-0.003688407	0.838104764	0.98455457	RP11-89K11.1
ENSG00000280187.1	-0.00622573	0.838109506	0.98455457	CTC-351M12.1
ENSG00000018699.13	0.001402453	0.83811972	0.98455457	TTC27
ENSG00000148400.13	-0.002386219	0.838226546	0.98455457	NOTCH1
ENSG00000144893.12	0.004178419	0.838302691	0.98455457	MED12L
ENSG00000170011.14	-0.008928993	0.838436958	0.98455457	MYRIP
ENSG00000160352.16	0.003943154	0.838457548	0.98455457	ZNF714
ENSG00000213759.10	0.007478783	0.838521536	0.98455457	UGT2B11
ENSG00000236670.1	0.005536133	0.838532428	0.98455457	KRT18P5
ENSG00000133597.11	0.001918655	0.838558131	0.98455457	ADCK2
ENSG00000243709.1	0.00653189	0.83859108	0.98455457	LEFTY1
ENSG00000221823.11	0.001440029	0.838637388	0.98455457	PPP3R1
ENSG00000196914.9	0.003518311	0.838681096	0.98455457	ARHGEF12
ENSG00000132563.16	-0.00507739	0.838689712	0.98455457	REEP2
ENSG00000275832.5	0.005043462	0.838728238	0.98455457	ARHGAP23
ENSG00000174306.22	-0.001683555	0.838755158	0.98455457	ZHX3
ENSG00000250159.7	0.004071682	0.838843875	0.98455457	RP11-381K20.2
ENSG00000107099.17	-0.002034464	0.83885345	0.98455457	DOCK8
ENSG00000175841.8	-0.007833462	0.838885801	0.98455457	FAM172BP
ENSG00000147526.20	0.001436064	0.838887077	0.98455457	TACC1
ENSG00000136270.14	0.001277009	0.838954737	0.98455457	TBRG4
ENSG00000148832.16	0.003412079	0.838983762	0.98455457	PAOX
ENSG00000214595.12	0.0059095	0.839013326	0.98455457	EML6
ENSG00000167258.15	0.001439514	0.83903264	0.98455457	CDK12
ENSG00000178971.16	0.00145004	0.839108631	0.98455457	CTC1
ENSG00000204634.13	0.00258941	0.839118556	0.98455457	TBC1D8
ENSG00000283537.2	-0.024069581	0.839124644	0.98455457	RP11-298A10.3
ENSG00000277566.1	0.009038076	0.839155734	0.98455457	RP11-545P7.9
ENSG00000186635.15	-0.001560777	0.839190455	0.98455457	ARAP1
ENSG00000287088.1	-0.015124818	0.839220053	0.98455457	AP001066.12
ENSG00000133105.8	-0.005935352	0.839253401	0.98455457	RXFP2

ENSG00000114626.18	-0.002060845	0.839364497	0.98455457	ABTB1
ENSG00000286220.1	-0.006227643	0.839369448	0.98455457	RP11-946P6.7
ENSG00000132326.12	0.001575685	0.839377419	0.98455457	PER2
ENSG00000181817.6	0.001685501	0.839546464	0.984650313	LSM10
ENSG00000197813.6	-0.003202036	0.83955507	0.984650313	CTC-301O7.4
ENSG00000142892.15	0.002349764	0.839671664	0.984665644	PIGK
ENSG00000261416.1	0.002024439	0.839711344	0.984665644	CORO1A-AS1
ENSG00000095574.12	0.002109983	0.839717455	0.984665644	IKZF5
ENSG00000109320.13	0.00147919	0.83991831	0.984665644	NFKB1
ENSG00000282024.1	-0.009593331	0.839924484	0.984665644	RP3-467D16.3
ENSG00000197081.16	0.002762138	0.839953586	0.984665644	IGF2R
ENSG00000235204.1	0.007135973	0.840018834	0.984665644	RP11-121A14.2
ENSG00000226329.2	0.004430957	0.840057443	0.984665644	AC005682.6
ENSG00000103199.14	0.001595639	0.840063201	0.984665644	ZNF500
ENSG00000110200.8	0.002124542	0.840071815	0.984665644	ANAPC15
ENSG00000244274.9	0.003880448	0.840181655	0.984665644	DBNDD2
ENSG00000145495.16	0.001396268	0.84018962	0.984665644	MARCHF6
ENSG00000100380.14	-0.001310297	0.840247427	0.984665644	ST13
ENSG00000161642.18	0.002489281	0.840354874	0.984665644	ZNF385A
ENSG00000286018.1	0.00304885	0.840400652	0.984665644	RP1-100J12.2
ENSG00000164031.17	-0.003132948	0.840403822	0.984665644	DNAJB14
ENSG00000266066.1	0.00995086	0.840419801	0.984665644	POLRMTP1
ENSG00000232859.10	0.00253183	0.84050814	0.984665644	LYRM9
ENSG00000242551.2	-0.009047371	0.840526909	0.984665644	POU5F1P6
ENSG00000123892.12	-0.006326973	0.840528416	0.984665644	RAB38
ENSG00000071242.12	0.002422766	0.840630439	0.984704817	RPS6KA2
ENSG00000211445.12	0.003979357	0.840657886	0.984704817	GPX3
ENSG00000271871.1	0.004072934	0.840730084	0.984730453	AC005740.6
ENSG00000001629.10	0.002393824	0.840908519	0.984730453	ANKIB1
ENSG00000255389.1	-0.003414213	0.841000981	0.984730453	C6orf3
ENSG00000213085.10	0.00381393	0.841073442	0.984730453	CFAP45
ENSG00000247400.4	-0.003307624	0.84109572	0.984730453	DNAJC3-DT
ENSG00000176641.11	0.008532047	0.841120522	0.984730453	RNF152
ENSG00000237651.8	0.004457048	0.841126317	0.984730453	C2orf74
ENSG00000115556.14	0.007495268	0.841134715	0.984730453	PLCD4
ENSG00000140474.14	0.001419106	0.841157072	0.984730453	ULK3
ENSG00000157240.4	0.002842775	0.841159941	0.984730453	FZD1
ENSG00000066322.15	-0.001375496	0.841290442	0.98482701	ELOVL1
ENSG00000141698.17	0.004058883	0.841424777	0.984872825	NT5C3B
ENSG00000197299.13	0.002039828	0.841425628	0.984872825	BLM
ENSG00000159212.13	-0.007627989	0.841523004	0.984930589	CLIC6
ENSG00000281831.1	-0.009574689	0.841591517	0.984951428	HCP5B
ENSG00000261056.2	0.008268361	0.841670931	0.984951428	RP11-454F8.2
ENSG00000244119.1	-0.003321782	0.841725317	0.984951428	PDCL3P4
ENSG00000246627.6	0.003091841	0.841779822	0.984951428	CACNA1C-AS1
ENSG00000170484.10	0.010653863	0.841780948	0.984951428	KRT74
ENSG00000174482.10	0.008119997	0.842009617	0.98509735	LINGO2

ENSG00000264520.1	-0.008245189	0.842053518	0.98509735	RP4-777O23.2
ENSG00000258365.1	0.003539152	0.842118051	0.98509735	RP11-1105G2.3
ENSG00000284308.2	0.003845398	0.842166697	0.98509735	C2orf81
ENSG00000260105.6	0.005727741	0.842210465	0.98509735	AOC4P
ENSG00000160688.19	-0.00127956	0.842281556	0.98509735	FLAD1
ENSG00000126215.14	0.001770061	0.842287247	0.98509735	XRCC3
ENSG00000174600.14	-0.003592171	0.842289937	0.98509735	CMKLR1
ENSG00000253368.4	-0.004909451	0.842370452	0.985135335	TRNP1
ENSG00000225953.3	0.006342616	0.842487039	0.985165835	SATB2-AS1
ENSG00000175711.8	-0.002291576	0.842503024	0.985165835	B3GNTL1
ENSG00000267895.1	0.003814472	0.842572266	0.985165835	SIGLEC19P
ENSG00000164040.18	-0.001001874	0.842588685	0.985165835	PGRMC2
ENSG00000145358.6	0.010190957	0.842784302	0.985315424	DDIT4L
ENSG00000124209.4	0.001150634	0.842821226	0.985315424	RAB22A
ENSG00000173875.14	-0.001252344	0.843031378	0.985315424	ZNF791
ENSG00000171097.14	-0.003417364	0.843050059	0.985315424	KYAT1
ENSG00000230918.2	0.004790115	0.843168971	0.985315424	DPP4-DT
ENSG00000106299.8	-0.001895161	0.843250387	0.985315424	WASL
ENSG00000143862.8	-0.001303689	0.843270422	0.985315424	ARL8A
ENSG00000262766.1	0.003026476	0.843281278	0.985315424	RP11-196G11.4
ENSG00000234175.1	-0.00482714	0.8433654	0.985315424	RP11-730A19.9
ENSG00000254739.1	-0.008849379	0.843623559	0.985315424	RP13-46H24.1
ENSG00000047365.12	-0.002665942	0.843696254	0.985315424	ARAP2
ENSG00000234160.1	0.00266547	0.843728784	0.985315424	RP11-613M10.6
ENSG00000288710.1	0.006499567	0.84373786	0.985315424	RP11-386G11.12
ENSG00000254851.1	-0.019449656	0.843754223	0.985315424	RP11-109L13.1
ENSG00000144161.13	-0.00153081	0.843858008	0.985315424	ZC3H8
ENSG00000223461.1	-0.006312906	0.843886829	0.985315424	AC004471.9
ENSG00000204922.5	-0.00222352	0.843914881	0.985315424	UQCC3
ENSG00000253878.5	0.006860466	0.843915419	0.985315424	RP11-347C18.3
ENSG00000246089.3	-0.002723164	0.84396876	0.985315424	RP11-115C21.2
ENSG00000186193.9	-0.007168308	0.843978353	0.985315424	SAPCD2
ENSG00000114857.18	-0.001970648	0.844013188	0.985315424	NKTR
ENSG00000131095.14	-0.006941652	0.84403795	0.985315424	GFAP
ENSG00000136108.15	0.002160537	0.844145593	0.985315424	CKAP2
ENSG00000131845.15	-0.002190719	0.844154024	0.985315424	ZNF304
ENSG00000141279.17	0.001100542	0.844266558	0.985315424	NPEPPS
ENSG00000284602.1	0.00291187	0.844281464	0.985315424	RP3-465N24.7
ENSG00000234043.3	-0.006884312	0.844284139	0.985315424	NUDT9P1
ENSG00000259225.7	-0.006405237	0.844314817	0.985315424	LINC02345
ENSG00000166575.17	0.002003472	0.844418326	0.985315424	TMEM135
ENSG00000083520.15	0.001743543	0.844551323	0.985315424	DIS3
ENSG00000268869.6	-0.009128536	0.84457613	0.985315424	ESPNP
ENSG00000162924.15	0.002117174	0.844602781	0.985315424	REL
ENSG00000146858.8	0.0057192	0.84472014	0.985315424	ZC3HAV1L
ENSG00000105514.8	0.001833956	0.844737145	0.985315424	RAB3D
ENSG00000277152.1	0.00589941	0.844820768	0.985315424	RP11-622C24.2

ENSG00000113761.12	-0.001143174	0.84483943	0.985315424	ZNF346
ENSG00000234663.7	0.003010159	0.844853301	0.985315424	LINC01934
ENSG00000092200.13	0.002646899	0.844884347	0.985315424	RPGRIP1
ENSG00000102710.20	0.00149524	0.844906834	0.985315424	SUPT20H
ENSG00000255045.1	0.004859138	0.844930444	0.985315424	RP11-677M14.2
ENSG00000169884.14	-0.003068119	0.844998747	0.985315424	WNT10B
ENSG00000225234.1	0.004391239	0.845032831	0.985315424	TRAPPC12-AS1
ENSG00000242622.2	-0.002460688	0.845061968	0.985315424	RP11-18H7.1
ENSG00000253234.1	-0.007131805	0.845111584	0.985315424	IGLV2-5
ENSG00000116337.20	-0.002014912	0.845116993	0.985315424	AMPD2
ENSG00000239521.9	-0.001701993	0.845238925	0.985315424	CASTOR3
ENSG00000234769.7	-0.003249151	0.845319849	0.985315424	WASH4P
ENSG00000175104.15	-0.001022213	0.845421021	0.985315424	TRAF6
ENSG00000126804.14	0.002121848	0.845426866	0.985315424	ZBTB1
ENSG00000273188.1	0.002529118	0.845471168	0.985315424	RP3-402G11.25
ENSG00000037280.16	0.004876397	0.845483847	0.985315424	FLT4
ENSG00000176108.9	-0.001865646	0.845494106	0.985315424	CHMP6
ENSG00000165915.14	-0.001308651	0.845646752	0.985315424	SLC39A13
ENSG00000255222.1	0.006661984	0.84572065	0.985315424	SETP17
ENSG00000156642.17	0.001300345	0.845748017	0.985315424	NPTN
ENSG00000165730.16	0.011424504	0.845765778	0.985315424	STOX1
ENSG00000117010.17	-0.003531618	0.845873781	0.985315424	ZNF684
ENSG00000146373.17	0.00357755	0.845881593	0.985315424	RNF217
ENSG00000238199.1	0.008801487	0.845883311	0.985315424	UBE2V2P3
ENSG00000164687.11	0.004416779	0.846102912	0.985315424	FABP5
ENSG00000137309.20	-0.001895201	0.846173078	0.985315424	HMGA1
ENSG00000075303.13	-0.002023168	0.846307943	0.985315424	SLC25A40
ENSG00000100197.23	-0.00407444	0.846325775	0.985315424	CYP2D6
ENSG00000188229.6	-0.001715131	0.84641341	0.985315424	TUBB4B
ENSG00000175643.10	-0.004353765	0.846420035	0.985315424	RMI2
ENSG00000165178.9	-0.003564974	0.846421054	0.985315424	NCF1C
ENSG00000286905.1	0.004920494	0.846482833	0.985315424	RP13-512J5.1
ENSG00000224796.1	0.007827488	0.846543526	0.985315424	RPL32P1
ENSG00000221926.13	-0.002242077	0.846609585	0.985315424	TRIM16
ENSG00000119147.10	-0.006211501	0.846624035	0.985315424	ECRG4
ENSG00000267767.3	0.003910779	0.84665294	0.985315424	LINC01801
ENSG00000259305.6	-0.004550108	0.846706142	0.985315424	ZHX1-C8orf76
ENSG00000134186.12	0.001771941	0.846752451	0.985315424	PRPF38B
ENSG00000102524.12	-0.003087953	0.846823421	0.985315424	TNFSF13B
ENSG00000075539.15	0.001618951	0.846833372	0.985315424	FRYL
ENSG00000163898.10	0.00704797	0.846886584	0.985315424	LIPH
ENSG00000175115.13	-0.001848941	0.846899096	0.985315424	PACS1
ENSG00000114268.12	0.001643226	0.846979684	0.985315424	PFKFB4
ENSG00000145348.17	-0.001534097	0.846994281	0.985315424	TBCK
ENSG00000181444.13	0.002514039	0.847009812	0.985315424	ZNF467
ENSG00000116560.11	-7.44E-04	0.847053121	0.985315424	SFPQ
ENSG00000162972.10	0.001644229	0.847092395	0.985315424	MAIP1

ENSG00000204304.12	-0.001265474	0.847122492	0.985315424	PBX2
ENSG00000139546.11	0.001428252	0.847136885	0.985315424	TARBP2
ENSG00000146587.18	-0.002481755	0.847254511	0.985315424	RBAK
ENSG00000272620.2	-0.00665011	0.847267518	0.985315424	AFAP1-AS1
ENSG00000072071.16	0.001850074	0.847408309	0.985315424	ADGRL1
ENSG00000062485.19	0.001199039	0.847520834	0.985315424	CS
ENSG00000142784.16	-0.001599295	0.84758693	0.985315424	WDTC1
ENSG00000176273.15	0.002955436	0.847591685	0.985315424	SLC35G1
ENSG00000227124.11	-0.002610539	0.847647691	0.985315424	ZNF717
ENSG00000150637.9	0.002617446	0.847673329	0.985315424	CD226
ENSG00000186625.14	-0.001190304	0.847711657	0.985315424	KATNA1
ENSG00000271503.6	-0.00394724	0.847722554	0.985315424	CCL5
ENSG00000230426.4	-0.007838463	0.847754072	0.985315424	LINC01036
ENSG00000240370.6	-0.002266232	0.847754234	0.985315424	RPL13P5
ENSG00000100417.12	0.001707935	0.847773016	0.985315424	PMM1
ENSG00000256155.1	-0.008463426	0.847821025	0.985315424	LINC02598
ENSG00000178996.14	-0.002025208	0.847821429	0.985315424	SNX18
ENSG00000135597.19	0.00129462	0.847855053	0.985315424	REPS1
ENSG00000215045.8	0.004345312	0.847903894	0.985315424	GRID2IP
ENSG00000040199.18	-0.002103211	0.84793418	0.985315424	PHLPP2
ENSG00000273424.1	0.003990708	0.848000019	0.985315424	CTA-223H9.9
ENSG00000105131.8	0.006417332	0.848104852	0.985315424	EPHX3
ENSG00000269399.2	0.002117121	0.848365094	0.985315424	CTD-3222D19.12
ENSG00000143167.12	-0.002948947	0.848374601	0.985315424	GPA33
ENSG00000258572.1	0.00623417	0.848390243	0.985315424	RP11-1070N10.3
ENSG00000101751.11	0.002638536	0.84848914	0.985315424	POLI
ENSG00000280122.1	0.008351552	0.848521249	0.985315424	RP11-87G24.2
ENSG00000164626.9	-0.00508326	0.84857792	0.985315424	KCNK5
ENSG00000113119.13	-0.00113962	0.848647999	0.985315424	TMCO6
ENSG00000138767.13	0.001525933	0.848689819	0.985315424	CNOT6L
ENSG00000136824.19	0.002239197	0.848709161	0.985315424	SMC2
ENSG00000119314.16	0.001469877	0.848808469	0.985315424	PTBP3
ENSG00000106588.12	-0.002865272	0.848851559	0.985315424	PSMA2
ENSG00000113504.21	-0.004374927	0.848918634	0.985315424	SLC12A7
ENSG00000211780.3	0.0051138	0.84895898	0.985315424	TRAV6
ENSG00000249492.2	0.004710346	0.84904138	0.985315424	RP11-159F24.3
ENSG00000146215.14	-0.00365515	0.849072035	0.985315424	CRIP3
ENSG00000137225.13	0.00976072	0.849090881	0.985315424	CAPN11
ENSG00000152932.8	0.008013794	0.849155009	0.985315424	RAB3C
ENSG00000104299.15	-0.001232645	0.84924548	0.985315424	INTS9
ENSG00000068971.14	0.001941169	0.849302022	0.985315424	PPP2R5B
ENSG00000140262.18	-0.001568472	0.849335326	0.985315424	TCF12
ENSG00000169635.10	-0.001453217	0.849364274	0.985315424	HIC2
ENSG00000096717.12	-0.001814604	0.849409303	0.985315424	SIRT1
ENSG00000217275.2	0.005245667	0.849497032	0.985315424	RP1-34B20.4
ENSG00000100764.14	0.001451195	0.849639773	0.985315424	PSMC1
ENSG00000141034.10	0.001605074	0.849712739	0.985315424	GID4

ENSG00000213542.3	0.00439634	0.849782862	0.985315424	RP11-467H10.1
ENSG00000279838.1	0.003749772	0.849790609	0.985315424	RP4-635A23.6
ENSG00000131242.18	0.001802064	0.849839925	0.985315424	RAB11FIP4
ENSG00000078070.13	0.001132899	0.849853068	0.985315424	MCCC1
ENSG00000157554.19	0.006713764	0.849893152	0.985315424	ERG
ENSG00000164729.7	0.010145385	0.84994965	0.985315424	SLC35G3
ENSG00000258682.1	-0.003559299	0.849988451	0.985315424	CTD-2002H8.2
ENSG00000286326.1	-0.007207827	0.850008509	0.985315424	CTA-445C9.17
ENSG00000168116.14	0.003073726	0.850105675	0.985315424	KIAA1586
ENSG00000048462.11	0.006516582	0.850109844	0.985315424	TNFRSF17
ENSG00000170653.19	-0.001200036	0.8501193	0.985315424	ATF7
ENSG00000168884.15	-0.001195452	0.850131132	0.985315424	TNIP2
ENSG00000250608.2	0.002556304	0.850148382	0.985315424	RP11-933H2.4
ENSG00000205084.11	0.003077688	0.850175691	0.985315424	TMEM231
ENSG00000232871.8	-0.004825937	0.850207792	0.985315424	SEC1P
ENSG00000285906.2	0.005089599	0.850262048	0.985315424	RP11-622I12.1
ENSG00000173193.15	0.003789615	0.850316279	0.985315424	PARP14
ENSG00000139505.12	-0.001981403	0.850337286	0.985315424	MTMR6
ENSG00000118655.7	-0.00145805	0.850352838	0.985315424	DCLRE1B
ENSG00000286116.1	0.00417884	0.850385852	0.985315424	FAS-AS1
ENSG00000173402.12	-0.001566094	0.850439226	0.985315424	DAG1
ENSG00000231123.1	0.005983755	0.850502527	0.985315424	SPATA20P1
ENSG00000288703.1	-0.006122636	0.850530285	0.985315424	RP11-91E8.2
ENSG00000165792.18	-0.001206923	0.850579975	0.985315424	METTL17
ENSG00000166266.14	0.002513866	0.850584151	0.985315424	CUL5
ENSG00000154263.17	-0.004011064	0.850584159	0.985315424	ABCA10
ENSG00000226496.3	-0.010265731	0.850596433	0.985315424	LINC00323
ENSG00000128694.12	0.001632331	0.850599551	0.985315424	OSGEPL1
ENSG00000267141.1	-0.004424751	0.850604744	0.985315424	CTB-31O20.8
ENSG00000196177.13	-0.002016145	0.850655268	0.985315424	ACADSB
ENSG00000135956.9	0.00132976	0.850734763	0.985315424	TMEM127
ENSG00000000460.17	0.00250395	0.850783101	0.985315424	C1orf112
ENSG00000231187.3	0.007230702	0.85086619	0.985315424	RP11-38L15.3
ENSG00000247121.7	-0.002159829	0.850872183	0.985315424	CTD-2260A17.2
ENSG00000267226.2	0.006200099	0.850913464	0.985315424	MALT1-AS1
ENSG00000113070.8	-0.003923056	0.850952908	0.985315424	HBEGF
ENSG00000041982.16	0.006769928	0.850989996	0.985315424	TNC
ENSG00000165868.16	0.005630611	0.851059445	0.985315424	HSPA12A
ENSG00000049246.15	0.003040077	0.851088232	0.985315424	PER3
ENSG00000105649.10	0.00238245	0.851178821	0.985315424	RAB3A
ENSG00000263466.1	0.006329031	0.851220594	0.985315424	RP1-56K13.2
ENSG00000167766.19	0.003213333	0.851301633	0.985315424	ZNF83
ENSG00000026950.17	0.001933732	0.851335752	0.985315424	BTN3A1
ENSG00000120057.5	0.008384209	0.851405308	0.985315424	SFRP5
ENSG00000261373.1	0.002775734	0.851479376	0.985315424	VPS9D1-AS1
ENSG00000197653.16	-0.005724271	0.851550006	0.985315424	DNAH10
ENSG00000094916.16	-0.001242377	0.85155001	0.985315424	CBX5

ENSG00000280387.1	-0.002956653	0.851572041	0.985315424	RP5-1153D9.5
ENSG00000278133.1	0.001297402	0.851621855	0.985315424	RP11-196G11.5
ENSG00000049883.15	-0.001715633	0.851700968	0.985315424	PTCD2
ENSG00000088298.13	0.001267938	0.851728469	0.985315424	EDEM2
ENSG00000277363.5	0.006504379	0.851767824	0.985315424	SRCIN1
ENSG00000174697.5	0.008363343	0.85207669	0.985315424	LEP
ENSG00000178980.15	0.001647417	0.852086811	0.985315424	SELENOW
ENSG00000133313.15	0.00174753	0.852112741	0.985315424	CNDP2
ENSG00000116194.13	0.009004093	0.8521578	0.985315424	ANGPTL1
ENSG00000110237.5	-0.007817659	0.852236857	0.985315424	ARHGEF17
ENSG00000113263.13	0.001708568	0.852240303	0.985315424	ITK
ENSG00000160216.21	-0.001393014	0.852418051	0.985315424	AGPAT3
ENSG00000224934.5	0.002496324	0.852457625	0.985315424	RP11-441O15.3
ENSG00000173862.3	0.006756764	0.852546997	0.985315424	RP11-89N17.1
ENSG00000112039.5	0.001373295	0.852559284	0.985315424	FANCE
ENSG00000256574.8	0.006427992	0.852584478	0.985315424	OR13A1
ENSG00000282416.1	-0.022379338	0.852643469	0.985315424	LLNLF-173C4.2
ENSG00000249485.1	0.004770319	0.852666101	0.985315424	RBBP4P1
ENSG00000248712.7	-0.002770979	0.852773488	0.985315424	CCDC153
ENSG00000171320.15	-0.005571245	0.852898578	0.985315424	ESCO2
ENSG00000101158.15	-0.00388353	0.852935857	0.985315424	NELFCD
ENSG00000095209.12	0.002350305	0.85295021	0.985315424	TMEM38B
ENSG00000279529.1	0.001869568	0.852956934	0.985315424	CTD-3222D19.8
ENSG00000128185.10	-0.001868524	0.853001265	0.985315424	DGCR6L
ENSG00000238197.6	0.002090498	0.853092999	0.985315424	PAXBP1-AS1
ENSG00000244926.7	-0.004176763	0.853193309	0.985315424	ALKBH3-AS1
ENSG00000175806.15	-0.002032899	0.853208145	0.985315424	MSRA
ENSG00000189195.15	0.003390986	0.853237554	0.985315424	BTBD8
ENSG00000268655.2	0.007550993	0.853265272	0.985315424	CTB-60B18.10
ENSG00000288663.1	-0.002204841	0.853280842	0.985315424	RP11-680A11.7
ENSG00000187984.13	-0.003101463	0.853289273	0.985315424	ANKRD19P
ENSG00000060656.20	-0.007257847	0.853293372	0.985315424	PTPRU
ENSG00000138593.9	-0.00220622	0.853486676	0.985315424	SECISBP2L
ENSG00000235770.6	-0.010899206	0.853491222	0.985315424	LINC00607
ENSG00000063761.16	0.002554131	0.853524414	0.985315424	ADCK1
ENSG00000260808.1	-0.004759422	0.853548434	0.985315424	CTD-2007L18.5
ENSG00000213782.7	0.002318815	0.853558912	0.985315424	DDX47
ENSG00000271046.1	-0.007768548	0.853616903	0.985315424	RP11-138I18.2
ENSG00000258875.6	0.002276233	0.85363426	0.985315424	CTD-2547L24.3
ENSG00000029363.17	-0.001645296	0.853678813	0.985315424	BCLAF1
ENSG00000160710.18	-0.001847933	0.853720966	0.985315424	ADAR
ENSG00000079263.19	-0.002395285	0.853861372	0.985315424	SP140
ENSG00000166763.8	0.005442586	0.853891023	0.985315424	STRCP1
ENSG00000255306.1	-0.0031321	0.854035632	0.985315424	RP5-901A4.1
ENSG00000147799.11	-0.005913059	0.854042113	0.985315424	ARHGAP39
ENSG00000241527.1	0.004226942	0.854138863	0.985315424	CA15P1
ENSG00000175730.8	0.004136056	0.854150245	0.985315424	BAK1P1

ENSG00000157306.14	0.002178824	0.85417436	0.985315424	ZFHX2-AS1
ENSG00000137177.20	0.001615844	0.854209899	0.985315424	KIF13A
ENSG00000242779.7	-0.004950109	0.854286946	0.985315424	ZNF702P
ENSG00000130300.9	-0.005866672	0.854289941	0.985315424	PLVAP
ENSG00000183161.6	-0.001150287	0.854296083	0.985315424	FANCF
ENSG00000232334.1	0.0044796	0.85431535	0.985315424	RP11-47G11.2
ENSG00000175984.15	0.003916515	0.85431879	0.985315424	DENND2C
ENSG00000128245.15	-0.001483911	0.854358523	0.985315424	YWHAH
ENSG00000236456.1	0.005568405	0.854382621	0.985315424	RP4-785G19.2
ENSG00000138316.11	0.005884417	0.854393858	0.985315424	ADAMTS14
ENSG00000165511.6	-0.00342641	0.854432564	0.985315424	ZNF22-AS1
ENSG00000267565.1	-0.003878595	0.854520802	0.985315424	CTC-559E9.8
ENSG00000284977.2	-0.003906634	0.854538282	0.985315424	RP11-281A20.2
ENSG00000171443.7	-0.002068911	0.854568282	0.985315424	ZNF524
ENSG00000229107.2	-0.009420677	0.854595214	0.985315424	ABHD17AP4
ENSG00000119906.13	0.001071924	0.854621758	0.985315424	SLF2
ENSG00000288018.1	0.006463409	0.85466244	0.985315424	RP11-657K20.3
ENSG00000162714.13	0.002311525	0.854680593	0.985315424	ZNF496
ENSG00000221947.8	0.010133625	0.85476173	0.985315424	XKR9
ENSG00000163820.15	-0.001748162	0.854777944	0.985315424	FYCO1
ENSG00000163577.8	-0.002601571	0.854779636	0.985315424	EIF5A2
ENSG00000228008.1	0.007281609	0.854800273	0.985315424	CTD-2330K9.3
ENSG00000173535.15	-0.002297097	0.854901873	0.985315424	TNFRSF10C
ENSG00000258810.1	0.004524297	0.854915821	0.985315424	RP11-219E7.1
ENSG00000211592.8	0.005775098	0.855020899	0.985315424	IGKC
ENSG00000131849.12	0.002891912	0.855149892	0.985315424	ZNF132
ENSG00000110777.12	-0.004193332	0.855272223	0.985315424	POU2AF1
ENSG00000204410.16	-0.002430896	0.855279011	0.985315424	MSH5
ENSG00000237135.1	-0.008676749	0.855280943	0.985315424	DDX10P1
ENSG00000196814.15	-0.002914471	0.85528208	0.985315424	MVB12B
ENSG00000269903.1	-0.004853492	0.855286159	0.985315424	RP11-571M6.18
ENSG00000231233.1	0.004287033	0.855336744	0.985315424	CFAP58-DT
ENSG00000137070.18	-0.002087064	0.855457407	0.985315424	IL11RA
ENSG00000169885.10	0.004809519	0.855517062	0.985315424	CALML6
ENSG00000197837.3	-0.003786201	0.855555405	0.985315424	H4-16
ENSG00000158828.8	0.002367075	0.855561746	0.985315424	PINK1
ENSG00000258471.2	-0.005655625	0.855568635	0.985315424	RP11-84C10.4
ENSG00000216863.10	-0.003341999	0.85563457	0.985315424	LY86-AS1
ENSG00000101146.13	-9.58E-04	0.855652526	0.985315424	RAE1
ENSG00000213763.4	0.004417786	0.855679772	0.985315424	ACTBP2
ENSG00000286507.1	-0.005666323	0.855723053	0.985315424	RP5-1007F24.2
ENSG00000140259.7	0.001050989	0.855764289	0.985315424	MFAP1
ENSG00000174799.11	0.002274633	0.855843598	0.985315424	CEP135
ENSG00000143970.18	0.001767904	0.855853415	0.985315424	ASXL2
ENSG00000284747.1	0.003241308	0.855878564	0.985315424	CTA-215D11.6
ENSG00000076826.10	-0.006470594	0.855903447	0.985315424	CAMSAP3
ENSG00000166592.12	-0.006565369	0.855954154	0.985315424	RRAD

ENSG00000041802.11	0.001441234	0.855979146	0.985315424	LSG1
ENSG00000100941.9	0.001408763	0.856033992	0.985315424	PNN
ENSG00000248527.1	-0.003318493	0.856035644	0.985315424	MTATP6P1
ENSG00000189007.16	-0.001783037	0.856049693	0.985315424	ADAT2
ENSG00000125877.13	0.001766317	0.856087934	0.985315424	ITPA
ENSG00000164051.14	0.001568164	0.856116682	0.985315424	CCDC51
ENSG00000231563.2	0.003496147	0.856117697	0.985315424	RP11-245P10.4
ENSG00000046604.14	-0.007781265	0.85628377	0.985315424	DSG2
ENSG00000279693.1	-0.005554038	0.856366781	0.985315424	RP11-71L14.3
ENSG00000171208.10	0.002298156	0.856372347	0.985315424	NETO2
ENSG00000286122.1	0.003704792	0.856423613	0.985315424	RP11-622O11.6
ENSG00000164327.13	-0.002711749	0.856487176	0.985315424	RICTOR
ENSG00000101452.15	-9.94E-04	0.856528382	0.985315424	DHX35
ENSG00000022567.10	0.002102303	0.856534411	0.985315424	SLC45A4
ENSG00000204632.12	-0.007020777	0.856653164	0.985315424	HLA-G
ENSG00000149231.16	0.003354939	0.856766791	0.985315424	CCDC82
ENSG00000081019.13	-0.001582328	0.856821254	0.985315424	RSBN1
ENSG00000259056.1	-0.009662143	0.856932791	0.985315424	DUXAP1
ENSG00000130703.17	0.001119904	0.857020981	0.985315424	OSBPL2
ENSG00000160113.6	0.003700354	0.857084343	0.985315424	NR2F6
ENSG00000166086.13	-0.003511156	0.85710859	0.985315424	JAM3
ENSG00000250565.7	0.001994403	0.857119425	0.985315424	ATP6V1E2
ENSG00000197713.15	0.001796057	0.857160548	0.985315424	RPE
ENSG00000230882.1	0.016742197	0.857227635	0.985315424	AC005077.14
ENSG00000196387.10	-0.002625338	0.857229756	0.985315424	ZNF140
ENSG00000010539.12	0.001510532	0.857348306	0.985315424	ZNF200
ENSG00000271967.1	0.005562109	0.857362175	0.985315424	RP11-134K13.4
ENSG00000227492.2	0.00287419	0.857372475	0.985315424	RP11-316M21.6
ENSG00000273320.1	-0.004367976	0.857536554	0.985315424	RP11-22N19.2
ENSG00000163121.10	0.007068325	0.857697252	0.985315424	NEURL3
ENSG00000167286.10	-0.00265356	0.857698964	0.985315424	CD3D
ENSG00000138073.14	0.001081815	0.857712903	0.985315424	PREB
ENSG00000172716.16	0.00165308	0.857789528	0.985315424	SLFN11
ENSG00000182628.13	-0.001840019	0.857803508	0.985315424	SKA2
ENSG00000156804.7	-0.001695028	0.85782726	0.985315424	FBXO32
ENSG00000144468.17	0.001211244	0.857850148	0.985315424	RHBDD1
ENSG00000154473.18	9.80E-04	0.857896339	0.985315424	BUB3
ENSG00000196704.14	-9.83E-04	0.857928553	0.985315424	AMZ2
ENSG00000288638.1	-0.023823622	0.857929766	0.985315424	LNCDAT
ENSG00000226180.3	0.003622621	0.857980949	0.985315424	RP11-278A23.1
ENSG00000068383.19	0.001342263	0.858027344	0.985315424	INPP5A
ENSG00000197629.6	-0.001813522	0.858034244	0.985315424	MPEG1
ENSG00000124780.14	0.008110546	0.858058547	0.985315424	KCNK17
ENSG00000028137.19	-0.001499063	0.85807528	0.985315424	TNFRSF1B
ENSG00000242686.4	0.006296714	0.858097374	0.985315424	PDE6B-AS1
ENSG00000137509.12	0.001266222	0.858195207	0.985315424	PRCP
ENSG00000237036.5	-0.002287224	0.858244411	0.985315424	ZEB1-AS1

ENSG00000133454.16	0.012394671	0.858312991	0.985315424	MYO18B
ENSG00000258102.5	0.002244485	0.858330484	0.985315424	MAP1LC3B2
ENSG00000126216.15	-8.79E-04	0.858354924	0.985315424	TUBGCP3
ENSG00000271119.1	0.006478288	0.858362608	0.985315424	CTD-2012J19.3
ENSG00000151576.10	-0.001103724	0.858398419	0.985315424	QTRT2
ENSG00000139053.3	-0.007620781	0.85843224	0.985315424	PDE6H
ENSG00000047188.16	0.001575761	0.858472433	0.985315424	YTHDC2
ENSG00000269910.1	0.003488871	0.858544465	0.985315424	RP11-73M18.10
ENSG00000151287.17	-0.002822031	0.858583552	0.985315424	TEX30
ENSG00000124215.17	-0.002275304	0.85863797	0.985315424	CDH26
ENSG00000158805.12	-0.001199897	0.858828576	0.985315424	ZNF276
ENSG00000250548.7	0.005349909	0.858872582	0.985315424	LINC01303
ENSG00000066427.24	-0.001226389	0.858904449	0.985315424	ATXN3
ENSG00000139112.11	-0.001542264	0.85896202	0.985315424	GABARAPL1
ENSG00000101474.12	0.001879251	0.858971246	0.985315424	APMAP
ENSG00000070526.15	0.004956137	0.858990133	0.985315424	ST6GALNAC1
ENSG00000187066.9	0.003216907	0.859001141	0.985315424	TMEM262
ENSG00000120253.14	-0.001155711	0.859020859	0.985315424	NUP43
ENSG00000162236.13	0.001399143	0.859224417	0.985315424	STX5
ENSG00000260803.1	0.004673694	0.859239521	0.985315424	Z84812.4
ENSG00000272512.1	-0.009509607	0.859283664	0.985315424	RP11-54O7.17
ENSG00000101574.15	-0.00170141	0.859302475	0.985315424	METTL4
ENSG00000161013.17	-0.001455184	0.859312799	0.985315424	MGAT4B
ENSG00000129317.15	0.002277128	0.859370725	0.985315424	PUS7L
ENSG00000124469.12	0.009080928	0.859434635	0.985315424	CEACAM8
ENSG00000115641.19	-0.002867844	0.859451076	0.985315424	FHL2
ENSG00000273507.5	0.005187287	0.859537196	0.985315424	RP11-50D16.4
ENSG00000230149.2	0.002341594	0.859603225	0.985315424	RP3-508I15.19
ENSG00000127022.16	0.001207694	0.859753209	0.985315424	CANX
ENSG00000136404.16	-0.001860079	0.859754309	0.985315424	TM6SF1
ENSG00000174292.12	0.00252408	0.859870605	0.985315424	TNK1
ENSG00000177239.16	-0.001254622	0.859925492	0.985315424	MAN1B1
ENSG00000204851.7	-0.007594523	0.859929563	0.985315424	PNMA8B
ENSG00000243989.9	-0.003464066	0.859944189	0.985315424	ACY1
ENSG00000111344.11	0.005924984	0.859969845	0.985315424	RASAL1
ENSG00000205089.8	-0.002794941	0.859984975	0.985315424	CCNI2
ENSG00000142279.13	-0.003756826	0.860012995	0.985315424	WTIP
ENSG00000167257.11	-0.001283664	0.860062807	0.985315424	RNF214
ENSG00000231887.7	0.00625711	0.860083121	0.985315424	PRH1
ENSG00000286162.2	0.003719645	0.860185268	0.985315424	RP11-574F11.4
ENSG00000065882.16	0.001328523	0.860247933	0.985315424	TBC1D1
ENSG00000128594.8	0.001972107	0.860278659	0.985315424	LRRC4
ENSG00000166313.19	-0.002121291	0.860282108	0.985315424	APBB1
ENSG00000253293.5	0.005566098	0.860288444	0.985315424	HOXA10
ENSG00000273702.1	0.005042719	0.860339169	0.985315424	RP11-758H9.2
ENSG00000176783.15	-0.001317619	0.860347482	0.985315424	RUFY1
ENSG00000184009.13	0.001231582	0.86036418	0.985315424	ACTG1

ENSG00000100225.18	0.004205701	0.860419367	0.985315424	FBXO7
ENSG00000175895.4	-0.002227386	0.860433764	0.985315424	PLEKHF2
ENSG00000268849.5	0.003018645	0.860439092	0.985315424	SIGLEC22P
ENSG00000248677.2	-0.00650463	0.860444711	0.985315424	LINC02102
ENSG00000106069.23	-0.00439475	0.860445384	0.985315424	CHN2
ENSG00000132199.20	-0.002219293	0.860531044	0.985358495	ENOSF1
ENSG00000113319.13	0.002325575	0.860683608	0.985478167	RASGRF2
ENSG00000176401.6	0.002343315	0.860793636	0.98549692	EID2B
ENSG00000266750.1	0.005776216	0.860838467	0.98549692	MIR4645
ENSG00000241014.2	-0.002760579	0.860906941	0.98549692	GPR199P
ENSG00000062598.18	0.001056546	0.860976269	0.98549692	ELMO2
ENSG00000203761.5	-0.001804567	0.860983179	0.98549692	MSTO2P
ENSG00000276916.1	-0.006715337	0.86102045	0.98549692	RP11-230F18.6
ENSG00000198453.13	-0.002025373	0.861106041	0.98549692	ZNF568
ENSG00000108395.14	-0.001577173	0.86112566	0.98549692	TRIM37
ENSG00000277715.1	-0.002669464	0.861132475	0.98549692	RP11-651L5.3
ENSG00000155561.15	0.001118208	0.86138846	0.985674511	NUP205
ENSG00000167085.13	-0.001272791	0.861429557	0.985674511	PHB
ENSG00000105778.19	-0.001182217	0.861431844	0.985674511	AVL9
ENSG00000254777.5	-0.004136842	0.861545824	0.985749931	AC022182.1
ENSG00000129422.14	0.005398488	0.86169348	0.985801395	MTUS1
ENSG00000204644.10	-0.023008628	0.861711999	0.985801395	ZFP57
ENSG00000226711.7	-0.002996908	0.861735011	0.985801395	FAM66C
ENSG00000120800.5	0.001476105	0.861843616	0.985870643	UTP20
ENSG00000127870.17	0.001643288	0.861940581	0.985926568	RNF6
ENSG00000259962.1	0.002955638	0.862018608	0.98593904	RP11-44F14.4
ENSG00000165621.9	-0.010744498	0.86210224	0.98593904	OXGR1
ENSG00000156172.6	0.003275586	0.862212499	0.98593904	C8orf37
ENSG00000117139.18	0.001277022	0.862244305	0.98593904	KDM5B
ENSG00000160271.16	-0.001641537	0.862260325	0.98593904	RALGDS
ENSG00000224863.3	0.006638634	0.862320348	0.98593904	LINC01398
ENSG00000144659.13	-0.001237053	0.862381199	0.98593904	SLC25A38
ENSG00000198356.12	0.001374142	0.862399076	0.98593904	GET3
ENSG00000235043.3	-0.003935177	0.862424559	0.98593904	TECRP1
ENSG00000271662.1	0.008007789	0.862458333	0.98593904	RP11-141C7.3
ENSG00000119285.11	0.001528006	0.862550586	0.98593904	HEATR1
ENSG00000234925.2	0.007239408	0.862553484	0.98593904	ATP5PDP4
ENSG00000105443.15	0.001231613	0.86275781	0.98593904	CYTH2
ENSG00000205423.12	0.001622655	0.862770017	0.98593904	CNEP1R1
ENSG00000287682.1	0.005144805	0.862833046	0.98593904	RP11-49I4.5
ENSG00000213888.3	-0.003818185	0.862833333	0.98593904	LINC01521
ENSG00000118260.15	-0.001347977	0.862849559	0.98593904	CREB1
ENSG00000205038.12	-0.004425415	0.862976514	0.98593904	PKHD1L1
ENSG00000234678.2	0.002369174	0.863031167	0.98593904	ELF3-AS1
ENSG00000152580.9	-0.010038428	0.863063481	0.98593904	IGSF10
ENSG00000140743.8	0.001723612	0.863069851	0.98593904	CDR2
ENSG00000082898.19	0.001385767	0.863153359	0.98593904	XPO1

ENSG00000149311.20	0.001913825	0.863158469	0.98593904	ATM
ENSG00000222335.1	-0.011153079	0.863162385	0.98593904	Y_RNA
ENSG00000089775.12	-0.001672694	0.863170544	0.98593904	ZBTB25
ENSG00000166927.13	-0.002743699	0.863225382	0.98593904	MS4A7
ENSG00000085832.17	0.001216778	0.863286688	0.98593904	EPS15
ENSG00000214535.3	0.005802312	0.863342387	0.98593904	RPS15AP1
ENSG00000170356.9	0.005743541	0.863345684	0.98593904	OR2A20P
ENSG00000184381.20	-0.001418526	0.863394577	0.985939973	PLA2G6
ENSG00000189045.15	-0.004978473	0.863669239	0.986160958	ANKDD1B
ENSG00000139697.14	-0.001011833	0.863693748	0.986160958	SBNO1
ENSG00000123240.17	-0.002210448	0.863768685	0.986160958	OPTN
ENSG00000263627.1	0.006502419	0.86382433	0.986160958	PPP4R1-AS1
ENSG00000288704.1	0.005692993	0.863828528	0.986160958	LLNLR-252D12.1
ENSG00000205730.7	0.002213307	0.864019352	0.986194691	ITPRIPL2
ENSG00000287061.1	-0.005307003	0.864045256	0.986194691	CTD-3094K11.3
ENSG00000235109.8	0.004222097	0.864049126	0.986194691	ZSCAN31
ENSG00000233695.2	0.004731874	0.86405043	0.986194691	GAS6-AS1
ENSG00000233198.4	0.006551313	0.864169397	0.986206368	RNF224
ENSG00000188076.3	0.009266802	0.864184475	0.986206368	SCGB1C1
ENSG00000182768.9	-0.001677072	0.864304849	0.986206368	NGRN
ENSG00000157869.16	0.001466963	0.864310526	0.986206368	RAB28
ENSG00000237638.2	-0.00260664	0.864353744	0.986206368	LINC02245
ENSG00000233916.1	0.006589521	0.86437212	0.986206368	ZDHHC20P1
ENSG00000145649.8	-0.003837114	0.864397282	0.986206368	GZMA
ENSG00000135926.15	0.001054363	0.864550012	0.986288062	TMBIM1
ENSG00000062725.10	-0.001230022	0.864694318	0.986288062	APPBP2
ENSG00000205643.11	0.001508874	0.864795924	0.986288062	CDPF1
ENSG00000096060.15	0.002792953	0.864846898	0.986288062	FKBP5
ENSG00000166527.8	0.004070847	0.864862815	0.986288062	CLEC4D
ENSG00000235530.7	-0.003341589	0.864890131	0.986288062	AC087294.2
ENSG00000244038.11	0.001026195	0.864911789	0.986288062	DDOST
ENSG00000225205.5	0.002347225	0.864966174	0.986288062	AC093818.1
ENSG00000106460.19	-0.002432751	0.864973228	0.986288062	TMEM106B
ENSG00000137818.12	0.002105297	0.865021044	0.986288062	RPLP1
ENSG00000179364.14	0.001156873	0.865207027	0.986288062	PACS2
ENSG00000226239.2	-0.005340662	0.865215423	0.986288062	RP1-310O13.7
ENSG00000135363.12	-0.001662854	0.865274279	0.986288062	LMO2
ENSG00000163295.5	-0.042443989	0.865348417	0.986288062	ALPI
ENSG00000188785.12	-0.00169806	0.865451928	0.986288062	ZNF548
ENSG00000286861.1	-0.004111797	0.865574137	0.986288062	RP11-1012A1.11
ENSG00000180229.12	0.009455542	0.865608402	0.986288062	HERC2P3
ENSG00000256612.7	-0.008744557	0.865720284	0.986288062	CYP2B7P
ENSG00000188859.7	0.006222711	0.865808983	0.986288062	FAM78B
ENSG00000164405.11	0.002849192	0.865814768	0.986288062	UQCRQ
ENSG00000224738.1	0.004498513	0.865853247	0.986288062	AC099850.1
ENSG00000198892.7	0.005559793	0.865858485	0.986288062	SHISA4
ENSG00000203780.12	-0.004832897	0.865859199	0.986288062	FANK1

ENSG00000143891.17	-0.002449105	0.865895159	0.986288062	GALM
ENSG00000186187.12	-0.001868834	0.865917273	0.986288062	ZNRF1
ENSG00000096070.19	0.001172488	0.865978882	0.986288062	BRPF3
ENSG00000145819.18	-0.00150423	0.866026172	0.986288062	ARHGAP26
ENSG00000137876.11	0.00435894	0.866077242	0.986288062	RSL24D1
ENSG00000275709.1	-0.005948835	0.866082292	0.986288062	RP11-96O20.5
ENSG00000174197.17	-0.001184966	0.866253537	0.986288062	MGA
ENSG00000128581.17	-0.002933314	0.866263783	0.986288062	IFT22
ENSG00000122188.13	-0.002007491	0.866304922	0.986288062	LAX1
ENSG00000227550.2	0.006388631	0.866428701	0.986288062	TRBV7-5
ENSG00000217702.3	0.006388261	0.86644853	0.986288062	RP11-287D1.4
ENSG00000196668.4	0.002094951	0.866450237	0.986288062	LINC00173
ENSG00000250155.2	-0.00544163	0.866495816	0.986288062	CTD-2353F22.1
ENSG00000256633.1	-0.002867111	0.866612742	0.986288062	PDE2A-AS2
ENSG00000165626.18	0.004289206	0.866616115	0.986288062	BEND7
ENSG00000112624.12	0.001357782	0.866629894	0.986288062	BICRAL
ENSG00000236287.8	0.001755698	0.866632714	0.986288062	ZBED5
ENSG00000172349.18	-0.001018743	0.866649989	0.986288062	IL16
ENSG00000246090.7	0.003485681	0.866773706	0.986288062	RP11-696N14.1
ENSG00000196757.8	-0.001493641	0.866855745	0.986288062	ZNF700
ENSG00000214655.11	-0.001437526	0.866855795	0.986288062	ZSWIM8
ENSG00000237510.7	0.0133062	0.866918999	0.986288062	GPAT2P1
ENSG00000162694.14	-0.002523647	0.866963863	0.986288062	EXTL2
ENSG00000118298.12	-0.003175101	0.867048038	0.986288062	CA14
ENSG00000186063.13	-0.001595392	0.867079784	0.986288062	AIDA
ENSG00000168060.16	0.001868699	0.867231436	0.986288062	NAALADL1
ENSG00000234996.4	-0.005370421	0.867240947	0.986288062	ACTG1P25
ENSG00000110721.12	-0.00134772	0.867243218	0.986288062	CHKA
ENSG00000135083.16	0.002478187	0.867264885	0.986288062	CCNJL
ENSG00000112659.14	0.001079106	0.867448617	0.986288062	CUL9
ENSG00000279801.1	-0.002473407	0.867492983	0.986288062	RP11-75C10.6
ENSG00000260917.1	0.001325263	0.867541549	0.986288062	RP11-57H14.4
ENSG00000187240.17	-0.002803898	0.867555863	0.986288062	DYNC2H1
ENSG00000230965.1	0.011788352	0.867678008	0.986288062	SNX18P13
ENSG00000165572.8	0.002172592	0.867759167	0.986288062	KBTBD6
ENSG00000278993.1	0.002620941	0.86782102	0.986288062	RP3-424M6.4
ENSG00000089902.10	0.001318065	0.867825446	0.986288062	RCOR1
ENSG00000223501.9	-9.30E-04	0.867854933	0.986288062	VPS52
ENSG00000239969.6	-0.004720015	0.867909358	0.986288062	RP11-163E9.2
ENSG00000228782.7	-0.003352783	0.867933186	0.986288062	MRPL45P2
ENSG00000211861.1	-0.007351465	0.867976237	0.986288062	TRAJ28
ENSG00000118922.18	-0.001808953	0.868000935	0.986288062	KLF12
ENSG00000126860.12	0.004033974	0.868086846	0.986288062	EVI2A
ENSG00000153989.8	-0.001720016	0.868110624	0.986288062	NUS1
ENSG00000119401.11	-0.001199432	0.868118729	0.986288062	TRIM32
ENSG00000101460.13	-0.003156033	0.86816585	0.986288062	MAP1LC3A
ENSG00000211745.3	-0.005149582	0.868206303	0.986288062	TRBV4-2

ENSG00000273151.1	0.001952643	0.868238416	0.986288062	RP11-449P15.2
ENSG00000236861.6	0.00491799	0.868267134	0.986288062	BET1-AS1
ENSG00000245694.10	-0.009114427	0.86828663	0.986288062	CRNDE
ENSG00000138741.11	-0.009310343	0.868321348	0.986288062	TRPC3
ENSG00000273669.1	-0.001845589	0.868358935	0.986288062	RP11-405M12.4
ENSG00000099256.19	0.004675827	0.868364325	0.986288062	PRTFDC1
ENSG00000143630.10	-0.00218669	0.868501564	0.986288062	HCN3
ENSG00000196187.12	-0.001254175	0.868523653	0.986288062	TMEM63A
ENSG00000266126.1	-0.006008901	0.868541241	0.986288062	RP11-209D14.4
ENSG00000197670.6	-0.004045909	0.868691063	0.986288062	RP4-724E16.2
ENSG00000128881.17	0.001469615	0.868713579	0.986288062	TTBK2
ENSG00000197993.9	0.004897358	0.868789968	0.986288062	KEL
ENSG00000140043.12	0.003897599	0.868834586	0.986288062	PTGR2
ENSG00000066827.16	-0.003699833	0.868871176	0.986288062	ZFAT
ENSG00000153936.17	0.001687832	0.869021615	0.986288062	HS2ST1
ENSG00000159640.17	0.00371382	0.869112215	0.986288062	ACE
ENSG00000272277.1	-0.006120314	0.869132169	0.986288062	RP1-40E16.12
ENSG00000185933.7	-0.004784293	0.869139655	0.986288062	CALHM1
ENSG00000129194.8	0.003332901	0.869145409	0.986288062	SOX15
ENSG00000197506.8	-0.005312435	0.869241061	0.986288062	SLC28A3
ENSG00000088854.13	0.00148533	0.869241641	0.986288062	C20orf194
ENSG00000232613.6	-0.006804394	0.869248899	0.986288062	LINC02576
ENSG00000259351.1	-0.004277995	0.869303434	0.986288062	RP11-111E14.1
ENSG00000144747.17	0.001972903	0.869381768	0.986288062	TMF1
ENSG00000118058.23	0.001448991	0.869417704	0.986288062	KMT2A
ENSG00000168078.10	0.008702293	0.869451866	0.986288062	PBK
ENSG00000243491.1	-0.009576934	0.869505626	0.986288062	RP11-521D12.5
ENSG00000261186.2	0.006692316	0.869527763	0.986288062	LINC01238
ENSG00000129003.19	-0.001884488	0.86962324	0.986288062	VPS13C
ENSG00000120519.15	0.001439036	0.869628725	0.986288062	SLC10A7
ENSG00000214192.3	0.006185958	0.869656623	0.986288062	UBE2V1P2
ENSG00000272195.1	-0.005286074	0.869665265	0.986288062	RP11-156E8.1
ENSG00000119703.15	-0.003729738	0.869727229	0.986288062	ZC2HC1C
ENSG00000272654.1	0.003013804	0.869872726	0.986288062	RP11-422P24.11
ENSG00000252620.1	0.007165275	0.869907573	0.986288062	RNU6ATAC24P
ENSG00000231476.1	-0.011860068	0.869911328	0.986288062	AC074389.5
ENSG00000174442.12	-0.001810475	0.869916463	0.986288062	ZWILCH
ENSG00000145476.16	0.002184431	0.870014616	0.986288062	CYP4V2
ENSG00000197859.11	-0.006130047	0.870108055	0.986288062	ADAMTSL2
ENSG00000172164.15	-0.001577223	0.870213208	0.986288062	SNTB1
ENSG00000153086.14	-0.005238463	0.870226758	0.986288062	ACMSD
ENSG00000123901.9	0.004738185	0.870263491	0.986288062	GPR83
ENSG00000112378.12	-0.003381717	0.870311128	0.986288062	PERP
ENSG00000136986.10	-7.62E-04	0.870352653	0.986288062	DERL1
ENSG00000143179.16	0.001428069	0.870378138	0.986288062	UCK2
ENSG00000169918.10	-0.002528786	0.870386341	0.986288062	OTUD7A
ENSG00000116954.8	-0.00125743	0.870412327	0.986288062	RRAGC

ENSG00000141294.10	-0.002687988	0.870420441	0.986288062	LRRC46
ENSG00000230561.6	0.005757914	0.870505307	0.986288062	CCDC192
ENSG00000180376.17	-0.001818571	0.870543668	0.986288062	CCDC66
ENSG00000114480.13	-0.001247427	0.870564484	0.986288062	GBE1
ENSG00000286065.2	0.003719088	0.870615845	0.986288062	SLFN12L
ENSG00000277744.1	0.003017549	0.870631306	0.986288062	CTC-435M10.12
ENSG00000270055.1	-0.003219732	0.870671992	0.986288062	CTD-3092A11.2
ENSG00000263711.6	-0.009710368	0.870790924	0.986288062	LINC02864
ENSG00000265798.6	0.006650317	0.870872283	0.986288062	RP11-271K11.5
ENSG00000138381.10	0.002257361	0.870914306	0.986288062	ASNSD1
ENSG00000263590.2	0.049599692	0.870960712	0.986288062	LINC02591
ENSG00000228172.5	0.003098227	0.870997911	0.986288062	RP1-317E23.3
ENSG00000170439.7	0.006526928	0.87101213	0.986288062	METTL7B
ENSG00000182224.12	-0.003439331	0.871037286	0.986288062	CYB5D1
ENSG00000169429.11	-0.004869182	0.871168354	0.986288062	CXCL8
ENSG00000275956.1	-0.006791886	0.871193696	0.986288062	RP5-966M1.7
ENSG00000148719.16	0.001103807	0.871303867	0.986288062	DNAJB12
ENSG00000226659.1	-0.005406163	0.871311946	0.986288062	RP11-137H2.4
ENSG00000203734.13	0.004473016	0.871379473	0.986288062	ECT2L
ENSG00000206341.7	-0.003907674	0.871435343	0.986288062	HLA-H
ENSG00000110536.14	0.001632458	0.871445144	0.986288062	PTPMT1
ENSG00000278627.1	0.004697884	0.871460369	0.986288062	RP11-506H21.5
ENSG00000186814.14	0.001316094	0.871479041	0.986288062	ZSCAN30
ENSG00000211967.3	0.005884243	0.871501343	0.986288062	IGHV3-53
ENSG00000034239.11	-0.007982095	0.871517596	0.986288062	EFCAB1
ENSG00000223573.7	0.003707993	0.87162648	0.986288062	TINCR
ENSG00000169016.17	0.001375442	0.871700596	0.986288062	E2F6
ENSG00000269487.1	-0.005223376	0.871764429	0.986288062	CTB-174O21.2
ENSG00000149548.15	0.002448621	0.871878075	0.986288062	CCDC15
ENSG00000072062.15	-0.001413721	0.87198311	0.986288062	PRKACA
ENSG00000171643.14	-0.002053038	0.871992886	0.986288062	S100Z
ENSG00000234917.2	0.00603133	0.872066343	0.986288062	RP5-994D16.3
ENSG00000140718.21	-0.001288346	0.872074754	0.986288062	FTO
ENSG00000148180.21	-0.002116282	0.872119434	0.986288062	GSN
ENSG00000225194.3	-0.003274634	0.872147107	0.986288062	LINC00092
ENSG00000151150.22	-0.00276162	0.872158832	0.986288062	ANK3
ENSG00000139970.17	-0.002253466	0.872210542	0.986288062	RTN1
ENSG00000163848.20	-0.001536097	0.872215582	0.986288062	ZNF148
ENSG00000150401.15	9.37E-04	0.872223598	0.986288062	DCUN1D2
ENSG00000186566.13	-0.001270662	0.872232808	0.986288062	GPATCH8
ENSG00000119977.22	0.001010694	0.872354563	0.986288062	TCTN3
ENSG00000163840.10	0.002130966	0.872362333	0.986288062	DTX3L
ENSG00000197927.13	0.007438191	0.872427703	0.986288062	NBEAP2
ENSG00000144028.15	-0.00111506	0.872467585	0.986288062	SNRNP200
ENSG00000099795.7	0.001948382	0.872558176	0.986288062	NDUFB7
ENSG00000242208.1	0.005531494	0.872578769	0.986288062	RPL5P29
ENSG00000167468.18	0.001842016	0.872601646	0.986288062	GPX4

ENSG00000140950.16	-0.001319645	0.872632512	0.986288062	MEAK7
ENSG00000205208.5	-0.001590268	0.872798881	0.986288062	C4orf46
ENSG00000280212.1	-0.003637174	0.872915749	0.986288062	RP11-49K24.3
ENSG00000273249.1	0.004906183	0.872932971	0.986288062	WDR5-DT
ENSG00000109084.14	0.002014727	0.872953622	0.986288062	TMEM97
ENSG00000170476.16	0.004219888	0.872979705	0.986288062	MZB1
ENSG00000041988.15	-0.001192925	0.873027	0.986288062	THAP3
ENSG00000139977.14	-0.001407186	0.873068369	0.986288062	NAA30
ENSG00000137145.21	-0.001830605	0.873142075	0.986288062	DENND4C
ENSG00000102445.20	0.002372099	0.873172648	0.986288062	RUBCNL
ENSG00000166436.17	-0.002122013	0.873263007	0.986288062	TRIM66
ENSG00000183955.13	-0.00104902	0.873295281	0.986288062	KMT5A
ENSG00000211595.2	-0.00585447	0.873315738	0.986288062	IGKJ3
ENSG00000235903.9	-0.003689428	0.873332961	0.986288062	CPB2-AS1
ENSG00000108932.12	0.001590182	0.873381192	0.986288062	SLC16A6
ENSG00000262370.6	0.002301774	0.873416261	0.986288062	RP11-473M20.9
ENSG00000169967.17	0.001707713	0.873436924	0.986288062	MAP3K2
ENSG00000263624.1	0.006235367	0.873439204	0.986288062	RP11-45M22.3
ENSG00000146540.15	-0.001935693	0.873453416	0.986288062	C7orf50
ENSG00000144711.16	-0.001194044	0.873467428	0.986288062	IQSEC1
ENSG00000248544.2	-0.003349282	0.873475675	0.986288062	CTB-47B11.3
ENSG00000234353.2	-0.00255163	0.87349186	0.986288062	AP000347.4
ENSG00000287123.1	-0.006713643	0.873494512	0.986288062	RP11-323F11.1
ENSG00000198734.12	-0.002589429	0.873510341	0.986288062	F5
ENSG00000161996.19	-0.001647569	0.873682964	0.986358169	WDR90
ENSG00000260572.1	-0.004017667	0.873684538	0.986358169	RP11-16N11.2
ENSG00000213693.4	0.004292834	0.873772444	0.986358169	SEC14L1P1
ENSG00000198492.16	-6.92E-04	0.873808514	0.986358169	YTHDF2
ENSG00000287419.1	-0.004866003	0.873969674	0.986358169	RP11-416N2.5
ENSG00000131634.14	-0.00274458	0.873972356	0.986358169	TMEM204
ENSG00000149599.16	0.011819948	0.874125586	0.986358169	DUSP15
ENSG00000213130.3	0.029226212	0.87418843	0.986358169	EEF1DP5
ENSG00000260075.1	-0.01211207	0.874219237	0.986358169	NSFP1
ENSG00000225489.7	-0.004764023	0.874251429	0.986358169	RP11-390F4.3
ENSG00000274949.1	0.004319705	0.874309095	0.986358169	RP5-1009E24.9
ENSG00000166997.8	0.001226985	0.874330792	0.986358169	CNPY4
ENSG00000255423.1	-0.004794646	0.874351827	0.986358169	EBLN2
ENSG00000137413.16	0.001175074	0.874352353	0.986358169	TAF8
ENSG00000272455.1	0.001770637	0.874363531	0.986358169	MRPL20-DT
ENSG00000204178.11	-9.38E-04	0.874439058	0.986358169	MACO1
ENSG00000240891.8	-0.002381256	0.874530819	0.986358169	PLCXD2
ENSG00000054965.11	0.00113962	0.8745393	0.986358169	FAM168A
ENSG00000127824.15	0.001664674	0.874597504	0.986358169	TUBA4A
ENSG00000285886.1	-0.004093185	0.874643668	0.986358169	CH17-264B6.5
ENSG00000198515.16	-0.007712571	0.874760852	0.986358169	CNGA1
ENSG00000280206.1	-0.001788567	0.874804417	0.986358169	CTB-193M12.5
ENSG00000101310.17	9.35E-04	0.874900986	0.986358169	SEC23B

ENSG00000111450.14	0.002202232	0.874980816	0.986358169	STX2
ENSG00000163913.13	0.001263433	0.875040387	0.986358169	IFT122
ENSG00000103507.14	-0.001491397	0.875045336	0.986358169	BCKDK
ENSG00000171224.9	0.002899156	0.875059251	0.986358169	FAM241B
ENSG00000226891.9	-0.002482976	0.875116526	0.986358169	LINC01359
ENSG00000111911.7	-0.001238169	0.875215866	0.986358169	HINT3
ENSG00000106328.10	0.004086469	0.875281086	0.986358169	FSCN3
ENSG00000132603.15	0.001011911	0.8752948	0.986358169	NIP7
ENSG00000214135.8	-0.001510097	0.875322553	0.986358169	AC024560.3
ENSG00000008300.17	0.003027554	0.875322656	0.986358169	CELSR3
ENSG00000165055.16	-0.001144981	0.875554267	0.986358169	METTL2B
ENSG00000198134.3	0.003994038	0.875558464	0.986358169	PTMAP9
ENSG00000224515.1	-0.013942112	0.87559723	0.986358169	RP11-5K23.5
ENSG00000112584.14	-8.92E-04	0.875652442	0.986358169	FAM120B
ENSG00000102796.12	0.002349883	0.875784197	0.986358169	DHRS12
ENSG00000204569.10	-0.00107092	0.875789236	0.986358169	PPP1R10
ENSG00000036448.10	0.009591641	0.87585967	0.986358169	MYOM2
ENSG00000116675.16	0.003578748	0.875882528	0.986358169	DNAJC6
ENSG00000197191.6	-0.004452513	0.875888051	0.986358169	CYSRT1
ENSG00000138119.17	-0.003052861	0.875967112	0.986358169	MYOF
ENSG00000164574.16	-0.001121493	0.875992808	0.986358169	GALNT10
ENSG00000183638.6	-0.010961792	0.876034159	0.986358169	RP1L1
ENSG00000197044.11	0.002806143	0.876062429	0.986358169	ZNF441
ENSG00000243806.1	-0.008395399	0.876163664	0.986358169	RPL7P18
ENSG00000164576.12	-7.56E-04	0.876283997	0.986358169	SAP30L
ENSG00000213918.11	-0.001137507	0.876363958	0.986358169	DNASE1
ENSG00000172059.11	-0.001802631	0.876472011	0.986358169	KLF11
ENSG00000103404.15	-0.001624744	0.876484914	0.986358169	USP31
ENSG00000196458.11	-0.002015544	0.876521839	0.986358169	ZNF605
ENSG00000234545.8	-0.001914816	0.876530807	0.986358169	FAM133B
ENSG00000166275.16	-0.002278257	0.876540116	0.986358169	BORCS7
ENSG00000286216.1	-0.011125745	0.876586184	0.986358169	RP11-433K2.8
ENSG00000200814.1	-0.005091677	0.876589553	0.986358169	RNU6-595P
ENSG00000142065.14	0.001807205	0.87661709	0.986358169	ZFP14
ENSG00000272269.1	0.003250879	0.876632671	0.986358169	NUP153-AS1
ENSG00000048162.21	0.00131689	0.876824526	0.986358169	NOP16
ENSG00000232124.3	0.007603818	0.876878412	0.986358169	AP001057.1
ENSG00000167112.10	-0.001079068	0.87691329	0.986358169	TRUB2
ENSG00000158480.11	-0.001135548	0.876931635	0.986358169	SPATA2
ENSG00000100226.16	-0.001255689	0.876963187	0.986358169	GTPBP1
ENSG00000174231.17	-0.001438595	0.87697003	0.986358169	PRPF8
ENSG00000168159.14	0.001805839	0.877052125	0.986358169	RNF187
ENSG00000165555.9	0.002526095	0.877116307	0.986358169	NOXRED1
ENSG00000260911.2	-0.002617854	0.877135547	0.986358169	RP11-196G11.2
ENSG00000140009.19	-0.002496737	0.877258766	0.986358169	ESR2
ENSG00000285159.1	-0.005223526	0.877285691	0.986358169	XXyac-YR21IF11.3
ENSG00000123384.14	0.002126841	0.877339118	0.986358169	LRP1

ENSG00000052344.16	-0.005101569	0.877343089	0.986358169	PRSS8
ENSG00000266651.1	-0.005387502	0.877344076	0.986358169	RP11-138I1.3
ENSG00000270164.1	0.003661995	0.877353199	0.986358169	LINC01480
ENSG00000197181.12	0.003364559	0.877445854	0.986358169	PIWIL2
ENSG00000107438.9	0.002588251	0.877448304	0.986358169	PDLIM1
ENSG00000250312.8	0.002833971	0.87749402	0.986358169	ZNF718
ENSG00000196476.12	-0.002030819	0.877509899	0.986358169	C20orf96
ENSG00000152193.8	-0.00214521	0.877557261	0.986358169	OBI1
ENSG00000138115.15	0.005087878	0.877569161	0.986358169	CYP2C8
ENSG00000130656.6	0.010807173	0.877571748	0.986358169	HBZ
ENSG00000107014.9	0.005033597	0.877656528	0.986358169	RLN2
ENSG00000157017.16	0.002270975	0.877715371	0.986358169	GHRL
ENSG00000052723.12	0.001446316	0.877902647	0.986358169	SIKE1
ENSG00000135637.14	0.001040889	0.878005884	0.986358169	CCDC142
ENSG00000166987.15	-0.001578866	0.878032189	0.986358169	MBD6
ENSG00000168824.14	0.004467739	0.878082364	0.986358169	NSG1
ENSG00000188529.15	-0.001510498	0.878179797	0.986358169	SRSF10
ENSG00000145414.9	-0.001279296	0.878198998	0.986358169	NAF1
ENSG00000069248.12	-8.11E-04	0.878243277	0.986358169	NUP133
ENSG00000225535.7	0.005391028	0.878250917	0.986358169	LINC01393
ENSG00000177352.10	-0.001152059	0.878261581	0.986358169	CCDC71
ENSG00000224888.4	-0.003211882	0.87836873	0.986358169	RP5-1142A6.2
ENSG00000172915.19	0.003402033	0.878390854	0.986358169	NBEA
ENSG00000100325.15	0.002725804	0.878403889	0.986358169	ASCC2
ENSG00000109906.15	-0.003157349	0.878421448	0.986358169	ZBTB16
ENSG00000260916.8	0.001514069	0.878448523	0.986358169	CCPG1
ENSG00000279722.1	-0.002344409	0.878468169	0.986358169	RP11-44F14.6
ENSG00000150961.15	0.001244804	0.878500168	0.986358169	SEC24D
ENSG00000170365.10	0.00236986	0.878542366	0.986358169	SMAD1
ENSG00000100036.13	0.002318607	0.878544456	0.986358169	SLC35E4
ENSG00000058091.17	0.002444461	0.878560216	0.986358169	CDK14
ENSG00000177272.9	-0.001548834	0.878576896	0.986358169	KCNA3
ENSG00000279162.1	0.004389724	0.878648829	0.986358169	CTD-3126B10.2
ENSG00000170430.10	-0.002216537	0.878682493	0.986358169	MGMT
ENSG00000258926.2	-0.004227355	0.878736066	0.986358169	RP11-47I22.1
ENSG00000006530.18	8.94E-04	0.878777085	0.986358169	AGK
ENSG00000168280.18	-0.002492464	0.878887446	0.986358169	KIF5C
ENSG00000172005.11	0.002977933	0.878887964	0.986358169	MAL
ENSG00000204947.9	0.002211169	0.878892661	0.986358169	ZNF425
ENSG00000135124.15	0.00141315	0.878929219	0.986358169	P2RX4
ENSG00000139405.16	-0.001207367	0.878956216	0.986358169	RITA1
ENSG00000179134.15	-0.00101899	0.878959213	0.986358169	SAMD4B
ENSG00000100592.16	-0.001727399	0.879187846	0.98647633	DAAM1
ENSG00000124214.21	-8.39E-04	0.879249762	0.98647633	STAU1
ENSG00000060982.15	-0.003881965	0.879291604	0.98647633	BCAT1
ENSG00000108528.14	0.001186759	0.879292078	0.98647633	SLC25A11
ENSG00000274414.1	0.005736138	0.879354488	0.98647633	RP5-965G21.4

ENSG00000180008.9	0.001760691	0.879422836	0.98647633	SOCS4
ENSG00000261663.1	0.002284948	0.879458576	0.98647633	RP11-304L19.11
ENSG00000259315.1	-0.002868822	0.879570922	0.98647633	ACTG1P17
ENSG00000239300.6	-0.003861714	0.879629083	0.98647633	RP11-400L8.2
ENSG00000273129.2	-0.003690523	0.879672428	0.98647633	PACERR
ENSG00000078319.10	-0.001090038	0.879682902	0.98647633	PMS2P1
ENSG00000115325.14	0.001243516	0.879811924	0.98647633	DOK1
ENSG00000183828.15	-0.001829541	0.879911832	0.98647633	NUDT14
ENSG00000176018.13	-0.002218128	0.879935119	0.98647633	LYSMD3
ENSG00000116991.11	-0.002581815	0.879939545	0.98647633	SIPA1L2
ENSG00000111886.11	-0.003026175	0.879973888	0.98647633	GABRR2
ENSG00000004139.14	0.001196306	0.879995521	0.98647633	SARM1
ENSG00000270011.8	0.004743371	0.880019151	0.98647633	ZNF559-ZNF177
ENSG00000116062.17	0.001076914	0.880038749	0.98647633	MSH6
ENSG00000136634.7	0.005205734	0.880099351	0.98647633	IL10
ENSG00000160013.9	0.002164054	0.880113681	0.98647633	PTGIR
ENSG00000256581.1	-0.003288434	0.880122752	0.98647633	NLRP9P1
ENSG00000112619.8	0.003833796	0.880426513	0.986631201	PRPH2
ENSG00000198964.14	0.001414029	0.880477692	0.986631201	SGMS1
ENSG00000168813.17	-0.00186215	0.880529261	0.986631201	ZNF507
ENSG00000163536.13	-0.002689599	0.880538457	0.986631201	SERPINI1
ENSG00000174885.13	-0.002800318	0.880621552	0.986631201	NLRP6
ENSG00000137038.8	0.001898919	0.880681527	0.986631201	DMAC1
ENSG00000258168.6	0.007161762	0.880780424	0.986631201	RP11-588H23.3
ENSG00000250318.1	-0.001843569	0.880791212	0.986631201	CTA-963H5.5
ENSG00000267551.3	-0.004163669	0.881088763	0.986631201	GNA15-DT
ENSG00000057593.14	-0.006748174	0.88109113	0.986631201	F7
ENSG00000237350.1	0.002498908	0.881180989	0.986631201	CDC42P6
ENSG00000117616.18	-0.001296793	0.881273814	0.986631201	RSRP1
ENSG00000127081.14	0.00221605	0.881275724	0.986631201	ZNF484
ENSG00000112697.17	-0.001470076	0.8812882	0.986631201	TMEM30A
ENSG00000265612.1	0.004594292	0.881319113	0.986631201	MIR4539
ENSG00000227199.1	0.006335238	0.881384044	0.986631201	ST7-AS1
ENSG00000224790.3	0.004435727	0.881450971	0.986631201	AP000704.5
ENSG00000167272.11	-0.001059458	0.881459197	0.986631201	POP5
ENSG00000266236.1	-0.002252379	0.881481556	0.986631201	NARF-IT1
ENSG00000268041.3	-0.002652784	0.881529728	0.986631201	ERFL
ENSG00000226380.9	-0.002477675	0.881552534	0.986631201	AC058791.1
ENSG00000224652.2	-0.00625315	0.881602448	0.986631201	LINC00885
ENSG00000150093.20	-0.001876268	0.88160705	0.986631201	ITGB1
ENSG00000185513.17	-0.001293494	0.88169787	0.986631201	L3MBTL1
ENSG00000229677.1	0.006287676	0.881756568	0.986631201	RP11-383F6.1
ENSG00000179091.5	-0.00124385	0.881763408	0.986631201	CYC1
ENSG00000141646.14	-7.30E-04	0.88178886	0.986631201	SMAD4
ENSG00000273329.1	0.001769045	0.881790477	0.986631201	RP11-448A19.1
ENSG00000277534.1	0.002807465	0.881803302	0.986631201	RP11-9E17.1
ENSG00000091651.9	-0.002736878	0.881849279	0.986631201	ORC6

ENSG00000131669.10	-0.001715044	0.88186126	0.986631201	NINJ1
ENSG00000066654.14	-0.001351144	0.88192155	0.986631201	THUMPD1
ENSG00000257594.4	0.005317357	0.881977068	0.986631201	GALNT4
ENSG00000180871.8	-0.001895396	0.882032287	0.986631201	CXCR2
ENSG00000213420.8	-0.002383563	0.882059035	0.986631201	GPC2
ENSG00000160049.12	-8.56E-04	0.882063691	0.986631201	DFFA
ENSG00000100218.12	0.003575739	0.882088386	0.986631201	RSPH14
ENSG00000174749.6	-0.001838368	0.882212046	0.986631201	FAM241A
ENSG00000134830.6	-0.001361896	0.882239715	0.986631201	C5AR2
ENSG00000158748.4	-0.005704252	0.882287333	0.986631201	HTR6
ENSG00000284922.2	0.002976286	0.882351872	0.986631201	RP11-807H22.10
ENSG00000217159.2	0.005133967	0.882383096	0.986631201	LARP1P1
ENSG00000186230.7	-0.001647378	0.882415828	0.986631201	ZNF749
ENSG00000043093.15	-0.001627234	0.882461482	0.986631201	DCUN1D1
ENSG00000146122.17	0.00656742	0.882599454	0.986631201	DAAM2
ENSG00000141506.15	-8.36E-04	0.882621003	0.986631201	PIK3R5
ENSG00000260329.1	-0.003592018	0.88267438	0.986631201	RP11-412D9.4
ENSG00000179477.11	0.004845801	0.882722617	0.986631201	ALOX12B
ENSG00000176105.14	0.002459529	0.882832087	0.986631201	YES1
ENSG00000049089.15	-0.002338912	0.882881157	0.986631201	COL9A2
ENSG00000237633.4	0.003510838	0.882971939	0.986631201	AC104623.2
ENSG00000232931.6	-0.001350602	0.882976824	0.986631201	LINC00342
ENSG00000166845.15	-0.002315007	0.882984018	0.986631201	C18orf54
ENSG00000239305.7	-0.0010807	0.883025767	0.986631201	RNF103
ENSG00000269713.7	-0.002004893	0.883053029	0.986631201	NBPF9
ENSG00000276651.1	0.004861214	0.883091521	0.986631201	AC007950.2
ENSG00000100364.19	-0.001362162	0.883095243	0.986631201	KIAA0930
ENSG00000215302.8	0.003946482	0.88311315	0.986631201	CTD-3092A11.1
ENSG00000151502.11	-8.07E-04	0.883211962	0.986631201	VPS26B
ENSG00000103966.11	-0.001175415	0.883289748	0.986631201	EHD4
ENSG00000269867.1	-0.00165314	0.883354663	0.986631201	CTD-2583A14.8
ENSG00000244071.1	-0.004157746	0.883367479	0.986631201	RPL9P33
ENSG00000271646.1	-0.001401199	0.883422633	0.986631201	IRF2-DT
ENSG00000108654.16	9.84E-04	0.883457693	0.986631201	DDX5
ENSG00000107249.24	-0.004853737	0.883611035	0.986631201	GLIS3
ENSG00000285632.1	-0.002570476	0.883649306	0.986631201	RP11-90P5.10
ENSG00000124743.6	-0.005586025	0.883656656	0.986631201	KLHL31
ENSG00000145214.14	-0.002174851	0.883671459	0.986631201	DGKQ
ENSG00000105137.13	-0.008303443	0.883770462	0.986631201	SYDE1
ENSG00000226629.1	0.015783032	0.88384943	0.986631201	LINC00974
ENSG00000269069.1	-0.004124218	0.883967872	0.986631201	CTC-471F3.5
ENSG00000236496.2	0.008037448	0.883981385	0.986631201	GPS2P1
ENSG00000198783.6	-8.85E-04	0.883996543	0.986631201	ZNF830
ENSG00000116747.13	0.00151334	0.884037833	0.986631201	RO60
ENSG00000273026.1	0.004044443	0.884083208	0.986631201	RP11-422P24.10
ENSG00000060138.13	-0.00395315	0.884279412	0.986631201	YBX3
ENSG00000286579.1	-0.005331578	0.884333293	0.986631201	RP11-453A3.1

ENSG00000141497.14	-0.002116878	0.884453037	0.986631201	ZMYND15
ENSG00000143185.4	0.004476282	0.884490558	0.986631201	XCL2
ENSG00000196427.14	-0.011678371	0.884532821	0.986631201	NBPF4
ENSG00000286689.1	-0.006689424	0.884625784	0.986631201	RP11-2B2.1
ENSG00000135677.11	-0.001283474	0.88476955	0.986631201	GNS
ENSG00000089123.16	0.001355248	0.884774071	0.986631201	TASP1
ENSG00000078053.17	-0.003165253	0.884866407	0.986631201	AMPH
ENSG00000088992.18	0.003384642	0.884881013	0.986631201	TESC
ENSG00000012223.13	-0.007591302	0.884920466	0.986631201	LTF
ENSG00000244538.1	0.006553355	0.884923993	0.986631201	RP11-10G12.1
ENSG00000166347.19	0.00161603	0.884945357	0.986631201	CYB5A
ENSG00000143772.10	8.61E-04	0.885048581	0.986631201	ITPKB
ENSG00000163734.4	0.004826534	0.885082525	0.986631201	CXCL3
ENSG00000226450.2	0.002598052	0.885207916	0.986631201	CYP2D8P
ENSG00000166337.10	9.79E-04	0.885239117	0.986631201	TAF10
ENSG00000224861.1	-0.002885861	0.885257873	0.986631201	YBX1P1
ENSG00000275468.1	0.005195522	0.885291434	0.986631201	LLNLR-304A6.2
ENSG00000271304.2	0.005053956	0.8852986	0.986631201	DPRXP2
ENSG00000232860.8	0.004235423	0.885343619	0.986631201	SMG7-AS1
ENSG00000269896.2	-0.002201692	0.885383292	0.986631201	RP4-740C4.5
ENSG00000278214.1	0.006195327	0.885545987	0.986631201	LINC02139
ENSG00000286912.1	-0.005894759	0.885556987	0.986631201	RP5-820A21.2
ENSG00000111962.8	-0.002010643	0.885582377	0.986631201	UST
ENSG00000146826.17	-0.001207509	0.885585952	0.986631201	TRAPPC14
ENSG00000272145.1	0.002441692	0.885595435	0.986631201	NFYC-AS1
ENSG00000198393.8	0.001222054	0.885621171	0.986631201	ZNF26
ENSG00000211697.4	-0.007102378	0.885644447	0.986631201	TRGV5
ENSG00000100324.14	-9.23E-04	0.885711274	0.986631201	TAB1
ENSG00000099204.20	0.001703455	0.8857224	0.986631201	ABLIM1
ENSG00000129315.11	9.09E-04	0.885812114	0.986631201	CCNT1
ENSG00000279748.1	0.00355413	0.885869965	0.986631201	CTD-3222D19.9
ENSG00000185386.15	-0.001979688	0.886041051	0.986631201	MAPK11
ENSG00000223884.7	0.00358598	0.886074379	0.986631201	AC019048.1
ENSG00000228857.2	0.003938459	0.886085084	0.986631201	AC104653.1
ENSG00000130035.9	-0.004167802	0.886193862	0.986631201	GALNT8
ENSG00000100104.13	0.001306093	0.886197977	0.986631201	SRRD
ENSG00000156869.14	-0.002630489	0.886203397	0.986631201	FRRS1
ENSG00000107282.8	-0.003215326	0.88632992	0.986631201	APBA1
ENSG00000187994.14	7.76E-04	0.886330226	0.986631201	RINL
ENSG00000162174.12	-0.00191276	0.886353601	0.986631201	ASRGL1
ENSG00000081189.16	-0.001601565	0.8864038	0.986631201	MEF2C
ENSG00000254799.1	0.006300985	0.886658167	0.986631201	SLC25A47P1
ENSG00000183439.9	-0.004458162	0.886715977	0.986631201	TRIM61
ENSG00000150907.10	-0.001269476	0.886735048	0.986631201	FOXO1
ENSG00000141753.7	-0.00309577	0.886766299	0.986631201	IGFBP4
ENSG00000145990.11	-0.002170536	0.886794664	0.986631201	GFOD1
ENSG00000231999.7	0.001684917	0.88680362	0.986631201	LRRC8C-DT

ENSG00000275743.1	-0.004304833	0.886820052	0.986631201	TRBV14
ENSG00000149577.16	0.001862884	0.886855462	0.986631201	SIDT2
ENSG00000112739.17	0.001148364	0.886952248	0.986631201	PRPF4B
ENSG00000105723.13	-0.00114764	0.887032278	0.986631201	GSK3A
ENSG00000211858.1	0.008487957	0.887086955	0.986631201	TRAJ31
ENSG00000084112.15	0.001242357	0.887090243	0.986631201	SSH1
ENSG00000137135.18	0.002082054	0.887093267	0.986631201	ARHGEF39
ENSG00000248092.8	0.001746033	0.887117937	0.986631201	NNT-AS1
ENSG00000229870.1	0.004909338	0.887130385	0.986631201	RPL21P89
ENSG00000130037.5	-0.004549092	0.887142742	0.986631201	KCNA5
ENSG00000260442.5	-0.005368752	0.887159017	0.986631201	ATP2A1-AS1
ENSG00000211793.2	-0.002556506	0.887172386	0.986631201	TRAV9-2
ENSG00000258768.2	-0.002305973	0.887179596	0.986631201	CTD-2292M16.8
ENSG00000237489.5	0.001696135	0.887207983	0.986631201	C10orf143
ENSG00000075420.13	-0.001638404	0.887215283	0.986631201	FNDC3B
ENSG00000087077.14	-0.001695597	0.887307356	0.986631201	TRIP6
ENSG00000280927.1	-0.001459646	0.887343297	0.986631201	CTBP1-AS
ENSG00000175105.8	0.001924851	0.88744138	0.986631201	ZNF654
ENSG00000152207.8	0.002279663	0.887444796	0.986631201	CYSLTR2
ENSG00000160323.19	-0.002246639	0.887504013	0.986631201	ADAMTS13
ENSG00000286535.1	0.003048586	0.887554138	0.986631201	CTD-3091F23.1
ENSG00000280401.1	-0.003081892	0.887582446	0.986631201	RP11-104F15.9
ENSG00000166479.10	-0.001963783	0.88759454	0.986631201	TMX3
ENSG00000134255.14	0.001396241	0.88761838	0.986631201	CEPT1
ENSG00000163918.12	0.001317462	0.887765187	0.986631201	RFC4
ENSG00000233554.6	-0.003123957	0.887817799	0.986631201	B4GALT1-AS1
ENSG00000272572.1	-0.004245445	0.887853896	0.986631201	RP11-179B2.2
ENSG00000086289.12	0.003328824	0.88787956	0.986631201	EPDR1
ENSG00000104687.14	8.36E-04	0.887887847	0.986631201	GSR
ENSG00000105851.11	-0.001096914	0.887898023	0.986631201	PIK3CG
ENSG00000119820.11	8.88E-04	0.887966054	0.986631201	YIPF4
ENSG00000286076.1	-0.005606828	0.888002956	0.986631201	CTB-30L5.2
ENSG00000228709.1	-0.008306334	0.888033203	0.986631201	LINC02575
ENSG00000260145.1	0.003657739	0.888069761	0.986631201	RP11-322D14.2
ENSG00000095321.18	0.001823733	0.888091553	0.986631201	CRAT
ENSG00000232934.8	0.002320452	0.888169084	0.986631201	RP11-324O2.3
ENSG00000070540.13	9.92E-04	0.888188338	0.986631201	WIPI1
ENSG00000162607.13	0.001603667	0.88825963	0.986631201	USP1
ENSG00000239948.2	-0.004961698	0.888356379	0.986631201	RN7SL368P
ENSG00000232909.1	-0.004120299	0.888395392	0.986631201	RP3-510O8.4
ENSG00000146232.17	0.001406279	0.888428924	0.986631201	NFKBIE
ENSG00000180139.11	-0.003430248	0.888458095	0.986631201	ACTA2-AS1
ENSG00000214700.6	0.003848854	0.888476176	0.986631201	C12orf71
ENSG00000173611.18	-0.001875908	0.888478048	0.986631201	SCAI
ENSG00000132205.11	-0.001667336	0.888502621	0.986631201	EMILIN2
ENSG00000262714.1	-0.003076019	0.888613299	0.986631201	RP11-44F14.8
ENSG00000234883.6	0.00391369	0.888642999	0.986631201	MIR155HG

ENSG00000177989.14	-0.002925704	0.888681083	0.986631201	ODF3B
ENSG00000138496.17	0.002381106	0.888690856	0.986631201	PARP9
ENSG00000132128.17	9.05E-04	0.888808584	0.986631201	LRRC41
ENSG00000123338.13	-8.88E-04	0.888852069	0.986631201	NCKAP1L
ENSG00000124233.12	0.00431	0.88886407	0.986631201	SEMG1
ENSG00000267855.6	-0.001937839	0.888919532	0.986631201	NDUFA7
ENSG00000139517.9	-0.001356898	0.888946633	0.986631201	LNX2
ENSG00000100219.16	0.001181839	0.888950874	0.986631201	XBP1
ENSG00000260618.1	-0.003722761	0.889013463	0.986631201	RP11-23N2.4
ENSG00000225614.4	-0.002154595	0.889043078	0.986631201	ZNF469
ENSG00000197429.11	-0.001219901	0.889064367	0.986631201	IPP
ENSG00000265800.1	0.002905356	0.88906498	0.986631201	RP11-649A18.5
ENSG00000227543.5	0.001671633	0.88919976	0.986672835	SPAG5-AS1
ENSG00000133256.13	0.001208603	0.889215287	0.986672835	PDE6B
ENSG00000231527.7	0.005674957	0.889246831	0.986672835	FAM27C
ENSG00000286786.1	-0.002835134	0.889414013	0.986705088	RP11-102L12.3
ENSG00000147669.11	-0.003400301	0.889488274	0.986705088	POLR2K
ENSG00000232713.2	-0.003150352	0.889494214	0.986705088	RPS12P3
ENSG00000131409.13	-0.004433052	0.88951801	0.986705088	LRRC4B
ENSG00000235217.6	0.001713268	0.889521376	0.986705088	TSPY26P
ENSG00000229140.11	-0.004949915	0.889628059	0.986705088	CCDC26
ENSG00000114656.12	-0.00154045	0.889697684	0.986705088	CFAP92
ENSG00000242588.6	0.003206231	0.889737232	0.986705088	RP11-274B21.14
ENSG00000088888.18	8.34E-04	0.889771742	0.986705088	MAVS
ENSG00000246366.6	-0.003310115	0.889823314	0.986705088	LACTB2-AS1
ENSG00000198265.12	0.001115133	0.889845199	0.986705088	HELZ
ENSG00000100982.12	-0.001055503	0.889853257	0.986705088	PCIF1
ENSG00000170442.12	0.00582749	0.889998294	0.986784313	KRT86
ENSG00000164754.15	9.96E-04	0.89002094	0.986784313	RAD21
ENSG00000108848.16	0.001348641	0.89022507	0.986926162	LUC7L3
ENSG00000284753.2	0.002027539	0.890245127	0.986926162	EEF1AKMT4
ENSG00000276337.1	0.003501244	0.890308311	0.986942856	RP11-106D4.2
ENSG00000171812.13	0.002317832	0.890425307	0.986990102	COL8A2
ENSG00000048649.14	0.001033215	0.890590597	0.986990102	RSF1
ENSG00000256973.1	-0.005871215	0.890597952	0.986990102	RP11-359J14.2
ENSG00000158485.11	0.004018074	0.89061592	0.986990102	CD1B
ENSG00000254362.1	-0.004936004	0.890618267	0.986990102	RP11-14I17.3
ENSG00000050767.18	0.004414571	0.890822324	0.986990102	COL23A1
ENSG00000260660.4	-0.003152813	0.890823341	0.986990102	RP11-69H7.2
ENSG00000169131.13	0.002292486	0.890966387	0.986990102	ZNF354A
ENSG00000153914.16	0.00157112	0.890979825	0.986990102	SREK1
ENSG00000233191.2	-0.004646847	0.890980836	0.986990102	AC006372.6
ENSG00000162923.17	8.80E-04	0.891024969	0.986990102	WDR26
ENSG00000187778.14	-8.91E-04	0.891031212	0.986990102	MCRS1
ENSG00000100221.11	-7.70E-04	0.891141457	0.986990102	JOSD1
ENSG00000253540.5	-0.002933755	0.891263008	0.986990102	FAM86HP
ENSG00000164404.8	0.003895871	0.891315609	0.986990102	GDF9

ENSG00000107819.13	9.89E-04	0.891407966	0.986990102	SFXN3
ENSG00000228839.5	-0.002615602	0.891482997	0.986990102	PIK3IP1-DT
ENSG00000248487.9	-0.001470794	0.891502338	0.986990102	ABHD14A
ENSG00000184792.16	-0.004003332	0.891581149	0.986990102	OSBP2
ENSG00000134070.5	0.001567549	0.891587572	0.986990102	IRAK2
ENSG00000152291.14	-8.39E-04	0.89158787	0.986990102	TGOLN2
ENSG00000235419.5	-0.005140711	0.891679372	0.986990102	AC010149.4
ENSG00000236525.1	0.004303774	0.891714274	0.986990102	AC007278.2
ENSG00000108349.17	9.44E-04	0.891767989	0.986990102	CASC3
ENSG00000125871.14	-0.001170301	0.891834635	0.986990102	MGME1
ENSG00000198055.11	9.58E-04	0.89188228	0.986990102	GRK6
ENSG00000211883.1	0.005265348	0.891901963	0.986990102	TRAJ6
ENSG00000254911.3	-0.004526361	0.891923338	0.986990102	SCARNA9
ENSG00000150995.21	0.001525975	0.891931362	0.986990102	ITPR1
ENSG00000233665.8	-0.002636174	0.891946463	0.986990102	RP11-523O18.5
ENSG00000203797.12	-0.003796372	0.891976349	0.986990102	DDO
ENSG00000278195.2	0.003245399	0.892015799	0.986990102	SSTR3
ENSG00000279088.1	-0.00133329	0.892086875	0.986990102	RP11-574K11.24
ENSG00000162415.7	-0.002132824	0.89221842	0.986990102	ZSWIM5
ENSG00000197323.12	0.001308571	0.892258415	0.986990102	TRIM33
ENSG00000211869.1	-0.007000453	0.892272965	0.986990102	TRAJ20
ENSG00000166128.13	0.001529435	0.892274492	0.986990102	RAB8B
ENSG00000197969.14	0.001650034	0.892374349	0.986990102	VPS13A
ENSG00000243926.2	-0.003756752	0.892472178	0.986990102	TIPARP-AS1
ENSG00000197601.13	-0.001760239	0.892502085	0.986990102	FAR1
ENSG00000286989.1	-0.001974054	0.89250652	0.986990102	RP11-345P4.12
ENSG00000258565.1	0.004912608	0.892564582	0.986990102	BLZF2P
ENSG00000163960.12	9.05E-04	0.892568642	0.986990102	UBXN7
ENSG00000236140.1	-0.004215952	0.892582956	0.986990102	RP11-89F3.2
ENSG00000168421.13	-0.001043415	0.892801526	0.986990102	RHOH
ENSG00000179630.11	-0.002089428	0.892802698	0.986990102	LACC1
ENSG00000275383.1	-0.004845321	0.892809074	0.986990102	RP11-615I2.6
ENSG00000205940.8	-0.005843105	0.892943509	0.986990102	HSP90AB2P
ENSG00000151474.23	0.001678855	0.893009746	0.986990102	FRMD4A
ENSG00000167183.3	0.0058243	0.893068685	0.986990102	PRR15L
ENSG00000224373.3	-0.004926767	0.89307726	0.986990102	IGHV4-59
ENSG00000134532.19	0.007116743	0.893134454	0.986990102	SOX5
ENSG00000163599.17	-0.001892506	0.893144282	0.986990102	CTLA4
ENSG00000206418.5	0.001425042	0.893153631	0.986990102	RAB12
ENSG00000267519.6	-0.001846029	0.893175759	0.986990102	MIR23AHG
ENSG00000263015.1	-0.008367001	0.893189503	0.986990102	RP5-1029F21.3
ENSG00000124557.14	0.003369494	0.893202108	0.986990102	BTN1A1
ENSG00000164938.14	0.001501943	0.89323449	0.986990102	TP53INP1
ENSG00000115414.21	0.004248159	0.893256472	0.986990102	FN1
ENSG00000260908.1	0.004885332	0.893261667	0.986990102	CTB-134H23.3
ENSG00000131408.15	-0.001152053	0.893286682	0.986990102	NR1H2
ENSG00000267744.1	0.005895381	0.893447837	0.986994123	RP11-799D4.1

ENSG00000157450.16	0.001160453	0.893448988	0.986994123	RNF111
ENSG00000230537.2	-0.004368075	0.893485862	0.986994123	RP11-305L7.1
ENSG00000099942.13	-7.81E-04	0.893735386	0.986994123	CRKL
ENSG00000280173.1	-0.002163832	0.893739705	0.986994123	RP11-447D11.3
ENSG00000144362.12	0.003459574	0.89374168	0.986994123	PHOSPHO2
ENSG00000101695.9	-9.90E-04	0.893772092	0.986994123	RNF125
ENSG00000103042.9	0.001197804	0.893785184	0.986994123	SLC38A7
ENSG00000110048.12	7.05E-04	0.893800321	0.986994123	OSBP
ENSG00000160818.17	0.004267203	0.893800947	0.986994123	GPATCH4
ENSG00000132661.4	0.001329539	0.893854469	0.986994123	NXT1
ENSG00000134917.10	0.004037808	0.893908641	0.986994123	ADAMTS8
ENSG00000179387.10	-0.001704481	0.893915976	0.986994123	ELMOD2
ENSG00000149182.15	-8.18E-04	0.894017539	0.987053119	ARFGAP2
ENSG00000277383.1	0.003828047	0.894067654	0.987055311	CTD-3001H11.2
ENSG00000248673.2	-0.004753681	0.894196836	0.987087387	LINC01331
ENSG00000159459.12	0.001357823	0.894366599	0.987087387	UBR1
ENSG00000238025.1	0.001470286	0.894382709	0.987087387	ZDHHC4P1
ENSG00000096746.18	0.001114432	0.894478023	0.987087387	HNRNPH3
ENSG00000122643.23	-0.001745702	0.894608709	0.987087387	NT5C3A
ENSG00000211647.1	0.009735092	0.894712801	0.987087387	IGLV5-48
ENSG00000120137.7	-0.001473374	0.894748755	0.987087387	PANK3
ENSG00000234492.4	-0.00411404	0.894787153	0.987087387	RPL34-DT
ENSG00000214548.18	-0.006535793	0.894796946	0.987087387	MEG3
ENSG00000183542.5	-0.004583536	0.894797716	0.987087387	KLRC4
ENSG00000173825.7	0.002000589	0.894809137	0.987087387	TIGD3
ENSG00000170270.5	0.001969282	0.8948429	0.987087387	GON7
ENSG00000042832.12	0.002522243	0.894974471	0.987087387	TG
ENSG00000186908.15	0.002006613	0.894987106	0.987087387	ZDHHC17
ENSG00000285517.1	-0.007016129	0.89506102	0.987087387	LINC00941
ENSG00000189164.15	-0.001489821	0.895075452	0.987087387	ZNF527
ENSG00000005469.12	-0.001184495	0.895078525	0.987087387	CROT
ENSG00000087338.5	0.001261837	0.89508047	0.987087387	GMCL1
ENSG00000116649.10	-0.001327863	0.895089135	0.987087387	SRM
ENSG00000158636.16	-0.001049759	0.895094015	0.987087387	EMSY
ENSG00000254413.8	-0.002250714	0.895190057	0.987087387	CHKB-CPT1B
ENSG00000124198.10	0.001410037	0.895191592	0.987087387	ARFGEF2
ENSG00000187147.18	7.11E-04	0.89520374	0.987087387	RNF220
ENSG00000173083.16	-0.001628203	0.895374052	0.987214981	HPSE
ENSG00000163867.17	-0.001066222	0.895415733	0.987214981	ZMYM6
ENSG00000269680.1	-0.002369475	0.895555313	0.987315792	CTD-3128G10.6
ENSG00000145451.13	0.007266056	0.895694879	0.987346777	GLRA3
ENSG00000227372.12	9.29E-04	0.895721159	0.987346777	TP73-AS1
ENSG00000077092.19	0.003874663	0.895727852	0.987346777	RARB
ENSG00000267264.1	0.005281686	0.895943344	0.987531231	CTC-459F4.6
ENSG00000137962.13	0.003405905	0.896039043	0.987583635	ARHGAP29
ENSG00000258302.2	0.002810141	0.896206033	0.98762411	RP11-981P6.1
ENSG00000067704.10	-7.53E-04	0.896285775	0.98762411	IARS2

ENSG00000197696.10	-0.002128965	0.896417992	0.98762411	NMB
ENSG00000287771.1	-0.003660809	0.896430244	0.98762411	RP11-4B12.1
ENSG00000095794.19	-0.001419775	0.896431975	0.98762411	CREM
ENSG00000167291.16	-0.002080244	0.896462526	0.98762411	TBC1D16
ENSG00000162733.19	0.003682843	0.896523559	0.98762411	DDR2
ENSG00000070366.14	-0.001412277	0.896546252	0.98762411	SMG6
ENSG00000186918.14	-9.96E-04	0.896547431	0.98762411	ZNF395
ENSG00000117009.12	0.002191157	0.896557346	0.98762411	KMO
ENSG00000186487.20	-0.007785849	0.896717523	0.987650145	MYT1L
ENSG00000255237.1	0.003071539	0.896760683	0.987650145	RP13-317D12.3
ENSG00000102189.17	-0.001823908	0.896763358	0.987650145	EEA1
ENSG00000279794.1	0.003659246	0.896773618	0.987650145	CTD-2621I17.3
ENSG00000230732.4	0.004245971	0.8968236	0.987652153	AC127904.2
ENSG00000198626.17	-0.005354264	0.896930697	0.987699245	RYR2
ENSG00000095539.15	0.001485128	0.896996419	0.987699245	SEMA4G
ENSG00000167216.18	-0.001805563	0.897010847	0.987699245	KATNAL2
ENSG00000143257.12	-0.002895557	0.897253034	0.987860735	NR1I3
ENSG00000087266.17	-7.89E-04	0.897302131	0.987860735	SH3BP2
ENSG00000246084.5	0.001824529	0.897403053	0.987860735	LINC02325
ENSG00000065308.5	0.001060925	0.897415025	0.987860735	TRAM2
ENSG00000253716.6	0.001861954	0.897421339	0.987860735	MINCR
ENSG00000189227.6	-0.001401254	0.897446526	0.987860735	C15orf61
ENSG00000169071.15	0.007811718	0.897590337	0.987900602	ROR2
ENSG00000087303.18	-0.002588094	0.897664024	0.987900602	NID2
ENSG00000153006.16	-0.001281745	0.897695501	0.987900602	SREK1IP1
ENSG00000204052.4	-0.010095309	0.897728387	0.987900602	LRRC73
ENSG00000203709.12	0.001516518	0.897732622	0.987900602	MIR29B2CHG
ENSG00000100814.18	0.001138848	0.897771773	0.987900602	CCNB1IP1
ENSG00000130844.19	-0.001276187	0.897862307	0.987938059	ZNF331
ENSG00000033050.9	-0.001016183	0.897902159	0.987938059	ABCF2
ENSG00000267568.6	-0.006087538	0.897979889	0.987970578	RP11-87G24.3
ENSG00000204421.3	-0.004774978	0.898058397	0.988003949	LY6G6C
ENSG00000279281.1	0.010168701	0.89823382	0.988107388	RP11-226M10.3
ENSG00000256690.1	0.003188995	0.898248783	0.988107388	RP11-727F15.9
ENSG00000197841.15	-0.00216368	0.898343884	0.988132452	ZNF181
ENSG00000266934.1	-0.003459467	0.898469017	0.988132452	RP11-332H18.3
ENSG00000188026.13	-0.001905511	0.898491289	0.988132452	RILPL1
ENSG00000286063.1	0.006935798	0.898503182	0.988132452	RP5-1050C22.1
ENSG00000254859.1	0.006237624	0.898542983	0.988132452	RP11-661A12.5
ENSG00000188886.4	0.002720153	0.898589256	0.988132452	ASTL
ENSG00000257557.2	-0.003708502	0.898608846	0.988132452	PPP1R12A-AS1
ENSG00000157227.14	0.002410768	0.898781294	0.988213758	MMP14
ENSG00000254858.10	-0.001157218	0.898839975	0.988213758	MPV17L2
ENSG00000230736.2	0.003262322	0.898882549	0.988213758	RP1-149A16.3
ENSG00000141956.14	0.001010443	0.898922508	0.988213758	PRDM15
ENSG00000048140.18	-0.001014039	0.899008143	0.988213758	TSPAN17
ENSG00000124091.9	0.002628499	0.899011617	0.988213758	GCNT7

ENSG00000109787.13	-0.001121524	0.899020094	0.988213758	KLF3
ENSG00000226396.1	-0.002009637	0.899136675	0.988261824	RP5-1056L3.3
ENSG00000198730.9	7.70E-04	0.899170717	0.988261824	CTR9
ENSG00000244723.3	-0.002711749	0.899208389	0.988261824	ASLP1
ENSG00000119899.13	7.84E-04	0.899257253	0.988262566	SLC17A5
ENSG00000279161.1	-0.004980026	0.899340073	0.988299292	CTB-12A17.2
ENSG00000258457.5	-0.004951539	0.899387053	0.988299292	RP11-298I3.4
ENSG00000111615.14	0.001335677	0.89949017	0.98831176	KRR1
ENSG00000211795.3	0.002368066	0.899552581	0.98831176	TRAV8-6
ENSG00000278834.1	-0.003526343	0.89959413	0.98831176	RP11-458J1.1
ENSG00000235531.11	0.003697041	0.899626501	0.98831176	MSC-AS1
ENSG00000282572.2	0.002434616	0.899639357	0.98831176	RP11-482G13.1
ENSG00000254826.1	-0.004720169	0.899725128	0.988338848	CTD-2530H12.2
ENSG00000256249.1	-0.004081057	0.899864445	0.988338848	RP11-324E6.6
ENSG00000233476.3	0.001825754	0.899913094	0.988338848	EEF1A1P6
ENSG00000236882.8	-0.003931494	0.899940555	0.988338848	LINC01554
ENSG00000140280.14	-0.001585437	0.899961212	0.988338848	LYSMD2
ENSG00000159784.18	-0.002729601	0.899977258	0.988338848	FAM131B
ENSG00000173480.11	0.001149999	0.900001832	0.988338848	ZNF417
ENSG00000205890.3	-0.002079667	0.900049557	0.988338848	RP11-473M20.5
ENSG00000169554.22	0.001221961	0.900161027	0.988403224	ZEB2
ENSG00000140632.17	6.00E-04	0.900232775	0.988403224	GLYR1
ENSG00000234498.3	0.002188309	0.900380256	0.988403224	RPL13AP20
ENSG00000152520.14	-9.98E-04	0.900429441	0.988403224	PAN3
ENSG00000163946.14	0.001108665	0.900490266	0.988403224	TASOR
ENSG00000266235.1	0.003301087	0.900493309	0.988403224	MIR3176
ENSG00000258476.6	-0.003128033	0.900694292	0.988403224	LINC02207
ENSG00000132305.21	-8.33E-04	0.900713223	0.988403224	IMMT
ENSG00000123095.6	-0.003731447	0.9008142	0.988403224	BHLHE41
ENSG00000198826.11	0.00256556	0.900846987	0.988403224	ARHGAP11A
ENSG00000163870.16	-0.001151094	0.900901628	0.988403224	TPRA1
ENSG00000160695.15	-8.17E-04	0.90094885	0.988403224	VPS11
ENSG00000214652.6	0.005158115	0.900969248	0.988403224	ZNF727
ENSG00000187037.9	-0.001498384	0.901006519	0.988403224	GPR141
ENSG00000239819.2	-0.004852983	0.901038334	0.988403224	IGKV1D-8
ENSG00000281344.1	-0.004035369	0.901063575	0.988403224	HELLPAR
ENSG00000131323.16	-7.40E-04	0.90106761	0.988403224	TRAF3
ENSG00000175938.7	0.001182787	0.901135652	0.988403224	ORAI3
ENSG00000205209.8	-0.003181732	0.90113768	0.988403224	SCGB2B2
ENSG00000155034.19	-0.001067667	0.901184863	0.988403224	FBXL18
ENSG00000101079.21	-7.55E-04	0.901186719	0.988403224	NDRG3
ENSG00000150873.12	0.003152817	0.901207025	0.988403224	C2orf50
ENSG00000121210.16	8.55E-04	0.901229873	0.988403224	TMEM131L
ENSG00000132740.10	0.002090834	0.901264886	0.988403224	IGHMBP2
ENSG00000230487.8	-0.00124501	0.901427865	0.988453848	PSMG3-AS1
ENSG00000255624.3	0.002944299	0.901543136	0.988453848	C10orf88B
ENSG00000074621.14	0.001036082	0.901558944	0.988453848	SLC24A1

ENSG00000109133.13	-7.55E-04	0.901576432	0.988453848	TMEM33
ENSG00000184575.12	8.04E-04	0.901579336	0.988453848	XPOT
ENSG00000276931.1	0.00378896	0.901618098	0.988453848	RP11-161M6.6
ENSG00000232702.3	-0.003054493	0.901677565	0.988453848	RP3-437C15.1
ENSG00000174373.17	-0.001412156	0.901726261	0.988453848	RALGAPA1
ENSG00000081307.15	9.24E-04	0.901763766	0.988453848	UBA5
ENSG00000179397.18	0.00326732	0.901793031	0.988453848	CATSPERE
ENSG00000079462.8	-0.001448055	0.901886849	0.988456961	PAFAH1B3
ENSG00000136003.16	9.84E-04	0.901930051	0.988456961	ISCU
ENSG00000157045.9	-0.001273039	0.901951639	0.988456961	NTAN1
ENSG00000253853.3	-0.007122241	0.901988666	0.988456961	GS1-57L11.1
ENSG00000279923.1	0.003206141	0.902103219	0.988487369	CTD-2008E3.1
ENSG00000127526.15	-6.59E-04	0.902118212	0.988487369	SLC35E1
ENSG00000131844.17	5.96E-04	0.902204857	0.988487369	MCCC2
ENSG00000287043.1	-0.009594426	0.902209214	0.988487369	RP11-29A19.3
ENSG00000173715.18	-0.001656322	0.902337692	0.98850774	C11orf80
ENSG00000245156.1	0.001724741	0.902430049	0.98850774	RP11-867G23.3
ENSG00000262039.1	-0.00229212	0.9024609	0.98850774	RP11-81K2.1
ENSG00000274605.2	0.003252705	0.902473713	0.98850774	PCCA-DT
ENSG00000107882.12	-8.76E-04	0.902507633	0.98850774	SUFU
ENSG00000132623.16	0.001449486	0.902564087	0.98850774	ANKEF1
ENSG00000231167.3	-0.002849639	0.902633163	0.98850774	YBX1P2
ENSG00000100387.9	0.001808577	0.902789353	0.98850774	RBX1
ENSG00000180822.12	0.001051379	0.902797494	0.98850774	PSMG4
ENSG00000187134.14	0.003412492	0.902912115	0.98850774	AKR1C1
ENSG00000282639.1	-0.008164956	0.902961922	0.98850774	IGHV3-64D
ENSG00000089063.15	-7.94E-04	0.903026726	0.98850774	TMEM230
ENSG00000178297.14	-0.003469411	0.903068473	0.98850774	TMPRSS9
ENSG00000183172.9	0.001701673	0.903144522	0.98850774	SMDT1
ENSG00000196943.14	-6.96E-04	0.903276572	0.98850774	NOP9
ENSG00000114770.17	-0.001419869	0.903330957	0.98850774	ABCC5
ENSG00000230387.4	0.006147643	0.903378317	0.98850774	RP4-737E23.7
ENSG00000257800.1	-0.004264451	0.903411598	0.98850774	FNBP1P1
ENSG00000274134.1	0.005648457	0.903461641	0.98850774	MIR6774
ENSG00000271781.1	0.003383626	0.903615033	0.98850774	CTD-2589H19.6
ENSG00000101447.15	-0.00234414	0.903649085	0.98850774	FAM83D
ENSG00000104863.12	0.001564796	0.903702937	0.98850774	LIN7B
ENSG00000124587.14	0.001985873	0.903743652	0.98850774	PEX6
ENSG00000204138.13	-7.49E-04	0.903803581	0.98850774	PHACTR4
ENSG00000261002.5	0.004935316	0.903928707	0.98850774	VPS39-DT
ENSG00000223294.1	0.005196241	0.903930279	0.98850774	SNORD83
ENSG00000133678.14	8.67E-04	0.903935987	0.98850774	TMEM254
ENSG00000005812.11	-0.001269009	0.903956216	0.98850774	FBXL3
ENSG00000145725.20	0.001511066	0.903961826	0.98850774	PPIP5K2
ENSG00000237440.9	0.001599637	0.904011073	0.98850774	ZNF737
ENSG00000225213.2	0.004372451	0.904011315	0.98850774	RP11-197M22.2
ENSG00000132906.18	8.65E-04	0.90406813	0.98850774	CASP9

ENSG00000128590.5	-0.001281039	0.904071117	0.98850774	DNAJB9
ENSG00000135587.9	0.001115526	0.904073399	0.98850774	SMPD2
ENSG00000174721.10	-0.001772096	0.904232534	0.98850774	FGFBP3
ENSG00000182531.7	-0.008569707	0.904290492	0.98850774	OR7E115P
ENSG00000133226.17	-6.97E-04	0.904301012	0.98850774	SRRM1
ENSG00000183628.14	-0.001891094	0.904301505	0.98850774	DGCR6
ENSG00000211739.4	0.010460222	0.904312758	0.98850774	TRBV12-2
ENSG00000224003.1	-0.003790391	0.904346351	0.98850774	YES1P1
ENSG00000116525.14	-7.86E-04	0.904367302	0.98850774	TRIM62
ENSG00000170325.16	0.001151195	0.904391998	0.98850774	PRDM10
ENSG00000084070.12	0.001260617	0.904394718	0.98850774	SMAP2
ENSG00000196876.18	-0.003314497	0.904430328	0.98850774	SCN8A
ENSG00000072609.18	-5.78E-04	0.904498362	0.98850774	CHFR
ENSG00000185305.11	-8.66E-04	0.904513952	0.98850774	ARL15
ENSG00000068097.15	0.001055782	0.904535659	0.98850774	HEATR6
ENSG00000285923.1	0.003746016	0.904541459	0.98850774	RP11-256E16.3
ENSG00000110104.13	-0.00115355	0.904608784	0.988527786	CCDC86
ENSG00000132704.16	-0.003682168	0.904656206	0.988527786	FCRL2
ENSG00000133805.15	-9.58E-04	0.904740017	0.988554452	AMPD3
ENSG00000262061.6	-0.005942021	0.904832935	0.988554452	RPH3AL-AS1
ENSG00000069849.11	9.87E-04	0.904864727	0.988554452	ATP1B3
ENSG00000114805.18	0.00430219	0.904903416	0.988554452	PLCH1
ENSG00000072518.22	8.11E-04	0.904989849	0.988554452	MARK2
ENSG00000232680.2	0.002575461	0.905009553	0.988554452	AC002511.3
ENSG00000211819.3	0.004412406	0.905018034	0.988554452	TRAV40
ENSG00000196656.7	0.00734758	0.905111337	0.988603712	AC004057.1
ENSG00000087085.16	0.005097882	0.905238404	0.988636746	ACHE
ENSG00000138735.16	0.00178091	0.905259118	0.988636746	PDE5A
ENSG00000256427.2	-0.003343462	0.905286203	0.988636746	RP11-118B22.2
ENSG00000104974.12	0.00107444	0.905422293	0.988651764	LILRA1
ENSG00000080189.15	-7.16E-04	0.905435606	0.988651764	SLC35C2
ENSG00000211714.3	0.004438122	0.905476023	0.988651764	TRBV7-3
ENSG00000124422.12	-7.17E-04	0.905509029	0.988651764	USP22
ENSG00000114999.8	6.74E-04	0.905587182	0.988651764	TTL
ENSG00000181790.11	-0.00196897	0.905639429	0.988651764	ADGRB1
ENSG00000185220.12	-0.001020104	0.90565467	0.988651764	PGBD2
ENSG00000225032.5	-0.001548949	0.905755028	0.988651764	RP11-228B15.4
ENSG00000188596.11	0.003326991	0.905769053	0.988651764	CFAP54
ENSG00000164654.16	-8.57E-04	0.905851527	0.988651764	MIOS
ENSG00000233038.6	-0.002196901	0.90589525	0.988651764	PTPRN2-AS1
ENSG00000171720.10	7.22E-04	0.905961928	0.988651764	HDAC3
ENSG00000196396.10	6.74E-04	0.905966026	0.988651764	PTPN1
ENSG00000204622.11	-0.003966083	0.905974869	0.988651764	HLA-J
ENSG00000170364.12	0.001110923	0.90612948	0.988682924	SETMAR
ENSG00000206527.10	9.82E-04	0.906210468	0.988682924	HACD2
ENSG00000089234.16	5.58E-04	0.906271516	0.988682924	BRAP
ENSG00000177853.14	-0.001634089	0.906304306	0.988682924	ZNF518A

ENSG00000155858.6	9.59E-04	0.90631295	0.988682924	LSM11
ENSG00000282320.1	0.002583119	0.906339992	0.988682924	TRBJ1-1
ENSG00000269646.1	0.004227035	0.90634089	0.988682924	CTD-2331H12.7
ENSG00000159921.20	-8.14E-04	0.906441224	0.988722449	GNE
ENSG00000267698.1	-0.003166372	0.906521864	0.988722449	AC002116.7
ENSG00000224531.6	0.001435428	0.9066028	0.988722449	SMIM13
ENSG00000254317.1	0.002430102	0.9066379	0.988722449	RP11-473O4.5
ENSG00000160293.17	-0.001330861	0.906662096	0.988722449	VAV2
ENSG00000140382.15	0.001090459	0.906666392	0.988722449	HMG20A
ENSG00000166073.11	0.005937485	0.906749665	0.988749249	GPR176
ENSG00000026036.22	0.001611771	0.906787394	0.988749249	RTEL1-TNFRSF6B
ENSG00000176410.8	-0.001214808	0.906862793	0.988778891	DNAJC30
ENSG00000282870.1	-0.003828277	0.906948771	0.988820063	FRG1DP
ENSG00000274376.4	0.003234709	0.907173427	0.988926095	ADAMTS7P1
ENSG00000137672.13	0.004200803	0.907199112	0.988926095	TRPC6
ENSG00000198369.10	0.0016146	0.907214479	0.988926095	SPRED2
ENSG00000136153.20	0.001699883	0.907238909	0.988926095	LMO7
ENSG00000158987.22	0.001225913	0.907288018	0.988927062	RAPGEF6
ENSG00000243772.8	0.003824772	0.907342502	0.988933888	KIR2DL3
ENSG00000279159.1	0.001549217	0.907422359	0.988951525	RP3-394A18.1
ENSG00000250397.2	0.002142485	0.907517827	0.988951525	RP11-1391J7.1
ENSG00000125454.12	0.001162618	0.907607392	0.988951525	SLC25A19
ENSG00000172967.8	-0.006382646	0.907607431	0.988951525	XKR3
ENSG00000203721.7	-0.002501823	0.907633004	0.988951525	LINC00862
ENSG00000112655.16	0.002025448	0.907659105	0.988951525	PTK7
ENSG00000166189.8	-9.22E-04	0.907747871	0.988951525	HPS6
ENSG00000185633.10	0.004229215	0.907759688	0.988951525	NDUFA4L2
ENSG00000113300.13	9.26E-04	0.907872429	0.988951525	CNOT6
ENSG00000273062.1	0.003979797	0.907927944	0.988951525	RALGPS2-AS1
ENSG00000227082.3	0.005777748	0.907930704	0.988951525	LINC02798
ENSG00000188693.8	-0.008953699	0.907937357	0.988951525	CYP51A1-AS1
ENSG00000163071.11	-0.006284552	0.908116328	0.989016211	SPATA18
ENSG00000164305.19	-0.00169914	0.908184542	0.989016211	CASP3
ENSG00000231613.1	0.004545925	0.908214184	0.989016211	RP5-943J3.1
ENSG00000285669.1	0.001983542	0.908362361	0.989016211	RP11-51J9.7
ENSG00000198056.15	-0.001229445	0.908382984	0.989016211	PRIM1
ENSG00000115211.16	7.86E-04	0.908394763	0.989016211	EIF2B4
ENSG00000282173.1	0.001895073	0.908435435	0.989016211	TRBJ1-5
ENSG00000151131.11	-9.50E-04	0.908497257	0.989016211	C12orf45
ENSG00000270542.1	-0.005352961	0.908513204	0.989016211	RP11-311H10.7
ENSG00000276952.1	-0.002629973	0.908528287	0.989016211	RP5-965G21.6
ENSG00000196329.12	-0.001443851	0.908544215	0.989016211	GIMAP5
ENSG00000227630.4	0.003867396	0.908627794	0.989016211	LINC01132
ENSG00000197013.10	0.00212935	0.908666002	0.989016211	ZNF429
ENSG00000211677.2	0.004102453	0.908714223	0.989016211	IGLC2
ENSG00000135048.14	-0.001035292	0.908720132	0.989016211	CEMIP2
ENSG00000213923.13	-7.84E-04	0.908819799	0.989061958	CSNK1E

ENSG00000162433.15	-0.002731457	0.908917752	0.989061958	AK4
ENSG00000246174.9	0.003627148	0.908954377	0.989061958	KCTD21-AS1
ENSG00000003393.16	0.001042895	0.908994581	0.989061958	ALS2
ENSG00000023909.10	0.001183833	0.909003306	0.989061958	GCLM
ENSG00000089050.16	-8.78E-04	0.909054372	0.989065047	RBBP9
ENSG00000155099.8	0.001050843	0.909212487	0.989160391	PIP4P2
ENSG00000269553.1	0.003481974	0.909364121	0.989160391	U62631.5
ENSG00000216921.9	-0.005295293	0.909364784	0.989160391	FAM240C
ENSG00000132424.17	0.001219973	0.909396308	0.989160391	PNISR
ENSG00000149571.12	-0.003024455	0.909401922	0.989160391	KIRREL3
ENSG00000124688.14	9.92E-04	0.909537502	0.989160391	MAD2L1BP
ENSG00000146409.12	-0.001172634	0.909599131	0.989160391	SLC18B1
ENSG00000151835.17	-0.001426061	0.909679634	0.989160391	SACS
ENSG00000143889.16	-0.001167198	0.909681767	0.989160391	HNRNPLL
ENSG00000173011.12	-5.49E-04	0.909688543	0.989160391	TADA2B
ENSG00000204528.3	0.006176744	0.909788073	0.989160391	PSORS1C3
ENSG00000178809.11	-0.002521031	0.909827738	0.989160391	TRIM73
ENSG00000136243.17	-0.001027485	0.90989758	0.989160391	NUP42
ENSG00000269386.5	0.001617164	0.909922236	0.989160391	RAB11B-AS1
ENSG00000127483.19	6.88E-04	0.90993038	0.989160391	HP1BP3
ENSG00000100354.21	8.53E-04	0.909987898	0.989160391	TNRC6B
ENSG00000228486.10	-0.001907897	0.910000897	0.989160391	C2orf92
ENSG00000108592.17	-8.10E-04	0.910010196	0.989160391	FTSJ3
ENSG00000107669.18	-0.001058526	0.910108532	0.989213938	ATE1
ENSG00000189077.11	0.001084042	0.910219718	0.989213938	TMEM120A
ENSG00000151376.18	0.002382082	0.910246673	0.989213938	ME3
ENSG00000213551.7	9.24E-04	0.910262694	0.989213938	DNAJC9
ENSG00000236723.2	0.002865458	0.910336161	0.989213938	RP5-1024G6.2
ENSG00000189325.7	0.005406515	0.910348871	0.989213938	BNIP5
ENSG00000269984.1	-0.002734606	0.910412112	0.989230242	RP11-362K14.5
ENSG00000184828.10	-0.004388969	0.91052385	0.989299238	ZBTB7C
ENSG00000138606.19	-0.00166838	0.910643569	0.989317511	SHF
ENSG00000157741.15	-9.14E-04	0.910674195	0.989317511	UBN2
ENSG00000204172.12	-0.002715929	0.91068539	0.989317511	AGAP9
ENSG00000168016.15	-0.001415718	0.91082214	0.989380453	TRANK1
ENSG00000169981.11	0.00116339	0.910904139	0.989380453	ZNF35
ENSG00000091181.20	-0.003012315	0.910935527	0.989380453	IL5RA
ENSG00000077152.11	-0.002256907	0.910936303	0.989380453	UBE2T
ENSG00000218809.1	0.002190845	0.911080263	0.989449261	RP1-229K20.5
ENSG00000080603.17	0.00145599	0.911118935	0.989449261	SRCAP
ENSG00000204228.4	-0.001357529	0.911185745	0.989449261	HSD17B8
ENSG00000288701.1	0.001191122	0.9112107	0.989449261	PRRC2B
ENSG00000009413.16	-0.0011613	0.911240891	0.989449261	REV3L
ENSG00000116157.6	0.001276391	0.911348858	0.989514103	GPX7
ENSG00000104529.18	-7.38E-04	0.911548515	0.989517417	EEF1D
ENSG00000246203.2	-0.001519275	0.91155096	0.989517417	RP11-29H23.5
ENSG00000171492.14	-8.00E-04	0.911632612	0.989517417	LRRC8D

ENSG00000131876.17	-6.68E-04	0.911718376	0.989517417	SNRPA1
ENSG00000235078.1	0.002791484	0.911764201	0.989517417	AC142528.1
ENSG00000136144.12	0.001298711	0.911778796	0.989517417	RCBTB1
ENSG00000154305.18	6.44E-04	0.911840186	0.989517417	MIA3
ENSG00000185340.15	0.001907404	0.911862613	0.989517417	GAS2L1
ENSG00000233025.1	0.004539797	0.911871265	0.989517417	CRYZP1
ENSG00000239713.9	-0.001353678	0.911963799	0.989517417	APOBEC3G
ENSG00000092094.11	8.38E-04	0.911981614	0.989517417	OSGEP
ENSG00000129295.9	-0.002755763	0.912043289	0.989517417	LRRC6
ENSG00000143702.16	0.001229002	0.912052394	0.989517417	CEP170
ENSG00000255819.7	0.003288654	0.912053376	0.989517417	KLRC4-KLRK1
ENSG00000270165.1	0.003671603	0.912103871	0.989517417	RP11-167P11.2
ENSG00000137760.15	0.001093288	0.912138501	0.989517417	ALKBH8
ENSG00000249931.4	0.004503936	0.912172166	0.989517417	GOLGA8K
ENSG00000287091.1	-0.001581161	0.912419664	0.989599582	RP11-357L2.2
ENSG00000243749.1	-0.001244346	0.912430208	0.989599582	TMEM35B
ENSG00000171316.12	-0.001148893	0.912446837	0.989599582	CHD7
ENSG00000230076.1	-0.012522134	0.912474459	0.989599582	RPL10P6
ENSG00000197217.13	6.92E-04	0.912503252	0.989599582	ENTPD4
ENSG00000060558.4	-0.001202509	0.912537434	0.989599582	GNA15
ENSG00000234171.3	-0.001331486	0.912623555	0.989640645	RNASEH1-AS1
ENSG00000228217.1	-0.006190622	0.912764343	0.989740979	PNRC2P1
ENSG00000130584.12	-0.001171832	0.912917801	0.989774377	ZBTB46
ENSG00000131943.19	7.83E-04	0.91293573	0.989774377	C19orf12
ENSG00000260059.1	-0.003533206	0.912972867	0.989774377	RP11-231E19.1
ENSG00000115318.12	-0.00113884	0.913077187	0.989774377	LOXL3
ENSG00000010322.16	6.33E-04	0.913090125	0.989774377	NISCH
ENSG00000080822.17	0.001160388	0.913117808	0.989774377	CLDND1
ENSG00000143409.16	-0.001000055	0.913132983	0.989774377	MINDY1
ENSG00000092439.16	0.00104409	0.913372194	0.989823849	TRPM7
ENSG00000241566.1	-0.00497226	0.913446601	0.989823849	IGKV2D-24
ENSG00000106799.13	0.001317131	0.913480993	0.989823849	TGFBR1
ENSG00000235192.1	-0.001961216	0.913524249	0.989823849	AC009495.2
ENSG00000267666.2	0.004153405	0.913550482	0.989823849	AC004156.3
ENSG00000235944.8	-0.001285393	0.913563213	0.989823849	ZNF815P
ENSG00000105193.9	0.001255812	0.913619578	0.989823849	RPS16
ENSG00000136051.15	0.001655407	0.913647429	0.989823849	WASHC4
ENSG00000288398.1	-0.001208177	0.913764092	0.989823849	RP4-733M16.8
ENSG00000122733.12	-0.002976732	0.913777556	0.989823849	PHF24
ENSG00000153786.13	6.57E-04	0.913788062	0.989823849	ZDHHC7
ENSG00000197062.12	-8.42E-04	0.91380085	0.989823849	ZSCAN26
ENSG00000073417.15	-7.73E-04	0.913824275	0.989823849	PDE8A
ENSG00000225864.1	0.003573862	0.913854338	0.989823849	HCG4P11
ENSG00000154040.21	-0.00345367	0.914133707	0.990074151	CABYR
ENSG00000075651.16	0.001169632	0.914210644	0.990074575	PLD1
ENSG00000171970.13	-0.002379778	0.914230653	0.990074575	ZNF57
ENSG00000131746.13	0.002871999	0.914300714	0.990098165	TNS4

ENSG00000278107.1	-0.002205934	0.914357954	0.990107868	RP11-162A12.4
ENSG00000147689.16	0.006159042	0.914604558	0.990322612	FAM83A
ENSG00000271843.1	-0.002882718	0.914711604	0.990325467	RP11-245J9.5
ENSG00000196436.8	0.007329923	0.914745277	0.990325467	NPIPB15
ENSG00000187239.17	-6.73E-04	0.914802184	0.990325467	FNBP1
ENSG00000146476.11	-0.001347673	0.914896529	0.990325467	ARMT1
ENSG00000157570.12	0.001481184	0.91491371	0.990325467	TSPAN18
ENSG00000078177.14	0.001668553	0.914948441	0.990325467	N4BP2
ENSG00000087263.17	6.04E-04	0.914984385	0.990325467	OGFOD1
ENSG00000174827.14	-0.00241632	0.914993512	0.990325467	PDZK1
ENSG00000015171.20	-0.001029649	0.915096019	0.990384144	ZMYND11
ENSG00000240057.6	-0.001769098	0.915182411	0.990409176	RP11-572M11.4
ENSG00000067066.17	-1.00E-03	0.915313242	0.990409176	SP100
ENSG00000168268.11	-0.001945708	0.915324262	0.990409176	NT5DC2
ENSG00000286064.1	0.003454667	0.915431079	0.990409176	RP4-657E11.11
ENSG00000271857.1	0.001798916	0.91544063	0.990409176	RP1-244F24.1
ENSG00000006125.18	-6.75E-04	0.91545596	0.990409176	AP2B1
ENSG00000280721.1	-0.002620099	0.915480426	0.990409176	LINC01943
ENSG00000239857.7	0.001207501	0.915505498	0.990409176	GET4
ENSG00000099875.16	-9.10E-04	0.915579385	0.990436862	MKNK2
ENSG00000236432.8	-0.002763962	0.91575151	0.990522202	MFF-DT
ENSG00000152242.11	0.001033407	0.915778156	0.990522202	C18orf25
ENSG00000283341.2	-0.001454731	0.91581764	0.990522202	RP11-472I20.4
ENSG00000108821.14	-0.005185347	0.915851473	0.990522202	COL1A1
ENSG00000249189.1	0.013774505	0.915947856	0.990532845	AC010492.4
ENSG00000164172.20	0.001015449	0.915972667	0.990532845	MOCS2
ENSG00000234648.1	-0.002890853	0.916079597	0.990532845	AL162151.3
ENSG00000267385.1	0.00226307	0.916099576	0.990532845	CTB-50L17.14
ENSG00000116212.15	-7.60E-04	0.916173969	0.990532845	LRRC42
ENSG00000111537.5	-0.003377797	0.91621223	0.990532845	IFNG
ENSG00000223820.5	-0.002711541	0.916335176	0.990532845	CFL1P1
ENSG00000104549.12	-0.001306515	0.916340421	0.990532845	SQLE
ENSG00000186577.14	-9.77E-04	0.916405306	0.990532845	SMIM29
ENSG00000197253.13	-0.007902448	0.916426361	0.990532845	TPSB2
ENSG00000144868.14	-0.003878916	0.916455195	0.990532845	TMEM108
ENSG00000153531.14	-0.00211024	0.91646039	0.990532845	ADPRHL1
ENSG00000143457.11	-6.58E-04	0.916489211	0.990532845	GOLPH3L
ENSG00000100316.16	0.001103026	0.916654174	0.990658928	RPL3
ENSG00000279447.1	-0.002413848	0.916832498	0.99069637	RP4-737E23.6
ENSG00000224032.7	0.001252908	0.916865119	0.99069637	EPB41L4A-AS1
ENSG00000130733.11	9.39E-04	0.916886276	0.99069637	YIPF2
ENSG00000267152.1	-0.002123415	0.91689164	0.99069637	CTD-2528L19.6
ENSG00000280161.1	0.003005609	0.916955981	0.99069637	CTC-205M6.1
ENSG00000171681.13	-7.13E-04	0.916978667	0.99069637	ATF7IP
ENSG00000179532.13	-9.60E-04	0.917061668	0.990733851	DNHD1
ENSG00000096696.15	0.007942941	0.917197086	0.990827952	DSP
ENSG00000211893.4	0.004174086	0.917268909	0.990853346	IGHG2

ENSG00000266865.6	0.001544635	0.917541207	0.991008752	RP11-848P1.9
ENSG00000198003.12	0.002701242	0.917541647	0.991008752	ODAD3
ENSG00000272909.1	0.002898123	0.917557742	0.991008752	CTD-2555O16.4
ENSG00000106633.18	0.004295724	0.917676644	0.991029235	GCK
ENSG00000106565.18	-0.005826789	0.917741464	0.991029235	TMEM176B
ENSG00000196455.8	7.49E-04	0.917745151	0.991029235	PIK3R4
ENSG00000275329.1	-0.003270865	0.917795194	0.991029235	RP11-83N9.6
ENSG00000113360.17	-7.40E-04	0.917839448	0.991029235	DROSHA
ENSG00000153885.15	0.001042336	0.917909678	0.991029235	KCTD15
ENSG00000178694.10	7.33E-04	0.917914975	0.991029235	NSUN3
ENSG00000037042.9	-0.001128533	0.918023949	0.991040232	TUBG2
ENSG00000104970.11	-0.003323369	0.918030345	0.991040232	KIR3DX1
ENSG00000163435.16	0.002492432	0.918124578	0.991040232	ELF3
ENSG00000125447.18	-5.46E-04	0.918153929	0.991040232	GGA3
ENSG00000279611.1	-0.002098694	0.918166784	0.991040232	CTD-2619J13.5
ENSG00000282164.3	-0.001275318	0.918298521	0.991067019	PEG13
ENSG00000227863.3	0.004523827	0.918335684	0.991067019	AC002383.2
ENSG00000173517.11	0.001147003	0.918515998	0.991067019	PEAK1
ENSG00000143786.8	0.002213586	0.918661502	0.991067019	CNIH3
ENSG00000260729.1	0.002514369	0.918691937	0.991067019	RP11-106M3.2
ENSG00000140263.15	-0.00122457	0.918698911	0.991067019	SORD
ENSG00000131730.16	-0.003592149	0.918723534	0.991067019	CKMT2
ENSG00000122068.13	8.04E-04	0.918774916	0.991067019	FYTTD1
ENSG00000287680.1	-0.003780825	0.918901993	0.991067019	RP11-862L9.4
ENSG00000135823.14	-5.34E-04	0.918917483	0.991067019	STX6
ENSG00000182771.19	-0.005497463	0.918954506	0.991067019	GRID1
ENSG00000066651.20	0.001415344	0.919079797	0.991067019	TRMT11
ENSG00000197142.11	6.53E-04	0.919107139	0.991067019	ACSL5
ENSG00000186105.8	0.004119516	0.91910755	0.991067019	LRRC70
ENSG00000168672.4	-0.001260405	0.919137763	0.991067019	LRATD2
ENSG00000143258.16	-5.63E-04	0.91921047	0.991067019	USP21
ENSG00000072135.13	-8.14E-04	0.919230166	0.991067019	PTPN18
ENSG00000081760.17	-7.46E-04	0.919237901	0.991067019	AACS
ENSG00000223935.3	-0.002479833	0.919274377	0.991067019	LGALSL-DT
ENSG00000188313.13	-0.002215206	0.919290696	0.991067019	PLSCR1
ENSG00000244005.13	-6.80E-04	0.919305597	0.991067019	NFS1
ENSG00000100038.20	-0.002477796	0.919336651	0.991067019	TOP3B
ENSG00000160445.11	-0.001083147	0.919373783	0.991067019	ZER1
ENSG00000196275.15	-0.001374127	0.919401301	0.991067019	GTF2IRD2
ENSG00000254288.1	0.002532517	0.919423666	0.991067019	RP11-6I2.3
ENSG00000203880.12	-0.001234469	0.91949034	0.991067019	PCMTD2
ENSG00000114346.14	-0.001649305	0.919519179	0.991067019	ECT2
ENSG00000172432.19	7.74E-04	0.919544726	0.991067019	GTPBP2
ENSG00000283399.1	0.002862974	0.919604453	0.991079306	AC004381.8
ENSG00000254783.1	-0.001626282	0.919812789	0.991087874	RP11-320L11.2
ENSG00000107745.19	8.45E-04	0.919990409	0.991087874	MICU1
ENSG00000196782.12	0.001126841	0.920190146	0.991087874	MAML3

ENSG00000050344.9	0.001200781	0.920212012	0.991087874	NFE2L3
ENSG00000204842.18	-6.16E-04	0.920295012	0.991087874	ATXN2
ENSG00000236911.6	-0.003423193	0.920368315	0.991087874	RP11-78B10.2
ENSG00000155189.12	0.001154948	0.920394772	0.991087874	AGPAT5
ENSG00000197362.15	8.34E-04	0.920444799	0.991087874	ZNF786
ENSG00000164597.15	9.64E-04	0.920464656	0.991087874	COG5
ENSG00000143314.12	9.93E-04	0.920473481	0.991087874	MRPL24
ENSG00000178695.6	-0.001121501	0.920492597	0.991087874	KCTD12
ENSG00000116898.12	7.67E-04	0.920506522	0.991087874	MRPS15
ENSG00000117643.15	-0.001451185	0.92050956	0.991087874	MAN1C1
ENSG00000075413.19	5.87E-04	0.920576608	0.991087874	MARK3
ENSG00000151208.17	-0.00257849	0.92058494	0.991087874	DLG5
ENSG00000196218.13	0.001646217	0.920590997	0.991087874	RYR1
ENSG00000253394.6	-0.003836454	0.920598855	0.991087874	LINC00534
ENSG00000189292.16	-0.004000471	0.920645605	0.991087874	ALKAL2
ENSG00000126351.13	8.44E-04	0.920741617	0.991087874	THRA
ENSG00000182108.11	0.001043984	0.920797973	0.991087874	DEXI
ENSG00000151779.13	8.28E-04	0.920825413	0.991087874	NBAS
ENSG00000104883.8	0.002044376	0.920903403	0.991087874	PEX11G
ENSG00000106571.15	0.00397375	0.920978041	0.991087874	GLI3
ENSG00000235602.5	-0.001486652	0.92098336	0.991087874	POU5F1P3
ENSG00000248538.8	-0.007260615	0.921017023	0.991087874	RP11-115J16.1
ENSG00000270933.1	-0.003248446	0.92114731	0.991087874	CTD-2227E11.1
ENSG00000167733.14	0.001452522	0.921213847	0.991087874	HSD11B1L
ENSG00000127423.11	-0.002072926	0.921319679	0.991087874	AUNIP
ENSG00000265666.1	-0.001017135	0.921405122	0.991087874	RARA-AS1
ENSG00000205534.6	-0.001715008	0.92140956	0.991087874	SMG1P2
ENSG00000155530.3	-0.003779032	0.921420406	0.991087874	LRGUK
ENSG00000164251.5	0.001877983	0.921432485	0.991087874	F2RL1
ENSG00000124151.19	7.89E-04	0.921558306	0.991087874	NCOA3
ENSG00000168269.10	0.005610303	0.92156282	0.991087874	FOXI1
ENSG00000233775.1	0.003340574	0.92162263	0.991087874	RP4-811H24.9
ENSG00000188878.19	-9.60E-04	0.921696943	0.991087874	FBF1
ENSG00000254554.1	0.003023051	0.92182675	0.991087874	RP11-351I24.1
ENSG00000280143.1	-0.001605257	0.921828303	0.991087874	AP000892.6
ENSG00000236710.1	0.00411802	0.921860254	0.991087874	AC108448.2
ENSG00000213025.2	0.012083186	0.921860291	0.991087874	COX20P1
ENSG00000069667.16	-0.001330666	0.92186413	0.991087874	RORA
ENSG00000138795.10	0.001548772	0.92186683	0.991087874	LEF1
ENSG00000108384.15	0.001295182	0.9219158	0.991087874	RAD51C
ENSG00000196335.13	0.003294059	0.921924317	0.991087874	STK31
ENSG00000258017.2	-0.002253321	0.921950642	0.991087874	RP11-386G11.10
ENSG00000186710.12	-0.002427752	0.922030575	0.991087874	CFAP73
ENSG00000105656.13	-8.42E-04	0.92208668	0.991087874	ELL
ENSG00000086062.13	-6.93E-04	0.922113397	0.991087874	B4GALT1
ENSG00000279058.2	-0.003052917	0.922187652	0.991087874	AGAP14P
ENSG00000211513.5	0.002431102	0.922194028	0.991087874	MIR320E

ENSG00000171115.4	9.39E-04	0.922206448	0.991087874	GIMAP8
ENSG00000176749.9	-0.001586652	0.922212085	0.991087874	CDK5R1
ENSG00000267274.1	-0.001973869	0.922213604	0.991087874	CTD-2006C1.12
ENSG00000148606.13	-5.51E-04	0.922230157	0.991087874	POLR3A
ENSG00000163104.18	0.001016453	0.922270382	0.991087874	SMARCAD1
ENSG00000214046.9	-8.68E-04	0.92235075	0.991122305	SMIM7
ENSG00000253409.1	0.002693586	0.922499916	0.991204084	TRBV7-4
ENSG00000220586.2	-0.040178636	0.92252352	0.991204084	TUBBP9
ENSG00000152102.18	5.39E-04	0.922629909	0.991266367	FAM168B
ENSG00000116641.18	0.00129229	0.92278584	0.991266367	DOCK7
ENSG00000167302.11	6.64E-04	0.922792329	0.991266367	TEPSIN
ENSG00000166896.9	-0.001885083	0.922924802	0.991266367	ATP23
ENSG00000198816.7	0.001666006	0.922929837	0.991266367	ZNF358
ENSG00000211720.3	0.003388446	0.923057254	0.991266367	TRBV11-1
ENSG00000244357.3	-0.003427795	0.923134334	0.991266367	RN7SL145P
ENSG00000183092.17	0.004041138	0.923154735	0.991266367	BEGAIN
ENSG00000105647.19	0.002409448	0.923214558	0.991266367	PIK3R2
ENSG00000262703.1	-0.004058524	0.923237081	0.991266367	RP11-485G7.6
ENSG00000205413.8	0.001779949	0.923246026	0.991266367	SAMD9
ENSG00000161381.14	0.001690385	0.92327759	0.991266367	PLXDC1
ENSG00000198242.14	0.00104992	0.923347154	0.991266367	RPL23A
ENSG00000198742.10	5.00E-04	0.923355091	0.991266367	SMURF1
ENSG00000168026.19	0.001804619	0.923396512	0.991266367	TTC21A
ENSG00000160796.18	-9.14E-04	0.923445923	0.991266367	NBEAL2
ENSG00000116824.5	0.001121342	0.923504913	0.991266367	CD2
ENSG00000114353.17	-7.32E-04	0.923526538	0.991266367	GNAI2
ENSG00000132832.10	0.003650306	0.923549842	0.991266367	RP11-445H22.5
ENSG00000188647.13	-0.001664765	0.923567589	0.991266367	PTAR1
ENSG00000196116.8	-0.001116378	0.923630918	0.991266367	TDRD7
ENSG00000068903.20	-7.04E-04	0.923700063	0.991266367	SIRT2
ENSG00000180921.7	0.001242154	0.923771739	0.991266367	FAM83H
ENSG00000153201.16	8.34E-04	0.923787457	0.991266367	RANBP2
ENSG00000103544.15	6.83E-04	0.923806872	0.991266367	VPS35L
ENSG00000275665.1	-0.003418161	0.923881779	0.991266367	CTB-58E17.9
ENSG00000113966.10	0.001631746	0.924010509	0.991266367	ARL6
ENSG00000086666.19	8.06E-04	0.924014982	0.991266367	ZFAND6
ENSG00000099139.14	0.001200417	0.924083767	0.991266367	PCSK5
ENSG00000117385.16	5.62E-04	0.924180355	0.991266367	P3H1
ENSG00000196591.12	5.04E-04	0.924221185	0.991266367	HDAC2
ENSG00000099385.12	0.001038758	0.924222386	0.991266367	BCL7C
ENSG00000171497.5	5.67E-04	0.924246149	0.991266367	PPID
ENSG00000278864.1	0.001797749	0.924274031	0.991266367	RP11-45M22.2
ENSG00000180015.12	0.00372255	0.924302718	0.991266367	RP11-756P10.3
ENSG00000175489.10	9.07E-04	0.924321569	0.991266367	LRRC25
ENSG00000168306.13	-0.002737226	0.924377386	0.99127439	ACOX2
ENSG00000244716.3	-0.004688813	0.924507675	0.99136227	RP11-20O24.4
ENSG00000004487.17	-5.01E-04	0.924709525	0.991445821	KDM1A

ENSG00000113721.14	0.002542214	0.92482933	0.991445821	PDGFRB
ENSG00000211598.2	-0.003231891	0.924952367	0.991445821	IGKV4-1
ENSG00000168961.17	0.001586008	0.924966041	0.991445821	LGALS9
ENSG00000177663.14	7.72E-04	0.924982094	0.991445821	IL17RA
ENSG00000250031.1	-0.002674733	0.925102265	0.991445821	RP11-114M5.1
ENSG00000197093.11	-0.001710006	0.925211867	0.991445821	GAL3ST4
ENSG00000163161.14	-4.15E-04	0.925265231	0.991445821	ERCC3
ENSG00000239827.8	0.002440535	0.925271395	0.991445821	SUGT1P3
ENSG00000143324.14	-6.12E-04	0.92529422	0.991445821	XPR1
ENSG00000185477.5	-9.44E-04	0.925462945	0.991445821	GPRIN3
ENSG00000178927.18	6.96E-04	0.925471828	0.991445821	CYBC1
ENSG00000116754.13	9.87E-04	0.925579113	0.991445821	SRSF11
ENSG00000187713.7	-6.60E-04	0.925612033	0.991445821	TMEM203
ENSG00000111291.8	0.004105966	0.92577632	0.991445821	GPRC5D
ENSG00000121716.20	0.002809325	0.925887761	0.991445821	PILRB
ENSG00000172000.7	0.003777742	0.925992784	0.991445821	ZNF556
ENSG00000138386.17	0.001223179	0.926043523	0.991445821	NAB1
ENSG00000152760.10	-0.004980628	0.926240021	0.991445821	DYNLT5
ENSG00000172830.13	-8.85E-04	0.926246142	0.991445821	SSH3
ENSG00000269176.2	-0.0019739	0.926367371	0.991445821	RP11-727F15.12
ENSG00000231993.1	0.003249178	0.926410414	0.991445821	EP300-AS1
ENSG00000223834.5	-0.002614594	0.926436899	0.991445821	RP11-472N13.2
ENSG00000104689.10	8.49E-04	0.926470478	0.991445821	TNFRSF10A
ENSG00000275278.1	-0.003183484	0.926478718	0.991445821	RP11-946L16.2
ENSG00000101188.5	0.002768391	0.92649657	0.991445821	NTSR1
ENSG00000173113.7	9.18E-04	0.926521367	0.991445821	TRMT112
ENSG00000230091.6	0.002419609	0.926554212	0.991445821	TMEM254-AS1
ENSG00000176095.12	6.69E-04	0.926560877	0.991445821	IP6K1
ENSG00000225992.1	0.002628213	0.92658421	0.991445821	TRGVA
ENSG00000256913.1	-0.00353687	0.926687304	0.991445821	RP1-102E24.6
ENSG00000116661.11	0.002192759	0.926737598	0.991445821	FBXO2
ENSG00000140598.15	5.86E-04	0.926792423	0.991445821	EFL1
ENSG00000118113.12	0.004839635	0.926830061	0.991445821	MMP8
ENSG00000228434.1	0.001945117	0.926866798	0.991445821	AC004951.6
ENSG00000213390.11	7.58E-04	0.926887417	0.991445821	ARHGAP19
ENSG00000276449.1	0.002151336	0.926914459	0.991445821	AC004076.5
ENSG00000164241.14	0.001177289	0.92691735	0.991445821	C5orf63
ENSG00000065413.20	0.001005803	0.926924614	0.991445821	ANKRD44
ENSG00000145220.14	-9.00E-04	0.926941428	0.991445821	LYAR
ENSG00000181991.16	6.24E-04	0.927011771	0.991445821	MRPS11
ENSG00000251333.3	-0.002320769	0.927047978	0.991445821	RTN3P1
ENSG00000197124.12	0.001628092	0.927087894	0.991445821	ZNF682
ENSG00000235084.3	0.002539658	0.927131129	0.991445821	CHCHD2P6
ENSG00000148660.21	5.55E-04	0.927144355	0.991445821	CAMK2G
ENSG00000272163.1	0.002447781	0.927205488	0.991445821	GS1-72M22.1
ENSG00000021300.14	-0.001364768	0.927266343	0.991445821	PLEKHB1
ENSG00000204120.16	-6.31E-04	0.927283128	0.991445821	GIGYF2

ENSG00000285967.1	0.001191615	0.927284487	0.991445821	NIPBL-DT
ENSG00000259309.2	0.003919064	0.927338519	0.991445821	RP11-19J5.2
ENSG00000188001.10	0.002267456	0.927354611	0.991445821	TPRG1
ENSG00000286223.1	-0.001693533	0.927419791	0.991445821	RP11-686O6.3
ENSG00000229502.5	-0.01590444	0.927519472	0.991445821	RP11-52J3.2
ENSG00000177181.15	-0.002737062	0.9275256	0.991445821	RIMKLA
ENSG00000180509.13	-0.00140825	0.927596641	0.991445821	KCNE1
ENSG00000125484.12	-6.67E-04	0.92789968	0.991445821	GTF3C4
ENSG00000135052.16	0.001077491	0.927913772	0.991445821	GOLM1
ENSG00000160856.21	-0.001822385	0.927915284	0.991445821	FCRL3
ENSG00000103353.16	-4.58E-04	0.927928991	0.991445821	UBFD1
ENSG00000273855.1	-0.001841081	0.928057846	0.991445821	RP11-133K1.12
ENSG00000137496.18	-7.66E-04	0.928072667	0.991445821	IL18BP
ENSG00000275022.1	0.001660481	0.928094965	0.991445821	MIR6753
ENSG00000053371.13	-8.92E-04	0.92816912	0.991445821	AKR7A2
ENSG00000161526.15	-5.31E-04	0.928206752	0.991445821	SAP30BP
ENSG00000281649.2	-0.001076679	0.928259703	0.991445821	EBLN3P
ENSG00000172243.18	-9.33E-04	0.928273071	0.991445821	CLEC7A
ENSG00000226259.10	0.00330384	0.928278768	0.991445821	GTF2H2B
ENSG00000154642.11	0.001140847	0.928297511	0.991445821	C21orf91
ENSG00000170473.17	-7.62E-04	0.928307425	0.991445821	PYM1
ENSG00000100027.17	-0.001291001	0.92837112	0.991445821	YPEL1
ENSG00000077458.13	-0.001426452	0.928468373	0.991445821	FAM76B
ENSG00000214022.12	6.20E-04	0.928469972	0.991445821	REPIN1
ENSG00000268799.4	-0.004374967	0.928574245	0.991445821	H3Y2
ENSG00000114779.20	8.23E-04	0.928587574	0.991445821	ABHD14B
ENSG00000114737.16	-0.001058438	0.928589258	0.991445821	CISH
ENSG00000105669.14	-8.54E-04	0.928674368	0.991445821	COPE
ENSG00000267016.1	0.002619476	0.928689427	0.991445821	RP11-75C10.9
ENSG00000131115.16	6.37E-04	0.928759475	0.991445821	ZNF227
ENSG00000124802.12	0.001816668	0.928863787	0.991445821	EEF1E1
ENSG00000039600.11	0.003271067	0.928888013	0.991445821	SOX30
ENSG00000185905.4	-6.79E-04	0.928896158	0.991445821	C16orf54
ENSG00000111875.8	8.26E-04	0.92893153	0.991445821	ASF1A
ENSG00000285416.1	0.001404472	0.928969089	0.991445821	RP13-131K19.8
ENSG00000027001.10	-7.98E-04	0.92911368	0.991445821	MIPEP
ENSG00000243431.1	-0.00223767	0.929147136	0.991445821	RPL5P30
ENSG00000273133.1	0.002345471	0.929158882	0.991445821	RP11-799M12.2
ENSG00000284633.1	0.002425838	0.929188016	0.991445821	RP3-333H23.10
ENSG00000156232.7	-4.21E-04	0.929236305	0.991445821	WHAMM
ENSG00000003402.20	6.43E-04	0.929241378	0.991445821	CFLAR
ENSG00000261798.1	0.002617474	0.929306486	0.991445821	RP1-118J21.25
ENSG00000083312.19	8.86E-04	0.929345669	0.991445821	TNPO1
ENSG00000149531.15	-0.001773412	0.929392958	0.991445821	FRG1BP
ENSG00000148248.14	-4.69E-04	0.929447907	0.991445821	SURF4
ENSG00000251532.1	-0.004921973	0.929497882	0.991445821	CTD-2245E15.3
ENSG00000248489.2	0.001495686	0.929504075	0.991445821	LINC02062

ENSG00000221944.9	0.001363252	0.92957628	0.991445821	TIGD1
ENSG00000268240.1	0.002837197	0.929597928	0.991445821	RP11-678G14.4
ENSG00000214021.16	-7.40E-04	0.929600155	0.991445821	TTLL3
ENSG00000279884.1	-0.001184025	0.929655022	0.991445821	RP11-483E17.1
ENSG00000264548.1	-0.002750382	0.929659313	0.991445821	RP13-516M14.2
ENSG00000207802.1	-0.003667734	0.929679901	0.991445821	MIR646
ENSG00000171596.7	-0.001859025	0.929685452	0.991445821	NMUR1
ENSG00000111266.9	8.68E-04	0.929764964	0.991445821	DUSP16
ENSG00000254756.1	0.002458239	0.92980466	0.991445821	RP11-867G23.12
ENSG00000260920.2	0.001070545	0.929850593	0.991445821	RP1-228H13.5
ENSG00000111237.19	-8.37E-04	0.92985245	0.991445821	VPS29
ENSG00000178917.17	0.001346295	0.929854977	0.991445821	ZNF852
ENSG00000183688.4	-0.001050333	0.92986179	0.991445821	RFLNB
ENSG00000166685.12	6.21E-04	0.92987589	0.991445821	COG1
ENSG00000120279.6	-0.002297826	0.929936369	0.991445821	MYCT1
ENSG00000204650.14	0.001060326	0.929951814	0.991445821	LINC02210
ENSG00000158865.12	0.004464476	0.930078438	0.991480362	SLC5A11
ENSG00000242609.1	0.003552398	0.930105038	0.991480362	CCDC137P
ENSG00000171847.11	0.003074506	0.930129251	0.991480362	FAM90A1
ENSG00000250893.1	-0.002631333	0.930228762	0.991534899	RP11-588L15.2
ENSG00000167671.12	-0.001909443	0.93036571	0.991619936	UBXN6
ENSG00000279179.1	-0.001321814	0.930494906	0.991619936	RP11-131M11.3
ENSG00000163507.14	0.001703017	0.93060074	0.991619936	CIP2A
ENSG00000108309.14	0.002828011	0.930606344	0.991619936	RUNDC3A
ENSG00000232546.1	0.002852808	0.930620756	0.991619936	RP11-458F8.1
ENSG00000226800.5	-0.00167261	0.93064441	0.991619936	CACTIN-AS1
ENSG00000131023.13	-8.56E-04	0.930741299	0.991619936	LATS1
ENSG00000156876.10	-0.00127376	0.930887201	0.991619936	SASS6
ENSG00000233214.1	0.001684447	0.930943478	0.991619936	AC002511.2
ENSG00000164180.14	-0.001160513	0.93097833	0.991619936	TMEM161B
ENSG00000223466.2	0.003156233	0.930997693	0.991619936	LINC01825
ENSG00000228835.1	-0.004786249	0.931008663	0.991619936	AC012123.1
ENSG00000100934.15	8.15E-04	0.931012231	0.991619936	SEC23A
ENSG00000159403.18	-0.001286594	0.931029022	0.991619936	C1R
ENSG00000215424.10	-7.76E-04	0.931033834	0.991619936	MCM3AP-AS1
ENSG00000240429.1	0.003766532	0.931123565	0.991630522	LRRFIP1P1
ENSG00000258439.1	-0.002247057	0.93114048	0.991630522	RP11-173A8.2
ENSG00000198408.14	5.72E-04	0.931226648	0.991670791	OGA
ENSG00000149609.6	0.003184061	0.931361971	0.991701522	C20orf144
ENSG00000125388.20	-0.001262335	0.93140833	0.991701522	GRK4
ENSG00000199906.1	0.001932001	0.931419256	0.991701522	RNU5B-2P
ENSG00000211844.1	-0.003477163	0.93146749	0.991701522	TRAJ45
ENSG00000070915.10	-0.002218637	0.931691305	0.991701522	SLC12A3
ENSG00000267265.5	-0.001859731	0.931900574	0.991701522	GP6-AS1
ENSG00000258116.1	0.004271646	0.931940107	0.991701522	PPIAP45
ENSG00000013441.16	-0.001124733	0.931977447	0.991701522	CLK1
ENSG00000172888.12	-9.28E-04	0.931992784	0.991701522	ZNF621

ENSG00000158552.13	-7.05E-04	0.932014542	0.991701522	ZFAND2B
ENSG00000265018.6	0.005027196	0.932165939	0.991701522	AGAP12P
ENSG00000090382.7	0.001802377	0.932212343	0.991701522	LYZ
ENSG00000127249.15	0.002376411	0.932261567	0.991701522	ATP13A4
ENSG00000126821.8	-0.001146263	0.93226482	0.991701522	SGPP1
ENSG00000121680.16	-7.54E-04	0.93236031	0.991701522	PEX16
ENSG00000255328.1	0.003063792	0.932362857	0.991701522	RP11-326C3.12
ENSG00000184787.19	3.91E-04	0.932482612	0.991701522	UBE2G2
ENSG00000164024.12	3.89E-04	0.932503763	0.991701522	METAP1
ENSG00000108950.12	0.001961209	0.932533061	0.991701522	FAM20A
ENSG00000239419.3	-0.002252315	0.932620238	0.991701522	RN7SL535P
ENSG00000136161.13	6.29E-04	0.932635419	0.991701522	RCBTB2
ENSG00000152270.9	0.001114392	0.932832582	0.991701522	PDE3B
ENSG00000169715.15	0.002614802	0.932837916	0.991701522	MT1E
ENSG00000173548.9	-8.51E-04	0.932873117	0.991701522	SNX33
ENSG00000131848.10	8.87E-04	0.932895813	0.991701522	ZSCAN5A
ENSG00000267100.1	6.77E-04	0.932920547	0.991701522	ILF3-DT
ENSG00000159231.6	-0.002196897	0.933029712	0.991701522	CBR3
ENSG00000267673.6	-0.002877056	0.933050117	0.991701522	FDX2
ENSG00000280214.1	0.001465879	0.933062474	0.991701522	CTD-2012K14.5
ENSG00000119686.10	-0.00104994	0.933066415	0.991701522	FLVCR2
ENSG00000257027.1	7.79E-04	0.933092857	0.991701522	RP11-705C15.3
ENSG00000204219.11	0.001712828	0.933096817	0.991701522	TCEA3
ENSG00000260078.3	-0.0015056	0.933225232	0.991701522	RP11-44F14.1
ENSG00000101425.14	0.003382148	0.933229439	0.991701522	BPI
ENSG00000116016.14	0.001660236	0.933282362	0.991701522	EPAS1
ENSG00000224892.6	0.001664454	0.933384249	0.991701522	RPS4XP16
ENSG00000254030.1	-0.003816163	0.933484799	0.991701522	IGLC5
ENSG00000100697.15	-8.70E-04	0.933514113	0.991701522	DICER1
ENSG00000179855.9	-0.002026113	0.933538424	0.991701522	GIPC3
ENSG00000227674.3	-0.003293392	0.933569243	0.991701522	LINC00355
ENSG00000220205.9	-6.64E-04	0.933602058	0.991701522	VAMP2
ENSG00000168556.7	8.87E-04	0.933686984	0.991701522	ING2
ENSG00000205220.12	8.77E-04	0.933688965	0.991701522	PSMB10
ENSG00000143537.14	-9.56E-04	0.933754112	0.991701522	ADAM15
ENSG00000170558.10	-0.002979036	0.933767572	0.991701522	CDH2
ENSG00000167046.4	-0.003233734	0.933804293	0.991701522	RP11-93B14.6
ENSG00000086200.17	7.12E-04	0.933805239	0.991701522	IPO11
ENSG00000131503.21	-9.04E-04	0.933859603	0.991701522	ANKHD1
ENSG00000051620.11	6.77E-04	0.933916043	0.991701522	HEBP2
ENSG00000164211.13	0.001384105	0.933931618	0.991701522	STARD4
ENSG00000008277.15	-0.001201889	0.933967578	0.991701522	ADAM22
ENSG00000118496.5	-0.001043213	0.933986197	0.991701522	FBXO30
ENSG00000272417.1	-0.002849033	0.93399473	0.991701522	CTD-2199O4.6
ENSG00000262488.1	0.003627128	0.933996985	0.991701522	RP11-876N24.1
ENSG00000172367.16	0.002040524	0.934038106	0.991701522	PDZD3
ENSG00000089169.15	-0.003021838	0.934051287	0.991701522	RPH3A

ENSG00000173261.9	-0.002426615	0.934072876	0.991701522	PLAC8L1
ENSG00000157593.19	4.88E-04	0.934080418	0.991701522	SLC35B2
ENSG00000261342.1	-0.00187599	0.934112807	0.991701522	AC006538.1
ENSG00000120802.14	-6.46E-04	0.934233928	0.991701522	TMPO
ENSG00000197329.12	9.81E-04	0.934284342	0.991701522	PELI1
ENSG00000145715.15	-8.61E-04	0.934361692	0.991701522	RASA1
ENSG00000139132.16	0.001070296	0.934409576	0.991701522	FGD4
ENSG00000115760.14	-7.39E-04	0.934454692	0.991701522	BIRC6
ENSG00000117983.17	-0.003362966	0.93457586	0.991701522	MUC5B
ENSG00000283475.1	-0.00210027	0.934603947	0.991701522	MIR1244-4
ENSG00000173409.14	9.08E-04	0.934609863	0.991701522	ARV1
ENSG00000143590.14	0.001259078	0.934626001	0.991701522	EFNA3
ENSG00000065970.9	5.73E-04	0.93467109	0.991701522	FOXJ2
ENSG00000213801.4	0.001048708	0.934688339	0.991701522	ZNF321P
ENSG00000146828.18	6.53E-04	0.934734748	0.991701522	SLC12A9
ENSG00000169964.8	6.91E-04	0.934753333	0.991701522	TMEM42
ENSG00000267317.2	8.75E-04	0.934785553	0.991701522	CTB-25B13.12
ENSG00000137843.12	-0.001710875	0.934991482	0.991801888	PAK6
ENSG00000139178.12	-6.38E-04	0.935026847	0.991801888	C1RL
ENSG00000272888.7	-8.71E-04	0.93505134	0.991801888	CHASERR
ENSG00000000419.14	9.65E-04	0.935086506	0.991801888	DPM1
ENSG00000267296.2	-0.001513321	0.935148741	0.991801888	CEBPA-DT
ENSG00000280149.1	0.001291868	0.935170329	0.991801888	RP4-751H13.5
ENSG00000165698.16	-0.001112764	0.935439316	0.992013589	SPACA9
ENSG00000273447.1	-0.001473742	0.935508326	0.992013589	AC004067.5
ENSG00000107929.15	3.82E-04	0.935515058	0.992013589	LARP4B
ENSG00000196267.13	5.94E-04	0.935578321	0.992029378	ZNF836
ENSG00000197561.7	0.004167176	0.935664351	0.992069306	ELANE
ENSG00000183018.9	-0.001363699	0.935762611	0.992122195	SPNS2
ENSG00000122390.19	-6.24E-04	0.935934093	0.992252707	NAA60
ENSG00000171428.15	0.001325158	0.936004875	0.992276452	NAT1
ENSG00000104442.10	-7.11E-04	0.936168495	0.992306827	ARMC1
ENSG00000101126.18	5.01E-04	0.936280405	0.992306827	ADNP
ENSG00000044090.12	-5.48E-04	0.936382178	0.992306827	CUL7
ENSG00000139567.13	0.001765001	0.936419375	0.992306827	ACVRL1
ENSG00000251675.1	-0.002436513	0.936422197	0.992306827	CTC-458I2.2
ENSG00000182544.9	7.29E-04	0.936452272	0.992306827	MFSD5
ENSG00000171634.18	7.43E-04	0.936586436	0.992306827	BPTF
ENSG00000229405.1	-0.00327963	0.936593107	0.992306827	AC092580.1
ENSG00000215769.8	-6.00E-04	0.936601446	0.992306827	ARHGAP27P1-BPTFP1-KPNA2P3
ENSG00000258561.2	0.002168806	0.936676657	0.992306827	RP11-72M17.1
ENSG00000062524.16	0.002128388	0.936725326	0.992306827	LTK
ENSG00000138061.12	0.001561487	0.936792197	0.992306827	CYP1B1
ENSG00000113141.18	4.51E-04	0.936820393	0.992306827	IK
ENSG00000179562.3	-4.41E-04	0.936918432	0.992306827	GCC1
ENSG00000260979.1	-0.002490178	0.936947228	0.992306827	USP7-AS1
ENSG00000171940.14	8.10E-04	0.93705417	0.992306827	ZNF217

ENSG00000249662.6	0.001539609	0.937119406	0.992306827	LINC02218
ENSG00000274918.1	0.003532857	0.937197368	0.992306827	RP11-54G14.1
ENSG00000183655.13	-8.06E-04	0.937250375	0.992306827	KLHL25
ENSG00000149488.14	0.001945113	0.937329881	0.992306827	TMC2
ENSG00000182810.7	5.35E-04	0.937556381	0.992306827	DDX28
ENSG00000186073.14	9.04E-04	0.937576989	0.992306827	CDIN1
ENSG00000174483.20	0.001071556	0.937616142	0.992306827	BBS1
ENSG00000109171.15	4.98E-04	0.937661248	0.992306827	SLAIN2
ENSG00000162302.13	7.87E-04	0.937675047	0.992306827	RPS6KA4
ENSG00000106089.12	9.11E-04	0.937712668	0.992306827	STX1A
ENSG00000166148.4	-0.002426081	0.937755905	0.992306827	AVPR1A
ENSG00000258732.1	-0.002494409	0.937770331	0.992306827	RP11-603B24.1
ENSG00000266907.1	-0.003079045	0.937815368	0.992306827	AC006116.22
ENSG00000151012.13	-0.001776271	0.937901356	0.992306827	SLC7A11
ENSG00000057663.16	9.23E-04	0.937933197	0.992306827	ATG5
ENSG00000204020.6	-0.001931896	0.937955371	0.992306827	LIPN
ENSG00000136738.15	4.94E-04	0.938013464	0.992306827	STAM
ENSG00000180488.15	-9.44E-04	0.93803996	0.992306827	MIGA1
ENSG00000124942.14	-9.01E-04	0.93804011	0.992306827	AHNAK
ENSG00000139687.16	-8.31E-04	0.93808697	0.992306827	RB1
ENSG00000172403.11	-0.002161048	0.938112427	0.992306827	SYNPO2
ENSG00000260051.1	-0.001335026	0.938152011	0.992306827	LA16c-390E6.4
ENSG00000164323.14	8.58E-04	0.938164243	0.992306827	CFAP97
ENSG00000226445.2	0.002028818	0.938186435	0.992306827	XXyac-YX65C7_A.2
ENSG00000277778.2	0.001922076	0.938201737	0.992306827	PGM5P2
ENSG00000180316.13	0.001188873	0.938280801	0.992306827	PNPLA1
ENSG00000249096.7	0.001283803	0.938288379	0.992306827	LINC02362
ENSG00000214439.4	-0.00174382	0.938344188	0.992306827	FAM185BP
ENSG00000236104.3	-4.76E-04	0.938432323	0.992306827	ZBTB22
ENSG00000244754.9	-7.67E-04	0.938445695	0.992306827	N4BP2L2
ENSG00000183873.18	0.003069328	0.938452251	0.992306827	SCN5A
ENSG00000134539.17	-0.001518371	0.938477472	0.992306827	KLRD1
ENSG00000164187.7	-0.001141302	0.938485335	0.992306827	LMBRD2
ENSG00000226609.2	0.002176308	0.938495887	0.992306827	RP11-276E15.4
ENSG00000063978.16	-3.66E-04	0.93850123	0.992306827	RNF4
ENSG00000153714.6	0.003338331	0.938618629	0.992379793	LURAP1L
ENSG00000213809.9	0.001527296	0.938688761	0.992402779	KLRK1
ENSG00000186265.10	0.001391146	0.938799779	0.992438246	BTLA
ENSG00000284726.1	-0.002631333	0.938851985	0.992438246	RP5-1057J7.8
ENSG00000245910.8	-7.17E-04	0.938867486	0.992438246	SNHG6
ENSG00000186056.10	-8.78E-04	0.938935467	0.992458951	MATN1-AS1
ENSG00000206535.8	-0.001742013	0.939197155	0.992638558	LNP1
ENSG00000264781.1	0.002398174	0.939271011	0.992638558	MIR4537
ENSG00000260271.3	-0.003213933	0.939281027	0.992638558	RP1-45N11.1
ENSG00000140650.13	-8.26E-04	0.939298998	0.992638558	PMM2
ENSG00000257953.1	0.002125909	0.939543384	0.992773736	RP11-620J15.1
ENSG00000218175.2	-0.002249892	0.939582563	0.992773736	AC016739.2

ENSG00000162944.11	0.001214694	0.939642193	0.992773736	RFTN2
ENSG00000108556.10	0.001770202	0.939649913	0.992773736	CHRNE
ENSG00000228325.5	0.003764034	0.939687663	0.992773736	IGKV3D-7
ENSG00000163596.17	-0.001142405	0.939744673	0.992773736	ICA1L
ENSG00000185404.17	5.32E-04	0.93982335	0.992773736	SP140L
ENSG00000177370.5	-5.83E-04	0.939861149	0.992773736	TIMM22
ENSG00000286017.1	0.002814612	0.939866817	0.992773736	RP11-21N3.2
ENSG00000133935.7	-6.10E-04	0.939911003	0.992773736	ERG28
ENSG00000149923.14	-7.10E-04	0.939980148	0.992795637	PPP4C
ENSG00000160404.18	-6.09E-04	0.940176365	0.99289737	TOR2A
ENSG00000104427.12	0.001594451	0.940279444	0.99289737	ZC2HC1A
ENSG00000113555.6	-7.34E-04	0.94032628	0.99289737	PCDH12
ENSG00000273419.1	-0.001889464	0.940352255	0.99289737	RP4-751H13.7
ENSG00000066468.24	-0.002988212	0.940360534	0.99289737	FGFR2
ENSG00000174348.14	-0.002187583	0.94036696	0.99289737	PODN
ENSG00000197353.4	0.002849332	0.940483461	0.992931114	LYPD2
ENSG00000166974.13	4.24E-04	0.940512559	0.992931114	MAPRE2
ENSG00000256262.1	0.00109561	0.940544169	0.992931114	USP30-AS1
ENSG00000146094.16	-7.47E-04	0.940645172	0.992943414	DOK3
ENSG00000197102.14	-6.72E-04	0.940664472	0.992943414	DYNC1H1
ENSG00000173457.11	8.71E-04	0.940795201	0.992943414	PPP1R14B
ENSG00000273489.1	0.001894205	0.940810392	0.992943414	RP11-180C16.1
ENSG00000213023.11	0.004945941	0.940816144	0.992943414	SYT3
ENSG00000184428.13	-8.79E-04	0.940846325	0.992943414	TOP1MT
ENSG00000160703.16	-7.06E-04	0.940942683	0.992994008	NLRX1
ENSG00000135749.19	-6.83E-04	0.941041689	0.992997506	PCNX2
ENSG00000249695.6	-0.0045367	0.941096415	0.992997506	RP11-598F7.4
ENSG00000061273.18	-5.89E-04	0.941153028	0.992997506	HDAC7
ENSG00000136237.19	-0.002846999	0.94116488	0.992997506	RAPGEF5
ENSG00000258388.7	0.00101255	0.941188098	0.992997506	PPT2-EGFL8
ENSG00000125462.19	-0.001880657	0.941436754	0.993079049	MIR9-1HG
ENSG00000089159.17	-5.98E-04	0.941592791	0.993079049	PXN
ENSG00000143756.12	6.10E-04	0.941641475	0.993079049	FBXO28
ENSG00000260844.2	0.002028251	0.941649065	0.993079049	RP11-578F21.9
ENSG00000187627.17	-0.003080661	0.941702753	0.993079049	RGPD1
ENSG00000287202.1	0.001397566	0.941743761	0.993079049	RP11-951I11.5
ENSG00000275074.2	8.17E-04	0.941767173	0.993079049	NUDT18
ENSG00000221500.1	-0.002337228	0.941871806	0.993079049	SNORD100
ENSG00000267119.1	0.003210624	0.941919049	0.993079049	RPL10P15
ENSG00000108797.12	0.001293049	0.941940528	0.993079049	CNTNAP1
ENSG00000262539.1	-0.011521546	0.941961764	0.993079049	RP11-259G18.3
ENSG00000187912.12	-0.001374081	0.942001362	0.993079049	CLEC17A
ENSG00000105427.10	0.002077867	0.942007154	0.993079049	CNFN
ENSG00000244701.1	-0.001261075	0.942071868	0.993079049	RP5-894A10.2
ENSG00000124107.5	0.002783362	0.94208694	0.993079049	SLPI
ENSG00000206172.8	0.002710643	0.942098448	0.993079049	HBA1
ENSG00000159140.21	5.95E-04	0.942125257	0.993079049	SON

ENSG00000213433.5	-0.001801204	0.94220363	0.993079049	RPLP1P6
ENSG00000177337.8	8.30E-04	0.942240251	0.993079049	DLGAP1-AS1
ENSG00000174628.16	-0.001854871	0.942352785	0.993079049	IQCK
ENSG00000180891.13	7.10E-04	0.942413096	0.993079049	CUEDC1
ENSG00000140931.20	-5.83E-04	0.942413672	0.993079049	CMTM3
ENSG00000087299.12	0.001012801	0.94244044	0.993079049	L2HGDH
ENSG00000260279.4	-0.001530613	0.942472144	0.993079049	AC137932.5
ENSG00000273724.1	0.001430877	0.942605867	0.993079049	RP11-347C12.12
ENSG00000242612.7	9.66E-04	0.942626293	0.993079049	DECR2
ENSG00000122678.17	-5.51E-04	0.942682518	0.993079049	POLM
ENSG00000173253.16	-0.006558599	0.942696593	0.993079049	DMRT2
ENSG00000103245.14	-6.28E-04	0.942717108	0.993079049	CIAO3
ENSG00000100433.16	0.002075549	0.942828644	0.993079049	KCNK10
ENSG00000267493.4	8.98E-04	0.942928412	0.993079049	CIRBP-AS1
ENSG00000105048.17	-0.002888055	0.942932618	0.993079049	TNNT1
ENSG00000280411.1	0.002872904	0.942990217	0.993079049	IGHV1-69D
ENSG00000185619.18	7.85E-04	0.943058059	0.993079049	PCGF3
ENSG00000183077.15	7.51E-04	0.94306048	0.993079049	AFMID
ENSG00000107651.14	-4.60E-04	0.94321516	0.993079049	SEC23IP
ENSG00000112308.13	5.37E-04	0.9432192	0.993079049	C6orf62
ENSG00000162368.14	-6.92E-04	0.943247217	0.993079049	CMPK1
ENSG00000187792.5	-8.28E-04	0.943392365	0.993079049	ZNF70
ENSG00000255153.1	0.001585334	0.943415985	0.993079049	TOLLIP-AS1
ENSG00000154978.13	-5.26E-04	0.943449443	0.993079049	VOPP1
ENSG00000259660.2	-0.002185507	0.943566959	0.993079049	DNM1P47
ENSG00000204220.12	-5.73E-04	0.943646804	0.993079049	PFDN6
ENSG00000137171.15	-4.56E-04	0.943648837	0.993079049	KLC4
ENSG00000278867.1	8.81E-04	0.943690788	0.993079049	RP11-640N20.4
ENSG00000204310.13	6.16E-04	0.943691488	0.993079049	AGPAT1
ENSG00000215559.8	-0.00412897	0.943708431	0.993079049	ANKRD20A11P
ENSG00000279901.1	-0.0023659	0.94376888	0.993079049	CTD-2270P14.2
ENSG00000177406.5	-9.18E-04	0.943814574	0.993079049	NINJ2-AS1
ENSG00000153066.13	3.85E-04	0.943854186	0.993079049	TXNDC11
ENSG00000141404.17	-6.78E-04	0.943868582	0.993079049	GNAL
ENSG00000179151.13	4.22E-04	0.943879876	0.993079049	EDC3
ENSG00000004059.11	-6.58E-04	0.943897034	0.993079049	ARF5
ENSG00000134419.15	-0.001533415	0.944038129	0.993079049	RPS15A
ENSG00000288559.1	-0.00115056	0.944089748	0.993079049	RP11-152N13.10
ENSG00000272056.1	-0.002220146	0.944133584	0.993079049	RP11-503P10.1
ENSG00000241975.1	0.00243132	0.944140725	0.993079049	ELOCP19
ENSG00000118418.14	-6.17E-04	0.944148089	0.993079049	HMGN3
ENSG00000188779.12	-0.001751891	0.944168984	0.993079049	SKOR1
ENSG00000186204.15	-0.001603379	0.944242985	0.993079049	CYP4F12
ENSG00000132196.16	-5.17E-04	0.944303861	0.993079049	HSD17B7
ENSG00000258769.1	0.00258733	0.944354048	0.993079049	RAP1AP
ENSG00000165617.15	-0.002615041	0.94437066	0.993079049	DACT1
ENSG00000207741.1	-0.001817138	0.944371002	0.993079049	MIR590

ENSG00000182858.14	-5.45E-04	0.944588328	0.993079049	ALG12
ENSG00000231437.3	-0.00269659	0.94464506	0.993079049	LINC01750
ENSG00000228057.1	-0.001905692	0.944648478	0.993079049	SEC63P1
ENSG00000132002.9	-4.49E-04	0.944678229	0.993079049	DNAJB1
ENSG00000100296.14	-5.21E-04	0.944719259	0.993079049	THOC5
ENSG00000229321.2	0.001980357	0.944741626	0.993079049	AC008269.2
ENSG00000287611.1	-0.0026305	0.944773813	0.993079049	RP11-178F10.5
ENSG00000074935.14	-8.95E-04	0.94477429	0.993079049	TUBE1
ENSG00000100300.18	9.42E-04	0.944800338	0.993079049	TSPO
ENSG00000115425.14	-5.82E-04	0.944904603	0.993097435	PECR
ENSG00000198837.10	-3.83E-04	0.945001911	0.993097435	DENND4B
ENSG00000025770.19	-5.18E-04	0.945016707	0.993097435	NCAPH2
ENSG00000141505.12	0.001036188	0.94503347	0.993097435	ASGR1
ENSG00000112394.17	0.001588837	0.945087215	0.993097435	SLC16A10
ENSG00000272834.1	-0.001437477	0.945199221	0.993097435	RP5-1042K10.13
ENSG00000163788.14	6.61E-04	0.945224777	0.993097435	SNRK
ENSG00000236008.3	0.001306394	0.945251187	0.993097435	LINC01814
ENSG00000010361.14	-7.53E-04	0.945330563	0.993097435	FUZ
ENSG00000163166.15	-3.41E-04	0.945438007	0.993097435	IWS1
ENSG00000211451.12	-9.23E-04	0.945468292	0.993097435	GNRHR2
ENSG00000177706.9	0.001193214	0.945527466	0.993097435	FAM20C
ENSG00000225075.1	0.002007783	0.945629107	0.993097435	RP11-426L16.3
ENSG00000179833.4	5.77E-04	0.945722087	0.993097435	SERTAD2
ENSG00000107175.12	-5.08E-04	0.945914622	0.993097435	CREB3
ENSG00000280739.3	-9.51E-04	0.946076109	0.993097435	EIF1B-AS1
ENSG00000169379.17	-0.001090918	0.946091153	0.993097435	ARL13B
ENSG00000135503.13	4.99E-04	0.946098639	0.993097435	ACVR1B
ENSG00000138821.14	-6.73E-04	0.946220359	0.993097435	SLC39A8
ENSG00000245958.6	-6.72E-04	0.946221955	0.993097435	SEPTIN7P14
ENSG00000250116.2	0.001315251	0.9462943	0.993097435	RHOQ-AS1
ENSG00000116127.20	-6.59E-04	0.946357705	0.993097435	ALMS1
ENSG00000182923.20	6.23E-04	0.946467197	0.993097435	CEP63
ENSG00000096080.12	6.76E-04	0.946473263	0.993097435	MRPS18A
ENSG00000211962.2	-0.002591977	0.946479616	0.993097435	IGHV1-46
ENSG00000007944.15	6.48E-04	0.946490724	0.993097435	MYLIP
ENSG00000181523.13	5.26E-04	0.946556889	0.993097435	SGSH
ENSG00000102893.16	-4.30E-04	0.946658806	0.993097435	PHKB
ENSG00000259236.1	-0.001853307	0.946665284	0.993097435	GOLGA8VP
ENSG00000260496.3	0.001303665	0.946694359	0.993097435	RP11-161M6.3
ENSG00000168944.17	7.74E-04	0.94673091	0.993097435	CEP120
ENSG00000273962.2	0.002969821	0.946738328	0.993097435	IGKV2-40
ENSG00000245164.8	-5.40E-04	0.946796369	0.993097435	LINC00861
ENSG00000267868.1	0.00235421	0.946822986	0.993097435	RP11-120K24.3
ENSG00000174579.4	-4.15E-04	0.946835668	0.993097435	MSL2
ENSG00000259326.1	0.001732305	0.946899615	0.993097435	RP11-102L12.2
ENSG00000006576.16	-9.93E-04	0.94690687	0.993097435	PHTF2
ENSG00000171444.18	-0.001066381	0.946941247	0.993097435	MCC

ENSG00000101596.16	-8.20E-04	0.946962041	0.993097435	SMCHD1
ENSG00000058056.10	6.55E-04	0.947021041	0.993097435	USP13
ENSG00000177200.18	-8.85E-04	0.947032357	0.993097435	CHD9
ENSG00000242082.2	-0.003849907	0.947060446	0.993097435	SLC5A4-AS1
ENSG00000241484.10	-0.00285747	0.94708518	0.993097435	ARHGAP8
ENSG00000271601.4	-3.96E-04	0.947119206	0.993097435	LIX1L
ENSG00000198870.8	-0.001499909	0.947231155	0.993097435	STKLD1
ENSG00000231466.2	0.001624519	0.947247415	0.993097435	CTA-246H3.11
ENSG00000133657.16	-7.28E-04	0.947284764	0.993097435	ATP13A3
ENSG00000164209.17	7.83E-04	0.947324339	0.993097435	SLC25A46
ENSG00000228037.1	-0.001211659	0.947332969	0.993097435	RP3-395M20.9
ENSG00000271607.1	-0.001617722	0.947355176	0.993097435	RP3-418A9.3
ENSG00000283199.3	-0.001390242	0.947395448	0.993097435	C13orf46
ENSG00000237372.4	-0.002148292	0.947419681	0.993097435	UNQ6494
ENSG00000067167.8	-5.99E-04	0.947427883	0.993097435	TRAM1
ENSG00000231940.1	-0.002043235	0.947432773	0.993097435	RPS7P3
ENSG00000179388.9	0.001702528	0.947553698	0.993128469	EGR3
ENSG00000253438.4	0.001166004	0.947559233	0.993128469	PCAT1
ENSG00000280047.1	-9.95E-04	0.947715458	0.993216097	CTC-463A16.1
ENSG00000173926.6	0.001125439	0.947776091	0.993216097	MARCHF3
ENSG00000102882.12	6.56E-04	0.947807863	0.993216097	MAPK3
ENSG00000107581.13	5.16E-04	0.947836563	0.993216097	EIF3A
ENSG00000135457.10	-3.69E-04	0.948078985	0.993371644	TFCP2
ENSG00000011275.19	3.32E-04	0.94808188	0.993371644	RNF216
ENSG00000226945.1	0.00138879	0.948184195	0.993384537	RP11-564A8.4
ENSG00000227113.2	0.002564496	0.948231489	0.993384537	RP11-460N20.4
ENSG00000261770.1	-0.001577904	0.948254067	0.993384537	CTC-459F4.1
ENSG00000144034.16	6.99E-04	0.948411182	0.993384537	TPRKB
ENSG00000264204.2	-0.001490534	0.948466119	0.993384537	AGAP7P
ENSG00000235605.1	-0.001979131	0.948544194	0.993384537	RP5-827C21.1
ENSG00000142657.21	6.21E-04	0.948555958	0.993384537	PGD
ENSG00000181885.18	0.001271655	0.948557702	0.993384537	CLDN7
ENSG00000090686.16	3.47E-04	0.948562237	0.993384537	USP48
ENSG00000204253.4	5.83E-04	0.948578574	0.993384537	HNRNPCP2
ENSG00000021574.13	6.39E-04	0.948640378	0.993392263	SPAST
ENSG00000105755.8	7.02E-04	0.948683879	0.993392263	ETHE1
ENSG00000143748.18	3.68E-04	0.948731269	0.993392263	NVL
ENSG00000237200.1	-0.002625823	0.94885317	0.993440521	ZBTB40-IT1
ENSG00000245904.4	0.00100578	0.948874241	0.993440521	RP11-796E2.4
ENSG00000162878.13	-0.003462413	0.9489416	0.993453986	PKDCC
ENSG00000134802.18	6.88E-04	0.949013058	0.993453986	SLC43A3
ENSG00000197714.9	-0.00102785	0.949032428	0.993453986	ZNF460
ENSG00000105865.11	-7.74E-04	0.949095508	0.993469307	DUS4L
ENSG00000172687.14	-8.56E-04	0.949248098	0.993570858	ZNF738
ENSG00000173153.17	-4.60E-04	0.94929781	0.993570858	ESRRA
ENSG00000151553.15	8.21E-04	0.94950838	0.993570858	FAM160B1
ENSG00000120370.13	8.49E-04	0.949553396	0.993570858	GORAB

ENSG00000113163.19	-5.23E-04	0.949635031	0.993570858	CERT1
ENSG00000182199.11	-5.28E-04	0.949646465	0.993570858	SHMT2
ENSG00000114166.8	7.24E-04	0.949673103	0.993570858	KAT2B
ENSG00000119707.14	5.57E-04	0.949735503	0.993570858	RBM25
ENSG00000228655.7	-0.001689091	0.949746212	0.993570858	AC096558.1
ENSG00000172331.12	-0.001780734	0.949805996	0.993570858	BPGM
ENSG00000124613.9	0.001245888	0.949920706	0.993570858	ZNF391
ENSG00000267523.1	7.52E-04	0.949952028	0.993570858	CTD-2537I9.12
ENSG00000145901.16	-5.43E-04	0.949982114	0.993570858	TNIP1
ENSG00000168765.17	-0.001490796	0.949993656	0.993570858	GSTM4
ENSG00000238005.4	-0.001234892	0.950009427	0.993570858	RP11-443B7.1
ENSG00000263606.1	-0.002090964	0.950040345	0.993570858	CHORDC1P4
ENSG00000184113.10	-0.001815863	0.950086218	0.993570858	CLDN5
ENSG00000135424.18	-0.001248394	0.950093431	0.993570858	ITGA7
ENSG00000115649.16	-7.17E-04	0.950113034	0.993570858	CNPPD1
ENSG00000237286.1	-0.002205148	0.950193811	0.993602801	CARD11-AS1
ENSG00000100266.19	4.96E-04	0.950283449	0.993602801	PACSIN2
ENSG00000148175.13	8.19E-04	0.950307515	0.993602801	STOM
ENSG00000121753.13	-0.001231427	0.950337378	0.993602801	ADGRB2
ENSG00000168350.8	-0.001508169	0.950387553	0.993604606	DEGS2
ENSG00000185495.10	-8.96E-04	0.950458674	0.993628307	SEPTIN7P13
ENSG00000128271.22	9.11E-04	0.950553187	0.993676459	ADORA2A
ENSG00000140968.11	-6.20E-04	0.95075878	0.993734356	IRF8
ENSG00000162604.12	-4.63E-04	0.950763895	0.993734356	TM2D1
ENSG00000181896.12	-5.53E-04	0.95078147	0.993734356	ZNF101
ENSG00000215481.9	0.001875875	0.95089416	0.993734356	BCRP3
ENSG00000173868.12	0.001036719	0.951000284	0.993734356	PHOSPHO1
ENSG00000247363.2	-0.001433964	0.951083449	0.993734356	RP11-637A17.2
ENSG00000280129.1	-0.00125082	0.951095941	0.993734356	RP11-298I3.3
ENSG00000085433.16	5.46E-04	0.95116924	0.993734356	WDR47
ENSG00000176153.12	-0.001374928	0.951348253	0.993734356	GPX2
ENSG00000125686.12	4.90E-04	0.951438915	0.993734356	MED1
ENSG00000172171.11	8.27E-04	0.951453062	0.993734356	TEFM
ENSG00000237254.2	-0.002552809	0.95152897	0.993734356	TRBV30
ENSG00000186501.15	4.57E-04	0.951571563	0.993734356	TMEM222
ENSG00000175287.19	0.001385902	0.951747678	0.993734356	PHYHD1
ENSG00000143368.10	-5.31E-04	0.951753642	0.993734356	SF3B4
ENSG00000273442.1	-0.003051154	0.951864234	0.993734356	AC006946.17
ENSG00000197128.12	-0.0011026	0.951917024	0.993734356	ZNF772
ENSG00000162997.15	-8.51E-04	0.951931609	0.993734356	PRORSD1P
ENSG00000250305.9	-0.003426708	0.951982576	0.993734356	TRMT9B
ENSG00000119403.15	3.95E-04	0.952009012	0.993734356	PHF19
ENSG00000269720.2	-0.002276921	0.952019535	0.993734356	CCDC194
ENSG00000229848.1	0.00197973	0.952049176	0.993734356	RP13-766D20.2
ENSG00000233621.2	7.93E-04	0.952129803	0.993734356	LINC01137
ENSG00000152990.14	0.001587457	0.952162381	0.993734356	ADGRA3
ENSG00000075643.6	-0.003377847	0.952174651	0.993734356	MOCOS

ENSG00000128617.3	-0.008432247	0.95217522	0.993734356	OPN1SW
ENSG00000286236.1	0.001486137	0.952266507	0.993734356	LLNLR-470E3.2
ENSG00000235072.2	-9.44E-04	0.952293827	0.993734356	ARNILA
ENSG00000189068.11	0.001552386	0.952325796	0.993734356	VSTM1
ENSG00000254006.5	0.001927101	0.95243731	0.993734356	RP11-1D12.2
ENSG00000239900.14	4.17E-04	0.952442588	0.993734356	ADSL
ENSG00000100365.16	-5.94E-04	0.95245021	0.993734356	NCF4
ENSG00000172824.16	-7.16E-04	0.952493272	0.993734356	CES4A
ENSG00000171159.5	-7.53E-04	0.952674246	0.993734356	C9orf16
ENSG00000137312.15	6.21E-04	0.952813364	0.993734356	FLOT1
ENSG00000270124.1	-0.001701482	0.95282357	0.993734356	RP11-118F19.1
ENSG00000253250.3	-0.001841208	0.952825655	0.993734356	C8orf88
ENSG00000177150.13	-5.16E-04	0.952834206	0.993734356	FAM210A
ENSG00000111726.13	-4.62E-04	0.952861384	0.993734356	CMAS
ENSG00000162910.19	-5.84E-04	0.952872083	0.993734356	MRPL55
ENSG00000143248.13	0.00165946	0.952901576	0.993734356	RGS5
ENSG00000259212.1	0.001982278	0.95291039	0.993734356	CTD-3065B20.2
ENSG00000149273.15	-6.72E-04	0.952932595	0.993734356	RPS3
ENSG00000064115.11	3.33E-04	0.953033154	0.993734356	TM7SF3
ENSG00000175768.13	-8.61E-04	0.953045896	0.993734356	TOMM5
ENSG00000171121.16	0.001197128	0.953121655	0.993734356	KCNMB3
ENSG00000129667.13	6.72E-04	0.95321064	0.993734356	RHBDF2
ENSG00000268379.1	0.003238736	0.953362733	0.993734356	CTC-360J11.4
ENSG00000254685.8	-9.10E-04	0.953393039	0.993734356	FPGT
ENSG00000155636.15	-4.39E-04	0.953434772	0.993734356	RBM45
ENSG00000237422.2	0.002192285	0.953471305	0.993734356	RP11-305L7.3
ENSG00000108107.15	7.38E-04	0.953566363	0.993734356	RPL28
ENSG00000091513.16	0.001509967	0.953571942	0.993734356	TF
ENSG00000178338.11	-6.27E-04	0.953575883	0.993734356	ZNF354B
ENSG00000158019.21	-5.61E-04	0.953624888	0.993734356	BABAM2
ENSG00000110446.12	-5.94E-04	0.953625813	0.993734356	SLC15A3
ENSG00000064313.12	-5.05E-04	0.953670171	0.993734356	TAF2
ENSG00000135698.10	-7.11E-04	0.953710835	0.993734356	MPHOSPH6
ENSG00000143379.12	-3.31E-04	0.953723713	0.993734356	SETDB1
ENSG00000284543.1	0.001900752	0.953762653	0.993734356	LINC01226
ENSG00000196670.14	6.21E-04	0.953877163	0.993734356	ZFP62
ENSG00000113460.13	5.78E-04	0.953879882	0.993734356	BRIX1
ENSG00000100347.15	-4.14E-04	0.95394777	0.993734356	SAMM50
ENSG00000104805.16	-6.35E-04	0.954042246	0.993734356	NUCB1
ENSG00000266643.1	-0.001599797	0.954050261	0.993734356	MIR3677
ENSG00000100393.14	7.21E-04	0.954052337	0.993734356	EP300
ENSG00000198252.12	5.78E-04	0.954136575	0.993734356	STYX
ENSG00000033178.13	-0.000603151	0.954142862	0.993734356	UBA6
ENSG00000162650.17	-4.28E-04	0.954238455	0.993734356	ATXN7L2
ENSG00000197933.13	8.59E-04	0.954240316	0.993734356	ZNF823
ENSG00000136732.16	-0.001389281	0.954246729	0.993734356	GYPC
ENSG00000187257.16	4.07E-04	0.954299429	0.993734356	RSBN1L

ENSG00000117500.13	6.24E-04	0.954351381	0.993734356	TMED5
ENSG00000116906.13	2.62E-04	0.95444182	0.993734356	GNPAT
ENSG00000161544.10	0.001972126	0.954468155	0.993734356	CYGB
ENSG00000279164.1	0.001913041	0.954661244	0.993734356	RP4-737E23.5
ENSG00000237950.3	-7.41E-04	0.954682989	0.993734356	RP11-7O11.9
ENSG00000279858.1	0.001668972	0.954717554	0.993734356	RP11-618M23.5
ENSG00000240291.1	9.94E-04	0.954751375	0.993734356	RP11-499P20.2
ENSG00000179934.7	0.001644865	0.954785364	0.993734356	CCR8
ENSG00000185607.5	-0.001699601	0.954810889	0.993734356	ACTBP7
ENSG00000159714.12	0.00105632	0.954824393	0.993734356	ZDHHC1
ENSG00000144749.14	-5.56E-04	0.954841573	0.993734356	LRIG1
ENSG00000226699.1	0.002008773	0.954887374	0.993734356	RP11-122K13.7
ENSG00000165806.21	-7.01E-04	0.954896765	0.993734356	CASP7
ENSG00000108604.16	-3.81E-04	0.954901663	0.993734356	SMARCD2
ENSG00000187954.13	3.97E-04	0.954904056	0.993734356	CYHR1
ENSG00000243224.1	0.001083089	0.95491259	0.993734356	RP5-1157M23.2
ENSG00000287778.1	-0.001833116	0.95503014	0.993734356	RP11-281P23.3
ENSG00000138600.10	3.90E-04	0.955059556	0.993734356	SPPL2A
ENSG00000166920.13	0.002186546	0.955119467	0.993734356	C15orf48
ENSG00000249859.12	7.33E-04	0.955137491	0.993734356	PVT1
ENSG00000278330.1	-8.55E-04	0.955138124	0.993734356	RP11-4B16.4
ENSG00000176834.14	-0.001439457	0.955225491	0.993734356	VSIG10
ENSG00000166913.13	3.23E-04	0.955290572	0.993734356	YWHAB
ENSG00000170581.14	-9.08E-04	0.955297139	0.993734356	STAT2
ENSG00000259635.1	-0.001725216	0.955478908	0.993734356	AC100830.3
ENSG00000161395.14	-4.57E-04	0.955486256	0.993734356	PGAP3
ENSG00000116791.14	9.95E-04	0.955519157	0.993734356	CRYZ
ENSG00000205981.8	5.42E-04	0.955611864	0.993734356	DNAJC19
ENSG00000120210.8	0.001980652	0.955625865	0.993734356	INSL6
ENSG00000138623.10	-8.68E-04	0.955628407	0.993734356	SEMA7A
ENSG00000159723.5	-0.001980589	0.955671345	0.993734356	AGRP
ENSG00000149636.16	4.24E-04	0.955719661	0.993734356	DSN1
ENSG00000154134.15	-0.001058618	0.955750304	0.993734356	ROBO3
ENSG00000214425.8	0.001881855	0.955829485	0.993734356	LRRC37A4P
ENSG00000263155.6	0.002123913	0.955840861	0.993734356	MYZAP
ENSG00000229931.1	0.001325801	0.955841813	0.993734356	ATXN1-AS1
ENSG00000165782.11	-3.22E-04	0.956004265	0.993799011	PIP4P1
ENSG00000284138.1	0.003184832	0.956079066	0.993799011	ATP6V0CP4
ENSG00000137218.10	-5.05E-04	0.956087104	0.993799011	FRS3
ENSG00000258655.2	-0.001420088	0.956181544	0.993799011	ARHGAP5-AS1
ENSG00000110172.12	5.82E-04	0.956184576	0.993799011	CHORDC1
ENSG00000269893.8	-8.11E-04	0.956194757	0.993799011	SNHG8
ENSG00000144591.20	4.58E-04	0.95634134	0.993900988	GMPPA
ENSG00000226423.1	-0.001916099	0.95646667	0.993942362	AC093642.4
ENSG00000140564.13	5.02E-04	0.956649027	0.993942362	FURIN
ENSG00000267322.2	9.28E-04	0.956695451	0.993942362	SNHG22
ENSG00000131379.10	0.001660269	0.956712606	0.993942362	C3orf20

ENSG00000153162.9	-9.56E-04	0.956732464	0.993942362	BMP6
ENSG00000136840.19	0.001129769	0.956782981	0.993942362	ST6GALNAC4
ENSG00000101361.17	-3.99E-04	0.956854047	0.993942362	NOP56
ENSG00000188938.17	2.18E-04	0.956885758	0.993942362	FAM120AOS
ENSG00000106665.16	-6.25E-04	0.956949301	0.993942362	CLIP2
ENSG00000248019.3	4.56E-04	0.956975663	0.993942362	FAM13A-AS1
ENSG00000120832.10	-5.57E-04	0.95699811	0.993942362	MTERF2
ENSG00000188559.15	-4.37E-04	0.957009009	0.993942362	RALGAPA2
ENSG00000133422.14	4.28E-04	0.957046344	0.993942362	MORC2
ENSG00000139679.16	0.001206085	0.957107659	0.993942362	LPAR6
ENSG00000198939.8	-0.001443887	0.957141123	0.993942362	ZFP2
ENSG00000272529.1	-8.87E-04	0.957156607	0.993942362	RP11-415F23.4
ENSG00000105676.14	4.18E-04	0.957238853	0.993977438	ARMC6
ENSG00000181847.12	-8.07E-04	0.95738812	0.9939939	TIGIT
ENSG00000180340.7	8.15E-04	0.957404729	0.9939939	FZD2
ENSG00000168876.9	7.39E-04	0.957431059	0.9939939	ANKRD49
ENSG00000179841.8	-9.06E-04	0.957448581	0.9939939	AKAP5
ENSG00000225101.6	-0.001074684	0.957552468	0.99405143	OR52K3P
ENSG00000188283.12	4.49E-04	0.957683887	0.994077154	ZNF383
ENSG00000100731.16	4.45E-04	0.957824092	0.994077154	PCNX1
ENSG00000142025.16	0.001783237	0.957869053	0.994077154	DMRTC2
ENSG00000165895.19	0.001429008	0.957900501	0.994077154	ARHGAP42
ENSG00000148824.19	5.70E-04	0.957947886	0.994077154	MTG1
ENSG00000183604.14	0.001108662	0.957954435	0.994077154	SMG1P5
ENSG00000124523.17	3.06E-04	0.957994036	0.994077154	SIRT5
ENSG00000272420.1	-0.001402237	0.958037493	0.994077154	RP4-740C4.7
ENSG00000255224.1	0.001175656	0.95805354	0.994077154	CTD-3065J16.9
ENSG00000015676.18	-2.99E-04	0.958061973	0.994077154	NUDCD3
ENSG00000111640.15	-4.80E-04	0.958123047	0.994087944	GAPDH
ENSG00000167780.12	-0.001864317	0.958232878	0.994087944	SOAT2
ENSG00000213058.3	-0.003840419	0.958374314	0.994087944	RP4-765C7.2
ENSG00000132507.18	4.32E-04	0.958402981	0.994087944	EIF5A
ENSG00000258317.1	-0.00123894	0.958458431	0.994087944	RP11-603J24.5
ENSG00000103150.7	2.64E-04	0.958551623	0.994087944	MLYCD
ENSG00000146707.15	-0.001222475	0.958552322	0.994087944	POMZP3
ENSG00000130363.13	5.48E-04	0.958565533	0.994087944	RSPH3
ENSG00000230869.1	0.001233445	0.958634754	0.994087944	AGAP10P
ENSG00000240489.1	-0.001515929	0.958647402	0.994087944	SETP14
ENSG00000125510.18	-7.40E-04	0.958650986	0.994087944	OPRL1
ENSG00000120215.10	9.07E-04	0.958654051	0.994087944	MLANA
ENSG00000172366.20	-4.94E-04	0.958946298	0.994338529	MCRIP2
ENSG00000188493.15	4.75E-04	0.958992675	0.994338529	C19orf54
ENSG00000279207.1	5.75E-04	0.95914181	0.994442884	RP11-159D12.8
ENSG00000111653.20	3.51E-04	0.959240486	0.994494913	ING4
ENSG00000140545.15	6.89E-04	0.959292302	0.994498359	MFGE8
ENSG00000103313.13	-5.10E-04	0.959462127	0.994552656	MEFV
ENSG00000224699.9	6.73E-04	0.959467489	0.994552656	LAMTOR5-AS1

ENSG00000116096.6	-9.01E-04	0.959493378	0.994552656	SPR
ENSG00000267336.1	0.002045787	0.959538661	0.994552656	EIF4A2P1
ENSG00000160746.13	4.33E-04	0.959623314	0.994590131	ANO10
ENSG00000143850.16	-0.001086369	0.959807832	0.994653574	PLEKHA6
ENSG00000171862.11	-4.47E-04	0.959814911	0.994653574	PTEN
ENSG00000103275.21	3.11E-04	0.959830028	0.994653574	UBE2I
ENSG00000214941.8	-3.82E-04	0.95989043	0.994665906	ZSWIM7
ENSG00000244515.1	-0.001316616	0.959963379	0.994682749	KRT18P34
ENSG00000227540.1	-0.001112681	0.960003689	0.994682749	DNAJC9-AS1
ENSG00000140400.17	-5.86E-04	0.960249604	0.994887283	MAN2C1
ENSG00000123444.14	-3.58E-04	0.96038154	0.994933673	KBTBD4
ENSG00000245060.8	-4.27E-04	0.960391407	0.994933673	LINC00847
ENSG00000268230.5	9.52E-04	0.960492927	0.994935552	CTD-2619J13.8
ENSG00000280367.1	0.001761033	0.960523698	0.994935552	RP11-121L10.2
ENSG00000239961.3	-0.001131527	0.960598782	0.994935552	LILRA4
ENSG00000224812.3	-0.001310267	0.960650515	0.994935552	TMEM72-AS1
ENSG00000148057.16	4.57E-04	0.960654477	0.994935552	IDNK
ENSG00000132475.10	4.02E-04	0.960717552	0.994935552	H3-3B
ENSG00000163785.13	4.26E-04	0.960786023	0.994935552	RYK
ENSG00000183011.14	6.59E-04	0.960827383	0.994935552	NAA38
ENSG00000273111.6	-0.00204484	0.960829852	0.994935552	LYPD4
ENSG00000160325.15	5.67E-04	0.961020254	0.994954771	CACFD1
ENSG00000285106.2	-8.08E-04	0.961057344	0.994954771	RP11-36B6.2
ENSG00000145757.16	0.001319726	0.961058443	0.994954771	SPATA9
ENSG00000212939.2	-0.002319418	0.961112953	0.994954771	RP1-29C18.10
ENSG00000274461.2	0.001645506	0.961149227	0.994954771	RP11-323P17.2
ENSG00000165131.8	0.001322273	0.961178173	0.994954771	LLCFC1
ENSG00000183160.9	-0.00158244	0.96118802	0.994954771	TMEM119
ENSG00000252503.1	0.00183803	0.961617405	0.995307608	RNU6-531P
ENSG00000108443.14	3.98E-04	0.961734447	0.995307608	RPS6KB1
ENSG00000107890.17	-7.49E-04	0.961781345	0.995307608	ANKRD26
ENSG00000177700.6	-6.20E-04	0.961826808	0.995307608	POLR2L
ENSG00000104343.21	-4.81E-04	0.961925966	0.995307608	UBE2W
ENSG00000112578.10	5.00E-04	0.961933898	0.995307608	BYSL
ENSG00000283234.1	-0.001881117	0.961991733	0.995307608	RP11-26H16.4
ENSG00000227398.4	6.24E-04	0.962000359	0.995307608	KIF9-AS1
ENSG00000166578.10	-0.001201575	0.962015227	0.995307608	IQCD
ENSG00000204396.11	7.55E-04	0.962089083	0.995307608	VWA7
ENSG00000170231.16	-0.001135378	0.962134977	0.995307608	FABP6
ENSG00000211683.3	0.001548953	0.962145166	0.995307608	KB-1572G7.3
ENSG00000215388.3	-0.001592207	0.962161246	0.995307608	ACTG1P3
ENSG00000249412.1	0.001567751	0.962210198	0.995307608	CTC-563A5.2
ENSG00000263072.8	-3.75E-04	0.962256873	0.995307608	ZNF213-AS1
ENSG00000231609.7	9.55E-04	0.962498634	0.99543115	AC009501.4
ENSG00000117480.16	-7.17E-04	0.962560958	0.99543115	FAAH
ENSG00000150625.16	0.002912499	0.962599358	0.99543115	GPM6A
ENSG00000240207.7	-0.001226124	0.962771426	0.99543115	RP11-379F4.4

ENSG00000165752.17	-6.63E-04	0.96282084	0.99543115	STK32C
ENSG00000279469.1	0.001329586	0.962868516	0.99543115	RP11-215P8.2
ENSG00000197279.5	0.001153828	0.962876182	0.99543115	ZNF165
ENSG00000135624.16	3.00E-04	0.963010355	0.99543115	CCT7
ENSG00000180694.14	5.68E-04	0.96303288	0.99543115	TMEM64
ENSG00000225022.1	-0.001117244	0.963058451	0.99543115	UBE2D3P1
ENSG00000279811.1	0.001826102	0.96309591	0.99543115	RP11-862L9.2
ENSG00000166716.10	3.39E-04	0.963123383	0.99543115	ZNF592
ENSG00000119950.21	-9.05E-04	0.963136659	0.99543115	MXI1
ENSG00000108829.10	-3.28E-04	0.963142032	0.99543115	LRRC59
ENSG00000269050.1	-0.001448696	0.963186301	0.99543115	CTC-360G5.6
ENSG00000156050.10	4.77E-04	0.963221799	0.99543115	FAM161B
ENSG00000115827.14	4.02E-04	0.963239768	0.99543115	DCAF17
ENSG00000164171.11	0.001062679	0.963364047	0.99543115	ITGA2
ENSG00000019186.10	0.002252553	0.963474512	0.99543115	CYP24A1
ENSG00000246596.7	-0.001335683	0.963493177	0.99543115	RP11-1277A3.2
ENSG00000166503.9	-6.37E-04	0.963540444	0.99543115	HDGFL3
ENSG00000002726.21	-0.002472175	0.963563089	0.99543115	AOC1
ENSG00000218186.2	0.001798658	0.963569534	0.99543115	KRT8P43
ENSG00000204832.10	-0.001296394	0.963614511	0.99543115	ST8SIA6-AS1
ENSG00000160685.13	3.20E-04	0.963748746	0.99543115	ZBTB7B
ENSG00000196950.14	-5.46E-04	0.963862254	0.99543115	SLC39A10
ENSG00000276115.1	0.001331736	0.96391764	0.99543115	RP11-817I4.2
ENSG00000143493.13	-3.20E-04	0.963994707	0.99543115	INTS7
ENSG00000257008.6	-0.002568912	0.964030051	0.99543115	GPR142
ENSG00000189308.11	4.40E-04	0.964072773	0.99543115	LIN54
ENSG00000198860.14	3.46E-04	0.964082978	0.99543115	TSEN15
ENSG00000188312.14	6.88E-04	0.964182973	0.99543115	CENPP
ENSG00000106560.11	6.52E-04	0.964280029	0.99543115	GIMAP2
ENSG00000160055.20	-3.50E-04	0.964350977	0.99543115	TMEM234
ENSG00000277443.3	5.96E-04	0.964361383	0.99543115	MARCKS
ENSG00000286757.2	7.66E-04	0.96438543	0.99543115	RP11-134O15.3
ENSG00000146530.14	-0.002016437	0.964415272	0.99543115	VWDE
ENSG00000154153.15	-6.34E-04	0.96446234	0.99543115	RETREG1
ENSG00000084073.10	-3.29E-04	0.964524253	0.99543115	ZMPSTE24
ENSG00000147642.17	-0.001271826	0.964539027	0.99543115	SYBU
ENSG00000232615.4	0.002263184	0.964681136	0.99543115	CTD-2012J19.1
ENSG00000269892.1	6.89E-04	0.964694035	0.99543115	RP4-761J14.10
ENSG00000169251.13	-6.59E-04	0.964703298	0.99543115	NMD3
ENSG00000113532.13	-4.42E-04	0.964704405	0.99543115	ST8SIA4
ENSG00000164219.10	-4.10E-04	0.964711821	0.99543115	PGGT1B
ENSG00000114982.19	2.08E-04	0.964711863	0.99543115	KANSL3
ENSG00000227370.1	0.001088652	0.964806543	0.99543115	RP4-669P10.19
ENSG00000239569.3	5.89E-04	0.964834712	0.99543115	KMT2E-AS1
ENSG00000237593.1	-0.001593518	0.964846116	0.99543115	RP11-317B3.2
ENSG00000226137.5	-5.65E-04	0.964874452	0.99543115	BAIAP2-DT
ENSG00000143603.19	-0.00151242	0.964881009	0.99543115	KCNN3

ENSG00000226049.3	-0.001194458	0.964920034	0.99543115	TLK2P1
ENSG00000157856.12	0.001486494	0.964992161	0.99543115	DRC1
ENSG00000170486.11	0.001942806	0.9649974	0.99543115	KRT72
ENSG00000111361.13	-1.86E-04	0.965086072	0.995472546	EIF2B1
ENSG00000129595.14	0.001309764	0.965139738	0.995477833	EPB41L4A
ENSG00000280353.1	-0.001466785	0.965309372	0.995554786	CTC-453G23.7
ENSG00000109680.11	-6.82E-04	0.965311436	0.995554786	TBC1D19
ENSG00000233337.1	8.54E-04	0.965589932	0.995736786	UBE2FP3
ENSG00000143851.15	3.95E-04	0.965629008	0.995736786	PTPN7
ENSG00000162777.17	-2.89E-04	0.965650509	0.995736786	DENND2D
ENSG00000160190.14	3.12E-04	0.965722378	0.995736786	SLC37A1
ENSG00000037897.17	-5.11E-04	0.965861981	0.995736786	METTL1
ENSG00000260261.2	-8.01E-04	0.965873251	0.995736786	RP11-480A16.2
ENSG00000077232.19	4.04E-04	0.965879265	0.995736786	DNAJC10
ENSG00000185129.8	3.76E-04	0.965903245	0.995736786	PURA
ENSG00000211802.3	-0.001029325	0.965924888	0.995736786	TRAV22
ENSG00000285847.2	0.001716499	0.96606713	0.99583336	RP4-768P8.2
ENSG00000237990.3	-0.001895274	0.966329245	0.995861836	CNTN4-AS1
ENSG00000258376.2	-8.84E-04	0.966345361	0.995861836	RP4-647C14.2
ENSG00000110844.14	-6.34E-04	0.966359543	0.995861836	PRPF40B
ENSG00000245552.7	6.64E-04	0.966517064	0.995861836	RP11-712B9.2
ENSG00000085719.13	3.02E-04	0.96659354	0.995861836	CPNE3
ENSG00000135426.16	4.90E-04	0.966656966	0.995861836	TESPA1
ENSG00000091127.14	5.30E-04	0.966726341	0.995861836	PUS7
ENSG00000107021.16	2.55E-04	0.966751048	0.995861836	TBC1D13
ENSG00000111145.8	-3.25E-04	0.966756033	0.995861836	ELK3
ENSG00000110013.13	-3.95E-04	0.966762067	0.995861836	SIAE
ENSG00000228352.2	0.001146407	0.966827022	0.995861836	RP11-537H15.3
ENSG00000274383.1	-0.0011419	0.966834581	0.995861836	CTD-2017F17.2
ENSG00000212658.1	0.001880989	0.966950592	0.995861836	KRTAP29-1
ENSG00000049239.13	3.45E-04	0.966976777	0.995861836	H6PD
ENSG00000262902.1	0.002140227	0.967049752	0.995861836	MTCO1P40
ENSG00000175582.20	-2.59E-04	0.967091253	0.995861836	RAB6A
ENSG00000197405.8	4.08E-04	0.967104911	0.995861836	C5AR1
ENSG00000241163.8	-5.04E-04	0.967121792	0.995861836	LINC00877
ENSG00000138764.15	-4.79E-04	0.967134873	0.995861836	CCNG2
ENSG00000110274.16	-2.61E-04	0.967182753	0.995861836	CEP164
ENSG00000125944.21	-1.90E-04	0.967197971	0.995861836	HNRNPR
ENSG00000160563.14	-3.79E-04	0.967255085	0.995861836	MED27
ENSG00000140992.19	3.27E-04	0.967274232	0.995861836	PDPK1
ENSG00000127080.10	4.64E-04	0.967537513	0.995861836	IPPK
ENSG00000198399.15	-3.46E-04	0.967583329	0.995861836	ITSN2
ENSG00000179526.17	-6.17E-04	0.967605714	0.995861836	SHARPIN
ENSG00000261889.1	-0.001381591	0.967641921	0.995861836	RP11-473M20.16
ENSG00000127084.19	3.26E-04	0.967717761	0.995861836	FGD3
ENSG00000173041.12	-7.75E-04	0.967740429	0.995861836	ZNF680
ENSG00000272256.1	-0.001336746	0.96777717	0.995861836	RP11-489E7.4

ENSG00000242371.1	0.001334456	0.967818702	0.995861836	IGKV1-39
ENSG00000261052.6	0.001353688	0.967827125	0.995861836	SULT1A3
ENSG00000130956.14	3.96E-04	0.96784084	0.995861836	HABP4
ENSG00000138792.10	-0.004170361	0.967884732	0.995861836	ENPEP
ENSG00000266074.10	-5.98E-04	0.967908216	0.995861836	BAHCC1
ENSG00000166848.7	2.06E-04	0.968028202	0.995861836	TERF2IP
ENSG00000121864.10	-3.46E-04	0.968044402	0.995861836	ZNF639
ENSG00000163083.6	-0.001070202	0.96807293	0.995861836	INHBB
ENSG00000115274.15	-5.21E-04	0.968080433	0.995861836	INO80B
ENSG00000170786.13	-0.00236467	0.968104607	0.995861836	SDR16C5
ENSG00000104714.14	3.54E-04	0.968151245	0.995861836	ERICH1
ENSG00000198719.9	0.001054577	0.968168615	0.995861836	DLL1
ENSG00000134061.5	-6.90E-04	0.968182821	0.995861836	CD180
ENSG00000248925.1	-0.001419493	0.968302439	0.995934922	CTD-2083E4.6
ENSG00000230358.5	0.001146631	0.968402647	0.995944626	SPDYE21
ENSG00000204954.10	4.67E-04	0.968427952	0.995944626	C12orf73
ENSG00000178074.6	-5.36E-04	0.968483962	0.995944626	C2orf69
ENSG00000144724.20	-0.001707528	0.968641373	0.995944626	PTPRG
ENSG00000205959.4	-7.78E-04	0.968736749	0.995944626	RP11-689P11.2
ENSG00000283031.2	-0.001215773	0.96875712	0.995944626	RP11-557N21.1
ENSG00000133619.17	3.09E-04	0.968829557	0.995944626	KRBA1
ENSG00000213512.3	-0.001805308	0.968875078	0.995944626	GBP7
ENSG00000019582.16	-4.72E-04	0.968912213	0.995944626	CD74
ENSG00000003987.14	5.13E-04	0.968965688	0.995944626	MTMR7
ENSG00000104290.11	-5.95E-04	0.968975972	0.995944626	FZD3
ENSG00000251143.1	6.13E-04	0.96898467	0.995944626	RP11-849H4.4
ENSG00000229619.4	6.38E-04	0.969005321	0.995944626	MBNL1-AS1
ENSG00000204217.15	4.26E-04	0.96901696	0.995944626	BMPR2
ENSG00000164106.8	0.001270891	0.969040329	0.995944626	SCRG1
ENSG00000099817.12	-3.33E-04	0.96918373	0.995957971	POLR2E
ENSG00000272103.2	-0.001019489	0.969184577	0.995957971	CTD-2653M23.3
ENSG00000138660.12	5.14E-04	0.969199006	0.995957971	AP1AR
ENSG00000130943.7	-0.001231551	0.969365674	0.996062467	PKDREJ
ENSG00000261864.1	-0.001007123	0.969397834	0.996062467	RP11-67A1.2
ENSG00000137878.17	-0.001556563	0.969693238	0.996310745	GCOM1
ENSG00000197223.12	4.59E-04	0.969892087	0.996310745	C1D
ENSG00000132406.12	4.08E-04	0.969911256	0.996310745	TMEM128
ENSG00000197586.13	2.48E-04	0.970015537	0.996310745	ENTPD6
ENSG00000005810.19	3.59E-04	0.970028134	0.996310745	MYCBP2
ENSG00000189079.17	3.13E-04	0.970123376	0.996310745	ARID2
ENSG00000126500.4	0.001077706	0.97013157	0.996310745	FLRT1
ENSG00000179057.14	-7.78E-04	0.970154256	0.996310745	IGSF22
ENSG00000172361.6	9.37E-04	0.970173138	0.996310745	CFAP53
ENSG00000165269.13	0.001301357	0.970268709	0.996310745	AQP7
ENSG00000091986.16	0.001448603	0.97029419	0.996310745	CCDC80
ENSG00000117523.17	-3.44E-04	0.970329058	0.996310745	PRRC2C
ENSG00000234478.1	-0.001735593	0.970384166	0.996310745	ACBD3-AS1

ENSG00000105889.16	-0.001296703	0.970411037	0.996310745	STEAP1B
ENSG00000266824.1	8.40E-04	0.970464676	0.996310745	RP11-599B13.7
ENSG00000146414.16	6.23E-04	0.970503762	0.996310745	SHPRH
ENSG00000103316.12	-0.002009944	0.970507228	0.996310745	CRYM
ENSG00000256660.7	0.001101722	0.970555588	0.996310745	CLEC12B
ENSG00000108840.16	-3.13E-04	0.970567678	0.996310745	HDAC5
ENSG00000065357.20	-4.09E-04	0.970611097	0.996310745	DGKA
ENSG00000018280.17	-4.55E-04	0.970673773	0.996325212	SLC11A1
ENSG00000067369.14	-3.41E-04	0.970774577	0.99636875	TP53BP1
ENSG00000213047.13	5.73E-04	0.970813359	0.99636875	DENND1B
ENSG00000145730.21	-7.90E-04	0.971049315	0.996401045	PAM
ENSG00000075089.10	6.97E-04	0.971053297	0.996401045	ACTR6
ENSG00000089248.7	2.95E-04	0.971067873	0.996401045	ERP29
ENSG00000249846.7	0.001015388	0.971157805	0.996401045	LINC02021
ENSG00000116771.6	4.99E-04	0.971207264	0.996401045	AGMAT
ENSG00000143751.10	-3.36E-04	0.97125915	0.996401045	SDE2
ENSG00000139990.18	1.58E-04	0.971326589	0.996401045	DCAF5
ENSG00000286619.1	-0.002897437	0.971368218	0.996401045	RP4-681L3.3
ENSG00000072682.20	7.20E-04	0.971454455	0.996401045	P4HA2
ENSG00000111907.21	-0.001029453	0.97170104	0.996401045	TPD52L1
ENSG00000137801.11	-0.0010873	0.97171482	0.996401045	THBS1
ENSG00000136891.14	1.74E-04	0.971716799	0.996401045	TEX10
ENSG00000237886.1	6.71E-04	0.971740719	0.996401045	NALT1
ENSG00000100288.20	3.32E-04	0.971745904	0.996401045	CHKB
ENSG00000250896.1	-0.001025879	0.971755436	0.996401045	RNPS1P1
ENSG00000240399.1	7.72E-04	0.971759996	0.996401045	RP1-228P16.1
ENSG00000160410.15	4.71E-04	0.971813248	0.996401045	SHKBP1
ENSG00000232811.1	5.97E-04	0.971828277	0.996401045	RP11-96K19.2
ENSG00000249738.10	0.001128142	0.971866072	0.996401045	AC008697.1
ENSG00000271870.1	4.14E-04	0.971902383	0.996401045	RP11-97C16.1
ENSG00000267474.1	6.94E-04	0.971906975	0.996401045	CTC-548K16.6
ENSG00000200737.1	-0.001130171	0.97199076	0.996401045	Y_RNA
ENSG00000085788.14	2.52E-04	0.97199875	0.996401045	DDHD2
ENSG00000157106.17	3.36E-04	0.972031681	0.996401045	SMG1
ENSG00000100888.15	0.000263991	0.972071485	0.996401045	CHD8
ENSG00000255446.1	-0.004311715	0.97210806	0.996401045	CTD-2531D15.4
ENSG00000215421.10	2.66E-04	0.972210734	0.996456481	ZNF407
ENSG00000263400.8	-9.72E-04	0.972284809	0.996482602	TMEM220-AS1
ENSG00000128040.11	0.00103492	0.972505816	0.996627724	SPINK2
ENSG00000139218.18	2.91E-04	0.972567092	0.996627724	SCAF11
ENSG00000176563.10	-3.61E-04	0.972616072	0.996627724	CNTD1
ENSG00000224420.3	-7.93E-04	0.972620795	0.996627724	ADM5
ENSG00000012983.12	3.89E-04	0.972822412	0.996741435	MAP4K5
ENSG00000222179.1	-0.00119955	0.972828972	0.996741435	RN7SKP26
ENSG00000135446.17	-2.56E-04	0.972883777	0.996747789	CDK4
ENSG00000114654.8	-0.001137784	0.972953833	0.996758638	EFCC1
ENSG00000133731.10	-4.74E-04	0.972991573	0.996758638	IMPA1

ENSG00000169231.13	-3.24E-04	0.973228468	0.99695152	THBS3
ENSG00000186407.7	4.76E-04	0.973460704	0.997139609	CD300E
ENSG00000228334.2	0.002429087	0.973519491	0.997150022	AC024028.1
ENSG00000286167.1	-0.001219051	0.973704091	0.997289292	RP11-1415G13.1
ENSG00000273375.1	0.001290781	0.973795772	0.99730129	RP11-803P9.1
ENSG00000064300.9	-8.23E-04	0.973829867	0.99730129	NGFR
ENSG00000100503.26	-2.99E-04	0.973928175	0.99730129	NIN
ENSG00000170906.16	3.63E-04	0.973928561	0.99730129	NDUFA3
ENSG00000179240.13	3.98E-04	0.974008341	0.99730129	GVQW3
ENSG00000260903.3	-5.94E-04	0.974033417	0.99730129	XKR7
ENSG00000128604.20	-3.33E-04	0.97408465	0.99730129	IRF5
ENSG00000242866.10	-0.001189253	0.974112779	0.99730129	STRC
ENSG00000188681.11	0.001065056	0.974239136	0.99730129	TEKT4P2
ENSG00000164362.21	0.001415295	0.974250285	0.99730129	TERT
ENSG00000131127.14	2.76E-04	0.974484903	0.99730129	ZNF141
ENSG00000109445.11	-1.90E-04	0.974688197	0.99730129	ZNF330
ENSG00000075340.23	-5.35E-04	0.974723964	0.99730129	ADD2
ENSG00000108262.16	-2.26E-04	0.974771546	0.99730129	GIT1
ENSG00000225973.4	-3.70E-04	0.974783477	0.99730129	PIGBOS1
ENSG00000080573.7	0.001624787	0.974816487	0.99730129	COL5A3
ENSG00000229559.2	-0.001970054	0.974857758	0.99730129	RP1-153P14.3
ENSG00000161638.11	-2.93E-04	0.97489058	0.99730129	ITGA5
ENSG00000157193.18	3.34E-04	0.974893169	0.99730129	LRP8
ENSG00000170899.11	6.08E-04	0.975020075	0.99730129	GSTA4
ENSG00000287100.1	-8.91E-04	0.975049685	0.99730129	RP1-193N13.1
ENSG00000112218.9	-0.001020618	0.975144097	0.99730129	GPR63
ENSG00000130038.10	-2.18E-04	0.975332686	0.99730129	CRACR2A
ENSG00000150045.12	7.02E-04	0.975447984	0.99730129	KLRF1
ENSG00000188536.13	-9.72E-04	0.975499092	0.99730129	HBA2
ENSG00000048991.17	-1.84E-04	0.975536465	0.99730129	R3HDM1
ENSG00000284634.1	7.19E-04	0.975551056	0.99730129	RP11-726G1.2
ENSG00000175161.14	-0.002110283	0.975555211	0.99730129	CADM2
ENSG00000263006.6	0.001443869	0.975564316	0.99730129	ROCK1P1
ENSG00000168803.16	-3.30E-04	0.975627209	0.99730129	ADAL
ENSG00000249173.6	-0.001242094	0.97568157	0.99730129	LINC01093
ENSG00000144451.19	-5.01E-04	0.975767578	0.99730129	SPAG16
ENSG00000188573.8	0.002708033	0.97591802	0.99730129	FBLL1
ENSG00000147996.17	4.59E-04	0.975921896	0.99730129	CBWD5
ENSG00000257127.6	-0.001159685	0.976074881	0.99730129	CLLU1
ENSG00000259953.2	3.11E-04	0.976145154	0.99730129	RP11-4O1.2
ENSG00000127328.22	3.25E-04	0.976196775	0.99730129	RAB3IP
ENSG00000005961.19	-0.001003699	0.976210321	0.99730129	ITGA2B
ENSG00000112320.12	-9.94E-04	0.976289811	0.99730129	SOBP
ENSG00000089220.5	2.39E-04	0.976291684	0.99730129	PEBP1
ENSG00000104998.4	-1.88E-04	0.97634391	0.99730129	IL27RA
ENSG00000273272.2	6.18E-04	0.976388996	0.99730129	CTA-384D8.34
ENSG00000226321.5	8.31E-04	0.97645685	0.99730129	CROCC2

ENSG00000157168.20	0.00100259	0.976534331	0.99730129	NRG1
ENSG00000242553.1	-0.001009489	0.976570424	0.99730129	AP001432.14
ENSG00000148444.16	-3.51E-04	0.976592006	0.99730129	COMMD3
ENSG00000259529.2	4.53E-04	0.976604065	0.99730129	RP11-468E2.4
ENSG00000063177.13	3.33E-04	0.976700509	0.99730129	RPL18
ENSG00000242265.6	-8.55E-04	0.976738602	0.99730129	PEG10
ENSG00000119242.9	2.29E-04	0.976744407	0.99730129	CCDC92
ENSG00000071894.17	4.00E-04	0.976774327	0.99730129	CPSF1
ENSG00000224034.1	-0.001214444	0.976855077	0.99730129	LINC02561
ENSG00000116652.6	-0.00106155	0.976870265	0.99730129	DLEU2L
ENSG00000197324.9	-2.67E-04	0.976877774	0.99730129	LRP10
ENSG00000249741.2	-0.001100734	0.976907134	0.99730129	RP11-673E1.3
ENSG00000131931.8	-2.77E-04	0.976992592	0.99730129	THAP1
ENSG00000271856.1	3.60E-04	0.977009204	0.99730129	LINC01215
ENSG00000036054.13	-2.96E-04	0.977104983	0.99730129	TBC1D23
ENSG00000262155.2	-5.60E-04	0.977163476	0.99730129	LINC02175
ENSG00000168903.9	-0.003586965	0.977220595	0.99730129	BTNL3
ENSG00000204439.4	-1.70E-04	0.977245136	0.99730129	C6orf47
ENSG00000184185.10	-0.001626143	0.977367443	0.99730129	KCNJ12
ENSG00000106336.13	-5.17E-04	0.977373712	0.99730129	FBXO24
ENSG00000176428.6	0.001058429	0.977381183	0.99730129	VPS37D
ENSG00000228668.1	-0.001390411	0.977396246	0.99730129	TRGV5P
ENSG00000179583.20	3.52E-04	0.977499961	0.99730129	CIITA
ENSG00000286330.1	-6.89E-04	0.977552816	0.99730129	RP11-512C24.4
ENSG00000175395.16	3.06E-04	0.977619728	0.99730129	ZNF25
ENSG00000038382.20	-3.28E-04	0.977694188	0.99730129	TRIO
ENSG00000284862.3	-8.02E-04	0.977701651	0.99730129	CCDC39
ENSG00000132612.16	-1.95E-04	0.977748122	0.99730129	VPS4A
ENSG00000204356.14	2.40E-04	0.977765478	0.99730129	NELFE
ENSG00000110324.11	1.86E-04	0.977826149	0.99730129	IL10RA
ENSG00000280099.1	-7.68E-04	0.977891013	0.99730129	RP11-33E12.2
ENSG00000107554.17	-3.61E-04	0.977949194	0.99730129	DNMBP
ENSG00000108582.12	-3.11E-04	0.977954491	0.99730129	CPD
ENSG00000223318.1	0.001915481	0.977970548	0.99730129	RNA5SP111
ENSG00000152061.24	2.34E-04	0.97800182	0.99730129	RABGAP1L
ENSG00000204869.8	-0.001213865	0.978006195	0.99730129	IGFL4
ENSG00000278974.1	-0.001067521	0.9780282	0.99730129	RP11-756P10.6
ENSG00000103510.20	-1.68E-04	0.978064133	0.99730129	KAT8
ENSG00000117266.16	4.44E-04	0.978091431	0.99730129	CDK18
ENSG00000223298.1	0.001362236	0.978203826	0.99730129	RNY3P8
ENSG00000165512.5	3.94E-04	0.978335753	0.99730129	ZNF22
ENSG00000132471.12	2.69E-04	0.978338526	0.99730129	WBP2
ENSG00000157343.9	4.85E-04	0.978415484	0.99730129	ARMC12
ENSG00000227063.5	8.08E-04	0.978434952	0.99730129	RPL41P1
ENSG00000164808.17	1.55E-04	0.97845838	0.99730129	SPIDR
ENSG00000177888.8	-4.75E-04	0.97846591	0.99730129	ZBTB41
ENSG00000100234.12	-0.001336945	0.978554473	0.99730129	TIMP3

ENSG00000280033.1	4.43E-04	0.978589963	0.99730129	CTD-2095E4.3
ENSG00000120029.13	-1.44E-04	0.978659156	0.99730129	ARMH3
ENSG00000233834.6	-0.001214526	0.978669503	0.99730129	AC005083.1
ENSG00000173120.15	1.54E-04	0.978684173	0.99730129	KDM2A
ENSG00000251637.6	6.87E-04	0.978721876	0.99730129	LINC02754
ENSG00000230099.2	-6.47E-04	0.978724714	0.99730129	TRBV5-4
ENSG00000187951.11	3.66E-04	0.978796071	0.99730129	RP5-1086D14.6
ENSG00000157827.20	-5.21E-04	0.978905133	0.99730129	FMNL2
ENSG00000276136.1	-3.57E-04	0.978909067	0.99730129	RP11-50I19.2
ENSG00000079931.15	7.57E-04	0.978953755	0.99730129	MOXD1
ENSG00000184517.12	2.98E-04	0.978983299	0.99730129	ZFP1
ENSG00000092096.18	6.16E-04	0.97900795	0.99730129	SLC22A17
ENSG00000105355.9	-2.43E-04	0.979031358	0.99730129	PLIN3
ENSG00000166682.13	-4.93E-04	0.979070567	0.99730129	TMPRSS5
ENSG00000177409.12	-5.64E-04	0.979117511	0.99730129	SAMD9L
ENSG00000153902.14	-7.32E-04	0.979131475	0.99730129	LGI4
ENSG00000280231.1	6.06E-04	0.979193828	0.99730129	LA16c-380F5.3
ENSG00000284820.1	6.38E-04	0.979224709	0.99730129	RP11-803J6.1
ENSG00000107262.26	5.34E-04	0.979258021	0.99730129	BAG1
ENSG00000275210.1	-4.83E-04	0.979353188	0.99730129	AC008984.2
ENSG00000140367.12	2.44E-04	0.979355391	0.99730129	UBE2Q2
ENSG00000102908.22	-4.25E-04	0.979362153	0.99730129	NFAT5
ENSG00000185442.13	-4.20E-04	0.979409559	0.99730129	FAM174B
ENSG00000166452.12	2.61E-04	0.979433078	0.99730129	AKIP1
ENSG00000102763.18	-2.05E-04	0.97951215	0.99730129	VWA8
ENSG00000198160.15	-2.98E-04	0.97954801	0.99730129	MIER1
ENSG00000233822.5	-4.98E-04	0.979627735	0.99730129	H2BC15
ENSG00000181315.11	4.33E-04	0.979672548	0.99730129	ZNF322
ENSG00000169442.9	-5.10E-04	0.979775692	0.99730129	CD52
ENSG00000104388.15	-1.77E-04	0.979861836	0.99730129	RAB2A
ENSG00000225439.3	3.43E-04	0.97993063	0.99730129	BOLA3-AS1
ENSG00000277301.1	6.26E-04	0.979967234	0.99730129	RP5-1184F4.7
ENSG00000271461.1	0.001371463	0.979976195	0.99730129	CKAP2LP1
ENSG00000287574.1	-7.48E-04	0.980036985	0.99730129	RP5-971C3.1
ENSG00000124535.16	-1.26E-04	0.980067207	0.99730129	WRNIP1
ENSG00000108823.17	-6.86E-04	0.980085768	0.99730129	SGCA
ENSG00000173599.15	-3.39E-04	0.980090791	0.99730129	PC
ENSG00000176101.12	-2.19E-04	0.980123305	0.99730129	SSNA1
ENSG00000125629.15	2.67E-04	0.980134055	0.99730129	INSIG2
ENSG00000137161.17	2.09E-04	0.980156014	0.99730129	CNPY3
ENSG00000084731.15	2.91E-04	0.980165625	0.99730129	KIF3C
ENSG00000144290.17	-0.00102579	0.980192602	0.99730129	SLC4A10
ENSG00000224424.8	3.23E-04	0.98020711	0.99730129	PRKAR2A-AS1
ENSG00000130517.14	-1.65E-04	0.980232207	0.99730129	PGPEP1
ENSG00000156928.5	2.22E-04	0.980421342	0.997403842	MALSU1
ENSG00000124275.15	-2.19E-04	0.980430273	0.997403842	MTRR
ENSG00000005381.8	-8.67E-04	0.980574592	0.997490342	MPO

ENSG00000265681.8	2.76E-04	0.980657294	0.997490342	RPL17
ENSG00000215193.14	1.26E-04	0.980678348	0.997490342	PEX26
ENSG00000140022.13	4.41E-04	0.980739488	0.997490342	STON2
ENSG00000267547.1	5.25E-04	0.980758497	0.997490342	RP11-686D22.4
ENSG00000103067.14	-5.02E-04	0.981146711	0.997822991	ESRP2
ENSG00000196169.15	8.31E-04	0.981209025	0.997822991	KIF19
ENSG00000128159.12	1.83E-04	0.981289238	0.997822991	TUBGCP6
ENSG00000206053.13	-1.18E-04	0.98135974	0.997822991	JPT2
ENSG00000136271.11	-1.46E-04	0.981374331	0.997822991	DDX56
ENSG00000176731.12	-2.98E-04	0.981472743	0.997822991	RBIS
ENSG00000286550.1	0.001000697	0.981514817	0.997822991	RP11-527B17.3
ENSG00000076555.15	-2.58E-04	0.981542305	0.997822991	ACACB
ENSG00000175773.13	4.97E-04	0.981586715	0.997822991	ZBTB44-DT
ENSG00000125457.15	-1.84E-04	0.981594504	0.997822991	MIF4GD
ENSG00000244952.2	6.80E-04	0.981620775	0.997822991	RP11-1000B6.5
ENSG00000151743.11	-2.95E-04	0.981673452	0.997827079	AMN1
ENSG00000047457.14	8.22E-04	0.981803688	0.997872493	CP
ENSG00000165113.13	-3.52E-04	0.981850707	0.997872493	GKAP1
ENSG00000265678.1	-3.79E-04	0.981893242	0.997872493	RP11-1376P16.2
ENSG00000011295.16	-1.73E-04	0.982034853	0.997872493	TTC19
ENSG00000270115.1	6.97E-04	0.982036294	0.997872493	RP11-415J8.7
ENSG00000111731.13	-2.12E-04	0.982096598	0.997872493	C2CD5
ENSG00000279744.1	-3.75E-04	0.982211679	0.997872493	RP13-20L14.10
ENSG00000075073.15	-8.65E-04	0.982232929	0.997872493	TACR2
ENSG00000253269.1	7.86E-04	0.982250695	0.997872493	CTB-37A13.1
ENSG00000165661.17	2.13E-04	0.982290211	0.997872493	QSOX2
ENSG00000188760.11	-6.58E-04	0.982470403	0.997872493	TMEM198
ENSG00000266910.2	-4.72E-04	0.982482873	0.997872493	CTC-448F2.6
ENSG00000009790.15	1.41E-04	0.982498151	0.997872493	TRAF3IP3
ENSG00000157999.6	-5.39E-04	0.982563137	0.997872493	ANKRD61
ENSG00000223804.5	-3.81E-04	0.982636035	0.997872493	CH17-472G23.2
ENSG00000121903.15	2.30E-04	0.982700742	0.997872493	ZSCAN20
ENSG00000158863.22	-1.54E-04	0.982703462	0.997872493	FAM160B2
ENSG00000169288.18	-4.15E-04	0.982780544	0.997872493	MRPL1
ENSG00000139629.16	1.99E-04	0.98278518	0.997872493	GALNT6
ENSG00000132591.12	1.68E-04	0.98283321	0.997872493	ERAL1
ENSG00000217128.12	2.23E-04	0.982867946	0.997872493	FNIP1
ENSG00000235527.7	6.79E-04	0.982873425	0.997872493	HIPK1-AS1
ENSG00000163872.16	-1.42E-04	0.982928055	0.997872493	YEATS2
ENSG00000172250.16	0.00136063	0.983018029	0.997872493	SERHL
ENSG00000122483.17	-3.15E-04	0.983022307	0.997872493	CCDC18
ENSG00000198894.8	2.00E-04	0.983063942	0.997872493	CIPC
ENSG00000197965.12	-3.60E-04	0.983082498	0.997872493	MPZL1
ENSG00000268836.1	4.90E-04	0.983126835	0.997872493	LA16c-OS12.2
ENSG00000215533.8	0.001113395	0.983129204	0.997872493	LINC00189
ENSG00000143569.19	1.43E-04	0.983300946	0.997891658	UBAP2L
ENSG00000253194.2	8.55E-04	0.98331592	0.997891658	RP11-351A11.1

ENSG00000173598.14	1.81E-04	0.983336278	0.997891658	NUDT4
ENSG00000287033.1	7.37E-04	0.983342721	0.997891658	RP11-318L16.8
ENSG00000149346.15	1.85E-04	0.983429544	0.997913256	SLX4IP
ENSG00000088256.9	3.42E-04	0.983461323	0.997913256	GNA11
ENSG00000166888.12	-1.33E-04	0.983535882	0.997935878	STAT6
ENSG00000277147.6	-2.01E-04	0.983580939	0.997935878	LINC00869
ENSG00000196126.11	3.42E-04	0.983730492	0.998038237	HLA-DRB1
ENSG00000160298.18	-2.22E-04	0.98382925	0.998084611	C21orf58
ENSG00000246225.7	-7.39E-04	0.983873537	0.998084611	RP11-17A1.3
ENSG00000145362.21	-0.003476359	0.983973507	0.998120865	ANK2
ENSG00000108510.10	-2.14E-04	0.984006614	0.998120865	MED13
ENSG00000174527.10	-6.80E-04	0.984075515	0.998141385	MYO1H
ENSG00000112144.16	1.84E-04	0.984284535	0.998257551	CILK1
ENSG00000151136.15	-2.56E-04	0.984466313	0.998257551	BTBD11
ENSG00000255521.2	-7.05E-04	0.984660105	0.998257551	RP4-607I7.1
ENSG00000264304.1	-3.89E-04	0.984676979	0.998257551	RP11-20B24.7
ENSG00000105221.18	1.10E-04	0.984743393	0.998257551	AKT2
ENSG00000122335.17	-2.04E-04	0.984833118	0.998257551	SERAC1
ENSG00000152601.18	2.46E-04	0.984837194	0.998257551	MBNL1
ENSG00000080166.16	-4.43E-04	0.984848587	0.998257551	DCT
ENSG00000169427.8	0.001650682	0.98495122	0.998257551	KCNK9
ENSG00000280184.2	-5.25E-04	0.98506117	0.998257551	RP3-466P17.3
ENSG00000274515.1	-4.14E-04	0.985063461	0.998257551	CTD-2026K11.5
ENSG00000163565.19	-2.36E-04	0.9850641	0.998257551	IFI16
ENSG00000181409.14	2.86E-04	0.98510011	0.998257551	AATK
ENSG00000178607.17	-2.39E-04	0.985219962	0.998257551	ERN1
ENSG00000178732.5	3.96E-04	0.9852222	0.998257551	GP5
ENSG00000225190.11	-1.31E-04	0.985228205	0.998257551	PLEKHM1
ENSG00000134545.14	6.64E-04	0.985289606	0.998257551	KLRC1
ENSG00000272325.2	1.92E-04	0.985373722	0.998257551	NUDT3
ENSG00000197372.10	2.95E-04	0.985405256	0.998257551	ZNF675
ENSG00000172869.15	2.26E-04	0.985433739	0.998257551	DMXL1
ENSG00000197768.10	-5.37E-04	0.985512362	0.998257551	STPG3
ENSG00000285231.1	-5.63E-04	0.985539913	0.998257551	OOSP3
ENSG00000048707.15	1.32E-04	0.985567575	0.998257551	VPS13D
ENSG00000105483.18	1.44E-04	0.985644369	0.998257551	CARD8
ENSG00000239665.8	-1.36E-04	0.985683299	0.998257551	RP11-295P9.3
ENSG00000205537.2	4.64E-04	0.985684734	0.998257551	RP11-89H19.1
ENSG00000232125.5	-6.01E-04	0.985686038	0.998257551	DYTN
ENSG00000286599.1	3.16E-04	0.985813672	0.998257551	RP11-345F18.3
ENSG00000152409.9	1.84E-04	0.985879068	0.998257551	JMY
ENSG00000270972.1	-6.31E-04	0.985955126	0.998257551	RP11-326C3.15
ENSG00000065243.20	-2.54E-04	0.985970244	0.998257551	PKN2
ENSG00000116903.7	-2.02E-04	0.985977424	0.998257551	EXOC8
ENSG00000227212.3	-8.86E-04	0.986089027	0.998257551	PFN1P6
ENSG00000170502.13	1.18E-04	0.986108529	0.998257551	NUDT9
ENSG00000182568.17	-1.86E-04	0.9861397	0.998257551	SATB1

ENSG00000175279.22	4.81E-04	0.986147355	0.998257551	CENPS
ENSG00000237927.1	-4.68E-04	0.986261946	0.998257551	RP3-393E18.2
ENSG00000051825.15	-1.78E-04	0.986327433	0.998257551	MPHOSPH9
ENSG00000104679.12	-1.27E-04	0.986359707	0.998257551	R3HCC1
ENSG00000107560.12	2.31E-04	0.986365315	0.998257551	RAB11FIP2
ENSG00000131400.8	-3.06E-04	0.986387168	0.998257551	NAPSA
ENSG00000164458.10	1.91E-05	0.986445909	0.998257551	TBXT
ENSG00000228696.10	6.84E-04	0.98646582	0.998257551	ARL17B
ENSG00000185127.6	1.64E-04	0.986487447	0.998257551	C6orf120
ENSG00000169813.17	7.55E-05	0.986507865	0.998257551	HNRNPF
ENSG00000285410.1	-1.23E-04	0.986526636	0.998257551	GABPB1-IT1
ENSG00000020256.20	-1.27E-04	0.986560934	0.998257551	ZFP64
ENSG00000187837.4	3.20E-04	0.986565922	0.998257551	H1-2
ENSG00000204524.7	-1.34E-04	0.986582114	0.998257551	ZNF805
ENSG00000287625.1	1.99E-04	0.986623869	0.998257551	RP11-617F9.2
ENSG00000243725.7	-1.88E-04	0.986944344	0.998436552	TTC4
ENSG00000117899.11	-7.54E-05	0.987007221	0.998436552	MESD
ENSG00000136895.19	3.28E-04	0.987017089	0.998436552	GARNL3
ENSG00000116221.15	1.33E-04	0.98703344	0.998436552	MRPL37
ENSG00000131187.10	-2.78E-04	0.987115916	0.998436552	F12
ENSG00000287170.1	2.90E-04	0.987162269	0.998436552	CTD-2587H24.16
ENSG00000267543.1	-4.59E-04	0.987174779	0.998436552	RP11-666A8.7
ENSG00000168679.18	3.31E-04	0.987190266	0.998436552	SLC16A4
ENSG00000112312.10	2.30E-04	0.987263105	0.998442528	GMNN
ENSG00000170390.16	-6.75E-04	0.987326457	0.998442528	DCLK2
ENSG00000203710.12	2.03E-04	0.987364123	0.998442528	CR1
ENSG00000198182.13	-1.80E-04	0.987390916	0.998442528	ZNF607
ENSG00000178222.13	4.08E-04	0.987595824	0.998543511	RNF212
ENSG00000023228.14	6.88E-05	0.987609839	0.998543511	NDUFS1
ENSG00000226245.1	-3.58E-04	0.987660651	0.998543511	ZNF32-AS1
ENSG00000257696.1	-4.96E-04	0.987685543	0.998543511	RP11-175P13.2
ENSG00000269688.1	3.01E-04	0.987861121	0.998627778	AC008982.2
ENSG00000261461.1	4.03E-04	0.987882005	0.998627778	UBE2MP1
ENSG00000122042.10	1.58E-04	0.987914978	0.998627778	UBL3
ENSG00000216490.4	-1.94E-04	0.988046181	0.998711178	IFI30
ENSG00000170802.16	2.05E-04	0.988286061	0.998712054	FOXN2
ENSG00000213213.14	-2.34E-04	0.988296155	0.998712054	CCDC183
ENSG00000135945.10	-1.19E-04	0.988380937	0.998712054	REV1
ENSG00000040275.17	1.84E-04	0.9883927	0.998712054	SPDL1
ENSG00000285791.1	5.50E-04	0.988406319	0.998712054	RP11-346I3.5
ENSG00000158270.12	-7.80E-04	0.988408688	0.998712054	COLEC12
ENSG00000178458.5	-3.46E-04	0.988529823	0.998712054	H3P16
ENSG00000100321.15	-2.42E-04	0.988532435	0.998712054	SYNGR1
ENSG00000117155.17	-1.97E-04	0.988583037	0.998712054	SSX2IP
ENSG00000226084.5	-3.98E-04	0.988584109	0.998712054	RP4-706A16.3
ENSG00000171241.9	2.86E-04	0.988716118	0.998712054	SHCBP1
ENSG00000101298.15	-2.15E-04	0.988761398	0.998712054	SNPH

ENSG00000166788.10	-1.21E-04	0.988824553	0.998712054	SAAL1
ENSG00000166135.14	-5.71E-05	0.988966582	0.998712054	HIF1AN
ENSG00000163464.8	1.83E-04	0.98900383	0.998712054	CXCR1
ENSG00000228050.1	-0.001734884	0.989021184	0.998712054	TOP3BP1
ENSG00000203865.11	1.16E-04	0.989039201	0.998712054	ATP1A1-AS1
ENSG00000239789.6	1.33E-04	0.989058716	0.998712054	MRPS17
ENSG00000053918.18	-1.20E-04	0.98909099	0.998712054	KCNQ1
ENSG00000234327.8	-2.07E-04	0.989101239	0.998712054	ZNF232-AS1
ENSG00000212541.1	-5.81E-04	0.989230254	0.998712054	RNU6-510P
ENSG00000162373.13	2.81E-04	0.989234417	0.998712054	BEND5
ENSG00000067113.17	-1.81E-04	0.989242225	0.998712054	PLPP1
ENSG00000143224.18	-1.03E-04	0.98935714	0.998712054	PPOX
ENSG00000187535.14	-1.49E-04	0.989368822	0.998712054	IFT140
ENSG00000178935.5	-9.75E-05	0.989410357	0.998712054	ZNF552
ENSG00000089154.11	8.65E-05	0.989516498	0.998712054	GCN1
ENSG00000128805.15	-2.74E-04	0.989523025	0.998712054	ARHGAP22
ENSG00000165731.21	-2.81E-04	0.989547918	0.998712054	RET
ENSG00000181074.5	3.53E-04	0.989610236	0.998712054	OR52N4
ENSG00000241511.1	5.42E-04	0.989660782	0.998712054	RPS15AP24
ENSG00000180353.11	-1.06E-04	0.989681574	0.998712054	HCLS1
ENSG00000160714.10	-6.01E-05	0.989745226	0.998712054	UBE2Q1
ENSG00000074410.14	-4.56E-04	0.989760129	0.998712054	CA12
ENSG00000023902.14	1.14E-04	0.989816074	0.998712054	PLEKHO1
ENSG00000138829.12	3.07E-04	0.989834732	0.998712054	FBN2
ENSG00000283103.3	9.79E-05	0.989854568	0.998712054	LLNLR-245B6.1
ENSG00000184508.12	1.21E-04	0.989928492	0.998712054	HDDC3
ENSG00000204130.14	-1.23E-04	0.990026959	0.998712054	RUFY2
ENSG00000211688.1	-4.24E-04	0.990122818	0.998712054	TRGJP2
ENSG00000257743.8	-3.24E-04	0.990126424	0.998712054	MGAM2
ENSG00000157303.11	1.55E-04	0.99021921	0.998712054	SUSD3
ENSG00000261115.6	-5.33E-04	0.990235855	0.998712054	TMEM178B
ENSG00000272573.6	-3.13E-04	0.99030568	0.998712054	MUSTN1
ENSG00000182557.8	2.43E-04	0.990390772	0.998712054	SPNS3
ENSG00000152689.18	-2.38E-04	0.990409355	0.998712054	RASGRP3
ENSG00000247572.8	-1.78E-04	0.990493141	0.998712054	CKMT2-AS1
ENSG00000112514.18	-1.25E-04	0.99049854	0.998712054	CUTA
ENSG00000166411.14	-1.06E-04	0.990535331	0.998712054	IDH3A
ENSG00000270108.1	-2.78E-04	0.990586488	0.998712054	RP11-73M18.6
ENSG00000113615.13	1.24E-04	0.990644881	0.998712054	SEC24A
ENSG00000176624.12	1.31E-04	0.990681045	0.998712054	MEX3C
ENSG00000105793.16	-1.12E-04	0.990710122	0.998712054	GTPBP10
ENSG00000149451.18	-6.71E-04	0.99076337	0.998712054	ADAM33
ENSG00000204277.1	-3.94E-04	0.990777069	0.998712054	LINC01993
ENSG00000184811.4	0.000407296	0.990881708	0.998712054	TRARG1
ENSG00000184106.8	4.31E-04	0.990993269	0.998712054	TREML3P
ENSG00000196371.4	9.96E-05	0.991005253	0.998712054	FUT4
ENSG00000241351.3	-3.31E-04	0.991051792	0.998712054	IGKV3-11

ENSG00000286602.1	-4.09E-04	0.99110323	0.998712054	RP11-227H4.9
ENSG00000287250.1	4.70E-04	0.991147562	0.998712054	RP11-51I5.3
ENSG00000237441.10	-7.91E-05	0.991150298	0.998712054	RGL2
ENSG00000084693.16	6.98E-05	0.991162142	0.998712054	AGBL5
ENSG00000227740.2	-3.31E-04	0.991209884	0.998712054	LINC02803
ENSG00000282936.2	-3.84E-04	0.991212461	0.998712054	CTC-281F24.5
ENSG00000129354.12	3.26E-04	0.991418255	0.998735768	AP1M2
ENSG00000151876.13	1.44E-04	0.991450783	0.998735768	FBXO4
ENSG00000164398.15	1.52E-04	0.991527212	0.998735768	ACSL6
ENSG00000239556.4	1.63E-04	0.99153913	0.998735768	AC004951.5
ENSG00000286299.1	6.50E-04	0.991545254	0.998735768	RP1-167O5.3
ENSG00000065060.17	1.24E-04	0.991573051	0.998735768	UHRF1BP1
ENSG00000215483.11	-2.69E-04	0.99166398	0.998735768	LINC00598
ENSG00000158286.13	2.08E-04	0.991708399	0.998735768	RNF207
ENSG00000096006.12	-5.11E-04	0.991755292	0.998735768	CRISP3
ENSG00000188365.3	-2.94E-04	0.99177452	0.998735768	AC092171.2
ENSG00000166164.16	-5.47E-05	0.991870622	0.998735768	BRD7
ENSG00000273448.1	2.77E-04	0.991951208	0.998735768	RP11-166O4.6
ENSG00000258890.7	5.97E-05	0.991981876	0.998735768	CEP95
ENSG00000136960.13	1.93E-04	0.991998139	0.998735768	ENPP2
ENSG00000211899.10	2.74E-04	0.992057767	0.998735768	IGHM
ENSG00000164512.18	2.26E-04	0.992069992	0.998735768	ANKRD55
ENSG00000164167.12	9.85E-05	0.992138725	0.998735768	LSM6
ENSG00000180914.11	3.21E-04	0.992194283	0.998735768	OXTR
ENSG00000271427.1	-2.43E-04	0.992228337	0.998735768	RP11-188D8.1
ENSG00000271849.1	-3.03E-04	0.992275091	0.998735768	MAN2A1-DT
ENSG00000100578.18	6.70E-05	0.992306416	0.998735768	KIAA0586
ENSG00000214870.9	-2.27E-04	0.992307393	0.998735768	AC004540.5
ENSG00000165943.5	4.39E-05	0.992386236	0.998766105	MOAP1
ENSG00000011566.15	1.52E-04	0.992577229	0.998783238	MAP4K3
ENSG00000134086.8	-5.41E-05	0.992608137	0.998783238	VHL
ENSG00000287382.1	-2.52E-04	0.99271127	0.998783238	RP11-345F18.2
ENSG00000026652.15	-1.18E-04	0.992714958	0.998783238	AGPAT4
ENSG00000196502.12	-1.98E-04	0.992725631	0.998783238	SULT1A1
ENSG00000164011.18	6.76E-05	0.9927555	0.998783238	ZNF691
ENSG00000259823.6	2.94E-04	0.99278638	0.998783238	LYPD8
ENSG00000196872.12	1.52E-04	0.992926755	0.998783238	CRACDL
ENSG00000106609.16	-3.75E-05	0.993001788	0.998783238	TMEM248
ENSG00000116199.12	-5.90E-05	0.99304292	0.998783238	FAM20B
ENSG00000174206.13	7.21E-05	0.993110858	0.998783238	C12orf66
ENSG00000163481.8	6.72E-05	0.993143286	0.998783238	RNF25
ENSG00000149313.11	-7.07E-05	0.993205671	0.998783238	AASDHPPT
ENSG00000172748.14	1.34E-04	0.993217576	0.998783238	ZNF596
ENSG00000104341.17	1.48E-04	0.993270737	0.998783238	LAPTM4B
ENSG00000198520.12	1.15E-04	0.99329612	0.998783238	ARMH1
ENSG00000121579.13	6.33E-05	0.993355018	0.998783238	NAA50
ENSG00000172123.13	1.20E-04	0.993415189	0.998783238	SLFN12

ENSG00000215126.11	1.50E-04	0.993441884	0.998783238	CBWD6
ENSG00000174365.20	7.54E-05	0.993591031	0.998783238	SNHG11
ENSG00000157837.16	-5.58E-05	0.993613427	0.998783238	SPPL3
ENSG00000047249.18	5.01E-05	0.99367119	0.998783238	ATP6V1H
ENSG00000204475.10	-1.31E-04	0.993687258	0.998783238	NCR3
ENSG00000188002.10	1.21E-04	0.993691884	0.998783238	RP11-43F13.1
ENSG00000099330.9	8.26E-05	0.993718574	0.998783238	OCEL1
ENSG00000178999.13	1.26E-04	0.993726054	0.998783238	AURKB
ENSG00000054967.13	-5.59E-05	0.993755549	0.998783238	RELT
ENSG00000115295.20	8.28E-05	0.993829663	0.998783238	CLIP4
ENSG00000146070.17	-1.46E-04	0.993845371	0.998783238	PLA2G7
ENSG00000095970.17	-5.71E-04	0.993908458	0.998783238	TREM2
ENSG00000134709.12	-1.50E-04	0.994011586	0.998783238	HOOK1
ENSG00000137880.6	-1.19E-04	0.994097695	0.998783238	GCHFR
ENSG00000186638.17	-1.38E-04	0.994102616	0.998783238	KIF24
ENSG00000169679.15	-1.71E-04	0.994108571	0.998783238	BUB1
ENSG00000034677.13	8.21E-05	0.994116805	0.998783238	RNF19A
ENSG00000204933.3	1.89E-04	0.994156536	0.998783238	CD177P1
ENSG00000275791.1	-1.66E-04	0.994282852	0.99886121	TRBV10-3
ENSG00000171204.13	9.11E-05	0.994505373	0.999035817	TMEM126B
ENSG00000269967.1	1.51E-04	0.994586974	0.999068851	RP11-84A19.4
ENSG00000117600.14	4.82E-04	0.994678944	0.999070717	PLPPR4
ENSG00000178201.5	1.98E-04	0.994686264	0.999070717	VN1R1
ENSG00000149177.13	-5.54E-05	0.994793936	0.999096568	PTPRJ
ENSG00000268895.6	-8.64E-05	0.994848877	0.999096568	A1BG-AS1
ENSG00000282995.1	1.92E-04	0.99494468	0.999096568	FRG1EP
ENSG00000187630.17	-7.65E-05	0.994970493	0.999096568	DHRS4L2
ENSG00000013288.9	-4.15E-05	0.995008891	0.999096568	MAN2B2
ENSG00000253919.1	2.20E-04	0.995051947	0.999096568	THAP12P7
ENSG00000273314.1	-1.09E-04	0.995174667	0.999096568	RP5-1136G13.2
ENSG00000211803.2	-1.70E-04	0.995197067	0.999096568	TRAV23DV6
ENSG00000205832.8	-1.92E-04	0.995210738	0.999096568	C16orf96
ENSG00000089335.21	-1.01E-04	0.995220683	0.999096568	ZNF302
ENSG00000102781.14	-7.39E-05	0.99529602	0.999096568	KATNAL1
ENSG00000110218.9	6.03E-05	0.995314686	0.999096568	PANX1
ENSG00000256069.7	1.46E-04	0.995345328	0.999096568	A2MP1
ENSG00000136682.15	7.75E-05	0.995523673	0.999189685	CBWD2
ENSG00000275645.1	1.25E-04	0.995547487	0.999189685	RP11-817O13.9
ENSG00000189419.8	1.02E-04	0.995584261	0.999189685	SPATA41
ENSG00000136381.13	4.49E-05	0.995816477	0.999322695	IREB2
ENSG00000215190.9	1.04E-04	0.995958945	0.999322695	LINC00680
ENSG00000103194.16	4.14E-05	0.996038963	0.999322695	USP10
ENSG00000127837.10	-3.77E-05	0.996082566	0.999322695	AAMP
ENSG00000164530.15	-1.25E-04	0.996088247	0.999322695	PI16
ENSG00000234073.1	7.59E-05	0.996134019	0.999322695	AC011816.1
ENSG00000091592.16	-3.74E-05	0.996140583	0.999322695	NLRP1
ENSG00000111961.18	1.25E-04	0.996143568	0.999322695	SASH1

ENSG00000178965.14	-6.45E-05	0.99615747	0.999322695	ERICH3
ENSG00000229127.2	-8.56E-05	0.996204076	0.999322695	KANSL1L-AS1
ENSG00000088812.18	-3.84E-05	0.996311995	0.999342287	ATRN
ENSG00000105072.9	5.16E-05	0.996321065	0.999342287	C19orf44
ENSG00000107854.6	3.51E-05	0.996399129	0.999371709	TNKS2
ENSG00000168734.14	-8.27E-05	0.99649564	0.999402696	PKIG
ENSG00000286830.1	3.63E-04	0.996527489	0.999402696	RP11-577H5.6
ENSG00000186715.11	1.41E-04	0.996614246	0.999430089	MST1L
ENSG00000142227.11	-4.85E-05	0.996652269	0.999430089	EMP3
ENSG00000185627.18	-2.99E-05	0.996893437	0.999474074	PSMD13
ENSG00000235191.1	2.03E-04	0.996916098	0.999474074	NUCB1-AS1
ENSG00000230409.3	-6.74E-05	0.997002371	0.999474074	TCEA1P2
ENSG00000258101.2	-5.59E-05	0.99703297	0.999474074	RP11-977B10.2
ENSG00000267149.2	1.45E-04	0.997120028	0.999474074	CTC-550B14.6
ENSG00000166860.3	-2.73E-05	0.997123523	0.999474074	ZBTB39
ENSG00000143570.19	-5.37E-05	0.997147598	0.999474074	SLC39A1
ENSG00000224992.2	1.34E-04	0.997149666	0.999474074	RP11-295G24.5
ENSG00000136536.15	3.52E-05	0.997159396	0.999474074	MARCHF7
ENSG00000229191.1	8.46E-05	0.997229776	0.999474074	RP11-168O16.1
ENSG00000136541.15	3.87E-05	0.997239965	0.999474074	ERMN
ENSG00000198040.11	3.65E-05	0.997280962	0.999474074	ZNF84
ENSG00000259548.1	2.39E-04	0.997477848	0.999555412	RP11-38G5.2
ENSG00000136169.16	-2.21E-05	0.997504073	0.999555412	SETDB2
ENSG00000086548.9	-1.83E-04	0.997543822	0.999555412	CEACAM6
ENSG00000226849.1	-9.52E-05	0.997650471	0.999555412	RP4-635E18.7
ENSG00000135250.17	-2.24E-05	0.997687421	0.999555412	SRPK2
ENSG00000237073.1	-5.14E-05	0.99768789	0.999555412	RP11-168K11.2
ENSG00000204991.11	5.93E-05	0.997714106	0.999555412	SPIRE2
ENSG00000099282.10	-6.96E-05	0.997792567	0.999555412	TSPAN15
ENSG00000254997.4	-3.93E-05	0.99783188	0.999555412	KRTAP5-9
ENSG00000196420.7	-4.92E-05	0.99784952	0.999555412	S100A5
ENSG00000183111.12	1.53E-04	0.998129723	0.999726433	ARHGEF37
ENSG00000140157.16	-1.31E-05	0.998130678	0.999726433	NIPA2
ENSG00000026751.17	3.53E-05	0.998230965	0.999726433	SLAMF7
ENSG00000135253.15	3.15E-05	0.998235554	0.999726433	KCP
ENSG00000145734.19	-3.24E-05	0.998263989	0.999726433	BDP1
ENSG00000189046.11	2.14E-05	0.998428764	0.999842624	ALKBH2
ENSG00000170144.21	1.00E-05	0.998595514	0.999946322	HNRNPA3
ENSG00000158163.15	8.17E-06	0.998651711	0.999946322	DZIP1L
ENSG00000158792.16	-1.95E-05	0.998678592	0.999946322	SPATA2L
ENSG00000109618.13	1.71E-05	0.998784168	0.999957152	SEPSECS
ENSG00000175309.15	9.15E-06	0.998786928	0.999957152	PHYKPL
ENSG00000118369.13	1.08E-05	0.998864332	0.99998583	USP35
ENSG00000155744.10	2.20E-05	0.998954889	1	FAM126B
ENSG00000155093.19	-2.66E-05	0.999066676	1	PTPRN2
ENSG00000274756.1	-9.62E-05	0.999195685	1	CTB-75G16.1
ENSG00000274341.1	8.50E-06	0.999198811	1	RP11-227G15.10

ENSG00000148842.18	7.69E-06	0.999203953	1	CNNM2
ENSG00000257489.6	-6.91E-05	0.99927669	1	RP11-175P13.3
ENSG00000185650.10	-7.54E-06	0.999339276	1	ZFP36L1
ENSG00000259917.1	1.19E-05	0.999394147	1	HNRNPLP2
ENSG00000110756.18	5.02E-06	0.999474826	1	HPS5
ENSG00000196305.19	3.79E-06	0.99949201	1	IARS1
ENSG00000127364.4	-1.88E-05	0.999521247	1	TAS2R4
ENSG00000287097.1	-1.04E-04	0.999536351	1	RP3-383B8.1
ENSG00000230438.7	-9.57E-06	0.999575237	1	SERPINB9P1
ENSG00000266601.1	-3.51E-05	0.999587752	1	RP11-6N17.3
ENSG00000261208.1	-6.81E-05	0.999851213	1	RP11-452D12.1
ENSG00000064607.17	-8.96E-07	0.999872306	1	SUGP2
ENSG00000143514.17	-9.16E-07	0.99989702	1	TP53BP2
ENSG00000100813.15	5.14E-07	0.999906237	1	ACIN1
ENSG00000160193.12	-1.69E-06	0.999914301	1	WDR4
ENSG00000161653.11	1.83E-07	0.999926987	1	NAGS
ENSG00000151962.8	-0.053140165	1	1	RBM46
ENSG00000254132.1	0.030966147	1	1	MTND6P3
ENSG00000100448.4	0.007777136	1	1	CTSG

Supplemental Table 1.2: Association between black carbon and gene expression in the Rotterdam Study

Geneid	logFC	P-value	Adjusted p-value	Gene name
ENSG00000225630.1	0.943631455	2.35E-06	0.048091722	MTND2P28
ENSG00000275004.4	-0.363013805	1.68E-05	0.145734384	ZNF280B
ENSG00000275294.4	-1.171045908	2.44E-05	0.145734384	LINC02340
ENSG00000227678.8	-0.416076814	3.57E-05	0.145734384	RP11-73O6.3
ENSG00000272491.1	0.788865356	3.88E-05	0.145734384	RP5-1024N4.4
ENSG00000142973.14	1.678602463	4.77E-05	0.145734384	CYP4B1
ENSG00000172673.11	-0.378947595	5.39E-05	0.145734384	THEMIS
ENSG00000244921.2	2.632118876	6.59E-05	0.145734384	MTCYBP18
ENSG00000272825.1	0.350853919	7.33E-05	0.145734384	LL21NC02-1C16.2
ENSG00000168026.19	0.441819626	7.53E-05	0.145734384	TTC21A
ENSG00000159189.12	1.011534049	7.82E-05	0.145734384	C1QC
ENSG00000164283.13	-0.961045654	9.77E-05	0.166958687	ESM1
ENSG00000258891.1	-0.853303223	1.29E-04	0.18172552	RP5-1021I20.5
ENSG00000010278.15	0.582332895	1.35E-04	0.18172552	CD9
ENSG00000134324.12	-0.195198378	1.43E-04	0.18172552	LPIN1
ENSG00000169016.17	-0.195555325	1.49E-04	0.18172552	E2F6
ENSG00000144354.14	-0.466932357	1.63E-04	0.18172552	CDCA7
ENSG00000122986.14	0.23285265	1.67E-04	0.18172552	HVCN1
ENSG00000104218.16	-0.19107217	1.68E-04	0.18172552	CSPP1
ENSG00000169398.19	0.28785667	1.84E-04	0.188822247	PTK2
ENSG00000110492.16	0.450853922	2.53E-04	0.233867222	MDK
ENSG00000250909.1	-0.496343877	2.62E-04	0.233867222	RP11-843P14.2
ENSG00000211747.3	0.370834499	2.62E-04	0.233867222	TRBV20-1
ENSG00000011600.12	0.215329713	3.13E-04	0.252040922	TYROBP
ENSG00000205763.13	0.433737122	3.14E-04	0.252040922	RP9P
ENSG00000196323.14	-0.173864441	3.20E-04	0.252040922	ZBTB44
ENSG00000121413.13	0.29694918	3.41E-04	0.259303022	ZSCAN18
ENSG00000101017.14	0.352226204	3.72E-04	0.269563872	CD40
ENSG00000173369.17	0.852195512	3.85E-04	0.269563872	C1QB
ENSG00000155463.14	0.134321117	3.94E-04	0.269563872	OXA1L
ENSG00000126458.4	0.290274236	4.45E-04	0.294332405	RRAS
ENSG00000169246.16	-0.475570399	5.23E-04	0.300688982	NPIPB3
ENSG00000104325.7	0.149109703	5.59E-04	0.300688982	DECR1
ENSG00000288611.1	-1.165482957	5.60E-04	0.300688982	NPBWR1
ENSG00000158517.15	0.298157133	5.78E-04	0.300688982	NCF1
ENSG00000085382.12	-0.291955989	5.88E-04	0.300688982	HACE1
ENSG00000108622.11	0.181562051	6.10E-04	0.300688982	ICAM2
ENSG00000157212.19	-0.15449234	6.36E-04	0.300688982	PAXIP1
ENSG00000255240.6	0.488774042	6.55E-04	0.300688982	RP11-142C4.6
ENSG00000287920.1	-0.4536189	6.69E-04	0.300688982	RP3-510L9.2
ENSG00000143110.12	0.193117118	6.74E-04	0.300688982	C1orf162
ENSG00000162688.17	-0.276350156	6.74E-04	0.300688982	AGL
ENSG00000168994.14	0.376652208	6.82E-04	0.300688982	PXDC1
ENSG00000225556.1	-0.594201227	6.83E-04	0.300688982	C2CD4D

ENSG00000164976.9	-0.353863444	6.87E-04	0.300688982	MYORG
ENSG00000228981.3	0.612454494	7.01E-04	0.300688982	RP11-364L4.1
ENSG00000092068.20	0.770179955	7.24E-04	0.300688982	SLC7A8
ENSG00000077348.9	0.18980775	7.35E-04	0.300688982	EXOSC5
ENSG00000151612.18	-0.24926282	7.67E-04	0.300688982	ZNF827
ENSG00000247853.2	-0.428065884	7.76E-04	0.300688982	RP5-940J5.6
ENSG00000268743.1	-0.620500939	7.92E-04	0.300688982	CTD-2538G9.5
ENSG00000135317.14	-0.214278469	7.96E-04	0.300688982	SNX14
ENSG00000235162.9	-0.343210223	8.50E-04	0.300688982	C12orf75
ENSG00000272799.1	0.520374682	8.55E-04	0.300688982	RP11-474N24.6
ENSG00000167608.12	-0.491178261	8.56E-04	0.300688982	TMC4
ENSG00000236308.1	-0.464370557	8.68E-04	0.300688982	RP11-316M21.7
ENSG00000131069.20	0.153112332	8.91E-04	0.300688982	ACSS2
ENSG00000143036.17	0.799860227	8.92E-04	0.300688982	SLC44A3
ENSG00000097046.13	-0.303349354	9.16E-04	0.300688982	CDC7
ENSG00000272808.4	0.958895616	9.58E-04	0.300688982	RP11-66B24.7
ENSG00000100796.18	-0.105899162	9.60E-04	0.300688982	PPP4R3A
ENSG00000179195.16	-0.135832504	9.64E-04	0.300688982	ZNF664
ENSG00000136490.9	0.168627515	9.68E-04	0.300688982	LIMD2
ENSG00000158793.14	0.107441411	9.93E-04	0.300688982	NIT1
ENSG00000185480.12	-0.416103863	0.00100425	0.300688982	PARPBP
ENSG00000246250.3	-0.708476272	0.001016558	0.300688982	RP11-613D13.5
ENSG00000108387.16	0.595838717	0.001019579	0.300688982	SEPTIN4
ENSG00000119655.11	0.222054925	0.001033996	0.300688982	NPC2
ENSG00000132622.11	-1.301595054	0.001067941	0.300688982	HSPA12B
ENSG00000183255.12	0.108166443	0.001088557	0.300688982	PTTG1IP
ENSG00000280157.1	0.340600744	0.001096709	0.300688982	RP11-520H14.7
ENSG00000231128.6	-0.602429002	0.001103875	0.300688982	RP5-1073O3.2
ENSG00000166866.13	0.378780613	0.001111142	0.300688982	MYO1A
ENSG00000070759.17	0.15586488	0.001158253	0.300688982	TESK2
ENSG00000159433.12	-0.232730025	0.00116092	0.300688982	STARD9
ENSG00000119969.15	-0.283120177	0.001168476	0.300688982	HELLS
ENSG00000126070.20	-0.16214109	0.001173309	0.300688982	AGO3
ENSG00000174501.14	-0.415538992	0.001176698	0.300688982	ANKRD36C
ENSG00000166228.9	0.2240134	0.001208951	0.300688982	PCBD1
ENSG00000165125.22	0.571848388	0.001212862	0.300688982	TRPV6
ENSG00000166822.13	-0.117221801	0.00122356	0.300688982	TMEM170A
ENSG00000163879.11	0.736973151	0.001241524	0.300688982	DNALI1
ENSG00000073050.12	0.1562502	0.001279765	0.300688982	XRCC1
ENSG00000287733.1	0.423864737	0.001280104	0.300688982	RP11-134L10.2
ENSG00000259150.6	1.173217161	0.001295669	0.300688982	LINC00929
ENSG00000185339.9	0.400711627	0.001305149	0.300688982	TCN2
ENSG00000115255.12	0.308902919	0.001314462	0.300688982	REEP6
ENSG00000251330.3	-0.625098069	0.001328659	0.300688982	CTD-2283N19.1
ENSG00000235508.3	0.435120405	0.001329467	0.300688982	RPS2P7
ENSG00000163156.12	0.162824429	0.001365824	0.300688982	SCNM1
ENSG00000265458.2	0.282564509	0.001367432	0.300688982	NARF-AS2

ENSG00000107745.19	0.160816625	0.001380072	0.300688982	MICU1
ENSG00000167850.4	0.244013663	0.001430694	0.300688982	CD300C
ENSG00000105499.14	0.78168263	0.001432466	0.300688982	PLA2G4C
ENSG00000099203.7	0.172643701	0.001439255	0.300688982	TMED1
ENSG00000173372.17	0.598515731	0.001441991	0.300688982	C1QA
ENSG00000108639.8	0.167161756	0.001464789	0.300688982	SYNGR2
ENSG00000110446.12	0.19373344	0.001471257	0.300688982	SLC15A3
ENSG00000047365.12	-0.25663417	0.001508143	0.300688982	ARAP2
ENSG00000126773.13	-0.178347952	0.001511383	0.300688982	PCNX4
ENSG00000164062.13	0.119326293	0.001513588	0.300688982	APEH
ENSG00000211660.3	0.675373089	0.001516527	0.300688982	IGLV2-23
ENSG00000159596.7	0.110690963	0.001518435	0.300688982	TMEM69
ENSG00000172840.7	-0.175582438	0.001549485	0.300688982	PDP2
ENSG00000134882.16	0.085101158	0.001570507	0.300688982	UBAC2
ENSG00000116786.13	0.137781779	0.001580862	0.300688982	PLEKHM2
ENSG00000115998.8	0.117284746	0.001612558	0.300688982	C2orf42
ENSG00000123815.13	0.124468953	0.001613086	0.300688982	COQ8B
ENSG00000146677.7	0.462232602	0.001615556	0.300688982	RPL32P18
ENSG00000123201.14	1.046153617	0.00161971	0.300688982	GUCY1B2
ENSG00000101363.13	0.133618149	0.001628507	0.300688982	MANBAL
ENSG00000078081.8	0.757148157	0.001669116	0.300688982	LAMP3
ENSG00000160233.8	0.323691486	0.001705504	0.300688982	LRRC3
ENSG00000187024.15	-0.903493465	0.001713732	0.300688982	PTRH1
ENSG00000255760.2	0.305083352	0.001739341	0.300688982	LINC02422
ENSG00000177311.12	-0.261713482	0.00174376	0.300688982	ZBTB38
ENSG00000198324.14	0.205958283	0.001744704	0.300688982	PHETA1
ENSG00000001631.16	-0.185937215	0.001763794	0.300688982	KRIT1
ENSG00000162430.18	0.154668312	0.001793425	0.300688982	SELENON
ENSG00000158773.14	0.135108744	0.00179476	0.300688982	USF1
ENSG00000048405.10	-0.123737688	0.001832198	0.300688982	ZNF800
ENSG00000172273.13	0.102681461	0.001860759	0.300688982	HINFP
ENSG00000171827.11	-0.20822821	0.001868842	0.300688982	ZNF570
ENSG00000139842.15	-0.101501707	0.001888701	0.300688982	CUL4A
ENSG00000104894.12	0.175237362	0.00189599	0.300688982	CD37
ENSG00000149476.16	0.160442279	0.00190065	0.300688982	TKFC
ENSG00000263916.1	-0.455493512	0.001914057	0.300688982	RP11-110H1.4
ENSG00000259098.1	1.380902531	0.001915837	0.300688982	RP11-603B24.2
ENSG00000155850.8	-0.205243245	0.001931072	0.300688982	SLC26A2
ENSG00000219747.1	0.869243678	0.001934902	0.300688982	RPL32P16
ENSG00000182718.18	0.189500231	0.001934983	0.300688982	ANXA2
ENSG00000163154.6	0.155756422	0.001943447	0.300688982	TNFAIP8L2
ENSG00000138621.12	0.185484825	0.00195005	0.300688982	PPCDC
ENSG00000237214.3	0.432306402	0.001981201	0.302845689	RP11-12M9.3
ENSG00000188460.4	0.649942076	0.00200137	0.302845689	ACTBP11
ENSG00000148835.11	-0.129906702	0.002012147	0.302845689	TAF5
ENSG00000175800.5	0.567633254	0.002023106	0.302845689	OR52B3P
ENSG00000125430.9	-0.189429545	0.002052398	0.305004143	HS3ST3B1

ENSG00000019169.11	0.51213498	0.002079409	0.306795171	MARCO
ENSG00000118960.13	0.138443942	0.002144758	0.307867107	HS1BP3
ENSG00000113088.6	-0.473217037	0.002154993	0.307867107	GZMK
ENSG00000151835.17	-0.232556401	0.002174815	0.307867107	SACS
ENSG00000255920.3	-0.495022604	0.002176504	0.307867107	CCND2-AS1
ENSG00000226976.3	0.33698363	0.002180845	0.307867107	COX6A1P2
ENSG00000287644.1	0.400870147	0.00220586	0.307867107	CTD-2267D19.8
ENSG00000204231.11	0.100523558	0.002219481	0.307867107	RXRB
ENSG00000117305.15	0.130883771	0.002221538	0.307867107	HMGCL
ENSG00000253676.1	0.323927845	0.002221783	0.307867107	TAGLN2P1
ENSG00000255776.1	-0.736784113	0.002283252	0.311803165	VDAC2P2
ENSG00000231889.7	-0.237276157	0.002283342	0.311803165	TRAF3IP2-AS1
ENSG00000160352.16	-0.354334019	0.002307291	0.311803165	ZNF714
ENSG00000133641.18	-0.269185817	0.002312455	0.311803165	C12orf29
ENSG00000268858.2	0.144010514	0.002334422	0.311803165	RP4-591C20.9
ENSG00000163719.20	0.117771987	0.002341412	0.311803165	MTMR14
ENSG00000269720.2	0.700752364	0.002379439	0.314822811	CCDC194
ENSG00000170264.13	-0.428241193	0.002440891	0.317680572	FAM161A
ENSG00000131459.13	-0.909098012	0.002454997	0.317680572	GFPT2
ENSG00000101457.13	0.148171613	0.002459252	0.317680572	DNTTIP1
ENSG00000154473.18	-0.099489233	0.002465761	0.317680572	BUB3
ENSG00000280828.1	-0.544645792	0.002490689	0.317680572	RP11-274B21.1
ENSG00000054116.12	0.112653025	0.002501595	0.317680572	TRAPPC3
ENSG00000134531.10	0.436212088	0.002509472	0.317680572	EMP1
ENSG00000172987.13	-1.066630601	0.002563814	0.321002317	HPSE2
ENSG00000259212.1	-0.652938376	0.002594713	0.321002317	CTD-3065B20.2
ENSG00000109113.20	0.246661847	0.002606277	0.321002317	RAB34
ENSG00000234663.7	-0.280379291	0.002616057	0.321002317	LINC01934
ENSG00000160948.14	0.196384316	0.002620461	0.321002317	VPS28
ENSG00000223496.3	-0.165733322	0.002632784	0.321002317	EXOSC6
ENSG00000258057.6	-0.325527674	0.002649259	0.321002317	BCDIN3D-AS1
ENSG00000176371.14	-0.136801282	0.002673639	0.321002317	ZSCAN2
ENSG00000212694.8	0.198827621	0.002676585	0.321002317	LINC01089
ENSG00000151445.16	0.099379182	0.00273936	0.326620938	VIPAS39
ENSG00000198400.13	0.613393179	0.002768905	0.32677487	NTRK1
ENSG00000188000.6	-2.647889222	0.002782381	0.32677487	OR7D2
ENSG00000254531.1	0.273883013	0.002794449	0.32677487	FLJ20021
ENSG00000278238.1	0.698549332	0.002839533	0.32677487	RP11-245D16.4
ENSG00000122966.17	-0.347860334	0.00290302	0.32677487	CIT
ENSG00000228804.5	-0.670550499	0.002930193	0.32677487	RP11-211G3.3
ENSG00000111644.8	0.308617001	0.002938455	0.32677487	ACRBP
ENSG00000279277.2	-0.246879628	0.002942111	0.32677487	RP11-861A13.2
ENSG00000182179.13	0.141457921	0.00298576	0.32677487	UBA7
ENSG00000165140.12	0.219220656	0.002990856	0.32677487	FBP1
ENSG00000089327.15	0.188021327	0.002996622	0.32677487	FXYD5
ENSG00000240891.8	-0.270979803	0.002997842	0.32677487	PLCXD2
ENSG00000188677.15	0.249421253	0.003004247	0.32677487	PARVB

ENSG00000122188.13	-0.212800299	0.003040016	0.32677487	LAX1
ENSG00000187260.16	0.257843678	0.003040369	0.32677487	WDR86
ENSG00000130653.16	0.313474458	0.003048706	0.32677487	PNPLA7
ENSG00000132600.17	0.12766159	0.003052004	0.32677487	PRMT7
ENSG00000130433.8	0.564839177	0.003087156	0.32677487	CACNG6
ENSG00000175054.16	-0.138740248	0.003089392	0.32677487	ATR
ENSG00000126262.5	0.24762443	0.003165207	0.32677487	FFAR2
ENSG00000165475.15	0.15281081	0.003191851	0.32677487	CRYL1
ENSG00000244720.1	-0.636852036	0.003218904	0.32677487	NT5C3AP2
ENSG00000080839.12	-0.154751779	0.003238277	0.32677487	RBL1
ENSG00000106701.13	-0.357305256	0.003270145	0.32677487	FSD1L
ENSG00000019582.16	0.210933967	0.003272058	0.32677487	CD74
ENSG00000259793.1	0.592406248	0.003286257	0.32677487	RP11-400N9.1
ENSG00000248124.8	-0.186690894	0.003304932	0.32677487	RRN3P1
ENSG00000160593.19	0.129987205	0.003316619	0.32677487	JAML
ENSG00000106077.19	0.190719649	0.003317381	0.32677487	ABHD11
ENSG00000105649.10	0.224003002	0.003416321	0.32677487	RAB3A
ENSG00000267279.2	0.477804649	0.003440084	0.32677487	RP11-879F14.2
ENSG00000226686.8	0.854349224	0.003456566	0.32677487	LINC01535
ENSG00000237886.1	0.327376904	0.003474771	0.32677487	NALT1
ENSG00000112242.15	-0.20458246	0.003475564	0.32677487	E2F3
ENSG00000163389.12	-0.134641937	0.003479555	0.32677487	POGLUT1
ENSG00000164082.15	-0.390270971	0.003484355	0.32677487	GRM2
ENSG00000121864.10	-0.15210948	0.00348739	0.32677487	ZNF639
ENSG00000160325.15	0.20431142	0.003506067	0.32677487	CACFD1
ENSG00000104979.9	0.172040193	0.003509666	0.32677487	C19orf53
ENSG00000286545.1	-0.392974865	0.00351301	0.32677487	RP11-399B17.2
ENSG00000229325.1	-0.490391932	0.003531492	0.32677487	ACAP2-IT1
ENSG00000124762.14	0.281092432	0.003558761	0.32677487	CDKN1A
ENSG00000277496.1	0.184882643	0.003569111	0.32677487	RP11-93B14.9
ENSG00000278259.5	-0.126113746	0.003591024	0.32677487	MYO19
ENSG00000137814.12	-0.127432098	0.003633047	0.32677487	HAUS2
ENSG00000134905.17	0.141839583	0.0036574	0.32677487	CARS2
ENSG00000174791.11	0.278250853	0.003663186	0.32677487	RIN1
ENSG00000172766.19	-0.171505246	0.003679709	0.32677487	NAA16
ENSG00000260417.1	-0.722235799	0.003687856	0.32677487	CTD-2542L18.1
ENSG00000066279.19	-0.548073963	0.003726206	0.32677487	ASPM
ENSG00000204922.5	0.197636872	0.003730235	0.32677487	UQCC3
ENSG00000163558.13	-0.182031474	0.003758271	0.32677487	PRKCI
ENSG00000204576.12	0.102092536	0.003764304	0.32677487	PRR3
ENSG00000240671.4	0.692147681	0.003785302	0.32677487	IGKV1-8
ENSG00000153933.10	-0.163482234	0.003795865	0.32677487	DGKE
ENSG00000233223.3	0.235316616	0.003802113	0.32677487	AC113189.5
ENSG00000211946.3	0.8656652	0.003807572	0.32677487	IGHV3-20
ENSG00000263590.2	-2.365821955	0.003823729	0.32677487	LINC02591
ENSG00000163378.14	-0.195813146	0.0038437	0.32677487	EOGT
ENSG00000147601.15	-0.120121072	0.003847895	0.32677487	TERF1

ENSG00000138942.16	0.119230194	0.003879714	0.32677487	RNF185
ENSG00000169908.12	0.983020349	0.003920856	0.32677487	TM4SF1
ENSG00000138180.16	-0.519489764	0.003921159	0.32677487	CEP55
ENSG00000182013.18	-2.38793993	0.00393626	0.32677487	PNMA8A
ENSG00000130529.16	0.392868255	0.00394761	0.32677487	TRPM4
ENSG00000175482.9	0.145381744	0.0039847	0.32677487	POLD4
ENSG00000124209.4	-0.100436182	0.004024349	0.32677487	RAB22A
ENSG00000211640.4	0.820237253	0.004032958	0.32677487	IGLV6-57
ENSG00000183250.12	0.230335044	0.004041108	0.32677487	LINC01547
ENSG00000204386.12	0.119837825	0.00406671	0.32677487	NEU1
ENSG00000247828.8	-0.170055221	0.004073025	0.32677487	TMEM161B-AS1
ENSG00000234630.1	-0.65791206	0.004076376	0.32677487	LL22NC03-2H8.4
ENSG00000211651.3	0.692756148	0.004082269	0.32677487	IGLV1-44
ENSG00000124120.11	-0.110021746	0.004100564	0.32677487	TTPAL
ENSG00000162676.12	-0.323253291	0.00411834	0.32677487	GFI1
ENSG00000115204.15	0.103187447	0.004121002	0.32677487	MPV17
ENSG00000242766.1	-1.767733543	0.004135755	0.32677487	IGKV1D-17
ENSG00000107938.18	-0.125223947	0.004138	0.32677487	EDRF1
ENSG00000230438.7	0.295804114	0.004205617	0.32677487	SERPINB9P1
ENSG00000240382.3	0.652315759	0.00424933	0.32677487	IGKV1-17
ENSG00000162511.8	0.112900352	0.004253147	0.32677487	LAPTM5
ENSG00000170627.11	0.42713941	0.004266199	0.32677487	GTSF1
ENSG00000232630.1	-0.392848289	0.004273922	0.32677487	PRPS1P2
ENSG00000123353.10	0.148472249	0.004276267	0.32677487	ORMDL2
ENSG00000278390.5	-0.384068522	0.004283293	0.32677487	RP11-74J13.8
ENSG00000052723.12	-0.161600092	0.004299818	0.32677487	SIKE1
ENSG00000257221.4	0.35365013	0.004310364	0.32677487	RP11-689B22.2
ENSG00000275344.1	0.511803879	0.004315381	0.32677487	MIR6503
ENSG00000146433.10	-0.1965919	0.004316674	0.32677487	TMEM181
ENSG00000079459.13	0.130682872	0.004338681	0.32677487	FDFT1
ENSG00000215375.6	0.21195284	0.00434004	0.32677487	MYL5
ENSG00000270755.1	0.529188951	0.004348776	0.32677487	RP11-138E2.1
ENSG00000146540.15	0.209720677	0.004359832	0.32677487	C7orf50
ENSG00000109016.17	0.141282766	0.004365379	0.32677487	DHRS7B
ENSG00000260461.1	-0.542220118	0.004373086	0.32677487	RP11-541N10.3
ENSG00000229769.2	-0.818464494	0.004387398	0.32677487	TRBV10-2
ENSG00000261499.2	-1.156432077	0.004390745	0.32677487	CH17-260O16.1
ENSG00000139977.14	-0.151546096	0.004394745	0.32677487	NAA30
ENSG00000101190.13	-0.159693143	0.004417835	0.32677487	TCFL5
ENSG00000116586.12	0.173639667	0.00443546	0.32677487	LAMTOR2
ENSG00000100029.18	0.143993603	0.004443858	0.32677487	PES1
ENSG00000108604.16	0.114372141	0.004450244	0.32677487	SMARCD2
ENSG00000119004.16	-0.119516871	0.004455775	0.32677487	CYP20A1
ENSG00000169189.17	0.166016702	0.00446345	0.32677487	NSMCE1
ENSG00000159403.18	0.255225422	0.004465601	0.32677487	C1R
ENSG00000178184.16	-0.374415777	0.004521447	0.32677487	PARD6G
ENSG00000079332.15	-0.074117897	0.004566485	0.32677487	SAR1A

ENSG00000196422.11	0.215637256	0.004586976	0.32677487	PPP1R26
ENSG00000185088.14	-0.231897696	0.004609732	0.32677487	RPS27L
ENSG00000052126.15	-0.382032182	0.004611665	0.32677487	PLEKHA5
ENSG00000255189.1	0.654125188	0.004617513	0.32677487	GLYATL1P1
ENSG00000172432.19	0.130194607	0.004673629	0.32677487	GTPBP2
ENSG00000258515.1	-0.333169095	0.004677263	0.32677487	RP11-203M5.7
ENSG00000174744.14	0.135784465	0.00467797	0.32677487	BRMS1
ENSG00000244038.11	0.102102482	0.004679849	0.32677487	DDOST
ENSG00000152785.8	0.994263769	0.004717862	0.32677487	BMP3
ENSG00000103490.14	0.198676416	0.004719601	0.32677487	PYCARD
ENSG00000167904.15	-0.207752349	0.004753494	0.32677487	TMEM68
ENSG00000171953.16	0.133825405	0.00475843	0.32677487	ATPAF2
ENSG00000167984.18	-0.130807796	0.004762223	0.32677487	NLRC3
ENSG00000251706.1	-0.682459304	0.0047764	0.32677487	RNU7-61P
ENSG00000211679.2	0.666063278	0.00481083	0.32677487	IGLC3
ENSG00000111679.17	0.143608615	0.004827232	0.32677487	PTPN6
ENSG00000066654.14	-0.154512369	0.004834714	0.32677487	THUMPD1
ENSG00000103274.11	0.147300624	0.004853754	0.32677487	NUBP1
ENSG00000117016.10	0.183748765	0.00486536	0.32677487	RIMS3
ENSG00000197520.10	0.442551473	0.004897979	0.32677487	FAM177B
ENSG00000134242.16	-0.211203339	0.004924764	0.32677487	PTPN22
ENSG00000164074.16	-0.155957177	0.004931739	0.32677487	ABHD18
ENSG00000147853.17	-0.139258566	0.00494081	0.32677487	AK3
ENSG00000223534.1	0.668047637	0.004952104	0.32677487	HLA-DQB1-AS1
ENSG00000257108.2	0.235122971	0.004960318	0.32677487	NHLRC4
ENSG00000204257.15	0.193768818	0.004967143	0.32677487	HLA-DMA
ENSG00000230303.6	-0.545041308	0.004973673	0.32677487	RP11-213G2.2
ENSG00000144820.8	-4.34193966	0.00498864	0.32677487	ADGRG7
ENSG00000198520.12	0.231833341	0.005019432	0.32677487	ARMH1
ENSG00000147872.10	0.161870104	0.005022932	0.32677487	PLIN2
ENSG00000115084.15	-0.154232142	0.005042674	0.32677487	SLC35F5
ENSG00000163945.19	-0.194928261	0.005045918	0.32677487	UVSSA
ENSG00000232656.9	-0.406771306	0.005064837	0.32677487	IDI2-AS1
ENSG00000139537.11	-0.180294177	0.005081144	0.32677487	CCDC65
ENSG00000198598.7	0.32614364	0.005099578	0.32677487	MMP17
ENSG00000228382.1	0.268755136	0.005145045	0.32677487	ITPKB-IT1
ENSG00000211815.3	0.463357423	0.005158293	0.32677487	TRAV36DV7
ENSG00000261019.1	-0.529319088	0.005162282	0.32677487	RP11-111K18.2
ENSG00000211952.3	-0.748498468	0.005164261	0.32677487	IGHV4-28
ENSG00000143590.14	0.257066319	0.005167999	0.32677487	EFNA3
ENSG00000251136.9	-0.233119456	0.005191966	0.32677487	RP11-37B2.1
ENSG00000258904.1	-0.509040284	0.00520334	0.32677487	RP11-638I2.2
ENSG00000171617.15	-0.455676405	0.005230449	0.32677487	ENC1
ENSG00000183625.16	0.388162571	0.005296918	0.32677487	CCR3
ENSG00000205791.3	0.575333082	0.005310756	0.32677487	LOH12CR2
ENSG00000110442.12	0.126352511	0.005317967	0.32677487	COMMD9
ENSG00000197363.10	0.132737664	0.005356803	0.32677487	ZNF517

ENSG00000167264.18	0.153591124	0.005366505	0.32677487	DUS2
ENSG00000025772.8	0.170405423	0.005375674	0.32677487	TOMM34
ENSG00000182150.19	-0.119808633	0.005400125	0.32677487	ERCC6L2
ENSG00000213399.3	0.500970564	0.005422536	0.32677487	RPS2P17
ENSG00000200087.1	-0.822559277	0.005437962	0.32677487	SNORA73B
ENSG00000162882.15	0.213808501	0.00544126	0.32677487	HAAO
ENSG00000119402.18	-0.08804582	0.005476486	0.32677487	FBXW2
ENSG00000162694.14	-0.251850902	0.005488636	0.32677487	EXTL2
ENSG00000088298.13	0.112391186	0.005496833	0.32677487	EDEM2
ENSG00000276672.1	-0.364998309	0.005498395	0.32677487	RP11-142E9.1
ENSG00000137161.17	0.139463085	0.005507904	0.32677487	CNPY3
ENSG00000237491.10	-0.288522932	0.005524033	0.32677487	LINC01409
ENSG00000243547.1	0.353317406	0.00553196	0.32677487	HNRNPKP4
ENSG00000286952.1	0.474711712	0.005533134	0.32677487	RP11-241K3.5
ENSG00000164733.22	0.169051545	0.00553873	0.32677487	CTSB
ENSG00000221817.10	-0.13213401	0.005557041	0.32677487	PPP3CB-AS1
ENSG00000105053.11	0.12020911	0.005562892	0.32677487	VRK3
ENSG00000149311.20	-0.1846092	0.00557842	0.32677487	ATM
ENSG00000133943.21	0.135374337	0.005581267	0.32677487	DGLUCY
ENSG00000131203.13	0.531550081	0.005607965	0.32677487	IDO1
ENSG00000160602.14	0.177835653	0.005631948	0.32677487	NEK8
ENSG00000260518.2	2.258085976	0.005638212	0.32677487	BMS1P8
ENSG00000121644.19	-0.144836711	0.005641811	0.32677487	DESI2
ENSG00000205155.8	0.200138929	0.005648658	0.32677487	PSENEN
ENSG00000242086.8	-0.279346996	0.005662627	0.32677487	MUC20-OT1
ENSG00000159479.17	0.09793449	0.005667599	0.32677487	MED8
ENSG00000270240.2	-0.559120788	0.005667795	0.32677487	AC015849.2
ENSG00000170891.11	0.598982656	0.005679521	0.32677487	CYTL1
ENSG00000114745.14	0.107530135	0.005699368	0.32677487	GORASP1
ENSG00000259330.3	0.125009168	0.005717771	0.32677487	INAFM2
ENSG00000142252.11	0.189656565	0.005727706	0.32677487	GEMIN7
ENSG00000257086.1	0.513826998	0.005746953	0.32677487	RP11-783K16.13
ENSG00000151552.12	0.199852863	0.0057482	0.32677487	QDPR
ENSG00000172667.11	-0.136586566	0.005752632	0.32677487	ZMAT3
ENSG00000105866.15	-0.197852338	0.00576147	0.32677487	SP4
ENSG00000268401.2	-0.469451528	0.00579157	0.32677487	CTC-344H19.4
ENSG00000240376.1	0.432367012	0.005812037	0.32677487	RP11-36C20.1
ENSG00000271789.1	-0.489449352	0.00582116	0.32677487	RP5-1112D6.7
ENSG00000137145.21	-0.190940628	0.005828014	0.32677487	DENND4C
ENSG00000106415.13	-0.184635921	0.005858973	0.32677487	GLCCI1
ENSG00000223705.9	0.190033349	0.005859009	0.32677487	NSUN5P1
ENSG00000105048.17	0.674125761	0.005875136	0.32677487	TNNT1
ENSG00000281912.1	0.388423039	0.005879653	0.32677487	LINC01144
ENSG00000107669.18	-0.155629736	0.005903601	0.326909336	ATE1
ENSG00000231731.7	-0.239761892	0.005954098	0.326909336	AC010976.2
ENSG00000213398.8	0.154872884	0.005962331	0.326909336	LCAT
ENSG00000184787.19	-0.076651603	0.005962418	0.326909336	UBE2G2

ENSG00000121579.13	-0.125136141	0.00599064	0.326909336	NAA50
ENSG00000162775.17	-0.120554299	0.005997007	0.326909336	RBM15
ENSG00000145358.6	-0.91562468	0.006028668	0.326909336	DDIT4L
ENSG00000122417.15	-0.188613457	0.006031542	0.326909336	ODF2L
ENSG00000185201.17	0.232970474	0.006044092	0.326909336	IFITM2
ENSG00000125869.10	0.382021862	0.006069846	0.326909336	LAMP5
ENSG00000114857.18	-0.164309941	0.006071665	0.326909336	NKTR
ENSG00000237356.7	-0.373446573	0.00607336	0.326909336	AL163953.3
ENSG00000152953.13	0.362934217	0.006101596	0.327103511	STK32B
ENSG00000166863.13	0.757682972	0.006108867	0.327103511	TAC3
ENSG00000285979.1	-0.308911019	0.006175512	0.329810927	RP11-407G23.8
ENSG00000177692.12	-0.393116185	0.006205157	0.330140892	DNAJC28
ENSG00000117419.16	0.14146611	0.006213886	0.330140892	ERI3
ENSG00000006534.16	0.159612049	0.006241872	0.330549827	ALDH3B1
ENSG00000102780.17	-0.260976625	0.00625382	0.330549827	DGKH
ENSG00000101473.17	0.115635436	0.006331575	0.331607523	ACOT8
ENSG00000053524.13	-0.336154733	0.006347866	0.331607523	MCF2L2
ENSG00000172183.16	0.215301578	0.006348813	0.331607523	ISG20
ENSG00000232940.6	-0.264826829	0.006353656	0.331607523	HCG25
ENSG00000050730.16	-0.543707129	0.006398757	0.331607523	TNIP3
ENSG00000173875.14	-0.104330356	0.006413685	0.331607523	ZNF791
ENSG00000172345.14	0.218254573	0.006417173	0.331607523	STARD5
ENSG00000133612.19	0.14312913	0.006436176	0.331607523	AGAP3
ENSG00000228929.1	0.265437581	0.006446099	0.331607523	RPS13P2
ENSG00000237980.2	0.679107512	0.006446127	0.331607523	LINC02773
ENSG00000173171.15	0.134877122	0.006451697	0.331607523	MTX1
ENSG00000172725.14	0.133915086	0.006488485	0.332513487	CORO1B
ENSG00000274184.1	-0.436681352	0.006516992	0.332513487	RP11-158H5.8
ENSG00000243056.2	0.216030023	0.006528346	0.332513487	EIF4EBP3
ENSG00000198081.11	-0.13143606	0.006534179	0.332513487	ZBTB14
ENSG00000109065.12	0.120662498	0.00656412	0.333170273	NAT9
ENSG00000142731.11	-0.333695097	0.006614372	0.333170273	PLK4
ENSG00000106078.19	0.644366247	0.006624733	0.333170273	COBL
ENSG00000148335.15	0.134923444	0.006628062	0.333170273	NTMT1
ENSG00000278740.1	-0.46543753	0.006633042	0.333170273	RP11-147L13.14
ENSG00000151292.17	-0.220373069	0.00664456	0.333170273	CSNK1G3
ENSG00000143994.14	-0.270995872	0.006675309	0.333895688	ABHD1
ENSG00000108107.15	0.205700026	0.006720799	0.334513773	RPL28
ENSG00000083520.15	-0.145251803	0.00673129	0.334513773	DIS3
ENSG00000134852.15	-0.164383999	0.006747666	0.334513773	CLOCK
ENSG00000108854.16	-0.133905813	0.006752911	0.334513773	SMURF2
ENSG00000196312.15	-0.136182026	0.006787217	0.335402998	MFSD14C
ENSG00000126226.22	-0.106573946	0.006831104	0.336760307	PCID2
ENSG00000185607.5	0.489250652	0.006875407	0.337210982	ACTBP7
ENSG00000138182.15	-0.262167488	0.006882482	0.337210982	KIF20B
ENSG00000285737.1	-0.442423305	0.006903849	0.337210982	LINC02680
ENSG00000202538.1	-1.992497813	0.006906018	0.337210982	RNU4-2

ENSG00000258926.2	-0.451253802	0.00692364	0.337258804	RP11-47I22.1
ENSG00000104805.16	0.177524011	0.006948445	0.337258804	NUCB1
ENSG00000245205.3	0.506805598	0.007012268	0.337258804	EEF1A1P4
ENSG00000178776.5	1.077390893	0.007037933	0.337258804	C5orf46
ENSG00000132603.15	-0.104914032	0.007044428	0.337258804	NIP7
ENSG00000113716.13	0.079357579	0.007045894	0.337258804	HMGXB3
ENSG00000196141.14	0.514777439	0.007063579	0.337258804	SPATS2L
ENSG00000152582.14	-0.220527157	0.007064988	0.337258804	SPEF2
ENSG00000139726.11	-0.112274174	0.007070323	0.337258804	DENR
ENSG00000172301.11	0.208615318	0.007071449	0.337258804	COPRS
ENSG00000185246.18	-0.190275247	0.007103807	0.338015943	PRPF39
ENSG00000138778.13	-0.439306486	0.007127102	0.338339391	CENPE
ENSG00000162738.6	-1.145510614	0.007146016	0.338453787	VANGL2
ENSG00000258682.1	-0.30890994	0.007163864	0.338517338	CTD-2002H8.2
ENSG00000165929.13	-0.210278596	0.007238872	0.339514414	TC2N
ENSG00000287895.1	-0.519008786	0.007270318	0.339514414	RP5-881P19.8
ENSG00000251247.11	-0.187710791	0.007270473	0.339514414	ZNF345
ENSG00000135976.20	-0.325176031	0.007284626	0.339514414	ANKRD36
ENSG00000168591.16	0.089304979	0.00732977	0.339514414	TMUB2
ENSG00000148343.19	0.10044056	0.007331707	0.339514414	MIGA2
ENSG00000109680.11	-0.260386247	0.007331974	0.339514414	TBC1D19
ENSG00000164164.17	-0.154783512	0.007333617	0.339514414	OTUD4
ENSG00000263574.2	-0.692918115	0.007334269	0.339514414	CTD-2532D12.4
ENSG00000170989.10	-0.128104297	0.007369829	0.339514414	S1PR1
ENSG00000281490.1	-0.287033185	0.007396598	0.339514414	CICP14
ENSG00000101213.7	0.236177879	0.007397519	0.339514414	PTK6
ENSG00000139116.19	-0.300333494	0.007404567	0.339514414	KIF21A
ENSG00000114166.8	-0.183857191	0.007416738	0.339514414	KAT2B
ENSG00000089060.12	0.114739656	0.007465868	0.341002273	SLC8B1
ENSG00000223901.2	-0.602605567	0.007543289	0.342849943	AP001469.5
ENSG00000167552.15	0.183476465	0.007557465	0.342849943	TUBA1A
ENSG00000188603.22	0.123610877	0.007568319	0.342849943	CLN3
ENSG00000135740.17	-0.227412717	0.007573192	0.342849943	SLC9A5
ENSG00000178162.8	-0.746207856	0.007608845	0.343510483	FAR2P2
ENSG00000100523.16	-0.15834996	0.007621283	0.343510483	DDHD1
ENSG00000138160.7	-0.280199573	0.007651314	0.344107763	KIF11
ENSG00000130147.16	0.282305891	0.007700257	0.344407519	SH3BP4
ENSG00000198744.5	1.995786532	0.007709317	0.344407519	MTCO3P12
ENSG00000229870.1	0.55572823	0.007711507	0.344407519	RPL21P89
ENSG00000211643.2	0.852923202	0.007747752	0.344407519	IGLV5-52
ENSG00000145022.4	0.111076145	0.007754279	0.344407519	TCTA
ENSG00000224097.5	0.482080698	0.007758742	0.344407519	RP11-472B18.1
ENSG00000175182.15	0.103664729	0.007808638	0.344530496	FAM131A
ENSG00000053918.18	0.139492481	0.007838582	0.344530496	KCNQ1
ENSG00000035499.13	-0.480859051	0.007846175	0.344530496	DEPDC1B
ENSG00000165025.15	0.12215116	0.007852039	0.344530496	SYK
ENSG00000100889.12	0.148936292	0.007892045	0.344530496	PCK2

ENSG00000160410.15	0.214296625	0.007950539	0.344530496	SHKBP1
ENSG00000157191.20	0.105436532	0.007957556	0.344530496	NECAP2
ENSG00000243663.1	0.601246527	0.00798479	0.344530496	RPS4XP14
ENSG00000237286.1	-0.568946574	0.008007641	0.344530496	CARD11-AS1
ENSG00000146263.12	-0.165307789	0.00801798	0.344530496	MMS22L
ENSG00000108443.14	-0.132285634	0.008040402	0.344530496	RPS6KB1
ENSG00000273802.2	0.377818449	0.00807739	0.344530496	H2BC8
ENSG00000153551.14	0.137801909	0.008122746	0.344530496	CMTM7
ENSG00000149131.17	0.653213383	0.008129492	0.344530496	SERPING1
ENSG00000225921.7	0.108572273	0.008141411	0.344530496	NOL7
ENSG00000214029.6	-0.210693439	0.008160732	0.344530496	ZNF891
ENSG00000135587.9	0.14667833	0.008166123	0.344530496	SMPD2
ENSG00000078098.14	-1.054499412	0.008169232	0.344530496	FAP
ENSG00000141543.12	0.114116597	0.008179682	0.344530496	EIF4A3
ENSG00000131408.15	0.136311942	0.008191458	0.344530496	NR1H2
ENSG00000184575.12	-0.103551888	0.008199508	0.344530496	XPOT
ENSG00000153944.12	-0.106037016	0.008202922	0.344530496	MSI2
ENSG00000034677.13	-0.176585088	0.008209149	0.344530496	RNF19A
ENSG00000167107.13	0.169283772	0.008243145	0.344530496	ACSF2
ENSG00000213160.10	-0.374464676	0.008278785	0.344530496	KLHL23
ENSG00000232216.1	0.637072044	0.008295602	0.344530496	IGHV3-43
ENSG00000107874.11	0.146753715	0.008342587	0.344530496	CUEDC2
ENSG00000273759.1	0.24032593	0.008381712	0.344530496	RP4-563E14.1
ENSG00000128283.7	0.466348841	0.008413137	0.344530496	CDC42EP1
ENSG00000162078.11	0.426855035	0.008416228	0.344530496	ZG16B
ENSG00000231246.2	0.467624665	0.008449804	0.344530496	LINC02884
ENSG00000109103.12	0.108460665	0.008452144	0.344530496	UNC119
ENSG00000069702.11	-0.306062895	0.008458885	0.344530496	TGFBR3
ENSG00000248240.1	-0.664723883	0.008462603	0.344530496	RP11-159F24.5
ENSG00000229358.3	-0.62347407	0.008463289	0.344530496	DPY19L1P1
ENSG00000174353.17	-0.19830092	0.008474678	0.344530496	STAG3L3
ENSG00000230068.2	-0.52435676	0.008485419	0.344530496	CDC42-IT1
ENSG00000104324.16	0.145233323	0.00851306	0.344530496	CPQ
ENSG00000165813.20	-0.192340917	0.008532702	0.344530496	CCDC186
ENSG00000241764.4	0.253268154	0.008533446	0.344530496	AC002467.7
ENSG00000131061.14	0.125179027	0.008560899	0.344530496	ZNF341
ENSG00000135916.16	0.232417403	0.008562601	0.344530496	ITM2C
ENSG00000100220.12	0.115743965	0.008564225	0.344530496	RTCB
ENSG00000125814.17	-0.142213662	0.008569373	0.344530496	NAPB
ENSG00000236876.3	0.514293608	0.008574921	0.344530496	TMSB4XP1
ENSG00000171522.6	-0.149933757	0.008608664	0.344530496	PTGER4
ENSG00000251578.1	0.437849042	0.008611635	0.344530496	TRBV21-1
ENSG00000163961.4	-0.113986916	0.008656433	0.344530496	RNF168
ENSG00000229852.4	-0.205630871	0.008668784	0.344530496	RP11-398K22.12
ENSG00000114346.14	-0.26066969	0.00868588	0.344530496	ECT2
ENSG00000171135.15	0.132513469	0.008727876	0.344530496	JAGN1
ENSG00000183087.15	0.236891121	0.008734685	0.344530496	GAS6

ENSG00000231389.7	0.201301158	0.008740372	0.344530496	HLA-DPA1
ENSG00000160446.19	0.160420682	0.00874616	0.344530496	ZDHHC12
ENSG00000169660.17	0.155510748	0.008783173	0.344530496	HEXD
ENSG00000186395.9	0.174489123	0.008801431	0.344530496	KRT10
ENSG00000166548.17	0.120091664	0.008818956	0.344530496	TK2
ENSG00000242372.9	0.155070986	0.008820525	0.344530496	EIF6
ENSG00000128805.15	0.328681882	0.008839678	0.344530496	ARHGAP22
ENSG00000277954.1	-0.634252917	0.00885749	0.344530496	RP11-679B19.1
ENSG00000177042.16	0.140418714	0.008875887	0.344530496	TMEM80
ENSG00000260219.2	0.228688636	0.008891604	0.344530496	CD2BP2-DT
ENSG00000184209.15	0.13202004	0.008893347	0.344530496	SNRNP35
ENSG00000255970.1	-0.522067333	0.00889482	0.344530496	LINC02421
ENSG00000243406.6	-0.387860216	0.008899519	0.344530496	MRPS31P5
ENSG00000173889.16	-0.168605803	0.008981409	0.344530496	PHC3
ENSG00000280721.1	-0.390907987	0.008997539	0.344530496	LINC01943
ENSG00000168283.14	-0.185818771	0.00902714	0.344530496	BMI1
ENSG00000172159.16	0.36621845	0.009055425	0.344530496	FRMD3
ENSG00000111537.5	-0.515666716	0.009092222	0.344530496	IFNG
ENSG00000139636.16	0.088143448	0.009125626	0.344530496	LMBR1L
ENSG00000164073.11	-0.128840022	0.009140521	0.344530496	MFSD8
ENSG00000159761.15	0.183039774	0.009156801	0.344530496	C16orf86
ENSG00000153147.6	-0.130617368	0.00916879	0.344530496	SMARCA5
ENSG00000053501.13	0.149949387	0.00918092	0.344530496	USE1
ENSG00000244041.7	0.204740957	0.009188443	0.344530496	LINC01011
ENSG00000119737.6	-0.404279347	0.009205384	0.344530496	GPR75
ENSG00000273076.1	-0.311229157	0.009227338	0.344530496	RP3-508I15.22
ENSG00000242375.1	-0.823890167	0.009234607	0.344530496	RP11-498P14.3
ENSG00000132694.19	0.138285756	0.009254126	0.344530496	ARHGEF11
ENSG00000135968.21	-0.200600939	0.009291983	0.344530496	GCC2
ENSG00000151690.15	-0.138632615	0.009304321	0.344530496	MFSD6
ENSG00000225972.1	0.72071306	0.009313736	0.344530496	MTND1P23
ENSG00000108679.13	0.32390631	0.009329259	0.344530496	LGALS3BP
ENSG00000172575.12	-0.149783474	0.009330125	0.344530496	RASGRP1
ENSG00000103066.13	0.154697018	0.009448122	0.344530496	PLA2G15
ENSG00000178234.13	-0.107494995	0.009469548	0.344530496	GALNT11
ENSG00000158715.6	0.49123081	0.009514625	0.344530496	SLC45A3
ENSG00000143228.13	-0.405478705	0.009547597	0.344530496	NUF2
ENSG00000251593.1	0.404021938	0.00955255	0.344530496	MSNP1
ENSG00000187653.11	0.248862012	0.009571638	0.344530496	TMSB4XP8
ENSG00000144843.11	0.172689911	0.009582103	0.344530496	ADPRH
ENSG00000188404.10	0.182728922	0.009582597	0.344530496	SELL
ENSG00000255987.1	-0.301705864	0.009585211	0.344530496	TOMM20P2
ENSG00000122481.17	-0.27162524	0.009642418	0.344530496	RWDD3
ENSG00000189308.11	-0.152398392	0.009646995	0.344530496	LIN54
ENSG00000280287.1	-0.232708891	0.009647338	0.344530496	RP13-554M15.7
ENSG00000065054.14	0.259846099	0.009651635	0.344530496	SLC9A3R2
ENSG00000023902.14	0.139295246	0.009668368	0.344530496	PLEKHO1

ENSG00000180385.8	-0.291439094	0.009678901	0.344530496	EMC3-AS1
ENSG00000155903.13	-0.185255116	0.009682662	0.344530496	RASA2
ENSG00000204301.6	0.27047669	0.009689611	0.344530496	NOTCH4
ENSG00000167468.18	0.178503574	0.009696326	0.344530496	GPX4
ENSG00000231494.1	-0.518981041	0.009736464	0.344530496	RPL21P35
ENSG00000137656.12	0.081417901	0.009745988	0.344530496	BUD13
ENSG00000225544.1	0.956568863	0.009762978	0.344530496	KB-1027C11.4
ENSG00000198502.6	0.403870662	0.00980087	0.344530496	HLA-DRB5
ENSG00000127483.19	-0.09435245	0.009835923	0.344530496	HP1BP3
ENSG00000237493.3	-0.546135343	0.009858202	0.344530496	RP11-603J24.7
ENSG00000121577.14	0.17271958	0.009889739	0.344530496	POPDC2
ENSG00000248544.2	-0.333641317	0.009927559	0.344530496	CTB-47B11.3
ENSG00000185129.8	-0.136747728	0.00993491	0.344530496	PURA
ENSG00000168002.12	0.117472122	0.009946599	0.344530496	POLR2G
ENSG00000261857.7	0.538375746	0.009963879	0.344530496	MIA
ENSG00000235859.5	-0.134970257	0.009971172	0.344530496	AC006978.6
ENSG00000111348.9	0.119803912	0.009973342	0.344530496	ARHGDIB
ENSG00000126838.10	-0.390867901	0.009983044	0.344530496	PZP
ENSG00000162695.12	-0.154787215	0.010037833	0.344530496	SLC30A7
ENSG00000066322.15	0.105791119	0.010047955	0.344530496	ELOVL1
ENSG00000172500.13	0.134545376	0.01012231	0.344530496	FIBP
ENSG00000114423.23	-0.135977081	0.010143778	0.344530496	CBLB
ENSG00000170266.16	0.118246159	0.010151647	0.344530496	GLB1
ENSG00000285578.1	0.523845602	0.010175468	0.344530496	RP3-416J7.6
ENSG00000112144.16	-0.144566333	0.010182541	0.344530496	CILK1
ENSG00000163320.12	-0.15051496	0.010216954	0.344530496	CGGBP1
ENSG00000074603.19	-0.116181207	0.010237284	0.344530496	DPP8
ENSG00000250535.1	1.224261024	0.010252157	0.344530496	STK19B
ENSG00000125885.13	-0.188076084	0.010263743	0.344530496	MCM8
ENSG00000106346.11	-0.117486937	0.010274842	0.344530496	USP42
ENSG00000276968.1	0.476721673	0.010296295	0.344530496	RP11-90M2.5
ENSG00000204287.14	0.1839129	0.0102998	0.344530496	HLA-DRA
ENSG00000115685.15	0.113323039	0.01032469	0.344530496	PPP1R7
ENSG00000287771.1	-0.434970306	0.01032919	0.344530496	RP11-4B12.1
ENSG00000089248.7	0.124799932	0.010366716	0.344530496	ERP29
ENSG00000135047.16	0.313595028	0.010393688	0.344530496	CTSL
ENSG00000168298.7	-0.717047918	0.010398704	0.344530496	H1-4
ENSG00000229390.1	0.677632189	0.010424158	0.344530496	MICD
ENSG00000069956.13	-0.187199757	0.010468247	0.344530496	MAPK6
ENSG00000177034.17	-0.16921746	0.010489364	0.344530496	MTX3
ENSG00000174840.9	-0.088603051	0.010494864	0.344530496	PDE12
ENSG00000111725.11	0.085946834	0.010500453	0.344530496	PRKAB1
ENSG00000211976.2	0.726921039	0.010508227	0.344530496	IGHV3-73
ENSG00000186469.8	-0.155012926	0.010527656	0.344530496	GNG2
ENSG00000135617.4	0.173950839	0.01054015	0.344530496	PRADC1
ENSG00000001084.13	-0.125632546	0.010551557	0.344530496	GCLC
ENSG00000237476.1	0.334119909	0.010558626	0.344530496	LINC01637

ENSG00000112210.12	-0.399919403	0.010589913	0.344530496	RAB23
ENSG00000026025.16	0.16253453	0.010610658	0.344530496	VIM
ENSG00000081019.13	-0.134707193	0.010628577	0.344530496	RSBN1
ENSG00000279568.1	-0.506731256	0.010661622	0.344530496	RP11-20I23.5
ENSG00000139200.14	0.285106568	0.010676887	0.344530496	PIANP
ENSG00000120662.16	-0.138970997	0.010694623	0.344530496	MTRF1
ENSG00000251819.1	-0.548445797	0.010695702	0.344530496	RNU6-322P
ENSG00000261609.8	-0.15169685	0.010746807	0.344530496	GAN
ENSG00000131778.19	-0.208701673	0.010753571	0.344530496	CHD1L
ENSG00000197020.11	-0.263127532	0.010769279	0.344530496	ZNF100
ENSG00000127081.14	-0.230386098	0.010781409	0.344530496	ZNF484
ENSG00000159199.14	0.155612306	0.010785348	0.344530496	ATP5MC1
ENSG00000125498.20	0.559484599	0.010816162	0.344530496	KIR2DL1
ENSG00000176340.4	0.178981733	0.010859726	0.344530496	COX8A
ENSG00000121621.7	-0.374239086	0.010874273	0.344530496	KIF18A
ENSG00000105865.11	-0.189543236	0.010909867	0.344530496	DUS4L
ENSG00000286299.1	-0.691764572	0.010915163	0.344530496	RP1-167O5.3
ENSG00000094975.14	-0.188708722	0.010930687	0.344530496	SUCO
ENSG00000212541.1	-0.615903001	0.010968453	0.344530496	RNU6-510P
ENSG00000180104.16	0.105144226	0.010975997	0.344530496	EXOC3
ENSG00000165886.5	0.174579226	0.011003717	0.344530496	UBTD1
ENSG00000265625.1	-0.317652137	0.011011098	0.344530496	RP11-68I3.11
ENSG00000117569.19	-0.176910491	0.011011798	0.344530496	PTBP2
ENSG00000161048.12	-0.120531089	0.011045278	0.344530496	NAPEPLD
ENSG00000176624.12	-0.171334214	0.011069939	0.344530496	MEX3C
ENSG00000129562.11	0.140524617	0.01110222	0.344530496	DAD1
ENSG00000151233.11	-0.156672833	0.011104856	0.344530496	GXYLT1
ENSG00000260252.1	-0.439003799	0.011108297	0.344530496	RP11-384M15.3
ENSG00000288096.1	0.394659524	0.01111734	0.344530496	CTD-3065J16.12
ENSG00000117640.18	0.089625714	0.011126093	0.344530496	MTFR1L
ENSG00000186818.12	0.214684183	0.011134918	0.344530496	LILRB4
ENSG00000214212.9	0.138050906	0.011147117	0.344530496	C19orf38
ENSG00000244754.9	-0.151168351	0.011162849	0.344530496	N4BP2L2
ENSG00000100068.14	0.161161124	0.011171155	0.344530496	LRP5L
ENSG00000213722.10	0.168656525	0.011178007	0.344530496	DDAH2
ENSG00000173465.8	0.150117496	0.011183932	0.344530496	ZNRD2
ENSG00000130856.17	-0.160942506	0.011227366	0.344530496	ZNF236
ENSG00000071894.17	0.210633423	0.011252813	0.344530496	CPSF1
ENSG00000148308.18	0.101798861	0.011297365	0.344530496	GTF3C5
ENSG00000133302.13	-0.1967679	0.01130928	0.344530496	SLF1
ENSG00000060971.19	0.100399635	0.011318017	0.344530496	ACAA1
ENSG00000267493.4	-0.195488341	0.011328156	0.344530496	CIRBP-AS1
ENSG00000145916.19	0.096430196	0.011335143	0.344530496	RMND5B
ENSG00000115232.14	-0.199169332	0.011341764	0.344530496	ITGA4
ENSG00000114353.17	0.115616809	0.011343604	0.344530496	GNAI2
ENSG00000166803.14	-0.6010173	0.011359406	0.344530496	PCLAF
ENSG00000175567.11	0.165856753	0.011360397	0.344530496	UCP2

ENSG00000136379.12	-0.264588413	0.011371229	0.344530496	ABHD17C
ENSG00000165650.12	-0.191636587	0.011376248	0.344530496	PDZD8
ENSG00000161958.11	-0.540166413	0.011439297	0.344530496	FGF11
ENSG00000177963.15	0.094382122	0.011445109	0.344530496	RIC8A
ENSG00000104442.10	-0.135620665	0.011447082	0.344530496	ARMC1
ENSG00000080298.16	-0.182092841	0.011466194	0.344530496	RFX3
ENSG00000172927.8	0.464141963	0.011497632	0.344530496	MYEOV
ENSG00000204619.8	0.107651753	0.011501404	0.344530496	PPP1R11
ENSG00000254901.8	0.11203072	0.011503323	0.344530496	BORCS8
ENSG00000072163.20	0.299027031	0.011505307	0.344530496	LIMS2
ENSG00000271751.1	-0.602166257	0.01150567	0.344530496	RP11-110I1.14
ENSG00000117594.10	0.607583591	0.01153982	0.344530496	HSD11B1
ENSG00000100225.18	-0.363936402	0.011540654	0.344530496	FBXO7
ENSG00000267023.5	-0.187970859	0.011555918	0.344530496	LRRC37A16P
ENSG00000165507.9	0.411316681	0.011582344	0.344530496	DEPP1
ENSG00000127334.11	-0.122508215	0.011584541	0.344530496	DYRK2
ENSG00000015475.19	0.120771973	0.01158627	0.344530496	BID
ENSG00000100934.15	-0.142555257	0.011595255	0.344530496	SEC23A
ENSG00000211887.2	-0.533155622	0.01159716	0.344530496	TRAJ2
ENSG00000128789.21	0.102520997	0.011628787	0.344530496	PSMG2
ENSG00000227155.7	-0.322536539	0.011628848	0.344530496	RP11-165F24.3
ENSG00000091986.16	-0.71581695	0.011662542	0.344530496	CCDC80
ENSG00000169026.13	0.183234563	0.01169914	0.344530496	SLC49A3
ENSG00000204356.14	0.129561229	0.011702587	0.344530496	NELFE
ENSG00000092470.12	-0.150530139	0.011717777	0.344530496	WDR76
ENSG00000120159.13	-0.161826426	0.011728601	0.344530496	CAAP1
ENSG00000211669.3	0.725524162	0.011755324	0.344530496	IGLV3-10
ENSG00000255423.1	-0.465529243	0.011757836	0.344530496	EBLN2
ENSG00000184752.14	0.164512328	0.011773166	0.344530496	NDUFA12
ENSG00000105671.12	0.119553983	0.011800426	0.344530496	DDX49
ENSG00000285774.3	0.431944654	0.01181783	0.344530496	RP11-724F15.2
ENSG00000196705.8	-0.172859766	0.011819986	0.344530496	ZNF431
ENSG00000196811.13	-0.55920391	0.011828633	0.344530496	CHRNG
ENSG00000189068.11	0.386305591	0.011829089	0.344530496	VSTM1
ENSG00000196810.5	-0.130888154	0.011881214	0.344530496	CTBP1-DT
ENSG00000128524.5	0.152139017	0.011887641	0.344530496	ATP6V1F
ENSG00000166860.3	-0.115535852	0.011910307	0.344530496	ZBTB39
ENSG00000213442.5	0.22056509	0.011910317	0.344530496	RPL18AP3
ENSG00000270587.1	-0.324346309	0.011913926	0.344530496	RP11-51F16.9
ENSG00000088179.9	-0.177347193	0.011930037	0.344530496	PTPN4
ENSG00000106123.12	0.172128489	0.011931426	0.344530496	EPHB6
ENSG00000240535.8	-0.340407612	0.011948016	0.344530496	CTD-2313F11.1
ENSG00000144357.18	-0.132367567	0.011974295	0.344530496	UBR3
ENSG00000011009.11	0.138846188	0.012005113	0.344530496	LYPLA2
ENSG00000170629.14	-0.246228094	0.012007819	0.344530496	DPY19L2P2
ENSG00000174373.17	-0.173692023	0.012009824	0.344530496	RALGAPA1
ENSG00000124357.13	0.163702309	0.012047364	0.344530496	NAGK

ENSG00000287269.1	-0.440428098	0.012052595	0.344530496	RP1-68O2.5
ENSG00000196233.14	-0.105814208	0.01205325	0.344530496	LCOR
ENSG00000068120.15	0.101946808	0.012058577	0.344530496	COASY
ENSG00000261253.2	0.369142138	0.012067638	0.344530496	AC137932.6
ENSG00000149531.15	0.302455343	0.012074002	0.344530496	FRG1BP
ENSG00000283602.1	0.234421924	0.012086855	0.344530496	AC011380.8
ENSG00000196865.4	-0.158053429	0.01211554	0.344530496	NHLRC2
ENSG00000214078.13	0.158983501	0.01212567	0.344530496	CPNE1
ENSG00000140525.19	-0.132908709	0.012134826	0.344530496	FANCI
ENSG00000127863.16	-0.680866315	0.012160123	0.344530496	TNFRSF19
ENSG00000164265.9	0.781284985	0.012167016	0.344530496	SCGB3A2
ENSG00000155363.18	0.211235069	0.012181217	0.344530496	MOV10
ENSG00000231767.4	0.459350499	0.012188477	0.344530496	RPS27AP5
ENSG00000103540.16	-0.169322445	0.012189831	0.344530496	CCP110
ENSG00000128245.15	0.120698288	0.012191116	0.344530496	YWHAH
ENSG00000104823.9	0.146507157	0.012197405	0.344530496	ECH1
ENSG00000158019.21	0.144909521	0.012198301	0.344530496	BABAM2
ENSG00000146453.13	0.675141662	0.012209857	0.344530496	PNLDC1
ENSG00000134748.13	-0.062839268	0.012221525	0.344530496	PRPF38A
ENSG00000259826.1	0.283051903	0.012230297	0.344530496	RP11-467D6.1
ENSG00000150459.12	0.092550951	0.012294331	0.344530496	SAP18
ENSG00000162994.16	-0.358343968	0.012333626	0.344530496	CLHC1
ENSG00000115419.13	-0.149746075	0.012341494	0.344530496	GLS
ENSG00000196123.13	0.20281426	0.012345326	0.344530496	KIAA0895L
ENSG00000105438.9	0.126921549	0.012373216	0.344530496	KDELR1
ENSG00000112282.18	-0.136990996	0.012388403	0.344530496	MED23
ENSG00000102763.18	-0.12046396	0.012401789	0.344530496	VWA8
ENSG00000163006.12	-0.213188149	0.012402366	0.344530496	CCDC138
ENSG00000234745.13	0.157682593	0.012405666	0.344530496	HLA-B
ENSG00000163508.13	-0.279496075	0.012423915	0.344530496	EOMES
ENSG00000156970.13	-0.447140772	0.012427744	0.344530496	BUB1B
ENSG00000231991.4	0.188778396	0.012451422	0.344530496	ANXA2P2
ENSG00000134255.14	-0.148402463	0.012452697	0.344530496	CEPT1
ENSG00000179455.10	-0.673317351	0.012474674	0.344530496	MKRN3
ENSG00000123685.9	0.270636896	0.012478491	0.344530496	BATF3
ENSG00000179935.10	-0.441119805	0.012528221	0.344530496	LINC00652
ENSG00000257322.6	0.607721622	0.012540433	0.344530496	RP11-511B23.2
ENSG00000115216.14	0.084181386	0.012549534	0.344530496	NRBP1
ENSG00000234337.4	0.453379119	0.012605964	0.344530496	RP11-297L17.6
ENSG00000158711.14	-0.136788589	0.012606911	0.344530496	ELK4
ENSG00000168961.17	0.250343616	0.012616912	0.344530496	LGALS9
ENSG00000106799.13	-0.182008862	0.012619647	0.344530496	TGFBR1
ENSG00000151846.9	0.315058847	0.012623456	0.344530496	PABPC3
ENSG00000114902.14	0.112177071	0.012630587	0.344530496	SPCS1
ENSG00000064726.10	-0.11920138	0.012668236	0.344530496	BTBD1
ENSG00000113300.13	-0.119779816	0.012681768	0.344530496	CNOT6
ENSG00000211790.2	0.297276845	0.012696716	0.344530496	TRAV8-4

ENSG00000230224.1	-0.293804221	0.012711496	0.344530496	PHBP9
ENSG00000049089.15	0.23742697	0.012713021	0.344530496	COL9A2
ENSG00000164066.13	0.679293978	0.012743358	0.344530496	INTU
ENSG00000065883.16	-0.085949544	0.012747051	0.344530496	CDK13
ENSG00000010017.13	-0.087883334	0.012761099	0.344530496	RANBP9
ENSG00000160991.17	0.163819722	0.01278175	0.344530496	ORAI2
ENSG00000003987.14	-0.199502207	0.012785186	0.344530496	MTMR7
ENSG00000066427.24	-0.10347748	0.012785657	0.344530496	ATXN3
ENSG00000166278.15	0.471541358	0.01279697	0.344530496	C2
ENSG00000203363.2	0.712534802	0.012824922	0.344530496	GPR160P1
ENSG00000186481.16	-0.427105401	0.012904669	0.344530496	ANKRD20A5P
ENSG00000280173.1	-0.244122002	0.012914086	0.344530496	RP11-447D11.3
ENSG00000091483.8	0.096837833	0.012926305	0.344530496	FH
ENSG00000259856.1	0.206897693	0.012932334	0.344530496	RAB43P1
ENSG00000169131.13	-0.250974879	0.012934799	0.344530496	ZNF354A
ENSG00000136381.13	-0.127703625	0.01295253	0.344530496	IREB2
ENSG00000164342.13	-0.360292068	0.012962566	0.344530496	TLR3
ENSG00000224805.2	0.429810201	0.012975695	0.344530496	LINC00853
ENSG00000257103.9	-0.067253075	0.013028025	0.344530496	LSM14A
ENSG00000204482.11	0.151991004	0.013052712	0.344530496	LST1
ENSG00000198743.7	-0.188083402	0.01305756	0.344530496	SLC5A3
ENSG00000275763.3	0.415502852	0.013064554	0.344530496	ZNF516-DT
ENSG00000107223.13	0.143752774	0.013067961	0.344530496	EDF1
ENSG00000122741.16	-0.124782948	0.01308032	0.344530496	DCAF10
ENSG00000138964.17	0.100850844	0.013084999	0.344530496	PARVG
ENSG00000040531.16	0.109820219	0.013097953	0.344530496	CTNS
ENSG00000099337.5	0.124632714	0.01309991	0.344530496	KCNK6
ENSG00000110799.14	0.397696798	0.013105201	0.344530496	VWF
ENSG00000116649.10	0.149338529	0.013137661	0.344530496	SRM
ENSG00000247903.1	-0.329554675	0.013137863	0.344530496	RP11-421F16.3
ENSG00000127884.5	0.14011642	0.013148146	0.344530496	ECHS1
ENSG00000054611.14	0.108618744	0.013160628	0.344530496	TBC1D22A
ENSG00000117280.13	-0.107343901	0.013177867	0.344530496	RAB29
ENSG00000244879.8	-0.423903484	0.013183124	0.344530496	GABPB1-AS1
ENSG00000129315.11	-0.094144007	0.013191202	0.344530496	CCNT1
ENSG00000197122.12	0.186057548	0.013233195	0.344530496	SRC
ENSG00000277075.2	0.403301879	0.013243706	0.344530496	H2AC8
ENSG00000154134.15	0.284730999	0.013250445	0.344530496	ROBO3
ENSG00000127080.10	-0.173091197	0.013262342	0.344530496	IPPK
ENSG00000182985.18	-0.433525502	0.013276043	0.344530496	CADM1
ENSG00000205611.5	0.56071169	0.013291598	0.344530496	LINC01597
ENSG00000213047.13	-0.233930704	0.01332174	0.344530496	DENND1B
ENSG00000197747.9	0.170863117	0.013322254	0.344530496	S100A10
ENSG00000162368.14	-0.144769698	0.013324774	0.344530496	CMPK1
ENSG00000230749.5	0.649496353	0.013358487	0.344530496	MEIS1-AS2
ENSG00000224870.7	0.091634942	0.013382281	0.344530496	MRPL20-AS1
ENSG00000141504.12	0.144011795	0.01341031	0.344530496	SAT2

ENSG00000153558.16	-0.243362102	0.013417144	0.344530496	FBXL2
ENSG00000150637.9	-0.203690372	0.013462124	0.344530496	CD226
ENSG00000163539.18	-0.118685188	0.013478615	0.344530496	CLASP2
ENSG00000090104.12	-0.475276873	0.013489838	0.344530496	RGS1
ENSG00000179639.10	0.389175662	0.013513952	0.344530496	FCER1A
ENSG00000197471.12	-0.143780365	0.01352141	0.344530496	SPN
ENSG00000162552.15	0.261128667	0.013557659	0.344530496	WNT4
ENSG00000188282.12	0.328877162	0.013564912	0.344530496	RUFY4
ENSG00000075711.21	-0.128292372	0.013603459	0.344530496	DLG1
ENSG00000151135.10	-0.170592899	0.013608674	0.344530496	TMEM263
ENSG00000094804.12	-0.345390747	0.013621226	0.344530496	CDC6
ENSG00000254162.1	-0.591938447	0.01362242	0.344530496	RP11-48B3.3
ENSG00000141349.9	0.144513927	0.013630387	0.344530496	G6PC3
ENSG00000161904.12	0.072377909	0.013638671	0.344530496	LEMD2
ENSG00000137207.12	0.124212158	0.01364612	0.344530496	YIPF3
ENSG00000163428.4	-0.163458768	0.013655517	0.344530496	LRRC58
ENSG00000256525.8	-0.102807747	0.013658245	0.344530496	POLG2
ENSG00000126787.13	-0.546787679	0.013691876	0.344954538	DLGAP5
ENSG00000168404.13	0.123235397	0.013720242	0.34504589	MLKL
ENSG00000254030.1	0.693385486	0.013752109	0.34504589	IGLC5
ENSG00000145375.9	-0.143690714	0.013752155	0.34504589	SPATA5
ENSG00000085365.18	-0.164526752	0.013778845	0.34504589	SCAMP1
ENSG00000111530.13	-0.098781492	0.013784734	0.34504589	CAND1
ENSG00000125970.12	0.120111293	0.013796452	0.34504589	RALY
ENSG00000238390.1	-0.520433922	0.013915061	0.346851757	ENSG10010138890.1
ENSG00000175573.7	0.136591744	0.014002432	0.346851757	C11orf68
ENSG00000092439.16	-0.141518801	0.014008144	0.346851757	TRPM7
ENSG00000184517.12	-0.167735738	0.01401244	0.346851757	ZFP1
ENSG00000257511.1	-0.270385539	0.014054684	0.346851757	RP11-278C7.1
ENSG00000160305.18	-0.227330952	0.014082066	0.346851757	DIP2A
ENSG00000162601.11	-0.197366672	0.014103687	0.346851757	MYSM1
ENSG00000147955.18	0.106779541	0.014114862	0.346851757	SIGMAR1
ENSG00000186081.12	0.54230274	0.014122295	0.346851757	KRT5
ENSG00000136451.9	-0.096930611	0.014124158	0.346851757	VEZF1
ENSG00000134186.12	-0.135209058	0.01412777	0.346851757	PRPF38B
ENSG00000286207.1	0.300139697	0.014182627	0.346851757	RP11-17G11.2
ENSG00000105583.11	0.550917367	0.014185338	0.346851757	WDR83OS
ENSG00000174842.17	-0.16696327	0.014203576	0.346851757	GLMN
ENSG00000246451.2	-0.227681743	0.014233994	0.346851757	RP11-894P9.1
ENSG00000273796.1	0.872931798	0.014235328	0.346851757	LL21NC02-21A1.1
ENSG00000172995.17	0.781849843	0.01423797	0.346851757	ARPP21
ENSG00000169682.18	-0.191033872	0.01428346	0.346851757	SPNS1
ENSG00000185885.17	0.253781162	0.014295861	0.346851757	IFITM1
ENSG00000259959.1	-0.247405006	0.014299284	0.346851757	RP11-121C2.2
ENSG00000143499.14	-0.118457937	0.014299631	0.346851757	SMYD2
ENSG00000164114.19	-0.299491812	0.014330674	0.346851757	MAP9
ENSG00000273576.1	0.12605598	0.014331021	0.346851757	RP11-390P24.1

ENSG00000169914.6	-0.120488411	0.014343899	0.346851757	OTUD3
ENSG00000169855.20	-0.722895764	0.014366743	0.346851757	ROBO1
ENSG00000123374.11	-0.097695357	0.014378777	0.346851757	CDK2
ENSG00000174885.13	0.275637389	0.014385421	0.346851757	NLRP6
ENSG00000140265.13	-0.132779401	0.014396755	0.346851757	ZSCAN29
ENSG00000185482.8	0.19027902	0.01440319	0.346851757	STAC3
ENSG00000228695.10	1.440052589	0.014404966	0.346851757	CES1P1
ENSG00000153339.15	-0.133873292	0.014450988	0.346851757	TRAPPC8
ENSG00000196735.13	0.417177069	0.014459172	0.346851757	HLA-DQA1
ENSG00000287868.1	-0.358705693	0.014478757	0.346851757	RP11-392A14.13
ENSG00000264695.1	0.503036104	0.014478907	0.346851757	RP11-178F10.2
ENSG00000204859.13	0.100132753	0.014502383	0.346851757	ZBTB48
ENSG00000162543.6	-0.342176163	0.014517655	0.346851757	UBXN10
ENSG00000196792.12	-0.162057363	0.014520853	0.346851757	STRN3
ENSG00000164162.14	-0.327083652	0.014527815	0.346851757	ANAPC10
ENSG00000266028.8	0.159582539	0.014528265	0.346851757	SRGAP2
ENSG00000263370.1	-0.448086727	0.014614724	0.347503978	RP11-68I3.5
ENSG00000111790.14	-0.190851205	0.014636028	0.347503978	FGFR1OP2
ENSG00000155621.15	-0.128468125	0.014641964	0.347503978	C9orf85
ENSG00000181827.16	-0.15558183	0.01465758	0.347503978	RFX7
ENSG00000023892.11	0.115891138	0.014674819	0.347503978	DEF6
ENSG00000196177.13	-0.157666551	0.014690391	0.347503978	ACADSB
ENSG00000163931.16	0.129994641	0.014718255	0.347503978	TKT
ENSG00000100462.16	0.12965573	0.014729165	0.347503978	PRMT5
ENSG00000086504.18	0.143189016	0.014747911	0.347503978	MRPL28
ENSG00000117834.13	0.255750347	0.014755469	0.347503978	SLC5A9
ENSG00000198879.13	-0.160201062	0.014779541	0.347503978	SFMBT2
ENSG00000088280.19	0.295298422	0.014788139	0.347503978	ASAP3
ENSG00000105983.23	-0.098629108	0.014789208	0.347503978	LMBR1
ENSG00000099910.17	0.10567831	0.014792811	0.347503978	KLHL22
ENSG00000129925.11	0.145658025	0.01485946	0.348670259	PGAP6
ENSG00000167633.18	0.505103303	0.014894213	0.348944755	KIR3DL1
ENSG00000263033.2	0.370879403	0.014910058	0.348944755	RP11-396B14.2
ENSG00000215481.9	0.442212762	0.014937807	0.348944755	BCRP3
ENSG00000146833.16	-0.104076185	0.014951316	0.348944755	TRIM4
ENSG00000275052.5	-0.109746786	0.014979297	0.348944755	PPP4R3B
ENSG00000152944.9	-0.19088766	0.014984559	0.348944755	MED21
ENSG00000183347.15	0.525398505	0.015004997	0.348944755	GBP6
ENSG00000261512.2	0.182069113	0.015033261	0.348944755	RP11-46D6.1
ENSG00000170190.16	0.122111267	0.015061652	0.348944755	SLC16A5
ENSG00000211924.1	0.653995816	0.01506746	0.348944755	IGHD3-9
ENSG00000139211.7	-0.14170327	0.015073136	0.348944755	AMIGO2
ENSG00000125952.20	0.090924342	0.015081445	0.348944755	MAX
ENSG00000170312.16	-0.475828683	0.015092354	0.348944755	CDK1
ENSG00000064309.16	-0.425053625	0.015154729	0.349992331	CDON
ENSG00000271730.1	-0.501788574	0.015234312	0.351376754	RP11-787I22.3
ENSG00000178691.11	-0.164981637	0.015253007	0.351376754	SUZ12

ENSG00000197272.2	0.326147332	0.0152715	0.351376754	IL27
ENSG00000080189.15	0.087955338	0.015289301	0.351376754	SLC35C2
ENSG00000197566.10	-0.224172786	0.015343519	0.351376754	ZNF624
ENSG00000129465.16	0.105487215	0.015345213	0.351376754	RIPK3
ENSG00000214517.10	0.088758779	0.015376115	0.351376754	PPME1
ENSG00000149418.11	0.220890388	0.015378685	0.351376754	ST14
ENSG00000225675.2	-0.635909121	0.015382791	0.351376754	LINC01771
ENSG00000128607.14	-0.073901979	0.015405345	0.351376754	KLHDC10
ENSG00000091157.14	-0.136023224	0.015411328	0.351376754	WDR7
ENSG00000155307.19	-0.200038915	0.015420279	0.351376754	SAMSN1
ENSG00000187193.9	0.216537854	0.015478027	0.352090523	MT1X
ENSG00000164031.17	-0.226605997	0.015509032	0.352090523	DNAJB14
ENSG00000100106.22	0.12932553	0.015509872	0.352090523	TRIOBP
ENSG00000228594.4	0.21042853	0.015520277	0.352090523	FNDC10
ENSG00000160791.13	-0.288443315	0.015570087	0.352519111	CCR5
ENSG00000139684.15	0.115793934	0.015577781	0.352519111	ESD
ENSG00000141698.17	0.294827517	0.015593233	0.352519111	NT5C3B
ENSG00000101166.16	-0.113998966	0.01560809	0.352519111	PRELID3B
ENSG00000108469.15	0.111911626	0.015649957	0.352519111	RECQL5
ENSG00000130724.9	0.156620396	0.015653901	0.352519111	CHMP2A
ENSG00000155465.20	0.142534933	0.015659494	0.352519111	SLC7A7
ENSG00000268938.2	0.245190407	0.015696645	0.352879165	AC005387.3
ENSG00000133657.16	-0.160117514	0.015717368	0.352879165	ATP13A3
ENSG00000182511.12	0.13170144	0.015754298	0.352879165	FES
ENSG00000158710.15	0.181468788	0.015787923	0.352879165	TAGLN2
ENSG00000148606.13	-0.082624357	0.015809523	0.352879165	POLR3A
ENSG00000133874.2	0.143658656	0.015813476	0.352879165	RNF122
ENSG00000247287.3	-0.153130948	0.015829268	0.352879165	RP11-902B17.1
ENSG00000177030.18	0.113208063	0.015865754	0.352879165	DEAF1
ENSG00000260279.4	-0.307798792	0.01586803	0.352879165	AC137932.5
ENSG00000088833.18	0.113010542	0.015874247	0.352879165	NSFL1C
ENSG00000129255.16	0.134373459	0.015874762	0.352879165	MPDU1
ENSG00000101126.18	-0.090509862	0.015895685	0.352879165	ADNP
ENSG00000171720.10	0.088297448	0.015911702	0.352879165	HDAC3
ENSG00000273343.1	-0.243157635	0.015916385	0.352879165	XXbac-B444P24.13
ENSG00000066136.20	0.092260013	0.015960627	0.353395751	NFYC
ENSG00000112159.12	-0.128565388	0.016015035	0.353395751	MDN1
ENSG00000137103.20	0.147142253	0.016018746	0.353395751	TMEM8B
ENSG00000103187.8	0.129858162	0.016022363	0.353395751	COTL1
ENSG00000160055.20	0.11297708	0.016025846	0.353395751	TMEM234
ENSG00000146414.16	-0.245005281	0.016051929	0.353519722	SHPRH
ENSG00000110721.12	0.116716665	0.016086519	0.353519722	CHKA
ENSG00000034510.6	0.20322116	0.016099711	0.353519722	TMSB10
ENSG00000135596.18	0.117745945	0.01610042	0.353519722	MICAL1
ENSG00000141401.12	0.172085827	0.016149619	0.35409577	IMPA2
ENSG00000090060.19	-0.110051441	0.016172037	0.35409577	PAPOLA
ENSG00000204248.12	0.292789883	0.016178454	0.35409577	COL11A2

ENSG00000277595.1	-0.314534949	0.016269443	0.355107009	RP11-946P6.6
ENSG00000286691.1	-0.419891351	0.016287295	0.355107009	RP11-181P4.2
ENSG00000023909.10	-0.150173716	0.016328905	0.355107009	GCLM
ENSG00000069667.16	-0.195702597	0.016338894	0.355107009	RORA
ENSG00000187147.18	0.077775462	0.016339305	0.355107009	RNF220
ENSG00000129354.12	0.42454277	0.016344365	0.355107009	AP1M2
ENSG00000170185.11	-0.116003558	0.016355211	0.355107009	USP38
ENSG00000117528.14	-0.145162695	0.016371671	0.355107009	ABCD3
ENSG00000104643.10	-0.122527367	0.016391189	0.355107009	MTMR9
ENSG00000163902.12	0.090816519	0.016397812	0.355107009	RPN1
ENSG00000110955.9	0.089013738	0.016436696	0.355343366	ATP5F1B
ENSG00000103043.15	0.1002232	0.016446911	0.355343366	VAC14
ENSG00000183207.14	0.126485817	0.016475405	0.355343366	RUVBL2
ENSG00000253729.8	-0.145498405	0.016497098	0.355343366	PRKDC
ENSG00000168255.20	0.291317045	0.016497668	0.355343366	POLR2J3
ENSG00000109321.11	0.733337158	0.016520659	0.355343366	AREG
ENSG00000155876.6	0.078195002	0.01653811	0.355343366	RRAGA
ENSG00000123607.16	-0.126615976	0.016550091	0.355343366	TTC21B
ENSG00000281195.1	-0.417855147	0.01656467	0.355343366	RP11-236P2.2
ENSG00000211850.1	-0.558020835	0.016594856	0.355618929	TRAJ39
ENSG00000177076.6	-0.330111661	0.016621009	0.35565213	ACER2
ENSG00000122705.17	0.121655734	0.01663109	0.35565213	CLTA
ENSG00000001561.7	-0.269454123	0.01668066	0.35573255	ENPP4
ENSG00000187607.16	-0.148954273	0.01669015	0.35573255	ZNF286A
ENSG00000117448.14	0.123766834	0.016692879	0.35573255	AKR1A1
ENSG00000064651.14	-0.160189837	0.016704235	0.35573255	SLC12A2
ENSG00000107175.12	0.107841446	0.016744252	0.355750027	CREB3
ENSG00000107738.20	0.127990245	0.016758693	0.355750027	VSIR
ENSG00000140905.11	-0.509701243	0.016804649	0.355750027	GCSH
ENSG00000162928.9	-0.111659995	0.016814365	0.355750027	PEX13
ENSG00000117410.14	0.135781846	0.016826818	0.355750027	ATP6V0B
ENSG00000250479.9	0.187854499	0.016844726	0.355750027	CHCHD10
ENSG00000168995.13	0.176836538	0.016859963	0.355750027	SIGLEC7
ENSG00000188994.13	-0.227500106	0.016889029	0.355750027	ZNF292
ENSG00000167925.16	0.118031538	0.016900212	0.355750027	GHDC
ENSG00000110917.8	0.074935388	0.016904055	0.355750027	MLEC
ENSG00000234769.7	0.238689114	0.016925935	0.355750027	WASH4P
ENSG00000120805.14	-0.101882925	0.01694258	0.355750027	ARL1
ENSG00000127952.17	0.180191257	0.016946103	0.355750027	STYXL1
ENSG00000173200.13	-0.186752308	0.016957592	0.355750027	PARP15
ENSG00000236088.10	-0.17431663	0.016965259	0.355750027	COX10-AS1
ENSG00000184117.12	0.090842205	0.017057086	0.356854243	NIPSNAP1
ENSG00000132356.11	-0.139884188	0.017078684	0.356854243	PRKAA1
ENSG00000162949.16	0.696502545	0.017079976	0.356854243	CAPN13
ENSG00000228049.7	0.382489948	0.017108368	0.356854243	POLR2J2
ENSG00000184602.6	0.107916048	0.017120564	0.356854243	SNN
ENSG00000065518.8	0.127233294	0.017123168	0.356854243	NDUFB4

ENSG00000259205.2	-0.496996996	0.017139723	0.356854243	PRKXP1
ENSG00000100417.12	0.127547519	0.017177453	0.357179445	PMM1
ENSG00000177105.10	0.150323163	0.017190175	0.357179445	RHOG
ENSG00000196126.11	0.239179854	0.01724945	0.357604555	HLA-DRB1
ENSG00000248514.1	0.489330829	0.017255024	0.357604555	CTC-338M12.1
ENSG00000211872.1	-0.530549202	0.017262947	0.357604555	TRAJ17
ENSG00000188529.15	-0.140923144	0.017292781	0.35786111	SRSF10
ENSG00000170113.16	-0.149487704	0.017337797	0.358106887	NIPA1
ENSG00000163848.20	-0.136767485	0.017340454	0.358106887	ZNF148
ENSG00000196172.9	-0.256911226	0.01736387	0.358106887	ZNF681
ENSG00000198576.4	0.592266102	0.017374505	0.358106887	ARC
ENSG00000158089.15	0.406744896	0.017419502	0.358345892	GALNT14
ENSG00000116830.12	-0.131884001	0.017453202	0.358345892	TTF2
ENSG00000003249.15	0.348082367	0.017454962	0.358345892	DBNDD1
ENSG00000227507.3	0.186908592	0.017455995	0.358345892	LTB
ENSG00000247271.7	0.158148468	0.017508458	0.358602416	ZBED5-AS1
ENSG00000135404.12	0.143445572	0.017523127	0.358602416	CD63
ENSG00000171067.11	0.125769869	0.017533747	0.358602416	C11orf24
ENSG00000196353.12	0.74852209	0.017538435	0.358602416	CPNE4
ENSG00000158825.6	0.219217147	0.017618694	0.359526806	CDA
ENSG00000076321.11	-0.12361537	0.017620989	0.359526806	KLHL20
ENSG00000099330.9	0.147888769	0.017692507	0.359526806	OCEL1
ENSG00000260103.2	-0.513564612	0.017692549	0.359526806	RP11-10O17.1
ENSG00000033867.16	-0.229710026	0.017695685	0.359526806	SLC4A7
ENSG00000170500.13	-0.739428577	0.017702242	0.359526806	LONRF2
ENSG00000259792.1	0.435639295	0.017707305	0.359526806	RP11-114H24.6
ENSG00000213362.3	0.406863003	0.017723893	0.359526806	FTH1P12
ENSG00000253590.1	-0.74363959	0.017768723	0.359735394	IGLV3-13
ENSG00000151881.15	-0.231529683	0.017769259	0.359735394	TMEM267
ENSG00000255274.9	-0.229235793	0.017842633	0.360398803	SMIM35
ENSG00000138107.13	0.091998073	0.017850013	0.360398803	ACTR1A
ENSG00000285331.2	0.229412281	0.017854749	0.360398803	RP11-323F24.5
ENSG00000232912.6	0.166849952	0.017909649	0.360456573	RERE-AS1
ENSG00000273026.1	0.399225845	0.017917975	0.360456573	RP11-422P24.10
ENSG00000170043.12	0.141267663	0.017921744	0.360456573	TRAPPC1
ENSG00000105085.11	0.09952973	0.017955246	0.360456573	MED26
ENSG00000162390.18	0.304859406	0.01796308	0.360456573	ACOT11
ENSG00000236977.1	-0.363475197	0.01799479	0.360456573	ANKRD44-IT1
ENSG00000111907.21	-0.428058205	0.018006071	0.360456573	TPD52L1
ENSG00000160789.24	0.283911312	0.018053858	0.360456573	LMNA
ENSG00000138821.14	-0.14182507	0.018053904	0.360456573	SLC39A8
ENSG00000120262.10	0.257023949	0.018079365	0.360456573	CCDC170
ENSG00000126267.11	0.165988282	0.018083408	0.360456573	COX6B1
ENSG00000272810.1	0.393758979	0.018088337	0.360456573	U91328.22
ENSG00000104147.9	-0.422930407	0.018103331	0.360456573	OIP5
ENSG00000214140.11	0.272311099	0.01810368	0.360456573	PRCD
ENSG00000196118.12	0.242855549	0.018170837	0.360605394	CCDC189

ENSG00000173391.9	-0.740226636	0.018191902	0.360605394	OLR1
ENSG00000175985.10	0.653098519	0.018283467	0.360605394	PLEKHD1
ENSG00000118596.12	-0.199900334	0.018307497	0.360605394	SLC16A7
ENSG00000129450.9	0.116980785	0.018315289	0.360605394	SIGLEC9
ENSG00000236278.2	-0.418703293	0.018317003	0.360605394	PEBP1P3
ENSG00000267731.1	-0.354061277	0.018327532	0.360605394	RP11-147L13.8
ENSG00000226816.3	0.62092188	0.018332026	0.360605394	AC005082.12
ENSG00000262312.2	-0.258831034	0.018346362	0.360605394	LA16c-390H2.4
ENSG00000133703.13	-0.117638727	0.018357356	0.360605394	KRAS
ENSG00000163946.14	-0.125359708	0.018380984	0.360605394	TASOR
ENSG00000183258.12	0.106092625	0.018396647	0.360605394	DDX41
ENSG00000110057.8	0.168266647	0.018402149	0.360605394	UNC93B1
ENSG00000169231.13	0.136931153	0.018410309	0.360605394	THBS3
ENSG00000148834.13	0.16592888	0.018420773	0.360605394	GSTO1
ENSG00000087077.14	0.16965497	0.018449787	0.360605394	TRIP6
ENSG00000132254.13	0.088422764	0.018451801	0.360605394	ARFIP2
ENSG00000115307.17	0.089517373	0.018455803	0.360605394	AUP1
ENSG00000279253.1	0.153800987	0.018459823	0.360605394	RP4-614O4.13
ENSG00000111371.16	-0.127365815	0.018462827	0.360605394	SLC38A1
ENSG00000261671.1	-0.597411449	0.018483728	0.360649106	RP11-573G6.6
ENSG00000113384.14	-0.081342354	0.018500237	0.360649106	GOLPH3
ENSG00000154265.16	-0.203286365	0.018527397	0.360835573	ABCA5
ENSG00000171763.20	-0.136782084	0.018573555	0.360943025	SPATA5L1
ENSG00000135945.10	-0.115426832	0.018598931	0.360943025	REV1
ENSG00000165178.9	0.26294009	0.018627147	0.360943025	NCF1C
ENSG00000186074.19	0.115939889	0.018641269	0.360943025	CD300LF
ENSG00000263417.4	0.499009255	0.018664607	0.360943025	GTSCR1
ENSG00000006042.12	0.979299979	0.018671639	0.360943025	TMEM98
ENSG00000225733.6	-0.106130182	0.01870539	0.360943025	FGD5-AS1
ENSG00000154727.11	-0.183449939	0.018718323	0.360943025	GABPA
ENSG00000125122.16	0.155527377	0.018748157	0.360943025	LRRC29
ENSG00000108953.17	0.086991078	0.018817441	0.360943025	YWHAE
ENSG00000164045.12	-0.454615994	0.018823021	0.360943025	CDC25A
ENSG00000122783.17	0.141965667	0.018838006	0.360943025	CYREN
ENSG00000013364.19	0.119431075	0.018876935	0.360943025	MVP
ENSG00000148803.12	0.201907085	0.018935616	0.360943025	FUOM
ENSG00000039523.20	0.124315357	0.018945879	0.360943025	RIPOR1
ENSG00000116120.11	0.096251117	0.018957865	0.360943025	FARSB
ENSG00000285518.1	0.358882668	0.018979153	0.360943025	RP4-816G1.2
ENSG00000127603.31	-0.138032368	0.018998871	0.360943025	MACF1
ENSG00000211933.2	0.491781457	0.019040636	0.360943025	IGHV6-1
ENSG00000261693.1	0.592232356	0.019060558	0.360943025	RP13-467H17.1
ENSG00000181467.5	-0.093457484	0.01907981	0.360943025	RAP2B
ENSG00000153898.13	-0.246006916	0.019101886	0.360943025	MCOLN2
ENSG00000089022.14	-0.081710316	0.019111914	0.360943025	MAPKAPK5
ENSG00000263155.6	0.541724795	0.019114317	0.360943025	MYZAP
ENSG00000258405.11	0.338146312	0.01913357	0.360943025	ZNF578

ENSG00000179344.16	0.501659843	0.019162267	0.360943025	HLA-DQB1
ENSG00000108771.13	0.281936985	0.019188519	0.360943025	DHX58
ENSG00000252391.1	-0.475773273	0.019193885	0.360943025	RNU6-638P
ENSG00000167747.15	0.145455069	0.019195161	0.360943025	C19orf48
ENSG00000214160.10	0.123879242	0.019200473	0.360943025	ALG3
ENSG00000177239.16	0.099903074	0.019209228	0.360943025	MAN1B1
ENSG00000070882.13	-0.120778761	0.019250442	0.360943025	OSBPL3
ENSG00000103966.11	0.111422418	0.019285661	0.360943025	EHD4
ENSG00000125445.11	0.110494889	0.019288925	0.360943025	MRPS7
ENSG00000170412.18	0.516266799	0.019307002	0.360943025	GPRC5C
ENSG00000086189.11	-0.126943128	0.019323584	0.360943025	DIMT1
ENSG00000226608.3	0.247421238	0.019334736	0.360943025	FTLP3
ENSG00000182551.14	0.10703055	0.019360159	0.360943025	ADI1
ENSG00000198860.14	-0.108508653	0.019397803	0.360943025	TSEN15
ENSG00000042493.16	0.193757979	0.019404983	0.360943025	CAPG
ENSG00000145734.19	-0.211800586	0.019407609	0.360943025	BDP1
ENSG00000280649.2	0.226893818	0.01941814	0.360943025	CH17-172B3.1
ENSG00000104969.11	0.10764495	0.019425189	0.360943025	SGTA
ENSG00000160326.14	0.164132642	0.019455177	0.360943025	SLC2A6
ENSG00000111727.12	-0.159069175	0.01946581	0.360943025	HCFC2
ENSG00000237637.1	0.492044315	0.019469169	0.360943025	FRY-AS1
ENSG00000176124.15	-0.149570328	0.01947237	0.360943025	DLEU1
ENSG00000111358.14	-0.115306399	0.019473876	0.360943025	GTF2H3
ENSG00000170234.13	-0.097252177	0.01947753	0.360943025	PWWP2A
ENSG00000155761.14	0.583157742	0.019483652	0.360943025	SPAG17
ENSG00000121964.15	-0.111521616	0.01948873	0.360943025	GTDC1
ENSG00000180488.15	-0.171719105	0.019495715	0.360943025	MIGA1
ENSG00000121350.16	-0.174400472	0.019538077	0.360943025	PYROXD1
ENSG00000155367.15	0.293442516	0.019556775	0.360943025	PPM1J
ENSG00000146054.18	0.207958887	0.019578469	0.360943025	TRIM7
ENSG00000261490.1	-0.144798861	0.019598327	0.360943025	RP11-448G15.3
ENSG00000128604.20	0.143058635	0.019600892	0.360943025	IRF5
ENSG00000158477.7	0.244225319	0.019602001	0.360943025	CD1A
ENSG00000223878.1	-0.352093974	0.019602105	0.360943025	PPIAP53
ENSG00000198482.14	-0.134944727	0.019611614	0.360943025	ZNF808
ENSG00000198658.4	0.460262161	0.01963764	0.360943025	ABHD17AP1
ENSG00000204308.8	0.129763273	0.019662667	0.360943025	RNF5
ENSG00000253755.1	0.617439211	0.019668872	0.360943025	IGHGP
ENSG00000258301.3	0.256756611	0.019678058	0.360943025	VASH1-AS1
ENSG00000177453.7	-0.434346989	0.01968546	0.360943025	NIM1K
ENSG00000197956.10	0.167283203	0.019694521	0.360943025	S100A6
ENSG00000211677.2	0.511776203	0.019732369	0.361274164	IGLC2
ENSG00000279369.1	0.122563873	0.01976356	0.361274164	RP11-51F16.1
ENSG00000138073.14	0.084379386	0.019773473	0.361274164	PREB
ENSG00000164151.12	-0.102589992	0.019837084	0.361274164	ICE1
ENSG00000167889.13	0.649804003	0.019841001	0.361274164	MGAT5B
ENSG00000146700.9	0.372998838	0.019863412	0.361274164	SSC4D

ENSG00000254461.1	-0.360192484	0.01986618	0.361274164	RP11-755F10.3
ENSG00000198835.4	-0.366255965	0.019886654	0.361274164	GJC2
ENSG00000119650.13	0.151916321	0.019888922	0.361274164	IFT43
ENSG00000167642.13	0.153812264	0.019910136	0.361274164	SPINT2
ENSG00000272599.2	-0.291512353	0.019933892	0.361274164	RP11-152N13.16
ENSG00000058673.17	-0.361228047	0.01999462	0.361274164	ZC3H11A
ENSG00000053371.13	0.137545347	0.020022306	0.361274164	AKR7A2
ENSG00000170802.16	-0.196885977	0.020032575	0.361274164	FOXN2
ENSG00000118276.12	-0.325986112	0.020035917	0.361274164	B4GALT6
ENSG00000225465.10	-0.358611504	0.020037059	0.361274164	RFPL1S
ENSG00000174442.12	-0.156239573	0.020050752	0.361274164	ZWILCH
ENSG00000284600.1	1.286117135	0.020086785	0.361274164	RP11-755H23.1
ENSG00000099341.12	0.104467806	0.020101199	0.361274164	PSMD8
ENSG00000187837.4	0.264149508	0.020142735	0.361274164	H1-2
ENSG00000154529.15	0.851311024	0.020147411	0.361274164	CNTNAP3B
ENSG00000154258.17	0.635209003	0.020172854	0.361274164	ABCA9
ENSG00000101695.9	-0.103755455	0.020179346	0.361274164	RNF125
ENSG00000196843.17	0.137699807	0.020181587	0.361274164	ARID5A
ENSG00000173276.14	-0.155057706	0.020191983	0.361274164	ZBTB21
ENSG00000140939.14	0.210103655	0.02020318	0.361274164	NOL3
ENSG00000164463.12	-0.147618354	0.020226653	0.361274164	CREBRF
ENSG00000120725.13	0.155168808	0.020227531	0.361274164	SIL1
ENSG00000112077.17	-0.650089833	0.020228134	0.361274164	RHAG
ENSG00000249396.2	-0.455413674	0.020241077	0.361274164	LINC02212
ENSG00000214797.3	0.395462987	0.020265041	0.36138735	RP11-1036E20.9
ENSG00000198961.10	-0.152729004	0.020312352	0.361916346	PJA2
ENSG00000061938.21	0.124895966	0.020335123	0.362007554	TNK2
ENSG00000172845.18	-0.15715285	0.020446386	0.362803947	SP3
ENSG00000158636.16	-0.110524637	0.020455986	0.362803947	EMSY
ENSG00000160007.19	-0.115703771	0.020458959	0.362803947	ARHGAP35
ENSG00000197056.11	-0.152735482	0.020470114	0.362803947	ZMYM1
ENSG00000275665.1	-0.523320833	0.020484349	0.362803947	CTB-58E17.9
ENSG00000137166.17	0.152547669	0.020494792	0.362803947	FOXP4
ENSG00000007541.17	0.131925595	0.020503695	0.362803947	PIGQ
ENSG00000101115.13	0.610945147	0.020523728	0.362845354	SALL4
ENSG00000161642.18	0.171130007	0.020653598	0.364491221	ZNF385A
ENSG00000072756.17	-0.140266272	0.020662972	0.364491221	TRNT1
ENSG00000143443.10	0.126864082	0.020672235	0.364491221	C1orf56
ENSG00000277999.1	0.283643603	0.020692767	0.364491221	RP11-426C22.8
ENSG00000213654.10	0.135327791	0.020707295	0.364491221	GPSM3
ENSG00000180233.11	-0.154455108	0.020735763	0.364491221	ZNRF2
ENSG00000145725.20	-0.173890162	0.020745849	0.364491221	PPIP5K2
ENSG00000177885.15	0.082465523	0.020759008	0.364491221	GRB2
ENSG00000162373.13	0.284116355	0.02084104	0.365102308	BEND5
ENSG00000165702.15	0.274804943	0.020846263	0.365102308	GFI1B
ENSG00000078674.18	-0.111661526	0.020847221	0.365102308	PCM1
ENSG00000183317.17	-0.768091489	0.020880201	0.365270723	EPHA10

ENSG00000232859.10	0.175108166	0.020897278	0.365270723	LYRM9
ENSG00000040933.16	-0.085707255	0.020910459	0.365270723	INPP4A
ENSG00000167863.12	0.134179949	0.020956632	0.365270723	ATP5PD
ENSG00000166575.17	-0.141890951	0.020957505	0.365270723	TMEM135
ENSG00000051825.15	-0.145181142	0.020963704	0.365270723	MPHOSPH9
ENSG00000276663.1	-0.483170467	0.021041322	0.366015231	RP11-407G23.7
ENSG00000225698.4	0.634505513	0.021055305	0.366015231	IGHV3-72
ENSG00000166734.20	-0.144414068	0.021081165	0.366015231	GOLM2
ENSG00000074416.15	0.267853288	0.021092534	0.366015231	MGLL
ENSG00000253710.5	-0.188597534	0.02109567	0.366015231	ALG11
ENSG00000223519.8	0.413842029	0.021194827	0.367424777	KIF28P
ENSG00000281103.2	-0.177635341	0.021235227	0.367546663	TRG-AS1
ENSG00000241484.10	-0.716924903	0.021253809	0.367546663	ARHGAP8
ENSG00000174483.20	-0.193668724	0.021279879	0.367546663	BBS1
ENSG00000196268.12	-0.173940785	0.021294885	0.367546663	ZNF493
ENSG00000100599.16	0.124280965	0.021319497	0.367546663	RIN3
ENSG00000175938.7	0.130632444	0.0213453	0.367546663	ORAI3
ENSG00000107890.17	-0.219038375	0.021364256	0.367546663	ANKRD26
ENSG00000257176.3	-0.287878365	0.021369953	0.367546663	RP11-996F15.2
ENSG00000150991.15	0.112529628	0.021405074	0.367546663	UBC
ENSG00000118707.11	0.093423771	0.021416964	0.367546663	TGIF2
ENSG00000149547.15	0.075100493	0.021452627	0.367546663	EI24
ENSG00000168612.5	0.107115555	0.021455648	0.367546663	ZSWIM1
ENSG00000077312.9	0.119288056	0.02148062	0.367546663	SNRPA
ENSG00000160213.9	0.159487968	0.021497334	0.367546663	CSTB
ENSG00000116791.14	-0.247872563	0.021515274	0.367546663	CRYZ
ENSG00000136100.14	-0.132929269	0.021568151	0.367546663	VPS36
ENSG00000154518.10	0.125606662	0.021582293	0.367546663	ATP5MC3
ENSG00000131165.16	0.115329718	0.021589561	0.367546663	CHMP1A
ENSG00000223547.10	0.175438148	0.021591329	0.367546663	ZNF844
ENSG00000084072.17	0.167547295	0.021594489	0.367546663	PPIE
ENSG00000111669.15	0.11725747	0.021628951	0.367546663	TPI1
ENSG00000103653.17	0.118839203	0.021654892	0.367546663	CSK
ENSG00000086544.3	0.112925758	0.021669076	0.367546663	ITPKC
ENSG00000267904.1	-0.307973377	0.021707521	0.367546663	CTC-429P9.5
ENSG00000067221.14	0.146326925	0.021722772	0.367546663	STOML1
ENSG00000179091.5	0.115093461	0.021740526	0.367546663	CYC1
ENSG00000171169.9	0.090298918	0.02176295	0.367546663	NAIF1
ENSG00000130487.9	0.239081208	0.021765178	0.367546663	KLHDC7B
ENSG00000176871.9	-0.129369052	0.021766202	0.367546663	WSB2
ENSG00000214787.10	0.190843092	0.021775901	0.367546663	MS4A4E
ENSG00000206417.8	0.247942476	0.021784969	0.367546663	H1-10-AS1
ENSG00000137767.14	0.152995309	0.02178659	0.367546663	SQOR
ENSG00000261332.1	-0.522891585	0.021795417	0.367546663	RP11-297C4.1
ENSG00000175550.8	0.183196293	0.02181121	0.367546663	DRAP1
ENSG00000159307.19	0.498228948	0.021852773	0.367572674	SCUBE1
ENSG00000175505.11	-0.196495893	0.021868507	0.367572674	CLCF1

ENSG00000260331.1	-0.531970752	0.021877263	0.367572674	RP11-111J6.2
ENSG00000184402.15	-0.124071461	0.021884447	0.367572674	SS18L1
ENSG00000271554.1	-0.401690501	0.021946003	0.367993457	RP4-665N4.8
ENSG00000125449.7	0.115162629	0.021985699	0.367993457	ARMC7
ENSG00000262339.5	-0.495543284	0.022009369	0.367993457	RP11-1197K16.2
ENSG00000162639.16	-0.145902607	0.022011132	0.367993457	HENMT1
ENSG00000233967.7	-0.308394187	0.022029252	0.367993457	RP11-250B2.3
ENSG00000001629.10	-0.164178808	0.022036537	0.367993457	ANKIB1
ENSG00000100578.18	-0.095734183	0.022061141	0.367993457	KIAA0586
ENSG00000181143.15	-0.754427183	0.022077273	0.367993457	MUC16
ENSG00000109805.10	-0.422025216	0.022078381	0.367993457	NCAPG
ENSG00000104219.13	-0.179849688	0.022104121	0.367993457	ZDHHC2
ENSG00000169139.12	-0.131777466	0.022106882	0.367993457	UBE2V2
ENSG00000112679.15	0.090567798	0.02213827	0.368217068	DUSP22
ENSG00000139737.22	-0.110605327	0.022168954	0.368247334	SLAIN1
ENSG00000160883.11	0.134669215	0.02221136	0.368247334	HK3
ENSG00000280202.1	-0.190465599	0.022224048	0.368247334	RP5-1180D12.1
ENSG00000081087.15	-0.144449814	0.02222924	0.368247334	OSTM1
ENSG00000253194.2	-0.518132135	0.02224107	0.368247334	RP11-351A11.1
ENSG00000108055.10	-0.114820323	0.022255736	0.368247334	SMC3
ENSG00000105711.14	0.259677058	0.022265784	0.368247334	SCN1B
ENSG00000115155.19	0.75114122	0.022312459	0.368721922	OTOF
ENSG00000120896.14	0.166681294	0.022333616	0.368774393	SORBS3
ENSG00000148290.10	0.11697028	0.022358812	0.368893422	SURF1
ENSG00000226648.2	-0.224286302	0.022396893	0.369224661	PLCG1-AS1
ENSG00000226318.1	-0.442723981	0.022435764	0.369335127	RPS3AP38
ENSG00000142227.11	0.158338087	0.022460407	0.369335127	EMP3
ENSG00000279789.1	-0.224940462	0.02246387	0.369335127	CTD-2574D22.3
ENSG00000267080.6	0.100189248	0.022475631	0.369335127	ASB16-AS1
ENSG00000278023.7	-0.383438258	0.022514359	0.369675312	RDM1
ENSG00000105669.14	0.131223872	0.02257575	0.370134691	COPE
ENSG00000235568.7	0.120773473	0.022600572	0.370134691	NFAM1
ENSG00000168564.6	-0.112024978	0.022605565	0.370134691	CDKN2AIP
ENSG00000154001.14	-0.088872827	0.022614529	0.370134691	PPP2R5E
ENSG00000177427.13	0.101496025	0.02263515	0.370176765	MIEF2
ENSG00000164967.10	0.139177887	0.022663321	0.370342148	RPP25L
ENSG00000266714.9	0.190700595	0.022715436	0.370624796	MYO15B
ENSG00000077150.20	0.128838994	0.022717257	0.370624796	NFKB2
ENSG00000131042.15	0.13023612	0.022734835	0.370624796	LILRB2
ENSG00000145287.11	0.173889958	0.022801259	0.371170561	PLAC8
ENSG00000120899.18	0.090461946	0.022804511	0.371170561	PTK2B
ENSG00000062725.10	-0.098775274	0.022879133	0.371956795	APPBP2
ENSG00000250420.8	-2.383847317	0.022916788	0.371956795	AACSP1
ENSG00000218018.3	0.133506754	0.022918611	0.371956795	RBM38-AS1
ENSG00000226261.1	0.441442897	0.022925365	0.371956795	PIMREGP1
ENSG00000166263.13	-0.194994332	0.022967376	0.371975718	STXBP4
ENSG00000274471.2	-0.377638639	0.022985155	0.371975718	HERC2P7

ENSG00000168890.14	0.102988208	0.023026272	0.371975718	TMEM150A
ENSG00000112576.13	0.120754937	0.02302867	0.371975718	CCND3
ENSG00000128534.8	-0.166611936	0.023031756	0.371975718	LSM8
ENSG00000116918.15	-0.174033461	0.02303536	0.371975718	TSNAX
ENSG00000108582.12	-0.152903232	0.023090081	0.372440001	CPD
ENSG00000203667.10	-0.213611301	0.023100433	0.372440001	COX20
ENSG00000031698.13	0.090882024	0.023142423	0.372737382	SARS1
ENSG00000157483.9	0.22682489	0.023155228	0.372737382	MYO1E
ENSG00000274383.1	0.380575631	0.023180496	0.372803822	CTD-2017F17.2
ENSG00000110011.13	0.124444832	0.023237248	0.372803822	DNAJC4
ENSG00000161835.11	0.192814331	0.023286259	0.372803822	TAMALIN
ENSG00000169919.17	0.100619146	0.023296332	0.372803822	GUSB
ENSG00000206567.10	0.157899905	0.023316018	0.372803822	AC022007.5
ENSG00000256720.1	-0.37815537	0.023317421	0.372803822	BTG1P1
ENSG00000265452.1	-0.406548766	0.023319795	0.372803822	MIR3682
ENSG00000220472.1	0.497811845	0.023322401	0.372803822	RP11-69L16.5
ENSG00000274124.1	-0.326809882	0.023379638	0.372803822	RP11-474C8.8
ENSG00000137675.5	-1.711142492	0.023408424	0.372803822	MMP27
ENSG00000159176.14	0.094277937	0.023410055	0.372803822	CSRP1
ENSG00000119707.14	-0.120337249	0.02344363	0.372803822	RBM25
ENSG00000108592.17	0.097749179	0.023447867	0.372803822	FTSJ3
ENSG00000111254.8	0.322515708	0.023450563	0.372803822	AKAP3
ENSG00000221500.1	-0.438186176	0.023464481	0.372803822	SNORD100
ENSG00000078304.19	-0.085895084	0.023476708	0.372803822	PPP2R5C
ENSG00000253616.5	0.246358218	0.023493974	0.372803822	RP11-875O11.3
ENSG00000122359.18	0.118203398	0.023499328	0.372803822	ANXA11
ENSG00000164134.13	-0.114269173	0.023504747	0.372803822	NAA15
ENSG00000183773.16	0.240237785	0.023532602	0.372918273	AIFM3
ENSG00000144224.17	-0.131638367	0.023548331	0.372918273	UBXN4
ENSG00000243989.9	0.270621123	0.023628166	0.373452925	ACY1
ENSG00000116962.15	0.270884238	0.023656096	0.373452925	NID1
ENSG00000109107.14	0.146698371	0.023670161	0.373452925	ALDOC
ENSG00000160752.15	0.088164961	0.023670467	0.373452925	FDPS
ENSG00000180921.7	0.176912178	0.023708347	0.373452925	FAM83H
ENSG00000029363.17	-0.121000386	0.023710706	0.373452925	BCLAF1
ENSG00000242445.1	0.442538291	0.023721036	0.373452925	RPL7AP11
ENSG00000182487.12	0.205888559	0.023735773	0.373452925	NCF1B
ENSG00000198585.12	0.100847586	0.023765075	0.373452925	NUDT16
ENSG00000070061.16	-0.078628599	0.023806061	0.373452925	ELP1
ENSG00000286365.1	-0.507505041	0.023833792	0.373452925	KB-1440D3.21
ENSG00000137504.15	-0.178391142	0.023837079	0.373452925	CREBZF
ENSG00000100982.12	0.103340655	0.023855468	0.373452925	PCIF1
ENSG00000263843.1	-0.25893194	0.023858934	0.373452925	MIF4GD-DT
ENSG00000137492.9	-0.143222085	0.02386076	0.373452925	THAP12
ENSG00000100802.15	0.099945669	0.023873454	0.373452925	C14orf93
ENSG00000239282.8	0.183910724	0.023929208	0.373950557	CASTOR1
ENSG00000251411.1	0.387298353	0.023941734	0.373950557	RP11-397E7.4

ENSG00000177556.12	0.128599658	0.02398193	0.374059754	ATOX1
ENSG00000132664.12	-0.121742444	0.023985205	0.374059754	POLR3F
ENSG00000113391.19	-0.126099783	0.02410114	0.374731321	FAM172A
ENSG00000124251.11	-0.242847267	0.024102291	0.374731321	TP53TG5
ENSG00000015171.20	-0.131544715	0.024103064	0.374731321	ZMYND11
ENSG00000162894.12	0.152343301	0.024110974	0.374731321	FCMR
ENSG00000099953.10	0.252134272	0.024119629	0.374731321	MMP11
ENSG00000166250.12	-0.321820111	0.024168927	0.375088857	CLMP
ENSG00000105643.11	0.077734163	0.024179221	0.375088857	ARRDC2
ENSG00000118985.16	-0.149165503	0.024237354	0.375706465	ELL2
ENSG00000287837.1	-0.286120216	0.024295009	0.375806711	RP11-471M10.3
ENSG00000058668.15	-0.14295942	0.024329847	0.375806711	ATP2B4
ENSG00000182199.11	0.112325112	0.024331423	0.375806711	SHMT2
ENSG00000105443.15	0.096558464	0.024339121	0.375806711	CYTH2
ENSG00000151414.15	-0.190471969	0.024350428	0.375806711	NEK7
ENSG00000113569.16	-0.079771958	0.024365262	0.375806711	NUP155
ENSG00000105223.20	0.14311065	0.024372095	0.375806711	PLD3
ENSG00000128262.8	1.330024008	0.024428542	0.376194743	POM121L9P
ENSG00000271868.1	-0.469070972	0.024434111	0.376194743	RP11-1293J14.1
ENSG00000277791.5	0.140997323	0.024468199	0.376194743	PSMB3
ENSG00000250859.1	0.271112723	0.02448109	0.376194743	HNRNPKP1
ENSG00000148019.14	-0.191292832	0.024488979	0.376194743	CEP78
ENSG00000187730.9	0.93896206	0.024531632	0.376322044	GABRD
ENSG00000072135.13	0.108141764	0.024546434	0.376322044	PTPN18
ENSG00000113595.15	-0.20323131	0.024559114	0.376322044	TRIM23
ENSG00000118058.23	-0.118461413	0.024570666	0.376322044	KMT2A
ENSG00000099219.14	-0.141903505	0.024609457	0.376634878	ERMP1
ENSG00000142687.18	0.114114966	0.024651403	0.376995504	KIAA0319L
ENSG00000102910.14	-0.093621665	0.024691308	0.377324393	LONP2
ENSG00000182154.8	0.188691698	0.024726057	0.377574067	MRPL41
ENSG00000189079.17	-0.112961549	0.024759294	0.377800299	ARID2
ENSG00000137270.11	0.267161414	0.024794073	0.378049698	GCM1
ENSG00000111615.14	-0.143428934	0.024812756	0.378053495	KRR1
ENSG00000276231.5	0.232130241	0.024946756	0.379171266	PIK3R6
ENSG00000139793.19	-0.182633199	0.02495051	0.379171266	MBNL2
ENSG00000278129.2	-0.102324979	0.025010657	0.379171266	ZNF8
ENSG00000253522.6	-0.201356541	0.025024705	0.379171266	MIR3142HG
ENSG00000225331.1	-0.536359511	0.025027484	0.379171266	LINC01678
ENSG00000230982.1	-0.295443154	0.025028464	0.379171266	DSTNP1
ENSG00000176463.14	0.102646897	0.02503161	0.379171266	SLCO3A1
ENSG00000169136.12	0.177296762	0.02503403	0.379171266	ATF5
ENSG00000167770.12	0.11056298	0.025058807	0.379200583	OTUB1
ENSG00000004700.16	-0.186278465	0.025072947	0.379200583	RECQL
ENSG00000211654.2	0.939736805	0.025119158	0.379619525	IGLV5-37
ENSG00000148690.12	-0.150933329	0.025153241	0.379679005	FRA10AC1
ENSG00000286136.2	-0.374652953	0.025160121	0.379679005	RP11-348N12.2
ENSG00000245156.1	-0.193330882	0.025198884	0.379984343	RP11-867G23.3

ENSG00000092140.16	-0.175012337	0.025233197	0.380222199	G2E3
ENSG00000205426.10	0.547926623	0.025303039	0.380326946	KRT81
ENSG00000111785.21	-0.085886011	0.025329523	0.380326946	RIC8B
ENSG00000260911.2	0.22840042	0.025333988	0.380326946	RP11-196G11.2
ENSG00000176018.13	-0.198019581	0.025361736	0.380326946	LYSMD3
ENSG00000175106.17	-0.161351714	0.025382507	0.380326946	TVP23C
ENSG00000085265.11	0.17143335	0.025397247	0.380326946	FCN1
ENSG00000279880.1	-0.45529943	0.025410751	0.380326946	RP11-855A2.2
ENSG00000065427.15	0.094165018	0.025413524	0.380326946	KARS1
ENSG00000078687.17	-0.11509224	0.025415027	0.380326946	TNRC6C
ENSG00000260361.1	0.241419089	0.025438006	0.380326946	CTD-2313J17.5
ENSG00000260924.2	0.24546211	0.025466219	0.380326946	LINC01311
ENSG00000109089.7	0.462309954	0.025502811	0.380326946	CDR2L
ENSG00000108528.14	0.104623241	0.025510023	0.380326946	SLC25A11
ENSG00000229405.1	-0.586303554	0.025537149	0.380326946	AC092580.1
ENSG00000169926.11	-0.091206441	0.02557704	0.380326946	KLF13
ENSG00000163191.6	0.166294727	0.025593579	0.380326946	S100A11
ENSG00000205593.12	0.165704996	0.025610983	0.380326946	DENND6B
ENSG00000254595.1	-0.262350304	0.025613197	0.380326946	CTD-2010I16.1
ENSG00000108828.16	0.128572309	0.025632527	0.380326946	VAT1
ENSG00000189227.6	0.147687409	0.025641097	0.380326946	C15orf61
ENSG00000180479.14	-0.178875427	0.025688426	0.380326946	ZNF571
ENSG00000268034.1	0.396175661	0.025725294	0.380326946	AC005795.1
ENSG00000154016.14	0.176900609	0.025727654	0.380326946	GRAP
ENSG00000284770.2	-0.186283684	0.025777882	0.380326946	TBCE
ENSG00000249087.7	0.18729079	0.02578009	0.380326946	ZNF436-AS1
ENSG00000077254.14	-0.103735356	0.025788944	0.380326946	USP33
ENSG00000060339.14	-0.113168751	0.025791698	0.380326946	CCAR1
ENSG00000105793.16	-0.130607958	0.025805397	0.380326946	GTPBP10
ENSG00000168411.14	-0.086956827	0.025811997	0.380326946	RFWD3
ENSG00000008735.14	0.41772061	0.025829962	0.380326946	MAPK8IP2
ENSG00000156017.13	-0.171251174	0.025831854	0.380326946	CARNMT1
ENSG00000173545.5	0.098128803	0.025851304	0.380326946	ZNF622
ENSG00000162461.8	0.214019542	0.025862098	0.380326946	SLC25A34
ENSG00000007520.4	0.124717384	0.025870689	0.380326946	TSR3
ENSG00000102879.16	0.104582986	0.025912579	0.380341511	CORO1A
ENSG00000119421.7	0.135862983	0.025952952	0.380341511	NDUFA8
ENSG00000106348.18	0.117173127	0.025997978	0.380341511	IMPDH1
ENSG00000278535.5	0.16407144	0.026007942	0.380341511	DHRS11
ENSG00000108784.10	0.10890245	0.026008656	0.380341511	NAGLU
ENSG00000168724.17	-0.102668283	0.026026019	0.380341511	DNAJC21
ENSG00000110074.11	0.122241528	0.02603032	0.380341511	FOXRED1
ENSG00000047056.16	-0.059367914	0.026035703	0.380341511	WDR37
ENSG00000102878.18	0.190848102	0.026043131	0.380341511	HSF4
ENSG00000104951.16	0.306005106	0.02609898	0.380341511	IL4I1
ENSG00000174606.14	-0.127636817	0.026102729	0.380341511	ANGEL2
ENSG00000082153.18	-0.093122067	0.026108898	0.380341511	BZW1

ENSG00000122679.8	0.611713157	0.026112778	0.380341511	RAMP3
ENSG00000243389.1	0.353132282	0.026146473	0.380415736	AC012442.5
ENSG00000177156.11	0.139612508	0.026172651	0.380415736	TALDO1
ENSG00000139567.13	0.288152131	0.026173523	0.380415736	ACVRL1
ENSG00000148634.16	-0.104618233	0.026216843	0.38073581	HERC4
ENSG00000270804.1	0.195928188	0.026250239	0.38073581	CTD-2583A14.11
ENSG00000203910.9	-0.560941023	0.02625124	0.38073581	C1orf146
ENSG00000185507.21	0.272515941	0.026296202	0.381118381	IRF7
ENSG00000006062.18	0.096314549	0.026406847	0.381747414	MAP3K14
ENSG00000175376.9	-0.061782303	0.026433059	0.381747414	EIF1AD
ENSG00000116016.14	0.265313024	0.026472338	0.381747414	EPAS1
ENSG00000105568.18	0.112490249	0.026481382	0.381747414	PPP2R1A
ENSG00000277998.1	0.414777238	0.026496132	0.381747414	RP11-452N17.1
ENSG00000187608.10	0.607728079	0.026504247	0.381747414	ISG15
ENSG00000125734.15	0.107122932	0.026515909	0.381747414	GPR108
ENSG00000033627.17	0.118914542	0.02651695	0.381747414	ATP6V0A1
ENSG00000171130.19	0.155679312	0.026519682	0.381747414	ATP6V0E2
ENSG00000164169.13	-0.125668983	0.026533165	0.381747414	PRMT9
ENSG00000127995.17	-0.163645284	0.026544364	0.381747414	CASD1
ENSG00000071073.13	-0.135582512	0.026591321	0.382154742	MGAT4A
ENSG00000278876.1	0.374621986	0.026644058	0.382554284	RP11-498C9.4
ENSG00000223387.6	0.932422333	0.02665643	0.382554284	LINC02068
ENSG00000165304.8	-0.426191263	0.026695459	0.382811581	MELK
ENSG00000064313.12	-0.116397139	0.026711692	0.382811581	TAF2
ENSG00000142408.7	0.414685884	0.026736547	0.382900215	CACNG8
ENSG00000163069.13	-0.18863279	0.026865117	0.384230991	SGCB
ENSG00000152413.15	-0.342780372	0.026868531	0.384230991	HOMER1
ENSG00000124635.9	0.280635927	0.026885677	0.384230991	H2BC11
ENSG00000167118.11	0.108318666	0.026937691	0.384706245	URM1
ENSG00000115652.15	0.089202063	0.026976722	0.384982698	UXS1
ENSG00000156990.14	0.099598554	0.027031959	0.384982698	RPUSD3
ENSG00000139372.15	-0.128013024	0.027038757	0.384982698	TDG
ENSG00000173511.10	0.150229926	0.027050544	0.384982698	VEGFB
ENSG00000118492.18	0.932655496	0.02705091	0.384982698	ADGB
ENSG00000007312.13	0.266551562	0.027081236	0.385147013	CD79B
ENSG00000131747.15	-0.33989669	0.027118199	0.385405416	TOP2A
ENSG00000139697.14	-0.078222783	0.027166679	0.3856112	SBNO1
ENSG00000273749.5	0.145449325	0.027170284	0.3856112	CYFIP1
ENSG00000287978.1	0.367826412	0.027211946	0.385935394	CH17-408M7.2
ENSG00000258811.1	0.453332391	0.027277336	0.386458102	CTD-3051D23.1
ENSG00000125835.19	0.129984389	0.02728649	0.386458102	SNRPB
ENSG00000164543.7	-0.088947386	0.027319261	0.386634113	STK17A
ENSG00000197969.14	-0.162125072	0.027338708	0.386634113	VPS13A
ENSG00000100804.19	0.114047972	0.027363249	0.386634113	PSMB5
ENSG00000183475.13	-0.095796776	0.027382529	0.386634113	ASB7
ENSG00000178149.17	0.103530915	0.027397315	0.386634113	DALRD3
ENSG00000272636.4	-0.692178699	0.027427804	0.386634113	DOC2B

ENSG00000111057.11	0.242892885	0.02743541	0.386634113	KRT18
ENSG00000163104.18	-0.138386488	0.027464312	0.386634113	SMARCAD1
ENSG00000287095.1	-0.293396938	0.027475839	0.386634113	CTC-215C12.2
ENSG00000263465.5	-0.085243538	0.027487446	0.386634113	SRSF8
ENSG00000169379.17	-0.215649721	0.02751349	0.386701454	ARL13B
ENSG00000155755.19	-0.194885421	0.027529946	0.386701454	TMEM237
ENSG00000137507.11	0.358526335	0.02757307	0.387042109	LRRC32
ENSG00000246705.4	0.137379251	0.027626126	0.387128951	H2AJ
ENSG00000159063.14	0.095908024	0.027633178	0.387128951	ALG8
ENSG00000276568.1	0.503525325	0.02764691	0.387128951	Metazoa_SRP
ENSG00000076641.4	-0.151147077	0.027663427	0.387128951	PAG1
ENSG00000156802.13	-0.159494438	0.02767796	0.387128951	ATAD2
ENSG00000159147.18	-0.115740253	0.027715267	0.387128951	DONSON
ENSG00000197448.14	0.105842403	0.027724977	0.387128951	GSTK1
ENSG00000160993.4	0.110965127	0.027731031	0.387128951	ALKBH4
ENSG00000115020.17	-0.145887096	0.027758516	0.387128951	PIKFYVE
ENSG00000179981.11	-0.131033889	0.027772819	0.387128951	TSHZ1
ENSG00000171735.19	0.12049191	0.027786903	0.387128951	CAMTA1
ENSG00000232748.3	-0.33614289	0.027850729	0.387512071	RP11-196G11.6
ENSG00000270903.1	-0.487280578	0.027852194	0.387512071	HNRNPA3P9
ENSG00000147813.16	0.216704138	0.027882953	0.387677019	NAPRT
ENSG00000163481.8	0.103139425	0.027922757	0.387967416	RNF25
ENSG00000127084.19	0.106830722	0.027960291	0.388097687	FGD3
ENSG00000211897.9	0.521140196	0.027977476	0.388097687	IGHG3
ENSG00000007255.10	0.142946728	0.028005512	0.388097687	TRAPPC6A
ENSG00000122477.13	-0.228323739	0.028093934	0.388097687	LRRC39
ENSG00000120137.7	-0.146911365	0.02811788	0.388097687	PANK3
ENSG00000244687.12	-0.144623635	0.028122456	0.388097687	UBE2V1
ENSG00000276168.1	-0.305597027	0.028156153	0.388097687	RN7SL1
ENSG00000156521.14	0.083930079	0.028167978	0.388097687	TYSND1
ENSG00000244627.5	-0.14552464	0.028180462	0.388097687	TPTEP2
ENSG00000197343.11	-0.072688547	0.028205178	0.388097687	ZNF655
ENSG00000134077.16	-0.100453006	0.028218965	0.388097687	THUMPD3
ENSG00000279722.1	-0.203063643	0.028255667	0.388097687	RP11-44F14.6
ENSG00000211771.1	-0.214505041	0.028261513	0.388097687	TRBJ2-7
ENSG00000242574.9	0.135635883	0.028264326	0.388097687	HLA-DMB
ENSG00000135220.11	1.753843936	0.028269358	0.388097687	UGT2A3
ENSG00000117650.13	-0.485209321	0.028307437	0.388097687	NEK2
ENSG00000139734.19	-0.573851137	0.028319316	0.388097687	DIAPH3
ENSG00000219470.1	0.257777181	0.028328044	0.388097687	RP3-337H4.6
ENSG00000260277.2	-0.29454893	0.028335596	0.388097687	RP11-101E7.2
ENSG00000143119.14	0.073168585	0.028347421	0.388097687	CD53
ENSG00000100721.11	0.424603759	0.028383451	0.388097687	TCL1A
ENSG00000196358.11	0.211334553	0.028412756	0.388097687	NTNG2
ENSG00000166770.11	0.331536945	0.028425224	0.388097687	ZNF667-AS1
ENSG00000118600.12	-0.121336291	0.028430314	0.388097687	RXYLT1
ENSG00000140287.11	0.547910664	0.028436027	0.388097687	HDC

ENSG00000178917.17	-0.204212372	0.02844869	0.388097687	ZNF852
ENSG00000103510.20	0.080393113	0.028462504	0.388097687	KAT8
ENSG00000171160.18	0.313927842	0.028470141	0.388097687	MORN4
ENSG00000102854.16	-1.521728853	0.02848628	0.388097687	MSLN
ENSG00000241506.1	0.256295862	0.028499859	0.388097687	PSMC1P1
ENSG00000080819.9	-0.117467387	0.028533923	0.388303713	CPOX
ENSG00000101160.15	0.104600261	0.028564791	0.388466009	CTSZ
ENSG00000174106.3	-0.160462655	0.028612986	0.38886356	LEMD3
ENSG00000198799.12	-0.077698275	0.028676509	0.389468774	LRIG2
ENSG00000211647.1	0.903293882	0.028722685	0.389837744	IGLV5-48
ENSG00000182175.14	-0.517395106	0.028773969	0.389956925	RGMA
ENSG00000175309.15	0.078906335	0.028809626	0.389956925	PHYKPL
ENSG00000176087.16	0.092368602	0.028810872	0.389956925	SLC35A4
ENSG00000115234.11	0.099280725	0.028831488	0.389956925	SNX17
ENSG00000170296.10	0.125893681	0.028866085	0.389956925	GABARAP
ENSG00000084207.18	0.152103646	0.028876625	0.389956925	GSTP1
ENSG00000114107.9	-0.2187994	0.028879972	0.389956925	CEP70
ENSG00000005469.12	-0.118943131	0.028897062	0.389956925	CROT
ENSG00000177479.20	-0.057045412	0.0289026	0.389956925	ARIH2
ENSG00000287900.1	-1.247353612	0.028942771	0.38998697	AC061961.2
ENSG00000272693.4	-0.407696785	0.02894286	0.38998697	RP11-479O9.4
ENSG00000168944.17	-0.152239103	0.02897592	0.390176079	CEP120
ENSG00000174405.14	-0.20473418	0.029030906	0.390417755	LIG4
ENSG00000244462.8	-0.060588959	0.029042987	0.390417755	RBM12
ENSG00000072849.11	0.073921788	0.02905779	0.390417755	DERL2
ENSG00000116198.14	-0.100521037	0.029070017	0.390417755	CEP104
ENSG00000231628.1	-0.292295161	0.029099729	0.390447621	RP3-355L5.4
ENSG00000196968.11	-0.135865197	0.029110319	0.390447621	FUT11
ENSG00000261218.5	0.196793537	0.029236702	0.39146628	RP11-960L18.1
ENSG00000132294.15	-0.13932064	0.029288328	0.39146628	EFR3A
ENSG00000122694.16	0.100045203	0.029290196	0.39146628	GLIPR2
ENSG00000100991.12	0.078625561	0.029295188	0.39146628	TRPC4AP
ENSG00000260487.1	-0.444168579	0.029323969	0.39146628	RP11-297C4.3
ENSG00000211460.12	-0.070932287	0.029327131	0.39146628	TSN
ENSG00000267317.2	0.139874928	0.029402635	0.39146628	CTB-25B13.12
ENSG00000115827.14	-0.114501513	0.029409161	0.39146628	DCAF17
ENSG00000092051.17	0.228277178	0.029417952	0.39146628	JPH4
ENSG00000257703.6	-0.632627747	0.029428553	0.39146628	RP11-328J6.1
ENSG00000149231.16	-0.243490758	0.029472612	0.39146628	CCDC82
ENSG00000141959.17	0.111194796	0.029481693	0.39146628	PFKL
ENSG00000233191.2	0.442693704	0.029508566	0.39146628	AC006372.6
ENSG00000129003.19	-0.149559328	0.029545768	0.39146628	VPS13C
ENSG00000100983.12	0.098180581	0.029557256	0.39146628	GSS
ENSG00000183309.12	-0.083138072	0.029558005	0.39146628	ZNF623
ENSG00000087884.14	0.191173106	0.029560508	0.39146628	AAMDC
ENSG00000135720.13	-0.160975596	0.029613566	0.39146628	DYNC1LI2
ENSG00000166887.16	0.067393875	0.029651274	0.39146628	VPS39

ENSG00000149541.10	0.124216614	0.029721114	0.39146628	B3GAT3
ENSG00000257261.6	0.30028506	0.0297468	0.39146628	RP11-96H19.1
ENSG00000136243.17	-0.118986954	0.029753453	0.39146628	NUP42
ENSG00000085514.16	0.1291496	0.02975718	0.39146628	PILRA
ENSG00000103507.14	0.123567494	0.029759433	0.39146628	BCKDK
ENSG00000186523.14	-0.46110441	0.029780978	0.39146628	FAM86B1
ENSG00000182141.10	-0.201528207	0.029798593	0.39146628	ZNF708
ENSG00000119632.4	0.182957785	0.02979886	0.39146628	IFI27L2
ENSG00000126804.14	-0.141801191	0.029800267	0.39146628	ZBTB1
ENSG00000177303.10	-0.369119117	0.029816974	0.39146628	CASKIN2
ENSG00000188211.9	-0.370618072	0.029840632	0.39146628	NCR3LG1
ENSG00000134283.18	-0.074269494	0.029879408	0.39146628	PPHLN1
ENSG00000124198.10	-0.139939093	0.029925659	0.39146628	ARFGEF2
ENSG00000288064.1	-0.593386766	0.029953901	0.39146628	RP11-629B4.1
ENSG00000159166.15	1.133995366	0.029981837	0.39146628	LAD1
ENSG00000168894.10	0.124099982	0.029997576	0.39146628	RNF181
ENSG00000228998.4	-0.378595424	0.030021355	0.39146628	RP11-697E2.7
ENSG00000183718.5	-0.139862379	0.030028206	0.39146628	TRIM52
ENSG00000165685.9	-0.795053877	0.03003641	0.39146628	TMEM52B
ENSG00000177989.14	0.275689935	0.030069787	0.39146628	ODF3B
ENSG00000232442.1	0.155505049	0.030090758	0.39146628	MHENCR
ENSG00000255135.4	0.23811194	0.03009209	0.39146628	RP11-111M22.3
ENSG00000184939.16	-0.117501723	0.030119798	0.39146628	ZFP90
ENSG00000108021.20	-0.142553828	0.03013255	0.39146628	TASOR2
ENSG00000137501.17	-0.236186908	0.030135811	0.39146628	SYTL2
ENSG00000077063.11	-0.621544235	0.030160611	0.39146628	CTTNBP2
ENSG00000256705.3	-0.289399902	0.030178513	0.39146628	CTD-2017C7.1
ENSG00000105321.14	0.134089531	0.030202663	0.39146628	CCDC9
ENSG00000266993.3	-0.278876722	0.030211705	0.39146628	RP4-657D16.3
ENSG00000278638.2	-0.481998872	0.030212765	0.39146628	CTC-268N12.2
ENSG00000263220.1	-0.46705501	0.030224528	0.39146628	RP11-420A6.2
ENSG00000140995.17	0.10322105	0.030258781	0.39146628	DEF8
ENSG00000274370.1	0.247368336	0.030273904	0.39146628	AC144831.3
ENSG00000127947.16	-0.128834135	0.030286852	0.39146628	PTPN12
ENSG00000256862.3	0.317889623	0.030308219	0.39146628	PARP11-AS1
ENSG00000121966.7	0.115942865	0.030365332	0.39146628	CXCR4
ENSG00000213465.8	0.15428504	0.030377593	0.39146628	ARL2
ENSG00000143761.16	0.075677771	0.030387738	0.39146628	ARF1
ENSG00000245648.2	-0.247399014	0.0303891	0.39146628	KLRK1-AS1
ENSG00000272831.1	0.362785042	0.03041026	0.39146628	RP11-792A8.4
ENSG00000163807.6	-0.086919412	0.030415001	0.39146628	KIAA1143
ENSG00000181481.14	0.103148477	0.030426109	0.39146628	RNF135
ENSG00000185658.14	-0.139649051	0.030444898	0.39146628	BRWD1
ENSG00000168813.17	-0.161537276	0.030455013	0.39146628	ZNF507
ENSG00000119689.15	0.043899757	0.030472955	0.39146628	DLST
ENSG00000270589.1	-0.187833992	0.030557164	0.39146628	RP11-348N5.7
ENSG00000174903.16	0.11052979	0.030567966	0.39146628	RAB1B

ENSG00000233850.1	0.323223149	0.030576307	0.39146628	AC103563.8
ENSG00000148935.11	-0.2419702	0.030578448	0.39146628	GAS2
ENSG00000161547.17	-0.077315234	0.030581946	0.39146628	SRSF2
ENSG00000213463.5	-0.132368365	0.030588151	0.39146628	SYNJ2BP
ENSG00000267632.1	-0.30548782	0.030594604	0.39146628	RP11-400F19.18
ENSG00000162378.13	-0.118825347	0.030616144	0.39146628	ZYG11B
ENSG00000110987.9	0.263002975	0.030630789	0.39146628	BCL7A
ENSG00000259318.1	-0.631193969	0.030638632	0.39146628	HMGN1P1
ENSG00000233297.4	0.487721159	0.030685775	0.39146628	RASA4DP
ENSG00000005436.14	-0.120193095	0.030709279	0.39146628	GCFC2
ENSG00000276317.1	0.182945324	0.030726833	0.39146628	RP11-93B14.10
ENSG00000125895.6	0.474442249	0.030733884	0.39146628	TMEM74B
ENSG00000133065.11	-0.089055345	0.030736029	0.39146628	SLC41A1
ENSG00000143815.15	-0.138782504	0.030743324	0.39146628	LBR
ENSG00000164081.13	0.103781085	0.030779512	0.39146628	TEX264
ENSG00000180694.14	-0.159226372	0.030800756	0.39146628	TMEM64
ENSG00000223553.6	0.501942975	0.030826055	0.39146628	SMPD4P1
ENSG00000242689.3	-0.382787947	0.030830824	0.39146628	CNTF
ENSG00000108788.12	0.065858464	0.03085868	0.39146628	MLX
ENSG00000070729.14	0.551182809	0.030858795	0.39146628	CNGB1
ENSG00000100060.18	0.092479986	0.03089019	0.39146628	MFNG
ENSG00000276529.1	0.246991009	0.030916515	0.39146628	AP001505.10
ENSG00000157540.22	-0.090523707	0.030917782	0.39146628	DYRK1A
ENSG00000167261.14	0.128474475	0.030919927	0.39146628	DPEP2
ENSG00000175455.16	-0.145029078	0.030923316	0.39146628	CCDC14
ENSG00000227671.4	-0.221588955	0.030990894	0.391977245	ZNF731P
ENSG00000260992.1	0.25591606	0.031017452	0.391977245	DOCK9-DT
ENSG00000111875.8	-0.120760876	0.031028306	0.391977245	ASF1A
ENSG00000163956.13	0.112563525	0.031042653	0.391977245	LRPAP1
ENSG00000226029.1	-0.196861328	0.031059246	0.391977245	LINC01772
ENSG00000170325.16	-0.12390298	0.031079928	0.39199703	PRDM10
ENSG00000163751.4	0.489702542	0.031138757	0.392140094	CPA3
ENSG00000136824.19	-0.152134771	0.031143051	0.392140094	SMC2
ENSG00000047346.13	-0.140367565	0.031151811	0.392140094	FAM214A
ENSG00000132792.19	0.101282748	0.031167757	0.392140094	CTNNBL1
ENSG00000256673.1	-0.495261585	0.03128052	0.392816292	RP11-599J14.2
ENSG00000159720.12	0.129692149	0.031287985	0.392816292	ATP6V0D1
ENSG00000157106.17	-0.123814442	0.031303426	0.392816292	SMG1
ENSG00000184350.11	-1.084062609	0.031303483	0.392816292	MRGPRE
ENSG00000168269.10	0.74923253	0.03133045	0.392816292	FOXI1
ENSG00000136159.4	-0.149427395	0.03134394	0.392816292	NUDT15
ENSG00000030582.18	0.162141099	0.031384911	0.392816292	GRN
ENSG00000048028.11	-0.144629288	0.031398651	0.392816292	USP28
ENSG00000095383.20	0.123990596	0.031421501	0.392816292	TBC1D2
ENSG00000066557.6	-0.188412823	0.031429935	0.392816292	LRRC40
ENSG00000020577.14	0.294272598	0.031432199	0.392816292	SAMD4A
ENSG00000103035.11	0.063778846	0.031459306	0.392915623	PSMD7

ENSG00000100426.7	-0.086910909	0.031503412	0.393227011	ZBED4
ENSG00000131748.16	0.087756188	0.031610741	0.394326683	STARD3
ENSG00000108840.16	0.109536877	0.031697964	0.395174371	HDAC5
ENSG00000156671.15	-0.146211129	0.031740812	0.395185331	SAMD8
ENSG00000102977.17	0.113529346	0.031756146	0.395185331	ACD
ENSG00000206573.9	-0.113082696	0.031756652	0.395185331	THUMPD3-AS1
ENSG00000218073.1	-0.335831554	0.031812831	0.395595625	RP1-13D10.2
ENSG00000267787.7	0.135268037	0.031828872	0.395595625	RP11-35G9.5
ENSG00000113615.13	-0.135833942	0.031859737	0.395595625	SEC24A
ENSG00000104856.14	0.157769611	0.031866782	0.395595625	RELB
ENSG00000141452.10	0.075508848	0.031929844	0.396074412	RMC1
ENSG00000173327.8	0.122809759	0.031943977	0.396074412	MAP3K11
ENSG00000013810.21	0.118414636	0.031969934	0.396156744	TACC3
ENSG00000245025.3	-0.136449564	0.032073488	0.397199939	RP11-875O11.1
ENSG00000286786.1	-0.266209189	0.032110875	0.397418467	RP11-102L12.3
ENSG00000185527.12	0.208575345	0.032129892	0.397418467	PDE6G
ENSG00000204227.5	0.096291253	0.032154626	0.397484671	RING1
ENSG00000273218.1	0.293259947	0.032210202	0.397577026	LLNLR-246C6.1
ENSG00000112033.14	0.095459333	0.032210937	0.397577026	PPARD
ENSG00000286024.1	-0.48971861	0.032238314	0.397577026	CTD-2639E6.11
ENSG00000278239.1	0.665837965	0.032239643	0.397577026	AC020956.3
ENSG00000161671.17	0.096334461	0.032290719	0.397696604	EMC10
ENSG00000140006.12	-0.175910016	0.03230382	0.397696604	WDR89
ENSG00000085274.16	-0.188883226	0.032316579	0.397696604	MYNN
ENSG00000251474.6	-0.115978192	0.032332478	0.397696604	RPL32P3
ENSG00000141552.18	0.131290649	0.032357407	0.397696604	ANAPC11
ENSG00000224531.6	-0.158618392	0.032365693	0.397696604	SMIM13
ENSG00000143367.16	0.154529116	0.032394891	0.397735421	TUFT1
ENSG00000182810.7	0.087896686	0.03240764	0.397735421	DDX28
ENSG00000067601.8	0.231954984	0.032475132	0.398325367	PMS2P4
ENSG00000171148.14	0.100204237	0.032502633	0.398424384	TADA3
ENSG00000159352.16	0.108204509	0.032522332	0.398427703	PSMD4
ENSG00000177150.13	-0.113365775	0.032630304	0.399050133	FAM210A
ENSG00000276023.5	-0.163406202	0.032641207	0.399050133	DUSP14
ENSG00000225663.8	0.142175624	0.032658984	0.399050133	MCRIP1
ENSG00000288704.1	0.434333096	0.032692414	0.399050133	LLNLR-252D12.1
ENSG00000281128.2	0.553268237	0.032695848	0.399050133	PTENP1-AS
ENSG00000149932.17	0.134781184	0.032706581	0.399050133	TMEM219
ENSG00000237073.1	-0.337606896	0.032751038	0.399050133	RP11-168K11.2
ENSG00000204392.11	0.125697813	0.032767178	0.399050133	LSM2
ENSG00000175213.3	0.104190796	0.032779826	0.399050133	ZNF408
ENSG00000114738.11	0.09652691	0.03279392	0.399050133	MAPKAPK3
ENSG00000211898.8	0.417760358	0.032795476	0.399050133	IGHD
ENSG00000227946.1	-0.202186946	0.032806638	0.399050133	AC007383.3
ENSG00000051180.17	-0.290783349	0.032828238	0.39907617	RAD51
ENSG00000186020.13	-0.128261735	0.032881573	0.399298878	ZNF529
ENSG00000005075.15	0.129654394	0.032901124	0.399298878	POLR2J

ENSG00000021826.17	-0.463710594	0.032904969	0.399298878	CPS1
ENSG00000211934.3	0.629471205	0.032926489	0.39932373	IGHV1-2
ENSG00000168256.18	0.082258299	0.032987895	0.39959094	NKIRAS2
ENSG00000127831.11	0.381269182	0.033001313	0.39959094	VIL1
ENSG00000286909.1	-0.376201457	0.033013341	0.39959094	RP11-496H1.3
ENSG00000261476.1	-0.505226972	0.03302646	0.39959094	RP11-77K12.3
ENSG00000184730.11	0.186670657	0.033057354	0.399728897	APOBR
ENSG00000114956.20	0.107012143	0.033130047	0.400371835	DGUOK
ENSG00000172819.17	0.107866104	0.033163607	0.400541377	RARG
ENSG00000137449.17	-0.223305945	0.033183994	0.400551704	CPEB2
ENSG00000142039.4	0.087635087	0.033206819	0.400591436	CCDC97
ENSG00000196220.16	0.228217097	0.033255747	0.400601574	SRGAP3
ENSG00000177432.8	-0.215771095	0.03326533	0.400601574	NAP1L5
ENSG00000204264.12	0.137858892	0.033279437	0.400601574	PSMB8
ENSG00000146802.13	-0.133659972	0.033285795	0.400601574	TMEM168
ENSG00000064601.21	0.129449645	0.033465169	0.402386679	CTSA
ENSG00000261801.6	-0.16266054	0.03347336	0.402386679	LOXL1-AS1
ENSG00000260917.1	-0.10197127	0.033517517	0.402681454	RP11-57H14.4
ENSG00000012983.12	-0.146399995	0.03354147	0.402733296	MAP4K5
ENSG00000066422.5	-0.160501549	0.033594788	0.403135068	ZBTB11
ENSG00000110042.8	0.180722085	0.033614246	0.403135068	DTX4
ENSG00000248592.7	0.160754286	0.033642199	0.403234494	STIMATE-MUSTN1
ENSG00000165506.15	-0.132375469	0.033669202	0.403322432	DNAAF2
ENSG00000137409.20	0.067777816	0.033710572	0.403582264	MTCH1
ENSG00000104870.13	0.127231146	0.033745968	0.403625794	FCGRT
ENSG00000156256.15	-0.155347007	0.033753571	0.403625794	USP16
ENSG00000161999.13	0.11182112	0.033827516	0.403852397	JMJD8
ENSG00000055609.21	-0.157402975	0.03382921	0.403852397	KMT2C
ENSG00000117620.15	-0.152802708	0.033831598	0.403852397	SLC35A3
ENSG00000228887.3	0.385848017	0.033901025	0.404445739	EEF1DP1
ENSG00000137845.15	-0.122526876	0.033938085	0.404527174	ADAM10
ENSG00000270681.1	0.324038431	0.033947302	0.404527174	RP11-372K14.2
ENSG00000264773.1	0.419618631	0.034065921	0.405403422	MIR4420
ENSG00000138867.17	0.082741162	0.034067581	0.405403422	GUCD1
ENSG00000167216.18	0.179022394	0.034116475	0.405403422	KATNAL2
ENSG00000174485.16	-0.113288668	0.034132241	0.405403422	DENND4A
ENSG00000146005.4	-0.451102812	0.034148439	0.405403422	PSD2
ENSG00000161405.17	-0.120812065	0.034151189	0.405403422	IKZF3
ENSG00000114812.13	0.156640476	0.034181088	0.405403422	VIPR1
ENSG00000274523.5	0.098475509	0.034205443	0.405403422	RCC1L
ENSG00000103599.20	-0.415260239	0.034210451	0.405403422	IQCH
ENSG00000167325.15	-0.094618945	0.034218516	0.405403422	RRM1
ENSG00000151883.19	-0.112173785	0.034255531	0.405508119	PARP8
ENSG00000159140.21	-0.103827479	0.034266899	0.405508119	SON
ENSG00000038945.15	0.443764326	0.034290941	0.405558606	MSR1
ENSG00000255837.2	-0.227301821	0.034314329	0.405601302	TAS2R20
ENSG00000118620.13	-0.162733798	0.034390885	0.406272042	ZNF430

ENSG00000168876.9	-0.175974549	0.034435612	0.406300181	ANKRD49
ENSG00000157224.16	-0.293475993	0.034459482	0.406300181	CLDN12
ENSG00000140398.14	0.1814289	0.034469042	0.406300181	NEIL1
ENSG00000051523.11	0.129886219	0.034472514	0.406300181	CYBA
ENSG00000100056.12	0.100763608	0.034513181	0.40654585	ESS2
ENSG00000108852.15	0.326989919	0.034551862	0.406634858	MPP2
ENSG00000158092.7	-0.097966288	0.034560394	0.406634858	NCK1
ENSG00000254703.2	0.177065	0.03458981	0.406747601	SENCR
ENSG00000136982.6	-0.403972329	0.034622864	0.406902977	DSCC1
ENSG00000224272.2	-0.824901584	0.034715623	0.407521036	AC114730.3
ENSG00000211713.3	0.476076905	0.034717842	0.407521036	TRBV6-4
ENSG00000166619.15	0.06088716	0.034743302	0.407521036	BLCAP
ENSG00000250658.1	1.300538699	0.034754939	0.407521036	RP11-138B4.1
ENSG00000075303.13	-0.13272819	0.0347766	0.407542007	SLC25A40
ENSG00000104921.15	0.38237689	0.034805502	0.407647767	FCER2
ENSG00000171443.7	0.143246629	0.034879135	0.408204414	ZNF524
ENSG00000129538.14	0.518310737	0.034902184	0.408204414	RNASE1
ENSG00000273375.1	0.52242855	0.034913291	0.408204414	RP11-803P9.1
ENSG00000270000.1	0.566410567	0.034932648	0.408204414	RP3-449M8.9
ENSG00000204311.15	-0.224462597	0.035022335	0.409019388	PJVK
ENSG00000099817.12	0.109462363	0.035089012	0.409418686	POLR2E
ENSG00000138802.11	-0.093349183	0.035096453	0.409418686	SEC24B
ENSG00000173214.7	-0.124673182	0.035122326	0.409487579	MFSD4B
ENSG00000279089.1	-0.242535334	0.035151857	0.409599023	RP11-481C4.2
ENSG00000176208.9	-0.154207089	0.035220122	0.410161419	ATAD5
ENSG00000138594.14	-0.082466299	0.035243079	0.41019584	TMOD3
ENSG00000147439.13	0.108911393	0.035266915	0.410240441	BIN3
ENSG00000232855.7	-0.302575626	0.035375839	0.410883881	AF131217.1
ENSG00000066336.12	0.137679084	0.035387385	0.410883881	SPI1
ENSG00000245571.7	-0.117688435	0.035387835	0.410883881	FAM111A-DT
ENSG00000248712.7	0.188656996	0.03540491	0.410883881	CCDC153
ENSG00000005189.20	0.246231918	0.035460394	0.410883881	REXO5
ENSG00000179348.12	0.485017583	0.035461497	0.410883881	GATA2
ENSG00000144674.16	-0.146754744	0.035489298	0.410883881	GOLGA4
ENSG00000235081.1	-0.380113948	0.035505791	0.410883881	AC010492.2
ENSG00000253451.1	0.746006947	0.035520221	0.410883881	IGLV2-28
ENSG00000175066.16	-0.193896882	0.035527691	0.410883881	GK5
ENSG00000147799.11	0.424387502	0.035542618	0.410883881	ARHGAP39
ENSG00000287855.1	-0.265784884	0.035592622	0.410917097	LA16c-360B4.2
ENSG00000139405.16	0.09988506	0.035617774	0.410917097	RITA1
ENSG00000116539.14	-0.119267033	0.03562245	0.410917097	ASH1L
ENSG00000103355.14	0.421330958	0.035644102	0.410917097	PRSS33
ENSG00000168003.18	0.109807792	0.035650314	0.410917097	SLC3A2
ENSG00000173145.11	-0.17697626	0.035665713	0.410917097	NOC3L
ENSG00000249667.1	-0.263299167	0.035703334	0.410985626	LINC01259
ENSG00000130787.14	0.139966317	0.035714877	0.410985626	HIP1R
ENSG00000273174.1	0.201423921	0.035746966	0.410985626	RP11-434H6.6

ENSG00000272145.1	-0.216396264	0.035765129	0.410985626	NFYC-AS1
ENSG00000185697.17	-0.255666351	0.035783193	0.410985626	MYBL1
ENSG00000212232.1	-0.70597008	0.035791902	0.410985626	SNORD17
ENSG00000148719.16	0.085505347	0.035845376	0.411092958	DNAJB12
ENSG00000136689.19	0.196968524	0.035868108	0.411092958	IL1RN
ENSG00000188493.15	0.115746378	0.035901322	0.411092958	C19orf54
ENSG00000083099.11	-0.139713033	0.035925272	0.411092958	LYRM2
ENSG00000114062.22	-0.113835163	0.035953128	0.411092958	UBE3A
ENSG00000067225.19	0.117639881	0.035984474	0.411092958	PKM
ENSG00000138642.15	0.252897251	0.035984725	0.411092958	HERC6
ENSG00000184979.10	0.517488631	0.036009284	0.411092958	USP18
ENSG00000171161.13	0.096508914	0.03601038	0.411092958	ZNF672
ENSG00000181104.7	-0.261633814	0.036021306	0.411092958	F2R
ENSG00000281691.1	-0.348217495	0.036069371	0.411092958	RBM5-AS1
ENSG00000146215.14	0.241873821	0.036076247	0.411092958	CRIP3
ENSG00000157741.15	-0.102184773	0.036112968	0.411092958	UBN2
ENSG00000177096.9	0.152061393	0.036114072	0.411092958	PHETA2
ENSG00000166479.10	-0.175245404	0.036128553	0.411092958	TMX3
ENSG00000184840.12	0.096950386	0.036132792	0.411092958	TMED9
ENSG00000185624.16	0.091498077	0.036153483	0.411092958	P4HB
ENSG00000120129.6	0.174571833	0.036179207	0.411092958	DUSP1
ENSG00000161955.17	0.124866408	0.03618496	0.411092958	TNFSF13
ENSG00000205220.12	0.133064634	0.036207938	0.411092958	PSMB10
ENSG00000168913.7	0.29853053	0.036239286	0.411092958	ENHO
ENSG00000287593.1	0.505609209	0.036260403	0.411092958	RP3-413H6.3
ENSG00000060558.4	0.137449232	0.036294324	0.411092958	GNA15
ENSG00000040341.18	-0.101724265	0.036306832	0.411092958	STAU2
ENSG00000160570.14	0.104927547	0.036338388	0.411092958	DEDD2
ENSG00000179119.15	-0.105169134	0.03634211	0.411092958	SPTY2D1
ENSG00000012061.16	0.093020625	0.036342478	0.411092958	ERCC1
ENSG00000065485.20	0.151010619	0.036386241	0.4113611	PDIA5
ENSG00000204954.10	-0.149998781	0.036410502	0.411408582	C12orf73
ENSG00000268157.1	1.079857354	0.036459085	0.411730682	AC010524.4
ENSG00000125971.17	0.149406268	0.036487954	0.411829919	DYNLRB1
ENSG00000148498.16	0.327293364	0.036509046	0.411841323	PARD3
ENSG00000167565.13	0.102804896	0.036559277	0.411866278	SERTAD3
ENSG00000197323.12	-0.121491471	0.036584263	0.411866278	TRIM33
ENSG00000106113.19	-0.571785019	0.036602844	0.411866278	CRHR2
ENSG00000100246.13	0.097089099	0.036674119	0.411866278	DNAL4
ENSG00000207547.1	0.217777008	0.036676086	0.411866278	MIR25
ENSG00000090530.10	0.325626462	0.036677696	0.411866278	P3H2
ENSG00000102804.15	0.145222867	0.036683539	0.411866278	TSC22D1
ENSG00000145782.13	-0.099036465	0.036684774	0.411866278	ATG12
ENSG00000260793.2	0.135201813	0.036706901	0.411866278	RP5-882C2.2
ENSG00000211644.3	0.487166887	0.036712091	0.411866278	IGLV1-51
ENSG00000211809.2	0.293611939	0.03676885	0.412277516	TRAV27
ENSG00000196074.13	-0.267168358	0.036893251	0.413446337	SYCP2

ENSG00000204390.10	0.112080392	0.036941126	0.413447519	HSPA1L
ENSG00000110711.11	0.104279046	0.036948325	0.413447519	AIP
ENSG00000058063.16	-0.142415691	0.036953838	0.413447519	ATP11B
ENSG00000196437.12	-0.182508768	0.036980021	0.413514869	ZNF569
ENSG00000072195.15	0.269792511	0.037044867	0.41401424	SPEG
ENSG00000055957.11	0.269966296	0.037067991	0.414047041	ITIH1
ENSG00000036054.13	-0.129489766	0.037123693	0.414274674	TBC1D23
ENSG00000006744.19	0.085735051	0.037128772	0.414274674	ELAC2
ENSG00000139218.18	-0.105844928	0.037155614	0.41434874	SCAF11
ENSG00000232053.7	-0.406118092	0.037240442	0.4149095	AC009784.3
ENSG00000274808.6	0.539102517	0.037246362	0.4149095	TBC1D3B
ENSG00000155287.11	0.09203417	0.037277937	0.415035796	SLC25A28
ENSG00000015532.10	0.086350368	0.037326816	0.415354498	XYLT2
ENSG00000179913.11	0.521386005	0.037393749	0.41536106	B3GNT3
ENSG00000164896.20	0.105064983	0.037426097	0.41536106	FASTK
ENSG00000196655.12	0.107488362	0.037435068	0.41536106	TRAPPC4
ENSG00000040275.17	-0.156008014	0.037515147	0.41536106	SPDL1
ENSG00000244161.1	0.431262675	0.037515228	0.41536106	FLNB-AS1
ENSG00000249846.7	-0.347646822	0.03753444	0.41536106	LINC02021
ENSG00000169251.13	-0.188267028	0.037535045	0.41536106	NMD3
ENSG00000250348.1	0.419916622	0.037572637	0.41536106	RP11-466P24.6
ENSG00000006530.18	-0.073236306	0.037576627	0.41536106	AGK
ENSG00000139514.13	-0.112125647	0.0375902	0.41536106	SLC7A1
ENSG00000111412.6	-0.06191445	0.037592206	0.41536106	SPRING1
ENSG00000108510.10	-0.133247476	0.03759594	0.41536106	MED13
ENSG00000196152.10	-0.115209442	0.037605725	0.41536106	ZNF79
ENSG00000170100.14	-0.085498013	0.037610956	0.41536106	ZNF778
ENSG00000281649.2	-0.149370489	0.037674578	0.415553718	EBLN3P
ENSG00000158486.13	0.383608994	0.037699749	0.415553718	DNAH3
ENSG00000232729.8	-0.535673202	0.037720961	0.415553718	AC083884.8
ENSG00000284882.1	0.203117232	0.037726765	0.415553718	RP11-118B23.6
ENSG00000242960.1	0.267746138	0.037729716	0.415553718	FTH1P23
ENSG00000107643.16	-0.103781829	0.037793087	0.415904648	MAPK8
ENSG00000010072.16	-0.106176259	0.037802139	0.415904648	SPRTN
ENSG00000054654.19	-0.142716881	0.03787702	0.416256185	SYNE2
ENSG00000140464.20	0.207091842	0.037902729	0.416256185	PML
ENSG00000186815.13	0.114794076	0.037908433	0.416256185	TPCN1
ENSG00000118007.13	-0.129194208	0.03791528	0.416256185	STAG1
ENSG00000067955.15	-0.09366066	0.037943609	0.416334955	CBFB
ENSG00000213341.11	-0.131400232	0.037969538	0.416334955	CHUK
ENSG00000123268.9	-0.114282987	0.037991577	0.416334955	ATF1
ENSG00000173930.9	-0.203193984	0.038036699	0.416334955	SLCO4C1
ENSG00000265590.10	-0.284800259	0.038039952	0.416334955	CFAP298-TCP10L
ENSG00000015479.20	-0.217169281	0.038074697	0.416334955	MATR3
ENSG00000154945.7	-0.068964432	0.03807598	0.416334955	ANKRD40
ENSG00000090372.15	0.092792668	0.038103554	0.416334955	STRN4
ENSG00000185238.13	-0.110189793	0.038105164	0.416334955	PRMT3

ENSG00000118242.16	-0.141474098	0.038145633	0.41636283	MREG
ENSG00000154642.11	-0.158116991	0.038164768	0.41636283	C21orf91
ENSG00000280081.4	-1.407320342	0.038180389	0.41636283	LINC01667
ENSG00000128881.17	-0.111654946	0.038188925	0.41636283	TTBK2
ENSG00000180875.5	0.395804045	0.038249603	0.416802794	GREM2
ENSG00000242071.3	0.203823262	0.038289729	0.417018466	RPL7AP6
ENSG00000143970.18	-0.120709921	0.038314409	0.41706576	ASXL2
ENSG00000271662.1	-0.595362815	0.038417222	0.417537846	RP11-141C7.3
ENSG00000115524.17	-0.119944774	0.038419497	0.417537846	SF3B1
ENSG00000117020.19	-0.103079653	0.038428247	0.417537846	AKT3
ENSG00000093217.11	0.22142993	0.038453205	0.417537846	XYLB
ENSG00000231551.8	-0.149896202	0.038481043	0.417537846	PDE4DIPP6
ENSG00000139921.13	-0.188684087	0.038484272	0.417537846	TMX1
ENSG00000110367.13	-0.070126934	0.038545708	0.417537846	DDX6
ENSG00000157601.15	0.485048693	0.038559998	0.417537846	MX1
ENSG00000121895.8	0.146595662	0.038592621	0.417537846	TMEM156
ENSG00000161036.13	0.155254272	0.038606026	0.417537846	LRWD1
ENSG00000125826.21	0.132257252	0.038606831	0.417537846	RBCK1
ENSG00000080371.6	-0.090846364	0.038608873	0.417537846	RAB21
ENSG00000224745.1	-0.23955239	0.038636757	0.417537846	RP11-380G5.2
ENSG00000168385.18	-0.091388281	0.038642814	0.417537846	SEPTIN2
ENSG00000213809.9	-0.249124478	0.038724842	0.417915044	KLRK1
ENSG00000237702.2	0.304571862	0.03872951	0.417915044	TRBV3-1
ENSG00000176953.12	-0.059419741	0.038738858	0.417915044	NFATC2IP
ENSG00000279649.1	-0.242482864	0.038817597	0.418159628	RP11-96D1.8
ENSG00000057663.16	-0.147807781	0.038826003	0.418159628	ATG5
ENSG00000078053.17	-0.275118367	0.038840079	0.418159628	AMPH
ENSG00000231856.2	0.140014861	0.038853648	0.418159628	RP11-327P2.5
ENSG00000179950.14	0.099762601	0.038867281	0.418159628	PUF60
ENSG00000047617.16	0.683017287	0.038901117	0.418159628	ANO2
ENSG00000130255.13	0.156202259	0.038934059	0.418159628	RPL36
ENSG00000130429.15	0.109546232	0.038936026	0.418159628	ARPC1B
ENSG00000143436.11	0.086798813	0.038953562	0.418159628	MRPL9
ENSG00000196911.11	-0.205867361	0.038981423	0.418159628	KPNA5
ENSG00000124406.16	-0.185111624	0.039077674	0.418159628	ATP8A1
ENSG00000162971.11	-0.113674564	0.039087469	0.418159628	TYW5
ENSG00000154832.16	0.092093949	0.039087742	0.418159628	CXXC1
ENSG00000055917.16	-0.102281777	0.039091475	0.418159628	PUM2
ENSG00000288156.1	0.118774528	0.039099766	0.418159628	AC104530.1
ENSG00000105755.8	0.134640554	0.039111482	0.418159628	ETHE1
ENSG00000227057.10	0.094458491	0.039116932	0.418159628	WDR46
ENSG00000279759.1	-0.21392543	0.039128551	0.418159628	CTC-425O23.5
ENSG00000129535.13	0.150226025	0.039183685	0.418530737	NRL
ENSG00000240445.6	-0.180254674	0.039260913	0.418827375	FOXO3B
ENSG00000116406.19	-0.173227654	0.039294203	0.418827375	EDEM3
ENSG00000224093.5	0.48815744	0.039319364	0.418827375	BCAR3-AS1
ENSG00000169045.17	-0.073587299	0.039347035	0.418827375	HNRNPH1

ENSG00000087111.21	0.074743648	0.039381613	0.418827375	PIGS
ENSG00000144579.8	0.094630878	0.039412645	0.418827375	CTDSP1
ENSG00000142875.19	-0.169987171	0.039421309	0.418827375	PRKACB
ENSG00000137992.15	-0.106711565	0.039425096	0.418827375	DBT
ENSG00000169018.6	-0.102738698	0.039448274	0.418827375	FEM1B
ENSG00000215030.5	0.221781229	0.039460742	0.418827375	RPL13P12
ENSG00000205423.12	-0.116121825	0.039467824	0.418827375	CNEP1R1
ENSG00000287387.1	-0.229783068	0.039503563	0.418827375	RP11-339H12.2
ENSG00000232303.2	-0.390972939	0.039514791	0.418827375	DFFBP1
ENSG00000230262.8	-0.410870119	0.039531752	0.418827375	LINC02603
ENSG00000273002.1	0.141947058	0.039573624	0.418827375	ARHGEF2-AS2
ENSG00000050767.18	0.402301826	0.039575823	0.418827375	COL23A1
ENSG00000250365.7	-0.286887008	0.039592647	0.418827375	CTD-2213F21.2
ENSG00000160094.15	0.090819642	0.039599407	0.418827375	ZNF362
ENSG00000159377.11	0.100771234	0.039599487	0.418827375	PSMB4
ENSG00000249565.2	0.345168283	0.039665376	0.419025525	SERBP1P5
ENSG00000280385.1	-0.223033803	0.039677743	0.419025525	AP000648.5
ENSG00000113810.16	-0.188618909	0.039679519	0.419025525	SMC4
ENSG00000143194.13	1.079694009	0.039733615	0.419028576	MAEL
ENSG00000067900.8	-0.17852177	0.039772799	0.419028576	ROCK1
ENSG00000196821.10	0.08106239	0.039792593	0.419028576	ILRUN
ENSG00000131779.11	0.07751306	0.039794623	0.419028576	PEX11B
ENSG00000149600.12	0.102575017	0.039818032	0.419028576	COMMD7
ENSG00000100503.26	-0.113447774	0.039826772	0.419028576	NIN
ENSG00000196743.9	0.210868797	0.039829801	0.419028576	GM2A
ENSG00000116205.14	-0.077357014	0.039843267	0.419028576	TCEANC2
ENSG00000131023.13	-0.121679646	0.039892804	0.419063076	LATS1
ENSG00000175294.6	0.175701205	0.03990051	0.419063076	CATSPER1
ENSG00000211893.4	0.499204171	0.03990785	0.419063076	IGHG2
ENSG00000134308.14	-0.093135267	0.03995874	0.419098826	YWHAQ
ENSG00000152969.20	-0.326086745	0.039961924	0.419098826	JAKMIP1
ENSG00000115339.14	-0.161505105	0.039972562	0.419098826	GALNT3
ENSG00000155366.17	0.173594439	0.040001698	0.419189992	RHOC
ENSG00000235579.1	-0.300079749	0.04005834	0.419331516	RPL38P5
ENSG00000183696.14	0.115971371	0.040091085	0.419331516	UPP1
ENSG00000110344.10	-0.152171149	0.040122141	0.419331516	UBE4A
ENSG00000139531.13	0.112367734	0.040124477	0.419331516	SUOX
ENSG00000013275.8	0.094866485	0.040139592	0.419331516	PSMC4
ENSG00000131475.7	0.101279176	0.04014143	0.419331516	VPS25
ENSG00000171700.14	0.105078047	0.040160671	0.419331516	RGS19
ENSG00000198736.12	0.127978724	0.040202125	0.419331516	MSRB1
ENSG00000171812.13	0.206152878	0.040210893	0.419331516	COL8A2
ENSG00000144426.19	-0.190564335	0.040244514	0.419331516	NBEAL1
ENSG00000197713.15	-0.123963653	0.04025454	0.419331516	RPE
ENSG00000115325.14	0.101577584	0.040274626	0.419331516	DOK1
ENSG00000214975.4	0.287929482	0.04030397	0.419331516	PPIAP29
ENSG00000181472.5	-0.070408347	0.040387728	0.419331516	ZBTB2

ENSG00000070367.16	-0.132192414	0.04039933	0.419331516	EXOC5
ENSG00000160014.17	0.090496049	0.040421575	0.419331516	CALM3
ENSG00000267470.6	0.315004155	0.040422651	0.419331516	ZNF571-AS1
ENSG00000178502.6	-0.250007131	0.040430702	0.419331516	KLHL11
ENSG00000013583.10	0.137479907	0.040455388	0.419331516	HEBP1
ENSG00000170421.12	0.341157046	0.040460428	0.419331516	KRT8
ENSG00000257365.8	0.138356379	0.040465916	0.419331516	FNTB
ENSG00000156873.16	0.099510192	0.040479425	0.419331516	PHKG2
ENSG00000105705.16	0.078914289	0.040485489	0.419331516	SUGP1
ENSG00000276334.1	-0.414756458	0.040522691	0.41950497	AL133243.1
ENSG00000011426.11	-0.391320231	0.04055789	0.419513092	ANLN
ENSG00000274294.1	-0.349578799	0.040564388	0.419513092	RP11-20G13.5
ENSG00000167740.10	0.097093101	0.040603849	0.419584094	CYB5D2
ENSG00000238246.1	0.370488781	0.040621763	0.419584094	RP11-575A19.2
ENSG00000174886.14	0.133076586	0.040648647	0.419584094	NDUFA11
ENSG00000159082.18	-0.120458486	0.040672338	0.419584094	SYNJ1
ENSG00000135506.16	0.097524134	0.040673551	0.419584094	OS9
ENSG00000152234.16	0.065935449	0.0406945	0.419589148	ATP5F1A
ENSG00000075420.13	-0.141426915	0.040724322	0.419685625	FNDC3B
ENSG00000138138.14	-0.141423367	0.040749261	0.419731713	ATAD1
ENSG00000089916.18	-0.084531002	0.040849666	0.419961666	GPATCH2L
ENSG00000175130.7	0.145366391	0.040889522	0.419961666	MARCKSL1
ENSG00000107951.15	-0.073992736	0.040898925	0.419961666	MTPAP
ENSG00000072736.19	-0.121868248	0.040899682	0.419961666	NFATC3
ENSG00000163939.18	-0.097242926	0.040900116	0.419961666	PBRM1
ENSG00000213231.13	0.650800226	0.040901753	0.419961666	TCL1B
ENSG00000198826.11	-0.257504392	0.040914931	0.419961666	ARHGAP11A
ENSG00000137747.16	0.157431142	0.041001603	0.42012699	TMPRSS13
ENSG00000164088.18	0.087009424	0.041005593	0.42012699	PPM1M
ENSG00000172932.16	0.085727548	0.041015933	0.42012699	ANKRD13D
ENSG00000239388.9	-0.222235763	0.041080543	0.42012699	ASB14
ENSG00000287312.1	-0.736419794	0.041086519	0.42012699	RP13-726E6.4
ENSG00000132424.17	-0.131395784	0.041168877	0.42012699	PNISR
ENSG00000234282.1	-0.437934347	0.041192225	0.42012699	RP4-673D20.4
ENSG00000174197.17	-0.086201447	0.041193189	0.42012699	MGA
ENSG00000122042.10	-0.128119666	0.04120032	0.42012699	UBL3
ENSG00000155256.18	0.063637418	0.041224438	0.42012699	ZFYVE27
ENSG00000066777.9	-0.086504838	0.041244001	0.42012699	ARFGEF1
ENSG00000079819.19	0.152264427	0.041259863	0.42012699	EPB41L2
ENSG00000166483.12	-0.173768178	0.041266454	0.42012699	WEE1
ENSG00000279570.1	-0.378506271	0.041311372	0.42012699	CTD-2357A8.2
ENSG00000278206.1	-0.354780334	0.041318562	0.42012699	RP1-20N2.8
ENSG00000282608.2	0.325798577	0.041342246	0.42012699	ADORA3
ENSG00000223509.8	-0.20637241	0.04139614	0.42012699	RP11-632K20.7
ENSG00000149534.9	0.491511747	0.041408929	0.42012699	MS4A2
ENSG00000164080.14	-0.073276949	0.04142587	0.42012699	RAD54L2
ENSG00000213186.8	-0.142374429	0.041442517	0.42012699	TRIM59

ENSG00000275395.6	-0.309532203	0.041445911	0.42012699	FCGBP
ENSG00000185019.17	0.106845901	0.041455448	0.42012699	UBOX5
ENSG00000226233.1	0.753171266	0.041460355	0.42012699	RP1-151B14.9
ENSG00000122257.20	-0.078061091	0.041486298	0.42012699	RBBP6
ENSG00000287049.1	-0.444175513	0.041500153	0.42012699	RP11-27M15.2
ENSG00000106635.8	0.071165612	0.041523794	0.42012699	BCL7B
ENSG00000286616.1	-0.444413431	0.041525229	0.42012699	RP3-487J7.4
ENSG00000080345.18	-0.140976185	0.041546179	0.42012699	RIF1
ENSG00000159917.17	-0.132461995	0.041549076	0.42012699	ZNF235
ENSG00000211698.2	-0.4245367	0.041567082	0.42012699	TRGV4
ENSG00000108839.12	0.169806227	0.041576422	0.42012699	ALOX12
ENSG00000173272.16	0.182498685	0.04158659	0.42012699	MZT2A
ENSG00000235782.1	0.709830151	0.041699392	0.420147904	RP3-333A15.1
ENSG00000163349.22	-0.10803867	0.041701251	0.420147904	HIPK1
ENSG00000112759.19	0.287627915	0.04170829	0.420147904	SLC29A1
ENSG00000100147.14	-0.132223336	0.041735981	0.420147904	CCDC134
ENSG00000146842.17	-0.121397909	0.041740044	0.420147904	TMEM209
ENSG00000280383.1	-0.28540414	0.041755042	0.420147904	CTA-941F9.10
ENSG00000120832.10	-0.127383938	0.041781565	0.420147904	MTERF2
ENSG00000177200.18	-0.163592602	0.04178567	0.420147904	CHD9
ENSG00000235192.1	-0.225729535	0.041787917	0.420147904	AC009495.2
ENSG00000039600.11	0.477454037	0.04179353	0.420147904	SOX30
ENSG00000174365.20	0.114422936	0.041861582	0.420431826	SNHG11
ENSG00000273173.5	-0.358079572	0.041881381	0.420431826	SNURF
ENSG00000224420.3	0.280405785	0.041883276	0.420431826	ADM5
ENSG00000048649.14	-0.091971066	0.041960248	0.420975337	RSF1
ENSG00000068079.8	0.253640574	0.041978475	0.420975337	IFI35
ENSG00000197841.15	-0.209867499	0.042021643	0.42105066	ZNF181
ENSG00000061273.18	0.097676818	0.042027048	0.42105066	HDAC7
ENSG00000165181.17	-0.260133909	0.042060442	0.421179469	SHOC1
ENSG00000228476.2	-0.742714086	0.042092027	0.421290036	CSTP1
ENSG00000244165.2	0.134827063	0.042197293	0.421451218	P2RY11
ENSG00000100614.18	-0.129978248	0.042226238	0.421451218	PPM1A
ENSG00000164754.15	-0.087507995	0.04223242	0.421451218	RAD21
ENSG00000146232.17	0.122969859	0.042249729	0.421451218	NFKBIE
ENSG00000121680.16	0.10851773	0.042277979	0.421451218	PEX16
ENSG00000158615.9	-0.094620593	0.042284286	0.421451218	PPP1R15B
ENSG00000254598.3	0.358615414	0.04230205	0.421451218	CSNK2A3
ENSG00000059758.8	-0.158555625	0.042319207	0.421451218	CDK17
ENSG00000170619.10	0.081924555	0.042332251	0.421451218	COMMD5
ENSG00000211772.11	0.146174514	0.0423441	0.421451218	TRBC2
ENSG00000174738.13	-0.12314565	0.042355944	0.421451218	NR1D2
ENSG00000105176.18	-0.083548576	0.042366273	0.421451218	URI1
ENSG00000137171.15	0.078480721	0.042401958	0.421451218	KLC4
ENSG00000161011.20	0.101597119	0.04241471	0.421451218	SQSTM1
ENSG00000162927.14	-0.141290452	0.042416389	0.421451218	PUS10
ENSG00000085733.16	0.28596356	0.042467824	0.421713043	CTTN

ENSG00000265678.1	-0.203808092	0.042486396	0.421713043	RP11-1376P16.2
ENSG00000075643.6	-0.664164884	0.04250443	0.421713043	MOCOS
ENSG00000162517.13	0.093925549	0.042540947	0.421871247	PEF1
ENSG00000286885.1	-0.258267805	0.042575943	0.42201423	RP11-14H3.7
ENSG00000114544.17	0.086576591	0.042626221	0.422241441	SLC41A3
ENSG00000173153.17	0.088575099	0.042659608	0.422241441	ESRRA
ENSG00000072954.7	0.169441713	0.042671721	0.422241441	TMEM38A
ENSG00000135763.10	-0.110917362	0.042687091	0.422241441	URB2
ENSG00000243243.5	-0.363905356	0.042705808	0.422241441	AC073130.3
ENSG00000204650.14	-0.148282329	0.042722401	0.422241441	LINC02210
ENSG00000152455.16	-0.157112834	0.042857301	0.422317951	SUV39H2
ENSG00000160703.16	0.116236884	0.042904385	0.422317951	NLRX1
ENSG00000100227.18	0.064276259	0.042924302	0.422317951	POLDIP3
ENSG00000152213.4	0.095843798	0.042951533	0.422317951	ARL11
ENSG00000175164.16	0.541206635	0.042959214	0.422317951	ABO
ENSG00000162065.14	-0.087348987	0.04297577	0.422317951	TBC1D24
ENSG00000141985.9	0.090681008	0.042987822	0.422317951	SH3GL1
ENSG00000136536.15	-0.120219975	0.042995139	0.422317951	MARCHF7
ENSG00000239305.7	-0.08978141	0.043072006	0.422317951	RNF103
ENSG00000261371.6	0.102614473	0.043088818	0.422317951	PECAM1
ENSG00000176903.5	-0.099547293	0.043123882	0.422317951	PNMA1
ENSG00000144214.10	0.186603239	0.043178893	0.422317951	LYG1
ENSG00000076944.16	0.12530812	0.043205165	0.422317951	STXBP2
ENSG00000262223.8	-0.605874618	0.043228116	0.422317951	RP11-1055B8.3
ENSG00000041982.16	-0.444737404	0.043228686	0.422317951	TNC
ENSG00000163714.18	-0.116624616	0.04326005	0.422317951	U2SURP
ENSG00000224478.3	0.272054614	0.043261933	0.422317951	RP11-13P5.1
ENSG00000170677.6	-0.176370719	0.043268973	0.422317951	SOCS6
ENSG00000100439.10	0.101754281	0.043323907	0.422317951	ABHD4
ENSG00000131375.10	-0.128045378	0.043361195	0.422317951	CAPN7
ENSG00000151923.18	-0.048130868	0.043370361	0.422317951	TIAL1
ENSG00000088832.18	0.086941465	0.043376797	0.422317951	FKBP1A
ENSG00000273145.1	0.277453263	0.043396146	0.422317951	CITF22-92A6.1
ENSG00000112406.5	-0.101918041	0.043404047	0.422317951	HECA
ENSG00000125651.14	0.084166476	0.043411147	0.422317951	GTF2F1
ENSG00000185052.13	0.256739291	0.043418502	0.422317951	SLC24A3
ENSG00000196227.11	-0.111019409	0.043426768	0.422317951	FAM217B
ENSG00000262089.1	0.156067698	0.043471522	0.422317951	RP11-589P10.5
ENSG00000211658.2	0.651564234	0.043479351	0.422317951	IGLV3-27
ENSG00000211961.3	0.606476937	0.043527119	0.422317951	IGHV1-45
ENSG00000126261.13	-0.075112275	0.043529183	0.422317951	UBA2
ENSG00000113108.20	0.08893464	0.043531698	0.422317951	APBB3
ENSG00000229628.1	-0.48363528	0.043532411	0.422317951	AC073115.7
ENSG00000186615.11	0.252863416	0.043544075	0.422317951	KTN1-AS1
ENSG00000267737.1	0.33891414	0.043601376	0.422317951	AC061992.2
ENSG00000061987.16	-0.100261087	0.043645376	0.422317951	MON2
ENSG00000213516.10	-0.08271994	0.043647949	0.422317951	RBMXL1

ENSG00000182685.8	0.161987844	0.043663071	0.422317951	BRICD5
ENSG00000145332.14	-0.130247458	0.043664916	0.422317951	KLHL8
ENSG00000241157.1	-0.686494246	0.043691928	0.422317951	RPL32P32
ENSG00000156973.14	0.082248565	0.043699671	0.422317951	PDE6D
ENSG00000211456.13	-0.124059048	0.043705655	0.422317951	SACM1L
ENSG00000149357.10	0.107446637	0.043718622	0.422317951	LAMTOR1
ENSG00000203757.2	0.663876958	0.043727538	0.422317951	OR6K3
ENSG00000242220.9	-0.43963279	0.043730514	0.422317951	TCP10L
ENSG00000026652.15	-0.157448552	0.04378768	0.422317951	AGPAT4
ENSG00000139163.16	-0.172068806	0.043811468	0.422317951	ETNK1
ENSG00000147854.17	-0.082296168	0.043816242	0.422317951	UHRF2
ENSG00000100027.17	-0.174654638	0.043835957	0.422317951	YPEL1
ENSG00000240893.1	0.65242652	0.043846501	0.422317951	LINC02042
ENSG00000102753.10	-0.092996245	0.043879326	0.422317951	KPNA3
ENSG00000197568.14	0.178619669	0.043885269	0.422317951	HHLA3
ENSG00000154269.15	0.420541488	0.043921629	0.422317951	ENPP3
ENSG00000260075.1	-0.954326137	0.043934464	0.422317951	NSFP1
ENSG00000136156.15	0.095636117	0.04393877	0.422317951	ITM2B
ENSG00000119383.21	0.106416546	0.043942933	0.422317951	PTPA
ENSG00000286416.1	-0.325853582	0.043952606	0.422317951	RP11-128N14.6
ENSG00000196923.14	0.147020057	0.04395937	0.422317951	PDLIM7
ENSG00000272211.1	-0.264538641	0.043977869	0.422317951	RP11-347P5.1
ENSG00000169885.10	0.32623503	0.043981998	0.422317951	CALML6
ENSG00000151229.13	-0.204884952	0.043986305	0.422317951	SLC2A13
ENSG00000112514.18	0.126368631	0.044030566	0.42254509	CUTA
ENSG00000156030.13	-0.098899597	0.044097586	0.422858075	MIDEAS
ENSG00000153391.16	0.114647722	0.044104419	0.422858075	INO80C
ENSG00000162063.13	-0.138489243	0.044172644	0.423156043	CCNF
ENSG00000152332.16	-0.127318604	0.044196269	0.423156043	UHMK1
ENSG00000263528.8	0.096917227	0.044219411	0.423156043	IKBKE
ENSG00000152409.9	-0.126173356	0.044237957	0.423156043	JMY
ENSG00000141295.14	0.127131935	0.044238666	0.423156043	SCRN2
ENSG00000145386.11	-0.230396681	0.044275644	0.423312307	CCNA2
ENSG00000076924.12	0.095088616	0.044314171	0.423313535	XAB2
ENSG00000139146.14	-0.119194507	0.044327259	0.423313535	SINHCAF
ENSG00000181904.9	-0.158567464	0.044337696	0.423313535	C5orf24
ENSG00000204525.17	0.174561402	0.044358889	0.423318799	HLA-C
ENSG00000020181.18	0.238906978	0.044419401	0.423382926	ADGRA2
ENSG00000085719.13	-0.087278729	0.044423831	0.423382926	CPNE3
ENSG00000140025.16	0.167999105	0.044455287	0.423382926	EFCAB11
ENSG00000179295.18	-0.105565506	0.044487907	0.423382926	PTPN11
ENSG00000152443.13	-0.116667524	0.044504507	0.423382926	ZNF776
ENSG00000259315.1	-0.232731928	0.044532338	0.423382926	ACTG1P17
ENSG00000105726.17	0.100536101	0.044541526	0.423382926	ATP13A1
ENSG00000234127.9	0.097314895	0.044557106	0.423382926	TRIM26
ENSG00000230306.1	0.376917868	0.044571744	0.423382926	BANF1P2
ENSG00000104332.12	-0.85251724	0.044572057	0.423382926	SFRP1

ENSG00000251323.3	0.4337435	0.044614841	0.423593132	LINC02728	
ENSG00000205537.2	0.306613654	0.044750235	0.423681293	RP11-89H19.1	
ENSG00000127838.14	0.128818529	0.044785669	0.423681293	PNKD	
ENSG00000286423.1	-0.713531819	0.044850102	0.423681293	RP6-91H8.6	
ENSG00000158864.13	0.082198073	0.044870216	0.423681293	NDUFS2	
ENSG00000172403.11	0.339697883	0.044903332	0.423681293	SYNPO2	
ENSG00000021574.13	-0.119932994	0.044905516	0.423681293	SPAST	
ENSG00000104524.14	0.137013407	0.044914004	0.423681293	PYCR3	
ENSG00000271109.2	0.15174772	0.044954436	0.423681293	CTC-523E23.11	
ENSG00000143368.10	0.104948028	0.044990649	0.423681293	SF3B4	
ENSG00000167604.15	0.103998061	0.0449995	0.423681293	NFKBID	
ENSG00000235172.8	0.253045434	0.045016772	0.423681293	LINC01366	
ENSG00000162236.13	0.094649582	0.045022073	0.423681293	STX5	
ENSG00000179454.14	-0.165531669	0.045042235	0.423681293	KLHL28	
ENSG00000138029.14	0.065138028	0.045065619	0.423681293	HADHB	
ENSG00000215458.8	0.16188411	0.04507188	0.423681293	AATBC	
ENSG00000160218.13	-0.147552692	0.045072933	0.423681293	TRAPPC10	
ENSG00000226054.2	-0.195474773	0.045088045	0.423681293	MEMO1P1	
ENSG00000205710.4	-0.29744874	0.045088733	0.423681293	C17orf107	
ENSG00000204310.13	0.104666082	0.045090768	0.423681293	AGPAT1	
ENSG00000188647.13	-0.208424515	0.045092604	0.423681293	PTAR1	
ENSG00000054967.13	0.086348306	0.04509814	0.423681293	RELT	
ENSG00000023516.9	-0.145223681	0.045206675	0.423681293	AKAP11	
ENSG00000112742.10	-0.389140849	0.045206776	0.423681293	TTK	
ENSG00000074695.6	-0.116783424	0.045228846	0.423681293	LMAN1	
ENSG00000244437.1	0.360180127	0.045242537	0.423681293	IGKV3-15	
ENSG00000112739.17	-0.09716853	0.045267335	0.423681293	PRPF4B	
ENSG00000214331.8	-0.140464241	0.045273451	0.423681293	PDPR2P	
ENSG00000145604.17	-0.116617194	0.045282557	0.423681293	SKP2	
ENSG00000109927.11	0.238726037	0.045287364	0.423681293	TECTA	
ENSG00000170909.13	0.155839622	0.045314388	0.423681293	OSCAR	
ENSG00000169826.8	-0.131351549	0.045338938	0.423681293	CSGALNACT2	
ENSG00000110717.13	0.116343868	0.045362952	0.423681293	NDUFS8	
ENSG00000127980.16	-0.108159733	0.045387806	0.423681293	PEX1	
ENSG00000232450.1	0.259758843	0.045451587	0.423681293	RP4-730K3.3	
ENSG00000204872.4	0.358106556	0.045452995	0.423681293	NAT8B	
ENSG00000135341.18	-0.095865017	0.045457277	0.423681293	MAP3K7	
ENSG00000185338.7	0.271075983	0.045489893	0.423681293	SOCS1	
ENSG00000102897.10	0.105694144	0.045508114	0.423681293	LYRM1	
ENSG00000163159.14	0.088872009	0.045512693	0.423681293	VPS72	
ENSG00000138381.10	-0.168095741	0.045535209	0.423681293	ASNSD1	
ENSG00000100591.8	0.082212364	0.045563937	0.423681293	AHSA1	
ENSG00000247626.5	-0.102782684	0.045615196	0.423681293		mar.02
ENSG00000015153.15	-0.109950168	0.045719909	0.423681293	YAF2	
ENSG00000251259.1	0.203765387	0.045726842	0.423681293	AC004069.2	
ENSG00000101444.13	0.092954868	0.045735732	0.423681293	AHCY	
ENSG00000211802.3	-0.291042283	0.045760413	0.423681293	TRAV22	

ENSG00000232931.6	-0.110179451	0.045765994	0.423681293	LINC00342
ENSG00000165102.15	0.086697525	0.045787994	0.423681293	HGSNAT
ENSG00000164284.15	-0.136098423	0.045788849	0.423681293	GRPEL2
ENSG00000074219.14	0.33403759	0.045789542	0.423681293	TEAD2
ENSG00000162923.17	-0.07704776	0.045823956	0.423681293	WDR26
ENSG00000120837.8	-0.198838702	0.045831039	0.423681293	NFYB
ENSG00000068796.19	-0.098730459	0.045841199	0.423681293	KIF2A
ENSG00000135164.19	-0.110206248	0.045913278	0.423681293	DMTF1
ENSG00000234465.11	-0.341100719	0.045915979	0.423681293	PINLYP
ENSG00000186501.15	0.090255808	0.04598994	0.423681293	TMEM222
ENSG00000122008.15	-0.180044545	0.046012045	0.423681293	POLK
ENSG00000158062.20	0.115105363	0.046016273	0.423681293	UBXN11
ENSG00000268240.1	-0.408722982	0.046028965	0.423681293	RP11-678G14.4
ENSG00000143891.17	0.172012691	0.046033941	0.423681293	GALM
ENSG00000180011.7	-0.096888668	0.046084755	0.423681293	ZADH2
ENSG00000100483.14	-0.124839779	0.046110527	0.423681293	VCPKMT
ENSG00000268093.1	-0.261229239	0.046155341	0.423681293	AC022154.7
ENSG00000243491.1	0.653334162	0.046170509	0.423681293	RP11-521D12.5
ENSG00000159423.17	0.130645364	0.046182599	0.423681293	ALDH4A1
ENSG00000105926.16	-0.206115921	0.046184648	0.423681293	MPP6
ENSG00000213513.3	-0.60210409	0.046192268	0.423681293	IMPDH1P5
ENSG00000165113.13	-0.191253783	0.046202479	0.423681293	GKAP1
ENSG00000226002.1	0.485264388	0.046224966	0.423681293	GTF2IP14
ENSG00000106803.10	0.111383088	0.046232295	0.423681293	SEC61B
ENSG00000125249.7	-0.203143292	0.046241282	0.423681293	RAP2A
ENSG00000009724.17	-0.195030451	0.046248526	0.423681293	MASP2
ENSG00000109390.13	0.115781235	0.046258846	0.423681293	NDUFC1
ENSG00000163576.18	-0.570404655	0.046267808	0.423681293	EFHB
ENSG00000181588.17	-0.181216943	0.046276777	0.423681293	MEX3D
ENSG00000280734.3	0.152628892	0.046277107	0.423681293	LINC01232
ENSG00000160229.13	-0.294686081	0.046277739	0.423681293	ZNF66
ENSG00000115942.9	-0.074048854	0.046308894	0.423681293	ORC2
ENSG00000104863.12	0.15585736	0.046325192	0.423681293	LIN7B
ENSG00000169727.12	0.094234285	0.046329152	0.423681293	GPS1
ENSG00000070950.10	-0.080302677	0.046329539	0.423681293	RAD18
ENSG00000142657.21	0.115238627	0.046336393	0.423681293	PGD
ENSG00000002822.16	0.201079855	0.04633885	0.423681293	MAD1L1
ENSG00000230267.7	0.87889109	0.046417573	0.424211934	HERC2P4
ENSG00000135624.16	0.077102511	0.046443732	0.424261938	CCT7
ENSG00000204852.17	0.150401273	0.046496214	0.424428588	TCTN1
ENSG00000286067.1	-0.203123682	0.046503366	0.424428588	RP1-278C19.9
ENSG00000124019.10	0.217242743	0.04654119	0.424493807	FAM124B
ENSG00000101096.20	-0.12046195	0.046570754	0.424493807	NFATC2
ENSG00000285416.1	0.191822832	0.046572609	0.424493807	RP13-131K19.8
ENSG00000271680.1	0.257679028	0.04664744	0.424633844	RP11-564A8.8
ENSG00000185728.17	-0.104839884	0.046663882	0.424633844	YTHDF3
ENSG00000014123.10	-0.161817604	0.046669092	0.424633844	UFL1

ENSG00000156162.16	-0.165768132	0.046676781	0.424633844	DPY19L4
ENSG00000122068.13	-0.094529024	0.046692689	0.424633844	FYTTD1
ENSG00000163697.17	0.339949639	0.04671983	0.424633844	APBB2
ENSG00000117533.15	-0.121373315	0.046740508	0.424633844	VAMP4
ENSG00000110172.12	-0.126940601	0.046764957	0.424633844	CHORDC1
ENSG00000165916.9	0.096634751	0.046774505	0.424633844	PSMC3
ENSG00000101361.17	0.087844435	0.046804801	0.424633844	NOP56
ENSG00000141232.5	-0.113385382	0.04687266	0.424633844	TOB1
ENSG00000142556.19	-0.131796942	0.046893214	0.424633844	ZNF614
ENSG00000176396.11	-0.089195064	0.046944671	0.424633844	EID2
ENSG00000249685.1	-0.364201537	0.046945377	0.424633844	RP11-360F5.3
ENSG00000276754.1	0.518860514	0.046972906	0.424633844	RP11-461A8.5
ENSG00000161204.11	0.07591106	0.046979534	0.424633844	ABCF3
ENSG00000108344.15	0.081521922	0.046996977	0.424633844	PSMD3
ENSG00000169955.7	0.089807236	0.047015422	0.424633844	ZNF747
ENSG00000109920.13	-0.079203962	0.047056096	0.424633844	FNBP4
ENSG00000263412.1	0.2755142	0.047070011	0.424633844	NFE2L1-DT
ENSG00000184293.7	-0.30576064	0.047077353	0.424633844	CLECL1
ENSG00000120370.13	-0.161157317	0.047078236	0.424633844	GORAB
ENSG00000103037.12	-0.089661489	0.047079726	0.424633844	SETD6
ENSG00000270344.3	-0.149303557	0.047106819	0.424633844	POC1B-AS1
ENSG00000255495.1	-0.582387574	0.047159005	0.424633844	AC145124.2
ENSG00000280614.1	-2.651864306	0.047162379	0.424633844	CH507-513H4.4
ENSG00000139746.16	-0.095480231	0.047166412	0.424633844	RBM26
ENSG00000160408.16	-0.191730884	0.047167734	0.424633844	ST6GALNAC6
ENSG00000287454.1	-0.392765481	0.047194559	0.424688906	RP11-394J1.3
ENSG00000049239.13	-0.099406727	0.047246562	0.424773517	H6PD
ENSG00000109670.16	-0.093016682	0.047246783	0.424773517	FBXW7
ENSG00000276564.1	-0.409744807	0.047303855	0.424773517	CTA-276F8.1
ENSG00000106462.12	-0.10718314	0.047317539	0.424773517	EZH2
ENSG00000104915.15	0.108242334	0.047323172	0.424773517	STX10
ENSG00000091009.8	-0.10820208	0.047329161	0.424773517	RBM27
ENSG00000108829.10	0.084185829	0.04739879	0.424773517	LRRC59
ENSG00000047579.20	0.108518353	0.047414192	0.424773517	DTNBP1
ENSG00000101335.10	0.45610745	0.047426151	0.424773517	MYL9
ENSG00000115211.16	0.081493421	0.047452998	0.424773517	EIF2B4
ENSG00000117385.16	0.070515269	0.04747144	0.424773517	P3H1
ENSG00000260257.2	0.150334789	0.047476291	0.424773517	RP5-1085F17.3
ENSG00000162734.13	0.095517479	0.04752071	0.424773517	PEA15
ENSG00000241547.1	-0.417380696	0.047521911	0.424773517	ACTG1P20
ENSG00000154040.21	0.386023461	0.047527229	0.424773517	CABYR
ENSG00000261644.2	0.124290251	0.047535363	0.424773517	CYLD-AS1
ENSG00000164512.18	0.271320754	0.047564986	0.424779231	ANKRD55
ENSG00000162616.9	-0.215071667	0.047577702	0.424779231	DNAJB4
ENSG00000272106.1	0.114058309	0.047598141	0.424779231	RP11-345P4.9
ENSG00000115705.22	0.53581796	0.047660576	0.42515141	TPO
ENSG00000064961.19	0.095003099	0.047687053	0.425202644	HMG20B

ENSG00000104064.18	-0.082402845	0.047732571	0.425249479	GABPB1
ENSG00000169087.11	0.099996962	0.047733777	0.425249479	HSPBAP1
ENSG00000134294.14	-0.132837132	0.047794641	0.425606815	SLC38A2
ENSG00000162819.12	-0.101648063	0.047868278	0.425828064	BROX
ENSG00000154319.16	-0.410690878	0.047889075	0.425828064	FAM167A
ENSG00000188060.8	-0.322208606	0.047890618	0.425828064	RAB42
ENSG00000134780.10	0.176439734	0.047975095	0.425828064	DAGLA
ENSG00000066379.15	0.117641992	0.048013829	0.425828064	POLR1H
ENSG00000141971.13	0.115754042	0.048017552	0.425828064	MVB12A
ENSG00000278784.1	0.123385853	0.048023725	0.425828064	RP11-468E2.11
ENSG00000126088.14	0.094452603	0.04805277	0.425828064	UROD
ENSG00000169758.13	-0.247718189	0.048065563	0.425828064	TMEM266
ENSG00000196305.19	-0.070025045	0.048071062	0.425828064	IARS1
ENSG00000114988.12	0.080779381	0.04809974	0.425828064	LMAN2L
ENSG00000197054.12	0.158412508	0.048180133	0.425828064	ZNF763
ENSG00000179240.13	-0.146361967	0.048205207	0.425828064	GVQW3
ENSG00000131943.19	-0.085779783	0.048207711	0.425828064	C19orf12
ENSG00000070087.14	0.22371675	0.048210815	0.425828064	PFN2
ENSG00000107854.6	-0.092179596	0.048226342	0.425828064	TNKS2
ENSG00000163605.15	-0.163195226	0.048258611	0.425828064	PPP4R2
ENSG00000118922.18	-0.129521881	0.048260818	0.425828064	KLF12
ENSG00000159214.13	0.203832159	0.048276272	0.425828064	CCDC24
ENSG00000240322.3	-0.373779159	0.048297556	0.425828064	RN7SL481P
ENSG00000198796.7	-0.558288891	0.04831163	0.425828064	ALPK2
ENSG00000068383.19	0.088743273	0.048330779	0.425828064	INPP5A
ENSG00000101336.18	0.109231098	0.048377168	0.425828064	HCK
ENSG00000100079.7	0.390596815	0.048387709	0.425828064	LGALS2
ENSG00000188452.15	-0.229915763	0.048388289	0.425828064	CERKL
ENSG00000234432.4	-0.31790438	0.04839257	0.425828064	RP11-1275H24.1
ENSG00000263006.6	-0.578785103	0.048396645	0.425828064	ROCK1P1
ENSG00000211792.2	-0.329604316	0.048400879	0.425828064	TRAV14DV4
ENSG00000144320.14	-0.106748905	0.048469127	0.426039341	LNPK
ENSG00000260853.2	-0.143932159	0.048483497	0.426039341	RP11-264B17.2
ENSG00000134265.13	-0.10757843	0.048489954	0.426039341	NAPG
ENSG00000221968.9	0.158475768	0.04850799	0.426039341	FADS3
ENSG00000143847.15	0.32274271	0.048536565	0.426099934	PPFIA4
ENSG00000238057.10	0.282320446	0.048600405	0.426099934	ZEB2-AS1
ENSG00000163781.14	-0.078341865	0.048650538	0.426099934	TOPBP1
ENSG00000274922.1	0.158931129	0.048664443	0.426099934	RP11-88E10.5
ENSG00000268785.1	-0.488404499	0.04872868	0.426099934	RPL7P50
ENSG00000235531.11	-0.352298431	0.048739435	0.426099934	MSC-AS1
ENSG00000105472.13	-0.292466458	0.048751958	0.426099934	CLEC11A
ENSG00000162144.10	0.112778536	0.048766732	0.426099934	CYB561A3
ENSG00000265195.1	-0.515652203	0.048783516	0.426099934	MIR4312
ENSG00000188636.4	-0.122340989	0.048786737	0.426099934	RTL6
ENSG00000258376.2	0.247756066	0.048790384	0.426099934	RP4-647C14.2
ENSG00000151240.17	-0.216663808	0.048809503	0.426099934	DIP2C

ENSG00000133313.15	0.110368288	0.048851879	0.426099934	CNDP2
ENSG00000242866.10	0.45409663	0.048870229	0.426099934	STRC
ENSG00000047188.16	-0.104768312	0.048886864	0.426099934	YTHDC2
ENSG00000112796.10	-0.249051737	0.048901294	0.426099934	ENPP5
ENSG00000126453.10	0.120539022	0.048913819	0.426099934	BCL2L12
ENSG00000211648.2	0.398778632	0.048915966	0.426099934	IGLV1-47
ENSG00000095066.12	0.110027295	0.048924464	0.426099934	HOOK2
ENSG00000134996.12	0.081831337	0.048947069	0.426099934	OSTF1
ENSG00000211750.2	0.38841838	0.048951211	0.426099934	TRBV24-1
ENSG00000249896.2	-0.402658928	0.049018088	0.426499383	LINC02495
ENSG00000171848.16	-0.329607228	0.049062624	0.426499383	RRM2
ENSG00000169223.15	0.090839146	0.049075351	0.426499383	LMAN2
ENSG00000140948.13	-0.10138353	0.049106052	0.426499383	ZCCHC14
ENSG00000138078.16	-0.123229204	0.049137941	0.426499383	PREPL
ENSG00000234420.7	-0.091054811	0.049145799	0.426499383	ZNF37BP
ENSG00000280351.2	-0.204136986	0.049156999	0.426499383	CTD-2561B21.8
ENSG00000078699.22	-0.060910308	0.049175925	0.426499383	CBFA2T2
ENSG00000181215.15	-0.373837657	0.049193652	0.426499383	C4orf50
ENSG00000234009.1	0.348015091	0.049205068	0.426499383	RPL5P34
ENSG00000166454.10	-0.059089481	0.049231042	0.426544236	ATMIN
ENSG00000073737.17	0.254298479	0.049284926	0.426830768	DHRS9
ENSG00000196372.13	0.10762848	0.049337439	0.426937577	ASB13
ENSG00000168427.9	0.613100218	0.049338895	0.426937577	KLHL30
ENSG00000130811.12	0.104631004	0.049428688	0.427046129	EIF3G
ENSG00000090581.11	0.077489793	0.049493709	0.427046129	GNPTG
ENSG00000237719.1	-0.372882571	0.049506204	0.427046129	RP1-179N16.3
ENSG00000237471.2	-0.408672629	0.049510842	0.427046129	AC073115.6
ENSG00000243607.3	0.423858752	0.049518367	0.427046129	RPL35AP26
ENSG00000132128.17	0.076082401	0.049536923	0.427046129	LRRC41
ENSG00000231645.3	1.07912911	0.049556749	0.427046129	KRT17P6
ENSG00000117676.14	0.085534235	0.049578302	0.427046129	RPS6KA1
ENSG00000250990.1	-0.450715214	0.049603818	0.427046129	AC073635.5
ENSG00000123901.9	-0.346288748	0.049615393	0.427046129	GPR83
ENSG00000104518.11	0.118167313	0.049629246	0.427046129	GSDMD
ENSG00000288009.1	-0.427102236	0.049631959	0.427046129	RP11-63L7.6
ENSG00000172053.18	0.070243697	0.049636181	0.427046129	QARS1
ENSG00000108309.14	-0.39517251	0.049649261	0.427046129	RUNDC3A
ENSG00000155304.6	-0.171194345	0.049677595	0.427046129	HSPA13
ENSG00000157184.7	0.075409825	0.049690726	0.427046129	CPT2
ENSG00000124256.15	0.226242655	0.049730823	0.427046129	ZBP1
ENSG00000161638.11	0.109309704	0.049761367	0.427046129	ITGA5
ENSG00000187257.16	-0.083648661	0.049785435	0.427046129	RSBN1L
ENSG00000124789.12	-0.098898697	0.049786205	0.427046129	NUP153
ENSG00000270871.1	-0.231521609	0.049821014	0.427046129	AC015849.19
ENSG00000188186.10	0.135897807	0.049822374	0.427046129	LAMTOR4
ENSG00000120458.13	-0.084134012	0.049891369	0.427046129	MSANTD2
ENSG00000198830.11	0.084870868	0.049897326	0.427046129	HMGN2

ENSG00000160613.14	-0.108790672	0.049902196	0.427046129	PCSK7
ENSG00000100347.15	0.08473627	0.049906199	0.427046129	SAMM50
ENSG00000129559.13	0.125133301	0.049958281	0.427046129	NEDD8
ENSG00000180767.10	0.335394992	0.049965719	0.427046129	CHST13
ENSG00000157873.18	0.093154931	0.049992319	0.427046129	TNFRSF14
ENSG00000135547.9	0.430048953	0.050019011	0.427046129	HEY2
ENSG00000211584.14	0.138170228	0.050025915	0.427046129	SLC48A1
ENSG00000143294.15	0.083997623	0.05002763	0.427046129	PRCC
ENSG00000133574.10	0.094380008	0.050061362	0.427046129	GIMAP4
ENSG00000198721.13	0.092595361	0.050088417	0.427046129	ECI2
ENSG00000157368.11	0.400683925	0.050104463	0.427046129	IL34
ENSG00000236830.7	0.204255866	0.050118826	0.427046129	CBR3-AS1
ENSG00000175894.18	0.42680001	0.050121905	0.427046129	TSPEAR
ENSG00000287926.1	0.251847062	0.05020754	0.427475429	RP11-69H7.5
ENSG00000177738.4	-0.088060801	0.05021398	0.427475429	CTD-2201E18.3
ENSG00000287697.1	0.480238213	0.050270514	0.427779132	RP1-102G20.8
ENSG00000106628.12	0.112657969	0.05035039	0.428139157	POLD2
ENSG00000283646.2	-0.644228236	0.050357404	0.428139157	LINC02009
ENSG00000184361.13	-0.342558622	0.050418655	0.428139157	SPATA32
ENSG00000072958.9	0.091693783	0.050425338	0.428139157	AP1M1
ENSG00000108256.9	-0.108187172	0.050425407	0.428139157	NUFIP2
ENSG00000246859.3	-0.251559573	0.050468448	0.428139157	STARD4-AS1
ENSG00000147419.19	-0.099134586	0.050489998	0.428139157	CCDC25
ENSG00000049247.14	0.582814349	0.050491524	0.428139157	UTS2
ENSG00000131095.14	-0.42618408	0.050500713	0.428139157	GFAP
ENSG00000109787.13	-0.10401029	0.050554664	0.428417357	KLF3
ENSG00000171045.16	0.11569789	0.050601242	0.428417357	TSNARE1
ENSG00000286410.1	0.404312242	0.050622309	0.428417357	RP11-798P24.2
ENSG00000175582.20	-0.07395944	0.050623721	0.428417357	RAB6A
ENSG00000073756.13	-0.201377467	0.050637979	0.428417357	PTGS2
ENSG00000182544.9	0.1066881	0.050712707	0.428454201	MFSD5
ENSG00000117519.16	0.267736735	0.050749	0.428454201	CNN3
ENSG00000226979.9	0.161274764	0.050771219	0.428454201	LTA
ENSG00000116903.7	-0.135029733	0.050787282	0.428454201	EXOC8
ENSG00000253476.1	-0.22768212	0.050801338	0.428454201	RP11-395I14.2
ENSG00000164975.15	-0.097226413	0.050839167	0.428454201	SNAPC3
ENSG00000261423.1	-0.274005424	0.050865095	0.428454201	TMEM202-AS1
ENSG00000259146.4	0.351773155	0.050922194	0.428454201	RP1-261D10.2
ENSG00000140262.18	-0.097235303	0.050922386	0.428454201	TCF12
ENSG00000188385.12	-0.322959937	0.0509253	0.428454201	JAKMIP3
ENSG00000031003.10	-0.10244297	0.050974483	0.428454201	FAM13B
ENSG00000185361.9	0.09107004	0.050998025	0.428454201	TNFAIP8L1
ENSG00000144591.20	0.098108661	0.051001578	0.428454201	GMPPA
ENSG00000170906.16	0.130190876	0.051001932	0.428454201	NDUFA3
ENSG00000116266.11	-0.111267313	0.051038033	0.428454201	STXBP3
ENSG00000176978.14	0.118460666	0.051057034	0.428454201	DPP7
ENSG00000167645.17	0.118733913	0.051093485	0.428454201	YIF1B

ENSG00000163959.10	-0.130658417	0.051099526	0.428454201	SLC51A
ENSG00000183298.5	0.433121351	0.051108876	0.428454201	RPSAP19
ENSG00000128285.4	0.503634823	0.051119406	0.428454201	MCHR1
ENSG00000115694.15	0.065161891	0.051149498	0.428454201	STK25
ENSG00000106133.18	-0.318713001	0.051158265	0.428454201	NSUN5P2
ENSG00000131437.16	-0.145547879	0.051166374	0.428454201	KIF3A
ENSG00000107485.18	-0.124754835	0.051177957	0.428454201	GATA3
ENSG00000133884.10	0.075994253	0.051247246	0.428454201	DPF2
ENSG00000092096.18	0.272317775	0.051256391	0.428454201	SLC22A17
ENSG00000197586.13	0.077145671	0.051259273	0.428454201	ENTPD6
ENSG00000249806.1	0.329450144	0.051284956	0.428454201	RP11-223C24.1
ENSG00000267065.2	-0.575461583	0.051290372	0.428454201	LINC02080
ENSG00000173890.17	-0.190467858	0.051308424	0.428454201	GPR160
ENSG00000284688.1	0.503730477	0.051314773	0.428454201	BTN1A1P1
ENSG00000233178.7	-0.492510097	0.051365933	0.428454201	RP11-88I18.2
ENSG00000180008.9	-0.136254542	0.051391735	0.428454201	SOCS4
ENSG00000226564.1	0.191075022	0.051410454	0.428454201	FTH1P20
ENSG00000166529.15	-0.109382079	0.05142347	0.428454201	ZSCAN21
ENSG00000101019.22	0.056868837	0.05142661	0.428454201	UQCC1
ENSG00000130775.16	0.093627521	0.051428501	0.428454201	THEMIS2
ENSG00000137098.14	0.171928435	0.05145458	0.428454201	SPAG8
ENSG00000100307.13	0.078531981	0.051457124	0.428454201	CBX7
ENSG00000277053.4	0.18979247	0.05148404	0.428504341	GTF2IP1
ENSG00000260807.7	0.289700728	0.05154639	0.428594928	CEROX1
ENSG00000119900.9	-0.137675676	0.051558516	0.428594928	OGFRL1
ENSG00000254132.1	1.093755705	0.05156673	0.428594928	MTND6P3
ENSG00000169696.16	0.115304131	0.05157852	0.428594928	ASPSCR1
ENSG00000163382.13	0.114947269	0.051684623	0.429153078	NAXE
ENSG00000136450.13	-0.080486461	0.051687542	0.429153078	SRSF1
ENSG00000157020.18	0.068270521	0.051724454	0.429285759	SEC13
ENSG00000134690.11	-0.154013058	0.051753432	0.4293525	CDCA8
ENSG00000136930.13	0.072471319	0.051776571	0.429370768	PSMB7
ENSG00000099899.15	0.086988775	0.051931425	0.429930266	TRMT2A
ENSG00000280213.1	-0.217799292	0.051955568	0.429930266	UCKL1-AS1
ENSG00000255503.1	0.327179303	0.051980517	0.429930266	RP11-113K21.4
ENSG00000140839.12	0.347554963	0.052033529	0.429930266	CLEC18B
ENSG00000112146.17	-0.136388173	0.052059881	0.429930266	FBXO9
ENSG00000023191.17	0.121919616	0.052094948	0.429930266	RNH1
ENSG00000275432.1	-0.386295458	0.052105572	0.429930266	CTD-3093B17.2
ENSG00000180914.11	0.379896794	0.052187425	0.429930266	OXTR
ENSG00000091732.17	0.082405017	0.05220274	0.429930266	ZC3HC1
ENSG00000152601.18	-0.150938017	0.052218794	0.429930266	MBNL1
ENSG00000167635.12	-0.102609864	0.052243933	0.429930266	ZNF146
ENSG00000166037.11	-0.13549692	0.052250556	0.429930266	CEP57
ENSG00000144468.17	0.079241014	0.052300887	0.429930266	RHBDD1
ENSG00000146576.13	0.088258307	0.052314685	0.429930266	C7orf26
ENSG00000088726.16	0.262433636	0.05232755	0.429930266	TMEM40

ENSG00000165914.15	0.1996514	0.052342254	0.429930266	TTC7B
ENSG00000172466.16	-0.099352359	0.052356552	0.429930266	ZNF24
ENSG00000159625.15	0.285420508	0.052400415	0.429930266	DRC7
ENSG00000137713.16	-0.076006621	0.052424091	0.429930266	PPP2R1B
ENSG00000124733.5	0.09810731	0.052436273	0.429930266	MEA1
ENSG00000227508.6	0.198978331	0.052437343	0.429930266	LINC01624
ENSG00000269416.6	1.337298454	0.05243904	0.429930266	LINC01224
ENSG00000184232.9	0.127714695	0.052449235	0.429930266	OAF
ENSG00000225131.2	0.247626792	0.052450941	0.429930266	PSME2P2
ENSG00000213613.2	-0.390683134	0.052483896	0.429930266	RPL11P3
ENSG00000198836.11	-0.09378932	0.052489052	0.429930266	OPA1
ENSG00000170458.14	0.162565807	0.052560377	0.429930266	CD14
ENSG00000123240.17	-0.150379783	0.052581245	0.429930266	OPTN
ENSG00000236439.4	-0.427749955	0.052582006	0.429930266	RP11-175B9.3
ENSG00000151689.13	0.10410114	0.052597552	0.429930266	INPP1
ENSG00000159459.12	-0.119177534	0.052598209	0.429930266	UBR1
ENSG00000196576.16	0.156612779	0.052612026	0.429930266	PLXNB2
ENSG00000173786.17	0.116189534	0.052617841	0.429930266	CNP
ENSG00000189144.15	-0.256582514	0.052636107	0.429930266	ZNF573
ENSG00000141424.13	-0.092714316	0.052648994	0.429930266	SLC39A6
ENSG00000043093.15	-0.129020084	0.052657143	0.429930266	DCUN1D1
ENSG00000212978.6	0.130654521	0.052660327	0.429930266	AC016747.3
ENSG00000204839.9	0.190526919	0.052660898	0.429930266	MROH6
ENSG00000154920.15	-0.252139433	0.052678158	0.429930266	EME1
ENSG00000250506.8	0.166220029	0.0526826	0.429930266	CDK3
ENSG00000213171.3	0.6085283	0.052715558	0.429984313	LINGO4
ENSG00000129691.16	0.054629271	0.052768761	0.429984313	ASH2L
ENSG00000178538.10	0.397723636	0.052792058	0.429984313	CA8
ENSG00000141101.13	0.077869704	0.052808423	0.429984313	NOB1
ENSG00000152133.16	-0.142968971	0.052821589	0.429984313	GPATCH11
ENSG00000004864.14	0.084776232	0.052842671	0.429984313	SLC25A13
ENSG00000213593.10	0.081125484	0.052875908	0.429984313	TMX2
ENSG00000100379.17	0.101896455	0.052890532	0.429984313	KCTD17
ENSG00000235917.1	0.500426631	0.052912239	0.429984313	MTCO2P11
ENSG00000166349.10	-0.296879746	0.052921578	0.429984313	RAG1
ENSG00000177674.16	0.110442527	0.052937666	0.429984313	AGTRAP
ENSG00000109667.12	0.134198439	0.052944803	0.429984313	SLC2A9
ENSG00000180773.15	-0.125777339	0.052966023	0.429984313	SLC36A4
ENSG00000121210.16	0.0798427	0.052982756	0.429984313	TMEM131L
ENSG00000130706.13	0.104947001	0.053031124	0.430032259	ADRM1
ENSG00000197070.14	0.102011638	0.053045247	0.430032259	ARRDC1
ENSG00000198846.6	-0.169043342	0.053062019	0.430032259	TOX
ENSG00000074842.8	0.112003385	0.053123419	0.430032259	MYDGF
ENSG00000254064.1	-0.204424128	0.053125615	0.430032259	CTD-2530N21.4
ENSG00000108039.18	0.068254398	0.053131295	0.430032259	XPNPEP1
ENSG00000273456.1	-0.243621449	0.053135447	0.430032259	RP11-686O6.2
ENSG00000136854.24	0.180332179	0.053176288	0.430156096	STXBP1

ENSG00000114948.13	0.376591344	0.053192698	0.430156096	ADAM23
ENSG00000113119.13	0.069550277	0.053230984	0.430159629	TMCO6
ENSG00000172936.16	0.08526734	0.053235086	0.430159629	MYD88
ENSG00000178057.15	0.131667291	0.053292233	0.430445545	NDUFAF3
ENSG00000225632.1	0.455869048	0.053312448	0.430445545	RP5-997D24.3
ENSG00000035687.10	-0.076794421	0.053342994	0.430522675	ADSS2
ENSG00000163412.13	-0.122327576	0.053371739	0.430585217	EIF4E3
ENSG00000258441.1	-0.129438071	0.053408687	0.430637958	LINC00641
ENSG00000223891.6	0.116990222	0.053424888	0.430637958	OSER1-DT
ENSG00000213204.8	-0.372909256	0.053441272	0.430637958	RP3-382I10.7
ENSG00000173585.17	-0.365872136	0.053512344	0.430790698	CCR9
ENSG00000090273.14	0.102469931	0.05352591	0.430790698	NUDC
ENSG00000108846.16	0.229438637	0.053533225	0.430790698	ABCC3
ENSG00000165795.24	0.138690497	0.053544251	0.430790698	NDRG2
ENSG00000277586.4	0.327824873	0.053597432	0.431049464	NEFL
ENSG00000168067.12	0.074384912	0.053654064	0.431335771	MAP4K2
ENSG00000250848.1	-0.44146094	0.053700891	0.431427197	CTD-2083E4.5
ENSG00000259326.1	-0.304448405	0.053711387	0.431427197	RP11-102L12.2
ENSG00000178694.10	-0.082741438	0.053734508	0.431427197	NSUN3
ENSG00000277363.5	-0.411383919	0.053749585	0.431427197	SRCIN1
ENSG00000198146.4	-0.20239523	0.05377493	0.431461764	ZNF770
ENSG00000111640.15	0.105660136	0.053799358	0.431488943	GAPDH
ENSG00000182240.16	0.259132089	0.053837175	0.431623446	BACE2
ENSG00000234911.2	-0.248098881	0.05387567	0.431655676	TEX21P
ENSG00000130222.11	0.223578677	0.053923905	0.431655676	GADD45G
ENSG00000142669.15	0.134578837	0.053927061	0.431655676	SH3BGRL3
ENSG00000123485.12	-0.331833686	0.053936483	0.431655676	HJURP
ENSG00000245105.4	-0.253346516	0.053946435	0.431655676	A2M-AS1
ENSG00000138767.13	-0.092325842	0.053978705	0.431745428	CNOT6L
ENSG00000262999.1	-0.40192591	0.054023614	0.431936171	CTD-3088G3.6
ENSG00000140030.6	-0.142933697	0.0540774	0.432064812	GPR65
ENSG00000284606.1	-0.434860292	0.054095685	0.432064812	RP11-556O5.7
ENSG00000174021.11	0.128223288	0.054102908	0.432064812	GNG5
ENSG00000128513.16	-0.131751888	0.054124159	0.432066273	POT1
ENSG00000104388.15	-0.08135525	0.054180379	0.432260571	RAB2A
ENSG00000273243.1	-0.255650925	0.054202255	0.432260571	CTA-217C2.2
ENSG00000101246.20	0.100828554	0.054282139	0.432260571	ARFRP1
ENSG00000274736.5	0.561299533	0.054287542	0.432260571	CCL23
ENSG00000279561.3	-0.344049659	0.054320577	0.432260571	RP11-4L24.4
ENSG00000169217.9	0.073904662	0.054360793	0.432260571	CD2BP2
ENSG00000201544.1	-0.424653022	0.054367327	0.432260571	SNORA16B
ENSG00000071537.14	-0.10028901	0.054368439	0.432260571	SEL1L
ENSG00000188001.10	-0.291214768	0.054389623	0.432260571	TPRG1
ENSG00000267136.1	-0.436425834	0.054401078	0.432260571	RP11-53B2.3
ENSG00000181652.19	0.195212022	0.054438635	0.432260571	ATG9B
ENSG00000106588.12	-0.175524802	0.054445425	0.432260571	PSMA2
ENSG00000163681.16	-0.115790286	0.054461942	0.432260571	SLMAP

ENSG00000274963.1	-0.286417294	0.054473823	0.432260571	RN7SL600P
ENSG00000278195.2	0.278616825	0.054474482	0.432260571	SSTR3
ENSG00000059122.17	-0.198052512	0.05449048	0.432260571	FLYWCH1
ENSG00000051341.15	-0.303847147	0.054514224	0.432260571	POLQ
ENSG00000173846.13	0.101100745	0.054561424	0.432260571	PLK3
ENSG00000255737.2	-0.2429428	0.054592383	0.432260571	AGAP2-AS1
ENSG00000211899.10	0.315679519	0.054625244	0.432260571	IGHM
ENSG00000157111.13	-0.575678419	0.054631354	0.432260571	TMEM171
ENSG00000165689.17	0.077721194	0.054640662	0.432260571	ENTR1
ENSG00000122557.11	-0.065544113	0.054641369	0.432260571	HERPUD2
ENSG00000101294.18	0.097664114	0.054672277	0.432260571	HM13
ENSG00000264553.1	0.415797184	0.05473902	0.432260571	MIR4257
ENSG00000120798.17	-0.090090731	0.054773881	0.432260571	NR2C1
ENSG00000161664.7	0.144158855	0.054787678	0.432260571	ASB16
ENSG00000269044.2	-0.101953912	0.054790172	0.432260571	CTC-429P9.3
ENSG00000231437.3	0.437226857	0.054801514	0.432260571	LINC01750
ENSG00000112299.8	-0.297138674	0.054832013	0.432260571	VNN1
ENSG00000258659.7	-0.269331218	0.054851889	0.432260571	TRIM34
ENSG00000153914.16	-0.132353622	0.054858268	0.432260571	SREK1
ENSG00000144642.22	0.413718672	0.054859093	0.432260571	RBMS3
ENSG00000100359.21	0.108833395	0.054865139	0.432260571	SGSM3
ENSG00000103269.14	0.226435403	0.054898962	0.432360947	RHBDL1
ENSG00000116001.17	-0.122892935	0.055108762	0.432963979	TIA1
ENSG00000167766.19	-0.19682131	0.055122712	0.432963979	ZNF83
ENSG00000148950.11	-0.241286033	0.055129829	0.432963979	IMMP1L
ENSG00000172167.8	-0.157588668	0.0551505	0.432963979	MTBP
ENSG00000177370.5	0.088813914	0.055154091	0.432963979	TIMM22
ENSG00000163808.17	-0.29248523	0.055197446	0.432963979	KIF15
ENSG00000085552.17	0.460222356	0.055209227	0.432963979	IGSF9
ENSG00000260456.7	0.334970829	0.055215294	0.432963979	C16orf95
ENSG00000106526.11	-0.235214542	0.055220331	0.432963979	ACTR3C
ENSG00000072062.15	0.100632403	0.05523319	0.432963979	PRKACA
ENSG00000166897.16	0.301921721	0.055239891	0.432963979	ELFN2
ENSG00000177463.15	-0.125998287	0.05524679	0.432963979	NR2C2
ENSG00000263606.1	-0.407492062	0.055249987	0.432963979	CHORDC1P4
ENSG00000040487.13	0.100841655	0.055306217	0.433049549	SLC66A1
ENSG00000129911.9	0.121339749	0.055323264	0.433049549	KLF16
ENSG00000137101.13	0.269540121	0.05533131	0.433049549	CD72
ENSG00000213300.5	0.283403607	0.055347366	0.433049549	HNRNPA3P6
ENSG00000022277.13	0.077301334	0.055366487	0.433049549	RTF2
ENSG00000259719.6	0.26964299	0.055418059	0.433287665	LINC02284
ENSG00000279656.1	0.362213955	0.05544168	0.433307154	RP11-298I3.6
ENSG00000135148.12	0.124416349	0.055505072	0.433637343	TRAFD1
ENSG00000015676.18	0.065322015	0.055553194	0.433742507	NUDCD3
ENSG00000223865.11	0.14839917	0.055560833	0.433742507	HLA-DPB1
ENSG00000288075.1	0.306590175	0.055596669	0.433857113	RP11-1396O13.23
ENSG00000008382.16	0.111704986	0.055661678	0.433981602	MPND

ENSG00000143995.20	0.248385669	0.055661772	0.433981602	MEIS1
ENSG00000198356.12	0.091131248	0.055676107	0.433981602	GET3
ENSG00000257337.7	-0.180809969	0.055708012	0.434065315	RP11-983P16.4
ENSG00000122547.11	0.12587167	0.055811529	0.43470674	EEPD1
ENSG00000162522.11	0.191084584	0.055860232	0.434845823	KIAA1522
ENSG00000270362.1	-0.343202657	0.055871794	0.434845823	HMGN3-AS1
ENSG00000058272.19	-0.150994422	0.055961639	0.435379854	PPP1R12A
ENSG00000198816.7	0.197487237	0.056033998	0.435596057	ZNF358
ENSG00000136732.16	-0.282741606	0.056099449	0.435596057	GYPC
ENSG00000074696.13	-0.079550523	0.056102412	0.435596057	HACD3
ENSG00000176973.8	0.129049664	0.056141159	0.435596057	FAM89B
ENSG00000055950.16	0.09171043	0.056155716	0.435596057	MRPL43
ENSG00000245468.4	0.346813787	0.056179006	0.435596057	LINC02447
ENSG00000101544.9	-0.078425039	0.056179325	0.435596057	ADNP2
ENSG00000100142.16	0.106869788	0.056184778	0.435596057	POLR2F
ENSG00000164669.13	-0.567473663	0.056213915	0.435596057	INTS4P1
ENSG00000139546.11	0.085294257	0.056225747	0.435596057	TARBP2
ENSG00000170921.15	-0.134140084	0.056232536	0.435596057	TANC2
ENSG00000166971.17	-0.102387601	0.056244312	0.435596057	AKTIP
ENSG00000079387.14	-0.075902529	0.056366832	0.436230108	SENP1
ENSG00000204592.9	0.088886442	0.056368724	0.436230108	HLA-E
ENSG00000173812.11	0.093297038	0.0564256	0.436367366	EIF1
ENSG00000110075.15	-0.064197536	0.056431959	0.436367366	PPP6R3
ENSG00000137310.12	-0.139778974	0.056469295	0.436367366	TCF19
ENSG00000197019.5	0.122727551	0.056507814	0.436367366	SERTAD1
ENSG00000056097.16	-0.094661518	0.056508856	0.436367366	ZFR
ENSG00000206503.13	0.139747678	0.05655912	0.436367366	HLA-A
ENSG00000159593.15	-0.106848442	0.056573384	0.436367366	NAE1
ENSG00000269836.1	-0.385821439	0.056587134	0.436367366	CTD-3032J10.4
ENSG00000163864.17	0.179652003	0.056598624	0.436367366	NMNAT3
ENSG00000154025.16	0.163821751	0.056599239	0.436367366	SLC5A10
ENSG00000077549.19	0.079769927	0.056655491	0.436517937	CAPZB
ENSG00000206527.10	-0.095753174	0.056661339	0.436517937	HACD2
ENSG00000130396.21	-0.277665259	0.056785302	0.437005639	AFDN
ENSG00000172940.13	-0.454834093	0.056811773	0.437005639	SLC22A13
ENSG00000184162.15	0.09676747	0.056831076	0.437005639	NR2C2AP
ENSG00000184900.16	0.072150573	0.056853833	0.437005639	SUMO3
ENSG00000244731.8	0.344783491	0.056908193	0.437005639	C4A
ENSG00000092148.13	-0.091448978	0.056913901	0.437005639	HECTD1
ENSG00000258551.6	0.560614639	0.056916483	0.437005639	CRAT37
ENSG00000054267.22	-0.157773577	0.056982959	0.437005639	ARID4B
ENSG00000182774.13	0.093769528	0.05701255	0.437005639	RPS17
ENSG00000175387.16	-0.047332094	0.057019404	0.437005639	SMAD2
ENSG00000138085.17	0.091688067	0.057035953	0.437005639	ATRAID
ENSG00000206418.5	-0.122117059	0.057042622	0.437005639	RAB12
ENSG00000135776.5	-0.129092525	0.05707493	0.437005639	ABCB10
ENSG00000184489.13	0.183037862	0.05708869	0.437005639	PTP4A3

ENSG00000114126.18	-0.108060817	0.057090013	0.437005639	TFDP2
ENSG00000177675.9	0.409839648	0.057092918	0.437005639	CD163L1
ENSG00000240409.1	0.78038238	0.057122005	0.437005639	MTATP8P1
ENSG00000149182.15	0.070164619	0.057137496	0.437005639	ARFGAP2
ENSG00000233806.7	-0.262001894	0.057146179	0.437005639	LINC01237
ENSG00000142186.17	0.095926852	0.057150825	0.437005639	SCYL1
ENSG00000074319.13	0.066798246	0.057185091	0.437104677	TSG101
ENSG00000169957.10	0.11014536	0.057260287	0.437516378	ZNF768
ENSG00000103160.12	-0.084209476	0.057295206	0.437620145	HSDL1
ENSG00000272205.1	0.288536364	0.057412656	0.438312083	POU2F1-DT
ENSG00000177000.13	0.094620167	0.057428543	0.438312083	MTHFR
ENSG00000211749.1	-0.289790313	0.057457283	0.438368288	TRBV23-1
ENSG00000188177.14	-0.100789804	0.057516652	0.438658055	ZC3H6
ENSG00000134061.5	0.196593282	0.057546415	0.438693676	CD180
ENSG00000223117.1	-0.451471282	0.057564106	0.438693676	RN7SKP296
ENSG00000167005.14	-0.12691614	0.057600359	0.438806893	NUDT21
ENSG00000174851.16	0.11097253	0.05765818	0.439081526	YIF1A
ENSG00000227500.10	0.097526935	0.057687066	0.439081526	SCAMP4
ENSG00000171928.14	-0.168756136	0.057700639	0.439081526	TVP23B
ENSG00000143183.18	-0.187843511	0.057742454	0.439236735	TMCO1
ENSG00000256937.1	-0.499877255	0.057781716	0.439372422	KRT17P8
ENSG00000072415.10	-0.106666748	0.057842888	0.439570221	MPP5
ENSG00000092295.12	0.169888446	0.05785526	0.439570221	TGM1
ENSG00000104691.16	-0.152786448	0.05787203	0.439570221	UBXN8
ENSG00000096717.12	-0.108495417	0.05790471	0.43965561	SIRT1
ENSG00000100519.12	-0.177996086	0.057940132	0.439660213	PSMC6
ENSG00000197506.8	-0.384830121	0.057989628	0.439660213	SLC28A3
ENSG00000131126.19	0.597294757	0.057990266	0.439660213	TEX101
ENSG00000173253.16	-1.024858133	0.05806001	0.439660213	DMRT2
ENSG00000149743.14	0.097511584	0.058062995	0.439660213	TRPT1
ENSG00000169230.10	0.108238389	0.058075317	0.439660213	PRELID1
ENSG00000164209.17	-0.13538314	0.058077453	0.439660213	SLC25A46
ENSG00000163584.18	-0.335829874	0.058117618	0.439660213	RPL22L1
ENSG00000104756.16	-0.111287283	0.058130891	0.439660213	KCTD9
ENSG00000102908.22	-0.187687085	0.058135869	0.439660213	NFAT5
ENSG00000112685.14	-0.078463506	0.05814114	0.439660213	EXOC2
ENSG00000137040.10	-0.143979133	0.058207605	0.440000574	RANBP6
ENSG00000109171.15	-0.072684172	0.058251868	0.440172918	SLAIN2
ENSG00000166016.6	0.216653766	0.05832412	0.440474829	ABTB2
ENSG00000105552.15	0.111493135	0.058334778	0.440474829	BCAT2
ENSG00000115750.17	-0.123733311	0.058411147	0.440770675	TAF1B
ENSG00000204628.12	0.091548649	0.058424262	0.440770675	RACK1
ENSG00000273073.1	-0.599511259	0.058449202	0.440770675	RP11-109E12.1
ENSG00000236861.6	-0.347276736	0.058460869	0.440770675	BET1-AS1
ENSG00000116922.15	-0.081854632	0.058481422	0.440770675	C1orf109
ENSG00000227028.6	-0.425840426	0.058514191	0.440850447	SLC8A1-AS1
ENSG00000276744.1	0.309228785	0.058558273	0.440850447	CTD-2562G15.3

ENSG00000037637.11	0.059172273	0.05857253	0.440850447	FBXO42
ENSG00000101421.4	0.08516877	0.058619929	0.440850447	CHMP4B
ENSG00000162571.14	-0.494111674	0.05862559	0.440850447	TTLL10
ENSG00000138395.17	0.49423669	0.058677286	0.440850447	CDK15
ENSG00000157326.19	0.134534845	0.058697975	0.440850447	DHRS4
ENSG00000286001.1	-0.428405045	0.058703192	0.440850447	CTD-2546F11.1
ENSG00000271849.1	0.415527046	0.058734702	0.440850447	MAN2A1-DT
ENSG00000132661.4	0.113250684	0.058771751	0.440850447	NXT1
ENSG00000259129.6	-1.855933517	0.058778175	0.440850447	LINC00648
ENSG00000134317.18	0.276448584	0.058791964	0.440850447	GRHL1
ENSG00000172262.12	-0.098150104	0.058792153	0.440850447	ZNF131
ENSG00000197599.12	0.209815972	0.058825652	0.440850447	CCDC154
ENSG00000242588.6	-0.262425158	0.058835926	0.440850447	RP11-274B21.14
ENSG00000272343.1	0.236936453	0.058840257	0.440850447	RP11-140I16.3
ENSG00000267751.5	-0.265101412	0.058857447	0.440850447	BSG-AS1
ENSG00000263961.8	0.440929929	0.058902427	0.44087328	RHEX
ENSG00000166348.19	-0.112756087	0.058903491	0.44087328	USP54
ENSG00000156471.13	0.060136014	0.058929752	0.440900943	PTDSS1
ENSG00000239203.1	-0.271676671	0.058950185	0.440900943	AC093484.4
ENSG00000155254.13	0.151359088	0.05900094	0.441119678	MARVELD1
ENSG00000142065.14	-0.13335767	0.05907394	0.441325991	ZFP14
ENSG00000167930.17	0.099132491	0.059112456	0.441325991	FAM234A
ENSG00000104517.13	-0.103966881	0.059127981	0.441325991	UBR5
ENSG00000101084.18	0.095607588	0.059132194	0.441325991	RAB5IF
ENSG00000068903.20	0.083284807	0.059199757	0.441325991	SIRT2
ENSG00000116747.13	-0.117532382	0.059206813	0.441325991	RO60
ENSG00000165275.10	-0.075884328	0.059222907	0.441325991	TRMT10B
ENSG00000125846.16	0.074399688	0.059225335	0.441325991	ZNF133
ENSG00000176101.12	0.100050497	0.059226058	0.441325991	SSNA1
ENSG00000249637.2	-0.229440307	0.059243732	0.441325991	CTC-329D1.2
ENSG00000107036.12	-0.107046954	0.059268626	0.441351121	RIC1
ENSG00000211637.2	0.404713647	0.059345556	0.441569961	IGLV4-69
ENSG00000179918.19	0.059865066	0.059357005	0.441569961	SEPHS2
ENSG00000253251.3	-0.25525929	0.059362609	0.441569961	SHLD3
ENSG00000008394.13	0.160814587	0.059458011	0.441901575	MGST1
ENSG00000129515.20	-0.088207001	0.059530099	0.441901575	SNX6
ENSG00000139832.5	0.187735281	0.059538812	0.441901575	RAB20
ENSG00000118508.5	0.116360599	0.059543792	0.441901575	RAB32
ENSG00000073670.14	0.243871358	0.059574207	0.441901575	ADAM11
ENSG00000115289.14	0.086812218	0.05957472	0.441901575	PCGF1
ENSG00000265817.4	-0.346074153	0.059587315	0.441901575	FSBP
ENSG00000174500.13	0.153561119	0.059664319	0.441901575	GCSAM
ENSG00000112561.19	0.108498682	0.059684923	0.441901575	TFEB
ENSG00000213523.12	0.109205517	0.059734323	0.441901575	SRA1
ENSG00000103174.13	0.098813663	0.059737251	0.441901575	NAGPA
ENSG00000269403.1	-0.373287899	0.059744002	0.441901575	CTD-2616J11.11
ENSG00000163788.14	-0.108585593	0.05974516	0.441901575	SNRK

ENSG00000255438.2	-0.362714801	0.059753919	0.441901575	CTD-2653D5.1
ENSG00000028203.18	-0.102773224	0.059757784	0.441901575	VEZT
ENSG00000124570.21	0.114528762	0.059785485	0.441901575	SERPINB6
ENSG00000100949.15	0.094106829	0.059787437	0.441901575	RABGGTA
ENSG00000143476.18	-0.326032033	0.059806415	0.441901575	DTL
ENSG00000285967.1	-0.147599498	0.059839308	0.441901575	NIPBL-DT
ENSG00000112799.9	0.156248841	0.059841571	0.441901575	LY86
ENSG00000268278.1	-0.598229866	0.05987959	0.441901575	RP11-420K14.1
ENSG00000130303.14	0.182996687	0.059907467	0.441901575	BST2
ENSG00000279488.1	-0.298635087	0.059928298	0.441901575	AC004623.3
ENSG00000120334.16	-0.127964012	0.059932482	0.441901575	CENPL
ENSG00000275582.1	-0.184218604	0.059945884	0.441901575	RP4-681N20.5
ENSG00000171860.5	0.224512777	0.059996924	0.441964234	C3AR1
ENSG00000124171.9	-0.196234948	0.060016399	0.441964234	PARD6B
ENSG00000157954.15	0.066630118	0.060120218	0.441964234	WIPI2
ENSG00000148773.14	-0.282542686	0.060122107	0.441964234	MKI67
ENSG00000215845.11	0.115794688	0.060146942	0.441964234	TSTD1
ENSG00000105928.16	0.348899063	0.060182229	0.441964234	GSDME
ENSG00000121741.17	-0.114520927	0.060195106	0.441964234	ZMYM2
ENSG00000103145.11	0.133147791	0.060199631	0.441964234	HCFC1R1
ENSG00000145882.11	0.090103822	0.06021732	0.441964234	PCYOX1L
ENSG00000130731.16	0.133167427	0.060244671	0.441964234	METTL26
ENSG00000141380.14	-0.071569381	0.060305965	0.441964234	SS18
ENSG00000197714.9	-0.182545059	0.06035441	0.441964234	ZNF460
ENSG00000062370.17	0.387568135	0.060388298	0.441964234	ZNF112
ENSG00000211879.1	-0.488903866	0.060390684	0.441964234	TRAJ10
ENSG00000172974.11	0.197380916	0.060396238	0.441964234	VDAC2P5
ENSG00000284138.1	-0.548401489	0.060398607	0.441964234	ATP6V0CP4
ENSG00000131791.8	-0.103532193	0.060398927	0.441964234	PRKAB2
ENSG00000233820.2	-0.433297915	0.060425733	0.441964234	RP11-535M15.2
ENSG00000089012.14	0.15333433	0.06044919	0.441964234	SIRPG
ENSG00000163930.10	0.059487348	0.060486194	0.441964234	BAP1
ENSG00000173442.13	0.100520072	0.060489782	0.441964234	EHBP1L1
ENSG00000249348.1	0.246331503	0.060517231	0.441964234	UGDH-AS1
ENSG00000152242.11	-0.110832035	0.060526657	0.441964234	C18orf25
ENSG00000102882.12	0.112895415	0.060559985	0.441964234	MAPK3
ENSG00000164109.14	-0.191387489	0.060570645	0.441964234	MAD2L1
ENSG00000197372.10	-0.18215171	0.060586415	0.441964234	ZNF675
ENSG00000100342.21	0.195687708	0.060617298	0.441964234	APOL1
ENSG00000166800.10	-0.342300808	0.060620254	0.441964234	LDHAL6A
ENSG00000282780.1	-0.192176229	0.060625496	0.441964234	TRBJ1-6
ENSG00000128656.15	0.272527554	0.060630345	0.441964234	CHN1
ENSG00000231125.2	-0.229646493	0.06063773	0.441964234	AF129075.5
ENSG00000164506.14	-0.130830681	0.06065235	0.441964234	STXBP5
ENSG00000237973.1	0.201533809	0.060665561	0.441964234	MTCO1P12
ENSG00000105778.19	-0.076636982	0.06068796	0.441970414	AVL9
ENSG00000169247.14	0.288091891	0.060740066	0.442192856	SH3TC2

ENSG00000271888.1	0.31365468	0.060803119	0.442485728	JARID2-DT
ENSG00000138443.17	-0.088223867	0.060831921	0.442485728	ABI2
ENSG00000037897.17	0.133858455	0.060883275	0.442485728	METTL1
ENSG00000131495.9	0.12385173	0.060895891	0.442485728	NDUFA2
ENSG00000188549.12	0.130113542	0.060902245	0.442485728	CCDC9B
ENSG00000134444.15	-0.127176863	0.060914461	0.442485728	RELCH
ENSG00000258674.5	0.284787562	0.060931329	0.442485728	CTC-260F20.3
ENSG00000259984.1	-0.375679025	0.061007552	0.442816186	RP11-335G20.7
ENSG00000187902.13	-0.46617323	0.061020019	0.442816186	SHISA7
ENSG00000165322.18	-0.154238643	0.061091298	0.44317663	ARHGAP12
ENSG00000127824.15	0.118322441	0.061121604	0.443239694	TUBA4A
ENSG00000165879.9	0.118285201	0.061143798	0.443243907	FRAT1
ENSG00000197249.14	0.125920528	0.061204658	0.443528316	SERPINA1
ENSG00000110429.14	-0.098710876	0.061243775	0.443543727	FBXO3
ENSG00000183735.11	-0.124984702	0.061264066	0.443543727	TBK1
ENSG00000273061.1	-0.328949537	0.06128956	0.443543727	CDC37L1-DT
ENSG00000106261.17	-0.078174036	0.06131169	0.443543727	ZKSCAN1
ENSG00000084234.18	0.098958896	0.061334245	0.443543727	APLP2
ENSG00000279348.1	-0.158283408	0.061336552	0.443543727	RP11-566E18.1
ENSG00000213672.8	0.099066809	0.061396578	0.443821298	NCKIPSD
ENSG00000174004.6	0.110779733	0.061439048	0.443971808	NRROS
ENSG00000253111.2	0.59799852	0.061497423	0.444237112	RP11-136O12.2
ENSG00000165410.15	-0.154094312	0.061544552	0.444369064	CFL2
ENSG00000095370.20	0.095102365	0.061559026	0.444369064	SH2D3C
ENSG00000112039.5	0.083348034	0.061614165	0.44461059	FANCE
ENSG00000211947.2	0.386425805	0.061647893	0.4446975	IGHV3-21
ENSG00000232133.1	-0.381077611	0.061673197	0.4447236	IMPDH1P10
ENSG00000242247.11	-0.066142253	0.061708778	0.444823765	ARFGAP3
ENSG00000104343.21	-0.113395908	0.061841036	0.444928344	UBE2W
ENSG00000011114.15	-0.088130993	0.061843112	0.444928344	BTBD7
ENSG00000205269.6	-0.172942998	0.061861478	0.444928344	TMEM170B
ENSG00000258315.5	0.148383368	0.061884665	0.444928344	C17orf49
ENSG00000167778.9	0.091265802	0.061918755	0.444928344	SPRYD3
ENSG00000266401.2	0.314734824	0.061930398	0.444928344	RP11-874J12.4
ENSG00000165637.13	0.079464608	0.061938408	0.444928344	VDAC2
ENSG00000155749.13	-0.184923996	0.061957013	0.444928344	FLACC1
ENSG00000072201.14	-0.181877034	0.061963168	0.444928344	LNX1
ENSG00000186407.7	0.159784213	0.062030363	0.444928344	CD300E
ENSG00000080493.18	-0.271668693	0.062040979	0.444928344	SLC4A4
ENSG00000164466.13	-0.075533241	0.062047018	0.444928344	SFXN1
ENSG00000198225.5	0.195352939	0.062068696	0.444928344	FKBP1C
ENSG00000211813.2	0.459211202	0.062073172	0.444928344	TRAV34
ENSG00000232546.1	-0.395225146	0.062074284	0.444928344	RP11-458F8.1
ENSG00000116521.11	0.097649223	0.062084817	0.444928344	SCAMP3
ENSG00000075142.14	0.083790839	0.062110199	0.444928344	SRI
ENSG00000155545.20	-0.184182986	0.062113802	0.444928344	MIER3
ENSG00000188037.13	0.483003952	0.062175883	0.445142921	CLCN1

ENSG00000127054.21	0.073644279	0.062192719	0.445142921	INTS11
ENSG00000273899.5	0.094923364	0.062231255	0.445142921	NOL12
ENSG00000228409.6	-0.11843992	0.062236703	0.445142921	CCT6P1
ENSG00000080503.24	-0.080460863	0.062252287	0.445142921	SMARCA2
ENSG00000169245.6	0.495804749	0.062374728	0.445404531	CXCL10
ENSG00000119820.11	-0.070706241	0.062377964	0.445404531	YIPF4
ENSG00000135083.16	0.164539393	0.062404542	0.445404531	CCNJL
ENSG00000165898.14	0.106527669	0.062406053	0.445404531	ISCA2
ENSG00000156735.11	-0.100620558	0.062428938	0.445404531	BAG4
ENSG00000128708.13	-0.208185116	0.06243328	0.445404531	HAT1
ENSG00000272512.1	0.563340258	0.062440902	0.445404531	RP11-54O7.17
ENSG00000114737.16	0.132768929	0.062512136	0.445623657	CISH
ENSG00000165863.17	0.633399317	0.06251508	0.445623657	C10orf82
ENSG00000285420.1	-0.144484758	0.062571369	0.445718319	PDGFRL2P
ENSG00000113263.13	-0.103100296	0.062571828	0.445718319	ITK
ENSG00000224513.2	-1.109892724	0.06261981	0.445905227	AC109309.4
ENSG00000244480.1	-0.115865272	0.062667773	0.445997249	AC005154.7
ENSG00000112081.17	-0.060201469	0.062676227	0.445997249	SRSF3
ENSG00000212127.6	-0.244297045	0.0627964	0.446005832	TAS2R14
ENSG00000143401.15	-0.134211642	0.062861142	0.446005832	ANP32E
ENSG00000214413.9	-0.124814678	0.062928839	0.446005832	BBIP1
ENSG00000115592.11	-0.478175269	0.063016735	0.446005832	PRKAG3
ENSG00000165487.14	-0.110278449	0.063034171	0.446005832	MICU2
ENSG00000126353.3	0.214555003	0.063046903	0.446005832	CCR7
ENSG00000205181.7	-0.222691553	0.063058926	0.446005832	LINC00654
ENSG00000112697.17	-0.109975239	0.063077137	0.446005832	TMEM30A
ENSG00000135211.6	0.111526999	0.063089934	0.446005832	TMEM60
ENSG00000108946.15	-0.11036297	0.06310272	0.446005832	PRKAR1A
ENSG00000106976.21	0.194099681	0.063108276	0.446005832	DNM1
ENSG00000230006.7	-0.45790707	0.063134981	0.446005832	ANKRD36BP2
ENSG00000286244.1	-0.340094345	0.063136572	0.446005832	RP11-51I5.2
ENSG00000161911.11	0.319006676	0.063154006	0.446005832	TREML1
ENSG00000036565.15	-0.456661043	0.063191207	0.446005832	SLC18A1
ENSG00000169967.17	-0.120012496	0.063211928	0.446005832	MAP3K2
ENSG00000070731.11	0.170505633	0.063233845	0.446005832	ST6GALNAC2
ENSG00000211930.1	0.477828169	0.063250993	0.446005832	IGHD3-3
ENSG00000229754.1	0.234916051	0.063254523	0.446005832	CXCR2P1
ENSG00000204179.11	0.565668546	0.063263571	0.446005832	PTPN20
ENSG00000070610.14	0.067193029	0.063289578	0.446005832	GBA2
ENSG00000121152.10	-0.212462116	0.063300654	0.446005832	NCAPH
ENSG00000250656.2	-0.600045358	0.063310095	0.446005832	ST3GAL1P1
ENSG00000105971.15	0.402508259	0.063314632	0.446005832	CAV2
ENSG00000133422.14	0.088949155	0.063322922	0.446005832	MORC2
ENSG00000168300.14	-0.160029203	0.06332699	0.446005832	PCMTD1
ENSG00000117266.16	0.178916257	0.063329623	0.446005832	CDK18
ENSG00000134463.15	0.191289372	0.063357802	0.446005832	ECHDC3
ENSG00000266896.1	-0.189843097	0.063374329	0.446005832	RP1-266L20.9

ENSG00000139687.16	-0.11916586	0.063391869	0.446005832	RB1
ENSG00000113597.18	-0.145437165	0.063404756	0.446005832	TRAPPC13
ENSG00000226312.7	-0.201218232	0.063408003	0.446005832	CFLAR-AS1
ENSG00000166311.10	0.10547891	0.063425582	0.446005832	SMPD1
ENSG00000065150.21	-0.05659048	0.063427215	0.446005832	IPO5
ENSG00000156587.16	0.205403182	0.063439349	0.446005832	UBE2L6
ENSG00000286599.1	-0.206014682	0.063460358	0.446005832	RP11-345F18.3
ENSG00000150782.12	0.135234507	0.063511139	0.446209814	IL18
ENSG00000179869.15	-0.507199379	0.063551704	0.446341902	ABCA13
ENSG00000092345.14	0.704062117	0.06358014	0.446388742	DAZL
ENSG00000129317.15	-0.143777605	0.06361687	0.446483922	PUS7L
ENSG00000172590.18	0.082429248	0.063722835	0.446483922	MRPL52
ENSG00000157259.8	-0.067933305	0.063746085	0.446483922	GATAD1
ENSG00000180257.14	-0.153953179	0.063793857	0.446483922	ZNF816
ENSG00000023287.13	-0.16795293	0.063796722	0.446483922	RB1CC1
ENSG00000143344.16	0.175043639	0.063803738	0.446483922	RGL1
ENSG00000133739.16	-0.249843165	0.063850928	0.446483922	LRRCC1
ENSG00000169738.8	0.153651969	0.063891037	0.446483922	DCXR
ENSG00000100281.14	-0.08287646	0.063917281	0.446483922	HMGXB4
ENSG00000242616.4	-0.229414157	0.063942072	0.446483922	GNG10
ENSG00000133317.16	0.212447912	0.063981549	0.446483922	LGALS12
ENSG00000279699.1	0.429337899	0.063983703	0.446483922	RP1-102K2.9
ENSG00000141376.23	0.092553094	0.063989354	0.446483922	BCAS3
ENSG00000167964.13	-0.393292192	0.063995509	0.446483922	RAB26
ENSG00000101365.21	0.081715261	0.064074536	0.446483922	IDH3B
ENSG00000168259.17	0.060472666	0.064083983	0.446483922	DNAJC7
ENSG00000101220.18	0.183881026	0.064109945	0.446483922	C20orf27
ENSG00000237499.7	-0.141027689	0.06411099	0.446483922	WAKMAR2
ENSG00000227495.1	0.238327087	0.064126103	0.446483922	KIF1C-AS1
ENSG00000246526.2	-0.199719142	0.064136429	0.446483922	LINC02481
ENSG00000035862.12	0.094430427	0.064143828	0.446483922	TIMP2
ENSG00000127220.6	0.123837645	0.064153458	0.446483922	ABHD8
ENSG00000099992.16	0.076215403	0.064189956	0.446483922	TBC1D10A
ENSG00000288573.1	-0.274950511	0.064219963	0.446483922	RP11-7O11.3
ENSG00000179862.7	0.218189242	0.064228452	0.446483922	CITED4
ENSG00000287975.1	0.24781654	0.064352751	0.446483922	RP11-33I11.4
ENSG00000287104.1	0.131712394	0.064397714	0.446483922	RP11-326F6.1
ENSG00000184860.10	-0.163508421	0.064431379	0.446483922	SDR42E1
ENSG00000142794.19	-0.260845111	0.064493771	0.446483922	NBPF3
ENSG00000120437.9	0.096050071	0.064495267	0.446483922	ACAT2
ENSG00000204767.4	-0.275963366	0.064521342	0.446483922	INSYN2B
ENSG00000172830.13	0.106440224	0.064526334	0.446483922	SSH3
ENSG00000115107.20	0.217011768	0.064557404	0.446483922	STEAP3
ENSG00000136213.10	-0.138912486	0.064567165	0.446483922	CHST12
ENSG00000286159.1	-0.229085261	0.064581322	0.446483922	RP5-998C11.1
ENSG00000136279.21	0.066287887	0.064598262	0.446483922	DBNL
ENSG00000129951.19	-0.617264746	0.064621399	0.446483922	PLPPR3

ENSG00000164111.15	0.117792942	0.064634911	0.446483922	ANXA5
ENSG00000144589.22	0.08313381	0.064670567	0.446483922	STK11IP
ENSG00000100365.16	0.110408186	0.064764312	0.446483922	NCF4
ENSG00000130313.7	0.11114844	0.06480865	0.446483922	PGLS
ENSG00000146416.19	0.105383905	0.064822577	0.446483922	AIG1
ENSG00000144741.18	0.08987955	0.064826666	0.446483922	SLC25A26
ENSG00000198707.17	-0.166276026	0.064832023	0.446483922	CEP290
ENSG00000182400.15	-0.210456915	0.064868304	0.446483922	TRAPPC6B
ENSG00000152684.11	0.10221543	0.064877919	0.446483922	PELO
ENSG00000196407.12	-0.195553872	0.064892944	0.446483922	THEM5
ENSG00000114933.16	-0.088481451	0.064904255	0.446483922	INO80D
ENSG00000087053.20	-0.081828481	0.064908984	0.446483922	MTMR2
ENSG00000135390.21	0.106975913	0.06491148	0.446483922	ATP5MC2
ENSG00000110719.10	0.111712631	0.064924256	0.446483922	TCIRG1
ENSG00000135094.11	0.293251836	0.064932481	0.446483922	SDS
ENSG00000206113.11	0.327510355	0.065006539	0.446483922	CFAP99
ENSG00000225200.2	0.245825285	0.065009901	0.446483922	AB019441.29
ENSG00000168899.5	0.210596043	0.065022723	0.446483922	VAMP5
ENSG00000205352.11	0.100958085	0.06503984	0.446483922	PRR13
ENSG00000226137.5	0.141699456	0.065060892	0.446483922	BAIAP2-DT
ENSG00000244026.6	0.215641933	0.065077516	0.446483922	FAM86DP
ENSG00000160695.15	0.072594184	0.065090916	0.446483922	VPS11
ENSG00000280206.1	0.126066446	0.065170489	0.446483922	CTB-193M12.5
ENSG00000184678.10	0.164464888	0.065173767	0.446483922	H2BC21
ENSG00000260947.1	0.439817856	0.06517802	0.446483922	RP11-384P7.7
ENSG00000096696.15	0.830189268	0.06518354	0.446483922	DSP
ENSG00000269514.3	0.178614088	0.065231475	0.446483922	RP11-370I10.12
ENSG00000166136.16	0.107663612	0.065241061	0.446483922	NDUFB8
ENSG00000198042.11	-0.078618781	0.065268199	0.446483922	MAK16
ENSG00000163249.13	0.071294867	0.06528051	0.446483922	CCNYL1
ENSG00000187735.14	-0.078735595	0.065288269	0.446483922	TCEA1
ENSG00000167797.8	0.121419821	0.06532915	0.446483922	CDK2AP2
ENSG00000103245.14	0.097438998	0.065329584	0.446483922	CIAO3
ENSG00000116750.14	-0.106330508	0.065331221	0.446483922	UCHL5
ENSG00000286707.1	0.223821934	0.065337769	0.446483922	RP11-499P9.1
ENSG00000197859.11	0.420351798	0.065343716	0.446483922	ADAMTSL2
ENSG00000161920.11	0.086674472	0.06538125	0.446483922	MED11
ENSG00000251580.3	-0.31152989	0.065383695	0.446483922	LINC02482
ENSG00000179562.3	0.061643669	0.065453614	0.446483922	GCC1
ENSG00000176055.10	-0.163504793	0.065475686	0.446483922	MBLAC2
ENSG00000153214.11	-0.105241645	0.065488107	0.446483922	TMEM87B
ENSG00000179085.8	0.146755068	0.065490863	0.446483922	DPM3
ENSG00000246363.3	0.385736399	0.06550742	0.446483922	LINC02458
ENSG00000205890.3	0.185792276	0.065557105	0.446483922	RP11-473M20.5
ENSG00000125967.17	0.110345878	0.065620412	0.446483922	NECAB3
ENSG00000111880.16	-0.094147858	0.065633045	0.446483922	RNGTT
ENSG00000132467.4	0.077902796	0.065649421	0.446483922	UTP3

ENSG00000091972.18	0.308516117	0.065674673	0.446483922	CD200
ENSG00000088808.18	-0.092364118	0.065701164	0.446483922	PPP1R13B
ENSG00000198252.12	-0.111857429	0.065702629	0.446483922	STYX
ENSG00000198885.10	-0.182490892	0.065707128	0.446483922	ITPRIPL1
ENSG00000123395.15	0.107439355	0.065726886	0.446483922	ATG101
ENSG00000125863.20	-0.113145156	0.065729711	0.446483922	MKKS
ENSG00000260018.1	0.41957891	0.065730859	0.446483922	RP11-505K9.1
ENSG00000139323.14	-0.10170323	0.065816432	0.446483922	POC1B
ENSG00000175334.8	0.109013102	0.065825358	0.446483922	BANF1
ENSG00000141469.18	-0.231959352	0.065852324	0.446483922	SLC14A1
ENSG00000151553.15	-0.143839034	0.065866046	0.446483922	FAM160B1
ENSG00000135999.12	-0.095844796	0.065969263	0.446483922	EPC2
ENSG00000131652.14	0.103553536	0.065969883	0.446483922	THOC6
ENSG00000157227.14	0.209534317	0.06597546	0.446483922	MMP14
ENSG00000171150.9	-0.113123924	0.066014945	0.446483922	SOCS5
ENSG00000110274.16	0.070171959	0.066022881	0.446483922	CEP164
ENSG00000106003.13	0.092773341	0.066026611	0.446483922	LFNG
ENSG00000125484.12	-0.081583871	0.066035334	0.446483922	GTF3C4
ENSG00000244045.13	0.059894676	0.066058127	0.446483922	TMEM199
ENSG00000136011.15	-0.373491463	0.066063871	0.446483922	STAB2
ENSG00000215021.10	0.076658903	0.06606587	0.446483922	PHB2
ENSG00000251393.4	0.184737625	0.06607167	0.446483922	RP1-240K6.3
ENSG00000089351.15	0.102198156	0.066073617	0.446483922	GRAMD1A
ENSG00000118894.15	0.1067221	0.066073823	0.446483922	EEF2KMT
ENSG00000128040.11	-0.395508212	0.066110361	0.446483922	SPINK2
ENSG00000258572.1	0.360456556	0.066142155	0.446483922	RP11-1070N10.3
ENSG00000132953.17	-0.082716518	0.066143529	0.446483922	XPO4
ENSG00000155100.11	-0.187405137	0.066154552	0.446483922	OTUD6B
ENSG00000256155.1	-0.501997828	0.066181763	0.446483922	LINC02598
ENSG00000172354.10	0.105598872	0.066211354	0.446483922	GNB2
ENSG00000117362.13	0.067186884	0.066242821	0.446483922	APH1A
ENSG00000153560.12	-0.057472153	0.066259605	0.446483922	UBP1
ENSG00000135245.10	0.143527564	0.066282563	0.446483922	HILPDA
ENSG00000164970.15	0.061732677	0.066287604	0.446483922	FAM219A
ENSG00000121940.16	-0.081115196	0.066304905	0.446483922	CLCC1
ENSG00000115368.10	-0.093982582	0.066309522	0.446483922	WDR75
ENSG00000139826.6	-0.160975112	0.066351816	0.446483922	ABHD13
ENSG00000113580.15	-0.102542611	0.066372809	0.446483922	NR3C1
ENSG00000164323.14	-0.121585346	0.066417297	0.446483922	CFAP97
ENSG00000196862.10	-0.502248888	0.066420934	0.446483922	RGPD4
ENSG00000143258.16	0.061208917	0.06644139	0.446483922	USP21
ENSG00000166199.13	0.092081984	0.066445099	0.446483922	ALKBH3
ENSG00000189164.15	-0.126411583	0.066469956	0.446483922	ZNF527
ENSG00000125944.21	-0.050973894	0.066477133	0.446483922	HNRNPR
ENSG00000106367.15	0.11254988	0.066485557	0.446483922	AP1S1
ENSG00000127870.17	-0.104461831	0.066502414	0.446483922	RNF6
ENSG00000137871.21	-0.18199563	0.066511676	0.446483922	ZNF280D

ENSG00000105516.11	0.108390201	0.066540127	0.446483922	DBP
ENSG00000112200.17	-0.105485151	0.066591495	0.446483922	ZNF451
ENSG00000164483.17	-0.16527956	0.066593035	0.446483922	SAMD3
ENSG00000136169.16	-0.079427261	0.066607418	0.446483922	SETDB2
ENSG00000165264.11	0.120918867	0.066631308	0.446483922	NDUFB6
ENSG00000138593.9	-0.132145864	0.066670994	0.446483922	SECISBP2L
ENSG00000083312.19	-0.11020474	0.066690312	0.446483922	TNPO1
ENSG00000105723.13	0.08893152	0.066699425	0.446483922	GSK3A
ENSG00000144407.10	0.425917306	0.066699822	0.446483922	PTH2R
ENSG00000182621.18	-0.20293857	0.066705674	0.446483922	PLCB1
ENSG00000196209.13	0.123151158	0.066707501	0.446483922	SIRPB2
ENSG00000272908.1	-0.22546839	0.066709293	0.446483922	RP11-121A8.1
ENSG00000114268.12	0.093655111	0.066740585	0.446483922	PFKFB4
ENSG00000133101.10	0.481518564	0.066770235	0.446483922	CCNA1
ENSG00000278970.2	0.108594186	0.066772293	0.446483922	HEIH
ENSG00000184903.10	0.173778492	0.066828448	0.446503864	IMMP2L
ENSG00000136542.9	-0.539189431	0.066834889	0.446503864	GALNT5
ENSG00000115760.14	-0.098900472	0.066840592	0.446503864	BIRC6
ENSG00000084754.12	0.066899395	0.067085508	0.447598427	HADHA
ENSG00000262484.2	0.262401213	0.067094078	0.447598427	CCER2
ENSG00000082258.13	-0.151567944	0.067110862	0.447598427	CCNT2
ENSG00000165716.11	0.217102291	0.067136066	0.447598427	DIPK1B
ENSG00000108395.14	-0.099055814	0.067151995	0.447598427	TRIM37
ENSG00000171311.13	0.074067756	0.067161289	0.447598427	EXOSC1
ENSG00000109689.19	-0.065190057	0.06721283	0.447598427	STIM2
ENSG00000239791.2	-0.317002176	0.067219904	0.447598427	AC002310.7
ENSG00000254858.10	0.100047948	0.067236833	0.447598427	MPV17L2
ENSG00000255200.1	0.203320838	0.067256263	0.447598427	PGAM1P8
ENSG00000069329.18	0.055335156	0.067285065	0.447598427	VPS35
ENSG00000237248.5	-0.266402945	0.067291124	0.447598427	LINC00987
ENSG00000236304.1	0.410595435	0.067309281	0.447598427	AP001189.4
ENSG00000181751.10	-0.13153278	0.067325086	0.447598427	MACIR
ENSG00000256913.1	-0.434935503	0.067348134	0.447598427	RP1-102E24.6
ENSG00000066117.15	0.064229522	0.067353654	0.447598427	SMARCD1
ENSG00000171790.15	-0.241280673	0.067448985	0.44794772	SLFNL1
ENSG00000286721.1	-0.370319635	0.067469356	0.44794772	CTD-2093G19.1
ENSG00000245149.4	-0.119723214	0.067498716	0.44794772	RNF139-AS1
ENSG00000125991.20	0.082175959	0.067501712	0.44794772	ERGIC3
ENSG00000115421.13	-0.082281659	0.067515428	0.44794772	PAPOLG
ENSG00000165156.15	-0.130017523	0.067603001	0.448105131	ZHX1
ENSG00000178226.11	0.143387964	0.067639606	0.448105131	PRSS36
ENSG00000182359.15	-0.2051426	0.067681045	0.448105131	KBTBD3
ENSG00000265749.6	-0.32320469	0.06768636	0.448105131	RP11-849F2.5
ENSG00000225234.1	-0.250071221	0.067692484	0.448105131	TRAPPC12-AS1
ENSG00000100865.15	0.069854191	0.067698776	0.448105131	CINP
ENSG00000115365.12	-0.090268297	0.067701067	0.448105131	LANCL1
ENSG00000122420.10	-0.612404086	0.067713956	0.448105131	PTGFR

ENSG00000219665.8	-0.130451371	0.067793628	0.44848765	ZNF433-AS1
ENSG00000148426.13	0.199858678	0.067830208	0.448584941	PROSER2
ENSG00000230872.2	-0.323549628	0.067875434	0.44873933	MFSD13B
ENSG00000264546.1	0.558639938	0.067908259	0.448811661	RP11-464D20.6
ENSG00000136237.19	0.414544636	0.067979551	0.449138091	RAPGEF5
ENSG00000278864.1	-0.210510968	0.068039523	0.449224061	RP11-45M22.2
ENSG00000111605.18	-0.075176441	0.068051298	0.449224061	CPSF6
ENSG00000101350.8	-0.077827865	0.068058278	0.449224061	KIF3B
ENSG00000112249.14	-0.094145775	0.068110422	0.449303687	ASCC3
ENSG00000197037.11	-0.059179822	0.068174211	0.449303687	ZSCAN25
ENSG00000079263.19	0.142182093	0.068175684	0.449303687	SP140
ENSG00000151849.16	-0.104166945	0.068182627	0.449303687	CENPJ
ENSG00000228334.2	-0.701559317	0.068203364	0.449303687	AC024028.1
ENSG00000168172.9	-0.084106166	0.068234818	0.449303687	HOOK3
ENSG00000231043.3	0.163130476	0.068268439	0.449303687	AC007238.1
ENSG00000010610.10	0.114256149	0.068283225	0.449303687	CD4
ENSG00000232956.9	0.084316589	0.068300246	0.449303687	SNHG15
ENSG00000017260.20	-0.070468972	0.068326536	0.449303687	ATP2C1
ENSG00000179364.14	0.074998086	0.068363897	0.449303687	PACS2
ENSG00000126883.17	0.066952968	0.06837283	0.449303687	NUP214
ENSG00000093072.18	0.112796751	0.068373051	0.449303687	ADA2
ENSG00000224470.9	-0.079369441	0.068377063	0.449303687	ATXN1L
ENSG00000188761.13	-0.192791672	0.068461067	0.44955919	BCL2L15
ENSG00000100297.16	0.093424931	0.068475431	0.44955919	MCM5
ENSG00000198718.13	-0.149089149	0.068488565	0.44955919	TOGARAM1
ENSG00000286488.1	0.145590112	0.068507782	0.44955919	RP11-761I4.6
ENSG00000124788.19	-0.087527657	0.068541277	0.44955919	ATXN1
ENSG00000186603.7	-0.328718753	0.068547474	0.44955919	HPDL
ENSG00000132300.19	-0.057532485	0.068597344	0.449637526	PTCD3
ENSG00000173020.11	0.075662918	0.068603268	0.449637526	GRK2
ENSG00000272084.1	-0.235866612	0.068660136	0.44976468	RP5-1126H10.2
ENSG00000021488.13	-0.33310792	0.068709374	0.44976468	SLC7A9
ENSG00000118762.8	-0.142122193	0.068739383	0.44976468	PKD2
ENSG00000173457.11	0.128097586	0.068740669	0.44976468	PPP1R14B
ENSG00000165633.13	-0.650461907	0.068749957	0.44976468	VSTM4
ENSG00000109519.13	0.064136185	0.068772888	0.44976468	GRPEL1
ENSG00000174038.13	-0.427593982	0.068793203	0.44976468	C9orf131
ENSG00000112578.10	0.114741253	0.068798118	0.44976468	BYSL
ENSG00000166794.6	0.090661457	0.068901524	0.450297148	PPIB
ENSG00000151773.13	0.303956985	0.068949985	0.450339446	CCDC122
ENSG00000251369.9	-0.148199898	0.068978815	0.450339446	ZNF550
ENSG00000100075.10	0.118663431	0.068985544	0.450339446	SLC25A1
ENSG00000104872.11	0.086276693	0.068995833	0.450339446	PIH1D1
ENSG00000248275.2	0.153230901	0.069060519	0.450569077	TRIM52-AS1
ENSG00000132471.12	0.108085592	0.069074955	0.450569077	WBP2
ENSG00000126062.4	0.087159089	0.069113062	0.4506743	TMEM115
ENSG00000231329.8	-0.297140514	0.069171704	0.450803308	RP1-225E12.2

ENSG00000184897.6	0.139251887	0.06917681	0.450803308	H1-10
ENSG00000170242.19	-0.112764919	0.06926726	0.451042594	USP47
ENSG00000244116.3	0.364152844	0.069271647	0.451042594	IGKV2-28
ENSG00000244005.13	0.073468516	0.069279509	0.451042594	NFS1
ENSG00000131043.13	0.075418799	0.069344959	0.451325429	AAR2
ENSG00000085563.15	-0.183297284	0.069457429	0.451775914	ABCB1
ENSG00000143882.12	-0.159315228	0.069485632	0.451775914	ATP6V1C2
ENSG00000204681.11	0.111809841	0.069487288	0.451775914	GABBR1
ENSG00000146674.16	-0.253739405	0.069502292	0.451775914	IGFBP3
ENSG00000117036.12	-0.068454851	0.069533843	0.451837787	ETV3
ENSG00000138658.16	-0.15901	0.069606881	0.451937817	ZGRF1
ENSG00000010244.19	-0.050182111	0.069607403	0.451937817	ZNF207
ENSG00000042753.12	0.124963716	0.069630886	0.451937817	AP2S1
ENSG00000233337.1	-0.221153199	0.069637386	0.451937817	UBE2FP3
ENSG00000269072.1	0.194010338	0.069672761	0.452024355	CTD-3187F8.14
ENSG00000125454.12	0.110041778	0.069758949	0.452100743	SLC25A19
ENSG00000259834.1	-0.127336514	0.069771945	0.452100743	RP11-284N8.3
ENSG00000130592.17	0.119568756	0.06977941	0.452100743	LSP1
ENSG00000260293.2	-0.314719864	0.069786837	0.452100743	RP11-715J22.6
ENSG00000107371.14	-0.080279878	0.069882991	0.452100743	EXOSC3
ENSG00000234776.5	-0.415809282	0.069886578	0.452100743	C11orf94
ENSG00000163947.12	-0.087970983	0.069888932	0.452100743	ARHGEF3
ENSG00000149016.16	0.09653438	0.069893999	0.452100743	TUT1
ENSG00000164713.10	0.113533582	0.069919167	0.452100743	BRI3
ENSG00000226252.1	0.335348249	0.069923214	0.452100743	RP1-18D14.7
ENSG00000089693.11	0.100516774	0.069927031	0.452100743	MLF2
ENSG00000197548.12	0.076064714	0.069956931	0.452151508	ATG7
ENSG00000126231.15	-0.271560347	0.070056722	0.452466107	PROZ
ENSG00000115756.13	0.092040383	0.070060901	0.452466107	HPCAL1
ENSG00000167664.8	0.226621674	0.07009602	0.452466107	TMIGD2
ENSG00000023171.19	0.156926991	0.070116953	0.452466107	GRAMD1B
ENSG00000183808.12	-0.116235545	0.070127693	0.452466107	RBM12B
ENSG00000198677.12	-0.096829568	0.070168296	0.452466107	TTC37
ENSG00000130340.17	0.109558631	0.070171823	0.452466107	SNX9
ENSG00000235191.1	0.394114272	0.070192455	0.452466107	NUCB1-AS1
ENSG00000070761.8	0.069895919	0.070224125	0.452466107	CFAP20
ENSG00000099958.15	0.278472919	0.07025437	0.452466107	DERL3
ENSG00000117505.13	-0.075859806	0.070308624	0.452466107	DR1
ENSG00000107796.13	0.234717205	0.070336304	0.452466107	ACTA2
ENSG00000168036.18	-0.048264037	0.070367534	0.452466107	CTNNB1
ENSG00000182957.16	-0.114603856	0.070398269	0.452466107	SPATA13
ENSG00000101460.13	0.207988529	0.07040824	0.452466107	MAP1LC3A
ENSG00000160447.7	-0.196769547	0.070410679	0.452466107	PKN3
ENSG00000254428.1	-0.212978114	0.070431074	0.452466107	RP11-110I1.11
ENSG00000166004.15	-0.170492444	0.070443526	0.452466107	CEP295
ENSG00000237991.3	0.305466673	0.070492562	0.452466107	RPL35P1
ENSG00000130544.12	-0.076250182	0.07049464	0.452466107	ZNF557

ENSG00000196110.8	-0.121328025	0.070504154	0.452466107	ZNF699
ENSG00000269404.7	0.209812245	0.070505523	0.452466107	SPIB
ENSG00000185669.6	0.119525187	0.070517549	0.452466107	SNAI3
ENSG00000171051.9	0.126911674	0.070560171	0.452466107	FPR1
ENSG00000260816.2	0.552169993	0.07056892	0.452466107	RP11-319G9.3
ENSG00000257596.1	0.20034365	0.070579241	0.452466107	SCAT2
ENSG00000111300.10	-0.089997821	0.070601361	0.452466472	NAA25
ENSG00000127837.10	0.083377663	0.070684307	0.452758366	AAMP
ENSG00000008516.18	0.207515916	0.070705564	0.452758366	MMP25
ENSG00000144043.12	0.055221009	0.070713139	0.452758366	TEX261
ENSG00000134072.11	0.144275067	0.070789853	0.452767695	CAMK1
ENSG00000241556.1	0.354750962	0.07080149	0.452767695	RP11-490G8.1
ENSG00000114779.20	0.099474428	0.070806432	0.452767695	ABHD14B
ENSG00000250959.2	-0.135447804	0.070811758	0.452767695	GLUD1P3
ENSG00000121380.12	0.4698471	0.070824984	0.452767695	BCL2L14
ENSG00000010256.11	0.098212734	0.070869731	0.45289829	UQCRC1
ENSG00000198951.12	0.104034327	0.070899231	0.45289829	NAGA
ENSG00000172671.20	-0.09011734	0.070915598	0.45289829	ZFAND4
ENSG00000137309.20	0.10569507	0.070935757	0.45289829	HMGA1
ENSG00000256250.1	-0.637773908	0.070955832	0.45289829	RP11-989F5.1
ENSG00000163281.12	-0.134551703	0.071055154	0.45317532	GNPDA2
ENSG00000159314.11	0.06608263	0.071061264	0.45317532	ARHGAP27
ENSG00000072778.20	0.08870198	0.071094153	0.45317532	ACADVL
ENSG00000173209.23	-0.093484571	0.071121113	0.45317532	AHSA2P
ENSG00000233913.7	0.594088971	0.071128369	0.45317532	RPL10P9
ENSG00000103489.12	-0.111165943	0.071131819	0.45317532	XYLT1
ENSG00000270108.1	-0.277590923	0.071227901	0.453374941	RP11-73M18.6
ENSG00000146247.14	-0.163706211	0.071236373	0.453374941	PHIP
ENSG00000157653.12	0.312372414	0.071291084	0.453374941	C9orf43
ENSG00000108819.11	0.083421183	0.071295518	0.453374941	PPP1R9B
ENSG00000013441.16	-0.143260237	0.07130403	0.453374941	CLK1
ENSG00000287839.1	0.152698485	0.071357163	0.453374941	RP11-29H23.8
ENSG00000185946.16	-0.129318228	0.071362894	0.453374941	RNPC3
ENSG00000160867.15	0.228982196	0.071379646	0.453374941	FGFR4
ENSG00000145780.8	-0.118254262	0.071380787	0.453374941	FEM1C
ENSG00000133835.18	0.076155376	0.071391918	0.453374941	HSD17B4
ENSG00000123908.12	-0.087943031	0.071420539	0.453374941	AGO2
ENSG00000185215.11	0.088788421	0.071428439	0.453374941	TNFAIP2
ENSG00000267244.5	-0.152259313	0.071481169	0.453569247	CTB-31O20.4
ENSG00000176476.9	0.114943234	0.07151324	0.453619316	SGF29
ENSG00000138757.15	-0.072778105	0.071602517	0.453619316	G3BP2
ENSG00000119986.7	0.210399552	0.071639613	0.453619316	AVPI1
ENSG00000158796.17	0.064260688	0.071674008	0.453619316	DEDD
ENSG00000105556.12	0.097970362	0.071682408	0.453619316	MIER2
ENSG00000288093.1	-0.3562761	0.071690398	0.453619316	RP11-122G18.13
ENSG00000257052.1	0.298035278	0.07170413	0.453619316	RP11-881M11.2
ENSG00000113649.13	-0.069432055	0.071737484	0.453619316	TCERG1

ENSG00000237264.4	0.198861823	0.071740287	0.453619316	FTH1P11
ENSG00000150093.20	-0.136698536	0.071752699	0.453619316	ITGB1
ENSG00000023041.12	-0.050449655	0.071762966	0.453619316	ZDHHC6
ENSG00000232344.2	-0.363672834	0.071775357	0.453619316	AC087163.2
ENSG00000196776.17	-0.086949287	0.071776608	0.453619316	CD47
ENSG00000114270.18	0.203267667	0.071807392	0.453674056	COL7A1
ENSG00000198730.9	-0.065767359	0.071854605	0.453709663	CTR9
ENSG00000128311.14	0.142153235	0.071857274	0.453709663	TST
ENSG00000074356.17	-0.053161854	0.071897348	0.453822962	NCBP3
ENSG00000165244.7	-0.159106441	0.071944491	0.453980808	ZNF367
ENSG00000110583.13	-0.076610865	0.072086788	0.454631352	NAA40
ENSG00000155636.15	-0.08236878	0.072091923	0.454631352	RBM45
ENSG00000040199.18	-0.119875395	0.072122909	0.454686941	PHLPP2
ENSG00000086548.9	-0.569680321	0.072183071	0.454716699	CEACAM6
ENSG00000137955.16	-0.094609963	0.072204669	0.454716699	RABGGTB
ENSG00000168060.16	0.121820807	0.072223904	0.454716699	NAALADL1
ENSG00000168734.14	0.208877117	0.072239542	0.454716699	PKIG
ENSG00000131398.15	0.162745714	0.072265987	0.454716699	KCNC3
ENSG00000063180.9	0.111071061	0.072286372	0.454716699	CA11
ENSG00000267305.2	1.294154737	0.072288114	0.454716699	RP11-378J18.10
ENSG00000261346.1	-0.293286079	0.072323519	0.454716699	RP11-297C4.2
ENSG00000112208.11	-0.137322861	0.072327183	0.454716699	BAG2
ENSG00000167483.19	0.246428078	0.07235288	0.454738848	NIBAN3
ENSG00000164638.11	-0.2899348	0.072494529	0.455303202	SLC29A4
ENSG00000270039.1	-0.210469859	0.072529549	0.455303202	RP11-571M6.17
ENSG00000225871.2	-0.179414769	0.072534802	0.455303202	RP11-495P10.10
ENSG00000279314.1	-0.283353546	0.072540195	0.455303202	RP1-257C22.2
ENSG00000166527.8	-0.259576689	0.07255368	0.455303202	CLEC4D
ENSG00000031691.7	-0.284737556	0.072597542	0.455351815	CENPQ
ENSG00000113558.19	0.073228601	0.072611722	0.455351815	SKP1
ENSG00000131018.25	-0.109004352	0.072628037	0.455351815	SYNE1
ENSG00000106244.13	0.0632314	0.072721546	0.455481098	PDAP1
ENSG00000111667.14	0.086048198	0.072724341	0.455481098	USP5
ENSG00000115207.13	0.057041131	0.072758233	0.455481098	GTF3C2
ENSG00000135926.15	0.066517672	0.072758516	0.455481098	TMBIM1
ENSG00000198924.8	-0.135060836	0.072775617	0.455481098	DCLRE1A
ENSG00000138709.19	-0.115585142	0.072781917	0.455481098	LARP1B
ENSG00000122971.9	0.102655881	0.072814551	0.455546312	ACADS
ENSG00000180573.10	0.121003706	0.072848584	0.455620235	H2AC6
ENSG00000105186.16	-0.057465708	0.072888971	0.455733844	ANKRD27
ENSG00000235527.7	0.356272941	0.072968213	0.455770854	HIPK1-AS1
ENSG00000140367.12	-0.101907353	0.072984186	0.455770854	UBE2Q2
ENSG00000164983.8	-0.111723693	0.072987431	0.455770854	TMEM65
ENSG00000075884.14	0.084887672	0.072994206	0.455770854	ARHGAP15
ENSG00000182557.8	0.220257961	0.073006521	0.455770854	SPNS3
ENSG00000244187.8	0.112971134	0.073028234	0.455770854	TMEM141
ENSG00000168062.10	0.423885113	0.07307562	0.455782577	BATF2

ENSG00000047249.18	0.067809203	0.073106587	0.455782577	ATP6V1H
ENSG00000266947.1	0.149726687	0.07312077	0.455782577	RP11-799D4.4
ENSG00000160075.12	0.076229919	0.07315939	0.455782577	SSU72
ENSG00000143369.15	0.245586523	0.073172779	0.455782577	ECM1
ENSG00000011638.11	0.089900354	0.073173607	0.455782577	TMEM159
ENSG00000071189.21	-0.135375393	0.073204816	0.455782577	SNX13
ENSG00000272386.1	0.296452337	0.073207909	0.455782577	RP11-666A8.12
ENSG00000104549.12	0.133914899	0.073271911	0.455998302	SQLE
ENSG00000277149.5	0.295172885	0.073287933	0.455998302	TYW1B
ENSG00000100258.18	0.092734847	0.073324453	0.455998302	LMF2
ENSG00000198931.11	0.108650912	0.0733315	0.455998302	APRT
ENSG00000101138.12	0.049040302	0.073409446	0.456228759	CSTF1
ENSG00000177853.14	-0.149240263	0.073413054	0.456228759	ZNF518A
ENSG00000279930.1	0.294058363	0.073485929	0.456235991	LA16c-312E8.4
ENSG00000268069.2	-0.285062446	0.073486097	0.456235991	RP5-1057I20.4
ENSG00000211845.1	-0.481484512	0.073498706	0.456235991	TRAJ44
ENSG00000184436.12	0.101848849	0.073508953	0.456235991	THAP7
ENSG00000256128.6	-0.305366642	0.073525451	0.456235991	LINC00944
ENSG00000127903.14	0.248241816	0.07357418	0.45636493	ZNF835
ENSG00000102531.16	-0.132658987	0.073610873	0.45636493	FNDC3A
ENSG00000275441.1	0.396737848	0.073612989	0.45636493	RP11-666O2.2
ENSG00000184470.21	0.095581748	0.073672101	0.456593367	TXNRD2
ENSG00000173852.15	-0.12974792	0.073754364	0.456752515	DPY19L1
ENSG00000106299.8	-0.10389695	0.073761161	0.456752515	WASL
ENSG00000171649.12	0.139932255	0.073789715	0.456752515	ZIK1
ENSG00000198580.7	0.34334475	0.073792912	0.456752515	RP11-611L7.2
ENSG00000214530.10	0.146927121	0.073829811	0.456752515	STARD10
ENSG00000253716.6	0.156051554	0.073853792	0.456752515	MINCR
ENSG00000033178.13	-0.113133681	0.073895185	0.456752515	UBA6
ENSG00000274276.4	-0.8274333	0.073911483	0.456752515	CBSL
ENSG00000164933.12	-0.279354567	0.073916317	0.456752515	SLC25A32
ENSG00000132718.9	-0.126562993	0.073925369	0.456752515	SYT11
ENSG00000119878.6	-0.081146168	0.073943158	0.456752515	CRIPT
ENSG00000196670.14	-0.116386098	0.073981134	0.456752515	ZFP62
ENSG00000257093.7	-0.100503561	0.074012747	0.456752515	DENND11
ENSG00000267048.1	-0.277624932	0.074025828	0.456752515	RP11-566K11.7
ENSG00000110888.17	-0.083417948	0.074031859	0.456752515	CAPRIN2
ENSG00000139289.14	-0.169480418	0.074081017	0.456753276	PHLDA1
ENSG00000166105.16	-0.187787812	0.074089491	0.456753276	GLB1L3
ENSG00000143319.17	0.051685475	0.074163036	0.456753276	ISG20L2
ENSG00000182919.15	-0.108429905	0.074181236	0.456753276	C11orf54
ENSG00000136021.19	-0.107992267	0.074200859	0.456753276	SCYL2
ENSG00000133424.22	0.26426608	0.074206404	0.456753276	LARGE1
ENSG00000100575.14	0.100219593	0.07426096	0.456753276	TIMM9
ENSG00000185122.11	0.086213005	0.074263791	0.456753276	HSF1
ENSG00000160299.17	-0.092832033	0.074270453	0.456753276	PCNT
ENSG00000276180.1	0.177549865	0.074272071	0.456753276	H4C9

ENSG00000198853.12	0.132370192	0.074276974	0.456753276	RUSC2
ENSG00000151006.7	-0.128961108	0.074353095	0.456781066	PRSS53
ENSG00000134243.12	0.11408462	0.074364881	0.456781066	SORT1
ENSG00000175224.17	0.067380997	0.07436956	0.456781066	ATG13
ENSG00000176125.7	0.245136662	0.074370586	0.456781066	UFSP1
ENSG00000218052.5	0.340924568	0.074466838	0.457148801	ADAMTS7P4
ENSG00000116005.12	-0.082184629	0.074495864	0.457148801	PCYOX1
ENSG00000173588.15	-0.148880773	0.074497332	0.457148801	CEP83
ENSG00000182504.11	-0.136495909	0.074541706	0.457267005	CEP97
ENSG00000174951.12	-0.470697164	0.074598751	0.457267005	FUT1
ENSG00000108700.5	0.813003055	0.074615942	0.457267005	CCL8
ENSG00000275791.1	-0.240039439	0.074657754	0.457267005	TRBV10-3
ENSG00000104976.12	0.106525335	0.074658607	0.457267005	SNAPC2
ENSG00000251000.1	-0.252493884	0.074663778	0.457267005	GGCTP1
ENSG00000189241.8	-0.053074873	0.074687838	0.457267005	TSPYL1
ENSG00000269194.1	0.232793768	0.074694971	0.457267005	AC006942.4
ENSG00000197601.13	-0.139849534	0.074772832	0.457601013	FAR1
ENSG00000172795.17	-0.105577876	0.074794158	0.457601013	DCP2
ENSG00000176170.14	0.16958432	0.074834967	0.457714137	SPHK1
ENSG00000271092.5	-0.325717579	0.074894157	0.457934755	TLCD4-RWDD3
ENSG00000071243.16	-0.072412072	0.0749386	0.457934755	ING3
ENSG00000230266.1	0.331218362	0.07495958	0.457934755	XXYLT1-AS2
ENSG00000132612.16	0.074736015	0.074972617	0.457934755	VPS4A
ENSG00000106211.10	0.16099097	0.074982685	0.457934755	HSPB1
ENSG00000285728.2	-0.118562852	0.075007474	0.457949772	RP5-994D16.13
ENSG00000197930.13	-0.113144155	0.075080185	0.458150737	ERO1A
ENSG00000287608.1	-0.345655312	0.075085071	0.458150737	CTD-2224J9.11
ENSG00000163728.11	-0.120715634	0.075116346	0.458205244	TTC14
ENSG00000133026.13	-0.147506819	0.075198609	0.458570645	MYH10
ENSG00000111737.12	0.067369176	0.075277229	0.458646366	RAB35
ENSG00000178177.16	-0.132403985	0.075311044	0.458646366	LCORL
ENSG00000268041.3	0.191012258	0.075325187	0.458646366	ERFL
ENSG00000080947.15	-0.106181304	0.075355285	0.458646366	CROCCP3
ENSG00000095932.7	-0.370474909	0.075359747	0.458646366	SMIM24
ENSG00000064102.15	-0.083618501	0.07536345	0.458646366	INTS13
ENSG00000267680.5	-0.095269094	0.075367576	0.458646366	ZNF224
ENSG00000111731.13	-0.101282358	0.075473934	0.459150689	C2CD5
ENSG00000198171.13	0.100079994	0.075495227	0.459150689	DDRGK1
ENSG00000228606.3	-0.219044112	0.075558979	0.459242873	RP11-574F21.2
ENSG00000279554.1	-0.485297728	0.07556927	0.459242873	CTA-113A6.2
ENSG00000143393.17	0.052628235	0.075577565	0.459242873	PI4KB
ENSG00000101745.17	-0.152361174	0.075630611	0.459429079	ANKRD12
ENSG00000139187.10	-0.247992397	0.075659341	0.459467508	KLRG1
ENSG00000143514.17	-0.081752602	0.075712187	0.45953631	TP53BP2
ENSG00000189077.11	0.10245203	0.075715486	0.45953631	TMEM120A
ENSG00000187391.22	0.304381291	0.075745527	0.459582624	MAGI2
ENSG00000239830.1	0.455762712	0.075777738	0.459642076	RPS4XP22

ENSG00000131013.4	-0.106239515	0.075993278	0.460542578	PPIL4
ENSG00000113272.14	0.087379955	0.076026216	0.460542578	THG1L
ENSG00000147649.10	-0.068905598	0.076027945	0.460542578	MTDH
ENSG00000173825.7	0.162115986	0.076046181	0.460542578	TIGD3
ENSG00000197057.10	-0.247813024	0.076091897	0.460542578	DTHD1
ENSG00000108799.13	0.054769045	0.076093709	0.460542578	EZH1
ENSG00000163257.11	-0.0749267	0.076170621	0.460542578	DCAF16
ENSG00000069493.15	-0.078737648	0.076199096	0.460542578	CLEC2D
ENSG00000179967.11	0.148623215	0.076213772	0.460542578	PPP1R14BP3
ENSG00000171517.6	-0.453637686	0.076229139	0.460542578	LPAR3
ENSG00000163660.12	-0.093131812	0.076301116	0.460542578	CCNL1
ENSG00000260285.1	0.118375671	0.076346638	0.460542578	RP11-600F24.7
ENSG00000118961.15	-0.089356464	0.076348654	0.460542578	LDAH
ENSG00000182307.14	0.058013813	0.076356504	0.460542578	C8orf33
ENSG00000161929.15	0.117861511	0.076370462	0.460542578	SCIMP
ENSG00000258769.1	-0.448039356	0.076384393	0.460542578	RAP1AP
ENSG00000152240.13	-0.206445909	0.076388682	0.460542578	HAUS1
ENSG00000204472.13	0.145132096	0.076403685	0.460542578	AIF1
ENSG00000154102.11	0.184920142	0.076412375	0.460542578	C16orf74
ENSG00000169258.7	-0.204180286	0.076442566	0.460542578	GPRIN1
ENSG00000105771.14	0.082901519	0.076442834	0.460542578	SMG9
ENSG00000105287.13	0.109517169	0.076480173	0.460542578	PRKD2
ENSG00000104783.14	0.192365902	0.076502466	0.460542578	KCNN4
ENSG00000229422.2	-0.49583744	0.076526768	0.460542578	RP11-262H14.13
ENSG00000155980.13	-0.22313547	0.076558912	0.460542578	KIF5A
ENSG00000158747.15	0.270848462	0.076564334	0.460542578	NBL1
ENSG00000234199.3	0.249579416	0.076564554	0.460542578	LINC01191
ENSG00000231858.5	-0.15811607	0.0766743	0.460542578	AC067945.4
ENSG00000239819.2	0.417421461	0.076677765	0.460542578	IGKV1D-8
ENSG00000176108.9	0.102412034	0.076686303	0.460542578	CHMP6
ENSG00000188811.14	-0.0887662	0.076688797	0.460542578	NHLRC3
ENSG00000244273.1	0.478561271	0.076705189	0.460542578	PGBD4P1
ENSG00000231528.3	0.394586623	0.076706858	0.460542578	FAM225A
ENSG00000260286.4	-0.26272401	0.076709224	0.460542578	ARMH2
ENSG00000174718.12	-0.130517106	0.076749017	0.460542578	RESF1
ENSG00000076382.17	-0.137673318	0.076752344	0.460542578	SPAG5
ENSG00000245904.4	0.166285204	0.076757096	0.460542578	RP11-796E2.4
ENSG00000146281.6	-0.138560168	0.076795516	0.460638326	PM20D2
ENSG00000265100.1	-0.264481386	0.076870813	0.460955156	RP11-147L13.2
ENSG00000109272.4	0.454376177	0.076916254	0.46105093	PF4V1
ENSG00000121274.14	-0.115687715	0.076931748	0.46105093	TENT4B
ENSG00000226212.2	0.495131001	0.076983605	0.461099779	TRGV6
ENSG00000269737.2	-0.300231853	0.076984867	0.461099779	RP11-345P4.6
ENSG00000163820.15	-0.101266639	0.077045694	0.461329367	FYCO1
ENSG00000136710.10	0.057183213	0.077078162	0.461331736	CCDC115
ENSG00000101752.12	-0.151477665	0.077123976	0.461331736	MIB1
ENSG00000280347.1	-0.134823132	0.077138918	0.461331736	AC000123.2

ENSG00000170385.10	-0.133623438	0.077176803	0.461331736	SLC30A1
ENSG00000072274.13	-0.096313378	0.077194399	0.461331736	TFRC
ENSG00000127993.16	-0.055440432	0.077199714	0.461331736	RBM48
ENSG00000271743.1	0.446334026	0.077203556	0.461331736	CTD-2541M15.3
ENSG00000281183.1	-0.311480679	0.077273306	0.46161403	NPTN-IT1
ENSG00000169359.16	-0.097679572	0.077373789	0.462020252	SLC33A1
ENSG00000126457.22	0.076808697	0.077386365	0.462020252	PRMT1
ENSG00000149923.14	0.100081779	0.077447665	0.462156868	PPP4C
ENSG00000028528.15	0.039222915	0.077454318	0.462156868	SNX1
ENSG00000065268.11	0.1336401	0.077501514	0.462178565	WDR18
ENSG00000186567.13	0.20593198	0.077510263	0.462178565	CEACAM19
ENSG00000167302.11	0.07279184	0.077525564	0.462178565	TEPSIN
ENSG00000103381.12	0.106372742	0.077594902	0.462457497	CPPED1
ENSG00000109919.10	0.072566308	0.077660474	0.462597948	MTCH2
ENSG00000151715.8	0.22671646	0.077663582	0.462597948	TMEM45B
ENSG00000253626.4	0.387779094	0.077778252	0.462965071	EIF5AL1
ENSG00000111834.13	-0.271373976	0.077791616	0.462965071	RSPH4A
ENSG00000160323.19	0.169598135	0.077828539	0.462965071	ADAMTS13
ENSG00000075539.15	-0.088742435	0.077841657	0.462965071	FRYL
ENSG00000282164.3	-0.131304058	0.077857798	0.462965071	PEG13
ENSG00000249353.2	0.165339049	0.077860666	0.462965071	NPM1P27
ENSG00000141294.10	0.175016563	0.077890481	0.462999491	LRRC46
ENSG00000059377.18	0.084218637	0.077917123	0.462999491	TBXAS1
ENSG00000167112.10	0.074075265	0.077961336	0.462999491	TRUB2
ENSG00000279613.1	0.415206652	0.07798448	0.462999491	RP13-638C3.5
ENSG00000136425.14	0.198594564	0.07798704	0.462999491	CIB2
ENSG00000123119.12	-0.282397246	0.078001913	0.462999491	NECAB1
ENSG00000129353.15	0.093732989	0.078194093	0.463494288	SLC44A2
ENSG00000278739.1	0.479798209	0.078194235	0.463494288	MIR6859-4
ENSG00000273213.4	-0.4989902	0.078227964	0.463494288	H3-2
ENSG00000230565.1	0.2522344	0.078234552	0.463494288	ZNF32-AS2
ENSG00000244405.8	-0.235283365	0.078239078	0.463494288	ETV5
ENSG00000253986.1	-0.542115697	0.078257302	0.463494288	CTC-756D1.3
ENSG00000099622.14	0.069747971	0.078263276	0.463494288	CIRBP
ENSG00000140807.7	0.170628033	0.078281057	0.463494288	NKD1
ENSG00000178904.19	-0.117574418	0.078288678	0.463494288	DPY19L3
ENSG00000100605.17	0.111219165	0.078320841	0.463550881	ITPK1
ENSG00000100652.5	-0.320617637	0.078346951	0.463571631	SLC10A1
ENSG00000083093.10	-0.084091635	0.078374133	0.46359871	PALB2
ENSG00000122545.20	-0.081008215	0.078463116	0.463991229	SEPTIN7
ENSG00000101639.18	-0.127411283	0.078563028	0.464071081	CEP192
ENSG00000198113.3	0.105659147	0.078639872	0.464071081	TOR4A
ENSG00000129158.11	0.103090633	0.078641182	0.464071081	SERGEF
ENSG00000107338.10	0.244784844	0.078651133	0.464071081	SHB
ENSG00000172613.8	0.083257689	0.078661811	0.464071081	RAD9A
ENSG00000277072.5	-0.133519659	0.078673754	0.464071081	STAG3L2
ENSG00000183604.14	-0.228440342	0.078679653	0.464071081	SMG1P5

ENSG00000267796.8	0.096084923	0.078700947	0.464071081	LIN37
ENSG00000117000.9	-0.101008947	0.078709666	0.464071081	RLF
ENSG00000271933.1	-0.34467478	0.078719827	0.464071081	RP11-338I21.1
ENSG00000128340.15	0.089947437	0.078768952	0.464071081	RAC2
ENSG00000175467.15	0.099906469	0.078785443	0.464071081	SART1
ENSG00000187778.14	0.068815801	0.078791413	0.464071081	MCRS1
ENSG00000172005.11	0.204267393	0.078814321	0.464071081	MAL
ENSG00000150764.14	0.246946611	0.078816051	0.464071081	DIXDC1
ENSG00000137817.17	0.049408047	0.07885494	0.464166792	PARP6
ENSG00000226174.7	0.164398584	0.078992052	0.464273641	TEX22
ENSG00000163870.16	0.097633961	0.078994721	0.464273641	TPRA1
ENSG00000166333.14	0.117074012	0.078996225	0.464273641	ILK
ENSG00000088325.16	-0.247626254	0.079126336	0.464273641	TPX2
ENSG00000185344.15	-0.070114832	0.079135935	0.464273641	ATP6V0A2
ENSG00000117586.11	0.161739263	0.079149906	0.464273641	TNFSF4
ENSG00000138760.11	0.106882592	0.079155665	0.464273641	SCARB2
ENSG00000162222.14	0.056336539	0.079159329	0.464273641	TTC9C
ENSG00000272541.1	0.486023431	0.079244493	0.464273641	XXbac-BPGBPG55C20.1
ENSG00000184007.22	-0.064985221	0.079252647	0.464273641	PTP4A2
ENSG00000115138.11	0.313961154	0.079258115	0.464273641	POMC
ENSG00000135312.7	0.580002506	0.079278614	0.464273641	HTR1B
ENSG00000110104.13	0.101127604	0.079290684	0.464273641	CCDC86
ENSG00000114861.23	0.101783285	0.079319102	0.464273641	FOXP1
ENSG00000168291.13	0.067321702	0.079371969	0.464273641	PDHB
ENSG00000168056.17	0.173115663	0.079387816	0.464273641	LTBP3
ENSG00000163468.15	0.070508315	0.079397236	0.464273641	CCT3
ENSG00000171777.16	0.089070711	0.079414474	0.464273641	RASGRP4
ENSG00000249207.2	-0.307534219	0.079416926	0.464273641	RP11-360F5.1
ENSG00000130558.20	0.373703131	0.0794514	0.464273641	OLFM1
ENSG00000106344.8	-0.055158198	0.079452982	0.464273641	RBM28
ENSG00000182993.5	0.211694368	0.079467811	0.464273641	C12orf60
ENSG00000078808.19	0.068602945	0.079475103	0.464273641	SDF4
ENSG00000286733.1	0.579447336	0.079475891	0.464273641	LINC02348
ENSG00000040633.13	0.074534835	0.07948375	0.464273641	PHF23
ENSG00000149575.9	0.537325196	0.079488021	0.464273641	SCN2B
ENSG00000213015.9	0.11339383	0.079492122	0.464273641	ZNF580
ENSG00000277425.1	-0.377766779	0.079506974	0.464273641	RP5-1116H23.3
ENSG00000085832.17	-0.074403607	0.079599446	0.464414214	EPS15
ENSG00000117308.15	0.091220678	0.07962024	0.464414214	GALE
ENSG00000132824.14	-0.065037232	0.079647175	0.464414214	SERINC3
ENSG00000157350.13	0.093118919	0.079653217	0.464414214	ST3GAL2
ENSG00000142961.15	0.085106423	0.079665425	0.464414214	MOB3C
ENSG00000268006.1	-0.274501458	0.07966692	0.464414214	PTOV1-AS1
ENSG00000204778.4	0.285399136	0.07971469	0.464522511	CBWD4P
ENSG00000171109.19	-0.088593105	0.079733665	0.464522511	MFN1
ENSG00000115464.15	-0.155444306	0.07975345	0.464522511	USP34
ENSG00000166377.20	0.068362837	0.079801069	0.464588309	ATP9B

ENSG00000261002.5	-0.458976763	0.079866248	0.464588309	VPS39-DT
ENSG00000124203.7	-0.094340009	0.079897984	0.464588309	ZNF831
ENSG00000128272.17	0.064567437	0.079915801	0.464588309	ATF4
ENSG00000166439.6	-0.164356146	0.079915925	0.464588309	RNF169
ENSG00000114796.16	-0.077492853	0.079936715	0.464588309	KLHL24
ENSG00000176597.12	-0.188459207	0.079957841	0.464588309	B3GNT5
ENSG00000188611.17	-0.462963773	0.079964957	0.464588309	ASAH2
ENSG00000228444.2	0.24568526	0.080003718	0.464588309	RP11-173B14.4
ENSG00000146350.14	-0.172838216	0.080054019	0.464588309	TBC1D32
ENSG00000117245.13	0.355172104	0.080057214	0.464588309	KIF17
ENSG00000276953.1	-0.320740431	0.080058841	0.464588309	TRBV12-4
ENSG00000286219.1	-0.2917026	0.080087051	0.464588309	NOTCH2NLC
ENSG00000139324.12	-0.155607462	0.080117842	0.464588309	TMTC3
ENSG00000008300.17	-0.204667355	0.080131706	0.464588309	CELSR3
ENSG00000164024.12	-0.048560645	0.080179118	0.464588309	METAP1
ENSG00000176909.12	-0.215442761	0.080200424	0.464588309	MAMSTR
ENSG00000164548.11	-0.070447131	0.080205917	0.464588309	TRA2A
ENSG00000214753.4	0.077855768	0.080226299	0.464588309	HNRNPUL2
ENSG00000286808.1	-0.153816334	0.080235356	0.464588309	RP11-21M24.6
ENSG00000196497.17	0.155944695	0.080240481	0.464588309	IPO4
ENSG00000175899.15	-0.260895388	0.080307093	0.464746566	A2M
ENSG00000019102.12	0.247268161	0.080370393	0.464746566	VSIG2
ENSG00000198815.9	-0.062334681	0.080378609	0.464746566	FOXJ3
ENSG00000173080.6	-0.361702657	0.080400416	0.464746566	RXFP4
ENSG00000184076.13	0.111421028	0.08044132	0.464746566	UQCR10
ENSG00000168090.10	0.079922075	0.080460475	0.464746566	COPS6
ENSG00000018510.18	-0.090053954	0.080485773	0.464746566	AGPS
ENSG00000158874.11	0.353125154	0.080530856	0.464746566	APOA2
ENSG00000167232.14	-0.111414507	0.080554752	0.464746566	ZNF91
ENSG00000254717.2	0.419761334	0.080561	0.464746566	GLYATL1P2
ENSG00000169519.21	-0.114000128	0.080582956	0.464746566	METTL15
ENSG00000273017.1	0.254582225	0.080598066	0.464746566	AP000240.9
ENSG00000169918.10	-0.164678423	0.080606765	0.464746566	OTUD7A
ENSG00000163510.14	-0.093495276	0.080615741	0.464746566	CWC22
ENSG00000284642.1	-0.238162844	0.080629185	0.464746566	RP4-736L20.5
ENSG00000161013.17	0.086633599	0.080630402	0.464746566	MGAT4B
ENSG00000164053.22	0.073002302	0.080681095	0.46490809	ATRIP
ENSG00000130413.15	-0.371713982	0.080715642	0.464976513	STK33
ENSG00000149925.22	0.134283876	0.080822587	0.465063041	ALDOA
ENSG00000181444.13	0.136739371	0.080828887	0.465063041	ZNF467
ENSG00000169155.10	-0.068517508	0.080832665	0.465063041	ZBTB43
ENSG00000137404.15	0.102562722	0.080834624	0.465063041	NRM
ENSG00000273521.1	-0.338873226	0.08086077	0.465063041	RP11-45A17.4
ENSG00000187621.15	0.331714322	0.080866725	0.465063041	TCL6
ENSG00000149781.13	0.095552778	0.080896093	0.465101509	FERMT3
ENSG00000149657.20	0.060223178	0.080945252	0.46518116	LSM14B
ENSG00000262246.6	0.106036464	0.080955313	0.46518116	CORO7

ENSG00000110455.14	0.255755375	0.081030761	0.46548427	ACCS
ENSG00000144747.17	-0.126126761	0.081114379	0.465579081	TMF1
ENSG00000114923.17	0.232706299	0.081131452	0.465579081	SLC4A3
ENSG00000175792.12	0.077619618	0.081156714	0.465579081	RUVBL1
ENSG00000100030.15	-0.075068719	0.081156925	0.465579081	MAPK1
ENSG00000185627.18	0.080681201	0.081182097	0.465579081	PSMD13
ENSG00000224203.2	0.416675918	0.081183479	0.465579081	RPS23P10
ENSG00000167851.15	0.101859216	0.081249044	0.465595725	CD300A
ENSG00000168827.15	-0.065499623	0.081261254	0.465595725	GFM1
ENSG00000169021.6	0.091299225	0.08126513	0.465595725	UQCRFS1
ENSG00000253328.2	-0.240856542	0.081310142	0.465595725	SUMO2P19
ENSG00000170946.15	-0.099691736	0.081321649	0.465595725	DNAJC24
ENSG00000273084.1	0.286340957	0.081372978	0.465595725	RP11-1275H24.3
ENSG00000167985.7	0.100618304	0.081381215	0.465595725	SDHAF2
ENSG00000280069.1	-0.382066357	0.081387869	0.465595725	CTD-2349P21.3
ENSG00000138101.19	0.092500703	0.08139071	0.465595725	DTNB
ENSG00000152932.8	0.437521817	0.081429903	0.465690033	RAB3C
ENSG00000025770.19	0.078605942	0.081469297	0.465719833	NCAPH2
ENSG00000116754.13	-0.110583316	0.081492618	0.465719833	SRSF11
ENSG00000088766.12	-0.157879544	0.081508452	0.465719833	CRLS1
ENSG00000088387.20	-0.121535654	0.081550921	0.465719833	DOCK9
ENSG00000175203.17	0.060988175	0.081565972	0.465719833	DCTN2
ENSG00000119927.14	-0.133617081	0.081601322	0.465719833	GPAM
ENSG00000269903.1	-0.283103796	0.081628208	0.465719833	RP11-571M6.18
ENSG00000229191.1	0.218592153	0.081643793	0.465719833	RP11-168O16.1
ENSG00000233953.1	0.530320215	0.081643908	0.465719833	AC009970.1
ENSG00000211598.2	0.36426631	0.081680944	0.465719833	IGKV4-1
ENSG00000219200.12	0.094138461	0.081684915	0.465719833	RNASEK
ENSG00000013374.17	0.104337845	0.081756083	0.465996038	NUB1
ENSG00000261740.6	-0.315641823	0.081815071	0.466202689	BOLA2-SMG1P6
ENSG00000086730.17	0.079207041	0.081858778	0.46632217	LAT2
ENSG00000138623.10	0.164969228	0.081946374	0.466618929	SEMA7A
ENSG00000197579.8	-0.10470142	0.081956377	0.466618929	TOPORS
ENSG00000126107.15	0.07158357	0.081983679	0.466627644	HECTD3
ENSG00000126215.14	0.09265092	0.082040746	0.466627644	XRCC3
ENSG00000234782.3	0.50775896	0.08204332	0.466627644	TPT1P9
ENSG00000240970.1	-0.242749119	0.082053176	0.466627644	RPL23AP64
ENSG00000164327.13	-0.156805191	0.082131611	0.466627644	RICTOR
ENSG00000170265.12	0.0884685	0.082149612	0.466627644	ZNF282
ENSG00000002919.15	0.075348624	0.082154425	0.466627644	SNX11
ENSG00000285676.1	-0.247206164	0.082182198	0.466627644	RP11-431N8.1
ENSG00000168397.17	0.075152981	0.082243341	0.466627644	ATG4B
ENSG00000163737.4	0.293220695	0.082254229	0.466627644	PF4
ENSG00000229344.1	0.628115661	0.08225785	0.466627644	MTCO2P12
ENSG00000135272.12	-0.119557054	0.082266863	0.466627644	MDFIC
ENSG00000152253.9	-0.486886246	0.082279939	0.466627644	SPC25
ENSG00000060491.17	0.102135145	0.082307324	0.466627644	OGFR

ENSG00000196235.14	0.08216712	0.082372367	0.466627644	SUPT5H
ENSG00000215148.11	0.386240264	0.082387069	0.466627644	PRSS41
ENSG00000069974.16	-0.088508145	0.082390843	0.466627644	RAB27A
ENSG00000267074.1	-0.158507104	0.082413555	0.466627644	RP11-1094M14.5
ENSG00000164039.15	-0.167303961	0.082415138	0.466627644	BDH2
ENSG00000278987.1	-0.382723642	0.082419626	0.466627644	LA16c-431H6.7
ENSG00000213228.5	0.590409749	0.082450746	0.466627644	RPL12P38
ENSG00000284948.1	-0.344925203	0.082476194	0.466627644	RP11-875O11.5
ENSG00000174780.17	-0.080802164	0.082481237	0.466627644	SRP72
ENSG00000273245.1	-0.257079934	0.082536208	0.46680986	RP11-434P11.2
ENSG00000198538.11	-0.14480769	0.082560204	0.466816837	ZNF28
ENSG00000106070.20	0.166213843	0.082625576	0.46699769	GRB10
ENSG00000227082.3	0.513410753	0.082637732	0.46699769	LINC02798
ENSG00000115866.11	-0.076735761	0.082781194	0.467670169	DARS1
ENSG00000141837.22	0.201346625	0.08280234	0.467670169	CACNA1A
ENSG00000119777.20	0.079053192	0.082912236	0.467700295	TMEM214
ENSG00000179218.15	0.09563267	0.082928263	0.467700295	CALR
ENSG00000162551.14	0.229506876	0.082933596	0.467700295	ALPL
ENSG00000133030.22	-0.073467891	0.08300954	0.467700295	MPRIP
ENSG00000186010.19	0.115000916	0.083021949	0.467700295	NDUFA13
ENSG00000138081.22	-0.128420061	0.083031723	0.467700295	FBXO11
ENSG00000104907.13	0.082361035	0.08304033	0.467700295	TRMT1
ENSG00000116667.15	-0.212450103	0.083095373	0.467700295	C1orf21
ENSG00000158488.16	0.246934835	0.083106318	0.467700295	CD1E
ENSG00000068885.15	-0.145247178	0.083113246	0.467700295	IFT80
ENSG00000198791.12	-0.07277809	0.083144025	0.467700295	CNOT7
ENSG00000092067.6	0.249795339	0.083154196	0.467700295	CEBPE
ENSG00000032219.19	-0.126147598	0.083170995	0.467700295	ARID4A
ENSG00000120616.16	-0.053614494	0.083190008	0.467700295	EPC1
ENSG00000234263.2	0.339980209	0.08320439	0.467700295	MAP3K5-AS1
ENSG00000239763.2	-0.273691827	0.083220539	0.467700295	AC009120.3
ENSG00000214026.11	0.106858852	0.083222529	0.467700295	MRPL23
ENSG00000160654.11	-0.112221685	0.083224059	0.467700295	CD3G
ENSG00000254101.7	-0.979198221	0.083257686	0.467700295	LINC02055
ENSG00000103534.17	-0.276492186	0.083282155	0.467700295	TMC5
ENSG00000196428.12	-0.096142829	0.083286594	0.467700295	TSC22D2
ENSG00000211650.2	0.424832655	0.08331139	0.467700336	IGLV5-45
ENSG00000229017.7	0.299958551	0.083357777	0.467700336	LINC01277
ENSG00000170791.18	-0.072326449	0.083388804	0.467700336	CHCHD7
ENSG00000135837.16	-0.099610182	0.083414364	0.467700336	CEP350
ENSG00000198265.12	-0.083834094	0.083435303	0.467700336	HELZ
ENSG00000163346.17	0.105668974	0.083469118	0.467700336	PBXIP1
ENSG00000277161.2	-0.110685424	0.083470229	0.467700336	PIGW
ENSG00000124795.17	-0.131531171	0.083482675	0.467700336	DEK
ENSG00000267296.2	0.195514909	0.083491853	0.467700336	CEBPA-DT
ENSG00000254536.2	-0.177555652	0.083653251	0.468476482	RP11-108K14.8
ENSG00000185924.7	0.274551517	0.083680922	0.468503506	RTN4RL1

ENSG00000264188.1	-0.29405082	0.083729149	0.468645575	RP11-13N13.5
ENSG00000170430.10	0.151259063	0.083783295	0.468664211	MGMT
ENSG00000163932.15	0.075821811	0.083796768	0.468664211	PRKCD
ENSG00000105220.17	0.10627831	0.083815562	0.468664211	GPI
ENSG00000220201.7	0.160085605	0.083835881	0.468664211	ZGLP1
ENSG00000130717.13	0.065621076	0.083873574	0.468664211	UCK1
ENSG00000130813.18	0.117363713	0.083875645	0.468664211	SHFL
ENSG00000265491.5	-0.049639897	0.083892717	0.468664211	RNF115
ENSG00000100918.13	0.14349842	0.083915301	0.468664211	REC8
ENSG00000177125.5	-0.106345052	0.083945939	0.46870768	ZBTB34
ENSG00000182149.21	0.046640474	0.084011989	0.46871383	IST1
ENSG00000188986.9	0.075566538	0.084012124	0.46871383	NELFB
ENSG00000196876.18	-0.288744263	0.084015606	0.46871383	SCN8A
ENSG00000112782.19	-0.160410955	0.084104267	0.469008721	CLIC5
ENSG00000117155.17	-0.144814089	0.084114203	0.469008721	SSX2IP
ENSG00000136936.11	-0.08989639	0.084326984	0.469415788	XPA
ENSG00000261754.2	-0.210668535	0.084333885	0.469415788	CTC-523E23.1
ENSG00000272841.1	-0.253524303	0.08435021	0.469415788	MAP3K4-AS1
ENSG00000272501.1	0.136545279	0.084352954	0.469415788	XXbac-BPG299F13.17
ENSG00000185090.15	-0.183074889	0.084364816	0.469415788	MANEAL
ENSG00000215493.3	-0.324399823	0.084383633	0.469415788	XXbac-B562F10.11
ENSG00000149577.16	0.137685741	0.084384692	0.469415788	SIDT2
ENSG00000189233.12	-0.247999746	0.084386818	0.469415788	NUGGC
ENSG00000139410.15	0.220073336	0.084402883	0.469415788	SDSL
ENSG00000204252.14	0.176792074	0.084416102	0.469415788	HLA-DOA
ENSG00000274818.1	0.281130091	0.084478271	0.469623543	RP1-292L20.3
ENSG00000099940.12	0.069678011	0.084559589	0.469623543	SNAP29
ENSG00000081692.13	0.103366824	0.084564192	0.469623543	JMJD4
ENSG00000164296.7	0.128940569	0.084630328	0.469623543	TIGD6
ENSG00000215187.12	0.314241991	0.084646009	0.469623543	FAM166B
ENSG00000136937.13	-0.057375821	0.08465522	0.469623543	NCBP1
ENSG00000269821.1	0.097318636	0.084663689	0.469623543	KCNQ1OT1
ENSG00000149269.10	0.082398239	0.084672828	0.469623543	PAK1
ENSG00000162980.17	-0.141595479	0.08467602	0.469623543	ARL5A
ENSG00000132963.8	0.121036178	0.084682459	0.469623543	POMP
ENSG00000030110.13	0.124779051	0.084729604	0.469757969	BAK1
ENSG00000110931.19	0.0727942	0.084786917	0.469844231	CAMKK2
ENSG00000287356.1	0.238991115	0.084790984	0.469844231	RP11-181G12.8
ENSG00000176160.11	-0.212262104	0.084865957	0.470082539	HSF5
ENSG00000256069.7	-0.288349287	0.084897964	0.470082539	A2MP1
ENSG00000162402.14	-0.06524056	0.084967126	0.470082539	USP24
ENSG00000254363.6	-0.182093447	0.08497561	0.470082539	CTB-131B5.5
ENSG00000145545.12	0.087851949	0.085025834	0.470082539	SRD5A1
ENSG00000155542.12	-0.242046075	0.085030342	0.470082539	SETD9
ENSG00000185920.17	-0.180523734	0.085037987	0.470082539	PTCH1
ENSG00000075568.17	0.084090049	0.085094873	0.470082539	TMEM131
ENSG00000185404.17	0.073019473	0.085139203	0.470082539	SP140L

ENSG00000111664.11	0.293002682	0.085163069	0.470082539	GNB3
ENSG00000112851.15	-0.116405811	0.085173898	0.470082539	ERBIN
ENSG00000109220.11	0.081354442	0.085192889	0.470082539	CHIC2
ENSG00000232653.8	-0.22248828	0.085276737	0.470082539	GOLGA8N
ENSG00000124225.16	0.200206952	0.085336808	0.470082539	PMEPA1
ENSG00000278727.1	0.212509719	0.085373779	0.470082539	AC000403.4
ENSG00000089685.15	-0.312031165	0.085387544	0.470082539	BIRC5
ENSG00000151725.12	-0.188926007	0.085397663	0.470082539	CENPU
ENSG00000127328.22	-0.113274791	0.085433948	0.470082539	RAB3IP
ENSG00000106605.11	0.151394225	0.085434334	0.470082539	BLVRA
ENSG00000197982.14	0.122373294	0.085512735	0.470082539	C1orf122
ENSG00000075914.13	0.089654357	0.085524107	0.470082539	EXOSC7
ENSG00000275318.1	-0.187813246	0.085537687	0.470082539	RP11-526D8.11
ENSG00000132109.10	0.108681788	0.085551451	0.470082539	TRIM21
ENSG00000170236.16	-0.187350413	0.085556687	0.470082539	USP50
ENSG00000113763.12	0.218322912	0.085562861	0.470082539	UNC5A
ENSG00000164211.13	-0.172885775	0.085564735	0.470082539	STARD4
ENSG00000171314.9	0.071667063	0.08556553	0.470082539	PGAM1
ENSG00000064787.13	-0.208852869	0.085605702	0.470082539	BCAS1
ENSG00000131236.18	0.057840716	0.085650177	0.470082539	CAP1
ENSG00000131634.14	0.179572615	0.085661209	0.470082539	TMEM204
ENSG00000153989.8	-0.107607928	0.085663098	0.470082539	NUS1
ENSG00000281021.1	-0.403563558	0.085669306	0.470082539	RP1-12G14.9
ENSG00000120063.10	-0.099662961	0.085676094	0.470082539	GNA13
ENSG00000099246.18	-0.128021993	0.085716287	0.470082539	RAB18
ENSG00000077238.14	0.136767957	0.085743876	0.470082539	IL4R
ENSG00000153130.18	-0.233754344	0.085754676	0.470082539	SCOC
ENSG00000161813.22	-0.110861782	0.085803664	0.470082539	LARP4
ENSG00000204130.14	-0.103953532	0.085819959	0.470082539	RUFY2
ENSG00000260022.1	-0.165525785	0.085843173	0.470082539	LA16c-306A4.1
ENSG00000229334.1	-0.313249842	0.085845477	0.470082539	AC046143.3
ENSG00000183486.14	0.145114871	0.085848208	0.470082539	MX2
ENSG00000220842.6	-0.362962216	0.085887991	0.470082539	RPL21P16
ENSG00000229671.3	0.28523615	0.085902939	0.470082539	LINC01150
ENSG00000075945.13	-0.071156747	0.085934803	0.470082539	KIFAP3
ENSG00000239961.3	0.241941119	0.08593794	0.470082539	LILRA4
ENSG00000197183.14	-0.071032429	0.085961764	0.470082539	NOL4L
ENSG00000186017.15	-0.104470147	0.08600191	0.470082539	ZNF566
ENSG00000108506.13	-0.117160206	0.086005682	0.470082539	INTS2
ENSG00000164244.21	-0.079962002	0.086028033	0.470082539	PRRC1
ENSG00000213676.13	0.062862472	0.086028789	0.470082539	ATF6B
ENSG00000254860.6	-0.222335866	0.086068279	0.470082539	TMEM9B-AS1
ENSG00000233547.1	0.398225296	0.086151663	0.470082539	RP11-57H14.2
ENSG00000249790.3	-0.546012747	0.086203646	0.470082539	RP11-20D14.6
ENSG00000159164.10	-0.150419693	0.086228493	0.470082539	SV2A
ENSG00000196586.16	-0.246124336	0.08623784	0.470082539	MYO6
ENSG00000032389.13	0.081558409	0.086265386	0.470082539	EIPR1

ENSG00000224376.1	0.22961566	0.086279632	0.470082539	AC017104.6
ENSG00000257345.2	0.424226968	0.086291086	0.470082539	LINC02413
ENSG00000148341.18	0.078759243	0.086294506	0.470082539	SH3GLB2
ENSG00000224080.1	0.291000788	0.08630123	0.470082539	UBE2FP1
ENSG00000116701.15	0.071623477	0.086303484	0.470082539	NCF2
ENSG00000153902.14	0.287704711	0.086304099	0.470082539	LGI4
ENSG00000113732.9	0.095568982	0.086339661	0.470082539	ATP6V0E1
ENSG00000120549.18	0.611588535	0.086351674	0.470082539	KIAA1217
ENSG00000185432.12	0.095485811	0.086365508	0.470082539	METTL7A
ENSG00000196588.20	0.086211662	0.086389836	0.470082539	MRTFA
ENSG00000095574.12	-0.107423567	0.086444838	0.470082539	IKZF5
ENSG00000074800.16	0.10194868	0.086446013	0.470082539	ENO1
ENSG00000182903.16	-0.135796126	0.086451039	0.470082539	ZNF721
ENSG00000237094.12	-0.217139947	0.086475685	0.470082539	RP4-669L17.4
ENSG00000272320.1	0.442092813	0.086476785	0.470082539	RP11-506K6.4
ENSG00000123992.20	0.084734588	0.086485242	0.470082539	DNPEP
ENSG00000254777.5	0.243605984	0.086544872	0.470082539	AC022182.1
ENSG00000062822.15	0.095750254	0.086604413	0.470082539	POLD1
ENSG00000175414.7	-0.111952933	0.086621633	0.470082539	ARL10
ENSG00000262700.1	-0.440567445	0.08663359	0.470082539	RP11-266L9.3
ENSG00000196510.14	0.050721095	0.086652012	0.470082539	ANAPC7
ENSG00000104679.12	0.076824652	0.086666432	0.470082539	R3HCC1
ENSG00000140400.17	0.121871336	0.086675475	0.470082539	MAN2C1
ENSG00000211956.2	0.391281862	0.086693423	0.470082539	IGHV4-34
ENSG00000155744.10	-0.167469767	0.08669593	0.470082539	FAM126B
ENSG00000231073.1	-0.335258504	0.086713587	0.470082539	RP11-316M1.3
ENSG00000204104.12	-0.101649554	0.086825607	0.470488139	TRAF3IP1
ENSG00000240253.6	0.728525701	0.086834289	0.470488139	FAR2P3
ENSG00000155158.20	-0.146929157	0.086861485	0.470511184	TTC39B
ENSG00000205045.11	-0.122407453	0.086984768	0.470964158	SLFN12L
ENSG00000147443.13	0.102550643	0.086991039	0.470964158	DOK2
ENSG00000013375.16	-0.089703905	0.087024423	0.471020553	PGM3
ENSG00000211803.2	0.289665733	0.087082483	0.471181822	TRAV23DV6
ENSG00000214870.9	0.351710613	0.08710017	0.471181822	AC004540.5
ENSG00000146477.6	0.369340574	0.087149042	0.47129855	SLC22A3
ENSG00000119906.13	-0.060369947	0.087169147	0.47129855	SLF2
ENSG00000153201.16	-0.089355332	0.087232614	0.47129855	RANBP2
ENSG00000156113.24	0.515552915	0.087253199	0.47129855	KCNMA1
ENSG00000080815.19	0.059374897	0.087346005	0.47129855	PSEN1
ENSG00000120992.18	-0.11730218	0.08735762	0.47129855	LYPLA1
ENSG00000267288.2	0.197263835	0.08736445	0.47129855	RP13-890H12.2
ENSG00000286132.1	0.411175851	0.08736561	0.47129855	CTC-359D24.5
ENSG00000145715.15	-0.10752522	0.087375485	0.47129855	RASA1
ENSG00000175595.16	-0.098192873	0.087380374	0.47129855	ERCC4
ENSG00000140992.19	-0.081990339	0.087395871	0.47129855	PDPK1
ENSG00000100348.10	0.087836906	0.087417871	0.47129855	TXN2
ENSG00000137200.13	0.115263856	0.087420503	0.47129855	CMTR1

ENSG00000258940.2	0.330606503	0.087495392	0.471324105	RP11-407N17.5
ENSG00000116863.11	0.081175214	0.08753355	0.471324105	ADPRS
ENSG00000053770.12	-0.089524999	0.087547049	0.471324105	AP5M1
ENSG00000063177.13	0.117212479	0.087590645	0.471324105	RPL18
ENSG00000173530.6	0.10740944	0.087598445	0.471324105	TNFRSF10D
ENSG00000229474.8	-0.181656817	0.087654095	0.471324105	PATL2
ENSG00000065413.20	-0.112743667	0.087662118	0.471324105	ANKRD44
ENSG00000271795.1	-0.16777541	0.087685093	0.471324105	CTC-251D13.1
ENSG00000237441.10	0.073486759	0.087694219	0.471324105	RGL2
ENSG00000100280.17	0.086667329	0.087694452	0.471324105	AP1B1
ENSG00000125656.11	0.102416966	0.087700667	0.471324105	CLPP
ENSG00000266718.2	0.249396394	0.087721604	0.471324105	RP11-466A19.1
ENSG00000225783.8	-0.207476184	0.087732832	0.471324105	MIAT
ENSG00000279072.1	0.371633411	0.087746998	0.471324105	RP13-580B18.4
ENSG00000205790.1	-0.180645748	0.087811555	0.471547362	DPP9-AS1
ENSG00000273008.1	0.26526574	0.087854721	0.471655657	CSGALNACT2-DT
ENSG00000105677.12	0.104263058	0.087881818	0.471677655	TMEM147
ENSG00000113520.11	0.357471783	0.087919874	0.471758446	IL4
ENSG00000081760.17	0.0754286	0.088008221	0.472094527	AACS
ENSG00000100376.12	0.348686488	0.088033289	0.472094527	FAM118A
ENSG00000085644.14	0.092742347	0.088051568	0.472094527	ZNF213
ENSG00000211955.2	0.348304344	0.088141772	0.47226685	IGHV3-33
ENSG00000128923.11	-0.147569667	0.088150512	0.47226685	MINDY2
ENSG00000228403.1	-0.358396127	0.088156218	0.47226685	RP11-563N6.6
ENSG00000157869.16	-0.088584713	0.088175822	0.47226685	RAB28
ENSG00000121775.18	0.080876499	0.088204445	0.472296805	TMEM39B
ENSG00000225648.5	-0.117904544	0.088281122	0.472514346	SBDSP1
ENSG00000072571.20	-0.36829419	0.088291153	0.472514346	HMMR
ENSG00000150594.7	0.367183488	0.08831539	0.472520745	ADRA2A
ENSG00000006016.12	-0.162358146	0.088342283	0.472541351	CRLF1
ENSG00000288253.1	-0.234198208	0.088411109	0.472721822	CTD-3018O17.7
ENSG00000249695.6	-0.654532823	0.088425	0.472721822	RP11-598F7.4
ENSG00000227953.7	0.295435925	0.088445174	0.472721822	LINC01341
ENSG00000279689.1	-0.118356199	0.088498836	0.472782346	RP11-574K11.32
ENSG00000165521.15	-0.338947365	0.088502605	0.472782346	EML5
ENSG00000147684.10	0.104368304	0.088565642	0.472995883	NDUFB9
ENSG00000259381.2	-0.412351275	0.088649463	0.473226694	RP11-192M23.1
ENSG00000270184.1	-0.275727831	0.088655011	0.473226694	RP11-568J23.5
ENSG00000254413.8	0.174907794	0.088719351	0.473446906	CHKB-CPT1B
ENSG00000197756.10	0.124426432	0.088779707	0.473571849	RPL37A
ENSG00000139946.10	-0.085327558	0.088814097	0.473571849	PELI2
ENSG00000156603.19	0.110685666	0.088858921	0.473571849	MED19
ENSG00000285938.1	-0.255878562	0.088871848	0.473571849	BCL6-AS1
ENSG00000168936.11	0.067248407	0.088882886	0.473571849	TMEM129
ENSG00000143167.12	0.157459616	0.088902958	0.473571849	GPA33
ENSG00000106266.11	0.107510366	0.088904409	0.473571849	SNX8
ENSG00000167173.19	0.111921881	0.088976601	0.473573877	C15orf39

ENSG00000283633.1	0.270222778	0.088994002	0.473573877	AC005301.9
ENSG00000120800.5	-0.087226154	0.088994082	0.473573877	UTP20
ENSG00000171425.10	0.108046065	0.088997158	0.473573877	ZNF581
ENSG00000237883.2	-0.128294604	0.089021058	0.473578172	DGUOK-AS1
ENSG00000105369.10	0.268887749	0.089101118	0.473843892	CD79A
ENSG00000063761.16	0.139973245	0.089168945	0.473843892	ADCK1
ENSG00000211817.2	0.253809425	0.089171023	0.473843892	TRAV38-2DV8
ENSG00000270921.1	0.497994472	0.089188037	0.473843892	CICP23
ENSG00000256039.2	-0.331501147	0.089218155	0.473843892	LINC02446
ENSG00000039319.17	-0.155871595	0.089220536	0.473843892	ZFYVE16
ENSG00000100211.10	0.107059841	0.089234037	0.473843892	CBY1
ENSG00000134987.12	-0.09525208	0.089255849	0.473843892	WDR36
ENSG00000105640.13	0.124566134	0.089295183	0.473930025	RPL18A
ENSG00000269825.2	-0.21386426	0.08932544	0.473967949	CTD-3099C6.13
ENSG00000129204.17	0.294590954	0.089374991	0.47405365	USP6
ENSG00000143549.21	0.049618568	0.089410459	0.47405365	TPM3
ENSG00000118640.11	0.11961786	0.089410938	0.47405365	VAMP8
ENSG00000113240.13	-0.145150601	0.089472017	0.47406578	CLK4
ENSG00000096433.11	-0.130441153	0.089479345	0.47406578	ITPR3
ENSG00000138074.15	0.075181322	0.089482574	0.47406578	SLC5A6
ENSG00000198420.10	-0.156997526	0.08954965	0.47429861	TCAF1
ENSG00000168209.6	0.177287344	0.089608218	0.474486273	DDIT4
ENSG00000139154.16	-0.098816564	0.089643173	0.474548839	AEBP2
ENSG00000111647.13	-0.108769241	0.089669298	0.474564634	UHRF1BP1L
ENSG00000111581.10	-0.07226764	0.089741404	0.474818772	NUP107
ENSG00000131149.19	-0.097566355	0.089769643	0.474818772	GSE1
ENSG00000162888.4	0.159493814	0.089790603	0.474818772	C1orf147
ENSG00000204435.15	0.097212709	0.089816246	0.474818772	CSNK2B
ENSG00000119280.17	0.14795126	0.089833082	0.474818772	C1orf198
ENSG00000104998.4	0.064584763	0.089931342	0.475215654	IL27RA
ENSG00000151876.13	-0.135874449	0.090082146	0.475815114	FBXO4
ENSG00000116560.11	-0.039193466	0.090099641	0.475815114	SFPQ
ENSG00000122435.10	-0.160172909	0.090115327	0.475815114	TRMT13
ENSG00000226935.6	0.423738716	0.090202983	0.475815114	LINC00161
ENSG00000067560.14	0.065468631	0.09021694	0.475815114	RHOA
ENSG00000196712.18	-0.088118704	0.090218088	0.475815114	NF1
ENSG00000188157.15	0.316095905	0.090225719	0.475815114	AGRN
ENSG00000272255.1	0.366135525	0.090232873	0.475815114	CTD-3224K15.3
ENSG00000197603.16	-0.130616301	0.090275701	0.475815114	CPLANE1
ENSG00000106603.20	-0.088574789	0.0902768	0.475815114	COA1
ENSG00000169718.18	0.075661483	0.090308639	0.475860628	DUS1L
ENSG00000220875.1	0.254290595	0.090463751	0.476324843	H3C9P
ENSG00000109854.14	0.075196224	0.090464434	0.476324843	HTATIP2
ENSG00000250254.2	-0.259220085	0.09054373	0.476324843	PTTG2
ENSG00000177494.6	-0.337828433	0.090544425	0.476324843	ZBED2
ENSG00000047315.17	-0.062746684	0.090585809	0.476324843	POLR2B
ENSG00000234477.1	-0.365015442	0.090587086	0.476324843	AC004231.2

ENSG00000159200.18	-0.080165536	0.090599653	0.476324843	RCAN1
ENSG00000228486.10	-0.172239513	0.090657163	0.476324843	C2orf92
ENSG00000145113.22	-0.481218837	0.090669234	0.476324843	MUC4
ENSG00000100296.14	0.076313446	0.090677174	0.476324843	THOC5
ENSG00000085063.17	0.101871385	0.090687564	0.476324843	CD59
ENSG00000131016.17	0.416425364	0.090706097	0.476324843	AKAP12
ENSG00000087470.19	-0.066457614	0.090718131	0.476324843	DNM1L
ENSG00000100201.23	-0.077642487	0.090728207	0.476324843	DDX17
ENSG00000120686.12	-0.10738163	0.090786696	0.476324843	UFM1
ENSG00000258920.1	0.18556015	0.09081892	0.476324843	FOXN3-AS1
ENSG00000007376.8	0.106173756	0.090965882	0.476324843	RPUSD1
ENSG00000250415.1	-0.411427217	0.090966802	0.476324843	CTC-461F20.1
ENSG00000231890.8	0.148723561	0.090977937	0.476324843	DARS1-AS1
ENSG00000107736.22	0.102811408	0.090980972	0.476324843	CDH23
ENSG00000272316.1	-0.186396869	0.091027845	0.476324843	XXbac-BPGBPG55C20.2
ENSG00000285534.1	-0.498876796	0.091030537	0.476324843	RP11-358F13.1
ENSG00000055044.11	-0.104165475	0.09104019	0.476324843	NOP58
ENSG00000100744.15	-0.153417201	0.091045917	0.476324843	GSKIP
ENSG00000134825.16	0.121541246	0.091049901	0.476324843	TMEM258
ENSG00000261136.1	-0.113740876	0.091055971	0.476324843	RP11-37C7.3
ENSG00000023697.13	0.073398608	0.091070018	0.476324843	DERA
ENSG00000213079.10	-0.075227903	0.091078688	0.476324843	SCAF8
ENSG00000167397.15	0.106558667	0.091126938	0.476324843	VKORC1
ENSG00000248866.1	-0.29840508	0.091130624	0.476324843	USP46-DT
ENSG00000166128.13	-0.115446899	0.091139569	0.476324843	RAB8B
ENSG00000000419.14	-0.121182724	0.091139979	0.476324843	DPM1
ENSG00000174579.4	-0.063242059	0.091207197	0.476554701	MSL2
ENSG00000143322.21	-0.076321913	0.09127006	0.476761687	ABL2
ENSG00000144395.18	0.315910443	0.091389463	0.476973701	CCDC150
ENSG00000214135.8	-0.097880796	0.091394334	0.476973701	AC024560.3
ENSG00000181274.7	0.111389328	0.09139679	0.476973701	FRAT2
ENSG00000254676.1	-0.201982356	0.091406612	0.476973701	RP11-727A23.4
ENSG00000272345.1	0.149700182	0.091440282	0.476973701	RP1-30M3.5
ENSG00000161944.16	0.20061146	0.091497653	0.476973701	ASGR2
ENSG00000087074.8	0.101648673	0.091518415	0.476973701	PPP1R15A
ENSG00000076662.10	0.110171928	0.091548956	0.476973701	ICAM3
ENSG00000267551.3	0.282451278	0.091577459	0.476973701	GNA15-DT
ENSG00000005812.11	-0.106994033	0.091597778	0.476973701	FBXL3
ENSG00000249673.7	0.082745685	0.091623668	0.476973701	NOP14-AS1
ENSG00000113013.16	-0.047026278	0.091666712	0.476973701	HSPA9
ENSG00000169607.13	-0.304784246	0.091675497	0.476973701	CKAP2L
ENSG00000057294.16	0.248460429	0.091694679	0.476973701	PKP2
ENSG00000065548.18	-0.130203723	0.091719708	0.476973701	ZC3H15
ENSG00000268658.5	-0.491531176	0.091731842	0.476973701	LINC00664
ENSG00000246263.3	-0.158178931	0.091737189	0.476973701	UBR5-AS1
ENSG00000173338.13	0.16002634	0.091745925	0.476973701	KCNK7
ENSG00000262678.1	-0.275388314	0.091752548	0.476973701	CAMTA2-AS1

ENSG00000170448.12	-0.300647256	0.091824554	0.477227054	NFXL1
ENSG00000161526.15	0.059761521	0.09188258	0.477357218	SAP30BP
ENSG00000204524.7	-0.082048785	0.091896153	0.477357218	ZNF805
ENSG00000105341.19	0.069390702	0.091935996	0.477443251	DMAC2
ENSG00000153037.15	-0.084012878	0.091961283	0.477453671	SRP19
ENSG00000138175.9	0.098356721	0.092054765	0.47767456	ARL3
ENSG00000100036.13	0.15468668	0.092069455	0.47767456	SLC35E4
ENSG00000198246.9	0.095260629	0.092089629	0.47767456	SLC29A3
ENSG00000151151.6	-0.182829142	0.092096997	0.47767456	IPMK
ENSG00000137710.17	-0.113278517	0.092175444	0.477813739	RDX
ENSG00000172716.16	-0.093135672	0.092182085	0.477813739	SLFN11
ENSG00000213281.5	-0.094943402	0.092193728	0.477813739	NRAS
ENSG00000103502.14	0.085730822	0.092318839	0.478119222	CDIPT
ENSG00000249825.6	-0.182972266	0.092344606	0.478119222	THBS4-AS1
ENSG00000152127.9	0.086109096	0.092401772	0.478119222	MGAT5
ENSG00000100292.18	0.130822266	0.092403033	0.478119222	HMOX1
ENSG00000181856.15	-0.406192696	0.092425876	0.478119222	SLC2A4
ENSG00000135940.7	0.119405359	0.092438086	0.478119222	COX5B
ENSG00000275160.1	-0.348331546	0.092441539	0.478119222	RP11-187C18.3
ENSG00000101199.13	0.068812803	0.092455359	0.478119222	ARFGAP1
ENSG00000089775.12	-0.098363684	0.092487672	0.478119222	ZBTB25
ENSG00000240616.1	-0.249054834	0.092501736	0.478119222	RPS6P25
ENSG00000248015.7	-0.308445438	0.092509122	0.478119222	AC005329.7
ENSG00000275056.1	-0.366574536	0.092540566	0.478152776	RP11-127I20.8
ENSG00000243433.1	-0.293681788	0.092574331	0.478152776	RP11-148K1.10
ENSG00000116984.15	-0.09123818	0.092632269	0.478152776	MTR
ENSG00000250539.1	-0.295697543	0.092645168	0.478152776	KRT8P33
ENSG00000176783.15	0.075488007	0.092660473	0.478152776	RUFY1
ENSG00000070669.17	-0.195161895	0.092709036	0.478152776	ASNS
ENSG00000204262.14	-0.438623582	0.092729483	0.478152776	COL5A2
ENSG00000105205.7	0.313879443	0.092746174	0.478152776	CLC
ENSG00000120802.14	-0.079207163	0.092764854	0.478152776	TMPO
ENSG00000287555.1	-0.389419105	0.092788522	0.478152776	RP11-555K12.4
ENSG00000157470.12	0.280309406	0.09280975	0.478152776	FAM81A
ENSG00000110218.9	-0.104787661	0.092826148	0.478152776	PANX1
ENSG00000169951.10	-0.068329474	0.092830221	0.478152776	ZNF764
ENSG00000177628.16	0.092900365	0.092842886	0.478152776	GBA
ENSG00000156886.12	-0.349770822	0.092876167	0.478152776	ITGAD
ENSG00000139132.16	-0.131993597	0.092888661	0.478152776	FGD4
ENSG00000189159.16	0.099372342	0.092991637	0.478562732	JPT1
ENSG00000088256.9	0.166672999	0.09302634	0.478587422	GNA11
ENSG00000135124.15	0.094003016	0.093043108	0.478587422	P2RX4
ENSG00000156831.8	-0.165151106	0.093104841	0.478695129	NSMCE2
ENSG00000284691.1	0.109707376	0.093122104	0.478695129	RP11-511P7.5
ENSG00000267397.1	0.366050837	0.09314765	0.478695129	RP11-873E20.1
ENSG00000276289.5	0.408508511	0.093177593	0.478695129	KCNE1B
ENSG00000177565.18	-0.1022439	0.09319878	0.478695129	TBL1XR1

ENSG00000134982.17	-0.101454353	0.093204098	0.478695129	APC
ENSG00000005486.17	0.088498719	0.093235508	0.478736553	RHBDD2
ENSG00000262655.4	0.18521932	0.093325525	0.478970609	SPON1
ENSG00000142546.14	0.123593281	0.093327802	0.478970609	NOSIP
ENSG00000120942.14	0.06390126	0.093419455	0.479063445	UBIAD1
ENSG00000142945.13	-0.211590832	0.093427905	0.479063445	KIF2C
ENSG00000049618.24	-0.079145617	0.093428129	0.479063445	ARID1B
ENSG00000213614.11	0.062693354	0.093442904	0.479063445	HEXA
ENSG00000116353.16	0.091338684	0.09346269	0.479063445	MECR
ENSG00000166833.22	-0.311140835	0.093509089	0.479119473	NAV2
ENSG00000246308.2	0.228249639	0.093542598	0.479119473	RP11-685M7.3
ENSG00000116574.6	-0.147240894	0.093565713	0.479119473	RHOU
ENSG00000272870.3	-0.18711637	0.093567071	0.479119473	SAP30-DT
ENSG00000124466.9	0.196168828	0.093654689	0.47944842	LYPD3
ENSG00000152284.5	0.125019369	0.093704474	0.479583565	TCF7L1
ENSG00000172244.9	-0.179505294	0.09375083	0.47966589	C5orf34
ENSG00000164037.17	-0.220251131	0.093767337	0.47966589	SLC9B1
ENSG00000238249.2	0.174627301	0.09383195	0.479840203	HMGN2P17
ENSG00000099994.11	0.213187773	0.093853015	0.479840203	SUSD2
ENSG00000275367.1	-0.28146622	0.093871606	0.479840203	RP11-266K4.14
ENSG00000156876.10	-0.148860226	0.093921374	0.479974965	SASS6
ENSG00000107902.14	0.123277312	0.093948558	0.479994275	LHPP
ENSG00000066468.24	0.422455062	0.094018594	0.480190564	FGFR2
ENSG00000205583.13	0.273570122	0.094033807	0.480190564	STAG3L1
ENSG00000272777.1	-0.266811657	0.094126228	0.480478647	RP11-571L19.8
ENSG00000112996.11	-0.061162085	0.094137078	0.480478647	MRPS30
ENSG00000173451.7	-0.122883527	0.094202255	0.480520686	THAP2
ENSG00000143702.16	-0.111978994	0.094223087	0.480520686	CEP170
ENSG00000007866.22	0.329574634	0.094272475	0.480520686	TEAD3
ENSG00000176946.12	0.079708591	0.094281719	0.480520686	THAP4
ENSG00000138670.18	0.123136146	0.094296022	0.480520686	RASGEF1B
ENSG00000136098.17	0.105342534	0.094311696	0.480520686	NEK3
ENSG00000111181.13	0.189538445	0.09431933	0.480520686	SLC6A12
ENSG00000268520.1	-0.249347923	0.094343832	0.480520686	CTD-2616J11.10
ENSG00000100294.13	0.10062717	0.094356193	0.480520686	MCAT
ENSG00000140299.13	-0.085033651	0.094485734	0.481060932	BNIP2
ENSG00000226415.1	0.165521684	0.094615733	0.481382602	TPI1P1
ENSG00000197858.11	0.090465619	0.094623126	0.481382602	GPAA1
ENSG00000174891.14	-0.089672504	0.094659217	0.481382602	RSRC1
ENSG00000215790.8	-0.151488101	0.094661837	0.481382602	SLC35E2A
ENSG00000119559.17	0.076289218	0.09472024	0.481382602	C19orf25
ENSG00000264357.1	-0.366406359	0.094720728	0.481382602	MIR4648
ENSG00000173653.8	0.083456896	0.094754678	0.481382602	RCE1
ENSG00000117118.10	0.06637478	0.09476116	0.481382602	SDHB
ENSG00000153006.16	-0.100777902	0.094787874	0.481382602	SREK1IP1
ENSG00000169241.19	0.075101582	0.094818494	0.481382602	SLC50A1
ENSG00000133731.10	-0.140926366	0.09484858	0.481382602	IMPA1

ENSG00000158411.11	-0.080173519	0.094871	0.481382602	MITD1
ENSG00000244515.1	-0.271453745	0.094903863	0.481382602	KRT18P34
ENSG00000169704.5	0.288281337	0.094906276	0.481382602	GP9
ENSG00000113812.14	-0.055657726	0.094941527	0.481382602	ACTR8
ENSG00000126522.17	0.085271419	0.094944574	0.481382602	ASL
ENSG00000101439.9	0.151589311	0.094951328	0.481382602	CST3
ENSG00000134802.18	0.107154021	0.095002334	0.481382602	SLC43A3
ENSG00000110660.15	-0.111579227	0.095004111	0.481382602	SLC35F2
ENSG00000198208.12	0.219954392	0.095018372	0.481382602	RPS6KL1
ENSG00000230897.1	0.409472774	0.095104499	0.481699944	RPS18P12
ENSG00000173809.18	0.503883518	0.095174454	0.481802444	TDRD12
ENSG00000178971.16	-0.071924073	0.095187327	0.481802444	CTC1
ENSG00000187840.5	0.125306185	0.095195217	0.481802444	EIF4EBP1
ENSG00000196684.12	0.113995185	0.095224573	0.48183211	HSH2D
ENSG00000123473.17	-0.177310694	0.095273316	0.481920209	STIL
ENSG00000256171.1	0.279189839	0.095288982	0.481920209	GCSHP4
ENSG00000214367.9	-0.097280306	0.095341984	0.481963453	HAUS3
ENSG00000186806.6	0.205595994	0.095358738	0.481963453	VSIG10L
ENSG00000205978.6	-0.253910572	0.095370161	0.481963453	NYNRIN
ENSG00000151575.14	-0.345976575	0.095423219	0.481963453	TEX9
ENSG00000184731.6	-0.303107648	0.095427694	0.481963453	FAM110C
ENSG00000108848.16	-0.097897302	0.09543854	0.481963453	LUC7L3
ENSG00000119965.13	-0.103815093	0.09548903	0.482099712	C10orf88
ENSG00000158156.9	0.091887345	0.095550018	0.482146954	XKR8
ENSG00000065809.14	-0.072002701	0.095564739	0.482146954	FAM107B
ENSG00000249923.1	-0.929705022	0.095568918	0.482146954	LINC02891
ENSG00000205959.4	-0.199306596	0.095607031	0.482166394	RP11-689P11.2
ENSG00000055070.17	0.055365688	0.095633663	0.482166394	SZRD1
ENSG00000100181.22	0.288148489	0.095643305	0.482166394	TPTEP1
ENSG00000245954.8	0.30628888	0.095704975	0.482358718	LINC02273
ENSG00000211886.1	-0.319947962	0.095826682	0.482735134	TRAJ3
ENSG00000156395.14	-0.373471891	0.095826737	0.482735134	SORCS3
ENSG00000167965.18	0.087625536	0.095928923	0.483131226	MLST8
ENSG00000135913.11	-0.179644143	0.09599919	0.483210605	USP37
ENSG00000273424.1	0.211391216	0.096001279	0.483210605	CTA-223H9.9
ENSG00000130766.5	-0.108195137	0.096039912	0.483210605	SESN2
ENSG00000146701.12	0.083761122	0.096048116	0.483210605	MDH2
ENSG00000049323.16	0.257864506	0.096082252	0.483210605	LTBP1
ENSG00000172375.14	0.073334125	0.096086057	0.483210605	C2CD2L
ENSG00000159086.15	-0.105876924	0.096134415	0.483251715	PAXBP1
ENSG00000148334.16	0.077398728	0.096141359	0.483251715	PTGES2
ENSG00000099365.11	0.195290289	0.096245012	0.48356342	STX1B
ENSG00000175489.10	0.095581772	0.096268308	0.48356342	LRRC25
ENSG00000168917.9	-0.313774361	0.096296904	0.48356342	SLC35G2
ENSG00000146834.14	0.078675291	0.096297689	0.48356342	MEPCE
ENSG00000218226.1	-0.207353233	0.096333915	0.48362691	TATDN2P2
ENSG00000108061.12	-0.091101974	0.09641865	0.483756845	SHOC2

ENSG00000100577.19	0.119186918	0.096426103	0.483756845	GSTZ1
ENSG00000272361.2	-0.35757264	0.096430563	0.483756845	GS1-166A23.2
ENSG00000197744.5	0.21256618	0.096462278	0.483791777	PTMAP2
ENSG00000258730.1	-0.306225667	0.096510052	0.483791777	ITPK1-AS1
ENSG00000280183.1	-0.377772561	0.096527176	0.483791777	RP11-400F19.12
ENSG00000125827.9	-0.089051918	0.09661799	0.483791777	TMX4
ENSG00000185262.9	0.112888523	0.096657851	0.483791777	UBALD2
ENSG00000141034.10	-0.084856522	0.096670722	0.483791777	GID4
ENSG00000144566.11	-0.071060871	0.096693847	0.483791777	RAB5A
ENSG00000124780.14	0.466551443	0.09669629	0.483791777	KCNK17
ENSG00000272341.1	-0.154179944	0.096761292	0.483791777	RP1-151F17.2
ENSG00000141446.11	-0.131151517	0.096763646	0.483791777	ESCO1
ENSG00000146856.14	-0.191973413	0.096771663	0.483791777	AGBL3
ENSG00000188566.15	0.08938466	0.096782165	0.483791777	NDOR1
ENSG00000141458.13	-0.098748626	0.096786103	0.483791777	NPC1
ENSG00000127666.10	0.094348438	0.096801032	0.483791777	TICAM1
ENSG00000197816.16	-0.134609169	0.09681439	0.483791777	CCDC180
ENSG00000126247.11	0.102659757	0.096814972	0.483791777	CAPNS1
ENSG00000174917.9	0.10993555	0.096850743	0.483852628	MICOS13
ENSG00000198711.5	-0.180860747	0.096889486	0.483928293	SSBP3-AS1
ENSG00000169242.12	0.248691869	0.096986786	0.484296325	EFNA1
ENSG00000135932.11	-0.080831205	0.097063491	0.484385874	CAB39
ENSG00000249646.2	0.273955778	0.097071198	0.484385874	OR7E94P
ENSG00000197191.6	0.291648123	0.097084902	0.484385874	CYSRT1
ENSG00000100726.15	0.098224991	0.097099197	0.484385874	TELO2
ENSG00000176454.14	-0.068698251	0.097134351	0.484403748	LPCAT4
ENSG00000168137.19	-0.059617944	0.097151904	0.484403748	SETD5
ENSG00000108587.16	-0.043664469	0.097173641	0.484403748	GOSR1
ENSG00000188152.13	-0.240580145	0.097204888	0.484410672	NUTM2G
ENSG00000081870.12	-0.106329752	0.097222271	0.484410672	HSPB11
ENSG00000105355.9	0.0922213	0.09728792	0.484509834	PLIN3
ENSG00000118292.9	-0.147617141	0.097293603	0.484509834	C1orf54
ENSG00000253552.8	0.291043942	0.097347709	0.484509834	HOXA-AS2
ENSG00000138646.9	0.402748306	0.097351402	0.484509834	HERC5
ENSG00000183785.16	-0.284054507	0.0973603	0.484509834	TUBA8
ENSG00000100600.15	0.13774626	0.097478316	0.484686275	LGMN
ENSG00000187790.11	-0.157309762	0.097489674	0.484686275	FANCM
ENSG00000173914.12	0.066229004	0.097490027	0.484686275	RBM4B
ENSG00000165997.5	-0.128821882	0.097509747	0.484686275	ARL5B
ENSG00000263001.7	-0.132921229	0.097540413	0.484686275	GTF2I
ENSG00000276250.1	0.337890881	0.097545938	0.484686275	CTD-2349P21.12
ENSG00000107864.15	-0.151635087	0.097565168	0.484686275	CPEB3
ENSG00000183562.3	-0.299235306	0.097598446	0.484686275	CTC-343N3.1
ENSG00000149679.11	-0.082413456	0.097617262	0.484686275	CABLES2
ENSG00000165417.12	-0.090478116	0.097657222	0.484686275	GTF2A1
ENSG00000162377.6	-0.092330733	0.097688091	0.484686275	COA7
ENSG00000286061.1	0.217549574	0.097716225	0.484686275	RCAN3AS

ENSG00000133027.18	0.112733178	0.097725517	0.484686275	PEMT
ENSG00000128394.17	0.10135749	0.097726631	0.484686275	APOBEC3F
ENSG00000111364.16	-0.067468722	0.097762241	0.484693605	DDX55
ENSG00000182054.10	0.095568924	0.097775378	0.484693605	IDH2
ENSG00000269246.1	0.331489546	0.097825672	0.484825731	CTC-246B18.10
ENSG00000115486.12	-0.066522237	0.097867438	0.484915541	GGCX
ENSG00000150477.15	-0.092038185	0.097907957	0.484999127	KIAA1328
ENSG00000272669.1	0.146492606	0.097976072	0.48521934	RP3-508I15.21
ENSG00000187189.11	-0.078634338	0.098059807	0.485480812	TSPYL4
ENSG00000198728.11	0.054851967	0.098095772	0.485480812	LDB1
ENSG00000178988.11	-0.085516009	0.098119202	0.485480812	MRFAP1L1
ENSG00000253047.1	-0.251268366	0.098134049	0.485480812	ENSG10010137222.1
ENSG00000085788.14	-0.071478505	0.09815855	0.485480812	DDHD2
ENSG00000119401.11	-0.072096521	0.098170905	0.485480812	TRIM32
ENSG00000058804.12	-0.076197701	0.098204526	0.485529996	NDC1
ENSG00000184060.11	0.115519774	0.098241882	0.48559762	ADAP2
ENSG00000103995.14	-0.118125399	0.098270197	0.485620529	CEP152
ENSG00000280099.1	-0.260700444	0.098314389	0.485721871	RP11-33E12.2
ENSG00000167196.14	-0.079834901	0.098367645	0.485867932	FBXO22
ENSG00000119411.11	0.242058758	0.098431465	0.485953696	BSPRY
ENSG00000144152.13	0.188135048	0.098438118	0.485953696	FBLN7
ENSG00000285672.1	0.347765006	0.098456096	0.485953696	RP11-569D9.7
ENSG00000285448.1	0.381223519	0.098512777	0.486116467	RP11-178C10.4
ENSG00000140740.11	0.057119547	0.098542032	0.486118322	UQCRC2
ENSG00000163157.15	-0.093336157	0.098612835	0.486118322	TMOD4
ENSG00000185862.7	0.063856196	0.098640075	0.486118322	EVI2B
ENSG00000110876.10	0.091765809	0.09869644	0.486118322	SELPLG
ENSG00000161653.11	0.108640709	0.098703901	0.486118322	NAGS
ENSG00000149289.11	-0.203755173	0.098706087	0.486118322	ZC3H12C
ENSG00000251229.1	-0.3219771	0.098707065	0.486118322	RP11-503N18.5
ENSG00000198040.11	-0.103801241	0.098708028	0.486118322	ZNF84
ENSG00000115966.17	-0.115420219	0.098726488	0.486118322	ATF2
ENSG00000105221.18	0.057386116	0.098756317	0.486135429	AKT2
ENSG00000252620.1	-0.442330743	0.098788328	0.486135429	RNU6ATAC24P
ENSG00000119227.8	0.182344603	0.098801076	0.486135429	PIGZ
ENSG00000170667.15	0.194582609	0.098871035	0.486362963	RASA4B
ENSG00000139083.11	0.061622253	0.098952061	0.48647097	ETV6
ENSG00000164172.20	-0.096486939	0.09896735	0.48647097	MOCS2
ENSG00000134056.12	0.100020328	0.098971939	0.48647097	MRPS36
ENSG00000218048.2	0.732411886	0.098987876	0.48647097	RP3-407E4.4
ENSG00000237945.7	-0.169976691	0.099077129	0.486562275	LINC00649
ENSG00000263004.2	0.227481976	0.099117435	0.486562275	AKAP1-DT
ENSG00000120868.14	-0.112599328	0.099158108	0.486562275	APAF1
ENSG00000168807.16	-0.097670601	0.099180455	0.486562275	SNTB2
ENSG00000131051.24	-0.070516907	0.099181279	0.486562275	RBM39
ENSG00000166394.15	0.322552248	0.0992348	0.486562275	CYB5R2
ENSG00000099800.8	0.106618535	0.099264589	0.486562275	TIMM13

ENSG00000213585.11	0.070836889	0.099331724	0.486562275	VDAC1
ENSG00000237101.1	0.315047823	0.099337729	0.486562275	RP11-365O16.6
ENSG00000113851.15	-0.118506139	0.099373406	0.486562275	CRBN
ENSG00000272886.6	-0.059261419	0.099415233	0.486562275	DCP1A
ENSG00000134668.12	0.309422137	0.099452106	0.486562275	SPOCD1
ENSG00000179115.11	0.088183505	0.099495414	0.486562275	FARSA
ENSG00000173992.9	0.098876323	0.099509888	0.486562275	CCS
ENSG00000181885.18	0.196716025	0.09952317	0.486562275	CLDN7
ENSG00000068724.17	0.080234506	0.099527538	0.486562275	TTC7A
ENSG00000184792.16	-0.292174555	0.099539258	0.486562275	OSBP2
ENSG00000103051.20	0.058941248	0.099544406	0.486562275	COG4
ENSG00000269713.7	0.134808417	0.099583275	0.486562275	NBPF9
ENSG00000169925.17	-0.089756494	0.099592233	0.486562275	BRD3
ENSG00000260428.3	-0.303962298	0.099601479	0.486562275	SCX
ENSG00000171634.18	-0.092330959	0.09961469	0.486562275	BPTF
ENSG00000085465.13	0.143968168	0.099615733	0.486562275	OVGP1
ENSG00000270504.1	0.335766453	0.099643023	0.486562275	RP11-420L9.5
ENSG00000184524.6	-0.288921302	0.099644261	0.486562275	CEND1
ENSG00000270379.6	-0.216907323	0.099646661	0.486562275	HEATR9
ENSG00000122515.15	0.083402448	0.099647043	0.486562275	ZMIZ2
ENSG00000096063.16	-0.107337191	0.09968441	0.486628868	SRPK1
ENSG00000114982.19	-0.046678391	0.099755619	0.486637169	KANSL3
ENSG00000135801.10	-0.062265713	0.099758753	0.486637169	TAF5L
ENSG00000255310.2	-0.125572987	0.099777996	0.486637169	AF131215.2
ENSG00000178338.11	-0.107784203	0.099817883	0.486637169	ZNF354B
ENSG00000011021.23	0.080053757	0.099820918	0.486637169	CLCN6
ENSG00000064933.18	-0.111860119	0.099828485	0.486637169	PMS1
ENSG00000119778.15	-0.112665267	0.099884762	0.486694344	ATAD2B
ENSG00000177700.6	0.128304114	0.099898574	0.486694344	POLR2L
ENSG00000134153.10	0.06424503	0.09991141	0.486694344	EMC7
ENSG00000151239.14	-0.159810799	0.099959607	0.486746483	TWF1
ENSG00000088356.6	0.073353865	0.099969582	0.486746483	PDRG1
ENSG00000116809.12	0.069124611	0.100004134	0.486795131	ZBTB17
ENSG00000004897.12	-0.073265664	0.100072101	0.486795131	CDC27
ENSG00000064607.17	-0.055615536	0.100080264	0.486795131	SUGP2
ENSG00000118946.12	-0.859809975	0.100101815	0.486795131	PCDH17
ENSG00000056586.16	-0.068203968	0.100112027	0.486795131	RC3H2
ENSG00000081237.20	-0.101281835	0.100125594	0.486795131	PTPRC
ENSG00000259935.1	-0.393312903	0.100159314	0.486795131	RP11-519C12.1
ENSG00000005100.13	-0.074340407	0.100169468	0.486795131	DHX33
ENSG00000023228.14	-0.044009271	0.100194732	0.48680255	NDUFS1
ENSG00000197746.14	0.098989973	0.100262166	0.486832984	PSAP
ENSG00000252965.1	0.24251499	0.10033922	0.486832984	Y_RNA
ENSG00000119041.11	-0.083084356	0.100343952	0.486832984	GTF3C3
ENSG00000172869.15	-0.122235648	0.100393012	0.486832984	DMXL1
ENSG00000096080.12	0.100374348	0.100423319	0.486832984	MRPS18A
ENSG00000123870.10	-0.150791855	0.100470335	0.486832984	ZNF137P

ENSG00000105607.13	0.077605373	0.100525584	0.486832984	GCDH
ENSG00000117906.14	-0.13516214	0.100531806	0.486832984	RCN2
ENSG00000196411.10	0.175307317	0.100543677	0.486832984	EPHB4
ENSG00000198039.11	-0.15782665	0.100552357	0.486832984	ZNF273
ENSG00000188710.3	0.293983015	0.10055286	0.486832984	QRFP
ENSG00000146676.10	-0.080485426	0.100566047	0.486832984	PURB
ENSG00000114999.8	-0.056345925	0.100577046	0.486832984	TTL
ENSG00000100095.19	0.218133825	0.100625386	0.486832984	SEZ6L
ENSG00000103647.13	0.387492955	0.100704269	0.486832984	CORO2B
ENSG00000111011.18	-0.054610203	0.100759402	0.486832984	RSRC2
ENSG00000272455.1	0.1106842	0.100821525	0.486832984	MRPL20-DT
ENSG00000213965.4	-0.07772024	0.100866667	0.486832984	NUDT19
ENSG00000234614.1	-0.222482102	0.100871316	0.486832984	C2CD4D-AS1
ENSG00000123091.5	-0.139766103	0.100902859	0.486832984	RNF11
ENSG00000135452.10	0.061945243	0.100930121	0.486832984	TSPAN31
ENSG00000253234.1	0.359140113	0.10095528	0.486832984	IGLV2-5
ENSG00000154485.5	-0.274838403	0.100956646	0.486832984	MMP21
ENSG00000273604.2	-0.230175012	0.100964838	0.486832984	EPOP
ENSG00000262692.1	-0.126029638	0.100970557	0.486832984	CTD-3195I5.3
ENSG00000261235.1	0.572411109	0.100987947	0.486832984	RP11-510J16.5
ENSG00000174720.16	-0.148908367	0.101007774	0.486832984	LARP7
ENSG00000172757.13	0.094457829	0.101010501	0.486832984	CFL1
ENSG00000227076.1	0.232037045	0.101026551	0.486832984	RP11-4C20.4
ENSG00000146909.8	-0.06867895	0.101027182	0.486832984	NOM1
ENSG00000137509.12	0.069784808	0.101073859	0.486832984	PRCP
ENSG00000129245.12	0.066470035	0.101074194	0.486832984	FXR2
ENSG00000203697.12	-0.256927604	0.101102577	0.486832984	CAPN8
ENSG00000197961.12	-0.080479365	0.101110064	0.486832984	ZNF121
ENSG00000185477.5	-0.099342248	0.101154048	0.486832984	GPRIN3
ENSG00000151665.13	-0.108420607	0.101162558	0.486832984	PIGF
ENSG00000173456.5	0.075883607	0.101173815	0.486832984	RNF26
ENSG00000219682.4	-0.403800257	0.101205605	0.486832984	RP3-425P12.4
ENSG00000204455.7	0.483014691	0.101213683	0.486832984	TRIM51BP
ENSG00000130518.17	0.16222189	0.101255652	0.486832984	IQCN
ENSG00000118113.12	-0.53308831	0.101292167	0.486832984	MMP8
ENSG00000149187.18	-0.057972022	0.101348063	0.486832984	CELF1
ENSG00000265511.1	0.140438893	0.101381481	0.486832984	RP11-524F11.1
ENSG00000163002.13	-0.120641422	0.101400101	0.486832984	NUP35
ENSG00000184584.13	0.082862522	0.101403266	0.486832984	STING1
ENSG00000110077.15	0.119175629	0.101404374	0.486832984	MS4A6A
ENSG00000083123.15	-0.091945834	0.1014104	0.486832984	BCKDHB
ENSG00000183444.10	0.262198799	0.10143665	0.486832984	OR7E38P
ENSG00000229036.7	-0.125724373	0.101443388	0.486832984	VDAC1P8
ENSG00000138796.18	0.052409913	0.101471518	0.486832984	HADH
ENSG00000081913.14	0.13222609	0.101503273	0.486832984	PHLPP1
ENSG00000102901.13	0.07616455	0.101542161	0.486832984	CENPT
ENSG00000185219.17	-0.072834507	0.101584898	0.486832984	ZNF445

ENSG00000227939.1	0.254196355	0.101603314	0.486832984	RPL3P2
ENSG00000158604.15	-0.029651238	0.101606184	0.486832984	TMED4
ENSG00000143862.8	0.06497798	0.101609632	0.486832984	ARL8A
ENSG00000244459.2	0.140511087	0.101623916	0.486832984	RP11-1398P2.1
ENSG00000068878.15	-0.057763076	0.101626835	0.486832984	PSME4
ENSG00000132436.12	-0.140756094	0.101645351	0.486832984	FIGNL1
ENSG00000145495.16	-0.068096713	0.101670083	0.486832984	MARCHF6
ENSG00000257495.5	0.315430994	0.101683434	0.486832984	KRT73-AS1
ENSG00000143178.13	0.09225806	0.1017046	0.486832984	TBX19
ENSG00000284931.1	0.663575003	0.101739839	0.486832984	CTD-2643I7.4
ENSG00000163554.15	-0.319620583	0.101759524	0.486832984	SPTA1
ENSG00000145740.19	-0.066611536	0.101775108	0.486832984	SLC30A5
ENSG00000283063.1	-0.303751721	0.101780709	0.486832984	TRBV6-2
ENSG00000155760.3	-0.334267378	0.101791488	0.486832984	FZD7
ENSG00000080822.17	-0.104071504	0.10196648	0.487556209	CLDND1
ENSG00000168228.15	-0.084718523	0.102053891	0.487860421	ZCCHC4
ENSG00000113389.16	-0.348554532	0.102095484	0.48794551	NPR3
ENSG00000151746.15	-0.115587789	0.102168319	0.488179841	BICD1
ENSG00000131401.11	0.231269833	0.102207684	0.488254176	NAPSB
ENSG00000251661.3	0.215475311	0.102266065	0.488254242	RP11-326C3.11
ENSG00000100362.13	0.490403106	0.102266592	0.488254242	PVALB
ENSG00000112893.10	-0.089109688	0.102294562	0.488254242	MAN2A1
ENSG00000173862.3	0.361554443	0.102305625	0.488254242	RP11-89N17.1
ENSG00000269388.1	-0.373078763	0.102358879	0.488254242	AC018755.16
ENSG00000224712.12	-0.784473338	0.102361389	0.488254242	NPIPA3
ENSG00000232811.1	-0.171621782	0.102395905	0.488254242	RP11-96K19.2
ENSG00000119203.14	0.056924693	0.102411398	0.488254242	CPSF3
ENSG00000166166.13	0.101806996	0.102436206	0.488254242	TRMT61A
ENSG00000244661.1	0.704274487	0.102445777	0.488254242	TRBV12-1
ENSG00000167280.17	0.110671396	0.102490186	0.488352402	ENGASE
ENSG00000275066.5	-0.070409211	0.102525876	0.48840898	SYNRG
ENSG00000184156.17	0.436004999	0.102625519	0.488585489	KCNQ3
ENSG00000009413.16	-0.102834494	0.102628097	0.488585489	REV3L
ENSG00000274105.1	-0.276876589	0.102634401	0.488585489	RP11-278C7.3
ENSG00000161040.16	-0.214171401	0.102687257	0.488723666	FBXL13
ENSG00000197935.7	0.289752458	0.102766162	0.488748017	ZNF311
ENSG00000187535.14	0.110213606	0.102766836	0.488748017	IFT140
ENSG00000105698.16	0.074522834	0.10277721	0.488748017	USF2
ENSG00000261211.1	0.280663799	0.102809992	0.488748017	RP1-80N2.3
ENSG00000162607.13	-0.112024851	0.102811534	0.488748017	USP1
ENSG00000101182.15	0.070991158	0.102910165	0.489087457	PSMA7
ENSG00000076650.7	0.070809719	0.102930635	0.489087457	GPATCH1
ENSG00000158985.14	-0.068631733	0.102985161	0.489131928	CDC42SE2
ENSG00000278601.1	-0.229083764	0.102987696	0.489131928	RP11-348N5.9
ENSG00000089091.16	-0.11801444	0.1030233	0.489187739	DZANK1
ENSG00000162881.6	0.160438307	0.103076819	0.489328567	OXER1
ENSG00000143942.5	-0.184912586	0.103127293	0.489454878	CHAC2

ENSG00000164104.12	-0.154724584	0.103170316	0.489538217	HMGB2
ENSG00000037749.13	-0.083500123	0.103206758	0.489538217	MFAP3
ENSG00000143256.5	0.12206438	0.103216464	0.489538217	PFDN2
ENSG00000118873.16	-0.094089589	0.103271945	0.489576684	RAB3GAP2
ENSG00000122692.9	0.043955454	0.103277328	0.489576684	SMU1
ENSG00000275106.1	-0.37711665	0.103296192	0.489576684	RP11-309L24.10
ENSG00000106105.15	0.056938992	0.103331644	0.489588239	GARS1
ENSG00000125945.15	-0.07361782	0.103346376	0.489588239	ZNF436
ENSG00000152348.16	0.158711572	0.103390821	0.489685673	ATG10
ENSG00000211678.2	0.467861412	0.103417991	0.489701263	IGLJ3
ENSG00000246985.8	0.161079752	0.103464438	0.489808101	SOCS2-AS1
ENSG00000165915.14	0.066116142	0.103521181	0.489915301	SLC39A13
ENSG00000182986.15	-0.126641025	0.103627418	0.489915301	ZNF320
ENSG00000184984.10	-0.230726858	0.103649403	0.489915301	CHRM5
ENSG00000099956.20	0.082973827	0.103693838	0.489915301	SMARCB1
ENSG00000179627.10	0.113503017	0.103719945	0.489915301	ZBTB42
ENSG00000072422.17	0.224033217	0.103748342	0.489915301	RHOBTB1
ENSG00000162244.12	0.101905978	0.103748456	0.489915301	RPL29
ENSG00000234389.1	-0.38521583	0.103751512	0.489915301	AC007278.3
ENSG00000217128.12	-0.101150382	0.10378462	0.489915301	FNIP1
ENSG00000180353.11	0.079995583	0.103845256	0.489915301	HCLS1
ENSG00000228434.1	-0.213611341	0.103868262	0.489915301	AC004951.6
ENSG00000147457.14	0.082818979	0.1038742	0.489915301	CHMP7
ENSG00000090971.5	0.137820359	0.103877804	0.489915301	NAT14
ENSG00000140990.15	0.0979993	0.103878978	0.489915301	NDUFB10
ENSG00000136492.10	-0.200182181	0.103906747	0.489915301	BRIP1
ENSG00000150768.17	-0.062957031	0.103918633	0.489915301	DLAT
ENSG00000162892.16	0.136200004	0.103953966	0.489915301	IL24
ENSG00000205771.6	-0.238530041	0.103975831	0.489915301	CATSPER2P1
ENSG00000172172.8	-0.204894266	0.10399761	0.489915301	MRPL13
ENSG00000275180.1	0.17336419	0.104037574	0.489915301	RP11-631N16.4
ENSG00000127314.18	-0.101932803	0.104038701	0.489915301	RAP1B
ENSG00000130725.8	0.092408814	0.104069544	0.489915301	UBE2M
ENSG00000093009.11	-0.289060673	0.104091338	0.489915301	CDC45
ENSG00000129167.10	-0.216939003	0.104131885	0.489915301	TPH1
ENSG00000248485.2	-0.314590019	0.104156209	0.489915301	PCP4L1
ENSG00000115318.12	0.101710427	0.104234211	0.489915301	LOXL3
ENSG00000153814.13	-0.093967263	0.104253045	0.489915301	JAZF1
ENSG00000198586.14	-0.093663126	0.104263913	0.489915301	TLK1
ENSG00000120306.11	0.148308916	0.104266796	0.489915301	CYSTM1
ENSG00000141161.12	-0.253749758	0.10427078	0.489915301	UNC45B
ENSG00000169814.16	0.052669204	0.104283068	0.489915301	BTD
ENSG00000278985.1	-0.284693799	0.104306696	0.489915301	RP11-303E16.9
ENSG00000236349.1	0.311067651	0.104319246	0.489915301	SUCLG2P2
ENSG00000241666.2	0.299735965	0.104319541	0.489915301	RP3-455J7.4
ENSG00000138386.17	-0.128669709	0.104323197	0.489915301	NAB1
ENSG00000086666.19	-0.082835933	0.104397488	0.490055381	ZFAND6

ENSG00000170476.16	0.260097895	0.104424157	0.490055381	MZB1
ENSG00000160013.9	0.138088438	0.104424713	0.490055381	PTGIR
ENSG00000140548.10	0.094171005	0.104471321	0.490161941	ZNF710
ENSG00000110435.13	-0.073445535	0.104539716	0.490335166	PDHX
ENSG00000151116.17	-0.090791863	0.104595615	0.490335166	UEVLD
ENSG00000261556.10	-0.110478639	0.104613729	0.490335166	SMG1P7
ENSG00000233822.5	0.190380406	0.104619449	0.490335166	H2BC15
ENSG00000128218.8	0.273559654	0.104627789	0.490335166	VPREB3
ENSG00000198382.9	0.054501587	0.104720452	0.490635823	UVRAG
ENSG00000144455.14	0.085488873	0.104756383	0.490635823	SUMF1
ENSG00000215005.2	0.935927222	0.104772284	0.490635823	HSPD1P7
ENSG00000178741.12	0.091416106	0.104803646	0.490635823	COX5A
ENSG00000136830.12	0.113437379	0.104811563	0.490635823	NIBAN2
ENSG00000278745.1	0.615250969	0.104857991	0.490716588	RP1-229K20.9
ENSG00000125845.7	-0.430264501	0.104876673	0.490716588	BMP2
ENSG00000115875.19	-0.065407181	0.104930685	0.490783111	SRSF7
ENSG00000177888.8	-0.172679465	0.104978376	0.490783111	ZBTB41
ENSG00000121058.5	-0.060831319	0.10497998	0.490783111	COIL
ENSG00000285581.1	-0.408573755	0.105032265	0.490783111	RP11-371C18.3
ENSG00000125503.13	0.072735834	0.105063187	0.490783111	PPP1R12C
ENSG00000100319.13	0.087194944	0.105140581	0.490783111	ZMAT5
ENSG00000172456.18	0.132418819	0.105156125	0.490783111	FGGY
ENSG00000148180.21	0.12819004	0.105233706	0.490783111	GSN
ENSG00000079134.12	-0.054957061	0.10524652	0.490783111	THOC1
ENSG00000185716.12	-0.123454245	0.105247648	0.490783111	MOSMO
ENSG00000178685.14	0.140086677	0.105259171	0.490783111	PARP10
ENSG00000215126.11	-0.179350511	0.105263279	0.490783111	CBWD6
ENSG00000127311.10	-0.182358851	0.105266019	0.490783111	HELB
ENSG00000271857.1	0.165557006	0.105268709	0.490783111	RP1-244F24.1
ENSG00000198176.13	-0.103869145	0.105277027	0.490783111	TFDP1
ENSG00000242477.1	0.300655253	0.10530186	0.490783111	CTD-2161E19.1
ENSG00000138032.21	-0.116774644	0.105313338	0.490783111	PPM1B
ENSG00000197465.14	-0.329659742	0.105361312	0.490783111	GYPE
ENSG00000207741.1	-0.256066054	0.105363708	0.490783111	MIR590
ENSG00000269646.1	0.346126651	0.105391346	0.490783111	CTD-2331H12.7
ENSG00000114331.15	-0.096232555	0.105475065	0.490783111	ACAP2
ENSG00000107672.15	-0.074277274	0.105508566	0.490783111	NSMCE4A
ENSG00000145996.11	-0.063203615	0.105556529	0.490783111	CDKAL1
ENSG00000143458.12	-0.067382064	0.105556673	0.490783111	GABPB2
ENSG00000089847.12	0.213811113	0.105560508	0.490783111	ANKRD24
ENSG00000272505.1	-0.166621473	0.105587031	0.490783111	RP11-981G7.6
ENSG00000231074.9	-0.069282596	0.105593986	0.490783111	HCG18
ENSG00000143147.15	0.321621711	0.105603935	0.490783111	GPR161
ENSG00000227544.10	0.34951342	0.105620909	0.490783111	AC018647.3
ENSG00000111361.13	0.041524443	0.105642072	0.490783111	EIF2B1
ENSG00000136514.3	0.238701734	0.105645404	0.490783111	RTP4
ENSG00000100811.14	-0.03629842	0.105664593	0.490783111	YY1

ENSG00000117500.13	-0.106170523	0.105696101	0.490783111	TMED5
ENSG00000235419.5	-0.384610299	0.105727602	0.490783111	AC010149.4
ENSG00000247596.10	0.083908101	0.105728486	0.490783111	TWF2
ENSG00000114120.14	-0.112630436	0.105760795	0.490809185	SLC25A36
ENSG00000237298.10	0.125530274	0.105811261	0.490809185	TTN-AS1
ENSG00000253230.9	-1.202599667	0.105840156	0.490809185	MIR124-1HG
ENSG00000255568.3	0.372445205	0.105882833	0.490809185	BRWD1-AS2
ENSG00000115540.15	-0.141674976	0.105885804	0.490809185	MOB4
ENSG00000136603.14	-0.15281489	0.105906684	0.490809185	SKIL
ENSG00000178567.8	-0.080467521	0.105912958	0.490809185	EPM2AIP1
ENSG00000134884.15	-0.153813245	0.105934494	0.490809185	ARGLU1
ENSG00000218283.2	0.073013776	0.105949496	0.490809185	MORF4L1P1
ENSG00000167711.14	0.180413844	0.106010024	0.490978674	SERPINF2
ENSG00000172340.15	0.074375611	0.106038457	0.490999473	SUCLG2
ENSG00000160563.14	0.090018913	0.106085793	0.491107776	MED27
ENSG00000265293.2	-0.328535613	0.106152602	0.491144917	ARGFXP2
ENSG00000272049.1	0.449735965	0.106153009	0.491144917	RP11-480D4.6
ENSG00000079335.20	-0.130095872	0.106183243	0.491144917	CDC14A
ENSG00000242550.6	-0.4540349	0.106227183	0.491144917	SERPINB10
ENSG00000211937.3	0.379843178	0.10624095	0.491144917	IGHV2-5
ENSG00000136099.13	-1.278012946	0.106264371	0.491144917	PCDH8
ENSG00000110422.12	-0.132279273	0.106278432	0.491144917	HIPK3
ENSG00000188760.11	0.310634855	0.106285408	0.491144917	TMEM198
ENSG00000107897.20	-0.081386687	0.106360931	0.491261508	ACBD5
ENSG00000135722.9	0.130515951	0.106384228	0.491261508	FBXL8
ENSG00000078246.17	-0.079753286	0.106424183	0.491261508	TULP3
ENSG00000246477.3	-0.319011597	0.106425551	0.491261508	AF131216.6
ENSG00000262703.1	0.397056786	0.106444201	0.491261508	RP11-485G7.6
ENSG00000257704.4	0.14586698	0.106482084	0.491261508	INAFM1
ENSG00000139899.11	0.256468155	0.106488114	0.491261508	CBLN3
ENSG00000108599.15	-0.070113871	0.106511916	0.491261508	AKAP10
ENSG00000095585.17	0.181960972	0.10652623	0.491261508	BLNK
ENSG00000127578.7	0.25461309	0.10655578	0.491276507	WFIKKN1
ENSG00000286216.1	0.649329791	0.106577393	0.491276507	RP11-433K2.8
ENSG00000278341.1	-0.210263144	0.106607828	0.491306367	RP5-1142A6.10
ENSG00000187118.14	-0.14934989	0.106691194	0.491320644	CMC1
ENSG00000143575.15	0.079689142	0.10671589	0.491320644	HAX1
ENSG00000170852.11	-0.071884629	0.106746317	0.491320644	KBTBD2
ENSG00000171658.8	-0.397423204	0.106807001	0.491320644	NMRAL2P
ENSG00000114631.11	0.200779245	0.106813177	0.491320644	PODXL2
ENSG00000134571.11	0.132193238	0.106817136	0.491320644	MYBPC3
ENSG00000162191.14	0.087472402	0.106859488	0.491320644	UBXN1
ENSG00000168874.13	0.449018185	0.106892683	0.491320644	ATOH8
ENSG00000233975.2	0.608116731	0.106916985	0.491320644	LINC02574
ENSG00000220785.7	0.243831104	0.106950975	0.491320644	MTMR9LP
ENSG00000117791.16	0.270706761	0.106958843	0.491320644	MTARC2
ENSG00000081154.12	-0.099881116	0.106961515	0.491320644	PCNP

ENSG00000152270.9	-0.128216952	0.106962714	0.491320644	PDE3B
ENSG00000105700.11	0.055353557	0.107043162	0.491320644	KXD1
ENSG00000165959.12	0.0960598	0.10704457	0.491320644	CLMN
ENSG00000198898.14	-0.153898927	0.107065042	0.491320644	CAPZA2
ENSG00000164187.7	-0.144424432	0.107072659	0.491320644	LMBRD2
ENSG00000170759.11	-0.119100539	0.107081998	0.491320644	KIF5B
ENSG00000275393.1	0.258316933	0.107094439	0.491320644	RP11-568J23.8
ENSG00000149761.9	0.084282858	0.10710897	0.491320644	NUDT22
ENSG00000228716.7	-0.135551363	0.107114034	0.491320644	DHFR
ENSG00000118496.5	-0.12238146	0.107215492	0.491676055	FBXO30
ENSG00000137100.16	0.070329613	0.107295821	0.491876618	DCTN3
ENSG00000108423.15	-0.081621738	0.107316381	0.491876618	TUBD1
ENSG00000203485.14	0.158734903	0.107331181	0.491876618	INF2
ENSG00000272812.1	-0.256421686	0.107504795	0.492552146	RP5-855D21.3
ENSG00000138758.12	-0.091208139	0.107532082	0.492552146	SEPTIN11
ENSG00000125148.7	0.257007815	0.107552853	0.492552146	MT2A
ENSG00000134030.14	0.099318996	0.107593195	0.492552146	CTIF
ENSG00000179988.15	-0.103517745	0.107598674	0.492552146	PSTK
ENSG00000111247.15	-0.286884004	0.107642695	0.492643694	RAD51AP1
ENSG00000267892.1	0.255847655	0.107697145	0.49272385	CTD-2540F13.2
ENSG00000139496.16	-0.084316117	0.107708261	0.49272385	NUP58
ENSG00000260792.1	0.384391245	0.107765455	0.492875548	LINC02280
ENSG00000241490.1	-0.296012047	0.107824865	0.492892007	RP11-553L6.2
ENSG00000166002.7	0.13911298	0.107834539	0.492892007	SMCO4
ENSG00000096654.16	-0.110954161	0.107847309	0.492892007	ZNF184
ENSG00000120708.17	0.11929159	0.107868783	0.492892007	TGFBI
ENSG00000095794.19	-0.106213158	0.107889225	0.492892007	CREM
ENSG00000231507.2	0.240612311	0.10794426	0.493018921	LINC01353
ENSG00000250654.7	-0.188339963	0.108040094	0.493018921	RP11-834C11.7
ENSG00000107560.12	-0.13011305	0.108042804	0.493018921	RAB11FIP2
ENSG00000143756.12	-0.080666231	0.10806995	0.493018921	FBXO28
ENSG00000123095.6	-0.287230294	0.108131102	0.493018921	BHLHE41
ENSG00000263366.2	-0.431953357	0.108175044	0.493018921	ABHD17AP5
ENSG00000163867.17	-0.078833667	0.108185267	0.493018921	ZMYM6
ENSG00000077463.15	0.091207487	0.108215669	0.493018921	SIRT6
ENSG00000164609.10	-0.087565367	0.108260052	0.493018921	SLU7
ENSG00000088035.18	-0.074551813	0.10826743	0.493018921	ALG6
ENSG00000011523.14	-0.081036487	0.108287842	0.493018921	CEP68
ENSG00000102786.15	-0.069712072	0.108316816	0.493018921	INTS6
ENSG00000169371.14	0.072056949	0.108330524	0.493018921	SNUPN
ENSG00000102898.12	0.074858526	0.108336233	0.493018921	NUTF2
ENSG00000185386.15	0.134280994	0.108406925	0.493018921	MAPK11
ENSG00000233901.6	0.13032644	0.108411274	0.493018921	LINC01503
ENSG00000139505.12	-0.101455083	0.10841488	0.493018921	MTMR6
ENSG00000273348.1	-0.25027201	0.10843345	0.493018921	RP11-535A5.1
ENSG00000128585.18	-0.097458133	0.108498606	0.493018921	MKLN1
ENSG00000167695.15	0.166458301	0.108520348	0.493018921	TLCD3A

ENSG00000204344.15	0.069230922	0.108527925	0.493018921	STK19
ENSG00000267809.1	0.292327285	0.108528392	0.493018921	NDUFV2P1
ENSG00000078369.18	0.050692769	0.108528574	0.493018921	GNB1
ENSG00000164078.13	0.216175527	0.108537479	0.493018921	MST1R
ENSG00000006712.15	0.08495647	0.108537569	0.493018921	PAF1
ENSG00000177683.14	-0.136127174	0.108542053	0.493018921	THAP5
ENSG00000176715.17	0.063398226	0.108586626	0.493029348	ACSF3
ENSG00000148700.15	-0.102350728	0.108603109	0.493029348	ADD3
ENSG00000254395.1	-0.505428527	0.108639495	0.493029348	IGHV4-55
ENSG00000258725.2	-0.250170155	0.108724201	0.493029348	PRC1-AS1
ENSG00000130300.9	-0.313169187	0.108738473	0.493029348	PLVAP
ENSG00000272682.1	0.33206464	0.108764162	0.493029348	AC004471.10
ENSG00000258890.7	-0.057478954	0.108764936	0.493029348	CEP95
ENSG00000071539.14	-0.262664477	0.108770565	0.493029348	TRIP13
ENSG00000084093.19	-0.073459352	0.10880319	0.493029348	REST
ENSG00000120875.9	-0.214642552	0.108830447	0.493029348	DUSP4
ENSG00000261441.1	-0.343768586	0.108846802	0.493029348	POLG-DT
ENSG00000011105.14	0.23730512	0.108858838	0.493029348	TSPAN9
ENSG00000180867.11	0.10800604	0.108872016	0.493029348	PDIA3P1
ENSG00000118482.12	-0.075745486	0.108899929	0.493029348	PHF3
ENSG00000115073.8	0.047945909	0.108936345	0.493029348	ACTR1B
ENSG00000184381.20	0.080052367	0.108939155	0.493029348	PLA2G6
ENSG00000067064.12	-0.12943847	0.108953043	0.493029348	IDI1
ENSG00000167995.17	0.090447079	0.108981643	0.493049976	BEST1
ENSG00000271659.1	-0.317054416	0.109069041	0.493336547	RP11-435O5.4
ENSG00000122484.9	-0.082862752	0.109132168	0.493482715	RPAP2
ENSG00000231940.1	0.310896368	0.109166152	0.493482715	RPS7P3
ENSG00000198393.8	-0.081997944	0.109173668	0.493482715	ZNF26
ENSG00000129473.11	0.079913217	0.109197609	0.493482715	BCL2L2
ENSG00000158552.13	0.07952841	0.109268891	0.493512791	ZFAND2B
ENSG00000131446.17	0.078686095	0.109276842	0.493512791	MGAT1
ENSG00000247095.3	0.23438124	0.109313065	0.493512791	MIR210HG
ENSG00000185499.16	0.20189719	0.10931342	0.493512791	MUC1
ENSG00000157823.17	0.078465523	0.109399635	0.493512791	AP3S2
ENSG00000001460.18	0.111838756	0.109400341	0.493512791	STPG1
ENSG00000107758.16	-0.05485889	0.109447585	0.493512791	PPP3CB
ENSG00000116793.16	-0.090475775	0.109510854	0.493512791	PHTF1
ENSG00000145220.14	-0.094566811	0.109520247	0.493512791	LYAR
ENSG00000070214.16	-0.081714949	0.109546846	0.493512791	SLC44A1
ENSG00000260145.1	-0.254531919	0.109563106	0.493512791	RP11-322D14.2
ENSG00000224418.1	0.33008835	0.10958165	0.493512791	STK24-AS1
ENSG00000131143.10	0.093111414	0.109582415	0.493512791	COX4I1
ENSG00000230362.1	0.269054763	0.109585351	0.493512791	ACTG1P23
ENSG00000168297.16	-0.080240576	0.109591658	0.493512791	PXK
ENSG00000075415.15	0.064479935	0.109619049	0.493512791	SLC25A3
ENSG00000116885.18	0.224911532	0.109621535	0.493512791	OSCP1
ENSG00000020922.13	-0.072096472	0.109637423	0.493512791	MRE11

ENSG00000100749.9	-0.090135104	0.109693591	0.493637926	VRK1
ENSG00000164038.16	-0.090645032	0.109737519	0.493637926	SLC9B2
ENSG00000205339.10	-0.08222138	0.109751014	0.493637926	IPO7
ENSG00000133393.13	-0.095913914	0.109761505	0.493637926	CEP20
ENSG00000171853.16	0.066167591	0.109834247	0.493856772	TRAPPC12
ENSG00000106258.15	-0.123940075	0.109971574	0.494036054	CYP3A5
ENSG00000196505.11	-0.065147926	0.109971685	0.494036054	GDAP2
ENSG00000100324.14	0.061754713	0.109980676	0.494036054	TAB1
ENSG00000008323.16	-0.206897353	0.110030892	0.494036054	PLEKHG6
ENSG00000141574.8	0.146179226	0.110051811	0.494036054	SECTM1
ENSG00000280181.1	-0.293407725	0.110082561	0.494036054	RP11-629N8.4
ENSG00000227719.1	0.212662909	0.110105961	0.494036054	AC006042.6
ENSG00000123416.15	0.088545549	0.110112873	0.494036054	TUBA1B
ENSG00000231062.1	0.212042176	0.110158606	0.494036054	AC103563.9
ENSG00000168575.10	-0.059156874	0.110166465	0.494036054	SLC20A2
ENSG00000272507.1	0.341343353	0.110168486	0.494036054	RNU6-88P
ENSG00000100911.16	0.133056127	0.110220382	0.494036054	PSME2
ENSG00000173762.8	0.142698855	0.110228353	0.494036054	CD7
ENSG00000224578.5	0.149990381	0.110265218	0.494036054	HNRNPA1P48
ENSG00000239883.8	-0.196253558	0.110270591	0.494036054	PARGP1
ENSG00000230551.4	-0.130012019	0.110272816	0.494036054	CTB-89H12.4
ENSG00000092330.18	0.054431901	0.110311938	0.494036054	TINF2
ENSG00000168140.5	0.12797965	0.110335081	0.494036054	VASN
ENSG00000121879.6	-0.109811251	0.110337971	0.494036054	PIK3CA
ENSG00000154146.13	0.22299409	0.11039874	0.494036054	NRGN
ENSG00000165061.15	-0.495008189	0.110475922	0.494036054	ZMAT4
ENSG00000123106.10	-0.089293997	0.110505794	0.494036054	CCDC91
ENSG00000100814.18	-0.085691341	0.110571504	0.494036054	CCNB1IP1
ENSG00000152683.14	-0.068383248	0.110601291	0.494036054	SLC30A6
ENSG00000243970.3	-0.169459434	0.11063953	0.494036054	PPIEL
ENSG00000196504.19	-0.086946968	0.110659946	0.494036054	PRPF40A
ENSG00000237350.1	0.161689609	0.11067366	0.494036054	CDC42P6
ENSG00000118579.13	-0.059826457	0.110743346	0.494036054	MED28
ENSG00000214708.4	0.363943662	0.110744771	0.494036054	AC090616.2
ENSG00000181126.13	0.617502998	0.110763468	0.494036054	HLA-V
ENSG00000088986.11	0.092574106	0.110800862	0.494036054	DYNLL1
ENSG00000241839.10	0.084692988	0.11080889	0.494036054	PLEKHO2
ENSG00000114626.18	0.097593512	0.110830396	0.494036054	ABTB1
ENSG00000168081.9	0.210083028	0.110849666	0.494036054	PNOC
ENSG00000241134.3	-0.376984386	0.110863302	0.494036054	BET1P1
ENSG00000108773.11	0.075379961	0.110970904	0.494036054	KAT2A
ENSG00000239445.6	0.345417687	0.110971312	0.494036054	ST3GAL6-AS1
ENSG00000136931.10	0.395933615	0.110993608	0.494036054	NR5A1
ENSG00000081307.15	-0.072149645	0.110999191	0.494036054	UBA5
ENSG00000178467.18	0.059798629	0.11099957	0.494036054	P4HTM
ENSG00000187531.14	0.061914925	0.111020227	0.494036054	SIRT7
ENSG00000164331.10	-0.102544419	0.111025101	0.494036054	ANKRA2

ENSG00000089199.10	0.624773215	0.111025512	0.494036054	CHGB	
ENSG00000113328.19	-0.080173256	0.111033693	0.494036054	CCNG1	
ENSG00000151079.7	-0.282815969	0.111035854	0.494036054	KCNA6	
ENSG00000138378.19	-0.078275071	0.111050118	0.494036054	STAT4	
ENSG00000172831.14	0.057960037	0.11106076	0.494036054	CES2	
ENSG00000245008.4	0.386718364	0.111084487	0.494036054	RP11-744N12.3	
ENSG00000179978.11	-0.226694927	0.111100483	0.494036054	NAIPP2	
ENSG00000164291.17	-0.144800356	0.111113598	0.494036054	ARSK	
ENSG00000248213.3	-0.35248457	0.111132485	0.494036054	CICP16	
ENSG00000197670.6	-0.234171798	0.11114846	0.494036054	RP4-724E16.2	
ENSG00000100138.15	0.069012957	0.111150885	0.494036054	SNU13	
ENSG00000140950.16	0.079235633	0.111204751	0.494125084	MEAK7	
ENSG00000124469.12	-0.493505371	0.111219104	0.494125084	CEACAM8	
ENSG00000084623.12	0.06753709	0.111344038	0.494374898	EIF3I	
ENSG00000135114.13	0.322425061	0.11134924	0.494374898	OASL	
ENSG00000121067.19	0.037865257	0.111382359	0.494374898	SPOP	
ENSG00000225079.2	-0.254981	0.11140757	0.494374898	FTH1P22	
ENSG00000175305.18	-0.118836977	0.111421271	0.494374898	CCNE2	
ENSG00000179044.16	0.33953796	0.111436661	0.494374898	EXOC3L1	
ENSG00000146109.5	0.061452464	0.111444078	0.494374898	ABT1	
ENSG00000157693.15	0.107939104	0.111600182	0.494960321	TMEM268	
ENSG00000260910.2	0.226804256	0.111658866	0.495111167	LINC00565	
ENSG00000138814.17	-0.078117646	0.111682478	0.495111167	PPP3CA	
ENSG00000170892.13	0.095624919	0.111788294	0.495270673	TSEN34	
ENSG00000111481.10	0.069071243	0.111807771	0.495270673	COPZ1	
ENSG00000164180.14	-0.128796793	0.111813517	0.495270673	TMEM161B	
ENSG00000166986.15	0.047982055	0.111815058	0.495270673		mar.01
ENSG00000136152.15	-0.066460422	0.111926436	0.495582303	COG3	
ENSG00000183111.12	-0.313850058	0.111933744	0.495582303	ARHGEF37	
ENSG00000074582.15	0.074633226	0.112054949	0.495726803	BCS1L	
ENSG00000146757.14	-0.165837867	0.112072523	0.495726803	ZNF92	
ENSG00000184990.13	0.082710566	0.112077493	0.495726803	SIVA1	
ENSG00000131759.18	0.090876743	0.112093364	0.495726803	RARA	
ENSG00000201217.1	-0.473043891	0.112107469	0.495726803	Y_RNA	
ENSG00000175756.13	0.110232442	0.112124392	0.495726803	AURKAIP1	
ENSG00000112367.12	0.062482922	0.112135588	0.495726803	FIG4	
ENSG00000086200.17	-0.0828943	0.112174364	0.495791348	IPO11	
ENSG00000148090.12	-0.088695661	0.112242616	0.495986117	AUH	
ENSG00000144560.15	-0.090809973	0.112357108	0.496280403	VGLL4	
ENSG00000280135.1	-0.117434972	0.112357612	0.496280403	RP1-47M23.3	
ENSG00000140396.13	-0.111517472	0.11242213	0.496389389	NCOA2	
ENSG00000116815.16	-0.115764098	0.112447047	0.496389389	CD58	
ENSG00000180891.13	0.093888569	0.112462724	0.496389389	CUEDC1	
ENSG00000152223.15	-0.074179951	0.112479105	0.496389389	EPG5	
ENSG00000176853.16	-0.101987061	0.112552091	0.496489819	FAM91A1	
ENSG00000170779.11	-0.088803495	0.112617186	0.496489819	CDCA4	
ENSG00000115604.12	-0.147166372	0.112619069	0.496489819	IL18R1	

ENSG00000132002.9	0.061921445	0.112620853	0.496489819	DNAJB1
ENSG00000229539.1	0.266361713	0.11262291	0.496489819	PANK2-AS1
ENSG00000175262.14	0.201117696	0.11266626	0.496574179	C1orf127
ENSG00000255328.1	0.335852249	0.112742817	0.496619654	RP11-326C3.12
ENSG00000160179.19	0.168477052	0.11274869	0.496619654	ABCG1
ENSG00000059588.10	-0.084402812	0.11278319	0.496619654	TARBP1
ENSG00000129667.13	0.109474025	0.112796695	0.496619654	RHBDF2
ENSG00000188599.17	0.166388491	0.112797657	0.496619654	NPIPP1
ENSG00000111846.19	0.153591828	0.112950674	0.497186609	GCNT2
ENSG00000153936.17	-0.098066614	0.112995233	0.49727377	HS2ST1
ENSG00000139908.15	-0.138551948	0.11303096	0.49727377	TSSK4
ENSG00000095787.24	-0.050700652	0.113043218	0.49727377	WAC
ENSG00000105401.9	0.068713906	0.113132944	0.497561746	CDC37
ENSG00000100154.14	0.195543869	0.113254119	0.497925562	TTC28
ENSG00000176659.9	-0.142570068	0.11328639	0.497925562	C20orf197
ENSG00000267096.1	-0.191242045	0.113288506	0.497925562	CTD-2537I9.13
ENSG00000211949.3	0.31574183	0.113319605	0.497955531	IGHV3-23
ENSG00000039123.16	-0.090286528	0.113359841	0.498019343	MTREX
ENSG00000175707.9	0.179198145	0.113382695	0.498019343	KDF1
ENSG00000176142.13	-0.055167796	0.113448859	0.49812805	TMEM39A
ENSG00000106952.8	0.091251854	0.113496972	0.49812805	TNFSF8
ENSG00000134291.12	-0.079152562	0.113504456	0.49812805	TMEM106C
ENSG00000181666.18	0.081040684	0.113526215	0.49812805	ZNF875
ENSG00000142892.15	-0.111063393	0.113528891	0.49812805	PIGK
ENSG00000259865.1	-0.179615997	0.113571811	0.498209775	RP11-488L18.10
ENSG00000149929.16	0.095241213	0.113649009	0.498361348	HIRIP3
ENSG00000170298.16	0.440765815	0.113667987	0.498361348	LGALS9B
ENSG00000092853.14	-0.204948336	0.113679266	0.498361348	CLSPN
ENSG00000085998.14	0.060969645	0.113731598	0.498407123	POMGNT1
ENSG00000138448.12	-0.169936225	0.113761556	0.498407123	ITGAV
ENSG00000223855.2	0.256042033	0.113762617	0.498407123	HRAT92
ENSG00000152942.19	-0.059537875	0.113802232	0.498444742	RAD17
ENSG00000180139.11	-0.237376926	0.113820115	0.498444742	ACTA2-AS1
ENSG00000005194.15	0.060664852	0.113854347	0.498444742	CIAPIN1
ENSG00000279662.1	-0.341598043	0.113868423	0.498444742	RP11-609N14.4
ENSG00000196421.9	-0.309116935	0.11397069	0.498635914	C20orf204
ENSG00000232818.2	0.208690539	0.11404816	0.498635914	RPS2P32
ENSG00000144306.15	-0.092514716	0.114113455	0.498635914	SCRN3
ENSG00000157303.11	0.119704078	0.114144276	0.498635914	SUSD3
ENSG00000161203.13	0.07876608	0.114170821	0.498635914	AP2M1
ENSG00000285053.1	-0.157263911	0.114181819	0.498635914	TBCE
ENSG00000189042.14	-0.141345526	0.114223643	0.498635914	ZNF567
ENSG00000124702.18	0.069763505	0.114235202	0.498635914	KLHDC3
ENSG00000154188.10	-0.269617024	0.114237366	0.498635914	ANGPT1
ENSG00000233527.9	0.116523674	0.114238349	0.498635914	ZNF529-AS1
ENSG00000133454.16	-0.679655262	0.114250342	0.498635914	MYO18B
ENSG00000257242.8	-0.160094636	0.114290214	0.498635914	LINC01619

ENSG00000280231.1	0.218803982	0.11431202	0.498635914	LA16c-380F5.3
ENSG00000242259.9	-0.04682441	0.114317196	0.498635914	C22orf39
ENSG00000035681.9	-0.050032474	0.114334418	0.498635914	NSMAF
ENSG00000079974.18	0.061464086	0.114352765	0.498635914	RABL2B
ENSG00000177051.6	0.076222544	0.11435505	0.498635914	FBXO46
ENSG00000267422.1	0.217272675	0.114359821	0.498635914	CTD-2554C21.1
ENSG00000282965.1	1.028587844	0.114410946	0.498635914	RP11-683L23.7
ENSG00000273265.1	0.178791645	0.11441635	0.498635914	CNNM3-DT
ENSG00000279283.1	-0.215375756	0.114422694	0.498635914	RP11-417L19.5
ENSG00000106636.9	0.044723936	0.114507573	0.498893814	YKT6
ENSG00000271646.1	0.091576191	0.114546948	0.498893814	IRF2-DT
ENSG00000135049.16	-0.088155594	0.114555776	0.498893814	AGTPBP1
ENSG00000175634.15	0.065114292	0.11458016	0.498893814	RPS6KB2
ENSG00000063176.16	0.088418658	0.114664574	0.498893814	SPHK2
ENSG00000104738.18	-0.114523635	0.114709351	0.498893814	MCM4
ENSG00000103496.15	0.069009881	0.114719352	0.498893814	STX4
ENSG00000171204.13	-0.125323803	0.114737377	0.498893814	TMEM126B
ENSG00000080007.8	-0.332660846	0.11473823	0.498893814	DDX43
ENSG00000266777.1	-0.138322598	0.114741609	0.498893814	SH3GL1P1
ENSG00000178202.13	-0.109273298	0.114756031	0.498893814	POGLUT3
ENSG00000116489.13	-0.12003218	0.114773796	0.498893814	CAPZA1
ENSG00000204438.11	0.072780238	0.114817022	0.498975946	GPANK1
ENSG00000151702.17	-0.055083529	0.114907862	0.499176361	FLI1
ENSG00000106069.23	-0.24137528	0.11491182	0.499176361	CHN2
ENSG00000124459.12	-0.079811673	0.11498555	0.499390862	ZNF45
ENSG00000268618.1	-0.336477988	0.115045674	0.499546195	AC092316.1
ENSG00000213453.3	0.185495978	0.115168146	0.499729662	FTH1P3
ENSG00000139131.13	0.069464148	0.115175888	0.499729662	YARS2
ENSG00000221510.1	0.388372077	0.115179214	0.499729662	MIR548O
ENSG00000062485.19	0.058986385	0.115185397	0.499729662	CS
ENSG00000136933.17	0.077605055	0.115234206	0.49976278	RABEPK
ENSG00000258826.6	-0.232174786	0.115270072	0.49976278	LINC01147
ENSG00000269656.1	-0.26051398	0.115277102	0.49976278	NOP53-AS1
ENSG00000137876.11	-0.248103852	0.115304067	0.49976278	RSL24D1
ENSG00000125388.20	-0.142078348	0.115314876	0.49976278	GRK4
ENSG00000167703.15	0.098578729	0.115414748	0.500068166	SLC43A2
ENSG00000171488.15	-0.070666873	0.115434108	0.500068166	LRRC8C
ENSG00000087191.13	0.070808936	0.115529531	0.500360032	PSMC5
ENSG00000070756.17	0.051004988	0.115550278	0.500360032	PABPC1
ENSG00000226471.6	-0.171620348	0.115680076	0.500586291	CTA-292E10.6
ENSG00000276103.1	-0.328123278	0.115695159	0.500586291	U4
ENSG00000163322.14	-0.111026546	0.115717666	0.500586291	ABRAXAS1
ENSG00000198589.14	-0.092246599	0.11571861	0.500586291	LRBA
ENSG00000107438.9	0.157955621	0.115724576	0.500586291	PDLIM1
ENSG00000104299.15	0.061333604	0.115770608	0.500624306	INTS9
ENSG00000254999.4	0.084847999	0.115816566	0.500624306	BRK1
ENSG00000048392.12	-0.120988803	0.115842279	0.500624306	RRM2B

ENSG00000106546.14	-0.124431387	0.115883576	0.500624306	AHR
ENSG00000272568.6	0.416165156	0.115927598	0.500624306	CTB-113D17.1
ENSG00000186283.14	0.065561254	0.11593439	0.500624306	TOR3A
ENSG00000147889.18	-0.187622562	0.115964011	0.500624306	CDKN2A
ENSG00000198162.12	-0.083706058	0.115986981	0.500624306	MAN1A2
ENSG00000227598.1	-0.228336523	0.115989328	0.500624306	RP1-167A14.2
ENSG00000100485.12	-0.092152269	0.115989489	0.500624306	SOS2
ENSG00000283312.1	-0.372398984	0.116001887	0.500624306	RP11-562I5.1
ENSG00000184863.11	-0.062057822	0.116058452	0.500763041	RBM33
ENSG00000254098.1	0.511833819	0.116114395	0.500861236	IGKV2-26
ENSG00000007047.15	0.111605379	0.116130055	0.500861236	MARK4
ENSG00000175087.10	-0.121947048	0.116167879	0.500902837	PDIK1L
ENSG00000228079.2	-0.270567954	0.116192385	0.500902837	AC012368.1
ENSG00000237877.7	-0.297505507	0.116237497	0.500902837	LINC01473
ENSG00000151117.9	0.12974055	0.116259099	0.500902837	TMEM86A
ENSG00000120885.22	0.246306631	0.116261825	0.500902837	CLU
ENSG00000121797.10	0.125220105	0.116297942	0.500941177	CCRL2
ENSG00000165119.21	0.041642864	0.116319577	0.500941177	HNRNPK
ENSG00000136051.15	-0.144159948	0.116372438	0.501063606	WASHC4
ENSG00000273015.2	-0.089726503	0.116458313	0.501104884	RP11-352M15.2
ENSG00000130726.12	0.084438872	0.116470301	0.501104884	TRIM28
ENSG00000115594.12	-0.130136692	0.1164774	0.501104884	IL1R1
ENSG00000264522.6	-0.098918501	0.116492033	0.501104884	OTUD7B
ENSG00000184545.11	-0.201534143	0.116504198	0.501104884	DUSP8
ENSG00000278949.1	-0.172667469	0.116576792	0.501311986	RP11-46H11.11
ENSG00000228589.3	0.171323369	0.116722083	0.501831547	SPCS2P4
ENSG00000105058.12	0.057885912	0.116780575	0.50197779	FAM32A
ENSG00000110321.19	-0.052129674	0.116858914	0.502022521	EIF4G2
ENSG00000177359.20	-0.662215914	0.116901269	0.502022521	OVOS2
ENSG00000104918.8	-0.269717929	0.11690398	0.502022521	RETN
ENSG00000181524.6	0.201907038	0.116906564	0.502022521	RPL24P4
ENSG00000258655.2	0.236357319	0.116913378	0.502022521	ARHGAP5-AS1
ENSG00000213939.4	0.257566055	0.116943894	0.502048436	RP11-314A20.1
ENSG00000081026.19	-0.129930763	0.116991228	0.502146527	MAGI3
ENSG00000135324.6	-0.979409239	0.117088249	0.502402129	MRAP2
ENSG00000203965.13	-0.164155817	0.117126133	0.502402129	EFCAB7
ENSG00000020129.16	0.083162986	0.117160433	0.502402129	NCDN
ENSG00000115904.14	-0.075150665	0.117203652	0.502402129	SOS1
ENSG00000237510.7	0.780333953	0.117208187	0.502402129	GPAT2P1
ENSG00000275700.6	0.077959804	0.117258095	0.502402129	AATF
ENSG00000106780.9	-0.097107246	0.117297641	0.502402129	MEGF9
ENSG00000110031.13	0.061910014	0.117349348	0.502402129	LPXN
ENSG00000121454.6	-0.191801859	0.117367534	0.502402129	LHX4
ENSG00000158023.10	0.192885008	0.117399739	0.502402129	CFAP251
ENSG00000157429.16	0.12129167	0.11740261	0.502402129	ZNF19
ENSG00000259436.1	0.108584008	0.117418622	0.502402129	CTC-378H22.2
ENSG00000238278.3	-0.26757435	0.117433404	0.502402129	ALG1L6P

ENSG00000170390.16	0.379929979	0.117447109	0.502402129	DCLK2
ENSG00000181619.11	-0.233523235	0.117463959	0.502402129	GPR135
ENSG00000179958.10	0.093158082	0.117467611	0.502402129	DCTPP1
ENSG00000146112.12	0.07631252	0.117489023	0.502402129	PPP1R18
ENSG00000146828.18	0.07527039	0.117506332	0.502402129	SLC12A9
ENSG00000122482.21	-0.142834024	0.117516238	0.502402129	ZNF644
ENSG00000197535.15	-0.082132299	0.117644511	0.502845691	MYO5A
ENSG00000189013.14	0.323619784	0.117734797	0.503123734	KIR2DL4
ENSG00000068697.7	0.064757606	0.117758627	0.503123734	LAPTM4A
ENSG00000171606.18	0.053182892	0.117828231	0.503162475	ZNF274
ENSG00000104983.8	0.086704159	0.117859159	0.503162475	CCDC61
ENSG00000084073.10	-0.069905074	0.117880991	0.503162475	ZMPSTE24
ENSG00000173113.7	0.093273362	0.117886607	0.503162475	TRMT112
ENSG00000163933.10	0.066100219	0.11792592	0.503162475	RFT1
ENSG00000095564.14	-0.132909807	0.117969721	0.503162475	BTAF1
ENSG00000248554.1	0.305658901	0.117979628	0.503162475	C5orf34-AS1
ENSG00000197562.10	0.080068392	0.118005431	0.503162475	RAB40C
ENSG00000270562.1	0.132781894	0.118008072	0.503162475	RP11-154H23.3
ENSG00000058729.11	-0.107284919	0.118013044	0.503162475	RIOK2
ENSG00000106327.13	-0.137001533	0.118079235	0.50330922	TFR2
ENSG00000006695.12	0.05739335	0.118096546	0.50330922	COX10
ENSG00000094914.14	0.066050152	0.118155031	0.503411515	AAAS
ENSG00000185278.15	-0.100053625	0.118178144	0.503411515	ZBTB37
ENSG00000059769.20	-0.13306286	0.118200552	0.503411515	DNAJC25
ENSG00000129292.21	-0.117825165	0.118218737	0.503411515	PHF20L1
ENSG00000203907.10	-0.525891361	0.118289057	0.503548065	OOEP
ENSG00000140859.16	0.162085334	0.118299911	0.503548065	KIFC3
ENSG00000254165.1	-0.300793831	0.118357447	0.503677162	RP11-503E24.2
ENSG00000180376.17	-0.105708761	0.118380927	0.503677162	CCDC66
ENSG00000100906.11	0.098553639	0.118439163	0.503677162	NFKBIA
ENSG00000229222.1	-0.282369277	0.118464038	0.503677162	KRT18P4
ENSG00000058799.15	0.077721992	0.118481909	0.503677162	YIPF1
ENSG00000211789.2	-0.205444455	0.118514992	0.503677162	TRAV12-2
ENSG00000273987.1	-0.306188241	0.118522426	0.503677162	RP11-585P4.6
ENSG00000170881.5	-0.081738561	0.11852672	0.503677162	RNF139
ENSG00000151332.19	-0.100337759	0.118601452	0.503727382	MBIP
ENSG00000287586.1	0.236773637	0.118602016	0.503727382	RP1-77N19.1
ENSG00000065609.14	1.395810713	0.118612226	0.503727382	SNAP91
ENSG00000144597.14	-0.060311081	0.118672869	0.503790277	EAF1
ENSG00000246627.6	-0.147084697	0.118693786	0.503790277	CACNA1C-AS1
ENSG00000120696.9	-0.12512093	0.118719441	0.503790277	KBTBD7
ENSG00000248636.7	0.293811084	0.118789097	0.503790277	RP11-768F21.1
ENSG00000287543.1	0.189637017	0.118822611	0.503790277	RP11-941F15.2
ENSG00000196787.3	0.181651782	0.118840126	0.503790277	H2AC11
ENSG00000271122.1	-0.119627793	0.118845355	0.503790277	HERPUD2-AS1
ENSG00000014919.13	0.051369686	0.118846612	0.503790277	COX15
ENSG00000028137.19	0.078332894	0.118848126	0.503790277	TNFRSF1B

ENSG00000164535.15	0.07316656	0.118899223	0.50389159	DAGLB
ENSG00000236772.1	-0.263937608	0.119002142	0.50389159	RP5-1184F4.5
ENSG00000091136.15	-0.136336855	0.11901877	0.50389159	LAMB1
ENSG00000143553.11	0.068002513	0.119032316	0.50389159	SNAPIN
ENSG00000103811.18	0.109362363	0.119035897	0.50389159	CTSH
ENSG00000121073.15	0.05571586	0.119056341	0.50389159	SLC35B1
ENSG00000184220.12	0.106947866	0.119109367	0.50389159	CMSS1
ENSG00000121769.8	-0.154567528	0.119126462	0.50389159	FABP3
ENSG00000131876.17	-0.056988076	0.119143514	0.50389159	SNRPA1
ENSG00000001167.15	-0.06274072	0.119145981	0.50389159	NFYA
ENSG00000186827.11	0.201686667	0.119149327	0.50389159	TNFRSF4
ENSG00000177272.9	-0.09540868	0.119172324	0.50389159	KCNA3
ENSG00000197608.12	-0.08352184	0.119191442	0.50389159	ZNF841
ENSG00000005238.20	0.068560552	0.119244797	0.503915217	FAM214B
ENSG00000170954.12	0.319503736	0.119274105	0.503915217	ZNF415
ENSG00000122870.12	0.402500456	0.119283769	0.503915217	BICC1
ENSG00000065882.16	0.070908955	0.119295318	0.503915217	TBC1D1
ENSG00000215835.2	0.217488747	0.119380071	0.503988231	RP11-9L18.2
ENSG00000062524.16	0.248418976	0.119402381	0.503988231	LTK
ENSG00000143457.11	-0.059681701	0.119405109	0.503988231	GOLPH3L
ENSG00000115526.11	-0.122214214	0.119472978	0.503988231	CHST10
ENSG00000105135.16	0.079153488	0.119475837	0.503988231	ILVBL
ENSG00000197980.13	0.384188277	0.119480538	0.503988231	LEKR1
ENSG00000206177.7	-0.325140696	0.119484629	0.503988231	HBM
ENSG00000272077.1	-0.176534166	0.119516073	0.504017195	RP11-348P10.2
ENSG00000213178.3	0.280393793	0.119568967	0.50403531	RPL22P1
ENSG00000273249.1	0.289730855	0.119569523	0.50403531	WDR5-DT
ENSG00000162772.17	0.276995971	0.119595804	0.504042488	ATF3
ENSG00000167130.18	0.064798822	0.1196593	0.504079472	DOLPP1
ENSG00000035928.16	-0.049557515	0.119678492	0.504079472	RFC1
ENSG00000149809.17	0.101427494	0.11970274	0.504079472	TM7SF2
ENSG00000198862.14	-0.1391668	0.119702898	0.504079472	LTN1
ENSG00000283297.2	-0.146288728	0.119792325	0.504140138	TEX52
ENSG00000228653.2	-0.317827696	0.119818732	0.504140138	HNRNPCP7
ENSG00000164221.13	-0.129029421	0.119860022	0.504140138	CCDC112
ENSG00000227097.5	0.495918713	0.119882508	0.504140138	RPS28P7
ENSG00000179528.16	-0.264097961	0.11988713	0.504140138	LBX2
ENSG00000272325.2	0.099873127	0.119890638	0.504140138	NUDT3
ENSG00000234936.1	0.145307738	0.119933645	0.504140138	AC010883.5
ENSG00000031823.14	0.057070836	0.11998262	0.504140138	RANBP3
ENSG00000268362.6	0.168449446	0.120032151	0.504140138	CTD-2017D11.1
ENSG00000178982.10	0.083381911	0.120033289	0.504140138	EIF3K
ENSG00000166428.14	0.169729117	0.120045152	0.504140138	PLD4
ENSG00000145912.9	0.089674591	0.120048236	0.504140138	NHP2
ENSG00000204315.4	0.10517936	0.120050684	0.504140138	FKBPL
ENSG00000107262.26	-0.192223858	0.120114783	0.504140138	BAG1
ENSG00000225476.1	0.468740976	0.120123436	0.504140138	MTCO3P5

ENSG00000101146.13	0.049061285	0.120130408	0.504140138	RAE1
ENSG00000180245.7	-0.355577404	0.120135208	0.504140138	RRH
ENSG00000238231.2	0.355749529	0.120265971	0.504206812	RP11-460I13.6
ENSG00000151093.8	-0.102286148	0.120266789	0.504206812	OXSM
ENSG00000198160.15	-0.108889222	0.120270349	0.504206812	MIER1
ENSG00000185340.15	0.16221556	0.120280903	0.504206812	GAS2L1
ENSG00000154118.13	-0.341214222	0.120365701	0.504206812	JPH3
ENSG00000156384.15	-0.127730607	0.120401552	0.504206812	SFR1
ENSG00000275183.2	0.115183248	0.120432275	0.504206812	LENG9
ENSG00000124006.15	0.283172792	0.120466076	0.504206812	OBSL1
ENSG00000138326.21	-0.126918526	0.120478919	0.504206812	RPS24
ENSG00000242611.2	0.608451571	0.12048636	0.504206812	AC093627.8
ENSG00000161643.13	0.148800159	0.120499244	0.504206812	SIGLEC16
ENSG00000168040.5	0.097218847	0.120519551	0.504206812	FADD
ENSG00000114026.22	0.051631284	0.120559764	0.504206812	OGG1
ENSG00000128000.17	-0.111202018	0.12056077	0.504206812	ZNF780B
ENSG00000251450.2	-0.227568877	0.120585843	0.504206812	RASGRF2-AS1
ENSG00000259972.3	-0.07236254	0.120588296	0.504206812	AC009120.6
ENSG00000260001.7	0.351993348	0.120611471	0.504206812	TGFBR3L
ENSG00000244575.3	-0.381105088	0.120627476	0.504206812	IGKV1-27
ENSG00000100300.18	0.127767295	0.120649661	0.504206812	TSPO
ENSG00000213918.11	-0.068522236	0.12066515	0.504206812	DNASE1
ENSG00000107651.14	-0.060353362	0.120678402	0.504206812	SEC23IP
ENSG00000138376.11	-0.135753478	0.120691986	0.504206812	BARD1
ENSG00000196693.15	-0.080589224	0.120735655	0.504286518	ZNF33B
ENSG00000100284.22	0.099947225	0.120804617	0.504404322	TOM1
ENSG00000134371.14	-0.072090886	0.12081305	0.504404322	CDC73
ENSG00000269892.1	0.14437139	0.120855311	0.50447806	RP4-761J14.10
ENSG00000166503.9	-0.131339476	0.12090174	0.504569166	HDGFL3
ENSG00000159685.11	0.124921854	0.120994455	0.504780109	CHCHD6
ENSG00000175806.15	0.101442374	0.121002431	0.504780109	MSRA
ENSG00000152782.17	0.21534276	0.121026126	0.504780109	PANK1
ENSG00000280411.1	0.377596271	0.12105817	0.504811091	IGHV1-69D
ENSG00000187534.6	0.112748297	0.121170265	0.505085179	PRR13P5
ENSG00000213977.8	0.105068131	0.121207891	0.505085179	TAX1BP3
ENSG00000178440.8	-0.288780892	0.121210505	0.505085179	TIMM23B-AGAP6
ENSG00000182247.10	0.092248824	0.121239181	0.505085179	UBE2E2
ENSG00000163141.20	-0.116483045	0.121250366	0.505085179	BNIPL
ENSG00000168374.11	0.05204329	0.121271671	0.505085179	ARF4
ENSG00000100353.18	0.056098981	0.121317701	0.505138841	EIF3D
ENSG00000060140.9	-0.296441532	0.121339642	0.505138841	STYK1
ENSG00000091947.10	0.073629422	0.121358449	0.505138841	TMEM101
ENSG00000232545.1	-0.340067209	0.121501619	0.505524503	KB-318B8.7
ENSG00000126777.18	-0.130226277	0.121547908	0.505524503	KTN1
ENSG00000145476.16	-0.124826288	0.121548874	0.505524503	CYP4V2
ENSG00000135446.17	0.07010464	0.121557695	0.505524503	CDK4
ENSG00000143643.13	-0.063061172	0.121616097	0.505524503	TTC13

ENSG00000256987.1	-0.31944213	0.121623943	0.505524503	RP11-428G5.7
ENSG00000126467.11	0.420480804	0.121647817	0.505524503	TSKS
ENSG00000172086.9	-0.141644144	0.121672192	0.505524503	KRCC1
ENSG00000227036.8	0.345374185	0.121681598	0.505524503	LINC00511
ENSG00000227063.5	-0.287860234	0.121703005	0.505524503	RPL41P1
ENSG00000163577.8	-0.13462634	0.121722255	0.505524503	EIF5A2
ENSG00000078177.14	-0.146653361	0.121814133	0.505803653	N4BP2
ENSG00000170471.15	-0.05648874	0.12188266	0.505985746	RALGAPB
ENSG00000158863.22	0.066357587	0.121912893	0.506008828	FAM160B2
ENSG00000271151.1	0.150015154	0.121956137	0.506085889	RP11-394I13.2
ENSG00000250144.1	0.380438608	0.12201006	0.506104609	RP11-553P9.1
ENSG00000170293.9	0.213251152	0.122040056	0.506104609	CMTM8
ENSG00000075131.10	-0.136737945	0.12207068	0.506104609	TIPIN
ENSG00000138119.17	0.181027477	0.122091004	0.506104609	MYOF
ENSG00000168653.11	0.164300559	0.122102454	0.506104609	NDUFS5
ENSG00000106682.16	0.038237698	0.122110917	0.506104609	EIF4H
ENSG00000133048.13	0.277183576	0.122133397	0.506104609	CHI3L1
ENSG00000131653.13	0.05910356	0.122183855	0.506134961	TRAF7
ENSG00000155093.19	0.200292886	0.122210833	0.506134961	PTPRN2
ENSG00000255284.2	-0.14654923	0.122214762	0.506134961	AP006621.5
ENSG00000124562.10	0.089583084	0.122370684	0.506643086	SNRPC
ENSG00000088827.13	0.447377087	0.12238851	0.506643086	SIGLEC1
ENSG00000257027.1	-0.08593169	0.122412376	0.506643086	RP11-705C15.3
ENSG00000121022.14	-0.065645882	0.122436275	0.506643086	COPS5
ENSG00000204220.12	0.076008974	0.122537303	0.506879155	PFDN6
ENSG00000126561.18	0.065687573	0.122551871	0.506879155	STAT5A
ENSG00000288558.2	0.086110906	0.122589083	0.506879155	DUS4L-BCAP29
ENSG00000158669.11	0.043447126	0.122592189	0.506879155	GPAT4
ENSG00000211645.2	0.478773089	0.122623486	0.506906358	IGLV1-50
ENSG00000104129.10	0.069386228	0.122704021	0.507137052	DNAJC17
ENSG00000272054.1	-0.198677655	0.122815835	0.507496907	RP11-423P10.2
ENSG00000163535.18	-0.189294801	0.122859415	0.507529654	SGO2
ENSG00000270259.2	0.546985211	0.122873256	0.507529654	RP13-122B23.9
ENSG00000158445.10	-0.159043033	0.122900143	0.507538488	KCNB1
ENSG00000173575.22	-0.063275076	0.123017104	0.507743297	CHD2
ENSG00000146670.10	-0.203673606	0.123018235	0.507743297	CDCA5
ENSG00000103479.17	-0.085896113	0.123024012	0.507743297	RBL2
ENSG00000143314.12	0.092644389	0.123077217	0.507860679	MRPL24
ENSG00000153767.10	-0.085703329	0.123215483	0.508107908	GTF2E1
ENSG00000107372.13	-0.070745662	0.123232226	0.508107908	ZFAND5
ENSG00000172531.16	0.076912	0.12323545	0.508107908	PPP1CA
ENSG00000140545.15	0.124421021	0.12324099	0.508107908	MFGE8
ENSG00000257267.3	-0.097059873	0.123261012	0.508107908	ZNF271P
ENSG00000134489.7	0.250651158	0.123309362	0.508111644	HRH4
ENSG00000272849.1	0.107650668	0.123371495	0.508111644	RP11-347I19.8
ENSG00000275772.1	-0.259172822	0.123392984	0.508111644	LL22NC03-24A12.9
ENSG00000259479.6	0.258385713	0.123433079	0.508111644	SORD2P

ENSG00000205208.5	-0.093233131	0.123436494	0.508111644	C4orf46
ENSG00000137497.18	0.077162844	0.12345303	0.508111644	NUMA1
ENSG00000142507.10	0.097262361	0.123467373	0.508111644	PSMB6
ENSG00000166947.15	-0.341255706	0.123475088	0.508111644	EPB42
ENSG00000145979.18	0.107300437	0.123484905	0.508111644	TBC1D7
ENSG00000028277.21	0.080552934	0.123564236	0.508336079	POU2F2
ENSG00000153066.13	0.050456173	0.123650208	0.508533285	TXNDC11
ENSG00000259709.1	-0.45524685	0.123667645	0.508533285	CTD-2184D3.7
ENSG00000166796.12	-1.151854481	0.123686563	0.508533285	LDHC
ENSG00000288109.1	0.566102173	0.123746036	0.508675827	RP11-360N9.5
ENSG00000166762.19	-0.111726503	0.123775081	0.508693258	CATSPER2
ENSG00000188011.5	-0.326296278	0.123848891	0.508894621	RTP5
ENSG00000285669.1	-0.162438827	0.123893056	0.508922262	RP11-51J9.7
ENSG00000056736.10	-0.143567973	0.123917209	0.508922262	IL17RB
ENSG00000254389.3	0.327089712	0.123930065	0.508922262	RHPN1-AS1
ENSG00000241057.2	0.243554468	0.124050835	0.509295619	AC004985.12
ENSG00000085491.17	-0.127755198	0.124070651	0.509295619	SLC25A24
ENSG00000169403.12	0.085378053	0.12411444	0.509342585	PTAFR
ENSG00000168101.14	0.084914037	0.124131765	0.509342585	NUDT16L1
ENSG00000119917.15	0.344268313	0.124166387	0.509382728	IFIT3
ENSG00000267575.7	0.070732185	0.124256642	0.509434528	CTC-459F4.3
ENSG00000126878.13	0.369814541	0.124261476	0.509434528	AIF1L
ENSG00000138660.12	-0.123866693	0.124290675	0.509434528	AP1AR
ENSG00000165732.13	-0.064091645	0.124295132	0.509434528	DDX21
ENSG00000199082.1	0.281186395	0.124312288	0.509434528	MIR342
ENSG00000025434.19	0.107110922	0.124340978	0.509434528	NR1H3
ENSG00000254332.1	0.24527417	0.124354755	0.509434528	GS1-44D20.1
ENSG00000269858.6	0.0730893	0.124387422	0.509434528	EGLN2
ENSG00000005801.18	-0.0907521	0.124451437	0.509434528	ZNF195
ENSG00000118217.7	0.069573986	0.124454996	0.509434528	ATF6
ENSG00000197870.12	0.469006991	0.124466195	0.509434528	PRB3
ENSG00000275807.1	0.24095094	0.124477103	0.509434528	RP11-1348G14.8
ENSG00000275549.1	0.272143587	0.124503966	0.509442806	STPG3-AS1
ENSG00000092010.15	0.104787081	0.124566738	0.509597976	PSME1
ENSG00000118939.18	0.179427382	0.124621636	0.509602161	UCHL3
ENSG00000149451.18	-0.496182534	0.124630324	0.509602161	ADAM33
ENSG00000140022.13	0.167019277	0.124651728	0.509602161	STON2
ENSG00000188747.9	0.136691273	0.124693009	0.509602161	NOXA1
ENSG00000117477.12	-0.236361941	0.124703365	0.509602161	CCDC181
ENSG00000075618.18	0.148893396	0.124751504	0.509602161	FSCN1
ENSG00000236901.6	0.169124183	0.124757684	0.509602161	MIR600HG
ENSG00000008405.12	-0.083492405	0.124766552	0.509602161	CRY1
ENSG00000277246.1	0.231804371	0.124812211	0.509687141	RP11-153I24.4
ENSG00000270959.1	0.137222251	0.124893437	0.509917303	LPP-AS2
ENSG00000141956.14	-0.07381681	0.124926782	0.509951919	PRDM15
ENSG00000134323.12	-0.435499009	0.124982814	0.510079114	MYCN
ENSG00000218357.3	0.403524469	0.12503483	0.510108509	LINC01644

ENSG00000196565.15	0.60663537	0.125063747	0.510108509	HBG2
ENSG00000137760.15	-0.092366928	0.125066502	0.510108509	ALKBH8
ENSG00000119541.10	-0.065463003	0.12509841	0.510108509	VPS4B
ENSG00000261098.1	-0.149720189	0.125127705	0.510108509	RP11-819C21.1
ENSG00000109943.9	-0.172374093	0.125157481	0.510108509	CRTAM
ENSG00000211892.4	-0.454692824	0.125169717	0.510108509	IGHG4
ENSG00000181804.15	0.075549803	0.125241961	0.510108509	SLC9A9
ENSG00000158481.13	0.142930205	0.125253767	0.510108509	CD1C
ENSG00000164163.11	-0.095137596	0.12526311	0.510108509	ABCE1
ENSG00000131941.8	-0.245388203	0.12526467	0.510108509	RHPN2
ENSG00000197077.14	-0.253311712	0.1252885	0.510108509	KIAA1671
ENSG00000257924.1	0.541904487	0.1253634	0.510312148	LINC02416
ENSG00000100354.21	-0.069279192	0.125426856	0.510469135	TNRC6B
ENSG00000115977.21	-0.064075887	0.125530237	0.510788511	AAK1
ENSG00000141499.17	0.074359817	0.125571854	0.510856494	WRAP53
ENSG00000114098.18	-0.04696274	0.12563662	0.511006614	ARMC8
ENSG00000249141.1	0.297776918	0.125725448	0.511006614	RP11-514O12.4
ENSG00000101425.14	-0.372467776	0.125735368	0.511006614	BPI
ENSG00000259728.5	0.388396166	0.125736172	0.511006614	LINC00933
ENSG00000248874.5	1.414718055	0.125756745	0.511006614	C5orf17
ENSG00000226856.7	0.552729015	0.125792217	0.511006614	THORLNC
ENSG00000185721.13	0.071454937	0.125797947	0.511006614	DRG1
ENSG00000073584.20	-0.072851849	0.125808094	0.511006614	SMARCE1
ENSG00000188277.10	-0.085986333	0.125902971	0.511183587	C15orf62
ENSG00000225963.8	-0.222109929	0.125911587	0.511183587	AC009950.2
ENSG00000181026.15	0.071382955	0.125926443	0.511183587	AEN
ENSG00000164603.12	-0.110203772	0.126024636	0.511431698	BMT2
ENSG00000253326.2	0.325786492	0.126037439	0.511431698	RP11-261C10.7
ENSG00000203875.13	0.17610857	0.126088175	0.511536359	SNHG5
ENSG00000239559.2	0.369666423	0.126163222	0.511616781	RPL37P2
ENSG00000158296.14	-0.269970834	0.126183446	0.511616781	SLC13A3
ENSG00000258130.8	-0.36237379	0.126196731	0.511616781	RP11-347C12.3
ENSG00000100226.16	0.074377752	0.126244999	0.511616781	GTPBP1
ENSG00000244300.3	0.391288223	0.126261152	0.511616781	GATA2-AS1
ENSG00000213983.12	0.072838342	0.126276816	0.511616781	AP1G2
ENSG00000284428.1	-0.119564683	0.126282628	0.511616781	RP11-15H20.6
ENSG00000181315.11	-0.15762172	0.126329789	0.511701193	ZNF322
ENSG00000136731.13	-0.063205269	0.126353901	0.511701193	UGGT1
ENSG00000148219.18	-0.129580262	0.126378318	0.511701193	ASTN2
ENSG00000246100.4	0.313329502	0.126405272	0.5117093	LINC00900
ENSG00000186130.5	-0.106764372	0.126435556	0.511730883	ZBTB6
ENSG00000160783.19	0.086819055	0.126475948	0.511762687	PMF1
ENSG00000162702.8	-0.109514487	0.126493323	0.511762687	ZNF281
ENSG00000074071.15	0.094375848	0.12664111	0.512259543	MRPS34
ENSG00000145936.9	0.128793133	0.126674661	0.512294212	KCNMB1
ENSG00000140263.15	0.112005367	0.126755779	0.512436939	SORD
ENSG00000133111.4	-0.092419016	0.126759927	0.512436939	RFXAP

ENSG00000189195.15	-0.171333453	0.126821198	0.512583589	BTBD8
ENSG00000273340.1	-0.193837492	0.126936799	0.512683636	MICE
ENSG00000211945.2	0.323966016	0.126997649	0.512683636	IGHV1-18
ENSG00000138380.18	-0.12423607	0.126997808	0.512683636	CARF
ENSG00000241657.1	0.2454052	0.12700372	0.512683636	TRBV11-2
ENSG00000103415.12	0.075280379	0.127011615	0.512683636	HMOX2
ENSG00000125753.14	0.092313981	0.127025572	0.512683636	VASP
ENSG00000251669.6	0.185960858	0.127038813	0.512683636	FAM86EP
ENSG00000134986.14	0.14259449	0.127045945	0.512683636	NREP
ENSG00000175166.17	0.062300821	0.127085592	0.512739333	PSMD2
ENSG00000235194.9	0.086721005	0.127109751	0.512739333	PPP1R3E
ENSG00000167625.11	0.068352939	0.127139183	0.512757203	ZNF526
ENSG00000140497.16	0.068607096	0.127214141	0.512771542	SCAMP2
ENSG00000131148.9	0.063323121	0.12721705	0.512771542	EMC8
ENSG00000135218.19	0.124083816	0.127217749	0.512771542	CD36
ENSG00000012124.17	0.228198879	0.12725307	0.512813118	CD22
ENSG00000130005.13	0.132156275	0.127338412	0.51295207	GAMT
ENSG00000096006.12	-0.414824849	0.127351689	0.51295207	CRISP3
ENSG00000167562.13	-0.082048494	0.127362587	0.51295207	ZNF701
ENSG00000177483.12	-0.240210692	0.127478251	0.513149298	RBM44
ENSG00000213790.2	0.259586779	0.127488519	0.513149298	OLA1P1
ENSG00000123342.16	0.226471546	0.12752647	0.513149298	MMP19
ENSG00000138433.16	0.09631822	0.12755658	0.513149298	CIR1
ENSG00000197451.12	0.057236206	0.127609927	0.513149298	HNRNPAB
ENSG00000047932.14	-0.105468794	0.127640705	0.513149298	GOPC
ENSG00000206384.10	-0.360732889	0.127657314	0.513149298	COL6A6
ENSG00000221821.4	0.136238355	0.127665783	0.513149298	C6orf226
ENSG00000183624.14	0.067366375	0.127685658	0.513149298	HMCES
ENSG00000174469.23	0.406368391	0.127688895	0.513149298	CNTNAP2
ENSG00000129197.15	-0.076341475	0.127720539	0.513149298	RPAIN
ENSG00000004455.17	0.039080828	0.127723981	0.513149298	AK2
ENSG00000132535.22	0.095815116	0.127736843	0.513149298	DLG4
ENSG00000172113.10	0.055196476	0.127785625	0.513244733	NME6
ENSG00000284735.1	-0.260348691	0.127862499	0.513274483	RP4-736L20.4
ENSG00000240024.5	-0.157493395	0.12788449	0.513274483	LINC00888
ENSG00000110013.13	0.087204672	0.127917733	0.513274483	SIAE
ENSG00000140459.18	0.452524488	0.12793102	0.513274483	CYP11A1
ENSG00000184005.11	-0.167697409	0.127954904	0.513274483	ST6GALNAC3
ENSG00000240065.8	0.136174467	0.127958095	0.513274483	PSMB9
ENSG00000143033.18	-0.086341544	0.127978134	0.513274483	MTF2
ENSG00000246130.1	-0.309656198	0.127993257	0.513274483	RP11-875O11.2
ENSG00000258790.1	-0.326252985	0.128033276	0.513334588	RP11-561B11.2
ENSG00000108468.15	0.053577696	0.128261513	0.514096834	CBX1
ENSG00000008277.15	-0.133307856	0.128346394	0.514096834	ADAM22
ENSG00000213639.10	-0.106684398	0.128369935	0.514096834	PPP1CB
ENSG00000287063.1	0.410755498	0.128376671	0.514096834	RP11-384P7.10
ENSG00000105732.13	0.080029246	0.128383196	0.514096834	ZNF574

ENSG00000259881.1	0.147011633	0.128388208	0.514096834	RP11-830F9.5
ENSG00000068650.19	-0.096758191	0.128398868	0.514096834	ATP11A
ENSG00000137942.18	0.157875937	0.128475883	0.514304785	FNBP1L
ENSG00000283384.1	0.348128509	0.128504171	0.51431763	RP11-153I24.5
ENSG00000101608.13	0.080432693	0.128635715	0.514561555	MYL12A
ENSG00000115414.21	0.291007454	0.128641088	0.514561555	FN1
ENSG00000272476.1	0.341495566	0.128652676	0.514561555	RP1-191J18.66
ENSG00000227006.2	-0.345153777	0.12866548	0.514561555	RP5-956O18.2
ENSG00000279432.1	-0.21212608	0.128693854	0.514574684	RP11-135N5.3
ENSG00000167193.8	-0.077975214	0.128737719	0.514649736	CRK
ENSG00000168952.15	-0.514147868	0.128878938	0.51511387	STXBP6
ENSG00000272040.1	-0.139118653	0.128907567	0.515127898	CTC-366B18.4
ENSG00000176236.6	0.373034324	0.128961783	0.515189068	RPP38-DT
ENSG00000241553.13	0.094643201	0.128973117	0.515189068	ARPC4
ENSG00000147874.11	-0.093781276	0.129021629	0.515282485	HAUS6
ENSG00000183401.12	0.081119153	0.129113237	0.51554795	CCDC159
ENSG00000106609.16	0.038875952	0.129144046	0.515566724	TMEM248
ENSG00000167208.15	0.057183018	0.129182491	0.515566724	SNX20
ENSG00000146574.16	-0.145641796	0.129193358	0.515566724	CCZ1B
ENSG00000123329.20	0.079679539	0.129262647	0.515635432	ARHGAP9
ENSG00000164823.11	-0.114853216	0.129282021	0.515635432	OSGIN2
ENSG00000196591.12	-0.048659444	0.129286005	0.515635432	HDAC2
ENSG00000133250.14	0.106800906	0.129323377	0.515684195	ZNF414
ENSG00000171940.14	-0.093398087	0.12941234	0.515862409	ZNF217
ENSG00000065613.15	-0.099190604	0.129424041	0.515862409	SLK
ENSG00000134152.11	-0.131074364	0.129446992	0.515862409	KATNBL1
ENSG00000137486.17	0.072542783	0.129468686	0.515862409	ARRB1
ENSG00000232533.1	0.109007324	0.129527212	0.515995351	AC093673.5
ENSG00000164338.10	-0.08277562	0.129582431	0.516073479	UTP15
ENSG00000100097.12	0.119891913	0.129597153	0.516073479	LGALS1
ENSG00000104164.12	-0.097953435	0.129687124	0.516196466	BLOC1S6
ENSG00000113387.12	-0.164320511	0.129704064	0.516196466	SUB1
ENSG00000114904.13	-0.089870061	0.129717477	0.516196466	NEK4
ENSG00000264235.6	-0.210020051	0.12972872	0.516196466	MYL12-AS1
ENSG00000179921.15	0.13657622	0.129756023	0.516204951	GPBAR1
ENSG00000204410.16	-0.123935521	0.129892031	0.516555055	MSH5
ENSG00000143387.14	-0.107979742	0.12990528	0.516555055	CTSK
ENSG00000130175.10	0.076166242	0.129919591	0.516555055	PRKCSH
ENSG00000116685.16	0.072441454	0.129974139	0.516614426	KIAA2013
ENSG00000134460.18	0.136541307	0.130005105	0.516614426	IL2RA
ENSG00000134146.12	-0.100563901	0.130022864	0.516614426	DPH6
ENSG00000287763.1	0.664530958	0.130082263	0.516614426	RP11-153P14.1
ENSG00000286887.1	0.362422596	0.130084848	0.516614426	RP3-528L19.1
ENSG00000224067.2	0.242415212	0.130085669	0.516614426	RP11-430K21.2
ENSG00000129993.15	0.095857406	0.13014115	0.516732472	CBFA2T3
ENSG00000183977.14	-0.222608635	0.130186526	0.516732472	PP2D1
ENSG00000178700.9	-0.099281031	0.130260332	0.516732472	DHFR2

ENSG00000260828.1	-0.194182509	0.130271105	0.516732472	HMGB3P32
ENSG00000235488.1	-0.256755397	0.130292278	0.516732472	JARID2-AS1
ENSG00000172530.21	0.074296108	0.130301419	0.516732472	BANP
ENSG00000231365.6	-0.096596889	0.130306213	0.516732472	WARS2-AS1
ENSG00000214544.7	0.383798681	0.130316966	0.516732472	GTF2IRD2P1
ENSG00000196756.13	0.092436368	0.130353924	0.516779097	SNHG17
ENSG00000176182.6	0.094769904	0.130389667	0.516820892	MYPOP
ENSG00000178105.11	-0.066677407	0.130428344	0.516874294	DDX10
ENSG00000099834.19	0.223989092	0.130480179	0.516979815	CDHR5
ENSG00000050555.19	0.346944341	0.13060612	0.517378851	LAMC3
ENSG00000140350.15	0.051868506	0.130639956	0.517412942	ANP32A
ENSG00000141026.6	0.058889254	0.130674664	0.517450477	MED9
ENSG00000256427.2	-0.260494878	0.130774531	0.517691529	RP11-118B22.2
ENSG00000162572.21	0.133603788	0.130786026	0.517691529	SCNN1D
ENSG00000259236.1	0.246579546	0.130832968	0.517721647	GOLGA8VP
ENSG00000186281.12	0.359667095	0.130900245	0.517721647	GPAT2
ENSG00000182253.15	-0.3052597	0.130912659	0.517721647	SYNM
ENSG00000160404.18	0.073843489	0.130918295	0.517721647	TOR2A
ENSG00000166444.19	0.250422824	0.130954501	0.517721647	DENND2B
ENSG00000137714.3	-0.090037686	0.130956704	0.517721647	FDX1
ENSG00000196419.13	0.06702516	0.130982171	0.517721647	XRCC6
ENSG00000116396.15	-0.105762539	0.130995593	0.517721647	KCNC4
ENSG00000267100.1	0.073592101	0.131041206	0.517802131	ILF3-DT
ENSG00000144677.15	0.202042904	0.131135444	0.518074685	CTDSPL
ENSG00000185475.11	0.078098388	0.131230388	0.518349923	TMEM179B
ENSG00000163909.8	-0.337834144	0.131281825	0.518405206	HEYL
ENSG00000143554.14	0.100375221	0.131310127	0.518405206	SLC27A3
ENSG00000172775.17	0.040565274	0.131339432	0.518405206	PSME3IP1
ENSG00000225643.1	-0.221858842	0.131345497	0.518405206	RP11-70P17.1
ENSG00000287884.1	-0.305173418	0.131380677	0.518444282	LLNLR-229A6.1
ENSG00000279803.1	-0.275167889	0.131416687	0.51846006	RP11-407G23.5
ENSG00000272072.1	-0.217773866	0.131464272	0.51846006	CTA-363E19.2
ENSG00000163449.11	0.108914341	0.131470651	0.51846006	TMEM169
ENSG00000260641.1	0.253946801	0.131497571	0.51846006	RP11-1299A16.3
ENSG00000127948.16	0.09113142	0.13151108	0.51846006	POR
ENSG00000102543.14	-0.081537183	0.13162476	0.518808489	CDADC1
ENSG00000162614.19	0.203442988	0.131668915	0.518839068	NEXN
ENSG00000241464.3	-0.296011818	0.131712256	0.518839068	RPL39P38
ENSG00000138018.18	-0.087753936	0.131716368	0.518839068	SELENOI
ENSG00000188092.15	-0.087783408	0.131733715	0.518839068	GPR89B
ENSG00000282122.1	0.887636183	0.131828475	0.51911259	IGHV7-4-1
ENSG00000183978.8	0.097578502	0.131863662	0.519151464	COA3
ENSG00000284526.1	0.146837396	0.132094421	0.519960152	RP11-666A8.13
ENSG00000119396.11	-0.050134682	0.132124203	0.519977577	RAB14
ENSG00000184445.12	-0.08953453	0.132162153	0.520027136	KNTC1
ENSG00000101188.5	0.267364561	0.132257748	0.520242228	NTSR1
ENSG00000261114.1	-0.192202111	0.132281022	0.520242228	RP11-325K4.2

ENSG00000119314.16	-0.069801123	0.132301509	0.520242228	PTBP3
ENSG00000224078.15	-0.107535673	0.132318289	0.520242228	SNHG14
ENSG00000176533.13	0.138468406	0.132402404	0.520473165	GNG7
ENSG00000119953.14	-0.062269028	0.132463722	0.520507946	SMNDC1
ENSG00000258443.1	-0.369418142	0.132495548	0.520507946	RP3-414A15.11
ENSG00000118454.13	-0.092032249	0.132495718	0.520507946	ANKRD13C
ENSG00000075218.19	-0.203682752	0.132512775	0.520507946	GTSE1
ENSG00000271843.1	-0.246892141	0.132609346	0.520543319	RP11-245J9.5
ENSG00000182732.18	0.210422047	0.132720888	0.520543319	RGS6
ENSG00000083642.19	-0.070663397	0.132721708	0.520543319	PDS5B
ENSG00000145241.11	-0.079396743	0.132731009	0.520543319	CENPC
ENSG00000115241.11	0.071069858	0.132741556	0.520543319	PPM1G
ENSG00000113532.13	-0.090147863	0.132770152	0.520543319	ST8SIA4
ENSG00000139239.7	0.108446183	0.132820084	0.520543319	RPL14P1
ENSG00000144815.16	-0.076877164	0.132820377	0.520543319	NXPE3
ENSG00000188820.13	0.291925771	0.13282119	0.520543319	CALHM6
ENSG00000106333.13	0.11429945	0.13283562	0.520543319	PCOLCE
ENSG00000170442.12	0.373927071	0.132839349	0.520543319	KRT86
ENSG00000167967.16	0.074512869	0.132896497	0.520543319	E4F1
ENSG00000198130.16	0.070589975	0.132930772	0.520543319	HIBCH
ENSG00000189423.13	-0.251726743	0.132981004	0.520543319	USP32P3
ENSG00000196642.19	0.07204376	0.13300572	0.520543319	RABL6
ENSG00000235173.7	0.076709275	0.133022353	0.520543319	HGH1
ENSG00000188229.6	0.080420527	0.133069693	0.520543319	TUBB4B
ENSG00000160799.12	0.093544149	0.133070346	0.520543319	CCDC12
ENSG00000215417.13	-0.161436544	0.133110024	0.520543319	MIR17HG
ENSG00000235750.10	0.073388257	0.133121616	0.520543319	KIAA0040
ENSG00000260868.1	-0.299162484	0.133143378	0.520543319	LINC01960
ENSG00000285280.2	0.202986723	0.133152789	0.520543319	RP11-139E24.1
ENSG00000163795.14	0.065628013	0.13317425	0.520543319	ZNF513
ENSG00000286848.1	-0.441218012	0.133190316	0.520543319	RP11-46J23.2
ENSG00000119509.13	0.077149988	0.133195512	0.520543319	INVS
ENSG00000261613.2	0.742904203	0.133206098	0.520543319	RP11-20I23.13
ENSG00000266604.1	0.297438741	0.133212354	0.520543319	LINC01887
ENSG00000260953.1	-0.223537493	0.133232489	0.520543319	RP11-426C22.6
ENSG00000034693.15	-0.086566425	0.133445228	0.521144187	PEX3
ENSG00000286629.1	-0.346379016	0.133447215	0.521144187	RP11-214C8.6
ENSG00000092421.17	-0.118918268	0.133499095	0.521144187	SEMA6A
ENSG00000196083.10	-0.142119755	0.133505997	0.521144187	IL1RAP
ENSG00000077380.16	-0.061442436	0.133513339	0.521144187	DYNC1I2
ENSG00000211904.2	0.487931009	0.133590849	0.521310193	IGHJ2
ENSG00000260671.2	-0.535871149	0.133615057	0.521310193	RP11-278A23.2
ENSG00000008952.17	-0.093021842	0.133657397	0.521310193	SEC62
ENSG00000261684.2	-0.225807208	0.133657548	0.521310193	RP11-265N6.1
ENSG00000129636.13	0.050408935	0.133829769	0.521811442	ITFG1
ENSG00000180667.11	-0.132621235	0.133836951	0.521811442	YOD1
ENSG00000005448.17	0.09238051	0.133894958	0.521938378	WDR54

ENSG00000138592.14	-0.064761825	0.134009539	0.522285751	USP8
ENSG00000179023.9	0.505786131	0.1340679	0.522413925	KLHDC7A
ENSG00000186204.15	0.211216208	0.134125947	0.522540828	CYP4F12
ENSG00000251637.6	0.25332064	0.134166576	0.522547294	LINC02754
ENSG00000224138.1	-0.140202237	0.134178567	0.522547294	AC000123.4
ENSG00000122299.12	-0.060575163	0.134277823	0.522834552	ZC3H7A
ENSG00000145439.12	-0.0802859	0.134313073	0.522872531	CBR4
ENSG00000269918.1	0.112301047	0.134367423	0.522891622	AF131215.9
ENSG00000100271.17	0.090891821	0.13439136	0.522891622	TTLL1
ENSG00000139133.7	-0.137955422	0.134395933	0.522891622	ALG10
ENSG00000268218.1	-0.108651319	0.134448173	0.522891622	AC137932.4
ENSG00000212864.3	0.223899612	0.134469388	0.522891622	RNF208
ENSG00000185043.12	0.092539004	0.134470959	0.522891622	CIB1
ENSG00000213212.3	-0.257134974	0.134555347	0.523097166	NCLP1
ENSG00000090447.12	0.078106083	0.13461244	0.523097166	TFAP4
ENSG00000108591.10	0.056945629	0.134623259	0.523097166	DRG2
ENSG00000183814.16	0.167482731	0.134654678	0.523097166	LIN9
ENSG00000136270.14	0.056551597	0.134694631	0.523097166	TBRG4
ENSG00000108262.16	0.064245543	0.134695406	0.523097166	GIT1
ENSG00000283486.2	0.196337452	0.134724446	0.523097166	FAM95C
ENSG00000157379.14	0.081162863	0.134727874	0.523097166	DHRS1
ENSG00000204577.11	0.090973945	0.134795897	0.523249678	LILRB3
ENSG00000130204.13	0.073818386	0.134818183	0.523249678	TOMM40
ENSG00000145191.15	0.043655481	0.134868302	0.523268651	EIF2B5
ENSG00000163635.20	-0.091555986	0.134895337	0.523268651	ATXN7
ENSG00000262879.6	-0.109214398	0.134899618	0.523268651	RP11-156P1.3
ENSG00000132394.11	0.081410815	0.135008083	0.523449669	EEFSEC
ENSG00000132879.14	0.085647228	0.135020752	0.523449669	FBXO44
ENSG00000269845.1	-0.353280466	0.135022857	0.523449669	CCNYL6
ENSG00000198231.13	-0.042270047	0.135099514	0.523647862	DDX42
ENSG00000172992.12	0.076074587	0.135148126	0.523737295	DCAKD
ENSG00000241644.2	0.289203089	0.135184327	0.52377861	INMT
ENSG00000228113.7	-0.256487641	0.135216469	0.523804182	AC003991.3
ENSG00000144228.9	-0.113901945	0.135255394	0.523856018	SPOPL
ENSG00000065717.15	0.141121686	0.135400653	0.524319597	TLE2
ENSG00000159873.10	-0.067568729	0.135443635	0.524387023	CCDC117
ENSG00000186577.14	0.083611554	0.135513888	0.524559989	SMIM29
ENSG00000285863.1	-0.276681309	0.135680621	0.525015154	RP11-16B9.3
ENSG00000213930.12	0.062674402	0.135686881	0.525015154	GALT
ENSG00000168701.19	0.092717076	0.135766104	0.525015154	TMEM208
ENSG00000166266.14	-0.119831724	0.135799111	0.525015154	CUL5
ENSG00000058262.10	0.055255244	0.13581067	0.525015154	SEC61A1
ENSG00000107771.17	-0.077809724	0.135838707	0.525015154	CCSER2
ENSG00000058335.16	0.46896617	0.135839661	0.525015154	RASGRF1
ENSG00000136261.15	0.060248445	0.135858411	0.525015154	BZW2
ENSG00000183684.8	0.084580273	0.13586188	0.525015154	ALYREF
ENSG00000171456.20	-0.045460384	0.135954069	0.525272427	ASXL1

ENSG00000048740.18	-0.051558403	0.135990545	0.525314389	CELF2
ENSG00000258744.2	-0.22409544	0.136057962	0.525458185	RP11-80A15.1
ENSG00000164054.16	0.113046741	0.136122294	0.525458185	SHISA5
ENSG00000116731.22	-0.058450747	0.136139656	0.525458185	PRDM2
ENSG00000242208.1	-0.319953128	0.136165498	0.525458185	RPL5P29
ENSG00000148356.15	0.061664736	0.136176701	0.525458185	LRSAM1
ENSG00000091106.19	0.084445489	0.136181502	0.525458185	NLRC4
ENSG00000050405.13	-0.098267185	0.13622982	0.525469866	LIMA1
ENSG00000110906.13	-0.072714241	0.136252291	0.525469866	KCTD10
ENSG00000113593.12	-0.087935171	0.136261398	0.525469866	PPWD1
ENSG00000169118.18	-0.059681377	0.136331115	0.525639878	CSNK1G1
ENSG00000119522.16	0.089752852	0.136384427	0.525714321	DENND1A
ENSG00000134057.15	-0.154742798	0.136429066	0.525714321	CCNB1
ENSG00000136436.15	0.052822264	0.13643167	0.525714321	CALCOCO2
ENSG00000130176.8	0.49773242	0.136452961	0.525714321	CNN1
ENSG00000172809.13	0.100450741	0.136559974	0.525926219	RPL38
ENSG00000185614.7	0.150636906	0.136572863	0.525926219	INKA1
ENSG00000069188.17	0.258704768	0.136584896	0.525926219	SDK2
ENSG00000174371.17	-0.257655993	0.136618711	0.525957672	EXO1
ENSG00000149308.17	-0.077758146	0.13667402	0.525968443	NPAT
ENSG00000135074.16	0.10016974	0.136716644	0.525968443	ADAM19
ENSG00000174953.14	-0.066501549	0.136730669	0.525968443	DHX36
ENSG00000228305.2	0.219493343	0.136746233	0.525968443	PRELID1P6
ENSG00000236114.1	-0.284472289	0.136749743	0.525968443	RP11-517P14.7
ENSG00000203880.12	-0.109530704	0.136830366	0.526123497	PCMTD2
ENSG00000136319.12	-0.0619797	0.136841366	0.526123497	TTC5
ENSG00000128915.12	-0.07816563	0.137002024	0.526642458	ICE2
ENSG00000285043.3	0.208237595	0.137032856	0.52666226	CTD-2515O10.6
ENSG00000103275.21	0.055054577	0.137073154	0.526706765	UBE2I
ENSG00000187522.16	-0.106538578	0.137111953	0.526706765	HSPA14
ENSG00000175073.8	-0.100374667	0.137162808	0.526706765	VCPIP1
ENSG00000175854.14	0.081947174	0.137175078	0.526706765	SWI5
ENSG00000133962.8	-0.180012125	0.137180245	0.526706765	CATSPERB
ENSG00000270127.2	-0.238050086	0.137210646	0.526706765	RP11-526I2.5
ENSG00000273824.1	-0.318270733	0.137224217	0.526706765	RP11-81H14.1
ENSG00000180279.7	0.347609171	0.137274157	0.526799851	LINC01869
ENSG00000189376.12	0.090730695	0.137372888	0.527080111	C8orf76
ENSG00000166595.12	0.084596981	0.137400873	0.52708887	CIAO2B
ENSG00000263968.2	-0.362669994	0.137481311	0.52729881	RN7SL381P
ENSG00000051128.19	0.124288767	0.137534892	0.527405677	HOMER3
ENSG00000054983.17	-0.07316929	0.137584456	0.527497108	GALC
ENSG00000144824.21	-0.165385545	0.137653612	0.527663602	PHLDB2
ENSG00000125844.16	0.101145649	0.137754291	0.52781623	RRBP1
ENSG00000204305.15	0.100202686	0.137784336	0.52781623	AGER
ENSG00000141526.18	0.090452164	0.137794972	0.52781623	SLC16A3
ENSG00000256249.1	-0.293116687	0.137796377	0.52781623	RP11-324E6.6
ENSG00000197013.10	-0.168783965	0.137866303	0.527968575	ZNF429

ENSG00000186153.18	-0.084951477	0.137887638	0.527968575	WWOX
ENSG00000081386.13	-0.077412952	0.137967839	0.528105344	ZNF510
ENSG00000086288.12	-0.148334964	0.138006244	0.528105344	NME8
ENSG00000265194.1	-0.295142503	0.138006582	0.528105344	RP11-70L8.4
ENSG00000143595.13	0.319879947	0.138026363	0.528105344	AQP10
ENSG00000100418.8	0.05601061	0.13805674	0.528123039	DESI1
ENSG00000251988.1	-0.313856566	0.138102766	0.528200583	RNU4ATAC18P
ENSG00000273355.1	0.283480899	0.138142009	0.528252157	RP11-672L10.6
ENSG00000196935.9	-0.194385863	0.138300439	0.528706894	SRGAP1
ENSG00000247796.3	-0.166157961	0.138313151	0.528706894	CTD-2366F13.1
ENSG00000142347.19	0.081075713	0.138338268	0.528706894	MYO1F
ENSG00000131153.9	-0.224242824	0.138372601	0.528739575	GINS2
ENSG00000105486.14	0.071715422	0.138464542	0.528992331	LIG1
ENSG00000082781.12	0.211174691	0.138503049	0.529040889	ITGB5
ENSG00000135407.10	-0.123936736	0.138546495	0.529108289	AVIL
ENSG00000065328.17	-0.309617832	0.138588752	0.529171127	MCM10
ENSG00000273319.1	-0.335516759	0.138751256	0.529620138	RP11-138A9.2
ENSG00000125995.16	0.126490899	0.138757997	0.529620138	ROMO1
ENSG00000171115.4	0.08501383	0.138821081	0.529762325	GIMAP8
ENSG00000152503.10	0.18957607	0.138873276	0.529859273	TRIM36
ENSG00000271781.1	0.250527458	0.138898159	0.529859273	CTD-2589H19.6
ENSG00000094880.11	-0.043490104	0.138998533	0.529961542	CDC23
ENSG00000275413.1	-0.2537871	0.139019897	0.529961542	CTC-529I10.1
ENSG00000099377.14	0.196064539	0.13902881	0.529961542	HSD3B7
ENSG00000162836.12	-0.097645343	0.139044658	0.529961542	ACP6
ENSG00000180776.16	-0.110923388	0.139054177	0.529961542	ZDHHC20
ENSG00000173598.14	-0.077559585	0.139091088	0.530003723	NUDT4
ENSG00000243943.10	0.056218182	0.139169788	0.530122261	ZNF512
ENSG00000259116.2	-0.323108075	0.139173896	0.530122261	RP11-973N13.4
ENSG00000184635.16	0.119632652	0.139241989	0.530126912	ZNF93
ENSG00000128059.8	-0.102272283	0.139276182	0.530126912	PPAT
ENSG00000130643.9	0.24752588	0.139276578	0.530126912	CALY
ENSG00000251075.2	-0.346625096	0.139278516	0.530126912	HTT-AS
ENSG00000205929.11	0.242988403	0.139426942	0.530567274	C21orf62
ENSG00000068831.19	0.059096794	0.139465529	0.530567274	RASGRP2
ENSG00000249264.1	0.152563837	0.139471824	0.530567274	EEF1A1P9
ENSG00000025293.17	-0.052479963	0.139557742	0.530761214	PHF20
ENSG00000131899.11	-0.058119751	0.139574567	0.530761214	LLGL1
ENSG00000104970.11	-0.290893659	0.139620122	0.530836015	KIR3DX1
ENSG00000125534.10	0.168377708	0.139658318	0.530882815	PPDPF
ENSG00000160961.12	-0.073428909	0.139702038	0.530950592	ZNF333
ENSG00000100823.12	0.062975071	0.139750045	0.531003222	APEX1
ENSG00000249309.1	-0.284431791	0.139796422	0.531003222	RP11-153M7.5
ENSG00000100416.15	0.060765427	0.139818381	0.531003222	TRMU
ENSG00000216895.9	-0.101064246	0.139819456	0.531003222	AC009403.2
ENSG00000145901.16	0.076766683	0.139849797	0.531020115	TNIP1
ENSG00000177548.13	0.085973308	0.13988905	0.531070833	RABEP2

ENSG00000090432.7	0.067060549	0.140008936	0.531212138	MUL1
ENSG00000150556.17	0.441022769	0.140009088	0.531212138	LYPD6B
ENSG00000246375.3	0.472136047	0.140077782	0.531212138	PPM1K-DT
ENSG00000255529.9	-0.100255221	0.140081763	0.531212138	POLR2M
ENSG00000235522.6	-0.342900316	0.140108461	0.531212138	AC009505.2
ENSG00000137776.17	-0.048002128	0.140195182	0.531212138	SLTM
ENSG00000183323.13	0.089340392	0.140219739	0.531212138	CCDC125
ENSG00000198373.13	0.075285979	0.140231547	0.531212138	WWP2
ENSG00000128322.7	0.423754573	0.140234123	0.531212138	IGLL1
ENSG00000151208.17	-0.232459182	0.140235542	0.531212138	DLG5
ENSG00000186174.13	-0.121622368	0.140241605	0.531212138	BCL9L
ENSG00000243238.1	-0.375185157	0.140262011	0.531212138	IGKV2-30
ENSG00000136274.9	-0.433044836	0.140276152	0.531212138	NACAD
ENSG00000100058.12	0.085914576	0.140313497	0.531212138	CRYBB2P1
ENSG00000204351.12	0.064383012	0.140314811	0.531212138	SKIV2L
ENSG00000163617.11	0.106609597	0.140344486	0.531214085	CCDC191
ENSG00000205084.11	-0.14608864	0.140367131	0.531214085	TMEM231
ENSG00000204186.10	-0.187961806	0.140407108	0.531267336	ZDBF2
ENSG00000213793.5	-0.105923497	0.140444175	0.53130108	ZNF888
ENSG00000184616.9	-0.208794107	0.140478921	0.53130108	SPDYE12P
ENSG00000265743.1	0.170454237	0.140500539	0.53130108	RP11-848P1.3
ENSG00000266750.1	0.290261598	0.140539094	0.53130108	MIR4645
ENSG00000162396.6	0.09048159	0.140545561	0.53130108	PARS2
ENSG00000125510.18	0.126751999	0.140644667	0.531564291	OPRL1
ENSG00000169862.19	0.421806027	0.140667028	0.531564291	CTNND2
ENSG00000144909.8	-0.074754296	0.140758606	0.53181236	OSBPL11
ENSG00000123200.17	-0.074836001	0.140796702	0.53185831	ZC3H13
ENSG00000246334.2	-0.103405109	0.140834299	0.531860066	PRR7-AS1
ENSG00000189362.12	-0.072804596	0.140855219	0.531860066	NEMP2
ENSG00000286872.1	-0.229697701	0.140908728	0.531860066	RP11-382A20.8
ENSG00000116095.11	-0.076594068	0.140924667	0.531860066	PLEKHA3
ENSG00000114520.11	-0.145280756	0.140926838	0.531860066	SNX4
ENSG00000204421.3	0.339829685	0.140981993	0.531876388	LY6G6C
ENSG00000154174.8	-0.055972075	0.141006119	0.531876388	TOMM70
ENSG00000196189.13	0.113735092	0.141008968	0.531876388	SEMA4A
ENSG00000009954.11	-0.062571825	0.141057726	0.531891073	BAZ1B
ENSG00000139547.8	-0.115961428	0.141150018	0.531891073	RDH16
ENSG00000159708.18	0.241362778	0.141166751	0.531891073	LRRC36
ENSG00000270392.2	-0.243148521	0.141178745	0.531891073	PFN1P2
ENSG00000135966.13	-0.048275621	0.141189652	0.531891073	TGFBRAP1
ENSG00000111696.12	-0.172654836	0.141191649	0.531891073	NT5DC3
ENSG00000254427.2	0.242441324	0.141194412	0.531891073	LINC02696
ENSG00000170571.12	-0.081309369	0.141273285	0.532012116	EMB
ENSG00000066651.20	-0.123913365	0.14130106	0.532012116	TRMT11
ENSG00000273272.2	0.184852581	0.141304369	0.532012116	CTA-384D8.34
ENSG00000134321.13	0.523499721	0.141366196	0.532147199	RSAD2
ENSG00000265975.1	0.114257753	0.141421524	0.5321935	CTB-41I6.2

ENSG00000101132.10	-0.287711529	0.141460868	0.5321935	PFDN4
ENSG00000234336.7	-0.28849872	0.141470933	0.5321935	JAZF1-AS1
ENSG00000010361.14	0.0981497	0.141482298	0.5321935	FUZ
ENSG00000211667.3	-0.653655463	0.141533067	0.532286839	IGLV3-12
ENSG00000120833.14	0.118017425	0.141628055	0.532432359	SOCS2
ENSG00000228474.6	0.113587007	0.141651449	0.532432359	OST4
ENSG00000134313.16	-0.091237459	0.141655327	0.532432359	KIDINS220
ENSG00000002549.13	0.216387821	0.141675609	0.532432359	LAP3
ENSG00000197879.17	0.104802223	0.14174473	0.532594528	MYO1C
ENSG00000189403.15	-0.098827289	0.141780001	0.532629466	HMGB1
ENSG00000229409.1	0.313126846	0.141834942	0.532738276	RP11-494O16.3
ENSG00000101557.15	-0.055165796	0.141926911	0.532986102	USP14
ENSG00000099998.19	0.319206866	0.141959653	0.53298789	GGT5
ENSG00000128185.10	0.089023384	0.141979366	0.53298789	DGCR6L
ENSG00000178585.15	0.06607643	0.142008506	0.532999717	CTNNBIP1
ENSG00000279255.1	0.307854865	0.142113898	0.533297681	LA16c-380A1.2
ENSG00000178397.13	-0.058879027	0.142169972	0.533410498	FAM220A
ENSG00000211805.1	-0.290362215	0.142231097	0.533542224	TRAV24
ENSG00000105379.10	0.090552658	0.142312534	0.533608023	ETFB
ENSG00000177352.10	0.06625307	0.142315213	0.533608023	CCDC71
ENSG00000259612.1	0.353649081	0.142326696	0.533608023	EEF1A1P22
ENSG00000255899.1	-0.401379508	0.14243017	0.53389836	XXbac-BPG299F13.16
ENSG00000035720.8	0.181461352	0.142527788	0.533913948	STAP1
ENSG00000225282.1	0.434107013	0.142559388	0.533913948	AP000350.6
ENSG00000182636.8	0.349021692	0.142572305	0.533913948	NDN
ENSG00000008256.16	-0.096918128	0.142575842	0.533913948	CYTH3
ENSG00000274987.1	-0.264135037	0.142580915	0.533913948	RP11-295I5.3
ENSG00000110619.18	0.049098513	0.142616523	0.533913948	CARS1
ENSG00000106692.15	-0.089015106	0.142617791	0.533913948	FKTN
ENSG00000164867.11	0.158423498	0.142673469	0.533913948	NOS3
ENSG00000272666.1	0.203202575	0.142708572	0.533913948	KLHDC7B-DT
ENSG00000286084.2	-0.368178136	0.142717627	0.533913948	RP3-359N14.3
ENSG00000225880.5	-0.154614454	0.142720707	0.533913948	LINC00115
ENSG00000114302.16	-0.044516177	0.142887962	0.53438821	PRKAR2A
ENSG00000159231.6	0.233477874	0.142929958	0.53438821	CBR3
ENSG00000068912.14	-0.080307118	0.14294535	0.53438821	ERLEC1
ENSG00000198794.12	0.186262645	0.142951713	0.53438821	SCAMP5
ENSG00000262587.2	-0.13454369	0.143002851	0.534481953	RP11-266L9.4
ENSG00000267508.6	0.194652359	0.143094451	0.53472686	ZNF285
ENSG00000270647.6	0.116047879	0.143146821	0.53476386	TAF15
ENSG00000189171.15	0.139094611	0.143156504	0.53476386	S100A13
ENSG00000177173.5	-0.315196033	0.143272664	0.53500203	NAP1L4P1
ENSG00000168246.6	-0.08339661	0.143284589	0.53500203	UBTD2
ENSG00000253853.3	0.538710246	0.143298525	0.53500203	GS1-57L11.1
ENSG00000104635.15	-0.089305545	0.143391025	0.535249936	SLC39A14
ENSG00000215039.7	0.072602202	0.143490279	0.535256493	CD27-AS1
ENSG00000196653.12	-0.211878653	0.143517585	0.535256493	ZNF502

ENSG00000251230.6	0.211404687	0.143548201	0.535256493	MIR3945HG
ENSG00000167785.9	-0.0839734	0.143553489	0.535256493	ZNF558
ENSG00000101412.13	-0.180649496	0.143558346	0.535256493	E2F1
ENSG00000186517.14	0.050252193	0.143561042	0.535256493	ARHGAP30
ENSG00000087116.16	-0.603397723	0.143575481	0.535256493	ADAMTS2
ENSG00000261360.1	0.201863564	0.143637109	0.535298173	OTULIN-DT
ENSG00000127743.6	0.255890869	0.143638865	0.535298173	IL17B
ENSG00000123405.14	0.105795846	0.14368551	0.535374717	NFE2
ENSG00000106290.15	0.073424038	0.143867034	0.535933329	TAF6
ENSG00000143412.10	0.092307381	0.143887698	0.535933329	ANXA9
ENSG00000122376.12	-0.113382176	0.143940999	0.536034502	SHLD2
ENSG00000166747.13	-0.047819285	0.144014972	0.536197291	AP1G1
ENSG00000256582.1	-0.234525906	0.14406185	0.536197291	LINC02390
ENSG00000162521.19	-0.035321169	0.14406315	0.536197291	RBBP4
ENSG00000243244.7	-0.130609005	0.144131749	0.536355272	STON1
ENSG00000120526.12	-0.15904848	0.14425741	0.536583813	NUDCD1
ENSG00000205659.11	-0.089556624	0.144290545	0.536583813	LIN52
ENSG00000143222.12	0.084802159	0.144309262	0.536583813	UFC1
ENSG00000232884.9	0.391491718	0.144320762	0.536583813	AF127936.3
ENSG00000215440.12	0.082442871	0.144350733	0.536583813	NPEPL1
ENSG00000188931.4	-0.145629738	0.144353081	0.536583813	CFAP126
ENSG00000165555.9	0.142603362	0.144425786	0.536583813	NOXRED1
ENSG00000105339.11	0.068285656	0.14443881	0.536583813	DENND3
ENSG00000279412.1	-0.235765103	0.144466081	0.536583813	RP11-394B2.5
ENSG00000182552.15	-0.090108949	0.144493974	0.536583813	RWDD4
ENSG00000158158.12	-0.063456255	0.144520679	0.536583813	CNNM4
ENSG00000104231.11	-0.109778911	0.144533564	0.536583813	ZFAND1
ENSG00000105258.9	0.135987414	0.144556342	0.536583813	POLR2I
ENSG00000070961.16	-0.087197801	0.144559468	0.536583813	ATP2B1
ENSG00000276471.1	0.298801875	0.144638023	0.536584905	RP11-398A8.5
ENSG00000105655.19	0.100777264	0.144666325	0.536584905	ISYNA1
ENSG00000242028.8	-0.091752526	0.144674853	0.536584905	HYPK
ENSG00000104331.9	-0.063539708	0.144683815	0.536584905	BPNT2
ENSG00000259337.4	0.794443949	0.144709099	0.536584905	IGHV1OR15-2
ENSG00000235052.1	0.323364589	0.14471675	0.536584905	RP1-150O5.3
ENSG00000159399.10	-0.106970112	0.144814391	0.536758402	HK2
ENSG00000196639.7	-0.410043618	0.144828918	0.536758402	HRH1
ENSG00000147421.18	-0.0581104	0.144930186	0.536758402	HMBOX1
ENSG00000152439.13	0.083375861	0.14493596	0.536758402	ZNF773
ENSG00000211941.3	0.343212016	0.144948325	0.536758402	IGHV3-11
ENSG00000165502.7	0.134228023	0.144990142	0.536758402	RPL36AL
ENSG00000273980.1	-0.422237847	0.145028377	0.536758402	RP13-49I15.6
ENSG00000218809.1	-0.174086916	0.145044879	0.536758402	RP1-229K20.5
ENSG00000267082.1	-0.323687375	0.14509341	0.536758402	CTC-510F12.2
ENSG00000254681.6	-0.526815257	0.145109332	0.536758402	PKD1P5
ENSG00000272129.1	0.311089472	0.145118837	0.536758402	RP11-250B2.6
ENSG00000142327.13	0.081709124	0.145128813	0.536758402	RNPEPL1

ENSG00000205356.10	0.072209036	0.145130141	0.536758402	TECPR1
ENSG00000286813.1	-0.212074582	0.145137818	0.536758402	RP11-81A1.10
ENSG00000183856.11	-0.366938854	0.145156139	0.536758402	IQGAP3
ENSG00000140830.9	0.051341451	0.145302331	0.536916394	TXNL4B
ENSG00000140307.11	0.078877606	0.145302444	0.536916394	GTF2A2
ENSG00000263874.2	-0.104956162	0.145305222	0.536916394	LINC00672
ENSG00000176700.20	-0.101287617	0.145327239	0.536916394	SCAND2P
ENSG00000127507.18	0.14289336	0.145350256	0.536916394	ADGRE2
ENSG00000235079.1	-0.222074546	0.145445941	0.536916394	ZRANB2-AS1
ENSG00000036549.13	-0.097889744	0.145446769	0.536916394	ZZZ3
ENSG00000272941.1	0.124595254	0.145455685	0.536916394	RP11-134L10.1
ENSG00000198521.12	-0.078219041	0.145459216	0.536916394	ZNF43
ENSG00000123636.18	-0.120553308	0.145460673	0.536916394	BAZ2B
ENSG00000140455.17	0.04468547	0.14553	0.53700278	USP3
ENSG00000160208.13	-0.055474401	0.145565781	0.53700278	RRP1B
ENSG00000136367.14	0.234036927	0.145585067	0.53700278	ZFHX2
ENSG00000105011.9	0.117663417	0.145649848	0.53700278	ASF1B
ENSG00000285589.1	-0.08024945	0.145659393	0.53700278	CTD-2192J16.27
ENSG00000160223.17	0.500796065	0.145672537	0.53700278	ICOSLG
ENSG00000052749.14	0.134823819	0.145764375	0.53700278	RRP12
ENSG00000078814.17	0.140434653	0.145773327	0.53700278	MYH7B
ENSG00000198746.13	0.065156426	0.145794	0.53700278	GPATCH3
ENSG00000085721.13	-0.062496342	0.145807683	0.53700278	RRN3
ENSG00000122912.15	-0.059502647	0.145823791	0.53700278	SLC25A16
ENSG00000211445.12	0.168874138	0.145847613	0.53700278	GPX3
ENSG00000278989.1	-0.332762055	0.145850883	0.53700278	RP11-762L8.6
ENSG00000228663.1	-0.207705074	0.145889106	0.53700278	PSMD10P1
ENSG00000169554.22	-0.08562227	0.145890801	0.53700278	ZEB2
ENSG00000198355.5	0.10614986	0.145903037	0.53700278	PIM3
ENSG00000235308.1	-0.189188598	0.145948964	0.537075429	RP11-82K18.2
ENSG00000272182.1	-0.20691476	0.146002884	0.537177456	RP11-802O23.3
ENSG00000204138.13	-0.054273523	0.146059468	0.537289249	PHACTR4
ENSG00000237133.1	0.270297973	0.146095849	0.5373267	RASGRP3-AS1
ENSG00000081014.11	-0.076153489	0.146239974	0.53765424	AP4E1
ENSG00000227288.3	0.222585719	0.146250821	0.53765424	ARID3BP1
ENSG00000205133.12	-0.101079351	0.146301815	0.53765424	TRIQK
ENSG00000106268.16	0.102973962	0.146317162	0.53765424	NUDT1
ENSG00000205250.9	0.049270746	0.146327463	0.53765424	E2F4
ENSG00000131669.10	0.100800242	0.146342206	0.53765424	NINJ1
ENSG00000243335.10	-0.059769247	0.146415679	0.537782908	KCTD7
ENSG00000154736.6	-0.23370482	0.146429674	0.537782908	ADAMTS5
ENSG00000100916.14	-0.13391273	0.146576418	0.538189707	BRMS1L
ENSG00000144369.13	-0.350639314	0.146592925	0.538189707	FAM171B
ENSG00000164458.10	1.008953412	0.146650655	0.538217891	TBXT
ENSG00000257621.8	-0.082116347	0.14665309	0.538217891	PSMA3-AS1
ENSG00000143537.14	0.100455291	0.146683663	0.538233772	ADAM15
ENSG00000135414.10	-0.065430213	0.146729916	0.538282292	GDF11

ENSG00000214432.9	0.354554694	0.146749381	0.538282292	VPS33B-DT
ENSG00000158106.14	0.144720775	0.146823952	0.538341899	RHPN1
ENSG00000140105.18	0.153353361	0.146833003	0.538341899	WARS1
ENSG00000132386.11	0.104369895	0.146844382	0.538341899	SERPINF1
ENSG00000033030.15	-0.038735522	0.146911567	0.538491942	ZCCHC8
ENSG00000132185.18	0.230556929	0.146944732	0.538517256	FCRLA
ENSG00000125124.14	-0.085420404	0.14711626	0.538980768	BBS2
ENSG00000260196.1	-0.152437484	0.147187932	0.538980768	RP1-239B22.5
ENSG00000231560.1	0.350413084	0.147273421	0.538980768	CLEC12A-AS1
ENSG00000181852.18	0.036761063	0.147286348	0.538980768	RNF41
ENSG00000169994.19	0.176580539	0.147293183	0.538980768	MYO7B
ENSG00000164219.10	-0.080955094	0.147394787	0.538980768	PGGT1B
ENSG00000215199.3	-0.279737005	0.147402337	0.538980768	YWHAZP6
ENSG00000214562.15	0.192431932	0.147457827	0.538980768	NUTM2D
ENSG00000157837.16	0.060693382	0.147521305	0.538980768	SPPL3
ENSG00000239306.5	0.058828253	0.147533791	0.538980768	RBM14
ENSG00000140749.9	0.069347102	0.147561127	0.538980768	IGSF6
ENSG00000245812.3	0.349195463	0.147577372	0.538980768	LINC02202
ENSG00000162910.19	0.086284794	0.14761026	0.538980768	MRPL55
ENSG00000268500.6	0.160454346	0.147622037	0.538980768	SIGLEC5
ENSG00000129128.13	-0.052660302	0.147631453	0.538980768	SPCS3
ENSG00000134970.14	-0.092402959	0.147639868	0.538980768	TMED7
ENSG00000143924.19	-0.071309388	0.147652276	0.538980768	EML4
ENSG00000183844.17	0.733834239	0.147654181	0.538980768	FAM3B
ENSG00000186260.17	-0.097997439	0.147656531	0.538980768	MRTFB
ENSG00000134363.12	0.942049017	0.147694414	0.538980768	FST
ENSG00000215712.11	-0.068559403	0.147727172	0.538980768	TMEM242
ENSG00000164308.17	0.322522668	0.147727356	0.538980768	ERAP2
ENSG00000196329.12	-0.109910077	0.147746048	0.538980768	GIMAP5
ENSG00000222037.5	0.386192839	0.14775031	0.538980768	IGLC6
ENSG00000138792.10	0.692878505	0.147823781	0.538980768	ENPEP
ENSG00000285888.1	-0.366133974	0.147854654	0.538980768	RP3-431A14.7
ENSG00000129534.14	-0.0983993	0.147866866	0.538980768	MIS18BP1
ENSG00000198169.9	-0.066005213	0.147871266	0.538980768	ZNF251
ENSG00000173163.10	0.087956917	0.147889375	0.538980768	COMMD1
ENSG00000261105.6	-0.257782218	0.147889387	0.538980768	LMO7-AS1
ENSG00000142920.17	0.103949994	0.147900954	0.538980768	AZIN2
ENSG00000144852.20	0.270933546	0.147914408	0.538980768	NR1I2
ENSG00000277200.1	0.379974175	0.147966376	0.538980768	RP11-74E22.8
ENSG00000130772.14	0.068840725	0.148002449	0.538980768	MED18
ENSG00000235159.1	0.328073984	0.148017891	0.538980768	RP6-109B7.4
ENSG00000054219.11	-0.092370312	0.148079194	0.538980768	LY75
ENSG00000231584.8	0.108054453	0.148102671	0.538980768	FAHD2CP
ENSG00000248559.1	-0.265940167	0.148119746	0.538980768	RP11-215P8.3
ENSG00000132874.15	0.27353978	0.148125223	0.538980768	SLC14A2
ENSG00000090339.9	0.103037167	0.148164271	0.538980768	ICAM1
ENSG00000075089.10	-0.168289615	0.148183952	0.538980768	ACTR6

ENSG00000198648.11	-0.074962251	0.148244587	0.538980768	STK39
ENSG00000085978.22	-0.0365244	0.148277453	0.538980768	ATG16L1
ENSG00000181061.14	-0.123658202	0.148281598	0.538980768	HIGD1A
ENSG00000211938.2	0.315349606	0.148295138	0.538980768	IGHV3-7
ENSG00000225756.1	0.212490376	0.148360549	0.538980768	DBH-AS1
ENSG00000145833.16	-0.049875925	0.148370125	0.538980768	DDX46
ENSG00000032444.17	0.08171323	0.148375419	0.538980768	PNPLA6
ENSG00000095321.18	0.112864907	0.14838403	0.538980768	CRAT
ENSG00000187240.17	-0.148598526	0.148411141	0.538980768	DYNC2H1
ENSG00000148175.13	-0.114504043	0.148411566	0.538980768	STOM
ENSG00000218426.5	0.118795002	0.14849259	0.538989338	RP11-475C16.1
ENSG00000116906.13	-0.040218447	0.148554165	0.538989338	GNPAT
ENSG00000169203.16	-0.314021551	0.148556698	0.538989338	NPIPB12
ENSG00000107551.21	0.084824413	0.148563345	0.538989338	RASSF4
ENSG00000171566.12	-0.070865105	0.148569513	0.538989338	PLRG1
ENSG00000163655.16	-0.041715724	0.148611274	0.538989338	GMPS
ENSG00000152256.14	-0.102962964	0.148650253	0.538989338	PDK1
ENSG00000233057.1	0.323337501	0.148680878	0.538989338	EEF1A1P14
ENSG00000146409.12	0.089685354	0.14872117	0.538989338	SLC18B1
ENSG00000184988.8	0.078497601	0.148731245	0.538989338	TMEM106A
ENSG00000221946.8	0.178125172	0.148740698	0.538989338	FXYD7
ENSG00000104450.13	-0.139398279	0.148744316	0.538989338	SPAG1
ENSG00000004059.11	0.081563452	0.148784465	0.538989338	ARF5
ENSG00000082996.20	-0.079432417	0.148794421	0.538989338	RNF13
ENSG00000197880.9	0.189619434	0.1488132	0.538989338	MDS2
ENSG00000144713.13	0.113147549	0.148901426	0.538989338	RPL32
ENSG00000180071.20	0.319560202	0.148934754	0.538989338	ANKRD18A
ENSG00000241294.1	0.328252914	0.148970465	0.538989338	IGKV2-24
ENSG00000163806.16	-0.184714749	0.148978336	0.538989338	SPDYA
ENSG00000106080.11	-0.107465755	0.148983273	0.538989338	FKBP14
ENSG00000198795.11	0.2806938	0.148987783	0.538989338	ZNF521
ENSG00000233885.7	-0.143493936	0.148992128	0.538989338	YEATS2-AS1
ENSG00000135052.16	0.102350321	0.149020295	0.538996157	GOLM1
ENSG00000272140.2	-0.179474462	0.149088547	0.539147933	RP11-574K11.29
ENSG00000235532.1	0.188819075	0.149127633	0.5391942	LINC00402
ENSG00000179941.9	-0.121132617	0.14915902	0.539212619	BBS10
ENSG00000243264.3	0.48390478	0.149188792	0.539225192	IGKV2D-29
ENSG00000207721.1	-0.304220057	0.149271721	0.539375077	MIR186
ENSG00000273437.1	0.182180664	0.149386602	0.539375077	RP11-434H6.7
ENSG00000169429.11	-0.257194053	0.14941212	0.539375077	CXCL8
ENSG00000023318.8	0.049722638	0.149456595	0.539375077	ERP44
ENSG00000286305.1	0.2355647	0.149460503	0.539375077	RP4-808A1.2
ENSG00000185986.12	0.091269326	0.149490332	0.539375077	SDHAP3
ENSG00000134864.11	0.092950738	0.149495666	0.539375077	GGACT
ENSG00000166523.8	0.127298346	0.149509539	0.539375077	CLEC4E
ENSG00000271447.6	0.286097292	0.14956366	0.539375077	MMP28
ENSG00000130475.14	0.079906046	0.149576575	0.539375077	FCHO1

ENSG00000125378.16	0.434644369	0.149595117	0.539375077	BMP4
ENSG00000250548.7	0.26748697	0.14962082	0.539375077	LINC01303
ENSG00000102871.16	0.090301501	0.149636476	0.539375077	TRADD
ENSG00000126749.16	-0.070024426	0.149640072	0.539375077	EMG1
ENSG00000257122.5	-0.074639951	0.149671969	0.539375077	RRN3P3
ENSG00000145907.16	-0.038827617	0.149676314	0.539375077	G3BP1
ENSG00000170801.10	-0.468198171	0.149677373	0.539375077	HTRA3
ENSG00000244124.1	-0.264560527	0.149738972	0.539440355	ATP1B3-AS1
ENSG00000128228.5	0.11264453	0.149748095	0.539440355	SDF2L1
ENSG00000120925.16	-0.059873935	0.149877938	0.53981327	RNF170
ENSG00000095637.23	-0.181338972	0.149976367	0.539953435	SORBS1
ENSG00000173818.17	0.122771571	0.150026227	0.539953435	ENDOV
ENSG00000124177.16	-0.07721373	0.150039105	0.539953435	CHD6
ENSG00000165568.18	0.267861232	0.150065569	0.539953435	AKR1E2
ENSG00000204228.4	0.105867906	0.150075113	0.539953435	HSD17B8
ENSG00000139651.11	0.037133102	0.150101144	0.539953435	ZNF740
ENSG00000143382.16	0.113139104	0.150155087	0.539953435	ADAMTSL4
ENSG00000228008.1	0.361378968	0.1501688	0.539953435	CTD-2330K9.3
ENSG00000148488.17	-0.175719629	0.150189897	0.539953435	ST8SIA6
ENSG00000272463.1	-0.231462346	0.150216135	0.539953435	RP11-532F6.3
ENSG00000180992.7	0.104034927	0.150226279	0.539953435	MRPL14
ENSG00000213235.3	0.320211356	0.150282851	0.539953435	EEF1A1P16
ENSG00000261560.1	-0.255563295	0.15030199	0.539953435	RP11-166B2.3
ENSG00000135018.14	-0.047975352	0.150320086	0.539953435	UBQLN1
ENSG00000130520.11	0.070040884	0.150404762	0.539953435	LSM4
ENSG00000181090.21	0.056304995	0.150443464	0.539953435	EHMT1
ENSG00000179476.8	-0.112275978	0.150480804	0.539953435	C14orf28
ENSG00000149716.13	-0.046906612	0.150487185	0.539953435	LTO1
ENSG00000174326.14	0.18188601	0.150489244	0.539953435	SLC16A11
ENSG00000125447.18	0.045945133	0.150506604	0.539953435	GGA3
ENSG00000228205.1	0.189963337	0.150528295	0.539953435	RP11-778D9.4
ENSG00000269958.1	0.063322864	0.150528692	0.539953435	RP11-73M18.8
ENSG00000167515.10	0.074296033	0.150542266	0.539953435	TRAPPC2L
ENSG00000078403.17	-0.069881203	0.150565914	0.539953435	MLLT10
ENSG00000209042.1	0.355645562	0.150584728	0.539953435	SNORD12C
ENSG00000119787.14	-0.079529485	0.150601407	0.539953435	ATL2
ENSG00000139644.13	0.042017982	0.150648177	0.540026712	TMBIM6
ENSG00000268996.3	0.152264632	0.150721673	0.540077147	MAN1B1-DT
ENSG00000267758.1	-0.414741217	0.150741816	0.540077147	RP11-358B23.5
ENSG00000197768.10	0.236323522	0.150795506	0.540077147	STPG3
ENSG00000230615.7	-0.289903759	0.150807353	0.540077147	RP5-1198O20.4
ENSG00000153406.14	0.084892491	0.150810593	0.540077147	NMRAL1
ENSG00000066739.12	-0.068580245	0.150877466	0.540077147	ATG2B
ENSG00000126705.15	-0.095682783	0.150878472	0.540077147	AHDC1
ENSG00000152104.12	-0.253259362	0.150880095	0.540077147	PTPN14
ENSG00000163541.12	0.058875814	0.150914654	0.540077147	SUCLG1
ENSG00000130560.9	0.056664868	0.150949566	0.540077147	UBAC1

ENSG00000128253.15	0.256644606	0.150951931	0.540077147	RFPL2
ENSG00000197124.12	-0.154892262	0.15102313	0.540201885	ZNF682
ENSG00000203797.12	-0.243017299	0.151042062	0.540201885	DDO
ENSG00000130703.17	0.053743562	0.151065819	0.540201885	OSBPL2
ENSG00000184389.9	-0.377805852	0.151114752	0.540220231	A3GALT2
ENSG00000228408.6	-0.426930816	0.151123633	0.540220231	RP1-111D6.3
ENSG00000213600.3	-0.167973321	0.151256687	0.540575471	XXcos-LUCA16.1
ENSG00000009335.18	-0.044090477	0.151276574	0.540575471	UBE3C
ENSG00000105509.11	-0.406144176	0.151302087	0.540575471	HAS1
ENSG00000125898.13	0.11576945	0.151375217	0.54070088	FAM110A
ENSG00000224046.2	0.240823455	0.151389919	0.54070088	AC005076.5
ENSG00000250722.6	-0.226077806	0.151468265	0.540804283	SELENOP
ENSG00000161277.11	0.080935118	0.151471611	0.540804283	THAP8
ENSG00000118965.16	-0.091525352	0.1515216	0.540888596	WDR35
ENSG00000133392.18	-0.145880674	0.151648392	0.54115838	MYH11
ENSG00000137474.22	0.167370643	0.151649952	0.54115838	MYO7A
ENSG00000179715.13	0.10789226	0.151706326	0.541207271	PCED1B
ENSG00000270923.1	-0.326169537	0.151739455	0.541207271	TAS2R6P
ENSG00000177455.15	0.231251858	0.151797749	0.541207271	CD19
ENSG00000132464.13	-0.469012441	0.151803216	0.541207271	ENAM
ENSG00000166855.9	-0.040843466	0.151825678	0.541207271	CLPX
ENSG00000180539.8	0.080008446	0.151828221	0.541207271	C9orf139
ENSG00000108556.10	-0.204107595	0.15187035	0.541207271	CHRNE
ENSG00000258839.4	-0.134619259	0.151874773	0.541207271	MC1R
ENSG00000152620.13	-0.089350344	0.15190649	0.541226251	NADK2
ENSG00000225978.3	0.181293844	0.15193829	0.541245519	HAR1A
ENSG00000164631.19	-0.087948814	0.151994904	0.54135316	ZNF12
ENSG00000116478.12	0.03864537	0.152046407	0.541442562	HDAC1
ENSG00000231752.6	0.248814903	0.152111424	0.54148609	EMBP1
ENSG00000184677.18	-0.058628093	0.152125826	0.54148609	ZBTB40
ENSG00000124535.16	-0.043420082	0.152137841	0.54148609	WRNIP1
ENSG00000187569.3	0.543018669	0.152179134	0.541490708	DPPA3
ENSG00000130164.14	0.13526931	0.152191946	0.541490708	LDLR
ENSG00000286086.1	-0.405833665	0.152292732	0.541625518	RP11-110C15.5
ENSG00000272369.1	0.356994166	0.152330395	0.541625518	RP11-446N19.1
ENSG00000105851.11	-0.066913666	0.152409644	0.541625518	PIK3CG
ENSG00000116221.15	0.070210248	0.152418327	0.541625518	MRPL37
ENSG00000172031.7	-0.394397536	0.152475838	0.541625518	EPHX4
ENSG00000260803.1	0.228170171	0.152495465	0.541625518	Z84812.4
ENSG00000134900.12	-0.067539527	0.152513938	0.541625518	TPP2
ENSG00000174839.13	-0.068326149	0.152521411	0.541625518	DENND6A
ENSG00000124155.18	0.057311064	0.152525848	0.541625518	PIGT
ENSG00000266601.1	-0.171510837	0.152527583	0.541625518	RP11-6N17.3
ENSG00000105717.14	-0.09741437	0.152554254	0.541625518	PBX4
ENSG00000260645.2	-0.275230985	0.152559051	0.541625518	RP11-250B2.5
ENSG00000156374.16	-0.115747685	0.152573172	0.541625518	PCGF6
ENSG00000119946.11	0.364791672	0.152633444	0.541745701	CNNM1

ENSG00000161800.13	-0.069286346	0.152752486	0.542022912	RACGAP1
ENSG00000152518.8	-0.091528221	0.152764406	0.542022912	ZFP36L2
ENSG00000103150.7	0.043954527	0.152791777	0.542026252	MLYCD
ENSG00000148655.15	0.131070916	0.152859649	0.542110127	LRMDA
ENSG00000258559.2	-0.126003408	0.152896038	0.542110127	AC005519.4
ENSG00000131242.18	0.081964673	0.152930012	0.542110127	RAB11FIP4
ENSG00000074935.14	-0.112133697	0.152976935	0.542110127	TUBE1
ENSG00000132581.10	0.065478452	0.152976999	0.542110127	SDF2
ENSG00000223834.5	0.247300744	0.153021608	0.542110127	RP11-472N13.2
ENSG00000156136.10	-0.084986507	0.153032137	0.542110127	DCK
ENSG00000119866.22	0.121703853	0.153040955	0.542110127	BCL11A
ENSG00000126602.11	0.099300771	0.153053327	0.542110127	TRAP1
ENSG00000143612.21	0.050032155	0.153178239	0.542442215	C1orf43
ENSG00000105366.15	0.256585256	0.153199986	0.542442215	SIGLEC8
ENSG00000135241.17	-0.116395318	0.153279903	0.54254413	PNPLA8
ENSG00000140577.16	0.064958363	0.15328168	0.54254413	CRTC3
ENSG00000135925.9	0.165994738	0.153359275	0.54272511	WNT10A
ENSG00000138071.14	-0.066938675	0.153409017	0.542807473	ACTR2
ENSG00000204439.4	0.05122093	0.153497086	0.54298546	C6orf47
ENSG00000246596.7	-0.252563786	0.153534992	0.54298546	RP11-1277A3.2
ENSG00000261056.2	-0.364764912	0.15353875	0.54298546	RP11-454F8.2
ENSG00000149485.19	0.129227772	0.153631092	0.543035886	FADS1
ENSG00000099991.18	0.08464391	0.153634288	0.543035886	CABIN1
ENSG00000260588.1	0.227767988	0.153651488	0.543035886	RP11-930P14.2
ENSG00000287261.1	0.210459705	0.153658926	0.543035886	RP11-439D8.4
ENSG00000179933.6	0.05735367	0.153724359	0.543106202	C14orf119
ENSG00000183066.14	-0.160728286	0.153732671	0.543106202	WBP2NL
ENSG00000257150.3	0.734930441	0.15375827	0.543106202	PGAM1P5
ENSG00000176105.14	-0.145271003	0.153802498	0.543117013	YES1
ENSG00000205581.11	-0.062241352	0.153814297	0.543117013	HMGN1
ENSG00000284734.1	-0.160201267	0.153918484	0.543391335	RP4-620F22.3
ENSG00000272711.1	0.136114167	0.153974223	0.543440906	HK2-DT
ENSG00000108298.12	0.086565529	0.154005945	0.543440906	RPL19
ENSG00000198887.9	-0.081180348	0.154012022	0.543440906	SMC5
ENSG00000224738.1	0.233217252	0.154082579	0.54359634	AC099850.1
ENSG00000187954.13	0.06029743	0.154269532	0.544162292	CYHR1
ENSG00000162627.17	0.52882258	0.154309773	0.544173197	SNX7
ENSG00000260563.3	0.095637517	0.154325693	0.544173197	RP13-516M14.1
ENSG00000261819.1	0.229437474	0.154402456	0.544260481	RP11-680G24.4
ENSG00000204583.10	0.427544877	0.154403524	0.544260481	LRCOL1
ENSG00000115295.20	-0.092245894	0.154454576	0.544346872	CLIP4
ENSG00000182376.2	-0.266073291	0.154613688	0.544814006	RP5-1142A6.8
ENSG00000196155.13	0.101153948	0.1546583	0.544865411	PLEKHG4
ENSG00000146955.11	-0.148547372	0.154752643	0.544865411	RAB19
ENSG00000163464.8	0.112442256	0.154786258	0.544865411	CXCR1
ENSG00000115677.18	0.067129448	0.154807766	0.544865411	HDLBP
ENSG00000087586.18	-0.122965334	0.154809809	0.544865411	AURKA

ENSG00000155792.10	0.177468027	0.154811337	0.544865411	DEPTOR
ENSG00000130045.16	-0.52958106	0.154814255	0.544865411	NXNL2
ENSG00000025708.14	0.145202686	0.154863152	0.544943983	TYMP
ENSG00000183647.11	0.073933084	0.154969343	0.545074721	ZNF530
ENSG00000204613.11	-0.266340255	0.15497863	0.545074721	TRIM10
ENSG00000119760.16	-0.034673327	0.154980042	0.545074721	SUPT7L
ENSG00000244055.2	-0.137496341	0.155237294	0.545805238	AC007566.10
ENSG00000085871.9	0.110356543	0.155240977	0.545805238	MGST2
ENSG00000262118.1	-0.47160724	0.155312336	0.545822568	MTCO1P28
ENSG00000198911.12	0.063381679	0.155322519	0.545822568	SREBF2
ENSG00000247081.8	0.240178745	0.155352753	0.545822568	BAALC-AS1
ENSG00000133103.17	-0.106006512	0.155365022	0.545822568	COG6
ENSG00000189350.13	-0.141425523	0.155387192	0.545822568	TOGARAM2
ENSG00000141449.15	-0.276492432	0.155431773	0.545822568	GREB1L
ENSG00000166816.15	0.221672312	0.155432212	0.545822568	LDHD
ENSG00000115239.24	-0.05683161	0.155628135	0.546333807	ASB3
ENSG00000170085.18	0.067410445	0.155640211	0.546333807	SIMC1
ENSG00000165392.11	-0.076256092	0.155703362	0.546333807	WRN
ENSG00000113494.17	0.205410789	0.155708959	0.546333807	PRLR
ENSG00000165572.8	-0.112701075	0.155710996	0.546333807	KBTBD6
ENSG00000197696.10	0.141053605	0.155743312	0.546353718	NMB
ENSG00000105610.6	-0.301046498	0.155837614	0.546417299	KLF1
ENSG00000092929.12	0.071271686	0.155849918	0.546417299	UNC13D
ENSG00000214652.6	0.333160208	0.155852201	0.546417299	ZNF727
ENSG00000133704.10	-0.056811164	0.155868012	0.546417299	IPO8
ENSG00000170260.9	0.055586941	0.156053599	0.546962404	ZNF212
ENSG00000241468.8	0.089091303	0.156076848	0.546962404	ATP5MF
ENSG00000152315.5	0.144683206	0.156141366	0.54700475	KCNK13
ENSG00000235298.1	-0.289333115	0.156142277	0.54700475	RP11-575L7.8
ENSG00000122378.14	0.129324372	0.156184599	0.547059564	PRXL2A
ENSG00000205930.10	-0.130119569	0.156284072	0.547314505	C21orf62-AS1
ENSG00000177600.9	0.115080644	0.156347387	0.547421112	RPLP2
ENSG00000132912.13	-0.05422525	0.156392729	0.547421112	DCTN4
ENSG00000152382.6	-0.071567719	0.156439675	0.547421112	TADA1
ENSG00000101442.10	0.055517343	0.156458015	0.547421112	ACTR5
ENSG00000132275.11	0.049023431	0.156471656	0.547421112	RRP8
ENSG00000234571.2	0.420982963	0.156474671	0.547421112	H2BP2
ENSG00000078618.22	0.05044941	0.156518836	0.547449399	NRDC
ENSG00000198106.8	0.200150012	0.156554702	0.547449399	RP11-231C14.7
ENSG00000231964.1	0.095604834	0.15659719	0.547449399	RP11-67C2.2
ENSG00000130348.11	0.080631052	0.156646314	0.547449399	QRSL1
ENSG00000279059.1	-0.09490131	0.156682107	0.547449399	RP11-257O5.2
ENSG00000143442.22	-0.046143111	0.156703264	0.547449399	POGZ
ENSG00000166126.11	0.175980706	0.156731659	0.547449399	AMN
ENSG00000251364.7	0.138034197	0.156739549	0.547449399	CTD-2516F10.2
ENSG00000162496.9	0.109430091	0.156749984	0.547449399	DHRS3
ENSG00000104897.10	0.093365796	0.156787162	0.547449399	SF3A2

ENSG00000006194.10	0.037687373	0.156821106	0.547449399	ZNF263
ENSG00000251297.1	0.973994549	0.156850631	0.547449399	TUBB7P
ENSG00000167555.14	-0.096761798	0.156857962	0.547449399	ZNF528
ENSG00000113407.14	-0.050488566	0.156877677	0.547449399	TARS1
ENSG00000196263.8	0.282982171	0.156927063	0.547449399	ZNF471
ENSG00000280128.1	-0.08229575	0.156950232	0.547449399	XXbac-BPG283O16.9
ENSG00000151012.13	-0.19430256	0.156975751	0.547449399	SLC7A11
ENSG00000082641.16	0.064966169	0.156987489	0.547449399	NFE2L1
ENSG00000108576.10	0.214242893	0.157012376	0.547449399	SLC6A4
ENSG00000162337.12	0.169243086	0.157016645	0.547449399	LRP5
ENSG00000106049.9	-0.067720899	0.157194102	0.547919943	HIBADH
ENSG00000117601.15	-0.291394733	0.157205039	0.547919943	SERPINC1
ENSG00000204176.14	0.322161624	0.157234432	0.547929266	SYT15
ENSG00000104381.14	0.111330645	0.157291074	0.548033527	GDAP1
ENSG00000183049.13	0.095645224	0.157346215	0.548132526	CAMK1D
ENSG00000148660.21	0.051609005	0.157406737	0.548250231	CAMK2G
ENSG00000169291.10	-0.331599397	0.157459767	0.548262056	SHE
ENSG00000158163.15	0.300762652	0.157500767	0.548262056	DZIP1L
ENSG00000053438.11	-0.180250747	0.157513834	0.548262056	NNAT
ENSG00000160741.17	0.059269245	0.15752126	0.548262056	CRTC2
ENSG00000197024.9	-0.050662993	0.157543802	0.548262056	ZNF398
ENSG00000265206.5	0.110996931	0.157663881	0.548506904	RP5-1171I10.5
ENSG00000171611.10	0.197614183	0.157667652	0.548506904	PTCRA
ENSG00000237575.4	0.281855249	0.157767391	0.548741151	PYY2
ENSG00000286065.2	-0.196791149	0.1577885	0.548741151	SLFN12L
ENSG00000248455.6	-0.296929203	0.157825409	0.548776447	LINC02217
ENSG00000264924.1	-0.257629161	0.157857611	0.548795369	RP11-799B12.2
ENSG00000143870.13	0.059564325	0.157942135	0.548910212	PDIA6
ENSG00000280149.1	0.138852005	0.157944176	0.548910212	RP4-751H13.5
ENSG00000132681.17	0.767014429	0.158060439	0.548994534	ATP1A4
ENSG00000076826.10	0.298387873	0.158066474	0.548994534	CAMSAP3
ENSG00000279672.2	-0.163150511	0.158067862	0.548994534	CMB9-55F22.1
ENSG00000177103.15	0.354960816	0.158101123	0.548994534	DSCAML1
ENSG00000125817.8	0.075879503	0.158102288	0.548994534	CENPB
ENSG00000155097.12	-0.06877352	0.158187342	0.549196888	ATP6V1C1
ENSG00000287769.1	-0.207743041	0.158323565	0.549576789	RP11-317B17.5
ENSG00000143198.13	0.102980623	0.158373656	0.549657632	MGST3
ENSG00000273444.1	0.298938453	0.158418809	0.54972131	RP5-1147A1.2
ENSG00000130304.17	0.084299339	0.158490095	0.549875633	SLC27A1
ENSG00000211966.2	0.296634826	0.158580857	0.549958826	IGHV5-51
ENSG00000277879.1	-0.207351165	0.158604544	0.549958826	RP11-129M16.4
ENSG00000248727.6	0.131563509	0.158607706	0.549958826	LINC01948
ENSG00000173728.11	-0.241595066	0.158621341	0.549958826	C1orf100
ENSG00000196975.16	0.071346445	0.158695099	0.550042633	ANXA4
ENSG00000107164.16	-0.062759196	0.158699154	0.550042633	FUBP3
ENSG00000183426.17	0.182448843	0.158773946	0.55020887	NPIPA1
ENSG00000154310.17	-0.075276078	0.158851938	0.550344526	TNIK

ENSG00000237352.4	-0.237068941	0.158866764	0.550344526	LINC01358
ENSG00000272906.1	0.148016279	0.158901055	0.550370348	RP11-533E19.7
ENSG00000211794.3	-0.214018076	0.158969178	0.550513322	TRAV12-3
ENSG00000241014.2	-0.136983766	0.159017968	0.550589311	GPR199P
ENSG00000107829.15	0.068021169	0.159101565	0.550736471	FBXW4
ENSG00000172538.7	-0.435804722	0.159114179	0.550736471	FAM170B
ENSG00000241860.7	-0.211008794	0.159162201	0.550809723	RP11-34P13.13
ENSG00000158887.19	0.08800438	0.159216749	0.550815517	MPZ
ENSG00000169105.8	0.075716413	0.159259558	0.550815517	CHST14
ENSG00000172315.6	-0.053929367	0.159275957	0.550815517	TP53RK
ENSG00000164626.9	-0.227742012	0.159296331	0.550815517	KCNK5
ENSG00000172426.16	0.191127882	0.159298168	0.550815517	RSPH9
ENSG00000198399.15	-0.072123246	0.159346107	0.550828321	ITSN2
ENSG00000158816.16	-0.371751997	0.15935559	0.550828321	VWA5B1
ENSG00000170956.17	0.089046062	0.159446936	0.550921427	CEACAM3
ENSG00000225137.1	0.228687788	0.15945992	0.550921427	DYNC1I2P1
ENSG00000167632.18	0.082905137	0.159463116	0.550921427	TRAPPC9
ENSG00000166342.19	-0.323355376	0.159516413	0.550964029	NETO1
ENSG00000123338.13	0.05371188	0.159529179	0.550964029	NCKAP1L
ENSG00000141425.18	-0.061554744	0.159642515	0.551262621	RPRD1A
ENSG00000088836.14	-0.23418642	0.159680805	0.551302013	SLC4A11
ENSG00000188878.19	0.083109135	0.159922843	0.551946259	FBF1
ENSG00000164845.16	0.186634147	0.159951725	0.551946259	FAM86FP
ENSG00000211856.1	-0.400707838	0.159970914	0.551946259	TRAJ33
ENSG00000242580.1	0.405469748	0.159987809	0.551946259	IGKV1D-43
ENSG00000228013.1	0.196866563	0.160021572	0.551946259	IL6R-AS1
ENSG00000133114.18	-0.066981024	0.160046704	0.551946259	GPALPP1
ENSG00000167508.12	0.098826822	0.160079358	0.551946259	MVD
ENSG00000111405.9	0.290971754	0.160082717	0.551946259	ENDOU
ENSG00000214353.8	0.285135191	0.160194068	0.551994891	VAC14-AS1
ENSG00000087338.5	-0.080996914	0.160203728	0.551994891	GMCL1
ENSG00000128463.13	0.057793235	0.160215825	0.551994891	EMC4
ENSG00000248571.1	-0.241382392	0.1602182	0.551994891	RP11-768B22.2
ENSG00000088882.8	-0.326981366	0.160261352	0.551994891	CPXM1
ENSG00000149591.17	0.164943173	0.160269262	0.551994891	TAGLN
ENSG00000253764.3	0.334601972	0.160285234	0.551994891	RP11-439C15.4
ENSG00000288610.1	0.598357422	0.16043793	0.552427985	RP11-736N17.12
ENSG00000161677.12	0.123405097	0.16048405	0.552494023	JOSD2
ENSG00000159579.14	-0.048082365	0.160519797	0.552524335	RSPRY1
ENSG00000138028.16	-0.357577863	0.160578753	0.55261711	CGREF1
ENSG00000242802.9	0.072757852	0.160642455	0.55261711	AP5Z1
ENSG00000058056.10	-0.084071446	0.160646473	0.55261711	USP13
ENSG00000106665.16	0.097420337	0.160673782	0.55261711	CLIP2
ENSG00000066084.13	-0.070763269	0.160733776	0.55261711	DIP2B
ENSG00000255002.2	-0.372770594	0.160735036	0.55261711	LINC02324
ENSG00000075292.19	-0.067306425	0.160749141	0.55261711	ZNF638
ENSG00000213020.10	-0.092291045	0.160781102	0.55261711	ZNF611

ENSG00000165660.8	-0.043875595	0.160789267	0.55261711	ABRAXAS2
ENSG00000127152.18	-0.077987316	0.160941834	0.552967918	BCL11B
ENSG00000082269.17	-0.128865499	0.160965893	0.552967918	FAM135A
ENSG00000135519.8	0.155076246	0.160972229	0.552967918	KCNH3
ENSG00000167637.18	-0.102598803	0.161037308	0.553098831	ZNF283
ENSG00000156858.12	0.068123953	0.161118959	0.553244281	PRR14
ENSG00000169220.18	0.061674629	0.161133611	0.553244281	RGS14
ENSG00000005108.16	0.415008773	0.161251957	0.55336846	THSD7A
ENSG00000104824.18	0.032710419	0.161262379	0.55336846	HNRNPL
ENSG00000198912.12	-0.05703575	0.161270871	0.55336846	C1orf174
ENSG00000213839.4	-0.093484711	0.16127771	0.55336846	TMX2P1
ENSG00000085662.14	0.069281797	0.161317121	0.55337891	AKR1B1
ENSG00000107949.17	0.06617281	0.161334723	0.55337891	BCCIP
ENSG00000224051.7	0.093094585	0.161407938	0.553527557	CPTP
ENSG00000242485.6	0.064810423	0.161461745	0.553527557	MRPL20
ENSG00000196218.13	0.140520738	0.161515579	0.553527557	RYR1
ENSG00000260034.1	-0.344147526	0.161524884	0.553527557	LCMT1-AS2
ENSG00000078747.16	-0.063257307	0.161531253	0.553527557	ITCH
ENSG00000105088.9	0.110163308	0.161549336	0.553527557	OLFM2
ENSG00000143155.13	-0.060012903	0.161566996	0.553527557	TIPRL
ENSG00000231616.9	-0.35255625	0.161621277	0.553621038	RP11-575L7.4
ENSG00000132434.10	0.064607255	0.161735338	0.553877529	LANCL2
ENSG00000198553.9	-0.244870465	0.161750691	0.553877529	KCNRG
ENSG00000253106.1	-0.09010814	0.161784237	0.553877529	RP11-158K1.3
ENSG00000106723.17	-0.066347726	0.161827613	0.553877529	SPIN1
ENSG00000270264.1	-0.197869924	0.161905126	0.553877529	NDUFB8P2
[truncated: 3,438,594 more chars]
